# Proteomic Profiling of Major Peanut Allergens and Their Post-Translational Modifications Affected by Roasting

**DOI:** 10.3390/foods11243993

**Published:** 2022-12-09

**Authors:** Teodora Đukić, Katarina Smiljanić, Jelena Mihailović, Ivana Prodić, Danijela Apostolović, Shu-Hua Liu, Michelle M. Epstein, Marianne van Hage, Dragana Stanić-Vučinić, Tanja Ćirković Veličković

**Affiliations:** 1 University of Belgrade—Faculty of Chemistry, Department of Biochemistry and Centre of Excellence for Molecular Food Sciences, Studentski Trg 12–16, 11000 Belgrade, Serbia; 2 University of Belgrade—Faculty of Chemistry, Innovation Center Ltd., Studentski Trg 12-16, 11000 Belgrade, Serbia; 3 Department of Medicine Solna, Division of Immunology and Allergy, Karolinska Institutet and Karolinska University Hospital, SE-171 76 Stockholm, Sweden; 4 Medical University of Vienna Department of Dermatology, Experimental Allergy Laboratory, Waehringer Guertel 18–20, 1090 Vienna, Austria; 5 Ghent University Global Campus, Incheon 406-840, Korea; 6 Faculty of Bioscience Engineering, Ghent University, Coupure Links 653, 9000 Ghent, Belgium; 7 Serbian Academy of Sciences and Arts, Kneza Mihaila 35, 1100 Belgrade, Serbia

**Keywords:** peanut allergen profiling, roasting, allergy, shotgun proteomics, high resolution mass spectrometry (HRMS), post-translational modifications, PTM profiling, western blot

## Abstract

Post-translational modifications (PTMs) are covalent changes occurring on amino acid side chains of proteins and yet are neglected structural and functional aspects of protein architecture. The objective was to detect differences in PTM profiles that take place after roasting using open PTM search. We conducted a bottom-up proteomic study to investigate the impact of peanut roasting on readily soluble allergens and their PTM profiles. Proteomic PTM profiling of certain modifications was confirmed by Western blotting with a series of PTM-specific antibodies. In addition to inducing protein aggregation and denaturation, roasting may facilitate change in their PTM pattern and relative profiling. We have shown that Ara h 1 is the most modified major allergen in both samples in terms of modification versatility and extent. The most frequent PTM was methionine oxidation, especially in roasted samples. PTMs uniquely found in roasted samples were hydroxylation (Trp), formylation (Arg/Lys), and oxidation or hydroxylation (Asn). Raw and roasted peanut extracts did not differ in the binding of IgE from the serum of peanut-sensitised individuals done by ELISA. This study provides a better understanding of how roasting impacts the PTM profile of major peanut allergens and provides a good foundation for further exploration of PTMs.

## 1. Introduction

Post-translational modifications (PTMs) are covalent changes occurring on amino acid (AA) side chains of proteins via enzymatic action or spontaneously. They also occur during food storage, influenced by environmental factors and food processing. The AA side chains can be enzymatically or chemically modified in a positive or negative delta mass manner (e.g., through a mass gain or a mass loss). Certain modifications are reversible, such as the single methionine (M) oxidation, while others are irreversible, e.g., sulfone, which is double-oxidized (M). The most modified AA side chains reported from major PTM databases in descending manner are the hydroxyl groups of serine and threonine, the ε-amino group of lysine, the phenolic group of tyrosine, the thiol group of cysteine, guanidino group of arginine, and carboxamide group of asparagine, while methionine is the last AA with a somewhat higher modification frequency compared to the median and average values [1]. More than 400 different modifications [2] and over 300 entries are registered in mass-spectrometry-devoted PTMs databases (e.g., Unimod, http://www.unimod.org/modifications_list.php?, accessed on 22 November 2021). Despite this general data, the PTMs still represent “The Dark matter of the proteomics” [3] and a highly neglected structural and functional aspect of protein architecture, especially their open, quantitative profiling across proteomes [4,5].

Ara h 1 allergen from raw peanuts is a glycoprotein containing one N-glycosylation site due to enzymatic glycosylation by α-mannosidase II [6]. Ara h 6 does not contain an N-linked glycosylation consensus sequence and is not regarded as a glycoprotein [7]. Findings that Ara h 2 isoforms are glycosylated and possess the putative N-glycosylation consensus sequence were not confirmed by Li et al. (2010) [8], using high-resolution mass spectrometry (HRMS) on another commercial cultivar of peanut with the same runner variety. On the other hand, Ara h 2 possesses 3 sites of proline (P) hydroxylation in heavier and 2 sites in the lighter version of isoforms [8]. It is not clear whether these site-specific P hydroxylations happen at 100% saturation or partially. The absence of P hydroxylation at linear Ara h 2 epitopes results in lower IgE binding [9].

Roasted peanut proteins undergo a Maillard reaction with advanced glycation end (AGE) product formation and concomitant protein oligomerisation and have a more substantial allergenic potential than fried or boiled peanuts [10]. AGE modifications were found on Ara h 1 and Ara h 3 in raw and roasted peanut extracts, such as carboxymethyl lysine (CML), but no AGE modifications were found on Ara h 2 [11]. CML, malondialdehyde (MDA), and hydroxy-nonenal (HNE) adducts were all present in raw and roasted peanuts, with roasted peanuts exhibiting a higher level of AGE and MDA adducts than raw peanuts [12].

Unspecified (unrestrictive, open) PTMs search, as a mass spectrometry-proteomic approach has already been applied [5], while we initiated relative, quantitative, unspecified PTMs profiling without immunoprecipitation [4] Moreover, the semi-quantitative profiling of PTMs on allergens from raw and thermally processed peanuts is still lacking in current literature. Therefore, proteomic, and immunological methods were applied to qualitatively and semi-quantitatively profile proteins and their PTMs, focusing on major peanut allergens (Ara h 1–3 and Ara h 6) in raw and roasted peanut extracts (PEs). The goal was to obtain proteins under mild, aqueous conditions similar to those employed when isolating allergens for diagnostic skin prick testing to reduce PTMs from being introduced during the extraction phase. With one-dimensional and two-dimensional SDS-PAGE (1D and 2D) and 1D gel-based bottom-up proteomics approach, the protein profiles’ comparison of the raw and roasted PEs was achieved. We determined PTM patterns and PTM profiling of raw and roasted peanut allergens with PEAKS Studio XPro. PTMs-specific antibodies (AbPTM) were used in Western blotting to confirm the presence of several PTMs detected on major allergens in both extracts. In addition, a competitive enzyme-linked immunosorbent assay (ELISA) was used to compare IgE binding response to raw and roasted peanuts.

We hypothesize that roasted peanuts have different protein extraction and PTM profiles compared to raw peanuts, whether they contain a unique set of PTMs induced by roasting or differ in the arrangement or frequency of common PTMs along the AA sequence. Significant PTM heterogeneity of peanut proteins results in myriads of proteoforms of major peanut allergens and could contribute to epitope diversity.

PTMs are still neglected, since their open, quantitative search by mass spectrometry-based proteomics is just emerging. In addition, different PTMs could affect digestion efficiencies of major gastric and intestinal peptidases. Looking at how PTMs affect digestion enzymes is the next step to uncovering one of their possible effects.

## 2. Materials and Methods

### 2.1. Peanut Thermal Treatment and Extract Preparation

Raw peanuts (*Arachis hypogea* L.) were obtained from a local grocery. The peanuts were roasted for 20 min at 175 °C. Raw and roasted peanut kernels were milled in a food processor. Milled peanut paste was defatted with *n*-hexane (1:6 *w/v*) by washing at room temperature (RT). Peanut paste was mixed with a glass stick and then left to settle. This action was repeated 3 times before *n*-hexane was drained using a cloth to squeeze out the remaining liquid. Defatted peanut flour was left to dry at RT for 3 h. Aqueous PEs were obtained in mild conditions, so to preserve as much of PTMs and to prevent the introduction of artificial modifications when using urea or other chaotropic substances [13] as well as to minimise further oxidative modifications by introducing excessive molecular oxygen during vigorous and prolonged vortexing.

Therefore, PEs from defatted flour were prepared in phosphate buffer saline (PBS, 1/10, *v/v*) by medium-to-intense magnetic stirring for 1.5 h at 4 °C with a protease inhibitor cocktail (P2714, Sigma-Aldrich, Taufkirchen Germany). The PEs were defatted with tetrachloroethylene (1:3 *v/v*) by vortexing the mixture for 3 × 5 min and then separating the organic and inorganic fractions by centrifuging at 13,000 rpm for 10 min. The organic layer was discarded. Defatted peanut extract was used in further experiments. Preparation of the insoluble fractions of raw and roasted peanuts was carried out as described in the Supplementary Material. Protein concentration was determined using the Bicinchoninic acid assay (Thermo Fischer, Munich, Germany).

### 2.2. 1D and 2D Electrophoresis

1D SDS-PAGE was carried out on a Hoefer scientific instrumentation apparatus (Amersham Biosciences, Uppsala, Sweden) with a discontinuous buffer system. To compare PEs protein profiles, 100 µg of each extract was applied per lane. Proteins were resolved on hand-cast 12% and 14% polyacrylamide (PAA) gels under reducing conditions. Isoelectric focusing was performed on rehydrated Immobiline strips IPG 3-10NL 13 cm in Ettan IPGPhor 3 (GE Healthcare, Chicago, IL, USA). Proteins were focused with a seven-step program mounting to 17,000 Vh, according to the in-house protocol [14]. The second dimension was carried out on 14% PAA gels. All gels (1DE and 2DE) were stained with Coomassie Brilliant Blue R-250 (Serva, Heidelberg, Germany). Raw and roasted PE 2DE gels were scanned with Typhoon FLA 7000 (GE Healthcare, Chicago, IL, USA), and protein spots were matched and quantified using Image Master 2DE Platinum 7.0 software (GE Healthcare, Chicago, IL, USA) (for more details, see Supplementary Materials).

### 2.3. Sample Preparation for Nano Liquid Chromatography Coupled to Tandem Mass Spectrometry (nLC-MS/MS)

**Figure 1:**
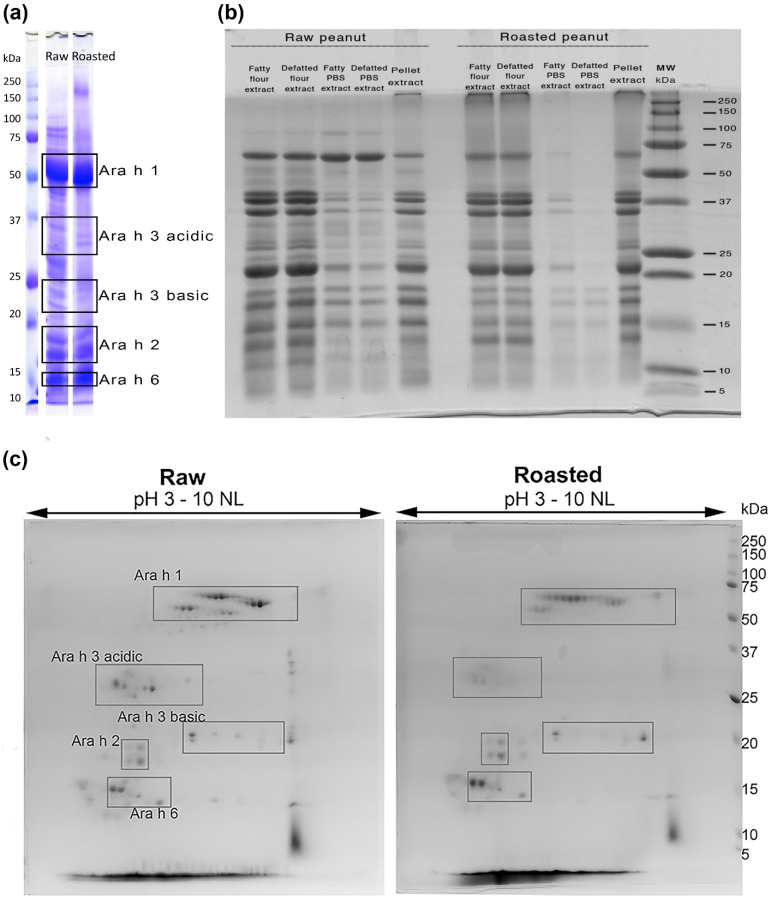
Raw and roasted PBS-based peanut extract (PEs) protein profiles. (**a**) Sodium dodecyl sulfate polyacrylamide gel electrophoresis (SDS-PAGE) of peanut extracts (100 μg per lane) resolved on 12% polyacrylamide gel in reducing conditions. The grey boxes denote gel pieces excised and analyzed by nLC-MS/MS. (**b**) SDS-PAGE profiles of insoluble and soluble fractions analyzed with the equal volumes of raw and roasted peanuts. SDS-PAGE of the non-defatted and defatted crude peanut paste was obtained in reducing conditions by Laemmli buffer, phosphate-buffered saline (PBS) extracts were prepared from non-defatted and defatted peanut flour, while pellet extract was obtained by pellet extraction with denaturing buffer. (**c**) 2D SDS-PAGE protein profiles of raw and roasted peanut extracts.

Major protein gel bands of major peanut allergens were excised, and in-gel trypsin digested as previously described [15]. Briefly, protein bands were resolved on 12% PAA gels and excised at positions corresponding to the four major peanut allergens (Figure 1a). Proteins in excised bands had their disulphide bridges reduced by 10 mM dithiothreitol, and cysteines alkylated with 55 mM iodoacetamide. Both samples were digested with proteomics-grade trypsin (Sigma-Aldrich, Taufkirchen, Germany) in a 1:50 enzyme-to-substrate ratio overnight at 37 °C. After trypsin digestion, sample cleanup with C18 ZipTips (Sigma-Aldrich, Taufkirchen, Germany) was done, and samples were ready for HRMS analysis.

### 2.4. nLC-MS/MS

As previously described, all digested samples were analyzed by nLC-MS/MS [16]. Briefly, peptides were chromatographically separated using the EASY-nLC II system (Thermo Fisher Scientific, Waltham, MA, USA) using A: 0.1% formic acid in water and B: 0.1% formic acid in acetonitrile as the mobile phase. Samples were chromatographically resolved using a 5–70–95% B gradient for 80 min at a flow rate of 300 nL/min. Peptides were analyzed using LTQ Orbitrap XL (Thermo Fisher Scientific Inc., Bremen, Germany) in a data-dependent mode with the 10 most intense precursors subjected to fragmentation by collision-induced dissociation.

### 2.5. Identification and PTM Profiling of Major Peanut Allergens

Peanut proteins were identified using the PEAKS XPro platform (BioinformaticsSolutions Inc., Waterloo, ON, Canada). Signature MS/MS spectra were searched using the PEAKS DB and PTM algorithms against a database consisting of UniProtKB (http://www.uniprot.org/, accessed on 18 October 2019) *Arachis hypogaea* entries (taxon ID 3818, 98,981 sequences, accessed on 18 October 2019) and contamination database common Repository of Adventitious Protein entries (http://www.thegpm.org/) (116 sequences, accessed on 18 October 2019). Protein groups, reported by the PEAKS PTM algorithm with at least two unique peptides, and contaminant hits were filtered out and the peptide false discovery rate (FDR) was less than 0.5%. In addition, the PEAKS platform included 313 post-translational and chemical modifications in the search space, regularly updated from the Unimod web-based database. The term “PTM profiling” means the relative quantification or extent of the modifications within a single sample. The mass spectrometry proteomics data have been deposited to the ProteomeXchange Consortium via the PRIDE [17] with the dataset identifier PXD033166 and doi:10.6019/PXD033166 Username: review-er_pxd033166@ebi.ac.uk, Password: GaJmjXJS.

### 2.6. Western Blot Analysis with Antibodies Specific to Modified AAs

PEs and their purified allergens [18], serving as controls (Ara h 1, Ara h 2, and Ara h 6), were electrophoretically resolved. Proteins were transferred from the gel to 0.2 µm nitrocellulose membranes (Bio-Rad, Hercules, CA, USA) using a semi-dry Nova-Blot system (GE Healthcare, Chicago, IL, USA) with a membrane current of 1 mA/cm^2^. The membranes were blocked with 1% bovine serum albumin (BSA) (Sigma-Aldrich, Taufkirchen, Germany) in 30 mM Tris-buffered saline of pH 7.5 containing 0.1% Tween 20 (1× TTBS) for 1 h at RT and subsequently washed three times with TTBS. Membranes were incubated for 3 h at RT with AbPTM (all polyclonal IgG, raised in a rabbit). Anti-methionine sulfoxide antibody (600160 Cayman Chemical, Ann Arbor, MI, USA) was diluted to 1:200 (*v/v*), while anti-acetyl-L-lysine (ab42789, Abcam, Branford, CT, USA), anti-hydroxy-proline (ab37067, Abcam, Branford, CT, USA), anti-carbamyl lysine (STA-078, Cell Biolabs, San Diego, CA, USA), anti-methyl-lysine (NB600-824, Novus Biologicals, Centennial, CO, USA), pan-anti-propionyl-lysine (PTM-201, PTM Biolab, Chicago, IL, USA), and anti-pyroglutamic acid (ABIN5662172, Antibodies online, Pottstown, PA, USA) were diluted to 1:1000 (*v/v*) in 0.5% BSA in 1× TTBS. BSA in 1× TTBS (0.5%) was used as the negative control. Alkaline phosphatase-conjugated (ALP) goat anti-rabbit IgG (111-055-045, Jackson ImmunoResearch, West Grove, PA, USA) was applied as a secondary antibody diluted to 1:1000 (*v/v*) in TTBS. Nitro-blue tetrazolium chloride and 5-bromo-4-chloro-3′-indolyl-phosphate were used to detect protein bands with modified AAs.

### 2.7. IgE Binding in ELISA

Raw and roasted PEs were diluted with coating buffer (100 mM NaHCO_3_ with Na_2_CO_3_, pH 9.6) to reach the concentration of 10 µg/mL, after which ELISA plates (Nunc, Roskilde, Denmark) were coated with 1 µg of protein per well. ELISA plates were incubated overnight at 4 °C. Coated plates were washed with 1× TTBS and blocked with 1% BSA in 1× TTBS for 1.5 h at RT. Sera of 10 peanut-sensitized patients (Table S1) were pooled and used for competitive ELISA. Raw and roasted PEs in a concentration range from 100–0.04 µg/mL with 3-fold dilution. 100 µL of a mixture of raw or roasted PE and the serum pool was added to the plate and incubated for 1 h at RT. To detect the remaining IgE binding to raw and roasted PE after the washing step, plates were incubated with anti-human IgE labelled with ALP (dilution 1:2000; MIAB, Uppsala, Sweden) for 1 h at RT. Color development was performed using p-Nitrophenyl Phosphate (Sigma-Aldrich, Taufkirchen, Germany) in detection buffer (diethanolamine 10 mM, MgCl_2_ 0.5 mM, pH 9.5) for 1.5 h. Inhibition of IgE binding was calculated as ((OD no inhibitor − OD inhibitor)/OD no inhibitor) × 100, and the concentration needed to inhibit 50% of this signal was calculated (IC50).

### 2.8. Statistical Analysis

Protein concentration differences were assessed with an unpaired two-tailed *t*-test using GraphPad Prism 7.00 software (GraphPad, San Diego, CA, USA) to search for significant differences between raw and roasted PEs and descriptive statistics.

## 3. Results and Discussion

### 3.1. Protein Electrophoretic and Mass Spectrometry Qualitative Comparison of Allergen Profiles in Raw and Roasted Peanut Samples

Raw and roasted PEs showed significant differences in protein concentrations (raw PE, 18.8 mg/mL vs. roasted PE, 4.8 mg/mL, at *p* = 0.003). This aligns with previous studies, where lower protein concentration of roasted PE results from decreased extractability due to protein aggregation promoted by the Maillard reaction during roasting and the formation of AGEs [16,19,20]. This was illustrated in Figure 1b, where the same volumes of raw and roasted PEs were resolved on reducing 1D SDS-PAGE, with a very faint roasted PE profile, compared to the raw counterpart. The water-soluble fraction is only a small part of the total peanut proteins from both PEs (Figure 1b). Profiles and levels of PTMs in the soluble fractions do not represent PTMs profiles of total protein content and, therefore, cannot provide the whole picture of protein PTMs induced by roasting. Consequently, it can be expected that PTM profiles in insoluble fractions are dramatically different between raw and roasted peanuts. Further investigation of PTM profiles of raw and roasted peanut soluble fractions obtained under simulated gastric and intestinal extraction conditions is warranted to provide a more relevant PTM picture.

Figure 1a,c show that Ara h 3 prevails in raw PE, like Ara h 1, while the opposite is true for Ara h 6, which is enriched in roasted PE; Ara h 2 bands are almost the same intensity. The presence of the four major peanut allergens at gel positions framed in Figure 1a correspond to full-length versions of Ara h 1, Ara h 2, Ara h 6, and Ara h 3 acidic and basic subunits, which HRMS confirmed. However, their actual distribution is more complex, and the identity of each major allergen or fragment has been found throughout several bands (Report S1). These differences in protein identification, as non-match between positions in mass segments on the gel and database-reported MW, were prominent in roasted samples, implying excessive proteolytic processing due to the roasting (MS identifications from the 1D SDS-PAGE, Report S1). For example, it could be inaccurately concluded that the relative content of Ara h 3 is lower in roasted compared to the raw PE according to the gels in Figure 1a,c, but that is not the case. Once mass spectrometry data were reviewed (Report S1), plenty of Ara h 3 was found in the lower-mass gel parts, marked as Ara h 2 and Ara h 6 segments (Figure 1a) in contrast to the same parts of the gel taken from the raw PE where such fragments were not found. Most likely, these fragments were formed as a result of thermal processing. The identity of the double band at ~85 kDa, observed in Figure 1a, was previously revealed, and corresponds to lipoxygenase (Uniprot entry Q9M5D3, Figure 1a and Table S2 in Prodic et al. (2019)) [16].

The high molecular weight band of ~200 kDa that can be noticed on the 1D gel (Figure 1a, lane roasted) is missing from the 2D gel corresponding to the roasted peanut extract (Figure 1c). The most probable reason is that the oligomerized portion of Ara h 1 did not transfer from the IPG strip onto the gel during the second dimension of 2D SDS-PAGE, which often happens with such large molecules. The raw peanut 2D profile in Figure 1c was similar to the profile of the standard raw PE by Prodic et al. (2018) [21]. The increased abundance of Ara h 1 and Ara h 6 and decreased share of Ara h 3 compared to the 2D map of standard peanut extract obtained by Prodic et al. (2018) [21], probably due to the different composition and pH of the buffers and the length of the extraction’s time.

Comparing the mass spectrometry results obtained from the in-gel analysis, 40 unique proteins were found in raw PE, 33 in both samples, and 13 unique proteins in roasted (Figure S1). Among the unique proteins found in raw PE, the following were allergens: Ara h 8 (non-specific lipid transfer proteins) and Ara h 10 (plant defensins) (Report S2). Among unique proteins, Ara h 1 specific isoform was found in a roasted peanut sample. Allergen groups shared by both extracts were Ara h 1–3, Ara h 6, and Ara h Agglutinin (Report S2). The mature seed of *Arachis hypogea* holds only a few dozen expressed proteins, precisely 32 groups of proteins [22]. These proteins and their isoforms can result in up to 500 spots on a high-resolution 2D silver-stained gel [23]. Even though the extraction conditions were mild and short, the versatility of proteins, especially in the case of raw PE, covered approximately one-third of the overall protein repertoire since more than 180 spots were detected (Figure S2), and 73 proteins were found in a raw peanut sample. In addition, Johnson et al., (2016) found 123 proteins with unique accession numbers, under harsh extraction conditions and by applying data-independent ion mobility MS [24].

Although we only analyzed 1D SDS-PAGE bands, and those masses corresponded to the main peanut allergens, other non-allergenic proteins were found. For example, in raw gel bands, according to the sum of areas under the curves of extracted ion chromatography (XIC), 89.8% of proteins detected were allergens, while in roasted, 97.81% (data not shown).

### 3.2. PTM Patterns of Raw and Roasted Allergen Peanut Samples

The most frequent modifications identified by the PEAKS PTM search algorithm and MudPIT crunching mode were oxidation (MHW), deamidated asparagine or glutamine (NQ), hydroxylated P (HyP), methylated lysine or arginine (KR), carbamoylation and two protons replaced by iron at aspartic acid and glutamic acid (DE) in descending manner and having roughly the same order in both sets of samples (Table S2). The majority of the peptides in both sets of samples were unmodified, and less than 3% of peptides were modified. HRMS detected more than 40 different types of modification in raw and roasted samples (Table S2). Out of all modifications, oxidized (M) was the most frequent, and it comprised 27.8% in raw and 41.8% in roasted; up to 18% for the deamidated (NQ) in raw samples and 6% in roasted; 2.5% for methylated (KR) in the raw sample and 9.3% in roasted (Table S2). Other modifications accounted for less than 6% of both samples’ overall modification abundances. These percentages are expressed as modification XIC curve area sums divided by the sum of all-modified peptides XIC curve areas (Table S2). The less frequent and abundant PTMs involved carbamoylation (KR), dehydration (STD), oxidation of histidine and tryptophan (HW), dihydroxylation (W), loss of ammonia from N, dimethylation (KR) and pyroglutamic acid from Q (Pyro-Glu from Q) at few relevant N-term sites and K/R formylation (Table S2). The allergen with the highest number of uniquely modified peptides was Ara h 1 followed by Ara h 3, which is not surprising since the highest number of peptides was detected for these two proteins (Report S1).

Table 1 depicts all confident and sensible site-specific PTM differences in patterns between raw and roasted samples that are not primarily the consequence of chemical opportunities brought by the trypsin digestion of peptide bonds and the liberation of N-terminal amino groups. Therefore, only the cases of carbamoylation of the Lys/Arg ε-amino group, including their methylation and formylation, were treated as relevant. In addition, we searched via www.iedb.org to ascertain if modified amino acids were a part of linear epitopes, as they could influence IgE-binding. This was carried out because many of these linear epitopes fall into a consensus of linear and conformational IgE binding epitopes derived experimentally and in silico [25].

Looking at Table 1, PTMs predominantly found in roasted samples are oxidation of (MHW), HyP, deamidation (NQ), methylation (R), dihydroxylation (W), and formylation (KR). The most frequent modification found in the Ara h 1 roasted sample was oxidation (MHW) which is a sign of oxidative stress, as methionine is a direct target of radical formation. While reduction of single oxidized M (OxM) is possible, OxM to methionine sulfone is a spontaneous and irreversible process that occurs through food processing or prolonged storage [26].

Deamidation (NQ) is an irreversible, non-enzymatic PTM, and it has been related to protein degradation and ageing, in this case, prolonged food storage [27]. Even if deamidation (NQ) can be introduced by sample preparation, due to basic conditions, we cannot fail to mention its increase in roasted Ara h 1 and Ara h 3 allergens [27].

In addition, unique for roasted samples are dihydroxylation (W), also a marker of intensive oxidation via hydroxyl radical [26,28], formylation (KR), and oxidation or hydroxylation (N) (Table 1). Non-enzymatic formylation of K, R side-chain residues is considered a marker of AGE in glycation processes [29].

**Figure 2:**
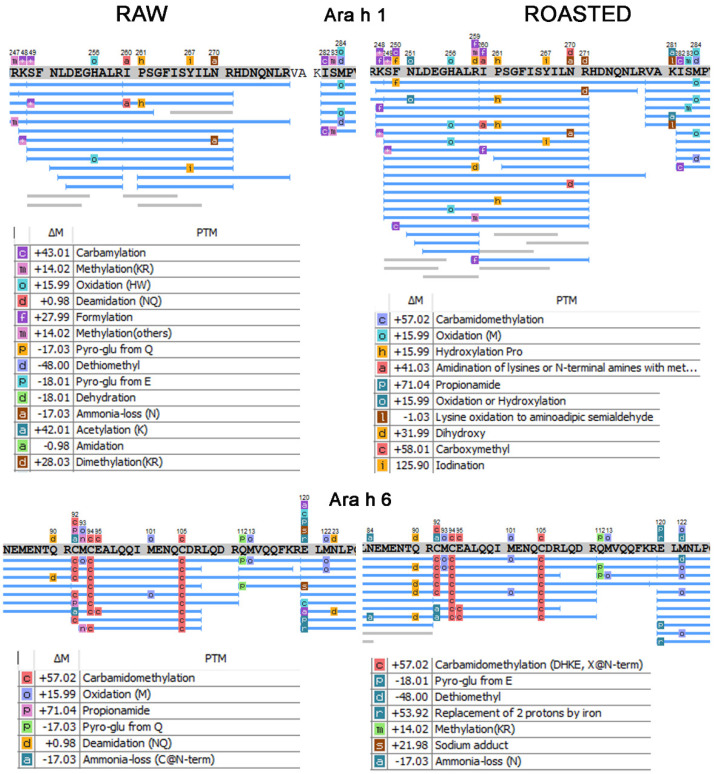
Selected sequence stretches mapped as immunodominant epitopes of Ara h 1 (accession #P43237) and Ara h 6 (accession #Q647G9), with supporting peptides and their confident PTMs shown on the top of the sequence (AScore > 50 and manually inspected MS2 spectra).

Looking at Figure 2, we can see selected examples of Ara h 1 and Ara h 6 sequence stretches that encompass immune, dominant epitopes mapped via www.iedb.org and a graphical presentation of PTM patterns from which one can intuitively comprehend the relationship between uniquely positioned PTM and those that are comparable, with higher or lower frequency. Many features can be grasped from Figure 2, like higher frequency of peptides with OxM (both Ara h 1 and 6), HyP, and oxidised (H) (Ara h 1) in roasted PE than in the raw PE. There is also a higher frequency of N-deamidated events at both allergen stretches in the roasted PE and several unique PTMs, such as K281 acetylation, K248, and R259 formylations on roasted Ara h 1, that are absent in the raw counterpart, including many more (Figure 2, Table S2, Report S1).

### 3.3. 1D Western Blot with Anti-PTM Antibodies in Relation to Mass Spectrometry Relative to PTM-Profiling

**Figure 3:**
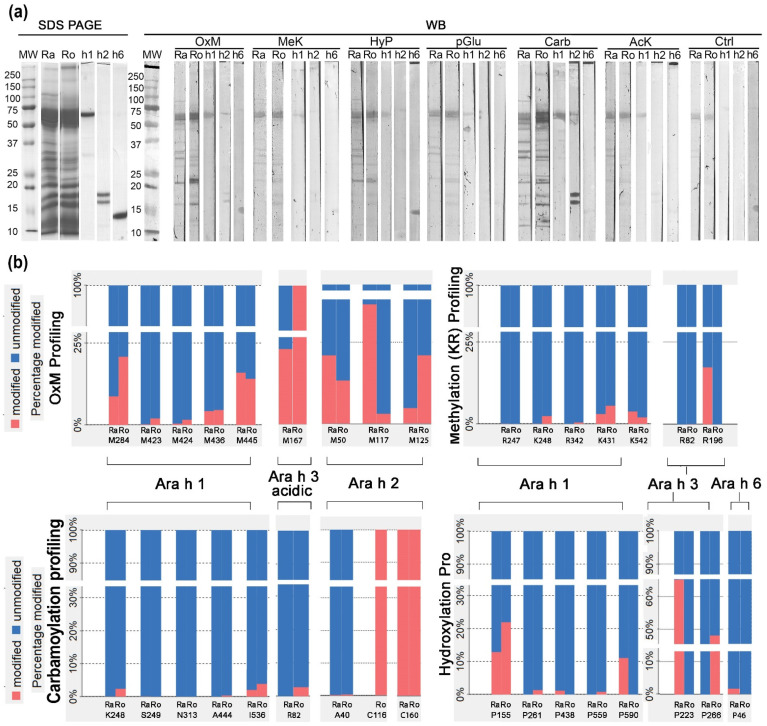
1D immunoblots of protein post-translational modifications (PTMs) of the raw and roasted peanut extracts, purified allergens, and representative examples of relative PTM profiling by tandem mass spectrometry and PEAKS PTM relative profiling tool. (**a**) Immunoblot with rabbit IgG antibodies specific to methionine sulfoxide (OxM), methyl-lysine (MeK), hydroxy-proline (HyP), pyroglutamate (pGlu), carbamyl-lysine (Carb), and acetyl-lysine (AcK). Proteins were resolved on 14% polyacrylamide gel in reducing conditions. Goat anti-rabbit IgG conjugated with alkaline phosphatase was used as the secondary antibody. (**b**) PTM-profiling tool that delivers relative saturation of site-specific PTMs by PEAKS X Pro Studio. Legend: WB—western blot, Ra—raw PE, Ro—roasted PE, h1—Ara h 1, h2—Ara h 2, h6—Ara h 6, Ctrl—control without primary antibody.

Electrophoretically resolved raw, roasted PEs and purified peanut allergens (Ara h 1, 2, and 6) were probed with different and commercially available antibodies against specific PTMs via 1D Western blot (Figure 3a). In Figure 3a, the strongest signal of binding was seen in the cases of carbamoylation (K) (CarbK), oxidation of (M) (OxM), and HyP, out of the six different AbPTM applied. However, we can also see non-specific binding in control samples of raw and roasted Pes of the same intensity, in the range of total monomer of Ara h 1 (~65 kDa), yet less intense than in the rest of Ara h 1 AbPTM-reactive bands (Figure 3a); therefore, an offset in judgment was considered.

From Figure 3b, we can see which AAs are prone to being modified during the roasting process. This is important for several reasons. For example, as mentioned above, deamidation (NQ) can lead to degradation [30]; PTMs positioned on K and R residues can affect trypsin’s ability to cut after these residues [5]; hydroxylation P within DPYSP (OH) motif of Ara h 2 linear epitopes has been shown to contribute significantly to the IgE reactivity of this allergen when this PTM is absent, IgE binding is decreased [9]. Let us not forget that a single phosphorylation on P53 regulates cell cycle checkpoint which depicts the significance of PTM profiling [31].

The representative examples for observing OxM on Ara h 1 allergen are sites M284, M423, and M424, which are more prone to oxidation in roasted samples, contrary to M445, which is less prone to oxidation by oxidation by roasting. In addition, the Ara h 2 M125 site seems more prone to oxidation during roasting than the other two shown in Figure 3b.

Looking at both figures together, we see that PTMs obtained by Western blotting at positions with masses corresponding to specific allergens (Figure 3a) closely match with PTMs identified by HRMS (Figure 3b). Relative PTM profiling covers all cases of open PTMs search and has a representation of saturation levels of modified peptides vs unmodified within PE samples’ (Figure 3b). PTM profiling graphs in Figure 3b represent the relative abundance of confidently identified modified and unmodified peptides within a specific raw and roasted peanut gel band, respectively.

We have found an accurate match regarding the confirmation of respective PTMs found by MS (Figure 3b) and their signal intensity relationship seen on the blot (Figure 3a). Figure 3b depicts the PTM profile of four PTMs which we chose to compare to the 1D Western blot (Figure 3a). For example, OxM is an ideal PTM for validation since it involves a single AA, sulfoxide form of M, and has a specific AbPTM. Furthermore, OxM is independent of amino group chemistry liberated after trypsin digestion. Therefore, this PTM served as a tool to study the effects of oxidative stress in certain conditions when its threshold levels via controls were established [4,32]. We can see from Figure 3 a precise match between OxM profiles on the blot, and selected representative examples of PEAKS software identified OxM profiling on certain isoforms of Ara h 1, 2, and 3. A good match was replicated in the rest of the profiling comparisons: methylation (K)(MeK), HyP, and CarbK. While CarbK and OxM showed clear signs of a stronger binding signal in roasted PE, it seems that for HyP there is no noticeable difference in overall intensity; however, there is a slight difference among the patterns (Figure 3a). The signal intensity of the other 3 PTMs is fainter, with MeK antibody binding being stronger in the roasted PE, while acetylation (K)(AcK) and pyroglutamic acid (pGlu) had profiles of the rather similar intensity of binding between the extracts. The source of the non-specific binding seen in the control extracts is probably due to vast amounts of Ara h 1 protein in the extract compared to the amount of purified Ara h 1 applied to the control.

While OxM, MeK, and HyP were straightforward for comparison between Western blot and MS data, carbamoylation imposed some extra work to enable a fair process. Namely, carbamoylation events discovered by MS had to be differentiated into K, R side chain carbamoylation events and N-term peptide carbamoylations, which could happen as post-tryptic digestion events [13]. Furthermore, the carbamoylation reaction has a higher rate on primary amines than the Lys ε-NH_2_ group [13]. However, in our study, carbamoylation was studied only from the 1D gel, and urea was not used in any step. In connection to this, Kollipara et al. (2013) have shown that when using control (in 0.1% TFA *v/v*) or 0.1 M urea, 50 mM ammonium bicarbonate, pH 7.8, there is no in vitro carbamoylation of Lys/Arg side chains, while there is up to 2% of carbamoylated primary NH_2_ on post-tryptic peptides. This is the limiting factor for analyzing PTMs; their chemistry is associated with amino group reactivity and application of trypsin in bottom-up proteomics. However, in Figure S3, there is proof that the carbamoylation event existed even before trypsin digestion due to position in the sequence DKD, which most likely prevented the scission of the peptide bond by trypsin. Although protein carbamoylation is rare in plants, it seems that it could occur in legumes. Legumes, such as peanuts, transport fixed nitrogen from nodules to upper parts in the form of ureides, such as allantoin, synthesized de novo in the nodule. After their transformation to ureidoglycolate, ureidoglycolate urea-lyase releases urea [33]. This results in Lys carbamoylation, similar to animal tissue events [34]. During roasting, high temperatures lead to an additional urea generation by degradation of ureidoglycolate, which could also occur spontaneously [35], resulting in more intense carbamoylation.

Reassessed data from our previous study [21] showed that Ara h 2 from raw peanuts had 6 HyP sites with a double HyP at the linear epitope DP(OH)YSP(OH). In this study, a mixed situation was encountered with the rest of the major allergens regarding HyP presence or absence at specific P sites in the raw and the roasted PEs, where no HyP site on Ara h 2 was found (Table 1). More important is the fact that there is a non-enzymatic way of proline hydroxylation [25,36]. The research on the roles of proline in oxidative stress survival [35] allows the possibility of direct proline involvement as a ROS scavenger and being hydroxylated via OH radical attack while remaining in the protein backbone peptide chain as hydroxyprolyl residue. Therefore, a possible explanation for the absence of HyP in the roasted PE could be the engagement of these hydroxyl groups into further stages of proline oxidation and transformation into 2 pyrrolidone [37], leading to peptide backbone chain breakage, which we were unable to detect.

The main limitation regarding this attempt to confirm MS-discovered PTMs by Western blot is that many PTMs found by MS could not be confirmed due to the lack of appropriate antibodies. However, complementing each other, these two methods could provide a more precise and reliable picture of the semi-quantitative and qualitative profile of PTMs.

### 3.4. Effects of Thermal Processing on IgE Binding to Raw and Roasted PEs

**Figure 4:**
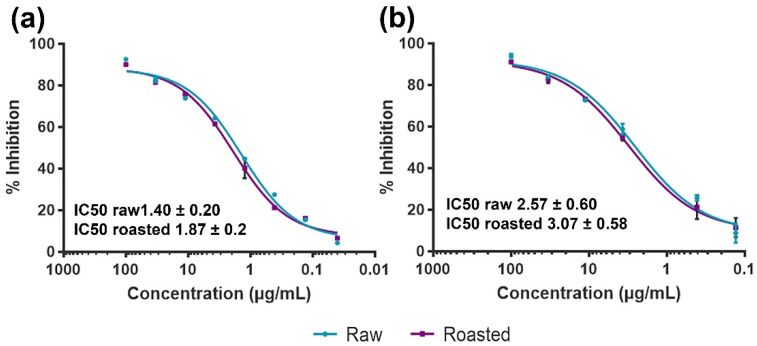
IgE binding from the pool of patient sera to peanut extracts (PEs) in ELISA. (**a**) Inhibition of peanut-allergic patient IgE binding to raw PE (base-coat) by raw PE (turquoise line) and roasted PE (purple line). (**b**) Inhibition of peanut-allergic patient IgE binding to roasted PE (base-coat) by raw PE (turquoise line) and roasted PE (purple line).

ELISA inhibition was performed to test the IgE binding potency of raw and roasted PEs. When raw PE was bound to the plate, the binding of IgE was similarly inhibited by raw and roasted extracts. The sigmoid curves for the two inhibitors overlapped (Figure 4a), and there were no statistically significant differences between the IC50 values (1.40 ± 0.20 µg/mL for raw as an inhibitor and 1.87 ± 0.2 µg/mL for roasted peanuts as inhibitors). The same was noticed for roasted PE (bound to the plate) with IC50 2.57 (±0.60) µg/mL for inhibition by raw PE and 3.07 (±0.58) µg/mL for homologous inhibition (Figure 4b). Considering that the same amount of protein was applied in both cases and on the same microtiter plate, it was evident that the IC50 values of both inhibitors were twofold higher for roasted PE as matrices compared to raw PE, but the difference in IC50 values between raw and roasted PEs was not statistically significant. The narrow range of IC50 values between raw and roasted PE indicated a highly similar IgE-binding potency in these samples. Raw and roasted PEs obtained in the study of Maleki et al. (2000) [10], under unknown extraction conditions, have shown different IgE binding properties in direct competitive ELISA, with the roasted PE being 90 times more potent than the raw one, explaining this by the formation of Maillard reaction products during the roasting process. On the other hand, in the study by Di Stasio et al. (2020), where raw and roasted peanuts were subjected to the Infogest protocol [38], no difference was found in eliciting the response in the degranulation assay. It was even found that roasted peanuts were more prone to degradation during digestion [39].

The probable reason for the lack of immune difference between the peanut samples that significantly differed in allergen content and PTMs profiles is the IgE binding properties of the serum pool. Patients’ sera highly favor Ara h 2 binding (Table S1) instead of Ara h 1. The predominant allergen among the four major allergens in PEs is Ara h 1, while Ara h 2 is the least abundant (Figure 1c). In addition, in both raw and roasted PE, the crucial PTM for IgE binding of Ara h 2, HyPs located within immunodominant DPYSPS epitopes [9] was absent, thus resulting in a lack of significant IgE binding difference between raw and roasted PEs.

## 4. Conclusions

Here we conducted a bottom-up proteomic study that compares allergens and their PTM profiles of aqueous PEs obtained in mild extraction conditions, similar to allergenic extracts for skin prick testing, before and after roasting. After roasting, peanut proteins are almost 4 times less soluble under mild aqueous extraction conditions. Although the most soluble protein fraction of roasted peanuts contains a slightly higher share of allergens than raw peanuts, roasted peanuts would possibly release a lower quantity of allergens during the gastric phase of digestion due to the inefficient extractability of proteins. Among four major peanut allergen groups, we found that Ara h 3 prevails in raw PE, similar to Ara h 1, while the opposite is true for Ara h 6, which is enriched in roasted PE; Ara h 2 bands are near the same intensity.

HRMS detected more than 40 different types of modification in raw and roasted samples. There were evident differences in types and the presence of specific AA modifications between allergens in raw and roasted samples. Roasting affected the most frequent modifications by enrichment of OxM, HyP, carbamoylation (KR), and deamidation (NQ). The modifications could also be mapped to the regions of IgE-binding epitopes of Ara h 1–3 and Ara h 6, where most modified sites were a part of linear immuno-epitopes deposited on IEDB (www.iedb.org accessed on 9 November 2021) Ara h 1 was the most versatile modified allergen, and its PTM profile resembled the overall samples PTM profile.

Western blot with specific AbPTM showed a good match with our MS profiling PTMs data. In addition, a precise match was observed between OxM, CarbK, HyP, and MeK. CarbK and OxM showed clear signs of a stronger binding signal in roasted PE, while for HyP there was no noticeable difference in overall intensity; however, there was a slight difference in signal pattern.

The effects of roasting were assessed with competitive ELISA, and no differences between the potency of raw and roasted PEs were observed.

This study provides insights into the modifications of peanut allergens affected by roasting and their allergenic properties. Our results can contribute to a better understanding of allergenic proteoforms readily available for interaction with the immune system. In addition, the results presented in this paper can contribute to developing the methodology for the risk assessment of allergen contamination and the effects of thermal processing on allergen properties. The open, quantitative PTMs proteomic search that we presented in this manuscript can serve as a starting point for deciphering the deeper roles of specific PTMs or starting to look at PTMs as biomarkers of certain physiological states. Incorporating INFOGEST 2.0 protocol in this equation can also shed light on how PTMs affect the specificity of digestion enzymes. This kind of approach can be replicated and applied to any other field involving specific or wide protein characterization, for example the environmental sciences.

## Figures and Tables

**Table 1:** Sites of modifications’ qualitative differences between raw and roasted samples of major peanut allergens summarized on dominant UniProt accession entries. Ara h 1—P43237 ^a^; E5G076 *; P43238 ^b^; Q6PSU3 ^c^; Ara h 3—A1DZF0 ^d^; Q6IWG5 ^e^; Q0GM57 ^f^; Q6T2T4 ^g^; Q6 T2T4 ^g^; Q647H3 ^h^; Ara h 2—Q6PSU2 ^j^; Q647G9 ^k^; Ara h 6—Q647G9.

Modification	Δ Mass (Da)	Ara h 1		Ara h 3		Ara h 2		Ara h 6	
		Raw	Roasted	Raw	Roasted	Raw	Roasted	Raw	Roasted
Acetylation (K)	42.0106	**K222 ^a^**	/	/	/	/	/	/	/
Amidation (K)	−0.9840	**K222 ^a^**	/	/	/	/	/	/	/
Carbamoylation	43.0058	**H223 ^a^**	/	/	/	/	/	/	/
		**K542 ^a^**	/	/	/	/	/	/	/
		**K457** *	/	/	/	/	/	/	/
Carboxylation (D)	43.9898	/	/	/	/	/	/	**D81**	/
Deamidation (NQ)	0.984	N216 ^a^	/	/	**N57 ^d^**	/	/	/	/
		/	**N270 ^a^**	/	/	/	/	/	/
		**Q303 ^a^**	/	/	**Q61 ^d^**	/	/	/	/
		**N313 ^a^**	/	/	**N496 ^d^**	/	/	/	/
		**N324 ^a^**	/	/	**N503 ^d^**	/	/	/	/
		/	**N334 ^a^**	N381 ^e^	/	/	/	/	/
		/	**N388 ^a^**	N383 ^f^	/	/	/	/	/
		/	**N415 ^a^**	/	/	/	/	/	/
		/	**Q417 ^a^**	/	/	/	/	/	/
		/	**Q552 ^a^**	/	/	/	/	/	/
		/	**Q150 ^c^**	/	/	/	/	/	/
Dehydration (QT)	−18.0106	**Q234 ^a^**	/	/	/	/	/	/	/
		T223 *	/	/	/	/	/	/	/
		Q240 *	/	/	/	/	/	/	/
Dihydroxy (W)	31.9898	/	/	/	**W388 ^d^**	/	/	/	/
		/	/	/	**W395 ^g^**	/	/	/	/
Dimethylation (R)	28.0313	**R214 ^a^**	/	/	/	/	/	/	/
Formylation (KR)	27.9949	/	**K248 ^a^**	/	/	/	/	/	/
		/	**R259 ^a^**	/	/	/	/	/	/
Hydroxylation (P)	15.9949	**P221 ^a^**	/	/	/	/	/	/	/
		/	**P559 ^a^**	/	/	/	/	/	/
		/	P161 *	/	/	/	/	/	/
		/	P564 *	/	/	/	/	/	/
Methylation (R)	14.0157	/	**R342 ^a^**	R196 ^h^	/	/	/	/	/
		/	R345 *	R195 ^d^	/	/	/	/	/
Oxidation (MHW)	15.9949	/	**W152 ^a^**	/	**W388 ^d^**	/	/	/	M56
		/	**H206 ^a^**	/	**W396 ^h^**	/	/	/	/
		**H223 ^a^**	/	/	/	/	**M125 ^j^**	/	/
		/	**H256 ^a^**	M167 ^h^	/	/	/	**M113**	/
		/	W476 ^a^	/	/	/	/	/	/
		/	W158 *	/	/	/	/	/	/
		/	H369 *	/	/	/	/	/	/
		/	W481 *	/	/	/	/	/	/
		/	**M431 ^b^**	/	/	/	/	/	/
		/	**M432 ^b^**	/	/	/	/	/	/
		/	**M444 ^b^**	/	/	/	/	/	/
		/	**M290 ^b^**	/	/	/	/	/	/
		/	**M284 ^a^**	/	/	/	/	/	/
Oxidation or Hydroxylation (N)	15.9949	/	**N251 ^a^**	/	/	/	/	/	/
Pyro-glu from E	−18.0106	/	**E432 ^a^**	/	/	/	/	/	E120
		/	E437 *	/	/	/	/	/	/
Pyro-glu from Q	−17.0265	/	**Q552 ^a^**	/	**Q25 ^d^**	/	/	/	/
		/	Q557 *	/	/	/	/	/	/
Replacement of 2 protons by iron (E)	59.9193	**E563 ^a^**	/	/	E380 ^d^	/	/	/	/
		E568 *	/	/	E387 ^g^	/	/	/	/

Amino acid residues that are a part of known linear epitopes are designated with bolded characters. Letters in the superscript represent the most dominant UniProt accession numbers. * Denote additional sites within E05G76 allergen isoform with the difference in PTM site between raw and roasted variant, not observed in P43237 Ara h 1 allergen isoform or primary sequence homologous sites with a different outcome in modification presence/absence in respect to P43237 allergen isoform.

## Data Availability

The mass spectrometry proteomics data have been deposited to the ProteomeXchange Consortium via the PRIDE partner repository with the dataset identifier PXD033166 and doi:10.6019/PXD033166 Username: reviewer_pxd033166@ebi.ac.uk, Password: GaJmjXJS.

## Supplementary Materials

The following supporting information can be downloaded at: https://www.mdpi.com/article/10.3390/foods11243993/s1, Figure S1: Venn diagram of all raw and roasted peanut gel bands that were processed in MudPIT manner, showing the number of unique proteins found in each sample, including the shared ones; Figure S2: 2D SDS-PAGE protein profiles of raw (**a**) and roasted (**b**) peanut extracts with spots and spot matches numbered; Figure S3: Overview of MS2 spectrum of the peptide with K542 carbamoylation modification on Ara h 1 isoform E5G076; Report S1: Allergen gel bands processed/crunched each as a separate sample; Report S2: All raw allergen 1D SDS-PAGE bands processed together as one crunch/sample (pages 1–143). All roasted allergen 1D SDS-PAGE bands processed together as one crunch/sample (pages144–325); Table S1: IgE levels of peanut-sensitised patients determined by ImmunoCAP in kUA/; Table S2 (excel file): Peanut allergen modification profiling—PTM profiles of peanut major allergen modifications from raw and roasted peanut samples.
