# Supplementary material for: Proteomic Profiling of Major Peanut Allergens and Their Post-Translational Modifications Affected by Roasting
Source: Foods. 2022 Dec 9;11(24):3993. doi: 10.3390/foods11243993 (PMC9778155; doi:10.3390/foods11243993)
Supplement: Supplementary file 1 [file foods-11-03993-s001.zip › Report S1.pdf]

Summary ALLERGEN GEL BANDS PROCESSED/CRUNCHED EACH AS A SEPARATE SAMPLE

1. Notes

Ara h 1 GEL BAND RAW

2. Result Statistics

Figure 1. False discovery rate (FDR) curve. X axis is the number of peptides being kept. Y axis is the corresponding FDR.

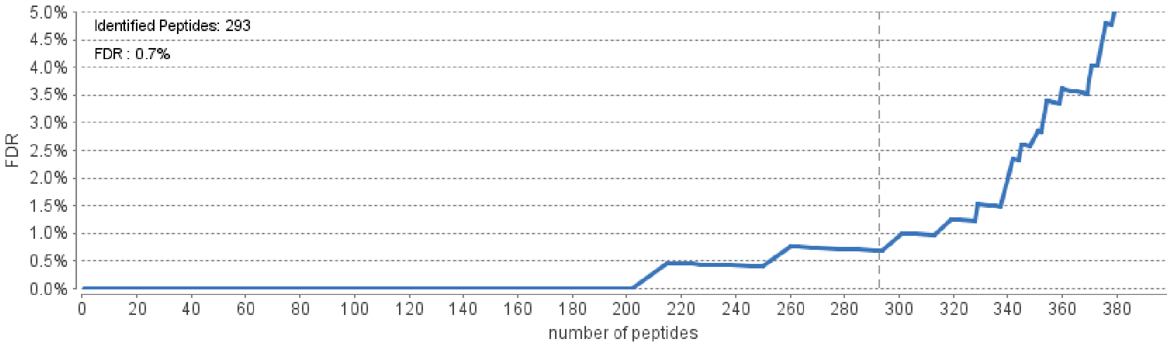

Figure 2. PSM score distribution. **(a)** Distribution of PEAKS peptide score; **(b)** Scatterplot of PEAKS peptide score versus precursor mass error.

(a)

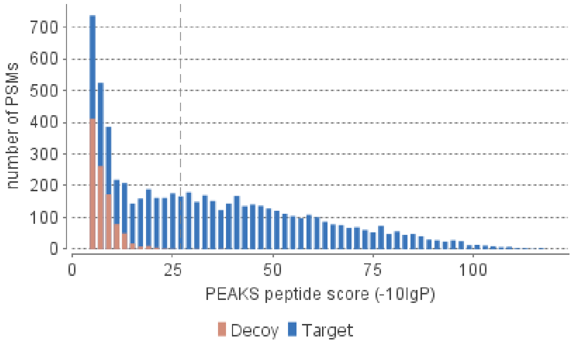

(b)

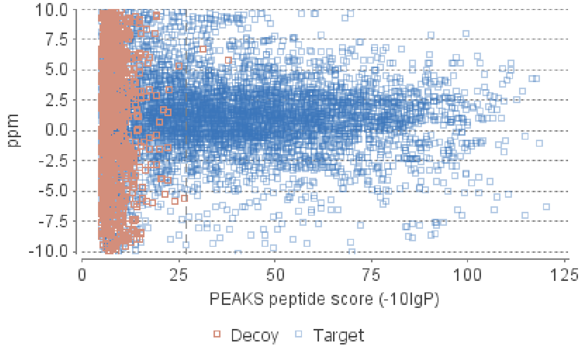

Table 1. Statistics of data.

	#Scans			#Features	Identified			#Peptides	#Sequences	#Proteins*		
	MS1	MS/MS	#Chimera		#PSMs	#Scans	#Features**			Groups	All	Top
Total	4978	7621	1342	11980	3438	3305	877	293	123	3	5	4
raw peanut	4978	7621	1342	11980	3438	3305	877	293	123	3	5	4

* proteins with significant peptides are used in counts.
** features are identified by DB search only.

Figure 3. Sample overlap for Proteins and Peptides (up to 8 samples). **(a)** All Proteins; **(b)** Top Proteins; **(c)** Peptides;

(a) **Not applicable to only one sample** **(b)** **Not applicable to only one sample** **(c)** **Not applicable to only one sample**

Figure 4. Distribution of peptide feature detection. **(a)** Feature m/z distribution; **(b)** Feature RT distribution.

(a)

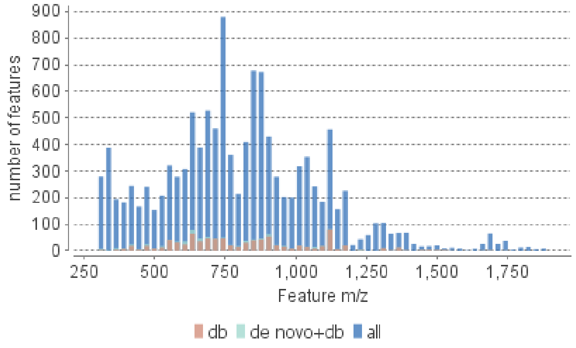

(b)

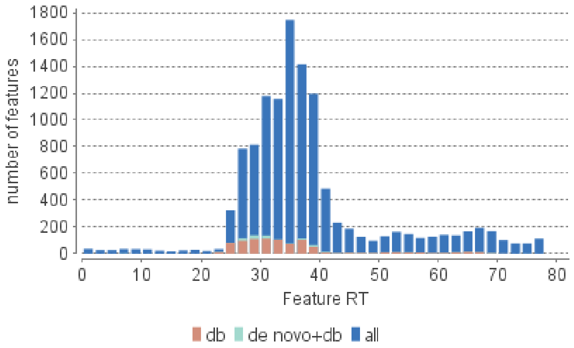

Figure 5. Distribution of identified peptide features. **(a)** Feature abundance distribution; **(b)** *De novo* sequencing validation.

(a) **(b)**

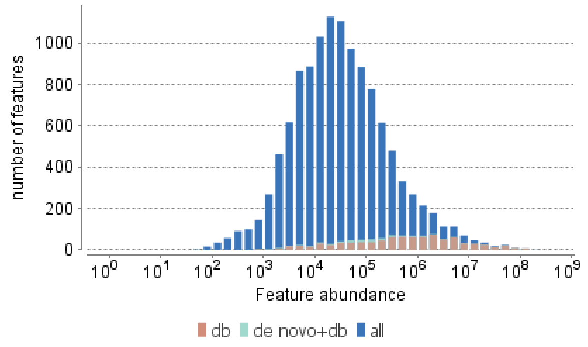

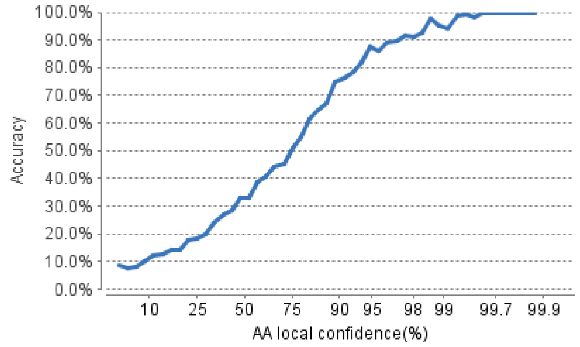

Table 2. Result filtration parameters.

Peptide -10lgP	≥26.8
PTM Ascore	≥50
Protein -10lgP	≥20
Proteins unique peptides	≥2
De novo score(%)	≥50%

Table 3. Statistics of filtered result.

FDR (Peptide-Spectrum Matches)	0.1%
FDR (Peptide Sequences)	0.7%
FDR (Protein Group)	0.0%
De Novo Only Spectra	368

Table 4. PTM profile.

Name	ΔMass	Position	#PSM	-10lgP	Abundance	AScore
Deamidation	.98	NQ	250	111.24	6.8E7	25.66
Carbamidomethyl	57.02	C	190	120.22	7.23E6	1000.00
Oxidation	15.99	M	184	110.46		30.46
HydPro	15.99	P	47	65.62	2.67E6	1000.00
Dethiomethyl	-48.00	M	36	96.95	4.28E6	1000.00
Carbamylation	43.01	K,N-term	33	80.77	1.79E6	252.86
Amidine	41.03	N-term	31	59.92	2.02E6	1000.00
Methylation(KR)	14.02	KR	29	64.78	2.03E6	69.38
Methylation(others)	14.02	CDHST	25	88.98		33.98
Dehydration	-18.01	DST,C-term	24	52.23	6.18E6	1000.00
Ammonia loss	-17.03	N	14	99.83	6.34E5	11.10
Pyro-glu from Q	-17.03	N-term	8	65.38	9.23E5	1000.00
Oxidation	15.99	HW	7	56.30		1000.00
Acetylation	42.01	K	5	41.87	1.17E6	13.38
Methyl+Deamidated	15.00	Q	4	43.55	5.72E6	121.92

3. Experiment Control

Figure 6. Precursor mass error of peptide-spectrum matches (PSM) in filtered result. **(a)** Distribution of precursor mass error in ppm; **(b)** Scatterplot of precursor m/z versus precursor mass error in ppm.

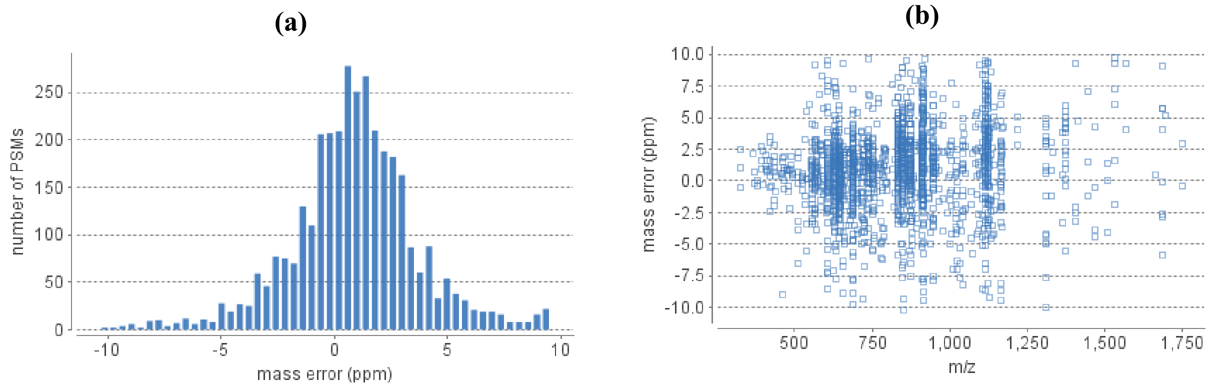

Table 5. Number of identified peptides in each sample by the number of missed cleavages.

Missed Cleavages	0	1	2	3	4+
raw peanut	93	152	48	0	0

4. Other Information

Table 6. Search parameters.

Search Engine Name: PEAKS
Parent Mass Error Tolerance: 10.0 ppm
Fragment Mass Error Tolerance: 0.5 Da
Precursor Mass Search Type: monoisotopic
Enzyme: Trypsin
Max Missed Cleavages: 2
Digest Mode: Unspecific
Fixed Modifications:
  Carbamidomethylation: 57.02
Variable Modifications:
  Deamidation (NQ): 0.98
  Oxidation (M): 15.99
  Hydroxylation Pro: 15.99
  Acetylation (K): 42.01
  Acetylation (Protein N-term): 42.01
  Acetylation (N-term): 42.01

Table 7. Instrument parameters.

Fractions: OB5939 H1 raw.raw, OB5940 H1 raw.raw, OB5941 H1 raw.raw
Ion Source: ESI(nano-spray)
Fragmentation Mode: CID, CAD(y and b ions)
MS Scan Mode: FT-ICR/Orbitrap
MS/MS Scan Mode: Linear Ion Trap

Amidation: -0.98
Beta-methylthiolation: 45.99
and 305 more...

Max Variable PTM Per Peptide: 5
Database: Uniprot_Peanut-3818_Jul18
Taxon: All
Contaminant Database: contaminantsMQ_mar19
Searched Entry: 1723
FDR Estimation: Enabled
De novo score(%) threshold: 15
Peptide hit threshold (-10logP): 30.0
Peaks run ID: 12
Merge Options: no merge
Precursor Options: corrected
Charge Options: no correction
Filter Charge: 2 - 15
Process: true
Associate chimera: yes

Protein List

Protein Accession Contains:

Protein Description Contains:

Protein Sample Area >=

Protein PTM Contains:

Protein Group	Protein ID	Accession	-10lgP	Coverage (%)	Coverage (%) raw peanut	Area raw peanut	#Peptides	#Unique	#Spec raw peanut	PTM	Avg. Mass	Description
**1**	3	P43237|ALL11_ARAHY	509.70	67	67	3.3474E8	97	11	3098	Y	70283	Allergen Ara h 1, clone P17 OS=Arachis hypogaea OX=3818 PE=1 SV=1
**1**	2	tr|B3IXL2|B3IXL2_ARAHY	509.70	67	67	3.3474E8	97	11	3098	Y	70283	Main allergen Ara h1 OS=Arachis hypogaea OX=3818 PE=2 SV=1
**5**	7	tr|E5G076|E5G076_ARAHY	456.46	57	57	1.3782E7	63	2	1104	Y	70788	Ara h 1 allergen OS=Arachis hypogaea OX=3818 GN=ara h 1 PE=4 SV=1
**7**	12	tr|A0A290FZZ3|A0A290FZZ3_ARAHY	118.71	11	11	1.9568E5	2	2	2	N	41532	Resistance protein (Fragment) OS=Arachis hypogaea OX=3818 PE=2 SV=1
**total 4 proteins**												

P43237|ALL11_ARAHY

back to list

| **Protein Coverage** | **Supporting Peptides** |

Protein Coverage:

1 MRGRVSPLML LLGILVLASV SATQAKSPYR KTENPCAQRC LQSCQQEPDD LKQKACESRC TKLEYDPRCV YDTGATNQRH
81 PPGERTRGRQ PGDYDDDRRQ PRREEGGR**WG PAEPR**ER**ERE EDWRQPREDW RRPSHQQPR**K **IRPEGREGEQ EWGTPGSEVR**

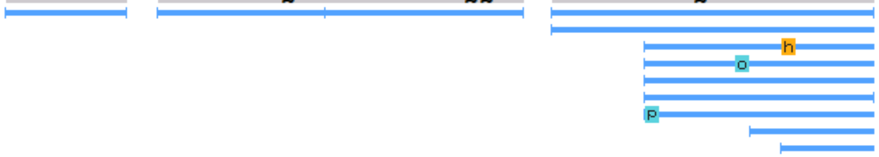

a Acetylation (K) (+42.01)
a Amidination of lysines or N-terminal amines with methyl acetimidate (+41.03)
a Ammonia-loss (N) (-17.03)
c Carbamidomethylation (+57.02)
c Carbamylation (+43.01)
c Carboxymethyl (+58.01)
d Deamidation (NQ) (+0.98)
d Dehydration (-18.01)
d Dimethylation(KR) (+28.03)
d Deamidation followed by a methylation (+15.00)
d Dethiomethyl (-48.00)
f Formylation (+27.99)

161 EETSRNNPFY FPSRRFSTRY GNQNGRIRVL QRFDQRSKQF QNLQNHRIVQ IEARPNTLVL PKHADADNIL VIQQGQATVT

241 VANGNNRKSF NLDEGHALRI PSGFISYILN RHDNQNLRVA KISMPVNTPG QFEDFFPASS RDQSSYLQGF SRNTLEAAFN

321 AEFNEIRRVL LEENAGGEQE ERGQRRRSTR SSDNEGVIVK VSKEHVQELT KHAKSVSKKG SEEEDITNPI NLRDGEPDLS

401 NNFGRLFEVK PDKKNPQLQD LDMMLTCVEI KEGALMLPHF NSKAMVIVVV NKGTGNLELV AVRKEQQQRG RREQEWEEEE

481 EDEEEEGSNR EVRRYTARLK EGDVFIMPAA HPVAINASSE LHLLGFGINA ENNHRIFLAG DKDNVIDQIE KQAKDLAFPG

- f Formylation (+27.99)
- h Hydroxylation Pro (+15.99)
- i Iodination (+125.90)
- l Lysine oxidation to aminoadipic semialdehyde (-1.03)
- m Methylation(KR) (+14.02)
- m Methylation(others) (+14.02)
- o Oxidation (M) (+15.99)
- o Oxidation (HW) (+15.99)
- p Pyro-glu from E (-18.01)
- p Pyro-glu from Q (-17.03)
- s Sulphone (+31.99)
- * Acetylation (N-term) (+42.01), Oxidation (HW) (+15.99)
- * Amidation (-0.98), Acetylation (K) (+42.01)
- * Carbamylation (+43.01), Methylation(KR) (+14.02)
- * Carbamylation (+43.01), Methylation(others) (+14.02)
- * Methylation(C-term) (+14.02), Carbamylation (+43.01)

561 SGEQVEKLIK NQRESHFVSA RPQSQSPSSP EKEDQEEENQ GGKGPLLSIL KAFN

Supporting Peptides:

Peptide	Uniq	-10lgP	Mass	Length	ppm	m/z	z	RT	Fraction	Scan	Source File	Area raw peanut	#Feature	#Feature raw peanut	Start	End	PTM	AScore	Found By
K.NPQLQDLDMMLTC(+57.02)VEIK.E	N	120.22	2046.9788	17	-6.3	1024.4902	2	37.10	1	F1:1864	OB5939 H1 raw.raw	1.3638E7	5	5	415	431	Carbamidomethylation	C13:Carbamidomethylation:1000.00	PEAKS DB
K.KNPQLQDLDMMLTC(+57.02)VEIK.E	N	118.64	2175.0737	18	-1.1	1088.5430	2	34.52	2	F2:1751	OB5940 H1 raw.raw	4.131E6	3	3	414	431	Carbamidomethylation	C14:Carbamidomethylation:1000.00	PEAKS DB
R.EQEWEEEEEDEEEEGSNR.E	N	117.85	2280.8413	18	4.3	1141.4329	2	28.23	1	F1:1323	OB5939 H1 raw.raw	4.2916E6	3	3	473	490			PEAKS DB
R.NTLEAAFNAEFNEIR.R	N	114.83	1737.8322	15	-0.4	869.9230	2	34.89	2	F2:1770	OB5940 H1 raw.raw	1.3513E7	6	6	313	327			PEAKS DB
K.HADADNILVIQQGQATVTVANGNNRK.S	N	114.12	2746.4111	26	3.2	916.4806	3	29.15	1	F1:1378	OB5939 H1 raw.raw	4.7574E7	8	8	223	248			PEAKS DB
K.HADADNILVIQQGQATVTVAN(+.98)GNNRK.S	N	111.24	2747.3950	26	5.5	916.8107	3	29.51	1	F1:1400	OB5939 H1 raw.raw	3.8297E8	20	20	223	248		N21:Deamidation (NQ):26.31	PEAKS DB
K.HADADNILVIQQGQATVTVANGNNR.K	N	111.11	2618.3162	25	5.0	1310.1719	2	30.00	2	F2:1473	OB5940 H1 raw.raw	6.2545E6	3	3	223	247			PEAKS DB
K.NPQLQDLDM(+15.99)MLTC(+57.02)VEIK.E	N	110.46	2062.9736	17	-5.3	1032.4886	2	36.07	2	F2:1840	OB5940 H1 raw.raw	3.4606E5	2	2	415	431	Carbamidomethylation	M9:Oxidation (M):30.46;C13:Carbamidomethylation:1000.00	PEAKS DB
K.ISM(+15.99)PVNTPGQFEDFFPASSR.D	N	109.22	2242.0364	20	-1.8	1122.0234	2	33.79	2	F2:1706	OB5940 H1 raw.raw	1.7538E7	15	15	282	301	Oxidation (M)	M3:Oxidation (M):1000.00	PEAKS DB
K.ISMPVNTPGQFEDFFPASSR.D	N	107.66	2226.0415	20	1.7	1114.0299	2	35.27	2	F2:1792	OB5940 H1 raw.raw	2.1717E8	29	29	282	301			PEAKS DB
R.IFLAGDKDNVIDQIEK.Q	N	103.70	1816.9570	16	-1.1	909.4848	2	32.03	1	F1:1588	OB5939 H1 raw.raw	7.5151E8	30	30	536	551			PEAKS DB
K.HADADNILVIQQGQATVTVAN(-17.03)GNNRK.S	N	99.83	2729.3845	26	0.5	1365.7002	2	28.96	2	F2:1400	OB5940 H1 raw.raw	9.5179E6	6	6	223	248		N21:Ammonia-loss (N):18.53	PEAKS PTM
R.IFLAGDKDNVIDQIEKQAK.D	N	99.61	2144.1477	19	1.7	715.7244	3	32.37	1	F1:1571	OB5939 H1 raw.raw	5.6465E7	7	7	536	554			PEAKS DB

total 247 peptides

Peptide	Uniq	-10lgP	Mass	Length	ppm	m/z	z	RT	Fraction	Scan	Source File	Area raw peanut	#Feature	#Feature raw peanut	Start	End	PTM	AScore	Found By
K.AMVIVVVNKGTGNLELVAVR.K	N	99.39	2081.2031	20	-3.5	694.7392	3	33.79	2	F2:1711	OB5940 H1 raw.raw	6.8972E7	9	9	444	463			PEAKS DB
K.GSEEEDITNPINLRDGEPDLSNNFGR.L	N	98.56	2887.3220	26	2.3	963.4502	3	32.73	1	F1:1590	OB5939 H1 raw.raw	4.549E7	8	8	380	405			PEAKS DB
K.SFNLDEGHALRIPSGFISYILNR.H	N	98.27	2618.3604	23	-1.1	873.7931	3	37.47	2	F2:1880	OB5940 H1 raw.raw	1.7034E8	24	23	249	271			PEAKS DB
R.KSFNLDEGHALRIPSGFISYILNR.H	N	97.91	2746.4553	24	-2.1	916.4905	3	36.16	1	F1:1802	OB5939 H1 raw.raw	8.5492E6	5	5	248	271			PEAKS DB
R.VLLEENAGGEQEER.G	N	97.22	1571.7427	14	2.5	786.8806	2	26.75	1	F1:1224	OB5939 H1 raw.raw	1.2414E7	3	3	329	342			PEAKS DB
R.DQSSYLQGFSR.N	N	97.09	1286.5891	11	-3.0	644.2999	2	30.18	2	F2:1484	OB5940 H1 raw.raw	6.1544E7	13	13	302	312			PEAKS DB
K.AM(-48.00)VIVVVNKGTGNLELVAVR.K	N	96.95	2033.1997	20	1.4	678.7415	3	30.71	2	F2:1519	OB5940 H1 raw.raw	1.7497E7	6	6	444	463	Dethiomethyl	M2:Dethiomethyl:1000.00	PEAKS PTM
K.SFNLDEGHALR.I	N	96.68	1257.6101	11	1.8	629.8135	2	27.87	1	F1:1301	OB5939 H1 raw.raw	7.3788E7	15	15	249	259			PEAKS DB
K.AMVIVVVNKGTGNLELVAVRK.E	N	96.39	2209.2981	21	-2.2	737.4384	3	31.97	2	F2:1628	OB5940 H1 raw.raw	2.8068E8	12	12	444	464			PEAKS DB
K.AM(+15.99)VIVVVNKGTGNLELVAVR.K	N	95.84	2097.1980	20	-2.2	1049.6040	2	32.73	3	F3:1638	OB5941 H1 raw.raw	3.1624E7	6	6	444	463	Oxidation (M)	M2:Oxidation (M):1000.00	PEAKS DB
R.IPSGFISYILNR.H	N	93.86	1378.7609	12	1.2	690.3885	2	36.74	2	F2:1881	OB5940 H1 raw.raw	1.7713E8	5	5	260	271			PEAKS DB
R.RVLLEENAGGEQEER.G	N	93.51	1727.8438	15	0.4	576.9554	3	24.77	2	F2:1156	OB5940 H1 raw.raw	1.346E5	5	5	328	342			PEAKS DB
K.NPQLQDLDMMLTC(+57.02)VEIKEGALMLPHFNSK.A	N	92.80	3371.6387	29	-2.9	1686.8218	2	37.92	1	F1:1910	OB5939 H1 raw.raw	1.8404E8	11	11	415	443	Carbamidomethylation	C13:Carbamidomethylation:1000.00	PEAKS DB
R.KSFNLDEGHALR.I	N	89.31	1385.7051	12	1.9	693.8611	2	25.26	2	F2:1181	OB5940 H1 raw.raw	3.3181E6	6	6	248	259			PEAKS DB
K.KGSEEEDITNPINLRDGEPDLSNNFGR.L	N	89.20	3015.4170	27	-0.8	1006.1455	3	31.46	1	F1:1545	OB5939 H1 raw.raw	7.9044E7	10	10	379	405			PEAKS DB
K.NPQLQDLDMMLTC(+14.02)VEIK.E	N	88.98	2003.9730	17	-4.9	1002.9888	2	37.72	2	F2:1938	OB5940 H1 raw.raw	6.7012E5	2	2	415	431		C13:Methylation(others):33.98	PEAKS PTM
R.NTLEAAFNAEFNEIRR.V	N	86.44	1893.9332	16	-3.6	947.9705	2	35.28	1	F1:1741	OB5939 H1 raw.raw	3.2153E8	22	22	313	328			PEAKS DB
K.HADADNILVIQQGQATVTVANGN(-17.03)NRK.S	N	85.41	2729.3845	26	-3.3	1365.6951	2	29.38	3	F3:1432	OB5941 H1 raw.raw	2.4846E6	2	2	223	248		N23:Ammonia-loss (N):0.00	PEAKS PTM

total 247 peptides

Peptide	Uniq	-10lgP	Mass	Length	ppm	m/z	z	RT	Fraction	Scan	Source File	Area raw peanut	#Feature	#Feature raw peanut	Start	End	PTM	AScore	Found By
K.DLAFPGSGEQVEK.L	N	85.12	1375.6619	13	1.9	688.8395	2	29.66	2	F2:1451	OB5940 H1 raw.raw	6.5078E7	3	3	555	567			PEAKS DB
K.NPQLQDLDMM(-48.00)LTC(+57.02)VEIK.E	N	85.01	1998.9755	17	0.5	1000.4955	2	32.73	1	F1:1599	OB5939 H1 raw.raw	1.7365E7	6	6	415	431	Carbamidomethylation	M10:Dethiomethyl:27.96;C13:Carbamidomethylation:1000.00	PEAKS PTM
R.VLLEENAGGEQEERGQR.R	N	84.92	1912.9238	17	1.8	957.4709	2	25.31	3	F3:1182	OB5941 H1 raw.raw	1.9929E6	6	6	329	345			PEAKS DB
R.SSDNEGVIVKVSKEHVQELTK.H	N	84.86	2325.2175	21	1.3	776.0808	3	28.31	3	F3:1362	OB5941 H1 raw.raw	1.2881E5	2	2	351	371			PEAKS DB
K.KGSEEEDITNPINLR.D	N	84.79	1713.8533	15	3.6	857.9370	2	28.42	2	F2:1374	OB5940 H1 raw.raw	1.1329E7	7	7	379	393			PEAKS DB
K.KNPQLQDLDMM(+15.99)LTC(+57.02)VEIKEGALMLPHFNSK.A	N	84.57	3515.7285	30	-2.7	879.9370	4	34.52	2	F2:1755	OB5940 H1 raw.raw	2.6704E6	3	3	414	443	Carbamidomethylation	M11:Oxidation (M):30.46;C14:Carbamidomethylation:1000.00	PEAKS DB
R.DGEPDLSNNFGR.L	N	84.53	1319.5742	12	0.4	660.7947	2	29.14	1	F1:1381	OB5939 H1 raw.raw	3.3217E6	3	3	394	405			PEAKS DB
K.AM(+15.99)VIVVVNKGTGNLELVAVRK.E	N	83.52	2225.2930	21	-2.3	1113.6512	2	31.28	1	F1:1523	OB5939 H1 raw.raw	4.3919E7	8	8	444	464	Oxidation (M)	M2:Oxidation (M):1000.00	PEAKS DB
R.EGEQEWGTPGSEVREETSR.N	Y	82.53	2161.9512	19	4.3	1081.9875	2	27.69	1	F1:1291	OB5939 H1 raw.raw	2.0963E7	6	6	147	165			PEAKS DB
K.HADADNILVIQQGQATVTVAN(+.98)GNNR.K	N	82.15	2619.3000	25	-1.2	1310.6558	2	30.35	2	F2:1535	OB5940 H1 raw.raw	5.2956E6	1	1	223	247		N21:Deamidation (NQ):7.32	PEAKS DB
R.EGEQEWGTPGSEVR.E	Y	81.97	1559.6852	14	1.4	780.8510	2	27.35	2	F2:1310	OB5940 H1 raw.raw	8.022E5	3	3	147	160			PEAKS DB
K.KNPQLQDLDMM(+15.99)LTC(+57.02)VEIK.E	N	81.97	2191.0686	18	0.5	1096.5421	2	32.43	2	F2:1621	OB5940 H1 raw.raw	1.9806E5	1	1	414	431	Carbamidomethylation	M11:Oxidation (M):9.34;C14:Carbamidomethylation:1000.00	PEAKS DB
K.EGALMLPHFNSK.A	N	81.44	1342.6703	12	4.0	672.3451	2	30.40	1	F1:1456	OB5939 H1 raw.raw	9.7706E6	3	3	432	443			PEAKS DB
K.QAKDLAFPGSGEQVEK.L	N	81.31	1702.8525	16	1.8	852.4351	2	26.85	3	F3:1278	OB5941 H1 raw.raw	1.3516E6	7	7	552	567			PEAKS DB
K.GSEEEDITNPINLR.D	N	80.86	1585.7583	14	2.4	793.8883	2	30.52	2	F2:1506	OB5940 H1 raw.raw	3.0736E6	3	3	380	393			PEAKS DB

total 247 peptides

Peptide	Uniq	-10lgP	Mass	Length	ppm	m/z	z	RT	Fraction	Scan	Source File	Area raw peanut	#Feature	#Feature raw peanut	Start	End	PTM	AScore	Found By
K.H(+43.01)(+14.02)ADADNILVIQQGQATVTVANGNNRK.S	N	80.77	2803.4324	26	-1.3	935.4835	3	29.92	3	F3:1452	OB5941H1raw.raw	2.1221E6	2	2	223	248		H1:Carbamylation:252.86;H1:Methylation(others):25.66	PEAKS PTM
K.AMVIVVVNK.G	N	78.49	971.5837	9	0.9	486.7996	2	29.32	2	F2:1448	OB5940H1raw.raw	9.4803E6	3	3	444	452			PEAKS DB
F.LAGDKDNVIDQIEK.Q	N	78.09	1556.8046	14	-6.1	779.4048	2	31.62	2	F2:1564	OB5940H1raw.raw	4.6126E6	2	2	538	551			PEAKS DB
K.KNPQLQDLDM(+15.99)MLTC(+57.02)VEIK.E	N	77.76	2191.0686	18	0.6	1096.5422	2	33.03	1	F1:1615	OB5939H1raw.raw	0	0	0	414	431	Carbamidomethylation	M10:Oxidation(M):14.02;C14:Carbamidomethylation:1000.00	PEAKS DB
R.IPSGFISYILNRHDNQNLR.V	N	77.66	2256.1763	19	3.4	1129.0992	2	33.47	1	F1:1643	OB5939H1raw.raw	3.1228E8	24	24	260	278			PEAKS DB
R.GRREQEWEEEEEDEEEEGSNR.E	N	77.42	2650.0649	21	0.3	884.3625	3	25.64	2	F2:1205	OB5940H1raw.raw	2.3251E6	7	7	470	490			PEAKS DB
K.NPQLQDLDM(+15.99)MLTC(+57.02)VEIKEGALMLPHFNSK.A	N	75.51	3387.6335	29	-1.5	1130.2168	3	36.17	3	F3:1926	OB5941H1raw.raw	2.5418E7	3	3	415	443	Carbamidomethylation	M9:Oxidation(M):10.11;C13:Carbamidomethylation:1000.00	PEAKS DB
K.EGALM(+15.99)LPHFNSK.A	N	74.93	1358.6653	12	2.1	680.3414	2	29.74	3	F3:1443	OB5941H1raw.raw	2.0704E6	3	3	432	443	Oxidation (M)	M5:Oxidation(M):1000.00	PEAKS DB
R.LFEVKPDKKNPQLQDLDMMLTC(+57.02)VEIK.E	N	74.78	3131.6069	26	3.7	1044.8801	3	33.97	2	F2:1716	OB5940H1raw.raw	3.7773E6	3	3	406	431	Carbamidomethylation	C22:Carbamidomethylation:1000.00	PEAKS DB
K.KNPQLQDLDMMLTC(+57.02)VEIKEGALM(+15.99)LPHFNSK.A	N	74.26	3515.7285	30	-0.8	879.9387	4	35.84	1	F1:1790	OB5939H1raw.raw	5.1448E6	2	2	414	443	Carbamidomethylation; Oxidation (M)	C14:Carbamidomethylation:1000.00;M23:Oxidation(M):69.38	PEAKS DB
K.HADADNILVIQQGQATVTVANGNN(+.98)RK.S	N	73.98	2747.3950	26	1.9	916.8074	3	39.56	2	F2:2095	OB5940H1raw.raw	5.4145E5	4	4	223	248		N24:Deamidation (NQ):14.02	PEAKS DB
K.GTGNLELVAVRK.E	N	73.67	1255.7249	12	0.9	628.8702	2	26.85	3	F3:1263	OB5941H1raw.raw	1.0553E7	7	7	453	464			PEAKS DB

total 247 peptides

Peptide	Uniq	-10lgP	Mass	Length	ppm	m/z	z	RT	Fraction	Scan	Source File	Area raw peanut	#Feature	#Feature raw peanut	Start	End	PTM	AScore	Found By
K.NPQLQDLDMM(+15.99)LTC(+57.02)VEIKEGALMLPHFNSK.A	N	72.71	3387.6335	29	3.8	1130.2228	3	37.84	2	F2:1902	OB5940 H1 raw.raw	8.789E6	3	3	415	443	Carbamidomethylation	M10:Oxidation (M):14.02;C13:Carbamidomethylation:1000.00	PEAKS DB
K.GTGNLELVAVR.K	N	71.83	1127.6299	11	0.2	564.8223	2	29.32	2	F2:1429	OB5940 H1 raw.raw	1.0095E7	3	3	453	463			PEAKS DB
R.NNPFYFPSR.R	N	71.25	1140.5352	9	0.9	571.2754	2	30.89	2	F2:1528	OB5940 H1 raw.raw	1.2382E7	4	4	166	174			PEAKS DB
R.VLLEENAGGEQEERGQRR.R	N	71.19	2069.0249	18	2.1	690.6837	3	25.31	3	F3:1186	OB5941 H1 raw.raw	1.1654E6	2	2	329	346			PEAKS DB
I.PSGFISYILNR.H	N	70.27	1265.6768	11	2.4	633.8472	2	36.17	2	F2:1844	OB5940 H1 raw.raw	2.1876E5	2	2	261	271			PEAKS DB
R.EETSRNNPFYFPSRR.F	N	69.88	1898.9023	15	1.2	633.9755	3	27.17	2	F2:1289	OB5940 H1 raw.raw	7.2799E6	5	5	161	175			PEAKS DB
K.NPQLQDLDMMLTC(+57.02)VEIKEGALM(+15.99)LPHFNSK.A	N	69.58	3387.6335	29	-9.3	1130.2080	3	36.62	2	F2:1874	OB5940 H1 raw.raw	2.6984E7	4	4	415	443	Carbamidomethylation; Oxidation (M)	C13:Carbamidomethylation:1000.00; M22:Oxidation (M):69.89	PEAKS DB
K.ISMPVNTPGQ(+.98)FEDFFPASSR.D	N	68.89	2227.0254	20	6.4	1114.5271	2	63.55	3	F3:3427	OB5941 H1 raw.raw	9.613E3	2	2	282	301		Q10:Deamidation (NQ):33.26	PEAKS DB
R.REQEWEEEEEDEEEEGSNR.E	N	68.88	2436.9424	19	2.8	1219.4819	2	26.47	3	F3:1258	OB5941 H1 raw.raw	3.0427E4	2	2	472	490			PEAKS DB
R.IPSGFISY(+125.90)ILNR.H	N	68.75	1504.6575	12	1.5	753.3372	2	37.84	2	F2:1946	OB5940 H1 raw.raw	9.8548E5	2	2	260	271	Iodination	Y8:Iodination:1000.00	PEAKS PTM
R.NTLEAAFNAEFN(+.98)EIRR.V	N	68.73	1894.9172	16	-3.7	948.4624	2	32.73	3	F3:1640	OB5941 H1 raw.raw	4.5098E6	3	3	313	328	Deamidation (NQ)	N12:Deamidation (NQ):55.21	PEAKS DB
R.N(+.98)TLEAAFNAEFNEIRR.V	N	67.69	1894.9172	16	2.2	948.4680	2	35.88	1	F1:1787	OB5939 H1 raw.raw	6.2604E7	1	1	313	328	Deamidation (NQ)	N1:Deamidation (NQ): 61.82	PEAKS DB
R.NNPFYFPSRR.F	N	67.53	1296.6364	10	1.5	649.3264	2	28.95	1	F1:1356	OB5939 H1 raw.raw	1.2601E8	9	9	166	175			PEAKS DB

total 247 peptides

Peptide	Uniq	-10lgP	Mass	Length	ppm	m/z	z	RT	Fraction	Scan	Source File	Area raw peanut	#Feature	#Feature raw peanut	Start	End	PTM	AScore	Found By
K.KNPQLQDLDM(+15.99)MLTC(+57.02)VEIKEGALMLPHFNSK.A	N	66.89	3515.7285	30	-3.1	879.9366	4	35.44	2	F2:1830	OB5940 H1 raw.raw	7.3362E6	2	2	414	443	Carbamidomethylation	M10:Oxidation (M):11.06;C14:Carbamidomethylation:1000.00	PEAKS DB
K.HADADNILVIQQGQATVTVANGN(+.98)NRK.S	N	66.81	2747.3950	26	3.3	916.8087	3	44.43	3	F3:2322	OB5941 H1 raw.raw	2.5002E7	10	10	223	248		N23:Deamidation (NQ):17.01	PEAKS DB
K.KNPQLQDLDMMLTC(+57.02)VEIKEGALMLPHFNSK.A	N	66.20	3499.7336	30	-0.1	875.9406	4	36.52	3	F3:1868	OB5941 H1 raw.raw	1.1425E7	3	3	414	443	Carbamidomethylation	C14:Carbamidomethylation:1000.00	PEAKS DB
K.HADADNILVIQQGQ(+.98)ATVTVANGNNRK.S	N	66.16	2747.3950	26	5.1	916.8103	3	44.47	1	F1:2283	OB5939 H1 raw.raw	5.1832E5	2	2	223	248		Q14:Deamidation (NQ):17.37	PEAKS DB
K.SVSKKGSEEEDITNPINLR.D	N	66.11	2115.0808	19	2.6	706.0361	3	27.22	3	F3:1299	OB5941 H1 raw.raw	6.5352E4	2	2	375	393			PEAKS DB
R.VLLEEN(+.98)AGGEQEER.G	N	66.02	1572.7267	14	-3.7	787.3677	2	26.82	2	F2:1276	OB5940 H1 raw.raw	0	0	0	329	342	Deamidation (NQ)	N6:Deamidation (NQ):71.64	PEAKS DB
R.IVQIEARPNTLVLPK.H	Y	65.77	1690.0140	15	-3.0	846.0117	2	30.24	3	F3:1474	OB5941 H1 raw.raw	2.8055E8	18	18	208	222			PEAKS DB
R.EGEQEWGTP(+15.99)GSEVREETSR.N	Y	65.62	2177.9460	19	1.3	726.9902	3	26.28	3	F3:1241	OB5941 H1 raw.raw	4.9462E6	6	6	147	165	Hydroxylation Pro	P9:Hydroxylation Pro:1000.00	PEAKS DB
K.Q(-17.03)FQNLQNHR.I	N	65.38	1166.5581	9	2.4	584.2877	2	28.05	1	F1:1306	OB5939 H1 raw.raw	3.4214E6	3	3	199	207	Pyro-glu from Q	Q1:Pyro-glu from Q:1000.00	PEAKS PTM
K.NPQLQDLDMMLTCVEIK(+14.02)EGALMLPHFNSK.A	N	64.78	3328.6328	29	1.8	1110.5536	3	38.03	2	F2:1959	OB5940 H1 raw.raw	1.6164E7	6	6	415	443	Methylation(KR)	K17:Methylation(KR):77.78	PEAKS PTM
I.FLAGDKDNVIDQIEK.Q	N	64.24	1703.8729	15	-1.7	852.9423	2	32.19	1	F1:1574	OB5939 H1 raw.raw	1.36E6	3	3	537	551			PEAKS DB
R.EETSRNNPFYFPSR.R	N	63.62	1742.8011	14	-0.9	581.9405	3	29.89	1	F1:1419	OB5939 H1 raw.raw	6.2933E4	1	1	161	174			PEAKS DB
R.I(+27.99)FLAGDKDNVIDQIEK.Q	N	63.40	1844.9519	16	5.2	923.4880	2	36.16	3	F3:1844	OB5941 H1 raw.raw	0	0	0	536	551	Formylation	I1:Formylation:77.93	PEAKS PTM
L.AGDKDNVIDQIEK.Q	N	63.17	1443.7205	13	-3.1	722.8653	2	32.19	1	F1:1560	OB5939 H1 raw.raw	9.2796E5	2	2	539	551			PEAKS DB

total 247 peptides

Peptide	Uniq	-10lgP	Mass	Length	ppm	m/z	z	RT	Fraction	Scan	Source File	Area raw peanut	#Feature	#Feature raw peanut	Start	End	PTM	AScore	Found By
K.I(+43.01)S(+14.02)MPVNTPGQFEDFFPASSR.D	N	62.82	2283.0630	20	-0.1	1142.5387	2	35.84	1	F1:1793	OB5939 H1 raw.raw	7.6784E5	1	1	282	301	Carbamylation; Methylation(others)	I1:Carbamylation:1000.00;S2:Methylation(others):86.16	PEAKS PTM
R.IPSGFISYILN(-17.03)R.H	N	62.55	1361.7343	12	1.4	681.8754	2	37.47	2	F2:1926	OB5940 H1 raw.raw	5.558E5	2	2	260	271	Ammonia-loss (N)	N11:Ammonia-loss (N):1000.00	PEAKS PTM
K.HADADNILVIQQGQ(+.98)ATVTVAN(+.98)GNNRK.S	N	62.38	2748.3792	26	2.7	917.1361	3	32.55	3	F3:1635	OB5941 H1 raw.raw	5.5427E5	2	2	223	248		Q14:Deamidation (NQ):32.28;N21:Deamidation (NQ):14.04	PEAKS DB
R.SSDNEGVIVK.V	N	62.38	1046.5244	10	0.3	524.2697	2	22.95	2	F2:1057	OB5940 H1 raw.raw	2.5465E5	3	3	351	360			PEAKS DB
K.AM(+15.99)VIVVVNK.G	N	62.22	987.5787	9	0.3	494.7968	2	26.41	2	F2:1234	OB5940 H1 raw.raw	8.6851E5	3	3	444	452	Oxidation (M)	M2:Oxidation (M):1000.00	PEAKS DB
A.GDKDNVIDQIEK.Q	N	62.19	1372.6833	12	-2.8	687.3470	2	32.19	1	F1:1561	OB5939 H1 raw.raw	1.5319E6	3	3	540	551			PEAKS DB
F.NLDEGHALR.I	N	62.08	1023.5097	9	1.8	512.7631	2	27.87	1	F1:1303	OB5939 H1 raw.raw	1.5945E6	3	3	251	259			PEAKS DB
Q.GQATVTVAN(+.98)GNNRK.S	N	62.04	1429.7273	14	-1.0	715.8702	2	28.79	2	F2:1402	OB5940 H1 raw.raw	6.6423E5	3	3	235	248		N9:Deamidation (NQ):41.83	PEAKS DB
A.FPGSGEQVEK.L	N	61.40	1076.5138	10	5.5	539.2672	2	30.23	1	F1:1449	OB5939 H1 raw.raw	6.3559E4	1	1	558	567			PEAKS DB
K.HADADNILVIQ(+.98)Q(+.98)GQATVTVAN(+.98)GNNRK.S	N	60.41	2749.3630	26	9.6	917.4704	3	30.18	2	F2:1540	OB5940 H1 raw.raw	4.3875E6	1	1	223	248		Q11:Deamidation (NQ):26.52;Q12:Deamidation (NQ):18.53;N21:Deamidation (NQ):14.04	PEAKS DB
K.SFNLDEGHALRIPSGFISYILNRHDNQNLR.V	N	60.12	3495.7759	30	-1.0	700.1617	5	36.35	2	F2:1862	OB5940 H1 raw.raw	7.0033E7	8	8	249	278			PEAKS DB

total 247 peptides

Peptide	Uniq	-10lgP	Mass	Length	ppm	m/z	z	RT	Fraction	Scan	Source File	Area raw peanut	#Feature	#Feature raw peanut	Start	End	PTM	AScore	Found By
K.NPQLQ(+.98)DLDMMLTC(+57.02)VEIKEGALM(+15.99)LPHFNSK.A	N	60.12	3388.6174	29	-2.4	1130.5437	3	36.35	2	F2:1874	OB5940 H1 raw.raw	6.4476E5	1	1	415	443	Carbamidomethylation; Oxidation (M)	Q5:Deamidation (NQ):9.86;C13:Carbamidomethylation:1000.00;M22:Oxidation (M):54.40	PEAKS DB
K.NPQLQDLDM(+15.99)M(+15.99)LTC(+57.02)VEIKEGALMLPHFNSK.A	N	60.05	3403.6284	29	-4.3	1135.5452	3	35.63	3	F3:1818	OB5941 H1 raw.raw	7.9921E5	1	1	415	443	Oxidation (M); Carbamidomethylation	M9:Oxidation (M):49.25;M10:Oxidation (M):53.09;C13:Carbamidomethylation:1000.00	PEAKS DB
K.NPQLQ(+.98)DLDMMLTC(+57.02)VEIKEGALMLPHFNSK.A	N	59.95	3372.6226	29	5.7	1125.2212	3	37.84	2	F2:1936	OB5940 H1 raw.raw	5.4487E7	3	3	415	443	Carbamidomethylation	Q5:Deamidation (NQ):32.28;C13:Carbamidomethylation:1000.00	PEAKS DB
R.I(+41.03)P(+15.99)SGFISYILNR.H	N	59.92	1435.7823	12	-3.1	718.8962	2	36.92	1	F1:1841	OB5939 H1 raw.raw	1.5727E7	4	4	260	271	Amidination of lysines or N-terminal amines with methyl acetimidate; Hydroxylation Pro	I1:Amidination of lysines or N-terminal amines with methyl acetimidate:1000.00;P2:Hydroxylation Pro:1000.00	PEAKS PTM
K.A(+41.03)M(+15.99)VIVVVNKGTGNLELVAVR.K	N	59.12	2138.2246	20	-4.5	1070.1147	2	33.90	2	F2:1710	OB5940 H1 raw.raw	5.8929E6	3	3	444	463	Amidination of lysines or N-terminal amines with methyl acetimidate; Oxidation (M)	A1:Amidination of lysines or N-terminal amines with methyl acetimidate:170.86;M2:Oxidation (M):1000.00	PEAKS PTM
K.IRPEGREGEQEWGTPGSEVREETSR.N	Y	58.78	2870.3542	25	1.1	957.7931	3	27.23	3	F3:1290	OB5941 H1 raw.raw	4.3127E6	4	4	141	165			PEAKS DB

total 247 peptides

Peptide	Uniq	-10lgP	Mass	Length	ppm	m/z	z	RT	Fraction	Scan	Source File	Area raw peanut	#Feature	#Feature raw peanut	Start	End	PTM	AScore	Found By
E.PDLSNNFGR.L	N	58.76	1018.4832	9	-0.1	510.2488	2	31.24	3	F3:1545	OB5941 H1 raw.raw	4.0306E6	3	3	397	405			PEAKS DB
K.HADADNILVIQQ(+.98)GQ(+.98)ATVTVAN(+.98)GNNRK.S	N	56.82	2749.3630	26	9.6	917.4704	3	30.18	2	F2:1487	OB5940 H1 raw.raw	4.3875E6	1	1	223	248		Q12:Deamidation (NQ):14.02;Q14:Deamidation (NQ):13.03;N21:Deamidation (NQ):11.10	PEAKS DB
K.ISMPVNTPGQFEDFFPASSRDQSSYLQGFSR.N	N	56.52	3494.6201	31	3.0	1748.3225	2	35.84	1	F1:1789	OB5939 H1 raw.raw	9.3656E7	16	16	282	312			PEAKS DB
K.H(+42.01)(+15.99)ADADNILVIQQGQATVTVANGNNRK.S	N	56.30	2804.4165	26	-2.5	1403.2120	2	29.79	1	F1:1416	OB5939 H1 raw.raw	2.4794E5	1	1	223	248	Acetylation (N-term); Oxidation (HW)	H1:Acetylation (N-term):1000.00;H1:Oxidation (HW):1000.00	PEAKS PTM
K.HADADN(+.98)ILVIQQGQATVTVANGNNRK.S	N	55.97	2747.3950	26	3.7	916.8090	3	50.62	1	F1:2616	OB5939 H1 raw.raw	5.4571E5	5	5	223	248		N6:Deamidation (NQ):42.57	PEAKS DB
K.ISMP(+15.99)VNTPGQFEDFFPASSRDQSSYLQGFSR.N	N	55.83	3510.6150	31	2.3	1171.2150	3	34.95	1	F1:1731	OB5939 H1 raw.raw	0	0	0	282	312		P4:Hydroxylation Pro:49.79	PEAKS DB
K.ISM(-48.00)PVNTPGQFEDFFPASSR.D	N	55.62	2178.0381	20	0.2	727.0201	3	32.37	1	F1:1577	OB5939 H1 raw.raw	3.0139E7	6	6	282	301	Dethiomethyl	M3:Dethiomethyl:1000.00	PEAKS PTM
K.N(+.98)PQLQDLDMMLTC(+57.02)VEIKEGALMLPHFNSK.A	N	55.55	3372.6226	29	5.7	1125.2212	3	37.84	2	F2:1983	OB5940 H1 raw.raw	1.3378E7	1	1	415	443	Carbamidomethylation	N1:Deamidation (NQ):14.04;C13:Carbamidomethylation:1000.00	PEAKS DB
K.HADADNILVIQ(+.98)QGQATVTVAN(+.98)GNNRK.S	N	55.03	2748.3792	26	5.1	1375.2039	2	31.28	1	F1:1539	OB5939 H1 raw.raw	2.1986E5	2	2	223	248		Q11:Deamidation (NQ):15.10;N21:Deamidation (NQ):17.01	PEAKS DB
K.HADADNILVIQ(+.98)QGQATVTVANGNNRK.S	N	55.00	2747.3950	26	5.9	916.8110	3	45.04	3	F3:2357	OB5941 H1 raw.raw	2.5167E4	1	1	223	248		Q11:Deamidation (NQ):9.34	PEAKS DB

total 247 peptides

Peptide	Uniq	-10lgP	Mass	Length	ppm	m/z	z	RT	Fraction	Scan	Source File	Area raw peanut	#Feature	#Feature raw peanut	Start	End	PTM	AScore	Found By
K.NPQLQDLDMM(+15.99)LTC(+57.02)VEIKEGALM(+15.99)LPHFNSK.A	N	54.50	3403.6284	29	4.5	1135.5552	3	36.52	3	F3:1873	OB5941 H1 raw.raw	6.8814E5	2	2	415	443	Carbamidomethylation	M10:Oxidation (M):12.28;C13:Carbamidomethylation:1000.00;M22:Oxidation (M):28.29	PEAKS DB
R.EQEWEEEEEDEEEEGSNREVR.R	N	54.08	2665.0535	21	2.4	889.3606	3	27.87	1	F1:1308	OB5939 H1 raw.raw	4.4193E5	2	2	473	493			PEAKS DB
K.HADADNILVIQQ(+.98)GQATVTVANGNNRK.S	N	53.63	2747.3950	26	1.9	916.8074	3	39.56	2	F2:2225	OB5940 H1 raw.raw	5.559E5	4	4	223	248		Q12:Deamidation (NQ):11.24	PEAKS DB
N.PFYFPSRR.F	N	53.53	1068.5504	8	3.3	535.2842	2	28.95	1	F1:1360	OB5939 H1 raw.raw	3.1024E6	3	3	168	175			PEAKS DB
R.LFEVKPDKKNPQLQDLDMM(+15.99)LTC(+57.02)VEIK.E	N	53.37	3147.6018	26	-4.4	787.9042	4	32.55	3	F3:1633	OB5941 H1 raw.raw	2.1681E6	3	3	406	431	Carbamidomethylation	M19:Oxidation (M):17.01;C22:Carbamidomethylation:1000.00	PEAKS DB
K.HADADNILVIQQ(+.98)GQATVTVAN(+.98)GNNRK.S	N	52.71	2748.3792	26	3.1	1375.2010	2	30.08	3	F3:1495	OB5941 H1 raw.raw	1.5758E6	1	1	223	248		Q12:Deamidation (NQ):27.58;N21:Deamidation (NQ):15.73	PEAKS DB
K.NPQLQDLDMMLTCVEIK(+14.02)EGALM(+15.99)LPHFNSK.A	N	52.56	3344.6277	29	4.1	837.1677	4	37.13	2	F2:1903	OB5940 H1 raw.raw	1.5437E6	1	1	415	443	Oxidation (M)	K17:Methylation(KR):24.93;M22:Oxidation (M):51.99	PEAKS PTM
K.ISM(+15.99)PVNTPGQFEDFFPASSRDQ(+.98)SSYLQGFSR.N	N	52.28	3511.5989	31	4.3	1171.5453	3	34.92	1	F1:1731	OB5939 H1 raw.raw	1.6252E6	1	1	282	312	Oxidation (M)	M3:Oxidation (M):1000.00;Q22:Deamidation (NQ):20.93	PEAKS DB
R.NT(-18.01)LEAAFNAEFNEIRR.V	N	52.23	1875.9227	16	-2.4	626.3134	3	33.43	2	F2:1683	OB5940 H1 raw.raw	1.354E7	3	3	313	328	Dehydration	T2:Dehydration:1000.00	PEAKS PTM
L.EAAFNAEFNEIRR.V	N	52.09	1565.7585	13	-4.7	783.8829	2	34.06	1	F1:1677	OB5939 H1 raw.raw	1.7113E6	2	2	316	328			PEAKS DB

total 247 peptides

Peptide	Uniq	-10lgP	Mass	Length	ppm	m/z	z	RT	Fraction	Scan	Source File	Area raw peanut	#Feature	#Feature raw peanut	Start	End	PTM	AScore	Found By
R.LFEVKPDK.K	N	51.87	974.5436	8	0.9	488.2795	2	25.40	1	F1:1153	OB5939 H1 raw.raw	7.0978E5	6	6	406	413			PEAKS DB
R.WGPAEPR.E	Y	51.81	811.3976	7	1.2	406.7066	2	25.31	3	F3:1167	OB5941 H1 raw.raw	1.2136E6	3	3	109	115			PEAKS DB
K.AM(-48.00)VIVVVNKGTGNLELVAVRK.E	N	51.58	2161.2947	21	1.4	1081.6561	2	29.80	3	F3:1453	OB5941 H1 raw.raw	6.785E6	4	4	444	464	Dethiomethyl	M2:Dethiomethyl:1000.00	PEAKS PTM
R.IFLAGDKDNVIDQIEK(+14.02)(+43.01).Q	N	51.42	1873.9785	16	1.0	937.9974	2	32.37	1	F1:1563	OB5939 H1 raw.raw	1.103E7	1	1	536	551	Methylation(C-term); Carbamylation	K16:Methylation(C-term):1000.00;K16:Carbamylation:164.73	PEAKS PTM
K.HADADNILVIQQGQAT(-18.01)VTVAN(+.98)GNNRK.S	N	51.33	2729.3845	26	-1.3	910.8009	3	31.10	3	F3:1500	OB5941 H1 raw.raw	8.3833E5	2	2	223	248		T16:Dehydration:11.99;N21:Deamidation(NQ):13.67	PEAKS PTM
K.S(+43.01)(+14.02)FNLDEGHALR.I	N	51.03	1314.6316	11	0.8	658.3236	2	27.58	3	F3:1340	OB5941 H1 raw.raw	4.5043E6	4	4	249	259	Carbamylation; Methylation(others)	S1:Carbamylation:1000.00;S1:Methylation(others):109.97	PEAKS PTM
K.NPQLQDLDMM(+15.99)LTCVEIK(+14.02).E	N	51.01	2019.9679	17	-7.0	1010.9842	2	37.36	1	F1:1877	OB5939 H1 raw.raw	0	0	0	415	431	Methylation(KR)	M10:Oxidation(M):11.06;K17:Methylation(KR):1000.00	PEAKS PTM
R.RVLLEENAGGEQEERGQR.R	N	50.12	2069.0249	18	2.1	690.6837	3	24.88	2	F2:1165	OB5940 H1 raw.raw	2.8524E5	1	1	328	345			PEAKS DB
K.KNPQLQDLDMM(-48.00)LTC(+57.02)VEIK.E	N	49.96	2127.0703	18	1.7	710.0319	3	30.71	2	F2:1521	OB5940 H1 raw.raw	3.3467E6	2	2	414	431	Carbamidomethylation	M11:Dethiomethyl:14.02;C14:Carbamidomethylation:1000.00	PEAKS PTM
N.LDEGHALR.I	N	49.09	909.4668	8	0.6	455.7409	2	27.35	2	F2:1312	OB5940 H1 raw.raw	3.1446E4	1	1	252	259			PEAKS DB
K.KNPQLQDLDMMLTCVEIK(+14.02)EGALMLPHFNSK.A	N	49.06	3456.7278	30	2.7	865.1915	4	36.90	2	F2:1892	OB5940 H1 raw.raw	3.1386E5	1	1	414	443	Methylation(KR)	K18:Methylation(KR):50.90	PEAKS PTM

total 247 peptides

Peptide	Uniq	-10lgP	Mass	Length	ppm	m/z	z	RT	Fraction	Scan	Source File	Area raw peanut	#Feature	#Feature raw peanut	Start	End	PTM	AScore	Found By
K.NPQLQDLDMM(+15.99)LTC(+57.02)VEIKEGALMLPHFN(+.98)SK.A	N	48.99	3388.6174	29	5.0	848.1659	4	36.17	3	F3:1849	OB5941 H1 raw.raw	7.0919E6	2	2	415	443	Carbamidomethylation; Deamidation (NQ)	M10:Oxidation (M):0.00;C13:Carbamidomethylation:1000.00;N27:Deamidation (NQ):51.54	PEAKS DB
K.HADADNILVIQQ(+.98)GQATVTVANGNN(+.98)RK.S	N	48.74	2748.3792	26	8.9	917.1418	3	48.01	1	F1:2471	OB5939 H1 raw.raw	2.2044E4	1	1	223	248		Q12:Deamidation (NQ):23.10;N24:Deamidation (NQ):0.00	PEAKS DB
R.LFEVKPDKKNPQLQDLDM(+15.99)M(+15.99)LTC(+57.02)VEIK.E	N	48.67	3163.5967	26	2.1	791.9081	4	32.37	1	F1:1585	OB5939 H1 raw.raw	6.4006E5	1	1	406	431	Oxidation (M); Carbamidomethylation	M18:Oxidation (M):1000.00; M19:Oxidation (M):1000.00; C22:Carbamidomethylation:1000.00	PEAKS DB
K.NPQLQDLDM(+15.99)MLTC(+57.02)VEIKEGALM(+15.99)LPHFNSK.A	N	48.60	3403.6284	29	2.2	851.9163	4	36.24	2	F2:1851	OB5940 H1 raw.raw	3.928E6	2	2	415	443	Carbamidomethylation; Oxidation (M)	M9:Oxidation (M):10.11;C13:Carbamidomethylation:1000.00; M22:Oxidation (M):53.09	PEAKS DB
N.TPGQFEDFFPASSR.D	N	48.49	1584.7208	14	-0.2	793.3676	2	35.63	3	F3:1821	OB5941 H1 raw.raw	2.7376E5	1	1	288	301			PEAKS DB
K.ISMP(+15.99)VNTPGQFEDFFPASSR.D	N	48.39	2242.0364	20	6.5	1122.0327	2	45.36	1	F1:2277	OB5939 H1 raw.raw	8.1169E4	2	2	282	301		P4:Hydroxylation Pro: 33.81	PEAKS DB
R.NTLEAAFN(+.98)AEFNEIRR.V	N	48.19	1894.9172	16	-0.3	948.4656	2	32.92	1	F1:1570	OB5939 H1 raw.raw	1.5926E6	1	1	313	328		N8:Deamidation (NQ): 8.26	PEAKS DB
K.NPQLQDLDMMLTC(+57.02)VEIKEGALMLPHFN(+.98)SK.A	N	47.92	3372.6226	29	7.4	844.1691	4	37.85	2	F2:1914	OB5940 H1 raw.raw	5.3901E7	2	2	415	443	Carbamidomethylation; Deamidation (NQ)	C13:Carbamidomethylation:1000.00; N27:Deamidation (NQ):61.84	PEAKS DB

total 247 peptides

Peptide	Uniq	-10lgP	Mass	Length	ppm	m/z	z	RT	Fraction	Scan	Source File	Area raw peanut	#Feature	#Feature raw peanut	Start	End	PTM	AScore	Found By
K.ISMP(+15.99)VNTPGQ(+.98)FEDFFPASSR.D	N	47.82	2243.0205	20	6.2	1122.5245	2	50.73	1	F1:2643	OB5939 H1 raw.raw	0	0	0	282	301		P4:Hydroxylation Pro:38.03;Q10:Deamidation (NQ):19.56	PEAKS DB
R.SKQFQNLQNHR.I	Y	47.59	1398.7117	11	1.0	467.2450	3	24.31	2	F2:1132	OB5940 H1 raw.raw	2.9256E4	3	3	197	207			PEAKS DB
R.K(+43.01)(+14.02)SFNLDEGHALR.I	N	47.09	1442.7266	12	-0.4	481.9159	3	25.70	3	F3:1209	OB5941 H1 raw.raw	2.261E5	3	3	248	259	Carbamylation; Methylation(KR)	K1:Carbamylation:1000.00;K1:Methylation(KR):160.69	PEAKS PTM
K.ISM(+15.99)PVNTPGQFEDFFPASSRDQSSYLQGFSR.N	N	47.05	3510.6150	31	4.2	1171.2172	3	34.34	2	F2:1739	OB5940 H1 raw.raw	6.0177E6	2	2	282	312	Oxidation (M)	M3:Oxidation (M):1000.00	PEAKS DB
K.QFQNLQNHR.I	N	46.78	1183.5846	9	0.4	592.7998	2	23.96	2	F2:1106	OB5940 H1 raw.raw	7.7423E4	5	5	199	207			PEAKS DB
D.KDNVIDQIEK.Q	N	46.62	1200.6350	10	-0.8	601.3243	2	25.89	3	F3:1225	OB5941 H1 raw.raw	4.877E4	2	2	542	551			PEAKS DB
K.AMVIVVVN(+.98)KGTGNLELVAVRK.E	N	46.54	2210.2820	21	9.6	737.7750	3	33.10	3	F3:1695	OB5941 H1 raw.raw	8.2125E6	2	2	444	464		N8:Deamidation (NQ):41.87	PEAKS DB
Q.GQATVTVANGNNRK.S	N	46.45	1428.7433	14	-0.2	715.3788	2	29.02	3	F3:1412	OB5941 H1 raw.raw	1.3861E5	2	2	235	248			PEAKS DB
R.EQEWEEEEEDEEEEGSNREVRR.Y	N	46.42	2821.1545	22	1.8	941.3939	3	26.75	1	F1:1227	OB5939 H1 raw.raw	3.455E6	3	3	473	494			PEAKS DB
K.Q(-17.03)AKDLAFPGSGEQVEK.L	N	45.78	1685.8260	16	0.9	843.9211	2	29.97	1	F1:1427	OB5939 H1 raw.raw	0	0	0	552	567	Pyro-glu from Q	Q1:Pyro-glu from Q:1000.00	PEAKS PTM
K.HADAD(-18.01)NILVIQQGQATVTVANGNNRK.S	N	45.35	2728.4004	26	3.5	910.4772	3	28.70	2	F2:1390	OB5940 H1 raw.raw	0	0	0	223	248		D5:Dehydration:30.36	PEAKS PTM
K.KN(+.98)PQLQDLDMM(+15.99)LTC(+57.02)VEIK.E	N	44.87	2192.0527	18	9.2	1097.0437	2	32.33	2	F2:1621	OB5940 H1 raw.raw	8.5764E4	1	1	414	431	Carbamidomethylation	N2:Deamidation (NQ):14.04;M11:Oxidation (M):0.00;C14:Carbamidomethylation:1000.00	PEAKS DB
R.KSFNLDEGHALRIPSGFISYILN(+.98)R.H	N	44.70	2747.4395	24	4.5	687.8702	4	36.15	3	F3:1837	OB5941 H1 raw.raw	1.1013E6	1	1	248	271		N23:Deamidation (NQ):0.00	PEAKS DB

total 247 peptides

Peptide	Uniq	-10lgP	Mass	Length	ppm	m/z	z	RT	Fraction	Scan	Source File	Area raw peanut	#Feature	#Feature raw peanut	Start	End	PTM	AScore	Found By
K.IRPEGREGEQEWGTPGSEVR.E	Y	44.50	2268.0881	20	1.1	757.0375	3	26.28	3	F3:1248	OB5941 H1 raw.raw	7.4174E4	2	2	141	160			PEAKS DB
W.GTPGSEVREETSR.N	Y	44.35	1403.6641	13	3.5	702.8417	2	27.60	3	F3:1327	OB5941 H1 raw.raw	7.5188E4	1	1	153	165			PEAKS DB
K.A(+41.03)M(+15.99)VIVVVNKGTGNLELVAVRK.E	N	44.32	2266.3196	21	-1.6	1134.1653	2	32.33	2	F2:1602	OB5940 H1 raw.raw	5.361E7	7	7	444	464	Amidination of lysines or N-terminal amines with methyl acetimidate; Oxidation (M)	A1:Amidination of lysines or N-terminal amines with methyl acetimidate:161.20; M2:Oxidation (M):1000.00	PEAKS PTM
V.QIEARPNTLVLPK.H	Y	43.15	1477.8616	13	-3.7	739.9353	2	30.24	3	F3:1485	OB5941 H1 raw.raw	1.0391E6	3	3	210	222			PEAKS DB
R.LFEVKPDKK.N	N	43.08	1102.6385	9	1.1	552.3271	2	24.94	3	F3:1179	OB5941 H1 raw.raw	2.3087E5	2	2	406	414			PEAKS DB
N.TLEAAFNAEFNEIRR.V	N	43.00	1779.8904	15	-3.0	890.9498	2	32.91	3	F3:1655	OB5941 H1 raw.raw	4.3913E5	1	1	314	328			PEAKS DB
K.NPQLQDLDMMLTC(+57.02)VEIKEGALM(-48.00)LPHFNSK.A	N	42.94	3323.6353	29	-1.0	831.9153	4	35.81	3	F3:1829	OB5941 H1 raw.raw	2.5446E6	1	1	415	443	Carbamidomethylation	C13:Carbamidomethylation:1000.00; M22:Dethiomethyl:30.24	PEAKS PTM
K.ISM(+15.99)PVN(+.98)TPGQFEDFFPASSR.D	N	42.69	2243.0205	20	7.6	1122.5260	2	49.72	2	F2:2625	OB5940 H1 raw.raw	0	0	0	282	301	Oxidation (M)	M3:Oxidation (M):1000.00; N6:Deamidation (NQ):25.70	PEAKS DB
K.KNPQLQ(+.98)DLDMMLTC(+57.02)VEIKEGALMLPHFNSK.A	N	42.58	3500.7175	30	0.4	1167.9136	3	36.34	2	F2:1850	OB5940 H1 raw.raw	5.0176E5	1	1	414	443	Carbamidomethylation	Q6:Deamidation (NQ):14.04; C14:Carbamidomethylation:1000.00	PEAKS DB
R.REQEWEEEEEDEEEEGSN(+.98)R.E	N	42.55	2437.9265	19	7.6	813.6556	3	26.55	1	F1:1226	OB5939 H1 raw.raw	3.9265E4	1	1	472	490		N18:Deamidation (NQ):0.00	PEAKS DB

total 247 peptides

Peptide	Uniq	-10lgP	Mass	Length	ppm	m/z	z	RT	Fraction	Scan	Source File	Area raw peanut	#Feature	#Feature raw peanut	Start	End	PTM	AScore	Found By
K.NPQLQDLDMMLTC(+57.02)VEIK(+43.01)EGALMLPHFNSK(+14.02).A	N	42.54	3428.6602	29	-4.2	1143.8892	3	37.82	3	F3:1939	OB5941 H1 raw.raw	1.0346E6	1	1	415	443	Carbamidomethylation	C13:Carbamidomethylation:1000.00;K17:Carbamylation:10.80;K29:Methylation(KR):10.80	PEAKS PTM
K.HADADNILVIQ(+.98)QGQ(+.98)ATVTVANGNNRK.S	N	42.52	2748.3792	26	3.1	917.1365	3	57.24	2	F2:3064	OB5940 H1 raw.raw	0	0	0	223	248		Q11:Deamidation (NQ):0.00;Q14:Deamidation (NQ):0.00	PEAKS DB
R.IFLAGDKDNVIDQIEK(+43.01)QAK(+14.02).D	N	42.41	2201.1692	19	2.7	734.7323	3	32.55	1	F1:1581	OB5939 H1 raw.raw	3.5153E6	1	1	536	554		K16:Carbamylation:0.00;K19:Methylation (KR):0.00	PEAKS PTM
K.GTGNLELVAVRKEQQQR.G	N	41.95	1925.0442	17	1.8	642.6898	3	26.36	1	F1:1214	OB5939 H1 raw.raw	1.4197E5	3	3	453	469			PEAKS DB
R.IFLAGDKDNVIDQIEK(+14.02)Q(+.98)AK(+42.01).D	N	41.87	2201.1580	19	3.1	734.7289	3	31.83	2	F2:1588	OB5940 H1 raw.raw	3.595E6	2	2	536	554		K16:Methylation(KR):13.38;Q17:Deamidation (NQ):25.11;K19:Acetylation (K):13.38	PEAKS PTM
K.D(+43.01)(+14.02)LAFPGSGEQVEK.L	N	41.75	1432.6833	13	3.8	717.3517	2	30.92	1	F1:1485	OB5939 H1 raw.raw	2.8695E5	1	1	555	567	Carbamylation; Methylation(others)	D1:Carbamylation:166.34;D1:Methylation(others):102.87	PEAKS PTM
R.IFLAGDKDNVIDQ(+.98)IEK.Q	N	41.66	1817.9410	16	9.1	909.9861	2	66.49	2	F2:3489	OB5940 H1 raw.raw	4.5271E4	3	3	536	551		Q13:Deamidation (NQ):38.67	PEAKS DB
L.DEGHALR.I	N	40.78	796.3828	7	2.3	399.1996	2	27.48	2	F2:1315	OB5940 H1 raw.raw	2.4528E4	1	1	253	259			PEAKS DB

**total 247 peptides**

Peptide	Uniq	-10lgP	Mass	Length	ppm	m/z	z	RT	Fraction	Scan	Source File	Area raw peanut	#Feature	#Feature raw peanut	Start	End	PTM	AScore	Found By
K.HADADNILVIQ(+.98)QGQATVTVANGN(+.98)NRK.S	N	40.70	2748.3792	26	8.8	917.1417	3	55.82	2	F2:2975	OB5940 H1 raw.raw	2.5591E3	1	1	223	248		Q11:Deamidation (NQ):0.00;N23:Deamidation (NQ):0.00	PEAKS DB
K.ISMPVN(+.98)TPGQFEDFFPASSR.D	N	40.63	2227.0254	20	9.3	1114.5303	2	58.23	3	F3:3170	OB5941 H1 raw.raw	2.6294E4	2	2	282	301		N6:Deamidation (NQ):42.89	PEAKS DB
R.IFLAGD(+14.02)K(+43.01)DNVIDQIEK.Q	N	40.36	1873.9785	16	-5.0	937.9919	2	32.19	3	F3:1583	OB5941 H1 raw.raw	4.0088E7	4	4	536	551	Carbamylation	D6:Methylation (others):24.32;K7:Carbamylation:57.71	PEAKS PTM
K.KN(+.98)PQLQDLDMMLTC(+57.02)VEIKEGALMLPHFNSK.A	N	40.34	3500.7175	30	7.1	876.1929	4	36.26	2	F2:1847	OB5940 H1 raw.raw	2.213E6	1	1	414	443	Carbamidomethylation	N2:Deamidation (NQ):0.00;C14:Carbamidomethylation:1000.00	PEAKS DB
K.NPQLQDLDM(+15.99)MLTC(+57.02)VEIKEGALMLP(+15.99)HFNSK.A	N	40.24	3403.6284	29	-10.2	851.9057	4	36.72	1	F1:1838	OB5939 H1 raw.raw	0	0	0	415	443	Carbamidomethylation; Hydroxylation Pro	M9:Oxidation (M):26.02;C13:Carbamidomethylation:1000.00;P24:Hydroxylation Pro:63.93	PEAKS DB
R.ESHFVSARPQSQSP(+15.99)SSPEKEDQEEEN.Q	N	40.09	2973.2859	26	-2.5	992.1001	3	25.16	1	F1:1143	OB5939 H1 raw.raw	0	0	0	574	599		P14:Hydroxylation Pro:29.32	PEAKS DB
K.SFN(+.98)LDEGHALRIPSGFISYILNR.H	N	39.87	2619.3445	23	9.3	655.8495	4	38.22	1	F1:1947	OB5939 H1 raw.raw	1.0439E6	2	2	249	271		N3:Deamidation (NQ):39.81	PEAKS DB
T.PGQFEDFFPASSR.D	N	39.65	1483.6731	13	-1.3	742.8429	2	40.34	2	F2:2103	OB5940 H1 raw.raw	3.5787E3	1	1	289	301			PEAKS DB
K.EGALM(-48.00)LPHFNSK.A	N	38.72	1294.6670	12	2.0	432.5638	3	28.31	3	F3:1348	OB5941 H1 raw.raw	1.3523E6	1	1	432	443	Dethiomethyl	M5:Dethiomethyl:1000.00	PEAKS PTM
K.ISMPVNTPGQ(+.98)FEDFFPASSRDQSSYLQGFSR.N	N	38.62	3495.6040	31	6.7	1166.2164	3	54.16	3	F3:2883	OB5941 H1 raw.raw	0	0	0	282	312		Q10:Deamidation (NQ):5.38	PEAKS DB
R.DQS(-18.01)SYLQGFSR.N	N	38.22	1268.5785	11	7.0	635.3010	2	30.87	1	F1:1484	OB5939 H1 raw.raw	0	0	0	302	312		S3:Dehydration:33.98	PEAKS PTM

total 247 peptides

Peptide	Uniq	-10lgP	Mass	Length	ppm	m/z	z	RT	Fraction	Scan	Source File	Area raw peanut	#Feature	#Feature raw peanut	Start	End	PTM	AScore	Found By
K.N(+.98)PQ(+.98)LQDLDMMLTC(+57.02)VEIKEGALM(+15.99)LPHFNSK.A	N	37.95	3389.6016	29	9.0	848.4153	4	36.92	1	F1:1851	OB5939 H1 raw.raw	8.517E6	2	2	415	443	Carbamidomethylation	N1:Deamidation (NQ): 11.12; Q3:Deamidation (NQ): 0.00;C13:Carbamidomethylation:1000.00; M22:Oxidation (M):24.93	PEAKS DB
K.SFN(+.98)LDEGHALRIPSGFISYILNRHDNQNLR.V	N	37.76	3496.7600	30	-3.9	700.3566	5	36.38	1	F1:1849	OB5939 H1 raw.raw	7.9247E5	1	1	249	278		N3:Deamidation (NQ): 20.27	PEAKS DB
R.E(-18.01)GEQEWGTPGSEVREETSR.N	Y	37.06	2143.9407	19	2.9	715.6562	3	27.17	2	F2:1294	OB5940 H1 raw.raw	9.0561E4	1	1	147	165	Pyro-glu from E	E1:Pyro-glu from E:1000.00	PEAKS PTM
K.NPQLQDLDMM(-48.00)LTC(+57.02)VEIKEGALMLPHFNSK.A	N	36.81	3323.6353	29	0.8	1108.8866	3	35.97	3	F3:1833	OB5941 H1 raw.raw	5.0189E6	2	2	415	443	Carbamidomethylation	M10:Dethiomethyl:8.14;C13:Carbamidomethylation:1000.00	PEAKS PTM
R.LFEVKPDKKN(+.98)PQLQDLDMMLTC(+57.02)VEIK.E	N	36.71	3132.5908	26	9.3	1567.3173	2	34.35	3	F3:1745	OB5941 H1 raw.raw	2.2872E5	1	1	406	431	Carbamidomethylation	N10:Deamidation (NQ):14.04;C22:Carbamidomethylation:1000.00	PEAKS DB
R.IVQIEAR(+28.03)PN(+.98)TLVLPK.H	Y	36.70	1719.0294	15	0.1	860.5220	2	30.00	2	F2:1445	OB5940 H1 raw.raw	1.1278E7	2	2	208	222	Dimethylation(KR); Deamidation (NQ)	R7:Dimethylation(KR): 71.07; N9:Deamidation (NQ): 90.21	PEAKS PTM
R.ESHFVSARPQSQSP(+15.99)SSPEKEDQEEE.N	N	36.67	2859.2429	25	2.6	954.0907	3	24.51	2	F2:1151	OB5940 H1 raw.raw	1.0898E4	1	1	574	598		P14:Hydroxylation Pro:12.33	PEAKS DB
K.NPQLQDLDM(+15.99)M(+15.99)LTC(+57.02)VEIK.E	N	36.40	2078.9688	17	1.1	1040.4928	2	33.80	3	F3:1701	OB5941 H1 raw.raw	0	0	0	415	431	Oxidation (M); Carbamidomethylation	M9:Oxidation (M):1000.00; M10:Oxidation (M):1000.00; C13:Carbamidomethylation:1000.00	PEAKS DB
**total 247 peptides**																			

Peptide	Uniq	-10lgP	Mass	Length	ppm	m/z	z	RT	Fraction	Scan	Source File	Area raw peanut	#Feature	#Feature raw peanut	Start	End	PTM	AScore	Found By
R.IVQIEARPNTLVLP(+15.99)K(-.98)(+42.01).H	Y	36.38	1747.0355	15	-2.4	874.5229	2	30.35	2	F2:1460	OB5940 H1 raw.raw	8.4907E6	1	1	208	222	Hydroxylation Pro; Amidation; Acetylation (K)	P14:Hydroxylation Pro:74.55;K15:Amidation:1000.00;K15:Acetylation (K):1000.00	PEAKS PTM
R.N(-17.03)NPFYFPSRR.F	N	36.19	1279.6097	10	-0.4	427.5437	3	28.25	2	F2:1369	OB5940 H1 raw.raw	1.5243E5	1	1	166	175		N1:Ammonia-loss (N):0.00	PEAKS PTM
K.AM(+31.99)VIVVVNKGTGNLELVAVRK.E	N	36.16	2241.2878	21	1.1	748.1041	3	31.46	1	F1:1540	OB5939 H1 raw.raw	1.7223E5	1	1	444	464	Sulphone	M2:Sulphone:1000.00	PEAKS PTM
K.NPQ(+.98)LQDLDMMLTC(+57.02)VEIKEGALMLPHFNSK.A	N	36.11	3372.6226	29	9.4	1125.2253	3	40.75	1	F1:2064	OB5939 H1 raw.raw	5.8612E5	1	1	415	443	Carbamidomethylation	Q3:Deamidation (NQ):0.00;C13:Carbamidomethylation:1000.00	PEAKS DB
K.N(+.98)PQ(+.98)LQDLDMMLTC(+57.02)VEIKEGALMLPHFNSK.A	N	35.87	3373.6067	29	9.1	1687.8259	2	38.03	2	F2:1999	OB5940 H1 raw.raw	2.685E5	1	1	415	443	Carbamidomethylation	N1:Deamidation (NQ):3.13;Q3:Deamidation (NQ):5.47;C13:Carbamidomethylation:1000.00	PEAKS DB
T.PGSEVREETSR.N	Y	35.66	1245.5948	11	1.9	623.8059	2	27.69	1	F1:1287	OB5939 H1 raw.raw	8.4353E4	2	2	155	165			PEAKS DB
K.EGALMLPH(+14.02)FNSK.A	N	35.50	1356.6860	12	1.3	679.3512	2	30.49	3	F3:1499	OB5941 H1 raw.raw	2.5433E4	1	1	432	443	Methylation(others)	H8:Methylation(others):53.53	PEAKS PTM
I.PSGFISYILNRHDNQNLR.V	N	35.49	2143.0923	18	4.0	715.3742	3	33.53	1	F1:1645	OB5939 H1 raw.raw	0	0	0	261	278			PEAKS DB
R.VLLE(+21.98)ENAGGEQEER.G	N	35.40	1593.7246	14	-0.7	797.8690	2	26.03	2	F2:1239	OB5940 H1 raw.raw	1.529E5	3	3	329	342		E4:Sodium adduct:40.00	PEAKS PTM
K.KGSEEEDIT(-18.01)N(+.98)PINLR.D	N	35.32	1696.8268	15	0.0	566.6162	3	29.14	1	F1:1369	OB5939 H1 raw.raw	6.926E4	1	1	379	393		T9:Dehydration:11.50;N10:Deamidation (NQ):25.67	PEAKS PTM

total 247 peptides

Peptide	Uniq	-10lgP	Mass	Length	ppm	m/z	z	RT	Fraction	Scan	Source File	Area raw peanut	#Feature	#Feature raw peanut	Start	End	PTM	AScore	Found By
K.NP(+15.99)QLQDLDMM(+15.99)LTC(+57.02)VEIKEGALMLPHFNSK.A	N	35.20	3403.6284	29	-3.4	851.9115	4	35.28	1	F1:1792	OB5939 H1 raw.raw	1.7685E6	1	1	415	443	Carbamidomethylation	P2:Hydroxylation Pro:48.46;M10:Oxidation (M):11.06;C13:Carbamidomethylation:1000.00	PEAKS DB
K.SFNLDEGH(+15.99)ALRIPSGFISYILNR.H	N	35.13	2634.3555	23	-0.4	879.1254	3	38.33	2	F2:1974	OB5940 H1 raw.raw	0	0	0	249	271	Oxidation (HW)	H8:Oxidation (HW):1000.00	PEAKS PTM
K.KGSEEEDITN(-17.03)PINLR.D	N	34.98	1696.8268	15	3.3	566.6180	3	28.85	3	F3:1397	OB5941 H1 raw.raw	3.0405E5	1	1	379	393		N10:Ammonia-loss (N):40.63	PEAKS PTM
K.HADADNILVIQQ(-18.01).G	N	34.95	1317.6677	12	-4.9	659.8379	2	29.56	3	F3:1421	OB5941 H1 raw.raw	1.0066E5	1	1	223	234	Dehydration	Q12:Dehydration:76.18	PEAKS PTM
K.GT(-18.01)GNLELVAVRK.E	N	34.88	1237.7142	12	0.9	413.5790	3	26.41	2	F2:1259	OB5940 H1 raw.raw	7.6712E5	3	3	453	464	Dehydration	T2:Dehydration:1000.00	PEAKS PTM
K.NPQLQDLDM(-48.00)MLTC(+57.02)VEIKEGALMLPHFNSK.A	N	34.70	3323.6353	29	8.1	831.9229	4	35.72	2	F2:1816	OB5940 H1 raw.raw	1.6745E6	2	2	415	443	Carbamidomethylation	M9:Dethiomethyl:17.01;C13:Carbamidomethylation:1000.00	PEAKS PTM
R.IFLAGDKDNVIDQ(+15.00)IEK(+42.01).Q	N	34.39	1873.9673	16	-4.1	937.9871	2	31.58	2	F2:1569	OB5940 H1 raw.raw	0	0	0	536	551	Deamidation followed by a methylation; Acetylation (K)	Q13:Deamidation followed by a methylation:72.09;K16:Acetylation (K):97.49	PEAKS PTM
K.HADADN(+.98)ILVIQQGQATVTVAN(+.98)GNNRK.S	N	34.16	2748.3792	26	8.9	917.1418	3	48.01	1	F1:2528	OB5939 H1 raw.raw	2.2044E4	1	1	223	248		N6:Deamidation (NQ):10.34;N21:Deamidation (NQ):14.04	PEAKS DB
N.PQLQDLDMMLTC(+57.02)VEIKEGALMLPHFNSK.A	N	34.02	3257.5957	28	0.9	815.4069	4	37.83	3	F3:1952	OB5941 H1 raw.raw	2.5417E5	1	1	416	443	Carbamidomethylation	C12:Carbamidomethylation:1000.00	PEAKS DB
R.PNTLVLPK.H	N	33.50	880.5382	8	0.4	441.2766	2	27.53	2	F2:1313	OB5940 H1 raw.raw	8.6509E4	1	1	215	222			PEAKS DB

total 247 peptides

Peptide	Uniq	-10lgP	Mass	Length	ppm	m/z	z	RT	Fraction	Scan	Source File	Area raw peanut	#Feature	#Feature raw peanut	Start	End	PTM	AScore	Found By
R.IFLAGD(-18.01)KDNVIDQIEK.Q	N	33.20	1798.9464	16	-2.5	600.6546	3	31.45	2	F2:1554	OB5940 H1 raw.raw	1.217E6	1	1	536	551		D6:Dehydration:31.02	PEAKS PTM
K.G(+43.01)T(+14.02)GNLELVAVRK.E	N	33.06	1312.7462	12	-0.8	657.3799	2	26.85	3	F3:1280	OB5941 H1 raw.raw	3.165E5	2	2	453	464	Carbamylation; Methylation(others)	G1:Carbamylation:148.28;T2:Methylation(others):59.30	PEAKS PTM
R.IPSGFISYILN(+.98)RHDNQNLR.V	N	33.06	2257.1604	19	9.2	565.3026	4	35.09	3	F3:1797	OB5941 H1 raw.raw	1.752E6	1	1	260	278		N11:Deamidation (NQ):16.76	PEAKS DB
R.REQEWEEEEEDEEEEGSNREVR.R	N	33.06	2821.1545	22	3.9	941.3958	3	26.47	3	F3:1247	OB5941 H1 raw.raw	1.8551E6	1	1	472	493			PEAKS DB
R.IPSGFISYILN(+.98)R(+14.02)H(+42.01)DNQNLR.V	N	32.77	2313.1865	19	6.8	579.3079	4	33.29	1	F1:1633	OB5939 H1 raw.raw	2.3452E7	3	3	260	278		N11:Deamidation (NQ):35.13;R12:Methylation(KR):45.25;H13:Acetylation (TSCYH):27.86	PEAKS PTM
Q.IEARPNTLVLPK.H	Y	32.24	1349.8030	12	2.2	675.9103	2	30.40	1	F1:1461	OB5939 H1 raw.raw	7.0908E5	2	2	211	222			PEAKS DB
K.HADADNILVIQQGQ(+.98)ATVTVANGNN(+.98)RK.S	N	32.00	2748.3792	26	7.7	917.1407	3	55.56	2	F2:2966	OB5940 H1 raw.raw	0	0	0	223	248		Q14:Deamidation (NQ):19.05;N24:Deamidation (NQ):0.00	PEAKS DB
R.EGEQEW(+15.99)GTPGSEVREETSR.N	Y	31.83	2177.9460	19	2.6	1089.9832	2	26.55	1	F1:1197	OB5939 H1 raw.raw	2.78E4	1	1	147	165	Oxidation (HW)	W6:Oxidation (HW):1000.00	PEAKS PTM
K.AMVIVVVNK(-1.03)GTGNLELVAVR.K	N	31.46	2080.1714	20	4.3	1041.0974	2	33.79	2	F2:1738	OB5940 H1 raw.raw	2.5754E7	1	1	444	463	Lysine oxidation to aminoadipic semialdehyde	K9:Lysine oxidation to aminoadipic semialdehyde:1000.00	PEAKS PTM
K.HADADNILVIQQGQATVTVANGN(+.98)N(+.98)RK.S	N	31.37	2748.3792	26	5.7	917.1389	3	45.07	1	F1:2318	OB5939 H1 raw.raw	5.4428E3	1	1	223	248		N23:Deamidation (NQ):0.00;N24:Deamidation (NQ):0.00	PEAKS DB

total 247 peptides

Peptide	Uniq	-10lgP	Mass	Length	ppm	m/z	z	RT	Fraction	Scan	Source File	Area raw peanut	#Feature	#Feature raw peanut	Start	End	PTM	AScore	Found By
R.IFLAGDKDNVID.Q	N	31.18	1318.6769	12	1.8	660.3469	2	31.65	1	F1:1537	OB5939 H1 raw.raw	9.3082E4	1	1	536	547			PEAKS DB
R.EREEDWRQPR.E	N	31.07	1399.6592	10	0.1	467.5604	3	24.77	3	F3:1154	OB5941 H1 raw.raw	2.8345E4	1	1	118	127			PEAKS DB
K.S(-18.01)FNLDEGHALRIPSGFISYILNR.H	N	30.52	2600.3499	23	-0.3	651.0945	4	37.85	1	F1:1904	OB5939 H1 raw.raw	4.9973E5	1	1	249	271		S1:Dehydration:11.12	PEAKS PTM
R.EGEQEWGT(-18.01)PGSEVREETSR.N	Y	30.04	2143.9407	19	2.2	1072.9800	2	27.69	1	F1:1293	OB5939 H1 raw.raw	4.5095E4	1	1	147	165		T8:Dehydration:20.70	PEAKS PTM
K.SFNLDEGHALRIPS.G	N	29.93	1554.7791	14	-4.8	778.3931	2	30.79	2	F2:1515	OB5940 H1 raw.raw	5.213E3	1	1	249	262			PEAKS DB
K.HADADNILVIQQGQATVT(-18.01)VANGNNR.K	N	29.88	2600.3054	25	-2.7	867.7734	3	30.76	3	F3:1503	OB5941 H1 raw.raw	3.2843E5	1	1	223	247		T18:Dehydration:22.85	PEAKS PTM
K.NPQLQDLDM(-48.00)MLTC(+57.02)VEIK.E	N	29.75	1998.9755	17	-0.6	667.3320	3	34.07	2	F2:1713	OB5940 H1 raw.raw	8.006E5	1	1	415	431	Carbamidomethylation	M9:Dethiomethyl:0.00;C13:Carbamidomethylation:1000.00	PEAKS PTM
R.EDWRRPSHQQPR.K	N	29.71	1590.7764	12	-0.3	398.7013	4	24.94	3	F3:1169	OB5941 H1 raw.raw	1.2481E4	1	1	128	139			PEAKS DB
R.SSDNEGVIVK(+43.01)VSK(+14.02)EHVQELTK.H	N	29.63	2382.2390	21	0.9	795.0876	3	28.31	3	F3:1368	OB5941 H1 raw.raw	5.0291E4	1	1	351	371		K10:Carbamylation:5.08;K13:Methylation(KR):5.08	PEAKS PTM
N.NPFYFPSRR.F	N	29.59	1182.5934	9	1.6	395.2057	3	28.08	2	F2:1358	OB5940 H1 raw.raw	2.5271E4	1	1	167	175			PEAKS DB
R.IVQIEARPNTLVLP(+15.99)K.H	Y	29.48	1706.0090	15	1.6	854.0131	2	29.82	1	F1:1418	OB5939 H1 raw.raw	0	0	0	208	222		P14:Hydroxylation Pro:43.89	PEAKS DB

total 247 peptides

Peptide	Uniq	-10lgP	Mass	Length	ppm	m/z	z	RT	Fraction	Scan	Source File	Area raw peanut	#Feature	#Feature raw peanut	Start	End	PTM	AScore	Found By
K.N(+.98)PQ(+.98)LQ(+.98)DLDMM(+15.99)LTC(+57.02)VEIKEGALMLPHFNSK.A	N	29.44	3390.5854	29	3.7	848.6568	4	38.47	3	F3:1982	OB5941 H1 raw.raw	0	0	0	415	443	Deamidation (NQ); Carbamidomethylation	N1:Deamidation (NQ): 62.42; Q3:Deamidation (NQ): 65.92; Q5:Deamidation (NQ): 60.92; M10:Oxidation (M):12.28;C13:Carbamidomethylation:1000.00	PEAKS DB
K.HADADNILVIQQGQATVTVANGNNR(+14.02)K(+43.99).S	N	29.12	2804.4165	26	-3.2	1403.2111	2	29.38	3	F3:1435	OB5941 H1 raw.raw	2.4694E5	1	1	223	248	Methylation(KR)	R25:Methylation(KR): 1000.00;K26: Carboxylation (DKW): 41.02	PEAKS PTM
R.EQEW(+15.99)EEEEEDEEEEGSNR.E	N	29.12	2296.8362	18	4.4	1149.4304	2	27.05	1	F1:1252	OB5939 H1 raw.raw	0	0	0	473	490	Oxidation (HW)	W4:Oxidation (HW):1000.00	PEAKS PTM
K.HADADNILVIQQGQAT(-18.01)VTVANGNNR.K	N	29.07	2600.3054	25	-1.0	867.7748	3	30.11	2	F2:1479	OB5940 H1 raw.raw	0	0	0	223	247		T16:Dehydration:0.00	PEAKS PTM
R.E(-18.01)QEWEEEEEDEEEEGSNREVRR.Y	N	28.92	2803.1440	22	2.8	935.3912	3	28.31	3	F3:1363	OB5941 H1 raw.raw	2.9497E4	1	1	473	494	Pyro-glu from E	E1:Pyro-glu from E:1000.00	PEAKS PTM
K.KN(+.98)P(+15.99)Q(+.98)LQ(+.98)DLDMMLTC(+57.02)VEIKEGALMLPHFNSK.A	N	28.66	3518.6804	30	-5.1	880.6729	4	37.59	1	F1:1890	OB5939 H1 raw.raw	0	0	0	414	443	Deamidation (NQ); Carbamidomethylation	N2:Deamidation (NQ): 55.07;P3:Hydroxylation Pro:43.60;Q4:Deamidation (NQ): 57.68; Q6:Deamidation (NQ): 58.78; C14:Carbamidomethylation:1000.00	PEAKS DB

total 247 peptides

Peptide	Uniq	-10lgP	Mass	Length	ppm	m/z	z	RT	Fraction	Scan	Source File	Area raw peanut	#Feature	#Feature raw peanut	Start	End	PTM	AScore	Found By
K.H(+42.01)(+15.99)ADADNILVIQQGQATVTVAN(+.98)GNNRK.S	N	28.60	2805.4006	26	9.3	1403.7207	2	30.57	1	F1:1450	OB5939 H1 raw.raw	9.6068E4	1	1	223	248	Acetylation (N-term); Oxidation (HW)	H1:Acetylation (N-term):1000.00;H1:Oxidation (HW):1000.00;N21:Deamidation (NQ):14.04	PEAKS PTM
R.NTLEAAFNAEFNEIRRVLLEENAGGEQEER.G	N	28.52	3447.6653	30	-1.2	1150.2277	3	38.03	2	F2:1966	OB5940 H1 raw.raw	1.3831E5	1	1	313	342			PEAKS DB
R.ESHFVSARPQSQSP(+15.99)SSP(+15.99)EKEDQEEEN.Q	N	28.43	2989.2808	26	0.3	997.4344	3	24.81	3	F3:1156	OB5941 H1 raw.raw	0	0	0	574	599		P14:Hydroxylation Pro:17.91;P17:Hydroxylation Pro:28.14	PEAKS DB
K.NPQLQDLDMM(+15.99)LTC(+58.01)VEIK.E	N	28.36	2063.9578	17	-3.4	1032.9827	2	37.19	1	F1:1881	OB5939 H1 raw.raw	2.5393E4	1	1	415	431	Carboxymethyl	M10:Oxidation (M):8.69;C13:Carboxymethyl:1000.00	PEAKS PTM
K.DLAFPGSGEQVEK(+14.02)LIK(+43.01).N	N	28.34	1786.9464	16	-3.8	894.4771	2	32.91	3	F3:1654	OB5941 H1 raw.raw	6.9695E5	1	1	555	570		K13:Methylation(KR):4.52;K16:Carbamylation:4.52	PEAKS PTM
K.KN(+.98)PQ(+.98)LQDLDMMLTC(+57.02)VEIKEGALMLPHFNSK.A	N	28.34	3501.7017	30	6.9	1168.2493	3	36.92	1	F1:1845	OB5939 H1 raw.raw	2.5153E5	1	1	414	443	Carbamidomethylation	N2:Deamidation (NQ):0.00;Q4:Deamidation (NQ):0.00;C14:Carbamidomethylation:1000.00	PEAKS DB
K.N(+.98)PQLQDLDM(+15.99)MLTC(+57.02)VEIKEGALMLPHFNSK.A	N	27.69	3388.6174	29	8.6	1130.5562	3	39.33	3	F3:2085	OB5941 H1 raw.raw	8.4125E5	1	1	415	443	Carbamidomethylation	N1:Deamidation (NQ):0.00;M9:Oxidation (M):0.00;C13:Carbamidomethylation:1000.00	PEAKS DB

total 247 peptides

Peptide	Uniq	-10lgP	Mass	Length	ppm	m/z	z	RT	Fraction	Scan	Source File	Area raw peanut	#Feature	#Feature raw peanut	Start	End	PTM	AScore	Found By
R.SKQ(+.98)FQN(+.98)LQ(+.98)NHR.I	Y	27.06	1401.6637	11	2.0	468.2294	3	24.91	1	F1:1129	OB5939 H1 raw.raw	2.0347E3	1	1	197	207		Q3:Deamidation (NQ): 14.04; N6:Deamidation (NQ): 11.06; Q8:Deamidation (NQ): 16.66	PEAKS DB
K.SFNLD(+53.92)EGHALR.I	N	26.85	1311.5294	11	-0.8	438.1834	3	27.41	2	F2:1311	OB5940 H1 raw.raw	0	0	0	249	259		D5:Replacement of 2 protons by iron: 20.92	PEAKS PTM
**total 247 peptides**																			

tr|B3IXL2|B3IXL2_ARAHY

back to list

| Protein Coverage | Supporting Peptides |

Protein Coverage:

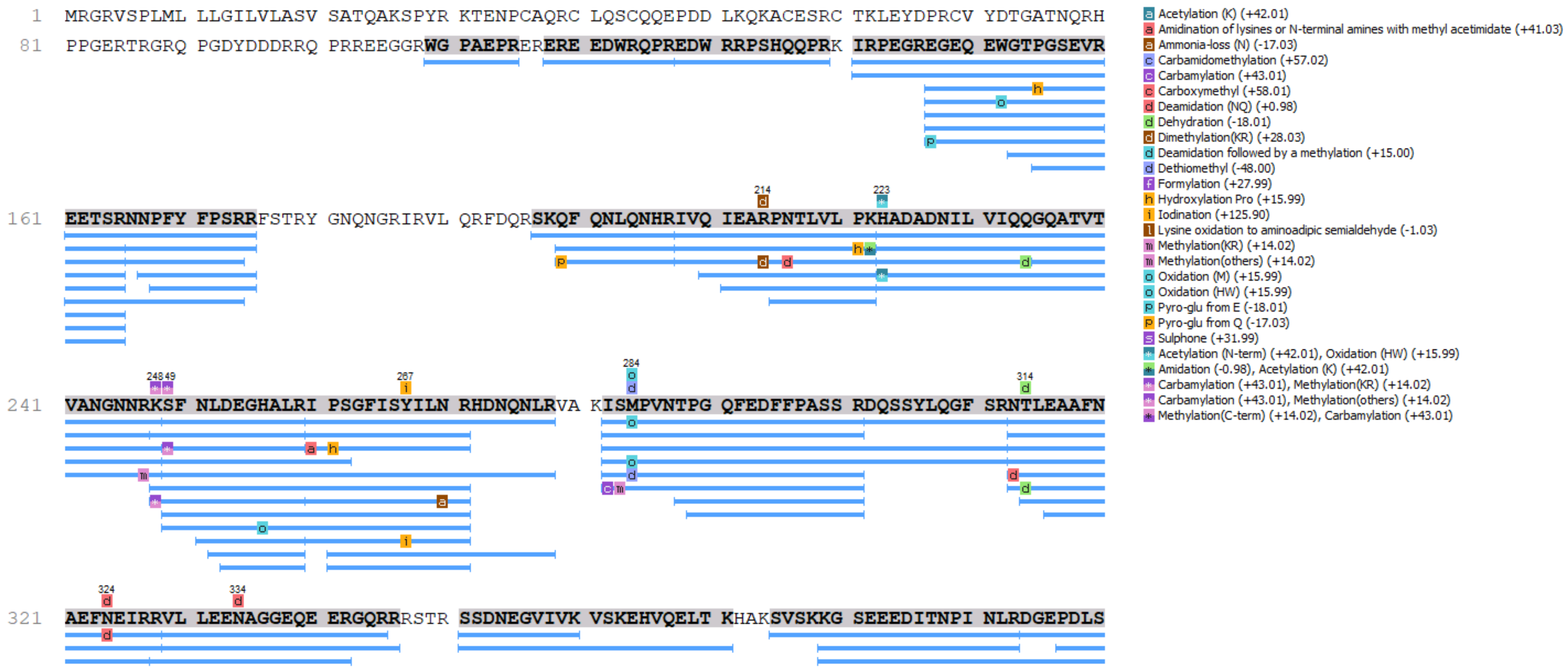

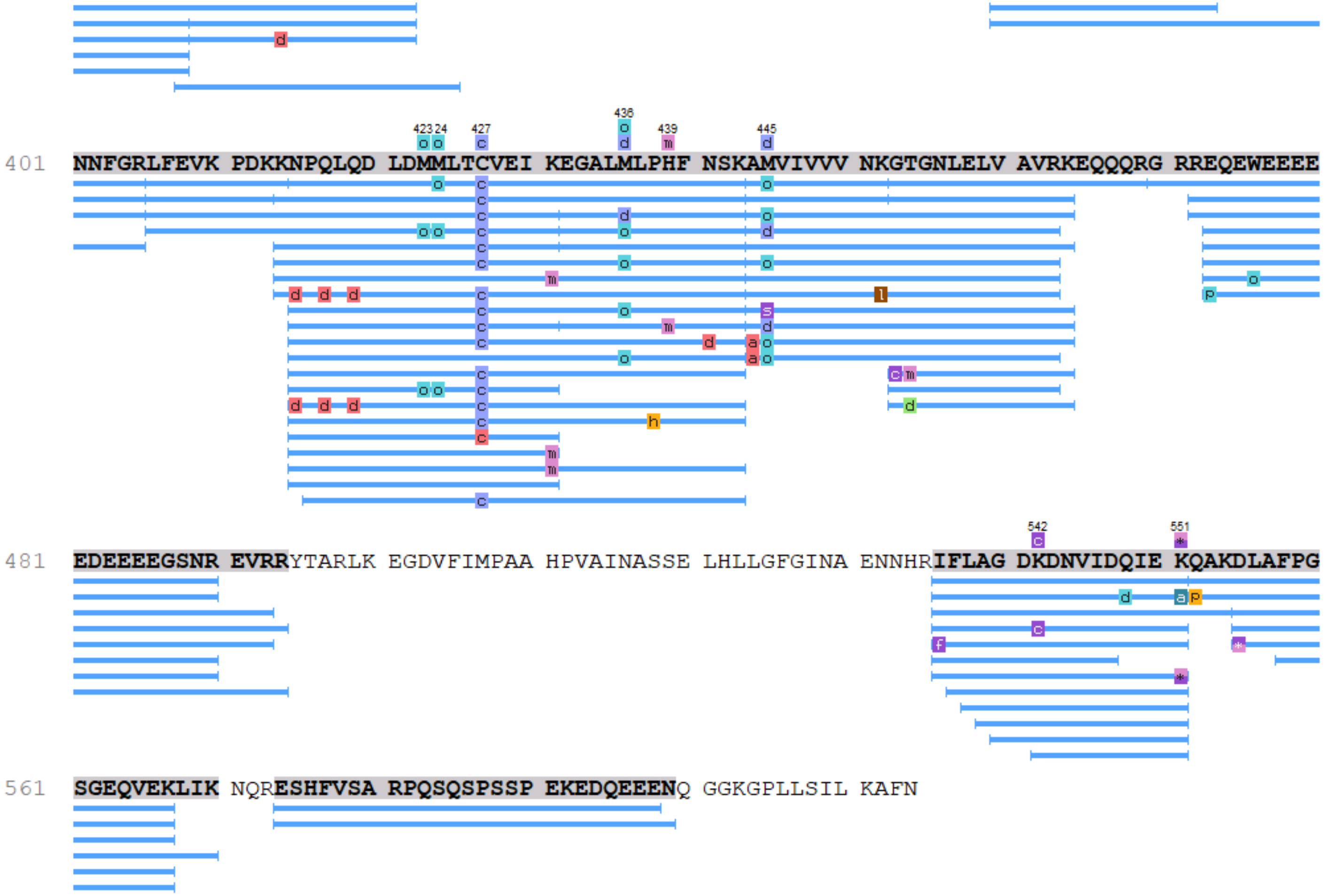

Supporting Peptides:

Peptide	Uniq	-10lgP	Mass	Length	ppm	m/z	z	RT	Fraction	Scan	Source File	Area raw peanut	#Feature	#Feature raw peanut	Start	End	PTM	AScore	Found By
K.NPQLQDLDMMLTC(+57.02)VEIK.E	N	120.22	2046.9788	17	-6.3	1024.4902	2	37.10	1	F1:1864	OB5939H1 raw.raw	1.3638E7	5	5	415	431	Carbamidomethylation	C13:Carbamidomethylation:1000.00	PEAKS DB
K.KNPQLQDLDMMLTC(+57.02)VEIK.E	N	118.64	2175.0737	18	-1.1	1088.5430	2	34.52	2	F2:1751	OB5940H1 raw.raw	4.131E6	3	3	414	431	Carbamidomethylation	C14:Carbamidomethylation:1000.00	PEAKS DB
R.EQEWEEEEEDEEEEGSNR.E	N	117.85	2280.8413	18	4.3	1141.4329	2	28.23	1	F1:1323	OB5939H1 raw.raw	4.2916E6	3	3	473	490			PEAKS DB

total 247 peptides

Peptide	Uniq	-10lgP	Mass	Length	ppm	m/z	z	RT	Fraction	Scan	Source File	Area raw peanut	#Feature	#Feature raw peanut	Start	End	PTM	AScore	Found By
R.NTLEAAFNAEFNEIR.R	N	114.83	1737.8322	15	-0.4	869.9230	2	34.89	2	F2:1770	OB5940 H1 raw.raw	1.3513E7	6	6	313	327			PEAKS DB
K.HADADNILVIQQGQATVTVANGNNRK.S	N	114.12	2746.4111	26	3.2	916.4806	3	29.15	1	F1:1378	OB5939 H1 raw.raw	4.7574E7	8	8	223	248			PEAKS DB
K.HADADNILVIQQGQATVTVAN(+.98)GNNRK.S	N	111.24	2747.3950	26	5.5	916.8107	3	29.51	1	F1:1400	OB5939 H1 raw.raw	3.8297E8	20	20	223	248		N21:Deamidation (NQ):26.31	PEAKS DB
K.HADADNILVIQQGQATVTVANGNNR.K	N	111.11	2618.3162	25	5.0	1310.1719	2	30.00	2	F2:1473	OB5940 H1 raw.raw	6.2545E6	3	3	223	247			PEAKS DB
K.NPQLQDLDM(+15.99)MLTC(+57.02)VEIK.E	N	110.46	2062.9736	17	-5.3	1032.4886	2	36.07	2	F2:1840	OB5940 H1 raw.raw	3.4606E5	2	2	415	431	Carbamidomethylation	M9:Oxidation (M):30.46;C13:Carbamidomethylation:1000.00	PEAKS DB
K.ISM(+15.99)PVNTPGQFEDFFPASSR.D	N	109.22	2242.0364	20	-1.8	1122.0234	2	33.79	2	F2:1706	OB5940 H1 raw.raw	1.7538E7	15	15	282	301	Oxidation (M)	M3:Oxidation (M):1000.00	PEAKS DB
K.ISMPVNTPGQFEDFFPASSR.D	N	107.66	2226.0415	20	1.7	1114.0299	2	35.27	2	F2:1792	OB5940 H1 raw.raw	2.1717E8	29	29	282	301			PEAKS DB
R.IFLAGDKDNVIDQIEK.Q	N	103.70	1816.9570	16	-1.1	909.4848	2	32.03	1	F1:1588	OB5939 H1 raw.raw	7.5151E8	30	30	536	551			PEAKS DB
K.HADADNILVIQQGQATVTVAN(-17.03)GNNRK.S	N	99.83	2729.3845	26	0.5	1365.7002	2	28.96	2	F2:1400	OB5940 H1 raw.raw	9.5179E6	6	6	223	248		N21:Ammonia-loss (N):18.53	PEAKS PTM
R.IFLAGDKDNVIDQIEKQAK.D	N	99.61	2144.1477	19	1.7	715.7244	3	32.37	1	F1:1571	OB5939 H1 raw.raw	5.6465E7	7	7	536	554			PEAKS DB
K.AMVIVVVNKGTGNLELVAVR.K	N	99.39	2081.2031	20	-3.5	694.7392	3	33.79	2	F2:1711	OB5940 H1 raw.raw	6.8972E7	9	9	444	463			PEAKS DB
K.GSEEEDITNPINLRDGEPDLSNNFGR.L	N	98.56	2887.3220	26	2.3	963.4502	3	32.73	1	F1:1590	OB5939 H1 raw.raw	4.549E7	8	8	380	405			PEAKS DB
K.SFNLDEGHALRIPSGFISYILNR.H	N	98.27	2618.3604	23	-1.1	873.7931	3	37.47	2	F2:1880	OB5940 H1 raw.raw	1.7034E8	24	23	249	271			PEAKS DB
R.KSFNLDEGHALRIPSGFISYILNR.H	N	97.91	2746.4553	24	-2.1	916.4905	3	36.16	1	F1:1802	OB5939 H1 raw.raw	8.5492E6	5	5	248	271			PEAKS DB
R.VLLEENAGGEQEER.G	N	97.22	1571.7427	14	2.5	786.8806	2	26.75	1	F1:1224	OB5939 H1 raw.raw	1.2414E7	3	3	329	342			PEAKS DB
R.DQSSYLQGFSR.N	N	97.09	1286.5891	11	-3.0	644.2999	2	30.18	2	F2:1484	OB5940 H1 raw.raw	6.1544E7	13	13	302	312			PEAKS DB
K.AM(-48.00)VIVVVNKGTGNLELVAVR.K	N	96.95	2033.1997	20	1.4	678.7415	3	30.71	2	F2:1519	OB5940 H1 raw.raw	1.7497E7	6	6	444	463	Dethiomethyl	M2:Dethiomethyl:1000.00	PEAKS PTM

total 247 peptides

Peptide	Uniq	-10lgP	Mass	Length	ppm	m/z	z	RT	Fraction	Scan	Source File	Area raw peanut	#Feature	#Feature raw peanut	Start	End	PTM	AScore	Found By
K.SFNLDEGHALR.I	N	96.68	1257.6101	11	1.8	629.8135	2	27.87	1	F1:1301	OB5939 H1 raw.raw	7.3788E7	15	15	249	259			PEAKS DB
K.AMVIVVVNKGTGNLELVAVRK.E	N	96.39	2209.2981	21	-2.2	737.4384	3	31.97	2	F2:1628	OB5940 H1 raw.raw	2.8068E8	12	12	444	464			PEAKS DB
K.AM(+15.99)VIVVVNKGTGNLELVAVR.K	N	95.84	2097.1980	20	-2.2	1049.6040	2	32.73	3	F3:1638	OB5941 H1 raw.raw	3.1624E7	6	6	444	463	Oxidation (M)	M2:Oxidation (M):1000.00	PEAKS DB
R.IPSGFISYILNR.H	N	93.86	1378.7609	12	1.2	690.3885	2	36.74	2	F2:1881	OB5940 H1 raw.raw	1.7713E8	5	5	260	271			PEAKS DB
R.RVLLEENAGGEQEER.G	N	93.51	1727.8438	15	0.4	576.9554	3	24.77	2	F2:1156	OB5940 H1 raw.raw	1.346E5	5	5	328	342			PEAKS DB
K.NPQLQDLDMMLTC(+57.02)VEIKEGALMLPHFNSK.A	N	92.80	3371.6387	29	-2.9	1686.8218	2	37.92	1	F1:1910	OB5939 H1 raw.raw	1.8404E8	11	11	415	443	Carbamidomethylation	C13:Carbamidomethylation:1000.00	PEAKS DB
R.KSFNLDEGHALR.I	N	89.31	1385.7051	12	1.9	693.8611	2	25.26	2	F2:1181	OB5940 H1 raw.raw	3.3181E6	6	6	248	259			PEAKS DB
K.KGSEEEDITNPINLRDGEPDLSNNFGR.L	N	89.20	3015.4170	27	-0.8	1006.1455	3	31.46	1	F1:1545	OB5939 H1 raw.raw	7.9044E7	10	10	379	405			PEAKS DB
K.NPQLQDLDMMLTC(+14.02)VEIK.E	N	88.98	2003.9730	17	-4.9	1002.9888	2	37.72	2	F2:1938	OB5940 H1 raw.raw	6.7012E5	2	2	415	431		C13:Methylation(others):33.98	PEAKS PTM
R.NTLEAAFNAEFNEIRR.V	N	86.44	1893.9332	16	-3.6	947.9705	2	35.28	1	F1:1741	OB5939 H1 raw.raw	3.2153E8	22	22	313	328			PEAKS DB
K.HADADNILVIQQGQATVTVANGN(-17.03)NRK.S	N	85.41	2729.3845	26	-3.3	1365.6951	2	29.38	3	F3:1432	OB5941 H1 raw.raw	2.4846E6	2	2	223	248		N23:Ammonia-loss (N):0.00	PEAKS PTM
K.DLAFPGSGEQVEK.L	N	85.12	1375.6619	13	1.9	688.8395	2	29.66	2	F2:1451	OB5940 H1 raw.raw	6.5078E7	3	3	555	567			PEAKS DB
K.NPQLQDLDMM(-48.00)LTC(+57.02)VEIK.E	N	85.01	1998.9755	17	0.5	1000.4955	2	32.73	1	F1:1599	OB5939 H1 raw.raw	1.7365E7	6	6	415	431	Carbamidomethylation	M10:Dethiomethyl:27.96;C13:Carbamidomethylation:1000.00	PEAKS PTM
R.VLLEENAGGEQEERGQR.R	N	84.92	1912.9238	17	1.8	957.4709	2	25.31	3	F3:1182	OB5941 H1 raw.raw	1.9929E6	6	6	329	345			PEAKS DB
R.SSDNEGVIVKVSKEHVQELTK.H	N	84.86	2325.2175	21	1.3	776.0808	3	28.31	3	F3:1362	OB5941 H1 raw.raw	1.2881E5	2	2	351	371			PEAKS DB
K.KGSEEEDITNPINLR.D	N	84.79	1713.8533	15	3.6	857.9370	2	28.42	2	F2:1374	OB5940 H1 raw.raw	1.1329E7	7	7	379	393			PEAKS DB

total 247 peptides

Peptide	Uniq	-10lgP	Mass	Length	ppm	m/z	z	RT	Fraction	Scan	Source File	Area raw peanut	#Feature	#Feature raw peanut	Start	End	PTM	AScore	Found By
K.KNPQLQDLDMM(+15.99)LTC(+57.02)VEIKEGALMLPHFNSK.A	N	84.57	3515.7285	30	-2.7	879.9370	4	34.52	2	F2:1755	OB5940 H1 raw.raw	2.6704E6	3	3	414	443	Carbamidomethylation	M11:Oxidation (M):30.46;C14:Carbamidomethylation:1000.00	PEAKS DB
R.DGEPDLSNNFGR.L	N	84.53	1319.5742	12	0.4	660.7947	2	29.14	1	F1:1381	OB5939 H1 raw.raw	3.3217E6	3	3	394	405			PEAKS DB
K.AM(+15.99)VIVVVNKGTGNLELVAVRK.E	N	83.52	2225.2930	21	-2.3	1113.6512	2	31.28	1	F1:1523	OB5939 H1 raw.raw	4.3919E7	8	8	444	464	Oxidation (M)	M2:Oxidation (M):1000.00	PEAKS DB
R.EGEQEWGTPGSEVREETSR.N	Y	82.53	2161.9512	19	4.3	1081.9875	2	27.69	1	F1:1291	OB5939 H1 raw.raw	2.0963E7	6	6	147	165			PEAKS DB
K.HADADNILVIQQGQATVTVAN(+.98)GNNR.K	N	82.15	2619.3000	25	-1.2	1310.6558	2	30.35	2	F2:1535	OB5940 H1 raw.raw	5.2956E6	1	1	223	247		N21:Deamidation (NQ):7.32	PEAKS DB
R.EGEQEWGTPGSEVR.E	Y	81.97	1559.6852	14	1.4	780.8510	2	27.35	2	F2:1310	OB5940 H1 raw.raw	8.022E5	3	3	147	160			PEAKS DB
K.KNPQLQDLDMM(+15.99)LTC(+57.02)VEIK.E	N	81.97	2191.0686	18	0.5	1096.5421	2	32.43	2	F2:1621	OB5940 H1 raw.raw	1.9806E5	1	1	414	431	Carbamidomethylation	M11:Oxidation (M):9.34;C14:Carbamidomethylation:1000.00	PEAKS DB
K.EGALMLPHFNSK.A	N	81.44	1342.6703	12	4.0	672.3451	2	30.40	1	F1:1456	OB5939 H1 raw.raw	9.7706E6	3	3	432	443			PEAKS DB
K.QAKDLAFPGSGEQVEK.L	N	81.31	1702.8525	16	1.8	852.4351	2	26.85	3	F3:1278	OB5941 H1 raw.raw	1.3516E6	7	7	552	567			PEAKS DB
K.GSEEEDITNPINLR.D	N	80.86	1585.7583	14	2.4	793.8883	2	30.52	2	F2:1506	OB5940 H1 raw.raw	3.0736E6	3	3	380	393			PEAKS DB
K.H(+43.01)(+14.02)ADADNILVIQQGQATVTVANGNNRK.S	N	80.77	2803.4324	26	-1.3	935.4835	3	29.92	3	F3:1452	OB5941 H1 raw.raw	2.1221E6	2	2	223	248		H1:Carbamylation:252.86;H1:Methylation (others):25.66	PEAKS PTM
K.AMVIVVVNK.G	N	78.49	971.5837	9	0.9	486.7996	2	29.32	2	F2:1448	OB5940 H1 raw.raw	9.4803E6	3	3	444	452			PEAKS DB
F.LAGDKDNVIDQIEK.Q	N	78.09	1556.8046	14	-6.1	779.4048	2	31.62	2	F2:1564	OB5940 H1 raw.raw	4.6126E6	2	2	538	551			PEAKS DB

total 247 peptides

Peptide	Uniq	-10lgP	Mass	Length	ppm	m/z	z	RT	Fraction	Scan	Source File	Area raw peanut	#Feature	#Feature raw peanut	Start	End	PTM	AScore	Found By
K.KNPQLQDLDM(+15.99)MLTC(+57.02)VEIK.E	N	77.76	2191.0686	18	0.6	1096.5422	2	33.03	1	F1:1615	OB5939 H1 raw.raw	0	0	0	414	431	Carbamidomethylation	M10:Oxidation (M):14.02;C14:Carbamidomethylation:1000.00	PEAKS DB
R.IPSGFISYILNRHDNQNLR.V	N	77.66	2256.1763	19	3.4	1129.0992	2	33.47	1	F1:1643	OB5939 H1 raw.raw	3.1228E8	24	24	260	278			PEAKS DB
R.GRREQEWEEEEEDEEEEGSNR.E	N	77.42	2650.0649	21	0.3	884.3625	3	25.64	2	F2:1205	OB5940 H1 raw.raw	2.3251E6	7	7	470	490			PEAKS DB
K.NPQLQDLDM(+15.99)MLTC(+57.02)VEIKEGALMLPHFNSK.A	N	75.51	3387.6335	29	-1.5	1130.2168	3	36.17	3	F3:1926	OB5941 H1 raw.raw	2.5418E7	3	3	415	443	Carbamidomethylation	M9:Oxidation (M):10.11;C13:Carbamidomethylation:1000.00	PEAKS DB
K.EGALM(+15.99)LPHFNSK.A	N	74.93	1358.6653	12	2.1	680.3414	2	29.74	3	F3:1443	OB5941 H1 raw.raw	2.0704E6	3	3	432	443	Oxidation (M)	M5:Oxidation (M):1000.00	PEAKS DB
R.LFEVKPDKKNPQLQDLDMMLTC(+57.02)VEIK.E	N	74.78	3131.6069	26	3.7	1044.8801	3	33.97	2	F2:1716	OB5940 H1 raw.raw	3.7773E6	3	3	406	431	Carbamidomethylation	C22:Carbamidomethylation:1000.00	PEAKS DB
K.KNPQLQDLDMMLTC(+57.02)VEIKEGALM(+15.99)LPHFNSK.A	N	74.26	3515.7285	30	-0.8	879.9387	4	35.84	1	F1:1790	OB5939 H1 raw.raw	5.1448E6	2	2	414	443	Carbamidomethylation; Oxidation (M)	C14:Carbamidomethylation:1000.00; M23:Oxidation (M):69.38	PEAKS DB
K.HADADNILVIQQGQATVTVANGNN(+.98)RK.S	N	73.98	2747.3950	26	1.9	916.8074	3	39.56	2	F2:2095	OB5940 H1 raw.raw	5.4145E5	4	4	223	248		N24:Deamidation (NQ):14.02	PEAKS DB
K.GTGNLELVAVRK.E	N	73.67	1255.7249	12	0.9	628.8702	2	26.85	3	F3:1263	OB5941 H1 raw.raw	1.0553E7	7	7	453	464			PEAKS DB
K.NPQLQDLDMM(+15.99)LTC(+57.02)VEIKEGALMLPHFNSK.A	N	72.71	3387.6335	29	3.8	1130.2228	3	37.84	2	F2:1902	OB5940 H1 raw.raw	8.789E6	3	3	415	443	Carbamidomethylation	M10:Oxidation (M):14.02;C13:Carbamidomethylation:1000.00	PEAKS DB
K.GTGNLELVAVR.K	N	71.83	1127.6299	11	0.2	564.8223	2	29.32	2	F2:1429	OB5940 H1 raw.raw	1.0095E7	3	3	453	463			PEAKS DB
R.NNPFYFPSR.R	N	71.25	1140.5352	9	0.9	571.2754	2	30.89	2	F2:1528	OB5940 H1 raw.raw	1.2382E7	4	4	166	174			PEAKS DB

total 247 peptides

Peptide	Uniq	-10lgP	Mass	Length	ppm	m/z	z	RT	Fraction	Scan	Source File	Area raw peanut	#Feature	#Feature raw peanut	Start	End	PTM	AScore	Found By
R.VLLEENAGGEQEERGQRR.R	N	71.19	2069.0249	18	2.1	690.6837	3	25.31	3	F3:1186	OB5941 H1 raw.raw	1.1654E6	2	2	329	346			PEAKS DB
I.PSGFISYILNR.H	N	70.27	1265.6768	11	2.4	633.8472	2	36.17	2	F2:1844	OB5940 H1 raw.raw	2.1876E5	2	2	261	271			PEAKS DB
R.EETSRNNPFYFPSRR.F	N	69.88	1898.9023	15	1.2	633.9755	3	27.17	2	F2:1289	OB5940 H1 raw.raw	7.2799E6	5	5	161	175			PEAKS DB
K.NPQLQDLDMMLTC(+57.02)VEIKEGALM(+15.99)LPHFNSK.A	N	69.58	3387.6335	29	-9.3	1130.2080	3	36.62	2	F2:1874	OB5940 H1 raw.raw	2.6984E7	4	4	415	443	Carbamidomethylation; Oxidation (M)	C13:Carbamidomethylation:1000.00; M22:Oxidation (M):69.89	PEAKS DB
K.ISMPVNTPGQ(+.98)FEDFFPASSR.D	N	68.89	2227.0254	20	6.4	1114.5271	2	63.55	3	F3:3427	OB5941 H1 raw.raw	9.613E3	2	2	282	301		Q10:Deamidation (NQ):33.26	PEAKS DB
R.REQEWEEEEEDEEEEGSNR.E	N	68.88	2436.9424	19	2.8	1219.4819	2	26.47	3	F3:1258	OB5941 H1 raw.raw	3.0427E4	2	2	472	490			PEAKS DB
R.IPSGFISY(+125.90)ILNR.H	N	68.75	1504.6575	12	1.5	753.3372	2	37.84	2	F2:1946	OB5940 H1 raw.raw	9.8548E5	2	2	260	271	Iodination	Y8:Iodination:1000.00	PEAKS PTM
R.NTLEAAFNAEFN(+.98)EIRR.V	N	68.73	1894.9172	16	-3.7	948.4624	2	32.73	3	F3:1640	OB5941 H1 raw.raw	4.5098E6	3	3	313	328	Deamidation (NQ)	N12:Deamidation (NQ):55.21	PEAKS DB
R.N(+.98)TLEAAFNAEFNEIRR.V	N	67.69	1894.9172	16	2.2	948.4680	2	35.88	1	F1:1787	OB5939 H1 raw.raw	6.2604E7	1	1	313	328	Deamidation (NQ)	N1:Deamidation (NQ):61.82	PEAKS DB
R.NNPFYFPSRR.F	N	67.53	1296.6364	10	1.5	649.3264	2	28.95	1	F1:1356	OB5939 H1 raw.raw	1.2601E8	9	9	166	175			PEAKS DB
K.KNPQLQDLDM(+15.99)MLTC(+57.02)VEIKEGALMLPHFNSK.A	N	66.89	3515.7285	30	-3.1	879.9366	4	35.44	2	F2:1830	OB5940 H1 raw.raw	7.3362E6	2	2	414	443	Carbamidomethylation	M10:Oxidation (M):11.06;C14:Carbamidomethylation:1000.00	PEAKS DB
K.HADADNILVIQQGQATVTVANGN(+.98)NRK.S	N	66.81	2747.3950	26	3.3	916.8087	3	44.43	3	F3:2322	OB5941 H1 raw.raw	2.5002E7	10	10	223	248		N23:Deamidation (NQ):17.01	PEAKS DB
K.KNPQLQDLDMMLTC(+57.02)VEIKEGALMLPHFNSK.A	N	66.20	3499.7336	30	-0.1	875.9406	4	36.52	3	F3:1868	OB5941 H1 raw.raw	1.1425E7	3	3	414	443	Carbamidomethylation	C14:Carbamidomethylation:1000.00	PEAKS DB

total 247 peptides

Peptide	Uniq	-10lgP	Mass	Length	ppm	m/z	z	RT	Fraction	Scan	Source File	Area raw peanut	#Feature	#Feature raw peanut	Start	End	PTM	AScore	Found By
K.HADADNILVIQQGQ(+.98)ATVTVANGNNRK.S	N	66.16	2747.3950	26	5.1	916.8103	3	44.47	1	F1:2283	OB5939 H1 raw.raw	5.1832E5	2	2	223	248		Q14:Deamidation (NQ):17.37	PEAKS DB
K.SVSKKGSEEEDITNPINLR.D	N	66.11	2115.0808	19	2.6	706.0361	3	27.22	3	F3:1299	OB5941 H1 raw.raw	6.5352E4	2	2	375	393			PEAKS DB
R.VLLEEN(+.98)AGGEQEER.G	N	66.02	1572.7267	14	-3.7	787.3677	2	26.82	2	F2:1276	OB5940 H1 raw.raw	0	0	0	329	342	Deamidation (NQ)	N6:Deamidation (NQ): 71.64	PEAKS DB
R.IVQIEARPNTLVLPK.H	Y	65.77	1690.0140	15	-3.0	846.0117	2	30.24	3	F3:1474	OB5941 H1 raw.raw	2.8055E8	18	18	208	222			PEAKS DB
R.EGEQEWGTP(+15.99)GSEVREETSR.N	Y	65.62	2177.9460	19	1.3	726.9902	3	26.28	3	F3:1241	OB5941 H1 raw.raw	4.9462E6	6	6	147	165	Hydroxylation Pro	P9:Hydroxylation Pro: 1000.00	PEAKS DB
K.Q(-17.03)FQNLQNHR.I	N	65.38	1166.5581	9	2.4	584.2877	2	28.05	1	F1:1306	OB5939 H1 raw.raw	3.4214E6	3	3	199	207	Pyro-glu from Q	Q1:Pyro-glu from Q:1000.00	PEAKS PTM
K.NPQLQDLDMMLTCVEIK(+14.02)EGALMLPHFNSK.A	N	64.78	3328.6328	29	1.8	1110.5536	3	38.03	2	F2:1959	OB5940 H1 raw.raw	1.6164E7	6	6	415	443	Methylation(KR)	K17:Methylation(KR): 77.78	PEAKS PTM
I.FLAGDKDNVIDQIEK.Q	N	64.24	1703.8729	15	-1.7	852.9423	2	32.19	1	F1:1574	OB5939 H1 raw.raw	1.36E6	3	3	537	551			PEAKS DB
R.EETSRNNPFYFPSR.R	N	63.62	1742.8011	14	-0.9	581.9405	3	29.89	1	F1:1419	OB5939 H1 raw.raw	6.2933E4	1	1	161	174			PEAKS DB
R.I(+27.99)FLAGDKDNVIDQIEK.Q	N	63.40	1844.9519	16	5.2	923.4880	2	36.16	3	F3:1844	OB5941 H1 raw.raw	0	0	0	536	551	Formylation	I1:Formylation:77.93	PEAKS PTM
L.AGDKDNVIDQIEK.Q	N	63.17	1443.7205	13	-3.1	722.8653	2	32.19	1	F1:1560	OB5939 H1 raw.raw	9.2796E5	2	2	539	551			PEAKS DB
K.I(+43.01)S(+14.02)MPVNTPGQFEDFFPASSR.D	N	62.82	2283.0630	20	-0.1	1142.5387	2	35.84	1	F1:1793	OB5939 H1 raw.raw	7.6784E5	1	1	282	301	Carbamylation; Methylation(others)	I1:Carbamylation:1000.00;S2:Methylation(others):86.16	PEAKS PTM
R.IPSGFISYILN(-17.03)R.H	N	62.55	1361.7343	12	1.4	681.8754	2	37.47	2	F2:1926	OB5940 H1 raw.raw	5.558E5	2	2	260	271	Ammonia-loss (N)	N11:Ammonia-loss (N):1000.00	PEAKS PTM
K.HADADNILVIQQGQ(+.98)ATVTVAN(+.98)GNNRK.S	N	62.38	2748.3792	26	2.7	917.1361	3	32.55	3	F3:1635	OB5941 H1 raw.raw	5.5427E5	2	2	223	248		Q14:Deamidation (NQ):32.28;N21:Deamidation (NQ):14.04	PEAKS DB

total 247 peptides

Peptide	Uniq	-10lgP	Mass	Length	ppm	m/z	z	RT	Fraction	Scan	Source File	Area raw peanut	#Feature	#Feature raw peanut	Start	End	PTM	AScore	Found By
R.SSDNEGVIVK.V	N	62.38	1046.5244	10	0.3	524.2697	2	22.95	2	F2:1057	OB5940 H1 raw.raw	2.5465E5	3	3	351	360			PEAKS DB
K.AM(+15.99)VIVVVNK.G	N	62.22	987.5787	9	0.3	494.7968	2	26.41	2	F2:1234	OB5940 H1 raw.raw	8.6851E5	3	3	444	452	Oxidation (M)	M2:Oxidation (M):1000.00	PEAKS DB
A.GDKDNVIDQIEK.Q	N	62.19	1372.6833	12	-2.8	687.3470	2	32.19	1	F1:1561	OB5939 H1 raw.raw	1.5319E6	3	3	540	551			PEAKS DB
F.NLDEGHALR.I	N	62.08	1023.5097	9	1.8	512.7631	2	27.87	1	F1:1303	OB5939 H1 raw.raw	1.5945E6	3	3	251	259			PEAKS DB
Q.GQATVTVAN(+.98)GNNRK.S	N	62.04	1429.7273	14	-1.0	715.8702	2	28.79	2	F2:1402	OB5940 H1 raw.raw	6.6423E5	3	3	235	248		N9:Deamidation (NQ):41.83	PEAKS DB
A.FPGSGEQVEK.L	N	61.40	1076.5138	10	5.5	539.2672	2	30.23	1	F1:1449	OB5939 H1 raw.raw	6.3559E4	1	1	558	567			PEAKS DB
K.HADADNILVIQ(+.98)Q(+.98)GQATVTVAN(+.98)GNNRK.S	N	60.41	2749.3630	26	9.6	917.4704	3	30.18	2	F2:1540	OB5940 H1 raw.raw	4.3875E6	1	1	223	248		Q11:Deamidation (NQ):26.52;Q12:Deamidation (NQ):18.53;N21:Deamidation (NQ):14.04	PEAKS DB
K.SFNLDEGHALRIPSGFISYILNRHDNQNLR.V	N	60.12	3495.7759	30	-1.0	700.1617	5	36.35	2	F2:1862	OB5940 H1 raw.raw	7.0033E7	8	8	249	278			PEAKS DB
K.NPQLQ(+.98)DLDMMLTC(+57.02)VEIKEGALM(+15.99)LPHFNSK.A	N	60.12	3388.6174	29	-2.4	1130.5437	3	36.35	2	F2:1874	OB5940 H1 raw.raw	6.4476E5	1	1	415	443	Carbamidomethylation; Oxidation (M)	Q5:Deamidation (NQ):9.86;C13:Carbamidomethylation:1000.00;M22:Oxidation (M):54.40	PEAKS DB
K.NPQLQDLDM(+15.99)M(+15.99)LTC(+57.02)VEIKEGALMLPHFNSK.A	N	60.05	3403.6284	29	-4.3	1135.5452	3	35.63	3	F3:1818	OB5941 H1 raw.raw	7.9921E5	1	1	415	443	Oxidation (M); Carbamidomethylation	M9:Oxidation (M):49.25;M10:Oxidation (M):53.09;C13:Carbamidomethylation:1000.00	PEAKS DB

total 247 peptides

Peptide	Uniq	-10lgP	Mass	Length	ppm	m/z	z	RT	Fraction	Scan	Source File	Area raw peanut	#Feature	#Feature raw peanut	Start	End	PTM	AScore	Found By
K.NPQLQ(+.98)DLDMMLTC(+57.02)VEIKEGALMLPHFNSK.A	N	59.95	3372.6226	29	5.7	1125.2212	3	37.84	2	F2:1936	OB5940 H1 raw.raw	5.4487E7	3	3	415	443	Carbamidomethylation	Q5:Deamidation (NQ):32.28;C13:Carbamidomethylation:1000.00	PEAKS DB
R.I(+41.03)P(+15.99)SGFISYILNR.H	N	59.92	1435.7823	12	-3.1	718.8962	2	36.92	1	F1:1841	OB5939 H1 raw.raw	1.5727E7	4	4	260	271	Amidination of lysines or N-terminal amines with methyl acetimidate; Hydroxylation Pro	I1:Amidination of lysines or N-terminal amines with methyl acetimidate:1000.00;P2:Hydroxylation Pro:1000.00	PEAKS PTM
K.A(+41.03)M(+15.99)VIVVVNKGTGNLELVAVR.K	N	59.12	2138.2246	20	-4.5	1070.1147	2	33.90	2	F2:1710	OB5940 H1 raw.raw	5.8929E6	3	3	444	463	Amidination of lysines or N-terminal amines with methyl acetimidate; Oxidation (M)	A1:Amidination of lysines or N-terminal amines with methyl acetimidate:170.86;M2:Oxidation (M):1000.00	PEAKS PTM
K.IRPEGREGEQEWGTPGSEVREETSR.N	Y	58.78	2870.3542	25	1.1	957.7931	3	27.23	3	F3:1290	OB5941 H1 raw.raw	4.3127E6	4	4	141	165			PEAKS DB
E.PDLSNNFGR.L	N	58.76	1018.4832	9	-0.1	510.2488	2	31.24	3	F3:1545	OB5941 H1 raw.raw	4.0306E6	3	3	397	405			PEAKS DB
K.HADADNILVIQQ(+.98)GQ(+.98)ATVTVAN(+.98)GNNRK.S	N	56.82	2749.3630	26	9.6	917.4704	3	30.18	2	F2:1487	OB5940 H1 raw.raw	4.3875E6	1	1	223	248		Q12:Deamidation (NQ):14.02;Q14:Deamidation (NQ):13.03;N21:Deamidation (NQ):11.10	PEAKS DB
K.ISMPVNTPGQFEDFFPASSRDQSSYLQGFSR.N	N	56.52	3494.6201	31	3.0	1748.3225	2	35.84	1	F1:1789	OB5939 H1 raw.raw	9.3656E7	16	16	282	312			PEAKS DB

total 247 peptides

Peptide	Uniq	-10lgP	Mass	Length	ppm	m/z	z	RT	Fraction	Scan	Source File	Area raw peanut	#Feature	#Feature raw peanut	Start	End	PTM	AScore	Found By
K.H(+42.01)(+15.99)ADADNILVIQQGQATVTVANGNNRK.S	N	56.30	2804.4165	26	-2.5	1403.2120	2	29.79	1	F1:1416	OB5939 H1 raw.raw	2.4794E5	1	1	223	248	Acetylation (N-term); Oxidation (HW)	H1:Acetylation (N-term):1000.00;H1:Oxidation (HW):1000.00	PEAKS PTM
K.HADADN(+.98)ILVIQQGQATVTVANGNNRK.S	N	55.97	2747.3950	26	3.7	916.8090	3	50.62	1	F1:2616	OB5939 H1 raw.raw	5.4571E5	5	5	223	248		N6:Deamidation (NQ):42.57	PEAKS DB
K.ISMP(+15.99)VNTPGQFEDFFPASSRDQSSYLQGFSR.N	N	55.83	3510.6150	31	2.3	1171.2150	3	34.95	1	F1:1731	OB5939 H1 raw.raw	0	0	0	282	312		P4:Hydroxylation Pro:49.79	PEAKS DB
K.ISM(-48.00)PVNTPGQFEDFFPASSR.D	N	55.62	2178.0381	20	0.2	727.0201	3	32.37	1	F1:1577	OB5939 H1 raw.raw	3.0139E7	6	6	282	301	Dethiomethyl	M3:Dethiomethyl:1000.00	PEAKS PTM
K.N(+.98)PQLQDLDMMLTC(+57.02)VEIKEGALMLPHFNSK.A	N	55.55	3372.6226	29	5.7	1125.2212	3	37.84	2	F2:1983	OB5940 H1 raw.raw	1.3378E7	1	1	415	443	Carbamidomethylation	N1:Deamidation (NQ):14.04;C13:Carbamidomethylation:1000.00	PEAKS DB
K.HADADNILVIQ(+.98)QGQATVTVAN(+.98)GNNRK.S	N	55.03	2748.3792	26	5.1	1375.2039	2	31.28	1	F1:1539	OB5939 H1 raw.raw	2.1986E5	2	2	223	248		Q11:Deamidation (NQ):15.10;N21:Deamidation (NQ):17.01	PEAKS DB
K.HADADNILVIQ(+.98)QGQATVTVANGNNRK.S	N	55.00	2747.3950	26	5.9	916.8110	3	45.04	3	F3:2357	OB5941 H1 raw.raw	2.5167E4	1	1	223	248		Q11:Deamidation (NQ):9.34	PEAKS DB
K.NPQLQDLDMM(+15.99)LTC(+57.02)VEIKEGALM(+15.99)LPHFNSK.A	N	54.50	3403.6284	29	4.5	1135.5552	3	36.52	3	F3:1873	OB5941 H1 raw.raw	6.8814E5	2	2	415	443	Carbamidomethylation	M10:Oxidation (M):12.28;C13:Carbamidomethylation:1000.00;M22:Oxidation (M):28.29	PEAKS DB
R.EQEWEEEEEDEEEEGSNREVR.R	N	54.08	2665.0535	21	2.4	889.3606	3	27.87	1	F1:1308	OB5939 H1 raw.raw	4.4193E5	2	2	473	493			PEAKS DB
K.HADADNILVIQQ(+.98)GQATVTVANGNNRK.S	N	53.63	2747.3950	26	1.9	916.8074	3	39.56	2	F2:2225	OB5940 H1 raw.raw	5.559E5	4	4	223	248		Q12:Deamidation (NQ):11.24	PEAKS DB
N.PFYFPSRR.F	N	53.53	1068.5504	8	3.3	535.2842	2	28.95	1	F1:1360	OB5939 H1 raw.raw	3.1024E6	3	3	168	175			PEAKS DB

total 247 peptides

Peptide	Uniq	-10lgP	Mass	Length	ppm	m/z	z	RT	Fraction	Scan	Source File	Area raw peanut	#Feature	#Feature raw peanut	Start	End	PTM	AScore	Found By
R.LFEVKPDKKNPQLQDLDMM(+15.99)LTC(+57.02)VEIK.E	N	53.37	3147.6018	26	-4.4	787.9042	4	32.55	3	F3:1633	OB5941 H1 raw.raw	2.1681E6	3	3	406	431	Carbamidomethylation	M19:Oxidation (M):17.01;C22:Carbamidomethylation:1000.00	PEAKS DB
K.HADADNILVIQQ(+.98)GQATVTVAN(+.98)GNNRK.S	N	52.71	2748.3792	26	3.1	1375.2010	2	30.08	3	F3:1495	OB5941 H1 raw.raw	1.5758E6	1	1	223	248		Q12:Deamidation (NQ):27.58;N21:Deamidation (NQ):15.73	PEAKS DB
K.NPQLQDLDMMLTCVEIK(+14.02)EGALM(+15.99)LPHFNSK.A	N	52.56	3344.6277	29	4.1	837.1677	4	37.13	2	F2:1903	OB5940 H1 raw.raw	1.5437E6	1	1	415	443	Oxidation (M)	K17:Methylation(KR):24.93;M22:Oxidation (M):51.99	PEAKS PTM
K.ISM(+15.99)PVNTPGQFEDFFPASSRDQ(+.98)SSYLQGFSR.N	N	52.28	3511.5989	31	4.3	1171.5453	3	34.92	1	F1:1731	OB5939 H1 raw.raw	1.6252E6	1	1	282	312	Oxidation (M)	M3:Oxidation (M):1000.00;Q22:Deamidation (NQ):20.93	PEAKS DB
R.NT(-18.01)LEAAFNAEFNEIRR.V	N	52.23	1875.9227	16	-2.4	626.3134	3	33.43	2	F2:1683	OB5940 H1 raw.raw	1.354E7	3	3	313	328	Dehydration	T2:Dehydration:1000.00	PEAKS PTM
L.EAAFNAEFNEIRR.V	N	52.09	1565.7585	13	-4.7	783.8829	2	34.06	1	F1:1677	OB5939 H1 raw.raw	1.7113E6	2	2	316	328			PEAKS DB
R.LFEVKPDK.K	N	51.87	974.5436	8	0.9	488.2795	2	25.40	1	F1:1153	OB5939 H1 raw.raw	7.0978E5	6	6	406	413			PEAKS DB
R.WGPAEPR.E	Y	51.81	811.3976	7	1.2	406.7066	2	25.31	3	F3:1167	OB5941 H1 raw.raw	1.2136E6	3	3	109	115			PEAKS DB
K.AM(-48.00)VIVVVNKGTGNLELVAVRK.E	N	51.58	2161.2947	21	1.4	1081.6561	2	29.80	3	F3:1453	OB5941 H1 raw.raw	6.785E6	4	4	444	464	Dethiomethyl	M2:Dethiomethyl:1000.00	PEAKS PTM
R.IFLAGDKDNVIDQIEK(+14.02)(+43.01).Q	N	51.42	1873.9785	16	1.0	937.9974	2	32.37	1	F1:1563	OB5939 H1 raw.raw	1.103E7	1	1	536	551	Methylation(C-term); Carbamylation	K16:Methylation(C-term):1000.00;K16:Carbamylation:164.73	PEAKS PTM

total 247 peptides

Peptide	Uniq	-10lgP	Mass	Length	ppm	m/z	z	RT	Fraction	Scan	Source File	Area raw peanut	#Feature	#Feature raw peanut	Start	End	PTM	AScore	Found By
K.HADADNILVIQQGQAT(-18.01)VTVAN(+.98)GNNRK.S	N	51.33	2729.3845	26	-1.3	910.8009	3	31.10	3	F3:1500	OB5941 H1 raw.raw	8.3833E5	2	2	223	248		T16:Dehydration:11.99;N21:Deamidation (NQ):13.67	PEAKS PTM
K.S(+43.01)(+14.02)FNLDEGHALR.I	N	51.03	1314.6316	11	0.8	658.3236	2	27.58	3	F3:1340	OB5941 H1 raw.raw	4.5043E6	4	4	249	259	Carbamylation; Methylation(others)	S1:Carbamylation:1000.00; S1:Methylation(others):109.97	PEAKS PTM
K.NPQLQDLDMM(+15.99)LTCVEIK(+14.02).E	N	51.01	2019.9679	17	-7.0	1010.9842	2	37.36	1	F1:1877	OB5939 H1 raw.raw	0	0	0	415	431	Methylation(KR)	M10:Oxidation (M):11.06;K17:Methylation (KR):1000.00	PEAKS PTM
R.RVLLEENAGGEQEERGQR.R	N	50.12	2069.0249	18	2.1	690.6837	3	24.88	2	F2:1165	OB5940 H1 raw.raw	2.8524E5	1	1	328	345			PEAKS DB
K.KNPQLQDLDMM(-48.00)LTC(+57.02)VEIK.E	N	49.96	2127.0703	18	1.7	710.0319	3	30.71	2	F2:1521	OB5940 H1 raw.raw	3.3467E6	2	2	414	431	Carbamidomethylation	M11:Dethiomethyl:14.02;C14:Carbamidomethylation:1000.00	PEAKS PTM
N.LDEGHALR.I	N	49.09	909.4668	8	0.6	455.7409	2	27.35	2	F2:1312	OB5940 H1 raw.raw	3.1446E4	1	1	252	259			PEAKS DB
K.KNPQLQDLDMMLTCVEIK(+14.02)EGALMLPHFNSK.A	N	49.06	3456.7278	30	2.7	865.1915	4	36.90	2	F2:1892	OB5940 H1 raw.raw	3.1386E5	1	1	414	443	Methylation(KR)	K18:Methylation(KR):50.90	PEAKS PTM
K.NPQLQDLDMM(+15.99)LTC(+57.02)VEIKEGALMLPHFN(+.98)SK.A	N	48.99	3388.6174	29	5.0	848.1659	4	36.17	3	F3:1849	OB5941 H1 raw.raw	7.0919E6	2	2	415	443	Carbamidomethylation; Deamidation (NQ)	M10:Oxidation (M):0.00;C13:Carbamidomethylation:1000.00;N27:Deamidation (NQ):51.54	PEAKS DB
K.HADADNILVIQQ(+.98)GQATVTVANGNN(+.98)RK.S	N	48.74	2748.3792	26	8.9	917.1418	3	48.01	1	F1:2471	OB5939 H1 raw.raw	2.2044E4	1	1	223	248		Q12:Deamidation (NQ):23.10;N24:Deamidation (NQ):0.00	PEAKS DB

total 247 peptides

Peptide	Uniq	-10lgP	Mass	Length	ppm	m/z	z	RT	Fraction	Scan	Source File	Area raw peanut	#Feature	#Feature raw peanut	Start	End	PTM	AScore	Found By
R.LFEVKPDKKNPQLQDLDM(+15.99)M(+15.99)LTC(+57.02)VEIK.E	N	48.67	3163.5967	26	2.1	791.9081	4	32.37	1	F1:1585	OB5939 H1 raw.raw	6.4006E5	1	1	406	431	Oxidation (M); Carbamidomethylation	M18:Oxidation (M):1000.00; M19:Oxidation (M):1000.00; C22:Carbamidomethylation:1000.00	PEAKS DB
K.NPQLQDLDM(+15.99)MLTC(+57.02)VEIKEGALM(+15.99)LPHFNSK.A	N	48.60	3403.6284	29	2.2	851.9163	4	36.24	2	F2:1851	OB5940 H1 raw.raw	3.928E6	2	2	415	443	Carbamidomethylation; Oxidation (M)	M9:Oxidation (M):10.11;C13:Carbamidomethylation:1000.00;M22:Oxidation (M):53.09	PEAKS DB
N.TPGQFEDFFPASSR.D	N	48.49	1584.7208	14	-0.2	793.3676	2	35.63	3	F3:1821	OB5941 H1 raw.raw	2.7376E5	1	1	288	301			PEAKS DB
K.ISMP(+15.99)VNTPGQFEDFFPASSR.D	N	48.39	2242.0364	20	6.5	1122.0327	2	45.36	1	F1:2277	OB5939 H1 raw.raw	8.1169E4	2	2	282	301		P4:Hydroxylation on Pro:33.81	PEAKS DB
R.NTLEAAFN(+.98)AEFNEIRR.V	N	48.19	1894.9172	16	-0.3	948.4656	2	32.92	1	F1:1570	OB5939 H1 raw.raw	1.5926E6	1	1	313	328		N8:Deamidation (NQ):8.26	PEAKS DB
K.NPQLQDLDMMLTC(+57.02)VEIKEGALMLPHFN(+.98)SK.A	N	47.92	3372.6226	29	7.4	844.1691	4	37.85	2	F2:1914	OB5940 H1 raw.raw	5.3901E7	2	2	415	443	Carbamidomethylation; Deamidation (NQ)	C13:Carbamidomethylation:1000.00; N27:Deamidation (NQ):61.84	PEAKS DB
K.ISMP(+15.99)VNTPGQ(+.98)FEDFFPASSR.D	N	47.82	2243.0205	20	6.2	1122.5245	2	50.73	1	F1:2643	OB5939 H1 raw.raw	0	0	0	282	301		P4:Hydroxylation on Pro:38.03; Q10:Deamidation (NQ):19.56	PEAKS DB
R.SKQFQNLQNHR.I	Y	47.59	1398.7117	11	1.0	467.2450	3	24.31	2	F2:1132	OB5940 H1 raw.raw	2.9256E4	3	3	197	207			PEAKS DB
R.K(+43.01)(+14.02)SFNLDEGHALR.I	N	47.09	1442.7266	12	-0.4	481.9159	3	25.70	3	F3:1209	OB5941 H1 raw.raw	2.261E5	3	3	248	259	Carbamylation; Methylation(KR)	K1:Carbamylation:1000.00; K1:Methylation (KR):160.69	PEAKS PTM

total 247 peptides

Peptide	Uniq	-10lgP	Mass	Length	ppm	m/z	z	RT	Fraction	Scan	Source File	Area raw peanut	#Feature	#Feature raw peanut	Start	End	PTM	AScore	Found By
K.ISM(+15.99)PVNTPGQFEDFFPASSRDQSSYLQGFSR.N	N	47.05	3510.6150	31	4.2	1171.2172	3	34.34	2	F2:1739	OB5940 H1 raw.raw	6.0177E6	2	2	282	312	Oxidation (M)	M3:Oxidation (M):1000.00	PEAKS DB
K.QFQNLQNHR.I	N	46.78	1183.5846	9	0.4	592.7998	2	23.96	2	F2:1106	OB5940 H1 raw.raw	7.7423E4	5	5	199	207			PEAKS DB
D.KDNVIDQIEK.Q	N	46.62	1200.6350	10	-0.8	601.3243	2	25.89	3	F3:1225	OB5941 H1 raw.raw	4.877E4	2	2	542	551			PEAKS DB
K.AMVIVVVN(+.98)KGTGNLELVAVRK.E	N	46.54	2210.2820	21	9.6	737.7750	3	33.10	3	F3:1695	OB5941 H1 raw.raw	8.2125E6	2	2	444	464		N8:Deamidation (NQ):41.87	PEAKS DB
Q.GQATVTVANGNNRK.S	N	46.45	1428.7433	14	-0.2	715.3788	2	29.02	3	F3:1412	OB5941 H1 raw.raw	1.3861E5	2	2	235	248			PEAKS DB
R.EQEWEEEEEDEEEEGSNREVRR.Y	N	46.42	2821.1545	22	1.8	941.3939	3	26.75	1	F1:1227	OB5939 H1 raw.raw	3.455E6	3	3	473	494			PEAKS DB
K.Q(-17.03)AKDLAFPGSGEQVEK.L	N	45.78	1685.8260	16	0.9	843.9211	2	29.97	1	F1:1427	OB5939 H1 raw.raw	0	0	0	552	567	Pyro-glu from Q	Q1:Pyro-glu from Q:1000.00	PEAKS PTM
K.HADAD(-18.01)NILVIQQGQATVTVANGNNRK.S	N	45.35	2728.4004	26	3.5	910.4772	3	28.70	2	F2:1390	OB5940 H1 raw.raw	0	0	0	223	248		D5:Dehydration:30.36	PEAKS PTM
K.KN(+.98)PQLQDLDMM(+15.99)LTC(+57.02)VEIK.E	N	44.87	2192.0527	18	9.2	1097.0437	2	32.33	2	F2:1621	OB5940 H1 raw.raw	8.5764E4	1	1	414	431	Carbamidomethylation	N2:Deamidation (NQ):14.04;M11:Oxidation (M):0.00;C14:Carbamidomethylation:1000.00	PEAKS DB
R.KSFNLDEGHALRIPSGFISYILN(+.98)R.H	N	44.70	2747.4395	24	4.5	687.8702	4	36.15	3	F3:1837	OB5941 H1 raw.raw	1.1013E6	1	1	248	271		N23:Deamidation (NQ):0.00	PEAKS DB
K.IRPEGREGEQEWGTPGSEVR.E	Y	44.50	2268.0881	20	1.1	757.0375	3	26.28	3	F3:1248	OB5941 H1 raw.raw	7.4174E4	2	2	141	160			PEAKS DB
W.GTPGSEVREETSR.N	Y	44.35	1403.6641	13	3.5	702.8417	2	27.60	3	F3:1327	OB5941 H1 raw.raw	7.5188E4	1	1	153	165			PEAKS DB
K.A(+41.03)M(+15.99)VIVVVNKGTGNLELVAVRK.E	N	44.32	2266.3196	21	-1.6	1134.1653	2	32.33	2	F2:1602	OB5940 H1 raw.raw	5.361E7	7	7	444	464	Amidination of lysines or N-terminal amines with methyl acetimidate; Oxidation (M)	A1:Amidination of lysines or N-terminal amines with methyl acetimidate:161.20;M2:Oxidation (M):1000.00	PEAKS PTM

total 247 peptides

Peptide	Uniq	-10lgP	Mass	Length	ppm	m/z	z	RT	Fraction	Scan	Source File	Area raw peanut	#Feature	#Feature raw peanut	Start	End	PTM	AScore	Found By
V.QIEARPNTLVLPK.H	Y	43.15	1477.8616	13	-3.7	739.9353	2	30.24	3	F3:1485	OB5941 H1 raw.raw	1.0391E6	3	3	210	222			PEAKS DB
R.LFEVKPDKK.N	N	43.08	1102.6385	9	1.1	552.3271	2	24.94	3	F3:1179	OB5941 H1 raw.raw	2.3087E5	2	2	406	414			PEAKS DB
N.TLEAAFNAEFNEIRR.V	N	43.00	1779.8904	15	-3.0	890.9498	2	32.91	3	F3:1655	OB5941 H1 raw.raw	4.3913E5	1	1	314	328			PEAKS DB
K.NPQLQDLDMMLTC(+57.02)VEIKEGALM(-48.00)LPHFNSK.A	N	42.94	3323.6353	29	-1.0	831.9153	4	35.81	3	F3:1829	OB5941 H1 raw.raw	2.5446E6	1	1	415	443	Carbamidomethylation	C13:Carbamidomethylation:1000.00; M22:Dethiomethyl:30.24	PEAKS PTM
K.ISM(+15.99)PVN(+.98)TPGQFEDFFPASSR.D	N	42.69	2243.0205	20	7.6	1122.5260	2	49.72	2	F2:2625	OB5940 H1 raw.raw	0	0	0	282	301	Oxidation (M)	M3:Oxidation (M):1000.00; N6:Deamidation (NQ): 25.70	PEAKS DB
K.KNPQLQ(+.98)DLDMMLTC(+57.02)VEIKEGALMLPHFNSK.A	N	42.58	3500.7175	30	0.4	1167.9136	3	36.34	2	F2:1850	OB5940 H1 raw.raw	5.0176E5	1	1	414	443	Carbamidomethylation	Q6:Deamidation (NQ): 14.04; C14:Carbamidomethylation:1000.00	PEAKS DB
R.REQEWEEEEEDEEEEGSN(+.98)R.E	N	42.55	2437.9265	19	7.6	813.6556	3	26.55	1	F1:1226	OB5939 H1 raw.raw	3.9265E4	1	1	472	490		N18:Deamidation (NQ):0.00	PEAKS DB
K.NPQLQDLDMMLTC(+57.02)VEIK(+43.01)EGALMLPHFNSK(+14.02).A	N	42.54	3428.6602	29	-4.2	1143.8892	3	37.82	3	F3:1939	OB5941 H1 raw.raw	1.0346E6	1	1	415	443	Carbamidomethylation	C13:Carbamidomethylation:1000.00; K17:Carbamylation:10.80;K29:Methylation (KR):10.80	PEAKS PTM
K.HADADNILVIQ(+.98)QGQ(+.98)ATVTVANGNNRK.S	N	42.52	2748.3792	26	3.1	917.1365	3	57.24	2	F2:3064	OB5940 H1 raw.raw	0	0	0	223	248		Q11:Deamidation (NQ):0.00;Q14:Deamidation (NQ):0.00	PEAKS DB

total 247 peptides

Peptide	Uniq	-10lgP	Mass	Length	ppm	m/z	z	RT	Fraction	Scan	Source File	Area raw peanut	#Feature	#Feature raw peanut	Start	End	PTM	AScore	Found By
R.IFLAGDKDNVIDQIEK(+43.01)QAK(+14.02).D	N	42.41	2201.1692	19	2.7	734.7323	3	32.55	1	F1:1581	OB5939 H1 raw.raw	3.5153E6	1	1	536	554		K16:Carbamylation:0.00;K19:Methylation(KR):0.00	PEAKS PTM
K.GTGNLELVAVRKEQQQR.G	N	41.95	1925.0442	17	1.8	642.6898	3	26.36	1	F1:1214	OB5939 H1 raw.raw	1.4197E5	3	3	453	469			PEAKS DB
R.IFLAGDKDNVIDQIEK(+14.02)Q(+.98)AK(+42.01).D	N	41.87	2201.1580	19	3.1	734.7289	3	31.83	2	F2:1588	OB5940 H1 raw.raw	3.595E6	2	2	536	554		K16:Methylation(KR):13.38;Q17:Deamidation (NQ):25.11;K19:Acetylation (K):13.38	PEAKS PTM
K.D(+43.01)(+14.02)LAFPGSGEQVEK.L	N	41.75	1432.6833	13	3.8	717.3517	2	30.92	1	F1:1485	OB5939 H1 raw.raw	2.8695E5	1	1	555	567	Carbamylation; Methylation(others)	D1:Carbamylation:166.34;D1:Methylation(others):102.87	PEAKS PTM
R.IFLAGDKDNVIDQ(+.98)IEK.Q	N	41.66	1817.9410	16	9.1	909.9861	2	66.49	2	F2:3489	OB5940 H1 raw.raw	4.5271E4	3	3	536	551		Q13:Deamidation (NQ):38.67	PEAKS DB
L.DEGHALR.I	N	40.78	796.3828	7	2.3	399.1996	2	27.48	2	F2:1315	OB5940 H1 raw.raw	2.4528E4	1	1	253	259			PEAKS DB
K.HADADNILVIQ(+.98)QGQATVTVANGN(+.98)NRK.S	N	40.70	2748.3792	26	8.8	917.1417	3	55.82	2	F2:2975	OB5940 H1 raw.raw	2.5591E3	1	1	223	248		Q11:Deamidation (NQ):0.00;N23:Deamidation (NQ):0.00	PEAKS DB
K.ISMPVN(+.98)TPGQFEDFFPASSR.D	N	40.63	2227.0254	20	9.3	1114.5303	2	58.23	3	F3:3170	OB5941 H1 raw.raw	2.6294E4	2	2	282	301		N6:Deamidation (NQ):42.89	PEAKS DB
R.IFLAGD(+14.02)K(+43.01)DNVIDQIEK.Q	N	40.36	1873.9785	16	-5.0	937.9919	2	32.19	3	F3:1583	OB5941 H1 raw.raw	4.0088E7	4	4	536	551	Carbamylation	D6:Methylation(others):24.32;K7:Carbamylation:57.71	PEAKS PTM

total 247 peptides

Peptide	Uniq	-10lgP	Mass	Length	ppm	m/z	z	RT	Fraction	Scan	Source File	Area raw peanut	#Feature	#Feature raw peanut	Start	End	PTM	AScore	Found By
K.KN(+.98)PQLQDLDMMLTC(+57.02)VEIKEGALMLPHFNSK.A	N	40.34	3500.7175	30	7.1	876.1929	4	36.26	2	F2:1847	OB5940 H1 raw.raw	2.213E6	1	1	414	443	Carbamidomethylation	N2:Deamidation (NQ): 0.00;C14:Carbamidomethylation:1000.00	PEAKS DB
K.NPQLQDLDM(+15.99)MLTC(+57.02)VEIKEGALMLP(+15.99)HFNSK.A	N	40.24	3403.6284	29	-10.2	851.9057	4	36.72	1	F1:1838	OB5939 H1 raw.raw	0	0	0	415	443	Carbamidomethylation; Hydroxylation Pro	M9:Oxidation (M):26.02;C13:Carbamidomethylation:1000.00; P24:Hydroxylation Pro:63.93	PEAKS DB
R.ESHFVSARPQSQSP(+15.99)SSPEKEDQEEEN.Q	N	40.09	2973.2859	26	-2.5	992.1001	3	25.16	1	F1:1143	OB5939 H1 raw.raw	0	0	0	574	599		P14:Hydroxylation Pro:29.32	PEAKS DB
K.SFN(+.98)LDEGHALRIPSGFISYILNR.H	N	39.87	2619.3445	23	9.3	655.8495	4	38.22	1	F1:1947	OB5939 H1 raw.raw	1.0439E6	2	2	249	271		N3:Deamidation (NQ): 39.81	PEAKS DB
T.PGQFEDFFPASSR.D	N	39.65	1483.6731	13	-1.3	742.8429	2	40.34	2	F2:2103	OB5940 H1 raw.raw	3.5787E3	1	1	289	301			PEAKS DB
K.EGALM(-48.00)LPHFNSK.A	N	38.72	1294.6670	12	2.0	432.5638	3	28.31	3	F3:1348	OB5941 H1 raw.raw	1.3523E6	1	1	432	443	Dethiomethyl	M5:Dethiomethyl:1000.00	PEAKS PTM
K.ISMPVNTPGQ(+.98)FEDFFPASSRDQSSYLQGFSR.N	N	38.62	3495.6040	31	6.7	1166.2164	3	54.16	3	F3:2883	OB5941 H1 raw.raw	0	0	0	282	312		Q10:Deamidation (NQ):5.38	PEAKS DB
R.DQS(-18.01)SYLQGFSR.N	N	38.22	1268.5785	11	7.0	635.3010	2	30.87	1	F1:1484	OB5939 H1 raw.raw	0	0	0	302	312		S3:Dehydration:33.98	PEAKS PTM
K.N(+.98)PQ(+.98)LQDLDMMLTC(+57.02)VEIKEGALM(+15.99)LPHFNSK.A	N	37.95	3389.6016	29	9.0	848.4153	4	36.92	1	F1:1851	OB5939 H1 raw.raw	8.517E6	2	2	415	443	Carbamidomethylation	N1:Deamidation (NQ): 11.12; Q3:Deamidation (NQ): 0.00;C13:Carbamidomethylation:1000.00; M22:Oxidation (M):24.93	PEAKS DB
K.SFN(+.98)LDEGHALRIPSGFISYILNRHDNQNLR.V	N	37.76	3496.7600	30	-3.9	700.3566	5	36.38	1	F1:1849	OB5939 H1 raw.raw	7.9247E5	1	1	249	278		N3:Deamidation (NQ): 20.27	PEAKS DB

total 247 peptides

Peptide	Uniq	-10lgP	Mass	Length	ppm	m/z	z	RT	Fraction	Scan	Source File	Area raw peanut	#Feature	#Feature raw peanut	Start	End	PTM	AScore	Found By
R.E(-18.01)GEQEWGTPGSEVREETSR.N	Y	37.06	2143.9407	19	2.9	715.6562	3	27.17	2	F2:1294	OB5940 H1 raw.raw	9.0561E4	1	1	147	165	Pyro-glu from E	E1:Pyro-glu from E:1000.00	PEAKS PTM
K.NPQLQDLDMM(-48.00)LTC(+57.02)VEIKEGALMLPHFNSK.A	N	36.81	3323.6353	29	0.8	1108.8866	3	35.97	3	F3:1833	OB5941 H1 raw.raw	5.0189E6	2	2	415	443	Carbamidomethylation	M10:Dethiomethyl:8.14;C13:Carbamidomethylation:1000.00	PEAKS PTM
R.LFEVKPDKKN(+.98)PQLQDLDMMLTC(+57.02)VEIK.E	N	36.71	3132.5908	26	9.3	1567.3173	2	34.35	3	F3:1745	OB5941 H1 raw.raw	2.2872E5	1	1	406	431	Carbamidomethylation	N10:Deamidation (NQ):14.04;C22:Carbamidomethylation:1000.00	PEAKS DB
R.IVQIEAR(+28.03)PN(+.98)TLVLPK.H	Y	36.70	1719.0294	15	0.1	860.5220	2	30.00	2	F2:1445	OB5940 H1 raw.raw	1.1278E7	2	2	208	222	Dimethylation(KR); Deamidation (NQ)	R7:Dimethylation(KR):71.07; N9:Deamidation (NQ):90.21	PEAKS PTM
R.ESHFVSARPQSQSP(+15.99)SSPEKEDQEEE.N	N	36.67	2859.2429	25	2.6	954.0907	3	24.51	2	F2:1151	OB5940 H1 raw.raw	1.0898E4	1	1	574	598		P14:Hydroxylation Pro:12.33	PEAKS DB
K.NPQLQDLDM(+15.99)M(+15.99)LTC(+57.02)VEIK.E	N	36.40	2078.9688	17	1.1	1040.4928	2	33.80	3	F3:1701	OB5941 H1 raw.raw	0	0	0	415	431	Oxidation (M); Carbamidomethylation	M9:Oxidation (M):1000.00; M10:Oxidation (M):1000.00; C13:Carbamidomethylation:1000.00	PEAKS DB
R.IVQIEARPNTLVLP(+15.99)K(-.98)(+42.01).H	Y	36.38	1747.0355	15	-2.4	874.5229	2	30.35	2	F2:1460	OB5940 H1 raw.raw	8.4907E6	1	1	208	222	Hydroxylation Pro; Amidation; Acetylation (K)	P14:Hydroxylation Pro:74.55;K15:Amidation:1000.00;K15:Acetylation (K):1000.00	PEAKS PTM
R.N(-17.03)NPFYFPSRR.F	N	36.19	1279.6097	10	-0.4	427.5437	3	28.25	2	F2:1369	OB5940 H1 raw.raw	1.5243E5	1	1	166	175		N1:Ammonia-loss (N):0.00	PEAKS PTM

total 247 peptides

Peptide	Uniq	-10lgP	Mass	Length	ppm	m/z	z	RT	Fraction	Scan	Source File	Area raw peanut	#Feature	#Feature raw peanut	Start	End	PTM	AScore	Found By
K.AM(+31.99)VIVVVNKGTGNLELVAVRK.E	N	36.16	2241.2878	21	1.1	748.1041	3	31.46	1	F1:1540	OB5939 H1 raw.raw	1.7223E5	1	1	444	464	Sulphone	M2:Sulphone:1000.00	PEAKS PTM
K.NPQ(+.98)LQDLDMMLTC(+57.02)VEIKEGALMLPHFNSK.A	N	36.11	3372.6226	29	9.4	1125.2253	3	40.75	1	F1:2064	OB5939 H1 raw.raw	5.8612E5	1	1	415	443	Carbamidomethylation	Q3:Deamidation (NQ):0.00;C13:Carbamidomethylation:1000.00	PEAKS DB
K.N(+.98)PQ(+.98)LQDLDMMLTC(+57.02)VEIKEGALMLPHFNSK.A	N	35.87	3373.6067	29	9.1	1687.8259	2	38.03	2	F2:1999	OB5940 H1 raw.raw	2.685E5	1	1	415	443	Carbamidomethylation	N1:Deamidation (NQ):3.13;Q3:Deamidation (NQ):5.47;C13:Carbamidomethylation:1000.00	PEAKS DB
T.PGSEVREETSR.N	Y	35.66	1245.5948	11	1.9	623.8059	2	27.69	1	F1:1287	OB5939 H1 raw.raw	8.4353E4	2	2	155	165			PEAKS DB
K.EGALMLPH(+14.02)FNSK.A	N	35.50	1356.6860	12	1.3	679.3512	2	30.49	3	F3:1499	OB5941 H1 raw.raw	2.5433E4	1	1	432	443	Methylation(others)	H8:Methylation(others):53.53	PEAKS PTM
I.PSGFISYILNRHDNQNLR.V	N	35.49	2143.0923	18	4.0	715.3742	3	33.53	1	F1:1645	OB5939 H1 raw.raw	0	0	0	261	278			PEAKS DB
R.VLLE(+21.98)ENAGGEQEER.G	N	35.40	1593.7246	14	-0.7	797.8690	2	26.03	2	F2:1239	OB5940 H1 raw.raw	1.529E5	3	3	329	342		E4:Sodium adduct:40.00	PEAKS PTM
K.KGSEEEDIT(-18.01)N(+.98)PINLR.D	N	35.32	1696.8268	15	0.0	566.6162	3	29.14	1	F1:1369	OB5939 H1 raw.raw	6.926E4	1	1	379	393		T9:Dehydration:11.50;N10:Deamidation (NQ):25.67	PEAKS PTM
K.NP(+15.99)QLQDLDMM(+15.99)LTC(+57.02)VEIKEGALMLPHFNSK.A	N	35.20	3403.6284	29	-3.4	851.9115	4	35.28	1	F1:1792	OB5939 H1 raw.raw	1.7685E6	1	1	415	443	Carbamidomethylation	P2:Hydroxylation on Pro:48.46;M10:Oxidation (M):11.06;C13:Carbamidomethylation:1000.00	PEAKS DB
K.SFNLDEGH(+15.99)ALRIPSGFISYILNR.H	N	35.13	2634.3555	23	-0.4	879.1254	3	38.33	2	F2:1974	OB5940 H1 raw.raw	0	0	0	249	271	Oxidation (HW)	H8:Oxidation (HW):1000.00	PEAKS PTM

total 247 peptides

Peptide	Uniq	-10lgP	Mass	Length	ppm	m/z	z	RT	Fraction	Scan	Source File	Area raw peanut	#Feature	#Feature raw peanut	Start	End	PTM	AScore	Found By
K.KGSEEEDITN(-17.03)PINLR.D	N	34.98	1696.8268	15	3.3	566.6180	3	28.85	3	F3:1397	OB5941 H1 raw.raw	3.0405E5	1	1	379	393		N10:Ammonia-loss (N):40.63	PEAKS PTM
K.HADADNILVIQQ(-18.01).G	N	34.95	1317.6677	12	-4.9	659.8379	2	29.56	3	F3:1421	OB5941 H1 raw.raw	1.0066E5	1	1	223	234	Dehydration	Q12:Dehydration:76.18	PEAKS PTM
K.GT(-18.01)GNLELVAVRK.E	N	34.88	1237.7142	12	0.9	413.5790	3	26.41	2	F2:1259	OB5940 H1 raw.raw	7.6712E5	3	3	453	464	Dehydration	T2:Dehydration:1000.00	PEAKS PTM
K.NPQLQDLDM(-48.00)MLTC(+57.02)VEIKEGALMLPHFNSK.A	N	34.70	3323.6353	29	8.1	831.9229	4	35.72	2	F2:1816	OB5940 H1 raw.raw	1.6745E6	2	2	415	443	Carbamidomethylation	M9:Dethiomethyl:17.01;C13:Carbamidomethylation:1000.00	PEAKS PTM
R.IFLAGDKDNVIDQ(+15.00)IEK(+42.01).Q	N	34.39	1873.9673	16	-4.1	937.9871	2	31.58	2	F2:1569	OB5940 H1 raw.raw	0	0	0	536	551	Deamidation followed by a methylation; Acetylation (K)	Q13:Deamidation followed by a methylation:72.09;K16:Acetylation (K):97.49	PEAKS PTM
K.HADADN(+.98)ILVIQQGQATVTVAN(+.98)GNNRK.S	N	34.16	2748.3792	26	8.9	917.1418	3	48.01	1	F1:2528	OB5939 H1 raw.raw	2.2044E4	1	1	223	248		N6:Deamidation (NQ):10.34;N21:Deamidation (NQ):14.04	PEAKS DB
N.PQLQDLDMMLTC(+57.02)VEIKEGALMLPHFNSK.A	N	34.02	3257.5957	28	0.9	815.4069	4	37.83	3	F3:1952	OB5941 H1 raw.raw	2.5417E5	1	1	416	443	Carbamidomethylation	C12:Carbamidomethylation:1000.00	PEAKS DB
R.PNTLVLPK.H	N	33.50	880.5382	8	0.4	441.2766	2	27.53	2	F2:1313	OB5940 H1 raw.raw	8.6509E4	1	1	215	222			PEAKS DB
R.IFLAGD(-18.01)KDNVIDQIEK.Q	N	33.20	1798.9464	16	-2.5	600.6546	3	31.45	2	F2:1554	OB5940 H1 raw.raw	1.217E6	1	1	536	551		D6:Dehydration:31.02	PEAKS PTM
K.G(+43.01)T(+14.02)GNLELVAVRK.E	N	33.06	1312.7462	12	-0.8	657.3799	2	26.85	3	F3:1280	OB5941 H1 raw.raw	3.165E5	2	2	453	464	Carbamylation; Methylation(others)	G1:Carbamylation:148.28;T2:Methylation (others):59.30	PEAKS PTM
R.IPSGFISYILN(+.98)RHDNQNLR.V	N	33.06	2257.1604	19	9.2	565.3026	4	35.09	3	F3:1797	OB5941 H1 raw.raw	1.752E6	1	1	260	278		N11:Deamidation (NQ):16.76	PEAKS DB

total 247 peptides

Peptide	Uniq	-10lgP	Mass	Length	ppm	m/z	z	RT	Fraction	Scan	Source File	Area raw peanut	#Feature	#Feature raw peanut	Start	End	PTM	AScore	Found By
R.REQEWEEEEEDEEEEGSNREVR.R	N	33.06	2821.1545	22	3.9	941.3958	3	26.47	3	F3:1247	OB5941 H1 raw.raw	1.8551E6	1	1	472	493			PEAKS DB
R.IPSGFISYILN(+.98)R(+14.02)H(+42.01)DNQNLR.V	N	32.77	2313.1865	19	6.8	579.3079	4	33.29	1	F1:1633	OB5939 H1 raw.raw	2.3452E7	3	3	260	278		N11:Deamidation (NQ):35.13;R12:Methylation(KR):45.25;H13:Acetylation (TSCYH):27.86	PEAKS PTM
Q.IEARPNTLVLPK.H	Y	32.24	1349.8030	12	2.2	675.9103	2	30.40	1	F1:1461	OB5939 H1 raw.raw	7.0908E5	2	2	211	222			PEAKS DB
K.HADADNILVIQQGQ(+.98)ATVTVANGNN(+.98)RK.S	N	32.00	2748.3792	26	7.7	917.1407	3	55.56	2	F2:2966	OB5940 H1 raw.raw	0	0	0	223	248		Q14:Deamidation (NQ):19.05;N24:Deamidation (NQ):0.00	PEAKS DB
R.EGEQEW(+15.99)GTPGSEVREETSR.N	Y	31.83	2177.9460	19	2.6	1089.9832	2	26.55	1	F1:1197	OB5939 H1 raw.raw	2.78E4	1	1	147	165	Oxidation (HW)	W6:Oxidation (HW):1000.00	PEAKS PTM
K.AMVIVVVNK(-1.03)GTGNLELVAVR.K	N	31.46	2080.1714	20	4.3	1041.0974	2	33.79	2	F2:1738	OB5940 H1 raw.raw	2.5754E7	1	1	444	463	Lysine oxidation to aminoadipic semialdehyde	K9:Lysine oxidation to aminoadipic semialdehyde:1000.00	PEAKS PTM
K.HADADNILVIQQGQATVTVANGN(+.98)N(+.98)RK.S	N	31.37	2748.3792	26	5.7	917.1389	3	45.07	1	F1:2318	OB5939 H1 raw.raw	5.4428E3	1	1	223	248		N23:Deamidation (NQ):0.00;N24:Deamidation (NQ):0.00	PEAKS DB
R.IFLAGDKDNVID.Q	N	31.18	1318.6769	12	1.8	660.3469	2	31.65	1	F1:1537	OB5939 H1 raw.raw	9.3082E4	1	1	536	547			PEAKS DB
R.EREEDWRQPR.E	N	31.07	1399.6592	10	0.1	467.5604	3	24.77	3	F3:1154	OB5941 H1 raw.raw	2.8345E4	1	1	118	127			PEAKS DB
K.S(-18.01)FNLDEGHALRIPSGFISYILNR.H	N	30.52	2600.3499	23	-0.3	651.0945	4	37.85	1	F1:1904	OB5939 H1 raw.raw	4.9973E5	1	1	249	271		S1:Dehydration:11.12	PEAKS PTM
R.EGEQEWGT(-18.01)PGSEVREETSR.N	Y	30.04	2143.9407	19	2.2	1072.9800	2	27.69	1	F1:1293	OB5939 H1 raw.raw	4.5095E4	1	1	147	165		T8:Dehydration:20.70	PEAKS PTM
K.SFNLDEGHALRIPS.G	N	29.93	1554.7791	14	-4.8	778.3931	2	30.79	2	F2:1515	OB5940 H1 raw.raw	5.213E3	1	1	249	262			PEAKS DB

total 247 peptides

Peptide	Uniq	-10lgP	Mass	Length	ppm	m/z	z	RT	Fraction	Scan	Source File	Area raw peanut	#Feature	#Feature raw peanut	Start	End	PTM	AScore	Found By
K.HADADNILVIQQGQATVT(-18.01)VANGNNR.K	N	29.88	2600.3054	25	-2.7	867.7734	3	30.76	3	F3:1503	OB5941 H1 raw.raw	3.2843E5	1	1	223	247		T18:Dehydration:22.85	PEAKS PTM
K.NPQLQDLDM(-48.00)MLTC(+57.02)VEIK.E	N	29.75	1998.9755	17	-0.6	667.3320	3	34.07	2	F2:1713	OB5940 H1 raw.raw	8.006E5	1	1	415	431	Carbamidomethylation	M9:Dethiomethyl:0.00;C13:Carbamidomethylation:1000.00	PEAKS PTM
R.EDWRRPSHQQPR.K	N	29.71	1590.7764	12	-0.3	398.7013	4	24.94	3	F3:1169	OB5941 H1 raw.raw	1.2481E4	1	1	128	139			PEAKS DB
R.SSDNEGVIVK(+43.01)VSK(+14.02)EHVQELTK.H	N	29.63	2382.2390	21	0.9	795.0876	3	28.31	3	F3:1368	OB5941 H1 raw.raw	5.0291E4	1	1	351	371		K10:Carbamylation:5.08;K13:Methylation (KR):5.08	PEAKS PTM
N.NPFYFPSRR.F	N	29.59	1182.5934	9	1.6	395.2057	3	28.08	2	F2:1358	OB5940 H1 raw.raw	2.5271E4	1	1	167	175			PEAKS DB
R.IVQIEARPNTLVLP(+15.99)K.H	Y	29.48	1706.0090	15	1.6	854.0131	2	29.82	1	F1:1418	OB5939 H1 raw.raw	0	0	0	208	222		P14:Hydroxylation Pro:43.89	PEAKS DB
K.N(+.98)PQ(+.98)LQ(+.98)DLDMM(+15.99)LTC(+57.02)VEIKEGALMLPHFNSK.A	N	29.44	3390.5854	29	3.7	848.6568	4	38.47	3	F3:1982	OB5941 H1 raw.raw	0	0	0	415	443	Deamidation (NQ); Carbamidomethylation	N1:Deamidation (NQ):62.42;Q3:Deamidation (NQ):65.92;Q5:Deamidation (NQ):60.92;M10:Oxidation (M):12.28;C13:Carbamidomethylation:1000.00	PEAKS DB
K.HADADNILVIQQGQATVTVANGNNR(+14.02)K(+43.99).S	N	29.12	2804.4165	26	-3.2	1403.2111	2	29.38	3	F3:1435	OB5941 H1 raw.raw	2.4694E5	1	1	223	248	Methylation(KR)	R25:Methylation(KR):1000.00;K26:Carboxylation (DKW):41.02	PEAKS PTM
R.EQEW(+15.99)EEEEEDEEEEGSNR.E	N	29.12	2296.8362	18	4.4	1149.4304	2	27.05	1	F1:1252	OB5939 H1 raw.raw	0	0	0	473	490	Oxidation (HW)	W4:Oxidation (HW):1000.00	PEAKS PTM

total 247 peptides

Peptide	Uniq	-10lgP	Mass	Length	ppm	m/z	z	RT	Fraction	Scan	Source File	Area raw peanut	#Feature	#Feature raw peanut	Start	End	PTM	AScore	Found By
K.HADADNILVIQQGQAT(-18.01)VTVANGNNR.K	N	29.07	2600.3054	25	-1.0	867.7748	3	30.11	2	F2:1479	OB5940 H1 raw.raw	0	0	0	223	247		T16:Dehydration:0.00	PEAKS PTM
R.E(-18.01)QEWEEEEEDEEEEGSNREVRR.Y	N	28.92	2803.1440	22	2.8	935.3912	3	28.31	3	F3:1363	OB5941 H1 raw.raw	2.9497E4	1	1	473	494	Pyro-glu from E	E1:Pyro-glu from E:1000.00	PEAKS PTM
K.KN(+.98)P(+15.99)Q(+.98)LQ(+.98)DLDMMLTC(+57.02)VEIKEGALMLPHFNSK.A	N	28.66	3518.6804	30	-5.1	880.6729	4	37.59	1	F1:1890	OB5939 H1 raw.raw	0	0	0	414	443	Deamidation (NQ); Carbamidomethylation	N2:Deamidation (NQ):55.07;P3:Hydroxylation Pro:43.60;Q4:Deamidation (NQ):57.68;Q6:Deamidation (NQ):58.78;C14:Carbamidomethylation:1000.00	PEAKS DB
K.H(+42.01)(+15.99)ADADNILVIQQGQATVTVAN(+.98)GNNRK.S	N	28.60	2805.4006	26	9.3	1403.7207	2	30.57	1	F1:1450	OB5939 H1 raw.raw	9.6068E4	1	1	223	248	Acetylation (N-term); Oxidation (HW)	H1:Acetylation (N-term):1000.00;H1:Oxidation (HW):1000.00;N21:Deamidation (NQ):14.04	PEAKS PTM
R.NTLEAAFNAEFNEIRRVLLEENAGGEQEER.G	N	28.52	3447.6653	30	-1.2	1150.2277	3	38.03	2	F2:1966	OB5940 H1 raw.raw	1.3831E5	1	1	313	342			PEAKS DB
R.ESHFVSARPQSQSP(+15.99)SSP(+15.99)EKEDQEEEN.Q	N	28.43	2989.2808	26	0.3	997.4344	3	24.81	3	F3:1156	OB5941 H1 raw.raw	0	0	0	574	599		P14:Hydroxylation Pro:17.91;P17:Hydroxylation Pro:28.14	PEAKS DB
K.NPQLQDLDMM(+15.99)LTC(+58.01)VEIK.E	N	28.36	2063.9578	17	-3.4	1032.9827	2	37.19	1	F1:1881	OB5939 H1 raw.raw	2.5393E4	1	1	415	431	Carboxymethyl	M10:Oxidation (M):8.69;C13:Carboxymethyl:1000.00	PEAKS PTM
K.DLAFPGSGEQVEK(+14.02)LIK(+43.01).N	N	28.34	1786.9464	16	-3.8	894.4771	2	32.91	3	F3:1654	OB5941 H1 raw.raw	6.9695E5	1	1	555	570		K13:Methylation(KR):4.52;K16:Carbamylation:4.52	PEAKS PTM

total 247 peptides

Peptide	Uniq	-10lgP	Mass	Length	ppm	m/z	z	RT	Fraction	Scan	Source File	Area raw peanut	#Feature	#Feature raw peanut	Start	End	PTM	AScore	Found By
K.KN(+.98)PQ(+.98)LQDLDMMLTC(+57.02)VEIKEGALMLPHFNSK.A	N	28.34	3501.7017	30	6.9	1168.2493	3	36.92	1	F1:1845	OB5939 H1 raw.raw	2.5153E5	1	1	414	443	Carbamidomethylation	N2:Deamidation (NQ):0.00;Q4:Deamidation (NQ):0.00;C14:Carbamidomethylation:1000.00	PEAKS DB
K.N(+.98)PQLQDLDM(+15.99)MLTC(+57.02)VEIKEGALMLPHFNSK.A	N	27.69	3388.6174	29	8.6	1130.5562	3	39.33	3	F3:2085	OB5941 H1 raw.raw	8.4125E5	1	1	415	443	Carbamidomethylation	N1:Deamidation (NQ):0.00;M9:Oxidation (M):0.00;C13:Carbamidomethylation:1000.00	PEAKS DB
R.SKQ(+.98)FQN(+.98)LQ(+.98)NHR.I	Y	27.06	1401.6637	11	2.0	468.2294	3	24.91	1	F1:1129	OB5939 H1 raw.raw	2.0347E3	1	1	197	207		Q3:Deamidation (NQ):14.04;N6:Deamidation (NQ):11.06;Q8:Deamidation (NQ):16.66	PEAKS DB
K.SFNLD(+53.92)EGHALR.I	N	26.85	1311.5294	11	-0.8	438.1834	3	27.41	2	F2:1311	OB5940 H1 raw.raw	0	0	0	249	259		D5:Replacement of 2 protons by iron:20.92	PEAKS PTM
**total 247 peptides**																			

tr|E5G076|E5G076_ARAHY

back to list

| Protein Coverage | Supporting Peptides |

Protein Coverage:

1 MRGRVSPLML LLGILVLASV SATHAKSSPY QKKTENPCAQ RCLQSCQQEP DDLKQKACES RCTKLEYDPR CVYDPRGHTG

81 TTNQRSPPGE RTRGRQPGDY DDDRRQPRRE EGGR**WGPAGP R**ER**EREEDWR QPREDWRRPS HQQPR**K**IRPE GREGEQEWGT**

221

161 **PGSHVREETS RNNPFYFPSR R**FSTRYGNQN GRIRVLQRFD QR**SRQFQNLQ NHRIVQIEAK PNTLVLPKHA DADNILVIQQ**

- a Acetylation (N-term) (+42.01)
- c Carbamidomethylation (+57.02)
- c Carbamylation (+43.01)
- c Carboxymethyl (+58.01)
- c Carboxymethyl (KW, X@N-term) (+58.01)
- d Deamidation (NQ) (+0.98)
- d Dehydration (-18.01)
- d Deamidation followed by a methylation (+15.00)
- d Dethiomethyl (-48.00)
- h Hydroxylation Pro (+15.99)
- m Methylation(KR) (+14.02)
- m Methylation(others) (+14.02)
- o Oxidation (M) (+15.99)
- o Oxidation (HW) (+15.99)
- p Pyro-glu from E (-18.01)
- p Pyro-glu from Q (-17.03)
- * Amidation (-0.98), Acetylation (K) (+42.01)
- * Carbamylation (+43.01), Methylation(KR) (+14.02)

Carbamylation (+43.01), Methylation(others) (+14.02)

241 GQATVTVANG NNRRALILTR AMHSESHPFH FLHLDDMTPE LRVAKSHAVN TPGQFEDFFP ASSRDQSSYL QGFSRNTLEA

321 AFNAEFNEIR RVLLEENAGG EQEERGQRRW STRSSENNEG VIVEVSKEHV EELTKHAKSV SKKGSEEEGD ITNPINLREG

401 EPDLSDNFGR LFEVKPDKKN PQLQDLDMML TCVEIKEGAL MLPHFNSKAM VIVVINKGTG NLELVAVRKE QQQRGRREQE

481 WEEEEEDEEE EGSNREVRRY TARLKEGDVF IMPAAHPVAI NASSELHLLG FGINAENNHR IFLAGDKDNV VDQIEKQAKD

561 LAFPGSGEQV EKLIKNQRES HFVSARPQSQ SPSSPEKEDQ EEENQGGKGP LLSILKAFN

Supporting Peptides:

Peptide	Uniq	-10lgP	Mass	Length	ppm	m/z	z	RT	Fraction	Scan	Source File	Area raw peanut	#Feature	#Feature raw peanut	Start	End	PTM	AScore	Found By

total 145 peptides

Peptide	Uniq	-10lgP	Mass	Length	ppm	m/z	z	RT	Fraction	Scan	Source File	Area raw peanut	#Feature	#Feature raw peanut	Start	End	PTM	AScore	Found By
K.NPQLQDLDMMLTC(+57.02)VEIK.E	N	120.22	2046.9788	17	-6.3	1024.4902	2	37.10	1	F1:1864	OB5939 H1 raw.raw	1.3638E7	5	5	420	436	Carbamidomethylation	C13:Carbamidomethylation:1000.00	PEAKS DB
K.KNPQLQDLDMMLTC(+57.02)VEIK.E	N	118.64	2175.0737	18	-1.1	1088.5430	2	34.52	2	F2:1751	OB5940 H1 raw.raw	4.131E6	3	3	419	436	Carbamidomethylation	C14:Carbamidomethylation:1000.00	PEAKS DB
R.EQEWEEEEEDEEEEGSNR.E	N	117.85	2280.8413	18	4.3	1141.4329	2	28.23	1	F1:1323	OB5939 H1 raw.raw	4.2916E6	3	3	478	495			PEAKS DB
R.NTLEAAFNAEFNEIR.R	N	114.83	1737.8322	15	-0.4	869.9230	2	34.89	2	F2:1770	OB5940 H1 raw.raw	1.3513E7	6	6	316	330			PEAKS DB
K.HADADNILVIQQGQATVTVANGNNR.R	N	111.11	2618.3162	25	5.0	1310.1719	2	30.00	2	F2:1473	OB5940 H1 raw.raw	6.2545E6	3	3	229	253			PEAKS DB
K.NPQLQDLDM(+15.99)MLTC(+57.02)VEIK.E	N	110.46	2062.9736	17	-5.3	1032.4886	2	36.07	2	F2:1840	OB5940 H1 raw.raw	3.4606E5	2	2	420	436	Carbamidomethylation	M9:Oxidation (M):30.46;C13:Carbamidomethylation:1000.00	PEAKS DB
K.KGSEEEGDITNPINLR.E	N	104.81	1770.8748	16	3.1	886.4474	2	28.08	2	F2:1364	OB5940 H1 raw.raw	2.2478E7	7	7	383	398			PEAKS DB
R.VLLEENAGGEQEER.G	N	97.22	1571.7427	14	2.5	786.8806	2	26.75	1	F1:1224	OB5939 H1 raw.raw	1.2414E7	3	3	332	345			PEAKS DB
R.DQSSYLQGFSR.N	N	97.09	1286.5891	11	-3.0	644.2999	2	30.18	2	F2:1484	OB5940 H1 raw.raw	6.1544E7	13	13	305	315			PEAKS DB
R.RVLLEENAGGEQEER.G	N	93.51	1727.8438	15	0.4	576.9554	3	24.77	2	F2:1156	OB5940 H1 raw.raw	1.346E5	5	5	331	345			PEAKS DB
K.NPQLQDLDMMLTC(+57.02)VEIKEGALMLPHFNSK.A	N	92.80	3371.6387	29	-2.9	1686.8218	2	37.92	1	F1:1910	OB5939 H1 raw.raw	1.8404E8	11	11	420	448	Carbamidomethylation	C13:Carbamidomethylation:1000.00	PEAKS DB
K.GSEEEGDITNPINLR.E	N	92.57	1642.7798	15	-1.0	822.3964	2	30.18	2	F2:1508	OB5940 H1 raw.raw	9.2673E6	3	3	384	398			PEAKS DB
K.NPQLQDLDMMLTC(+14.02)VEIK.E	N	88.98	2003.9730	17	-4.9	1002.9888	2	37.72	2	F2:1938	OB5940 H1 raw.raw	6.7012E5	2	2	420	436		C13:Methylation(others):33.98	PEAKS PTM
R.EGEQEWGTPGSHVR.E	N	87.00	1567.7015	14	0.5	784.8585	2	25.51	3	F3:1193	OB5941 H1 raw.raw	5.178E5	6	6	153	166			PEAKS DB
R.NTLEAAFNAEFNEIRR.V	N	86.44	1893.9332	16	-3.6	947.9705	2	35.28	1	F1:1741	OB5939 H1 raw.raw	3.2153E8	22	22	316	331			PEAKS DB
K.DLAFPGSGEQVEK.L	N	85.12	1375.6619	13	1.9	688.8395	2	29.66	2	F2:1451	OB5940 H1 raw.raw	6.5078E7	3	3	560	572			PEAKS DB

total 145 peptides

Peptide	Uniq	-10lgP	Mass	Length	ppm	m/z	z	RT	Fraction	Scan	Source File	Area raw peanut	#Feature	#Feature raw peanut	Start	End	PTM	AScore	Found By
K.NPQLQDLDMM(-48.00)LTC(+57.02)VEIK.E	N	85.01	1998.9755	17	0.5	1000.4955	2	32.73	1	F1:1599	OB5939 H1 raw.raw	1.7365E7	6	6	420	436	Carbamidomethylation	M10:Dethiomethyl:27.96;C13:Carbamidomethylation:1000.00	PEAKS PTM
R.VLLEENAGGEQEERGQR.R	N	84.92	1912.9238	17	1.8	957.4709	2	25.31	3	F3:1182	OB5941 H1 raw.raw	1.9929E6	6	6	332	348			PEAKS DB
K.KNPQLQDLDMM(+15.99)LTC(+57.02)VEIKEGALMLPHFNSK.A	N	84.57	3515.7285	30	-2.7	879.9370	4	34.52	2	F2:1755	OB5940 H1 raw.raw	2.6704E6	3	3	419	448	Carbamidomethylation	M11:Oxidation (M):30.46;C14:Carbamidomethylation:1000.00	PEAKS DB
K.SVSKKGSEEEGDITNPINLR.E	N	83.99	2172.1023	20	0.7	725.0419	3	27.21	3	F3:1286	OB5941 H1 raw.raw	1.3763E5	2	2	379	398			PEAKS DB
K.HADADNILVIQQGQATVTVAN(+.98)GNNR.R	N	82.15	2619.3000	25	-1.2	1310.6558	2	30.35	2	F2:1535	OB5940 H1 raw.raw	5.2956E6	1	1	229	253		N21:Deamidation (NQ):7.32	PEAKS DB
K.KNPQLQDLDMM(+15.99)LTC(+57.02)VEIK.E	N	81.97	2191.0686	18	0.5	1096.5421	2	32.43	2	F2:1621	OB5940 H1 raw.raw	1.9806E5	1	1	419	436	Carbamidomethylation	M11:Oxidation (M):9.34;C14:Carbamidomethylation:1000.00	PEAKS DB
K.EGALMLPHFNSK.A	N	81.44	1342.6703	12	4.0	672.3451	2	30.40	1	F1:1456	OB5939 H1 raw.raw	9.7706E6	3	3	437	448			PEAKS DB
K.QAKDLAFPGSGEQVEK.L	N	81.31	1702.8525	16	1.8	852.4351	2	26.85	3	F3:1278	OB5941 H1 raw.raw	1.3516E6	7	7	557	572			PEAKS DB
K.KNPQLQDLDM(+15.99)MLTC(+57.02)VEIK.E	N	77.76	2191.0686	18	0.6	1096.5422	2	33.03	1	F1:1615	OB5939 H1 raw.raw	0	0	0	419	436	Carbamidomethylation	M10:Oxidation (M):14.02;C14:Carbamidomethylation:1000.00	PEAKS DB
R.GRREQEWEEEEEDEEEEGSNR.E	N	77.42	2650.0649	21	0.3	884.3625	3	25.64	2	F2:1205	OB5940 H1 raw.raw	2.3251E6	7	7	475	495			PEAKS DB
R.IVQIEAKPNTLVLPK.H	N	76.21	1662.0079	15	5.0	832.0154	2	29.90	2	F2:1463	OB5940 H1 raw.raw	3.0833E8	29	29	214	228			PEAKS DB

total 145 peptides

Peptide	Uniq	-10lgP	Mass	Length	ppm	m/z	z	RT	Fraction	Scan	Source File	Area raw peanut	#Feature	#Feature raw peanut	Start	End	PTM	AScore	Found By
K.NPQLQDLDM(+15.99)MLTC(+57.02)VEIKEGALMLPHFNSK.A	N	75.51	3387.6335	29	-1.5	1130.2168	3	36.17	3	F3:1926	OB5941 H1 raw.raw	2.5418E7	3	3	420	448	Carbamidomethylation	M9:Oxidation (M):10.11;C13:Carbamidomethylation:1000.00	PEAKS DB
K.EGALM(+15.99)LPHFNSK.A	N	74.93	1358.6653	12	2.1	680.3414	2	29.74	3	F3:1443	OB5941 H1 raw.raw	2.0704E6	3	3	437	448	Oxidation (M)	M5:Oxidation (M):1000.00	PEAKS DB
R.LFEVKPDKKNPQLQDLDMMLTC(+57.02)VEIK.E	N	74.78	3131.6069	26	3.7	1044.8801	3	33.97	2	F2:1716	OB5940 H1 raw.raw	3.7773E6	3	3	411	436	Carbamidomethylation	C22:Carbamidomethylation:1000.00	PEAKS DB
K.KNPQLQDLDMMLTC(+57.02)VEIKEGALM(+15.99)LPHFNSK.A	N	74.26	3515.7285	30	-0.8	879.9387	4	35.84	1	F1:1790	OB5939 H1 raw.raw	5.1448E6	2	2	419	448	Carbamidomethylation; Oxidation (M)	C14:Carbamidomethylation:1000.00; M23:Oxidation (M):69.38	PEAKS DB
K.GTGNLELVAVRK.E	N	73.67	1255.7249	12	0.9	628.8702	2	26.85	3	F3:1263	OB5941 H1 raw.raw	1.0553E7	7	7	458	469			PEAKS DB
K.NPQLQDLDMM(+15.99)LTC(+57.02)VEIKEGALMLPHFNSK.A	N	72.71	3387.6335	29	3.8	1130.2228	3	37.84	2	F2:1902	OB5940 H1 raw.raw	8.789E6	3	3	420	448	Carbamidomethylation	M10:Oxidation (M):14.02;C13:Carbamidomethylation:1000.00	PEAKS DB
K.GTGNLELVAVR.K	N	71.83	1127.6299	11	0.2	564.8223	2	29.32	2	F2:1429	OB5940 H1 raw.raw	1.0095E7	3	3	458	468			PEAKS DB
R.NNPFYFPSR.R	N	71.25	1140.5352	9	0.9	571.2754	2	30.89	2	F2:1528	OB5940 H1 raw.raw	1.2382E7	4	4	172	180			PEAKS DB
R.VLLEENAGGEQEERGQRR.W	N	71.19	2069.0249	18	2.1	690.6837	3	25.31	3	F3:1186	OB5941 H1 raw.raw	1.1654E6	2	2	332	349			PEAKS DB
R.EETSRNNPFYFPSRR.F	N	69.88	1898.9023	15	1.2	633.9755	3	27.17	2	F2:1289	OB5940 H1 raw.raw	7.2799E6	5	5	167	181			PEAKS DB
K.NPQLQDLDMMLTC(+57.02)VEIKEGALM(+15.99)LPHFNSK.A	N	69.58	3387.6335	29	-9.3	1130.2080	3	36.62	2	F2:1874	OB5940 H1 raw.raw	2.6984E7	4	4	420	448	Carbamidomethylation; Oxidation (M)	C13:Carbamidomethylation:1000.00; M22:Oxidation (M):69.89	PEAKS DB
R.REQEWEEEEEDEEEEGSNR.E	N	68.88	2436.9424	19	2.8	1219.4819	2	26.47	3	F3:1258	OB5941 H1 raw.raw	3.0427E4	2	2	477	495			PEAKS DB

total 145 peptides

Peptide	Uniq	-10lgP	Mass	Length	ppm	m/z	z	RT	Fraction	Scan	Source File	Area raw peanut	#Feature	#Feature raw peanut	Start	End	PTM	AScore	Found By
R.NTLEAAFNAEFN(+.98)EIRR.V	N	68.73	1894.9172	16	-3.7	948.4624	2	32.73	3	F3:1640	OB5941 H1 raw.raw	4.5098E6	3	3	316	331	Deamidation (NQ)	N12:Deamidation (NQ):55.21	PEAKS DB
R.N(+.98)TLEAAFNAEFNEIRR.V	N	67.69	1894.9172	16	2.2	948.4680	2	35.88	1	F1:1787	OB5939 H1 raw.raw	6.2604E7	1	1	316	331	Deamidation (NQ)	N1:Deamidation (NQ): 61.82	PEAKS DB
R.NNPFYFPSRR.F	N	67.53	1296.6364	10	1.5	649.3264	2	28.95	1	F1:1356	OB5939 H1 raw.raw	1.2601E8	9	9	172	181			PEAKS DB
K.KNPQLQDLDM(+15.99)MLTC(+57.02)VEIKEGALMLPHFNSK.A	N	66.89	3515.7285	30	-3.1	879.9366	4	35.44	2	F2:1830	OB5940 H1 raw.raw	7.3362E6	2	2	419	448	Carbamidomethylation	M10:Oxidation (M):11.06;C14:Carbamidomethylation:1000.00	PEAKS DB
K.KNPQLQDLDMMLTC(+57.02)VEIKEGALMLPHFNSK.A	N	66.20	3499.7336	30	-0.1	875.9406	4	36.52	3	F3:1868	OB5941 H1 raw.raw	1.1425E7	3	3	419	448	Carbamidomethylation	C14:Carbamidomethylation:1000.00	PEAKS DB
R.VLLEEN(+.98)AGGEQEER.G	N	66.02	1572.7267	14	-3.7	787.3677	2	26.82	2	F2:1276	OB5940 H1 raw.raw	0	0	0	332	345	Deamidation (NQ)	N6:Deamidation (NQ): 71.64	PEAKS DB
R.Q(-17.03)FQNLQNHR.I	N	65.38	1166.5581	9	2.4	584.2877	2	28.05	1	F1:1306	OB5939 H1 raw.raw	3.4214E6	3	3	205	213	Pyro-glu from Q	Q1:Pyro-glu from Q:1000.00	PEAKS PTM
K.NPQLQDLDMMLTCVEIK(+14.02)EGALMLPHFNSK.A	N	64.78	3328.6328	29	1.8	1110.5536	3	38.03	2	F2:1959	OB5940 H1 raw.raw	1.6164E7	6	6	420	448	Methylation(KR)	K17:Methylation(KR): 77.78	PEAKS PTM
E.VSKEHVEELTK.H	N	64.44	1297.6877	11	0.8	433.5702	3	24.82	1	F1:1120	OB5939 H1 raw.raw	1.1352E5	6	6	365	375			PEAKS DB
R.EETSRNNPFYFPSR.R	N	63.62	1742.8011	14	-0.9	581.9405	3	29.89	1	F1:1419	OB5939 H1 raw.raw	6.2933E4	1	1	167	180			PEAKS DB
A.FPGSGEQVEK.L	N	61.40	1076.5138	10	5.5	539.2672	2	30.23	1	F1:1449	OB5939 H1 raw.raw	6.3559E4	1	1	563	572			PEAKS DB
K.NPQLQ(+.98)DLDMMLTC(+57.02)VEIKEGALM(+15.99)LPHFNSK.A	N	60.12	3388.6174	29	-2.4	1130.5437	3	36.35	2	F2:1874	OB5940 H1 raw.raw	6.4476E5	1	1	420	448	Carbamidomethylation; Oxidation (M)	Q5:Deamidation (NQ): 9.86;C13:Carbamidomethylation:1000.00; M22:Oxidation (M):54.40	PEAKS DB

total 145 peptides

Peptide	Uniq	-10lgP	Mass	Length	ppm	m/z	z	RT	Fraction	Scan	Source File	Area raw peanut	#Feature	#Feature raw peanut	Start	End	PTM	AScore	Found By
K.NPQLQDLDM(+15.99)M(+15.99)LTC(+57.02)VEIKEGALMLPHFNSK.A	N	60.05	3403.6284	29	-4.3	1135.5452	3	35.63	3	F3:1818	OB5941 H1 raw.raw	7.9921E5	1	1	420	448	Oxidation (M); Carbamidomethylation	M9:Oxidation (M):49.25;M10:Oxidation (M):53.09;C13:Carbamidomethylation:1000.00	PEAKS DB
R.SRQFQNLQNHR.I	N	59.95	1426.7178	11	1.0	476.5803	3	24.41	2	F2:1135	OB5940 H1 raw.raw	1.5724E4	2	2	203	213			PEAKS DB
K.NPQLQ(+.98)DLDMMLTC(+57.02)VEIKEGALMLPHFNSK.A	N	59.95	3372.6226	29	5.7	1125.2212	3	37.84	2	F2:1936	OB5940 H1 raw.raw	5.4487E7	3	3	420	448	Carbamidomethylation	Q5:Deamidation (NQ): 32.28; C13:Carbamidomethylation:1000.00	PEAKS DB
K.N(+.98)PQLQDLDMMLTC(+57.02)VEIKEGALMLPHFNSK.A	N	55.55	3372.6226	29	5.7	1125.2212	3	37.84	2	F2:1983	OB5940 H1 raw.raw	1.3378E7	1	1	420	448	Carbamidomethylation	N1:Deamidation (NQ): 14.04; C13:Carbamidomethylation:1000.00	PEAKS DB
R.EGEQEWGTPGSHVREETSR.N	N	55.42	2169.9675	19	1.4	724.3308	3	26.17	1	F1:1192	OB5939 H1 raw.raw	2.1848E6	5	5	153	171			PEAKS DB
K.NPQLQDLDMM(+15.99)LTC(+57.02)VEIKEGALM(+15.99)LPHFNSK.A	N	54.50	3403.6284	29	4.5	1135.5552	3	36.52	3	F3:1873	OB5941 H1 raw.raw	6.8814E5	2	2	420	448	Carbamidomethylation	M10:Oxidation (M):12.28;C13:Carbamidomethylation:1000.00;M22:Oxidation (M):28.29	PEAKS DB
R.EQEWEEEEEDEEEEGSNREVR.R	N	54.08	2665.0535	21	2.4	889.3606	3	27.87	1	F1:1308	OB5939 H1 raw.raw	4.4193E5	2	2	478	498			PEAKS DB
R.WGPAGPR.E	N	53.83	739.3765	7	-0.2	370.6955	2	24.50	2	F2:1143	OB5940 H1 raw.raw	2.1628E5	2	2	115	121			PEAKS DB
N.PFYFPSRR.F	N	53.53	1068.5504	8	3.3	535.2842	2	28.95	1	F1:1360	OB5939 H1 raw.raw	3.1024E6	3	3	174	181			PEAKS DB

total 145 peptides

Peptide	Uniq	-10lgP	Mass	Length	ppm	m/z	z	RT	Fraction	Scan	Source File	Area raw peanut	#Feature	#Feature raw peanut	Start	End	PTM	AScore	Found By
R.LFEVKPDKKNPQLQDLDMM(+15.99)LTC(+57.02)VEIK.E	N	53.37	3147.6018	26	-4.4	787.9042	4	32.55	3	F3:1633	OB5941 H1 raw.raw	2.1681E6	3	3	411	436	Carbamidomethylation	M19:Oxidation (M):17.01;C22:Carbamidomethylation:1000.00	PEAKS DB
K.NPQLQDLDMMLTCVEIK(+14.02)EGALM(+15.99)LPHFNSK.A	N	52.56	3344.6277	29	4.1	837.1677	4	37.13	2	F2:1903	OB5940 H1 raw.raw	1.5437E6	1	1	420	448	Oxidation (M)	K17:Methylation(KR): 24.93; M22:Oxidation (M):51.99	PEAKS PTM
R.NT(-18.01)LEAAFNAEFNEIRR.V	N	52.23	1875.9227	16	-2.4	626.3134	3	33.43	2	F2:1683	OB5940 H1 raw.raw	1.354E7	3	3	316	331	Dehydration	T2:Dehydration:1000.00	PEAKS PTM
L.EAAFNAEFNEIRR.V	N	52.09	1565.7585	13	-4.7	783.8829	2	34.06	1	F1:1677	OB5939 H1 raw.raw	1.7113E6	2	2	319	331			PEAKS DB
K.IRPEGREGEQEWGTPGSHVR.E	N	51.92	2276.1045	20	0.7	570.0338	4	26.47	3	F3:1255	OB5941 H1 raw.raw	1.9287E5	1	1	147	166			PEAKS DB
R.LFEVKPDK.K	N	51.87	974.5436	8	0.9	488.2795	2	25.40	1	F1:1153	OB5939 H1 raw.raw	7.0978E5	6	6	411	418			PEAKS DB
R.IVQIEAKP(+15.99)NTLVLPK.H	N	51.31	1678.0028	15	-2.8	840.0063	2	29.56	3	F3:1444	OB5941 H1 raw.raw	9.9803E5	2	2	214	228	Hydroxylation Pro	P8:Hydroxylation Pro: 65.06	PEAKS DB
K.K(+43.01)(+14.02)GSEEEGDITNPINLR.E	N	51.26	1827.8962	16	0.6	914.9559	2	28.50	3	F3:1391	OB5941 H1 raw.raw	2.757E5	2	2	383	398	Carbamylation; Methylation(KR)	K1:Carbamylation:1000.00; K1:Methylation (KR):168.07	PEAKS PTM
K.NPQLQDLDMM(+15.99)LTCVEIK(+14.02).E	N	51.01	2019.9679	17	-7.0	1010.9842	2	37.36	1	F1:1877	OB5939 H1 raw.raw	0	0	0	420	436	Methylation(KR)	M10:Oxidation (M):11.06;K17:Methylation (KR):1000.00	PEAKS PTM
R.RVLLEENAGGEQEERGQR.R	N	50.12	2069.0249	18	2.1	690.6837	3	24.88	2	F2:1165	OB5940 H1 raw.raw	2.8524E5	1	1	331	348			PEAKS DB
K.KNPQLQDLDMM(-48.00)LTC(+57.02)VEIK.E	N	49.96	2127.0703	18	1.7	710.0319	3	30.71	2	F2:1521	OB5940 H1 raw.raw	3.3467E6	2	2	419	436	Carbamidomethylation	M11:Dethiomethyl:14.02;C14:Carbamidomethylation:1000.00	PEAKS PTM
K.KNPQLQDLDMMLTCVEIK(+14.02)EGALMLPHFNSK.A	N	49.06	3456.7278	30	2.7	865.1915	4	36.90	2	F2:1892	OB5940 H1 raw.raw	3.1386E5	1	1	419	448	Methylation(KR)	K18:Methylation(KR): 50.90	PEAKS PTM

total 145 peptides

Peptide	Uniq	-10lgP	Mass	Length	ppm	m/z	z	RT	Fraction	Scan	Source File	Area raw peanut	#Feature	#Feature raw peanut	Start	End	PTM	AScore	Found By
K.NPQLQDLDMM(+15.99)LTC(+57.02)VEIKEGALMLPHFN(+.98)SK.A	N	48.99	3388.6174	29	5.0	848.1659	4	36.17	3	F3:1849	OB5941 H1 raw.raw	7.0919E6	2	2	420	448	Carbamidomethylation; Deamidation (NQ)	M10:Oxidation (M):0.00;C13:Carbamidomethylation:1000.00;N27:Deamidation (NQ):51.54	PEAKS DB
R.LFEVKPDKKNPQLQDLDM(+15.99)M(+15.99)LTC(+57.02)VEIK.E	N	48.67	3163.5967	26	2.1	791.9081	4	32.37	1	F1:1585	OB5939 H1 raw.raw	6.4006E5	1	1	411	436	Oxidation (M); Carbamidomethylation	M18:Oxidation (M):1000.00;M19:Oxidation (M):1000.00;C22:Carbamidomethylation:1000.00	PEAKS DB
K.NPQLQDLDM(+15.99)MLTC(+57.02)VEIKEGALM(+15.99)LPHFNSK.A	N	48.60	3403.6284	29	2.2	851.9163	4	36.24	2	F2:1851	OB5940 H1 raw.raw	3.928E6	2	2	420	448	Carbamidomethylation; Oxidation (M)	M9:Oxidation (M):10.11;C13:Carbamidomethylation:1000.00;M22:Oxidation (M):53.09	PEAKS DB
N.TPGQFEDFFPASSR.D	N	48.49	1584.7208	14	-0.2	793.3676	2	35.63	3	F3:1821	OB5941 H1 raw.raw	2.7376E5	1	1	291	304			PEAKS DB
R.NTLEAAFN(+.98)AEFNEIRR.V	N	48.19	1894.9172	16	-0.3	948.4656	2	32.92	1	F1:1570	OB5939 H1 raw.raw	1.5926E6	1	1	316	331		N8:Deamidation (NQ):8.26	PEAKS DB
K.NPQLQDLDMMLTC(+57.02)VEIKEGALMLPHFN(+.98)SK.A	N	47.92	3372.6226	29	7.4	844.1691	4	37.85	2	F2:1914	OB5940 H1 raw.raw	5.3901E7	2	2	420	448	Carbamidomethylation; Deamidation (NQ)	C13:Carbamidomethylation:1000.00;N27:Deamidation (NQ):61.84	PEAKS DB
R.QFQNLQNHR.I	N	46.78	1183.5846	9	0.4	592.7998	2	23.96	2	F2:1106	OB5940 H1 raw.raw	7.7423E4	5	5	205	213			PEAKS DB
R.EQEWEEEEEDEEEEGSNREVRR.Y	N	46.42	2821.1545	22	1.8	941.3939	3	26.75	1	F1:1227	OB5939 H1 raw.raw	3.455E6	3	3	478	499			PEAKS DB
K.Q(-17.03)AKDLAFPGSGEQVEK.L	N	45.78	1685.8260	16	0.9	843.9211	2	29.97	1	F1:1427	OB5939 H1 raw.raw	0	0	0	557	572	Pyro-glu from Q	Q1:Pyro-glu from Q:1000.00	PEAKS PTM

total 145 peptides

Peptide	Uniq	-10lgP	Mass	Length	ppm	m/z	z	RT	Fraction	Scan	Source File	Area raw peanut	#Feature	#Feature raw peanut	Start	End	PTM	AScore	Found By
V.QIEAKPNTLVLPK.H	N	45.72	1449.8555	13	-3.5	725.9325	2	29.92	3	F3:1467	OB5941 H1 raw.raw	1.2647E6	3	3	216	228			PEAKS DB
K.KN(+.98)PQLQDLDMM(+15.99)LTC(+57.02)VEIK.E	N	44.87	2192.0527	18	9.2	1097.0437	2	32.33	2	F2:1621	OB5940 H1 raw.raw	8.5764E4	1	1	419	436	Carbamidomethylation	N2:Deamidation (NQ):14.04;M11:Oxidation (M):0.00;C14:Carbamidomethylation:1000.00	PEAKS DB
R.I(+42.01)VQ(+15.00)IEAKPNTLVLPK.H	N	43.55	1719.0182	15	6.6	860.5220	2	30.00	2	F2:1474	OB5940 H1 raw.raw	1.6151E7	3	3	214	228	Acetylation (N-term); Deamidation followed by a methylation	I1:Acetylation (N-term):1000.00;Q3:Deamidation followed by a methylation:121.92	PEAKS PTM
R.LFEVKPDKK.N	N	43.08	1102.6385	9	1.1	552.3271	2	24.94	3	F3:1179	OB5941 H1 raw.raw	2.3087E5	2	2	411	419			PEAKS DB
N.TLEAAFNAEFNEIRR.V	N	43.00	1779.8904	15	-3.0	890.9498	2	32.91	3	F3:1655	OB5941 H1 raw.raw	4.3913E5	1	1	317	331			PEAKS DB
K.NPQLQDLDMMLTC(+57.02)VEIKEGALM(-48.00)LPHFNSK.A	N	42.94	3323.6353	29	-1.0	831.9153	4	35.81	3	F3:1829	OB5941 H1 raw.raw	2.5446E6	1	1	420	448	Carbamidomethylation	C13:Carbamidomethylation:1000.00;M22:Dethiomethyl:30.24	PEAKS PTM
R.WGP(+15.99)AGPR.E	N	42.77	755.3714	7	-0.4	378.6928	2	22.57	2	F2:1037	OB5940 H1 raw.raw	3.1259E3	1	1	115	121		P3:Hydroxylation Pro:22.34	PEAKS DB
K.KNPQLQ(+.98)DLDMMLTC(+57.02)VEIKEGALMLPHFNSK.A	N	42.58	3500.7175	30	0.4	1167.9136	3	36.34	2	F2:1850	OB5940 H1 raw.raw	5.0176E5	1	1	419	448	Carbamidomethylation	Q6:Deamidation (NQ):14.04;C14:Carbamidomethylation:1000.00	PEAKS DB
R.REQEWEEEEEDEEEEGSN(+.98)R.E	N	42.55	2437.9265	19	7.6	813.6556	3	26.55	1	F1:1226	OB5939 H1 raw.raw	3.9265E4	1	1	477	495		N18:Deamidation (NQ):0.00	PEAKS DB

total 145 peptides

Peptide	Uniq	-10lgP	Mass	Length	ppm	m/z	z	RT	Fraction	Scan	Source File	Area raw peanut	#Feature	#Feature raw peanut	Start	End	PTM	AScore	Found By
K.NPQLQDLDMMLTC(+57.02)VEIK(+43.01)EGALMLPHFNSK(+14.02).A	N	42.54	3428.6602	29	-4.2	1143.8892	3	37.82	3	F3:1939	OB5941 H1 raw.raw	1.0346E6	1	1	420	448	Carbamidomethylation	C13:Carbamidomethylation:1000.00;K17:Carbamylation:10.80;K29:Methylation(KR):10.80	PEAKS PTM
K.GTGNLELVAVRKEQQQR.G	N	41.95	1925.0442	17	1.8	642.6898	3	26.36	1	F1:1214	OB5939 H1 raw.raw	1.4197E5	3	3	458	474			PEAKS DB
K.D(+43.01)(+14.02)LAFPGSGEQVEK.L	N	41.75	1432.6833	13	3.8	717.3517	2	30.92	1	F1:1485	OB5939 H1 raw.raw	2.8695E5	1	1	560	572	Carbamylation; Methylation(others)	D1:Carbamylation:166.34;D1:Methylation(others):102.87	PEAKS PTM
K.KN(+.98)PQLQDLDMMLTC(+57.02)VEIKEGALMLPHFNSK.A	N	40.34	3500.7175	30	7.1	876.1929	4	36.26	2	F2:1847	OB5940 H1 raw.raw	2.213E6	1	1	419	448	Carbamidomethylation	N2:Deamidation (NQ):0.00;C14:Carbamidomethylation:1000.00	PEAKS DB
K.NPQLQDLDM(+15.99)MLTC(+57.02)VEIKEGALMLP(+15.99)HFNSK.A	N	40.24	3403.6284	29	-10.2	851.9057	4	36.72	1	F1:1838	OB5939 H1 raw.raw	0	0	0	420	448	Carbamidomethylation; Hydroxylation Pro	M9:Oxidation (M):26.02;C13:Carbamidomethylation:1000.00;P24:Hydroxylation Pro:63.93	PEAKS DB
R.ESHFVSARPQSQSP(+15.99)SSPEKEDQEEEN.Q	N	40.09	2973.2859	26	-2.5	992.1001	3	25.16	1	F1:1143	OB5939 H1 raw.raw	0	0	0	579	604		P14:Hydroxylation Pro:29.32	PEAKS DB
T.PGQFEDFFPASSR.D	N	39.65	1483.6731	13	-1.3	742.8429	2	40.34	2	F2:2103	OB5940 H1 raw.raw	3.5787E3	1	1	292	304			PEAKS DB
K.EGALM(-48.00)LPHFNSK.A	N	38.72	1294.6670	12	2.0	432.5638	3	28.31	3	F3:1348	OB5941 H1 raw.raw	1.3523E6	1	1	437	448	Dethiomethyl	M5:Dethiomethyl:1000.00	PEAKS PTM
R.DQS(-18.01)SYLQGFSR.N	N	38.22	1268.5785	11	7.0	635.3010	2	30.87	1	F1:1484	OB5939 H1 raw.raw	0	0	0	305	315		S3:Dehydration:33.98	PEAKS PTM

total 145 peptides

Peptide	Uniq	-10lgP	Mass	Length	ppm	m/z	z	RT	Fraction	Scan	Source File	Area raw peanut	#Feature	#Feature raw peanut	Start	End	PTM	AScore	Found By
K.N(+.98)PQ(+.98)LQDLDMMLTC(+57.02)VEIKEGALM(+15.99)LPHFNSK.A	N	37.95	3389.6016	29	9.0	848.4153	4	36.92	1	F1:1851	OB5939 H1 raw.raw	8.517E6	2	2	420	448	Carbamidomethylation	N1:Deamidation (NQ):11.12;Q3:Deamidation (NQ):0.00;C13:Carbamidomethylation:1000.00;M22:Oxidation (M):24.93	PEAKS DB
R.WGPAGP(+15.99)R.E	N	37.74	755.3714	7	0.0	378.6930	2	23.87	3	F3:1097	OB5941 H1 raw.raw	7.3229E3	1	1	115	121		P6:Hydroxylation Pro:32.97	PEAKS DB
K.NPQLQDLDMM(-48.00)LTC(+57.02)VEIKEGALMLPHFNSK.A	N	36.81	3323.6353	29	0.8	1108.8866	3	35.97	3	F3:1833	OB5941 H1 raw.raw	5.0189E6	2	2	420	448	Carbamidomethylation	M10:Dethiomethyl:8.14;C13:Carbamidomethylation:1000.00	PEAKS PTM
R.LFEVKPDKKN(+.98)PQLQDLDMMLTC(+57.02)VEIK.E	N	36.71	3132.5908	26	9.3	1567.3173	2	34.35	3	F3:1745	OB5941 H1 raw.raw	2.2872E5	1	1	411	436	Carbamidomethylation	N10:Deamidation (NQ):14.04;C22:Carbamidomethylation:1000.00	PEAKS DB
R.ESHFVSARPQSQSP(+15.99)SSPEKEDQEEE.N	N	36.67	2859.2429	25	2.6	954.0907	3	24.51	2	F2:1151	OB5940 H1 raw.raw	1.0898E4	1	1	579	603		P14:Hydroxylation Pro:12.33	PEAKS DB
R.E(-18.01)GEQEWGTPGSHVR.E	N	36.58	1549.6909	14	-1.7	775.8514	2	27.60	3	F3:1325	OB5941 H1 raw.raw	5.8319E4	1	1	153	166	Pyro-glu from E	E1:Pyro-glu from E:1000.00	PEAKS PTM
K.NPQLQDLDM(+15.99)M(+15.99)LTC(+57.02)VEIK.E	N	36.40	2078.9688	17	1.1	1040.4928	2	33.80	3	F3:1701	OB5941 H1 raw.raw	0	0	0	420	436	Oxidation (M); Carbamidomethylation	M9:Oxidation (M):1000.00;M10:Oxidation (M):1000.00;C13:Carbamidomethylation:1000.00	PEAKS DB
R.N(-17.03)NPFYFPSRR.F	N	36.19	1279.6097	10	-0.4	427.5437	3	28.25	2	F2:1369	OB5940 H1 raw.raw	1.5243E5	1	1	172	181		N1:Ammonia-loss (N):0.00	PEAKS PTM

total 145 peptides

Peptide	Uniq	-10lgP	Mass	Length	ppm	m/z	z	RT	Fraction	Scan	Source File	Area raw peanut	#Feature	#Feature raw peanut	Start	End	PTM	AScore	Found By
K.NPQ(+.98)LQDLDMMLTC(+57.02)VEIKEGALMLPHFNSK.A	N	36.11	3372.6226	29	9.4	1125.2253	3	40.75	1	F1:2064	OB5939 H1 raw.raw	5.8612E5	1	1	420	448	Carbamidomethylation	Q3:Deamidation (NQ):0.00;C13:Carbamidomethylation:1000.00	PEAKS DB
K.N(+.98)PQ(+.98)LQDLDMMLTC(+57.02)VEIKEGALMLPHFNSK.A	N	35.87	3373.6067	29	9.1	1687.8259	2	38.03	2	F2:1999	OB5940 H1 raw.raw	2.685E5	1	1	420	448	Carbamidomethylation	N1:Deamidation (NQ):3.13;Q3:Deamidation (NQ):5.47;C13:Carbamidomethylation:1000.00	PEAKS DB
Q.IEAKPNTLVLPK.H	N	35.55	1321.7969	12	-4.5	661.9028	2	29.65	2	F2:1449	OB5940 H1 raw.raw	6.4226E5	2	2	217	228			PEAKS DB
K.EGALMLPH(+14.02)FNSK.A	N	35.50	1356.6860	12	1.3	679.3512	2	30.49	3	F3:1499	OB5941 H1 raw.raw	2.5433E4	1	1	437	448	Methylation(others)	H8:Methylation(others):53.53	PEAKS PTM
R.VLLE(+21.98)ENAGGEQEER.G	N	35.40	1593.7246	14	-0.7	797.8690	2	26.03	2	F2:1239	OB5940 H1 raw.raw	1.529E5	3	3	332	345		E4:Sodium adduct:40.00	PEAKS PTM
K.NP(+15.99)QLQDLDMM(+15.99)LTC(+57.02)VEIKEGALMLPHFNSK.A	N	35.20	3403.6284	29	-3.4	851.9115	4	35.28	1	F1:1792	OB5939 H1 raw.raw	1.7685E6	1	1	420	448	Carbamidomethylation	P2:Hydroxylation Pro:48.46;M10:Oxidation (M):11.06;C13:Carbamidomethylation:1000.00	PEAKS DB
K.HADADNILVIQQ(-18.01).G	N	34.95	1317.6677	12	-4.9	659.8379	2	29.56	3	F3:1421	OB5941 H1 raw.raw	1.0066E5	1	1	229	240	Dehydration	Q12:Dehydration:76.18	PEAKS PTM
K.GT(-18.01)GNLELVAVRK.E	N	34.88	1237.7142	12	0.9	413.5790	3	26.41	2	F2:1259	OB5940 H1 raw.raw	7.6712E5	3	3	458	469	Dehydration	T2:Dehydration:1000.00	PEAKS PTM
K.NPQLQDLDM(-48.00)MLTC(+57.02)VEIKEGALMLPHFNSK.A	N	34.70	3323.6353	29	8.1	831.9229	4	35.72	2	F2:1816	OB5940 H1 raw.raw	1.6745E6	2	2	420	448	Carbamidomethylation	M9:Dethiomethyl:17.01;C13:Carbamidomethylation:1000.00	PEAKS PTM

total 145 peptides

Peptide	Uniq	-10lgP	Mass	Length	ppm	m/z	z	RT	Fraction	Scan	Source File	Area raw peanut	#Feature	#Feature raw peanut	Start	End	PTM	AScore	Found By
N.PQLQDLDMMLTC(+57.02)VEIKEGALMLPHFNSK.A	N	34.02	3257.5957	28	0.9	815.4069	4	37.83	3	F3:1952	OB5941 H1 raw.raw	2.5417E5	1	1	421	448	Carbamidomethylation	C12:Carbamidomethylation:1000.00	PEAKS DB
R.IVQ(+.98)IEAKPNTLVLPK.H	N	33.90	1662.9919	15	6.7	832.5088	2	31.45	2	F2:1578	OB5940 H1 raw.raw	3.0057E5	1	1	214	228		Q3:Deamidation (NQ):13.11	PEAKS DB
K.AMVIVVINK(+43.01)GTGNLELVAVRK.E	Y	33.56	2266.3196	21	-2.3	756.4454	3	32.33	2	F2:1600	OB5940 H1 raw.raw	9.4705E6	1	1	449	469	Carbamylation	K9:Carbamylation:73.08	PEAKS PTM
K.PNTLVLPK.H	N	33.50	880.5382	8	0.4	441.2766	2	27.53	2	F2:1313	OB5940 H1 raw.raw	8.6509E4	1	1	221	228			PEAKS DB
K.G(+43.01)T(+14.02)GNLELVAVRK.E	N	33.06	1312.7462	12	-0.8	657.3799	2	26.85	3	F3:1280	OB5941 H1 raw.raw	3.165E5	2	2	458	469	Carbamylation; Methylation(others)	G1:Carbamylation:148.28;T2:Methylation(others):59.30	PEAKS PTM
R.REQEWEEEEEDEEEEGSNREVR.R	N	33.06	2821.1545	22	3.9	941.3958	3	26.47	3	F3:1247	OB5941 H1 raw.raw	1.8551E6	1	1	477	498			PEAKS DB
K.KGSEEEGDIT(-18.01)N(+.98)PINLR.E	N	33.05	1753.8483	16	2.8	585.6250	3	28.08	2	F2:1354	OB5940 H1 raw.raw	8.0018E5	2	2	383	398		T10:Dehydration:25.86;N11:Deamidation (NQ):32.46	PEAKS PTM
R.EREEDWRQPR.E	N	31.07	1399.6592	10	0.1	467.5604	3	24.77	3	F3:1154	OB5941 H1 raw.raw	2.8345E4	1	1	124	133			PEAKS DB
R.EGEQEW(+15.99)GTPGSHVREETSR.N	N	30.97	2185.9624	19	1.0	729.6621	3	25.31	3	F3:1191	OB5941 H1 raw.raw	3.6705E4	1	1	153	171		W6:Oxidation (HW):12.65	PEAKS PTM
K.HADADNILVIQQGQATVT(-18.01)VANGNNR.R	N	29.88	2600.3054	25	-2.7	867.7734	3	30.76	3	F3:1503	OB5941 H1 raw.raw	3.2843E5	1	1	229	253		T18:Dehydration:22.85	PEAKS PTM
K.NPQLQDLDM(-48.00)MLTC(+57.02)VEIK.E	N	29.75	1998.9755	17	-0.6	667.3320	3	34.07	2	F2:1713	OB5940 H1 raw.raw	8.006E5	1	1	420	436	Carbamidomethylation	M9:Dethiomethyl:0.00;C13:Carbamidomethylation:1000.00	PEAKS PTM
R.EDWRRPSHQQPR.K	N	29.71	1590.7764	12	-0.3	398.7013	4	24.94	3	F3:1169	OB5941 H1 raw.raw	1.2481E4	1	1	134	145			PEAKS DB
N.NPFYFPSRR.F	N	29.59	1182.5934	9	1.6	395.2057	3	28.08	2	F2:1358	OB5940 H1 raw.raw	2.5271E4	1	1	173	181			PEAKS DB

total 145 peptides

Peptide	Uniq	-10lgP	Mass	Length	ppm	m/z	z	RT	Fraction	Scan	Source File	Area raw peanut	#Feature	#Feature raw peanut	Start	End	PTM	AScore	Found By
R.IVQIEAKPNTLVLP(+15.99)K(-.98)(+42.01).H	N	29.49	1719.0294	15	3.0	860.5245	2	30.75	1	F1:1437	OB5939 H1 raw.raw	4.8723E6	1	1	214	228	Hydroxylation Pro; Amidation; Acetylation (K)	P14:Hydroxylation Pro:62.39;K15:Amidation:1000.00;K15:Acetylation (K):66.64	PEAKS PTM
K.N(+.98)PQ(+.98)LQ(+.98)DLDMM(+15.99)LTC(+57.02)VEIKEGALMLPHFNSK.A	N	29.44	3390.5854	29	3.7	848.6568	4	38.47	3	F3:1982	OB5941 H1 raw.raw	0	0	0	420	448	Deamidation (NQ); Carbamidomethylation	N1:Deamidation (NQ): 62.42; Q3:Deamidation (NQ): 65.92; Q5:Deamidation (NQ): 60.92; M10:Oxidation (M):12.28;C13:Carbamidomethylation:1000.00	PEAKS DB
R.EQEW(+15.99)EEEEEDEEEEGSNR.E	N	29.12	2296.8362	18	4.4	1149.4304	2	27.05	1	F1:1252	OB5939 H1 raw.raw	0	0	0	478	495	Oxidation (HW)	W4:Oxidation (HW):1000.00	PEAKS PTM
K.HADADNILVIQQGQAT(-18.01)VTVANGNNR.R	N	29.07	2600.3054	25	-1.0	867.7748	3	30.11	2	F2:1479	OB5940 H1 raw.raw	0	0	0	229	253		T16:Dehydration:0.00	PEAKS PTM
R.E(-18.01)QEWEEEEEDEEEEGSNREVRR.Y	N	28.92	2803.1440	22	2.8	935.3912	3	28.31	3	F3:1363	OB5941 H1 raw.raw	2.9497E4	1	1	478	499	Pyro-glu from E	E1:Pyro-glu from E:1000.00	PEAKS PTM
K.KN(+.98)P(+15.99)Q(+.98)LQ(+.98)DLDMMLTC(+57.02)VEIKEGALMLPHFNSK.A	N	28.66	3518.6804	30	-5.1	880.6729	4	37.59	1	F1:1890	OB5939 H1 raw.raw	0	0	0	419	448	Deamidation (NQ); Carbamidomethylation	N2:Deamidation (NQ): 55.07;P3:Hydroxylation Pro:43.60;Q4:Deamidation (NQ): 57.68; Q6:Deamidation (NQ): 58.78; C14:Carbamidomethylation:1000.00	PEAKS DB
R.NTLEAAFNAEFNEIRRVLLEENAGGEQEER.G	N	28.52	3447.6653	30	-1.2	1150.2277	3	38.03	2	F2:1966	OB5940 H1 raw.raw	1.3831E5	1	1	316	345			PEAKS DB

total 145 peptides

Peptide	Uniq	-10lgP	Mass	Length	ppm	m/z	z	RT	Fraction	Scan	Source File	Area raw peanut	#Feature	#Feature raw peanut	Start	End	PTM	AScore	Found By
R.ESHFVSARPQSQSP(+15.99)SSP(+15.99)EKEDQEEEN.Q	N	28.43	2989.2808	26	0.3	997.4344	3	24.81	3	F3:1156	OB5941 H1 raw.raw	0	0	0	579	604		P14:Hydroxylation Pro:17.91;P17:Hydroxylation Pro:28.14	PEAKS DB
K.NPQLQDLDMM(+15.99)LTC(+58.01)VEIK.E	N	28.36	2063.9578	17	-3.4	1032.9827	2	37.19	1	F1:1881	OB5939 H1 raw.raw	2.5393E4	1	1	420	436	Carboxymethyl	M10:Oxidation (M):8.69;C13:Carboxymethyl:1000.00	PEAKS PTM
K.DLAFPGSGEQVEK(+14.02)LIK(+43.01).N	N	28.34	1786.9464	16	-3.8	894.4771	2	32.91	3	F3:1654	OB5941 H1 raw.raw	6.9695E5	1	1	560	575		K13:Methylation(KR):4.52;K16:Carbamylation:4.52	PEAKS PTM
K.KN(+.98)PQ(+.98)LQDLDMMLTC(+57.02)VEIKEGALMLPHFNSK.A	N	28.34	3501.7017	30	6.9	1168.2493	3	36.92	1	F1:1845	OB5939 H1 raw.raw	2.5153E5	1	1	419	448	Carbamidomethylation	N2:Deamidation (NQ):0.00;Q4:Deamidation (NQ):0.00;C14:Carbamidomethylation:1000.00	PEAKS DB
K.N(+.98)PQLQDLDM(+15.99)MLTC(+57.02)VEIKEGALMLPHFNSK.A	N	27.69	3388.6174	29	8.6	1130.5562	3	39.33	3	F3:2085	OB5941 H1 raw.raw	8.4125E5	1	1	420	448	Carbamidomethylation	N1:Deamidation (NQ):0.00;M9:Oxidation (M):0.00;C13:Carbamidomethylation:1000.00	PEAKS DB
R.IFLAGDKDNVVDQIEK(+71.04).Q	Y	27.67	1873.9785	16	-7.1	625.6624	3	32.19	3	F3:1586	OB5941 H1 raw.raw	4.3111E6	1	1	541	556		K16:Propionamide (K, X@N-term):31.37	PEAKS PTM
R.EGEQEWGTP(+15.99)GSHVREETSR.N	N	27.48	2185.9624	19	1.1	729.6622	3	24.88	2	F2:1169	OB5940 H1 raw.raw	1.6768E4	1	1	153	171	Hydroxylation Pro	P9:Hydroxylation Pro:1000.00	PEAKS DB
R.IVQIEAKPNT(-18.01)LVLPK.H	N	27.41	1643.9974	15	6.1	549.0098	3	30.06	1	F1:1436	OB5939 H1 raw.raw	1.3913E6	1	1	214	228	Dehydration	T10:Dehydration:1000.00	PEAKS PTM

total 145 peptides

Peptide	Uniq	-10lgP	Mass	Length	ppm	m/z	z	RT	Fraction	Scan	Source File	Area raw peanut	#Feature	#Feature raw peanut	Start	End	PTM	AScore	Found By
K.K(+58.01)GSEEEGDITNPINLR.E	N	27.35	1828.8802	16	6.8	915.4536	2	28.67	3	F3:1391	OB5941 H1 raw.raw	8.3578E4	1	1	383	398	Carboxymethyl (KW, X@N-term)	K1:Carboxymethyl (KW, X@N-term): 1000.00	PEAKS PTM
total 145 peptides																			

tr|A0A290FZZ3|A0A290FZZ3_ARAHY

back to list

| Protein Coverage | Supporting Peptides |

Protein Coverage:

```
  1 SDPSGMLSKW IRGTDCCTWP GLNCLFENKR VTSISIAGQP DQPNSFLSGT ISSSLSKLQF LDGIYFTNLR NISGPFPGFL
 81 LNMPNLEYIY IENSQISGRI PDSFGNSTRK FGAFSFQGNR LTGTVPSSLS LLTQLTQLKL GDNLLTGAIP DGIRNLKNLT
161 YLSLQGNQLS GNIPDFFTSL KNLRILELSR NKFSGTIPAS IATLAPTLGY LELGHNSLSG KIPDFLGKMK ALDTLDLSSN
241 RFTGSVPQSF KNLTKIFNLD LSNNLLVDPF PEMNVKGIES LDLSNNNLHL GTIPKWVTSS PIIYSLKLAK CGIRMKLDDW
321 KPSETYFYDY IDLSGNDISG SAIGLLNRTD YLVGFWASGN KLKFDMGGLR IVEKLKY
```

Supporting Peptides:

Peptide	Uniq	-10lgP	Mass	Length	ppm	m/z	z	RT	Fraction	Scan	Source File	Area raw peanut	#Feature	#Feature raw peanut	Start	End	PTM	AScore	Found By
R.LTGTVPSSLSLLTQLTQLK.L	Y	87.74	1999.1565	19	-0.9	1000.5846	2	38.02	1	F1:1918	OB5939 H1 raw.raw	1.9568E5	1	1	121	139			PEAKS DB
K.IFNLDLSNNLLVDPFPEMNVK.G	Y	42.96	2431.2456	21	4.1	1216.6350	2	39.67	1	F1:2010	OB5939 H1 raw.raw	0	0	0	256	276			PEAKS DB
total 2 peptides																			

Peptide List

Prepared with PEAKS ™ (bioinfor.com)

Summary

1. Notes

Ara h 1 GEL BAND ROASTED

2. Result Statistics

Figure 1. False discovery rate (FDR) curve. X axis is the number of peptides being kept. Y axis is the corresponding FDR.

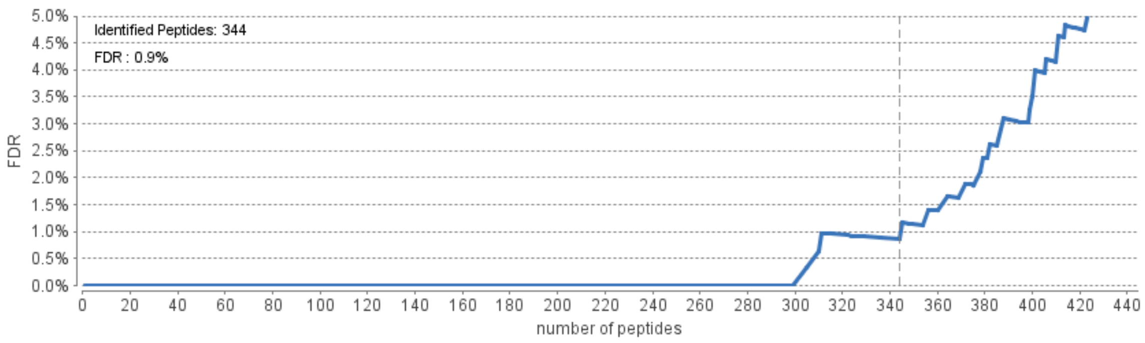

Figure 2. PSM score distribution. **(a)** Distribution of PEAKS peptide score; **(b)** Scatterplot of PEAKS peptide score versus precursor mass error.

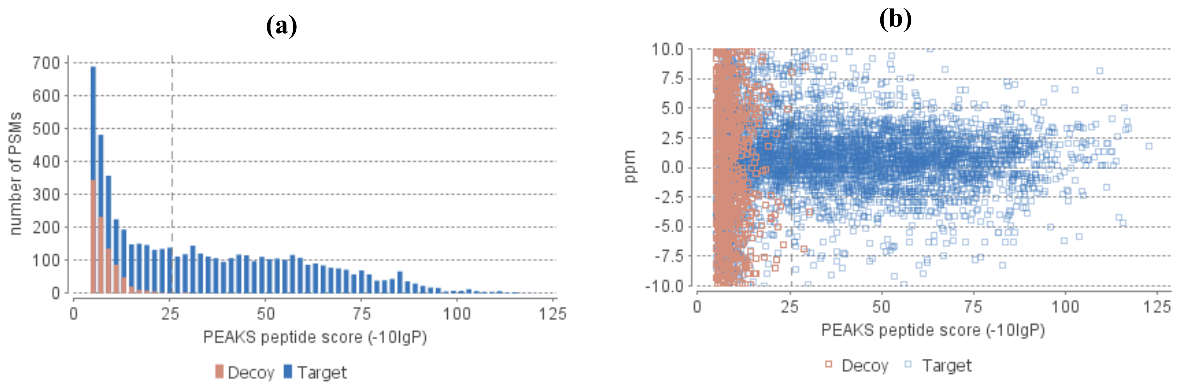

Table 1. Statistics of data.

	#Scans	#Features	Identified	#Peptides	#Sequences	

											#Proteins*		
	MS1	MS/MS	#Chimera		#PSMs	#Scans	#Features**				Groups	All	Top
Total	5509	7162	1123	12747	3058	2940	918	344	126		2	4	3
roasted peanut	5509	7162	1123	12747	3058	2940	918	344	126		2	4	3

* proteins with significant peptides are used in counts.
** features are identified by DB search only.

Figure 3. Sample overlap for Proteins and Peptides (up to 8 samples). **(a)** All Proteins; **(b)** Top Proteins; **(c)** Peptides;

(a)
Not applicable to only one sample

(b)
Not applicable to only one sample

(c)
Not applicable to only one sample

Figure 4. Distribution of peptide feature detection. **(a)** Feature m/z distribution; **(b)** Feature RT distribution.

(a)

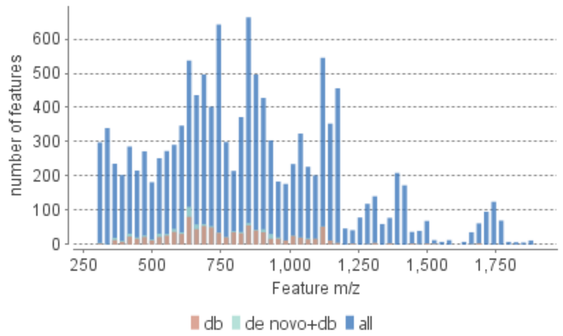

(b)

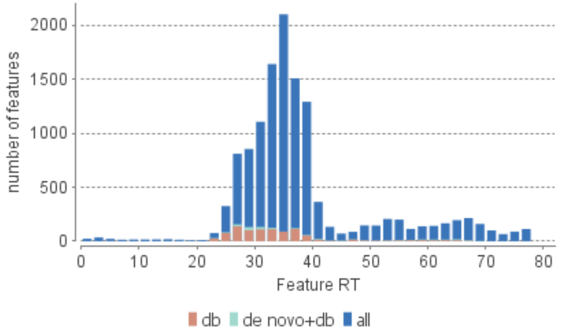

Figure 5. Distribution of identified peptide features. **(a)** Feature abundance distribution; **(b)** *De novo* sequencing validation.

(a)

(b)

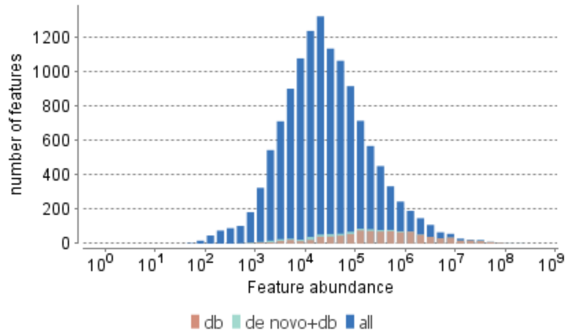

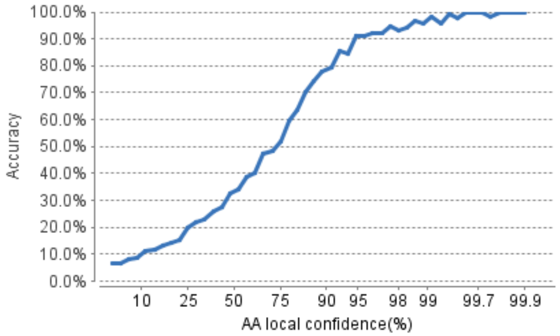

Table 2. Result filtration parameters.

Peptide -10lgP	≥25.5
PTM Ascore	≥50
Protein -10lgP	≥20
Proteins unique peptides	≥2
De novo score(%)	≥50%

Table 3. Statistics of filtered result.

FDR (Peptide-Spectrum Matches)	0.1%
FDR (Peptide Sequences)	0.9%
FDR (Protein Group)	0.0%
De Novo Only Spectra	608

Table 4. PTM profile.

Name	ΔMass	Position	#PSM	-10lgP	Abundance	AScore
Carbamidomethyl	57.02	C	303	116.47	4.22E6	1000.00
Oxidation	15.99	M	232	116.10	3.41E6	26.02
Deamidation	.98	NQ	114	101.58	2.7E6	24.32
HydPro	15.99	P	104	84.52	1.07E7	41.53
Methylation(KR)	14.02	KR	46	85.42	3.72E5	1000.00
Carbamylation	43.01	K,N-term	39	55.95	3.83E4	1000.00
Oxidation	15.99	HW	34	86.71	3.36E5	1000.00
Amidine	41.03	N-term	34	59.87	1.04E7	1000.00
Methylation(others)	14.02	CDEHST	31	93.65	1.36E6	33.98
Dehydration	-18.01	DSTY	27	68.93	1.92E5	40.00
Oxidation	15.99	DKNRY	19	57.68	3.67E5	51.51
Dethiomethyl	-48.00	M	19	76.61	1.43E6	14.02
Formylation	27.99	K,N-term	15	78.41	1.02E6	98.75
Pyro-glu from E	-18.01	N-term	12	60.61	1.12E5	1000.00
Dihydroxy	31.99	RW	10	60.54		21.02
Cation:Fe[II]	53.92	DE	9	50.61		20.92
Pyro-glu from Q	-17.03	N-term	9	65.43	2.88E4	1000.00
Ammonia loss	-17.03	N	8	72.17	7.61E5	1000.00
Sodium	21.98	E	7	48.97	1.59E5	9.34
Acetylation	42.01	K	5	77.03		1000.00

3. Experiment Control

Figure 6. Precursor mass error of peptide-spectrum matches (PSM) in filtered result. **(a)** Distribution of precursor mass error in ppm; **(b)** Scatterplot of precursor m/z versus precursor mass error in ppm.

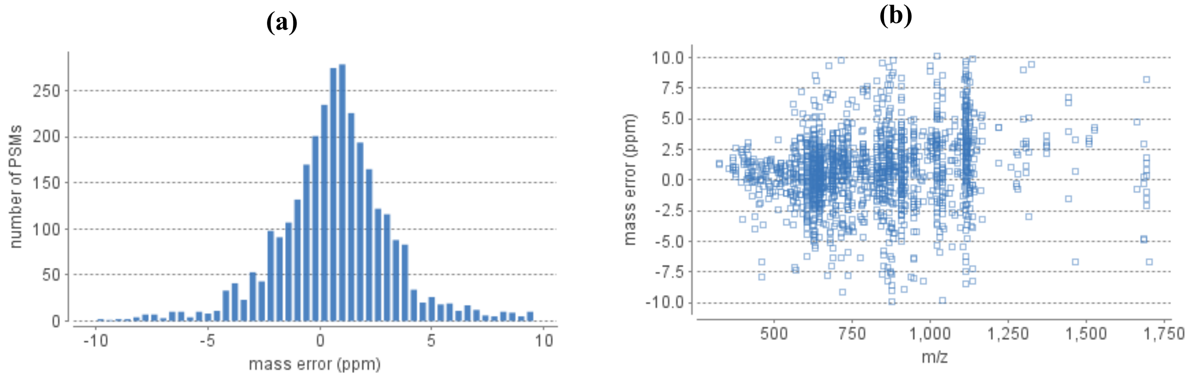

Table 5. Number of identified peptides in each sample by the number of missed cleavages.

Missed Cleavages	0	1	2	3	4+
roasted peanut	143	154	47	0	0

4. Other Information

Table 6. Search parameters.

Search Engine Name: PEAKS
Parent Mass Error Tolerance: 10.0 ppm
Fragment Mass Error Tolerance: 0.5 Da
Precursor Mass Search Type: monoisotopic
Enzyme: Trypsin
Max Missed Cleavages: 2
Digest Mode: Unspecific
Fixed Modifications:
  Carbamidomethylation: 57.02
Variable Modifications:
  Deamidation (NQ): 0.98
  Oxidation (M): 15.99
  Hydroxylation Pro: 15.99

Table 7. Instrument parameters.

Fractions: OB5954 H1 Ro.raw, OB5955 H1 Ro.raw, OB5956 H1 Ro.raw

Ion Source: ESI(nano-spray)
Fragmentation Mode: CID, CAD(y and b ions)
MS Scan Mode: FT-ICR/Orbitrap
MS/MS Scan Mode: Linear Ion Trap

Acetylation (K): 42.01
Acetylation (Protein N-term): 42.01
Acetylation (N-term): 42.01
Amidation: -0.98
Beta-methylthiolation: 45.99
and 305 more...

Max Variable PTM Per Peptide: 5
Database: Uniprot_Peanut-3818_Jul18
Taxon: All
Contaminant Database: contaminantsMQ_mar19
Searched Entry: 1723
FDR Estimation: Enabled
De novo score(%) threshold: 15
Peptide hit threshold (-10logP): 30.0
Peaks run ID: 15
Merge Options: no merge
Precursor Options: corrected
Charge Options: no correction
Filter Charge: 2 - 15
Process: true
Associate chimera: yes

Protein List

Protein Accession Contains:
Protein Description Contains:
Protein Sample Area >=
Protein PTM Contains:

Protein Group	Protein ID	Accession	-10lgP	Coverage (%)	Coverage (%) roasted peanut	Area roasted peanut	#Peptides	#Unique	#Spec roasted peanut	PTM	Avg. Mass	Description
**2**	3	P43237|ALL11_ARAHY	493.02	64	64	1.2143E8	93	10	2819	Y	70283	Allergen Ara h 1, clone P17 OS=Arachis hypogaea OX=3818 PE=1 SV=1
**2**	2	tr|B3IXL2|B3IXL2_ARAHY	493.02	64	64	1.2143E8	93	10	2819	Y	70283	Main allergen Ara h1 OS=Arachis hypogaea OX=3818 PE=2 SV=1
**4**	7	tr|E5G076|E5G076_ARAHY	440.15	48	48	3.4977E6	58	3	1319	Y	70788	Ara h 1 allergen OS=Arachis hypogaea OX=3818 GN=ara h 1 PE=4 SV=1
**total 3 proteins**												

P43237|ALL11_ARAHY

back to list

| **Protein Coverage** | **Supporting Peptides** |

Protein Coverage:

1 MRGRVSPLML LLGILVLASV SATQAKSPYR KTENPCAQRC LQSCQQEPDD LKQKACESRC TKLEYDPRCV YDTGATNQRH

147 P, 152 O

81 PPGERTRGRQ PGDYDDDRRQ PRREEGGRWG PAEPRERERE EDWRQPREDW RRPSHQQPRK IRPEGREGEQ EWGTPGSEVR

a Acetylation (K) (+42.01)
a Amidation (-0.98)
a Amidination of lysines or N-terminal amines with methyl acetimidate (+41.03)
a Ammonia-loss (N) (-17.03)
b Biotinylation (+226.08)
c Carbamidomethylation (+57.02)
c Carbamylation (+43.01)
c Carboxymethyl (+58.01)
d Deamidation (NQ) (+0.98)
d Dehydration (-18.01)
d Dimethylation(KR) (+28.03)

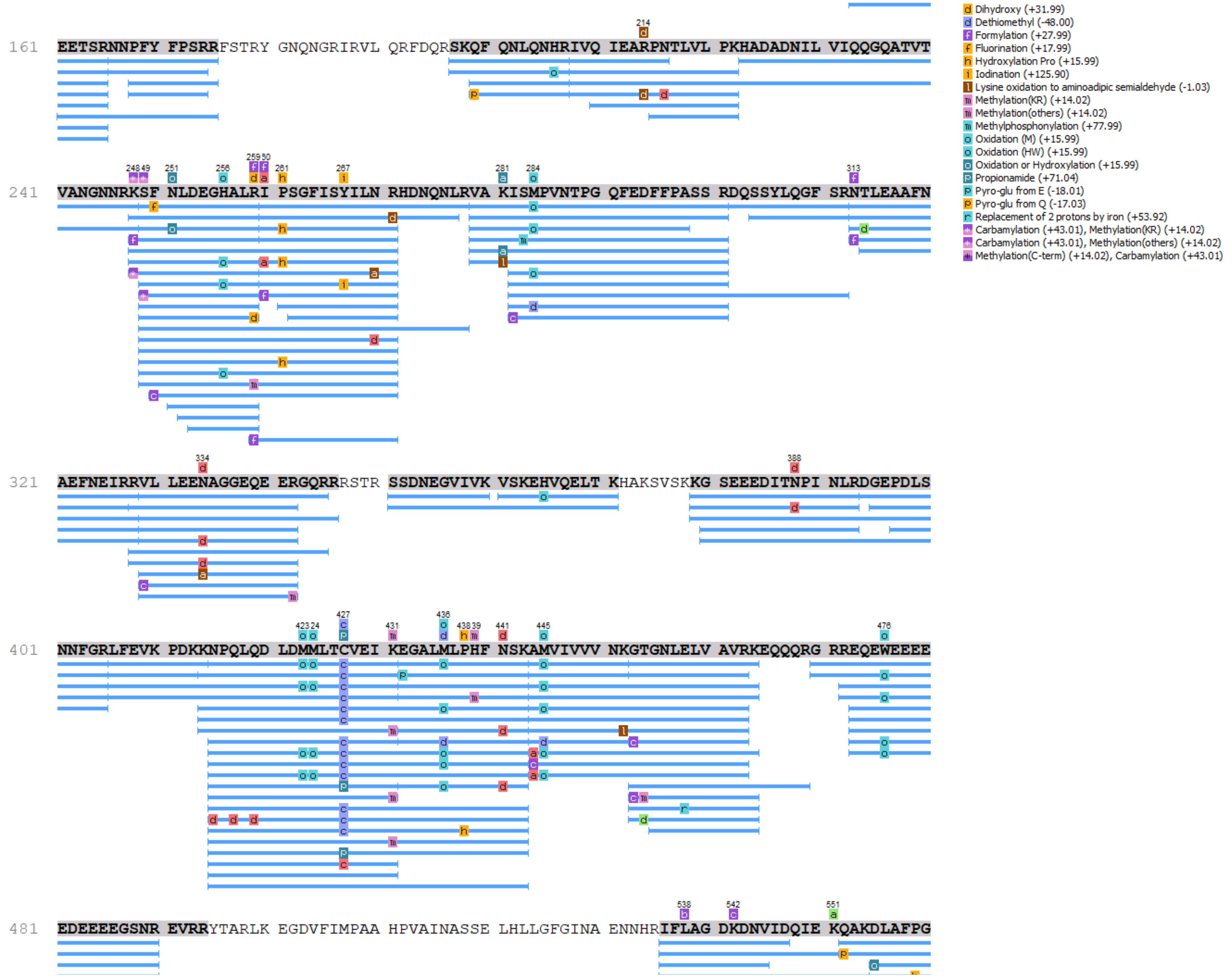
161 EETSRNNPFY FPSRRFSTRY GNQNGRIRVL QRFDQRSKQF QNLQNHRIVQ IEARPNTLVL PKHADADNIL VIQQGQATVT
241 VANGNNRKSF NLDEGHALRI PSGFISYILN RHDNQNLRVA KISMPVNTPG QFEDFFPASS RDQSSYLQGF SRNTLEAAFN
321 AEFNEIRRVL LEENAGGEQE ERGQRRRSTR SSDNEGVIVK VSKEHVQELT KHAKSVSKKG SEEEDITNPI NLRDGEPDLS
401 NNFGRLFEVK PDKKNPQLQD LDMMLTCVEI KEGALMLPHF NSKAMVIVVV NKGTGNLELV AVRKEQQQRG RREQEWEEEE
481 EDEEEEGSNR EVRRYTARLK EGDVFIMPAA HPVAINASSE LHLLGFGINA ENNHRIFLAG DKDNVIDQIE KQAKDLAFPG
d Dihydroxy (+31.99)
d Dethiomethyl (-48.00)
f Formylation (+27.99)
f Fluorination (+17.99)
h Hydroxylation Pro (+15.99)
i Iodination (+125.90)
l Lysine oxidation to aminoadipic semialdehyde (-1.03)
m Methylation(KR) (+14.02)
m Methylation(others) (+14.02)
m Methylphosphonylation (+77.99)
o Oxidation (M) (+15.99)
o Oxidation (HW) (+15.99)
o Oxidation or Hydroxylation (+15.99)
p Propionamide (+71.04)
p Pyro-glu from E (-18.01)
p Pyro-glu from Q (-17.03)
r Replacement of 2 protons by iron (+53.92)
Carbamylation (+43.01), Methylation(KR) (+14.02)
Carbamylation (+43.01), Methylation(others) (+14.02)
Methylation(C-term) (+14.02), Carbamylation (+43.01)

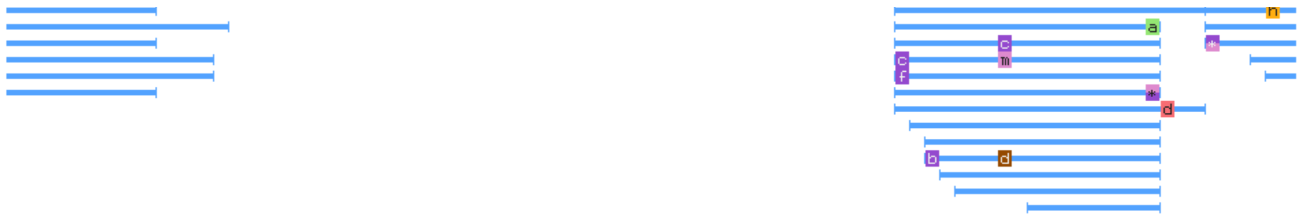

561 SGEQVEKLIK NQRESHFVSA RPQSQSPSSP EKEDQEEENQ GGKGPLLSIL KAFN

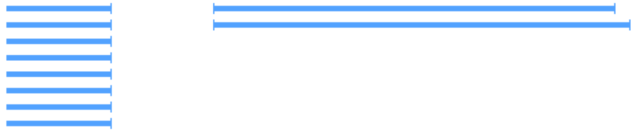

Supporting Peptides:

Peptide	Uniq	-10lgP	Mass	Length	ppm	m/z	z	RT	Fraction	Scan	Source File	Area roasted peanut	#Feature	#Feature roasted peanut	Start	End	PTM	AScore	Found By
R.EQEWEEEEEDEEEEGSNR.E	N	117.09	2280.8413	18	3.3	1141.4316	2	28.28	5	F5:1371	OB5955 H1 Ro.raw	4.6914E6	7	7	473	490			PEAKS DB
K.KNPQLQDLDMMLTC(+57.02)VEIK.E	N	116.47	2175.0737	18	3.2	1088.5476	2	35.21	5	F5:1788	OB5955 H1 Ro.raw	1.5503E7	6	6	414	431	Carbamidomethylation	C14:Carbamidomethylation:1000.00	PEAKS DB
K.NPQLQDLDMM(+15.99)LTC(+57.02)VEIK.E	N	116.10	2062.9736	17	5.0	1032.4993	2	34.71	6	F6:1780	OB5956 H1 Ro.raw	1.155E7	4	4	415	431	Carbamidomethylation	M10:Oxidation (M):30.46;C13:Carbamidomethylation:1000.00	PEAKS DB
K.NPQLQDLDM(+15.99)MLTC(+57.02)VEIK.E	N	115.70	2062.9736	17	-4.7	1032.4893	2	36.62	4	F4:1873	OB5954 H1 Ro.raw	3.8486E6	3	3	415	431	Carbamidomethylation	M9:Oxidation (M):30.46;C13:Carbamidomethylation:1000.00	PEAKS DB
K.NPQLQDLDMMLTC(+57.02)VEIK.E	N	112.86	2046.9788	17	0.2	1024.4968	2	37.11	6	F6:1959	OB5956 H1 Ro.raw	3.5075E7	10	10	415	431	Carbamidomethylation	C13:Carbamidomethylation:1000.00	PEAKS DB
R.NTLEAAFNAEFNEIR.R	N	112.69	1737.8322	15	1.4	869.9246	2	35.64	6	F6:1825	OB5956 H1 Ro.raw	1.2023E7	6	6	313	327			PEAKS DB
K.ISM(+15.99)PVNTPGQFEDFFPASSR.D	N	109.35	2242.0364	20	8.1	1122.0345	2	34.52	6	F6:1771	OB5956 H1 Ro.raw	2.1717E7	8	8	282	301	Oxidation (M)	M3:Oxidation (M):1000.00	PEAKS DB
K.ISMPVNTPGQFEDFFPASSR.D	N	107.77	2226.0415	20	0.9	1114.0291	2	35.86	4	F4:1823	OB5954 H1 Ro.raw	8.7375E7	15	15	282	301			PEAKS DB
K.HADADNILVIQQGQATVTVANGNNRK.S	N	104.88	2746.4111	26	3.3	916.4807	3	29.36	4	F4:1435	OB5954 H1 Ro.raw	3.9035E6	4	4	223	248			PEAKS DB

total 287 peptides

Peptide	Uniq	-10lgP	Mass	Length	ppm	m/z	z	RT	Fraction	Scan	Source File	Area roasted peanut	#Feature	#Feature roasted peanut	Start	End	PTM	AScore	Found By
K.KGSEEEDITNPINLRDGEPDLSNNFGR.L	N	103.39	3015.4170	27	3.4	1006.1497	3	31.62	4	F4:1565	OB5954 H1 Ro.raw	1.1819E7	10	10	379	405			PEAKS DB
K.KNPQLQDLDM(+15.99)MLTC(+57.02)VEIK.E	N	103.13	2191.0686	18	3.6	1096.5455	2	34.71	6	F6:1781	OB5956 H1 Ro.raw	1.4412E6	4	4	414	431	Carbamidomethylation	M10:Oxidation (M):27.96;C14:Carbamidomethylation:1000.00	PEAKS DB
R.IFLAGDKDNVIDQIEK.Q	N	102.67	1816.9570	16	6.5	909.4917	2	32.54	4	F4:1630	OB5954 H1 Ro.raw	4.3939E8	47	47	536	551			PEAKS DB
K.HADADNILVIQQGQATVTVAN(+.98)GNNRK.S	N	101.58	2747.3950	26	3.3	916.8086	3	29.66	4	F4:1454	OB5954 H1 Ro.raw	4.7756E6	7	7	223	248		N21:Deamidation (N Q):31.91	PEAKS DB
K.AMVIVVVNKGTGNLELVAVR.K	N	99.57	2081.2031	20	-0.4	1041.6084	2	34.57	4	F4:1751	OB5954 H1 Ro.raw	3.4777E7	8	8	444	463			PEAKS DB
K.GSEEEDITNPINLRDGEPDLSNNFGR.L	N	98.43	2887.3220	26	-0.7	963.4473	3	32.67	6	F6:1660	OB5956 H1 Ro.raw	1.2511E7	5	5	380	405			PEAKS DB
K.SFNLDEGHALR.I	N	97.07	1257.6101	11	3.0	629.8142	2	28.04	4	F4:1388	OB5954 H1 Ro.raw	1.1757E8	21	21	249	259			PEAKS DB
K.NPQLQDLDM(+15.99)M(+15.99)LTC(+57.02)VEIK.E	N	96.89	2078.9688	17	6.6	1040.4985	2	33.82	6	F6:1726	OB5956 H1 Ro.raw	7.6578E4	2	2	415	431	Oxidation (M); Carbamidomethylation	M9:Oxidation (M):1000.00; M10:Oxidation (M):1000.00; C13:Carbamidomethylation:1000.00	PEAKS DB
K.AM(+15.99)VIVVVNKGTGNLELVAVR.K	N	96.06	2097.1980	20	3.4	1049.6099	2	32.98	5	F5:1656	OB5955 H1 Ro.raw	2.4028E7	6	6	444	463	Oxidation (M)	M2:Oxidation (M):1000.00	PEAKS DB
R.VLLEENAGGEQEER.G	N	95.38	1571.7427	14	2.2	786.8804	2	26.92	4	F4:1300	OB5954 H1 Ro.raw	3.043E7	6	6	329	342			PEAKS DB
K.KNPQLQDLDMM(+15.99)LTC(+57.02)VEIK.E	N	95.29	2191.0686	18	3.1	1096.5449	2	32.98	5	F5:1657	OB5955 H1 Ro.raw	1.9358E6	4	4	414	431	Carbamidomethylation	M11:Oxidation (M):23.10;C14:Carbamidomethylation:1000.00	PEAKS DB
K.SFNLDEGHALRIPSGFISYILNR.H	N	95.27	2618.3604	23	1.9	873.7957	3	37.65	6	F6:1935	OB5956 H1 Ro.raw	7.1821E7	11	11	249	271			PEAKS DB
R.DQSSYLQGFSR.N	N	94.26	1286.5891	11	-0.9	644.3013	2	30.91	4	F4:1531	OB5954 H1 Ro.raw	9.6573E7	19	19	302	312			PEAKS DB
R.RVLLEENAGGEQEER.G	N	93.77	1727.8438	15	0.2	576.9553	3	25.30	5	F5:1185	OB5955 H1 Ro.raw	8.7628E5	6	6	328	342			PEAKS DB

total 287 peptides

Peptide	Uniq	-10lgP	Mass	Length	ppm	m/z	z	RT	Fraction	Scan	Source File	Area roasted peanut	#Feature	#Feature roasted peanut	Start	End	PTM	AScore	Found By
K.NPQLQDLDMMLTC(+14.02)VEIK.E	N	93.65	2003.9730	17	2.7	1002.9965	2	38.33	5	F5:1965	OB5955 H1 Ro.raw	5.8314E6	4	4	415	431		C13:Methylation(others):33.98	PEAKS PTM
R.IFLAGDKDNVIDQIEKQAK.D	N	92.18	2144.1477	19	-2.1	715.7217	3	32.42	5	F5:1625	OB5955 H1 Ro.raw	1.615E6	3	3	536	554			PEAKS DB
R.IPSGFISYILNR.H	N	92.13	1378.7609	12	1.1	690.3885	2	37.47	6	F6:1945	OB5956 H1 Ro.raw	4.5588E8	9	9	260	271			PEAKS DB
K.AMVIVVVNKGTGNLELVAVRK.E	N	91.52	2209.2981	21	4.7	737.4434	3	32.59	4	F4:1633	OB5954 H1 Ro.raw	1.2306E8	14	14	444	464			PEAKS DB
R.KSFNLDEGHALR.I	N	88.41	1385.7051	12	1.3	693.8607	2	26.06	5	F5:1228	OB5955 H1 Ro.raw	5.4977E6	6	6	248	259			PEAKS DB
R.REQEWEEEEEDEEEEGSNR.E	N	88.08	2436.9424	19	0.6	813.3219	3	26.73	4	F4:1278	OB5954 H1 Ro.raw	9.3919E5	7	7	472	490			PEAKS DB
R.EQEW(+15.99)EEEEEDEEEEGSNR.E	N	86.71	2296.8362	18	2.5	1149.4282	2	27.64	6	F6:1309	OB5956 H1 Ro.raw	1.0675E6	3	3	473	490	Oxidation (HW)	W4:Oxidation (HW):1000.00	PEAKS PTM
K.KGSEEEDITNPINLR.D	N	86.46	1713.8533	15	4.2	857.9375	2	29.29	4	F4:1432	OB5954 H1 Ro.raw	8.9159E6	6	6	379	393			PEAKS DB
K.NPQLQDLDM(+15.99)MLTCVEIK(+14.02).E	N	85.42	2019.9679	17	2.2	1010.9934	2	37.33	4	F4:1920	OB5954 H1 Ro.raw	7.9299E5	2	2	415	431	Methylation(KR)	M9:Oxidation (M):21.94;K17:Methylation (KR):1000.00	PEAKS PTM
K.DLAFPGSGEQVEK.L	N	85.27	1375.6619	13	-0.7	688.8377	2	30.39	4	F4:1498	OB5954 H1 Ro.raw	9.2216E7	3	3	555	567			PEAKS DB
R.VLLEEN(+.98)AGGEQEER.G	N	85.22	1572.7267	14	2.8	787.3728	2	27.46	6	F6:1340	OB5956 H1 Ro.raw	6.8644E5	3	3	329	342	Deamidation (NQ)	N6:Deamidation (NQ):121.37	PEAKS DB
K.NPQLQDLDMMLTC(+57.02)VEIKEGALMLPHFNSK.A	N	85.17	3371.6387	29	-4.9	1686.8184	2	38.00	5	F5:1955	OB5955 H1 Ro.raw	1.5541E8	10	10	415	443	Carbamidomethylation	C13:Carbamidomethylation:1000.00	PEAKS DB
R.DGEPDLSNNFGR.L	N	84.59	1319.5742	12	0.6	660.7948	2	29.29	4	F4:1422	OB5954 H1 Ro.raw	7.8399E6	3	3	394	405			PEAKS DB
K.ISMP(+15.99)VNTPGQFEDFFPASSR.D	N	84.52	2242.0364	20	-2.0	1122.0232	2	34.47	5	F5:1814	OB5955 H1 Ro.raw	1.0743E7	2	2	282	301		P4:Hydroxylation Pro:42.89	PEAKS DB
R.EGEQEWGTPGSEVREETSR.N	Y	84.24	2161.9512	19	4.4	1081.9877	2	27.86	4	F4:1344	OB5954 H1 Ro.raw	4.0932E7	7	7	147	165			PEAKS DB
K.NPQLQDLDM(+15.99)MLTC(+57.02)VEIKEGALMLPHFNSK.A	N	83.17	3387.6335	29	-0.6	1130.2178	3	37.70	4	F4:1939	OB5954 H1 Ro.raw	9.055E7	6	6	415	443	Carbamidomethylation	M9:Oxidation (M):26.02;C13:Carbamidomethylation:1000.00	PEAKS DB

total 287 peptides

Peptide	Uniq	-10lgP	Mass	Length	ppm	m/z	z	RT	Fraction	Scan	Source File	Area roasted peanut	#Feature	#Feature roasted peanut	Start	End	PTM	AScore	Found By
R.NTLEAAFNAEFNEIRR.V	N	81.08	1893.9332	16	-2.6	947.9714	2	34.11	5	F5:1711	OB5955 H1 Ro.raw	2.8495E8	15	15	313	328			PEAKS DB
R.GRREQEWEEEEEDEEEEGSNR.E	N	80.86	2650.0649	21	1.1	884.3632	3	26.73	4	F4:1280	OB5954 H1 Ro.raw	8.7144E5	6	6	470	490			PEAKS DB
R.EGEQEWGTPGSEVR.E	Y	80.82	1559.6852	14	1.4	780.8510	2	28.04	4	F4:1358	OB5954 H1 Ro.raw	3.4069E6	3	3	147	160			PEAKS DB
K.EGALMLPHFNSK.A	N	80.31	1342.6703	12	2.3	672.3440	2	30.63	5	F5:1504	OB5955 H1 Ro.raw	1.4317E7	5	5	432	443			PEAKS DB
K.SFNLDEGH(+15.99)ALR.I	N	80.04	1273.6051	11	-0.5	637.8095	2	30.29	4	F4:1491	OB5954 H1 Ro.raw	9.1981E5	2	2	249	259	Oxidation (HW)	H8:Oxidation (HW):1000.00	PEAKS PTM
R.LFEVKPDKKNPQLQDLDMMLTC(+57.02)VEIK.E	N	78.90	3131.6069	26	-4.0	1044.8721	3	34.65	5	F5:1756	OB5955 H1 Ro.raw	5.2558E6	4	4	406	431	Carbamidomethylation	C22:Carbamidomethylation:1000.00	PEAKS DB
R.I(+27.99)FLAGDKDNVIDQIEK.Q	N	78.41	1844.9519	16	-2.1	923.4813	2	36.23	4	F4:1853	OB5954 H1 Ro.raw	2.4413E6	3	3	536	551	Formylation	I1:Formylation:98.75	PEAKS PTM
K.GSEEEDITNPINLR.D	N	78.13	1585.7583	14	2.3	793.8882	2	31.16	5	F5:1548	OB5955 H1 Ro.raw	3.4694E6	3	3	380	393			PEAKS DB
F.LAGDKDNVIDQIEK.Q	N	78.02	1556.8046	14	-6.3	779.4047	2	32.21	6	F6:1629	OB5956 H1 Ro.raw	5.4762E6	4	4	538	551			PEAKS DB
K.AMVIVVVNK.G	N	77.85	971.5837	9	-0.8	486.7987	2	30.10	5	F5:1540	OB5955 H1 Ro.raw	7.1402E6	3	3	444	452			PEAKS DB
R.VAK(+42.01)ISMPVNTPGQFEDFFPASSR.D	N	77.03	2566.2524	23	0.5	1284.1342	2	36.43	6	F6:1881	OB5956 H1 Ro.raw	0	0	0	279	301	Acetylation (K)	K3:Acetylation (K):1000.00	PEAKS PTM
K.NPQLQDLDMM(-48.00)LTC(+57.02)VEIK.E	N	76.61	1998.9755	17	1.9	1000.4969	2	32.85	6	F6:1670	OB5956 H1 Ro.raw	6.204E6	3	3	415	431	Carbamidomethylation	M10:Dethiomethyl:14.02;C13:Carbamidomethylation:1000.00	PEAKS PTM
K.ISMPVNTP(+15.99)GQFEDFFPASSR.D	N	75.57	2242.0364	20	1.5	1122.0271	2	35.64	6	F6:1836	OB5956 H1 Ro.raw	1.0022E6	1	1	282	301		P8:Hydroxylation Pro:10.93	PEAKS DB
K.EGALMLPHFN(+.98)SK.A	N	75.32	1343.6543	12	2.6	672.8362	2	31.66	6	F6:1595	OB5956 H1 Ro.raw	1.7726E5	2	2	432	443	Deamidation (NQ)	N10:Deamidation (NQ):1000.00	PEAKS DB
R.VAKISMPVNTPGQFEDFFPASSR.D	N	75.04	2524.2419	23	-2.9	842.4188	3	34.29	5	F5:1735	OB5955 H1 Ro.raw	1.671E6	3	3	279	301			PEAKS DB
K.KNPQLQDLDMMLTC(+57.02)VEIKEGALMLPHFNSK.A	N	74.64	3499.7336	30	-3.9	875.9373	4	36.80	4	F4:1884	OB5954 H1 Ro.raw	6.9756E6	4	4	414	443	Carbamidomethylation	C14:Carbamidomethylation:1000.00	PEAKS DB

total 287 peptides

Peptide	Uniq	-10lgP	Mass	Length	ppm	m/z	z	RT	Fraction	Scan	Source File	Area roasted peanut	#Feature	#Feature roasted peanut	Start	End	PTM	AScore	Found By
R.NTLEAAFN(+.98)AEFNEIRR.V	N	74.52	1894.9172	16	-2.2	948.4638	2	32.35	4	F4:1616	OB5954 H1 Ro.raw	1.3754E6	3	3	313	328		N8:Deamidation (NQ): 48.12	PEAKS DB
R.KSFNLDEGHALRIPSGFISYILNR.H	N	73.99	2746.4553	24	0.1	687.6212	4	36.45	5	F5:1860	OB5955 H1 Ro.raw	1.0287E6	2	2	248	271			PEAKS DB
R.EETSRN(+.98)NPFYFPSRR.F	N	73.61	1899.8864	15	-0.2	634.3026	3	28.39	4	F4:1381	OB5954 H1 Ro.raw	5.4651E5	3	3	161	175		N6:Deamidation (NQ): 33.98	PEAKS DB
R.RVLLEENAGGEQEERGQR.R	N	73.30	2069.0249	18	1.9	690.6835	3	25.60	4	F4:1205	OB5954 H1 Ro.raw	1.8274E6	3	3	328	345			PEAKS DB
K.GTGNLELVAVR.K	N	72.98	1127.6299	11	-1.3	564.8215	2	30.21	4	F4:1476	OB5954 H1 Ro.raw	2.4766E7	4	4	453	463			PEAKS DB
K.EGALM(+15.99)LPHFNSK.A	N	72.82	1358.6653	12	-0.5	680.3396	2	30.10	5	F5:1469	OB5955 H1 Ro.raw	3.1504E6	5	5	432	443	Oxidation (M)	M5:Oxidation (M):1000.00	PEAKS DB
R.IPSGFISYILN(-17.03)R.H	N	72.17	1361.7343	12	-0.6	681.8740	2	38.07	4	F4:1963	OB5954 H1 Ro.raw	1.5056E6	3	3	260	271	Ammonia-loss (N)	N11:Ammonia-loss (N):1000.00	PEAKS PTM
R.IFLAGDKDNVIDQIEKQ(+.98)AK.D	N	71.42	2145.1316	19	1.5	716.0522	3	33.08	5	F5:1661	OB5955 H1 Ro.raw	0	0	0	536	554		Q17:Deamidation (NQ):48.83	PEAKS DB
R.IPSGFISYILNRHDNQNLR.V	N	71.19	2256.1763	19	6.5	753.0709	3	33.61	6	F6:1714	OB5956 H1 Ro.raw	5.9166E7	10	10	260	278			PEAKS DB
K.GTGNLELVAVRK.E	N	70.92	1255.7249	12	0.7	628.8701	2	27.30	4	F4:1290	OB5954 H1 Ro.raw	2.2945E7	7	7	453	464			PEAKS DB
K.KNPQLQDLDMMLTC(+57.02)VEIKEGALM(+15.99)LPHFNSK.A	N	70.54	3515.7285	30	-1.9	879.9377	4	36.03	6	F6:1857	OB5956 H1 Ro.raw	6.9145E6	3	3	414	443	Carbamidomethylation; Oxidation (M)	C14:Carbamidomethylation:1000.00;M23:Oxidation (M):107.40	PEAKS DB
K.NPQLQDLDM(+15.99)MLTC(+57.02)VEIKEGALM(+15.99)LPHFNSK.A	N	70.15	3403.6284	29	1.1	851.9153	4	37.51	4	F4:1883	OB5954 H1 Ro.raw	7.9309E6	6	6	415	443	Carbamidomethylation; Oxidation (M)	M9:Oxidation (M):27.96;C13:Carbamidomethylation:1000.00;M22:Oxidation (M):71.31	PEAKS DB
R.VLLEENAGGEQEERGQR.R	N	69.54	1912.9238	17	0.6	957.4698	2	25.30	5	F5:1191	OB5955 H1 Ro.raw	3.4466E6	6	6	329	345			PEAKS DB
I.PSGFISYILNR.H	N	69.26	1265.6768	11	-5.5	633.8422	2	36.68	4	F4:1876	OB5954 H1 Ro.raw	3.6428E5	2	2	261	271			PEAKS DB

total 287 peptides

Peptide	Uniq	-10lgP	Mass	Length	ppm	m/z	z	RT	Fraction	Scan	Source File	Area roasted peanut	#Feature	#Feature roasted peanut	Start	End	PTM	AScore	Found By
R.DQS(-18.01)SYLQGFSR.N	N	68.93	1268.5785	11	0.2	635.2966	2	30.91	4	F4:1537	OB5954 H1 Ro.raw	6.7379E5	3	3	302	312		S3:Dehydration:40.00	PEAKS PTM
R.IP(+15.99)SGFISYILNR.H	N	68.69	1394.7557	12	1.5	698.3862	2	37.24	5	F5:1913	OB5955 H1 Ro.raw	6.9724E6	9	9	260	271	Hydroxylation Pro	P2:Hydroxylation Pro:1000.00	PEAKS DB
K.NPQLQDLDMM(+15.99)LTC(+57.02)VEIKEGALMLPHFNSK.A	N	68.62	3387.6335	29	-0.8	1130.2175	3	36.42	4	F4:1862	OB5954 H1 Ro.raw	7.1368E7	6	6	415	443	Carbamidomethylation	M10:Oxidation (M):24.44;C13:Carbamidomethylation:1000.00	PEAKS DB
R.RVLLEEN(+.98)AGGEQEER.G	N	68.51	1728.8278	15	-0.8	577.2827	3	25.96	5	F5:1233	OB5955 H1 Ro.raw	1.3591E4	2	2	328	342	Deamidation (NQ)	N7:Deamidation (NQ):77.53	PEAKS DB
R.VAKISM(+15.99)PVNTPGQFEDFFPASSR.D	N	68.50	2540.2368	23	2.1	847.7546	3	32.98	5	F5:1665	OB5955 H1 Ro.raw	5.6098E5	2	2	279	301	Oxidation (M)	M6:Oxidation (M):1000.00	PEAKS DB
R.NNPFYFPSR.R	N	68.49	1140.5352	9	0.9	571.2754	2	31.81	4	F4:1575	OB5954 H1 Ro.raw	1.7544E7	5	5	166	174			PEAKS DB
K.NPQLQDLDMMLTCVEIK(+14.02)EGALMLPHFNSK.A	N	68.29	3328.6328	29	0.9	833.1663	4	38.52	5	F5:1986	OB5955 H1 Ro.raw	1.7448E7	6	6	415	443	Methylation(KR)	K17:Methylation(KR):98.15	PEAKS PTM
R.NNPFYFPSRR.F	N	67.62	1296.6364	10	0.0	649.3254	2	28.95	4	F4:1567	OB5954 H1 Ro.raw	1.3432E8	8	8	166	175			PEAKS DB
R.EETSRNNPFYFPSRR.F	N	67.58	1898.9023	15	1.8	633.9759	3	27.75	5	F5:1339	OB5955 H1 Ro.raw	9.3426E5	3	3	161	175			PEAKS DB
R.IVQIEARPNTLVLPK.H	Y	67.30	1690.0140	15	0.3	846.0145	2	30.46	5	F5:1502	OB5955 H1 Ro.raw	4.7655E7	8	8	208	222			PEAKS DB
K.KNPQLQDLDMMLTCVEIK(+14.02).E	N	66.83	2132.0679	18	-0.6	1067.0405	2	36.31	4	F4:1858	OB5954 H1 Ro.raw	6.1629E5	2	2	414	431	Methylation(KR)	K18:Methylation(KR):130.57	PEAKS PTM
R.IPSGFISY(+125.90)ILNR.H	N	65.90	1504.6575	12	-1.9	753.3346	2	38.39	6	F6:2001	OB5956 H1 Ro.raw	2.2019E5	2	2	260	271	Iodination	Y8:Iodination:1000.00	PEAKS PTM
R.VLLEEN(-17.03)AGGEQEER.G	N	65.50	1554.7162	14	1.2	778.3663	2	27.73	5	F5:1335	OB5955 H1 Ro.raw	0	0	0	329	342	Ammonia-loss (N)	N6:Ammonia-loss (N):1000.00	PEAKS PTM
K.Q(-17.03)AKDLAFPGSGEQVEK.L	N	65.43	1685.8260	16	0.1	843.9204	2	29.98	6	F6:1503	OB5956 H1 Ro.raw	1.0547E5	3	3	552	567	Pyro-glu from Q	Q1:Pyro-glu from Q:1000.00	PEAKS PTM

total 287 peptides

Peptide	Uniq	-10lgP	Mass	Length	ppm	m/z	z	RT	Fraction	Scan	Source File	Area roasted peanut	#Feature	#Feature roasted peanut	Start	End	PTM	AScore	Found By
K.NPQLQDLDM(+15.99)M(+15.99)LTC(+57.02)VEIKEGALMLPHFNSK.A	N	64.96	3403.6284	29	-3.9	1135.5457	3	35.83	6	F6:1849	OB5956 H1 Ro.raw	8.47E6	5	5	415	443	Oxidation (M); Carbamidomethylation	M9:Oxidation (M):54.18;M10:Oxidation (M):63.99;C13:Carbamidomethylation:1000.00	PEAKS DB
R.VLLEENAGGEQEER(+14.02).G	N	64.84	1585.7583	14	0.7	793.8870	2	27.86	4	F4:1339	OB5954 H1 Ro.raw	1.7453E5	3	3	329	342	Methylation(KR)	R14:Methylation(KR):1000.00	PEAKS PTM
I.FLAGDKDNVIDQIEK.Q	N	64.23	1703.8729	15	-5.7	852.9388	2	32.22	6	F6:1635	OB5956 H1 Ro.raw	3.2256E5	3	3	537	551			PEAKS DB
L.AGDKDNVIDQIEK.Q	N	63.80	1443.7205	13	-1.1	722.8667	2	32.43	4	F4:1623	OB5954 H1 Ro.raw	3.3427E5	1	1	539	551			PEAKS DB
K.HADADNILVIQQGQ(+.98)ATVTVANGNNRK.S	N	63.07	2747.3950	26	0.8	916.8063	3	31.39	4	F4:1560	OB5954 H1 Ro.raw	0	0	0	223	248		Q14:Deamidation (N Q):0.00	PEAKS DB
K.AM(+15.99)VIVVVNK.G	N	63.02	987.5787	9	1.1	494.7972	2	27.11	4	F4:1283	OB5954 H1 Ro.raw	1.9071E6	3	3	444	452	Oxidation (M)	M2:Oxidation (M):1000.00	PEAKS DB
K.DLAFP(+15.99)GSGEQVEK.L	N	62.87	1391.6569	13	0.4	696.8360	2	29.40	5	F5:1432	OB5955 H1 Ro.raw	8.022E5	3	3	555	567	Hydroxylation Pro	P5:Hydroxylation Pro:1000.00	PEAKS DB
K.AM(+15.99)VIVVVNKGTGNLELVAVRK.E	N	62.80	2225.2930	21	-4.1	742.7686	3	32.36	6	F6:1577	OB5956 H1 Ro.raw	1.1837E6	2	2	444	464	Oxidation (M)	M2:Oxidation (M):1000.00	PEAKS DB
K.NPQLQDLDMMLTC(+57.02)VEIKEGALM(+15.99)LPHFNSK.A	N	62.74	3387.6335	29	-1.0	1130.2173	3	37.10	5	F5:1900	OB5955 H1 Ro.raw	9.055E7	6	6	415	443	Carbamidomethylation; Oxidation (M)	C13:Carbamidomethylation:1000.00;M22:Oxidation (M):82.05	PEAKS DB
R.SSDNEGVIVK.V	N	62.02	1046.5244	10	0.4	524.2697	2	23.40	5	F5:1065	OB5955 H1 Ro.raw	3.7744E5	3	3	351	360			PEAKS DB
K.QAKDLAFPGSGEQVEK.L	N	61.87	1702.8525	16	1.2	568.6255	3	27.08	6	F6:1321	OB5956 H1 Ro.raw	2.8725E5	6	6	552	567			PEAKS DB
K.SFNLD(-18.01)EGHALR.I	N	61.63	1239.5996	11	2.9	620.8089	2	28.79	4	F4:1400	OB5954 H1 Ro.raw	0	0	0	249	259		D5:Dehydration:49.79	PEAKS PTM
K.SFNLDEGHALR(+31.99)IPSGFISYILNR.H	N	60.54	2650.3503	23	0.1	884.4575	3	39.14	5	F5:2021	OB5955 H1 Ro.raw	5.6521E5	1	1	249	271		R11:Dihydroxy:46.57	PEAKS PTM
Q.SSYLQGFSR.N	N	60.35	1043.5035	9	0.6	522.7593	2	29.24	6	F6:1453	OB5956 H1 Ro.raw	6.7195E4	3	3	304	312			PEAKS DB

total 287 peptides

Peptide	Uniq	-10lgP	Mass	Length	ppm	m/z	z	RT	Fraction	Scan	Source File	Area roasted peanut	#Feature	#Feature roasted peanut	Start	End	PTM	AScore	Found By
K.NPQLQDLDMM(+15.99)LTC(+57.02)VEIKEGALM(+15.99)LPHFNSK.A	N	60.02	3403.6284	29	-8.2	1135.5408	3	36.74	5	F5:1878	OB5955 H1 Ro.raw	6.6522E6	6	6	415	443	Carbamidomethylation; Oxidation (M)	M10:Oxidation (M):21.94;C13:Carbamidomethylation:1000.00;M22:Oxidation (M):61.26	PEAKS DB
K.QFQNLQNHR.I	N	59.95	1183.5846	9	0.0	592.7996	2	23.81	5	F5:1102	OB5955 H1 Ro.raw	1.3905E5	5	5	199	207			PEAKS DB
K.Q(-17.03)FQNLQNHR.I	N	59.90	1166.5581	9	3.5	584.2884	2	28.04	4	F4:1346	OB5954 H1 Ro.raw	5.0983E6	3	3	199	207	Pyro-glu from Q	Q1:Pyro-glu from Q:1000.00	PEAKS PTM
R.I(+41.03)P(+15.99)SGFISYILNR.H	N	59.87	1435.7823	12	-3.6	718.8959	2	36.92	6	F6:1901	OB5956 H1 Ro.raw	1.7838E7	3	3	260	271	Amidination of lysines or N-terminal amines with methyl acetimidate; Hydroxylation Pro	I1:Amidination of lysines or N-terminal amines with methyl acetimidate:1000.00;P2:Hydroxylation Pro:1000.00	PEAKS PTM
K.KNPQLQDLDM(+15.99)MLTC(+57.02)VEIKEGALM(+15.99)LPHFNSK.A	N	59.67	3531.7234	30	-1.9	883.9364	4	35.86	4	F4:1821	OB5954 H1 Ro.raw	2.0004E6	3	3	414	443	Carbamidomethylation; Oxidation (M)	M10:Oxidation (M):17.01;C14:Carbamidomethylation:1000.00;M23:Oxidation (M):101.58	PEAKS DB
K.NPQLQ(+.98)DLDMMLTC(+57.02)VEIK.E	N	59.63	2047.9629	17	1.5	1024.9902	2	44.46	6	F6:2348	OB5956 H1 Ro.raw	0	0	0	415	431	Carbamidomethylation	Q5:Deamidation (NQ):19.68;C13:Carbamidomethylation:1000.00	PEAKS DB
R.IPSGFISYILN(+.98)RHDNQNLR.V	N	59.62	2257.1604	19	4.7	753.3976	3	34.29	5	F5:1740	OB5955 H1 Ro.raw	6.672E5	1	1	260	278		N11:Deamidation (NQ):36.24	PEAKS DB
K.EGALMLPH(+14.02)FNSK.A	N	59.24	1356.6860	12	-3.0	679.3483	2	30.80	5	F5:1531	OB5955 H1 Ro.raw	3.037E5	3	3	432	443	Methylation(others)	H8:Methylation(others):65.81	PEAKS PTM

total 287 peptides

Peptide	Uniq	-10lgP	Mass	Length	ppm	m/z	z	RT	Fraction	Scan	Source File	Area roasted peanut	#Feature	#Feature roasted peanut	Start	End	PTM	AScore	Found By
A.FPGSGEQVEK.L	N	58.82	1076.5138	10	-3.4	539.2623	2	30.34	6	F6:1519	OB5956 H1 Ro.raw	4.3607E5	3	3	558	567			PEAKS DB
K.ISM(-48.00)PVNTPGQFEDFFPASSR.D	N	58.66	2178.0381	20	2.8	727.0220	3	32.48	6	F6:1652	OB5956 H1 Ro.raw	6.4552E6	4	4	282	301	Dethiomethyl	M3:Dethiomethyl:1000.00	PEAKS PTM
R.I(+27.99)PSGFISYILNR.H	N	58.63	1406.7557	12	0.7	704.3856	2	42.27	5	F5:2199	OB5955 H1 Ro.raw	0	0	0	260	271	Formylation	I1:Formylation:1000.00	PEAKS PTM
R.LFEVKPDKKNPQLQDLDMM(+15.99)LTC(+57.02)VEIK.E	N	58.02	3147.6018	26	2.2	787.9095	4	33.24	5	F5:1670	OB5955 H1 Ro.raw	2.286E6	2	2	406	431	Carbamidomethylation	M19:Oxidation (M):40.00;C22:Carbamidomethylation:1000.00	PEAKS DB
R.EGEQEWGTPGS(+42.01)EVR.E	Y	57.75	1601.6958	14	7.6	801.8613	2	27.46	6	F6:1334	OB5956 H1 Ro.raw	1.0047E5	3	3	147	160		S11:Acetylation (TSCYH):32.97	PEAKS PTM
K.SFN(+15.99)LDEGHALR.I	N	57.68	1273.6051	11	0.9	637.8104	2	27.50	4	F4:1324	OB5954 H1 Ro.raw	9.739E5	3	3	249	259	Oxidation or Hydroxylation	N3:Oxidation or Hydroxylation:55.92	PEAKS PTM
F.NLDEGHALR.I	N	57.57	1023.5097	9	0.6	512.7625	2	27.93	5	F5:1349	OB5955 H1 Ro.raw	2.6681E6	3	3	251	259			PEAKS DB
K.A(+41.03)M(+15.99)VIVVVNKGTGNLELVAVR.K	N	57.55	2138.2246	20	-5.4	1070.1138	2	34.47	5	F5:1739	OB5955 H1 Ro.raw	4.5183E6	3	3	444	463	Amidination of lysines or N-terminal amines with methyl acetimidate; Oxidation (M)	A1:Amidination of lysines or N-terminal amines with methyl acetimidate:207.56;M2:Oxidation (M):1000.00	PEAKS PTM
K.NPQLQDLDMMLTC(+71.04)VEIK.E	N	57.51	2060.9944	17	3.0	1031.5076	2	37.24	5	F5:1915	OB5955 H1 Ro.raw	1.057E5	2	2	415	431	Propionamide	C13:Propionamide:1000.00	PEAKS PTM
A.GDKDNVIDQIEK.Q	N	57.39	1372.6833	12	-1.5	687.3479	2	32.35	4	F4:1613	OB5954 H1 Ro.raw	1.1865E6	3	3	540	551			PEAKS DB
R.K(+43.01)(+14.02)SFNLDEGHALR.I	N	55.95	1442.7266	12	0.0	722.3705	2	26.25	5	F5:1250	OB5955 H1 Ro.raw	4.2824E5	6	6	248	259	Carbamylation; Methylation(KR)	K1:Carbamylation:1000.00;K1:Methylation(KR):156.90	PEAKS PTM
R.IPSGFISYILNRHDN(+.98)QNLR.V	N	55.73	2257.1604	19	-0.7	753.3936	3	34.26	4	F4:1732	OB5954 H1 Ro.raw	0	0	0	260	278		N15:Deamidation (NQ):0.00	PEAKS DB

total 287 peptides

Peptide	Uniq	-10lgP	Mass	Length	ppm	m/z	z	RT	Fraction	Scan	Source File	Area roasted peanut	#Feature	#Feature roasted peanut	Start	End	PTM	AScore	Found By
K.ISMPVN(+.98)TPGQFEDFFPASSR.D	N	54.95	2227.0254	20	6.6	1114.5273	2	36.77	4	F4:1885	OB5954 H1 Ro.raw	1.011E5	2	2	282	301		N6:Deamidation (NQ): 42.68	PEAKS DB
R.K(+27.99)SFNLDEGHALR.I	N	54.77	1413.7001	12	1.4	707.8583	2	28.46	5	F5:1387	OB5955 H1 Ro.raw	5.5831E4	2	2	248	259	Formylation	K1:Formylation:1000.00	PEAKS PTM
R.LFEVKPDKK.N	N	54.58	1102.6385	9	1.1	552.3271	2	24.84	4	F4:1157	OB5954 H1 Ro.raw	1.1453E6	6	6	406	414			PEAKS DB
K.NPQLQDLDMMLTC(+57.02)VEIKEGALMLP(+15.99)HFNSK.A	N	54.18	3387.6335	29	-0.6	1130.2178	3	37.70	4	F4:2016	OB5954 H1 Ro.raw	4.3311E7	3	3	415	443	Carbamidomethylation; Hydroxylation Pro	C13:Carbamidomethylation:1000.00; P24:Hydroxylation Pro:122.99	PEAKS DB
R.EGEQEWGTP(+15.99)GSEVREETSR.N	Y	54.09	2177.9460	19	2.3	1089.9828	2	26.43	5	F5:1263	OB5955 H1 Ro.raw	1.3271E7	6	6	147	165	Hydroxylation Pro	P9:Hydroxylation Pro: 1000.00	PEAKS DB
R.IFLAGDKDNVIDQIEK(+42.01)Q(+.98)AK(+14.02).D	N	53.39	2201.1580	19	2.9	734.7288	3	32.42	5	F5:1627	OB5955 H1 Ro.raw	4.6872E5	1	1	536	554		K16:Acetylation (K): 4.05;Q17:Deamidation (NQ): 20.26; K19:Methylation(KR): 4.05	PEAKS PTM
E.PDLSNNFGR.L	N	53.18	1018.4832	9	0.9	510.2493	2	31.57	6	F6:1584	OB5956 H1 Ro.raw	1.3909E6	4	4	397	405			PEAKS DB
K.NPQLQDLDMM(+15.99)LTCVEIK(+14.02).E	N	53.12	2019.9679	17	1.7	1010.9930	2	35.46	5	F5:1802	OB5955 H1 Ro.raw	4.2793E5	1	1	415	431	Methylation(KR)	M10:Oxidation (M):8.14;K17: Methylation(KR):1000.00	PEAKS PTM
R.IFLAGDKDNVIDQ(+.98)IEKQAK.D	N	53.10	2145.1316	19	1.4	716.0521	3	33.04	6	F6:1686	OB5956 H1 Ro.raw	1.2146E5	1	1	536	554		Q13:Deamidation (NQ):25.18	PEAKS DB
K.SFNLDEGHALRIP(+15.99)SGFISYILNR.H	N	53.04	2634.3552	23	-3.9	879.1223	3	38.76	6	F6:2023	OB5956 H1 Ro.raw	1.5218E6	3	3	249	271	Hydroxylation Pro	P13:Hydroxylation Pro:1000.00	PEAKS DB
R.VAKIS(+77.99)MPVNTPGQFEDFFPASSR.D	N	52.77	2602.2290	23	2.9	1302.1256	2	38.06	5	F5:1958	OB5955 H1 Ro.raw	0	0	0	279	301	Methylphosphonylation	S5:Methylphosphonylation:56.99	PEAKS PTM
R.EQEWEEEEEDEEEEGSNREVRR.Y	N	52.77	2821.1545	22	1.7	941.3937	3	26.62	5	F5:1273	OB5955 H1 Ro.raw	8.8078E5	3	3	473	494			PEAKS DB

total 287 peptides

Peptide	Uniq	-10lgP	Mass	Length	ppm	m/z	z	RT	Fraction	Scan	Source File	Area roasted peanut	#Feature	#Feature roasted peanut	Start	End	PTM	AScore	Found By
K.QFQNLQN(+.98)HR.I	N	52.53	1184.5686	9	5.4	593.2948	2	23.60	6	F6:1114	OB5956 H1 Ro.raw	8.0383E2	1	1	199	207		N7:Deamidation (NQ): 0.00	PEAKS DB
N.PFYFPSRR.F	N	52.44	1068.5504	8	-0.1	535.2824	2	28.89	6	F6:1431	OB5956 H1 Ro.raw	4.3491E6	4	4	168	175			PEAKS DB
K.NPQLQDLDM(+15.99)M(+15.99)LTC(+57.02)VEIKEGALM(+15.99)LPHFNSK.A	N	52.35	3419.6233	29	-0.9	855.9124	4	34.83	5	F5:1766	OB5955 H1 Ro.raw	1.7196E6	3	3	415	443	Oxidation (M); Carbamidomethylation	M9:Oxidation (M):1000.00; M10:Oxidation (M):1000.00; C13:Carbamidomethylation:1000.00; M22:Oxidation (M):1000.00	PEAKS DB
R.VAK(+27.99)ISMPVNTPGQFEDFFPASSR.D	N	52.16	2552.2368	23	-0.3	1277.1252	2	36.45	6	F6:1882	OB5956 H1 Ro.raw	0	0	0	279	301		K3:Formylation:9.40	PEAKS PTM
K.ISMPVNTPGQFEDFFPASSRDQSSYLQGFSR.N	N	52.15	3494.6201	31	-0.9	1165.8796	3	36.01	6	F6:1851	OB5956 H1 Ro.raw	4.0537E6	2	2	282	312			PEAKS DB
R.V(+27.99)AKISMPVNTPGQFEDFFPASSR.D	N	51.59	2552.2368	23	-0.5	1277.1250	2	36.43	4	F4:1865	OB5954 H1 Ro.raw	7.7611E5	1	1	279	301		V1:Formylation:0.00	PEAKS PTM
R.WGPAEPR.E	Y	51.54	811.3976	7	1.8	406.7068	2	25.60	4	F4:1224	OB5954 H1 Ro.raw	1.6393E6	3	3	109	115			PEAKS DB
K.SFNLDEGHALR(+31.99).I	N	50.82	1289.6000	11	-1.4	645.8063	2	30.41	6	F6:1520	OB5956 H1 Ro.raw	0	0	0	249	259	Dihydroxy	R11:Dihydroxy:122.75	PEAKS PTM
R.IFLAGD(+15.99)KDNVIDQIEK.Q	N	50.66	1832.9519	16	-1.9	917.4814	2	31.99	4	F4:1600	OB5954 H1 Ro.raw	1.5111E6	3	3	536	551		D6:Oxidation or Hydroxylation:12.60	PEAKS PTM
R.VLLEE(+53.92)NAGGEQEER.G	N	50.61	1625.6620	14	-1.0	542.8940	3	26.76	5	F5:1278	OB5955 H1 Ro.raw	0	0	0	329	342		E5:Replacement of 2 protons by iron: 20.92	PEAKS PTM
P.SGFISYILNR.H	N	50.48	1168.6240	10	1.5	585.3202	2	35.08	6	F6:1803	OB5956 H1 Ro.raw	3.0349E5	1	1	262	271			PEAKS DB
R.IFLAGDKDNVIDQIEK(+14.02)Q(+.98)AK(+42.01).D	N	50.18	2201.1580	19	5.4	734.7306	3	32.48	6	F6:1657	OB5956 H1 Ro.raw	3.9073E5	1	1	536	554	Deamidation (NQ)	K16:Methylation(KR): 7.64;Q17:Deamidation (NQ): 58.99; K19:Acetylation (K): 7.64	PEAKS PTM

total 287 peptides

Peptide	Uniq	-10lgP	Mass	Length	ppm	m/z	z	RT	Fraction	Scan	Source File	Area roasted peanut	#Feature	#Feature roasted peanut	Start	End	PTM	AScore	Found By
K.S(+43.01)(+14.02)FNLDEGHALR.I	N	50.01	1314.6316	11	-0.5	658.3228	2	28.11	5	F5:1359	OB5955 H1 Ro.raw	1.9424E6	1	1	249	259	Carbamylation; Methylation(others)	S1:Carbamylation:1000.00; S1:Methylation(others):88.98	PEAKS PTM
N.LDEGHALR.I	N	49.91	909.4668	8	0.0	455.7407	2	27.93	5	F5:1350	OB5955 H1 Ro.raw	1.94E5	3	3	252	259			PEAKS DB
K.SFNLDEGH(+15.99)ALR(+31.99)IPSGFISYILNR.H	N	49.58	2666.3452	23	1.2	889.7900	3	39.33	6	F6:2062	OB5956 H1 Ro.raw	3.3065E5	2	2	249	271	Oxidation (HW)	H8:Oxidation (HW):1000.00; R11:Dihydroxy:45.01	PEAKS PTM
K.SFNLDEGH(+15.99)ALRIPSGFISYILNR.H	N	49.38	2634.3555	23	2.3	1318.1880	2	38.82	5	F5:2002	OB5955 H1 Ro.raw	9.0457E5	1	1	249	271	Oxidation (HW)	H8:Oxidation (HW):1000.00	PEAKS PTM
R.ESHFVSARPQSQSP(+15.99)SSPEKEDQEEEN.Q	N	49.34	2973.2859	26	1.6	992.1041	3	24.81	6	F6:1186	OB5956 H1 Ro.raw	3.8098E5	2	2	574	599		P14:Hydroxylation Pro:11.12	PEAKS DB
R.VLLE(+21.98)ENAGGEQEER.G	N	48.97	1593.7246	14	-0.5	797.8692	2	26.81	5	F5:1275	OB5955 H1 Ro.raw	4.3339E5	3	3	329	342		E4:Sodium adduct:40.00	PEAKS PTM
R.LFEVKPDK.K	N	48.69	974.5436	8	1.7	488.2799	2	25.30	5	F5:1183	OB5955 H1 Ro.raw	1.7748E6	6	6	406	413			PEAKS DB
K.NPQLQDLDMM(+15.99)LTC(+57.02)VEIKEGALMLPHFN(+.98)SK.A	N	48.63	3388.6174	29	6.2	848.1669	4	36.39	6	F6:1880	OB5956 H1 Ro.raw	2.0276E6	1	1	415	443	Carbamidomethylation	M10:Oxidation (M):27.96;C13:Carbamidomethylation:1000.00; N27:Deamidation (NQ):18.27	PEAKS DB
R.SKQFQNLQNHR.I	Y	48.58	1398.7117	11	-0.5	467.2443	3	24.46	4	F4:1146	OB5954 H1 Ro.raw	1.2409E5	3	3	197	207			PEAKS DB
R.IVQIEARPN.T	Y	48.42	1038.5822	9	0.7	520.2987	2	25.99	4	F4:1238	OB5954 H1 Ro.raw	4.8023E4	3	3	208	216			PEAKS DB
R.N(+27.99)TLEAAFNAEFNEIRR.V	N	48.34	1921.9282	16	-1.7	961.9697	2	37.51	4	F4:1908	OB5954 H1 Ro.raw	4.2206E4	1	1	313	328	Formylation	N1:Formylation:1000.00	PEAKS PTM
R.NT(-18.01)LEAAFNAEFNEIRR.V	N	48.27	1875.9227	16	-1.8	626.3137	3	34.20	4	F4:1731	OB5954 H1 Ro.raw	1.108E7	3	3	313	328	Dehydration	T2:Dehydration:1000.00	PEAKS PTM

total 287 peptides

Peptide	Uniq	-10lgP	Mass	Length	ppm	m/z	z	RT	Fraction	Scan	Source File	Area roasted peanut	#Feature	#Feature roasted peanut	Start	End	PTM	AScore	Found By
R.LFEVKPDKKNPQLQDLDM(+15.99)MLTC(+57.02)VEIK.E	N	48.12	3147.6018	26	-1.9	787.9062	4	34.20	4	F4:1734	OB5954 H1 Ro.raw	2.5552E6	4	4	406	431	Carbamidomethylation	M18:Oxidation (M):33.98;C22:Carbamidomethylation:1000.00	PEAKS DB
K.KNPQLQDLDMM(+15.99)LTC(+57.02)VEIKEGALM(+15.99)LPHFNSK.A	N	47.73	3531.7234	30	-1.1	883.9372	4	35.80	5	F5:1822	OB5955 H1 Ro.raw	0	0	0	414	443	Carbamidomethylation	M11:Oxidation (M):0.00;C14:Carbamidomethylation:1000.00;M23:Oxidation (M):45.87	PEAKS DB
R.DQSSY(-18.01)LQGFSR.N	N	47.62	1268.5785	11	-0.6	635.2961	2	30.85	6	F6:1579	OB5956 H1 Ro.raw	2.6456E5	1	1	302	312		Y5:Dehydration:0.00	PEAKS PTM
K.KGSEEEDIT(-18.01)N(+.98)PINLR.D	N	47.57	1696.8268	15	2.3	566.6175	3	29.07	6	F6:1442	OB5956 H1 Ro.raw	3.8587E5	3	3	379	393	Deamidation (NQ)	T9:Dehydration:45.01;N10:Deamidation (NQ):59.10	PEAKS PTM
R.NTLEAAFNAEFN(+.98)EIR.R	N	47.43	1738.8162	15	9.2	870.4233	2	44.36	5	F5:2303	OB5955 H1 Ro.raw	5.1969E3	1	1	313	327		N12:Deamidation (NQ):0.00	PEAKS DB
K.SFNLDEGHALRIPSGFISYILNRHDNQNLR.V	N	47.02	3495.7759	30	-2.1	700.1610	5	35.83	6	F6:1842	OB5956 H1 Ro.raw	2.2604E6	2	2	249	278			PEAKS DB
K.NPQLQ(+.98)DLDM(+15.99)MLTC(+57.02)VEIKEGALM(+15.99)LPHFNSK.A	N	47.00	3404.6125	29	-0.4	1135.8777	3	36.69	5	F5:1878	OB5955 H1 Ro.raw	3.8413E5	1	1	415	443	Carbamidomethylation	Q5:Deamidation (NQ):45.47;M9:Oxidation (M):0.00;C13:Carbamidomethylation:1000.00;M22:Oxidation (M):38.75	PEAKS DB
K.D(+43.01)(+14.02)LAFPGSGEQVEK.L	N	46.93	1432.6833	13	-0.1	717.3489	2	31.08	4	F4:1535	OB5954 H1 Ro.raw	9.9373E5	3	3	555	567	Carbamylation; Methylation(others)	D1:Carbamylation:220.79;D1:Methylation (others):132.53	PEAKS PTM

total 287 peptides

Peptide	Uniq	-10lgP	Mass	Length	ppm	m/z	z	RT	Fraction	Scan	Source File	Area roasted peanut	#Feature	#Feature roasted peanut	Start	End	PTM	AScore	Found By
K.SFNLDEGHALR(+14.02)IPSGFISYILN(+.98)R.H	N	46.86	2633.3601	23	-6.4	878.7883	3	36.34	5	F5:1848	OB5955 H1 Ro.raw	1.8431E5	2	2	249	271	Deamidation (NQ)	R11:Methylation(KR):28.64;N22:Deamidation (NQ):57.71	PEAKS PTM
F.PGSGEQVEK.L	N	46.51	929.4454	9	-3.7	465.7282	2	30.34	6	F6:1524	OB5956 H1 Ro.raw	5.467E4	1	1	559	567			PEAKS DB
R.ESHFVSARPQSQSP(+15.99)SSPEKEDQEEE.N	N	46.47	2859.2429	25	2.4	954.0906	3	24.92	5	F5:1173	OB5955 H1 Ro.raw	7.3448E4	1	1	574	598		P14:Hydroxylation Pro:26.52	PEAKS DB
R.DQSSYLQGFSR(+15.99).N	N	46.23	1302.5840	11	2.5	652.3009	2	30.63	5	F5:1518	OB5955 H1 Ro.raw	1.9563E5	4	4	302	312		R11:Oxidation or Hydroxylation:42.88	PEAKS PTM
L.DEGHALR.I	N	45.97	796.3828	7	2.5	399.1996	2	27.93	5	F5:1351	OB5955 H1 Ro.raw	9.4873E4	3	3	253	259			PEAKS DB
R.LFEVKPDKKNPQLQ(+.98)DLDMM(+15.99)LTC(+57.02)VEIK.E	N	45.61	3148.5857	26	7.4	788.1595	4	33.17	5	F5:1670	OB5955 H1 Ro.raw	4.4314E5	1	1	406	431	Carbamidomethylation	Q14:Deamidation (NQ):0.00;M19:Oxidation (M):23.10;C22:Carbamidomethylation:1000.00	PEAKS DB
S.F(+43.01)NLDE(+21.98)GHALRIPSGFISYILNR.H	N	45.55	2596.3162	22	7.8	866.4528	3	40.28	6	F6:2103	OB5956 H1 Ro.raw	2.2619E6	3	3	250	271	Carbamylation	F1:Carbamylation:1000.00;E5:Sodium adduct:0.00	PEAKS PTM
R.IFLAGDKDNVID.Q	N	45.24	1318.6769	12	0.0	660.3457	2	31.81	4	F4:1586	OB5954 H1 Ro.raw	1.1587E5	3	3	536	547			PEAKS DB
K.KNPQLQDLDM(+15.99)MLTC(+57.02)VEIKEGALMLPHFNSK.A	N	45.13	3515.7285	30	-1.9	879.9377	4	35.30	4	F4:1798	OB5954 H1 Ro.raw	6.4172E5	1	1	414	443	Carbamidomethylation	M10:Oxidation (M):14.02;C14:Carbamidomethylation:1000.00	PEAKS DB
R.NTLEAAFNAEFN(+.98)EIRR.V	N	45.08	1894.9172	16	-4.2	948.4619	2	33.08	4	F4:1691	OB5954 H1 Ro.raw	2.2628E6	3	3	313	328		N12:Deamidation (NQ):39.02	PEAKS DB
R.IFLAGDK(+15.99)DNVIDQIEK.Q	N	44.85	1832.9519	16	0.9	917.4841	2	31.94	6	F6:1617	OB5956 H1 Ro.raw	3.7677E5	1	1	536	551		K7:Oxidation or Hydroxylation:17.01	PEAKS PTM
V.QIEARPNTLVLPK.H	Y	44.77	1477.8616	13	-1.8	739.9368	2	30.56	4	F4:1513	OB5954 H1 Ro.raw	1.5072E5	3	3	210	222			PEAKS DB
**total 287 peptides**																			

Peptide	Uniq	-10lgP	Mass	Length	ppm	m/z	z	RT	Fraction	Scan	Source File	Area roasted peanut	#Feature	#Feature roasted peanut	Start	End	PTM	AScore	Found By
R.KSFNLDEGH(+15.99)ALR.I	N	44.62	1401.7001	12	3.9	701.8600	2	27.64	6	F6:1349	OB5956 H1 Ro.raw	7.3368E4	2	2	248	259	Oxidation (HW)	H9:Oxidation (HW):1000.00	PEAKS PTM
R.N(+.98)TLEAAFNAEFNEIRR.V	N	44.42	1894.9172	16	3.8	948.4695	2	36.66	6	F6:1890	OB5956 H1 Ro.raw	1.2834E6	2	2	313	328		N1:Deamidation (NQ): 41.52	PEAKS DB
R.ESHFVSARP(+15.99)QSQSPSSPEKEDQEEEN.Q	N	44.15	2973.2859	26	2.8	992.1053	3	24.92	5	F5:1161	OB5955 H1 Ro.raw	1.8315E5	1	1	574	599		P9:Hydroxylation Pro: 18.35	PEAKS DB
K.AMVIVVVN(+.98)KGTGNLELVAVRK.E	N	43.52	2210.2820	21	1.9	1106.1504	2	34.35	5	F5:1720	OB5955 H1 Ro.raw	6.7558E5	2	2	444	464		N8:Deamidation (NQ): 28.79	PEAKS DB
R.IFLAGDKDNVIDQIEK(+14.02)(+43.01).Q	N	43.38	1873.9785	16	-1.3	937.9953	2	32.42	5	F5:1608	OB5955 H1 Ro.raw	8.0571E6	1	1	536	551	Methylation(C-term); Carbamylation	K16:Methylation(C-term):1000.00;K16:Carbamylation:128.00	PEAKS PTM
R.D(-18.01)QSSYLQGFSR.N	N	43.26	1268.5785	11	1.6	635.2975	2	29.92	6	F6:1489	OB5956 H1 Ro.raw	0	0	0	302	312		D1:Dehydration:0.00	PEAKS PTM
R.LFEVKPDKKNPQLQDLDM(+15.99)M(+15.99)LTC(+57.02)VEIK.E	N	43.03	3163.5967	26	-3.4	791.9037	4	32.67	6	F6:1663	OB5956 H1 Ro.raw	2.1471E6	3	3	406	431	Oxidation (M); Carbamidomethylation	M18:Oxidation (M):1000.00; M19:Oxidation (M):1000.00; C22:Carbamidomethylation:1000.00	PEAKS DB
T.GNLELVAVRK.E	N	43.00	1097.6556	10	0.5	549.8354	2	27.00	5	F5:1296	OB5955 H1 Ro.raw	3.6527E5	3	3	455	464			PEAKS DB
K.KN(+.98)PQLQ(+.98)DLDMMLTC(+57.02)VEIKEGALMLPHFNSK.A	N	42.92	3501.7017	30	7.8	876.4395	4	36.93	6	F6:1903	OB5956 H1 Ro.raw	5.6427E6	1	1	414	443	Carbamidomethylation	N2:Deamidation (NQ): 14.04; Q6:Deamidation (NQ): 0.00;C14:Carbamidomethylation:1000.00	PEAKS DB
R.IFLAGDKDNVIDQIEK(+43.01)QAK(+14.02).D	N	42.77	2201.1692	19	0.9	734.7310	3	32.71	4	F4:1644	OB5954 H1 Ro.raw	3.8417E5	1	1	536	554		K16:Carbamylation:0.00;K19:Methylation (KR):0.00	PEAKS PTM

total 287 peptides

Peptide	Uniq	-10lgP	Mass	Length	ppm	m/z	z	RT	Fraction	Scan	Source File	Area roasted peanut	#Feature	#Feature roasted peanut	Start	End	PTM	AScore	Found By
K.S(+43.01)(+14.02)FNLDEGHALRIPSGFISYILNR.H	N	42.43	2675.3818	23	-1.9	892.7996	3	37.80	6	F6:1964	OB5956 H1 Ro.raw	0	0	0	249	271		S1:Carbamylation:1000.00; S1:Methylation(others):34.30	PEAKS PTM
K.GSEEEDITNPIN(+.98)LRDGEPDLSNNFGR.L	N	42.34	2888.3059	26	6.7	1445.1699	2	32.90	4	F4:1642	OB5954 H1 Ro.raw	1.8658E5	1	1	380	405		N12:Deamidation (NQ):12.33	PEAKS DB
R.V(+43.01)LLE(+14.02)ENAGGEQEER.G	N	42.24	1628.7642	14	1.7	815.3907	2	27.00	5	F5:1297	OB5955 H1 Ro.raw	4.2233E5	3	3	329	342	Carbamylation	V1:Carbamylation:1000.00;E4:Methylation(others):30.46	PEAKS PTM
R.I(+43.01)FLAGDK(+14.02)DNVIDQIEK.Q	N	42.15	1873.9785	16	4.9	938.0011	2	32.57	4	F4:1632	OB5954 H1 Ro.raw	0	0	0	536	551	Carbamylation; Methylation(KR)	I1:Carbamylation:135.80;K7:Methylation(KR):195.94	PEAKS PTM
K.D(+15.99)LAFPGSGEQVEK.L	N	42.08	1391.6569	13	0.4	696.8360	2	29.40	5	F5:1368	OB5955 H1 Ro.raw	5.511E5	2	2	555	567	Oxidation or Hydroxylation	D1:Oxidation or Hydroxylation:77.13	PEAKS PTM
K.KNPQLQDLDMM(+15.99)LTC(+57.02)VEIKEGALMLPHFNSK.A	N	42.02	3515.7285	30	1.3	879.9406	4	35.25	5	F5:1790	OB5955 H1 Ro.raw	0	0	0	414	443	Carbamidomethylation	M11:Oxidation (M):12.28;C14:Carbamidomethylation:1000.00	PEAKS DB
K.A(+41.03)M(+15.99)VIVVVNKGTGNLELVAVRK.E	N	41.92	2266.3196	21	0.6	1134.1677	2	32.98	5	F5:1660	OB5955 H1 Ro.raw	1.5326E7	6	6	444	464	Amidination of lysines or N-terminal amines with methyl acetimidate; Oxidation (M)	A1:Amidination of lysines or N-terminal amines with methyl acetimidate:131.34;M2:Oxidation (M):1000.00	PEAKS PTM
K.EGALM(+15.99)LPHFN(+.98)SK.A	N	41.90	1359.6493	12	2.8	680.8338	2	31.22	4	F4:1550	OB5954 H1 Ro.raw	0	0	0	432	443	Oxidation (M); Deamidation (NQ)	M5:Oxidation (M):1000.00;N10:Deamidation (NQ):1000.00	PEAKS DB

total 287 peptides

Peptide	Uniq	-10lgP	Mass	Length	ppm	m/z	z	RT	Fraction	Scan	Source File	Area roasted peanut	#Feature	#Feature roasted peanut	Start	End	PTM	AScore	Found By
K.NPQLQDLDM(-48.00)MLTC(+57.02)VEIK.E	N	41.66	1998.9755	17	6.7	667.3369	3	34.52	6	F6:1773	OB5956 H1 Ro.raw	2.0007E6	2	2	415	431	Carbamidomethylation	M9:Dethiomethyl:11.06;C13:Carbamidomethylation:1000.00	PEAKS PTM
K.GTGNLELVAVRKEQQQR.G	N	41.33	1925.0442	17	1.4	642.6896	3	26.67	6	F6:1294	OB5956 H1 Ro.raw	2.686E4	1	1	453	469			PEAKS DB
K.NPQLQDLDMMLTC(+57.02)VEIK(+43.01)EGALMLPHFNSK(+14.02).A	N	41.19	3428.6602	29	-1.5	858.1710	4	37.84	6	F6:1971	OB5956 H1 Ro.raw	2.9855E6	1	1	415	443	Carbamidomethylation	C13:Carbamidomethylation:1000.00;K17:Carbamylation:11.67;K29:Methylation(KR):11.67	PEAKS PTM
R.IFLAGDKDN(+.98)VIDQIEKQ(+.98)AK.D	N	41.17	2146.1157	19	9.8	716.3862	3	32.98	5	F5:1661	OB5955 H1 Ro.raw	1.2092E5	1	1	536	554		N9:Deamidation (NQ):13.83;Q17:Deamidation (NQ):17.65	PEAKS DB
K.NPQ(+.98)LQDLDMMLTC(+57.02)VEIK.E	N	41.15	2047.9629	17	2.9	1024.9917	2	46.45	4	F4:2441	OB5954 H1 Ro.raw	0	0	0	415	431	Carbamidomethylation	Q3:Deamidation (NQ):14.04;C13:Carbamidomethylation:1000.00	PEAKS DB
R.EQEWEEEEEDEEEEGSNREVR.R	N	41.08	2665.0535	21	2.4	889.3605	3	27.88	4	F4:1353	OB5954 H1 Ro.raw	4.8884E4	1	1	473	493			PEAKS DB
R.VLLEEN(+.98)AGGEQEERGQR.R	N	40.76	1913.9078	17	-1.6	638.9755	3	25.99	4	F4:1234	OB5954 H1 Ro.raw	0	0	0	329	345		N6:Deamidation (NQ):28.79	PEAKS DB
K.GT(-18.01)GNLELVAVRK.E	N	40.55	1237.7142	12	2.6	413.5797	3	27.00	5	F5:1287	OB5955 H1 Ro.raw	9.9167E5	2	2	453	464	Dehydration	T2:Dehydration:1000.00	PEAKS PTM
K.KNPQLQ(+.98)DLDMMLTC(+57.02)VEIKEGALMLPHFNSK.A	N	40.26	3500.7175	30	3.4	876.1896	4	37.51	4	F4:1896	OB5954 H1 Ro.raw	1.6398E6	1	1	414	443	Carbamidomethylation	Q6:Deamidation (NQ):12.19;C14:Carbamidomethylation:1000.00	PEAKS DB
R.EGEQEW(+15.99)GTPGSEVREETSR.N	Y	39.91	2177.9460	19	1.6	726.9905	3	26.43	5	F5:1304	OB5955 H1 Ro.raw	1.2886E7	4	4	147	165	Oxidation (HW)	W6:Oxidation (HW):1000.00	PEAKS PTM

total 287 peptides

Peptide	Uniq	-10lgP	Mass	Length	ppm	m/z	z	RT	Fraction	Scan	Source File	Area roasted peanut	#Feature	#Feature roasted peanut	Start	End	PTM	AScore	Found By
R.IFLAGD(+14.02)K(+43.01)DNVIDQIEK.Q	N	39.86	1873.9785	16	1.7	937.9982	2	32.48	6	F6:1653	OB5956 H1 Ro.raw	9.6228E6	3	3	536	551	Carbamylation	D6:Methylation (others):18.53;K7:Carbamylation:62.41	PEAKS PTM
R.E(-18.01)GEQEWGTPGSEVREETSR.N	Y	39.24	2143.9407	19	2.7	1072.9805	2	29.74	5	F5:1461	OB5955 H1 Ro.raw	2.5488E5	3	3	147	165	Pyro-glu from E	E1:Pyro-glu from E:1000.00	PEAKS PTM
R.EEDWRQPR.E	N	38.91	1114.5155	8	1.8	558.2661	2	23.91	4	F4:1112	OB5954 H1 Ro.raw	0	0	0	120	127			PEAKS DB
R.SKQFQNLQNH(+15.99)R.I	Y	38.88	1414.7065	11	2.4	708.3622	2	24.12	5	F5:1124	OB5955 H1 Ro.raw	1.2982E3	1	1	197	207	Oxidation (HW)	H10:Oxidation (HW):1000.00	PEAKS PTM
K.A(+43.01)MVIVVVNKGTGNLELVAVR.K	N	38.81	2124.2090	20	0.7	1063.1125	2	33.65	4	F4:1688	OB5954 H1 Ro.raw	6.7878E5	3	3	444	463	Carbamylation	A1:Carbamylation:161.20	PEAKS PTM
K.EGALM(-48.00)LPHFNSK.A	N	38.72	1294.6670	12	1.5	432.5636	3	28.65	5	F5:1381	OB5955 H1 Ro.raw	3.9929E6	3	3	432	443	Dethiomethyl	M5:Dethiomethyl:1000.00	PEAKS PTM
R.PNTLVLPK.H	N	38.45	880.5382	8	-2.9	441.2751	2	28.00	6	F6:1374	OB5956 H1 Ro.raw	1.9367E5	2	2	215	222			PEAKS DB
K.S(+42.01)(+14.02)FNLDEGHALR.I	N	38.42	1313.6364	11	-3.6	657.8231	2	28.09	5	F5:1359	OB5955 H1 Ro.raw	1.5617E6	1	1	249	259		S1:Acetylation (N-term):1000.00;S1:Methylation (others):49.79	PEAKS PTM
R.IFLAGDKDN(+.98)VIDQIEK(-.98).Q	N	38.33	1816.9570	16	0.5	606.6599	3	57.76	5	F5:3342	OB5955 H1 Ro.raw	3.1778E5	3	3	536	551	Amidation	N9:Deamidation (NQ):29.57; K16:Amidation:1000.00	PEAKS PTM
K.S(-18.01)FNLD(-18.01)EGHALR(+14.02)IPSGFISYILNR.H	N	37.89	2596.3550	23	-7.1	866.4528	3	40.28	6	F6:2110	OB5956 H1 Ro.raw	1.4449E6	1	1	249	271	Methylation(KR)	S1:Dehydration:19.61;D5:Dehydration:34.08;R11:Methylation (KR):157.81	PEAKS PTM
K.SFNLD(+53.92)EGHALR.I	N	37.31	1311.5294	11	1.0	438.1842	3	28.04	4	F4:1362	OB5954 H1 Ro.raw	1.3891E5	2	2	249	259		D5:Replacement of 2 protons by iron:18.17	PEAKS PTM
R.N(+.98)TLEAAFNAEFNEIR.R	N	36.98	1738.8162	15	9.2	870.4233	2	44.36	5	F5:2311	OB5955 H1 Ro.raw	5.1969E3	1	1	313	327		N1:Deamidation (NQ):21.41	PEAKS DB
**total 287 peptides**																			

Peptide	Uniq	-10lgP	Mass	Length	ppm	m/z	z	RT	Fraction	Scan	Source File	Area roasted peanut	#Feature	#Feature roasted peanut	Start	End	PTM	AScore	Found By
R.REQEW(+15.99)EEEEEDEEEEGSNR.E	N	36.76	2452.9375	19	-0.5	818.6527	3	25.98	4	F4:1239	OB5954 H1 Ro.raw	6.7106E4	2	2	472	490	Oxidation (HW)	W5:Oxidation (HW):1000.00	PEAKS PTM
R.IFLAGDK(+27.99)DNVIDQIEK.Q	N	36.69	1844.9519	16	-0.6	923.4827	2	34.83	5	F5:1775	OB5955 H1 Ro.raw	1.1863E5	1	1	536	551		K7:Formylation:17.54	PEAKS PTM
K.SFNLDE(+53.92)GHALR.I	N	36.63	1311.5294	11	-0.2	438.1837	3	27.93	5	F5:1353	OB5955 H1 Ro.raw	5.2242E4	1	1	249	259		E6:Replacement of 2 protons by iron:16.90	PEAKS PTM
R.IFLAGDKDNVID(-18.01)QIEK.Q	N	36.38	1798.9464	16	0.7	900.4811	2	31.39	6	F6:1591	OB5956 H1 Ro.raw	6.7841E4	1	1	536	551		D12:Dehydration:0.00	PEAKS PTM
K.NPQLQ(+.98)DLDMM(+15.99)LTC(+57.02)VEIKEGALM(+15.99)LPHFNSK.A	N	36.05	3404.6125	29	-1.8	852.1589	4	38.22	6	F6:1993	OB5956 H1 Ro.raw	8.1948E5	1	1	415	443	Carbamidomethylation	Q5:Deamidation (NQ):30.14;M10:Oxidation (M):0.00;C13:Carbamidomethylation:1000.00;M22:Oxidation (M):14.60	PEAKS DB
K.NPQLQDLDMMLTCVEIK(+14.02)EGALM(+15.99)LPHFNSK.A	N	36.00	3344.6277	29	-0.2	837.1640	4	37.84	6	F6:1961	OB5956 H1 Ro.raw	7.3561E6	2	2	415	443		K17:Methylation(KR):22.74;M22:Oxidation (M):20.37	PEAKS PTM
K.AMVIVVVNKGTGN(+.98)LELVAVRK.E	N	35.97	2210.2820	21	6.2	737.7725	3	40.10	5	F5:2113	OB5955 H1 Ro.raw	2.1847E5	1	1	444	464		N13:Deamidation (NQ):18.40	PEAKS DB
W.GTPGSEVREETSR.N	Y	35.79	1403.6641	13	3.8	702.8420	2	27.77	5	F5:1332	OB5955 H1 Ro.raw	2.4946E4	1	1	153	165			PEAKS DB
K.NPQLQDLDMMLTC(+71.04)VEIKEGALMLPHFNSK.A	N	35.62	3385.6543	29	-1.5	1129.5570	3	38.07	4	F4:1978	OB5954 H1 Ro.raw	1.5713E5	1	1	415	443	Propionamide	C13:Propionamide:1000.00	PEAKS PTM
R.N(-17.03)NPFYFPSRR.F	N	35.47	1279.6097	10	1.1	427.5443	3	28.89	6	F6:1434	OB5956 H1 Ro.raw	5.7683E5	3	3	166	175		N1:Ammonia-loss (N):9.34	PEAKS PTM
R.NTLEAAFNAEFN(+15.99)EIRR.V	N	35.08	1909.9282	16	0.9	955.9723	2	32.98	5	F5:1662	OB5955 H1 Ro.raw	2.4059E6	2	2	313	328		N12:Oxidation or Hydroxylation:9.28	PEAKS PTM
R.EGEQEW(+31.99)GTPGSEVREETSR.N	Y	34.83	2193.9409	19	0.5	732.3213	3	25.57	6	F6:1219	OB5956 H1 Ro.raw	2.9433E5	1	1	147	165		W6:Dihydroxy:28.36	PEAKS PTM

total 287 peptides

Peptide	Uniq	-10lgP	Mass	Length	ppm	m/z	z	RT	Fraction	Scan	Source File	Area roasted peanut	#Feature	#Feature roasted peanut	Start	End	PTM	AScore	Found By
R.IFLAGDKD(-18.01)NVIDQIEK.Q	N	34.72	1798.9464	16	-0.9	600.6555	3	32.15	6	F6:1589	OB5956 H1 Ro.raw	3.8289E5	1	1	536	551		D8:Dehydration:30.83	PEAKS PTM
K.SFNLDEGHALRIP(+15.99)SGFISYILN(+.98)R.H	N	34.44	2635.3394	23	8.6	879.4612	3	36.15	5	F5:1848	OB5955 H1 Ro.raw	1.0826E5	1	1	249	271	Hydroxylation Pro	P13:Hydroxylation Pro:1000.00;N22:Deamidation (NQ):32.85	PEAKS DB
R.NNPFYFP(+15.99)SRR.F	N	34.32	1312.6312	10	-0.5	657.3226	2	28.74	5	F5:1397	OB5955 H1 Ro.raw	7.8182E5	1	1	166	175		P7:Hydroxylation Pro:32.48	PEAKS DB
K.NPQLQ(+.98)DLDMM(-48.00)LTC(+57.02)VEIK.E	N	34.13	1999.9595	17	9.3	1000.9963	2	32.90	4	F4:1653	OB5954 H1 Ro.raw	1.9871E6	1	1	415	431	Carbamidomethylation	Q5:Deamidation (NQ):11.12;M10:Dethiomethyl:0.00;C13:Carbamidomethylation:1000.00	PEAKS PTM
K.DLAFPGSGE(+53.92)QVEK.L	N	33.99	1429.5812	13	-6.3	715.7933	2	30.34	6	F6:1518	OB5956 H1 Ro.raw	6.355E5	2	2	555	567		E9:Replacement of 2 protons by iron:37.54	PEAKS PTM
K.NPQ(+.98)LQDLDMMLTC(+57.02)VEIKEGALM(+15.99)LPHFNSK.A	N	33.64	3388.6174	29	6.0	1130.5532	3	38.52	5	F5:2024	OB5955 H1 Ro.raw	7.1577E5	1	1	415	443	Carbamidomethylation	Q3:Deamidation (NQ):0.00;C13:Carbamidomethylation:1000.00;M22:Oxidation (M):34.40	PEAKS DB
R.VAKISM(+15.99)PVNTPGQFEDFFP(+15.99)A.S	N	33.59	2226.0667	20	-7.4	1114.0323	2	51.06	5	F5:2785	OB5955 H1 Ro.raw	2.817E4	1	1	279	298	Oxidation (M)	M6:Oxidation (M):1000.00;P19:Hydroxylation Pro:21.81	PEAKS DB
N.PFYFPSR.R	N	33.58	912.4493	7	-0.1	457.2319	2	31.70	5	F5:1584	OB5955 H1 Ro.raw	8.1854E4	1	1	168	174			PEAKS DB
K.D(+53.92)LAFPGSGEQVEK.L	N	33.35	1429.5812	13	-4.3	715.7948	2	30.56	4	F4:1500	OB5954 H1 Ro.raw	3.8253E5	1	1	555	567		D1:Replacement of 2 protons by iron:0.00	PEAKS PTM

total 287 peptides

Peptide	Uniq	-10lgP	Mass	Length	ppm	m/z	z	RT	Fraction	Scan	Source File	Area roasted peanut	#Feature	#Feature roasted peanut	Start	End	PTM	AScore	Found By
K.N(+.98)PQLQDLDMMLTC(+57.02)VEIKEGALM(+15.99)LPHFNSK.A	N	33.25	3388.6174	29	6.0	1130.5532	3	38.52	5	F5:2054	OB5955 H1 Ro.raw	7.1577E5	1	1	415	443	Carbamidomethylation	N1:Deamidation (NQ): 0.00;C13:Carbamidomethylation:1000.00; M22:Oxidation (M):0.00	PEAKS DB
K.Q(+.98)FQNLQNHR.I	N	33.25	1184.5686	9	4.6	593.2943	2	23.68	5	F5:1098	OB5955 H1 Ro.raw	0	0	0	199	207		Q1:Deamidation (NQ): 25.21	PEAKS DB
K.ISMPVNTP(+15.99)GQ(+.98)FEDFFPASSR.D	N	33.02	2243.0205	20	5.1	1122.5232	2	47.86	4	F4:2523	OB5954 H1 Ro.raw	0	0	0	282	301		P8:Hydroxylation Pro: 0.00;Q10:Deamidation (NQ): 31.27	PEAKS DB
R.IFLAGDKDNVIDQ(+.98)IEK.Q	N	33.02	1817.9410	16	7.6	909.9847	2	63.08	6	F6:3435	OB5956 H1 Ro.raw	3.476E3	1	1	536	551		Q13:Deamidation (NQ):11.12	PEAKS DB
K.N(+.98)PQLQDLDM(+15.99)MLTC(+57.02)VEIKEGALMLPHFNSK.A	N	33.01	3388.6174	29	8.2	1695.3300	2	37.72	4	F4:1941	OB5954 H1 Ro.raw	7.0286E5	1	1	415	443	Carbamidomethylation	N1:Deamidation (NQ): 0.00;M9:Oxidation (M):0.00;C13: Carbamidomethylation:1000.00	PEAKS DB
K.E(-18.01)GALMLPHFNSK.A	N	32.81	1324.6598	12	0.0	442.5605	3	30.63	5	F5:1521	OB5955 H1 Ro.raw	2.0893E5	2	2	432	443	Pyro-glu from E	E1:Pyro-glu from E:1000.00	PEAKS PTM
R.E(-18.01)GEQEWGTP(+15.99)GSEVREETSR.N	Y	32.75	2159.9355	19	1.6	1080.9768	2	28.57	4	F4:1386	OB5954 H1 Ro.raw	1.3772E5	2	2	147	165	Pyro-glu from E; Hydroxylation Pro	E1:Pyro-glu from E:1000.00;P9:Hydroxylation Pro:1000.00	PEAKS PTM
K.KNPQLQDLDM(-48.00)MLTC(+57.02)VEIK.E	N	32.73	2127.0703	18	1.8	710.0320	3	33.04	6	F6:1688	OB5956 H1 Ro.raw	7.9877E5	1	1	414	431	Carbamidomethylation	M10:Dethiomethyl:8.14;C14: Carbamidomethylation:1000.00	PEAKS PTM

total 287 peptides

Peptide	Uniq	-10lgP	Mass	Length	ppm	m/z	z	RT	Fraction	Scan	Source File	Area roasted peanut	#Feature	#Feature roasted peanut	Start	End	PTM	AScore	Found By
K.KNPQLQ(+.98)DLDMM(+15.99)LTC(+57.02)VEIKEGALMLPHFNSK.A	N	32.59	3516.7124	30	3.3	880.1883	4	35.48	4	F4:1798	OB5954H1Ro.raw	7.7853E5	1	1	414	443	Carbamidomethylation	Q6:Deamidation (NQ):23.13;M11:Oxidation (M):14.02;C14:Carbamidomethylation:1000.00	PEAKS DB
K.G(+43.01)T(+14.02)GNLELVAVRK.E	N	32.58	1312.7462	12	-0.4	657.3801	2	27.30	4	F4:1303	OB5954H1Ro.raw	3.8825E5	2	2	453	464	Carbamylation; Methylation(others)	G1:Carbamylation:147.00;T2:Methylation(others):68.07	PEAKS PTM
R.IPSGFISYILNR(+28.03)HDN(+162.05)QNL.R	N	32.30	2290.1592	18	5.9	764.3982	3	34.37	5	F5:1737	OB5955H1Ro.raw	0	0	0	260	277	Dimethylation(KR)	R12:Dimethylation(KR):1000.00;N15:Hexose (NSY):17.01	PEAKS PTM
K.G(+43.01)T(+14.02)GNLELVAVR.K	N	32.00	1184.6514	11	-2.2	593.3317	2	30.21	4	F4:1493	OB5954H1Ro.raw	1.7455E5	2	2	453	463	Carbamylation	G1:Carbamylation:1000.00;T2:Methylation(others):46.31	PEAKS PTM
K.ISMP(+15.99)VNTPGQ(+.98)FEDFFPASSR.D	N	31.78	2243.0205	20	5.7	1122.5239	2	46.64	5	F5:2451	OB5955H1Ro.raw	0	0	0	282	301		P4:Hydroxylation Pro:24.67;Q10:Deamidation (NQ):21.91	PEAKS DB
D.GEPDLSNNFGR.L	N	31.47	1204.5472	11	3.0	603.2827	2	28.20	4	F4:1371	OB5954H1Ro.raw	1.6138E4	1	1	395	405			PEAKS DB
K.NPQLQ(+.98)DLDMMLTC(+57.02)VEIKEGALMLPHFNSK.A	N	31.45	3372.6226	29	7.2	844.1690	4	45.92	6	F6:2433	OB5956H1Ro.raw	0	0	0	415	443	Carbamidomethylation	Q5:Deamidation (NQ):14.19;C13:Carbamidomethylation:1000.00	PEAKS DB
K.KN(+.98)PQLQDLDMMLTC(+57.02)VEIKEGALMLPHFNSK.A	N	31.43	3500.7175	30	4.4	1167.9182	3	36.77	4	F4:1901	OB5954H1Ro.raw	9.2406E4	1	1	414	443	Carbamidomethylation	N2:Deamidation (NQ):14.04;C14:Carbamidomethylation:1000.00	PEAKS DB

total 287 peptides

Peptide	Uniq	-10lgP	Mass	Length	ppm	m/z	z	RT	Fraction	Scan	Source File	Area roasted peanut	#Feature	#Feature roasted peanut	Start	End	PTM	AScore	Found By
K.IRPEGREGEQEWGTPGSEVREETSR.N	Y	31.36	2870.3542	25	2.3	718.5975	4	27.83	6	F6:1355	OB5956 H1 Ro.raw	1.3652E5	1	1	141	165			PEAKS DB
K.VSKEH(+15.99)VQELTK.H	N	31.09	1312.6986	11	1.8	657.3578	2	23.73	5	F5:1100	OB5955 H1 Ro.raw	1.5735E3	1	1	361	371	Oxidation (HW)	H5:Oxidation (HW):1000.00	PEAKS PTM
K.N(+.98)PQ(+.98)LQ(+.98)DLDM(+15.99)MLTC(+57.02)VEIKEGALM(+15.99)LPHFNSK.A	N	30.70	3406.5806	29	9.9	1136.5454	3	36.75	6	F6:1904	OB5956 H1 Ro.raw	3.0926E5	1	1	415	443	Deamidation (NQ); Carbamidomethylation	N1:Deamidation (NQ): 69.03; Q3:Deamidation (NQ): 71.61; Q5:Deamidation (NQ): 53.75; M9:Oxidation (M):17.01;C13:Carbamidomethylation:1000.00; M22:Oxidation (M):16.22	PEAKS DB
R.WGP(+15.99)AEPR.E	Y	30.70	827.3926	7	0.0	414.7036	2	24.54	5	F5:1155	OB5955 H1 Ro.raw	3.9908E3	1	1	109	115		P3:Hydroxylation Pro: 36.24	PEAKS DB
F.L(+226.08)AGDK(+28.03)DNVIDQIEK.Q	N	30.44	1810.9135	14	-0.8	906.4633	2	39.29	5	F5:2033	OB5955 H1 Ro.raw	7.8052E4	1	1	538	551	Biotinylation; Dimethylation(KR)	L1:Biotinylation:152.87;K5:Dimethylation(KR):178.84	PEAKS PTM
L.R(+27.99)IPSGFISYILNR.H	N	29.85	1562.8568	13	-2.0	782.4341	2	37.84	6	F6:1976	OB5956 H1 Ro.raw	1.6397E5	1	1	259	271	Formylation	R1:Formylation:1000.00	PEAKS PTM
R.IVQIEAR(+28.03)PN(+.98)TLVLPK.H	Y	29.66	1719.0294	15	-1.6	860.5206	2	30.29	6	F6:1512	OB5956 H1 Ro.raw	0	0	0	208	222	Dimethylation(KR); Deamidation (NQ)	R7:Dimethylation(KR): 66.64; N9:Deamidation (NQ): 90.21	PEAKS PTM
R.LFEVKPDKKNPQLQDLDMM(+31.99)LTC(+57.02)VEIK.E	N	29.63	3163.5967	26	-2.8	791.9042	4	32.90	4	F4:1683	OB5954 H1 Ro.raw	1.0179E6	1	1	406	431	Carbamidomethylation	M19:Sulphone: 14.02; C22:Carbamidomethylation:1000.00	PEAKS PTM
K.ISMPVNTPGQ(+.98)FEDFFPASSR.D	N	29.56	2227.0254	20	9.7	1114.5308	2	54.22	4	F4:2901	OB5954 H1 Ro.raw	0	0	0	282	301		Q10:Deamidation (NQ):14.58	PEAKS DB

total 287 peptides

Peptide	Uniq	-10lgP	Mass	Length	ppm	m/z	z	RT	Fraction	Scan	Source File	Area roasted peanut	#Feature	#Feature roasted peanut	Start	End	PTM	AScore	Found By
R.GRREQEW(+15.99)EEEEEDEEEEGSNR.E	N	29.48	2666.0601	21	0.8	889.6946	3	26.10	4	F4:1240	OB5954 H1 Ro.raw	0	0	0	470	490	Oxidation (HW)	W7:Oxidation (HW):1000.00	PEAKS PTM
K.N(+.98)PQLQ(+.98)DLDMMLTC(+57.02)VEIKEGALM(+15.99)LPHFNSK.A	N	29.27	3389.6016	29	9.6	848.4158	4	37.65	6	F6:2047	OB5956 H1 Ro.raw	8.4697E6	1	1	415	443	Carbamidomethylation	N1:Deamidation (NQ):0.00;Q5:Deamidation (NQ):14.04;C13:Carbamidomethylation:1000.00;M22:Oxidation (M):0.00	PEAKS DB
N.TLEAAFNAEFNEIRR.V	N	28.88	1779.8904	15	3.5	890.9556	2	33.23	6	F6:1696	OB5956 H1 Ro.raw	1.5612E5	1	1	314	328			PEAKS DB
K.SFN(+.98)LDEGHALR(+31.99)IPSGFISYILNR.H	N	28.71	2651.3342	23	9.4	1326.6869	2	39.13	4	F4:2021	OB5954 H1 Ro.raw	0	0	0	249	271		N3:Deamidation (NQ):38.55;R11:Dihydroxy:14.78	PEAKS PTM
K.ISM(+15.99)PVN(+.98)TPGQFEDFFPASSR.D	N	28.37	2243.0205	20	8.5	1122.5271	2	44.80	6	F6:2368	OB5956 H1 Ro.raw	0	0	0	282	301	Oxidation (M)	M3:Oxidation (M):1000.00;N6:Deamidation (NQ):0.00	PEAKS DB
K.GTGNLE(+53.92)LVAVRK.E	N	28.35	1309.6440	12	-0.1	437.5553	3	27.29	4	F4:1309	OB5954 H1 Ro.raw	2.3239E4	1	1	453	464	Replacement of 2 protons by iron	E6:Replacement of 2 protons by iron:60.54	PEAKS PTM
D.NVIDQIEK.Q	N	28.35	957.5131	8	1.6	479.7646	2	28.19	5	F5:1367	OB5955 H1 Ro.raw	2.9963E3	1	1	544	551			PEAKS DB
K.I(+43.01)S(+14.02)MPVNTPGQFEDFFPASSR.D	N	28.31	2283.0630	20	-0.7	1142.5380	2	36.01	6	F6:1860	OB5956 H1 Ro.raw	2.4015E5	1	1	282	301	Carbamylation	I1:Carbamylation:1000.00;S2:Methylation (others):39.44	PEAKS PTM
R.VLLEENAGGEQEERGQRR.R	N	28.30	2069.0249	18	1.0	518.2640	4	25.49	5	F5:1205	OB5955 H1 Ro.raw	1.3378E5	1	1	329	346			PEAKS DB
K.NP(+15.99)QLQDLDMMLTC(+57.02)VEIKEGALMLPHFNSK.A	N	28.11	3387.6335	29	3.2	1130.2220	3	40.44	5	F5:2090	OB5955 H1 Ro.raw	3.3176E4	1	1	415	443	Carbamidomethylation	P2:Hydroxylation Pro:14.98;C13:Carbamidomethylation:1000.00	PEAKS DB

total 287 peptides

Peptide	Uniq	-10lgP	Mass	Length	ppm	m/z	z	RT	Fraction	Scan	Source File	Area roasted peanut	#Feature	#Feature roasted peanut	Start	End	PTM	AScore	Found By
K.AMVIVVVNK(-1.03)GTGNLELVAVR.K	N	27.94	2080.1714	20	8.5	1041.1018	2	34.56	4	F4:1751	OB5954 H1 Ro.raw	2.9653E7	1	1	444	463	Lysine oxidation to aminoadipic semialdehyde	K9:Lysine oxidation to aminoadipic semialdehyde:1000.00	PEAKS PTM
K.SF(+17.99)NLDEGHALR.I	N	27.90	1275.6007	11	8.1	638.8128	2	26.62	5	F5:1267	OB5955 H1 Ro.raw	9.5818E3	1	1	249	259	Fluorination	F2:Fluorination:1000.00	PEAKS PTM
K.SFNLDEGHALRIP(+15.99)SGF(+17.99)ISYILNR.H	N	27.40	2652.3459	23	8.7	885.1303	3	38.95	5	F5:2010	OB5955 H1 Ro.raw	0	0	0	249	271	Hydroxylation Pro	P13:Hydroxylation Pro:1000.00;F16:Fluorination:38.16	PEAKS PTM
R.VLLEENAGGEQEER(+15.99).G	N	27.33	1587.7375	14	1.8	794.8775	2	26.70	4	F4:1275	OB5954 H1 Ro.raw	0	0	0	329	342		R14:Oxidation or Hydroxylation:20.88	PEAKS PTM
K.NPQLQ(+.98)DLDMM(+15.99)LTC(+57.02)VEIKEGALMLPHFNSK.A	N	27.25	3388.6174	29	8.0	1130.5554	3	37.44	5	F5:1884	OB5955 H1 Ro.raw	6.7537E6	1	1	415	443	Carbamidomethylation	Q5:Deamidation (NQ):0.00;M10:Oxidation (M):12.28;C13:Carbamidomethylation:1000.00	PEAKS DB
R.N(+42.01)(+15.00)NPFYFPSRR.F	N	27.12	1353.6465	10	9.4	677.8369	2	29.74	5	F5:1453	OB5955 H1 Ro.raw	3.0438E5	1	1	166	175		N1:Acetylation (N-term):1000.00;N1:Deamidation followed by a methylation:12.28	PEAKS PTM
R.VAK(-1.03)ISMPVNTPGQFEDFFPASSR.D	N	27.11	2523.2104	23	1.1	1262.6139	2	36.53	6	F6:1887	OB5956 H1 Ro.raw	0	0	0	279	301	Lysine oxidation to aminoadipic semialdehyde	K3:Lysine oxidation to aminoadipic semialdehyde:1000.00	PEAKS PTM
K.NPQLQDLDMMLTC(+58.01)VEIK.E	N	26.84	2047.9629	17	-3.6	1024.9850	2	46.58	4	F4:2449	OB5954 H1 Ro.raw	0	0	0	415	431	Carboxymethyl	C13:Carboxymethyl:1000.00	PEAKS PTM
R.EQEW(+15.99)EEEEEDEEEEGSNREVR.R	N	26.79	2681.0483	21	1.1	894.6910	3	26.92	4	F4:1316	OB5954 H1 Ro.raw	7.9753E4	1	1	473	493	Oxidation (HW)	W4:Oxidation (HW):1000.00	PEAKS PTM

total 287 peptides

Peptide	Uniq	-10lgP	Mass	Length	ppm	m/z	z	RT	Fraction	Scan	Source File	Area roasted peanut	#Feature	#Feature roasted peanut	Start	End	PTM	AScore	Found By
K.NPQLQDLDMM(+15.99)LTC(+57.02)VEIKEGALMLP(+15.99)HFNSK.A	N	26.70	3403.6284	29	-6.6	1702.8102	2	36.88	6	F6:1908	OB5956 H1 Ro.raw	0	0	0	415	443	Carbamidomethylation	M10:Oxidation (M):7.65;C13:Carbamidomethylation:1000.00;P24:Hydroxylation Pro:30.14	PEAKS DB
R.IFLAGDKD(-18.01)NV.I	N	26.68	1072.5553	10	0.4	537.2852	2	32.21	4	F4:1610	OB5954 H1 Ro.raw	2.1773E6	1	1	536	545		D8:Dehydration:45.01	PEAKS PTM
K.KNPQLQDLDMM(-48.00)LTC(+57.02)VEIK.E	N	26.57	2127.0703	18	-0.4	710.0304	3	31.57	6	F6:1598	OB5956 H1 Ro.raw	1.2455E5	1	1	414	431	Carbamidomethylation	M11:Dethiomethyl:0.00;C14:Carbamidomethylation:1000.00	PEAKS PTM
K.KNPQ(+.98)LQ(+.98)DLDM(+15.99)MLTC(+57.02)VEIKEGALM(+15.99)LPHFNSK.A	N	26.17	3533.6914	30	9.7	884.4387	4	35.77	5	F5:1822	OB5955 H1 Ro.raw	2.4464E5	1	1	414	443	Carbamidomethylation	Q4:Deamidation (NQ):9.40;Q6:Deamidation (NQ):6.65;M10:Oxidation (M):0.00;C14:Carbamidomethylation:1000.00;M23:Oxidation (M):40.04	PEAKS DB
K.NPQLQ(+.98)DLDM(+15.99)M(+15.99)LTC(+57.02)VEIKEGALM(+15.99)LPHFNSK.A	N	26.02	3420.6074	29	5.5	1141.2161	3	34.83	5	F5:1769	OB5955 H1 Ro.raw	2.4925E5	1	1	415	443	Oxidation (M); Carbamidomethylation	Q5:Deamidation (NQ):11.12;M9:Oxidation (M):1000.00;M10:Oxidation (M):1000.00;C13:Carbamidomethylation:1000.00;M22:Oxidation (M):1000.00	PEAKS DB
K.AM(-48.00)VIVVVNK.G	N	25.96	923.5804	9	0.0	462.7975	2	25.00	6	F6:1192	OB5956 H1 Ro.raw	6.3039E3	1	1	444	452	Dethiomethyl	M2:Dethiomethyl:1000.00	PEAKS PTM

total 287 peptides

Peptide	Uniq	-10lgP	Mass	Length	ppm	m/z	z	RT	Fraction	Scan	Source File	Area roasted peanut	#Feature	#Feature roasted peanut	Start	End	PTM	AScore	Found By
R.IVQIEARPNTLVLP(+15.99)KHADADNILVIQQGQ(+.98)ATVTVAN(+.98)GNNRK.S	Y	25.90	4436.3774	41	6.0	1110.1083	4	32.89	4	F4:1658	OB5954 H1 Ro.raw	3.5945E5	1	1	208	248		P14:Hydroxylation Pro:1.99;Q29:Deamidation (NQ):0.00;N36:Deamidation (NQ):14.04	PEAKS DB
R.SSDNEGVIVKVSKEHVQELTK.H	N	25.71	2325.2175	21	0.9	776.0804	3	28.86	4	F4:1404	OB5954 H1 Ro.raw	0	0	0	351	371			PEAKS DB
R.DQSSY(+15.99)LQGFSR.N	N	25.52	1302.5840	11	1.6	652.3003	2	29.24	6	F6:1470	OB5956 H1 Ro.raw	7.3464E4	1	1	302	312		Y5:Oxidation or Hydroxylation: 26.36	PEAKS PTM
**total 287 peptides**																			

tr|B3IXL2|B3IXL2_ARAHY

back to list

| Protein Coverage | Supporting Peptides |

Protein Coverage:

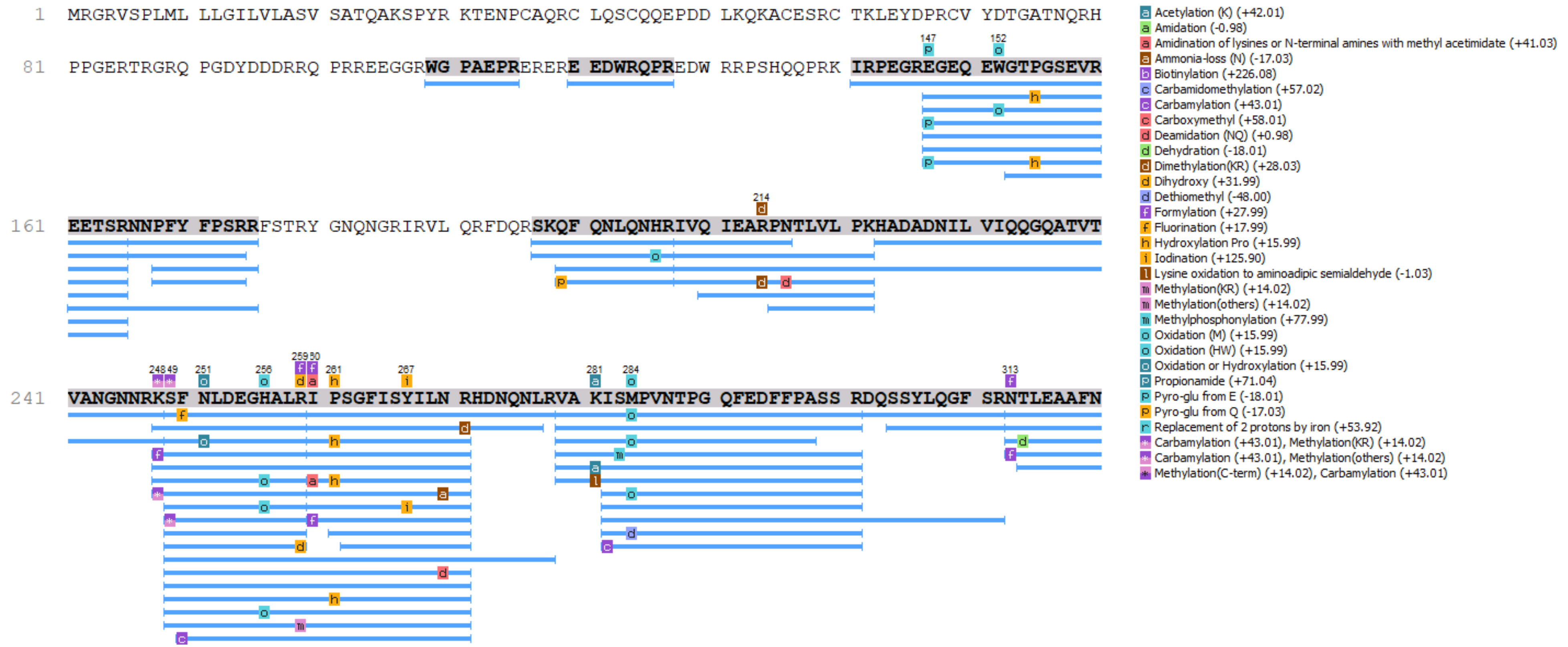

321 AEFNEIRRVL LEENAGGEQE ERGQRRRSTR SSDNEGVIVK VSKEHVQELT KHAKSVSKKG SEEEDITNPI NLRDGEPDLS

401 NNFGRLFEVK PDKKNPQLQD LDMMLTCVEI KEGALMLPHF NSKAMVIVVV NKGTGNLELV AVRKEQQQRG RREQEWEEEE

481 EDEEEEGSNR EVRRYTARLK EGDVFIMPAA HPVAINASSE LHLLGFGINA ENNHRIFLAG DKDNVIDQIE KQAKDLAFPG

561 SGEQVEKLIK NQRESHFVSA RPQSQSPSSP EKEDQEEENQ GGKGPLLSIL KAFN

Supporting Peptides:

Peptide	Uniq	-10lgP	Mass	Length	ppm	m/z	z	RT	Fraction	Scan	Source File	Area roasted peanut	#Feature	#Feature roasted peanut	Start	End	PTM	AScore	Found By
R.EQEWEEEEEDEEEEGSNR.E	N	117.09	2280.8413	18	3.3	1141.4316	2	28.28	5	F5:1371	OB5955 H1 Ro.raw	4.6914E6	7	7	473	490			PEAKS DB
total 287 peptides																			

Peptide	Uniq	-10lgP	Mass	Length	ppm	m/z	z	RT	Fraction	Scan	Source File	Area roasted peanut	#Feature	#Feature roasted peanut	Start	End	PTM	AScore	Found By
K.KNPQLQDLDMMLTC(+57.02)VEIK.E	N	116.47	2175.0737	18	3.2	1088.5476	2	35.21	5	F5:1788	OB5955 H1 Ro.raw	1.5503E7	6	6	414	431	Carbamidomethylation	C14:Carbamidomethylation:1000.00	PEAKS DB
K.NPQLQDLDMM(+15.99)LTC(+57.02)VEIK.E	N	116.10	2062.9736	17	5.0	1032.4993	2	34.71	6	F6:1780	OB5956 H1 Ro.raw	1.155E7	4	4	415	431	Carbamidomethylation	M10:Oxidation (M):30.46;C13:Carbamidomethylation:1000.00	PEAKS DB
K.NPQLQDLDM(+15.99)MLTC(+57.02)VEIK.E	N	115.70	2062.9736	17	-4.7	1032.4893	2	36.62	4	F4:1873	OB5954 H1 Ro.raw	3.8486E6	3	3	415	431	Carbamidomethylation	M9:Oxidation (M):30.46;C13:Carbamidomethylation:1000.00	PEAKS DB
K.NPQLQDLDMMLTC(+57.02)VEIK.E	N	112.86	2046.9788	17	0.2	1024.4968	2	37.11	6	F6:1959	OB5956 H1 Ro.raw	3.5075E7	10	10	415	431	Carbamidomethylation	C13:Carbamidomethylation:1000.00	PEAKS DB
R.NTLEAAFNAEFNEIR.R	N	112.69	1737.8322	15	1.4	869.9246	2	35.64	6	F6:1825	OB5956 H1 Ro.raw	1.2023E7	6	6	313	327			PEAKS DB
K.ISM(+15.99)PVNTPGQFEDFFPASSR.D	N	109.35	2242.0364	20	8.1	1122.0345	2	34.52	6	F6:1771	OB5956 H1 Ro.raw	2.1717E7	8	8	282	301	Oxidation (M)	M3:Oxidation (M):1000.00	PEAKS DB
K.ISMPVNTPGQFEDFFPASSR.D	N	107.77	2226.0415	20	0.9	1114.0291	2	35.86	4	F4:1823	OB5954 H1 Ro.raw	8.7375E7	15	15	282	301			PEAKS DB
K.HADADNILVIQQGQATVTVANGNNRK.S	N	104.88	2746.4111	26	3.3	916.4807	3	29.36	4	F4:1435	OB5954 H1 Ro.raw	3.9035E6	4	4	223	248			PEAKS DB
K.KGSEEEDITNPINLRDGEPDLSNNFGR.L	N	103.39	3015.4170	27	3.4	1006.1497	3	31.62	4	F4:1565	OB5954 H1 Ro.raw	1.1819E7	10	10	379	405			PEAKS DB
K.KNPQLQDLDM(+15.99)MLTC(+57.02)VEIK.E	N	103.13	2191.0686	18	3.6	1096.5455	2	34.71	6	F6:1781	OB5956 H1 Ro.raw	1.4412E6	4	4	414	431	Carbamidomethylation	M10:Oxidation (M):27.96;C14:Carbamidomethylation:1000.00	PEAKS DB
R.IFLAGDKDNVIDQIEK.Q	N	102.67	1816.9570	16	6.5	909.4917	2	32.54	4	F4:1630	OB5954 H1 Ro.raw	4.3939E8	47	47	536	551			PEAKS DB
K.HADADNILVIQQGQATVTVAN(+.98)GNNRK.S	N	101.58	2747.3950	26	3.3	916.8086	3	29.66	4	F4:1454	OB5954 H1 Ro.raw	4.7756E6	7	7	223	248		N21:Deamidation (NQ):31.91	PEAKS DB
K.AMVIVVVNKGTGNLELVAVR.K	N	99.57	2081.2031	20	-0.4	1041.6084	2	34.57	4	F4:1751	OB5954 H1 Ro.raw	3.4777E7	8	8	444	463			PEAKS DB
K.GSEEEDITNPINLRDGEPDLSNNFGR.L	N	98.43	2887.3220	26	-0.7	963.4473	3	32.67	6	F6:1660	OB5956 H1 Ro.raw	1.2511E7	5	5	380	405			PEAKS DB

total 287 peptides

Peptide	Uniq	-10lgP	Mass	Length	ppm	m/z	z	RT	Fraction	Scan	Source File	Area roasted peanut	#Feature	#Feature roasted peanut	Start	End	PTM	AScore	Found By
K.SFNLDEGHALR.I	N	97.07	1257.6101	11	3.0	629.8142	2	28.04	4	F4:1388	OB5954 H1 Ro.raw	1.1757E8	21	21	249	259			PEAKS DB
K.NPQLQDLDM(+15.99)M(+15.99)LTC(+57.02)VEIK.E	N	96.89	2078.9688	17	6.6	1040.4985	2	33.82	6	F6:1726	OB5956 H1 Ro.raw	7.6578E4	2	2	415	431	Oxidation (M); Carbamidomethylation	M9:Oxidation (M):1000.00; M10:Oxidation (M):1000.00; C13:Carbamidomethylation:1000.00	PEAKS DB
K.AM(+15.99)VIVVVNKGTGNLELVAVR.K	N	96.06	2097.1980	20	3.4	1049.6099	2	32.98	5	F5:1656	OB5955 H1 Ro.raw	2.4028E7	6	6	444	463	Oxidation (M)	M2:Oxidation (M):1000.00	PEAKS DB
R.VLLEENAGGEQEER.G	N	95.38	1571.7427	14	2.2	786.8804	2	26.92	4	F4:1300	OB5954 H1 Ro.raw	3.043E7	6	6	329	342			PEAKS DB
K.KNPQLQDLDMM(+15.99)LTC(+57.02)VEIK.E	N	95.29	2191.0686	18	3.1	1096.5449	2	32.98	5	F5:1657	OB5955 H1 Ro.raw	1.9358E6	4	4	414	431	Carbamidomethylation	M11:Oxidation (M):23.10;C14:Carbamidomethylation:1000.00	PEAKS DB
K.SFNLDEGHALRIPSGFISYILNR.H	N	95.27	2618.3604	23	1.9	873.7957	3	37.65	6	F6:1935	OB5956 H1 Ro.raw	7.1821E7	11	11	249	271			PEAKS DB
R.DQSSYLQGFSR.N	N	94.26	1286.5891	11	-0.9	644.3013	2	30.91	4	F4:1531	OB5954 H1 Ro.raw	9.6573E7	19	19	302	312			PEAKS DB
R.RVLLEENAGGEQEER.G	N	93.77	1727.8438	15	0.2	576.9553	3	25.30	5	F5:1185	OB5955 H1 Ro.raw	8.7628E5	6	6	328	342			PEAKS DB
K.NPQLQDLDMMLTC(+14.02)VEIK.E	N	93.65	2003.9730	17	2.7	1002.9965	2	38.33	5	F5:1965	OB5955 H1 Ro.raw	5.8314E6	4	4	415	431		C13:Methylation(others):33.98	PEAKS PTM
R.IFLAGDKDNVIDQIEKQAK.D	N	92.18	2144.1477	19	-2.1	715.7217	3	32.42	5	F5:1625	OB5955 H1 Ro.raw	1.615E6	3	3	536	554			PEAKS DB
R.IPSGFISYILNR.H	N	92.13	1378.7609	12	1.1	690.3885	2	37.47	6	F6:1945	OB5956 H1 Ro.raw	4.5588E8	9	9	260	271			PEAKS DB
K.AMVIVVVNKGTGNLELVAVRK.E	N	91.52	2209.2981	21	4.7	737.4434	3	32.59	4	F4:1633	OB5954 H1 Ro.raw	1.2306E8	14	14	444	464			PEAKS DB
R.KSFNLDEGHALR.I	N	88.41	1385.7051	12	1.3	693.8607	2	26.06	5	F5:1228	OB5955 H1 Ro.raw	5.4977E6	6	6	248	259			PEAKS DB
R.REQEWEEEEEDEEEEGSNR.E	N	88.08	2436.9424	19	0.6	813.3219	3	26.73	4	F4:1278	OB5954 H1 Ro.raw	9.3919E5	7	7	472	490			PEAKS DB
R.EQEW(+15.99)EEEEEDEEEEGSNR.E	N	86.71	2296.8362	18	2.5	1149.4282	2	27.64	6	F6:1309	OB5956 H1 Ro.raw	1.0675E6	3	3	473	490	Oxidation (HW)	W4:Oxidation (HW):1000.00	PEAKS PTM

total 287 peptides

Peptide	Uniq	-10lgP	Mass	Length	ppm	m/z	z	RT	Fraction	Scan	Source File	Area roasted peanut	#Feature	#Feature roasted peanut	Start	End	PTM	AScore	Found By
K.KGSEEEDITNPINLR.D	N	86.46	1713.8533	15	4.2	857.9375	2	29.29	4	F4:1432	OB5954 H1 Ro.raw	8.9159E6	6	6	379	393			PEAKS DB
K.NPQLQDLDM(+15.99)MLTCVEIK(+14.02).E	N	85.42	2019.9679	17	2.2	1010.9934	2	37.33	4	F4:1920	OB5954 H1 Ro.raw	7.9299E5	2	2	415	431	Methylation(KR)	M9:Oxidation (M):21.94;K17:Methylation (KR):1000.00	PEAKS PTM
K.DLAFPGSGEQVEK.L	N	85.27	1375.6619	13	-0.7	688.8377	2	30.39	4	F4:1498	OB5954 H1 Ro.raw	9.2216E7	3	3	555	567			PEAKS DB
R.VLLEEN(+.98)AGGEQEER.G	N	85.22	1572.7267	14	2.8	787.3728	2	27.46	6	F6:1340	OB5956 H1 Ro.raw	6.8644E5	3	3	329	342	Deamidation (NQ)	N6:Deamidation (NQ): 121.37	PEAKS DB
K.NPQLQDLDMMLTC(+57.02)VEIKEGALMLPHFNSK.A	N	85.17	3371.6387	29	-4.9	1686.8184	2	38.00	5	F5:1955	OB5955 H1 Ro.raw	1.5541E8	10	10	415	443	Carbamidomethylation	C13:Carbamidomethylation:1000.00	PEAKS DB
R.DGEPDLSNNFGR.L	N	84.59	1319.5742	12	0.6	660.7948	2	29.29	4	F4:1422	OB5954 H1 Ro.raw	7.8399E6	3	3	394	405			PEAKS DB
K.ISMP(+15.99)VNTPGQFEDFFPASSR.D	N	84.52	2242.0364	20	-2.0	1122.0232	2	34.47	5	F5:1814	OB5955 H1 Ro.raw	1.0743E7	2	2	282	301		P4:Hydroxylation Pro: 42.89	PEAKS DB
R.EGEQEWGTPGSEVREETSR.N	Y	84.24	2161.9512	19	4.4	1081.9877	2	27.86	4	F4:1344	OB5954 H1 Ro.raw	4.0932E7	7	7	147	165			PEAKS DB
K.NPQLQDLDM(+15.99)MLTC(+57.02)VEIKEGALMLPHFNSK.A	N	83.17	3387.6335	29	-0.6	1130.2178	3	37.70	4	F4:1939	OB5954 H1 Ro.raw	9.055E7	6	6	415	443	Carbamidomethylation	M9:Oxidation (M):26.02;C13:Carbamidomethylation:1000.00	PEAKS DB
R.NTLEAAFNAEFNEIRR.V	N	81.08	1893.9332	16	-2.6	947.9714	2	34.11	5	F5:1711	OB5955 H1 Ro.raw	2.8495E8	15	15	313	328			PEAKS DB
R.GRREQEWEEEEEDEEEEGSNR.E	N	80.86	2650.0649	21	1.1	884.3632	3	26.73	4	F4:1280	OB5954 H1 Ro.raw	8.7144E5	6	6	470	490			PEAKS DB
R.EGEQEWGTPGSEVR.E	Y	80.82	1559.6852	14	1.4	780.8510	2	28.04	4	F4:1358	OB5954 H1 Ro.raw	3.4069E6	3	3	147	160			PEAKS DB
K.EGALMLPHFNSK.A	N	80.31	1342.6703	12	2.3	672.3440	2	30.63	5	F5:1504	OB5955 H1 Ro.raw	1.4317E7	5	5	432	443			PEAKS DB
K.SFNLDEGH(+15.99)ALR.I	N	80.04	1273.6051	11	-0.5	637.8095	2	30.29	4	F4:1491	OB5954 H1 Ro.raw	9.1981E5	2	2	249	259	Oxidation (HW)	H8:Oxidation (HW):1000.00	PEAKS PTM
R.LFEVKPDKKNPQLQDLDMMLTC(+57.02)VEIK.E	N	78.90	3131.6069	26	-4.0	1044.8721	3	34.65	5	F5:1756	OB5955 H1 Ro.raw	5.2558E6	4	4	406	431	Carbamidomethylation	C22:Carbamidomethylation:1000.00	PEAKS DB
R.I(+27.99)FLAGDKDNVIDQIEK.Q	N	78.41	1844.9519	16	-2.1	923.4813	2	36.23	4	F4:1853	OB5954 H1 Ro.raw	2.4413E6	3	3	536	551	Formylation	I1:Formylation:98.75	PEAKS PTM

total 287 peptides

Peptide	Uniq	-10lgP	Mass	Length	ppm	m/z	z	RT	Fraction	Scan	Source File	Area roasted peanut	#Feature	#Feature roasted peanut	Start	End	PTM	AScore	Found By
K.GSEEEDITNPINLR.D	N	78.13	1585.7583	14	2.3	793.8882	2	31.16	5	F5:1548	OB5955 H1 Ro.raw	3.4694E6	3	3	380	393			PEAKS DB
F.LAGDKDNVIDQIEK.Q	N	78.02	1556.8046	14	-6.3	779.4047	2	32.21	6	F6:1629	OB5956 H1 Ro.raw	5.4762E6	4	4	538	551			PEAKS DB
K.AMVIVVVNK.G	N	77.85	971.5837	9	-0.8	486.7987	2	30.10	5	F5:1540	OB5955 H1 Ro.raw	7.1402E6	3	3	444	452			PEAKS DB
R.VAK(+42.01)ISMPVNTPGQFEDFFPASSR.D	N	77.03	2566.2524	23	0.5	1284.1342	2	36.43	6	F6:1881	OB5956 H1 Ro.raw	0	0	0	279	301	Acetylation (K)	K3:Acetylation (K):1000.00	PEAKS PTM
K.NPQLQDLDMM(-48.00)LTC(+57.02)VEIK.E	N	76.61	1998.9755	17	1.9	1000.4969	2	32.85	6	F6:1670	OB5956 H1 Ro.raw	6.204E6	3	3	415	431	Carbamidomethylation	M10:Dethiomethyl:14.02;C13:Carbamidomethylation:1000.00	PEAKS PTM
K.ISMPVNTP(+15.99)GQFEDFFPASSR.D	N	75.57	2242.0364	20	1.5	1122.0271	2	35.64	6	F6:1836	OB5956 H1 Ro.raw	1.0022E6	1	1	282	301		P8:Hydroxylation Pro: 10.93	PEAKS DB
K.EGALMLPHFN(+.98)SK.A	N	75.32	1343.6543	12	2.6	672.8362	2	31.66	6	F6:1595	OB5956 H1 Ro.raw	1.7726E5	2	2	432	443	Deamidation (NQ)	N10:Deamidation (NQ):1000.00	PEAKS DB
R.VAKISMPVNTPGQFEDFFPASSR.D	N	75.04	2524.2419	23	-2.9	842.4188	3	34.29	5	F5:1735	OB5955 H1 Ro.raw	1.671E6	3	3	279	301			PEAKS DB
K.KNPQLQDLDMMLTC(+57.02)VEIKEGALMLPHFNSK.A	N	74.64	3499.7336	30	-3.9	875.9373	4	36.80	4	F4:1884	OB5954 H1 Ro.raw	6.9756E6	4	4	414	443	Carbamidomethylation	C14:Carbamidomethylation:1000.00	PEAKS DB
R.NTLEAAFN(+.98)AEFNEIRR.V	N	74.52	1894.9172	16	-2.2	948.4638	2	32.35	4	F4:1616	OB5954 H1 Ro.raw	1.3754E6	3	3	313	328		N8:Deamidation (NQ): 48.12	PEAKS DB
R.KSFNLDEGHALRIPSGFISYILNR.H	N	73.99	2746.4553	24	0.1	687.6212	4	36.45	5	F5:1860	OB5955 H1 Ro.raw	1.0287E6	2	2	248	271			PEAKS DB
R.EETSRN(+.98)NPFYFPSRR.F	N	73.61	1899.8864	15	-0.2	634.3026	3	28.39	4	F4:1381	OB5954 H1 Ro.raw	5.4651E5	3	3	161	175		N6:Deamidation (NQ): 33.98	PEAKS DB
R.RVLLEENAGGEQEERGQR.R	N	73.30	2069.0249	18	1.9	690.6835	3	25.60	4	F4:1205	OB5954 H1 Ro.raw	1.8274E6	3	3	328	345			PEAKS DB
K.GTGNLELVAVR.K	N	72.98	1127.6299	11	-1.3	564.8215	2	30.21	4	F4:1476	OB5954 H1 Ro.raw	2.4766E7	4	4	453	463			PEAKS DB
K.EGALM(+15.99)LPHFNSK.A	N	72.82	1358.6653	12	-0.5	680.3396	2	30.10	5	F5:1469	OB5955 H1 Ro.raw	3.1504E6	5	5	432	443	Oxidation (M)	M5:Oxidation (M):1000.00	PEAKS DB
R.IPSGFISYILN(-17.03)R.H	N	72.17	1361.7343	12	-0.6	681.8740	2	38.07	4	F4:1963	OB5954 H1 Ro.raw	1.5056E6	3	3	260	271	Ammonia-loss (N)	N11:Ammonia-loss (N):1000.00	PEAKS PTM

total 287 peptides

Peptide	Uniq	-10lgP	Mass	Length	ppm	m/z	z	RT	Fraction	Scan	Source File	Area roasted peanut	#Feature	#Feature roasted peanut	Start	End	PTM	AScore	Found By
R.IFLAGDKDNVIDQIEKQ(+.98)AK.D	N	71.42	2145.1316	19	1.5	716.0522	3	33.08	5	F5:1661	OB5955 H1 Ro.raw	0	0	0	536	554		Q17:Deamidation (NQ):48.83	PEAKS DB
R.IPSGFISYILNRHDNQNLR.V	N	71.19	2256.1763	19	6.5	753.0709	3	33.61	6	F6:1714	OB5956 H1 Ro.raw	5.9166E7	10	10	260	278			PEAKS DB
K.GTGNLELVAVRK.E	N	70.92	1255.7249	12	0.7	628.8701	2	27.30	4	F4:1290	OB5954 H1 Ro.raw	2.2945E7	7	7	453	464			PEAKS DB
K.KNPQLQDLDMMLTC(+57.02)VEIKEGALM(+15.99)LPHFNSK.A	N	70.54	3515.7285	30	-1.9	879.9377	4	36.03	6	F6:1857	OB5956 H1 Ro.raw	6.9145E6	3	3	414	443	Carbamidomethylation; Oxidation (M)	C14:Carbamidomethylation:1000.00;M23:Oxidation (M):107.40	PEAKS DB
K.NPQLQDLDM(+15.99)MLTC(+57.02)VEIKEGALM(+15.99)LPHFNSK.A	N	70.15	3403.6284	29	1.1	851.9153	4	37.51	4	F4:1883	OB5954 H1 Ro.raw	7.9309E6	6	6	415	443	Carbamidomethylation; Oxidation (M)	M9:Oxidation (M):27.96;C13:Carbamidomethylation:1000.00;M22:Oxidation (M):71.31	PEAKS DB
R.VLLEENAGGEQEERGQR.R	N	69.54	1912.9238	17	0.6	957.4698	2	25.30	5	F5:1191	OB5955 H1 Ro.raw	3.4466E6	6	6	329	345			PEAKS DB
I.PSGFISYILNR.H	N	69.26	1265.6768	11	-5.5	633.8422	2	36.68	4	F4:1876	OB5954 H1 Ro.raw	3.6428E5	2	2	261	271			PEAKS DB
R.DQS(-18.01)SYLQGFSR.N	N	68.93	1268.5785	11	0.2	635.2966	2	30.91	4	F4:1537	OB5954 H1 Ro.raw	6.7379E5	3	3	302	312		S3:Dehydration:40.00	PEAKS PTM
R.IP(+15.99)SGFISYILNR.H	N	68.69	1394.7557	12	1.5	698.3862	2	37.24	5	F5:1913	OB5955 H1 Ro.raw	6.9724E6	9	9	260	271	Hydroxylation Pro	P2:Hydroxylation Pro:1000.00	PEAKS DB
K.NPQLQDLDMM(+15.99)LTC(+57.02)VEIKEGALMLPHFNSK.A	N	68.62	3387.6335	29	-0.8	1130.2175	3	36.42	4	F4:1862	OB5954 H1 Ro.raw	7.1368E7	6	6	415	443	Carbamidomethylation	M10:Oxidation (M):24.44;C13:Carbamidomethylation:1000.00	PEAKS DB
R.RVLLEEN(+.98)AGGEQEER.G	N	68.51	1728.8278	15	-0.8	577.2827	3	25.96	5	F5:1233	OB5955 H1 Ro.raw	1.3591E4	2	2	328	342	Deamidation (NQ)	N7:Deamidation (NQ):77.53	PEAKS DB
R.VAKISM(+15.99)PVNTPGQFEDFFPASSR.D	N	68.50	2540.2368	23	2.1	847.7546	3	32.98	5	F5:1665	OB5955 H1 Ro.raw	5.6098E5	2	2	279	301	Oxidation (M)	M6:Oxidation (M):1000.00	PEAKS DB
R.NNPFYFPSR.R	N	68.49	1140.5352	9	0.9	571.2754	2	31.81	4	F4:1575	OB5954 H1 Ro.raw	1.7544E7	5	5	166	174			PEAKS DB

total 287 peptides

Peptide	Uniq	-10lgP	Mass	Length	ppm	m/z	z	RT	Fraction	Scan	Source File	Area roasted peanut	#Feature	#Feature roasted peanut	Start	End	PTM	AScore	Found By
K.NPQLQDLDMMLTCVEIK(+14.02)EGALMLPHFNSK.A	N	68.29	3328.6328	29	0.9	833.1663	4	38.52	5	F5:1986	OB5955H1 Ro.raw	1.7448E7	6	6	415	443	Methylation(KR)	K17:Methylation(KR): 98.15	PEAKS PTM
R.NNPFYFPSRR.F	N	67.62	1296.6364	10	0.0	649.3254	2	28.95	4	F4:1567	OB5954H1 Ro.raw	1.3432E8	8	8	166	175			PEAKS DB
R.EETSRNNPFYFPSRR.F	N	67.58	1898.9023	15	1.8	633.9759	3	27.75	5	F5:1339	OB5955H1 Ro.raw	9.3426E5	3	3	161	175			PEAKS DB
R.IVQIEARPNTLVLPK.H	Y	67.30	1690.0140	15	0.3	846.0145	2	30.46	5	F5:1502	OB5955H1 Ro.raw	4.7655E7	8	8	208	222			PEAKS DB
K.KNPQLQDLDMMLTCVEIK(+14.02).E	N	66.83	2132.0679	18	-0.6	1067.0405	2	36.31	4	F4:1858	OB5954H1 Ro.raw	6.1629E5	2	2	414	431	Methylation(KR)	K18:Methylation(KR): 130.57	PEAKS PTM
R.IPSGFISY(+125.90)ILNR.H	N	65.90	1504.6575	12	-1.9	753.3346	2	38.39	6	F6:2001	OB5956H1 Ro.raw	2.2019E5	2	2	260	271	Iodination	Y8:Iodination: 1000.00	PEAKS PTM
R.VLLEEN(-17.03)AGGEQEER.G	N	65.50	1554.7162	14	1.2	778.3663	2	27.73	5	F5:1335	OB5955H1 Ro.raw	0	0	0	329	342	Ammonia-loss (N)	N6:Ammonia-loss (N):1000.00	PEAKS PTM
K.Q(-17.03)AKDLAFPGSGEQVEK.L	N	65.43	1685.8260	16	0.1	843.9204	2	29.98	6	F6:1503	OB5956H1 Ro.raw	1.0547E5	3	3	552	567	Pyro-glu from Q	Q1:Pyro-glu from Q:1000.00	PEAKS PTM
K.NPQLQDLDM(+15.99)M(+15.99)LTC(+57.02)VEIKEGALMLPHFNSK.A	N	64.96	3403.6284	29	-3.9	1135.5457	3	35.83	6	F6:1849	OB5956H1 Ro.raw	8.47E6	5	5	415	443	Oxidation (M); Carbamidomethylation	M9:Oxidation (M):54.18;M10:Oxidation (M):63.99;C13:Carbamidomethylation:1000.00	PEAKS DB
R.VLLEENAGGEQEER(+14.02).G	N	64.84	1585.7583	14	0.7	793.8870	2	27.86	4	F4:1339	OB5954H1 Ro.raw	1.7453E5	3	3	329	342	Methylation(KR)	R14:Methylation(KR): 1000.00	PEAKS PTM
I.FLAGDKDNVIDQIEK.Q	N	64.23	1703.8729	15	-5.7	852.9388	2	32.22	6	F6:1635	OB5956H1 Ro.raw	3.2256E5	3	3	537	551			PEAKS DB
L.AGDKDNVIDQIEK.Q	N	63.80	1443.7205	13	-1.1	722.8667	2	32.43	4	F4:1623	OB5954H1 Ro.raw	3.3427E5	1	1	539	551			PEAKS DB
K.HADADNILVIQQGQ(+.98)ATVTVANGNNRK.S	N	63.07	2747.3950	26	0.8	916.8063	3	31.39	4	F4:1560	OB5954H1 Ro.raw	0	0	0	223	248		Q14:Deamidation (NQ):0.00	PEAKS DB
K.AM(+15.99)VIVVVNK.G	N	63.02	987.5787	9	1.1	494.7972	2	27.11	4	F4:1283	OB5954H1 Ro.raw	1.9071E6	3	3	444	452	Oxidation (M)	M2:Oxidation (M):1000.00	PEAKS DB
K.DLAFP(+15.99)GSGEQVEK.L	N	62.87	1391.6569	13	0.4	696.8360	2	29.40	5	F5:1432	OB5955H1 Ro.raw	8.022E5	3	3	555	567	Hydroxylation Pro	P5:Hydroxylation Pro: 1000.00	PEAKS DB

total 287 peptides

Peptide	Uniq	-10lgP	Mass	Length	ppm	m/z	z	RT	Fraction	Scan	Source File	Area roasted peanut	#Feature	#Feature roasted peanut	Start	End	PTM	AScore	Found By
K.AM(+15.99)VIVVVNKGTGNLELVAVRK.E	N	62.80	2225.2930	21	-4.1	742.7686	3	32.36	6	F6:1577	OB5956 H1 Ro.raw	1.1837E6	2	2	444	464	Oxidation (M)	M2:Oxidation (M):1000.00	PEAKS DB
K.NPQLQDLDMMLTC(+57.02)VEIKEGALM(+15.99)LPHFNSK.A	N	62.74	3387.6335	29	-1.0	1130.2173	3	37.10	5	F5:1900	OB5955 H1 Ro.raw	9.055E7	6	6	415	443	Carbamidomethylation; Oxidation (M)	C13:Carbamidomethylation:1000.00; M22:Oxidation (M):82.05	PEAKS DB
R.SSDNEGVIVK.V	N	62.02	1046.5244	10	0.4	524.2697	2	23.40	5	F5:1065	OB5955 H1 Ro.raw	3.7744E5	3	3	351	360			PEAKS DB
K.QAKDLAFPGSGEQVEK.L	N	61.87	1702.8525	16	1.2	568.6255	3	27.08	6	F6:1321	OB5956 H1 Ro.raw	2.8725E5	6	6	552	567			PEAKS DB
K.SFNLD(-18.01)EGHALR.I	N	61.63	1239.5996	11	2.9	620.8089	2	28.79	4	F4:1400	OB5954 H1 Ro.raw	0	0	0	249	259		D5:Dehydration:49.79	PEAKS PTM
K.SFNLDEGHALR(+31.99)IPSGFISYILNR.H	N	60.54	2650.3503	23	0.1	884.4575	3	39.14	5	F5:2021	OB5955 H1 Ro.raw	5.6521E5	1	1	249	271		R11:Dihydroxy:46.57	PEAKS PTM
Q.SSYLQGFSR.N	N	60.35	1043.5035	9	0.6	522.7593	2	29.24	6	F6:1453	OB5956 H1 Ro.raw	6.7195E4	3	3	304	312			PEAKS DB
K.NPQLQDLDMM(+15.99)LTC(+57.02)VEIKEGALM(+15.99)LPHFNSK.A	N	60.02	3403.6284	29	-8.2	1135.5408	3	36.74	5	F5:1878	OB5955 H1 Ro.raw	6.6522E6	6	6	415	443	Carbamidomethylation; Oxidation (M)	M10:Oxidation (M):21.94;C13:Carbamidomethylation:1000.00; M22:Oxidation (M):61.26	PEAKS DB
K.QFQNLQNHR.I	N	59.95	1183.5846	9	0.0	592.7996	2	23.81	5	F5:1102	OB5955 H1 Ro.raw	1.3905E5	5	5	199	207			PEAKS DB
K.Q(-17.03)FQNLQNHR.I	N	59.90	1166.5581	9	3.5	584.2884	2	28.04	4	F4:1346	OB5954 H1 Ro.raw	5.0983E6	3	3	199	207	Pyro-glu from Q	Q1:Pyro-glu from Q:1000.00	PEAKS PTM
R.I(+41.03)P(+15.99)SGFISYILNR.H	N	59.87	1435.7823	12	-3.6	718.8959	2	36.92	6	F6:1901	OB5956 H1 Ro.raw	1.7838E7	3	3	260	271	Amidination of lysines or N-terminal amines with methyl acetimidate; Hydroxylation Pro	I1:Amidination of lysines or N-terminal amines with methyl acetimidate:1000.00;P2:Hydroxylation Pro:1000.00	PEAKS PTM

total 287 peptides

Peptide	Uniq	-10lgP	Mass	Length	ppm	m/z	z	RT	Fraction	Scan	Source File	Area roasted peanut	#Feature	#Feature roasted peanut	Start	End	PTM	AScore	Found By
K.KNPQLQDLDM(+15.99)MLTC(+57.02)VEIKEGALM(+15.99)LPHFNSK.A	N	59.67	3531.7234	30	-1.9	883.9364	4	35.86	4	F4:1821	OB5954 H1 Ro.raw	2.0004E6	3	3	414	443	Carbamidomethylation; Oxidation (M)	M10:Oxidation (M):17.01;C14:Carbamidomethylation:1000.00;M23:Oxidation (M):101.58	PEAKS DB
K.NPQLQ(+.98)DLDMMLTC(+57.02)VEIK.E	N	59.63	2047.9629	17	1.5	1024.9902	2	44.46	6	F6:2348	OB5956 H1 Ro.raw	0	0	0	415	431	Carbamidomethylation	Q5:Deamidation (NQ):19.68;C13:Carbamidomethylation:1000.00	PEAKS DB
R.IPSGFISYILN(+.98)RHDNQNLR.V	N	59.62	2257.1604	19	4.7	753.3976	3	34.29	5	F5:1740	OB5955 H1 Ro.raw	6.672E5	1	1	260	278		N11:Deamidation (NQ):36.24	PEAKS DB
K.EGALMLPH(+14.02)FNSK.A	N	59.24	1356.6860	12	-3.0	679.3483	2	30.80	5	F5:1531	OB5955 H1 Ro.raw	3.037E5	3	3	432	443	Methylation(others)	H8:Methylation(others):65.81	PEAKS PTM
A.FPGSGEQVEK.L	N	58.82	1076.5138	10	-3.4	539.2623	2	30.34	6	F6:1519	OB5956 H1 Ro.raw	4.3607E5	3	3	558	567			PEAKS DB
K.ISM(-48.00)PVNTPGQFEDFFPASSR.D	N	58.66	2178.0381	20	2.8	727.0220	3	32.48	6	F6:1652	OB5956 H1 Ro.raw	6.4552E6	4	4	282	301	Dethiomethyl	M3:Dethiomethyl:1000.00	PEAKS PTM
R.I(+27.99)PSGFISYILNR.H	N	58.63	1406.7557	12	0.7	704.3856	2	42.27	5	F5:2199	OB5955 H1 Ro.raw	0	0	0	260	271	Formylation	I1:Formylation:1000.00	PEAKS PTM
R.LFEVKPDKKNPQLQDLDMM(+15.99)LTC(+57.02)VEIK.E	N	58.02	3147.6018	26	2.2	787.9095	4	33.24	5	F5:1670	OB5955 H1 Ro.raw	2.286E6	2	2	406	431	Carbamidomethylation	M19:Oxidation (M):40.00;C22:Carbamidomethylation:1000.00	PEAKS DB
R.EGEQEWGTPGS(+42.01)EVR.E	Y	57.75	1601.6958	14	7.6	801.8613	2	27.46	6	F6:1334	OB5956 H1 Ro.raw	1.0047E5	3	3	147	160		S11:Acetylation (TSCYH):32.97	PEAKS PTM
K.SFN(+15.99)LDEGHALR.I	N	57.68	1273.6051	11	0.9	637.8104	2	27.50	4	F4:1324	OB5954 H1 Ro.raw	9.739E5	3	3	249	259	Oxidation or Hydroxylation	N3:Oxidation or Hydroxylation:55.92	PEAKS PTM
F.NLDEGHALR.I	N	57.57	1023.5097	9	0.6	512.7625	2	27.93	5	F5:1349	OB5955 H1 Ro.raw	2.6681E6	3	3	251	259			PEAKS DB

total 287 peptides

Peptide	Uniq	-10lgP	Mass	Length	ppm	m/z	z	RT	Fraction	Scan	Source File	Area roasted peanut	#Feature	#Feature roasted peanut	Start	End	PTM	AScore	Found By
K.A(+41.03)M(+15.99)VIVVVNKGTGNLELVAVR.K	N	57.55	2138.2246	20	-5.4	1070.1138	2	34.47	5	F5:1739	OB5955 H1 Ro.raw	4.5183E6	3	3	444	463	Amidination of lysines or N-terminal amines with methyl acetimidate; Oxidation (M)	A1:Amidination of lysines or N-terminal amines with methyl acetimidate:207.56; M2:Oxidation (M):1000.00	PEAKS PTM
K.NPQLQDLDMMLTC(+71.04)VEIK.E	N	57.51	2060.9944	17	3.0	1031.5076	2	37.24	5	F5:1915	OB5955 H1 Ro.raw	1.057E5	2	2	415	431	Propionamide	C13:Propionamide:1000.00	PEAKS PTM
A.GDKDNVIDQIEK.Q	N	57.39	1372.6833	12	-1.5	687.3479	2	32.35	4	F4:1613	OB5954 H1 Ro.raw	1.1865E6	3	3	540	551			PEAKS DB
R.K(+43.01)(+14.02)SFNLDEGHALR.I	N	55.95	1442.7266	12	0.0	722.3705	2	26.25	5	F5:1250	OB5955 H1 Ro.raw	4.2824E5	6	6	248	259	Carbamylation; Methylation(KR)	K1:Carbamylation:1000.00; K1:Methylation (KR):156.90	PEAKS PTM
R.IPSGFISYILNRHDN(+.98)QNLR.V	N	55.73	2257.1604	19	-0.7	753.3936	3	34.26	4	F4:1732	OB5954 H1 Ro.raw	0	0	0	260	278		N15:Deamidation (NQ):0.00	PEAKS DB
K.ISMPVN(+.98)TPGQFEDFFPASSR.D	N	54.95	2227.0254	20	6.6	1114.5273	2	36.77	4	F4:1885	OB5954 H1 Ro.raw	1.011E5	2	2	282	301		N6:Deamidation (NQ):42.68	PEAKS DB
R.K(+27.99)SFNLDEGHALR.I	N	54.77	1413.7001	12	1.4	707.8583	2	28.46	5	F5:1387	OB5955 H1 Ro.raw	5.5831E4	2	2	248	259	Formylation	K1:Formylation:1000.00	PEAKS PTM
R.LFEVKPDKK.N	N	54.58	1102.6385	9	1.1	552.3271	2	24.84	4	F4:1157	OB5954 H1 Ro.raw	1.1453E6	6	6	406	414			PEAKS DB
K.NPQLQDLDMMLTC(+57.02)VEIKEGALMLP(+15.99)HFNSK.A	N	54.18	3387.6335	29	-0.6	1130.2178	3	37.70	4	F4:2016	OB5954 H1 Ro.raw	4.3311E7	3	3	415	443	Carbamidomethylation; Hydroxylation Pro	C13:Carbamidomethylation:1000.00; P24:Hydroxylation Pro:122.99	PEAKS DB
R.EGEQEWGTP(+15.99)GSEVREETSR.N	Y	54.09	2177.9460	19	2.3	1089.9828	2	26.43	5	F5:1263	OB5955 H1 Ro.raw	1.3271E7	6	6	147	165	Hydroxylation Pro	P9:Hydroxylation Pro:1000.00	PEAKS DB

total 287 peptides

Peptide	Uniq	-10lgP	Mass	Length	ppm	m/z	z	RT	Fraction	Scan	Source File	Area roasted peanut	#Feature	#Feature roasted peanut	Start	End	PTM	AScore	Found By
R.IFLAGDKDNVIDQIEK(+42.01)Q(+.98)AK(+14.02).D	N	53.39	2201.1580	19	2.9	734.7288	3	32.42	5	F5:1627	OB5955 H1 Ro.raw	4.6872E5	1	1	536	554		K16:Acetylation (K): 4.05;Q17:Deamidation (NQ): 20.26; K19:Methylation(KR): 4.05	PEAKS PTM
E.PDLSNNFGR.L	N	53.18	1018.4832	9	0.9	510.2493	2	31.57	6	F6:1584	OB5956 H1 Ro.raw	1.3909E6	4	4	397	405			PEAKS DB
K.NPQLQDLDMM(+15.99)LTCVEIK(+14.02).E	N	53.12	2019.9679	17	1.7	1010.9930	2	35.46	5	F5:1802	OB5955 H1 Ro.raw	4.2793E5	1	1	415	431	Methylation(KR)	M10:Oxidation (M):8.14;K17: Methylation(KR):1000.00	PEAKS PTM
R.IFLAGDKDNVIDQ(+.98)IEKQAK.D	N	53.10	2145.1316	19	1.4	716.0521	3	33.04	6	F6:1686	OB5956 H1 Ro.raw	1.2146E5	1	1	536	554		Q13:Deamidation (NQ):25.18	PEAKS DB
K.SFNLDEGHALRIP(+15.99)SGFISYILNR.H	N	53.04	2634.3552	23	-3.9	879.1223	3	38.76	6	F6:2023	OB5956 H1 Ro.raw	1.5218E6	3	3	249	271	Hydroxylation Pro	P13:Hydroxylation Pro:1000.00	PEAKS DB
R.VAKIS(+77.99)MPVNTPGQFEDFFPASSR.D	N	52.77	2602.2290	23	2.9	1302.1256	2	38.06	5	F5:1958	OB5955 H1 Ro.raw	0	0	0	279	301	Methylphosphonylation	S5:Methylphosphonylation:56.99	PEAKS PTM
R.EQEWEEEEEDEEEEGSNREVRR.Y	N	52.77	2821.1545	22	1.7	941.3937	3	26.62	5	F5:1273	OB5955 H1 Ro.raw	8.8078E5	3	3	473	494			PEAKS DB
K.QFQNLQN(+.98)HR.I	N	52.53	1184.5686	9	5.4	593.2948	2	23.60	6	F6:1114	OB5956 H1 Ro.raw	8.0383E2	1	1	199	207		N7:Deamidation (NQ): 0.00	PEAKS DB
N.PFYFPSRR.F	N	52.44	1068.5504	8	-0.1	535.2824	2	28.89	6	F6:1431	OB5956 H1 Ro.raw	4.3491E6	4	4	168	175			PEAKS DB
K.NPQLQDLDM(+15.99)M(+15.99)LTC(+57.02)VEIKEGALM(+15.99)LPHFNSK.A	N	52.35	3419.6233	29	-0.9	855.9124	4	34.83	5	F5:1766	OB5955 H1 Ro.raw	1.7196E6	3	3	415	443	Oxidation (M); Carbamidomethylation	M9:Oxidation (M):1000.00; M10:Oxidation (M):1000.00; C13:Carbamidomethylation:1000.00; M22:Oxidation (M):1000.00	PEAKS DB
R.VAK(+27.99)ISMPVNTPGQFEDFFPASSR.D	N	52.16	2552.2368	23	-0.3	1277.1252	2	36.45	6	F6:1882	OB5956 H1 Ro.raw	0	0	0	279	301		K3:Formylation:9.40	PEAKS PTM

total 287 peptides

Peptide	Uniq	-10lgP	Mass	Length	ppm	m/z	z	RT	Fraction	Scan	Source File	Area roasted peanut	#Feature	#Feature roasted peanut	Start	End	PTM	AScore	Found By
K.ISMPVNTPGQFEDFFPASSRDQSSYLQGFSR.N	N	52.15	3494.6201	31	-0.9	1165.8796	3	36.01	6	F6:1851	OB5956 H1 Ro.raw	4.0537E6	2	2	282	312			PEAKS DB
R.V(+27.99)AKISMPVNTPGQFEDFFPASSR.D	N	51.59	2552.2368	23	-0.5	1277.1250	2	36.43	4	F4:1865	OB5954 H1 Ro.raw	7.7611E5	1	1	279	301		V1:Formylation:0.00	PEAKS PTM
R.WGPAEPR.E	Y	51.54	811.3976	7	1.8	406.7068	2	25.60	4	F4:1224	OB5954 H1 Ro.raw	1.6393E6	3	3	109	115			PEAKS DB
K.SFNLDEGHALR(+31.99).I	N	50.82	1289.6000	11	-1.4	645.8063	2	30.41	6	F6:1520	OB5956 H1 Ro.raw	0	0	0	249	259	Dihydroxy	R11:Dihydroxy:122.75	PEAKS PTM
R.IFLAGD(+15.99)KDNVIDQIEK.Q	N	50.66	1832.9519	16	-1.9	917.4814	2	31.99	4	F4:1600	OB5954 H1 Ro.raw	1.5111E6	3	3	536	551		D6:Oxidation or Hydroxylation:12.60	PEAKS PTM
R.VLLEE(+53.92)NAGGEQEER.G	N	50.61	1625.6620	14	-1.0	542.8940	3	26.76	5	F5:1278	OB5955 H1 Ro.raw	0	0	0	329	342		E5:Replacement of 2 protons by iron:20.92	PEAKS PTM
P.SGFISYILNR.H	N	50.48	1168.6240	10	1.5	585.3202	2	35.08	6	F6:1803	OB5956 H1 Ro.raw	3.0349E5	1	1	262	271			PEAKS DB
R.IFLAGDKDNVIDQIEK(+14.02)Q(+.98)AK(+42.01).D	N	50.18	2201.1580	19	5.4	734.7306	3	32.48	6	F6:1657	OB5956 H1 Ro.raw	3.9073E5	1	1	536	554	Deamidation (NQ)	K16:Methylation(KR):7.64;Q17:Deamidation (NQ):58.99;K19:Acetylation (K):7.64	PEAKS PTM
K.S(+43.01)(+14.02)FNLDEGHALR.I	N	50.01	1314.6316	11	-0.5	658.3228	2	28.11	5	F5:1359	OB5955 H1 Ro.raw	1.9424E6	1	1	249	259	Carbamylation; Methylation(others)	S1:Carbamylation:1000.00;S1:Methylation(others):88.98	PEAKS PTM
N.LDEGHALR.I	N	49.91	909.4668	8	0.0	455.7407	2	27.93	5	F5:1350	OB5955 H1 Ro.raw	1.94E5	3	3	252	259			PEAKS DB
K.SFNLDEGH(+15.99)ALR(+31.99)IPSGFISYILNR.H	N	49.58	2666.3452	23	1.2	889.7900	3	39.33	6	F6:2062	OB5956 H1 Ro.raw	3.3065E5	2	2	249	271	Oxidation (HW)	H8:Oxidation (HW):1000.00;R11:Dihydroxy:45.01	PEAKS PTM
K.SFNLDEGH(+15.99)ALRIPSGFISYILNR.H	N	49.38	2634.3555	23	2.3	1318.1880	2	38.82	5	F5:2002	OB5955 H1 Ro.raw	9.0457E5	1	1	249	271	Oxidation (HW)	H8:Oxidation (HW):1000.00	PEAKS PTM
R.ESHFVSARPQSQSP(+15.99)SSPEKEDQEEEN.Q	N	49.34	2973.2859	26	1.6	992.1041	3	24.81	6	F6:1186	OB5956 H1 Ro.raw	3.8098E5	2	2	574	599		P14:Hydroxylation Pro:11.12	PEAKS DB

total 287 peptides

Peptide	Uniq	-10lgP	Mass	Length	ppm	m/z	z	RT	Fraction	Scan	Source File	Area roasted peanut	#Feature	#Feature roasted peanut	Start	End	PTM	AScore	Found By
R.VLLE(+21.98)ENAGGEQEER.G	N	48.97	1593.7246	14	-0.5	797.8692	2	26.81	5	F5:1275	OB5955 H1 Ro.raw	4.3339E5	3	3	329	342		E4:Sodium adduct:40.00	PEAKS PTM
R.LFEVKPDK.K	N	48.69	974.5436	8	1.7	488.2799	2	25.30	5	F5:1183	OB5955 H1 Ro.raw	1.7748E6	6	6	406	413			PEAKS DB
K.NPQLQDLDMM(+15.99)LTC(+57.02)VEIKEGALMLPHFN(+.98)SK.A	N	48.63	3388.6174	29	6.2	848.1669	4	36.39	6	F6:1880	OB5956 H1 Ro.raw	2.0276E6	1	1	415	443	Carbamidomethylation	M10:Oxidation (M):27.96;C13:Carbamidomethylation:1000.00;N27:Deamidation (NQ):18.27	PEAKS DB
R.SKQFQNLQNHR.I	Y	48.58	1398.7117	11	-0.5	467.2443	3	24.46	4	F4:1146	OB5954 H1 Ro.raw	1.2409E5	3	3	197	207			PEAKS DB
R.IVQIEARPN.T	Y	48.42	1038.5822	9	0.7	520.2987	2	25.99	4	F4:1238	OB5954 H1 Ro.raw	4.8023E4	3	3	208	216			PEAKS DB
R.N(+27.99)TLEAAFNAEFNEIRR.V	N	48.34	1921.9282	16	-1.7	961.9697	2	37.51	4	F4:1908	OB5954 H1 Ro.raw	4.2206E4	1	1	313	328	Formylation	N1:Formylation:1000.00	PEAKS PTM
R.NT(-18.01)LEAAFNAEFNEIRR.V	N	48.27	1875.9227	16	-1.8	626.3137	3	34.20	4	F4:1731	OB5954 H1 Ro.raw	1.108E7	3	3	313	328	Dehydration	T2:Dehydration:1000.00	PEAKS PTM
R.LFEVKPDKKNPQLQDLDM(+15.99)MLTC(+57.02)VEIK.E	N	48.12	3147.6018	26	-1.9	787.9062	4	34.20	4	F4:1734	OB5954 H1 Ro.raw	2.5552E6	4	4	406	431	Carbamidomethylation	M18:Oxidation (M):33.98;C22:Carbamidomethylation:1000.00	PEAKS DB
K.KNPQLQDLDMM(+15.99)LTC(+57.02)VEIKEGALM(+15.99)LPHFNSK.A	N	47.73	3531.7234	30	-1.1	883.9372	4	35.80	5	F5:1822	OB5955 H1 Ro.raw	0	0	0	414	443	Carbamidomethylation	M11:Oxidation (M):0.00;C14:Carbamidomethylation:1000.00;M23:Oxidation (M):45.87	PEAKS DB
R.DQSSY(-18.01)LQGFSR.N	N	47.62	1268.5785	11	-0.6	635.2961	2	30.85	6	F6:1579	OB5956 H1 Ro.raw	2.6456E5	1	1	302	312		Y5:Dehydration:0.00	PEAKS PTM
K.KGSEEEDIT(-18.01)N(+.98)PINLR.D	N	47.57	1696.8268	15	2.3	566.6175	3	29.07	6	F6:1442	OB5956 H1 Ro.raw	3.8587E5	3	3	379	393	Deamidation (NQ)	T9:Dehydration:45.01;N10:Deamidation (NQ):59.10	PEAKS PTM

total 287 peptides

Peptide	Uniq	-10lgP	Mass	Length	ppm	m/z	z	RT	Fraction	Scan	Source File	Area roasted peanut	#Feature	#Feature roasted peanut	Start	End	PTM	AScore	Found By
R.NTLEAAFNAEFN(+.98)EIR.R	N	47.43	1738.8162	15	9.2	870.4233	2	44.36	5	F5:2303	OB5955 H1 Ro.raw	5.1969E3	1	1	313	327		N12:Deamidation (NQ):0.00	PEAKS DB
K.SFNLDEGHALRIPSGFISYILNRHDNQNLR.V	N	47.02	3495.7759	30	-2.1	700.1610	5	35.83	6	F6:1842	OB5956 H1 Ro.raw	2.2604E6	2	2	249	278			PEAKS DB
K.NPQLQ(+.98)DLDM(+15.99)MLTC(+57.02)VEIKEGALM(+15.99)LPHFNSK.A	N	47.00	3404.6125	29	-0.4	1135.8777	3	36.69	5	F5:1878	OB5955 H1 Ro.raw	3.8413E5	1	1	415	443	Carbamidomethylation	Q5:Deamidation (NQ): 45.47;M9:Oxidation (M):0.00;C13:Carbamidomethylation:1000.00;M22:Oxidation (M):38.75	PEAKS DB
K.D(+43.01)(+14.02)LAFPGSGEQVEK.L	N	46.93	1432.6833	13	-0.1	717.3489	2	31.08	4	F4:1535	OB5954 H1 Ro.raw	9.9373E5	3	3	555	567	Carbamylation; Methylation(others)	D1:Carbamylation:220.79;D1:Methylation(others):132.53	PEAKS PTM
K.SFNLDEGHALR(+14.02)IPSGFISYILN(+.98)R.H	N	46.86	2633.3601	23	-6.4	878.7883	3	36.34	5	F5:1848	OB5955 H1 Ro.raw	1.8431E5	2	2	249	271	Deamidation (NQ)	R11:Methylation(KR): 28.64;N22:Deamidation (NQ):57.71	PEAKS PTM
F.PGSGEQVEK.L	N	46.51	929.4454	9	-3.7	465.7282	2	30.34	6	F6:1524	OB5956 H1 Ro.raw	5.467E4	1	1	559	567			PEAKS DB
R.ESHFVSARPQSQSP(+15.99)SSPEKEDQEEE.N	N	46.47	2859.2429	25	2.4	954.0906	3	24.92	5	F5:1173	OB5955 H1 Ro.raw	7.3448E4	1	1	574	598		P14:Hydroxylation Pro:26.52	PEAKS DB
R.DQSSYLQGFSR(+15.99).N	N	46.23	1302.5840	11	2.5	652.3009	2	30.63	5	F5:1518	OB5955 H1 Ro.raw	1.9563E5	4	4	302	312		R11:Oxidation or Hydroxylation:42.88	PEAKS PTM
L.DEGHALR.I	N	45.97	796.3828	7	2.5	399.1996	2	27.93	5	F5:1351	OB5955 H1 Ro.raw	9.4873E4	3	3	253	259			PEAKS DB
R.LFEVKPDKKNPQLQ(+.98)DLDMM(+15.99)LTC(+57.02)VEIK.E	N	45.61	3148.5857	26	7.4	788.1595	4	33.17	5	F5:1670	OB5955 H1 Ro.raw	4.4314E5	1	1	406	431	Carbamidomethylation	Q14:Deamidation (NQ):0.00;M19:Oxidation (M): 23.10;C22:Carbamidomethylation:1000.00	PEAKS DB

total 287 peptides

Peptide	Uniq	-10lgP	Mass	Length	ppm	m/z	z	RT	Fraction	Scan	Source File	Area roasted peanut	#Feature	#Feature roasted peanut	Start	End	PTM	AScore	Found By
S.F(+43.01)NLDE(+21.98)GHALRIPSGFISYILNR.H	N	45.55	2596.3162	22	7.8	866.4528	3	40.28	6	F6:2103	OB5956 H1 Ro.raw	2.2619E6	3	3	250	271	Carbamylation	F1:Carbamylation:1000.00;E5:Sodium adduct:0.00	PEAKS PTM
R.IFLAGDKDNVID.Q	N	45.24	1318.6769	12	0.0	660.3457	2	31.81	4	F4:1586	OB5954 H1 Ro.raw	1.1587E5	3	3	536	547			PEAKS DB
K.KNPQLQDLDM(+15.99)MLTC(+57.02)VEIKEGALMLPHFNSK.A	N	45.13	3515.7285	30	-1.9	879.9377	4	35.30	4	F4:1798	OB5954 H1 Ro.raw	6.4172E5	1	1	414	443	Carbamidomethylation	M10:Oxidation (M):14.02;C14:Carbamidomethylation:1000.00	PEAKS DB
R.NTLEAAFNAEFN(+.98)EIRR.V	N	45.08	1894.9172	16	-4.2	948.4619	2	33.08	4	F4:1691	OB5954 H1 Ro.raw	2.2628E6	3	3	313	328		N12:Deamidation (NQ):39.02	PEAKS DB
R.IFLAGDK(+15.99)DNVIDQIEK.Q	N	44.85	1832.9519	16	0.9	917.4841	2	31.94	6	F6:1617	OB5956 H1 Ro.raw	3.7677E5	1	1	536	551		K7:Oxidation or Hydroxylation:17.01	PEAKS PTM
V.QIEARPNTLVLPK.H	Y	44.77	1477.8616	13	-1.8	739.9368	2	30.56	4	F4:1513	OB5954 H1 Ro.raw	1.5072E5	3	3	210	222			PEAKS DB
R.KSFNLDEGH(+15.99)ALR.I	N	44.62	1401.7001	12	3.9	701.8600	2	27.64	6	F6:1349	OB5956 H1 Ro.raw	7.3368E4	2	2	248	259	Oxidation (HW)	H9:Oxidation (HW):1000.00	PEAKS PTM
R.N(+.98)TLEAAFNAEFNEIRR.V	N	44.42	1894.9172	16	3.8	948.4695	2	36.66	6	F6:1890	OB5956 H1 Ro.raw	1.2834E6	2	2	313	328		N1:Deamidation (NQ):41.52	PEAKS DB
R.ESHFVSARP(+15.99)QSQSPSSPEKEDQEEEN.Q	N	44.15	2973.2859	26	2.8	992.1053	3	24.92	5	F5:1161	OB5955 H1 Ro.raw	1.8315E5	1	1	574	599		P9:Hydroxylation Pro:18.35	PEAKS DB
K.AMVIVVVN(+.98)KGTGNLELVAVRK.E	N	43.52	2210.2820	21	1.9	1106.1504	2	34.35	5	F5:1720	OB5955 H1 Ro.raw	6.7558E5	2	2	444	464		N8:Deamidation (NQ):28.79	PEAKS DB
R.IFLAGDKDNVIDQIEK(+14.02)(+43.01).Q	N	43.38	1873.9785	16	-1.3	937.9953	2	32.42	5	F5:1608	OB5955 H1 Ro.raw	8.0571E6	1	1	536	551	Methylation(C-term); Carbamylation	K16:Methylation(C-term):1000.00;K16:Carbamylation:128.00	PEAKS PTM
R.D(-18.01)QSSYLQGFSR.N	N	43.26	1268.5785	11	1.6	635.2975	2	29.92	6	F6:1489	OB5956 H1 Ro.raw	0	0	0	302	312		D1:Dehydration:0.00	PEAKS PTM

total 287 peptides

Peptide	Uniq	-10lgP	Mass	Length	ppm	m/z	z	RT	Fraction	Scan	Source File	Area roasted peanut	#Feature	#Feature roasted peanut	Start	End	PTM	AScore	Found By
R.LFEVKPDKKNPQLQDLDM(+15.99)M(+15.99)LTC(+57.02)VEIK.E	N	43.03	3163.5967	26	-3.4	791.9037	4	32.67	6	F6:1663	OB5956 H1 Ro.raw	2.1471E6	3	3	406	431	Oxidation (M); Carbamidomethylation	M18:Oxidation (M):1000.00; M19:Oxidation (M):1000.00; C22:Carbamidomethylation:1000.00	PEAKS DB
T.GNLELVAVRK.E	N	43.00	1097.6556	10	0.5	549.8354	2	27.00	5	F5:1296	OB5955 H1 Ro.raw	3.6527E5	3	3	455	464			PEAKS DB
K.KN(+.98)PQLQ(+.98)DLDMMLTC(+57.02)VEIKEGALMLPHFNSK.A	N	42.92	3501.7017	30	7.8	876.4395	4	36.93	6	F6:1903	OB5956 H1 Ro.raw	5.6427E6	1	1	414	443	Carbamidomethylation	N2:Deamidation (NQ):14.04; Q6:Deamidation (NQ):0.00;C14:Carbamidomethylation:1000.00	PEAKS DB
R.IFLAGDKDNVIDQIEK(+43.01)QAK(+14.02).D	N	42.77	2201.1692	19	0.9	734.7310	3	32.71	4	F4:1644	OB5954 H1 Ro.raw	3.8417E5	1	1	536	554		K16:Carbamylation:0.00;K19:Methylation (KR):0.00	PEAKS PTM
K.S(+43.01)(+14.02)FNLDEGHALRIPSGFISYILNR.H	N	42.43	2675.3818	23	-1.9	892.7996	3	37.80	6	F6:1964	OB5956 H1 Ro.raw	0	0	0	249	271		S1:Carbamylation:1000.00; S1:Methylation (others):34.30	PEAKS PTM
K.GSEEEDITNPIN(+.98)LRDGEPDLSNNFGR.L	N	42.34	2888.3059	26	6.7	1445.1699	2	32.90	4	F4:1642	OB5954 H1 Ro.raw	1.8658E5	1	1	380	405		N12:Deamidation (NQ):12.33	PEAKS DB
R.V(+43.01)LLE(+14.02)ENAGGEQEER.G	N	42.24	1628.7642	14	1.7	815.3907	2	27.00	5	F5:1297	OB5955 H1 Ro.raw	4.2233E5	3	3	329	342	Carbamylation	V1:Carbamylation:1000.00;E4:Methylation (others):30.46	PEAKS PTM
R.I(+43.01)FLAGDK(+14.02)DNVIDQIEK.Q	N	42.15	1873.9785	16	4.9	938.0011	2	32.57	4	F4:1632	OB5954 H1 Ro.raw	0	0	0	536	551	Carbamylation; Methylation(KR)	I1:Carbamylation:135.80;K7:Methylation(KR):195.94	PEAKS PTM

total 287 peptides

Peptide	Uniq	-10lgP	Mass	Length	ppm	m/z	z	RT	Fraction	Scan	Source File	Area roasted peanut	#Feature	#Feature roasted peanut	Start	End	PTM	AScore	Found By
K.D(+15.99)LAFPGSGEQVEK.L	N	42.08	1391.6569	13	0.4	696.8360	2	29.40	5	F5:1368	OB5955H1Ro.raw	5.511E5	2	2	555	567	Oxidation or Hydroxylation	D1:Oxidation or Hydroxylation:77.13	PEAKS PTM
K.KNPQLQDLDMM(+15.99)LTC(+57.02)VEIKEGALMLPHFNSK.A	N	42.02	3515.7285	30	1.3	879.9406	4	35.25	5	F5:1790	OB5955H1Ro.raw	0	0	0	414	443	Carbamidomethylation	M11:Oxidation (M):12.28;C14:Carbamidomethylation:1000.00	PEAKS DB
K.A(+41.03)M(+15.99)VIVVVNKGTGNLELVAVRK.E	N	41.92	2266.3196	21	0.6	1134.1677	2	32.98	5	F5:1660	OB5955H1Ro.raw	1.5326E7	6	6	444	464	Amidination of lysines or N-terminal amines with methyl acetimidate; Oxidation (M)	A1:Amidination of lysines or N-terminal amines with methyl acetimidate:131.34;M2:Oxidation (M):1000.00	PEAKS PTM
K.EGALM(+15.99)LPHFN(+.98)SK.A	N	41.90	1359.6493	12	2.8	680.8338	2	31.22	4	F4:1550	OB5954H1Ro.raw	0	0	0	432	443	Oxidation (M); Deamidation (NQ)	M5:Oxidation (M):1000.00;N10:Deamidation (NQ):1000.00	PEAKS DB
K.NPQLQDLDM(-48.00)MLTC(+57.02)VEIK.E	N	41.66	1998.9755	17	6.7	667.3369	3	34.52	6	F6:1773	OB5956H1Ro.raw	2.0007E6	2	2	415	431	Carbamidomethylation	M9:Dethiomethyl:11.06;C13:Carbamidomethylation:1000.00	PEAKS PTM
K.GTGNLELVAVRKEQQQR.G	N	41.33	1925.0442	17	1.4	642.6896	3	26.67	6	F6:1294	OB5956H1Ro.raw	2.686E4	1	1	453	469			PEAKS DB
K.NPQLQDLDMMLTC(+57.02)VEIK(+43.01)EGALMLPHFNSK(+14.02).A	N	41.19	3428.6602	29	-1.5	858.1710	4	37.84	6	F6:1971	OB5956H1Ro.raw	2.9855E6	1	1	415	443	Carbamidomethylation	C13:Carbamidomethylation:1000.00;K17:Carbamylation:11.67;K29:Methylation(KR):11.67	PEAKS PTM

total 287 peptides

Peptide	Uniq	-10lgP	Mass	Length	ppm	m/z	z	RT	Fraction	Scan	Source File	Area roasted peanut	#Feature	#Feature roasted peanut	Start	End	PTM	AScore	Found By
R.IFLAGDKDN(+.98)VIDQIEKQ(+.98)AK.D	N	41.17	2146.1157	19	9.8	716.3862	3	32.98	5	F5:1661	OB5955 H1 Ro.raw	1.2092E5	1	1	536	554		N9:Deamidation (NQ):13.83;Q17:Deamidation (NQ):17.65	PEAKS DB
K.NPQ(+.98)LQDLDMMLTC(+57.02)VEIK.E	N	41.15	2047.9629	17	2.9	1024.9917	2	46.45	4	F4:2441	OB5954 H1 Ro.raw	0	0	0	415	431	Carbamidomethylation	Q3:Deamidation (NQ):14.04;C13:Carbamidomethylation:1000.00	PEAKS DB
R.EQEWEEEEEDEEEEGSNREVR.R	N	41.08	2665.0535	21	2.4	889.3605	3	27.88	4	F4:1353	OB5954 H1 Ro.raw	4.8884E4	1	1	473	493			PEAKS DB
R.VLLEEN(+.98)AGGEQEERGQR.R	N	40.76	1913.9078	17	-1.6	638.9755	3	25.99	4	F4:1234	OB5954 H1 Ro.raw	0	0	0	329	345		N6:Deamidation (NQ):28.79	PEAKS DB
K.GT(-18.01)GNLELVAVRK.E	N	40.55	1237.7142	12	2.6	413.5797	3	27.00	5	F5:1287	OB5955 H1 Ro.raw	9.9167E5	2	2	453	464	Dehydration	T2:Dehydration:1000.00	PEAKS PTM
K.KNPQLQ(+.98)DLDMMLTC(+57.02)VEIKEGALMLPHFNSK.A	N	40.26	3500.7175	30	3.4	876.1896	4	37.51	4	F4:1896	OB5954 H1 Ro.raw	1.6398E6	1	1	414	443	Carbamidomethylation	Q6:Deamidation (NQ):12.19;C14:Carbamidomethylation:1000.00	PEAKS DB
R.EGEQEW(+15.99)GTPGSEVREETSR.N	Y	39.91	2177.9460	19	1.6	726.9905	3	26.43	5	F5:1304	OB5955 H1 Ro.raw	1.2886E7	4	4	147	165	Oxidation (HW)	W6:Oxidation (HW):1000.00	PEAKS PTM
R.IFLAGD(+14.02)K(+43.01)DNVIDQIEK.Q	N	39.86	1873.9785	16	1.7	937.9982	2	32.48	6	F6:1653	OB5956 H1 Ro.raw	9.6228E6	3	3	536	551	Carbamylation	D6:Methylation (others):18.53;K7:Carbamylation:62.41	PEAKS PTM
R.E(-18.01)GEQEWGTPGSEVREETSR.N	Y	39.24	2143.9407	19	2.7	1072.9805	2	29.74	5	F5:1461	OB5955 H1 Ro.raw	2.5488E5	3	3	147	165	Pyro-glu from E	E1:Pyro-glu from E:1000.00	PEAKS PTM
R.EEDWRQPR.E	N	38.91	1114.5155	8	1.8	558.2661	2	23.91	4	F4:1112	OB5954 H1 Ro.raw	0	0	0	120	127			PEAKS DB
R.SKQFQNLQNH(+15.99)R.I	Y	38.88	1414.7065	11	2.4	708.3622	2	24.12	5	F5:1124	OB5955 H1 Ro.raw	1.2982E3	1	1	197	207	Oxidation (HW)	H10:Oxidation (HW):1000.00	PEAKS PTM
K.A(+43.01)MVIVVVNKGTGNLELVAVR.K	N	38.81	2124.2090	20	0.7	1063.1125	2	33.65	4	F4:1688	OB5954 H1 Ro.raw	6.7878E5	3	3	444	463	Carbamylation	A1:Carbamylation:161.20	PEAKS PTM

total 287 peptides

Peptide	Uniq	-10lgP	Mass	Length	ppm	m/z	z	RT	Fraction	Scan	Source File	Area roasted peanut	#Feature	#Feature roasted peanut	Start	End	PTM	AScore	Found By
K.EGALM(-48.00)LPHFNSK.A	N	38.72	1294.6670	12	1.5	432.5636	3	28.65	5	F5:1381	OB5955 H1 Ro.raw	3.9929E6	3	3	432	443	Dethiomethyl	M5:Dethiomethyl:1000.00	PEAKS PTM
R.PNTLVLPK.H	N	38.45	880.5382	8	-2.9	441.2751	2	28.00	6	F6:1374	OB5956 H1 Ro.raw	1.9367E5	2	2	215	222			PEAKS DB
K.S(+42.01)(+14.02)FNLDEGHALR.I	N	38.42	1313.6364	11	-3.6	657.8231	2	28.09	5	F5:1359	OB5955 H1 Ro.raw	1.5617E6	1	1	249	259		S1:Acetylation (N-term):1000.00;S1:Methylation (others):49.79	PEAKS PTM
R.IFLAGDKDN(+.98)VIDQIEK(-.98).Q	N	38.33	1816.9570	16	0.5	606.6599	3	57.76	5	F5:3342	OB5955 H1 Ro.raw	3.1778E5	3	3	536	551	Amidation	N9:Deamidation (NQ):29.57;K16:Amidation:1000.00	PEAKS PTM
K.S(-18.01)FNLD(-18.01)EGHALR(+14.02)IPSGFISYILNR.H	N	37.89	2596.3550	23	-7.1	866.4528	3	40.28	6	F6:2110	OB5956 H1 Ro.raw	1.4449E6	1	1	249	271	Methylation(KR)	S1:Dehydration:19.61;D5:Dehydration:34.08;R11:Methylation (KR):157.81	PEAKS PTM
K.SFNLD(+53.92)EGHALR.I	N	37.31	1311.5294	11	1.0	438.1842	3	28.04	4	F4:1362	OB5954 H1 Ro.raw	1.3891E5	2	2	249	259		D5:Replacement of 2 protons by iron:18.17	PEAKS PTM
R.N(+.98)TLEAAFNAEFNEIR.R	N	36.98	1738.8162	15	9.2	870.4233	2	44.36	5	F5:2311	OB5955 H1 Ro.raw	5.1969E3	1	1	313	327		N1:Deamidation (NQ):21.41	PEAKS DB
R.REQEW(+15.99)EEEEEDEEEEGSNR.E	N	36.76	2452.9375	19	-0.5	818.6527	3	25.98	4	F4:1239	OB5954 H1 Ro.raw	6.7106E4	2	2	472	490	Oxidation (HW)	W5:Oxidation (HW):1000.00	PEAKS PTM
R.IFLAGDK(+27.99)DNVIDQIEK.Q	N	36.69	1844.9519	16	-0.6	923.4827	2	34.83	5	F5:1775	OB5955 H1 Ro.raw	1.1863E5	1	1	536	551		K7:Formylation:17.54	PEAKS PTM
K.SFNLDE(+53.92)GHALR.I	N	36.63	1311.5294	11	-0.2	438.1837	3	27.93	5	F5:1353	OB5955 H1 Ro.raw	5.2242E4	1	1	249	259		E6:Replacement of 2 protons by iron:16.90	PEAKS PTM
R.IFLAGDKDNVID(-18.01)QIEK.Q	N	36.38	1798.9464	16	0.7	900.4811	2	31.39	6	F6:1591	OB5956 H1 Ro.raw	6.7841E4	1	1	536	551		D12:Dehydration:0.00	PEAKS PTM

total 287 peptides

Peptide	Uniq	-10lgP	Mass	Length	ppm	m/z	z	RT	Fraction	Scan	Source File	Area roasted peanut	#Feature	#Feature roasted peanut	Start	End	PTM	AScore	Found By
K.NPQLQ(+.98)DLDMM(+15.99)LTC(+57.02)VEIKEGALM(+15.99)LPHFNSK.A	N	36.05	3404.6125	29	-1.8	852.1589	4	38.22	6	F6:1993	OB5956 H1 Ro.raw	8.1948E5	1	1	415	443	Carbamidomethylation	Q5:Deamidation (NQ): 30.14; M10:Oxidation (M):0.00;C13: Carbamidomethylation:1000.00;M22:Oxidation (M):14.60	PEAKS DB
K.NPQLQDLDMMLTCVEIK(+14.02)EGALM(+15.99)LPHFNSK.A	N	36.00	3344.6277	29	-0.2	837.1640	4	37.84	6	F6:1961	OB5956 H1 Ro.raw	7.3561E6	2	2	415	443		K17:Methylation(KR): 22.74; M22:Oxidation (M):20.37	PEAKS PTM
K.AMVIVVVNKGTGN(+.98)LELVAVRK.E	N	35.97	2210.2820	21	6.2	737.7725	3	40.10	5	F5:2113	OB5955 H1 Ro.raw	2.1847E5	1	1	444	464		N13:Deamidation (N Q):18.40	PEAKS DB
W.GTPGSEVREETSR.N	Y	35.79	1403.6641	13	3.8	702.8420	2	27.77	5	F5:1332	OB5955 H1 Ro.raw	2.4946E4	1	1	153	165			PEAKS DB
K.NPQLQDLDMMLTC(+71.04)VEIKEGALMLPHFNSK.A	N	35.62	3385.6543	29	-1.5	1129.5570	3	38.07	4	F4:1978	OB5954 H1 Ro.raw	1.5713E5	1	1	415	443	Propionamide	C13:Propionamide:1000.00	PEAKS PTM
R.N(-17.03)NPFYFPSRR.F	N	35.47	1279.6097	10	1.1	427.5443	3	28.89	6	F6:1434	OB5956 H1 Ro.raw	5.7683E5	3	3	166	175		N1:Ammonia-loss (N):9.34	PEAKS PTM
R.NTLEAAFNAEFN(+15.99)EIRR.V	N	35.08	1909.9282	16	0.9	955.9723	2	32.98	5	F5:1662	OB5955 H1 Ro.raw	2.4059E6	2	2	313	328		N12:Oxidation or Hydroxylation:9.28	PEAKS PTM
R.EGEQEW(+31.99)GTPGSEVREETSR.N	Y	34.83	2193.9409	19	0.5	732.3213	3	25.57	6	F6:1219	OB5956 H1 Ro.raw	2.9433E5	1	1	147	165		W6:Dihydroxy: 28.36	PEAKS PTM
R.IFLAGDKD(-18.01)NVIDQIEK.Q	N	34.72	1798.9464	16	-0.9	600.6555	3	32.15	6	F6:1589	OB5956 H1 Ro.raw	3.8289E5	1	1	536	551		D8:Dehydration:30.83	PEAKS PTM
K.SFNLDEGHALRIP(+15.99)SGFISYILN(+.98)R.H	N	34.44	2635.3394	23	8.6	879.4612	3	36.15	5	F5:1848	OB5955 H1 Ro.raw	1.0826E5	1	1	249	271	Hydroxylation Pro	P13:Hydroxylation Pro:1000.00;N22:Deamidation (NQ): 32.85	PEAKS DB
R.NNPFYFP(+15.99)SRR.F	N	34.32	1312.6312	10	-0.5	657.3226	2	28.74	5	F5:1397	OB5955 H1 Ro.raw	7.8182E5	1	1	166	175		P7:Hydroxylation Pro: 32.48	PEAKS DB

total 287 peptides

Peptide	Uniq	-10lgP	Mass	Length	ppm	m/z	z	RT	Fraction	Scan	Source File	Area roasted peanut	#Feature	#Feature roasted peanut	Start	End	PTM	AScore	Found By
K.NPQLQ(+.98)DLDMM(-48.00)LTC(+57.02)VEIK.E	N	34.13	1999.9595	17	9.3	1000.9963	2	32.90	4	F4:1653	OB5954 H1 Ro.raw	1.9871E6	1	1	415	431	Carbamidomethylation	Q5:Deamidation (NQ):11.12;M10:Dethiomethyl:0.00;C13:Carbamidomethylation:1000.00	PEAKS PTM
K.DLAFPGSGE(+53.92)QVEK.L	N	33.99	1429.5812	13	-6.3	715.7933	2	30.34	6	F6:1518	OB5956 H1 Ro.raw	6.355E5	2	2	555	567		E9:Replacement of 2 protons by iron:37.54	PEAKS PTM
K.NPQ(+.98)LQDLDMMLTC(+57.02)VEIKEGALM(+15.99)LPHFNSK.A	N	33.64	3388.6174	29	6.0	1130.5532	3	38.52	5	F5:2024	OB5955 H1 Ro.raw	7.1577E5	1	1	415	443	Carbamidomethylation	Q3:Deamidation (NQ):0.00;C13:Carbamidomethylation:1000.00;M22:Oxidation (M):34.40	PEAKS DB
R.VAKISM(+15.99)PVNTPGQFEDFFP(+15.99)A.S	N	33.59	2226.0667	20	-7.4	1114.0323	2	51.06	5	F5:2785	OB5955 H1 Ro.raw	2.817E4	1	1	279	298	Oxidation (M)	M6:Oxidation (M):1000.00;P19:Hydroxylation on Pro:21.81	PEAKS DB
N.PFYFPSR.R	N	33.58	912.4493	7	-0.1	457.2319	2	31.70	5	F5:1584	OB5955 H1 Ro.raw	8.1854E4	1	1	168	174			PEAKS DB
K.D(+53.92)LAFPGSGEQVEK.L	N	33.35	1429.5812	13	-4.3	715.7948	2	30.56	4	F4:1500	OB5954 H1 Ro.raw	3.8253E5	1	1	555	567		D1:Replacement of 2 protons by iron:0.00	PEAKS PTM
K.N(+.98)PQLQDLDMMLTC(+57.02)VEIKEGALM(+15.99)LPHFNSK.A	N	33.25	3388.6174	29	6.0	1130.5532	3	38.52	5	F5:2054	OB5955 H1 Ro.raw	7.1577E5	1	1	415	443	Carbamidomethylation	N1:Deamidation (NQ):0.00;C13:Carbamidomethylation:1000.00;M22:Oxidation (M):0.00	PEAKS DB
K.Q(+.98)FQNLQNHR.I	N	33.25	1184.5686	9	4.6	593.2943	2	23.68	5	F5:1098	OB5955 H1 Ro.raw	0	0	0	199	207		Q1:Deamidation (NQ):25.21	PEAKS DB

total 287 peptides

Peptide	Uniq	-10lgP	Mass	Length	ppm	m/z	z	RT	Fraction	Scan	Source File	Area roasted peanut	#Feature	#Feature roasted peanut	Start	End	PTM	AScore	Found By
K.ISMPVNTP(+15.99)GQ(+.98)FEDFFPASSR.D	N	33.02	2243.0205	20	5.1	1122.5232	2	47.86	4	F4:2523	OB5954 H1 Ro.raw	0	0	0	282	301		P8:Hydroxylation Pro:0.00;Q10:Deamidation (NQ):31.27	PEAKS DB
R.IFLAGDKDNVIDQ(+.98)IEK.Q	N	33.02	1817.9410	16	7.6	909.9847	2	63.08	6	F6:3435	OB5956 H1 Ro.raw	3.476E3	1	1	536	551		Q13:Deamidation (NQ):11.12	PEAKS DB
K.N(+.98)PQLQDLDM(+15.99)MLTC(+57.02)VEIKEGALMLPHFNSK.A	N	33.01	3388.6174	29	8.2	1695.3300	2	37.72	4	F4:1941	OB5954 H1 Ro.raw	7.0286E5	1	1	415	443	Carbamidomethylation	N1:Deamidation (NQ):0.00;M9:Oxidation (M):0.00;C13:Carbamidomethylation:1000.00	PEAKS DB
K.E(-18.01)GALMLPHFNSK.A	N	32.81	1324.6598	12	0.0	442.5605	3	30.63	5	F5:1521	OB5955 H1 Ro.raw	2.0893E5	2	2	432	443	Pyro-glu from E	E1:Pyro-glu from E:1000.00	PEAKS PTM
R.E(-18.01)GEQEWGTP(+15.99)GSEVREETSR.N	Y	32.75	2159.9355	19	1.6	1080.9768	2	28.57	4	F4:1386	OB5954 H1 Ro.raw	1.3772E5	2	2	147	165	Pyro-glu from E; Hydroxylation Pro	E1:Pyro-glu from E:1000.00;P9:Hydroxylation Pro:1000.00	PEAKS PTM
K.KNPQLQDLDM(-48.00)MLTC(+57.02)VEIK.E	N	32.73	2127.0703	18	1.8	710.0320	3	33.04	6	F6:1688	OB5956 H1 Ro.raw	7.9877E5	1	1	414	431	Carbamidomethylation	M10:Dethiomethyl:8.14;C14:Carbamidomethylation:1000.00	PEAKS PTM
K.KNPQLQ(+.98)DLDMM(+15.99)LTC(+57.02)VEIKEGALMLPHFNSK.A	N	32.59	3516.7124	30	3.3	880.1883	4	35.48	4	F4:1798	OB5954 H1 Ro.raw	7.7853E5	1	1	414	443	Carbamidomethylation	Q6:Deamidation (NQ):23.13;M11:Oxidation (M):14.02;C14:Carbamidomethylation:1000.00	PEAKS DB
K.G(+43.01)T(+14.02)GNLELVAVRK.E	N	32.58	1312.7462	12	-0.4	657.3801	2	27.30	4	F4:1303	OB5954 H1 Ro.raw	3.8825E5	2	2	453	464	Carbamylation; Methylation(others)	G1:Carbamylation:147.00;T2:Methylation(others):68.07	PEAKS PTM

total 287 peptides

Peptide	Uniq	-10lgP	Mass	Length	ppm	m/z	z	RT	Fraction	Scan	Source File	Area roasted peanut	#Feature	#Feature roasted peanut	Start	End	PTM	AScore	Found By
R.IPSGFISYILNR(+28.03)HDN(+162.05)QNL.R	N	32.30	2290.1592	18	5.9	764.3982	3	34.37	5	F5:1737	OB5955 H1 Ro.raw	0	0	0	260	277	Dimethylation(KR)	R12:Dimethylation(KR):1000.00;N15:Hexose (NSY):17.01	PEAKS PTM
K.G(+43.01)T(+14.02)GNLELVAVR.K	N	32.00	1184.6514	11	-2.2	593.3317	2	30.21	4	F4:1493	OB5954 H1 Ro.raw	1.7455E5	2	2	453	463	Carbamylation	G1:Carbamylation:1000.00;T2:Methylation (others):46.31	PEAKS PTM
K.ISMP(+15.99)VNTPGQ(+.98)FEDFFPASSR.D	N	31.78	2243.0205	20	5.7	1122.5239	2	46.64	5	F5:2451	OB5955 H1 Ro.raw	0	0	0	282	301		P4:Hydroxylation Pro: 24.67; Q10:Deamidation (NQ):21.91	PEAKS DB
D.GEPDLSNNFGR.L	N	31.47	1204.5472	11	3.0	603.2827	2	28.20	4	F4:1371	OB5954 H1 Ro.raw	1.6138E4	1	1	395	405			PEAKS DB
K.NPQLQ(+.98)DLDMMLTC(+57.02)VEIKEGALMLPHFNSK.A	N	31.45	3372.6226	29	7.2	844.1690	4	45.92	6	F6:2433	OB5956 H1 Ro.raw	0	0	0	415	443	Carbamidomethylation	Q5:Deamidation (NQ): 14.19; C13:Carbamidomethylation:1000.00	PEAKS DB
K.KN(+.98)PQLQDLDMMLTC(+57.02)VEIKEGALMLPHFNSK.A	N	31.43	3500.7175	30	4.4	1167.9182	3	36.77	4	F4:1901	OB5954 H1 Ro.raw	9.2406E4	1	1	414	443	Carbamidomethylation	N2:Deamidation (NQ): 14.04; C14:Carbamidomethylation:1000.00	PEAKS DB
K.IRPEGREGEQEWGTPGSEVREETSR.N	Y	31.36	2870.3542	25	2.3	718.5975	4	27.83	6	F6:1355	OB5956 H1 Ro.raw	1.3652E5	1	1	141	165			PEAKS DB
K.VSKEH(+15.99)VQELTK.H	N	31.09	1312.6986	11	1.8	657.3578	2	23.73	5	F5:1100	OB5955 H1 Ro.raw	1.5735E3	1	1	361	371	Oxidation (HW)	H5:Oxidation (HW):1000.00	PEAKS PTM

total 287 peptides

Peptide	Uniq	-10lgP	Mass	Length	ppm	m/z	z	RT	Fraction	Scan	Source File	Area roasted peanut	#Feature	#Feature roasted peanut	Start	End	PTM	AScore	Found By
K.N(+.98)PQ(+.98)LQ(+.98)DLDM(+15.99)MLTC(+57.02)VEIKEGALM(+15.99)LPHFNSK.A	N	30.70	3406.5806	29	9.9	1136.5454	3	36.75	6	F6:1904	OB5956 H1 Ro.raw	3.0926E5	1	1	415	443	Deamidation (NQ); Carbamidomethylation	N1:Deamidation (NQ): 69.03; Q3:Deamidation (NQ): 71.61; Q5:Deamidation (NQ): 53.75; M9:Oxidation (M):17.01;C13:Carbamidomethylation:1000.00; M22:Oxidation (M):16.22	PEAKS DB
R.WGP(+15.99)AEPR.E	Y	30.70	827.3926	7	0.0	414.7036	2	24.54	5	F5:1155	OB5955 H1 Ro.raw	3.9908E3	1	1	109	115		P3:Hydroxylation on Pro: 36.24	PEAKS DB
F.L(+226.08)AGDK(+28.03)DNVIDQIEK.Q	N	30.44	1810.9135	14	-0.8	906.4633	2	39.29	5	F5:2033	OB5955 H1 Ro.raw	7.8052E4	1	1	538	551	Biotinylation; Dimethylation(KR)	L1:Biotinylation:152.87;K5:Dimethylation(KR):178.84	PEAKS PTM
L.R(+27.99)IPSGFISYILNR.H	N	29.85	1562.8568	13	-2.0	782.4341	2	37.84	6	F6:1976	OB5956 H1 Ro.raw	1.6397E5	1	1	259	271	Formylation	R1:Formylation:1000.00	PEAKS PTM
R.IVQIEAR(+28.03)PN(+.98)TLVLPK.H	Y	29.66	1719.0294	15	-1.6	860.5206	2	30.29	6	F6:1512	OB5956 H1 Ro.raw	0	0	0	208	222	Dimethylation(KR); Deamidation (NQ)	R7:Dimethylation(KR): 66.64; N9:Deamidation (NQ): 90.21	PEAKS PTM
R.LFEVKPDKKNPQLQDLDMM(+31.99)LTC(+57.02)VEIK.E	N	29.63	3163.5967	26	-2.8	791.9042	4	32.90	4	F4:1683	OB5954 H1 Ro.raw	1.0179E6	1	1	406	431	Carbamidomethylation	M19:Sulphone: 14.02; C22:Carbamidomethylation:1000.00	PEAKS PTM
K.ISMPVNTPGQ(+.98)FEDFFPASSR.D	N	29.56	2227.0254	20	9.7	1114.5308	2	54.22	4	F4:2901	OB5954 H1 Ro.raw	0	0	0	282	301		Q10:Deamidation (NQ):14.58	PEAKS DB
R.GRREQEW(+15.99)EEEEEDEEEEGSNR.E	N	29.48	2666.0601	21	0.8	889.6946	3	26.10	4	F4:1240	OB5954 H1 Ro.raw	0	0	0	470	490	Oxidation (HW)	W7:Oxidation (HW):1000.00	PEAKS PTM

total 287 peptides

Peptide	Uniq	-10lgP	Mass	Length	ppm	m/z	z	RT	Fraction	Scan	Source File	Area roasted peanut	#Feature	#Feature roasted peanut	Start	End	PTM	AScore	Found By
K.N(+.98)PQLQ(+.98)DLDMMLTC(+57.02)VEIKEGALM(+15.99)LPHFNSK.A	N	29.27	3389.6016	29	9.6	848.4158	4	37.65	6	F6:2047	OB5956 H1 Ro.raw	8.4697E6	1	1	415	443	Carbamidomethylation	N1:Deamidation (NQ): 0.00;Q5:Deamidation (NQ): 14.04; C13:Carbamidomethylation:1000.00; M22:Oxidation (M):0.00	PEAKS DB
N.TLEAAFNAEFNEIRR.V	N	28.88	1779.8904	15	3.5	890.9556	2	33.23	6	F6:1696	OB5956 H1 Ro.raw	1.5612E5	1	1	314	328			PEAKS DB
K.SFN(+.98)LDEGHALR(+31.99)IPSGFISYILNR.H	N	28.71	2651.3342	23	9.4	1326.6869	2	39.13	4	F4:2021	OB5954 H1 Ro.raw	0	0	0	249	271		N3:Deamidation (NQ): 38.55; R11:Dihydroxy:14.78	PEAKS PTM
K.ISM(+15.99)PVN(+.98)TPGQFEDFFPASSR.D	N	28.37	2243.0205	20	8.5	1122.5271	2	44.80	6	F6:2368	OB5956 H1 Ro.raw	0	0	0	282	301	Oxidation (M)	M3:Oxidation (M):1000.00; N6:Deamidation (NQ): 0.00	PEAKS DB
K.GTGNLE(+53.92)LVAVRK.E	N	28.35	1309.6440	12	-0.1	437.5553	3	27.29	4	F4:1309	OB5954 H1 Ro.raw	2.3239E4	1	1	453	464	Replacement of 2 protons by iron	E6:Replacement of 2 protons by iron: 60.54	PEAKS PTM
D.NVIDQIEK.Q	N	28.35	957.5131	8	1.6	479.7646	2	28.19	5	F5:1367	OB5955 H1 Ro.raw	2.9963E3	1	1	544	551			PEAKS DB
K.I(+43.01)S(+14.02)MPVNTPGQFEDFFPASSR.D	N	28.31	2283.0630	20	-0.7	1142.5380	2	36.01	6	F6:1860	OB5956 H1 Ro.raw	2.4015E5	1	1	282	301	Carbamylation	I1:Carbamylation:1000.00;S2:Methylation (others):39.44	PEAKS PTM
R.VLLEENAGGEQEERGQRR.R	N	28.30	2069.0249	18	1.0	518.2640	4	25.49	5	F5:1205	OB5955 H1 Ro.raw	1.3378E5	1	1	329	346			PEAKS DB
K.NP(+15.99)QLQDLDMMLTC(+57.02)VEIKEGALMLPHFNSK.A	N	28.11	3387.6335	29	3.2	1130.2220	3	40.44	5	F5:2090	OB5955 H1 Ro.raw	3.3176E4	1	1	415	443	Carbamidomethylation	P2:Hydroxylation Pro: 14.98; C13:Carbamidomethylation:1000.00	PEAKS DB

total 287 peptides

Peptide	Uniq	-10lgP	Mass	Length	ppm	m/z	z	RT	Fraction	Scan	Source File	Area roasted peanut	#Feature	#Feature roasted peanut	Start	End	PTM	AScore	Found By
K.AMVIVVVNK(-1.03)GTGNLELVAVR.K	N	27.94	2080.1714	20	8.5	1041.1018	2	34.56	4	F4:1751	OB5954 H1 Ro.raw	2.9653E7	1	1	444	463	Lysine oxidation to aminoadipic semialdehyde	K9:Lysine oxidation to aminoadipic semialdehyde:1000.00	PEAKS PTM
K.SF(+17.99)NLDEGHALR.I	N	27.90	1275.6007	11	8.1	638.8128	2	26.62	5	F5:1267	OB5955 H1 Ro.raw	9.5818E3	1	1	249	259	Fluorination	F2:Fluorination:1000.00	PEAKS PTM
K.SFNLDEGHALRIP(+15.99)SGF(+17.99)ISYILNR.H	N	27.40	2652.3459	23	8.7	885.1303	3	38.95	5	F5:2010	OB5955 H1 Ro.raw	0	0	0	249	271	Hydroxylation Pro	P13:Hydroxylation Pro:1000.00;F16:Fluorination:38.16	PEAKS PTM
R.VLLEENAGGEQEER(+15.99).G	N	27.33	1587.7375	14	1.8	794.8775	2	26.70	4	F4:1275	OB5954 H1 Ro.raw	0	0	0	329	342		R14:Oxidation or Hydroxylation:20.88	PEAKS PTM
K.NPQLQ(+.98)DLDMM(+15.99)LTC(+57.02)VEIKEGALMLPHFNSK.A	N	27.25	3388.6174	29	8.0	1130.5554	3	37.44	5	F5:1884	OB5955 H1 Ro.raw	6.7537E6	1	1	415	443	Carbamidomethylation	Q5:Deamidation (NQ):0.00;M10:Oxidation (M):12.28;C13:Carbamidomethylation:1000.00	PEAKS DB
R.N(+42.01)(+15.00)NPFYFPSRR.F	N	27.12	1353.6465	10	9.4	677.8369	2	29.74	5	F5:1453	OB5955 H1 Ro.raw	3.0438E5	1	1	166	175		N1:Acetylation (N-term):1000.00;N1:Deamidation followed by a methylation:12.28	PEAKS PTM
R.VAK(-1.03)ISMPVNTPGQFEDFFPASSR.D	N	27.11	2523.2104	23	1.1	1262.6139	2	36.53	6	F6:1887	OB5956 H1 Ro.raw	0	0	0	279	301	Lysine oxidation to aminoadipic semialdehyde	K3:Lysine oxidation to aminoadipic semialdehyde:1000.00	PEAKS PTM
K.NPQLQDLDMMLTC(+58.01)VEIK.E	N	26.84	2047.9629	17	-3.6	1024.9850	2	46.58	4	F4:2449	OB5954 H1 Ro.raw	0	0	0	415	431	Carboxymethyl	C13:Carboxymethyl:1000.00	PEAKS PTM
R.EQEW(+15.99)EEEEEDEEEEGSNREVR.R	N	26.79	2681.0483	21	1.1	894.6910	3	26.92	4	F4:1316	OB5954 H1 Ro.raw	7.9753E4	1	1	473	493	Oxidation (HW)	W4:Oxidation (HW):1000.00	PEAKS PTM

total 287 peptides

Peptide	Uniq	-10lgP	Mass	Length	ppm	m/z	z	RT	Fraction	Scan	Source File	Area roasted peanut	#Feature	#Feature roasted peanut	Start	End	PTM	AScore	Found By
K.NPQLQDLDMM(+15.99)LTC(+57.02)VEIKEGALMLP(+15.99)HFNSK.A	N	26.70	3403.6284	29	-6.6	1702.8102	2	36.88	6	F6:1908	OB5956 H1 Ro.raw	0	0	0	415	443	Carbamidomethylation	M10:Oxidation (M):7.65;C13:Carbamidomethylation:1000.00;P24:Hydroxylation Pro:30.14	PEAKS DB
R.IFLAGDKD(-18.01)NV.I	N	26.68	1072.5553	10	0.4	537.2852	2	32.21	4	F4:1610	OB5954 H1 Ro.raw	2.1773E6	1	1	536	545		D8:Dehydration:45.01	PEAKS PTM
K.KNPQLQDLDMM(-48.00)LTC(+57.02)VEIK.E	N	26.57	2127.0703	18	-0.4	710.0304	3	31.57	6	F6:1598	OB5956 H1 Ro.raw	1.2455E5	1	1	414	431	Carbamidomethylation	M11:Dethiomethyl:0.00;C14:Carbamidomethylation:1000.00	PEAKS PTM
K.KNPQ(+.98)LQ(+.98)DLDM(+15.99)MLTC(+57.02)VEIKEGALM(+15.99)LPHFNSK.A	N	26.17	3533.6914	30	9.7	884.4387	4	35.77	5	F5:1822	OB5955 H1 Ro.raw	2.4464E5	1	1	414	443	Carbamidomethylation	Q4:Deamidation (NQ):9.40;Q6:Deamidation (NQ):6.65;M10:Oxidation (M):0.00;C14:Carbamidomethylation:1000.00;M23:Oxidation (M):40.04	PEAKS DB
K.NPQLQ(+.98)DLDM(+15.99)M(+15.99)LTC(+57.02)VEIKEGALM(+15.99)LPHFNSK.A	N	26.02	3420.6074	29	5.5	1141.2161	3	34.83	5	F5:1769	OB5955 H1 Ro.raw	2.4925E5	1	1	415	443	Oxidation (M); Carbamidomethylation	Q5:Deamidation (NQ):11.12;M9:Oxidation (M):1000.00;M10:Oxidation (M):1000.00;C13:Carbamidomethylation:1000.00;M22:Oxidation (M):1000.00	PEAKS DB
K.AM(-48.00)VIVVVNK.G	N	25.96	923.5804	9	0.0	462.7975	2	25.00	6	F6:1192	OB5956 H1 Ro.raw	6.3039E3	1	1	444	452	Dethiomethyl	M2:Dethiomethyl:1000.00	PEAKS PTM

total 287 peptides

Peptide	Uniq	-10lgP	Mass	Length	ppm	m/z	z	RT	Fraction	Scan	Source File	Area roasted peanut	#Feature	#Feature roasted peanut	Start	End	PTM	AScore	Found By
R.IVQIEARPNTLVLP(+15.99)KHADADNILVIQQGQ(+.98)ATVTVAN(+.98)GNNRK.S	Y	25.90	4436.3774	41	6.0	1110.1083	4	32.89	4	F4:1658	OB5954 H1 Ro.raw	3.5945E5	1	1	208	248		P14:Hydroxylation Pro:1.99;Q29:Deamidation (NQ):0.00;N36:Deamidation (NQ):14.04	PEAKS DB
R.SSDNEGVIVKVSKEHVQELTK.H	N	25.71	2325.2175	21	0.9	776.0804	3	28.86	4	F4:1404	OB5954 H1 Ro.raw	0	0	0	351	371			PEAKS DB
R.DQSSY(+15.99)LQGFSR.N	N	25.52	1302.5840	11	1.6	652.3003	2	29.24	6	F6:1470	OB5956 H1 Ro.raw	7.3464E4	1	1	302	312		Y5:Oxidation or Hydroxylation:26.36	PEAKS PTM
**total 287 peptides**																			

tr|E5G076|E5G076_ARAHY

back to list

| Protein Coverage | Supporting Peptides |

Protein Coverage:

1 MRGRVSPLML LLGILVLASV SATHAKSSPY QKKTENPCAQ RCLQSCQQEP DDLKQKACES RCTKLEYDPR CVYDPRGHTG

81 TTNQRSPPGE RTRGRQPGDY DDDRRQPRRE EGGRWGPAGP REREREEDWR QPREDWRRPS HQQPRKIRPE GREGEQEWGT

161 PGSHVREETS RNNPFYFPSR RFSTRYGNQN GRIRVLQRFD QRSRQFQNLQ NHRIVQIEAK PNTLVLPKHA DADNILVIQQ

241 GQATVTVANG NNRRALILTR AMHSESHPFH FLHLDDMTPE LRVAKSHAVN TPGQFEDFFP ASSRDQSSYL QGFSRNTLEA

321 AFNAEFNEIR RVLLEENAGG EQEERGQRRW STRSSENNEG VIVEVSKEHV EELTKHAKSV SKKGSEEEGD ITNPINLREG

- a Acetylation (N-term) (+42.01)
- a Ammonia-loss (N) (-17.03)
- c Carbamidomethylation (+57.02)
- c Carbamylation (+43.01)
- c Carboxymethyl (+58.01)
- d Deamidation (NQ) (+0.98)
- d Dehydration (-18.01)
- d Deamidation followed by a methylation (+15.00)
- d Dethiomethyl (-48.00)
- f Formylation (+27.99)
- h Hydroxylation Pro (+15.99)
- m Methylation(KR) (+14.02)
- m Methylation(others) (+14.02)
- o Oxidation (M) (+15.99)
- o Oxidation (HW) (+15.99)
- o Oxidation or Hydroxylation (+15.99)
- p Propionamide (+71.04)
- p Pyro-glu from E (-18.01)
- p Pyro-glu from Q (-17.03)
- r Replacement of 2 protons by iron (+53.92)
- * Carbamylation (+43.01), Methylation(KR) (+14.02)
- * Carbamylation (+43.01), Methylation(others) (+14.02)

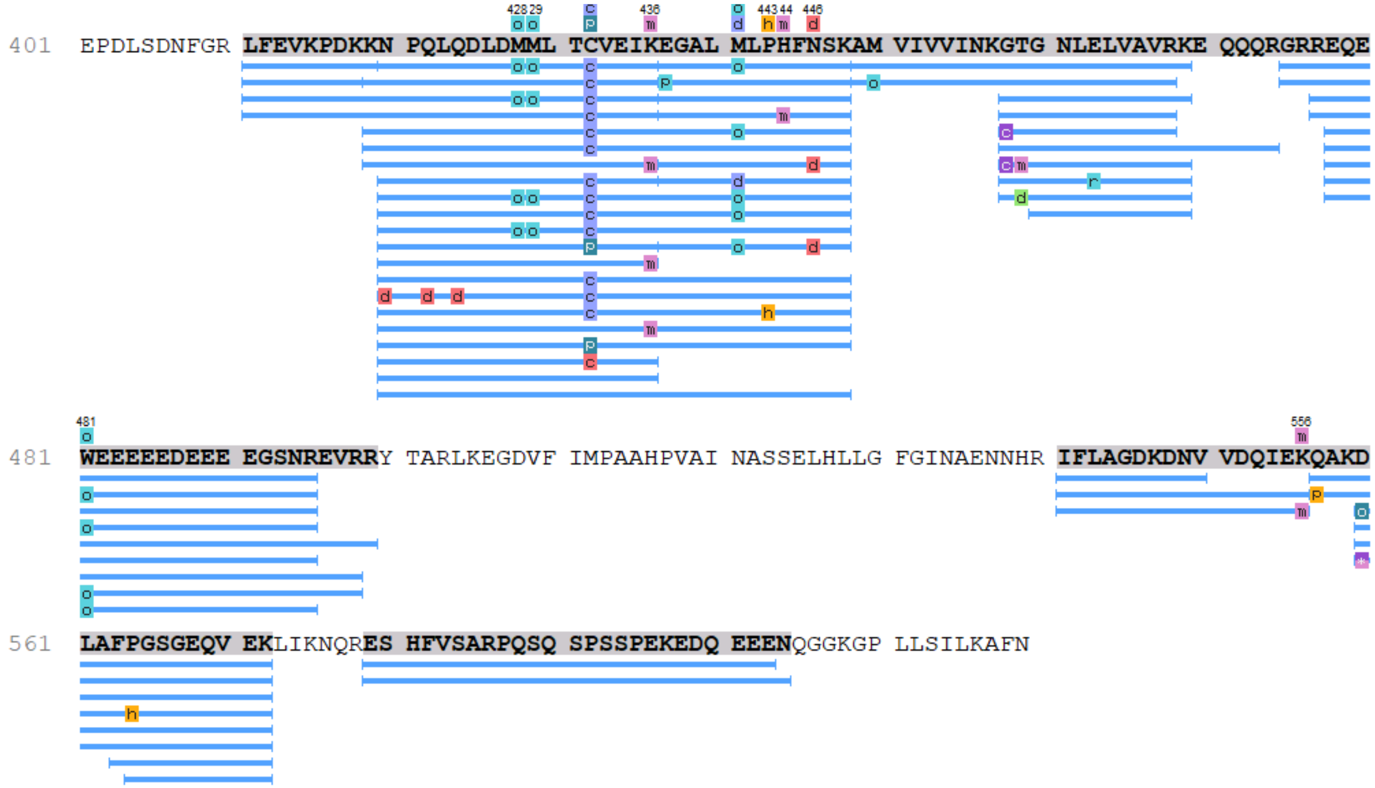

Supporting Peptides:

Peptide	Uniq	-10lgP	Mass	Length	ppm	m/z	z	RT	Fraction	Scan	Source File	Area roasted peanut	#Feature	#Feature roasted peanut	Start	End	PTM	AScore	Found By
R.EQEWEEEEEDEEEEGSNR.E	N	117.09	2280.8413	18	3.3	1141.4316	2	28.28	5	F5:1371	OB5955 H1 Ro.raw	4.6914E6	7	7	478	495			PEAKS DB
K.KNPQLQDLDMMLTC(+57.02)VEIK.E	N	116.47	2175.0737	18	3.2	1088.5476	2	35.21	5	F5:1788	OB5955 H1 Ro.raw	1.5503E7	6	6	419	436	Carbamidomethylation	C14:Carbamidomethylation:1000.00	PEAKS DB
K.NPQLQDLDMM(+15.99)LTC(+57.02)VEIK.E	N	116.10	2062.9736	17	5.0	1032.4993	2	34.71	6	F6:1780	OB5956 H1 Ro.raw	1.155E7	4	4	420	436	Carbamidomethylation	M10:Oxidation(M):30.46;C13:Carbamidomethylation:1000.00	PEAKS DB
K.NPQLQDLDM(+15.99)MLTC(+57.02)VEIK.E	N	115.70	2062.9736	17	-4.7	1032.4893	2	36.62	4	F4:1873	OB5954 H1 Ro.raw	3.8486E6	3	3	420	436	Carbamidomethylation	M9:Oxidation(M):30.46;C13:Carbamidomethylation:1000.00	PEAKS DB

total 184 peptides

Peptide	Uniq	-10lgP	Mass	Length	ppm	m/z	z	RT	Fraction	Scan	Source File	Area roasted peanut	#Feature	#Feature roasted peanut	Start	End	PTM	AScore	Found By
K.NPQLQDLDMMLTC(+57.02)VEIK.E	N	112.86	2046.9788	17	0.2	1024.4968	2	37.11	6	F6:1959	OB5956 H1 Ro.raw	3.5075E7	10	10	420	436	Carbamidomethylation	C13:Carbamidomethylation:1000.00	PEAKS DB
R.NTLEAAFNAEFNEIR.R	N	112.69	1737.8322	15	1.4	869.9246	2	35.64	6	F6:1825	OB5956 H1 Ro.raw	1.2023E7	6	6	316	330			PEAKS DB
K.KGSEEEGDITNPINLR.E	N	105.71	1770.8748	16	0.6	886.4452	2	28.91	6	F6:1429	OB5956 H1 Ro.raw	3.1807E7	6	6	383	398			PEAKS DB
K.KNPQLQDLDM(+15.99)MLTC(+57.02)VEIK.E	N	103.13	2191.0686	18	3.6	1096.5455	2	34.71	6	F6:1781	OB5956 H1 Ro.raw	1.4412E6	4	4	419	436	Carbamidomethylation	M10:Oxidation (M):27.96;C14:Carbamidomethylation:1000.00	PEAKS DB
K.NPQLQDLDM(+15.99)M(+15.99)LTC(+57.02)VEIK.E	N	96.89	2078.9688	17	6.6	1040.4985	2	33.82	6	F6:1726	OB5956 H1 Ro.raw	7.6578E4	2	2	420	436	Oxidation (M); Carbamidomethylation	M9:Oxidation (M):1000.00; M10:Oxidation (M):1000.00; C13:Carbamidomethylation:1000.00	PEAKS DB
R.EGEQEWGTPGSHVR.E	N	95.59	1567.7015	14	2.9	784.8604	2	25.79	4	F4:1223	OB5954 H1 Ro.raw	3.5597E6	6	6	153	166			PEAKS DB
R.VLLEENAGGEQEER.G	N	95.38	1571.7427	14	2.2	786.8804	2	26.92	4	F4:1300	OB5954 H1 Ro.raw	3.043E7	6	6	332	345			PEAKS DB
K.KNPQLQDLDMM(+15.99)LTC(+57.02)VEIK.E	N	95.29	2191.0686	18	3.1	1096.5449	2	32.98	5	F5:1657	OB5955 H1 Ro.raw	1.9358E6	4	4	419	436	Carbamidomethylation	M11:Oxidation (M):23.10;C14:Carbamidomethylation:1000.00	PEAKS DB
R.DQSSYLQGFSR.N	N	94.26	1286.5891	11	-0.9	644.3013	2	30.91	4	F4:1531	OB5954 H1 Ro.raw	9.6573E7	19	19	305	315			PEAKS DB
K.GSEEEGDITNPINLR.E	N	94.26	1642.7798	15	-1.1	822.3963	2	30.91	4	F4:1555	OB5954 H1 Ro.raw	1.1859E7	3	3	384	398			PEAKS DB
R.RVLLEENAGGEQEER.G	N	93.77	1727.8438	15	0.2	576.9553	3	25.30	5	F5:1185	OB5955 H1 Ro.raw	8.7628E5	6	6	331	345			PEAKS DB
K.NPQLQDLDMMLTC(+14.02)VEIK.E	N	93.65	2003.9730	17	2.7	1002.9965	2	38.33	5	F5:1965	OB5955 H1 Ro.raw	5.8314E6	4	4	420	436		C13:Methylation(others):33.98	PEAKS PTM
R.REQEWEEEEEDEEEEGSNR.E	N	88.08	2436.9424	19	0.6	813.3219	3	26.73	4	F4:1278	OB5954 H1 Ro.raw	9.3919E5	7	7	477	495			PEAKS DB

total 184 peptides

Peptide	Uniq	-10lgP	Mass	Length	ppm	m/z	z	RT	Fraction	Scan	Source File	Area roasted peanut	#Feature	#Feature roasted peanut	Start	End	PTM	AScore	Found By
R.EQEW(+15.99)EEEEEDEEEEGSNR.E	N	86.71	2296.8362	18	2.5	1149.4282	2	27.64	6	F6:1309	OB5956 H1 Ro.raw	1.0675E6	3	3	478	495	Oxidation (HW)	W4:Oxidation (HW):1000.00	PEAKS PTM
K.NPQLQDLDM(+15.99)MLTCVEIK(+14.02).E	N	85.42	2019.9679	17	2.2	1010.9934	2	37.33	4	F4:1920	OB5954 H1 Ro.raw	7.9299E5	2	2	420	436	Methylation(KR)	M9:Oxidation (M):21.94;K17:Methylation (KR):1000.00	PEAKS PTM
K.DLAFPGSGEQVEK.L	N	85.27	1375.6619	13	-0.7	688.8377	2	30.39	4	F4:1498	OB5954 H1 Ro.raw	9.2216E7	3	3	560	572			PEAKS DB
R.VLLEEN(+.98)AGGEQEER.G	N	85.22	1572.7267	14	2.8	787.3728	2	27.46	6	F6:1340	OB5956 H1 Ro.raw	6.8644E5	3	3	332	345	Deamidation (NQ)	N6:Deamidation (NQ):121.37	PEAKS DB
K.NPQLQDLDMMLTC(+57.02)VEIKEGALMLPHFNSK.A	N	85.17	3371.6387	29	-4.9	1686.8184	2	38.00	5	F5:1955	OB5955 H1 Ro.raw	1.5541E8	10	10	420	448	Carbamidomethylation	C13:Carbamidomethylation:1000.00	PEAKS DB
K.NPQLQDLDM(+15.99)MLTC(+57.02)VEIKEGALMLPHFNSK.A	N	83.17	3387.6335	29	-0.6	1130.2178	3	37.70	4	F4:1939	OB5954 H1 Ro.raw	9.055E7	6	6	420	448	Carbamidomethylation	M9:Oxidation (M):26.02;C13:Carbamidomethylation:1000.00	PEAKS DB
R.NTLEAAFNAEFNEIRR.V	N	81.08	1893.9332	16	-2.6	947.9714	2	34.11	5	F5:1711	OB5955 H1 Ro.raw	2.8495E8	15	15	316	331			PEAKS DB
R.GRREQEWEEEEEDEEEEGSNR.E	N	80.86	2650.0649	21	1.1	884.3632	3	26.73	4	F4:1280	OB5954 H1 Ro.raw	8.7144E5	6	6	475	495			PEAKS DB
K.EGALMLPHFNSK.A	N	80.31	1342.6703	12	2.3	672.3440	2	30.63	5	F5:1504	OB5955 H1 Ro.raw	1.4317E7	5	5	437	448			PEAKS DB
R.LFEVKPDKKNPQLQDLDMMLTC(+57.02)VEIK.E	N	78.90	3131.6069	26	-4.0	1044.8721	3	34.65	5	F5:1756	OB5955 H1 Ro.raw	5.2558E6	4	4	411	436	Carbamidomethylation	C22:Carbamidomethylation:1000.00	PEAKS DB
K.NPQLQDLDMM(-48.00)LTC(+57.02)VEIK.E	N	76.61	1998.9755	17	1.9	1000.4969	2	32.85	6	F6:1670	OB5956 H1 Ro.raw	6.204E6	3	3	420	436	Carbamidomethylation	M10:Dethiomethyl:14.02;C13:Carbamidomethylation:1000.00	PEAKS PTM
R.IVQIEAKPNTLVLPK.H	N	75.36	1662.0079	15	-1.3	832.0101	2	30.39	4	F4:1488	OB5954 H1 Ro.raw	2.8512E7	17	17	214	228			PEAKS DB
K.EGALMLPHFN(+.98)SK.A	N	75.32	1343.6543	12	2.6	672.8362	2	31.66	6	F6:1595	OB5956 H1 Ro.raw	1.7726E5	2	2	437	448	Deamidation (NQ)	N10:Deamidation (NQ):1000.00	PEAKS DB

total 184 peptides

Peptide	Uniq	-10lgP	Mass	Length	ppm	m/z	z	RT	Fraction	Scan	Source File	Area roasted peanut	#Feature	#Feature roasted peanut	Start	End	PTM	AScore	Found By
K.KNPQLQDLDMMLTC(+57.02)VEIKEGALMLPHFNSK.A	N	74.64	3499.7336	30	-3.9	875.9373	4	36.80	4	F4:1884	OB5954 H1 Ro.raw	6.9756E6	4	4	419	448	Carbamidomethylation	C14:Carbamidomethylation:1000.00	PEAKS DB
R.NTLEAAFN(+.98)AEFNEIRR.V	N	74.52	1894.9172	16	-2.2	948.4638	2	32.35	4	F4:1616	OB5954 H1 Ro.raw	1.3754E6	3	3	316	331		N8:Deamidation (NQ):48.12	PEAKS DB
R.EETSRN(+.98)NPFYFPSRR.F	N	73.61	1899.8864	15	-0.2	634.3026	3	28.39	4	F4:1381	OB5954 H1 Ro.raw	5.4651E5	3	3	167	181		N6:Deamidation (NQ):33.98	PEAKS DB
R.RVLLEENAGGEQEERGQR.R	N	73.30	2069.0249	18	1.9	690.6835	3	25.60	4	F4:1205	OB5954 H1 Ro.raw	1.8274E6	3	3	331	348			PEAKS DB
K.GTGNLELVAVR.K	N	72.98	1127.6299	11	-1.3	564.8215	2	30.21	4	F4:1476	OB5954 H1 Ro.raw	2.4766E7	4	4	458	468			PEAKS DB
K.EGALM(+15.99)LPHFNSK.A	N	72.82	1358.6653	12	-0.5	680.3396	2	30.10	5	F5:1469	OB5955 H1 Ro.raw	3.1504E6	5	5	437	448	Oxidation (M)	M5:Oxidation (M):1000.00	PEAKS DB
K.GTGNLELVAVRK.E	N	70.92	1255.7249	12	0.7	628.8701	2	27.30	4	F4:1290	OB5954 H1 Ro.raw	2.2945E7	7	7	458	469			PEAKS DB
K.KNPQLQDLDMMLTC(+57.02)VEIKEGALM(+15.99)LPHFNSK.A	N	70.54	3515.7285	30	-1.9	879.9377	4	36.03	6	F6:1857	OB5956 H1 Ro.raw	6.9145E6	3	3	419	448	Carbamidomethylation; Oxidation (M)	C14:Carbamidomethylation:1000.00;M23:Oxidation (M):107.40	PEAKS DB
K.NPQLQDLDM(+15.99)MLTC(+57.02)VEIKEGALM(+15.99)LPHFNSK.A	N	70.15	3403.6284	29	1.1	851.9153	4	37.51	4	F4:1883	OB5954 H1 Ro.raw	7.9309E6	6	6	420	448	Carbamidomethylation; Oxidation (M)	M9:Oxidation (M):27.96;C13:Carbamidomethylation:1000.00;M22:Oxidation (M):71.31	PEAKS DB
R.EGEQEWGTP(+15.99)GSHVR.E	N	69.74	1583.6964	14	0.8	792.8561	2	27.64	6	F6:1358	OB5956 H1 Ro.raw	1.3284E5	4	4	153	166	Hydroxylation Pro	P9:Hydroxylation Pro:1000.00	PEAKS DB
R.VLLEENAGGEQEERGQR.R	N	69.54	1912.9238	17	0.6	957.4698	2	25.30	5	F5:1191	OB5955 H1 Ro.raw	3.4466E6	6	6	332	348			PEAKS DB
R.DQS(-18.01)SYLQGFSR.N	N	68.93	1268.5785	11	0.2	635.2966	2	30.91	4	F4:1537	OB5954 H1 Ro.raw	6.7379E5	3	3	305	315		S3:Dehydration:40.00	PEAKS PTM
K.NPQLQDLDMM(+15.99)LTC(+57.02)VEIKEGALMLPHFNSK.A	N	68.62	3387.6335	29	-0.8	1130.2175	3	36.42	4	F4:1862	OB5954 H1 Ro.raw	7.1368E7	6	6	420	448	Carbamidomethylation	M10:Oxidation (M):24.44;C13:Carbamidomethylation:1000.00	PEAKS DB

total 184 peptides

Peptide	Uniq	-10lgP	Mass	Length	ppm	m/z	z	RT	Fraction	Scan	Source File	Area roasted peanut	#Feature	#Feature roasted peanut	Start	End	PTM	AScore	Found By
R.RVLLEEN(+.98)AGGEQEER.G	N	68.51	1728.8278	15	-0.8	577.2827	3	25.96	5	F5:1233	OB5955 H1 Ro.raw	1.3591E4	2	2	331	345	Deamidation (NQ)	N7:Deamidation (NQ): 77.53	PEAKS DB
R.NNPFYFPSR.R	N	68.49	1140.5352	9	0.9	571.2754	2	31.81	4	F4:1575	OB5954 H1 Ro.raw	1.7544E7	5	5	172	180			PEAKS DB
K.NPQLQDLDMMLTCVEIK(+14.02)EGALMLPHFNSK.A	N	68.29	3328.6328	29	0.9	833.1663	4	38.52	5	F5:1986	OB5955 H1 Ro.raw	1.7448E7	6	6	420	448	Methylation(KR)	K17:Methylation(KR): 98.15	PEAKS PTM
R.EGEQEWGTPGSHVREETSR.N	N	67.84	2169.9675	19	2.2	724.3314	3	26.51	6	F6:1287	OB5956 H1 Ro.raw	5.9384E5	3	3	153	171			PEAKS DB
R.NNPFYFPSRR.F	N	67.62	1296.6364	10	0.0	649.3254	2	28.95	4	F4:1567	OB5954 H1 Ro.raw	1.3432E8	8	8	172	181			PEAKS DB
R.EETSRNNPFYFPSRR.F	N	67.58	1898.9023	15	1.8	633.9759	3	27.75	5	F5:1339	OB5955 H1 Ro.raw	9.3426E5	3	3	167	181			PEAKS DB
K.KNPQLQDLDMMLTCVEIK(+14.02).E	N	66.83	2132.0679	18	-0.6	1067.0405	2	36.31	4	F4:1858	OB5954 H1 Ro.raw	6.1629E5	2	2	419	436	Methylation(KR)	K18:Methylation(KR): 130.57	PEAKS PTM
E.VSKEHVEELTK.H	N	66.65	1297.6877	11	-0.3	649.8510	2	24.21	4	F4:1125	OB5954 H1 Ro.raw	2.2976E5	5	5	365	375			PEAKS DB
R.VLLEEN(-17.03)AGGEQEER.G	N	65.50	1554.7162	14	1.2	778.3663	2	27.73	5	F5:1335	OB5955 H1 Ro.raw	0	0	0	332	345	Ammonia-loss (N)	N6:Ammonia-loss (N):1000.00	PEAKS PTM
K.Q(-17.03)AKDLAFPGSGEQVEK.L	N	65.43	1685.8260	16	0.1	843.9204	2	29.98	6	F6:1503	OB5956 H1 Ro.raw	1.0547E5	3	3	557	572	Pyro-glu from Q	Q1:Pyro-glu from Q:1000.00	PEAKS PTM
K.NPQLQDLDM(+15.99)M(+15.99)LTC(+57.02)VEIKEGALMLPHFNSK.A	N	64.96	3403.6284	29	-3.9	1135.5457	3	35.83	6	F6:1849	OB5956 H1 Ro.raw	8.47E6	5	5	420	448	Oxidation (M); Carbamidomethylation	M9:Oxidation (M):54.18;M10:Oxidation (M):63.99;C13:Carbamidomethylation:1000.00	PEAKS DB
R.VLLEENAGGEQEER(+14.02).G	N	64.84	1585.7583	14	0.7	793.8870	2	27.86	4	F4:1339	OB5954 H1 Ro.raw	1.7453E5	3	3	332	345	Methylation(KR)	R14:Methylation(KR): 1000.00	PEAKS PTM
K.SVSKKGSEEEGDITNPINLR.E	N	63.28	2172.1023	20	0.9	725.0420	3	27.48	4	F4:1325	OB5954 H1 Ro.raw	1.9445E5	2	2	379	398			PEAKS DB
K.DLAFP(+15.99)GSGEQVEK.L	N	62.87	1391.6569	13	0.4	696.8360	2	29.40	5	F5:1432	OB5955 H1 Ro.raw	8.022E5	3	3	560	572	Hydroxylation Pro	P5:Hydroxylation Pro: 1000.00	PEAKS DB

total 184 peptides

Peptide	Uniq	-10lgP	Mass	Length	ppm	m/z	z	RT	Fraction	Scan	Source File	Area roasted peanut	#Feature	#Feature roasted peanut	Start	End	PTM	AScore	Found By
K.NPQLQDLDMMLTC(+57.02)VEIKEGALM(+15.99)LPHFNSK.A	N	62.74	3387.6335	29	-1.0	1130.2173	3	37.10	5	F5:1900	OB5955 H1 Ro.raw	9.055E7	6	6	420	448	Carbamidomethylation; Oxidation (M)	C13:Carbamidomethylation:1000.00; M22:Oxidation (M):82.05	PEAKS DB
K.QAKDLAFPGSGEQVEK.L	N	61.87	1702.8525	16	1.2	568.6255	3	27.08	6	F6:1321	OB5956 H1 Ro.raw	2.8725E5	6	6	557	572			PEAKS DB
R.E(-18.01)GEQEWGTPGSHVR.E	N	60.61	1549.6909	14	0.7	775.8533	2	27.75	5	F5:1342	OB5955 H1 Ro.raw	4.4086E5	4	4	153	166	Pyro-glu from E	E1:Pyro-glu from E:1000.00	PEAKS PTM
Q.SSYLQGFSR.N	N	60.35	1043.5035	9	0.6	522.7593	2	29.24	6	F6:1453	OB5956 H1 Ro.raw	6.7195E4	3	3	307	315			PEAKS DB
K.NPQLQDLDMM(+15.99)LTC(+57.02)VEIKEGALM(+15.99)LPHFNSK.A	N	60.02	3403.6284	29	-8.2	1135.5408	3	36.74	5	F5:1878	OB5955 H1 Ro.raw	6.6522E6	6	6	420	448	Carbamidomethylation; Oxidation (M)	M10:Oxidation (M):21.94;C13:Carbamidomethylation:1000.00; M22:Oxidation (M):61.26	PEAKS DB
R.QFQNLQNHR.I	N	59.95	1183.5846	9	0.0	592.7996	2	23.81	5	F5:1102	OB5955 H1 Ro.raw	1.3905E5	5	5	205	213			PEAKS DB
R.Q(-17.03)FQNLQNHR.I	N	59.90	1166.5581	9	3.5	584.2884	2	28.04	4	F4:1346	OB5954 H1 Ro.raw	5.0983E6	3	3	205	213	Pyro-glu from Q	Q1:Pyro-glu from Q:1000.00	PEAKS PTM
K.KNPQLQDLDM(+15.99)MLTC(+57.02)VEIKEGALM(+15.99)LPHFNSK.A	N	59.67	3531.7234	30	-1.9	883.9364	4	35.86	4	F4:1821	OB5954 H1 Ro.raw	2.0004E6	3	3	419	448	Carbamidomethylation; Oxidation (M)	M10:Oxidation (M):17.01;C14:Carbamidomethylation:1000.00; M23:Oxidation (M):101.58	PEAKS DB
K.NPQLQ(+.98)DLDMMLTC(+57.02)VEIK.E	N	59.63	2047.9629	17	1.5	1024.9902	2	44.46	6	F6:2348	OB5956 H1 Ro.raw	0	0	0	420	436	Carbamidomethylation	Q5:Deamidation (NQ):19.68; C13:Carbamidomethylation:1000.00	PEAKS DB
K.EGALMLPH(+14.02)FNSK.A	N	59.24	1356.6860	12	-3.0	679.3483	2	30.80	5	F5:1531	OB5955 H1 Ro.raw	3.037E5	3	3	437	448	Methylation(others)	H8:Methylation(others):65.81	PEAKS PTM
A.FPGSGEQVEK.L	N	58.82	1076.5138	10	-3.4	539.2623	2	30.34	6	F6:1519	OB5956 H1 Ro.raw	4.3607E5	3	3	563	572			PEAKS DB

total 184 peptides

Peptide	Uniq	-10lgP	Mass	Length	ppm	m/z	z	RT	Fraction	Scan	Source File	Area roasted peanut	#Feature	#Feature roasted peanut	Start	End	PTM	AScore	Found By
K.IRPEGREGEQEWGTPGSHVR.E	N	58.19	2276.1045	20	0.5	570.0337	4	27.56	5	F5:1329	OB5955 H1 Ro.raw	9.9199E4	2	2	147	166			PEAKS DB
R.LFEVKPDKKNPQLQDLDMM(+15.99)LTC(+57.02)VEIK.E	N	58.02	3147.6018	26	2.2	787.9095	4	33.24	5	F5:1670	OB5955 H1 Ro.raw	2.286E6	2	2	411	436	Carbamidomethylation	M19:Oxidation (M):40.00;C22:Carbamidomethylation:1000.00	PEAKS DB
K.NPQLQDLDMMLTC(+71.04)VEIK.E	N	57.51	2060.9944	17	3.0	1031.5076	2	37.24	5	F5:1915	OB5955 H1 Ro.raw	1.057E5	2	2	420	436	Propionamide	C13:Propionamide:1000.00	PEAKS PTM
K.K(+43.01)(+14.02)GSEEEGDITNPINLR.E	N	55.92	1827.8962	16	-0.5	914.9550	2	28.83	5	F5:1409	OB5955 H1 Ro.raw	1.3944E5	2	2	383	398	Carbamylation; Methylation(KR)	K1:Carbamylation:1000.00; K1:Methylation (KR):151.56	PEAKS PTM
R.WGPAGPR.E	N	54.94	739.3765	7	1.0	370.6959	2	25.22	4	F4:1191	OB5954 H1 Ro.raw	3.1963E5	3	3	115	121			PEAKS DB
R.LFEVKPDKK.N	N	54.58	1102.6385	9	1.1	552.3271	2	24.84	4	F4:1157	OB5954 H1 Ro.raw	1.1453E6	6	6	411	419			PEAKS DB
K.NPQLQDLDMMLTC(+57.02)VEIKEGALMLP(+15.99)HFNSK.A	N	54.18	3387.6335	29	-0.6	1130.2178	3	37.70	4	F4:2016	OB5954 H1 Ro.raw	4.3311E7	3	3	420	448	Carbamidomethylation; Hydroxylation Pro	C13:Carbamidomethylation:1000.00; P24:Hydroxylation Pro:122.99	PEAKS DB
R.WGPAGP(+15.99)R.E	N	53.23	755.3714	7	2.6	378.6940	2	23.83	4	F4:1105	OB5954 H1 Ro.raw	2.9481E4	3	3	115	121		P6:Hydroxylation Pro:38.16	PEAKS DB
K.NPQLQDLDMM(+15.99)LTCVEIK(+14.02).E	N	53.12	2019.9679	17	1.7	1010.9930	2	35.46	5	F5:1802	OB5955 H1 Ro.raw	4.2793E5	1	1	420	436	Methylation(KR)	M10:Oxidation (M):8.14;K17:Methylation(KR):1000.00	PEAKS PTM
R.EQEWEEEEEDEEEEGSNREVRR.Y	N	52.77	2821.1545	22	1.7	941.3937	3	26.62	5	F5:1273	OB5955 H1 Ro.raw	8.8078E5	3	3	478	499			PEAKS DB
R.QFQNLQN(+.98)HR.I	N	52.53	1184.5686	9	5.4	593.2948	2	23.60	6	F6:1114	OB5956 H1 Ro.raw	8.0383E2	1	1	205	213		N7:Deamidation (NQ):0.00	PEAKS DB
N.PFYFPSRR.F	N	52.44	1068.5504	8	-0.1	535.2824	2	28.89	6	F6:1431	OB5956 H1 Ro.raw	4.3491E6	4	4	174	181			PEAKS DB

total 184 peptides

Peptide	Uniq	-10lgP	Mass	Length	ppm	m/z	z	RT	Fraction	Scan	Source File	Area roasted peanut	#Feature	#Feature roasted peanut	Start	End	PTM	AScore	Found By
K.NPQLQDLDM(+15.99)M(+15.99)LTC(+57.02)VEIKEGALM(+15.99)LPHFNSK.A	N	52.35	3419.6233	29	-0.9	855.9124	4	34.83	5	F5:1766	OB5955 H1 Ro.raw	1.7196E6	3	3	420	448	Oxidation (M); Carbamidomethylation	M9:Oxidation (M):1000.00; M10:Oxidation (M):1000.00; C13:Carbamidomethylation:1000.00; M22:Oxidation (M):1000.00	PEAKS DB
R.VLLEE(+53.92)NAGGEQEER.G	N	50.61	1625.6620	14	-1.0	542.8940	3	26.76	5	F5:1278	OB5955 H1 Ro.raw	0	0	0	332	345		E5:Replacement of 2 protons by iron:20.92	PEAKS PTM
R.SRQFQNLQNHR.I	N	50.36	1426.7178	11	0.3	476.5800	3	24.16	6	F6:1148	OB5956 H1 Ro.raw	6.1256E4	3	3	203	213			PEAKS DB
R.EGEQEW(+15.99)GTPGSHVR.E	N	49.49	1583.6964	14	-0.6	792.8550	2	25.22	4	F4:1196	OB5954 H1 Ro.raw	3.4731E4	2	2	153	166	Oxidation (HW)	W6:Oxidation (HW):72.73	PEAKS PTM
R.ESHFVSARPQSQSP(+15.99)SSPEKEDQEEEN.Q	N	49.34	2973.2859	26	1.6	992.1041	3	24.81	6	F6:1186	OB5956 H1 Ro.raw	3.8098E5	2	2	579	604		P14:Hydroxylation Pro:11.12	PEAKS DB
R.VLLE(+21.98)ENAGGEQEER.G	N	48.97	1593.7246	14	-0.5	797.8692	2	26.81	5	F5:1275	OB5955 H1 Ro.raw	4.3339E5	3	3	332	345		E4:Sodium adduct:40.00	PEAKS PTM
R.LFEVKPDK.K	N	48.69	974.5436	8	1.7	488.2799	2	25.30	5	F5:1183	OB5955 H1 Ro.raw	1.7748E6	6	6	411	418			PEAKS DB
K.NPQLQDLDMM(+15.99)LTC(+57.02)VEIKEGALMLPHFN(+.98)SK.A	N	48.63	3388.6174	29	6.2	848.1669	4	36.39	6	F6:1880	OB5956 H1 Ro.raw	2.0276E6	1	1	420	448	Carbamidomethylation	M10:Oxidation (M):27.96;C13:Carbamidomethylation:1000.00; N27:Deamidation (NQ):18.27	PEAKS DB
R.N(+27.99)TLEAAFNAEFNEIRR.V	N	48.34	1921.9282	16	-1.7	961.9697	2	37.51	4	F4:1908	OB5954 H1 Ro.raw	4.2206E4	1	1	316	331	Formylation	N1:Formylation:1000.00	PEAKS PTM
R.NT(-18.01)LEAAFNAEFNEIRR.V	N	48.27	1875.9227	16	-1.8	626.3137	3	34.20	4	F4:1731	OB5954 H1 Ro.raw	1.108E7	3	3	316	331	Dehydration	T2:Dehydration:1000.00	PEAKS PTM

total 184 peptides

Peptide	Uniq	-10lgP	Mass	Length	ppm	m/z	z	RT	Fraction	Scan	Source File	Area roasted peanut	#Feature	#Feature roasted peanut	Start	End	PTM	AScore	Found By
R.LFEVKPDKKNPQLQDLDM(+15.99)MLTC(+57.02)VEIK.E	N	48.12	3147.6018	26	-1.9	787.9062	4	34.20	4	F4:1734	OB5954 H1 Ro.raw	2.5552E6	4	4	411	436	Carbamidomethylation	M18:Oxidation (M):33.98;C22:Carbamidomethylation:1000.00	PEAKS DB
K.KNPQLQDLDMM(+15.99)LTC(+57.02)VEIKEGALM(+15.99)LPHFNSK.A	N	47.73	3531.7234	30	-1.1	883.9372	4	35.80	5	F5:1822	OB5955 H1 Ro.raw	0	0	0	419	448	Carbamidomethylation	M11:Oxidation (M):0.00;C14:Carbamidomethylation:1000.00;M23:Oxidation (M):45.87	PEAKS DB
R.DQSSY(-18.01)LQGFSR.N	N	47.62	1268.5785	11	-0.6	635.2961	2	30.85	6	F6:1579	OB5956 H1 Ro.raw	2.6456E5	1	1	305	315		Y5:Dehydration:0.00	PEAKS PTM
R.NTLEAAFNAEFN(+.98)EIR.R	N	47.43	1738.8162	15	9.2	870.4233	2	44.36	5	F5:2303	OB5955 H1 Ro.raw	5.1969E3	1	1	316	330		N12:Deamidation (NQ):0.00	PEAKS DB
K.NPQLQ(+.98)DLDM(+15.99)MLTC(+57.02)VEIKEGALM(+15.99)LPHFNSK.A	N	47.00	3404.6125	29	-0.4	1135.8777	3	36.69	5	F5:1878	OB5955 H1 Ro.raw	3.8413E5	1	1	420	448	Carbamidomethylation	Q5:Deamidation (NQ): 45.47; M9:Oxidation (M):0.00;C13:Carbamidomethylation:1000.00;M22:Oxidation (M):38.75	PEAKS DB
K.D(+43.01)(+14.02)LAFPGSGEQVEK.L	N	46.93	1432.6833	13	-0.1	717.3489	2	31.08	4	F4:1535	OB5954 H1 Ro.raw	9.9373E5	3	3	560	572	Carbamylation; Methylation(others)	D1:Carbamylation:220.79;D1:Methylation(others):132.53	PEAKS PTM
F.PGSGEQVEK.L	N	46.51	929.4454	9	-3.7	465.7282	2	30.34	6	F6:1524	OB5956 H1 Ro.raw	5.467E4	1	1	564	572			PEAKS DB
R.ESHFVSARPQSQSP(+15.99)SSPEKEDQEEE.N	N	46.47	2859.2429	25	2.4	954.0906	3	24.92	5	F5:1173	OB5955 H1 Ro.raw	7.3448E4	1	1	579	603		P14:Hydroxylation Pro:26.52	PEAKS DB
R.DQSSYLQGFSR(+15.99).N	N	46.23	1302.5840	11	2.5	652.3009	2	30.63	5	F5:1518	OB5955 H1 Ro.raw	1.9563E5	4	4	305	315		R11:Oxidation or Hydroxylation:42.88	PEAKS PTM

total 184 peptides

Peptide	Uniq	-10lgP	Mass	Length	ppm	m/z	z	RT	Fraction	Scan	Source File	Area roasted peanut	#Feature	#Feature roasted peanut	Start	End	PTM	AScore	Found By
R.LFEVKPDKKNPQLQ(+.98)DLDMM(+15.99)LTC(+57.02)VEIK.E	N	45.61	3148.5857	26	7.4	788.1595	4	33.17	5	F5:1670	OB5955 H1 Ro.raw	4.4314E5	1	1	411	436	Carbamidomethylation	Q14:Deamidation (NQ):0.00;M19:Oxidation (M):23.10;C22:Carbamidomethylation:1000.00	PEAKS DB
K.KNPQLQDLDM(+15.99)MLTC(+57.02)VEIKEGALMLPHFNSK.A	N	45.13	3515.7285	30	-1.9	879.9377	4	35.30	4	F4:1798	OB5954 H1 Ro.raw	6.4172E5	1	1	419	448	Carbamidomethylation	M10:Oxidation (M):14.02;C14:Carbamidomethylation:1000.00	PEAKS DB
R.NTLEAAFNAEFN(+.98)EIRR.V	N	45.08	1894.9172	16	-4.2	948.4619	2	33.08	4	F4:1691	OB5954 H1 Ro.raw	2.2628E6	3	3	316	331		N12:Deamidation (NQ):39.02	PEAKS DB
R.N(+.98)TLEAAFNAEFNEIRR.V	N	44.42	1894.9172	16	3.8	948.4695	2	36.66	6	F6:1890	OB5956 H1 Ro.raw	1.2834E6	2	2	316	331		N1:Deamidation (NQ):41.52	PEAKS DB
R.ESHFVSARP(+15.99)QSQSPSSPEKEDQEEEN.Q	N	44.15	2973.2859	26	2.8	992.1053	3	24.92	5	F5:1161	OB5955 H1 Ro.raw	1.8315E5	1	1	579	604		P9:Hydroxylation Pro:18.35	PEAKS DB
R.IVQIEAKPNTLVLP(+15.99)K.H	N	43.33	1678.0028	15	-1.5	840.0074	2	30.25	5	F5:1488	OB5955 H1 Ro.raw	0	0	0	214	228		P14:Hydroxylation Pro:0.00	PEAKS DB
R.D(-18.01)QSSYLQGFSR.N	N	43.26	1268.5785	11	1.6	635.2975	2	29.92	6	F6:1489	OB5956 H1 Ro.raw	0	0	0	305	315		D1:Dehydration:0.00	PEAKS PTM
R.IVQIEAKP(+15.99)NTLVLPK.H	N	43.09	1678.0028	15	-0.7	840.0081	2	30.35	4	F4:1495	OB5954 H1 Ro.raw	1.2596E5	3	3	214	228		P8:Hydroxylation Pro:25.21	PEAKS DB
R.LFEVKPDKKNPQLQDLDM(+15.99)M(+15.99)LTC(+57.02)VEIK.E	N	43.03	3163.5967	26	-3.4	791.9037	4	32.67	6	F6:1663	OB5956 H1 Ro.raw	2.1471E6	3	3	411	436	Oxidation (M); Carbamidomethylation	M18:Oxidation (M):1000.00;M19:Oxidation (M):1000.00;C22:Carbamidomethylation:1000.00	PEAKS DB
T.GNLELVAVRK.E	N	43.00	1097.6556	10	0.5	549.8354	2	27.00	5	F5:1296	OB5955 H1 Ro.raw	3.6527E5	3	3	460	469			PEAKS DB

total 184 peptides

Peptide	Uniq	-10lgP	Mass	Length	ppm	m/z	z	RT	Fraction	Scan	Source File	Area roasted peanut	#Feature	#Feature roasted peanut	Start	End	PTM	AScore	Found By
K.KN(+.98)PQLQ(+.98)DLDMMLTC(+57.02)VEIKEGALMLPHFNSK.A	N	42.92	3501.7017	30	7.8	876.4395	4	36.93	6	F6:1903	OB5956 H1 Ro.raw	5.6427E6	1	1	419	448	Carbamidomethylation	N2:Deamidation (NQ): 14.04; Q6:Deamidation (NQ): 0.00;C14:Carbamidomethylation:1000.00	PEAKS DB
R.V(+43.01)LLE(+14.02)ENAGGEQEER.G	N	42.24	1628.7642	14	1.7	815.3907	2	27.00	5	F5:1297	OB5955 H1 Ro.raw	4.2233E5	3	3	332	345	Carbamylation	V1:Carbamylation:1000.00;E4:Methylation (others):30.46	PEAKS PTM
K.D(+15.99)LAFPGSGEQVEK.L	N	42.08	1391.6569	13	0.4	696.8360	2	29.40	5	F5:1368	OB5955 H1 Ro.raw	5.511E5	2	2	560	572	Oxidation or Hydroxylation	D1:Oxidation or Hydroxylation:77.13	PEAKS PTM
K.KNPQLQDLDMM(+15.99)LTC(+57.02)VEIKEGALMLPHFNSK.A	N	42.02	3515.7285	30	1.3	879.9406	4	35.25	5	F5:1790	OB5955 H1 Ro.raw	0	0	0	419	448	Carbamidomethylation	M11:Oxidation (M):12.28;C14:Carbamidomethylation:1000.00	PEAKS DB
K.EGALM(+15.99)LPHFN(+.98)SK.A	N	41.90	1359.6493	12	2.8	680.8338	2	31.22	4	F4:1550	OB5954 H1 Ro.raw	0	0	0	437	448	Oxidation (M); Deamidation (NQ)	M5:Oxidation (M):1000.00; N10:Deamidation (NQ):1000.00	PEAKS DB
K.NPQLQDLDM(-48.00)MLTC(+57.02)VEIK.E	N	41.66	1998.9755	17	6.7	667.3369	3	34.52	6	F6:1773	OB5956 H1 Ro.raw	2.0007E6	2	2	420	436	Carbamidomethylation	M9:Dethiomethyl:11.06;C13:Carbamidomethylation:1000.00	PEAKS PTM
K.GTGNLELVAVRKEQQQR.G	N	41.33	1925.0442	17	1.4	642.6896	3	26.67	6	F6:1294	OB5956 H1 Ro.raw	2.686E4	1	1	458	474			PEAKS DB
K.NPQLQDLDMMLTC(+57.02)VEIK(+43.01)EGALMLPHFNSK(+14.02).A	N	41.19	3428.6602	29	-1.5	858.1710	4	37.84	6	F6:1971	OB5956 H1 Ro.raw	2.9855E6	1	1	420	448	Carbamidomethylation	C13:Carbamidomethylation:1000.00;K17:Carbamylation:11.67;K29:Methylation (KR):11.67	PEAKS PTM

total 184 peptides

Peptide	Uniq	-10lgP	Mass	Length	ppm	m/z	z	RT	Fraction	Scan	Source File	Area roasted peanut	#Feature	#Feature roasted peanut	Start	End	PTM	AScore	Found By
K.NPQ(+.98)LQDLDMMLTC(+57.02)VEIK.E	N	41.15	2047.9629	17	2.9	1024.9917	2	46.45	4	F4:2441	OB5954 H1 Ro.raw	0	0	0	420	436	Carbamidomethylation	Q3:Deamidation (NQ):14.04;C13:Carbamidomethylation:1000.00	PEAKS DB
R.EQEWEEEEEDEEEEGSNREVR.R	N	41.08	2665.0535	21	2.4	889.3605	3	27.88	4	F4:1353	OB5954 H1 Ro.raw	4.8884E4	1	1	478	498			PEAKS DB
R.VLLEEN(+.98)AGGEQEERGQR.R	N	40.76	1913.9078	17	-1.6	638.9755	3	25.99	4	F4:1234	OB5954 H1 Ro.raw	0	0	0	332	348		N6:Deamidation (NQ):28.79	PEAKS DB
R.EGE(+21.98)QEWGTPGSHVR.E	N	40.62	1589.6835	14	1.4	795.8501	2	25.83	4	F4:1231	OB5954 H1 Ro.raw	3.6173E3	1	1	153	166		E3:Sodium adduct:9.74	PEAKS PTM
K.GT(-18.01)GNLELVAVRK.E	N	40.55	1237.7142	12	2.6	413.5797	3	27.00	5	F5:1287	OB5955 H1 Ro.raw	9.9167E5	2	2	458	469	Dehydration	T2:Dehydration:1000.00	PEAKS PTM
K.KNPQLQ(+.98)DLDMMLTC(+57.02)VEIKEGALMLPHFNSK.A	N	40.26	3500.7175	30	3.4	876.1896	4	37.51	4	F4:1896	OB5954 H1 Ro.raw	1.6398E6	1	1	419	448	Carbamidomethylation	Q6:Deamidation (NQ):12.19;C14:Carbamidomethylation:1000.00	PEAKS DB
V.QIEAKPNTLVLPK.H	N	39.98	1449.8555	13	-3.8	725.9323	2	30.34	6	F6:1513	OB5956 H1 Ro.raw	4.8564E4	1	1	216	228			PEAKS DB
R.IVQIEAKPN.T	N	39.98	1010.5760	9	-0.3	506.2951	2	25.40	5	F5:1199	OB5955 H1 Ro.raw	0	0	0	214	222			PEAKS DB
R.I(+42.01)VQ(+15.00)IEAKPNTLVLPK.H	N	38.96	1719.0182	15	2.8	860.5188	2	30.87	6	F6:1547	OB5956 H1 Ro.raw	4.4118E5	2	2	214	228	Acetylation (N-term); Deamidation followed by a methylation	I1:Acetylation (N-term):1000.00;Q3:Deamidation followed by a methylation:129.72	PEAKS PTM
R.EEDWRQPR.E	N	38.91	1114.5155	8	1.8	558.2661	2	23.91	4	F4:1112	OB5954 H1 Ro.raw	0	0	0	126	133			PEAKS DB
K.EGALM(-48.00)LPHFNSK.A	N	38.72	1294.6670	12	1.5	432.5636	3	28.65	5	F5:1381	OB5955 H1 Ro.raw	3.9929E6	3	3	437	448	Dethiomethyl	M5:Dethiomethyl:1000.00	PEAKS PTM
K.PNTLVLPK.H	N	38.45	880.5382	8	-2.9	441.2751	2	28.00	6	F6:1374	OB5956 H1 Ro.raw	1.9367E5	2	2	221	228			PEAKS DB
E.VSKEH(+15.99)VEELTK.H	N	37.60	1313.6826	11	0.8	657.8491	2	24.35	6	F6:1164	OB5956 H1 Ro.raw	1.7908E4	3	3	365	375	Oxidation (HW)	H5:Oxidation (HW):1000.00	PEAKS PTM

total 184 peptides

Peptide	Uniq	-10lgP	Mass	Length	ppm	m/z	z	RT	Fraction	Scan	Source File	Area roasted peanut	#Feature	#Feature roasted peanut	Start	End	PTM	AScore	Found By
R.EGEQEWGTP(+15.99)GSHVREETSR.N	N	37.53	2185.9624	19	0.3	729.6616	3	25.63	4	F4:1213	OB5954 H1 Ro.raw	4.5841E4	1	1	153	171	Hydroxylation Pro	P9:Hydroxylation Pro:1000.00	PEAKS DB
K.KGSEEEGDIT(-18.01)N(+.98)PINLR.E	N	37.46	1753.8483	16	-1.2	585.6227	3	28.83	5	F5:1398	OB5955 H1 Ro.raw	7.9917E5	2	2	383	398	Deamidation (NQ)	T10:Dehydration:19.68;N11:Deamidation (NQ):77.24	PEAKS PTM
R.N(+.98)TLEAAFNAEFNEIR.R	N	36.98	1738.8162	15	9.2	870.4233	2	44.36	5	F5:2311	OB5955 H1 Ro.raw	5.1969E3	1	1	316	330		N1:Deamidation (NQ):21.41	PEAKS DB
R.REQEW(+15.99)EEEEEDEEEEGSNR.E	N	36.76	2452.9375	19	-0.5	818.6527	3	25.98	4	F4:1239	OB5954 H1 Ro.raw	6.7106E4	2	2	477	495	Oxidation (HW)	W5:Oxidation (HW):1000.00	PEAKS PTM
K.AMVIVVINK(+43.01)GTGNLELVAVRK.E	Y	36.30	2266.3196	21	1.3	756.4481	3	33.23	6	F6:1673	OB5956 H1 Ro.raw	3.1381E6	1	1	449	469		K9:Carbamylation:49.34	PEAKS PTM
K.NPQLQ(+.98)DLDMM(+15.99)LTC(+57.02)VEIKEGALM(+15.99)LPHFNSK.A	N	36.05	3404.6125	29	-1.8	852.1589	4	38.22	6	F6:1993	OB5956 H1 Ro.raw	8.1948E5	1	1	420	448	Carbamidomethylation	Q5:Deamidation (NQ):30.14;M10:Oxidation (M):0.00;C13:Carbamidomethylation:1000.00;M22:Oxidation (M):14.60	PEAKS DB
K.NPQLQDLDMMLTCVEIK(+14.02)EGALM(+15.99)LPHFNSK.A	N	36.00	3344.6277	29	-0.2	837.1640	4	37.84	6	F6:1961	OB5956 H1 Ro.raw	7.3561E6	2	2	420	448		K17:Methylation(KR):22.74;M22:Oxidation (M):20.37	PEAKS PTM
K.NPQLQDLDMMLTC(+71.04)VEIKEGALMLPHFNSK.A	N	35.62	3385.6543	29	-1.5	1129.5570	3	38.07	4	F4:1978	OB5954 H1 Ro.raw	1.5713E5	1	1	420	448	Propionamide	C13:Propionamide:1000.00	PEAKS PTM
R.N(-17.03)NPFYFPSRR.F	N	35.47	1279.6097	10	1.1	427.5443	3	28.89	6	F6:1434	OB5956 H1 Ro.raw	5.7683E5	3	3	172	181		N1:Ammonia-loss (N):9.34	PEAKS PTM
R.NTLEAAFNAEFN(+15.99)EIRR.V	N	35.08	1909.9282	16	0.9	955.9723	2	32.98	5	F5:1662	OB5955 H1 Ro.raw	2.4059E6	2	2	316	331		N12:Oxidation or Hydroxylation:9.28	PEAKS PTM
R.NNPFYFP(+15.99)SRR.F	N	34.32	1312.6312	10	-0.5	657.3226	2	28.74	5	F5:1397	OB5955 H1 Ro.raw	7.8182E5	1	1	172	181		P7:Hydroxylation Pro:32.48	PEAKS DB

total 184 peptides

Peptide	Uniq	-10lgP	Mass	Length	ppm	m/z	z	RT	Fraction	Scan	Source File	Area roasted peanut	#Feature	#Feature roasted peanut	Start	End	PTM	AScore	Found By
K.NPQLQ(+.98)DLDMM(-48.00)LTC(+57.02)VEIK.E	N	34.13	1999.9595	17	9.3	1000.9963	2	32.90	4	F4:1653	OB5954 H1 Ro.raw	1.9871E6	1	1	420	436	Carbamidomethylation	Q5:Deamidation (NQ): 11.12; M10:Dethiomethyl:0.00;C13:Carbamidomethylation:1000.00	PEAKS PTM
K.DLAFPGSGE(+53.92)QVEK.L	N	33.99	1429.5812	13	-6.3	715.7933	2	30.34	6	F6:1518	OB5956 H1 Ro.raw	6.355E5	2	2	560	572		E9:Replacement of 2 protons by iron: 37.54	PEAKS PTM
K.NPQ(+.98)LQDLDMMLTC(+57.02)VEIKEGALM(+15.99)LPHFNSK.A	N	33.64	3388.6174	29	6.0	1130.5532	3	38.52	5	F5:2024	OB5955 H1 Ro.raw	7.1577E5	1	1	420	448	Carbamidomethylation	Q3:Deamidation (NQ): 0.00;C13:Carbamidomethylation:1000.00; M22:Oxidation (M):34.40	PEAKS DB
N.PFYFPSR.R	N	33.58	912.4493	7	-0.1	457.2319	2	31.70	5	F5:1584	OB5955 H1 Ro.raw	8.1854E4	1	1	174	180			PEAKS DB
R.IVQ(+.98)IEAKPNTLVLPK.H	N	33.40	1662.9919	15	8.1	832.5100	2	30.29	5	F5:1525	OB5955 H1 Ro.raw	3.5708E6	1	1	214	228		Q3:Deamidation (NQ): 22.65	PEAKS DB
K.D(+53.92)LAFPGSGEQVEK.L	N	33.35	1429.5812	13	-4.3	715.7948	2	30.56	4	F4:1500	OB5954 H1 Ro.raw	3.8253E5	1	1	560	572		D1:Replacement of 2 protons by iron: 0.00	PEAKS PTM
K.N(+.98)PQLQDLDMMLTC(+57.02)VEIKEGALM(+15.99)LPHFNSK.A	N	33.25	3388.6174	29	6.0	1130.5532	3	38.52	5	F5:2054	OB5955 H1 Ro.raw	7.1577E5	1	1	420	448	Carbamidomethylation	N1:Deamidation (NQ): 0.00;C13:Carbamidomethylation:1000.00; M22:Oxidation (M):0.00	PEAKS DB
R.Q(+.98)FQNLQNHR.I	N	33.25	1184.5686	9	4.6	593.2943	2	23.68	5	F5:1098	OB5955 H1 Ro.raw	0	0	0	205	213		Q1:Deamidation (NQ): 25.21	PEAKS DB
R.IFLAGDKDNVVDQIEK(+14.02).Q	Y	33.10	1816.9570	16	0.9	606.6602	3	61.67	6	F6:3449	OB5956 H1 Ro.raw	4.4688E4	2	2	541	556	Methylation(KR)	K16:Methylation(KR): 61.14	PEAKS PTM

total 184 peptides

Peptide	Uniq	-10lgP	Mass	Length	ppm	m/z	z	RT	Fraction	Scan	Source File	Area roasted peanut	#Feature	#Feature roasted peanut	Start	End	PTM	AScore	Found By
K.N(+.98)PQLQDLDM(+15.99)MLTC(+57.02)VEIKEGALMLPHFNSK.A	N	33.01	3388.6174	29	8.2	1695.3300	2	37.72	4	F4:1941	OB5954 H1 Ro.raw	7.0286E5	1	1	420	448	Carbamidomethylation	N1:Deamidation (NQ):0.00;M9:Oxidation (M):0.00;C13:Carbamidomethylation:1000.00	PEAKS DB
K.E(-18.01)GALMLPHFNSK.A	N	32.81	1324.6598	12	0.0	442.5605	3	30.63	5	F5:1521	OB5955 H1 Ro.raw	2.0893E5	2	2	437	448	Pyro-glu from E	E1:Pyro-glu from E:1000.00	PEAKS PTM
K.KNPQLQDLDM(-48.00)MLTC(+57.02)VEIK.E	N	32.73	2127.0703	18	1.8	710.0320	3	33.04	6	F6:1688	OB5956 H1 Ro.raw	7.9877E5	1	1	419	436	Carbamidomethylation	M10:Dethiomethyl:8.14;C14:Carbamidomethylation:1000.00	PEAKS PTM
K.G(+43.01)S(+14.02)EEEGDITNPINLR.E	N	32.64	1699.8013	15	-3.0	850.9054	2	30.85	6	F6:1556	OB5956 H1 Ro.raw	4.7917E4	1	1	384	398	Carbamylation	G1:Carbamylation:1000.00;S2:Methylation(others):0.00	PEAKS PTM
K.KNPQLQ(+.98)DLDMM(+15.99)LTC(+57.02)VEIKEGALMLPHFNSK.A	N	32.59	3516.7124	30	3.3	880.1883	4	35.48	4	F4:1798	OB5954 H1 Ro.raw	7.7853E5	1	1	419	448	Carbamidomethylation	Q6:Deamidation (NQ):23.13;M11:Oxidation (M):14.02;C14:Carbamidomethylation:1000.00	PEAKS DB
K.G(+43.01)T(+14.02)GNLELVAVRK.E	N	32.58	1312.7462	12	-0.4	657.3801	2	27.30	4	F4:1303	OB5954 H1 Ro.raw	3.8825E5	2	2	458	469	Carbamylation; Methylation(others)	G1:Carbamylation:147.00;T2:Methylation(others):68.07	PEAKS PTM
H.RIVQIEAKPNTLVLPK.H	N	32.22	1818.1090	16	0.6	607.0439	3	29.55	5	F5:1454	OB5955 H1 Ro.raw	8.094E4	1	1	213	228			PEAKS DB
K.G(+43.01)T(+14.02)GNLELVAVR.K	N	32.00	1184.6514	11	-2.2	593.3317	2	30.21	4	F4:1493	OB5954 H1 Ro.raw	1.7455E5	2	2	458	468	Carbamylation	G1:Carbamylation:1000.00;T2:Methylation(others):46.31	PEAKS PTM

total 184 peptides

Peptide	Uniq	-10lgP	Mass	Length	ppm	m/z	z	RT	Fraction	Scan	Source File	Area roasted peanut	#Feature	#Feature roasted peanut	Start	End	PTM	AScore	Found By
K.NPQLQ(+.98)DLDMMLTC(+57.02)VEIKEGALMLPHFNSK.A	N	31.45	3372.6226	29	7.2	844.1690	4	45.92	6	F6:2433	OB5956 H1 Ro.raw	0	0	0	420	448	Carbamidomethylation	Q5:Deamidation (NQ): 14.19; C13:Carbamidomethylation:1000.00	PEAKS DB
K.KN(+.98)PQLQDLDMMLTC(+57.02)VEIKEGALMLPHFNSK.A	N	31.43	3500.7175	30	4.4	1167.9182	3	36.77	4	F4:1901	OB5954 H1 Ro.raw	9.2406E4	1	1	419	448	Carbamidomethylation	N2:Deamidation (NQ): 14.04; C14:Carbamidomethylation:1000.00	PEAKS DB
R.IFLAGDK(+14.02)DNVVDQIEK.Q	Y	30.97	1816.9570	16	8.3	909.4933	2	55.20	6	F6:2979	OB5956 H1 Ro.raw	7.7523E4	2	2	541	556		K7:Methylation (KR):18.04	PEAKS PTM
R.WGP(+15.99)AGPR.E	N	30.77	755.3714	7	1.0	378.6934	2	23.04	6	F6:1080	OB5956 H1 Ro.raw	1.1224E4	3	3	115	121		P3:Hydroxylation on Pro: 30.72	PEAKS DB
K.N(+.98)PQ(+.98)LQ(+.98)DLDM(+15.99)MLTC(+57.02)VEIKEGALM(+15.99)LPHFNSK.A	N	30.70	3406.5806	29	9.9	1136.5454	3	36.75	6	F6:1904	OB5956 H1 Ro.raw	3.0926E5	1	1	420	448	Deamidation (NQ); Carbamidomethylation	N1:Deamidation (NQ): 69.03; Q3:Deamidation (NQ): 71.61; Q5:Deamidation (NQ): 53.75; M9:Oxidation (M):17.01;C13:Carbamidomethylation:1000.00; M22:Oxidation (M):16.22	PEAKS DB
R.LFEVKPDKKNPQLQDLDMM(+31.99)LTC(+57.02)VEIK.E	N	29.63	3163.5967	26	-2.8	791.9042	4	32.90	4	F4:1683	OB5954 H1 Ro.raw	1.0179E6	1	1	411	436	Carbamidomethylation	M19:Sulphone: 14.02; C22:Carbamidomethylation:1000.00	PEAKS PTM
R.GRREQEW(+15.99)EEEEEDEEEEGSNR.E	N	29.48	2666.0601	21	0.8	889.6946	3	26.10	4	F4:1240	OB5954 H1 Ro.raw	0	0	0	475	495	Oxidation (HW)	W7:Oxidation (HW):1000.00	PEAKS PTM

total 184 peptides

Peptide	Uniq	-10lgP	Mass	Length	ppm	m/z	z	RT	Fraction	Scan	Source File	Area roasted peanut	#Feature	#Feature roasted peanut	Start	End	PTM	AScore	Found By
K.N(+.98)PQLQ(+.98)DLDMMLTC(+57.02)VEIKEGALM(+15.99)LPHFNSK.A	N	29.27	3389.6016	29	9.6	848.4158	4	37.65	6	F6:2047	OB5956 H1 Ro.raw	8.4697E6	1	1	420	448	Carbamidomethylation	N1:Deamidation (NQ): 0.00;Q5:Deamidation (NQ): 14.04; C13:Carbamidomethylation:1000.00; M22:Oxidation (M):0.00	PEAKS DB
N.TLEAAFNAEFNEIRR.V	N	28.88	1779.8904	15	3.5	890.9556	2	33.23	6	F6:1696	OB5956 H1 Ro.raw	1.5612E5	1	1	317	331			PEAKS DB
K.GTGNLE(+53.92)LVAVRK.E	N	28.35	1309.6440	12	-0.1	437.5553	3	27.29	4	F4:1309	OB5954 H1 Ro.raw	2.3239E4	1	1	458	469	Replacement of 2 protons by iron	E6:Replacement of 2 protons by iron: 60.54	PEAKS PTM
R.VLLEENAGGEQEERGQRR.W	N	28.30	2069.0249	18	1.0	518.2640	4	25.49	5	F5:1205	OB5955 H1 Ro.raw	1.3378E5	1	1	332	349			PEAKS DB
K.AM(+15.99)VIVVINK(+43.01)GTGNLELVAVR.K	Y	28.14	2154.2195	20	2.8	1078.1200	2	32.98	5	F5:1664	OB5955 H1 Ro.raw	2.3746E5	1	1	449	468	Oxidation (M)	M2:Oxidation (M):1000.00; K9:Carbamylation:40.94	PEAKS PTM
K.NP(+15.99)QLQDLDMMLTC(+57.02)VEIKEGALMLPHFNSK.A	N	28.11	3387.6335	29	3.2	1130.2220	3	40.44	5	F5:2090	OB5955 H1 Ro.raw	3.3176E4	1	1	420	448	Carbamidomethylation	P2:Hydroxylation on Pro: 14.98; C13:Carbamidomethylation:1000.00	PEAKS DB
R.VLLEENAGGEQEER(+15.99).G	N	27.33	1587.7375	14	1.8	794.8775	2	26.70	4	F4:1275	OB5954 H1 Ro.raw	0	0	0	332	345		R14:Oxidation or Hydroxylation:20.88	PEAKS PTM
K.NPQLQ(+.98)DLDMM(+15.99)LTC(+57.02)VEIKEGALMLPHFNSK.A	N	27.25	3388.6174	29	8.0	1130.5554	3	37.44	5	F5:1884	OB5955 H1 Ro.raw	6.7537E6	1	1	420	448	Carbamidomethylation	Q5:Deamidation (NQ): 0.00;M10:Oxidation (M):12.28;C13:Carbamidomethylation:1000.00	PEAKS DB

total 184 peptides

Peptide	Uniq	-10lgP	Mass	Length	ppm	m/z	z	RT	Fraction	Scan	Source File	Area roasted peanut	#Feature	#Feature roasted peanut	Start	End	PTM	AScore	Found By
R.N(+42.01)(+15.00)NPFYFPSRR.F	N	27.12	1353.6465	10	9.4	677.8369	2	29.74	5	F5:1453	OB5955 H1 Ro.raw	3.0438E5	1	1	172	181		N1:Acetylation (N-term):1000.00;N1:Deamidation followed by a methylation:12.28	PEAKS PTM
K.NPQLQDLDMMLTC(+58.01)VEIK.E	N	26.84	2047.9629	17	-3.6	1024.9850	2	46.58	4	F4:2449	OB5954 H1 Ro.raw	0	0	0	420	436	Carboxymethyl	C13:Carboxymethyl:1000.00	PEAKS PTM
R.EQEW(+15.99)EEEEEDEEEEGSNREVR.R	N	26.79	2681.0483	21	1.1	894.6910	3	26.92	4	F4:1316	OB5954 H1 Ro.raw	7.9753E4	1	1	478	498	Oxidation (HW)	W4:Oxidation (HW):1000.00	PEAKS PTM
K.NPQLQDLDMM(+15.99)LTC(+57.02)VEIKEGALMLP(+15.99)HFNSK.A	N	26.70	3403.6284	29	-6.6	1702.8102	2	36.88	6	F6:1908	OB5956 H1 Ro.raw	0	0	0	420	448	Carbamidomethylation	M10:Oxidation (M):7.65;C13:Carbamidomethylation:1000.00;P24:Hydroxylation Pro:30.14	PEAKS DB
R.IFLAGDKD(-18.01)NV.V	N	26.68	1072.5553	10	0.4	537.2852	2	32.21	4	F4:1610	OB5954 H1 Ro.raw	2.1773E6	1	1	541	550		D8:Dehydration:45.01	PEAKS PTM
R.IVQIEAKPNTLVLP(+13.98)K.H	N	26.67	1675.9872	15	-0.2	839.0007	2	30.09	5	F5:1488	OB5955 H1 Ro.raw	7.1099E4	1	1	214	228		P14:Proline oxidation to pyroglutamic acid:39.25	PEAKS PTM
K.KNPQLQDLDMM(-48.00)LTC(+57.02)VEIK.E	N	26.57	2127.0703	18	-0.4	710.0304	3	31.57	6	F6:1598	OB5956 H1 Ro.raw	1.2455E5	1	1	419	436	Carbamidomethylation	M11:Dethiomethyl:0.00;C14:Carbamidomethylation:1000.00	PEAKS PTM

total 184 peptides

Peptide	Uniq	-10lgP	Mass	Length	ppm	m/z	z	RT	Fraction	Scan	Source File	Area roasted peanut	#Feature	#Feature roasted peanut	Start	End	PTM	AScore	Found By
K.KNPQ(+.98)LQ(+.98)DLDM(+15.99)MLTC(+57.02)VEIKEGALM(+15.99)LPHFNSK.A	N	26.17	3533.6914	30	9.7	884.4387	4	35.77	5	F5:1822	OB5955 H1 Ro.raw	2.4464E5	1	1	419	448	Carbamidomethylation	Q4:Deamidation (NQ):9.40;Q6:Deamidation (NQ):6.65;M10:Oxidation (M):0.00;C14:Carbamidomethylation:1000.00;M23:Oxidation (M):40.04	PEAKS DB
R.IVQIEAKPNT(-18.01)LVLPK.H	N	26.13	1643.9974	15	1.4	549.0072	3	30.16	6	F6:1509	OB5956 H1 Ro.raw	2.0726E5	1	1	214	228	Dehydration	T10:Dehydration:1000.00	PEAKS PTM
K.NPQLQ(+.98)DLDM(+15.99)M(+15.99)LTC(+57.02)VEIKEGALM(+15.99)LPHFNSK.A	N	26.02	3420.6074	29	5.5	1141.2161	3	34.83	5	F5:1769	OB5955 H1 Ro.raw	2.4925E5	1	1	420	448	Oxidation (M); Carbamidomethylation	Q5:Deamidation (NQ):11.12;M9:Oxidation (M):1000.00;M10:Oxidation (M):1000.00;C13:Carbamidomethylation:1000.00;M22:Oxidation (M):1000.00	PEAKS DB
R.DQSSY(+15.99)LQGFSR.N	N	25.52	1302.5840	11	1.6	652.3003	2	29.24	6	F6:1470	OB5956 H1 Ro.raw	7.3464E4	1	1	305	315		Y5:Oxidation or Hydroxylation:26.36	PEAKS PTM
**total 184 peptides**																			

Peptide List

Prepared with PEAKS ™ (bioinfor.com)

Summary

Ara h 2 GEL BAND RAW

1. Notes

```
raw Ara h 2 band that contains a lot of Ara h 3 and first 6 the most abundant proteins are some Ara h 2 isoforms. Conglu
tin Q6PSU2 is on 7 th place.
```

2. Result Statistics

Figure 1. False discovery rate (FDR) curve. X axis is the number of peptides being kept. Y axis is the corresponding FDR.

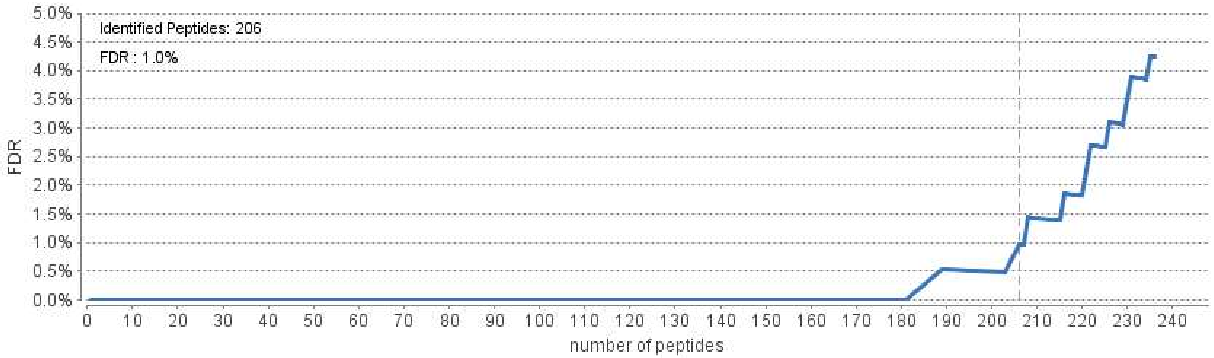

Figure 2. PSM score distribution. **(a)** Distribution of PEAKS peptide score; **(b)** Scatterplot of PEAKS peptide score versus precursor mass error.

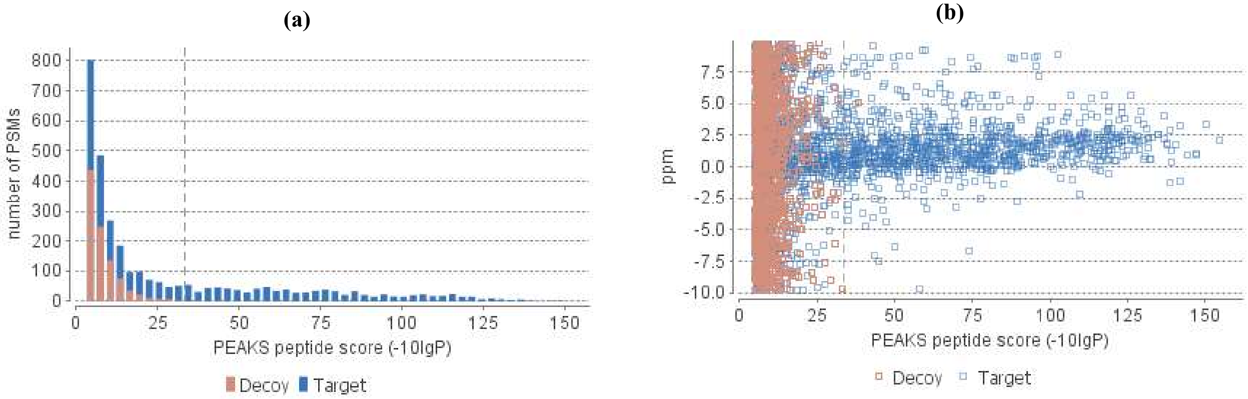

Table 1. Statistics of data.

	#Scans	#Features	Identified	#Peptides	#Sequences	

										#Proteins*		
	MS1	MS/MS	#Chimera		#PSMs	#Scans	#Features**			Groups	All	Top
Total	6768	4921	666	9374	944	913	489	206	130	13	20	16
Raw Ara h 2	6768	4921	666	9374	944	913	489	206	130	13	20	16

* proteins with significant peptides are used in counts.
** features are identified by DB search only.

Figure 3. Sample overlap for Proteins and Peptides (up to 8 samples). **(a)** All Proteins; **(b)** Top Proteins; **(c)** Peptides;

(a)
Not applicable to only one sample

(b)
Not applicable to only one sample

(c)
Not applicable to only one sample

Figure 4. Distribution of peptide feature detection. **(a)** Feature m/z distribution; **(b)** Feature RT distribution.

(a)

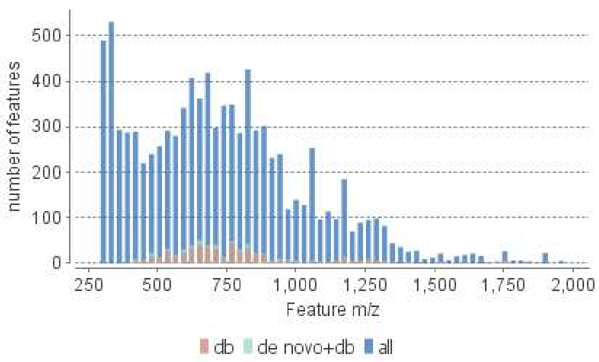

(b)

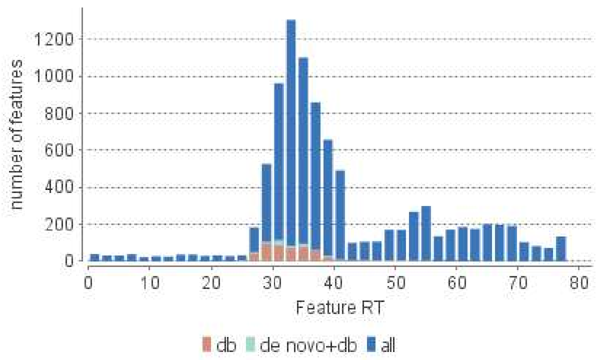

Figure 5. Distribution of identified peptide features. **(a)** Feature abundance distribution; **(b)** *De novo* sequencing validation.

(a)

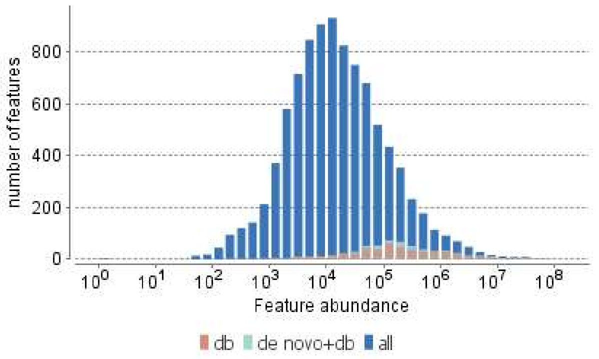

(b)

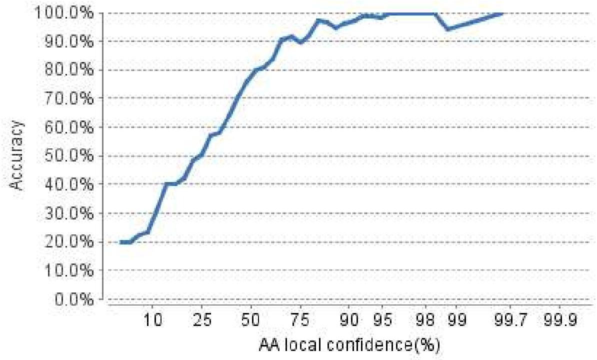

Table 2. Result filtration parameters.

Peptide -10lgP	≥33.5
PTM Ascore	≥50
Protein -10lgP	≥20
Proteins unique peptides	≥2
De novo score(%)	≥50%

Table 3. Statistics of filtered result.

FDR (Peptide-Spectrum Matches)	0.2%
FDR (Peptide Sequences)	1.0%
FDR (Protein Group)	0.0%
De Novo Only Spectra	132

Table 4. PTM profile.

Name	ΔMass	Position	#PSM	-10lgP	Abundance	AScore
Carbamidomethyl	57.02	C	134	136.92	1.56E6	1000.00
Deamidation	.98	NQ	91	154.33		164.73
Oxidation	15.99	M	38	130.39	3.12E5	1000.00
Dimethylation(KR)	28.03	R	20	123.01	3.83E7	1000.00
Carbamidomethyl	57.02	EK,N-term	16	77.15	8.18E3	30.83
Methylation(others)	14.02	DH	10	52.59	1.42E5	18.00
Propionamide	71.04	C	7	78.59	7.6E4	0.00
Cation:Fe[II]	53.92	DE	5	45.19	6.96E4	87.12
Ethyl	28.03	D,N-term	4	74.14	2.35E6	7.21
Diethylation	56.06	K	3	40.01	1.37E6	88.84
Oxidation	15.99	P	2	55.11	4.72E5	7.65
Dihydroxy	31.99	W	2	66.16	1.02E5	24.67
Phosphorylation	79.97	T	2	42.79	1.27E6	71.87
BITC	149.03	K,N-term	2	44.84	1.2E5	4.73
Deoxy	-15.99	S	1	48.15	4.33E4	24.44
Amidine	41.03	N-term	1	37.87	1.3E5	1000.00
DehydroalaY	-94.04	Y	1	42.79	1.27E6	69.38
ethylamino	27.05	S	1	58.10	1.33E5	119.64
Ammonia loss	-17.03	N-term	1	42.30		1000.00
Pyro-glu from Q	-17.03	N-term	1	35.41	1.24E4	1000.00
Acetylation	42.01	K	1	39.62		1000.00

3. Experiment Control

Figure 6. Precursor mass error of peptide-spectrum matches (PSM) in filtered result. **(a)** Distribution of precursor mass error in ppm; **(b)** Scatterplot of precursor m/z versus precursor mass error in ppm.

(a)

(b)

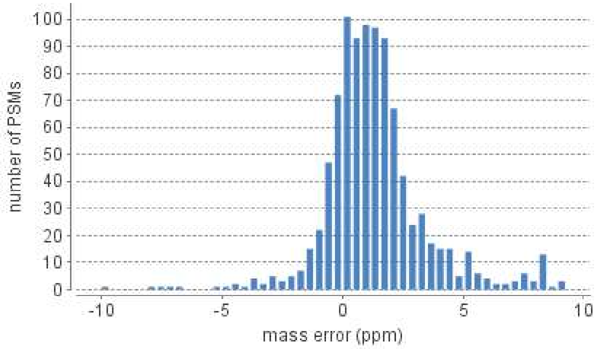

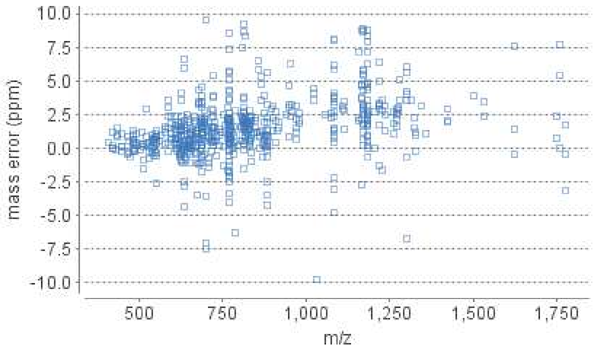

Table 5. Number of identified peptides in each sample by the number of missed cleavages.

Missed Cleavages	0	1	2	3	4+
Raw Ara h 2	135	64	7	0	0

4. Other Information

Table 6. Search parameters.

PEAKS Version: PEAKS Studio 10.6 build 20201015
Search Engine Name: PEAKS
Parent Mass Error Tolerance: 10.0 ppm
Fragment Mass Error Tolerance: 0.5 Da
Precursor Mass Search Type: monoisotopic
Enzyme: Trypsin
Max Missed Cleavages: 2
Digest Mode: Semispecific
Fixed Modifications:
  Carbamidomethylation: 57.02
Variable Modifications:
  Oxidation (M): 15.99
  Deamidation (NQ): 0.98
  Acetylation (K): 42.01
  Acetylation (Protein N-term): 42.01
  Acetylation (N-term): 42.01
  Amidation: -0.98
  Beta-methylthiolation: 45.99
  Biotinylation: 226.08
  and 303 more...
Max Variable PTM Per Peptide: 5
Database: Uniprot_Peanut-3818_Jul18
Taxon: All
Contaminant Database: contam MQ
Searched Entry: 1723
FDR Estimation: Enabled
De novo score(%) threshold: 15
Peptide hit threshold (-10logP): 30.0

Table 7. Instrument parameters.

Fractions: OB5921 H2 raw.raw, OB5932 H2 raw.raw, OB5933 H2 raw.raw
Ion Source: ESI(nano-spray)
Fragmentation Mode: CID, CAD(y and b ions)
MS Scan Mode: FT-ICR/Orbitrap
MS/MS Scan Mode: FT-ICR/Orbitrap

Peaks run ID: 27
Merge Options: no merge
Precursor Options: corrected
Charge Options: no correction
Filter m/z: 200.0 - 2000.0
Filter Charge: 2 - 12
Process: true
Associate chimera: yes

Protein List

Protein Accession Contains:
Protein Description Contains:
Protein Sample Area >=
Protein PTM Contains:

Protein Group	Protein ID	Accession	-10lgP	Coverage (%)	Coverage (%) Raw Ara h 2	Area Raw Ara h 2	#Peptides	#Unique	#Spec Raw Ara h 2	PTM	Avg. Mass	Description
**1**	3	A1DZF0|A1DZF0_ARAHY	434.34	33	33	3.8197E6	17	2	259	Y	60375	Arachin 6 OS=Arachis hypogaea OX=3818 PE=2 SV=1
**8**	1	Q6IWG5|Q6IWG5_ARAHY	415.78	35	35	2.5322E8	17	15	153	Y	58061	Glycinin (Fragment) OS=Arachis hypogaea OX=3818 PE=2 SV=1
**8**	2	Q0GM57|Q0GM57_ARAHY	415.78	34	34	2.5322E8	17	15	153	Y	58263	Iso-Ara h3 OS=Arachis hypogaea OX=3818 PE=2 SV=1
**2**	5	Q6T2T4|Q6T2T4_ARAHY	415.12	32	32	6.5207E6	15	4	248	Y	61499	Storage protein OS=Arachis hypogaea OX=3818 PE=2 SV=1
**2**	6	Q647H4|Q647H4_ARAHY	415.12	32	32	6.5207E6	15	4	248	Y	61506	Arachin Ahy-1 OS=Arachis hypogaea OX=3818 PE=2 SV=1
**7**	12	E9LFE8|E9LFE8_ARAHY	399.56	55	55	1.3004E8	15	15	173	Y	28290	11S arachin (Fragment) OS=Arachis hypogaea OX=3818 PE=2 SV=1
**5**	10	Q9FZ11|Q9FZ11_ARAHY	386.55	26	26	1.9336E6	15	2	176	Y	60449	Gly1 OS=Arachis hypogaea OX=3818 GN=Gly1 PE=2 SV=1
**10**	25	A1E2B1|A1E2B1_ARAHY	360.17	53	53	2.2172E7	13	13	55	Y	31268	11S seed storage globulin B2 OS=Arachis hypogaea OX=3818 PE=2 SV=1
**11**	8	sp|Q647H2|AHY3_ARAHY	342.76	30	30	1.854E7	12	10	49	Y	54569	Arachin Ahy-3 OS=Arachis hypogaea OX=3818 PE=1 SV=1
**15**	14	Q647H1|Q647H1_ARAHY	245.82	10	10	8.3606E6	5	5	15	Y	75933	Conarachin OS=Arachis hypogaea OX=3818 PE=2 SV=1
**12**	24	sp|Q6PSU2|CONG7_ARAHY	235.40	48	48	7.5984E6	8	8	39	Y	20114	Conglutin-7 OS=Arachis hypogaea OX=3818 PE=1 SV=2
**17**	27	A5Z1R0|A5Z1R0_ARAHY	165.46	18	18	2.8784E5	2	2	6	Y	16920	Ara h 6 allergen OS=Arachis hypogaea OX=3818 GN=Ara h 6 PE=4 SV=1
**17**	28	sp|Q647G9|CONG_ARAHY	165.46	18	18	2.8784E5	2	2	6	Y	16920	Conglutin OS=Arachis hypogaea OX=3818 PE=1 SV=1
**18**	415	N1NJN8|N1NJN8_ARAHY	127.73	16	16	3.5194E5	3	3	4	Y	23506	Proteasome subunit OS=Arachis hypogaea OX=3818 GN=ARAX_AHF417E0 7-015 PE=4 SV=1
**16**	29	#CONTAM#P00761	109.64	15	15	3.7364E5	3	3	13	N	24409	SWISS-PROT:#CONTAM#P00761|TRYP_PIG Trypsin - Sus scrofa (Pig).
**19**	421	Q1PCR5|Q1PCR5_ARAHY	93.41	13	13	9.4732E4	2	2	3	N	24047	20S proteasome beta subunit (Fragment) OS=Arachis hypogaea OX=3818 PE=2 SV=1
**total 16 proteins**												

A1DZF0|A1DZF0_ARAHY

back to list

| Protein Coverage | Supporting Peptides |

Protein Coverage:

```
1   MAKLLELSFC FCFLVLGASS ISFRQQPEEN ACQFQRLNAQ RPDNRIESEG GYIETWNPNN QEFECAGVAL SRLVLRRNAL
81  RRPFYSNAPQ EIFIQQGRGY FGLIFPGCPS TYEEPAQQGR RYQSQRPPRR LQEEDQSQQQ QDSHQKVHRF NEGDLIAVPT
161 GVAFWLYNDH DTDVVAVSLT DTNNNDNQLD QFPRRFNLAG NHEQEFLRYQ QQSRQSRRRS LPLSPYSPQP GQEDREFSPQ
241 GQHGRRERAG QEQENEGGNI FSGFTSEFLA QAFQVDDRQI VQNLRGENES EEQGAIVTVK GGLRILSPDR KSPDEEEEYD
                                       351
                                        c
321 EDEYAEEERQ QDRRRGRGSR GSGNGIEETI CTATVKKNIG RNRSPDIYNP QAGSLKTANE LNLLILRWLG LSAEYGNLYR
401 NALFVPHYNT NAHSIIYALR GRAHVQVVDS NGNRVYDEEL QEGHVLVVPQ NFAVAGKSQS ENFEYVAFKT DSRPSIANLA
                   496
                    d
481 GENSFIDNLP EEVVANSYGL PREQARQLKN NNPFKFFVPP FQQSPRAVA
```

c Carbamidomethylation (+57.02)
d Deamidation (NQ) (+0.98)
P Pyro-glu from Q (-17.03)
r Replacement of 2 protons by iron (+53.92)

Supporting Peptides:

Peptide	Uniq	-10lgP	Mass	Length	ppm	m/z	z	RT	Fraction	Scan	Source File	Area Raw Ara h 2	#Feature	#Feature Raw Ara h 2	Start	End	PTM	AScore	Found By
K.TDSRPSIANLAGENSFIDNLPEEVVANSYGLPR.E	N	147.12	3544.7434	33	1.0	1182.5896	3	37.44	2	F2:1890	OB5932 H2 raw.raw	1.1607E8	11	11	470	502			PEAKS DB
R.SPDIYNPQAGSLKTANELNLLILR.W	N	144.08	2639.4282	24	0.9	880.8175	3	35.79	2	F2:1765	OB5932 H2 raw.raw	1.8094E6	3	3	364	387			PEAKS DB
R.WLGLSAEYGNLYR.N	N	135.04	1540.7673	13	2.6	771.3929	2	34.80	1	F1:1640	OB5921 H2 raw.raw	3.956E7	16	15	388	400			PEAKS DB
R.NALFVPHYNTNAHSIIYALR.G	N	133.14	2313.2019	20	2.3	1157.6108	2	33.37	2	F2:1607	OB5932 H2 raw.raw	3.0446E7	14	13	401	420			PEAKS DB
K.SQSENFEYVAFK.T	N	122.47	1447.6619	12	2.4	724.8400	2	31.43	1	F1:1392	OB5921 H2 raw.raw	1.027E7	4	4	458	469			PEAKS DB
R.VYDEELQEGHVLVVPQNFAVAGK.S	N	118.55	2540.2910	23	2.1	847.7727	3	32.93	1	F1:1498	OB5921 H2 raw.raw	1.5993E6	6	6	435	457			PEAKS DB
K.FFVPPFQQSPR.A	Y	117.36	1348.6927	11	1.2	675.3545	2	33.11	1	F1:1496	OB5921 H2 raw.raw	2.8823E6	3	3	516	526			PEAKS DB
R.NRSPDIYNPQAGSLK.T	N	115.99	1658.8376	15	0.4	830.4265	2	27.80	2	F2:1285	OB5932 H2 raw.raw	8.3597E5	6	6	362	376			PEAKS DB
K.TDSRPSIANLAGENSFIDNLPEEVVAN(+.98)SYGLPR.E	N	110.77	3545.7273	33	0.2	1182.9166	3	36.74	2	F2:1825	OB5932 H2 raw.raw	7.8948E3	1	1	470	502	Deamidation (NQ)	N27:Deamidation (NQ):118.18	PEAKS DB
R.SPDIYNPQAGSLK.T	N	106.60	1388.6936	13	1.9	695.3554	2	28.54	3	F3:1354	OB5933 H2 raw.raw	1.3705E6	3	3	364	376			PEAKS DB
N.GIEETIC(+57.02)TATVK.K	N	103.68	1320.6595	12	0.2	661.3372	2	29.46	3	F3:1405	OB5933 H2 raw.raw	2.7846E5	3	3	345	356	Carbamidomethylation	C7:Carbamidomethylation:1000.00	PEAKS DB

total 29 peptides

Peptide	Uniq	-10lgP	Mass	Length	ppm	m/z	z	RT	Fraction	Scan	Source File	Area Raw Ara h 2	#Feature	#Feature Raw Ara h 2	Start	End	PTM	AScore	Found By
N.GIEETIC(+57.02)TATVKK.N	N	87.79	1448.7545	13	0.2	725.3847	2	27.80	2	F2:1296	OB5932 H2 raw.raw	5.8408E5	5	5	345	357	Carbamidomethylation	C7:Carbamidomethylation:1000.00	PEAKS DB
R.AHVQVVDSNGNRVYDEELQEGHVLVVPQNFAVAGK.S	N	86.70	3816.9182	35	2.8	1273.3169	3	32.06	3	F3:1574	OB5933 H2 raw.raw	3.9867E6	2	2	423	457			PEAKS DB
K.FFVPPFQQSPRA.V	Y	85.28	1419.7299	12	0.8	710.8728	2	33.11	1	F1:1502	OB5921 H2 raw.raw	4.6593E5	3	3	516	527			PEAKS DB
K.TANELNLLILR.W	N	82.01	1268.7452	11	0.7	635.3803	2	34.98	1	F1:1608	OB5921 H2 raw.raw	2.5833E7	5	5	377	387			PEAKS DB
R.S(+57.02)PDIYNPQAGSLK.T	N	77.15	1445.7150	13	5.4	723.8687	2	28.54	3	F3:1379	OB5933 H2 raw.raw	8.1843E3	1	1	364	376		S1:Carbamidomethylation (DHKE, X@N-term):30.83	PEAKS PTM
V.PQNFAVAGK.S	N	71.84	930.4923	9	0.8	466.2538	2	32.44	2	F2:1562	OB5932 H2 raw.raw	4.3278E6	2	2	449	457			PEAKS DB
R.NRSPDIYNPQAGSLKTANELNLLILR.W	N	64.74	2909.5723	26	1.5	970.8661	3	34.50	2	F2:1698	OB5932 H2 raw.raw	6.2575E4	1	1	362	387			PEAKS DB
K.FFVPPFQQSP(+15.99)R.A	Y	55.11	1364.6876	11	0.7	683.3516	2	32.74	1	F1:1483	OB5921 H2 raw.raw	4.7156E5	1	1	516	526		P10:Oxidation or Hydroxylation:7.65	PEAKS PTM
R.NALFVPH(+14.02)YNTNAHSIIYALR.G	N	52.59	2327.2175	20	0.3	776.7467	3	35.36	1	F1:1633	OB5921 H2 raw.raw	1.4175E5	1	1	401	420		H7:Methylation(others):18.00	PEAKS PTM
K.TDSRPSIANLAGEN(+.98)SFIDNLPEEVVANSYGLPR.E	N	46.28	3545.7273	33	6.3	1182.9238	3	48.72	1	F1:2418	OB5921 H2 raw.raw	9.731E3	2	2	470	502		N14:Deamidation (NQ):29.85	PEAKS DB
R.QLKNNNPFK.F	N	45.67	1101.5930	9	-0.6	551.8035	2	27.25	3	F3:1282	OB5933 H2 raw.raw	1.2465E4	2	2	507	515			PEAKS DB
R.WLGLSAE(+53.92)YGNLYR.N	N	45.19	1594.6866	13	0.4	532.5697	3	34.87	2	F2:1718	OB5932 H2 raw.raw	1.8492E5	2	2	388	400	Replacement of 2 protons by iron	E7:Replacement of 2 protons by iron:156.85	PEAKS PTM
N.G(+57.02)IEETIC(+57.02)TATVK.K	N	44.11	1377.6809	12	0.2	689.8479	2	29.76	1	F1:1306	OB5921 H2 raw.raw	0	0	0	345	356	Carbamidomethylation	G1:Carbamidomethylation (DHKE, X@N-term):5.03; C7:Carbamidomethylation:1000.00	PEAKS PTM
R.N(+.98)ALFVPHYNTNAHSIIYALR.G	N	41.98	2314.1858	20	3.6	772.4053	3	45.28	3	F3:2182	OB5933 H2 raw.raw	6.3088E4	1	1	401	420		N1:Deamidation (NQ):32.94	PEAKS DB
N.G(+57.02)IEETIC(+57.02)TATVKK.N	N	37.32	1505.7759	13	2.5	753.8971	2	28.15	1	F1:1210	OB5921 H2 raw.raw	1.685E4	1	1	345	357	Carbamidomethylation	G1:Carbamidomethylation (DHKE, X@N-term):26.31;C7:Carbamidomethylation:1000.00	PEAKS PTM
R.WLGLSAEYGN(+.98)LYR.N	N	36.96	1541.7513	13	7.3	771.8886	2	52.84	1	F1:2630	OB5921 H2 raw.raw	0	0	0	388	400	Deamidation (NQ)	N10:Deamidation (NQ):1000.00	PEAKS DB
K.SQSE(+53.92)NFEYVAFK.T	N	36.67	1501.5812	12	-1.3	751.7969	2	31.69	2	F2:1525	OB5932 H2 raw.raw	2.6345E5	1	1	458	469		E4:Replacement of 2 protons by iron:32.69	PEAKS PTM
R.Q(-17.03)LKNNNPFK.F	N	35.41	1084.5665	9	0.4	543.2908	2	28.36	3	F3:1357	OB5933 H2 raw.raw	1.237E4	1	1	507	515	Pyro-glu from Q	Q1:Pyro-glu from Q:1000.00	PEAKS PTM
**total 29 peptides**																			

Q6IWG5|Q6IWG5_ARAHY

back to list

| Protein Coverage | Supporting Peptides |

Protein Coverage:

1 KLLALSLCFC VLVLGASSVT FRQGGEENEC QFQRLNAQRP DNRIESEGGY IETWNPNNQE FQCAGVALSR TVLRRNALRR
81 PFYSNAPLEI YVQQGSGYFG LIFPGCPSTY EEPAQEGRRY QSQKPSRRFQ VGQDDPSQQQ QDSHQKVHRF DEGDLIAVPT
161 GVAFWMYNDE DTDVVTVTLS DTSSIHNQLD QFPRRFYLAG NQEQEFLRYQ QQQGSRPHYR QISPRVRGDE QENEGSNIFS
241 GFAQEFLQHA FQVDRQTVEN LRGENEREEQ GAIVTVKGGL RILSPDEEDE SSRSPPSRRE EFDEDRSRPQ QRGKYDENRR
321 GYKNGIEETI CSASVKKNLG RSSNPDIYNP QAGSLRSVNE LDLPILGWLG LSAQHGTIYR NAMFVPHYTL NAHTIVVALN
401 GRAHVQVVDS NGNRVYDEEL QEGHVLVVPQ NFAVAAKAQS ENYEYLAFKT DSRPSIANLA GENSIIDNLP EEVVANSYRL
481 PREQARQLKN NNPFKFFVPP FDHQSMREVA

C Carbamidomethylation (+57.02)
d Deamidation (NQ) (+0.98)
o Oxidation (M) (+15.99)
P Pyro-glu from Q (-17.03)

Supporting Peptides:

Peptide	Uniq	-10lgP	Mass	Length	ppm	m/z	z	RT	Fraction	Scan	Source File	Area Raw Ara h 2	#Feature	#Feature Raw Ara h 2	Start	End	PTM	AScore	Found By
R.NAMFVPHYTLNAHTIVVALN(+.98)GR.A	Y	154.33	2438.2529	22	2.1	813.7599	3	33.95	3	F3:1683	OB5933 H2 raw.raw	5.0871E6	4	4	381	402	Deamidation (NQ)	N20:Deamidation (NQ):218.32	PEAKS DB
R.SVNELDLPILGWLGLSAQHGTIYR.N	Y	147.15	2651.4070	24	1.0	1326.7120	2	39.90	1	F1:1894	OB5921 H2 raw.raw	6.1669E7	7	7	357	380			PEAKS DB
R.NAMFVPHYTLNAHTIVVALNGR.A	Y	140.28	2437.2688	22	1.7	1219.6438	2	35.24	2	F2:1717	OB5932 H2 raw.raw	1.1969E7	6	6	381	402			PEAKS DB
K.TDSRPSIANLAGENSIIDNLPEEVVANSYR.L	Y	128.13	3243.6006	30	3.7	1082.2114	3	36.34	2	F2:1837	OB5932 H2 raw.raw	1.7742E7	6	6	450	479			PEAKS DB
K.TDSRPSIANLAGENSIIDNLPEEVVAN(+.98)SYR.L	Y	123.94	3244.5847	30	3.8	1082.5397	3	35.65	2	F2:1760	OB5932 H2 raw.raw	0	0	0	450	479	Deamidation (NQ)	N27:Deamidation (NQ):81.59	PEAKS DB
K.AQSENYEYLAFK.T	Y	123.69	1461.6776	12	0.8	731.8467	2	31.43	1	F1:1390	OB5921 H2 raw.raw	5.3867E6	3	3	438	449			PEAKS DB
R.NAM(+15.99)FVPHYTLNAHTIVVALNGR.A	Y	121.47	2453.2637	22	2.2	818.7636	3	33.56	3	F3:1651	OB5933 H2 raw.raw	1.2362E6	2	2	381	402	Oxidation (M)	M3:Oxidation (M):1000.00	PEAKS DB
R.SSNPDIYNPQAGSLR.S	Y	121.43	1617.7747	15	0.5	809.8950	2	28.51	1	F1:1221	OB5921 H2 raw.raw	1.8561E6	3	3	342	356			PEAKS DB

total 35 peptides

Peptide	Uniq	-10lgP	Mass	Length	ppm	m/z	z	RT	Fraction	Scan	Source File	Area Raw Ara h 2	#Feature	#Feature Raw Ara h 2	Start	End	PTM	AScore	Found By
R.N(+.98)AMFVPHYTLNAHTIVVALNGR.A	Y	118.49	2438.2529	22	2.9	1220.1373	2	34.78	2	F2:1708	OB5932 H2 raw.raw	0	0	0	381	402	Deamidation (NQ)	N1:Deamidation (NQ):79.74	PEAKS DB
L.PILGWLGLSAQHGTIYR.N	Y	111.93	1881.0261	17	2.5	941.5227	2	39.89	3	F3:2036	OB5933 H2 raw.raw	1.5582E5	1	1	364	380			PEAKS DB
R.NAM(+15.99)FVPHYTLNAHTIVVALN(+.98)GR.A	Y	110.86	2454.2478	22	2.8	1228.1346	2	32.81	3	F3:1623	OB5933 H2 raw.raw	1.7713E6	6	6	381	402	Oxidation (M); Deamidation (NQ)	M3:Oxidation (M):1000.00;N20:Deamidation (NQ):123.21	PEAKS DB
R.AHVQVVDSN(+.98)GNRVYDEELQEGHVLVVPQNFAVAAK.A	Y	98.59	3831.9180	35	3.6	1278.3179	3	32.25	3	F3:1584	OB5933 H2 raw.raw	9.4081E6	2	2	403	437		N9:Deamidation (NQ):9.40	PEAKS DB
N.GIEETIC(+57.02)SASVK.K	Y	95.93	1292.6282	12	2.2	647.3228	2	29.07	1	F1:1261	OB5921 H2 raw.raw	1.1569E5	3	3	325	336	Carbamidomethylation	C7:Carbamidomethylation:1000.00	PEAKS DB
K.TDSRPSIANLAGEN(+.98)SIIDNLPEEVVANSYR.L	Y	95.61	3244.5847	30	8.0	1082.5442	3	36.12	3	F3:1826	OB5933 H2 raw.raw	5.3609E7	2	2	450	479		N14:Deamidation (NQ):31.31	PEAKS DB
R.N(+.98)AMFVPHYTLNAHTIVVALN(+.98)GR.A	Y	87.68	2439.2368	22	8.7	814.0933	3	34.13	3	F3:1683	OB5933 H2 raw.raw	4.359E6	1	1	381	402	Deamidation (NQ)	N1:Deamidation (NQ):53.47;N20:Deamidation (NQ):61.12	PEAKS DB
K.FFVPPFDHQSMRE.V	Y	82.77	1635.7504	13	1.4	818.8836	2	32.55	1	F1:1469	OB5921 H2 raw.raw	1.5304E6	4	4	496	508			PEAKS DB
R.NAMFVPHYTLN(+.98)AHTIVVALNGR.A	Y	81.31	2438.2529	22	3.7	813.7613	3	35.31	1	F1:1635	OB5921 H2 raw.raw	0	0	0	381	402		N11:Deamidation (NQ):33.51	PEAKS DB
R.VYDEELQEGHVLVVPQNFAVAAK.A	Y	74.42	2554.3066	23	1.2	852.4438	3	33.17	1	F1:1510	OB5921 H2 raw.raw	0	0	0	415	437			PEAKS DB
R.SVNE(+57.02)LDLPILGWLGLSAQHGTIYR.N	Y	72.38	2708.4285	24	1.0	1355.2229	2	40.09	2	F2:2024	OB5932 H2 raw.raw	1.9952E5	1	1	357	380		E4:Carbamidomethylation (DHKE, X@N-term):5.03	PEAKS PTM
N.GIEETIC(+57.02)SASVKK.N	Y	70.24	1420.7231	13	0.8	711.3694	2	27.44	2	F2:1263	OB5932 H2 raw.raw	3.7518E5	3	3	325	337	Carbamidomethylation	C7:Carbamidomethylation:1000.00	PEAKS DB
K.TDSRPSIANLAGENSIIDNLPEEVVANSYRLPR.E	Y	68.47	3609.8386	33	2.2	903.4689	4	36.12	3	F3:1817	OB5933 H2 raw.raw	2.0614E7	5	5	450	482			PEAKS DB
R.N(+.98)AM(+15.99)FVPHYTLNAHTIVVALN(+.98)GR.A	Y	68.22	2455.2317	22	8.4	819.4247	3	34.50	2	F2:1707	OB5932 H2 raw.raw	1.9858E5	1	1	381	402	Oxidation (M)	N1:Deamidation (NQ):27.37;M3:Oxidation (M):1000.00;N20:Deamidation (NQ):39.72	PEAKS DB
R.SVNELDLPILGW(+31.99)LGLSAQHGTIYR.N	Y	66.16	2683.3970	24	3.1	1342.7100	2	38.93	3	F3:1986	OB5933 H2 raw.raw	6.9221E5	2	2	357	380		W12:Dihydroxy:33.65	PEAKS PTM
K.FFVPPFDHQS.M	Y	63.99	1219.5662	10	0.3	610.7905	2	33.29	1	F1:1544	OB5921 H2 raw.raw	2.015E5	1	1	496	505			PEAKS DB
R.N(+.98)AMFVPHYTLN(+.98)AHTIVVALNGR.A	Y	59.69	2439.2368	22	9.3	814.0938	3	35.76	3	F3:1773	OB5933 H2 raw.raw	7.1072E5	1	1	381	402		N1:Deamidation (NQ):40.15;N11:Deamidation (NQ):28.29	PEAKS DB
V.PQNFAVAAK.A	N	51.53	944.5079	9	0.5	473.2615	2	32.25	3	F3:1586	OB5933 H2 raw.raw	2.3208E6	2	2	429	437			PEAKS DB

total 35 peptides

Peptide	Uniq	-10lgP	Mass	Length	ppm	m/z	z	RT	Fraction	Scan	Source File	Area Raw Ara h 2	#Feature	#Feature Raw Ara h 2	Start	End	PTM	AScore	Found By
K.TDSRPSIANLAGENSIIDN(+.98)LPEEVVANSYR.L	Y	49.89	3244.5847	30	8.0	1082.5442	3	36.12	3	F3:1810	OB5933 H2 raw.raw	5.3512E7	1	1	450	479		N19:Deamidation (NQ):6.49	PEAKS DB
R.AHVQVVDSNGN(+.98)RVYDEELQEGHVLVVPQNFAVAAK.A	Y	49.15	3831.9180	35	1.8	767.3923	5	32.25	3	F3:1589	OB5933 H2 raw.raw	2.7476E5	1	1	403	437		N11:Deamidation (NQ):0.00	PEAKS DB
R.QLKNNNPFK.F	N	45.67	1101.5930	9	-0.6	551.8035	2	27.25	3	F3:1282	OB5933 H2 raw.raw	1.2465E4	2	2	487	495			PEAKS DB
K.FFVPPFDHQSM(+15.99).R	Y	44.78	1366.6016	11	-0.2	684.3079	2	33.49	1	F1:1527	OB5921 H2 raw.raw	4.1366E4	1	1	496	506	Oxidation (M)	M11:Oxidation (M):1000.00	PEAKS DB
K.FFVPPFDHQSM.R	Y	42.50	1350.6067	11	-0.5	676.3103	2	34.43	1	F1:1571	OB5921 H2 raw.raw	4.9902E4	1	1	496	506			PEAKS DB
K.FFVPPFDHQSM(+15.99)RE.V	Y	37.46	1651.7452	13	0.6	551.5894	3	31.99	1	F1:1457	OB5921 H2 raw.raw	2.1439E5	1	1	496	508	Oxidation (M)	M11:Oxidation (M):1000.00	PEAKS DB
R.Q(-17.03)LKNNNPFK.F	N	35.41	1084.5665	9	0.4	543.2908	2	28.36	3	F3:1357	OB5933 H2 raw.raw	1.237E4	1	1	487	495	Pyro-glu from Q	Q1:Pyro-glu from Q:1000.00	PEAKS PTM
R.AHVQ(+.98)VVDSNGNRVYDEELQEGHVLVVPQNFAVAAK.A	Y	35.00	3831.9180	35	2.1	767.3925	5	32.58	2	F2:1579	OB5932 H2 raw.raw	0	0	0	403	437		Q4:Deamidation (NQ):0.00	PEAKS DB
K.AQSENYEYLAFKTDSRPSIANLAGEN(+.98)SIIDNLPEEVVANSYR.L	Y	34.68	4688.2515	42	4.7	1173.0757	4	36.70	2	F2:1816	OB5932 H2 raw.raw	2.4176E5	1	1	438	479		N26:Deamidation (NQ):0.00	PEAKS DB
**total 35 peptides**																			

Q0GM57|Q0GM57_ARAHY

back to list

| Protein Coverage | Supporting Peptides |

Protein Coverage:

1 MAKLLALSLC FCVLVLGASS VTFRQGGEEN ECQFQRLNAQ RPDNRIESEG GYIETWNPNN QEFQCAGVAL SRTVLRRNAL
81 RRPFYSNAPL EIYVQQGSGY FGLIFPGCPS TYEEPAQEGR RYQSQKPSRR FQVGQDDPSQ QQQDSHQKVH RFDEGDLIAV
161 PTGVAFWMYN DEDTDVVTVT LSDTSSIHNQ LDQFPRRFYL AGNQEQEFLR YQQQQGSRPH YRQISPRVRG DEQENEGSNI
241 FSGFAQEFLQ HAFQVDRQTV ENLRGENERE EQGAIVTVKG GLRILSPDEE DESSRSPPSR REEFDEDRSR PQQRGKYDEN
321 RRGYKNGIEE TICSASVKKN LGRSSNPDIY NPQAGSLRSV NELDLPILGW LGLSAQHGTI YRNAMFVPHY TLNAHTIVVA
401 LNGRAHVQVV DSNGNRVYDE ELQEGHVLVV PQNFAVAAKA QSENYEYLAF KTDSRPSIAN LAGENSIIDN LPEEVVANSY
481 RLPREQARQL KNNPFKFFV PPFDHQSMRE VA

c Carbamidomethylation (+57.02)
d Deamidation (NQ) (+0.98)
o Oxidation (M) (+15.99)
p Pyro-glu from Q (-17.03)

Supporting Peptides:

Peptide	Uniq	-10lgP	Mass	Length	ppm	m/z	z	RT	Fraction	Scan	Source File	Area Raw Ara h 2	#Feature	#Feature Raw Ara h 2	Start	End	PTM	AScore	Found By
R.NAMFVPHYTLNAHTIVVALN(+.98)GR.A	Y	154.33	2438.2529	22	2.1	813.7599	3	33.95	3	F3:1683	OB5933 H2 raw.raw	5.0871E6	4	4	383	404	Deamidation (NQ)	N20:Deamidation (NQ):218.32	PEAKS DB
R.SVNELDLPILGWLGLSAQHGTIYR.N	Y	147.15	2651.4070	24	1.0	1326.7120	2	39.90	1	F1:1894	OB5921 H2 raw.raw	6.1669E7	7	7	359	382			PEAKS DB
R.NAMFVPHYTLNAHTIVVALNGR.A	Y	140.28	2437.2688	22	1.7	1219.6438	2	35.24	2	F2:1717	OB5932 H2 raw.raw	1.1969E7	6	6	383	404			PEAKS DB
K.TDSRPSIANLAGENSIIDNLPEEVVANSYR.L	Y	128.13	3243.6006	30	3.7	1082.2114	3	36.34	2	F2:1837	OB5932 H2 raw.raw	1.7742E7	6	6	452	481			PEAKS DB
K.TDSRPSIANLAGENSIIDNLPEEVVAN(+.98)SYR.L	Y	123.94	3244.5847	30	3.8	1082.5397	3	35.65	2	F2:1760	OB5932 H2 raw.raw	0	0	0	452	481	Deamidation (NQ)	N27:Deamidation (NQ):81.59	PEAKS DB
K.AQSENYEYLAFK.T	Y	123.69	1461.6776	12	0.8	731.8467	2	31.43	1	F1:1390	OB5921 H2 raw.raw	5.3867E6	3	3	440	451			PEAKS DB
R.NAM(+15.99)FVPHYTLNAHTIVVALNGR.A	Y	121.47	2453.2637	22	2.2	818.7636	3	33.56	3	F3:1651	OB5933 H2 raw.raw	1.2362E6	2	2	383	404	Oxidation (M)	M3:Oxidation (M):1000.00	PEAKS DB
R.SSNPDIYNPQAGSLR.S	Y	121.43	1617.7747	15	0.5	809.8950	2	28.51	1	F1:1221	OB5921 H2 raw.raw	1.8561E6	3	3	344	358			PEAKS DB
R.N(+.98)AMFVPHYTLNAHTIVVALNGR.A	Y	118.49	2438.2529	22	2.9	1220.1373	2	34.78	2	F2:1708	OB5932 H2 raw.raw	0	0	0	383	404	Deamidation (NQ)	N1:Deamidation (NQ):79.74	PEAKS DB

total 35 peptides

Peptide	Uniq	-10lgP	Mass	Length	ppm	m/z	z	RT	Fraction	Scan	Source File	Area Raw Ara h 2	#Feature	#Feature Raw Ara h 2	Start	End	PTM	AScore	Found By
L.PILGWLGLSAQHGTIYR.N	Y	111.93	1881.0261	17	2.5	941.5227	2	39.89	3	F3:2036	OB5933 H2 raw.raw	1.5582E5	1	1	366	382			PEAKS DB
R.NAM(+15.99)FVPHYTLNAHTIVVALN(+.98)GR.A	Y	110.86	2454.2478	22	2.8	1228.1346	2	32.81	3	F3:1623	OB5933 H2 raw.raw	1.7713E6	6	6	383	404	Oxidation (M); Deamidation (NQ)	M3:Oxidation (M):1000.00;N20:Deamidation (NQ):123.21	PEAKS DB
R.AHVQVVDSN(+.98)GNRVYDEELQEGHVLVVPQNFAVAAK.A	Y	98.59	3831.9180	35	3.6	1278.3179	3	32.25	3	F3:1584	OB5933 H2 raw.raw	9.4081E6	2	2	405	439		N9:Deamidation (NQ):9.40	PEAKS DB
N.GIEETIC(+57.02)SASVK.K	Y	95.93	1292.6282	12	2.2	647.3228	2	29.07	1	F1:1261	OB5921 H2 raw.raw	1.1569E5	3	3	327	338	Carbamidomethylation	C7:Carbamidomethylation:1000.00	PEAKS DB
K.TDSRPSIANLAGEN(+.98)SIIDNLPEEVVANSYR.L	Y	95.61	3244.5847	30	8.0	1082.5442	3	36.12	3	F3:1826	OB5933 H2 raw.raw	5.3609E7	2	2	452	481		N14:Deamidation (NQ):31.31	PEAKS DB
R.N(+.98)AMFVPHYTLNAHTIVVALN(+.98)GR.A	Y	87.68	2439.2368	22	8.7	814.0933	3	34.13	3	F3:1683	OB5933 H2 raw.raw	4.359E6	1	1	383	404	Deamidation (NQ)	N1:Deamidation (NQ):53.47;N20:Deamidation (NQ):61.12	PEAKS DB
K.FFVPPFDHQSMRE.V	Y	82.77	1635.7504	13	1.4	818.8836	2	32.55	1	F1:1469	OB5921 H2 raw.raw	1.5304E6	4	4	498	510			PEAKS DB
R.NAMFVPHYTLN(+.98)AHTIVVALNGR.A	Y	81.31	2438.2529	22	3.7	813.7613	3	35.31	1	F1:1635	OB5921 H2 raw.raw	0	0	0	383	404		N11:Deamidation (NQ):33.51	PEAKS DB
R.VYDEELQEGHVLVVPQNFAVAAK.A	Y	74.42	2554.3066	23	1.2	852.4438	3	33.17	1	F1:1510	OB5921 H2 raw.raw	0	0	0	417	439			PEAKS DB
R.SVNE(+57.02)LDLPILGWLGLSAQHGTIYR.N	Y	72.38	2708.4285	24	1.0	1355.2229	2	40.09	2	F2:2024	OB5932 H2 raw.raw	1.9952E5	1	1	359	382		E4:Carbamidomethylation (DHKE, X@N-term):5.03	PEAKS PTM
N.GIEETIC(+57.02)SASVKK.N	Y	70.24	1420.7231	13	0.8	711.3694	2	27.44	2	F2:1263	OB5932 H2 raw.raw	3.7518E5	3	3	327	339	Carbamidomethylation	C7:Carbamidomethylation:1000.00	PEAKS DB
K.TDSRPSIANLAGENSIIDNLPEEVVANSYRLPR.E	Y	68.47	3609.8386	33	2.2	903.4689	4	36.12	3	F3:1817	OB5933 H2 raw.raw	2.0614E7	5	5	452	484			PEAKS DB
R.N(+.98)AM(+15.99)FVPHYTLNAHTIVVALN(+.98)GR.A	Y	68.22	2455.2317	22	8.4	819.4247	3	34.50	2	F2:1707	OB5932 H2 raw.raw	1.9858E5	1	1	383	404	Oxidation (M)	N1:Deamidation (NQ):27.37;M3:Oxidation (M):1000.00;N20:Deamidation (NQ):39.72	PEAKS DB
R.SVNELDLPILGW(+31.99)LGLSAQHGTIYR.N	Y	66.16	2683.3970	24	3.1	1342.7100	2	38.93	3	F3:1986	OB5933 H2 raw.raw	6.9221E5	2	2	359	382		W12:Dihydroxy:33.65	PEAKS PTM
K.FFVPPFDHQS.M	Y	63.99	1219.5662	10	0.3	610.7905	2	33.29	1	F1:1544	OB5921 H2 raw.raw	2.015E5	1	1	498	507			PEAKS DB
R.N(+.98)AMFVPHYTLN(+.98)AHTIVVALNGR.A	Y	59.69	2439.2368	22	9.3	814.0938	3	35.76	3	F3:1773	OB5933 H2 raw.raw	7.1072E5	1	1	383	404		N1:Deamidation (NQ):40.15;N11:Deamidation (NQ):28.29	PEAKS DB
V.PQNFAVAAK.A	N	51.53	944.5079	9	0.5	473.2615	2	32.25	3	F3:1586	OB5933 H2 raw.raw	2.3208E6	2	2	431	439			PEAKS DB
K.TDSRPSIANLAGENSIIDN(+.98)LPEEVVANSYR.L	Y	49.89	3244.5847	30	8.0	1082.5442	3	36.12	3	F3:1810	OB5933 H2 raw.raw	5.3512E7	1	1	452	481		N19:Deamidation (NQ):6.49	PEAKS DB

total 35 peptides

Peptide	Uniq	-10lgP	Mass	Length	ppm	m/z	z	RT	Fraction	Scan	Source File	Area Raw Ara h 2	#Feature	#Feature Raw Ara h 2	Start	End	PTM	AScore	Found By
R.AHVQVVDSNGN(+.98)RVYDEELQEGHVLVVPQNFAVAAK.A	Y	49.15	3831.9180	35	1.8	767.3923	5	32.25	3	F3:1589	OB5933 H2 raw.raw	2.7476E5	1	1	405	439		N11:Deamidation (NQ):0.00	PEAKS DB
R.QLKNNNPFK.F	N	45.67	1101.5930	9	-0.6	551.8035	2	27.25	3	F3:1282	OB5933 H2 raw.raw	1.2465E4	2	2	489	497			PEAKS DB
K.FFVPPFDHQSM(+15.99).R	Y	44.78	1366.6016	11	-0.2	684.3079	2	33.49	1	F1:1527	OB5921 H2 raw.raw	4.1366E4	1	1	498	508	Oxidation (M)	M11:Oxidation (M):1000.00	PEAKS DB
K.FFVPPFDHQSM.R	Y	42.50	1350.6067	11	-0.5	676.3103	2	34.43	1	F1:1571	OB5921 H2 raw.raw	4.9902E4	1	1	498	508			PEAKS DB
K.FFVPPFDHQSM(+15.99)RE.V	Y	37.46	1651.7452	13	0.6	551.5894	3	31.99	1	F1:1457	OB5921 H2 raw.raw	2.1439E5	1	1	498	510	Oxidation (M)	M11:Oxidation (M):1000.00	PEAKS DB
R.Q(-17.03)LKNNNPFK.F	N	35.41	1084.5665	9	0.4	543.2908	2	28.36	3	F3:1357	OB5933 H2 raw.raw	1.237E4	1	1	489	497	Pyro-glu from Q	Q1:Pyro-glu from Q:1000.00	PEAKS PTM
R.AHVQ(+.98)VVDSNGNRVYDEELQEGHVLVVPQNFAVAAK.A	Y	35.00	3831.9180	35	2.1	767.3925	5	32.58	2	F2:1579	OB5932 H2 raw.raw	0	0	0	405	439		Q4:Deamidation (NQ):0.00	PEAKS DB
K.AQSENYEYLAFKTDSRPSIANLAGEN(+.98)SIIDNLPEEVVANSYR.L	Y	34.68	4688.2515	42	4.7	1173.0757	4	36.70	2	F2:1816	OB5932 H2 raw.raw	2.4176E5	1	1	440	481		N26:Deamidation (NQ):0.00	PEAKS DB
**total 35 peptides**																			

Q6T2T4|Q6T2T4_ARAHY

back to list

| Protein Coverage | Supporting Peptides |

Protein Coverage:

1 MGKLLALSVC FCFLVLGASS ISFRQQPEEN ACQFQRLNAQ RPDNRIESEG GYIETWNPNN QEFECAGVAL SRLVLRRNAL
81 RRPFYSNAPQ EIFIQQGRAY FGLIFLGCPS TYEEPAQQGR RHQSQRPPRR FQGQDQSQQQ QDSHQKVHRF DEGDLIAVPT
161 GVAFWMYNDH DTDVVAVSLT DTNNNDNQLD QFPRRFNLAG NHEQEFLRYQ QQSRRRSLPY SPYSPQTQPK QEDREFSPRG
241 QHGRRERAGQ EQENEGGNIF SGFTPEFLAQ AFQVDDRQIL QNLRGENESD EQGAIVTVRG GLRILSPDRK KRQQYERPDE

358 c c p

321 EEEYDEDEYE YDEEERQQDR RRGRGSRGSG NGIEETICTA SFKKNIGRNR SPDIYNPQAG SLKTANELNL LILRWLGLSA

401 EYGNLYRNAL FVPHYNTNAH SIIYALRGRA HVQVVDSNGD RVFDEELQEG HVLVVPQNFA VAGKSQSENF EYVAFKTDSR

r d

503 d

481 PSIANLAGEN SFIDNLPEEV VANSYGLPRE QARQLKNNNP FKFFVPPSEQ SLRAVA

d p

c Carbamidomethylation (+57.02)
d Deamidation (NQ) (+0.98)
p Propionamide (+71.04)
p Pyro-glu from Q (-17.03)
r Replacement of 2 protons by iron (+53.92)

Supporting Peptides:

Peptide	Uniq	-10lgP	Mass	Length	ppm	m/z	z	RT	Fraction	Scan	Source File	Area Raw Ara h 2	#Feature	#Feature Raw Ara h 2	Start	End	PTM	AScore	Found By
**total 27 peptides**																			

Peptide	Uniq	-10lgP	Mass	Length	ppm	m/z	z	RT	Fraction	Scan	Source File	Area Raw Ara h 2	#Feature	#Feature Raw Ara h 2	Start	End	PTM	AScore	Found By
K.TDSRPSIANLAGENSFIDNLPEEVVANSYGLPR.E	N	147.12	3544.7434	33	1.0	1182.5896	3	37.44	2	F2:1890	OB5932 H2 raw.raw	1.1607E8	11	11	477	509			PEAKS DB
R.SPDIYNPQAGSLKTANELNLLILR.W	N	144.08	2639.4282	24	0.9	880.8175	3	35.79	2	F2:1765	OB5932 H2 raw.raw	1.8094E6	3	3	371	394			PEAKS DB
R.WLGLSAEYGNLYR.N	N	135.04	1540.7673	13	2.6	771.3929	2	34.80	1	F1:1640	OB5921 H2 raw.raw	3.956E7	16	15	395	407			PEAKS DB
R.NALFVPHYNTNAHSIIYALR.G	N	133.14	2313.2019	20	2.3	1157.6108	2	33.37	2	F2:1607	OB5932 H2 raw.raw	3.0446E7	14	13	408	427			PEAKS DB
K.SQSENFEYVAFK.T	N	122.47	1447.6619	12	2.4	724.8400	2	31.43	1	F1:1392	OB5921 H2 raw.raw	1.027E7	4	4	465	476			PEAKS DB
R.NRSPDIYNPQAGSLK.T	N	115.99	1658.8376	15	0.4	830.4265	2	27.80	2	F2:1285	OB5932 H2 raw.raw	8.3597E5	6	6	369	383			PEAKS DB
K.TDSRPSIANLAGENSFIDNLPEEVVAN(+.98)SYGLPR.E	N	110.77	3545.7273	33	0.2	1182.9166	3	36.74	2	F2:1825	OB5932 H2 raw.raw	7.8948E3	1	1	477	509	Deamidation (NQ)	N27:Deamidation (N Q):118.18	PEAKS DB
R.AHVQVVDSNGDRVFDEELQEGHVLVVPQNFAVAGK.S	Y	109.59	3801.9075	35	2.2	1268.3125	3	33.37	2	F2:1626	OB5932 H2 raw.raw	3.9148E6	2	2	430	464			PEAKS DB
R.SPDIYNPQAGSLK.T	N	106.60	1388.6936	13	1.9	695.3554	2	28.54	3	F3:1354	OB5933 H2 raw.raw	1.3705E6	3	3	371	383			PEAKS DB
K.FFVPPSEQSLRA.V	Y	86.01	1376.7087	12	1.8	689.3629	2	31.25	1	F1:1399	OB5921 H2 raw.raw	1.104E6	3	3	523	534			PEAKS DB
K.TANELNLLILR.W	N	82.01	1268.7452	11	0.7	635.3803	2	34.98	1	F1:1608	OB5921 H2 raw.raw	2.5833E7	5	5	384	394			PEAKS DB
N.GIEETIC(+57.02)TASFKK.N	Y	81.85	1482.7388	13	1.0	742.3774	2	29.09	2	F2:1362	OB5932 H2 raw.raw	7.7376E5	5	5	352	364	Carbamidomethylation	C7:Carbamidomethyla tion:1000.00	PEAKS DB
K.FFVPPSEQSLR.A	Y	77.46	1305.6716	11	-0.5	653.8428	2	30.96	2	F2:1479	OB5932 H2 raw.raw	5.8436E5	3	3	523	533			PEAKS DB
R.S(+57.02)PDIYNPQAGSLK.T	N	77.15	1445.7150	13	5.4	723.8687	2	28.54	3	F3:1379	OB5933 H2 raw.raw	8.1843E3	1	1	371	383		S1:Carbamidomethyla tion (DHKE, X@N-ter m):30.83	PEAKS PTM
V.PQNFAVAGK.S	N	71.84	930.4923	9	0.8	466.2538	2	32.44	2	F2:1562	OB5932 H2 raw.raw	4.3278E6	2	2	456	464			PEAKS DB
N.G(+57.02)IEETIC(+57.02)TASFKK.N	Y	66.26	1539.7603	13	0.5	770.8878	2	29.27	2	F2:1382	OB5932 H2 raw.raw	6.2038E4	2	2	352	364	Carbamidomethylation	G1:Carbamidomethyl ation (DHKE, X@N-ter m):22.85;C7:Carbami domethylation:1000.0 0	PEAKS PTM
R.NRSPDIYNPQAGSLKTANELNLLILR.W	N	64.74	2909.5723	26	1.5	970.8661	3	34.50	2	F2:1698	OB5932 H2 raw.raw	6.2575E4	1	1	369	394			PEAKS DB
R.AHVQVVDSNGDRVFDEELQ(+.98)EGHVLVVPQNFAVAGK.S	Y	64.57	3802.8914	35	6.3	951.7361	4	33.37	2	F2:1633	OB5932 H2 raw.raw	6.1141E4	1	1	430	464		Q19:Deamidation (N Q):0.00	PEAKS DB
R.NALFVPH(+14.02)YNTNAHSIIYALR.G	N	52.59	2327.2175	20	0.3	776.7467	3	35.36	1	F1:1633	OB5921 H2 raw.raw	1.4175E5	1	1	408	427		H7:Methylation(other s):18.00	PEAKS PTM

total 27 peptides

Peptide	Uniq	-10lgP	Mass	Length	ppm	m/z	z	RT	Fraction	Scan	Source File	Area Raw Ara h 2	#Feature	#Feature Raw Ara h 2	Start	End	PTM	AScore	Found By
K.TDSRPSIANLAGEN(+.98)SFIDNLPEEVVANSYGLPR.E	N	46.28	3545.7273	33	6.3	1182.9238	3	48.72	1	F1:2418	OB5921 H2 raw.raw	9.731E3	2	2	477	509		N14:Deamidation (N Q):29.85	PEAKS DB
R.QLKNNNPFK.F	N	45.67	1101.5930	9	-0.6	551.8035	2	27.25	3	F3:1282	OB5933 H2 raw.raw	1.2465E4	2	2	514	522			PEAKS DB
R.WLGLSAE(+53.92)YGNLYR.N	N	45.19	1594.6866	13	0.4	532.5697	3	34.87	2	F2:1718	OB5932 H2 raw.raw	1.8492E5	2	2	395	407	Replacement of 2 protons by iron	E7:Replacement of 2 protons by iron:156.8 5	PEAKS PTM
R.N(+.98)ALFVPHYNTNAHSIIYALR.G	N	41.98	2314.1858	20	3.6	772.4053	3	45.28	3	F3:2182	OB5933 H2 raw.raw	6.3088E4	1	1	408	427		N1:Deamidation (N Q):32.94	PEAKS DB
R.WLGLSAEYGN(+.98)LYR.N	N	36.96	1541.7513	13	7.3	771.8886	2	52.84	1	F1:2630	OB5921 H2 raw.raw	0	0	0	395	407	Deamidation (NQ)	N10:Deamidation (N Q):1000.00	PEAKS DB
N.GIEETIC(+71.04)TASFKK.N	Y	36.68	1496.7544	13	1.2	749.3854	2	29.27	2	F2:1378	OB5932 H2 raw.raw	2.0631E4	1	1	352	364	Propionamide	C7:Propionamide:100 0.00	PEAKS PTM
K.SQSE(+53.92)NFEYVAFK.T	N	36.67	1501.5812	12	-1.3	751.7969	2	31.69	2	F2:1525	OB5932 H2 raw.raw	2.6345E5	1	1	465	476		E4:Replacement of 2 protons by iron:32.69	PEAKS PTM
R.Q(-17.03)LKNNNPFK.F	N	35.41	1084.5665	9	0.4	543.2908	2	28.36	3	F3:1357	OB5933 H2 raw.raw	1.237E4	1	1	514	522	Pyro-glu from Q	Q1:Pyro-glu from Q:1 000.00	PEAKS PTM
**total 27 peptides**																			

Q647H4|Q647H4_ARAHY

back to list

| Protein Coverage | Supporting Peptides |

Protein Coverage:

```
  1 MGKLLALSVC FCFLVLGASS ISFRQQPEEN ACQFQRLNAQ RPDNRIESEG GYIETWNPNN QEFECAGVAL SRLVLRRNAL
 81 RRPFYSNAPQ EIFIQQGRGY FGLIFPGCPS TYEEPAQQGR RHQSQRPPRR FQGQDQSQQQ QDSHQKVHRF DEGDLIAVPT
161 GVAFWMYNDH DTDVVAVSLT DTNNNDNQLD QFPRRFNLAG NHEQEFLRYQ QQSRRRSLPY SPYSPQTQPK QEDREFSPRG
241 QHGRRERAGQ EQENEGGNIF SGFTPEFLAQ AFQVDDRQIL QNLRGENESD EQGAIVTVRG GLRILSPDRK RRQQYERPDE
321 EEEYDEDEYE YDEEERQHDR RRGRGSRGSG NGIEETICTA SFKKNIGRNR SPDIYNPQAG SLKTANELNL LILRWLGLSA
401 EYGNLYRNAL FVPHYNTNAH SIIYALRGRA HVQVVDSNGD RVFDEELQEG HVLVVPQNFA VAGKSQSENF EYVAFKTDSR
481 PSIANLAGEN SFIDNLPEEV VANSYGLPRE QARQLKNNNP FKFFVPPSEQ SLRAVA
```

358 c; 503 d

- c Carbamidomethylation (+57.02)
- d Deamidation (NQ) (+0.98)
- p Propionamide (+71.04)
- p Pyro-glu from Q (-17.03)
- r Replacement of 2 protons by iron (+53.92)

Supporting Peptides:

Peptide	Uniq	-10lgP	Mass	Length	ppm	m/z	z	RT	Fraction	Scan	Source File	Area Raw Ara h 2	#Feature	#Feature Raw Ara h 2	Start	End	PTM	AScore	Found By
**total 27 peptides**																			

Peptide	Uniq	-10lgP	Mass	Length	ppm	m/z	z	RT	Fraction	Scan	Source File	Area Raw Ara h 2	#Feature	#Feature Raw Ara h 2	Start	End	PTM	AScore	Found By
K.TDSRPSIANLAGENSFIDNLPEEVVANSYGLPR.E	N	147.12	3544.7434	33	1.0	1182.5896	3	37.44	2	F2:1890	OB5932 H2 raw.raw	1.1607E8	11	11	477	509			PEAKS DB
R.SPDIYNPQAGSLKTANELNLLILR.W	N	144.08	2639.4282	24	0.9	880.8175	3	35.79	2	F2:1765	OB5932 H2 raw.raw	1.8094E6	3	3	371	394			PEAKS DB
R.WLGLSAEYGNLYR.N	N	135.04	1540.7673	13	2.6	771.3929	2	34.80	1	F1:1640	OB5921 H2 raw.raw	3.956E7	16	15	395	407			PEAKS DB
R.NALFVPHYNTNAHSIIYALR.G	N	133.14	2313.2019	20	2.3	1157.6108	2	33.37	2	F2:1607	OB5932 H2 raw.raw	3.0446E7	14	13	408	427			PEAKS DB
K.SQSENFEYVAFK.T	N	122.47	1447.6619	12	2.4	724.8400	2	31.43	1	F1:1392	OB5921 H2 raw.raw	1.027E7	4	4	465	476			PEAKS DB
R.NRSPDIYNPQAGSLK.T	N	115.99	1658.8376	15	0.4	830.4265	2	27.80	2	F2:1285	OB5932 H2 raw.raw	8.3597E5	6	6	369	383			PEAKS DB
K.TDSRPSIANLAGENSFIDNLPEEVVAN(+.98)SYGLPR.E	N	110.77	3545.7273	33	0.2	1182.9166	3	36.74	2	F2:1825	OB5932 H2 raw.raw	7.8948E3	1	1	477	509	Deamidation (NQ)	N27:Deamidation (N Q):118.18	PEAKS DB
R.AHVQVVDSNGDRVFDEELQEGHVLVVPQNFAVAGK.S	Y	109.59	3801.9075	35	2.2	1268.3125	3	33.37	2	F2:1626	OB5932 H2 raw.raw	3.9148E6	2	2	430	464			PEAKS DB
R.SPDIYNPQAGSLK.T	N	106.60	1388.6936	13	1.9	695.3554	2	28.54	3	F3:1354	OB5933 H2 raw.raw	1.3705E6	3	3	371	383			PEAKS DB
K.FFVPPSEQSLRA.V	Y	86.01	1376.7087	12	1.8	689.3629	2	31.25	1	F1:1399	OB5921 H2 raw.raw	1.104E6	3	3	523	534			PEAKS DB
K.TANELNLLILR.W	N	82.01	1268.7452	11	0.7	635.3803	2	34.98	1	F1:1608	OB5921 H2 raw.raw	2.5833E7	5	5	384	394			PEAKS DB
N.GIEETIC(+57.02)TASFKK.N	Y	81.85	1482.7388	13	1.0	742.3774	2	29.09	2	F2:1362	OB5932 H2 raw.raw	7.7376E5	5	5	352	364	Carbamidomethylation	C7:Carbamidomethyla tion:1000.00	PEAKS DB
K.FFVPPSEQSLR.A	Y	77.46	1305.6716	11	-0.5	653.8428	2	30.96	2	F2:1479	OB5932 H2 raw.raw	5.8436E5	3	3	523	533			PEAKS DB
R.S(+57.02)PDIYNPQAGSLK.T	N	77.15	1445.7150	13	5.4	723.8687	2	28.54	3	F3:1379	OB5933 H2 raw.raw	8.1843E3	1	1	371	383		S1:Carbamidomethyla tion (DHKE, X@N-ter m):30.83	PEAKS PTM
V.PQNFAVAGK.S	N	71.84	930.4923	9	0.8	466.2538	2	32.44	2	F2:1562	OB5932 H2 raw.raw	4.3278E6	2	2	456	464			PEAKS DB
N.G(+57.02)IEETIC(+57.02)TASFKK.N	Y	66.26	1539.7603	13	0.5	770.8878	2	29.27	2	F2:1382	OB5932 H2 raw.raw	6.2038E4	2	2	352	364	Carbamidomethylation	G1:Carbamidomethyl ation (DHKE, X@N-ter m):22.85;C7:Carbami domethylation:1000.0 0	PEAKS PTM
R.NRSPDIYNPQAGSLKTANELNLLILR.W	N	64.74	2909.5723	26	1.5	970.8661	3	34.50	2	F2:1698	OB5932 H2 raw.raw	6.2575E4	1	1	369	394			PEAKS DB
R.AHVQVVDSNGDRVFDEELQ(+.98)EGHVLVVPQNFAVAGK.S	Y	64.57	3802.8914	35	6.3	951.7361	4	33.37	2	F2:1633	OB5932 H2 raw.raw	6.1141E4	1	1	430	464		Q19:Deamidation (N Q):0.00	PEAKS DB
R.NALFVPH(+14.02)YNTNAHSIIYALR.G	N	52.59	2327.2175	20	0.3	776.7467	3	35.36	1	F1:1633	OB5921 H2 raw.raw	1.4175E5	1	1	408	427		H7:Methylation(other s):18.00	PEAKS PTM

total 27 peptides

Peptide	Uniq	-10lgP	Mass	Length	ppm	m/z	z	RT	Fraction	Scan	Source File	Area Raw Ara h 2	#Feature	#Feature Raw Ara h 2	Start	End	PTM	AScore	Found By
K.TDSRPSIANLAGEN(+.98)SFIDNLPEEVVANSYGLPR.E	N	46.28	3545.7273	33	6.3	1182.9238	3	48.72	1	F1:2418	OB5921 H2 raw.raw	9.731E3	2	2	477	509		N14:Deamidation (N Q):29.85	PEAKS DB
R.QLKNNNPFK.F	N	45.67	1101.5930	9	-0.6	551.8035	2	27.25	3	F3:1282	OB5933 H2 raw.raw	1.2465E4	2	2	514	522			PEAKS DB
R.WLGLSAE(+53.92)YGNLYR.N	N	45.19	1594.6866	13	0.4	532.5697	3	34.87	2	F2:1718	OB5932 H2 raw.raw	1.8492E5	2	2	395	407	Replacement of 2 protons by iron	E7:Replacement of 2 protons by iron:156.8 5	PEAKS PTM
R.N(+.98)ALFVPHYNTNAHSIIYALR.G	N	41.98	2314.1858	20	3.6	772.4053	3	45.28	3	F3:2182	OB5933 H2 raw.raw	6.3088E4	1	1	408	427		N1:Deamidation (N Q):32.94	PEAKS DB
R.WLGLSAEYGN(+.98)LYR.N	N	36.96	1541.7513	13	7.3	771.8886	2	52.84	1	F1:2630	OB5921 H2 raw.raw	0	0	0	395	407	Deamidation (NQ)	N10:Deamidation (N Q):1000.00	PEAKS DB
N.GIEETIC(+71.04)TASFKK.N	Y	36.68	1496.7544	13	1.2	749.3854	2	29.27	2	F2:1378	OB5932 H2 raw.raw	2.0631E4	1	1	352	364	Propionamide	C7:Propionamide:100 0.00	PEAKS PTM
K.SQSE(+53.92)NFEYVAFK.T	N	36.67	1501.5812	12	-1.3	751.7969	2	31.69	2	F2:1525	OB5932 H2 raw.raw	2.6345E5	1	1	465	476		E4:Replacement of 2 protons by iron:32.69	PEAKS PTM
R.Q(-17.03)LKNNNPFK.F	N	35.41	1084.5665	9	0.4	543.2908	2	28.36	3	F3:1357	OB5933 H2 raw.raw	1.237E4	1	1	514	522	Pyro-glu from Q	Q1:Pyro-glu from Q:1 000.00	PEAKS PTM

total 27 peptides

E9LFE8|E9LFE8_ARAHY

back to list

| Protein Coverage | Supporting Peptides |

Protein Coverage:

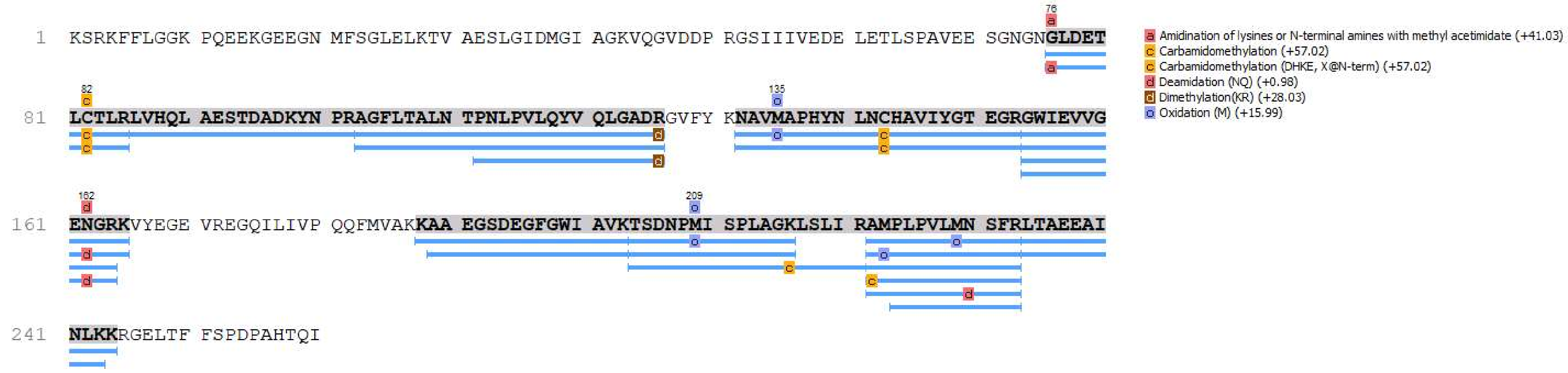

Supporting Peptides:

Peptide	Uniq	-10lgP	Mass	Length	ppm	m/z	z	RT	Fraction	Scan	Source File	Area Raw Ara h 2	#Feature	#Feature Raw Ara h 2	Start	End	PTM	AScore	Found By

total 29 peptides

Peptide	Uniq	-10lgP	Mass	Length	ppm	m/z	z	RT	Fraction	Scan	Source File	Area Raw Ara h 2	#Feature	#Feature Raw Ara h 2	Start	End	PTM	AScore	Found By
K.KAAEGSDEGFGWIAVK.T	Y	138.77	1663.8206	16	2.8	832.9199	2	31.50	3	F3:1541	OB5933 H2 raw.raw	1.5376E6	4	4	188	203			PEAKS DB
K.NAVMAPHYNLNC(+57.02)HAVIYGTEGR.G	Y	136.92	2486.1582	22	2.2	829.7285	3	30.40	2	F2:1439	OB5932 H2 raw.raw	4.9994E6	6	6	132	153	Carbamidomethylation	C12:Carbamidomethylatio n:1000.00	PEAKS DB
K.AAEGSDEGFGWIAVK.T	Y	131.37	1535.7256	15	2.3	768.8718	2	33.49	1	F1:1529	OB5921 H2 raw.raw	7.8688E6	4	4	189	203			PEAKS DB
K.NAVM(+15.99)APHYNLNC(+57.02)HAVIYGTEGR.G	Y	130.39	2502.1533	22	0.8	835.0590	3	29.65	3	F3:1430	OB5933 H2 raw.raw	6.8396E5	4	4	132	153	Oxidation (M); Carbamidomethylation	M4:Oxidation (M):1000.00; C12:Carbamidomethylatio n:1000.00	PEAKS DB
R.AGFLTALNTPNLPVLQYVQLGADR(+28.03).G	Y	123.01	2598.4170	24	4.3	1300.2213	2	39.90	2	F2:2005	OB5932 H2 raw.raw	7.247E7	7	7	103	126	Dimethylation(KR)	R24:Dimethylation(KR):100 0.00	PEAKS PTM
R.AMPLPVLMN(+.98)SFR.L	Y	119.74	1375.6992	12	1.0	688.8575	2	35.55	1	F1:1651	OB5921 H2 raw.raw	2.0235E5	2	2	222	233	Deamidation (NQ)	N9:Deamidation (NQ):100 0.00	PEAKS DB
K.TSDNPMISPLAGK.L	Y	118.92	1329.6598	13	2.4	665.8387	2	29.79	1	F1:1298	OB5921 H2 raw.raw	2.7907E6	3	3	204	216			PEAKS DB
R.AMPLPVLMNSFR.L	Y	117.01	1374.7152	12	1.8	688.3661	2	36.30	1	F1:1687	OB5921 H2 raw.raw	1.702E7	4	4	222	233			PEAKS DB
R.LVHQLAESTDADKYNPR.A	Y	112.35	1955.9701	17	2.6	978.9949	2	28.36	3	F3:1344	OB5933 H2 raw.raw	2.5664E6	9	9	86	102			PEAKS DB
R.AMPLPVLM(+15.99)NSFR.L	Y	108.13	1390.7101	12	1.9	696.3636	2	34.43	1	F1:1551	OB5921 H2 raw.raw	2.5374E6	3	3	222	233	Oxidation (M)	M8:Oxidation (M):151.86	PEAKS DB
R.GWIEVVGENGR.K	Y	107.73	1214.6044	11	0.0	608.3094	2	31.69	2	F2:1532	OB5932 H2 raw.raw	6.914E5	3	3	154	164			PEAKS DB
R.AM(+15.99)PLPVLMNSFR.L	Y	100.79	1390.7101	12	1.9	696.3636	2	34.43	1	F1:1586	OB5921 H2 raw.raw	2.5374E6	3	3	222	233	Oxidation (M)	M2:Oxidation (M):90.85	PEAKS DB
R.GWIEVVGEN(+.98)GR.K	Y	98.37	1215.5884	11	0.7	608.8019	2	32.18	1	F1:1446	OB5921 H2 raw.raw	9.7206E4	1	1	154	164	Deamidation (NQ)	N9:Deamidation (NQ):100 0.00	PEAKS DB
R.GWIEVVGEN(+.98)GRK.V	Y	87.28	1343.6833	12	0.4	672.8492	2	30.33	1	F1:1342	OB5921 H2 raw.raw	6.6322E5	4	4	154	165	Deamidation (NQ)	N9:Deamidation (NQ):100 0.00	PEAKS DB
N.GLDETLC(+57.02)TLR.L	Y	86.99	1176.5808	10	3.0	589.2994	2	30.96	2	F2:1472	OB5932 H2 raw.raw	1.5884E6	5	5	76	85	Carbamidomethylation	C7:Carbamidomethylation: 1000.00	PEAKS DB
R.GWIEVVGENGRK.V	Y	85.25	1342.6993	12	0.8	672.3575	2	30.17	1	F1:1331	OB5921 H2 raw.raw	3.292E5	3	3	154	165			PEAKS DB
R.LTAEEAINLK.K	Y	80.99	1100.6077	10	-0.1	551.3110	2	29.82	2	F2:1410	OB5932 H2 raw.raw	2.6604E5	3	3	234	243			PEAKS DB
R.LTAEEAINLKK.R	Y	79.43	1228.7026	11	0.0	615.3586	2	27.27	1	F1:1147	OB5921 H2 raw.raw	3.3199E5	4	4	234	244			PEAKS DB
R.AGFLTALNTPNLPVLQYVQLGAD(+28.03)R.G	Y	74.14	2598.4170	24	4.8	867.1504	3	39.70	3	F3:2024	OB5933 H2 raw.raw	2.3507E6	1	1	103	126		D23:Ethylation:7.21	PEAKS PTM
K.TSDNPM(+15.99)ISPLAGK.L	Y	68.00	1345.6548	13	0.6	673.8351	2	27.45	1	F1:1162	OB5921 H2 raw.raw	1.2503E5	4	4	204	216	Oxidation (M)	M6:Oxidation (M):1000.00	PEAKS DB
**total 29 peptides**																			

Peptide	Uniq	-10lgP	Mass	Length	ppm	m/z	z	RT	Fraction	Scan	Source File	Area Raw Ara h 2	#Feature	#Feature Raw Ara h 2	Start	End	PTM	AScore	Found By
T.PNLPVLQYVQLGADR(+28.03).G	Y	64.66	1709.9464	15	6.6	855.9861	2	39.90	2	F2:2015	OB5932 H2 raw.raw	3.524E5	2	2	112	126	Dimethylation(KR)	R15:Dimethylation(KR):1000.00	PEAKS PTM
R.A(+57.02)MPLPVLMNSFR.L	Y	58.83	1431.7367	12	1.0	716.8763	2	36.48	1	F1:1707	OB5921 H2 raw.raw	1.3263E5	2	2	222	233	Carbamidomethylation (DHKE, X@N-term)	A1:Carbamidomethylation (DHKE, X@N-term):1000.00	PEAKS PTM
M.PLPVLMNSFR.L	Y	54.32	1172.6376	10	-0.2	587.3259	2	36.30	1	F1:1699	OB5921 H2 raw.raw	2.1961E4	1	1	224	233			PEAKS DB
N.GLD(+14.02)ETLC(+57.02)TLR.L	Y	51.77	1190.5966	10	2.3	596.3069	2	31.14	2	F2:1483	OB5932 H2 raw.raw	7.3643E6	3	3	76	85	Carbamidomethylation	D3:Methylation(others):33.98;C7:Carbamidomethylation:1000.00	PEAKS PTM
K.TSDNPMISPLAGK(+57.02)LSLIR.A	Y	48.87	1969.0667	18	0.0	657.3629	3	33.74	2	F2:1656	OB5932 H2 raw.raw	6.7977E4	1	1	204	221	Carbamidomethylation (DHKE, X@N-term)	K13:Carbamidomethylation (DHKE, X@N-term):74.64	PEAKS PTM
N.G(+57.02)LD(+14.02)ETLC(+57.02)TLR.L	Y	42.96	1247.6179	10	-0.3	624.8160	2	31.32	2	F2:1510	OB5932 H2 raw.raw	3.1525E5	3	3	76	85	Carbamidomethylation	G1:Carbamidomethylation (DHKE, X@N-term):49.37; D3:Methylation(others):33.98;C7:Carbamidomethylation:1000.00	PEAKS PTM
R.AMPLPVLM(+15.99)N(+.98)SFR.L	Y	41.81	1391.6941	12	-0.5	696.8540	2	33.83	1	F1:1548	OB5921 H2 raw.raw	0	0	0	222	233	Deamidation (NQ)	M8:Oxidation (M):37.35;N9:Deamidation (NQ):1000.00	PEAKS DB
N.G(+41.03)LDETLC(+57.02)TLR.L	Y	37.87	1217.6074	10	0.7	609.8114	2	31.34	2	F2:1497	OB5932 H2 raw.raw	1.3034E5	1	1	76	85	Amidination of lysines or N-terminal amines with methyl acetimidate; Carbamidomethylation	G1:Amidination of lysines or N-terminal amines with methyl acetimidate:1000.00; C7:Carbamidomethylation:1000.00	PEAKS PTM
K.AAEGSD(+53.92)EGFGWIAVK.T	Y	37.12	1589.6449	15	0.2	795.8299	2	33.60	3	F3:1663	OB5933 H2 raw.raw	0	0	0	189	203		D6:Replacement of 2 protons by iron:33.98	PEAKS PTM
**total 29 peptides**																			

Q9FZ11|Q9FZ11_ARAHY

back to list

| Protein Coverage | Supporting Peptides |

Protein Coverage:

1 MIRGRLALSV CFCFLVLGAS SISFRQQPEE NACQFQRLNA QRPDNRLESE GGYIETWNPN NQEFECAGVA LSRLVLRRNA
81 LRRPFYSNAP QEIFIQQGRG YFGLIFPGCP STYEEPAQQG RRHQSQRAPR RFEGEDQSQQ QQQDSHQKVR RFDEGDLIAV
161 PTGVALWMFN DHDTDVVAVS LTDTNNNDNQ LDQFPRRFNL AGNHEQEFLR YQQQSRRRSL PYSPYSPQSQ PRQEEREFSP
241 RGQHSRRERA GQEEENEGGN IFSGFTPEFL AQAFQVDDRQ IVQNLRGENE SEEEGAIVTV KGGLRILSPD RKRGADEEEE
351 379
321 YDEDEYEYDE EDRRRGRGSR GRGN**GIEETI CTASVKK**NIG R**NRSPDIYNP QAGSLKTAND LNLLILRWLG LSAEYGNLYR**
401 **NALFVPHYNT NAHSIIYALR** GR**AHVQVVDS NGNRVYDEEL QEGHVLVVPQ NFAVAGKSQS DNFEYVAFK**T DSRPNIANFA
481 GENSIIDNLP EEVVANSYGL PREQAR**QLKN NNPFKFFVPP SQQSLRA**VA

c Carbamidomethylation (+57.02)
d Deamidation (NQ) (+0.98)
P Pyro-glu from Q (-17.03)
r Replacement of 2 protons by iron (+53.92)

Supporting Peptides:

Peptide	Uniq	-10lgP	Mass	Length	ppm	m/z	z	RT	Fraction	Scan	Source File	Area Raw Ara h 2	#Feature	#Feature Raw Ara h 2	Start	End	PTM	AScore	Found By
R.WLGLSAEYGNLYR.N	N	135.04	1540.7673	13	2.6	771.3929	2	34.80	1	F1:1640	OB5921 H2 raw.raw	3.956E7	16	15	388	400			PEAKS DB
R.NALFVPHYNTNAHSIIYALR.G	N	133.14	2313.2019	20	2.3	1157.6108	2	33.37	2	F2:1607	OB5932 H2 raw.raw	3.0446E7	14	13	401	420			PEAKS DB
K.SQSDNFEYVAFK.T	N	122.97	1433.6462	12	1.6	717.8315	2	31.69	3	F3:1543	OB5933 H2 raw.raw	4.4734E6	3	3	458	469			PEAKS DB
R.VYDEELQEGHVLVVPQNFAVAGK.S	N	118.55	2540.2910	23	2.1	847.7727	3	32.93	1	F1:1498	OB5921 H2 raw.raw	1.5993E6	6	6	435	457			PEAKS DB
R.NRSPDIYNPQAGSLK.T	N	115.99	1658.8376	15	0.4	830.4265	2	27.80	2	F2:1285	OB5932 H2 raw.raw	8.3597E5	6	6	362	376			PEAKS DB
R.SPDIYNPQAGSLK.T	N	106.60	1388.6936	13	1.9	695.3554	2	28.54	3	F3:1354	OB5933 H2 raw.raw	1.3705E6	3	3	364	376			PEAKS DB
K.FFVPPSQQSLRA.V	Y	101.65	1375.7247	12	1.8	688.8708	2	30.77	3	F3:1487	OB5933 H2 raw.raw	1.3188E6	3	3	516	527			PEAKS DB
N.GIEETIC(+57.02)TASVK.K	N	90.00	1306.6438	12	0.0	654.3292	2	28.91	3	F3:1386	OB5933 H2 raw.raw	2.32E5	2	2	345	356	Carbamidomethylation	C7:Carbamidomethylation: 1000.00	PEAKS DB
R.SPDIYNPQAGSLKTANDLNLLILR.W	N	89.73	2625.4126	24	0.2	876.1450	3	35.24	2	F2:1743	OB5932 H2 raw.raw	1.5604E5	1	1	364	387			PEAKS DB
K.FFVPPSQQSLR.A	Y	88.50	1304.6876	11	0.4	653.3513	2	30.22	3	F3:1455	OB5933 H2 raw.raw	6.1475E5	3	3	516	526			PEAKS DB
R.AHVQVVDSNGNRVYDEELQEGHVLVVPQNFAVAGK.S	N	86.70	3816.9182	35	2.8	1273.3169	3	32.06	3	F3:1574	OB5933 H2 raw.raw	3.9867E6	2	2	423	457			PEAKS DB
N.GIEETIC(+57.02)TASVKK.N	N	79.22	1434.7388	13	1.2	718.3775	2	27.26	2	F2:1253	OB5932 H2 raw.raw	2.7175E5	3	3	345	357	Carbamidomethylation	C7:Carbamidomethylation: 1000.00	PEAKS DB
K.TANDLNLLILR.W	N	79.10	1254.7296	11	1.2	628.3729	2	34.87	2	F2:1737	OB5932 H2 raw.raw	1.5481E7	5	5	377	387			PEAKS DB
R.S(+57.02)PDIYNPQAGSLK.T	N	77.15	1445.7150	13	5.4	723.8687	2	28.54	3	F3:1379	OB5933 H2 raw.raw	8.1843E3	1	1	364	376		S1:Carbamidomethylation (DHKE, X@N-term):30.83	PEAKS PTM
V.PQNFAVAGK.S	N	71.84	930.4923	9	0.8	466.2538	2	32.44	2	F2:1562	OB5932 H2 raw.raw	4.3278E6	2	2	449	457			PEAKS DB
K.TAN(+.98)DLNLLILR.W	N	67.54	1255.7136	11	-1.0	628.8635	2	35.40	3	F3:1771	OB5933 H2 raw.raw	0	0	0	377	387	Deamidation (NQ)	N3:Deamidation (NQ):68.3 1	PEAKS DB
R.NALFVPH(+14.02)YNTNAHSIIYALR.G	N	52.59	2327.2175	20	0.3	776.7467	3	35.36	1	F1:1633	OB5921 H2 raw.raw	1.4175E5	1	1	401	420		H7:Methylation(others):18. 00	PEAKS PTM
R.QLKNNNPFK.F	N	45.67	1101.5930	9	-0.6	551.8035	2	27.25	3	F3:1282	OB5933 H2 raw.raw	1.2465E4	2	2	507	515			PEAKS DB
R.WLGLSAE(+53.92)YGNLYR.N	N	45.19	1594.6866	13	0.4	532.5697	3	34.87	2	F2:1718	OB5932 H2 raw.raw	1.8492E5	2	2	388	400	Replacement of 2 protons by iron	E7:Replacement of 2 proto ns by iron:156.85	PEAKS PTM

total 24 peptides

Peptide	Uniq	-10lgP	Mass	Length	ppm	m/z	z	RT	Fraction	Scan	Source File	Area Raw Ara h 2	#Feature	#Feature Raw Ara h 2	Start	End	PTM	AScore	Found By
R.N(+.98)ALFVPHYNTNAHSIIYALR.G	N	41.98	2314.1858	20	3.6	772.4053	3	45.28	3	F3:2182	OB5933 H2 raw.raw	6.3088E4	1	1	401	420		N1:Deamidation (NQ):32.94	PEAKS DB
K.TAND(+53.92)LNLLILR.W	N	41.37	1308.6488	11	-1.1	655.3309	2	34.62	1	F1:1597	OB5921 H2 raw.raw	1.4902E5	1	1	377	387	Replacement of 2 protons by iron	D4:Replacement of 2 protons by iron:128.79	PEAKS PTM
N.G(+57.02)IEETIC(+57.02)TASVKK.N	N	37.32	1491.7603	13	1.2	746.8883	2	27.43	3	F3:1293	OB5933 H2 raw.raw	2.0504E4	1	1	345	357	Carbamidomethylation	G1:Carbamidomethylation (DHKE, X@N-term):30.83; C7:Carbamidomethylation:1000.00	PEAKS PTM
R.WLGLSAEYGN(+.98)LYR.N	N	36.96	1541.7513	13	7.3	771.8886	2	52.84	1	F1:2630	OB5921 H2 raw.raw	0	0	0	388	400	Deamidation (NQ)	N10:Deamidation (NQ):1000.00	PEAKS DB
R.Q(-17.03)LKNNNPFK.F	N	35.41	1084.5665	9	0.4	543.2908	2	28.36	3	F3:1357	OB5933 H2 raw.raw	1.237E4	1	1	507	515	Pyro-glu from Q	Q1:Pyro-glu from Q:1000.00	PEAKS PTM

total 24 peptides

A1E2B1|A1E2B1_ARAHY

back to list

| Protein Coverage | Supporting Peptides |

Protein Coverage:

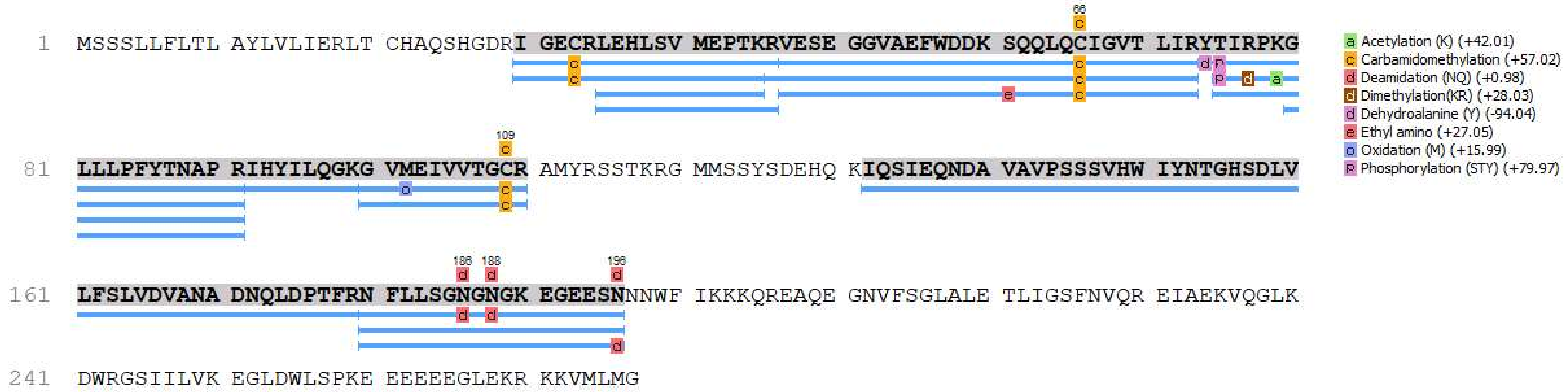

Supporting Peptides:

Peptide	Uniq	-10lgP	Mass	Length	ppm	m/z	z	RT	Fraction	Scan	Source File	Area Raw Ara h 2	#Feature	#Feature Raw Ara h 2	Start	End	PTM	AScore	Found By
K.GLLLPFYTNAPR.I	Y	141.76	1360.7502	12	-1.2	681.3816	2	35.39	3	F3:1765	OB5933 H2 raw.raw	3.6865E6	3	3	80	91			PEAKS DB
R.VESEGGVAEFWDDKSQQLQC(+57.02)IGVTLIR.Y	Y	136.52	3063.4971	27	4.1	1022.1771	3	36.71	2	F2:1824	OB5932 H2 raw.raw	9.8845E6	5	5	47	73	Carbamidomethylation	C20:Carbamidomethylation:1000.00	PEAKS DB
K.RVESEGGVAEFWDDKSQQLQC(+57.02)IGVTLIR.Y	Y	122.38	3219.5981	28	2.3	1074.2091	3	35.76	3	F3:1794	OB5933 H2 raw.raw	2.9779E6	4	4	46	73	Carbamidomethylation	C21:Carbamidomethylation:1000.00	PEAKS DB

total 21 peptides

Peptide	Uniq	-10lgP	Mass	Length	ppm	m/z	z	RT	Fraction	Scan	Source File	Area Raw Ara h 2	#Feature	#Feature Raw Ara h 2	Start	End	PTM	AScore	Found By
K.GVMEIVVTGC(+57.02)R.A	Y	109.83	1219.6053	11	1.8	610.8110	2	31.25	1	F1:1386	OB5921 H2 raw.raw	9.381E5	3	3	100	110	Carbamidomethylation	C10:Carbamidomethylation:1000.00	PEAKS DB
R.LEHLSVMEPTK.R	Y	103.36	1282.6591	11	-0.9	642.3362	2	27.79	3	F3:1323	OB5933 H2 raw.raw	1.5006E4	2	2	35	45			PEAKS DB
K.GVM(+15.99)EIVVTGC(+57.02)R.A	Y	91.34	1235.6002	11	2.0	618.8086	2	28.16	2	F2:1326	OB5932 H2 raw.raw	5.5468E3	1	1	100	110	Oxidation (M); Carbamidomethylation	M3:Oxidation (M):1000.00; C10:Carbamidomethylation:1000.00	PEAKS DB
R.NFLLSGN(+.98)GN(+.98)GKEGEESN.N	Y	84.92	1766.7594	17	0.2	884.3871	2	30.77	2	F2:1501	OB5932 H2 raw.raw	8.8395E4	1	1	180	196	Deamidation (NQ)	N7:Deamidation (NQ):96.06;N9:Deamidation (NQ):72.89	PEAKS DB
R.NFLLSGNGN(+.98)GKEGEESN.N	Y	79.73	1765.7754	17	-0.1	883.8949	2	29.97	1	F1:1326	OB5921 H2 raw.raw	8.6902E4	2	2	180	196		N9:Deamidation (NQ):39.76	PEAKS DB
R.IHYILQGK.G	Y	75.66	970.5600	8	0.7	486.2876	2	27.62	2	F2:1288	OB5932 H2 raw.raw	3.1727E4	2	2	92	99			PEAKS DB
R.LEHLSVMEPTKR.V	Y	75.18	1438.7603	12	0.4	720.3877	2	28.17	3	F3:1338	OB5933 H2 raw.raw	4.4747E3	1	1	35	46			PEAKS DB
R.NFLLSGN(+.98)GNGKEGEESN(+.98).N	Y	72.91	1766.7594	17	0.2	884.3871	2	30.77	2	F2:1462	OB5932 H2 raw.raw	1.261E5	2	2	180	196		N7:Deamidation (NQ):39.76;N17:Deamidation (NQ):47.16	PEAKS DB
R.IGEC(+57.02)RLEHLSVMEPTKR.V	Y	71.33	2054.0400	17	-1.6	514.5165	4	30.02	2	F2:1433	OB5932 H2 raw.raw	7.9524E4	3	3	30	46	Carbamidomethylation	C4:Carbamidomethylation:1000.00	PEAKS DB
R.IGEC(+57.02)RLEHLSVMEPTK.R	Y	67.02	1897.9390	16	0.1	633.6537	3	30.21	2	F2:1441	OB5932 H2 raw.raw	3.8072E5	4	4	30	45	Carbamidomethylation	C4:Carbamidomethylation:1000.00	PEAKS DB
R.NFLLSGNGNGKEGEESN(+.98).N	Y	62.96	1765.7754	17	1.0	883.8958	2	30.21	2	F2:1414	OB5932 H2 raw.raw	9.4247E4	2	2	180	196		N17:Deamidation (NQ):23.01	PEAKS DB
R.NFLLSGNGN(+.98)GKEGEESN(+.98).N	Y	61.89	1766.7594	17	0.4	884.3873	2	30.59	3	F3:1477	OB5933 H2 raw.raw	8.6083E4	1	1	180	196	Deamidation (NQ)	N9:Deamidation (NQ):30.36;N17:Deamidation (NQ):65.92	PEAKS DB
R.VESEGGVAEFWDDKS(+27.05)QQLQC(+57.02)IGVTLIR.Y	Y	58.10	3090.5444	27	-9.7	1031.1787	3	35.92	1	F1:1673	OB5921 H2 raw.raw	1.3332E5	1	1	47	73	Ethyl amino; Carbamidomethylation	S15:Ethyl amino:119.64;C20:Carbamidomethylation:1000.00	PEAKS PTM
K.IQSIEQNDAVAVPSSSVHWIYNTGHSDLVLFSLVDVANADNQLDPTFR.N	Y	57.23	5281.5957	48	1.5	1321.4082	4	38.94	2	F2:1947	OB5932 H2 raw.raw	2.1676E6	2	2	132	179			PEAKS DB
Y.TIRPK(+149.03)GLLLPFYTNAPR.I	Y	44.84	2105.1606	17	-7.5	702.7222	3	34.98	1	F1:1620	OB5921 H2 raw.raw	1.195E5	1	1	75	91		K5:Benzyl isothiocyanate:4.73	PEAKS PTM
Y.T(+149.03)IRPKGLLLPFYTNAPR.I	Y	43.47	2105.1606	17	-7.1	702.7225	3	34.97	2	F2:1719	OB5932 H2 raw.raw	0	0	0	75	91		T1:Benzyl isothiocyanate:5.74	PEAKS PTM

total 21 peptides

Peptide	Uniq	-10lgP	Mass	Length	ppm	m/z	z	RT	Fraction	Scan	Source File	Area Raw Ara h 2	#Feature	#Feature Raw Ara h 2	Start	End	PTM	AScore	Found By
R.Y(-94.04)T(+79.97)IRPKGLLLPFYTNAPR.I	Y	42.79	2105.1187	18	9.6	702.7202	3	34.85	3	F3:1730	OB5933 H2 raw.raw	1.2657E6	1	1	74	91	Dehydroalanine (Y); Phosphorylation (STY)	Y1:Dehydroalanine (Y):69.38;T2:Phosphorylation (STY):71.87	PEAKS PTM
Y.T(+79.97)IR(+28.03)PK(+42.01)GLLLPFYTNAPR.I	Y	39.62	2106.1389	17	-3.6	703.0510	3	35.25	3	F3:1762	OB5933 H2 raw.raw	0	0	0	75	91	Phosphorylation (STY); Dimethylation(KR); Acetylation (K)	T1:Phosphorylation (STY):88.25;R3:Dimethylation(KR):153.36;K5:Acetylation (K):1000.00	PEAKS PTM

total 21 peptides

sp|Q647H2|AHY3_ARAHY

back to list

| Protein Coverage | Supporting Peptides |

Protein Coverage:

```
  1 MAKLLALSVC FCFLVLGASS VTFRQQGEEN ECQFQRLNAQ RPDNCIESEG GYIETWNPNN QEFQCAGVAL SRFVLRRNAL
 81 RRPFYSNAPQ EIFIYQGSGY FGLIFPGCPG TFEEPIQGSE QFQRPSRHFQ GQDQSQRPLD THQKVHGFRE GDLIAVPHGV
161 AFWIYNDQDT DVVAISVLHT NSLHNQLDQF PRRFNLAGKQ EQEFLRYQQR SGRQSPKGEE QEQEQENEGG NVFSGFSTEF
241 LSHGFQVNED IVRNLRGENE REEQGAIVTV KGGLSILVPP EWRQSYQQPG RGDKDFNNGI EETICTATVK MNIGKSTSAD
321 IYNPQAGSVR TVNELDLPIL NRLGLSAEYG SIHRDAMFVP HYNMNANSMI YALHGGAHVQ VVDCNGNRVF DEELQEGQSL
401 VVPQNFAVAA KSQSEHFLYV AFKTNSRASI SNLAGKNSYM WNLPEDVVAN SYGLQYEQAR QLKNNNPFTF LVPPQDSQMI
481 RTVA
```

305

C Carbamidomethylation (+57.02)
C Carbamidomethylation (DHKE, X@N-term) (+57.02)

Supporting Peptides:

Peptide	Uniq	-10lgP	Mass	Length	ppm	m/z	z	RT	Fraction	Scan	Source File	Area Raw Ara h 2	#Feature	#Feature Raw Ara h 2	Start	End	PTM	AScore	Found By
K.NSYMWNLPEDVVANSYGLQYEQAR.Q	Y	129.97	2846.2969	24	2.1	1424.1587	2	36.85	3	F3:1864	OB5933 H2 raw.raw	8.6777E5	4	4	437	460			PEAKS DB
K.SQSEHFLYVAFK.T	Y	127.24	1454.7194	12	2.0	728.3684	2	31.80	1	F1:1430	OB5921 H2 raw.raw	2.9709E6	6	6	412	423			PEAKS DB
R.TVNELDLPILNR.L	Y	113.80	1395.7721	12	1.8	698.8946	2	34.50	2	F2:1692	OB5932 H2 raw.raw	1.2891E7	3	3	331	342			PEAKS DB
R.LGLSAEYGSIHR.D	Y	113.18	1301.6727	12	1.1	651.8444	2	28.72	3	F3:1375	OB5933 H2 raw.raw	6.496E5	5	5	343	354			PEAKS DB

total 14 peptides

Peptide	Uniq	-10lgP	Mass	Length	ppm	m/z	z	RT	Fraction	Scan	Source File	Area Raw Ara h 2	#Feature	#Feature Raw Ara h 2	Start	End	PTM	AScore	Found By
N.GIEETIC(+57.02)TATVK.M	N	103.68	1320.6595	12	0.2	661.3372	2	29.46	3	F3:1405	OB5933 H2 raw.raw	2.7846E5	3	3	299	310	Carbamidomethylation	C7:Carbamidomethylation:1000.00	PEAKS DB
K.STSADIYNPQAGSVR.T	Y	100.14	1564.7482	15	0.1	783.3814	2	27.43	3	F3:1288	OB5933 H2 raw.raw	2.6144E5	3	3	316	330			PEAKS DB
R.VFDEELQEGQSLVVPQNFAVAAK.S	Y	98.07	2517.2751	23	2.7	1259.6482	2	35.02	3	F3:1751	OB5933 H2 raw.raw	1.6786E5	1	1	389	411			PEAKS DB
K.NNNPFTFLVPPQDSQMIR.T	Y	83.39	2117.0364	18	2.5	1059.5281	2	36.56	2	F2:1814	OB5932 H2 raw.raw	0	0	0	464	481			PEAKS DB
K.NNNPFTFLVPPQDSQMIRT.V	Y	69.26	2218.0840	19	2.9	1110.0525	2	36.48	1	F1:1705	OB5921 H2 raw.raw	3.5597E5	2	2	464	482			PEAKS DB
R.RFNLAGKQEQEFLR.Y	Y	57.99	1734.9165	14	0.4	579.3130	3	30.03	3	F3:1459	OB5933 H2 raw.raw	8.9519E4	2	2	193	206			PEAKS DB
R.L(+57.02)GLSAEYGSIHR.D	Y	53.80	1358.6942	12	0.7	680.3549	2	29.11	2	F2:1370	OB5932 H2 raw.raw	2.142E4	1	1	343	354	Carbamidomethylation (DHKE, X@N-term)	L1:Carbamidomethylation (DHKE, X@ N-term):52.68	PEAKS PTM
V.PQNFAVAAK.S	N	51.53	944.5079	9	0.5	473.2615	2	32.25	3	F3:1586	OB5933 H2 raw.raw	2.3208E6	2	2	403	411			PEAKS DB
N.G(+57.02)IEETIC(+57.02)TATVK.M	N	44.11	1377.6809	12	0.2	689.8479	2	29.76	1	F1:1306	OB5921 H2 raw.raw	0	0	0	299	310	Carbamidomethylation	G1:Carbamidomethylation (DHKE, X@ N-term):5.03;C7:Carbamidomethylati on:1000.00	PEAKS PTM
R.QLKNNNPFTFLVPPQDSQMIRT.V	Y	34.68	2587.3218	22	0.0	863.4479	3	35.02	3	F3:1753	OB5933 H2 raw.raw	2.6528E5	1	1	461	482			PEAKS DB
**total 14 peptides**																			

Q647H1|Q647H1_ARAHY

back to list

| Protein Coverage | Supporting Peptides |

Protein Coverage:

```
1   MVIGPFRLSL CVCLVFLTSA CFGTRLEESF NECQLDRLNA LTPDNRIESQ GGITETWNSN HPELRCAGVT LLKRTIFPNG
81  FHLPSYANYP QLIFIAQGNG VFGVSLPGCP VTYEEAESQS REDRRQRIVI KRESEQEQEQ QGDSHHKIYH FRQGHLLAIP
161 AGVPYWSFNY GNEPIVAITL LDTSNLDNQL DPSPRRFYLA GNPEEEHPET QQQQPQTRRR HGQHQQDEYG SQGEEEGNNV
241 LSGFSTQLLA HAFGVDEEIA RILQNPPEQT KDQIVRVEGG FRDVISPRWG EGKQYEDELE ERQRQPPRRD EQGKGYDYDD
321 DRRPRHRQDP YREGDEDDRR PRGSRQGQGR GYDDDDRRPG QYEEGEEDDR RPRRSSRPKR QGRRHDDDDR RADEDDRRGY
401 DDDERRPDED DRRGYDDDER RPDDDDRQGY DDDDRRPRWS SRPKGQGRNG VEETLCSPTL VEDIARPSRA DFYNPAAGRI
481 SSANSLTFPI LRWFQLSAEH VLLYRNGIYS PHWNNNANSI IYGLRGEGRI QVVNSQGNAV FNGVLREGQI LLVPQNFAVG
561 KQAGNEGFEY VAFKTADRAS PATSSKCLGE SPLMFSSMLL AFEIIKSVLS NTMETRPLWS LLMIPSMGLN VVINLNHNNN
    d
641 AQVDSKNNDG SRLWWPSSII IK
```

d Diethylation (+56.06)

Supporting Peptides:

Peptide	Uniq	-10lgP	Mass	Length	ppm	m/z	z	RT	Fraction	Scan	Source File	Area Raw Ara h 2	#Feature	#Feature Raw Ara h 2	Start	End	PTM	AScore	Found By
R.IQVVNSQGNAVFNGVLR.E	Y	123.06	1813.9799	17	2.0	907.9990	2	32.81	2	F2:1595	OB5932 H2 raw.raw	4.7427E5	2	2	530	546			PEAKS DB
R.WFQLSAEHVLLYR.N	Y	113.97	1660.8726	13	0.1	831.4436	2	34.98	1	F1:1614	OB5921 H2 raw.raw	2.1298E5	2	2	493	505			PEAKS DB
R.ISSANSLTFPILR.W	Y	87.74	1417.7928	13	1.5	709.9048	2	34.24	1	F1:1575	OB5921 H2 raw.raw	6.7566E5	3	3	480	492			PEAKS DB
R.ADFYNPAAGR.I	Y	86.77	1080.4988	10	1.0	541.2572	2	28.15	1	F1:1213	OB5921 H2 raw.raw	4.2854E3	1	1	470	479			PEAKS DB
R.EGQILLVPQNFAVGK.Q	Y	74.17	1611.8984	15	0.5	806.9569	2	34.30	3	F3:1712	OB5933 H2 raw.raw	5.2907E5	3	3	547	561			PEAKS DB
R.IQVVNSQGN(+.98)AVFNGVLR.E	Y	72.74	1814.9639	17	0.5	908.4896	2	33.38	2	F2:1634	OB5932 H2 raw.raw	1.7282E4	1	1	530	546		N9:Deamidation (NQ):2 6.38	PEAKS DB
R.EGQILLVPQNFAVGK(+56.06).Q	Y	40.01	1667.9609	15	2.1	834.9895	2	34.69	2	F2:1704	OB5932 H2 raw.raw	6.4471E6	3	3	547	561	Diethylation	K15:Diethylation:121.27	PEAKS PTM
total 7 peptides																			

sp|Q6PSU2|CONG7_ARAHY

back to list

| Protein Coverage | Supporting Peptides |

Protein Coverage:

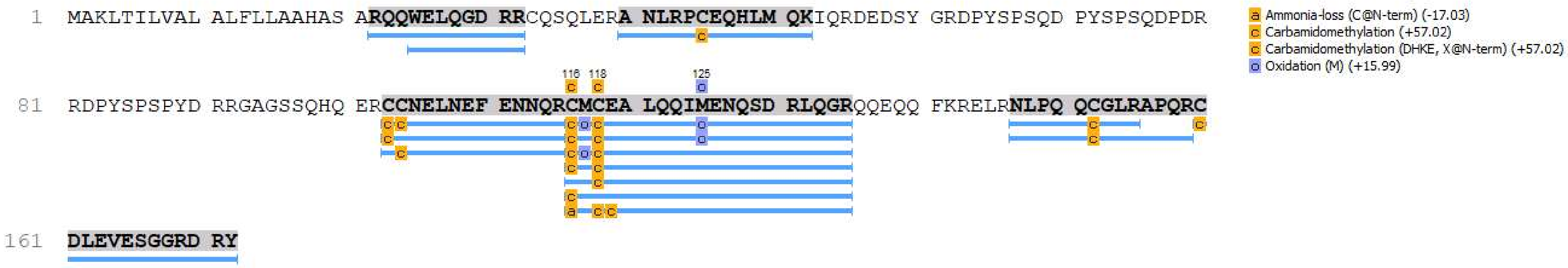

Supporting Peptides:

Peptide	Uniq	-10lgP	Mass	Length	ppm	m/z	z	RT	Fraction	Scan	Source File	Area Raw Ara h 2	#Feature	#Feature Raw Ara h 2	Start	End	PTM	AScore	Found By
R.C(+57.02)C(+57.02)NELNEFENNQR.C	Y	115.71	1725.6835	13	0.8	863.8497	2	28.73	3	F3:1378	OB5933 H2 raw.raw	2.2936E5	3	3	103	115	Carbamidomethylation	C1:Carbamidomethylation:1000.00;C2:Carbamidomethylation:1000.00	PEAKS DB
R.C(+57.02)MC(+57.02)EALQQIMENQSDRLQGR.Q	Y	98.71	2466.0872	20	1.8	823.0378	3	33.56	3	F3:1662	OB5933 H2 raw.raw	4.5608E6	6	6	116	135	Carbamidomethylation	C1:Carbamidomethylation:1000.00;C3:Carbamidomethylation:1000.00	PEAKS DB
R.ANLRPC(+57.02)EQHLMQK.I	Y	87.54	1623.7974	13	-0.3	542.2729	3	28.69	1	F1:1233	OB5921 H2 raw.raw	2.0501E5	3	3	40	52	Carbamidomethylation	C6:Carbamidomethylation:1000.00	PEAKS DB

total 16 peptides

Peptide	Uniq	-10lgP	Mass	Length	ppm	m/z	z	RT	Fraction	Scan	Source File	Area Raw Ara h 2	#Feature	#Feature Raw Ara h 2	Start	End	PTM	AScore	Found By
R.C(+71.04)C(+57.02)NELNEFENNQR.C	Y	78.59	1739.6992	13	1.2	870.8580	2	28.91	2	F2:1352	OB5932 H2 raw.raw	7.6E4	1	1	103	115	Carbamidomethylation	C1:Propionamide:0.00;C2:Carbamidomethylation:1000.00	PEAKS PTM
R.C(+57.02)M(+15.99)C(+57.02)EALQQIMENQSDRLQGR.Q	Y	73.14	2482.0820	20	2.0	828.3696	3	31.87	3	F3:1563	OB5933 H2 raw.raw	9.6142E5	3	3	116	135	Carbamidomethylation; Oxidation (M)	C1:Carbamidomethylation:1000.00;M2:Oxidation (M):73.16;C3:Carbamidomethylation:1000.00	PEAKS DB
R.C(+57.02)C(+71.04)NELNEFENNQR.C	Y	70.79	1739.6992	13	-0.6	870.8563	2	28.69	1	F1:1238	OB5921 H2 raw.raw	1.1347E5	2	2	103	115	Carbamidomethylation	C1:Carbamidomethylation:1000.00;C2:Propionamide:0.00	PEAKS PTM
R.C(+57.02)MC(+57.02)EALQQIM(+15.99)ENQSDRLQGR.Q	Y	65.02	2482.0820	20	1.4	828.3691	3	30.77	3	F3:1501	OB5933 H2 raw.raw	2.8999E5	2	2	116	135	Carbamidomethylation; Oxidation (M)	C1:Carbamidomethylation:1000.00;C3:Carbamidomethylation:1000.00;M10:Oxidation (M):139.69	PEAKS DB
R.C(+57.02)M(+15.99)C(+57.02)EALQQIM(+15.99)ENQSDRLQGR.Q	Y	61.41	2498.0771	20	1.7	833.7010	3	29.28	3	F3:1401	OB5933 H2 raw.raw	1.0641E5	2	2	116	135	Carbamidomethylation; Oxidation (M)	C1:Carbamidomethylation:1000.00;M2:Oxidation (M):1000.00;C3:Carbamidomethylation:1000.00;M10:Oxidation (M):1000.00	PEAKS DB
R.C(+71.04)MC(+57.02)EALQQIMENQSDRLQGR.Q	Y	59.16	2480.1028	20	0.4	827.7086	3	33.68	1	F1:1538	OB5921 H2 raw.raw	1.409E5	1	1	116	135	Carbamidomethylation	C1:Propionamide:32.28;C3:Carbamidomethylation:1000.00	PEAKS PTM
R.C(+57.02)DLEVESGGRDRY	Y	56.07	1554.6732	13	1.2	778.3448	2	27.26	2	F2:1261	OB5932 H2 raw.raw	3.8274E4	2	2	160	172	Carbamidomethylation	C1:Carbamidomethylation:1000.00	PEAKS DB
R.C(+57.02)MC(+71.04)EALQQIMENQSDRLQGR.Q	Y	54.75	2480.1028	20	1.1	827.7091	3	33.56	3	F3:1664	OB5933 H2 raw.raw	7.0183E5	2	2	116	135	Carbamidomethylation	C1:Carbamidomethylation:1000.00;C3:Propionamide:13.45	PEAKS PTM
R.NLPQQC(+57.02)GLR.A	Y	53.37	1084.5448	9	0.2	543.2798	2	25.65	2	F2:1166	OB5932 H2 raw.raw	7.6965E4	2	2	147	155	Carbamidomethylation	C6:Carbamidomethylation:1000.00	PEAKS DB

total 16 peptides

Peptide	Uniq	-10lgP	Mass	Length	ppm	m/z	z	RT	Fraction	Scan	Source File	Area Raw Ara h 2	#Feature	#Feature Raw Ara h 2	Start	End	PTM	AScore	Found By
R.C(-17.03)MC(+57.02)E(+57.02)ALQQIMENQSDRLQGR.Q	Y	42.30	2449.0608	20	-1.7	1225.5356	2	37.66	2	F2:1881	OB5932 H2 raw.raw	0	0	0	116	135	Ammonia-loss (C@N-term); Carbamidomethylation; Carbamidomethylation (DHKE, X@N-term)	C1:Ammonia-loss (C@N-term):1000.00;C3:Carbamidomethylation:1000.00;E4:Carbamidomethylation (DHKE, X@N-term):72.07	PEAKS PTM
R.NLPQQC(+57.02)GLRAPQR.C	Y	41.36	1536.7943	13	0.3	513.2722	3	27.16	2	F2:1257	OB5932 H2 raw.raw	2.6104E4	2	2	147	159	Carbamidomethylation	C6:Carbamidomethylation:1000.00	PEAKS DB
Q.WELQGDRR.C	Y	37.54	1058.5258	8	-0.7	530.2698	2	27.08	2	F2:1254	OB5932 H2 raw.raw	1.2218E4	2	2	25	32			PEAKS DB
A.RQQWELQGDRR.C	Y	33.61	1470.7440	11	1.1	491.2558	3	27.61	3	F3:1301	OB5933 H2 raw.raw	5.9603E4	1	1	22	32			PEAKS DB
**total 16 peptides**																			

A5Z1R0|A5Z1R0_ARAHY

back to list

| Protein Coverage | Supporting Peptides |

Protein Coverage:

1 MAKSTILVAL LALVLVAHAS AMRRERGRQG DSSSCERQVD RVNLKPCEQH IMQRIMGEQE QYDSYDIRST RSSDQQQRCC

81 DELNEMENTQ RCMCEALQQI MENQCDRLQD RQMVQQFKRE LMNLPQQCNF RAPQRCDLDV SGGRC

C Carbamidomethylation (+57.02)

Supporting Peptides:

Peptide	Uniq	-10lgP	Mass	Length	ppm	m/z	z	RT	Fraction	Scan	Source File	Area Raw Ara h 2	#Feature	#Feature Raw Ara h 2	Start	End	PTM	AScore	Found By
R.ELMNLPQQC(+57.02)NFR.A	Y	111.29	1548.7177	12	1.2	775.3671	2	31.14	3	F3:1524	OB5933 H2 raw.raw	1.8769E5	3	3	120	131	Carbamidomethylation	C9:Carbamidomethylation:1000.00	PEAKS DB
R.IMGEQEQYDSYDIR.S	Y	108.32	1745.7566	14	0.6	873.8861	2	30.21	2	F2:1444	OB5932 H2 raw.raw	1.0015E5	3	3	55	68			PEAKS DB
**total 2 peptides**																			

sp|Q647G9|CONG_ARAHY

back to list

| Protein Coverage | Supporting Peptides |

Protein Coverage:

1 MAKSTILVAL LALVLVAHAS AMRRERGRQG DSSSCERQVD RVNLKPCEQH IMQRIMGEQE QYDSYDIRST RSSDQQQRCC

81 DELNEMENTQ RCMCEALQQI MENQCDRLQD RQMVQQFKRE LMNLPQQCNF RAPQRCDLDV SGGRC

C Carbamidomethylation (+57.02)

Supporting Peptides:

Peptide	Uniq	-10lgP	Mass	Length	ppm	m/z	z	RT	Fraction	Scan	Source File	Area Raw Ara h 2	#Feature	#Feature Raw Ara h 2	Start	End	PTM	AScore	Found By
**total 2 peptides**																			

Peptide	Uniq	-10lgP	Mass	Length	ppm	m/z	z	RT	Fraction	Scan	Source File	Area Raw Ara h 2	#Feature	#Feature Raw Ara h 2	Start	End	PTM	AScore	Found By
R.ELMNLPQQC(+57.02)NFR.A	Y	111.29	1548.7177	12	1.2	775.3671	2	31.14	3	F3:1524	OB5933 H2 raw.raw	1.8769E5	3	3	120	131	Carbamidomethylation	C9:Carbamidomethylation:1000.00	PEAKS DB
R.IMGEQEQYDSYDIR.S	Y	108.32	1745.7566	14	0.6	873.8861	2	30.21	2	F2:1444	OB5932 H2 raw.raw	1.0015E5	3	3	55	68			PEAKS DB
total 2 peptides																			

N1NJN8|N1NJN8_ARAHY

back to list

| Protein Coverage | Supporting Peptides |

Protein Coverage:

```
  1  MECVFGLVGK GFAIVVADTS AVHGILVHKT NEEKIMKLDS HKLMAASGEG GDRVQFTEYI LKNVALYQFR NGIPLTTSAA
 81  ANFTRAELAA ALRKLVGVVF VQNPYAVNIL LAGYDNHTGP SLYYIDYLAT LHKVDKAAFG YASYFSLSIM DTHYHAGMSL
161  QEAIDLVDKC ITEIRSRLVV APPNFVIKIV DQHGARECAW RQSLPSASPT FIHN
```

d Deamidation (NQ) (+0.98)

Supporting Peptides:

Peptide	Uniq	-10lgP	Mass	Length	ppm	m/z	z	RT	Fraction	Scan	Source File	Area Raw Ara h 2	#Feature	#Feature Raw Ara h 2	Start	End	PTM	AScore	Found By
K.NVALYQFR.N	Y	79.34	1009.5345	8	-0.1	505.7745	2	30.58	2	F2:1470	OB5932 H2 raw.raw	1.2272E4	1	1	63	70			PEAKS DB
R.LVVAPPNFVIK.I	Y	64.66	1195.7328	11	0.7	598.8741	2	33.55	2	F2:1641	OB5932 H2 raw.raw	2.964E5	2	2	178	188			PEAKS DB
R.N(+.98)GIPLTTS(-15.99)AAANFTR.A	Y	48.15	1517.7838	15	1.8	759.9005	2	33.86	1	F1:1554	OB5921 H2 raw.raw	4.327E4	1	1	71	85	Deamidation (NQ)	N1:Deamidation (NQ):81.94;S8:Deoxy:24.44	PEAKS PTM
total 3 peptides																			

#CONTAM#P00761

back to list

| Protein Coverage | Supporting Peptides |

Protein Coverage:

```
  1  FPTDDDDKIV GGYTCAANSI PYQVSLNSGS HFCGGSLINS QWVVSAAHCY KSRIQVRLGE HNIDVLEGNE QFINAAKIIT
 81  HPNFNGNTLD NDIMLIKLSS PATLNSRVAT VSLPRSCAAA GTECLISGWG NTKSSGSSYP SLLQCLKAPV LSDSSCKSSY
161  PGQITGNMIC VGFLEGGKDS CQGDSGGPVV CNGQLQGIVS WGYGCAQKNK PGVYTKVCNY VNWIQQTIAA N
```

Supporting Peptides:

Peptide	Uniq	-10lgP	Mass	Length	ppm	m/z	z	RT	Fraction	Scan	Source File	Area Raw Ara h 2	#Feature	#Feature Raw Ara h 2	Start	End	PTM	AScore	Found By
R.LGEHNIDVLEGNEQFIN.A	Y	60.70	1939.9275	17	3.1	970.9741	2	33.42	1	F1:1524	OB5921 H2 raw.raw	1.7023E5	2	2	58	74			PEAKS DB
K.LSSPATLNSR.V	Y	60.35	1044.5564	10	0.9	523.2859	2	25.26	1	F1:1054	OB5921 H2 raw.raw	1.392E4	3	3	98	107			PEAKS DB
R.VATVSLPR.S	Y	56.28	841.5021	8	0.0	421.7584	2	27.44	2	F2:1287	OB5932 H2 raw.raw	1.8949E5	3	3	108	115			PEAKS DB
total 3 peptides																			

Q1PCR5|Q1PCR5_ARAHY

back to list

| Protein Coverage | Supporting Peptides |

Protein Coverage:

1 SDRSHAPPVV MAAAGFRLAD TFHTGASMGG YISSQSVKKI IEINPYMLGT MAGGAADCQF WHRNLGIKCR LHELANKRRI
81 SVTGASK**LLA NILYSYR**GMG LSVGTMIAGW DETGPGLYYV DSEGGRLKGT RFSVGSGSPY AYGILDSGYR YDLSVDEAVE
161 LGRRAIYHAT FR**DGASGGVV SVYHVGPEGW KK**WTGDDVGD LHYQYYPVVP STVEQEMAEA TGA

Supporting Peptides:

Peptide	Uniq	-10lgP	Mass	Length	ppm	m/z	z	RT	Fraction	Scan	Source File	Area Raw Ara h 2	#Feature	#Feature Raw Ara h 2	Start	End	PTM	AScore	Found By
K.LLANILYSYR.G	Y	76.10	1224.6866	10	1.0	613.3512	2	34.31	2	F2:1690	OB5932 H2 raw.raw	7.006E4	2	2	88	97			PEAKS DB
R.DGASGGVVSVYHVGPEGWKK.W	Y	34.60	2028.0065	20	-1.1	677.0087	3	30.03	3	F3:1461	OB5933 H2 raw.raw	2.4672E4	1	1	173	192			PEAKS DB
**total 2 peptides**																			

Peptide List

Prepared with PEAKS ™ (bioinfor.com)

Summary

1. Notes

Ara h 2 GEL BAND ROASTED

```
Roasted Ara h 2 band also conatins a lot of  Ara h 3 allergens and Ara h 2 is alos on the sixts lace per CIX  cruve area
s as in raw  arah 2 file.
```

2. Result Statistics

Figure 1. False discovery rate (FDR) curve. X axis is the number of peptides being kept. Y axis is the corresponding FDR.

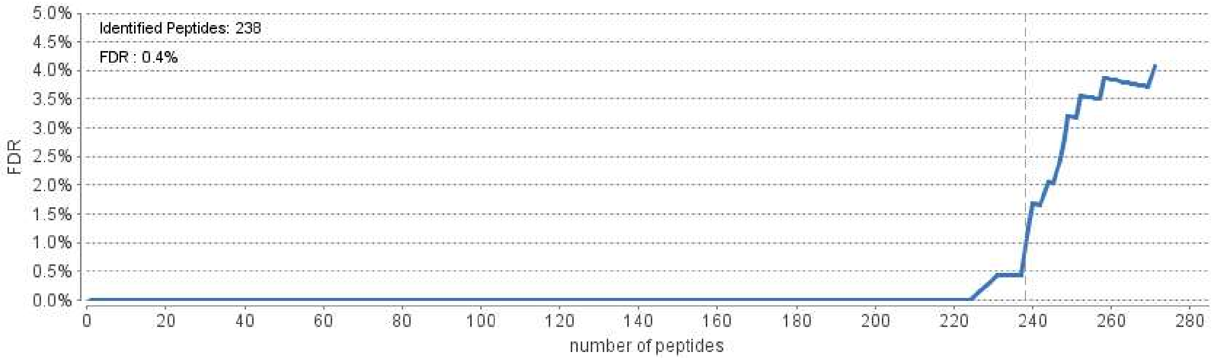

Figure 2. PSM score distribution. **(a)** Distribution of PEAKS peptide score; **(b)** Scatterplot of PEAKS peptide score versus precursor mass error.

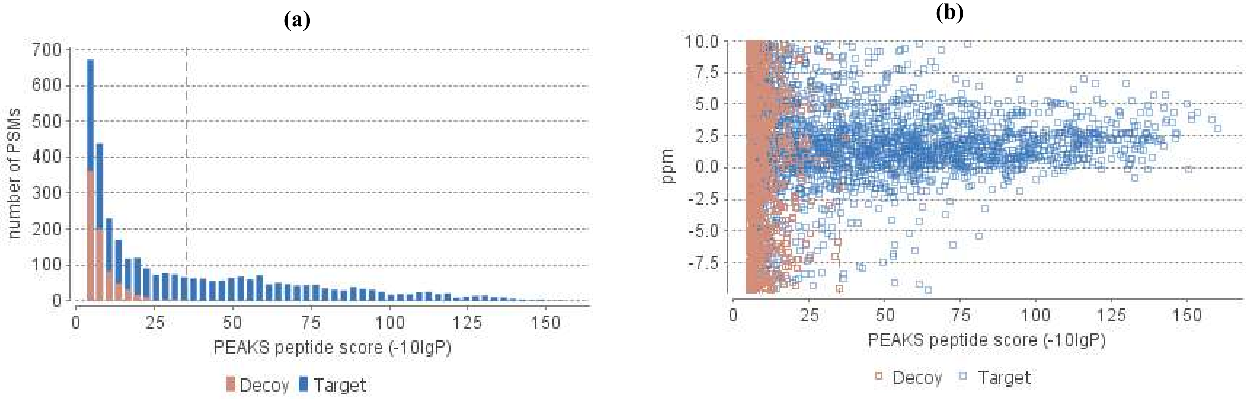

Table 1. Statistics of data.

	#Scans	#Features	Identified	#Peptides	#Sequences	

										#Proteins*		
	MS1	MS/MS	#Chimera		#PSMs	#Scans	#Features**			Groups	All	Top
Total	6754	4932	742	8080	1299	1283	558	238	141	12	17	14
Roast Ara h 2	6754	4932	742	8080	1299	1283	558	238	141	12	17	14

* proteins with significant peptides are used in counts.

** features are identified by DB search only.

Figure 3. Sample overlap for Proteins and Peptides (up to 8 samples). **(a)** All Proteins; **(b)** Top Proteins; **(c)** Peptides;

(a)
Not applicable to only one sample

(b)
Not applicable to only one sample

(c)
Not applicable to only one sample

Figure 4. Distribution of peptide feature detection. **(a)** Feature m/z distribution; **(b)** Feature RT distribution.

(a)

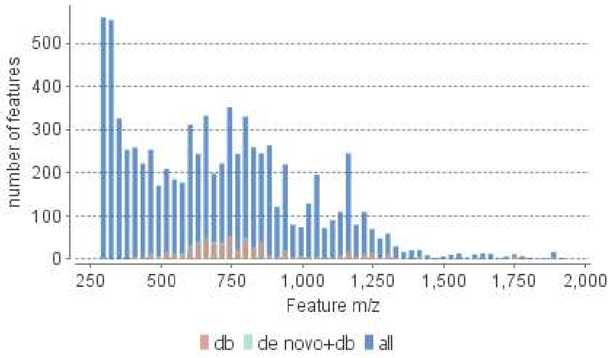

(b)

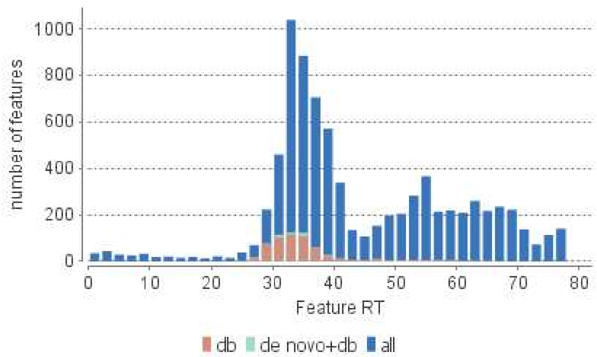

Figure 5. Distribution of identified peptide features. **(a)** Feature abundance distribution; **(b)** *De novo* sequencing validation.

(a)

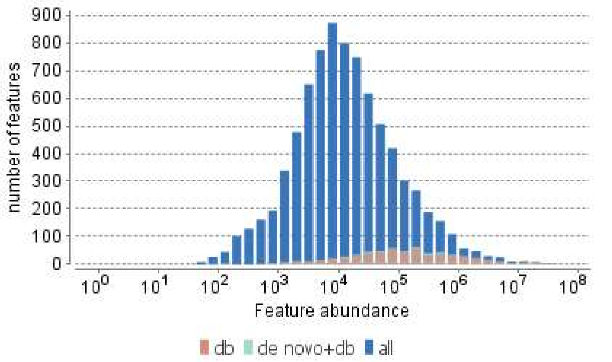

(b)

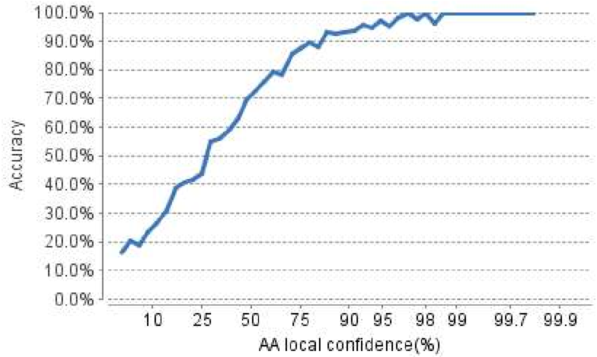

Table 2. Result filtration parameters.

Peptide -10lgP	≥35.1
PTM Ascore	≥50
Protein -10lgP	≥20
Proteins unique peptides	≥2
De novo score(%)	≥50%

Table 3. Statistics of filtered result.

FDR (Peptide-Spectrum Matches)	0.1%
FDR (Peptide Sequences)	0.4%
FDR (Protein Group)	0.0%
De Novo Only Spectra	52

Table 4. PTM profile.

Name	ΔMass	Position	#PSM	-10lgP	Abundance	AScore
Carbamidomethyl	57.02	C	131	121.98	1.31E5	1000.00
Deamidation	.98	NQ	71	160.37	9.54E5	180.29
Oxidation	15.99	M	42	126.44	1.15E6	1000.00
Carbamidomethyl	57.02	DEH,N-term	41	132.85	4.74E5	0.00
Propionamide	71.04	C	14	94.71	9.26E3	9.34
Dimethylation(KR)	28.03	KR	13	151.29	3.06E6	1000.00
Methylation(others)	14.02	DH	12	93.74	5.81E5	18.00
Ethyl	28.03	E,N-term	10	74.92	8.8E5	42.22
Cation:Fe[II]	53.92	DE	9	69.70	2.33E5	154.98
Oxidation	15.99	PR	8	76.27	8.19E5	0.00
Amidine	41.03	N-term	6	63.11	1.55E4	96.48
Sodium	21.98	DE	6	60.96	2.76E4	0.00
Ammonia loss	-17.03	N-term	5	61.66	2.21E5	1000.00
Pyro-glu from Q	-17.03	N-term	5	67.76	4.21E3	1000.00
Dihydroxy	31.99	WY	4	103.31	7.44E4	44.28
Acetylation	42.01	N-term	2	42.71	2.74E5	1000.00
Diethylation	56.06	K	2	48.11	4.01E5	121.40
Dehydration	-18.01	S	1	52.68	3.08E4	14.04
Phosphorylation	79.97	T	1	41.04	1.88E5	71.87
BITC	149.03	K	1	52.86	5.13E4	22.37
Carboxylation	43.99	E	1	39.76		1000.00
Methylation(KR)	14.02	R	1	42.71	2.74E5	1000.00

3. Experiment Control

Figure 6. Precursor mass error of peptide-spectrum matches (PSM) in filtered result. **(a)** Distribution of precursor mass error in ppm; **(b)** Scatterplot of precursor m/z versus precursor mass error in ppm.

(a)

(b)

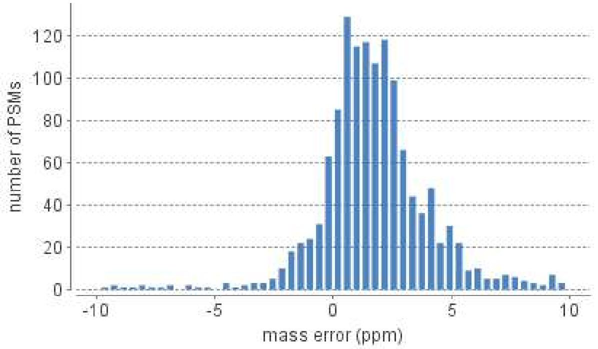

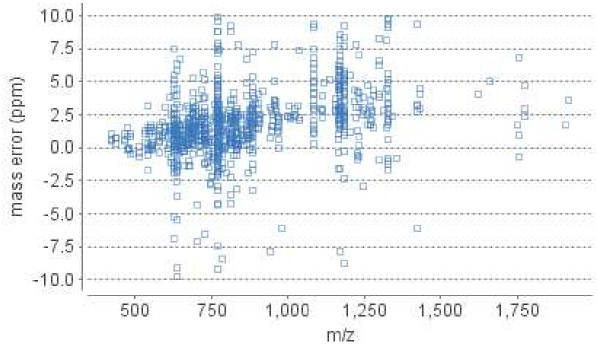

Table 5. Number of identified peptides in each sample by the number of missed cleavages.

Missed Cleavages	0	1	2	3	4+
Roast Ara h 2	147	76	15	0	0

4. Other Information

Table 6. Search parameters.

PEAKS Version: PEAKS Studio 10.6 build 20201015
Search Engine Name: PEAKS
Parent Mass Error Tolerance: 10.0 ppm
Fragment Mass Error Tolerance: 0.5 Da
Precursor Mass Search Type: monoisotopic
Enzyme: Trypsin
Max Missed Cleavages: 2
Digest Mode: Semispecific
Fixed Modifications:
  Carbamidomethylation: 57.02
Variable Modifications:
  Oxidation (M): 15.99
  Deamidation (NQ): 0.98
  Acetylation (K): 42.01
  Acetylation (Protein N-term): 42.01
  Acetylation (N-term): 42.01
  Amidation: -0.98
  Beta-methylthiolation: 45.99
  Biotinylation: 226.08
  and 303 more...
Max Variable PTM Per Peptide: 5
Database: Uniprot_Peanut-3818_Jul18
Taxon: All
Contaminant Database: contam MQ
Searched Entry: 1723
FDR Estimation: Enabled
De novo score(%) threshold: 15
Peptide hit threshold (-10logP): 30.0

Table 7. Instrument parameters.

Fractions: OB5922 H2 Ro.raw, OB5945 H2 Ro.raw, OB5946 H2 Ro.raw

Ion Source: ESI(nano-spray)
Fragmentation Mode: CID, CAD(y and b ions)
MS Scan Mode: FT-ICR/Orbitrap
MS/MS Scan Mode: FT-ICR/Orbitrap

Peaks run ID: 30
Merge Options: no merge
Precursor Options: corrected
Charge Options: no correction
Filter m/z: 200.0 - 2000.0
Filter Charge: 2 - 12
Process: true
Associate chimera: yes

Protein List

Protein Accession Contains:
Protein Description Contains:
Protein Sample Area >=
Protein PTM Contains:

Protein Group	Protein ID	Accession	-10lgP	Coverage (%)	Coverage (%) Roast Ara h 2	Area Roast Ara h 2	#Peptides	#Unique	#Spec Roast Ara h 2	PTM	Avg. Mass	Description
1	3	A1DZF0|A1DZF0_ARAHY	485.56	41	41	8.3772E6	28	6	497	Y	60375	Arachin 6 OS=Arachis hypogaea OX=3818 PE=2 SV=1
8	1	Q6IWG5|Q6IWG5_ARAHY	464.33	42	42	1.4872E8	21	19	273	Y	58061	Glycinin (Fragment) OS=Arachis hypogaea OX=3818 PE=2 SV=1
8	2	Q0GM57|Q0GM57_ARAHY	464.33	42	42	1.4872E8	21	19	273	Y	58263	Iso-Ara h3 OS=Arachis hypogaea OX=3818 PE=2 SV=1
6	10	Q9FZ11|Q9FZ11_ARAHY	460.73	34	34	2.2384E6	22	3	367	Y	60449	Gly1 OS=Arachis hypogaea OX=3818 GN=Gly1 PE=2 SV=1
3	9	Q647H3|Q647H3_ARAHY	459.83	40	40	6.7933E7	23	4	412	Y	61532	Arachin Ahy-2 OS=Arachis hypogaea OX=3818 PE=2 SV=1
5	7	Q8LKN1|Q8LKN1_ARAHY	435.43	40	40	2.7383E5	21	2	368	Y	61738	Allergen Arah3/Arah4 OS=Arachis hypogaea OX=3818 PE=3 SV=1
10	12	E9LFE8|E9LFE8_ARAHY	407.17	55	55	2.2478E7	14	14	95	Y	28290	11S arachin (Fragment) OS=Arachis hypogaea OX=3818 PE=2 SV=1
11	8	sp|Q647H2|AHY3_ARAHY	371.36	33	33	9.1524E6	13	11	70	Y	54569	Arachin Ahy-3 OS=Arachis hypogaea OX=3818 PE=1 SV=1
15	14	Q647H1|Q647H1_ARAHY	313.27	15	15	2.7735E6	8	8	25	Y	75933	Conarachin OS=Arachis hypogaea OX=3818 PE=2 SV=1
16	25	A1E2B1|A1E2B1_ARAHY	258.34	49	49	1.8619E6	7	7	17	Y	31268	11S seed storage globulin B2 OS=Arachis hypogaea OX=3818 PE=2 SV=1
17	27	A5Z1R0|A5Z1R0_ARAHY	220.47	34	34	6.8329E5	4	4	16	Y	16920	Ara h 6 allergen OS=Arachis hypogaea OX=3818 GN=Ara h 6 PE=4 SV=1
17	28	sp|Q647G9|CONG_ARAHY	220.47	34	34	6.8329E5	4	4	16	Y	16920	Conglutin OS=Arachis hypogaea OX=3818 PE=1 SV=1
12	24	sp|Q6PSU2|CONG7_ARAHY	203.38	38	38	4.8478E6	5	5	40	Y	20114	Conglutin-7 OS=Arachis hypogaea OX=3818 PE=1 SV=2
18	29	#CONTAM#P00761	107.34	11	11	3.2741E5	2	2	10	N	24409	SWISS-PROT:#CONTAM#P00761|TRYP_PIG Trypsin - Sus scrofa (Pig).
**total 14 proteins**												

A1DZF0|A1DZF0_ARAHY

back to list

| Protein Coverage | Supporting Peptides |

Protein Coverage:

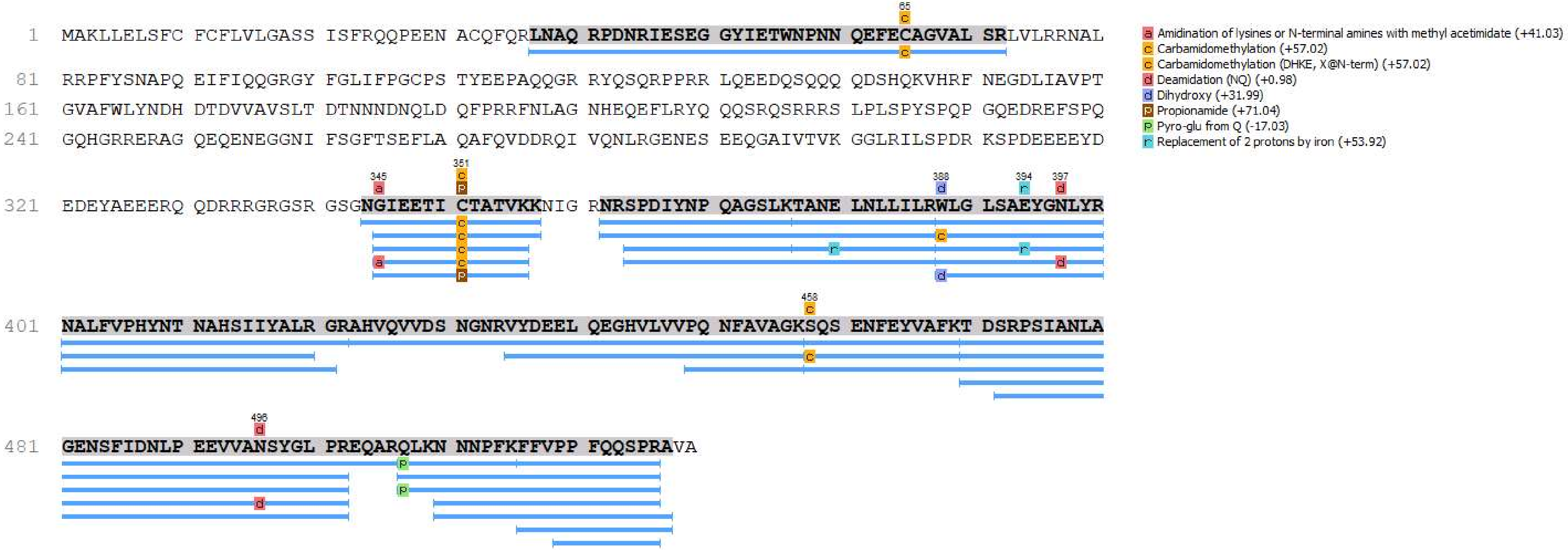

Supporting Peptides:

Peptide	Uniq	-10lgP	Mass	Length	ppm	m/z	z	RT	Fraction	Scan	Source File	Area Roast Ara h 2	#Feature	#Feature Roast Ara h 2	Start	End	PTM	AScore	Found By
R.NALFVPHYNTNAHSIIYALR.G	N	151.62	2313.2019	20	3.7	1157.6125	2	33.79	4	F4:1486	OB5922 H2 Ro.raw	4.79E7	11	11	401	420			PEAKS DB
R.WLGLSAEYGNLYR.N	N	145.22	1540.7673	13	3.5	771.3937	2	36.40	4	F4:1654	OB5922 H2 Ro.raw	5.6282E7	22	22	388	400			PEAKS DB
K.TDSRPSIANLAGENSFIDNLPEEVVANSYGLPR.E	N	142.57	3544.7434	33	4.7	1182.5940	3	38.01	6	F6:1881	OB5946 H2 Ro.raw	5.7129E7	11	11	470	502			PEAKS DB
R.SPDIYNPQAGSLKTANELNLLILR.W	N	135.56	2639.4282	24	1.4	880.8179	3	36.01	4	F4:1634	OB5922 H2 Ro.raw	6.2632E6	5	4	364	387			PEAKS DB
R.NRSPDIYNPQAGSLKTANELNLLILR.W	N	134.91	2909.5723	26	2.3	970.8669	3	34.91	4	F4:1560	OB5922 H2 Ro.raw	3.3615E6	6	6	362	387			PEAKS DB
K.SQSENFEYVAFK.T	N	130.69	1447.6619	12	2.8	724.8402	2	31.92	4	F4:1368	OB5922 H2 Ro.raw	1.7167E7	4	4	458	469			PEAKS DB
K.FFVPPFQQSPR.A	Y	127.31	1348.6927	11	2.2	675.3551	2	33.42	4	F4:1469	OB5922 H2 Ro.raw	3.074E6	3	3	516	526			PEAKS DB
K.FFVPPFQQSPRA.V	Y	116.09	1419.7299	12	2.0	710.8737	2	33.08	6	F6:1597	OB5946 H2 Ro.raw	1.5948E6	3	3	516	527			PEAKS DB

total 58 peptides

Peptide	Uniq	-10lgP	Mass	Length	ppm	m/z	z	RT	Fraction	Scan	Source File	Area Roast Ara h 2	#Feature	#Feature Roast Ara h 2	Start	End	PTM	AScore	Found By
K.NNNPFKFFVPPFQQSPR.A	Y	113.79	2063.0376	17	1.8	688.6877	3	35.64	4	F4:1615	OB5922 H2 Ro.raw	2.4453E5	5	5	510	526			PEAKS DB
K.TDSRPSIANLAGENSFIDNLPEEVVAN(+.98)SYGLPR.E	N	112.33	3545.7273	33	1.8	1182.9185	3	36.63	5	F5:1807	OB5945 H2 Ro.raw	0	0	0	470	502	Deamidation (NQ)	N27:Deamidation (NQ):103.10	PEAKS DB
N.GIEETIC(+57.02)TATVK.K	N	109.07	1320.6595	12	-0.2	661.3369	2	29.91	4	F4:1260	OB5922 H2 Ro.raw	2.1822E5	3	3	345	356	Carbamidomethylation	C7:Carbamidomethylation:1000.00	PEAKS DB
R.VYDEELQEGHVLVVPQNFAVAGK.S	N	102.31	2540.2910	23	1.4	847.7721	3	33.42	4	F4:1478	OB5922 H2 Ro.raw	1.4453E6	6	6	435	457			PEAKS DB
R.SPDIYNPQAGSLK.T	N	95.77	1388.6936	13	1.7	695.3552	2	28.58	5	F5:1323	OB5945 H2 Ro.raw	5.949E5	3	3	364	376			PEAKS DB
R.NALFVPH(+14.02)YNTNAHSIIYALR.G	N	93.74	2327.2175	20	0.7	776.7470	3	34.91	4	F4:1561	OB5922 H2 Ro.raw	1.3103E6	3	3	401	420		H7:Methylation(others):22.45	PEAKS PTM
R.NRSPDIYNPQAGSLK.T	N	91.67	1658.8376	15	0.8	830.4268	2	28.33	6	F6:1298	OB5946 H2 Ro.raw	4.0221E5	6	6	362	376			PEAKS DB
R.AHVQVVDSNGNRVYDEELQEGHVLVVPQNFAVAGK.S	N	90.86	3816.9182	35	3.6	955.2402	4	32.32	6	F6:1544	OB5946 H2 Ro.raw	8.2893E6	8	8	423	457			PEAKS DB
K.TANELNLLILR.W	N	90.00	1268.7452	11	0.6	635.3802	2	35.27	4	F4:1655	OB5922 H2 Ro.raw	2.0517E7	4	4	377	387			PEAKS DB
V.PQNFAVAGK.S	N	85.45	930.4923	9	1.0	466.2539	2	32.32	6	F6:1542	OB5946 H2 Ro.raw	3.3444E6	3	3	449	457			PEAKS DB
R.QLKNNNPFKFFVPPFQQSPR.A	Y	82.81	2432.2751	20	0.9	609.0766	4	34.45	5	F5:1679	OB5945 H2 Ro.raw	6.0459E5	3	3	507	526			PEAKS DB
R.W(+31.99)LGLSAEYGNLYR.N	N	77.74	1572.7572	13	-1.6	787.3846	2	33.64	6	F6:1624	OB5946 H2 Ro.raw	8.5976E4	2	2	388	400	Dihydroxy	W1:Dihydroxy:62.93	PEAKS PTM
K.S(+57.02)QSENFEYVAFK.T	N	77.12	1504.6833	12	0.0	753.3489	2	31.77	6	F6:1521	OB5946 H2 Ro.raw	4.3047E5	3	3	458	469	Carbamidomethylation (DHKE, X@N-term)	S1:Carbamidomethylation (DHKE, X@N-term):56.34	PEAKS PTM
K.FFVPPFQQSP(+15.99)R.A	Y	76.27	1364.6876	11	0.9	683.3517	2	33.23	4	F4:1459	OB5922 H2 Ro.raw	1.4813E6	3	3	516	526		P10:Oxidation or Hydroxylation:12.28	PEAKS PTM
R.AHVQVVDSN(+.98)GNRVYDEELQEGHVLVVPQNFAVAGK.S	N	75.25	3817.9023	35	5.0	955.4877	4	33.04	4	F4:1438	OB5922 H2 Ro.raw	1.3721E6	1	1	423	457		N9:Deamidation (NQ):4.64	PEAKS DB
N.G(+57.02)IEETIC(+57.02)TATVK.K	N	70.02	1377.6809	12	2.0	689.8491	2	30.19	4	F4:1285	OB5922 H2 Ro.raw	5.5841E3	1	1	345	356	Carbamidomethylation	G1:Carbamidomethylation (DHKE, X@N-term):30.83;C7:Carbamidomethylation:1000.00	PEAKS PTM
K.TANE(+53.92)LNLLILR.W	N	69.70	1322.6646	11	-1.4	662.3386	2	35.27	4	F4:1590	OB5922 H2 Ro.raw	2.333E5	1	1	377	387	Replacement of 2 protons by iron	E4:Replacement of 2 protons by iron:154.98	PEAKS PTM

total 58 peptides

Peptide	Uniq	-10lgP	Mass	Length	ppm	m/z	z	RT	Fraction	Scan	Source File	Area Roast Ara h 2	#Feature	#Feature Roast Ara h 2	Start	End	PTM	AScore	Found By
R.AHVQVVDSNGN(+.98)RVYDEELQEGHVLVVPQNFAVAGK.S	N	68.98	3817.9023	35	3.2	955.4859	4	32.51	5	F5:1567	OB5945 H2 Ro.raw	0	0	0	423	457		N11:Deamidation (N Q):8.22	PEAKS DB
K.NNNPFKFFVPPFQQSPRA.V	Y	68.01	2134.0747	18	0.0	712.3655	3	35.15	6	F6:1723	OB5946 H2 Ro.raw	8.836E4	2	2	510	527			PEAKS DB
R.Q(-17.03)LKNNNPFK.F	N	67.76	1084.5665	9	1.6	543.2914	2	28.75	5	F5:1346	OB5945 H2 Ro.raw	1.0606E4	2	2	507	515	Pyro-glu from Q	Q1:Pyro-glu from Q:1000.00	PEAKS PTM
N.GIEETIC(+57.02)TATVKK.N	N	66.30	1448.7545	13	0.5	725.3849	2	27.89	6	F6:1301	OB5946 H2 Ro.raw	3.1708E5	3	3	345	357	Carbamidomethylation	C7:Carbamidomethylation:1000.00	PEAKS DB
R.W(+57.02)LGLSAEYGNLYR.N	N	65.45	1597.7888	13	0.6	799.9022	2	35.09	5	F5:1725	OB5945 H2 Ro.raw	1.848E4	1	1	388	400	Carbamidomethylation (DHKE, X@N-term)	W1:Carbamidomethylation (DHKE, X@N-term): 62.19	PEAKS PTM
N.GIEETIC(+71.04)TATVK.K	N	65.44	1334.6752	12	3.2	668.3470	2	29.62	5	F5:1395	OB5945 H2 Ro.raw	7.3781E3	1	1	345	356	Propionamide	C7:Propionamide:1000.00	PEAKS PTM
R.WLGLSAE(+53.92)YGNLYR.N	N	65.30	1594.6866	13	1.3	532.5702	3	34.72	5	F5:1698	OB5945 H2 Ro.raw	6.0447E5	3	3	388	400	Replacement of 2 protons by iron	E7:Replacement of 2 protons by iron:123.05	PEAKS PTM
R.S(+57.02)PDIYNPQAGSLK.T	N	64.83	1445.7150	13	5.7	723.8689	2	28.94	5	F5:1350	OB5945 H2 Ro.raw	6.4489E3	2	2	364	376		S1:Carbamidomethylation (DHKE, X@N-term): 14.04	PEAKS PTM
R.NALFVPHYNTNAHSIIYALRG.R	N	61.88	2370.2231	21	1.2	791.0826	3	33.77	5	F5:1624	OB5945 H2 Ro.raw	4.2894E6	2	2	401	421			PEAKS DB
K.SQSE(+21.98)NFEYVAFK.T	N	60.96	1469.6438	12	-0.6	735.8287	2	31.51	5	F5:1513	OB5945 H2 Ro.raw	2.7649E4	1	1	458	469		E4:Sodium adduct:0.00	PEAKS PTM
K.SQSENFE(+21.98)YVAFK.T	N	59.61	1469.6438	12	-1.1	735.8284	2	31.92	4	F4:1388	OB5922 H2 Ro.raw	4.36E4	1	1	458	469		E7:Sodium adduct:14.84	PEAKS PTM
R.N(+57.02)ALFVPHYNTNAHSIIYALR.G	N	59.27	2370.2231	20	-0.1	791.0816	3	34.53	4	F4:1557	OB5922 H2 Ro.raw	3.2966E6	1	1	401	420		N1:Carbamidomethylation (DHKE, X@N-term): 10.82	PEAKS PTM
K.SQSE(+53.92)NFEYVAFK.T	N	58.53	1501.5812	12	-2.3	751.7961	2	31.58	6	F6:1507	OB5946 H2 Ro.raw	9.4575E4	1	1	458	469		E4:Replacement of 2 protons by iron:34.87	PEAKS PTM
K.TDSRPSIANLAGENSFIDNLPEEVVANSYGLPREQAR.Q	N	58.03	4028.9827	37	2.2	1008.2552	4	36.41	5	F5:1777	OB5945 H2 Ro.raw	1.9797E6	2	2	470	506			PEAKS DB
K.TDSRPSIANLAGENSFIDN(+.98)LPEEVVANSYGLPR.E	N	57.24	3545.7273	33	5.5	1182.9229	3	37.60	4	F4:1724	OB5922 H2 Ro.raw	0	0	0	470	502		N19:Deamidation (N Q):20.10	PEAKS DB
R.SPD(+57.02)IYNPQAGSLK.T	N	55.35	1445.7150	13	2.2	723.8663	2	29.51	4	F4:1244	OB5922 H2 Ro.raw	0	0	0	364	376		D3:Carbamidomethylation (DHKE, X@N-term): 0.00	PEAKS PTM
K.SQSENFEYVAFKTDSRPSIANLAGENSFIDNLPEEVVANSYGLPR.E	N	54.99	4974.3945	45	2.5	1244.6090	4	38.26	4	F4:1777	OB5922 H2 Ro.raw	3.3359E6	3	3	458	502			PEAKS DB

total 58 peptides

Peptide	Uniq	-10lgP	Mass	Length	ppm	m/z	z	RT	Fraction	Scan	Source File	Area Roast Ara h 2	#Feature	#Feature Roast Ara h 2	Start	End	PTM	AScore	Found By
R.AHVQVVDSNGNRVYDEELQ(+.98)EGHVLVVPQNFAVAGK.S	N	53.98	3817.9023	35	7.9	955.4904	4	33.43	6	F6:1615	OB5946 H2 Ro.raw	0	0	0	423	457		Q19:Deamidation (NQ):4.91	PEAKS DB
K.SQS(-18.01)ENFEYVAFK.T	N	52.68	1429.6514	12	1.9	715.8344	2	31.92	4	F4:1380	OB5922 H2 Ro.raw	3.0777E4	1	1	458	469		S3:Dehydration:14.04	PEAKS PTM
V.PPFQQSPR.A	Y	50.68	955.4875	8	-0.2	478.7510	2	35.15	6	F6:1720	OB5946 H2 Ro.raw	1.8433E5	2	2	519	526			PEAKS DB
R.LNAQRPDNRIESEGGYIETWNPNNQEFEC(+57.02)AGVALSR.L	N	50.20	4133.9248	36	7.4	1034.4961	4	33.23	4	F4:1476	OB5922 H2 Ro.raw	5.4856E4	1	1	37	72	Carbamidomethylation	C29:Carbamidomethylation:1000.00	PEAKS DB
K.FFVPPFQQSPR(+15.99)A.V	Y	49.54	1435.7247	12	2.2	718.8712	2	33.04	4	F4:1458	OB5922 H2 Ro.raw	9.538E5	2	2	516	527		R11:Oxidation or Hydroxylation:12.28	PEAKS PTM
R.NALFVPH(+57.02)YNTNAHSIIYALR.G	N	49.15	2370.2231	20	0.1	791.0817	3	34.03	6	F6:1601	OB5946 H2 Ro.raw	8.9747E5	1	1	401	420		H7:Carbamidomethylation (DHKE, X@N-term):20.35	PEAKS PTM
D.SRPSIANLAGENSFIDNLPEEVVANSYGLPR.E	N	48.34	3328.6687	31	3.9	1110.5679	3	37.36	5	F5:1841	OB5945 H2 Ro.raw	3.0619E5	2	2	472	502			PEAKS DB
K.TDSRPSIANLAGEN(+.98)SFIDNLPEEVVANSYGLPR.E	N	47.42	3545.7273	33	9.2	1182.9272	3	48.53	5	F5:2493	OB5945 H2 Ro.raw	0	0	0	470	502		N14:Deamidation (NQ):19.39	PEAKS DB
R.WLGLSAEYGN(+.98)LYR.N	N	47.40	1541.7513	13	5.4	771.8871	2	54.24	4	F4:2679	OB5922 H2 Ro.raw	0	0	0	388	400	Deamidation (NQ)	N10:Deamidation (NQ):1000.00	PEAKS DB
N.G(+41.03)IEETIC(+57.02)TATVK.K	N	47.00	1361.6860	12	0.7	681.8508	2	29.66	5	F5:1390	OB5945 H2 Ro.raw	4.1987E4	2	2	345	356	Amidination of lysines or N-terminal amines with methyl acetimidate; Carbamidomethylation	G1:Amidination of lysines or N-terminal amines with methyl acetimidate:96.48;C7:Carbamidomethylation:1000.00	PEAKS PTM
R.NALFVPHYNTNAHSIIYALRGR.A	N	46.11	2526.3245	22	0.6	843.1160	3	33.60	4	F4:1492	OB5922 H2 Ro.raw	7.6168E5	2	2	401	422			PEAKS DB
R.Q(-17.03)LKNNNPFKFFVPPFQQSPR.A	Y	45.85	2415.2488	20	1.8	806.0917	3	36.41	5	F5:1798	OB5945 H2 Ro.raw	1.5157E5	1	1	507	526	Pyro-glu from Q	Q1:Pyro-glu from Q:1000.00	PEAKS PTM
K.SQSEN(+.98)FEYVAFKTDSRPSIANLAGENSFIDNLPEEVVANSYGLPR.E	N	45.32	4975.3789	45	2.8	1244.8555	4	39.75	5	F5:1987	OB5945 H2 Ro.raw	0	0	0	458	502		N5:Deamidation (NQ):0.00	PEAKS DB
G.NGIEETIC(+57.02)TATVKK.N	N	42.60	1562.7974	14	0.1	782.4060	2	29.85	5	F5:1417	OB5945 H2 Ro.raw	8.2899E3	2	2	344	357	Carbamidomethylation	C8:Carbamidomethylation:1000.00	PEAKS DB
R.N(+.98)ALFVPHYNTNAHSIIYALR.G	N	38.37	2314.1858	20	7.3	772.4082	3	50.66	4	F4:2450	OB5922 H2 Ro.raw	1.0573E4	1	1	401	420		N1:Deamidation (NQ):32.94	PEAKS DB
R.WLGLSAE(+21.98)YGNLYR.N	N	36.57	1562.7494	13	-8.4	782.3754	2	35.27	4	F4:1583	OB5922 H2 Ro.raw	5.0047E4	2	2	388	400		E7:Sodium adduct:42.78	PEAKS PTM
**total 58 peptides**																			

Q6IWG5|Q6IWG5_ARAHY

back to list

| Protein Coverage | Supporting Peptides |

Protein Coverage:

```
1   KLLALSLCFC VLVLGASSVT FRQGGEENEC QFQRLNAQRP DNRIESEGGY IETWNPNNQE FQCAGVALSR TVLRRNALRR
81  PFYSNAPLEI YVQQGSGYFG LIFPGCPSTY EEPAQEGRRY QSQKPSRRFQ VGQDDPSQQQ QDSHQKVHRF DEGDLIAVPT
161 GVAFWMYNDE DTDVVTVTLS DTSSIHNQLD QFPRRFYLAG NQEQEFLRYQ QQQGSRPHYR QISPRVRGDE QENEGSNIFS
241 GFAQEFLQHA FQVDRQTVEN LRGENEREEQ GAIVTKGGL RILSPDEEDE SSRSPPSRRE EFDEDRSRPQ QRGKYDENRR
321 GYKNGIEETI CSASVKKNLG RSSNPDIYNP QAGSLRSVNE LDLPILGWLG LSAQHGTIYR NAMFVPHYTL NAHTIVVALN
401 GRAHVQVVDS NGNRVYDEEL QEGHVLVVPQ NFAVAAKAQS ENYEYLAFKT DSRPSIANLA GENSIIDNLP EEVVANSYRL
481 PREQARQLKN NNPFKFFVPP FDHQSMREVA
```

Modification positions marked: 63 (c), 325 (a), 331 (c), 383 (o), 400 (d), 438 (c), 476 (d)

- a Amidination of lysines or N-terminal amines with methyl acetimidate (+41.03)
- c Carbamidomethylation (+57.02)
- c Carbamidomethylation (DHKE, X@N-term) (+57.02)
- d Deamidation (NQ) (+0.98)
- o Oxidation (M) (+15.99)
- p Propionamide (+71.04)
- p Pyro-glu from Q (-17.03)

Supporting Peptides:

Peptide	Uniq	-10lgP	Mass	Length	ppm	m/z	z	RT	Fraction	Scan	Source File	Area Roast Ara h 2	#Feature	#Feature Roast Ara h 2	Start	End	PTM	AScore	Found By
R.NAMFVPHYTLNAHTIVVALN(+.98)GR.A	Y	160.37	2438.2529	22	3.0	1220.1375	2	33.96	5	F5:1643	OB5945 H2 Ro.raw	9.2221E6	5	5	381	402	Deamidation (NQ)	N20:Deamidation (NQ):180.29	PEAKS DB
R.SVNELDLPILGWLGLSAQHGTIYR.N	Y	158.35	2651.4070	24	3.8	1326.7158	2	39.91	6	F6:1991	OB5946 H2 Ro.raw	6.2356E7	15	15	357	380			PEAKS DB
K.TDSRPSIANLAGENSIIDNLPEEVVANSYR.L	Y	150.05	3243.6006	30	5.0	1082.2129	3	36.29	6	F6:1782	OB5946 H2 Ro.raw	1.2988E7	7	7	450	479			PEAKS DB
R.NAMFVPHYTLNAHTIVVALNGR.A	Y	142.76	2437.2688	22	3.5	1219.6460	2	34.54	5	F5:1687	OB5945 H2 Ro.raw	7.7824E6	7	7	381	402			PEAKS DB
K.AQSENYEYLAFK.T	Y	135.05	1461.6776	12	0.6	731.8465	2	31.92	4	F4:1369	OB5922 H2 Ro.raw	6.5522E6	3	3	438	449			PEAKS DB
R.S(+57.02)VNELDLPILGWLGLSAQHGTIYR.N	Y	132.85	2708.4285	24	-0.8	1355.2205	2	40.55	4	F4:1898	OB5922 H2 Ro.raw	4.7421E5	1	1	357	380		S1:Carbamidomethylation (DHKE, X@N-term): 0.00	PEAKS PTM
R.SSNPDIYNPQAGSLR.S	Y	129.02	1617.7747	15	0.7	809.8952	2	29.12	4	F4:1205	OB5922 H2 Ro.raw	1.2321E6	3	3	342	356			PEAKS DB

total 46 peptides

Peptide	Uniq	-10lgP	Mass	Length	ppm	m/z	z	RT	Fraction	Scan	Source File	Area Roast Ara h 2	#Feature	#Feature Roast Ara h 2	Start	End	PTM	AScore	Found By
R.NAM(+15.99)FVPHYTLNAHTIVVALNGR.A	Y	126.44	2453.2637	22	1.4	818.7629	3	34.16	4	F4:1519	OB5922 H2 Ro.raw	2.32E6	3	3	381	402	Oxidation (M)	M3:Oxidation (M):1000.00	PEAKS DB
L.PILGWLGLSAQHGTIYR.N	Y	124.94	1881.0261	17	0.7	941.5210	2	40.57	4	F4:1889	OB5922 H2 Ro.raw	2.9616E5	2	2	364	380			PEAKS DB
R.AHVQVVDSNGN(+.98)RVYDEELQEGHVLVVPQNFAVAAK.A	Y	113.48	3831.9180	35	4.0	1278.3184	3	32.50	6	F6:1562	OB5946 H2 Ro.raw	3.2728E6	4	4	403	437		N11:Deamidation (NQ):15.73	PEAKS DB
R.NAM(+15.99)FVPHYTLNAHTIVVALN(+.98)GR.A	Y	106.88	2454.2478	22	0.3	819.0901	3	32.82	5	F5:1580	OB5945 H2 Ro.raw	1.0956E6	4	4	381	402	Oxidation (M); Deamidation (NQ)	M3:Oxidation (M):1000.00;N20:Deamidation (NQ):104.25	PEAKS DB
R.AHVQVVDSN(+.98)GNRVYDEELQEGHVLVVPQNFAVAAK.A	Y	104.42	3831.9180	35	4.4	1278.3188	3	32.26	5	F5:1553	OB5945 H2 Ro.raw	5.1124E6	4	4	403	437		N9:Deamidation (NQ):39.44	PEAKS DB
R.SVNELDLPILGW(+31.99)LGLSAQHGTIYR.N	Y	103.31	2683.3970	24	2.4	1342.7090	2	39.79	4	F4:1852	OB5922 H2 Ro.raw	7.442E4	1	1	357	380		W12:Dihydroxy:44.28	PEAKS PTM
K.TDSRPSIANLAGENSIIDNLPEEVVANSYRLPR.E	Y	99.31	3609.8386	33	3.4	903.4700	4	37.52	4	F4:1753	OB5922 H2 Ro.raw	2.5873E7	4	4	450	482			PEAKS DB
N.GIEETIC(+57.02)SASVK.K	Y	94.29	1292.6282	12	0.9	647.3220	2	29.26	6	F6:1364	OB5946 H2 Ro.raw	6.9435E4	3	3	325	336	Carbamidomethylation	C7:Carbamidomethylation:1000.00	PEAKS DB
K.FFVPPFDHQSMRE.V	Y	93.17	1635.7504	13	0.3	818.8827	2	32.86	4	F4:1440	OB5922 H2 Ro.raw	2.6869E6	5	5	496	508			PEAKS DB
K.A(+57.02)QSENYEYLAFK.T	Y	91.59	1518.6990	12	-0.7	760.3562	2	31.51	5	F5:1505	OB5945 H2 Ro.raw	1.9478E5	3	3	438	449	Carbamidomethylation (DHKE, X@N-term)	A1:Carbamidomethylation (DHKE, X@N-term):56.34	PEAKS PTM
K.TDSRPSIANLAGENSIIDNLPEEVVAN(+.98)SYR.L	Y	82.09	3244.5847	30	1.7	1082.5374	3	35.58	5	F5:1746	OB5945 H2 Ro.raw	0	0	0	450	479	Deamidation (NQ)	N27:Deamidation (NQ):78.75	PEAKS DB
R.NAM(+15.99)FVPHYTLN(+.98)AHTIVVALNGR.A	Y	76.23	2454.2478	22	1.2	819.0908	3	33.30	5	F5:1613	OB5945 H2 Ro.raw	0	0	0	381	402	Oxidation (M)	M3:Oxidation (M):1000.00;N11:Deamidation (NQ):0.00	PEAKS DB
R.VYDEELQEGHVLVVPQNFAVAAK.A	Y	75.46	2554.3066	23	0.5	852.4432	3	33.26	6	F6:1608	OB5946 H2 Ro.raw	2.0809E4	1	1	415	437			PEAKS DB
R.LNAQRPDNRIESEGGYIETWNPNNQEFQC(+57.02)AGVALSR.T	Y	73.52	4132.9409	36	2.1	1034.2446	4	32.69	6	F6:1579	OB5946 H2 Ro.raw	1.2843E5	1	1	35	70	Carbamidomethylation	C29:Carbamidomethylation:1000.00	PEAKS DB
N.GIEETIC(+57.02)SASVKK.N	Y	72.67	1420.7231	13	1.3	711.3698	2	28.13	4	F4:1149	OB5922 H2 Ro.raw	1.93E5	3	3	325	337	Carbamidomethylation	C7:Carbamidomethylation:1000.00	PEAKS DB
K.FFVPPFDHQSM(+15.99)RE.V	Y	67.96	1651.7452	13	1.7	826.8813	2	32.49	4	F4:1425	OB5922 H2 Ro.raw	7.5314E5	4	4	496	508	Oxidation (M)	M11:Oxidation (M):1000.00	PEAKS DB
R.Q(-17.03)LKNNNPFK.F	N	67.76	1084.5665	9	1.6	543.2914	2	28.75	5	F5:1346	OB5945 H2 Ro.raw	1.0606E4	2	2	487	495	Pyro-glu from Q	Q1:Pyro-glu from Q:1000.00	PEAKS PTM
R.AHVQVVDSN(+.98)GN(+.98)RVYDEELQEGHVLVVPQNFAVAAK.A	Y	66.65	3832.9019	35	7.9	767.5937	5	32.26	5	F5:1556	OB5945 H2 Ro.raw	3.9346E5	1	1	403	437		N9:Deamidation (NQ):5.83;N11:Deamidation (NQ):8.28	PEAKS DB

total 46 peptides

Peptide	Uniq	-10lgP	Mass	Length	ppm	m/z	z	RT	Fraction	Scan	Source File	Area Roast Ara h 2	#Feature	#Feature Roast Ara h 2	Start	End	PTM	AScore	Found By
R.N(+.98)AMFVPHYTLNAHTIVVALNGR.A	Y	66.31	2438.2529	22	3.0	1220.1375	2	33.96	5	F5:1680	OB5945 H2 Ro.raw	9.5447E5	1	1	381	402	Deamidation (NQ)	N1:Deamidation (NQ):63.03	PEAKS DB
V.PQNFAVAAK.A	N	63.04	944.5079	9	0.9	473.2617	2	32.86	4	F4:1447	OB5922 H2 Ro.raw	2.0461E6	3	3	429	437			PEAKS DB
R.NAMFVPHYTLNAH(+57.02)TIVVALNGR.A	Y	62.29	2494.2903	22	-3.1	832.4348	3	34.84	5	F5:1702	OB5945 H2 Ro.raw	0	0	0	381	402		H13:Carbamidomethylation (DHKE, X@N-term):5.30	PEAKS PTM
K.FFVPPFDHQSM.R	Y	62.13	1350.6067	11	0.3	676.3108	2	34.60	6	F6:1680	OB5946 H2 Ro.raw	2.36E5	3	3	496	506			PEAKS DB
K.AQSENYEYLAFKTDSRPSIANLAGENSIIDNLPEEVVANSYR.L	Y	61.90	4687.2676	42	3.1	1172.8278	4	36.79	5	F5:1810	OB5945 H2 Ro.raw	2.0852E6	1	1	438	479			PEAKS DB
D.LPILGWLGLSAQHGTIYR.N	Y	60.41	1994.1101	18	2.4	998.0647	2	40.55	4	F4:1901	OB5922 H2 Ro.raw	1.1908E5	1	1	363	380			PEAKS DB
K.FFVPPFDHQS.M	Y	57.76	1219.5662	10	1.5	610.7913	2	33.58	5	F5:1616	OB5945 H2 Ro.raw	2.2184E5	2	2	496	505			PEAKS DB
R.SVN(+.98)ELDLPILGWLGLSAQHGTIYR.N	Y	54.93	2652.3911	24	9.1	1327.2150	2	46.79	4	F4:2254	OB5922 H2 Ro.raw	0	0	0	357	380	Deamidation (NQ)	N3:Deamidation (NQ):57.31	PEAKS DB
K.NNNPFKFFVPPFDHQSMRE.V	Y	53.67	2350.0952	19	1.4	784.3734	3	34.54	5	F5:1693	OB5945 H2 Ro.raw	4.2082E5	2	2	490	508			PEAKS DB
N.G(+57.02)IEETIC(+57.02)SASVKK.N	Y	52.58	1477.7446	13	-0.5	739.8792	2	28.58	4	F4:1182	OB5922 H2 Ro.raw	1.8561E4	2	2	325	337	Carbamidomethylation	G1:Carbamidomethylation (DHKE, X@N-term):22.85;C7:Carbamidomethylation:1000.00	PEAKS PTM
R.AHVQ(+.98)VVDSNGNRVYDEELQEGHVLVVPQNFAVAAK.A	Y	49.71	3831.9180	35	2.7	767.3929	5	32.86	4	F4:1451	OB5922 H2 Ro.raw	3.9416E5	1	1	403	437		Q4:Deamidation (NQ):0.00	PEAKS DB
N.G(+41.03)IEETIC(+57.02)SASVK.K	Y	48.35	1333.6548	12	0.4	667.8350	2	29.26	6	F6:1372	OB5946 H2 Ro.raw	8.2083E3	1	1	325	336	Amidination of lysines or N-terminal amines with methyl acetimidate; Carbamidomethylation	G1:Amidination of lysines or N-terminal amines with methyl acetimidate:71.87; C7:Carbamidomethylation:1000.00	PEAKS PTM
R.NAMFVPHYTLNAH(+57.02)TIVVALN(+.98)GR.A	Y	47.39	2495.2744	22	0.0	832.7654	3	33.96	5	F5:1660	OB5945 H2 Ro.raw	1.2865E5	1	1	381	402		H13:Carbamidomethylation (DHKE, X@N-term):0.00;N20:Deamidation (NQ):43.52	PEAKS PTM
K.FFVPPFDHQSM(+15.99).R	Y	45.84	1366.6016	11	1.6	684.3091	2	33.79	4	F4:1494	OB5922 H2 Ro.raw	5.6927E4	1	1	496	506	Oxidation (M)	M11:Oxidation (M):1000.00	PEAKS DB
K.TDSRPSIANLAGENSIIDNLPEEVVAN(+.98)SYRLPR.E	Y	45.48	3610.8225	33	5.4	1204.6213	3	35.38	5	F5:1734	OB5945 H2 Ro.raw	0	0	0	450	482		N27:Deamidation (NQ):32.94	PEAKS DB
R.S(+57.02)SNPDIYNPQAGSLR.S	Y	39.95	1674.7961	15	7.9	838.4119	2	29.48	4	F4:1242	OB5922 H2 Ro.raw	0	0	0	342	356		S1:Carbamidomethylation (DHKE, X@N-term):11.12	PEAKS PTM

total 46 peptides

Peptide	Uniq	-10lgP	Mass	Length	ppm	m/z	z	RT	Fraction	Scan	Source File	Area Roast Ara h 2	#Feature	#Feature Roast Ara h 2	Start	End	PTM	AScore	Found By
R.SSNPD(+21.98)IYNPQAGSLR.S	Y	39.67	1639.7566	15	4.2	820.8890	2	29.21	4	F4:1226	OB5922 H2 Ro.raw	0	0	0	342	356		D5:Sodium adduct:43.70	PEAKS PTM
K.AQSENYE(+21.98)YLAFK.T	Y	37.74	1483.6595	12	-1.5	742.8359	2	31.58	6	F6:1513	OB5946 H2 Ro.raw	8.2984E3	1	1	438	449		E7:Sodium adduct:0.00	PEAKS PTM
K.NGIEETIC(+71.04)SASVKK.N	Y	36.28	1548.7817	14	3.3	775.4007	2	29.48	5	F5:1385	OB5945 H2 Ro.raw	2.1762E3	1	1	324	337	Propionamide	C8:Propionamide:1000.00	PEAKS PTM
R.SVNELDLPILGWLGLSAQHGTIY(+31.99)R.N	Y	36.02	2683.3970	24	3.4	895.4760	3	39.79	4	F4:1960	OB5922 H2 Ro.raw	9.7778E5	1	1	357	380		Y23:Dihydroxy:0.00	PEAKS PTM
K.TDSRPSIAN(+.98)LAGEN(+.98)SIIDNLPEEVVANSYR.L	Y	35.41	3245.5686	30	4.2	1082.8680	3	47.69	4	F4:2306	OB5922 H2 Ro.raw	0	0	0	450	479		N9:Deamidation (NQ):12.20;N14:Deamidation (NQ):10.19	PEAKS DB
total 46 peptides																			

Q0GM57|Q0GM57_ARAHY

back to list

| Protein Coverage | Supporting Peptides |

Protein Coverage:

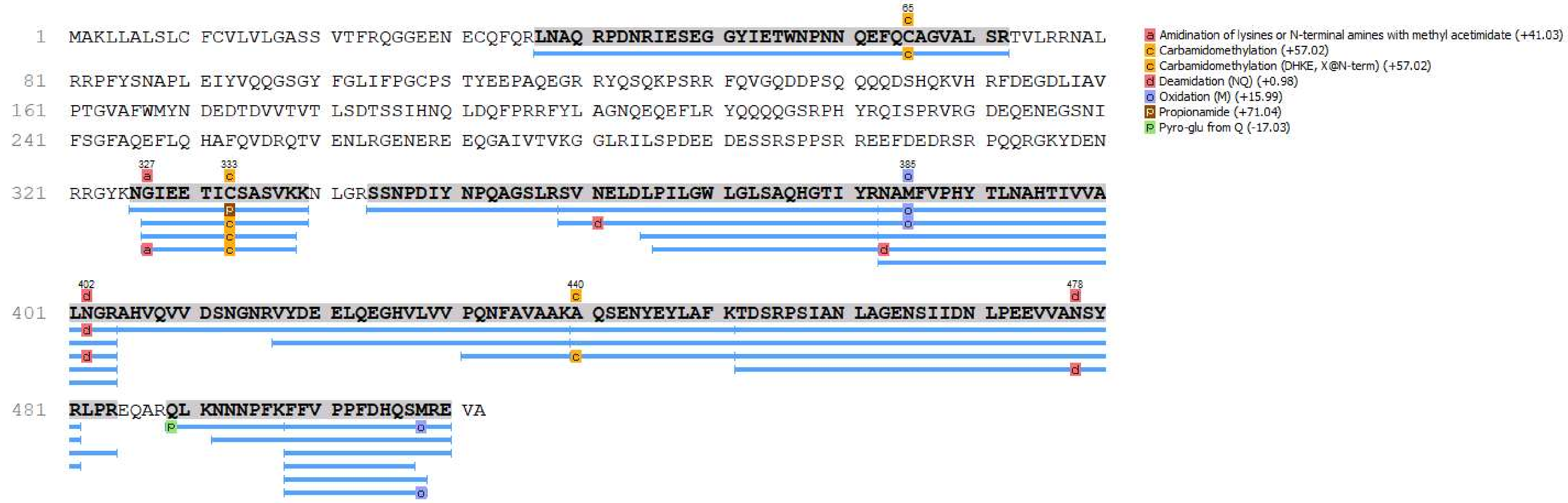

Supporting Peptides:

Peptide	Uniq	-10lgP	Mass	Length	ppm	m/z	z	RT	Fraction	Scan	Source File	Area Roast Ara h 2	#Feature	#Feature Roast Ara h 2	Start	End	PTM	AScore	Found By
total 46 peptides																			

Peptide	Uniq	-10lgP	Mass	Length	ppm	m/z	z	RT	Fraction	Scan	Source File	Area Roast Ara h 2	#Feature	#Feature Roast Ara h 2	Start	End	PTM	AScore	Found By
R.NAMFVPHYTLNAHTIVVALN(+.98)GR.A	Y	160.37	2438.2529	22	3.0	1220.1375	2	33.96	5	F5:1643	OB5945 H2 Ro.raw	9.2221E6	5	5	383	404	Deamidation (NQ)	N20:Deamidation (NQ):180.29	PEAKS DB
R.SVNELDLPILGWLGLSAQHGTIYR.N	Y	158.35	2651.4070	24	3.8	1326.7158	2	39.91	6	F6:1991	OB5946 H2 Ro.raw	6.2356E7	15	15	359	382			PEAKS DB
K.TDSRPSIANLAGENSIIDNLPEEVVANSYR.L	Y	150.05	3243.6006	30	5.0	1082.2129	3	36.29	6	F6:1782	OB5946 H2 Ro.raw	1.2988E7	7	7	452	481			PEAKS DB
R.NAMFVPHYTLNAHTIVVALNGR.A	Y	142.76	2437.2688	22	3.5	1219.6460	2	34.54	5	F5:1687	OB5945 H2 Ro.raw	7.7824E6	7	7	383	404			PEAKS DB
K.AQSENYEYLAFK.T	Y	135.05	1461.6776	12	0.6	731.8465	2	31.92	4	F4:1369	OB5922 H2 Ro.raw	6.5522E6	3	3	440	451			PEAKS DB
R.S(+57.02)VNELDLPILGWLGLSAQHGTIYR.N	Y	132.85	2708.4285	24	-0.8	1355.2205	2	40.55	4	F4:1898	OB5922 H2 Ro.raw	4.7421E5	1	1	359	382		S1:Carbamidomethylation (DHKE, X@N-term): 0.00	PEAKS PTM
R.SSNPDIYNPQAGSLR.S	Y	129.02	1617.7747	15	0.7	809.8952	2	29.12	4	F4:1205	OB5922 H2 Ro.raw	1.2321E6	3	3	344	358			PEAKS DB
R.NAM(+15.99)FVPHYTLNAHTIVVALNGR.A	Y	126.44	2453.2637	22	1.4	818.7629	3	34.16	4	F4:1519	OB5922 H2 Ro.raw	2.32E6	3	3	383	404	Oxidation (M)	M3:Oxidation (M):1000.00	PEAKS DB
L.PILGWLGLSAQHGTIYR.N	Y	124.94	1881.0261	17	0.7	941.5210	2	40.57	4	F4:1889	OB5922 H2 Ro.raw	2.9616E5	2	2	366	382			PEAKS DB
R.AHVQVVDSNGN(+.98)RVYDEELQEGHVLVVPQNFAVAAK.A	Y	113.48	3831.9180	35	4.0	1278.3184	3	32.50	6	F6:1562	OB5946 H2 Ro.raw	3.2728E6	4	4	405	439		N11:Deamidation (NQ):15.73	PEAKS DB
R.NAM(+15.99)FVPHYTLNAHTIVVALN(+.98)GR.A	Y	106.88	2454.2478	22	0.3	819.0901	3	32.82	5	F5:1580	OB5945 H2 Ro.raw	1.0956E6	4	4	383	404	Oxidation (M); Deamidation (NQ)	M3:Oxidation (M):1000.00;N20:Deamidation (NQ):104.25	PEAKS DB
R.AHVQVVDSN(+.98)GNRVYDEELQEGHVLVVPQNFAVAAK.A	Y	104.42	3831.9180	35	4.4	1278.3188	3	32.26	5	F5:1553	OB5945 H2 Ro.raw	5.1124E6	4	4	405	439		N9:Deamidation (NQ):39.44	PEAKS DB
R.SVNELDLPILGW(+31.99)LGLSAQHGTIYR.N	Y	103.31	2683.3970	24	2.4	1342.7090	2	39.79	4	F4:1852	OB5922 H2 Ro.raw	7.442E4	1	1	359	382		W12:Dihydroxy: 44.28	PEAKS PTM
K.TDSRPSIANLAGENSIIDNLPEEVVANSYRLPR.E	Y	99.31	3609.8386	33	3.4	903.4700	4	37.52	4	F4:1753	OB5922 H2 Ro.raw	2.5873E7	4	4	452	484			PEAKS DB
N.GIEETIC(+57.02)SASVK.K	Y	94.29	1292.6282	12	0.9	647.3220	2	29.26	6	F6:1364	OB5946 H2 Ro.raw	6.9435E4	3	3	327	338	Carbamidomethylation	C7:Carbamidomethylation:1000.00	PEAKS DB
K.FFVPPFDHQSMRE.V	Y	93.17	1635.7504	13	0.3	818.8827	2	32.86	4	F4:1440	OB5922 H2 Ro.raw	2.6869E6	5	5	498	510			PEAKS DB
K.A(+57.02)QSENYEYLAFK.T	Y	91.59	1518.6990	12	-0.7	760.3562	2	31.51	5	F5:1505	OB5945 H2 Ro.raw	1.9478E5	3	3	440	451	Carbamidomethylation (DHKE, X@N-term)	A1:Carbamidomethylation (DHKE, X@N-term):56.34	PEAKS PTM
K.TDSRPSIANLAGENSIIDNLPEEVVAN(+.98)SYR.L	Y	82.09	3244.5847	30	1.7	1082.5374	3	35.58	5	F5:1746	OB5945 H2 Ro.raw	0	0	0	452	481	Deamidation (NQ)	N27:Deamidation (NQ):78.75	PEAKS DB

total 46 peptides

Peptide	Uniq	-10lgP	Mass	Length	ppm	m/z	z	RT	Fraction	Scan	Source File	Area Roast Ara h 2	#Feature	#Feature Roast Ara h 2	Start	End	PTM	AScore	Found By
R.NAM(+15.99)FVPHYTLN(+.98)AHTIVVALNGR.A	Y	76.23	2454.2478	22	1.2	819.0908	3	33.30	5	F5:1613	OB5945 H2 Ro.raw	0	0	0	383	404	Oxidation (M)	M3:Oxidation (M):1000.00;N11:Deamidation (NQ):0.00	PEAKS DB
R.VYDEELQEGHVLVVPQNFAVAAK.A	Y	75.46	2554.3066	23	0.5	852.4432	3	33.26	6	F6:1608	OB5946 H2 Ro.raw	2.0809E4	1	1	417	439			PEAKS DB
R.LNAQRPDNRIESEGGYIETWNPNNQEFQC(+57.02)AGVALSR.T	Y	73.52	4132.9409	36	2.1	1034.2446	4	32.69	6	F6:1579	OB5946 H2 Ro.raw	1.2843E5	1	1	37	72	Carbamidomethylation	C29:Carbamidomethylation:1000.00	PEAKS DB
N.GIEETIC(+57.02)SASVKK.N	Y	72.67	1420.7231	13	1.3	711.3698	2	28.13	4	F4:1149	OB5922 H2 Ro.raw	1.93E5	3	3	327	339	Carbamidomethylation	C7:Carbamidomethylation:1000.00	PEAKS DB
K.FFVPPFDHQSM(+15.99)RE.V	Y	67.96	1651.7452	13	1.7	826.8813	2	32.49	4	F4:1425	OB5922 H2 Ro.raw	7.5314E5	4	4	498	510	Oxidation (M)	M11:Oxidation (M):1000.00	PEAKS DB
R.Q(-17.03)LKNNNPFK.F	N	67.76	1084.5665	9	1.6	543.2914	2	28.75	5	F5:1346	OB5945 H2 Ro.raw	1.0606E4	2	2	489	497	Pyro-glu from Q	Q1:Pyro-glu from Q:1000.00	PEAKS PTM
R.AHVQVVDSN(+.98)GN(+.98)RVYDEELQEGHVLVVPQNFAVAAK.A	Y	66.65	3832.9019	35	7.9	767.5937	5	32.26	5	F5:1556	OB5945 H2 Ro.raw	3.9346E5	1	1	405	439		N9:Deamidation (NQ):5.83;N11:Deamidation (NQ):8.28	PEAKS DB
R.N(+.98)AMFVPHYTLNAHTIVVALNGR.A	Y	66.31	2438.2529	22	3.0	1220.1375	2	33.96	5	F5:1680	OB5945 H2 Ro.raw	9.5447E5	1	1	383	404	Deamidation (NQ)	N1:Deamidation (NQ):63.03	PEAKS DB
V.PQNFAVAAK.A	N	63.04	944.5079	9	0.9	473.2617	2	32.86	4	F4:1447	OB5922 H2 Ro.raw	2.0461E6	3	3	431	439			PEAKS DB
R.NAMFVPHYTLNAH(+57.02)TIVVALNGR.A	Y	62.29	2494.2903	22	-3.1	832.4348	3	34.84	5	F5:1702	OB5945 H2 Ro.raw	0	0	0	383	404		H13:Carbamidomethylation (DHKE, X@N-term):5.30	PEAKS PTM
K.FFVPPFDHQSM.R	Y	62.13	1350.6067	11	0.3	676.3108	2	34.60	6	F6:1680	OB5946 H2 Ro.raw	2.36E5	3	3	498	508			PEAKS DB
K.AQSENYEYLAFKTDSRPSIANLAGENSIIDNLPEEVVANSYR.L	Y	61.90	4687.2676	42	3.1	1172.8278	4	36.79	5	F5:1810	OB5945 H2 Ro.raw	2.0852E6	1	1	440	481			PEAKS DB
D.LPILGWLGLSAQHGTIYR.N	Y	60.41	1994.1101	18	2.4	998.0647	2	40.55	4	F4:1901	OB5922 H2 Ro.raw	1.1908E5	1	1	365	382			PEAKS DB
K.FFVPPFDHQS.M	Y	57.76	1219.5662	10	1.5	610.7913	2	33.58	5	F5:1616	OB5945 H2 Ro.raw	2.2184E5	2	2	498	507			PEAKS DB
R.SVN(+.98)ELDLPILGWLGLSAQHGTIYR.N	Y	54.93	2652.3911	24	9.1	1327.2150	2	46.79	4	F4:2254	OB5922 H2 Ro.raw	0	0	0	359	382	Deamidation (NQ)	N3:Deamidation (NQ):57.31	PEAKS DB
K.NNNPFKFFVPPFDHQSMRE.V	Y	53.67	2350.0952	19	1.4	784.3734	3	34.54	5	F5:1693	OB5945 H2 Ro.raw	4.2082E5	2	2	492	510			PEAKS DB
N.G(+57.02)IEETIC(+57.02)SASVKK.N	Y	52.58	1477.7446	13	-0.5	739.8792	2	28.58	4	F4:1182	OB5922 H2 Ro.raw	1.8561E4	2	2	327	339	Carbamidomethylation	G1:Carbamidomethylation (DHKE, X@N-term):22.85;C7:Carbamidomethylation:1000.00	PEAKS PTM
R.AHVQ(+.98)VVDSNGNRVYDEELQEGHVLVVPQNFAVAAK.A	Y	49.71	3831.9180	35	2.7	767.3929	5	32.86	4	F4:1451	OB5922 H2 Ro.raw	3.9416E5	1	1	405	439		Q4:Deamidation (NQ):0.00	PEAKS DB

total 46 peptides

Peptide	Uniq	-10lgP	Mass	Length	ppm	m/z	z	RT	Fraction	Scan	Source File	Area Roast Ara h 2	#Feature	#Feature Roast Ara h 2	Start	End	PTM	AScore	Found By
N.G(+41.03)IEETIC(+57.02)SASVK.K	Y	48.35	1333.6548	12	0.4	667.8350	2	29.26	6	F6:1372	OB5946 H2 Ro.raw	8.2083E3	1	1	327	338	Amidination of lysines or N-terminal amines with methyl acetimidate; Carbamidomethylation	G1:Amidination of lysines or N-terminal amines with methyl acetimidate:71.87; C7:Carbamidomethylation:1000.00	PEAKS PTM
R.NAMFVPHYTLNAH(+57.02)TIVVALN(+.98)GR.A	Y	47.39	2495.2744	22	0.0	832.7654	3	33.96	5	F5:1660	OB5945 H2 Ro.raw	1.2865E5	1	1	383	404		H13:Carbamidomethylation (DHKE, X@N-term):0.00;N20:Deamidation (NQ):43.52	PEAKS PTM
K.FFVPPFDHQSM(+15.99).R	Y	45.84	1366.6016	11	1.6	684.3091	2	33.79	4	F4:1494	OB5922 H2 Ro.raw	5.6927E4	1	1	498	508	Oxidation (M)	M11:Oxidation (M):1000.00	PEAKS DB
K.TDSRPSIANLAGENSIIDNLPEEVVAN(+.98)SYRLPR.E	Y	45.48	3610.8225	33	5.4	1204.6213	3	35.38	5	F5:1734	OB5945 H2 Ro.raw	0	0	0	452	484		N27:Deamidation (NQ):32.94	PEAKS DB
R.S(+57.02)SNPDIYNPQAGSLR.S	Y	39.95	1674.7961	15	7.9	838.4119	2	29.48	4	F4:1242	OB5922 H2 Ro.raw	0	0	0	344	358		S1:Carbamidomethylation (DHKE, X@N-term):11.12	PEAKS PTM
R.SSNPD(+21.98)IYNPQAGSLR.S	Y	39.67	1639.7566	15	4.2	820.8890	2	29.21	4	F4:1226	OB5922 H2 Ro.raw	0	0	0	344	358		D5:Sodium adduct:43.70	PEAKS PTM
K.AQSENYE(+21.98)YLAFK.T	Y	37.74	1483.6595	12	-1.5	742.8359	2	31.58	6	F6:1513	OB5946 H2 Ro.raw	8.2984E3	1	1	440	451		E7:Sodium adduct:0.00	PEAKS PTM
K.NGIEETIC(+71.04)SASVKK.N	Y	36.28	1548.7817	14	3.3	775.4007	2	29.48	5	F5:1385	OB5945 H2 Ro.raw	2.1762E3	1	1	326	339	Propionamide	C8:Propionamide:1000.00	PEAKS PTM
R.SVNELDLPILGWLGLSAQHGTIY(+31.99)R.N	Y	36.02	2683.3970	24	3.4	895.4760	3	39.79	4	F4:1960	OB5922 H2 Ro.raw	9.7778E5	1	1	359	382		Y23:Dihydroxy:0.00	PEAKS PTM
K.TDSRPSIAN(+.98)LAGEN(+.98)SIIDNLPEEVVANSYR.L	Y	35.41	3245.5686	30	4.2	1082.8680	3	47.69	4	F4:2306	OB5922 H2 Ro.raw	0	0	0	452	481		N9:Deamidation (NQ):12.20;N14:Deamidation (NQ):10.19	PEAKS DB
**total 46 peptides**																			

Q9FZ11|Q9FZ11_ARAHY

back to list

| Protein Coverage | Supporting Peptides |

Protein Coverage:

1 MIRGRLALSV CFCFLVLGAS SISFRQQPEE NACQFQRLNA QRPDNRLESE GGYIETWNPN NQEFECAGVA LSRLVLRRNA
81 LRRPFYSNAP QEIFIQQGRG YFGLIFPGCP STYEEPAQQG RRHQSQRAPR RFEGEDQSQQ QQDSHQKVR RFDEGDLIAV
161 PTGVALWMFN DHDTDVVAVS LTDTNNNDNQ LDQFPRRFNL AGNHEQEFLR YQQQSRRRSL PYSPYSPQSQ PRQEEREFSP
241 RGQHSRRERA GQEEENEGGN IFSGFTPEFL AQAFQVDDRQ IVQNLRGENE SEEEGAIVTV KGGLRILSPD RKRGADEEEE
321 YDEDEYEYDE EDRRRGRGSR GRGNGIEETI CTASVKKNIG RNRSPDIYNP QAGSLKTAND LNLLILRWLG LSAEYGNLYR
401 NALFVPHYNT NAHSIIYALR GRAHVQVVDS NGNRVYDEEL QEGHVLVVPQ NFAVAGKSQS DNFEYVAFKT DSRPNIANFA
481 GENSIIDNLP EEVVANSYGL PREQARQLKN NNPFKFFVPP SQQSLRAVA

a Amidination of lysines or N-terminal amines with methyl acetimidate (+41.03)
c Carbamidomethylation (+57.02)
c Carbamidomethylation (DHKE, X@N-term) (+57.02)
d Deamidation (NQ) (+0.98)
d Dihydroxy (+31.99)
p Pyro-glu from Q (-17.03)
r Replacement of 2 protons by iron (+53.92)

Supporting Peptides:

Peptide	Uniq	-10lgP	Mass	Length	ppm	m/z	z	RT	Fraction	Scan	Source File	Area Roast Ara h 2	#Feature	#Feature Roast Ara h 2	Start	End	PTM	AScore	Found By
R.NALFVPHYNTNAHSIIYALR.G	N	151.62	2313.2019	20	3.7	1157.6125	2	33.79	4	F4:1486	OB5922 H2 Ro.raw	4.79E7	11	11	401	420			PEAKS DB
R.SPDIYNPQAGSLKTANDLNLLILR.W	N	150.50	2625.4126	24	-0.1	876.1447	3	35.61	4	F4:1607	OB5922 H2 Ro.raw	1.276E6	3	3	364	387			PEAKS DB
R.WLGLSAEYGNLYR.N	N	145.22	1540.7673	13	3.5	771.3937	2	36.40	4	F4:1654	OB5922 H2 Ro.raw	5.6282E7	22	22	388	400			PEAKS DB
K.SQSDNFEYVAFK.T	N	136.19	1433.6462	12	2.6	717.8323	2	31.76	6	F6:1518	OB5946 H2 Ro.raw	5.1214E6	3	3	458	469			PEAKS DB
R.NRSPDIYNPQAGSLKTANDLNLLILR.W	N	129.49	2895.5566	26	1.6	966.1943	3	34.35	4	F4:1530	OB5922 H2 Ro.raw	2.2791E6	6	6	362	387			PEAKS DB
K.FFVPPSQQSLRA.V	Y	119.32	1375.7247	12	2.6	688.8715	2	30.85	6	F6:1464	OB5946 H2 Ro.raw	1.4487E6	3	3	516	527			PEAKS DB
K.FFVPPSQQSLR.A	Y	110.32	1304.6876	11	0.7	653.3516	2	30.28	6	F6:1421	OB5946 H2 Ro.raw	4.7622E5	3	3	516	526			PEAKS DB
R.VYDEELQEGHVLVVPQNFAVAGK.S	N	102.31	2540.2910	23	1.4	847.7721	3	33.42	4	F4:1478	OB5922 H2 Ro.raw	1.4453E6	6	6	435	457			PEAKS DB
R.SPDIYNPQAGSLK.T	N	95.77	1388.6936	13	1.7	695.3552	2	28.58	5	F5:1323	OB5945 H2 Ro.raw	5.949E5	3	3	364	376			PEAKS DB
N.GIEETIC(+57.02)TASVK.K	N	94.89	1306.6438	12	0.9	654.3298	2	29.08	6	F6:1349	OB5946 H2 Ro.raw	1.2427E5	3	3	345	356	Carbamidomethylation	C7:Carbamidomethylation:1000.00	PEAKS DB

total 42 peptides

Peptide	Uniq	-10lgP	Mass	Length	ppm	m/z	z	RT	Fraction	Scan	Source File	Area Roast Ara h 2	#Feature	#Feature Roast Ara h 2	Start	End	PTM	AScore	Found By
R.NALFVPH(+14.02)YNTNAHSIIYALR.G	N	93.74	2327.2175	20	0.7	776.7470	3	34.91	4	F4:1561	OB5922 H2 Ro.raw	1.3103E6	3	3	401	420		H7:Methylation(others):22.45	PEAKS PTM
R.NRSPDIYNPQAGSLK.T	N	91.67	1658.8376	15	0.8	830.4268	2	28.33	6	F6:1298	OB5946 H2 Ro.raw	4.0221E5	6	6	362	376			PEAKS DB
R.AHVQVVDSNGNRVYDEELQEGHVLVVPQNFAVAGK.S	N	90.86	3816.9182	35	3.6	955.2402	4	32.32	6	F6:1544	OB5946 H2 Ro.raw	8.2893E6	8	8	423	457			PEAKS DB
R.QLKNNNPFKFFVPPSQQSLR.A	Y	86.55	2388.2703	20	2.2	797.0991	3	33.23	4	F4:1468	OB5922 H2 Ro.raw	1.3469E5	1	1	507	526			PEAKS DB
K.TANDLNLLILR.W	N	85.96	1254.7296	11	1.8	628.3732	2	34.79	6	F6:1750	OB5946 H2 Ro.raw	9.2784E6	3	3	377	387			PEAKS DB
V.PQNFAVAGK.S	N	85.45	930.4923	9	1.0	466.2539	2	32.32	6	F6:1542	OB5946 H2 Ro.raw	3.3444E6	3	3	449	457			PEAKS DB
N.GIEETIC(+57.02)TASVKK.N	N	84.54	1434.7388	13	0.2	718.3768	2	28.07	4	F4:1177	OB5922 H2 Ro.raw	2.0536E5	4	4	345	357	Carbamidomethylation	C7:Carbamidomethylation:1000.00	PEAKS DB
R.W(+31.99)LGLSAEYGNLYR.N	N	77.74	1572.7572	13	-1.6	787.3846	2	33.64	6	F6:1624	OB5946 H2 Ro.raw	8.5976E4	2	2	388	400	Dihydroxy	W1:Dihydroxy:62.93	PEAKS PTM
R.S(+57.02)PDIYNPQAGSLKTANDLNLLILR.W	N	75.36	2682.4341	24	1.5	895.1533	3	35.28	5	F5:1735	OB5945 H2 Ro.raw	9.5441E4	1	1	364	387		S1:Carbamidomethylation (DHKE, X@N-term):14.04	PEAKS PTM
R.AHVQVVDSN(+.98)GNRVYDEELQEGHVLVVPQNFAVAGK.S	N	75.25	3817.9023	35	5.0	955.4877	4	33.04	4	F4:1438	OB5922 H2 Ro.raw	1.3721E6	1	1	423	457		N9:Deamidation (NQ):4.64	PEAKS DB
K.FFVPPSQQ(+.98)SLR.A	Y	69.30	1305.6716	11	0.6	653.8435	2	31.03	6	F6:1505	OB5946 H2 Ro.raw	1.7874E5	1	1	516	526		Q8:Deamidation (NQ):12.79	PEAKS DB
R.AHVQVVDSNGN(+.98)RVYDEELQEGHVLVVPQNFAVAGK.S	N	68.98	3817.9023	35	3.2	955.4859	4	32.51	5	F5:1567	OB5945 H2 Ro.raw	0	0	0	423	457		N11:Deamidation (NQ):8.22	PEAKS DB
R.Q(-17.03)LKNNNPFK.F	N	67.76	1084.5665	9	1.6	543.2914	2	28.75	5	F5:1346	OB5945 H2 Ro.raw	1.0606E4	2	2	507	515	Pyro-glu from Q	Q1:Pyro-glu from Q:1000.00	PEAKS PTM
R.W(+57.02)LGLSAEYGNLYR.N	N	65.45	1597.7888	13	0.6	799.9022	2	35.09	5	F5:1725	OB5945 H2 Ro.raw	1.848E4	1	1	388	400	Carbamidomethylation (DHKE, X@N-term)	W1:Carbamidomethylation (DHKE, X@N-term):62.19	PEAKS PTM
R.WLGLSAE(+53.92)YGNLYR.N	N	65.30	1594.6866	13	1.3	532.5702	3	34.72	5	F5:1698	OB5945 H2 Ro.raw	6.0447E5	3	3	388	400	Replacement of 2 protons by iron	E7:Replacement of 2 protons by iron:123.05	PEAKS PTM
R.S(+57.02)PDIYNPQAGSLK.T	N	64.83	1445.7150	13	5.7	723.8689	2	28.94	5	F5:1350	OB5945 H2 Ro.raw	6.4489E3	2	2	364	376		S1:Carbamidomethylation (DHKE, X@N-term):14.04	PEAKS PTM
N.G(+41.03)IEETIC(+57.02)TASVK.K	N	63.11	1347.6704	12	0.3	674.8427	2	29.12	5	F5:1370	OB5945 H2 Ro.raw	1.5496E4	1	1	345	356	Amidination of lysines or N-terminal amines with methyl acetimidate; Carbamidomethylation	G1:Amidination of lysines or N-terminal amines with methyl acetimidate:96.48;C7:Carbamidomethylation:1000.00	PEAKS PTM
R.NALFVPHYNTNAHSIIYALRG.R	N	61.88	2370.2231	21	1.2	791.0826	3	33.77	5	F5:1624	OB5945 H2 Ro.raw	4.2894E6	2	2	401	421			PEAKS DB

total 42 peptides

Peptide	Uniq	-10lgP	Mass	Length	ppm	m/z	z	RT	Fraction	Scan	Source File	Area Roast Ara h 2	#Feature	#Feature Roast Ara h 2	Start	End	PTM	AScore	Found By
R.N(+57.02)ALFVPHYNTNAHSIIYALR.G	N	59.27	2370.2231	20	-0.1	791.0816	3	34.53	4	F4:1557	OB5922 H2 Ro.raw	3.2966E6	1	1	401	420		N1:Carbamidomethylation (DHKE, X@ N-term):10.82	PEAKS PTM
R.SPD(+57.02)IYNPQAGSLK.T	N	55.35	1445.7150	13	2.2	723.8663	2	29.51	4	F4:1244	OB5922 H2 Ro.raw	0	0	0	364	376		D3:Carbamidomethylation (DHKE, X@ N-term):0.00	PEAKS PTM
R.AHVQVVDSNGNRVYDEELQ(+.98)EGHVLVVPQNFAVAGK.S	N	53.98	3817.9023	35	7.9	955.4904	4	33.43	6	F6:1615	OB5946 H2 Ro.raw	0	0	0	423	457		Q19:Deamidation (NQ):4.91	PEAKS DB
K.SQSD(+53.92)NFEYVAFK.T	N	53.30	1487.5656	12	-2.8	744.7880	2	32.03	4	F4:1395	OB5922 H2 Ro.raw	0	0	0	458	469		D4:Replacement of 2 protons by iron:20.50	PEAKS PTM
K.NNNPFKFFVPPSQQS.L	N	52.69	1749.8474	15	1.0	875.9319	2	34.91	4	F4:1573	OB5922 H2 Ro.raw	4.7342E4	1	1	510	524			PEAKS DB
R.QLKNNNPFKFFVPPSQQS.L	N	51.09	2119.0850	18	1.9	707.3703	3	33.27	6	F6:1604	OB5946 H2 Ro.raw	6.7737E4	1	1	507	524			PEAKS DB
K.TAND(+53.92)LNLLILR.W	N	50.78	1308.6488	11	0.2	655.3318	2	35.09	4	F4:1584	OB5922 H2 Ro.raw	8.7715E4	1	1	377	387	Replacement of 2 protons by iron	D4:Replacement of 2 protons by iron:55.73	PEAKS PTM
R.LNAQRPDNRLESEGGYIETWNPNNQEFEC(+57.02)AGVALSR.L	N	50.20	4133.9248	36	7.4	1034.4961	4	33.23	4	F4:1476	OB5922 H2 Ro.raw	5.4856E4	1	1	38	73	Carbamidomethylation	C29:Carbamidomethylation:1000.00	PEAKS DB
R.NALFVPH(+57.02)YNTNAHSIIYALR.G	N	49.15	2370.2231	20	0.1	791.0817	3	34.03	6	F6:1601	OB5946 H2 Ro.raw	8.9747E5	1	1	401	420		H7:Carbamidomethylation (DHKE, X@ N-term):20.35	PEAKS PTM
R.WLGLSAEYGN(+.98)LYR.N	N	47.40	1541.7513	13	5.4	771.8871	2	54.24	4	F4:2679	OB5922 H2 Ro.raw	0	0	0	388	400	Deamidation (NQ)	N10:Deamidation (NQ):1000.00	PEAKS DB
R.NALFVPHYNTNAHSIIYALRGR.A	N	46.11	2526.3245	22	0.6	843.1160	3	33.60	4	F4:1492	OB5922 H2 Ro.raw	7.6168E5	2	2	401	422			PEAKS DB
N.G(+57.02)IEETIC(+57.02)TASVKK.N	N	41.72	1491.7603	13	2.0	746.8889	2	28.52	4	F4:1178	OB5922 H2 Ro.raw	9.4457E3	1	1	345	357	Carbamidomethylation	G1:Carbamidomethylation (DHKE, X@ N-term):12.81;C7:Carbamidomethylation:1000.00	PEAKS PTM
R.N(+.98)ALFVPHYNTNAHSIIYALR.G	N	38.37	2314.1858	20	7.3	772.4082	3	50.66	4	F4:2450	OB5922 H2 Ro.raw	1.0573E4	1	1	401	420		N1:Deamidation (NQ):32.94	PEAKS DB
R.WLGLSAE(+21.98)YGNLYR.N	N	36.57	1562.7494	13	-8.4	782.3754	2	35.27	4	F4:1583	OB5922 H2 Ro.raw	5.0047E4	2	2	388	400		E7:Sodium adduct:42.78	PEAKS PTM
**total 42 peptides**																			

Q647H3|Q647H3_ARAHY

back to list

| Protein Coverage | Supporting Peptides |

Protein Coverage:

```
  1 MAKLLALSVC FCFLVLGASS ISFRQQPEEN ACQFQRLNAQ RPDNRLESEG GYIETWNPNN QEFECAGVAL SRLVLRRNAL
 81 RRPFYSNAPQ EIFIQQGRGY FGLIFPGCPS TYEEPAQQGR RHQSQRAPRR FEGEDQSQQQ QQDSHQKVRR FDEGDLIAVP
161 TGVALWMYND HDTDVVAVSL TDTNNNDNQL DQFPRRFNLA GNHEQEFLRY QQQSRRRSLP YSPYSPQSQP RQEEREFSPR
241 GQHSRRERAG QEQENEGGNI FSGFTPEFLA QAFQVDDRQI LQNLRGENES DEQGAIVTVR GGLRILSPDR KRRQQYERPD
321 EEEEYDEDEY EYDEEERQQD RRRGRGSRGR GNGIEETICT ASVKKNIGRN RSPDIYNPQA GSLKTANDLN LLILRWLGLS
401 AEYGNLYRNA LFVPHYNTNA HSIIYALRGR AHVQVVDSNG NRVYDEELQE GHVLVVPQNF AVAGKSQSDN FEYVAFKTDS
481 RPSIANLAGE NSIIDNLPEE VVANSYGLPR EQARQLKNNN PFKFFVPPSQ QSLGAVA
```

a Amidination of lysines or N-terminal amines with methyl acetimidate (+41.03)
c Carbamidomethylation (+57.02)
c Carbamidomethylation (DHKE, X@N-term) (+57.02)
d Deamidation (NQ) (+0.98)
d Dihydroxy (+31.99)
p Pyro-glu from Q (-17.03)
r Replacement of 2 protons by iron (+53.92)

Supporting Peptides:

Peptide	Uniq	-10lgP	Mass	Length	ppm	m/z	z	RT	Fraction	Scan	Source File	Area Roast Ara h 2	#Feature	#Feature Roast Ara h 2	Start	End	PTM	AScore	Found By
R.NALFVPHYNTNAHSIIYALR.G	N	151.62	2313.2019	20	3.7	1157.6125	2	33.79	4	F4:1486	OB5922 H2 Ro.raw	4.79E7	11	11	409	428			PEAKS DB
R.SPDIYNPQAGSLKTANDLNLLILR.W	N	150.50	2625.4126	24	-0.1	876.1447	3	35.61	4	F4:1607	OB5922 H2 Ro.raw	1.276E6	3	3	372	395			PEAKS DB
R.WLGLSAEYGNLYR.N	N	145.22	1540.7673	13	3.5	771.3937	2	36.40	4	F4:1654	OB5922 H2 Ro.raw	5.6282E7	22	22	396	408			PEAKS DB
K.TDSRPSIANLAGENSIIDNLPEEVVANSYGLPR.E	Y	143.06	3510.7590	33	5.7	1171.2670	3	37.70	4	F4:1764	OB5922 H2 Ro.raw	6.2773E7	10	10	478	510			PEAKS DB
K.SQSDNFEYVAFK.T	N	136.19	1433.6462	12	2.6	717.8323	2	31.76	6	F6:1518	OB5946 H2 Ro.raw	5.1214E6	3	3	466	477			PEAKS DB
R.NRSPDIYNPQAGSLKTANDLNLLILR.W	N	129.49	2895.5566	26	1.6	966.1943	3	34.35	4	F4:1530	OB5922 H2 Ro.raw	2.2791E6	6	6	370	395			PEAKS DB
R.VYDEELQEGHVLVVPQNFAVAGK.S	N	102.31	2540.2910	23	1.4	847.7721	3	33.42	4	F4:1478	OB5922 H2 Ro.raw	1.4453E6	6	6	443	465			PEAKS DB
R.SPDIYNPQAGSLK.T	N	95.77	1388.6936	13	1.7	695.3552	2	28.58	5	F5:1323	OB5945 H2 Ro.raw	5.949E5	3	3	372	384			PEAKS DB
N.GIEETIC(+57.02)TASVK.K	N	94.89	1306.6438	12	0.9	654.3298	2	29.08	6	F6:1349	OB5946 H2 Ro.raw	1.2427E5	3	3	353	364	Carbamidomethylation	C7:Carbamidomethylation:1000.00	PEAKS DB

total 44 peptides

Peptide	Uniq	-10lgP	Mass	Length	ppm	m/z	z	RT	Fraction	Scan	Source File	Area Roast Ara h 2	#Feature	#Feature Roast Ara h 2	Start	End	PTM	AScore	Found By
R.NALFVPH(+14.02)YNTNAHSIIYALR.G	N	93.74	2327.2175	20	0.7	776.7470	3	34.91	4	F4:1561	OB5922 H2 Ro.raw	1.3103E6	3	3	409	428		H7:Methylation(others):22.45	PEAKS PTM
R.NRSPDIYNPQAGSLK.T	N	91.67	1658.8376	15	0.8	830.4268	2	28.33	6	F6:1298	OB5946 H2 Ro.raw	4.0221E5	6	6	370	384			PEAKS DB
R.AHVQVVDSNGNRVYDEELQEGHVLVVPQNFAVAGK.S	N	90.86	3816.9182	35	3.6	955.2402	4	32.32	6	F6:1544	OB5946 H2 Ro.raw	8.2893E6	8	8	431	465			PEAKS DB
K.TANDLNLLILR.W	N	85.96	1254.7296	11	1.8	628.3732	2	34.79	6	F6:1750	OB5946 H2 Ro.raw	9.2784E6	3	3	385	395			PEAKS DB
V.PQNFAVAGK.S	N	85.45	930.4923	9	1.0	466.2539	2	32.32	6	F6:1542	OB5946 H2 Ro.raw	3.3444E6	3	3	457	465			PEAKS DB
N.GIEETIC(+57.02)TASVKK.N	N	84.54	1434.7388	13	0.2	718.3768	2	28.07	4	F4:1177	OB5922 H2 Ro.raw	2.0536E5	4	4	353	365	Carbamidomethylation	C7:Carbamidomethylation:1000.00	PEAKS DB
R.W(+31.99)LGLSAEYGNLYR.N	N	77.74	1572.7572	13	-1.6	787.3846	2	33.64	6	F6:1624	OB5946 H2 Ro.raw	8.5976E4	2	2	396	408	Dihydroxy	W1:Dihydroxy:62.93	PEAKS PTM
R.S(+57.02)PDIYNPQAGSLKTANDLNLLILR.W	N	75.36	2682.4341	24	1.5	895.1533	3	35.28	5	F5:1735	OB5945 H2 Ro.raw	9.5441E4	1	1	372	395		S1:Carbamidomethylation (DHKE, X @N-term):14.04	PEAKS PTM
R.AHVQVVDSN(+.98)GNRVYDEELQEGHVLVVPQNFAVAGK.S	N	75.25	3817.9023	35	5.0	955.4877	4	33.04	4	F4:1438	OB5922 H2 Ro.raw	1.3721E6	1	1	431	465		N9:Deamidation (NQ):4.64	PEAKS DB
R.AHVQVVDSNGN(+.98)RVYDEELQEGHVLVVPQNFAVAGK.S	N	68.98	3817.9023	35	3.2	955.4859	4	32.51	5	F5:1567	OB5945 H2 Ro.raw	0	0	0	431	465		N11:Deamidation (NQ):8.22	PEAKS DB
R.Q(-17.03)LKNNNPFK.F	N	67.76	1084.5665	9	1.6	543.2914	2	28.75	5	F5:1346	OB5945 H2 Ro.raw	1.0606E4	2	2	515	523	Pyro-glu from Q	Q1:Pyro-glu from Q:1000.00	PEAKS PTM
R.W(+57.02)LGLSAEYGNLYR.N	N	65.45	1597.7888	13	0.6	799.9022	2	35.09	5	F5:1725	OB5945 H2 Ro.raw	1.848E4	1	1	396	408	Carbamidomethylation (DHKE, X@N-term)	W1:Carbamidomethylation (DHKE, X @N-term):62.19	PEAKS PTM
R.WLGLSAE(+53.92)YGNLYR.N	N	65.30	1594.6866	13	1.3	532.5702	3	34.72	5	F5:1698	OB5945 H2 Ro.raw	6.0447E5	3	3	396	408	Replacement of 2 protons by iron	E7:Replacement of 2 protons by iron: 123.05	PEAKS PTM
R.S(+57.02)PDIYNPQAGSLK.T	N	64.83	1445.7150	13	5.7	723.8689	2	28.94	5	F5:1350	OB5945 H2 Ro.raw	6.4489E3	2	2	372	384		S1:Carbamidomethylation (DHKE, X @N-term):14.04	PEAKS PTM
N.G(+41.03)IEETIC(+57.02)TASVK.K	N	63.11	1347.6704	12	0.3	674.8427	2	29.12	5	F5:1370	OB5945 H2 Ro.raw	1.5496E4	1	1	353	364	Amidination of lysines or N-terminal amines with methyl acetimidate; Carbamidomethylation	G1:Amidination of lysines or N-terminal amines with methyl acetimidate: 96.48;C7:Carbamidomethylation:1000.00	PEAKS PTM
R.NALFVPHYNTNAHSIIYALRG.R	N	61.88	2370.2231	21	1.2	791.0826	3	33.77	5	F5:1624	OB5945 H2 Ro.raw	4.2894E6	2	2	409	429			PEAKS DB
R.N(+57.02)ALFVPHYNTNAHSIIYALR.G	N	59.27	2370.2231	20	-0.1	791.0816	3	34.53	4	F4:1557	OB5922 H2 Ro.raw	3.2966E6	1	1	409	428		N1:Carbamidomethylation (DHKE, X @N-term):10.82	PEAKS PTM
R.SPD(+57.02)IYNPQAGSLK.T	N	55.35	1445.7150	13	2.2	723.8663	2	29.51	4	F4:1244	OB5922 H2 Ro.raw	0	0	0	372	384		D3:Carbamidomethylation (DHKE, X @N-term):0.00	PEAKS PTM

total 44 peptides

Peptide	Uniq	-10lgP	Mass	Length	ppm	m/z	z	RT	Fraction	Scan	Source File	Area Roast Ara h 2	#Feature	#Feature Roast Ara h 2	Start	End	PTM	AScore	Found By
R.AHVQVVDSNGNRVYDEELQ(+.98)EGHVLVVPQNFAVAGK.S	N	53.98	3817.9023	35	7.9	955.4904	4	33.43	6	F6:1615	OB5946 H2 Ro.raw	0	0	0	431	465		Q19:Deamidation (NQ):4.91	PEAKS DB
R.QLKNNNPFKFFVPPSQQSLGAV.A	Y	53.42	2459.2961	22	3.6	820.7756	3	33.50	4	F4:1470	OB5922 H2 Ro.raw	5.0433E5	1	1	515	536			PEAKS DB
K.SQSD(+53.92)NFEYVAFK.T	N	53.30	1487.5656	12	-2.8	744.7880	2	32.03	4	F4:1395	OB5922 H2 Ro.raw	0	0	0	466	477		D4:Replacement of 2 protons by iron:20.50	PEAKS PTM
K.NNNPFKFFVPPSQQS.L	N	52.69	1749.8474	15	1.0	875.9319	2	34.91	4	F4:1573	OB5922 H2 Ro.raw	4.7342E4	1	1	518	532			PEAKS DB
R.QLKNNNPFKFFVPPSQQS.L	N	51.09	2119.0850	18	1.9	707.3703	3	33.27	6	F6:1604	OB5946 H2 Ro.raw	6.7737E4	1	1	515	532			PEAKS DB
K.TAND(+53.92)LNLLILR.W	N	50.78	1308.6488	11	0.2	655.3318	2	35.09	4	F4:1584	OB5922 H2 Ro.raw	8.7715E4	1	1	385	395	Replacement of 2 protons by iron	D4:Replacement of 2 protons by iron:55.73	PEAKS PTM
R.LNAQRPDNRLESEGGYIETWNPNNQEFEC(+57.02)AGVALSR.L	N	50.20	4133.9248	36	7.4	1034.4961	4	33.23	4	F4:1476	OB5922 H2 Ro.raw	5.4856E4	1	1	37	72	Carbamidomethylation	C29:Carbamidomethylation:1000.00	PEAKS DB
R.NALFVPH(+57.02)YNTNAHSIIYALR.G	N	49.15	2370.2231	20	0.1	791.0817	3	34.03	6	F6:1601	OB5946 H2 Ro.raw	8.9747E5	1	1	409	428		H7:Carbamidomethylation (DHKE, X @N-term):20.35	PEAKS PTM
K.SQSDNFEYVAFKTDSRPSIANLAGENSIIDNLPEEVVANSYGLPR.E	Y	48.98	4926.3945	45	-0.7	1232.6051	4	37.16	5	F5:1856	OB5945 H2 Ro.raw	4.0502E6	2	2	466	510			PEAKS DB
R.WLGLSAEYGN(+.98)LYR.N	N	47.40	1541.7513	13	5.4	771.8871	2	54.24	4	F4:2679	OB5922 H2 Ro.raw	0	0	0	396	408	Deamidation (NQ)	N10:Deamidation (NQ):1000.00	PEAKS DB
R.NALFVPHYNTNAHSIIYALRGR.A	N	46.11	2526.3245	22	0.6	843.1160	3	33.60	4	F4:1492	OB5922 H2 Ro.raw	7.6168E5	2	2	409	430			PEAKS DB
K.TDSRPSIANLAGENSIIDNLPEEVVAN(+.98)SYGLPR.E	Y	42.62	3511.7429	33	5.1	1171.5942	3	46.13	5	F5:2348	OB5945 H2 Ro.raw	4.9547E4	2	2	478	510		N27:Deamidation (NQ):43.76	PEAKS DB
N.G(+57.02)IEETIC(+57.02)TASVKK.N	N	41.72	1491.7603	13	2.0	746.8889	2	28.52	4	F4:1178	OB5922 H2 Ro.raw	9.4457E3	1	1	353	365	Carbamidomethylation	G1:Carbamidomethylation (DHKE, X @N-term):12.81;C7:Carbamidomethylation:1000.00	PEAKS PTM
K.TDSRPSIANLAGEN(+.98)SIIDNLPEEVVANSYGLPR.E	Y	40.49	3511.7429	33	8.6	1171.5983	3	48.17	4	F4:2321	OB5922 H2 Ro.raw	2.3111E4	2	2	478	510		N14:Deamidation (NQ):5.79	PEAKS DB
R.N(+.98)ALFVPHYNTNAHSIIYALR.G	N	38.37	2314.1858	20	7.3	772.4082	3	50.66	4	F4:2450	OB5922 H2 Ro.raw	1.0573E4	1	1	409	428		N1:Deamidation (NQ):32.94	PEAKS DB
R.WLGLSAE(+21.98)YGNLYR.N	N	36.57	1562.7494	13	-8.4	782.3754	2	35.27	4	F4:1583	OB5922 H2 Ro.raw	5.0047E4	2	2	396	408		E7:Sodium adduct:42.78	PEAKS PTM
K.TDSRPSIANLAGENSIIDNLPEEVVANSYGLPREQAR.Q	Y	35.32	3994.9983	37	2.4	999.7593	4	35.84	5	F5:1758	OB5945 H2 Ro.raw	5.332E5	1	1	478	514			PEAKS DB
**total 44 peptides**																			

Q8LKN1|Q8LKN1_ARAHY

back to list

| Protein Coverage | Supporting Peptides |

Protein Coverage:

1 MGKLLALSVC FCFLVLGASS ISFRQQPEEN ACQFQRLNAQ RPDNRIESEG GYIETWNPNN QEFECAGVAL SRLVLRRNAL
81 RRPFYSNAPQ EIFIQQGRGY FGLIFPGCPS TYEEPAQQGR RHQSQRPPRR FQGQDQSQQQ QDSHQKVHRF DEGDLIAVPT
161 GVAFWMYNDH DTDVVAVSLT DTNNNDNQLD QFPRRFNLAG NHEQEFLRYQ QQSRRRSLPY SPYSPQTQPK QEDREFSPRG
241 QHGRRERAGQ EQENEGGNIF SGFTPEFLAQ AFQVDDRQIL QNLRGENESD EQGAIVTVRG GLRILSPDRK RRQQYERPDE
321 EEEYDEDEYE YDEEERQQDR RRGRGSRGSG NGIEETICTA SFKKNIGRNR SPDIYNPQAG SLKTANELQL NLLILRWLGL
401 SAEYGNLYRN ALFVPHYNTN AHSIIYALRG RAHVQVVDSN GDRVFDEELQ EGHVLVVPQN FAVAGKSQSE NFEYVAFKTD
481 SRPSIANLAG ENSFIDNLPE EVVANSYGLP REQARQLKNN NPFKFFVPPS EQSLRAVA

a Acetylation (N-term) (+42.01)
c Carbamidomethylation (+57.02)
c Carboxylation (E) (+43.99)
c Carbamidomethylation (DHKE, X@N-term) (+57.02)
d Deamidation (NQ) (+0.98)
d Dihydroxy (+31.99)
m Methylation(KR) (+14.02)
p Propionamide (+71.04)
p Pyro-glu from Q (-17.03)
r Replacement of 2 protons by iron (+53.92)

Supporting Peptides:

Peptide	Uniq	-10lgP	Mass	Length	ppm	m/z	z	RT	Fraction	Scan	Source File	Area Roast Ara h 2	#Feature	#Feature Roast Ara h 2	Start	End	PTM	AScore	Found By
R.NALFVPHYNTNAHSIIYALR.G	N	151.62	2313.2019	20	3.7	1157.6125	2	33.79	4	F4:1486	OB5922 H2 Ro.raw	4.79E7	11	11	410	429			PEAKS DB
R.WLGLSAEYGNLYR.N	N	145.22	1540.7673	13	3.5	771.3937	2	36.40	4	F4:1654	OB5922 H2 Ro.raw	5.6282E7	22	22	397	409			PEAKS DB
K.TDSRPSIANLAGENSFIDNLPEEVVANSYGLPR.E	N	142.57	3544.7434	33	4.7	1182.5940	3	38.01	6	F6:1881	OB5946 H2 Ro.raw	5.7129E7	11	11	479	511			PEAKS DB
K.SQSENFEYVAFK.T	N	130.69	1447.6619	12	2.8	724.8402	2	31.92	4	F4:1368	OB5922 H2 Ro.raw	1.7167E7	4	4	467	478			PEAKS DB
K.TDSRPSIANLAGENSFIDNLPEEVVAN(+.98)SYGLPR.E	N	112.33	3545.7273	33	1.8	1182.9185	3	36.63	5	F5:1807	OB5945 H2 Ro.raw	0	0	0	479	511	Deamidation (NQ)	N27:Deamidation (N Q):103.10	PEAKS DB
N.GIEETIC(+57.02)TASFKK.N	N	110.54	1482.7388	13	0.4	742.3770	2	29.26	6	F6:1358	OB5946 H2 Ro.raw	7.064E5	3	3	352	364	Carbamidomethylation	C7:Carbamidomethylation:1000.00	PEAKS DB
R.AHVQVVDSNGDRVFDEELQEGHVLVVPQNFAVAGK.S	N	107.17	3801.9075	35	3.6	1268.3143	3	33.20	5	F5:1608	OB5945 H2 Ro.raw	1.4284E6	3	3	432	466			PEAKS DB
N.GIEETIC(+57.02)TASFK.K	N	100.90	1354.6438	12	2.0	678.3305	2	31.03	6	F6:1474	OB5946 H2 Ro.raw	5.4233E4	3	3	352	363	Carbamidomethylation	C7:Carbamidomethylation:1000.00	PEAKS DB

total 43 peptides

Peptide	Uniq	-10lgP	Mass	Length	ppm	m/z	z	RT	Fraction	Scan	Source File	Area Roast Ara h 2	#Feature	#Feature Roast Ara h 2	Start	End	PTM	AScore	Found By
K.FFVPPSEQSLRA.V	N	99.04	1376.7087	12	2.6	689.3635	2	31.58	6	F6:1497	OB5946 H2 Ro.raw	2.1983E6	3	3	525	536			PEAKS DB
R.SPDIYNPQAGSLK.T	N	95.77	1388.6936	13	1.7	695.3552	2	28.58	5	F5:1323	OB5945 H2 Ro.raw	5.949E5	3	3	371	383			PEAKS DB
R.NALFVPH(+14.02)YNTNAHSIIYALR.G	N	93.74	2327.2175	20	0.7	776.7470	3	34.91	4	F4:1561	OB5922 H2 Ro.raw	1.3103E6	3	3	410	429		H7:Methylation(others): 22.45	PEAKS PTM
R.NRSPDIYNPQAGSLK.T	N	91.67	1658.8376	15	0.8	830.4268	2	28.33	6	F6:1298	OB5946 H2 Ro.raw	4.0221E5	6	6	369	383			PEAKS DB
K.FFVPPSEQSLR.A	N	90.90	1305.6716	11	0.4	653.8433	2	30.78	5	F5:1466	OB5945 H2 Ro.raw	4.1482E5	3	3	525	535			PEAKS DB
V.PQNFAVAGK.S	N	85.45	930.4923	9	1.0	466.2539	2	32.32	6	F6:1542	OB5946 H2 Ro.raw	3.3444E6	3	3	458	466			PEAKS DB
R.W(+31.99)LGLSAEYGNLYR.N	N	77.74	1572.7572	13	-1.6	787.3846	2	33.64	6	F6:1624	OB5946 H2 Ro.raw	8.5976E4	2	2	397	409	Dihydroxy	W1:Dihydroxy:62.93	PEAKS PTM
K.S(+57.02)QSENFEYVAFK.T	N	77.12	1504.6833	12	0.0	753.3489	2	31.77	6	F6:1521	OB5946 H2 Ro.raw	4.3047E5	3	3	467	478	Carbamidomethylation (DHKE, X@N-term)	S1:Carbamidomethylation (DHKE, X@N-term): 56.34	PEAKS PTM
R.Q(-17.03)LKNNNPFK.F	N	67.76	1084.5665	9	1.6	543.2914	2	28.75	5	F5:1346	OB5945 H2 Ro.raw	1.0606E4	2	2	516	524	Pyro-glu from Q	Q1:Pyro-glu from Q:100 0.00	PEAKS PTM
R.W(+57.02)LGLSAEYGNLYR.N	N	65.45	1597.7888	13	0.6	799.9022	2	35.09	5	F5:1725	OB5945 H2 Ro.raw	1.848E4	1	1	397	409	Carbamidomethylation (DHKE, X@N-term)	W1:Carbamidomethylation (DHKE, X@N-term): 62.19	PEAKS PTM
R.WLGLSAE(+53.92)YGNLYR.N	N	65.30	1594.6866	13	1.3	532.5702	3	34.72	5	F5:1698	OB5945 H2 Ro.raw	6.0447E5	3	3	397	409	Replacement of 2 protons by iron	E7:Replacement of 2 protons by iron:123.05	PEAKS PTM
R.S(+57.02)PDIYNPQAGSLK.T	N	64.83	1445.7150	13	5.7	723.8689	2	28.94	5	F5:1350	OB5945 H2 Ro.raw	6.4489E3	2	2	371	383		S1:Carbamidomethylation (DHKE, X@N-term): 14.04	PEAKS PTM
R.NALFVPHYNTNAHSIIYALRG.R	N	61.88	2370.2231	21	1.2	791.0826	3	33.77	5	F5:1624	OB5945 H2 Ro.raw	4.2894E6	2	2	410	430			PEAKS DB
K.SQSE(+21.98)NFEYVAFK.T	N	60.96	1469.6438	12	-0.6	735.8287	2	31.51	5	F5:1513	OB5945 H2 Ro.raw	2.7649E4	1	1	467	478		E4:Sodium adduct:0.00	PEAKS PTM
K.SQSENFE(+21.98)YVAFK.T	N	59.61	1469.6438	12	-1.1	735.8284	2	31.92	4	F4:1388	OB5922 H2 Ro.raw	4.36E4	1	1	467	478		E7:Sodium adduct:14.8 4	PEAKS PTM
R.N(+57.02)ALFVPHYNTNAHSIIYALR.G	N	59.27	2370.2231	20	-0.1	791.0816	3	34.53	4	F4:1557	OB5922 H2 Ro.raw	3.2966E6	1	1	410	429		N1:Carbamidomethylation (DHKE, X@N-term): 10.82	PEAKS PTM
K.SQSE(+53.92)NFEYVAFK.T	N	58.53	1501.5812	12	-2.3	751.7961	2	31.58	6	F6:1507	OB5946 H2 Ro.raw	9.4575E4	1	1	467	478		E4:Replacement of 2 protons by iron:34.87	PEAKS PTM

total 43 peptides

Peptide	Uniq	-10lgP	Mass	Length	ppm	m/z	z	RT	Fraction	Scan	Source File	Area Roast Ara h 2	#Feature	#Feature Roast Ara h 2	Start	End	PTM	AScore	Found By
K.TDSRPSIANLAGENSFIDNLPEEVVANSYGLPREQAR.Q	N	58.03	4028.9827	37	2.2	1008.2552	4	36.41	5	F5:1777	OB5945 H2 Ro.raw	1.9797E6	2	2	479	515			PEAKS DB
K.TDSRPSIANLAGENSFIDN(+.98)LPEEVVANSYGLPR.E	N	57.24	3545.7273	33	5.5	1182.9229	3	37.60	4	F4:1724	OB5922 H2 Ro.raw	0	0	0	479	511		N19:Deamidation (N Q):20.10	PEAKS DB
R.SPD(+57.02)IYNPQAGSLK.T	N	55.35	1445.7150	13	2.2	723.8663	2	29.51	4	F4:1244	OB5922 H2 Ro.raw	0	0	0	371	383		D3:Carbamidomethylation (DHKE, X@N-term): 0.00	PEAKS PTM
K.SQSENFEYVAFKTDSRPSIANLAGENSFIDNLPEEVVANSYGLPR.E	N	54.99	4974.3945	45	2.5	1244.6090	4	38.26	4	F4:1777	OB5922 H2 Ro.raw	3.3359E6	3	3	467	511			PEAKS DB
K.SQS(-18.01)ENFEYVAFK.T	N	52.68	1429.6514	12	1.9	715.8344	2	31.92	4	F4:1380	OB5922 H2 Ro.raw	3.0777E4	1	1	467	478		S3:Dehydration:14.04	PEAKS PTM
R.LNAQRPDNRIESEGGYIETWNPNNQEFEC(+57.02)AGVALSR.L	N	50.20	4133.9248	36	7.4	1034.4961	4	33.23	4	F4:1476	OB5922 H2 Ro.raw	5.4856E4	1	1	37	72	Carbamidomethylation	C29:Carbamidomethylation:1000.00	PEAKS DB
R.NALFVPH(+57.02)YNTNAHSIIYALR.G	N	49.15	2370.2231	20	0.1	791.0817	3	34.03	6	F6:1601	OB5946 H2 Ro.raw	8.9747E5	1	1	410	429		H7:Carbamidomethylation (DHKE, X@N-term): 20.35	PEAKS PTM
N.GIEETIC(+71.04)TASFKK.N	N	48.49	1496.7544	13	0.4	749.3848	2	29.91	4	F4:1267	OB5922 H2 Ro.raw	6.4028E4	3	3	352	364	Propionamide	C7:Propionamide:1000.00	PEAKS PTM
D.SRPSIANLAGENSFIDNLPEEVVANSYGLPR.E	N	48.34	3328.6687	31	3.9	1110.5679	3	37.36	5	F5:1841	OB5945 H2 Ro.raw	3.0619E5	2	2	481	511			PEAKS DB
K.TDSRPSIANLAGEN(+.98)SFIDNLPEEVVANSYGLPR.E	N	47.42	3545.7273	33	9.2	1182.9272	3	48.53	5	F5:2493	OB5945 H2 Ro.raw	0	0	0	479	511		N14:Deamidation (N Q):19.39	PEAKS DB
R.WLGLSAEYGN(+.98)LYR.N	N	47.40	1541.7513	13	5.4	771.8871	2	54.24	4	F4:2679	OB5922 H2 Ro.raw	0	0	0	397	409	Deamidation (NQ)	N10:Deamidation (N Q):1000.00	PEAKS DB
R.NALFVPHYNTNAHSIIYALRGR.A	N	46.11	2526.3245	22	0.6	843.1160	3	33.60	4	F4:1492	OB5922 H2 Ro.raw	7.6168E5	2	2	410	431			PEAKS DB
K.SQSEN(+.98)FEYVAFKTDSRPSIANLAGENSFIDNLPEEVVANSYGLPR.E	N	45.32	4975.3789	45	2.8	1244.8555	4	39.75	5	F5:1987	OB5945 H2 Ro.raw	0	0	0	467	511		N5:Deamidation (NQ): 0.00	PEAKS DB
N.G(+57.02)IEETIC(+57.02)TASFKK.N	N	43.30	1539.7603	13	-0.3	770.8872	2	30.37	4	F4:1296	OB5922 H2 Ro.raw	0	0	0	352	364	Carbamidomethylation	G1:Carbamidomethylation (DHKE, X@N-term): 14.04;C7:Carbamidomethylation:1000.00	PEAKS PTM
N.E(+42.01)LQLNLLILR(+14.02)WLGLSAEYGNLYR(+14.02).N	Y	42.71	2816.5588	23	-7.9	939.8528	3	40.93	4	F4:1919	OB5922 H2 Ro.raw	2.7383E5	1	1	387	409	Acetylation (N-term); Methylation(KR)	E1:Acetylation (N-term):1000.00;R10:Methylation(KR): 1000.00;R23:Methylation(KR):1000.00	PEAKS PTM

total 43 peptides

Peptide	Uniq	-10lgP	Mass	Length	ppm	m/z	z	RT	Fraction	Scan	Source File	Area Roast Ara h 2	#Feature	#Feature Roast Ara h 2	Start	End	PTM	AScore	Found By
N.E(+43.99)LQ(+.98)LNLLILR.W	Y	39.76	1268.7340	10	-1.9	635.3730	2	40.91	4	F4:1916	OB5922 H2 Ro.raw	0	0	0	387	396	Carboxylation (E)	E1:Carboxylation (E):1000.00;Q3:Deamidation (NQ):44.72	PEAKS PTM
R.N(+.98)ALFVPHYNTNAHSIIYALR.G	N	38.37	2314.1858	20	7.3	772.4082	3	50.66	4	F4:2450	OB5922 H2 Ro.raw	1.0573E4	1	1	410	429		N1:Deamidation (NQ): 32.94	PEAKS DB
R.WLGLSAE(+21.98)YGNLYR.N	N	36.57	1562.7494	13	-8.4	782.3754	2	35.27	4	F4:1583	OB5922 H2 Ro.raw	5.0047E4	2	2	397	409		E7:Sodium adduct:42.78	PEAKS PTM
**total 43 peptides**																			

E9LFE8|E9LFE8_ARAHY

back to list

| Protein Coverage | Supporting Peptides |

Protein Coverage:

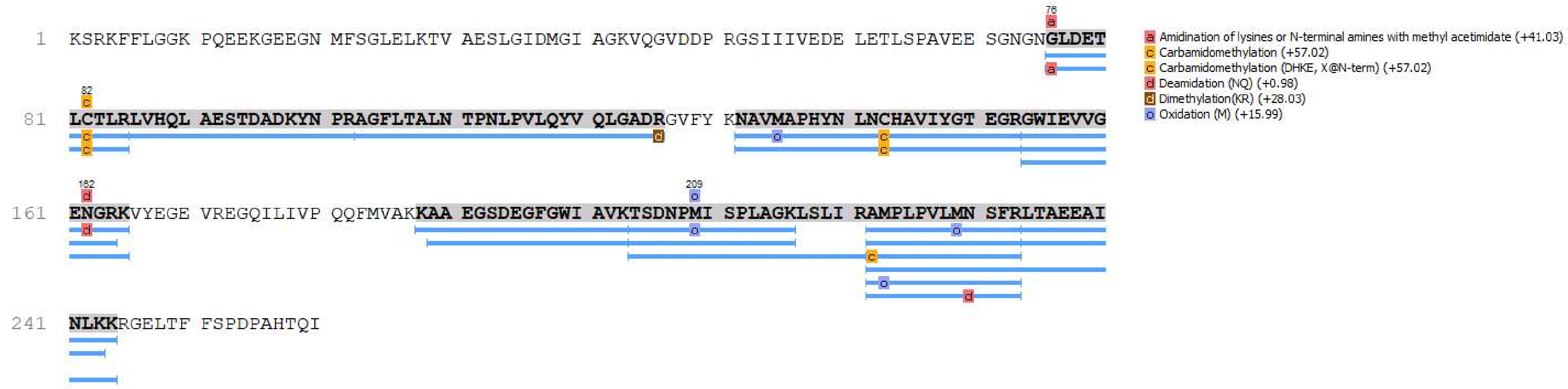

Supporting Peptides:

Peptide	Uniq	-10lgP	Mass	Length	ppm	m/z	z	RT	Fraction	Scan	Source File	Area Roast Ara h 2	#Feature	#Feature Roast Ara h 2	Start	End	PTM	AScore	Found By
R.AGFLTALNTPNLPVLQYVQLGADR(+28.03).G	Y	151.29	2598.4170	24	4.1	1300.2211	2	40.36	4	F4:1874	OB5922 H2 Ro.raw	8.4413E6	6	6	103	126	Dimethylation(KR)	R24:Dimethylation(KR):1000.00	PEAKS PTM
K.KAAEGSDEGFGWIAVK.T	Y	146.19	1663.8206	16	2.8	832.9199	2	32.11	4	F4:1402	OB5922 H2 Ro.raw	5.9962E5	5	5	188	203			PEAKS DB
K.AAEGSDEGFGWIAVK.T	Y	137.31	1535.7256	15	2.4	768.8719	2	33.64	6	F6:1628	OB5946 H2 Ro.raw	4.1039E6	3	3	189	203			PEAKS DB
R.AMPLPVLMNSFR.L	Y	122.04	1374.7152	12	2.5	688.3666	2	36.76	4	F4:1666	OB5922 H2 Ro.raw	4.2211E6	4	4	222	233			PEAKS DB

total 26 peptides

Peptide	Uniq	-10lgP	Mass	Length	ppm	m/z	z	RT	Fraction	Scan	Source File	Area Roast Ara h 2	#Feature	#Feature Roast Ara h 2	Start	End	PTM	AScore	Found By
R.AMPLPVLM(+15.99)NSFR.L	Y	113.73	1390.7101	12	2.1	696.3638	2	34.40	4	F4:1535	OB5922 H2 Ro.raw	4.0273E5	1	1	222	233	Oxidation (M)	M8:Oxidation (M):146.62	PEAKS DB
K.TSDNPMISPLAGK.L	Y	113.60	1329.6598	13	0.5	665.8375	2	29.91	6	F6:1397	OB5946 H2 Ro.raw	4.6371E5	3	3	204	216			PEAKS DB
R.GWIEVVGENGR.K	Y	109.17	1214.6044	11	0.0	608.3094	2	31.76	6	F6:1523	OB5946 H2 Ro.raw	6.5203E4	2	2	154	164			PEAKS DB
K.NAVMAPHYNLNC(+57.02)HAVIYGTEGR.G	Y	98.11	2486.1582	22	1.2	829.7277	3	31.22	6	F6:1485	OB5946 H2 Ro.raw	3.5159E5	2	2	132	153	Carbamidomethylation	C12:Carbamidomethylation:1000.00	PEAKS DB
K.TSDNPMISPLAGKLSLIR.A	Y	95.78	1912.0452	18	-0.1	638.3556	3	34.16	4	F4:1525	OB5922 H2 Ro.raw	1.9762E5	2	2	204	221			PEAKS DB
N.GLDETLC(+57.02)TLR.L	Y	92.72	1176.5808	10	0.9	589.2982	2	30.78	5	F5:1456	OB5945 H2 Ro.raw	4.2059E5	3	3	76	85	Carbamidomethylation	C7:Carbamidomethylation:1000.00	PEAKS DB
K.TSDNPM(+15.99)ISPLAGK.L	Y	91.04	1345.6548	13	-1.0	673.8340	2	28.04	4	F4:1158	OB5922 H2 Ro.raw	8.6931E3	3	3	204	216	Oxidation (M)	M6:Oxidation (M):1000.00	PEAKS DB
R.LTAEEAINLK.K	Y	83.63	1100.6077	10	1.8	551.3121	2	29.67	5	F5:1400	OB5945 H2 Ro.raw	2.6983E4	3	3	234	243			PEAKS DB
K.A(+57.02)AEGSDEGFGWIAVK.T	Y	80.99	1592.7471	15	1.2	797.3818	2	33.97	4	F4:1507	OB5922 H2 Ro.raw	2.9688E4	1	1	189	203		A1:Carbamidomethylation (DHKE, X@N-term):11.10	PEAKS PTM
N.G(+57.02)LDETLC(+57.02)TLR.L	Y	78.72	1233.6023	10	2.6	617.8100	2	30.88	5	F5:1470	OB5945 H2 Ro.raw	0	0	0	76	85	Carbamidomethylation	G1:Carbamidomethylation (DHKE, X@N-term):26.31; C7:Carbamidomethylation:1000.00	PEAKS PTM
R.GWIEVVGEN(+.98)GRK.V	Y	74.66	1343.6833	12	0.3	672.8492	2	30.41	5	F5:1444	OB5945 H2 Ro.raw	1.4478E4	1	1	154	165	Deamidation (NQ)	N9:Deamidation (NQ):1000.00	PEAKS DB
R.GWIEVVGENGRK.V	Y	73.34	1342.6993	12	0.6	672.3574	2	30.50	6	F6:1442	OB5946 H2 Ro.raw	0	0	0	154	165			PEAKS DB
R.A(+57.02)MPLPVLMNSFR.L	Y	73.22	1431.7367	12	1.5	716.8767	2	36.95	4	F4:1692	OB5922 H2 Ro.raw	1.0591E5	3	3	222	233	Carbamidomethylation (DHKE, X@N-term)	A1:Carbamidomethylation (DHKE, X@N-term):1000.00	PEAKS PTM
R.LVHQLAESTDADKYNPR.A	Y	72.60	1955.9701	17	-6.1	978.9863	2	30.10	4	F4:1292	OB5922 H2 Ro.raw	7.2507E4	3	3	86	102			PEAKS DB
N.GLD(+14.02)ETLC(+57.02)TLR.L	Y	70.01	1190.5966	10	3.1	596.3074	2	31.37	4	F4:1347	OB5922 H2 Ro.raw	1.792E6	3	3	76	85	Carbamidomethylation	D3:Methylation(others):33.98;C7:Carbamidomethylation:1000.00	PEAKS PTM
K.NAVM(+15.99)APHYNLNC(+57.02)HAVIYGTEGR.G	Y	69.81	2502.1533	22	1.1	835.0593	3	30.66	6	F6:1454	OB5946 H2 Ro.raw	9.2873E4	3	3	132	153	Oxidation (M); Carbamidomethylation	M4:Oxidation (M):1000.00; C12:Carbamidomethylation:1000.00	PEAKS DB
R.LTAEEAINLKK.R	Y	67.83	1228.7026	11	1.1	615.3593	2	28.04	4	F4:1144	OB5922 H2 Ro.raw	4.2354E4	3	3	234	244			PEAKS DB
N.G(+57.02)LD(+14.02)ETLC(+57.02)TLR.L	Y	58.35	1247.6179	10	1.2	624.8170	2	31.22	6	F6:1488	OB5946 H2 Ro.raw	4.2493E4	2	2	76	85	Carbamidomethylation	G1:Carbamidomethylation (DHKE, X@N-term):49.37; D3:Methylation(others):27.96;C7:Carbamidomethylation:1000.00	PEAKS PTM
R.AMPLPVLMN(+.98)SFR.L	Y	57.25	1375.6992	12	1.7	688.8580	2	36.11	4	F4:1637	OB5922 H2 Ro.raw	0	0	0	222	233	Deamidation (NQ)	N9:Deamidation (NQ):1000.00	PEAKS DB

total 26 peptides

Peptide	Uniq	-10lgP	Mass	Length	ppm	m/z	z	RT	Fraction	Scan	Source File	Area Roast Ara h 2	#Feature	#Feature Roast Ara h 2	Start	End	PTM	AScore	Found By
R.AM(+15.99)PLPVLMNSFR.L	Y	57.04	1390.7101	12	3.1	696.3645	2	34.72	4	F4:1571	OB5922 H2 Ro.raw	5.1224E5	2	2	222	233	Oxidation (M)	M2:Oxidation (M):71.27	PEAKS DB
N.G(+41.03)LDETLC(+57.02)TLR.L	Y	43.33	1217.6074	10	0.9	609.8115	2	31.33	5	F5:1490	OB5945 H2 Ro.raw	2.7231E4	1	1	76	85	Amidination of lysines or N-terminal amines with methyl acetimidate; Carbamidomethylation	G1:Amidination of lysines or N-terminal amines with methyl acetimidate:1000.00; C7:Carbamidomethylation: 1000.00	PEAKS PTM
R.AMPLPVLMNSFRLTAEEAINLKK.R	Y	40.26	2585.4072	23	2.5	862.8118	3	39.79	4	F4:1844	OB5922 H2 Ro.raw	4.4272E5	1	1	222	244			PEAKS DB

total 26 peptides

sp|Q647H2|AHY3_ARAHY

back to list

| **Protein Coverage** | **Supporting Peptides** |

Protein Coverage:

```
  1  MAKLLALSVC FCFLVLGASS VTFRQQGEEN ECQFQRLNAQ RPDNCIESEG GYIETWNPNN QEFQCAGVAL SRFVLRRNAL
 81  RRPFYSNAPQ EIFIYQGSGY FGLIFPGCPG TFEEPIQGSE QFQRPSRHFQ GQDQSQRPLD THQKVHGFRE GDLIAVPHGV
161  AFWIYNDQDT DVVAISVLHT NSLHNQLDQF PRRFNLAGKQ EQEFLRYQQR SGRQSPKGEE QEQEQENEGG NVFSGFSTEF
241  LSHGFQVNED IVRNLRGENE REEQGAIVTV KGGLSILVPP EWRQSYQQPG RGDKDFNNGI EETICTATVK MNIGKSTSAD
321  IYNPQAGSVR TVNELDLPIL NRLGLSAEYG SIHRDAMFVP HYNMNANSMI YALHGGAHVQ VVDCNGNRVF DEELQEGQSL
401  VVPQNFAVAA KSQSEHFLYV AFKTNSRASI SNLAGKNSYM WNLPEDVVAN SYGLQYEQAR QLKNNNPFTF LVPPQDSQMI
481  RTVA
```

299 a; 305 c p

a Amidination of lysines or N-terminal amines with methyl acetimidate (+41.03)
c Carbamidomethylation (+57.02)
o Oxidation (M) (+15.99)
p Propionamide (+71.04)

Supporting Peptides:

Peptide	Uniq	-10lgP	Mass	Length	ppm	m/z	z	RT	Fraction	Scan	Source File	Area Roast Ara h 2	#Feature	#Feature Roast Ara h 2	Start	End	PTM	AScore	Found By
K.NSYMWNLPEDVVANSYGLQYEQAR.Q	Y	139.65	2846.2969	24	3.2	1424.1603	2	37.32	4	F4:1709	OB5922 H2 Ro.raw	9.5352E5	4	4	437	460			PEAKS DB
K.SQSEHFLYVAFK.T	Y	132.94	1454.7194	12	1.5	728.3680	2	32.30	4	F4:1412	OB5922 H2 Ro.raw	2.2082E6	6	6	412	423			PEAKS DB
R.TVNELDLPILNR.L	Y	119.74	1395.7721	12	1.9	698.8947	2	34.41	6	F6:1672	OB5946 H2 Ro.raw	4.4893E6	3	3	331	342			PEAKS DB
R.ASISNLAGKNSYMWNLPEDVVANSYGLQYEQAR.Q	Y	115.74	3687.7627	33	7.0	1230.2701	3	36.76	4	F4:1683	OB5922 H2 Ro.raw	3.0022E5	3	3	428	460			PEAKS DB

total 19 peptides

Peptide	Uniq	-10lgP	Mass	Length	ppm	m/z	z	RT	Fraction	Scan	Source File	Area Roast Ara h 2	#Feature	#Feature Roast Ara h 2	Start	End	PTM	AScore	Found By
K.NSYM(+15.99)WNLPEDVVANSYGLQYEQAR.Q	Y	114.80	2862.2917	24	4.0	1432.1589	2	36.20	4	F4:1647	OB5922 H2 Ro.raw	5.9092E4	2	2	437	460	Oxidation (M)	M4:Oxidation (M):1000.00	PEAKS DB
R.LGLSAEYGSIHR.D	Y	112.58	1301.6727	12	0.5	651.8440	2	29.26	6	F6:1369	OB5946 H2 Ro.raw	2.0752E5	6	6	343	354			PEAKS DB
K.SQSEHFLYVAFKTNSR.A	Y	109.97	1912.9431	16	1.0	638.6556	3	31.92	4	F4:1392	OB5922 H2 Ro.raw	1.6409E5	3	3	412	427			PEAKS DB
N.GIEETIC(+57.02)TATVK.M	N	109.07	1320.6595	12	-0.2	661.3369	2	29.91	4	F4:1260	OB5922 H2 Ro.raw	2.1822E5	3	3	299	310	Carbamidomethylation	C7:Carbamidomethylation:1000.00	PEAKS DB
K.STSADIYNPQAGSVR.T	Y	83.45	1564.7482	15	2.2	783.3831	2	27.91	4	F4:1134	OB5922 H2 Ro.raw	4.5802E4	3	3	316	330			PEAKS DB
R.VFDEELQEGQSLVVPQNFAVAAK.S	Y	78.21	2517.2751	23	4.7	1259.6508	2	35.09	5	F5:1723	OB5945 H2 Ro.raw	7.46E4	3	3	389	411			PEAKS DB
N.G(+57.02)IEETIC(+57.02)TATVK.M	N	70.02	1377.6809	12	2.0	689.8491	2	30.19	4	F4:1285	OB5922 H2 Ro.raw	5.5841E3	1	1	299	310	Carbamidomethylation	G1:Carbamidomethylation (DHKE, X@N-term):30.83;C7:Carbamidomethylation:1000.00	PEAKS PTM
K.S(+57.02)QSEHFLYVAFK.T	Y	66.19	1511.7408	12	0.6	756.8782	2	32.49	4	F4:1431	OB5922 H2 Ro.raw	4.3132E4	1	1	412	423		S1:Carbamidomethylation (DHKE, X@N-term):9.42	PEAKS PTM
N.GIEETIC(+71.04)TATVK.M	N	65.44	1334.6752	12	3.2	668.3470	2	29.62	5	F5:1395	OB5945 H2 Ro.raw	7.3781E3	1	1	299	310	Propionamide	C7:Propionamide:1000.00	PEAKS PTM
V.PQNFAVAAK.S	N	63.04	944.5079	9	0.9	473.2617	2	32.86	4	F4:1447	OB5922 H2 Ro.raw	2.0461E6	3	3	403	411			PEAKS DB
R.QLKNNNPFTFLVPPQDSQMIRT.V	Y	61.56	2587.3218	22	0.9	863.4487	3	35.50	4	F4:1601	OB5922 H2 Ro.raw	5.4546E5	2	2	461	482			PEAKS DB
R.RFNLAGKQEQEFLR.Y	Y	60.95	1734.9165	14	0.4	579.3130	3	31.51	5	F5:1507	OB5945 H2 Ro.raw	6.1511E4	2	2	193	206			PEAKS DB
K.N(+.98)SYMWNLPEDVVANSYGLQYEQAR.Q	Y	56.66	2847.2810	24	9.4	1424.6611	2	39.12	6	F6:1945	OB5946 H2 Ro.raw	0	0	0	437	460		N1:Deamidation (NQ):7.38	PEAKS DB
K.NNNPFTFLVPPQDSQMIRT.V	Y	50.17	2218.0840	19	2.9	1110.0525	2	37.02	4	F4:1690	OB5922 H2 Ro.raw	0	0	0	464	482			PEAKS DB
N.G(+41.03)IEETIC(+57.02)TATVK.M	N	47.00	1361.6860	12	0.7	681.8508	2	29.66	5	F5:1390	OB5945 H2 Ro.raw	4.1987E4	2	2	299	310	Amidination of lysines or N-terminal amines with methyl acetimidate; Carbamidomethylation	G1:Amidination of lysines or N-terminal amines with methyl acetimidate:96.48;C7:Carbamidomethylation:1000.00	PEAKS PTM
total 19 peptides																			

Q647H1|Q647H1_ARAHY

back to list

| Protein Coverage | Supporting Peptides |

Protein Coverage:

```
  1 MVIGPFRLSL CVCLVFLTSA CFGTRLEESF NECQLDRLNA LTPDNRIESQ GGITETWNSN HPELRCAGVT LLKRTIFPNG
 81 FHLPSYANYP QLIFIAQGNG VFGVSLPGCP VTYEEAESQS REDRRQRIVI KRESEQEQEQ QGDSHHKIYH FRQGHLLAIP
161 AGVPYWSFNY GNEPIVAITL LDTSNLDNQL DPSPRRFYLA GNPEEEHPET QQQQPQTRRR HGQHQQDEYG SQGEEEGNNV
241 LSGFSTQLLA HAFGVDEEIA RILQNPPEQT KDQIVRVEGG FRDVISPRWG EGKQYEDELE ERQRQPPRRD EQGKGYDYDD
321 DRRPRHRQDP YREGDEDDRR PRGSRQGQGR GYDDDDRRPG QYEEGEEDDR RPRRSSRPKR QGRRHDDDDR RADEDDRRGY
401 DDDERRPDED DRRGYDDDER RPDDDDRQGY DDDDRRPRWS SRPKGQGRNG VEETLCSPTL VEDIARPSRA DFYNPAAGRI
481 SSANSLTFPI LRWFQLSAEH VLLYRNGIYS PHWNNNANSI IYGLRGEGRI QVVNSQGNAV FNGVLREGQI LLVPQNFAVG
561 KQAGNEGFEY VAFKTADRAS PATSSKCLGE SPLMFSSMLL AFEIIKSVLS NTMETRPLWS LLMIPSMGLN VVINLNHNNN
641 AQVDSKNNDG SRLWWPSSII IK
```

456 c (Carbamidomethylation), d (Diethylation) markers

c Carbamidomethylation (+57.02)
d Diethylation (+56.06)

Supporting Peptides:

Peptide	Uniq	-10lgP	Mass	Length	ppm	m/z	z	RT	Fraction	Scan	Source File	Area Roast Ara h 2	#Feature	#Feature Roast Ara h 2	Start	End	PTM	AScore	Found By
R.IQVVNSQGNAVFNGVLR.E	Y	138.92	1813.9799	17	2.5	907.9995	2	33.04	4	F4:1457	OB5922 H2 Ro.raw	3.9071E5	3	3	530	546			PEAKS DB
R.WFQLSAEHVLLYR.N	Y	130.13	1660.8726	13	-0.1	831.4435	2	34.60	6	F6:1686	OB5946 H2 Ro.raw	5.0508E5	4	4	493	505			PEAKS DB
R.ISSANSLTFPILR.W	Y	104.78	1417.7928	13	0.5	709.9041	2	34.35	5	F5:1668	OB5945 H2 Ro.raw	4.7913E5	3	3	480	492			PEAKS DB
R.EGQILLVPQNFAVGK.Q	Y	100.83	1611.8984	15	1.4	806.9576	2	34.53	4	F4:1550	OB5922 H2 Ro.raw	3.6726E5	3	3	547	561			PEAKS DB
K.QAGNEGFEYVAFK.T	Y	91.27	1458.6779	13	0.7	730.3467	2	32.31	4	F4:1403	OB5922 H2 Ro.raw	2.1182E5	2	2	562	574			PEAKS DB
R.ADFYNPAAGR.I	Y	89.68	1080.4988	10	-0.6	541.2563	2	28.20	5	F5:1311	OB5945 H2 Ro.raw	7.0309E3	3	3	470	479			PEAKS DB
R.IQVVNSQGNAVFNGVLREGQILLVPQNFAVGK.Q	Y	67.67	3407.8677	32	5.1	1136.9690	3	38.18	6	F6:1888	OB5946 H2 Ro.raw	3.4122E4	1	1	530	561			PEAKS DB
N.GVEETLC(+57.02)SPTLVEDIARPSR.A	Y	50.19	2228.1106	20	2.1	743.7124	3	32.69	6	F6:1580	OB5946 H2 Ro.raw	1.5861E5	2	2	450	469	Carbamidomethylation	C7:Carbamidomethylation:1000.00	PEAKS DB
R.EGQILLVPQNFAVGK(+56.06).Q	Y	48.11	1667.9609	15	0.8	834.9884	2	34.91	4	F4:1568	OB5922 H2 Ro.raw	6.1967E5	2	2	547	561	Diethylation	K15:Diethylation:121.40	PEAKS PTM
**total 9 peptides**																			

A1E2B1|A1E2B1_ARAHY

back to list

| Protein Coverage | Supporting Peptides |

Protein Coverage:

```
1   MSSSLLFLTL AYLVLIERLT CHAQSHGDRI GECRLEHLSV MEPTKRVESE GGVAEFWDDK SQQLQCIGVT LIRYTIRPKG
81  LLLPFYTNAP RIHYILQGKG VMEIVVTGCR AMYRSSTKRG MMSSYSDEHQ KIQSIEQNDA VAVPSSSVHW IYNTGHSDLV
161 LFSLVDVANA DNQLDPTFRN FLLSGNGNGK EGEESNNNWF IKKKQREAQE GNVFSGLALE TLIGSFNVQR EIAEKVQGLK
241 DWRGSIILVK EGLDWLSPKE EEEEEGLEKR KKVMLMG
```

66 C, 109 C

C Carbamidomethylation (+57.02)
C Carbamidomethylation (DHKE, X@N-term) (+57.02)
* Acetylation (N-term) (+42.01), Phosphorylation (STY) (+79.97)

Supporting Peptides:

Peptide	Uniq	-10lgP	Mass	Length	ppm	m/z	z	RT	Fraction	Scan	Source File	Area Roast Ara h 2	#Feature	#Feature Roast Ara h 2	Start	End	PTM	AScore	Found By
K.GLLLPFYTNAPR.I	Y	131.34	1360.7502	12	1.9	681.3837	2	35.83	4	F4:1614	OB5922 H2 Ro.raw	8.7006E5	3	3	80	91			PEAKS DB
K.GVMEIVVTGC(+57.02)R.A	Y	116.98	1219.6053	11	0.2	610.8101	2	31.40	6	F6:1498	OB5946 H2 Ro.raw	1.3757E5	2	2	100	110	Carbamidomethylation	C10:Carbamidomethylation:1000.00	PEAKS DB
R.VESEGGVAEFWDDKSQQLQC(+57.02)IGVTLIR.Y	Y	98.95	3063.4971	27	2.5	1022.1755	3	37.32	4	F4:1703	OB5922 H2 Ro.raw	5.5365E5	3	3	47	73	Carbamidomethylation	C20:Carbamidomethylation:1000.00	PEAKS DB
Y.TIRPK(+149.03)GLLLPFYTNAPR.I	Y	52.86	2105.1606	17	-7.1	702.7225	3	34.93	5	F5:1700	OB5945 H2 Ro.raw	5.1304E4	1	1	75	91		K5:Benzyl isothiocyanate:22.37	PEAKS PTM
K.G(+57.02)LLLPFYTNAPR.I	Y	49.75	1417.7717	12	3.7	709.8958	2	35.99	4	F4:1630	OB5922 H2 Ro.raw	0	0	0	80	91	Carbamidomethylation (DHKE, X@N-term)	G1:Carbamidomethylation (DHKE, X@N-term):1000.00	PEAKS PTM
R.NFLLSGN(+.98)GNGKEGEESN.N	Y	47.77	1765.7754	17	2.2	883.8969	2	30.89	4	F4:1327	OB5922 H2 Ro.raw	0	0	0	180	196		N7:Deamidation (NQ):18.53	PEAKS DB
R.IGEC(+57.02)RLEHLSVMEPTK.R	Y	45.16	1897.9390	16	1.1	633.6543	3	31.15	5	F5:1494	OB5945 H2 Ro.raw	1.9364E4	1	1	30	45	Carbamidomethylation	C4:Carbamidomethylation:1000.00	PEAKS DB

total 9 peptides

Peptide	Uniq	-10lgP	Mass	Length	ppm	m/z	z	RT	Fraction	Scan	Source File	Area Roast Ara h 2	#Feature	#Feature Roast Ara h 2	Start	End	PTM	AScore	Found By
Y.T(+42.01)(+79.97)IRPK(+28.03)GLLLPFYTNAPR.I	Y	41.04	2106.1389	17	-4.3	703.0505	3	35.64	4	F4:1612	OB5922 H2 Ro.raw	1.8812E5	1	1	75	91	Acetylation (N-term); Phosphorylation (STY)	T1:Acetylation (N-term):1000.00;T1:Phosphorylation (STY):71.87;K5:Dimethylation (KR):0.00	PEAKS PTM
K.IQSIEQNDAVAVPSSSVHWIYNTGHSDLVLFSLVDVAN(+.98)ADNQLDPTFR.N	Y	36.60	5282.5796	48	8.2	1321.6630	4	39.07	5	F5:1954	OB5945 H2 Ro.raw	4.1788E4	1	1	132	179		N38:Deamidation (NQ):22.45	PEAKS DB
**total 9 peptides**																			

A5Z1R0|A5Z1R0_ARAHY

back to list

| Protein Coverage | Supporting Peptides |

Protein Coverage:

1 MAKSTILVAL LALVLVAHAS AMRRERGRQG DSSSCERQVD RVNLKPCEQH IMQRIMGEQE QYDSYDIRST RSSDQQQRCC

81 DELNEMENTQ RCMCEALQQI MENQCDRLQD RQMVQQFKRE LMNLPQQCNF RAPQRCDLDV SGGRC

C Carbamidomethylation (+57.02)
O Oxidation (M) (+15.99)

Supporting Peptides:

Peptide	Uniq	-10lgP	Mass	Length	ppm	m/z	z	RT	Fraction	Scan	Source File	Area Roast Ara h 2	#Feature	#Feature Roast Ara h 2	Start	End	PTM	AScore	Found By
R.ELMNLPQQC(+57.02)NFR.A	Y	121.98	1548.7177	12	1.9	775.3676	2	31.56	4	F4:1364	OB5922 H2 Ro.raw	2.7372E5	3	3	120	131	Carbamidomethylation	C9:Carbamidomethylation:1000.00	PEAKS DB
R.IMGEQEQYDSYDIR.S	Y	120.66	1745.7566	14	2.9	873.8881	2	30.67	4	F4:1316	OB5922 H2 Ro.raw	2.1664E5	3	3	55	68			PEAKS DB
R.ELMNLPQQC(+57.02)NFRAPQR.C	Y	68.46	2000.9673	16	0.5	667.9967	3	30.28	6	F6:1430	OB5946 H2 Ro.raw	5.4257E4	1	1	120	135	Carbamidomethylation	C9:Carbamidomethylation:1000.00	PEAKS DB
R.C(+57.02)MC(+57.02)EALQQIMENQC(+57.02)DRLQDR.Q	Y	61.33	2597.0913	20	0.2	866.7046	3	33.64	6	F6:1634	OB5946 H2 Ro.raw	1.3866E5	2	2	92	111	Carbamidomethylation	C1:Carbamidomethylation:1000.00;C3:Carbamidomethylation:1000.00;C14:Carbamidomethylation:1000.00	PEAKS DB
R.IM(+15.99)GEQEQYDSYDIR.S	Y	56.40	1761.7516	14	3.0	881.8857	2	28.72	5	F5:1341	OB5945 H2 Ro.raw	0	0	0	55	68	Oxidation (M)	M2:Oxidation (M):1000.00	PEAKS DB
R.ELM(+15.99)NLPQQC(+57.02)NFR.A	Y	46.49	1564.7126	12	1.3	783.3646	2	29.68	4	F4:1254	OB5922 H2 Ro.raw	0	0	0	120	131	Oxidation (M); Carbamidomethylation	M3:Oxidation (M):1000.00;C9:Carbamidomethylation:1000.00	PEAKS DB
**total 6 peptides**																			

sp|Q647G9|CONG_ARAHY

back to list

| **Protein Coverage** | **Supporting Peptides** |

Protein Coverage:

1 MAKSTILVAL LALVLVAHAS AMRRERGRQG DSSSCERQVD RVNLKPCEQH IMQRIMGEQE QYDSYDIRST RSSDQQQRCC

81 DELNEMENTQ RCMCEALQQI MENQCDRLQD RQMVQQFKRE LMNLPQQCNF RAPQRCDLDV SGGRC

94

C Carbamidomethylation (+57.02)
O Oxidation (M) (+15.99)

Supporting Peptides:

Peptide	Uniq	-10lgP	Mass	Length	ppm	m/z	z	RT	Fraction	Scan	Source File	Area Roast Ara h 2	#Feature	#Feature Roast Ara h 2	Start	End	PTM	AScore	Found By
R.ELMNLPQQC(+57.02)NFR.A	Y	121.98	1548.7177	12	1.9	775.3676	2	31.56	4	F4:1364	OB5922 H2 Ro.raw	2.7372E5	3	3	120	131	Carbamidomethylation	C9:Carbamidomethylation:1000.00	PEAKS DB
R.IMGEQEQYDSYDIR.S	Y	120.66	1745.7566	14	2.9	873.8881	2	30.67	4	F4:1316	OB5922 H2 Ro.raw	2.1664E5	3	3	55	68			PEAKS DB
R.ELMNLPQQC(+57.02)NFRAPQR.C	Y	68.46	2000.9673	16	0.5	667.9967	3	30.28	6	F6:1430	OB5946 H2 Ro.raw	5.4257E4	1	1	120	135	Carbamidomethylation	C9:Carbamidomethylation:1000.00	PEAKS DB
R.C(+57.02)MC(+57.02)EALQQIMENQC(+57.02)DRLQDR.Q	Y	61.33	2597.0913	20	0.2	866.7046	3	33.64	6	F6:1634	OB5946 H2 Ro.raw	1.3866E5	2	2	92	111	Carbamidomethylation	C1:Carbamidomethylation:1000.00;C3:Carbamidomethylation:1000.00;C14:Carbamidomethylation:1000.00	PEAKS DB
R.IM(+15.99)GEQEQYDSYDIR.S	Y	56.40	1761.7516	14	3.0	881.8857	2	28.72	5	F5:1341	OB5945 H2 Ro.raw	0	0	0	55	68	Oxidation (M)	M2:Oxidation (M):1000.00	PEAKS DB
R.ELM(+15.99)NLPQQC(+57.02)NFR.A	Y	46.49	1564.7126	12	1.3	783.3646	2	29.68	4	F4:1254	OB5922 H2 Ro.raw	0	0	0	120	131	Oxidation (M); Carbamidomethylation	M3:Oxidation (M):1000.00;C9:Carbamidomethylation:1000.00	PEAKS DB
**total 6 peptides**																			

sp|Q6PSU2|CONG7_ARAHY

back to list

| **Protein Coverage** | **Supporting Peptides** |

Protein Coverage:

1 MAKLTILVAL ALFLLAAHAS ARQQWELQGD RRCQSQLERA NLRPCEQHLM QKIQRDEDSY GRDPYSPSQD PYSPSQDPDR

81 RDPYSPSPYD RRGAGSSQHQ ERCCNELNEF ENNQRCMCEA LQQIMENQSD RLQGRQQEQQ FKRELRNLPQ QCGLRAPQRC

104 118

161 DLEVESGGRD RY

a Ammonia-loss (C@N-term) (-17.03)
C Carbamidomethylation (+57.02)
C Carbamidomethylation (DHKE, X@N-term) (+57.02)
O Oxidation (M) (+15.99)

Supporting Peptides:

Peptide	Uniq	-10lgP	Mass	Length	ppm	m/z	z	RT	Fraction	Scan	Source File	Area Roast Ara h 2	#Feature	#Feature Roast Ara h 2	Start	End	PTM	AScore	Found By
R.C(+57.02)MC(+57.02)EALQQIMENQSDRLQGR.Q	Y	114.89	2466.0872	20	1.5	823.0375	3	33.97	4	F4:1513	OB5922 H2 Ro.raw	2.638E6	4	4	116	135	Carbamidomethylation	C1:Carbamidomethylation:1000.00;C3:Carbamidomethylation:1000.00	PEAKS DB
R.C(+57.02)C(+57.02)NELNEFENNQR.C	Y	97.90	1725.6835	13	1.4	863.8502	2	29.25	4	F4:1229	OB5922 H2 Ro.raw	7.9798E4	3	3	103	115	Carbamidomethylation	C1:Carbamidomethylation:1000.00;C2:Carbamidomethylation:1000.00	PEAKS DB
R.C(+71.04)C(+57.02)NELNEFENNQR.C	Y	94.71	1739.6992	13	-1.9	870.8552	2	29.24	4	F4:1230	OB5922 H2 Ro.raw	2.3314E4	2	2	103	115	Carbamidomethylation	C1:Propionamide:17.01;C2:Carbamidomethylation:1000.00	PEAKS PTM
R.C(+57.02)C(+71.04)NELNEFENNQR.C	Y	92.10	1739.6992	13	1.3	870.8580	2	28.90	6	F6:1332	OB5946 H2 Ro.raw	2.335E4	2	2	103	115	Carbamidomethylation	C1:Carbamidomethylation:1000.00;C2:Propionamide:0.00	PEAKS PTM
R.C(+57.02)MC(+57.02)EALQQIM(+15.99)ENQSDRLQGR.Q	Y	86.98	2482.0820	20	2.2	828.3698	3	31.37	4	F4:1354	OB5922 H2 Ro.raw	4.9214E5	3	3	116	135	Carbamidomethylation; Oxidation (M)	C1:Carbamidomethylation:1000.00;C3:Carbamidomethylation:1000.00;M10:Oxidation (M):99.62	PEAKS DB
R.C(+57.02)M(+15.99)C(+57.02)EALQQIMENQSDRLQGR.Q	Y	81.90	2482.0820	20	2.7	828.3702	3	32.38	4	F4:1416	OB5922 H2 Ro.raw	5.0048E5	3	3	116	135	Carbamidomethylation; Oxidation (M)	C1:Carbamidomethylation:1000.00;M2:Oxidation (M):113.22;C3:Carbamidomethylation:1000.00	PEAKS DB
R.C(+57.02)MC(+71.04)EALQQIMENQSDRLQGR.Q	Y	72.98	2480.1028	20	0.2	827.7084	3	33.64	6	F6:1632	OB5946 H2 Ro.raw	1.3204E5	1	1	116	135	Carbamidomethylation	C1:Carbamidomethylation:1000.00;C3:Propionamide:0.00	PEAKS PTM
R.C(-17.03)MC(+57.02)E(+57.02)ALQQIMENQSDRLQGR.Q	Y	61.66	2449.0608	20	-1.0	1225.5365	2	37.89	4	F4:1744	OB5922 H2 Ro.raw	8.4646E5	4	4	116	135	Ammonia-loss (C@N-term); Carbamidomethylation; Carbamidomethylation (DHKE, X@N-term)	C1:Ammonia-loss (C@N-term):1000.00;C3:Carbamidomethylation:1000.00;E4:Carbamidomethylation (DHKE, X@N-term):83.89	PEAKS PTM
R.C(+57.02)DLEVESGGRDRY	Y	55.61	1554.6732	13	1.2	778.3448	2	28.32	4	F4:1171	OB5922 H2 Ro.raw	0	0	0	160	172	Carbamidomethylation	C1:Carbamidomethylation:1000.00	PEAKS DB
**total 14 peptides**																			

Peptide	Uniq	-10lgP	Mass	Length	ppm	m/z	z	RT	Fraction	Scan	Source File	Area Roast Ara h 2	#Feature	#Feature Roast Ara h 2	Start	End	PTM	AScore	Found By
A.RQQWELQGDRR.C	Y	52.37	1470.7440	11	-0.4	491.2551	3	29.55	4	F4:1249	OB5922 H2 Ro.raw	1.0511E4	1	1	22	32			PEAKS DB
R.C(+57.02)M(+15.99)C(+57.02)EALQQIM(+15.99)ENQSDRLQGR.Q	Y	43.84	2498.0771	20	-0.1	833.6996	3	29.67	5	F5:1392	OB5945 H2 Ro.raw	6.8064E4	2	2	116	135	Carbamidomethylation; Oxidation (M)	C1:Carbamidomethylation:1000.00;M2:Oxidation (M):1000.00;C3:Carbamidomethylation:1000.00;M10:Oxidation (M):1000.00	PEAKS DB
R.C(-17.03)C(+57.02)NE(+57.02)LNEFENNQR.C	Y	42.16	1708.6570	13	0.2	855.3359	2	32.82	5	F5:1595	OB5945 H2 Ro.raw	1.523E4	1	1	103	115	Ammonia-loss (C@N-term); Carbamidomethylation	C1:Ammonia-loss (C@N-term):1000.00;C2:Carbamidomethylation:1000.00;E4:Carbamidomethylation (DHKE, X@N-term):38.16	PEAKS PTM
R.NLPQQC(+57.02)GLR.A	Y	39.49	1084.5448	9	3.1	543.2814	2	26.19	4	F4:1068	OB5922 H2 Ro.raw	2.4455E3	1	1	147	155	Carbamidomethylation	C6:Carbamidomethylation:1000.00	PEAKS DB
R.C(+71.04)MC(+57.02)EALQQIM(+15.99)ENQSDRLQGR.Q	Y	35.15	2496.0979	20	2.9	833.0424	3	31.03	6	F6:1469	OB5946 H2 Ro.raw	1.5949E4	1	1	116	135	Carbamidomethylation; Oxidation (M)	C1:Propionamide:15.21;C3:Carbamidomethylation:1000.00;M10:Oxidation (M):63.64	PEAKS PTM
**total 14 peptides**																			

#CONTAM#P00761

back to list

| Protein Coverage | Supporting Peptides |

Protein Coverage:

```
  1  FPTDDDDKIV GGYTCAANSI PYQVSLNSGS HFCGGSLINS QWVVSAAHCY KSRIQVRLGE HNIDVLEGNE QFINAAKIIT
 81  HPNFNGNTLD NDIMLIKLSS PATLNSRVAT VSLPRSCAAA GTECLISGWG NTKSSGSSYP SLLQCLKAPV LSDSSCKSSY
161  PGQITGNMIC VGFLEGGKDS CQGDSGGPVV CNGQLQGIVS WGYGCAQKNK PGVYTKVCNY VNWIQQTIAA N
```

Supporting Peptides:

Peptide	Uniq	-10lgP	Mass	Length	ppm	m/z	z	RT	Fraction	Scan	Source File	Area Roast Ara h 2	#Feature	#Feature Roast Ara h 2	Start	End	PTM	AScore	Found By
R.LGEHNIDVLEGNEQFIN.A	Y	81.75	1939.9275	17	0.7	970.9717	2	33.79	4	F4:1501	OB5922 H2 Ro.raw	2.411E5	3	3	58	74			PEAKS DB

total 2 peptides

Peptide	Uniq	-10lgP	Mass	Length	ppm	m/z	z	RT	Fraction	Scan	Source File	Area Roast Ara h 2	#Feature	#Feature Roast Ara h 2	Start	End	PTM	AScore	Found By
R.VATVSLPR.S	Y	51.16	841.5021	8	0.6	421.7586	2	27.54	6	F6:1247	OB5946 H2 Ro.raw	8.6311E4	3	3	108	115			PEAKS DB
**total 2 peptides**																			

Peptide List

Prepared with PEAKS ™ (bioinfor.com)

Summary

Ara h 3 GEL BAND RAW

1. Notes

Verz verz faint Ara h 3 isoform in this project dedicated to Ara h 3 and this is sample RAW Ara h 3. PTM66

2. Result Statistics

Figure 1. False discovery rate (FDR) curve. X axis is the number of peptides being kept. Y axis is the corresponding FDR.

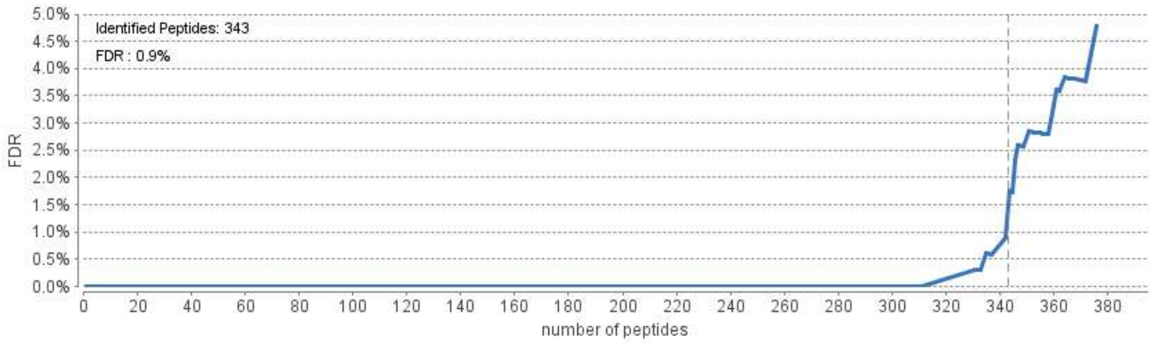

Figure 2. PSM score distribution. **(a)** Distribution of PEAKS peptide score; **(b)** Scatterplot of PEAKS peptide score versus precursor mass error.

(a)

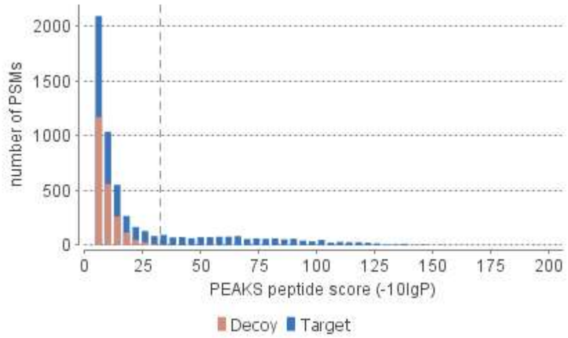

(b)

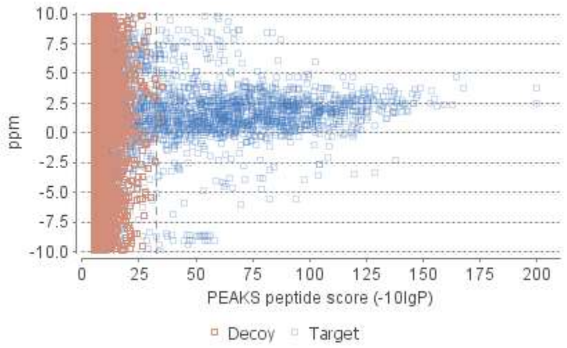

Table 1. Statistics of data.

	#Scans			#Features	Identified			#Peptides	#Sequences	#Proteins*		
	MS1	MS/MS	#Chimera		#PSMs	#Scans	#Features**			Groups	All	Top
Total	13358	10233	1612	27139	1390	1352	935	343	275	27	42	34

Raw Ara h 3	13358	10233	1612	27139	1390	1352	935	343	275	27	42	34

* proteins with significant peptides are used in counts.

** features are identified by DB search only.

Figure 3. Sample overlap for Proteins and Peptides (up to 8 samples). **(a)** All Proteins; **(b)** Top Proteins; **(c)** Peptides;

(a)
Not applicable to only one sample

(b)
Not applicable to only one sample

(c)
Not applicable to only one sample

Figure 4. Distribution of peptide feature detection. **(a)** Feature m/z distribution; **(b)** Feature RT distribution.

(a)

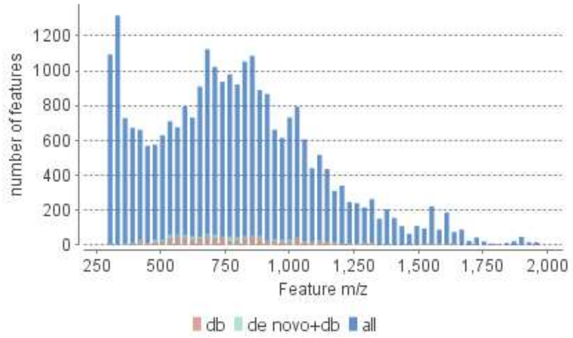

(b)

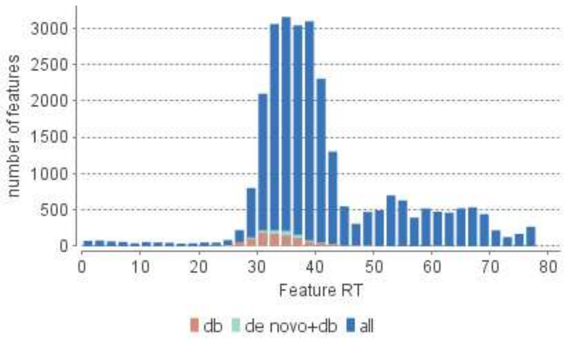

Figure 5. Distribution of identified peptide features. **(a)** Feature abundance distribution; **(b)** *De novo* sequencing validation.

(a)

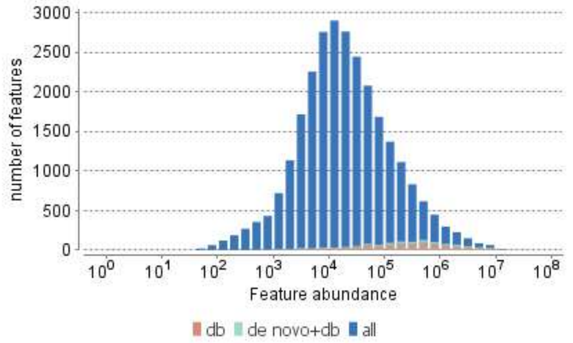

(b)

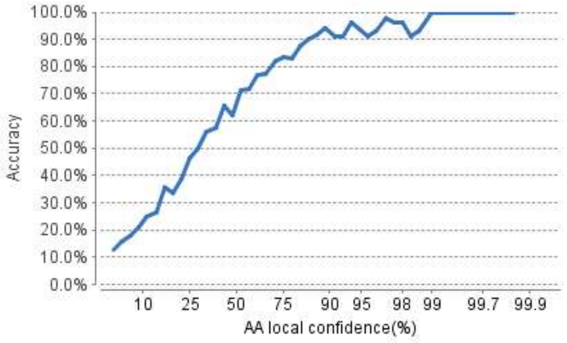

Table 2. Result filtration parameters.

Peptide -10lgP	≥32.7
PTM Ascore	≥50
Protein -10lgP	≥20
Proteins unique peptides	≥2
De novo score(%)	≥50%

Table 4. PTM profile.

Name	ΔMass	Position	#PSM	-10lgP	Abundance	AScore
Carbamidomethyl	57.02	C	147	156.27	1.03E5	1000.00
Oxidation	15.99	M	65	159.13	6.58E5	1000.00
Deamidation	.98	NQ	44	138.99	6.29E4	102.87

Table 3. Statistics of filtered result.

FDR (Peptide-Spectrum Matches)	0.2%
FDR (Peptide Sequences)	0.9%
FDR (Protein Group)	0.0%
De Novo Only Spectra	301

Acetylation	42.01	Protein N-term	29	103.49	6.15E4	1000.00
SMA	127.06	N-term	23	58.01	6.87E6	1000.00
Carbamidomethyl	57.02	DE,N-term	21	96.93	7.12E4	30.02
Methylation(others)	14.02	DHST	5	58.57	6.15E5	41.83
Methylation(KR)	14.02	KR	5	61.96		1000.00
Ammonia loss	-17.03	N-term	4	67.01	4.09E5	1000.00
Dihydroxy	31.99	PY	4	58.57	6.15E5	104.80
Oxidation	15.99	PR	3	47.01	1.8E4	20.78
Deoxy	-15.99	S	1	36.45	1.67E4	40.00
Biotin	226.08	N-term	1	33.77		1000.00
Dimethylation(KR)	28.03	R	1	89.94	3.05E5	1000.00
Acetyl	42.01	S	1	70.57	1.3E6	0.00
Benzoyl	104.03	N-term	1	38.94	1.27E5	124.10
MethylamineST	13.03	S	1	46.36		16.65
Iodination	125.90	Y	1	42.00	2.41E4	1000.00
B-methylthiol	45.99	D	1	64.39	6.35E5	71.27

3. Experiment Control

Figure 6. Precursor mass error of peptide-spectrum matches (PSM) in filtered result. **(a)** Distribution of precursor mass error in ppm; **(b)** Scatterplot of precursor m/z versus precursor mass error in ppm.

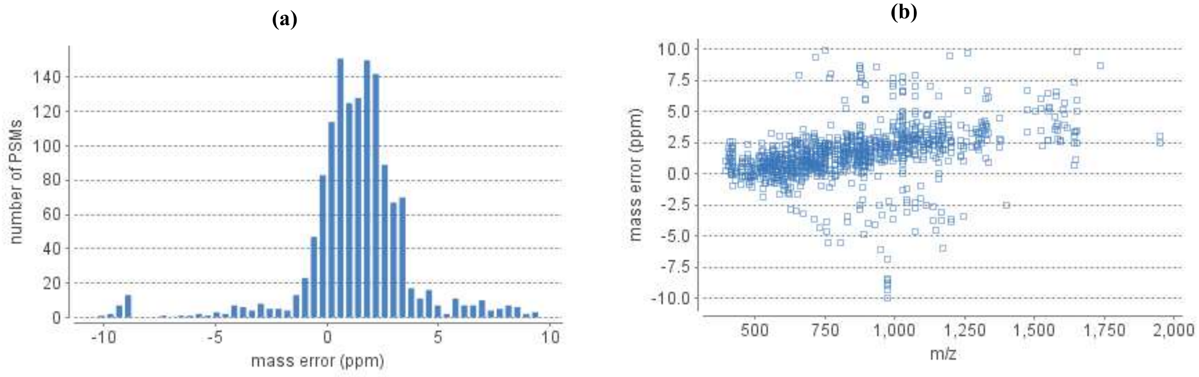

Table 5. Number of identified peptides in each sample by the number of missed cleavages.

Missed Cleavages	0	1	2	3	4+
Raw Ara h 3	166	136	41	0	0

4. Other Information

Table 6. Search parameters.

PEAKS Version: PEAKS Studio 10.6 build 20201015
Search Engine Name: PEAKS
Parent Mass Error Tolerance: 10.0 ppm
Fragment Mass Error Tolerance: 0.5 Da
Precursor Mass Search Type: monoisotopic
Enzyme: Trypsin
Max Missed Cleavages: 2
Digest Mode: Semispecific
Fixed Modifications:
 Carbamidomethylation: 57.02
Variable Modifications:
 Oxidation (M): 15.99
 Deamidation (NQ): 0.98
 Acetylation (K): 42.01
 Acetylation (Protein N-term): 42.01
 Acetylation (N-term): 42.01
 Amidation: -0.98
 Beta-methylthiolation: 45.99
 Biotinylation: 226.08
 and 303 more...
Max Variable PTM Per Peptide: 5
Database: Uniprot_Peanut-3818_Jul18
Taxon: All
Contaminant Database: contam MQ
Searched Entry: 1723
FDR Estimation: Enabled
De novo score(%) threshold: 15
Peptide hit threshold (-10logP): 30.0
Peaks run ID: 65
Merge Options: no merge
Precursor Options: corrected
Charge Options: no correction
Filter m/z: 200.0 - 2000.0
Filter Charge: 2 - 12
Process: true
Associate chimera: yes

Table 7. Instrument parameters.

Fractions: OB5936 H3A raw.raw, OB5937 H3A raw.raw, OB5938 H3A raw.raw, OB5925 H3B raw.raw, OB5934 H3B raw.raw, OB5935 H3B raw.raw
Ion Source: ESI(nano-spray)
Fragmentation Mode: CID, CAD(y and b ions)
MS Scan Mode: FT-ICR/Orbitrap
MS/MS Scan Mode: FT-ICR/Orbitrap

Protein List

Protein Accession Contains:
Protein Description Contains:
Protein Sample Area >=
Protein PTM Contains:

Protein Group	Protein ID	Accession	-10lgP	Coverage (%)	Coverage (%) Raw Ara h 3	Area Raw Ara h 3	#Peptides	#Unique	#Spec Raw Ara h 3	PTM	Avg. Mass	Description
1	5	N1NG13|N1NG13_ARAHY	542.39	47	47	5.9226E6	38	8	351	Y	71345	Seed storage protein Ara h1 OS=Arachis hypogaea OX=3818 GN=ARAX_AHF417E07-017 PE=4 SV=1
1	4	sp|P43238|ALL12_ARAHY	542.39	47	47	5.9226E6	38	8	351	Y	71345	Allergen Ara h 1, clone P41B OS=Arachis hypogaea OX=3818 PE=1 SV=1
2	1	Q6PSU3|Q6PSU3_ARAHY	534.59	52	52	5.5582E6	38	3	342	Y	66575	Conarachin (Fragment) OS=Arachis hypogaea OX=3818 PE=4 SV=1
2	3	sp|P43237|ALL11_ARAHY	534.59	49	49	5.5582E6	38	3	342	Y	70283	Allergen Ara h 1, clone P17 OS=Arachis hypogaea OX=3818 PE=1 SV=1

total 34 proteins

Protein Group	Protein ID	Accession	-10lgP	Coverage (%)	Coverage (%) Raw Ara h 3	Area Raw Ara h 3	#Peptides	#Unique	#Spec Raw Ara h 3	PTM	Avg. Mass	Description
**2**	2	B3IXL2|B3IXL2_ARAHY	534.59	49	49	5.5582E6	38	3	342	Y	70283	Main allergen Ara h1 OS=Arachis hypogaea OX=3818 PE=2 SV=1
**6**	20	Q38711|Q38711_ARAHY	479.22	80	80	6.0276E6	18	2	146	Y	29134	Galactose-binding lectin (Fragment) OS=Arachis hypogaea OX=3818 GN=lec PE=2 SV =1
**7**	6	T2B9M0|T2B9M0_ARAHY	474.60	85	85	5.4325E7	30	30	138	Y	38383	Fructose-bisphosphate aldolase OS=Arachis hypogaea OX=3818 PE=2 SV=1
**8**	21	A0A089ZXL7|A0A089ZXL7_ARAHY	467.31	83	83	4.7657E5	19	3	125	Y	29407	Peanut agglutinin variant OS=Arachis hypogaea OX=3818 PE=2 SV=1
**4**	25	A1E2B0|A1E2B0_ARAHY	455.92	63	63	8.6911E7	18	16	163	Y	33520	11S seed storage globulin B1 OS=Arachis hypogaea OX=3818 PE=2 SV=1
**10**	11	A0A0F6VX63|A0A0F6VX63_ARAHY	387.54	66	66	4.6311E7	22	22	72	Y	36080	Annexin OS=Arachis hypogaea OX=3818 PE=2 SV=1
**9**	29	A0A0A6ZDP1|A0A0A6ZDP1_ARAHY	384.00	70	70	8.9057E7	14	5	97	Y	20101	Glyceraldehyde-3-phosphate dehydrogenase C2 OS=Arachis hypogaea OX=3818 PE= 2 SV=1
**15**	8	Q647H3|Q647H3_ARAHY	337.54	39	39	5.4041E5	13	2	52	Y	61532	Arachin Ahy-2 OS=Arachis hypogaea OX=3818 PE=2 SV=1
**13**	41	E9LFE8|E9LFE8_ARAHY	318.55	36	36	8.4221E6	6	4	54	Y	28290	11S arachin (Fragment) OS=Arachis hypogaea OX=3818 PE=2 SV=1
**19**	35	A0A0A6ZDT0|A0A0A6ZDT0_ARAHY	316.95	45	45	1.1133E6	11	2	40	Y	20099	Glyceraldehyde-3-phosphate dehydrogenase C2 OS=Arachis hypogaea OX=3818 PE= 4 SV=1
**11**	42	E9LFE9|E9LFE9_ARAHY	299.25	45	45	2.7097E7	9	9	56	Y	28300	11S arachin OS=Arachis hypogaea OX=3818 PE=2 SV=1
**23**	24	A0A0F7GF62|A0A0F7GF62_ARAHY	262.78	40	40	1.6839E6	8	8	16	Y	36134	Annexin OS=Arachis hypogaea OX=3818 GN=AnnAh6 PE=2 SV=1
**21**	27	Q6IWG5|Q6IWG5_ARAHY	242.80	38	38	7.7017E6	9	9	18	Y	58061	Glycinin (Fragment) OS=Arachis hypogaea OX=3818 PE=2 SV=1
**21**	28	Q0GM57|Q0GM57_ARAHY	242.80	38	38	7.7017E6	9	9	18	Y	58263	Iso-Ara h3 OS=Arachis hypogaea OX=3818 PE=2 SV=1
**26**	31	D8KXZ8|D8KXZ8_ARAHY	233.90	37	37	1.6879E4	9	2	13	Y	41475	Enoyl-ACP reductase 1-3 OS=Arachis hypogaea OX=3818 GN=ENR1-3 PE=2 SV=1
**24**	51	H6U596|H6U596_ARAHY	220.07	31	31	2.0502E7	6	6	15	N	19370	Alcohol dehydrogenase (Fragment) OS=Arachis hypogaea OX=3818 PE=2 SV=1
**20**	48	B4UW81|B4UW81_ARAHY	218.20	46	46	1.0555E7	5	5	23	Y	20343	Glutathione S-transferase 2 OS=Arachis hypogaea OX=3818 PE=2 SV=1
**22**	49	N1NKG9|N1NKG9_ARAHY	213.01	36	36	2.7737E6	4	4	17	N	26041	Seed maturation protein OS=Arachis hypogaea OX=3818 GN=ARAX_AHF417E07-003 PE=4 SV=1
**30**	46	Q42515|Q42515_ARAHY	201.87	21	21	9.0898E5	3	3	8	Y	28924	Chitinase (Class II) OS=Arachis hypogaea OX=3818 GN=chi2;2 PE=2 SV=1
**28**	37	A0A075M981|A0A075M981_ARAHY	199.37	16	16	1.3383E6	6	6	11	Y	51912	Ribulose bisphosphate carboxylase large chain (Fragment) OS=Arachis hypogaea OX= 3818 GN=rbcL PE=3 SV=1
**28**	38	A0A191UJ50|A0A191UJ50_ARAHY	199.37	16	16	1.3383E6	6	6	11	Y	52587	Ribulose bisphosphate carboxylase large chain OS=Arachis hypogaea OX=3818 GN=r bcL PE=3 SV=1
**28**	36	O20356|O20356_ARAHY	199.37	17	17	1.3383E6	6	6	11	Y	51526	Ribulose bisphosphate carboxylase large chain (Fragment) OS=Arachis hypogaea OX= 3818 GN=rbcL PE=3 SV=1
**31**	43	A0A0R4UXQ1|A0A0R4UXQ1_ARAHY	184.49	20	20	9.4117E5	4	4	7	Y	48348	3-ketoacyl-CoA thiolase OS=Arachis hypogaea OX=3818 GN=PKT PE=2 SV=1
**32**	50	sp|Q647H2|AHY3_ARAHY	155.59	3	3	5.4508E5	2	2	5	N	54569	Arachin Ahy-3 OS=Arachis hypogaea OX=3818 PE=1 SV=1
**37**	80	B4UWD5|B4UWD5_ARAHY	142.60	26	26	1.1738E6	2	2	4	N	15013	Proteasome subunit alpha type (Fragment) OS=Arachis hypogaea OX=3818 PE=2 SV =1
**33**	53	A5Z1R0|A5Z1R0_ARAHY	128.86	23	23	3.4109E5	2	2	5	Y	16920	Ara h 6 allergen OS=Arachis hypogaea OX=3818 GN=Ara h 6 PE=4 SV=1
**33**	52	sp|Q647G9|CONG_ARAHY	128.86	23	23	3.4109E5	2	2	5	Y	16920	Conglutin OS=Arachis hypogaea OX=3818 PE=1 SV=1
**25**	58	#CONTAM#P00761	123.14	15	15	2.8404E5	3	3	14	N	24409	SWISS-PROT:#CONTAM#P00761|TRYP_PIG Trypsin - Sus scrofa (Pig).
**39**	59	E5FHZ2|E5FHZ2_ARAHY	120.16	20	20	7.7408E5	3	3	3	N	27370	Late embryogenesis abundant protein group 5 protein OS=Arachis hypogaea OX=381 8 GN=LEA5-2 PE=2 SV=1
**35**	120	Q06H31|Q06H31_ARAHY	106.48	26	26	9.4736E5	2	2	5	Y	19128	Translationally controlled tumor-like protein OS=Arachis hypogaea OX=3818 PE=2 SV =1
**total 34 proteins**												

N1NG13|N1NG13_ARAHY

back to list

| Protein Coverage | Supporting Peptides |

Protein Coverage:

1 MRGRVSPLML LLGILVLASV SATHAKSSPY QKKTENPCAQ RCLQSCQQEP DDLKQKACES RCTKLEYDPR CVYDPRGHTG
81 TTNQRSPPGE RTRGRQPGDY DDDRRQPRRE EGGRWGPAGP REREREEDWR QPREDWRRPS HQQPRKIRPE GREGEQEWGT
161 PGSHVR**EETS RNNPFYFPSR R**FSTRYGNQN GRIRVLQRFD QRSRQFQNLQ NHR**IVQIEAK PNTLVLPKHA DADNILVIQQ**
241 **GQATVTVANG NNRKSFNLDE GHALRIPSGF ISYILNRHDN QNLR**VAK**ISM PVNTPGQFED FFPASSRDQS SYLQGFSRNT**
321 **LEAAFNAEFN EIRRVLLEEN AGGEQEERGQ RR**WSTR**SSEN NEGVIVKVSK EHVEELTK**HA KSVSK**KGSEE EGDITNPINL**
435
401 **REGEPDLSNN FGKLFEVKPD KKNPQLQDLD MMLTCVEIKE GALMLPHFNS KAMVIVVVNK GTGNLELVAV RK**EQQQRGRR
481 EEEEDEDEEE EGSNREVRRY TARLKEGDVF IMPAAHPVAI NASSELHLLG FGINAENNHR **IFLAGDKDNV IDQIEKQAKD**
561 **LAFPGSGEQV EKLIK**NQKES HFVSARPQSQ SQSPSSPEKE SPEKEDQEEE NQGGKGPLLS ILKAFN

C Carbamidomethylation (+57.02)
O Oxidation (M) (+15.99)

Supporting Peptides:

Peptide	Uniq	-10lgP	Mass	Length	ppm	m/z	z	RT	Fraction	Scan	Source File	Area Raw Ara h 3	#Feature	#Feature Raw Ara h 3	Start	End	PTM	AScore	Found By
K.ISMPVNTPGQFEDFFPASSR.D	N	164.25	2226.0415	20	4.6	1114.0332	2	35.91	4	F4:1601	OB5925 H3B raw.raw	7.0048E6	6	6	288	307			PEAKS DB
K.ISM(+15.99)PVNTPGQFEDFFPASSR.D	N	159.13	2242.0364	20	2.3	1122.0281	2	34.42	4	F4:1522	OB5925 H3B raw.raw	1.6427E6	5	5	288	307	Oxidation (M)	M3:Oxidation (M):1000.00	PEAKS DB
K.NPQLQDLDMMLTC(+57.02)VEIK.E	N	156.27	2046.9788	17	2.3	1024.4990	2	37.39	5	F5:1843	OB5934 H3B raw.raw	3.0752E5	3	3	423	439	Carbamidomethylation	C13:Carbamidomethylation:1000.00	PEAKS DB
K.KGSEEEGDITNPINLREGEPDLSNNFGK.L	Y	143.03	3058.4478	28	1.8	1020.4917	3	31.22	5	F5:1480	OB5934 H3B raw.raw	4.8628E5	7	7	386	413			PEAKS DB
K.HADADNILVIQQGQATVTVAN(+.98)GNNRK.S	N	134.38	2747.3950	26	2.1	916.8075	3	29.70	2	F2:1436	OB5937 H3A raw.raw	1.18E6	5	5	229	254		N21:Deamidation (NQ): 15.73	PEAKS DB
R.IFLAGDKDNVIDQIEKQAK.D	N	133.54	2144.1477	19	1.1	715.7240	3	32.93	5	F5:1578	OB5934 H3B raw.raw	3.7212E6	6	6	541	559			PEAKS DB
R.IPSGFISYILNR.H	N	131.66	1378.7609	12	1.8	690.3889	2	37.02	5	F5:1820	OB5934 H3B raw.raw	2.3128E7	6	6	266	277			PEAKS DB
K.SFNLDEGHALRIPSGFISYILNR.H	N	130.89	2618.3604	23	2.5	873.7963	3	40.61	4	F4:1864	OB5925 H3B raw.raw	4.4362E7	16	16	255	277			PEAKS DB
R.NTLEAAFNAEFNEIR.R	N	130.33	1737.8322	15	2.0	869.9250	2	35.72	4	F4:1599	OB5925 H3B raw.raw	4.8629E5	2	2	319	333			PEAKS DB

total 52 peptides

Peptide	Uniq	-10lgP	Mass	Length	ppm	m/z	z	RT	Fraction	Scan	Source File	Area Raw Ara h 3	#Feature	#Feature Raw Ara h 3	Start	End	PTM	AScore	Found By
R.IFLAGDKDNVIDQIEK.Q	N	122.64	1816.9570	16	2.7	909.4882	2	32.53	5	F5:1557	OB5934 H3B raw.raw	2.0088E7	12	12	541	556			PEAKS DB
K.SFNLDEGHALR.I	N	115.46	1257.6101	11	-0.1	629.8123	2	28.39	2	F2:1360	OB5937 H3A raw.raw	8.0983E5	11	11	255	265			PEAKS DB
R.DQSSYLQGFSR.N	N	113.23	1286.5891	11	2.4	644.3033	2	31.02	2	F2:1506	OB5937 H3A raw.raw	1.7114E6	5	5	308	318			PEAKS DB
K.GSEEEGDITNPINLREGEPDLSNNFGK.L	Y	109.87	2930.3530	27	1.6	977.7932	3	32.18	4	F4:1388	OB5925 H3B raw.raw	6.897E5	3	3	387	413			PEAKS DB
K.HADADNILVIQQGQATVTVANGNNRK.S	N	109.04	2746.4111	26	1.7	916.4792	3	29.52	2	F2:1425	OB5937 H3A raw.raw	1.1363E6	5	5	229	254			PEAKS DB
K.AMVIVVVNKGTGNLELVAVRK.E	N	105.72	2209.2981	21	0.6	737.4404	3	34.43	1	F1:1679	OB5936 H3A raw.raw	1.0985E7	7	7	452	472			PEAKS DB
R.IPSGFISYILNRHDNQNLR.V	N	105.41	2256.1763	19	1.1	753.0669	3	34.19	2	F2:1689	OB5937 H3A raw.raw	3.796E7	18	18	266	284			PEAKS DB
K.KGSEEEGDITNPINLR.E	Y	103.49	1770.8748	16	0.6	886.4452	2	28.95	2	F2:1396	OB5937 H3A raw.raw	1.4319E5	11	11	386	401			PEAKS DB
K.DLAFPGSGEQVEKLIK.N	N	102.68	1729.9249	16	1.0	865.9706	2	33.30	5	F5:1601	OB5934 H3B raw.raw	6.2551E5	3	3	560	575			PEAKS DB
R.VLLEENAGGEQEER.G	N	97.57	1571.7427	14	0.2	786.8788	2	27.11	2	F2:1274	OB5937 H3A raw.raw	1.1444E5	4	4	335	348			PEAKS DB
R.IVQIEAKPNTLVLPK.H	Y	92.83	1662.0079	15	2.0	832.0129	2	30.46	2	F2:1476	OB5937 H3A raw.raw	4.4939E6	12	12	214	228			PEAKS DB
R.NTLEAAFNAEFNEIRR.V	N	92.46	1893.9332	16	2.6	947.9763	2	34.61	5	F5:1666	OB5934 H3B raw.raw	5.3025E7	15	15	319	334			PEAKS DB
K.AM(+15.99)VIVVVNKGTGNLELVAVR.K	N	91.27	2097.1980	20	1.9	1049.6083	2	33.11	5	F5:1594	OB5934 H3B raw.raw	2.0031E6	4	4	452	471	Oxidation (M)	M2:Oxidation (M):1000.00	PEAKS DB
K.NPQLQDLDMMLTC(+57.02)VEIKEGALMLPHFNSK.A	N	90.42	3371.6387	29	0.9	843.9177	4	38.28	2	F2:1944	OB5937 H3A raw.raw	7.6836E6	5	5	423	451	Carbamidomethylation	C13:Carbamidomethylation:1000.00	PEAKS DB
K.DLAFPGSGEQVEK.L	N	90.32	1375.6619	13	1.8	688.8395	2	30.46	2	F2:1473	OB5937 H3A raw.raw	8.5836E5	6	6	560	572			PEAKS DB
K.AMVIVVVNKGTGNLELVAVR.K	N	88.82	2081.2031	20	2.3	1041.6112	2	34.47	3	F3:1725	OB5938 H3A raw.raw	3.2769E6	3	3	452	471			PEAKS DB
R.NNPFYFPSRR.F	N	88.17	1296.6364	10	1.1	649.3262	2	29.14	3	F3:1401	OB5938 H3A raw.raw	2.2468E6	9	9	172	181			PEAKS DB
K.ISMPVNTPGQFEDFFPASSRDQSSYLQGFSR.N	N	84.22	3494.6201	31	2.7	1165.8838	3	36.29	4	F4:1620	OB5925 H3B raw.raw	1.0081E7	6	6	288	318			PEAKS DB
K.HADADNILVIQQGQATVTVANGNN(+.98)RK.S	N	79.42	2747.3950	26	4.5	1374.7109	2	29.70	2	F2:1444	OB5937 H3A raw.raw	1.1778E4	1	1	229	254		N24:Deamidation (NQ):7.21	PEAKS DB
R.SSENNEGVIVK.V	Y	77.21	1174.5830	11	0.7	588.2992	2	23.63	3	F3:1098	OB5938 H3A raw.raw	1.1897E4	3	3	357	367			PEAKS DB
K.SFNLDEGHALRIPSGFISYILNRHDNQNLR.V	N	70.02	3495.7759	30	0.6	700.1629	5	38.32	1	F1:1912	OB5936 H3A raw.raw	2.2417E7	7	7	255	284			PEAKS DB

total 52 peptides

Peptide	Uniq	-10lgP	Mass	Length	ppm	m/z	z	RT	Fraction	Scan	Source File	Area Raw Ara h 3	#Feature	#Feature Raw Ara h 3	Start	End	PTM	AScore	Found By
R.VLLEENAGGEQEERGQR.R	N	65.63	1912.9238	17	0.0	638.6486	3	29.50	1	F1:1389	OB5936 H3A raw.raw	4.0372E4	2	2	335	351			PEAKS DB
R.IFLAGDKDNVIDQ(+.98)IEKQAK.D	N	64.02	2145.1316	19	9.3	716.0578	3	32.93	4	F4:1432	OB5925 H3B raw.raw	1.5596E6	1	1	541	559		Q13:Deamidation (NQ): 0.00	PEAKS DB
K.GTGNLELVAVR.K	N	62.22	1127.6299	11	0.1	564.8223	2	29.90	3	F3:1460	OB5938 H3A raw.raw	1.5877E5	3	3	461	471			PEAKS DB
K.AMVIVVVNK.G	N	61.23	971.5837	9	-0.3	486.7990	2	29.89	4	F4:1251	OB5925 H3B raw.raw	0	0	0	452	460			PEAKS DB
R.NNPFYFPSR.R	N	60.62	1140.5352	9	-0.1	571.2748	2	31.58	3	F3:1564	OB5938 H3A raw.raw	4.133E4	1	1	172	180			PEAKS DB
K.ISM(+15.99)PVNTPGQFEDFFPASSRDQSSYLQGFSR.N	N	59.85	3510.6150	31	1.9	1171.2145	3	34.93	3	F3:1753	OB5938 H3A raw.raw	4.1096E6	6	6	288	318	Oxidation (M)	M3:Oxidation (M):1000.00	PEAKS DB
K.HADADNILVIQQGQ(+.98)ATVTVANGNNRK.S	N	57.58	2747.3950	26	0.5	687.8564	4	29.52	3	F3:1436	OB5938 H3A raw.raw	1.466E5	1	1	229	254		Q14:Deamidation (NQ): 5.77	PEAKS DB
K.NPQLQDLDMMLTC(+57.02)VEIKEGALM(+15.99)LPHFNSK.A	N	54.78	3387.6335	29	2.9	847.9181	4	37.33	2	F2:1882	OB5937 H3A raw.raw	2.6128E6	2	2	423	451	Carbamidomethylation	C13:Carbamidomethylation:1000.00;M22:Oxidation (M):19.27	PEAKS DB
R.IPSGFISYILN(+.98)RHDNQNLR.V	N	53.84	2257.1604	19	9.9	753.4015	3	37.61	4	F4:1698	OB5925 H3B raw.raw	2.7971E5	1	1	266	284		N11:Deamidation (NQ): 33.06	PEAKS DB
R.EETSRNNPFYFPSRR.F	N	53.22	1898.9023	15	-0.2	633.9746	3	28.39	3	F3:1372	OB5938 H3A raw.raw	2.391E4	1	1	167	181			PEAKS DB
R.I(+57.02)FLAGDKDNVIDQIEK.Q	N	51.72	1873.9785	16	1.3	937.9977	2	32.56	4	F4:1413	OB5925 H3B raw.raw	1.5532E5	1	1	541	556		I1:Carbamidomethylation (DHKE, X@N-term):32.60	PEAKS PTM
K.GSEEEGDITNPINLR.E	Y	50.96	1642.7798	15	0.4	822.3975	2	32.95	1	F1:1607	OB5936 H3A raw.raw	7.6191E3	1	1	387	401			PEAKS DB
K.GTGNLELVAVRK.E	N	49.78	1255.7249	12	0.1	628.8698	2	27.83	2	F2:1327	OB5937 H3A raw.raw	1.327E4	2	2	461	472			PEAKS DB
K.LFEVKPDK.K	N	47.93	974.5436	8	0.9	488.2795	2	26.83	2	F2:1264	OB5937 H3A raw.raw	1.0234E3	1	1	414	421			PEAKS DB
K.KGSEEEGDITNPINLREGEPDLSNN(+.98)FGK.L	Y	46.27	3059.4319	28	7.6	765.8711	4	31.46	6	F6:1493	OB5935 H3B raw.raw	3.4976E4	1	1	386	413		N25:Deamidation (NQ): 0.00	PEAKS DB
K.NPQLQDLDM(+15.99)MLTC(+57.02)VEIKEGALMLPHFNSK.A	N	45.78	3387.6335	29	-0.7	847.9150	4	39.43	5	F5:1972	OB5934 H3B raw.raw	2.2084E6	1	1	423	451	Carbamidomethylation	M9:Oxidation (M):0.00;C13:Carbamidomethylation:1000.00	PEAKS DB
R.VLLEENAGGEQEERGQRR.W	N	45.71	2069.0249	18	0.0	690.6823	3	27.83	2	F2:1326	OB5937 H3A raw.raw	3.3003E4	1	1	335	352			PEAKS DB
K.VSKEHVEELTK.H	Y	42.15	1297.6877	11	-3.0	649.8492	2	26.38	2	F2:1236	OB5937 H3A raw.raw	0	0	0	368	378			PEAKS DB
K.LFEVKPDKK.N	N	40.23	1102.6385	9	-0.7	552.3262	2	29.58	1	F1:1398	OB5936 H3A raw.raw	0	0	0	414	422			PEAKS DB
G.SEEEGDITNPINLREGEPDLSNNFGK.L	Y	39.45	2873.3315	26	2.9	958.7872	3	32.34	5	F5:1553	OB5934 H3B raw.raw	5.5034E4	1	1	388	413			PEAKS DB
K.HADADN(+.98)ILVIQQGQATVTVANGNNRK.S	N	37.99	2747.3950	26	2.5	916.8079	3	30.09	5	F5:1426	OB5934 H3B raw.raw	9.4717E4	1	1	229	254		N6:Deamidation (NQ): 0.00	PEAKS DB

total 52 peptides

Peptide	Uniq	-10lgP	Mass	Length	ppm	m/z	z	RT	Fraction	Scan	Source File	Area Raw Ara h 3	#Feature	#Feature Raw Ara h 3	Start	End	PTM	AScore	Found By
K.SFN(+.98)LDEGHALRIPSGFISYILNR.H	N	36.85	2619.3445	23	8.5	874.1295	3	48.22	4	F4:2372	OB5925 H3B raw.raw	1.3134E4	1	1	255	277		N3:Deamidation (NQ):32.08	PEAKS DB
**total 52 peptides**																			

sp|P43238|ALL12_ARAHY

back to list

| Protein Coverage | Supporting Peptides |

Protein Coverage:

```
1   MRGRVSPLML LLGILVLASV SATHAKSSPY QKKTENPCAQ RCLQSCQQEP DDLKQKACES RCTKLEYDPR CVYDPRGHTG
81  TTNQRSPPGE RTRGRQPGDY DDDRRQPRRE EGGRWGPAGP REREREEDWR QPREDWRRPS HQQPRKIRPE GREGEQEWGT
161 PGSHVREETS RNNPFYFPSR RFSTRYGNQN GRIRVLQRFD QRSRQFQNLQ NHRIVQIEAK PNTLVLPKHA DADNILVIQQ
241 GQATVTVANG NNRKSFNLDE GHALRIPSGF ISYILNRHDN QNLRVAKISM PVNTPGQFED FFPASSRDQS SYLQGFSRNT
321 LEAAFNAEFN EIRRVLLEEN AGGEQEERGQ RRWSTRSSEN NEGVIVKVSK EHVEELTKHA KSVSKKGSEE EGDITNPINL
401 REGEPDLSNN FGKLFEVKPD KKNPQLQDLD MMLTCVEIKE GALMLPHFNS KAMVIVVVNK GTGNLELVAV RKEQQQRGRR
481 EEEEDEDEEE EGSNREVRRY TARLKEGDVF IMPAAHPVAI NASSELHLLG FGINAENNHR IFLAGDKDNV IDQIEKQAKD
561 LAFPGSGEQV EKLIKNQKES HFVSARPQSQ SQSPSSPEKE SPEKEDQEEE NQGGKGPLLS ILKAFN
```

C Carbamidomethylation (+57.02)
O Oxidation (M) (+15.99)

Supporting Peptides:

Peptide	Uniq	-10lgP	Mass	Length	ppm	m/z	z	RT	Fraction	Scan	Source File	Area Raw Ara h 3	#Feature	#Feature Raw Ara h 3	Start	End	PTM	AScore	Found By
K.ISMPVNTPGQFEDFFPASSR.D	N	164.25	2226.0415	20	4.6	1114.0332	2	35.91	4	F4:1601	OB5925 H3B raw.raw	7.0048E6	6	6	288	307			PEAKS DB
K.ISM(+15.99)PVNTPGQFEDFFPASSR.D	N	159.13	2242.0364	20	2.3	1122.0281	2	34.42	4	F4:1522	OB5925 H3B raw.raw	1.6427E6	5	5	288	307	Oxidation (M)	M3:Oxidation (M):1000.00	PEAKS DB
K.NPQLQDLDMMLTC(+57.02)VEIK.E	N	156.27	2046.9788	17	2.3	1024.4990	2	37.39	5	F5:1843	OB5934 H3B raw.raw	3.0752E5	3	3	423	439	Carbamidomethylation	C13:Carbamidomethylation:1000.00	PEAKS DB
K.KGSEEEGDITNPINLREGEPDLSNNFGK.L	Y	143.03	3058.4478	28	1.8	1020.4917	3	31.22	5	F5:1480	OB5934 H3B raw.raw	4.8628E5	7	7	386	413			PEAKS DB
K.HADADNILVIQQGQATVTVAN(+.98)GNNRK.S	N	134.38	2747.3950	26	2.1	916.8075	3	29.70	2	F2:1436	OB5937 H3A raw.raw	1.18E6	5	5	229	254		N21:Deamidation (NQ):15.73	PEAKS DB

total 52 peptides

Peptide	Uniq	-10lgP	Mass	Length	ppm	m/z	z	RT	Fraction	Scan	Source File	Area Raw Ara h 3	#Feature	#Feature Raw Ara h 3	Start	End	PTM	AScore	Found By
R.IFLAGDKDNVIDQIEKQAK.D	N	133.54	2144.1477	19	1.1	715.7240	3	32.93	5	F5:1578	OB5934 H3B raw.raw	3.7212E6	6	6	541	559			PEAKS DB
R.IPSGFISYILNR.H	N	131.66	1378.7609	12	1.8	690.3889	2	37.02	5	F5:1820	OB5934 H3B raw.raw	2.3128E7	6	6	266	277			PEAKS DB
K.SFNLDEGHALRIPSGFISYILNR.H	N	130.89	2618.3604	23	2.5	873.7963	3	40.61	4	F4:1864	OB5925 H3B raw.raw	4.4362E7	16	16	255	277			PEAKS DB
R.NTLEAAFNAEFNEIR.R	N	130.33	1737.8322	15	2.0	869.9250	2	35.72	4	F4:1599	OB5925 H3B raw.raw	4.8629E5	2	2	319	333			PEAKS DB
R.IFLAGDKDNVIDQIEK.Q	N	122.64	1816.9570	16	2.7	909.4882	2	32.53	5	F5:1557	OB5934 H3B raw.raw	2.0088E7	12	12	541	556			PEAKS DB
K.SFNLDEGHALR.I	N	115.46	1257.6101	11	-0.1	629.8123	2	28.39	2	F2:1360	OB5937 H3A raw.raw	8.0983E5	11	11	255	265			PEAKS DB
R.DQSSYLQGFSR.N	N	113.23	1286.5891	11	2.4	644.3033	2	31.02	2	F2:1506	OB5937 H3A raw.raw	1.7114E6	5	5	308	318			PEAKS DB
K.GSEEEGDITNPINLREGEPDLSNNFGK.L	Y	109.87	2930.3530	27	1.6	977.7932	3	32.18	4	F4:1388	OB5925 H3B raw.raw	6.897E5	3	3	387	413			PEAKS DB
K.HADADNILVIQQGQATVTVANGNNRK.S	N	109.04	2746.4111	26	1.7	916.4792	3	29.52	2	F2:1425	OB5937 H3A raw.raw	1.1363E6	5	5	229	254			PEAKS DB
K.AMVIVVVNKGTGNLELVAVRK.E	N	105.72	2209.2981	21	0.6	737.4404	3	34.43	1	F1:1679	OB5936 H3A raw.raw	1.0985E7	7	7	452	472			PEAKS DB
R.IPSGFISYILNRHDNQNLR.V	N	105.41	2256.1763	19	1.1	753.0669	3	34.19	2	F2:1689	OB5937 H3A raw.raw	3.796E7	18	18	266	284			PEAKS DB
K.KGSEEEGDITNPINLR.E	Y	103.49	1770.8748	16	0.6	886.4452	2	28.95	2	F2:1396	OB5937 H3A raw.raw	1.4319E5	11	11	386	401			PEAKS DB
K.DLAFPGSGEQVEKLIK.N	N	102.68	1729.9249	16	1.0	865.9706	2	33.30	5	F5:1601	OB5934 H3B raw.raw	6.2551E5	3	3	560	575			PEAKS DB
R.VLLEENAGGEQEER.G	N	97.57	1571.7427	14	0.2	786.8788	2	27.11	2	F2:1274	OB5937 H3A raw.raw	1.1444E5	4	4	335	348			PEAKS DB
R.IVQIEAKPNTLVLPK.H	Y	92.83	1662.0079	15	2.0	832.0129	2	30.46	2	F2:1476	OB5937 H3A raw.raw	4.4939E6	12	12	214	228			PEAKS DB
R.NTLEAAFNAEFNEIRR.V	N	92.46	1893.9332	16	2.6	947.9763	2	34.61	5	F5:1666	OB5934 H3B raw.raw	5.3025E7	15	15	319	334			PEAKS DB
K.AM(+15.99)VIVVVNKGTGNLELVAVR.K	N	91.27	2097.1980	20	1.9	1049.6083	2	33.11	5	F5:1594	OB5934 H3B raw.raw	2.0031E6	4	4	452	471	Oxidation (M)	M2:Oxidation (M):1000.00	PEAKS DB
K.NPQLQDLDMMLTC(+57.02)VEIKEGALMLPHFNSK.A	N	90.42	3371.6387	29	0.9	843.9177	4	38.28	2	F2:1944	OB5937 H3A raw.raw	7.6836E6	5	5	423	451	Carbamidomethylation	C13:Carbamidomethylation:1000.00	PEAKS DB
K.DLAFPGSGEQVEK.L	N	90.32	1375.6619	13	1.8	688.8395	2	30.46	2	F2:1473	OB5937 H3A raw.raw	8.5836E5	6	6	560	572			PEAKS DB
K.AMVIVVVNKGTGNLELVAVR.K	N	88.82	2081.2031	20	2.3	1041.6112	2	34.47	3	F3:1725	OB5938 H3A raw.raw	3.2769E6	3	3	452	471			PEAKS DB
R.NNPFYFPSRR.F	N	88.17	1296.6364	10	1.1	649.3262	2	29.14	3	F3:1401	OB5938 H3A raw.raw	2.2468E6	9	9	172	181			PEAKS DB

total 52 peptides

Peptide	Uniq	-10lgP	Mass	Length	ppm	m/z	z	RT	Fraction	Scan	Source File	Area Raw Ara h 3	#Feature	#Feature Raw Ara h 3	Start	End	PTM	AScore	Found By
K.ISMPVNTPGQFEDFFPASSRDQSSYLQGFSR.N	N	84.22	3494.6201	31	2.7	1165.8838	3	36.29	4	F4:1620	OB5925 H3B raw.raw	1.0081E7	6	6	288	318			PEAKS DB
K.HADADNILVIQQGQATVTVANGNN(+.98)RK.S	N	79.42	2747.3950	26	4.5	1374.7109	2	29.70	2	F2:1444	OB5937 H3A raw.raw	1.1778E4	1	1	229	254		N24:Deamidation (NQ): 7.21	PEAKS DB
R.SSENNEGVIVK.V	Y	77.21	1174.5830	11	0.7	588.2992	2	23.63	3	F3:1098	OB5938 H3A raw.raw	1.1897E4	3	3	357	367			PEAKS DB
K.SFNLDEGHALRIPSGFISYILNRHDNQNLR.V	N	70.02	3495.7759	30	0.6	700.1629	5	38.32	1	F1:1912	OB5936 H3A raw.raw	2.2417E7	7	7	255	284			PEAKS DB
R.VLLEENAGGEQEERGQR.R	N	65.63	1912.9238	17	0.0	638.6486	3	29.50	1	F1:1389	OB5936 H3A raw.raw	4.0372E4	2	2	335	351			PEAKS DB
R.IFLAGDKDNVIDQ(+.98)IEKQAK.D	N	64.02	2145.1316	19	9.3	716.0578	3	32.93	4	F4:1432	OB5925 H3B raw.raw	1.5596E6	1	1	541	559		Q13:Deamidation (NQ): 0.00	PEAKS DB
K.GTGNLELVAVR.K	N	62.22	1127.6299	11	0.1	564.8223	2	29.90	3	F3:1460	OB5938 H3A raw.raw	1.5877E5	3	3	461	471			PEAKS DB
K.AMVIVVVNK.G	N	61.23	971.5837	9	-0.3	486.7990	2	29.89	4	F4:1251	OB5925 H3B raw.raw	0	0	0	452	460			PEAKS DB
R.NNPFYFPSR.R	N	60.62	1140.5352	9	-0.1	571.2748	2	31.58	3	F3:1564	OB5938 H3A raw.raw	4.133E4	1	1	172	180			PEAKS DB
K.ISM(+15.99)PVNTPGQFEDFFPASSRDQSSYLQGFSR.N	N	59.85	3510.6150	31	1.9	1171.2145	3	34.93	3	F3:1753	OB5938 H3A raw.raw	4.1096E6	6	6	288	318	Oxidation (M)	M3:Oxidation (M):1000.00	PEAKS DB
K.HADADNILVIQQGQ(+.98)ATVTVANGNNRK.S	N	57.58	2747.3950	26	0.5	687.8564	4	29.52	3	F3:1436	OB5938 H3A raw.raw	1.466E5	1	1	229	254		Q14:Deamidation (NQ): 5.77	PEAKS DB
K.NPQLQDLDMMLTC(+57.02)VEIKEGALM(+15.99)LPHFNSK.A	N	54.78	3387.6335	29	2.9	847.9181	4	37.33	2	F2:1882	OB5937 H3A raw.raw	2.6128E6	2	2	423	451	Carbamidomethylation	C13:Carbamidomethylation:1000.00;M22:Oxidation (M):19.27	PEAKS DB
R.IPSGFISYILN(+.98)RHDNQNLR.V	N	53.84	2257.1604	19	9.9	753.4015	3	37.61	4	F4:1698	OB5925 H3B raw.raw	2.7971E5	1	1	266	284		N11:Deamidation (NQ): 33.06	PEAKS DB
R.EETSRNNPFYFPSRR.F	N	53.22	1898.9023	15	-0.2	633.9746	3	28.39	3	F3:1372	OB5938 H3A raw.raw	2.391E4	1	1	167	181			PEAKS DB
R.I(+57.02)FLAGDKDNVIDQIEK.Q	N	51.72	1873.9785	16	1.3	937.9977	2	32.56	4	F4:1413	OB5925 H3B raw.raw	1.5532E5	1	1	541	556		I1:Carbamidomethylation (DHKE, X@N-term):32.60	PEAKS PTM
K.GSEEEGDITNPINLR.E	Y	50.96	1642.7798	15	0.4	822.3975	2	32.95	1	F1:1607	OB5936 H3A raw.raw	7.6191E3	1	1	387	401			PEAKS DB
K.GTGNLELVAVRK.E	N	49.78	1255.7249	12	0.1	628.8698	2	27.83	2	F2:1327	OB5937 H3A raw.raw	1.327E4	2	2	461	472			PEAKS DB
K.LFEVKPDK.K	N	47.93	974.5436	8	0.9	488.2795	2	26.83	2	F2:1264	OB5937 H3A raw.raw	1.0234E3	1	1	414	421			PEAKS DB
K.KGSEEEGDITNPINLREGEPDLSNN(+.98)FGK.L	Y	46.27	3059.4319	28	7.6	765.8711	4	31.46	6	F6:1493	OB5935 H3B raw.raw	3.4976E4	1	1	386	413		N25:Deamidation (NQ): 0.00	PEAKS DB
K.NPQLQDLDM(+15.99)MLTC(+57.02)VEIKEGALMLPHFNSK.A	N	45.78	3387.6335	29	-0.7	847.9150	4	39.43	5	F5:1972	OB5934 H3B raw.raw	2.2084E6	1	1	423	451	Carbamidomethylation	M9:Oxidation (M):0.00; C13:Carbamidomethylation:1000.00	PEAKS DB
R.VLLEENAGGEQEERGQRR.W	N	45.71	2069.0249	18	0.0	690.6823	3	27.83	2	F2:1326	OB5937 H3A raw.raw	3.3003E4	1	1	335	352			PEAKS DB

total 52 peptides

Peptide	Uniq	-10lgP	Mass	Length	ppm	m/z	z	RT	Fraction	Scan	Source File	Area Raw Ara h 3	#Feature	#Feature Raw Ara h 3	Start	End	PTM	AScore	Found By
K.VSKEHVEELTK.H	Y	42.15	1297.6877	11	-3.0	649.8492	2	26.38	2	F2:1236	OB5937 H3A raw.raw	0	0	0	368	378			PEAKS DB
K.LFEVKPDKK.N	N	40.23	1102.6385	9	-0.7	552.3262	2	29.58	1	F1:1398	OB5936 H3A raw.raw	0	0	0	414	422			PEAKS DB
G.SEEEGDITNPINLREGEPDLSNNFGK.L	Y	39.45	2873.3315	26	2.9	958.7872	3	32.34	5	F5:1553	OB5934 H3B raw.raw	5.5034E4	1	1	388	413			PEAKS DB
K.HADADN(+.98)ILVIQQGQATVTVANGNNRK.S	N	37.99	2747.3950	26	2.5	916.8079	3	30.09	5	F5:1426	OB5934 H3B raw.raw	9.4717E4	1	1	229	254		N6:Deamidation (NQ): 0.00	PEAKS DB
K.SFN(+.98)LDEGHALRIPSGFISYILNR.H	N	36.85	2619.3445	23	8.5	874.1295	3	48.22	4	F4:2372	OB5925 H3B raw.raw	1.3134E4	1	1	255	277		N3:Deamidation (NQ):3 2.08	PEAKS DB

total 52 peptides

Q6PSU3|Q6PSU3_ARAHY

back to list

| Protein Coverage | Supporting Peptides |

Protein Coverage:

1 MRGRVSPLML LLGILVLASV SATQAKSPYR KTENPCAQRC LQSCQQEPDD LKQKACESRC TKLEYDPRCV YDTGATNQRH

81 PPGERTRGRQ PGDYDDDRRQ PRREEGGRWG PAEPRERERE EDWRQPREDW RRPSHQQPRK IRPEGREGEQ EWGTPGSEVR

161 EETSRNNPFY FPSRRFSTRY GNQNGRIRVL QRFDQRSKQF QNLQNHRIVQ IEARPNTLVL PKHADADNIL VIQQGQATVT

241 VANGNNRKSF NLDEGHALRI PSGFISYILN RHDNQNLRVA KISMPVNTPG QFEDFFPASS RDQSSYLQGF SRNTLEAAFN

321 AEFNEIRRVL LEENAGGEQE ERGQRRRSTR SSDNEGVIVK VSKEHVQELT KHAKSVSKKG SEEEDITNPI NLRDGEPDLS

427

401 NNFGRLFEVK PDKKNPQLQD LDMMLTCVEI KEGALMLPHF NSKAMVIVVV NKGTGNLELV AVRKEQQQRG RREQEWEEEE

481 EDEEEEGSNR EVRRYTARLK EGDVFIMPAA HPVAINASSE LHLLGFGINA ENNHRIFLAG DKDNVIDQIE KQAKDLAFPG

561 SGEQVEKLIK NQRESHFVSA

C Carbamidomethylation (+57.02)
O Oxidation (M) (+15.99)

Supporting Peptides:

Peptide	Uniq	-10lgP	Mass	Length	ppm	m/z	z	RT	Fraction	Scan	Source File	Area Raw Ara h 3	#Feature	#Feature Raw Ara h 3	Start	End	PTM	AScore	Found By
K.ISMPVNTPGQFEDFFPASSR.D	N	164.25	2226.0415	20	4.6	1114.0332	2	35.91	4	F4:1601	OB5925 H3B raw.raw	7.0048E6	6	6	282	301			PEAKS DB

total 51 peptides

Peptide	Uniq	-10lgP	Mass	Length	ppm	m/z	z	RT	Fraction	Scan	Source File	Area Raw Ara h 3	#Feature	#Feature Raw Ara h 3	Start	End	PTM	AScore	Found By
K.ISM(+15.99)PVNTPGQFEDFFPASSR.D	N	159.13	2242.0364	20	2.3	1122.0281	2	34.42	4	F4:1522	OB5925 H3B raw.raw	1.6427E6	5	5	282	301	Oxidation (M)	M3:Oxidation (M):1000.00	PEAKS DB
K.NPQLQDLDMMLTC(+57.02)VEIK.E	N	156.27	2046.9788	17	2.3	1024.4990	2	37.39	5	F5:1843	OB5934 H3B raw.raw	3.0752E5	3	3	415	431	Carbamidomethylation	C13:Carbamidomethylation:1000.00	PEAKS DB
K.HADADNILVIQQGQATVTVAN(+.98)GNNRK.S	N	134.38	2747.3950	26	2.1	916.8075	3	29.70	2	F2:1436	OB5937 H3A raw.raw	1.18E6	5	5	223	248		N21:Deamidation (NQ):15.73	PEAKS DB
R.IFLAGDKDNVIDQIEKQAK.D	N	133.54	2144.1477	19	1.1	715.7240	3	32.93	5	F5:1578	OB5934 H3B raw.raw	3.7212E6	6	6	536	554			PEAKS DB
R.IPSGFISYILNR.H	N	131.66	1378.7609	12	1.8	690.3889	2	37.02	5	F5:1820	OB5934 H3B raw.raw	2.3128E7	6	6	260	271			PEAKS DB
K.SFNLDEGHALRIPSGFISYILNR.H	N	130.89	2618.3604	23	2.5	873.7963	3	40.61	4	F4:1864	OB5925 H3B raw.raw	4.4362E7	16	16	249	271			PEAKS DB
R.NTLEAAFNAEFNEIR.R	N	130.33	1737.8322	15	2.0	869.9250	2	35.72	4	F4:1599	OB5925 H3B raw.raw	4.8629E5	2	2	313	327			PEAKS DB
R.IFLAGDKDNVIDQIEK.Q	N	122.64	1816.9570	16	2.7	909.4882	2	32.53	5	F5:1557	OB5934 H3B raw.raw	2.0088E7	12	12	536	551			PEAKS DB
K.SFNLDEGHALR.I	N	115.46	1257.6101	11	-0.1	629.8123	2	28.39	2	F2:1360	OB5937 H3A raw.raw	8.0983E5	11	11	249	259			PEAKS DB
R.DQSSYLQGFSR.N	N	113.23	1286.5891	11	2.4	644.3033	2	31.02	2	F2:1506	OB5937 H3A raw.raw	1.7114E6	5	5	302	312			PEAKS DB
K.HADADNILVIQQGQATVTVANGNNRK.S	N	109.04	2746.4111	26	1.7	916.4792	3	29.52	2	F2:1425	OB5937 H3A raw.raw	1.1363E6	5	5	223	248			PEAKS DB
K.KGSEEEDITNPINLRDGEPDLSNNFGR.L	N	108.20	3015.4170	27	-0.3	754.8613	4	31.59	5	F5:1505	OB5934 H3B raw.raw	6.3819E5	7	7	379	405			PEAKS DB
K.AMVIVVVNKGTGNLELVAVRK.E	N	105.72	2209.2981	21	0.6	737.4404	3	34.43	1	F1:1679	OB5936 H3A raw.raw	1.0985E7	7	7	444	464			PEAKS DB
R.IPSGFISYILNRHDNQNLR.V	N	105.41	2256.1763	19	1.1	753.0669	3	34.19	2	F2:1689	OB5937 H3A raw.raw	3.796E7	18	18	260	278			PEAKS DB
K.DLAFPGSGEQVEKLIK.N	N	102.68	1729.9249	16	1.0	865.9706	2	33.30	5	F5:1601	OB5934 H3B raw.raw	6.2551E5	3	3	555	570			PEAKS DB
R.VLLEENAGGEQEER.G	N	97.57	1571.7427	14	0.2	786.8788	2	27.11	2	F2:1274	OB5937 H3A raw.raw	1.1444E5	4	4	329	342			PEAKS DB
R.NTLEAAFNAEFNEIRR.V	N	92.46	1893.9332	16	2.6	947.9763	2	34.61	5	F5:1666	OB5934 H3B raw.raw	5.3025E7	15	15	313	328			PEAKS DB
K.AM(+15.99)VIVVVNKGTGNLELVAVR.K	N	91.27	2097.1980	20	1.9	1049.6083	2	33.11	5	F5:1594	OB5934 H3B raw.raw	2.0031E6	4	4	444	463	Oxidation (M)	M2:Oxidation (M):1000.00	PEAKS DB
K.NPQLQDLDMMLTC(+57.02)VEIKEGALMLPHFNSK.A	N	90.42	3371.6387	29	0.9	843.9177	4	38.28	2	F2:1944	OB5937 H3A raw.raw	7.6836E6	5	5	415	443	Carbamidomethylation	C13:Carbamidomethylation:1000.00	PEAKS DB
K.DLAFPGSGEQVEK.L	N	90.32	1375.6619	13	1.8	688.8395	2	30.46	2	F2:1473	OB5937 H3A raw.raw	8.5836E5	6	6	555	567			PEAKS DB
K.AMVIVVVNKGTGNLELVAVR.K	N	88.82	2081.2031	20	2.3	1041.6112	2	34.47	3	F3:1725	OB5938 H3A raw.raw	3.2769E6	3	3	444	463			PEAKS DB

total 51 peptides

Peptide	Uniq	-10lgP	Mass	Length	ppm	m/z	z	RT	Fraction	Scan	Source File	Area Raw Ara h 3	#Feature	#Feature Raw Ara h 3	Start	End	PTM	AScore	Found By
R.NNPFYFPSRR.F	N	88.17	1296.6364	10	1.1	649.3262	2	29.14	3	F3:1401	OB5938 H3A raw.raw	2.2468E6	9	9	166	175			PEAKS DB
K.GSEEEDITNPINLRDGEPDLSNNFGR.L	N	86.64	2887.3220	26	1.0	963.4489	3	32.96	6	F6:1577	OB5935 H3B raw.raw	4.0621E5	3	3	380	405			PEAKS DB
R.IVQIEARPNTLVLPK.H	Y	84.29	1690.0140	15	2.2	846.0161	2	30.65	5	F5:1446	OB5934 H3B raw.raw	5.4418E6	12	12	208	222			PEAKS DB
K.ISMPVNTPGQFEDFFPASSRDQSSYLQGFSR.N	N	84.22	3494.6201	31	2.7	1165.8838	3	36.29	4	F4:1620	OB5925 H3B raw.raw	1.0081E7	6	6	282	312			PEAKS DB
R.SSDNEGVIVK.V	N	81.08	1046.5244	10	1.8	524.2704	2	23.93	2	F2:1106	OB5937 H3A raw.raw	1.3145E4	3	3	351	360			PEAKS DB
K.HADADNILVIQQGQATVTVANGNN(+.98)RK.S	N	79.42	2747.3950	26	4.5	1374.7109	2	29.70	2	F2:1444	OB5937 H3A raw.raw	1.1778E4	1	1	223	248		N24:Deamidation (NQ): 7.21	PEAKS DB
G.SEEEDITNPINLRDGEPDLSNNFGR.L	N	76.97	2830.3005	25	1.2	944.4419	3	32.96	6	F6:1576	OB5935 H3B raw.raw	5.6172E5	3	3	381	405			PEAKS DB
R.EGEQEWGTPGSEVREETSR.N	Y	74.60	2161.9512	19	2.0	721.6591	3	30.32	1	F1:1445	OB5936 H3A raw.raw	1.0653E5	3	3	147	165			PEAKS DB
K.SFNLDEGHALRIPSGFISYILNRHDNQNLR.V	N	70.02	3495.7759	30	0.6	700.1629	5	38.32	1	F1:1912	OB5936 H3A raw.raw	2.2417E7	7	7	249	278			PEAKS DB
R.VLLEENAGGEQEERGQR.R	N	65.63	1912.9238	17	0.0	638.6486	3	29.50	1	F1:1389	OB5936 H3A raw.raw	4.0372E4	2	2	329	345			PEAKS DB
R.IFLAGDKDNVIDQ(+.98)IEKQAK.D	N	64.02	2145.1316	19	9.3	716.0578	3	32.93	4	F4:1432	OB5925 H3B raw.raw	1.5596E6	1	1	536	554		Q13:Deamidation (NQ): 0.00	PEAKS DB
K.GTGNLELVAVR.K	N	62.22	1127.6299	11	0.1	564.8223	2	29.90	3	F3:1460	OB5938 H3A raw.raw	1.5877E5	3	3	453	463			PEAKS DB
K.AMVIVVVNK.G	N	61.23	971.5837	9	-0.3	486.7990	2	29.89	4	F4:1251	OB5925 H3B raw.raw	0	0	0	444	452			PEAKS DB
R.NNPFYFPSR.R	N	60.62	1140.5352	9	-0.1	571.2748	2	31.58	3	F3:1564	OB5938 H3A raw.raw	4.133E4	1	1	166	174			PEAKS DB
K.KGSEEEDITNPINLR.D	N	60.41	1713.8533	15	1.6	857.9352	2	31.45	1	F1:1515	OB5936 H3A raw.raw	9.8097E3	1	1	379	393			PEAKS DB
K.ISM(+15.99)PVNTPGQFEDFFPASSRDQSSYLQGFSR.N	N	59.85	3510.6150	31	1.9	1171.2145	3	34.93	3	F3:1753	OB5938 H3A raw.raw	4.1096E6	6	6	282	312	Oxidation (M)	M3:Oxidation (M):1000.00	PEAKS DB
K.HADADNILVIQQGQ(+.98)ATVTVANGNNRK.S	N	57.58	2747.3950	26	0.5	687.8564	4	29.52	3	F3:1436	OB5938 H3A raw.raw	1.466E5	1	1	223	248		Q14:Deamidation (NQ): 5.77	PEAKS DB
K.NPQLQDLDMMLTC(+57.02)VEIKEGALM(+15.99)LPHFNSK.A	N	54.78	3387.6335	29	2.9	847.9181	4	37.33	2	F2:1882	OB5937 H3A raw.raw	2.6128E6	2	2	415	443	Carbamidomethylation	C13:Carbamidomethylation:1000.00;M22:Oxidation (M):19.27	PEAKS DB
R.IPSGFISYILN(+.98)RHDNQNLR.V	N	53.84	2257.1604	19	9.9	753.4015	3	37.61	4	F4:1698	OB5925 H3B raw.raw	2.7971E5	1	1	260	278		N11:Deamidation (NQ): 33.06	PEAKS DB
R.EETSRNNPFYFPSRR.F	N	53.22	1898.9023	15	-0.2	633.9746	3	28.39	3	F3:1372	OB5938 H3A raw.raw	2.391E4	1	1	161	175			PEAKS DB
R.I(+57.02)FLAGDKDNVIDQIEK.Q	N	51.72	1873.9785	16	1.3	937.9977	2	32.56	4	F4:1413	OB5925 H3B raw.raw	1.5532E5	1	1	536	551		I1:Carbamidomethylation (DHKE, X@N-term):32.60	PEAKS PTM

total 51 peptides

Peptide	Uniq	-10lgP	Mass	Length	ppm	m/z	z	RT	Fraction	Scan	Source File	Area Raw Ara h 3	#Feature	#Feature Raw Ara h 3	Start	End	PTM	AScore	Found By
K.GTGNLELVAVRK.E	N	49.78	1255.7249	12	0.1	628.8698	2	27.83	2	F2:1327	OB5937 H3A raw.raw	1.327E4	2	2	453	464			PEAKS DB
R.LFEVKPDK.K	N	47.93	974.5436	8	0.9	488.2795	2	26.83	2	F2:1264	OB5937 H3A raw.raw	1.0234E3	1	1	406	413			PEAKS DB
K.NPQLQDLDM(+15.99)MLTC(+57.02)VEIKEGALMLPHFNSK.A	N	45.78	3387.6335	29	-0.7	847.9150	4	39.43	5	F5:1972	OB5934 H3B raw.raw	2.2084E6	1	1	415	443	Carbamidomethylation	M9:Oxidation (M):0.00; C13:Carbamidomethylation:1000.00	PEAKS DB
R.VLLEENAGGEQEERGQRR.R	N	45.71	2069.0249	18	0.0	690.6823	3	27.83	2	F2:1326	OB5937 H3A raw.raw	3.3003E4	1	1	329	346			PEAKS DB
R.WGPAEPR.E	Y	42.35	811.3976	7	0.6	406.7063	2	26.18	2	F2:1219	OB5937 H3A raw.raw	9.9043E3	2	2	109	115			PEAKS DB
R.LFEVKPDKK.N	N	40.23	1102.6385	9	-0.7	552.3262	2	29.58	1	F1:1398	OB5936 H3A raw.raw	0	0	0	406	414			PEAKS DB
K.HADADN(+.98)ILVIQQGQATVTVANGNNRK.S	N	37.99	2747.3950	26	2.5	916.8079	3	30.09	5	F5:1426	OB5934 H3B raw.raw	9.4717E4	1	1	223	248		N6:Deamidation (NQ): 0.00	PEAKS DB
K.SFN(+.98)LDEGHALRIPSGFISYILNR.H	N	36.85	2619.3445	23	8.5	874.1295	3	48.22	4	F4:2372	OB5925 H3B raw.raw	1.3134E4	1	1	249	271		N3:Deamidation (NQ):32.08	PEAKS DB
**total 51 peptides**																			

sp|P43237|ALL11_ARAHY

back to list

| Protein Coverage | Supporting Peptides |

Protein Coverage:

```
  1 MRGRVSPLML LLGILVLASV SATQAKSPYR KTENPCAQRC LQSCQQEPDD LKQKACESRC TKLEYDPRCV YDTGATNQRH
 81 PPGERTRGRQ PGDYDDDRRQ PRREEGGRWG PAEPRERERE EDWRQPREDW RRPSHQQPRK IRPEGREGEQ EWGTPGSEVR
161 EETSRNNPFY FPSRRFSTRY GNQNGRIRVL QRFDQRSKQF QNLQNHRIVQ IEARPNTLVL PKHADADNIL VIQQGQATVT
241 VANGNNRKSF NLDEGHALRI PSGFISYILN RHDNQNLRVA KISMPVNTPG QFEDFFPASS RDQSSYLQGF SRNTLEAAFN
321 AEFNEIRRVL LEENAGGEQE ERGQRRRSTR SSDNEGVIVK VSKEHVQELT KHAKSVSKKG SEEEDITNPI NLRDGEPDLS
401 NNFGRLFEVK PDKKNPQLQD LDMMLTCVEI KEGALMLPHF NSKAMVIVVV NKGTGNLELV AVRKEQQQRG RREQEWEEEE
481 EDEEEEGSNR EVRRYTARLK EGDVFIMPAA HPVAINASSE LHLLGFGINA ENNHRIFLAG DKDNVIDQIE KQAKDLAFPG
561 SGEQVEKLIK NQRESHFVSA RPQSQSPSSP EKEDQEEENQ GGKGPLLSIL KAFN
```

427

C Carbamidomethylation (+57.02)
O Oxidation (M) (+15.99)

Supporting Peptides:

Peptide	Uniq	-10lgP	Mass	Length	ppm	m/z	z	RT	Fraction	Scan	Source File	Area Raw Ara h 3	#Feature	#Feature Raw Ara h 3	Start	End	PTM	AScore	Found By
K.ISMPVNTPGQFEDFFPASSR.D	N	164.25	2226.0415	20	4.6	1114.0332	2	35.91	4	F4:1601	OB5925 H3B raw.raw	7.0048E6	6	6	282	301			PEAKS DB
K.ISM(+15.99)PVNTPGQFEDFFPASSR.D	N	159.13	2242.0364	20	2.3	1122.0281	2	34.42	4	F4:1522	OB5925 H3B raw.raw	1.6427E6	5	5	282	301	Oxidation (M)	M3:Oxidation (M):1000.00	PEAKS DB
K.NPQLQDLDMMLTC(+57.02)VEIK.E	N	156.27	2046.9788	17	2.3	1024.4990	2	37.39	5	F5:1843	OB5934 H3B raw.raw	3.0752E5	3	3	415	431	Carbamidomethylation	C13:Carbamidomethylation:1000.00	PEAKS DB
K.HADADNILVIQQGQATVTVAN(+.98)GNNRK.S	N	134.38	2747.3950	26	2.1	916.8075	3	29.70	2	F2:1436	OB5937 H3A raw.raw	1.18E6	5	5	223	248		N21:Deamidation (NQ):15.73	PEAKS DB
R.IFLAGDKDNVIDQIEKQAK.D	N	133.54	2144.1477	19	1.1	715.7240	3	32.93	5	F5:1578	OB5934 H3B raw.raw	3.7212E6	6	6	536	554			PEAKS DB
R.IPSGFISYILNR.H	N	131.66	1378.7609	12	1.8	690.3889	2	37.02	5	F5:1820	OB5934 H3B raw.raw	2.3128E7	6	6	260	271			PEAKS DB
K.SFNLDEGHALRIPSGFISYILNR.H	N	130.89	2618.3604	23	2.5	873.7963	3	40.61	4	F4:1864	OB5925 H3B raw.raw	4.4362E7	16	16	249	271			PEAKS DB
R.NTLEAAFNAEFNEIR.R	N	130.33	1737.8322	15	2.0	869.9250	2	35.72	4	F4:1599	OB5925 H3B raw.raw	4.8629E5	2	2	313	327			PEAKS DB
R.IFLAGDKDNVIDQIEK.Q	N	122.64	1816.9570	16	2.7	909.4882	2	32.53	5	F5:1557	OB5934 H3B raw.raw	2.0088E7	12	12	536	551			PEAKS DB
K.SFNLDEGHALR.I	N	115.46	1257.6101	11	-0.1	629.8123	2	28.39	2	F2:1360	OB5937 H3A raw.raw	8.0983E5	11	11	249	259			PEAKS DB
R.DQSSYLQGFSR.N	N	113.23	1286.5891	11	2.4	644.3033	2	31.02	2	F2:1506	OB5937 H3A raw.raw	1.7114E6	5	5	302	312			PEAKS DB
K.HADADNILVIQQGQATVTVANGNNRK.S	N	109.04	2746.4111	26	1.7	916.4792	3	29.52	2	F2:1425	OB5937 H3A raw.raw	1.1363E6	5	5	223	248			PEAKS DB
K.KGSEEEDITNPINLRDGEPDLSNNFGR.L	N	108.20	3015.4170	27	-0.3	754.8613	4	31.59	5	F5:1505	OB5934 H3B raw.raw	6.3819E5	7	7	379	405			PEAKS DB
K.AMVIVVVNKGTGNLELVAVRK.E	N	105.72	2209.2981	21	0.6	737.4404	3	34.43	1	F1:1679	OB5936 H3A raw.raw	1.0985E7	7	7	444	464			PEAKS DB
R.IPSGFISYILNRHDNQNLR.V	N	105.41	2256.1763	19	1.1	753.0669	3	34.19	2	F2:1689	OB5937 H3A raw.raw	3.796E7	18	18	260	278			PEAKS DB
K.DLAFPGSGEQVEKLIK.N	N	102.68	1729.9249	16	1.0	865.9706	2	33.30	5	F5:1601	OB5934 H3B raw.raw	6.2551E5	3	3	555	570			PEAKS DB
R.VLLEENAGGEQEER.G	N	97.57	1571.7427	14	0.2	786.8788	2	27.11	2	F2:1274	OB5937 H3A raw.raw	1.1444E5	4	4	329	342			PEAKS DB
R.NTLEAAFNAEFNEIRR.V	N	92.46	1893.9332	16	2.6	947.9763	2	34.61	5	F5:1666	OB5934 H3B raw.raw	5.3025E7	15	15	313	328			PEAKS DB
K.AM(+15.99)VIVVVNKGTGNLELVAVR.K	N	91.27	2097.1980	20	1.9	1049.6083	2	33.11	5	F5:1594	OB5934 H3B raw.raw	2.0031E6	4	4	444	463	Oxidation (M)	M2:Oxidation (M):1000.00	PEAKS DB
K.NPQLQDLDMMLTC(+57.02)VEIKEGALMLPHFNSK.A	N	90.42	3371.6387	29	0.9	843.9177	4	38.28	2	F2:1944	OB5937 H3A raw.raw	7.6836E6	5	5	415	443	Carbamidomethylation	C13:Carbamidomethylation:1000.00	PEAKS DB

total 51 peptides

Peptide	Uniq	-10lgP	Mass	Length	ppm	m/z	z	RT	Fraction	Scan	Source File	Area Raw Ara h 3	#Feature	#Feature Raw Ara h 3	Start	End	PTM	AScore	Found By
K.DLAFPGSGEQVEK.L	N	90.32	1375.6619	13	1.8	688.8395	2	30.46	2	F2:1473	OB5937 H3A raw.raw	8.5836E5	6	6	555	567			PEAKS DB
K.AMVIVVVNKGTGNLELVAVR.K	N	88.82	2081.2031	20	2.3	1041.6112	2	34.47	3	F3:1725	OB5938 H3A raw.raw	3.2769E6	3	3	444	463			PEAKS DB
R.NNPFYFPSRR.F	N	88.17	1296.6364	10	1.1	649.3262	2	29.14	3	F3:1401	OB5938 H3A raw.raw	2.2468E6	9	9	166	175			PEAKS DB
K.GSEEEDITNPINLRDGEPDLSNNFGR.L	N	86.64	2887.3220	26	1.0	963.4489	3	32.96	6	F6:1577	OB5935 H3B raw.raw	4.0621E5	3	3	380	405			PEAKS DB
R.IVQIEARPNTLVLPK.H	Y	84.29	1690.0140	15	2.2	846.0161	2	30.65	5	F5:1446	OB5934 H3B raw.raw	5.4418E6	12	12	208	222			PEAKS DB
K.ISMPVNTPGQFEDFFPASSRDQSSYLQGFSR.N	N	84.22	3494.6201	31	2.7	1165.8838	3	36.29	4	F4:1620	OB5925 H3B raw.raw	1.0081E7	6	6	282	312			PEAKS DB
R.SSDNEGVIVK.V	N	81.08	1046.5244	10	1.8	524.2704	2	23.93	2	F2:1106	OB5937 H3A raw.raw	1.3145E4	3	3	351	360			PEAKS DB
K.HADADNILVIQQGQATVTVANGNN(+.98)RK.S	N	79.42	2747.3950	26	4.5	1374.7109	2	29.70	2	F2:1444	OB5937 H3A raw.raw	1.1778E4	1	1	223	248		N24:Deamidation (NQ): 7.21	PEAKS DB
G.SEEEDITNPINLRDGEPDLSNNFGR.L	N	76.97	2830.3005	25	1.2	944.4419	3	32.96	6	F6:1576	OB5935 H3B raw.raw	5.6172E5	3	3	381	405			PEAKS DB
R.EGEQEWGTPGSEVREETSR.N	Y	74.60	2161.9512	19	2.0	721.6591	3	30.32	1	F1:1445	OB5936 H3A raw.raw	1.0653E5	3	3	147	165			PEAKS DB
K.SFNLDEGHALRIPSGFISYILNRHDNQNLR.V	N	70.02	3495.7759	30	0.6	700.1629	5	38.32	1	F1:1912	OB5936 H3A raw.raw	2.2417E7	7	7	249	278			PEAKS DB
R.VLLEENAGGEQEERGQR.R	N	65.63	1912.9238	17	0.0	638.6486	3	29.50	1	F1:1389	OB5936 H3A raw.raw	4.0372E4	2	2	329	345			PEAKS DB
R.IFLAGDKDNVIDQ(+.98)IEKQAK.D	N	64.02	2145.1316	19	9.3	716.0578	3	32.93	4	F4:1432	OB5925 H3B raw.raw	1.5596E6	1	1	536	554		Q13:Deamidation (NQ): 0.00	PEAKS DB
K.GTGNLELVAVR.K	N	62.22	1127.6299	11	0.1	564.8223	2	29.90	3	F3:1460	OB5938 H3A raw.raw	1.5877E5	3	3	453	463			PEAKS DB
K.AMVIVVVNK.G	N	61.23	971.5837	9	-0.3	486.7990	2	29.89	4	F4:1251	OB5925 H3B raw.raw	0	0	0	444	452			PEAKS DB
R.NNPFYFPSR.R	N	60.62	1140.5352	9	-0.1	571.2748	2	31.58	3	F3:1564	OB5938 H3A raw.raw	4.133E4	1	1	166	174			PEAKS DB
K.KGSEEEDITNPINLR.D	N	60.41	1713.8533	15	1.6	857.9352	2	31.45	1	F1:1515	OB5936 H3A raw.raw	9.8097E3	1	1	379	393			PEAKS DB
K.ISM(+15.99)PVNTPGQFEDFFPASSRDQSSYLQGFSR.N	N	59.85	3510.6150	31	1.9	1171.2145	3	34.93	3	F3:1753	OB5938 H3A raw.raw	4.1096E6	6	6	282	312	Oxidation (M)	M3:Oxidation (M):1000.00	PEAKS DB
K.HADADNILVIQQGQ(+.98)ATVTVANGNNRK.S	N	57.58	2747.3950	26	0.5	687.8564	4	29.52	3	F3:1436	OB5938 H3A raw.raw	1.466E5	1	1	223	248		Q14:Deamidation (NQ): 5.77	PEAKS DB
K.NPQLQDLDMMLTC(+57.02)VEIKEGALM(+15.99)LPHFNSK.A	N	54.78	3387.6335	29	2.9	847.9181	4	37.33	2	F2:1882	OB5937 H3A raw.raw	2.6128E6	2	2	415	443	Carbamidomethylation	C13:Carbamidomethylation:1000.00;M22:Oxidation (M):19.27	PEAKS DB
R.IPSGFISYILN(+.98)RHDNQNLR.V	N	53.84	2257.1604	19	9.9	753.4015	3	37.61	4	F4:1698	OB5925 H3B raw.raw	2.7971E5	1	1	260	278		N11:Deamidation (NQ): 33.06	PEAKS DB

total 51 peptides

Peptide	Uniq	-10lgP	Mass	Length	ppm	m/z	z	RT	Fraction	Scan	Source File	Area Raw Ara h 3	#Feature	#Feature Raw Ara h 3	Start	End	PTM	AScore	Found By
R.EETSRNNPFYFPSRR.F	N	53.22	1898.9023	15	-0.2	633.9746	3	28.39	3	F3:1372	OB5938 H3A raw.raw	2.391E4	1	1	161	175			PEAKS DB
R.I(+57.02)FLAGDKDNVIDQIEK.Q	N	51.72	1873.9785	16	1.3	937.9977	2	32.56	4	F4:1413	OB5925 H3B raw.raw	1.5532E5	1	1	536	551		I1:Carbamidomethylation (DHKE, X@N-term):32.60	PEAKS PTM
K.GTGNLELVAVRK.E	N	49.78	1255.7249	12	0.1	628.8698	2	27.83	2	F2:1327	OB5937 H3A raw.raw	1.327E4	2	2	453	464			PEAKS DB
R.LFEVKPDK.K	N	47.93	974.5436	8	0.9	488.2795	2	26.83	2	F2:1264	OB5937 H3A raw.raw	1.0234E3	1	1	406	413			PEAKS DB
K.NPQLQDLDM(+15.99)MLTC(+57.02)VEIKEGALMLPHFNSK.A	N	45.78	3387.6335	29	-0.7	847.9150	4	39.43	5	F5:1972	OB5934 H3B raw.raw	2.2084E6	1	1	415	443	Carbamidomethylation	M9:Oxidation (M):0.00; C13:Carbamidomethylation:1000.00	PEAKS DB
R.VLLEENAGGEQEERGQRR.R	N	45.71	2069.0249	18	0.0	690.6823	3	27.83	2	F2:1326	OB5937 H3A raw.raw	3.3003E4	1	1	329	346			PEAKS DB
R.WGPAEPR.E	Y	42.35	811.3976	7	0.6	406.7063	2	26.18	2	F2:1219	OB5937 H3A raw.raw	9.9043E3	2	2	109	115			PEAKS DB
R.LFEVKPDKK.N	N	40.23	1102.6385	9	-0.7	552.3262	2	29.58	1	F1:1398	OB5936 H3A raw.raw	0	0	0	406	414			PEAKS DB
K.HADADN(+.98)ILVIQQGQATVTVANGNNRK.S	N	37.99	2747.3950	26	2.5	916.8079	3	30.09	5	F5:1426	OB5934 H3B raw.raw	9.4717E4	1	1	223	248		N6:Deamidation (NQ): 0.00	PEAKS DB
K.SFN(+.98)LDEGHALRIPSGFISYILNR.H	N	36.85	2619.3445	23	8.5	874.1295	3	48.22	4	F4:2372	OB5925 H3B raw.raw	1.3134E4	1	1	249	271		N3:Deamidation (NQ):32.08	PEAKS DB
**total 51 peptides**																			

B3IXL2|B3IXL2_ARAHY

back to list

| Protein Coverage | Supporting Peptides |

Protein Coverage:

C Carbamidomethylation (+57.02)
O Oxidation (M) (+15.99)

1 MRGRVSPLML LLGILVLASV SATQAKSPYR KTENPCAQRC LQSCQQEPDD LKQKACESRC TKLEYDPRCV YDTGATNQRH
81 PPGERTRGRQ PGDYDDDRRQ PRREEGGRWG PAEPRERERE EDWRQPREDW RRPSHQQPRK IRPEGREGEQ EWGTPGSEVR
161 EETSRNNPFY FPSRRFSTRY GNQNGRIRVL QRFDQRSKQF QNLQNHRIVQ IEARPNTLVL PKHADADNIL VIQQGQATVT
241 VANGNNRKSF NLDEGHALRI PSGFISYILN RHDNQNLRVA KISMPVNTPG QFEDFFPASS RDQSSYLQGF SRNTLEAAFN
321 AEFNEIRRVL LEENAGGEQE ERGQRRRSTR SSDNEGVIVK VSKEHVQELT KHAKSVSKKG SEEEDITNPI NLRDGEPDLS
427
401 NNFGRLFEVK PDKKNPQLQD LDMMLTCVEI KEGALMLPHF NSKAMVIVVV NKGTGNLELV AVRKEQQQRG RREQEWEEEE
481 EDEEEEGSNR EVRRYTARLK EGDVFIMPAA HPVAINASSE LHLLGFGINA ENNHRIFLAG DKDNVIDQIE KQAKDLAFPG
561 SGEQVEKLIK NQRESHFVSA RPQSQSPSSP EKEDQEEENQ GGKGPLLSIL KAFN

Supporting Peptides:

Peptide	Uniq	-10lgP	Mass	Length	ppm	m/z	z	RT	Fraction	Scan	Source File	Area Raw Ara h 3	#Feature	#Feature Raw Ara h 3	Start	End	PTM	AScore	Found By
K.ISMPVNTPGQFEDFFPASSR.D	N	164.25	2226.0415	20	4.6	1114.0332	2	35.91	4	F4:1601	OB5925 H3B raw.raw	7.0048E6	6	6	282	301			PEAKS DB
K.ISM(+15.99)PVNTPGQFEDFFPASSR.D	N	159.13	2242.0364	20	2.3	1122.0281	2	34.42	4	F4:1522	OB5925 H3B raw.raw	1.6427E6	5	5	282	301	Oxidation (M)	M3:Oxidation (M):1000.00	PEAKS DB
K.NPQLQDLDMMLTC(+57.02)VEIK.E	N	156.27	2046.9788	17	2.3	1024.4990	2	37.39	5	F5:1843	OB5934 H3B raw.raw	3.0752E5	3	3	415	431	Carbamidomethylation	C13:Carbamidomethylation:1000.00	PEAKS DB
K.HADADNILVIQQGQATVTVAN(+.98)GNNRK.S	N	134.38	2747.3950	26	2.1	916.8075	3	29.70	2	F2:1436	OB5937 H3A raw.raw	1.18E6	5	5	223	248		N21:Deamidation (NQ):15.73	PEAKS DB
R.IFLAGDKDNVIDQIEKQAK.D	N	133.54	2144.1477	19	1.1	715.7240	3	32.93	5	F5:1578	OB5934 H3B raw.raw	3.7212E6	6	6	536	554			PEAKS DB
R.IPSGFISYILNR.H	N	131.66	1378.7609	12	1.8	690.3889	2	37.02	5	F5:1820	OB5934 H3B raw.raw	2.3128E7	6	6	260	271			PEAKS DB
K.SFNLDEGHALRIPSGFISYILNR.H	N	130.89	2618.3604	23	2.5	873.7963	3	40.61	4	F4:1864	OB5925 H3B raw.raw	4.4362E7	16	16	249	271			PEAKS DB
R.NTLEAAFNAEFNEIR.R	N	130.33	1737.8322	15	2.0	869.9250	2	35.72	4	F4:1599	OB5925 H3B raw.raw	4.8629E5	2	2	313	327			PEAKS DB
R.IFLAGDKDNVIDQIEK.Q	N	122.64	1816.9570	16	2.7	909.4882	2	32.53	5	F5:1557	OB5934 H3B raw.raw	2.0088E7	12	12	536	551			PEAKS DB

total 51 peptides

Peptide	Uniq	-10lgP	Mass	Length	ppm	m/z	z	RT	Fraction	Scan	Source File	Area Raw Ara h 3	#Feature	#Feature Raw Ara h 3	Start	End	PTM	AScore	Found By
K.SFNLDEGHALR.I	N	115.46	1257.6101	11	-0.1	629.8123	2	28.39	2	F2:1360	OB5937 H3A raw.raw	8.0983E5	11	11	249	259			PEAKS DB
R.DQSSYLQGFSR.N	N	113.23	1286.5891	11	2.4	644.3033	2	31.02	2	F2:1506	OB5937 H3A raw.raw	1.7114E6	5	5	302	312			PEAKS DB
K.HADADNILVIQQGQATVTVANGNNRK.S	N	109.04	2746.4111	26	1.7	916.4792	3	29.52	2	F2:1425	OB5937 H3A raw.raw	1.1363E6	5	5	223	248			PEAKS DB
K.KGSEEEDITNPINLRDGEPDLSNNFGR.L	N	108.20	3015.4170	27	-0.3	754.8613	4	31.59	5	F5:1505	OB5934 H3B raw.raw	6.3819E5	7	7	379	405			PEAKS DB
K.AMVIVVVNKGTGNLELVAVRK.E	N	105.72	2209.2981	21	0.6	737.4404	3	34.43	1	F1:1679	OB5936 H3A raw.raw	1.0985E7	7	7	444	464			PEAKS DB
R.IPSGFISYILNRHDNQNLR.V	N	105.41	2256.1763	19	1.1	753.0669	3	34.19	2	F2:1689	OB5937 H3A raw.raw	3.796E7	18	18	260	278			PEAKS DB
K.DLAFPGSGEQVEKLIK.N	N	102.68	1729.9249	16	1.0	865.9706	2	33.30	5	F5:1601	OB5934 H3B raw.raw	6.2551E5	3	3	555	570			PEAKS DB
R.VLLEENAGGEQEER.G	N	97.57	1571.7427	14	0.2	786.8788	2	27.11	2	F2:1274	OB5937 H3A raw.raw	1.1444E5	4	4	329	342			PEAKS DB
R.NTLEAAFNAEFNEIRR.V	N	92.46	1893.9332	16	2.6	947.9763	2	34.61	5	F5:1666	OB5934 H3B raw.raw	5.3025E7	15	15	313	328			PEAKS DB
K.AM(+15.99)VIVVVNKGTGNLELVAVR.K	N	91.27	2097.1980	20	1.9	1049.6083	2	33.11	5	F5:1594	OB5934 H3B raw.raw	2.0031E6	4	4	444	463	Oxidation (M)	M2:Oxidation (M):1000.00	PEAKS DB
K.NPQLQDLDMMLTC(+57.02)VEIKEGALMLPHFNSK.A	N	90.42	3371.6387	29	0.9	843.9177	4	38.28	2	F2:1944	OB5937 H3A raw.raw	7.6836E6	5	5	415	443	Carbamidomethylation	C13:Carbamidomethylation:1000.00	PEAKS DB
K.DLAFPGSGEQVEK.L	N	90.32	1375.6619	13	1.8	688.8395	2	30.46	2	F2:1473	OB5937 H3A raw.raw	8.5836E5	6	6	555	567			PEAKS DB
K.AMVIVVVNKGTGNLELVAVR.K	N	88.82	2081.2031	20	2.3	1041.6112	2	34.47	3	F3:1725	OB5938 H3A raw.raw	3.2769E6	3	3	444	463			PEAKS DB
R.NNPFYFPSRR.F	N	88.17	1296.6364	10	1.1	649.3262	2	29.14	3	F3:1401	OB5938 H3A raw.raw	2.2468E6	9	9	166	175			PEAKS DB
K.GSEEEDITNPINLRDGEPDLSNNFGR.L	N	86.64	2887.3220	26	1.0	963.4489	3	32.96	6	F6:1577	OB5935 H3B raw.raw	4.0621E5	3	3	380	405			PEAKS DB
R.IVQIEARPNTLVLPK.H	Y	84.29	1690.0140	15	2.2	846.0161	2	30.65	5	F5:1446	OB5934 H3B raw.raw	5.4418E6	12	12	208	222			PEAKS DB
K.ISMPVNTPGQFEDFFPASSRDQSSYLQGFSR.N	N	84.22	3494.6201	31	2.7	1165.8838	3	36.29	4	F4:1620	OB5925 H3B raw.raw	1.0081E7	6	6	282	312			PEAKS DB
R.SSDNEGVIVK.V	N	81.08	1046.5244	10	1.8	524.2704	2	23.93	2	F2:1106	OB5937 H3A raw.raw	1.3145E4	3	3	351	360			PEAKS DB
K.HADADNILVIQQGQATVTVANGNN(+.98)RK.S	N	79.42	2747.3950	26	4.5	1374.7109	2	29.70	2	F2:1444	OB5937 H3A raw.raw	1.1778E4	1	1	223	248		N24:Deamidation (NQ):7.21	PEAKS DB
G.SEEEDITNPINLRDGEPDLSNNFGR.L	N	76.97	2830.3005	25	1.2	944.4419	3	32.96	6	F6:1576	OB5935 H3B raw.raw	5.6172E5	3	3	381	405			PEAKS DB
R.EGEQEWGTPGSEVREETSR.N	Y	74.60	2161.9512	19	2.0	721.6591	3	30.32	1	F1:1445	OB5936 H3A raw.raw	1.0653E5	3	3	147	165			PEAKS DB

total 51 peptides

Peptide	Uniq	-10lgP	Mass	Length	ppm	m/z	z	RT	Fraction	Scan	Source File	Area Raw Ara h 3	#Feature	#Feature Raw Ara h 3	Start	End	PTM	AScore	Found By
K.SFNLDEGHALRIPSGFISYILNRHDNQNLR.V	N	70.02	3495.7759	30	0.6	700.1629	5	38.32	1	F1:1912	OB5936 H3A raw.raw	2.2417E7	7	7	249	278			PEAKS DB
R.VLLEENAGGEQEERGQR.R	N	65.63	1912.9238	17	0.0	638.6486	3	29.50	1	F1:1389	OB5936 H3A raw.raw	4.0372E4	2	2	329	345			PEAKS DB
R.IFLAGDKDNVIDQ(+.98)IEKQAK.D	N	64.02	2145.1316	19	9.3	716.0578	3	32.93	4	F4:1432	OB5925 H3B raw.raw	1.5596E6	1	1	536	554		Q13:Deamidation (NQ): 0.00	PEAKS DB
K.GTGNLELVAVR.K	N	62.22	1127.6299	11	0.1	564.8223	2	29.90	3	F3:1460	OB5938 H3A raw.raw	1.5877E5	3	3	453	463			PEAKS DB
K.AMVIVVVNK.G	N	61.23	971.5837	9	-0.3	486.7990	2	29.89	4	F4:1251	OB5925 H3B raw.raw	0	0	0	444	452			PEAKS DB
R.NNPFYFPSR.R	N	60.62	1140.5352	9	-0.1	571.2748	2	31.58	3	F3:1564	OB5938 H3A raw.raw	4.133E4	1	1	166	174			PEAKS DB
K.KGSEEEDITNPINLR.D	N	60.41	1713.8533	15	1.6	857.9352	2	31.45	1	F1:1515	OB5936 H3A raw.raw	9.8097E3	1	1	379	393			PEAKS DB
K.ISM(+15.99)PVNTPGQFEDFFPASSRDQSSYLQGFSR.N	N	59.85	3510.6150	31	1.9	1171.2145	3	34.93	3	F3:1753	OB5938 H3A raw.raw	4.1096E6	6	6	282	312	Oxidation (M)	M3:Oxidation (M):1000.00	PEAKS DB
K.HADADNILVIQQGQ(+.98)ATVTVANGNNRK.S	N	57.58	2747.3950	26	0.5	687.8564	4	29.52	3	F3:1436	OB5938 H3A raw.raw	1.466E5	1	1	223	248		Q14:Deamidation (NQ): 5.77	PEAKS DB
K.NPQLQDLDMMLTC(+57.02)VEIKEGALM(+15.99)LPHFNSK.A	N	54.78	3387.6335	29	2.9	847.9181	4	37.33	2	F2:1882	OB5937 H3A raw.raw	2.6128E6	2	2	415	443	Carbamidomethylation	C13:Carbamidomethylation:1000.00;M22:Oxidation (M):19.27	PEAKS DB
R.IPSGFISYILN(+.98)RHDNQNLR.V	N	53.84	2257.1604	19	9.9	753.4015	3	37.61	4	F4:1698	OB5925 H3B raw.raw	2.7971E5	1	1	260	278		N11:Deamidation (NQ): 33.06	PEAKS DB
R.EETSRNNPFYFPSRR.F	N	53.22	1898.9023	15	-0.2	633.9746	3	28.39	3	F3:1372	OB5938 H3A raw.raw	2.391E4	1	1	161	175			PEAKS DB
R.I(+57.02)FLAGDKDNVIDQIEK.Q	N	51.72	1873.9785	16	1.3	937.9977	2	32.56	4	F4:1413	OB5925 H3B raw.raw	1.5532E5	1	1	536	551		I1:Carbamidomethylation (DHKE, X@N-term):32.60	PEAKS PTM
K.GTGNLELVAVRK.E	N	49.78	1255.7249	12	0.1	628.8698	2	27.83	2	F2:1327	OB5937 H3A raw.raw	1.327E4	2	2	453	464			PEAKS DB
R.LFEVKPDK.K	N	47.93	974.5436	8	0.9	488.2795	2	26.83	2	F2:1264	OB5937 H3A raw.raw	1.0234E3	1	1	406	413			PEAKS DB
K.NPQLQDLDM(+15.99)MLTC(+57.02)VEIKEGALMLPHFNSK.A	N	45.78	3387.6335	29	-0.7	847.9150	4	39.43	5	F5:1972	OB5934 H3B raw.raw	2.2084E6	1	1	415	443	Carbamidomethylation	M9:Oxidation (M):0.00;C13:Carbamidomethylation:1000.00	PEAKS DB
R.VLLEENAGGEQEERGQRR.R	N	45.71	2069.0249	18	0.0	690.6823	3	27.83	2	F2:1326	OB5937 H3A raw.raw	3.3003E4	1	1	329	346			PEAKS DB
R.WGPAEPR.E	Y	42.35	811.3976	7	0.6	406.7063	2	26.18	2	F2:1219	OB5937 H3A raw.raw	9.9043E3	2	2	109	115			PEAKS DB
R.LFEVKPDKK.N	N	40.23	1102.6385	9	-0.7	552.3262	2	29.58	1	F1:1398	OB5936 H3A raw.raw	0	0	0	406	414			PEAKS DB
K.HADADN(+.98)ILVIQQGQATVTVANGNNRK.S	N	37.99	2747.3950	26	2.5	916.8079	3	30.09	5	F5:1426	OB5934 H3B raw.raw	9.4717E4	1	1	223	248		N6:Deamidation (NQ): 0.00	PEAKS DB
K.SFN(+.98)LDEGHALRIPSGFISYILNR.H	N	36.85	2619.3445	23	8.5	874.1295	3	48.22	4	F4:2372	OB5925 H3B raw.raw	1.3134E4	1	1	249	271		N3:Deamidation (NQ):32.08	PEAKS DB
**total 51 peptides**																			

Q38711|Q38711_ARAHY

back to list

| Protein Coverage | Supporting Peptides |

Protein Coverage:

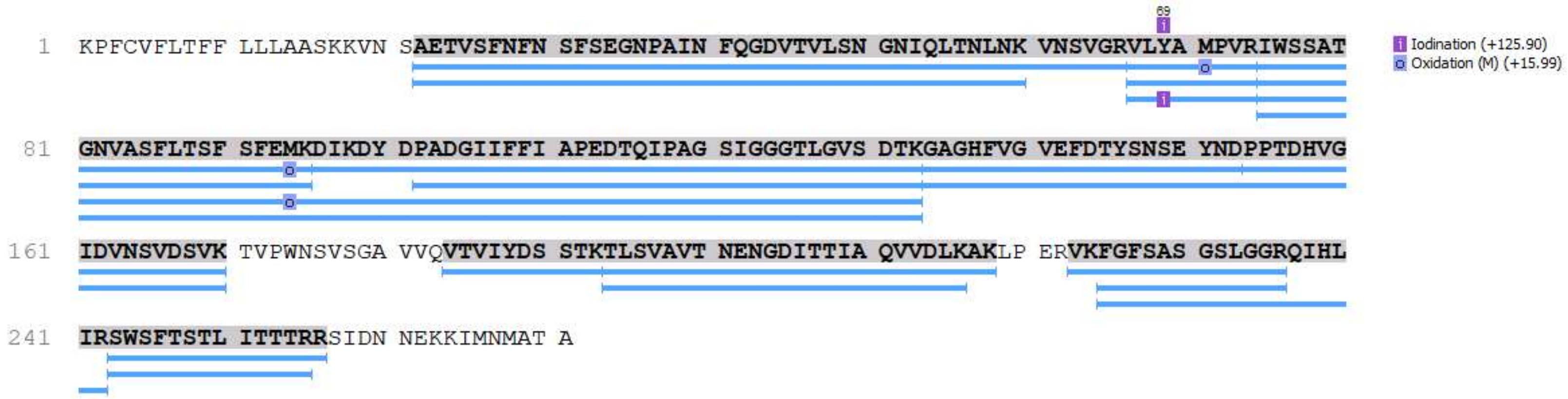

Supporting Peptides:

Peptide	Uniq	-10lgP	Mass	Length	ppm	m/z	z	RT	Fraction	Scan	Source File	Area Raw Ara h 3	#Feature	#Feature Raw Ara h 3	Start	End	PTM	AScore	Found By
R.IWSSATGNVASFLTSFSFEMK.D	N	200.00	2309.1038	21	3.7	1155.5635	2	40.56	5	F5:2025	OB5934 H3B raw.raw	1.0023E7	3	3	75	95			PEAKS DB
R.SWSFTSTLITTTR.R	N	140.96	1499.7620	13	1.8	750.8896	2	34.42	4	F4:1520	OB5925 H3B raw.raw	9.5864E6	3	3	243	255			PEAKS DB
K.DIKDYDPADGIIFFIAPEDTQIPAGSIGGGTLGVSDTK.G	N	140.91	3892.9258	38	2.7	1298.6527	3	39.11	4	F4:1787	OB5925 H3B raw.raw	1.6354E7	5	5	96	133			PEAKS DB
R.IWSSATGNVASFLTSFSFEM(+15.99)K.D	N	137.48	2325.0986	21	1.6	1163.5585	2	39.06	5	F5:1947	OB5934 H3B raw.raw	1.6326E6	3	3	75	95	Oxidation (M)	M20:Oxidation (M):1000.00	PEAKS DB
K.TLSVAVTNENGDITTIAQVVDLK.A	Y	135.88	2400.2747	23	2.6	1201.1477	2	36.27	5	F5:1788	OB5934 H3B raw.raw	4.3282E6	3	3	194	216			PEAKS DB
K.FGFSASGSLGGR.Q	N	125.50	1141.5515	12	1.4	571.7838	2	30.50	4	F4:1278	OB5925 H3B raw.raw	1.5431E6	3	3	225	236			PEAKS DB
R.VKFGFSASGSLGGR.Q	N	124.68	1368.7150	14	1.1	685.3655	2	30.27	5	F5:1424	OB5934 H3B raw.raw	1.6529E5	4	4	223	236			PEAKS DB
K.GAGHFVGVEFDTYSNSEYNDPPTDHVGIDVNSVDSVK.T	N	111.11	3966.7820	37	3.1	1323.2721	3	33.12	4	F4:1445	OB5925 H3B raw.raw	1.9838E7	7	7	134	170			PEAKS DB
D.PPTDHVGIDVNSVDSVK.T	N	110.01	1777.8846	17	-0.4	889.9493	2	33.30	4	F4:1450	OB5925 H3B raw.raw	1.6838E6	7	7	154	170			PEAKS DB
R.VLYAMPVR.I	N	93.30	947.5262	8	2.3	474.7715	2	30.09	5	F5:1402	OB5934 H3B raw.raw	9.4625E5	3	3	67	74			PEAKS DB
K.GAGHFVGVEFDTYSNSEYND.P	N	92.68	2206.9080	20	2.4	1104.4639	2	32.91	5	F5:1579	OB5934 H3B raw.raw	1.1587E6	3	3	134	153			PEAKS DB
K.TLSVAVTNENGDITTIAQVVDLKAK.L	Y	75.41	2599.4067	25	0.1	867.4763	3	34.79	5	F5:1695	OB5934 H3B raw.raw	1.6993E6	3	3	194	218			PEAKS DB

total 23 peptides

Peptide	Uniq	-10lgP	Mass	Length	ppm	m/z	z	RT	Fraction	Scan	Source File	Area Raw Ara h 3	#Feature	#Feature Raw Ara h 3	Start	End	PTM	AScore	Found By
R.SWSFTSTLITTTRR.S	N	74.70	1655.8632	14	1.5	828.9401	2	32.96	6	F6:1573	OB5935 H3B raw.raw	1.7686E7	6	6	243	256			PEAKS DB
K.FGFSASGSLGGRQIHLIR.S	N	74.68	1902.0223	18	0.5	635.0150	3	32.37	4	F4:1405	OB5925 H3B raw.raw	2.3617E5	2	2	225	242			PEAKS DB
D.P(+57.02)PTDHVGIDVNSVDSVK.T	N	70.75	1834.9061	17	0.0	612.6426	3	30.08	6	F6:1405	OB5935 H3B raw.raw	0	0	0	154	170		P1:Carbamidomethylation (DHKE, X@N-term):28.36	PEAKS PTM
R.VLYAM(+15.99)PVR.I	N	61.38	963.5212	8	-0.9	482.7674	2	29.11	5	F5:1353	OB5934 H3B raw.raw	1.9715E4	2	2	67	74	Oxidation (M)	M5:Oxidation (M):1000.00	PEAKS DB
D.PADGIIFFIAPEDTQIPAGSIGGGTLGVSDTK.G	N	48.65	3143.6025	32	4.6	1572.8158	2	38.57	6	F6:1906	OB5935 H3B raw.raw	6.2609E5	2	2	102	133			PEAKS DB
Q.VTVIYDSSTK.T	N	46.28	1111.5760	10	0.7	556.7957	2	26.75	5	F5:1237	OB5934 H3B raw.raw	6.5025E2	1	1	184	193			PEAKS DB
S.AETVSFNFNSFSEGNPAINFQGDVTVLSNGNIQLTNLNKVNSVGR.V	N	45.30	4812.3740	45	5.6	1605.1410	3	39.31	6	F6:1951	OB5935 H3B raw.raw	1.7542E7	3	3	22	66			PEAKS DB
R.IWSSATGNVASFLTSFSFEMKDIKDYDPADGIIFFIAPEDTQIPAGSIGGGTLGVSDTK.G	N	44.58	6184.0190	59	6.4	1547.0220	4	42.84	5	F5:2162	OB5934 H3B raw.raw	5.5947E6	1	1	75	133			PEAKS DB
R.VLY(+125.90)AMPVR.I	N	42.00	1073.4229	8	0.8	537.7191	2	32.78	6	F6:1571	OB5935 H3B raw.raw	2.4073E4	1	1	67	74	Iodination	Y3:Iodination: 1000.00	PEAKS PTM
R.IWSSATGNVASFLTSFSFEM(+15.99)KDIKDYDPADGIIFFIAPEDTQIPAGSIGGGTLGVSDTK.G	N	40.85	6200.0137	59	5.3	1551.0189	4	41.88	5	F5:2118	OB5934 H3B raw.raw	1.2633E7	1	1	75	133	Oxidation (M)	M20:Oxidation (M):1000.00	PEAKS DB
S.AETVSFNFNSFSEGNPAINFQGDVTVLSNGNIQLTNLNK.V	N	32.70	4200.0400	39	-2.5	1401.0171	3	45.67	6	F6:2317	OB5935 H3B raw.raw	0	0	0	22	60			PEAKS DB
**total 23 peptides**																			

T2B9M0|T2B9M0_ARAHY

back to list

| Protein Coverage | Supporting Peptides |

Protein Coverage:

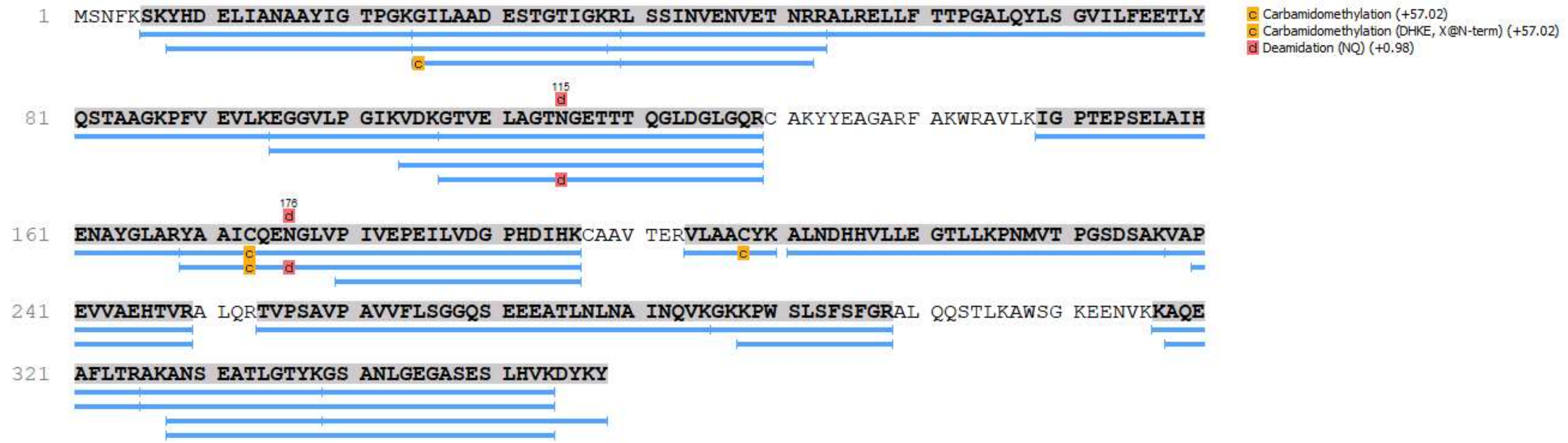

Supporting Peptides:

Peptide	Uniq	-10lgP	Mass	Length	ppm	m/z	z	RT	Fraction	Scan	Source File	Area Raw Ara h 3	#Feature	#Feature Raw Ara h 3	Start	End	PTM	AScore	Found By
K.GTVELAGTNGETTTQGLDGLGQR.C	Y	147.43	2274.1086	23	3.2	1138.0652	2	31.01	2	F2:1514	OB5937 H3A raw.raw	1.1133E5	3	3	107	129			PEAKS DB
K.GTVELAGTN(+.98)GETTTQGLDGLGQR.C	Y	138.99	2275.0928	23	3.3	1138.5574	2	31.59	2	F2:1543	OB5937 H3A raw.raw	1.6073E5	3	3	107	129	Deamidation (NQ)	N9:Deamidation (NQ):102.87	PEAKS DB
K.IGPTEPSELAIHENAYGLAR.Y	Y	126.96	2137.0803	20	2.9	1069.5505	2	31.02	3	F3:1523	OB5938 H3A raw.raw	8.8583E6	6	6	149	168			PEAKS DB
K.EGGVLPGIKVDKGTVELAGTNGETTTQGLDGLGQR.C	Y	125.17	3466.7903	35	2.3	1156.6067	3	32.71	2	F2:1614	OB5937 H3A raw.raw	1.4131E6	3	3	95	129			PEAKS DB
R.TVPSAVPAVVFLSGGQSEEEATLNLNAINQVK.G	Y	122.93	3281.7144	32	3.4	1641.8700	2	37.33	2	F2:1887	OB5937 H3A raw.raw	2.1228E7	9	9	254	285			PEAKS DB
K.SKYHDELIANAAYIGTPGK.G	Y	121.92	2047.0374	19	1.6	683.3541	3	29.90	2	F2:1442	OB5937 H3A raw.raw	3.3341E5	4	4	6	24			PEAKS DB
K.VAPEVVAEHTVR.A	Y	120.72	1305.7041	12	0.4	653.8596	2	26.96	2	F2:1262	OB5937 H3A raw.raw	3.6523E5	5	5	238	249			PEAKS DB
K.KPWSLSFSFGR.A	Y	118.79	1310.6771	11	0.5	656.3462	2	33.46	2	F2:1658	OB5937 H3A raw.raw	6.1097E5	2	2	288	298			PEAKS DB
K.GILAADESTGTIGKR.L	Y	118.11	1487.7943	15	0.0	744.9044	2	27.45	3	F3:1307	OB5938 H3A raw.raw	3.9441E5	4	4	25	39			PEAKS DB
R.YAAIC(+57.02)QEN(+.98)GLVPIVEPEILVDGPHDIHK.C	Y	118.04	3126.5696	28	3.9	1043.2012	3	34.93	2	F2:1744	OB5937 H3A raw.raw	0	0	0	169	196	Carbamidomethylation; Deamidation (NQ)	C5:Carbamidomethylation:100 0.00;N8:Deamidation (NQ):5 2.04	PEAKS DB
K.GKKPWSLSFSFGR.A	Y	114.49	1495.7936	13	-0.2	499.6050	3	32.14	3	F3:1581	OB5938 H3A raw.raw	4.526E6	6	6	286	298			PEAKS DB
K.YHDELIANAAYIGTPGK.G	Y	113.25	1831.9104	17	1.7	916.9640	2	30.83	2	F2:1510	OB5937 H3A raw.raw	6.0481E4	1	1	8	24			PEAKS DB

total 37 peptides

Peptide	Uniq	-10lgP	Mass	Length	ppm	m/z	z	RT	Fraction	Scan	Source File	Area Raw Ara h 3	#Feature	#Feature Raw Ara h 3	Start	End	PTM	AScore	Found By
K.ANSEATLGTYKGSANLGEGASESLHVK.D	Y	109.58	2690.3147	27	1.6	897.7803	3	28.95	2	F2:1392	OB5937 H3A raw.raw	1.356E5	2	2	328	354			PEAKS DB
R.YAAIC(+57.02)QENGLVPIVEPEILVDGPHDIHK.C	Y	107.85	3125.5854	28	-2.1	1042.8669	3	34.56	3	F3:1733	OB5938 H3A raw.raw	7.5947E6	3	3	169	196	Carbamidomethylation	C5:Carbamidomethylation:100 0.00	PEAKS DB
K.GILAADESTGTIGK.R	Y	102.63	1331.6932	14	0.1	666.8539	2	31.07	1	F1:1493	OB5936 H3A raw.raw	2.8213E4	2	2	25	38			PEAKS DB
R.LSSINVENVETNR.R	Y	101.65	1473.7423	13	-1.0	737.8777	2	28.94	2	F2:1383	OB5937 H3A raw.raw	1.8416E5	3	3	40	52			PEAKS DB
K.VDKGTVELAGTNGETTTQGLDGLGQR.C	Y	98.71	2616.2991	26	0.9	873.1078	3	29.90	3	F3:1453	OB5938 H3A raw.raw	6.3702E4	2	2	104	129			PEAKS DB
K.GSANLGEGASESLHVKDYKY	Y	98.27	2124.0122	20	2.8	709.0133	3	31.07	1	F1:1488	OB5936 H3A raw.raw	1.9959E6	6	6	339	358			PEAKS DB
K.ALNDHHVLLEGTLLKPNMVTPGSDSAK.V	Y	95.79	2856.4802	27	0.9	953.1682	3	31.77	2	F2:1566	OB5937 H3A raw.raw	2.319E5	1	1	211	237			PEAKS DB
K.AQEAFLTR.A	Y	88.96	934.4872	8	0.9	468.2513	2	27.27	2	F2:1294	OB5937 H3A raw.raw	1.5187E5	3	3	318	325			PEAKS DB
K.GSANLGEGASESLHVK.D	Y	84.69	1554.7638	16	0.0	778.3892	2	26.95	2	F2:1269	OB5937 H3A raw.raw	1.9867E4	3	3	339	354			PEAKS DB
K.ANSEATLGTYK.G	Y	79.54	1153.5615	11	1.6	577.7889	2	25.17	3	F3:1176	OB5938 H3A raw.raw	6.2011E3	2	2	328	338			PEAKS DB
R.AKANSEATLGTYK.G	Y	78.09	1352.6936	13	-0.3	677.3539	2	27.72	1	F1:1294	OB5936 H3A raw.raw	8.8393E3	3	3	326	338			PEAKS DB
K.EGGVLPGIKVDK.G	Y	73.84	1210.6921	12	-0.6	606.3530	2	28.38	2	F2:1357	OB5937 H3A raw.raw	9.8645E4	3	3	95	106			PEAKS DB
V.PIVEPEILVDGPHDIHK.C	Y	73.22	1907.0151	17	2.3	954.5170	2	34.91	2	F2:1729	OB5937 H3A raw.raw	4.6955E6	2	2	180	196			PEAKS DB
R.AKANSEATLGTYKGSANLGEGASESLHVK.D	Y	66.86	2889.4468	29	0.7	723.3695	4	28.76	2	F2:1381	OB5937 H3A raw.raw	1.705E5	3	3	326	354			PEAKS DB
K.KAQEAFLTR.A	Y	66.61	1062.5822	9	0.9	532.2988	2	26.54	3	F3:1258	OB5938 H3A raw.raw	1.0907E4	3	3	317	325			PEAKS DB
K.VDKGTVELAGTN(+.98)GETTTQGLDGLGQR.C	Y	64.49	2617.2830	26	1.4	873.4362	3	32.69	1	F1:1582	OB5936 H3A raw.raw	1.5447E5	1	1	104	129		N12:Deamidation (NQ):41.57	PEAKS DB
K.VDKGTVELAGTNGETTTQ(+.98)GLDGLGQR.C	Y	63.32	2617.2830	26	1.5	873.4363	3	30.46	2	F2:1482	OB5937 H3A raw.raw	1.4142E5	1	1	104	129		Q18:Deamidation (NQ):0.00	PEAKS DB
R.LSSINVENVETNRR.A	Y	58.53	1629.8434	14	1.7	544.2893	3	27.29	2	F2:1293	OB5937 H3A raw.raw	5.2708E5	4	4	40	53			PEAKS DB
R.VLAAC(+57.02)YK.A	Y	48.46	823.4262	7	1.2	412.7209	2	24.59	3	F3:1143	OB5938 H3A raw.raw	8.3473E3	2	2	204	210	Carbamidomethylation	C5:Carbamidomethylation:100 0.00	PEAKS DB
K.RLSSINVENVETNRR.A	Y	47.68	1785.9446	15	0.4	596.3224	3	27.45	3	F3:1317	OB5938 H3A raw.raw	1.6138E4	2	2	39	53			PEAKS DB
K.G(+57.02)ILAADESTGTIGKR.L	Y	47.28	1544.8158	15	3.9	773.4182	2	27.74	3	F3:1329	OB5938 H3A raw.raw	0	0	0	25	39	Carbamidomethylation (DHKE, X@N-term)	G1:Carbamidomethylation (DH KE, X@N-term):61.30	PEAKS PTM

total 37 peptides

Peptide	Uniq	-10lgP	Mass	Length	ppm	m/z	z	RT	Fraction	Scan	Source File	Area Raw Ara h 3	#Feature	#Feature Raw Ara h 3	Start	End	PTM	AScore	Found By
A.PEVVAEHTVR.A	Y	43.61	1135.5985	10	0.5	568.8068	2	27.03	2	F2:1276	OB5937 H3A raw.raw	2.1131E3	1	1	240	249			PEAKS DB
K.V(+57.02)APEVVAEHTVR.A	Y	43.26	1362.7256	12	3.0	682.3721	2	27.40	2	F2:1299	OB5937 H3A raw.raw	0	0	0	238	249		V1:Carbamidomethylation (DHKE, X@N-term):22.45	PEAKS PTM
R.LS(-15.99)SINVENVETNRR.A	Y	36.45	1613.8485	14	0.0	538.9568	3	29.95	1	F1:1425	OB5936 H3A raw.raw	1.6662E4	1	1	40	53		S2:Deoxy:40.00	PEAKS PTM
R.ALRELLFTTPGALQYLSGVILFEETLYQSTAAGKPFVEVLK.E	Y	34.13	4512.4561	41	7.0	1129.1292	4	43.45	3	F3:2247	OB5938 H3A raw.raw	0	0	0	54	94			PEAKS DB

total 37 peptides

A0A089ZXL7|A0A089ZXL7_ARAHY

back to list

| Protein Coverage | Supporting Peptides |

Protein Coverage:

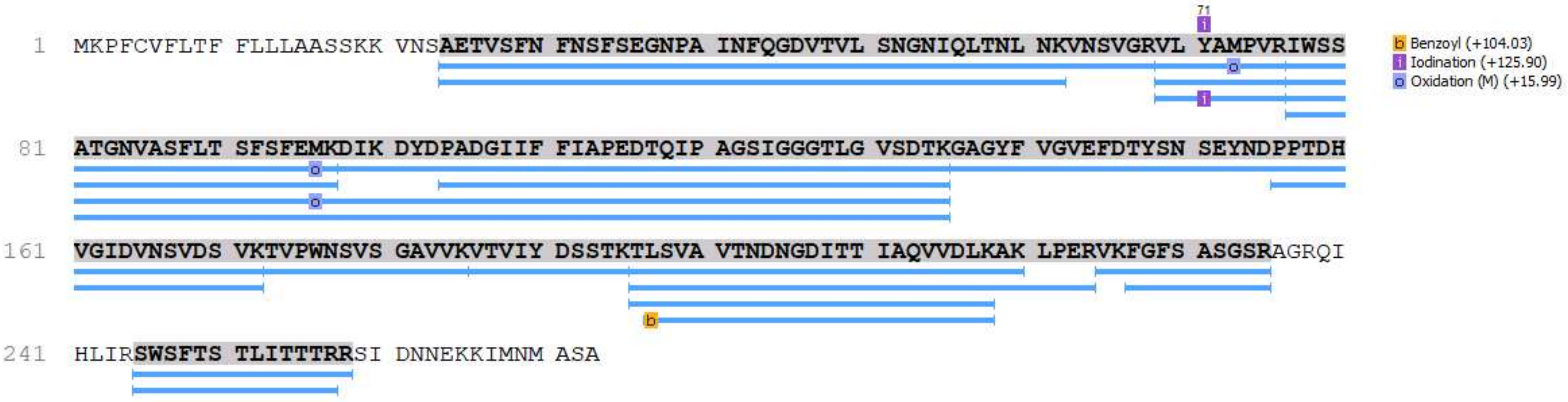

Supporting Peptides:

Peptide	Uniq	-10lgP	Mass	Length	ppm	m/z	z	RT	Fraction	Scan	Source File	Area Raw Ara h 3	#Feature	#Feature Raw Ara h 3	Start	End	PTM	AScore	Found By
R.IWSSATGNVASFLTSFSFEMK.D	N	200.00	2309.1038	21	3.7	1155.5635	2	40.56	5	F5:2025	OB5934 H3B raw.raw	1.0023E7	3	3	77	97			PEAKS DB
K.TLSVAVTNDNGDITTIAQVVDLK.A	N	146.70	2386.2590	23	2.9	1194.1403	2	35.91	4	F4:1609	OB5925 H3B raw.raw	1.7984E7	4	4	196	218			PEAKS DB
R.SWSFTSTLITTTR.R	N	140.96	1499.7620	13	1.8	750.8896	2	34.42	4	F4:1520	OB5925 H3B raw.raw	9.5864E6	3	3	245	257			PEAKS DB
K.DIKDYDPADGIIFFIAPEDTQIPAGSIGGGTLGVSDTK.G	N	140.91	3892.9258	38	2.7	1298.6527	3	39.11	4	F4:1787	OB5925 H3B raw.raw	1.6354E7	5	5	98	135			PEAKS DB
R.IWSSATGNVASFLTSFSFEM(+15.99)K.D	N	137.48	2325.0986	21	1.6	1163.5585	2	39.06	5	F5:1947	OB5934 H3B raw.raw	1.6326E6	3	3	77	97	Oxidation (M)	M20:Oxidation (M):1000.00	PEAKS DB
K.TVPWNSVSGAVVK.V	N	119.50	1342.7245	13	2.9	672.3715	2	31.21	5	F5:1468	OB5934 H3B raw.raw	1.6677E6	3	3	173	185			PEAKS DB

total 26 peptides

Peptide	Uniq	-10lgP	Mass	Length	ppm	m/z	z	RT	Fraction	Scan	Source File	Area Raw Ara h 3	#Feature	#Feature Raw Ara h 3	Start	End	PTM	AScore	Found By
D.PPTDHVGIDVNSVDSVK.T	N	110.01	1777.8846	17	-0.4	889.9493	2	33.30	4	F4:1450	OB5925 H3B raw.raw	1.6838E6	7	7	156	172			PEAKS DB
K.TLSVAVTNDNGDITTIAQVVDLKAK.L	N	94.67	2585.3911	25	2.1	1293.7056	2	34.66	6	F6:1679	OB5935 H3B raw.raw	8.0711E6	5	5	196	220			PEAKS DB
R.VLYAMPVR.I	N	93.30	947.5262	8	2.3	474.7715	2	30.09	5	F5:1402	OB5934 H3B raw.raw	9.4625E5	3	3	69	76			PEAKS DB
K.FGFSASGSR.A	Y	78.76	914.4246	9	-0.4	458.2194	2	27.93	4	F4:1143	OB5925 H3B raw.raw	1.3943E4	3	3	227	235			PEAKS DB
R.SWSFTSTLITTTRR.S	N	74.70	1655.8632	14	1.5	828.9401	2	32.96	6	F6:1573	OB5935 H3B raw.raw	1.7686E7	6	6	245	258			PEAKS DB
D.P(+57.02)PTDHVGIDVNSVDSVK.T	N	70.75	1834.9061	17	0.0	612.6426	3	30.08	6	F6:1405	OB5935 H3B raw.raw	0	0	0	156	172		P1:Carbamidomethylation (DHKE, X@N-term):28.36	PEAKS PTM
R.VLYAM(+15.99)PVR.I	N	61.38	963.5212	8	-0.9	482.7674	2	29.11	5	F5:1353	OB5934 H3B raw.raw	1.9715E4	2	2	69	76	Oxidation (M)	M5:Oxidation (M):1000.00	PEAKS DB
K.GAGYFVGVEFDTYSNSEYNDPPTDHVGIDVNSVDSVK.T	Y	54.24	3992.7864	37	2.7	1331.9397	3	37.42	4	F4:1595	OB5925 H3B raw.raw	4.597E5	1	1	136	172			PEAKS DB
R.VKFGFSASGSR.A	Y	49.56	1141.5880	11	-0.8	571.8008	2	29.33	6	F6:1359	OB5935 H3B raw.raw	1.1968E3	1	1	225	235			PEAKS DB
D.PADGIIFFIAPEDTQIPAGSIGGGTLGVSDTK.G	N	48.65	3143.6025	32	4.6	1572.8158	2	38.57	6	F6:1906	OB5935 H3B raw.raw	6.2609E5	2	2	104	135			PEAKS DB
K.VTVIYDSSTK.T	N	46.28	1111.5760	10	0.7	556.7957	2	26.75	5	F5:1237	OB5934 H3B raw.raw	6.5025E2	1	1	186	195			PEAKS DB
S.AETVSFNFNSFSEGNPAINFQGDVTVLSNGNIQLTNLNKVNSVGR.V	N	45.30	4812.3740	45	5.6	1605.1410	3	39.31	6	F6:1951	OB5935 H3B raw.raw	1.7542E7	3	3	24	68			PEAKS DB
R.IWSSATGNVASFLTSFSFEMKDIKDYDPADGIIFFIAPEDTQIPAGSIGGGTLGVSDTK.G	N	44.58	6184.0190	59	6.4	1547.0220	4	42.84	5	F5:2162	OB5934 H3B raw.raw	5.5947E6	1	1	77	135			PEAKS DB
R.VLY(+125.90)AMPVR.I	N	42.00	1073.4229	8	0.8	537.7191	2	32.78	6	F6:1571	OB5935 H3B raw.raw	2.4073E4	1	1	69	76	Iodination	Y3:Iodination: 1000.00	PEAKS PTM
R.IWSSATGNVASFLTSFSFEM(+15.99)KDIKDYDPADGIIFFIAPEDTQIPAGSIGGGTLGVSDTK.G	N	40.85	6200.0137	59	5.3	1551.0189	4	41.88	5	F5:2118	OB5934 H3B raw.raw	1.2633E7	1	1	77	135	Oxidation (M)	M20:Oxidation (M):1000.00	PEAKS DB
K.TLSVAVTNDNGDITTIAQVVDLKAKLPER.V	N	39.09	3080.6716	29	2.1	771.1768	4	36.14	6	F6:1745	OB5935 H3B raw.raw	1.01E6	1	1	196	224			PEAKS DB
T.L(+104.03)SVAVTNDNGDITTIAQVVDLK.A	N	38.94	2389.2375	22	9.4	1195.6373	2	36.47	4	F4:1654	OB5925 H3B raw.raw	1.2686E5	1	1	197	218	Benzoyl	L1:Benzoyl:124.10	PEAKS PTM
K.TLSVAVTNDN(+.98)GDITTIAQVVDLKAKLPER.V	N	38.41	3081.6558	29	8.0	771.4274	4	35.96	6	F6:1745	OB5935 H3B raw.raw	2.6837E5	1	1	196	224		N10:Deamidation (NQ):14.04	PEAKS DB
K.GAGYFVGVEFDTYSNSEYNDPPTDHVGIDVN(+.98)SVDSVK.T	Y	35.37	3993.7703	37	3.8	1332.2690	3	48.53	5	F5:2486	OB5934 H3B raw.raw	1.7305E3	1	1	136	172		N31:Deamidation (NQ):10.83	PEAKS DB
S.AETVSFNFNSFSEGNPAINFQGDVTVLSNGNIQLTNLNK.V	N	32.70	4200.0400	39	-2.5	1401.0171	3	45.67	6	F6:2317	OB5935 H3B raw.raw	0	0	0	24	62			PEAKS DB
**total 26 peptides**																			

A1E2B0|A1E2B0_ARAHY back to list

| Protein Coverage | Supporting Peptides |

Protein Coverage:

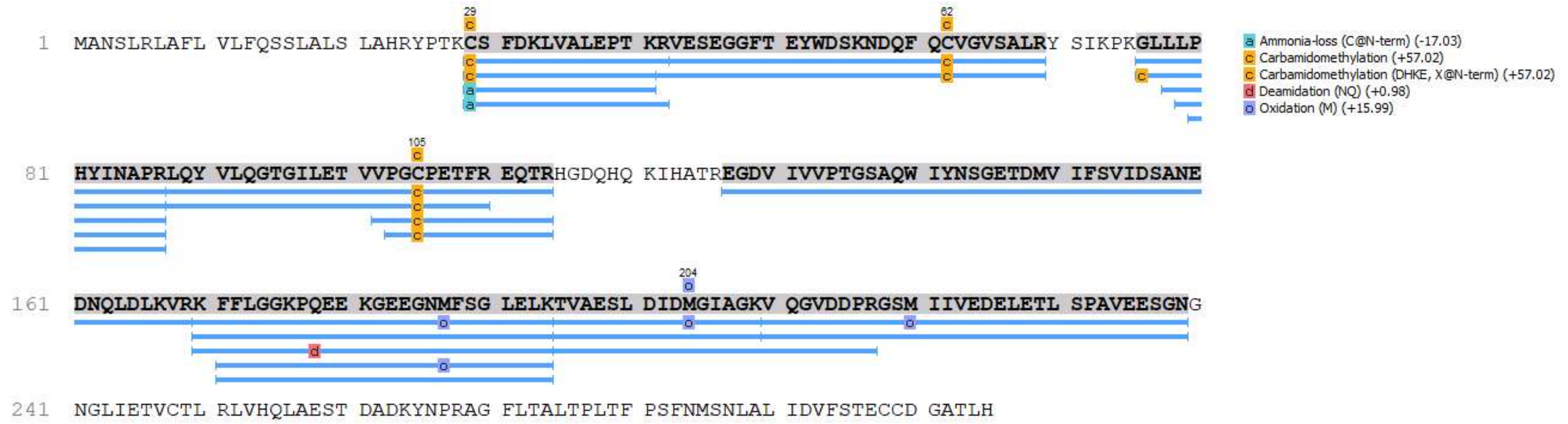

Supporting Peptides:

Peptide	Uniq	-10lgP	Mass	Length	ppm	m/z	z	RT	Fraction	Scan	Source File	Area Raw Ara h 3	#Feature	#Feature Raw Ara h 3	Start	End	PTM	AScore	Found By
K.FFLGGKPQEEKGEEGNMFSGLELK.T	N	162.92	2670.2998	24	2.4	891.1094	3	34.78	4	F4:1553	OB5925 H3B raw.raw	1.858E7	6	6	171	194			PEAKS DB
R.VESEGGFTEYWDSKNDQFQC(+57.02)VGVSALR.Y	Y	145.04	3107.3931	27	3.3	1036.8083	3	34.05	4	F4:1498	OB5925 H3B raw.raw	5.0527E6	4	4	43	69	Carbamidomethylation	C20:Carbamidomethylation:1000.00	PEAKS DB
K.TVAESLDIDMGIAGK.V	Y	137.21	1518.7599	15	2.3	760.3890	2	33.12	4	F4:1443	OB5925 H3B raw.raw	5.1238E6	3	3	195	209			PEAKS DB
R.LQYVLQGTGILETVVPGC(+57.02)PETFR.E	Y	137.04	2576.3308	23	3.4	1289.1770	2	36.84	5	F5:1810	OB5934 H3B raw.raw	1.1189E6	3	3	88	110	Carbamidomethylation	C18:Carbamidomethylation:1000.00	PEAKS DB
K.RVESEGGFTEYWDSKNDQFQC(+57.02)VGVSALR.Y	Y	136.17	3263.4941	28	3.1	1088.8420	3	33.12	4	F4:1439	OB5925 H3B raw.raw	7.9145E5	5	5	42	69	Carbamidomethylation	C21:Carbamidomethylation:1000.00	PEAKS DB
K.GLLLPHYINAPR.L	Y	122.78	1362.7771	12	0.3	682.3960	2	31.41	5	F5:1486	OB5934 H3B raw.raw	8.3765E6	6	6	76	87			PEAKS DB
R.KFFLGGKPQEEKGEEGNMFSGLELK.T	N	122.64	2798.3948	25	2.1	933.8075	3	34.42	4	F4:1518	OB5925 H3B raw.raw	1.4026E7	9	9	170	194			PEAKS DB
R.LQYVLQGTGILETVVPGC(+57.02)PETFREQTR.H	Y	118.53	3090.5808	27	3.1	1031.2041	3	35.35	4	F4:1702	OB5925 H3B raw.raw	5.1222E7	15	15	88	114	Carbamidomethylation	C18:Carbamidomethylation:1000.00	PEAKS DB
K.C(+57.02)SFDKLVALEPTK.R	Y	111.77	1506.7751	13	1.5	754.3960	2	31.13	5	F5:1476	OB5934 H3B raw.raw	1.4434E5	4	4	29	41	Carbamidomethylation	C1:Carbamidomethylation:1000.00	PEAKS DB
K.C(+57.02)SFDKLVALEPTKR.V	Y	103.22	1662.8762	14	1.5	555.3002	3	30.69	4	F4:1301	OB5925 H3B raw.raw	5.6961E4	1	1	29	42	Carbamidomethylation	C1:Carbamidomethylation:1000.00	PEAKS DB
K.FFLGGKPQEEKGEEGNM(+15.99)FSGLELK.T	N	102.01	2686.2947	24	1.7	896.4404	3	32.74	4	F4:1411	OB5925 H3B raw.raw	2.6867E6	4	4	171	194	Oxidation (M)	M17:Oxidation (M):1000.00	PEAKS DB

total 27 peptides

Peptide	Uniq	-10lgP	Mass	Length	ppm	m/z	z	RT	Fraction	Scan	Source File	Area Raw Ara h 3	#Feature	#Feature Raw Ara h 3	Start	End	PTM	AScore	Found By
R.KFFLGGKPQEEKGEEGNM(+15.99)FSGLELK.T	N	101.19	2814.3896	25	0.9	704.6053	4	32.34	5	F5:1545	OB5934 H3B raw.raw	1.3174E6	4	4	170	194	Oxidation (M)	M18:Oxidation (M):1000.00	PEAKS DB
K.TVAESLDIDM(+15.99)GIAGK.V	Y	99.20	1534.7549	15	2.8	768.3869	2	30.87	4	F4:1309	OB5925 H3B raw.raw	5.2987E4	2	2	195	209	Oxidation (M)	M10:Oxidation (M):1000.00	PEAKS DB
R.EGDVIVVPTGSAQWIYNSGETDMVIFSVIDSANEDNQLDLKVR.K	Y	92.51	4723.2964	43	3.8	1575.4454	3	41.12	5	F5:2051	OB5934 H3B raw.raw	4.2033E6	3	3	127	169			PEAKS DB
K.G(+57.02)LLLPHYINAPR.L	Y	88.07	1419.7986	12	0.8	710.9072	2	31.80	4	F4:1367	OB5925 H3B raw.raw	6.0002E5	6	6	76	87	Carbamidomethylation (DHKE, X@N-term)	G1:Carbamidomethylation (DHKE, X@N-term):80.35	PEAKS PTM
K.VQGVDDPRGSMIIVEDELETLSPAVEESGN.G	Y	77.82	3184.5081	30	2.6	1593.2655	2	38.69	5	F5:1920	OB5934 H3B raw.raw	9.8208E5	2	2	210	239			PEAKS DB
L.PHYINAPR.L	Y	67.39	966.5035	8	-0.3	484.2589	2	31.65	6	F6:1495	OB5935 H3B raw.raw	3.8685E5	3	3	80	87			PEAKS DB
K.C(-17.03)SFD(+57.02)KLVALEPTK.R	Y	67.01	1489.7487	13	2.1	745.8832	2	34.42	4	F4:1510	OB5925 H3B raw.raw	8.2628E5	2	2	29	41	Ammonia-loss (C@N-term)	C1:Ammonia-loss (C@N-term):1000.00;D4:Carbamidomethylation (DHKE, X@N-term):14.02	PEAKS PTM
L.LLPHYINAPR.L	Y	62.87	1192.6716	10	0.0	597.3431	2	31.41	5	F5:1491	OB5934 H3B raw.raw	8.2176E5	3	3	78	87			PEAKS DB
K.C(-17.03)SFD(+57.02)KLVALEPTKR.V	Y	59.13	1645.8497	14	0.3	549.6240	3	32.55	4	F4:1406	OB5925 H3B raw.raw	5.152E5	2	2	29	42	Ammonia-loss (C@N-term)	C1:Ammonia-loss (C@N-term):1000.00;D4:Carbamidomethylation (DHKE, X@N-term):20.00	PEAKS PTM
L.LPHYINAPR.L	Y	58.80	1079.5875	9	0.0	540.8010	2	31.65	6	F6:1499	OB5935 H3B raw.raw	1.0935E5	2	2	79	87			PEAKS DB
R.KFFLGGKPQ(+.98)EEKGEEGNMFSGLELK.T	N	54.88	2799.3789	25	8.5	934.1415	3	34.08	5	F5:1645	OB5934 H3B raw.raw	0	0	0	170	194	Deamidation (NQ)	Q9:Deamidation (NQ):56.68	PEAKS DB
K.TVAESLDIDMGIAGKVQGVDDPR.G	Y	53.61	2385.1846	23	3.9	1193.6042	2	33.53	6	F6:1612	OB5935 H3B raw.raw	2.104E5	2	2	195	217			PEAKS DB
V.VPGC(+57.02)PETFREQTR.H	Y	52.87	1575.7463	13	2.4	788.8823	2	35.35	5	F5:1724	OB5934 H3B raw.raw	1.1594E6	1	1	102	114	Carbamidomethylation	C4:Carbamidomethylation:1000.00	PEAKS DB
K.VQGVDDPRGSM(+15.99)IIVEDELETLSPAVEESGN.G	Y	50.43	3200.5029	30	5.1	1601.2670	2	36.46	5	F5:1790	OB5934 H3B raw.raw	8.8212E4	1	1	210	239	Oxidation (M)	M11:Oxidation (M):1000.00	PEAKS DB
V.PGC(+57.02)PETFREQTR.H	Y	49.55	1476.6780	12	2.0	739.3478	2	35.59	6	F6:1753	OB5935 H3B raw.raw	5.0655E6	2	2	103	114	Carbamidomethylation	C3:Carbamidomethylation:1000.00	PEAKS DB
R.LQ(+.98)YVLQGTGILETVVPGC(+57.02)PETFREQTR.H	Y	44.26	3091.5647	27	7.8	1031.5369	3	47.69	6	F6:2433	OB5935 H3B raw.raw	3.862E3	1	1	88	114	Carbamidomethylation	Q2:Deamidation (NQ):31.56;C18:Carbamidomethylation:1000.00	PEAKS DB
**total 27 peptides**																			

A0A0F6VX63|A0A0F6VX63_ARAHY

back to list

| Protein Coverage | Supporting Peptides |

Protein Coverage:

```
1   MSTLRIPQPV PPVADDCEQL RKAFAGWGTN EDLIISILGH RNAAQRKLIR ETYFETYGED LLKALDKELS NDFERLVHLW
81  TLDPAERDAF LANEATKRWT SSNQVLMEIA CTRSSDQLLF ARKAYHARYK KSLEEDVAHH TTGEFRKLLL PLVSSYRYEG
161 DEVNLTLAKT EAKLLHEKIS KKAYSDDELI RILATRSKPQ INATLNHYKD EFGKDINKDL KADPKDVFLA LLRSTVKCLV
241 RTERYFEKAA RLAINKRGTD EGALTRVVAT RAEIDLKIIA DEYQRRSSVP LDKAVAKDTT GDYEKMLLAI LGHDDV
```

a Acetylation (Protein N-term) (+42.01)
c Carbamidomethylation (+57.02)
o Oxidation (M) (+15.99)

Supporting Peptides:

Peptide	Uniq	-10lgP	Mass	Length	ppm	m/z	z	RT	Fraction	Scan	Source File	Area Raw Ara h 3	#Feature	#Feature Raw Ara h 3	Start	End	PTM	AScore	Found By
K.LLLPLVSSYRYEGDEVNLTLAK.T	Y	128.62	2492.3525	22	-3.4	1247.1793	2	35.69	2	F2:1789	OB5937 H3A raw.raw	1.2701E7	5	5	148	169			PEAKS DB
K.AFAGWGTNEDLIISILGHR.N	Y	118.74	2069.0693	19	2.6	1035.5447	2	37.54	3	F3:1909	OB5938 H3A raw.raw	2.0436E7	6	6	23	41			PEAKS DB
K.DTTGDYEKM(+15.99)LLAILGHDDV	Y	117.44	2120.9937	19	2.8	1061.5071	2	37.52	2	F2:1899	OB5937 H3A raw.raw	1.7507E6	3	3	298	316	Oxidation (M)	M9:Oxidation (M):1000.00	PEAKS DB
K.DTTGDYEKMLLAILGHDDV	Y	114.87	2104.9985	19	2.6	1053.5093	2	39.41	2	F2:2009	OB5937 H3A raw.raw	2.9926E6	3	3	298	316			PEAKS DB
R.KAFAGWGTNEDLIISILGHR.N	Y	110.17	2197.1643	20	1.8	733.3967	3	36.03	3	F3:1820	OB5938 H3A raw.raw	1.1777E6	3	3	22	41			PEAKS DB
K.ALDKELSNDFER.L	Y	98.61	1435.6942	12	0.7	718.8549	2	28.58	2	F2:1373	OB5937 H3A raw.raw	8.7943E4	3	3	64	75			PEAKS DB
R.IPQPVPPVADDC(+57.02)EQLRK.A	Y	96.06	1961.0040	17	1.9	654.6765	3	28.76	2	F2:1372	OB5937 H3A raw.raw	8.3045E5	3	3	6	22	Carbamidomethylation	C12:Carbamidomethylation:1000.00	PEAKS DB
K.SLEEDVAHHTTGEFR.K	Y	93.56	1726.7910	15	0.2	576.6044	3	30.69	1	F1:1462	OB5936 H3A raw.raw	3.5275E5	3	3	132	146			PEAKS DB
K.MLLAILGHDDV	Y	91.59	1195.6271	11	1.2	598.8215	2	35.57	2	F2:1777	OB5937 H3A raw.raw	1.4911E6	3	3	306	316			PEAKS DB
K.KAYSDDELIR.I	Y	91.36	1208.6036	10	-0.8	605.3086	2	26.68	2	F2:1249	OB5937 H3A raw.raw	2.4558E4	3	3	182	191			PEAKS DB
K.SLEEDVAHHTTGEFRK.L	Y	89.54	1854.8860	16	0.2	619.3027	3	28.20	2	F2:1348	OB5937 H3A raw.raw	1.8944E5	2	2	132	147			PEAKS DB
R.KLLLPLVSSYRYEGDEVNLTLAK.T	Y	88.72	2620.4475	23	1.9	874.4915	3	34.56	2	F2:1726	OB5937 H3A raw.raw	1.5288E6	3	3	147	169			PEAKS DB
R.ETYFETYGEDLLKALDKELSNDFER.L	Y	83.82	3024.4238	25	2.4	1009.1509	3	40.92	2	F2:2098	OB5937 H3A raw.raw	9.7793E5	2	2	51	75			PEAKS DB
R.SSDQLLFAR.K	Y	82.53	1035.5349	9	1.4	518.7755	2	30.46	2	F2:1477	OB5937 H3A raw.raw	3.9148E5	3	3	114	122			PEAKS DB

total 25 peptides

Peptide	Uniq	-10lgP	Mass	Length	ppm	m/z	z	RT	Fraction	Scan	Source File	Area Raw Ara h 3	#Feature	#Feature Raw Ara h 3	Start	End	PTM	AScore	Found By
R.ETYFETYGEDLLK.A	Y	82.24	1606.7402	13	-5.6	804.3729	2	36.83	1	F1:1834	OB5936 H3A raw.raw	3.134E5	2	2	51	63			PEAKS DB
R.WTSSNQVLMEIAC(+57.02)TR.S	Y	79.90	1794.8394	15	1.8	898.4286	2	36.10	1	F1:1791	OB5936 H3A raw.raw	2.4676E5	1	1	99	113	Carbamidomethylation	C13:Carbamidomethylation:1000.00	PEAKS DB
R.WTSSNQVLM(+15.99)EIAC(+57.02)TR.S	Y	76.72	1810.8342	15	1.3	906.4255	2	35.18	1	F1:1732	OB5936 H3A raw.raw	7.2086E4	2	2	99	113	Oxidation (M); Carbamidomethylation	M9:Oxidation (M):1000.00;C13:Carbamidomethylation:1000.00	PEAKS DB
R.YEGDEVNLTLAK.T	Y	76.44	1350.6666	12	1.3	676.3415	2	29.90	3	F3:1464	OB5938 H3A raw.raw	1.4769E4	1	1	158	169			PEAKS DB
R.LVHLWTLDPAERDAFLANEATKR.W	Y	74.26	2665.3975	23	-0.7	667.3562	4	33.46	2	F2:1665	OB5937 H3A raw.raw	4.8836E5	3	3	76	98			PEAKS DB
M.S(+42.01)TLRIPQPVPPVADDC(+57.02)EQLRK.A	Y	69.22	2460.2795	21	0.9	821.1012	3	33.70	1	F1:1644	OB5936 H3A raw.raw	1.4069E5	1	1	2	22	Acetylation (Protein N-term); Carbamidomethylation	S1:Acetylation (Protein N-term):1000.00;C16:Carbamidomethylation:1000.00	PEAKS PTM
R.VVATRAEIDLK.I	Y	42.22	1213.7030	11	-0.2	607.8586	2	27.11	2	F2:1285	OB5937 H3A raw.raw	4.1122E3	1	1	267	277			PEAKS DB
R.DAFLANEATKR.W	Y	41.43	1234.6306	11	-0.8	618.3221	2	26.52	3	F3:1255	OB5938 H3A raw.raw	0	0	0	88	98			PEAKS DB
R.SSVPLDKAVAK.D	Y	35.59	1113.6393	11	-0.7	557.8265	2	25.57	2	F2:1192	OB5937 H3A raw.raw	0	0	0	287	297			PEAKS DB
R.AEIDLKIIADEYQRR.S	Y	35.59	1831.9791	15	0.3	611.6672	3	33.70	1	F1:1650	OB5936 H3A raw.raw	8.875E4	1	1	272	286			PEAKS DB
R.IPQPVPPVAD(+14.02)DC(+57.02)EQLRK.A	Y	34.81	1975.0197	17	1.3	659.3480	3	31.07	1	F1:1494	OB5936 H3A raw.raw	1.0353E4	1	1	6	22	Carbamidomethylation	D10:Methylation(others):17.01;C12:Carbamidomethylation:1000.00	PEAKS PTM
**total 25 peptides**																			

A0A0A6ZDP1|A0A0A6ZDP1_ARAHY

back to list

| Protein Coverage | Supporting Peptides |

Protein Coverage:

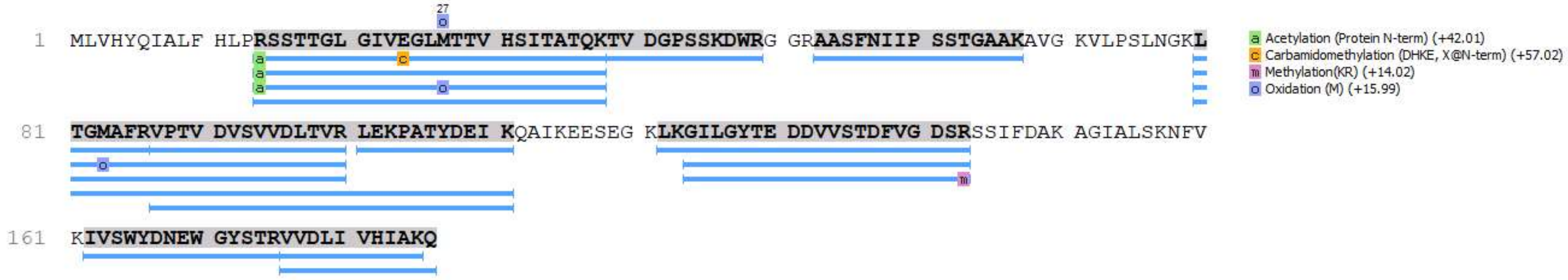

Supporting Peptides:

Peptide	Uniq	-10lgP	Mass	Length	ppm	m/z	z	RT	Fraction	Scan	Source File	Area Raw Ara h 3	#Feature	#Feature Raw Ara h 3	Start	End	PTM	AScore	Found By
**total 20 peptides**																			

Peptide	Uniq	-10lgP	Mass	Length	ppm	m/z	z	RT	Fraction	Scan	Source File	Area Raw Ara h 3	#Feature	#Feature Raw Ara h 3	Start	End	PTM	AScore	Found By
K.GILGYTEDDVVSTDFVGDSR.S	Y	153.49	2143.9910	20	3.6	1073.0066	2	34.92	2	F2:1742	OB5937 H3A raw.raw	1.9792E7	9	9	124	143			PEAKS DB
K.IVSWYDNEWGYSTR.V	N	134.23	1774.7950	14	1.2	888.4058	2	34.01	2	F2:1691	OB5937 H3A raw.raw	2.0685E6	3	3	162	175			PEAKS DB
R.VPTVDVSVVDLTVR.L	N	121.52	1497.8402	14	1.3	749.9283	2	34.19	2	F2:1704	OB5937 H3A raw.raw	2.6396E6	3	3	87	100			PEAKS DB
R.VVDLIVHIAKQ	Y	113.45	1233.7445	11	1.0	617.8801	2	30.64	2	F2:1484	OB5937 H3A raw.raw	1.4659E6	6	6	176	186			PEAKS DB
K.LTGMAFRVPTVDVSVVDLTVR.L	N	104.04	2274.2405	21	-2.6	1138.1245	2	36.04	2	F2:1813	OB5937 H3A raw.raw	1.7435E6	4	4	80	100			PEAKS DB
R.AASFNIIPSSTGAAK.A	N	101.66	1433.7513	15	2.0	717.8844	2	31.01	2	F2:1503	OB5937 H3A raw.raw	5.125E6	3	3	53	67			PEAKS DB
K.LKGILGYTEDDVVSTDFVGDSR.S	Y	100.93	2385.1699	22	1.5	796.0651	3	33.83	3	F3:1688	OB5938 H3A raw.raw	1.1517E6	2	2	122	143			PEAKS DB
K.G(+57.02)ILGYTEDDVVSTDFVGDSR.S	Y	96.93	2201.0125	20	1.2	1101.5148	2	34.92	2	F2:1752	OB5937 H3A raw.raw	7.1244E4	1	1	124	143		G1:Carbamidomethylation (DHKE, X@N-term):30.02	PEAKS PTM
K.LTGM(+15.99)AFRVPTVDVSVVDLTVR.L	N	93.82	2290.2354	21	-5.6	764.4148	3	36.83	1	F1:1833	OB5936 H3A raw.raw	1.375E6	4	4	80	100	Oxidation (M)	M4:Oxidation (M):1000.00	PEAKS DB
P.R(+42.01)SSTTGLGIVEGLM(+15.99)TTVHSITATQK.T	Y	87.21	2645.3694	25	7.9	882.8040	3	34.56	2	F2:1722	OB5937 H3A raw.raw	1.6313E7	3	3	14	38	Acetylation (Protein N-term); Oxidation (M)	R1:Acetylation (Protein N-term):1000.00;M14:Oxidation (M):1000.00	PEAKS PTM
P.R(+42.01)SSTTGLGIVEGLMTTVHSITATQK.T	Y	83.95	2629.3745	25	8.4	877.4728	3	36.96	2	F2:1855	OB5937 H3A raw.raw	4.5719E7	4	4	14	38	Acetylation (Protein N-term)	R1:Acetylation (Protein N-term):1000.00	PEAKS PTM
P.R(+57.02)S(+42.01)STTGLGIVEGLMTTVHSITATQK.T	Y	70.57	2686.3960	25	5.9	896.4779	3	38.70	1	F1:1935	OB5936 H3A raw.raw	1.2969E6	1	1	14	38		R1:Carbamidomethylation (DHKE, X@N-term):29.04;S2:Acetylation (TSCYH):0.00	PEAKS PTM
R.LEKPATYDEIK.Q	N	68.98	1305.6816	11	-1.7	653.8470	2	26.49	2	F2:1243	OB5937 H3A raw.raw	0	0	0	101	111			PEAKS DB
R.VVDLIVHIAK.Q	Y	67.01	1105.6859	10	0.2	553.8503	2	30.46	2	F2:1480	OB5937 H3A raw.raw	6.1004E4	3	3	176	185			PEAKS DB
P.R(+42.01)SSTTGLGIVE(+57.02)GLMTTVHSITATQK.T	Y	62.66	2686.3960	25	6.1	896.4780	3	37.14	2	F2:1876	OB5937 H3A raw.raw	3.1868E6	2	2	14	38	Acetylation (Protein N-term); Carbamidomethylation (DHKE, X@N-term)	R1:Acetylation (Protein N-term):1000.00;E11:Carbamidomethylation (DHKE, X@N-term):201.30	PEAKS PTM
K.GILGYTEDDVVSTDFVGDSR(+14.02).S	Y	61.96	2158.0066	20	2.8	1080.0135	2	35.93	3	F3:1812	OB5938 H3A raw.raw	0	0	0	124	143	Methylation(KR)	R20:Methylation(KR):1000.00	PEAKS PTM
R.VPTVDVSVVDLTVRLEKPATYDEIK.Q	N	61.92	2785.5112	25	-4.1	929.5072	3	36.47	1	F1:1815	OB5936 H3A raw.raw	3.1435E6	4	4	87	111			PEAKS DB
K.LTGMAFRVPTVDVSVVDLTVRLEKPATYDEIK.Q	N	53.47	3561.9116	32	-4.9	891.4808	4	37.76	1	F1:1887	OB5936 H3A raw.raw	1.7224E6	2	2	80	111			PEAKS DB
K.TVDGPSSKDWR.G	N	51.15	1246.5942	11	-1.7	624.3033	2	26.08	2	F2:1212	OB5937 H3A raw.raw	1.1675E4	2	2	39	49			PEAKS DB
K.LTGMAFR.V	N	42.85	794.4109	7	1.0	398.2131	2	30.69	1	F1:1449	OB5936 H3A raw.raw	9.0748E3	1	1	80	86			PEAKS DB
**total 20 peptides**																			

Q647H3|Q647H3_ARAHY

back to list

| **Protein Coverage** | **Supporting Peptides** |

Protein Coverage:

```
        32
        C
1   MAKLLALSVC FCFLVLGASS ISFRQQPEEN ACQFQRLNAQ RPDNRLESEG GYIETWNPNN QEFECAGVAL SRLVLRRNAL

        108
        C
81  RRPFYSNAPQ EIFIQQGRGY FGLIFPGCPS TYEEPAQQGR RHQSQRAPRR FEGEDQSQQQ QQDSHQKVRR FDEGDLIAVP

161 TGVALWMYND HDTDVVAVSL TDTNNNDNQL DQFPRRFNLA GNHEQEFLRY QQQSRRRSLP YSPYSPQSQP RQEEREFSPR

241 GQHSRRERAG QEQENEGGNI FSGFTPEFLA QAFQVDDRQI LQNLRGENES DEQGAIVTVR GGLRILSPDR KRRQQYERPD

321 EEEEYDEDEY EYDEEERQQD RRRGRGSRGR GNGIEETICT ASVKKNIGRN RSPDIYNPQA GSLKTANDLN LLILRWLGLS

401 AEYGNLYRNA LFVPHYNTNA HSIIYALRGR AHVQVVDSNG NRVYDEELQE GHVLVVPQNF AVAGKSQSDN FEYVAFKTDS

481 RPSIANLAGE NSIIDNLPEE VVANSYGLPR EQARQLKNNN PFKFFVPPSQ QSLGAVA
```

C Carbamidomethylation (+57.02)
m Methylation(KR) (+14.02)
O Oxidation (M) (+15.99)

Supporting Peptides:

Peptide	Uniq	-10lgP	Mass	Length	ppm	m/z	z	RT	Fraction	Scan	Source File	Area Raw Ara h 3	#Feature	#Feature Raw Ara h 3	Start	End	PTM	AScore	Found By
R.GYFGLIFPGC(+57.02)PSTYEEPAQQGR.R	N	135.05	2473.1372	22	3.9	1237.5807	2	36.47	4	F4:1635	OB5925 H3B raw.raw	5.6724E5	5	5	99	120	Carbamidomethylation	C10:Carbamidomethylation:1000.00	PEAKS DB
R.RPFYSNAPQEIFIQQGR.G	N	119.14	2050.0383	17	2.3	1026.0288	2	31.40	3	F3:1547	OB5938 H3A raw.raw	1.5008E6	8	8	82	98			PEAKS DB
R.RFNLAGNHEQEFLR.Y	N	113.49	1729.8647	14	1.5	577.6297	3	30.08	2	F2:1438	OB5937 H3A raw.raw	6.1619E6	6	6	196	209			PEAKS DB
R.AGQ(+.98)EQENEGGNIFSGFTPEFLAQAFQVDDR.Q	N	102.24	3301.4800	30	5.9	1651.7571	2	39.98	2	F2:2041	OB5937 H3A raw.raw	3.2739E5	1	1	249	278		Q3:Deamidation (NQ): 14.04	PEAKS DB
S.ISFRQQPEENAC(+57.02)QFQR.L	N	92.73	2036.9486	16	0.8	679.9907	3	27.97	3	F3:1335	OB5938 H3A raw.raw	1.7296E5	3	3	21	36	Carbamidomethylation	C12:Carbamidomethylation:1000.00	PEAKS DB
R.WLGLSAEYGNLYR.N	N	90.48	1540.7673	13	0.4	771.3912	2	34.97	4	F4:1558	OB5925 H3B raw.raw	1.2371E5	1	1	396	408			PEAKS DB
R.AGQEQENEGGNIFSGFTPEFLAQAFQVDDR.Q	N	87.17	3300.4958	30	2.5	1651.2593	2	39.97	3	F3:2048	OB5938 H3A raw.raw	0	0	0	249	278			PEAKS DB
R.NALRRPFYSNAPQEIFIQQGR.G	N	82.56	2504.3037	21	1.7	835.7766	3	33.51	1	F1:1633	OB5936 H3A raw.raw	9.3975E5	4	4	78	98			PEAKS DB
R.GYFGLIFPGC(+57.02)PSTYEEPAQQGRR.H	N	81.37	2629.2383	23	1.6	877.4214	3	34.78	4	F4:1543	OB5925 H3B raw.raw	1.2146E6	3	3	99	121	Carbamidomethylation	C10:Carbamidomethylation:1000.00	PEAKS DB

total 18 peptides

Peptide	Uniq	-10lgP	Mass	Length	ppm	m/z	z	RT	Fraction	Scan	Source File	Area Raw Ara h 3	#Feature	#Feature Raw Ara h 3	Start	End	PTM	AScore	Found By
R.FNLAGNHEQEFLR.Y	N	68.89	1573.7637	13	1.4	787.8902	2	30.26	3	F3:1483	OB5938 H3A raw.raw	1.9024E5	5	5	197	209			PEAKS DB
R.QQPEENAC(+57.02)QFQR.L	N	54.65	1533.6630	12	1.7	767.8401	2	25.17	2	F2:1164	OB5937 H3A raw.raw	6.6881E3	2	2	25	36	Carbamidomethylation	C8:Carbamidomethylation:1000.00	PEAKS DB
R.SLPYSPY(+31.99)SPQSQPR.Q	N	54.08	1637.7686	14	1.0	819.8923	2	29.76	1	F1:1401	OB5936 H3A raw.raw	3.8534E4	1	1	218	231		Y7:Dihydroxy:0.00	PEAKS PTM
R.FNLAGNH(+14.02)EQEFLR.Y	N	44.51	1587.7793	13	1.9	530.2681	3	32.39	1	F1:1568	OB5936 H3A raw.raw	1.4807E5	2	2	197	209		H7:Methylation(others):33.98	PEAKS PTM
R.R(+14.02)FNLAGNHEQEFLR.Y	N	43.10	1743.8805	14	-0.1	582.3007	3	32.19	1	F1:1569	OB5936 H3A raw.raw	1.2906E6	2	2	196	209	Methylation(KR)	R1:Methylation(KR):67.11	PEAKS PTM
K.TDSRPSIANLAGENSIIDNLPEEVVANSYGLPR.E	Y	38.26	3510.7590	33	4.1	1171.2651	3	37.58	5	F5:1869	OB5934 H3B raw.raw	4.1382E5	1	1	478	510			PEAKS DB
R.RFDEGDLIAVPTGVALWM(+15.99)YN(+.98)DHDTDVVAVSLTDTNNNDNQ(+.98)LDQFPR.R	Y	35.22	5208.3892	46	8.7	1737.1521	3	38.89	1	F1:1958	OB5936 H3A raw.raw	1.266E5	1	1	150	195	Oxidation (M)	M18:Oxidation(M):1000.00;N20:Deamidation (NQ):12.67;Q40:Deamidation(NQ):0.00	PEAKS DB
R.SLPYSP(+31.99)YSPQSQPR.Q	N	35.06	1637.7686	14	1.7	819.8929	2	27.29	2	F2:1288	OB5937 H3A raw.raw	7.4071E4	1	1	218	231		P6:Dihydroxy:0.00	PEAKS PTM
R.R(+57.02)FNLAGNHEQEFLR.Y	N	32.70	1786.8862	14	-0.5	596.6357	3	32.39	1	F1:1563	OB5936 H3A raw.raw	6.2203E4	1	1	196	209		R1:Carbamidomethylation (DHKE,X@N-term):9.75	PEAKS PTM
**total 18 peptides**																			

E9LFE8|E9LFE8_ARAHY

back to list

| Protein Coverage | Supporting Peptides |

Protein Coverage:

38

1 KSRKFFLGGK PQEEKGEEGN MFSGLELKTV AESLGIDMGI AGKVQGVDDP RGSIIIVEDE LETLSPAVEE SGNGNGLDET

81 LCTLRLVHQL AESTDADKYN PRAGFLTALN TPNLPVLQYV QLGADRGVFY KNAVMAPHYN LNCHAVIYGT EGRGWIEVVG

161 ENGRKVYEGE VREGQILIVP QQFMVAKKAA EGSDEGFGWI AVKTSDNPMI SPLAGKLSLI RAMPLPVLMN SFRLTAEEAI

241 NLKKRGELTF FSPDPAHTQI

d Deamidation (NQ) (+0.98)
d Dimethylation(KR) (+28.03)
o Oxidation (M) (+15.99)

Supporting Peptides:

Peptide	Uniq	-10lgP	Mass	Length	ppm	m/z	z	RT	Fraction	Scan	Source File	Area Raw Ara h 3	#Feature	#Feature Raw Ara h 3	Start	End	PTM	AScore	Found By
K.FFLGGKPQEEKGEEGNMFSGLELK.T	N	162.92	2670.2998	24	2.4	891.1094	3	34.78	4	F4:1553	OB5925 H3B raw.raw	1.858E7	6	6	5	28			PEAKS DB
K.TVAESLGIDMGIAGK.V	Y	139.60	1460.7544	15	2.2	731.3861	2	33.48	5	F5:1604	OB5934 H3B raw.raw	4.8068E6	3	3	29	43			PEAKS DB
R.KFFLGGKPQEEKGEEGNMFSGLELK.T	N	122.64	2798.3948	25	2.1	933.8075	3	34.42	4	F4:1518	OB5925 H3B raw.raw	1.4026E7	9	9	4	28			PEAKS DB
K.TVAESLGIDM(+15.99)GIAGK.V	Y	104.94	1476.7494	15	1.8	739.3833	2	30.84	5	F5:1465	OB5934 H3B raw.raw	3.5299E4	3	3	29	43	Oxidation (M)	M10:Oxidation (M):100 0.00	PEAKS DB
K.FFLGGKPQEEKGEEGNM(+15.99)FSGLELK.T	N	102.01	2686.2947	24	1.7	896.4404	3	32.74	4	F4:1411	OB5925 H3B raw.raw	2.6867E6	4	4	5	28	Oxidation (M)	M17:Oxidation (M):100 0.00	PEAKS DB
R.KFFLGGKPQEEKGEEGNM(+15.99)FSGLELK.T	N	101.19	2814.3896	25	0.9	704.6053	4	32.34	5	F5:1545	OB5934 H3B raw.raw	1.3174E6	4	4	4	28	Oxidation (M)	M18:Oxidation (M):100 0.00	PEAKS DB
R.AGFLTALNTPNLPVLQYVQLGADR(+28.03).G	Y	89.94	2598.4170	24	3.2	1300.2200	2	40.37	5	F5:2024	OB5934 H3B raw.raw	3.0506E5	1	1	103	126	Dimethylation(KR)	R24:Dimethylation(KR): 1000.00	PEAKS PTM
K.VQGVDDPRGSIIIVEDELETLSPAVEESGN.G	Y	79.95	3166.5518	30	3.5	1584.2887	2	39.25	5	F5:1952	OB5934 H3B raw.raw	3.2404E6	3	3	44	73			PEAKS DB
R.KFFLGGKPQ(+.98)EEKGEEGNMFSGLELK.T	N	54.88	2799.3789	25	8.5	934.1415	3	34.08	5	F5:1645	OB5934 H3B raw.raw	0	0	0	4	28	Deamidation (NQ)	Q9:Deamidation (NQ):5 6.68	PEAKS DB
K.TVAESLGIDMGIAGKVQGVDDPR.G	Y	38.80	2327.1790	23	3.2	1164.6005	2	33.53	6	F6:1613	OB5935 H3B raw.raw	3.4542E4	1	1	29	51			PEAKS DB
**total 10 peptides**																			

A0A0A6ZDT0|A0A0A6ZDT0_ARAHY

back to list

| Protein Coverage | Supporting Peptides |

Protein Coverage:

1 MLVHYQIALF HLPRSSTTGL GIVEGLMTAV HSITATQKTV DGPSSKDWRG GRAASFNIIP SSTGAAKAVG KVLPSLNGKL

81 TGMAFRVPTV DVSVVDLTVR LEKPATYDEI KQAIKEESEG KLKVILGYTE DDVVSTDFVG DSRSSIFDAK AGIALSKNFV

161 KIVSWYDNEW GYSTRVVDLV VHIAKQ

o Oxidation (M) (+15.99)

Supporting Peptides:

Peptide	Uniq	-10lgP	Mass	Length	ppm	m/z	z	RT	Fraction	Scan	Source File	Area Raw Ara h 3	#Feature	#Feature Raw Ara h 3	Start	End	PTM	AScore	Found By
**total 12 peptides**																			

Peptide	Uniq	-10lgP	Mass	Length	ppm	m/z	z	RT	Fraction	Scan	Source File	Area Raw Ara h 3	#Feature	#Feature Raw Ara h 3	Start	End	PTM	AScore	Found By
K.IVSWYDNEWGYSTR.V	N	134.23	1774.7950	14	1.2	888.4058	2	34.01	2	F2:1691	OB5937 H3A raw.raw	2.0685E6	3	3	162	175			PEAKS DB
R.VPTVDVSVVDLTVR.L	N	121.52	1497.8402	14	1.3	749.9283	2	34.19	2	F2:1704	OB5937 H3A raw.raw	2.6396E6	3	3	87	100			PEAKS DB
K.LTGMAFRVPTVDVSVVDLTVR.L	N	104.04	2274.2405	21	-2.6	1138.1245	2	36.04	2	F2:1813	OB5937 H3A raw.raw	1.7435E6	4	4	80	100			PEAKS DB
R.AASFNIIPSSTGAAK.A	N	101.66	1433.7513	15	2.0	717.8844	2	31.01	2	F2:1503	OB5937 H3A raw.raw	5.125E6	3	3	53	67			PEAKS DB
R.VVDLVVHIAKQ	Y	98.43	1219.7289	11	0.6	610.8721	2	29.51	2	F2:1414	OB5937 H3A raw.raw	1.0958E6	6	6	176	186			PEAKS DB
K.LTGM(+15.99)AFRVPTVDVSVVDLTVR.L	N	93.82	2290.2354	21	-5.6	764.4148	3	36.83	1	F1:1833	OB5936 H3A raw.raw	1.375E6	4	4	80	100	Oxidation (M)	M4:Oxidation (M):1000.00	PEAKS DB
R.LEKPATYDEIK.Q	N	68.98	1305.6816	11	-1.7	653.8470	2	26.49	2	F2:1243	OB5937 H3A raw.raw	0	0	0	101	111			PEAKS DB
R.VPTVDVSVVDLTVRLEKPATYDEIK.Q	N	61.92	2785.5112	25	-4.1	929.5072	3	36.47	1	F1:1815	OB5936 H3A raw.raw	3.1435E6	4	4	87	111			PEAKS DB
R.VVDLVVHIAK.Q	Y	55.01	1091.6703	10	0.4	546.8427	2	29.14	3	F3:1409	OB5938 H3A raw.raw	1.7498E4	2	2	176	185			PEAKS DB
K.LTGMAFRVPTVDVSVVDLTVRLEKPATYDEIK.Q	N	53.47	3561.9116	32	-4.9	891.4808	4	37.76	1	F1:1887	OB5936 H3A raw.raw	1.7224E6	2	2	80	111			PEAKS DB
K.TVDGPSSKDWR.G	N	51.15	1246.5942	11	-1.7	624.3033	2	26.08	2	F2:1212	OB5937 H3A raw.raw	1.1675E4	2	2	39	49			PEAKS DB
K.LTGMAFR.V	N	42.85	794.4109	7	1.0	398.2131	2	30.69	1	F1:1449	OB5936 H3A raw.raw	9.0748E3	1	1	80	86			PEAKS DB

total 12 peptides

E9LFE9|E9LFE9_ARAHY

back to list

| Protein Coverage | Supporting Peptides |

Protein Coverage:

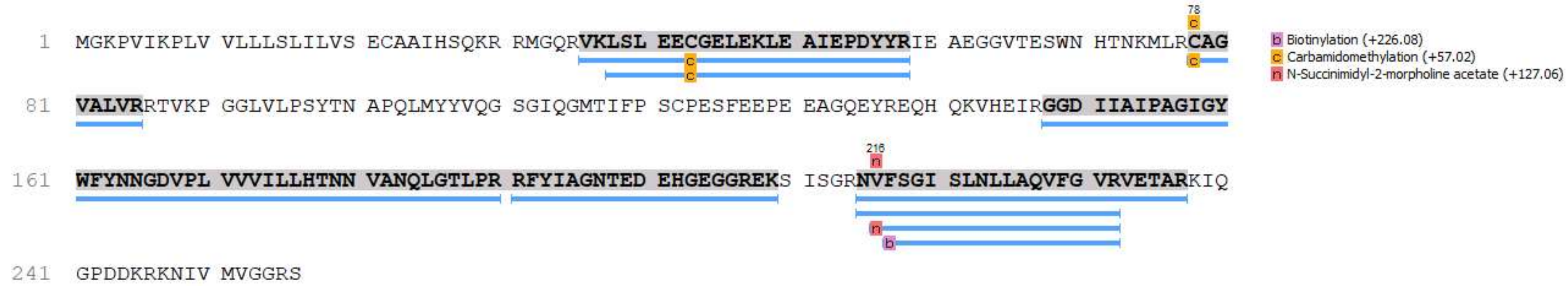

Supporting Peptides:

Peptide	Uniq	-10lgP	Mass	Length	ppm	m/z	z	RT	Fraction	Scan	Source File	Area Raw Ara h 3	#Feature	#Feature Raw Ara h 3	Start	End	PTM	AScore	Found By
K.LSLEEC(+57.02)GELEKLEAIEPDYYR.I	Y	136.26	2555.2100	21	1.7	852.7454	3	34.79	5	F5:1692	OB5934 H3B raw.raw	1.2508E6	4	4	38	58	Carbamidomethylation	C6:Carbamidomethylation:1000.00	PEAKS DB
R.NVFSGISLNLLAQVFGVR.V	Y	122.96	1933.0785	18	2.8	967.5492	2	43.21	5	F5:2172	OB5934 H3B raw.raw	1.5453E6	5	5	215	232			PEAKS DB
R.VKLSLEEC(+57.02)GELEKLEAIEPDYYR.I	Y	120.68	2782.3735	23	1.8	928.4668	3	34.24	5	F5:1649	OB5934 H3B raw.raw	7.0271E5	2	2	36	58	Carbamidomethylation	C8:Carbamidomethylation:1000.00	PEAKS DB

total 11 peptides

Peptide	Uniq	-10lgP	Mass	Length	ppm	m/z	z	RT	Fraction	Scan	Source File	Area Raw Ara h 3	#Feature	#Feature Raw Ara h 3	Start	End	PTM	AScore	Found By
R.NVFSGISLNLLAQVFGVRVETAR.K	Y	84.03	2489.3755	23	3.0	830.8016	3	42.69	4	F4:1993	OB5925 H3B raw.raw	4.1048E6	4	4	215	237			PEAKS DB
R.C(+57.02)AGVALVR.R	Y	76.26	844.4589	8	1.7	423.2374	2	26.94	4	F4:1100	OB5925 H3B raw.raw	1.0611E3	1	1	78	85	Carbamidomethylation	C1:Carbamidomethylation:1000.00	PEAKS DB
N.V(+127.06)FSGISLNLLAQVFGVR.V	Y	58.01	1946.0989	17	-9.0	974.0479	2	41.75	6	F6:2092	OB5935 H3B raw.raw	1.8558E7	5	5	216	232	N-Succinimidyl-2-morpholine acetate	V1:N-Succinimidyl-2-morpholine acetate:1000.00	PEAKS PTM
R.GGDIIAIPAGIGYWFYNNGDVPLVVVILLHTNNVANQLGTLPR.R	Y	50.04	4573.4482	43	3.5	1525.4954	3	44.60	4	F4:2114	OB5925 H3B raw.raw	9.2311E5	3	3	148	190			PEAKS DB
R.NVFSGIS(+13.03)LNLLAQVFGVR.V	Y	46.36	1946.1101	18	-9.4	974.0532	2	47.20	5	F5:2410	OB5934 H3B raw.raw	0	0	0	215	232		S7:Michael addition with methylamine:16.65	PEAKS PTM
R.NVFSGIS(+14.02)LNLLAQVFGVR.V	Y	42.16	1947.0941	18	2.0	974.5563	2	47.47	4	F4:2277	OB5925 H3B raw.raw	0	0	0	215	232		S7:Methylation(others):15.62	PEAKS PTM
R.RFYIAGNTEDEHGEGGREK.S	Y	35.61	2163.9932	19	-0.6	542.0052	4	29.79	6	F6:1390	OB5935 H3B raw.raw	1.1167E4	1	1	191	209			PEAKS DB
V.F(+226.08)SGISLNLLAQVFGVR.V	Y	33.77	1946.0447	16	2.4	974.0319	2	50.95	4	F4:2479	OB5925 H3B raw.raw	0	0	0	217	232	Biotinylation	F1:Biotinylation:1000.00	PEAKS PTM
**total 11 peptides**																			

A0A0F7GF62|A0A0F7GF62_ARAHY

back to list

| Protein Coverage | Supporting Peptides |

Protein Coverage:

```
   2
   a
  1 MATLIAPSNH SSAEDAEALQ KAFKGWGADD KTIIAILGHR NVHQRQQIRK AYEELHQEDL IKRLESEISG DFERAMYRWM
     a
 81 LEPADRDAVL ANVAIRNGKK DLHVIAEIAC VLSAEELLAV RRAYRHRYKR SLEEDVAANT TGHLRQLLVG LVSSYRYEGD
                                   c
161 EINARLAQSE ANILHETVKE KKGNYEEAIR ILTTRSKTQL VATFNRYRDE HAISISKKLL DNQASDDFYK ALHTAIRCIN
241 DHKKYYEKVL RNAIKKVGTD EDALSRVVVT RAEKDLRDIK ELYYKRNSVH LEDAVAKETS GDYKKFLLTL LGKQD
```

a Acetylation (Protein N-term) (+42.01)
c Carbamidomethylation (+57.02)

Supporting Peptides:

Peptide	Uniq	-10lgP	Mass	Length	ppm	m/z	z	RT	Fraction	Scan	Source File	Area Raw Ara h 3	#Feature	#Feature Raw Ara h 3	Start	End	PTM	AScore	Found By
R.SLEEDVAANTTGHLR.Q	Y	128.71	1611.7853	15	0.6	806.9004	2	29.32	2	F2:1409	OB5937 H3A raw.raw	2.1959E5	3	3	131	145			PEAKS DB
M.A(+42.01)TLIAPSNHSSAEDAEALQK.A	Y	103.49	2094.0229	20	1.7	1048.0205	2	30.83	2	F2:1507	OB5937 H3A raw.raw	1.9399E5	3	3	2	21	Acetylation (Protein N-term)	A1:Acetylation (Protein N-term):1000.00	PEAKS PTM
R.SKTQLVATFNR.Y	Y	96.50	1263.6935	11	0.6	632.8544	2	27.65	2	F2:1310	OB5937 H3A raw.raw	1.9511E3	1	1	196	206			PEAKS DB
K.FLLTLLGKQD	Y	81.14	1146.6648	10	0.9	574.3402	2	35.29	3	F3:1769	OB5938 H3A raw.raw	1.1511E6	2	2	306	315			PEAKS DB
K.AFKGWGADDKTIIAILGHR.N	Y	59.59	2068.1218	19	-0.4	518.0375	4	33.81	3	F3:1685	OB5938 H3A raw.raw	0	0	0	22	40			PEAKS DB
K.LLDNQASDDFYK.A	Y	50.72	1427.6569	12	0.8	714.8362	2	30.00	2	F2:1465	OB5937 H3A raw.raw	2.1343E4	2	2	219	230			PEAKS DB
K.DLHVIAEIAC(+57.02)VLSAEELLAVRR.A	Y	35.66	2476.3472	22	3.2	826.4590	3	41.50	1	F1:2111	OB5936 H3A raw.raw	8.5296E4	1	1	101	122	Carbamidomethylation	C10:Carbamidomethylation:1000.00	PEAKS DB

total 8 peptides

Peptide	Uniq	-10lgP	Mass	Length	ppm	m/z	z	RT	Fraction	Scan	Source File	Area Raw Ara h 3	#Feature	#Feature Raw Ara h 3	Start	End	PTM	AScore	Found By
R.LAQSEANILHETVKEK.K	Y	35.22	1808.9631	16	0.6	603.9954	3	28.02	2	F2:1341	OB5937 H3A raw.raw	1.063E4	1	1	166	181			PEAKS DB
**total 8 peptides**																			

Q6IWG5|Q6IWG5_ARAHY

back to list

| Protein Coverage | Supporting Peptides |

Protein Coverage:

```
                                         30                                                         63
  1  KLLALSLCFC VLVLGASSVT FRQGGEENEC QFQRLNAQRP DNRIESEGGY IETWNPNNQE FQCAGVALSR TVLRRNALRR   C Carbamidomethylation (+57.02)
                                    106
 81  PFYSNAPLEI YVQQGSGYFG LIFPGCPSTY EEPAQEGRRY QSQKPSRRFQ VGQDDPSQQQ QDSHQKVHRF DEGDLIAVPT
161  GVAFWMYNDE DTDVVTVTLS DTSSIHNQLD QFPRRFYLAG NQEQEFLRYQ QQQGSRPHYR QISPRVRGDE QENEGSNIFS
241  GFAQEFLQHA FQVDRQTVEN LRGENEREEQ GAIVTVKGGL RILSPDEEDE SSRSPPSRRE EFDEDRSRPQ QRGKYDENRR
321  GYKNGIEETI CSASVKKNLG RSSNPDIYNP QAGSLRSVNE LDLPILGWLG LSAQHGTIYR NAMFVPHYTL NAHTIVVALN
401  GRAHVQVVDS NGNRVYDEEL QEGHVLVVPQ NFAVAAKAQS ENYEYLAFKT DSRPSIANLA GENSIIDNLP EEVVANSYRL
481  PREQARQLKN NNPFKFFVPP FDHQSMREVA
```

Supporting Peptides:

Peptide	Uniq	-10lgP	Mass	Length	ppm	m/z	z	RT	Fraction	Scan	Source File	Area Raw Ara h 3	#Feature	#Feature Raw Ara h 3	Start	End	PTM	AScore	Found By
R.LNAQRPDNRIESEGGYIETWNPNNQEFQC(+57.02)AGVALSR.T	Y	116.63	4132.9409	36	3.0	1034.2456	4	33.11	5	F5:1590	OB5934 H3B raw.raw	2.0092E6	3	3	35	70	Carbamidomethylation	C29:Carbamidomethylation: 1000.00	PEAKS DB
K.TDSRPSIANLAGENSIIDNLPEEVVANSYR.L	Y	83.57	3243.6006	30	2.9	1082.2106	3	36.46	5	F5:1789	OB5934 H3B raw.raw	2.8671E5	1	1	450	479			PEAKS DB
R.SVNELDLPILGWLGLSAQHGTIYR.N	Y	77.09	2651.4070	24	1.8	884.8112	3	41.50	5	F5:2076	OB5934 H3B raw.raw	1.8337E6	4	4	357	380			PEAKS DB
R.RPFYSNAPLEIYVQQGSGYFGLIFPGC(+57.02)PSTYEEPAQEGR.R	Y	76.78	4424.0845	39	5.2	1475.7097	3	38.66	2	F2:1955	OB5937 H3A raw.raw	1.3954E6	3	3	80	118	Carbamidomethylation	C27:Carbamidomethylation: 1000.00	PEAKS DB
R.FYLAGNQEQEFLR.Y	Y	73.60	1613.7837	13	1.1	807.9000	2	33.08	2	F2:1641	OB5937 H3A raw.raw	2.0837E4	1	1	196	208			PEAKS DB
R.VRGDEQENEGSNIFSGFAQEFLQHAFQVDR.Q	Y	54.63	3453.5974	30	2.2	864.4086	4	40.19	1	F1:2028	OB5936 H3A raw.raw	1.0226E6	2	2	226	255			PEAKS DB
S.VTFRQGGEENEC(+57.02)QFQR.L	Y	52.22	1983.8857	16	2.4	662.3041	3	27.37	2	F2:1301	OB5937 H3A raw.raw	3.4333E3	1	1	19	34	Carbamidomethylation	C12:Carbamidomethylation: 1000.00	PEAKS DB
R.RPFYSNAPLEIYVQQGSGYFGLIFPGC(+57.02)PSTYEEPAQEGRR.Y	Y	35.79	4580.1860	40	2.2	1527.7394	3	37.52	2	F2:1900	OB5937 H3A raw.raw	6.9008E5	1	1	80	119	Carbamidomethylation	C27:Carbamidomethylation: 1000.00	PEAKS DB

total 9 peptides

Peptide	Uniq	-10lgP	Mass	Length	ppm	m/z	z	RT	Fraction	Scan	Source File	Area Raw Ara h 3	#Feature	#Feature Raw Ara h 3	Start	End	PTM	AScore	Found By
R.NALRRPFYSNAPLEIYVQ(+.98)QGSGYFGLIFPGC(+57.02)PSTYEEPAQEGRR.Y	Y	33.49	5035.4351	44	6.6	1259.8744	4	38.70	1	F1:1944	OB5936 H3A raw.raw	4.3989E5	1	1	76	119	Carbamidomethylation	Q18:Deamidation (NQ):0.00; C31:Carbamidomethylation: 1000.00	PEAKS DB
**total 9 peptides**																			

Q0GM57|Q0GM57_ARAHY

back to list

| Protein Coverage | Supporting Peptides |

Protein Coverage:

C Carbamidomethylation (+57.02)

```
  1 MAKLLALSLC FCVLVLGASS VTFRQGGEEN ECQFQRLNAQ RPDNRIESEG GYIETWNPNN QEFQCAGVAL SRTVLRRNAL
 81 RRPFYSNAPL EIYVQQGSGY FGLIFPGCPS TYEEPAQEGR RYQSQKPSRR FQVGQDDPSQ QQQDSHQKVH RFDEGDLIAV
161 PTGVAFWMYN DEDTDVVTVT LSDTSSIHNQ LDQFPRRFYL AGNQEQEFLR YQQQQGSRPH YRQISPRVRG DEQENEGSNI
241 FSGFAQEFLQ HAFQVDRQTV ENLRGENERE EQGAIVTVKG GLRILSPDEE DESSRSPPSR REEFDEDRSR PQQRGKYDEN
321 RRGYKNGIEE TICSASVKKN LGRSSNPDIY NPQAGSLRSV NELDLPILGW LGLSAQHGTI YRNAMFVPHY TLNAHTIVVA
401 LNGRAHVQVV DSNGNRVYDE ELQEGHVLVV PQNFAVAAKA QSENYEYLAF KTDSRPSIAN LAGENSIIDN LPEEVVANSY
481 RLPREQARQL KNNNPFKFFV PPFDHQSMRE VA
```

(Modification sites marked: 32 C, 65 C, 108 C)

Supporting Peptides:

Peptide	Uniq	-10lgP	Mass	Length	ppm	m/z	z	RT	Fraction	Scan	Source File	Area Raw Ara h 3	#Feature	#Feature Raw Ara h 3	Start	End	PTM	AScore	Found By
R.LNAQRPDNRIESEGGYIETWNPNNQEFQC(+57.02)AGVALSR.T	Y	116.63	4132.9409	36	3.0	1034.2456	4	33.11	5	F5:1590	OB5934 H3B raw.raw	2.0092E6	3	3	37	72	Carbamidomethylation	C29:Carbamidomethylation: 1000.00	PEAKS DB
K.TDSRPSIANLAGENSIIDNLPEEVVANSYR.L	Y	83.57	3243.6006	30	2.9	1082.2106	3	36.46	5	F5:1789	OB5934 H3B raw.raw	2.8671E5	1	1	452	481			PEAKS DB
R.SVNELDLPILGWLGLSAQHGTIYR.N	Y	77.09	2651.4070	24	1.8	884.8112	3	41.50	5	F5:2076	OB5934 H3B raw.raw	1.8337E6	4	4	359	382			PEAKS DB
R.RPFYSNAPLEIYVQQGSGYFGLIFPGC(+57.02)PSTYEEPAQEGR.R	Y	76.78	4424.0845	39	5.2	1475.7097	3	38.66	2	F2:1955	OB5937 H3A raw.raw	1.3954E6	3	3	82	120	Carbamidomethylation	C27:Carbamidomethylation: 1000.00	PEAKS DB
R.FYLAGNQEQEFLR.Y	Y	73.60	1613.7837	13	1.1	807.9000	2	33.08	2	F2:1641	OB5937 H3A raw.raw	2.0837E4	1	1	198	210			PEAKS DB
R.VRGDEQENEGSNIFSGFAQEFLQHAFQVDR.Q	Y	54.63	3453.5974	30	2.2	864.4086	4	40.19	1	F1:2028	OB5936 H3A raw.raw	1.0226E6	2	2	228	257			PEAKS DB
S.VTFRQGGEENEC(+57.02)QFQR.L	Y	52.22	1983.8857	16	2.4	662.3041	3	27.37	2	F2:1301	OB5937 H3A raw.raw	3.4333E3	1	1	21	36	Carbamidomethylation	C12:Carbamidomethylation: 1000.00	PEAKS DB

total 9 peptides

Peptide	Uniq	-10lgP	Mass	Length	ppm	m/z	z	RT	Fraction	Scan	Source File	Area Raw Ara h 3	#Feature	#Feature Raw Ara h 3	Start	End	PTM	AScore	Found By
R.RPFYSNAPLEIYVQQGSGYFGLIFPGC(+57.02)PSTYEEPAQEGRR.Y	Y	35.79	4580.1860	40	2.2	1527.7394	3	37.52	2	F2:1900	OB5937 H3A raw.raw	6.9008E5	1	1	82	121	Carbamidomethylation	C27:Carbamidomethylation: 1000.00	PEAKS DB
R.NALRRPFYSNAPLEIYVQ(+.98)QGSGYFGLIFPGC(+57.02)PSTYEEPAQEGRR.Y	Y	33.49	5035.4351	44	6.6	1259.8744	4	38.70	1	F1:1944	OB5936 H3A raw.raw	4.3989E5	1	1	78	121	Carbamidomethylation	Q18:Deamidation (NQ):0.00; C31:Carbamidomethylation: 1000.00	PEAKS DB
**total 9 peptides**																			

D8KXZ8|D8KXZ8_ARAHY

back to list

| Protein Coverage | Supporting Peptides |

Protein Coverage:

```
  1 MATTPFSGLQ TAMSRSCIPS SQRSNVMAFS GKSMVGSWNK LASVCHVSSV QPFQRVFTSS TIKFERIVTK AMSDSSQKSL
 81 VSGLPIDLKG KRAFIAGVAD DNGYGWAIAK ALAAAGAQIL VGTWVPALNI FESSLRRGKF DESRVLPDGS LMEITKVYPL
161 DAVFDNPEDV PEDIKTNKRY AGSSKWTVQE VAESVKEDFG TIDILVHSLA NGPEVTKPLL ETSRNGYLAA LSASSYSYVS
241 LLKHFLPIMN PGGSSISLTY IASEKIIPGY GGGMSSAKAA LESDTRVLAF EAGRKRKIRV NTISAGPLRS RAAKAIGFID
321 MMIDYSIANA PLQKELSADE VGNSAAFLAS PLASAITGTV LYVDNGLNAM GVGVDSPVFK DLDIPKDQH
```

d Deamidation (NQ) (+0.98)

Supporting Peptides:

Peptide	Uniq	-10lgP	Mass	Length	ppm	m/z	z	RT	Fraction	Scan	Source File	Area Raw Ara h 3	#Feature	#Feature Raw Ara h 3	Start	End	PTM	AScore	Found By
K.AIGFIDMMIDYSIANAPLQK.E	N	121.19	2210.1116	20	3.4	1106.0668	2	40.60	3	F3:2085	OB5938 H3A raw.raw	0	0	0	315	334			PEAKS DB
K.ALAAAGAQILVGTWVPALNIFESSLRR.G	Y	73.91	2823.5759	27	3.3	942.2024	3	42.17	1	F1:2142	OB5936 H3A raw.raw	0	0	0	111	137			PEAKS DB
K.VYPLDAVFDNPEDVPEDIKTNKR.Y	N	72.98	2673.3286	23	0.9	892.1176	3	33.08	3	F3:1637	OB5938 H3A raw.raw	5.7671E5	3	3	157	179			PEAKS DB
K.ALAAAGAQILVGTWVPALNIFESSLR.R	Y	67.95	2667.4749	26	3.2	1334.7490	2	42.57	2	F2:2189	OB5937 H3A raw.raw	1.6879E4	2	2	111	136			PEAKS DB
R.VNTISAGPLR.S	N	62.49	1026.5822	10	1.4	514.2991	2	27.48	2	F2:1306	OB5937 H3A raw.raw	3.2787E3	1	1	300	309			PEAKS DB
K.SLVSGLPIDLK.G	N	47.68	1140.6754	11	-0.5	571.3447	2	34.19	2	F2:1708	OB5937 H3A raw.raw	2.8994E5	1	1	79	89			PEAKS DB
W.VPALN(+.98)IFESSLR.R	N	43.00	1345.7241	12	1.0	673.8700	2	33.70	1	F1:1639	OB5936 H3A raw.raw	1.1143E4	1	1	125	136	Deamidation (NQ)	N5:Deamidation (NQ): 1000.00	PEAKS DB
K.WTVQEVAESVKEDFGTIDILVHSLANGPEVTKPLLETSR.N	N	33.27	4307.2324	39	1.2	1077.8167	4	43.60	1	F1:2217	OB5936 H3A raw.raw	9.6238E5	1	1	186	224			PEAKS DB
R.VLPDGSLMEITK.V	N	33.18	1301.6901	12	1.5	651.8533	2	32.33	3	F3:1607	OB5938 H3A raw.raw	1.2455E5	1	1	145	156			PEAKS DB
**total 9 peptides**																			

H6U596|H6U596_ARAHY

back to list

| Protein Coverage | Supporting Peptides |

Protein Coverage:

1 SPLFPRIFGH EAGGIVESVG EGVTHLKPGD HALPVFTGEC GECPHCKSEE SNMCDLLR**IN TDRGVMIHDQ ESRFSIK**GKP

81 IYHFVGTSTF SEYTVVHAGC VAKINPEAPL DKVCVLSCGI CTGLGATINV AKPK**PGSSVA VFGLGAVGLA AAEGAR**ISGA

161 SR**IIGVDLVS SRFELAKK**FG VNEFV

Supporting Peptides:

Peptide	Uniq	-10lgP	Mass	Length	ppm	m/z	z	RT	Fraction	Scan	Source File	Area Raw Ara h 3	#Feature	#Feature Raw Ara h 3	Start	End	PTM	AScore	Found By
R.IIGVDLVSSRFELAK.K	Y	122.53	1645.9402	15	1.1	823.9783	2	33.84	2	F2:1678	OB5937 H3A raw.raw	1.8835E7	6	6	163	177			PEAKS DB
R.IIGVDLVSSR.F	Y	83.02	1057.6132	10	0.2	529.8140	2	31.58	2	F2:1551	OB5937 H3A raw.raw	1.959E5	2	2	163	172			PEAKS DB
R.INTDRGVMIHDQESRFSIK.G	Y	80.10	2245.1272	19	0.2	562.2892	4	29.32	2	F2:1415	OB5937 H3A raw.raw	4.7416E4	1	1	59	77			PEAKS DB
K.PGSSVAVFGLGAVGLAAAEGAR.I	Y	60.25	1956.0428	22	-2.3	979.0265	2	37.95	1	F1:1901	OB5936 H3A raw.raw	1.1681E5	1	1	135	156			PEAKS DB
R.I(+57.02)IGVDLVSSRFELAK.K	Y	55.15	1702.9617	15	0.3	852.4884	2	36.07	1	F1:1782	OB5936 H3A raw.raw	0	0	0	163	177		I1:Carbamidomethylation (DHKE, X@N-term): 41.53	PEAKS PTM
I.GVDLVSSRFELAK.K	Y	41.78	1419.7721	13	0.6	710.8937	2	33.65	3	F3:1682	OB5938 H3A raw.raw	3.3254E5	2	2	165	177			PEAKS DB
R.IIGVDLVSSRFELAKK.F	Y	35.47	1774.0352	16	1.6	592.3533	3	32.33	2	F2:1590	OB5937 H3A raw.raw	9.7384E5	1	1	163	178			PEAKS DB

total 7 peptides

B4UW81|B4UW81_ARAHY

back to list

| Protein Coverage | Supporting Peptides |

Protein Coverage:

1 MASDTKKLKL YSYWRSSCSF RVRIALNLKE FLKLNPVGFV PVLLDGDLVL ADSLAIIMYL DDKYPQHPLL PSDIHKR**AIN**

81 **FQATHIVSSS IQPLQNISFL NYIGEKVGPD EKLPWVQSVL RK**GFTALEKL LKDHTGR**YAT GDEIFLADVF LAPQLHAAFT**

161 **RFNIPMNEFP IMSR**LHGNV

b Beta-methylthiolation (ND) (+45.99)
c Dihydroxy (+31.99)
m Methylation(KR) (+14.02)

Supporting Peptides:

Peptide	Uniq	-10lgP	Mass	Length	ppm	m/z	z	RT	Fraction	Scan	Source File	Area Raw Ara h 3	#Feature	#Feature Raw Ara h 3	Start	End	PTM	AScore	Found By
K.VGPDEKLPWVQSVLR.K	Y	109.83	1721.9464	15	1.4	574.9902	3	34.05	5	F5:1636	OB5934 H3B raw.raw	1.7988E6	5	5	107	121			PEAKS DB
R.AINFQATHIVSSSIQPLQNISFLNYIGEK.V	Y	104.21	3231.6929	29	2.8	1078.2412	3	38.13	5	F5:1882	OB5934 H3B raw.raw	2.7951E6	4	4	78	106			PEAKS DB
R.YATGDEIFLADVFLAPQLHAAFTR.F	Y	91.73	2665.3540	24	2.2	889.4606	3	40.56	5	F5:2030	OB5934 H3B raw.raw	3.0891E6	4	4	138	161			PEAKS DB
R.FNIPMNEFPIMSR.L	Y	73.26	1594.7635	13	2.9	798.3914	2	35.91	4	F4:1610	OB5925 H3B raw.raw	1.1285E6	3	3	162	174			PEAKS DB
R.YATGD(+45.99)EIFLADVFLAPQLHAAFTR.F	Y	64.39	2711.3416	24	1.0	904.7887	3	40.37	5	F5:2016	OB5934 H3B raw.raw	6.3461E5	1	1	138	161	Beta-methylthiolation (ND)	D5:Beta-methylthiolation (ND):71.27	PEAKS PTM

total 8 peptides

Peptide	Uniq	-10lgP	Mass	Length	ppm	m/z	z	RT	Fraction	Scan	Source File	Area Raw Ara h 3	#Feature	#Feature Raw Ara h 3	Start	End	PTM	AScore	Found By
R.Y(+31.99)AT(+14.02)GDEIFLADVFLAPQLHAAFTR.F	Y	58.57	2711.3594	24	-4.0	904.7901	3	40.81	6	F6:2032	OB5935 H3B raw.raw	6.1471E5	1	1	138	161	Dihydroxy	Y1:Dihydroxy:104.80;T3:Methylation(others):41.83	PEAKS PTM
K.VGPDEKLPWVQSVLRK.G	Y	36.83	1850.0414	16	-1.4	617.6869	3	32.38	4	F4:1404	OB5925 H3B raw.raw	5.764E4	1	1	107	122			PEAKS DB
K.VGP(+15.99)DEK(+14.02)LPWVQSVLR.K	Y	35.41	1751.9569	15	1.0	584.9935	3	33.91	6	F6:1628	OB5935 H3B raw.raw	4.3613E5	1	1	107	121	Methylation(KR)	P3:Oxidation or Hydroxylation:0.00; K6:Methylation(KR):145.00	PEAKS PTM
**total 8 peptides**																			

N1NKG9|N1NKG9_ARAHY

back to list

| Protein Coverage | Supporting Peptides |

Protein Coverage:

1 MSQEQHRRPE ESDPIKYGDV FNVSGELAQK PVKPEDAAMM QSAETRVLGK TQPGGAAAAM QSAATLNEQA GLVGHHDVNE
81 VAGDRGVTVT DMQAPGRRIV TESVGGQVVG QYVEPTPVQS SRASAVRESA ITIGEALEAT AQTVGSKPVE QSDAAAIQAA
161 EVRATGSNVI QPGGLAAMAQ SAVAFNAGCR NPRDKIKLAD ILTGATAKLP ADKAATHQDA EGVASAEARN NTGGTTPGGV
241 AASVAAAARL NENVNV

Supporting Peptides:

Peptide	Uniq	-10lgP	Mass	Length	ppm	m/z	z	RT	Fraction	Scan	Source File	Area Raw Ara h 3	#Feature	#Feature Raw Ara h 3	Start	End	PTM	AScore	Found By
K.LADILTGATAK.L	Y	109.92	1072.6128	11	0.5	537.3140	2	29.90	2	F2:1453	OB5937 H3A raw.raw	1.4089E5	3	3	198	208			PEAKS DB
R.ESAITIGEALEATAQTVGSKPVEQSDAAAIQAAEVR.A	Y	104.28	3610.8325	36	1.7	1204.6201	3	35.85	3	F3:1809	OB5938 H3A raw.raw	2.358E6	4	4	128	163			PEAKS DB
R.IVTESVGGQVVGQYVEPTPVQSSR.A	Y	94.88	2515.2917	24	3.6	1258.6577	2	31.77	2	F2:1561	OB5937 H3A raw.raw	1.1935E5	2	2	99	122			PEAKS DB
R.NNTGGTTPGGVAASVAAAAR.L	Y	77.27	1741.8707	20	1.0	871.9435	2	32.21	1	F1:1555	OB5936 H3A raw.raw	9.5216E4	3	3	230	249			PEAKS DB
R.IVTESVGGQ(+.98)VVGQYVEPTPVQSSR.A	Y	60.10	2516.2759	24	9.6	1259.1573	2	31.77	5	F5:1521	OB5934 H3B raw.raw	9.01E3	1	1	99	122		Q9:Deamidation (NQ):48.43	PEAKS DB
R.NNTGGTTPGGVAASVAAAAR(+15.99).L	Y	47.01	1757.8656	20	2.7	879.9424	2	30.14	1	F1:1433	OB5936 H3A raw.raw	1.8033E4	1	1	230	249		R20:Oxidation or Hydroxylation:20.78	PEAKS PTM
R.NNTGGTTP(+15.99)GGVAASVAAAAR.L	Y	44.57	1757.8656	20	1.2	879.9412	2	27.83	2	F2:1320	OB5937 H3A raw.raw	3.3239E4	1	1	230	249		P8:Oxidation or Hydroxylation: 20.77	PEAKS PTM
**total 7 peptides**																			

Q42515|Q42515_ARAHY

back to list

| Protein Coverage | Supporting Peptides |

Protein Coverage:

C Carbamidomethylation (+57.02)

1 MKFCNFFTFT LALIVVLQAL GASADDAGTI ITQPLYNEFL KHLTDSRCEA HGFYTYNAFV TAARAFPAFG TTGDDVTRKR
81 ELAAFFGQTS HETTGGTTNA PDEFEWGYCF LREQTKEQHC DSTQAPCPAG KQYYGRGPIQ LTSNSNYQLA GQAIKADLIN
161 NPDLVATDAV ISFKTAIWFW MTPQGNKPSC HDVITNAWRP TATDSAAGRA PGYGVITNII NGGIECGKGA NTNSNNRIGF
241 YKRYCDILKI GYGSNLDCAN QQHF

Supporting Peptides:

Peptide	Uniq	-10lgP	Mass	Length	ppm	m/z	z	RT	Fraction	Scan	Source File	Area Raw Ara h 3	#Feature	#Feature Raw Ara h 3	Start	End	PTM	AScore	Found By

Peptide	Uniq	-10lgP	Mass	Length	ppm	m/z	z	RT	Fraction	Scan	Source File	Area Raw Ara h 3	#Feature	#Feature Raw Ara h 3	Start	End	PTM	AScore	Found By
K.ADLINNPDLVATDAVISFK.T	Y	120.18	2015.0575	19	2.8	1008.5388	2	36.32	6	F6:1776	OB5935 H3B raw.raw	3.6486E5	3	3	156	174			PEAKS DB
A.DDAGTIITQPLYNEFLK.H	Y	112.05	1936.9781	17	1.8	969.4980	2	37.02	5	F5:1823	OB5934 H3B raw.raw	3.7509E5	3	3	25	41			PEAKS DB
R.APGYGVITNIINGGIEC(+57.02)GK.G	Y	76.97	1931.9774	19	2.8	966.9987	2	35.96	6	F6:1755	OB5935 H3B raw.raw	1.6903E5	2	2	210	228	Carbamidomethylation	C17:Carbamidomethylation:1000.00	PEAKS DB
total 3 peptides																			

A0A075M981|A0A075M981_ARAHY

back to list

| Protein Coverage | Supporting Peptides |

Protein Coverage:

C Carbamidomethylation (+57.02)

1 FMLPQTETKA SVGFKAGVKD YKLTYYTPEY ETKDTDILAA FRVTPQPGVP PEEAGAAVAA ESSTGTWTTV WTDGLTSLDR
81 YKGRCYNIEP VAGEENQYIA YVAYPLDLFE EGSVTNMFTS IVGNVFGFKA LR**ALRLEDLR IPISYIKTFQ GPPHGIQVER**

222 C
161 DKLNKYGRPL LGCTIKPKLG LSAKNYGRAV YECLRGGLDF TKDDENVNSQ PFMRWRDR**FL FCAEAIFK**SQ AETGEIKGHY
241 LNATAGTCEE MIKRAVFARG LGAPIVMHDY LTGGFTANTS LAHYCR**DNGL LLHIHR**AMHA VIDRQKNHGM HFRVLAKALR
321 LSGGDHIHAG TVVGKLEGER **DITLGFVDLL RDDFIEKDR**S RGIYFTQDWV SLPGVLPVAS GGIHVWHMPA LTEIFGDDSV

428 C
401 LQFGGGTLGH PWGNAPGAVA NR**VALEACVQ AR**NEGRDLAR EGNEIIREAS KWSPELAAAC EVWKAIKFE

Supporting Peptides:

Peptide	Uniq	-10lgP	Mass	Length	ppm	m/z	z	RT	Fraction	Scan	Source File	Area Raw Ara h 3	#Feature	#Feature Raw Ara h 3	Start	End	PTM	AScore	Found By
R.DITLGFVDLLRDDFIEKDR.S	Y	96.63	2279.1797	19	2.4	760.7357	3	38.30	3	F3:1951	OB5938 H3A raw.raw	2.0066E5	1	1	341	359			PEAKS DB
R.ALRLEDLRIPISYIK.T	Y	86.70	1799.0669	15	0.5	450.7742	4	34.01	3	F3:1706	OB5938 H3A raw.raw	1.0366E6	3	3	133	147			PEAKS DB
R.VALEAC(+57.02)VQAR.N	Y	84.77	1115.5757	10	3.3	558.7969	2	26.89	3	F3:1278	OB5938 H3A raw.raw	0	0	0	423	432	Carbamidomethylation	C6:Carbamidomethylation:1000.00	PEAKS DB
K.TFQGPPHGIQVER.D	Y	55.73	1464.7473	13	0.5	489.2566	3	30.19	1	F1:1435	OB5936 H3A raw.raw	2.0466E4	2	2	148	160			PEAKS DB
R.FLFC(+57.02)AEAIFK.S	Y	53.81	1244.6263	10	0.8	623.3209	2	36.22	3	F3:1836	OB5938 H3A raw.raw	8.051E4	1	1	219	228	Carbamidomethylation	C4:Carbamidomethylation:1000.00	PEAKS DB
R.DNGLLLHIHR.A	Y	38.59	1186.6571	10	0.2	396.5597	3	28.88	3	F3:1396	OB5938 H3A raw.raw	0	0	0	287	296			PEAKS DB
total 6 peptides																			

A0A191UJ50|A0A191UJ50_ARAHY

back to list

| Protein Coverage | Supporting Peptides |

Protein Coverage:

1 MSPQTETKAS VGFKAGVKDY KLTYYTPEYE TKDTDILAAF RVTPQPGVPP EEAGAAVAAE SSTGTWTTVW TDGLTSLDRY
81 KGRCYNIEPV AGEENQYIAY VAYPLDLFEE GSVTNMFTSI VGNVFGFKAL RALRLEDLRI PISYIKTFQG PPHGIQVERD
161 KLNKYGRPLL GCTIKPKLGL SAKNYGRAVY ECLRGGLDFT KDDENVNSQP FMRWRDRFLF CAEAIFKSQA ETGEIKGHYL
241 NATAGTCEEM IKRAVFAREL GAPIVMHDYL TGGFTANTSL AHYCRDNGLL LHIHRAMHAV IDRQKNHGMH FRVLAKALRL
321 SGGDHIHAGT VVGKLEGERD ITLGFVDLLR DDFIEKDRSR GIYFTQDWVS LPGVLPVASG GIHVWHMPAL TEIFGDDSVL
401 QFGGGTLGHP WGNAPGAVAN RVALEACVQA RNEGRDLARE GNEIIREASK WSPELAACE VWKAIKFEFP AMDTL

C Carbamidomethylation (+57.02)

Supporting Peptides:

Peptide	Uniq	-10lgP	Mass	Length	ppm	m/z	z	RT	Fraction	Scan	Source File	Area Raw Ara h 3	#Feature	#Feature Raw Ara h 3	Start	End	PTM	AScore	Found By
R.DITLGFVDLLRDDFIEKDR.S	Y	96.63	2279.1797	19	2.4	760.7357	3	38.30	3	F3:1951	OB5938 H3A raw.raw	2.0066E5	1	1	340	358			PEAKS DB
R.ALRLEDLRIPISYIK.T	Y	86.70	1799.0669	15	0.5	450.7742	4	34.01	3	F3:1706	OB5938 H3A raw.raw	1.0366E6	3	3	132	146			PEAKS DB
R.VALEAC(+57.02)VQAR.N	Y	84.77	1115.5757	10	3.3	558.7969	2	26.89	3	F3:1278	OB5938 H3A raw.raw	0	0	0	422	431	Carbamidomethylation	C6:Carbamidomethylation:100 0.00	PEAKS DB
K.TFQGPPHGIQVER.D	Y	55.73	1464.7473	13	0.5	489.2566	3	30.19	1	F1:1435	OB5936 H3A raw.raw	2.0466E4	2	2	147	159			PEAKS DB
R.FLFC(+57.02)AEAIFK.S	Y	53.81	1244.6263	10	0.8	623.3209	2	36.22	3	F3:1836	OB5938 H3A raw.raw	8.051E4	1	1	218	227	Carbamidomethylation	C4:Carbamidomethylation:100 0.00	PEAKS DB
R.DNGLLLHIHR.A	Y	38.59	1186.6571	10	0.2	396.5597	3	28.88	3	F3:1396	OB5938 H3A raw.raw	0	0	0	286	295			PEAKS DB

total 6 peptides

O20356|O20356_ARAHY

back to list

| Protein Coverage | Supporting Peptides |

Protein Coverage:

1 VGFKAGVKDY KLTYYTPEYE TKDTDILAAF RVTPQPGVPP EEAGAAVAAE SSTGTWTTVW TDGLTSLDRY KGRCYNIEPV
81 AGEENQYIAY VAYPLDLFEE GSVTNMFTSI VGNVFGFKAL RALRLEDLRI PISYIKTFQG PPHGIQVERD KLNKYGRPLL
161 GCTIKPKLGL SAKNYGRAVY ECLRGGLDFT KDDENVNSQP FMRWRDRFLF CAEAIFKSQA ETGEIKGHYL NATAGTCEEM
241 IKRAVFAREL GAPIVMHDYL TGGFTANTSL AHYCRDNGLL LHIHRAMHAV IDRQKNHGMH FRVLAKALRL SGGDHIHAGT
321 VVGKLEGERD ITLGFVDLLR DDFIEKDRSR GIYFTQDWVS LPGVLPVASG GIHVWHMPAL TEIFGDDSVL QFGGGTLGHP
401 WGNAPGAVAN RVALEACVQA RNEGRDLARE GNEIIREASK WSPELAACE VWKAIKFEFP AMDTL

C Carbamidomethylation (+57.02)

Supporting Peptides:

Peptide	Uniq	-10lgP	Mass	Length	ppm	m/z	z	RT	Fraction	Scan	Source File	Area Raw Ara h 3	#Feature	#Feature Raw Ara h 3	Start	End	PTM	AScore	Found By

total 6 peptides

Peptide	Uniq	-10lgP	Mass	Length	ppm	m/z	z	RT	Fraction	Scan	Source File	Area Raw Ara h 3	#Feature	#Feature Raw Ara h 3	Start	End	PTM	AScore	Found By
R.DITLGFVDLLRDDFIEKDR.S	Y	96.63	2279.1797	19	2.4	760.7357	3	38.30	3	F3:1951	OB5938 H3A raw.raw	2.0066E5	1	1	330	348			PEAKS DB
R.ALRLEDLRIPISYIK.T	Y	86.70	1799.0669	15	0.5	450.7742	4	34.01	3	F3:1706	OB5938 H3A raw.raw	1.0366E6	3	3	122	136			PEAKS DB
R.VALEAC(+57.02)VQAR.N	Y	84.77	1115.5757	10	3.3	558.7969	2	26.89	3	F3:1278	OB5938 H3A raw.raw	0	0	0	412	421	Carbamidomethylation	C6:Carbamidomethylation:1000.00	PEAKS DB
K.TFQGPPHGIQVER.D	Y	55.73	1464.7473	13	0.5	489.2566	3	30.19	1	F1:1435	OB5936 H3A raw.raw	2.0466E4	2	2	137	149			PEAKS DB
R.FLFC(+57.02)AEAIFK.S	Y	53.81	1244.6263	10	0.8	623.3209	2	36.22	3	F3:1836	OB5938 H3A raw.raw	8.051E4	1	1	208	217	Carbamidomethylation	C4:Carbamidomethylation:1000.00	PEAKS DB
R.DNGLLLHIHR.A	Y	38.59	1186.6571	10	0.2	396.5597	3	28.88	3	F3:1396	OB5938 H3A raw.raw	0	0	0	276	285			PEAKS DB
**total 6 peptides**																			

A0A0R4UXQ1|A0A0R4UXQ1_ARAHY

back to list

| Protein Coverage | Supporting Peptides |

Protein Coverage:

Carbamidomethylation (+57.02)

1 MEKAINRQKV LLHHLNPSSS NVDLSSSSSI FASVCLAGDS AAYQRTGVFG DDVVIVAAYR TAICKAKR**GG FKDTPADDLL**
81 **APVLK**AVVEK **TNLNPSEVGD IVVGSVLAPG ALR**ATECRMA AFYAGFPETV PVRTVNRQCS SGLQAVADVA ASIRAGFYEI
161 GIGAGLESMS TNAMGWDGDV NPKVKQFEQA RNCLLPMGVT SENVAQRFGV SRKEQDQAAV ESHRRAAAAT AAGKFKDEII
241 PVSTKIVDPK **TGDETPVTIS VDDGIRPNAS VADLGK**LKPV FKKDGSTTAG NSSQVSDGAG AVLLMKRSTA MQKGLPILGV
321 FRSFSAVGVD PAIMGVGPAA AIPVAVK**AAG LELDDIDLFE INEAFASQFV YCR**NKLGLDA EKINVNGGAM ALGHPLGATG
401 ARCTATLLHE MKRRGKDCRF GVISMCIGTG MGAAAVFERG DGVDELSNAR KVATNNLLSK DAR

Supporting Peptides:

Peptide	Uniq	-10lgP	Mass	Length	ppm	m/z	z	RT	Fraction	Scan	Source File	Area Raw Ara h 3	#Feature	#Feature Raw Ara h 3	Start	End	PTM	AScore	Found By
K.TNLNPSEVGDIVVGSVLAPGALR.A	Y	120.04	2277.2329	23	1.4	1139.6254	2	37.36	3	F3:1898	OB5938 H3A raw.raw	4.468E5	2	2	91	113			PEAKS DB
K.AAGLELDDIDLFEINEAFASQFVYC(+57.02)R.N	Y	65.66	3005.4116	26	5.1	1503.7207	2	42.64	1	F1:2172	OB5936 H3A raw.raw	2.4389E4	1	1	348	373	Carbamidomethylation	C25:Carbamidomethylation:1000.00	PEAKS DB
K.TGDETPVTISVDDGIRPNASVADLGK.L	Y	64.47	2626.3086	26	3.0	876.4461	3	32.33	2	F2:1599	OB5937 H3A raw.raw	4.6998E5	2	2	251	276			PEAKS DB
R.GGFKDTPADDLLAPVLK.A	Y	40.52	1755.9407	17	0.1	878.9777	2	35.69	1	F1:1759	OB5936 H3A raw.raw	0	0	0	69	85			PEAKS DB
**total 4 peptides**																			

sp|Q647H2|AHY3_ARAHY

back to list

| Protein Coverage | Supporting Peptides |

Protein Coverage:

1 MAKLLALSVC FCFLVLGASS VTFRQQGEEN ECQFQRLNAQ RPDNCIESEG GYIETWNPNN QEFQCAGVAL SRFVLRRNAL
81 RRPFYSNAPQ EIFIYQGSGY FGLIFPGCPG TFEEPIQGSE QFQRPSRHFQ GQDQSQRPLD THQKVHGFRE GDLIAVPHGV
161 AFWIYNDQDT DVVAISVLHT NSLHNQLDQF PR**RFNLAGKQ EQEFLR**YQQR SGRQSPKGEE QEQEQENEGG NVFSGFSTEF
241 LSHGFQVNED IVRNLRGENE REEQGAIVTV KGGLSILVPP EWRQSYQQPG RGDKDFNNGI EETICTATVK MNIGKSTSAD
321 IYNPQAGSVR TVNELDLPIL NRLGLSAEYG SIHRDAMFVP HYNMNANSMI YALHGGAHVQ VVDCNGNRVF DEELQEGQSL
401 VVPQNFAVAA KSQSEHFLYV AFKTNSRASI SNLAGKNSYM WNLPEDVVAN SYGLQYEQAR QLKNNNPFTF LVPPQDSQMI
481 RTVA

Supporting Peptides:

Peptide	Uniq	-10lgP	Mass	Length	ppm	m/z	z	RT	Fraction	Scan	Source File	Area Raw Ara h 3	#Feature	#Feature Raw Ara h 3	Start	End	PTM	AScore	Found By
R.RFNLAGKQEQEFLR.Y	Y	119.18	1734.9165	14	-0.3	579.3126	3	30.84	5	F5:1458	OB5934 H3B raw.raw	5.0305E5	3	3	193	206			PEAKS DB
R.FNLAGKQEQEFLR.Y	Y	72.82	1578.8154	13	-0.1	790.4149	2	30.90	6	F6:1458	OB5935 H3B raw.raw	4.203E4	2	2	194	206			PEAKS DB
**total 2 peptides**																			

B4UWD5|B4UWD5_ARAHY

back to list

| Protein Coverage | Supporting Peptides |

Protein Coverage:

1 MARYDR**AITV FSPDGHLFQV EYALEAVR**KG NAAVGVRGTD NVVLGVEKKS TAKLQDSRTV RKIVNLDDHI ALACAGLKAD
81 ARVLINRARV ECQSHR**LTVE DPVTVEYITR** YIAGLHQSYT QSGGVRPFGL STLIVGF

Supporting Peptides:

Peptide	Uniq	-10lgP	Mass	Length	ppm	m/z	z	RT	Fraction	Scan	Source File	Area Raw Ara h 3	#Feature	#Feature Raw Ara h 3	Start	End	PTM	AScore	Found By
R.LTVEDPVTVEYITR.Y	Y	112.55	1633.8563	14	2.5	817.9375	2	33.34	6	F6:1599	OB5935 H3B raw.raw	9.0801E5	3	3	97	110			PEAKS DB
R.AITVFSPDGHLFQVEYALEAVR.K	Y	60.08	2461.2642	22	5.9	821.4335	3	38.74	4	F4:1769	OB5925 H3B raw.raw	2.6576E5	1	1	7	28			PEAKS DB
**total 2 peptides**																			

A5Z1R0|A5Z1R0_ARAHY

back to list

| Protein Coverage | Supporting Peptides |

Protein Coverage:

1 MAKSTILVAL LALVLVAHAS AMRRERGRQG DSSSCERQVD RVNLKPCEQH IMQR**IMGEQE QYDSYDIR**ST RSSDQQQRCC

C Carbamidomethylation (+57.02)

92 94
81 DELNEMENTQ R**CMCEALQQI MENQCDRLQD R**QMVQQFKRE LMNLPQQCNF RAPQRCDLDV SGGRC

Supporting Peptides:

Peptide	Uniq	-10lgP	Mass	Length	ppm	m/z	z	RT	Fraction	Scan	Source File	Area Raw Ara h 3	#Feature	#Feature Raw Ara h 3	Start	End	PTM	AScore	Found By
R.IMGEQEQYDSYDIR.S	Y	87.94	1745.7566	14	2.0	873.8873	2	30.24	4	F4:1275	OB5925 H3B raw.raw	2.7489E4	2	2	55	68			PEAKS DB
R.C(+57.02)MC(+57.02)EALQQIMENQC(+57.02)DRLQDR.Q	Y	81.82	2597.0913	20	1.1	866.7053	3	33.67	4	F4:1480	OB5925 H3B raw.raw	3.136E5	3	3	92	111	Carbamidomethylation	C1:Carbamidomethylation:1000.00;C3:Carbamidomethylation:1000.00;C14:Carbamidomethylation:1000.00	PEAKS DB
**total 2 peptides**																			

sp|Q647G9|CONG_ARAHY

back to list

| Protein Coverage | Supporting Peptides |

Protein Coverage:

```
 1  MAKSTILVAL LALVLVAHAS AMRRERGRQG DSSSCERQVD RVNLKPCEQH IMQRIMGEQE QYDSYDIRST RSSDQQQRCC
81  DELNEMENTQ RCMCEALQQI MENQCDRLQD RQMVQQFKRE LMNLPQQCNF RAPQRCDLDV SGGRC
```

C Carbamidomethylation (+57.02)

92 94

Supporting Peptides:

Peptide	Uniq	-10lgP	Mass	Length	ppm	m/z	z	RT	Fraction	Scan	Source File	Area Raw Ara h 3	#Feature	#Feature Raw Ara h 3	Start	End	PTM	AScore	Found By
R.IMGEQEQYDSYDIR.S	Y	87.94	1745.7566	14	2.0	873.8873	2	30.24	4	F4:1275	OB5925 H3B raw.raw	2.7489E4	2	2	55	68			PEAKS DB
R.C(+57.02)MC(+57.02)EALQQIMENQC(+57.02)DRLQDR.Q	Y	81.82	2597.0913	20	1.1	866.7053	3	33.67	4	F4:1480	OB5925 H3B raw.raw	3.136E5	3	3	92	111	Carbamidomethylation	C1:Carbamidomethylation:1000.00;C3:Carbamidomethylation:1000.00;C14:Carbamidomethylation:1000.00	PEAKS DB
total 2 peptides																			

#CONTAM#P00761

back to list

| Protein Coverage | Supporting Peptides |

Protein Coverage:

```
  1  FPTDDDDKIV GGYTCAANSI PYQVSLNSGS HFCGGSLINS QWVVSAAHCY KSRIQVRLGE HNIDVLEGNE QFINAAKIIT
 81  HPNFNGNTLD NDIMLIKLSS PATLNSRVAT VSLPRSCAAA GTECLISGWG NTKSSGSSYP SLLQCLKAPV LSDSSCKSSY
161  PGQITGNMIC VGFLEGGKDS CQGDSGGPVV CNGQLQGIVS WGYGCAQKNK PGVYTKVCNY VNWIQQTIAA N
```

Supporting Peptides:

Peptide	Uniq	-10lgP	Mass	Length	ppm	m/z	z	RT	Fraction	Scan	Source File	Area Raw Ara h 3	#Feature	#Feature Raw Ara h 3	Start	End	PTM	AScore	Found By
R.LGEHNIDVLEGNEQFIN.A	Y	70.56	1939.9275	17	2.6	970.9735	2	33.72	6	F6:1622	OB5935 H3B raw.raw	1.059E5	1	1	58	74			PEAKS DB
K.LSSPATLNSR.V	Y	67.10	1044.5564	10	0.1	523.2855	2	25.44	2	F2:1182	OB5937 H3A raw.raw	1.0806E4	3	3	98	107			PEAKS DB
R.VATVSLPR.S	Y	57.08	841.5021	8	2.0	421.7592	2	29.77	1	F1:1403	OB5936 H3A raw.raw	1.6733E5	5	5	108	115			PEAKS DB
total 3 peptides																			

E5FHZ2|E5FHZ2_ARAHY

back to list

| Protein Coverage | Supporting Peptides |

Protein Coverage:

```
  1  MSQQQTPRRP QEEGQEHKDP IKYGDIFNVS GDLASEPIMP QDAALMQATE NQALGQTQKG GPASVMQSAA VTNVSAGLVG
 81  RGDVSSVAKD QGICVLESKI GVNRVITESV GEQVVGEFVE PNVPMKTPGS ALDKDAITIG EALEATTLAP ASDKPVDQSD
161  AAAIQAAEMR ATGKNEIQPG GLAAVAQSAA TKNLRTMGDF QKTTLSDVLT DAKEKLPSDK AVTREDAEGV IGAEIRNKAD
241  MKTTPGGVAA SLAAAATLNQ NKSSS
```

Supporting Peptides:

Peptide	Uniq	-10lgP	Mass	Length	ppm	m/z	z	RT	Fraction	Scan	Source File	Area Raw Ara h 3	#Feature	#Feature Raw Ara h 3	Start	End	PTM	AScore	Found By

Peptide	Uniq	-10lgP	Mass	Length	ppm	m/z	z	RT	Fraction	Scan	Source File	Area Raw Ara h 3	#Feature	#Feature Raw Ara h 3	Start	End	PTM	AScore	Found By
K.TTPGGVAASLAAAATLNQNK.S	Y	90.39	1854.9799	20	2.2	928.4993	2	33.30	4	F4:1460	OB5925 H3B raw.raw	6.4018E4	1	1	243	262			PEAKS DB
K.TTLSDVLTDAKEKLPSDK.A	Y	35.73	1960.0364	18	0.4	654.3530	3	32.33	2	F2:1595	OB5937 H3A raw.raw	7.1006E5	1	1	203	220			PEAKS DB
K.AVTREDAEGVIGAEIR.N	Y	35.69	1684.8744	16	1.9	843.4461	2	29.50	3	F3:1432	OB5938 H3A raw.raw	0	0	0	221	236			PEAKS DB
**total 3 peptides**																			

Q06H31|Q06H31_ARAHY

back to list

| Protein Coverage | Supporting Peptides |

Protein Coverage:

```
  1 MLVYQDLLTG DELLSDSFPY KEIENGILWE VEGKWVIQGA INVDIGANPS AEGGEEDESV DDQAVKVVDI VDTFRLQEQP
 81 AFDKKQFVTY MKRYIKLLTA KLEPEQQEHF KKNIEGATKF LLSKLSDLQF FVGESMHDDG SLVFAYYKEG ATDPTFLYFA
161 HGLKEIKC
```

o Oxidation (M) (+15.99)

Supporting Peptides:

Peptide	Uniq	-10lgP	Mass	Length	ppm	m/z	z	RT	Fraction	Scan	Source File	Area Raw Ara h 3	#Feature	#Feature Raw Ara h 3	Start	End	PTM	AScore	Found By
M(+15.99)LVYQDLLTGDELLSDSFPYKEIENGILWEVEGK.W	Y	73.25	3958.9436	34	3.4	1320.6597	3	42.89	6	F6:2152	OB5935 H3B raw.raw	4.2504E5	2	2	1	34	Oxidation (M)	M1:Oxidation (M):1000.00	PEAKS DB
MLVYQDLLTGDELLSDSFPYKEIENGILWEVEGK.W	Y	70.50	3942.9487	34	3.9	1315.3286	3	43.02	5	F5:2174	OB5934 H3B raw.raw	5.0903E5	2	2	1	34			PEAKS DB
K.VVDIVDTFR.L	Y	66.45	1062.5709	9	-0.3	532.2926	2	32.96	6	F6:1559	OB5935 H3B raw.raw	1.3289E4	1	1	67	75			PEAKS DB
**total 3 peptides**																			

Peptide List

Prepared with PEAKS ™ (bioinfor.com)

Summary

Ara h 3 GEL BAND ROASTED

1. Notes

```
Ara h 3 project, sample ROAST Ara h 3 with 6 files, node PTM69.
The most abundant is Ara h 3 isoform! via XIC aspect.
```

2. Result Statistics

Figure 1. False discovery rate (FDR) curve. X axis is the number of peptides being kept. Y axis is the corresponding FDR.

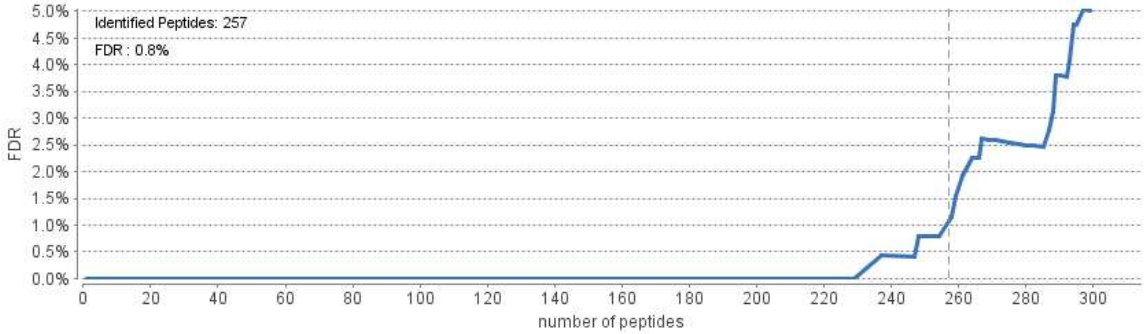

Figure 2. PSM score distribution. **(a)** Distribution of PEAKS peptide score; **(b)** Scatterplot of PEAKS peptide score versus precursor mass error.

(a)

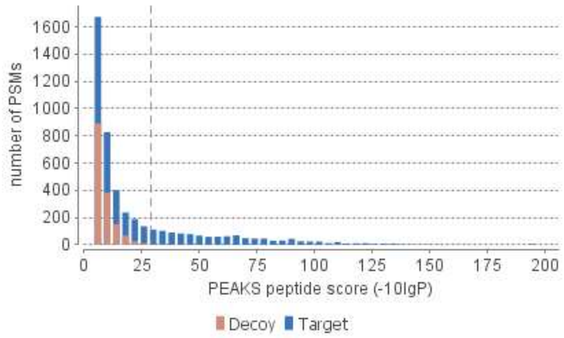

(b)

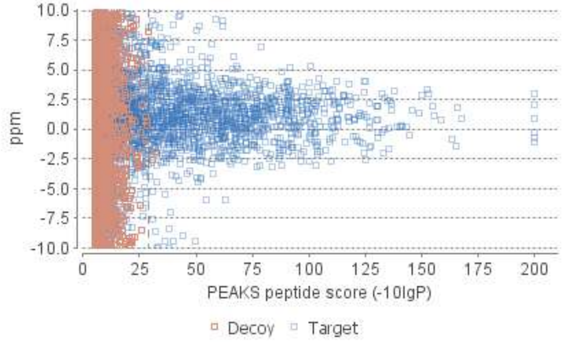

Table 1. Statistics of data.

	#Scans			#Features	Identified			#Peptides	#Sequences	#Proteins*		
	MS1	MS/MS	#Chimera		#PSMs	#Scans	#Features**			Groups	All	Top
Total	14350	8619	1176	15108	1216	1191	672	257	175	20	30	26

Roast Ara h 3	14350	8619	1176	15108	1216	1191	672	257	175	20	30	26

* proteins with significant peptides are used in counts.

** features are identified by DB search only.

Figure 3. Sample overlap for Proteins and Peptides (up to 8 samples). **(a)** All Proteins; **(b)** Top Proteins; **(c)** Peptides;

(a) **Not applicable to only one sample**

(b) **Not applicable to only one sample**

(c) **Not applicable to only one sample**

Figure 4. Distribution of peptide feature detection. **(a)** Feature m/z distribution; **(b)** Feature RT distribution.

(a) **(b)**

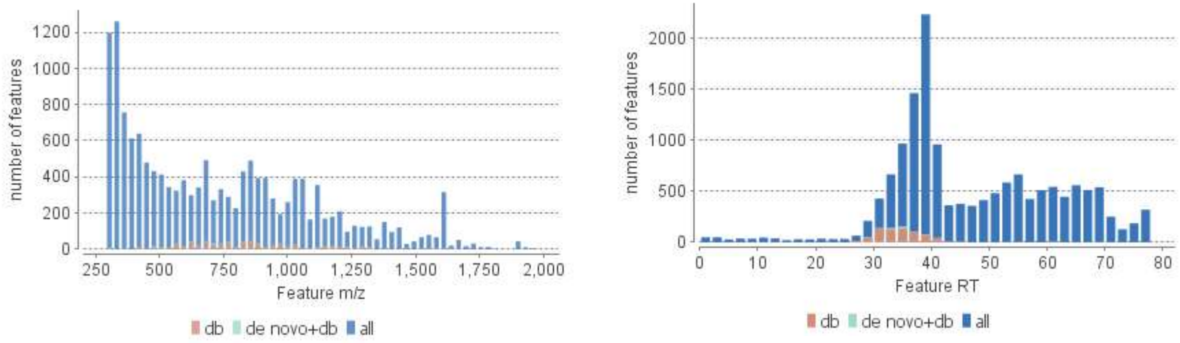

Figure 5. Distribution of identified peptide features. **(a)** Feature abundance distribution; **(b)** *De novo* sequencing validation.

(a) **(b)**

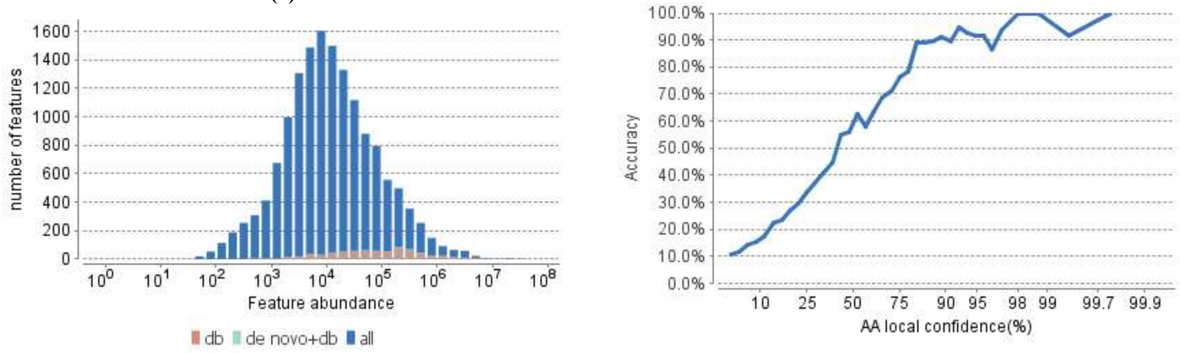

Table 2. Result filtration parameters.

Peptide -10lgP	≥29.1
PTM Ascore	≥50
Protein -10lgP	≥20
Proteins unique peptides	≥2
De novo score(%)	≥50%

Table 4. PTM profile.

Name	ΔMass	Position	#PSM	-10lgP	Abundance	AScore
Carbamidomethyl	57.02	C	173	200.00	3.47E5	1000.00
Oxidation	15.99	M	79	154.77	2.76E5	1000.00
Deamidation	.98	NQ	57	78.87	5.78E4	39.44

proteins

Table 3. Statistics of filtered result.

FDR (Peptide-Spectrum Matches)	0.2%
FDR (Peptide Sequences)	0.8%
FDR (Protein Group)	0.0%
De Novo Only Spectra	95

Carbamidomethyl	57.02	DE,N-term	22	91.10	1.24E4	1000.00
Dimethylation(KR)	28.03	R	5	90.94	1.34E5	1000.00
SMA	127.06	N-term	5	49.60	5.17E5	1000.00
Propionamide	71.04	C	4	72.57	1.78E4	1000.00
Methylation(others)	14.02	H	3	41.89	1.36E5	17.01
Dihydroxy	31.99	PW	3	46.85	3.33E3	17.01
Methylation(KR)	14.02	R	3	32.14	2.89E5	67.58
Ammonia loss	-17.03	N-term	2	30.70	1.28E4	1000.00
Pyro-glu from Q	-17.03	N-term	2	55.02		1000.00
Oxidation	15.99	P	1	34.29	4.33E4	32.28
HNE	156.12	H	1	31.09	2.48E5	1000.00
Ammonia loss	-17.03	N	1	37.32		0.00

3. Experiment Control

Figure 6. Precursor mass error of peptide-spectrum matches (PSM) in filtered result. **(a)** Distribution of precursor mass error in ppm; **(b)** Scatterplot of precursor m/z versus precursor mass error in ppm.

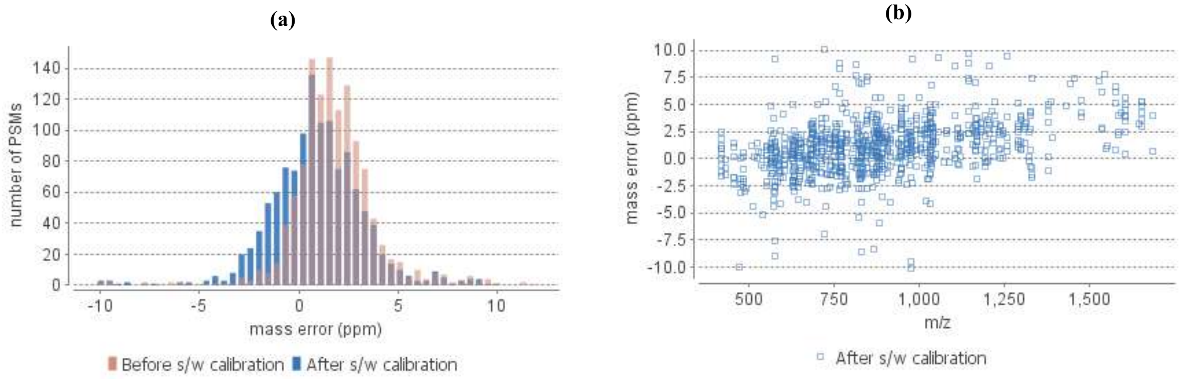

Table 5. Number of identified peptides in each sample by the number of missed cleavages.

Missed Cleavages	0	1	2	3	4+
Roast Ara h 3	117	110	30	0	0

4. Other Information

Table 6. Search parameters.

PEAKS Version: PEAKS Studio 10.6 build 20201015
Search Engine Name: PEAKS

Table 7. Instrument parameters.

Fractions: OB5951 H3A Ro.raw, OB5952 H3A Ro.raw, OB5953 H3A Ro.raw, OB5926 H3B Ro.raw, OB5948 H3B Ro.raw, OB5949 H3B Ro.raw

Parent Mass Error Tolerance: 10.0 ppm
Fragment Mass Error Tolerance: 0.5 Da
Precursor Mass Search Type: monoisotopic
Enzyme: Trypsin
Max Missed Cleavages: 2
Digest Mode: Semispecific
Fixed Modifications:
Carbamidomethylation: 57.02
Variable Modifications:
Oxidation (M): 15.99
Deamidation (NQ): 0.98
Acetylation (K): 42.01
Acetylation (Protein N-term): 42.01
Acetylation (N-term): 42.01
Amidation: -0.98
Beta-methylthiolation: 45.99
Biotinylation: 226.08
and 303 more...
Max Variable PTM Per Peptide: 5
Database: Uniprot_Peanut-3818_Jul18
Taxon: All
Contaminant Database: contam MQ
Searched Entry: 1723
FDR Estimation: Enabled
De novo score(%) threshold: 15
Peptide hit threshold (-10logP): 30.0
Peaks run ID: 68
Merge Options: no merge
Precursor Options: corrected
Charge Options: no correction
Filter m/z: 200.0 - 2000.0
Filter Charge: 2 - 12
Process: true
Associate chimera: yes

Ion Source: ESI(nano-spray)
Fragmentation Mode: CID, CAD(y and b ions)
MS Scan Mode: FT-ICR/Orbitrap
MS/MS Scan Mode: FT-ICR/Orbitrap

Protein List

Protein Accession Contains:
Protein Description Contains:
Protein Sample Area >=
Protein PTM Contains:

Protein Group	Protein ID	Accession	-10lgP	Coverage (%)	Coverage (%) Roast Ara h 3	Area Roast Ara h 3	#Peptides	#Unique	#Spec Roast Ara h 3	PTM	Avg. Mass	Description
**2**	3	sp|P43237|ALL11_ARAHY	561.12	50	50	1.0349E6	39	2	449	Y	70283	Allergen Ara h 1, clone P17 OS=Arachis hypogaea OX=3818 PE=1 SV=1
**2**	2	B3IXL2|B3IXL2_ARAHY	561.12	50	50	1.0349E6	39	2	449	Y	70283	Main allergen Ara h1 OS=Arachis hypogaea OX=3818 PE=2 SV=1
**2**	1	Q6PSU3|Q6PSU3_ARAHY	561.12	53	53	1.0349E6	39	2	449	Y	66575	Conarachin (Fragment) OS=Arachis hypogaea OX=3818 PE=4 SV=1
**1**	5	N1NG13|N1NG13_ARAHY	558.94	47	47	9.6993E5	37	6	452	Y	71345	Seed storage protein Ara h1 OS=Arachis hypogaea OX=3818 GN=ARAX_AHF417E07-017 PE=4 SV=1
**1**	4	sp|P43238|ALL12_ARAHY	558.94	47	47	9.6993E5	37	6	452	Y	71345	Allergen Ara h 1, clone P41B OS=Arachis hypogaea OX=3818 PE=1 SV=1
**5**	9	A1DZF0|A1DZF0_ARAHY	491.39	60	60	4.4788E6	20	3	166	Y	60375	Arachin 6 OS=Arachis hypogaea OX=3818 PE=2 SV=1
**11**	20	Q38711|Q38711_ARAHY	488.55	85	85	3.3979E5	16	2	128	Y	29134	Galactose-binding lectin (Fragment) OS=Arachis hypogaea OX=3818 GN=lec PE=2 SV=1
**13**	21	A0A089ZXL7|A0A089ZXL7_ARAHY	466.25	77	77	2.7476E5	14	2	90	Y	29407	Peanut agglutinin variant OS=Arachis hypogaea OX=3818 PE=2 SV=1
**9**	17	Q5I6T2|Q5I6T2_ARAHY	453.37	43	43	4.3973E5	16	2	145	Y	60736	Arachin Ahy-4 OS=Arachis hypogaea OX=3818 PE=2 SV=1
**12**	25	A1E2B0|A1E2B0_ARAHY	350.42	63	63	1.3012E7	12	10	91	Y	33520	11S seed storage globulin B1 OS=Arachis hypogaea OX=3818 PE=2 SV=1

total 26 proteins

Protein Group	Protein ID	Accession	-10lgP	Coverage (%)	Coverage (%) Roast Ara h 3	Area Roast Ara h 3	#Peptides	#Unique	#Spec Roast Ara h 3	PTM	Avg. Mass	Description
**16**	41	E9LFE8|E9LFE8_ARAHY	336.44	47	47	2.9081E6	9	7	37	Y	28290	11S arachin (Fragment) OS=Arachis hypogaea OX=3818 PE=2 SV=1
**14**	27	Q6IWG5|Q6IWG5_ARAHY	310.98	35	35	2.7033E7	12	11	72	Y	58061	Glycinin (Fragment) OS=Arachis hypogaea OX=3818 PE=2 SV=1
**14**	28	Q0GM57|Q0GM57_ARAHY	310.98	35	35	2.7033E7	12	11	72	Y	58263	Iso-Ara h3 OS=Arachis hypogaea OX=3818 PE=2 SV=1
**19**	42	E9LFE9|E9LFE9_ARAHY	228.62	26	26	1.9022E6	4	4	23	Y	28300	11S arachin OS=Arachis hypogaea OX=3818 PE=2 SV=1
**17**	53	A5Z1R0|A5Z1R0_ARAHY	216.49	34	34	1.4416E5	4	4	37	Y	16920	Ara h 6 allergen OS=Arachis hypogaea OX=3818 GN=Ara h 6 PE=4 SV=1
**17**	52	sp|Q647G9|CONG_ARAHY	216.49	34	34	1.4416E5	4	4	37	Y	16920	Conglutin OS=Arachis hypogaea OX=3818 PE=1 SV=1
**21**	44	#CONTAM#P04264	191.54	10	10	1.9341E5	4	3	12	N	66018	SWISS-PROT:#CONTAM#P04264 Tax_Id=9606 Gene_Symbol=KRT1 Keratin, type II cytoskeletal 1
**23**	50	sp|Q647H2|AHY3_ARAHY	181.13	11	11	3.8077E5	4	3	9	Y	54569	Arachin Ahy-3 OS=Arachis hypogaea OX=3818 PE=1 SV=1
**22**	45	#CONTAM#P13645	175.75	15	15	1.1312E6	4	4	10	Y	59511	SWISS-PROT:#CONTAM#P13645 Tax_Id=9606 Gene_Symbol=KRT10 Keratin, type I cytoskeletal 10
**24**	56	E5FHZ1|E5FHZ1_ARAHY	163.46	27	27	9.4991E5	2	2	8	N	22636	Late embryogenesis abundant protein group 5 protein OS=Arachis hypogaea OX=3818 GN=LEA5-1 PE=2 SV=1
**24**	49	N1NKG9|N1NKG9_ARAHY	163.46	23	23	9.4991E5	2	2	8	N	26041	Seed maturation protein OS=Arachis hypogaea OX=3818 GN=ARAX_AHF417E07-003 PE=4 SV=1
**18**	47	sp|Q6PSU2|CONG7_ARAHY	143.91	33	33	1.0478E6	4	4	30	Y	20114	Conglutin-7 OS=Arachis hypogaea OX=3818 PE=1 SV=2
**27**	55	#CONTAM#P35908	129.42	4	4	3.8507E4	2	2	4	N	65865	SWISS-PROT:#CONTAM#P35908 Tax_Id=9606 Gene_Symbol=KRT2 Keratin, type II cytoskeletal 2 epidermal
**20**	58	#CONTAM#P00761	127.73	12	12	3.0738E5	3	3	16	N	24409	SWISS-PROT:#CONTAM#P00761|TRYP_PIG Trypsin - Sus scrofa (Pig).
**25**	105	A1E2B1|A1E2B1_ARAHY	126.24	13	13	1.1946E5	2	2	6	N	31268	11S seed storage globulin B2 OS=Arachis hypogaea OX=3818 PE=2 SV=1
**31**	46	Q42515|Q42515_ARAHY	90.44	14	14	2.7447E4	2	2	2	N	28924	Chitinase (Class II) OS=Arachis hypogaea OX=3818 GN=chi2;2 PE=2 SV=1
**total 26 proteins**												

sp|P43237|ALL11_ARAHY

back to list

| Protein Coverage | Supporting Peptides |

Protein Coverage:

1 MRGRVSPLML LLGILVLASV SATQAKSPYR KTENPCAQRC LQSCQQEPDD LKQKACESRC TKLEYDPRCV YDTGATNQRH
81 PPGERTRGRQ PGDYDDDRRQ PRREEGGRWG PAEPRERERE EDWRQPREDW RRPSHQQPRK IRPEGREGEQ EWGTPGSEVR
161 EETSRNNPFY FPSRRFSTRY GNQNGRIRVL QRFDQRSKQF QNLQNHRIVQ IEARPNTLVL PKHADADNIL VIQQGQATVT
241 VANGNNRKSF NLDEGHALRI PSGFISYILN RHDNQNLRVA KISMPVNTPG QFEDFFPASS RDQSSYLQGF SRNTLEAAFN
321 AEFNEIRRVL LEENAGGEQE ERGQRRRSTR SSDNEGVIVK VSKEHVQELT KHAKSVSKKG SEEEDITNPI NLRDGEPDLS
401 NNFGRLFEVK PDKKNPQLQD LDMMLTCVEI KEGALMLPHF NSKAMVIVVV NKGTGNLELV AVRKEQQQRG RREQEWEEEE
481 EDEEEEGSNR EVRRYTARLK EGDVFIMPAA HPVAINASSE LHLLGFGINA ENNHRIFLAG DKDNVIDQIE KQAKDLAFPG
561 SGEQVEKLIK NQRESHFVSA RPQSQSPSSP EKEDQEEENQ GGKGPLLSIL KAFN

C Carbamidomethylation (+57.02)
C Carbamidomethylation (DHKE, X@N-term) (+57.02)
d Deamidation (NQ) (+0.98)
o Oxidation (M) (+15.99)
P Pyro-glu from Q (-17.03)

Supporting Peptides:

Peptide	Uniq	-10lgP	Mass	Length	ppm	m/z	z	RT	Fraction	Scan	Source File	Area Roast Ara h 3	#Feature	#Feature Roast Ara h 3	Start	End	PTM	AScore	Found By
K.ISMPVNTPGQFEDFFPASSR.D	N	200.00	2226.0415	20	2.0	1114.0303	2	35.53	7	F7:1725	OB5951 H3A Ro.raw	8.7598E5	6	6	282	301			PEAKS DB
R.NTLEAAFNAEFNEIR.R	N	165.72	1737.8322	15	1.8	869.9249	2	35.34	7	F7:1714	OB5951 H3A Ro.raw	1.3226E6	6	6	313	327			PEAKS DB
K.ISM(+15.99)PVNTPGQFEDFFPASSR.D	N	154.77	2242.0364	20	1.9	1122.0276	2	34.42	9	F9:1655	OB5953 H3A Ro.raw	9.4493E5	6	5	282	301	Oxidation (M)	M3:Oxidation (M):1000.00	PEAKS DB
R.IPSGFISYILNR.H	N	136.44	1378.7609	12	2.2	690.3892	2	37.11	9	F9:1819	OB5953 H3A Ro.raw	5.8838E6	6	6	260	271			PEAKS DB
K.SFNLDEGHALRIPSGFISYILNR.H	N	130.32	2618.3604	23	0.7	873.7969	3	39.49	10	F10:1795	OB5926 H3B Ro.raw	2.1577E7	15	15	249	271			PEAKS DB
R.IFLAGDKDNVIDQIEK.Q	N	127.66	1816.9570	16	-1.4	909.4865	2	33.07	11	F11:1576	OB5948 H3B Ro.raw	3.3211E6	12	11	536	551			PEAKS DB
R.DQSSYLQGFSR.N	N	125.12	1286.5891	11	-2.6	644.3015	2	30.77	11	F11:1443	OB5948 H3B Ro.raw	3.7573E5	6	6	302	312			PEAKS DB

total 63 peptides

Peptide	Uniq	-10lgP	Mass	Length	ppm	m/z	z	RT	Fraction	Scan	Source File	Area Roast Ara h 3	#Feature	#Feature Roast Ara h 3	Start	End	PTM	AScore	Found By
K.KGSEEEDITNPINLRDGEPDLSNNFGR.L	N	113.18	3015.4170	27	1.8	754.8629	4	31.56	9	F9:1480	OB5953 H3A Ro.raw	4.7042E5	11	11	379	405			PEAKS DB
K.SFNLDEGHALR.I	N	110.09	1257.6101	11	-2.6	629.8120	2	29.12	11	F11:1344	OB5948 H3B Ro.raw	1.3894E5	10	10	249	259			PEAKS DB
K.DLAFPGSGEQVEKLIK.N	N	103.34	1729.9249	16	-1.4	865.9703	2	33.26	11	F11:1597	OB5948 H3B Ro.raw	3.9305E5	5	4	555	570			PEAKS DB
K.GSEEEDITNPINLRDGEPDLSNNFGR.L	N	102.75	2887.3220	26	1.4	963.4493	3	32.29	7	F7:1538	OB5951 H3A Ro.raw	4.5249E5	6	6	380	405			PEAKS DB
R.VLLEENAGGEQEER.G	N	102.67	1571.7427	14	2.2	786.8804	2	27.19	9	F9:1251	OB5953 H3A Ro.raw	9.5555E3	3	3	329	342			PEAKS DB
R.IFLAGDKDNVIDQIEKQAK.D	N	100.84	2144.1477	19	-0.2	715.7245	3	33.83	11	F11:1613	OB5948 H3B Ro.raw	1.8641E6	6	6	536	554			PEAKS DB
R.NTLEAAFNAEFNEIRR.V	N	96.95	1893.9332	16	2.8	947.9765	2	34.01	7	F7:1637	OB5951 H3A Ro.raw	3.4558E7	12	11	313	328			PEAKS DB
K.NPQLQDLDMMLTC(+57.02)VEIK.E	N	94.07	2046.9788	17	3.3	1024.5000	2	36.94	7	F7:1805	OB5951 H3A Ro.raw	0	0	0	415	431	Carbamidomethylation	C13:Carbamidomethylation:1000.00	PEAKS DB
R.IPSGFISYILNRHDNQNLR.V	N	93.37	2256.1763	19	0.1	565.0514	4	35.73	7	F7:1716	OB5951 H3A Ro.raw	8.2074E6	11	11	260	278			PEAKS DB
R.I(+57.02)PSGFISYILNR.H	N	91.10	1435.7823	12	0.7	718.8989	2	36.87	7	F7:1795	OB5951 H3A Ro.raw	7.2345E4	3	3	260	271	Carbamidomethylation (DHKE, X@N-term)	I1:Carbamidomethylation (DHKE, X@N-term):1000.00	PEAKS PTM
R.NNPFYFPSRR.F	N	90.91	1296.6364	10	0.1	649.3255	2	30.10	9	F9:1398	OB5953 H3A Ro.raw	4.2765E5	8	8	166	175			PEAKS DB
K.GTGNLELVAVR.K	N	87.71	1127.6299	11	4.8	564.8250	2	30.09	9	F9:1403	OB5953 H3A Ro.raw	2.2478E3	1	1	453	463			PEAKS DB
K.AM(+15.99)VIVVVNKGTGNLELVAVR.K	N	87.55	2097.1980	20	1.6	1049.6079	2	32.67	7	F7:1560	OB5951 H3A Ro.raw	2.6865E5	3	3	444	463	Oxidation (M)	M2:Oxidation (M):1000.00	PEAKS DB
K.DLAFPGSGEQVEK.L	N	85.86	1375.6619	13	-0.7	688.8392	2	30.28	11	F11:1414	OB5948 H3B Ro.raw	1.4145E5	4	4	555	567			PEAKS DB
K.AMVIVVVNKGTGNLELVAVR.K	N	83.81	2081.2031	20	1.7	1041.6106	2	34.96	7	F7:1704	OB5951 H3A Ro.raw	2.013E6	4	4	444	463			PEAKS DB
R.NNPFYFPSR.R	N	81.75	1140.5352	9	0.1	571.2749	2	31.76	8	F8:1498	OB5952 H3A Ro.raw	1.1774E5	3	3	166	174			PEAKS DB
K.SFNLDEGHALRIPSGFISYILNRHDNQNLR.V	N	79.13	3495.7759	30	1.5	700.1635	5	39.97	9	F9:1987	OB5953 H3A Ro.raw	2.0697E7	15	15	249	278			PEAKS DB
K.HADADNILVIQQGQATVTVAN(+.98)GNNRK.S	N	78.05	2747.3950	26	1.8	916.8073	3	30.31	8	F8:1428	OB5952 H3A Ro.raw	8.0166E4	3	3	223	248		N21:Deamidation (NQ):14.04	PEAKS DB
G.SEEEDITNPINLRDGEPDLSNNFGR.L	N	77.06	2830.3005	25	-1.5	944.4417	3	33.02	10	F10:1421	OB5926 H3B Ro.raw	1.3758E5	3	3	381	405			PEAKS DB
K.AMVIVVVNKGTGNLELVAVRK.E	N	75.76	2209.2981	21	-1.5	737.4407	3	35.66	10	F10:1586	OB5926 H3B Ro.raw	3.5137E6	8	8	444	464			PEAKS DB
K.ISMPVNTPGQFEDFFPASSRDQSSYLQGFSR.N	N	74.46	3494.6201	31	0.8	1165.8840	3	36.12	11	F11:1762	OB5948 H3B Ro.raw	2.9084E6	5	5	282	312			PEAKS DB
**total 63 peptides**																			

Peptide	Uniq	-10lgP	Mass	Length	ppm	m/z	z	RT	Fraction	Scan	Source File	Area Roast Ara h 3	#Feature	#Feature Roast Ara h 3	Start	End	PTM	AScore	Found By
R.IVQIEARPNTLVLPK.H	Y	72.30	1690.0140	15	1.0	564.3458	3	30.81	8	F8:1446	OB5952 H3A Ro.raw	1.034E6	12	12	208	222			PEAKS DB
R.KSFNLDEGHALRIPSGFISYILNR.H	N	70.24	2746.4553	24	1.8	687.6223	4	39.03	8	F8:1914	OB5952 H3A Ro.raw	3.7974E5	2	2	248	271			PEAKS DB
R.I(+57.02)FLAGDKDNVIDQIEK.Q	N	66.79	1873.9785	16	-2.1	937.9969	2	33.39	10	F10:1458	OB5926 H3B Ro.raw	2.2898E4	1	1	536	551	Carbamidomethylation (DHKE, X@N-term)	I1:Carbamidomethylation (DHKE, X@N-term):56.99	PEAKS PTM
K.HADADNILVIQQGQATVTVANGNNR.K	N	65.50	2618.3162	25	0.5	873.7798	3	30.34	7	F7:1426	OB5951 H3A Ro.raw	1.8654E3	1	1	223	247			PEAKS DB
K.HADADNILVIQQGQATVTVANGNNRK.S	N	62.55	2746.4111	26	0.2	916.4778	3	29.66	7	F7:1385	OB5951 H3A Ro.raw	6.7701E4	2	2	223	248			PEAKS DB
K.ISM(+15.99)PVNTPGQFEDFFPASSRDQSSYLQGFSR.N	N	62.16	3510.6150	31	-1.8	1171.2126	3	35.35	11	F11:1707	OB5948 H3B Ro.raw	2.4799E6	5	5	282	312	Oxidation (M)	M3:Oxidation (M):1000.00	PEAKS DB
R.EETSRNNPFYFPSRR.F	N	62.14	1898.9023	15	-0.7	633.9758	3	30.73	10	F10:1288	OB5926 H3B Ro.raw	1.7594E4	1	1	161	175			PEAKS DB
K.NPQLQDLDMMLTC(+57.02)VEIKEGALMLPHFNSK.A	N	59.27	3371.6387	29	-0.3	843.9188	4	39.49	10	F10:1807	OB5926 H3B Ro.raw	8.6484E5	3	3	415	443	Carbamidomethylation	C13:Carbamidomethylation:1000.00	PEAKS DB
K.KGSEEEDITNPINLR.D	N	58.67	1713.8533	15	2.3	572.2930	3	29.61	9	F9:1375	OB5953 H3A Ro.raw	1.9283E3	1	1	379	393			PEAKS DB
K.NPQLQDLDMMLTC(+57.02)VEIKEGALM(+15.99)LPHFNSK.A	N	58.64	3387.6335	29	1.2	847.9185	4	38.07	11	F11:1874	OB5948 H3B Ro.raw	3.9631E5	2	2	415	443	Carbamidomethylation; Oxidation (M)	C13:Carbamidomethylation:1000.00;M22:Oxidation (M):51.99	PEAKS DB
K.NPQLQDLDM(+15.99)MLTC(+57.02)VEIK.E	N	57.29	2062.9736	17	2.8	1032.4969	2	36.63	9	F9:1780	OB5953 H3A Ro.raw	0	0	0	415	431	Carbamidomethylation	M9:Oxidation (M):24.44;C13:Carbamidomethylation:1000.00	PEAKS DB
R.NTLEAAFN(+.98)AEFNEIRR.V	N	56.23	1894.9172	16	0.4	948.4663	2	32.13	9	F9:1524	OB5953 H3A Ro.raw	1.3345E4	2	2	313	328		N8:Deamidation (NQ):20.76	PEAKS DB
R.VLLEENAGGEQEERGQR.R	N	52.47	1912.9238	17	-2.1	638.6472	3	28.03	8	F8:1297	OB5952 H3A Ro.raw	8.2166E3	2	2	329	345			PEAKS DB
R.NTLEAAFNAEFN(+.98)EIRR.V	N	50.81	1894.9172	16	1.9	948.4677	2	32.67	7	F7:1563	OB5951 H3A Ro.raw	9.2576E4	2	2	313	328		N12:Deamidation (NQ):8.26	PEAKS DB
K.HADADNILVIQQGQATVTVANGN(+.98)NRK.S	N	50.48	2747.3950	26	-4.1	916.8038	3	30.36	11	F11:1426	OB5948 H3B Ro.raw	0	0	0	223	248		N23:Deamidation (NQ):9.40	PEAKS DB
K.S(+57.02)FNLDEGHALRIPSGFISYILNR.H	N	49.55	2675.3818	23	2.3	892.8033	3	39.35	7	F7:1901	OB5951 H3A Ro.raw	6.7035E5	1	1	249	271	Carbamidomethylation (DHKE, X@N-term)	S1:Carbamidomethylation (DHKE, X@N-term):63.97	PEAKS PTM
R.IFLAGD(+57.02)KDNVIDQIEK.Q	N	45.63	1873.9785	16	1.4	937.9978	2	33.53	12	F12:1622	OB5949 H3B Ro.raw	3.5356E3	1	1	536	551		D6:Carbamidomethylation (DHKE, X@N-term):15.57	PEAKS PTM
K.HADADNILVIQQGQ(+.98)ATVTVANGNNRK.S	N	45.21	2747.3950	26	2.6	916.8103	3	30.46	10	F10:1280	OB5926 H3B Ro.raw	0	0	0	223	248		Q14:Deamidation (NQ):0.00	PEAKS DB
K.AM(+15.99)VIVVVNKGTGNLELVAVRK.E	N	45.01	2225.2930	21	-1.5	742.7723	3	33.77	10	F10:1559	OB5926 H3B Ro.raw	4.0307E5	2	2	444	464	Oxidation (M)	M2:Oxidation (M):1000.00	PEAKS DB

total 63 peptides

Peptide	Uniq	-10lgP	Mass	Length	ppm	m/z	z	RT	Fraction	Scan	Source File	Area Roast Ara h 3	#Feature	#Feature Roast Ara h 3	Start	End	PTM	AScore	Found By
R.VAKISM(+15.99)PVNTPGQFEDFFPASSR.D	N	44.60	2540.2368	23	3.4	847.7558	3	33.41	8	F8:1598	OB5952 H3A Ro.raw	0	0	0	279	301	Oxidation (M)	M6:Oxidation (M):1000.00	PEAKS DB
K.AMVIVVVNK.G	N	42.66	971.5837	9	-3.0	486.7977	2	29.40	7	F7:1370	OB5951 H3A Ro.raw	0	0	0	444	452			PEAKS DB
R.N(+.98)TLEAAFNAEFNEIRR.V	N	42.24	1894.9172	16	3.2	948.4689	2	33.46	8	F8:1601	OB5952 H3A Ro.raw	5.9553E4	1	1	313	328		N1:Deamidation (NQ):34.75	PEAKS DB
K.NPQLQDLDM(+15.99)M(+15.99)LTC(+57.02)VEIKEGALM(+15.99)LPHFNSK.A	N	39.18	3419.6233	29	1.7	855.9146	4	35.97	8	F8:1751	OB5952 H3A Ro.raw	6.0894E4	1	1	415	443	Oxidation (M); Carbamidomethylation	M9:Oxidation (M):1000.00;M10:Oxidation (M):1000.00;C13:Carbamidomethylation:1000.00;M22:Oxidation (M):1000.00	PEAKS DB
R.EGEQEWGTPGSEVREETSR.N	Y	38.57	2161.9512	19	-7.0	721.6542	3	28.61	11	F11:1321	OB5948 H3B Ro.raw	0	0	0	147	165			PEAKS DB
E.PDLSNNFGR.L	N	38.29	1018.4832	9	1.2	510.2495	2	31.26	7	F7:1478	OB5951 H3A Ro.raw	0	0	0	397	405			PEAKS DB
K.GTGNLELVAVRK.E	N	37.48	1255.7249	12	-1.1	628.8690	2	28.81	8	F8:1332	OB5952 H3A Ro.raw	1.1804E3	2	2	453	464			PEAKS DB
K.NPQLQDLDMM(+15.99)LTC(+57.02)VEIKEGALM(+15.99)LPHFNSK.A	N	36.15	3403.6284	29	4.5	851.9182	4	36.31	7	F7:1742	OB5951 H3A Ro.raw	1.3711E5	1	1	415	443	Carbamidomethylation	M10:Oxidation (M):0.00;C13:Carbamidomethylation:1000.00;M22:Oxidation (M):40.85	PEAKS DB
R.IPSGFISYILN(+.98)RHDNQNLR.V	N	35.18	2257.1604	19	6.7	753.4007	3	36.32	11	F11:1765	OB5948 H3B Ro.raw	5.0814E4	1	1	260	278		N11:Deamidation (NQ):11.12	PEAKS DB
K.SFNLDEGHALRIPSGFISYILNRHDNQN(+.98)LR.V	N	34.85	3496.7600	30	4.9	700.3627	5	37.51	8	F8:1925	OB5952 H3A Ro.raw	2.7478E5	1	1	249	278		N28:Deamidation (NQ):10.11	PEAKS DB
K.NPQLQDLDM(+15.99)MLTC(+57.02)VEIKEGALMLPHFNSK.A	N	34.78	3387.6335	29	4.0	847.9191	4	38.06	9	F9:1825	OB5953 H3A Ro.raw	2.0615E5	1	1	415	443	Carbamidomethylation	M9:Oxidation (M):0.00;C13:Carbamidomethylation:1000.00	PEAKS DB
K.NPQLQDLDM(+15.99)MLTC(+57.02)VEIKEGALM(+15.99)LPHFNSK.A	N	34.77	3403.6284	29	0.8	851.9150	4	37.70	8	F8:1849	OB5952 H3A Ro.raw	1.9344E5	1	1	415	443	Carbamidomethylation	M9:Oxidation (M):24.44;C13:Carbamidomethylation:1000.00;M22:Oxidation (M):19.27	PEAKS DB
R.SSDNEGVIVKVSKEHVQELTK.H	N	34.18	2325.2175	21	-0.9	776.0791	3	30.32	9	F9:1416	OB5953 H3A Ro.raw	4.7496E3	1	1	351	371			PEAKS DB
K.NPQLQDLDM(+15.99)M(+15.99)LTC(+57.02)VEIKEGALMLPHFNSK.A	N	32.60	3403.6284	29	1.4	851.9156	4	37.49	9	F9:1800	OB5953 H3A Ro.raw	2.8417E5	1	1	415	443	Carbamidomethylation	M9:Oxidation (M):0.00;M10:Oxidation (M):0.00;C13:Carbamidomethylation:1000.00	PEAKS DB
R.EGEQ(+.98)EWGTPGSEVREETSR.N	Y	32.39	2162.9351	19	10.0	721.9944	3	28.65	11	F11:1321	OB5948 H3B Ro.raw	9.7072E2	1	1	147	165	Deamidation (NQ)	Q4:Deamidation (NQ):1000.00	PEAKS DB
K.Q(-17.03)FQNLQNHR.I	N	31.50	1166.5581	9	-0.6	584.2859	2	28.13	7	F7:1303	OB5951 H3A Ro.raw	4.0835E2	1	1	199	207	Pyro-glu from Q	Q1:Pyro-glu from Q:1000.00	PEAKS PTM
**total 63 peptides**																			

B3IXL2|B3IXL2_ARAHY

back to list

| Protein Coverage | Supporting Peptides |

Protein Coverage:

1 MRGRVSPLML LLGILVLASV SATQAKSPYR KTENPCAQRC LQSCQQEPDD LKQKACESRC TKLEYDPRCV YDTGATNQRH

81 PPGERTRGRQ PGDYDDDRRQ PRREEGGRWG PAEPRERERE EDWRQPREDW RRPSHQQPRK IRPEGREGEQ EWGTPGSEVR

161 EETSRNNPFY FPSRRFSTRY GNQNGRIRVL QRFDQRSKQF QNLQNHRIVQ IEARPNTLVL PKHADADNIL VIQQGQATVT

249 260 284

241 VANGNNRKSF NLDEGHALRI PSGFISYILN RHDNQNLRVA KISMPVNTPG QFEDFFPASS RDQSSYLQGF SRNTLEAAFN

321 AEFNEIRRVL LEENAGGEQE ERGQRRRSTR SSDNEGVIVK VSKEHVQELT KHAKSVSKKG SEEEDITNPI NLRDGEPDLS

427

401 NNFGRLFEVK PDKKNPQLQD LDMMLTCVEI KEGALMLPHF NSKAMVIVVV NKGTGNLELV AVRKEQQQRG RREQEWEEEE

536

481 EDEEEEGSNR EVRRYTARLK EGDVFIMPAA HPVAINASSE LHLLGFGINA ENNHRIFLAG DKDNVIDQIE KQAKDLAFPG

561 SGEQVEKLIK NQRESHFVSA RPQSQSPSSP EKEDQEEENQ GGKGPLLSIL KAFN

C Carbamidomethylation (+57.02)
C Carbamidomethylation (DHKE, X@N-term) (+57.02)
d Deamidation (NQ) (+0.98)
o Oxidation (M) (+15.99)
p Pyro-glu from Q (-17.03)

Supporting Peptides:

Peptide	Uniq	-10lgP	Mass	Length	ppm	m/z	z	RT	Fraction	Scan	Source File	Area Roast Ara h 3	#Feature	#Feature Roast Ara h 3	Start	End	PTM	AScore	Found By
K.ISMPVNTPGQFEDFFPASSR.D	N	200.00	2226.0415	20	2.0	1114.0303	2	35.53	7	F7:1725	OB5951 H3A Ro.raw	8.7598E5	6	6	282	301			PEAKS DB
R.NTLEAAFNAEFNEIR.R	N	165.72	1737.8322	15	1.8	869.9249	2	35.34	7	F7:1714	OB5951 H3A Ro.raw	1.3226E6	6	6	313	327			PEAKS DB
K.ISM(+15.99)PVNTPGQFEDFFPASSR.D	N	154.77	2242.0364	20	1.9	1122.0276	2	34.42	9	F9:1655	OB5953 H3A Ro.raw	9.4493E5	6	5	282	301	Oxidation (M)	M3:Oxidation (M):1000.00	PEAKS DB
R.IPSGFISYILNR.H	N	136.44	1378.7609	12	2.2	690.3892	2	37.11	9	F9:1819	OB5953 H3A Ro.raw	5.8838E6	6	6	260	271			PEAKS DB
K.SFNLDEGHALRIPSGFISYILNR.H	N	130.32	2618.3604	23	0.7	873.7969	3	39.49	10	F10:1795	OB5926 H3B Ro.raw	2.1577E7	15	15	249	271			PEAKS DB

total 63 peptides

Peptide	Uniq	-10lgP	Mass	Length	ppm	m/z	z	RT	Fraction	Scan	Source File	Area Roast Ara h 3	#Feature	#Feature Roast Ara h 3	Start	End	PTM	AScore	Found By
R.IFLAGDKDNVIDQIEK.Q	N	127.66	1816.9570	16	-1.4	909.4865	2	33.07	11	F11:1576	OB5948 H3B Ro.raw	3.3211E6	12	11	536	551			PEAKS DB
R.DQSSYLQGFSR.N	N	125.12	1286.5891	11	-2.6	644.3015	2	30.77	11	F11:1443	OB5948 H3B Ro.raw	3.7573E5	6	6	302	312			PEAKS DB
K.KGSEEEDITNPINLRDGEPDLSNNFGR.L	N	113.18	3015.4170	27	1.8	754.8629	4	31.56	9	F9:1480	OB5953 H3A Ro.raw	4.7042E5	11	11	379	405			PEAKS DB
K.SFNLDEGHALR.I	N	110.09	1257.6101	11	-2.6	629.8120	2	29.12	11	F11:1344	OB5948 H3B Ro.raw	1.3894E5	10	10	249	259			PEAKS DB
K.DLAFPGSGEQVEKLIK.N	N	103.34	1729.9249	16	-1.4	865.9703	2	33.26	11	F11:1597	OB5948 H3B Ro.raw	3.9305E5	5	4	555	570			PEAKS DB
K.GSEEEDITNPINLRDGEPDLSNNFGR.L	N	102.75	2887.3220	26	1.4	963.4493	3	32.29	7	F7:1538	OB5951 H3A Ro.raw	4.5249E5	6	6	380	405			PEAKS DB
R.VLLEENAGGEQEER.G	N	102.67	1571.7427	14	2.2	786.8804	2	27.19	9	F9:1251	OB5953 H3A Ro.raw	9.5555E3	3	3	329	342			PEAKS DB
R.IFLAGDKDNVIDQIEKQAK.D	N	100.84	2144.1477	19	-0.2	715.7245	3	33.83	11	F11:1613	OB5948 H3B Ro.raw	1.8641E6	6	6	536	554			PEAKS DB
R.NTLEAAFNAEFNEIRR.V	N	96.95	1893.9332	16	2.8	947.9765	2	34.01	7	F7:1637	OB5951 H3A Ro.raw	3.4558E7	12	11	313	328			PEAKS DB
K.NPQLQDLDMMLTC(+57.02)VEIK.E	N	94.07	2046.9788	17	3.3	1024.5000	2	36.94	7	F7:1805	OB5951 H3A Ro.raw	0	0	0	415	431	Carbamidomethylation	C13:Carbamidomethylation:1000.00	PEAKS DB
R.IPSGFISYILNRHDNQNLR.V	N	93.37	2256.1763	19	0.1	565.0514	4	35.73	7	F7:1716	OB5951 H3A Ro.raw	8.2074E6	11	11	260	278			PEAKS DB
R.I(+57.02)PSGFISYILNR.H	N	91.10	1435.7823	12	0.7	718.8989	2	36.87	7	F7:1795	OB5951 H3A Ro.raw	7.2345E4	3	3	260	271	Carbamidomethylation (DHKE, X@N-term)	I1:Carbamidomethylation (DHKE, X@N-term):1000.00	PEAKS PTM
R.NNPFYFPSRR.F	N	90.91	1296.6364	10	0.1	649.3255	2	30.10	9	F9:1398	OB5953 H3A Ro.raw	4.2765E5	8	8	166	175			PEAKS DB
K.GTGNLELVAVR.K	N	87.71	1127.6299	11	4.8	564.8250	2	30.09	9	F9:1403	OB5953 H3A Ro.raw	2.2478E3	1	1	453	463			PEAKS DB
K.AM(+15.99)VIVVVNKGTGNLELVAVR.K	N	87.55	2097.1980	20	1.6	1049.6079	2	32.67	7	F7:1560	OB5951 H3A Ro.raw	2.6865E5	3	3	444	463	Oxidation (M)	M2:Oxidation (M):1000.00	PEAKS DB
K.DLAFPGSGEQVEK.L	N	85.86	1375.6619	13	-0.7	688.8392	2	30.28	11	F11:1414	OB5948 H3B Ro.raw	1.4145E5	4	4	555	567			PEAKS DB
K.AMVIVVVNKGTGNLELVAVR.K	N	83.81	2081.2031	20	1.7	1041.6106	2	34.96	7	F7:1704	OB5951 H3A Ro.raw	2.013E6	4	4	444	463			PEAKS DB
R.NNPFYFPSR.R	N	81.75	1140.5352	9	0.1	571.2749	2	31.76	8	F8:1498	OB5952 H3A Ro.raw	1.1774E5	3	3	166	174			PEAKS DB
K.SFNLDEGHALRIPSGFISYILNRHDNQNLR.V	N	79.13	3495.7759	30	1.5	700.1635	5	39.97	9	F9:1987	OB5953 H3A Ro.raw	2.0697E7	15	15	249	278			PEAKS DB
K.HADADNILVIQQGQATVTVAN(+.98)GNNRK.S	N	78.05	2747.3950	26	1.8	916.8073	3	30.31	8	F8:1428	OB5952 H3A Ro.raw	8.0166E4	3	3	223	248		N21:Deamidation (NQ):14.04	PEAKS DB
G.SEEEDITNPINLRDGEPDLSNNFGR.L	N	77.06	2830.3005	25	-1.5	944.4417	3	33.02	10	F10:1421	OB5926 H3B Ro.raw	1.3758E5	3	3	381	405			PEAKS DB
**total 63 peptides**																			

Peptide	Uniq	-10lgP	Mass	Length	ppm	m/z	z	RT	Fraction	Scan	Source File	Area Roast Ara h 3	#Feature	#Feature Roast Ara h 3	Start	End	PTM	AScore	Found By
K.AMVIVVVNKGTGNLELVAVRK.E	N	75.76	2209.2981	21	-1.5	737.4407	3	35.66	10	F10:1586	OB5926 H3B Ro.raw	3.5137E6	8	8	444	464			PEAKS DB
K.ISMPVNTPGQFEDFFPASSRDQSSYLQGFSR.N	N	74.46	3494.6201	31	0.8	1165.8840	3	36.12	11	F11:1762	OB5948 H3B Ro.raw	2.9084E6	5	5	282	312			PEAKS DB
R.IVQIEARPNTLVLPK.H	Y	72.30	1690.0140	15	1.0	564.3458	3	30.81	8	F8:1446	OB5952 H3A Ro.raw	1.034E6	12	12	208	222			PEAKS DB
R.KSFNLDEGHALRIPSGFISYILNR.H	N	70.24	2746.4553	24	1.8	687.6223	4	39.03	8	F8:1914	OB5952 H3A Ro.raw	3.7974E5	2	2	248	271			PEAKS DB
R.I(+57.02)FLAGDKDNVIDQIEK.Q	N	66.79	1873.9785	16	-2.1	937.9969	2	33.39	10	F10:1458	OB5926 H3B Ro.raw	2.2898E4	1	1	536	551	Carbamidomethylation (DHKE, X@N-term)	I1:Carbamidomethylation (DHKE, X@N-term):56.99	PEAKS PTM
K.HADADNILVIQQGQATVTVANGNNR.K	N	65.50	2618.3162	25	0.5	873.7798	3	30.34	7	F7:1426	OB5951 H3A Ro.raw	1.8654E3	1	1	223	247			PEAKS DB
K.HADADNILVIQQGQATVTVANGNNRK.S	N	62.55	2746.4111	26	0.2	916.4778	3	29.66	7	F7:1385	OB5951 H3A Ro.raw	6.7701E4	2	2	223	248			PEAKS DB
K.ISM(+15.99)PVNTPGQFEDFFPASSRDQSSYLQGFSR.N	N	62.16	3510.6150	31	-1.8	1171.2126	3	35.35	11	F11:1707	OB5948 H3B Ro.raw	2.4799E6	5	5	282	312	Oxidation (M)	M3:Oxidation (M):1000.00	PEAKS DB
R.EETSRNNPFYFPSRR.F	N	62.14	1898.9023	15	-0.7	633.9758	3	30.73	10	F10:1288	OB5926 H3B Ro.raw	1.7594E4	1	1	161	175			PEAKS DB
K.NPQLQDLDMMLTC(+57.02)VEIKEGALMLPHFNSK.A	N	59.27	3371.6387	29	-0.3	843.9188	4	39.49	10	F10:1807	OB5926 H3B Ro.raw	8.6484E5	3	3	415	443	Carbamidomethylation	C13:Carbamidomethylation:1000.00	PEAKS DB
K.KGSEEEDITNPINLR.D	N	58.67	1713.8533	15	2.3	572.2930	3	29.61	9	F9:1375	OB5953 H3A Ro.raw	1.9283E3	1	1	379	393			PEAKS DB
K.NPQLQDLDMMLTC(+57.02)VEIKEGALM(+15.99)LPHFNSK.A	N	58.64	3387.6335	29	1.2	847.9185	4	38.07	11	F11:1874	OB5948 H3B Ro.raw	3.9631E5	2	2	415	443	Carbamidomethylation; Oxidation (M)	C13:Carbamidomethylation:1000.00;M22:Oxidation (M):51.99	PEAKS DB
K.NPQLQDLDM(+15.99)MLTC(+57.02)VEIK.E	N	57.29	2062.9736	17	2.8	1032.4969	2	36.63	9	F9:1780	OB5953 H3A Ro.raw	0	0	0	415	431	Carbamidomethylation	M9:Oxidation (M):24.44;C13:Carbamidomethylation:1000.00	PEAKS DB
R.NTLEAAFN(+.98)AEFNEIRR.V	N	56.23	1894.9172	16	0.4	948.4663	2	32.13	9	F9:1524	OB5953 H3A Ro.raw	1.3345E4	2	2	313	328		N8:Deamidation (NQ):20.76	PEAKS DB
R.VLLEENAGGEQEERGQR.R	N	52.47	1912.9238	17	-2.1	638.6472	3	28.03	8	F8:1297	OB5952 H3A Ro.raw	8.2166E3	2	2	329	345			PEAKS DB
R.NTLEAAFNAEFN(+.98)EIRR.V	N	50.81	1894.9172	16	1.9	948.4677	2	32.67	7	F7:1563	OB5951 H3A Ro.raw	9.2576E4	2	2	313	328		N12:Deamidation (NQ):8.26	PEAKS DB
K.HADADNILVIQQGQATVTVANGN(+.98)NRK.S	N	50.48	2747.3950	26	-4.1	916.8038	3	30.36	11	F11:1426	OB5948 H3B Ro.raw	0	0	0	223	248		N23:Deamidation (NQ):9.40	PEAKS DB
K.S(+57.02)FNLDEGHALRIPSGFISYILNR.H	N	49.55	2675.3818	23	2.3	892.8033	3	39.35	7	F7:1901	OB5951 H3A Ro.raw	6.7035E5	1	1	249	271	Carbamidomethylation (DHKE, X@N-term)	S1:Carbamidomethylation (DHKE, X@N-term):63.97	PEAKS PTM
R.IFLAGD(+57.02)KDNVIDQIEK.Q	N	45.63	1873.9785	16	1.4	937.9978	2	33.53	12	F12:1622	OB5949 H3B Ro.raw	3.5356E3	1	1	536	551		D6:Carbamidomethylation (DHKE, X@N-term):15.57	PEAKS PTM

total 63 peptides

Peptide	Uniq	-10lgP	Mass	Length	ppm	m/z	z	RT	Fraction	Scan	Source File	Area Roast Ara h 3	#Feature	#Feature Roast Ara h 3	Start	End	PTM	AScore	Found By
K.HADADNILVIQQGQ(+.98)ATVTVANGNNRK.S	N	45.21	2747.3950	26	2.6	916.8103	3	30.46	10	F10:1280	OB5926 H3B Ro.raw	0	0	0	223	248		Q14:Deamidation (NQ):0.00	PEAKS DB
K.AM(+15.99)VIVVVNKGTGNLELVAVRK.E	N	45.01	2225.2930	21	-1.5	742.7723	3	33.77	10	F10:1559	OB5926 H3B Ro.raw	4.0307E5	2	2	444	464	Oxidation (M)	M2:Oxidation (M):1000.00	PEAKS DB
R.VAKISM(+15.99)PVNTPGQFEDFFPASSR.D	N	44.60	2540.2368	23	3.4	847.7558	3	33.41	8	F8:1598	OB5952 H3A Ro.raw	0	0	0	279	301	Oxidation (M)	M6:Oxidation (M):1000.00	PEAKS DB
K.AMVIVVVNK.G	N	42.66	971.5837	9	-3.0	486.7977	2	29.40	7	F7:1370	OB5951 H3A Ro.raw	0	0	0	444	452			PEAKS DB
R.N(+.98)TLEAAFNAEFNEIRR.V	N	42.24	1894.9172	16	3.2	948.4689	2	33.46	8	F8:1601	OB5952 H3A Ro.raw	5.9553E4	1	1	313	328		N1:Deamidation (NQ):34.75	PEAKS DB
K.NPQLQDLDM(+15.99)M(+15.99)LTC(+57.02)VEIKEGALM(+15.99)LPHFNSK.A	N	39.18	3419.6233	29	1.7	855.9146	4	35.97	8	F8:1751	OB5952 H3A Ro.raw	6.0894E4	1	1	415	443	Oxidation (M); Carbamidomethylation	M9:Oxidation (M):1000.00;M10:Oxidation (M):1000.00;C13:Carbamidomethylation:1000.00;M22:Oxidation (M):1000.00	PEAKS DB
R.EGEQEWGTPGSEVREETSR.N	Y	38.57	2161.9512	19	-7.0	721.6542	3	28.61	11	F11:1321	OB5948 H3B Ro.raw	0	0	0	147	165			PEAKS DB
E.PDLSNNFGR.L	N	38.29	1018.4832	9	1.2	510.2495	2	31.26	7	F7:1478	OB5951 H3A Ro.raw	0	0	0	397	405			PEAKS DB
K.GTGNLELVAVRK.E	N	37.48	1255.7249	12	-1.1	628.8690	2	28.81	8	F8:1332	OB5952 H3A Ro.raw	1.1804E3	2	2	453	464			PEAKS DB
K.NPQLQDLDMM(+15.99)LTC(+57.02)VEIKEGALM(+15.99)LPHFNSK.A	N	36.15	3403.6284	29	4.5	851.9182	4	36.31	7	F7:1742	OB5951 H3A Ro.raw	1.3711E5	1	1	415	443	Carbamidomethylation	M10:Oxidation (M):0.00;C13:Carbamidomethylation:1000.00;M22:Oxidation (M):40.85	PEAKS DB
R.IPSGFISYILN(+.98)RHDNQNLR.V	N	35.18	2257.1604	19	6.7	753.4007	3	36.32	11	F11:1765	OB5948 H3B Ro.raw	5.0814E4	1	1	260	278		N11:Deamidation (NQ):11.12	PEAKS DB
K.SFNLDEGHALRIPSGFISYILNRHDNQN(+.98)LR.V	N	34.85	3496.7600	30	4.9	700.3627	5	37.51	8	F8:1925	OB5952 H3A Ro.raw	2.7478E5	1	1	249	278		N28:Deamidation (NQ):10.11	PEAKS DB
K.NPQLQDLDM(+15.99)MLTC(+57.02)VEIKEGALMLPHFNSK.A	N	34.78	3387.6335	29	4.0	847.9191	4	38.06	9	F9:1825	OB5953 H3A Ro.raw	2.0615E5	1	1	415	443	Carbamidomethylation	M9:Oxidation (M):0.00;C13:Carbamidomethylation:1000.00	PEAKS DB
K.NPQLQDLDM(+15.99)MLTC(+57.02)VEIKEGALM(+15.99)LPHFNSK.A	N	34.77	3403.6284	29	0.8	851.9150	4	37.70	8	F8:1849	OB5952 H3A Ro.raw	1.9344E5	1	1	415	443	Carbamidomethylation	M9:Oxidation (M):24.44;C13:Carbamidomethylation:1000.00;M22:Oxidation (M):19.27	PEAKS DB
R.SSDNEGVIVKVSKEHVQELTK.H	N	34.18	2325.2175	21	-0.9	776.0791	3	30.32	9	F9:1416	OB5953 H3A Ro.raw	4.7496E3	1	1	351	371			PEAKS DB
K.NPQLQDLDM(+15.99)M(+15.99)LTC(+57.02)VEIKEGALMLPHFNSK.A	N	32.60	3403.6284	29	1.4	851.9156	4	37.49	9	F9:1800	OB5953 H3A Ro.raw	2.8417E5	1	1	415	443	Carbamidomethylation	M9:Oxidation (M):0.00;M10:Oxidation (M):0.00;C13:Carbamidomethylation:1000.00	PEAKS DB

total 63 peptides

Peptide	Uniq	-10lgP	Mass	Length	ppm	m/z	z	RT	Fraction	Scan	Source File	Area Roast Ara h 3	#Feature	#Feature Roast Ara h 3	Start	End	PTM	AScore	Found By
R.EGEQ(+.98)EWGTPGSEVREETSR.N	Y	32.39	2162.9351	19	10.0	721.9944	3	28.65	11	F11:1321	OB5948 H3B Ro.raw	9.7072E2	1	1	147	165	Deamidation (NQ)	Q4:Deamidation (NQ):1000.00	PEAKS DB
K.Q(-17.03)FQNLQNHR.I	N	31.50	1166.5581	9	-0.6	584.2859	2	28.13	7	F7:1303	OB5951 H3A Ro.raw	4.0835E2	1	1	199	207	Pyro-glu from Q	Q1:Pyro-glu from Q:1000.00	PEAKS PTM
**total 63 peptides**																			

Q6PSU3|Q6PSU3_ARAHY

back to list

| Protein Coverage | Supporting Peptides |

Protein Coverage:

```
  1 MRGRVSPLML LLGILVLASV SATQAKSPYR KTENPCAQRC LQSCQQEPDD LKQKACESRC TKLEYDPRCV YDTGATNQRH
 81 PPGERTRGRQ PGDYDDDRRQ PRREEGGRWG PAEPRERERE EDWRQPREDW RRPSHQQPRK IRPEGREGEQ EWGTPGSEVR
161 EETSRNNPFY FPSRRFSTRY GNQNGRIRVL QRFDQRSKQF QNLQNHRIVQ IEARPNTLVL PKHADADNIL VIQQGQATVT
241 VANGNNRKSF NLDEGHALRI PSGFISYILN RHDNQNLRVA KISMPVNTPG QFEDFFPASS RDQSSYLQGF SRNTLEAAFN
321 AEFNEIRRVL LEENAGGEQE ERGQRRRSTR SSDNEGVIVK VSKEHVQELT KHAKSVSKKG SEEEDITNPI NLRDGEPDLS
401 NNFGRLFEVK PDKKNPQLQD LDMMLTCVEI KEGALMLPHF NSKAMVIVVV NKGTGNLELV AVRKEQQQRG RREQEWEEEE
481 EDEEEEGSNR EVRRYTARLK EGDVFIMPAA HPVAINASSE LHLLGFGINA ENNHRIFLAG DKDNVIDQIE KQAKDLAFPG
561 SGEQVEKLIK NQRESHFVSA
```

C Carbamidomethylation (+57.02)
C Carbamidomethylation (DHKE, X@N-term) (+57.02)
d Deamidation (NQ) (+0.98)
o Oxidation (M) (+15.99)
P Pyro-glu from Q (-17.03)

Supporting Peptides:

Peptide	Uniq	-10lgP	Mass	Length	ppm	m/z	z	RT	Fraction	Scan	Source File	Area Roast Ara h 3	#Feature	#Feature Roast Ara h 3	Start	End	PTM	AScore	Found By
K.ISMPVNTPGQFEDFFPASSR.D	N	200.00	2226.0415	20	2.0	1114.0303	2	35.53	7	F7:1725	OB5951 H3A Ro.raw	8.7598E5	6	6	282	301			PEAKS DB

total 63 peptides

Peptide	Uniq	-10lgP	Mass	Length	ppm	m/z	z	RT	Fraction	Scan	Source File	Area Roast Ara h 3	#Feature	#Feature Roast Ara h 3	Start	End	PTM	AScore	Found By
R.NTLEAAFNAEFNEIR.R	N	165.72	1737.8322	15	1.8	869.9249	2	35.34	7	F7:1714	OB5951 H3A Ro.raw	1.3226E6	6	6	313	327			PEAKS DB
K.ISM(+15.99)PVNTPGQFEDFFPASSR.D	N	154.77	2242.0364	20	1.9	1122.0276	2	34.42	9	F9:1655	OB5953 H3A Ro.raw	9.4493E5	6	5	282	301	Oxidation (M)	M3:Oxidation (M):1000.00	PEAKS DB
R.IPSGFISYILNR.H	N	136.44	1378.7609	12	2.2	690.3892	2	37.11	9	F9:1819	OB5953 H3A Ro.raw	5.8838E6	6	6	260	271			PEAKS DB
K.SFNLDEGHALRIPSGFISYILNR.H	N	130.32	2618.3604	23	0.7	873.7969	3	39.49	10	F10:1795	OB5926 H3B Ro.raw	2.1577E7	15	15	249	271			PEAKS DB
R.IFLAGDKDNVIDQIEK.Q	N	127.66	1816.9570	16	-1.4	909.4865	2	33.07	11	F11:1576	OB5948 H3B Ro.raw	3.3211E6	12	11	536	551			PEAKS DB
R.DQSSYLQGFSR.N	N	125.12	1286.5891	11	-2.6	644.3015	2	30.77	11	F11:1443	OB5948 H3B Ro.raw	3.7573E5	6	6	302	312			PEAKS DB
K.KGSEEEDITNPINLRDGEPDLSNNFGR.L	N	113.18	3015.4170	27	1.8	754.8629	4	31.56	9	F9:1480	OB5953 H3A Ro.raw	4.7042E5	11	11	379	405			PEAKS DB
K.SFNLDEGHALR.I	N	110.09	1257.6101	11	-2.6	629.8120	2	29.12	11	F11:1344	OB5948 H3B Ro.raw	1.3894E5	10	10	249	259			PEAKS DB
K.DLAFPGSGEQVEKLIK.N	N	103.34	1729.9249	16	-1.4	865.9703	2	33.26	11	F11:1597	OB5948 H3B Ro.raw	3.9305E5	5	4	555	570			PEAKS DB
K.GSEEEDITNPINLRDGEPDLSNNFGR.L	N	102.75	2887.3220	26	1.4	963.4493	3	32.29	7	F7:1538	OB5951 H3A Ro.raw	4.5249E5	6	6	380	405			PEAKS DB
R.VLLEENAGGEQEER.G	N	102.67	1571.7427	14	2.2	786.8804	2	27.19	9	F9:1251	OB5953 H3A Ro.raw	9.5555E3	3	3	329	342			PEAKS DB
R.IFLAGDKDNVIDQIEKQAK.D	N	100.84	2144.1477	19	-0.2	715.7245	3	33.83	11	F11:1613	OB5948 H3B Ro.raw	1.8641E6	6	6	536	554			PEAKS DB
R.NTLEAAFNAEFNEIRR.V	N	96.95	1893.9332	16	2.8	947.9765	2	34.01	7	F7:1637	OB5951 H3A Ro.raw	3.4558E7	12	11	313	328			PEAKS DB
K.NPQLQDLDMMLTC(+57.02)VEIK.E	N	94.07	2046.9788	17	3.3	1024.5000	2	36.94	7	F7:1805	OB5951 H3A Ro.raw	0	0	0	415	431	Carbamidomethylation	C13:Carbamidomethylation:1000.00	PEAKS DB
R.IPSGFISYILNRHDNQNLR.V	N	93.37	2256.1763	19	0.1	565.0514	4	35.73	7	F7:1716	OB5951 H3A Ro.raw	8.2074E6	11	11	260	278			PEAKS DB
R.I(+57.02)PSGFISYILNR.H	N	91.10	1435.7823	12	0.7	718.8989	2	36.87	7	F7:1795	OB5951 H3A Ro.raw	7.2345E4	3	3	260	271	Carbamidomethylation (DHKE, X@N-term)	I1:Carbamidomethylation (DHKE, X@N-term):1000.00	PEAKS PTM
R.NNPFYFPSRR.F	N	90.91	1296.6364	10	0.1	649.3255	2	30.10	9	F9:1398	OB5953 H3A Ro.raw	4.2765E5	8	8	166	175			PEAKS DB
K.GTGNLELVAVR.K	N	87.71	1127.6299	11	4.8	564.8250	2	30.09	9	F9:1403	OB5953 H3A Ro.raw	2.2478E3	1	1	453	463			PEAKS DB
K.AM(+15.99)VIVVVNKGTGNLELVAVR.K	N	87.55	2097.1980	20	1.6	1049.6079	2	32.67	7	F7:1560	OB5951 H3A Ro.raw	2.6865E5	3	3	444	463	Oxidation (M)	M2:Oxidation (M):1000.00	PEAKS DB
K.DLAFPGSGEQVEK.L	N	85.86	1375.6619	13	-0.7	688.8392	2	30.28	11	F11:1414	OB5948 H3B Ro.raw	1.4145E5	4	4	555	567			PEAKS DB
K.AMVIVVVNKGTGNLELVAVR.K	N	83.81	2081.2031	20	1.7	1041.6106	2	34.96	7	F7:1704	OB5951 H3A Ro.raw	2.013E6	4	4	444	463			PEAKS DB
**total 63 peptides**																			

Peptide	Uniq	-10lgP	Mass	Length	ppm	m/z	z	RT	Fraction	Scan	Source File	Area Roast Ara h 3	#Feature	#Feature Roast Ara h 3	Start	End	PTM	AScore	Found By
R.NNPFYFPSR.R	N	81.75	1140.5352	9	0.1	571.2749	2	31.76	8	F8:1498	OB5952 H3A Ro.raw	1.1774E5	3	3	166	174			PEAKS DB
K.SFNLDEGHALRIPSGFISYILNRHDNQNLR.V	N	79.13	3495.7759	30	1.5	700.1635	5	39.97	9	F9:1987	OB5953 H3A Ro.raw	2.0697E7	15	15	249	278			PEAKS DB
K.HADADNILVIQQGQATVTVAN(+.98)GNNRK.S	N	78.05	2747.3950	26	1.8	916.8073	3	30.31	8	F8:1428	OB5952 H3A Ro.raw	8.0166E4	3	3	223	248		N21:Deamidation (NQ):14.04	PEAKS DB
G.SEEEDITNPINLRDGEPDLSNNFGR.L	N	77.06	2830.3005	25	-1.5	944.4417	3	33.02	10	F10:1421	OB5926 H3B Ro.raw	1.3758E5	3	3	381	405			PEAKS DB
K.AMVIVVVNKGTGNLELVAVRK.E	N	75.76	2209.2981	21	-1.5	737.4407	3	35.66	10	F10:1586	OB5926 H3B Ro.raw	3.5137E6	8	8	444	464			PEAKS DB
K.ISMPVNTPGQFEDFFPASSRDQSSYLQGFSR.N	N	74.46	3494.6201	31	0.8	1165.8840	3	36.12	11	F11:1762	OB5948 H3B Ro.raw	2.9084E6	5	5	282	312			PEAKS DB
R.IVQIEARPNTLVLPK.H	Y	72.30	1690.0140	15	1.0	564.3458	3	30.81	8	F8:1446	OB5952 H3A Ro.raw	1.034E6	12	12	208	222			PEAKS DB
R.KSFNLDEGHALRIPSGFISYILNR.H	N	70.24	2746.4553	24	1.8	687.6223	4	39.03	8	F8:1914	OB5952 H3A Ro.raw	3.7974E5	2	2	248	271			PEAKS DB
R.I(+57.02)FLAGDKDNVIDQIEK.Q	N	66.79	1873.9785	16	-2.1	937.9969	2	33.39	10	F10:1458	OB5926 H3B Ro.raw	2.2898E4	1	1	536	551	Carbamidomethylation (DHKE, X@N-term)	I1:Carbamidomethylation (DHKE, X@N-term):56.99	PEAKS PTM
K.HADADNILVIQQGQATVTVANGNNR.K	N	65.50	2618.3162	25	0.5	873.7798	3	30.34	7	F7:1426	OB5951 H3A Ro.raw	1.8654E3	1	1	223	247			PEAKS DB
K.HADADNILVIQQGQATVTVANGNNRK.S	N	62.55	2746.4111	26	0.2	916.4778	3	29.66	7	F7:1385	OB5951 H3A Ro.raw	6.7701E4	2	2	223	248			PEAKS DB
K.ISM(+15.99)PVNTPGQFEDFFPASSRDQSSYLQGFSR.N	N	62.16	3510.6150	31	-1.8	1171.2126	3	35.35	11	F11:1707	OB5948 H3B Ro.raw	2.4799E6	5	5	282	312	Oxidation (M)	M3:Oxidation (M):1000.00	PEAKS DB
R.EETSRNNPFYFPSRR.F	N	62.14	1898.9023	15	-0.7	633.9758	3	30.73	10	F10:1288	OB5926 H3B Ro.raw	1.7594E4	1	1	161	175			PEAKS DB
K.NPQLQDLDMMLTC(+57.02)VEIKEGALMLPHFNSK.A	N	59.27	3371.6387	29	-0.3	843.9188	4	39.49	10	F10:1807	OB5926 H3B Ro.raw	8.6484E5	3	3	415	443	Carbamidomethylation	C13:Carbamidomethylation:1000.00	PEAKS DB
K.KGSEEEDITNPINLR.D	N	58.67	1713.8533	15	2.3	572.2930	3	29.61	9	F9:1375	OB5953 H3A Ro.raw	1.9283E3	1	1	379	393			PEAKS DB
K.NPQLQDLDMMLTC(+57.02)VEIKEGALM(+15.99)LPHFNSK.A	N	58.64	3387.6335	29	1.2	847.9185	4	38.07	11	F11:1874	OB5948 H3B Ro.raw	3.9631E5	2	2	415	443	Carbamidomethylation; Oxidation (M)	C13:Carbamidomethylation:1000.00;M22:Oxidation (M):51.99	PEAKS DB
K.NPQLQDLDM(+15.99)MLTC(+57.02)VEIK.E	N	57.29	2062.9736	17	2.8	1032.4969	2	36.63	9	F9:1780	OB5953 H3A Ro.raw	0	0	0	415	431	Carbamidomethylation	M9:Oxidation (M):24.44;C13:Carbamidomethylation:1000.00	PEAKS DB
R.NTLEAAFN(+.98)AEFNEIRR.V	N	56.23	1894.9172	16	0.4	948.4663	2	32.13	9	F9:1524	OB5953 H3A Ro.raw	1.3345E4	2	2	313	328		N8:Deamidation (NQ):20.76	PEAKS DB
R.VLLEENAGGEQEERGQR.R	N	52.47	1912.9238	17	-2.1	638.6472	3	28.03	8	F8:1297	OB5952 H3A Ro.raw	8.2166E3	2	2	329	345			PEAKS DB

total 63 peptides

Peptide	Uniq	-10lgP	Mass	Length	ppm	m/z	z	RT	Fraction	Scan	Source File	Area Roast Ara h 3	#Feature	#Feature Roast Ara h 3	Start	End	PTM	AScore	Found By
R.NTLEAAFNAEFN(+.98)EIRR.V	N	50.81	1894.9172	16	1.9	948.4677	2	32.67	7	F7:1563	OB5951 H3A Ro.raw	9.2576E4	2	2	313	328		N12:Deamidation (NQ):8.26	PEAKS DB
K.HADADNILVIQQGQATVTVANGN(+.98)NRK.S	N	50.48	2747.3950	26	-4.1	916.8038	3	30.36	11	F11:1426	OB5948 H3B Ro.raw	0	0	0	223	248		N23:Deamidation (NQ):9.40	PEAKS DB
K.S(+57.02)FNLDEGHALRIPSGFISYILNR.H	N	49.55	2675.3818	23	2.3	892.8033	3	39.35	7	F7:1901	OB5951 H3A Ro.raw	6.7035E5	1	1	249	271	Carbamidomethylation (DHKE, X@N-term)	S1:Carbamidomethylation (DHKE, X@N-term):63.97	PEAKS PTM
R.IFLAGD(+57.02)KDNVIDQIEK.Q	N	45.63	1873.9785	16	1.4	937.9978	2	33.53	12	F12:1622	OB5949 H3B Ro.raw	3.5356E3	1	1	536	551		D6:Carbamidomethylation (DHKE, X@N-term):15.57	PEAKS PTM
K.HADADNILVIQQGQ(+.98)ATVTVANGNNRK.S	N	45.21	2747.3950	26	2.6	916.8103	3	30.46	10	F10:1280	OB5926 H3B Ro.raw	0	0	0	223	248		Q14:Deamidation (NQ):0.00	PEAKS DB
K.AM(+15.99)VIVVVNKGTGNLELVAVRK.E	N	45.01	2225.2930	21	-1.5	742.7723	3	33.77	10	F10:1559	OB5926 H3B Ro.raw	4.0307E5	2	2	444	464	Oxidation (M)	M2:Oxidation (M):1000.00	PEAKS DB
R.VAKISM(+15.99)PVNTPGQFEDFFPASSR.D	N	44.60	2540.2368	23	3.4	847.7558	3	33.41	8	F8:1598	OB5952 H3A Ro.raw	0	0	0	279	301	Oxidation (M)	M6:Oxidation (M):1000.00	PEAKS DB
K.AMVIVVVNK.G	N	42.66	971.5837	9	-3.0	486.7977	2	29.40	7	F7:1370	OB5951 H3A Ro.raw	0	0	0	444	452			PEAKS DB
R.N(+.98)TLEAAFNAEFNEIRR.V	N	42.24	1894.9172	16	3.2	948.4689	2	33.46	8	F8:1601	OB5952 H3A Ro.raw	5.9553E4	1	1	313	328		N1:Deamidation (NQ):34.75	PEAKS DB
K.NPQLQDLDM(+15.99)M(+15.99)LTC(+57.02)VEIKEGALM(+15.99)LPHFNSK.A	N	39.18	3419.6233	29	1.7	855.9146	4	35.97	8	F8:1751	OB5952 H3A Ro.raw	6.0894E4	1	1	415	443	Oxidation (M); Carbamidomethylation	M9:Oxidation (M):1000.00;M10:Oxidation (M):1000.00;C13:Carbamidomethylation:1000.00;M22:Oxidation (M):1000.00	PEAKS DB
R.EGEQEWGTPGSEVREETSR.N	Y	38.57	2161.9512	19	-7.0	721.6542	3	28.61	11	F11:1321	OB5948 H3B Ro.raw	0	0	0	147	165			PEAKS DB
E.PDLSNNFGR.L	N	38.29	1018.4832	9	1.2	510.2495	2	31.26	7	F7:1478	OB5951 H3A Ro.raw	0	0	0	397	405			PEAKS DB
K.GTGNLELVAVRK.E	N	37.48	1255.7249	12	-1.1	628.8690	2	28.81	8	F8:1332	OB5952 H3A Ro.raw	1.1804E3	2	2	453	464			PEAKS DB
K.NPQLQDLDMM(+15.99)LTC(+57.02)VEIKEGALM(+15.99)LPHFNSK.A	N	36.15	3403.6284	29	4.5	851.9182	4	36.31	7	F7:1742	OB5951 H3A Ro.raw	1.3711E5	1	1	415	443	Carbamidomethylation	M10:Oxidation (M):0.00;C13:Carbamidomethylation:1000.00;M22:Oxidation (M):40.85	PEAKS DB
R.IPSGFISYILN(+.98)RHDNQNLR.V	N	35.18	2257.1604	19	6.7	753.4007	3	36.32	11	F11:1765	OB5948 H3B Ro.raw	5.0814E4	1	1	260	278		N11:Deamidation (NQ):11.12	PEAKS DB
K.SFNLDEGHALRIPSGFISYILNRHDNQN(+.98)LR.V	N	34.85	3496.7600	30	4.9	700.3627	5	37.51	8	F8:1925	OB5952 H3A Ro.raw	2.7478E5	1	1	249	278		N28:Deamidation (NQ):10.11	PEAKS DB
K.NPQLQDLDM(+15.99)MLTC(+57.02)VEIKEGALMLPHFNSK.A	N	34.78	3387.6335	29	4.0	847.9191	4	38.06	9	F9:1825	OB5953 H3A Ro.raw	2.0615E5	1	1	415	443	Carbamidomethylation	M9:Oxidation (M):0.00;C13:Carbamidomethylation:1000.00	PEAKS DB

total 63 peptides

Peptide	Uniq	-10lgP	Mass	Length	ppm	m/z	z	RT	Fraction	Scan	Source File	Area Roast Ara h 3	#Feature	#Feature Roast Ara h 3	Start	End	PTM	AScore	Found By
K.NPQLQDLDM(+15.99)MLTC(+57.02)VEIKEGALM(+15.99)LPHFNSK.A	N	34.77	3403.6284	29	0.8	851.9150	4	37.70	8	F8:1849	OB5952 H3A Ro.raw	1.9344E5	1	1	415	443	Carbamidomethylation	M9:Oxidation (M):24.44;C13:Carbamidomethylation:1000.00;M22:Oxidation (M):19.27	PEAKS DB
R.SSDNEGVIVKVSKEHVQELTK.H	N	34.18	2325.2175	21	-0.9	776.0791	3	30.32	9	F9:1416	OB5953 H3A Ro.raw	4.7496E3	1	1	351	371			PEAKS DB
K.NPQLQDLDM(+15.99)M(+15.99)LTC(+57.02)VEIKEGALMLPHFNSK.A	N	32.60	3403.6284	29	1.4	851.9156	4	37.49	9	F9:1800	OB5953 H3A Ro.raw	2.8417E5	1	1	415	443	Carbamidomethylation	M9:Oxidation (M):0.00;M10:Oxidation (M):0.00;C13:Carbamidomethylation:1000.00	PEAKS DB
R.EGEQ(+.98)EWGTPGSEVREETSR.N	Y	32.39	2162.9351	19	10.0	721.9944	3	28.65	11	F11:1321	OB5948 H3B Ro.raw	9.7072E2	1	1	147	165	Deamidation (NQ)	Q4:Deamidation (NQ):1000.00	PEAKS DB
K.Q(-17.03)FQNLQNHR.I	N	31.50	1166.5581	9	-0.6	584.2859	2	28.13	7	F7:1303	OB5951 H3A Ro.raw	4.0835E2	1	1	199	207	Pyro-glu from Q	Q1:Pyro-glu from Q:1000.00	PEAKS PTM
**total 63 peptides**																			

N1NG13|N1NG13_ARAHY

back to list

| Protein Coverage | Supporting Peptides |

Protein Coverage:

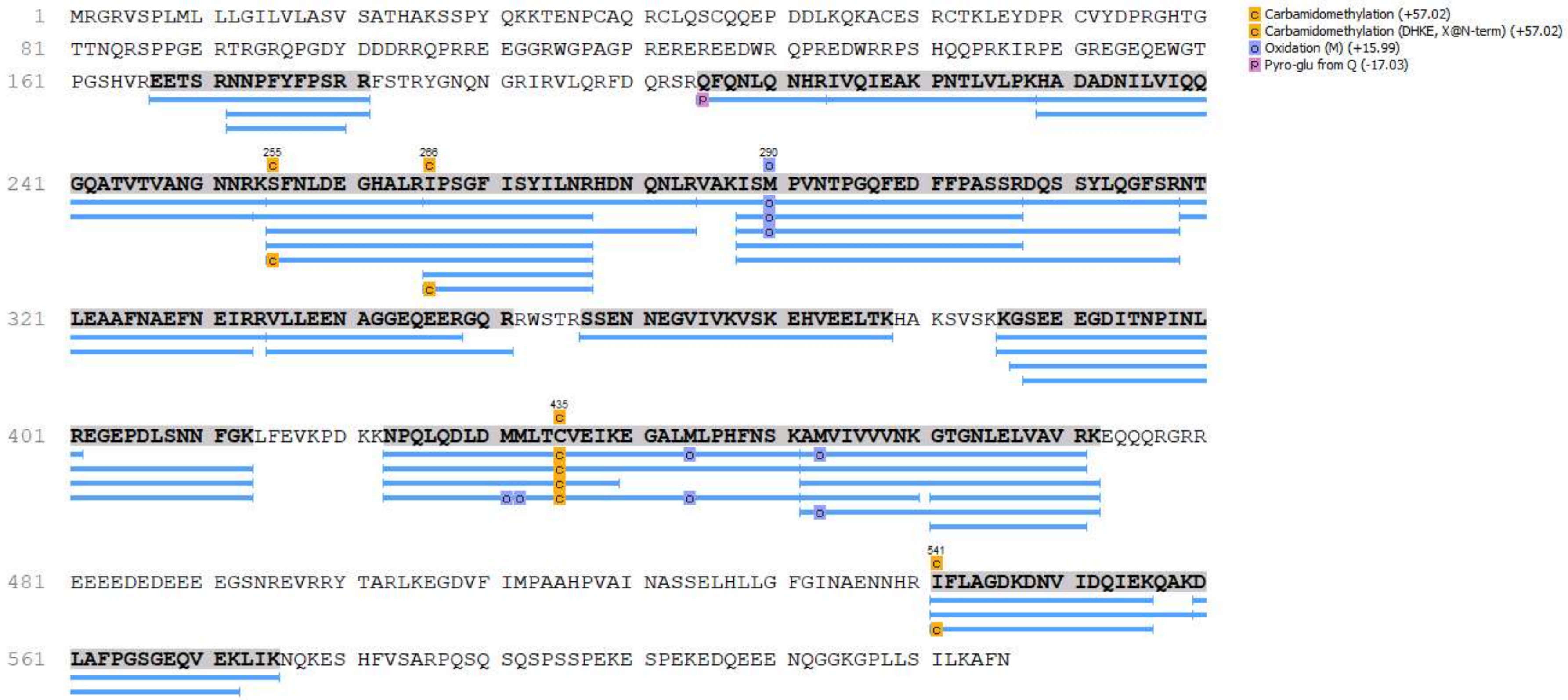

Supporting Peptides:

Peptide	Uniq	-10lgP	Mass	Length	ppm	m/z	z	RT	Fraction	Scan	Source File	Area Roast Ara h 3	#Feature	#Feature Roast Ara h 3	Start	End	PTM	AScore	Found By
K.ISMPVNTPGQFEDFFPASSR.D	N	200.00	2226.0415	20	2.0	1114.0303	2	35.53	7	F7:1725	OB5951 H3A Ro.raw	8.7598E5	6	6	288	307			PEAKS DB
R.NTLEAAFNAEFNEIR.R	N	165.72	1737.8322	15	1.8	869.9249	2	35.34	7	F7:1714	OB5951 H3A Ro.raw	1.3226E6	6	6	319	333			PEAKS DB
K.ISM(+15.99)PVNTPGQFEDFFPASSR.D	N	154.77	2242.0364	20	1.9	1122.0276	2	34.42	9	F9:1655	OB5953 H3A Ro.raw	9.4493E5	6	5	288	307	Oxidation (M)	M3:Oxidation (M):1000.00	PEAKS DB
R.IPSGFISYILNR.H	N	136.44	1378.7609	12	2.2	690.3892	2	37.11	9	F9:1819	OB5953 H3A Ro.raw	5.8838E6	6	6	266	277			PEAKS DB
K.SFNLDEGHALRIPSGFISYILNR.H	N	130.32	2618.3604	23	0.7	873.7969	3	39.49	10	F10:1795	OB5926 H3B Ro.raw	2.1577E7	15	15	255	277			PEAKS DB
R.IFLAGDKDNVIDQIEK.Q	N	127.66	1816.9570	16	-1.4	909.4865	2	33.07	11	F11:1576	OB5948 H3B Ro.raw	3.3211E6	12	11	541	556			PEAKS DB
R.DQSSYLQGFSR.N	N	125.12	1286.5891	11	-2.6	644.3015	2	30.77	11	F11:1443	OB5948 H3B Ro.raw	3.7573E5	6	6	308	318			PEAKS DB

total 66 peptides

Peptide	Uniq	-10lgP	Mass	Length	ppm	m/z	z	RT	Fraction	Scan	Source File	Area Roast Ara h 3	#Feature	#Feature Roast Ara h 3	Start	End	PTM	AScore	Found By
K.GSEEEGDITNPINLREGEPDLSNNFGK.L	Y	111.31	2930.3530	27	2.1	977.7936	3	32.13	9	F9:1522	OB5953 H3A Ro.raw	2.0907E5	5	5	387	413			PEAKS DB
K.SFNLDEGHALR.I	N	110.09	1257.6101	11	-2.6	629.8120	2	29.12	11	F11:1344	OB5948 H3B Ro.raw	1.3894E5	10	10	255	265			PEAKS DB
K.DLAFPGSGEQVEKLIK.N	N	103.34	1729.9249	16	-1.4	865.9703	2	33.26	11	F11:1597	OB5948 H3B Ro.raw	3.9305E5	5	4	560	575			PEAKS DB
R.VLLEENAGGEQEER.G	N	102.67	1571.7427	14	2.2	786.8804	2	27.19	9	F9:1251	OB5953 H3A Ro.raw	9.5555E3	3	3	335	348			PEAKS DB
R.IFLAGDKDNVIDQIEKQAK.D	N	100.84	2144.1477	19	-0.2	715.7245	3	33.83	11	F11:1613	OB5948 H3B Ro.raw	1.8641E6	6	6	541	559			PEAKS DB
R.NTLEAAFNAEFNEIRR.V	N	96.95	1893.9332	16	2.8	947.9765	2	34.01	7	F7:1637	OB5951 H3A Ro.raw	3.4558E7	12	11	319	334			PEAKS DB
R.IVQIEAKPNTLVLPK.H	Y	96.59	1662.0079	15	0.5	832.0117	2	30.42	9	F9:1413	OB5953 H3A Ro.raw	6.1515E5	10	10	214	228			PEAKS DB
K.NPQLQDLDMMLTC(+57.02)VEIK.E	N	94.07	2046.9788	17	3.3	1024.5000	2	36.94	7	F7:1805	OB5951 H3A Ro.raw	0	0	0	423	439	Carbamidomethylation	C13:Carbamidomethylation:1000.00	PEAKS DB
R.IPSGFISYILNRHDNQNLR.V	N	93.37	2256.1763	19	0.1	565.0514	4	35.73	7	F7:1716	OB5951 H3A Ro.raw	8.2074E6	11	11	266	284			PEAKS DB
R.I(+57.02)PSGFISYILNR.H	N	91.10	1435.7823	12	0.7	718.8989	2	36.87	7	F7:1795	OB5951 H3A Ro.raw	7.2345E4	3	3	266	277	Carbamidomethylation (DHKE, X@N-term)	I1:Carbamidomethylation (DHKE, X@N-term):1000.00	PEAKS PTM
R.NNPFYFPSRR.F	N	90.91	1296.6364	10	0.1	649.3255	2	30.10	9	F9:1398	OB5953 H3A Ro.raw	4.2765E5	8	8	172	181			PEAKS DB
K.GTGNLELVAVR.K	N	87.71	1127.6299	11	4.8	564.8250	2	30.09	9	F9:1403	OB5953 H3A Ro.raw	2.2478E3	1	1	461	471			PEAKS DB
K.AM(+15.99)VIVVVNKGTGNLELVAVR.K	N	87.55	2097.1980	20	1.6	1049.6079	2	32.67	7	F7:1560	OB5951 H3A Ro.raw	2.6865E5	3	3	452	471	Oxidation (M)	M2:Oxidation (M):1000.00	PEAKS DB
K.DLAFPGSGEQVEK.L	N	85.86	1375.6619	13	-0.7	688.8392	2	30.28	11	F11:1414	OB5948 H3B Ro.raw	1.4145E5	4	4	560	572			PEAKS DB
K.AMVIVVVNKGTGNLELVAVR.K	N	83.81	2081.2031	20	1.7	1041.6106	2	34.96	7	F7:1704	OB5951 H3A Ro.raw	2.013E6	4	4	452	471			PEAKS DB
R.NNPFYFPSR.R	N	81.75	1140.5352	9	0.1	571.2749	2	31.76	8	F8:1498	OB5952 H3A Ro.raw	1.1774E5	3	3	172	180			PEAKS DB
K.SFNLDEGHALRIPSGFISYILNRHDNQNLR.V	N	79.13	3495.7759	30	1.5	700.1635	5	39.97	9	F9:1987	OB5953 H3A Ro.raw	2.0697E7	15	15	255	284			PEAKS DB
K.HADADNILVIQQGQATVTVAN(+.98)GNNRK.S	N	78.05	2747.3950	26	1.8	916.8073	3	30.31	8	F8:1428	OB5952 H3A Ro.raw	8.0166E4	3	3	229	254		N21:Deamidation (NQ):14.04	PEAKS DB
K.KGSEEEGDITNPINLR.E	Y	75.97	1770.8748	16	-1.1	591.2982	3	29.41	9	F9:1365	OB5953 H3A Ro.raw	5.9957E3	3	3	386	401			PEAKS DB
K.AMVIVVVNKGTGNLELVAVRK.E	N	75.76	2209.2981	21	-1.5	737.4407	3	35.66	10	F10:1586	OB5926 H3B Ro.raw	3.5137E6	8	8	452	472			PEAKS DB
K.ISMPVNTPGQFEDFFPASSRDQSSYLQGFSR.N	N	74.46	3494.6201	31	0.8	1165.8840	3	36.12	11	F11:1762	OB5948 H3B Ro.raw	2.9084E6	5	5	288	318			PEAKS DB

total 66 peptides

Peptide	Uniq	-10lgP	Mass	Length	ppm	m/z	z	RT	Fraction	Scan	Source File	Area Roast Ara h 3	#Feature	#Feature Roast Ara h 3	Start	End	PTM	AScore	Found By
G.SEEEGDITNPINLREGEPDLSNNFGK.L	Y	71.24	2873.3315	26	0.5	958.7869	3	32.19	11	F11:1534	OB5948 H3B Ro.raw	1.9904E4	1	1	388	413			PEAKS DB
R.KSFNLDEGHALRIPSGFISYILNR.H	N	70.24	2746.4553	24	1.8	687.6223	4	39.03	8	F8:1914	OB5952 H3A Ro.raw	3.7974E5	2	2	254	277			PEAKS DB
R.I(+57.02)FLAGDKDNVIDQIEK.Q	N	66.79	1873.9785	16	-2.1	937.9969	2	33.39	10	F10:1458	OB5926 H3B Ro.raw	2.2898E4	1	1	541	556	Carbamidomethylation (DHKE, X@N-term)	I1:Carbamidomethylation (DHKE, X@N-term):56.99	PEAKS PTM
K.HADADNILVIQQGQATVTVANGNNR.K	N	65.50	2618.3162	25	0.5	873.7798	3	30.34	7	F7:1426	OB5951 H3A Ro.raw	1.8654E3	1	1	229	253			PEAKS DB
K.KGSEEEGDITNPINLREGEPDLSNNFGK.L	Y	62.77	3058.4478	28	4.3	1020.4943	3	31.17	9	F9:1469	OB5953 H3A Ro.raw	8.012E4	3	3	386	413			PEAKS DB
K.HADADNILVIQQGQATVTVANGNNRK.S	N	62.55	2746.4111	26	0.2	916.4778	3	29.66	7	F7:1385	OB5951 H3A Ro.raw	6.7701E4	2	2	229	254			PEAKS DB
K.ISM(+15.99)PVNTPGQFEDFFPASSRDQSSYLQGFSR.N	N	62.16	3510.6150	31	-1.8	1171.2126	3	35.35	11	F11:1707	OB5948 H3B Ro.raw	2.4799E6	5	5	288	318	Oxidation (M)	M3:Oxidation (M):1000.00	PEAKS DB
R.EETSRNNPFYFPSRR.F	N	62.14	1898.9023	15	-0.7	633.9758	3	30.73	10	F10:1288	OB5926 H3B Ro.raw	1.7594E4	1	1	167	181			PEAKS DB
K.GSEEEGDITN(+.98)PINLREGEPDLSNNFGK.L	Y	61.63	2931.3369	27	9.2	978.1306	3	32.21	11	F11:1532	OB5948 H3B Ro.raw	9.2634E3	1	1	387	413		N10:Deamidation (NQ):25.20	PEAKS DB
K.NPQLQDLDMMLTC(+57.02)VEIKEGALMLPHFNSK.A	N	59.27	3371.6387	29	-0.3	843.9188	4	39.49	10	F10:1807	OB5926 H3B Ro.raw	8.6484E5	3	3	423	451	Carbamidomethylation	C13:Carbamidomethylation:1000.00	PEAKS DB
K.NPQLQDLDMMLTC(+57.02)VEIKEGALM(+15.99)LPHFNSK.A	N	58.64	3387.6335	29	1.2	847.9185	4	38.07	11	F11:1874	OB5948 H3B Ro.raw	3.9631E5	2	2	423	451	Carbamidomethylation; Oxidation (M)	C13:Carbamidomethylation:1000.00;M22:Oxidation (M):51.99	PEAKS DB
K.NPQLQDLDM(+15.99)MLTC(+57.02)VEIK.E	N	57.29	2062.9736	17	2.8	1032.4969	2	36.63	9	F9:1780	OB5953 H3A Ro.raw	0	0	0	423	439	Carbamidomethylation	M9:Oxidation (M):24.44;C13:Carbamidomethylation:1000.00	PEAKS DB
R.NTLEAAFN(+.98)AEFNEIRR.V	N	56.23	1894.9172	16	0.4	948.4663	2	32.13	9	F9:1524	OB5953 H3A Ro.raw	1.3345E4	2	2	319	334		N8:Deamidation (NQ):20.76	PEAKS DB
R.SSENNEGVIVKVSKEHVEELTK.H	Y	54.00	2454.2603	22	1.5	819.0952	3	30.81	8	F8:1445	OB5952 H3A Ro.raw	1.1749E4	1	1	357	378			PEAKS DB
R.VLLEENAGGEQEERGQR.R	N	52.47	1912.9238	17	-2.1	638.6472	3	28.03	8	F8:1297	OB5952 H3A Ro.raw	8.2166E3	2	2	335	351			PEAKS DB
R.NTLEAAFNAEFN(+.98)EIRR.V	N	50.81	1894.9172	16	1.9	948.4677	2	32.67	7	F7:1563	OB5951 H3A Ro.raw	9.2576E4	2	2	319	334		N12:Deamidation (NQ):8.26	PEAKS DB
K.HADADNILVIQQGQATVTVANGN(+.98)NRK.S	N	50.48	2747.3950	26	-4.1	916.8038	3	30.36	11	F11:1426	OB5948 H3B Ro.raw	0	0	0	229	254		N23:Deamidation (NQ):9.40	PEAKS DB
K.S(+57.02)FNLDEGHALRIPSGFISYILNR.H	N	49.55	2675.3818	23	2.3	892.8033	3	39.35	7	F7:1901	OB5951 H3A Ro.raw	6.7035E5	1	1	255	277	Carbamidomethylation (DHKE, X@N-term)	S1:Carbamidomethylation (DHKE, X@N-term):63.97	PEAKS PTM
R.IFLAGD(+57.02)KDNVIDQIEK.Q	N	45.63	1873.9785	16	1.4	937.9978	2	33.53	12	F12:1622	OB5949 H3B Ro.raw	3.5356E3	1	1	541	556		D6:Carbamidomethylation (DHKE, X@N-term):15.57	PEAKS PTM

total 66 peptides

Peptide	Uniq	-10lgP	Mass	Length	ppm	m/z	z	RT	Fraction	Scan	Source File	Area Roast Ara h 3	#Feature	#Feature Roast Ara h 3	Start	End	PTM	AScore	Found By
K.HADADNILVIQQGQ(+.98)ATVTVANGNNRK.S	N	45.21	2747.3950	26	2.6	916.8103	3	30.46	10	F10:1280	OB5926 H3B Ro.raw	0	0	0	229	254		Q14:Deamidation (NQ):0.00	PEAKS DB
K.AM(+15.99)VIVVVNKGTGNLELVAVRK.E	N	45.01	2225.2930	21	-1.5	742.7723	3	33.77	10	F10:1559	OB5926 H3B Ro.raw	4.0307E5	2	2	452	472	Oxidation (M)	M2:Oxidation (M):1000.00	PEAKS DB
R.VAKISM(+15.99)PVNTPGQFEDFFPASSR.D	N	44.60	2540.2368	23	3.4	847.7558	3	33.41	8	F8:1598	OB5952 H3A Ro.raw	0	0	0	285	307	Oxidation (M)	M6:Oxidation (M):1000.00	PEAKS DB
K.AMVIVVVNK.G	N	42.66	971.5837	9	-3.0	486.7977	2	29.40	7	F7:1370	OB5951 H3A Ro.raw	0	0	0	452	460			PEAKS DB
R.N(+.98)TLEAAFNAEFNEIRR.V	N	42.24	1894.9172	16	3.2	948.4689	2	33.46	8	F8:1601	OB5952 H3A Ro.raw	5.9553E4	1	1	319	334		N1:Deamidation (NQ):34.75	PEAKS DB
R.I(+57.02)VQIEAKPNTLVLPK.H	Y	39.31	1719.0294	15	0.6	860.5225	2	30.81	8	F8:1447	OB5952 H3A Ro.raw	7.5816E3	1	1	214	228		I1:Carbamidomethylation (DHKE, X@N-term):28.70	PEAKS PTM
K.NPQLQDLDM(+15.99)M(+15.99)LTC(+57.02)VEIKEGALM(+15.99)LPHFNSK.A	N	39.18	3419.6233	29	1.7	855.9146	4	35.97	8	F8:1751	OB5952 H3A Ro.raw	6.0894E4	1	1	423	451	Oxidation (M); Carbamidomethylation	M9:Oxidation (M):1000.00;M10:Oxidation (M):1000.00;C13:Carbamidomethylation:1000.00;M22:Oxidation (M):1000.00	PEAKS DB
K.KGSEEEGDITNPINLREGEPDLSN(+.98)NFGK.L	Y	39.05	3059.4319	28	0.6	765.8674	4	31.36	11	F11:1485	OB5948 H3B Ro.raw	0	0	0	386	413		N24:Deamidation (NQ):0.00	PEAKS DB
K.GTGNLELVAVRK.E	N	37.48	1255.7249	12	-1.1	628.8690	2	28.81	8	F8:1332	OB5952 H3A Ro.raw	1.1804E3	2	2	461	472			PEAKS DB
K.NPQLQDLDMM(+15.99)LTC(+57.02)VEIKEGALM(+15.99)LPHFNSK.A	N	36.15	3403.6284	29	4.5	851.9182	4	36.31	7	F7:1742	OB5951 H3A Ro.raw	1.3711E5	1	1	423	451	Carbamidomethylation	M10:Oxidation (M):0.00;C13:Carbamidomethylation:1000.00;M22:Oxidation (M):40.85	PEAKS DB
G.SEEEGDITNPIN(+.98)LREGEPDLSNNFGK.L	Y	35.57	2874.3154	26	4.6	959.1188	3	32.12	11	F11:1534	OB5948 H3B Ro.raw	1.1098E4	1	1	388	413		N12:Deamidation (NQ):0.00	PEAKS DB
R.IPSGFISYILN(+.98)RHDNQNLR.V	N	35.18	2257.1604	19	6.7	753.4007	3	36.32	11	F11:1765	OB5948 H3B Ro.raw	5.0814E4	1	1	266	284		N11:Deamidation (NQ):11.12	PEAKS DB
K.SFNLDEGHALRIPSGFISYILNRHDNQN(+.98)LR.V	N	34.85	3496.7600	30	4.9	700.3627	5	37.51	8	F8:1925	OB5952 H3A Ro.raw	2.7478E5	1	1	255	284		N28:Deamidation (NQ):10.11	PEAKS DB
K.NPQLQDLDM(+15.99)MLTC(+57.02)VEIKEGALMLPHFNSK.A	N	34.78	3387.6335	29	4.0	847.9191	4	38.06	9	F9:1825	OB5953 H3A Ro.raw	2.0615E5	1	1	423	451	Carbamidomethylation	M9:Oxidation (M):0.00;C13:Carbamidomethylation:1000.00	PEAKS DB
K.NPQLQDLDM(+15.99)MLTC(+57.02)VEIKEGALM(+15.99)LPHFNSK.A	N	34.77	3403.6284	29	0.8	851.9150	4	37.70	8	F8:1849	OB5952 H3A Ro.raw	1.9344E5	1	1	423	451	Carbamidomethylation	M9:Oxidation (M):24.44;C13:Carbamidomethylation:1000.00;M22:Oxidation (M):19.27	PEAKS DB
R.IVQ(+.98)IEAKPNTLVLPK.H	Y	32.72	1662.9919	15	6.9	832.5090	2	31.77	12	F12:1516	OB5949 H3B Ro.raw	0	0	0	214	228		Q3:Deamidation (NQ):22.09	PEAKS DB

total 66 peptides

Peptide	Uniq	-10lgP	Mass	Length	ppm	m/z	z	RT	Fraction	Scan	Source File	Area Roast Ara h 3	#Feature	#Feature Roast Ara h 3	Start	End	PTM	AScore	Found By
K.NPQLQDLDM(+15.99)M(+15.99)LTC(+57.02)VEIKEGALMLPHFNSK.A	N	32.60	3403.6284	29	1.4	851.9156	4	37.49	9	F9:1800	OB5953 H3A Ro.raw	2.8417E5	1	1	423	451	Carbamidomethylation	M9:Oxidation (M):0.00;M10: Oxidation (M): 0.00;C13:Carb amidomethylati on:1000.00	PEAKS DB
K.KGSEEEGDITNPINLREGEPDLSNN(+.98)FGK.L	Y	32.29	3059.4319	28	8.8	765.8720	4	31.05	7	F7:1466	OB5951 H3A Ro.raw	0	0	0	386	413		N25:Deamidati on (NQ):0.00	PEAKS DB
R.Q(-17.03)FQNLQNHR.I	N	31.50	1166.5581	9	-0.6	584.2859	2	28.13	7	F7:1303	OB5951 H3A Ro.raw	4.0835E2	1	1	205	213	Pyro-glu from Q	Q1:Pyro-glu fr om Q:1000.00	PEAKS PTM
**total 66 peptides**																			

sp|P43238|ALL12_ARAHY

back to list

| Protein Coverage | Supporting Peptides |

Protein Coverage:

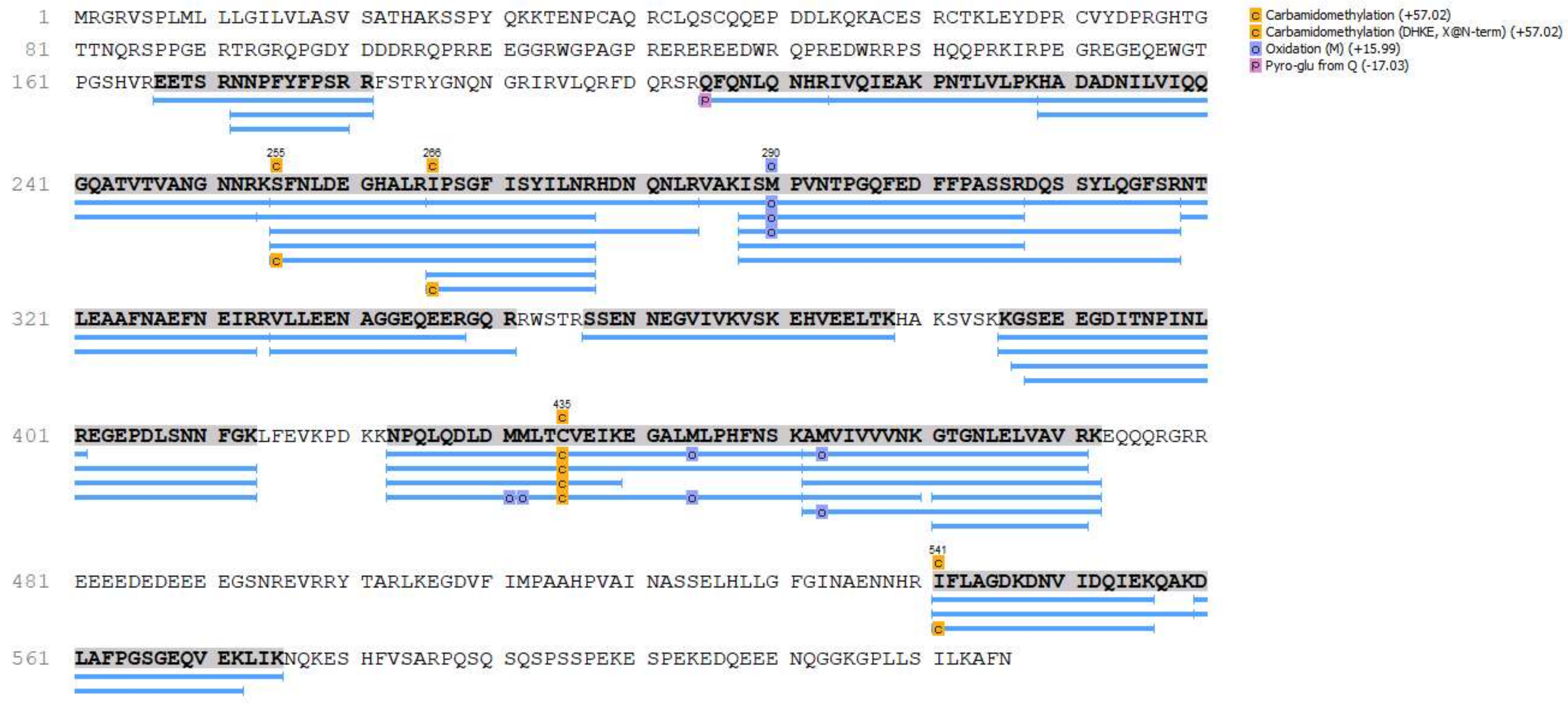

Supporting Peptides:

Peptide	Uniq	-10lgP	Mass	Length	ppm	m/z	z	RT	Fraction	Scan	Source File	Area Roast Ara h 3	#Feature	#Feature Roast Ara h 3	Start	End	PTM	AScore	Found By

total 66 peptides

Peptide	Uniq	-10lgP	Mass	Length	ppm	m/z	z	RT	Fraction	Scan	Source File	Area Roast Ara h 3	#Feature	#Feature Roast Ara h 3	Start	End	PTM	AScore	Found By
K.ISMPVNTPGQFEDFFPASSR.D	N	200.00	2226.0415	20	2.0	1114.0303	2	35.53	7	F7:1725	OB5951 H3A Ro.raw	8.7598E5	6	6	288	307			PEAKS DB
R.NTLEAAFNAEFNEIR.R	N	165.72	1737.8322	15	1.8	869.9249	2	35.34	7	F7:1714	OB5951 H3A Ro.raw	1.3226E6	6	6	319	333			PEAKS DB
K.ISM(+15.99)PVNTPGQFEDFFPASSR.D	N	154.77	2242.0364	20	1.9	1122.0276	2	34.42	9	F9:1655	OB5953 H3A Ro.raw	9.4493E5	6	5	288	307	Oxidation (M)	M3:Oxidation (M):1000.00	PEAKS DB
R.IPSGFISYILNR.H	N	136.44	1378.7609	12	2.2	690.3892	2	37.11	9	F9:1819	OB5953 H3A Ro.raw	5.8838E6	6	6	266	277			PEAKS DB
K.SFNLDEGHALRIPSGFISYILNR.H	N	130.32	2618.3604	23	0.7	873.7969	3	39.49	10	F10:1795	OB5926 H3B Ro.raw	2.1577E7	15	15	255	277			PEAKS DB
R.IFLAGDKDNVIDQIEK.Q	N	127.66	1816.9570	16	-1.4	909.4865	2	33.07	11	F11:1576	OB5948 H3B Ro.raw	3.3211E6	12	11	541	556			PEAKS DB
R.DQSSYLQGFSR.N	N	125.12	1286.5891	11	-2.6	644.3015	2	30.77	11	F11:1443	OB5948 H3B Ro.raw	3.7573E5	6	6	308	318			PEAKS DB
K.GSEEEGDITNPINLREGEPDLSNNFGK.L	Y	111.31	2930.3530	27	2.1	977.7936	3	32.13	9	F9:1522	OB5953 H3A Ro.raw	2.0907E5	5	5	387	413			PEAKS DB
K.SFNLDEGHALR.I	N	110.09	1257.6101	11	-2.6	629.8120	2	29.12	11	F11:1344	OB5948 H3B Ro.raw	1.3894E5	10	10	255	265			PEAKS DB
K.DLAFPGSGEQVEKLIK.N	N	103.34	1729.9249	16	-1.4	865.9703	2	33.26	11	F11:1597	OB5948 H3B Ro.raw	3.9305E5	5	4	560	575			PEAKS DB
R.VLLEENAGGEQEER.G	N	102.67	1571.7427	14	2.2	786.8804	2	27.19	9	F9:1251	OB5953 H3A Ro.raw	9.5555E3	3	3	335	348			PEAKS DB
R.IFLAGDKDNVIDQIEKQAK.D	N	100.84	2144.1477	19	-0.2	715.7245	3	33.83	11	F11:1613	OB5948 H3B Ro.raw	1.8641E6	6	6	541	559			PEAKS DB
R.NTLEAAFNAEFNEIRR.V	N	96.95	1893.9332	16	2.8	947.9765	2	34.01	7	F7:1637	OB5951 H3A Ro.raw	3.4558E7	12	11	319	334			PEAKS DB
R.IVQIEAKPNTLVLPK.H	Y	96.59	1662.0079	15	0.5	832.0117	2	30.42	9	F9:1413	OB5953 H3A Ro.raw	6.1515E5	10	10	214	228			PEAKS DB
K.NPQLQDLDMMLTC(+57.02)VEIK.E	N	94.07	2046.9788	17	3.3	1024.5000	2	36.94	7	F7:1805	OB5951 H3A Ro.raw	0	0	0	423	439	Carbamidomethylation	C13:Carbamidomethylation:1000.00	PEAKS DB
R.IPSGFISYILNRHDNQNLR.V	N	93.37	2256.1763	19	0.1	565.0514	4	35.73	7	F7:1716	OB5951 H3A Ro.raw	8.2074E6	11	11	266	284			PEAKS DB
R.I(+57.02)PSGFISYILNR.H	N	91.10	1435.7823	12	0.7	718.8989	2	36.87	7	F7:1795	OB5951 H3A Ro.raw	7.2345E4	3	3	266	277	Carbamidomethylation (DHKE, X@N-term)	I1:Carbamidomethylation (DHKE, X@N-term):1000.00	PEAKS PTM
R.NNPFYFPSRR.F	N	90.91	1296.6364	10	0.1	649.3255	2	30.10	9	F9:1398	OB5953 H3A Ro.raw	4.2765E5	8	8	172	181			PEAKS DB
K.GTGNLELVAVR.K	N	87.71	1127.6299	11	4.8	564.8250	2	30.09	9	F9:1403	OB5953 H3A Ro.raw	2.2478E3	1	1	461	471			PEAKS DB
K.AM(+15.99)VIVVVNKGTGNLELVAVR.K	N	87.55	2097.1980	20	1.6	1049.6079	2	32.67	7	F7:1560	OB5951 H3A Ro.raw	2.6865E5	3	3	452	471	Oxidation (M)	M2:Oxidation (M):1000.00	PEAKS DB
K.DLAFPGSGEQVEK.L	N	85.86	1375.6619	13	-0.7	688.8392	2	30.28	11	F11:1414	OB5948 H3B Ro.raw	1.4145E5	4	4	560	572			PEAKS DB

total 66 peptides

Peptide	Uniq	-10lgP	Mass	Length	ppm	m/z	z	RT	Fraction	Scan	Source File	Area Roast Ara h 3	#Feature	#Feature Roast Ara h 3	Start	End	PTM	AScore	Found By
K.AMVIVVVNKGTGNLELVAVR.K	N	83.81	2081.2031	20	1.7	1041.6106	2	34.96	7	F7:1704	OB5951 H3A Ro.raw	2.013E6	4	4	452	471			PEAKS DB
R.NNPFYFPSR.R	N	81.75	1140.5352	9	0.1	571.2749	2	31.76	8	F8:1498	OB5952 H3A Ro.raw	1.1774E5	3	3	172	180			PEAKS DB
K.SFNLDEGHALRIPSGFISYILNRHDNQNLR.V	N	79.13	3495.7759	30	1.5	700.1635	5	39.97	9	F9:1987	OB5953 H3A Ro.raw	2.0697E7	15	15	255	284			PEAKS DB
K.HADADNILVIQQGQATVTVAN(+.98)GNNRK.S	N	78.05	2747.3950	26	1.8	916.8073	3	30.31	8	F8:1428	OB5952 H3A Ro.raw	8.0166E4	3	3	229	254		N21:Deamidation (NQ):14.04	PEAKS DB
K.KGSEEEGDITNPINLR.E	Y	75.97	1770.8748	16	-1.1	591.2982	3	29.41	9	F9:1365	OB5953 H3A Ro.raw	5.9957E3	3	3	386	401			PEAKS DB
K.AMVIVVVNKGTGNLELVAVRK.E	N	75.76	2209.2981	21	-1.5	737.4407	3	35.66	10	F10:1586	OB5926 H3B Ro.raw	3.5137E6	8	8	452	472			PEAKS DB
K.ISMPVNTPGQFEDFFPASSRDQSSYLQGFSR.N	N	74.46	3494.6201	31	0.8	1165.8840	3	36.12	11	F11:1762	OB5948 H3B Ro.raw	2.9084E6	5	5	288	318			PEAKS DB
G.SEEEGDITNPINLREGEPDLSNNFGK.L	Y	71.24	2873.3315	26	0.5	958.7869	3	32.19	11	F11:1534	OB5948 H3B Ro.raw	1.9904E4	1	1	388	413			PEAKS DB
R.KSFNLDEGHALRIPSGFISYILNR.H	N	70.24	2746.4553	24	1.8	687.6223	4	39.03	8	F8:1914	OB5952 H3A Ro.raw	3.7974E5	2	2	254	277			PEAKS DB
R.I(+57.02)FLAGDKDNVIDQIEK.Q	N	66.79	1873.9785	16	-2.1	937.9969	2	33.39	10	F10:1458	OB5926 H3B Ro.raw	2.2898E4	1	1	541	556	Carbamidomethylation (DHKE, X@N-term)	I1:Carbamidomethylation (DHKE, X@N-term):56.99	PEAKS PTM
K.HADADNILVIQQGQATVTVANGNNR.K	N	65.50	2618.3162	25	0.5	873.7798	3	30.34	7	F7:1426	OB5951 H3A Ro.raw	1.8654E3	1	1	229	253			PEAKS DB
K.KGSEEEGDITNPINLREGEPDLSNNFGK.L	Y	62.77	3058.4478	28	4.3	1020.4943	3	31.17	9	F9:1469	OB5953 H3A Ro.raw	8.012E4	3	3	386	413			PEAKS DB
K.HADADNILVIQQGQATVTVANGNNRK.S	N	62.55	2746.4111	26	0.2	916.4778	3	29.66	7	F7:1385	OB5951 H3A Ro.raw	6.7701E4	2	2	229	254			PEAKS DB
K.ISM(+15.99)PVNTPGQFEDFFPASSRDQSSYLQGFSR.N	N	62.16	3510.6150	31	-1.8	1171.2126	3	35.35	11	F11:1707	OB5948 H3B Ro.raw	2.4799E6	5	5	288	318	Oxidation (M)	M3:Oxidation (M):1000.00	PEAKS DB
R.EETSRNNPFYFPSRR.F	N	62.14	1898.9023	15	-0.7	633.9758	3	30.73	10	F10:1288	OB5926 H3B Ro.raw	1.7594E4	1	1	167	181			PEAKS DB
K.GSEEEGDITN(+.98)PINLREGEPDLSNNFGK.L	Y	61.63	2931.3369	27	9.2	978.1306	3	32.21	11	F11:1532	OB5948 H3B Ro.raw	9.2634E3	1	1	387	413		N10:Deamidation (NQ):25.20	PEAKS DB
K.NPQLQDLDMMLTC(+57.02)VEIKEGALMLPHFNSK.A	N	59.27	3371.6387	29	-0.3	843.9188	4	39.49	10	F10:1807	OB5926 H3B Ro.raw	8.6484E5	3	3	423	451	Carbamidomethylation	C13:Carbamidomethylation:1000.00	PEAKS DB
K.NPQLQDLDMMLTC(+57.02)VEIKEGALM(+15.99)LPHFNSK.A	N	58.64	3387.6335	29	1.2	847.9185	4	38.07	11	F11:1874	OB5948 H3B Ro.raw	3.9631E5	2	2	423	451	Carbamidomethylation; Oxidation (M)	C13:Carbamidomethylation:1000.00;M22:Oxidation (M):51.99	PEAKS DB
K.NPQLQDLDM(+15.99)MLTC(+57.02)VEIK.E	N	57.29	2062.9736	17	2.8	1032.4969	2	36.63	9	F9:1780	OB5953 H3A Ro.raw	0	0	0	423	439	Carbamidomethylation	M9:Oxidation (M):24.44;C13:Carbamidomethylation:1000.00	PEAKS DB

total 66 peptides

Peptide	Uniq	-10lgP	Mass	Length	ppm	m/z	z	RT	Fraction	Scan	Source File	Area Roast Ara h 3	#Feature	#Feature Roast Ara h 3	Start	End	PTM	AScore	Found By
R.NTLEAAFN(+.98)AEFNEIRR.V	N	56.23	1894.9172	16	0.4	948.4663	2	32.13	9	F9:1524	OB5953 H3A Ro.raw	1.3345E4	2	2	319	334		N8:Deamidation (NQ):20.76	PEAKS DB
R.SSENNEGVIVKVSKEHVEELTK.H	Y	54.00	2454.2603	22	1.5	819.0952	3	30.81	8	F8:1445	OB5952 H3A Ro.raw	1.1749E4	1	1	357	378			PEAKS DB
R.VLLEENAGGEQEERGQR.R	N	52.47	1912.9238	17	-2.1	638.6472	3	28.03	8	F8:1297	OB5952 H3A Ro.raw	8.2166E3	2	2	335	351			PEAKS DB
R.NTLEAAFNAEFN(+.98)EIRR.V	N	50.81	1894.9172	16	1.9	948.4677	2	32.67	7	F7:1563	OB5951 H3A Ro.raw	9.2576E4	2	2	319	334		N12:Deamidation (NQ):8.26	PEAKS DB
K.HADADNILVIQQGQATVTVANGN(+.98)NRK.S	N	50.48	2747.3950	26	-4.1	916.8038	3	30.36	11	F11:1426	OB5948 H3B Ro.raw	0	0	0	229	254		N23:Deamidation (NQ):9.40	PEAKS DB
K.S(+57.02)FNLDEGHALRIPSGFISYILNR.H	N	49.55	2675.3818	23	2.3	892.8033	3	39.35	7	F7:1901	OB5951 H3A Ro.raw	6.7035E5	1	1	255	277	Carbamidomethylation (DHKE, X@N-term)	S1:Carbamidomethylation (DHKE, X@N-term):63.97	PEAKS PTM
R.IFLAGD(+57.02)KDNVIDQIEK.Q	N	45.63	1873.9785	16	1.4	937.9978	2	33.53	12	F12:1622	OB5949 H3B Ro.raw	3.5356E3	1	1	541	556		D6:Carbamidomethylation (DHKE, X@N-term):15.57	PEAKS PTM
K.HADADNILVIQQGQ(+.98)ATVTVANGNNRK.S	N	45.21	2747.3950	26	2.6	916.8103	3	30.46	10	F10:1280	OB5926 H3B Ro.raw	0	0	0	229	254		Q14:Deamidation (NQ):0.00	PEAKS DB
K.AM(+15.99)VIVVVNKGTGNLELVAVRK.E	N	45.01	2225.2930	21	-1.5	742.7723	3	33.77	10	F10:1559	OB5926 H3B Ro.raw	4.0307E5	2	2	452	472	Oxidation (M)	M2:Oxidation (M):1000.00	PEAKS DB
R.VAKISM(+15.99)PVNTPGQFEDFFPASSR.D	N	44.60	2540.2368	23	3.4	847.7558	3	33.41	8	F8:1598	OB5952 H3A Ro.raw	0	0	0	285	307	Oxidation (M)	M6:Oxidation (M):1000.00	PEAKS DB
K.AMVIVVVNK.G	N	42.66	971.5837	9	-3.0	486.7977	2	29.40	7	F7:1370	OB5951 H3A Ro.raw	0	0	0	452	460			PEAKS DB
R.N(+.98)TLEAAFNAEFNEIRR.V	N	42.24	1894.9172	16	3.2	948.4689	2	33.46	8	F8:1601	OB5952 H3A Ro.raw	5.9553E4	1	1	319	334		N1:Deamidation (NQ):34.75	PEAKS DB
R.I(+57.02)VQIEAKPNTLVLPK.H	Y	39.31	1719.0294	15	0.6	860.5225	2	30.81	8	F8:1447	OB5952 H3A Ro.raw	7.5816E3	1	1	214	228		I1:Carbamidomethylation (DHKE, X@N-term):28.70	PEAKS PTM
K.NPQLQDLDM(+15.99)M(+15.99)LTC(+57.02)VEIKEGALM(+15.99)LPHFNSK.A	N	39.18	3419.6233	29	1.7	855.9146	4	35.97	8	F8:1751	OB5952 H3A Ro.raw	6.0894E4	1	1	423	451	Oxidation (M); Carbamidomethylation	M9:Oxidation (M):1000.00;M10:Oxidation (M):1000.00;C13:Carbamidomethylation:1000.00;M22:Oxidation (M):1000.00	PEAKS DB
K.KGSEEEGDITNPINLREGEPDLSN(+.98)NFGK.L	Y	39.05	3059.4319	28	0.6	765.8674	4	31.36	11	F11:1485	OB5948 H3B Ro.raw	0	0	0	386	413		N24:Deamidation (NQ):0.00	PEAKS DB
K.GTGNLELVAVRK.E	N	37.48	1255.7249	12	-1.1	628.8690	2	28.81	8	F8:1332	OB5952 H3A Ro.raw	1.1804E3	2	2	461	472			PEAKS DB
K.NPQLQDLDMM(+15.99)LTC(+57.02)VEIKEGALM(+15.99)LPHFNSK.A	N	36.15	3403.6284	29	4.5	851.9182	4	36.31	7	F7:1742	OB5951 H3A Ro.raw	1.3711E5	1	1	423	451	Carbamidomethylation	M10:Oxidation (M):0.00;C13:Carbamidomethylation:1000.00;M22:Oxidation (M):40.85	PEAKS DB

total 66 peptides

Peptide	Uniq	-10lgP	Mass	Length	ppm	m/z	z	RT	Fraction	Scan	Source File	Area Roast Ara h 3	#Feature	#Feature Roast Ara h 3	Start	End	PTM	AScore	Found By
G.SEEEGDITNPIN(+.98)LREGEPDLSNNFGK.L	Y	35.57	2874.3154	26	4.6	959.1188	3	32.12	11	F11:1534	OB5948 H3B Ro.raw	1.1098E4	1	1	388	413		N12:Deamidation (NQ):0.00	PEAKS DB
R.IPSGFISYILN(+.98)RHDNQNLR.V	N	35.18	2257.1604	19	6.7	753.4007	3	36.32	11	F11:1765	OB5948 H3B Ro.raw	5.0814E4	1	1	266	284		N11:Deamidation (NQ):11.12	PEAKS DB
K.SFNLDEGHALRIPSGFISYILNRHDNQN(+.98)LR.V	N	34.85	3496.7600	30	4.9	700.3627	5	37.51	8	F8:1925	OB5952 H3A Ro.raw	2.7478E5	1	1	255	284		N28:Deamidation (NQ):10.11	PEAKS DB
K.NPQLQDLDM(+15.99)MLTC(+57.02)VEIKEGALMLPHFNSK.A	N	34.78	3387.6335	29	4.0	847.9191	4	38.06	9	F9:1825	OB5953 H3A Ro.raw	2.0615E5	1	1	423	451	Carbamidomethylation	M9:Oxidation (M):0.00;C13:Carbamidomethylation:1000.00	PEAKS DB
K.NPQLQDLDM(+15.99)MLTC(+57.02)VEIKEGALM(+15.99)LPHFNSK.A	N	34.77	3403.6284	29	0.8	851.9150	4	37.70	8	F8:1849	OB5952 H3A Ro.raw	1.9344E5	1	1	423	451	Carbamidomethylation	M9:Oxidation (M):24.44;C13:Carbamidomethylation:1000.00;M22:Oxidation (M):19.27	PEAKS DB
R.IVQ(+.98)IEAKPNTLVLPK.H	Y	32.72	1662.9919	15	6.9	832.5090	2	31.77	12	F12:1516	OB5949 H3B Ro.raw	0	0	0	214	228		Q3:Deamidation (NQ):22.09	PEAKS DB
K.NPQLQDLDM(+15.99)M(+15.99)LTC(+57.02)VEIKEGALMLPHFNSK.A	N	32.60	3403.6284	29	1.4	851.9156	4	37.49	9	F9:1800	OB5953 H3A Ro.raw	2.8417E5	1	1	423	451	Carbamidomethylation	M9:Oxidation (M):0.00;M10:Oxidation (M):0.00;C13:Carbamidomethylation:1000.00	PEAKS DB
K.KGSEEEGDITNPINLREGEPDLSNN(+.98)FGK.L	Y	32.29	3059.4319	28	8.8	765.8720	4	31.05	7	F7:1466	OB5951 H3A Ro.raw	0	0	0	386	413		N25:Deamidation (NQ):0.00	PEAKS DB
R.Q(-17.03)FQNLQNHR.I	N	31.50	1166.5581	9	-0.6	584.2859	2	28.13	7	F7:1303	OB5951 H3A Ro.raw	4.0835E2	1	1	205	213	Pyro-glu from Q	Q1:Pyro-glu from Q:1000.00	PEAKS PTM
**total 66 peptides**																			

A1DZF0|A1DZF0_ARAHY

back to list

| Protein Coverage | Supporting Peptides |

Protein Coverage:

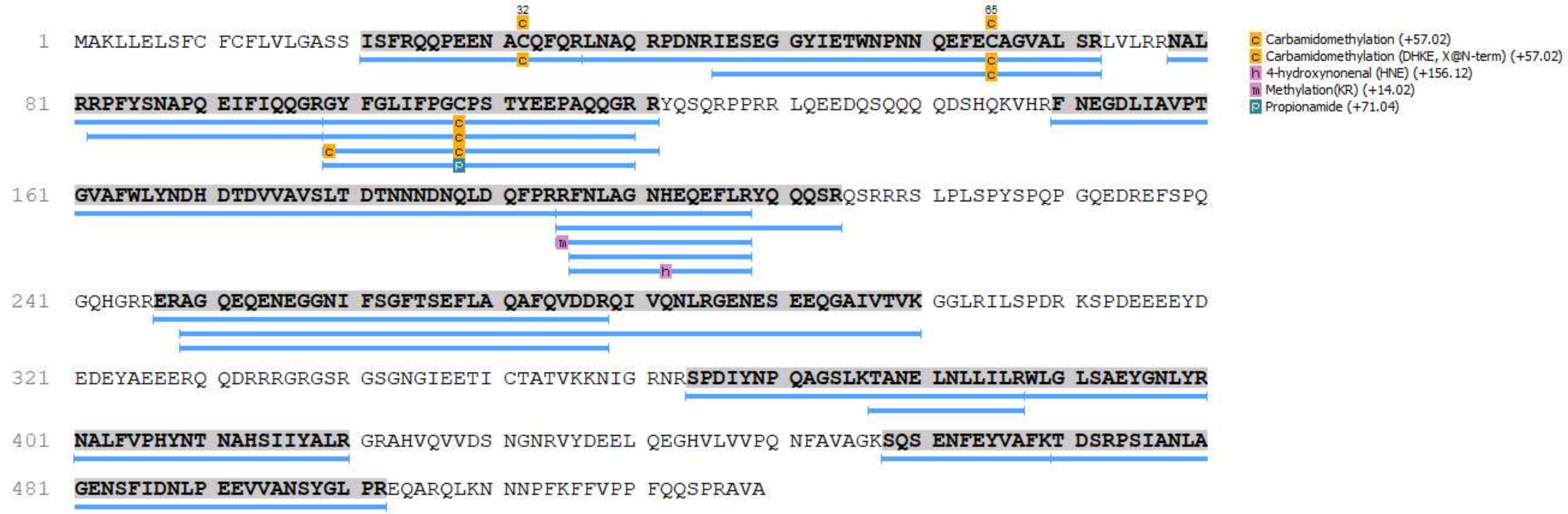

Supporting Peptides:

Peptide	Uniq	-10lgP	Mass	Length	ppm	m/z	z	RT	Fraction	Scan	Source File	Area Roast Ara h 3	#Feature	#Feature Roast Ara h 3	Start	End	PTM	AScore	Found By
R.GYFGLIFPGC(+57.02)PSTYEEPAQQGR.R	N	200.00	2473.1372	22	3.0	1237.5796	2	36.11	7	F7:1750	OB5951 H3A Ro.raw	1.587E6	6	6	99	120	Carbamidomethylation	C10:Carbamidomethylation: 1000.00	PEAKS DB
R.WLGLSAEYGNLYR.N	N	163.36	1540.7673	13	-0.8	771.3920	2	34.97	11	F11:1697	OB5948 H3B Ro.raw	6.3564E5	6	6	388	400			PEAKS DB
R.RPFYSNAPQEIFIQQGR.G	N	137.88	2050.0383	17	3.2	684.3556	3	31.52	7	F7:1483	OB5951 H3A Ro.raw	5.9353E6	9	9	82	98			PEAKS DB
K.TDSRPSIANLAGENSFIDNLPEEVVANSYGLPR.E	N	129.79	3544.7434	33	0.8	1182.5919	3	37.65	11	F11:1851	OB5948 H3B Ro.raw	2.2816E6	5	5	470	502			PEAKS DB
R.NALRRPFYSNAPQEIFIQQGR.G	N	125.40	2504.3037	21	1.0	835.7760	3	32.71	9	F9:1535	OB5953 H3A Ro.raw	6.1298E6	10	10	78	98			PEAKS DB
R.FNLAGNHEQEFLR.Y	N	104.44	1573.7637	13	4.3	787.8925	2	30.57	7	F7:1440	OB5951 H3A Ro.raw	1.243E5	7	7	196	208			PEAKS DB
K.SQSENFEYVAFK.T	N	103.88	1447.6619	12	2.4	724.8400	2	31.14	7	F7:1469	OB5951 H3A Ro.raw	3.2277E4	3	3	458	469			PEAKS DB
R.RFNLAGNHEQEFLR.Y	N	99.39	1729.8647	14	1.5	577.6297	3	31.36	9	F9:1478	OB5953 H3A Ro.raw	1.8255E6	6	6	195	208			PEAKS DB
R.GYFGLIFPGC(+57.02)PSTYEEPAQQGRR.Y	N	99.33	2629.2383	23	2.4	877.4221	3	35.18	9	F9:1676	OB5953 H3A Ro.raw	4.5018E6	7	7	99	121	Carbamidomethylation	C10:Carbamidomethylation: 1000.00	PEAKS DB
K.TANELNLLILR.W	N	92.46	1268.7452	11	-0.4	635.3796	2	34.99	9	F9:1688	OB5953 H3A Ro.raw	2.3728E5	3	3	377	387			PEAKS DB
R.AGQEQENEGGNIFSGFTSEFLAQAFQVDDR.Q	Y	86.95	3290.4751	30	3.2	1646.2535	2	40.50	11	F11:2017	OB5948 H3B Ro.raw	5.1868E4	1	1	249	278			PEAKS DB

total 30 peptides

Peptide	Uniq	-10lgP	Mass	Length	ppm	m/z	z	RT	Fraction	Scan	Source File	Area Roast Ara h 3	#Feature	#Feature Roast Ara h 3	Start	End	PTM	AScore	Found By
R.GYFGLIFPGC(+71.04)PSTYEEPAQQGR.R	N	72.57	2487.1528	22	2.6	1244.5869	2	36.34	9	F9:1767	OB5953 H3A Ro.raw	2.3282E4	2	2	99	120	Propionamide	C10:Propiona mide:1000.00	PEAKS PTM
R.FNEGDLIAVPTGVAFWLYNDHDTDVVAVSLTDTNNNDNQLDQFPR.R	N	69.73	5049.3691	45	0.7	1684.1357	3	40.24	10	F10:1843	OB5926 H3B Ro.raw	1.1398E6	2	2	150	194			PEAKS DB
R.SPDIYNPQAGSLKTANELNLLILR.W	N	66.95	2639.4282	24	2.2	880.8186	3	36.15	9	F9:1756	OB5953 H3A Ro.raw	1.5334E5	4	4	364	387			PEAKS DB
R.IESEGGYIETWNPNNQEFEC(+57.02)AGVALSR.L	N	66.94	3069.3774	27	6.2	1535.7054	2	34.89	8	F8:1684	OB5952 H3A Ro.raw	0	0	0	46	72	Carbamidomethylation	C20:Carbamid omethylation: 1000.00	PEAKS DB
R.RFNLAGNHEQEFLRYQQQSR.Q	N	66.29	2520.2371	20	-2.2	631.0667	4	34.12	10	F10:1496	OB5926 H3B Ro.raw	7.5688E4	2	2	195	214			PEAKS DB
R.NALFVPHYNTNAHSIIYALR.G	N	63.14	2313.2019	20	0.2	772.0747	3	35.15	7	F7:1685	OB5951 H3A Ro.raw	9.0952E5	5	5	401	420			PEAKS DB
S.ISFRQQPEENAC(+57.02)QFQR.L	N	45.61	2036.9486	16	-1.4	679.9907	3	28.84	11	F11:1329	OB5948 H3B Ro.raw	4.7949E4	5	5	21	36	Carbamidomethylation	C12:Carbamid omethylation: 1000.00	PEAKS DB
R.AGQEQENEGGNIFSGFTSEFLAQAFQVDDRQIVQNLRGENESEEQGAIVTVK.G	Y	44.21	5712.7202	52	4.9	1429.1943	4	39.90	12	F12:1989	OB5949 H3B Ro.raw	4.1738E6	1	1	249	300			PEAKS DB
R.ERAGQEQENEGGNIFSGFTSEFLAQAFQVDDR.Q	Y	44.17	3575.6189	32	-0.4	1192.8823	3	39.35	11	F11:1955	OB5948 H3B Ro.raw	5.3613E4	1	1	247	278			PEAKS DB
R.RFNLAGNH(+14.02)EQEFLR.Y	N	41.89	1743.8805	14	0.5	582.3011	3	31.14	7	F7:1453	OB5951 H3A Ro.raw	4.477E5	2	2	195	208		H8:Methylatio n(others):40.0 0	PEAKS PTM
R.G(+57.02)YFGLIFPGC(+57.02)PSTYEEPAQQGRR.Y	N	35.90	2686.2598	23	1.6	896.4286	3	35.57	8	F8:1718	OB5952 H3A Ro.raw	5.4933E4	1	1	99	121	Carbamidomethylation (DHKE, X@N-term); Carbamidomethylation	G1:Carbamido methylation (D HKE, X@N-ter m):130.55;C1 0:Carbamidom ethylation:100 0.00	PEAKS PTM
R.LNAQRPDNRIESEGGYIETWNPNNQEFEC(+57.02)AGVALSR.L	N	35.63	4133.9248	36	4.2	1034.4928	4	36.08	8	F8:1752	OB5952 H3A Ro.raw	1.6394E5	1	1	37	72	Carbamidomethylation	C29:Carbamid omethylation: 1000.00	PEAKS DB
R.RPFYSNAP(+15.99)QEIFIQQGR.G	N	34.29	2066.0332	17	0.2	689.6852	3	31.94	9	F9:1497	OB5953 H3A Ro.raw	4.3282E4	1	1	82	98		P8:Oxidation o r Hydroxylatio n:32.28	PEAKS PTM
R.R(+14.02)FNLAGNHEQEFLR.Y	N	32.14	1743.8805	14	0.9	582.3013	3	31.38	8	F8:1472	OB5952 H3A Ro.raw	2.9356E5	2	2	195	208	Methylation(KR)	R1:Methylation (KR):67.58	PEAKS PTM
R.RFNLAGNHEQ(+.98)EFLRYQQQSR.Q	N	31.27	2521.2209	20	5.0	631.3173	4	34.33	10	F10:1528	OB5926 H3B Ro.raw	4.7768E4	1	1	195	214		Q10:Deamidati on (NQ):0.00	PEAKS DB
R.FNLAGNH(+14.02)EQEFLR.Y	N	31.24	1587.7793	13	0.7	530.2674	3	30.81	8	F8:1450	OB5952 H3A Ro.raw	6.1216E4	1	1	196	208		H7:Methylatio n(others):14.0 2	PEAKS PTM
R.FNLAGNH(+156.12)EQEFLR.Y	N	31.09	1729.8788	13	-7.6	577.6292	3	31.13	7	F7:1444	OB5951 H3A Ro.raw	2.4781E5	1	1	196	208	4-hydroxynonenal (HNE)	H7:4-hydroxyn onenal (HNE): 1000.00	PEAKS PTM
R.RFN(+.98)LAGNHEQEFLR.Y	N	30.90	1730.8488	14	9.2	577.9634	3	32.85	11	F11:1572	OB5948 H3B Ro.raw	0	0	0	195	208		N3:Deamidatio n (NQ):17.65	PEAKS DB
R.AGQEQEN(+.98)EGGNIFSGFTSEFLAQAFQVDDRQIVQNLRGENESEEQGAIVTVK.G	Y	30.03	5713.7041	52	7.4	1143.7566	5	39.90	12	F12:2004	OB5949 H3B Ro.raw	1.9949E5	1	1	249	300		N7:Deamidatio n (NQ):8.26	PEAKS DB
**total 30 peptides**																			

Q38711|Q38711_ARAHY

back to list

| **Protein Coverage** | **Supporting Peptides** |

Protein Coverage:

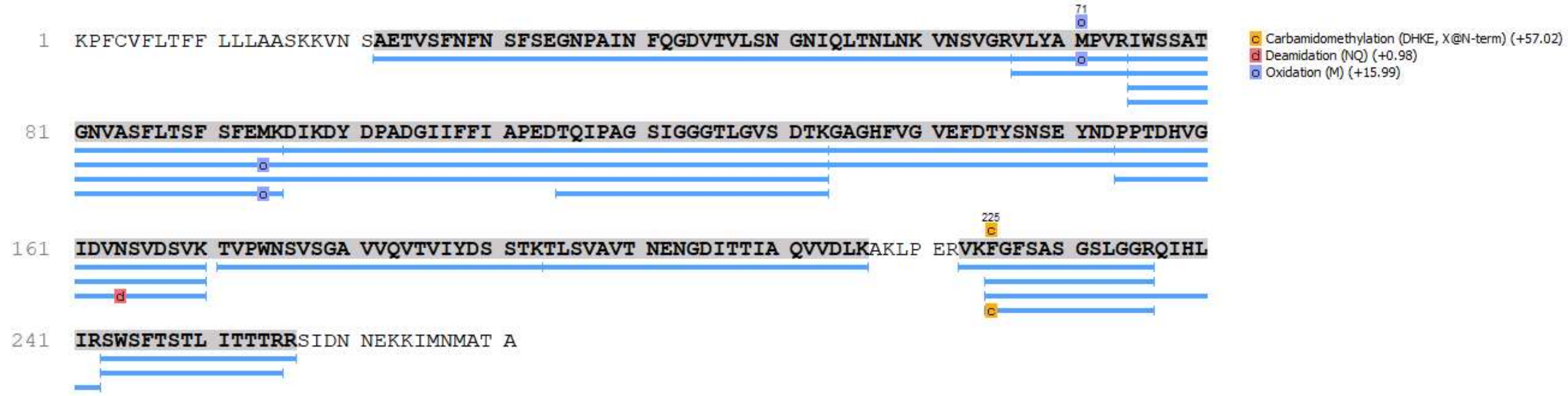

Supporting Peptides:

Peptide	Uniq	-10lgP	Mass	Length	ppm	m/z	z	RT	Fraction	Scan	Source File	Area Roast Ara h 3	#Feature	#Feature Roast Ara h 3	Start	End	PTM	AScore	Found By
R.SWSFTSTLITTTR.R	N	200.00	1499.7620	13	-1.1	750.8890	2	34.40	11	F11:1663	OB5948 H3B Ro.raw	1.0426E6	6	6	243	255			PEAKS DB
R.IWSSATGNVASFLTSFSFEMK.D	N	200.00	2309.1038	21	-0.7	1155.5608	2	39.93	11	F11:1986	OB5948 H3B Ro.raw	5.3682E5	3	3	75	95			PEAKS DB
K.TLSVAVTNENGDITTIAQVVDLK.A	Y	130.69	2400.2747	23	-0.3	1201.1469	2	35.93	11	F11:1752	OB5948 H3B Ro.raw	2.3058E5	4	4	194	216			PEAKS DB
K.DIKDYDPADGIIFFIAPEDTQIPAGSIGGGTLGVSDTK.G	N	120.14	3892.9258	38	0.9	1298.6531	3	38.97	11	F11:1923	OB5948 H3B Ro.raw	1.1326E6	3	3	96	133			PEAKS DB
R.IWSSATGNVASFLTSFSFEM(+15.99)K.D	N	119.39	2325.0986	21	-0.6	1163.5583	2	38.94	11	F11:1921	OB5948 H3B Ro.raw	4.3252E4	1	1	75	95	Oxidation (M)	M20:Oxidation (M):1000.00	PEAKS DB
D.PPTDHVGIDVNSVDSVK.T	N	115.65	1777.8846	17	-1.1	593.6361	3	29.68	11	F11:1382	OB5948 H3B Ro.raw	1.08E6	10	10	154	170			PEAKS DB
K.TVPWNSVSGAVVQVTVIYDSSTK.T	Y	111.07	2436.2537	23	0.2	1219.1370	2	37.45	11	F11:1834	OB5948 H3B Ro.raw	1.092E5	3	3	171	193			PEAKS DB
K.FGFSASGSLGGR.Q	N	103.60	1141.5515	12	-1.3	571.7837	2	30.73	10	F10:1279	OB5926 H3B Ro.raw	4.8005E5	3	3	225	236			PEAKS DB
R.VLYAMPVR.I	N	103.56	947.5262	8	-1.9	474.7705	2	29.80	11	F11:1400	OB5948 H3B Ro.raw	5.0266E5	3	3	67	74			PEAKS DB
K.GAGHFVGVEFDTYSNSEYND.P	N	102.79	2206.9080	20	0.5	1104.4641	2	32.88	11	F11:1577	OB5948 H3B Ro.raw	3.4015E5	3	3	134	153			PEAKS DB
R.VKFGFSASGSLGGR.Q	N	87.36	1368.7150	14	-1.8	685.3650	2	30.41	11	F11:1429	OB5948 H3B Ro.raw	2.1689E4	3	3	223	236			PEAKS DB

**total 24 peptides**

Peptide	Uniq	-10lgP	Mass	Length	ppm	m/z	z	RT	Fraction	Scan	Source File	Area Roast Ara h 3	#Feature	#Feature Roast Ara h 3	Start	End	PTM	AScore	Found By
K.GAGHFVGVEFDTYSNSEYNDPPTDHVGIDVNSVDSVK.T	N	79.48	3966.7820	37	1.1	992.7060	4	33.64	11	F11:1609	OB5948 H3B Ro.raw	4.277E6	6	6	134	170			PEAKS DB
K.F(+57.02)GFSASGSLGGR.Q	N	66.26	1198.5730	12	4.1	600.2977	2	30.99	10	F10:1312	OB5926 H3B Ro.raw	0	0	0	225	236	Carbamidomethylation (DHKE, X@N-term)	F1:Carbamidomethylation (DHKE, X@N-term):1000.00	PEAKS PTM
R.SWSFTSTLITTTRR.S	N	62.89	1655.8632	14	-1.8	828.9391	2	33.07	11	F11:1575	OB5948 H3B Ro.raw	1.4683E6	5	5	243	256			PEAKS DB
R.IWSSATGNVASFLTSFSFEM(+15.99)KDIKDYDPADGIIFFIAPEDTQIPAGSIGGGTLGVSDTK.G	N	52.25	6200.0137	59	1.2	1551.0165	4	41.20	10	F10:1895	OB5926 H3B Ro.raw	7.8846E5	1	1	75	133	Oxidation (M)	M20:Oxidation (M):1000.00	PEAKS DB
R.VLYAM(+15.99)PVR.I	N	51.60	963.5212	8	-1.9	482.7680	2	27.02	11	F11:1243	OB5948 H3B Ro.raw	1.3575E4	3	3	67	74	Oxidation (M)	M5:Oxidation (M):1000.00	PEAKS DB
K.FGFSASGSLGGRQIHLIR.S	N	51.02	1902.0223	18	-1.6	635.0153	3	35.47	10	F10:1558	OB5926 H3B Ro.raw	1.7216E5	2	2	225	242			PEAKS DB
S.AETVSFNFNSFSEGNPAINFQGDVTVLSNGNIQLTNLNKVNSVGR.V	N	49.99	4812.3740	45	4.5	1605.1392	3	40.87	12	F12:2044	OB5949 H3B Ro.raw	5.6245E6	3	3	22	66			PEAKS DB
R.SW(+31.99)SFTSTLITTTR.R	N	46.85	1531.7518	13	-1.2	766.8839	2	33.45	11	F11:1617	OB5948 H3B Ro.raw	3.3337E3	1	1	243	255		W2:Dihydroxy:17.01	PEAKS PTM
D.PPTDHVGIDVN(+.98)SVDSVK.T	N	45.57	1778.8687	17	2.6	593.9665	3	29.71	10	F10:1235	OB5926 H3B Ro.raw	0	0	0	154	170	Deamidation (NQ)	N11:Deamidation (NQ):1000.00	PEAKS DB
R.IWSSATGNVASFLTSFSFEMKDIKDYDPADGIIFFIAPEDTQIPAGSIGGGTLGVSDTK.G	N	40.18	6184.0190	59	0.9	1547.0173	4	42.35	10	F10:1960	OB5926 H3B Ro.raw	8.2055E5	1	1	75	133			PEAKS DB
S.AETVSFNFNSFSEGNPAINFQGDVTVLSNGNIQLTNLN(+.98)KVNSVGR.V	N	33.54	4813.3584	45	8.6	1204.3572	4	38.44	12	F12:1904	OB5949 H3B Ro.raw	0	0	0	22	66		N38:Deamidation (NQ):0.00	PEAKS DB
D.TQIPAGSIGGGTLGVSDTK.G	N	31.05	1757.9159	19	0.6	879.9658	2	30.69	12	F12:1451	OB5949 H3B Ro.raw	0	0	0	115	133			PEAKS DB
K.GAGHFVGVEFDTYSNSEYN(+.98)D.P	N	29.27	2207.8918	20	8.6	1104.9626	2	34.00	12	F12:1651	OB5949 H3B Ro.raw	2.0669E4	1	1	134	153		N19:Deamidation (NQ):0.00	PEAKS DB
**total 24 peptides**																			

A0A089ZXL7|A0A089ZXL7_ARAHY

back to list

| Protein Coverage | Supporting Peptides |

Protein Coverage:

73

1 MKPFCVFLTF FLLLAASSKK VNSAETVSFN FNSFSEGNPA INFQGDVTVL SNGNIQLTNL NKVNSVGRVL YAMPVRIWSS

81 ATGNVASFLT SFSFEMKDIK DYDPADGIIF FIAPEDTQIP AGSIGGGTLG VSDTKGAGYF VGVEFDTYSN SEYNDPPTDH

161 VGIDVNSVDS VKTVPWNSVS GAVVKVTVIY DSSTKTLSVA VTNDNGDITT IAQVVDLKAK LPERVKFGFS ASGSRAGRQI

241 HLIRSWSFTS TLITTTRRSI DNNEKKIMNM ASA

d Deamidation (NQ) (+0.98)
o Oxidation (M) (+15.99)

Supporting Peptides:

Peptide	Uniq	-10lgP	Mass	Length	ppm	m/z	z	RT	Fraction	Scan	Source File	Area Roast Ara h 3	#Feature	#Feature Roast Ara h 3	Start	End	PTM	AScore	Found By
R.SWSFTSTLITTTR.R	N	200.00	1499.7620	13	-1.1	750.8890	2	34.40	11	F11:1663	OB5948 H3B Ro.raw	1.0426E6	6	6	245	257			PEAKS DB
R.IWSSATGNVASFLTSFSFEMK.D	N	200.00	2309.1038	21	-0.7	1155.5608	2	39.93	11	F11:1986	OB5948 H3B Ro.raw	5.3682E5	3	3	77	97			PEAKS DB
K.TLSVAVTNDNGDITTIAQVVDLK.A	N	126.86	2386.2590	23	0.6	1194.1405	2	36.05	10	F10:1608	OB5926 H3B Ro.raw	7.7468E5	3	3	196	218			PEAKS DB
K.DIKDYDPADGIIFFIAPEDTQIPAGSIGGGTLGVSDTK.G	N	120.14	3892.9258	38	0.9	1298.6531	3	38.97	11	F11:1923	OB5948 H3B Ro.raw	1.1326E6	3	3	98	135			PEAKS DB
R.IWSSATGNVASFLTSFSFEM(+15.99)K.D	N	119.39	2325.0986	21	-0.6	1163.5583	2	38.94	11	F11:1921	OB5948 H3B Ro.raw	4.3252E4	1	1	77	97	Oxidation (M)	M20:Oxidation (M): 1000.00	PEAKS DB
D.PPTDHVGIDVNSVDSVK.T	N	115.65	1777.8846	17	-1.1	593.6361	3	29.68	11	F11:1382	OB5948 H3B Ro.raw	1.08E6	10	10	156	172			PEAKS DB
K.TVPWNSVSGAVVK.V	N	110.22	1342.7245	13	-0.7	672.3705	2	30.95	11	F11:1462	OB5948 H3B Ro.raw	1.4337E5	3	3	173	185			PEAKS DB
R.VLYAMPVR.I	N	103.56	947.5262	8	-1.9	474.7705	2	29.80	11	F11:1400	OB5948 H3B Ro.raw	5.0266E5	3	3	69	76			PEAKS DB
K.TLSVAVTNDNGDITTIAQVVDLKAK.L	N	67.79	2585.3911	25	-1.3	862.8051	3	34.40	11	F11:1665	OB5948 H3B Ro.raw	1.5947E4	1	1	196	220			PEAKS DB
K.GAGYFVGVEFDTYSNSEYNDPPTDHVGIDVNSVDSVK.T	Y	64.56	3992.7864	37	-1.8	1331.9365	3	35.53	11	F11:1727	OB5948 H3B Ro.raw	6.5603E4	1	1	136	172			PEAKS DB
R.SWSFTSTLITTTRR.S	N	62.89	1655.8632	14	-1.8	828.9391	2	33.07	11	F11:1575	OB5948 H3B Ro.raw	1.4683E6	5	5	245	258			PEAKS DB
K.FGFSASGSR.A	Y	58.08	914.4246	9	1.0	458.2212	2	27.95	10	F10:1148	OB5926 H3B Ro.raw	0	0	0	227	235			PEAKS DB
R.IWSSATGNVASFLTSFSFEM(+15.99)KDIKDYDPADGIIFFIAPEDTQIPAGSIGGGTLGVSDTK.G	N	52.25	6200.0137	59	1.2	1551.0165	4	41.20	10	F10:1895	OB5926 H3B Ro.raw	7.8846E5	1	1	77	135	Oxidation (M)	M20:Oxidation (M): 1000.00	PEAKS DB
R.VLYAM(+15.99)PVR.I	N	51.60	963.5212	8	-1.9	482.7680	2	27.02	11	F11:1243	OB5948 H3B Ro.raw	1.3575E4	3	3	69	76	Oxidation (M)	M5:Oxidation (M): 1000.00	PEAKS DB

total 22 peptides

Peptide	Uniq	-10lgP	Mass	Length	ppm	m/z	z	RT	Fraction	Scan	Source File	Area Roast Ara h 3	#Feature	#Feature Roast Ara h 3	Start	End	PTM	AScore	Found By
K.TLSVAVTNDN(+.98)GDITTIAQVVDLK.A	N	51.13	2387.2432	23	-0.7	1194.6310	2	36.59	10	F10:1638	OB5926 H3B Ro.raw	0	0	0	196	218		N10:Deamidation (NQ):8.22	PEAKS DB
S.AETVSFNFNSFSEGNPAINFQGDVTVLSNGNIQLTNLNKVNSVGR.V	N	49.99	4812.3740	45	4.5	1605.1392	3	40.87	12	F12:2044	OB5949 H3B Ro.raw	5.6245E6	3	3	24	68			PEAKS DB
R.SW(+31.99)SFTSTLITTTR.R	N	46.85	1531.7518	13	-1.2	766.8839	2	33.45	11	F11:1617	OB5948 H3B Ro.raw	3.3337E3	1	1	245	257		W2:Dihydroxy:17.01	PEAKS PTM
D.PPTDHVGIDVN(+.98)SVDSVK.T	N	45.57	1778.8687	17	2.6	593.9665	3	29.71	10	F10:1235	OB5926 H3B Ro.raw	0	0	0	156	172	Deamidation (NQ)	N11:Deamidation (NQ):1000.00	PEAKS DB
K.GAGYFVGVEFDTYSN(+.98)SEYNDPPTDHVGIDVNSVDSVK.T	Y	40.96	3993.7703	37	7.4	1332.2772	3	35.85	10	F10:1577	OB5926 H3B Ro.raw	2.0916E5	1	1	136	172		N15:Deamidation (NQ):25.70	PEAKS DB
R.IWSSATGNVASFLTSFSFEMKDIKDYDPADGIIFFIAPEDTQIPAGSIGGGTLGVSDTK.G	N	40.18	6184.0190	59	0.9	1547.0173	4	42.35	10	F10:1960	OB5926 H3B Ro.raw	8.2055E5	1	1	77	135			PEAKS DB
S.AETVSFNFNSFSEGNPAINFQGDVTVLSNGNIQLTNLN(+.98)KVNSVGR.V	N	33.54	4813.3584	45	8.6	1204.3572	4	38.44	12	F12:1904	OB5949 H3B Ro.raw	0	0	0	24	68		N38:Deamidation (NQ):0.00	PEAKS DB
D.TQIPAGSIGGGTLGVSDTK.G	N	31.05	1757.9159	19	0.6	879.9658	2	30.69	12	F12:1451	OB5949 H3B Ro.raw	0	0	0	117	135			PEAKS DB
**total 22 peptides**																			

Q5I6T2|Q5I6T2_ARAHY

back to list

| Protein Coverage | Supporting Peptides |

Protein Coverage:

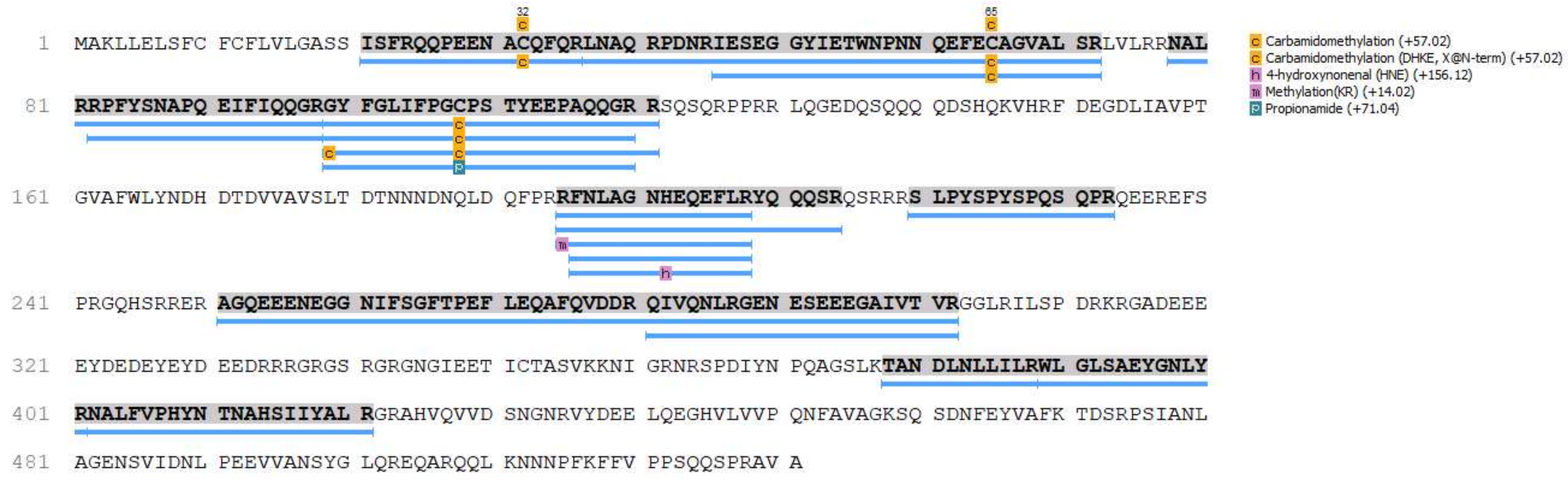

Supporting Peptides:

Peptide	Uniq	-10lgP	Mass	Length	ppm	m/z	z	RT	Fraction	Scan	Source File	Area Roast Ara h 3	#Feature	#Feature Roast Ara h 3	Start	End	PTM	AScore	Found By
R.GYFGLIFPGC(+57.02)PSTYEEPAQQGR.R	N	200.00	2473.1372	22	3.0	1237.5796	2	36.11	7	F7:1750	OB5951 H3A Ro.raw	1.587E6	6	6	99	120	Carbamidomethylation	C10:Carbamidomethylation:1000.00	PEAKS DB
R.WLGLSAEYGNLYR.N	N	163.36	1540.7673	13	-0.8	771.3920	2	34.97	11	F11:1697	OB5948 H3B Ro.raw	6.3564E5	6	6	389	401			PEAKS DB
R.RPFYSNAPQEIFIQQGR.G	N	137.88	2050.0383	17	3.2	684.3556	3	31.52	7	F7:1483	OB5951 H3A Ro.raw	5.9353E6	9	9	82	98			PEAKS DB
R.NALRRPFYSNAPQEIFIQQGR.G	N	125.40	2504.3037	21	1.0	835.7760	3	32.71	9	F9:1535	OB5953 H3A Ro.raw	6.1298E6	10	10	78	98			PEAKS DB
R.FNLAGNHEQEFLR.Y	N	104.44	1573.7637	13	4.3	787.8925	2	30.57	7	F7:1440	OB5951 H3A Ro.raw	1.243E5	7	7	196	208			PEAKS DB
R.RFNLAGNHEQEFLR.Y	N	99.39	1729.8647	14	1.5	577.6297	3	31.36	9	F9:1478	OB5953 H3A Ro.raw	1.8255E6	6	6	195	208			PEAKS DB
R.GYFGLIFPGC(+57.02)PSTYEEPAQQGRR.S	N	99.33	2629.2383	23	2.4	877.4221	3	35.18	9	F9:1676	OB5953 H3A Ro.raw	4.5018E6	7	7	99	121	Carbamidomethylation	C10:Carbamidomethylation:1000.00	PEAKS DB
K.TANDLNLLILR.W	N	75.51	1254.7296	11	1.1	628.3727	2	34.39	7	F7:1659	OB5951 H3A Ro.raw	1.8375E5	4	4	378	388			PEAKS DB
R.GYFGLIFPGC(+71.04)PSTYEEPAQQGR.R	N	72.57	2487.1528	22	2.6	1244.5869	2	36.34	9	F9:1767	OB5953 H3A Ro.raw	2.3282E4	2	2	99	120	Propionamide	C10:Propionamide:1000.00	PEAKS PTM
R.IESEGGYIETWNPNNQEFEC(+57.02)AGVALSR.L	N	66.94	3069.3774	27	6.2	1535.7054	2	34.89	8	F8:1684	OB5952 H3A Ro.raw	0	0	0	46	72	Carbamidomethylation	C20:Carbamidomethylation:1000.00	PEAKS DB
R.RFNLAGNHEQEFLRYQQQSR.Q	N	66.29	2520.2371	20	-2.2	631.0667	4	34.12	10	F10:1496	OB5926 H3B Ro.raw	7.5688E4	2	2	195	214			PEAKS DB
R.NALFVPHYNTNAHSIIYALR.G	N	63.14	2313.2019	20	0.2	772.0747	3	35.15	7	F7:1685	OB5951 H3A Ro.raw	9.0952E5	5	5	402	421			PEAKS DB
S.ISFRQQPEENAC(+57.02)QFQR.L	N	45.61	2036.9486	16	-1.4	679.9907	3	28.84	11	F11:1329	OB5948 H3B Ro.raw	4.7949E4	5	5	21	36	Carbamidomethylation	C12:Carbamidomethylation:1000.00	PEAKS DB
R.RFNLAGNH(+14.02)EQEFLR.Y	N	41.89	1743.8805	14	0.5	582.3011	3	31.14	7	F7:1453	OB5951 H3A Ro.raw	4.477E5	2	2	195	208		H8:Methylation (others):40.00	PEAKS PTM
R.QIVQNLRGENESEEEGAIVTVR.G	Y	38.40	2469.2458	22	7.0	824.0950	3	30.09	7	F7:1410	OB5951 H3A Ro.raw	0	0	0	281	302			PEAKS DB
R.G(+57.02)YFGLIFPGC(+57.02)PSTYEEPAQQGRR.S	N	35.90	2686.2598	23	1.6	896.4286	3	35.57	8	F8:1718	OB5952 H3A Ro.raw	5.4933E4	1	1	99	121	Carbamidomethylation (DHKE, X@N-term); Carbamidomethylation	G1:Carbamidomethylation (DHKE, X@N-term):130.55;C10:Carbamidomethylation:1000.00	PEAKS PTM
R.LNAQRPDNRIESEGGYIETWNPNNQEFEC(+57.02)AGVALSR.L	N	35.63	4133.9248	36	4.2	1034.4928	4	36.08	8	F8:1752	OB5952 H3A Ro.raw	1.6394E5	1	1	37	72	Carbamidomethylation	C29:Carbamidomethylation:1000.00	PEAKS DB
R.RPFYSNAP(+15.99)QEIFIQQGR.G	N	34.29	2066.0332	17	0.2	689.6852	3	31.94	9	F9:1497	OB5953 H3A Ro.raw	4.3282E4	1	1	82	98		P8:Oxidation or Hydroxylation:32.28	PEAKS PTM
R.SLPYSPYSP(+31.99)QSQPR.Q	N	33.78	1637.7686	14	3.2	819.8942	2	27.07	7	F7:1250	OB5951 H3A Ro.raw	6.6096E3	1	1	220	233		P9:Dihydroxy:9.42	PEAKS PTM
R.R(+14.02)FNLAGNHEQEFLR.Y	N	32.14	1743.8805	14	0.9	582.3013	3	31.38	8	F8:1472	OB5952 H3A Ro.raw	2.9356E5	2	2	195	208	Methylation(KR)	R1:Methylation (KR):67.58	PEAKS PTM

total 25 peptides

Peptide	Uniq	-10lgP	Mass	Length	ppm	m/z	z	RT	Fraction	Scan	Source File	Area Roast Ara h 3	#Feature	#Feature Roast Ara h 3	Start	End	PTM	AScore	Found By
R.RFNLAGNHEQ(+.98)EFLRYQQQSR.Q	N	31.27	2521.2209	20	5.0	631.3173	4	34.33	10	F10:1528	OB5926 H3B Ro.raw	4.7768E4	1	1	195	214		Q10:Deamidation (NQ):0.00	PEAKS DB
R.FNLAGNH(+14.02)EQEFLR.Y	N	31.24	1587.7793	13	0.7	530.2674	3	30.81	8	F8:1450	OB5952 H3A Ro.raw	6.1216E4	1	1	196	208		H7:Methylation (others):14.02	PEAKS PTM
R.FNLAGNH(+156.12)EQEFLR.Y	N	31.09	1729.8788	13	-7.6	577.6292	3	31.13	7	F7:1444	OB5951 H3A Ro.raw	2.4781E5	1	1	196	208	4-hydroxynonenal (HNE)	H7:4-hydroxynonenal (HNE):1000.00	PEAKS PTM
R.AGQEEENEGGN(+.98)IFSGFTPEFLEQAFQVDDRQIVQNLRGENESEEEGAIVTVR.G	Y	30.95	5811.7046	52	7.3	1453.9441	4	39.59	9	F9:1932	OB5953 H3A Ro.raw	4.3973E5	1	1	251	302		N11:Deamidation (NQ):0.00	PEAKS DB
R.RFN(+.98)LAGNHEQEFLR.Y	N	30.90	1730.8488	14	9.2	577.9634	3	32.85	11	F11:1572	OB5948 H3B Ro.raw	0	0	0	195	208		N3:Deamidation (NQ):17.65	PEAKS DB
**total 25 peptides**																			

A1E2B0|A1E2B0_ARAHY

back to list

| Protein Coverage | Supporting Peptides |

Protein Coverage:

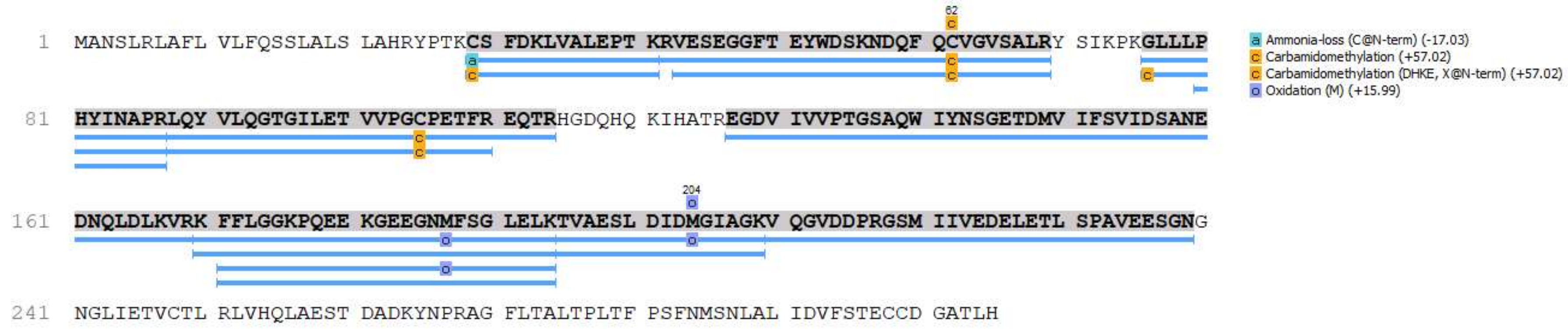

Supporting Peptides:

Peptide	Uniq	-10lgP	Mass	Length	ppm	m/z	z	RT	Fraction	Scan	Source File	Area Roast Ara h 3	#Feature	#Feature Roast Ara h 3	Start	End	PTM	AScore	Found By
K.TVAESLDIDMGIAGK.V	Y	135.15	1518.7599	15	-1.6	760.3879	2	33.39	10	F10:1446	OB5926 H3B Ro.raw	8.1424E4	2	2	195	209			PEAKS DB
K.GLLLPHYINAPR.L	Y	133.17	1362.7771	12	-2.0	682.3959	2	32.12	11	F11:1521	OB5948 H3B Ro.raw	2.2835E6	7	7	76	87			PEAKS DB
R.LQYVLQGTGILETVVPGC(+57.02)PETFR.E	Y	122.40	2576.3308	23	1.3	1289.1771	2	36.71	11	F11:1795	OB5948 H3B Ro.raw	6.8807E4	2	2	88	110	Carbamidomethylation	C18:Carbamidomethylation:1000.00	PEAKS DB
R.KFFLGGKPQEEKGEEGNMFSGLELK.T	N	102.43	2798.3948	25	-0.4	933.8075	3	36.62	10	F10:1634	OB5926 H3B Ro.raw	2.112E5	1	1	170	194			PEAKS DB
R.LQYVLQGTGILETVVPGC(+57.02)PETFREQTR.H	Y	100.71	3090.5808	27	2.4	1031.2056	3	35.54	11	F11:1773	OB5948 H3B Ro.raw	8.3413E6	6	6	88	114	Carbamidomethylation	C18:Carbamidomethylation:1000.00	PEAKS DB
K.TVAESLDIDM(+15.99)GIAGK.V	Y	98.92	1534.7549	15	-1.2	768.3854	2	30.91	11	F11:1459	OB5948 H3B Ro.raw	5.6446E3	1	1	195	209	Oxidation (M)	M10:Oxidation (M):1000.00	PEAKS DB
**total 20 peptides**																			

Peptide	Uniq	-10lgP	Mass	Length	ppm	m/z	z	RT	Fraction	Scan	Source File	Area Roast Ara h 3	#Feature	#Feature Roast Ara h 3	Start	End	PTM	AScore	Found By
R.VESEGGFTEYWDSKNDQFQC(+57.02)VGVSALR.Y	Y	97.89	3107.3931	27	2.8	1036.8079	3	34.76	12	F12:1692	OB5949 H3B Ro.raw	9.1132E5	3	3	43	69	Carbamidomethylation	C20:Carbamidomethylation:1000.00	PEAKS DB
K.FFLGGKPQEEKGEEGNMFSGLELK.T	N	92.04	2670.2998	24	-0.7	891.1088	3	36.43	10	F10:1612	OB5926 H3B Ro.raw	1.0891E6	5	5	171	194			PEAKS DB
K.G(+57.02)LLLPHYINAPR.L	Y	87.45	1419.7986	12	-1.2	710.9072	2	32.70	11	F11:1554	OB5948 H3B Ro.raw	1.9861E5	3	3	76	87	Carbamidomethylation (DHKE, X@N-term)	G1:Carbamidomethylation (DHKE, X@N-term):71.64	PEAKS PTM
R.EGDVIVVPTGSAQWIYNSGETDMVIFSVIDSANEDNQLDLKVR.K	Y	78.53	4723.2964	43	0.8	1575.4441	3	40.31	11	F11:1999	OB5948 H3B Ro.raw	4.2652E5	2	2	127	169			PEAKS DB
K.RVESEGGFTEYWDSKNDQFQC(+57.02)VGVSALR.Y	Y	77.67	3263.4941	28	-0.3	1088.8411	3	34.90	10	F10:1540	OB5926 H3B Ro.raw	7.5628E4	1	1	42	69	Carbamidomethylation	C21:Carbamidomethylation:1000.00	PEAKS DB
K.FFLGGKPQEEKGEEGNM(+15.99)FSGLELK.T	N	67.27	2686.2947	24	-0.9	896.4402	3	34.90	10	F10:1542	OB5926 H3B Ro.raw	3.9762E5	3	3	171	194	Oxidation (M)	M17:Oxidation (M):1000.00	PEAKS DB
K.VQGVDDPRGSMIIVEDELETLSPAVEESGN.G	Y	62.82	3184.5081	30	4.2	1593.2681	2	38.96	12	F12:1934	OB5949 H3B Ro.raw	0	0	0	210	239			PEAKS DB
K.C(+57.02)SFDKLVALEPTK.R	Y	51.24	1506.7751	13	-0.8	754.3959	2	31.21	11	F11:1476	OB5948 H3B Ro.raw	0	0	0	29	41	Carbamidomethylation	C1:Carbamidomethylation:1000.00	PEAKS DB
L.PHYINAPR.L	Y	39.30	966.5035	8	-2.1	484.2592	2	33.21	10	F10:1450	OB5926 H3B Ro.raw	4.9369E4	3	3	80	87			PEAKS DB
R.KFFLGGKPQEEKGEEGNM(+15.99)FSGLELK.T	N	38.49	2814.3896	25	1.5	939.1409	3	35.47	10	F10:1579	OB5926 H3B Ro.raw	8.019E4	1	1	170	194	Oxidation (M)	M18:Oxidation (M):1000.00	PEAKS DB
R.EGDVIVVPTGSAQ(+.98)WIYNSGETDMVIFSVIDSANEDNQLDLKVR.K	Y	36.81	4724.2803	43	5.1	1575.7793	3	40.63	10	F10:1862	OB5926 H3B Ro.raw	2.2969E5	1	1	127	169		Q13:Deamidation (NQ):34.15	PEAKS DB
R.LQYVLQGTGILETVVPGC(+57.02)PETFREQ(+.98)TR.H	Y	35.03	3091.5647	27	1.2	1031.5322	3	43.45	11	F11:2184	OB5948 H3B Ro.raw	0	0	0	88	114	Carbamidomethylation	C18:Carbamidomethylation:1000.00;Q25:Deamidation (NQ):42.82	PEAKS DB
K.C(-17.03)SFD(+57.02)KLVALEPTK.R	Y	30.70	1489.7487	13	0.1	745.8835	2	34.51	10	F10:1523	OB5926 H3B Ro.raw	1.2812E4	1	1	29	41	Ammonia-loss (C@N-term)	C1:Ammonia-loss (C@N-term):1000.00;D4:Carbamidomethylation (DHKE, X@N-term):17.01	PEAKS PTM
R.LQYVLQGTGILETVVPGC(+57.02)PETFR(+14.02)EQTR.H	Y	29.28	3104.5964	27	0.7	1035.8757	3	35.54	11	F11:1791	OB5948 H3B Ro.raw	3.2709E5	1	1	88	114	Carbamidomethylation	C18:Carbamidomethylation:1000.00;R23:Methylation(KR):11.01	PEAKS PTM
**total 20 peptides**																			

E9LFE8|E9LFE8_ARAHY

back to list

| Protein Coverage | Supporting Peptides |

Protein Coverage:

1 KSR**KFFLGGK PQEEKGEEGN MFSGLELKTV AESLGIDMGI AGKVQGVDDP RGSIIIVEDE LETLSPAVEE SGN**GNGLDET

81 LCTLRLVHQL AESTDADKYN PR**AGFLTALN TPNLPVLQYV QLGADR**GVFY KNAVMAPHYN LNCHAVIYGT EGRGWIEVVG

161 ENGRKVYEGE VREGQILIVP QQFMVAKK**AA EGSDEGFGWI AVK**TSDNPMI SPLAGKLSLI R**AMPLPVLMN SFR**LTAEEAI

241 NLKKRGELTF FSPDPAHTQI

d Deamidation (NQ) (+0.98)
d Dimethylation(KR) (+28.03)
o Oxidation (M) (+15.99)

Supporting Peptides:

Peptide	Uniq	-10lgP	Mass	Length	ppm	m/z	z	RT	Fraction	Scan	Source File	Area Roast Ara h 3	#Feature	#Feature Roast Ara h 3	Start	End	PTM	AScore	Found By
K.TVAESLGIDMGIAGK.V	Y	164.96	1460.7544	15	-1.4	731.3850	2	33.26	11	F11:1598	OB5948 H3B Ro.raw	1.9292E5	4	4	29	43			PEAKS DB
K.AAEGSDEGFGWIAVK.T	Y	126.69	1535.7256	15	0.1	768.8702	2	33.25	7	F7:1594	OB5951 H3A Ro.raw	2.9372E4	3	3	189	203			PEAKS DB
K.VQGVDDPRGSIIIVEDELETLSPAVEESGN.G	Y	105.29	3166.5518	30	2.1	1584.2899	2	39.16	11	F11:1927	OB5948 H3B Ro.raw	1.8789E6	3	3	44	73			PEAKS DB
R.KFFLGGKPQEEKGEEGNMFSGLELK.T	N	102.43	2798.3948	25	-0.4	933.8075	3	36.62	10	F10:1634	OB5926 H3B Ro.raw	2.112E5	1	1	4	28			PEAKS DB
K.TVAESLGIDM(+15.99)GIAGK.V	Y	99.82	1476.7494	15	-2.7	739.3818	2	30.86	10	F10:1300	OB5926 H3B Ro.raw	3.516E3	1	1	29	43	Oxidation (M)	M10:Oxidation (M):1000.00	PEAKS DB
K.FFLGGKPQEEKGEEGNMFSGLELK.T	N	92.04	2670.2998	24	-0.7	891.1088	3	36.43	10	F10:1612	OB5926 H3B Ro.raw	1.0891E6	5	5	5	28			PEAKS DB
R.AGFLTALNTPNLPVLQYVQLGADR(+28.03).G	Y	90.94	2598.4170	24	4.0	1300.2209	2	39.97	9	F9:1967	OB5953 H3A Ro.raw	5.2434E5	5	5	103	126	Dimethylation(KR)	R24:Dimethylation(KR):1000.00	PEAKS PTM
K.FFLGGKPQEEKGEEGNM(+15.99)FSGLELK.T	N	67.27	2686.2947	24	-0.9	896.4402	3	34.90	10	F10:1542	OB5926 H3B Ro.raw	3.9762E5	3	3	5	28	Oxidation (M)	M17:Oxidation (M):1000.00	PEAKS DB
K.VQGVDDPRGSIIIVEDELETLSPAVEESGN(+.98).G	Y	58.55	3167.5356	30	9.3	1056.8623	3	39.51	12	F12:1974	OB5949 H3B Ro.raw	3.4995E4	1	1	44	73	Deamidation (NQ)	N30:Deamidation (NQ):53.58	PEAKS DB
R.AMPLPVLM(+15.99)NSFR.L	Y	42.81	1390.7101	12	1.6	696.3635	2	34.42	9	F9:1639	OB5953 H3A Ro.raw	1.9478E4	1	1	222	233		M8:Oxidation (M):25.69	PEAKS DB
R.KFFLGGKPQEEKGEEGNM(+15.99)FSGLELK.T	N	38.49	2814.3896	25	1.5	939.1409	3	35.47	10	F10:1579	OB5926 H3B Ro.raw	8.019E4	1	1	4	28	Oxidation (M)	M18:Oxidation (M):1000.00	PEAKS DB
K.TVAESLGIDM(+15.99)GIAGKVQ(+.98)GVDDPRGSIIIVEDELETLSPAVEESGN.G	Y	34.42	4626.2744	45	7.8	1543.1107	3	39.51	12	F12:1964	OB5949 H3B Ro.raw	3.4721E4	1	1	29	73	Oxidation (M); Deamidation (NQ)	M10:Oxidation (M):1000.00;Q17:Deamidation (NQ):73.94	PEAKS DB
K.FFLGGKPQEEKGEEGNMFSGLELKTVAESLGIDMGIAGK.V	Y	30.89	4113.0439	39	-1.9	1029.2689	4	40.43	10	F10:1855	OB5926 H3B Ro.raw	1.8985E5	1	1	5	43			PEAKS DB
**total 13 peptides**																			

Q6IWG5|Q6IWG5_ARAHY

back to list

| Protein Coverage | Supporting Peptides |

Protein Coverage:

63

1 KLLALSLCFC VLVLGASSVT FRQGGEENEC QFQR**LNAQRP DNRIESEGGY IETWNPNNQE FQCAGVALSR** TVLRR**NALRR**

81 **PFYSNAPLEI YVQQGSGYFG LIFPGCPSTY EEPAQEGRR**Y QSQKPSRRFQ VGQDDPSQQQ QDSHQKVHRF DEGDLIAVPT

161 GVAFWMYNDE DTDVVTVTLS DTSSIHNQLD QFPR**RFYLAG NQEQEFLR**YQ QQQGSRPHYR QISPR**VRGDE QENEGSNIFS**

241 **GFAQEFLQHA FQVDR**QTVEN LRGENEREEQ GAIVTVKGGL RILSPDEEDE SSRSPPSRRE EFDEDRSRPQ QRGKYDENRR

321 GYKNGIEETI CSASVKKNLG RSSNPDIYNP QAGSLR**SVNE LDLPILGWLG LSAQHGTIYR** NAMFVPHYTL NAHTIVVALN

401 GRAHVQVVDS NGNRVYDEEL QEGHVLVVPQ NFAVAAKAQS ENYEYLAFK**T DSRPSIANLA GENSIIDNLP EEVVANSYRL**

481 **PR**EQARQLKN NNPFKFFVPP FDHQSMREVA

C Carbamidomethylation (+57.02)

Supporting Peptides:

Peptide	Uniq	-10lgP	Mass	Length	ppm	m/z	z	RT	Fraction	Scan	Source File	Area Roast Ara h 3	#Feature	#Feature Roast Ara h 3	Start	End	PTM	AScore	Found By
R.FYLAGNQEQEFLR.Y	Y	135.23	1613.7837	13	0.1	807.8992	2	32.67	7	F7:1561	OB5951 H3A Ro.raw	5.1792E4	4	4	196	208			PEAKS DB
K.TDSRPSIANLAGENSIIDNLPEEVVANSYR.L	Y	112.03	3243.6006	30	0.9	1082.2107	3	36.51	11	F11:1779	OB5948 H3B Ro.raw	3.6943E5	4	4	450	479			PEAKS DB
R.SVNELDLPILGWLGLSAQHGTIYR.N	Y	105.06	2651.4070	24	0.2	884.8116	3	40.31	11	F11:1994	OB5948 H3B Ro.raw	2.2662E6	12	12	357	380			PEAKS DB
R.LNAQRPDNRIESEGGYIETWNPNNQEFQC(+57.02)AGVALSR.T	Y	102.77	4132.9409	36	-0.3	1034.2444	4	33.64	11	F11:1603	OB5948 H3B Ro.raw	1.9614E7	8	8	35	70	Carbamidomethylation	C29:Carbamidomethylation:1000.00	PEAKS DB
R.RFYLAGNQEQEFLR.Y	Y	74.35	1769.8849	14	-1.3	590.9681	3	31.33	7	F7:1486	OB5951 H3A Ro.raw	6.7764E4	3	3	195	208			PEAKS DB
R.VRGDEQENEGSNIFSGFAQEFLQHAFQVDR.Q	Y	69.72	3453.5974	30	4.1	864.4102	4	39.78	9	F9:1952	OB5953 H3A Ro.raw	3.9334E5	2	2	226	255			PEAKS DB
R.RPFYSNAPLEIYVQQGSGYFGLIFPGC(+57.02)PSTYEEPAQEGR.R	Y	67.79	4424.0845	39	5.3	1475.7100	3	38.84	8	F8:1912	OB5952 H3A Ro.raw	1.5644E6	3	3	80	118	Carbamidomethylation	C27:Carbamidomethylation:1000.00	PEAKS DB
R.RPFYSNAPLEIYVQQGSGYFGLIFPGC(+57.02)PSTYEEPAQEGRR.Y	Y	58.89	4580.1860	40	3.1	1146.0574	4	38.30	8	F8:1880	OB5952 H3A Ro.raw	1.9768E5	1	1	80	119	Carbamidomethylation	C27:Carbamidomethylation:1000.00	PEAKS DB
R.NALRRPFYSNAPLEIYVQQGSGYFGLIFPGC(+57.02)PSTYEEPAQEGR.R	Y	47.55	4878.3501	43	2.7	1220.5981	4	38.66	9	F9:1897	OB5953 H3A Ro.raw	5.9202E5	2	2	76	118	Carbamidomethylation	C31:Carbamidomethylation:1000.00	PEAKS DB
K.TDSRPSIANLAGENSIIDNLPEEVVANSYRLPR.E	Y	45.23	3609.8386	33	2.2	903.4689	4	36.50	7	F7:1770	OB5951 H3A Ro.raw	5.4722E5	3	3	450	482			PEAKS DB

total 15 peptides

Peptide	Uniq	-10lgP	Mass	Length	ppm	m/z	z	RT	Fraction	Scan	Source File	Area Roast Ara h 3	#Feature	#Feature Roast Ara h 3	Start	End	PTM	AScore	Found By
R.RPFYSNAPLEIYVQQGSGYFGLIFPGC(+57.02)PSTYEEPAQ(+.98)EGRR.Y	Y	43.08	4581.1699	40	9.6	1146.3108	4	38.25	9	F9:1866	OB5953 H3A Ro.raw	4.8906E5	2	2	80	119	Carbamidomethylation	C27:Carbamidomethylation:1000.00;Q36:Deamidation (NQ):12.58	PEAKS DB
R.LNAQRPDNRIESEGGYIETWN(+.98)PNNQEFQC(+57.02)AGVALSR.T	Y	33.79	4133.9248	36	-0.7	1034.4878	4	43.56	8	F8:2183	OB5952 H3A Ro.raw	0	0	0	35	70	Carbamidomethylation	N21:Deamidation (NQ):7.58;C29:Carbamidomethylation:1000.00	PEAKS DB
R.IESEGGYIETWNPNNQEFQC(+57.02)AGVALSR.T	N	33.38	3068.3933	27	2.9	1535.2084	2	34.64	8	F8:1684	OB5952 H3A Ro.raw	7.7336E3	1	1	44	70	Carbamidomethylation	C20:Carbamidomethylation:1000.00	PEAKS DB
R.NALRRPFYSNAPLEIYVQQGSGYFGLIFPGC(+57.02)PSTYEEPAQEGRR.Y	Y	31.63	5034.4512	44	1.2	1259.6216	4	37.82	7	F7:1841	OB5951 H3A Ro.raw	8.5213E5	1	1	76	119	Carbamidomethylation	C31:Carbamidomethylation:1000.00	PEAKS DB
R.LNAQRPDNRIESEGGYIETWNPNN(+.98)QEFQC(+57.02)AGVALSR.T	Y	30.18	4133.9248	36	3.3	1378.9868	3	34.37	12	F12:1670	OB5949 H3B Ro.raw	2.7728E4	1	1	35	70	Carbamidomethylation	N24:Deamidation (NQ):0.00;C29:Carbamidomethylation:1000.00	PEAKS DB

total 15 peptides

Q0GM57|Q0GM57_ARAHY

back to list

| Protein Coverage | Supporting Peptides |

Protein Coverage:

65 C — C Carbamidomethylation (+57.02)

1 MAKLLALSLC FCVLVLGASS VTFRQGGEEN ECQFQR**LNAQ RPDNRIESEG GYIETWNPNN QEFQCAGVAL SR**TVLRR**NAL**

81 **RRPFYSNAPL EIYVQQGSGY FGLIFPGCPS TYEEPAQEGR R**YQSQKPSRR FQVGQDDPSQ QQQDSHQKVH RFDEGDLIAV

161 PTGVAFWMYN DEDTDVVTVT LSDTSSIHNQ LDQFPR**RFYL AGNQEQEFLR** YQQQQGSRPH YRQISPR**VRG DEQENEGSNI**

241 **FSGFAQEFLQ HAFQVDR**QTV ENLRGENERE EQGAIVTVKG GLRILSPDEE DESSRSPPSR REEFDEDRSR PQQRGKYDEN

321 RRGYKNGIEE TICSASVKKN LGRSSNPDIY NPQAGSLR**SV NELDLPILGW LGLSAQHGTI YR**NAMFVPHY TLNAHTIVVA

401 LNGRAHVQVV DSNGNRVYDE ELQEGHVLVV PQNFAVAAKA QSENYEYLAF K**TDSRPSIAN LAGENSIIDN LPEEVVANSY**

481 **RLPR**EQARQL KNNNPFKFFV PPFDHQSMRE VA

Supporting Peptides:

Peptide	Uniq	-10lgP	Mass	Length	ppm	m/z	z	RT	Fraction	Scan	Source File	Area Roast Ara h 3	#Feature	#Feature Roast Ara h 3	Start	End	PTM	AScore	Found By
R.FYLAGNQEQEFLR.Y	Y	135.23	1613.7837	13	0.1	807.8992	2	32.67	7	F7:1561	OB5951 H3A Ro.raw	5.1792E4	4	4	198	210			PEAKS DB
K.TDSRPSIANLAGENSIIDNLPEEVVANSYR.L	Y	112.03	3243.6006	30	0.9	1082.2107	3	36.51	11	F11:1779	OB5948 H3B Ro.raw	3.6943E5	4	4	452	481			PEAKS DB

total 15 peptides

Peptide	Uniq	-10lgP	Mass	Length	ppm	m/z	z	RT	Fraction	Scan	Source File	Area Roast Ara h 3	#Feature	#Feature Roast Ara h 3	Start	End	PTM	AScore	Found By
R.SVNELDLPILGWLGLSAQHGTIYR.N	Y	105.06	2651.4070	24	0.2	884.8116	3	40.31	11	F11:1994	OB5948 H3B Ro.raw	2.2662E6	12	12	359	382			PEAKS DB
R.LNAQRPDNRIESEGGYIETWNPNNQEFQC(+57.02)AGVALSR.T	Y	102.77	4132.9409	36	-0.3	1034.2444	4	33.64	11	F11:1603	OB5948 H3B Ro.raw	1.9614E7	8	8	37	72	Carbamidomethylation	C29:Carbamidomethylation:1000.00	PEAKS DB
R.RFYLAGNQEQEFLR.Y	Y	74.35	1769.8849	14	-1.3	590.9681	3	31.33	7	F7:1486	OB5951 H3A Ro.raw	6.7764E4	3	3	197	210			PEAKS DB
R.VRGDEQENEGSNIFSGFAQEFLQHAFQVDR.Q	Y	69.72	3453.5974	30	4.1	864.4102	4	39.78	9	F9:1952	OB5953 H3A Ro.raw	3.9334E5	2	2	228	257			PEAKS DB
R.RPFYSNAPLEIYVQQGSGYFGLIFPGC(+57.02)PSTYEEPAQEGR.R	Y	67.79	4424.0845	39	5.3	1475.7100	3	38.84	8	F8:1912	OB5952 H3A Ro.raw	1.5644E6	3	3	82	120	Carbamidomethylation	C27:Carbamidomethylation:1000.00	PEAKS DB
R.RPFYSNAPLEIYVQQGSGYFGLIFPGC(+57.02)PSTYEEPAQEGRR.Y	Y	58.89	4580.1860	40	3.1	1146.0574	4	38.30	8	F8:1880	OB5952 H3A Ro.raw	1.9768E5	1	1	82	121	Carbamidomethylation	C27:Carbamidomethylation:1000.00	PEAKS DB
R.NALRRPFYSNAPLEIYVQQGSGYFGLIFPGC(+57.02)PSTYEEPAQEGR.R	Y	47.55	4878.3501	43	2.7	1220.5981	4	38.66	9	F9:1897	OB5953 H3A Ro.raw	5.9202E5	2	2	78	120	Carbamidomethylation	C31:Carbamidomethylation:1000.00	PEAKS DB
K.TDSRPSIANLAGENSIIDNLPEEVVANSYRLPR.E	Y	45.23	3609.8386	33	2.2	903.4689	4	36.50	7	F7:1770	OB5951 H3A Ro.raw	5.4722E5	3	3	452	484			PEAKS DB
R.RPFYSNAPLEIYVQQGSGYFGLIFPGC(+57.02)PSTYEEPAQ(+.98)EGRR.Y	Y	43.08	4581.1699	40	9.6	1146.3108	4	38.25	9	F9:1866	OB5953 H3A Ro.raw	4.8906E5	2	2	82	121	Carbamidomethylation	C27:Carbamidomethylation:1000.00;Q36:Deamidation (NQ):12.58	PEAKS DB
R.LNAQRPDNRIESEGGYIETWN(+.98)PNNQEFQC(+57.02)AGVALSR.T	Y	33.79	4133.9248	36	-0.7	1034.4878	4	43.56	8	F8:2183	OB5952 H3A Ro.raw	0	0	0	37	72	Carbamidomethylation	N21:Deamidation (NQ):7.58;C29:Carbamidomethylation:1000.00	PEAKS DB
R.IESEGGYIETWNPNNQEFQC(+57.02)AGVALSR.T	N	33.38	3068.3933	27	2.9	1535.2084	2	34.64	8	F8:1684	OB5952 H3A Ro.raw	7.7336E3	1	1	46	72	Carbamidomethylation	C20:Carbamidomethylation:1000.00	PEAKS DB
R.NALRRPFYSNAPLEIYVQQGSGYFGLIFPGC(+57.02)PSTYEEPAQEGRR.Y	Y	31.63	5034.4512	44	1.2	1259.6216	4	37.82	7	F7:1841	OB5951 H3A Ro.raw	8.5213E5	1	1	78	121	Carbamidomethylation	C31:Carbamidomethylation:1000.00	PEAKS DB
R.LNAQRPDNRIESEGGYIETWNPNN(+.98)QEFQC(+57.02)AGVALSR.T	Y	30.18	4133.9248	36	3.3	1378.9868	3	34.37	12	F12:1670	OB5949 H3B Ro.raw	2.7728E4	1	1	37	72	Carbamidomethylation	N24:Deamidation (NQ):0.00;C29:Carbamidomethylation:1000.00	PEAKS DB
**total 15 peptides**																			

E9LFE9|E9LFE9_ARAHY

back to list

| Protein Coverage | Supporting Peptides |

Protein Coverage:

n N-Succinimidyl-2-morpholine acetate (+127.06)

```
  1 MGKPVIKPLV VLLLSLILVS ECAAIHSQKR RMGQRVKLSL EECGELEKLE AIEPDYYRIE AEGGVTESWN HTNKMLRCAG
 81 VALVRRTVKP GGLVLPSYTN APQLMYYVQG SGIQGMTIFP SCPESFEEPE EAGQEYREQH QKVHEIRGGD IIAIPAGIGY
                                                              216
161 WFYNNGDVPL VVVILLHTNN VANQLGTLPR RFYIAGNTED EHGEGGREKS ISGRNVFSGI SLNLLAQVFG VRVETARKIQ
241 GPDDKRKNIV MVGGRS
```

Supporting Peptides:

Peptide	Uniq	-10lgP	Mass	Length	ppm	m/z	z	RT	Fraction	Scan	Source File	Area Roast Ara h 3	#Feature	#Feature Roast Ara h 3	Start	End	PTM	AScore	Found By
R.NVFSGISLNLLAQVFGVR.V	Y	158.60	1933.0785	18	0.8	967.5493	2	42.42	11	F11:2125	OB5948 H3B Ro.raw	1.2301E5	3	3	215	232			PEAKS DB
R.NVFSGISLNLLAQVFGVRVETAR.K	Y	90.51	2489.3755	23	0.2	830.8013	3	41.58	10	F10:1917	OB5926 H3B Ro.raw	1.1995E6	5	5	215	237			PEAKS DB
N.V(+127.06)FSGISLNLLAQVFGVR.V	Y	49.60	1946.0989	17	-9.4	974.0475	2	41.23	12	F12:2066	OB5949 H3B Ro.raw	5.4396E5	2	2	216	232	N-Succinimidyl-2-morpholine acetate	V1:N-Succinimidyl-2-morpholine acetate:1000.00	PEAKS PTM
R.GGDIIAIPAGIGYWFYN(+.98)NGDVPLVVVILLHTNNVANQLGTLPR.R	Y	32.90	4574.4326	43	7.1	1525.8328	3	44.05	10	F10:2070	OB5926 H3B Ro.raw	3.5735E4	1	1	148	190		N17:Deamidation (NQ): 30.46	PEAKS DB
**total 4 peptides**																			

A5Z1R0|A5Z1R0_ARAHY

back to list

| Protein Coverage | Supporting Peptides |

Protein Coverage:

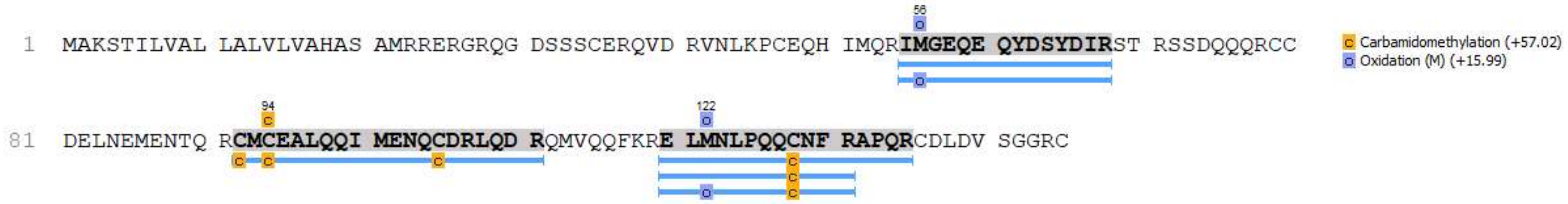

Supporting Peptides:

Peptide	Uniq	-10lgP	Mass	Length	ppm	m/z	z	RT	Fraction	Scan	Source File	Area Roast Ara h 3	#Feature	#Feature Roast Ara h 3	Start	End	PTM	AScore	Found By
R.ELMNLPQQC(+57.02)NFR.A	Y	130.50	1548.7177	12	-0.7	775.3672	2	31.27	11	F11:1474	OB5948 H3B Ro.raw	5.3223E4	6	6	120	131	Carbamidomethylation	C9:Carbamidomethylation:1000.00	PEAKS DB
R.IM(+15.99)GEQEQYDSYDIR.S	Y	103.58	1761.7516	14	-1.0	881.8840	2	28.71	11	F11:1332	OB5948 H3B Ro.raw	1.9449E4	4	4	55	68	Oxidation (M)	M2:Oxidation (M):1000.00	PEAKS DB
R.IMGEQEQYDSYDIR.S	Y	90.26	1745.7566	14	-1.3	873.8866	2	30.26	10	F10:1263	OB5926 H3B Ro.raw	4.6099E4	5	5	55	68			PEAKS DB
R.ELM(+15.99)NLPQQC(+57.02)NFR.A	Y	71.68	1564.7126	12	-2.7	783.3632	2	28.90	11	F11:1336	OB5948 H3B Ro.raw	7.1508E3	3	3	120	131	Oxidation (M); Carbamidomethylation	M3:Oxidation (M):1000.00;C9:Carbamidomethylation:1000.00	PEAKS DB
R.ELMNLPQQC(+57.02)NFRAPQR.C	Y	70.25	2000.9673	16	0.2	667.9965	3	30.17	9	F9:1409	OB5953 H3A Ro.raw	7.0151E3	3	3	120	135	Carbamidomethylation	C9:Carbamidomethylation:1000.00	PEAKS DB
R.C(+57.02)MC(+57.02)EALQQIMENQC(+57.02)DRLQDR.Q	Y	43.12	2597.0913	20	1.2	866.7054	3	33.96	8	F8:1628	OB5952 H3A Ro.raw	1.1224E4	1	1	92	111	Carbamidomethylation	C1:Carbamidomethylation:1000.00;C3:Carbamidomethylation:1000.00;C14:Carbamidomethylation:1000.00	PEAKS DB
R.C(+57.02)MC(+57.02)EALQQ(+.98)IMENQC(+57.02)DRLQDR.Q	Y	37.92	2598.0754	20	-8.3	867.0270	3	33.99	11	F11:1638	OB5948 H3B Ro.raw	0	0	0	92	111	Carbamidomethylation	C1:Carbamidomethylation:1000.00;C3:Carbamidomethylation:1000.00;Q8:Deamidation (NQ):0.00;C14:Carbamidomethylation:1000.00	PEAKS DB
R.IM(+15.99)GEQ(+.98)EQYDSYDIR.S	Y	34.46	1762.7356	14	4.0	882.3805	2	28.56	11	F11:1318	OB5948 H3B Ro.raw	0	0	0	55	68	Oxidation (M)	M2:Oxidation (M):1000.00;Q5:Deamidation (NQ):0.00	PEAKS DB
**total 8 peptides**																			

sp|Q647G9|CONG_ARAHY

back to list

| Protein Coverage | Supporting Peptides |

Protein Coverage:

56 O

1 MAKSTILVAL LALVLVAHAS AMRRERGRQG DSSSCERQVD RVNLKPCEQH IMQR**IMGEQE QYDSYDIR**ST RSSDQQQRCC

C Carbamidomethylation (+57.02)
O Oxidation (M) (+15.99)

94 C, 122 O

81 DELNEMENTQ R**CMCEALQQI MENQCDRLQD R**QMVQQFKR**E LMNLPQQCNF RAPQR**CDLDV SGGRC

Supporting Peptides:

Peptide	Uniq	-10lgP	Mass	Length	ppm	m/z	z	RT	Fraction	Scan	Source File	Area Roast Ara h 3	#Feature	#Feature Roast Ara h 3	Start	End	PTM	AScore	Found By
R.ELMNLPQQC(+57.02)NFR.A	Y	130.50	1548.7177	12	-0.7	775.3672	2	31.27	11	F11:1474	OB5948 H3B Ro.raw	5.3223E4	6	6	120	131	Carbamidomethylation	C9:Carbamidomethylation:1000.00	PEAKS DB
R.IM(+15.99)GEQEQYDSYDIR.S	Y	103.58	1761.7516	14	-1.0	881.8840	2	28.71	11	F11:1332	OB5948 H3B Ro.raw	1.9449E4	4	4	55	68	Oxidation (M)	M2:Oxidation (M):1000.00	PEAKS DB
R.IMGEQEQYDSYDIR.S	Y	90.26	1745.7566	14	-1.3	873.8866	2	30.26	10	F10:1263	OB5926 H3B Ro.raw	4.6099E4	5	5	55	68			PEAKS DB
R.ELM(+15.99)NLPQQC(+57.02)NFR.A	Y	71.68	1564.7126	12	-2.7	783.3632	2	28.90	11	F11:1336	OB5948 H3B Ro.raw	7.1508E3	3	3	120	131	Oxidation (M); Carbamidomethylation	M3:Oxidation (M):1000.00;C9:Carbamidomethylation:1000.00	PEAKS DB
R.ELMNLPQQC(+57.02)NFRAPQR.C	Y	70.25	2000.9673	16	0.2	667.9965	3	30.17	9	F9:1409	OB5953 H3A Ro.raw	7.0151E3	3	3	120	135	Carbamidomethylation	C9:Carbamidomethylation:1000.00	PEAKS DB
R.C(+57.02)MC(+57.02)EALQQIMENQC(+57.02)DRLQDR.Q	Y	43.12	2597.0913	20	1.2	866.7054	3	33.96	8	F8:1628	OB5952 H3A Ro.raw	1.1224E4	1	1	92	111	Carbamidomethylation	C1:Carbamidomethylation:1000.00;C3:Carbamidomethylation:1000.00;C14:Carbamidomethylation:1000.00	PEAKS DB
R.C(+57.02)MC(+57.02)EALQQ(+.98)IMENQC(+57.02)DRLQDR.Q	Y	37.92	2598.0754	20	-8.3	867.0270	3	33.99	11	F11:1638	OB5948 H3B Ro.raw	0	0	0	92	111	Carbamidomethylation	C1:Carbamidomethylation:1000.00;C3:Carbamidomethylation:1000.00;Q8:Deamidation (NQ):0.00;C14:Carbamidomethylation:1000.00	PEAKS DB
R.IM(+15.99)GEQ(+.98)EQYDSYDIR.S	Y	34.46	1762.7356	14	4.0	882.3805	2	28.56	11	F11:1318	OB5948 H3B Ro.raw	0	0	0	55	68	Oxidation (M)	M2:Oxidation (M):1000.00;Q5:Deamidation (NQ):0.00	PEAKS DB
**total 8 peptides**																			

#CONTAM#P04264

back to list

| Protein Coverage | Supporting Peptides |

Protein Coverage:

```
  1 MSRQFSSRSG YRSGGGFSSG SAGIINYQRR TTSSSTRRSG GGGGRFSSCG GGGGSFGAGG GFGSRSLVNL GGSKSISISV
 81 ARGGGRGSGF GGGYGGGGFG GGGFGGGGFG GGGIGGGGFG GFGSGGGGFG GGGFGGGGYG GGYGPVCPPG GIQEVTINQS
161 LLQPLNVEID PEIQKVKSRE REQIKSLNNQ FASFIDKVRF LEQQNQVLQT KWELLQQVDT STRTHNLEPY FESFINNLRR
241 RVDQLKSDQS RLDSELKNMQ DMVEDYRNKY EDEINKRTNA ENEFVTIKKD VDGAYMTKVD LQAKLDNLQQ EIDFLTALYQ
321 AELSQMQTQI SETNVILSMD NNRSLDLDSI IAEVKAQNED IAQKSKAEAE SLYQSKYEEL QITAGRHGDS VRNSKIEISE
401 LNRVIQRLRS EIDNVKKQIS NLQQSISDAE QRGENALKDA KNKLNDLEDA LQQAKEDLAR LLRDYQELMN TKLALDLEIA
481 TYRTLLEGEE SRMSGECAPN VSVSVSTSHT TISGGGSRGG GGGGYGSGGS SYGSGGGSYG SGGGGGGGRG SYGSGGSSYG
561 SGGGSYGSGG GGGGHGSYGS GSSSGGYRGG SGGGGGGSSG GRGSGGGSSG GSIGGRGSSS GGVKSSGGSS SVRFVSTTYS
641 GVTR
```

Supporting Peptides:

Peptide	Uniq	-10lgP	Mass	Length	ppm	m/z	z	RT	Fraction	Scan	Source File	Area Roast Ara h 3	#Feature	#Feature Roast Ara h 3	Start	End	PTM	AScore	Found By
R.SLDLDSIIAEVK.A	N	117.78	1301.7078	12	1.0	651.8618	2	36.11	7	F7:1758	OB5951 H3A Ro.raw	8.8459E4	3	3	344	355			PEAKS DB
R.FLEQQNQVLQTKWELLQQVDTSTR.T	Y	74.98	2931.5090	24	1.7	978.1786	3	35.80	12	F12:1751	OB5949 H3B Ro.raw	1.6833E5	3	3	200	223			PEAKS DB
K.SLNNQFASFIDKVR.F	Y	66.52	1637.8525	14	2.9	819.9359	2	33.55	7	F7:1610	OB5951 H3A Ro.raw	1.4106E4	2	2	186	199			PEAKS DB
R.THNLEPYFESFINNLRR.R	Y	56.37	2149.0703	17	1.7	717.3652	3	37.33	8	F8:1829	OB5952 H3A Ro.raw	1.0967E4	1	1	224	240			PEAKS DB
**total 4 peptides**																			

sp|Q647H2|AHY3_ARAHY

back to list

| Protein Coverage | Supporting Peptides |

Protein Coverage:

```
  1 MAKLLALSVC FCFLVLGASS VTFRQQGEEN ECQFQRLNAQ RPDNCIESEG GYIETWNPNN QEFQCAGVAL SRFVLRRNAL
 81 RRPFYSNAPQ EIFIYQGSGY FGLIFPGCPG TFEEPIQGSE QFQRPSRHFQ GQDQSQRPLD THQKVHGFRE GDLIAVPHGV
161 AFWIYNDQDT DVVAISVLHT NSLHNQLDQF PRRFNLAGKQ EQEFLRYQQR SGRQSPKGEE QEQEQENEGG NVFSGFSTEF
241 LSHGFQVNED IVRNLRGENE REEQGAIVTV KGGLSILVPP EWRQSYQQPG RGDKDFNNGI EETICTATVK MNIGKSTSAD
321 IYNPQAGSVR TVNELDLPIL NRLGLSAEYG SIHRDAMFVP HYNMNANSMI YALHGGAHVQ VVDCNGNRVF DEELQEGQSL
401 VVPQNFAVAA KSQSEHFLYV AFKTNSRASI SNLAGKNSYM WNLPEDVVAN SYGLQYEQAR QLKNNNPFTF LVPPQDSQMI
481 RTVA
```

C Carbamidomethylation (+57.02)

Supporting Peptides:

Peptide	Uniq	-10lgP	Mass	Length	ppm	m/z	z	RT	Fraction	Scan	Source File	Area Roast Ara h 3	#Feature	#Feature Roast Ara h 3	Start	End	PTM	AScore	Found By
R.TVNELDLPILNR.L	Y	118.58	1395.7721	12	2.1	698.8948	2	34.42	9	F9:1647	OB5953 H3A Ro.raw	1.472E5	4	4	331	342			PEAKS DB
R.RFNLAGKQEQEFLR.Y	Y	68.54	1734.9165	14	-2.6	579.3125	3	31.75	11	F11:1467	OB5948 H3B Ro.raw	2.3357E5	2	2	193	206			PEAKS DB
R.FNLAGKQEQEFLR.Y	Y	59.79	1578.8154	13	-2.4	790.4151	2	31.75	10	F10:1357	OB5926 H3B Ro.raw	0	0	0	194	206			PEAKS DB
C.IESEGGYIETWNPNNQEFQC(+57.02)AGVALSR.F	N	33.38	3068.3933	27	2.9	1535.2084	2	34.64	8	F8:1684	OB5952 H3A Ro.raw	7.7336E3	1	1	46	72	Carbamidomethylation	C20:Carbamidomethylation:1000.00	PEAKS DB
**total 4 peptides**																			

#CONTAM#P13645

back to list

| Protein Coverage | Supporting Peptides |

Protein Coverage:

```
  1 MSVRYSSSKH YSSSRSGGGG GGGGCGGGGG VSSLRISSSK GSLGGGFSSG GFSGGSFSRG SSGGGCFGGS SGGYGGLGGF
 81 GGGSFRGSYG SSSFGGSYGG SFGGGSFGGG SFGGGSFGGG GFGGGGFGGG FGGGFGGDGG LLSGNEKVTM QNLNDRLASY
161 LDKVRALEES NYELEGKIKE WYEKHGNSHQ GEPRDYSKYY KTIDDLKNQI LNLTTDNANI LLQIDNARLA ADDFRLKYEN
241 EVALRQSVEA DINGLRRVLD ELTLTKADLE MQIESLTEEL AYLKKNHEEE MKDLRNVSTG DVNVEMNAAP GVDLTQLLNN
321 MRSQYEQLAE QNRKDAEAWF NEKSKELTTE IDNNIEQISS YKSEITELRR NVQALEIELQ SQLALKQSLE ASLAETEGRY
401 CVQLSQIQAQ ISALEEQLQQ IRAETECQNT EYQQLLDIKI RLENEIQTYR SLLEGEGSSG GGGRGGGSFG GGYGGGSSGG
481 GSSGGGYGGG HGGSSGGGYG GGSSGGGSSG GGYGGGSSSG GHGGGSSSGG HGGSSSGGYG GGSSGGGGGG YGGGSSGGGS
561 SSGGGYGGGS SSGGHKSSSS GSVGESSSKG PRY
```

Oxidation (M) (+15.99)

Supporting Peptides:

Peptide	Uniq	-10lgP	Mass	Length	ppm	m/z	z	RT	Fraction	Scan	Source File	Area Roast Ara h 3	#Feature	#Feature Roast Ara h 3	Start	End	PTM	AScore	Found By
K.TIDDLKNQILNLTTDNANILLQIDNAR.L	Y	117.47	3051.6201	27	2.8	1018.2168	3	38.78	7	F7:1915	OB5951 H3A Ro.raw	1.093E6	6	6	202	228			PEAKS DB
R.NVQALEIELQSQLALK.Q	Y	59.81	1796.0043	16	1.5	899.0108	2	36.15	9	F9:1759	OB5953 H3A Ro.raw	2.0759E4	1	1	371	386			PEAKS DB
K.VTMQNLNDRLASYLDKVR.A	Y	59.53	2135.1157	18	2.1	712.7140	3	35.40	8	F8:1723	OB5952 H3A Ro.raw	1.7452E4	1	1	148	165			PEAKS DB
R.NVSTGDVN(+.98)VEM(+15.99)NAAPGVDLTQLLNNMR.S	Y	34.11	2888.3645	27	6.9	1445.1995	2	37.14	9	F9:1809	OB5953 H3A Ro.raw	0	0	0	296	322	Oxidation (M)	N8:Deamidation (NQ):2.37;M11: Oxidation (M):55.60	PEAKS DB
**total 4 peptides**																			

E5FHZ1|E5FHZ1_ARAHY

back to list

| Protein Coverage | Supporting Peptides |

Protein Coverage:

```
  1 MSQEQHRRPE ESDPIKYGDV FNVSGELAQK PVKPEDAAMM QSAETRVLGK TQPGGAAAAM QSAATLNEQA GLVGHHDVNE
 81 VAGDRGVTVT DMQAPGRRIV TESVGGQVVG QYVEPTPVQS SRASAVRESA ITIGEALEAT AQTVGSKPVE QSDAAAIQAA
161 EVRATGSNVI QPGGLAAMAQ SAVAFNAGCR NPRDKIKLAD ILTGATAKLP ADKAATHQGC
```

Supporting Peptides:

Peptide	Uniq	-10lgP	Mass	Length	ppm	m/z	z	RT	Fraction	Scan	Source File	Area Roast Ara h 3	#Feature	#Feature Roast Ara h 3	Start	End	PTM	AScore	Found By
R.IVTESVGGQVVGQYVEPTPVQSSR.A	Y	117.93	2515.2917	24	3.8	1258.6580	2	31.22	7	F7:1476	OB5951 H3A Ro.raw	3.0299E4	1	1	99	122			PEAKS DB
R.ESAITIGEALEATAQTVGSKPVEQSDAAAIQAAEVR.A	Y	91.05	3610.8325	36	2.4	1204.6210	3	35.92	7	F7:1740	OB5951 H3A Ro.raw	8.9254E5	3	3	128	163			PEAKS DB
R.IVTESVGGQVVGQYVEPTPVQ(+.98)SSR.A	Y	42.31	2516.2759	24	9.4	1259.1571	2	31.76	8	F8:1505	OB5952 H3A Ro.raw	2.7069E4	1	1	99	122		Q21:Deamidation (NQ):0.00	PEAKS DB
**total 3 peptides**																			

N1NKG9|N1NKG9_ARAHY

back to list

| Protein Coverage | Supporting Peptides |

Protein Coverage:

1 MSQEQHRRPE ESDPIKYGDV FNVSGELAQK PVKPEDAAMM QSAETRVLGK TQPGGAAAAM QSAATLNEQA GLVGHHDVNE
81 VAGDRGVTVT DMQAPGRRIV TESVGGQVVG QYVEPTPVQS SRASAVRESA ITIGEALEAT AQTVGSKPVE QSDAAAIQAA
161 EVRATGSNVI QPGGLAAMAQ SAVAFNAGCR NPRDKIKLAD ILTGATAKLP ADKAATHQDA EGVASAEARN NTGGTTPGGV
241 AASVAAAARL NENVNV

Supporting Peptides:

Peptide	Uniq	-10lgP	Mass	Length	ppm	m/z	z	RT	Fraction	Scan	Source File	Area Roast Ara h 3	#Feature	#Feature Roast Ara h 3	Start	End	PTM	AScore	Found By
R.IVTESVGGQVVGQYVEPTPVQSSR.A	Y	117.93	2515.2917	24	3.8	1258.6580	2	31.22	7	F7:1476	OB5951 H3A Ro.raw	3.0299E4	1	1	99	122			PEAKS DB
R.ESAITIGEALEATAQTVGSKPVEQSDAAAIQAAEVR.A	Y	91.05	3610.8325	36	2.4	1204.6210	3	35.92	7	F7:1740	OB5951 H3A Ro.raw	8.9254E5	3	3	128	163			PEAKS DB
R.IVTESVGGQVVGQYVEPTPVQ(+.98)SSR.A	Y	42.31	2516.2759	24	9.4	1259.1571	2	31.76	8	F8:1505	OB5952 H3A Ro.raw	2.7069E4	1	1	99	122		Q21:Deamidation (NQ):0.00	PEAKS DB
**total 3 peptides**																			

sp|Q6PSU2|CONG7_ARAHY

back to list

| Protein Coverage | Supporting Peptides |

Protein Coverage:

1 MAKLTILVAL ALFLLAAHAS ARQQWELQGD RRCQSQLERA NLRPCEQHLM QKIQRDEDSY GRDPYSPSQD PYSPSQDPDR
81 RDPYSPSPYD RRGAGSSQHQ ERCCNELNEF ENNQRCMCEA LQQIMENQSD RLQGRQQEQQ FKRELRNLPQ QCGLRAPQRC
161 DLEVESGGRD RY

a Ammonia-loss (C@N-term) (-17.03)
c Carbamidomethylation (+57.02)
o Oxidation (M) (+15.99)

Supporting Peptides:

Peptide	Uniq	-10lgP	Mass	Length	ppm	m/z	z	RT	Fraction	Scan	Source File	Area Roast Ara h 3	#Feature	#Feature Roast Ara h 3	Start	End	PTM	AScore	Found By
R.C(+57.02)MC(+57.02)EALQQIMENQSDRLQGR.Q	Y	92.60	2466.0872	20	-1.5	823.0369	3	34.04	11	F11:1630	OB5948 H3B Ro.raw	6.8502E5	4	4	116	135	Carbamidomethylation	C1:Carbamidomethylation:1000.00;C3:Carbamidomethylation:1000.00	PEAKS DB
R.C(+57.02)M(+15.99)C(+57.02)EALQQIMENQSDRLQGR.Q	Y	75.48	2482.0820	20	-1.3	828.3687	3	32.12	11	F11:1531	OB5948 H3B Ro.raw	1.478E5	2	2	116	135	Carbamidomethylation; Oxidation (M)	C1:Carbamidomethylation:1000.00;M2:Oxidation (M):97.31;C3:Carbamidomethylation:1000.00	PEAKS DB
R.C(+57.02)C(+57.02)NELNEFENNQR.C	Y	67.01	1725.6835	13	1.3	863.8519	2	28.51	11	F11:1322	OB5948 H3B Ro.raw	3.7158E3	1	1	103	115	Carbamidomethylation	C1:Carbamidomethylation:1000.00;C2:Carbamidomethylation:1000.00	PEAKS DB
R.C(+57.02)M(+15.99)C(+57.02)EALQQIM(+15.99)ENQSDRLQGR.Q	Y	51.60	2498.0771	20	1.5	833.7009	3	30.07	12	F12:1419	OB5949 H3B Ro.raw	2.7424E4	2	2	116	135	Carbamidomethylation; Oxidation (M)	C1:Carbamidomethylation:1000.00;M2:Oxidation (M):1000.00;C3:Carbamidomethylation:1000.00;M10:Oxidation (M):1000.00	PEAKS DB
R.C(+57.02)MC(+57.02)EALQQIM(+15.99)ENQSDRLQGR.Q	Y	51.16	2482.0820	20	-0.8	828.3690	3	30.77	11	F11:1452	OB5948 H3B Ro.raw	4.9643E4	3	3	116	135	Carbamidomethylation; Oxidation (M)	C1:Carbamidomethylation:1000.00;C3:Carbamidomethylation:1000.00;M10:Oxidation (M):78.75	PEAKS DB

total 11 peptides

Peptide	Uniq	-10lgP	Mass	Length	ppm	m/z	z	RT	Fraction	Scan	Source File	Area Roast Ara h 3	#Feature	#Feature Roast Ara h 3	Start	End	PTM	AScore	Found By
R.C(+57.02)MC(+57.02)EALQQIMEN(+.98)QSDRLQGR.Q	Y	42.46	2467.0713	20	2.4	1234.5490	2	34.84	10	F10:1538	OB5926 H3B Ro.raw	0	0	0	116	135	Carbamidomethylation	C1:Carbamidomethylation:1000.00;C3:Carbamidomethylation:1000.00;N12:Deamidation (NQ):27.42	PEAKS DB
R.C(+57.02)C(+57.02)NELN(-17.03)EFENNQR.C	Y	37.32	1708.6570	13	-1.4	855.3364	2	32.73	11	F11:1565	OB5948 H3B Ro.raw	0	0	0	103	115	Carbamidomethylation	C1:Carbamidomethylation:1000.00;C2:Carbamidomethylation:1000.00;N6:Ammonia-loss (N):0.00	PEAKS PTM
R.C(+57.02)C(+71.04)NELNEFENNQR.C	Y	33.01	1739.6992	13	1.2	870.8579	2	29.23	12	F12:1363	OB5949 H3B Ro.raw	1.7958E3	1	1	103	115	Carbamidomethylation	C1:Carbamidomethylation:1000.00;C2:Propionamide:0.00	PEAKS PTM
R.ANLRPC(+57.02)EQHLMQK.I	Y	30.71	1623.7974	13	-5.2	542.2716	3	29.47	10	F10:1222	OB5926 H3B Ro.raw	0	0	0	40	52	Carbamidomethylation	C6:Carbamidomethylation:1000.00	PEAKS DB
A.RQQWELQGDRR.C	Y	30.25	1470.7440	11	-2.8	491.2552	3	29.32	10	F10:1217	OB5926 H3B Ro.raw	5.3324E3	1	1	22	32			PEAKS DB
R.C(-17.03)MC(+57.02)E(+57.02)ALQQIMENQSDRLQGR.Q	Y	29.27	2449.0608	20	3.2	1225.5416	2	38.18	12	F12:1892	OB5949 H3B Ro.raw	1.2706E5	1	1	116	135	Ammonia-loss (C@N-term); Carbamidomethylation	C1:Ammonia-loss (C@N-term):1000.00;C3:Carbamidomethylation:1000.00;E4:Carbamidomethylation (DHKE, X@N-term):36.07	PEAKS PTM
**total 11 peptides**																			

#CONTAM#P35908

back to list

| Protein Coverage | Supporting Peptides |

Protein Coverage:

```
  1 MSCQISCKSR GRGGGGGGFR GFSSGSAVVS GGSRRSTSSF SCLSRHGGGG GGFGGGGFGS RSLVGLGGTK SISISVAGGG
 81 GGFGAAGGFG GRGGGFGGGS GFGGGSGFGG GSGFSGGGFG GGGFGGGRFG GFGGPGGVGG LGGPGGFGPG GYPGGIHEVS
161 VNQSLLQPLN VKVDPEIQNV KAQEREQIKT LNNKFASFID KVRFLEQQNQ VLQTKWELLQ QMNVGTRPIN LEPIFQGYID
241 SLKRYLDGLT AERTSQNSEL NNMQDLVEDY KKKYEDEINK RTAAENDFVT LKKDVDNAYM IKVELQSKVD LLNQEIEFLK
321 VLYDAEISQI HQSVTDTNVI LSMDNSRNLD LDSIIAEVKA QYEEIAQRSK EEAEALYHSK YEELQVTVGR HGDSLKEIKI
401 EISELNRVIQ RLQGEIAHVK KQCKNVQDAI ADAEQRGEHA LKDARNKLND LEEALQQAKE DLARLLRDYQ ELMNVKLALD
481 VEIATYRKLL EGEECRMSGD LSSNVTVSVT SSTISSNVAS KAAFGGSGGR GSSSGGGYSS GSSSYGSGGR QSGSRGGSGG
561 GGSISGGGYG SGGGSGGRYG SGGGSKGGSI SGGGYGSGGG KHSSGGGSRG GSSSGGGYGS GGGGSSSVKG SSGEAFGSSV
641 TFSFR
```

Supporting Peptides:

Peptide	Uniq	-10lgP	Mass	Length	ppm	m/z	z	RT	Fraction	Scan	Source File	Area Roast Ara h 3	#Feature	#Feature Roast Ara h 3	Start	End	PTM	AScore	Found By
R.NLDLDSIIAEVK.A	Y	111.46	1328.7188	12	-0.1	665.3666	2	36.11	7	F7:1759	OB5951 H3A Ro.raw	3.8507E4	3	3	348	359			PEAKS DB
K.VDLLNQEIEFLK.V	Y	35.92	1459.7922	12	0.9	730.9041	2	36.29	8	F8:1764	OB5952 H3A Ro.raw	0	0	0	309	320			PEAKS DB
**total 2 peptides**																			

#CONTAM#P00761

back to list

| Protein Coverage | Supporting Peptides |

Protein Coverage:

1 FPTDDDDKIV GGYTCAANSI PYQVSLNSGS HFCGGSLINS QWVVSAAHCY KSRIQVR**LGE HNIDVLEGNE QFINAA**KIIT

81 HPNFNGNTLD NDIMLIKLSS PATLNSR**VAT VSLPR**SCAAA GTECLISGWG NTKSSGSSYP SLLQCLKAPV LSDSSCKSSY

161 PGQITGNMIC VGFLEGGKDS CQGDSGGPVV CNGQLQGIVS WGYGCAQKNK PGVYTKVCNY VNWIQQTIAA N

Supporting Peptides:

Peptide	Uniq	-10lgP	Mass	Length	ppm	m/z	z	RT	Fraction	Scan	Source File	Area Roast Ara h 3	#Feature	#Feature Roast Ara h 3	Start	End	PTM	AScore	Found By
R.LGEHNIDVLEGNEQFIN.A	Y	78.44	1939.9275	17	2.1	970.9730	2	33.47	9	F9:1599	OB5953 H3A Ro.raw	2.6295E5	6	6	58	74			PEAKS DB
R.LGEHNIDVLEGNEQFINAA.K	Y	62.93	2082.0017	19	2.3	1042.0105	2	34.27	8	F8:1648	OB5952 H3A Ro.raw	0	0	0	58	76			PEAKS DB
R.VATVSLPR.S	Y	53.48	841.5021	8	0.4	421.7585	2	27.07	7	F7:1253	OB5951 H3A Ro.raw	4.4431E4	5	5	108	115			PEAKS DB
**total 3 peptides**																			

A1E2B1|A1E2B1_ARAHY

back to list

| Protein Coverage | Supporting Peptides |

Protein Coverage:

1 MSSSLLFLTL AYLVLIERLT CHAQSHGDRI GECRLEHLSV MEPTKRVESE GGVAEFWDDK SQQLQCIGVT LIRYTIRPK**G**

81 **LLLPFYTNAP R**IHYILQGKG VMEIVVTGCR AMYRSSTKRG MMSSYSDEHQ KIQSIEQNDA VAVPSSSVHW IYNTGHSDLV

161 LFSLVDVANA DNQLDPTFRN FLLSGNGNGK EGEESNNNWF IKKKQR**EAQE GNVFSGLALE TLIGSFNVQR** EIAEKVQGLK

241 DWRGSIILVK EGLDWLSPKE EEEEEGLEKR KKVMLMG

Supporting Peptides:

Peptide	Uniq	-10lgP	Mass	Length	ppm	m/z	z	RT	Fraction	Scan	Source File	Area Roast Ara h 3	#Feature	#Feature Roast Ara h 3	Start	End	PTM	AScore	Found By
R.EAQEGNVFSGLALETLIGSFNVQR.E	Y	106.83	2578.3027	24	1.7	1290.1636	2	40.50	11	F11:2018	OB5948 H3B Ro.raw	1.1946E5	3	3	207	230			PEAKS DB
K.GLLLPFYTNAPR.I	Y	38.82	1360.7502	12	0.4	681.3827	2	35.77	8	F8:1734	OB5952 H3A Ro.raw	0	0	0	80	91			PEAKS DB
**total 2 peptides**																			

Q42515|Q42515_ARAHY

back to list

| Protein Coverage | Supporting Peptides |

Protein Coverage:

1 MKFCNFFTFT LALIVVLQAL GASA**DDAGTI ITQPLYNEFL K**HLTDSRCEA HGFYTYNAFV TAARAFPAFG TTGDDVTRKR

81 ELAAFFGQTS HETTGGTTNA PDEFEWGYCF LREQTKEQHC DSTQAPCPAG KQYYGR**GPIQ LTSNSNYQLA GQAIK**ADLIN

161 NPDLVATDAV ISFKTAIWFW MTPQGNKPSC HDVITNAWRP TATDSAAGRA PGYGVITNII NGGIECGKGA NTNSNNRIGF

241 YKRYCDILKI GYGSNLDCAN QQHF

Supporting Peptides:

Peptide	Uniq	-10lgP	Mass	Length	ppm	m/z	z	RT	Fraction	Scan	Source File	Area Roast Ara h 3	#Feature	#Feature Roast Ara h 3	Start	End	PTM	AScore	Found By
A.DDAGTIITQPLYNEFLK.H	Y	63.02	1936.9781	17	2.4	969.4987	2	37.23	12	F12:1836	OB5949 H3B Ro.raw	2.7447E4	1	1	25	41			PEAKS DB
R.GPIQLTSNSNYQLAGQAIK.A	Y	54.85	2002.0483	19	5.6	1002.0396	2	31.80	10	F10:1360	OB5926 H3B Ro.raw	0	0	0	137	155			PEAKS DB
**total 2 peptides**																			

Peptide List

Prepared with PEAKS ™ (bioinfor.com)

Summary

1. Notes

Ara h 6 GEL BAND RAW

2. Result Statistics

Figure 1. False discovery rate (FDR) curve. X axis is the number of peptides being kept. Y axis is the corresponding FDR.

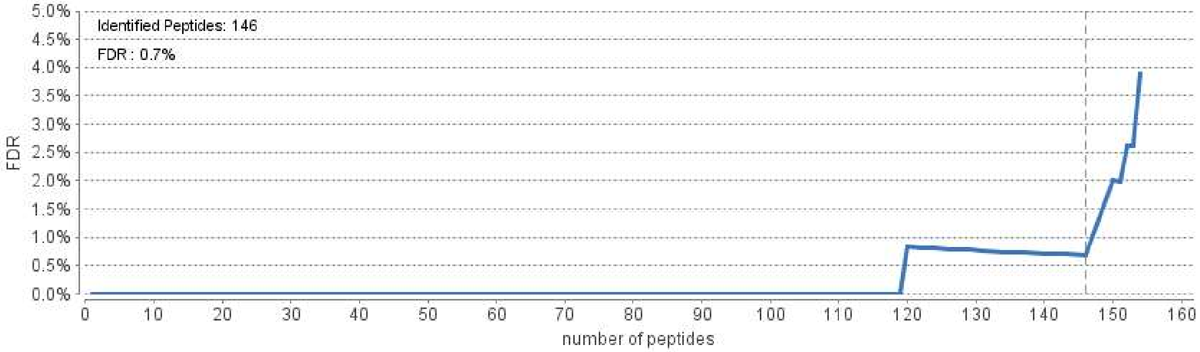

Figure 2. PSM score distribution. **(a)** Distribution of PEAKS peptide score; **(b)** Scatterplot of PEAKS peptide score versus precursor mass error.

(a)

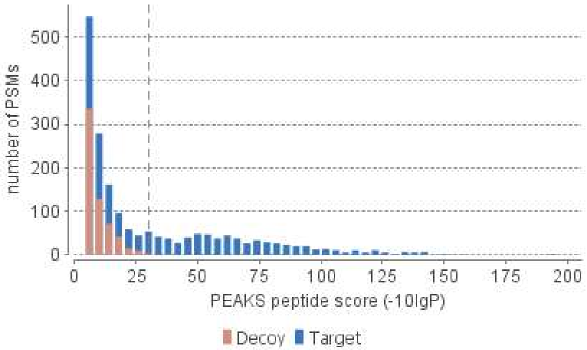

(b)

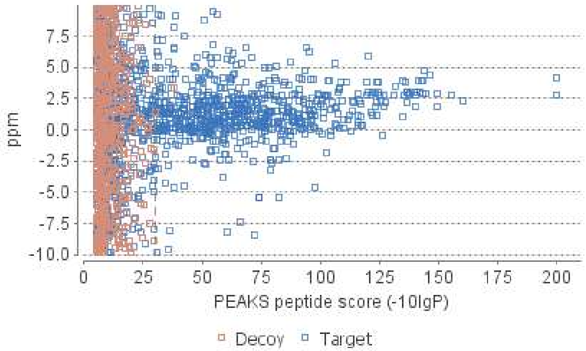

Table 1. Statistics of data.

	#Scans			#Features	Identified			#Peptides	#Sequences	#Proteins*		
	MS1	MS/MS	#Chimera		#PSMs	#Scans	#Features**			Groups	All	Top
Total	7599	3719	318	5330	677	665	246	146	90	13	30	20

Raw Ara h 6	7599	3719	318	5330	677	665	246	146	90	13	30	20

* proteins with significant peptides are used in counts.
** features are identified by DB search only.

Figure 3. Sample overlap for Proteins and Peptides (up to 8 samples). **(a)** All Proteins; **(b)** Top Proteins; **(c)** Peptides;

(a)
Not applicable to only one sample

(b)
Not applicable to only one sample

(c)
Not applicable to only one sample

Figure 4. Distribution of peptide feature detection. **(a)** Feature m/z distribution; **(b)** Feature RT distribution.

(a)

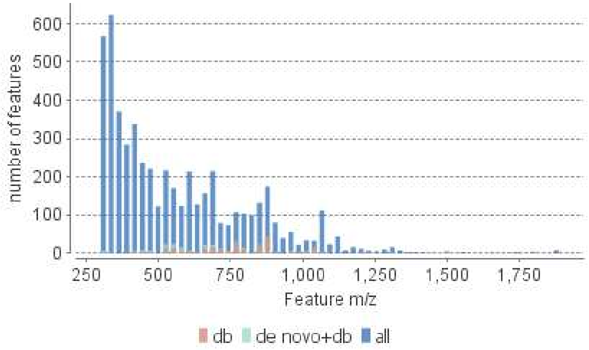

(b)

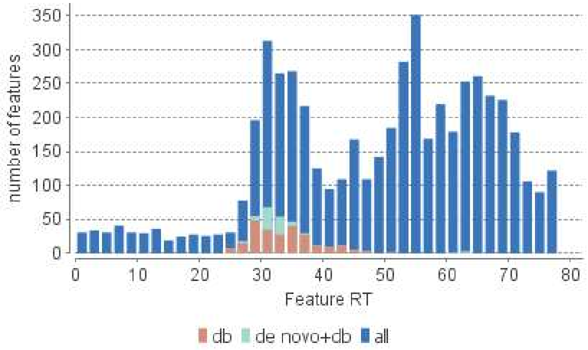

Figure 5. Distribution of identified peptide features. **(a)** Feature abundance distribution; **(b)** *De novo* sequencing validation.

(a)

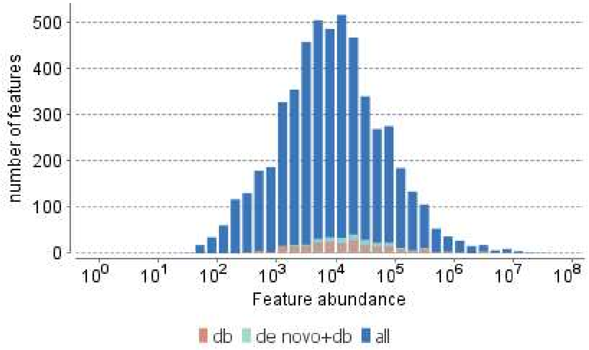

(b)

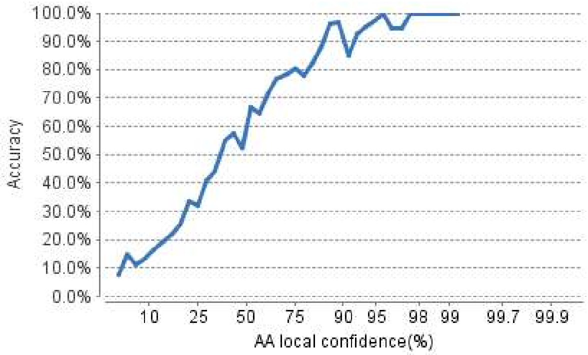

Table 2. Result filtration parameters.

Peptide -10lgP	≥30.4
PTM Ascore	≥50
Protein -10lgP	≥20

Table 4. PTM profile.

Name	ΔMass	Position	#PSM	-10lgP	Abundance	AScore
Carbamidomethyl	57.02	C	257	200.00	2.94E6	1000.00

proteins

Proteins unique peptides	≥2
De novo score(%)	≥50%

Table 3. Statistics of filtered result.

FDR (Peptide-Spectrum Matches)	0.1%
FDR (Peptide Sequences)	0.7%
FDR (Protein Group)	0.0%
De Novo Only Spectra	101

Oxidation	15.99	M	58	159.99	9.75E5	1000.00
Deamidation	.98	NQ	32	143.79		177.50
Propionamide	71.04	C	31	115.59	1.54E5	1000.00
Pyro-glu from Q	-17.03	N-term	8	81.09	6.97E4	1000.00
Ethyl	28.03	D,N-term	7	76.48	6.59E3	6.59
Sodium	21.98	DE	6	66.88		12.81
Carbamidomethyl	57.02	DEK	6	98.03	8.98E3	174.73
Dehydration	-18.01	DTY	5	51.30		0.00
Ammonia loss	-17.03	N-term	5	98.03	8.98E3	1000.00
Dimethylation(KR)	28.03	K	4	100.40	6.56E4	1000.00
Cation:Fe[II]	53.92	E	4	50.16		88.04
Acetylation	42.01	Protein N-term	3	70.92	1.19E4	1000.00
Carbamylation	43.01	N-term	2	32.96		1000.00
Methylation(KR)	14.02	K	2	78.26	1.55E4	1000.00
Acetylation	42.01	N-term	1	37.89		1000.00
Methylation(others)	14.02	E	1	33.33		7.32
NDA	175.04	N-term	1	30.83	1.81E3	1000.00
Pyro-glu from E	-18.01	N-term	1	61.73		1000.00
Carboxylation	43.99	D	1	35.98	3.94E3	1000.00

3. Experiment Control

Figure 6. Precursor mass error of peptide-spectrum matches (PSM) in filtered result. **(a)** Distribution of precursor mass error in ppm; **(b)** Scatterplot of precursor m/z versus precursor mass error in ppm.

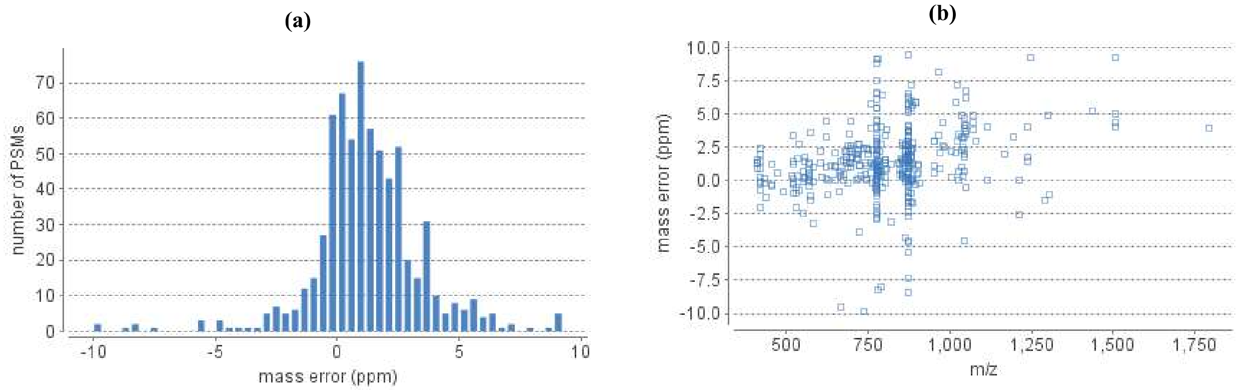

Table 5. Number of identified peptides in each sample by the number of missed cleavages.

Missed Cleavages	0	1	2	3	4+
Raw Ara h 6	112	32	2	0	0

4. Other Information

Table 6. Search parameters.

PEAKS Version: PEAKS Studio 10.6 build 20201015
Search Engine Name: PEAKS
Parent Mass Error Tolerance: 10.0 ppm
Fragment Mass Error Tolerance: 0.5 Da
Precursor Mass Search Type: monoisotopic
Enzyme: Trypsin
Max Missed Cleavages: 2
Digest Mode: Semispecific
Fixed Modifications:
  Carbamidomethylation: 57.02
Variable Modifications:
  Oxidation (M): 15.99
  Deamidation (NQ): 0.98
  Acetylation (K): 42.01
  Acetylation (Protein N-term): 42.01
  Acetylation (N-term): 42.01
  Amidation: -0.98
  Beta-methylthiolation: 45.99
  Biotinylation: 226.08
  and 303 more...
Max Variable PTM Per Peptide: 5
Database: Uniprot_Peanut-3818_Jul18
Taxon: All
Contaminant Database: contam MQ
Searched Entry: 1723
FDR Estimation: Enabled
De novo score(%) threshold: 15
Peptide hit threshold (-10logP): 30.0
Peaks run ID: 29
Merge Options: no merge
Precursor Options: corrected
Charge Options: no correction
Filter m/z: 200.0 - 2000.0
Filter Charge: 2 - 12
Process: true
Associate chimera: yes

Table 7. Instrument parameters.

Fractions: OB5918 H6 raw.raw, OB5930 H6 raw.raw, OB5931 H6 raw.raw
Ion Source: ESI(nano-spray)
Fragmentation Mode: CID, CAD(y and b ions)
MS Scan Mode: FT-ICR/Orbitrap
MS/MS Scan Mode: FT-ICR/Orbitrap

Protein List

Protein Accession Contains:

Protein Description Contains:

Protein Sample Area >=

Protein PTM Contains:

Protein Group	Protein ID	Accession	-10lgP	Coverage (%)	Coverage (%) Raw Ara h 6	Area Raw Ara h 6	#Peptides	#Unique	#Spec Raw Ara h 6	PTM	Avg. Mass	Description
**1**	1	A5Z1R0|A5Z1R0_ARAHY	474.75	67	67	3.7681E7	14	14	458	Y	16920	Ara h 6 allergen OS=Arachis hypogaea OX=3818 GN=Ara h 6 PE=4 SV=1
**1**	2	sp|Q647G9|CONG_ARAHY	474.75	67	67	3.7681E7	14	14	458	Y	16920	Conglutin OS=Arachis hypogaea OX=3818 PE=1 SV=1
**3**	6	B0YIU5|B0YIU5_ARAHY	280.47	59	59	3.25E5	5	5	30	Y	16413	Ara h 8 allergen isoform OS=Arachis hypogaea OX=3818 PE=2 SV=1
**2**	14	#CONTAM#P00761	271.18	29	29	2.4398E6	9	9	49	Y	24409	SWISS-PROT:#CONTAM#P00761|TRYP_PIG Trypsin - Sus scrofa (Pig).
**8**	18	Q2PK12|Q2PK12_ARAHY	257.59	60	60	1.581E5	5	5	16	Y	16090	Actin depolymerizing factor-like protein OS=Arachis hypogaea OX=3818 PE =2 SV=1
**9**	19	#CONTAM#P04264	224.47	15	15	1.7352E5	8	8	13	N	66018	SWISS-PROT:#CONTAM#P04264 Tax_Id=9606 Gene_Symbol=KRT1 Keratin, type II cytoskeletal 1
**14**	17	#CONTAM#P13645	192.47	12	12	2.1942E4	5	5	9	Y	59511	SWISS-PROT:#CONTAM#P13645 Tax_Id=9606 Gene_Symbol=KRT10 Kerati n, type I cytoskeletal 10
**12**	12	A0A0A6ZDP1|A0A0A6ZDP1_ARAHY	178.19	40	40	2.4038E4	5	5	11	N	20101	Glyceraldehyde-3-phosphate dehydrogenase C2 OS=Arachis hypogaea OX= 3818 PE=2 SV=1
**16**	25	E9LFE8|E9LFE8_ARAHY	174.91	15	15	7.8483E3	3	3	6	N	28290	11S arachin (Fragment) OS=Arachis hypogaea OX=3818 PE=2 SV=1
**13**	26	Q45W80|Q45W80_ARAHY	169.07	29	29	1.089E5	4	4	10	Y	16439	Nucleoside diphosphate kinase OS=Arachis hypogaea OX=3818 PE=2 SV=1
**19**	27	Q6PSU3|Q6PSU3_ARAHY	112.24	6	6	2.1534E4	2	2	4	N	66575	Conarachin (Fragment) OS=Arachis hypogaea OX=3818 PE=4 SV=1
**19**	29	sp|P43237|ALL11_ARAHY	112.24	5	5	2.1534E4	2	2	4	N	70283	Allergen Ara h 1, clone P17 OS=Arachis hypogaea OX=3818 PE=1 SV=1
**19**	28	B3IXL2|B3IXL2_ARAHY	112.24	5	5	2.1534E4	2	2	4	N	70283	Main allergen Ara h1 OS=Arachis hypogaea OX=3818 PE=2 SV=1
**19**	31	N1NG13|N1NG13_ARAHY	112.24	5	5	2.1534E4	2	2	4	N	71345	Seed storage protein Ara h1 OS=Arachis hypogaea OX=3818 GN=ARAX_AH F417E07-017 PE=4 SV=1
**19**	30	sp|P43238|ALL12_ARAHY	112.24	5	5	2.1534E4	2	2	4	N	71345	Allergen Ara h 1, clone P41B OS=Arachis hypogaea OX=3818 PE=1 SV=1
**19**	33	Q6PSU4|Q6PSU4_ARAHY	112.24	7	7	2.1534E4	2	2	4	N	48095	Conarachin (Fragment) OS=Arachis hypogaea OX=3818 PE=2 SV=1
**20**	48	K7PQ65|K7PQ65_ARAHY	102.66	13	13	1.6896E4	2	2	4	N	19193	Mother of FT and TFL1 OS=Arachis hypogaea OX=3818 GN=MFT PE=2 SV= 1
**17**	40	Q1HDS7|Q1HDS7_ARAHY	97.95	32	32	5.6334E5	2	2	5	N	15198	Superoxide dismutase [Cu-Zn] OS=Arachis hypogaea OX=3818 PE=2 SV=1
**17**	39	Q45W82|Q45W82_ARAHY	97.95	32	32	5.6334E5	2	2	5	N	15098	Cu-Zn superoxide dismutase OS=Arachis hypogaea OX=3818 PE=2 SV=1
**21**	49	#CONTAM#P35527	78.38	6	6	1.5851E4	2	2	3	N	62129	SWISS-PROT:#CONTAM#P35527 Tax_Id=9606 Gene_Symbol=KRT9 Keratin, type I cytoskeletal 9
**total 20 proteins**												

A5Z1R0|A5Z1R0_ARAHY

back to list

| Protein Coverage | Supporting Peptides |

Protein Coverage:

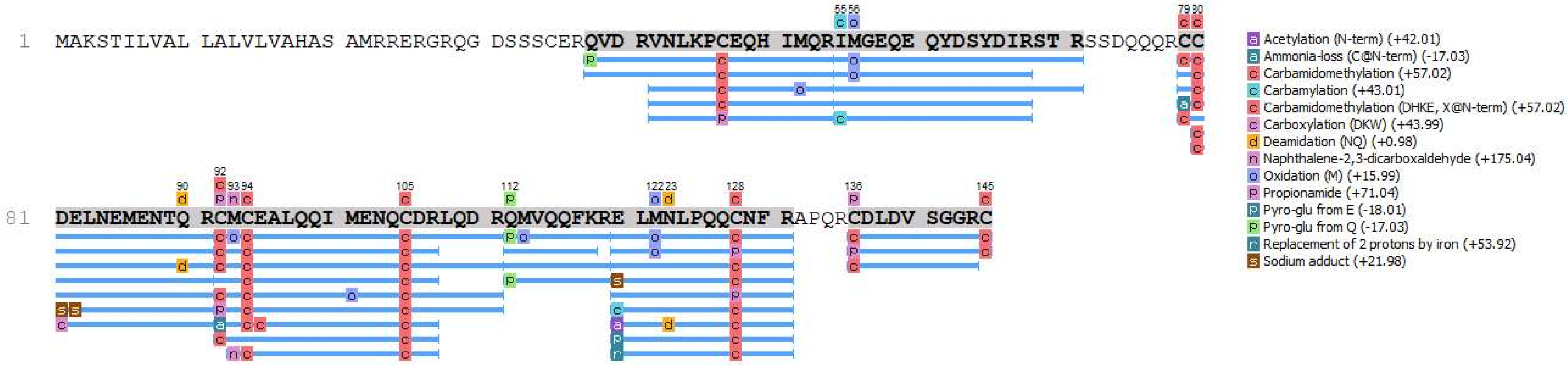

Supporting Peptides:

Peptide	Uniq	-10lgP	Mass	Length	ppm	m/z	z	RT	Fraction	Scan	Source File	Area Raw Ara h 6	#Feature	#Feature Raw Ara h 6	Start	End	PTM	AScore	Found By
R.ELMNLPQQC(+57.02)NFR.A	Y	200.00	1548.7177	12	2.7	775.3682	2	31.64	2	F2:1514	OB5930 H6 raw.raw	1.002E7	12	12	120	131	Carbamidomethylation	C9:Carbamidomethylation:1000.00	PEAKS DB
R.C(+57.02)MC(+57.02)EALQQIMENQC(+57.02)DR.L	Y	200.00	2084.8206	16	4.1	1043.4219	2	34.27	1	F1:1540	OB5918 H6 raw.raw	1.0292E5	5	5	92	107	Carbamidomethylation	C1:Carbamidomethylation:1000.00; C3:Carbamidomethylation:1000.00; C14:Carbamidomethylation:1000.00	PEAKS DB
R.IM(+15.99)GEQEQYDSYDIR.S	Y	159.99	1761.7516	14	2.3	881.8851	2	29.04	2	F2:1368	OB5930 H6 raw.raw	2.8909E6	4	4	55	68	Oxidation (M)	M2:Oxidation (M):1000.00	PEAKS DB
R.IMGEQEQYDSYDIR.S	Y	149.03	1745.7566	14	2.9	873.8881	2	30.57	2	F2:1471	OB5930 H6 raw.raw	1.5582E7	25	25	55	68			PEAKS DB
R.ELM(+15.99)NLPQQC(+57.02)NFR.A	Y	122.17	1564.7126	12	1.2	783.3645	2	29.23	2	F2:1369	OB5930 H6 raw.raw	1.279E6	4	4	120	131	Oxidation (M); Carbamidomethylation	M3:Oxidation (M):1000.00; C9:Carbamidomethylation:1000.00	PEAKS DB
R.ELMNLPQQC(+71.04)NFR.A	Y	115.59	1562.7334	12	0.5	782.3744	2	31.70	1	F1:1393	OB5918 H6 raw.raw	3.6232E5	3	3	120	131	Propionamide	C9:Propionamide:1000.00	PEAKS PTM
R.C(+57.02)C(+57.02)DELNEMENTQR.C	Y	112.33	1697.6444	13	1.2	849.8305	2	28.10	1	F1:1153	OB5918 H6 raw.raw	1.5792E5	3	3	79	91	Carbamidomethylation	C1:Carbamidomethylation:1000.00; C2:Carbamidomethylation:1000.00	PEAKS DB
R.C(+57.02)DLDVSGGRC(+57.02)	Y	106.51	1137.4543	10	0.3	569.7346	2	25.66	3	F3:1179	OB5931 H6 raw.raw	3.6673E4	3	3	136	145	Carbamidomethylation	C1:Carbamidomethylation:1000.00; C10:Carbamidomethylation:1000.00	PEAKS DB

total 61 peptides

Peptide	Uniq	-10lgP	Mass	Length	ppm	m/z	z	RT	Fraction	Scan	Source File	Area Raw Ara h 6	#Feature	#Feature Raw Ara h 6	Start	End	PTM	AScore	Found By
R.C(+57.02)MC(+57.02)EALQQIMENQC(+57.02)DRLQDR.Q	Y	104.61	2597.0913	20	1.7	866.7058	3	35.32	2	F2:1730	OB5930 H6 raw.raw	2.9307E6	5	5	92	111	Carbamidomethylation	C1:Carbamidomethylation:1000.00;C3:Carbamidomethylation:1000.00;C14:Carbamidomethylation:1000.00	PEAKS DB
R.VNLKPC(+57.02)EQHIMQR.I	Y	99.65	1651.8286	13	-0.1	551.6168	3	31.27	2	F2:1515	OB5930 H6 raw.raw	1.3033E6	6	6	42	54	Carbamidomethylation	C6:Carbamidomethylation:1000.00	PEAKS DB
R.C(-17.03)MC(+57.02)E(+57.02)ALQQIMENQC(+57.02)DR.L	Y	98.03	2067.7942	16	2.3	1034.9067	2	39.07	2	F2:1959	OB5930 H6 raw.raw	3.2701E4	3	3	92	107	Ammonia-loss (C@N-term); Carbamidomethylation; Carbamidomethylation (DHKE, X@N-term)	C1:Ammonia-loss (C@N-term):1000.00;C3:Carbamidomethylation:1000.00;E4:Carbamidomethylation (DHKE, X@N-term):194.40;C14:Carbamidomethylation:1000.00	PEAKS PTM
R.C(+57.02)M(+15.99)C(+57.02)EALQQIMENQC(+57.02)DRLQDR.Q	Y	97.13	2613.0862	20	1.6	872.0374	3	33.15	1	F1:1482	OB5918 H6 raw.raw	6.1802E5	3	3	92	111	Carbamidomethylation; Oxidation (M)	C1:Carbamidomethylation:1000.00;M2:Oxidation (M):131.34;C3:Carbamidomethylation:1000.00;C14:Carbamidomethylation:1000.00	PEAKS DB

total 61 peptides

Peptide	Uniq	-10lgP	Mass	Length	ppm	m/z	z	RT	Fraction	Scan	Source File	Area Raw Ara h 6	#Feature	#Feature Raw Ara h 6	Start	End	PTM	AScore	Found By
R.C(+71.04)MC(+57.02)EALQQIMENQC(+57.02)DR.L	Y	91.03	2098.8362	16	4.1	1050.4297	2	34.27	1	F1:1549	OB5918 H6 raw.raw	8.2569E3	1	1	92	107	Carbamidomethylation	C1:Propionamide:22.85;C3:Carbamidomethylation:1000.00;C14:Carbamidomethylation:1000.00	PEAKS PTM
R.C(+71.04)MC(+57.02)EALQQIMENQC(+57.02)DRLQDR.Q	Y	90.77	2611.1069	20	0.5	871.3766	3	34.84	1	F1:1571	OB5918 H6 raw.raw	3.7421E5	1	1	92	111	Propionamide; Carbamidomethylation	C1:Propionamide:54.01;C3:Carbamidomethylation:1000.00;C14:Carbamidomethylation:1000.00	PEAKS PTM
R.C(+57.02)C(+57.02)DELNEMENTQ(+.98)R.C	Y	90.49	1698.6284	13	1.2	850.3225	2	28.66	2	F2:1327	OB5930 H6 raw.raw	2.5004E5	3	3	79	91	Carbamidomethylation	C1:Carbamidomethylation:1000.00;C2:Carbamidomethylation:1000.00;Q12:Deamidation (NQ):36.05	PEAKS DB
R.C(+71.04)C(+57.02)DELNEMENTQR.C	Y	89.19	1711.6600	13	0.6	856.8378	2	28.19	2	F2:1295	OB5930 H6 raw.raw	1.4647E5	3	3	79	91	Carbamidomethylation	C1:Propionamide:10.11;C2:Carbamidomethylation:1000.00	PEAKS PTM
R.C(+71.04)DLDVSGGRC(+57.02)	Y	86.72	1151.4700	10	3.6	576.7443	2	25.80	1	F1:1058	OB5918 H6 raw.raw	7.0267E3	2	2	136	145	Propionamide; Carbamidomethylation	C1:Propionamide:123.21;C10:Carbamidomethylation:1000.00	PEAKS PTM
R.Q(-17.03)MVQQFKR.E	Y	81.09	1046.5331	8	1.5	524.2746	2	28.32	1	F1:1185	OB5918 H6 raw.raw	2.0304E5	3	3	112	119	Pyro-glu from Q	Q1:Pyro-glu from Q:1000.00	PEAKS PTM

total 61 peptides

Peptide	Uniq	-10lgP	Mass	Length	ppm	m/z	z	RT	Fraction	Scan	Source File	Area Raw Ara h 6	#Feature	#Feature Raw Ara h 6	Start	End	PTM	AScore	Found By
R.C(+57.02)(+28.03)C(+57.02)DELNEMENTQR.C	Y	76.48	1725.6757	13	0.1	863.8452	2	28.26	2	F2:1306	OB5930 H6 raw.raw	1.2726E4	2	2	79	91	Carbamidomethylation	C1:Carbamidomethylation:1000.00;C1:Ethylation:13.67;C2:Carbamidomethylation:1000.00	PEAKS PTM
R.C(+71.04)C(+57.02)DELNEMENTQ(+.98)R.C	Y	73.91	1712.6440	13	0.5	857.3297	2	28.89	3	F3:1347	OB5931 H6 raw.raw	2.4957E5	3	3	79	91	Carbamidomethylation; Deamidation (NQ)	C1:Propionamide:12.28;C2:Carbamidomethylation:1000.00;Q12:Deamidation (NQ):56.37	PEAKS PTM
R.C(+57.02)C(+71.04)DELNEMENTQR.C	Y	73.29	1711.6600	13	2.8	856.8397	2	28.39	2	F2:1328	OB5930 H6 raw.raw	5.119E4	1	1	79	91	Carbamidomethylation	C1:Carbamidomethylation:1000.00;C2:Propionamide:12.28	PEAKS PTM
R.C(-17.03)C(+57.02)D(+57.02)ELNEMENTQR.C	Y	72.79	1680.6178	13	1.3	841.3173	2	32.42	1	F1:1439	OB5918 H6 raw.raw	9.4578E3	1	1	79	91	Ammonia-loss (C@N-term); Carbamidomethylation	C1:Ammonia-loss (C@N-term):1000.00;C2:Carbamidomethylation:1000.00;D3:Carbamidomethylation (DHKE, X@N-term):12.28	PEAKS PTM
R.C(+57.02)DLDVSGGR.C	Y	69.89	977.4236	9	-0.8	489.7187	2	24.72	1	F1:1012	OB5918 H6 raw.raw	0	0	0	136	144	Carbamidomethylation	C1:Carbamidomethylation:1000.00	PEAKS DB
R.IMGEQEQYDSYDIRSTR.S	Y	68.55	2089.9375	17	2.6	697.6549	3	29.87	1	F1:1284	OB5918 H6 raw.raw	7.5645E4	3	3	55	71			PEAKS DB

**total 61 peptides**

Peptide	Uniq	-10lgP	Mass	Length	ppm	m/z	z	RT	Fraction	Scan	Source File	Area Raw Ara h 6	#Feature	#Feature Raw Ara h 6	Start	End	PTM	AScore	Found By
R.Q(-17.03)VDRVNLKPC(+57.02)EQHIMQR.I	Y	67.85	2133.0571	17	1.7	534.2725	4	31.34	1	F1:1372	OB5918 H6 raw.raw	3.0682E5	2	2	38	54	Pyro-glu from Q; Carbamidomethylation	Q1:Pyro-glu from Q:1000.00; C10:Carbamidomethylation:1000.00	PEAKS PTM
R.IMGEQE(+21.98)QYDSYDIR.S	Y	66.88	1767.7385	14	1.6	884.8780	2	30.42	2	F2:1450	OB5930 H6 raw.raw	0	0	0	55	68		E6:Sodium adduct:12.81	PEAKS PTM
R.ELM(+15.99)NLPQQC(+71.04)NFR.A	Y	64.44	1578.7283	12	0.7	790.3719	2	29.48	2	F2:1390	OB5930 H6 raw.raw	1.3436E4	2	2	120	131	Oxidation (M); Propionamide	M3:Oxidation (M):1000.00; C9:Propionamide:1000.00	PEAKS PTM
R.E(-18.01)LMNLPQQC(+57.02)NFR.A	Y	61.73	1530.7072	12	1.2	766.3618	2	35.00	2	F2:1722	OB5930 H6 raw.raw	0	0	0	120	131	Pyro-glu from E; Carbamidomethylation	E1:Pyro-glu from E:1000.00; C9:Carbamidomethylation:1000.00	PEAKS PTM
R.IMGEQEQ(+.98)YDSYDIR.S	Y	56.51	1746.7406	14	5.7	874.3826	2	40.55	3	F3:2044	OB5931 H6 raw.raw	0	0	0	55	68		Q7:Deamidation (NQ):19.68	PEAKS DB
R.C(+57.02)M(+15.99)C(+57.02)EALQQIMENQC(+57.02)DR.L	Y	54.61	2100.8157	16	6.8	1051.4222	2	32.37	3	F3:1570	OB5931 H6 raw.raw	0	0	0	92	107	Carbamidomethylation	C1:Carbamidomethylation:1000.00; M2:Oxidation (M):43.13;C3:Carbamidomethylation:1000.00;C14:Carbamidomethylation:1000.00	PEAKS DB
R.IMGEQ(+.98)EQYDSYDIR.S	Y	54.23	1746.7406	14	9.5	874.3859	2	40.80	1	F1:1928	OB5918 H6 raw.raw	1.3457E3	1	1	55	68		Q5:Deamidation (NQ):26.31	PEAKS DB
R.QMVQQFK.R	Y	53.65	907.4586	7	-0.3	454.7364	2	26.28	1	F1:1080	OB5918 H6 raw.raw	1.4384E4	3	3	112	118			PEAKS DB

total 61 peptides

Peptide	Uniq	-10lgP	Mass	Length	ppm	m/z	z	RT	Fraction	Scan	Source File	Area Raw Ara h 6	#Feature	#Feature Raw Ara h 6	Start	End	PTM	AScore	Found By
R.C(+57.02)(+28.03)C(+57.02)DELNEMENTQ(+.98)R.C	Y	53.00	1726.6597	13	2.3	864.3391	2	28.76	1	F1:1209	OB5918 H6 raw.raw	7.4015E3	1	1	79	91	Carbamidomethylation	C1:Carbamidomethylation:1000.00;C1:Ethylation:5.47;C2:Carbamidomethylation:1000.00;Q12:Deamidation (NQ):25.21	PEAKS PTM
R.VNLKPC(+57.02)EQHIM(+15.99)QR.I	Y	52.18	1667.8236	13	1.8	556.9495	3	28.32	3	F3:1320	OB5931 H6 raw.raw	3.5343E4	3	3	42	54	Carbamidomethylation; Oxidation (M)	C6:Carbamidomethylation:1000.00;M11:Oxidation (M):1000.00	PEAKS DB
R.C(+57.02)(+28.03)C(+57.02)DELNEMEN(+.98)TQR.C	Y	51.55	1726.6597	13	-0.1	864.3370	2	28.86	2	F2:1348	OB5930 H6 raw.raw	9.4354E3	1	1	79	91	Carbamidomethylation	C1:Carbamidomethylation:1000.00;C1:Ethylation:14.04;C2:Carbamidomethylation:1000.00;N10:Deamidation (NQ):27.19	PEAKS PTM
R.IMGEQEQYDSY(-18.01)DIR.S	Y	51.30	1727.7461	14	2.3	864.8823	2	31.01	2	F2:1485	OB5930 H6 raw.raw	0	0	0	55	68		Y11:Dehydration:0.00	PEAKS PTM
R.ELMNLPQQ(+.98)C(+57.02)NFR.A	Y	51.09	1549.7018	12	8.8	775.8650	2	43.21	1	F1:2062	OB5918 H6 raw.raw	5.8655E2	1	1	120	131	Carbamidomethylation	Q8:Deamidation (NQ):0.00;C9:Carbamidomethylation:1000.00	PEAKS DB
R.E(+53.92)LMNLPQQC(+57.02)NFR.A	Y	50.16	1602.6370	12	0.2	802.3259	2	31.56	1	F1:1384	OB5918 H6 raw.raw	0	0	0	120	131	Replacement of 2 protons by iron; Carbamidomethylation	E1:Replacement of 2 protons by iron:88.04;C9:Carbamidomethylation:1000.00	PEAKS PTM

total 61 peptides

Peptide	Uniq	-10lgP	Mass	Length	ppm	m/z	z	RT	Fraction	Scan	Source File	Area Raw Ara h 6	#Feature	#Feature Raw Ara h 6	Start	End	PTM	AScore	Found By
R.C(+57.02)MC(+71.04)EALQQIMENQC(+57.02)DR.L	Y	50.11	2098.8362	16	3.8	1050.4293	2	34.51	3	F3:1696	OB5931 H6 raw.raw	0	0	0	92	107	Carbamidomethylation	C1:Carbamidomethylation:1000.00;C3:Propionamide:0.00;C14:Carbamidomethylation:1000.00	PEAKS PTM
R.QVDRVNLKPC(+57.02)EQHIMQR.I	Y	50.11	2150.0837	17	-0.7	538.5278	4	33.49	2	F2:1625	OB5930 H6 raw.raw	2.7618E4	1	1	38	54	Carbamidomethylation	C10:Carbamidomethylation:1000.00	PEAKS DB
R.E(+21.98)LMNLPQQC(+57.02)NFR.A	Y	45.41	1570.6997	12	0.8	786.3577	2	31.52	1	F1:1386	OB5918 H6 raw.raw	9.3242E4	2	2	120	131	Sodium adduct; Carbamidomethylation	E1:Sodium adduct:57.72;C9:Carbamidomethylation:1000.00	PEAKS PTM
R.IMGEQEQYD(-18.01)SYDIR.S	Y	45.19	1727.7461	14	-0.1	864.8802	2	31.20	3	F3:1499	OB5931 H6 raw.raw	0	0	0	55	68		D9:Dehydration:8.69	PEAKS PTM
R.IMGE(+21.98)QEQYDSYDIR.S	Y	43.97	1767.7385	14	0.1	884.8766	2	30.72	3	F3:1472	OB5931 H6 raw.raw	2.0226E5	2	2	55	68		E4:Sodium adduct:17.01	PEAKS PTM
R.VNLKPC(+71.04)EQHIMQR.I	Y	42.26	1665.8444	13	0.2	556.2888	3	31.46	2	F2:1497	OB5930 H6 raw.raw	2.2279E4	1	1	42	54	Propionamide	C6:Propionamide:1000.00	PEAKS PTM
R.C(+57.02)C(+71.04)DELNEMENTQ(+.98)R.C	Y	40.31	1712.6440	13	0.5	857.3297	2	28.89	3	F3:1387	OB5931 H6 raw.raw	8.946E4	1	1	79	91	Carbamidomethylation	C1:Carbamidomethylation:1000.00;C2:Propionamide:0.00;Q12:Deamidation (NQ):28.86	PEAKS PTM
R.ELMNLPQ(+.98)QC(+57.02)NFR.A	Y	38.68	1549.7018	12	9.1	775.8652	2	43.03	1	F1:2056	OB5918 H6 raw.raw	0	0	0	120	131	Carbamidomethylation	Q7:Deamidation (NQ):12.28;C9:Carbamidomethylation:1000.00	PEAKS DB

total 61 peptides

Peptide	Uniq	-10lgP	Mass	Length	ppm	m/z	z	RT	Fraction	Scan	Source File	Area Raw Ara h 6	#Feature	#Feature Raw Ara h 6	Start	End	PTM	AScore	Found By
R.E(+42.01)LMN(+.98)LPQQC(+57.02)NFR.A	Y	37.89	1591.7123	12	6.4	796.8685	2	29.86	2	F2:1416	OB5930 H6 raw.raw	0	0	0	120	131	Acetylation (N-term); Deamidation (NQ); Carbamidomethylation	E1:Acetylation (N-term):1000.00;N4:Deamidation (NQ):56.34;C9:Carbamidomethylation:1000.00	PEAKS PTM
R.IMGEQE(+53.92)QYDSYDIR.S	Y	36.98	1799.6759	14	0.5	900.8456	2	31.02	1	F1:1351	OB5918 H6 raw.raw	0	0	0	55	68		E6:Replacement of 2 protons by iron:22.45	PEAKS PTM
R.IM(+15.99)GEQEQYDSYDIRSTR.S	Y	36.12	2105.9324	17	1.8	702.9860	3	28.23	2	F2:1323	OB5930 H6 raw.raw	1.5096E3	1	1	55	71	Oxidation (M)	M2:Oxidation (M):1000.00	PEAKS DB
C.C(+57.02)D(+43.99)ELNEMENTQ(+.98)R.C	Y	35.98	1582.5875	12	-8.0	792.2947	2	30.54	3	F3:1456	OB5931 H6 raw.raw	3.9371E3	1	1	80	91	Carbamidomethylation; Carboxylation (DKW)	C1:Carbamidomethylation:1000.00;D2:Carboxylation (DKW):1000.00;Q11:Deamidation (NQ):33.91	PEAKS PTM
R.C(+57.02)MC(+57.02)EALQ(+.98)QIMENQC(+57.02)DRLQDR.Q	Y	34.72	2598.0754	20	2.7	867.0348	3	39.74	2	F2:1998	OB5930 H6 raw.raw	0	0	0	92	111	Carbamidomethylation	C1:Carbamidomethylation:1000.00;C3:Carbamidomethylation:1000.00;Q7:Deamidation (NQ):0.00;C14:Carbamidomethylation:1000.00	PEAKS DB
R.QMVQQFKR.E	Y	34.28	1063.5597	8	-2.0	532.7861	2	27.21	1	F1:1126	OB5918 H6 raw.raw	2.4157E4	2	2	112	119			PEAKS DB

total 61 peptides

Peptide	Uniq	-10lgP	Mass	Length	ppm	m/z	z	RT	Fraction	Scan	Source File	Area Raw Ara h 6	#Feature	#Feature Raw Ara h 6	Start	End	PTM	AScore	Found By
R.IM(+15.99)GEQE(+53.92)QYDSYDIR.S	Y	33.87	1815.6708	14	-0.1	908.8425	2	29.10	3	F3:1374	OB5931 H6 raw.raw	0	0	0	55	68	Oxidation (M)	M2:Oxidation (M):1000.00;E6:Replacement of 2 protons by iron:33.18	PEAKS PTM
R.IMGE(+14.02)QEQ(+.98)YDSYDIR.S	Y	33.33	1760.7563	14	7.2	881.3918	2	31.49	1	F1:1379	OB5918 H6 raw.raw	0	0	0	55	68		E4:Methylation (others):7.32;Q7:Deamidation (NQ):14.04	PEAKS PTM
R.E(+43.01)LMNLPQQC(+57.02)NFR.A	Y	32.96	1591.7235	12	-0.5	796.8687	2	30.04	3	F3:1430	OB5931 H6 raw.raw	0	0	0	120	131	Carbamylation; Carbamidomethylation	E1:Carbamylation:1000.00; C9:Carbamidomethylation:1000.00	PEAKS PTM
R.Q(-17.03)M(+15.99)VQQFKR.E	Y	32.86	1062.5281	8	-0.5	532.2711	2	26.15	3	F3:1207	OB5931 H6 raw.raw	1.4877E3	1	1	112	119	Pyro-glu from Q; Oxidation (M)	Q1:Pyro-glu from Q:1000.00; M2:Oxidation (M):1000.00	PEAKS PTM
C.C(+57.02)D(+21.98)E(+21.98)LNEMENTQR.C	Y	32.78	1581.5776	12	4.1	791.7993	2	30.09	3	F3:1429	OB5931 H6 raw.raw	3.1759E3	1	1	80	91	Carbamidomethylation; Sodium adduct	C1:Carbamidomethylation:1000.00; D2:Sodium adduct:51.96;E3:Sodium adduct:65.81	PEAKS PTM
R.I(+43.01)MGEQEQYDSYDIR.S	Y	31.44	1788.7625	14	5.9	895.3937	2	29.69	3	F3:1409	OB5931 H6 raw.raw	0	0	0	55	68	Carbamylation	I1:Carbamylation:1000.00	PEAKS PTM
R.C(+71.04)C(+57.02)DELNEMEN(+.98)TQR.C	Y	30.87	1712.6440	13	0.3	857.3296	2	28.68	2	F2:1374	OB5930 H6 raw.raw	8.661E4	1	1	79	91	Carbamidomethylation	C1:Propionamide:7.65;C2:Carbamidomethylation:1000.00;N10:Deamidation (NQ):20.41	PEAKS PTM

total 61 peptides

Peptide	Uniq	-10lgP	Mass	Length	ppm	m/z	z	RT	Fraction	Scan	Source File	Area Raw Ara h 6	#Feature	#Feature Raw Ara h 6	Start	End	PTM	AScore	Found By
C.M(+175.04)C(+57.02)EALQQIMENQC(+57.02)DR.L	Y	30.83	2099.8323	15	6.2	1050.9299	2	34.47	2	F2:1687	OB5930 H6 raw.raw	1.8085E3	1	1	93	107	Naphthalene-2,3-dicarboxaldehyde; Carbamidomethylation	M1:Naphthalene-2,3-dicarboxaldehyde:1000.00;C2:Carbamidomethylation:1000.00;C13:Carbamidomethylation:1000.00	PEAKS PTM
R.C(+57.02)MC(+57.02)EALQQIM(+15.99)ENQC(+57.02)D(-18.01)RLQDR.Q	Y	30.45	2595.0757	20	2.6	866.0348	3	34.46	1	F1:1551	OB5918 H6 raw.raw	3.0045E4	1	1	92	111	Carbamidomethylation; Oxidation (M)	C1:Carbamidomethylation:1000.00;C3:Carbamidomethylation:1000.00;M10:Oxidation (M):99.62;C14:Carbamidomethylation:1000.00;D15:Dehydration:19.86	PEAKS PTM
**total 61 peptides**																			

sp|Q647G9|CONG_ARAHY

back to list

| Protein Coverage | Supporting Peptides |

Protein Coverage:

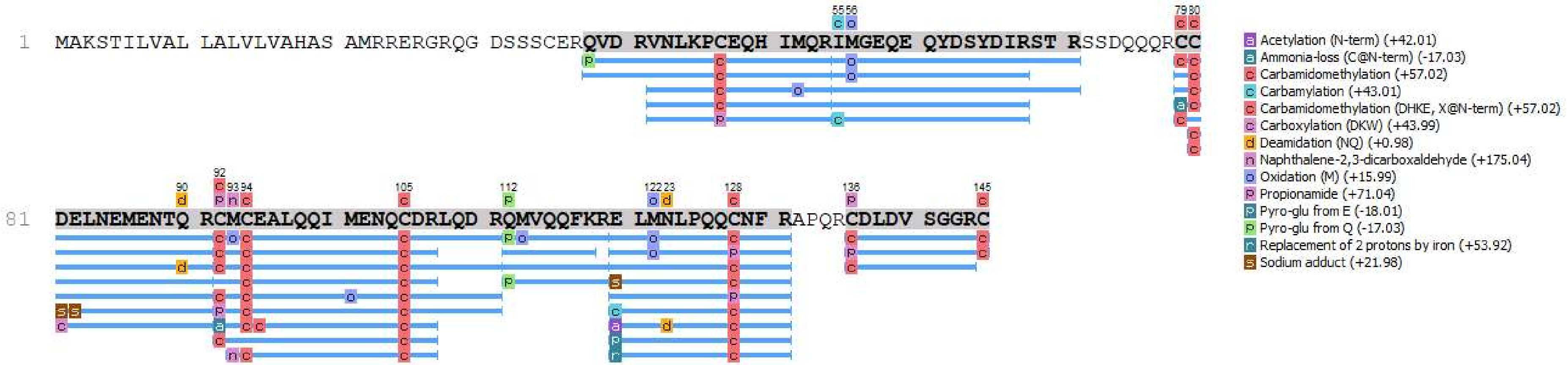

Supporting Peptides:

Peptide	Uniq	-10lgP	Mass	Length	ppm	m/z	z	RT	Fraction	Scan	Source File	Area Raw Ara h 6	#Feature	#Feature Raw Ara h 6	Start	End	PTM	AScore	Found By
R.ELMNLPQQC(+57.02)NFR.A	Y	200.00	1548.7177	12	2.7	775.3682	2	31.64	2	F2:1514	OB5930 H6 raw.raw	1.002E7	12	12	120	131	Carbamidomethylation	C9:Carbamidomethylation:1000.00	PEAKS DB
R.C(+57.02)MC(+57.02)EALQQIMENQC(+57.02)DR.L	Y	200.00	2084.8206	16	4.1	1043.4219	2	34.27	1	F1:1540	OB5918 H6 raw.raw	1.0292E5	5	5	92	107	Carbamidomethylation	C1:Carbamidomethylation:1000.00; C3:Carbamidomethylation:1000.00; C14:Carbamidomethylation:1000.00	PEAKS DB
R.IM(+15.99)GEQEQYDSYDIR.S	Y	159.99	1761.7516	14	2.3	881.8851	2	29.04	2	F2:1368	OB5930 H6 raw.raw	2.8909E6	4	4	55	68	Oxidation (M)	M2:Oxidation (M):1000.00	PEAKS DB
R.IMGEQEQYDSYDIR.S	Y	149.03	1745.7566	14	2.9	873.8881	2	30.57	2	F2:1471	OB5930 H6 raw.raw	1.5582E7	25	25	55	68			PEAKS DB
R.ELM(+15.99)NLPQQC(+57.02)NFR.A	Y	122.17	1564.7126	12	1.2	783.3645	2	29.23	2	F2:1369	OB5930 H6 raw.raw	1.279E6	4	4	120	131	Oxidation (M); Carbamidomethylation	M3:Oxidation (M):1000.00; C9:Carbamidomethylation:1000.00	PEAKS DB
R.ELMNLPQQC(+71.04)NFR.A	Y	115.59	1562.7334	12	0.5	782.3744	2	31.70	1	F1:1393	OB5918 H6 raw.raw	3.6232E5	3	3	120	131	Propionamide	C9:Propionamide:1000.00	PEAKS PTM

total 61 peptides

Peptide	Uniq	-10lgP	Mass	Length	ppm	m/z	z	RT	Fraction	Scan	Source File	Area Raw Ara h 6	#Feature	#Feature Raw Ara h 6	Start	End	PTM	AScore	Found By
R.C(+57.02)C(+57.02)DELNEMENTQR.C	Y	112.33	1697.6444	13	1.2	849.8305	2	28.10	1	F1:1153	OB5918 H6 raw.raw	1.5792E5	3	3	79	91	Carbamidomethylation	C1:Carbamidomethylation:1000.00;C2:Carbamidomethylation:1000.00	PEAKS DB
R.C(+57.02)DLDVSGGRC(+57.02)	Y	106.51	1137.4543	10	0.3	569.7346	2	25.66	3	F3:1179	OB5931 H6 raw.raw	3.6673E4	3	3	136	145	Carbamidomethylation	C1:Carbamidomethylation:1000.00;C10:Carbamidomethylation:1000.00	PEAKS DB
R.C(+57.02)MC(+57.02)EALQQIMENQC(+57.02)DRLQDR.Q	Y	104.61	2597.0913	20	1.7	866.7058	3	35.32	2	F2:1730	OB5930 H6 raw.raw	2.9307E6	5	5	92	111	Carbamidomethylation	C1:Carbamidomethylation:1000.00;C3:Carbamidomethylation:1000.00;C14:Carbamidomethylation:1000.00	PEAKS DB
R.VNLKPC(+57.02)EQHIMQR.I	Y	99.65	1651.8286	13	-0.1	551.6168	3	31.27	2	F2:1515	OB5930 H6 raw.raw	1.3033E6	6	6	42	54	Carbamidomethylation	C6:Carbamidomethylation:1000.00	PEAKS DB
R.C(-17.03)MC(+57.02)E(+57.02)ALQQIMENQC(+57.02)DR.L	Y	98.03	2067.7942	16	2.3	1034.9067	2	39.07	2	F2:1959	OB5930 H6 raw.raw	3.2701E4	3	3	92	107	Ammonia-loss (C@N-term); Carbamidomethylation; Carbamidomethylation (DHKE, X@N-term)	C1:Ammonia-loss (C@N-term):1000.00;C3:Carbamidomethylation:1000.00;E4:Carbamidomethylation (DHKE, X@N-term):194.40;C14:Carbamidomethylation:1000.00	PEAKS PTM

total 61 peptides

Peptide	Uniq	-10lgP	Mass	Length	ppm	m/z	z	RT	Fraction	Scan	Source File	Area Raw Ara h 6	#Feature	#Feature Raw Ara h 6	Start	End	PTM	AScore	Found By
R.C(+57.02)M(+15.99)C(+57.02)EALQQIMENQC(+57.02)DRLQDR.Q	Y	97.13	2613.0862	20	1.6	872.0374	3	33.15	1	F1:1482	OB5918 H6 raw.raw	6.1802E5	3	3	92	111	Carbamidomethylation; Oxidation (M)	C1:Carbamidomethylation:1000.00;M2:Oxidation (M):131.34;C3:Carbamidomethylation:1000.00;C14:Carbamidomethylation:1000.00	PEAKS DB
R.C(+71.04)MC(+57.02)EALQQIMENQC(+57.02)DR.L	Y	91.03	2098.8362	16	4.1	1050.4297	2	34.27	1	F1:1549	OB5918 H6 raw.raw	8.2569E3	1	1	92	107	Carbamidomethylation	C1:Propionamide:22.85;C3:Carbamidomethylation:1000.00;C14:Carbamidomethylation:1000.00	PEAKS PTM
R.C(+71.04)MC(+57.02)EALQQIMENQC(+57.02)DRLQDR.Q	Y	90.77	2611.1069	20	0.5	871.3766	3	34.84	1	F1:1571	OB5918 H6 raw.raw	3.7421E5	1	1	92	111	Propionamide; Carbamidomethylation	C1:Propionamide:54.01;C3:Carbamidomethylation:1000.00;C14:Carbamidomethylation:1000.00	PEAKS PTM
R.C(+57.02)C(+57.02)DELNEMENTQ(+.98)R.C	Y	90.49	1698.6284	13	1.2	850.3225	2	28.66	2	F2:1327	OB5930 H6 raw.raw	2.5004E5	3	3	79	91	Carbamidomethylation	C1:Carbamidomethylation:1000.00;C2:Carbamidomethylation:1000.00;Q12:Deamidation (NQ):36.05	PEAKS DB

total 61 peptides

Peptide	Uniq	-10lgP	Mass	Length	ppm	m/z	z	RT	Fraction	Scan	Source File	Area Raw Ara h 6	#Feature	#Feature Raw Ara h 6	Start	End	PTM	AScore	Found By
R.C(+71.04)C(+57.02)DELNEMENTQR.C	Y	89.19	1711.6600	13	0.6	856.8378	2	28.19	2	F2:1295	OB5930 H6 raw.raw	1.4647E5	3	3	79	91	Carbamidomethylation	C1:Propionamide:10.11;C2:Carbamidomethylation:1000.00	PEAKS PTM
R.C(+71.04)DLDVSGGRC(+57.02)	Y	86.72	1151.4700	10	3.6	576.7443	2	25.80	1	F1:1058	OB5918 H6 raw.raw	7.0267E3	2	2	136	145	Propionamide; Carbamidomethylation	C1:Propionamide:123.21;C10:Carbamidomethylation:1000.00	PEAKS PTM
R.Q(-17.03)MVQQFKR.E	Y	81.09	1046.5331	8	1.5	524.2746	2	28.32	1	F1:1185	OB5918 H6 raw.raw	2.0304E5	3	3	112	119	Pyro-glu from Q	Q1:Pyro-glu from Q:1000.00	PEAKS PTM
R.C(+57.02)(+28.03)C(+57.02)DELNEMENTQR.C	Y	76.48	1725.6757	13	0.1	863.8452	2	28.26	2	F2:1306	OB5930 H6 raw.raw	1.2726E4	2	2	79	91	Carbamidomethylation	C1:Carbamidomethylation:1000.00;C1:Ethylation:13.67;C2:Carbamidomethylation:1000.00	PEAKS PTM
R.C(+71.04)C(+57.02)DELNEMENTQ(+.98)R.C	Y	73.91	1712.6440	13	0.5	857.3297	2	28.89	3	F3:1347	OB5931 H6 raw.raw	2.4957E5	3	3	79	91	Carbamidomethylation; Deamidation (NQ)	C1:Propionamide:12.28;C2:Carbamidomethylation:1000.00;Q12:Deamidation (NQ):56.37	PEAKS PTM
R.C(+57.02)C(+71.04)DELNEMENTQR.C	Y	73.29	1711.6600	13	2.8	856.8397	2	28.39	2	F2:1328	OB5930 H6 raw.raw	5.119E4	1	1	79	91	Carbamidomethylation	C1:Carbamidomethylation:1000.00;C2:Propionamide:12.28	PEAKS PTM

total 61 peptides

Peptide	Uniq	-10lgP	Mass	Length	ppm	m/z	z	RT	Fraction	Scan	Source File	Area Raw Ara h 6	#Feature	#Feature Raw Ara h 6	Start	End	PTM	AScore	Found By
R.C(-17.03)C(+57.02)D(+57.02)ELNEMENTQR.C	Y	72.79	1680.6178	13	1.3	841.3173	2	32.42	1	F1:1439	OB5918 H6 raw.raw	9.4578E3	1	1	79	91	Ammonia-loss (C@N-term); Carbamidomethylation	C1:Ammonia-loss (C@N-term):1000.00;C2:Carbamidomethylation:1000.00; D3:Carbamidomethylation (DHKE, X@N-term):12.28	PEAKS PTM
R.C(+57.02)DLDVSGGR.C	Y	69.89	977.4236	9	-0.8	489.7187	2	24.72	1	F1:1012	OB5918 H6 raw.raw	0	0	0	136	144	Carbamidomethylation	C1:Carbamidomethylation:1000.00	PEAKS DB
R.IMGEQEQYDSYDIRSTR.S	Y	68.55	2089.9375	17	2.6	697.6549	3	29.87	1	F1:1284	OB5918 H6 raw.raw	7.5645E4	3	3	55	71			PEAKS DB
R.Q(-17.03)VDRVNLKPC(+57.02)EQHIMQR.I	Y	67.85	2133.0571	17	1.7	534.2725	4	31.34	1	F1:1372	OB5918 H6 raw.raw	3.0682E5	2	2	38	54	Pyro-glu from Q; Carbamidomethylation	Q1:Pyro-glu from Q:1000.00; C10:Carbamidomethylation:1000.00	PEAKS PTM
R.IMGEQE(+21.98)QYDSYDIR.S	Y	66.88	1767.7385	14	1.6	884.8780	2	30.42	2	F2:1450	OB5930 H6 raw.raw	0	0	0	55	68		E6:Sodium adduct:12.81	PEAKS PTM
R.ELM(+15.99)NLPQQC(+71.04)NFR.A	Y	64.44	1578.7283	12	0.7	790.3719	2	29.48	2	F2:1390	OB5930 H6 raw.raw	1.3436E4	2	2	120	131	Oxidation (M); Propionamide	M3:Oxidation (M):1000.00; C9:Propionamide:1000.00	PEAKS PTM
R.E(-18.01)LMNLPQQC(+57.02)NFR.A	Y	61.73	1530.7072	12	1.2	766.3618	2	35.00	2	F2:1722	OB5930 H6 raw.raw	0	0	0	120	131	Pyro-glu from E; Carbamidomethylation	E1:Pyro-glu from E:1000.00; C9:Carbamidomethylation:1000.00	PEAKS PTM
R.IMGEQEQ(+.98)YDSYDIR.S	Y	56.51	1746.7406	14	5.7	874.3826	2	40.55	3	F3:2044	OB5931 H6 raw.raw	0	0	0	55	68		Q7:Deamidation (NQ): 19.68	PEAKS DB

total 61 peptides

Peptide	Uniq	-10lgP	Mass	Length	ppm	m/z	z	RT	Fraction	Scan	Source File	Area Raw Ara h 6	#Feature	#Feature Raw Ara h 6	Start	End	PTM	AScore	Found By
R.C(+57.02)M(+15.99)C(+57.02)EALQQIMENQC(+57.02)DR.L	Y	54.61	2100.8157	16	6.8	1051.4222	2	32.37	3	F3:1570	OB5931 H6 raw.raw	0	0	0	92	107	Carbamidomethylation	C1:Carbamidomethylation:1000.00;M2:Oxidation(M):43.13;C3:Carbamidomethylation:1000.00;C14:Carbamidomethylation:1000.00	PEAKS DB
R.IMGEQ(+.98)EQYDSYDIR.S	Y	54.23	1746.7406	14	9.5	874.3859	2	40.80	1	F1:1928	OB5918 H6 raw.raw	1.3457E3	1	1	55	68		Q5:Deamidation (NQ): 26.31	PEAKS DB
R.QMVQQFK.R	Y	53.65	907.4586	7	-0.3	454.7364	2	26.28	1	F1:1080	OB5918 H6 raw.raw	1.4384E4	3	3	112	118			PEAKS DB
R.C(+57.02)(+28.03)C(+57.02)DELNEMENTQ(+.98)R.C	Y	53.00	1726.6597	13	2.3	864.3391	2	28.76	1	F1:1209	OB5918 H6 raw.raw	7.4015E3	1	1	79	91	Carbamidomethylation	C1:Carbamidomethylation:1000.00;C1:Ethylation:5.47;C2:Carbamidomethylation:1000.00;Q12:Deamidation (NQ):25.21	PEAKS PTM
R.VNLKPC(+57.02)EQHIM(+15.99)QR.I	Y	52.18	1667.8236	13	1.8	556.9495	3	28.32	3	F3:1320	OB5931 H6 raw.raw	3.5343E4	3	3	42	54	Carbamidomethylation; Oxidation (M)	C6:Carbamidomethylation:1000.00;M11:Oxidation(M):1000.00	PEAKS DB

total 61 peptides

Peptide	Uniq	-10lgP	Mass	Length	ppm	m/z	z	RT	Fraction	Scan	Source File	Area Raw Ara h 6	#Feature	#Feature Raw Ara h 6	Start	End	PTM	AScore	Found By
R.C(+57.02)(+28.03)C(+57.02)DELNEMEN(+.98)TQR.C	Y	51.55	1726.6597	13	-0.1	864.3370	2	28.86	2	F2:1348	OB5930 H6 raw.raw	9.4354E3	1	1	79	91	Carbamidomethylation	C1:Carbamidomethylation:1000.00;C1:Ethylation:14.04;C2:Carbamidomethylation:1000.00;N10:Deamidation (NQ):27.19	PEAKS PTM
R.IMGEQEQYDSY(-18.01)DIR.S	Y	51.30	1727.7461	14	2.3	864.8823	2	31.01	2	F2:1485	OB5930 H6 raw.raw	0	0	0	55	68		Y11:Dehydration:0.00	PEAKS PTM
R.ELMNLPQQ(+.98)C(+57.02)NFR.A	Y	51.09	1549.7018	12	8.8	775.8650	2	43.21	1	F1:2062	OB5918 H6 raw.raw	5.8655E2	1	1	120	131	Carbamidomethylation	Q8:Deamidation (NQ):0.00;C9:Carbamidomethylation:1000.00	PEAKS DB
R.E(+53.92)LMNLPQQC(+57.02)NFR.A	Y	50.16	1602.6370	12	0.2	802.3259	2	31.56	1	F1:1384	OB5918 H6 raw.raw	0	0	0	120	131	Replacement of 2 protons by iron; Carbamidomethylation	E1:Replacement of 2 protons by iron:88.04;C9:Carbamidomethylation:1000.00	PEAKS PTM
R.C(+57.02)MC(+71.04)EALQQIMENQC(+57.02)DR.L	Y	50.11	2098.8362	16	3.8	1050.4293	2	34.51	3	F3:1696	OB5931 H6 raw.raw	0	0	0	92	107	Carbamidomethylation	C1:Carbamidomethylation:1000.00;C3:Propionamide:0.00;C14:Carbamidomethylation:1000.00	PEAKS PTM
R.QVDRVNLKPC(+57.02)EQHIMQR.I	Y	50.11	2150.0837	17	-0.7	538.5278	4	33.49	2	F2:1625	OB5930 H6 raw.raw	2.7618E4	1	1	38	54	Carbamidomethylation	C10:Carbamidomethylation:1000.00	PEAKS DB

total 61 peptides

Peptide	Uniq	-10lgP	Mass	Length	ppm	m/z	z	RT	Fraction	Scan	Source File	Area Raw Ara h 6	#Feature	#Feature Raw Ara h 6	Start	End	PTM	AScore	Found By
R.E(+21.98)LMNLPQQC(+57.02)NFR.A	Y	45.41	1570.6997	12	0.8	786.3577	2	31.52	1	F1:1386	OB5918 H6 raw.raw	9.3242E4	2	2	120	131	Sodium adduct; Carbamidomethylation	E1:Sodium adduct:57.72;C9:Carbamidomethylation:1000.00	PEAKS PTM
R.IMGEQEQYD(-18.01)SYDIR.S	Y	45.19	1727.7461	14	-0.1	864.8802	2	31.20	3	F3:1499	OB5931 H6 raw.raw	0	0	0	55	68		D9:Dehydration:8.69	PEAKS PTM
R.IMGE(+21.98)QEQYDSYDIR.S	Y	43.97	1767.7385	14	0.1	884.8766	2	30.72	3	F3:1472	OB5931 H6 raw.raw	2.0226E5	2	2	55	68		E4:Sodium adduct:17.01	PEAKS PTM
R.VNLKPC(+71.04)EQHIMQR.I	Y	42.26	1665.8444	13	0.2	556.2888	3	31.46	2	F2:1497	OB5930 H6 raw.raw	2.2279E4	1	1	42	54	Propionamide	C6:Propionamide:1000.00	PEAKS PTM
R.C(+57.02)C(+71.04)DELNEMENTQ(+.98)R.C	Y	40.31	1712.6440	13	0.5	857.3297	2	28.89	3	F3:1387	OB5931 H6 raw.raw	8.946E4	1	1	79	91	Carbamidomethylation	C1:Carbamidomethylation:1000.00;C2:Propionamide:0.00;Q12:Deamidation (NQ):28.86	PEAKS PTM
R.ELMNLPQ(+.98)QC(+57.02)NFR.A	Y	38.68	1549.7018	12	9.1	775.8652	2	43.03	1	F1:2056	OB5918 H6 raw.raw	0	0	0	120	131	Carbamidomethylation	Q7:Deamidation (NQ):12.28;C9:Carbamidomethylation:1000.00	PEAKS DB
R.E(+42.01)LMN(+.98)LPQQC(+57.02)NFR.A	Y	37.89	1591.7123	12	6.4	796.8685	2	29.86	2	F2:1416	OB5930 H6 raw.raw	0	0	0	120	131	Acetylation (N-term); Deamidation (NQ); Carbamidomethylation	E1:Acetylation (N-term):1000.00;N4:Deamidation (NQ):56.34;C9:Carbamidomethylation:1000.00	PEAKS PTM
R.IMGEQE(+53.92)QYDSYDIR.S	Y	36.98	1799.6759	14	0.5	900.8456	2	31.02	1	F1:1351	OB5918 H6 raw.raw	0	0	0	55	68		E6:Replacement of 2 protons by iron:22.45	PEAKS PTM
R.IM(+15.99)GEQEQYDSYDIRSTR.S	Y	36.12	2105.9324	17	1.8	702.9860	3	28.23	2	F2:1323	OB5930 H6 raw.raw	1.5096E3	1	1	55	71	Oxidation (M)	M2:Oxidation (M):1000.00	PEAKS DB

total 61 peptides

Peptide	Uniq	-10lgP	Mass	Length	ppm	m/z	z	RT	Fraction	Scan	Source File	Area Raw Ara h 6	#Feature	#Feature Raw Ara h 6	Start	End	PTM	AScore	Found By
C.C(+57.02)D(+43.99)ELNEMENTQ(+.98)R.C	Y	35.98	1582.5875	12	-8.0	792.2947	2	30.54	3	F3:1456	OB5931 H6 raw.raw	3.9371E3	1	1	80	91	Carbamidomethylation; Carboxylation (DKW)	C1:Carbamidomethylation:1000.00;D2:Carboxylation (DKW):1000.00;Q11:Deamidation (NQ):33.91	PEAKS PTM
R.C(+57.02)MC(+57.02)EALQ(+.98)QIMENQC(+57.02)DRLQDR.Q	Y	34.72	2598.0754	20	2.7	867.0348	3	39.74	2	F2:1998	OB5930 H6 raw.raw	0	0	0	92	111	Carbamidomethylation	C1:Carbamidomethylation:1000.00;C3:Carbamidomethylation:1000.00;Q7:Deamidation (NQ):0.00;C14:Carbamidomethylation:1000.00	PEAKS DB
R.QMVQQFKR.E	Y	34.28	1063.5597	8	-2.0	532.7861	2	27.21	1	F1:1126	OB5918 H6 raw.raw	2.4157E4	2	2	112	119			PEAKS DB
R.IM(+15.99)GEQE(+53.92)QYDSYDIR.S	Y	33.87	1815.6708	14	-0.1	908.8425	2	29.10	3	F3:1374	OB5931 H6 raw.raw	0	0	0	55	68	Oxidation (M)	M2:Oxidation (M):1000.00;E6:Replacement of 2 protons by iron:33.18	PEAKS PTM
R.IMGE(+14.02)QEQ(+.98)YDSYDIR.S	Y	33.33	1760.7563	14	7.2	881.3918	2	31.49	1	F1:1379	OB5918 H6 raw.raw	0	0	0	55	68		E4:Methylation (others):7.32;Q7:Deamidation (NQ):14.04	PEAKS PTM
R.E(+43.01)LMNLPQQC(+57.02)NFR.A	Y	32.96	1591.7235	12	-0.5	796.8687	2	30.04	3	F3:1430	OB5931 H6 raw.raw	0	0	0	120	131	Carbamylation; Carbamidomethylation	E1:Carbamylation:1000.00;C9:Carbamidomethylation:1000.00	PEAKS PTM

total 61 peptides

Peptide	Uniq	-10lgP	Mass	Length	ppm	m/z	z	RT	Fraction	Scan	Source File	Area Raw Ara h 6	#Feature	#Feature Raw Ara h 6	Start	End	PTM	AScore	Found By
R.Q(-17.03)M(+15.99)VQQFKR.E	Y	32.86	1062.5281	8	-0.5	532.2711	2	26.15	3	F3:1207	OB5931 H6 raw.raw	1.4877E3	1	1	112	119	Pyro-glu from Q; Oxidation (M)	Q1:Pyro-glu from Q:1000.00;M2:Oxidation (M):1000.00	PEAKS PTM
C.C(+57.02)D(+21.98)E(+21.98)LNEMENTQR.C	Y	32.78	1581.5776	12	4.1	791.7993	2	30.09	3	F3:1429	OB5931 H6 raw.raw	3.1759E3	1	1	80	91	Carbamidomethylation; Sodium adduct	C1:Carbamidomethylation:1000.00;D2:Sodium adduct:51.96;E3:Sodium adduct:65.81	PEAKS PTM
R.I(+43.01)MGEQEQYDSYDIR.S	Y	31.44	1788.7625	14	5.9	895.3937	2	29.69	3	F3:1409	OB5931 H6 raw.raw	0	0	0	55	68	Carbamylation	I1:Carbamylation:1000.00	PEAKS PTM
R.C(+71.04)C(+57.02)DELNEMEN(+.98)TQR.C	Y	30.87	1712.6440	13	0.3	857.3296	2	28.68	2	F2:1374	OB5930 H6 raw.raw	8.661E4	1	1	79	91	Carbamidomethylation	C1:Propionamide:7.65;C2:Carbamidomethylation:1000.00;N10:Deamidation (NQ):20.41	PEAKS PTM
C.M(+175.04)C(+57.02)EALQQIMENQC(+57.02)DR.L	Y	30.83	2099.8323	15	6.2	1050.9299	2	34.47	2	F2:1687	OB5930 H6 raw.raw	1.8085E3	1	1	93	107	Naphthalene-2,3-dicarboxaldehyde; Carbamidomethylation	M1:Naphthalene-2,3-dicarboxaldehyde:1000.00;C2:Carbamidomethylation:1000.00;C13:Carbamidomethylation:1000.00	PEAKS PTM

total 61 peptides

Peptide	Uniq	-10lgP	Mass	Length	ppm	m/z	z	RT	Fraction	Scan	Source File	Area Raw Ara h 6	#Feature	#Feature Raw Ara h 6	Start	End	PTM	AScore	Found By
R.C(+57.02)MC(+57.02)EALQQIM(+15.99)ENQC(+57.02)D(-18.01)RLQDR.Q	Y	30.45	2595.0757	20	2.6	866.0348	3	34.46	1	F1:1551	OB5918 H6 raw.raw	3.0045E4	1	1	92	111	Carbamidomethylation; Oxidation (M)	C1:Carbamidomethylation:1000.00;C3:Carbamidomethylation:1000.00;M10:Oxidation (M):99.62;C14:Carbamidomethylation:1000.00;D15:Dehydration:19.86	PEAKS PTM
total 61 peptides																			

B0YIU5|B0YIU5_ARAHY

back to list

| Protein Coverage | Supporting Peptides |

Protein Coverage:

47 d

1 MGVHTFEEES TSPVPPAKLF KATVVDGDEL TPKLIPAIQS IEIVEGNGGP GTVKKVTAVE DGKTSYVLHK IDAIDEATYT

d Deamidation (NQ) (+0.98)

81 YDYTISGGTG FQEILEKVSF KTKLEAADGG SKIKVSVTFH TKGDAPLPDE VHQDVKQKSQ GIFKAIEGYV LSN

Supporting Peptides:

Peptide	Uniq	-10lgP	Mass	Length	ppm	m/z	z	RT	Fraction	Scan	Source File	Area Raw Ara h 6	#Feature	#Feature Raw Ara h 6	Start	End	PTM	AScore	Found By
K.IDAIDEATYTYDYTISGGTGFQEILEK.V	Y	146.27	3012.4126	27	4.4	1507.2202	2	38.52	2	F2:1926	OB5930 H6 raw.raw	7.9824E4	2	2	71	97			PEAKS DB
K.LIPAIQSIEIVEGN(+.98)GGPGTVK.K	Y	143.79	2092.1416	21	4.7	1047.0830	2	35.82	1	F1:1635	OB5918 H6 raw.raw	5.1573E4	1	1	34	54	Deamidation (NQ)	N14:Deamidation (NQ):177.50	PEAKS DB
K.LIPAIQSIEIVEGNGGPGTVK.K	Y	116.87	2091.1575	21	2.0	1046.5881	2	35.72	3	F3:1774	OB5931 H6 raw.raw	1.7378E5	3	3	34	54			PEAKS DB
K.GDAPLPDEVHQDVK.Q	Y	88.76	1518.7314	14	0.6	760.3735	2	27.51	1	F1:1143	OB5918 H6 raw.raw	3.5932E3	3	3	123	136			PEAKS DB
K.ATVVDGDELTPK.L	Y	74.60	1243.6295	12	1.0	622.8226	2	28.00	3	F3:1307	OB5931 H6 raw.raw	1.0017E4	2	2	22	33			PEAKS DB
M.GVHTFEEESTSPVPPAK.L	Y	70.31	1810.8737	17	0.6	906.4446	2	28.77	3	F3:1359	OB5931 H6 raw.raw	6.2111E3	3	3	2	18			PEAKS DB
total 6 peptides																			

#CONTAM#P00761

back to list

| Protein Coverage | Supporting Peptides |

Protein Coverage:

77

1 FPTDDDDKIV GGYTCAANSI PYQVSLNSGS HFCGGSLINS QWVVSAAHCY KSRIQVR**LGE HNIDVLEGNE QFINAAK**IIT

81 HPN**FNGNTLD NDIMLIKLSS PATLNSRVAT VSLPR**SCAAA GTECLISGWG NTKSSGSSYP SLLQCLKAPV LSDSSCKSSY

161 PGQITGNMIC VGFLEGGKDS CQGDSGGPVV CNGQLQGIVS WGYGCAQKNK PGVYTK**VCNY VNWIQQTIAA N**

c Carbamidomethylation (+57.02)
d Dimethylation(KR) (+28.03)
m Methylation(KR) (+14.02)

Supporting Peptides:

Peptide	Uniq	-10lgP	Mass	Length	ppm	m/z	z	RT	Fraction	Scan	Source File	Area Raw Ara h 6	#Feature	#Feature Raw Ara h 6	Start	End	PTM	AScore	Found By
K.VC(+57.02)NYVNWIQQTIAAN	Y	120.42	1792.8567	15	5.9	897.4409	2	38.51	1	F1:1793	OB5918 H6 raw.raw	0	0	0	217	231	Carbamidomethylation	C2:Carbamidomethylation:1000.00	PEAKS DB
R.LGEHNIDVLEGNEQFINAAK(+28.03).I	Y	100.40	2238.1279	20	2.5	747.0518	3	33.89	1	F1:1519	OB5918 H6 raw.raw	1.029E5	3	3	58	77	Dimethylation(KR)	K20:Dimethylation (KR):1000.00	PEAKS PTM
R.LGEHNIDVLEGNEQFIN.A	Y	100.26	1939.9275	17	2.1	970.9730	2	34.08	1	F1:1536	OB5918 H6 raw.raw	4.5041E5	3	3	58	74			PEAKS DB
K.LSSPATLNSR.V	Y	95.73	1044.5564	10	1.3	523.2861	2	25.64	1	F1:1052	OB5918 H6 raw.raw	7.8385E4	3	3	98	107			PEAKS DB
R.LGEHNIDVLEGNEQFINAAK(+14.02).I	Y	78.26	2224.1123	20	1.3	742.3790	3	34.01	2	F2:1668	OB5930 H6 raw.raw	7.5546E4	2	2	58	77	Methylation(KR)	K20:Methylation(KR):1000.00	PEAKS PTM
R.VATVSLPR.S	Y	68.83	841.5021	8	2.4	421.7593	2	27.95	3	F3:1328	OB5931 H6 raw.raw	1.1038E6	3	3	108	115			PEAKS DB
R.LGEHNIDVLEGNEQFINAA.K	Y	66.72	2082.0017	19	2.5	1042.0107	2	34.65	1	F1:1570	OB5918 H6 raw.raw	4.6459E4	3	3	58	76			PEAKS DB
R.LGEHNIDVLEGNEQ.F	Y	60.51	1565.7322	14	-8.2	783.8669	2	30.42	1	F1:1342	OB5918 H6 raw.raw	5.6676E3	1	1	58	71			PEAKS DB
R.V(+28.03)ATVSLPR.S	Y	52.23	869.5334	8	-0.2	435.7739	2	28.42	3	F3:1321	OB5931 H6 raw.raw	2.1492E4	2	2	108	115		V1:Ethylation:38.93	PEAKS PTM
R.LGEHNIDVLEGNEQFINA.A	Y	48.56	2010.9646	18	2.2	1006.4918	2	34.51	1	F1:1560	OB5918 H6 raw.raw	0	0	0	58	75			PEAKS DB
R.VAT(-18.01)VSLPR.S	Y	48.07	823.4916	8	1.3	412.7536	2	27.95	3	F3:1296	OB5931 H6 raw.raw	4.973E5	2	2	108	115		T3:Dehydration:0.00	PEAKS PTM
N.FNGNTLDND(+28.03)IMLIKLSSPATLNSR.V	Y	32.40	2661.3796	24	-0.1	888.1337	3	37.18	3	F3:1841	OB5931 H6 raw.raw	5.7847E4	1	1	84	107		D9:Ethylation:15.43	PEAKS PTM

total 12 peptides

Q2PK12|Q2PK12_ARAHY

back to list

| Protein Coverage | Supporting Peptides |

Protein Coverage:

1 MANAASGMAV HDDCKLRFLE LKAKRTHR**FI VFKIEENQK**Q VIVEK**LGEPA QGYEDFTACL PPNECRYAVY DFEYLTEGNV**

81 **PK**SR**IFFIAW SPDTSR**VRNK MIYASSKDRF KR**ELDGIQVE LQATDPTEMD LDVFK**SRAN

c Carbamidomethylation (+57.02)

Supporting Peptides:

Peptide	Uniq	-10lgP	Mass	Length	ppm	m/z	z	RT	Fraction	Scan	Source File	Area Raw Ara h 6	#Feature	#Feature Raw Ara h 6	Start	End	PTM	AScore	Found By

total 5 peptides

Peptide	Uniq	-10lgP	Mass	Length	ppm	m/z	z	RT	Fraction	Scan	Source File	Area Raw Ara h 6	#Feature	#Feature Raw Ara h 6	Start	End	PTM	AScore	Found By
R.YAVYDFEYLTEGNVPK.S	Y	136.28	1906.8988	16	3.0	954.4595	2	35.51	2	F2:1754	OB5930 H6 raw.raw	4.1839E4	3	3	67	82			PEAKS DB
R.IFFIAWSPDTSR.V	Y	127.03	1438.7245	12	2.8	720.3715	2	36.96	1	F1:1697	OB5918 H6 raw.raw	8.3888E4	3	3	85	96			PEAKS DB
R.FIVFKIEENQK.Q	Y	80.38	1393.7605	11	2.4	697.8892	2	34.12	3	F3:1671	OB5931 H6 raw.raw	3.2374E4	3	3	29	39			PEAKS DB
K.LGEPAQGYEDFTAC(+57.02)LPPNEC(+57.02)R.Y	Y	71.22	2423.0522	21	-2.6	1212.5303	2	33.24	2	F2:1619	OB5930 H6 raw.raw	0	0	0	46	66	Carbamidomethylation	C14:Carbamidomethylation:1000.00;C20:Carbamidomethylation:1000.00	PEAKS DB
R.ELDGIQVELQATDPTEMDLDVFK.S	Y	65.93	2605.2468	23	-1.1	1303.6293	2	38.15	3	F3:1905	OB5931 H6 raw.raw	0	0	0	113	135			PEAKS DB
**total 5 peptides**																			

#CONTAM#P04264

back to list

| Protein Coverage | Supporting Peptides |

Protein Coverage:

1 MSRQFSSRSG YRSGGGFSSG SAGIINYQRR TTSSSTRRSG GGGRFSSCG GGGGSFGAGG GFGSR**SLVNL GGSKSISIS**V
81 ARGGGRGSGF GGGYGGGGFG GGGFGGGGFG GGGIGGGGFG GFGSGGGGFG GGGFGGGGYG GGYGPVCPPG GIQEVTINQS
161 LLQPLNVEID PEIQKVKSRE REQIK**SLNNQ FASFIDKVRF LEQQNQVLQT KWELLQQVDT STR**THNLEPY FESFINNLRR
241 RVDQLKSDQS RLDSELKNMQ DMVEDYRNKY EDEINKR**TNA ENEFVTIKK**D VDGAYMTKVD LQAKLDNLQQ EIDFLTALYQ
321 AELSQMQTQI SETNVILSMD NNR**SLDLDSI IAEVKAQ**NED IAQKSKAEAE SLYQSKYEEL QITAGRHGDS VRNSKIEISE
401 LNRVIQRLRS EIDNVKKQIS NLQQSISDAE QRGENALKDA K**NKLNDLEDA LQQAKEDLAR** LLRDYQELMN TKLALDLEIA
481 TYRTLLEGEE SRMSGECAPN VSVSVSTSHT TISGGGSRGG GGGGYGSGGS SYGSGGGSYG SGGGGGGGRG SYGSGGSSYG
561 SGGGSYGSGG GGGGHGSYGS GSSSGGYRGG SGGGGGGSSG GRGSGGGSSG GSIGGRGSSS GGVKSSGGSS SVRFVSTTYS
641 GVTR

Supporting Peptides:

Peptide	Uniq	-10lgP	Mass	Length	ppm	m/z	z	RT	Fraction	Scan	Source File	Area Raw Ara h 6	#Feature	#Feature Raw Ara h 6	Start	End	PTM	AScore	Found By
R.SLDLDSIIAEVK.A	Y	123.83	1301.7078	12	1.1	651.8619	2	36.99	2	F2:1837	OB5930 H6 raw.raw	6.7781E4	2	2	344	355			PEAKS DB
K.NKLNDLEDALQQAKEDLAR.L	Y	73.20	2183.1182	19	0.4	728.7136	3	37.18	3	F3:1846	OB5931 H6 raw.raw	3.6534E4	1	1	442	460			PEAKS DB
R.FLEQQNQVLQTKWELLQQVDTSTR.T	Y	71.24	2931.5090	24	2.7	978.1796	3	36.22	3	F3:1790	OB5931 H6 raw.raw	5.1596E4	1	1	200	223			PEAKS DB
R.SLDLDSIIAEVKAQ.N	Y	62.74	1500.8035	14	2.3	751.4108	2	37.75	3	F3:1886	OB5931 H6 raw.raw	1.2762E4	1	1	344	357			PEAKS DB
K.WELLQQVDTSTR.T	Y	43.36	1474.7416	12	2.5	738.3799	2	33.91	3	F3:1661	OB5931 H6 raw.raw	0	0	0	212	223			PEAKS DB
R.TNAENEFVTIKK.D	Y	38.64	1392.7249	12	4.5	697.3728	2	27.57	2	F2:1278	OB5930 H6 raw.raw	0	0	0	278	289			PEAKS DB
K.SLNNQFASFIDKVR.F	Y	36.49	1637.8525	14	1.3	819.9346	2	35.54	1	F1:1619	OB5918 H6 raw.raw	0	0	0	186	199			PEAKS DB
R.SLVNLGGSKSISIS.V	Y	34.13	1360.7561	14	2.2	681.3868	2	32.06	1	F1:1422	OB5918 H6 raw.raw	4.8489E3	1	1	66	79			PEAKS DB
K.SLNN(+.98)QFASFIDKVR.F	Y	31.54	1638.8365	14	-3.1	820.4230	2	35.40	1	F1:1611	OB5918 H6 raw.raw	0	0	0	186	199		N4:Deamidation (NQ):0.00	PEAKS DB
**total 9 peptides**																			

#CONTAM#P13645

back to list

| Protein Coverage | Supporting Peptides |

Protein Coverage:

1 MSVRYSSSKH YSSSRSGGGG GGGGCGGGGG VSSLRISSSK **GSLGGGFSSG GFSGGSFSR**G SSGGGCFGGS SGGYGGLGGF

Oxidation (M) (+15.99)

81 GGGSFRGSYG SSSFGGSYGG SFGGGSFGGG SFGGGSFGGG GFGGGGFGGG FGGGFGGDGG LLSGNEKVTM QNLNDRLASY

161 LDKVRALEES NYELEGKIKE WYEKHGNSHQ GEPRDYSKYY KTIDDLKNQI LNLTTDNANI LLQIDNARLA ADDFRLKYEN

271

241 EVALRQSVEA DINGLRR**VLD ELTLTKADLE MQIESLTEEL AYLKK**NHEEE MKDLR**NVSTG DVNVEMNAAP GVDLTQLLNN**

321 **MR**SQYEQLAE QNRKDAEAWF NEKSKELTTE IDNNIEQISS YKSEITELRR NVQALEIELQ SQLALKQSLE ASLAETEGRY

401 CVQLSQIQAQ ISALEEQLQQ IRAETECQNT EYQQLLDIKI RLENEIQTYR SLLEGEGSSG GGGRGGGSFG GGYGGGSSGG

481 GSSGGGYGGG HGGSSGGGYG GGSSGGGSSG GGYGGGSSSG GHGGGSSSGG HGGSSSGGYG GGSSGGGGGG YGGGSSGGGS

561 SSGGGYGGGS SSGGHKSSSS GSVGESSSKG PRY

Supporting Peptides:

Peptide	Uniq	-10lgP	Mass	Length	ppm	m/z	z	RT	Fraction	Scan	Source File	Area Raw Ara h 6	#Feature	#Feature Raw Ara h 6	Start	End	PTM	AScore	Found By
N.AAPGVDLTQLLNNMR.S	Y	101.33	1611.8402	15	0.9	806.9281	2	37.36	3	F3:1861	OB5931 H6 raw.raw	1.1703E4	1	1	308	322			PEAKS DB
K.GSLGGGFSSGGFSGGSFSR.G	Y	88.34	1706.7648	19	1.3	854.3908	2	32.34	3	F3:1568	OB5931 H6 raw.raw	8.1302E3	2	2	41	59			PEAKS DB
R.VLDELTLTK.A	Y	66.95	1030.5911	9	0.3	516.3030	2	30.88	1	F1:1345	OB5918 H6 raw.raw	2.1085E3	1	1	258	266			PEAKS DB
R.NVSTGDVNVEMNAAPGVDLTQLLNNMR.S	Y	64.23	2871.3855	27	5.2	1436.7075	2	38.37	2	F2:1917	OB5930 H6 raw.raw	0	0	0	296	322			PEAKS DB
K.ADLEM(+15.99)QIESLTEELAYLKK.N	Y	42.92	2239.1294	19	0.1	747.3838	3	38.90	2	F2:1948	OB5930 H6 raw.raw	0	0	0	267	285	Oxidation (M)	M5:Oxidation (M):100 0.00	PEAKS DB
total 5 peptides																			

A0A0A6ZDP1|A0A0A6ZDP1_ARAHY

back to list

| Protein Coverage | Supporting Peptides |

Protein Coverage:

1 MLVHYQIALF HLPRSSTTGL GIVEGLMTTV HSITATQKTV DGPSSKDWRG GR**AASFNIIP SSTGAAK**AVG KVLPSLNGKL

81 TGMAFR**VPTV DVSVVDLTVR** LEKPATYDEI K**QAIKEESEG KLKGILGYTE DDVVSTDFVG DSR**SSIFDAK AGIALSKNFV

161 K**IVSWYDNEW GYSTR**VVDLI VHIAKQ

Supporting Peptides:

Peptide	Uniq	-10lgP	Mass	Length	ppm	m/z	z	RT	Fraction	Scan	Source File	Area Raw Ara h 6	#Feature	#Feature Raw Ara h 6	Start	End	PTM	AScore	Found By
R.VPTVDVSVVDLTVR.L	Y	95.12	1497.8402	14	2.0	749.9289	2	34.49	1	F1:1559	OB5918 H6 raw.raw	0	0	0	87	100			PEAKS DB

total 5 peptides

Peptide	Uniq	-10lgP	Mass	Length	ppm	m/z	z	RT	Fraction	Scan	Source File	Area Raw Ara h 6	#Feature	#Feature Raw Ara h 6	Start	End	PTM	AScore	Found By
K.IVSWYDNEWGYSTR.V	Y	75.46	1774.7950	14	2.5	888.4070	2	34.69	2	F2:1704	OB5930 H6 raw.raw	0	0	0	162	175			PEAKS DB
K.GILGYTEDDVVSTDFVGDSR.S	Y	68.51	2143.9910	20	3.8	1073.0068	2	35.45	3	F3:1754	OB5931 H6 raw.raw	9.6352E3	2	2	124	143			PEAKS DB
R.AASFNIIPSSTGAAK.A	Y	65.40	1433.7513	15	1.7	717.8842	2	31.18	2	F2:1493	OB5930 H6 raw.raw	1.4402E4	3	3	53	67			PEAKS DB
K.QAIK(+57.02)EESEGKLK(+57.02).G	Y	30.70	1472.7834	12	-9.8	737.3918	2	27.47	2	F2:1272	OB5930 H6 raw.raw	0	0	0	112	123		K4:Carbamidomethylation (DHKE, X@N-term):8.14;K12:Carbamidomethylation (DHKE, X@N-term):8.64	PEAKS PTM
**total 5 peptides**																			

E9LFE8|E9LFE8_ARAHY

back to list

| Protein Coverage | Supporting Peptides |

Protein Coverage:

```
  1 KSRKFFLGGK PQEEKGEEGN MFSGLELKTV AESLGIDMGI AGKVQGVDDP RGSIIIVEDE LETLSPAVEE SGNGNGLDET
 81 LCTLRLVHQL AESTDADKYN PRAGFLTALN TPNLPVLQYV QLGADRGVFY KNAVMAPHYN LNCHAVIYGT EGRGWIEVVG
161 ENGRKVYEGE VREGQILIVP QQFMVAKKAA EGSDEGFGWI AVKTSDNPMI SPLAGKLSLI RAMPLPVLMN SFRLTAEEAI
241 NLKKRGELTF FSPDPAHTQI
```

Supporting Peptides:

Peptide	Uniq	-10lgP	Mass	Length	ppm	m/z	z	RT	Fraction	Scan	Source File	Area Raw Ara h 6	#Feature	#Feature Raw Ara h 6	Start	End	PTM	AScore	Found By
K.AAEGSDEGFGWIAVK.T	Y	115.79	1535.7256	15	1.1	768.8709	2	34.20	1	F1:1542	OB5918 H6 raw.raw	2.877E3	1	1	189	203			PEAKS DB
K.TSDNPMISPLAGK.L	Y	93.15	1329.6598	13	0.3	665.8374	2	30.38	3	F3:1447	OB5931 H6 raw.raw	3.9471E3	2	2	204	216			PEAKS DB
R.AMPLPVLMNSFR.L	Y	37.60	1374.7152	12	-0.2	688.3647	2	37.09	1	F1:1708	OB5918 H6 raw.raw	1.0242E3	1	1	222	233			PEAKS DB
**total 3 peptides**																			

Q45W80|Q45W80_ARAHY

back to list

| Protein Coverage | Supporting Peptides |

Protein Coverage:

```
  1 MAEQTFIMIK PDGVQRGLVG EIISRFEKKG FYLKGLKLIT VDRPFAERHY SDLSAKPFFN GLVEYIISGP VVAMVWEGKG
 81 VVATGRKLIG ATNPLASEPG TIRGDFAIDI GRNVIHGSDS VESATKEIAL WFPEGPANWQ SSLHSWIYE
```

a Acetylation (Protein N-term) (+42.01)

Supporting Peptides:

Peptide	Uniq	-10lgP	Mass	Length	ppm	m/z	z	RT	Fraction	Scan	Source File	Area Raw Ara h 6	#Feature	#Feature Raw Ara h 6	Start	End	PTM	AScore	Found By
R.GLVGEIISRFEK.K	Y	83.87	1346.7557	12	0.4	674.3854	2	38.32	2	F2:1916	OB5930 H6 raw.raw	2.835E4	3	3	17	28			PEAKS DB
K.LIGATNPLASEPGTIR.G	Y	81.30	1608.8834	16	1.5	805.4502	2	31.70	1	F1:1395	OB5918 H6 raw.raw	1.5218E4	2	2	88	103			PEAKS DB
R.GLVGEIISR.F	Y	80.43	942.5498	9	0.6	472.2825	2	31.88	1	F1:1400	OB5918 H6 raw.raw	1.5932E4	1	1	17	25			PEAKS DB
M.A(+42.01)EQTFIMIKPDGVQR.G	Y	70.92	1773.9083	15	0.0	887.9614	2	34.01	2	F2:1657	OB5930 H6 raw.raw	4.9394E4	3	3	2	16	Acetylation (Protein N-term)	A1:Acetylation (Protein N-term):1000.00	PEAKS PTM
**total 4 peptides**																			

Q6PSU3|Q6PSU3_ARAHY

back to list

| Protein Coverage | Supporting Peptides |

Protein Coverage:

```
  1 MRGRVSPLML LLGILVLASV SATQAKSPYR KTENPCAQRC LQSCQQEPDD LKQKACESRC TKLEYDPRCV YDTGATNQRH
 81 PPGERTRGRQ PGDYDDDRRQ PRREEGGRWG PAEPRERERE EDWRQPREDW RRPSHQQPRK IRPEGREGEQ EWGTPGSEVR
161 EETSRNNPFY FPSRRFSTRY GNQNGRIRVL QRFDQRSKQF QNLQNHRIVQ IEARPNTLVL PKHADADNIL VIQQGQATVT
241 VANGNNRKSF NLDEGHALRI PSGFISYILN RHDNQNLRVA KISMPVNTPG QFEDFFPASS RDQSSYLQGF SRNTLEAAFN
321 AEFNEIRRVL LEENAGGEQE ERGQRRRSTR SSDNEGVIVK VSKEHVQELT KHAKSVSKKG SEEEDITNPI NLRDGEPDLS
401 NNFGRLFEVK PDKKNPQLQD LDMMLTCVEI KEGALMLPHF NSKAMVIVVV NKGTGNLELV AVRKEQQQRG RREQEWEEEE
481 EDEEEEGSNR EVRRYTARLK EGDVFIMPAA HPVAINASSE LHLLGFGINA ENNHRIFLAG DKDNVIDQIE KQAKDLAFPG
561 SGEQVEKLIK NQRESHFVSA
```

Supporting Peptides:

Peptide	Uniq	-10lgP	Mass	Length	ppm	m/z	z	RT	Fraction	Scan	Source File	Area Raw Ara h 6	#Feature	#Feature Raw Ara h 6	Start	End	PTM	AScore	Found By
R.IPSGFISYILNR.H	Y	81.38	1378.7609	12	2.6	690.3895	2	37.54	1	F1:1736	OB5918 H6 raw.raw	2.1534E4	2	2	260	271			PEAKS DB
K.ISMPVNTPGQFEDFFPASSR.D	Y	61.71	2226.0415	20	0.0	1114.0281	2	36.55	3	F3:1813	OB5931 H6 raw.raw	0	0	0	282	301			PEAKS DB
**total 2 peptides**																			

sp|P43237|ALL11_ARAHY

back to list

| Protein Coverage | Supporting Peptides |

Protein Coverage:

```
  1 MRGRVSPLML LLGILVLASV SATQAKSPYR KTENPCAQRC LQSCQQEPDD LKQKACESRC TKLEYDPRCV YDTGATNQRH
 81 PPGERTRGRQ PGDYDDDRRQ PRREEGGRWG PAEPRERERE EDWRQPREDW RRPSHQQPRK IRPEGREGEQ EWGTPGSEVR
161 EETSRNNPFY FPSRRFSTRY GNQNGRIRVL QRFDQRSKQF QNLQNHRIVQ IEARPNTLVL PKHADADNIL VIQQGQATVT
241 VANGNNRKSF NLDEGHALRI PSGFISYILN RHDNQNLRVA KISMPVNTPG QFEDFFPASS RDQSSYLQGF SRNTLEAAFN
321 AEFNEIRRVL LEENAGGEQE ERGQRRRSTR SSDNEGVIVK VSKEHVQELT KHAKSVSKKG SEEEDITNPI NLRDGEPDLS
401 NNFGRLFEVK PDKKNPQLQD LDMMLTCVEI KEGALMLPHF NSKAMVIVVV NKGTGNLELV AVRKEQQQRG RREQEWEEEE
481 EDEEEEGSNR EVRRYTARLK EGDVFIMPAA HPVAINASSE LHLLGFGINA ENNHRIFLAG DKDNVIDQIE KQAKDLAFPG
561 SGEQVEKLIK NQRESHFVSA RPQSQSPSSP EKEDQEEENQ GGKGPLLSIL KAFN
```

Supporting Peptides:

Peptide	Uniq	-10lgP	Mass	Length	ppm	m/z	z	RT	Fraction	Scan	Source File	Area Raw Ara h 6	#Feature	#Feature Raw Ara h 6	Start	End	PTM	AScore	Found By
R.IPSGFISYILNR.H	Y	81.38	1378.7609	12	2.6	690.3895	2	37.54	1	F1:1736	OB5918 H6 raw.raw	2.1534E4	2	2	260	271			PEAKS DB
K.ISMPVNTPGQFEDFFPASSR.D	Y	61.71	2226.0415	20	0.0	1114.0281	2	36.55	3	F3:1813	OB5931 H6 raw.raw	0	0	0	282	301			PEAKS DB
**total 2 peptides**																			

B3IXL2|B3IXL2_ARAHY

back to list

| Protein Coverage | Supporting Peptides |

Protein Coverage:

```
  1 MRGRVSPLML LLGILVLASV SATQAKSPYR KTENPCAQRC LQSCQQEPDD LKQKACESRC TKLEYDPRCV YDTGATNQRH
 81 PPGERTRGRQ PGDYDDDRRQ PRREEGGRWG PAEPRERERE EDWRQPREDW RRPSHQQPRK IRPEGREGEQ EWGTPGSEVR
161 EETSRNNPFY FPSRRFSTRY GNQNGRIRVL QRFDQRSKQF QNLQNHRIVQ IEARPNTLVL PKHADADNIL VIQQGQATVT
241 VANGNNRKSF NLDEGHALRI PSGFISYILN RHDNQNLRVA KISMPVNTPG QFEDFFPASS RDQSSYLQGF SRNTLEAAFN
321 AEFNEIRRVL LEENAGGEQE ERGQRRRSTR SSDNEGVIVK VSKEHVQELT KHAKSVSKKG SEEEDITNPI NLRDGEPDLS
401 NNFGRLFEVK PDKKNPQLQD LDMMLTCVEI KEGALMLPHF NSKAMVIVVV NKGTGNLELV AVRKEQQQRG RREQEWEEEE
481 EDEEEEGSNR EVRRYTARLK EGDVFIMPAA HPVAINASSE LHLLGFGINA ENNHRIFLAG DKDNVIDQIE KQAKDLAFPG
561 SGEQVEKLIK NQRESHFVSA RPQSQSPSSP EKEDQEEENQ GGKGPLLSIL KAFN
```

Supporting Peptides:

Peptide	Uniq	-10lgP	Mass	Length	ppm	m/z	z	RT	Fraction	Scan	Source File	Area Raw Ara h 6	#Feature	#Feature Raw Ara h 6	Start	End	PTM	AScore	Found By
R.IPSGFISYILNR.H	Y	81.38	1378.7609	12	2.6	690.3895	2	37.54	1	F1:1736	OB5918 H6 raw.raw	2.1534E4	2	2	260	271			PEAKS DB
K.ISMPVNTPGQFEDFFPASSR.D	Y	61.71	2226.0415	20	0.0	1114.0281	2	36.55	3	F3:1813	OB5931 H6 raw.raw	0	0	0	282	301			PEAKS DB
**total 2 peptides**																			

N1NG13|N1NG13_ARAHY

back to list

| Protein Coverage | Supporting Peptides |

Protein Coverage:

```
  1 MRGRVSPLML LLGILVLASV SATHAKSSPY QKKTENPCAQ RCLQSCQQEP DDLKQKACES RCTKLEYDPR CVYDPRGHTG
 81 TTNQRSPPGE RTRGRQPGDY DDDRRQPRRE EGGRWGPAGP RERERREEDWR QPREDWRRPS HQQPRKIRPE GREGEQEWGT
161 PGSHVREETS RNNPFYFPSR RFSTRYGNQN GRIRVLQRFD QRSRQFQNLQ NHRIVQIEAK PNTLVLPKHA DADNILVIQQ
241 GQATVTVANG NNRKSFNLDE GHALRIPSGF ISYILNRHDN QNLRVAKISM PVNTPGQFED FFPASSRDQS SYLQGFSRNT
321 LEAAFNAEFN EIRRVLLEEN AGGEQEERGQ RRWSTRSSEN NEGVIVKVSK EHVEELTKHA KSVSKKGSEE EGDITNPINL
401 REGEPDLSNN FGKLFEVKPD KKNPQLQDLD MMLTCVEIKE GALMLPHFNS KAMVIVVVNK GTGNLELVAV RKEQQQRGRR
481 EEEDEDEEE EGSNREVRRY TARLKEGDVF IMPAAHPVAI NASSELHLLG FGINAENNHR IFLAGDKDNV IDQIEKQAKD
561 LAFPGSGEQV EKLIKNQKES HFVSARPQSQ SQSPSSPEKE SPEKEDQEEE NQGGKGPLLS ILKAFN
```

Supporting Peptides:

Peptide	Uniq	-10lgP	Mass	Length	ppm	m/z	z	RT	Fraction	Scan	Source File	Area Raw Ara h 6	#Feature	#Feature Raw Ara h 6	Start	End	PTM	AScore	Found By
R.IPSGFISYILNR.H	Y	81.38	1378.7609	12	2.6	690.3895	2	37.54	1	F1:1736	OB5918 H6 raw.raw	2.1534E4	2	2	266	277			PEAKS DB
K.ISMPVNTPGQFEDFFPASSR.D	Y	61.71	2226.0415	20	0.0	1114.0281	2	36.55	3	F3:1813	OB5931 H6 raw.raw	0	0	0	288	307			PEAKS DB
**total 2 peptides**																			

sp|P43238|ALL12_ARAHY

back to list

| Protein Coverage | Supporting Peptides |

Protein Coverage:

1 MRGRVSPLML LLGILVLASV SATHAKSSPY QKKTENPCAQ RCLQSCQQEP DDLKQKACES RCTKLEYDPR CVYDPRGHTG
81 TTNQRSPPGE RTRGRQPGDY DDDRRQPRRE EGGRWGPAGP REREREEDWR QPREDWRRPS HQQPRKIRPE GREGEQEWGT
161 PGSHVREETS RNNPFYFPSR RFSTRYGNQN GRIRVLQRFD QRSRQFQNLQ NHRIVQIEAK PNTLVLPKHA DADNILVIQQ
241 GQATVTVANG NNRKSFNLDE GHALR**IPSGF ISYILNR**HDN QNLRVAK**ISM PVNTPGQFED FFPASSR**DQS SYLQGFSRNT
321 LEAAFNAEFN EIRRVLLEEN AGGEQEERGQ RRWSTRSSEN NEGVIVKVSK EHVEELTKHA KSVSKKGSEE EGDITNPINL
401 REGEPDLSNN FGKLFEVKPD KKNPQLQDLD MMLTCVEIKE GALMLPHFNS KAMVIVVVNK GTGNLELVAV RKEQQQRGRR
481 EEEEDEDEEE EGSNREVRRY TARLKEGDVF IMPAAHPVAI NASSELHLLG FGINAENNHR IFLAGDKDNV IDQIEKQAKD
561 LAFPGSGEQV EKLIKNQKES HFVSARPQSQ SQSPSSPEKE SPEKEDQEEE NQGGKGPLLS ILKAFN

Supporting Peptides:

Peptide	Uniq	-10lgP	Mass	Length	ppm	m/z	z	RT	Fraction	Scan	Source File	Area Raw Ara h 6	#Feature	#Feature Raw Ara h 6	Start	End	PTM	AScore	Found By
R.IPSGFISYILNR.H	Y	81.38	1378.7609	12	2.6	690.3895	2	37.54	1	F1:1736	OB5918 H6 raw.raw	2.1534E4	2	2	266	277			PEAKS DB
K.ISMPVNTPGQFEDFFPASSR.D	Y	61.71	2226.0415	20	0.0	1114.0281	2	36.55	3	F3:1813	OB5931 H6 raw.raw	0	0	0	288	307			PEAKS DB
total 2 peptides																			

Q6PSU4|Q6PSU4_ARAHY

back to list

| Protein Coverage | Supporting Peptides |

Protein Coverage:

1 GFDQRSRQFQ NLQNHRIVQI EAKPNTLVLP KHADADNILV IQQGQATVTV ANGNNRKSFN LDEGHALR**IP SGFISYILNR**
81 HDNQNLRVAK **ISMPVNTPGQ FEDFFPASSR** DQSSYLQGFS RNTLEAAFNA EFNEIRRVLL EENAGGEQEE RGQRRWSTRS
161 SENNEGVIVK VSKEHVEELT KHAKSVSKKG SEEGDITNPI NLREGEPDLS NNFGKLFEVK PDKKNPQLQD LDMMLTCVEI
241 KEGALMLPHF NSKAMVIVVV NKGTGNLELV AVRKEQQQRG RREEEEDEDE EEEGSNREVR RYTARLKEGD VFIMPAAHPV
321 AINASSELHL LGFGINAENN HRIFLAGDKD NVIDQIEKQA KDLAFPGSGE QVEKLIKNQK ESHFVSARPQ SQSQSPSSPE
401 KESPEKEDQE EENQGGKGPL LSILKAFN

Supporting Peptides:

Peptide	Uniq	-10lgP	Mass	Length	ppm	m/z	z	RT	Fraction	Scan	Source File	Area Raw Ara h 6	#Feature	#Feature Raw Ara h 6	Start	End	PTM	AScore	Found By
R.IPSGFISYILNR.H	Y	81.38	1378.7609	12	2.6	690.3895	2	37.54	1	F1:1736	OB5918 H6 raw.raw	2.1534E4	2	2	69	80			PEAKS DB
K.ISMPVNTPGQFEDFFPASSR.D	Y	61.71	2226.0415	20	0.0	1114.0281	2	36.55	3	F3:1813	OB5931 H6 raw.raw	0	0	0	91	110			PEAKS DB
total 2 peptides																			

K7PQ65|K7PQ65_ARAHY

back to list

| Protein Coverage | Supporting Peptides |

Protein Coverage:

1 MCMMGASVDP LVVGRVIGDV IDM**FVPSVGM SVYYGSK**HVT NGCDIKPSMA ISPPKVTLTG GNIHSLYTLV MTDPDAPSPS
81 EPTMREWLHW VVVDIPGGTN PTQGKEIVAY MGPRPPVGIH R**YILILFEQK** GVLGGVEQPA ARASFNTRYF ARQFNLGLPV
161 ATVYFNSQKE PASKRR

Supporting Peptides:

Peptide	Uniq	-10lgP	Mass	Length	ppm	m/z	z	RT	Fraction	Scan	Source File	Area Raw Ara h 6	#Feature	#Feature Raw Ara h 6	Start	End	PTM	AScore	Found By
R.YILILFEQK.G	Y	78.59	1165.6747	9	-3.3	583.8427	2	36.41	2	F2:1808	OB5930 H6 raw.raw	1.6896E4	3	3	122	130			PEAKS DB
M.FVPSVGMSVYYGSK.H	Y	48.13	1519.7380	14	1.3	760.8773	2	32.86	2	F2:1596	OB5930 H6 raw.raw	0	0	0	24	37			PEAKS DB
**total 2 peptides**																			

Q1HDS7|Q1HDS7_ARAHY

back to list

| Protein Coverage | Supporting Peptides |

Protein Coverage:

1 MVKAVAVLSS SEGVSGTIQF SQEGNGPTTV TGNLAGLKPG LHGFHVHALG DTTNGCLSTG PHFNPNNKEH GAPEDENRHA

81 GDLGNVNVGD DGTVSFSISD SQIPLSGPNS IVGRAVVVHA DPDDLGKGGH ELSKSTGNAG GRVACGIIGL QG

Supporting Peptides:

Peptide	Uniq	-10lgP	Mass	Length	ppm	m/z	z	RT	Fraction	Scan	Source File	Area Raw Ara h 6	#Feature	#Feature Raw Ara h 6	Start	End	PTM	AScore	Found By
R.AVVVHADPDDLGK.G	Y	73.16	1334.6830	13	0.7	668.3492	2	27.55	3	F3:1279	OB5931 H6 raw.raw	7.1935E2	1	1	115	127			PEAKS DB
R.HAGDLGNVNVGDDGTVSFSISDSQIPLSGPNSIVGR.A	Y	49.59	3580.7393	36	4.0	1791.3840	2	35.84	3	F3:1773	OB5931 H6 raw.raw	5.6262E5	2	2	79	114			PEAKS DB
**total 2 peptides**																			

Q45W82|Q45W82_ARAHY

back to list

| Protein Coverage | Supporting Peptides |

Protein Coverage:

1 MVKAVAVLSS SEGVSGTIQF SQEGNGPTTV TGNLAGLKPG LHGFHVHALG DTTNGCLSTG PHFNPNNKEH GAPEDENRHA

81 GDLGNVNVGD DGTVSFSISD SQIPLSGPNS IVGRAVVVHA DPDDLGKGGH ELSKSTGNAG GGVACGIIGL QG

Supporting Peptides:

Peptide	Uniq	-10lgP	Mass	Length	ppm	m/z	z	RT	Fraction	Scan	Source File	Area Raw Ara h 6	#Feature	#Feature Raw Ara h 6	Start	End	PTM	AScore	Found By
R.AVVVHADPDDLGK.G	Y	73.16	1334.6830	13	0.7	668.3492	2	27.55	3	F3:1279	OB5931 H6 raw.raw	7.1935E2	1	1	115	127			PEAKS DB
R.HAGDLGNVNVGDDGTVSFSISDSQIPLSGPNSIVGR.A	Y	49.59	3580.7393	36	4.0	1791.3840	2	35.84	3	F3:1773	OB5931 H6 raw.raw	5.6262E5	2	2	79	114			PEAKS DB
**total 2 peptides**																			

#CONTAM#P35527

back to list

| Protein Coverage | Supporting Peptides |

Protein Coverage:

1 MSCRQFSSSY LSRSGGGGGG GLGSGGSIRS SYSRFSSSGG RGGGGRFSSS SGYGGGSSRV CGRGGGGSFG YSYGGGSGGG
81 FSASSLGGGF GGGSRGFGGA SGGGYSSSGG FGGGFGGGSG GGFGGGYGSG FGGLGGFGGG AGGGDGGILT ANEKSTMQEL
161 NSR**LASYLDK VQAL**EEANND LENKIQDWYD KKGPAAIQK**N YSPYYNTIDD LKDQIVDLTV GNNK**TLLDID NTRMTLDDFR
241 IKFEMEQNLR QGVDADINGL RQVLDNLTME KSDLEMQYET LQEELMALKK NHKEEMSQLT GQNSGDVNVE INVAPGKDLT
321 KTLNDMRQEY EQLIAKNRKD IENQYETQIT QIEHEVSSSG QEVQSSAKEV TQLRHGVQEL EIELQSQLSK KAALEKSLED
401 TKNRYCGQLQ MIQEQISNLE AQITDVRQEI ECQNQEYSLL LSIKMRLEKE IETYHNLLEG GQEDFESSGA GKIGLGGRGG
481 SGGSYGRGSR GGSGGSYGGG GSGGGYGGGS GSRGGSGGSY GGGSGSGGGS GGGYGGGSGG GHSGGSGGGH SGGSGGNYGG
561 GSGSGGGSGG GYGGGSGSRG GSGGSHGGGS GFGGESGGSY GGGEEASGSG GGYGGGSGKS SHS

Supporting Peptides:

Peptide	Uniq	-10lgP	Mass	Length	ppm	m/z	z	RT	Fraction	Scan	Source File	Area Raw Ara h 6	#Feature	#Feature Raw Ara h 6	Start	End	PTM	AScore	Found By
K.NYSPYYNTIDDLKDQIVDLTVGNNK.T	Y	58.26	2901.4031	25	3.4	968.1449	3	38.27	3	F3:1912	OB5931 H6 raw.raw	0	0	0	200	224			PEAKS DB
R.LASYLDKVQAL.E	Y	40.23	1219.6812	11	0.3	610.8480	2	33.05	1	F1:1473	OB5918 H6 raw.raw	4.9064E3	1	1	164	174			PEAKS DB
K.N(+.98)YSPYYNTIDDLKDQIVDLTVGNNK.T	Y	38.28	2902.3872	25	8.1	968.4775	3	38.32	3	F3:1912	OB5931 H6 raw.raw	1.0944E4	1	1	200	224		N1:Deamidation (NQ):21.46	PEAKS DB
**total 3 peptides**																			

Peptide List

Prepared with PEAKS ™ (bioinfor.com)

Summary

1. Notes

Ara h 6 GEL BAND ROASTED

```
Project Ara h 6 gel PTM peanut; Sample: ROAST Ara h 6, PTM33 (node name).
However comparing the to RAW sample where  Ara h 6 allergen was on the first place in abundancy, here in ROAST sample, t
here is maple of Ara h1 and  Ara h 3 occupying the first 4 places.
```

2. Result Statistics

Figure 1. False discovery rate (FDR) curve. X axis is the number of peptides being kept. Y axis is the corresponding FDR.

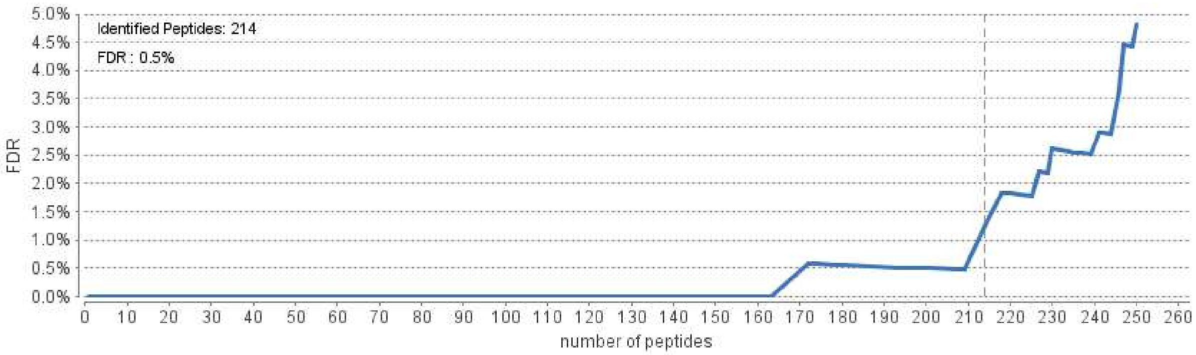

Figure 2. PSM score distribution. **(a)** Distribution of PEAKS peptide score; **(b)** Scatterplot of PEAKS peptide score versus precursor mass error.

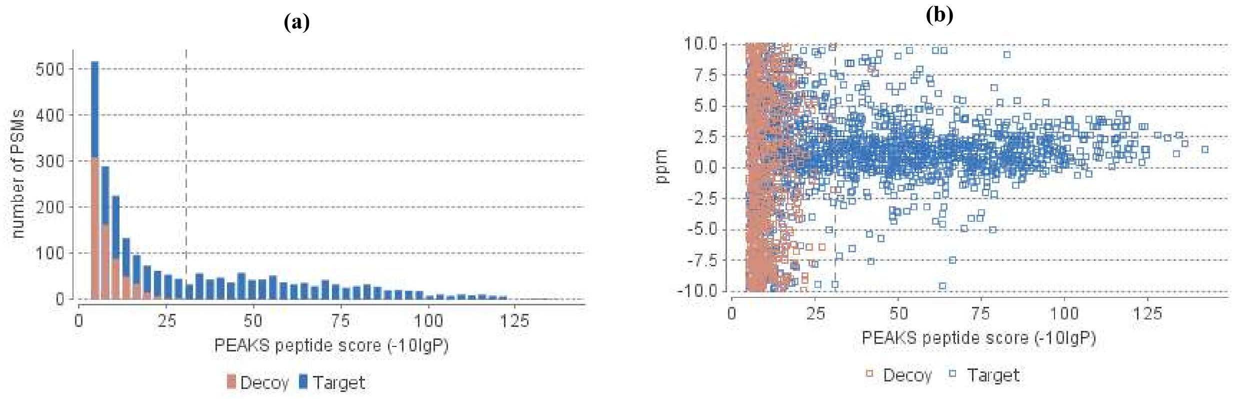

Table 1. Statistics of data.

	#Scans	#Features	Identified	#Peptides	#Sequences	

	MS1	MS/MS	#Chimera		#PSMs	#Scans	#Features**			#Proteins* Groups	All	Top
Total	7393	4181	330	3951	908	899	380	214	120	13	24	18
Roast Ara h 6	7393	4181	330	3951	908	899	380	214	120	13	24	18

* proteins with significant peptides are used in counts.
** features are identified by DB search only.

Figure 3. Sample overlap for Proteins and Peptides (up to 8 samples). **(a)** All Proteins; **(b)** Top Proteins; **(c)** Peptides;

(a) **Not applicable to only one sample**

(b) **Not applicable to only one sample**

(c) **Not applicable to only one sample**

Figure 4. Distribution of peptide feature detection. **(a)** Feature m/z distribution; **(b)** Feature RT distribution.

(a)

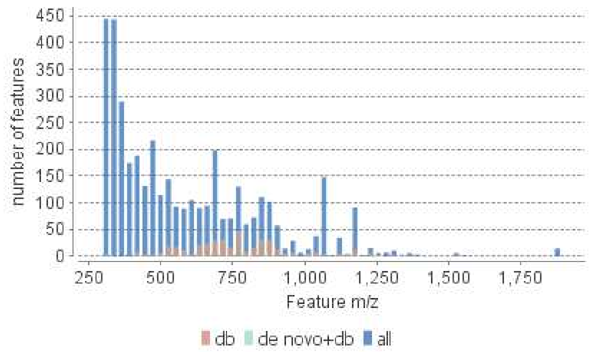

(b)

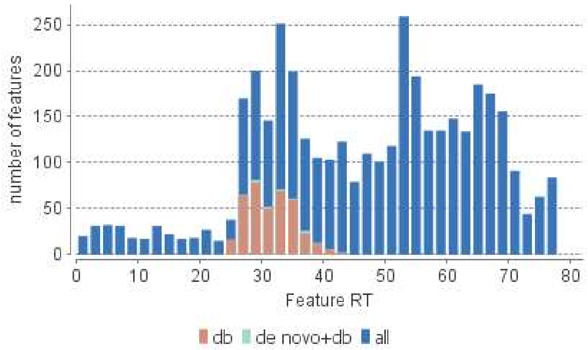

Figure 5. Distribution of identified peptide features. **(a)** Feature abundance distribution; **(b)** *De novo* sequencing validation.

(a)

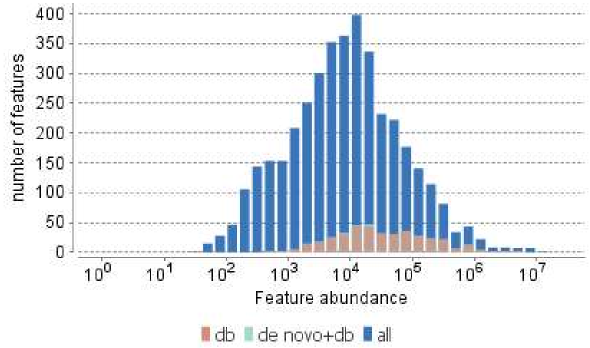

(b)

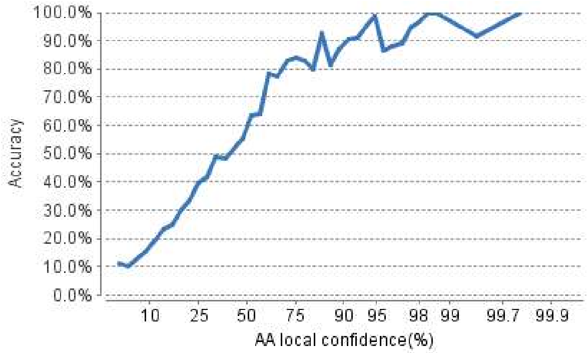

Table 2. Result filtration parameters.

Table 4. PTM profile.

Peptide -10lgP	≥30.8
PTM Ascore	≥50
Protein -10lgP	≥20
Proteins unique peptides	≥2
De novo score(%)	≥50%

Table 3. Statistics of filtered result.

FDR (Peptide-Spectrum Matches)	0.1%
FDR (Peptide Sequences)	0.5%
FDR (Protein Group)	0.0%
De Novo Only Spectra	21

Name	ΔMass	Position	#PSM	-10lgP	Abundance	AScore
Carbamidomethyl	57.02	C	319	142.29	3.56E5	1000.00
Oxidation	15.99	M	70	118.58	1.17E6	1000.00
Deamidation	.98	NQ	52	123.27		77.51
Propionamide	71.04	C	42	92.77	3.19E4	1000.00
Carbamidomethyl	57.02	DE,N-term	19	121.19	6.33E4	49.43
Pyro-glu from Q	-17.03	N-term	18	91.37	3.05E5	1000.00
Ethyl	28.03	DE,N-term	11	82.43	1.69E4	18.53
Ammonia loss	-17.03	N-term	8	88.83	1.5E4	1000.00
Dehydration	-18.01	DSTY	6	47.12		39.55
Cation:Fe[II]	53.92	E	5	48.83		14.63
Methylation(KR)	14.02	KR	4	42.80	2.44E4	1000.00
Dimethylation(KR)	28.03	K	3	63.33	2.88E4	1000.00
Ammonia loss	-17.03	N	3	58.39		67.21
Carboxymethyl	58.01	C	2	39.19		0.00
Sodium	21.98	DE	2	39.39	7.53E3	69.29
Pyro-glu from E	-18.01	N-term	2	38.68		1000.00
Dethiomethyl	-48.00	M	2	71.71	1.56E5	1000.00
Oxidation	15.99	P	1	41.41	4E4	0.00
Methylation(others)	14.02	H	1	36.41	1.57E4	4.52
Iodination	125.90	Y	1	43.24	6.88E3	41.59
Ammonium	17.03	E	1	48.35		18.53
Oxidation	15.99	W	1	37.59	3.89E3	1000.00
Carbamylation	43.01	N-term	1	37.45		1000.00
Amidine	41.03	K	1	59.14	4.17E3	38.16
Dihydroxy	31.99	F	1	63.29		0.00
Diethylation	56.06	K	1	41.08		88.07

3. Experiment Control

Figure 6. Precursor mass error of peptide-spectrum matches (PSM) in filtered result. **(a)** Distribution of precursor mass error in ppm; **(b)** Scatterplot of precursor m/z versus precursor mass error in ppm.

(a) **(b)**

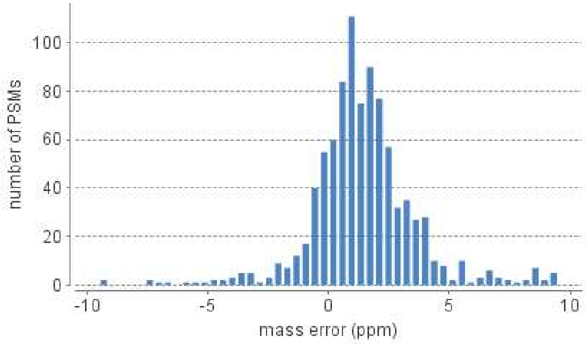

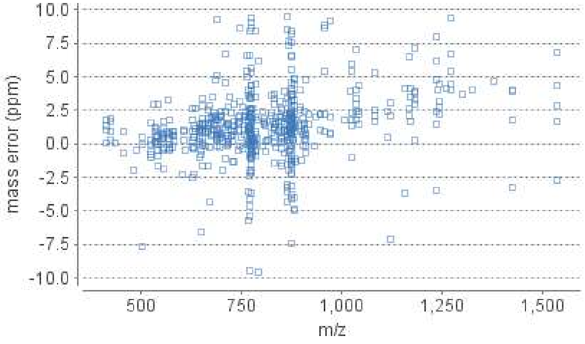

Table 5. Number of identified peptides in each sample by the number of missed cleavages.

Missed Cleavages	0	1	2	3	4+
Roast Ara h 6	148	61	5	0	0

4. Other Information

Table 6. Search parameters.

PEAKS Version: PEAKS Studio 10.6 build 20201015
Search Engine Name: PEAKS
Parent Mass Error Tolerance: 10.0 ppm
Fragment Mass Error Tolerance: 0.5 Da
Precursor Mass Search Type: monoisotopic
Enzyme: Trypsin
Max Missed Cleavages: 2
Digest Mode: Semispecific
Fixed Modifications:
  Carbamidomethylation: 57.02
Variable Modifications:
  Oxidation (M): 15.99
  Deamidation (NQ): 0.98
  Acetylation (K): 42.01
  Acetylation (Protein N-term): 42.01
  Acetylation (N-term): 42.01
  Amidation: -0.98
  Beta-methylthiolation: 45.99
  Biotinylation: 226.08
  and 303 more...
Max Variable PTM Per Peptide: 5
Database: Uniprot_Peanut-3818_Jul18
Taxon: All
Contaminant Database: contam MQ
Searched Entry: 1723
FDR Estimation: Enabled
De novo score(%) threshold: 15
Peptide hit threshold (-10logP): 30.0

Table 7. Instrument parameters.

Fractions: OB5942 H6 Ro.raw, OB5943 H6 Ro.raw, OB5944 H6 Ro.raw

Ion Source: ESI(nano-spray)
Fragmentation Mode: CID, CAD(y and b ions)
MS Scan Mode: FT-ICR/Orbitrap
MS/MS Scan Mode: FT-ICR/Orbitrap

Peaks run ID: 32
Merge Options: no merge
Precursor Options: corrected
Charge Options: no correction
Filter m/z: 200.0 - 2000.0
Filter Charge: 2 - 12
Process: true
Associate chimera: yes

Protein List

Protein Accession Contains:
Protein Description Contains:
Protein Sample Area >=
Protein PTM Contains:

Protein Group	Protein ID	Accession	-10lgP	Coverage (%)	Coverage (%) Roast Ara h 6	Area Roast Ara h 6	#Peptides	#Unique	#Spec Roast Ara h 6	PTM	Avg. Mass	Description
3	7	A1DZF0|A1DZF0_ARAHY	432.38	51	51	3.5979E5	23	2	230	Y	60375	Arachin 6 OS=Arachis hypogaea OX=3818 PE=2 SV=1
2	4	Q9FZ11|Q9FZ11_ARAHY	425.38	45	45	4.0221E5	22	2	232	Y	60449	Gly1 OS=Arachis hypogaea OX=3818 GN=Gly1 PE=2 SV=1
4	3	Q647H3|Q647H3_ARAHY	421.61	50	50	9.5117E5	22	2	230	Y	61532	Arachin Ahy-2 OS=Arachis hypogaea OX=3818 PE=2 SV=1
7	8	Q647H4|Q647H4_ARAHY	414.26	44	44	2.2999E5	20	3	199	Y	61506	Arachin Ahy-1 OS=Arachis hypogaea OX=3818 PE=2 SV=1
1	1	A5Z1R0|A5Z1R0_ARAHY	343.58	70	70	3.5903E7	15	15	352	Y	16920	Ara h 6 allergen OS=Arachis hypogaea OX=3818 GN=Ara h 6 PE=4 SV=1
1	2	sp|Q647G9|CONG_ARAHY	343.58	70	70	3.5903E7	15	15	352	Y	16920	Conglutin OS=Arachis hypogaea OX=3818 PE=1 SV=1
9	31	N1NG13|N1NG13_ARAHY	326.60	34	34	6.3611E4	17	2	65	Y	71345	Seed storage protein Ara h1 OS=Arachis hypogaea OX=3818 GN=ARAX_AHF4 17E07-017 PE=4 SV=1
9	30	sp|P43238|ALL12_ARAHY	326.60	34	34	6.3611E4	17	2	65	Y	71345	Allergen Ara h 1, clone P41B OS=Arachis hypogaea OX=3818 PE=1 SV=1
10	29	sp|P43237|ALL11_ARAHY	324.92	33	33	4.3126E4	17	2	63	Y	70283	Allergen Ara h 1, clone P17 OS=Arachis hypogaea OX=3818 PE=1 SV=1
10	28	B3IXL2|B3IXL2_ARAHY	324.92	33	33	4.3126E4	17	2	63	Y	70283	Main allergen Ara h1 OS=Arachis hypogaea OX=3818 PE=2 SV=1
10	27	Q6PSU3|Q6PSU3_ARAHY	324.92	35	35	4.3126E4	17	2	63	Y	66575	Conarachin (Fragment) OS=Arachis hypogaea OX=3818 PE=4 SV=1
12	305	Q647H1|Q647H1_ARAHY	280.08	15	15	3.6852E5	7	7	28	Y	75933	Conarachin OS=Arachis hypogaea OX=3818 PE=2 SV=1
15	976	sp|Q647H2|AHY3_ARAHY	254.10	28	28	1.0066E5	8	6	23	Y	54569	Arachin Ahy-3 OS=Arachis hypogaea OX=3818 PE=1 SV=1
14	60	Q6IWG5|Q6IWG5_ARAHY	241.18	30	30	1.6341E6	8	7	23	Y	58061	Glycinin (Fragment) OS=Arachis hypogaea OX=3818 PE=2 SV=1
14	61	Q0GM57|Q0GM57_ARAHY	241.18	30	30	1.6341E6	8	7	23	Y	58263	Iso-Ara h3 OS=Arachis hypogaea OX=3818 PE=2 SV=1
11	14	#CONTAM#P00761	196.82	23	23	2.0321E6	7	7	41	Y	24409	SWISS-PROT:#CONTAM#P00761|TRYP_PIG Trypsin - Sus scrofa (Pig).
16	25	E9LFE8|E9LFE8_ARAHY	189.73	35	35	6.6355E4	6	6	9	N	28290	11S arachin (Fragment) OS=Arachis hypogaea OX=3818 PE=2 SV=1
13	262	sp|Q6PSU2|CONG7_ARAHY	180.98	40	40	2.3053E5	5	5	25	Y	20114	Conglutin-7 OS=Arachis hypogaea OX=3818 PE=1 SV=2
**total 18 proteins**												

A1DZF0|A1DZF0_ARAHY

back to list

| Protein Coverage | Supporting Peptides |

Protein Coverage:

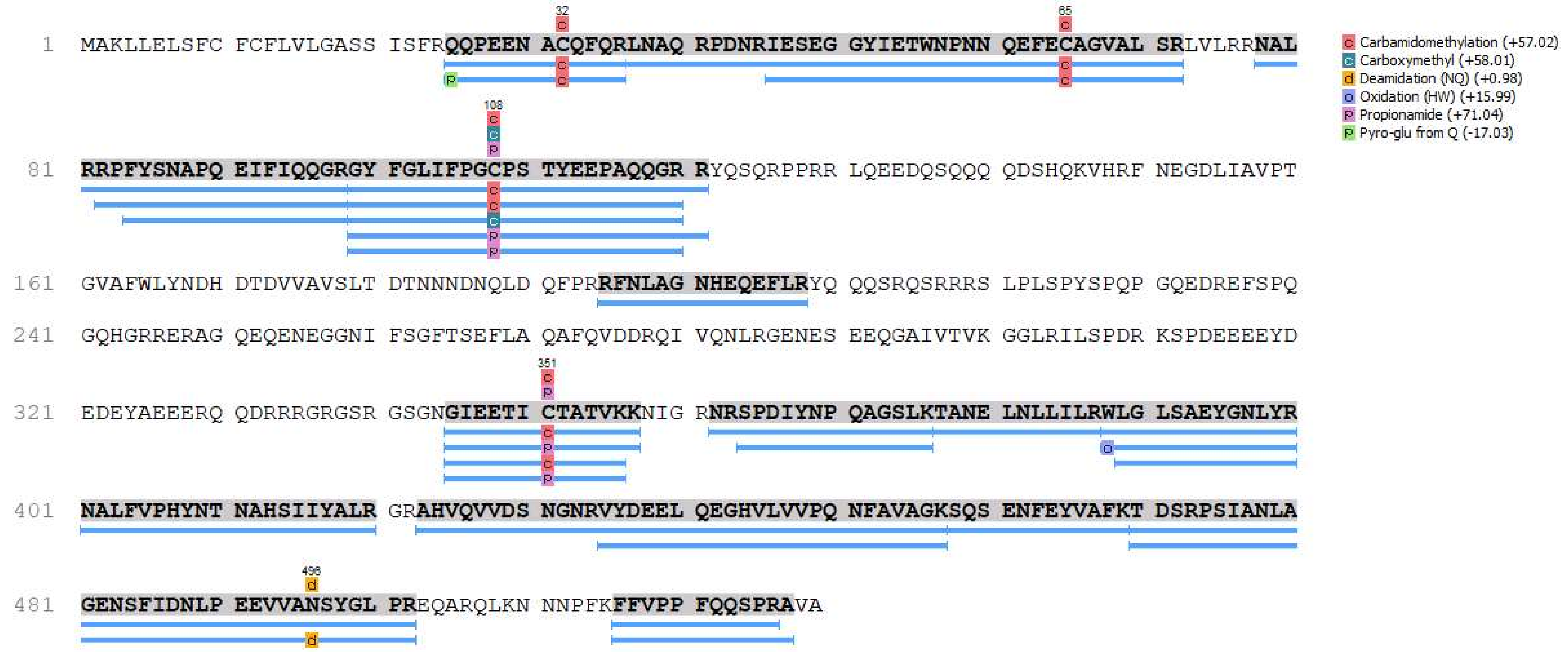

Supporting Peptides:

Peptide	Uniq	-10lgP	Mass	Length	ppm	m/z	z	RT	Fraction	Scan	Source File	Area Roast Ara h 6	#Feature	#Feature Roast Ara h 6	Start	End	PTM	AScore	Found By
R.GYFGLIFPGC(+57.02)PSTYEEPAQQGR.R	N	142.29	2473.1372	22	1.5	1237.5778	2	36.17	4	F4:1794	OB5942 H6 Ro.raw	1.0118E6	3	3	99	120	Carbamidomethylation	C10:Carbamidomethylation:1000.00	PEAKS DB
R.WLGLSAEYGNLYR.N	N	136.28	1540.7673	13	2.0	771.3925	2	34.64	4	F4:1712	OB5942 H6 Ro.raw	2.2268E6	6	6	388	400			PEAKS DB
K.SQSENFEYVAFK.T	N	124.82	1447.6619	12	1.0	724.8389	2	31.74	5	F5:1533	OB5943 H6 Ro.raw	9.8941E5	3	3	458	469			PEAKS DB
R.RPFYSNAPQEIFIQQGR.G	N	119.86	2050.0383	17	2.0	684.3547	3	32.67	5	F5:1597	OB5943 H6 Ro.raw	3.2279E6	6	6	82	98			PEAKS DB
K.FFVPPFQQSPR.A	Y	119.19	1348.6927	11	1.6	675.3547	2	33.24	5	F5:1632	OB5943 H6 Ro.raw	3.0083E5	3	3	516	526			PEAKS DB
R.NRSPDIYNPQAGSLK.T	N	114.42	1658.8376	15	0.1	830.4261	2	27.65	5	F5:1291	OB5943 H6 Ro.raw	4.04E5	6	6	362	376			PEAKS DB
R.SPDIYNPQAGSLK.T	N	102.05	1388.6936	13	0.9	695.3547	2	28.77	5	F5:1355	OB5943 H6 Ro.raw	7.3513E5	3	3	364	376			PEAKS DB
K.TDSRPSIANLAGENSFIDNLPEEVVANSYGLPR.E	N	101.93	3544.7434	33	3.9	1182.5930	3	37.73	6	F6:1866	OB5944 H6 Ro.raw	1.0339E6	4	4	470	502			PEAKS DB

total 45 peptides

Peptide	Uniq	-10lgP	Mass	Length	ppm	m/z	z	RT	Fraction	Scan	Source File	Area Roast Ara h 6	#Feature	#Feature Roast Ara h 6	Start	End	PTM	AScore	Found By
R.NALFVPHYNTNAHSIIYALR.G	N	97.15	2313.2019	20	-0.1	772.0745	3	35.38	6	F6:1747	OB5944 H6 Ro.raw	2.7972E6	8	8	401	420			PEAKS DB
R.GYFGLIFPGC(+71.04)PSTYEEPAQQGR.R	N	92.77	2487.1528	22	2.2	1244.5864	2	36.43	5	F5:1817	OB5943 H6 Ro.raw	8.0086E4	3	3	99	120	Propionamide	C10:Propionamide:1000.00	PEAKS PTM
N.GIEETIC(+57.02)TATVKK.N	N	91.54	1448.7545	13	0.7	725.3850	2	27.45	4	F4:1292	OB5942 H6 Ro.raw	1.4529E5	2	2	345	357	Carbamidomethylation	C7:Carbamidomethylation:1000.00	PEAKS DB
R.VYDEELQEGHVLVVPQNFAVAGK.S	N	89.66	2540.2910	23	2.7	847.7732	3	32.92	4	F4:1613	OB5942 H6 Ro.raw	4.6093E5	4	4	435	457			PEAKS DB
R.Q(-17.03)QPEENAC(+57.02)QFQR.L	N	88.35	1516.6365	12	2.5	759.3274	2	28.03	5	F5:1319	OB5943 H6 Ro.raw	7.279E3	2	2	25	36	Pyro-glu from Q; Carbamidomethylation	Q1:Pyro-glu from Q:1000.00;C8:Carbamidomethylation:1000.00	PEAKS PTM
R.IESEGGYIETWNPNNQEFEC(+57.02)AGVALSR.L	N	85.95	3069.3774	27	5.5	1024.1387	3	34.81	5	F5:1720	OB5943 H6 Ro.raw	1.0361E4	1	1	46	72	Carbamidomethylation	C20:Carbamidomethylation:1000.00	PEAKS DB
R.QQPEENAC(+57.02)QFQR.L	N	84.17	1533.6630	12	1.7	767.8400	2	25.24	5	F5:1157	OB5943 H6 Ro.raw	3.7544E4	3	3	25	36	Carbamidomethylation	C8:Carbamidomethylation:1000.00	PEAKS DB
K.TANELNLLILR.W	N	81.05	1268.7452	11	0.9	635.3805	2	34.83	4	F4:1715	OB5942 H6 Ro.raw	7.0668E5	3	3	377	387			PEAKS DB
N.GIEETIC(+57.02)TATVK.K	N	81.04	1320.6595	12	-0.1	661.3370	2	29.52	5	F5:1405	OB5943 H6 Ro.raw	2.3112E4	2	2	345	356	Carbamidomethylation	C7:Carbamidomethylation:1000.00	PEAKS DB
P.FYSNAPQEIFIQQGR.G	N	76.66	1796.8845	15	-1.2	899.4484	2	32.27	4	F4:1574	OB5942 H6 Ro.raw	1.5688E3	1	1	84	98			PEAKS DB
R.LNAQRPDNRIESEGGYIETWNPNNQEFEC(+57.02)AGVALSR.L	N	75.05	4133.9248	36	2.4	1034.4910	4	33.87	6	F6:1670	OB5944 H6 Ro.raw	1.3368E6	1	1	37	72	Carbamidomethylation	C29:Carbamidomethylation:1000.00	PEAKS DB
R.NALRRPFYSNAPQEIFIQQGR.G	N	74.35	2504.3037	21	0.3	627.0834	4	33.30	4	F4:1641	OB5942 H6 Ro.raw	1.8121E5	4	4	78	98			PEAKS DB
R.GYFGLIFPGC(+57.02)PSTYEEPAQQGRR.Y	N	73.07	2629.2383	23	1.4	877.4213	3	35.30	5	F5:1733	OB5943 H6 Ro.raw	1.2125E6	3	3	99	121	Carbamidomethylation	C10:Carbamidomethylation:1000.00	PEAKS DB
R.IESEGGYIETWNPNNQ(+.98)EFEC(+57.02)AGVALSR.L	N	62.09	3070.3613	27	6.0	1024.4672	3	34.92	5	F5:1736	OB5943 H6 Ro.raw	6.6817E3	1	1	46	72	Carbamidomethylation	Q16:Deamidation (NQ):0.00;C20:Carbamidomethylation:1000.00	PEAKS DB
R.VYDEELQEGHVLVVPQN(+.98)FAVAGK.S	N	61.03	2541.2751	23	9.5	1271.6569	2	32.92	4	F4:1618	OB5942 H6 Ro.raw	3.629E3	1	1	435	457		N17:Deamidation (NQ):14.02	PEAKS DB
K.FFVPPFQQSPRA.V	Y	60.07	1419.7299	12	-1.0	710.8715	2	33.42	5	F5:1644	OB5943 H6 Ro.raw	1.8963E4	1	1	516	527			PEAKS DB
K.TDSRPSIANLAGENSFIDNLPEEVVAN(+.98)SYGLPR.E	N	59.30	3545.7273	33	7.1	1182.9248	3	36.36	4	F4:1812	OB5942 H6 Ro.raw	3.983E3	1	1	470	502	Deamidation (NQ)	N27:Deamidation (NQ):51.97	PEAKS DB
R.AHVQVVDSNGN(+.98)RVYDEELQEGHVLVVPQNFAVAGK.S	N	57.69	3817.9023	35	5.5	955.4881	4	33.48	6	F6:1651	OB5944 H6 Ro.raw	7.3088E4	3	3	423	457		N11:Deamidation (NQ):0.00	PEAKS DB
N.GIEETIC(+71.04)TATVKK.N	N	51.71	1462.7701	13	0.9	732.3930	2	27.73	4	F4:1307	OB5942 H6 Ro.raw	5.283E3	2	2	345	357	Propionamide	C7:Propionamide:1000.00	PEAKS PTM

total 45 peptides

Peptide	Uniq	-10lgP	Mass	Length	ppm	m/z	z	RT	Fraction	Scan	Source File	Area Roast Ara h 6	#Feature	#Feature Roast Ara h 6	Start	End	PTM	AScore	Found By
R.AHVQ(+.98)VVDSNGNRVYDEELQEGHVLVVPQNFAVAGK.S	N	49.13	3817.9023	35	0.9	955.4838	4	33.12	4	F4:1624	OB5942 H6 Ro.raw	4.5408E4	1	1	423	457		Q4:Deamidation (NQ):25.58	PEAKS DB
R.RFNLAGNHEQEFLR.Y	N	45.43	1729.8647	14	0.9	577.6294	3	32.11	5	F5:1568	OB5943 H6 Ro.raw	3.821E3	1	1	195	208			PEAKS DB
W.LGLSAEYGNLYR.N	N	43.06	1354.6881	12	3.5	678.3537	2	31.68	4	F4:1539	OB5942 H6 Ro.raw	0	0	0	389	400			PEAKS DB
K.FFVPPFQQSP(+15.99)RA.V	Y	41.41	1435.7247	12	2.8	718.8716	2	32.73	6	F6:1598	OB5944 H6 Ro.raw	3.9991E4	1	1	516	527		P10:Oxidation or Hydroxylation: 0.00	PEAKS PTM
R.AHVQVVDSN(+.98)GN(+.98)RVYDEELQEGHVLVVPQNFAVAGK.S	N	41.04	3818.8862	35	8.6	955.7371	4	34.33	5	F5:1692	OB5943 H6 Ro.raw	0	0	0	423	457		N9:Deamidation (NQ):0.00;N11: Deamidation (NQ):0.00	PEAKS DB
R.AHVQ(+.98)VVDSNGNRVYDEELQEGHVLVVPQ(+.98)NFAVAGK.S	N	40.14	3818.8862	35	8.9	955.7373	4	33.12	4	F4:1624	OB5942 H6 Ro.raw	3.8034E4	1	1	423	457		Q4:Deamidation (NQ):8.81;Q28: Deamidation (NQ):12.28	PEAKS DB
R.IESEGGYIETWN(+.98)PNNQEFEC(+57.02)AGVALSR.L	N	40.05	3070.3613	27	6.9	1536.1985	2	34.73	5	F5:1729	OB5943 H6 Ro.raw	2.6575E4	1	1	46	72	Carbamidomethylation	N12:Deamidation (NQ):0.00;C20:Carbamidomethylation:1000.00	PEAKS DB
R.AHVQVVDSNGNRVYDEELQEGHVLVVPQNFAVAGK.S	N	39.89	3816.9182	35	-0.1	764.3909	5	33.91	5	F5:1668	OB5943 H6 Ro.raw	0	0	0	423	457			PEAKS DB
N.GIEETIC(+71.04)TATVK.K	N	37.92	1334.6752	12	1.9	668.3461	2	29.63	6	F6:1421	OB5944 H6 Ro.raw	0	0	0	345	356	Propionamide	C7:Propionamide:1000.00	PEAKS PTM
R.W(+15.99)LGLSAEYGNLYR.N	N	37.59	1556.7623	13	-0.8	779.3878	2	34.26	4	F4:1698	OB5942 H6 Ro.raw	3.8864E3	1	1	388	400	Oxidation (HW)	W1:Oxidation (HW):1000.00	PEAKS PTM
R.AHVQVVDSN(+.98)GNRVYDEELQEGHVLVVPQNFAVAGK.S	N	36.89	3817.9023	35	2.2	955.4850	4	33.52	5	F5:1663	OB5943 H6 Ro.raw	4.5408E4	1	1	423	457		N9:Deamidation (NQ):9.40	PEAKS DB
R.NALFVPH(+14.02)YNTNAHSIIYALR.G	N	36.41	2327.2175	20	2.7	776.7485	3	36.25	5	F5:1805	OB5943 H6 Ro.raw	1.569E4	1	1	401	420		H7:Methylation (others):4.52	PEAKS PTM
R.LNAQRPDNRIESEGGYIETWNPNN(+.98)QEFEC(+57.02)AGVALSR.L	N	36.07	4134.9087	36	4.7	1379.3167	3	33.85	6	F6:1671	OB5944 H6 Ro.raw	8.0788E4	1	1	37	72	Carbamidomethylation	N24:Deamidation (NQ):0.00;C29:Carbamidomethylation:1000.00	PEAKS DB
R.GYFGLIFPGC(+58.01)PSTYEEPAQQGR.R	N	35.93	2474.1211	22	4.1	1238.0729	2	38.39	6	F6:1932	OB5944 H6 Ro.raw	0	0	0	99	120	Carboxymethyl	C10:Carboxymethyl:1000.00	PEAKS PTM
R.GYFGLIFPGC(+71.04)PSTYEEPAQQGRR.Y	N	35.48	2643.2539	23	1.6	882.0933	3	34.83	4	F4:1720	OB5942 H6 Ro.raw	3.8119E4	1	1	99	121	Propionamide	C10:Propionamide:1000.00	PEAKS PTM
R.GYFGLIFPGC(+57.02)PSTYEEPAQQGR(+14.02)R.Y	N	34.44	2643.2539	23	1.0	882.0928	3	35.30	5	F5:1741	OB5943 H6 Ro.raw	3.3639E4	1	1	99	121	Carbamidomethylation	C10:Carbamidomethylation:1000.00;R22:Methylation(KR):0.00	PEAKS PTM
K.TDSRPSIANLAGENSFIDN(+.98)LPEEVVANSYGLPR.E	N	33.13	3545.7273	33	3.8	1182.9209	3	39.27	5	F5:1978	OB5943 H6 Ro.raw	0	0	0	470	502		N19:Deamidation (NQ):8.41	PEAKS DB

total 45 peptides

Peptide	Uniq	-10lgP	Mass	Length	ppm	m/z	z	RT	Fraction	Scan	Source File	Area Roast Ara h 6	#Feature	#Feature Roast Ara h 6	Start	End	PTM	AScore	Found By
K.SQSE(+53.92)NFEYVAFK.T	N	32.70	1501.5812	12	-0.3	751.7976	2	31.36	4	F4:1520	OB5942 H6 Ro.raw	0	0	0	458	469		E4:Replacement of 2 protons by iron:22.45	PEAKS PTM
**total 45 peptides**																			

Q9FZ11|Q9FZ11_ARAHY

back to list

| Protein Coverage | Supporting Peptides |

Protein Coverage:

1 MIRGRLALSV CFCFLVLGAS SISFRQQPEE NACQFQRLNA QRPDNRLESE GGYIETWNPN NQEFECAGVA LSRLVLRRNA

81 LRRPFYSNAP QEIFIQQGRG YFGLIFPGCP STYEEPAQQG RRHQSQRAPR RFEGEDQSQQ QQQDSHQKVR RFDEGDLIAV

161 PTGVALWMFN DHDTDVVAVS LTDTNNNDNQ LDQFPRRFNL AGNHEQEFLR YQQQSRRRSL PYSPYSPQSQ PRQEEREFSP

241 RGQHSRRERA GQEEENEGGN IFSGFTPEFL AQAFQVDDRQ IVQNLRGENE SEEEGAIVTV KGGLRILSPD RKRGADEEEE

321 YDEDEYEYDE EDRRRGRGSR GRGNGIEETI CTASVKKNIG RNRSPDIYNP QAGSLKTAND LNLLILRWLG LSAEYGNLYR

401 NALFVPHYNT NAHSIIYALR GRAHVQVVDS NGNRVYDEEL QEGHVLVVPQ NFAVAGKSQS DNFEYVAFKT DSRPNIANFA

481 GENSIIDNLP EEVVANSYGL PREQARQLKN NNPFKFFVPP SQQSLRAVA

(Modification markers: 33 c; 58 d; 66 c; 109 c, c, p; 351 c; 379 d)

- c Carbamidomethylation (+57.02)
- c Carboxymethyl (+58.01)
- d Deamidation (NQ) (+0.98)
- o Oxidation (HW) (+15.99)
- p Propionamide (+71.04)
- p Pyro-glu from Q (-17.03)

Supporting Peptides:

Peptide	Uniq	-10lgP	Mass	Length	ppm	m/z	z	RT	Fraction	Scan	Source File	Area Roast Ara h 6	#Feature	#Feature Roast Ara h 6	Start	End	PTM	AScore	Found By
R.GYFGLIFPGC(+57.02)PSTYEEPAQQGR.R	N	142.29	2473.1372	22	1.5	1237.5778	2	36.17	4	F4:1794	OB5942 H6 Ro.raw	1.0118E6	3	3	100	121	Carbamidomethylation	C10:Carbamido methylation:100 0.00	PEAKS DB
R.WLGLSAEYGNLYR.N	N	136.28	1540.7673	13	2.0	771.3925	2	34.64	4	F4:1712	OB5942 H6 Ro.raw	2.2268E6	6	6	388	400			PEAKS DB
K.SQSDNFEYVAFK.T	N	124.69	1433.6462	12	1.5	717.8315	2	31.43	4	F4:1526	OB5942 H6 Ro.raw	3.2229E5	3	3	458	469			PEAKS DB
R.RPFYSNAPQEIFIQQGR.G	N	119.86	2050.0383	17	2.0	684.3547	3	32.67	5	F5:1597	OB5943 H6 Ro.raw	3.2279E6	6	6	83	99			PEAKS DB
**total 41 peptides**																			

Peptide	Uniq	-10lgP	Mass	Length	ppm	m/z	z	RT	Fraction	Scan	Source File	Area Roast Ara h 6	#Feature	#Feature Roast Ara h 6	Start	End	PTM	AScore	Found By
R.NRSPDIYNPQAGSLK.T	N	114.42	1658.8376	15	0.1	830.4261	2	27.65	5	F5:1291	OB5943 H6 Ro.raw	4.04E5	6	6	362	376			PEAKS DB
R.SPDIYNPQAGSLK.T	N	102.05	1388.6936	13	0.9	695.3547	2	28.77	5	F5:1355	OB5943 H6 Ro.raw	7.3513E5	3	3	364	376			PEAKS DB
K.FFVPPSQQSLR.A	Y	100.14	1304.6876	11	0.4	653.3513	2	30.45	5	F5:1467	OB5943 H6 Ro.raw	1.2956E5	3	3	516	526			PEAKS DB
N.GIEETIC(+57.02)TASVK.K	N	98.34	1306.6438	12	-0.1	654.3291	2	29.14	5	F5:1389	OB5943 H6 Ro.raw	7.3439E4	3	3	345	356	Carbamidomethylation	C7:Carbamidomethylation:1000.00	PEAKS DB
R.NALFVPHYNTNAHSIIYALR.G	N	97.15	2313.2019	20	-0.1	772.0745	3	35.38	6	F6:1747	OB5944 H6 Ro.raw	2.7972E6	8	8	401	420			PEAKS DB
R.GYFGLIFPGC(+71.04)PSTYEEPAQQGR.R	N	92.77	2487.1528	22	2.2	1244.5864	2	36.43	5	F5:1817	OB5943 H6 Ro.raw	8.0086E4	3	3	100	121	Propionamide	C10:Propionamide:1000.00	PEAKS PTM
R.VYDEELQEGHVLVVPQNFAVAGK.S	N	89.66	2540.2910	23	2.7	847.7732	3	32.92	4	F4:1613	OB5942 H6 Ro.raw	4.6093E5	4	4	435	457			PEAKS DB
N.GIEETIC(+57.02)TASVKK.N	N	88.49	1434.7388	13	0.0	718.3766	2	27.14	5	F5:1263	OB5943 H6 Ro.raw	1.4573E5	4	4	345	357	Carbamidomethylation	C7:Carbamidomethylation:1000.00	PEAKS DB
R.Q(-17.03)QPEENAC(+57.02)QFQR.L	N	88.35	1516.6365	12	2.5	759.3274	2	28.03	5	F5:1319	OB5943 H6 Ro.raw	7.279E3	2	2	26	37	Pyro-glu from Q; Carbamidomethylation	Q1:Pyro-glu from Q:1000.00;C8:Carbamidomethylation:1000.00	PEAKS PTM
R.LESEGGYIETWNPNNQEFEC(+57.02)AGVALSR.L	N	85.95	3069.3774	27	5.5	1024.1387	3	34.81	5	F5:1720	OB5943 H6 Ro.raw	1.0361E4	1	1	47	73	Carbamidomethylation	C20:Carbamidomethylation:1000.00	PEAKS DB
R.QQPEENAC(+57.02)QFQR.L	N	84.17	1533.6630	12	1.7	767.8400	2	25.24	5	F5:1157	OB5943 H6 Ro.raw	3.7544E4	3	3	26	37	Carbamidomethylation	C8:Carbamidomethylation:1000.00	PEAKS DB
K.FFVPPSQQSLRA.V	Y	81.44	1375.7247	12	0.5	688.8700	2	31.01	5	F5:1500	OB5943 H6 Ro.raw	2.4638E5	3	3	516	527			PEAKS DB
K.TANDLNLLILR.W	N	76.99	1254.7296	11	-0.2	628.3719	2	34.73	5	F5:1761	OB5943 H6 Ro.raw	9.7454E5	3	3	377	387			PEAKS DB
P.FYSNAPQEIFIQQGR.G	N	76.66	1796.8845	15	-1.2	899.4484	2	32.27	4	F4:1574	OB5942 H6 Ro.raw	1.5688E3	1	1	85	99			PEAKS DB
R.LNAQRPDNRLESEGGYIETWNPNNQEFEC(+57.02)AGVALSR.L	N	75.05	4133.9248	36	2.4	1034.4910	4	33.87	6	F6:1670	OB5944 H6 Ro.raw	1.3368E6	1	1	38	73	Carbamidomethylation	C29:Carbamidomethylation:1000.00	PEAKS DB
R.NALRRPFYSNAPQEIFIQQGR.G	N	74.35	2504.3037	21	0.3	627.0834	4	33.30	4	F4:1641	OB5942 H6 Ro.raw	1.8121E5	4	4	79	99			PEAKS DB
R.GYFGLIFPGC(+57.02)PSTYEEPAQQGRR.H	N	73.07	2629.2383	23	1.4	877.4213	3	35.30	5	F5:1733	OB5943 H6 Ro.raw	1.2125E6	3	3	100	122	Carbamidomethylation	C10:Carbamidomethylation:1000.00	PEAKS DB
K.FFVPPSQQ(+.98)SLR.A	Y	67.31	1305.6716	11	2.9	653.8450	2	30.71	4	F4:1484	OB5942 H6 Ro.raw	2.6275E4	2	2	516	526		Q8:Deamidation (NQ):8.69	PEAKS DB
R.LESEGGYIETWNPNNQ(+.98)EFEC(+57.02)AGVALSR.L	N	62.09	3070.3613	27	6.0	1024.4672	3	34.92	5	F5:1736	OB5943 H6 Ro.raw	6.6817E3	1	1	47	73	Deamidation (NQ); Carbamidomethylation	Q16:Deamidation (NQ):1000.00;C20:Carbamidomethylation:1000.00	PEAKS DB

total 41 peptides

Peptide	Uniq	-10lgP	Mass	Length	ppm	m/z	z	RT	Fraction	Scan	Source File	Area Roast Ara h 6	#Feature	#Feature Roast Ara h 6	Start	End	PTM	AScore	Found By
R.VYDEELQEGHVLVVPQN(+.98)FAVAGK.S	N	61.03	2541.2751	23	9.5	1271.6569	2	32.92	4	F4:1618	OB5942 H6 Ro.raw	3.629E3	1	1	435	457		N17:Deamidation (NQ):14.02	PEAKS DB
R.AHVQVVDSNGN(+.98)RVYDEELQEGHVLVVPQNFAVAGK.S	N	57.69	3817.9023	35	5.5	955.4881	4	33.48	6	F6:1651	OB5944 H6 Ro.raw	7.3088E4	3	3	423	457		N11:Deamidation (NQ):0.00	PEAKS DB
K.TAN(+.98)DLNLLILR.W	N	56.97	1255.7136	11	2.6	628.8657	2	35.22	4	F4:1745	OB5942 H6 Ro.raw	0	0	0	377	387	Deamidation (NQ)	N3:Deamidation (NQ):68.31	PEAKS DB
R.AHVQ(+.98)VVDSNGNRVYDEELQEGHVLVVPQNFAVAGK.S	N	49.13	3817.9023	35	0.9	955.4838	4	33.12	4	F4:1624	OB5942 H6 Ro.raw	4.5408E4	1	1	423	457		Q4:Deamidation (NQ):25.58	PEAKS DB
R.RFNLAGNHEQEFLR.Y	N	45.43	1729.8647	14	0.9	577.6294	3	32.11	5	F5:1568	OB5943 H6 Ro.raw	3.821E3	1	1	197	210			PEAKS DB
W.LGLSAEYGNLYR.N	N	43.06	1354.6881	12	3.5	678.3537	2	31.68	4	F4:1539	OB5942 H6 Ro.raw	0	0	0	389	400			PEAKS DB
N.G(+57.02)IEETIC(+57.02)TASVKK.N	N	42.25	1491.7603	13	8.7	746.8939	2	26.96	4	F4:1261	OB5942 H6 Ro.raw	1.3952E4	2	2	345	357	Carbamidomethylation	G1:Carbamidomethylation (DHKE, X@N-term):32.28;C7:Carbamidomethylation:1000.00	PEAKS PTM
R.AHVQVVDSN(+.98)GN(+.98)RVYDEELQEGHVLVVPQNFAVAGK.S	N	41.04	3818.8862	35	8.6	955.7371	4	34.33	5	F5:1692	OB5943 H6 Ro.raw	0	0	0	423	457		N9:Deamidation (NQ):0.00;N11:Deamidation (NQ):0.00	PEAKS DB
R.AHVQ(+.98)VVDSNGNRVYDEELQEGHVLVVPQ(+.98)NFAVAGK.S	N	40.14	3818.8862	35	8.9	955.7373	4	33.12	4	F4:1624	OB5942 H6 Ro.raw	3.8034E4	1	1	423	457		Q4:Deamidation (NQ):8.81;Q28:Deamidation (NQ):12.28	PEAKS DB
R.LESEGGYIETWN(+.98)PNNQEFEC(+57.02)AGVALSR.L	N	40.05	3070.3613	27	6.9	1536.1985	2	34.73	5	F5:1729	OB5943 H6 Ro.raw	2.6575E4	1	1	47	73	Deamidation (NQ); Carbamidomethylation	N12:Deamidation (NQ):1000.00;C20:Carbamidomethylation:1000.00	PEAKS DB
R.AHVQVVDSNGNRVYDEELQEGHVLVVPQNFAVAGK.S	N	39.89	3816.9182	35	-0.1	764.3909	5	33.91	5	F5:1668	OB5943 H6 Ro.raw	0	0	0	423	457			PEAKS DB
R.W(+15.99)LGLSAEYGNLYR.N	N	37.59	1556.7623	13	-0.8	779.3878	2	34.26	4	F4:1698	OB5942 H6 Ro.raw	3.8864E3	1	1	388	400	Oxidation (HW)	W1:Oxidation (HW):1000.00	PEAKS PTM
R.AHVQVVDSN(+.98)GNRVYDEELQEGHVLVVPQNFAVAGK.S	N	36.89	3817.9023	35	2.2	955.4850	4	33.52	5	F5:1663	OB5943 H6 Ro.raw	4.5408E4	1	1	423	457		N9:Deamidation (NQ):9.40	PEAKS DB
R.NALFVPH(+14.02)YNTNAHSIIYALR.G	N	36.41	2327.2175	20	2.7	776.7485	3	36.25	5	F5:1805	OB5943 H6 Ro.raw	1.569E4	1	1	401	420		H7:Methylation (others):4.52	PEAKS PTM
R.LNAQRPDNRLESEGGYIETWNPNN(+.98)QEFEC(+57.02)AGVALSR.L	N	36.07	4134.9087	36	4.7	1379.3167	3	33.85	6	F6:1671	OB5944 H6 Ro.raw	8.0788E4	1	1	38	73	Deamidation (NQ); Carbamidomethylation	N24:Deamidation (NQ):1000.00;C29:Carbamidomethylation:1000.00	PEAKS DB
R.GYFGLIFPGC(+58.01)PSTYEEPAQQGR.R	N	35.93	2474.1211	22	4.1	1238.0729	2	38.39	6	F6:1932	OB5944 H6 Ro.raw	0	0	0	100	121	Carboxymethyl	C10:Carboxymethyl:1000.00	PEAKS PTM
R.GYFGLIFPGC(+71.04)PSTYEEPAQQGRR.H	N	35.48	2643.2539	23	1.6	882.0933	3	34.83	4	F4:1720	OB5942 H6 Ro.raw	3.8119E4	1	1	100	122	Propionamide	C10:Propionamide:1000.00	PEAKS PTM

total 41 peptides

Peptide	Uniq	-10lgP	Mass	Length	ppm	m/z	z	RT	Fraction	Scan	Source File	Area Roast Ara h 6	#Feature	#Feature Roast Ara h 6	Start	End	PTM	AScore	Found By
R.GYFGLIFPGC(+57.02)PSTYEEPAQQGR(+14.02)R.H	N	34.44	2643.2539	23	1.0	882.0928	3	35.30	5	F5:1741	OB5943 H6 Ro.raw	3.3639E4	1	1	100	122	Carbamidomethylation	C10:Carbamido methylation:100 0.00;R22:Methyl ation(KR):0.00	PEAKS PTM
total 41 peptides																			

Q647H3|Q647H3_ARAHY

back to list

| Protein Coverage | Supporting Peptides |

Protein Coverage:

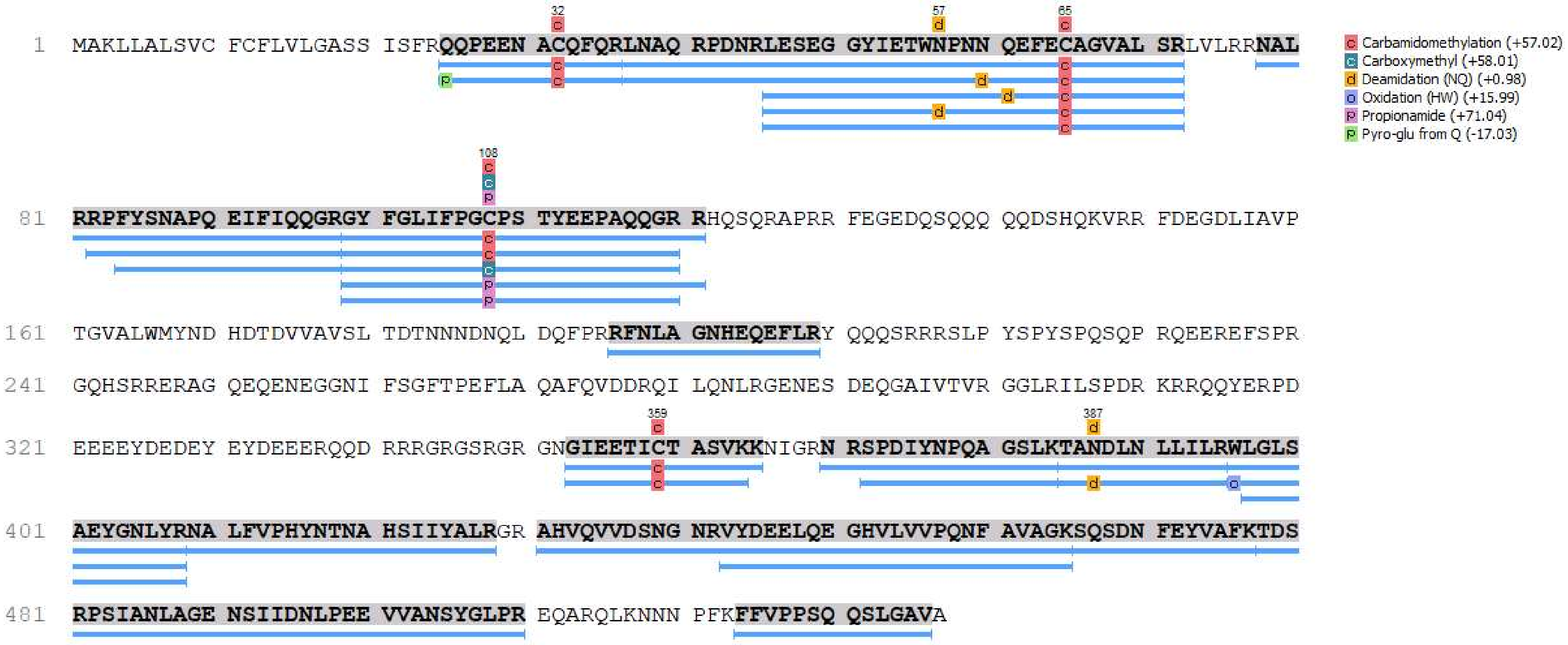

Supporting Peptides:

Peptide	Uniq	-10lgP	Mass	Length	ppm	m/z	z	RT	Fraction	Scan	Source File	Area Roast Ara h 6	#Feature	#Feature Roast Ara h 6	Start	End	PTM	AScore	Found By
R.GYFGLIFPGC(+57.02)PSTYEEPAQQGR.R	N	142.29	2473.1372	22	1.5	1237.5778	2	36.17	4	F4:1794	OB5942 H6 Ro.raw	1.0118E6	3	3	99	120	Carbamidomethylation	C10:Carbamido methylation:100 0.00	PEAKS DB
R.WLGLSAEYGNLYR.N	N	136.28	1540.7673	13	2.0	771.3925	2	34.64	4	F4:1712	OB5942 H6 Ro.raw	2.2268E6	6	6	396	408			PEAKS DB
K.SQSDNFEYVAFK.T	N	124.69	1433.6462	12	1.5	717.8315	2	31.43	4	F4:1526	OB5942 H6 Ro.raw	3.2229E5	3	3	466	477			PEAKS DB

total 40 peptides

Peptide	Uniq	-10lgP	Mass	Length	ppm	m/z	z	RT	Fraction	Scan	Source File	Area Roast Ara h 6	#Feature	#Feature Roast Ara h 6	Start	End	PTM	AScore	Found By
R.RPFYSNAPQEIFIQQGR.G	N	119.86	2050.0383	17	2.0	684.3547	3	32.67	5	F5:1597	OB5943 H6 Ro.raw	3.2279E6	6	6	82	98			PEAKS DB
R.NRSPDIYNPQAGSLK.T	N	114.42	1658.8376	15	0.1	830.4261	2	27.65	5	F5:1291	OB5943 H6 Ro.raw	4.04E5	6	6	370	384			PEAKS DB
R.SPDIYNPQAGSLK.T	N	102.05	1388.6936	13	0.9	695.3547	2	28.77	5	F5:1355	OB5943 H6 Ro.raw	7.3513E5	3	3	372	384			PEAKS DB
N.GIEETIC(+57.02)TASVK.K	N	98.34	1306.6438	12	-0.1	654.3291	2	29.14	5	F5:1389	OB5943 H6 Ro.raw	7.3439E4	3	3	353	364	Carbamidomethylation	C7:Carbamidomethylation:1000.00	PEAKS DB
R.NALFVPHYNTNAHSIIYALR.G	N	97.15	2313.2019	20	-0.1	772.0745	3	35.38	6	F6:1747	OB5944 H6 Ro.raw	2.7972E6	8	8	409	428			PEAKS DB
R.GYFGLIFPGC(+71.04)PSTYEEPAQQGR.R	N	92.77	2487.1528	22	2.2	1244.5864	2	36.43	5	F5:1817	OB5943 H6 Ro.raw	8.0086E4	3	3	99	120	Propionamide	C10:Propionamide:1000.00	PEAKS PTM
R.VYDEELQEGHVLVVPQNFAVAGK.S	N	89.66	2540.2910	23	2.7	847.7732	3	32.92	4	F4:1613	OB5942 H6 Ro.raw	4.6093E5	4	4	443	465			PEAKS DB
N.GIEETIC(+57.02)TASVKK.N	N	88.49	1434.7388	13	0.0	718.3766	2	27.14	5	F5:1263	OB5943 H6 Ro.raw	1.4573E5	4	4	353	365	Carbamidomethylation	C7:Carbamidomethylation:1000.00	PEAKS DB
R.Q(-17.03)QPEENAC(+57.02)QFQR.L	N	88.35	1516.6365	12	2.5	759.3274	2	28.03	5	F5:1319	OB5943 H6 Ro.raw	7.279E3	2	2	25	36	Pyro-glu from Q; Carbamidomethylation	Q1:Pyro-glu from Q:1000.00;C8:Carbamidomethylation:1000.00	PEAKS PTM
R.LESEGGYIETWNPNNQEFEC(+57.02)AGVALSR.L	N	85.95	3069.3774	27	5.5	1024.1387	3	34.81	5	F5:1720	OB5943 H6 Ro.raw	1.0361E4	1	1	46	72	Carbamidomethylation	C20:Carbamidomethylation:1000.00	PEAKS DB
R.QQPEENAC(+57.02)QFQR.L	N	84.17	1533.6630	12	1.7	767.8400	2	25.24	5	F5:1157	OB5943 H6 Ro.raw	3.7544E4	3	3	25	36	Carbamidomethylation	C8:Carbamidomethylation:1000.00	PEAKS DB
K.TDSRPSIANLAGENSIIDNLPEEVVANSYGLPR.E	Y	83.71	3510.7590	33	1.6	1171.2622	3	37.05	6	F6:1855	OB5944 H6 Ro.raw	8.6281E5	4	4	478	510			PEAKS DB
K.TANDLNLLILR.W	N	76.99	1254.7296	11	-0.2	628.3719	2	34.73	5	F5:1761	OB5943 H6 Ro.raw	9.7454E5	3	3	385	395			PEAKS DB
P.FYSNAPQEIFIQQGR.G	N	76.66	1796.8845	15	-1.2	899.4484	2	32.27	4	F4:1574	OB5942 H6 Ro.raw	1.5688E3	1	1	84	98			PEAKS DB
R.LNAQRPDNRLESEGGYIETWNPNNQEFEC(+57.02)AGVALSR.L	N	75.05	4133.9248	36	2.4	1034.4910	4	33.87	6	F6:1670	OB5944 H6 Ro.raw	1.3368E6	1	1	37	72	Carbamidomethylation	C29:Carbamidomethylation:1000.00	PEAKS DB
R.NALRRPFYSNAPQEIFIQQGR.G	N	74.35	2504.3037	21	0.3	627.0834	4	33.30	4	F4:1641	OB5942 H6 Ro.raw	1.8121E5	4	4	78	98			PEAKS DB
R.GYFGLIFPGC(+57.02)PSTYEEPAQQGRR.H	N	73.07	2629.2383	23	1.4	877.4213	3	35.30	5	F5:1733	OB5943 H6 Ro.raw	1.2125E6	3	3	99	121	Carbamidomethylation	C10:Carbamidomethylation:1000.00	PEAKS DB
R.LESEGGYIETWNPNNQ(+.98)EFEC(+57.02)AGVALSR.L	N	62.09	3070.3613	27	6.0	1024.4672	3	34.92	5	F5:1736	OB5943 H6 Ro.raw	6.6817E3	1	1	46	72	Deamidation (NQ); Carbamidomethylation	Q16:Deamidation (NQ):1000.00; C20:Carbamidomethylation:1000.00	PEAKS DB
R.VYDEELQEGHVLVVPQN(+.98)FAVAGK.S	N	61.03	2541.2751	23	9.5	1271.6569	2	32.92	4	F4:1618	OB5942 H6 Ro.raw	3.629E3	1	1	443	465		N17:Deamidation (NQ):14.02	PEAKS DB

total 40 peptides

Peptide	Uniq	-10lgP	Mass	Length	ppm	m/z	z	RT	Fraction	Scan	Source File	Area Roast Ara h 6	#Feature	#Feature Roast Ara h 6	Start	End	PTM	AScore	Found By
R.AHVQVVDSNGN(+.98)RVYDEELQEGHVLVVPQNFAVAGK.S	N	57.69	3817.9023	35	5.5	955.4881	4	33.48	6	F6:1651	OB5944 H6 Ro.raw	7.3088E4	3	3	431	465		N11:Deamidation (NQ):0.00	PEAKS DB
K.TAN(+.98)DLNLLILR.W	N	56.97	1255.7136	11	2.6	628.8657	2	35.22	4	F4:1745	OB5942 H6 Ro.raw	0	0	0	385	395	Deamidation (NQ)	N3:Deamidation (NQ):68.31	PEAKS DB
R.AHVQ(+.98)VVDSNGNRVYDEELQEGHVLVVPQNFAVAGK.S	N	49.13	3817.9023	35	0.9	955.4838	4	33.12	4	F4:1624	OB5942 H6 Ro.raw	4.5408E4	1	1	431	465		Q4:Deamidation (NQ):25.58	PEAKS DB
R.RFNLAGNHEQEFLR.Y	N	45.43	1729.8647	14	0.9	577.6294	3	32.11	5	F5:1568	OB5943 H6 Ro.raw	3.821E3	1	1	196	209			PEAKS DB
K.FFVPPSQQSLGAV.A	Y	44.36	1375.7135	13	9.3	688.8704	2	30.71	4	F4:1587	OB5942 H6 Ro.raw	8.8364E4	1	1	524	536			PEAKS DB
W.LGLSAEYGNLYR.N	N	43.06	1354.6881	12	3.5	678.3537	2	31.68	4	F4:1539	OB5942 H6 Ro.raw	0	0	0	397	408			PEAKS DB
N.G(+57.02)IEETIC(+57.02)TASVKK.N	N	42.25	1491.7603	13	8.7	746.8939	2	26.96	4	F4:1261	OB5942 H6 Ro.raw	1.3952E4	2	2	353	365	Carbamidomethylation	G1:Carbamidomethylation (DHKE, X@N-term):32.28;C7:Carbamidomethylation:1000.00	PEAKS PTM
R.AHVQVVDSN(+.98)GN(+.98)RVYDEELQEGHVLVVPQNFAVAGK.S	N	41.04	3818.8862	35	8.6	955.7371	4	34.33	5	F5:1692	OB5943 H6 Ro.raw	0	0	0	431	465		N9:Deamidation (NQ):0.00;N11:Deamidation (NQ):0.00	PEAKS DB
R.AHVQ(+.98)VVDSNGNRVYDEELQEGHVLVVPQ(+.98)NFAVAGK.S	N	40.14	3818.8862	35	8.9	955.7373	4	33.12	4	F4:1624	OB5942 H6 Ro.raw	3.8034E4	1	1	431	465		Q4:Deamidation (NQ):8.81;Q28:Deamidation (NQ):12.28	PEAKS DB
R.LESEGGYIETWN(+.98)PNNQEFEC(+57.02)AGVALSR.L	N	40.05	3070.3613	27	6.9	1536.1985	2	34.73	5	F5:1729	OB5943 H6 Ro.raw	2.6575E4	1	1	46	72	Deamidation (NQ); Carbamidomethylation	N12:Deamidation (NQ):1000.00;C20:Carbamidomethylation:1000.00	PEAKS DB
R.AHVQVVDSNGNRVYDEELQEGHVLVVPQNFAVAGK.S	N	39.89	3816.9182	35	-0.1	764.3909	5	33.91	5	F5:1668	OB5943 H6 Ro.raw	0	0	0	431	465			PEAKS DB
R.W(+15.99)LGLSAEYGNLYR.N	N	37.59	1556.7623	13	-0.8	779.3878	2	34.26	4	F4:1698	OB5942 H6 Ro.raw	3.8864E3	1	1	396	408	Oxidation (HW)	W1:Oxidation (HW):1000.00	PEAKS PTM
R.AHVQVVDSN(+.98)GNRVYDEELQEGHVLVVPQNFAVAGK.S	N	36.89	3817.9023	35	2.2	955.4850	4	33.52	5	F5:1663	OB5943 H6 Ro.raw	4.5408E4	1	1	431	465		N9:Deamidation (NQ):9.40	PEAKS DB
R.NALFVPH(+14.02)YNTNAHSIIYALR.G	N	36.41	2327.2175	20	2.7	776.7485	3	36.25	5	F5:1805	OB5943 H6 Ro.raw	1.569E4	1	1	409	428		H7:Methylation (others):4.52	PEAKS PTM
R.LNAQRPDNRLESEGGYIETWNPNN(+.98)QEFEC(+57.02)AGVALSR.L	N	36.07	4134.9087	36	4.7	1379.3167	3	33.85	6	F6:1671	OB5944 H6 Ro.raw	8.0788E4	1	1	37	72	Deamidation (NQ); Carbamidomethylation	N24:Deamidation (NQ):1000.00;C29:Carbamidomethylation:1000.00	PEAKS DB
R.GYFGLIFPGC(+58.01)PSTYEEPAQQGR.R	N	35.93	2474.1211	22	4.1	1238.0729	2	38.39	6	F6:1932	OB5944 H6 Ro.raw	0	0	0	99	120	Carboxymethyl	C10:Carboxymethyl:1000.00	PEAKS PTM
R.GYFGLIFPGC(+71.04)PSTYEEPAQQGRR.H	N	35.48	2643.2539	23	1.6	882.0933	3	34.83	4	F4:1720	OB5942 H6 Ro.raw	3.8119E4	1	1	99	121	Propionamide	C10:Propionamide:1000.00	PEAKS PTM

total 40 peptides

Peptide	Uniq	-10lgP	Mass	Length	ppm	m/z	z	RT	Fraction	Scan	Source File	Area Roast Ara h 6	#Feature	#Feature Roast Ara h 6	Start	End	PTM	AScore	Found By
R.GYFGLIFPGC(+57.02)PSTYEEPAQQGR(+14.02)R.H	N	34.44	2643.2539	23	1.0	882.0928	3	35.30	5	F5:1741	OB5943 H6 Ro.raw	3.3639E4	1	1	99	121	Carbamidomethylation	C10:Carbamidomethylation:1000.00;R22:Methylation(KR):0.00	PEAKS PTM
total 40 peptides																			

Q647H4|Q647H4_ARAHY

back to list

| Protein Coverage | Supporting Peptides |

Protein Coverage:

1 MGKLLALSVC FCFLVLGASS ISFRQQPEEN ACQFQRLNAQ RPDNRIESEG GYIETWNPNN QEFECAGVAL SRLVLRRNAL

81 RRPFYSNAPQ EIFIQQGRGY FGLIFPGCPS TYEEPAQQGR RHQSQRPPRR FQGQDQSQQQ QDSHQKVHRF DEGDLIAVPT

161 GVAFWMYNDH DTDVVAVSLT DTNNNDNQLD QFPRRFNLAG NHEQEFLRYQ QQSRRRSLPY SPYSPQTQPK QEDREFSPRG

241 QHGRRERAGQ EQENEGGNIF SGFTPEFLAQ AFQVDDRQIL QNLRGENESD EQGAIVTVRG GLRILSPDRK RRQQYERPDE

321 EEEYDEDEYE YDEEERQHDR RRGRGSRGSG NGIEETICTA SFKKNIGRNR SPDIYNPQAG SLKTANELNL LILRWLGLSA

401 EYGNLYRNAL FVPHYNTNAH SIIYALRGRA HVQVVDSNGD RVFDEELQEG HVLVVPQNFA VAGKSQSENF EYVAFKTDSR

481 PSIANLAGEN SFIDNLPEEV VANSYGLPRE QARQLKNNNP FKFFVPPSEQ SLRAVA

C Carbamidomethylation (+57.02)
C Carboxymethyl (+58.01)
d Deamidation (NQ) (+0.98)
o Oxidation (HW) (+15.99)
P Propionamide (+71.04)
P Pyro-glu from Q (-17.03)

Supporting Peptides:

Peptide	Uniq	-10lgP	Mass	Length	ppm	m/z	z	RT	Fraction	Scan	Source File	Area Roast Ara h 6	#Feature	#Feature Roast Ara h 6	Start	End	PTM	AScore	Found By
R.GYFGLIFPGC(+57.02)PSTYEEPAQQGR.R	N	142.29	2473.1372	22	1.5	1237.5778	2	36.17	4	F4:1794	OB5942 H6 Ro.raw	1.0118E6	3	3	99	120	Carbamidomethylation	C10:Carbamidomethylation:1000.00	PEAKS DB
R.WLGLSAEYGNLYR.N	N	136.28	1540.7673	13	2.0	771.3925	2	34.64	4	F4:1712	OB5942 H6 Ro.raw	2.2268E6	6	6	395	407			PEAKS DB
K.SQSENFEYVAFK.T	N	124.82	1447.6619	12	1.0	724.8389	2	31.74	5	F5:1533	OB5943 H6 Ro.raw	9.8941E5	3	3	465	476			PEAKS DB

total 33 peptides

Peptide	Uniq	-10lgP	Mass	Length	ppm	m/z	z	RT	Fraction	Scan	Source File	Area Roast Ara h 6	#Feature	#Feature Roast Ara h 6	Start	End	PTM	AScore	Found By
R.RPFYSNAPQEIFIQQGR.G	N	119.86	2050.0383	17	2.0	684.3547	3	32.67	5	F5:1597	OB5943 H6 Ro.raw	3.2279E6	6	6	82	98			PEAKS DB
R.NRSPDIYNPQAGSLK.T	N	114.42	1658.8376	15	0.1	830.4261	2	27.65	5	F5:1291	OB5943 H6 Ro.raw	4.04E5	6	6	369	383			PEAKS DB
R.SPDIYNPQAGSLK.T	N	102.05	1388.6936	13	0.9	695.3547	2	28.77	5	F5:1355	OB5943 H6 Ro.raw	7.3513E5	3	3	371	383			PEAKS DB
K.TDSRPSIANLAGENSFIDNLPEEVVANSYGLPR.E	N	101.93	3544.7434	33	3.9	1182.5930	3	37.73	6	F6:1866	OB5944 H6 Ro.raw	1.0339E6	4	4	477	509			PEAKS DB
R.NALFVPHYNTNAHSIIYALR.G	N	97.15	2313.2019	20	-0.1	772.0745	3	35.38	6	F6:1747	OB5944 H6 Ro.raw	2.7972E6	8	8	408	427			PEAKS DB
R.GYFGLIFPGC(+71.04)PSTYEEPAQQGR.R	N	92.77	2487.1528	22	2.2	1244.5864	2	36.43	5	F5:1817	OB5943 H6 Ro.raw	8.0086E4	3	3	99	120	Propionamide	C10:Propionamide:1000.00	PEAKS PTM
R.Q(-17.03)QPEENAC(+57.02)QFQR.L	N	88.35	1516.6365	12	2.5	759.3274	2	28.03	5	F5:1319	OB5943 H6 Ro.raw	7.279E3	2	2	25	36	Pyro-glu from Q; Carbamidomethylation	Q1:Pyro-glu from Q:1000.00;C8:Carbamidomethylation:1000.00	PEAKS PTM
R.IESEGGYIETWNPNNQEFEC(+57.02)AGVALSR.L	N	85.95	3069.3774	27	5.5	1024.1387	3	34.81	5	F5:1720	OB5943 H6 Ro.raw	1.0361E4	1	1	46	72	Carbamidomethylation	C20:Carbamidomethylation:1000.00	PEAKS DB
R.QQPEENAC(+57.02)QFQR.L	N	84.17	1533.6630	12	1.7	767.8400	2	25.24	5	F5:1157	OB5943 H6 Ro.raw	3.7544E4	3	3	25	36	Carbamidomethylation	C8:Carbamidomethylation:1000.00	PEAKS DB
K.FFVPPSEQSLR.A	Y	82.19	1305.6716	11	3.4	653.8453	2	30.79	6	F6:1485	OB5944 H6 Ro.raw	5.6571E3	1	1	523	533			PEAKS DB
K.TANELNLLILR.W	N	81.05	1268.7452	11	0.9	635.3805	2	34.83	4	F4:1715	OB5942 H6 Ro.raw	7.0668E5	3	3	384	394			PEAKS DB
P.FYSNAPQEIFIQQGR.G	N	76.66	1796.8845	15	-1.2	899.4484	2	32.27	4	F4:1574	OB5942 H6 Ro.raw	1.5688E3	1	1	84	98			PEAKS DB
N.GIEETIC(+57.02)TASFKK.N	Y	76.37	1482.7388	13	-0.4	742.3763	2	29.14	5	F5:1388	OB5943 H6 Ro.raw	5.037E4	3	3	352	364	Carbamidomethylation	C7:Carbamidomethylation:1000.00	PEAKS DB
R.LNAQRPDNRIESEGGYIETWNPNNQEFEC(+57.02)AGVALSR.L	N	75.05	4133.9248	36	2.4	1034.4910	4	33.87	6	F6:1670	OB5944 H6 Ro.raw	1.3368E6	1	1	37	72	Carbamidomethylation	C29:Carbamidomethylation:1000.00	PEAKS DB
R.NALRRPFYSNAPQEIFIQQGR.G	N	74.35	2504.3037	21	0.3	627.0834	4	33.30	4	F4:1641	OB5942 H6 Ro.raw	1.8121E5	4	4	78	98			PEAKS DB
R.GYFGLIFPGC(+57.02)PSTYEEPAQQGRR.H	N	73.07	2629.2383	23	1.4	877.4213	3	35.30	5	F5:1733	OB5943 H6 Ro.raw	1.2125E6	3	3	99	121	Carbamidomethylation	C10:Carbamidomethylation:1000.00	PEAKS DB
R.IESEGGYIETWNPNNQ(+.98)EFEC(+57.02)AGVALSR.L	N	62.09	3070.3613	27	6.0	1024.4672	3	34.92	5	F5:1736	OB5943 H6 Ro.raw	6.6817E3	1	1	46	72	Carbamidomethylation	Q16:Deamidation (NQ):0.00;C20:Carbamidomethylation:1000.00	PEAKS DB
K.TDSRPSIANLAGENSFIDNLPEEVVAN(+.98)SYGLPR.E	N	59.30	3545.7273	33	7.1	1182.9248	3	36.36	4	F4:1812	OB5942 H6 Ro.raw	3.983E3	1	1	477	509	Deamidation (NQ)	N27:Deamidation (NQ):51.97	PEAKS DB
K.FFVPPSEQSLRA.V	Y	56.98	1376.7087	12	0.2	689.3618	2	31.25	4	F4:1517	OB5942 H6 Ro.raw	1.7396E5	4	4	523	534			PEAKS DB

total 33 peptides

Peptide	Uniq	-10lgP	Mass	Length	ppm	m/z	z	RT	Fraction	Scan	Source File	Area Roast Ara h 6	#Feature	#Feature Roast Ara h 6	Start	End	PTM	AScore	Found By
R.RFNLAGNHEQEFLR.Y	N	45.43	1729.8647	14	0.9	577.6294	3	32.11	5	F5:1568	OB5943 H6 Ro.raw	3.821E3	1	1	195	208			PEAKS DB
W.LGLSAEYGNLYR.N	N	43.06	1354.6881	12	3.5	678.3537	2	31.68	4	F4:1539	OB5942 H6 Ro.raw	0	0	0	396	407			PEAKS DB
R.IESEGGYIETWN(+.98)PNNQEFEC(+57.02)AGVALSR.L	N	40.05	3070.3613	27	6.9	1536.1985	2	34.73	5	F5:1729	OB5943 H6 Ro.raw	2.6575E4	1	1	46	72	Carbamidomethylation	N12:Deamidation (NQ):0.00;C20:Carbamidomethylation:1000.00	PEAKS DB
R.W(+15.99)LGLSAEYGNLYR.N	N	37.59	1556.7623	13	-0.8	779.3878	2	34.26	4	F4:1698	OB5942 H6 Ro.raw	3.8864E3	1	1	395	407	Oxidation (HW)	W1:Oxidation (HW):1000.00	PEAKS PTM
R.NALFVPH(+14.02)YNTNAHSIIYALR.G	N	36.41	2327.2175	20	2.7	776.7485	3	36.25	5	F5:1805	OB5943 H6 Ro.raw	1.569E4	1	1	408	427		H7:Methylation (others):4.52	PEAKS PTM
R.LNAQRPDNRIESEGGYIETWNPNN(+.98)QEFEC(+57.02)AGVALSR.L	N	36.07	4134.9087	36	4.7	1379.3167	3	33.85	6	F6:1671	OB5944 H6 Ro.raw	8.0788E4	1	1	37	72	Carbamidomethylation	N24:Deamidation (NQ):0.00;C29:Carbamidomethylation:1000.00	PEAKS DB
R.GYFGLIFPGC(+58.01)PSTYEEPAQQGR.R	N	35.93	2474.1211	22	4.1	1238.0729	2	38.39	6	F6:1932	OB5944 H6 Ro.raw	0	0	0	99	120	Carboxymethyl	C10:Carboxymethyl:1000.00	PEAKS PTM
R.GYFGLIFPGC(+71.04)PSTYEEPAQQGRR.H	N	35.48	2643.2539	23	1.6	882.0933	3	34.83	4	F4:1720	OB5942 H6 Ro.raw	3.8119E4	1	1	99	121	Propionamide	C10:Propionamide:1000.00	PEAKS PTM
R.GYFGLIFPGC(+57.02)PSTYEEPAQQGR(+14.02)R.H	N	34.44	2643.2539	23	1.0	882.0928	3	35.30	5	F5:1741	OB5943 H6 Ro.raw	3.3639E4	1	1	99	121	Carbamidomethylation	C10:Carbamidomethylation:1000.00;R22:Methylation(KR):0.00	PEAKS PTM
K.TDSRPSIANLAGENSFIDN(+.98)LPEEVVANSYGLPR.E	N	33.13	3545.7273	33	3.8	1182.9209	3	39.27	5	F5:1978	OB5943 H6 Ro.raw	0	0	0	477	509		N19:Deamidation (NQ):8.41	PEAKS DB
K.SQSE(+53.92)NFEYVAFK.T	N	32.70	1501.5812	12	-0.3	751.7976	2	31.36	4	F4:1520	OB5942 H6 Ro.raw	0	0	0	465	476		E4:Replacement of 2 protons by iron:22.45	PEAKS PTM
**total 33 peptides**																			

A5Z1R0|A5Z1R0_ARAHY

back to list

| **Protein Coverage** | **Supporting Peptides** |

Protein Coverage:

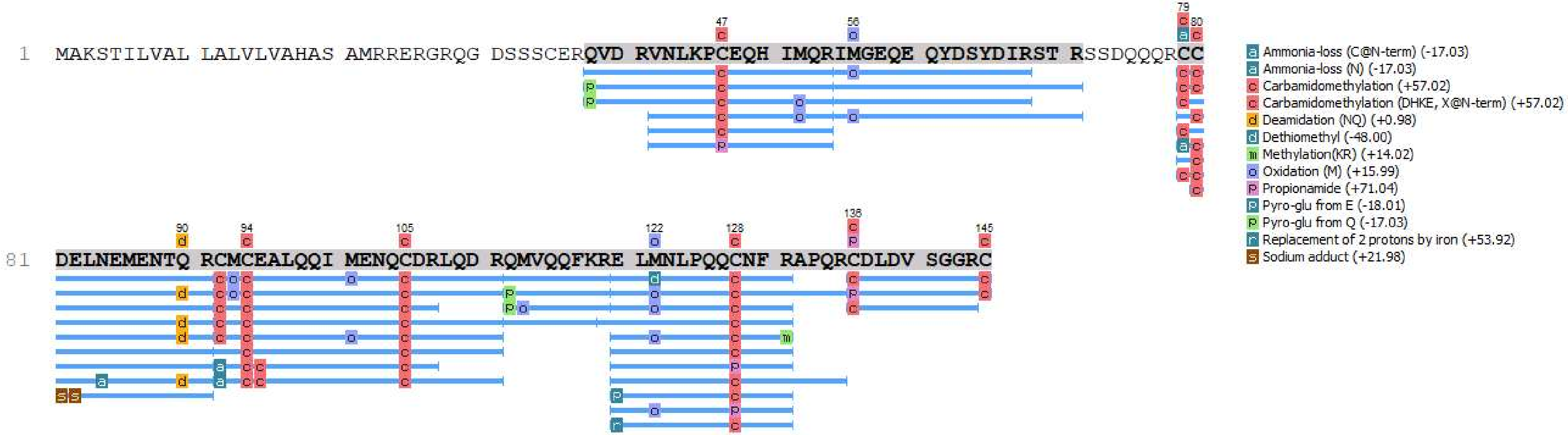

Supporting Peptides:

Peptide	Uniq	-10lgP	Mass	Length	ppm	m/z	z	RT	Fraction	Scan	Source File	Area Roast Ara h 6	#Feature	#Feature Roast Ara h 6	Start	End	PTM	AScore	Found By
R.IMGEQEQYDSYDIR.S	Y	124.33	1745.7566	14	3.3	873.8885	2	30.26	5	F5:1465	OB5943 H6 Ro.raw	1.4138E7	6	6	55	68			PEAKS DB
R.ELMNLPQQC(+57.02)NFR.A	Y	121.41	1548.7177	12	2.6	775.3681	2	31.38	5	F5:1532	OB5943 H6 Ro.raw	8.1025E6	8	8	120	131	Carbamidomethylation	C9:Carbamidomethylation:1000.00	PEAKS DB
R.I(+57.02)MGEQEQYDSYDIR.S	Y	121.19	1802.7781	14	1.9	902.3981	2	30.45	5	F5:1466	OB5943 H6 Ro.raw	1.078E5	3	3	55	68		I1:Carbamidomethylation (DHKE, X@N-term):49.43	PEAKS PTM
R.IM(+15.99)GEQEQYDSYDIR.S	Y	118.58	1761.7516	14	3.3	881.8860	2	28.96	5	F5:1366	OB5943 H6 Ro.raw	2.9554E6	4	4	55	68	Oxidation (M)	M2:Oxidation (M):1000.00	PEAKS DB
R.C(+57.02)MC(+57.02)EALQQIMENQC(+57.02)DR.L	Y	116.38	2084.8206	16	4.4	1043.4221	2	33.85	5	F5:1665	OB5943 H6 Ro.raw	3.1431E4	2	2	92	107	Carbamidomethylation	C1:Carbamidomethylation:1000.00;C3:Carbamidomethylation:1000.00;C14:Carbamidomethylation:1000.00	PEAKS DB

total 61 peptides

Peptide	Uniq	-10lgP	Mass	Length	ppm	m/z	z	RT	Fraction	Scan	Source File	Area Roast Ara h 6	#Feature	#Feature Roast Ara h 6	Start	End	PTM	AScore	Found By
R.ELM(+15.99)NLPQQC(+57.02)NFR.A	Y	109.46	1564.7126	12	0.9	783.3643	2	29.14	5	F5:1377	OB5943 H6 Ro.raw	9.4897E5	3	3	120	131	Oxidation (M); Carbamidomethylation	M3:Oxidation (M):1000.00; C9:Carbamidomethylation:1000.00	PEAKS DB
R.C(+57.02)C(+57.02)DELNEMENTQR.C	Y	102.36	1697.6444	13	0.6	849.8300	2	27.44	4	F4:1278	OB5942 H6 Ro.raw	3.0478E5	3	3	79	91	Carbamidomethylation	C1:Carbamidomethylation:1000.00; C2:Carbamidomethylation:1000.00	PEAKS DB
R.C(+57.02)C(+57.02)DELNEMENTQ(+.98)R.C	Y	100.88	1698.6284	13	1.6	850.3229	2	28.40	5	F5:1344	OB5943 H6 Ro.raw	3.601E5	3	3	79	91	Carbamidomethylation; Deamidation (NQ)	C1:Carbamidomethylation:1000.00; C2:Carbamidomethylation:1000.00; Q12:Deamidation (NQ):60.92	PEAKS DB
R.C(+57.02)MC(+57.02)EALQQIMENQC(+57.02)DRLQDR.Q	Y	97.66	2597.0913	20	3.1	866.7070	3	33.49	4	F4:1646	OB5942 H6 Ro.raw	2.7944E6	7	7	92	111	Carbamidomethylation	C1:Carbamidomethylation:1000.00; C3:Carbamidomethylation:1000.00; C14:Carbamidomethylation:1000.00	PEAKS DB
R.ELMNLPQQC(+71.04)NFR.A	Y	92.76	1562.7334	12	0.0	782.3740	2	31.56	5	F5:1534	OB5943 H6 Ro.raw	2.0625E5	3	3	120	131	Propionamide	C9:Propionamide:1000.00	PEAKS PTM
R.QVDRVNLKPC(+57.02)EQHIMQR.I	Y	91.79	2150.0837	17	0.0	538.5282	4	29.70	5	F5:1432	OB5943 H6 Ro.raw	4.1805E5	4	4	38	54	Carbamidomethylation	C10:Carbamidomethylation:1000.00	PEAKS DB
R.Q(-17.03)VDRVNLKPC(+57.02)EQHIMQR.I	Y	91.37	2133.0571	17	1.2	534.2722	4	30.44	5	F5:1454	OB5943 H6 Ro.raw	8.5431E5	5	5	38	54	Pyro-glu from Q; Carbamidomethylation	Q1:Pyro-glu from Q:1000.00; C10:Carbamidomethylation:1000.00	PEAKS PTM

total 61 peptides

Peptide	Uniq	-10lgP	Mass	Length	ppm	m/z	z	RT	Fraction	Scan	Source File	Area Roast Ara h 6	#Feature	#Feature Roast Ara h 6	Start	End	PTM	AScore	Found By
R.C(+71.04)C(+57.02)DELNEMENTQ(+.98)R.C	Y	91.34	1712.6440	13	1.5	857.3306	2	28.58	5	F5:1345	OB5943 H6 Ro.raw	2.2455E5	2	2	79	91	Carbamidomethylation; Deamidation (NQ)	C1:Propionamide:14.02;C2:Carbamidomethylation:1000.00;Q12:Deamidation (NQ):67.96	PEAKS PTM
R.VNLKPC(+57.02)EQHIMQR.I	Y	89.73	1651.8286	13	0.5	551.6171	3	28.40	5	F5:1356	OB5943 H6 Ro.raw	1.1244E6	6	6	42	54	Carbamidomethylation	C6:Carbamidomethylation:1000.00	PEAKS DB
R.C(-17.03)C(+57.02)D(+57.02)ELNEMENTQR.C	Y	88.83	1680.6178	13	1.0	841.3170	2	32.29	5	F5:1575	OB5943 H6 Ro.raw	3.9042E4	2	2	79	91	Ammonia-loss (C@N-term); Carbamidomethylation	C1:Ammonia-loss (C@N-term):1000.00;C2:Carbamidomethylation:1000.00;D3:Carbamidomethylation (DHKE, X@N-term):33.98	PEAKS PTM
R.C(-17.03)MC(+57.02)E(+57.02)ALQQIMENQC(+57.02)DR.L	Y	87.36	2067.7942	16	3.0	1034.9075	2	38.83	5	F5:1953	OB5943 H6 Ro.raw	2.7406E4	2	2	92	107	Ammonia-loss (C@N-term); Carbamidomethylation; Carbamidomethylation (DHKE, X@N-term)	C1:Ammonia-loss (C@N-term):1000.00;C3:Carbamidomethylation:1000.00;E4:Carbamidomethylation (DHKE, X@N-term):151.49;C14:Carbamidomethylation:1000.00	PEAKS PTM

total 61 peptides

Peptide	Uniq	-10lgP	Mass	Length	ppm	m/z	z	RT	Fraction	Scan	Source File	Area Roast Ara h 6	#Feature	#Feature Roast Ara h 6	Start	End	PTM	AScore	Found By
R.C(+57.02)C(+71.04)DELNEMENTQR.C	Y	86.36	1711.6600	13	1.0	856.8381	2	27.64	4	F4:1283	OB5942 H6 Ro.raw	2.8007E5	3	3	79	91	Carbamidomethylation	C1:Carbamidomethylation:1000.00;C2:Propionamide:10.11	PEAKS PTM
R.Q(-17.03)MVQQFKR.E	Y	84.77	1046.5331	8	0.0	524.2738	2	27.84	5	F5:1322	OB5943 H6 Ro.raw	3.5192E5	3	3	112	119	Pyro-glu from Q	Q1:Pyro-glu from Q:1000.00	PEAKS PTM
R.C(+57.02)DLDVSGGRC(+57.02)	Y	83.20	1137.4543	10	-0.5	569.7342	2	25.28	5	F5:1158	OB5943 H6 Ro.raw	4.867E4	3	3	136	145	Carbamidomethylation	C1:Carbamidomethylation:1000.00;C10:Carbamidomethylation:1000.00	PEAKS DB
R.C(+57.02)(+28.03)C(+57.02)DELNEMENTQR.C	Y	82.43	1725.6757	13	1.0	863.8460	2	28.03	5	F5:1316	OB5943 H6 Ro.raw	1.6949E4	1	1	79	91	Carbamidomethylation	C1:Carbamidomethylation:1000.00;C1:Ethylation:18.53;C2:Carbamidomethylation:1000.00	PEAKS PTM
R.C(+71.04)DLDVSGGRC(+57.02)	Y	81.95	1151.4700	10	0.9	576.7428	2	25.64	5	F5:1183	OB5943 H6 Ro.raw	1.3899E4	3	3	136	145	Propionamide; Carbamidomethylation	C1:Propionamide:156.52;C10:Carbamidomethylation:1000.00	PEAKS PTM
R.C(+71.04)MC(+57.02)EALQQIMENQC(+57.02)DRLQDR.Q	Y	81.33	2611.1069	20	1.1	871.3772	3	33.49	4	F4:1647	OB5942 H6 Ro.raw	6.2487E5	2	2	92	111	Carbamidomethylation	C1:Propionamide:20.41;C3:Carbamidomethylation:1000.00;C14:Carbamidomethylation:1000.00	PEAKS PTM

total 61 peptides

Peptide	Uniq	-10lgP	Mass	Length	ppm	m/z	z	RT	Fraction	Scan	Source File	Area Roast Ara h 6	#Feature	#Feature Roast Ara h 6	Start	End	PTM	AScore	Found By
R.C(+57.02)M(+15.99)C(+57.02)EALQQIMENQC(+57.02)DRLQDR.Q	Y	72.82	2613.0862	20	1.4	872.0372	3	31.19	5	F5:1576	OB5943 H6 Ro.raw	5.7636E5	5	5	92	111	Carbamidomethylation; Oxidation (M)	C1:Carbamidomethylation:1000.00;M2:Oxidation (M):89.48;C3:Carbamidomethylation:1000.00;C14:Carbamidomethylation:1000.00	PEAKS DB
R.ELM(-48.00)NLPQQC(+57.02)NFR.A	Y	71.71	1500.7144	12	-0.1	501.2454	3	27.84	5	F5:1311	OB5943 H6 Ro.raw	2.9967E5	2	2	120	131	Dethiomethyl; Carbamidomethylation	M3:Dethiomethyl:1000.00;C9:Carbamidomethylation:1000.00	PEAKS PTM
R.C(+57.02)DLDVSGGR.C	Y	64.95	977.4236	9	-0.5	489.7189	2	24.23	6	F6:1121	OB5944 H6 Ro.raw	8.8623E2	1	1	136	144	Carbamidomethylation	C1:Carbamidomethylation:1000.00	PEAKS DB
R.C(+71.04)C(+57.02)DELNEMENTQR.C	Y	63.78	1711.6600	13	1.0	856.8381	2	27.64	4	F4:1317	OB5942 H6 Ro.raw	1.8297E5	2	2	79	91	Carbamidomethylation	C1:Propionamide:8.69;C2:Carbamidomethylation:1000.00	PEAKS PTM
R.C(+57.02)(+28.03)C(+57.02)DELNEMENTQ(+.98)R.C	Y	63.72	1726.6597	13	9.5	864.3453	2	28.38	4	F4:1339	OB5942 H6 Ro.raw	2.3202E3	1	1	79	91	Carbamidomethylation	C1:Carbamidomethylation:1000.00;C1:Ethylation:22.85;C2:Carbamidomethylation:1000.00;Q12:Deamidation (NQ):20.63	PEAKS PTM
R.ELMNLPQQC(+57.02)NF(+31.99)R.A	Y	63.29	1580.7075	12	-9.5	791.3535	2	31.67	4	F4:1538	OB5942 H6 Ro.raw	0	0	0	120	131	Carbamidomethylation	C9:Carbamidomethylation:1000.00;F11:Dihydroxy:0.00	PEAKS PTM

total 61 peptides

Peptide	Uniq	-10lgP	Mass	Length	ppm	m/z	z	RT	Fraction	Scan	Source File	Area Roast Ara h 6	#Feature	#Feature Roast Ara h 6	Start	End	PTM	AScore	Found By
R.IM(+15.99)GE(+57.02)QEQYDSYDIR.S	Y	61.28	1818.7729	14	0.3	910.3940	2	29.25	5	F5:1394	OB5943 H6 Ro.raw	2.5217E3	1	1	55	68	Oxidation (M)	M2:Oxidation (M):1000.00;E4:Carbamidomethylation (DHKE, X@N-term):0.00	PEAKS PTM
R.ELMNLPQQC(+57.02)N(+.98)FRAPQR.C	Y	60.15	2001.9513	16	1.0	668.3250	3	30.88	5	F5:1490	OB5943 H6 Ro.raw	0	0	0	120	135	Carbamidomethylation	C9:Carbamidomethylation:1000.00; N10:Deamidation (NQ):24.24	PEAKS DB
R.C(+57.02)C(+71.04)DELNEMENTQ(+.98)R.C	Y	60.08	1712.6440	13	-0.7	857.3287	2	28.30	6	F6:1333	OB5944 H6 Ro.raw	1.8621E5	2	2	79	91	Carbamidomethylation; Deamidation (NQ)	C1:Carbamidomethylation:1000.00; C2:Propionamide:0.00;Q12:Deamidation (NQ):55.92	PEAKS PTM
R.VNLK(+41.03)PC(+57.02)EQHIMQR.I	Y	59.14	1692.8552	13	3.3	565.2942	3	29.51	5	F5:1408	OB5943 H6 Ro.raw	4.1722E3	1	1	42	54	Carbamidomethylation	K4:Amidination of lysines or N-terminal amines with methyl acetimidate:38.16; C6:Carbamidomethylation:1000.00	PEAKS PTM
R.ELM(+15.99)NLPQQC(+71.04)NFR.A	Y	59.00	1578.7283	12	1.8	790.3729	2	29.10	6	F6:1390	OB5944 H6 Ro.raw	0	0	0	120	131	Oxidation (M); Propionamide	M3:Oxidation (M):1000.00; C9:Propionamide:1000.00	PEAKS PTM
R.IMGEQEQYDSYDIRSTR.S	Y	56.36	2089.9375	17	2.0	697.6545	3	28.94	4	F4:1381	OB5942 H6 Ro.raw	7.2826E4	3	3	55	71			PEAKS DB

total 61 peptides

Peptide	Uniq	-10lgP	Mass	Length	ppm	m/z	z	RT	Fraction	Scan	Source File	Area Roast Ara h 6	#Feature	#Feature Roast Ara h 6	Start	End	PTM	AScore	Found By
R.C(+57.02)C(+57.02)DELN(+.98)EMENTQR.C	Y	52.99	1698.6284	13	-1.6	850.3201	2	29.09	4	F4:1387	OB5942 H6 Ro.raw	0	0	0	79	91	Carbamidomethylation	C1:Carbamidomethylation:1000.00;C2:Carbamidomethylation:1000.00;N6:Deamidation (NQ):31.56	PEAKS DB
R.VNLKPC(+57.02)EQHIM(+15.99)QR.I	Y	52.99	1667.8236	13	0.6	556.9488	3	26.91	4	F4:1258	OB5942 H6 Ro.raw	2.1143E4	1	1	42	54	Carbamidomethylation; Oxidation (M)	C6:Carbamidomethylation:1000.00;M11:Oxidation (M):1000.00	PEAKS DB
R.VNLKPC(+71.04)EQHIMQR.I	Y	52.61	1665.8444	13	-0.4	556.2885	3	28.77	5	F5:1372	OB5943 H6 Ro.raw	2.0373E4	1	1	42	54	Propionamide	C6:Propionamide:1000.00	PEAKS PTM
R.QMVQQFK.R	Y	51.26	907.4586	7	-0.6	454.7363	2	25.61	6	F6:1183	OB5944 H6 Ro.raw	8.0264E3	2	2	112	118			PEAKS DB
R.C(+57.02)M(+15.99)C(+57.02)EALQQIM(+15.99)ENQC(+57.02)DRLQDR.Q	Y	49.37	2629.0811	20	2.0	877.3694	3	29.88	5	F5:1425	OB5943 H6 Ro.raw	7.9642E3	1	1	92	111	Carbamidomethylation; Oxidation (M)	C1:Carbamidomethylation:1000.00;M2:Oxidation (M):1000.00;C3:Carbamidomethylation:1000.00;M10:Oxidation (M):1000.00;C14:Carbamidomethylation:1000.00	PEAKS DB
R.IMGEQE(+53.92)QYDSYDIR.S	Y	48.83	1799.6759	14	-2.3	600.8979	3	30.20	5	F5:1450	OB5943 H6 Ro.raw	0	0	0	55	68		E6:Replacement of 2 protons by iron:22.85	PEAKS PTM
R.ELMNLPQQC(+57.02)NFRAPQR.C	Y	48.77	2000.9673	16	0.9	667.9969	3	30.26	5	F5:1456	OB5943 H6 Ro.raw	6.8362E4	3	3	120	135	Carbamidomethylation	C9:Carbamidomethylation:1000.00	PEAKS DB

total 61 peptides

Peptide	Uniq	-10lgP	Mass	Length	ppm	m/z	z	RT	Fraction	Scan	Source File	Area Roast Ara h 6	#Feature	#Feature Roast Ara h 6	Start	End	PTM	AScore	Found By
R.Q(-17.03)M(+15.99)VQQFKR.E	Y	48.54	1062.5281	8	3.0	532.2729	2	25.67	5	F5:1189	OB5943 H6 Ro.raw	0	0	0	112	119	Pyro-glu from Q; Oxidation (M)	Q1:Pyro-glu from Q:1000.00;M2:Oxidation (M):1000.00	PEAKS PTM
R.IMGE(+17.03)QEQYDSYDIR.S	Y	48.35	1762.7832	14	-4.0	882.3953	2	30.83	4	F4:1488	OB5942 H6 Ro.raw	0	0	0	55	68		E4:Replacement of proton with ammonium ion:18.53	PEAKS PTM
K.RELMNLPQQC(+57.02)NFR.A	Y	48.08	1704.8188	13	0.8	569.2807	3	29.89	4	F4:1434	OB5942 H6 Ro.raw	0	0	0	119	131	Carbamidomethylation	C10:Carbamidomethylation:1000.00	PEAKS DB
R.C(+71.04)C(+57.02)DELNEMEN(+.98)TQR.C	Y	47.24	1712.6440	13	-0.7	857.3287	2	28.30	6	F6:1367	OB5944 H6 Ro.raw	9.9111E4	1	1	79	91	Carbamidomethylation	C1:Propionamide:0.00;C2:Carbamidomethylation:1000.00;N10:Deamidation (NQ):40.21	PEAKS PTM
R.IM(+15.99)GEQEQYDSYDIRSTR.S	Y	47.11	2105.9324	17	-1.9	702.9834	3	27.45	4	F4:1288	OB5942 H6 Ro.raw	2.3858E3	1	1	55	71	Oxidation (M)	M2:Oxidation (M):1000.00	PEAKS DB
R.IMGEQEQY(-18.01)DSYDIR.S	Y	46.28	1727.7461	14	3.4	864.8833	2	30.78	5	F5:1484	OB5943 H6 Ro.raw	0	0	0	55	68		Y8:Dehydration:0.00	PEAKS PTM
R.IMGEQEQY(+125.90)DSYDIR.S	Y	43.24	1871.6533	14	2.0	936.8358	2	32.29	5	F5:1560	OB5943 H6 Ro.raw	6.88E3	1	1	55	68		Y8:Iodination:41.59	PEAKS PTM
R.I(+57.02)M(+15.99)GEQEQYDSYDIR.S	Y	42.43	1818.7729	14	0.8	910.3945	2	28.84	4	F4:1372	OB5942 H6 Ro.raw	0	0	0	55	68	Oxidation (M)	I1:Carbamidomethylation (DHKE,X@N-term):17.32;M2:Oxidation (M):1000.00	PEAKS PTM

total 61 peptides

Peptide	Uniq	-10lgP	Mass	Length	ppm	m/z	z	RT	Fraction	Scan	Source File	Area Roast Ara h 6	#Feature	#Feature Roast Ara h 6	Start	End	PTM	AScore	Found By
R.C(+57.02)C(+57.02)DELN(-17.03)EMENTQ(+.98)R.C	Y	42.31	1681.6018	13	0.9	841.8090	2	32.39	6	F6:1584	OB5944 H6 Ro.raw	0	0	0	79	91	Carbamidomethylation; Ammonia-loss (N); Deamidation (NQ)	C1:Carbamidomethylation:1000.00;C2:Carbamidomethylation:1000.00;N6:Ammonia-loss (N):105.60;Q12:Deamidation (NQ):55.92	PEAKS PTM
R.C(-17.03)MC(+57.02)E(+57.02)ALQQIMENQC(+57.02)DRLQDR.Q	Y	42.03	2580.0647	20	2.2	861.0307	3	37.62	5	F5:1929	OB5943 H6 Ro.raw	1.6912E5	1	1	92	111	Ammonia-loss (C@N-term); Carbamidomethylation; Carbamidomethylation (DHKE, X@N-term)	C1:Ammonia-loss (C@N-term):1000.00;C3:Carbamidomethylation:1000.00;E4:Carbamidomethylation (DHKE, X@N-term):79.54;C14:Carbamidomethylation:1000.00	PEAKS PTM
R.C(+71.04)C(+57.02)DELN(+.98)EMENTQR.C	Y	40.31	1712.6440	13	-0.7	857.3287	2	28.30	6	F6:1382	OB5944 H6 Ro.raw	9.9111E4	1	1	79	91	Carbamidomethylation	C1:Propionamide:0.00;C2:Carbamidomethylation:1000.00;N6:Deamidation (NQ):0.00	PEAKS PTM
R.QMVQQFKR.E	Y	39.82	1063.5597	8	0.9	532.7876	2	26.84	5	F5:1239	OB5943 H6 Ro.raw	1.9987E4	2	2	112	119			PEAKS DB
R.E(+53.92)LMNLPQQC(+57.02)NFR.A	Y	39.79	1602.6370	12	-0.1	802.3257	2	31.08	4	F4:1503	OB5942 H6 Ro.raw	0	0	0	120	131	Replacement of 2 protons by iron; Carbamidomethylation	E1:Replacement of 2 protons by iron:76.82;C9:Carbamidomethylation:1000.00	PEAKS PTM
**total 61 peptides**																			

Peptide	Uniq	-10lgP	Mass	Length	ppm	m/z	z	RT	Fraction	Scan	Source File	Area Roast Ara h 6	#Feature	#Feature Roast Ara h 6	Start	End	PTM	AScore	Found By
C.C(+57.02)D(+21.98)E(+21.98)LNEMENTQR.C	Y	39.39	1581.5776	12	6.2	791.8010	2	29.69	5	F5:1412	OB5943 H6 Ro.raw	7.5318E3	1	1	80	91	Carbamidomethylation; Sodium adduct	C1:Carbamidomethylation:1000.00;D2:Sodium adduct:68.53;E3:Sodium adduct:69.29	PEAKS PTM
R.E(-18.01)LMNLPQQC(+57.02)NFR.A	Y	38.68	1530.7072	12	1.3	766.3618	2	34.58	4	F4:1708	OB5942 H6 Ro.raw	7.6045E3	1	1	120	131	Pyro-glu from E; Carbamidomethylation	E1:Pyro-glu from E:1000.00;C9:Carbamidomethylation:1000.00	PEAKS PTM
R.ELM(+15.99)NLPQQC(+57.02)NFR(+14.02).A	Y	35.11	1578.7283	12	0.4	790.3718	2	29.32	5	F5:1402	OB5943 H6 Ro.raw	9.8676E3	1	1	120	131	Oxidation (M); Carbamidomethylation; Methylation(KR)	M3:Oxidation (M):1000.00;C9:Carbamidomethylation:1000.00;R12:Methylation(KR):1000.00	PEAKS PTM
R.C(+57.02)MC(+57.02)EALQQIM(+15.99)ENQC(+57.02)D(-18.01)RLQDR.Q	Y	34.82	2595.0757	20	1.8	866.0341	3	33.29	4	F4:1627	OB5942 H6 Ro.raw	2.8862E4	1	1	92	111	Carbamidomethylation; Oxidation (M)	C1:Carbamidomethylation:1000.00;C3:Carbamidomethylation:1000.00;M10:Oxidation (M):73.05;C14:Carbamidomethylation:1000.00;D15:Dehydration:14.19	PEAKS PTM
R.ELM(+15.99)NLPQQC(+57.02)NFRAPQR.C	Y	33.01	2016.9622	16	2.0	673.3293	3	28.58	5	F5:1359	OB5943 H6 Ro.raw	7.79E3	1	1	120	135	Oxidation (M); Carbamidomethylation	M3:Oxidation (M):1000.00;C9:Carbamidomethylation:1000.00	PEAKS DB

total 61 peptides

Peptide	Uniq	-10lgP	Mass	Length	ppm	m/z	z	RT	Fraction	Scan	Source File	Area Roast Ara h 6	#Feature	#Feature Roast Ara h 6	Start	End	PTM	AScore	Found By
R.IMGE(+21.98)QEQYDSYDIR.S	Y	32.81	1767.7385	14	-1.3	884.8754	2	29.94	4	F4:1442	OB5942 H6 Ro.raw	1.7258E4	1	1	55	68		E4:Sodium adduct:0.00	PEAKS PTM
R.Q(-17.03)VDRVNLKPC(+57.02)EQHIM(+15.99)QR.I	Y	30.83	2149.0520	17	1.1	538.2709	4	28.97	4	F4:1380	OB5942 H6 Ro.raw	0	0	0	38	54	Pyro-glu from Q; Carbamidomethylation; Oxidation (M)	Q1:Pyro-glu from Q:1000.00; C10:Carbamidomethylation:1000.00; M15:Oxidation (M):1000.00	PEAKS PTM
**total 61 peptides**																			

sp|Q647G9|CONG_ARAHY

back to list

| Protein Coverage | Supporting Peptides |

Protein Coverage:

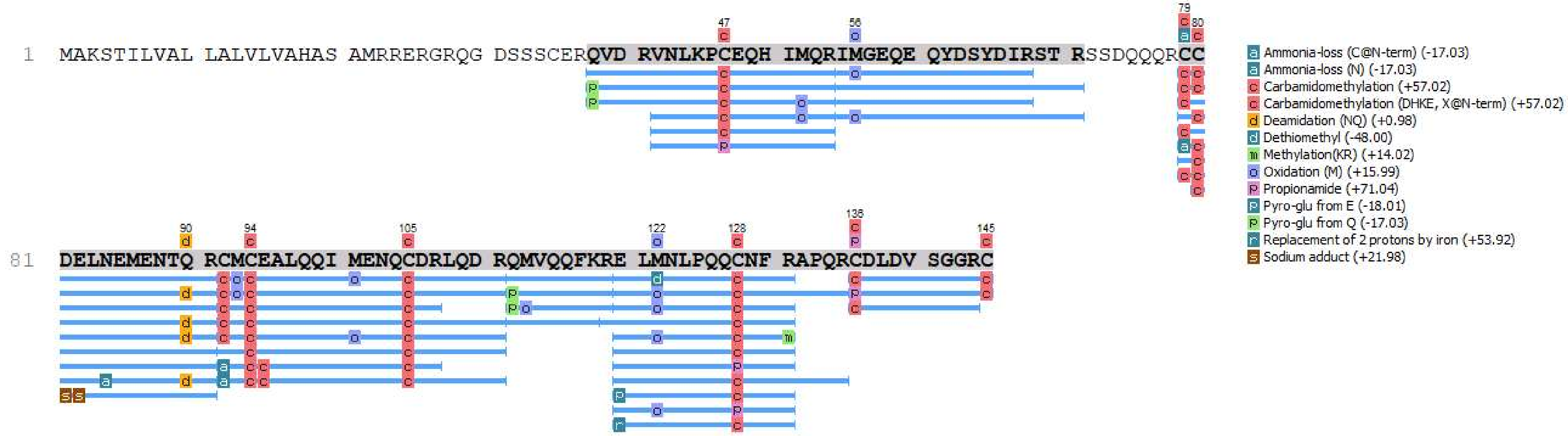

Supporting Peptides:

Peptide	Uniq	-10lgP	Mass	Length	ppm	m/z	z	RT	Fraction	Scan	Source File	Area Roast Ara h 6	#Feature	#Feature Roast Ara h 6	Start	End	PTM	AScore	Found By
R.IMGEQEQYDSYDIR.S	Y	124.33	1745.7566	14	3.3	873.8885	2	30.26	5	F5:1465	OB5943 H6 Ro.raw	1.4138E7	6	6	55	68			PEAKS DB
R.ELMNLPQQC(+57.02)NFR.A	Y	121.41	1548.7177	12	2.6	775.3681	2	31.38	5	F5:1532	OB5943 H6 Ro.raw	8.1025E6	8	8	120	131	Carbamidomethylation	C9:Carbamidomethylation:1000.00	PEAKS DB

total 61 peptides

Peptide	Uniq	-10lgP	Mass	Length	ppm	m/z	z	RT	Fraction	Scan	Source File	Area Roast Ara h 6	#Feature	#Feature Roast Ara h 6	Start	End	PTM	AScore	Found By
R.I(+57.02)MGEQEQYDSYDIR.S	Y	121.19	1802.7781	14	1.9	902.3981	2	30.45	5	F5:1466	OB5943 H6 Ro.raw	1.078E5	3	3	55	68		I1:Carbamidomethylation (DHKE, X@N-term):49.43	PEAKS PTM
R.IM(+15.99)GEQEQYDSYDIR.S	Y	118.58	1761.7516	14	3.3	881.8860	2	28.96	5	F5:1366	OB5943 H6 Ro.raw	2.9554E6	4	4	55	68	Oxidation (M)	M2:Oxidation (M):1000.00	PEAKS DB
R.C(+57.02)MC(+57.02)EALQQIMENQC(+57.02)DR.L	Y	116.38	2084.8206	16	4.4	1043.4221	2	33.85	5	F5:1665	OB5943 H6 Ro.raw	3.1431E4	2	2	92	107	Carbamidomethylation	C1:Carbamidomethylation:1000.00; C3:Carbamidomethylation:1000.00; C14:Carbamidomethylation:1000.00	PEAKS DB
R.ELM(+15.99)NLPQQC(+57.02)NFR.A	Y	109.46	1564.7126	12	0.9	783.3643	2	29.14	5	F5:1377	OB5943 H6 Ro.raw	9.4897E5	3	3	120	131	Oxidation (M); Carbamidomethylation	M3:Oxidation (M):1000.00; C9:Carbamidomethylation:1000.00	PEAKS DB
R.C(+57.02)C(+57.02)DELNEMENTQR.C	Y	102.36	1697.6444	13	0.6	849.8300	2	27.44	4	F4:1278	OB5942 H6 Ro.raw	3.0478E5	3	3	79	91	Carbamidomethylation	C1:Carbamidomethylation:1000.00; C2:Carbamidomethylation:1000.00	PEAKS DB
R.C(+57.02)C(+57.02)DELNEMENTQ(+.98)R.C	Y	100.88	1698.6284	13	1.6	850.3229	2	28.40	5	F5:1344	OB5943 H6 Ro.raw	3.601E5	3	3	79	91	Carbamidomethylation; Deamidation (NQ)	C1:Carbamidomethylation:1000.00; C2:Carbamidomethylation:1000.00; Q12:Deamidation (NQ):60.92	PEAKS DB

total 61 peptides

Peptide	Uniq	-10lgP	Mass	Length	ppm	m/z	z	RT	Fraction	Scan	Source File	Area Roast Ara h 6	#Feature	#Feature Roast Ara h 6	Start	End	PTM	AScore	Found By
R.C(+57.02)MC(+57.02)EALQQIMENQC(+57.02)DRLQDR.Q	Y	97.66	2597.0913	20	3.1	866.7070	3	33.49	4	F4:1646	OB5942 H6 Ro.raw	2.7944E6	7	7	92	111	Carbamidomethylation	C1:Carbamidomethylation:1000.00;C3:Carbamidomethylation:1000.00;C14:Carbamidomethylation:1000.00	PEAKS DB
R.ELMNLPQQC(+71.04)NFR.A	Y	92.76	1562.7334	12	0.0	782.3740	2	31.56	5	F5:1534	OB5943 H6 Ro.raw	2.0625E5	3	3	120	131	Propionamide	C9:Propionamide:1000.00	PEAKS PTM
R.QVDRVNLKPC(+57.02)EQHIMQR.I	Y	91.79	2150.0837	17	0.0	538.5282	4	29.70	5	F5:1432	OB5943 H6 Ro.raw	4.1805E5	4	4	38	54	Carbamidomethylation	C10:Carbamidomethylation:1000.00	PEAKS DB
R.Q(-17.03)VDRVNLKPC(+57.02)EQHIMQR.I	Y	91.37	2133.0571	17	1.2	534.2722	4	30.44	5	F5:1454	OB5943 H6 Ro.raw	8.5431E5	5	5	38	54	Pyro-glu from Q; Carbamidomethylation	Q1:Pyro-glu from Q:1000.00;C10:Carbamidomethylation:1000.00	PEAKS PTM
R.C(+71.04)C(+57.02)DELNEMENTQ(+.98)R.C	Y	91.34	1712.6440	13	1.5	857.3306	2	28.58	5	F5:1345	OB5943 H6 Ro.raw	2.2455E5	2	2	79	91	Carbamidomethylation; Deamidation (NQ)	C1:Propionamide:14.02;C2:Carbamidomethylation:1000.00;Q12:Deamidation (NQ):67.96	PEAKS PTM
R.VNLKPC(+57.02)EQHIMQR.I	Y	89.73	1651.8286	13	0.5	551.6171	3	28.40	5	F5:1356	OB5943 H6 Ro.raw	1.1244E6	6	6	42	54	Carbamidomethylation	C6:Carbamidomethylation:1000.00	PEAKS DB

total 61 peptides

Peptide	Uniq	-10lgP	Mass	Length	ppm	m/z	z	RT	Fraction	Scan	Source File	Area Roast Ara h 6	#Feature	#Feature Roast Ara h 6	Start	End	PTM	AScore	Found By
R.C(-17.03)C(+57.02)D(+57.02)ELNEMENTQR.C	Y	88.83	1680.6178	13	1.0	841.3170	2	32.29	5	F5:1575	OB5943 H6 Ro.raw	3.9042E4	2	2	79	91	Ammonia-loss (C@N-term); Carbamidomethylation	C1:Ammonia-loss (C@N-term):1000.00;C2:Carbamidomethylation:1000.00;D3:Carbamidomethylation (DHKE, X@N-term):33.98	PEAKS PTM
R.C(-17.03)MC(+57.02)E(+57.02)ALQQIMENQC(+57.02)DR.L	Y	87.36	2067.7942	16	3.0	1034.9075	2	38.83	5	F5:1953	OB5943 H6 Ro.raw	2.7406E4	2	2	92	107	Ammonia-loss (C@N-term); Carbamidomethylation; Carbamidomethylation (DHKE, X@N-term)	C1:Ammonia-loss (C@N-term):1000.00;C3:Carbamidomethylation:1000.00;E4:Carbamidomethylation (DHKE, X@N-term):151.49;C14:Carbamidomethylation:1000.00	PEAKS PTM
R.C(+57.02)C(+71.04)DELNEMENTQR.C	Y	86.36	1711.6600	13	1.0	856.8381	2	27.64	4	F4:1283	OB5942 H6 Ro.raw	2.8007E5	3	3	79	91	Carbamidomethylation	C1:Carbamidomethylation:1000.00;C2:Propionamide:10.11	PEAKS PTM
R.Q(-17.03)MVQQFKR.E	Y	84.77	1046.5331	8	0.0	524.2738	2	27.84	5	F5:1322	OB5943 H6 Ro.raw	3.5192E5	3	3	112	119	Pyro-glu from Q	Q1:Pyro-glu from Q:1000.00	PEAKS PTM
R.C(+57.02)DLDVSGGRC(+57.02)	Y	83.20	1137.4543	10	-0.5	569.7342	2	25.28	5	F5:1158	OB5943 H6 Ro.raw	4.867E4	3	3	136	145	Carbamidomethylation	C1:Carbamidomethylation:1000.00;C10:Carbamidomethylation:1000.00	PEAKS DB

total 61 peptides

Peptide	Uniq	-10lgP	Mass	Length	ppm	m/z	z	RT	Fraction	Scan	Source File	Area Roast Ara h 6	#Feature	#Feature Roast Ara h 6	Start	End	PTM	AScore	Found By
R.C(+57.02)(+28.03)C(+57.02)DELNEMENTQR.C	Y	82.43	1725.6757	13	1.0	863.8460	2	28.03	5	F5:1316	OB5943 H6 Ro.raw	1.6949E4	1	1	79	91	Carbamidomethylation	C1:Carbamidomethylation:1000.00;C1:Ethylation:18.53;C2:Carbamidomethylation:1000.00	PEAKS PTM
R.C(+71.04)DLDVSGGRC(+57.02)	Y	81.95	1151.4700	10	0.9	576.7428	2	25.64	5	F5:1183	OB5943 H6 Ro.raw	1.3899E4	3	3	136	145	Propionamide; Carbamidomethylation	C1:Propionamide:156.52;C10:Carbamidomethylation:1000.00	PEAKS PTM
R.C(+71.04)MC(+57.02)EALQQIMENQC(+57.02)DRLQDR.Q	Y	81.33	2611.1069	20	1.1	871.3772	3	33.49	4	F4:1647	OB5942 H6 Ro.raw	6.2487E5	2	2	92	111	Carbamidomethylation	C1:Propionamide:20.41;C3:Carbamidomethylation:1000.00;C14:Carbamidomethylation:1000.00	PEAKS PTM
R.C(+57.02)M(+15.99)C(+57.02)EALQQIMENQC(+57.02)DRLQDR.Q	Y	72.82	2613.0862	20	1.4	872.0372	3	31.19	5	F5:1576	OB5943 H6 Ro.raw	5.7636E5	5	5	92	111	Carbamidomethylation; Oxidation (M)	C1:Carbamidomethylation:1000.00;M2:Oxidation (M):89.48;C3:Carbamidomethylation:1000.00;C14:Carbamidomethylation:1000.00	PEAKS DB
R.ELM(-48.00)NLPQQC(+57.02)NFR.A	Y	71.71	1500.7144	12	-0.1	501.2454	3	27.84	5	F5:1311	OB5943 H6 Ro.raw	2.9967E5	2	2	120	131	Dethiomethyl; Carbamidomethylation	M3:Dethiomethyl:1000.00;C9:Carbamidomethylation:1000.00	PEAKS PTM

total 61 peptides

Peptide	Uniq	-10lgP	Mass	Length	ppm	m/z	z	RT	Fraction	Scan	Source File	Area Roast Ara h 6	#Feature	#Feature Roast Ara h 6	Start	End	PTM	AScore	Found By
R.C(+57.02)DLDVSGGR.C	Y	64.95	977.4236	9	-0.5	489.7189	2	24.23	6	F6:1121	OB5944 H6 Ro.raw	8.8623E2	1	1	136	144	Carbamidomethylation	C1:Carbamidomethylation:1000.00	PEAKS DB
R.C(+71.04)C(+57.02)DELNEMENTQR.C	Y	63.78	1711.6600	13	1.0	856.8381	2	27.64	4	F4:1317	OB5942 H6 Ro.raw	1.8297E5	2	2	79	91	Carbamidomethylation	C1:Propionamide:8.69;C2:Carbamidomethylation:1000.00	PEAKS PTM
R.C(+57.02)(+28.03)C(+57.02)DELNEMENTQ(+.98)R.C	Y	63.72	1726.6597	13	9.5	864.3453	2	28.38	4	F4:1339	OB5942 H6 Ro.raw	2.3202E3	1	1	79	91	Carbamidomethylation	C1:Carbamidomethylation:1000.00;C1:Ethylation:22.85;C2:Carbamidomethylation:1000.00;Q12:Deamidation (NQ):20.63	PEAKS PTM
R.ELMNLPQQC(+57.02)NF(+31.99)R.A	Y	63.29	1580.7075	12	-9.5	791.3535	2	31.67	4	F4:1538	OB5942 H6 Ro.raw	0	0	0	120	131	Carbamidomethylation	C9:Carbamidomethylation:1000.00;F11:Dihydroxy:0.00	PEAKS PTM
R.IM(+15.99)GE(+57.02)QEQYDSYDIR.S	Y	61.28	1818.7729	14	0.3	910.3940	2	29.25	5	F5:1394	OB5943 H6 Ro.raw	2.5217E3	1	1	55	68	Oxidation (M)	M2:Oxidation (M):1000.00;E4:Carbamidomethylation (DHKE, X@N-term):0.00	PEAKS PTM
R.ELMNLPQQC(+57.02)N(+.98)FRAPQR.C	Y	60.15	2001.9513	16	1.0	668.3250	3	30.88	5	F5:1490	OB5943 H6 Ro.raw	0	0	0	120	135	Carbamidomethylation	C9:Carbamidomethylation:1000.00;N10:Deamidation (NQ):24.24	PEAKS DB

total 61 peptides

Peptide	Uniq	-10lgP	Mass	Length	ppm	m/z	z	RT	Fraction	Scan	Source File	Area Roast Ara h 6	#Feature	#Feature Roast Ara h 6	Start	End	PTM	AScore	Found By
R.C(+57.02)C(+71.04)DELNEMENTQ(+.98)R.C	Y	60.08	1712.6440	13	-0.7	857.3287	2	28.30	6	F6:1333	OB5944 H6 Ro.raw	1.8621E5	2	2	79	91	Carbamidomethylation; Deamidation (NQ)	C1:Carbamidomethylation:1000.00; C2:Propionamide:0.00;Q12:Deamidation (NQ):55.92	PEAKS PTM
R.VNLK(+41.03)PC(+57.02)EQHIMQR.I	Y	59.14	1692.8552	13	3.3	565.2942	3	29.51	5	F5:1408	OB5943 H6 Ro.raw	4.1722E3	1	1	42	54	Carbamidomethylation	K4:Amidination of lysines or N-terminal amines with methyl acetimidate:38.16; C6:Carbamidomethylation:1000.00	PEAKS PTM
R.ELM(+15.99)NLPQQC(+71.04)NFR.A	Y	59.00	1578.7283	12	1.8	790.3729	2	29.10	6	F6:1390	OB5944 H6 Ro.raw	0	0	0	120	131	Oxidation (M); Propionamide	M3:Oxidation (M):1000.00; C9:Propionamide:1000.00	PEAKS PTM
R.IMGEQEQYDSYDIRSTR.S	Y	56.36	2089.9375	17	2.0	697.6545	3	28.94	4	F4:1381	OB5942 H6 Ro.raw	7.2826E4	3	3	55	71			PEAKS DB
R.C(+57.02)C(+57.02)DELN(+.98)EMENTQR.C	Y	52.99	1698.6284	13	-1.6	850.3201	2	29.09	4	F4:1387	OB5942 H6 Ro.raw	0	0	0	79	91	Carbamidomethylation	C1:Carbamidomethylation:1000.00; C2:Carbamidomethylation:1000.00; N6:Deamidation (NQ):31.56	PEAKS DB
R.VNLKPC(+57.02)EQHIM(+15.99)QR.I	Y	52.99	1667.8236	13	0.6	556.9488	3	26.91	4	F4:1258	OB5942 H6 Ro.raw	2.1143E4	1	1	42	54	Carbamidomethylation; Oxidation (M)	C6:Carbamidomethylation:1000.00; M11:Oxidation (M):1000.00	PEAKS DB
R.VNLKPC(+71.04)EQHIMQR.I	Y	52.61	1665.8444	13	-0.4	556.2885	3	28.77	5	F5:1372	OB5943 H6 Ro.raw	2.0373E4	1	1	42	54	Propionamide	C6:Propionamide:1000.00	PEAKS PTM

total 61 peptides

Peptide	Uniq	-10lgP	Mass	Length	ppm	m/z	z	RT	Fraction	Scan	Source File	Area Roast Ara h 6	#Feature	#Feature Roast Ara h 6	Start	End	PTM	AScore	Found By
R.QMVQQFK.R	Y	51.26	907.4586	7	-0.6	454.7363	2	25.61	6	F6:1183	OB5944 H6 Ro.raw	8.0264E3	2	2	112	118			PEAKS DB
R.C(+57.02)M(+15.99)C(+57.02)EALQQIM(+15.99)ENQC(+57.02)DRLQDR.Q	Y	49.37	2629.0811	20	2.0	877.3694	3	29.88	5	F5:1425	OB5943 H6 Ro.raw	7.9642E3	1	1	92	111	Carbamidomethylation; Oxidation (M)	C1:Carbamidomethylation:1000.00; M2:Oxidation (M):1000.00; C3:Carbamidomethylation:1000.00; M10:Oxidation (M):1000.00; C14:Carbamidomethylation:1000.00	PEAKS DB
R.IMGEQE(+53.92)QYDSYDIR.S	Y	48.83	1799.6759	14	-2.3	600.8979	3	30.20	5	F5:1450	OB5943 H6 Ro.raw	0	0	0	55	68		E6:Replacement of 2 protons by iron:22.85	PEAKS PTM
R.ELMNLPQQC(+57.02)NFRAPQR.C	Y	48.77	2000.9673	16	0.9	667.9969	3	30.26	5	F5:1456	OB5943 H6 Ro.raw	6.8362E4	3	3	120	135	Carbamidomethylation	C9:Carbamidomethylation:1000.00	PEAKS DB
R.Q(-17.03)M(+15.99)VQQFKR.E	Y	48.54	1062.5281	8	3.0	532.2729	2	25.67	5	F5:1189	OB5943 H6 Ro.raw	0	0	0	112	119	Pyro-glu from Q; Oxidation (M)	Q1:Pyro-glu from Q:1000.00; M2:Oxidation (M):1000.00	PEAKS PTM
R.IMGE(+17.03)QEQYDSYDIR.S	Y	48.35	1762.7832	14	-4.0	882.3953	2	30.83	4	F4:1488	OB5942 H6 Ro.raw	0	0	0	55	68		E4:Replacement of proton with ammonium ion:18.53	PEAKS PTM
K.RELMNLPQQC(+57.02)NFR.A	Y	48.08	1704.8188	13	0.8	569.2807	3	29.89	4	F4:1434	OB5942 H6 Ro.raw	0	0	0	119	131	Carbamidomethylation	C10:Carbamidomethylation:1000.00	PEAKS DB

total 61 peptides

Peptide	Uniq	-10lgP	Mass	Length	ppm	m/z	z	RT	Fraction	Scan	Source File	Area Roast Ara h 6	#Feature	#Feature Roast Ara h 6	Start	End	PTM	AScore	Found By
R.C(+71.04)C(+57.02)DELNEMEN(+.98)TQR.C	Y	47.24	1712.6440	13	-0.7	857.3287	2	28.30	6	F6:1367	OB5944 H6 Ro.raw	9.9111E4	1	1	79	91	Carbamidomethylation	C1:Propionamide:0.00;C2:Carbamidomethylation:1000.00;N10:Deamidation (NQ):40.21	PEAKS PTM
R.IM(+15.99)GEQEQYDSYDIRSTR.S	Y	47.11	2105.9324	17	-1.9	702.9834	3	27.45	4	F4:1288	OB5942 H6 Ro.raw	2.3858E3	1	1	55	71	Oxidation (M)	M2:Oxidation (M):1000.00	PEAKS DB
R.IMGEQEQY(-18.01)DSYDIR.S	Y	46.28	1727.7461	14	3.4	864.8833	2	30.78	5	F5:1484	OB5943 H6 Ro.raw	0	0	0	55	68		Y8:Dehydration:0.00	PEAKS PTM
R.IMGEQEQY(+125.90)DSYDIR.S	Y	43.24	1871.6533	14	2.0	936.8358	2	32.29	5	F5:1560	OB5943 H6 Ro.raw	6.88E3	1	1	55	68		Y8:Iodination:41.59	PEAKS PTM
R.I(+57.02)M(+15.99)GEQEQYDSYDIR.S	Y	42.43	1818.7729	14	0.8	910.3945	2	28.84	4	F4:1372	OB5942 H6 Ro.raw	0	0	0	55	68	Oxidation (M)	I1:Carbamidomethylation (DHKE, X@N-term):17.32;M2:Oxidation (M):1000.00	PEAKS PTM
R.C(+57.02)C(+57.02)DELN(-17.03)EMENTQ(+.98)R.C	Y	42.31	1681.6018	13	0.9	841.8090	2	32.39	6	F6:1584	OB5944 H6 Ro.raw	0	0	0	79	91	Carbamidomethylation; Ammonia-loss (N); Deamidation (NQ)	C1:Carbamidomethylation:1000.00;C2:Carbamidomethylation:1000.00;N6:Ammonia-loss (N):105.60;Q12:Deamidation (NQ):55.92	PEAKS PTM

total 61 peptides

Peptide	Uniq	-10lgP	Mass	Length	ppm	m/z	z	RT	Fraction	Scan	Source File	Area Roast Ara h 6	#Feature	#Feature Roast Ara h 6	Start	End	PTM	AScore	Found By
R.C(-17.03)MC(+57.02)E(+57.02)ALQQIMENQC(+57.02)DRLQDR.Q	Y	42.03	2580.0647	20	2.2	861.0307	3	37.62	5	F5:1929	OB5943 H6 Ro.raw	1.6912E5	1	1	92	111	Ammonia-loss (C@N-term); Carbamidomethylation; Carbamidomethylation (DHKE, X@N-term)	C1:Ammonia-loss (C@N-term):1000.00;C3:Carbamidomethylation:1000.00;E4:Carbamidomethylation (DHKE, X@N-term):79.54;C14:Carbamidomethylation:1000.00	PEAKS PTM
R.C(+71.04)C(+57.02)DELN(+.98)EMENTQR.C	Y	40.31	1712.6440	13	-0.7	857.3287	2	28.30	6	F6:1382	OB5944 H6 Ro.raw	9.9111E4	1	1	79	91	Carbamidomethylation	C1:Propionamide:0.00;C2:Carbamidomethylation:1000.00;N6:Deamidation (NQ):0.00	PEAKS PTM
R.QMVQQFKR.E	Y	39.82	1063.5597	8	0.9	532.7876	2	26.84	5	F5:1239	OB5943 H6 Ro.raw	1.9987E4	2	2	112	119			PEAKS DB
R.E(+53.92)LMNLPQQC(+57.02)NFR.A	Y	39.79	1602.6370	12	-0.1	802.3257	2	31.08	4	F4:1503	OB5942 H6 Ro.raw	0	0	0	120	131	Replacement of 2 protons by iron; Carbamidomethylation	E1:Replacement of 2 protons by iron:76.82;C9:Carbamidomethylation:1000.00	PEAKS PTM
C.C(+57.02)D(+21.98)E(+21.98)LNEMENTQR.C	Y	39.39	1581.5776	12	6.2	791.8010	2	29.69	5	F5:1412	OB5943 H6 Ro.raw	7.5318E3	1	1	80	91	Carbamidomethylation; Sodium adduct	C1:Carbamidomethylation:1000.00;D2:Sodium adduct:68.53;E3:Sodium adduct:69.29	PEAKS PTM

total 61 peptides

Peptide	Uniq	-10lgP	Mass	Length	ppm	m/z	z	RT	Fraction	Scan	Source File	Area Roast Ara h 6	#Feature	#Feature Roast Ara h 6	Start	End	PTM	AScore	Found By
R.E(-18.01)LMNLPQQC(+57.02)NFR.A	Y	38.68	1530.7072	12	1.3	766.3618	2	34.58	4	F4:1708	OB5942 H6 Ro.raw	7.6045E3	1	1	120	131	Pyro-glu from E; Carbamidomethylation	E1:Pyro-glu from E:1000.00; C9:Carbamidomethylation:1000.00	PEAKS PTM
R.ELM(+15.99)NLPQQC(+57.02)NFR(+14.02).A	Y	35.11	1578.7283	12	0.4	790.3718	2	29.32	5	F5:1402	OB5943 H6 Ro.raw	9.8676E3	1	1	120	131	Oxidation (M); Carbamidomethylation; Methylation(KR)	M3:Oxidation (M):1000.00; C9:Carbamidomethylation:1000.00; R12:Methylation(KR):1000.00	PEAKS PTM
R.C(+57.02)MC(+57.02)EALQQIM(+15.99)ENQC(+57.02)D(-18.01)RLQDR.Q	Y	34.82	2595.0757	20	1.8	866.0341	3	33.29	4	F4:1627	OB5942 H6 Ro.raw	2.8862E4	1	1	92	111	Carbamidomethylation; Oxidation (M)	C1:Carbamidomethylation:1000.00; C3:Carbamidomethylation:1000.00; M10:Oxidation (M):73.05;C14:Carbamidomethylation:1000.00; D15:Dehydration:14.19	PEAKS PTM
R.ELM(+15.99)NLPQQC(+57.02)NFRAPQR.C	Y	33.01	2016.9622	16	2.0	673.3293	3	28.58	5	F5:1359	OB5943 H6 Ro.raw	7.79E3	1	1	120	135	Oxidation (M); Carbamidomethylation	M3:Oxidation (M):1000.00; C9:Carbamidomethylation:1000.00	PEAKS DB
R.IMGE(+21.98)QEQYDSYDIR.S	Y	32.81	1767.7385	14	-1.3	884.8754	2	29.94	4	F4:1442	OB5942 H6 Ro.raw	1.7258E4	1	1	55	68		E4:Sodium adduct:0.00	PEAKS PTM

total 61 peptides

Peptide	Uniq	-10lgP	Mass	Length	ppm	m/z	z	RT	Fraction	Scan	Source File	Area Roast Ara h 6	#Feature	#Feature Roast Ara h 6	Start	End	PTM	AScore	Found By
R.Q(-17.03)VDRVNLKPC(+57.02)EQHIM(+15.99)QR.I	Y	30.83	2149.0520	17	1.1	538.2709	4	28.97	4	F4:1380	OB5942 H6 Ro.raw	0	0	0	38	54	Pyro-glu from Q; Carbamidomethylation; Oxidation (M)	Q1:Pyro-glu from Q:1000.00;C10:Carbamidomethylation:1000.00;M15:Oxidation (M):1000.00	PEAKS PTM
**total 61 peptides**																			

N1NG13|N1NG13_ARAHY

back to list

| Protein Coverage | Supporting Peptides |

Protein Coverage:

1 MRGRVSPLML LLGILVLASV SATHAKSSPY QKKTENPCAQ RCLQSCQQEP DDLKQKACES RCTKLEYDPR CVYDPRGHTG
81 TTNQRSPPGE RTRGRQPGDY DDDRRQPRRE EGGRWGPAGP REREREEDWR QPREDWRRPS HQQPRKIRPE GREGEQEWGT
161 PGSHVREETS RNNPFYFPSR RFSTRYGNQN GRIRVLQRFD QRSRQFQNLQ NHRIVQIEAK PNTLVLPKHA DADNILVIQQ
241 GQATVTVANG NNRKSFNLDE GHALRIPSGF ISYILNRHDN QNLRVAKISM PVNTPGQFED FFPASSRDQS SYLQGFSRNT
321 LEAAFNAEFN EIRRVLLEEN AGGEQEERGQ RRWSTRSSEN NEGVIVKVSK EHVEELTKHA KSVSKKGSEE EGDITNPINL
401 REGEPDLSNN FGKLFEVKPD KKNPQLQDLD MMLTCVEIKE GALMLPHFNS KAMVIVVVNK GTGNLELVAV RKEQQQRGRR
481 EEEEDEDEEE EGSNREVRRY TARLKEGDVF IMPAAHPVAI NASSELHLLG FGINAENNHR IFLAGDKDNV IDQIEKQAKD
561 LAFPGSGEQV EKLIKNQKES HFVSARPQSQ SQSPSSPEKE SPEKEDQEEE NQGGKGPLLS ILKAFN

C Carbamidomethylation (+57.02)
O Oxidation (M) (+15.99)

Supporting Peptides:

Peptide	Uniq	-10lgP	Mass	Length	ppm	m/z	z	RT	Fraction	Scan	Source File	Area Roast Ara h 6	#Feature	#Feature Roast Ara h 6	Start	End	PTM	AScore	Found By
R.IPSGFISYILNR.H	N	116.76	1378.7609	12	1.0	690.3884	2	36.77	4	F4:1833	OB5942 H6 Ro.raw	4.949E5	3	3	266	277			PEAKS DB
K.SFNLDEGHALR.I	N	105.18	1257.6101	11	0.5	629.8126	2	28.96	5	F5:1378	OB5943 H6 Ro.raw	6.7733E4	3	3	255	265			PEAKS DB
R.DQSSYLQGFSR.N	N	104.35	1286.5891	11	0.5	644.3021	2	30.70	6	F6:1482	OB5944 H6 Ro.raw	7.4069E4	3	3	308	318			PEAKS DB

total 20 peptides

Peptide	Uniq	-10lgP	Mass	Length	ppm	m/z	z	RT	Fraction	Scan	Source File	Area Roast Ara h 6	#Feature	#Feature Roast Ara h 6	Start	End	PTM	AScore	Found By
K.ISMPVNTPGQFEDFFPASSR.D	N	100.55	2226.0415	20	2.5	1114.0308	2	35.60	4	F4:1757	OB5942 H6 Ro.raw	1.7538E5	3	3	288	307			PEAKS DB
R.VLLEENAGGEQEER.G	N	100.30	1571.7427	14	0.7	786.8792	2	26.60	4	F4:1237	OB5942 H6 Ro.raw	6.3383E3	1	1	335	348			PEAKS DB
K.ISM(+15.99)PVNTPGQFEDFFPASSR.D	N	85.41	2242.0364	20	2.1	1122.0278	2	34.54	5	F5:1702	OB5943 H6 Ro.raw	2.1861E4	2	2	288	307	Oxidation (M)	M3:Oxidation (M):1000.00	PEAKS DB
R.NNPFYFPSRR.F	N	79.01	1296.6364	10	1.1	649.3262	2	30.07	5	F5:1443	OB5943 H6 Ro.raw	9.0774E4	3	3	172	181			PEAKS DB
R.NNPFYFPSR.R	N	78.65	1140.5352	9	1.8	571.2759	2	31.84	5	F5:1546	OB5943 H6 Ro.raw	5.7869E3	2	2	172	180			PEAKS DB
R.NTLEAAFNAEFNEIR.R	N	78.54	1737.8322	15	0.3	869.9236	2	35.52	4	F4:1762	OB5942 H6 Ro.raw	1.2135E3	1	1	319	333			PEAKS DB
R.IFLAGDKDNVIDQIEK.Q	N	78.42	1816.9570	16	-0.1	909.4857	2	33.42	5	F5:1643	OB5943 H6 Ro.raw	2.878E5	6	6	541	556			PEAKS DB
R.IVQIEAKPNTLVLPK.H	Y	77.37	1662.0079	15	2.9	832.0137	2	30.14	4	F4:1444	OB5942 H6 Ro.raw	6.3611E4	3	3	214	228			PEAKS DB
K.SFNLDEGHALRIPSGFISYILNR.H	N	64.24	2618.3604	23	0.8	873.7948	3	38.22	4	F4:1918	OB5942 H6 Ro.raw	1.6862E4	1	1	255	277			PEAKS DB
R.NTLEAAFNAEFNEIRR.V	N	54.71	1893.9332	16	-2.3	632.3169	3	34.83	5	F5:1724	OB5943 H6 Ro.raw	4.2865E5	5	5	319	334			PEAKS DB
K.DLAFPGSGEQVEK.L	N	54.25	1375.6619	13	0.6	688.8386	2	30.14	4	F4:1453	OB5942 H6 Ro.raw	1.9909E4	2	2	560	572			PEAKS DB
K.HADADNILVIQQGQATVTVANGNNRK.S	N	53.99	2746.4111	26	1.5	916.4790	3	30.13	5	F5:1446	OB5943 H6 Ro.raw	0	0	0	229	254			PEAKS DB
K.KGSEEEGDITNPINLREGEPDLSNNFGK.L	Y	49.97	3058.4478	28	4.0	1020.4939	3	30.91	4	F4:1493	OB5942 H6 Ro.raw	0	0	0	386	413			PEAKS DB
R.C(+57.02)LQS(-18.01)C(+57.02)Q(+.98)QEPDDLKQK.A	N	47.12	1858.8190	15	-0.1	930.4167	2	28.47	4	F4:1350	OB5942 H6 Ro.raw	0	0	0	42	56	Carbamidomethylation	C1:Carbamidomethylation:1000.00;S4:Dehydration:39.55;C5:Carbamidomethylation:1000.00;Q6:Deamidation (NQ):0.00	PEAKS PTM
K.HADADNILVIQQGQATVTVANGN(+.98)NRK.S	N	34.54	2747.3950	26	2.3	916.8077	3	29.76	4	F4:1422	OB5942 H6 Ro.raw	8.5956E3	1	1	229	254		N23:Deamidation (NQ):0.00	PEAKS DB
K.HADADNILVIQQGQATVTVAN(+.98)GNNRK.S	N	32.47	2747.3950	26	1.1	687.8568	4	30.07	5	F5:1448	OB5943 H6 Ro.raw	2.5891E3	1	1	229	254		N21:Deamidation (NQ):0.00	PEAKS DB
R.IPSGFISYILNRHDNQNLR.V	N	31.95	2256.1763	19	2.8	753.0681	3	34.64	4	F4:1759	OB5942 H6 Ro.raw	5.949E4	1	1	266	284			PEAKS DB
**total 20 peptides**																			

sp|P43238|ALL12_ARAHY

back to list

| Protein Coverage | Supporting Peptides |

Protein Coverage:

46

1 MRGRVSPLML LLGILVLASV SATHAKSSPY QKKTENPCAQ R**CLQSCQQEP DDLKQK**ACES RCTKLEYDPR CVYDPRGHTG

Carbamidomethylation (+57.02)
Oxidation (M) (+15.99)

81 TTNQRSPPGE RTRGRQPGDY DDDRRQPRRE EGGRWGPAGP REREREEDWR QPREDWRRPS HQQPRKIRPE GREGEQEWGT

161 PGSHVREETS R**NNPFYFPSR R**FSTRYGNQN GRIRVLQRFD QRSRQFQNLQ NHR**IVQIEAK PNTLVLPKHA DADNILVIQQ**

290

241 **GQATVTVANG NNRKSFNLDE GHALRIPSGF ISYILNRHDN QNLR**VAK**ISM PVNTPGQFED FFPASSRDQS SYLQGFSRNT**

321 **LEAAFNAEFN EIRRVLLEEN AGGEQEER**GQ RRWSTRSSEN NEGVIVKVSK EHVEELTKHA KSVSK**KGSEE EGDITNPINL**

401 **REGEPDLSNN FGK**LFEVKPD KKNPQLQDLD MMLTCVEIKE GALMLPHFNS KAMVIVVVNK GTGNLELVAV RKEQQQRGRR

481 EEEEDEDEEE EGSNREVRRY TARLKEGDVF IMPAAHPVAI NASSELHLLG FGINAENNHR **IFLAGDKDNV IDQIEK**QAK**D**

561 **LAFPGSGEQV EK**LIKNQKES HFVSARPQSQ SQSPSSPEKE SPEKEDQEEE NQGGKGPLLS ILKAFN

Supporting Peptides:

Peptide	Uniq	-10lgP	Mass	Length	ppm	m/z	z	RT	Fraction	Scan	Source File	Area Roast Ara h 6	#Feature	#Feature Roast Ara h 6	Start	End	PTM	AScore	Found By
R.IPSGFISYILNR.H	N	116.76	1378.7609	12	1.0	690.3884	2	36.77	4	F4:1833	OB5942 H6 Ro.raw	4.949E5	3	3	266	277			PEAKS DB
K.SFNLDEGHALR.I	N	105.18	1257.6101	11	0.5	629.8126	2	28.96	5	F5:1378	OB5943 H6 Ro.raw	6.7733E4	3	3	255	265			PEAKS DB
R.DQSSYLQGFSR.N	N	104.35	1286.5891	11	0.5	644.3021	2	30.70	6	F6:1482	OB5944 H6 Ro.raw	7.4069E4	3	3	308	318			PEAKS DB
K.ISMPVNTPGQFEDFFPASSR.D	N	100.55	2226.0415	20	2.5	1114.0308	2	35.60	4	F4:1757	OB5942 H6 Ro.raw	1.7538E5	3	3	288	307			PEAKS DB
R.VLLEENAGGEQEER.G	N	100.30	1571.7427	14	0.7	786.8792	2	26.60	4	F4:1237	OB5942 H6 Ro.raw	6.3383E3	1	1	335	348			PEAKS DB
K.ISM(+15.99)PVNTPGQFEDFFPASSR.D	N	85.41	2242.0364	20	2.1	1122.0278	2	34.54	5	F5:1702	OB5943 H6 Ro.raw	2.1861E4	2	2	288	307	Oxidation (M)	M3:Oxidation (M):1000.00	PEAKS DB
R.NNPFYFPSRR.F	N	79.01	1296.6364	10	1.1	649.3262	2	30.07	5	F5:1443	OB5943 H6 Ro.raw	9.0774E4	3	3	172	181			PEAKS DB
R.NNPFYFPSR.R	N	78.65	1140.5352	9	1.8	571.2759	2	31.84	5	F5:1546	OB5943 H6 Ro.raw	5.7869E3	2	2	172	180			PEAKS DB
R.NTLEAAFNAEFNEIR.R	N	78.54	1737.8322	15	0.3	869.9236	2	35.52	4	F4:1762	OB5942 H6 Ro.raw	1.2135E3	1	1	319	333			PEAKS DB
R.IFLAGDKDNVIDQIEK.Q	N	78.42	1816.9570	16	-0.1	909.4857	2	33.42	5	F5:1643	OB5943 H6 Ro.raw	2.878E5	6	6	541	556			PEAKS DB

total 20 peptides

Peptide	Uniq	-10lgP	Mass	Length	ppm	m/z	z	RT	Fraction	Scan	Source File	Area Roast Ara h 6	#Feature	#Feature Roast Ara h 6	Start	End	PTM	AScore	Found By
R.IVQIEAKPNTLVLPK.H	Y	77.37	1662.0079	15	2.9	832.0137	2	30.14	4	F4:1444	OB5942 H6 Ro.raw	6.3611E4	3	3	214	228			PEAKS DB
K.SFNLDEGHALRIPSGFISYILNR.H	N	64.24	2618.3604	23	0.8	873.7948	3	38.22	4	F4:1918	OB5942 H6 Ro.raw	1.6862E4	1	1	255	277			PEAKS DB
R.NTLEAAFNAEFNEIRR.V	N	54.71	1893.9332	16	-2.3	632.3169	3	34.83	5	F5:1724	OB5943 H6 Ro.raw	4.2865E5	5	5	319	334			PEAKS DB
K.DLAFPGSGEQVEK.L	N	54.25	1375.6619	13	0.6	688.8386	2	30.14	4	F4:1453	OB5942 H6 Ro.raw	1.9909E4	2	2	560	572			PEAKS DB
K.HADADNILVIQQGQATVTVANGNNRK.S	N	53.99	2746.4111	26	1.5	916.4790	3	30.13	5	F5:1446	OB5943 H6 Ro.raw	0	0	0	229	254			PEAKS DB
K.KGSEEEGDITNPINLREGEPDLSNNFGK.L	Y	49.97	3058.4478	28	4.0	1020.4939	3	30.91	4	F4:1493	OB5942 H6 Ro.raw	0	0	0	386	413			PEAKS DB
R.C(+57.02)LQS(-18.01)C(+57.02)Q(+.98)QEPDDLKQK.A	N	47.12	1858.8190	15	-0.1	930.4167	2	28.47	4	F4:1350	OB5942 H6 Ro.raw	0	0	0	42	56	Carbamidomethylation	C1:Carbamidomethylation:1000.00;S4:Dehydration:39.55;C5:Carbamidomethylation:1000.00;Q6:Deamidation (NQ):0.00	PEAKS PTM
K.HADADNILVIQQGQATVTVANGN(+.98)NRK.S	N	34.54	2747.3950	26	2.3	916.8077	3	29.76	4	F4:1422	OB5942 H6 Ro.raw	8.5956E3	1	1	229	254		N23:Deamidation (NQ):0.00	PEAKS DB
K.HADADNILVIQQGQATVTVAN(+.98)GNNRK.S	N	32.47	2747.3950	26	1.1	687.8568	4	30.07	5	F5:1448	OB5943 H6 Ro.raw	2.5891E3	1	1	229	254		N21:Deamidation (NQ):0.00	PEAKS DB
R.IPSGFISYILNRHDNQNLR.V	N	31.95	2256.1763	19	2.8	753.0681	3	34.64	4	F4:1759	OB5942 H6 Ro.raw	5.949E4	1	1	266	284			PEAKS DB
**total 20 peptides**																			

sp|P43237|ALL11_ARAHY

back to list

| **Protein Coverage** | **Supporting Peptides** |

Protein Coverage:

```
  1 MRGRVSPLML LLGILVLASV SATQAKSPYR KTENPCAQRC LQSCQQEPDD LKQKACESRC TKLEYDPRCV YDTGATNQRH
 81 PPGERTRGRQ PGDYDDDRRQ PRREEGGRWG PAEPRERERE EDWRQPREDW RRPSHQQPRK IRPEGREGEQ EWGTPGSEVR
161 EETSRNNPFY FPSRRFSTRY GNQNGRIRVL QRFDQRSKQF QNLQNHRIVQ IEARPNTLVL PKHADADNIL VIQQGQATVT
241 VANGNNRKSF NLDEGHALRI PSGFISYILN RHDNQNLRVA KISMPVNTPG QFEDFFPASS RDQSSYLQGF SRNTLEAAFN
321 AEFNEIRRVL LEENAGGEQE ERGQRRRSTR SSDNEGVIVK VSKEHVQELT KHAKSVSKKG SEEEDITNPI NLRDGEPDLS
401 NNFGRLFEVK PDKKNPQLQD LDMMLTCVEI KEGALMLPHF NSKAMVIVVV NKGTGNLELV AVRKEQQQRG RREQEWEEEE
481 EDEEEEGSNR EVRRYTARLK EGDVFIMPAA HPVAINASSE LHLLGFGINA ENNHRIFLAG DKDNVIDQIE KQAKDLAFPG
561 SGEQVEKLIK NQRESHFVSA RPQSQSPSSP EKEDQEEENQ GGKGPLLSIL KAFN
```

C Carbamidomethylation (+57.02)
O Oxidation (M) (+15.99)

44 C; 284 O

Supporting Peptides:

Peptide	Uniq	-10lgP	Mass	Length	ppm	m/z	z	RT	Fraction	Scan	Source File	Area Roast Ara h 6	#Feature	#Feature Roast Ara h 6	Start	End	PTM	AScore	Found By
R.IPSGFISYILNR.H	N	116.76	1378.7609	12	1.0	690.3884	2	36.77	4	F4:1833	OB5942 H6 Ro.raw	4.949E5	3	3	260	271			PEAKS DB
K.SFNLDEGHALR.I	N	105.18	1257.6101	11	0.5	629.8126	2	28.96	5	F5:1378	OB5943 H6 Ro.raw	6.7733E4	3	3	249	259			PEAKS DB
R.DQSSYLQGFSR.N	N	104.35	1286.5891	11	0.5	644.3021	2	30.70	6	F6:1482	OB5944 H6 Ro.raw	7.4069E4	3	3	302	312			PEAKS DB
K.ISMPVNTPGQFEDFFPASSR.D	N	100.55	2226.0415	20	2.5	1114.0308	2	35.60	4	F4:1757	OB5942 H6 Ro.raw	1.7538E5	3	3	282	301			PEAKS DB
R.VLLEENAGGEQEER.G	N	100.30	1571.7427	14	0.7	786.8792	2	26.60	4	F4:1237	OB5942 H6 Ro.raw	6.3383E3	1	1	329	342			PEAKS DB
K.ISM(+15.99)PVNTPGQFEDFFPASSR.D	N	85.41	2242.0364	20	2.1	1122.0278	2	34.54	5	F5:1702	OB5943 H6 Ro.raw	2.1861E4	2	2	282	301	Oxidation (M)	M3:Oxidation (M):1000.00	PEAKS DB
R.NNPFYFPSRR.F	N	79.01	1296.6364	10	1.1	649.3262	2	30.07	5	F5:1443	OB5943 H6 Ro.raw	9.0774E4	3	3	166	175			PEAKS DB
R.NNPFYFPSR.R	N	78.65	1140.5352	9	1.8	571.2759	2	31.84	5	F5:1546	OB5943 H6 Ro.raw	5.7869E3	2	2	166	174			PEAKS DB
R.NTLEAAFNAEFNEIR.R	N	78.54	1737.8322	15	0.3	869.9236	2	35.52	4	F4:1762	OB5942 H6 Ro.raw	1.2135E3	1	1	313	327			PEAKS DB
R.IFLAGDKDNVIDQIEK.Q	N	78.42	1816.9570	16	-0.1	909.4857	2	33.42	5	F5:1643	OB5943 H6 Ro.raw	2.878E5	6	6	536	551			PEAKS DB

total 20 peptides

Peptide	Uniq	-10lgP	Mass	Length	ppm	m/z	z	RT	Fraction	Scan	Source File	Area Roast Ara h 6	#Feature	#Feature Roast Ara h 6	Start	End	PTM	AScore	Found By
R.IVQIEARPNTLVLPK.H	Y	67.70	1690.0140	15	2.0	846.0160	2	30.33	4	F4:1460	OB5942 H6 Ro.raw	4.0157E4	2	2	208	222			PEAKS DB
K.SFNLDEGHALRIPSGFISYILNR.H	N	64.24	2618.3604	23	0.8	873.7948	3	38.22	4	F4:1918	OB5942 H6 Ro.raw	1.6862E4	1	1	249	271			PEAKS DB
R.NTLEAAFNAEFNEIRR.V	N	54.71	1893.9332	16	-2.3	632.3169	3	34.83	5	F5:1724	OB5943 H6 Ro.raw	4.2865E5	5	5	313	328			PEAKS DB
K.DLAFPGSGEQVEK.L	N	54.25	1375.6619	13	0.6	688.8386	2	30.14	4	F4:1453	OB5942 H6 Ro.raw	1.9909E4	2	2	555	567			PEAKS DB
K.HADADNILVIQQGQATVTVANGNNRK.S	N	53.99	2746.4111	26	1.5	916.4790	3	30.13	5	F5:1446	OB5943 H6 Ro.raw	0	0	0	223	248			PEAKS DB
R.C(+57.02)LQS(-18.01)C(+57.02)Q(+.98)QEPDDLKQK.A	N	47.12	1858.8190	15	-0.1	930.4167	2	28.47	4	F4:1350	OB5942 H6 Ro.raw	0	0	0	40	54	Carbamidomethylation	C1:Carbamidomethylation:1000.00;S4:Dehydration:39.55;C5:Carbamidomethylation:1000.00;Q6:Deamidation (NQ):0.00	PEAKS PTM
R.EGEQEWGTPGSEVREETSR.N	Y	38.79	2161.9512	19	3.5	721.6602	3	28.10	4	F4:1331	OB5942 H6 Ro.raw	2.9689E3	1	1	147	165			PEAKS DB
K.HADADNILVIQQGQATVTVANGN(+.98)NRK.S	N	34.54	2747.3950	26	2.3	916.8077	3	29.76	4	F4:1422	OB5942 H6 Ro.raw	8.5956E3	1	1	223	248		N23:Deamidation (NQ):0.00	PEAKS DB
K.HADADNILVIQQGQATVTVAN(+.98)GNNRK.S	N	32.47	2747.3950	26	1.1	687.8568	4	30.07	5	F5:1448	OB5943 H6 Ro.raw	2.5891E3	1	1	223	248		N21:Deamidation (NQ):0.00	PEAKS DB
R.IPSGFISYILNRHDNQNLR.V	N	31.95	2256.1763	19	2.8	753.0681	3	34.64	4	F4:1759	OB5942 H6 Ro.raw	5.949E4	1	1	260	278			PEAKS DB
**total 20 peptides**																			

B3IXL2|B3IXL2_ARAHY

back to list

| Protein Coverage | Supporting Peptides |

Protein Coverage:

44 C

1 MRGRVSPLML LLGILVLASV SATQAKSPYR KTENPCAQRC LQSCQQEPDD LKQKACESRC TKLEYDPRCV YDTGATNQRH

C Carbamidomethylation (+57.02)
O Oxidation (M) (+15.99)

81 PPGERTRGRQ PGDYDDDRRQ PRREEGGRWG PAEPRERERE EDWRQPREDW RRPSHQQPRK IRPEGREGEQ EWGTPGSEVR

161 EETSRNNPFY FPSRRFSTRY GNQNGRIRVL QRFDQRSKQF QNLQNHRIVQ IEARPNTLVL PKHADADNIL VIQQGQATVT

284 O

241 VANGNNRKSF NLDEGHALRI PSGFISYILN RHDNQNLRVA KISMPVNTPG QFEDFFPASS RDQSSYLQGF SRNTLEAAFN

321 AEFNEIRRVL LEENAGGEQE ERGQRRRSTR SSDNEGVIVK VSKEHVQELT KHAKSVSKKG SEEEDITNPI NLRDGEPDLS

401 NNFGRLFEVK PDKKNPQLQD LDMMLTCVEI KEGALMLPHF NSKAMVIVVV NKGTGNLELV AVRKEQQQRG RREQEWEEEE

481 EDEEEEGSNR EVRRYTARLK EGDVFIMPAA HPVAINASSE LHLLGFGINA ENNHRIFLAG DKDNVIDQIE KQAKDLAFPG

561 SGEQVEKLIK NQRESHFVSA RPQSQSPSSP EKEDQEEENQ GGKGPLLSIL KAFN

Supporting Peptides:

Peptide	Uniq	-10lgP	Mass	Length	ppm	m/z	z	RT	Fraction	Scan	Source File	Area Roast Ara h 6	#Feature	#Feature Roast Ara h 6	Start	End	PTM	AScore	Found By
R.IPSGFISYILNR.H	N	116.76	1378.7609	12	1.0	690.3884	2	36.77	4	F4:1833	OB5942 H6 Ro.raw	4.949E5	3	3	260	271			PEAKS DB
K.SFNLDEGHALR.I	N	105.18	1257.6101	11	0.5	629.8126	2	28.96	5	F5:1378	OB5943 H6 Ro.raw	6.7733E4	3	3	249	259			PEAKS DB
R.DQSSYLQGFSR.N	N	104.35	1286.5891	11	0.5	644.3021	2	30.70	6	F6:1482	OB5944 H6 Ro.raw	7.4069E4	3	3	302	312			PEAKS DB
K.ISMPVNTPGQFEDFFPASSR.D	N	100.55	2226.0415	20	2.5	1114.0308	2	35.60	4	F4:1757	OB5942 H6 Ro.raw	1.7538E5	3	3	282	301			PEAKS DB
R.VLLEENAGGEQEER.G	N	100.30	1571.7427	14	0.7	786.8792	2	26.60	4	F4:1237	OB5942 H6 Ro.raw	6.3383E3	1	1	329	342			PEAKS DB
K.ISM(+15.99)PVNTPGQFEDFFPASSR.D	N	85.41	2242.0364	20	2.1	1122.0278	2	34.54	5	F5:1702	OB5943 H6 Ro.raw	2.1861E4	2	2	282	301	Oxidation (M)	M3:Oxidation (M):1000.00	PEAKS DB
R.NNPFYFPSRR.F	N	79.01	1296.6364	10	1.1	649.3262	2	30.07	5	F5:1443	OB5943 H6 Ro.raw	9.0774E4	3	3	166	175			PEAKS DB
R.NNPFYFPSR.R	N	78.65	1140.5352	9	1.8	571.2759	2	31.84	5	F5:1546	OB5943 H6 Ro.raw	5.7869E3	2	2	166	174			PEAKS DB
R.NTLEAAFNAEFNEIR.R	N	78.54	1737.8322	15	0.3	869.9236	2	35.52	4	F4:1762	OB5942 H6 Ro.raw	1.2135E3	1	1	313	327			PEAKS DB
R.IFLAGDKDNVIDQIEK.Q	N	78.42	1816.9570	16	-0.1	909.4857	2	33.42	5	F5:1643	OB5943 H6 Ro.raw	2.878E5	6	6	536	551			PEAKS DB

total 20 peptides

Peptide	Uniq	-10lgP	Mass	Length	ppm	m/z	z	RT	Fraction	Scan	Source File	Area Roast Ara h 6	#Feature	#Feature Roast Ara h 6	Start	End	PTM	AScore	Found By
R.IVQIEARPNTLVLPK.H	Y	67.70	1690.0140	15	2.0	846.0160	2	30.33	4	F4:1460	OB5942 H6 Ro.raw	4.0157E4	2	2	208	222			PEAKS DB
K.SFNLDEGHALRIPSGFISYILNR.H	N	64.24	2618.3604	23	0.8	873.7948	3	38.22	4	F4:1918	OB5942 H6 Ro.raw	1.6862E4	1	1	249	271			PEAKS DB
R.NTLEAAFNAEFNEIRR.V	N	54.71	1893.9332	16	-2.3	632.3169	3	34.83	5	F5:1724	OB5943 H6 Ro.raw	4.2865E5	5	5	313	328			PEAKS DB
K.DLAFPGSGEQVEK.L	N	54.25	1375.6619	13	0.6	688.8386	2	30.14	4	F4:1453	OB5942 H6 Ro.raw	1.9909E4	2	2	555	567			PEAKS DB
K.HADADNILVIQQGQATVTVANGNNRK.S	N	53.99	2746.4111	26	1.5	916.4790	3	30.13	5	F5:1446	OB5943 H6 Ro.raw	0	0	0	223	248			PEAKS DB
R.C(+57.02)LQS(-18.01)C(+57.02)Q(+.98)QEPDDLKQK.A	N	47.12	1858.8190	15	-0.1	930.4167	2	28.47	4	F4:1350	OB5942 H6 Ro.raw	0	0	0	40	54	Carbamidomethylation	C1:Carbamidomethylation:1000.00;S4:Dehydration:39.55;C5:Carbamidomethylation:1000.00;Q6:Deamidation (NQ):0.00	PEAKS PTM
R.EGEQEWGTPGSEVREETSR.N	Y	38.79	2161.9512	19	3.5	721.6602	3	28.10	4	F4:1331	OB5942 H6 Ro.raw	2.9689E3	1	1	147	165			PEAKS DB
K.HADADNILVIQQGQATVTVANGN(+.98)NRK.S	N	34.54	2747.3950	26	2.3	916.8077	3	29.76	4	F4:1422	OB5942 H6 Ro.raw	8.5956E3	1	1	223	248		N23:Deamidation (NQ):0.00	PEAKS DB
K.HADADNILVIQQGQATVTVAN(+.98)GNNRK.S	N	32.47	2747.3950	26	1.1	687.8568	4	30.07	5	F5:1448	OB5943 H6 Ro.raw	2.5891E3	1	1	223	248		N21:Deamidation (NQ):0.00	PEAKS DB
R.IPSGFISYILNRHDNQNLR.V	N	31.95	2256.1763	19	2.8	753.0681	3	34.64	4	F4:1759	OB5942 H6 Ro.raw	5.949E4	1	1	260	278			PEAKS DB
**total 20 peptides**																			

Q6PSU3|Q6PSU3_ARAHY

back to list

| Protein Coverage | Supporting Peptides |

Protein Coverage:

1 MRGRVSPLML LLGILVLASV SATQAKSPYR KTENPCAQRC LQSCQQEPDD LKQKACESRC TKLEYDPRCV YDTGATNQRH

81 PPGERTRGRQ PGDYDDDRRQ PRREEGGRWG PAEPRERERE EDWRQPREDW RRPSHQQPRK IRPEGREGEQ EWGTPGSEVR

161 EETSRNNPFY FPSRRFSTRY GNQNGRIRVL QRFDQRSKQF QNLQNHRIVQ IEARPNTLVL PKHADADNIL VIQQGQATVT

241 VANGNNRKSF NLDEGHALRI PSGFISYILN RHDNQNLRVA KISMPVNTPG QFEDFFPASS RDQSSYLQGF SRNTLEAAFN

321 AEFNEIRRVL LEENAGGEQE ERGQRRRSTR SSDNEGVIVK VSKEHVQELT KHAKSVSKKG SEEEDITNPI NLRDGEPDLS

401 NNFGRLFEVK PDKKNPQLQD LDMMLTCVEI KEGALMLPHF NSKAMVIVVV NKGTGNLELV AVRKEQQQRG RREQEWEEEE

481 EDEEEEGSNR EVRRYTARLK EGDVFIMPAA HPVAINASSE LHLLGFGINA ENNHRIFLAG DKDNVIDQIE KQAKDLAFPG

561 SGEQVEKLIK NQRESHFVSA

C Carbamidomethylation (+57.02)
O Oxidation (M) (+15.99)

Supporting Peptides:

Peptide	Uniq	-10lgP	Mass	Length	ppm	m/z	z	RT	Fraction	Scan	Source File	Area Roast Ara h 6	#Feature	#Feature Roast Ara h 6	Start	End	PTM	AScore	Found By
R.IPSGFISYILNR.H	N	116.76	1378.7609	12	1.0	690.3884	2	36.77	4	F4:1833	OB5942 H6 Ro.raw	4.949E5	3	3	260	271			PEAKS DB
K.SFNLDEGHALR.I	N	105.18	1257.6101	11	0.5	629.8126	2	28.96	5	F5:1378	OB5943 H6 Ro.raw	6.7733E4	3	3	249	259			PEAKS DB
R.DQSSYLQGFSR.N	N	104.35	1286.5891	11	0.5	644.3021	2	30.70	6	F6:1482	OB5944 H6 Ro.raw	7.4069E4	3	3	302	312			PEAKS DB
K.ISMPVNTPGQFEDFFPASSR.D	N	100.55	2226.0415	20	2.5	1114.0308	2	35.60	4	F4:1757	OB5942 H6 Ro.raw	1.7538E5	3	3	282	301			PEAKS DB
R.VLLEENAGGEQEER.G	N	100.30	1571.7427	14	0.7	786.8792	2	26.60	4	F4:1237	OB5942 H6 Ro.raw	6.3383E3	1	1	329	342			PEAKS DB
K.ISM(+15.99)PVNTPGQFEDFFPASSR.D	N	85.41	2242.0364	20	2.1	1122.0278	2	34.54	5	F5:1702	OB5943 H6 Ro.raw	2.1861E4	2	2	282	301	Oxidation (M)	M3:Oxidation (M):1000.00	PEAKS DB
R.NNPFYFPSRR.F	N	79.01	1296.6364	10	1.1	649.3262	2	30.07	5	F5:1443	OB5943 H6 Ro.raw	9.0774E4	3	3	166	175			PEAKS DB
R.NNPFYFPSR.R	N	78.65	1140.5352	9	1.8	571.2759	2	31.84	5	F5:1546	OB5943 H6 Ro.raw	5.7869E3	2	2	166	174			PEAKS DB
R.NTLEAAFNAEFNEIR.R	N	78.54	1737.8322	15	0.3	869.9236	2	35.52	4	F4:1762	OB5942 H6 Ro.raw	1.2135E3	1	1	313	327			PEAKS DB
R.IFLAGDKDNVIDQIEK.Q	N	78.42	1816.9570	16	-0.1	909.4857	2	33.42	5	F5:1643	OB5943 H6 Ro.raw	2.878E5	6	6	536	551			PEAKS DB

total 20 peptides

Peptide	Uniq	-10lgP	Mass	Length	ppm	m/z	z	RT	Fraction	Scan	Source File	Area Roast Ara h 6	#Feature	#Feature Roast Ara h 6	Start	End	PTM	AScore	Found By
R.IVQIEARPNTLVLPK.H	Y	67.70	1690.0140	15	2.0	846.0160	2	30.33	4	F4:1460	OB5942 H6 Ro.raw	4.0157E4	2	2	208	222			PEAKS DB
K.SFNLDEGHALRIPSGFISYILNR.H	N	64.24	2618.3604	23	0.8	873.7948	3	38.22	4	F4:1918	OB5942 H6 Ro.raw	1.6862E4	1	1	249	271			PEAKS DB
R.NTLEAAFNAEFNEIRR.V	N	54.71	1893.9332	16	-2.3	632.3169	3	34.83	5	F5:1724	OB5943 H6 Ro.raw	4.2865E5	5	5	313	328			PEAKS DB
K.DLAFPGSGEQVEK.L	N	54.25	1375.6619	13	0.6	688.8386	2	30.14	4	F4:1453	OB5942 H6 Ro.raw	1.9909E4	2	2	555	567			PEAKS DB
K.HADADNILVIQQGQATVTVANGNNRK.S	N	53.99	2746.4111	26	1.5	916.4790	3	30.13	5	F5:1446	OB5943 H6 Ro.raw	0	0	0	223	248			PEAKS DB
R.C(+57.02)LQS(-18.01)C(+57.02)Q(+.98)QEPDDLKQK.A	N	47.12	1858.8190	15	-0.1	930.4167	2	28.47	4	F4:1350	OB5942 H6 Ro.raw	0	0	0	40	54	Carbamidomethylation	C1:Carbamidomethylation:1000.00;S4:Dehydration:39.55;C5:Carbamidomethylation:1000.00;Q6:Deamidation (NQ):0.00	PEAKS PTM
R.EGEQEWGTPGSEVREETSR.N	Y	38.79	2161.9512	19	3.5	721.6602	3	28.10	4	F4:1331	OB5942 H6 Ro.raw	2.9689E3	1	1	147	165			PEAKS DB
K.HADADNILVIQQGQATVTVANGN(+.98)NRK.S	N	34.54	2747.3950	26	2.3	916.8077	3	29.76	4	F4:1422	OB5942 H6 Ro.raw	8.5956E3	1	1	223	248		N23:Deamidation (NQ):0.00	PEAKS DB
K.HADADNILVIQQGQATVTVAN(+.98)GNNRK.S	N	32.47	2747.3950	26	1.1	687.8568	4	30.07	5	F5:1448	OB5943 H6 Ro.raw	2.5891E3	1	1	223	248		N21:Deamidation (NQ):0.00	PEAKS DB
R.IPSGFISYILNRHDNQNLR.V	N	31.95	2256.1763	19	2.8	753.0681	3	34.64	4	F4:1759	OB5942 H6 Ro.raw	5.949E4	1	1	260	278			PEAKS DB
**total 20 peptides**																			

Q647H1|Q647H1_ARAHY

back to list

| Protein Coverage | Supporting Peptides |

Protein Coverage:

1 MVIGPFRLSL CVCLVFLTSA CFGTRLEESF NECQLDRLNA LTPDNRIESQ GGITETWNSN HPELRCAGVT LLKRTIFPNG
81 FHLPSYANYP QLIFIAQGNG VFGVSLPGCP VTYEEAESQS REDRRQRIVI KRESEQEQEQ QGDSHHKIYH FRQGHLLAIP
161 AGVPYWSFNY GNEPIVAITL LDTSNLDNQL DPSPRRFYLA GNPEEEHPET QQQQPQTRRR HGQHQQDEYG SQGEEEGNNV
241 LSGFSTQLLA HAFGVDEEIA RILQNPPEQT KDQIVRVEGG FRDVISPRWG EGKQYEDELE ERQRQPPRRD EQGKGYDYDD
321 DRRPRHRQDP YREGDEDDRR PRGSRQGQGR GYDDDDRRPG QYEEGEEDDR RPRRSSRPKR QGRRHDDDDR RADEDDRRGY
401 DDDERRPDED DRRGYDDDER RPDDDDRQGY DDDDRRPRWS SRPKGQGRNG VEETLCSPTL VEDIARPSRA DFYNPAAGRI
542
481 SSANSLTFPI LRWFQLSAEH VLLYRNGIYS PHWNNNANSI IYGLRGEGRI QVVNSQGNAV FNGVLREGQI LLVPQNFAVG
561 KQAGNEGFEY VAFKTADRAS PATSSKCLGE SPLMFSSMLL AFEIIKSVLS NTMETRPLWS LLMIPSMGLN VVINLNHNNN
641 AQVDSKNNDG SRLWWPSSII IK

d Deamidation (NQ) (+0.98)
d Diethylation (+56.06)

Supporting Peptides:

Peptide	Uniq	-10lgP	Mass	Length	ppm	m/z	z	RT	Fraction	Scan	Source File	Area Roast Ara h 6	#Feature	#Feature Roast Ara h 6	Start	End	PTM	AScore	Found By
R.IQVVNSQGNAVFNGVLR.E	Y	133.65	1813.9799	17	1.1	907.9982	2	32.86	5	F5:1608	OB5943 H6 Ro.raw	4.365E4	3	3	530	546			PEAKS DB
R.IQVVNSQGNAVFN(+.98)GVLR.E	Y	123.27	1814.9639	17	0.5	908.4896	2	32.42	6	F6:1586	OB5944 H6 Ro.raw	0	0	0	530	546	Deamidation (NQ)	N13:Deamidation (NQ):77.51	PEAKS DB
R.WFQLSAEHVLLYR.N	Y	111.09	1660.8726	13	3.7	831.4467	2	35.21	4	F4:1749	OB5942 H6 Ro.raw	7.6112E4	5	5	493	505			PEAKS DB
R.ISSANSLTFPILR.W	Y	99.72	1417.7928	13	1.3	709.9046	2	34.07	4	F4:1680	OB5942 H6 Ro.raw	9.684E4	3	3	480	492			PEAKS DB
R.EGQILLVPQNFAVGK.Q	Y	95.83	1611.8984	15	1.4	806.9576	2	34.05	6	F6:1681	OB5944 H6 Ro.raw	1.1916E5	3	3	547	561			PEAKS DB
R.IQ(+.98)VVNSQGNAVFNGVLR.E	Y	81.15	1814.9639	17	2.4	908.4914	2	32.79	5	F5:1603	OB5943 H6 Ro.raw	0	0	0	530	546		Q2:Deamidation (NQ):37.78	PEAKS DB
R.ADFYNPAAGR.I	Y	75.66	1080.4988	10	0.7	541.2570	2	27.93	6	F6:1328	OB5944 H6 Ro.raw	2.9033E4	3	3	470	479			PEAKS DB
K.QAGNEGFEYVAFK.T	Y	71.35	1458.6779	13	1.7	730.3474	2	32.21	5	F5:1569	OB5943 H6 Ro.raw	0	0	0	562	574			PEAKS DB
R.IQVVN(+.98)SQGNAVFNGVLR.E	Y	49.07	1814.9639	17	1.6	908.4907	2	33.23	6	F6:1633	OB5944 H6 Ro.raw	0	0	0	530	546		N5:Deamidation (NQ):0.00	PEAKS DB
R.N(+.98)GIYSPHWNNNANSIIYGLR.G	Y	46.60	2303.1084	20	4.1	768.7132	3	34.70	6	F6:1718	OB5944 H6 Ro.raw	0	0	0	506	525		N1:Deamidation (NQ):32.94	PEAKS DB
R.EGQILLVPQNFAVGK(+56.06).Q	Y	41.08	1667.9609	15	1.4	834.9890	2	34.76	5	F5:1717	OB5943 H6 Ro.raw	0	0	0	547	561	Diethylation	K15:Diethylation:88.07	PEAKS PTM
R.N(+.98)GIYSPHWNNNAN(+.98)SIIYGLR.G	Y	35.88	2304.0923	20	8.5	769.0446	3	34.92	5	F5:1731	OB5943 H6 Ro.raw	3.732E3	1	1	506	525		N1:Deamidation (NQ):17.32;N13:Deamidation (NQ):6.63	PEAKS DB
R.NGIYSPHWNNNANSIIYGLR.G	Y	34.13	2302.1243	20	2.0	768.3835	3	34.36	4	F4:1695	OB5942 H6 Ro.raw	0	0	0	506	525			PEAKS DB
**total 13 peptides**																			

sp|Q647H2|AHY3_ARAHY

back to list

| Protein Coverage | Supporting Peptides |

Protein Coverage:

```
  1 MAKLLALSVC FCFLVLGASS VTFRQQGEEN ECQFQRLNAQ RPDNCIESEG GYIETWNPNN QEFQCAGVAL SRFVLRRNAL
 81 RRPFYSNAPQ EIFIYQGSGY FGLIFPGCPG TFEEPIQGSE QFQRPSRHFQ GQDQSQRPLD THQKVHGFRE GDLIAVPHGV
161 AFWIYNDQDT DVVAISVLHT NSLHNQLDQF PRRFNLAGKQ EQEFLRYQQR SGRQSPKGEE QEQEQENEGG NVFSGFSTEF
241 LSHGFQVNED IVRNLRGENE REEQGAIVTV KGGLSILVPP EWRQSYQQPG RGDKDFNNGI EETICTATVK MNIGKSTSAD
321 IYNPQAGSVR TVNELDLPIL NRLGLSAEYG SIHRDAMFVP HYNMNANSMI YALHGGAHVQ VVDCNGNRVF DEELQEGQSL
401 VVPQNFAVAA KSQSEHFLYV AFKTNSRASI SNLAGKNSYM WNLPEDVVAN SYGLQYEQAR QLKNNNPFTF LVPPQDSQMI
481 RTVA
```

305: C, P

C Carbamidomethylation (+57.02)
P Propionamide (+71.04)

Supporting Peptides:

Peptide	Uniq	-10lgP	Mass	Length	ppm	m/z	z	RT	Fraction	Scan	Source File	Area Roast Ara h 6	#Feature	#Feature Roast Ara h 6	Start	End	PTM	AScore	Found By
**total 9 peptides**																			

Peptide	Uniq	-10lgP	Mass	Length	ppm	m/z	z	RT	Fraction	Scan	Source File	Area Roast Ara h 6	#Feature	#Feature Roast Ara h 6	Start	End	PTM	AScore	Found By
R.TVNELDLPILNR.L	Y	110.86	1395.7721	12	2.4	698.8950	2	34.26	4	F4:1684	OB5942 H6 Ro.raw	5.2986E4	3	3	331	342			PEAKS DB
R.LGLSAEYGSIHR.D	Y	106.40	1301.6727	12	0.9	651.8442	2	29.01	5	F5:1379	OB5943 H6 Ro.raw	0	0	0	343	354			PEAKS DB
K.STSADIYNPQAGSVR.T	Y	93.73	1564.7482	15	-0.5	783.3810	2	27.48	5	F5:1285	OB5943 H6 Ro.raw	2.0503E4	3	3	316	330			PEAKS DB
N.GIEETIC(+57.02)TATVK.M	N	81.04	1320.6595	12	-0.1	661.3370	2	29.52	5	F5:1405	OB5943 H6 Ro.raw	2.3112E4	2	2	299	310	Carbamidomethylation	C7:Carbamidomethylation:1000.00	PEAKS DB
K.NSYMWNLPEDVVANSYGLQYEQAR.Q	Y	74.48	2846.2969	24	4.1	1424.1615	2	37.17	5	F5:1856	OB5943 H6 Ro.raw	0	0	0	437	460			PEAKS DB
K.SQSEHFLYVAFK.T	Y	72.80	1454.7194	12	0.2	728.3671	2	31.98	6	F6:1565	OB5944 H6 Ro.raw	2.7176E4	3	3	412	423			PEAKS DB
R.VFDEELQEGQSLVVPQNFAVAAK.S	Y	47.99	2517.2751	23	4.1	1259.6500	2	35.21	5	F5:1743	OB5943 H6 Ro.raw	0	0	0	389	411			PEAKS DB
N.GIEETIC(+71.04)TATVK.M	N	37.92	1334.6752	12	1.9	668.3461	2	29.63	6	F6:1421	OB5944 H6 Ro.raw	0	0	0	299	310	Propionamide	C7:Propionamide:1000.00	PEAKS PTM
C.IESEGGYIETWNPNNQEFQC(+57.02)AGVALSR.F	N	37.18	3068.3933	27	-0.9	1023.8041	3	34.37	6	F6:1699	OB5944 H6 Ro.raw	0	0	0	46	72	Carbamidomethylation	C20:Carbamidomethylation:1000.00	PEAKS DB
total 9 peptides																			

Q6IWG5|Q6IWG5_ARAHY

back to list

| Protein Coverage | Supporting Peptides |

Protein Coverage:

C Carbamidomethylation (+57.02)

```
1   KLLALSLCFC VLVLGASSVT FRQGGEENEC QFQRLNAQRP DNRIESEGGY IETWNPNNQE FQCAGVALSR TVLRRNALRR
81  PFYSNAPLEI YVQQGSGYFG LIFPGCPSTY EEPAQEGRRY QSQKPSRRFQ VGQDDPSQQQ QDSHQKVHRF DEGDLIAVPT
161 GVAFWMYNDE DTDVVTVTLS DTSSIHNQLD QFPRRFYLAG NQEQEFLRYQ QQQGSRPHYR QISPRVRGDE QENEGSNIFS
241 GFAQEFLQHA FQVDRQTVEN LRGENEREEQ GAIVTVKGGL RILSPDEEDE SSRSPPSRRE EFDEDRSRPQ QRGKYDENRR
321 GYKNGIEETI CSASVKKNLG RSSNPDIYNP QAGSLRSVNE LDLPILGWLG LSAQHGTIYR NAMFVPHYTL NAHTIVVALN
401 GRAHVQVVDS NGNRVYDEEL QEGHVLVVPQ NFAVAAKAQS ENYEYLAFKT DSRPSIANLA GENSIIDNLP EEVVANSYRL
481 PREQARQLKN NNPFKFFVPP FDHQSMREVA
```

(Modification marks: 63 C; 331 C)

Supporting Peptides:

Peptide	Uniq	-10lgP	Mass	Length	ppm	m/z	z	RT	Fraction	Scan	Source File	Area Roast Ara h 6	#Feature	#Feature Roast Ara h 6	Start	End	PTM	AScore	Found By
R.SSNPDIYNPQAGSLR.S	Y	107.40	1617.7747	15	-0.5	809.8942	2	28.38	4	F4:1346	OB5942 H6 Ro.raw	9.5502E4	3	3	342	356			PEAKS DB
K.AQSENYEYLAFK.T	Y	104.89	1461.6776	12	1.4	731.8471	2	31.44	6	F6:1523	OB5944 H6 Ro.raw	3.7693E4	3	3	438	449			PEAKS DB
K.TDSRPSIANLAGENSIIDNLPEEVVANSYR.L	Y	94.41	3243.6006	30	5.4	1082.2133	3	36.10	6	F6:1801	OB5944 H6 Ro.raw	7.4455E4	3	3	450	479			PEAKS DB
R.SVNELDLPILGWLGLSAQHGTIYR.N	Y	70.38	2651.4070	24	4.0	1326.7161	2	39.73	6	F6:2002	OB5944 H6 Ro.raw	1.8031E5	2	2	357	380			PEAKS DB

total 8 peptides

Peptide	Uniq	-10lgP	Mass	Length	ppm	m/z	z	RT	Fraction	Scan	Source File	Area Roast Ara h 6	#Feature	#Feature Roast Ara h 6	Start	End	PTM	AScore	Found By
N.GIEETIC(+57.02)SASVKK.N	Y	62.45	1420.7231	13	4.5	711.3721	2	27.29	5	F5:1274	OB5943 H6 Ro.raw	6.0887E3	1	1	325	337	Carbamidomethylation	C7:Carbamidomethylation:1000.00	PEAKS DB
R.LNAQRPDNRIESEGGYIETWNPNNQEFQC(+57.02)AGVALSR.T	Y	54.87	4132.9409	36	4.1	1034.2467	4	33.61	6	F6:1655	OB5944 H6 Ro.raw	1.24E6	1	1	35	70	Carbamidomethylation	C29:Carbamidomethylation:1000.00	PEAKS DB
R.NAMFVPHYTLNAHTIVVALN(+.98)GR.A	Y	41.82	2438.2529	22	0.3	813.7585	3	35.34	6	F6:1755	OB5944 H6 Ro.raw	0	0	0	381	402		N20:Deamidation (NQ):13.05	PEAKS DB
R.IESEGGYIETWNPNNQEFQC(+57.02)AGVALSR.T	N	37.18	3068.3933	27	-0.9	1023.8041	3	34.37	6	F6:1699	OB5944 H6 Ro.raw	0	0	0	44	70	Carbamidomethylation	C20:Carbamidomethylation:1000.00	PEAKS DB
**total 8 peptides**																			

Q0GM57|Q0GM57_ARAHY

back to list

| Protein Coverage | Supporting Peptides |

Protein Coverage:

1 MAKLLALSLC FCVLVLGASS VTFRQGGEEN ECQFQRLNAQ RPDNRIESEG GYIETWNPNN QEFQCAGVAL SRTVLRRNAL

C Carbamidomethylation (+57.02)

81 RRPFYSNAPL EIYVQQGSGY FGLIFPGCPS TYEEPAQEGR RYQSQKPSRR FQVGQDDPSQ QQQDSHQKVH RFDEGDLIAV

161 PTGVAFWMYN DEDTDVVTVT LSDTSSIHNQ LDQFPRRFYL AGNQEQEFLR YQQQQGSRPH YRQISPRVRG DEQENEGSNI

241 FSGFAQEFLQ HAFQVDRQTV ENLRGENERE EQGAIVTVKG GLRILSPDEE DESSRSPPSR REEFDEDRSR PQQRGKYDEN

321 RRGYKNGIEE TICSASVKKN LGRSSNPDIY NPQAGSLRSV NELDLPILGW LGLSAQHGTI YRNAMFVPHY TLNAHTIVVA

401 LNGRAHVQVV DSNGNRVYDE ELQEGHVLVV PQNFAVAAKA QSENYEYLAF KTDSRPSIAN LAGENSIIDN LPEEVVANSY

481 RLPREQARQL KNNNPFKFFV PPFDHQSMRE VA

Supporting Peptides:

Peptide	Uniq	-10lgP	Mass	Length	ppm	m/z	z	RT	Fraction	Scan	Source File	Area Roast Ara h 6	#Feature	#Feature Roast Ara h 6	Start	End	PTM	AScore	Found By
R.SSNPDIYNPQAGSLR.S	Y	107.40	1617.7747	15	-0.5	809.8942	2	28.38	4	F4:1346	OB5942 H6 Ro.raw	9.5502E4	3	3	344	358			PEAKS DB
K.AQSENYEYLAFK.T	Y	104.89	1461.6776	12	1.4	731.8471	2	31.44	6	F6:1523	OB5944 H6 Ro.raw	3.7693E4	3	3	440	451			PEAKS DB
K.TDSRPSIANLAGENSIIDNLPEEVVANSYR.L	Y	94.41	3243.6006	30	5.4	1082.2133	3	36.10	6	F6:1801	OB5944 H6 Ro.raw	7.4455E4	3	3	452	481			PEAKS DB
R.SVNELDLPILGWLGLSAQHGTIYR.N	Y	70.38	2651.4070	24	4.0	1326.7161	2	39.73	6	F6:2002	OB5944 H6 Ro.raw	1.8031E5	2	2	359	382			PEAKS DB
N.GIEETIC(+57.02)SASVKK.N	Y	62.45	1420.7231	13	4.5	711.3721	2	27.29	5	F5:1274	OB5943 H6 Ro.raw	6.0887E3	1	1	327	339	Carbamidomethylation	C7:Carbamidomethylation:1000.00	PEAKS DB
R.LNAQRPDNRIESEGGYIETWNPNNQEFQC(+57.02)AGVALSR.T	Y	54.87	4132.9409	36	4.1	1034.2467	4	33.61	6	F6:1655	OB5944 H6 Ro.raw	1.24E6	1	1	37	72	Carbamidomethylation	C29:Carbamidomethylation:1000.00	PEAKS DB

total 8 peptides

Peptide	Uniq	-10lgP	Mass	Length	ppm	m/z	z	RT	Fraction	Scan	Source File	Area Roast Ara h 6	#Feature	#Feature Roast Ara h 6	Start	End	PTM	AScore	Found By
R.NAMFVPHYTLNAHTIVVALN(+.98)GR.A	Y	41.82	2438.2529	22	0.3	813.7585	3	35.34	6	F6:1755	OB5944 H6 Ro.raw	0	0	0	383	404		N20:Deamidation (NQ):13.05	PEAKS DB
R.IESEGGYIETWNPNNQEFQC(+57.02)AGVALSR.T	N	37.18	3068.3933	27	-0.9	1023.8041	3	34.37	6	F6:1699	OB5944 H6 Ro.raw	0	0	0	46	72	Carbamidomethylation	C20:Carbamidomethylation:1000.00	PEAKS DB
**total 8 peptides**																			

#CONTAM#P00761

back to list

| Protein Coverage | Supporting Peptides |

Protein Coverage:

1 FPTDDDDKIV GGYTCAANSI PYQVSLNSGS HFCGGSLINS QWVVSAAHCY KSRIQVR**LGE HNIDVLEGNE QFINAAK**IIT

81 HPNFNGNTLD NDIMLIK**LSS PATLNSRVAT VSLPR**SCAAA GTECLISGWG NTKSSGSSYP SLLQCLKAPV LSDSSCKSSY

161 PGQITGNMIC VGFLEGGKDS CQGDSGGPVV CNGQLQGIVS WGYGCAQKNK PGVYTK**VCNY VNWIQQTIAA N**

c Carbamidomethylation (+57.02)
d Dimethylation(KR) (+28.03)
m Methylation(KR) (+14.02)

Supporting Peptides:

Peptide	Uniq	-10lgP	Mass	Length	ppm	m/z	z	RT	Fraction	Scan	Source File	Area Roast Ara h 6	#Feature	#Feature Roast Ara h 6	Start	End	PTM	AScore	Found By
K.VC(+57.02)NYVNWIQQTIAAN	Y	84.98	1792.8567	15	2.5	897.4379	2	37.98	6	F6:1908	OB5944 H6 Ro.raw	0	0	0	217	231	Carbamidomethylation	C2:Carbamidomethylation:1000.00	PEAKS DB
R.LGEHNIDVLEGNEQFIN.A	Y	84.29	1939.9275	17	0.8	970.9718	2	33.77	5	F5:1661	OB5943 H6 Ro.raw	2.2386E5	3	3	58	74			PEAKS DB
K.LSSPATLNSR.V	Y	84.13	1044.5564	10	-0.6	523.2852	2	25.39	5	F5:1175	OB5943 H6 Ro.raw	7.4318E4	3	3	98	107			PEAKS DB
R.VATVSLPR.S	Y	67.12	841.5021	8	1.6	421.7590	2	27.29	5	F5:1289	OB5943 H6 Ro.raw	1.1013E6	3	3	108	115			PEAKS DB
R.LGEHNIDVLEGNEQFINAAK(+28.03).I	Y	63.33	2238.1279	20	2.1	747.0515	3	33.05	5	F5:1620	OB5943 H6 Ro.raw	6.1249E4	3	3	58	77	Dimethylation(KR)	K20:Dimethylation(KR):1000.00	PEAKS PTM
R.V(+28.03)ATVSLPR.S	Y	48.52	869.5334	8	0.1	435.7740	2	27.64	4	F4:1296	OB5942 H6 Ro.raw	1.146E4	1	1	108	115		V1:Ethylation:27.75	PEAKS PTM
R.LGEHNIDVLEGNEQFINAA.K	Y	44.86	2082.0017	19	4.4	1042.0127	2	33.98	4	F4:1673	OB5942 H6 Ro.raw	2.6415E4	2	2	58	76			PEAKS DB
R.LGEHNIDVLEGNEQFINAAK(+14.02).I	Y	42.80	2224.1123	20	2.0	742.3795	3	32.54	4	F4:1595	OB5942 H6 Ro.raw	4.8247E4	2	2	58	77	Methylation(KR)	K20:Methylation(KR):1000.00	PEAKS PTM
R.VAT(-18.01)VSLPR.S	Y	38.92	823.4916	8	1.0	412.7535	2	27.07	6	F6:1282	OB5944 H6 Ro.raw	4.8523E5	2	2	108	115		T3:Dehydration:14.04	PEAKS PTM
R.LGEHNIDVLEGNEQFINA.A	Y	33.03	2010.9646	18	1.8	1006.4914	2	33.90	6	F6:1672	OB5944 H6 Ro.raw	0	0	0	58	75			PEAKS DB
**total 10 peptides**																			

E9LFE8|E9LFE8_ARAHY

back to list

| Protein Coverage | Supporting Peptides |

Protein Coverage:

1 KSRKFFLGGK PQEEKGEEGN MFSGLELKTV AESLGIDMGI AGKVQGVDDP RGSIIIVEDE LETLSPAVEE SGNGNGLDET

81 LCTLR**LVHQL AESTDADKYN PRAGFLTALN TPNLPVLQYV QLGADR**GVFY KNAVMAPHYN LNCHAVIYGT EGR**GWIEVVG**

161 **ENGR**KVYEGE VREGQILIVP QQFMVAKK**AA EGSDEGFGWI AVKTSDNPMI SPLAGK**LSLI R**AMPLPVLMN SFR**LTAEEAI

241 NLKKRGELTF FSPDPAHTQI

Supporting Peptides:

Peptide	Uniq	-10lgP	Mass	Length	ppm	m/z	z	RT	Fraction	Scan	Source File	Area Roast Ara h 6	#Feature	#Feature Roast Ara h 6	Start	End	PTM	AScore	Found By
K.AAEGSDEGFGWIAVK.T	Y	82.18	1535.7256	15	1.0	768.8708	2	33.77	5	F5:1664	OB5943 H6 Ro.raw	3.9296E3	1	1	189	203			PEAKS DB
K.TSDNPMISPLAGK.L	Y	82.04	1329.6598	13	1.5	665.8381	2	29.58	4	F4:1419	OB5942 H6 Ro.raw	3.9148E3	2	2	204	216			PEAKS DB
R.AMPLPVLMNSFR.L	Y	75.70	1374.7152	12	1.8	688.3661	2	36.29	6	F6:1813	OB5944 H6 Ro.raw	4.7937E3	1	1	222	233			PEAKS DB
R.AGFLTALNTPNLPVLQYVQLGAD(+28.03)R.G	Y	73.53	2598.4170	24	3.7	1300.2206	2	39.54	6	F6:2001	OB5944 H6 Ro.raw	3.6606E4	2	2	103	126		D23:Ethylation:8.14	PEAKS PTM
R.GWIEVVGENGR.K	Y	72.13	1214.6044	11	-0.4	608.3092	2	31.99	5	F5:1556	OB5943 H6 Ro.raw	0	0	0	154	164			PEAKS DB
R.LVHQLAESTDADKYNPR.A	Y	50.86	1955.9701	17	-1.7	652.9962	3	28.77	5	F5:1364	OB5943 H6 Ro.raw	1.711E4	1	1	86	102			PEAKS DB

total 6 peptides

sp|Q6PSU2|CONG7_ARAHY

back to list

| Protein Coverage | Supporting Peptides |

Protein Coverage:

1 MAKLTILVAL ALFLLAAHAS ARQQWELQGD RRCQSQLER**A NLRPCEQHLM QK**IQRDEDSY GRDPYSPSQD PYSPSQDPDR

81 RDPYSPSPYD RRGAGSSQHQ ER**CCNELNEF ENNQRCMCEA LQQIMENQSD RLQGR**QQEQQ FKRELR**NLPQ QCGLR**APQR**C**

161 **DLEVESGGRD RY**

45, 118

a Ammonia-loss (C@N-term) (-17.03)
a Ammonia-loss (N) (-17.03)
c Carbamidomethylation (+57.02)
c Carbamylation (+43.01)
c Carbamidomethylation (DHKE, X@N-term) (+57.02)

Supporting Peptides:

Peptide	Uniq	-10lgP	Mass	Length	ppm	m/z	z	RT	Fraction	Scan	Source File	Area Roast Ara h 6	#Feature	#Feature Roast Ara h 6	Start	End	PTM	AScore	Found By
R.C(+57.02)C(+57.02)NELNEFENNQR.C	Y	89.52	1725.6835	13	0.6	863.8495	2	28.77	5	F5:1368	OB5943 H6 Ro.raw	4.17E4	2	2	103	115	Carbamidomethylation	C1:Carbamidomethylation:1000.00;C2:Carbamidomethylation:1000.00	PEAKS DB
R.C(+57.02)C(+71.04)NELNEFENNQR.C	Y	86.91	1739.6992	13	1.9	870.8585	2	28.96	5	F5:1370	OB5943 H6 Ro.raw	1.9505E4	2	2	103	115	Carbamidomethylation	C1:Carbamidomethylation:1000.00;C2:Propionamide:0.00	PEAKS PTM
R.ANLRPC(+57.02)EQHLMQK.I	Y	75.34	1623.7974	13	2.9	542.2747	3	27.48	6	F6:1292	OB5944 H6 Ro.raw	1.3154E4	2	2	40	52	Carbamidomethylation	C6:Carbamidomethylation:1000.00	PEAKS DB

total 15 peptides

Peptide	Uniq	-10lgP	Mass	Length	ppm	m/z	z	RT	Fraction	Scan	Source File	Area Roast Ara h 6	#Feature	#Feature Roast Ara h 6	Start	End	PTM	AScore	Found By
R.C(+57.02)DLEVESGGRDRY	Y	75.20	1554.6732	13	-0.6	778.3434	2	27.14	5	F5:1269	OB5943 H6 Ro.raw	1.1586E4	3	3	160	172	Carbamidomethylation	C1:Carbamidomethylation:1000.00	PEAKS DB
R.C(+57.02)MC(+57.02)EALQQIMENQSDRLQGR.Q	Y	71.68	2466.0872	20	2.1	823.0381	3	33.87	6	F6:1661	OB5944 H6 Ro.raw	1.1688E5	2	2	116	135	Carbamidomethylation	C1:Carbamidomethylation:1000.00;C3:Carbamidomethylation:1000.00	PEAKS DB
R.C(+57.02)C(+57.02)N(+.98)ELNEFENNQR.C	Y	58.73	1726.6675	13	-3.2	864.3383	2	28.65	5	F5:1358	OB5943 H6 Ro.raw	0	0	0	103	115	Carbamidomethylation	C1:Carbamidomethylation:1000.00;C2:Carbamidomethylation:1000.00;N3:Deamidation (NQ):22.69	PEAKS DB
R.C(+57.02)C(+57.02)N(-17.03)ELNEFENNQR.C	Y	58.39	1708.6570	13	1.1	855.3367	2	32.90	5	F5:1610	OB5943 H6 Ro.raw	3.7737E3	1	1	103	115	Carbamidomethylation; Ammonia-loss (N)	C1:Carbamidomethylation:1000.00;C2:Carbamidomethylation:1000.00;N3:Ammonia-loss (N):67.21	PEAKS PTM
R.C(+57.02)M(+15.99)C(+57.02)EALQQIMENQSDRLQGR.Q	Y	56.92	2482.0820	20	1.7	828.3694	3	32.29	5	F5:1571	OB5943 H6 Ro.raw	1.1939E4	1	1	116	135	Carbamidomethylation	C1:Carbamidomethylation:1000.00;M2:Oxidation (M):49.31;C3:Carbamidomethylation:1000.00	PEAKS DB
R.NLPQQC(+57.02)GLR.A	Y	54.00	1084.5448	9	-1.6	543.2788	2	25.64	5	F5:1178	OB5943 H6 Ro.raw	7.5313E3	1	1	147	155	Carbamidomethylation	C6:Carbamidomethylation:1000.00	PEAKS DB
R.C(+57.02)C(+57.02)NELN(+.98)EFENNQR.C	Y	52.39	1726.6675	13	-3.5	864.3380	2	28.36	6	F6:1346	OB5944 H6 Ro.raw	0	0	0	103	115	Carbamidomethylation	C1:Carbamidomethylation:1000.00;C2:Carbamidomethylation:1000.00;N6:Deamidation (NQ):26.38	PEAKS DB
R.C(+57.02)C(+58.01)N(+.98)ELNEFENNQR.C	Y	39.19	1727.6515	13	8.5	864.8404	2	28.91	4	F4:1376	OB5942 H6 Ro.raw	0	0	0	103	115	Carbamidomethylation	C1:Carbamidomethylation:1000.00;C2:Carboxymethyl:0.00;N3:Deamidation (NQ):22.45	PEAKS PTM
R.C(+71.04)C(+57.02)NELNEFENNQR.C	Y	37.48	1739.6992	13	2.3	870.8589	2	28.64	4	F4:1360	OB5942 H6 Ro.raw	0	0	0	103	115	Carbamidomethylation	C1:Propionamide:14.02;C2:Carbamidomethylation:1000.00	PEAKS PTM
R.C(+43.01)DLEVESGGRDRY	Y	37.45	1540.6576	13	1.4	771.3372	2	26.82	6	F6:1254	OB5944 H6 Ro.raw	0	0	0	160	172	Carbamylation	C1:Carbamylation:1000.00	PEAKS PTM
R.C(-17.03)MC(+57.02)E(+57.02)ALQQIMENQSDRLQGR.Q	Y	35.18	2449.0608	20	0.3	817.3611	3	37.71	5	F5:1889	OB5943 H6 Ro.raw	4.4611E3	1	1	116	135	Ammonia-loss (C@N-term); Carbamidomethylation; Carbamidomethylation (DHKE, X@N-term)	C1:Ammonia-loss (C@N-term):1000.00;C3:Carbamidomethylation:1000.00;E4:Carbamidomethylation (DHKE, X@N-term):56.43	PEAKS PTM
R.C(+57.02)CNE(+57.02)LN(+.98)EFENNQR.C	Y	33.57	1726.6675	13	-3.1	864.3384	2	29.21	6	F6:1396	OB5944 H6 Ro.raw	0	0	0	103	115	Carbamidomethylation	C1:Carbamidomethylation:1000.00;E4:Carbamidomethylation (DHKE, X@N-term):47.36;N6:Deamidation (NQ):7.89	PEAKS PTM
**total 15 peptides**																			

Peptide List

Prepared with PEAKS ™ (bioinfor.com)
