# Supplementary material for: Proteomic Profiling of Major Peanut Allergens and Their Post-Translational Modifications Affected by Roasting
Source: Foods. 2022 Dec 9;11(24):3993. doi: 10.3390/foods11243993 (PMC9778155; doi:10.3390/foods11243993)
Supplement: Supplementary file 1 [file foods-11-03993-s001.zip › Report S2.pdf]

Summary ALL RAW ALLERGEN 1D SDS-PAGE BANDS PROCESSED TOGETHER AS ONE CRUNCH/SAMPLE (PAGES 1-143)
ALL ROASTED ALLERGEN 1D SDS-PAGE BANDS PROCESSED TOGETHER AS ONE CRUNCH/SAMPLE (PAGES144-325)

1. Notes

```
PEAKS PTM35
RAW peanut extract files.
All raw allergen bands from 1D SDS PAGE
```

2. Result Statistics

Figure 1. False discovery rate (FDR) curve. X axis is the number of peptides being kept. Y axis is the corresponding FDR.

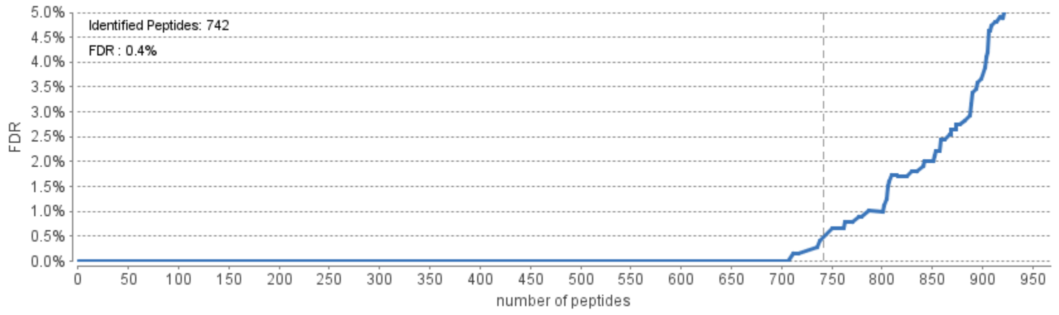

Figure 2. PSM score distribution. **(a)** Distribution of PEAKS peptide score; **(b)** Scatterplot of PEAKS peptide score versus precursor mass error.

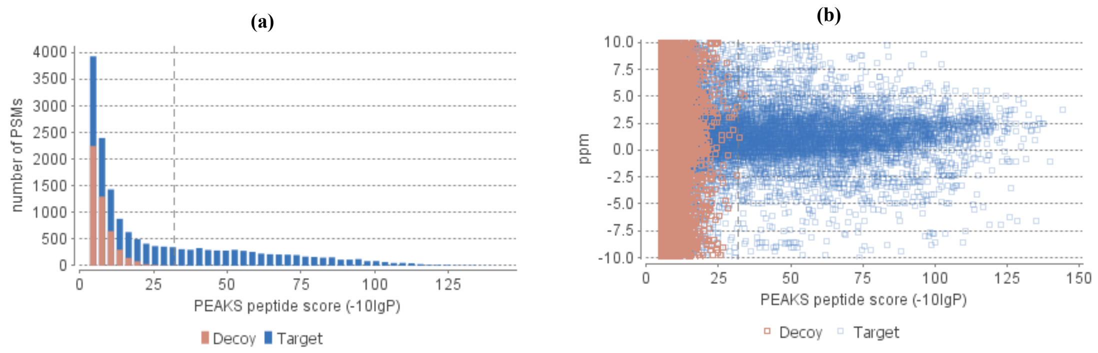

Table 1. Statistics of data.

	#Scans			#Features	Identified			#Peptides	#Sequences	#Proteins*		
	MS1	MS/MS	#Chimera		#PSMs	#Scans	#Features**			Groups	All	Top
Total	30104	25497	3827	51759	5546	5363	2290	742	467	59	94	73
Raw in gel	30104	25497	3827	51759	5546	5363	2290	742	467	59	94	73

* proteins with significant peptides are used in counts.

** features are identified by DB search only.

Figure 3. Sample overlap for Proteins and Peptides (up to 8 samples). **(a)** All Proteins; **(b)** Top Proteins; **(c)** Peptides;

(a)	(b)	(c)
**Not applicable to only one sample**	**Not applicable to only one sample**	**Not applicable to only one sample**

Figure 4. Distribution of peptide feature detection. **(a)** Feature m/z distribution; **(b)** Feature RT distribution.

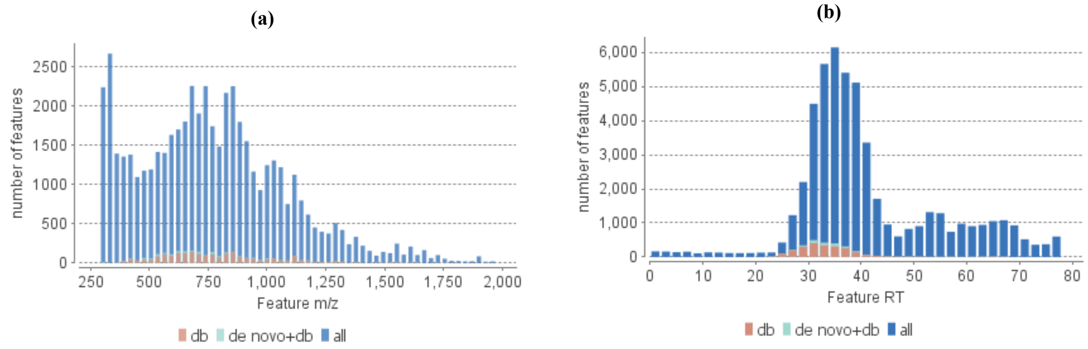

Figure 5. Distribution of identified peptide features. **(a)** Feature abundance distribution; **(b)** *De novo* sequencing validation.

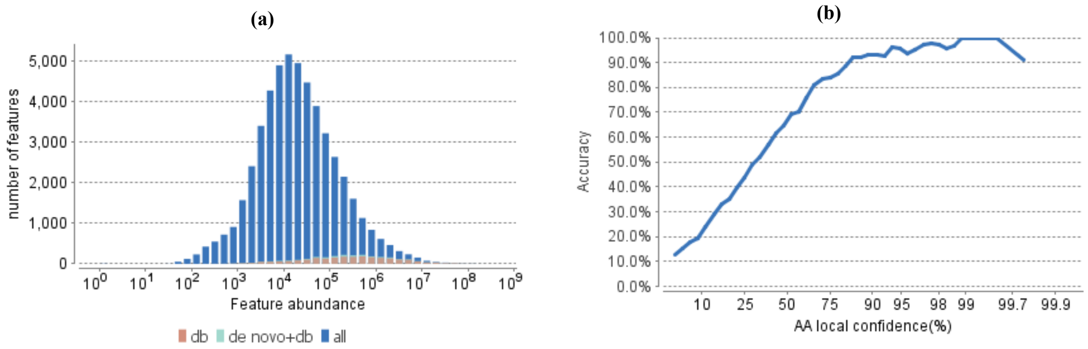

Table 2. Result filtration parameters.

Peptide -10lgP	≥31.8
PTM Ascore	≥50
Protein -10lgP	≥20
Proteins unique peptides	≥1
De novo score(%)	≥50%

Table 3. Statistics of filtered result.

FDR (Peptide-Spectrum Matches)	0.1%
FDR (Peptide Sequences)	0.4%
FDR (Protein Group)	1.7%
De Novo Only Spectra	516

Table 4. PTM profile.

Name	ΔMass	Position	#PSM	-10lgP	Abundance	AScore
Carbamidomethyl	57.02	C	575	137.14	1.51E6	1000.00
Deamidation	.98	NQ	290	140.09	1.13E8	15.73
Oxidation	15.99	M	288	134.77	6.58E5	1000.00
Carbamidomethyl	57.02	DEHK,N-term	147	104.65	7.25E6	1000.00
Acetylation	42.01	Protein N-term	31	96.71	6.15E4	1000.00
Dimethylation(KR)	28.03	KR	24	112.27	3.83E7	1000.00
Dethiomethyl	-48.00	M	24	93.52	3.52E6	26.02
SMA	127.06	N-term	21	53.35	3.56E6	1000.00

Dehydration	-18.01	DSTY,C-term	19	68.32	5.05E6	1000.00
Propionamide	71.04	C	14	68.93	7.6E4	0.00
Methylation(others)	14.02	DHST	13	55.85	1.42E5	18.00
Pyro-glu from Q	-17.03	N-term	12	76.65	1.56E6	1000.00
Methylation(KR)	14.02	KR	12	101.30	3.17E5	1000.00
Ammonia loss	-17.03	N	9	86.12	3.12E6	11.10
Ethyl	28.03	D,N-term	7	66.04	2.35E6	7.21
Oxidation	15.99	NPR	6	66.56	2.66E6	32.54
Cation:Fe[II]	53.92	DE	5	49.75	6.96E4	87.12
Sodium	21.98	E	5	52.44		12.81
Pyro-glu from E	-18.01	N-term	4	71.00	5.39E4	1000.00

3. Experiment Control

Figure 6. Precursor mass error of peptide-spectrum matches (PSM) in filtered result. **(a)** Distribution of precursor mass error in ppm; **(b)** Scatterplot of precursor m/z versus precursor mass error in ppm.

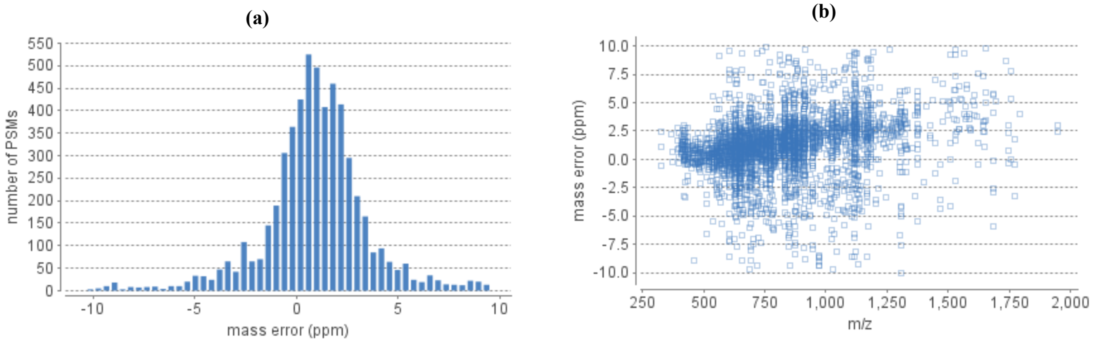

Table 5. Number of identified peptides in each sample by the number of missed cleavages.

Missed Cleavages	0	1	2	3	4+
Raw in gel	392	277	73	0	0

4. Other Information

Table 6. Search parameters.

PEAKS Version: PEAKS Studio 10.6 build 20201015
Search Engine Name: PEAKS
Parent Mass Error Tolerance: 10.0 ppm
Fragment Mass Error Tolerance: 0.5 Da
Precursor Mass Search Type: monoisotopic
Enzyme: Specified by each sample
Max Missed Cleavages: 2

Table 7. Instrument parameters.

Fractions: OB5921 H2 raw.raw, OB5925 H3B raw.raw, OB5930 H6 raw .raw, OB5931 H6 raw.raw, OB5932 H2 raw.raw, OB5933 H2 raw.raw , OB5934 H3B raw.raw, OB5935 H3B raw.raw, OB5936 H3A raw.raw , OB5937 H3A raw.raw, OB5938 H3A raw.raw, OB5939 H1 raw.raw, OB5940 H1 raw.raw, OB5941 H1 raw.raw
Ion Source: ESI(nano-spray)
Fragmentation Mode: CID, CAD(y and b ions)

Digest Mode: Unspecific
Fixed Modifications:
Carbamidomethylation: 57.02
Variable Modifications:
Oxidation (M): 15.99
Deamidation (NQ): 0.98
Acetylation (K): 42.01
Acetylation (Protein N-term): 42.01
Acetylation (N-term): 42.01
Amidation: -0.98
Beta-methylthiolation: 45.99
Biotinylation: 226.08
and 303 more...
Max Variable PTM Per Peptide: 3
Database: Uniprot_Peanut-3818_Jul18
Taxon: All
Contaminant Database: contam MQ
Searched Entry: 1723
FDR Estimation: Enabled
De novo score(%) threshold: 15
Peptide hit threshold (-10logP): 30.0
Peaks run ID: 16
Merge Options: no merge
Precursor Options: corrected
Charge Options: no correction
Filter m/z: 200.0 - 2000.0
Filter Charge: 2 - 8
Process: true
Associate chimera: yes

MS Scan Mode: FT-ICR/Orbitrap
MS/MS Scan Mode: FT-ICR/Orbitrap

Protein List

Protein Accession Contains:
Protein Description Contains:
Protein Sample Area >=
Protein PTM Contains:

Protein Group	Protein ID	Accession	-10lgP	Coverage (%)	Coverage (%) Raw in gel	Area Raw in gel	#Peptides	#Unique	#Spec Raw in gel	PTM	Avg. Mass	Description
**2**	7	sp|P43237|ALL11_ARAHY	588.42	63	63	3.5835E8	96	11	3030	Y	70283	Allergen Ara h 1, clone P17 OS=Arachis hypogaea OX=3818 PE=1 SV=1
**2**	6	B3IXL2|B3IXL2_ARAHY	588.42	63	63	3.5835E8	96	11	3030	Y	70283	Main allergen Ara h1 OS=Arachis hypogaea OX=3818 PE=2 SV=1
**2**	5	Q6PSU3|Q6PSU3_ARAHY	588.42	67	67	3.5835E8	96	11	3030	Y	66575	Conarachin (Fragment) OS=Arachis hypogaea OX=3818 PE=4 SV=1
**1**	3	N1NG13|N1NG13_ARAHY	582.61	61	61	7.4472E7	87	6	3116	Y	71345	Seed storage protein Ara h1 OS=Arachis hypogaea OX=3818 GN=ARAX_AHF417E0 7-017 PE=4 SV=1
**1**	2	sp|P43238|ALL12_ARAHY	582.61	61	61	7.4472E7	87	6	3116	Y	71345	Allergen Ara h 1, clone P41B OS=Arachis hypogaea OX=3818 PE=1 SV=1
**3**	8	Q6PSU6|Q6PSU6_ARAHY	513.69	70	70	5.0531E6	54	1	1284	Y	34133	Conarachin (Fragment) OS=Arachis hypogaea OX=3818 PE=2 SV=1
**5**	11	A1DZF0|A1DZF0_ARAHY	428.28	52	52	3.8796E6	26	3	312	Y	60375	Arachin 6 OS=Arachis hypogaea OX=3818 PE=2 SV=1
**7**	10	B5TYU1|B5TYU1_ARAHY	426.96	55	55	5.6625E7	28	1	268	Y	60624	Arachin Arah3 isoform OS=Arachis hypogaea OX=3818 PE=1 SV=1
**6**	14	Q647H4|Q647H4_ARAHY	420.67	52	52	6.4203E6	24	4	297	Y	61506	Arachin Ahy-1 OS=Arachis hypogaea OX=3818 PE=2 SV=1
**12**	25	E9LFE8|E9LFE8_ARAHY	412.93	82	82	1.3592E8	20	17	224	Y	28290	11S arachin (Fragment) OS=Arachis hypogaea OX=3818 PE=2 SV=1
**10**	13	Q9FZ11|Q9FZ11_ARAHY	405.27	53	53	2.2743E6	27	3	232	Y	60449	Gly1 OS=Arachis hypogaea OX=3818 GN=Gly1 PE=2 SV=1
**9**	9	Q647H3|Q647H3_ARAHY	401.77	61	61	8.8783E7	25	2	258	Y	61532	Arachin Ahy-2 OS=Arachis hypogaea OX=3818 PE=2 SV=1
**17**	15	T2B9M0|T2B9M0_ARAHY	399.21	74	74	5.3946E7	29	29	132	Y	38383	Fructose-bisphosphate aldolase OS=Arachis hypogaea OX=3818 PE=2 SV=1
**11**	18	Q5I6T2|Q5I6T2_ARAHY	394.77	47	47	0	25	1	223	Y	60736	Arachin Ahy-4 OS=Arachis hypogaea OX=3818 PE=2 SV=1
**15**	30	A1E2B0|A1E2B0_ARAHY	393.59	69	69	8.5236E7	19	16	157	Y	33520	11S seed storage globulin B1 OS=Arachis hypogaea OX=3818 PE=2 SV=1
**16**	26	Q38711|Q38711_ARAHY	383.89	80	80	6.0276E6	17	2	144	Y	29134	Galactose-binding lectin (Fragment) OS=Arachis hypogaea OX=3818 GN=lec PE=2 SV=1
**13**	19	Q6IWG5|Q6IWG5_ARAHY	377.88	61	61	2.596E8	23	21	157	Y	58061	Glycinin (Fragment) OS=Arachis hypogaea OX=3818 PE=2 SV=1
**13**	20	Q0GM57|Q0GM57_ARAHY	377.88	61	61	2.596E8	23	21	157	Y	58263	Iso-Ara h3 OS=Arachis hypogaea OX=3818 PE=2 SV=1

total 73 proteins

Protein Group	Protein ID	Accession	-10lgP	Coverage (%)	Coverage (%) Raw in gel	Area Raw in gel	#Peptides	#Unique	#Spec Raw in gel	PTM	Avg. Mass	Description
**18**	27	A0A089ZXL7|A0A089ZXL7_ARAHY	371.36	83	83	4.7484E5	18	3	119	Y	29407	Peanut agglutinin variant OS=Arachis hypogaea OX=3818 PE=2 SV=1
**24**	28	sp|Q647H2|AHY3_ARAHY	331.14	30	30	1.8822E7	12	10	53	Y	54569	Arachin Ahy-3 OS=Arachis hypogaea OX=3818 PE=1 SV=1
**21**	21	A0A0F6VX63|A0A0F6VX63_ARAHY	328.06	60	60	4.6212E7	20	20	67	Y	36080	Annexin OS=Arachis hypogaea OX=3818 PE=2 SV=1
**20**	32	A0A0A6ZDP1|A0A0A6ZDP1_ARAHY	323.84	70	70	8.8001E7	14	5	94	Y	20101	Glyceraldehyde-3-phosphate dehydrogenase C2 OS=Arachis hypogaea OX=3818 PE=2 SV=1
**19**	29	O82580|O82580_ARAHY	319.77	30	30	1.4756E6	16	1	99	Y	58350	Glycinin (Fragment) OS=Arachis hypogaea OX=3818 GN=Arah3 PE=2 SV=1
**8**	36	A5Z1R0|A5Z1R0_ARAHY	307.18	67	67	2.2488E7	11	11	263	Y	16920	Ara h 6 allergen OS=Arachis hypogaea OX=3818 GN=Ara h 6 PE=4 SV=1
**8**	35	sp|Q647G9|CONG_ARAHY	307.18	67	67	2.2488E7	11	11	263	Y	16920	Conglutin OS=Arachis hypogaea OX=3818 PE=1 SV=1
**25**	33	A1E2B1|A1E2B1_ARAHY	293.36	52	52	2.0862E7	12	12	52	Y	31268	11S seed storage globulin B2 OS=Arachis hypogaea OX=3818 PE=2 SV=1
**26**	34	A0A0A6ZDT0|A0A0A6ZDT0_ARAHY	277.30	45	45	1.1133E6	11	2	41	Y	20099	Glyceraldehyde-3-phosphate dehydrogenase C2 OS=Arachis hypogaea OX=3818 PE=4 SV=1
**23**	47	E9LFE9|E9LFE9_ARAHY	253.30	38	38	2.708E7	8	8	50	Y	28300	11S arachin OS=Arachis hypogaea OX=3818 PE=2 SV=1
**31**	40	Q647H1|Q647H1_ARAHY	233.97	10	10	4.7101E6	5	5	14	Y	75933	Conarachin OS=Arachis hypogaea OX=3818 PE=2 SV=1
**32**	37	A0A0F7GF62|A0A0F7GF62_ARAHY	224.59	28	28	1.588E6	6	6	14	Y	36134	Annexin OS=Arachis hypogaea OX=3818 GN=AnnAh6 PE=2 SV=1
**29**	50	B0YIU5|B0YIU5_ARAHY	203.22	59	59	2.7132E5	5	5	21	Y	16413	Ara h 8 allergen isoform OS=Arachis hypogaea OX=3818 PE=2 SV=1
**36**	48	Q2PK12|Q2PK12_ARAHY	196.35	60	60	9.4443E4	5	5	11	Y	16090	Actin depolymerizing factor-like protein OS=Arachis hypogaea OX=3818 PE=2 SV=1
**33**	63	H6U596|H6U596_ARAHY	192.07	30	30	1.9528E7	5	5	14	N	19370	Alcohol dehydrogenase (Fragment) OS=Arachis hypogaea OX=3818 PE=2 SV=1
**28**	52	B4UW81|B4UW81_ARAHY	192.06	46	46	1.0118E7	5	5	22	Y	20343	Glutathione S-transferase 2 OS=Arachis hypogaea OX=3818 PE=2 SV=1
**35**	38	D8KXZ8|D8KXZ8_ARAHY	187.85	23	23	1.6879E4	6	2	10	N	41475	Enoyl-ACP reductase 1-3 OS=Arachis hypogaea OX=3818 GN=ENR1-3 PE=2 SV=1
**27**	44	sp|Q6PSU2|CONG7_ARAHY	184.43	46	46	9.5425E6	7	7	39	Y	20114	Conglutin-7 OS=Arachis hypogaea OX=3818 PE=1 SV=2
**39**	39	E6Y9A9|E6Y9A9_ARAHY	175.27	23	23	1.1751E4	5	1	8	N	41491	Enoyl-ACP reductase OS=Arachis hypogaea OX=3818 GN=ER PE=2 SV=1
**38**	41	O20356|O20356_ARAHY	173.94	17	17	1.3383E6	6	6	11	Y	51526	Ribulose bisphosphate carboxylase large chain (Fragment) OS=Arachis hypogaea OX=3818 GN=rbcL PE=3 SV=1
**38**	42	A0A075M981|A0A075M981_ARAHY	173.94	16	16	1.3383E6	6	6	11	Y	51912	Ribulose bisphosphate carboxylase large chain (Fragment) OS=Arachis hypogaea OX=3818 GN=rbcL PE=3 SV=1
**38**	43	A0A191UJ50|A0A191UJ50_ARAHY	173.94	16	16	1.3383E6	6	6	11	Y	52587	Ribulose bisphosphate carboxylase large chain OS=Arachis hypogaea OX=3818 GN=rbcL PE=3 SV=1
**30**	54	N1NKG9|N1NKG9_ARAHY	173.74	36	36	2.7405E6	4	4	16	N	26041	Seed maturation protein OS=Arachis hypogaea OX=3818 GN=ARAX_AHF417E07-003 PE=4 SV=1
**41**	61	Q42515|Q42515_ARAHY	165.97	21	21	9.0898E5	3	3	8	Y	28924	Chitinase (Class II) OS=Arachis hypogaea OX=3818 GN=chi2;2 PE=2 SV=1
**37**	58	B4UW89|B4UW89_ARAHY	155.96	22	22	3.7088E4	4	4	10	Y	18100	Heat shock protein 2 OS=Arachis hypogaea OX=3818 PE=2 SV=1
**42**	53	A0A0R4UXQ1|A0A0R4UXQ1_ARAHY	155.35	16	16	9.4117E5	3	3	6	Y	48348	3-ketoacyl-CoA thiolase OS=Arachis hypogaea OX=3818 GN=PKT PE=2 SV=1
**47**	161	D4NXQ0|D4NXQ0_ARAHY	128.12	10	10	4.1064E5	1	1	5	Y	23448	Germin-like protein subfamily 3 member 3 OS=Arachis hypogaea OX=3818 GN=GLP1 PE=2 SV=1
**47**	173	A7LIS5|A7LIS5_ARAHY	128.12	10	10	4.1064E5	1	1	5	Y	23461	Oxalate oxidase OS=Arachis hypogaea OX=3818 GN=OxOxs PE=2 SV=1
**49**	90	B4UWD5|B4UWD5_ARAHY	120.07	26	26	1.1738E6	2	2	4	N	15013	Proteasome subunit alpha type (Fragment) OS=Arachis hypogaea OX=3818 PE=2 SV=1
**46**	96	Q45W80|Q45W80_ARAHY	106.45	29	29	4.3272E4	3	3	4	Y	16439	Nucleoside diphosphate kinase OS=Arachis hypogaea OX=3818 PE=2 SV=1
**54**	74	E5FHZ2|E5FHZ2_ARAHY	106.30	14	14	6.4018E4	2	2	2	N	27370	Late embryogenesis abundant protein group 5 protein OS=Arachis hypogaea OX=3818 GN=LEA5-2 PE=2 SV=1
**63**	109	A0A290FZZ3|A0A290FZZ3_ARAHY	99.74	5	5	1.8555E5	1	1	1	N	41532	Resistance protein (Fragment) OS=Arachis hypogaea OX=3818 PE=2 SV=1
**51**	122	N1NJN8|N1NJN8_ARAHY	96.65	9	9	3.0867E5	2	2	3	N	23506	Proteasome subunit OS=Arachis hypogaea OX=3818 GN=ARAX_AHF417E07-015 PE=4 SV=1
**45**	126	Q1PCR5|Q1PCR5_ARAHY	95.82	4	4	8.8119E5	1	1	5	N	24047	20S proteasome beta subunit (Fragment) OS=Arachis hypogaea OX=3818 PE=2 SV=1
**44**	121	Q06H31|Q06H31_ARAHY	94.32	26	26	6.6347E5	2	2	5	Y	19128	Translationally controlled tumor-like protein OS=Arachis hypogaea OX=3818 PE=2 SV=1
**62**	231	Q06H23|Q06H23_ARAHY	88.89	18	18	9.6761E4	1	1	2	Y	16492	Ubiquitin carrier-like protein OS=Arachis hypogaea OX=3818 PE=2 SV=1
**50**	117	Q1HDS7|Q1HDS7_ARAHY	84.00	32	32	1.9718E5	2	2	3	N	15198	Superoxide dismutase [Cu-Zn] OS=Arachis hypogaea OX=3818 PE=2 SV=1
**50**	116	Q45W82|Q45W82_ARAHY	84.00	32	32	1.9718E5	2	2	3	N	15098	Cu-Zn superoxide dismutase OS=Arachis hypogaea OX=3818 PE=2 SV=1
**52**	83	K7PQ65|K7PQ65_ARAHY	81.10	13	13	1.435E4	2	2	3	N	19193	Mother of FT and TFL1 OS=Arachis hypogaea OX=3818 GN=MFT PE=2 SV=1
**69**	232	B4UWB3|B4UWB3_ARAHY	80.51	8	8	0	1	1	1	N	16789	Putative L24 ribosomal protein OS=Arachis hypogaea OX=3818 PE=2 SV=1
**48**	98	Q647G5|Q647G5_ARAHY	79.23	7	7	4.7315E4	1	1	4	N	17753	Oleosin 17.8 OS=Arachis hypogaea OX=3818 PE=2 SV=2
**48**	105	Q647G4|Q647G4_ARAHY	79.23	7	7	4.7315E4	1	1	4	N	15527	Oleosin 2 (Fragment) OS=Arachis hypogaea OX=3818 PE=2 SV=1
**61**	215	B4UWC9|B4UWC9_ARAHY	67.57	16	16	1.231E5	1	1	2	N	14223	MCT-1 protein-like protein (Fragment) OS=Arachis hypogaea OX=3818 PE=2 SV=1
**53**	133	B4UWB2|B4UWB2_ARAHY	65.65	4	4	2.8581E5	1	1	2	N	21637	Kunitz trypsin inhibitor 4 OS=Arachis hypogaea OX=3818 PE=2 SV=1
**70**	71	A0A290GK88|A0A290GK88_ARAHY	65.30	12	12	2.2447E5	1	1	1	N	24410	Resistance protein (Fragment) OS=Arachis hypogaea OX=3818 PE=2 SV=1
**55**	235	A0A0A1EXN0|A0A0A1EXN0_ARAHY	65.01	9	9	7.7614E5	1	1	3	N	35871	Actin (Fragment) OS=Arachis hypogaea OX=3818 PE=3 SV=1

total 73 proteins

Protein Group	Protein ID	Accession	-10lgP	Coverage (%)	Coverage (%) Raw in gel	Area Raw in gel	#Peptides	#Unique	#Spec Raw in gel	PTM	Avg. Mass	Description
**55**	234	A0A0A1EUV7|A0A0A1EUV7_ARAHY	65.01	9	9	7.7614E5	1	1	3	N	35884	Actin (Fragment) OS=Arachis hypogaea OX=3818 PE=3 SV=1
**55**	233	A0A0A1ESG0|A0A0A1ESG0_ARAHY	65.01	9	9	7.7614E5	1	1	3	N	35855	Actin (Fragment) OS=Arachis hypogaea OX=3818 PE=3 SV=1
**74**	111	Q45W77|Q45W77_ARAHY	60.66	12	12	5.3162E3	1	1	1	N	17206	Ubiquitin-conjugating enzyme 1 OS=Arachis hypogaea OX=3818 PE=2 SV=1
**40**	152	F8UX79|F8UX79_ARAHY	57.87	2	2	1.0557E6	1	1	9	Y	43179	Glyceraldehyde-3-phosphate dehydrogenase OS=Arachis hypogaea OX=3818 PE=2 SV=1
**59**	118	B4UW57|B4UW57_ARAHY	53.39	6	6	3.2436E4	1	1	2	Y	24268	Putative dihydroflavonol reductase (Fragment) OS=Arachis hypogaea OX=3818 PE=2 SV=1
**76**	134	E9JSA9|E9JSA9_ARAHY	44.46	5	5	3.8207E4	1	1	1	N	46210	Acyl-[acyl-carrier-protein] desaturase OS=Arachis hypogaea OX=3818 GN=SAD1-2 PE=2 SV=1
**76**	136	E9JSA8|E9JSA8_ARAHY	44.46	5	5	3.8207E4	1	1	1	N	46324	Acyl-[acyl-carrier-protein] desaturase OS=Arachis hypogaea OX=3818 GN=SAD1-1 PE=2 SV=1
**76**	135	B6ECZ0|B6ECZ0_ARAHY	44.46	5	5	3.8207E4	1	1	1	N	46252	Acyl-[acyl-carrier-protein] desaturase OS=Arachis hypogaea OX=3818 PE=2 SV=1
**71**	110	U3MF97|U3MF97_ARAHY	33.42	19	19	0	1	1	1	Y	15748	40S ribosomal protein S23 OS=Arachis hypogaea OX=3818 PE=2 SV=1
**total 73 proteins**												

sp|P43237|ALL11_ARAHY

back to list

| Protein Coverage | Supporting Peptides |

Protein Coverage:

1 MRGRVSPLML LLGILVLASV SATQAKSPYR KTENPCAQRC LQSCQQEPDD LKQKACESRC TKLEYDPRCV YDTGATNQRH

81 PPGERTRGRQ PGDYDDDRRQ PRREEGGRWG PAEPRERERE EDWRQPREDW RRPSHQQPRK IRPEGREGEQ EWGTPGSEVR

161 EETSRNNPFY FPSRRFSTRY GNQNGRIRVL QRFDQRSKQF QNLQNHRIVQ IEARPNTLVL PKHADADNIL VIQQGQATVT

241 VANGNNRKSF NLDEGHALRI PSGFISYILN RHDNQNLRVA KISMPVNTPG QFEDFFPASS RDQSSYLQGF SRNTLEAAFN

321 AEFNEIRRVL LEENAGGEQE ERGQRRRSTR SSDNEGVIVK VSKEHVQELT KHAKSVSKKG SEEEDITNPI NLRDGEPDLS

401 NNFGRLFEVK PDKKNPQLQD LDMMLTCVEI KEGALMLPHF NSKAMVIVVV NKGTGNLELV AVRKEQQQRG RREQEWEEEE

481 EDEEEEGSNR EVRRYTARLK EGDVFIMPAA HPVAINASSE LHLLGFGINA ENNHRIFLAG DKDNVIDQIE KQAKDLAFPG

561 SGEQVEKLIK NQRESHFVSA RPQSQSPSSP EKEDQEEENQ GGKGPLLSIL KAFN

- a Ammonia-loss (N) (-17.03)
- a Acetaldehyde +26 (+26.02)
- c Carbamidomethylation (+57.02)
- c Carbamidomethylation (DHKE, X@N-term) (+57.02)
- d Deamidation (NQ) (+0.98)
- d Dethiomethyl (-48.00)
- d Dehydration (-18.01)
- f Formylation (+27.99)
- i Iodination (+125.90)
- l Lysine oxidation to aminoadipic semialdehyde (-1.03)
- m Methylation(KR) (+14.02)
- m Methylation(others) (+14.02)
- o Oxidation (M) (+15.99)
- p Pyro-glu from Q (-17.03)
- s 4-sulfophenyl isothiocyanate (+214.97)

Supporting Peptides:

Peptide	Uniq	-10lgP	Mass	Length	ppm	m/z	z	RT	Fraction	Scan	Source File	Area Raw in gel	#Feature	#Feature Raw in gel	Start	End	PTM	AScore	Found By
K.HADADNILVIQQGQATVTVAN(+.98)GNNRK.S	N	140.09	2747.3950	26	-1.1	916.8046	3	29.38	14	F14:1509	OB5941 H1 raw.raw	3.7837E8	20	20	223	248		N21:Deamidation (N Q):26.31	PEAKS DB
K.KNPQLQDLDMMLTC(+57.02)VEIK.E	N	137.14	2175.0737	18	2.4	1088.5468	2	34.90	14	F14:1774	OB5941 H1 raw.raw	4.1086E6	3	3	414	431	Carbamidomethylation	C14:Carbamidomethylation:1000.00	PEAKS DB
K.HADADNILVIQQGQATVTVANGNNR.K	N	135.84	2618.3162	25	2.9	1310.1692	2	30.42	14	F14:1494	OB5941 H1 raw.raw	5.8142E6	3	3	223	247			PEAKS DB
K.ISMPVNTPGQFEDFFPASSR.D	N	135.58	2226.0415	20	1.9	1114.0302	2	35.63	14	F14:1807	OB5941 H1 raw.raw	2.1661E8	32	26	282	301			PEAKS DB
K.ISM(+15.99)PVNTPGQFEDFFPASSR.D	N	134.77	2242.0364	20	2.3	1122.0281	2	34.42	2	F2:1522	OB5925 H3B raw.raw	1.6845E7	19	17	282	301	Oxidation (M)	M3:Oxidation (M):1000.00	PEAKS DB
K.NPQLQDLDMMLTC(+57.02)VEIK.E	N	131.65	2046.9788	17	-0.8	1024.4958	2	36.89	14	F14:1903	OB5941 H1 raw.raw	1.2559E7	8	8	415	431	Carbamidomethylation	C13:Carbamidomethylation:1000.00	PEAKS DB
R.EQEWEEEEEDEEEEGSNR.E	N	130.87	2280.8413	18	4.3	1141.4329	2	28.13	14	F14:1352	OB5941 H1 raw.raw	4.0373E6	3	3	473	490			PEAKS DB
K.NPQLQDLDM(+15.99)MLTC(+57.02)VEIK.E	N	128.59	2062.9736	17	-6.6	1032.4873	2	36.34	14	F14:1860	OB5941 H1 raw.raw	2.4371E5	2	2	415	431	Carbamidomethylation	M9:Oxidation (M):30.46;C13:Carbamidomethylation:1000.00	PEAKS DB
R.NTLEAAFNAEFNEIR.R	N	126.11	1737.8322	15	3.3	869.9262	2	35.27	14	F14:1792	OB5941 H1 raw.raw	1.3691E7	6	6	313	327			PEAKS DB
K.HADADNILVIQQGQATVTVANGNNRK.S	N	120.98	2746.4111	26	4.7	1374.2192	2	29.02	14	F14:1406	OB5941 H1 raw.raw	4.4412E7	13	13	223	248			PEAKS DB
K.SFNLDEGHALRIPSGFISYILNR.H	N	117.92	2618.3604	23	0.6	1310.1882	2	36.89	14	F14:1902	OB5941 H1 raw.raw	2.1092E8	40	39	249	271			PEAKS DB
R.VLLEENAGGEQEER.G	N	114.70	1571.7427	14	1.1	786.8795	2	26.65	14	F14:1250	OB5941 H1 raw.raw	1.2337E7	7	7	329	342			PEAKS DB
R.IFLAGDKDNVIDQIEKQAK.D	N	113.07	2144.1477	19	1.1	715.7240	3	32.93	7	F7:1578	OB5934 H3B raw.raw	5.566E7	12	12	536	554			PEAKS DB
K.AMVIVVVNKGTGNLELVAVR.K	N	112.17	2081.2031	20	1.5	694.7427	3	34.35	14	F14:1739	OB5941 H1 raw.raw	7.0931E7	12	12	444	463			PEAKS DB
R.KSFNLDEGHALR.I	N	112.03	1385.7051	12	1.1	693.8606	2	25.70	14	F14:1202	OB5941 H1 raw.raw	3.2948E6	6	6	248	259			PEAKS DB
R.IPSGFISYILNR.H	N	111.89	1378.7609	12	1.5	690.3887	2	43.03	14	F14:2242	OB5941 H1 raw.raw	2.1426E8	15	15	260	271			PEAKS DB
R.DQSSYLQGFSR.N	N	110.94	1286.5891	11	-1.4	644.3009	2	30.60	14	F14:1505	OB5941 H1 raw.raw	6.2405E7	21	21	302	312			PEAKS DB
K.KGSEEEDITNPINLR.D	N	110.67	1713.8533	15	3.2	857.9366	2	28.85	14	F14:1395	OB5941 H1 raw.raw	1.2495E7	8	8	379	393			PEAKS DB
K.NPQLQDLDMMLTC(+57.02)VEIKEGALMLPHFNSK.A	N	110.02	3371.6387	29	-0.1	1686.8264	2	37.85	14	F14:1946	OB5941 H1 raw.raw	1.7936E8	14	14	415	443	Carbamidomethylation	C13:Carbamidomethylation:1000.00	PEAKS DB
K.AMVIVVVNKGTGNLELVAVRK.E	N	109.98	2209.2981	21	-1.4	1105.6548	2	32.55	14	F14:1615	OB5941 H1 raw.raw	2.9136E8	18	18	444	464			PEAKS DB

total 219 peptides

Peptide	Uniq	-10lgP	Mass	Length	ppm	m/z	z	RT	Fraction	Scan	Source File	Area Raw in gel	#Feature	#Feature Raw in gel	Start	End	PTM	AScore	Found By
K.AM(+15.99)VIVVVNKGTGNLELVAVR.K	N	109.59	2097.1980	20	-2.2	1049.6040	2	32.73	14	F14:1638	OB5941 H1 raw.raw	3.1537E7	10	10	444	463	Oxidation (M)	M2:Oxidation (M):1000.00	PEAKS DB
K.SFNLDEGHALR.I	N	109.41	1257.6101	11	-3.3	629.8102	2	27.77	14	F14:1329	OB5941 H1 raw.raw	7.2473E7	30	30	249	259			PEAKS DB
R.IFLAGDKDNVIDQIEK.Q	N	108.13	1816.9570	16	0.2	909.4860	2	33.10	14	F14:1683	OB5941 H1 raw.raw	7.2865E8	41	41	536	551			PEAKS DB
R.KSFNLDEGHALRIPSGFISYILNR.H	N	107.80	2746.4553	24	-2.1	916.4905	3	36.16	12	F12:1802	OB5939 H1 raw.raw	7.186E6	4	4	248	271			PEAKS DB
K.GSEEEDITNPINLRDGEPDLSNNFGR.L	N	107.18	2887.3220	26	-1.7	963.4464	3	32.55	14	F14:1626	OB5941 H1 raw.raw	4.5198E7	9	9	380	405			PEAKS DB
R.EGEQEWGTPGSEVR.E	Y	105.02	1559.6852	14	-4.9	780.8460	2	27.77	14	F14:1333	OB5941 H1 raw.raw	7.2805E5	3	3	147	160			PEAKS DB
R.I(+57.02)PSGFISYILNR.H	N	104.65	1435.7823	12	-1.7	718.8972	2	36.52	14	F14:1869	OB5941 H1 raw.raw	1.3017E7	3	3	260	271	Carbamidomethylation (DHKE, X@N-term)	I1:Carbamidomethylation (DHKE, X@N-term):1000.00	PEAKS PTM
R.VLLEENAGGEQEERGQR.R	N	104.46	1912.9238	17	1.8	957.4709	2	25.31	14	F14:1182	OB5941 H1 raw.raw	1.9161E6	8	8	329	345			PEAKS DB
R.I(+57.02)FLAGDKDNVIDQIEK.Q	N	103.96	1873.9785	16	-5.0	937.9919	2	32.19	14	F14:1619	OB5941 H1 raw.raw	4.5184E7	7	7	536	551	Carbamidomethylation (DHKE, X@N-term)	I1:Carbamidomethylation (DHKE, X@N-term):90.91	PEAKS PTM
R.DGEPDLSNNFGR.L	N	102.95	1319.5742	12	0.8	660.7949	2	29.02	14	F14:1398	OB5941 H1 raw.raw	3.2103E6	3	3	394	405			PEAKS DB
K.QAKDLAFPGSGEQVEK.L	N	102.73	1702.8525	16	1.8	852.4351	2	26.85	14	F14:1278	OB5941 H1 raw.raw	1.286E6	6	6	552	567			PEAKS DB
K.H(+57.02)ADADNILVIQQGQATVTVANGNNRK.S	N	102.10	2803.4324	26	-1.3	935.4835	3	29.92	14	F14:1452	OB5941 H1 raw.raw	1.6897E6	1	1	223	248		H1:Carbamidomethylation (DHKE, X@N-term):39.76	PEAKS PTM
K.DLAFPGSGEQVEK.L	N	101.44	1375.6619	13	-2.0	688.8369	2	30.08	14	F14:1472	OB5941 H1 raw.raw	6.5563E7	9	9	555	567			PEAKS DB
K.NPQLQDLDMMLTCVEIK(+14.02).E	N	101.30	2003.9730	17	2.1	1002.9958	2	38.00	14	F14:1960	OB5941 H1 raw.raw	6.7851E5	2	2	415	431	Methylation(KR)	K17:Methylation(KR):1000.00	PEAKS PTM
K.S(+57.02)FNLDEGHALR.I	N	100.96	1314.6316	11	0.8	658.3236	2	27.59	14	F14:1340	OB5941 H1 raw.raw	5.6012E6	7	7	249	259	Carbamidomethylation (DHKE, X@N-term)	S1:Carbamidomethylation (DHKE, X@N-term):101.71	PEAKS PTM
R.RVLLEENAGGEQEER.G	N	98.06	1727.8438	15	0.4	576.9554	3	24.77	13	F13:1156	OB5940 H1 raw.raw	1.2517E5	5	5	328	342			PEAKS DB
R.IPSGFISYILNRHDNQNLR.V	N	97.42	2256.1763	19	0.5	565.0516	4	33.66	14	F14:1693	OB5941 H1 raw.raw	3.392E8	42	42	260	278			PEAKS DB
K.GSEEEDITNPINLR.D	N	97.01	1585.7583	14	2.4	793.8884	2	30.93	14	F14:1527	OB5941 H1 raw.raw	3.0498E6	3	3	380	393			PEAKS DB
K.AM(+15.99)VIVVVNKGTGNLELVAVRK.E	N	96.33	2225.2930	21	-0.4	1113.6533	2	30.76	14	F14:1520	OB5941 H1 raw.raw	4.0787E7	8	7	444	464	Oxidation (M)	M2:Oxidation (M):1000.00	PEAKS DB
R.EGEQEWGTPGSEVREETSR.N	Y	95.70	2161.9512	19	2.5	1081.9856	2	27.60	14	F14:1311	OB5941 H1 raw.raw	2.0479E7	9	9	147	165			PEAKS DB
**total 219 peptides**																			

Peptide	Uniq	-10lgP	Mass	Length	ppm	m/z	z	RT	Fraction	Scan	Source File	Area Raw in gel	#Feature	#Feature Raw in gel	Start	End	PTM	AScore	Found By
K.KNPQLQDLDMM(+15.99)LTC(+57.02)VEIK.E	N	95.01	2191.0686	18	-3.4	1096.5378	2	32.73	14	F14:1643	OB5941 H1 raw.raw	1.9787E5	1	1	414	431	Carbamidomethylation	M11:Oxidation (M):9.34;C14:Carbamidomethylation:1000.00	PEAKS DB
K.NPQLQDLDMM(-48.00)LTC(+57.02)VEIK.E	N	93.52	1998.9755	17	-2.5	1000.4926	2	32.55	14	F14:1628	OB5941 H1 raw.raw	1.3958E7	5	5	415	431	Carbamidomethylation	M10:Dethiomethyl:27.96;C13:Carbamidomethylation:1000.00	PEAKS PTM
K.NPQLQDLDM(+15.99)MLTC(+57.02)VEIKEGALMLPHFNSK.A	N	92.22	3387.6335	29	-1.5	1130.2168	3	36.17	14	F14:1926	OB5941 H1 raw.raw	2.8061E7	4	4	415	443	Carbamidomethylation	M9:Oxidation (M):10.11;C13:Carbamidomethylation:1000.00	PEAKS DB
R.NNPFYFPSRR.F	N	92.08	1296.6364	10	-3.0	649.3235	2	28.67	14	F14:1408	OB5941 H1 raw.raw	1.2788E8	21	21	166	175			PEAKS DB
K.EGALM(+15.99)LPHFNSK.A	N	91.98	1358.6653	12	2.1	680.3414	2	29.74	14	F14:1443	OB5941 H1 raw.raw	1.9561E6	3	3	432	443	Oxidation (M)	M5:Oxidation (M):1000.00	PEAKS DB
R.VLLEENAGGEQEERGQRR.R	N	91.09	2069.0249	18	2.1	690.6837	3	25.31	14	F14:1186	OB5941 H1 raw.raw	1.1888E6	3	3	329	346			PEAKS DB
R.SSDNEGVIVKVSKEHVQELTK.H	N	91.01	2325.2175	21	1.3	776.0808	3	28.31	14	F14:1362	OB5941 H1 raw.raw	1.0201E5	2	2	351	371			PEAKS DB
K.KNPQLQDLDMM(+15.99)LTC(+57.02)VEIKEGALMLPHFNSK.A	N	90.76	3515.7285	30	-2.7	879.9370	4	34.52	13	F13:1755	OB5940 H1 raw.raw	2.6989E6	3	3	414	443	Carbamidomethylation	M11:Oxidation (M):30.46;C14:Carbamidomethylation:1000.00	PEAKS DB
K.AM(-48.00)VIVVVNKGTGNLELVAVR.K	N	90.57	2033.1997	20	-3.4	1017.6036	2	31.46	14	F14:1539	OB5941 H1 raw.raw	1.6588E7	6	6	444	463	Dethiomethyl	M2:Dethiomethyl:1000.00	PEAKS PTM
K.A(+57.02)MVIVVVNKGTGNLELVAVR.K	N	90.54	2138.2246	20	-0.9	1070.1187	2	34.35	14	F14:1737	OB5941 H1 raw.raw	5.6414E6	3	3	444	463	Carbamidomethylation (DHKE, X@N-term)	A1:Carbamidomethylation (DHKE, X@N-term):207.56	PEAKS PTM
K.NPQLQDLDMMLTC(+57.02)VEIKEGALM(+15.99)LPHFNSK.A	N	90.37	3387.6335	29	-1.5	1130.2168	3	36.17	14	F14:1895	OB5941 H1 raw.raw	3.1109E7	7	7	415	443	Carbamidomethylation; Oxidation (M)	C13:Carbamidomethylation:1000.00;M22:Oxidation (M):69.89	PEAKS DB
K.H(+57.02)ADADNILVIQQGQATVTVAN(+.98)GNNRK.S	N	90.07	2804.4165	26	-1.3	935.8115	3	30.08	14	F14:1476	OB5941 H1 raw.raw	6.8084E6	2	2	223	248		H1:Carbamidomethylation (DHKE, X@N-term):45.01;N21:Deamidation (NQ):14.04	PEAKS PTM
K.KGSEEEDITNPINLRDGEPDLSNNFGR.L	N	89.87	3015.4170	27	-4.2	1006.1421	3	31.29	14	F14:1549	OB5941 H1 raw.raw	7.6E7	16	16	379	405			PEAKS DB
F.LAGDKDNVIDQIEK.Q	N	89.22	1556.8046	14	-3.2	779.4071	2	32.19	12	F12:1557	OB5939 H1 raw.raw	4.4864E6	2	2	538	551			PEAKS DB
K.I(+57.02)SMPVNTPGQFEDFFPASSR.D	N	88.49	2283.0630	20	-0.1	1142.5387	2	35.84	12	F12:1793	OB5939 H1 raw.raw	6.4772E5	1	1	282	301	Carbamidomethylation (DHKE, X@N-term)	I1:Carbamidomethylation (DHKE, X@N-term):156.03	PEAKS PTM
K.DLAFPGSGEQVEKLIK.N	N	87.96	1729.9249	16	1.0	865.9706	2	33.30	7	F7:1601	OB5934 H3B raw.raw	6.2551E5	3	3	555	570			PEAKS DB

total 219 peptides

Peptide	Uniq	-10lgP	Mass	Length	ppm	m/z	z	RT	Fraction	Scan	Source File	Area Raw in gel	#Feature	#Feature Raw in gel	Start	End	PTM	AScore	Found By
K.EGALMLPHFNSK.A	N	87.95	1342.6703	12	2.5	672.3441	2	29.83	13	F13:1462	OB5940 H1 raw.raw	9.3502E6	3	3	432	443			PEAKS DB
K.HADADNILVIQQGQATVTVAN(+.98)GNNR.K	N	87.06	2619.3000	25	-1.2	1310.6558	2	30.35	13	F13:1535	OB5940 H1 raw.raw	4.8683E6	1	1	223	247		N21:Deamidation (NQ):7.32	PEAKS DB
K.AMVIVVVNK.G	N	86.97	971.5837	9	0.9	486.7996	2	29.88	12	F12:1441	OB5939 H1 raw.raw	9.5124E6	3	3	444	452			PEAKS DB
K.GTGNLELVAVR.K	N	86.65	1127.6299	11	-0.8	564.8218	2	29.74	14	F14:1450	OB5941 H1 raw.raw	1.0907E7	6	6	453	463			PEAKS DB
R.NNPFYFPSR.R	N	86.37	1140.5352	9	-3.8	571.2727	2	31.46	14	F14:1552	OB5941 H1 raw.raw	1.201E7	5	5	166	174			PEAKS DB
K.HADADNILVIQQGQATVTVAN(-17.03)GNNRK.S	N	86.12	2729.3845	26	-1.1	910.8011	3	29.38	14	F14:1429	OB5941 H1 raw.raw	8.2265E6	4	4	223	248		N21:Ammonia-loss (N):18.53	PEAKS PTM
R.NTLEAAFNAEFNEIRR.V	N	86.03	1893.9332	16	-3.6	947.9705	2	35.28	12	F12:1741	OB5939 H1 raw.raw	3.4229E8	38	38	313	328			PEAKS DB
R.I(+27.99)FLAGDKDNVIDQIEK.Q	N	85.41	1844.9519	16	5.2	923.4880	2	36.16	14	F14:1844	OB5941 H1 raw.raw	0	0	0	536	551	Formylation	I1:Formylation:77.93	PEAKS PTM
K.SFNLDEGH(+57.02)ALR.I	N	84.87	1314.6316	11	-1.2	658.3223	2	28.05	12	F12:1298	OB5939 H1 raw.raw	5.1176E6	5	5	249	259		H8:Carbamidomethylation (DHKE, X@N-term):30.36	PEAKS PTM
R.K(+57.02)SFNLDEGHALR.I	N	83.09	1442.7266	12	-0.4	481.9159	3	25.70	14	F14:1209	OB5941 H1 raw.raw	2.2174E5	3	3	248	259	Carbamidomethylation (DHKE, X@N-term)	K1:Carbamidomethylation (DHKE, X@N-term):51.63	PEAKS PTM
K.KNPQLQDLDM(+15.99)MLTC(+57.02)VEIK.E	N	81.54	2191.0686	18	0.7	1096.5424	2	33.03	12	F12:1615	OB5939 H1 raw.raw	0	0	0	414	431	Carbamidomethylation	M10:Oxidation (M):14.02;C14:Carbamidomethylation:1000.00	PEAKS DB
K.KNPQLQDLDMMLTC(+57.02)VEIKEGALMLPHFNSK.A	N	81.26	3499.7336	30	-0.1	875.9406	4	36.52	14	F14:1868	OB5941 H1 raw.raw	1.091E7	3	3	414	443	Carbamidomethylation	C14:Carbamidomethylation:1000.00	PEAKS DB
K.GTGNLELVAVRK.E	N	79.30	1255.7249	12	0.9	628.8702	2	26.85	14	F14:1263	OB5941 H1 raw.raw	1.0437E7	9	9	453	464			PEAKS DB
R.GRREQEWEEEEEDEEEEGSNR.E	N	79.15	2650.0649	21	0.3	884.3625	3	25.64	13	F13:1205	OB5940 H1 raw.raw	2.2346E6	7	7	470	490			PEAKS DB
E.PDLSNNFGR.L	N	78.38	1018.4832	9	-0.1	510.2488	2	31.24	14	F14:1545	OB5941 H1 raw.raw	3.5959E6	3	3	397	405			PEAKS DB
R.EETSRNNPFYFPSRR.F	N	78.31	1898.9023	15	1.2	633.9755	3	27.17	13	F13:1289	OB5940 H1 raw.raw	6.7264E6	6	6	161	175			PEAKS DB
R.IFLAGDKDNVIDQIE(+57.02)K.Q	N	78.23	1873.9785	16	-5.0	937.9919	2	32.19	14	F14:1583	OB5941 H1 raw.raw	3.036E7	3	3	536	551		E15:Carbamidomethylation (DHKE, X@N-term):0.00	PEAKS PTM
I.PSGFISYILNR.H	N	77.77	1265.6768	11	2.4	633.8472	2	36.17	13	F13:1844	OB5940 H1 raw.raw	2.5204E5	2	2	261	271			PEAKS DB
I.FLAGDKDNVIDQIEK.Q	N	77.74	1703.8729	15	-9.3	852.9358	2	32.01	14	F14:1599	OB5941 H1 raw.raw	1.3574E6	3	3	537	551			PEAKS DB
K.D(+57.02)LAFPGSGEQVEK.L	N	77.21	1432.6833	13	3.8	717.3517	2	30.92	12	F12:1485	OB5939 H1 raw.raw	2.816E5	1	1	555	567	Carbamidomethylation (DHKE, X@N-term)	D1:Carbamidomethylation (DHKE,	PEAKS PTM

total 219 peptides

Peptide	Uniq	-10lgP	Mass	Length	ppm	m/z	z	RT	Fraction	Scan	Source File	Area Raw in gel	#Feature	#Feature Raw in gel	Start	End	PTM	AScore	Found By
																		X@N-ter m):93.18	
R.SSDNEGVIVK.V	N	76.72	1046.5244	10	0.3	524.2697	2	22.95	13	F13:1057	OB5940 H1 raw.raw	2.6758E5	6	6	351	360			PEAKS DB
K.Q(-17.03)FQNLQNHR.I	N	76.65	1166.5581	9	-1.5	584.2855	2	27.77	14	F14:1319	OB5941 H1 raw.raw	3.3996E6	3	3	199	207	Pyro-glu from Q	Q1:Pyro-glu from Q:1000.00	PEAKS PTM
K.HAD(+57.02)ADNILVIQQGQATVTVAN(+.98)GNNRK.S	N	74.85	2804.4165	26	-5.0	935.8081	3	28.96	13	F13:1511	OB5940 H1 raw.raw	3.7976E6	1	1	223	248		D3:Carbamidomethylation (DHKE, X@N-term):6.59;N21:Deamidation (NQ):14.04	PEAKS PTM
K.HADADNILVIQQGQATVTVANGN(-17.03)NRK.S	N	74.65	2729.3845	26	-3.3	1365.6951	2	29.38	14	F14:1432	OB5941 H1 raw.raw	5.4978E5	1	1	223	248		N23:Ammonia-loss (N):0.00	PEAKS PTM
K.ISMPVNTPGQFEDFFPASSRDQSSYLQGFSR.N	N	74.49	3494.6201	31	2.7	1165.8838	3	36.29	2	F2:1620	OB5925 H3B raw.raw	9.3886E7	15	15	282	312			PEAKS DB
R.NTLEAAFNAEFN(+.98)EIRR.V	N	74.39	1894.9172	16	-3.7	948.4624	2	32.73	14	F14:1640	OB5941 H1 raw.raw	4.113E6	3	3	313	328	Deamidation (NQ)	N12:Deamidation (NQ):55.21	PEAKS DB
R.LFEVKPDKKNPQLQDLDMMLTC(+57.02)VEIK.E	N	73.38	3131.6069	26	3.7	1044.8801	3	33.97	13	F13:1716	OB5940 H1 raw.raw	3.7487E6	3	3	406	431	Carbamidomethylation	C22:Carbamidomethylation:1000.00	PEAKS DB
K.NPQLQDLDMM(+15.99)LTC(+57.02)VEIKEGALMLPHFNSK.A	N	73.21	3387.6335	29	5.6	847.9204	4	35.82	13	F13:1825	OB5940 H1 raw.raw	9.6415E6	3	3	415	443	Carbamidomethylation	M10:Oxidation (M):16.90;C13:Carbamidomethylation:1000.00	PEAKS DB
A.GDKDNVIDQIEK.Q	N	73.04	1372.6833	12	-2.8	687.3470	2	32.19	12	F12:1561	OB5939 H1 raw.raw	1.4449E6	3	3	540	551			PEAKS DB
R.V(+57.02)LLEENAGGEQEER.G	N	73.00	1628.7642	14	1.1	815.3903	2	27.03	14	F14:1288	OB5941 H1 raw.raw	2.7852E5	3	3	329	342		V1:Carbamidomethylation (DHKE, X@N-term):44.44	PEAKS PTM
R.EETSRNNPFYFPSR.R	N	72.75	1742.8011	14	-0.9	581.9405	3	29.89	12	F12:1419	OB5939 H1 raw.raw	4.0306E4	1	1	161	174			PEAKS DB
R.IPSGFISYILN(-17.03)R.H	N	72.57	1361.7343	12	1.4	681.8754	2	37.47	13	F13:1926	OB5940 H1 raw.raw	5.3561E5	2	2	260	271	Ammonia-loss (N)	N11:Ammonia-loss (N):1000.00	PEAKS PTM
R.IPSGFISY(+125.90)ILNR.H	N	72.49	1504.6575	12	1.5	753.3372	2	37.84	13	F13:1946	OB5940 H1 raw.raw	9.8379E5	2	2	260	271	Iodination	Y8:Iodination:1000.00	PEAKS PTM
R.IPSGFISYILNRH(+57.02)DNQNLR.V	N	72.20	2313.1978	19	2.0	579.3079	4	33.29	12	F12:1633	OB5939 H1 raw.raw	3.81E7	4	4	260	278		H13:Carbamidomethylation (DHKE, X@N-term):9.34	PEAKS PTM
R.VLLEEN(+.98)AGGEQEER.G	N	71.59	1572.7267	14	-3.7	787.3677	2	26.82	13	F13:1276	OB5940 H1 raw.raw	0	0	0	329	342	Deamidation (NQ)	N6:Deamidation (NQ):71.64	PEAKS DB
A.FPGSGEQVEK.L	N	71.45	1076.5138	10	5.5	539.2672	2	30.23	12	F12:1449	OB5939 H1 raw.raw	6.1112E4	1	1	558	567			PEAKS DB
K.A(+57.02)MVIVVVNKGTGNLELVAVRK.E	N	70.77	2266.3196	21	-1.6	1134.1653	2	32.33	13	F13:1602	OB5940 H1 raw.raw	4.861E7	6	6	444	464	Carbamidomethylation (DHKE, X@N-term)	A1:Carbamidomethylation (DHKE, X@N-term):161.20	PEAKS PTM
**total 219 peptides**																			

Peptide	Uniq	-10lgP	Mass	Length	ppm	m/z	z	RT	Fraction	Scan	Source File	Area Raw in gel	#Feature	#Feature Raw in gel	Start	End	PTM	AScore	Found By
K.AM(+15.99)VIVVVNK.G	N	70.64	987.5787	9	0.7	494.7969	2	26.66	14	F14:1257	OB5941 H1 raw.raw	8.6431E5	3	3	444	452	Oxidation (M)	M2:Oxidation (M):1000.00	PEAKS DB
K.KNPQLQDLDMMLTC(+57.02)VEIKEGALM(+15.99)LPHFNSK.A	N	70.55	3515.7285	30	-0.8	879.9387	4	35.84	12	F12:1790	OB5939 H1 raw.raw	4.7958E6	2	2	414	443	Carbamidomethylation; Oxidation (M)	C14:Carbamidomethylation:1000.00;M23:Oxidation (M):69.38	PEAKS DB
K.SVSKKGSEEEDITNPINLR.D	N	69.76	2115.0808	19	2.6	706.0361	3	27.22	14	F14:1299	OB5941 H1 raw.raw	5.5052E4	1	1	375	393			PEAKS DB
Q.GQATVTVAN(+.98)GNNRK.S	N	69.60	1429.7273	14	-4.1	715.8680	2	29.38	14	F14:1424	OB5941 H1 raw.raw	5.134E5	2	2	235	248		N9:Deamidation (NQ):41.83	PEAKS DB
R.NT(-18.01)LEAAFNAEFNEIRR.V	N	68.32	1875.9227	16	-3.2	626.3129	3	33.81	14	F14:1704	OB5941 H1 raw.raw	1.3248E7	3	3	313	328	Dehydration	T2:Dehydration:1000.00	PEAKS PTM
G.SEEEDITNPINLRDGEPDLSNNFGR.L	N	67.86	2830.3005	25	1.2	944.4419	3	32.96	8	F8:1576	OB5935 H3B raw.raw	5.6172E5	3	3	381	405			PEAKS DB
R.IVQIEARPNTLVLPK.H	Y	67.84	1690.0140	15	2.2	846.0161	2	30.65	7	F7:1446	OB5934 H3B raw.raw	2.806E8	26	26	208	222			PEAKS DB
R.LFEVKPDKKNPQLQDLDM(+15.99)M(+15.99)LTC(+57.02)VEIK.E	N	67.75	3163.5967	26	2.1	791.9081	4	32.37	12	F12:1585	OB5939 H1 raw.raw	6.2675E5	1	1	406	431	Oxidation (M); Carbamidomethylation	M18:Oxidation (M):1000.00;M19:Oxidation (M):1000.00;C22:Carbamidomethylation:1000.00	PEAKS DB
L.AGDKDNVIDQIEK.Q	N	66.85	1443.7205	13	-3.1	722.8653	2	32.19	12	F12:1560	OB5939 H1 raw.raw	7.0907E5	2	2	539	551			PEAKS DB
K.G(+57.02)TGNLELVAVRK.E	N	66.68	1312.7462	12	-0.7	657.3799	2	26.85	14	F14:1280	OB5941 H1 raw.raw	3.417E5	3	3	453	464	Carbamidomethylation (DHKE, X@N-term)	G1:Carbamidomethylation (DHKE, X@N-term):56.51	PEAKS PTM
R.EGEQEWGTP(+15.99)GSEVREETSR.N	Y	66.56	2177.9460	19	1.3	726.9902	3	26.28	14	F14:1241	OB5941 H1 raw.raw	4.0056E6	2	2	147	165		P9:Oxidation or Hydroxylation:36.47	PEAKS PTM
K.SFNLDEGHALRIPSGFISYILNRHDNQNLR.V	N	66.45	3495.7759	30	-1.2	700.1616	5	36.31	13	F13:1855	OB5940 H1 raw.raw	1.2605E8	16	16	249	278			PEAKS DB
K.QFQNLQNHR.I	N	66.40	1183.5846	9	0.2	592.7997	2	24.53	14	F14:1134	OB5941 H1 raw.raw	7.3427E4	5	5	199	207			PEAKS DB
D.KDNVIDQIEK.Q	N	66.36	1200.6350	10	-0.8	601.3243	2	25.89	14	F14:1225	OB5941 H1 raw.raw	5.8609E4	3	3	542	551			PEAKS DB
K.NPQLQ(+.98)DLDMMLTC(+57.02)VEIKEGALMLPHFNSK.A	N	66.16	3372.6226	29	5.7	1125.2212	3	37.84	13	F13:1936	OB5940 H1 raw.raw	5.3127E7	3	3	415	443	Carbamidomethylation	Q5:Deamidation (NQ):32.28;C13:Carbamidomethylation:1000.00	PEAKS DB
K.HADADNILVIQQGQATVTVANGNN(+.98)RK.S	N	65.93	2747.3950	26	4.5	1374.7109	2	29.70	10	F10:1444	OB5937 H3A raw.raw	5.6818E5	3	3	223	248		N24:Deamidation (NQ):14.02	PEAKS DB
K.ISMPVNTPGQ(+.98)FEDFFPASSR.D	N	65.70	2227.0254	20	6.4	1114.5271	2	63.55	14	F14:3427	OB5941 H1 raw.raw	1.0155E4	2	2	282	301		Q10:Deamidation (NQ):33.26	PEAKS DB
R.N(+.98)TLEAAFNAEFNEIRR.V	N	65.61	1894.9172	16	2.2	948.4680	2	35.88	12	F12:1787	OB5939 H1 raw.raw	7.0859E7	1	1	313	328	Deamidation (NQ)	N1:Deamidation (NQ):61.82	PEAKS DB
**total 219 peptides**																			

Peptide	Uniq	-10lgP	Mass	Length	ppm	m/z	z	RT	Fraction	Scan	Source File	Area Raw in gel	#Feature	#Feature Raw in gel	Start	End	PTM	AScore	Found By
K.KNPQLQDLDM(+15.99)MLTC(+57.02)VEIKEGALMLPHFNSK.A	N	65.47	3515.7285	30	-3.1	879.9366	4	35.44	13	F13:1830	OB5940 H1 raw.raw	7.2415E6	3	3	414	443	Carbamidomethylation	M10:Oxidation (M):11.06;C14:Carbamidomethylation:1000.00	PEAKS DB
K.ISM(+15.99)PVNTPGQ(+.98)FEDFFPASSRDQSSYLQGFSR.N	N	65.25	3511.5989	31	4.3	1171.5453	3	34.92	12	F12:1731	OB5939 H1 raw.raw	1.4851E6	1	1	282	312	Oxidation (M)	M3:Oxidation (M):1000.00;Q10:Deamidation (NQ):27.62	PEAKS DB
K.NPQLQDLDM(+15.99)MLTC(+57.02)VEIKEGALM(+15.99)LPHFNSK.A	N	64.53	3403.6284	29	4.5	1135.5552	3	36.52	14	F14:1873	OB5941 H1 raw.raw	4.3451E6	3	3	415	443	Carbamidomethylation; Oxidation (M)	M9:Oxidation (M):10.11;C13:Carbamidomethylation:1000.00;M22:Oxidation (M):53.09	PEAKS DB
R.LFEVKPDK.K	N	63.66	974.5436	8	0.9	488.2795	2	25.40	12	F12:1153	OB5939 H1 raw.raw	6.8606E5	7	7	406	413			PEAKS DB
F.NLDEGHALR.I	N	63.51	1023.5097	9	2.3	512.7633	2	27.35	13	F13:1309	OB5940 H1 raw.raw	1.5878E6	3	3	251	259			PEAKS DB
R.WGPAEPR.E	Y	63.43	811.3976	7	1.2	406.7066	2	25.31	14	F14:1167	OB5941 H1 raw.raw	1.2167E6	5	5	109	115			PEAKS DB
R.EQEWEEEEEDEEEEGSNREVR.R	N	63.32	2665.0535	21	2.4	889.3606	3	27.87	12	F12:1308	OB5939 H1 raw.raw	4.1617E5	2	2	473	493			PEAKS DB
R.REQEWEEEEEDEEEEGSNR.E	N	63.17	2436.9424	19	2.8	1219.4819	2	26.47	14	F14:1258	OB5941 H1 raw.raw	2.6135E4	2	2	472	490			PEAKS DB
K.ISM(-48.00)PVNTPGQFEDFFPASSR.D	N	62.93	2178.0381	20	0.2	727.0201	3	32.37	12	F12:1577	OB5939 H1 raw.raw	2.6788E7	5	5	282	301	Dethiomethyl	M3:Dethiomethyl:1000.00	PEAKS PTM
N.TPGQFEDFFPASSR.D	N	62.85	1584.7208	14	-0.2	793.3676	2	35.63	14	F14:1821	OB5941 H1 raw.raw	2.1663E5	1	1	288	301			PEAKS DB
K.A(+57.02)M(+15.99)VIVVVNKGTGNLELVAVR.K	N	62.49	2154.2195	20	-0.1	1078.1169	2	32.92	12	F12:1614	OB5939 H1 raw.raw	4.5509E5	2	2	444	463	Carbamidomethylation (DHKE, X@N-term); Oxidation (M)	A1:Carbamidomethylation (DHKE, X@N-term):139.69;M2:Oxidation (M):1000.00	PEAKS PTM
L.EAAFNAEFNEIRR.V	N	62.30	1565.7585	13	-4.7	783.8829	2	34.06	12	F12:1677	OB5939 H1 raw.raw	6.1232E5	1	1	316	328			PEAKS DB
K.N(+.98)PQLQDLDMMLTC(+57.02)VEIKEGALMLPHFNSK.A	N	61.86	3372.6226	29	5.7	1125.2212	3	37.84	13	F13:1983	OB5940 H1 raw.raw	1.2903E7	1	1	415	443	Carbamidomethylation	N1:Deamidation (NQ):14.04;C13:Carbamidomethylation:1000.00	PEAKS DB
K.HADADNILVIQQGQ(+.98)ATVTVAN(+.98)GNNRK.S	N	61.75	2748.3792	26	1.4	1375.1987	2	29.55	13	F13:1443	OB5940 H1 raw.raw	4.856E5	1	1	223	248		Q14:Deamidation (NQ):32.28;N21:Deamidation (NQ):14.04	PEAKS DB
N.LDEGHALR.I	N	60.44	909.4668	8	0.6	455.7409	2	27.35	13	F13:1312	OB5940 H1 raw.raw	3.1152E4	1	1	252	259			PEAKS DB
R.IFLAGDKDNVIDQ(+.98)IEKQAK.D	N	58.72	2145.1316	19	9.3	716.0578	3	32.93	2	F2:1432	OB5925 H3B raw.raw	1.5596E6	1	1	536	554		Q13:Deamidation (NQ):0.00	PEAKS DB
K.HADADNILVIQ(+.98)Q(+.98)GQATVTVAN(+.98)GNNRK.S	N	58.62	2749.3630	26	9.6	917.4705	3	30.18	13	F13:1524	OB5940 H1 raw.raw	4.3812E6	1	1	223	248		Q11:Deamidation (NQ):26.52;Q	PEAKS DB
**total 219 peptides**																			

Peptide	Uniq	-10lgP	Mass	Length	ppm	m/z	z	RT	Fraction	Scan	Source File	Area Raw in gel	#Feature	#Feature Raw in gel	Start	End	PTM	AScore	Found By
																		12:Deamidation (NQ):18.53;N21:Deamidation (NQ):14.04	
R.D(+57.02)QSSYLQGFSR.N	N	58.49	1343.6106	11	-2.5	672.8109	2	31.19	12	F12:1506	OB5939 H1 raw.raw	3.7713E4	1	1	302	312	Carbamidomethylation (DHKE, X@N-term)	D1:Carbamidomethylation (DHKE, X@N-term):1000.00	PEAKS PTM
K.HADADNILVIQ(+.98)QGQATVTVAN(+.98)GNNRK.S	N	58.29	2748.3792	26	4.6	1375.2031	2	31.28	12	F12:1539	OB5939 H1 raw.raw	1.8636E5	1	1	223	248		Q11:Deamidation (NQ):15.10;N21:Deamidation (NQ):17.01	PEAKS DB
K.HADADN(+.98)ILVIQ(+.98)QGQATVTVANGNNRK.S	N	57.75	2748.3792	26	3.1	1375.2010	2	30.08	14	F14:1495	OB5941 H1 raw.raw	1.5756E6	1	1	223	248		N6:Deamidation (NQ):0.00;Q11:Deamidation (NQ):0.00	PEAKS DB
N.PFYFPSRR.F	N	57.72	1068.5504	8	3.3	535.2842	2	28.95	12	F12:1360	OB5939 H1 raw.raw	3.0845E6	3	3	168	175			PEAKS DB
R.LFEVKPDKKNPQLQDLDMM(+15.99)LTC(+57.02)VEIK.E	N	57.62	3147.6018	26	-4.4	787.9042	4	32.55	14	F14:1633	OB5941 H1 raw.raw	2.1102E6	3	3	406	431	Carbamidomethylation	M19:Oxidation (M):17.01;C22:Carbamidomethylation:1000.00	PEAKS DB
K.SFN(+.98)LDEGHALRIPSGFISYILNR.H	N	57.28	2619.3445	23	9.3	655.8495	4	38.22	12	F12:1947	OB5939 H1 raw.raw	1.0265E6	2	2	249	271		N3:Deamidation (NQ):32.08	PEAKS DB
K.AMVIVVVN(+.98)KGTGNLELVAVRK.E	N	56.84	2210.2820	21	9.6	737.7750	3	33.10	14	F14:1664	OB5941 H1 raw.raw	8.0625E6	2	2	444	464		N8:Deamidation (NQ):41.87	PEAKS DB
K.KNPQLQDLDMM(-48.00)LTC(+57.02)VEIK.E	N	56.43	2127.0703	18	0.8	710.0312	3	31.11	14	F14:1538	OB5941 H1 raw.raw	3.3192E6	2	2	414	431	Carbamidomethylation	M11:Dethiomethyl:14.02;C14:Carbamidomethylation:1000.00	PEAKS PTM
K.ISM(+15.99)PVNTPGQFEDFFPASSRDQSSYLQGFSR.N	N	56.19	3510.6150	31	2.9	1171.2157	3	34.90	14	F14:1765	OB5941 H1 raw.raw	1.0055E7	8	8	282	312	Oxidation (M)	M3:Oxidation (M):1000.00	PEAKS DB
V.QIEARPNTLVLPK.H	Y	55.52	1477.8616	13	-3.7	739.9353	2	30.24	14	F14:1485	OB5941 H1 raw.raw	9.708E5	3	3	210	222			PEAKS DB
R.N(+57.02)NPFYFPSRR.F	N	55.47	1353.6577	10	-2.6	677.8344	2	29.38	14	F14:1433	OB5941 H1 raw.raw	6.1234E5	2	2	166	175	Carbamidomethylation (DHKE, X@N-term)	N1:Carbamidomethylation (DHKE, X@N-term):1000.00	PEAKS PTM
R.SKQFQNLQNHR.I	Y	55.18	1398.7117	11	1.0	467.2450	3	24.31	13	F13:1132	OB5940 H1 raw.raw	2.6796E4	3	3	197	207			PEAKS DB
K.HADADNILVIQQGQATVTVANGN(+.98)NRK.S	N	55.13	2747.3950	26	4.2	687.8589	4	29.51	12	F12:1401	OB5939 H1 raw.raw	2.4633E7	5	5	223	248		N23:Deamidation (NQ):17.01	PEAKS DB
K.HADADNILVIQQ(+.98)GQ(+.98)ATVTVAN(+.98)GNNRK.S	N	55.12	2749.3630	26	9.6	917.4705	3	30.18	13	F13:1487	OB5940 H1 raw.raw	4.3812E6	1	1	223	248		Q12:Deamidation (NQ):14.02;Q14:Deamidation (NQ):13.03;N21:Deamidation (NQ):11.10	PEAKS DB
K.AM(-48.00)VIVVVNKGTGNLELVAVRK.E	N	54.93	2161.2947	21	1.4	1081.6561	2	29.80	14	F14:1453	OB5941 H1 raw.raw	1.7581E6	2	2	444	464	Dethiomethyl	M2:Dethiomethyl:1000.00	PEAKS PTM
**total 219 peptides**																			

Peptide	Uniq	-10lgP	Mass	Length	ppm	m/z	z	RT	Fraction	Scan	Source File	Area Raw in gel	#Feature	#Feature Raw in gel	Start	End	PTM	AScore	Found By
R.I(+57.02)VQIEARPNTLVLPK.H	Y	54.55	1747.0355	15	3.8	874.5283	2	30.92	12	F12:1489	OB5939 H1 raw.raw	3.7453E7	4	4	208	222	Carbamidomethylation (DHKE, X@N-term)	I1:Carbamidomethylation (DHKE, X@N-term):97.69	PEAKS PTM
K.NPQLQDLDM(+15.99)MLTC(+57.02)VEIKEGALMLPHFN(+.98)SK.A	N	54.06	3388.6174	29	5.0	848.1659	4	36.17	14	F14:1849	OB5941 H1 raw.raw	6.0156E6	2	2	415	443	Carbamidomethylation; Deamidation (NQ)	M9:Oxidation (M):14.02;C13:Carbamidomethylation:1000.00;N27:Deamidation (NQ):58.23	PEAKS DB
K.IRPEGREGEQEWGTPGSEVREETSR.N	Y	53.77	2870.3542	25	1.1	718.5966	4	27.49	12	F12:1279	OB5939 H1 raw.raw	3.216E6	3	3	141	165			PEAKS DB
R.IFLAGDKDNVIDQIE(+57.02)KQAK.D	N	52.99	2201.1692	19	-2.0	734.7289	3	31.83	13	F13:1588	OB5940 H1 raw.raw	3.3379E6	2	2	536	554		E15:Carbamidomethylation (DHKE, X@N-term):12.28	PEAKS PTM
K.NPQLQDLDMMLTC(+57.02)VEIKEGALMLPHFN(+.98)SK.A	N	52.89	3372.6226	29	7.4	844.1691	4	37.85	13	F13:1914	OB5940 H1 raw.raw	5.2564E7	2	2	415	443	Carbamidomethylation; Deamidation (NQ)	C13:Carbamidomethylation:1000.00;N27:Deamidation (NQ):61.84	PEAKS DB
K.HADADNILVIQQGQ(+.98)ATVTVANGNNRK.S	N	52.87	2747.3950	26	5.1	916.8103	3	44.47	12	F12:2283	OB5939 H1 raw.raw	1.466E5	1	1	223	248		Q14:Deamidation (NQ):14.04	PEAKS DB
K.NPQLQDLDM(+15.99)M(+15.99)LTC(+57.02)VEIKEGALMLPHFNSK.A	N	52.70	3403.6284	29	-4.3	1135.5452	3	35.63	14	F14:1818	OB5941 H1 raw.raw	1.9434E6	2	2	415	443	Oxidation (M); Carbamidomethylation	M9:Oxidation (M):49.75;M10:Oxidation (M):51.99;C13:Carbamidomethylation:1000.00	PEAKS DB
L.DEGHALR.I	N	52.68	796.3828	7	2.3	399.1996	2	27.87	12	F12:1309	OB5939 H1 raw.raw	2.418E4	1	1	253	259			PEAKS DB
K.DLAFPGSGEQVEK(+57.02)LIK.N	N	52.57	1786.9464	16	-3.8	894.4771	2	32.91	14	F14:1654	OB5941 H1 raw.raw	6.7177E5	1	1	555	570		K13:Carbamidomethylation (DHKE, X@N-term):27.96	PEAKS PTM
K.KN(+.98)PQLQDLDMM(+15.99)LTC(+57.02)VEIK.E	N	52.31	2192.0527	18	9.2	1097.0437	2	32.33	13	F13:1621	OB5940 H1 raw.raw	8.5775E4	1	1	414	431	Carbamidomethylation	N2:Deamidation (NQ):14.04;M11:Oxidation (M):0.00;C14:Carbamidomethylation:1000.00	PEAKS DB
Q.GQATVTVANGNNRK.S	N	51.97	1428.7433	14	-0.2	715.3788	2	29.02	14	F14:1412	OB5941 H1 raw.raw	1.2099E5	2	2	235	248			PEAKS DB
K.EGALMLPH(+14.02)FNSK.A	N	51.73	1356.6860	12	1.3	679.3512	2	30.49	14	F14:1499	OB5941 H1 raw.raw	2.5741E4	1	1	432	443	Methylation(others)	H8:Methylation(others):53.53	PEAKS PTM
K.N(+.98)PQLQ(+.98)DLDMMLTC(+57.02)VEIKEGALM(+15.99)LPHFNSK.A	N	51.60	3389.6016	29	9.0	848.4153	4	36.92	12	F12:1868	OB5939 H1 raw.raw	2.6873E6	1	1	415	443	Carbamidomethylation	N1:Deamidation (NQ):0.00;Q5:Deamidation (NQ):14.04;C13:Carbamidomethylation:1000.00;M22:Oxidation (M):18.84	PEAKS DB

total 219 peptides

Peptide	Uniq	-10lgP	Mass	Length	ppm	m/z	z	RT	Fraction	Scan	Source File	Area Raw in gel	#Feature	#Feature Raw in gel	Start	End	PTM	AScore	Found By
R.LFEVKPDKK.N	N	50.85	1102.6385	9	1.1	552.3271	2	24.94	14	F14:1179	OB5941 H1 raw.raw	2.3081E5	2	2	406	414			PEAKS DB
R.RVLLEENAGGEQEERGQR.R	N	50.67	2069.0249	18	2.1	690.6837	3	24.88	13	F13:1165	OB5940 H1 raw.raw	2.7227E5	1	1	328	345			PEAKS DB
K.N(+.98)PQLQDLDMMLTC(+57.02)VEIKEGALM(+15.99)LPHFNSK.A	N	50.17	3388.6174	29	-2.4	1130.5437	3	36.35	13	F13:1874	OB5940 H1 raw.raw	8.9006E5	1	1	415	443	Carbamidomethylation; Oxidation (M)	N1:Deamidation (NQ):0.00;C13:Carbamidomethylation:1000.00;M22:Oxidation (M):54.40	PEAKS DB
K.NPQLQDLDMM(+15.99)LTC(+57.02)VEIKEGALM(+15.99)LPHFNSK.A	N	48.97	3403.6284	29	2.7	1135.5531	3	35.09	14	F14:1786	OB5941 H1 raw.raw	3.2001E5	1	1	415	443	Carbamidomethylation	M10:Oxidation (M):12.28;C13:Carbamidomethylation:1000.00;M22:Oxidation (M):28.29	PEAKS DB
R.EQEWEEEEEDEEEEGSNREVRR.Y	N	48.85	2821.1545	22	1.8	941.3939	3	26.75	12	F12:1227	OB5939 H1 raw.raw	3.3887E6	3	3	473	494			PEAKS DB
K.KN(+.98)PQLQDLDMMLTC(+57.02)VEIKEGALMLPHFNSK.A	N	48.84	3500.7175	30	7.1	876.1929	4	36.26	13	F13:1847	OB5940 H1 raw.raw	2.2113E6	1	1	414	443	Carbamidomethylation	N2:Deamidation (NQ):0.00;C14:Carbamidomethylation:1000.00	PEAKS DB
R.IFLAGDKDNVIDQIEK(+57.02)QAK.D	N	48.82	2201.1692	19	2.7	734.7323	3	32.55	12	F12:1581	OB5939 H1 raw.raw	3.2958E6	1	1	536	554		K16:Carbamidomethylation (DHKE, X@N-term):15.91	PEAKS PTM
R.EREEDWRQPR.E	N	48.49	1399.6592	10	0.1	467.5604	3	24.77	14	F14:1154	OB5941 H1 raw.raw	2.5247E4	1	1	118	127			PEAKS DB
R.KSFN(+.98)LDEGHALRIPSGFISYILNR.H	N	47.51	2747.4395	24	4.5	687.8702	4	36.15	14	F14:1837	OB5941 H1 raw.raw	1.0524E6	1	1	248	271		N4:Deamidation (NQ):23.98	PEAKS DB
R.LFEVKPDKKN(+.98)PQLQDLDMMLTC(+57.02)VEIK.E	N	47.32	3132.5908	26	9.3	1567.3173	2	34.35	14	F14:1745	OB5941 H1 raw.raw	1.9315E5	1	1	406	431	Carbamidomethylation	N10:Deamidation (NQ):14.04;C22:Carbamidomethylation:1000.00	PEAKS DB
K.N(+.98)PQ(+.98)LQDLDMMLTC(+57.02)VEIKEGALM(+15.99)LPHFNSK.A	N	46.82	3389.6016	29	9.0	848.4153	4	36.92	12	F12:1851	OB5939 H1 raw.raw	7.3613E6	2	2	415	443	Carbamidomethylation	N1:Deamidation (NQ):11.12;Q3:Deamidation (NQ):0.00;C13:Carbamidomethylation:1000.00;M22:Oxidation (M):24.93	PEAKS DB
W.GTPGSEVREETSR.N	Y	46.79	1403.6641	13	3.5	702.8417	2	27.60	14	F14:1327	OB5941 H1 raw.raw	5.9917E4	1	1	153	165			PEAKS DB
N.PQLQDLDMMLTC(+57.02)VEIKEGALMLPHFNSK.A	N	46.63	3257.5957	28	0.9	815.4069	4	37.83	14	F14:1952	OB5941 H1 raw.raw	2.3692E5	1	1	416	443	Carbamidomethylation	C12:Carbamidomethylation:1000.00	PEAKS DB
K.Q(-17.03)AKDLAFPGSGEQVEK.L	N	46.49	1685.8260	16	0.9	843.9211	2	29.97	12	F12:1427	OB5939 H1 raw.raw	0	0	0	552	567	Pyro-glu from Q	Q1:Pyro-glu from Q:1000.00	PEAKS PTM
K.SFN(+.98)LDEGHALRIPSGFISYILNRHDNQNLR.V	N	46.45	3496.7600	30	-3.9	700.3566	5	36.38	12	F12:1849	OB5939 H1 raw.raw	6.986E5	1	1	249	278		N3:Deamidation (NQ):20.27	PEAKS DB

total 219 peptides

Peptide	Uniq	-10lgP	Mass	Length	ppm	m/z	z	RT	Fraction	Scan	Source File	Area Raw in gel	#Feature	#Feature Raw in gel	Start	End	PTM	AScore	Found By
R.REQ(+.98)EWEEEEEDEEEEGSNR.E	N	45.55	2437.9265	19	7.6	813.6556	3	26.55	12	F12:1226	OB5939 H1 raw.raw	4.2319E4	1	1	472	490		Q3:Deamidation (NQ): 42.90	PEAKS DB
T.PGQFEDFFPASSR.D	N	45.25	1483.6731	13	-1.3	742.8429	2	40.34	13	F13:2103	OB5940 H1 raw.raw	4.0298E3	1	1	289	301			PEAKS DB
R.IPSGFISYILN(+.98)RHDNQNLR.V	N	45.21	2257.1604	19	9.9	753.4015	3	37.61	2	F2:1698	OB5925 H3B raw.raw	2.0603E6	2	2	260	278		N11:Deamidation (N Q):33.06	PEAKS DB
T.PGSEVREETSR.N	Y	45.10	1245.5948	11	1.9	623.8059	2	27.69	12	F12:1287	OB5939 H1 raw.raw	4.1909E4	1	1	155	165			PEAKS DB
N.NPFYFPSRR.F	N	45.02	1182.5934	9	1.6	395.2057	3	28.08	13	F13:1358	OB5940 H1 raw.raw	2.9991E4	1	1	167	175			PEAKS DB
R.DQS(-18.01)SYLQGFSR.N	N	44.90	1268.5785	11	7.0	635.3010	2	30.87	12	F12:1484	OB5939 H1 raw.raw	0	0	0	302	312		S3:Dehydration:33.98	PEAKS PTM
K.GTGNLELVAVRKEQQQR.G	N	44.58	1925.0442	17	0.6	642.6890	3	25.89	14	F14:1220	OB5941 H1 raw.raw	1.1112E5	2	2	453	469			PEAKS DB
R.EDWRRPSHQQPR.K	N	43.98	1590.7764	12	-0.3	398.7012	4	24.94	14	F14:1169	OB5941 H1 raw.raw	1.0819E4	1	1	128	139			PEAKS DB
R.IVQIEARPNTLVLPK(+57.02).H	Y	43.35	1747.0355	15	-2.4	874.5229	2	30.35	13	F13:1460	OB5940 H1 raw.raw	8.446E6	1	1	208	222	Carbamidomethylation (DHKE, X@N-term)	K15:Carbamidomethylation (DHKE, X@N-term):110.46	PEAKS PTM
R.VLLE(+21.98)ENAGGEQEER.G	N	42.61	1593.7246	14	-0.7	797.8690	2	26.03	13	F13:1239	OB5940 H1 raw.raw	1.373E5	3	3	329	342		E4:Sodium adduct:40.00	PEAKS PTM
K.HADADNILVIQ(+.98)QGQATVTVANGNNRK.S	N	42.22	2747.3950	26	5.9	916.8110	3	45.04	14	F14:2357	OB5941 H1 raw.raw	0	0	0	223	248		Q11:Deamidation (N Q):9.34	PEAKS DB
K.IRPEGREGEQEWGTPGSEVR.E	Y	42.02	2268.0881	20	1.1	757.0375	3	26.28	14	F14:1248	OB5941 H1 raw.raw	5.4096E4	1	1	141	160			PEAKS DB
K.HADADNILVIQQGQAT(-18.01)VTVAN(+.98)GNNRK.S	N	41.92	2729.3845	26	0.3	683.3536	4	28.96	13	F13:1411	OB5940 H1 raw.raw	3.5545E5	1	1	223	248		T16:Dehydration:8.22; N21:Deamidation (N Q):6.08	PEAKS PTM
K.HADADN(+.98)ILVIQQGQATVTVANGNNRK.S	N	41.76	2747.3950	26	3.7	916.8090	3	50.62	12	F12:2616	OB5939 H1 raw.raw	1.1355E5	2	2	223	248		N6:Deamidation (NQ): 42.57	PEAKS DB
K.ISMPVN(+.98)TPGQFEDFFPASSR.D	N	41.75	2227.0254	20	9.3	1114.5303	2	58.23	14	F14:3138	OB5941 H1 raw.raw	2.4983E4	1	1	282	301		N6:Deamidation (NQ): 42.89	PEAKS DB
M.LPHFNSK.A	N	41.73	841.4446	7	-3.4	421.7281	2	30.24	14	F14:1484	OB5941 H1 raw.raw	1.8069E6	2	2	437	443			PEAKS DB
R.PNTLVLPK.H	N	41.66	880.5382	8	0.4	441.2766	2	27.53	13	F13:1313	OB5940 H1 raw.raw	8.5249E4	1	1	215	222			PEAKS DB
N.TLEAAFNAEFNEIRR.V	N	41.51	1779.8904	15	-3.0	890.9498	2	32.91	14	F14:1655	OB5941 H1 raw.raw	4.1608E5	1	1	314	328			PEAKS DB
K.KGSEEEDITN(-17.03)PINLR.D	N	41.49	1696.8268	15	3.3	566.6180	3	28.85	14	F14:1397	OB5941 H1 raw.raw	2.3089E5	1	1	379	393		N10:Ammonia-loss (N):40.63	PEAKS PTM
K.KNPQLQ(+.98)DLDMMLTC(+57.02)VEIKEGALMLPHFNSK.A	N	40.89	3500.7175	30	0.4	1167.9136	3	36.34	13	F13:1850	OB5940 H1 raw.raw	4.7608E5	1	1	414	443	Carbamidomethylation	Q6:Deamidation (NQ): 14.04;C14: Carbamidomethylation:1000.00	PEAKS DB
R.REQEWEEEEEDEEEEGSNREVR.R	N	40.76	2821.1545	22	3.9	941.3958	3	26.47	14	F14:1247	OB5941 H1 raw.raw	1.8468E6	1	1	472	493			PEAKS DB
**total 219 peptides**																			

Peptide	Uniq	-10lgP	Mass	Length	ppm	m/z	z	RT	Fraction	Scan	Source File	Area Raw in gel	#Feature	#Feature Raw in gel	Start	End	PTM	AScore	Found By
R.NTLEAAFN(+.98)AEFNEIRR.V	N	40.23	1894.9172	16	-0.3	948.4656	2	32.92	12	F12:1570	OB5939 H1 raw.raw	1.5322E6	1	1	313	328		N8:Deamidation (NQ): 8.26	PEAKS DB
K.NPQLQDLDM(+15.99)M(+15.99)LTC(+57.02)VEIK.E	N	39.57	2078.9688	17	1.1	1040.4928	2	33.80	14	F14:1701	OB5941 H1 raw.raw	0	0	0	415	431	Oxidation (M); Carbamidomethylation	M9:Oxidation (M):1000.00;M10: Oxidation (M):1000.00;C13:Carbamidomethylation:1000.00	PEAKS DB
R.IPS(+59.02)GFISYILNR.H	N	39.40	1437.7802	12	1.5	719.8984	2	36.96	12	F12:1870	OB5939 H1 raw.raw	4.3694E5	1	1	260	271		S3:Aminoethylcysteine:49.79	PEAKS PTM
K.HADADNILVIQQ(-18.01).G	N	38.68	1317.6677	12	-4.9	659.8379	2	29.56	14	F14:1421	OB5941 H1 raw.raw	1.6968E5	2	2	223	234	Dehydration	Q12:Dehydration:76.18	PEAKS PTM
K.AMVIVVVNK(-1.03)GTGNLELVAVR.K	N	37.92	2080.1714	20	4.3	1041.0974	2	33.79	13	F13:1738	OB5940 H1 raw.raw	2.1453E7	1	1	444	463	Lysine oxidation to aminoadipic semialdehyde	K9:Lysine oxidation to aminoadipic semialdehyde:1000.00	PEAKS PTM
I.PSGFISYILNRHDNQNLR.V	N	37.07	2143.0923	18	4.0	715.3742	3	33.53	12	F12:1645	OB5939 H1 raw.raw	0	0	0	261	278			PEAKS DB
K.EGALM(-48.00)LPHFNSK.A	N	36.97	1294.6670	12	2.0	432.5638	3	28.31	14	F14:1348	OB5941 H1 raw.raw	1.3683E6	1	1	432	443	Dethiomethyl	M5:Dethiomethyl:1000.00	PEAKS PTM
R.IFLAGDKDNVIDQ(+.98)IEK.Q	N	36.27	1817.9410	16	7.9	606.9924	3	63.32	14	F14:3434	OB5941 H1 raw.raw	1.3413E4	1	1	536	551		Q13:Deamidation (NQ):25.70	PEAKS DB
K.HAD(+57.02)ADNILVIQQGQATVTVANGN(+.98)NRK.S	N	35.92	2804.4165	26	-3.8	702.1088	4	28.95	13	F13:1413	OB5940 H1 raw.raw	2.7833E5	1	1	223	248		D3:Carbamidomethylation (DHKE, X@N-term):4.64;N23:Deamidation (NQ):0.00	PEAKS PTM
K.NPQ(+.98)LQDLDMMLTC(+57.02)VEIKEGALMLPHFNSK.A	N	35.84	3372.6226	29	9.4	1125.2253	3	40.73	12	F12:2064	OB5939 H1 raw.raw	5.6313E5	1	1	415	443	Carbamidomethylation	Q3:Deamidation (NQ): 0.00;C13:Carbamidomethylation: 1000.00	PEAKS DB
K.ISM(+15.99)PVNTPGQ(+.98)FEDFFPASSR.D	N	35.67	2243.0205	20	6.2	1122.5245	2	50.73	12	F12:2643	OB5939 H1 raw.raw	0	0	0	282	301	Oxidation (M)	M3:Oxidation (M):1000.00;Q10:Deamidation (NQ):22.37	PEAKS DB
K.GT(-18.01)GNLELVAVRK.E	N	35.64	1237.7142	12	0.9	413.5790	3	26.41	13	F13:1259	OB5940 H1 raw.raw	7.4255E5	3	3	453	464	Dehydration	T2:Dehydration:1000.00	PEAKS PTM
K.SFNLDEGHALRIPS.G	N	35.38	1554.7791	14	-4.8	778.3931	2	30.79	13	F13:1515	OB5940 H1 raw.raw	5.2113E3	1	1	249	262			PEAKS DB
R.DQ(+.98)SSYLQGFSR.N	N	34.61	1287.5731	11	-3.2	644.7917	2	63.83	14	F14:3449	OB5941 H1 raw.raw	0	0	0	302	312		Q2:Deamidation (NQ): 41.87	PEAKS DB
R.S(+57.02)SDNEGVIVK.V	N	34.54	1103.5459	10	0.3	552.7804	2	23.19	13	F13:1066	OB5940 H1 raw.raw	0	0	0	351	360		S1:Carbamidomethylation (DHKE, X@N-term):11.10	PEAKS PTM
Q.IEARPNTLVLPK.H	Y	34.19	1349.8030	12	2.2	675.9103	2	30.40	12	F12:1461	OB5939 H1 raw.raw	1.011E6	3	3	211	222			PEAKS DB
R.NN(-17.03)PFYFPSRR.F	N	34.10	1279.6097	10	-0.4	427.5437	3	28.25	13	F13:1369	OB5940 H1 raw.raw	1.47E5	1	1	166	175		N2:Ammonia-loss (N): 12.28	PEAKS PTM
**total 219 peptides**																			

Peptide	Uniq	-10lgP	Mass	Length	ppm	m/z	z	RT	Fraction	Scan	Source File	Area Raw in gel	#Feature	#Feature Raw in gel	Start	End	PTM	AScore	Found By
T.G(+214.97)NLELVAVRK.E	N	33.47	1312.6267	10	2.3	657.3221	2	27.95	14	F14:1280	OB5941 H1 raw.raw	2.4669E5	1	1	455	464	4-sulfophenyl isothiocyanate	G1:4-sulfophenyl isothiocyanate:144.45	PEAKS PTM
K.HADADNILVIQQ(+.98)GQATVTVANGNN(+.98)RK.S	N	33.28	2748.3792	26	7.6	917.1406	3	48.20	12	F12:2471	OB5939 H1 raw.raw	2.0672E4	1	1	223	248		Q12:Deamidation (NQ):23.10;N24:Deamidation (NQ):0.00	PEAKS DB
K.HADAD(-18.01)NILVIQQGQATVTVANGNNRK.S	N	33.25	2728.4004	26	3.5	910.4772	3	28.70	13	F13:1390	OB5940 H1 raw.raw	0	0	0	223	248		D5:Dehydration:30.36	PEAKS PTM
K.ISM(+15.99)PVN(+.98)TPGQFEDFFPASSR.D	N	33.05	2243.0205	20	7.6	1122.5260	2	49.72	13	F13:2625	OB5940 H1 raw.raw	0	0	0	282	301	Oxidation (M)	M3:Oxidation (M):1000.00;N6:Deamidation (NQ):25.70	PEAKS DB
R.IFLAGDK(+26.02)DNVIDQIEK.Q	N	32.97	1842.9727	16	-0.2	922.4934	2	35.63	14	F14:1844	OB5941 H1 raw.raw	1.4466E5	1	1	536	551	Acetaldehyde +26	K7:Acetaldehyde +26:74.34	PEAKS PTM
K.NPQLQ(+.98)DLDMM(+15.99)LTC(+57.02)VEIK.E	N	32.85	2063.9578	17	-3.4	1032.9827	2	37.19	12	F12:1881	OB5939 H1 raw.raw	2.2954E4	1	1	415	431	Carbamidomethylation	Q5:Deamidation (NQ):26.31;M10:Oxidation (M):0.00;C13:Carbamidomethylation:1000.00	PEAKS DB
K.HADADNILVIQ(+.98)QGQ(+.98)ATVTVANGNNRK.S	N	32.62	2748.3792	26	3.1	917.1365	3	57.24	13	F13:3064	OB5940 H1 raw.raw	0	0	0	223	248		Q11:Deamidation (NQ):0.00;Q14:Deamidation (NQ):0.00	PEAKS DB
K.N(+.98)PQ(+.98)LQ(+.98)DLDMM(+15.99)LTC(+57.02)VEIKEGALMLPHFNSK.A	N	32.23	3390.5854	29	3.7	848.6568	4	38.47	14	F14:1982	OB5941 H1 raw.raw	0	0	0	415	443	Deamidation (NQ); Carbamidomethylation	N1:Deamidation (NQ):62.42;Q3:Deamidation (NQ):65.92;Q5:Deamidation (NQ):60.92;M10:Oxidation (M):12.28;C13:Carbamidomethylation:1000.00	PEAKS DB
R.EGEQEWGT(-18.01)PGSEVREETSR.N	Y	32.15	2143.9407	19	2.2	1072.9800	2	27.69	12	F12:1293	OB5939 H1 raw.raw	4.1679E4	1	1	147	165		T8:Dehydration:20.70	PEAKS PTM
K.KN(+.98)PQ(+.98)LQ(+.98)DLDM(+15.99)MLTC(+57.02)VEIKEGALMLPHFNSK.A	N	31.81	3518.6804	30	-3.0	880.6747	4	37.45	12	F12:1882	OB5939 H1 raw.raw	0	0	0	414	443	Deamidation (NQ); Carbamidomethylation	N2:Deamidation (NQ):58.75;Q4:Deamidation (NQ):61.65;Q6:Deamidation (NQ):53.75;M10:Oxidation (M):0.00;C14:Carbamidomethylation:1000.00	PEAKS DB

total 219 peptides

B3IXL2|B3IXL2_ARAHY

back to list

| Protein Coverage | Supporting Peptides |

Protein Coverage:

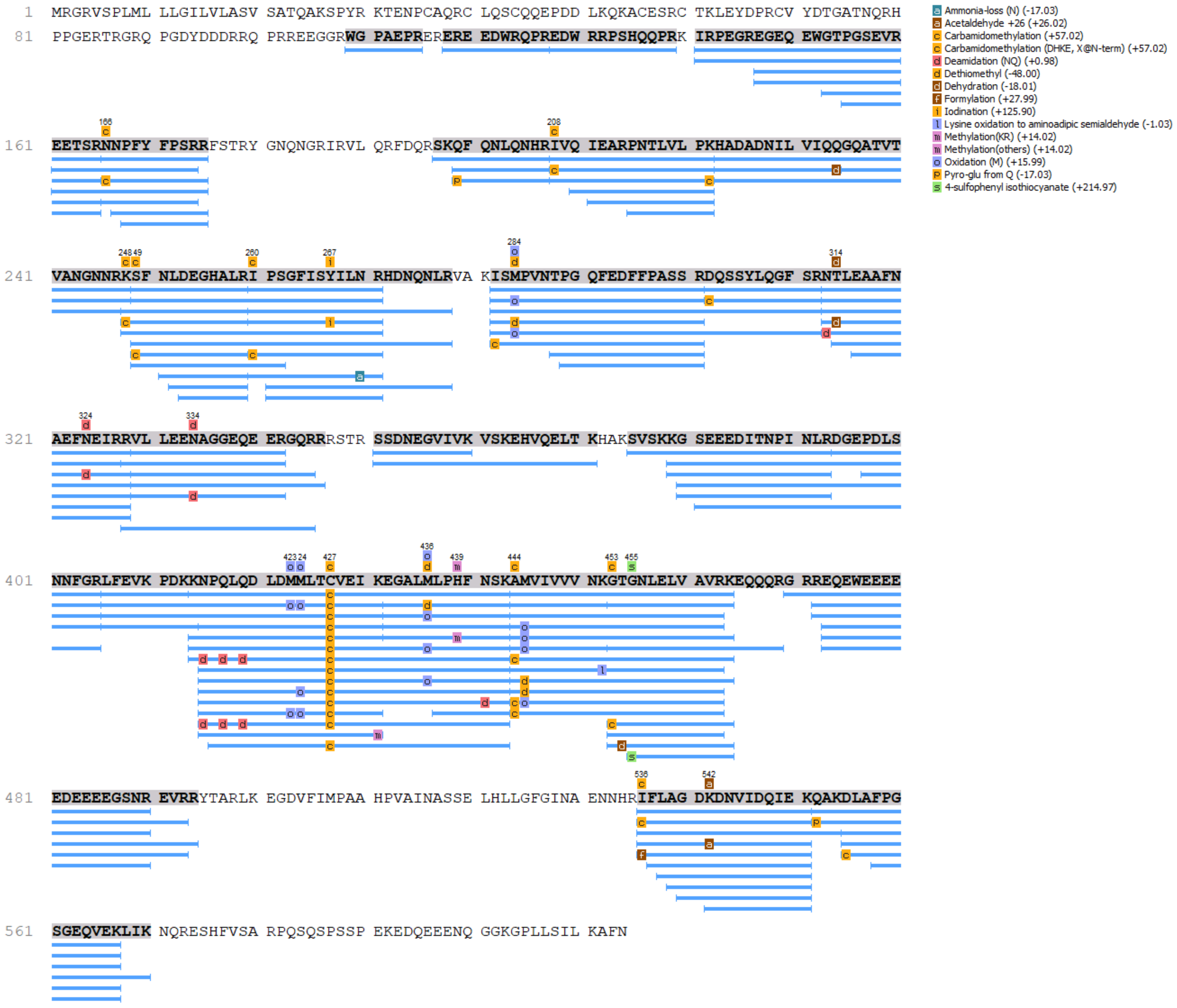
1 MRGRVSPLML LLGILVLASV SATQAKSPYR KTENPCAQRC LQSCQQEPDD LKQKACESRC TKLEYDPRCV YDTGATNQRH
81 PPGERTRGRQ PGDYDDDRRQ PRREEGGRWG PAEPRERERE EDWRQPREDW RRPSHQQPRK IRPEGREGEQ EWGTPGSEVR
161 EETSRNNPFY FPSRRFSTRY GNQNGRIRVL QRFDQRSKQF QNLQNHRIVQ IEARPNTLVL PKHADADNIL VIQQGQATVT
241 VANGNNRKSF NLDEGHALRI PSGFISYILN RHDNQNLRVA KISMPVNTPG QFEDFFPASS RDQSSYLQGF SRNTLEAAFN
321 AEFNEIRRVL LEENAGGEQE ERGQRRRSTR SSDNEGVIVK VSKEHVQELT KHAKSVSKKG SEEEDITNPI NLRDGEPDLS
401 NNFGRLFEVK PDKKNPQLQD LDMMLTCVEI KEGALMLPHF NSKAMVIVVV NKGTGNLELV AVRKEQQQRG RREQEWEEEE
481 EDEEEEGSNR EVRRYTARLK EGDVFIMPAA HPVAINASSE LHLLGFGINA ENNHRIFLAG DKDNVIDQIE KQAKDLAFPG
561 SGEQVEKLIK NQRESHFVSA RPQSQSPSSP EKEDQEEENQ GGKGPLLSIL KAFN
a Ammonia-loss (N) (-17.03)
a Acetaldehyde +26 (+26.02)
c Carbamidomethylation (+57.02)
c Carbamidomethylation (DHKE, X@N-term) (+57.02)
d Deamidation (NQ) (+0.98)
d Dethiomethyl (-48.00)
d Dehydration (-18.01)
f Formylation (+27.99)
i Iodination (+125.90)
l Lysine oxidation to aminoadipic semialdehyde (-1.03)
m Methylation(KR) (+14.02)
m Methylation(others) (+14.02)
o Oxidation (M) (+15.99)
p Pyro-glu from Q (-17.03)
s 4-sulfophenyl isothiocyanate (+214.97)

Supporting Peptides:

Peptide	Uniq	-10lgP	Mass	Length	ppm	m/z	z	RT	Fraction	Scan	Source File	Area Raw in gel	#Feature	#Feature Raw in gel	Start	End	PTM	AScore	Found By
K.HADADNILVIQQGQATVTVAN(+.98)GNNRK.S	N	140.09	2747.3950	26	-1.1	916.8046	3	29.38	14	F14:1509	OB5941 H1 raw.raw	3.7837E8	20	20	223	248		N21:Deamidation (N Q):26.31	PEAKS DB
K.KNPQLQDLDMMLTC(+57.02)VEIK.E	N	137.14	2175.0737	18	2.4	1088.5468	2	34.90	14	F14:1774	OB5941 H1 raw.raw	4.1086E6	3	3	414	431	Carbamidomethylation	C14:Carbamidomethylation:1000.00	PEAKS DB
K.HADADNILVIQQGQATVTVANGNNR.K	N	135.84	2618.3162	25	2.9	1310.1692	2	30.42	14	F14:1494	OB5941 H1 raw.raw	5.8142E6	3	3	223	247			PEAKS DB
K.ISMPVNTPGQFEDFFPASSR.D	N	135.58	2226.0415	20	1.9	1114.0302	2	35.63	14	F14:1807	OB5941 H1 raw.raw	2.1661E8	32	26	282	301			PEAKS DB
K.ISM(+15.99)PVNTPGQFEDFFPASSR.D	N	134.77	2242.0364	20	2.3	1122.0281	2	34.42	2	F2:1522	OB5925 H3B raw.raw	1.6845E7	19	17	282	301	Oxidation (M)	M3:Oxidation (M):1000.00	PEAKS DB
K.NPQLQDLDMMLTC(+57.02)VEIK.E	N	131.65	2046.9788	17	-0.8	1024.4958	2	36.89	14	F14:1903	OB5941 H1 raw.raw	1.2559E7	8	8	415	431	Carbamidomethylation	C13:Carbamidomethylation:1000.00	PEAKS DB
R.EQEWEEEEEDEEEEGSNR.E	N	130.87	2280.8413	18	4.3	1141.4329	2	28.13	14	F14:1352	OB5941 H1 raw.raw	4.0373E6	3	3	473	490			PEAKS DB
K.NPQLQDLDM(+15.99)MLTC(+57.02)VEIK.E	N	128.59	2062.9736	17	-6.6	1032.4873	2	36.34	14	F14:1860	OB5941 H1 raw.raw	2.4371E5	2	2	415	431	Carbamidomethylation	M9:Oxidation (M):30.46;C13:Carbamidomethylation:1000.00	PEAKS DB
R.NTLEAAFNAEFNEIR.R	N	126.11	1737.8322	15	3.3	869.9262	2	35.27	14	F14:1792	OB5941 H1 raw.raw	1.3691E7	6	6	313	327			PEAKS DB
K.HADADNILVIQQGQATVTVANGNNRK.S	N	120.98	2746.4111	26	4.7	1374.2192	2	29.02	14	F14:1406	OB5941 H1 raw.raw	4.4412E7	13	13	223	248			PEAKS DB
K.SFNLDEGHALRIPSGFISYILNR.H	N	117.92	2618.3604	23	0.6	1310.1882	2	36.89	14	F14:1902	OB5941 H1 raw.raw	2.1092E8	40	39	249	271			PEAKS DB
R.VLLEENAGGEQEER.G	N	114.70	1571.7427	14	1.1	786.8795	2	26.65	14	F14:1250	OB5941 H1 raw.raw	1.2337E7	7	7	329	342			PEAKS DB
R.IFLAGDKDNVIDQIEKQAK.D	N	113.07	2144.1477	19	1.1	715.7240	3	32.93	7	F7:1578	OB5934 H3B raw.raw	5.566E7	12	12	536	554			PEAKS DB
K.AMVIVVVNKGTGNLELVAVR.K	N	112.17	2081.2031	20	1.5	694.7427	3	34.35	14	F14:1739	OB5941 H1 raw.raw	7.0931E7	12	12	444	463			PEAKS DB
R.KSFNLDEGHALR.I	N	112.03	1385.7051	12	1.1	693.8606	2	25.70	14	F14:1202	OB5941 H1 raw.raw	3.2948E6	6	6	248	259			PEAKS DB
R.IPSGFISYILNR.H	N	111.89	1378.7609	12	1.5	690.3887	2	43.03	14	F14:2242	OB5941 H1 raw.raw	2.1426E8	15	15	260	271			PEAKS DB
R.DQSSYLQGFSR.N	N	110.94	1286.5891	11	-1.4	644.3009	2	30.60	14	F14:1505	OB5941 H1 raw.raw	6.2405E7	21	21	302	312			PEAKS DB
K.KGSEEEDITNPINLR.D	N	110.67	1713.8533	15	3.2	857.9366	2	28.85	14	F14:1395	OB5941 H1 raw.raw	1.2495E7	8	8	379	393			PEAKS DB
K.NPQLQDLDMMLTC(+57.02)VEIKEGALMLPHFNSK.A	N	110.02	3371.6387	29	-0.1	1686.8264	2	37.85	14	F14:1946	OB5941 H1 raw.raw	1.7936E8	14	14	415	443	Carbamidomethylation	C13:Carbamidomethylation:1000.00	PEAKS DB
K.AMVIVVVNKGTGNLELVAVRK.E	N	109.98	2209.2981	21	-1.4	1105.6548	2	32.55	14	F14:1615	OB5941 H1 raw.raw	2.9136E8	18	18	444	464			PEAKS DB

total 219 peptides

Peptide	Uniq	-10lgP	Mass	Length	ppm	m/z	z	RT	Fraction	Scan	Source File	Area Raw in gel	#Feature	#Feature Raw in gel	Start	End	PTM	AScore	Found By
K.AM(+15.99)VIVVVNKGTGNLELVAVR.K	N	109.59	2097.1980	20	-2.2	1049.6040	2	32.73	14	F14:1638	OB5941 H1 raw.raw	3.1537E7	10	10	444	463	Oxidation (M)	M2:Oxidation (M):1000.00	PEAKS DB
K.SFNLDEGHALR.I	N	109.41	1257.6101	11	-3.3	629.8102	2	27.77	14	F14:1329	OB5941 H1 raw.raw	7.2473E7	30	30	249	259			PEAKS DB
R.IFLAGDKDNVIDQIEK.Q	N	108.13	1816.9570	16	0.2	909.4860	2	33.10	14	F14:1683	OB5941 H1 raw.raw	7.2865E8	41	41	536	551			PEAKS DB
R.KSFNLDEGHALRIPSGFISYILNR.H	N	107.80	2746.4553	24	-2.1	916.4905	3	36.16	12	F12:1802	OB5939 H1 raw.raw	7.186E6	4	4	248	271			PEAKS DB
K.GSEEEDITNPINLRDGEPDLSNNFGR.L	N	107.18	2887.3220	26	-1.7	963.4464	3	32.55	14	F14:1626	OB5941 H1 raw.raw	4.5198E7	9	9	380	405			PEAKS DB
R.EGEQEWGTPGSEVR.E	Y	105.02	1559.6852	14	-4.9	780.8460	2	27.77	14	F14:1333	OB5941 H1 raw.raw	7.2805E5	3	3	147	160			PEAKS DB
R.I(+57.02)PSGFISYILNR.H	N	104.65	1435.7823	12	-1.7	718.8972	2	36.52	14	F14:1869	OB5941 H1 raw.raw	1.3017E7	3	3	260	271	Carbamidomethylation (DHKE, X@N-term)	I1:Carbamidomethylation (DHKE, X@N-term):1000.00	PEAKS PTM
R.VLLEENAGGEQEERGQR.R	N	104.46	1912.9238	17	1.8	957.4709	2	25.31	14	F14:1182	OB5941 H1 raw.raw	1.9161E6	8	8	329	345			PEAKS DB
R.I(+57.02)FLAGDKDNVIDQIEK.Q	N	103.96	1873.9785	16	-5.0	937.9919	2	32.19	14	F14:1619	OB5941 H1 raw.raw	4.5184E7	7	7	536	551	Carbamidomethylation (DHKE, X@N-term)	I1:Carbamidomethylation (DHKE, X@N-term):90.91	PEAKS PTM
R.DGEPDLSNNFGR.L	N	102.95	1319.5742	12	0.8	660.7949	2	29.02	14	F14:1398	OB5941 H1 raw.raw	3.2103E6	3	3	394	405			PEAKS DB
K.QAKDLAFPGSGEQVEK.L	N	102.73	1702.8525	16	1.8	852.4351	2	26.85	14	F14:1278	OB5941 H1 raw.raw	1.286E6	6	6	552	567			PEAKS DB
K.H(+57.02)ADADNILVIQQGQATVTVANGNNRK.S	N	102.10	2803.4324	26	-1.3	935.4835	3	29.92	14	F14:1452	OB5941 H1 raw.raw	1.6897E6	1	1	223	248		H1:Carbamidomethylation (DHKE, X@N-term):39.76	PEAKS PTM
K.DLAFPGSGEQVEK.L	N	101.44	1375.6619	13	-2.0	688.8369	2	30.08	14	F14:1472	OB5941 H1 raw.raw	6.5563E7	9	9	555	567			PEAKS DB
K.NPQLQDLDMMLTCVEIK(+14.02).E	N	101.30	2003.9730	17	2.1	1002.9958	2	38.00	14	F14:1960	OB5941 H1 raw.raw	6.7851E5	2	2	415	431	Methylation(KR)	K17:Methylation(KR):1000.00	PEAKS PTM
K.S(+57.02)FNLDEGHALR.I	N	100.96	1314.6316	11	0.8	658.3236	2	27.59	14	F14:1340	OB5941 H1 raw.raw	5.6012E6	7	7	249	259	Carbamidomethylation (DHKE, X@N-term)	S1:Carbamidomethylation (DHKE, X@N-term):101.71	PEAKS PTM
R.RVLLEENAGGEQEER.G	N	98.06	1727.8438	15	0.4	576.9554	3	24.77	13	F13:1156	OB5940 H1 raw.raw	1.2517E5	5	5	328	342			PEAKS DB
R.IPSGFISYILNRHDNQNLR.V	N	97.42	2256.1763	19	0.5	565.0516	4	33.66	14	F14:1693	OB5941 H1 raw.raw	3.392E8	42	42	260	278			PEAKS DB
K.GSEEEDITNPINLR.D	N	97.01	1585.7583	14	2.4	793.8884	2	30.93	14	F14:1527	OB5941 H1 raw.raw	3.0498E6	3	3	380	393			PEAKS DB
K.AM(+15.99)VIVVVNKGTGNLELVAVRK.E	N	96.33	2225.2930	21	-0.4	1113.6533	2	30.76	14	F14:1520	OB5941 H1 raw.raw	4.0787E7	8	7	444	464	Oxidation (M)	M2:Oxidation (M):1000.00	PEAKS DB
R.EGEQEWGTPGSEVREETSR.N	Y	95.70	2161.9512	19	2.5	1081.9856	2	27.60	14	F14:1311	OB5941 H1 raw.raw	2.0479E7	9	9	147	165			PEAKS DB

total 219 peptides

Peptide	Uniq	-10lgP	Mass	Length	ppm	m/z	z	RT	Fraction	Scan	Source File	Area Raw in gel	#Feature	#Feature Raw in gel	Start	End	PTM	AScore	Found By
K.KNPQLQDLDMM(+15.99)LTC(+57.02)VEIK.E	N	95.01	2191.0686	18	-3.4	1096.5378	2	32.73	14	F14:1643	OB5941 H1 raw.raw	1.9787E5	1	1	414	431	Carbamidomethylation	M11:Oxidation (M):9.34;C14:Carbamidomethylation:1000.00	PEAKS DB
K.NPQLQDLDMM(-48.00)LTC(+57.02)VEIK.E	N	93.52	1998.9755	17	-2.5	1000.4926	2	32.55	14	F14:1628	OB5941 H1 raw.raw	1.3958E7	5	5	415	431	Carbamidomethylation	M10:Dethiomethyl:27.96;C13:Carbamidomethylation:1000.00	PEAKS PTM
K.NPQLQDLDM(+15.99)MLTC(+57.02)VEIKEGALMLPHFNSK.A	N	92.22	3387.6335	29	-1.5	1130.2168	3	36.17	14	F14:1926	OB5941 H1 raw.raw	2.8061E7	4	4	415	443	Carbamidomethylation	M9:Oxidation (M):10.11;C13:Carbamidomethylation:1000.00	PEAKS DB
R.NNPFYFPSRR.F	N	92.08	1296.6364	10	-3.0	649.3235	2	28.67	14	F14:1408	OB5941 H1 raw.raw	1.2788E8	21	21	166	175			PEAKS DB
K.EGALM(+15.99)LPHFNSK.A	N	91.98	1358.6653	12	2.1	680.3414	2	29.74	14	F14:1443	OB5941 H1 raw.raw	1.9561E6	3	3	432	443	Oxidation (M)	M5:Oxidation (M):1000.00	PEAKS DB
R.VLLEENAGGEQEERGQRR.R	N	91.09	2069.0249	18	2.1	690.6837	3	25.31	14	F14:1186	OB5941 H1 raw.raw	1.1888E6	3	3	329	346			PEAKS DB
R.SSDNEGVIVKVSKEHVQELTK.H	N	91.01	2325.2175	21	1.3	776.0808	3	28.31	14	F14:1362	OB5941 H1 raw.raw	1.0201E5	2	2	351	371			PEAKS DB
K.KNPQLQDLDMM(+15.99)LTC(+57.02)VEIKEGALMLPHFNSK.A	N	90.76	3515.7285	30	-2.7	879.9370	4	34.52	13	F13:1755	OB5940 H1 raw.raw	2.6989E6	3	3	414	443	Carbamidomethylation	M11:Oxidation (M):30.46;C14:Carbamidomethylation:1000.00	PEAKS DB
K.AM(-48.00)VIVVVNKGTGNLELVAVR.K	N	90.57	2033.1997	20	-3.4	1017.6036	2	31.46	14	F14:1539	OB5941 H1 raw.raw	1.6588E7	6	6	444	463	Dethiomethyl	M2:Dethiomethyl:1000.00	PEAKS PTM
K.A(+57.02)MVIVVVNKGTGNLELVAVR.K	N	90.54	2138.2246	20	-0.9	1070.1187	2	34.35	14	F14:1737	OB5941 H1 raw.raw	5.6414E6	3	3	444	463	Carbamidomethylation (DHKE, X@N-term)	A1:Carbamidomethylation (DHKE, X@N-term):207.56	PEAKS PTM
K.NPQLQDLDMMLTC(+57.02)VEIKEGALM(+15.99)LPHFNSK.A	N	90.37	3387.6335	29	-1.5	1130.2168	3	36.17	14	F14:1895	OB5941 H1 raw.raw	3.1109E7	7	7	415	443	Carbamidomethylation; Oxidation (M)	C13:Carbamidomethylation:1000.00;M22:Oxidation (M):69.89	PEAKS DB
K.H(+57.02)ADADNILVIQQGQATVTVAN(+.98)GNNRK.S	N	90.07	2804.4165	26	-1.3	935.8115	3	30.08	14	F14:1476	OB5941 H1 raw.raw	6.8084E6	2	2	223	248		H1:Carbamidomethylation (DHKE, X@N-term):45.01;N21:Deamidation (NQ):14.04	PEAKS PTM
K.KGSEEEDITNPINLRDGEPDLSNNFGR.L	N	89.87	3015.4170	27	-4.2	1006.1421	3	31.29	14	F14:1549	OB5941 H1 raw.raw	7.6E7	16	16	379	405			PEAKS DB
F.LAGDKDNVIDQIEK.Q	N	89.22	1556.8046	14	-3.2	779.4071	2	32.19	12	F12:1557	OB5939 H1 raw.raw	4.4864E6	2	2	538	551			PEAKS DB
K.I(+57.02)SMPVNTPGQFEDFFPASSR.D	N	88.49	2283.0630	20	-0.1	1142.5387	2	35.84	12	F12:1793	OB5939 H1 raw.raw	6.4772E5	1	1	282	301	Carbamidomethylation (DHKE, X@N-term)	I1:Carbamidomethylation (DHKE, X@N-term):156.03	PEAKS PTM
K.DLAFPGSGEQVEKLIK.N	N	87.96	1729.9249	16	1.0	865.9706	2	33.30	7	F7:1601	OB5934 H3B raw.raw	6.2551E5	3	3	555	570			PEAKS DB

total 219 peptides

Peptide	Uniq	-10lgP	Mass	Length	ppm	m/z	z	RT	Fraction	Scan	Source File	Area Raw in gel	#Feature	#Feature Raw in gel	Start	End	PTM	AScore	Found By
K.EGALMLPHFNSK.A	N	87.95	1342.6703	12	2.5	672.3441	2	29.83	13	F13:1462	OB5940 H1 raw.raw	9.3502E6	3	3	432	443			PEAKS DB
K.HADADNILVIQQGQATVTVAN(+.98)GNNR.K	N	87.06	2619.3000	25	-1.2	1310.6558	2	30.35	13	F13:1535	OB5940 H1 raw.raw	4.8683E6	1	1	223	247		N21:Deamidation (NQ):7.32	PEAKS DB
K.AMVIVVVNK.G	N	86.97	971.5837	9	0.9	486.7996	2	29.88	12	F12:1441	OB5939 H1 raw.raw	9.5124E6	3	3	444	452			PEAKS DB
K.GTGNLELVAVR.K	N	86.65	1127.6299	11	-0.8	564.8218	2	29.74	14	F14:1450	OB5941 H1 raw.raw	1.0907E7	6	6	453	463			PEAKS DB
R.NNPFYFPSR.R	N	86.37	1140.5352	9	-3.8	571.2727	2	31.46	14	F14:1552	OB5941 H1 raw.raw	1.201E7	5	5	166	174			PEAKS DB
K.HADADNILVIQQGQATVTVAN(-17.03)GNNRK.S	N	86.12	2729.3845	26	-1.1	910.8011	3	29.38	14	F14:1429	OB5941 H1 raw.raw	8.2265E6	4	4	223	248		N21:Ammonia-loss (N):18.53	PEAKS PTM
R.NTLEAAFNAEFNEIRR.V	N	86.03	1893.9332	16	-3.6	947.9705	2	35.28	12	F12:1741	OB5939 H1 raw.raw	3.4229E8	38	38	313	328			PEAKS DB
R.I(+27.99)FLAGDKDNVIDQIEK.Q	N	85.41	1844.9519	16	5.2	923.4880	2	36.16	14	F14:1844	OB5941 H1 raw.raw	0	0	0	536	551	Formylation	I1:Formylation:77.93	PEAKS PTM
K.SFNLDEGH(+57.02)ALR.I	N	84.87	1314.6316	11	-1.2	658.3223	2	28.05	12	F12:1298	OB5939 H1 raw.raw	5.1176E6	5	5	249	259		H8:Carbamidomethylation (DHKE, X@N-term):30.36	PEAKS PTM
R.K(+57.02)SFNLDEGHALR.I	N	83.09	1442.7266	12	-0.4	481.9159	3	25.70	14	F14:1209	OB5941 H1 raw.raw	2.2174E5	3	3	248	259	Carbamidomethylation (DHKE, X@N-term)	K1:Carbamidomethylation (DHKE, X@N-term):51.63	PEAKS PTM
K.KNPQLQDLDM(+15.99)MLTC(+57.02)VEIK.E	N	81.54	2191.0686	18	0.7	1096.5424	2	33.03	12	F12:1615	OB5939 H1 raw.raw	0	0	0	414	431	Carbamidomethylation	M10:Oxidation (M):14.02;C14:Carbamidomethylation:1000.00	PEAKS DB
K.KNPQLQDLDMMLTC(+57.02)VEIKEGALMLPHFNSK.A	N	81.26	3499.7336	30	-0.1	875.9406	4	36.52	14	F14:1868	OB5941 H1 raw.raw	1.091E7	3	3	414	443	Carbamidomethylation	C14:Carbamidomethylation:1000.00	PEAKS DB
K.GTGNLELVAVRK.E	N	79.30	1255.7249	12	0.9	628.8702	2	26.85	14	F14:1263	OB5941 H1 raw.raw	1.0437E7	9	9	453	464			PEAKS DB
R.GRREQEWEEEEEDEEEEGSNR.E	N	79.15	2650.0649	21	0.3	884.3625	3	25.64	13	F13:1205	OB5940 H1 raw.raw	2.2346E6	7	7	470	490			PEAKS DB
E.PDLSNNFGR.L	N	78.38	1018.4832	9	-0.1	510.2488	2	31.24	14	F14:1545	OB5941 H1 raw.raw	3.5959E6	3	3	397	405			PEAKS DB
R.EETSRNNPFYFPSRR.F	N	78.31	1898.9023	15	1.2	633.9755	3	27.17	13	F13:1289	OB5940 H1 raw.raw	6.7264E6	6	6	161	175			PEAKS DB
R.IFLAGDKDNVIDQIE(+57.02)K.Q	N	78.23	1873.9785	16	-5.0	937.9919	2	32.19	14	F14:1583	OB5941 H1 raw.raw	3.036E7	3	3	536	551		E15:Carbamidomethylation (DHKE, X@N-term):0.00	PEAKS PTM
I.PSGFISYILNR.H	N	77.77	1265.6768	11	2.4	633.8472	2	36.17	13	F13:1844	OB5940 H1 raw.raw	2.5204E5	2	2	261	271			PEAKS DB
I.FLAGDKDNVIDQIEK.Q	N	77.74	1703.8729	15	-9.3	852.9358	2	32.01	14	F14:1599	OB5941 H1 raw.raw	1.3574E6	3	3	537	551			PEAKS DB
K.D(+57.02)LAFPGSGEQVEK.L	N	77.21	1432.6833	13	3.8	717.3517	2	30.92	12	F12:1485	OB5939 H1 raw.raw	2.816E5	1	1	555	567	Carbamidomethylation (DHKE, X@N-term)	D1:Carbamidomethylation (DHKE,	PEAKS PTM

total 219 peptides

Peptide	Uniq	-10lgP	Mass	Length	ppm	m/z	z	RT	Fraction	Scan	Source File	Area Raw in gel	#Feature	#Feature Raw in gel	Start	End	PTM	AScore	Found By
																		X@N-ter m):93.18	
R.SSDNEGVIVK.V	N	76.72	1046.5244	10	0.3	524.2697	2	22.95	13	F13:1057	OB5940 H1 raw.raw	2.6758E5	6	6	351	360			PEAKS DB
K.Q(-17.03)FQNLQNHR.I	N	76.65	1166.5581	9	-1.5	584.2855	2	27.77	14	F14:1319	OB5941 H1 raw.raw	3.3996E6	3	3	199	207	Pyro-glu from Q	Q1:Pyro-glu from Q:1000.00	PEAKS PTM
K.HAD(+57.02)ADNILVIQQGQATVTVAN(+.98)GNNRK.S	N	74.85	2804.4165	26	-5.0	935.8081	3	28.96	13	F13:1511	OB5940 H1 raw.raw	3.7976E6	1	1	223	248		D3:Carbamidomethylation (DHKE, X@N-term):6.59;N21:Deamidation (NQ):14.04	PEAKS PTM
K.HADADNILVIQQGQATVTVANGN(-17.03)NRK.S	N	74.65	2729.3845	26	-3.3	1365.6951	2	29.38	14	F14:1432	OB5941 H1 raw.raw	5.4978E5	1	1	223	248		N23:Ammonia-loss (N):0.00	PEAKS PTM
K.ISMPVNTPGQFEDFFPASSRDQSSYLQGFSR.N	N	74.49	3494.6201	31	2.7	1165.8838	3	36.29	2	F2:1620	OB5925 H3B raw.raw	9.3886E7	15	15	282	312			PEAKS DB
R.NTLEAAFNAEFN(+.98)EIRR.V	N	74.39	1894.9172	16	-3.7	948.4624	2	32.73	14	F14:1640	OB5941 H1 raw.raw	4.113E6	3	3	313	328	Deamidation (NQ)	N12:Deamidation (NQ):55.21	PEAKS DB
R.LFEVKPDKKNPQLQDLDMMLTC(+57.02)VEIK.E	N	73.38	3131.6069	26	3.7	1044.8801	3	33.97	13	F13:1716	OB5940 H1 raw.raw	3.7487E6	3	3	406	431	Carbamidomethylation	C22:Carbamidomethylation:1000.00	PEAKS DB
K.NPQLQDLDMM(+15.99)LTC(+57.02)VEIKEGALMLPHFNSK.A	N	73.21	3387.6335	29	5.6	847.9204	4	35.82	13	F13:1825	OB5940 H1 raw.raw	9.6415E6	3	3	415	443	Carbamidomethylation	M10:Oxidation (M):16.90;C13:Carbamidomethylation:1000.00	PEAKS DB
A.GDKDNVIDQIEK.Q	N	73.04	1372.6833	12	-2.8	687.3470	2	32.19	12	F12:1561	OB5939 H1 raw.raw	1.4449E6	3	3	540	551			PEAKS DB
R.V(+57.02)LLEENAGGEQEER.G	N	73.00	1628.7642	14	1.1	815.3903	2	27.03	14	F14:1288	OB5941 H1 raw.raw	2.7852E5	3	3	329	342		V1:Carbamidomethylation (DHKE, X@N-term):44.44	PEAKS PTM
R.EETSRNNPFYFPSR.R	N	72.75	1742.8011	14	-0.9	581.9405	3	29.89	12	F12:1419	OB5939 H1 raw.raw	4.0306E4	1	1	161	174			PEAKS DB
R.IPSGFISYILN(-17.03)R.H	N	72.57	1361.7343	12	1.4	681.8754	2	37.47	13	F13:1926	OB5940 H1 raw.raw	5.3561E5	2	2	260	271	Ammonia-loss (N)	N11:Ammonia-loss (N):1000.00	PEAKS PTM
R.IPSGFISY(+125.90)ILNR.H	N	72.49	1504.6575	12	1.5	753.3372	2	37.84	13	F13:1946	OB5940 H1 raw.raw	9.8379E5	2	2	260	271	Iodination	Y8:Iodination:1000.00	PEAKS PTM
R.IPSGFISYILNRH(+57.02)DNQNLR.V	N	72.20	2313.1978	19	2.0	579.3079	4	33.29	12	F12:1633	OB5939 H1 raw.raw	3.81E7	4	4	260	278		H13:Carbamidomethylation (DHKE, X@N-term):9.34	PEAKS PTM
R.VLLEEN(+.98)AGGEQEER.G	N	71.59	1572.7267	14	-3.7	787.3677	2	26.82	13	F13:1276	OB5940 H1 raw.raw	0	0	0	329	342	Deamidation (NQ)	N6:Deamidation (NQ):71.64	PEAKS DB
A.FPGSGEQVEK.L	N	71.45	1076.5138	10	5.5	539.2672	2	30.23	12	F12:1449	OB5939 H1 raw.raw	6.1112E4	1	1	558	567			PEAKS DB
K.A(+57.02)MVIVVVNKGTGNLELVAVRK.E	N	70.77	2266.3196	21	-1.6	1134.1653	2	32.33	13	F13:1602	OB5940 H1 raw.raw	4.861E7	6	6	444	464	Carbamidomethylation (DHKE, X@N-term)	A1:Carbamidomethylation (DHKE, X@N-term):161.20	PEAKS PTM
**total 219 peptides**																			

Peptide	Uniq	-10lgP	Mass	Length	ppm	m/z	z	RT	Fraction	Scan	Source File	Area Raw in gel	#Feature	#Feature Raw in gel	Start	End	PTM	AScore	Found By
K.AM(+15.99)VIVVVNK.G	N	70.64	987.5787	9	0.7	494.7969	2	26.66	14	F14:1257	OB5941 H1 raw.raw	8.6431E5	3	3	444	452	Oxidation (M)	M2:Oxidation (M):1000.00	PEAKS DB
K.KNPQLQDLDMMLTC(+57.02)VEIKEGALM(+15.99)LPHFNSK.A	N	70.55	3515.7285	30	-0.8	879.9387	4	35.84	12	F12:1790	OB5939 H1 raw.raw	4.7958E6	2	2	414	443	Carbamidomethylation; Oxidation (M)	C14:Carbamidomethylation:1000.00;M23:Oxidation (M):69.38	PEAKS DB
K.SVSKKGSEEEDITNPINLR.D	N	69.76	2115.0808	19	2.6	706.0361	3	27.22	14	F14:1299	OB5941 H1 raw.raw	5.5052E4	1	1	375	393			PEAKS DB
Q.GQATVTVAN(+.98)GNNRK.S	N	69.60	1429.7273	14	-4.1	715.8680	2	29.38	14	F14:1424	OB5941 H1 raw.raw	5.134E5	2	2	235	248		N9:Deamidation (NQ):41.83	PEAKS DB
R.NT(-18.01)LEAAFNAEFNEIRR.V	N	68.32	1875.9227	16	-3.2	626.3129	3	33.81	14	F14:1704	OB5941 H1 raw.raw	1.3248E7	3	3	313	328	Dehydration	T2:Dehydration:1000.00	PEAKS PTM
G.SEEEDITNPINLRDGEPDLSNNFGR.L	N	67.86	2830.3005	25	1.2	944.4419	3	32.96	8	F8:1576	OB5935 H3B raw.raw	5.6172E5	3	3	381	405			PEAKS DB
R.IVQIEARPNTLVLPK.H	Y	67.84	1690.0140	15	2.2	846.0161	2	30.65	7	F7:1446	OB5934 H3B raw.raw	2.806E8	26	26	208	222			PEAKS DB
R.LFEVKPDKKNPQLQDLDM(+15.99)M(+15.99)LTC(+57.02)VEIK.E	N	67.75	3163.5967	26	2.1	791.9081	4	32.37	12	F12:1585	OB5939 H1 raw.raw	6.2675E5	1	1	406	431	Oxidation (M); Carbamidomethylation	M18:Oxidation (M):1000.00;M19:Oxidation (M):1000.00;C22:Carbamidomethylation:1000.00	PEAKS DB
L.AGDKDNVIDQIEK.Q	N	66.85	1443.7205	13	-3.1	722.8653	2	32.19	12	F12:1560	OB5939 H1 raw.raw	7.0907E5	2	2	539	551			PEAKS DB
K.G(+57.02)TGNLELVAVRK.E	N	66.68	1312.7462	12	-0.7	657.3799	2	26.85	14	F14:1280	OB5941 H1 raw.raw	3.417E5	3	3	453	464	Carbamidomethylation (DHKE, X@N-term)	G1:Carbamidomethylation (DHKE, X@N-term):56.51	PEAKS PTM
R.EGEQEWGTP(+15.99)GSEVREETSR.N	Y	66.56	2177.9460	19	1.3	726.9902	3	26.28	14	F14:1241	OB5941 H1 raw.raw	4.0056E6	2	2	147	165		P9:Oxidation or Hydroxylation:36.47	PEAKS PTM
K.SFNLDEGHALRIPSGFISYILNRHDNQNLR.V	N	66.45	3495.7759	30	-1.2	700.1616	5	36.31	13	F13:1855	OB5940 H1 raw.raw	1.2605E8	16	16	249	278			PEAKS DB
K.QFQNLQNHR.I	N	66.40	1183.5846	9	0.2	592.7997	2	24.53	14	F14:1134	OB5941 H1 raw.raw	7.3427E4	5	5	199	207			PEAKS DB
D.KDNVIDQIEK.Q	N	66.36	1200.6350	10	-0.8	601.3243	2	25.89	14	F14:1225	OB5941 H1 raw.raw	5.8609E4	3	3	542	551			PEAKS DB
K.NPQLQ(+.98)DLDMMLTC(+57.02)VEIKEGALMLPHFNSK.A	N	66.16	3372.6226	29	5.7	1125.2212	3	37.84	13	F13:1936	OB5940 H1 raw.raw	5.3127E7	3	3	415	443	Carbamidomethylation	Q5:Deamidation (NQ):32.28;C13:Carbamidomethylation:1000.00	PEAKS DB
K.HADADNILVIQQGQATVTVANGNN(+.98)RK.S	N	65.93	2747.3950	26	4.5	1374.7109	2	29.70	10	F10:1444	OB5937 H3A raw.raw	5.6818E5	3	3	223	248		N24:Deamidation (NQ):14.02	PEAKS DB
K.ISMPVNTPGQ(+.98)FEDFFPASSR.D	N	65.70	2227.0254	20	6.4	1114.5271	2	63.55	14	F14:3427	OB5941 H1 raw.raw	1.0155E4	2	2	282	301		Q10:Deamidation (NQ):33.26	PEAKS DB
R.N(+.98)TLEAAFNAEFNEIRR.V	N	65.61	1894.9172	16	2.2	948.4680	2	35.88	12	F12:1787	OB5939 H1 raw.raw	7.0859E7	1	1	313	328	Deamidation (NQ)	N1:Deamidation (NQ):61.82	PEAKS DB
**total 219 peptides**																			

Peptide	Uniq	-10lgP	Mass	Length	ppm	m/z	z	RT	Fraction	Scan	Source File	Area Raw in gel	#Feature	#Feature Raw in gel	Start	End	PTM	AScore	Found By
K.KNPQLQDLDM(+15.99)MLTC(+57.02)VEIKEGALMLPHFNSK.A	N	65.47	3515.7285	30	-3.1	879.9366	4	35.44	13	F13:1830	OB5940 H1 raw.raw	7.2415E6	3	3	414	443	Carbamidomethylation	M10:Oxidation (M):11.06;C14:Carbamidomethylation:1000.00	PEAKS DB
K.ISM(+15.99)PVNTPGQ(+.98)FEDFFPASSRDQSSYLQGFSR.N	N	65.25	3511.5989	31	4.3	1171.5453	3	34.92	12	F12:1731	OB5939 H1 raw.raw	1.4851E6	1	1	282	312	Oxidation (M)	M3:Oxidation (M):1000.00;Q10:Deamidation (NQ):27.62	PEAKS DB
K.NPQLQDLDM(+15.99)MLTC(+57.02)VEIKEGALM(+15.99)LPHFNSK.A	N	64.53	3403.6284	29	4.5	1135.5552	3	36.52	14	F14:1873	OB5941 H1 raw.raw	4.3451E6	3	3	415	443	Carbamidomethylation; Oxidation (M)	M9:Oxidation (M):10.11;C13:Carbamidomethylation:1000.00;M22:Oxidation (M):53.09	PEAKS DB
R.LFEVKPDK.K	N	63.66	974.5436	8	0.9	488.2795	2	25.40	12	F12:1153	OB5939 H1 raw.raw	6.8606E5	7	7	406	413			PEAKS DB
F.NLDEGHALR.I	N	63.51	1023.5097	9	2.3	512.7633	2	27.35	13	F13:1309	OB5940 H1 raw.raw	1.5878E6	3	3	251	259			PEAKS DB
R.WGPAEPR.E	Y	63.43	811.3976	7	1.2	406.7066	2	25.31	14	F14:1167	OB5941 H1 raw.raw	1.2167E6	5	5	109	115			PEAKS DB
R.EQEWEEEEDEEEEGSNREVR.R	N	63.32	2665.0535	21	2.4	889.3606	3	27.87	12	F12:1308	OB5939 H1 raw.raw	4.1617E5	2	2	473	493			PEAKS DB
R.REQEWEEEEDEEEEGSNR.E	N	63.17	2436.9424	19	2.8	1219.4819	2	26.47	14	F14:1258	OB5941 H1 raw.raw	2.6135E4	2	2	472	490			PEAKS DB
K.ISM(-48.00)PVNTPGQFEDFFPASSR.D	N	62.93	2178.0381	20	0.2	727.0201	3	32.37	12	F12:1577	OB5939 H1 raw.raw	2.6788E7	5	5	282	301	Dethiomethyl	M3:Dethiomethyl:1000.00	PEAKS PTM
N.TPGQFEDFFPASSR.D	N	62.85	1584.7208	14	-0.2	793.3676	2	35.63	14	F14:1821	OB5941 H1 raw.raw	2.1663E5	1	1	288	301			PEAKS DB
K.A(+57.02)M(+15.99)VIVVVNKGTGNLELVAVR.K	N	62.49	2154.2195	20	-0.1	1078.1169	2	32.92	12	F12:1614	OB5939 H1 raw.raw	4.5509E5	2	2	444	463	Carbamidomethylation (DHKE, X@N-term); Oxidation (M)	A1:Carbamidomethylation (DHKE, X@N-term):139.69; M2:Oxidation (M):1000.00	PEAKS PTM
L.EAAFNAEFNEIRR.V	N	62.30	1565.7585	13	-4.7	783.8829	2	34.06	12	F12:1677	OB5939 H1 raw.raw	6.1232E5	1	1	316	328			PEAKS DB
K.N(+.98)PQLQDLDMMLTC(+57.02)VEIKEGALMLPHFNSK.A	N	61.86	3372.6226	29	5.7	1125.2212	3	37.84	13	F13:1983	OB5940 H1 raw.raw	1.2903E7	1	1	415	443	Carbamidomethylation	N1:Deamidation (NQ):14.04;C13:Carbamidomethylation:1000.00	PEAKS DB
K.HADADNILVIQQGQ(+.98)ATVTVAN(+.98)GNNRK.S	N	61.75	2748.3792	26	1.4	1375.1987	2	29.55	13	F13:1443	OB5940 H1 raw.raw	4.856E5	1	1	223	248		Q14:Deamidation (NQ):32.28;N21:Deamidation (NQ):14.04	PEAKS DB
N.LDEGHALR.I	N	60.44	909.4668	8	0.6	455.7409	2	27.35	13	F13:1312	OB5940 H1 raw.raw	3.1152E4	1	1	252	259			PEAKS DB
R.IFLAGDKDNVIDQ(+.98)IEKQAK.D	N	58.72	2145.1316	19	9.3	716.0578	3	32.93	2	F2:1432	OB5925 H3B raw.raw	1.5596E6	1	1	536	554		Q13:Deamidation (NQ):0.00	PEAKS DB
K.HADADNILVIQ(+.98)Q(+.98)GQATVTVAN(+.98)GNNRK.S	N	58.62	2749.3630	26	9.6	917.4705	3	30.18	13	F13:1524	OB5940 H1 raw.raw	4.3812E6	1	1	223	248		Q11:Deamidation (NQ):26.52;Q	PEAKS DB
**total 219 peptides**																			

Peptide	Uniq	-10lgP	Mass	Length	ppm	m/z	z	RT	Fraction	Scan	Source File	Area Raw in gel	#Feature	#Feature Raw in gel	Start	End	PTM	AScore	Found By
																		12:Deamidation (NQ):18.53;N21:Deamidation (NQ):14.04	
R.D(+57.02)QSSYLQGFSR.N	N	58.49	1343.6106	11	-2.5	672.8109	2	31.19	12	F12:1506	OB5939 H1 raw.raw	3.7713E4	1	1	302	312	Carbamidomethylation (DHKE, X@N-term)	D1:Carbamidomethylation (DHKE, X@N-term):1000.00	PEAKS PTM
K.HADADNILVIQ(+.98)QGQATVTVAN(+.98)GNNRK.S	N	58.29	2748.3792	26	4.6	1375.2031	2	31.28	12	F12:1539	OB5939 H1 raw.raw	1.8636E5	1	1	223	248		Q11:Deamidation (NQ):15.10;N21:Deamidation (NQ):17.01	PEAKS DB
K.HADADN(+.98)ILVIQ(+.98)QGQATVTVANGNNRK.S	N	57.75	2748.3792	26	3.1	1375.2010	2	30.08	14	F14:1495	OB5941 H1 raw.raw	1.5756E6	1	1	223	248		N6:Deamidation (NQ):0.00;Q11:Deamidation (NQ):0.00	PEAKS DB
N.PFYFPSRR.F	N	57.72	1068.5504	8	3.3	535.2842	2	28.95	12	F12:1360	OB5939 H1 raw.raw	3.0845E6	3	3	168	175			PEAKS DB
R.LFEVKPDKKNPQLQDLDMM(+15.99)LTC(+57.02)VEIK.E	N	57.62	3147.6018	26	-4.4	787.9042	4	32.55	14	F14:1633	OB5941 H1 raw.raw	2.1102E6	3	3	406	431	Carbamidomethylation	M19:Oxidation (M):17.01;C22:Carbamidomethylation:1000.00	PEAKS DB
K.SFN(+.98)LDEGHALRIPSGFISYILNR.H	N	57.28	2619.3445	23	9.3	655.8495	4	38.22	12	F12:1947	OB5939 H1 raw.raw	1.0265E6	2	2	249	271		N3:Deamidation (NQ):32.08	PEAKS DB
K.AMVIVVVN(+.98)KGTGNLELVAVRK.E	N	56.84	2210.2820	21	9.6	737.7750	3	33.10	14	F14:1664	OB5941 H1 raw.raw	8.0625E6	2	2	444	464		N8:Deamidation (NQ):41.87	PEAKS DB
K.KNPQLQDLDMM(-48.00)LTC(+57.02)VEIK.E	N	56.43	2127.0703	18	0.8	710.0312	3	31.11	14	F14:1538	OB5941 H1 raw.raw	3.3192E6	2	2	414	431	Carbamidomethylation	M11:Dethiomethyl:14.02;C14:Carbamidomethylation:1000.00	PEAKS PTM
K.ISM(+15.99)PVNTPGQFEDFFPASSRDQSSYLQGFSR.N	N	56.19	3510.6150	31	2.9	1171.2157	3	34.90	14	F14:1765	OB5941 H1 raw.raw	1.0055E7	8	8	282	312	Oxidation (M)	M3:Oxidation (M):1000.00	PEAKS DB
V.QIEARPNTLVLPK.H	Y	55.52	1477.8616	13	-3.7	739.9353	2	30.24	14	F14:1485	OB5941 H1 raw.raw	9.708E5	3	3	210	222			PEAKS DB
R.N(+57.02)NPFYFPSRR.F	N	55.47	1353.6577	10	-2.6	677.8344	2	29.38	14	F14:1433	OB5941 H1 raw.raw	6.1234E5	2	2	166	175	Carbamidomethylation (DHKE, X@N-term)	N1:Carbamidomethylation (DHKE, X@N-term):1000.00	PEAKS PTM
R.SKQFQNLQNHR.I	Y	55.18	1398.7117	11	1.0	467.2450	3	24.31	13	F13:1132	OB5940 H1 raw.raw	2.6796E4	3	3	197	207			PEAKS DB
K.HADADNILVIQQGQATVTVANGN(+.98)NRK.S	N	55.13	2747.3950	26	4.2	687.8589	4	29.51	12	F12:1401	OB5939 H1 raw.raw	2.4633E7	5	5	223	248		N23:Deamidation (NQ):17.01	PEAKS DB
K.HADADNILVIQQ(+.98)GQ(+.98)ATVTVAN(+.98)GNNRK.S	N	55.12	2749.3630	26	9.6	917.4705	3	30.18	13	F13:1487	OB5940 H1 raw.raw	4.3812E6	1	1	223	248		Q12:Deamidation (NQ):14.02;Q14:Deamidation (NQ):13.03;N21:Deamidation (NQ):11.10	PEAKS DB
K.AM(-48.00)VIVVVNKGTGNLELVAVRK.E	N	54.93	2161.2947	21	1.4	1081.6561	2	29.80	14	F14:1453	OB5941 H1 raw.raw	1.7581E6	2	2	444	464	Dethiomethyl	M2:Dethiomethyl:1000.00	PEAKS PTM

total 219 peptides

Peptide	Uniq	-10lgP	Mass	Length	ppm	m/z	z	RT	Fraction	Scan	Source File	Area Raw in gel	#Feature	#Feature Raw in gel	Start	End	PTM	AScore	Found By
R.I(+57.02)VQIEARPNTLVLPK.H	Y	54.55	1747.0355	15	3.8	874.5283	2	30.92	12	F12:1489	OB5939 H1 raw.raw	3.7453E7	4	4	208	222	Carbamidomethylation (DHKE, X@N-term)	I1:Carbamidomethylation (DHKE, X@N-term):97.69	PEAKS PTM
K.NPQLQDLDM(+15.99)MLTC(+57.02)VEIKEGALMLPHFN(+.98)SK.A	N	54.06	3388.6174	29	5.0	848.1659	4	36.17	14	F14:1849	OB5941 H1 raw.raw	6.0156E6	2	2	415	443	Carbamidomethylation; Deamidation (NQ)	M9:Oxidation (M):14.02;C13:Carbamidomethylation:1000.00;N27:Deamidation (NQ):58.23	PEAKS DB
K.IRPEGREGEQEWGTPGSEVREETSR.N	Y	53.77	2870.3542	25	1.1	718.5966	4	27.49	12	F12:1279	OB5939 H1 raw.raw	3.216E6	3	3	141	165			PEAKS DB
R.IFLAGDKDNVIDQIE(+57.02)KQAK.D	N	52.99	2201.1692	19	-2.0	734.7289	3	31.83	13	F13:1588	OB5940 H1 raw.raw	3.3379E6	2	2	536	554		E15:Carbamidomethylation (DHKE, X@N-term):12.28	PEAKS PTM
K.NPQLQDLDMMLTC(+57.02)VEIKEGALMLPHFN(+.98)SK.A	N	52.89	3372.6226	29	7.4	844.1691	4	37.85	13	F13:1914	OB5940 H1 raw.raw	5.2564E7	2	2	415	443	Carbamidomethylation; Deamidation (NQ)	C13:Carbamidomethylation:1000.00;N27:Deamidation (NQ):61.84	PEAKS DB
K.HADADNILVIQQGQ(+.98)ATVTVANGNNRK.S	N	52.87	2747.3950	26	5.1	916.8103	3	44.47	12	F12:2283	OB5939 H1 raw.raw	1.466E5	1	1	223	248		Q14:Deamidation (NQ):14.04	PEAKS DB
K.NPQLQDLDM(+15.99)M(+15.99)LTC(+57.02)VEIKEGALMLPHFNSK.A	N	52.70	3403.6284	29	-4.3	1135.5452	3	35.63	14	F14:1818	OB5941 H1 raw.raw	1.9434E6	2	2	415	443	Oxidation (M); Carbamidomethylation	M9:Oxidation (M):49.75;M10:Oxidation (M):51.99;C13:Carbamidomethylation:1000.00	PEAKS DB
L.DEGHALR.I	N	52.68	796.3828	7	2.3	399.1996	2	27.87	12	F12:1309	OB5939 H1 raw.raw	2.418E4	1	1	253	259			PEAKS DB
K.DLAFPGSGEQVEK(+57.02)LIK.N	N	52.57	1786.9464	16	-3.8	894.4771	2	32.91	14	F14:1654	OB5941 H1 raw.raw	6.7177E5	1	1	555	570		K13:Carbamidomethylation (DHKE, X@N-term):27.96	PEAKS PTM
K.KN(+.98)PQLQDLDMM(+15.99)LTC(+57.02)VEIK.E	N	52.31	2192.0527	18	9.2	1097.0437	2	32.33	13	F13:1621	OB5940 H1 raw.raw	8.5775E4	1	1	414	431	Carbamidomethylation	N2:Deamidation (NQ):14.04;M11:Oxidation (M):0.00;C14:Carbamidomethylation:1000.00	PEAKS DB
Q.GQATVTVANGNNRK.S	N	51.97	1428.7433	14	-0.2	715.3788	2	29.02	14	F14:1412	OB5941 H1 raw.raw	1.2099E5	2	2	235	248			PEAKS DB
K.EGALMLPH(+14.02)FNSK.A	N	51.73	1356.6860	12	1.3	679.3512	2	30.49	14	F14:1499	OB5941 H1 raw.raw	2.5741E4	1	1	432	443	Methylation(others)	H8:Methylation(others):53.53	PEAKS PTM
K.N(+.98)PQLQ(+.98)DLDMMLTC(+57.02)VEIKEGALM(+15.99)LPHFNSK.A	N	51.60	3389.6016	29	9.0	848.4153	4	36.92	12	F12:1868	OB5939 H1 raw.raw	2.6873E6	1	1	415	443	Carbamidomethylation	N1:Deamidation (NQ):0.00;Q5:Deamidation (NQ):14.04;C13:Carbamidomethylation:1000.00;M22:Oxidation (M):18.84	PEAKS DB
**total 219 peptides**																			

Peptide	Uniq	-10lgP	Mass	Length	ppm	m/z	z	RT	Fraction	Scan	Source File	Area Raw in gel	#Feature	#Feature Raw in gel	Start	End	PTM	AScore	Found By
R.LFEVKPDKK.N	N	50.85	1102.6385	9	1.1	552.3271	2	24.94	14	F14:1179	OB5941 H1 raw.raw	2.3081E5	2	2	406	414			PEAKS DB
R.RVLLEENAGGEQEERGQR.R	N	50.67	2069.0249	18	2.1	690.6837	3	24.88	13	F13:1165	OB5940 H1 raw.raw	2.7227E5	1	1	328	345			PEAKS DB
K.N(+.98)PQLQDLDMMLTC(+57.02)VEIKEGALM(+15.99)LPHFNSK.A	N	50.17	3388.6174	29	-2.4	1130.5437	3	36.35	13	F13:1874	OB5940 H1 raw.raw	8.9006E5	1	1	415	443	Carbamidomethylation; Oxidation (M)	N1:Deamidation (NQ):0.00;C13:Carbamidomethylation:1000.00;M22:Oxidation (M):54.40	PEAKS DB
K.NPQLQDLDMM(+15.99)LTC(+57.02)VEIKEGALM(+15.99)LPHFNSK.A	N	48.97	3403.6284	29	2.7	1135.5531	3	35.09	14	F14:1786	OB5941 H1 raw.raw	3.2001E5	1	1	415	443	Carbamidomethylation	M10:Oxidation (M):12.28;C13:Carbamidomethylation:1000.00;M22:Oxidation (M):28.29	PEAKS DB
R.EQEWEEEEEDEEEEGSNREVRR.Y	N	48.85	2821.1545	22	1.8	941.3939	3	26.75	12	F12:1227	OB5939 H1 raw.raw	3.3887E6	3	3	473	494			PEAKS DB
K.KN(+.98)PQLQDLDMMLTC(+57.02)VEIKEGALMLPHFNSK.A	N	48.84	3500.7175	30	7.1	876.1929	4	36.26	13	F13:1847	OB5940 H1 raw.raw	2.2113E6	1	1	414	443	Carbamidomethylation	N2:Deamidation (NQ):0.00;C14:Carbamidomethylation:1000.00	PEAKS DB
R.IFLAGDKDNVIDQIEK(+57.02)QAK.D	N	48.82	2201.1692	19	2.7	734.7323	3	32.55	12	F12:1581	OB5939 H1 raw.raw	3.2958E6	1	1	536	554		K16:Carbamidomethylation (DHKE, X@N-term):15.91	PEAKS PTM
R.EREEDWRQPR.E	N	48.49	1399.6592	10	0.1	467.5604	3	24.77	14	F14:1154	OB5941 H1 raw.raw	2.5247E4	1	1	118	127			PEAKS DB
R.KSFN(+.98)LDEGHALRIPSGFISYILNR.H	N	47.51	2747.4395	24	4.5	687.8702	4	36.15	14	F14:1837	OB5941 H1 raw.raw	1.0524E6	1	1	248	271		N4:Deamidation (NQ):23.98	PEAKS DB
R.LFEVKPDKKN(+.98)PQLQDLDMMLTC(+57.02)VEIK.E	N	47.32	3132.5908	26	9.3	1567.3173	2	34.35	14	F14:1745	OB5941 H1 raw.raw	1.9315E5	1	1	406	431	Carbamidomethylation	N10:Deamidation (NQ):14.04;C22:Carbamidomethylation:1000.00	PEAKS DB
K.N(+.98)PQ(+.98)LQDLDMMLTC(+57.02)VEIKEGALM(+15.99)LPHFNSK.A	N	46.82	3389.6016	29	9.0	848.4153	4	36.92	12	F12:1851	OB5939 H1 raw.raw	7.3613E6	2	2	415	443	Carbamidomethylation	N1:Deamidation (NQ):11.12;Q3:Deamidation (NQ):0.00;C13:Carbamidomethylation:1000.00;M22:Oxidation (M):24.93	PEAKS DB
W.GTPGSEVREETSR.N	Y	46.79	1403.6641	13	3.5	702.8417	2	27.60	14	F14:1327	OB5941 H1 raw.raw	5.9917E4	1	1	153	165			PEAKS DB
N.PQLQDLDMMLTC(+57.02)VEIKEGALMLPHFNSK.A	N	46.63	3257.5957	28	0.9	815.4069	4	37.83	14	F14:1952	OB5941 H1 raw.raw	2.3692E5	1	1	416	443	Carbamidomethylation	C12:Carbamidomethylation:1000.00	PEAKS DB
K.Q(-17.03)AKDLAFPGSGEQVEK.L	N	46.49	1685.8260	16	0.9	843.9211	2	29.97	12	F12:1427	OB5939 H1 raw.raw	0	0	0	552	567	Pyro-glu from Q	Q1:Pyro-glu from Q:1000.00	PEAKS PTM
K.SFN(+.98)LDEGHALRIPSGFISYILNRHDNQNLR.V	N	46.45	3496.7600	30	-3.9	700.3566	5	36.38	12	F12:1849	OB5939 H1 raw.raw	6.986E5	1	1	249	278		N3:Deamidation (NQ):20.27	PEAKS DB

total 219 peptides

Peptide	Uniq	-10lgP	Mass	Length	ppm	m/z	z	RT	Fraction	Scan	Source File	Area Raw in gel	#Feature	#Feature Raw in gel	Start	End	PTM	AScore	Found By
R.REQ(+.98)EWEEEEEDEEEEGSNR.E	N	45.55	2437.9265	19	7.6	813.6556	3	26.55	12	F12:1226	OB5939 H1 raw.raw	4.2319E4	1	1	472	490		Q3:Deamidation (NQ): 42.90	PEAKS DB
T.PGQFEDFFPASSR.D	N	45.25	1483.6731	13	-1.3	742.8429	2	40.34	13	F13:2103	OB5940 H1 raw.raw	4.0298E3	1	1	289	301			PEAKS DB
R.IPSGFISYILN(+.98)RHDNQNLR.V	N	45.21	2257.1604	19	9.9	753.4015	3	37.61	2	F2:1698	OB5925 H3B raw.raw	2.0603E6	2	2	260	278		N11:Deamidation (N Q):33.06	PEAKS DB
T.PGSEVREETSR.N	Y	45.10	1245.5948	11	1.9	623.8059	2	27.69	12	F12:1287	OB5939 H1 raw.raw	4.1909E4	1	1	155	165			PEAKS DB
N.NPFYFPSRR.F	N	45.02	1182.5934	9	1.6	395.2057	3	28.08	13	F13:1358	OB5940 H1 raw.raw	2.9991E4	1	1	167	175			PEAKS DB
R.DQS(-18.01)SYLQGFSR.N	N	44.90	1268.5785	11	7.0	635.3010	2	30.87	12	F12:1484	OB5939 H1 raw.raw	0	0	0	302	312		S3:Dehydration:33.98	PEAKS PTM
K.GTGNLELVAVRKEQQQR.G	N	44.58	1925.0442	17	0.6	642.6890	3	25.89	14	F14:1220	OB5941 H1 raw.raw	1.1112E5	2	2	453	469			PEAKS DB
R.EDWRRPSHQQPR.K	N	43.98	1590.7764	12	-0.3	398.7012	4	24.94	14	F14:1169	OB5941 H1 raw.raw	1.0819E4	1	1	128	139			PEAKS DB
R.IVQIEARPNTLVLPK(+57.02).H	Y	43.35	1747.0355	15	-2.4	874.5229	2	30.35	13	F13:1460	OB5940 H1 raw.raw	8.446E6	1	1	208	222	Carbamidomethylation (DHKE, X@N-term)	K15:Carbamidomethylation (DHKE, X@N-term):110.46	PEAKS PTM
R.VLLE(+21.98)ENAGGEQEER.G	N	42.61	1593.7246	14	-0.7	797.8690	2	26.03	13	F13:1239	OB5940 H1 raw.raw	1.373E5	3	3	329	342		E4:Sodium adduct:40.00	PEAKS PTM
K.HADADNILVIQ(+.98)QGQATVTVANGNNRK.S	N	42.22	2747.3950	26	5.9	916.8110	3	45.04	14	F14:2357	OB5941 H1 raw.raw	0	0	0	223	248		Q11:Deamidation (N Q):9.34	PEAKS DB
K.IRPEGREGEQEWGTPGSEVR.E	Y	42.02	2268.0881	20	1.1	757.0375	3	26.28	14	F14:1248	OB5941 H1 raw.raw	5.4096E4	1	1	141	160			PEAKS DB
K.HADADNILVIQQGQAT(-18.01)VTVAN(+.98)GNNRK.S	N	41.92	2729.3845	26	0.3	683.3536	4	28.96	13	F13:1411	OB5940 H1 raw.raw	3.5545E5	1	1	223	248		T16:Dehydration:8.22; N21:Deamidation (N Q):6.08	PEAKS PTM
K.HADADN(+.98)ILVIQQGQATVTVANGNNRK.S	N	41.76	2747.3950	26	3.7	916.8090	3	50.62	12	F12:2616	OB5939 H1 raw.raw	1.1355E5	2	2	223	248		N6:Deamidation (NQ): 42.57	PEAKS DB
K.ISMPVN(+.98)TPGQFEDFFPASSR.D	N	41.75	2227.0254	20	9.3	1114.5303	2	58.23	14	F14:3138	OB5941 H1 raw.raw	2.4983E4	1	1	282	301		N6:Deamidation (NQ): 42.89	PEAKS DB
M.LPHFNSK.A	N	41.73	841.4446	7	-3.4	421.7281	2	30.24	14	F14:1484	OB5941 H1 raw.raw	1.8069E6	2	2	437	443			PEAKS DB
R.PNTLVLPK.H	N	41.66	880.5382	8	0.4	441.2766	2	27.53	13	F13:1313	OB5940 H1 raw.raw	8.5249E4	1	1	215	222			PEAKS DB
N.TLEAAFNAEFNEIRR.V	N	41.51	1779.8904	15	-3.0	890.9498	2	32.91	14	F14:1655	OB5941 H1 raw.raw	4.1608E5	1	1	314	328			PEAKS DB
K.KGSEEEDITN(-17.03)PINLR.D	N	41.49	1696.8268	15	3.3	566.6180	3	28.85	14	F14:1397	OB5941 H1 raw.raw	2.3089E5	1	1	379	393		N10:Ammonia-loss (N):40.63	PEAKS PTM
K.KNPQLQ(+.98)DLDMMLTC(+57.02)VEIKEGALMLPHFNSK.A	N	40.89	3500.7175	30	0.4	1167.9136	3	36.34	13	F13:1850	OB5940 H1 raw.raw	4.7608E5	1	1	414	443	Carbamidomethylation	Q6:Deamidation (NQ): 14.04;C14: Carbamidomethylation:1000.00	PEAKS DB
R.REQEWEEEEEDEEEEGSNREVR.R	N	40.76	2821.1545	22	3.9	941.3958	3	26.47	14	F14:1247	OB5941 H1 raw.raw	1.8468E6	1	1	472	493			PEAKS DB
**total 219 peptides**																			

Peptide	Uniq	-10lgP	Mass	Length	ppm	m/z	z	RT	Fraction	Scan	Source File	Area Raw in gel	#Feature	#Feature Raw in gel	Start	End	PTM	AScore	Found By
R.NTLEAAFN(+.98)AEFNEIRR.V	N	40.23	1894.9172	16	-0.3	948.4656	2	32.92	12	F12:1570	OB5939H1 raw.raw	1.5322E6	1	1	313	328		N8:Deamidation (NQ): 8.26	PEAKS DB
K.NPQLQDLDM(+15.99)M(+15.99)LTC(+57.02)VEIK.E	N	39.57	2078.9688	17	1.1	1040.4928	2	33.80	14	F14:1701	OB5941H1 raw.raw	0	0	0	415	431	Oxidation (M); Carbamidomethylation	M9:Oxidation (M):1000.00;M10:Oxidation (M):1000.00;C13:Carbamidomethylation:1000.00	PEAKS DB
R.IPS(+59.02)GFISYILNR.H	N	39.40	1437.7802	12	1.5	719.8984	2	36.96	12	F12:1870	OB5939H1 raw.raw	4.3694E5	1	1	260	271		S3:Aminoethylcysteine:49.79	PEAKS PTM
K.HADADNILVIQQ(-18.01).G	N	38.68	1317.6677	12	-4.9	659.8379	2	29.56	14	F14:1421	OB5941H1 raw.raw	1.6968E5	2	2	223	234	Dehydration	Q12:Dehydration:76.18	PEAKS PTM
K.AMVIVVVNK(-1.03)GTGNLELVAVR.K	N	37.92	2080.1714	20	4.3	1041.0974	2	33.79	13	F13:1738	OB5940H1 raw.raw	2.1453E7	1	1	444	463	Lysine oxidation to aminoadipic semialdehyde	K9:Lysine oxidation to aminoadipic semialdehyde:1000.00	PEAKS PTM
I.PSGFISYILNRHDNQNLR.V	N	37.07	2143.0923	18	4.0	715.3742	3	33.53	12	F12:1645	OB5939H1 raw.raw	0	0	0	261	278			PEAKS DB
K.EGALM(-48.00)LPHFNSK.A	N	36.97	1294.6670	12	2.0	432.5638	3	28.31	14	F14:1348	OB5941H1 raw.raw	1.3683E6	1	1	432	443	Dethiomethyl	M5:Dethiomethyl:1000.00	PEAKS PTM
R.IFLAGDKDNVIDQ(+.98)IEK.Q	N	36.27	1817.9410	16	7.9	606.9924	3	63.32	14	F14:3434	OB5941H1 raw.raw	1.3413E4	1	1	536	551		Q13:Deamidation (NQ):25.70	PEAKS DB
K.HAD(+57.02)ADNILVIQQGQATVTVANGN(+.98)NRK.S	N	35.92	2804.4165	26	-3.8	702.1088	4	28.95	13	F13:1413	OB5940H1 raw.raw	2.7833E5	1	1	223	248		D3:Carbamidomethylation (DHKE, X@N-term):4.64;N23:Deamidation (NQ):0.00	PEAKS PTM
K.NPQ(+.98)LQDLDMMLTC(+57.02)VEIKEGALMLPHFNSK.A	N	35.84	3372.6226	29	9.4	1125.2253	3	40.73	12	F12:2064	OB5939H1 raw.raw	5.6313E5	1	1	415	443	Carbamidomethylation	Q3:Deamidation (NQ):0.00;C13:Carbamidomethylation:1000.00	PEAKS DB
K.ISM(+15.99)PVNTPGQ(+.98)FEDFFPASSR.D	N	35.67	2243.0205	20	6.2	1122.5245	2	50.73	12	F12:2643	OB5939H1 raw.raw	0	0	0	282	301	Oxidation (M)	M3:Oxidation (M):1000.00;Q10:Deamidation (NQ):22.37	PEAKS DB
K.GT(-18.01)GNLELVAVRK.E	N	35.64	1237.7142	12	0.9	413.5790	3	26.41	13	F13:1259	OB5940H1 raw.raw	7.4255E5	3	3	453	464	Dehydration	T2:Dehydration:1000.00	PEAKS PTM
K.SFNLDEGHALRIPS.G	N	35.38	1554.7791	14	-4.8	778.3931	2	30.79	13	F13:1515	OB5940H1 raw.raw	5.2113E3	1	1	249	262			PEAKS DB
R.DQ(+.98)SSYLQGFSR.N	N	34.61	1287.5731	11	-3.2	644.7917	2	63.83	14	F14:3449	OB5941H1 raw.raw	0	0	0	302	312		Q2:Deamidation (NQ): 41.87	PEAKS DB
R.S(+57.02)SDNEGVIVK.V	N	34.54	1103.5459	10	0.3	552.7804	2	23.19	13	F13:1066	OB5940H1 raw.raw	0	0	0	351	360		S1:Carbamidomethylation (DHKE, X@N-term):11.10	PEAKS PTM
Q.IEARPNTLVLPK.H	Y	34.19	1349.8030	12	2.2	675.9103	2	30.40	12	F12:1461	OB5939H1 raw.raw	1.011E6	3	3	211	222			PEAKS DB
R.NN(-17.03)PFYFPSRR.F	N	34.10	1279.6097	10	-0.4	427.5437	3	28.25	13	F13:1369	OB5940H1 raw.raw	1.47E5	1	1	166	175		N2:Ammonia-loss (N): 12.28	PEAKS PTM
**total 219 peptides**																			

Peptide	Uniq	-10lgP	Mass	Length	ppm	m/z	z	RT	Fraction	Scan	Source File	Area Raw in gel	#Feature	#Feature Raw in gel	Start	End	PTM	AScore	Found By
T.G(+214.97)NLELVAVRK.E	N	33.47	1312.6267	10	2.3	657.3221	2	27.95	14	F14:1280	OB5941 raw.raw	2.4669E5	1	1	455	464	4-sulfophenyl isothiocyanate	G1:4-sulfophenyl isothiocyanate:144.45	PEAKS PTM
K.HADADNILVIQQ(+.98)GQATVTVANGNN(+.98)RK.S	N	33.28	2748.3792	26	7.6	917.1406	3	48.20	12	F12:2471	OB5939 H1 raw.raw	2.0672E4	1	1	223	248		Q12:Deamidation (NQ):23.10;N24:Deamidation (NQ):0.00	PEAKS DB
K.HADAD(-18.01)NILVIQQGQATVTVANGNNRK.S	N	33.25	2728.4004	26	3.5	910.4772	3	28.70	13	F13:1390	OB5940 H1 raw.raw	0	0	0	223	248		D5:Dehydration:30.36	PEAKS PTM
K.ISM(+15.99)PVN(+.98)TPGQFEDFFPASSR.D	N	33.05	2243.0205	20	7.6	1122.5260	2	49.72	13	F13:2625	OB5940 H1 raw.raw	0	0	0	282	301	Oxidation (M)	M3:Oxidation (M):1000.00;N6:Deamidation (NQ):25.70	PEAKS DB
R.IFLAGDK(+26.02)DNVIDQIEK.Q	N	32.97	1842.9727	16	-0.2	922.4934	2	35.63	14	F14:1844	OB5941 H1 raw.raw	1.4466E5	1	1	536	551	Acetaldehyde +26	K7:Acetaldehyde +26:74.34	PEAKS PTM
K.NPQLQ(+.98)DLDMM(+15.99)LTC(+57.02)VEIK.E	N	32.85	2063.9578	17	-3.4	1032.9827	2	37.19	12	F12:1881	OB5939 H1 raw.raw	2.2954E4	1	1	415	431	Carbamidomethylation	Q5:Deamidation (NQ):26.31;M10:Oxidation (M):0.00;C13:Carbamidomethylation:1000.00	PEAKS DB
K.HADADNILVIQ(+.98)QGQ(+.98)ATVTVANGNNRK.S	N	32.62	2748.3792	26	3.1	917.1365	3	57.24	13	F13:3064	OB5940 H1 raw.raw	0	0	0	223	248		Q11:Deamidation (NQ):0.00;Q14:Deamidation (NQ):0.00	PEAKS DB
K.N(+.98)PQ(+.98)LQ(+.98)DLDMM(+15.99)LTC(+57.02)VEIKEGALMLPHFNSK.A	N	32.23	3390.5854	29	3.7	848.6568	4	38.47	14	F14:1982	OB5941 H1 raw.raw	0	0	0	415	443	Deamidation (NQ); Carbamidomethylation	N1:Deamidation (NQ):62.42;Q3:Deamidation (NQ):65.92;Q5:Deamidation (NQ):60.92;M10:Oxidation (M):12.28;C13:Carbamidomethylation:1000.00	PEAKS DB
R.EGEQEWGT(-18.01)PGSEVREETSR.N	Y	32.15	2143.9407	19	2.2	1072.9800	2	27.69	12	F12:1293	OB5939 H1 raw.raw	4.1679E4	1	1	147	165		T8:Dehydration:20.70	PEAKS PTM
K.KN(+.98)PQ(+.98)LQ(+.98)DLDM(+15.99)MLTC(+57.02)VEIKEGALMLPHFNSK.A	N	31.81	3518.6804	30	-3.0	880.6747	4	37.45	12	F12:1882	OB5939 H1 raw.raw	0	0	0	414	443	Deamidation (NQ); Carbamidomethylation	N2:Deamidation (NQ):58.75;Q4:Deamidation (NQ):61.65;Q6:Deamidation (NQ):53.75;M10:Oxidation (M):0.00;C14:Carbamidomethylation:1000.00	PEAKS DB
**total 219 peptides**																			

Q6PSU3|Q6PSU3_ARAHY

back to list

| Protein Coverage | Supporting Peptides |

Protein Coverage:

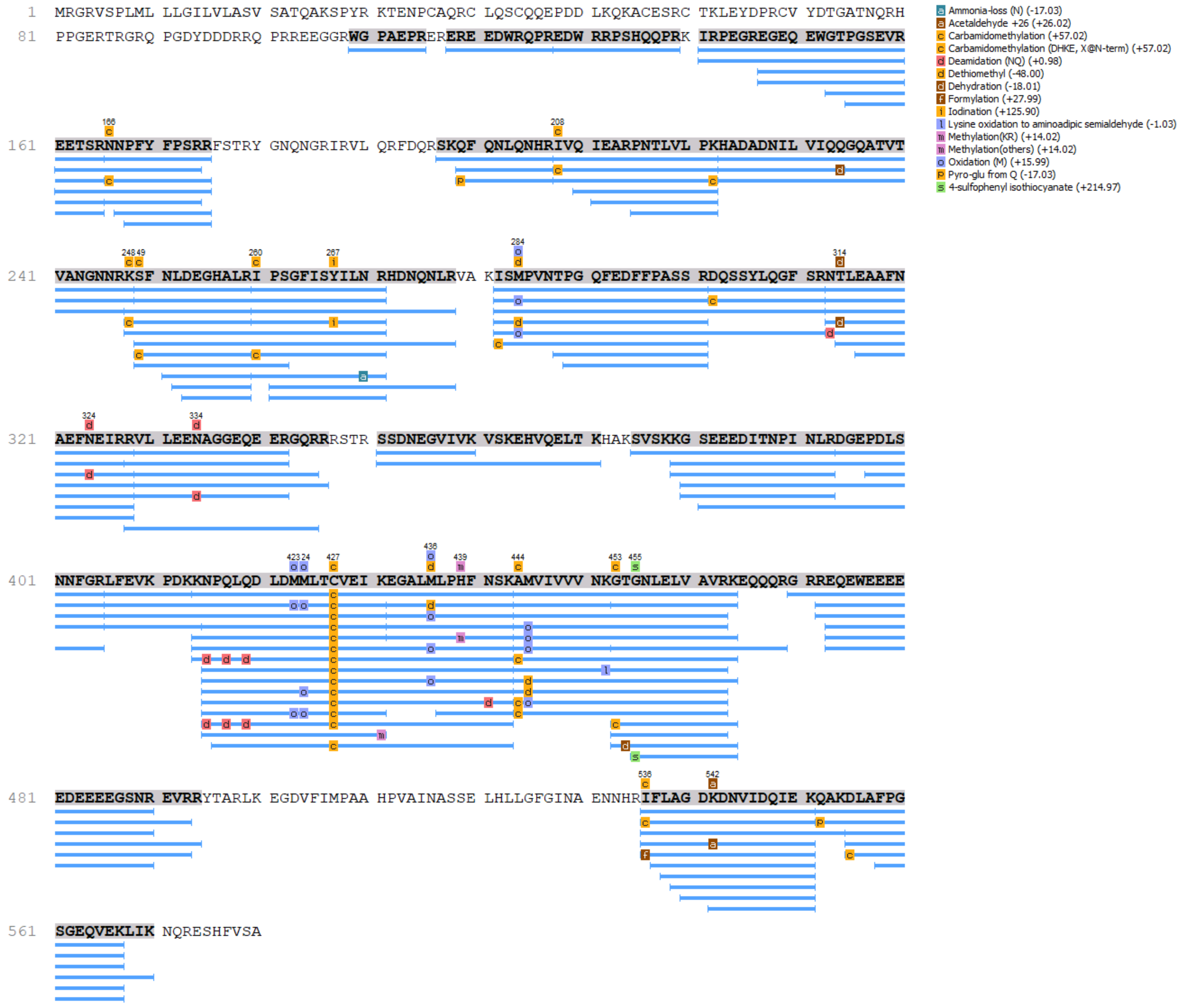

1 MRGRVSPLML LLGILVLASV SATQAKSPYR KTENPCAQRC LQSCQQEPDD LKQKACESRC TKLEYDPRCV YDTGATNQRH
81 PPGERTRGRQ PGDYDDDRRQ PRREEGGRWG PAEPRERERE EDWRQPREDW RRPSHQQPRK IRPEGREGEQ EWGTPGSEVR
166 208
161 EETSRNNPFY FPSRRFSTRY GNQNGRIRVL QRFDQRSKQF QNLQNHRIVQ IEARPNTLVL PKHADADNIL VIQQGQATVT
248 49 260 267 284 314
241 VANGNNRKSF NLDEGHALRI PSGFISYILN RHDNQNLRVA KISMPVNTPG QFEDFFPASS RDQSSYLQGF SRNTLEAAFN
324 334
321 AEFNEIRRVL LEENAGGEQE ERGQRRRSTR SSDNEGVIVK VSKEHVQELT KHAKSVSKKG SEEEDITNPI NLRDGEPDLS
423 24 427 436 439 444 453 455
401 NNFGRLFEVK PDKKNPQLQD LDMMLTCVEI KEGALMLPHF NSKAMVIVVV NKGTGNLELV AVRKEQQQRG RREQEWEEEE
536 542
481 EDEEEEGSNR EVRRYTARLK EGDVFIMPAA HPVAINASSE LHLLGFGINA ENNHRIFLAG DKDNVIDQIE KQAKDLAFPG
561 SGEQVEKLIK NQRESHFVSA
Ammonia-loss (N) (-17.03)
Acetaldehyde +26 (+26.02)
Carbamidomethylation (+57.02)
Carbamidomethylation (DHKE, X@N-term) (+57.02)
Deamidation (NQ) (+0.98)
Dethiomethyl (-48.00)
Dehydration (-18.01)
Formylation (+27.99)
Iodination (+125.90)
Lysine oxidation to aminoadipic semialdehyde (-1.03)
Methylation(KR) (+14.02)
Methylation(others) (+14.02)
Oxidation (M) (+15.99)
Pyro-glu from Q (-17.03)
4-sulfophenyl isothiocyanate (+214.97)

Supporting Peptides:

Peptide	Uniq	-10lgP	Mass	Length	ppm	m/z	z	RT	Fraction	Scan	Source File	Area Raw in gel	#Feature	#Feature Raw in gel	Start	End	PTM	AScore	Found By
K.HADADNILVIQQGQATVTVAN(+.98)GNNRK.S	N	140.09	2747.3950	26	-1.1	916.8046	3	29.38	14	F14:1509	OB5941 H1 raw.raw	3.7837E8	20	20	223	248		N21:Deamidation (NQ):26.31	PEAKS DB
K.KNPQLQDLDMMLTC(+57.02)VEIK.E	N	137.14	2175.0737	18	2.4	1088.5468	2	34.90	14	F14:1774	OB5941 H1 raw.raw	4.1086E6	3	3	414	431	Carbamidomethylation	C14:Carbamidomethylation:1000.00	PEAKS DB
K.HADADNILVIQQGQATVTVANGNNR.K	N	135.84	2618.3162	25	2.9	1310.1692	2	30.42	14	F14:1494	OB5941 H1 raw.raw	5.8142E6	3	3	223	247			PEAKS DB
K.ISMPVNTPGQFEDFFPASSR.D	N	135.58	2226.0415	20	1.9	1114.0302	2	35.63	14	F14:1807	OB5941 H1 raw.raw	2.1661E8	32	26	282	301			PEAKS DB
K.ISM(+15.99)PVNTPGQFEDFFPASSR.D	N	134.77	2242.0364	20	2.3	1122.0281	2	34.42	2	F2:1522	OB5925 H3B raw.raw	1.6845E7	19	17	282	301	Oxidation (M)	M3:Oxidation (M):1000.00	PEAKS DB
K.NPQLQDLDMMLTC(+57.02)VEIK.E	N	131.65	2046.9788	17	-0.8	1024.4958	2	36.89	14	F14:1903	OB5941 H1 raw.raw	1.2559E7	8	8	415	431	Carbamidomethylation	C13:Carbamidomethylation:1000.00	PEAKS DB
R.EQEWEEEEEDEEEEGSNR.E	N	130.87	2280.8413	18	4.3	1141.4329	2	28.13	14	F14:1352	OB5941 H1 raw.raw	4.0373E6	3	3	473	490			PEAKS DB
K.NPQLQDLDM(+15.99)MLTC(+57.02)VEIK.E	N	128.59	2062.9736	17	-6.6	1032.4873	2	36.34	14	F14:1860	OB5941 H1 raw.raw	2.4371E5	2	2	415	431	Carbamidomethylation	M9:Oxidation (M):30.46;C13:Carbamidomethylation:1000.00	PEAKS DB
R.NTLEAAFNAEFNEIR.R	N	126.11	1737.8322	15	3.3	869.9262	2	35.27	14	F14:1792	OB5941 H1 raw.raw	1.3691E7	6	6	313	327			PEAKS DB
K.HADADNILVIQQGQATVTVANGNNRK.S	N	120.98	2746.4111	26	4.7	1374.2192	2	29.02	14	F14:1406	OB5941 H1 raw.raw	4.4412E7	13	13	223	248			PEAKS DB
K.SFNLDEGHALRIPSGFISYILNR.H	N	117.92	2618.3604	23	0.6	1310.1882	2	36.89	14	F14:1902	OB5941 H1 raw.raw	2.1092E8	40	39	249	271			PEAKS DB
R.VLLEENAGGEQEER.G	N	114.70	1571.7427	14	1.1	786.8795	2	26.65	14	F14:1250	OB5941 H1 raw.raw	1.2337E7	7	7	329	342			PEAKS DB
R.IFLAGDKDNVIDQIEKQAK.D	N	113.07	2144.1477	19	1.1	715.7240	3	32.93	7	F7:1578	OB5934 H3B raw.raw	5.566E7	12	12	536	554			PEAKS DB
K.AMVIVVVNKGTGNLELVAVR.K	N	112.17	2081.2031	20	1.5	694.7427	3	34.35	14	F14:1739	OB5941 H1 raw.raw	7.0931E7	12	12	444	463			PEAKS DB
R.KSFNLDEGHALR.I	N	112.03	1385.7051	12	1.1	693.8606	2	25.70	14	F14:1202	OB5941 H1 raw.raw	3.2948E6	6	6	248	259			PEAKS DB
R.IPSGFISYILNR.H	N	111.89	1378.7609	12	1.5	690.3887	2	43.03	14	F14:2242	OB5941 H1 raw.raw	2.1426E8	15	15	260	271			PEAKS DB
R.DQSSYLQGFSR.N	N	110.94	1286.5891	11	-1.4	644.3009	2	30.60	14	F14:1505	OB5941 H1 raw.raw	6.2405E7	21	21	302	312			PEAKS DB
K.KGSEEEDITNPINLR.D	N	110.67	1713.8533	15	3.2	857.9366	2	28.85	14	F14:1395	OB5941 H1 raw.raw	1.2495E7	8	8	379	393			PEAKS DB
K.NPQLQDLDMMLTC(+57.02)VEIKEGALMLPHFNSK.A	N	110.02	3371.6387	29	-0.1	1686.8264	2	37.85	14	F14:1946	OB5941 H1 raw.raw	1.7936E8	14	14	415	443	Carbamidomethylation	C13:Carbamidomethylation:1000.00	PEAKS DB
K.AMVIVVVNKGTGNLELVAVRK.E	N	109.98	2209.2981	21	-1.4	1105.6548	2	32.55	14	F14:1615	OB5941 H1 raw.raw	2.9136E8	18	18	444	464			PEAKS DB

total 219 peptides

Peptide	Uniq	-10lgP	Mass	Length	ppm	m/z	z	RT	Fraction	Scan	Source File	Area Raw in gel	#Feature	#Feature Raw in gel	Start	End	PTM	AScore	Found By
K.AM(+15.99)VIVVVNKGTGNLELVAVR.K	N	109.59	2097.1980	20	-2.2	1049.6040	2	32.73	14	F14:1638	OB5941 H1 raw.raw	3.1537E7	10	10	444	463	Oxidation (M)	M2:Oxidation (M):1000.00	PEAKS DB
K.SFNLDEGHALR.I	N	109.41	1257.6101	11	-3.3	629.8102	2	27.77	14	F14:1329	OB5941 H1 raw.raw	7.2473E7	30	30	249	259			PEAKS DB
R.IFLAGDKDNVIDQIEK.Q	N	108.13	1816.9570	16	0.2	909.4860	2	33.10	14	F14:1683	OB5941 H1 raw.raw	7.2865E8	41	41	536	551			PEAKS DB
R.KSFNLDEGHALRIPSGFISYILNR.H	N	107.80	2746.4553	24	-2.1	916.4905	3	36.16	12	F12:1802	OB5939 H1 raw.raw	7.186E6	4	4	248	271			PEAKS DB
K.GSEEEDITNPINLRDGEPDLSNNFGR.L	N	107.18	2887.3220	26	-1.7	963.4464	3	32.55	14	F14:1626	OB5941 H1 raw.raw	4.5198E7	9	9	380	405			PEAKS DB
R.EGEQEWGTPGSEVR.E	Y	105.02	1559.6852	14	-4.9	780.8460	2	27.77	14	F14:1333	OB5941 H1 raw.raw	7.2805E5	3	3	147	160			PEAKS DB
R.I(+57.02)PSGFISYILNR.H	N	104.65	1435.7823	12	-1.7	718.8972	2	36.52	14	F14:1869	OB5941 H1 raw.raw	1.3017E7	3	3	260	271	Carbamidomethylation (DHKE, X@N-term)	I1:Carbamidomethylation (DHKE, X@N-term):1000.00	PEAKS PTM
R.VLLEENAGGEQEERGQR.R	N	104.46	1912.9238	17	1.8	957.4709	2	25.31	14	F14:1182	OB5941 H1 raw.raw	1.9161E6	8	8	329	345			PEAKS DB
R.I(+57.02)FLAGDKDNVIDQIEK.Q	N	103.96	1873.9785	16	-5.0	937.9919	2	32.19	14	F14:1619	OB5941 H1 raw.raw	4.5184E7	7	7	536	551	Carbamidomethylation (DHKE, X@N-term)	I1:Carbamidomethylation (DHKE, X@N-term):90.91	PEAKS PTM
R.DGEPDLSNNFGR.L	N	102.95	1319.5742	12	0.8	660.7949	2	29.02	14	F14:1398	OB5941 H1 raw.raw	3.2103E6	3	3	394	405			PEAKS DB
K.QAKDLAFPGSGEQVEK.L	N	102.73	1702.8525	16	1.8	852.4351	2	26.85	14	F14:1278	OB5941 H1 raw.raw	1.286E6	6	6	552	567			PEAKS DB
K.H(+57.02)ADADNILVIQQGQATVTVANGNNRK.S	N	102.10	2803.4324	26	-1.3	935.4835	3	29.92	14	F14:1452	OB5941 H1 raw.raw	1.6897E6	1	1	223	248		H1:Carbamidomethylation (DHKE, X@N-term):39.76	PEAKS PTM
K.DLAFPGSGEQVEK.L	N	101.44	1375.6619	13	-2.0	688.8369	2	30.08	14	F14:1472	OB5941 H1 raw.raw	6.5563E7	9	9	555	567			PEAKS DB
K.NPQLQDLDMMLTCVEIK(+14.02).E	N	101.30	2003.9730	17	2.1	1002.9958	2	38.00	14	F14:1960	OB5941 H1 raw.raw	6.7851E5	2	2	415	431	Methylation(KR)	K17:Methylation(KR):1000.00	PEAKS PTM
K.S(+57.02)FNLDEGHALR.I	N	100.96	1314.6316	11	0.8	658.3236	2	27.59	14	F14:1340	OB5941 H1 raw.raw	5.6012E6	7	7	249	259	Carbamidomethylation (DHKE, X@N-term)	S1:Carbamidomethylation (DHKE, X@N-term):101.71	PEAKS PTM
R.RVLLEENAGGEQEER.G	N	98.06	1727.8438	15	0.4	576.9554	3	24.77	13	F13:1156	OB5940 H1 raw.raw	1.2517E5	5	5	328	342			PEAKS DB
R.IPSGFISYILNRHDNQNLR.V	N	97.42	2256.1763	19	0.5	565.0516	4	33.66	14	F14:1693	OB5941 H1 raw.raw	3.392E8	42	42	260	278			PEAKS DB
K.GSEEEDITNPINLR.D	N	97.01	1585.7583	14	2.4	793.8884	2	30.93	14	F14:1527	OB5941 H1 raw.raw	3.0498E6	3	3	380	393			PEAKS DB
K.AM(+15.99)VIVVVNKGTGNLELVAVRK.E	N	96.33	2225.2930	21	-0.4	1113.6533	2	30.76	14	F14:1520	OB5941 H1 raw.raw	4.0787E7	8	7	444	464	Oxidation (M)	M2:Oxidation (M):1000.00	PEAKS DB
R.EGEQEWGTPGSEVREETSR.N	Y	95.70	2161.9512	19	2.5	1081.9856	2	27.60	14	F14:1311	OB5941 H1 raw.raw	2.0479E7	9	9	147	165			PEAKS DB
**total 219 peptides**																			

Peptide	Uniq	-10lgP	Mass	Length	ppm	m/z	z	RT	Fraction	Scan	Source File	Area Raw in gel	#Feature	#Feature Raw in gel	Start	End	PTM	AScore	Found By
K.KNPQLQDLDMM(+15.99)LTC(+57.02)VEIK.E	N	95.01	2191.0686	18	-3.4	1096.5378	2	32.73	14	F14:1643	OB5941 H1 raw.raw	1.9787E5	1	1	414	431	Carbamidomethylation	M11:Oxidation (M):9.34;C14:Carbamidomethylation:1000.00	PEAKS DB
K.NPQLQDLDMM(-48.00)LTC(+57.02)VEIK.E	N	93.52	1998.9755	17	-2.5	1000.4926	2	32.55	14	F14:1628	OB5941 H1 raw.raw	1.3958E7	5	5	415	431	Carbamidomethylation	M10:Dethiomethyl:27.96;C13:Carbamidomethylation:1000.00	PEAKS PTM
K.NPQLQDLDM(+15.99)MLTC(+57.02)VEIKEGALMLPHFNSK.A	N	92.22	3387.6335	29	-1.5	1130.2168	3	36.17	14	F14:1926	OB5941 H1 raw.raw	2.8061E7	4	4	415	443	Carbamidomethylation	M9:Oxidation (M):10.11;C13:Carbamidomethylation:1000.00	PEAKS DB
R.NNPFYFPSRR.F	N	92.08	1296.6364	10	-3.0	649.3235	2	28.67	14	F14:1408	OB5941 H1 raw.raw	1.2788E8	21	21	166	175			PEAKS DB
K.EGALM(+15.99)LPHFNSK.A	N	91.98	1358.6653	12	2.1	680.3414	2	29.74	14	F14:1443	OB5941 H1 raw.raw	1.9561E6	3	3	432	443	Oxidation (M)	M5:Oxidation (M):1000.00	PEAKS DB
R.VLLEENAGGEQEERGQRR.R	N	91.09	2069.0249	18	2.1	690.6837	3	25.31	14	F14:1186	OB5941 H1 raw.raw	1.1888E6	3	3	329	346			PEAKS DB
R.SSDNEGVIVKVSKEHVQELTK.H	N	91.01	2325.2175	21	1.3	776.0808	3	28.31	14	F14:1362	OB5941 H1 raw.raw	1.0201E5	2	2	351	371			PEAKS DB
K.KNPQLQDLDMM(+15.99)LTC(+57.02)VEIKEGALMLPHFNSK.A	N	90.76	3515.7285	30	-2.7	879.9370	4	34.52	13	F13:1755	OB5940 H1 raw.raw	2.6989E6	3	3	414	443	Carbamidomethylation	M11:Oxidation (M):30.46;C14:Carbamidomethylation:1000.00	PEAKS DB
K.AM(-48.00)VIVVVNKGTGNLELVAVR.K	N	90.57	2033.1997	20	-3.4	1017.6036	2	31.46	14	F14:1539	OB5941 H1 raw.raw	1.6588E7	6	6	444	463	Dethiomethyl	M2:Dethiomethyl:1000.00	PEAKS PTM
K.A(+57.02)MVIVVVNKGTGNLELVAVR.K	N	90.54	2138.2246	20	-0.9	1070.1187	2	34.35	14	F14:1737	OB5941 H1 raw.raw	5.6414E6	3	3	444	463	Carbamidomethylation (DHKE, X@N-term)	A1:Carbamidomethylation (DHKE, X@N-term):207.56	PEAKS PTM
K.NPQLQDLDMMLTC(+57.02)VEIKEGALM(+15.99)LPHFNSK.A	N	90.37	3387.6335	29	-1.5	1130.2168	3	36.17	14	F14:1895	OB5941 H1 raw.raw	3.1109E7	7	7	415	443	Carbamidomethylation; Oxidation (M)	C13:Carbamidomethylation:1000.00;M22:Oxidation (M):69.89	PEAKS DB
K.H(+57.02)ADADNILVIQQGQATVTVAN(+.98)GNNRK.S	N	90.07	2804.4165	26	-1.3	935.8115	3	30.08	14	F14:1476	OB5941 H1 raw.raw	6.8084E6	2	2	223	248		H1:Carbamidomethylation (DHKE, X@N-term):45.01;N21:Deamidation (NQ):14.04	PEAKS PTM
K.KGSEEEDITNPINLRDGEPDLSNNFGR.L	N	89.87	3015.4170	27	-4.2	1006.1421	3	31.29	14	F14:1549	OB5941 H1 raw.raw	7.6E7	16	16	379	405			PEAKS DB
F.LAGDKDNVIDQIEK.Q	N	89.22	1556.8046	14	-3.2	779.4071	2	32.19	12	F12:1557	OB5939 H1 raw.raw	4.4864E6	2	2	538	551			PEAKS DB
K.I(+57.02)SMPVNTPGQFEDFFPASSR.D	N	88.49	2283.0630	20	-0.1	1142.5387	2	35.84	12	F12:1793	OB5939 H1 raw.raw	6.4772E5	1	1	282	301	Carbamidomethylation (DHKE, X@N-term)	I1:Carbamidomethylation (DHKE, X@N-term):156.03	PEAKS PTM
K.DLAFPGSGEQVEKLIK.N	N	87.96	1729.9249	16	1.0	865.9706	2	33.30	7	F7:1601	OB5934 H3B raw.raw	6.2551E5	3	3	555	570			PEAKS DB
**total 219 peptides**																			

Peptide	Uniq	-10lgP	Mass	Length	ppm	m/z	z	RT	Fraction	Scan	Source File	Area Raw in gel	#Feature	#Feature Raw in gel	Start	End	PTM	AScore	Found By
K.EGALMLPHFNSK.A	N	87.95	1342.6703	12	2.5	672.3441	2	29.83	13	F13:1462	OB5940 H1 raw.raw	9.3502E6	3	3	432	443			PEAKS DB
K.HADADNILVIQQGQATVTVAN(+.98)GNNR.K	N	87.06	2619.3000	25	-1.2	1310.6558	2	30.35	13	F13:1535	OB5940 H1 raw.raw	4.8683E6	1	1	223	247		N21:Deamidation (NQ):7.32	PEAKS DB
K.AMVIVVVNK.G	N	86.97	971.5837	9	0.9	486.7996	2	29.88	12	F12:1441	OB5939 H1 raw.raw	9.5124E6	3	3	444	452			PEAKS DB
K.GTGNLELVAVR.K	N	86.65	1127.6299	11	-0.8	564.8218	2	29.74	14	F14:1450	OB5941 H1 raw.raw	1.0907E7	6	6	453	463			PEAKS DB
R.NNPFYFPSR.R	N	86.37	1140.5352	9	-3.8	571.2727	2	31.46	14	F14:1552	OB5941 H1 raw.raw	1.201E7	5	5	166	174			PEAKS DB
K.HADADNILVIQQGQATVTVAN(-17.03)GNNRK.S	N	86.12	2729.3845	26	-1.1	910.8011	3	29.38	14	F14:1429	OB5941 H1 raw.raw	8.2265E6	4	4	223	248		N21:Ammonia-loss (N):18.53	PEAKS PTM
R.NTLEAAFNAEFNEIRR.V	N	86.03	1893.9332	16	-3.6	947.9705	2	35.28	12	F12:1741	OB5939 H1 raw.raw	3.4229E8	38	38	313	328			PEAKS DB
R.I(+27.99)FLAGDKDNVIDQIEK.Q	N	85.41	1844.9519	16	5.2	923.4880	2	36.16	14	F14:1844	OB5941 H1 raw.raw	0	0	0	536	551	Formylation	I1:Formylation:77.93	PEAKS PTM
K.SFNLDEGH(+57.02)ALR.I	N	84.87	1314.6316	11	-1.2	658.3223	2	28.05	12	F12:1298	OB5939 H1 raw.raw	5.1176E6	5	5	249	259		H8:Carbamidomethylation (DHKE, X@N-term):30.36	PEAKS PTM
R.K(+57.02)SFNLDEGHALR.I	N	83.09	1442.7266	12	-0.4	481.9159	3	25.70	14	F14:1209	OB5941 H1 raw.raw	2.2174E5	3	3	248	259	Carbamidomethylation (DHKE, X@N-term)	K1:Carbamidomethylation (DHKE, X@N-term):51.63	PEAKS PTM
K.KNPQLQDLDM(+15.99)MLTC(+57.02)VEIK.E	N	81.54	2191.0686	18	0.7	1096.5424	2	33.03	12	F12:1615	OB5939 H1 raw.raw	0	0	0	414	431	Carbamidomethylation	M10:Oxidation (M):14.02;C14:Carbamidomethylation:1000.00	PEAKS DB
K.KNPQLQDLDMMLTC(+57.02)VEIKEGALMLPHFNSK.A	N	81.26	3499.7336	30	-0.1	875.9406	4	36.52	14	F14:1868	OB5941 H1 raw.raw	1.091E7	3	3	414	443	Carbamidomethylation	C14:Carbamidomethylation:1000.00	PEAKS DB
K.GTGNLELVAVRK.E	N	79.30	1255.7249	12	0.9	628.8702	2	26.85	14	F14:1263	OB5941 H1 raw.raw	1.0437E7	9	9	453	464			PEAKS DB
R.GRREQEWEEEEEDEEEEGSNR.E	N	79.15	2650.0649	21	0.3	884.3625	3	25.64	13	F13:1205	OB5940 H1 raw.raw	2.2346E6	7	7	470	490			PEAKS DB
E.PDLSNNFGR.L	N	78.38	1018.4832	9	-0.1	510.2488	2	31.24	14	F14:1545	OB5941 H1 raw.raw	3.5959E6	3	3	397	405			PEAKS DB
R.EETSRNNPFYFPSRR.F	N	78.31	1898.9023	15	1.2	633.9755	3	27.17	13	F13:1289	OB5940 H1 raw.raw	6.7264E6	6	6	161	175			PEAKS DB
R.IFLAGDKDNVIDQIE(+57.02)K.Q	N	78.23	1873.9785	16	-5.0	937.9919	2	32.19	14	F14:1583	OB5941 H1 raw.raw	3.036E7	3	3	536	551		E15:Carbamidomethylation (DHKE, X@N-term):0.00	PEAKS PTM
I.PSGFISYILNR.H	N	77.77	1265.6768	11	2.4	633.8472	2	36.17	13	F13:1844	OB5940 H1 raw.raw	2.5204E5	2	2	261	271			PEAKS DB
I.FLAGDKDNVIDQIEK.Q	N	77.74	1703.8729	15	-9.3	852.9358	2	32.01	14	F14:1599	OB5941 H1 raw.raw	1.3574E6	3	3	537	551			PEAKS DB
K.D(+57.02)LAFPGSGEQVEK.L	N	77.21	1432.6833	13	3.8	717.3517	2	30.92	12	F12:1485	OB5939 H1 raw.raw	2.816E5	1	1	555	567	Carbamidomethylation (DHKE, X@N-term)	D1:Carbamidomethylation (DHKE,	PEAKS PTM
**total 219 peptides**																			

Peptide	Uniq	-10lgP	Mass	Length	ppm	m/z	z	RT	Fraction	Scan	Source File	Area Raw in gel	#Feature	#Feature Raw in gel	Start	End	PTM	AScore	Found By
																		X@N-term):93.18	
R.SSDNEGVIVK.V	N	76.72	1046.5244	10	0.3	524.2697	2	22.95	13	F13:1057	OB5940 H1 raw.raw	2.6758E5	6	6	351	360			PEAKS DB
K.Q(-17.03)FQNLQNHR.I	N	76.65	1166.5581	9	-1.5	584.2855	2	27.77	14	F14:1319	OB5941 H1 raw.raw	3.3996E6	3	3	199	207	Pyro-glu from Q	Q1:Pyro-glu from Q:1000.00	PEAKS PTM
K.HAD(+57.02)ADNILVIQQGQATVTVAN(+.98)GNNRK.S	N	74.85	2804.4165	26	-5.0	935.8081	3	28.96	13	F13:1511	OB5940 H1 raw.raw	3.7976E6	1	1	223	248		D3:Carbamidomethylation (DHKE, X@N-term):6.59;N21:Deamidation (NQ):14.04	PEAKS PTM
K.HADADNILVIQQGQATVTVANGN(-17.03)NRK.S	N	74.65	2729.3845	26	-3.3	1365.6951	2	29.38	14	F14:1432	OB5941 H1 raw.raw	5.4978E5	1	1	223	248		N23:Ammonia-loss (N):0.00	PEAKS PTM
K.ISMPVNTPGQFEDFFPASSRDQSSYLQGFSR.N	N	74.49	3494.6201	31	2.7	1165.8838	3	36.29	2	F2:1620	OB5925 H3B raw.raw	9.3886E7	15	15	282	312			PEAKS DB
R.NTLEAAFNAEFN(+.98)EIRR.V	N	74.39	1894.9172	16	-3.7	948.4624	2	32.73	14	F14:1640	OB5941 H1 raw.raw	4.113E6	3	3	313	328	Deamidation (NQ)	N12:Deamidation (NQ):55.21	PEAKS DB
R.LFEVKPDKKNPQLQDLDMMLTC(+57.02)VEIK.E	N	73.38	3131.6069	26	3.7	1044.8801	3	33.97	13	F13:1716	OB5940 H1 raw.raw	3.7487E6	3	3	406	431	Carbamidomethylation	C22:Carbamidomethylation:1000.00	PEAKS DB
K.NPQLQDLDMM(+15.99)LTC(+57.02)VEIKEGALMLPHFNSK.A	N	73.21	3387.6335	29	5.6	847.9204	4	35.82	13	F13:1825	OB5940 H1 raw.raw	9.6415E6	3	3	415	443	Carbamidomethylation	M10:Oxidation (M):16.90;C13:Carbamidomethylation:1000.00	PEAKS DB
A.GDKDNVIDQIEK.Q	N	73.04	1372.6833	12	-2.8	687.3470	2	32.19	12	F12:1561	OB5939 H1 raw.raw	1.4449E6	3	3	540	551			PEAKS DB
R.V(+57.02)LLEENAGGEQEER.G	N	73.00	1628.7642	14	1.1	815.3903	2	27.03	14	F14:1288	OB5941 H1 raw.raw	2.7852E5	3	3	329	342		V1:Carbamidomethylation (DHKE, X@N-term):44.44	PEAKS PTM
R.EETSRNNPFYFPSR.R	N	72.75	1742.8011	14	-0.9	581.9405	3	29.89	12	F12:1419	OB5939 H1 raw.raw	4.0306E4	1	1	161	174			PEAKS DB
R.IPSGFISYILN(-17.03)R.H	N	72.57	1361.7343	12	1.4	681.8754	2	37.47	13	F13:1926	OB5940 H1 raw.raw	5.3561E5	2	2	260	271	Ammonia-loss (N)	N11:Ammonia-loss (N):1000.00	PEAKS PTM
R.IPSGFISY(+125.90)ILNR.H	N	72.49	1504.6575	12	1.5	753.3372	2	37.84	13	F13:1946	OB5940 H1 raw.raw	9.8379E5	2	2	260	271	Iodination	Y8:Iodination:1000.00	PEAKS PTM
R.IPSGFISYILNRH(+57.02)DNQNLR.V	N	72.20	2313.1978	19	2.0	579.3079	4	33.29	12	F12:1633	OB5939 H1 raw.raw	3.81E7	4	4	260	278		H13:Carbamidomethylation (DHKE, X@N-term):9.34	PEAKS PTM
R.VLLEEN(+.98)AGGEQEER.G	N	71.59	1572.7267	14	-3.7	787.3677	2	26.82	13	F13:1276	OB5940 H1 raw.raw	0	0	0	329	342	Deamidation (NQ)	N6:Deamidation (NQ):71.64	PEAKS DB
A.FPGSGEQVEK.L	N	71.45	1076.5138	10	5.5	539.2672	2	30.23	12	F12:1449	OB5939 H1 raw.raw	6.1112E4	1	1	558	567			PEAKS DB
K.A(+57.02)MVIVVVNKGTGNLELVAVRK.E	N	70.77	2266.3196	21	-1.6	1134.1653	2	32.33	13	F13:1602	OB5940 H1 raw.raw	4.861E7	6	6	444	464	Carbamidomethylation (DHKE, X@N-term)	A1:Carbamidomethylation (DHKE, X@N-term):161.20	PEAKS PTM
**total 219 peptides**																			

Peptide	Uniq	-10lgP	Mass	Length	ppm	m/z	z	RT	Fraction	Scan	Source File	Area Raw in gel	#Feature	#Feature Raw in gel	Start	End	PTM	AScore	Found By
K.AM(+15.99)VIVVVNK.G	N	70.64	987.5787	9	0.7	494.7969	2	26.66	14	F14:1257	OB5941 H1 raw.raw	8.6431E5	3	3	444	452	Oxidation (M)	M2:Oxidation (M):1000.00	PEAKS DB
K.KNPQLQDLDMMLTC(+57.02)VEIKEGALM(+15.99)LPHFNSK.A	N	70.55	3515.7285	30	-0.8	879.9387	4	35.84	12	F12:1790	OB5939 H1 raw.raw	4.7958E6	2	2	414	443	Carbamidomethylation; Oxidation (M)	C14:Carbamidomethylation:1000.00;M23:Oxidation (M):69.38	PEAKS DB
K.SVSKKGSEEEDITNPINLR.D	N	69.76	2115.0808	19	2.6	706.0361	3	27.22	14	F14:1299	OB5941 H1 raw.raw	5.5052E4	1	1	375	393			PEAKS DB
Q.GQATVTVAN(+.98)GNNRK.S	N	69.60	1429.7273	14	-4.1	715.8680	2	29.38	14	F14:1424	OB5941 H1 raw.raw	5.134E5	2	2	235	248		N9:Deamidation (NQ): 41.83	PEAKS DB
R.NT(-18.01)LEAAFNAEFNEIRR.V	N	68.32	1875.9227	16	-3.2	626.3129	3	33.81	14	F14:1704	OB5941 H1 raw.raw	1.3248E7	3	3	313	328	Dehydration	T2:Dehydration:1000.00	PEAKS PTM
G.SEEEDITNPINLRDGEPDLSNNFGR.L	N	67.86	2830.3005	25	1.2	944.4419	3	32.96	8	F8:1576	OB5935 H3B raw.raw	5.6172E5	3	3	381	405			PEAKS DB
R.IVQIEARPNTLVLPK.H	Y	67.84	1690.0140	15	2.2	846.0161	2	30.65	7	F7:1446	OB5934 H3B raw.raw	2.806E8	26	26	208	222			PEAKS DB
R.LFEVKPDKKNPQLQDLDM(+15.99)M(+15.99)LTC(+57.02)VEIK.E	N	67.75	3163.5967	26	2.1	791.9081	4	32.37	12	F12:1585	OB5939 H1 raw.raw	6.2675E5	1	1	406	431	Oxidation (M); Carbamidomethylation	M18:Oxidation (M):1000.00;M19: Oxidation (M):1000.00;C22:Carbamidomethylation:1000.00	PEAKS DB
L.AGDKDNVIDQIEK.Q	N	66.85	1443.7205	13	-3.1	722.8653	2	32.19	12	F12:1560	OB5939 H1 raw.raw	7.0907E5	2	2	539	551			PEAKS DB
K.G(+57.02)TGNLELVAVRK.E	N	66.68	1312.7462	12	-0.7	657.3799	2	26.85	14	F14:1280	OB5941 H1 raw.raw	3.417E5	3	3	453	464	Carbamidomethylation (DHKE, X@N-term)	G1:Carbamidomethylation (DHKE, X@N-term):56.51	PEAKS PTM
R.EGEQEWGTP(+15.99)GSEVREETSR.N	Y	66.56	2177.9460	19	1.3	726.9902	3	26.28	14	F14:1241	OB5941 H1 raw.raw	4.0056E6	2	2	147	165		P9:Oxidation or Hydroxylation:36.47	PEAKS PTM
K.SFNLDEGHALRIPSGFISYILNRHDNQNLR.V	N	66.45	3495.7759	30	-1.2	700.1616	5	36.31	13	F13:1855	OB5940 H1 raw.raw	1.2605E8	16	16	249	278			PEAKS DB
K.QFQNLQNHR.I	N	66.40	1183.5846	9	0.2	592.7997	2	24.53	14	F14:1134	OB5941 H1 raw.raw	7.3427E4	5	5	199	207			PEAKS DB
D.KDNVIDQIEK.Q	N	66.36	1200.6350	10	-0.8	601.3243	2	25.89	14	F14:1225	OB5941 H1 raw.raw	5.8609E4	3	3	542	551			PEAKS DB
K.NPQLQ(+.98)DLDMMLTC(+57.02)VEIKEGALMLPHFNSK.A	N	66.16	3372.6226	29	5.7	1125.2212	3	37.84	13	F13:1936	OB5940 H1 raw.raw	5.3127E7	3	3	415	443	Carbamidomethylation	Q5:Deamidation (NQ): 32.28;C13: Carbamidomethylation:1000.00	PEAKS DB
K.HADADNILVIQQGQATVTVANGNN(+.98)RK.S	N	65.93	2747.3950	26	4.5	1374.7109	2	29.70	10	F10:1444	OB5937 H3A raw.raw	5.6818E5	3	3	223	248		N24:Deamidation (N Q):14.02	PEAKS DB
K.ISMPVNTPGQ(+.98)FEDFFPASSR.D	N	65.70	2227.0254	20	6.4	1114.5271	2	63.55	14	F14:3427	OB5941 H1 raw.raw	1.0155E4	2	2	282	301		Q10:Deamidation (N Q):33.26	PEAKS DB
R.N(+.98)TLEAAFNAEFNEIRR.V	N	65.61	1894.9172	16	2.2	948.4680	2	35.88	12	F12:1787	OB5939 H1 raw.raw	7.0859E7	1	1	313	328	Deamidation (NQ)	N1:Deamidation (NQ): 61.82	PEAKS DB

total 219 peptides

Peptide	Uniq	-10lgP	Mass	Length	ppm	m/z	z	RT	Fraction	Scan	Source File	Area Raw in gel	#Feature	#Feature Raw in gel	Start	End	PTM	AScore	Found By
K.KNPQLQDLDM(+15.99)MLTC(+57.02)VEIKEGALMLPHFNSK.A	N	65.47	3515.7285	30	-3.1	879.9366	4	35.44	13	F13:1830	OB5940 H1 raw.raw	7.2415E6	3	3	414	443	Carbamidomethylation	M10:Oxidation (M):11.06;C14:Carbamidomethylation:1000.00	PEAKS DB
K.ISM(+15.99)PVNTPGQ(+.98)FEDFFPASSRDQSSYLQGFSR.N	N	65.25	3511.5989	31	4.3	1171.5453	3	34.92	12	F12:1731	OB5939 H1 raw.raw	1.4851E6	1	1	282	312	Oxidation (M)	M3:Oxidation (M):1000.00;Q10:Deamidation (NQ):27.62	PEAKS DB
K.NPQLQDLDM(+15.99)MLTC(+57.02)VEIKEGALM(+15.99)LPHFNSK.A	N	64.53	3403.6284	29	4.5	1135.5552	3	36.52	14	F14:1873	OB5941 H1 raw.raw	4.3451E6	3	3	415	443	Carbamidomethylation; Oxidation (M)	M9:Oxidation (M):10.11;C13:Carbamidomethylation:1000.00;M22:Oxidation (M):53.09	PEAKS DB
R.LFEVKPDK.K	N	63.66	974.5436	8	0.9	488.2795	2	25.40	12	F12:1153	OB5939 H1 raw.raw	6.8606E5	7	7	406	413			PEAKS DB
F.NLDEGHALR.I	N	63.51	1023.5097	9	2.3	512.7633	2	27.35	13	F13:1309	OB5940 H1 raw.raw	1.5878E6	3	3	251	259			PEAKS DB
R.WGPAEPR.E	Y	63.43	811.3976	7	1.2	406.7066	2	25.31	14	F14:1167	OB5941 H1 raw.raw	1.2167E6	5	5	109	115			PEAKS DB
R.EQEWEEEEEDEEEEGSNREVR.R	N	63.32	2665.0535	21	2.4	889.3606	3	27.87	12	F12:1308	OB5939 H1 raw.raw	4.1617E5	2	2	473	493			PEAKS DB
R.REQEWEEEEEDEEEEGSNR.E	N	63.17	2436.9424	19	2.8	1219.4819	2	26.47	14	F14:1258	OB5941 H1 raw.raw	2.6135E4	2	2	472	490			PEAKS DB
K.ISM(-48.00)PVNTPGQFEDFFPASSR.D	N	62.93	2178.0381	20	0.2	727.0201	3	32.37	12	F12:1577	OB5939 H1 raw.raw	2.6788E7	5	5	282	301	Dethiomethyl	M3:Dethiomethyl:1000.00	PEAKS PTM
N.TPGQFEDFFPASSR.D	N	62.85	1584.7208	14	-0.2	793.3676	2	35.63	14	F14:1821	OB5941 H1 raw.raw	2.1663E5	1	1	288	301			PEAKS DB
K.A(+57.02)M(+15.99)VIVVVNKGTGNLELVAVR.K	N	62.49	2154.2195	20	-0.1	1078.1169	2	32.92	12	F12:1614	OB5939 H1 raw.raw	4.5509E5	2	2	444	463	Carbamidomethylation (DHKE, X@N-term); Oxidation (M)	A1:Carbamidomethylation (DHKE, X@N-term):139.69; M2:Oxidation (M):1000.00	PEAKS PTM
L.EAAFNAEFNEIRR.V	N	62.30	1565.7585	13	-4.7	783.8829	2	34.06	12	F12:1677	OB5939 H1 raw.raw	6.1232E5	1	1	316	328			PEAKS DB
K.N(+.98)PQLQDLDMMLTC(+57.02)VEIKEGALMLPHFNSK.A	N	61.86	3372.6226	29	5.7	1125.2212	3	37.84	13	F13:1983	OB5940 H1 raw.raw	1.2903E7	1	1	415	443	Carbamidomethylation	N1:Deamidation (NQ):14.04;C13:Carbamidomethylation:1000.00	PEAKS DB
K.HADADNILVIQQGQ(+.98)ATVTVAN(+.98)GNNRK.S	N	61.75	2748.3792	26	1.4	1375.1987	2	29.55	13	F13:1443	OB5940 H1 raw.raw	4.856E5	1	1	223	248		Q14:Deamidation (NQ):32.28;N21:Deamidation (NQ):14.04	PEAKS DB
N.LDEGHALR.I	N	60.44	909.4668	8	0.6	455.7409	2	27.35	13	F13:1312	OB5940 H1 raw.raw	3.1152E4	1	1	252	259			PEAKS DB
R.IFLAGDKDNVIDQ(+.98)IEKQAK.D	N	58.72	2145.1316	19	9.3	716.0578	3	32.93	2	F2:1432	OB5925 H3B raw.raw	1.5596E6	1	1	536	554		Q13:Deamidation (NQ):0.00	PEAKS DB
K.HADADNILVIQ(+.98)Q(+.98)GQATVTVAN(+.98)GNNRK.S	N	58.62	2749.3630	26	9.6	917.4705	3	30.18	13	F13:1524	OB5940 H1 raw.raw	4.3812E6	1	1	223	248		Q11:Deamidation (NQ):26.52;Q	PEAKS DB

total 219 peptides

Peptide	Uniq	-10lgP	Mass	Length	ppm	m/z	z	RT	Fraction	Scan	Source File	Area Raw in gel	#Feature	#Feature Raw in gel	Start	End	PTM	AScore	Found By
																		12:Deamidation (NQ):18.53;N21:Deamidation (NQ):14.04	
R.D(+57.02)QSSYLQGFSR.N	N	58.49	1343.6106	11	-2.5	672.8109	2	31.19	12	F12:1506	OB5939 H1 raw.raw	3.7713E4	1	1	302	312	Carbamidomethylation (DHKE, X@N-term)	D1:Carbamidomethylation (DHKE, X@N-term):1000.00	PEAKS PTM
K.HADADNILVIQ(+.98)QGQATVTVAN(+.98)GNNRK.S	N	58.29	2748.3792	26	4.6	1375.2031	2	31.28	12	F12:1539	OB5939 H1 raw.raw	1.8636E5	1	1	223	248		Q11:Deamidation (NQ):15.10;N21:Deamidation (NQ):17.01	PEAKS DB
K.HADADN(+.98)ILVIQ(+.98)QGQATVTVANGNNRK.S	N	57.75	2748.3792	26	3.1	1375.2010	2	30.08	14	F14:1495	OB5941 H1 raw.raw	1.5756E6	1	1	223	248		N6:Deamidation (NQ):0.00;Q11:Deamidation (NQ):0.00	PEAKS DB
N.PFYFPSRR.F	N	57.72	1068.5504	8	3.3	535.2842	2	28.95	12	F12:1360	OB5939 H1 raw.raw	3.0845E6	3	3	168	175			PEAKS DB
R.LFEVKPDKKNPQLQDLDMM(+15.99)LTC(+57.02)VEIK.E	N	57.62	3147.6018	26	-4.4	787.9042	4	32.55	14	F14:1633	OB5941 H1 raw.raw	2.1102E6	3	3	406	431	Carbamidomethylation	M19:Oxidation (M):17.01;C22:Carbamidomethylation:1000.00	PEAKS DB
K.SFN(+.98)LDEGHALRIPSGFISYILNR.H	N	57.28	2619.3445	23	9.3	655.8495	4	38.22	12	F12:1947	OB5939 H1 raw.raw	1.0265E6	2	2	249	271		N3:Deamidation (NQ):32.08	PEAKS DB
K.AMVIVVVN(+.98)KGTGNLELVAVRK.E	N	56.84	2210.2820	21	9.6	737.7750	3	33.10	14	F14:1664	OB5941 H1 raw.raw	8.0625E6	2	2	444	464		N8:Deamidation (NQ):41.87	PEAKS DB
K.KNPQLQDLDMM(-48.00)LTC(+57.02)VEIK.E	N	56.43	2127.0703	18	0.8	710.0312	3	31.11	14	F14:1538	OB5941 H1 raw.raw	3.3192E6	2	2	414	431	Carbamidomethylation	M11:Dethiomethyl:14.02;C14:Carbamidomethylation:1000.00	PEAKS PTM
K.ISM(+15.99)PVNTPGQFEDFFPASSRDQSSYLQGFSR.N	N	56.19	3510.6150	31	2.9	1171.2157	3	34.90	14	F14:1765	OB5941 H1 raw.raw	1.0055E7	8	8	282	312	Oxidation (M)	M3:Oxidation (M):1000.00	PEAKS DB
V.QIEARPNTLVLPK.H	Y	55.52	1477.8616	13	-3.7	739.9353	2	30.24	14	F14:1485	OB5941 H1 raw.raw	9.708E5	3	3	210	222			PEAKS DB
R.N(+57.02)NPFYFPSRR.F	N	55.47	1353.6577	10	-2.6	677.8344	2	29.38	14	F14:1433	OB5941 H1 raw.raw	6.1234E5	2	2	166	175	Carbamidomethylation (DHKE, X@N-term)	N1:Carbamidomethylation (DHKE, X@N-term):1000.00	PEAKS PTM
R.SKQFQNLQNHR.I	Y	55.18	1398.7117	11	1.0	467.2450	3	24.31	13	F13:1132	OB5940 H1 raw.raw	2.6796E4	3	3	197	207			PEAKS DB
K.HADADNILVIQQGQATVTVANGN(+.98)NRK.S	N	55.13	2747.3950	26	4.2	687.8589	4	29.51	12	F12:1401	OB5939 H1 raw.raw	2.4633E7	5	5	223	248		N23:Deamidation (NQ):17.01	PEAKS DB
K.HADADNILVIQQ(+.98)GQ(+.98)ATVTVAN(+.98)GNNRK.S	N	55.12	2749.3630	26	9.6	917.4705	3	30.18	13	F13:1487	OB5940 H1 raw.raw	4.3812E6	1	1	223	248		Q12:Deamidation (NQ):14.02;Q14:Deamidation (NQ):13.03;N21:Deamidation (NQ):11.10	PEAKS DB
K.AM(-48.00)VIVVVNKGTGNLELVAVRK.E	N	54.93	2161.2947	21	1.4	1081.6561	2	29.80	14	F14:1453	OB5941 H1 raw.raw	1.7581E6	2	2	444	464	Dethiomethyl	M2:Dethiomethyl:1000.00	PEAKS PTM
**total 219 peptides**																			

Peptide	Uniq	-10lgP	Mass	Length	ppm	m/z	z	RT	Fraction	Scan	Source File	Area Raw in gel	#Feature	#Feature Raw in gel	Start	End	PTM	AScore	Found By
R.I(+57.02)VQIEARPNTLVLPK.H	Y	54.55	1747.0355	15	3.8	874.5283	2	30.92	12	F12:1489	OB5939 H1 raw.raw	3.7453E7	4	4	208	222	Carbamidomethylation (DHKE, X@N-term)	I1:Carbamidomethylation (DHKE, X@N-term):97.69	PEAKS PTM
K.NPQLQDLDM(+15.99)MLTC(+57.02)VEIKEGALMLPHFN(+.98)SK.A	N	54.06	3388.6174	29	5.0	848.1659	4	36.17	14	F14:1849	OB5941 H1 raw.raw	6.0156E6	2	2	415	443	Carbamidomethylation; Deamidation (NQ)	M9:Oxidation (M):14.02;C13:Carbamidomethylation:1000.00;N27:Deamidation (NQ):58.23	PEAKS DB
K.IRPEGREGEQEWGTPGSEVREETSR.N	Y	53.77	2870.3542	25	1.1	718.5966	4	27.49	12	F12:1279	OB5939 H1 raw.raw	3.216E6	3	3	141	165			PEAKS DB
R.IFLAGDKDNVIDQIE(+57.02)KQAK.D	N	52.99	2201.1692	19	-2.0	734.7289	3	31.83	13	F13:1588	OB5940 H1 raw.raw	3.3379E6	2	2	536	554		E15:Carbamidomethylation (DHKE, X@N-term):12.28	PEAKS PTM
K.NPQLQDLDMMLTC(+57.02)VEIKEGALMLPHFN(+.98)SK.A	N	52.89	3372.6226	29	7.4	844.1691	4	37.85	13	F13:1914	OB5940 H1 raw.raw	5.2564E7	2	2	415	443	Carbamidomethylation; Deamidation (NQ)	C13:Carbamidomethylation:1000.00;N27:Deamidation (NQ):61.84	PEAKS DB
K.HADADNILVIQQGQ(+.98)ATVTVANGNNRK.S	N	52.87	2747.3950	26	5.1	916.8103	3	44.47	12	F12:2283	OB5939 H1 raw.raw	1.466E5	1	1	223	248		Q14:Deamidation (NQ):14.04	PEAKS DB
K.NPQLQDLDM(+15.99)M(+15.99)LTC(+57.02)VEIKEGALMLPHFNSK.A	N	52.70	3403.6284	29	-4.3	1135.5452	3	35.63	14	F14:1818	OB5941 H1 raw.raw	1.9434E6	2	2	415	443	Oxidation (M); Carbamidomethylation	M9:Oxidation (M):49.75;M10:Oxidation (M):51.99;C13:Carbamidomethylation:1000.00	PEAKS DB
L.DEGHALR.I	N	52.68	796.3828	7	2.3	399.1996	2	27.87	12	F12:1309	OB5939 H1 raw.raw	2.418E4	1	1	253	259			PEAKS DB
K.DLAFPGSGEQVEK(+57.02)LIK.N	N	52.57	1786.9464	16	-3.8	894.4771	2	32.91	14	F14:1654	OB5941 H1 raw.raw	6.7177E5	1	1	555	570		K13:Carbamidomethylation (DHKE, X@N-term):27.96	PEAKS PTM
K.KN(+.98)PQLQDLDMM(+15.99)LTC(+57.02)VEIK.E	N	52.31	2192.0527	18	9.2	1097.0437	2	32.33	13	F13:1621	OB5940 H1 raw.raw	8.5775E4	1	1	414	431	Carbamidomethylation	N2:Deamidation (NQ):14.04;M11:Oxidation (M):0.00;C14:Carbamidomethylation:1000.00	PEAKS DB
Q.GQATVTVANGNNRK.S	N	51.97	1428.7433	14	-0.2	715.3788	2	29.02	14	F14:1412	OB5941 H1 raw.raw	1.2099E5	2	2	235	248			PEAKS DB
K.EGALMLPH(+14.02)FNSK.A	N	51.73	1356.6860	12	1.3	679.3512	2	30.49	14	F14:1499	OB5941 H1 raw.raw	2.5741E4	1	1	432	443	Methylation(others)	H8:Methylation(others):53.53	PEAKS PTM
K.N(+.98)PQLQ(+.98)DLDMMLTC(+57.02)VEIKEGALM(+15.99)LPHFNSK.A	N	51.60	3389.6016	29	9.0	848.4153	4	36.92	12	F12:1868	OB5939 H1 raw.raw	2.6873E6	1	1	415	443	Carbamidomethylation	N1:Deamidation (NQ):0.00;Q5:Deamidation (NQ):14.04;C13:Carbamidomethylation:1000.00;M22:Oxidation (M):18.84	PEAKS DB

total 219 peptides

Peptide	Uniq	-10lgP	Mass	Length	ppm	m/z	z	RT	Fraction	Scan	Source File	Area Raw in gel	#Feature	#Feature Raw in gel	Start	End	PTM	AScore	Found By
R.LFEVKPDKK.N	N	50.85	1102.6385	9	1.1	552.3271	2	24.94	14	F14:1179	OB5941 H1 raw.raw	2.3081E5	2	2	406	414			PEAKS DB
R.RVLLEENAGGEQEERGQR.R	N	50.67	2069.0249	18	2.1	690.6837	3	24.88	13	F13:1165	OB5940 H1 raw.raw	2.7227E5	1	1	328	345			PEAKS DB
K.N(+.98)PQLQDLDMMLTC(+57.02)VEIKEGALM(+15.99)LPHFNSK.A	N	50.17	3388.6174	29	-2.4	1130.5437	3	36.35	13	F13:1874	OB5940 H1 raw.raw	8.9006E5	1	1	415	443	Carbamidomethylation; Oxidation (M)	N1:Deamidation (NQ):0.00;C13:Carbamidomethylation:1000.00;M22:Oxidation (M):54.40	PEAKS DB
K.NPQLQDLDMM(+15.99)LTC(+57.02)VEIKEGALM(+15.99)LPHFNSK.A	N	48.97	3403.6284	29	2.7	1135.5531	3	35.09	14	F14:1786	OB5941 H1 raw.raw	3.2001E5	1	1	415	443	Carbamidomethylation	M10:Oxidation (M):12.28;C13:Carbamidomethylation:1000.00;M22:Oxidation (M):28.29	PEAKS DB
R.EQEWEEEEEDEEEEGSNREVRR.Y	N	48.85	2821.1545	22	1.8	941.3939	3	26.75	12	F12:1227	OB5939 H1 raw.raw	3.3887E6	3	3	473	494			PEAKS DB
K.KN(+.98)PQLQDLDMMLTC(+57.02)VEIKEGALMLPHFNSK.A	N	48.84	3500.7175	30	7.1	876.1929	4	36.26	13	F13:1847	OB5940 H1 raw.raw	2.2113E6	1	1	414	443	Carbamidomethylation	N2:Deamidation (NQ):0.00;C14:Carbamidomethylation:1000.00	PEAKS DB
R.IFLAGDKDNVIDQIEK(+57.02)QAK.D	N	48.82	2201.1692	19	2.7	734.7323	3	32.55	12	F12:1581	OB5939 H1 raw.raw	3.2958E6	1	1	536	554		K16:Carbamidomethylation (DHKE, X@N-term):15.91	PEAKS PTM
R.EREEDWRQPR.E	N	48.49	1399.6592	10	0.1	467.5604	3	24.77	14	F14:1154	OB5941 H1 raw.raw	2.5247E4	1	1	118	127			PEAKS DB
R.KSFN(+.98)LDEGHALRIPSGFISYILNR.H	N	47.51	2747.4395	24	4.5	687.8702	4	36.15	14	F14:1837	OB5941 H1 raw.raw	1.0524E6	1	1	248	271		N4:Deamidation (NQ):23.98	PEAKS DB
R.LFEVKPDKKN(+.98)PQLQDLDMMLTC(+57.02)VEIK.E	N	47.32	3132.5908	26	9.3	1567.3173	2	34.35	14	F14:1745	OB5941 H1 raw.raw	1.9315E5	1	1	406	431	Carbamidomethylation	N10:Deamidation (NQ):14.04;C22:Carbamidomethylation:1000.00	PEAKS DB
K.N(+.98)PQ(+.98)LQDLDMMLTC(+57.02)VEIKEGALM(+15.99)LPHFNSK.A	N	46.82	3389.6016	29	9.0	848.4153	4	36.92	12	F12:1851	OB5939 H1 raw.raw	7.3613E6	2	2	415	443	Carbamidomethylation	N1:Deamidation (NQ):11.12;Q3:Deamidation (NQ):0.00;C13:Carbamidomethylation:1000.00;M22:Oxidation (M):24.93	PEAKS DB
W.GTPGSEVREETSR.N	Y	46.79	1403.6641	13	3.5	702.8417	2	27.60	14	F14:1327	OB5941 H1 raw.raw	5.9917E4	1	1	153	165			PEAKS DB
N.PQLQDLDMMLTC(+57.02)VEIKEGALMLPHFNSK.A	N	46.63	3257.5957	28	0.9	815.4069	4	37.83	14	F14:1952	OB5941 H1 raw.raw	2.3692E5	1	1	416	443	Carbamidomethylation	C12:Carbamidomethylation:1000.00	PEAKS DB
K.Q(-17.03)AKDLAFPGSGEQVEK.L	N	46.49	1685.8260	16	0.9	843.9211	2	29.97	12	F12:1427	OB5939 H1 raw.raw	0	0	0	552	567	Pyro-glu from Q	Q1:Pyro-glu from Q:1000.00	PEAKS PTM
K.SFN(+.98)LDEGHALRIPSGFISYILNRHDNQNLR.V	N	46.45	3496.7600	30	-3.9	700.3566	5	36.38	12	F12:1849	OB5939 H1 raw.raw	6.986E5	1	1	249	278		N3:Deamidation (NQ):20.27	PEAKS DB

total 219 peptides

Peptide	Uniq	-10lgP	Mass	Length	ppm	m/z	z	RT	Fraction	Scan	Source File	Area Raw in gel	#Feature	#Feature Raw in gel	Start	End	PTM	AScore	Found By
R.REQ(+.98)EWEEEEEDEEEEGSNR.E	N	45.55	2437.9265	19	7.6	813.6556	3	26.55	12	F12:1226	OB5939 H1 raw.raw	4.2319E4	1	1	472	490		Q3:Deamidation (NQ): 42.90	PEAKS DB
T.PGQFEDFFPASSR.D	N	45.25	1483.6731	13	-1.3	742.8429	2	40.34	13	F13:2103	OB5940 H1 raw.raw	4.0298E3	1	1	289	301			PEAKS DB
R.IPSGFISYILN(+.98)RHDNQNLR.V	N	45.21	2257.1604	19	9.9	753.4015	3	37.61	2	F2:1698	OB5925 H3B raw.raw	2.0603E6	2	2	260	278		N11:Deamidation (NQ):33.06	PEAKS DB
T.PGSEVREETSR.N	Y	45.10	1245.5948	11	1.9	623.8059	2	27.69	12	F12:1287	OB5939 H1 raw.raw	4.1909E4	1	1	155	165			PEAKS DB
N.NPFYFPSRR.F	N	45.02	1182.5934	9	1.6	395.2057	3	28.08	13	F13:1358	OB5940 H1 raw.raw	2.9991E4	1	1	167	175			PEAKS DB
R.DQS(-18.01)SYLQGFSR.N	N	44.90	1268.5785	11	7.0	635.3010	2	30.87	12	F12:1484	OB5939 H1 raw.raw	0	0	0	302	312		S3:Dehydration:33.98	PEAKS PTM
K.GTGNLELVAVRKEQQQR.G	N	44.58	1925.0442	17	0.6	642.6890	3	25.89	14	F14:1220	OB5941 H1 raw.raw	1.1112E5	2	2	453	469			PEAKS DB
R.EDWRRPSHQQPR.K	N	43.98	1590.7764	12	-0.3	398.7012	4	24.94	14	F14:1169	OB5941 H1 raw.raw	1.0819E4	1	1	128	139			PEAKS DB
R.IVQIEARPNTLVLPK(+57.02).H	Y	43.35	1747.0355	15	-2.4	874.5229	2	30.35	13	F13:1460	OB5940 H1 raw.raw	8.446E6	1	1	208	222	Carbamidomethylation (DHKE, X@N-term)	K15:Carbamidomethylation (DHKE, X@N-term):110.46	PEAKS PTM
R.VLLE(+21.98)ENAGGEQEER.G	N	42.61	1593.7246	14	-0.7	797.8690	2	26.03	13	F13:1239	OB5940 H1 raw.raw	1.373E5	3	3	329	342		E4:Sodium adduct:40.00	PEAKS PTM
K.HADADNILVIQ(+.98)QGQATVTVANGNNRK.S	N	42.22	2747.3950	26	5.9	916.8110	3	45.04	14	F14:2357	OB5941 H1 raw.raw	0	0	0	223	248		Q11:Deamidation (NQ):9.34	PEAKS DB
K.IRPEGREGEQEWGTPGSEVR.E	Y	42.02	2268.0881	20	1.1	757.0375	3	26.28	14	F14:1248	OB5941 H1 raw.raw	5.4096E4	1	1	141	160			PEAKS DB
K.HADADNILVIQQGQAT(-18.01)VTVAN(+.98)GNNRK.S	N	41.92	2729.3845	26	0.3	683.3536	4	28.96	13	F13:1411	OB5940 H1 raw.raw	3.5545E5	1	1	223	248		T16:Dehydration:8.22; N21:Deamidation (NQ):6.08	PEAKS PTM
K.HADADN(+.98)ILVIQQGQATVTVANGNNRK.S	N	41.76	2747.3950	26	3.7	916.8090	3	50.62	12	F12:2616	OB5939 H1 raw.raw	1.1355E5	2	2	223	248		N6:Deamidation (NQ): 42.57	PEAKS DB
K.ISMPVN(+.98)TPGQFEDFFPASSR.D	N	41.75	2227.0254	20	9.3	1114.5303	2	58.23	14	F14:3138	OB5941 H1 raw.raw	2.4983E4	1	1	282	301		N6:Deamidation (NQ): 42.89	PEAKS DB
M.LPHFNSK.A	N	41.73	841.4446	7	-3.4	421.7281	2	30.24	14	F14:1484	OB5941 H1 raw.raw	1.8069E6	2	2	437	443			PEAKS DB
R.PNTLVLPK.H	N	41.66	880.5382	8	0.4	441.2766	2	27.53	13	F13:1313	OB5940 H1 raw.raw	8.5249E4	1	1	215	222			PEAKS DB
N.TLEAAFNAEFNEIRR.V	N	41.51	1779.8904	15	-3.0	890.9498	2	32.91	14	F14:1655	OB5941 H1 raw.raw	4.1608E5	1	1	314	328			PEAKS DB
K.KGSEEEDITN(-17.03)PINLR.D	N	41.49	1696.8268	15	3.3	566.6180	3	28.85	14	F14:1397	OB5941 H1 raw.raw	2.3089E5	1	1	379	393		N10:Ammonia-loss (N):40.63	PEAKS PTM
K.KNPQLQ(+.98)DLDMMLTC(+57.02)VEIKEGALMLPHFNSK.A	N	40.89	3500.7175	30	0.4	1167.9136	3	36.34	13	F13:1850	OB5940 H1 raw.raw	4.7608E5	1	1	414	443	Carbamidomethylation	Q6:Deamidation (NQ): 14.04;C14: Carbamidomethylation:1000.00	PEAKS DB
R.REQEWEEEEEDEEEEGSNREVR.R	N	40.76	2821.1545	22	3.9	941.3958	3	26.47	14	F14:1247	OB5941 H1 raw.raw	1.8468E6	1	1	472	493			PEAKS DB

total 219 peptides

Peptide	Uniq	-10lgP	Mass	Length	ppm	m/z	z	RT	Fraction	Scan	Source File	Area Raw in gel	#Feature	#Feature Raw in gel	Start	End	PTM	AScore	Found By
R.NTLEAAFN(+.98)AEFNEIRR.V	N	40.23	1894.9172	16	-0.3	948.4656	2	32.92	12	F12:1570	OB5939 H1 raw.raw	1.5322E6	1	1	313	328		N8:Deamidation (NQ): 8.26	PEAKS DB
K.NPQLQDLDM(+15.99)M(+15.99)LTC(+57.02)VEIK.E	N	39.57	2078.9688	17	1.1	1040.4928	2	33.80	14	F14:1701	OB5941 H1 raw.raw	0	0	0	415	431	Oxidation (M); Carbamidomethylation	M9:Oxidation (M):1000.00;M10: Oxidation (M):1000.00;C13:Carbamidomethylation:1000.00	PEAKS DB
R.IPS(+59.02)GFISYILNR.H	N	39.40	1437.7802	12	1.5	719.8984	2	36.96	12	F12:1870	OB5939 H1 raw.raw	4.3694E5	1	1	260	271		S3:Aminoethylcysteine:49.79	PEAKS PTM
K.HADADNILVIQQ(-18.01).G	N	38.68	1317.6677	12	-4.9	659.8379	2	29.56	14	F14:1421	OB5941 H1 raw.raw	1.6968E5	2	2	223	234	Dehydration	Q12:Dehydration:76.18	PEAKS PTM
K.AMVIVVVNK(-1.03)GTGNLELVAVR.K	N	37.92	2080.1714	20	4.3	1041.0974	2	33.79	13	F13:1738	OB5940 H1 raw.raw	2.1453E7	1	1	444	463	Lysine oxidation to aminoadipic semialdehyde	K9:Lysine oxidation to aminoadipic semialdehyde:1000.00	PEAKS PTM
I.PSGFISYILNRHDNQNLR.V	N	37.07	2143.0923	18	4.0	715.3742	3	33.53	12	F12:1645	OB5939 H1 raw.raw	0	0	0	261	278			PEAKS DB
K.EGALM(-48.00)LPHFNSK.A	N	36.97	1294.6670	12	2.0	432.5638	3	28.31	14	F14:1348	OB5941 H1 raw.raw	1.3683E6	1	1	432	443	Dethiomethyl	M5:Dethiomethyl:1000.00	PEAKS PTM
R.IFLAGDKDNVIDQ(+.98)IEK.Q	N	36.27	1817.9410	16	7.9	606.9924	3	63.32	14	F14:3434	OB5941 H1 raw.raw	1.3413E4	1	1	536	551		Q13:Deamidation (NQ):25.70	PEAKS DB
K.HAD(+57.02)ADNILVIQQGQATVTVANGN(+.98)NRK.S	N	35.92	2804.4165	26	-3.8	702.1088	4	28.95	13	F13:1413	OB5940 H1 raw.raw	2.7833E5	1	1	223	248		D3:Carbamidomethylation (DHKE, X@N-term):4.64;N23:Deamidation (NQ):0.00	PEAKS PTM
K.NPQ(+.98)LQDLDMMLTC(+57.02)VEIKEGALMLPHFNSK.A	N	35.84	3372.6226	29	9.4	1125.2253	3	40.73	12	F12:2064	OB5939 H1 raw.raw	5.6313E5	1	1	415	443	Carbamidomethylation	Q3:Deamidation (NQ): 0.00;C13:Carbamidomethylation: 1000.00	PEAKS DB
K.ISM(+15.99)PVNTPGQ(+.98)FEDFFPASSR.D	N	35.67	2243.0205	20	6.2	1122.5245	2	50.73	12	F12:2643	OB5939 H1 raw.raw	0	0	0	282	301	Oxidation (M)	M3:Oxidation (M):1000.00;Q10:Deamidation (NQ):22.37	PEAKS DB
K.GT(-18.01)GNLELVAVRK.E	N	35.64	1237.7142	12	0.9	413.5790	3	26.41	13	F13:1259	OB5940 H1 raw.raw	7.4255E5	3	3	453	464	Dehydration	T2:Dehydration:1000.00	PEAKS PTM
K.SFNLDEGHALRIPS.G	N	35.38	1554.7791	14	-4.8	778.3931	2	30.79	13	F13:1515	OB5940 H1 raw.raw	5.2113E3	1	1	249	262			PEAKS DB
R.DQ(+.98)SSYLQGFSR.N	N	34.61	1287.5731	11	-3.2	644.7917	2	63.83	14	F14:3449	OB5941 H1 raw.raw	0	0	0	302	312		Q2:Deamidation (NQ): 41.87	PEAKS DB
R.S(+57.02)SDNEGVIVK.V	N	34.54	1103.5459	10	0.3	552.7804	2	23.19	13	F13:1066	OB5940 H1 raw.raw	0	0	0	351	360		S1:Carbamidomethylation (DHKE, X@N-term):11.10	PEAKS PTM
Q.IEARPNTLVLPK.H	Y	34.19	1349.8030	12	2.2	675.9103	2	30.40	12	F12:1461	OB5939 H1 raw.raw	1.011E6	3	3	211	222			PEAKS DB
R.NN(-17.03)PFYFPSRR.F	N	34.10	1279.6097	10	-0.4	427.5437	3	28.25	13	F13:1369	OB5940 H1 raw.raw	1.47E5	1	1	166	175		N2:Ammonia-loss (N): 12.28	PEAKS PTM
**total 219 peptides**																			

Peptide	Uniq	-10lgP	Mass	Length	ppm	m/z	z	RT	Fraction	Scan	Source File	Area Raw in gel	#Feature	#Feature Raw in gel	Start	End	PTM	AScore	Found By
T.G(+214.97)NLELVAVRK.E	N	33.47	1312.6267	10	2.3	657.3221	2	27.95	14	F14:1280	OB5941 H1 raw.raw	2.4669E5	1	1	455	464	4-sulfophenyl isothiocyanate	G1:4-sulfophenyl isothiocyanate:144.45	PEAKS PTM
K.HADADNILVIQQ(+.98)GQATVTVANGNN(+.98)RK.S	N	33.28	2748.3792	26	7.6	917.1406	3	48.20	12	F12:2471	OB5939 H1 raw.raw	2.0672E4	1	1	223	248		Q12:Deamidation (NQ):23.10;N24:Deamidation (NQ):0.00	PEAKS DB
K.HADAD(-18.01)NILVIQQGQATVTVANGNNRK.S	N	33.25	2728.4004	26	3.5	910.4772	3	28.70	13	F13:1390	OB5940 H1 raw.raw	0	0	0	223	248		D5:Dehydration:30.36	PEAKS PTM
K.ISM(+15.99)PVN(+.98)TPGQFEDFFPASSR.D	N	33.05	2243.0205	20	7.6	1122.5260	2	49.72	13	F13:2625	OB5940 H1 raw.raw	0	0	0	282	301	Oxidation (M)	M3:Oxidation (M):1000.00;N6:Deamidation (NQ):25.70	PEAKS DB
R.IFLAGDK(+26.02)DNVIDQIEK.Q	N	32.97	1842.9727	16	-0.2	922.4934	2	35.63	14	F14:1844	OB5941 H1 raw.raw	1.4466E5	1	1	536	551	Acetaldehyde +26	K7:Acetaldehyde +26:74.34	PEAKS PTM
K.NPQLQ(+.98)DLDMM(+15.99)LTC(+57.02)VEIK.E	N	32.85	2063.9578	17	-3.4	1032.9827	2	37.19	12	F12:1881	OB5939 H1 raw.raw	2.2954E4	1	1	415	431	Carbamidomethylation	Q5:Deamidation (NQ):26.31;M10:Oxidation (M):0.00;C13:Carbamidomethylation:1000.00	PEAKS DB
K.HADADNILVIQ(+.98)QGQ(+.98)ATVTVANGNNRK.S	N	32.62	2748.3792	26	3.1	917.1365	3	57.24	13	F13:3064	OB5940 H1 raw.raw	0	0	0	223	248		Q11:Deamidation (NQ):0.00;Q14:Deamidation (NQ):0.00	PEAKS DB
K.N(+.98)PQ(+.98)LQ(+.98)DLDMM(+15.99)LTC(+57.02)VEIKEGALMLPHFNSK.A	N	32.23	3390.5854	29	3.7	848.6568	4	38.47	14	F14:1982	OB5941 H1 raw.raw	0	0	0	415	443	Deamidation (NQ); Carbamidomethylation	N1:Deamidation (NQ):62.42;Q3:Deamidation (NQ):65.92;Q5:Deamidation (NQ):60.92;M10:Oxidation (M):12.28;C13:Carbamidomethylation:1000.00	PEAKS DB
R.EGEQEWGT(-18.01)PGSEVREETSR.N	Y	32.15	2143.9407	19	2.2	1072.9800	2	27.69	12	F12:1293	OB5939 H1 raw.raw	4.1679E4	1	1	147	165		T8:Dehydration:20.70	PEAKS PTM
K.KN(+.98)PQ(+.98)LQ(+.98)DLDM(+15.99)MLTC(+57.02)VEIKEGALMLPHFNSK.A	N	31.81	3518.6804	30	-3.0	880.6747	4	37.45	12	F12:1882	OB5939 H1 raw.raw	0	0	0	414	443	Deamidation (NQ); Carbamidomethylation	N2:Deamidation (NQ):58.75;Q4:Deamidation (NQ):61.65;Q6:Deamidation (NQ):53.75;M10:Oxidation (M):0.00;C14:Carbamidomethylation:1000.00	PEAKS DB
**total 219 peptides**																			

N1NG13|N1NG13_ARAHY

back to list

| Protein Coverage | Supporting Peptides |

Protein Coverage:

1 MRGRVSPLML LLGILVLASV SATHAKSSPY QKKTENPCAQ RCLQSCQQEP DDLKQKACES RCTKLEYDPR CVYDPRGHTG

81 TTNQRSPPGE RTRGRQPGDY DDDRRQPRRE EGGRWGPAGP REREREEDWR QPREDWRRPS HQQPRKIRPE GREGEQEWGT

161 PGSHVREETS RNNPFYFPSR RFSTRYGNQN GRIRVLQRFD QRSRQFQNLQ NHRIVQIEAK PNTLVLPKHA DADNILVIQQ

241 GQATVTVANG NNRKSFNLDE GHALRIPSGF ISYILNRHDN QNLRVAKISM PVNTPGQFED FFPASSRDQS SYLQGFSRNT

321 LEAAFNAEFN EIRRVLLEEN AGGEQEERGQ RRWSTRSSEN NEGVIVKVSK EHVEELTKHA KSVSKKGSEE EGDITNPINL

401 REGEPDLSNN FGKLFEVKPD KKNPQLQDLD MMLTCVEIKE GALMLPHFNS KAMVIVVVNK GTGNLELVAV RKEQQQRGRR

481 EEEEDEDEEE EGSNREVRRY TARLKEGDVF IMPAAHPVAI NASSELHLLG FGINAENNHR IFLAGDKDNV IDQIEKQAKD

561 LAFPGSGEQV EKLIKNQKES HFVSARPQSQ SQSPSSPEKE SPEKEDQEEE NQGGKGPLLS ILKAFN

- a Ammonia-loss (N) (-17.03)
- a Acetaldehyde +26 (+26.02)
- c Carbamidomethylation (+57.02)
- c Carbamidomethylation (DHKE, X@N-term) (+57.02)
- d Deamidation (NQ) (+0.98)
- d Dethiomethyl (-48.00)
- d Dehydration (-18.01)
- f Formylation (+27.99)
- i Iodination (+125.90)
- l Lysine oxidation to aminoadipic semialdehyde (-1.03)
- m Methylation(KR) (+14.02)
- m Methylation(others) (+14.02)
- o Oxidation (M) (+15.99)
- p Pyro-glu from E (-18.01)
- p Pyro-glu from Q (-17.03)
- s 4-sulfophenyl isothiocyanate (+214.97)

Supporting Peptides:

Peptide	Uniq	-10lgP	Mass	Length	ppm	m/z	z	RT	Fraction	Scan	Source File	Area Raw in gel	#Feature	#Feature Raw in gel	Start	End	PTM	AScore	Found By
K.HADADNILVIQQGQATVTVAN(+.98)GNNRK.S	N	140.09	2747.3950	26	-1.1	916.8046	3	29.38	14	F14:1509	OB5941 H1 raw.raw	3.7837E8	20	20	229	254		N21:Deamidation (N Q):26.31	PEAKS DB
K.KNPQLQDLDMMLTC(+57.02)VEIK.E	N	137.14	2175.0737	18	2.4	1088.5468	2	34.90	14	F14:1774	OB5941 H1 raw.raw	4.1086E6	3	3	422	439	Carbamidomethylation	C14:Carbamidomethylation:1000.00	PEAKS DB
K.HADADNILVIQQGQATVTVANGNNR.K	N	135.84	2618.3162	25	2.9	1310.1692	2	30.42	14	F14:1494	OB5941 H1 raw.raw	5.8142E6	3	3	229	253			PEAKS DB
K.ISMPVNTPGQFEDFFPASSR.D	N	135.58	2226.0415	20	1.9	1114.0302	2	35.63	14	F14:1807	OB5941 H1 raw.raw	2.1661E8	32	26	288	307			PEAKS DB
K.ISM(+15.99)PVNTPGQFEDFFPASSR.D	N	134.77	2242.0364	20	2.3	1122.0281	2	34.42	2	F2:1522	OB5925 H3B raw.raw	1.6845E7	19	17	288	307	Oxidation (M)	M3:Oxidation (M):1000.00	PEAKS DB
K.NPQLQDLDMMLTC(+57.02)VEIK.E	N	131.65	2046.9788	17	-0.8	1024.4958	2	36.89	14	F14:1903	OB5941 H1 raw.raw	1.2559E7	8	8	423	439	Carbamidomethylation	C13:Carbamidomethylation:1000.00	PEAKS DB
K.NPQLQDLDM(+15.99)MLTC(+57.02)VEIK.E	N	128.59	2062.9736	17	-6.6	1032.4873	2	36.34	14	F14:1860	OB5941 H1 raw.raw	2.4371E5	2	2	423	439	Carbamidomethylation	M9:Oxidation (M):30.46;C13:Carbamidomethylation:1000.00	PEAKS DB
K.KGSEEEGDITNPINLREGEPDLSNNFGK.L	Y	126.17	3058.4478	28	-3.6	1020.4862	3	31.46	14	F14:1557	OB5941 H1 raw.raw	5.2061E7	16	16	386	413			PEAKS DB
R.NTLEAAFNAEFNEIR.R	N	126.11	1737.8322	15	3.3	869.9262	2	35.27	14	F14:1792	OB5941 H1 raw.raw	1.3691E7	6	6	319	333			PEAKS DB
K.HADADNILVIQQGQATVTVANGNNRK.S	N	120.98	2746.4111	26	4.7	1374.2192	2	29.02	14	F14:1406	OB5941 H1 raw.raw	4.4412E7	13	13	229	254			PEAKS DB
K.KGSEEEGDITNPINLR.E	N	117.96	1770.8748	16	-0.9	886.4438	2	28.67	14	F14:1384	OB5941 H1 raw.raw	2.1993E7	17	17	386	401			PEAKS DB
K.SFNLDEGHALRIPSGFISYILNR.H	N	117.92	2618.3604	23	0.6	1310.1882	2	36.89	14	F14:1902	OB5941 H1 raw.raw	2.1092E8	40	39	255	277			PEAKS DB
R.VLLEENAGGEQEER.G	N	114.70	1571.7427	14	1.1	786.8795	2	26.65	14	F14:1250	OB5941 H1 raw.raw	1.2337E7	7	7	335	348			PEAKS DB
K.GSEEEGDITNPINLR.E	N	114.17	1642.7798	15	1.7	822.3986	2	30.60	14	F14:1507	OB5941 H1 raw.raw	8.7873E6	4	4	387	401			PEAKS DB
R.IFLAGDKDNVIDQIEKQAK.D	N	113.07	2144.1477	19	1.1	715.7240	3	32.93	7	F7:1578	OB5934 H3B raw.raw	5.566E7	12	12	541	559			PEAKS DB
K.AMVIVVVNKGTGNLELVAVR.K	N	112.17	2081.2031	20	1.5	694.7427	3	34.35	14	F14:1739	OB5941 H1 raw.raw	7.0931E7	12	12	452	471			PEAKS DB
R.KSFNLDEGHALR.I	N	112.03	1385.7051	12	1.1	693.8606	2	25.70	14	F14:1202	OB5941 H1 raw.raw	3.2948E6	6	6	254	265			PEAKS DB
R.IPSGFISYILNR.H	N	111.89	1378.7609	12	1.5	690.3887	2	43.03	14	F14:2242	OB5941 H1 raw.raw	2.1426E8	15	15	266	277			PEAKS DB
R.DQSSYLQGFSR.N	N	110.94	1286.5891	11	-1.4	644.3009	2	30.60	14	F14:1505	OB5941 H1 raw.raw	6.2405E7	21	21	308	318			PEAKS DB
K.NPQLQDLDMMLTC(+57.02)VEIKEGALMLPHFNSK.A	N	110.02	3371.6387	29	-0.1	1686.8264	2	37.85	14	F14:1946	OB5941 H1 raw.raw	1.7936E8	14	14	423	451	Carbamidomethylation	C13:Carbamidomethylation:1000.00	PEAKS DB
K.AMVIVVVNKGTGNLELVAVRK.E	N	109.98	2209.2981	21	-1.4	1105.6548	2	32.55	14	F14:1615	OB5941 H1 raw.raw	2.9136E8	18	18	452	472			PEAKS DB
**total 217 peptides**																			

Peptide	Uniq	-10lgP	Mass	Length	ppm	m/z	z	RT	Fraction	Scan	Source File	Area Raw in gel	#Feature	#Feature Raw in gel	Start	End	PTM	AScore	Found By
K.AM(+15.99)VIVVVNKGTGNLELVAVR.K	N	109.59	2097.1980	20	-2.2	1049.6040	2	32.73	14	F14:1638	OB5941 H1 raw.raw	3.1537E7	10	10	452	471	Oxidation (M)	M2:Oxidation (M):1000.00	PEAKS DB
K.SFNLDEGHALR.I	N	109.41	1257.6101	11	-3.3	629.8102	2	27.77	14	F14:1329	OB5941 H1 raw.raw	7.2473E7	30	30	255	265			PEAKS DB
R.EGEQEWGTPGSHVR.E	N	108.42	1567.7015	14	0.5	784.8585	2	25.51	14	F14:1193	OB5941 H1 raw.raw	5.0052E5	6	6	153	166			PEAKS DB
R.IFLAGDKDNVIDQIEK.Q	N	108.13	1816.9570	16	0.2	909.4860	2	33.10	14	F14:1683	OB5941 H1 raw.raw	7.2865E8	41	41	541	556			PEAKS DB
R.KSFNLDEGHALRIPSGFISYILNR.H	N	107.80	2746.4553	24	-2.1	916.4905	3	36.16	12	F12:1802	OB5939 H1 raw.raw	7.186E6	4	4	254	277			PEAKS DB
R.I(+57.02)PSGFISYILNR.H	N	104.65	1435.7823	12	-1.7	718.8972	2	36.52	14	F14:1869	OB5941 H1 raw.raw	1.3017E7	3	3	266	277	Carbamidomethylation (DHKE, X@N-term)	I1:Carbamidomethylation (DHKE, X@N-term):1000.00	PEAKS PTM
R.VLLEENAGGEQEERGQR.R	N	104.46	1912.9238	17	1.8	957.4709	2	25.31	14	F14:1182	OB5941 H1 raw.raw	1.9161E6	8	8	335	351			PEAKS DB
R.I(+57.02)FLAGDKDNVIDQIEK.Q	N	103.96	1873.9785	16	-5.0	937.9919	2	32.19	14	F14:1619	OB5941 H1 raw.raw	4.5184E7	7	7	541	556	Carbamidomethylation (DHKE, X@N-term)	I1:Carbamidomethylation (DHKE, X@N-term):90.91	PEAKS PTM
R.EGEPDLSNNFGK.L	Y	103.27	1305.5836	12	0.4	653.7994	2	27.95	14	F14:1330	OB5941 H1 raw.raw	7.4849E6	3	3	402	413			PEAKS DB
K.QAKDLAFPGSGEQVEK.L	N	102.73	1702.8525	16	1.8	852.4351	2	26.85	14	F14:1278	OB5941 H1 raw.raw	1.286E6	6	6	557	572			PEAKS DB
K.H(+57.02)ADADNILVIQQGQATVTVANGNNRK.S	N	102.10	2803.4324	26	-1.3	935.4835	3	29.92	14	F14:1452	OB5941 H1 raw.raw	1.6897E6	1	1	229	254		H1:Carbamidomethylation (DHKE, X@N-term):39.76	PEAKS PTM
K.DLAFPGSGEQVEK.L	N	101.44	1375.6619	13	-2.0	688.8369	2	30.08	14	F14:1472	OB5941 H1 raw.raw	6.5563E7	9	9	560	572			PEAKS DB
K.GSEEEGDITNPINLREGEPDLSNNFGK.L	Y	101.39	2930.3530	27	-3.6	1466.1785	2	31.62	13	F13:1574	OB5940 H1 raw.raw	1.3299E7	8	8	387	413			PEAKS DB
K.NPQLQDLDMMLTCVEIK(+14.02).E	N	101.30	2003.9730	17	2.1	1002.9958	2	38.00	14	F14:1960	OB5941 H1 raw.raw	6.7851E5	2	2	423	439	Methylation(KR)	K17:Methylation(KR):1000.00	PEAKS PTM
K.S(+57.02)FNLDEGHALR.I	N	100.96	1314.6316	11	0.8	658.3236	2	27.59	14	F14:1340	OB5941 H1 raw.raw	5.6012E6	7	7	255	265	Carbamidomethylation (DHKE, X@N-term)	S1:Carbamidomethylation (DHKE, X@N-term):101.71	PEAKS PTM
R.RVLLEENAGGEQEER.G	N	98.06	1727.8438	15	0.4	576.9554	3	24.77	13	F13:1156	OB5940 H1 raw.raw	1.2517E5	5	5	334	348			PEAKS DB
R.IPSGFISYILNRHDNQNLR.V	N	97.42	2256.1763	19	0.5	565.0516	4	33.66	14	F14:1693	OB5941 H1 raw.raw	3.392E8	42	42	266	284			PEAKS DB
K.AM(+15.99)VIVVVNKGTGNLELVAVRK.E	N	96.33	2225.2930	21	-0.4	1113.6533	2	30.76	14	F14:1520	OB5941 H1 raw.raw	4.0787E7	8	7	452	472	Oxidation (M)	M2:Oxidation (M):1000.00	PEAKS DB
K.KNPQLQDLDMM(+15.99)LTC(+57.02)VEIK.E	N	95.01	2191.0686	18	-3.4	1096.5378	2	32.73	14	F14:1643	OB5941 H1 raw.raw	1.9787E5	1	1	422	439	Carbamidomethylation	M11:Oxidation (M):9.34;C14:Carbamidomethylation:1000.00	PEAKS DB
K.NPQLQDLDMM(-48.00)LTC(+57.02)VEIK.E	N	93.52	1998.9755	17	-2.5	1000.4926	2	32.55	14	F14:1628	OB5941 H1	1.3958E7	5	5	423	439	Carbamidomethylation	M10:Dethiomethyl:27.	PEAKS PTM

total 217 peptides

Peptide	Uniq	-10lgP	Mass	Length	ppm	m/z	z	RT	Fraction	Scan	Source File	Area Raw in gel	#Feature	#Feature Raw in gel	Start	End	PTM	AScore	Found By
											raw.raw							96;C13:Carbamidomethylation:1000.00	
K.NPQLQDLDM(+15.99)MLTC(+57.02)VEIKEGALMLPHFNSK.A	N	92.22	3387.6335	29	-1.5	1130.2168	3	36.17	14	F14:1926	OB5941 H1 raw.raw	2.8061E7	4	4	423	451	Carbamidomethylation	M9:Oxidation (M):10.11;C13:Carbamidomethylation:1000.00	PEAKS DB
R.NNPFYFPSRR.F	N	92.08	1296.6364	10	-3.0	649.3235	2	28.67	14	F14:1408	OB5941 H1 raw.raw	1.2788E8	21	21	172	181			PEAKS DB
K.EGALM(+15.99)LPHFNSK.A	N	91.98	1358.6653	12	2.1	680.3414	2	29.74	14	F14:1443	OB5941 H1 raw.raw	1.9561E6	3	3	440	451	Oxidation (M)	M5:Oxidation (M):1000.00	PEAKS DB
R.VLLEENAGGEQEERGQRR.W	N	91.09	2069.0249	18	2.1	690.6837	3	25.31	14	F14:1186	OB5941 H1 raw.raw	1.1888E6	3	3	335	352			PEAKS DB
K.KNPQLQDLDMM(+15.99)LTC(+57.02)VEIKEGALMLPHFNSK.A	N	90.76	3515.7285	30	-2.7	879.9370	4	34.52	13	F13:1755	OB5940 H1 raw.raw	2.6989E6	3	3	422	451	Carbamidomethylation	M11:Oxidation (M):30.46;C14:Carbamidomethylation:1000.00	PEAKS DB
K.AM(-48.00)VIVVVNKGTGNLELVAVR.K	N	90.57	2033.1997	20	-3.4	1017.6036	2	31.46	14	F14:1539	OB5941 H1 raw.raw	1.6588E7	6	6	452	471	Dethiomethyl	M2:Dethiomethyl:1000.00	PEAKS PTM
K.A(+57.02)MVIVVVNKGTGNLELVAVR.K	N	90.54	2138.2246	20	-0.9	1070.1187	2	34.35	14	F14:1737	OB5941 H1 raw.raw	5.6414E6	3	3	452	471	Carbamidomethylation (DHKE, X@N-term)	A1:Carbamidomethylation (DHKE, X@N-term):207.56	PEAKS PTM
K.NPQLQDLDMMLTC(+57.02)VEIKEGALM(+15.99)LPHFNSK.A	N	90.37	3387.6335	29	-1.5	1130.2168	3	36.17	14	F14:1895	OB5941 H1 raw.raw	3.1109E7	7	7	423	451	Carbamidomethylation; Oxidation (M)	C13:Carbamidomethylation:1000.00;M22:Oxidation (M):69.89	PEAKS DB
K.H(+57.02)ADADNILVIQQGQATVTVAN(+.98)GNNRK.S	N	90.07	2804.4165	26	-1.3	935.8115	3	30.08	14	F14:1476	OB5941 H1 raw.raw	6.8084E6	2	2	229	254		H1:Carbamidomethylation (DHKE, X@N-term):45.01;N21:Deamidation (NQ):14.04	PEAKS PTM
R.IVQIEAKPNTLVLPK.H	N	89.51	1662.0079	15	-3.4	832.0084	2	29.92	14	F14:1456	OB5941 H1 raw.raw	3.047E8	42	42	214	228			PEAKS DB
F.LAGDKDNVIDQIEK.Q	N	89.22	1556.8046	14	-3.2	779.4071	2	32.19	12	F12:1557	OB5939 H1 raw.raw	4.4864E6	2	2	543	556			PEAKS DB
K.I(+57.02)SMPVNTPGQFEDFFPASSR.D	N	88.49	2283.0630	20	-0.1	1142.5387	2	35.84	12	F12:1793	OB5939 H1 raw.raw	6.4772E5	1	1	288	307	Carbamidomethylation (DHKE, X@N-term)	I1:Carbamidomethylation (DHKE, X@N-term):156.03	PEAKS PTM
K.DLAFPGSGEQVEKLIK.N	N	87.96	1729.9249	16	1.0	865.9706	2	33.30	7	F7:1601	OB5934 H3B raw.raw	6.2551E5	3	3	560	575			PEAKS DB
K.EGALMLPHFNSK.A	N	87.95	1342.6703	12	2.5	672.3441	2	29.83	13	F13:1462	OB5940 H1 raw.raw	9.3502E6	3	3	440	451			PEAKS DB
K.HADADNILVIQQGQATVTVAN(+.98)GNNR.K	N	87.06	2619.3000	25	-1.2	1310.6558	2	30.35	13	F13:1535	OB5940 H1 raw.raw	4.8683E6	1	1	229	253		N21:Deamidation (NQ):7.32	PEAKS DB
K.AMVIVVVNK.G	N	86.97	971.5837	9	0.9	486.7996	2	29.88	12	F12:1441	OB5939 H1 raw.raw	9.5124E6	3	3	452	460			PEAKS DB

total 217 peptides

Peptide	Uniq	-10lgP	Mass	Length	ppm	m/z	z	RT	Fraction	Scan	Source File	Area Raw in gel	#Feature	#Feature Raw in gel	Start	End	PTM	AScore	Found By
R.SSENNEGVIVK.V	Y	86.86	1174.5830	11	-0.3	588.2986	2	23.31	14	F14:1084	OB5941 H1 raw.raw	3.5114E5	6	6	357	367			PEAKS DB
K.GTGNLELVAVR.K	N	86.65	1127.6299	11	-0.8	564.8218	2	29.74	14	F14:1450	OB5941 H1 raw.raw	1.0907E7	6	6	461	471			PEAKS DB
R.NNPFYFPSR.R	N	86.37	1140.5352	9	-3.8	571.2727	2	31.46	14	F14:1552	OB5941 H1 raw.raw	1.201E7	5	5	172	180			PEAKS DB
K.HADADNILVIQQGQATVTVAN(-17.03)GNNRK.S	N	86.12	2729.3845	26	-1.1	910.8011	3	29.38	14	F14:1429	OB5941 H1 raw.raw	8.2265E6	4	4	229	254		N21:Ammonia-loss (N):18.53	PEAKS PTM
R.NTLEAAFNAEFNEIRR.V	N	86.03	1893.9332	16	-3.6	947.9705	2	35.28	12	F12:1741	OB5939 H1 raw.raw	3.4229E8	38	38	319	334			PEAKS DB
R.I(+27.99)FLAGDKDNVIDQIEK.Q	N	85.41	1844.9519	16	5.2	923.4880	2	36.16	14	F14:1844	OB5941 H1 raw.raw	0	0	0	541	556	Formylation	I1:Formylation:77.93	PEAKS PTM
K.SFNLDEGH(+57.02)ALR.I	N	84.87	1314.6316	11	-1.2	658.3223	2	28.05	12	F12:1298	OB5939 H1 raw.raw	5.1176E6	5	5	255	265		H8:Carbamidomethylation (DHKE, X@N-term):30.36	PEAKS PTM
R.K(+57.02)SFNLDEGHALR.I	N	83.09	1442.7266	12	-0.4	481.9159	3	25.70	14	F14:1209	OB5941 H1 raw.raw	2.2174E5	3	3	254	265	Carbamidomethylation (DHKE, X@N-term)	K1:Carbamidomethylation (DHKE, X@N-term):51.63	PEAKS PTM
K.SVSKKGSEEEGDITNPINLR.E	N	83.00	2172.1023	20	0.7	725.0419	3	27.21	14	F14:1286	OB5941 H1 raw.raw	1.3065E5	2	2	382	401			PEAKS DB
K.KNPQLQDLDM(+15.99)MLTC(+57.02)VEIK.E	N	81.54	2191.0686	18	0.7	1096.5424	2	33.03	12	F12:1615	OB5939 H1 raw.raw	0	0	0	422	439	Carbamidomethylation	M10:Oxidation (M):14.02;C14:Carbamidomethylation:1000.00	PEAKS DB
K.KNPQLQDLDMMLTC(+57.02)VEIKEGALMLPHFNSK.A	N	81.26	3499.7336	30	-0.1	875.9406	4	36.52	14	F14:1868	OB5941 H1 raw.raw	1.091E7	3	3	422	451	Carbamidomethylation	C14:Carbamidomethylation:1000.00	PEAKS DB
K.GTGNLELVAVRK.E	N	79.30	1255.7249	12	0.9	628.8702	2	26.85	14	F14:1263	OB5941 H1 raw.raw	1.0437E7	9	9	461	472			PEAKS DB
R.I(+57.02)VQIEAKPNTLVLPK.H	N	78.65	1719.0294	15	3.0	860.5245	2	30.75	12	F12:1469	OB5939 H1 raw.raw	1.7854E7	5	5	214	228	Carbamidomethylation (DHKE, X@N-term)	I1:Carbamidomethylation (DHKE, X@N-term):97.69	PEAKS PTM
R.EETSRNNPFYFPSRR.F	N	78.31	1898.9023	15	1.2	633.9755	3	27.17	13	F13:1289	OB5940 H1 raw.raw	6.7264E6	6	6	167	181			PEAKS DB
R.IFLAGDKDNVIDQIE(+57.02)K.Q	N	78.23	1873.9785	16	-5.0	937.9919	2	32.19	14	F14:1583	OB5941 H1 raw.raw	3.036E7	3	3	541	556		E15:Carbamidomethylation (DHKE, X@N-term):0.00	PEAKS PTM
I.PSGFISYILNR.H	N	77.77	1265.6768	11	2.4	633.8472	2	36.17	13	F13:1844	OB5940 H1 raw.raw	2.5204E5	2	2	267	277			PEAKS DB
I.FLAGDKDNVIDQIEK.Q	N	77.74	1703.8729	15	-9.3	852.9358	2	32.01	14	F14:1599	OB5941 H1 raw.raw	1.3574E6	3	3	542	556			PEAKS DB
K.D(+57.02)LAFPGSGEQVEK.L	N	77.21	1432.6833	13	3.8	717.3517	2	30.92	12	F12:1485	OB5939 H1 raw.raw	2.816E5	1	1	560	572	Carbamidomethylation (DHKE, X@N-term)	D1:Carbamidomethylation (DHKE, X@N-term):93.18	PEAKS PTM
K.K(+57.02)GSEEEGDITNPINLR.E	N	77.19	1827.8962	16	-3.2	914.9525	2	28.80	14	F14:1391	OB5941 H1 raw.raw	0	0	0	386	401		K1:Carbamidomethylation (DHKE,	PEAKS PTM

total 217 peptides

Peptide	Uniq	-10lgP	Mass	Length	ppm	m/z	z	RT	Fraction	Scan	Source File	Area Raw in gel	#Feature	#Feature Raw in gel	Start	End	PTM	AScore	Found By
																		X@N-term):8.80	
R.Q(-17.03)FQNLQNHR.I	N	76.65	1166.5581	9	-1.5	584.2855	2	27.77	14	F14:1319	OB5941H1 raw.raw	3.3996E6	3	3	205	213	Pyro-glu from Q	Q1:Pyro-glu from Q:1000.00	PEAKS PTM
K.KGSEEEGDITN(+.98)PINLREGEPDLSNNFGK.L	Y	75.94	3059.4319	28	7.3	1530.7344	2	30.76	14	F14:1523	OB5941H1 raw.raw	5.8148E5	1	1	386	413		N11:Deamidation (NQ):10.86	PEAKS DB
K.HAD(+57.02)ADNILVIQQGQATVTVAN(+.98)GNNRK.S	N	74.85	2804.4165	26	-5.0	935.8081	3	28.96	13	F13:1511	OB5940H1 raw.raw	3.7976E6	1	1	229	254		D3:Carbamidomethylation (DHKE, X@N-term):6.59;N21:Deamidation (NQ):14.04	PEAKS PTM
K.HADADNILVIQQGQATVTVANGN(-17.03)NRK.S	N	74.65	2729.3845	26	-3.3	1365.6951	2	29.38	14	F14:1432	OB5941H1 raw.raw	5.4978E5	1	1	229	254		N23:Ammonia-loss (N):0.00	PEAKS PTM
K.ISMPVNTPGQFEDFFPASSRDQSSYLQGFSR.N	N	74.49	3494.6201	31	2.7	1165.8838	3	36.29	2	F2:1620	OB5925H3B raw.raw	9.3886E7	15	15	288	318			PEAKS DB
R.NTLEAAFNAEFN(+.98)EIRR.V	N	74.39	1894.9172	16	-3.7	948.4624	2	32.73	14	F14:1640	OB5941H1 raw.raw	4.113E6	3	3	319	334	Deamidation (NQ)	N12:Deamidation (NQ):55.21	PEAKS DB
K.LFEVKPDKKNPQLQDLDMMLTC(+57.02)VEIK.E	N	73.38	3131.6069	26	3.7	1044.8801	3	33.97	13	F13:1716	OB5940H1 raw.raw	3.7487E6	3	3	414	439	Carbamidomethylation	C22:Carbamidomethylation:1000.00	PEAKS DB
K.NPQLQDLDMM(+15.99)LTC(+57.02)VEIKEGALMLPHFNSK.A	N	73.21	3387.6335	29	5.6	847.9204	4	35.82	13	F13:1825	OB5940H1 raw.raw	9.6415E6	3	3	423	451	Carbamidomethylation	M10:Oxidation (M):16.90;C13:Carbamidomethylation:1000.00	PEAKS DB
A.GDKDNVIDQIEK.Q	N	73.04	1372.6833	12	-2.8	687.3470	2	32.19	12	F12:1561	OB5939H1 raw.raw	1.4449E6	3	3	545	556			PEAKS DB
R.V(+57.02)LLEENAGGEQEER.G	N	73.00	1628.7642	14	1.1	815.3903	2	27.03	14	F14:1288	OB5941H1 raw.raw	2.7852E5	3	3	335	348		V1:Carbamidomethylation (DHKE, X@N-term):44.44	PEAKS PTM
R.EETSRNNPFYFPSR.R	N	72.75	1742.8011	14	-0.9	581.9405	3	29.89	12	F12:1419	OB5939H1 raw.raw	4.0306E4	1	1	167	180			PEAKS DB
R.IPSGFISYILN(-17.03)R.H	N	72.57	1361.7343	12	1.4	681.8754	2	37.47	13	F13:1926	OB5940H1 raw.raw	5.3561E5	2	2	266	277	Ammonia-loss (N)	N11:Ammonia-loss (N):1000.00	PEAKS PTM
R.IPSGFISY(+125.90)ILNR.H	N	72.49	1504.6575	12	1.5	753.3372	2	37.84	13	F13:1946	OB5940H1 raw.raw	9.8379E5	2	2	266	277	Iodination	Y8:Iodination:1000.00	PEAKS PTM
R.IPSGFISYILNRH(+57.02)DNQNLR.V	N	72.20	2313.1978	19	2.0	579.3079	4	33.29	12	F12:1633	OB5939H1 raw.raw	3.81E7	4	4	266	284		H13:Carbamidomethylation (DHKE, X@N-term):9.34	PEAKS PTM
R.VLLEEN(+.98)AGGEQEER.G	N	71.59	1572.7267	14	-3.7	787.3677	2	26.82	13	F13:1276	OB5940H1 raw.raw	0	0	0	335	348	Deamidation (NQ)	N6:Deamidation (NQ):71.64	PEAKS DB
A.FPGSGEQVEK.L	N	71.45	1076.5138	10	5.5	539.2672	2	30.23	12	F12:1449	OB5939H1 raw.raw	6.1112E4	1	1	563	572			PEAKS DB
**total 217 peptides**																			

Peptide	Uniq	-10lgP	Mass	Length	ppm	m/z	z	RT	Fraction	Scan	Source File	Area Raw in gel	#Feature	#Feature Raw in gel	Start	End	PTM	AScore	Found By
R.E(-18.01)GE(+57.02)QEWGTPGSHVR.E	N	71.00	1606.7124	14	0.1	804.3635	2	27.42	14	F14:1300	OB5941 H1 raw.raw	8.0689E4	3	3	153	166	Pyro-glu from E	E1:Pyro-glu from E:1000.00;E3:Carbamidomethylation (DHKE, X@N-term):45.01	PEAKS PTM
K.A(+57.02)MVIVVVNKGTGNLELVAVRK.E	N	70.77	2266.3196	21	-1.6	1134.1653	2	32.33	13	F13:1602	OB5940 H1 raw.raw	4.861E7	6	6	452	472	Carbamidomethylation (DHKE, X@N-term)	A1:Carbamidomethylation (DHKE, X@N-term):161.20	PEAKS PTM
K.AM(+15.99)VIVVVNK.G	N	70.64	987.5787	9	0.7	494.7969	2	26.66	14	F14:1257	OB5941 H1 raw.raw	8.6431E5	3	3	452	460	Oxidation (M)	M2:Oxidation (M):1000.00	PEAKS DB
K.KNPQLQDLDMMLTC(+57.02)VEIKEGALM(+15.99)LPHFNSK.A	N	70.55	3515.7285	30	-0.8	879.9387	4	35.84	12	F12:1790	OB5939 H1 raw.raw	4.7958E6	2	2	422	451	Carbamidomethylation; Oxidation (M)	C14:Carbamidomethylation:1000.00;M23:Oxidation (M):69.38	PEAKS DB
K.VSKEHVEELTK.H	N	69.86	1297.6877	11	0.8	433.5702	3	24.82	12	F12:1120	OB5939 H1 raw.raw	1.1162E5	6	6	368	378			PEAKS DB
Q.GQATVTVAN(+.98)GNNRK.S	N	69.60	1429.7273	14	-4.1	715.8680	2	29.38	14	F14:1424	OB5941 H1 raw.raw	5.134E5	2	2	241	254		N9:Deamidation (NQ):41.83	PEAKS DB
R.NT(-18.01)LEAAFNAEFNEIRR.V	N	68.32	1875.9227	16	-3.2	626.3129	3	33.81	14	F14:1704	OB5941 H1 raw.raw	1.3248E7	3	3	319	334	Dehydration	T2:Dehydration:1000.00	PEAKS PTM
R.SSEN(+.98)NEGVIVK.V	Y	68.21	1175.5670	11	0.8	588.7913	2	24.17	14	F14:1126	OB5941 H1 raw.raw	1.8176E4	2	2	357	367		N4:Deamidation (NQ):20.00	PEAKS DB
R.SRQFQNLQNHR.I	N	67.97	1426.7178	11	-0.3	476.5797	3	24.94	14	F14:1159	OB5941 H1 raw.raw	1.4052E4	2	2	203	213			PEAKS DB
K.LFEVKPDKKNPQLQDLDM(+15.99)M(+15.99)LTC(+57.02)VEIK.E	N	67.75	3163.5967	26	2.1	791.9081	4	32.37	12	F12:1585	OB5939 H1 raw.raw	6.2675E5	1	1	414	439	Oxidation (M); Carbamidomethylation	M18:Oxidation (M):1000.00;M19:Oxidation (M):1000.00;C22:Carbamidomethylation:1000.00	PEAKS DB
L.AGDKDNVIDQIEK.Q	N	66.85	1443.7205	13	-3.1	722.8653	2	32.19	12	F12:1560	OB5939 H1 raw.raw	7.0907E5	2	2	544	556			PEAKS DB
K.G(+57.02)TGNLELVAVRK.E	N	66.68	1312.7462	12	-0.7	657.3799	2	26.85	14	F14:1280	OB5941 H1 raw.raw	3.417E5	3	3	461	472	Carbamidomethylation (DHKE, X@N-term)	G1:Carbamidomethylation (DHKE, X@N-term):56.51	PEAKS PTM
K.SFNLDEGHALRIPSGFISYILNRHDNQNLR.V	N	66.45	3495.7759	30	-1.2	700.1616	5	36.31	13	F13:1855	OB5940 H1 raw.raw	1.2605E8	16	16	255	284			PEAKS DB
R.QFQNLQNHR.I	N	66.40	1183.5846	9	0.2	592.7997	2	24.53	14	F14:1134	OB5941 H1 raw.raw	7.3427E4	5	5	205	213			PEAKS DB
D.KDNVIDQIEK.Q	N	66.36	1200.6350	10	-0.8	601.3243	2	25.89	14	F14:1225	OB5941 H1 raw.raw	5.8609E4	3	3	547	556			PEAKS DB
K.NPQLQ(+.98)DLDMMLTC(+57.02)VEIKEGALMLPHFNSK.A	N	66.16	3372.6226	29	5.7	1125.2212	3	37.84	13	F13:1936	OB5940 H1 raw.raw	5.3127E7	3	3	423	451	Carbamidomethylation	Q5:Deamidation (NQ):32.28;C13:Carbamidomethylation:1000.00	PEAKS DB
K.HADADNILVIQQGQATVTVANGNN(+.98)RK.S	N	65.93	2747.3950	26	4.5	1374.7109	2	29.70	10	F10:1444	OB5937 H3A raw.raw	5.6818E5	3	3	229	254		N24:Deamidation (NQ):14.02	PEAKS DB

total 217 peptides

Peptide	Uniq	-10lgP	Mass	Length	ppm	m/z	z	RT	Fraction	Scan	Source File	Area Raw in gel	#Feature	#Feature Raw in gel	Start	End	PTM	AScore	Found By
K.ISMPVNTPGQ(+.98)FEDFFPASSR.D	N	65.70	2227.0254	20	6.4	1114.5271	2	63.55	14	F14:3427	OB5941 H1 raw.raw	1.0155E4	2	2	288	307		Q10:Deamidation (NQ):33.26	PEAKS DB
R.N(+.98)TLEAAFNAEFNEIRR.V	N	65.61	1894.9172	16	2.2	948.4680	2	35.88	12	F12:1787	OB5939 H1 raw.raw	7.0859E7	1	1	319	334	Deamidation (NQ)	N1:Deamidation (NQ): 61.82	PEAKS DB
K.KNPQLQDLDM(+15.99)MLTC(+57.02)VEIKEGALMLPHFNSK.A	N	65.47	3515.7285	30	-3.1	879.9366	4	35.44	13	F13:1830	OB5940 H1 raw.raw	7.2415E6	3	3	422	451	Carbamidomethylation	M10:Oxidation (M):11.06;C14:Carbamidomethylation:1000.00	PEAKS DB
K.ISM(+15.99)PVNTPGQ(+.98)FEDFFPASSRDQSSYLQGFSR.N	N	65.25	3511.5989	31	4.3	1171.5453	3	34.92	12	F12:1731	OB5939 H1 raw.raw	1.4851E6	1	1	288	318	Oxidation (M)	M3:Oxidation (M):1000.00;Q10:Deamidation (NQ):27.62	PEAKS DB
K.NPQLQDLDM(+15.99)MLTC(+57.02)VEIKEGALM(+15.99)LPHFNSK.A	N	64.53	3403.6284	29	4.5	1135.5552	3	36.52	14	F14:1873	OB5941 H1 raw.raw	4.3451E6	3	3	423	451	Carbamidomethylation; Oxidation (M)	M9:Oxidation (M):10.11;C13:Carbamidomethylation:1000.00;M22:Oxidation (M):53.09	PEAKS DB
K.LFEVKPDK.K	N	63.66	974.5436	8	0.9	488.2795	2	25.40	12	F12:1153	OB5939 H1 raw.raw	6.8606E5	7	7	414	421			PEAKS DB
F.NLDEGHALR.I	N	63.51	1023.5097	9	2.3	512.7633	2	27.35	13	F13:1309	OB5940 H1 raw.raw	1.5878E6	3	3	257	265			PEAKS DB
V.QIEAKPNTLVLPK.H	N	63.24	1449.8555	13	-3.5	725.9325	2	29.92	14	F14:1467	OB5941 H1 raw.raw	1.2653E6	3	3	216	228			PEAKS DB
R.EGEQEWGTPGSHVREETSR.N	N	63.23	2169.9675	19	2.1	724.3313	3	25.88	14	F14:1208	OB5941 H1 raw.raw	2.2911E6	7	7	153	171			PEAKS DB
K.ISM(-48.00)PVNTPGQFEDFFPASSR.D	N	62.93	2178.0381	20	0.2	727.0201	3	32.37	12	F12:1577	OB5939 H1 raw.raw	2.6788E7	5	5	288	307	Dethiomethyl	M3:Dethiomethyl:1000.00	PEAKS PTM
N.TPGQFEDFFPASSR.D	N	62.85	1584.7208	14	-0.2	793.3676	2	35.63	14	F14:1821	OB5941 H1 raw.raw	2.1663E5	1	1	294	307			PEAKS DB
K.A(+57.02)M(+15.99)VIVVVNKGTGNLELVAVR.K	N	62.49	2154.2195	20	-0.1	1078.1169	2	32.92	12	F12:1614	OB5939 H1 raw.raw	4.5509E5	2	2	452	471	Carbamidomethylation (DHKE, X@N-term); Oxidation (M)	A1:Carbamidomethylation (DHKE, X@N-term):139.69;M2:Oxidation (M):1000.00	PEAKS PTM
L.EAAFNAEFNEIRR.V	N	62.30	1565.7585	13	-4.7	783.8829	2	34.06	12	F12:1677	OB5939 H1 raw.raw	6.1232E5	1	1	322	334			PEAKS DB
K.N(+.98)PQLQDLDMMLTC(+57.02)VEIKEGALMLPHFNSK.A	N	61.86	3372.6226	29	5.7	1125.2212	3	37.84	13	F13:1983	OB5940 H1 raw.raw	1.2903E7	1	1	423	451	Carbamidomethylation	N1:Deamidation (NQ):14.04;C13:Carbamidomethylation:1000.00	PEAKS DB
K.HADADNILVIQQGQ(+.98)ATVTVAN(+.98)GNNRK.S	N	61.75	2748.3792	26	1.4	1375.1987	2	29.55	13	F13:1443	OB5940 H1 raw.raw	4.856E5	1	1	229	254		Q14:Deamidation (NQ):32.28;N21:Deamidation (NQ): 14.04	PEAKS DB
N.LDEGHALR.I	N	60.44	909.4668	8	0.6	455.7409	2	27.35	13	F13:1312	OB5940 H1 raw.raw	3.1152E4	1	1	258	265			PEAKS DB
R.WGPAGPR.E	N	59.30	739.3765	7	-0.2	370.6955	2	24.50	13	F13:1143	OB5940 H1 raw.raw	2.137E5	2	2	115	121			PEAKS DB

total 217 peptides

Peptide	Uniq	-10lgP	Mass	Length	ppm	m/z	z	RT	Fraction	Scan	Source File	Area Raw in gel	#Feature	#Feature Raw in gel	Start	End	PTM	AScore	Found By
R.IFLAGDKDNVIDQ(+.98)IEKQAK.D	N	58.72	2145.1316	19	9.3	716.0578	3	32.93	2	F2:1432	OB5925H3B raw.raw	1.5596E6	1	1	541	559		Q13:Deamidation (N Q):0.00	PEAKS DB
K.HADADNILVIQ(+.98)Q(+.98)GQATVTVAN(+.98)GNNRK.S	N	58.62	2749.3630	26	9.6	917.4705	3	30.18	13	F13:1524	OB5940H1 raw.raw	4.3812E6	1	1	229	254		Q11:Deamidation (N Q):26.52;Q12:Deamidation (NQ): 18.53;N21:Deamidation (NQ):14.04	PEAKS DB
R.D(+57.02)QSSYLQGFSR.N	N	58.49	1343.6106	11	-2.5	672.8109	2	31.19	12	F12:1506	OB5939H1 raw.raw	3.7713E4	1	1	308	318	Carbamidomethylation (DHKE, X@N-term)	D1:Carbamidomethylation (DHKE, X@N-term):1000.00	PEAKS PTM
K.HADADNILVIQ(+.98)QGQATVTVAN(+.98)GNNRK.S	N	58.29	2748.3792	26	4.6	1375.2031	2	31.28	12	F12:1539	OB5939H1 raw.raw	1.8636E5	1	1	229	254		Q11:Deamidation (N Q):15.10;N21:Deamidation (NQ): 17.01	PEAKS DB
K.HADADN(+.98)ILVIQ(+.98)QGQATVTVANGNNRK.S	N	57.75	2748.3792	26	3.1	1375.2010	2	30.08	14	F14:1495	OB5941H1 raw.raw	1.5756E6	1	1	229	254		N6:Deamidation (NQ): 0.00;Q11:Deamidation (NQ):0.00	PEAKS DB
N.PFYFPSRR.F	N	57.72	1068.5504	8	3.3	535.2842	2	28.95	12	F12:1360	OB5939H1 raw.raw	3.0845E6	3	3	174	181			PEAKS DB
K.LFEVKPDKKNPQLQDLDMM(+15.99)LTC(+57.02)VEIK.E	N	57.62	3147.6018	26	-4.4	787.9042	4	32.55	14	F14:1633	OB5941H1 raw.raw	2.1102E6	3	3	414	439	Carbamidomethylation	M19:Oxidation (M):17.01;C22:Carbamidomethylation:1000.00	PEAKS DB
K.SFN(+.98)LDEGHALRIPSGFISYILNR.H	N	57.28	2619.3445	23	9.3	655.8495	4	38.22	12	F12:1947	OB5939H1 raw.raw	1.0265E6	2	2	255	277		N3:Deamidation (NQ): 32.08	PEAKS DB
K.KGSEEEGDITNPIN(+.98)LREGEPDLSNNFGK.L	Y	57.09	3059.4319	28	7.3	1530.7344	2	30.76	14	F14:1531	OB5941H1 raw.raw	5.8148E5	1	1	386	413		N14:Deamidation (N Q):35.60	PEAKS DB
K.AMVIVVVN(+.98)KGTGNLELVAVRK.E	N	56.84	2210.2820	21	9.6	737.7750	3	33.10	14	F14:1664	OB5941H1 raw.raw	8.0625E6	2	2	452	472		N8:Deamidation (NQ): 41.87	PEAKS DB
K.KNPQLQDLDMM(-48.00)LTC(+57.02)VEIK.E	N	56.43	2127.0703	18	0.8	710.0312	3	31.11	14	F14:1538	OB5941H1 raw.raw	3.3192E6	2	2	422	439	Carbamidomethylation	M11:Dethiomethyl:14.02;C14:Carbamidomethylation:1000.00	PEAKS PTM
K.ISM(+15.99)PVNTPGQFEDFFPASSRDQSSYLQGFSR.N	N	56.19	3510.6150	31	2.9	1171.2157	3	34.90	14	F14:1765	OB5941H1 raw.raw	1.0055E7	8	8	288	318	Oxidation (M)	M3:Oxidation (M):1000.00	PEAKS DB
R.IVQIEAK(+57.02)PNTLVLPK.H	N	56.13	1719.0294	15	-1.6	860.5206	2	30.60	14	F14:1466	OB5941H1 raw.raw	1.1048E7	2	2	214	228	Carbamidomethylation (DHKE, X@N-term)	K7:Carbamidomethylation (DHKE, X@N-term):51.51	PEAKS PTM
K.IRPEGREGEQEWGTPGSHVR.E	N	56.05	2276.1045	20	0.7	570.0338	4	26.47	14	F14:1255	OB5941H1 raw.raw	1.8795E5	1	1	147	166			PEAKS DB
R.N(+57.02)NPFYFPSRR.F	N	55.47	1353.6577	10	-2.6	677.8344	2	29.38	14	F14:1433	OB5941H1 raw.raw	6.1234E5	2	2	172	181	Carbamidomethylation (DHKE, X@N-term)	N1:Carbamidomethylation (DHKE, X@N-term):1000.00	PEAKS PTM
K.HADADNILVIQQGQATVTVANGN(+.98)NRK.S	N	55.13	2747.3950	26	4.2	687.8589	4	29.51	12	F12:1401	OB5939H1 raw.raw	2.4633E7	5	5	229	254		N23:Deamidation (N Q):17.01	PEAKS DB
**total 217 peptides**																			

Peptide	Uniq	-10lgP	Mass	Length	ppm	m/z	z	RT	Fraction	Scan	Source File	Area Raw in gel	#Feature	#Feature Raw in gel	Start	End	PTM	AScore	Found By
K.HADADNILVIQQ(+.98)GQ(+.98)ATVTVAN(+.98)GNNRK.S	N	55.12	2749.3630	26	9.6	917.4705	3	30.18	13	F13:1487	OB5940 H1 raw.raw	4.3812E6	1	1	229	254		Q12:Deamidation (NQ):14.02;Q14:Deamidation (NQ):13.03;N21:Deamidation (NQ):11.10	PEAKS DB
K.AM(-48.00)VIVVVNKGTGNLELVAVRK.E	N	54.93	2161.2947	21	1.4	1081.6561	2	29.80	14	F14:1453	OB5941 H1 raw.raw	1.7581E6	2	2	452	472	Dethiomethyl	M2:Dethiomethyl:1000.00	PEAKS PTM
K.NPQLQDLDM(+15.99)MLTC(+57.02)VEIKEGALMLPHFN(+.98)SK.A	N	54.06	3388.6174	29	5.0	848.1659	4	36.17	14	F14:1849	OB5941 H1 raw.raw	6.0156E6	2	2	423	451	Carbamidomethylation; Deamidation (NQ)	M9:Oxidation (M):14.02;C13:Carbamidomethylation:1000.00;N27:Deamidation (NQ):58.23	PEAKS DB
R.IFLAGDKDNVIDQIE(+57.02)KQAK.D	N	52.99	2201.1692	19	-2.0	734.7289	3	31.83	13	F13:1588	OB5940 H1 raw.raw	3.3379E6	2	2	541	559		E15:Carbamidomethylation (DHKE, X@N-term):12.28	PEAKS PTM
K.NPQLQDLDMMLTC(+57.02)VEIKEGALMLPHFN(+.98)SK.A	N	52.89	3372.6226	29	7.4	844.1691	4	37.85	13	F13:1914	OB5940 H1 raw.raw	5.2564E7	2	2	423	451	Carbamidomethylation; Deamidation (NQ)	C13:Carbamidomethylation:1000.00;N27:Deamidation (NQ):61.84	PEAKS DB
K.HADADNILVIQQGQ(+.98)ATVTVANGNNRK.S	N	52.87	2747.3950	26	5.1	916.8103	3	44.47	12	F12:2283	OB5939 H1 raw.raw	1.466E5	1	1	229	254		Q14:Deamidation (NQ):14.04	PEAKS DB
K.NPQLQDLDM(+15.99)M(+15.99)LTC(+57.02)VEIKEGALMLPHFNSK.A	N	52.70	3403.6284	29	-4.3	1135.5452	3	35.63	14	F14:1818	OB5941 H1 raw.raw	1.9434E6	2	2	423	451	Oxidation (M); Carbamidomethylation	M9:Oxidation (M):49.75;M10:Oxidation (M):51.99;C13:Carbamidomethylation:1000.00	PEAKS DB
L.DEGHALR.I	N	52.68	796.3828	7	2.3	399.1996	2	27.87	12	F12:1309	OB5939 H1 raw.raw	2.418E4	1	1	259	265			PEAKS DB
K.DLAFPGSGEQVEK(+57.02)LIK.N	N	52.57	1786.9464	16	-3.8	894.4771	2	32.91	14	F14:1654	OB5941 H1 raw.raw	6.7177E5	1	1	560	575		K13:Carbamidomethylation (DHKE, X@N-term):27.96	PEAKS PTM
K.KN(+.98)PQLQDLDMM(+15.99)LTC(+57.02)VEIK.E	N	52.31	2192.0527	18	9.2	1097.0437	2	32.33	13	F13:1621	OB5940 H1 raw.raw	8.5775E4	1	1	422	439	Carbamidomethylation	N2:Deamidation (NQ):14.04;M11:Oxidation (M):0.00;C14:Carbamidomethylation:1000.00	PEAKS DB
R.IVQIEAKPNTLVLPK(+57.02).H	N	52.13	1719.0294	15	3.0	860.5245	2	30.75	12	F12:1437	OB5939 H1 raw.raw	4.8623E6	1	1	214	228		K15:Carbamidomethylation (DHKE, X@N-term):25.69	PEAKS PTM
Q.GQATVTVANGNNRK.S	N	51.97	1428.7433	14	-0.2	715.3788	2	29.02	14	F14:1412	OB5941 H1 raw.raw	1.2099E5	2	2	241	254			PEAKS DB
K.EGALMLPH(+14.02)FNSK.A	N	51.73	1356.6860	12	1.3	679.3512	2	30.49	14	F14:1499	OB5941 H1 raw.raw	2.5741E4	1	1	440	451	Methylation(others)	H8:Methylation(others):53.53	PEAKS PTM
K.N(+.98)PQLQ(+.98)DLDMMLTC(+57.02)VEIKEGALM(+15.99)LPHFNSK.A	N	51.60	3389.6016	29	9.0	848.4153	4	36.92	12	F12:1868	OB5939 H1 raw.raw	2.6873E6	1	1	423	451	Carbamidomethylation	N1:Deamidation (NQ):0.00;Q5:De	PEAKS DB
**total 217 peptides**																			

Peptide	Uniq	-10lgP	Mass	Length	ppm	m/z	z	RT	Fraction	Scan	Source File	Area Raw in gel	#Feature	#Feature Raw in gel	Start	End	PTM	AScore	Found By
																		amidation (NQ):14.04;C13:Carbamidomethylation:1000.00;M22:Oxidation (M):18.84	
K.LFEVKPDKK.N	N	50.85	1102.6385	9	1.1	552.3271	2	24.94	14	F14:1179	OB5941 H1 raw.raw	2.3081E5	2	2	414	422			PEAKS DB
R.RVLLEENAGGEQEERGQR.R	N	50.67	2069.0249	18	2.1	690.6837	3	24.88	13	F13:1165	OB5940 H1 raw.raw	2.7227E5	1	1	334	351			PEAKS DB
K.N(+.98)PQLQDLDMMLTC(+57.02)VEIKEGALM(+15.99)LPHFNSK.A	N	50.17	3388.6174	29	-2.4	1130.5437	3	36.35	13	F13:1874	OB5940 H1 raw.raw	8.9006E5	1	1	423	451	Carbamidomethylation; Oxidation (M)	N1:Deamidation (NQ):0.00;C13:Carbamidomethylation:1000.00;M22:Oxidation (M):54.40	PEAKS DB
K.NPQLQDLDMM(+15.99)LTC(+57.02)VEIKEGALM(+15.99)LPHFNSK.A	N	48.97	3403.6284	29	2.7	1135.5531	3	35.09	14	F14:1786	OB5941 H1 raw.raw	3.2001E5	1	1	423	451	Carbamidomethylation	M10:Oxidation (M):12.28;C13:Carbamidomethylation:1000.00;M22:Oxidation (M):28.29	PEAKS DB
K.KN(+.98)PQLQDLDMMLTC(+57.02)VEIKEGALMLPHFNSK.A	N	48.84	3500.7175	30	7.1	876.1929	4	36.26	13	F13:1847	OB5940 H1 raw.raw	2.2113E6	1	1	422	451	Carbamidomethylation	N2:Deamidation (NQ):0.00;C14:Carbamidomethylation:1000.00	PEAKS DB
R.IFLAGDKDNVIDQIEK(+57.02)QAK.D	N	48.82	2201.1692	19	2.7	734.7323	3	32.55	12	F12:1581	OB5939 H1 raw.raw	3.2958E6	1	1	541	559		K16:Carbamidomethylation (DHKE, X@N-term):15.91	PEAKS PTM
R.EREEDWRQPR.E	N	48.49	1399.6592	10	0.1	467.5604	3	24.77	14	F14:1154	OB5941 H1 raw.raw	2.5247E4	1	1	124	133			PEAKS DB
R.KSFN(+.98)LDEGHALRIPSGFISYILNR.H	N	47.51	2747.4395	24	4.5	687.8702	4	36.15	14	F14:1837	OB5941 H1 raw.raw	1.0524E6	1	1	254	277		N4:Deamidation (NQ):23.98	PEAKS DB
K.LFEVKPDKKN(+.98)PQLQDLDMMLTC(+57.02)VEIK.E	N	47.32	3132.5908	26	9.3	1567.3173	2	34.35	14	F14:1745	OB5941 H1 raw.raw	1.9315E5	1	1	414	439	Carbamidomethylation	N10:Deamidation (NQ):14.04;C22:Carbamidomethylation:1000.00	PEAKS DB
K.N(+.98)PQ(+.98)LQDLDMMLTC(+57.02)VEIKEGALM(+15.99)LPHFNSK.A	N	46.82	3389.6016	29	9.0	848.4153	4	36.92	12	F12:1851	OB5939 H1 raw.raw	7.3613E6	2	2	423	451	Carbamidomethylation	N1:Deamidation (NQ):11.12;Q3:Deamidation (NQ):0.00;C13:Carbamidomethylation:1000.00;M22:Oxidation (M):24.93	PEAKS DB
N.PQLQDLDMMLTC(+57.02)VEIKEGALMLPHFNSK.A	N	46.63	3257.5957	28	0.9	815.4069	4	37.83	14	F14:1952	OB5941 H1 raw.raw	2.3692E5	1	1	424	451	Carbamidomethylation	C12:Carbamidomethylation:1000.00	PEAKS DB
R.S(+57.02)SENNEGVIVK.V	Y	46.57	1231.6044	11	-0.2	616.8093	2	23.54	14	F14:1091	OB5941 H1 raw.raw	1.0427E3	1	1	357	367		S1:Carbamidomethylation (DHKE, X@N-term):20.41	PEAKS PTM
**total 217 peptides**																			

Peptide	Uniq	-10lgP	Mass	Length	ppm	m/z	z	RT	Fraction	Scan	Source File	Area Raw in gel	#Feature	#Feature Raw in gel	Start	End	PTM	AScore	Found By
K.Q(-17.03)AKDLAFPGSGEQVEK.L	N	46.49	1685.8260	16	0.9	843.9211	2	29.97	12	F12:1427	OB5939 H1 raw.raw	0	0	0	557	572	Pyro-glu from Q	Q1:Pyro-glu from Q:1000.00	PEAKS PTM
K.SFN(+.98)LDEGHALRIPSGFISYILNRHDNQNLR.V	N	46.45	3496.7600	30	-3.9	700.3566	5	36.38	12	F12:1849	OB5939 H1 raw.raw	6.986E5	1	1	255	284		N3:Deamidation (NQ): 20.27	PEAKS DB
Q.IEAKPNTLVLPK.H	N	46.43	1321.7969	12	-4.5	661.9028	2	29.65	13	F13:1449	OB5940 H1 raw.raw	6.8969E5	2	2	217	228			PEAKS DB
T.PGQFEDFFPASSR.D	N	45.25	1483.6731	13	-1.3	742.8429	2	40.34	13	F13:2103	OB5940 H1 raw.raw	4.0298E3	1	1	295	307			PEAKS DB
R.IPSGFISYILN(+.98)RHDNQNLR.V	N	45.21	2257.1604	19	9.9	753.4015	3	37.61	2	F2:1698	OB5925 H3B raw.raw	2.0603E6	2	2	266	284		N11:Deamidation (NQ):33.06	PEAKS DB
N.NPFYFPSRR.F	N	45.02	1182.5934	9	1.6	395.2057	3	28.08	13	F13:1358	OB5940 H1 raw.raw	2.9991E4	1	1	173	181			PEAKS DB
R.DQS(-18.01)SYLQGFSR.N	N	44.90	1268.5785	11	7.0	635.3010	2	30.87	12	F12:1484	OB5939 H1 raw.raw	0	0	0	308	318		S3:Dehydration:33.98	PEAKS PTM
K.GTGNLELVAVRKEQQQR.G	N	44.58	1925.0442	17	0.6	642.6890	3	25.89	14	F14:1220	OB5941 H1 raw.raw	1.1112E5	2	2	461	477			PEAKS DB
R.EDWRRPSHQQPR.K	N	43.98	1590.7764	12	-0.3	398.7012	4	24.94	14	F14:1169	OB5941 H1 raw.raw	1.0819E4	1	1	134	145			PEAKS DB
R.VLLE(+21.98)ENAGGEQEER.G	N	42.61	1593.7246	14	-0.7	797.8690	2	26.03	13	F13:1239	OB5940 H1 raw.raw	1.373E5	3	3	335	348		E4:Sodium adduct:40.00	PEAKS PTM
K.HADADNILVIQ(+.98)QGQATVTVANGNNRK.S	N	42.22	2747.3950	26	5.9	916.8110	3	45.04	14	F14:2357	OB5941 H1 raw.raw	0	0	0	229	254		Q11:Deamidation (NQ):9.34	PEAKS DB
K.HADADNILVIQQGQAT(-18.01)VTVAN(+.98)GNNRK.S	N	41.92	2729.3845	26	0.3	683.3536	4	28.96	13	F13:1411	OB5940 H1 raw.raw	3.5545E5	1	1	229	254		T16:Dehydration:8.22; N21:Deamidation (NQ):6.08	PEAKS PTM
K.HADADN(+.98)ILVIQQGQATVTVANGNNRK.S	N	41.76	2747.3950	26	3.7	916.8090	3	50.62	12	F12:2616	OB5939 H1 raw.raw	1.1355E5	2	2	229	254		N6:Deamidation (NQ): 42.57	PEAKS DB
K.ISMPVN(+.98)TPGQFEDFFPASSR.D	N	41.75	2227.0254	20	9.3	1114.5303	2	58.23	14	F14:3138	OB5941 H1 raw.raw	2.4983E4	1	1	288	307		N6:Deamidation (NQ): 42.89	PEAKS DB
M.LPHFNSK.A	N	41.73	841.4446	7	-3.4	421.7281	2	30.24	14	F14:1484	OB5941 H1 raw.raw	1.8069E6	2	2	445	451			PEAKS DB
K.PNTLVLPK.H	N	41.66	880.5382	8	0.4	441.2766	2	27.53	13	F13:1313	OB5940 H1 raw.raw	8.5249E4	1	1	221	228			PEAKS DB
N.TLEAAFNAEFNEIRR.V	N	41.51	1779.8904	15	-3.0	890.9498	2	32.91	14	F14:1655	OB5941 H1 raw.raw	4.1608E5	1	1	320	334			PEAKS DB
K.KNPQLQ(+.98)DLDMMLTC(+57.02)VEIKEGALMLPHFNSK.A	N	40.89	3500.7175	30	0.4	1167.9136	3	36.34	13	F13:1850	OB5940 H1 raw.raw	4.7608E5	1	1	422	451	Carbamidomethylation	Q6:Deamidation (NQ): 14.04;C14: Carbamidomethylation:1000.00	PEAKS DB
R.NTLEAAFN(+.98)AEFNEIRR.V	N	40.23	1894.9172	16	-0.3	948.4656	2	32.92	12	F12:1570	OB5939 H1 raw.raw	1.5322E6	1	1	319	334		N8:Deamidation (NQ): 8.26	PEAKS DB
R.EGEQEWGT(-18.01)PGSHVR.E	N	39.67	1549.6909	14	-1.7	775.8514	2	27.60	14	F14:1325	OB5941 H1 raw.raw	5.8338E4	1	1	153	166		T8:Dehydration:47.09	PEAKS PTM
K.NPQLQDLDM(+15.99)M(+15.99)LTC(+57.02)VEIK.E	N	39.57	2078.9688	17	1.1	1040.4928	2	33.80	14	F14:1701	OB5941 H1 raw.raw	0	0	0	423	439	Oxidation (M); Carbamidomethylation	M9:Oxidation (M):1000.00;M10: Oxidation (M):1000.0	PEAKS DB
**total 217 peptides**																			

Peptide	Uniq	-10lgP	Mass	Length	ppm	m/z	z	RT	Fraction	Scan	Source File	Area Raw in gel	#Feature	#Feature Raw in gel	Start	End	PTM	AScore	Found By
																		0;C13:Carbamidomethylation:1000.00	
R.IPS(+59.02)GFISYILNR.H	N	39.40	1437.7802	12	1.5	719.8984	2	36.96	12	F12:1870	OB5939H1 raw.raw	4.3694E5	1	1	266	277		S3:Aminoethylcysteine:49.79	PEAKS PTM
K.HADADNILVIQQ(-18.01).G	N	38.68	1317.6677	12	-4.9	659.8379	2	29.56	14	F14:1421	OB5941H1 raw.raw	1.6968E5	2	2	229	240	Dehydration	Q12:Dehydration:76.18	PEAKS PTM
R.IVQ(+.98)IEAKPNTLVLPK.H	N	38.03	1662.9919	15	6.7	832.5088	2	31.45	13	F13:1578	OB5940H1 raw.raw	2.7795E5	1	1	214	228		Q3:Deamidation (NQ):13.11	PEAKS DB
K.AMVIVVVNK(-1.03)GTGNLELVAVR.K	N	37.92	2080.1714	20	4.3	1041.0974	2	33.79	13	F13:1738	OB5940H1 raw.raw	2.1453E7	1	1	452	471	Lysine oxidation to aminoadipic semialdehyde	K9:Lysine oxidation to aminoadipic semialdehyde:1000.00	PEAKS PTM
I.PSGFISYILNRHDNQNLR.V	N	37.07	2143.0923	18	4.0	715.3742	3	33.53	12	F12:1645	OB5939H1 raw.raw	0	0	0	267	284			PEAKS DB
K.EGALM(-48.00)LPHFNSK.A	N	36.97	1294.6670	12	2.0	432.5638	3	28.31	14	F14:1348	OB5941H1 raw.raw	1.3683E6	1	1	440	451	Dethiomethyl	M5:Dethiomethyl:1000.00	PEAKS PTM
R.IVQIEAKPN(+15.99)TLVLPK.H	N	36.93	1678.0028	15	-2.8	840.0063	2	29.56	14	F14:1444	OB5941H1 raw.raw	5.568E5	1	1	214	228		N9:Oxidation or Hydroxylation:0.00	PEAKS PTM
K.KGSEEEGDITNPINLREGEPDLSNN(+.98)FGK.L	Y	36.62	3059.4319	28	7.6	765.8711	4	31.46	8	F8:1493	OB5935H3B raw.raw	3.4976E4	1	1	386	413		N25:Deamidation (NQ):0.00	PEAKS DB
R.IFLAGDKDNVIDQ(+.98)IEK.Q	N	36.27	1817.9410	16	7.9	606.9924	3	63.32	14	F14:3434	OB5941H1 raw.raw	1.3413E4	1	1	541	556		Q13:Deamidation (NQ):25.70	PEAKS DB
K.HAD(+57.02)ADNILVIQQGQATVTVANGN(+.98)NRK.S	N	35.92	2804.4165	26	-3.8	702.1088	4	28.95	13	F13:1413	OB5940H1 raw.raw	2.7833E5	1	1	229	254		D3:Carbamidomethylation (DHKE, X@N-term):4.64;N23:Deamidation (NQ):0.00	PEAKS PTM
K.NPQ(+.98)LQDLDMMLTC(+57.02)VEIKEGALMLPHFNSK.A	N	35.84	3372.6226	29	9.4	1125.2253	3	40.73	12	F12:2064	OB5939H1 raw.raw	5.6313E5	1	1	423	451	Carbamidomethylation	Q3:Deamidation (NQ):0.00;C13:Carbamidomethylation:1000.00	PEAKS DB
K.ISM(+15.99)PVNTPGQ(+.98)FEDFFPASSR.D	N	35.67	2243.0205	20	6.2	1122.5245	2	50.73	12	F12:2643	OB5939H1 raw.raw	0	0	0	288	307	Oxidation (M)	M3:Oxidation (M):1000.00;Q10:Deamidation (NQ):22.37	PEAKS DB
K.GT(-18.01)GNLELVAVRK.E	N	35.64	1237.7142	12	0.9	413.5790	3	26.41	13	F13:1259	OB5940H1 raw.raw	7.4255E5	3	3	461	472	Dehydration	T2:Dehydration:1000.00	PEAKS PTM
K.KGSEEEGDIT(-18.01)N(+.98)PINLR.E	N	35.42	1753.8483	16	2.8	585.6250	3	28.08	13	F13:1354	OB5940H1 raw.raw	2.8958E5	1	1	386	401		T10:Dehydration:25.86;N11:Deamidation (NQ):32.46	PEAKS PTM
K.SFNLDEGHALRIPS.G	N	35.38	1554.7791	14	-4.8	778.3931	2	30.79	13	F13:1515	OB5940H1 raw.raw	5.2113E3	1	1	255	268			PEAKS DB
R.DQ(+.98)SSYLQGFSR.N	N	34.61	1287.5731	11	-3.2	644.7917	2	63.83	14	F14:3449	OB5941H1 raw.raw	0	0	0	308	318		Q2:Deamidation (NQ):41.87	PEAKS DB
R.NN(-17.03)PFYFPSRR.F	N	34.10	1279.6097	10	-0.4	427.5437	3	28.25	13	F13:1369	OB5940H1 raw.raw	1.47E5	1	1	172	181		N2:Ammonia-loss (N):12.28	PEAKS PTM

total 217 peptides

Peptide	Uniq	-10lgP	Mass	Length	ppm	m/z	z	RT	Fraction	Scan	Source File	Area Raw in gel	#Feature	#Feature Raw in gel	Start	End	PTM	AScore	Found By
G.SEEEGDITNPINLREGEPDLSNNFGK.L	Y	33.63	2873.3315	26	2.9	958.7872	3	32.34	7	F7:1553	OB5934 H3B raw.raw	5.5034E4	1	1	388	413			PEAKS DB
T.G(+214.97)NLELVAVRK.E	N	33.47	1312.6267	10	2.3	657.3221	2	27.95	14	F14:1280	OB5941 H1 raw.raw	2.4669E5	1	1	463	472	4-sulfophenyl isothiocyanate	G1:4-sulfophenyl isothiocyanate:144.45	PEAKS PTM
R.GRREEEEDEDEEEEGSNR.E	Y	33.44	2192.8689	18	0.0	731.9636	3	23.70	13	F13:1082	OB5940 H1 raw.raw	3.4989E3	1	1	478	495			PEAKS DB
K.HADADNILVIQQ(+.98)GQATVTVANGNN(+.98)RK.S	N	33.28	2748.3792	26	7.6	917.1406	3	48.20	12	F12:2471	OB5939 H1 raw.raw	2.0672E4	1	1	229	254		Q12:Deamidation (NQ):23.10;N24:Deamidation (NQ):0.00	PEAKS DB
K.HADAD(-18.01)NILVIQQGQATVTVANGNNRK.S	N	33.25	2728.4004	26	3.5	910.4772	3	28.70	13	F13:1390	OB5940 H1 raw.raw	0	0	0	229	254		D5:Dehydration:30.36	PEAKS PTM
K.ISM(+15.99)PVN(+.98)TPGQFEDFFPASSR.D	N	33.05	2243.0205	20	7.6	1122.5260	2	49.72	13	F13:2625	OB5940 H1 raw.raw	0	0	0	288	307	Oxidation (M)	M3:Oxidation (M):1000.00;N6:Deamidation (NQ):25.70	PEAKS DB
R.IFLAGDK(+26.02)DNVIDQIEK.Q	N	32.97	1842.9727	16	-0.2	922.4934	2	35.63	14	F14:1844	OB5941 H1 raw.raw	1.4466E5	1	1	541	556	Acetaldehyde +26	K7:Acetaldehyde +26:74.34	PEAKS PTM
K.NPQLQ(+.98)DLDMM(+15.99)LTC(+57.02)VEIK.E	N	32.85	2063.9578	17	-3.4	1032.9827	2	37.19	12	F12:1881	OB5939 H1 raw.raw	2.2954E4	1	1	423	439	Carbamidomethylation	Q5:Deamidation (NQ):26.31;M10:Oxidation (M):0.00;C13:Carbamidomethylation:1000.00	PEAKS DB
K.HADADNILVIQ(+.98)QGQ(+.98)ATVTVANGNNRK.S	N	32.62	2748.3792	26	3.1	917.1365	3	57.24	13	F13:3064	OB5940 H1 raw.raw	0	0	0	229	254		Q11:Deamidation (NQ):0.00;Q14:Deamidation (NQ):0.00	PEAKS DB
K.N(+.98)PQ(+.98)LQ(+.98)DLDMM(+15.99)LTC(+57.02)VEIKEGALMLPHFNSK.A	N	32.23	3390.5854	29	3.7	848.6568	4	38.47	14	F14:1982	OB5941 H1 raw.raw	0	0	0	423	451	Deamidation (NQ); Carbamidomethylation	N1:Deamidation (NQ):62.42;Q3:Deamidation (NQ):65.92;Q5:Deamidation (NQ):60.92;M10:Oxidation (M):12.28;C13:Carbamidomethylation:1000.00	PEAKS DB
K.KN(+.98)PQ(+.98)LQ(+.98)DLDM(+15.99)MLTC(+57.02)VEIKEGALMLPHFNSK.A	N	31.81	3518.6804	30	-3.0	880.6747	4	37.45	12	F12:1882	OB5939 H1 raw.raw	0	0	0	422	451	Deamidation (NQ); Carbamidomethylation	N2:Deamidation (NQ):58.75;Q4:Deamidation (NQ):61.65;Q6:Deamidation (NQ):53.75;M10:Oxidation (M):0.00;C14:Carbamidomethylation:1000.00	PEAKS DB
**total 217 peptides**																			

sp|P43238|ALL12_ARAHY

back to list

| Protein Coverage | Supporting Peptides |

Protein Coverage:

```
  1 MRGRVSPLML LLGILVLASV SATHAKSSPY QKKTENPCAQ RCLQSCQQEP DDLKQKACES RCTKLEYDPR CVYDPRGHTG
 81 TTNQRSPPGE RTRGRQPGDY DDDRRQPRRE EGGRWGPAGP REREREEDWR QPREDWRRPS HQQPRKIRPE GREGEQEWGT
161 PGSHVREETS RNNPFYFPSR RFSTRYGNQN GRIRVLQRFD QRSRQFQNLQ NHRIVQIEAK PNTLVLPKHA DADNILVIQQ
241 GQATVTVANG NNRKSFNLDE GHALRIPSGF ISYILNRHDN QNLRVAKISM PVNTPGQFED FFPASSRDQS SYLQGFSRNT
321 LEAAFNAEFN EIRRVLLEEN AGGEQEERGQ RRWSTRSSEN NEGVIVKVSK EHVEELTKHA KSVSKKGSEE EGDITNPINL
401 REGEPDLSNN FGKLFEVKPD KKNPQLQDLD MMLTCVEIKE GALMLPHFNS KAMVIVVVNK GTGNLELVAV RKEQQQRGRR
481 EEEEDEDEEE EGSNREVRRY TARLKEGDVF IMPAAHPVAI NASSELHLLG FGINAENNHR IFLAGDKDNV IDQIEKQAKD
561 LAFPGSGEQV EKLIKNQKES HFVSARPQSQ SQSPSSPEKE SPEKEDQEEE NQGGKGPLLS ILKAFN
```

- a Ammonia-loss (N) (-17.03)
- a Acetaldehyde +26 (+26.02)
- c Carbamidomethylation (+57.02)
- c Carbamidomethylation (DHKE, X@N-term) (+57.02)
- d Deamidation (NQ) (+0.98)
- d Dethiomethyl (-48.00)
- d Dehydration (-18.01)
- f Formylation (+27.99)
- i Iodination (+125.90)
- l Lysine oxidation to aminoadipic semialdehyde (-1.03)
- m Methylation(KR) (+14.02)
- m Methylation(others) (+14.02)
- o Oxidation (M) (+15.99)
- p Pyro-glu from E (-18.01)
- p Pyro-glu from Q (-17.03)
- s 4-sulfophenyl isothiocyanate (+214.97)

Supporting Peptides:

Peptide	Uniq	-10lgP	Mass	Length	ppm	m/z	z	RT	Fraction	Scan	Source File	Area Raw in gel	#Feature	#Feature Raw in gel	Start	End	PTM	AScore	Found By
K.HADADNILVIQQGQATVTVAN(+.98)GNNRK.S	N	140.09	2747.3950	26	-1.1	916.8046	3	29.38	14	F14:1509	OB5941 H1 raw.raw	3.7837E8	20	20	229	254		N21:Deamidation (N Q):26.31	PEAKS DB
K.KNPQLQDLDMMLTC(+57.02)VEIK.E	N	137.14	2175.0737	18	2.4	1088.5468	2	34.90	14	F14:1774	OB5941 H1 raw.raw	4.1086E6	3	3	422	439	Carbamidomethylation	C14:Carbamidomethylation:1000.00	PEAKS DB
K.HADADNILVIQQGQATVTVANGNNR.K	N	135.84	2618.3162	25	2.9	1310.1692	2	30.42	14	F14:1494	OB5941 H1 raw.raw	5.8142E6	3	3	229	253			PEAKS DB
K.ISMPVNTPGQFEDFFPASSR.D	N	135.58	2226.0415	20	1.9	1114.0302	2	35.63	14	F14:1807	OB5941 H1 raw.raw	2.1661E8	32	26	288	307			PEAKS DB
K.ISM(+15.99)PVNTPGQFEDFFPASSR.D	N	134.77	2242.0364	20	2.3	1122.0281	2	34.42	2	F2:1522	OB5925 H3B raw.raw	1.6845E7	19	17	288	307	Oxidation (M)	M3:Oxidation (M):1000.00	PEAKS DB
K.NPQLQDLDMMLTC(+57.02)VEIK.E	N	131.65	2046.9788	17	-0.8	1024.4958	2	36.89	14	F14:1903	OB5941 H1 raw.raw	1.2559E7	8	8	423	439	Carbamidomethylation	C13:Carbamidomethylation:1000.00	PEAKS DB
K.NPQLQDLDM(+15.99)MLTC(+57.02)VEIK.E	N	128.59	2062.9736	17	-6.6	1032.4873	2	36.34	14	F14:1860	OB5941 H1 raw.raw	2.4371E5	2	2	423	439	Carbamidomethylation	M9:Oxidation (M):30.46;C13:Carbamidomethylation:1000.00	PEAKS DB
K.KGSEEEGDITNPINLREGEPDLSNNFGK.L	Y	126.17	3058.4478	28	-3.6	1020.4862	3	31.46	14	F14:1557	OB5941 H1 raw.raw	5.2061E7	16	16	386	413			PEAKS DB
R.NTLEAAFNAEFNEIR.R	N	126.11	1737.8322	15	3.3	869.9262	2	35.27	14	F14:1792	OB5941 H1 raw.raw	1.3691E7	6	6	319	333			PEAKS DB
K.HADADNILVIQQGQATVTVANGNNRK.S	N	120.98	2746.4111	26	4.7	1374.2192	2	29.02	14	F14:1406	OB5941 H1 raw.raw	4.4412E7	13	13	229	254			PEAKS DB
K.KGSEEEGDITNPINLR.E	N	117.96	1770.8748	16	-0.9	886.4438	2	28.67	14	F14:1384	OB5941 H1 raw.raw	2.1993E7	17	17	386	401			PEAKS DB
K.SFNLDEGHALRIPSGFISYILNR.H	N	117.92	2618.3604	23	0.6	1310.1882	2	36.89	14	F14:1902	OB5941 H1 raw.raw	2.1092E8	40	39	255	277			PEAKS DB
R.VLLEENAGGEQEER.G	N	114.70	1571.7427	14	1.1	786.8795	2	26.65	14	F14:1250	OB5941 H1 raw.raw	1.2337E7	7	7	335	348			PEAKS DB
K.GSEEEGDITNPINLR.E	N	114.17	1642.7798	15	1.7	822.3986	2	30.60	14	F14:1507	OB5941 H1 raw.raw	8.7873E6	4	4	387	401			PEAKS DB
R.IFLAGDKDNVIDQIEKQAK.D	N	113.07	2144.1477	19	1.1	715.7240	3	32.93	7	F7:1578	OB5934 H3B raw.raw	5.566E7	12	12	541	559			PEAKS DB
K.AMVIVVVNKGTGNLELVAVR.K	N	112.17	2081.2031	20	1.5	694.7427	3	34.35	14	F14:1739	OB5941 H1 raw.raw	7.0931E7	12	12	452	471			PEAKS DB
R.KSFNLDEGHALR.I	N	112.03	1385.7051	12	1.1	693.8606	2	25.70	14	F14:1202	OB5941 H1 raw.raw	3.2948E6	6	6	254	265			PEAKS DB
R.IPSGFISYILNR.H	N	111.89	1378.7609	12	1.5	690.3887	2	43.03	14	F14:2242	OB5941 H1 raw.raw	2.1426E8	15	15	266	277			PEAKS DB
R.DQSSYLQGFSR.N	N	110.94	1286.5891	11	-1.4	644.3009	2	30.60	14	F14:1505	OB5941 H1 raw.raw	6.2405E7	21	21	308	318			PEAKS DB
K.NPQLQDLDMMLTC(+57.02)VEIKEGALMLPHFNSK.A	N	110.02	3371.6387	29	-0.1	1686.8264	2	37.85	14	F14:1946	OB5941 H1 raw.raw	1.7936E8	14	14	423	451	Carbamidomethylation	C13:Carbamidomethylation:1000.00	PEAKS DB

total 217 peptides

Peptide	Uniq	-10lgP	Mass	Length	ppm	m/z	z	RT	Fraction	Scan	Source File	Area Raw in gel	#Feature	#Feature Raw in gel	Start	End	PTM	AScore	Found By
K.AMVIVVVNKGTGNLELVAVRK.E	N	109.98	2209.2981	21	-1.4	1105.6548	2	32.55	14	F14:1615	OB5941 H1 raw.raw	2.9136E8	18	18	452	472			PEAKS DB
K.AM(+15.99)VIVVVNKGTGNLELVAVR.K	N	109.59	2097.1980	20	-2.2	1049.6040	2	32.73	14	F14:1638	OB5941 H1 raw.raw	3.1537E7	10	10	452	471	Oxidation (M)	M2:Oxidation (M):1000.00	PEAKS DB
K.SFNLDEGHALR.I	N	109.41	1257.6101	11	-3.3	629.8102	2	27.77	14	F14:1329	OB5941 H1 raw.raw	7.2473E7	30	30	255	265			PEAKS DB
R.EGEQEWGTPGSHVR.E	N	108.42	1567.7015	14	0.5	784.8585	2	25.51	14	F14:1193	OB5941 H1 raw.raw	5.0052E5	6	6	153	166			PEAKS DB
R.IFLAGDKDNVIDQIEK.Q	N	108.13	1816.9570	16	0.2	909.4860	2	33.10	14	F14:1683	OB5941 H1 raw.raw	7.2865E8	41	41	541	556			PEAKS DB
R.KSFNLDEGHALRIPSGFISYILNR.H	N	107.80	2746.4553	24	-2.1	916.4905	3	36.16	12	F12:1802	OB5939 H1 raw.raw	7.186E6	4	4	254	277			PEAKS DB
R.I(+57.02)PSGFISYILNR.H	N	104.65	1435.7823	12	-1.7	718.8972	2	36.52	14	F14:1869	OB5941 H1 raw.raw	1.3017E7	3	3	266	277	Carbamidomethylation (DHKE, X@N-term)	I1:Carbamidomethylation (DHKE, X@N-term):1000.00	PEAKS PTM
R.VLLEENAGGEQEERGQR.R	N	104.46	1912.9238	17	1.8	957.4709	2	25.31	14	F14:1182	OB5941 H1 raw.raw	1.9161E6	8	8	335	351			PEAKS DB
R.I(+57.02)FLAGDKDNVIDQIEK.Q	N	103.96	1873.9785	16	-5.0	937.9919	2	32.19	14	F14:1619	OB5941 H1 raw.raw	4.5184E7	7	7	541	556	Carbamidomethylation (DHKE, X@N-term)	I1:Carbamidomethylation (DHKE, X@N-term):90.91	PEAKS PTM
R.EGEPDLSNNFGK.L	Y	103.27	1305.5836	12	0.4	653.7994	2	27.95	14	F14:1330	OB5941 H1 raw.raw	7.4849E6	3	3	402	413			PEAKS DB
K.QAKDLAFPGSGEQVEK.L	N	102.73	1702.8525	16	1.8	852.4351	2	26.85	14	F14:1278	OB5941 H1 raw.raw	1.286E6	6	6	557	572			PEAKS DB
K.H(+57.02)ADADNILVIQQGQATVTVANGNNRK.S	N	102.10	2803.4324	26	-1.3	935.4835	3	29.92	14	F14:1452	OB5941 H1 raw.raw	1.6897E6	1	1	229	254		H1:Carbamidomethylation (DHKE, X@N-term):39.76	PEAKS PTM
K.DLAFPGSGEQVEK.L	N	101.44	1375.6619	13	-2.0	688.8369	2	30.08	14	F14:1472	OB5941 H1 raw.raw	6.5563E7	9	9	560	572			PEAKS DB
K.GSEEEGDITNPINLREGEPDLSNNFGK.L	Y	101.39	2930.3530	27	-3.6	1466.1785	2	31.62	13	F13:1574	OB5940 H1 raw.raw	1.3299E7	8	8	387	413			PEAKS DB
K.NPQLQDLDMMLTCVEIK(+14.02).E	N	101.30	2003.9730	17	2.1	1002.9958	2	38.00	14	F14:1960	OB5941 H1 raw.raw	6.7851E5	2	2	423	439	Methylation(KR)	K17:Methylation(KR):1000.00	PEAKS PTM
K.S(+57.02)FNLDEGHALR.I	N	100.96	1314.6316	11	0.8	658.3236	2	27.59	14	F14:1340	OB5941 H1 raw.raw	5.6012E6	7	7	255	265	Carbamidomethylation (DHKE, X@N-term)	S1:Carbamidomethylation (DHKE, X@N-term):101.71	PEAKS PTM
R.RVLLEENAGGEQEER.G	N	98.06	1727.8438	15	0.4	576.9554	3	24.77	13	F13:1156	OB5940 H1 raw.raw	1.2517E5	5	5	334	348			PEAKS DB
R.IPSGFISYILNRHDNQNLR.V	N	97.42	2256.1763	19	0.5	565.0516	4	33.66	14	F14:1693	OB5941 H1 raw.raw	3.392E8	42	42	266	284			PEAKS DB
K.AM(+15.99)VIVVVNKGTGNLELVAVRK.E	N	96.33	2225.2930	21	-0.4	1113.6533	2	30.76	14	F14:1520	OB5941 H1 raw.raw	4.0787E7	8	7	452	472	Oxidation (M)	M2:Oxidation (M):1000.00	PEAKS DB
K.KNPQLQDLDMM(+15.99)LTC(+57.02)VEIK.E	N	95.01	2191.0686	18	-3.4	1096.5378	2	32.73	14	F14:1643	OB5941 H1 raw.raw	1.9787E5	1	1	422	439	Carbamidomethylation	M11:Oxidation (M):9.34;C14:Carbamidomethylation:100	PEAKS DB

total 217 peptides

Peptide	Uniq	-10lgP	Mass	Length	ppm	m/z	z	RT	Fraction	Scan	Source File	Area Raw in gel	#Feature	#Feature Raw in gel	Start	End	PTM	AScore	Found By
																		0.00	
K.NPQLQDLDMM(-48.00)LTC(+57.02)VEIK.E	N	93.52	1998.9755	17	-2.5	1000.4926	2	32.55	14	F14:1628	OB5941 H1 raw.raw	1.3958E7	5	5	423	439	Carbamidomethylation	M10:Dethiomethyl:27.96;C13:Carbamidomethylation:1000.00	PEAKS PTM
K.NPQLQDLDM(+15.99)MLTC(+57.02)VEIKEGALMLPHFNSK.A	N	92.22	3387.6335	29	-1.5	1130.2168	3	36.17	14	F14:1926	OB5941 H1 raw.raw	2.8061E7	4	4	423	451	Carbamidomethylation	M9:Oxidation (M):10.11;C13:Carbamidomethylation:1000.00	PEAKS DB
R.NNPFYFPSRR.F	N	92.08	1296.6364	10	-3.0	649.3235	2	28.67	14	F14:1408	OB5941 H1 raw.raw	1.2788E8	21	21	172	181			PEAKS DB
K.EGALM(+15.99)LPHFNSK.A	N	91.98	1358.6653	12	2.1	680.3414	2	29.74	14	F14:1443	OB5941 H1 raw.raw	1.9561E6	3	3	440	451	Oxidation (M)	M5:Oxidation (M):1000.00	PEAKS DB
R.VLLEENAGGEQEERGQRR.W	N	91.09	2069.0249	18	2.1	690.6837	3	25.31	14	F14:1186	OB5941 H1 raw.raw	1.1888E6	3	3	335	352			PEAKS DB
K.KNPQLQDLDMM(+15.99)LTC(+57.02)VEIKEGALMLPHFNSK.A	N	90.76	3515.7285	30	-2.7	879.9370	4	34.52	13	F13:1755	OB5940 H1 raw.raw	2.6989E6	3	3	422	451	Carbamidomethylation	M11:Oxidation (M):30.46;C14:Carbamidomethylation:1000.00	PEAKS DB
K.AM(-48.00)VIVVVNKGTGNLELVAVR.K	N	90.57	2033.1997	20	-3.4	1017.6036	2	31.46	14	F14:1539	OB5941 H1 raw.raw	1.6588E7	6	6	452	471	Dethiomethyl	M2:Dethiomethyl:1000.00	PEAKS PTM
K.A(+57.02)MVIVVVNKGTGNLELVAVR.K	N	90.54	2138.2246	20	-0.9	1070.1187	2	34.35	14	F14:1737	OB5941 H1 raw.raw	5.6414E6	3	3	452	471	Carbamidomethylation (DHKE, X@N-term)	A1:Carbamidomethylation (DHKE, X@N-term):207.56	PEAKS PTM
K.NPQLQDLDMMLTC(+57.02)VEIKEGALM(+15.99)LPHFNSK.A	N	90.37	3387.6335	29	-1.5	1130.2168	3	36.17	14	F14:1895	OB5941 H1 raw.raw	3.1109E7	7	7	423	451	Carbamidomethylation; Oxidation (M)	C13:Carbamidomethylation:1000.00;M22:Oxidation (M):69.89	PEAKS DB
K.H(+57.02)ADADNILVIQQGQATVTVAN(+.98)GNNRK.S	N	90.07	2804.4165	26	-1.3	935.8115	3	30.08	14	F14:1476	OB5941 H1 raw.raw	6.8084E6	2	2	229	254		H1:Carbamidomethylation (DHKE, X@N-term):45.01;N21:Deamidation (NQ):14.04	PEAKS PTM
R.IVQIEAKPNTLVLPK.H	N	89.51	1662.0079	15	-3.4	832.0084	2	29.92	14	F14:1456	OB5941 H1 raw.raw	3.047E8	42	42	214	228			PEAKS DB
F.LAGDKDNVIDQIEK.Q	N	89.22	1556.8046	14	-3.2	779.4071	2	32.19	12	F12:1557	OB5939 H1 raw.raw	4.4864E6	2	2	543	556			PEAKS DB
K.I(+57.02)SMPVNTPGQFEDFFPASSR.D	N	88.49	2283.0630	20	-0.1	1142.5387	2	35.84	12	F12:1793	OB5939 H1 raw.raw	6.4772E5	1	1	288	307	Carbamidomethylation (DHKE, X@N-term)	I1:Carbamidomethylation (DHKE, X@N-term):156.03	PEAKS PTM
K.DLAFPGSGEQVEKLIK.N	N	87.96	1729.9249	16	1.0	865.9706	2	33.30	7	F7:1601	OB5934 H3B raw.raw	6.2551E5	3	3	560	575			PEAKS DB
K.EGALMLPHFNSK.A	N	87.95	1342.6703	12	2.5	672.3441	2	29.83	13	F13:1462	OB5940 H1 raw.raw	9.3502E6	3	3	440	451			PEAKS DB
K.HADADNILVIQQGQATVTVAN(+.98)GNNR.K	N	87.06	2619.3000	25	-1.2	1310.6558	2	30.35	13	F13:1535	OB5940 H1 raw.raw	4.8683E6	1	1	229	253		N21:Deamidation (NQ):7.32	PEAKS DB
**total 217 peptides**																			

Peptide	Uniq	-10lgP	Mass	Length	ppm	m/z	z	RT	Fraction	Scan	Source File	Area Raw in gel	#Feature	#Feature Raw in gel	Start	End	PTM	AScore	Found By
K.AMVIVVVNK.G	N	86.97	971.5837	9	0.9	486.7996	2	29.88	12	F12:1441	OB5939 H1 raw.raw	9.5124E6	3	3	452	460			PEAKS DB
R.SSENNEGVIVK.V	Y	86.86	1174.5830	11	-0.3	588.2986	2	23.31	14	F14:1084	OB5941 H1 raw.raw	3.5114E5	6	6	357	367			PEAKS DB
K.GTGNLELVAVR.K	N	86.65	1127.6299	11	-0.8	564.8218	2	29.74	14	F14:1450	OB5941 H1 raw.raw	1.0907E7	6	6	461	471			PEAKS DB
R.NNPFYFPSR.R	N	86.37	1140.5352	9	-3.8	571.2727	2	31.46	14	F14:1552	OB5941 H1 raw.raw	1.201E7	5	5	172	180			PEAKS DB
K.HADADNILVIQQGQATVTVAN(-17.03)GNNRK.S	N	86.12	2729.3845	26	-1.1	910.8011	3	29.38	14	F14:1429	OB5941 H1 raw.raw	8.2265E6	4	4	229	254		N21:Ammonia-loss (N):18.53	PEAKS PTM
R.NTLEAAFNAEFNEIRR.V	N	86.03	1893.9332	16	-3.6	947.9705	2	35.28	12	F12:1741	OB5939 H1 raw.raw	3.4229E8	38	38	319	334			PEAKS DB
R.I(+27.99)FLAGDKDNVIDQIEK.Q	N	85.41	1844.9519	16	5.2	923.4880	2	36.16	14	F14:1844	OB5941 H1 raw.raw	0	0	0	541	556	Formylation	I1:Formylation:77.93	PEAKS PTM
K.SFNLDEGH(+57.02)ALR.I	N	84.87	1314.6316	11	-1.2	658.3223	2	28.05	12	F12:1298	OB5939 H1 raw.raw	5.1176E6	5	5	255	265		H8:Carbamidomethylation (DHKE, X@N-term):30.36	PEAKS PTM
R.K(+57.02)SFNLDEGHALR.I	N	83.09	1442.7266	12	-0.4	481.9159	3	25.70	14	F14:1209	OB5941 H1 raw.raw	2.2174E5	3	3	254	265	Carbamidomethylation (DHKE, X@N-term)	K1:Carbamidomethylation (DHKE, X@N-term):51.63	PEAKS PTM
K.SVSKKGSEEEGDITNPINLR.E	N	83.00	2172.1023	20	0.7	725.0419	3	27.21	14	F14:1286	OB5941 H1 raw.raw	1.3065E5	2	2	382	401			PEAKS DB
K.KNPQLQDLDM(+15.99)MLTC(+57.02)VEIK.E	N	81.54	2191.0686	18	0.7	1096.5424	2	33.03	12	F12:1615	OB5939 H1 raw.raw	0	0	0	422	439	Carbamidomethylation	M10:Oxidation (M):14.02;C14:Carbamidomethylation:1000.00	PEAKS DB
K.KNPQLQDLDMMLTC(+57.02)VEIKEGALMLPHFNSK.A	N	81.26	3499.7336	30	-0.1	875.9406	4	36.52	14	F14:1868	OB5941 H1 raw.raw	1.091E7	3	3	422	451	Carbamidomethylation	C14:Carbamidomethylation:1000.00	PEAKS DB
K.GTGNLELVAVRK.E	N	79.30	1255.7249	12	0.9	628.8702	2	26.85	14	F14:1263	OB5941 H1 raw.raw	1.0437E7	9	9	461	472			PEAKS DB
R.I(+57.02)VQIEAKPNTLVLPK.H	N	78.65	1719.0294	15	3.0	860.5245	2	30.75	12	F12:1469	OB5939 H1 raw.raw	1.7854E7	5	5	214	228	Carbamidomethylation (DHKE, X@N-term)	I1:Carbamidomethylation (DHKE, X@N-term):97.69	PEAKS PTM
R.EETSRNNPFYFPSRR.F	N	78.31	1898.9023	15	1.2	633.9755	3	27.17	13	F13:1289	OB5940 H1 raw.raw	6.7264E6	6	6	167	181			PEAKS DB
R.IFLAGDKDNVIDQIE(+57.02)K.Q	N	78.23	1873.9785	16	-5.0	937.9919	2	32.19	14	F14:1583	OB5941 H1 raw.raw	3.036E7	3	3	541	556		E15:Carbamidomethylation (DHKE, X@N-term):0.00	PEAKS PTM
I.PSGFISYILNR.H	N	77.77	1265.6768	11	2.4	633.8472	2	36.17	13	F13:1844	OB5940 H1 raw.raw	2.5204E5	2	2	267	277			PEAKS DB
I.FLAGDKDNVIDQIEK.Q	N	77.74	1703.8729	15	-9.3	852.9358	2	32.01	14	F14:1599	OB5941 H1 raw.raw	1.3574E6	3	3	542	556			PEAKS DB
K.D(+57.02)LAFPGSGEQVEK.L	N	77.21	1432.6833	13	3.8	717.3517	2	30.92	12	F12:1485	OB5939 H1 raw.raw	2.816E5	1	1	560	572	Carbamidomethylation (DHKE, X@N-term)	D1:Carbamidomethylation (DHKE, X@N-term):93.18	PEAKS PTM
**total 217 peptides**																			

Peptide	Uniq	-10lgP	Mass	Length	ppm	m/z	z	RT	Fraction	Scan	Source File	Area Raw in gel	#Feature	#Feature Raw in gel	Start	End	PTM	AScore	Found By
K.K(+57.02)GSEEEGDITNPINLR.E	N	77.19	1827.8962	16	-3.2	914.9525	2	28.80	14	F14:1391	OB5941 H1 raw.raw	0	0	0	386	401		K1:Carbamidomethylation (DHKE, X@N-term):8.80	PEAKS PTM
R.Q(-17.03)FQNLQNHR.I	N	76.65	1166.5581	9	-1.5	584.2855	2	27.77	14	F14:1319	OB5941 H1 raw.raw	3.3996E6	3	3	205	213	Pyro-glu from Q	Q1:Pyro-glu from Q:1000.00	PEAKS PTM
K.KGSEEEGDITN(+.98)PINLREGEPDLSNNFGK.L	Y	75.94	3059.4319	28	7.3	1530.7344	2	30.76	14	F14:1523	OB5941 H1 raw.raw	5.8148E5	1	1	386	413		N11:Deamidation (NQ):10.86	PEAKS DB
K.HAD(+57.02)ADNILVIQQGQATVTVAN(+.98)GNNRK.S	N	74.85	2804.4165	26	-5.0	935.8081	3	28.96	13	F13:1511	OB5940 H1 raw.raw	3.7976E6	1	1	229	254		D3:Carbamidomethylation (DHKE, X@N-term):6.59;N21:Deamidation (NQ):14.04	PEAKS PTM
K.HADADNILVIQQGQATVTVANGN(-17.03)NRK.S	N	74.65	2729.3845	26	-3.3	1365.6951	2	29.38	14	F14:1432	OB5941 H1 raw.raw	5.4978E5	1	1	229	254		N23:Ammonia-loss (N):0.00	PEAKS PTM
K.ISMPVNTPGQFEDFFPASSRDQSSYLQGFSR.N	N	74.49	3494.6201	31	2.7	1165.8838	3	36.29	2	F2:1620	OB5925 H3B raw.raw	9.3886E7	15	15	288	318			PEAKS DB
R.NTLEAAFNAEFN(+.98)EIRR.V	N	74.39	1894.9172	16	-3.7	948.4624	2	32.73	14	F14:1640	OB5941 H1 raw.raw	4.113E6	3	3	319	334	Deamidation (NQ)	N12:Deamidation (NQ):55.21	PEAKS DB
K.LFEVKPDKKNPQLQDLDMMLTC(+57.02)VEIK.E	N	73.38	3131.6069	26	3.7	1044.8801	3	33.97	13	F13:1716	OB5940 H1 raw.raw	3.7487E6	3	3	414	439	Carbamidomethylation	C22:Carbamidomethylation:1000.00	PEAKS DB
K.NPQLQDLDMM(+15.99)LTC(+57.02)VEIKEGALMLPHFNSK.A	N	73.21	3387.6335	29	5.6	847.9204	4	35.82	13	F13:1825	OB5940 H1 raw.raw	9.6415E6	3	3	423	451	Carbamidomethylation	M10:Oxidation (M):16.90;C13:Carbamidomethylation:1000.00	PEAKS DB
A.GDKDNVIDQIEK.Q	N	73.04	1372.6833	12	-2.8	687.3470	2	32.19	12	F12:1561	OB5939 H1 raw.raw	1.4449E6	3	3	545	556			PEAKS DB
R.V(+57.02)LLEENAGGEQEER.G	N	73.00	1628.7642	14	1.1	815.3903	2	27.03	14	F14:1288	OB5941 H1 raw.raw	2.7852E5	3	3	335	348		V1:Carbamidomethylation (DHKE, X@N-term):44.44	PEAKS PTM
R.EETSRNNPFYFPSR.R	N	72.75	1742.8011	14	-0.9	581.9405	3	29.89	12	F12:1419	OB5939 H1 raw.raw	4.0306E4	1	1	167	180			PEAKS DB
R.IPSGFISYILN(-17.03)R.H	N	72.57	1361.7343	12	1.4	681.8754	2	37.47	13	F13:1926	OB5940 H1 raw.raw	5.3561E5	2	2	266	277	Ammonia-loss (N)	N11:Ammonia-loss (N):1000.00	PEAKS PTM
R.IPSGFISY(+125.90)ILNR.H	N	72.49	1504.6575	12	1.5	753.3372	2	37.84	13	F13:1946	OB5940 H1 raw.raw	9.8379E5	2	2	266	277	Iodination	Y8:Iodination:1000.00	PEAKS PTM
R.IPSGFISYILNRH(+57.02)DNQNLR.V	N	72.20	2313.1978	19	2.0	579.3079	4	33.29	12	F12:1633	OB5939 H1 raw.raw	3.81E7	4	4	266	284		H13:Carbamidomethylation (DHKE, X@N-term):9.34	PEAKS PTM
R.VLLEEN(+.98)AGGEQEER.G	N	71.59	1572.7267	14	-3.7	787.3677	2	26.82	13	F13:1276	OB5940 H1 raw.raw	0	0	0	335	348	Deamidation (NQ)	N6:Deamidation (NQ):71.64	PEAKS DB
A.FPGSGEQVEK.L	N	71.45	1076.5138	10	5.5	539.2672	2	30.23	12	F12:1449	OB5939 H1 raw.raw	6.1112E4	1	1	563	572			PEAKS DB
R.E(-18.01)GE(+57.02)QEWGTPGSHVR.E	N	71.00	1606.7124	14	0.1	804.3635	2	27.42	14	F14:1300	OB5941 H1 raw.raw	8.0689E4	3	3	153	166	Pyro-glu from E	E1:Pyro-glu from E:1000.00;E3:Carbamidome	PEAKS PTM

total 217 peptides

Peptide	Uniq	-10lgP	Mass	Length	ppm	m/z	z	RT	Fraction	Scan	Source File	Area Raw in gel	#Feature	#Feature Raw in gel	Start	End	PTM	AScore	Found By
																		thylation (DHKE, X@N-term):45.01	
K.A(+57.02)MVIVVVNKGTGNLELVAVRK.E	N	70.77	2266.3196	21	-1.6	1134.1653	2	32.33	13	F13:1602	OB5940H1 raw.raw	4.861E7	6	6	452	472	Carbamidomethylation (DHKE, X@N-term)	A1:Carbamidomethylation (DHKE, X@N-term):161.20	PEAKS PTM
K.AM(+15.99)VIVVVNK.G	N	70.64	987.5787	9	0.7	494.7969	2	26.66	14	F14:1257	OB5941H1 raw.raw	8.6431E5	3	3	452	460	Oxidation (M)	M2:Oxidation (M):1000.00	PEAKS DB
K.KNPQLQDLDMMLTC(+57.02)VEIKEGALM(+15.99)LPHFNSK.A	N	70.55	3515.7285	30	-0.8	879.9387	4	35.84	12	F12:1790	OB5939H1 raw.raw	4.7958E6	2	2	422	451	Carbamidomethylation; Oxidation (M)	C14:Carbamidomethylation:1000.00;M23:Oxidation (M):69.38	PEAKS DB
K.VSKEHVEELTK.H	N	69.86	1297.6877	11	0.8	433.5702	3	24.82	12	F12:1120	OB5939H1 raw.raw	1.1162E5	6	6	368	378			PEAKS DB
Q.GQATVTVAN(+.98)GNNRK.S	N	69.60	1429.7273	14	-4.1	715.8680	2	29.38	14	F14:1424	OB5941H1 raw.raw	5.134E5	2	2	241	254		N9:Deamidation (NQ):41.83	PEAKS DB
R.NT(-18.01)LEAAFNAEFNEIRR.V	N	68.32	1875.9227	16	-3.2	626.3129	3	33.81	14	F14:1704	OB5941H1 raw.raw	1.3248E7	3	3	319	334	Dehydration	T2:Dehydration:1000.00	PEAKS PTM
R.SSEN(+.98)NEGVIVK.V	Y	68.21	1175.5670	11	0.8	588.7913	2	24.17	14	F14:1126	OB5941H1 raw.raw	1.8176E4	2	2	357	367		N4:Deamidation (NQ):20.00	PEAKS DB
R.SRQFQNLQNHR.I	N	67.97	1426.7178	11	-0.3	476.5797	3	24.94	14	F14:1159	OB5941H1 raw.raw	1.4052E4	2	2	203	213			PEAKS DB
K.LFEVKPDKKNPQLQDLDM(+15.99)M(+15.99)LTC(+57.02)VEIK.E	N	67.75	3163.5967	26	2.1	791.9081	4	32.37	12	F12:1585	OB5939H1 raw.raw	6.2675E5	1	1	414	439	Oxidation (M); Carbamidomethylation	M18:Oxidation (M):1000.00;M19:Oxidation (M):1000.00;C22:Carbamidomethylation:1000.00	PEAKS DB
L.AGDKDNVIDQIEK.Q	N	66.85	1443.7205	13	-3.1	722.8653	2	32.19	12	F12:1560	OB5939H1 raw.raw	7.0907E5	2	2	544	556			PEAKS DB
K.G(+57.02)TGNLELVAVRK.E	N	66.68	1312.7462	12	-0.7	657.3799	2	26.85	14	F14:1280	OB5941H1 raw.raw	3.417E5	3	3	461	472	Carbamidomethylation (DHKE, X@N-term)	G1:Carbamidomethylation (DHKE, X@N-term):56.51	PEAKS PTM
K.SFNLDEGHALRIPSGFISYILNRHDNQNLR.V	N	66.45	3495.7759	30	-1.2	700.1616	5	36.31	13	F13:1855	OB5940H1 raw.raw	1.2605E8	16	16	255	284			PEAKS DB
R.QFQNLQNHR.I	N	66.40	1183.5846	9	0.2	592.7997	2	24.53	14	F14:1134	OB5941H1 raw.raw	7.3427E4	5	5	205	213			PEAKS DB
D.KDNVIDQIEK.Q	N	66.36	1200.6350	10	-0.8	601.3243	2	25.89	14	F14:1225	OB5941H1 raw.raw	5.8609E4	3	3	547	556			PEAKS DB
K.NPQLQ(+.98)DLDMMLTC(+57.02)VEIKEGALMLPHFNSK.A	N	66.16	3372.6226	29	5.7	1125.2212	3	37.84	13	F13:1936	OB5940H1 raw.raw	5.3127E7	3	3	423	451	Carbamidomethylation	Q5:Deamidation (NQ):32.28;C13:Carbamidomethylation:1000.00	PEAKS DB
K.HADADNILVIQQGQATVTVANGNN(+.98)RK.S	N	65.93	2747.3950	26	4.5	1374.7109	2	29.70	10	F10:1444	OB5937H3A raw.raw	5.6818E5	3	3	229	254		N24:Deamidation (NQ):14.02	PEAKS DB
K.ISMPVNTPGQ(+.98)FEDFFPASSR.D	N	65.70	2227.0254	20	6.4	1114.5271	2	63.55	14	F14:3427	OB5941H1 raw.raw	1.0155E4	2	2	288	307		Q10:Deamidation (NQ):33.26	PEAKS DB

total 217 peptides

Peptide	Uniq	-10lgP	Mass	Length	ppm	m/z	z	RT	Fraction	Scan	Source File	Area Raw in gel	#Feature	#Feature Raw in gel	Start	End	PTM	AScore	Found By
R.N(+.98)TLEAAFNAEFNEIRR.V	N	65.61	1894.9172	16	2.2	948.4680	2	35.88	12	F12:1787	OB5939 H1 raw.raw	7.0859E7	1	1	319	334	Deamidation (NQ)	N1:Deamidation (NQ):61.82	PEAKS DB
K.KNPQLQDLDM(+15.99)MLTC(+57.02)VEIKEGALMLPHFNSK.A	N	65.47	3515.7285	30	-3.1	879.9366	4	35.44	13	F13:1830	OB5940 H1 raw.raw	7.2415E6	3	3	422	451	Carbamidomethylation	M10:Oxidation (M):11.06;C14:Carbamidomethylation:1000.00	PEAKS DB
K.ISM(+15.99)PVNTPGQ(+.98)FEDFFPASSRDQSSYLQGFSR.N	N	65.25	3511.5989	31	4.3	1171.5453	3	34.92	12	F12:1731	OB5939 H1 raw.raw	1.4851E6	1	1	288	318	Oxidation (M)	M3:Oxidation (M):1000.00;Q10:Deamidation (NQ):27.62	PEAKS DB
K.NPQLQDLDM(+15.99)MLTC(+57.02)VEIKEGALM(+15.99)LPHFNSK.A	N	64.53	3403.6284	29	4.5	1135.5552	3	36.52	14	F14:1873	OB5941 H1 raw.raw	4.3451E6	3	3	423	451	Carbamidomethylation; Oxidation (M)	M9:Oxidation (M):10.11;C13:Carbamidomethylation:1000.00;M22:Oxidation (M):53.09	PEAKS DB
K.LFEVKPDK.K	N	63.66	974.5436	8	0.9	488.2795	2	25.40	12	F12:1153	OB5939 H1 raw.raw	6.8606E5	7	7	414	421			PEAKS DB
F.NLDEGHALR.I	N	63.51	1023.5097	9	2.3	512.7633	2	27.35	13	F13:1309	OB5940 H1 raw.raw	1.5878E6	3	3	257	265			PEAKS DB
V.QIEAKPNTLVLPK.H	N	63.24	1449.8555	13	-3.5	725.9325	2	29.92	14	F14:1467	OB5941 H1 raw.raw	1.2653E6	3	3	216	228			PEAKS DB
R.EGEQEWGTPGSHVREETSR.N	N	63.23	2169.9675	19	2.1	724.3313	3	25.88	14	F14:1208	OB5941 H1 raw.raw	2.2911E6	7	7	153	171			PEAKS DB
K.ISM(-48.00)PVNTPGQFEDFFPASSR.D	N	62.93	2178.0381	20	0.2	727.0201	3	32.37	12	F12:1577	OB5939 H1 raw.raw	2.6788E7	5	5	288	307	Dethiomethyl	M3:Dethiomethyl:1000.00	PEAKS PTM
N.TPGQFEDFFPASSR.D	N	62.85	1584.7208	14	-0.2	793.3676	2	35.63	14	F14:1821	OB5941 H1 raw.raw	2.1663E5	1	1	294	307			PEAKS DB
K.A(+57.02)M(+15.99)VIVVVNKGTGNLELVAVR.K	N	62.49	2154.2195	20	-0.1	1078.1169	2	32.92	12	F12:1614	OB5939 H1 raw.raw	4.5509E5	2	2	452	471	Carbamidomethylation (DHKE, X@N-term); Oxidation (M)	A1:Carbamidomethylation (DHKE, X@N-term):139.69; M2:Oxidation (M):1000.00	PEAKS PTM
L.EAAFNAEFNEIRR.V	N	62.30	1565.7585	13	-4.7	783.8829	2	34.06	12	F12:1677	OB5939 H1 raw.raw	6.1232E5	1	1	322	334			PEAKS DB
K.N(+.98)PQLQDLDMMLTC(+57.02)VEIKEGALMLPHFNSK.A	N	61.86	3372.6226	29	5.7	1125.2212	3	37.84	13	F13:1983	OB5940 H1 raw.raw	1.2903E7	1	1	423	451	Carbamidomethylation	N1:Deamidation (NQ):14.04;C13:Carbamidomethylation:1000.00	PEAKS DB
K.HADADNILVIQQGQ(+.98)ATVTVAN(+.98)GNNRK.S	N	61.75	2748.3792	26	1.4	1375.1987	2	29.55	13	F13:1443	OB5940 H1 raw.raw	4.856E5	1	1	229	254		Q14:Deamidation (NQ):32.28;N21:Deamidation (NQ):14.04	PEAKS DB
N.LDEGHALR.I	N	60.44	909.4668	8	0.6	455.7409	2	27.35	13	F13:1312	OB5940 H1 raw.raw	3.1152E4	1	1	258	265			PEAKS DB
R.WGPAGPR.E	N	59.30	739.3765	7	-0.2	370.6955	2	24.50	13	F13:1143	OB5940 H1 raw.raw	2.137E5	2	2	115	121			PEAKS DB
R.IFLAGDKDNVIDQ(+.98)IEKQAK.D	N	58.72	2145.1316	19	9.3	716.0578	3	32.93	2	F2:1432	OB5925 H3B raw.raw	1.5596E6	1	1	541	559		Q13:Deamidation (NQ):0.00	PEAKS DB
**total 217 peptides**																			

Peptide	Uniq	-10lgP	Mass	Length	ppm	m/z	z	RT	Fraction	Scan	Source File	Area Raw in gel	#Feature	#Feature Raw in gel	Start	End	PTM	AScore	Found By
K.HADADNILVIQ(+.98)Q(+.98)GQATVTVAN(+.98)GNNRK.S	N	58.62	2749.3630	26	9.6	917.4705	3	30.18	13	F13:1524	OB5940 H1 raw.raw	4.3812E6	1	1	229	254		Q11:Deamidation (NQ):26.52;Q12:Deamidation (NQ):18.53;N21:Deamidation (NQ):14.04	PEAKS DB
R.D(+57.02)QSSYLQGFSR.N	N	58.49	1343.6106	11	-2.5	672.8109	2	31.19	12	F12:1506	OB5939 H1 raw.raw	3.7713E4	1	1	308	318	Carbamidomethylation (DHKE, X@N-term)	D1:Carbamidomethylation (DHKE, X@N-term):1000.00	PEAKS PTM
K.HADADNILVIQ(+.98)QGQATVTVAN(+.98)GNNRK.S	N	58.29	2748.3792	26	4.6	1375.2031	2	31.28	12	F12:1539	OB5939 H1 raw.raw	1.8636E5	1	1	229	254		Q11:Deamidation (NQ):15.10;N21:Deamidation (NQ):17.01	PEAKS DB
K.HADADN(+.98)ILVIQ(+.98)QGQATVTVANGNNRK.S	N	57.75	2748.3792	26	3.1	1375.2010	2	30.08	14	F14:1495	OB5941 H1 raw.raw	1.5756E6	1	1	229	254		N6:Deamidation (NQ):0.00;Q11:Deamidation (NQ):0.00	PEAKS DB
N.PFYFPSRR.F	N	57.72	1068.5504	8	3.3	535.2842	2	28.95	12	F12:1360	OB5939 H1 raw.raw	3.0845E6	3	3	174	181			PEAKS DB
K.LFEVKPDKKNPQLQDLDMM(+15.99)LTC(+57.02)VEIK.E	N	57.62	3147.6018	26	-4.4	787.9042	4	32.55	14	F14:1633	OB5941 H1 raw.raw	2.1102E6	3	3	414	439	Carbamidomethylation	M19:Oxidation (M):17.01;C22:Carbamidomethylation:1000.00	PEAKS DB
K.SFN(+.98)LDEGHALRIPSGFISYILNR.H	N	57.28	2619.3445	23	9.3	655.8495	4	38.22	12	F12:1947	OB5939 H1 raw.raw	1.0265E6	2	2	255	277		N3:Deamidation (NQ):32.08	PEAKS DB
K.KGSEEEGDITNPIN(+.98)LREGEPDLSNNFGK.L	Y	57.09	3059.4319	28	7.3	1530.7344	2	30.76	14	F14:1531	OB5941 H1 raw.raw	5.8148E5	1	1	386	413		N14:Deamidation (NQ):35.60	PEAKS DB
K.AMVIVVVN(+.98)KGTGNLELVAVRK.E	N	56.84	2210.2820	21	9.6	737.7750	3	33.10	14	F14:1664	OB5941 H1 raw.raw	8.0625E6	2	2	452	472		N8:Deamidation (NQ):41.87	PEAKS DB
K.KNPQLQDLDMM(-48.00)LTC(+57.02)VEIK.E	N	56.43	2127.0703	18	0.8	710.0312	3	31.11	14	F14:1538	OB5941 H1 raw.raw	3.3192E6	2	2	422	439	Carbamidomethylation	M11:Dethiomethyl:14.02;C14:Carbamidomethylation:1000.00	PEAKS PTM
K.ISM(+15.99)PVNTPGQFEDFFPASSRDQSSYLQGFSR.N	N	56.19	3510.6150	31	2.9	1171.2157	3	34.90	14	F14:1765	OB5941 H1 raw.raw	1.0055E7	8	8	288	318	Oxidation (M)	M3:Oxidation (M):1000.00	PEAKS DB
R.IVQIEAK(+57.02)PNTLVLPK.H	N	56.13	1719.0294	15	-1.6	860.5206	2	30.60	14	F14:1466	OB5941 H1 raw.raw	1.1048E7	2	2	214	228	Carbamidomethylation (DHKE, X@N-term)	K7:Carbamidomethylation (DHKE, X@N-term):51.51	PEAKS PTM
K.IRPEGREGEQEWGTPGSHVR.E	N	56.05	2276.1045	20	0.7	570.0338	4	26.47	14	F14:1255	OB5941 H1 raw.raw	1.8795E5	1	1	147	166			PEAKS DB
R.N(+57.02)NPFYFPSRR.F	N	55.47	1353.6577	10	-2.6	677.8344	2	29.38	14	F14:1433	OB5941 H1 raw.raw	6.1234E5	2	2	172	181	Carbamidomethylation (DHKE, X@N-term)	N1:Carbamidomethylation (DHKE, X@N-term):1000.00	PEAKS PTM
K.HADADNILVIQQGQATVTVANGN(+.98)NRK.S	N	55.13	2747.3950	26	4.2	687.8589	4	29.51	12	F12:1401	OB5939 H1 raw.raw	2.4633E7	5	5	229	254		N23:Deamidation (NQ):17.01	PEAKS DB
K.HADADNILVIQQ(+.98)GQ(+.98)ATVTVAN(+.98)GNNRK.S	N	55.12	2749.3630	26	9.6	917.4705	3	30.18	13	F13:1487	OB5940 H1 raw.raw	4.3812E6	1	1	229	254		Q12:Deamidation (NQ):14.02;Q14:Deamid	PEAKS DB

total 217 peptides

Peptide	Uniq	-10lgP	Mass	Length	ppm	m/z	z	RT	Fraction	Scan	Source File	Area Raw in gel	#Feature	#Feature Raw in gel	Start	End	PTM	AScore	Found By
																		ation (NQ): 13.03;N21: Deamidation (NQ):11.10	
K.AM(-48.00)VIVVVNKGTGNLELVAVRK.E	N	54.93	2161.2947	21	1.4	1081.6561	2	29.80	14	F14:1453	OB5941 H1 raw.raw	1.7581E6	2	2	452	472	Dethiomethyl	M2:Dethiomethyl:1000.00	PEAKS PTM
K.NPQLQDLDM(+15.99)MLTC(+57.02)VEIKEGALMLPHFN(+.98)SK.A	N	54.06	3388.6174	29	5.0	848.1659	4	36.17	14	F14:1849	OB5941 H1 raw.raw	6.0156E6	2	2	423	451	Carbamidomethylation; Deamidation (NQ)	M9:Oxidation (M):14.02;C13:Carbamidomethylation:1000.00;N27:Deamidation (NQ):58.23	PEAKS DB
R.IFLAGDKDNVIDQIE(+57.02)KQAK.D	N	52.99	2201.1692	19	-2.0	734.7289	3	31.83	13	F13:1588	OB5940 H1 raw.raw	3.3379E6	2	2	541	559		E15:Carbamidomethylation (DHKE, X@N-term):12.28	PEAKS PTM
K.NPQLQDLDMMLTC(+57.02)VEIKEGALMLPHFN(+.98)SK.A	N	52.89	3372.6226	29	7.4	844.1691	4	37.85	13	F13:1914	OB5940 H1 raw.raw	5.2564E7	2	2	423	451	Carbamidomethylation; Deamidation (NQ)	C13:Carbamidomethylation:1000.00;N27:Deamidation (NQ):61.84	PEAKS DB
K.HADADNILVIQQGQ(+.98)ATVTVANGNNRK.S	N	52.87	2747.3950	26	5.1	916.8103	3	44.47	12	F12:2283	OB5939 H1 raw.raw	1.466E5	1	1	229	254		Q14:Deamidation (NQ):14.04	PEAKS DB
K.NPQLQDLDM(+15.99)M(+15.99)LTC(+57.02)VEIKEGALMLPHFNSK.A	N	52.70	3403.6284	29	-4.3	1135.5452	3	35.63	14	F14:1818	OB5941 H1 raw.raw	1.9434E6	2	2	423	451	Oxidation (M); Carbamidomethylation	M9:Oxidation (M):49.75;M10:Oxidation (M):51.99;C13:Carbamidomethylation:1000.00	PEAKS DB
L.DEGHALR.I	N	52.68	796.3828	7	2.3	399.1996	2	27.87	12	F12:1309	OB5939 H1 raw.raw	2.418E4	1	1	259	265			PEAKS DB
K.DLAFPGSGEQVEK(+57.02)LIK.N	N	52.57	1786.9464	16	-3.8	894.4771	2	32.91	14	F14:1654	OB5941 H1 raw.raw	6.7177E5	1	1	560	575		K13:Carbamidomethylation (DHKE, X@N-term):27.96	PEAKS PTM
K.KN(+.98)PQLQDLDMM(+15.99)LTC(+57.02)VEIK.E	N	52.31	2192.0527	18	9.2	1097.0437	2	32.33	13	F13:1621	OB5940 H1 raw.raw	8.5775E4	1	1	422	439	Carbamidomethylation	N2:Deamidation (NQ): 14.04;M11: Oxidation (M):0.00;C14:Carbamidomethylation:1000.00	PEAKS DB
R.IVQIEAKPNTLVLPK(+57.02).H	N	52.13	1719.0294	15	3.0	860.5245	2	30.75	12	F12:1437	OB5939 H1 raw.raw	4.8623E6	1	1	214	228		K15:Carbamidomethylation (DHKE, X@N-term):25.69	PEAKS PTM
Q.GQATVTVANGNNRK.S	N	51.97	1428.7433	14	-0.2	715.3788	2	29.02	14	F14:1412	OB5941 H1 raw.raw	1.2099E5	2	2	241	254			PEAKS DB
K.EGALMLPH(+14.02)FNSK.A	N	51.73	1356.6860	12	1.3	679.3512	2	30.49	14	F14:1499	OB5941 H1 raw.raw	2.5741E4	1	1	440	451	Methylation(others)	H8:Methylation(others):53.53	PEAKS PTM
K.N(+.98)PQLQ(+.98)DLDMMLTC(+57.02)VEIKEGALM(+15.99)LPHFNSK.A	N	51.60	3389.6016	29	9.0	848.4153	4	36.92	12	F12:1868	OB5939 H1 raw.raw	2.6873E6	1	1	423	451	Carbamidomethylation	N1:Deamidation (NQ): 0.00;Q5:Deamidation (NQ):14.04;C13:Carbamidomethylation:100	PEAKS DB
**total 217 peptides**																			

Peptide	Uniq	-10lgP	Mass	Length	ppm	m/z	z	RT	Fraction	Scan	Source File	Area Raw in gel	#Feature	#Feature Raw in gel	Start	End	PTM	AScore	Found By
																		0.00;M22:Oxidation (M):18.84	
K.LFEVKPDKK.N	N	50.85	1102.6385	9	1.1	552.3271	2	24.94	14	F14:1179	OB5941 H1 raw.raw	2.3081E5	2	2	414	422			PEAKS DB
R.RVLLEENAGGEQEERGQR.R	N	50.67	2069.0249	18	2.1	690.6837	3	24.88	13	F13:1165	OB5940 H1 raw.raw	2.7227E5	1	1	334	351			PEAKS DB
K.N(+.98)PQLQDLDMMLTC(+57.02)VEIKEGALM(+15.99)LPHFNSK.A	N	50.17	3388.6174	29	-2.4	1130.5437	3	36.35	13	F13:1874	OB5940 H1 raw.raw	8.9006E5	1	1	423	451	Carbamidomethylation; Oxidation (M)	N1:Deamidation (NQ):0.00;C13:Carbamidomethylation:1000.00;M22:Oxidation (M):54.40	PEAKS DB
K.NPQLQDLDMM(+15.99)LTC(+57.02)VEIKEGALM(+15.99)LPHFNSK.A	N	48.97	3403.6284	29	2.7	1135.5531	3	35.09	14	F14:1786	OB5941 H1 raw.raw	3.2001E5	1	1	423	451	Carbamidomethylation	M10:Oxidation (M):12.28;C13:Carbamidomethylation:1000.00;M22:Oxidation (M):28.29	PEAKS DB
K.KN(+.98)PQLQDLDMMLTC(+57.02)VEIKEGALMLPHFNSK.A	N	48.84	3500.7175	30	7.1	876.1929	4	36.26	13	F13:1847	OB5940 H1 raw.raw	2.2113E6	1	1	422	451	Carbamidomethylation	N2:Deamidation (NQ):0.00;C14:Carbamidomethylation:1000.00	PEAKS DB
R.IFLAGDKDNVIDQIEK(+57.02)QAK.D	N	48.82	2201.1692	19	2.7	734.7323	3	32.55	12	F12:1581	OB5939 H1 raw.raw	3.2958E6	1	1	541	559		K16:Carbamidomethylation (DHKE, X@N-term):15.91	PEAKS PTM
R.EREEDWRQPR.E	N	48.49	1399.6592	10	0.1	467.5604	3	24.77	14	F14:1154	OB5941 H1 raw.raw	2.5247E4	1	1	124	133			PEAKS DB
R.KSFN(+.98)LDEGHALRIPSGFISYILNR.H	N	47.51	2747.4395	24	4.5	687.8702	4	36.15	14	F14:1837	OB5941 H1 raw.raw	1.0524E6	1	1	254	277		N4:Deamidation (NQ):23.98	PEAKS DB
K.LFEVKPDKKN(+.98)PQLQDLDMMLTC(+57.02)VEIK.E	N	47.32	3132.5908	26	9.3	1567.3173	2	34.35	14	F14:1745	OB5941 H1 raw.raw	1.9315E5	1	1	414	439	Carbamidomethylation	N10:Deamidation (NQ):14.04;C22:Carbamidomethylation:1000.00	PEAKS DB
K.N(+.98)PQ(+.98)LQDLDMMLTC(+57.02)VEIKEGALM(+15.99)LPHFNSK.A	N	46.82	3389.6016	29	9.0	848.4153	4	36.92	12	F12:1851	OB5939 H1 raw.raw	7.3613E6	2	2	423	451	Carbamidomethylation	N1:Deamidation (NQ):11.12;Q3:Deamidation (NQ):0.00;C13:Carbamidomethylation:1000.00;M22:Oxidation (M):24.93	PEAKS DB
N.PQLQDLDMMLTC(+57.02)VEIKEGALMLPHFNSK.A	N	46.63	3257.5957	28	0.9	815.4069	4	37.83	14	F14:1952	OB5941 H1 raw.raw	2.3692E5	1	1	424	451	Carbamidomethylation	C12:Carbamidomethylation:1000.00	PEAKS DB
R.S(+57.02)SENNEGVIVK.V	Y	46.57	1231.6044	11	-0.2	616.8093	2	23.54	14	F14:1091	OB5941 H1 raw.raw	1.0427E3	1	1	357	367		S1:Carbamidomethylation (DHKE, X@N-term):20.41	PEAKS PTM
K.Q(-17.03)AKDLAFPGSGEQVEK.L	N	46.49	1685.8260	16	0.9	843.9211	2	29.97	12	F12:1427	OB5939 H1 raw.raw	0	0	0	557	572	Pyro-glu from Q	Q1:Pyro-glu from Q:1000.00	PEAKS PTM

total 217 peptides

Peptide	Uniq	-10lgP	Mass	Length	ppm	m/z	z	RT	Fraction	Scan	Source File	Area Raw in gel	#Feature	#Feature Raw in gel	Start	End	PTM	AScore	Found By
K.SFN(+.98)LDEGHALRIPSGFISYILNRHDNQNLR.V	N	46.45	3496.7600	30	-3.9	700.3566	5	36.38	12	F12:1849	OB5939 H1 raw.raw	6.986E5	1	1	255	284		N3:Deamidation (NQ): 20.27	PEAKS DB
Q.IEAKPNTLVLPK.H	N	46.43	1321.7969	12	-4.5	661.9028	2	29.65	13	F13:1449	OB5940 H1 raw.raw	6.8969E5	2	2	217	228			PEAKS DB
T.PGQFEDFFPASSR.D	N	45.25	1483.6731	13	-1.3	742.8429	2	40.34	13	F13:2103	OB5940 H1 raw.raw	4.0298E3	1	1	295	307			PEAKS DB
R.IPSGFISYILN(+.98)RHDNQNLR.V	N	45.21	2257.1604	19	9.9	753.4015	3	37.61	2	F2:1698	OB5925 H3B raw.raw	2.0603E6	2	2	266	284		N11:Deamidation (NQ):33.06	PEAKS DB
N.NPFYFPSRR.F	N	45.02	1182.5934	9	1.6	395.2057	3	28.08	13	F13:1358	OB5940 H1 raw.raw	2.9991E4	1	1	173	181			PEAKS DB
R.DQS(-18.01)SYLQGFSR.N	N	44.90	1268.5785	11	7.0	635.3010	2	30.87	12	F12:1484	OB5939 H1 raw.raw	0	0	0	308	318		S3:Dehydration:33.98	PEAKS PTM
K.GTGNLELVAVRKEQQQR.G	N	44.58	1925.0442	17	0.6	642.6890	3	25.89	14	F14:1220	OB5941 H1 raw.raw	1.1112E5	2	2	461	477			PEAKS DB
R.EDWRRPSHQQPR.K	N	43.98	1590.7764	12	-0.3	398.7012	4	24.94	14	F14:1169	OB5941 H1 raw.raw	1.0819E4	1	1	134	145			PEAKS DB
R.VLLE(+21.98)ENAGGEQEER.G	N	42.61	1593.7246	14	-0.7	797.8690	2	26.03	13	F13:1239	OB5940 H1 raw.raw	1.373E5	3	3	335	348		E4:Sodium adduct:40.00	PEAKS PTM
K.HADADNILVIQ(+.98)QGQATVTVANGNNRK.S	N	42.22	2747.3950	26	5.9	916.8110	3	45.04	14	F14:2357	OB5941 H1 raw.raw	0	0	0	229	254		Q11:Deamidation (NQ):9.34	PEAKS DB
K.HADADNILVIQQGQAT(-18.01)VTVAN(+.98)GNNRK.S	N	41.92	2729.3845	26	0.3	683.3536	4	28.96	13	F13:1411	OB5940 H1 raw.raw	3.5545E5	1	1	229	254		T16:Dehydration:8.22; N21:Deamidation (NQ):6.08	PEAKS PTM
K.HADADN(+.98)ILVIQQGQATVTVANGNNRK.S	N	41.76	2747.3950	26	3.7	916.8090	3	50.62	12	F12:2616	OB5939 H1 raw.raw	1.1355E5	2	2	229	254		N6:Deamidation (NQ): 42.57	PEAKS DB
K.ISMPVN(+.98)TPGQFEDFFPASSR.D	N	41.75	2227.0254	20	9.3	1114.5303	2	58.23	14	F14:3138	OB5941 H1 raw.raw	2.4983E4	1	1	288	307		N6:Deamidation (NQ): 42.89	PEAKS DB
M.LPHFNSK.A	N	41.73	841.4446	7	-3.4	421.7281	2	30.24	14	F14:1484	OB5941 H1 raw.raw	1.8069E6	2	2	445	451			PEAKS DB
K.PNTLVLPK.H	N	41.66	880.5382	8	0.4	441.2766	2	27.53	13	F13:1313	OB5940 H1 raw.raw	8.5249E4	1	1	221	228			PEAKS DB
N.TLEAAFNAEFNEIRR.V	N	41.51	1779.8904	15	-3.0	890.9498	2	32.91	14	F14:1655	OB5941 H1 raw.raw	4.1608E5	1	1	320	334			PEAKS DB
K.KNPQLQ(+.98)DLDMMLTC(+57.02)VEIKEGALMLPHFNSK.A	N	40.89	3500.7175	30	0.4	1167.9136	3	36.34	13	F13:1850	OB5940 H1 raw.raw	4.7608E5	1	1	422	451	Carbamidomethylation	Q6:Deamidation (NQ): 14.04;C14: Carbamidomethylation:1000.00	PEAKS DB
R.NTLEAAFN(+.98)AEFNEIRR.V	N	40.23	1894.9172	16	-0.3	948.4656	2	32.92	12	F12:1570	OB5939 H1 raw.raw	1.5322E6	1	1	319	334		N8:Deamidation (NQ): 8.26	PEAKS DB
R.EGEQEWGT(-18.01)PGSHVR.E	N	39.67	1549.6909	14	-1.7	775.8514	2	27.60	14	F14:1325	OB5941 H1 raw.raw	5.8338E4	1	1	153	166		T8:Dehydration:47.09	PEAKS PTM
K.NPQLQDLDM(+15.99)M(+15.99)LTC(+57.02)VEIK.E	N	39.57	2078.9688	17	1.1	1040.4928	2	33.80	14	F14:1701	OB5941 H1 raw.raw	0	0	0	423	439	Oxidation (M); Carbamidomethylation	M9:Oxidation (M):1000.00;M10: Oxidation (M):1000.00;C13:Carbamidomethylation:100	PEAKS DB

total 217 peptides

Peptide	Uniq	-10lgP	Mass	Length	ppm	m/z	z	RT	Fraction	Scan	Source File	Area Raw in gel	#Feature	#Feature Raw in gel	Start	End	PTM	AScore	Found By
																		0.00	
R.IPS(+59.02)GFISYILNR.H	N	39.40	1437.7802	12	1.5	719.8984	2	36.96	12	F12:1870	OB5939H1 raw.raw	4.3694E5	1	1	266	277		S3:Aminoethylcysteine:49.79	PEAKS PTM
K.HADADNILVIQQ(-18.01).G	N	38.68	1317.6677	12	-4.9	659.8379	2	29.56	14	F14:1421	OB5941H1 raw.raw	1.6968E5	2	2	229	240	Dehydration	Q12:Dehydration:76.18	PEAKS PTM
R.IVQ(+.98)IEAKPNTLVLPK.H	N	38.03	1662.9919	15	6.7	832.5088	2	31.45	13	F13:1578	OB5940H1 raw.raw	2.7795E5	1	1	214	228		Q3:Deamidation (NQ):13.11	PEAKS DB
K.AMVIVVVNK(-1.03)GTGNLELVAVR.K	N	37.92	2080.1714	20	4.3	1041.0974	2	33.79	13	F13:1738	OB5940H1 raw.raw	2.1453E7	1	1	452	471	Lysine oxidation to aminoadipic semialdehyde	K9:Lysine oxidation to aminoadipic semialdehyde:1000.00	PEAKS PTM
I.PSGFISYILNRHDNQNLR.V	N	37.07	2143.0923	18	4.0	715.3742	3	33.53	12	F12:1645	OB5939H1 raw.raw	0	0	0	267	284			PEAKS DB
K.EGALM(-48.00)LPHFNSK.A	N	36.97	1294.6670	12	2.0	432.5638	3	28.31	14	F14:1348	OB5941H1 raw.raw	1.3683E6	1	1	440	451	Dethiomethyl	M5:Dethiomethyl:1000.00	PEAKS PTM
R.IVQIEAKPN(+15.99)TLVLPK.H	N	36.93	1678.0028	15	-2.8	840.0063	2	29.56	14	F14:1444	OB5941H1 raw.raw	5.568E5	1	1	214	228		N9:Oxidation or Hydroxylation:0.00	PEAKS PTM
K.KGSEEEGDITNPINLREGEPDLSNN(+.98)FGK.L	Y	36.62	3059.4319	28	7.6	765.8711	4	31.46	8	F8:1493	OB5935H3B raw.raw	3.4976E4	1	1	386	413		N25:Deamidation (NQ):0.00	PEAKS DB
R.IFLAGDKDNVIDQ(+.98)IEK.Q	N	36.27	1817.9410	16	7.9	606.9924	3	63.32	14	F14:3434	OB5941H1 raw.raw	1.3413E4	1	1	541	556		Q13:Deamidation (NQ):25.70	PEAKS DB
K.HAD(+57.02)ADNILVIQQGQATVTVANGN(+.98)NRK.S	N	35.92	2804.4165	26	-3.8	702.1088	4	28.95	13	F13:1413	OB5940H1 raw.raw	2.7833E5	1	1	229	254		D3:Carbamidomethylation (DHKE, X@N-term):4.64;N23:Deamidation (NQ):0.00	PEAKS PTM
K.NPQ(+.98)LQDLDMMLTC(+57.02)VEIKEGALMLPHFNSK.A	N	35.84	3372.6226	29	9.4	1125.2253	3	40.73	12	F12:2064	OB5939H1 raw.raw	5.6313E5	1	1	423	451	Carbamidomethylation	Q3:Deamidation (NQ):0.00;C13:Carbamidomethylation:1000.00	PEAKS DB
K.ISM(+15.99)PVNTPGQ(+.98)FEDFFPASSR.D	N	35.67	2243.0205	20	6.2	1122.5245	2	50.73	12	F12:2643	OB5939H1 raw.raw	0	0	0	288	307	Oxidation (M)	M3:Oxidation (M):1000.00;Q10:Deamidation (NQ):22.37	PEAKS DB
K.GT(-18.01)GNLELVAVRK.E	N	35.64	1237.7142	12	0.9	413.5790	3	26.41	13	F13:1259	OB5940H1 raw.raw	7.4255E5	3	3	461	472	Dehydration	T2:Dehydration:1000.00	PEAKS PTM
K.KGSEEEGDIT(-18.01)N(+.98)PINLR.E	N	35.42	1753.8483	16	2.8	585.6250	3	28.08	13	F13:1354	OB5940H1 raw.raw	2.8958E5	1	1	386	401		T10:Dehydration:25.86;N11:Deamidation (NQ):32.46	PEAKS PTM
K.SFNLDEGHALRIPS.G	N	35.38	1554.7791	14	-4.8	778.3931	2	30.79	13	F13:1515	OB5940H1 raw.raw	5.2113E3	1	1	255	268			PEAKS DB
R.DQ(+.98)SSYLQGFSR.N	N	34.61	1287.5731	11	-3.2	644.7917	2	63.83	14	F14:3449	OB5941H1 raw.raw	0	0	0	308	318		Q2:Deamidation (NQ):41.87	PEAKS DB
R.NN(-17.03)PFYFPSRR.F	N	34.10	1279.6097	10	-0.4	427.5437	3	28.25	13	F13:1369	OB5940H1 raw.raw	1.47E5	1	1	172	181		N2:Ammonia-loss (N):12.28	PEAKS PTM
G.SEEEGDITNPINLREGEPDLSNNFGK.L	Y	33.63	2873.3315	26	2.9	958.7872	3	32.34	7	F7:1553	OB5934H3B raw.raw	5.5034E4	1	1	388	413			PEAKS DB

total 217 peptides

Peptide	Uniq	-10lgP	Mass	Length	ppm	m/z	z	RT	Fraction	Scan	Source File	Area Raw in gel	#Feature	#Feature Raw in gel	Start	End	PTM	AScore	Found By
T.G(+214.97)NLELVAVRK.E	N	33.47	1312.6267	10	2.3	657.3221	2	27.95	14	F14:1280	OB5941 H1 raw.raw	2.4669E5	1	1	463	472	4-sulfophenyl isothiocyanate	G1:4-sulfophenyl isothiocyanate:144.45	PEAKS PTM
R.GRREEEEDEDEEEEGSNR.E	Y	33.44	2192.8689	18	0.0	731.9636	3	23.70	13	F13:1082	OB5940 H1 raw.raw	3.4989E3	1	1	478	495			PEAKS DB
K.HADADNILVIQQ(+.98)GQATVTVANGNN(+.98)RK.S	N	33.28	2748.3792	26	7.6	917.1406	3	48.20	12	F12:2471	OB5939 H1 raw.raw	2.0672E4	1	1	229	254		Q12:Deamidation (NQ):23.10;N24:Deamidation (NQ):0.00	PEAKS DB
K.HADAD(-18.01)NILVIQQGQATVTVANGNNRK.S	N	33.25	2728.4004	26	3.5	910.4772	3	28.70	13	F13:1390	OB5940 H1 raw.raw	0	0	0	229	254		D5:Dehydration:30.36	PEAKS PTM
K.ISM(+15.99)PVN(+.98)TPGQFEDFFPASSR.D	N	33.05	2243.0205	20	7.6	1122.5260	2	49.72	13	F13:2625	OB5940 H1 raw.raw	0	0	0	288	307	Oxidation (M)	M3:Oxidation (M):1000.00;N6:Deamidation (NQ):25.70	PEAKS DB
R.IFLAGDK(+26.02)DNVIDQIEK.Q	N	32.97	1842.9727	16	-0.2	922.4934	2	35.63	14	F14:1844	OB5941 H1 raw.raw	1.4466E5	1	1	541	556	Acetaldehyde +26	K7:Acetaldehyde +26:74.34	PEAKS PTM
K.NPQLQ(+.98)DLDMM(+15.99)LTC(+57.02)VEIK.E	N	32.85	2063.9578	17	-3.4	1032.9827	2	37.19	12	F12:1881	OB5939 H1 raw.raw	2.2954E4	1	1	423	439	Carbamidomethylation	Q5:Deamidation (NQ):26.31;M10:Oxidation (M):0.00;C13:Carbamidomethylation:1000.00	PEAKS DB
K.HADADNILVIQ(+.98)QGQ(+.98)ATVTVANGNNRK.S	N	32.62	2748.3792	26	3.1	917.1365	3	57.24	13	F13:3064	OB5940 H1 raw.raw	0	0	0	229	254		Q11:Deamidation (NQ):0.00;Q14:Deamidation (NQ):0.00	PEAKS DB
K.N(+.98)PQ(+.98)LQ(+.98)DLDMM(+15.99)LTC(+57.02)VEIKEGALMLPHFNSK.A	N	32.23	3390.5854	29	3.7	848.6568	4	38.47	14	F14:1982	OB5941 H1 raw.raw	0	0	0	423	451	Deamidation (NQ); Carbamidomethylation	N1:Deamidation (NQ):62.42;Q3:Deamidation (NQ):65.92;Q5:Deamidation (NQ):60.92;M10:Oxidation (M):12.28;C13:Carbamidomethylation:1000.00	PEAKS DB
K.KN(+.98)PQ(+.98)LQ(+.98)DLDM(+15.99)MLTC(+57.02)VEIKEGALMLPHFNSK.A	N	31.81	3518.6804	30	-3.0	880.6747	4	37.45	12	F12:1882	OB5939 H1 raw.raw	0	0	0	422	451	Deamidation (NQ); Carbamidomethylation	N2:Deamidation (NQ):58.75;Q4:Deamidation (NQ):61.65;Q6:Deamidation (NQ):53.75;M10:Oxidation (M):0.00;C14:Carbamidomethylation:1000.00	PEAKS DB
**total 217 peptides**																			

Q6PSU6|Q6PSU6_ARAHY

back to list

| Protein Coverage | Supporting Peptides |

Protein Coverage:

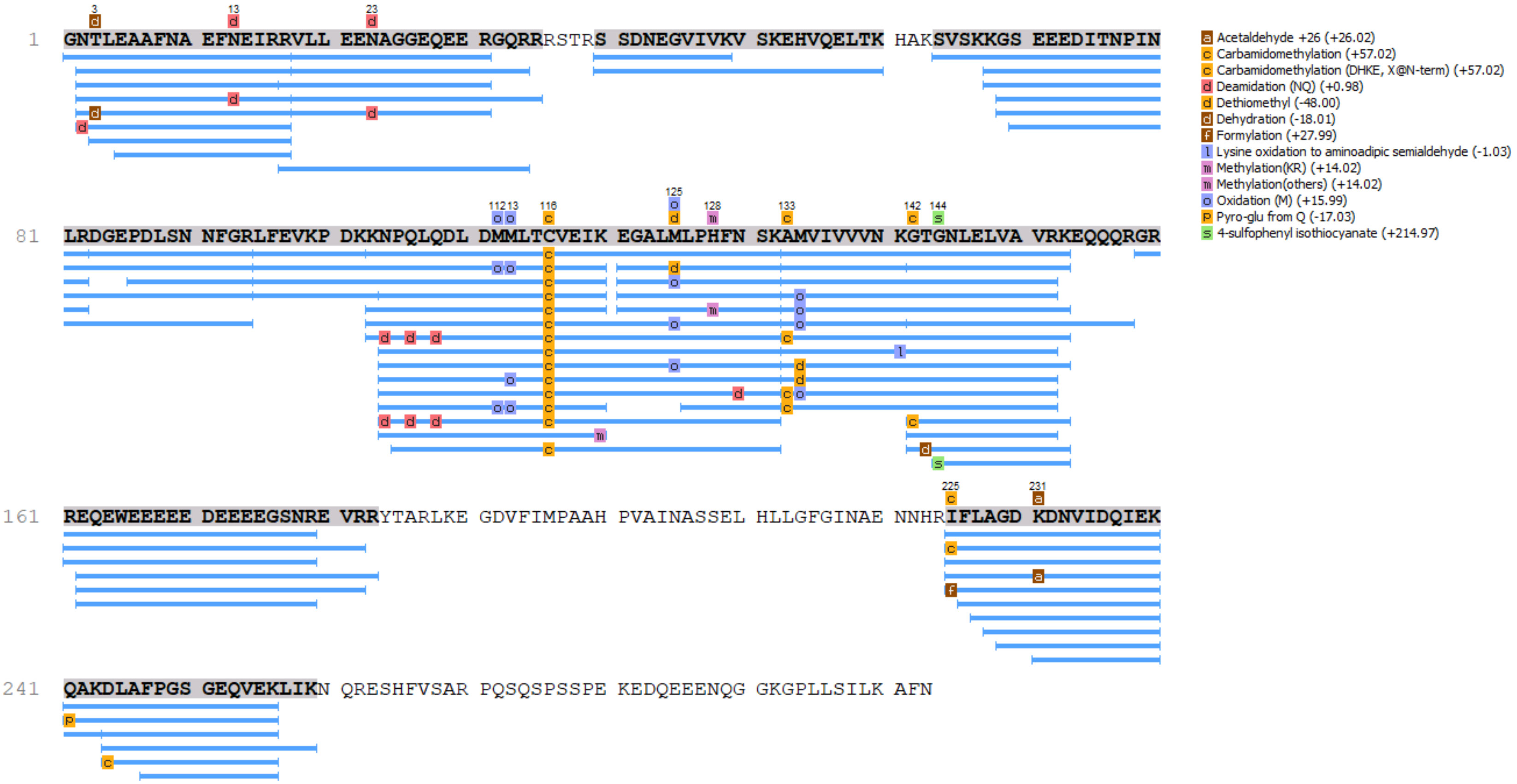

Supporting Peptides:

Peptide	Uniq	-10lgP	Mass	Length	ppm	m/z	z	RT	Fraction	Scan	Source File	Area Raw in gel	#Feature	#Feature Raw in gel	Start	End	PTM	AScore	Found By
K.KNPQLQDLDMMLTC(+57.02)VEIK.E	N	137.14	2175.0737	18	2.4	1088.5468	2	34.90	14	F14:1774	OB5941 H1 raw.raw	4.1086E6	3	3	103	120	Carbamidomethylation	C14:Carbamidomethylation:1000.00	PEAKS DB
K.NPQLQDLDMMLTC(+57.02)VEIK.E	N	131.65	2046.9788	17	-0.8	1024.4958	2	36.89	14	F14:1903	OB5941 H1 raw.raw	1.2559E7	8	8	104	120	Carbamidomethylation	C13:Carbamidomethylation:1000.00	PEAKS DB
R.EQEWEEEEEDEEEEGSNR.E	N	130.87	2280.8413	18	4.3	1141.4329	2	28.13	14	F14:1352	OB5941 H1 raw.raw	4.0373E6	3	3	162	179			PEAKS DB
K.NPQLQDLDM(+15.99)MLTC(+57.02)VEIK.E	N	128.59	2062.9736	17	-6.6	1032.4873	2	36.34	14	F14:1860	OB5941 H1 raw.raw	2.4371E5	2	2	104	120	Carbamidomethylation	M9:Oxidation (M):30.46;C13:Carbamidomethylation:1000.00	PEAKS DB
G.NTLEAAFNAEFNEIR.R	N	126.11	1737.8322	15	3.3	869.9262	2	35.27	14	F14:1792	OB5941 H1 raw.raw	1.3691E7	6	6	2	16			PEAKS DB
R.VLLEENAGGEQEER.G	N	114.70	1571.7427	14	1.1	786.8795	2	26.65	14	F14:1250	OB5941 H1 raw.raw	1.2337E7	7	7	18	31			PEAKS DB
**total 123 peptides**																			

Peptide	Uniq	-10lgP	Mass	Length	ppm	m/z	z	RT	Fraction	Scan	Source File	Area Raw in gel	#Feature	#Feature Raw in gel	Start	End	PTM	AScore	Found By
R.IFLAGDKDNVIDQIEKQAK.D	N	113.07	2144.1477	19	1.1	715.7240	3	32.93	7	F7:1578	OB5934 H3B raw.raw	5.566E7	12	12	225	243			PEAKS DB
K.AMVIVVVNKGTGNLELVAVR.K	N	112.17	2081.2031	20	1.5	694.7427	3	34.35	14	F14:1739	OB5941 H1 raw.raw	7.0931E7	12	12	133	152			PEAKS DB
K.KGSEEEDITNPINLR.D	N	110.67	1713.8533	15	3.2	857.9366	2	28.85	14	F14:1395	OB5941 H1 raw.raw	1.2495E7	8	8	68	82			PEAKS DB
K.NPQLQDLDMMLTC(+57.02)VEIKEGALMLPHFNSK.A	N	110.02	3371.6387	29	-0.1	1686.8264	2	37.85	14	F14:1946	OB5941 H1 raw.raw	1.7936E8	14	14	104	132	Carbamidomethylation	C13:Carbamidomethylation:1000.00	PEAKS DB
K.AMVIVVVNKGTGNLELVAVRK.E	N	109.98	2209.2981	21	-1.4	1105.6548	2	32.55	14	F14:1615	OB5941 H1 raw.raw	2.9136E8	18	18	133	153			PEAKS DB
K.AM(+15.99)VIVVVNKGTGNLELVAVR.K	N	109.59	2097.1980	20	-2.2	1049.6040	2	32.73	14	F14:1638	OB5941 H1 raw.raw	3.1537E7	10	10	133	152	Oxidation (M)	M2:Oxidation (M):1000.00	PEAKS DB
R.IFLAGDKDNVIDQIEK.Q	N	108.13	1816.9570	16	0.2	909.4860	2	33.10	14	F14:1683	OB5941 H1 raw.raw	7.2865E8	41	41	225	240			PEAKS DB
K.GSEEEDITNPINLRDGEPDLSNNFGR.L	N	107.18	2887.3220	26	-1.7	963.4464	3	32.55	14	F14:1626	OB5941 H1 raw.raw	4.5198E7	9	9	69	94			PEAKS DB
R.VLLEENAGGEQEERGQR.R	N	104.46	1912.9238	17	1.8	957.4709	2	25.31	14	F14:1182	OB5941 H1 raw.raw	1.9161E6	8	8	18	34			PEAKS DB
R.I(+57.02)FLAGDKDNVIDQIEK.Q	N	103.96	1873.9785	16	-5.0	937.9919	2	32.19	14	F14:1619	OB5941 H1 raw.raw	4.5184E7	7	7	225	240	Carbamidomethylation (DHKE, X@N-term)	I1:Carbamidomethylation (DHKE, X@N-term):90.91	PEAKS PTM
R.DGEPDLSNNFGR.L	N	102.95	1319.5742	12	0.8	660.7949	2	29.02	14	F14:1398	OB5941 H1 raw.raw	3.2103E6	3	3	83	94			PEAKS DB
K.QAKDLAFPGSGEQVEK.L	N	102.73	1702.8525	16	1.8	852.4351	2	26.85	14	F14:1278	OB5941 H1 raw.raw	1.286E6	6	6	241	256			PEAKS DB
K.DLAFPGSGEQVEK.L	N	101.44	1375.6619	13	-2.0	688.8369	2	30.08	14	F14:1472	OB5941 H1 raw.raw	6.5563E7	9	9	244	256			PEAKS DB
K.NPQLQDLDMMLTCVEIK(+14.02).E	N	101.30	2003.9730	17	2.1	1002.9958	2	38.00	14	F14:1960	OB5941 H1 raw.raw	6.7851E5	2	2	104	120	Methylation(KR)	K17:Methylation(KR):1000.00	PEAKS PTM
R.RVLLEENAGGEQEER.G	N	98.06	1727.8438	15	0.4	576.9554	3	24.77	13	F13:1156	OB5940 H1 raw.raw	1.2517E5	5	5	17	31			PEAKS DB
K.GSEEEDITNPINLR.D	N	97.01	1585.7583	14	2.4	793.8884	2	30.93	14	F14:1527	OB5941 H1 raw.raw	3.0498E6	3	3	69	82			PEAKS DB
K.AM(+15.99)VIVVVNKGTGNLELVAVRK.E	N	96.33	2225.2930	21	-0.4	1113.6533	2	30.76	14	F14:1520	OB5941 H1 raw.raw	4.0787E7	8	7	133	153	Oxidation (M)	M2:Oxidation (M):1000.00	PEAKS DB
K.KNPQLQDLDMM(+15.99)LTC(+57.02)VEIK.E	N	95.01	2191.0686	18	-3.4	1096.5378	2	32.73	14	F14:1643	OB5941 H1 raw.raw	1.9787E5	1	1	103	120	Carbamidomethylation	M11:Oxidation (M):9.34;C14:Carbamidomethylation:1000.00	PEAKS DB
K.NPQLQDLDMM(-48.00)LTC(+57.02)VEIK.E	N	93.52	1998.9755	17	-2.5	1000.4926	2	32.55	14	F14:1628	OB5941 H1 raw.raw	1.3958E7	5	5	104	120	Carbamidomethylation	M10:Dethiomethyl:27.96;C13:Carbamidomethylation:1000.00	PEAKS PTM
K.NPQLQDLDM(+15.99)MLTC(+57.02)VEIKEGALMLPHFNSK.A	N	92.22	3387.6335	29	-1.5	1130.2168	3	36.17	14	F14:1926	OB5941 H1 raw.raw	2.8061E7	4	4	104	132	Carbamidomethylation	M9:Oxidation (M):10.11;C13:Carbamidomet	PEAKS DB

total 123 peptides

Peptide	Uniq	-10lgP	Mass	Length	ppm	m/z	z	RT	Fraction	Scan	Source File	Area Raw in gel	#Feature	#Feature Raw in gel	Start	End	PTM	AScore	Found By
																		hylation:1000.00	
K.EGALM(+15.99)LPHFNSK.A	N	91.98	1358.6653	12	2.1	680.3414	2	29.74	14	F14:1443	OB5941 H1 raw.raw	1.9561E6	3	3	121	132	Oxidation (M)	M5:Oxidation (M):1000.00	PEAKS DB
R.VLLEENAGGEQEERGQRR.R	N	91.09	2069.0249	18	2.1	690.6837	3	25.31	14	F14:1186	OB5941 H1 raw.raw	1.1888E6	3	3	18	35			PEAKS DB
R.SSDNEGVIVKVSKEHVQELTK.H	N	91.01	2325.2175	21	1.3	776.0808	3	28.31	14	F14:1362	OB5941 H1 raw.raw	1.0201E5	2	2	40	60			PEAKS DB
K.KNPQLQDLDMM(+15.99)LTC(+57.02)VEIKEGALMLPHFNSK.A	N	90.76	3515.7285	30	-2.7	879.9370	4	34.52	13	F13:1755	OB5940 H1 raw.raw	2.6989E6	3	3	103	132	Carbamidomethylation	M11:Oxidation (M):30.46;C14:Carbamidomethylation:1000.00	PEAKS DB
K.AM(-48.00)VIVVVNKGTGNLELVAVR.K	N	90.57	2033.1997	20	-3.4	1017.6036	2	31.46	14	F14:1539	OB5941 H1 raw.raw	1.6588E7	6	6	133	152	Dethiomethyl	M2:Dethiomethyl:1000.00	PEAKS PTM
K.A(+57.02)MVIVVVNKGTGNLELVAVR.K	N	90.54	2138.2246	20	-0.9	1070.1187	2	34.35	14	F14:1737	OB5941 H1 raw.raw	5.6414E6	3	3	133	152	Carbamidomethylation (DHKE, X@N-term)	A1:Carbamidomethylation (DHKE, X@N-term):207.56	PEAKS PTM
K.NPQLQDLDMMLTC(+57.02)VEIKEGALM(+15.99)LPHFNSK.A	N	90.37	3387.6335	29	-1.5	1130.2168	3	36.17	14	F14:1895	OB5941 H1 raw.raw	3.1109E7	7	7	104	132	Carbamidomethylation; Oxidation (M)	C13:Carbamidomethylation:1000.00;M22:Oxidation (M):69.89	PEAKS DB
K.KGSEEEDITNPINLRDGEPDLSNNFGR.L	N	89.87	3015.4170	27	-4.2	1006.1421	3	31.29	14	F14:1549	OB5941 H1 raw.raw	7.6E7	16	16	68	94			PEAKS DB
F.LAGDKDNVIDQIEK.Q	N	89.22	1556.8046	14	-3.2	779.4071	2	32.19	12	F12:1557	OB5939 H1 raw.raw	4.4864E6	2	2	227	240			PEAKS DB
K.DLAFPGSGEQVEKLIK.N	N	87.96	1729.9249	16	1.0	865.9706	2	33.30	7	F7:1601	OB5934 H3B raw.raw	6.2551E5	3	3	244	259			PEAKS DB
K.EGALMLPHFNSK.A	N	87.95	1342.6703	12	2.5	672.3441	2	29.83	13	F13:1462	OB5940 H1 raw.raw	9.3502E6	3	3	121	132			PEAKS DB
K.AMVIVVVNK.G	N	86.97	971.5837	9	0.9	486.7996	2	29.88	12	F12:1441	OB5939 H1 raw.raw	9.5124E6	3	3	133	141			PEAKS DB
K.GTGNLELVAVR.K	N	86.65	1127.6299	11	-0.8	564.8218	2	29.74	14	F14:1450	OB5941 H1 raw.raw	1.0907E7	6	6	142	152			PEAKS DB
G.NTLEAAFNAEFNEIRR.V	N	86.03	1893.9332	16	-3.6	947.9705	2	35.28	12	F12:1741	OB5939 H1 raw.raw	3.4229E8	38	38	2	17			PEAKS DB
R.I(+27.99)FLAGDKDNVIDQIEK.Q	N	85.41	1844.9519	16	5.2	923.4880	2	36.16	14	F14:1844	OB5941 H1 raw.raw	0	0	0	225	240	Formylation	I1:Formylation:77.93	PEAKS PTM
GNTLEAAFNAEFNEIRR.V	Y	83.08	1950.9547	17	6.6	976.4910	2	34.72	14	F14:1759	OB5941 H1 raw.raw	5.0531E6	3	3	1	17			PEAKS DB
K.KNPQLQDLDM(+15.99)MLTC(+57.02)VEIK.E	N	81.54	2191.0686	18	0.7	1096.5424	2	33.03	12	F12:1615	OB5939 H1 raw.raw	0	0	0	103	120	Carbamidomethylation	M10:Oxidation (M):14.02;C14:Carbamidomethylation:1000.00	PEAKS DB
K.KNPQLQDLDMMLTC(+57.02)VEIKEGALMLPHFNSK.A	N	81.26	3499.7336	30	-0.1	875.9406	4	36.52	14	F14:1868	OB5941 H1 raw.raw	1.091E7	3	3	103	132	Carbamidomethylation	C14:Carbamidomethylation:1000.00	PEAKS DB
**total 123 peptides**																			

Peptide	Uniq	-10lgP	Mass	Length	ppm	m/z	z	RT	Fraction	Scan	Source File	Area Raw in gel	#Feature	#Feature Raw in gel	Start	End	PTM	AScore	Found By
K.GTGNLELVAVRK.E	N	79.30	1255.7249	12	0.9	628.8702	2	26.85	14	F14:1263	OB5941 H1 raw.raw	1.0437E7	9	9	142	153			PEAKS DB
R.GRREQEWEEEEEDEEEEGSNR.E	N	79.15	2650.0649	21	0.3	884.3625	3	25.64	13	F13:1205	OB5940 H1 raw.raw	2.2346E6	7	7	159	179			PEAKS DB
E.PDLSNNFGR.L	N	78.38	1018.4832	9	-0.1	510.2488	2	31.24	14	F14:1545	OB5941 H1 raw.raw	3.5959E6	3	3	86	94			PEAKS DB
R.IFLAGDKDNVIDQIE(+57.02)K.Q	N	78.23	1873.9785	16	-5.0	937.9919	2	32.19	14	F14:1583	OB5941 H1 raw.raw	3.036E7	3	3	225	240		E15:Carbamidomethylation (DHKE, X@N-term):0.00	PEAKS PTM
I.FLAGDKDNVIDQIEK.Q	N	77.74	1703.8729	15	-9.3	852.9358	2	32.01	14	F14:1599	OB5941 H1 raw.raw	1.3574E6	3	3	226	240			PEAKS DB
K.D(+57.02)LAFPGSGEQVEK.L	N	77.21	1432.6833	13	3.8	717.3517	2	30.92	12	F12:1485	OB5939 H1 raw.raw	2.816E5	1	1	244	256	Carbamidomethylation (DHKE, X@N-term)	D1:Carbamidomethylation (DHKE, X@N-term):93.18	PEAKS PTM
R.SSDNEGVIVK.V	N	76.72	1046.5244	10	0.3	524.2697	2	22.95	13	F13:1057	OB5940 H1 raw.raw	2.6758E5	6	6	40	49			PEAKS DB
G.NTLEAAFNAEFN(+.98)EIRR.V	N	74.39	1894.9172	16	-3.7	948.4624	2	32.73	14	F14:1640	OB5941 H1 raw.raw	4.113E6	3	3	2	17	Deamidation (NQ)	N12:Deamidation (NQ):55.21	PEAKS DB
R.LFEVKPDKKNPQLQDLDMMLTC(+57.02)VEIK.E	N	73.38	3131.6069	26	3.7	1044.8801	3	33.97	13	F13:1716	OB5940 H1 raw.raw	3.7487E6	3	3	95	120	Carbamidomethylation	C22:Carbamidomethylation:1000.00	PEAKS DB
K.NPQLQDLDMM(+15.99)LTC(+57.02)VEIKEGALMLPHFNSK.A	N	73.21	3387.6335	29	5.6	847.9204	4	35.82	13	F13:1825	OB5940 H1 raw.raw	9.6415E6	3	3	104	132	Carbamidomethylation	M10:Oxidation (M):16.90;C13:Carbamidomethylation:1000.00	PEAKS DB
A.GDKDNVIDQIEK.Q	N	73.04	1372.6833	12	-2.8	687.3470	2	32.19	12	F12:1561	OB5939 H1 raw.raw	1.4449E6	3	3	229	240			PEAKS DB
R.V(+57.02)LLEENAGGEQEER.G	N	73.00	1628.7642	14	1.1	815.3903	2	27.03	14	F14:1288	OB5941 H1 raw.raw	2.7852E5	3	3	18	31		V1:Carbamidomethylation (DHKE, X@N-term):44.44	PEAKS PTM
R.VLLEEN(+.98)AGGEQEER.G	N	71.59	1572.7267	14	-3.7	787.3677	2	26.82	13	F13:1276	OB5940 H1 raw.raw	0	0	0	18	31	Deamidation (NQ)	N6:Deamidation (NQ):71.64	PEAKS DB
A.FPGSGEQVEK.L	N	71.45	1076.5138	10	5.5	539.2672	2	30.23	12	F12:1449	OB5939 H1 raw.raw	6.1112E4	1	1	247	256			PEAKS DB
K.A(+57.02)MVIVVVNKGTGNLELVAVRK.E	N	70.77	2266.3196	21	-1.6	1134.1653	2	32.33	13	F13:1602	OB5940 H1 raw.raw	4.861E7	6	6	133	153	Carbamidomethylation (DHKE, X@N-term)	A1:Carbamidomethylation (DHKE, X@N-term):161.20	PEAKS PTM
K.AM(+15.99)VIVVVNK.G	N	70.64	987.5787	9	0.7	494.7969	2	26.66	14	F14:1257	OB5941 H1 raw.raw	8.6431E5	3	3	133	141	Oxidation (M)	M2:Oxidation (M):1000.00	PEAKS DB
K.KNPQLQDLDMMLTC(+57.02)VEIKEGALM(+15.99)LPHFNSK.A	N	70.55	3515.7285	30	-0.8	879.9387	4	35.84	12	F12:1790	OB5939 H1 raw.raw	4.7958E6	2	2	103	132	Carbamidomethylation; Oxidation (M)	C14:Carbamidomethylation:1000.00;M23:Oxidation (M):69.38	PEAKS DB
K.SVSKKGSEEEDITNPINLR.D	N	69.76	2115.0808	19	2.6	706.0361	3	27.22	14	F14:1299	OB5941 H1 raw.raw	5.5052E4	1	1	64	82			PEAKS DB

total 123 peptides

Peptide	Uniq	-10lgP	Mass	Length	ppm	m/z	z	RT	Fraction	Scan	Source File	Area Raw in gel	#Feature	#Feature Raw in gel	Start	End	PTM	AScore	Found By
G.NT(-18.01)LEAAFNAEFNEIRR.V	N	68.32	1875.9227	16	-3.2	626.3129	3	33.81	14	F14:1704	OB5941 H1 raw.raw	1.3248E7	3	3	2	17	Dehydration	T2:Dehydration:1000.00	PEAKS PTM
G.SEEEDITNPINLRDGEPDLSNNFGR.L	N	67.86	2830.3005	25	1.2	944.4419	3	32.96	8	F8:1576	OB5935 H3B raw.raw	5.6172E5	3	3	70	94			PEAKS DB
R.LFEVKPDKKNPQLQDLDM(+15.99)M(+15.99)LTC(+57.02)VEIK.E	N	67.75	3163.5967	26	2.1	791.9081	4	32.37	12	F12:1585	OB5939 H1 raw.raw	6.2675E5	1	1	95	120	Oxidation (M); Carbamidomethylation	M18:Oxidation (M):1000.00;M19:Oxidation (M):1000.00;C22:Carbamidomethylation:1000.00	PEAKS DB
L.AGDKDNVIDQIEK.Q	N	66.85	1443.7205	13	-3.1	722.8653	2	32.19	12	F12:1560	OB5939 H1 raw.raw	7.0907E5	2	2	228	240			PEAKS DB
K.G(+57.02)TGNLELVAVRK.E	N	66.68	1312.7462	12	-0.7	657.3799	2	26.85	14	F14:1280	OB5941 H1 raw.raw	3.417E5	3	3	142	153	Carbamidomethylation (DHKE, X@N-term)	G1:Carbamidomethylation (DHKE, X@N-term):56.51	PEAKS PTM
D.KDNVIDQIEK.Q	N	66.36	1200.6350	10	-0.8	601.3243	2	25.89	14	F14:1225	OB5941 H1 raw.raw	5.8609E4	3	3	231	240			PEAKS DB
K.NPQLQ(+.98)DLDMMLTC(+57.02)VEIKEGALMLPHFNSK.A	N	66.16	3372.6226	29	5.7	1125.2212	3	37.84	13	F13:1936	OB5940 H1 raw.raw	5.3127E7	3	3	104	132	Carbamidomethylation	Q5:Deamidation (NQ):32.28;C13:Carbamidomethylation:1000.00	PEAKS DB
G.N(+.98)TLEAAFNAEFNEIRR.V	N	65.61	1894.9172	16	2.2	948.4680	2	35.88	12	F12:1787	OB5939 H1 raw.raw	7.0859E7	1	1	2	17	Deamidation (NQ)	N1:Deamidation (NQ):61.82	PEAKS DB
K.KNPQLQDLDM(+15.99)MLTC(+57.02)VEIKEGALMLPHFNSK.A	N	65.47	3515.7285	30	-3.1	879.9366	4	35.44	13	F13:1830	OB5940 H1 raw.raw	7.2415E6	3	3	103	132	Carbamidomethylation	M10:Oxidation (M):11.06;C14:Carbamidomethylation:1000.00	PEAKS DB
K.NPQLQDLDM(+15.99)MLTC(+57.02)VEIKEGALM(+15.99)LPHFNSK.A	N	64.53	3403.6284	29	4.5	1135.5552	3	36.52	14	F14:1873	OB5941 H1 raw.raw	4.3451E6	3	3	104	132	Carbamidomethylation; Oxidation (M)	M9:Oxidation (M):10.11;C13:Carbamidomethylation:1000.00;M22:Oxidation (M):53.09	PEAKS DB
R.LFEVKPDK.K	N	63.66	974.5436	8	0.9	488.2795	2	25.40	12	F12:1153	OB5939 H1 raw.raw	6.8606E5	7	7	95	102			PEAKS DB
R.EQEWEEEEEDEEEEGSNREVR.R	N	63.32	2665.0535	21	2.4	889.3606	3	27.87	12	F12:1308	OB5939 H1 raw.raw	4.1617E5	2	2	162	182			PEAKS DB
R.REQEWEEEEEDEEEEGSNR.E	N	63.17	2436.9424	19	2.8	1219.4819	2	26.47	14	F14:1258	OB5941 H1 raw.raw	2.6135E4	2	2	161	179			PEAKS DB
K.A(+57.02)M(+15.99)VIVVVNKGTGNLELVAVR.K	N	62.49	2154.2195	20	-0.1	1078.1169	2	32.92	12	F12:1614	OB5939 H1 raw.raw	4.5509E5	2	2	133	152	Carbamidomethylation (DHKE, X@N-term); Oxidation (M)	A1:Carbamidomethylation (DHKE, X@N-term):139.69;M2:Oxidation (M):1000.00	PEAKS PTM
L.EAAFNAEFNEIRR.V	N	62.30	1565.7585	13	-4.7	783.8829	2	34.06	12	F12:1677	OB5939 H1 raw.raw	6.1232E5	1	1	5	17			PEAKS DB
K.N(+.98)PQLQDLDMMLTC(+57.02)VEIKEGALMLPHFNSK.A	N	61.86	3372.6226	29	5.7	1125.2212	3	37.84	13	F13:1983	OB5940 H1 raw.raw	1.2903E7	1	1	104	132	Carbamidomethylation	N1:Deamidation (NQ):14.04;C13:	PEAKS DB

total 123 peptides

Peptide	Uniq	-10lgP	Mass	Length	ppm	m/z	z	RT	Fraction	Scan	Source File	Area Raw in gel	#Feature	#Feature Raw in gel	Start	End	PTM	AScore	Found By
																		Carbamidomethylation:1000.00	
R.IFLAGDKDNVIDQ(+.98)IEKQAK.D	N	58.72	2145.1316	19	9.3	716.0578	3	32.93	2	F2:1432	OB5925 H3B raw.raw	1.5596E6	1	1	225	243		Q13:Deamidation (NQ):0.00	PEAKS DB
R.LFEVKPDKKNPQLQDLDMM(+15.99)LTC(+57.02)VEIK.E	N	57.62	3147.6018	26	-4.4	787.9042	4	32.55	14	F14:1633	OB5941 H1 raw.raw	2.1102E6	3	3	95	120	Carbamidomethylation	M19:Oxidation (M):17.01;C22:Carbamidomethylation:1000.00	PEAKS DB
K.AMVIVVVN(+.98)KGTGNLELVAVRK.E	N	56.84	2210.2820	21	9.6	737.7750	3	33.10	14	F14:1664	OB5941 H1 raw.raw	8.0625E6	2	2	133	153		N8:Deamidation (NQ):41.87	PEAKS DB
K.KNPQLQDLDMM(-48.00)LTC(+57.02)VEIK.E	N	56.43	2127.0703	18	0.8	710.0312	3	31.11	14	F14:1538	OB5941 H1 raw.raw	3.3192E6	2	2	103	120	Carbamidomethylation	M11:Dethiomethyl:14.02;C14:Carbamidomethylation:1000.00	PEAKS PTM
K.AM(-48.00)VIVVVNKGTGNLELVAVRK.E	N	54.93	2161.2947	21	1.4	1081.6561	2	29.80	14	F14:1453	OB5941 H1 raw.raw	1.7581E6	2	2	133	153	Dethiomethyl	M2:Dethiomethyl:1000.00	PEAKS PTM
K.NPQLQDLDM(+15.99)MLTC(+57.02)VEIKEGALMLPHFN(+.98)SK.A	N	54.06	3388.6174	29	5.0	848.1659	4	36.17	14	F14:1849	OB5941 H1 raw.raw	6.0156E6	2	2	104	132	Carbamidomethylation; Deamidation (NQ)	M9:Oxidation (M):14.02;C13:Carbamidomethylation:1000.00;N27:Deamidation (NQ):58.23	PEAKS DB
R.IFLAGDKDNVIDQIE(+57.02)KQAK.D	N	52.99	2201.1692	19	-2.0	734.7289	3	31.83	13	F13:1588	OB5940 H1 raw.raw	3.3379E6	2	2	225	243		E15:Carbamidomethylation (DHKE, X@N-term):12.28	PEAKS PTM
K.NPQLQDLDMMLTC(+57.02)VEIKEGALMLPHFN(+.98)SK.A	N	52.89	3372.6226	29	7.4	844.1691	4	37.85	13	F13:1914	OB5940 H1 raw.raw	5.2564E7	2	2	104	132	Carbamidomethylation; Deamidation (NQ)	C13:Carbamidomethylation:1000.00;N27:Deamidation (NQ):61.84	PEAKS DB
K.NPQLQDLDM(+15.99)M(+15.99)LTC(+57.02)VEIKEGALMLPHFNSK.A	N	52.70	3403.6284	29	-4.3	1135.5452	3	35.63	14	F14:1818	OB5941 H1 raw.raw	1.9434E6	2	2	104	132	Oxidation (M); Carbamidomethylation	M9:Oxidation (M):49.75;M10:Oxidation (M):51.99;C13:Carbamidomethylation:1000.00	PEAKS DB
K.DLAFPGSGEQVEK(+57.02)LIK.N	N	52.57	1786.9464	16	-3.8	894.4771	2	32.91	14	F14:1654	OB5941 H1 raw.raw	6.7177E5	1	1	244	259		K13:Carbamidomethylation (DHKE, X@N-term):27.96	PEAKS PTM
K.KN(+.98)PQLQDLDMM(+15.99)LTC(+57.02)VEIK.E	N	52.31	2192.0527	18	9.2	1097.0437	2	32.33	13	F13:1621	OB5940 H1 raw.raw	8.5775E4	1	1	103	120	Carbamidomethylation	N2:Deamidation (NQ):14.04;M11:Oxidation (M):0.00;C14:Carbamidomethylation:1000.00	PEAKS DB
K.EGALMLPH(+14.02)FNSK.A	N	51.73	1356.6860	12	1.3	679.3512	2	30.49	14	F14:1499	OB5941 H1 raw.raw	2.5741E4	1	1	121	132	Methylation(others)	H8:Methylation(others):53.53	PEAKS PTM
K.N(+.98)PQLQ(+.98)DLDMMLTC(+57.02)VEIKEGALM(+15.99)LPHFNSK.A	N	51.60	3389.6016	29	9.0	848.4153	4	36.92	12	F12:1868	OB5939 H1 raw.raw	2.6873E6	1	1	104	132	Carbamidomethylation	N1:Deamidation (NQ):0.00;Q5:Deamidation (NQ):14.04;C13:Carb	PEAKS DB
**total 123 peptides**																			

Peptide	Uniq	-10lgP	Mass	Length	ppm	m/z	z	RT	Fraction	Scan	Source File	Area Raw in gel	#Feature	#Feature Raw in gel	Start	End	PTM	AScore	Found By
																		amidomethylation:1000.00;M22:Oxidation (M):18.84	
R.LFEVKPDKK.N	N	50.85	1102.6385	9	1.1	552.3271	2	24.94	14	F14:1179	OB5941 H1 raw.raw	2.3081E5	2	2	95	103			PEAKS DB
R.RVLLEENAGGEQEERGQR.R	N	50.67	2069.0249	18	2.1	690.6837	3	24.88	13	F13:1165	OB5940 H1 raw.raw	2.7227E5	1	1	17	34			PEAKS DB
K.N(+.98)PQLQDLDMMLTC(+57.02)VEIKEGALM(+15.99)LPHFNSK.A	N	50.17	3388.6174	29	-2.4	1130.5437	3	36.35	13	F13:1874	OB5940 H1 raw.raw	8.9006E5	1	1	104	132	Carbamidomethylation; Oxidation (M)	N1:Deamidation (NQ):0.00;C13:Carbamidomethylation:1000.00;M22:Oxidation (M):54.40	PEAKS DB
K.NPQLQDLDMM(+15.99)LTC(+57.02)VEIKEGALM(+15.99)LPHFNSK.A	N	48.97	3403.6284	29	2.7	1135.5531	3	35.09	14	F14:1786	OB5941 H1 raw.raw	3.2001E5	1	1	104	132	Carbamidomethylation	M10:Oxidation (M):12.28;C13:Carbamidomethylation:1000.00;M22:Oxidation (M):28.29	PEAKS DB
R.EQEWEEEEEDEEEEGSNREVRR.Y	N	48.85	2821.1545	22	1.8	941.3939	3	26.75	12	F12:1227	OB5939 H1 raw.raw	3.3887E6	3	3	162	183			PEAKS DB
K.KN(+.98)PQLQDLDMMLTC(+57.02)VEIKEGALMLPHFNSK.A	N	48.84	3500.7175	30	7.1	876.1929	4	36.26	13	F13:1847	OB5940 H1 raw.raw	2.2113E6	1	1	103	132	Carbamidomethylation	N2:Deamidation (NQ):0.00;C14:Carbamidomethylation:1000.00	PEAKS DB
R.IFLAGDKDNVIDQIEK(+57.02)QAK.D	N	48.82	2201.1692	19	2.7	734.7323	3	32.55	12	F12:1581	OB5939 H1 raw.raw	3.2958E6	1	1	225	243		K16:Carbamidomethylation (DHKE, X@N-term):15.91	PEAKS PTM
R.LFEVKPDKKN(+.98)PQLQDLDMMLTC(+57.02)VEIK.E	N	47.32	3132.5908	26	9.3	1567.3173	2	34.35	14	F14:1745	OB5941 H1 raw.raw	1.9315E5	1	1	95	120	Carbamidomethylation	N10:Deamidation (NQ):14.04;C22:Carbamidomethylation:1000.00	PEAKS DB
K.N(+.98)PQ(+.98)LQDLDMMLTC(+57.02)VEIKEGALM(+15.99)LPHFNSK.A	N	46.82	3389.6016	29	9.0	848.4153	4	36.92	12	F12:1851	OB5939 H1 raw.raw	7.3613E6	2	2	104	132	Carbamidomethylation	N1:Deamidation (NQ):11.12;Q3:Deamidation (NQ):0.00;C13:Carbamidomethylation:1000.00;M22:Oxidation (M):24.93	PEAKS DB
N.PQLQDLDMMLTC(+57.02)VEIKEGALMLPHFNSK.A	N	46.63	3257.5957	28	0.9	815.4069	4	37.83	14	F14:1952	OB5941 H1 raw.raw	2.3692E5	1	1	105	132	Carbamidomethylation	C12:Carbamidomethylation:1000.00	PEAKS DB
K.Q(-17.03)AKDLAFPGSGEQVEK.L	N	46.49	1685.8260	16	0.9	843.9211	2	29.97	12	F12:1427	OB5939 H1 raw.raw	0	0	0	241	256	Pyro-glu from Q	Q1:Pyro-glu from Q:1000.00	PEAKS PTM
R.REQ(+.98)EWEEEEEDEEEEGSNR.E	N	45.55	2437.9265	19	7.6	813.6556	3	26.55	12	F12:1226	OB5939 H1 raw.raw	4.2319E4	1	1	161	179		Q3:Deamidation (NQ):42.90	PEAKS DB
K.GTGNLELVAVRKEQQQR.G	N	44.58	1925.0442	17	0.6	642.6890	3	25.89	14	F14:1220	OB5941 H1 raw.raw	1.1112E5	2	2	142	158			PEAKS DB

total 123 peptides

Peptide	Uniq	-10lgP	Mass	Length	ppm	m/z	z	RT	Fraction	Scan	Source File	Area Raw in gel	#Feature	#Feature Raw in gel	Start	End	PTM	AScore	Found By
R.VLLE(+21.98)ENAGGEQEER.G	N	42.61	1593.7246	14	-0.7	797.8690	2	26.03	13	F13:1239	OB5940 H1 raw.raw	1.373E5	3	3	18	31		E4:Sodium adduct:40.00	PEAKS PTM
M.LPHFNSK.A	N	41.73	841.4446	7	-3.4	421.7281	2	30.24	14	F14:1484	OB5941 H1 raw.raw	1.8069E6	2	2	126	132			PEAKS DB
N.TLEAAFNAEFNEIRR.V	N	41.51	1779.8904	15	-3.0	890.9498	2	32.91	14	F14:1655	OB5941 H1 raw.raw	4.1608E5	1	1	3	17			PEAKS DB
K.KGSEEEDITN(-17.03)PINLR.D	N	41.49	1696.8268	15	3.3	566.6180	3	28.85	14	F14:1397	OB5941 H1 raw.raw	2.3089E5	1	1	68	82		N10:Ammonia-loss (N):40.63	PEAKS PTM
K.KNPQLQ(+.98)DLDMMLTC(+57.02)VEIKEGALMLPHFNSK.A	N	40.89	3500.7175	30	0.4	1167.9136	3	36.34	13	F13:1850	OB5940 H1 raw.raw	4.7608E5	1	1	103	132	Carbamidomethylation	Q6:Deamidation (NQ):14.04;C14:Carbamidomethylation:1000.00	PEAKS DB
R.REQEWEEEEEDEEEEGSNREVR.R	N	40.76	2821.1545	22	3.9	941.3958	3	26.47	14	F14:1247	OB5941 H1 raw.raw	1.8468E6	1	1	161	182			PEAKS DB
G.NTLEAAFN(+.98)AEFNEIRR.V	N	40.23	1894.9172	16	-0.3	948.4656	2	32.92	12	F12:1570	OB5939 H1 raw.raw	1.5322E6	1	1	2	17		N8:Deamidation (NQ):8.26	PEAKS DB
K.NPQLQDLDM(+15.99)M(+15.99)LTC(+57.02)VEIK.E	N	39.57	2078.9688	17	1.1	1040.4928	2	33.80	14	F14:1701	OB5941 H1 raw.raw	0	0	0	104	120	Oxidation (M); Carbamidomethylation	M9:Oxidation (M):1000.00;M10:Oxidation (M):1000.00;C13:Carbamidomethylation:1000.00	PEAKS DB
K.AMVIVVVNK(-1.03)GTGNLELVAVR.K	N	37.92	2080.1714	20	4.3	1041.0974	2	33.79	13	F13:1738	OB5940 H1 raw.raw	2.1453E7	1	1	133	152	Lysine oxidation to aminoadipic semialdehyde	K9:Lysine oxidation to aminoadipic semialdehyde:1000.00	PEAKS PTM
K.EGALM(-48.00)LPHFNSK.A	N	36.97	1294.6670	12	2.0	432.5638	3	28.31	14	F14:1348	OB5941 H1 raw.raw	1.3683E6	1	1	121	132	Dethiomethyl	M5:Dethiomethyl:1000.00	PEAKS PTM
R.IFLAGDKDNVIDQ(+.98)IEK.Q	N	36.27	1817.9410	16	7.9	606.9924	3	63.32	14	F14:3434	OB5941 H1 raw.raw	1.3413E4	1	1	225	240		Q13:Deamidation (NQ):25.70	PEAKS DB
K.NPQ(+.98)LQDLDMMLTC(+57.02)VEIKEGALMLPHFNSK.A	N	35.84	3372.6226	29	9.4	1125.2253	3	40.73	12	F12:2064	OB5939 H1 raw.raw	5.6313E5	1	1	104	132	Carbamidomethylation	Q3:Deamidation (NQ):0.00;C13:Carbamidomethylation:1000.00	PEAKS DB
K.GT(-18.01)GNLELVAVRK.E	N	35.64	1237.7142	12	0.9	413.5790	3	26.41	13	F13:1259	OB5940 H1 raw.raw	7.4255E5	3	3	142	153	Dehydration	T2:Dehydration:1000.00	PEAKS PTM
R.S(+57.02)SDNEGVIVK.V	N	34.54	1103.5459	10	0.3	552.7804	2	23.19	13	F13:1066	OB5940 H1 raw.raw	0	0	0	40	49		S1:Carbamidomethylation (DHKE, X@N-term):11.10	PEAKS PTM
T.G(+214.97)NLELVAVRK.E	N	33.47	1312.6267	10	2.3	657.3221	2	27.95	14	F14:1280	OB5941 H1 raw.raw	2.4669E5	1	1	144	153	4-sulfophenyl isothiocyanate	G1:4-sulfophenyl isothiocyanate:144.45	PEAKS PTM
R.IFLAGDK(+26.02)DNVIDQIEK.Q	N	32.97	1842.9727	16	-0.2	922.4934	2	35.63	14	F14:1844	OB5941 H1 raw.raw	1.4466E5	1	1	225	240	Acetaldehyde +26	K7:Acetaldehyde +26:74.34	PEAKS PTM
K.NPQLQ(+.98)DLDMM(+15.99)LTC(+57.02)VEIK.E	N	32.85	2063.9578	17	-3.4	1032.9827	2	37.19	12	F12:1881	OB5939 H1 raw.raw	2.2954E4	1	1	104	120	Carbamidomethylation	Q5:Deamidation (NQ):26.31;M10:Oxidation (M):0.00;C13:Carbamidomethylati	PEAKS DB
**total 123 peptides**																			

Peptide	Uniq	-10lgP	Mass	Length	ppm	m/z	z	RT	Fraction	Scan	Source File	Area Raw in gel	#Feature	#Feature Raw in gel	Start	End	PTM	AScore	Found By
																		on:1000.00	
K.N(+.98)PQ(+.98)LQ(+.98)DLDMM(+15.99)LTC(+57.02)VEIKEGALMLPHFNSK.A	N	32.23	3390.5854	29	3.7	848.6568	4	38.47	14	F14:1982	OB5941 H1 raw.raw	0	0	0	104	132	Deamidation (NQ); Carbamidomethylation	N1:Deamidation (NQ): 62.42;Q3:Deamidation (NQ):65.92;Q5:Deamidation (NQ):60.92;M10:Oxidation (M):12.28;C13:Carbamidomethylation:1000.00	PEAKS DB
K.KN(+.98)PQ(+.98)LQ(+.98)DLDM(+15.99)MLTC(+57.02)VEIKEGALMLPHFNSK.A	N	31.81	3518.6804	30	-3.0	880.6747	4	37.45	12	F12:1882	OB5939 H1 raw.raw	0	0	0	103	132	Deamidation (NQ); Carbamidomethylation	N2:Deamidation (NQ): 58.75;Q4:Deamidation (NQ):61.65;Q6:Deamidation (NQ):53.75;M10:Oxidation (M):0.00;C14:Carbamidomethylation:1000.00	PEAKS DB
**total 123 peptides**																			

A1DZF0|A1DZF0_ARAHY

back to list

| Protein Coverage | Supporting Peptides |

Protein Coverage:

1 MAKLLELSFC FCFLVLGASS ISFRQQPEEN ACQFQRLNAQ RPDNRIESEG GYIETWNPNN QEFECAGVAL SRLVLRRNAL

81 RRPFYSNAPQ EIFIQQGRGY FGLIFPGCPS TYEEPAQQGR RYQSQRPPRR LQEEDQSQQQ QDSHQKVHRF NEGDLIAVPT

161 GVAFWLYNDH DTDVVAVSLT DTNNNDNQLD QFPRRFNLAG NHEQEFLRYQ QQSRQSRRRS LPLSPYSPQP GQEDREFSPQ

241 GQHGRRERAG QEQENEGGNI FSGFTSEFLA QAFQVDDRQI VQNLRGENES EEQGAIVTVK GGLRILSPDR KSPDEEEEYD

321 EDEYAEEERQ QDRRRGRGSR GSGNGIEETI CTATVKKNIG RNRSPDIYNP QAGSLKTANE LNLLILRWLG LSAEYGNLYR

401 NALFVPHYNT NAHSIIYALR GRAHVQVVDS NGNRVYDEEL QEGHVLVVPQ NFAVAGKSQS ENFEYVAFKT DSRPSIANLA

481 GENSFIDNLP EEVVANSYGL PREQARQLKN NNPFKFFVPP FQQSPRAVA

(32 C; 108 C; 351 C; 496 d)

- c Carbamidomethylation (+57.02)
- d Deamidation (NQ) (+0.98)
- m Methylation(KR) (+14.02)
- p Pyro-glu from Q (-17.03)
- r Replacement of 2 protons by iron (+53.92)

Supporting Peptides:

Peptide	Uniq	-10lgP	Mass	Length	ppm	m/z	z	RT	Fraction	Scan	Source File	Area Raw in gel	#Feature	#Feature Raw in gel	Start	End	PTM	AScore	Found By

Peptide	Uniq	-10lgP	Mass	Length	ppm	m/z	z	RT	Fraction	Scan	Source File	Area Raw in gel	#Feature	#Feature Raw in gel	Start	End	PTM	AScore	Found By
R.SPDIYNPQAGSLKTANELNLLILR.W	N	134.89	2639.4282	24	0.9	880.8175	3	35.79	5	F5:1765	OB5932 H2 raw.raw	1.8094E6	3	3	364	387			PEAKS DB
R.NALFVPHYNTNAHSIIYALR.G	N	123.74	2313.2019	20	2.3	1157.6108	2	33.37	5	F5:1607	OB5932 H2 raw.raw	3.0421E7	13	12	401	420			PEAKS DB
R.GYFGLIFPGC(+57.02)PSTYEEPAQQGR.R	N	122.36	2473.1372	22	3.9	1237.5807	2	36.47	2	F2:1635	OB5925 H3B raw.raw	6.1209E5	7	7	99	120	Carbamidomethylation	C10:Carbamidomethylation:1000.00	PEAKS DB
R.WLGLSAEYGNLYR.N	N	116.56	1540.7673	13	2.0	771.3925	2	34.87	5	F5:1714	OB5932 H2 raw.raw	3.9701E7	18	17	388	400			PEAKS DB
K.TDSRPSIANLAGENSFIDNLPEEVVANSYGLPR.E	N	114.51	3544.7434	33	1.0	1182.5896	3	37.44	5	F5:1890	OB5932 H2 raw.raw	1.1751E8	13	13	470	502			PEAKS DB
K.SQSENFEYVAFK.T	N	113.90	1447.6619	12	1.7	724.8394	2	31.69	5	F5:1516	OB5932 H2 raw.raw	1.027E7	4	4	458	469			PEAKS DB
R.NRSPDIYNPQAGSLK.T	N	110.81	1658.8376	15	0.4	830.4265	2	27.80	5	F5:1285	OB5932 H2 raw.raw	8.3597E5	6	6	362	376			PEAKS DB
R.RPFYSNAPQEIFIQQGR.G	N	110.68	2050.0383	17	2.3	1026.0288	2	31.40	11	F11:1547	OB5938 H3A raw.raw	1.6392E6	11	11	82	98			PEAKS DB
R.VYDEELQEGHVLVVPQNFAVAGK.S	N	103.27	2540.2910	23	2.1	847.7727	3	32.93	1	F1:1498	OB5921 H2 raw.raw	1.5993E6	6	6	435	457			PEAKS DB
R.RFNLAGNHEQEFLR.Y	N	100.69	1729.8647	14	1.5	577.6297	3	30.08	10	F10:1438	OB5937 H3A raw.raw	6.1619E6	6	6	195	208			PEAKS DB
N.GIEETIC(+57.02)TATVK.K	N	96.25	1320.6595	12	1.3	661.3379	2	29.46	5	F5:1390	OB5932 H2 raw.raw	2.7846E5	3	3	345	356	Carbamidomethylation	C7:Carbamidomethylation:1000.00	PEAKS DB
K.FFVPPFQQSPR.A	Y	95.76	1348.6927	11	1.2	675.3545	2	33.11	1	F1:1496	OB5921 H2 raw.raw	2.8823E6	3	3	516	526			PEAKS DB
K.TDSRPSIANLAGENSFIDNLPEEVVAN(+.98)SYGLPR.E	N	94.81	3545.7273	33	0.2	1182.9166	3	36.74	5	F5:1825	OB5932 H2 raw.raw	0	0	0	470	502	Deamidation (NQ)	N27:Deamidation (NQ):118.18	PEAKS DB
R.SPDIYNPQAGSLK.T	N	87.71	1388.6936	13	2.2	695.3556	2	28.73	5	F5:1375	OB5932 H2 raw.raw	1.3705E6	3	3	364	376			PEAKS DB
N.GIEETIC(+57.02)TATVKK.N	N	84.04	1448.7545	13	0.2	725.3847	2	27.80	5	F5:1296	OB5932 H2 raw.raw	5.4693E5	4	4	345	357	Carbamidomethylation	C7:Carbamidomethylation:1000.00	PEAKS DB
S.ISFRQQPEENAC(+57.02)QFQR.L	N	79.32	2036.9486	16	0.8	679.9907	3	27.97	11	F11:1335	OB5938 H3A raw.raw	1.7296E5	3	3	21	36	Carbamidomethylation	C12:Carbamidomethylation:1000.00	PEAKS DB
R.AHVQVVDSNGNRVYDEELQEGHVLVVPQNFAVAGK.S	N	72.39	3816.9182	35	3.0	1273.3171	3	32.25	5	F5:1561	OB5932 H2 raw.raw	3.9867E6	2	2	423	457			PEAKS DB
K.FFVPPFQQSPRA.V	Y	72.23	1419.7299	12	0.8	710.8728	2	33.11	1	F1:1502	OB5921 H2 raw.raw	4.6593E5	3	3	516	527			PEAKS DB
R.GYFGLIFPGC(+57.02)PSTYEEPAQQGRR.Y	N	71.34	2629.2383	23	1.6	877.4214	3	34.78	2	F2:1543	OB5925 H3B raw.raw	5.9452E5	2	2	99	121	Carbamidomethylation	C10:Carbamidomethylation:1000.00	PEAKS DB
K.TANELNLLILR.W	N	68.94	1268.7452	11	0.7	635.3803	2	34.98	1	F1:1608	OB5921 H2 raw.raw	2.5942E7	7	7	377	387			PEAKS DB
R.NALRRPFYSNAPQEIFIQQGR.G	N	68.46	2504.3037	21	1.1	835.7761	3	31.58	10	F10:1548	OB5937 H3A raw.raw	9.3975E5	4	4	78	98			PEAKS DB
V.PQNFAVAGK.S	N	67.74	930.4923	9	0.8	466.2538	2	32.44	5	F5:1562	OB5932 H2 raw.raw	4.3278E6	2	2	449	457			PEAKS DB
R.S(+57.02)PDIYNPQAGSLK.T	N	65.06	1445.7150	13	5.4	723.8687	2	28.54	6	F6:1379	OB5933 H2 raw.raw	8.1843E3	1	1	364	376		S1:Carbamidomethylation (DHKE, X@N-term):30.83	PEAKS PTM
R.FNLAGNHEQEFLR.Y	N	64.93	1573.7637	13	1.4	787.8902	2	30.26	11	F11:1483	OB5938 H3A raw.raw	1.9024E5	5	5	196	208			PEAKS DB
R.NRSPDIYNPQAGSLKTANELNLLILR.W	N	56.18	2909.5723	26	1.5	970.8661	3	34.50	5	F5:1698	OB5932 H2 raw.raw	6.2575E4	1	1	362	387			PEAKS DB
R.NALFVPH(+14.02)YNTNAHSIIYALR.G	N	55.85	2327.2175	20	0.3	776.7467	3	35.36	1	F1:1633	OB5921 H2 raw.raw	1.4175E5	1	1	401	420		H7:Methylation(others):18.00	PEAKS PTM
K.FFVPPFQQSP(+15.99)R.A	Y	54.39	1364.6876	11	0.7	683.3516	2	32.74	1	F1:1483	OB5921 H2 raw.raw	4.7156E5	1	1	516	526		P10:Oxidation or Hydroxylation:7.65	PEAKS PTM
R.WLGLSAE(+53.92)YGNLYR.N	N	49.75	1594.6866	13	0.4	532.5697	3	34.87	5	F5:1718	OB5932 H2 raw.raw	1.8492E5	2	2	388	400	Replacement of 2 protons by iron	E7:Replacement of 2 protons by iron:156.85	PEAKS PTM
N.G(+57.02)IEETIC(+57.02)TATVK.K	N	46.95	1377.6809	12	0.2	689.8479	2	29.76	1	F1:1306	OB5921 H2 raw.raw	0	0	0	345	356	Carbamidomethylation	G1:Carbamidomethylation (DHKE, X@N-term):5.03;C7:Carbamidomethylation:1000.00	PEAKS PTM
**total 42 peptides**																			

Peptide	Uniq	-10lgP	Mass	Length	ppm	m/z	z	RT	Fraction	Scan	Source File	Area Raw in gel	#Feature	#Feature Raw in gel	Start	End	PTM	AScore	Found By
R.QQPEENAC(+57.02)QFQR.L	N	45.72	1533.6630	12	1.7	767.8401	2	25.17	10	F10:1164	OB5937 H3A raw.raw	6.6881E3	2	2	25	36	Carbamidomethylation	C8:Carbamidomethylation:1000.00	PEAKS DB
R.AGQEQENEGGNIFSGFTSEFLAQAFQVDDR.Q	Y	45.58	3290.4751	30	1.1	1646.2466	2	41.04	7	F7:2056	OB5934 H3B raw.raw	0	0	0	249	278			PEAKS DB
N.G(+57.02)IEETIC(+57.02)TATVKK.N	N	44.53	1505.7759	13	2.5	753.8971	2	28.15	1	F1:1210	OB5921 H2 raw.raw	7.9254E4	3	3	345	357	Carbamidomethylation	G1:Carbamidomethylation (DHKE, X@N-term):26.31;C7:Carbamidomethylation:1000.00	PEAKS PTM
K.TDSRPSIANLAGEN(+.98)SFIDNLPEEVVANSYGLPR.E	N	43.89	3545.7273	33	6.3	1182.9238	3	48.72	1	F1:2418	OB5921 H2 raw.raw	9.731E3	2	2	470	502		N14:Deamidation (NQ):27.86	PEAKS DB
R.QLKNNNPFK.F	N	41.09	1101.5930	9	-0.6	551.8035	2	27.25	6	F6:1282	OB5933 H2 raw.raw	1.2465E4	2	2	507	515			PEAKS DB
R.AGQEQENEGGNIFSGFTSEFLAQ(+.98)AFQVDDR.Q	Y	41.09	3291.4592	30	3.4	1646.7424	2	41.11	7	F7:2056	OB5934 H3B raw.raw	5.9876E4	1	1	249	278		Q23:Deamidation (NQ):22.91	PEAKS DB
R.FNLAGNH(+14.02)EQEFLR.Y	N	40.10	1587.7793	13	1.9	530.2681	3	32.39	9	F9:1568	OB5936 H3A raw.raw	1.4807E5	2	2	196	208		H7:Methylation(others):33.98	PEAKS PTM
R.Q(-17.03)LKNNNPFK.F	N	37.25	1084.5665	9	0.4	543.2908	2	28.36	6	F6:1357	OB5933 H2 raw.raw	1.237E4	1	1	507	515	Pyro-glu from Q	Q1:Pyro-glu from Q:1000.00	PEAKS PTM
R.N(+.98)ALFVPHYNTNAHSIIYALR.G	N	36.85	2314.1858	20	3.6	772.4053	3	45.28	6	F6:2182	OB5933 H2 raw.raw	6.3088E4	1	1	401	420		N1:Deamidation (NQ):32.94	PEAKS DB
R.R(+14.02)FNLAGNHEQEFLR.Y	N	36.22	1743.8805	14	-0.1	582.3007	3	32.19	9	F9:1534	OB5936 H3A raw.raw	1.2906E6	2	2	195	208	Methylation(KR)	R1:Methylation(KR):67.11	PEAKS PTM
R.NALFVPH(+57.02)YNTNAHSIIYALR.G	N	32.65	2370.2231	20	-0.3	791.0814	3	33.37	5	F5:1616	OB5932 H2 raw.raw	6.404E5	1	1	401	420		H7:Carbamidomethylation (DHKE, X@N-term):11.86	PEAKS PTM
R.N(+57.02)ALFVPHYNTNAHSIIYALR.G	N	32.14	2370.2231	20	-0.5	791.0812	3	33.18	6	F6:1692	OB5933 H2 raw.raw	7.1997E5	1	1	401	420		N1:Carbamidomethylation (DHKE, X@N-term):42.70	PEAKS PTM
N.GIEETIC(+57.02)T(+14.02)ATVKK.N	N	31.99	1462.7701	13	4.7	732.3958	2	27.97	1	F1:1207	OB5921 H2 raw.raw	1.4542E3	1	1	345	357	Carbamidomethylation	C7:Carbamidomethylation:1000.00;T8:Methylation(others):6.59	PEAKS PTM
**total 42 peptides**																			

B5TYU1|B5TYU1_ARAHY

back to list

| Protein Coverage | Supporting Peptides |

Protein Coverage:

```
  1 MAKLLELSFC FCFLVLGASS ISFRQQPEEN ACQFQRLNAQ RPDNRIESEG GYIETWNPNN QEFECAGVAL SRLVLRRNAL
 81 RRPFYSNAPQ EIFIQQGRGY FGLIFPGCPS TYEEPAQQGR RYQSQRPPRR LQEEDQSQQQ QDSHQKVHRF NEGDLIAVPT
161 GVAFWLYNDH DTDVVAVSLT DTNNNDNQLD QFPRRFNLAG NHEQEFLRYQ QQSRQSRRRS LPYSPYSPQS QPRQEEREFS
241 PRGQHSRRER AGQEEENEGG NIFSGFTPEF LAQAFQVDDR QIVQNLRGEN ESEEQGAIVT VRGGLRILSP DRKRGADEEE
321 EYDEDEYEYD EEDRRRGRGS RGSGNGIEET ICTATVKKNI GRNRSPDIYN PQAGSLKTAN ELNLLILRWL GLSAEYGNLY
401 RNALFVPHYN TNAHSIIYAL RGRAHVQVVD SNGNRVYDEE LQEGHVLVVP QNFAVAGKSQ SDNFEYVAFK TDSRPSIANL
481 AGENSVIDNL PEEVVANSYG LPREQARQLK NNNPFKFFVP PSQQSPRAVA
```

Modification sites: 32 (C), 108 (C), 352 (C), 497 (d)

- C Carbamidomethylation (+57.02)
- d Deamidation (NQ) (+0.98)
- m Methylation(KR) (+14.02)
- P Pyro-glu from Q (-17.03)
- r Replacement of 2 protons by iron (+53.92)

Supporting Peptides:

Peptide	Uniq	-10lgP	Mass	Length	ppm	m/z	z	RT	Fraction	Scan	Source File	Area Raw in gel	#Feature	#Feature Raw in gel	Start	End	PTM	AScore	Found By
R.SPDIYNPQAGSLKTANELNLLILR.W	N	134.89	2639.4282	24	0.9	880.8175	3	35.79	5	F5:1765	OB5932 H2 raw.raw	1.8094E6	3	3	365	388			PEAKS DB
R.NALFVPHYNTNAHSIIYALR.G	N	123.74	2313.2019	20	2.3	1157.6108	2	33.37	5	F5:1607	OB5932 H2 raw.raw	3.0421E7	13	12	402	421			PEAKS DB
R.GYFGLIFPGC(+57.02)PSTYEEPAQQGR.R	N	122.36	2473.1372	22	3.9	1237.5807	2	36.47	2	F2:1635	OB5925 H3B raw.raw	6.1209E5	7	7	99	120	Carbamidomethylation	C10:Carbamidomethylation:1000.00	PEAKS DB
R.WLGLSAEYGNLYR.N	N	116.56	1540.7673	13	2.0	771.3925	2	34.87	5	F5:1714	OB5932 H2 raw.raw	3.9701E7	18	17	389	401			PEAKS DB
R.NRSPDIYNPQAGSLK.T	N	110.81	1658.8376	15	0.4	830.4265	2	27.80	5	F5:1285	OB5932 H2 raw.raw	8.3597E5	6	6	363	377			PEAKS DB
R.RPFYSNAPQEIFIQQGR.G	N	110.68	2050.0383	17	2.3	1026.0288	2	31.40	11	F11:1547	OB5938 H3A raw.raw	1.6392E6	11	11	82	98			PEAKS DB
K.SQSDNFEYVAFK.T	N	105.77	1433.6462	12	1.6	717.8315	2	31.87	5	F5:1530	OB5932 H2 raw.raw	4.4734E6	3	3	459	470			PEAKS DB
R.VYDEELQEGHVLVVPQNFAVAGK.S	N	103.27	2540.2910	23	2.1	847.7727	3	32.93	1	F1:1498	OB5921 H2 raw.raw	1.5993E6	6	6	436	458			PEAKS DB
K.TDSRPSIANLAGENSVIDNLPEEVVAN(+.98)SYGLPR.E	Y	100.92	3497.7273	33	2.2	1166.9189	3	35.99	5	F5:1780	OB5932 H2 raw.raw	9.4553E6	1	1	471	503	Deamidation (NQ)	N27:Deamidation (NQ):125.75	PEAKS DB
R.RFNLAGNHEQEFLR.Y	N	100.69	1729.8647	14	1.5	577.6297	3	30.08	10	F10:1438	OB5937 H3A raw.raw	6.1619E6	6	6	195	208			PEAKS DB
N.GIEETIC(+57.02)TATVK.K	N	96.25	1320.6595	12	1.3	661.3379	2	29.46	5	F5:1390	OB5932 H2 raw.raw	2.7846E5	3	3	346	357	Carbamidomethylation	C7:Carbamidomethylation:1000.00	PEAKS DB
K.TDSRPSIANLAGENSVIDNLPEEVVANSYGLPR.E	Y	94.24	3496.7434	33	4.7	1166.5939	3	36.68	6	F6:1848	OB5933 H2 raw.raw	2.8259E7	4	4	471	503			PEAKS DB
R.SPDIYNPQAGSLK.T	N	87.71	1388.6936	13	2.2	695.3556	2	28.73	5	F5:1375	OB5932 H2 raw.raw	1.3705E6	3	3	365	377			PEAKS DB
N.GIEETIC(+57.02)TATVKK.N	N	84.04	1448.7545	13	0.2	725.3847	2	27.80	5	F5:1296	OB5932 H2 raw.raw	5.4693E5	4	4	346	358	Carbamidomethylation	C7:Carbamidomethylation:1000.00	PEAKS DB
S.ISFRQQPEENAC(+57.02)QFQR.L	N	79.32	2036.9486	16	0.8	679.9907	3	27.97	11	F11:1335	OB5938 H3A raw.raw	1.7296E5	3	3	21	36	Carbamidomethylation	C12:Carbamidomethylation:1000.00	PEAKS DB

total 44 peptides

Peptide	Uniq	-10lgP	Mass	Length	ppm	m/z	z	RT	Fraction	Scan	Source File	Area Raw in gel	#Feature	#Feature Raw in gel	Start	End	PTM	AScore	Found By
K.TDSRPSIANLAGENSVIDN(+.98)LPEEVVANSYGLPR.E	Y	74.65	3497.7273	33	8.8	1166.9266	3	36.67	6	F6:1837	OB5933 H2 raw.raw	9.4553E6	1	1	471	503		N19:Deamidation (NQ):0.00	PEAKS DB
K.TDSRPSIANLAGEN(+.98)SVIDNLPEEVVANSYGLPR.E	Y	74.12	3497.7273	33	8.8	1166.9266	3	36.67	6	F6:1848	OB5933 H2 raw.raw	9.4553E6	1	1	471	503		N14:Deamidation (NQ):39.44	PEAKS DB
R.AGQEEENEGGNIFSGFTPEFLAQAFQVDDR.Q	N	73.71	3301.4800	30	5.0	1651.7555	2	41.34	9	F9:2094	OB5936 H3A raw.raw	3.0011E5	1	1	251	280			PEAKS DB
R.AHVQVVDSNGNRVYDEELQEGHVLVVPQNFAVAGK.S	N	72.39	3816.9182	35	3.0	1273.3171	3	32.25	5	F5:1561	OB5932 H2 raw.raw	3.9867E6	2	2	424	458			PEAKS DB
R.GYFGLIFPGC(+57.02)PSTYEEPAQQGRR.Y	N	71.34	2629.2383	23	1.6	877.4214	3	34.78	2	F2:1543	OB5925 H3B raw.raw	5.9452E5	2	2	99	121	Carbamidomethylation	C10:Carbamidomethylation:1000.00	PEAKS DB
K.TANELNLLILR.W	N	68.94	1268.7452	11	0.7	635.3803	2	34.98	1	F1:1608	OB5921 H2 raw.raw	2.5942E7	7	7	378	388			PEAKS DB
R.NALRRPFYSNAPQEIFIQQGR.G	N	68.46	2504.3037	21	1.1	835.7761	3	31.58	10	F10:1548	OB5937 H3A raw.raw	9.3975E5	4	4	78	98			PEAKS DB
V.PQNFAVAGK.S	N	67.74	930.4923	9	0.8	466.2538	2	32.44	5	F5:1562	OB5932 H2 raw.raw	4.3278E6	2	2	450	458			PEAKS DB
K.FFVPPSQQSPR.A	N	67.19	1288.6564	11	0.0	645.3354	2	28.51	1	F1:1229	OB5921 H2 raw.raw	9.0621E4	3	3	517	527			PEAKS DB
R.S(+57.02)PDIYNPQAGSLK.T	N	65.06	1445.7150	13	5.4	723.8687	2	28.54	6	F6:1379	OB5933 H2 raw.raw	8.1843E3	1	1	365	377		S1:Carbamidomethylation (DHKE, X@N-term):30.83	PEAKS PTM
R.FNLAGNHEQEFLR.Y	N	64.93	1573.7637	13	1.4	787.8902	2	30.26	11	F11:1483	OB5938 H3A raw.raw	1.9024E5	5	5	196	208			PEAKS DB
R.ERAGQEEENEGGNIFSGFTPEFLAQAFQVDDR.Q	N	57.80	3586.6235	32	3.1	1196.5521	3	38.65	10	F10:1961	OB5937 H3A raw.raw	5.3705E5	1	1	249	280			PEAKS DB
R.NRSPDIYNPQAGSLKTANELNLLILR.W	N	56.18	2909.5723	26	1.5	970.8661	3	34.50	5	F5:1698	OB5932 H2 raw.raw	6.2575E4	1	1	363	388			PEAKS DB
R.NALFVPH(+14.02)YNTNAHSIIYALR.G	N	55.85	2327.2175	20	0.3	776.7467	3	35.36	1	F1:1633	OB5921 H2 raw.raw	1.4175E5	1	1	402	421		H7:Methylation(others):18.00	PEAKS PTM
R.AGQEEENEGGN(+.98)IFSGFTPEFLAQAFQVDDR.Q	N	49.87	3302.4639	30	9.8	1652.2554	2	41.31	9	F9:2094	OB5936 H3A raw.raw	2.3662E5	1	1	251	280		N11:Deamidation (NQ):38.03	PEAKS DB
R.WLGLSAE(+53.92)YGNLYR.N	N	49.75	1594.6866	13	0.4	532.5697	3	34.87	5	F5:1718	OB5932 H2 raw.raw	1.8492E5	2	2	389	401	Replacement of 2 protons by iron	E7:Replacement of 2 protons by iron:156.85	PEAKS PTM
N.G(+57.02)IEETIC(+57.02)TATVK.K	N	46.95	1377.6809	12	0.2	689.8479	2	29.76	1	F1:1306	OB5921 H2 raw.raw	0	0	0	346	357	Carbamidomethylation	G1:Carbamidomethylation (DHKE, X@N-term):5.03;C7:Carbamidomethylation:1000.00	PEAKS PTM
R.SLPYSPY(+31.99)SPQSQPR.Q	N	46.01	1637.7686	14	1.0	819.8923	2	29.76	9	F9:1401	OB5936 H3A raw.raw	3.8534E4	1	1	220	233		Y7:Dihydroxy:0.00	PEAKS PTM
R.QQPEENAC(+57.02)QFQR.L	N	45.72	1533.6630	12	1.7	767.8401	2	25.17	10	F10:1164	OB5937 H3A raw.raw	6.6881E3	2	2	25	36	Carbamidomethylation	C8:Carbamidomethylation:1000.00	PEAKS DB
N.G(+57.02)IEETIC(+57.02)TATVKK.N	N	44.53	1505.7759	13	2.5	753.8971	2	28.15	1	F1:1210	OB5921 H2 raw.raw	7.9254E4	3	3	346	358	Carbamidomethylation	G1:Carbamidomethylation (DHKE, X@N-term):26.31;C7:Carbamidomethylation:1000.00	PEAKS PTM
K.FFVPPSQQSPRA.V	N	44.09	1359.6935	12	3.4	680.8563	2	29.07	1	F1:1270	OB5921 H2 raw.raw	1.1159E4	1	1	517	528			PEAKS DB
R.QLKNNNPFK.F	N	41.09	1101.5930	9	-0.6	551.8035	2	27.25	6	F6:1282	OB5933 H2 raw.raw	1.2465E4	2	2	508	516			PEAKS DB
R.FNLAGNH(+14.02)EQEFLR.Y	N	40.10	1587.7793	13	1.9	530.2681	3	32.39	9	F9:1568	OB5936 H3A raw.raw	1.4807E5	2	2	196	208		H7:Methylation(others):33.98	PEAKS PTM
R.Q(-17.03)LKNNNPFK.F	N	37.25	1084.5665	9	0.4	543.2908	2	28.36	6	F6:1357	OB5933 H2 raw.raw	1.237E4	1	1	508	516	Pyro-glu from Q	Q1:Pyro-glu from Q:1000.00	PEAKS PTM
R.N(+.98)ALFVPHYNTNAHSIIYALR.G	N	36.85	2314.1858	20	3.6	772.4053	3	45.28	6	F6:2182	OB5933 H2 raw.raw	6.3088E4	1	1	402	421		N1:Deamidation (NQ):32.94	PEAKS DB
R.R(+14.02)FNLAGNHEQEFLR.Y	N	36.22	1743.8805	14	-0.1	582.3007	3	32.19	9	F9:1534	OB5936 H3A raw.raw	1.2906E6	2	2	195	208	Methylation(KR)	R1:Methylation(KR):67.11	PEAKS PTM
R.NALFVPH(+57.02)YNTNAHSIIYALR.G	N	32.65	2370.2231	20	-0.3	791.0814	3	33.37	5	F5:1616	OB5932 H2 raw.raw	6.404E5	1	1	402	421		H7:Carbamidomethylation (DHKE, X@N-term):11.86	PEAKS PTM
**total 44 peptides**																			

Peptide	Uniq	-10lgP	Mass	Length	ppm	m/z	z	RT	Fraction	Scan	Source File	Area Raw in gel	#Feature	#Feature Raw in gel	Start	End	PTM	AScore	Found By
R.N(+57.02)ALFVPHYNTNAHSIIYALR.G	N	32.14	2370.2231	20	-0.5	791.0812	3	33.18	6	F6:1692	OB5933 H2 raw.raw	7.1997E5	1	1	402	421		N1:Carbamidomethylation (D HKE, X@N-term):42.70	PEAKS PTM
N.GIEETIC(+57.02)T(+14.02)ATVKK.N	N	31.99	1462.7701	13	4.7	732.3958	2	27.97	1	F1:1207	OB5921 H2 raw.raw	1.4542E3	1	1	346	358	Carbamidomethylation	C7:Carbamidomethylation:10 00.00;T8:Methylation(other s):6.59	PEAKS PTM

total 44 peptides

Q647H4|Q647H4_ARAHY

back to list

| Protein Coverage | Supporting Peptides |

Protein Coverage:

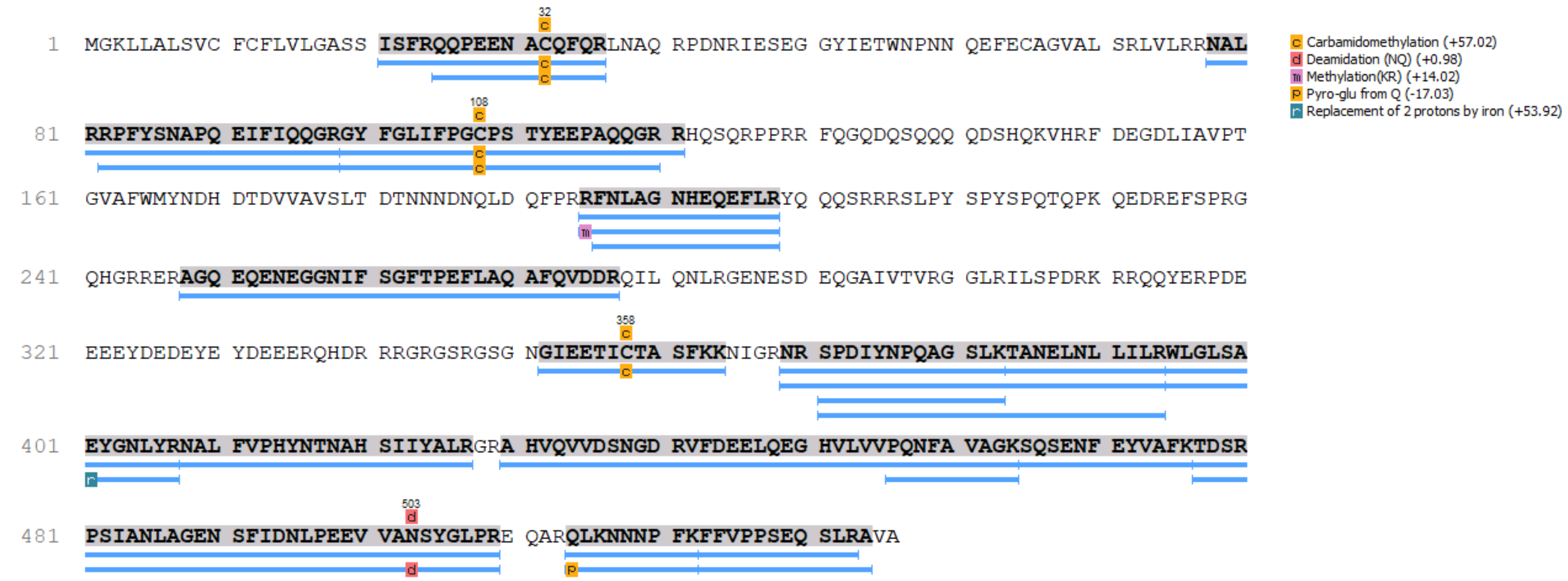

Supporting Peptides:

Peptide	Uniq	-10lgP	Mass	Length	ppm	m/z	z	RT	Fraction	Scan	Source File	Area Raw in gel	#Feature	#Feature Raw in gel	Start	End	PTM	AScore	Found By
R.SPDIYNPQAGSLKTANELNLLILR.W	N	134.89	2639.4282	24	0.9	880.8175	3	35.79	5	F5:1765	OB5932 H2 raw.raw	1.8094E6	3	3	371	394			PEAKS DB
R.NALFVPHYNTNAHSIIYALR.G	N	123.74	2313.2019	20	2.3	1157.6108	2	33.37	5	F5:1607	OB5932 H2 raw.raw	3.0421E7	13	12	408	427			PEAKS DB
R.GYFGLIFPGC(+57.02)PSTYEEPAQQGR.R	N	122.36	2473.1372	22	3.9	1237.5807	2	36.47	2	F2:1635	OB5925 H3B raw.raw	6.1209E5	7	7	99	120	Carbamidomethylation	C10:Carbamidomethylatio n:1000.00	PEAKS DB
R.WLGLSAEYGNLYR.N	N	116.56	1540.7673	13	2.0	771.3925	2	34.87	5	F5:1714	OB5932 H2 raw.raw	3.9701E7	18	17	395	407			PEAKS DB
K.TDSRPSIANLAGENSFIDNLPEEVVANSYGLPR.E	N	114.51	3544.7434	33	1.0	1182.5896	3	37.44	5	F5:1890	OB5932 H2 raw.raw	1.1751E8	13	13	477	509			PEAKS DB
K.SQSENFEYVAFK.T	N	113.90	1447.6619	12	1.7	724.8394	2	31.69	5	F5:1516	OB5932 H2 raw.raw	1.027E7	4	4	465	476			PEAKS DB
R.NRSPDIYNPQAGSLK.T	N	110.81	1658.8376	15	0.4	830.4265	2	27.80	5	F5:1285	OB5932 H2 raw.raw	8.3597E5	6	6	369	383			PEAKS DB

total 39 peptides

Peptide	Uniq	-10lgP	Mass	Length	ppm	m/z	z	RT	Fraction	Scan	Source File	Area Raw in gel	#Feature	#Feature Raw in gel	Start	End	PTM	AScore	Found By
R.RPFYSNAPQEIFIQQGR.G	N	110.68	2050.0383	17	2.3	1026.0288	2	31.40	11	F11:1547	OB5938 H3A raw.raw	1.6392E6	11	11	82	98			PEAKS DB
R.AHVQVVDSNGDRVFDEELQEGHVLVVPQNFAVAGK.S	Y	101.25	3801.9075	35	2.2	1268.3125	3	33.37	5	F5:1626	OB5932 H2 raw.raw	3.9148E6	2	2	430	464			PEAKS DB
R.RFNLAGNHEQEFLR.Y	N	100.69	1729.8647	14	1.5	577.6297	3	30.08	10	F10:1438	OB5937 H3A raw.raw	6.1619E6	6	6	195	208			PEAKS DB
K.TDSRPSIANLAGENSFIDNLPEEVVAN(+.98)SYGLPR.E	N	94.81	3545.7273	33	0.2	1182.9166	3	36.74	5	F5:1825	OB5932 H2 raw.raw	0	0	0	477	509	Deamidation (NQ)	N27:Deamidation (NQ):118.18	PEAKS DB
R.SPDIYNPQAGSLK.T	N	87.71	1388.6936	13	2.2	695.3556	2	28.73	5	F5:1375	OB5932 H2 raw.raw	1.3705E6	3	3	371	383			PEAKS DB
R.AGQ(+.98)EQENEGGNIFSGFTPEFLAQAFQVDDR.Q	N	84.98	3301.4800	30	5.9	1651.7571	2	39.98	10	F10:2041	OB5937 H3A raw.raw	3.2739E5	1	1	248	277		Q3:Deamidation (NQ):14.04	PEAKS DB
S.ISFRQQPEENAC(+57.02)QFQR.L	N	79.32	2036.9486	16	0.8	679.9907	3	27.97	11	F11:1335	OB5938 H3A raw.raw	1.7296E5	3	3	21	36	Carbamidomethylation	C12:Carbamidomethylation:1000.00	PEAKS DB
N.GIEETIC(+57.02)TASFKK.N	Y	76.16	1482.7388	13	1.0	742.3774	2	29.09	5	F5:1362	OB5932 H2 raw.raw	6.7331E5	3	3	352	364	Carbamidomethylation	C7:Carbamidomethylation:1000.00	PEAKS DB
R.AGQEQENEGGNIFSGFTPEFLAQAFQVDDR.Q	N	72.48	3300.4958	30	2.5	1651.2593	2	39.97	11	F11:2048	OB5938 H3A raw.raw	0	0	0	248	277			PEAKS DB
R.GYFGLIFPGC(+57.02)PSTYEEPAQQGRR.H	N	71.34	2629.2383	23	1.6	877.4214	3	34.78	2	F2:1543	OB5925 H3B raw.raw	5.9452E5	2	2	99	121	Carbamidomethylation	C10:Carbamidomethylation:1000.00	PEAKS DB
K.TANELNLLILR.W	N	68.94	1268.7452	11	0.7	635.3803	2	34.98	1	F1:1608	OB5921 H2 raw.raw	2.5942E7	7	7	384	394			PEAKS DB
K.FFVPPSEQSLRA.V	Y	68.71	1376.7087	12	1.8	689.3629	2	31.25	1	F1:1399	OB5921 H2 raw.raw	1.104E6	3	3	523	534			PEAKS DB
R.NALRRPFYSNAPQEIFIQQGR.G	N	68.46	2504.3037	21	1.1	835.7761	3	31.58	10	F10:1548	OB5937 H3A raw.raw	9.3975E5	4	4	78	98			PEAKS DB
K.FFVPPSEQSLR.A	Y	68.29	1305.6716	11	-0.5	653.8428	2	30.96	5	F5:1479	OB5932 H2 raw.raw	5.8436E5	3	3	523	533			PEAKS DB
V.PQNFAVAGK.S	N	67.74	930.4923	9	0.8	466.2538	2	32.44	5	F5:1562	OB5932 H2 raw.raw	4.3278E6	2	2	456	464			PEAKS DB
R.S(+57.02)PDIYNPQAGSLK.T	N	65.06	1445.7150	13	5.4	723.8687	2	28.54	6	F6:1379	OB5933 H2 raw.raw	8.1843E3	1	1	371	383		S1:Carbamidomethylation (DHKE, X@N-term):30.83	PEAKS PTM
R.FNLAGNHEQEFLR.Y	N	64.93	1573.7637	13	1.4	787.8902	2	30.26	11	F11:1483	OB5938 H3A raw.raw	1.9024E5	5	5	196	208			PEAKS DB
N.G(+57.02)IEETIC(+57.02)TASFKK.N	Y	60.94	1539.7603	13	0.5	770.8878	2	29.27	5	F5:1382	OB5932 H2 raw.raw	6.2038E4	2	2	352	364	Carbamidomethylation	G1:Carbamidomethylation (DHKE, X@N-term):22.85; C7:Carbamidomethylation:1000.00	PEAKS PTM
R.NRSPDIYNPQAGSLKTANELNLLILR.W	N	56.18	2909.5723	26	1.5	970.8661	3	34.50	5	F5:1698	OB5932 H2 raw.raw	6.2575E4	1	1	369	394			PEAKS DB
R.NALFVPH(+14.02)YNTNAHSIIYALR.G	N	55.85	2327.2175	20	0.3	776.7467	3	35.36	1	F1:1633	OB5921 H2 raw.raw	1.4175E5	1	1	408	427		H7:Methylation(others):18.00	PEAKS PTM
R.AHVQVVDSNGDRVFDEELQ(+.98)EGHVLVVPQNFAVAGK.S	Y	55.56	3802.8914	35	6.3	951.7361	4	33.37	5	F5:1633	OB5932 H2 raw.raw	6.1141E4	1	1	430	464		Q19:Deamidation (NQ):0.00	PEAKS DB
R.WLGLSAE(+53.92)YGNLYR.N	N	49.75	1594.6866	13	0.4	532.5697	3	34.87	5	F5:1718	OB5932 H2 raw.raw	1.8492E5	2	2	395	407	Replacement of 2 protons by iron	E7:Replacement of 2 protons by iron:156.85	PEAKS PTM
R.QQPEENAC(+57.02)QFQR.L	N	45.72	1533.6630	12	1.7	767.8401	2	25.17	10	F10:1164	OB5937 H3A raw.raw	6.6881E3	2	2	25	36	Carbamidomethylation	C8:Carbamidomethylation:1000.00	PEAKS DB

total 39 peptides

Peptide	Uniq	-10lgP	Mass	Length	ppm	m/z	z	RT	Fraction	Scan	Source File	Area Raw in gel	#Feature	#Feature Raw in gel	Start	End	PTM	AScore	Found By
K.TDSRPSIANLAGEN(+.98)SFIDNLPEEVVANSYGLPR.E	N	43.89	3545.7273	33	6.3	1182.9238	3	48.72	1	F1:2418	OB5921 H2 raw.raw	9.731E3	2	2	477	509		N14:Deamidation (NQ):27.86	PEAKS DB
R.QLKNNNPFK.F	N	41.09	1101.5930	9	-0.6	551.8035	2	27.25	6	F6:1282	OB5933 H2 raw.raw	1.2465E4	2	2	514	522			PEAKS DB
R.FNLAGNH(+14.02)EQEFLR.Y	N	40.10	1587.7793	13	1.9	530.2681	3	32.39	9	F9:1568	OB5936 H3A raw.raw	1.4807E5	2	2	196	208		H7:Methylation(others):33.98	PEAKS PTM
R.Q(-17.03)LKNNNPFK.F	N	37.25	1084.5665	9	0.4	543.2908	2	28.36	6	F6:1357	OB5933 H2 raw.raw	1.237E4	1	1	514	522	Pyro-glu from Q	Q1:Pyro-glu from Q:1000.00	PEAKS PTM
R.N(+.98)ALFVPHYNTNAHSIIYALR.G	N	36.85	2314.1858	20	3.6	772.4053	3	45.28	6	F6:2182	OB5933 H2 raw.raw	6.3088E4	1	1	408	427		N1:Deamidation (NQ):32.94	PEAKS DB
R.R(+14.02)FNLAGNHEQEFLR.Y	N	36.22	1743.8805	14	-0.1	582.3007	3	32.19	9	F9:1534	OB5936 H3A raw.raw	1.2906E6	2	2	195	208	Methylation(KR)	R1:Methylation(KR):67.11	PEAKS PTM
N.GIEETIC(+57.02)T(+14.02)ASFKK.N	Y	36.04	1496.7544	13	1.2	749.3854	2	29.27	5	F5:1378	OB5932 H2 raw.raw	2.0631E4	1	1	352	364	Carbamidomethylation	C7:Carbamidomethylation:1000.00;T8:Methylation(others):24.32	PEAKS PTM
R.NALFVPH(+57.02)YNTNAHSIIYALR.G	N	32.65	2370.2231	20	-0.3	791.0814	3	33.37	5	F5:1616	OB5932 H2 raw.raw	6.404E5	1	1	408	427		H7:Carbamidomethylation (DHKE, X@N-term):11.86	PEAKS PTM
R.N(+57.02)ALFVPHYNTNAHSIIYALR.G	N	32.14	2370.2231	20	-0.5	791.0812	3	33.18	6	F6:1692	OB5933 H2 raw.raw	7.1997E5	1	1	408	427		N1:Carbamidomethylation (DHKE, X@N-term):42.70	PEAKS PTM
**total 39 peptides**																			

E9LFE8|E9LFE8_ARAHY

back to list

| Protein Coverage | Supporting Peptides |

Protein Coverage:

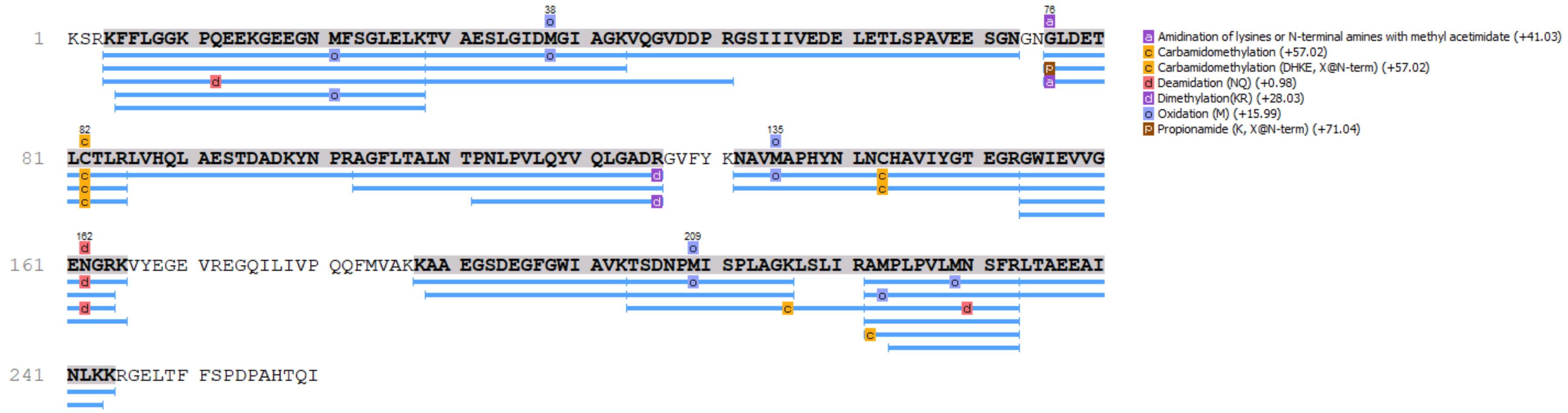

Supporting Peptides:

Peptide	Uniq	-10lgP	Mass	Length	ppm	m/z	z	RT	Fraction	Scan	Source File	Area Raw in gel	#Feature	#Feature Raw in gel	Start	End	PTM	AScore	Found By
K.FFLGGKPQEEKGEEGNMFSGLELK.T	N	136.55	2670.2998	24	2.4	891.1094	3	34.78	2	F2:1553	OB5925 H3B raw.raw	1.858E7	6	6	5	28			PEAKS DB
K.NAVMAPHYNLNC(+57.02)HAVIYGTEGR.G	Y	132.14	2486.1582	22	2.2	829.7285	3	30.40	5	F5:1439	OB5932 H2 raw.raw	4.9994E6	6	6	132	153	Carbamidomethylation	C12:Carbamidomethylation:1000.00	PEAKS DB
**total 38 peptides**																			

Peptide	Uniq	-10lgP	Mass	Length	ppm	m/z	z	RT	Fraction	Scan	Source File	Area Raw in gel	#Feature	#Feature Raw in gel	Start	End	PTM	AScore	Found By
K.KAAEGSDEGFGWIAVK.T	Y	128.06	1663.8206	16	1.7	832.9190	2	31.69	5	F5:1528	OB5932 H2 raw.raw	1.5376E6	4	4	188	203			PEAKS DB
K.TVAESLGIDMGIAGK.V	Y	119.07	1460.7544	15	2.2	731.3861	2	33.48	7	F7:1604	OB5934 H3B raw.raw	4.8068E6	3	3	29	43			PEAKS DB
K.AAEGSDEGFGWIAVK.T	Y	118.28	1535.7256	15	2.2	768.8718	2	33.74	5	F5:1648	OB5932 H2 raw.raw	7.8717E6	5	5	189	203			PEAKS DB
R.AGFLTALNTPNLPVLQYVQLGADR(+28.03).G	Y	112.27	2598.4170	24	4.3	1300.2213	2	39.90	5	F5:2005	OB5932 H2 raw.raw	7.2798E7	10	10	103	126	Dimethylation(KR)	R24:Dimethylation(KR):1000.00	PEAKS PTM
K.NAVM(+15.99)APHYNLNC(+57.02)HAVIYGTEGR.G	Y	108.73	2502.1533	22	1.0	835.0592	3	29.83	5	F5:1417	OB5932 H2 raw.raw	6.8396E5	4	4	132	153	Oxidation (M); Carbamidomethylation	M4:Oxidation (M):1000.00;C12:Carbamidomethylation:1000.00	PEAKS DB
R.KFFLGGKPQEEKGEEGNMFSGLELK.T	N	104.82	2798.3948	25	2.1	933.8075	3	34.42	2	F2:1518	OB5925 H3B raw.raw	1.4026E7	9	9	4	28			PEAKS DB
R.AMPLPVLMN(+.98)SFR.L	Y	104.49	1375.6992	12	1.0	688.8575	2	35.55	1	F1:1651	OB5921 H2 raw.raw	2.0235E5	2	2	222	233	Deamidation (NQ)	N9:Deamidation (NQ):1000.00	PEAKS DB
R.AMPLPVLMNSFR.L	Y	103.81	1374.7152	12	2.1	688.3663	2	36.34	5	F5:1802	OB5932 H2 raw.raw	1.702E7	4	4	222	233			PEAKS DB
R.GWIEVVGENGR.K	Y	103.10	1214.6044	11	0.0	608.3094	2	31.69	5	F5:1532	OB5932 H2 raw.raw	6.914E5	3	3	154	164			PEAKS DB
K.TVAESLGIDM(+15.99)GIAGK.V	Y	98.70	1476.7494	15	1.8	739.3833	2	30.84	7	F7:1465	OB5934 H3B raw.raw	3.5299E4	3	3	29	43	Oxidation (M)	M10:Oxidation (M):1000.00	PEAKS DB
K.TSDNPMISPLAGK.L	Y	96.67	1329.6598	13	2.4	665.8387	2	29.79	1	F1:1298	OB5921 H2 raw.raw	2.7946E6	5	5	204	216			PEAKS DB
R.AMPLPVLM(+15.99)NSFR.L	Y	95.24	1390.7101	12	1.9	696.3636	2	34.43	1	F1:1551	OB5921 H2 raw.raw	2.5374E6	3	3	222	233	Oxidation (M)	M8:Oxidation (M):151.86	PEAKS DB
R.GWIEVVGEN(+.98)GR.K	Y	92.90	1215.5884	11	0.7	608.8019	2	32.18	1	F1:1446	OB5921 H2 raw.raw	9.7206E4	1	1	154	164	Deamidation (NQ)	N9:Deamidation (NQ):1000.00	PEAKS DB
K.FFLGGKPQEEKGEEGNM(+15.99)FSGLELK.T	N	92.75	2686.2947	24	1.7	896.4404	3	32.74	2	F2:1411	OB5925 H3B raw.raw	2.6867E6	4	4	5	28	Oxidation (M)	M17:Oxidation (M):1000.00	PEAKS DB
R.LVHQLAESTDADKYNPR.A	N	86.93	1955.9701	17	1.5	652.9983	3	28.54	5	F5:1329	OB5932 H2 raw.raw	2.5664E6	9	9	86	102			PEAKS DB
R.AM(+15.99)PLPVLMNSFR.L	Y	85.64	1390.7101	12	1.9	696.3636	2	34.43	1	F1:1586	OB5921 H2 raw.raw	2.5374E6	3	3	222	233	Oxidation (M)	M2:Oxidation (M):90.85	PEAKS DB
N.GLDETLC(+57.02)TLR.L	Y	83.89	1176.5808	10	3.0	589.2994	2	30.96	5	F5:1472	OB5932 H2 raw.raw	1.5884E6	5	5	76	85	Carbamidomethylation	C7:Carbamidomethylation:1000.00	PEAKS DB
R.KFFLGGKPQEEKGEEGNM(+15.99)FSGLELK.T	N	80.26	2814.3896	25	0.9	704.6053	4	32.34	7	F7:1545	OB5934 H3B raw.raw	8.2213E5	2	2	4	28	Oxidation (M)	M18:Oxidation (M):1000.00	PEAKS DB
R.GWIEVVGEN(+.98)GRK.V	Y	80.12	1343.6833	12	0.4	672.8492	2	30.33	1	F1:1342	OB5921 H2 raw.raw	6.5174E5	3	3	154	165	Deamidation (NQ)	N9:Deamidation (NQ):1000.00	PEAKS DB
R.LTAEEAINLKK.R	Y	77.51	1228.7026	11	0.0	615.3586	2	27.27	1	F1:1147	OB5921 H2 raw.raw	3.3199E5	4	4	234	244			PEAKS DB
R.GWIEVVGENGRK.V	Y	76.86	1342.6993	12	0.8	672.3575	2	30.17	1	F1:1331	OB5921 H2 raw.raw	3.292E5	3	3	154	165			PEAKS DB
R.LTAEEAINLK.K	Y	73.12	1100.6077	10	-0.1	551.3110	2	29.82	5	F5:1410	OB5932 H2 raw.raw	2.6604E5	3	3	234	243			PEAKS DB
K.VQGVDDPRGSIIIVEDELETLSPAVEESGN.G	Y	68.06	3166.5518	30	3.5	1584.2887	2	39.25	7	F7:1952	OB5934 H3B raw.raw	3.2404E6	3	3	44	73			PEAKS DB

total 38 peptides

Peptide	Uniq	-10lgP	Mass	Length	ppm	m/z	z	RT	Fraction	Scan	Source File	Area Raw in gel	#Feature	#Feature Raw in gel	Start	End	PTM	AScore	Found By
R.AGFLTALNTPNLPVLQYVQLGAD(+28.03)R.G	Y	66.04	2598.4170	24	4.8	867.1504	3	39.70	6	F6:2024	OB5933 H2 raw.raw	2.3507E6	1	1	103	126		D23:Ethylation:7.21	PEAKS PTM
T.PNLPVLQYVQLGADR(+28.03).G	Y	64.22	1709.9464	15	6.6	855.9861	2	39.90	5	F5:2015	OB5932 H2 raw.raw	3.524E5	2	2	112	126	Dimethylation(KR)	R15:Dimethylation(KR):1000.00	PEAKS PTM
K.TSDNPM(+15.99)ISPLAGK.L	Y	62.31	1345.6548	13	0.6	673.8351	2	27.45	1	F1:1162	OB5921 H2 raw.raw	1.2503E5	4	4	204	216	Oxidation (M)	M6:Oxidation (M):1000.00	PEAKS DB
N.GLD(+14.02)ETLC(+57.02)TLR.L	Y	55.39	1190.5966	10	2.3	596.3069	2	31.14	5	F5:1483	OB5932 H2 raw.raw	7.3643E6	3	3	76	85	Carbamidomethylation	D3:Methylation(others):33.98;C7:Carbamidomethylation:1000.00	PEAKS PTM
R.KFFLGGKPQ(+.98)EEKGEEGNMFSGLELK.T	N	52.55	2799.3789	25	8.5	934.1415	3	34.08	7	F7:1645	OB5934 H3B raw.raw	0	0	0	4	28	Deamidation (NQ)	Q9:Deamidation (NQ):56.68	PEAKS DB
R.A(+57.02)MPLPVLMNSFR.L	Y	52.20	1431.7367	12	1.0	716.8763	2	36.48	1	F1:1707	OB5921 H2 raw.raw	1.3263E5	2	2	222	233	Carbamidomethylation (DHKE, X@N-term)	A1:Carbamidomethylation (DHKE, X@N-term):1000.00	PEAKS PTM
K.TSDNPMISPLAGK(+57.02)LSLIR.A	Y	51.17	1969.0667	18	0.0	657.3629	3	33.74	5	F5:1656	OB5932 H2 raw.raw	6.7977E4	1	1	204	221	Carbamidomethylation (DHKE, X@N-term)	K13:Carbamidomethylation (DHKE, X@N-term):74.64	PEAKS PTM
M.PLPVLMNSFR.L	Y	45.26	1172.6376	10	-0.2	587.3259	2	36.30	1	F1:1699	OB5921 H2 raw.raw	2.1961E4	1	1	224	233			PEAKS DB
N.G(+71.04)LDETLC(+57.02)TLR.L	Y	43.16	1247.6179	10	-0.3	624.8160	2	31.32	5	F5:1510	OB5932 H2 raw.raw	3.1525E5	3	3	76	85	Propionamide (K, X@N-term); Carbamidomethylation	G1:Propionamide (K, X@N-term):1000.00;C7:Carbamidomethylation:1000.00	PEAKS PTM
N.G(+41.03)LDETLC(+57.02)TLR.L	Y	36.86	1217.6074	10	0.7	609.8114	2	31.34	5	F5:1497	OB5932 H2 raw.raw	1.3034E5	1	1	76	85	Amidination of lysines or N-terminal amines with methyl acetimidate; Carbamidomethylation	G1:Amidination of lysines or N-terminal amines with methyl acetimidate:1000.00;C7:Carbamidomethylation:1000.00	PEAKS PTM
R.AMPLPVLM(+15.99)N(+.98)SFR.L	Y	36.60	1391.6941	12	-0.5	696.8540	2	33.83	1	F1:1548	OB5921 H2 raw.raw	0	0	0	222	233	Deamidation (NQ)	M8:Oxidation (M):37.35;N9:Deamidation (NQ):1000.00	PEAKS DB
K.AAEGSD(+53.92)EGFGWIAVK.T	Y	35.88	1589.6449	15	0.2	795.8299	2	33.60	6	F6:1663	OB5933 H2 raw.raw	0	0	0	189	203		D6:Replacement of 2 protons by iron:33.98	PEAKS PTM
K.TVAESLGIDMGIAGKVQGVDDPR.G	Y	31.82	2327.1790	23	3.2	1164.6005	2	33.53	8	F8:1613	OB5935 H3B raw.raw	3.4542E4	1	1	29	51			PEAKS DB
**total 38 peptides**																			

Q9FZ11|Q9FZ11_ARAHY

back to list

| Protein Coverage | Supporting Peptides |

Protein Coverage:

1 MIRGRLALSV CFCFLVLGAS SISFRQQPEE NACQFQRLNA QRPDNRLESE GGYIETWNPN NQEFECAGVA LSRLVLRRNA

81 LRRPFYSNAP QEIFIQQGRG YFGLIFPGCP STYEEPAQQG RRHQSQRAPR RFEGEDQSQQ QQQDSHQKVR RFDEGDLIAV

161 PTGVALWMFN DHDTDVVAVS LTDTNNNDNQ LDQFPRRFNL AGNHEQEFLR YQQQSRRRSL PYSPYSPQSQ PRQEEREFSP

241 RGQHSRRERA GQEEENEGGN IFSGFTPEFL AQAFQVDDRQ IVQNLRGENE SEEEGAIVTV KGGLRILSPD RKRGADEEEE

321 YDEDEYEYDE EDRRRGRGSR GRGNGIEETI CTASVKKNIG RNRSPDIYNP QAGSLKTAND LNLLILRWLG LSAEYGNLYR

401 NALFVPHYNT NAHSIIYALR GRAHVQVVDS NGNRVYDEEL QEGHVLVVPQ NFAVAGKSQS DNFEYVAFKT DSRPNIANFA

481 GENSIIDNLP EEVVANSYGL PREQARQLKN NNPFKFFVPP SQQSLRAVA

c Carbamidomethylation (+57.02)
d Deamidation (NQ) (+0.98)
m Methylation(KR) (+14.02)
p Pyro-glu from Q (-17.03)
r Replacement of 2 protons by iron (+53.92)

Supporting Peptides:

Peptide	Uniq	-10lgP	Mass	Length	ppm	m/z	z	RT	Fraction	Scan	Source File	Area Raw in gel	#Feature	#Feature Raw in gel	Start	End	PTM	AScore	Found By
R.NALFVPHYNTNAHSIIYALR.G	N	123.74	2313.2019	20	2.3	1157.6108	2	33.37	5	F5:1607	OB5932 H2 raw.raw	3.0421E7	13	12	401	420			PEAKS DB
R.GYFGLIFPGC(+57.02)PSTYEEPAQQGR.R	N	122.36	2473.1372	22	3.9	1237.5807	2	36.47	2	F2:1635	OB5925 H3B raw.raw	6.1209E5	7	7	100	121	Carbamidomethylation	C10:Carbamidomethylation:1000.00	PEAKS DB
R.WLGLSAEYGNLYR.N	N	116.56	1540.7673	13	2.0	771.3925	2	34.87	5	F5:1714	OB5932 H2 raw.raw	3.9701E7	18	17	388	400			PEAKS DB
R.NRSPDIYNPQAGSLK.T	N	110.81	1658.8376	15	0.4	830.4265	2	27.80	5	F5:1285	OB5932 H2 raw.raw	8.3597E5	6	6	362	376			PEAKS DB
R.RPFYSNAPQEIFIQQGR.G	N	110.68	2050.0383	17	2.3	1026.0288	2	31.40	11	F11:1547	OB5938 H3A raw.raw	1.6392E6	11	11	83	99			PEAKS DB
K.SQSDNFEYVAFK.T	N	105.77	1433.6462	12	1.6	717.8315	2	31.87	5	F5:1530	OB5932 H2 raw.raw	4.4734E6	3	3	458	469			PEAKS DB
R.VYDEELQEGHVLVVPQNFAVAGK.S	N	103.27	2540.2910	23	2.1	847.7727	3	32.93	1	F1:1498	OB5921 H2 raw.raw	1.5993E6	6	6	435	457			PEAKS DB
R.RFNLAGNHEQEFLR.Y	N	100.69	1729.8647	14	1.5	577.6297	3	30.08	10	F10:1438	OB5937 H3A raw.raw	6.1619E6	6	6	197	210			PEAKS DB
R.QIVQNLRGENESEEEGAIVTVK.G	Y	92.55	2441.2397	22	1.7	814.7552	3	29.14	11	F11:1413	OB5938 H3A raw.raw	3.4068E5	3	3	280	301			PEAKS DB
R.SPDIYNPQAGSLK.T	N	87.71	1388.6936	13	2.2	695.3556	2	28.73	5	F5:1375	OB5932 H2 raw.raw	1.3705E6	3	3	364	376			PEAKS DB
N.GIEETIC(+57.02)TASVK.K	N	85.93	1306.6438	12	1.2	654.3300	2	29.09	5	F5:1373	OB5932 H2 raw.raw	2.32E5	2	2	345	356	Carbamidomethylation	C7:Carbamidomethylation:1000.00	PEAKS DB
R.SPDIYNPQAGSLKTANDLNLLILR.W	N	81.29	2625.4126	24	0.2	876.1450	3	35.24	5	F5:1743	OB5932 H2 raw.raw	1.5604E5	1	1	364	387			PEAKS DB
S.ISFRQQPEENAC(+57.02)QFQR.L	N	79.32	2036.9486	16	0.8	679.9907	3	27.97	11	F11:1335	OB5938 H3A raw.raw	1.7296E5	3	3	22	37	Carbamidomethylation	C12:Carbamidomethylation:1000.00	PEAKS DB
K.FFVPPSQQSLRA.V	Y	75.54	1375.7247	12	1.8	688.8708	2	30.77	6	F6:1487	OB5933 H2 raw.raw	1.3188E6	3	3	516	527			PEAKS DB

total 40 peptides

Peptide	Uniq	-10lgP	Mass	Length	ppm	m/z	z	RT	Fraction	Scan	Source File	Area Raw in gel	#Feature	#Feature Raw in gel	Start	End	PTM	AScore	Found By
R.AGQEEENEGGNIFSGFTPEFLAQAFQVDDR.Q	N	73.71	3301.4800	30	5.0	1651.7555	2	41.34	9	F9:2094	OB5936 H3A raw.raw	3.0011E5	1	1	250	279			PEAKS DB
R.AHVQVVDSNGNRVYDEELQEGHVLVVPQNFAVAGK.S	N	72.39	3816.9182	35	3.0	1273.3171	3	32.25	5	F5:1561	OB5932 H2 raw.raw	3.9867E6	2	2	423	457			PEAKS DB
N.GIEETIC(+57.02)TASVKK.N	N	72.27	1434.7388	13	1.2	718.3775	2	27.26	5	F5:1253	OB5932 H2 raw.raw	2.7175E5	3	3	345	357	Carbamidomethylation	C7:Carbamidomethylation:1000.00	PEAKS DB
K.FFVPPSQQSLR.A	Y	72.07	1304.6876	11	0.5	653.3514	2	30.40	5	F5:1443	OB5932 H2 raw.raw	6.1475E5	3	3	516	526			PEAKS DB
R.GYFGLIFPGC(+57.02)PSTYEEPAQQGRR.H	N	71.34	2629.2383	23	1.6	877.4214	3	34.78	2	F2:1543	OB5925 H3B raw.raw	5.9452E5	2	2	100	122	Carbamidomethylation	C10:Carbamidomethylation:1000.00	PEAKS DB
R.NALRRPFYSNAPQEIFIQQGR.G	N	68.46	2504.3037	21	1.1	835.7761	3	31.58	10	F10:1548	OB5937 H3A raw.raw	9.3975E5	4	4	79	99			PEAKS DB
K.TANDLNLLILR.W	N	67.99	1254.7296	11	0.0	628.3721	2	35.65	4	F4:1764	OB5931 H6 raw.raw	1.549E7	6	6	377	387			PEAKS DB
V.PQNFAVAGK.S	N	67.74	930.4923	9	0.8	466.2538	2	32.44	5	F5:1562	OB5932 H2 raw.raw	4.3278E6	2	2	449	457			PEAKS DB
R.S(+57.02)PDIYNPQAGSLK.T	N	65.06	1445.7150	13	5.4	723.8687	2	28.54	6	F6:1379	OB5933 H2 raw.raw	8.1843E3	1	1	364	376		S1:Carbamidomethylation (DHKE, X@N-term):30.83	PEAKS PTM
R.FNLAGNHEQEFLR.Y	N	64.93	1573.7637	13	1.4	787.8902	2	30.26	11	F11:1483	OB5938 H3A raw.raw	1.9024E5	5	5	198	210			PEAKS DB
R.ERAGQEEENEGGNIFSGFTPEFLAQAFQVDDR.Q	N	57.80	3586.6235	32	3.1	1196.5521	3	38.65	10	F10:1961	OB5937 H3A raw.raw	5.3705E5	1	1	248	279			PEAKS DB
R.NALFVPH(+14.02)YNTNAHSIIYALR.G	N	55.85	2327.2175	20	0.3	776.7467	3	35.36	1	F1:1633	OB5921 H2 raw.raw	1.4175E5	1	1	401	420		H7:Methylation(others):18.00	PEAKS PTM
K.TAN(+.98)DLNLLILR.W	N	54.07	1255.7136	11	-1.0	628.8635	2	35.40	6	F6:1771	OB5933 H2 raw.raw	0	0	0	377	387	Deamidation (NQ)	N3:Deamidation (NQ):68.31	PEAKS DB
R.AGQEEENEGGN(+.98)IFSGFTPEFLAQAFQVDDR.Q	N	49.87	3302.4639	30	9.8	1652.2554	2	41.31	9	F9:2094	OB5936 H3A raw.raw	2.3662E5	1	1	250	279		N11:Deamidation (NQ):38.03	PEAKS DB
R.WLGLSAE(+53.92)YGNLYR.N	N	49.75	1594.6866	13	0.4	532.5697	3	34.87	5	F5:1718	OB5932 H2 raw.raw	1.8492E5	2	2	388	400	Replacement of 2 protons by iron	E7:Replacement of 2 protons by iron:156.85	PEAKS PTM
K.TAND(+53.92)LNLLILR.W	N	47.26	1308.6488	11	-1.1	655.3309	2	34.62	1	F1:1597	OB5921 H2 raw.raw	1.4902E5	1	1	377	387	Replacement of 2 protons by iron	D4:Replacement of 2 protons by iron:128.79	PEAKS PTM
R.SLPYSPY(+31.99)SPQSQPR.Q	N	46.01	1637.7686	14	1.0	819.8923	2	29.76	9	F9:1401	OB5936 H3A raw.raw	3.8534E4	1	1	219	232		Y7:Dihydroxy:0.00	PEAKS PTM
R.QQPEENAC(+57.02)QFQR.L	N	45.72	1533.6630	12	1.7	767.8401	2	25.17	10	F10:1164	OB5937 H3A raw.raw	6.6881E3	2	2	26	37	Carbamidomethylation	C8:Carbamidomethylation:1000.00	PEAKS DB
N.G(+57.02)IEETIC(+57.02)TASVKK.N	N	44.25	1491.7603	13	1.2	746.8883	2	27.43	6	F6:1293	OB5933 H2 raw.raw	2.0504E4	1	1	345	357	Carbamidomethylation	G1:Carbamidomethylation (DHKE, X@N-term):30.83;C7:Carbamidomethylation:1000.00	PEAKS PTM
R.QLKNNNPFK.F	N	41.09	1101.5930	9	-0.6	551.8035	2	27.25	6	F6:1282	OB5933 H2 raw.raw	1.2465E4	2	2	507	515			PEAKS DB
R.FNLAGNH(+14.02)EQEFLR.Y	N	40.10	1587.7793	13	1.9	530.2681	3	32.39	9	F9:1568	OB5936 H3A raw.raw	1.4807E5	2	2	198	210		H7:Methylation(others):33.98	PEAKS PTM
R.Q(-17.03)LKNNNPFK.F	N	37.25	1084.5665	9	0.4	543.2908	2	28.36	6	F6:1357	OB5933 H2 raw.raw	1.237E4	1	1	507	515	Pyro-glu from Q	Q1:Pyro-glu from Q:1000.00	PEAKS PTM
R.N(+.98)ALFVPHYNTNAHSIIYALR.G	N	36.85	2314.1858	20	3.6	772.4053	3	45.28	6	F6:2182	OB5933 H2 raw.raw	6.3088E4	1	1	401	420		N1:Deamidation (NQ):32.94	PEAKS DB
R.R(+14.02)FNLAGNHEQEFLR.Y	N	36.22	1743.8805	14	-0.1	582.3007	3	32.19	9	F9:1534	OB5936 H3A raw.raw	1.2906E6	2	2	197	210	Methylation(KR)	R1:Methylation(KR):67.11	PEAKS PTM
R.NALFVPH(+57.02)YNTNAHSIIYALR.G	N	32.65	2370.2231	20	-0.3	791.0814	3	33.37	5	F5:1616	OB5932 H2 raw.raw	6.404E5	1	1	401	420		H7:Carbamidomethylation (DHKE, X@N-term):11.86	PEAKS PTM
R.N(+57.02)ALFVPHYNTNAHSIIYALR.G	N	32.14	2370.2231	20	-0.5	791.0812	3	33.18	6	F6:1692	OB5933 H2 raw.raw	7.1997E5	1	1	401	420		N1:Carbamidomethylation (DHKE, X@N-term):42.70	PEAKS PTM
**total 40 peptides**																			

Q647H3|Q647H3_ARAHY

back to list

| Protein Coverage | Supporting Peptides |

Protein Coverage:

1 MAKLLALSVC FCFLVLGASS ISFRQQPEEN ACQFQRLNAQ RPDNRLESEG GYIETWNPNN QEFECAGVAL SRLVLRRNAL

81 RRPFYSNAPQ EIFIQQGRGY FGLIFPGCPS TYEEPAQQGR RHQSQRAPRR FEGEDQSQQQ QQDSHQKVRR FDEGDLIAVP

161 TGVALWMYND HDTDVVAVSL TDTNNNDNQL DQFPRRFNLA GNHEQEFLRY QQQSRRRSLP YSPYSPQSQP RQEEREFSPR

241 GQHSRRERAG QEQENEGGNI FSGFTPEFLA QAFQVDDRQI LQNLRGENES DEQGAIVTVR GGLRILSPDR KRRQQYERPD

321 EEEEYDEDEY EYDEEERQQD RRRGRGSRGR GNGIEETICT ASVKKNIGRN RSPDIYNPQA GSLKTANDLN LLILRWLGLS

401 AEYGNLYRNA LFVPHYNTNA HSIIYALRGR AHVQVVDSNG NRVYDEELQE GHVLVVPQNF AVAGKSQSDN FEYVAFKTDS

481 RPSIANLAGE NSIIDNLPEE VVANSYGLPR EQARQLKNNN PFKFFVPPSQ QSLGAVA

C Carbamidomethylation (+57.02)
d Deamidation (NQ) (+0.98)
m Methylation(KR) (+14.02)
o Oxidation (M) (+15.99)
P Pyro-glu from Q (-17.03)
r Replacement of 2 protons by iron (+53.92)

Supporting Peptides:

Peptide	Uniq	-10lgP	Mass	Length	ppm	m/z	z	RT	Fraction	Scan	Source File	Area Raw in gel	#Feature	#Feature Raw in gel	Start	End	PTM	AScore	Found By
R.NALFVPHYNTNAHSIIYALR.G	N	123.74	2313.2019	20	2.3	1157.6108	2	33.37	5	F5:1607	OB5932 H2 raw.raw	3.0421E7	13	12	409	428			PEAKS DB
R.GYFGLIFPGC(+57.02)PSTYEEPAQQGR.R	N	122.36	2473.1372	22	3.9	1237.5807	2	36.47	2	F2:1635	OB5925 H3B raw.raw	6.1209E5	7	7	99	120	Carbamidomethylation	C10:Carbamidomethylation:1000.00	PEAKS DB
R.WLGLSAEYGNLYR.N	N	116.56	1540.7673	13	2.0	771.3925	2	34.87	5	F5:1714	OB5932 H2 raw.raw	3.9701E7	18	17	396	408			PEAKS DB
K.TDSRPSIANLAGENSIIDNLPEEVVANSYGLPR.E	Y	111.57	3510.7590	33	3.0	1171.2638	3	37.25	5	F5:1848	OB5932 H2 raw.raw	7.7097E7	7	7	478	510			PEAKS DB
R.NRSPDIYNPQAGSLK.T	N	110.81	1658.8376	15	0.4	830.4265	2	27.80	5	F5:1285	OB5932 H2 raw.raw	8.3597E5	6	6	370	384			PEAKS DB
R.RPFYSNAPQEIFIQQGR.G	N	110.68	2050.0383	17	2.3	1026.0288	2	31.40	11	F11:1547	OB5938 H3A raw.raw	1.6392E6	11	11	82	98			PEAKS DB
K.SQSDNFEYVAFK.T	N	105.77	1433.6462	12	1.6	717.8315	2	31.87	5	F5:1530	OB5932 H2 raw.raw	4.4734E6	3	3	466	477			PEAKS DB
R.VYDEELQEGHVLVVPQNFAVAGK.S	N	103.27	2540.2910	23	2.1	847.7727	3	32.93	1	F1:1498	OB5921 H2 raw.raw	1.5993E6	6	6	443	465			PEAKS DB
R.RFNLAGNHEQEFLR.Y	N	100.69	1729.8647	14	1.5	577.6297	3	30.08	10	F10:1438	OB5937 H3A raw.raw	6.1619E6	6	6	196	209			PEAKS DB
**total 42 peptides**																			

Peptide	Uniq	-10lgP	Mass	Length	ppm	m/z	z	RT	Fraction	Scan	Source File	Area Raw in gel	#Feature	#Feature Raw in gel	Start	End	PTM	AScore	Found By
K.TDSRPSIANLAGENSIIDNLPEEVVAN(+.98)SYGLPR.E	Y	96.26	3511.7429	33	8.9	1171.5986	3	36.51	5	F5:1811	OB5932 H2 raw.raw	2.8767E6	1	1	478	510	Deamidation (NQ)	N27:Deamidation (NQ):131.72	PEAKS DB
R.SPDIYNPQAGSLK.T	N	87.71	1388.6936	13	2.2	695.3556	2	28.73	5	F5:1375	OB5932 H2 raw.raw	1.3705E6	3	3	372	384			PEAKS DB
N.GIEETIC(+57.02)TASVK.K	N	85.93	1306.6438	12	1.2	654.3300	2	29.09	5	F5:1373	OB5932 H2 raw.raw	2.32E5	2	2	353	364	Carbamidomethylation	C7:Carbamidomethylation:1000.00	PEAKS DB
R.AGQ(+.98)EQENEGGNIFSGFTPEFLAQAFQVDDR.Q	N	84.98	3301.4800	30	5.9	1651.7571	2	39.98	10	F10:2041	OB5937 H3A raw.raw	3.2739E5	1	1	249	278		Q3:Deamidation (NQ):14.04	PEAKS DB
K.TDSRPSIANLAGENSIIDN(+.98)LPEEVVANSYGLPR.E	Y	83.96	3511.7429	33	8.7	1171.5984	3	37.04	1	F1:1738	OB5921 H2 raw.raw	2.9292E6	2	2	478	510		N19:Deamidation (NQ):28.20	PEAKS DB
R.SPDIYNPQAGSLKTANDLNLLILR.W	N	81.29	2625.4126	24	0.2	876.1450	3	35.24	5	F5:1743	OB5932 H2 raw.raw	1.5604E5	1	1	372	395			PEAKS DB
S.ISFRQQPEENAC(+57.02)QFQR.L	N	79.32	2036.9486	16	0.8	679.9907	3	27.97	11	F11:1335	OB5938 H3A raw.raw	1.7296E5	3	3	21	36	Carbamidomethylation	C12:Carbamidomethylation:1000.00	PEAKS DB
R.AGQEQENEGGNIFSGFTPEFLAQAFQVDDR.Q	N	72.48	3300.4958	30	2.5	1651.2593	2	39.97	11	F11:2048	OB5938 H3A raw.raw	0	0	0	249	278			PEAKS DB
R.AHVQVVDSNGNRVYDEELQEGHVLVVPQNFAVAGK.S	N	72.39	3816.9182	35	3.0	1273.3171	3	32.25	5	F5:1561	OB5932 H2 raw.raw	3.9867E6	2	2	431	465			PEAKS DB
N.GIEETIC(+57.02)TASVKK.N	N	72.27	1434.7388	13	1.2	718.3775	2	27.26	5	F5:1253	OB5932 H2 raw.raw	2.7175E5	3	3	353	365	Carbamidomethylation	C7:Carbamidomethylation:1000.00	PEAKS DB
R.GYFGLIFPGC(+57.02)PSTYEEPAQQGRR.H	N	71.34	2629.2383	23	1.6	877.4214	3	34.78	2	F2:1543	OB5925 H3B raw.raw	5.9452E5	2	2	99	121	Carbamidomethylation	C10:Carbamidomethylation:1000.00	PEAKS DB
R.NALRRPFYSNAPQEIFIQQGR.G	N	68.46	2504.3037	21	1.1	835.7761	3	31.58	10	F10:1548	OB5937 H3A raw.raw	9.3975E5	4	4	78	98			PEAKS DB
K.TANDLNLLILR.W	N	67.99	1254.7296	11	0.0	628.3721	2	35.65	4	F4:1764	OB5931 H6 raw.raw	1.549E7	6	6	385	395			PEAKS DB
V.PQNFAVAGK.S	N	67.74	930.4923	9	0.8	466.2538	2	32.44	5	F5:1562	OB5932 H2 raw.raw	4.3278E6	2	2	457	465			PEAKS DB
R.S(+57.02)PDIYNPQAGSLK.T	N	65.06	1445.7150	13	5.4	723.8687	2	28.54	6	F6:1379	OB5933 H2 raw.raw	8.1843E3	1	1	372	384		S1:Carbamidomethylation (DHKE, X@N-term):30.83	PEAKS PTM
R.FNLAGNHEQEFLR.Y	N	64.93	1573.7637	13	1.4	787.8902	2	30.26	11	F11:1483	OB5938 H3A raw.raw	1.9024E5	5	5	197	209			PEAKS DB
R.NALFVPH(+14.02)YNTNAHSIIYALR.G	N	55.85	2327.2175	20	0.3	776.7467	3	35.36	1	F1:1633	OB5921 H2 raw.raw	1.4175E5	1	1	409	428		H7:Methylation (others):18.00	PEAKS PTM
**total 42 peptides**																			

Peptide	Uniq	-10lgP	Mass	Length	ppm	m/z	z	RT	Fraction	Scan	Source File	Area Raw in gel	#Feature	#Feature Raw in gel	Start	End	PTM	AScore	Found By
K.TAN(+.98)DLNLLILR.W	N	54.07	1255.7136	11	-1.0	628.8635	2	35.40	6	F6:1771	OB5933 H2 raw.raw	0	0	0	385	395	Deamidation (NQ)	N3:Deamidation (NQ):68.31	PEAKS DB
R.WLGLSAE(+53.92)YGNLYR.N	N	49.75	1594.6866	13	0.4	532.5697	3	34.87	5	F5:1718	OB5932 H2 raw.raw	1.8492E5	2	2	396	408	Replacement of 2 protons by iron	E7:Replacement of 2 protons by iron:156.85	PEAKS PTM
K.TAND(+53.92)LNLLILR.W	N	47.26	1308.6488	11	-1.1	655.3309	2	34.62	1	F1:1597	OB5921 H2 raw.raw	1.4902E5	1	1	385	395	Replacement of 2 protons by iron	D4:Replacement of 2 protons by iron:128.79	PEAKS PTM
R.SLPYSPY(+31.99)SPQSQPR.Q	N	46.01	1637.7686	14	1.0	819.8923	2	29.76	9	F9:1401	OB5936 H3A raw.raw	3.8534E4	1	1	218	231		Y7:Dihydroxy:0.00	PEAKS PTM
R.QQPEENAC(+57.02)QFQR.L	N	45.72	1533.6630	12	1.7	767.8401	2	25.17	10	F10:1164	OB5937 H3A raw.raw	6.6881E3	2	2	25	36	Carbamidomethylation	C8:Carbamidomethylation:1000.00	PEAKS DB
N.G(+57.02)IEETIC(+57.02)TASVKK.N	N	44.25	1491.7603	13	1.2	746.8883	2	27.43	6	F6:1293	OB5933 H2 raw.raw	2.0504E4	1	1	353	365	Carbamidomethylation	G1:Carbamidomethylation (DHKE, X@N-term):30.83;C7:Carbamidomethylation:1000.00	PEAKS PTM
R.QLKNNNPFK.F	N	41.09	1101.5930	9	-0.6	551.8035	2	27.25	6	F6:1282	OB5933 H2 raw.raw	1.2465E4	2	2	515	523			PEAKS DB
K.TDSRPSIANLAGEN(+.98)SIIDNLPEEVVANSYGLPR.E	Y	40.49	3511.7429	33	8.7	1171.5984	3	37.04	1	F1:1887	OB5921 H2 raw.raw	2.8767E6	1	1	478	510		N14:Deamidation (NQ):10.19	PEAKS DB
R.FNLAGNH(+14.02)EQEFLR.Y	N	40.10	1587.7793	13	1.9	530.2681	3	32.39	9	F9:1568	OB5936 H3A raw.raw	1.4807E5	2	2	197	209		H7:Methylation (others):33.98	PEAKS PTM
R.RFDEGDLIAVPTGVALWM(+15.99)YN(+.98)DHDTDVVAVSLTDTNNNDNQ(+.98)LDQFPR.R	Y	37.42	5208.3892	46	8.7	1737.1521	3	38.89	9	F9:1958	OB5936 H3A raw.raw	1.266E5	1	1	150	195	Oxidation (M)	M18:Oxidation (M):1000.00;N20:Deamidation (NQ):12.67;Q40:Deamidation (NQ):0.00	PEAKS DB
R.Q(-17.03)LKNNNPFK.F	N	37.25	1084.5665	9	0.4	543.2908	2	28.36	6	F6:1357	OB5933 H2 raw.raw	1.237E4	1	1	515	523	Pyro-glu from Q	Q1:Pyro-glu from Q:1000.00	PEAKS PTM
R.N(+.98)ALFVPHYNTNAHSIIYALR.G	N	36.85	2314.1858	20	3.6	772.4053	3	45.28	6	F6:2182	OB5933 H2 raw.raw	6.3088E4	1	1	409	428		N1:Deamidation (NQ):32.94	PEAKS DB
R.R(+14.02)FNLAGNHEQEFLR.Y	N	36.22	1743.8805	14	-0.1	582.3007	3	32.19	9	F9:1534	OB5936 H3A raw.raw	1.2906E6	2	2	196	209	Methylation(KR)	R1:Methylation (KR):67.11	PEAKS PTM

total 42 peptides

Peptide	Uniq	-10lgP	Mass	Length	ppm	m/z	z	RT	Fraction	Scan	Source File	Area Raw in gel	#Feature	#Feature Raw in gel	Start	End	PTM	AScore	Found By
K.TDSRPSIAN(+.98)LAGENSIIDNLPEEVVANSYGLPR.E	Y	33.62	3511.7429	33	8.7	1171.5984	3	37.04	1	F1:1940	OB5921 H2 raw.raw	2.8767E6	1	1	478	510		N9:Deamidation (NQ):13.89	PEAKS DB
R.NALFVPH(+57.02)YNTNAHSIIYALR.G	N	32.65	2370.2231	20	-0.3	791.0814	3	33.37	5	F5:1616	OB5932 H2 raw.raw	6.404E5	1	1	409	428		H7:Carbamidomethylation (DHKE, X@N-term):11.86	PEAKS PTM
R.N(+57.02)ALFVPHYNTNAHSIIYALR.G	N	32.14	2370.2231	20	-0.5	791.0812	3	33.18	6	F6:1692	OB5933 H2 raw.raw	7.1997E5	1	1	409	428		N1:Carbamidomethylation (DHKE, X@N-term):42.70	PEAKS PTM
**total 42 peptides**																			

T2B9M0|T2B9M0_ARAHY

back to list

| Protein Coverage | Supporting Peptides |

Protein Coverage:

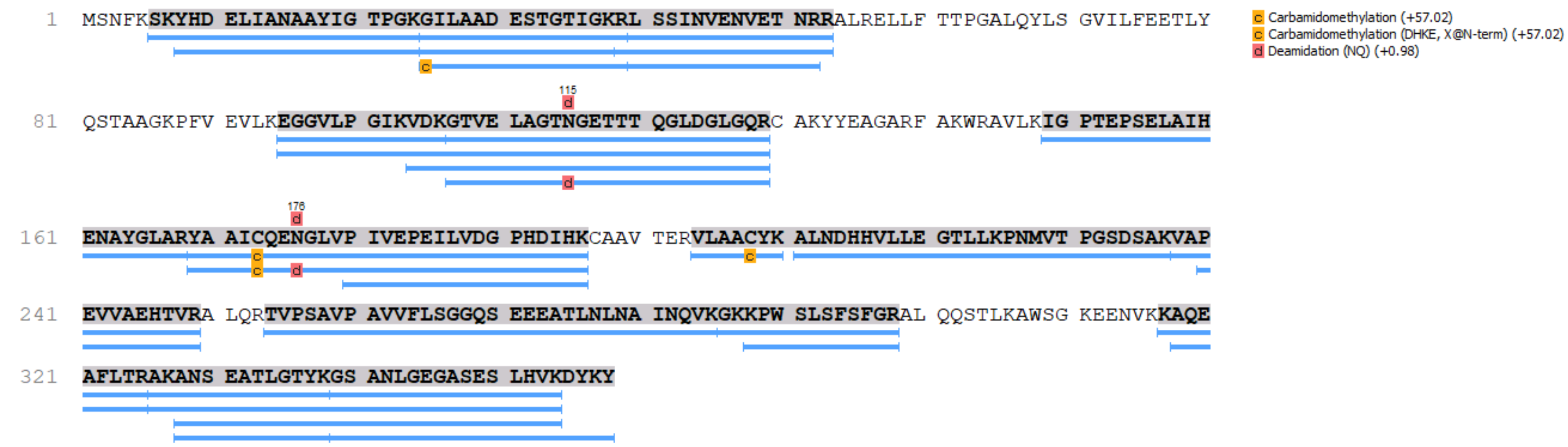

Supporting Peptides:

Peptide	Uniq	-10lgP	Mass	Length	ppm	m/z	z	RT	Fraction	Scan	Source File	Area Raw in gel	#Feature	#Feature Raw in gel	Start	End	PTM	AScore	Found By
K.GTVELAGTNGETTTQGLDGLGQR.C	Y	117.89	2274.1086	23	3.2	1138.0652	2	31.01	10	F10:1514	OB5937 H3A raw.raw	1.1133E5	3	3	107	129			PEAKS DB
K.GTVELAGTN(+.98)GETTTQGLDGLGQR.C	Y	113.53	2275.0928	23	3.0	1138.5571	2	31.39	11	F11:1541	OB5938 H3A raw.raw	1.6073E5	3	3	107	129	Deamidation (NQ)	N9:Deamidation (NQ):102.87	PEAKS DB
K.IGPTEPSELAIHENAYGLAR.Y	Y	113.22	2137.0803	20	2.9	1069.5505	2	31.02	11	F11:1523	OB5938 H3A raw.raw	8.8583E6	6	6	149	168			PEAKS DB
R.TVPSAVPAVVFLSGGQSEEEATLNLNAINQVK.G	Y	109.90	3281.7144	32	3.2	1094.9155	3	37.36	11	F11:1896	OB5938 H3A raw.raw	2.1228E7	9	9	254	285			PEAKS DB
**total 36 peptides**																			

Peptide	Uniq	-10lgP	Mass	Length	ppm	m/z	z	RT	Fraction	Scan	Source File	Area Raw in gel	#Feature	#Feature Raw in gel	Start	End	PTM	AScore	Found By
K.GILAADESTGTIGKR.L	Y	105.73	1487.7943	15	0.0	744.9044	2	27.45	11	F11:1307	OB5938 H3A raw.raw	3.9441E5	4	4	25	39			PEAKS DB
K.EGGVLPGIKVDKGTVELAGTNGETTTQGLDGLGQR.C	Y	102.77	3466.7903	35	2.3	1156.6067	3	32.71	10	F10:1614	OB5937 H3A raw.raw	1.4131E6	3	3	95	129			PEAKS DB
R.YAAIC(+57.02)QENGLVPIVEPEILVDGPHDIHK.C	Y	102.48	3125.5854	28	-2.1	1042.8669	3	34.56	11	F11:1733	OB5938 H3A raw.raw	7.5947E6	3	3	169	196	Carbamidomethylation	C5:Carbamidomethylation:1000.00	PEAKS DB
R.YAAIC(+57.02)QEN(+.98)GLVPIVEPEILVDGPHDIHK.C	Y	101.70	3126.5696	28	3.9	1043.2012	3	34.93	10	F10:1744	OB5937 H3A raw.raw	0	0	0	169	196	Carbamidomethylation; Deamidation (NQ)	C5:Carbamidomethylation:1000.00;N8:Deamidation (NQ):52.04	PEAKS DB
K.GKKPWSLSFSFGR.A	Y	99.46	1495.7936	13	-0.2	499.6050	3	32.14	11	F11:1581	OB5938 H3A raw.raw	4.526E6	6	6	286	298			PEAKS DB
K.KPWSLSFSFGR.A	Y	99.06	1310.6771	11	0.5	656.3462	2	33.46	10	F10:1658	OB5937 H3A raw.raw	6.1097E5	2	2	288	298			PEAKS DB
K.SKYHDELIANAAYIGTPGK.G	Y	98.40	2047.0374	19	1.6	683.3541	3	29.90	10	F10:1442	OB5937 H3A raw.raw	3.3341E5	4	4	6	24			PEAKS DB
K.YHDELIANAAYIGTPGK.G	Y	93.53	1831.9104	17	1.7	916.9640	2	30.83	10	F10:1510	OB5937 H3A raw.raw	6.0481E4	1	1	8	24			PEAKS DB
K.ANSEATLGTYKGSANLGEGASESLHVK.D	Y	91.88	2690.3147	27	1.6	897.7803	3	28.95	10	F10:1392	OB5937 H3A raw.raw	1.356E5	2	2	328	354			PEAKS DB
K.VDKGTVELAGTNGETTTQGLDGLGQR.C	Y	90.53	2616.2991	26	0.9	873.1078	3	29.90	11	F11:1453	OB5938 H3A raw.raw	6.3702E4	2	2	104	129			PEAKS DB
K.VAPEVVAEHTVR.A	Y	88.35	1305.7041	12	0.4	653.8596	2	26.96	10	F10:1262	OB5937 H3A raw.raw	3.6523E5	5	5	238	249			PEAKS DB
K.GILAADESTGTIGK.R	Y	88.05	1331.6932	14	0.1	666.8539	2	31.07	9	F9:1493	OB5936 H3A raw.raw	2.8213E4	2	2	25	38			PEAKS DB
R.LSSINVENVETNR.R	Y	85.55	1473.7423	13	0.1	737.8785	2	28.57	11	F11:1381	OB5938 H3A raw.raw	1.8416E5	3	3	40	52			PEAKS DB
K.ALNDHHVLLEGTLLKPNMVTPGSDSAK.V	Y	80.11	2856.4802	27	0.9	953.1682	3	31.77	10	F10:1566	OB5937 H3A raw.raw	2.319E5	1	1	211	237			PEAKS DB
K.AQEAFLTR.A	Y	73.62	934.4872	8	0.9	468.2513	2	27.27	10	F10:1294	OB5937 H3A raw.raw	1.5187E5	3	3	318	325			PEAKS DB
K.ANSEATLGTYK.G	Y	73.46	1153.5615	11	1.6	577.7889	2	25.17	11	F11:1176	OB5938 H3A raw.raw	6.2011E3	2	2	328	338			PEAKS DB
K.GSANLGEGASESLHVKDYKY	Y	73.40	2124.0122	20	2.8	709.0133	3	31.07	9	F9:1488	OB5936 H3A raw.raw	1.9959E6	6	6	339	358			PEAKS DB
K.GSANLGEGASESLHVK.D	Y	68.29	1554.7638	16	0.0	778.3892	2	26.95	10	F10:1269	OB5937 H3A raw.raw	1.9867E4	3	3	339	354			PEAKS DB
R.AKANSEATLGTYK.G	Y	64.49	1352.6936	13	-0.3	677.3539	2	27.72	9	F9:1294	OB5936 H3A raw.raw	8.8393E3	3	3	326	338			PEAKS DB
K.EGGVLPGIKVDK.G	Y	63.82	1210.6921	12	0.3	606.3535	2	28.10	11	F11:1351	OB5938 H3A raw.raw	9.8645E4	3	3	95	106			PEAKS DB
V.PIVEPEILVDGPHDIHK.C	Y	63.73	1907.0151	17	2.3	954.5170	2	34.91	10	F10:1729	OB5937 H3A raw.raw	4.6955E6	2	2	180	196			PEAKS DB
K.KAQEAFLTR.A	Y	60.63	1062.5822	9	0.9	532.2988	2	26.54	11	F11:1258	OB5938 H3A raw.raw	1.0907E4	3	3	317	325			PEAKS DB
K.VDKGTVELAGTNGETTTQ(+.98)GLDGLGQR.C	Y	54.47	2617.2830	26	1.5	873.4363	3	30.46	10	F10:1482	OB5937 H3A raw.raw	1.4142E5	1	1	104	129		Q18:Deamidation (NQ): 0.00	PEAKS DB
**total 36 peptides**																			

Peptide	Uniq	-10lgP	Mass	Length	ppm	m/z	z	RT	Fraction	Scan	Source File	Area Raw in gel	#Feature	#Feature Raw in gel	Start	End	PTM	AScore	Found By
K.VDKGTVELAGTN(+.98)GETTTQGLDGLGQR.C	Y	52.32	2617.2830	26	1.4	873.4362	3	32.69	9	F9:1582	OB5936 H3A raw.raw	1.5447E5	1	1	104	129		N12:Deamidation (NQ): 41.57	PEAKS DB
R.AKANSEATLGTYKGSANLGEGASESLHVK.D	Y	52.05	2889.4468	29	0.7	723.3695	4	28.76	10	F10:1381	OB5937 H3A raw.raw	1.2842E5	2	2	326	354			PEAKS DB
R.LSSINVENVETNRR.A	Y	47.58	1629.8434	14	1.7	544.2893	3	27.29	10	F10:1293	OB5937 H3A raw.raw	1.9075E5	1	1	40	53			PEAKS DB
R.VLAAC(+57.02)YK.A	Y	44.14	823.4262	7	1.2	412.7209	2	24.59	11	F11:1143	OB5938 H3A raw.raw	8.3473E3	2	2	204	210	Carbamidomethylation	C5:Carbamidomethylation:1000.00	PEAKS DB
K.G(+57.02)ILAADESTGTIGKR.L	Y	43.80	1544.8158	15	3.9	773.4182	2	27.74	11	F11:1329	OB5938 H3A raw.raw	0	0	0	25	39	Carbamidomethylation (DHKE, X@N-term)	G1:Carbamidomethylation (DHKE, X@N-term): 61.30	PEAKS PTM
K.RLSSINVENVETNRR.A	Y	42.99	1785.9446	15	0.4	596.3224	3	27.45	11	F11:1317	OB5938 H3A raw.raw	1.6138E4	2	2	39	53			PEAKS DB
A.PEVVAEHTVR.A	Y	40.67	1135.5985	10	0.5	568.8068	2	27.03	10	F10:1276	OB5937 H3A raw.raw	2.1131E3	1	1	240	249			PEAKS DB
R.LS(-15.99)SINVENVETNRR.A	Y	34.15	1613.8485	14	0.0	538.9568	3	29.95	9	F9:1425	OB5936 H3A raw.raw	1.6662E4	1	1	40	53		S2:Deoxy:40.00	PEAKS PTM
K.V(+57.02)APEVVAEHTVR.A	Y	33.55	1362.7256	12	3.0	682.3721	2	27.40	10	F10:1299	OB5937 H3A raw.raw	0	0	0	238	249		V1:Carbamidomethylation (DHKE, X@N-term): 22.45	PEAKS PTM

total 36 peptides

Q5I6T2|Q5I6T2_ARAHY

back to list

| Protein Coverage | Supporting Peptides |

Protein Coverage:

1 MAKLLELSFC FCFLVLGASS ISFRQQPEEN ACQFQRLNAQ RPDNRIESEG GYIETWNPNN QEFECAGVAL SRLVLRRNAL

81 RRPFYSNAPQ EIFIQQGRGY FGLIFPGCPS TYEEPAQQGR RSQSQRPPRR LQGEDQSQQQ QDSHQKVHRF DEGDLIAVPT

161 GVAFWLYNDH DTDVVAVSLT DTNNNDNQLD QFPRRFNLAG NHEQEFLRYQ QQSRQSRRRS LPYSPYSPQS QPRQEEREFS

241 PRGQHSRRER AGQEEENEGG NIFSGFTPEF LEQAFQVDDR QIVQNLRGEN ESEEEGAIVT VRGGLRILSP DRKRGADEEE

321 EYDEDEYEYD EEDRRRGRGS RGRGNGIEET ICTASVKKNI GRNRSPDIYN PQAGSLKTAN DLNLLILRWL GLSAEYGNLY

401 RNALFVPHYN TNAHSIIYAL RGRAHVQVVD SNGNRVYDEE LQEGHVLVVP QNFAVAGKSQ SDNFEYVAFK TDSRPSIANL

481 AGENSVIDNL PEEVVANSYG LQREQARQQL KNNNPFKFFV PPSQQSPRAV A

c Carbamidomethylation (+57.02)
d Deamidation (NQ) (+0.98)
m Methylation(KR) (+14.02)
p Pyro-glu from Q (-17.03)
r Replacement of 2 protons by iron (+53.92)

Supporting Peptides:

Peptide	Uniq	-10lgP	Mass	Length	ppm	m/z	z	RT	Fraction	Scan	Source File	Area Raw in gel	#Feature	#Feature Raw in gel	Start	End	PTM	AScore	Found By

total 37 peptides

Peptide	Uniq	-10lgP	Mass	Length	ppm	m/z	z	RT	Fraction	Scan	Source File	Area Raw in gel	#Feature	#Feature Raw in gel	Start	End	PTM	AScore	Found By
R.NALFVPHYNTNAHSIIYALR.G	N	123.74	2313.2019	20	2.3	1157.6108	2	33.37	5	F5:1607	OB5932 H2 raw.raw	3.0421E7	13	12	402	421			PEAKS DB
R.GYFGLIFPGC(+57.02)PSTYEEPAQQGR.R	N	122.36	2473.1372	22	3.9	1237.5807	2	36.47	2	F2:1635	OB5925 H3B raw.raw	6.1209E5	7	7	99	120	Carbamidomethylation	C10:Carbamidomethylation:1000.00	PEAKS DB
R.WLGLSAEYGNLYR.N	N	116.56	1540.7673	13	2.0	771.3925	2	34.87	5	F5:1714	OB5932 H2 raw.raw	3.9701E7	18	17	389	401			PEAKS DB
R.NRSPDIYNPQAGSLK.T	N	110.81	1658.8376	15	0.4	830.4265	2	27.80	5	F5:1285	OB5932 H2 raw.raw	8.3597E5	6	6	363	377			PEAKS DB
R.RPFYSNAPQEIFIQQGR.G	N	110.68	2050.0383	17	2.3	1026.0288	2	31.40	11	F11:1547	OB5938 H3A raw.raw	1.6392E6	11	11	82	98			PEAKS DB
K.SQSDNFEYVAFK.T	N	105.77	1433.6462	12	1.6	717.8315	2	31.87	5	F5:1530	OB5932 H2 raw.raw	4.4734E6	3	3	459	470			PEAKS DB
R.VYDEELQEGHVLVVPQNFAVAGK.S	N	103.27	2540.2910	23	2.1	847.7727	3	32.93	1	F1:1498	OB5921 H2 raw.raw	1.5993E6	6	6	436	458			PEAKS DB
R.RFNLAGNHEQEFLR.Y	N	100.69	1729.8647	14	1.5	577.6297	3	30.08	10	F10:1438	OB5937 H3A raw.raw	6.1619E6	6	6	195	208			PEAKS DB
R.SPDIYNPQAGSLK.T	N	87.71	1388.6936	13	2.2	695.3556	2	28.73	5	F5:1375	OB5932 H2 raw.raw	1.3705E6	3	3	365	377			PEAKS DB
N.GIEETIC(+57.02)TASVK.K	N	85.93	1306.6438	12	1.2	654.3300	2	29.09	5	F5:1373	OB5932 H2 raw.raw	2.32E5	2	2	346	357	Carbamidomethylation	C7:Carbamidomethylation:1000.00	PEAKS DB
R.SPDIYNPQAGSLKTANDLNLLILR.W	N	81.29	2625.4126	24	0.2	876.1450	3	35.24	5	F5:1743	OB5932 H2 raw.raw	1.5604E5	1	1	365	388			PEAKS DB
S.ISFRQQPEENAC(+57.02)QFQR.L	N	79.32	2036.9486	16	0.8	679.9907	3	27.97	11	F11:1335	OB5938 H3A raw.raw	1.7296E5	3	3	21	36	Carbamidomethylation	C12:Carbamidomethylation:1000.00	PEAKS DB
R.AHVQVVDSNGNRVYDEELQEGHVLVVPQNFAVAGK.S	N	72.39	3816.9182	35	3.0	1273.3171	3	32.25	5	F5:1561	OB5932 H2 raw.raw	3.9867E6	2	2	424	458			PEAKS DB
N.GIEETIC(+57.02)TASVKK.N	N	72.27	1434.7388	13	1.2	718.3775	2	27.26	5	F5:1253	OB5932 H2 raw.raw	2.7175E5	3	3	346	358	Carbamidomethylation	C7:Carbamidomethylation:1000.00	PEAKS DB
R.GYFGLIFPGC(+57.02)PSTYEEPAQQGRR.S	N	71.34	2629.2383	23	1.6	877.4214	3	34.78	2	F2:1543	OB5925 H3B raw.raw	5.9452E5	2	2	99	121	Carbamidomethylation	C10:Carbamidomethylation:1000.00	PEAKS DB
R.NALRRPFYSNAPQEIFIQQGR.G	N	68.46	2504.3037	21	1.1	835.7761	3	31.58	10	F10:1548	OB5937 H3A raw.raw	9.3975E5	4	4	78	98			PEAKS DB
K.TANDLNLLILR.W	N	67.99	1254.7296	11	0.0	628.3721	2	35.65	4	F4:1764	OB5931 H6 raw.raw	1.549E7	6	6	378	388			PEAKS DB
V.PQNFAVAGK.S	N	67.74	930.4923	9	0.8	466.2538	2	32.44	5	F5:1562	OB5932 H2 raw.raw	4.3278E6	2	2	450	458			PEAKS DB
K.FFVPPSQQSPR.A	N	67.19	1288.6564	11	0.0	645.3354	2	28.51	1	F1:1229	OB5921 H2 raw.raw	9.0621E4	3	3	518	528			PEAKS DB
R.S(+57.02)PDIYNPQAGSLK.T	N	65.06	1445.7150	13	5.4	723.8687	2	28.54	6	F6:1379	OB5933 H2 raw.raw	8.1843E3	1	1	365	377		S1:Carbamidomethylation (DHKE, X@N-term):30.83	PEAKS PTM
R.FNLAGNHEQEFLR.Y	N	64.93	1573.7637	13	1.4	787.8902	2	30.26	11	F11:1483	OB5938 H3A raw.raw	1.9024E5	5	5	196	208			PEAKS DB
R.NALFVPH(+14.02)YNTNAHSIIYALR.G	N	55.85	2327.2175	20	0.3	776.7467	3	35.36	1	F1:1633	OB5921 H2 raw.raw	1.4175E5	1	1	402	421		H7:Methylation(others):18.00	PEAKS PTM
K.TAN(+.98)DLNLLILR.W	N	54.07	1255.7136	11	-1.0	628.8635	2	35.40	6	F6:1771	OB5933 H2 raw.raw	0	0	0	378	388	Deamidation (NQ)	N3:Deamidation (NQ):68.31	PEAKS DB
R.WLGLSAE(+53.92)YGNLYR.N	N	49.75	1594.6866	13	0.4	532.5697	3	34.87	5	F5:1718	OB5932 H2 raw.raw	1.8492E5	2	2	389	401	Replacement of 2 protons by iron	E7:Replacement of 2 protons by iron:156.85	PEAKS PTM
K.TAND(+53.92)LNLLILR.W	N	47.26	1308.6488	11	-1.1	655.3309	2	34.62	1	F1:1597	OB5921 H2 raw.raw	1.4902E5	1	1	378	388	Replacement of 2 protons by iron	D4:Replacement of 2 protons by iron:128.79	PEAKS PTM
R.SLPYSPY(+31.99)SPQSQPR.Q	N	46.01	1637.7686	14	1.0	819.8923	2	29.76	9	F9:1401	OB5936 H3A raw.raw	3.8534E4	1	1	220	233		Y7:Dihydroxy:0.00	PEAKS PTM
R.QQPEENAC(+57.02)QFQR.L	N	45.72	1533.6630	12	1.7	767.8401	2	25.17	10	F10:1164	OB5937 H3A raw.raw	6.6881E3	2	2	25	36	Carbamidomethylation	C8:Carbamidomethylation:1000.00	PEAKS DB
N.G(+57.02)IEETIC(+57.02)TASVKK.N	N	44.25	1491.7603	13	1.2	746.8883	2	27.43	6	F6:1293	OB5933 H2 raw.raw	2.0504E4	1	1	346	358	Carbamidomethylation	G1:Carbamidomethylation (DHKE, X@N-term):30.83;C7:Carbamidomethylation:1000.00	PEAKS PTM

total 37 peptides

Peptide	Uniq	-10lgP	Mass	Length	ppm	m/z	z	RT	Fraction	Scan	Source File	Area Raw in gel	#Feature	#Feature Raw in gel	Start	End	PTM	AScore	Found By
K.FFVPPSQQSPRA.V	N	44.09	1359.6935	12	3.4	680.8563	2	29.07	1	F1:1270	OB5921 H2 raw.raw	1.1159E4	1	1	518	529			PEAKS DB
Q.QLKNNNPFK.F	N	41.09	1101.5930	9	-0.6	551.8035	2	27.25	6	F6:1282	OB5933 H2 raw.raw	1.2465E4	2	2	509	517			PEAKS DB
R.QIVQNLRGENESEEEGAIVTVR.G	Y	40.62	2469.2458	22	1.0	824.0900	3	29.85	10	F10:1443	OB5937 H3A raw.raw	0	0	0	281	302			PEAKS DB
R.FNLAGNH(+14.02)EQEFLR.Y	N	40.10	1587.7793	13	1.9	530.2681	3	32.39	9	F9:1568	OB5936 H3A raw.raw	1.4807E5	2	2	196	208		H7:Methylation(others):33.98	PEAKS PTM
Q.Q(-17.03)LKNNNPFK.F	N	37.25	1084.5665	9	0.4	543.2908	2	28.36	6	F6:1357	OB5933 H2 raw.raw	1.237E4	1	1	509	517	Pyro-glu from Q	Q1:Pyro-glu from Q:1000.00	PEAKS PTM
R.N(+.98)ALFVPHYNTNAHSIIYALR.G	N	36.85	2314.1858	20	3.6	772.4053	3	45.28	6	F6:2182	OB5933 H2 raw.raw	6.3088E4	1	1	402	421		N1:Deamidation (NQ):32.94	PEAKS DB
R.R(+14.02)FNLAGNHEQEFLR.Y	N	36.22	1743.8805	14	-0.1	582.3007	3	32.19	9	F9:1534	OB5936 H3A raw.raw	1.2906E6	2	2	195	208	Methylation(KR)	R1:Methylation(KR):67.11	PEAKS PTM
R.NALFVPH(+57.02)YNTNAHSIIYALR.G	N	32.65	2370.2231	20	-0.3	791.0814	3	33.37	5	F5:1616	OB5932 H2 raw.raw	6.404E5	1	1	402	421		H7:Carbamidomethylation (DHKE, X@N-term):11.86	PEAKS PTM
R.N(+57.02)ALFVPHYNTNAHSIIYALR.G	N	32.14	2370.2231	20	-0.5	791.0812	3	33.18	6	F6:1692	OB5933 H2 raw.raw	7.1997E5	1	1	402	421		N1:Carbamidomethylation (DHKE, X@N-term):42.70	PEAKS PTM
total 37 peptides																			

A1E2B0|A1E2B0_ARAHY

back to list

| Protein Coverage | Supporting Peptides |

Protein Coverage:

1 MANSLRLAFL VLFQSSLALS LAHRYPTKCS FDKLVALEPT KRVESEGGFT EYWDSKNDQF QCVGVSALRY SIKPKGLLLP

81 HYINAPRLQY VLQGTGILET VVPGCPETFR EQTRHGDQHQ KIHATREGDV IVVPTGSAQW IYNSGETDMV IFSVIDSANE

161 DNQLDLKVRK FFLGGKPQEE KGEEGNMFSG LELKTVAESL DIDMGIAGKV QGVDDPRGSM IIVEDELETL SPAVEESGNG

241 NGLIETVCTL RLVHQLAEST DADKYNPRAG FLTALTPLTF PSFNMSNLAL IDVFSTECCD GATLH

Modified positions: 29 (C), 62 (C), 105 (C), 204 (O)

- C Carbamidomethylation (+57.02)
- C Carbamidomethylation (DHKE, X@N-term) (+57.02)
- d Deamidation (NQ) (+0.98)
- O Oxidation (M) (+15.99)

Supporting Peptides:

Peptide	Uniq	-10lgP	Mass	Length	ppm	m/z	z	RT	Fraction	Scan	Source File	Area Raw in gel	#Feature	#Feature Raw in gel	Start	End	PTM	AScore	Found By
K.FFLGGKPQEEKGEEGNMFSGLELK.T	N	136.55	2670.2998	24	2.4	891.1094	3	34.78	2	F2:1553	OB5925 H3B raw.raw	1.858E7	6	6	171	194			PEAKS DB
R.VESEGGFTEYWDSKNDQFQC(+57.02)VGVSALR.Y	Y	124.63	3107.3931	27	3.3	1036.8083	3	34.05	2	F2:1498	OB5925 H3B raw.raw	5.0527E6	4	4	43	69	Carbamidomethylation	C20:Carbamidomethylation:1000.00	PEAKS DB
K.TVAESLDIDMGIAGK.V	Y	120.36	1518.7599	15	2.3	760.3890	2	33.12	2	F2:1443	OB5925 H3B raw.raw	5.1238E6	3	3	195	209			PEAKS DB
K.RVESEGGFTEYWDSKNDQFQC(+57.02)VGVSALR.Y	Y	115.64	3263.4941	28	3.1	1088.8420	3	33.12	2	F2:1439	OB5925 H3B raw.raw	7.9145E5	5	5	42	69	Carbamidomethylation	C21:Carbamidomethylation:1000.00	PEAKS DB
total 26 peptides																			

Peptide	Uniq	-10lgP	Mass	Length	ppm	m/z	z	RT	Fraction	Scan	Source File	Area Raw in gel	#Feature	#Feature Raw in gel	Start	End	PTM	AScore	Found By
K.GLLLPHYINAPR.L	Y	113.14	1362.7771	12	0.3	682.3960	2	31.41	7	F7:1486	OB5934 H3B raw.raw	8.3765E6	6	6	76	87			PEAKS DB
R.LQYVLQGTGILETVVPGC(+57.02)PETFR.E	Y	112.13	2576.3308	23	3.4	1289.1770	2	36.84	7	F7:1810	OB5934 H3B raw.raw	1.1189E6	3	3	88	110	Carbamidomethylation	C18:Carbamidomethylation:1000.00	PEAKS DB
R.KFFLGGKPQEEKGEEGNMFSGLELK.T	N	104.82	2798.3948	25	2.1	933.8075	3	34.42	2	F2:1518	OB5925 H3B raw.raw	1.4026E7	9	9	170	194			PEAKS DB
R.LQYVLQGTGILETVVPGC(+57.02)PETFREQTR.H	Y	98.38	3090.5808	27	3.6	1031.2046	3	35.35	7	F7:1755	OB5934 H3B raw.raw	5.1042E7	12	12	88	114	Carbamidomethylation	C18:Carbamidomethylation:1000.00	PEAKS DB
K.C(+57.02)SFDKLVALEPTKR.V	Y	93.33	1662.8762	14	1.5	555.3002	3	30.69	2	F2:1301	OB5925 H3B raw.raw	5.6961E4	1	1	29	42	Carbamidomethylation	C1:Carbamidomethylation:1000.00	PEAKS DB
K.FFLGGKPQEEKGEEGNM(+15.99)FSGLELK.T	N	92.75	2686.2947	24	1.7	896.4404	3	32.74	2	F2:1411	OB5925 H3B raw.raw	2.6867E6	4	4	171	194	Oxidation (M)	M17:Oxidation (M):1000.00	PEAKS DB
K.TVAESLDIDM(+15.99)GIAGK.V	Y	90.40	1534.7549	15	2.8	768.3869	2	30.87	2	F2:1309	OB5925 H3B raw.raw	5.2987E4	2	2	195	209	Oxidation (M)	M10:Oxidation (M):1000.00	PEAKS DB
K.C(+57.02)SFDKLVALEPTK.R	Y	86.99	1506.7751	13	1.5	754.3960	2	31.13	7	F7:1476	OB5934 H3B raw.raw	1.4434E5	4	4	29	41	Carbamidomethylation	C1:Carbamidomethylation:1000.00	PEAKS DB
R.LVHQLAESTDADKYNPR.A	N	86.93	1955.9701	17	1.5	652.9983	3	28.54	5	F5:1329	OB5932 H2 raw.raw	2.5664E6	9	9	252	268			PEAKS DB
R.KFFLGGKPQEEKGEEGNM(+15.99)FSGLELK.T	N	80.26	2814.3896	25	0.9	704.6053	4	32.34	7	F7:1545	OB5934 H3B raw.raw	8.2213E5	2	2	170	194	Oxidation (M)	M18:Oxidation (M):1000.00	PEAKS DB
K.G(+57.02)LLLPHYINAPR.L	Y	78.60	1419.7986	12	0.8	710.9072	2	31.80	2	F2:1367	OB5925 H3B raw.raw	5.831E5	5	5	76	87	Carbamidomethylation (DHKE, X@N-term)	G1:Carbamidomethylation (DHKE, X@N-term):80.35	PEAKS PTM
R.EGDVIVVPTGSAQWIYNSGETDMVIFSVIDSANEDNQLDLKVR.K	Y	74.54	4723.2964	43	3.8	1575.4454	3	41.12	7	F7:2051	OB5934 H3B raw.raw	4.2033E6	3	3	127	169			PEAKS DB
K.VQGVDDPRGSMIIVEDELETLSPAVEESGN.G	Y	61.95	3184.5081	30	2.6	1593.2654	2	38.94	8	F8:1922	OB5935 H3B raw.raw	9.8208E5	2	2	210	239			PEAKS DB
L.PHYINAPR.L	Y	60.86	966.5035	8	-0.3	484.2589	2	31.65	8	F8:1495	OB5935 H3B raw.raw	3.8685E5	3	3	80	87			PEAKS DB
L.LLPHYINAPR.L	Y	57.63	1192.6716	10	0.0	597.3431	2	31.41	7	F7:1491	OB5934 H3B raw.raw	8.2176E5	3	3	78	87			PEAKS DB
L.LPHYINAPR.L	Y	54.27	1079.5875	9	0.0	540.8010	2	31.65	8	F8:1499	OB5935 H3B raw.raw	1.0935E5	2	2	79	87			PEAKS DB
R.KFFLGGKPQ(+.98)EEKGEEGNMFSGLELK.T	N	52.55	2799.3789	25	8.5	934.1415	3	34.08	7	F7:1645	OB5934 H3B raw.raw	0	0	0	170	194	Deamidation (NQ)	Q9:Deamidation (NQ):56.68	PEAKS DB
K.VQGVDDPRGSM(+15.99)IIVEDELETLSPAVEESGN.G	Y	48.89	3200.5029	30	5.1	1601.2670	2	36.46	7	F7:1790	OB5934 H3B raw.raw	8.8212E4	1	1	210	239	Oxidation (M)	M11:Oxidation (M):1000.00	PEAKS DB
V.VPGC(+57.02)PETFREQTR.H	Y	45.84	1575.7463	13	2.4	788.8823	2	35.35	7	F7:1724	OB5934 H3B raw.raw	1.1594E6	1	1	102	114	Carbamidomethylation	C4:Carbamidomethylation:1000.00	PEAKS DB
V.PGC(+57.02)PETFREQTR.H	Y	45.04	1476.6780	12	2.0	739.3478	2	35.59	8	F8:1753	OB5935 H3B raw.raw	5.0655E6	2	2	103	114	Carbamidomethylation	C3:Carbamidomethylation:1000.00	PEAKS DB
K.TVAESLDIDMGIAGKVQGVDDPR.G	Y	44.86	2385.1846	23	3.9	1193.6042	2	33.53	8	F8:1612	OB5935 H3B raw.raw	7.2301E4	1	1	195	217			PEAKS DB
R.LQ(+.98)YVLQGTGILETVVPGC(+57.02)PETFREQTR.H	Y	37.95	3091.5647	27	7.8	1031.5369	3	47.69	8	F8:2433	OB5935 H3B raw.raw	3.862E3	1	1	88	114	Carbamidomethylation	Q2:Deamidation (NQ):31.56;C18:Carbamidomethylation:1000.00	PEAKS DB
**total 26 peptides**																			

Q38711|Q38711_ARAHY

back to list

| Protein Coverage | Supporting Peptides |

Protein Coverage:

```
  1  KPFCVFLTFF LLLAASKKVN SAETVSFNFN SFSEGNPAIN FQGDVTVLSN GNIQLTNLNK VNSVGRVLYA MPVRIWSSAT
 81  GNVASFLTSF SFEMKDIKDY DPADGIIFFI APEDTQIPAG SIGGGTLGVS DTKGAGHFVG VEFDTYSNSE YNDPPTDHVG
161  IDVNSVDSVK TVPWNSVSGA VVQVTVIYDS STKTLSVAVT NENGDITTIA QVVDLKAKLP ERVKFGFSAS GSLGGRQIHL
241  IRSWSFTSTL ITTTRRSIDN NEKKIMNMAT A
```

o Oxidation (M) (+15.99)

Supporting Peptides:

Peptide	Uniq	-10lgP	Mass	Length	ppm	m/z	z	RT	Fraction	Scan	Source File	Area Raw in gel	#Feature	#Feature Raw in gel	Start	End	PTM	AScore	Found By
R.IWSSATGNVASFLTSFSFEMK.D	N	144.16	2309.1038	21	3.7	1155.5635	2	40.56	7	F7:2025	OB5934 H3B raw.raw	1.0023E7	3	3	75	95			PEAKS DB
R.SWSFTSTLITTTR.R	N	124.66	1499.7620	13	2.5	750.8901	2	34.61	7	F7:1677	OB5934 H3B raw.raw	9.5864E6	3	3	243	255			PEAKS DB
K.TLSVAVTNENGDITTIAQVVDLK.A	Y	121.16	2400.2747	23	2.6	1201.1477	2	36.27	7	F7:1788	OB5934 H3B raw.raw	4.3282E6	3	3	194	216			PEAKS DB
K.FGFSASGSLGGR.Q	N	117.61	1141.5515	12	1.4	571.7838	2	30.50	2	F2:1278	OB5925 H3B raw.raw	1.5431E6	3	3	225	236			PEAKS DB
R.IWSSATGNVASFLTSFSFEM(+15.99)K.D	N	115.28	2325.0986	21	1.6	1163.5585	2	39.06	7	F7:1947	OB5934 H3B raw.raw	1.6326E6	3	3	75	95	Oxidation (M)	M20:Oxidation (M):1000.00	PEAKS DB
K.DIKDYDPADGIIFFIAPEDTQIPAGSIGGGTLGVSDTK.G	N	106.58	3892.9258	38	2.7	1298.6527	3	39.11	2	F2:1787	OB5925 H3B raw.raw	1.6354E7	5	5	96	133			PEAKS DB
R.VKFGFSASGSLGGR.Q	N	101.26	1368.7150	14	1.1	685.3655	2	30.27	7	F7:1424	OB5934 H3B raw.raw	1.6529E5	4	4	223	236			PEAKS DB
D.PPTDHVGIDVNSVDSVK.T	N	93.13	1777.8846	17	-0.4	889.9493	2	33.30	2	F2:1450	OB5925 H3B raw.raw	1.6838E6	7	7	154	170			PEAKS DB
K.GAGHFVGVEFDTYSNSEYNDPPTDHVGIDVNSVDSVK.T	N	88.70	3966.7820	37	3.1	1323.2721	3	33.12	2	F2:1445	OB5925 H3B raw.raw	1.9838E7	7	7	134	170			PEAKS DB
K.GAGHFVGVEFDTYSNSEYND.P	N	88.01	2206.9080	20	2.4	1104.4639	2	32.91	7	F7:1579	OB5934 H3B raw.raw	1.1587E6	3	3	134	153			PEAKS DB
R.VLYAMPVR.I	N	82.17	947.5262	8	2.3	474.7715	2	30.09	7	F7:1402	OB5934 H3B raw.raw	9.4625E5	3	3	67	74			PEAKS DB
R.SWSFTSTLITTTRR.S	N	69.66	1655.8632	14	1.5	828.9401	2	32.96	8	F8:1573	OB5935 H3B raw.raw	1.7686E7	6	6	243	256			PEAKS DB
K.TLSVAVTNENGDITTIAQVVDLKAK.L	Y	66.43	2599.4067	25	0.1	867.4763	3	34.79	7	F7:1695	OB5934 H3B raw.raw	1.6993E6	3	3	194	218			PEAKS DB
D.P(+57.02)PTDHVGIDVNSVDSVK.T	N	59.87	1834.9061	17	0.0	612.6426	3	30.08	8	F8:1405	OB5935 H3B raw.raw	0	0	0	154	170		P1:Carbamidomethylation (DHKE, X@N-term):28.36	PEAKS PTM
R.VLYAM(+15.99)PVR.I	N	59.45	963.5212	8	-0.9	482.7674	2	29.11	7	F7:1353	OB5934 H3B	1.9715E4	2	2	67	74	Oxidation (M)	M5:Oxidation (M):1000.00	PEAKS DB
**total 21 peptides**																			

Peptide	Uniq	-10lgP	Mass	Length	ppm	m/z	z	RT	Fraction	Scan	Source File	Area Raw in gel	#Feature	#Feature Raw in gel	Start	End	PTM	AScore	Found By
											raw.raw								
K.FGFSASGSLGGRQIHLIR.S	N	58.70	1902.0223	18	0.5	635.0150	3	32.37	2	F2:1405	OB5925 H3B raw.raw	1.9034E5	1	1	225	242			PEAKS DB
D.PADGIIFFIAPEDTQIPAGSIGGGTLGVSDTK.G	N	44.74	3143.6025	32	4.6	1572.8158	2	38.57	8	F8:1906	OB5935 H3B raw.raw	6.2609E5	2	2	102	133			PEAKS DB
R.IWSSATGNVASFLTSFSFEMKDIKDYDPADGIIFFIAPEDTQIPAGSIGGGTLGVSDTK.G	N	40.61	6184.0190	59	6.4	1547.0220	4	42.84	7	F7:2162	OB5934 H3B raw.raw	5.5947E6	1	1	75	133			PEAKS DB
S.AETVSFNFNSFSEGNPAINFQGDVTVLSNGNIQLTNLNKVNSVGR.V	N	40.54	4812.3740	45	3.9	1605.1382	3	39.43	7	F7:2007	OB5934 H3B raw.raw	1.7542E7	3	3	22	66			PEAKS DB
R.IWSSATGNVASFLTSFSFEM(+15.99)KDIKDYDPADGIIFFIAPEDTQIPAGSIGGGTLGVSDTK.G	N	37.46	6200.0137	59	5.3	1551.0189	4	41.88	7	F7:2118	OB5934 H3B raw.raw	1.2633E7	1	1	75	133	Oxidation (M)	M20:Oxidation (M):1000.00	PEAKS DB
Q.VTVIYDSSTK.T	N	37.41	1111.5760	10	0.7	556.7957	2	26.75	7	F7:1237	OB5934 H3B raw.raw	6.5025E2	1	1	184	193			PEAKS DB

total 21 peptides

Q6IWG5|Q6IWG5_ARAHY

back to list

| Protein Coverage | Supporting Peptides |

Protein Coverage:

```
  1 KLLALSLCFC VLVLGASSVT FRQGGEENEC QFQRLNAQRP DNRIESEGGY IETWNPNNQE FQCAGVALSR TVLRRNALRR
 81 PFYSNAPLEI YVQQGSGYFG LIFPGCPSTY EEPAQEGRRY QSQKPSRRFQ VGQDDPSQQQ QDSHQKVHRF DEGDLIAVPT
161 GVAFWMYNDE DTDVVTVTLS DTSSIHNQLD QFPRRFYLAG NQEQEFLRYQ QQQGSRPHYR QISPRVRGDE QENEGSNIFS
241 GFAQEFLQHA FQVDRQTVEN LRGENEREEQ GAIVTVKGGL RILSPDEEDE SSRSPPSRRE EFDEDRSRPQ QRGKYDENRR
321 GYKNGIEETI CSASVKKNLG RSSNPDIYNP QAGSLRSVNE LDLPILGWLG LSAQHGTIYR NAMFVPHYTL NAHTIVVALN
401 GRAHVQVVDS NGNRVYDEEL QEGHVLVVPQ NFAVAAKAQS ENYEYLAFKT DSRPSIANLA GENSIIDNLP EEVVANSYRL
481 PREQARQLKN NNPFKFFVPP FDHQSMREVA
```

Modification markers: 30 C, 63 C, 106 C, 331 C, 383 O, 400 d, 476 d

C Carbamidomethylation (+57.02)
d Deamidation (NQ) (+0.98)
O Oxidation (M) (+15.99)
P Pyro-glu from Q (-17.03)

Supporting Peptides:

Peptide	Uniq	-10lgP	Mass	Length	ppm	m/z	z	RT	Fraction	Scan	Source File	Area Raw in gel	#Feature	#Feature Raw in gel	Start	End	PTM	AScore	Found By
R.NAMFVPHYTLNAHTIVVALNGR.A	Y	133.55	2437.2688	22	1.7	1219.6438	2	35.24	5	F5:1717	OB5932 H2 raw.raw	1.1969E7	6	6	381	402			PEAKS DB

total 40 peptides

Peptide	Uniq	-10lgP	Mass	Length	ppm	m/z	z	RT	Fraction	Scan	Source File	Area Raw in gel	#Feature	#Feature Raw in gel	Start	End	PTM	AScore	Found By
R.NAMFVPHYTLNAHTIVVALN(+.98)GR.A	Y	132.37	2438.2529	22	3.2	1220.1377	2	34.12	5	F5:1671	OB5932 H2 raw.raw	5.0871E6	4	4	381	402	Deamidation (NQ)	N20:Deamidation (N Q):218.32	PEAKS DB
R.SVNELDLPILGWLGLSAQHGTIYR.N	Y	121.73	2651.4070	24	1.0	1326.7120	2	39.90	1	F1:1894	OB5921 H2 raw.raw	6.3358E7	10	10	357	380			PEAKS DB
K.TDSRPSIANLAGENSIIDNLPEEVVANSYR.L	Y	116.11	3243.6006	30	3.7	1082.2114	3	36.34	5	F5:1837	OB5932 H2 raw.raw	1.8021E7	7	7	450	479			PEAKS DB
R.N(+.98)AMFVPHYTLNAHTIVVALNGR.A	Y	114.32	2438.2529	22	2.9	1220.1373	2	34.78	5	F5:1708	OB5932 H2 raw.raw	0	0	0	381	402	Deamidation (NQ)	N1:Deamidation (NQ): 79.74	PEAKS DB
K.TDSRPSIANLAGENSIIDNLPEEVVAN(+.98)SYR.L	Y	114.06	3244.5847	30	3.8	1082.5397	3	35.65	5	F5:1760	OB5932 H2 raw.raw	0	0	0	450	479	Deamidation (NQ)	N27:Deamidation (N Q):81.59	PEAKS DB
K.AQSENYEYLAFK.T	Y	109.13	1461.6776	12	-0.5	731.8457	2	31.69	5	F5:1517	OB5932 H2 raw.raw	5.3867E6	3	3	438	449			PEAKS DB
R.SSNPDIYNPQAGSLR.S	Y	108.34	1617.7747	15	0.5	809.8950	2	28.51	1	F1:1221	OB5921 H2 raw.raw	1.8561E6	3	3	342	356			PEAKS DB
R.NAM(+15.99)FVPHYTLNAHTIVVALNGR.A	Y	101.30	2453.2637	22	1.2	818.7628	3	33.55	5	F5:1639	OB5932 H2 raw.raw	1.2362E6	2	2	381	402	Oxidation (M)	M3:Oxidation (M):100 0.00	PEAKS DB
R.LNAQRPDNRIESEGGYIETWNPNNQEFQC(+57.02)AGVALSR.T	Y	95.82	4132.9409	36	3.0	1034.2456	4	33.11	7	F7:1590	OB5934 H3B raw.raw	2.0092E6	3	3	35	70	Carbamidomethylation	C29:Carbamidomethyl ation:1000.00	PEAKS DB
R.NAM(+15.99)FVPHYTLNAHTIVVALN(+.98)GR.A	Y	95.09	2454.2478	22	1.8	819.0913	3	33.01	5	F5:1606	OB5932 H2 raw.raw	1.7713E6	6	6	381	402	Oxidation (M); Deamidation (NQ)	M3:Oxidation (M):100 0.00;N20:Deamidation (NQ):123.21	PEAKS DB
L.PILGWLGLSAQHGTIYR.N	Y	88.39	1881.0261	17	2.5	941.5227	2	39.89	6	F6:2036	OB5933 H2 raw.raw	1.5582E5	1	1	364	380			PEAKS DB
K.TDSRPSIANLAGEN(+.98)SIIDNLPEEVVANSYR.L	Y	79.83	3244.5847	30	8.0	1082.5442	3	36.12	6	F6:1826	OB5933 H2 raw.raw	5.3609E7	2	2	450	479		N14:Deamidation (N Q):31.31	PEAKS DB
N.GIEETIC(+57.02)SASVK.K	Y	77.84	1292.6282	12	2.2	647.3228	2	29.07	1	F1:1261	OB5921 H2 raw.raw	1.1569E5	3	3	325	336	Carbamidomethylation	C7:Carbamidomethylat ion:1000.00	PEAKS DB
R.SVNE(+57.02)LDLPILGWLGLSAQHGTIYR.N	Y	74.12	2708.4285	24	1.0	1355.2229	2	40.09	5	F5:2024	OB5932 H2 raw.raw	1.9952E5	1	1	357	380		E4:Carbamidomethylat ion (DHKE, X@N-ter m):5.03	PEAKS PTM
R.NAMFVPHYTLN(+.98)AHTIVVALNGR.A	Y	72.49	2438.2529	22	3.7	813.7613	3	35.31	1	F1:1635	OB5921 H2 raw.raw	0	0	0	381	402		N11:Deamidation (N Q):33.51	PEAKS DB
R.AHVQVVDSN(+.98)GNRVYDEELQEGHVLVVPQNFAVAAK.A	Y	72.14	3831.9180	35	3.6	1278.3179	3	32.25	6	F6:1584	OB5933 H2 raw.raw	9.4081E6	2	2	403	437		N9:Deamidation (NQ): 9.40	PEAKS DB
N.GIEETIC(+57.02)SASVKK.N	Y	70.86	1420.7231	13	0.8	711.3694	2	27.44	5	F5:1263	OB5932 H2 raw.raw	3.7518E5	3	3	325	337	Carbamidomethylation	C7:Carbamidomethylat ion:1000.00	PEAKS DB
R.N(+.98)AMFVPHYTLNAHTIVVALN(+.98)GR.A	Y	70.31	2439.2368	22	8.7	814.0933	3	34.13	6	F6:1683	OB5933 H2 raw.raw	4.359E6	1	1	381	402	Deamidation (NQ)	N1:Deamidation (NQ): 53.47;N20:Deamidatio n (NQ):61.12	PEAKS DB
K.FFVPPFDHQSMRE.V	Y	69.61	1635.7504	13	1.4	818.8836	2	32.55	1	F1:1469	OB5921 H2 raw.raw	1.1697E6	3	3	496	508			PEAKS DB
R.VYDEELQEGHVLVVPQNFAVAAK.A	Y	59.80	2554.3066	23	1.2	852.4438	3	33.17	1	F1:1510	OB5921 H2 raw.raw	0	0	0	415	437			PEAKS DB
R.RPFYSNAPLEIYVQQGSGYFGLIFPGC(+57.02)PSTYEEPAQEGR.R	Y	58.19	4424.0845	39	3.3	1475.7070	3	40.00	9	F9:2011	OB5936 H3A raw.raw	1.3954E6	3	3	80	118	Carbamidomethylation	C27:Carbamidomethyl ation:1000.00	PEAKS DB
R.N(+.98)AM(+15.99)FVPHYTLNAHTIVVALN(+.98)GR.A	Y	56.50	2455.2317	22	8.4	819.4247	3	34.50	5	F5:1707	OB5932 H2 raw.raw	1.9858E5	1	1	381	402	Oxidation (M)	N1:Deamidation (NQ): 27.37;M3:Oxidation (M):1000.00;N20:Dea midation (NQ):39.72	PEAKS DB
K.TDSRPSIANLAGENSIIDNLPEEVVANSYRLPR.E	Y	56.32	3609.8386	33	2.2	903.4689	4	36.12	6	F6:1817	OB5933 H2 raw.raw	2.0614E7	5	5	450	482			PEAKS DB

total 40 peptides

Peptide	Uniq	-10lgP	Mass	Length	ppm	m/z	z	RT	Fraction	Scan	Source File	Area Raw in gel	#Feature	#Feature Raw in gel	Start	End	PTM	AScore	Found By
R.FYLAGNQEQEFLR.Y	Y	55.86	1613.7837	13	1.1	807.9000	2	33.08	10	F10:1641	OB5937 H3A raw.raw	2.0837E4	1	1	196	208			PEAKS DB
V.PQNFAVAAK.A	N	51.55	944.5079	9	0.5	473.2615	2	32.25	6	F6:1586	OB5933 H2 raw.raw	2.3208E6	2	2	429	437			PEAKS DB
K.FFVPPFDHQS.M	Y	51.10	1219.5662	10	0.3	610.7905	2	33.29	1	F1:1544	OB5921 H2 raw.raw	2.015E5	1	1	496	505			PEAKS DB
R.N(+.98)AMFVPHYTLN(+.98)AHTIVVALNGR.A	Y	48.99	2439.2368	22	9.3	814.0938	3	35.76	6	F6:1773	OB5933 H2 raw.raw	7.1072E5	1	1	381	402		N1:Deamidation (NQ): 40.15;N11:Deamidation (NQ):28.29	PEAKS DB
R.VRGDEQENEGSNIFSGFAQEFLQHAFQVDR.Q	Y	46.71	3453.5974	30	2.1	864.4084	4	38.87	11	F11:1988	OB5938 H3A raw.raw	1.0226E6	2	2	226	255			PEAKS DB
K.TDSRPSIANLAGENSIIDN(+.98)LPEEVVANSYR.L	Y	46.23	3244.5847	30	8.0	1082.5442	3	36.12	6	F6:1810	OB5933 H2 raw.raw	5.3512E7	1	1	450	479		N19:Deamidation (NQ):6.49	PEAKS DB
R.SVNELDLPILGW(+31.99)LGLSAQHGTIYR.N	Y	45.20	2683.3970	24	2.9	895.4755	3	39.13	6	F6:1982	OB5933 H2 raw.raw	5.8987E5	1	1	357	380		W12:Dihydroxy:33.65	PEAKS PTM
R.AHVQVVDSNGN(+.98)RVYDEELQEGHVLVVPQNFAVAAK.A	Y	43.92	3831.9180	35	1.8	767.3923	5	32.25	6	F6:1589	OB5933 H2 raw.raw	2.7476E5	1	1	403	437		N11:Deamidation (NQ):0.00	PEAKS DB
S.VTFRQGGEENEC(+57.02)QFQR.L	Y	41.71	1983.8857	16	2.4	662.3041	3	27.37	10	F10:1301	OB5937 H3A raw.raw	3.4333E3	1	1	19	34	Carbamidomethylation	C12:Carbamidomethylation:1000.00	PEAKS DB
R.QLKNNNPFK.F	N	41.09	1101.5930	9	-0.6	551.8035	2	27.25	6	F6:1282	OB5933 H2 raw.raw	1.2465E4	2	2	487	495			PEAKS DB
R.Q(-17.03)LKNNNPFK.F	N	37.25	1084.5665	9	0.4	543.2908	2	28.36	6	F6:1357	OB5933 H2 raw.raw	1.237E4	1	1	487	495	Pyro-glu from Q	Q1:Pyro-glu from Q:1000.00	PEAKS PTM
K.AQSE(+53.92)NYEYLAFK.T	Y	36.12	1515.5968	12	0.0	758.8057	2	31.36	1	F1:1403	OB5921 H2 raw.raw	0	0	0	438	449		E4:Replacement of 2 protons by iron:37.81	PEAKS PTM
K.FFVPPFDHQSM(+15.99).R	Y	33.29	1366.6016	11	-0.2	684.3079	2	33.49	1	F1:1527	OB5921 H2 raw.raw	4.1366E4	1	1	496	506	Oxidation (M)	M11:Oxidation (M):1000.00	PEAKS DB
K.AQSENYEYLAFKTDSRPSIANLAGEN(+.98)SIIDNLPEEVVANSYR.L	Y	33.10	4688.2515	42	4.7	1173.0757	4	36.70	5	F5:1816	OB5932 H2 raw.raw	2.4176E5	1	1	438	479		N26:Deamidation (NQ):0.00	PEAKS DB
R.RPFYSNAPLEIYVQQGSGYFGLIFPGC(+57.02)PSTYEEPAQEGRR.Y	Y	31.99	4580.1860	40	2.2	1527.7394	3	37.52	10	F10:1900	OB5937 H3A raw.raw	6.9008E5	1	1	80	119	Carbamidomethylation	C27:Carbamidomethylation:1000.00	PEAKS DB
R.AHVQ(+.98)VVDSNGNRVYDEELQEGHVLVVPQNFAVAAK.A	Y	31.98	3831.9180	35	2.1	767.3925	5	32.58	5	F5:1579	OB5932 H2 raw.raw	0	0	0	403	437		Q4:Deamidation (NQ): 0.00	PEAKS DB
**total 40 peptides**																			

Q0GM57|Q0GM57_ARAHY

back to list

| Protein Coverage | Supporting Peptides |

Protein Coverage:

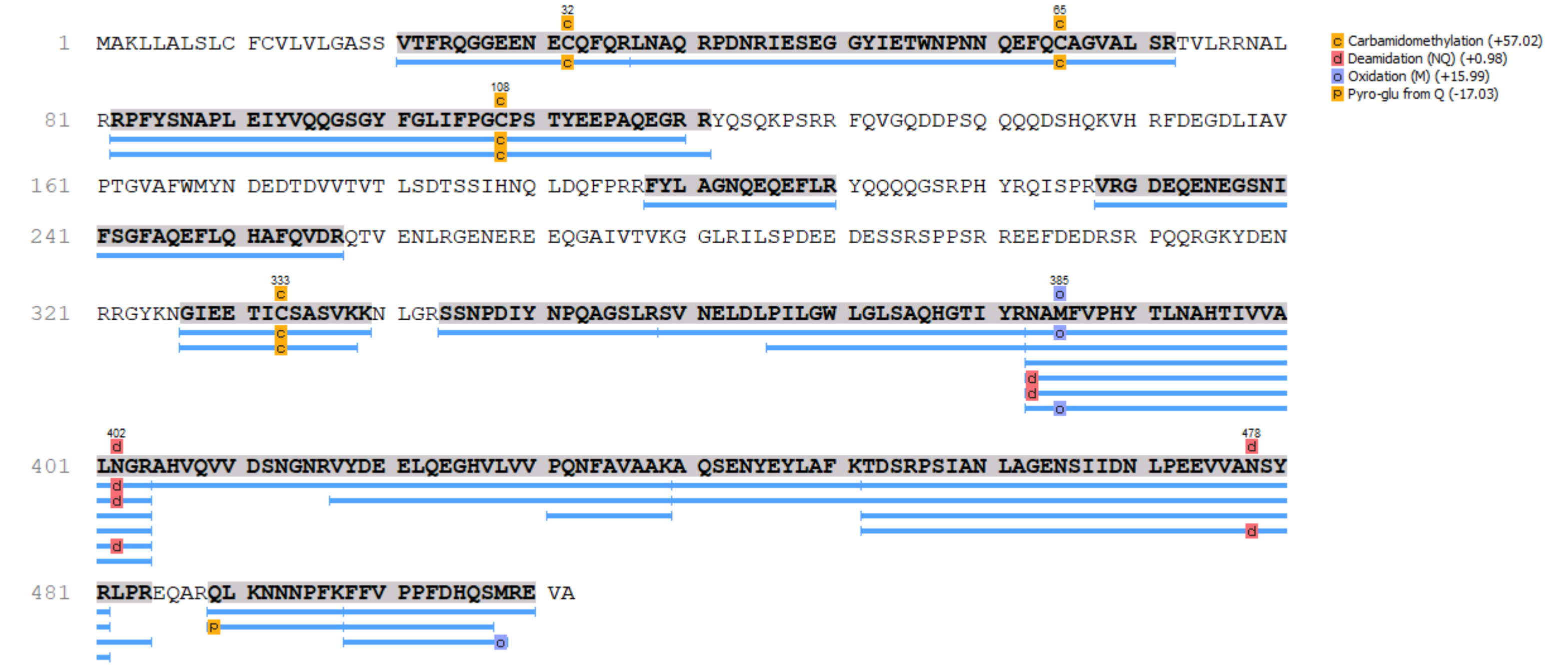

Supporting Peptides:

Peptide	Uniq	-10lgP	Mass	Length	ppm	m/z	z	RT	Fraction	Scan	Source File	Area Raw in gel	#Feature	#Feature Raw in gel	Start	End	PTM	AScore	Found By
R.NAMFVPHYTLNAHTIVVALNGR.A	Y	133.55	2437.2688	22	1.7	1219.6438	2	35.24	5	F5:1717	OB5932 H2 raw.raw	1.1969E7	6	6	383	404			PEAKS DB
R.NAMFVPHYTLNAHTIVVALN(+.98)GR.A	Y	132.37	2438.2529	22	3.2	1220.1377	2	34.12	5	F5:1671	OB5932 H2 raw.raw	5.0871E6	4	4	383	404	Deamidation (NQ)	N20:Deamidation (NQ):218.32	PEAKS DB
R.SVNELDLPILGWLGLSAQHGTIYR.N	Y	121.73	2651.4070	24	1.0	1326.7120	2	39.90	1	F1:1894	OB5921 H2 raw.raw	6.3358E7	10	10	359	382			PEAKS DB
K.TDSRPSIANLAGENSIIDNLPEEVVANSYR.L	Y	116.11	3243.6006	30	3.7	1082.2114	3	36.34	5	F5:1837	OB5932 H2 raw.raw	1.8021E7	7	7	452	481			PEAKS DB
R.N(+.98)AMFVPHYTLNAHTIVVALNGR.A	Y	114.32	2438.2529	22	2.9	1220.1373	2	34.78	5	F5:1708	OB5932 H2 raw.raw	0	0	0	383	404	Deamidation (NQ)	N1:Deamidation (NQ): 79.74	PEAKS DB
K.TDSRPSIANLAGENSIIDNLPEEVVAN(+.98)SYR.L	Y	114.06	3244.5847	30	3.8	1082.5397	3	35.65	5	F5:1760	OB5932 H2 raw.raw	0	0	0	452	481	Deamidation (NQ)	N27:Deamidation (NQ):81.59	PEAKS DB
K.AQSENYEYLAFK.T	Y	109.13	1461.6776	12	-0.5	731.8457	2	31.69	5	F5:1517	OB5932 H2 raw.raw	5.3867E6	3	3	440	451			PEAKS DB
R.SSNPDIYNPQAGSLR.S	Y	108.34	1617.7747	15	0.5	809.8950	2	28.51	1	F1:1221	OB5921 H2 raw.raw	1.8561E6	3	3	344	358			PEAKS DB
R.NAM(+15.99)FVPHYTLNAHTIVVALNGR.A	Y	101.30	2453.2637	22	1.2	818.7628	3	33.55	5	F5:1639	OB5932 H2 raw.raw	1.2362E6	2	2	383	404	Oxidation (M)	M3:Oxidation (M):1000.00	PEAKS DB
R.LNAQRPDNRIESEGGYIETWNPNNQEFQC(+57.02)AGVALSR.T	Y	95.82	4132.9409	36	3.0	1034.2456	4	33.11	7	F7:1590	OB5934 H3B raw.raw	2.0092E6	3	3	37	72	Carbamidomethylation	C29:Carbamidomethylation:1000.00	PEAKS DB
R.NAM(+15.99)FVPHYTLNAHTIVVALN(+.98)GR.A	Y	95.09	2454.2478	22	1.8	819.0913	3	33.01	5	F5:1606	OB5932 H2 raw.raw	1.7713E6	6	6	383	404	Oxidation (M); Deamidation (NQ)	M3:Oxidation (M):1000.00;N20:Deamidation (NQ):123.21	PEAKS DB

total 40 peptides

Peptide	Uniq	-10lgP	Mass	Length	ppm	m/z	z	RT	Fraction	Scan	Source File	Area Raw in gel	#Feature	#Feature Raw in gel	Start	End	PTM	AScore	Found By
L.PILGWLGLSAQHGTIYR.N	Y	88.39	1881.0261	17	2.5	941.5227	2	39.89	6	F6:2036	OB5933 H2 raw.raw	1.5582E5	1	1	366	382			PEAKS DB
K.TDSRPSIANLAGEN(+.98)SIIDNLPEEVVANSYR.L	Y	79.83	3244.5847	30	8.0	1082.5442	3	36.12	6	F6:1826	OB5933 H2 raw.raw	5.3609E7	2	2	452	481		N14:Deamidation (N Q):31.31	PEAKS DB
N.GIEETIC(+57.02)SASVK.K	Y	77.84	1292.6282	12	2.2	647.3228	2	29.07	1	F1:1261	OB5921 H2 raw.raw	1.1569E5	3	3	327	338	Carbamidomethylation	C7:Carbamidomethylation:1000.00	PEAKS DB
R.SVNE(+57.02)LDLPILGWLGLSAQHGTIYR.N	Y	74.12	2708.4285	24	1.0	1355.2229	2	40.09	5	F5:2024	OB5932 H2 raw.raw	1.9952E5	1	1	359	382		E4:Carbamidomethylation (DHKE, X@N-term):5.03	PEAKS PTM
R.NAMFVPHYTLN(+.98)AHTIVVALNGR.A	Y	72.49	2438.2529	22	3.7	813.7613	3	35.31	1	F1:1635	OB5921 H2 raw.raw	0	0	0	383	404		N11:Deamidation (N Q):33.51	PEAKS DB
R.AHVQVVDSN(+.98)GNRVYDEELQEGHVLVVPQNFAVAAK.A	Y	72.14	3831.9180	35	3.6	1278.3179	3	32.25	6	F6:1584	OB5933 H2 raw.raw	9.4081E6	2	2	405	439		N9:Deamidation (NQ): 9.40	PEAKS DB
N.GIEETIC(+57.02)SASVKK.N	Y	70.86	1420.7231	13	0.8	711.3694	2	27.44	5	F5:1263	OB5932 H2 raw.raw	3.7518E5	3	3	327	339	Carbamidomethylation	C7:Carbamidomethylation:1000.00	PEAKS DB
R.N(+.98)AMFVPHYTLNAHTIVVALN(+.98)GR.A	Y	70.31	2439.2368	22	8.7	814.0933	3	34.13	6	F6:1683	OB5933 H2 raw.raw	4.359E6	1	1	383	404	Deamidation (NQ)	N1:Deamidation (NQ): 53.47;N20:Deamidation (NQ):61.12	PEAKS DB
K.FFVPPFDHQSMRE.V	Y	69.61	1635.7504	13	1.4	818.8836	2	32.55	1	F1:1469	OB5921 H2 raw.raw	1.1697E6	3	3	498	510			PEAKS DB
R.VYDEELQEGHVLVVPQNFAVAAK.A	Y	59.80	2554.3066	23	1.2	852.4438	3	33.17	1	F1:1510	OB5921 H2 raw.raw	0	0	0	417	439			PEAKS DB
R.RPFYSNAPLEIYVQQGSGYFGLIFPGC(+57.02)PSTYEEPAQEGR.R	Y	58.19	4424.0845	39	3.3	1475.7070	3	40.00	9	F9:2011	OB5936 H3A raw.raw	1.3954E6	3	3	82	120	Carbamidomethylation	C27:Carbamidomethylation:1000.00	PEAKS DB
R.N(+.98)AM(+15.99)FVPHYTLNAHTIVVALN(+.98)GR.A	Y	56.50	2455.2317	22	8.4	819.4247	3	34.50	5	F5:1707	OB5932 H2 raw.raw	1.9858E5	1	1	383	404	Oxidation (M)	N1:Deamidation (NQ): 27.37;M3:Oxidation (M):1000.00;N20:Deamidation (NQ):39.72	PEAKS DB
K.TDSRPSIANLAGENSIIDNLPEEVVANSYRLPR.E	Y	56.32	3609.8386	33	2.2	903.4689	4	36.12	6	F6:1817	OB5933 H2 raw.raw	2.0614E7	5	5	452	484			PEAKS DB
R.FYLAGNQEQEFLR.Y	Y	55.86	1613.7837	13	1.1	807.9000	2	33.08	10	F10:1641	OB5937 H3A raw.raw	2.0837E4	1	1	198	210			PEAKS DB
V.PQNFAVAAK.A	N	51.55	944.5079	9	0.5	473.2615	2	32.25	6	F6:1586	OB5933 H2 raw.raw	2.3208E6	2	2	431	439			PEAKS DB
K.FFVPPFDHQS.M	Y	51.10	1219.5662	10	0.3	610.7905	2	33.29	1	F1:1544	OB5921 H2 raw.raw	2.015E5	1	1	498	507			PEAKS DB
R.N(+.98)AMFVPHYTLN(+.98)AHTIVVALNGR.A	Y	48.99	2439.2368	22	9.3	814.0938	3	35.76	6	F6:1773	OB5933 H2 raw.raw	7.1072E5	1	1	383	404		N1:Deamidation (NQ): 40.15;N11:Deamidation (NQ):28.29	PEAKS DB
R.VRGDEQENEGSNIFSGFAQEFLQHAFQVDR.Q	Y	46.71	3453.5974	30	2.1	864.4084	4	38.87	11	F11:1988	OB5938 H3A raw.raw	1.0226E6	2	2	228	257			PEAKS DB
K.TDSRPSIANLAGENSIIDN(+.98)LPEEVVANSYR.L	Y	46.23	3244.5847	30	8.0	1082.5442	3	36.12	6	F6:1810	OB5933 H2 raw.raw	5.3512E7	1	1	452	481		N19:Deamidation (N Q):6.49	PEAKS DB
R.SVNELDLPILGW(+31.99)LGLSAQHGTIYR.N	Y	45.20	2683.3970	24	2.9	895.4755	3	39.13	6	F6:1982	OB5933 H2 raw.raw	5.8987E5	1	1	359	382		W12:Dihydroxy:33.65	PEAKS PTM
R.AHVQVVDSNGN(+.98)RVYDEELQEGHVLVVPQNFAVAAK.A	Y	43.92	3831.9180	35	1.8	767.3923	5	32.25	6	F6:1589	OB5933 H2 raw.raw	2.7476E5	1	1	405	439		N11:Deamidation (N Q):0.00	PEAKS DB
S.VTFRQGGEENEC(+57.02)QFQR.L	Y	41.71	1983.8857	16	2.4	662.3041	3	27.37	10	F10:1301	OB5937 H3A raw.raw	3.4333E3	1	1	21	36	Carbamidomethylation	C12:Carbamidomethylation:1000.00	PEAKS DB
R.QLKNNNPFK.F	N	41.09	1101.5930	9	-0.6	551.8035	2	27.25	6	F6:1282	OB5933 H2 raw.raw	1.2465E4	2	2	489	497			PEAKS DB

total 40 peptides

Peptide	Uniq	-10lgP	Mass	Length	ppm	m/z	z	RT	Fraction	Scan	Source File	Area Raw in gel	#Feature	#Feature Raw in gel	Start	End	PTM	AScore	Found By
R.Q(-17.03)LKNNNPFK.F	N	37.25	1084.5665	9	0.4	543.2908	2	28.36	6	F6:1357	OB5933 H2 raw.raw	1.237E4	1	1	489	497	Pyro-glu from Q	Q1:Pyro-glu from Q:10 00.00	PEAKS PTM
K.AQSE(+53.92)NYEYLAFK.T	Y	36.12	1515.5968	12	0.0	758.8057	2	31.36	1	F1:1403	OB5921 H2 raw.raw	0	0	0	440	451		E4:Replacement of 2 p rotons by iron:37.81	PEAKS PTM
K.FFVPPFDHQSM(+15.99).R	Y	33.29	1366.6016	11	-0.2	684.3079	2	33.49	1	F1:1527	OB5921 H2 raw.raw	4.1366E4	1	1	498	508	Oxidation (M)	M11:Oxidation (M):10 00.00	PEAKS DB
K.AQSENYEYLAFKTDSRPSIANLAGEN(+.98)SIIDNLPEEVVANSYR.L	Y	33.10	4688.2515	42	4.7	1173.0757	4	36.70	5	F5:1816	OB5932 H2 raw.raw	2.4176E5	1	1	440	481		N26:Deamidation (N Q):0.00	PEAKS DB
R.RPFYSNAPLEIYVQQGSGYFGLIFPGC(+57.02)PSTYEEPAQEGRR.Y	Y	31.99	4580.1860	40	2.2	1527.7394	3	37.52	10	F10:1900	OB5937 H3A raw.raw	6.9008E5	1	1	82	121	Carbamidomethylation	C27:Carbamidomethyl ation:1000.00	PEAKS DB
R.AHVQ(+.98)VVDSNGNRVYDEELQEGHVLVVPQNFAVAAK.A	Y	31.98	3831.9180	35	2.1	767.3925	5	32.58	5	F5:1579	OB5932 H2 raw.raw	0	0	0	405	439		Q4:Deamidation (NQ): 0.00	PEAKS DB
**total 40 peptides**																			

A0A089ZXL7|A0A089ZXL7_ARAHY

back to list

| Protein Coverage | Supporting Peptides |

Protein Coverage:

```
  1  MKPFCVFLTF FLLLAASSKK VNSAETVSFN FNSFSEGNPA INFQGDVTVL SNGNIQLTNL NKVNSVGRVL YAMPVRIWSS
 81  ATGNVASFLT SFSFEMKDIK DYDPADGIIF FIAPEDTQIP AGSIGGGTLG VSDTKGAGYF VGVEFDTYSN SEYNDPPTDH
161  VGIDVNSVDS VKTVPWNSVS GAVVKVTVIY DSSTKTLSVA VTNDNGDITT IAQVVDLKAK LPERVKFGFS ASGSRAGRQI
241  HLIRSWSFTS TLITTTRRSI DNNEKKIMNM ASA
```

b Benzoyl (+104.03)
o Oxidation (M) (+15.99)

Supporting Peptides:

Peptide	Uniq	-10lgP	Mass	Length	ppm	m/z	z	RT	Fraction	Scan	Source File	Area Raw in gel	#Feature	#Feature Raw in gel	Start	End	PTM	AScore	Found By
R.IWSSATGNVASFLTSFSFEMK.D	N	144.16	2309.1038	21	3.7	1155.5635	2	40.56	7	F7:2025	OB5934 H3B raw.raw	1.0023E7	3	3	77	97			PEAKS DB
R.SWSFTSTLITTTR.R	N	124.66	1499.7620	13	2.5	750.8901	2	34.61	7	F7:1677	OB5934 H3B raw.raw	9.5864E6	3	3	245	257			PEAKS DB
K.TLSVAVTNDNGDITTIAQVVDLK.A	N	124.47	2386.2590	23	3.6	1194.1411	2	36.09	7	F7:1809	OB5934 H3B raw.raw	1.7984E7	4	4	196	218			PEAKS DB
R.IWSSATGNVASFLTSFSFEM(+15.99)K.D	N	115.28	2325.0986	21	1.6	1163.5585	2	39.06	7	F7:1947	OB5934 H3B raw.raw	1.6326E6	3	3	77	97	Oxidation (M)	M20:Oxidation (M):1000.00	PEAKS DB
K.DIKDYDPADGIIFFIAPEDTQIPAGSIGGGTLGVSDTK.G	N	106.58	3892.9258	38	2.7	1298.6527	3	39.11	2	F2:1787	OB5925 H3B raw.raw	1.6354E7	5	5	98	135			PEAKS DB
K.TVPWNSVSGAVVK.V	N	102.97	1342.7245	13	2.9	672.3715	2	31.21	7	F7:1468	OB5934 H3B raw.raw	1.6677E6	3	3	173	185			PEAKS DB
D.PPTDHVGIDVNSVDSVK.T	N	93.13	1777.8846	17	-0.4	889.9493	2	33.30	2	F2:1450	OB5925 H3B raw.raw	1.6838E6	7	7	156	172			PEAKS DB
**total 22 peptides**																			

Peptide	Uniq	-10lgP	Mass	Length	ppm	m/z	z	RT	Fraction	Scan	Source File	Area Raw in gel	#Feature	#Feature Raw in gel	Start	End	PTM	AScore	Found By
K.TLSVAVTNDNGDITTIAQVVDLKAK.L	N	82.35	2585.3911	25	2.1	1293.7056	2	34.66	8	F8:1679	OB5935 H3B raw.raw	8.0711E6	5	5	196	220			PEAKS DB
R.VLYAMPVR.I	N	82.17	947.5262	8	2.3	474.7715	2	30.09	7	F7:1402	OB5934 H3B raw.raw	9.4625E5	3	3	69	76			PEAKS DB
K.FGFSASGSR.A	Y	74.07	914.4246	9	-0.4	458.2194	2	27.93	2	F2:1143	OB5925 H3B raw.raw	1.3943E4	3	3	227	235			PEAKS DB
R.SWSFTSTLITTTRR.S	N	69.66	1655.8632	14	1.5	828.9401	2	32.96	8	F8:1573	OB5935 H3B raw.raw	1.7686E7	6	6	245	258			PEAKS DB
D.P(+57.02)PTDHVGIDVNSVDSVK.T	N	59.87	1834.9061	17	0.0	612.6426	3	30.08	8	F8:1405	OB5935 H3B raw.raw	0	0	0	156	172		P1:Carbamido methylation (D HKE, X@N-ter m):28.36	PEAKS PTM
R.VLYAM(+15.99)PVR.I	N	59.45	963.5212	8	-0.9	482.7674	2	29.11	7	F7:1353	OB5934 H3B raw.raw	1.9715E4	2	2	69	76	Oxidation (M)	M5:Oxidation (M):1000.00	PEAKS DB
D.PADGIIFFIAPEDTQIPAGSIGGGTLGVSDTK.G	N	44.74	3143.6025	32	4.6	1572.8158	2	38.57	8	F8:1906	OB5935 H3B raw.raw	6.2609E5	2	2	104	135			PEAKS DB
R.VKFGFSASGSR.A	Y	44.09	1141.5880	11	-0.8	571.8008	2	29.33	8	F8:1359	OB5935 H3B raw.raw	1.1968E3	1	1	225	235			PEAKS DB
K.GAGYFVGVEFDTYSNSEYNDPPTDHVGIDVNSVDSVK.T	Y	44.08	3992.7864	37	2.7	1331.9397	3	37.42	2	F2:1595	OB5925 H3B raw.raw	4.597E5	1	1	136	172			PEAKS DB
R.IWSSATGNVASFLTSFSFEMKDIKDYDPADGIIFFIAPEDTQIPAGSIGGGTLGVSDTK.G	N	40.61	6184.0190	59	6.4	1547.0220	4	42.84	7	F7:2162	OB5934 H3B raw.raw	5.5947E6	1	1	77	135			PEAKS DB
S.AETVSFNFNSFSEGNPAINFQGDVTVLSNGNIQLTNLNKVNSVGR.V	N	40.54	4812.3740	45	3.9	1605.1382	3	39.43	7	F7:2007	OB5934 H3B raw.raw	1.7542E7	3	3	24	68			PEAKS DB
T.L(+104.03)SVAVTNDNGDITTIAQVVDLK.A	N	40.14	2389.2375	22	9.4	1195.6373	2	36.47	2	F2:1654	OB5925 H3B raw.raw	1.2686E5	1	1	197	218	Benzoyl	L1:Benzoyl:12 4.10	PEAKS PTM
R.IWSSATGNVASFLTSFSFEM(+15.99)KDIKDYDPADGIIFFIAPEDTQIPAGSIGGGTLGVSDTK.G	N	37.46	6200.0137	59	5.3	1551.0189	4	41.88	7	F7:2118	OB5934 H3B raw.raw	1.2633E7	1	1	77	135	Oxidation (M)	M20:Oxidation (M):1000.00	PEAKS DB
K.VTVIYDSSTK.T	N	37.41	1111.5760	10	0.7	556.7957	2	26.75	7	F7:1237	OB5934 H3B raw.raw	6.5025E2	1	1	186	195			PEAKS DB
K.TLSVAVTNDNGDITTIAQVVDLKAKLPER.V	N	34.71	3080.6716	29	2.1	771.1768	4	36.14	8	F8:1745	OB5935 H3B raw.raw	1.01E6	1	1	196	224			PEAKS DB
**total 22 peptides**																			

sp|Q647H2|AHY3_ARAHY

back to list

| Protein Coverage | Supporting Peptides |

Protein Coverage:

```
  1 MAKLLALSVC FCFLVLGASS VTFRQQGEEN ECQFQRLNAQ RPDNCIESEG GYIETWNPNN QEFQCAGVAL SRFVLRRNAL
 81 RRPFYSNAPQ EIFIYQGSGY FGLIFPGCPG TFEEPIQGSE QFQRPSRHFQ GQDQSQRPLD THQKVHGFRE GDLIAVPHGV
161 AFWIYNDQDT DVVAISVLHT NSLHNQLDQF PRRFNLAGKQ EQEFLRYQQR SGRQSPKGEE QEQEQENEGG NVFSGFSTEF
                                                                          305
241 LSHGFQVNED IVRNLRGENE REEQGAIVTV KGGLSILVPP EWRQSYQQPG RGDKDFNNGI EETICTATVK MNIGKSTSAD
321 IYNPQAGSVR TVNELDLPIL NRLGLSAEYG SIHRDAMFVP HYNMNANSMI YALHGGAHVQ VVDCNGNRVF DEELQEGQSL
401 VVPQNFAVAA KSQSEHFLYV AFKTNSRASI SNLAGKNSYM WNLPEDVVAN SYGLQYEQAR QLKNNNPFTF LVPPQDSQMI
481 RTVA
```

C Carbamidomethylation (+57.02)
C Carbamidomethylation (DHKE, X@N-term) (+57.02)

Supporting Peptides:

Peptide	Uniq	-10lgP	Mass	Length	ppm	m/z	z	RT	Fraction	Scan	Source File	Area Raw in gel	#Feature	#Feature Raw in gel	Start	End	PTM	AScore	Found By
K.NSYMWNLPEDVVANSYGLQYEQAR.Q	Y	125.11	2846.2969	24	1.9	1424.1584	2	37.08	5	F5:1849	OB5932 H2 raw.raw	8.6777E5	4	4	437	460			PEAKS DB
K.SQSEHFLYVAFK.T	Y	117.09	1454.7194	12	2.0	728.3684	2	31.80	1	F1:1430	OB5921 H2 raw.raw	2.9709E6	6	6	412	423			PEAKS DB
R.RFNLAGKQEQEFLR.Y	Y	108.40	1734.9165	14	-0.3	579.3126	3	30.84	7	F7:1458	OB5934 H3B raw.raw	5.9257E5	5	5	193	206			PEAKS DB
R.TVNELDLPILNR.L	Y	102.21	1395.7721	12	1.8	698.8946	2	34.50	5	F5:1692	OB5932 H2 raw.raw	1.2891E7	3	3	331	342			PEAKS DB
N.GIEETIC(+57.02)TATVK.M	N	96.25	1320.6595	12	1.3	661.3379	2	29.46	5	F5:1390	OB5932 H2 raw.raw	2.7846E5	3	3	299	310	Carbamidomethylation	C7:Carbamidomethylation:1000.00	PEAKS DB
R.LGLSAEYGSIHR.D	Y	92.18	1301.6727	12	1.1	651.8444	2	28.72	6	F6:1375	OB5933 H2 raw.raw	6.496E5	5	5	343	354			PEAKS DB
K.STSADIYNPQAGSVR.T	Y	89.20	1564.7482	15	0.9	783.3821	2	27.61	5	F5:1266	OB5932 H2 raw.raw	2.6144E5	3	3	316	330			PEAKS DB
R.VFDEELQEGQSLVVPQNFAVAAK.S	Y	88.89	2517.2751	23	1.1	1259.6462	2	35.25	5	F5:1736	OB5932 H2 raw.raw	1.6786E5	1	1	389	411			PEAKS DB
K.NNNPFTFLVPPQDSQMIR.T	Y	74.25	2117.0364	18	2.5	1059.5281	2	36.56	5	F5:1814	OB5932 H2 raw.raw	0	0	0	464	481			PEAKS DB
K.NNNPFTFLVPPQDSQMIRT.V	Y	65.73	2218.0840	19	2.9	1110.0525	2	36.48	1	F1:1705	OB5921 H2 raw.raw	3.5597E5	2	2	464	482			PEAKS DB
R.FNLAGKQEQEFLR.Y	Y	65.24	1578.8154	13	-0.1	790.4149	2	30.90	8	F8:1458	OB5935 H3B raw.raw	2.0874E4	1	1	194	206			PEAKS DB
V.PQNFAVAAK.S	N	51.55	944.5079	9	0.5	473.2615	2	32.25	6	F6:1586	OB5933 H2 raw.raw	2.3208E6	2	2	403	411			PEAKS DB
R.L(+57.02)GLSAEYGSIHR.D	Y	50.42	1358.6942	12	0.7	680.3549	2	29.11	5	F5:1370	OB5932 H2 raw.raw	4.4143E4	2	2	343	354	Carbamidomethylation (DHKE, X@N-term)	L1:Carbamidomethylation (DHKE, X@ N-term):52.68	PEAKS PTM
N.G(+57.02)IEETIC(+57.02)TATVK.M	N	46.95	1377.6809	12	0.2	689.8479	2	29.76	1	F1:1306	OB5921 H2 raw.raw	0	0	0	299	310	Carbamidomethylation	G1:Carbamidomethylation (DHKE, X@ N-term):5.03;C7:Carbamidomethylation:1000.00	PEAKS PTM
total 14 peptides																			

A0A0F6VX63|A0A0F6VX63_ARAHY

back to list

| Protein Coverage | Supporting Peptides |

Protein Coverage:

1 MSTLRIPQPV PPVADDCEQL RKAFAGWGTN EDLIISILGH RNAAQRKLIR ETYFETYGED LLKALDKELS NDFERLVHLW

81 TLDPAERDAF LANEATKRWT SSNQVLMEIA CTRSSDQLLF ARKAYHARYK KSLEEDVAHH TTGEFRKLLL PLVSSYRYEG

161 DEVNLTLAKT EAKLLHEKIS KKAYSDDELI RILATRSKPQ INATLNHYKD EFGKDINKDL KADPKDVFLA LLRSTVKCLV

241 RTERYFEKAA RLAINKRGTD EGALTRVVAT RAEIDLKIIA DEYQRRSSVP LDKAVAKDTT GDYEKMLLAI LGHDDV

a Acetylation (Protein N-term) (+42.01)
c Carbamidomethylation (+57.02)
o Oxidation (M) (+15.99)

Supporting Peptides:

Peptide	Uniq	-10lgP	Mass	Length	ppm	m/z	z	RT	Fraction	Scan	Source File	Area Raw in gel	#Feature	#Feature Raw in gel	Start	End	PTM	AScore	Found By
K.AFAGWGTNEDLIISILGHR.N	Y	109.17	2069.0693	19	2.6	1035.5447	2	37.54	11	F11:1909	OB5938 H3A raw.raw	2.0436E7	6	6	23	41			PEAKS DB
K.LLLPLVSSYRYEGDEVNLTLAK.T	Y	106.30	2492.3525	22	-3.4	1247.1793	2	35.69	10	F10:1789	OB5937 H3A raw.raw	1.2701E7	5	5	148	169			PEAKS DB
R.KAFAGWGTNEDLIISILGHR.N	Y	98.47	2197.1643	20	1.8	733.3967	3	36.03	11	F11:1820	OB5938 H3A raw.raw	1.1777E6	3	3	22	41			PEAKS DB
K.DTTGDYEKM(+15.99)LLAILGHDDV	Y	89.67	2120.9937	19	2.8	1061.5071	2	37.52	10	F10:1899	OB5937 H3A raw.raw	1.7507E6	3	3	298	316	Oxidation (M)	M9:Oxidation (M):1000.00	PEAKS DB
K.DTTGDYEKMLLAILGHDDV	Y	88.82	2104.9985	19	2.6	1053.5093	2	39.41	10	F10:2009	OB5937 H3A raw.raw	2.9926E6	3	3	298	316			PEAKS DB
K.SLEEDVAHHTTGEFR.K	Y	80.77	1726.7910	15	0.2	576.6044	3	30.69	9	F9:1462	OB5936 H3A raw.raw	3.5275E5	3	3	132	146			PEAKS DB
K.KAYSDDELIR.I	Y	80.11	1208.6036	10	-0.8	605.3086	2	26.68	10	F10:1249	OB5937 H3A raw.raw	2.4558E4	3	3	182	191			PEAKS DB
R.KLLLPLVSSYRYEGDEVNLTLAK.T	Y	79.99	2620.4475	23	-2.9	874.4872	3	34.56	11	F11:1723	OB5938 H3A raw.raw	1.5288E6	3	3	147	169			PEAKS DB
R.IPQPVPPVADDC(+57.02)EQLRK.A	Y	77.43	1961.0040	17	1.9	654.6765	3	28.76	10	F10:1372	OB5937 H3A raw.raw	8.3045E5	3	3	6	22	Carbamidomethylation	C12:Carbamidomethylation:1000.00	PEAKS DB
K.ALDKELSNDFER.L	Y	76.95	1435.6942	12	0.7	718.8549	2	28.58	10	F10:1373	OB5937 H3A raw.raw	8.7943E4	3	3	64	75			PEAKS DB
K.SLEEDVAHHTTGEFRK.L	Y	75.68	1854.8860	16	0.2	619.3027	3	28.20	10	F10:1348	OB5937 H3A raw.raw	1.8944E5	2	2	132	147			PEAKS DB
K.MLLAILGHDDV	Y	74.32	1195.6271	11	1.2	598.8215	2	35.57	10	F10:1777	OB5937 H3A raw.raw	1.4911E6	3	3	306	316			PEAKS DB
R.SSDQLLFAR.K	Y	74.10	1035.5349	9	1.4	518.7755	2	30.46	10	F10:1477	OB5937 H3A raw.raw	3.9148E5	3	3	114	122			PEAKS DB
R.ETYFETYGEDLLKALDKELSNDFER.L	Y	70.40	3024.4238	25	2.4	1009.1509	3	40.92	10	F10:2098	OB5937 H3A raw.raw	9.7793E5	2	2	51	75			PEAKS DB
R.YEGDEVNLTLAK.T	Y	70.18	1350.6666	12	1.3	676.3415	2	29.90	11	F11:1464	OB5938 H3A raw.raw	1.4769E4	1	1	158	169			PEAKS DB
R.ETYFETYGEDLLK.A	Y	64.75	1606.7402	13	-5.6	804.3729	2	36.83	9	F9:1834	OB5936 H3A raw.raw	3.134E5	2	2	51	63			PEAKS DB
R.WTSSNQVLMEIAC(+57.02)TR.S	Y	64.68	1794.8394	15	1.8	898.4286	2	36.10	9	F9:1791	OB5936 H3A raw.raw	2.4676E5	1	1	99	113	Carbamidomethylation	C13:Carbamidomethylation:1000.00	PEAKS DB

total 22 peptides

Peptide	Uniq	-10lgP	Mass	Length	ppm	m/z	z	RT	Fraction	Scan	Source File	Area Raw in gel	#Feature	#Feature Raw in gel	Start	End	PTM	AScore	Found By
R.WTSSNQVLM(+15.99)EIAC(+57.02)TR.S	Y	60.30	1810.8342	15	1.3	906.4255	2	35.18	9	F9:1732	OB5936 H3A raw.raw	7.2086E4	2	2	99	113	Oxidation (M); Carbamidomethylation	M9:Oxidation (M):1000.00; C13:Carbamidomethylatio n:1000.00	PEAKS DB
R.LVHLWTLDPAERDAFLANEATKR.W	Y	60.13	2665.3975	23	-0.7	667.3562	4	33.46	10	F10:1665	OB5937 H3A raw.raw	4.8836E5	3	3	76	98			PEAKS DB
M.S(+42.01)TLRIPQPVPPVADDC(+57.02)EQLRK.A	Y	57.11	2460.2795	21	0.9	821.1012	3	33.70	9	F9:1644	OB5936 H3A raw.raw	1.4069E5	1	1	2	22	Acetylation (Protein N-term); Carbamidomethylation	S1:Acetylation (Protein N-t erm):1000.00;C16:Carbam idomethylation:1000.00	PEAKS PTM
R.VVATRAEIDLK.I	Y	39.64	1213.7030	11	-1.0	607.8582	2	27.01	11	F11:1285	OB5938 H3A raw.raw	4.1122E3	1	1	267	277			PEAKS DB
R.DAFLANEATKR.W	Y	36.75	1234.6306	11	-0.8	618.3221	2	26.52	11	F11:1255	OB5938 H3A raw.raw	0	0	0	88	98			PEAKS DB
**total 22 peptides**																			

A0A0A6ZDP1|A0A0A6ZDP1_ARAHY

back to list

| Protein Coverage | Supporting Peptides |

Protein Coverage:

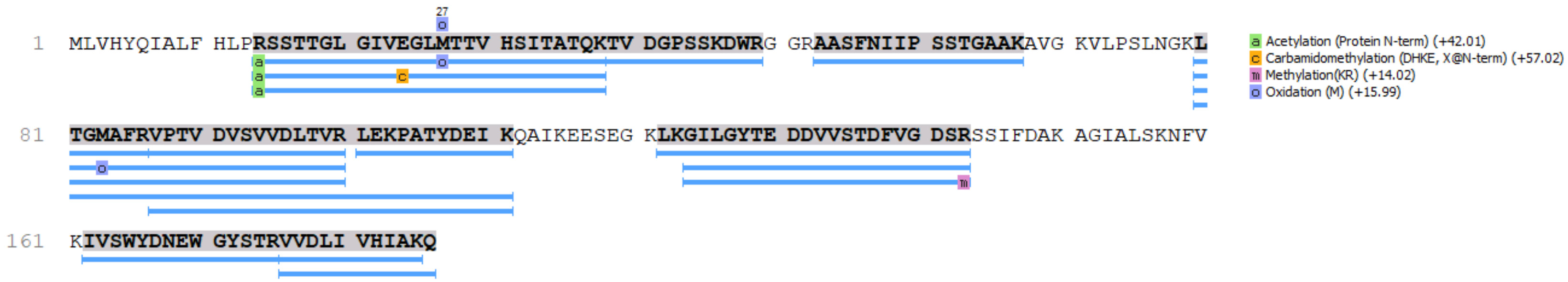

Supporting Peptides:

Peptide	Uniq	-10lgP	Mass	Length	ppm	m/z	z	RT	Fraction	Scan	Source File	Area Raw in gel	#Feature	#Feature Raw in gel	Start	End	PTM	AScore	Found By
K.GILGYTEDDVVSTDFVGDSR.S	Y	125.67	2143.9910	20	3.6	1073.0066	2	34.92	10	F10:1742	OB5937 H3A raw.raw	1.9799E7	10	10	124	143			PEAKS DB
K.IVSWYDNEWGYSTR.V	N	119.02	1774.7950	14	1.6	888.4062	2	33.83	11	F11:1692	OB5938 H3A raw.raw	2.0685E6	3	3	162	175			PEAKS DB
R.VPTVDVSVVDLTVR.L	N	109.01	1497.8402	14	0.8	749.9280	2	34.01	11	F11:1703	OB5938 H3A raw.raw	2.6396E6	3	3	87	100			PEAKS DB
R.VVDLIVHIAKQ	Y	91.25	1233.7445	11	1.5	617.8804	2	30.45	11	F11:1480	OB5938 H3A raw.raw	1.4659E6	6	6	176	186			PEAKS DB
K.LKGILGYTEDDVVSTDFVGDSR.S	Y	89.73	2385.1699	22	1.5	796.0651	3	33.83	11	F11:1688	OB5938 H3A raw.raw	1.1517E6	2	2	122	143			PEAKS DB
R.AASFNIIPSSTGAAK.A	N	84.99	1433.7513	15	2.0	717.8844	2	31.01	10	F10:1503	OB5937 H3A raw.raw	5.133E6	5	5	53	67			PEAKS DB
K.LTGMAFRVPTVDVSVVDLTVR.L	N	83.77	2274.2405	21	-2.6	1138.1245	2	36.04	10	F10:1813	OB5937 H3A raw.raw	1.7435E6	4	4	80	100			PEAKS DB
K.LTGM(+15.99)AFRVPTVDVSVVDLTVR.L	N	80.37	2290.2354	21	-5.6	764.4148	3	36.83	9	F9:1833	OB5936 H3A raw.raw	1.375E6	4	4	80	100	Oxidation (M)	M4:Oxidation (M):1000.0 0	PEAKS DB
K.G(+57.02)ILGYTEDDVVSTDFVGDSR.S	Y	75.68	2201.0125	20	1.2	1101.5148	2	34.92	10	F10:1752	OB5937 H3A raw.raw	7.1244E4	1	1	124	143		G1:Carbamidomethylatio n (DHKE, X@N-term):30. 02	PEAKS PTM
**total 19 peptides**																			

Peptide	Uniq	-10lgP	Mass	Length	ppm	m/z	z	RT	Fraction	Scan	Source File	Area Raw in gel	#Feature	#Feature Raw in gel	Start	End	PTM	AScore	Found By
P.R(+42.01)SSTTGLGIVEGLM(+15.99)TTVHSITATQK.T	Y	74.60	2645.3694	25	7.9	882.8040	3	34.56	10	F10:1722	OB5937 H3A raw.raw	1.6313E7	3	3	14	38	Acetylation (Protein N-term); Oxidation (M)	R1:Acetylation (Protein N -term):1000.00;M14:Oxi dation (M):1000.00	PEAKS PTM
P.R(+42.01)SSTTGLGIVEGLMTTVHSITATQK.T	Y	71.44	2629.3745	25	8.4	877.4728	3	36.96	10	F10:1968	OB5937 H3A raw.raw	4.5719E7	4	4	14	38	Acetylation (Protein N-term)	R1:Acetylation (Protein N -term):1000.00	PEAKS PTM
P.R(+42.01)SSTTGLGIVE(+57.02)GLMTTVHSITATQK.T	Y	56.13	2686.3960	25	5.9	896.4779	3	38.70	9	F9:1935	OB5936 H3A raw.raw	3.421E6	2	2	14	38	Acetylation (Protein N-term); Carbamidomethylation (DHKE, X@N-term)	R1:Acetylation (Protein N -term):1000.00;E11:Car bamidomethylation (DHK E, X@N-term):201.30	PEAKS PTM
R.LEKPATYDEIK.Q	N	55.56	1305.6816	11	-1.7	653.8470	2	26.49	10	F10:1243	OB5937 H3A raw.raw	0	0	0	101	111			PEAKS DB
R.VPTVDVSVVDLTVRLEKPATYDEIK.Q	N	54.73	2785.5112	25	2.7	929.5135	3	34.75	11	F11:1737	OB5938 H3A raw.raw	2.5869E6	3	3	87	111			PEAKS DB
K.GILGYTEDDVVSTDFVGDSR(+14.02).S	Y	54.39	2158.0066	20	2.8	1080.0135	2	35.93	11	F11:1812	OB5938 H3A raw.raw	0	0	0	124	143	Methylation(KR)	R20:Methylation(KR):100 0.00	PEAKS PTM
R.VVDLIVHIAK.Q	Y	50.87	1105.6859	10	0.2	553.8503	2	30.46	10	F10:1480	OB5937 H3A raw.raw	6.1004E4	3	3	176	185			PEAKS DB
K.LTGMAFRVPTVDVSVVDLTVRLEKPATYDEIK.Q	N	47.61	3561.9116	32	1.9	891.4869	4	36.22	11	F11:1821	OB5938 H3A raw.raw	1.7224E6	2	2	80	111			PEAKS DB
K.TVDGPSSKDWR.G	N	43.43	1246.5942	11	-1.7	624.3033	2	26.08	10	F10:1212	OB5937 H3A raw.raw	1.1675E4	2	2	39	49			PEAKS DB
K.LTGMAFR.V	N	35.18	794.4109	7	1.0	398.2131	2	30.69	9	F9:1449	OB5936 H3A raw.raw	9.0748E3	1	1	80	86			PEAKS DB
**total 19 peptides**																			

O82580|O82580_ARAHY

back to list

| Protein Coverage | Supporting Peptides |

Protein Coverage:

1 RQQPEENACQ FQRLNAQRPD NRIESEGGYI ETWNPNNQEF ECAGVALSRL VLRRNALRRP FYSNAPQEIF IQQGRGYFGL
81 IFPGCPRHYE EPHTQGRRSQ SQRPPRRLQG EDQSQQQRDS HQKVHRFDEG DLIAVPTGVA FWLYNDHDTD VVAVSLTDTN
161 NNDNQLDQFP RRFNLAGNTE QEFLRYQQQS RQSRRRSLPY SPYSPQSQPR QEEREFSPRG QHSRRERAGQ EEENEGGNIF
241 SGFTPEFLEQ AFQVDDRQIV QNLRGETESE EEGAIVTVRG GLRILSPDRK RRADEEEEYD EDEYEYDEED RRRGRGSRGR
357
321 GNGIEETICT ASAKKNIGRN RSPDIYNPQA GSLKTANDLN LLILRWLGPS AEYGNLYRNA LFVAHYNTNA HSIIYRLRGR
401 AHVQVVDSNG NRVYDEELQE GHVLVVPQNF AVAGKSQSEN FEYVAFKTDS RPSIANLAGE NSVIDNLPEE VVANSYGLQR
481 EQARQLKNNN PFKFFVPPSQ QSPRAVA

c Carbamidomethylation (+57.02)
d Deamidation (NQ) (+0.98)
e Ethylation (+28.03)
p Pyro-glu from Q (-17.03)
r Replacement of 2 protons by iron (+53.92)

Supporting Peptides:

Peptide	Uniq	-10lgP	Mass	Length	ppm	m/z	z	RT	Fraction	Scan	Source File	Area Raw in gel	#Feature	#Feature Raw in gel	Start	End	PTM	AScore	Found By
K.SQSENFEYVAFK.T	N	113.90	1447.6619	12	1.7	724.8394	2	31.69	5	F5:1516	OB5932 H2 raw.raw	1.027E7	4	4	436	447			PEAKS DB
**total 20 peptides**																			

Peptide	Uniq	-10lgP	Mass	Length	ppm	m/z	z	RT	Fraction	Scan	Source File	Area Raw in gel	#Feature	#Feature Raw in gel	Start	End	PTM	AScore	Found By
R.NRSPDIYNPQAGSLK.T	N	110.81	1658.8376	15	0.4	830.4265	2	27.80	5	F5:1285	OB5932 H2 raw.raw	8.3597E5	6	6	340	354			PEAKS DB
R.RPFYSNAPQEIFIQQGR.G	N	110.68	2050.0383	17	2.3	1026.0288	2	31.40	11	F11:1547	OB5938 H3A raw.raw	1.6392E6	11	11	59	75			PEAKS DB
R.VYDEELQEGHVLVVPQNFAVAGK.S	N	103.27	2540.2910	23	2.1	847.7727	3	32.93	1	F1:1498	OB5921 H2 raw.raw	1.5993E6	6	6	413	435			PEAKS DB
R.SPDIYNPQAGSLK.T	N	87.71	1388.6936	13	2.2	695.3556	2	28.73	5	F5:1375	OB5932 H2 raw.raw	1.3705E6	3	3	342	354			PEAKS DB
R.SPDIYNPQAGSLKTANDLNLLILR.W	N	81.29	2625.4126	24	0.2	876.1450	3	35.24	5	F5:1743	OB5932 H2 raw.raw	1.5604E5	1	1	342	365			PEAKS DB
R.AHVQVVDSNGNRVYDEELQEGHVLVVPQNFAVAGK.S	N	72.39	3816.9182	35	3.0	1273.3171	3	32.25	5	F5:1561	OB5932 H2 raw.raw	3.9867E6	2	2	401	435			PEAKS DB
R.NALRRPFYSNAPQEIFIQQGR.G	N	68.46	2504.3037	21	1.1	835.7761	3	31.58	10	F10:1548	OB5937 H3A raw.raw	9.3975E5	4	4	55	75			PEAKS DB
K.TANDLNLLILR.W	N	67.99	1254.7296	11	0.0	628.3721	2	35.65	4	F4:1764	OB5931 H6 raw.raw	1.549E7	6	6	355	365			PEAKS DB
V.PQNFAVAGK.S	N	67.74	930.4923	9	0.8	466.2538	2	32.44	5	F5:1562	OB5932 H2 raw.raw	4.3278E6	2	2	427	435			PEAKS DB
K.FFVPPSQQSPR.A	N	67.19	1288.6564	11	0.0	645.3354	2	28.51	1	F1:1229	OB5921 H2 raw.raw	9.0621E4	3	3	494	504			PEAKS DB
R.S(+57.02)PDIYNPQAGSLK.T	N	65.06	1445.7150	13	5.4	723.8687	2	28.54	6	F6:1379	OB5933 H2 raw.raw	8.1843E3	1	1	342	354		S1:Carbamidomethylation (DHKE, X@N-term):30.83	PEAKS PTM
R.W(+28.03)LGPSAEYGNLYR.N	Y	59.99	1552.7673	13	1.5	777.3921	2	35.79	5	F5:1772	OB5932 H2 raw.raw	1.4756E6	3	3	366	378	Ethylation	W1:Ethylation:51.01	PEAKS PTM
K.TAN(+.98)DLNLLILR.W	N	54.07	1255.7136	11	-1.0	628.8635	2	35.40	6	F6:1771	OB5933 H2 raw.raw	0	0	0	355	365	Deamidation (NQ)	N3:Deamidation (NQ):68.31	PEAKS DB
K.TAND(+53.92)LNLLILR.W	N	47.26	1308.6488	11	-1.1	655.3309	2	34.62	1	F1:1597	OB5921 H2 raw.raw	1.4902E5	1	1	355	365	Replacement of 2 protons by iron	D4:Replacement of 2 protons by iron:128.79	PEAKS PTM
R.SLPYSPY(+31.99)SPQSQPR.Q	N	46.01	1637.7686	14	1.0	819.8923	2	29.76	9	F9:1401	OB5936 H3A raw.raw	3.8534E4	1	1	197	210		Y7:Dihydroxy:0.00	PEAKS PTM
R.QQPEENAC(+57.02)QFQR.L	N	45.72	1533.6630	12	1.7	767.8401	2	25.17	10	F10:1164	OB5937 H3A raw.raw	6.6881E3	2	2	2	13	Carbamidomethylation	C8:Carbamidomethylation:1000.00	PEAKS DB
K.FFVPPSQQSPRA.V	N	44.09	1359.6935	12	3.4	680.8563	2	29.07	1	F1:1270	OB5921 H2 raw.raw	1.1159E4	1	1	494	505			PEAKS DB
R.QLKNNNPFK.F	N	41.09	1101.5930	9	-0.6	551.8035	2	27.25	6	F6:1282	OB5933 H2 raw.raw	1.2465E4	2	2	485	493			PEAKS DB
R.Q(-17.03)LKNNNPFK.F	N	37.25	1084.5665	9	0.4	543.2908	2	28.36	6	F6:1357	OB5933 H2 raw.raw	1.237E4	1	1	485	493	Pyro-glu from Q	Q1:Pyro-glu from Q:100.00	PEAKS PTM
total 20 peptides																			

A5Z1R0|A5Z1R0_ARAHY

back to list

| Protein Coverage | Supporting Peptides |

Protein Coverage:

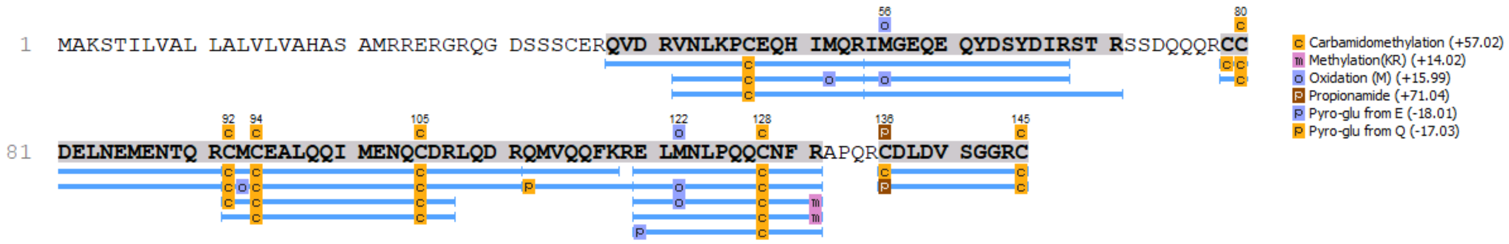

Supporting Peptides:

Peptide	Uniq	-10lgP	Mass	Length	ppm	m/z	z	RT	Fraction	Scan	Source File	Area Raw in gel	#Feature	#Feature Raw in gel	Start	End	PTM	AScore	Found By
total 30 peptides																			

Peptide	Uniq	-10lgP	Mass	Length	ppm	m/z	z	RT	Fraction	Scan	Source File	Area Raw in gel	#Feature	#Feature Raw in gel	Start	End	PTM	AScore	Found By
R.IM(+15.99)GEQEQYDSYDIR.S	Y	134.01	1761.7516	14	2.3	881.8851	2	29.04	3	F3:1368	OB5930 H6 raw.raw	1.9274E6	3	3	55	68	Oxidation (M)	M2:Oxidation (M):1000.00	PEAKS DB
R.IMGEQEQYDSYDIR.S	Y	115.59	1745.7566	14	3.8	873.8889	2	30.72	4	F4:1483	OB5931 H6 raw.raw	8.9499E6	22	22	55	68			PEAKS DB
R.ELMNLPQQC(+57.02)NFR.A	Y	114.35	1548.7177	12	2.7	775.3682	2	31.64	3	F3:1514	OB5930 H6 raw.raw	6.3372E6	11	11	120	131	Carbamidomethylation	C9:Carbamidomethylation:1000.00	PEAKS DB
R.C(+57.02)MC(+57.02)EALQQIMENQC(+57.02)DR.L	Y	106.38	2084.8206	16	1.8	1043.4194	2	34.38	3	F3:1680	OB5930 H6 raw.raw	4.3323E4	3	3	92	107	Carbamidomethylation	C1:Carbamidomethylation:1000.00;C3:Carbamidomethylation:1000.00;C14:Carbamidomethylation:1000.00	PEAKS DB
R.ELM(+15.99)NLPQQC(+57.02)NFR.A	Y	98.80	1564.7126	12	1.2	783.3645	2	29.23	3	F3:1369	OB5930 H6 raw.raw	8.8493E5	2	2	120	131	Oxidation (M); Carbamidomethylation	M3:Oxidation (M):1000.00;C9:Carbamidomethylation:1000.00	PEAKS DB
R.C(+57.02)C(+57.02)DELNEMENTQR.C	Y	87.55	1697.6444	13	0.4	849.8298	2	28.32	4	F4:1334	OB5931 H6 raw.raw	1.0555E5	2	2	79	91	Carbamidomethylation	C1:Carbamidomethylation:1000.00;C2:Carbamidomethylation:1000.00	PEAKS DB
R.VNLKPC(+57.02)EQHIMQR.I	Y	80.09	1651.8286	13	0.7	551.6172	3	31.53	4	F4:1544	OB5931 H6 raw.raw	1.0842E6	5	5	42	54	Carbamidomethylation	C6:Carbamidomethylation:1000.00	PEAKS DB
R.C(+57.02)DLDVSGGRC(+57.02)	Y	79.02	1137.4543	10	0.3	569.7346	2	25.66	4	F4:1179	OB5931 H6 raw.raw	1.5785E4	2	2	136	145	Carbamidomethylation	C1:Carbamidomethylation:1000.00;C10:Carbamidomethylation:1000.00	PEAKS DB
R.C(+57.02)C(+57.02)DELNEMENTQ(+.98)R.C	Y	74.70	1698.6284	13	1.2	850.3225	2	28.66	3	F3:1327	OB5930 H6 raw.raw	1.5687E5	2	2	79	91	Carbamidomethylation	C1:Carbamidomethylation:1000.00;C2:Carbamidomethylation:1000.00;Q12:Deamidation (NQ):36.05	PEAKS DB
R.C(+57.02)MC(+57.02)EALQQIMENQC(+57.02)DRLQDR.Q	Y	71.18	2597.0913	20	1.7	866.7058	3	35.32	3	F3:1730	OB5930 H6 raw.raw	2.1123E6	5	5	92	111	Carbamidomethylation	C1:Carbamidomethylation:1000.00;C3:Carbamidomethylation:1000.00;C14:Carbamidomethylation:1000.00	PEAKS DB
R.C(+71.04)C(+57.02)DELNEMENTQR.C	Y	68.78	1711.6600	13	0.6	856.8378	2	28.19	3	F3:1295	OB5930 H6 raw.raw	1.0694E5	2	2	79	91	Carbamidomethylation	C1:Propionamide:10.11;C2:Carbamidomethylation:1000.00	PEAKS PTM
R.C(+71.04)MC(+57.02)EALQQIMENQC(+57.02)DR.L	Y	59.70	2098.8362	16	3.5	1050.4291	2	34.40	3	F3:1687	OB5930 H6 raw.raw	0	0	0	92	107	Carbamidomethylation	C1:Propionamide:22.85;C3:Carbamidomethylation:1000.00;C14:Carbamidomethylation:1000.00	PEAKS PTM
R.C(+71.04)DLDVSGGRC(+57.02)	Y	58.84	1151.4700	10	-1.5	576.7414	2	25.98	4	F4:1198	OB5931 H6 raw.raw	2.0402E3	1	1	136	145	Propionamide; Carbamidomethylation	C1:Propionamide:106.68;C10:Carbamidomethylation:1000.00	PEAKS PTM
R.Q(-17.03)MVQQFKR.E	Y	54.35	1046.5331	8	-1.0	524.2733	2	28.52	4	F4:1323	OB5931 H6 raw.raw	1.3333E5	2	2	112	119	Pyro-glu from Q	Q1:Pyro-glu from Q:1000.00	PEAKS PTM
R.C(+57.02)(+28.03)C(+57.02)DELNEMENTQR.C	Y	53.62	1725.6757	13	0.1	863.8452	2	28.26	3	F3:1306	OB5930 H6 raw.raw	1.2726E4	2	2	79	91	Carbamidomethylation	C1:Carbamidomethylation:1000.00;C1:Ethylation:13.67;C2:Carbamidomethylation:1000.00	PEAKS PTM
R.IMGEQE(+21.98)QYDSYDIR.S	Y	52.44	1767.7385	14	1.6	884.8780	2	30.42	3	F3:1450	OB5930 H6 raw.raw	0	0	0	55	68		E6:Sodium adduct:12.81	PEAKS PTM
R.ELMNLPQQC(+57.02)NFR(+14.02).A	Y	46.74	1562.7334	12	1.5	782.3751	2	32.08	4	F4:1543	OB5931 H6 raw.raw	2.0814E5	2	2	120	131	Carbamidomethylation; Methylation(KR)	C9:Carbamidomethylation:1000.00;R12:Methylation(KR):1000.00	PEAKS PTM
R.IMGEQEQ(+.98)YDSYDIR.S	Y	46.05	1746.7406	14	5.7	874.3826	2	40.55	4	F4:2044	OB5931 H6 raw.raw	0	0	0	55	68		Q7:Deamidation (NQ):0.00	PEAKS DB
**total 30 peptides**																			

Peptide	Uniq	-10lgP	Mass	Length	ppm	m/z	z	RT	Fraction	Scan	Source File	Area Raw in gel	#Feature	#Feature Raw in gel	Start	End	PTM	AScore	Found By
R.QMVQQFK.R	Y	45.99	907.4586	7	-0.4	454.7364	2	26.12	3	F3:1199	OB5930 H6 raw.raw	9.8252E3	2	2	112	118			PEAKS DB
R.E(-18.01)LMNLPQQC(+57.02)NFR.A	Y	44.04	1530.7072	12	1.2	766.3618	2	35.00	3	F3:1722	OB5930 H6 raw.raw	0	0	0	120	131	Pyro-glu from E; Carbamidomethylation	E1:Pyro-glu from E:1000.00;C9:Carbamidomethylation:1000.00	PEAKS PTM
R.IMGEQEQYDSY(-18.01)DIR.S	Y	41.86	1727.7461	14	2.3	864.8823	2	31.01	3	F3:1485	OB5930 H6 raw.raw	0	0	0	55	68		Y11:Dehydration:0.00	PEAKS PTM
R.C(+57.02)M(+15.99)C(+57.02)EALQQIMENQC(+57.02)DR.L	Y	41.40	2100.8157	16	6.8	1051.4222	2	32.37	4	F4:1570	OB5931 H6 raw.raw	0	0	0	92	107	Carbamidomethylation	C1:Carbamidomethylation:1000.00;M2:Oxidation (M):43.13;C3:Carbamidomethylation:1000.00;C14:Carbamidomethylation:1000.00	PEAKS DB
R.VNLKPC(+57.02)EQHIM(+15.99)QR.I	Y	41.03	1667.8236	13	1.8	556.9495	3	28.32	4	F4:1320	OB5931 H6 raw.raw	3.1777E4	2	2	42	54	Carbamidomethylation; Oxidation (M)	C6:Carbamidomethylation:1000.00;M11:Oxidation (M):1000.00	PEAKS DB
R.IMGEQEQYDSYDIRSTR.S	Y	40.39	2089.9375	17	1.4	697.6541	3	30.22	4	F4:1428	OB5931 H6 raw.raw	2.3182E4	1	1	55	71			PEAKS DB
R.C(+57.02)M(+15.99)C(+57.02)EALQQIMENQC(+57.02)DRLQDR.Q	Y	38.06	2613.0862	20	-1.3	872.0349	3	33.74	4	F4:1642	OB5931 H6 raw.raw	2.8827E5	2	2	92	111	Carbamidomethylation; Oxidation (M)	C1:Carbamidomethylation:1000.00;M2:Oxidation (M):55.71;C3:Carbamidomethylation:1000.00;C14:Carbamidomethylation:1000.00	PEAKS DB
R.QVDRVNLKPC(+57.02)EQHIMQR.I	Y	36.67	2150.0837	17	-0.7	538.5278	4	33.49	3	F3:1625	OB5930 H6 raw.raw	2.7618E4	1	1	38	54	Carbamidomethylation	C10:Carbamidomethylation:1000.00	PEAKS DB
R.ELM(+15.99)NLPQQC(+57.02)NFR(+14.02).A	Y	35.50	1578.7283	12	0.4	790.3717	2	29.78	4	F4:1404	OB5931 H6 raw.raw	8.1416E3	1	1	120	131	Oxidation (M); Carbamidomethylation; Methylation(KR)	M3:Oxidation (M):1000.00;C9:Carbamidomethylation:1000.00;R12:Methylation(KR):1000.00	PEAKS PTM
R.C(+57.02)MC(+57.02)EALQ(+.98)QIMENQC(+57.02)DRLQDR.Q	Y	34.07	2598.0754	20	2.7	867.0348	3	39.74	3	F3:1998	OB5930 H6 raw.raw	0	0	0	92	111	Carbamidomethylation	C1:Carbamidomethylation:1000.00;C3:Carbamidomethylation:1000.00;Q7:Deamidation (NQ):0.00;C14:Carbamidomethylation:1000.00	PEAKS DB
R.IMGE(+21.98)QEQYDSYDIR.S	Y	33.97	1767.7385	14	0.1	884.8766	2	30.72	4	F4:1472	OB5931 H6 raw.raw	1.8041E4	1	1	55	68		E4:Sodium adduct:17.01	PEAKS PTM
R.IMGEQEQYD(-18.01)SYDIR.S	Y	33.03	1727.7461	14	-0.1	864.8802	2	31.20	4	F4:1499	OB5931 H6 raw.raw	0	0	0	55	68		D9:Dehydration:8.69	PEAKS PTM
**total 30 peptides**																			

sp|Q647G9|CONG_ARAHY

back to list

| Protein Coverage | Supporting Peptides |

Protein Coverage:

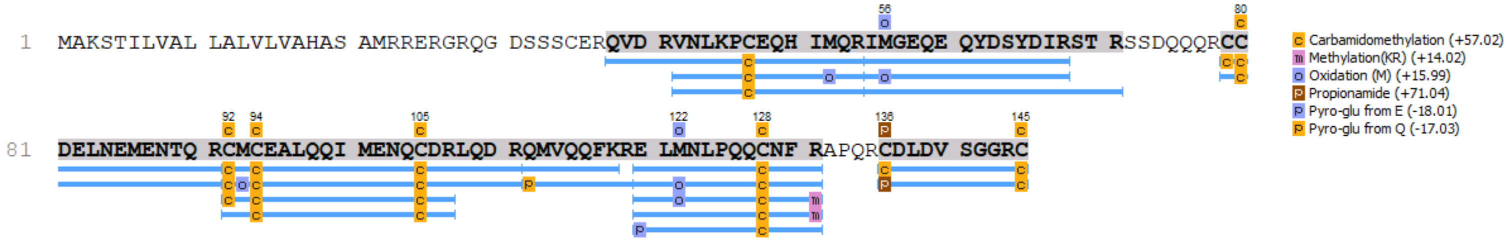

Supporting Peptides:

Peptide	Uniq	-10lgP	Mass	Length	ppm	m/z	z	RT	Fraction	Scan	Source File	Area Raw in gel	#Feature	#Feature Raw in gel	Start	End	PTM	AScore	Found By
R.IM(+15.99)GEQEQYDSYDIR.S	Y	134.01	1761.7516	14	2.3	881.8851	2	29.04	3	F3:1368	OB5930 H6 raw.raw	1.9274E6	3	3	55	68	Oxidation (M)	M2:Oxidation (M):1000.00	PEAKS DB
R.IMGEQEQYDSYDIR.S	Y	115.59	1745.7566	14	3.8	873.8889	2	30.72	4	F4:1483	OB5931 H6 raw.raw	8.9499E6	22	22	55	68			PEAKS DB
R.ELMNLPQQC(+57.02)NFR.A	Y	114.35	1548.7177	12	2.7	775.3682	2	31.64	3	F3:1514	OB5930 H6 raw.raw	6.3372E6	11	11	120	131	Carbamidomethylation	C9:Carbamidomethylation:1000.00	PEAKS DB
R.C(+57.02)MC(+57.02)EALQQIMENQC(+57.02)DR.L	Y	106.38	2084.8206	16	1.8	1043.4194	2	34.38	3	F3:1680	OB5930 H6 raw.raw	4.3323E4	3	3	92	107	Carbamidomethylation	C1:Carbamidomethylation:1000.00;C3:Carbamidomethylation:1000.00;C14:Carbamidomethylation:1000.00	PEAKS DB
R.ELM(+15.99)NLPQQC(+57.02)NFR.A	Y	98.80	1564.7126	12	1.2	783.3645	2	29.23	3	F3:1369	OB5930 H6 raw.raw	8.8493E5	2	2	120	131	Oxidation (M); Carbamidomethylation	M3:Oxidation (M):1000.00;C9:Carbamidomethylation:1000.00	PEAKS DB
R.C(+57.02)C(+57.02)DELNEMENTQR.C	Y	87.55	1697.6444	13	0.4	849.8298	2	28.32	4	F4:1334	OB5931 H6 raw.raw	1.0555E5	2	2	79	91	Carbamidomethylation	C1:Carbamidomethylation:1000.00;C2:Carbamidomethylation:1000.00	PEAKS DB
R.VNLKPC(+57.02)EQHIMQR.I	Y	80.09	1651.8286	13	0.7	551.6172	3	31.53	4	F4:1544	OB5931 H6 raw.raw	1.0842E6	5	5	42	54	Carbamidomethylation	C6:Carbamidomethylation:1000.00	PEAKS DB
R.C(+57.02)DLDVSGGRC(+57.02)	Y	79.02	1137.4543	10	0.3	569.7346	2	25.66	4	F4:1179	OB5931 H6 raw.raw	1.5785E4	2	2	136	145	Carbamidomethylation	C1:Carbamidomethylation:1000.00;C10:Carbamidomethylation:1000.00	PEAKS DB
R.C(+57.02)C(+57.02)DELNEMENTQ(+.98)R.C	Y	74.70	1698.6284	13	1.2	850.3225	2	28.66	3	F3:1327	OB5930 H6 raw.raw	1.5687E5	2	2	79	91	Carbamidomethylation	C1:Carbamidomethylation:1000.00;C2:Carbamidomethylation:1000.00;Q12:Deamidation (NQ):36.05	PEAKS DB
R.C(+57.02)MC(+57.02)EALQQIMENQC(+57.02)DRLQDR.Q	Y	71.18	2597.0913	20	1.7	866.7058	3	35.32	3	F3:1730	OB5930 H6 raw.raw	2.1123E6	5	5	92	111	Carbamidomethylation	C1:Carbamidomethylation:1000.00;C3:Carbamidomethylation:1000.00;C14:Carbamidomethylation:1000.00	PEAKS DB
R.C(+71.04)C(+57.02)DELNEMENTQR.C	Y	68.78	1711.6600	13	0.6	856.8378	2	28.19	3	F3:1295	OB5930 H6 raw.raw	1.0694E5	2	2	79	91	Carbamidomethylation	C1:Propionamide:10.11;C2:Carbamidomethylation:1000.00	PEAKS PTM
R.C(+71.04)MC(+57.02)EALQQIMENQC(+57.02)DR.L	Y	59.70	2098.8362	16	3.5	1050.4291	2	34.40	3	F3:1687	OB5930 H6 raw.raw	0	0	0	92	107	Carbamidomethylation	C1:Propionamide:22.85;C3:Carbamidomethylation:1000.00;C14:Carbamidomethylation:1000.00	PEAKS PTM
R.C(+71.04)DLDVSGGRC(+57.02)	Y	58.84	1151.4700	10	-1.5	576.7414	2	25.98	4	F4:1198	OB5931 H6 raw.raw	2.0402E3	1	1	136	145	Propionamide; Carbamidomethylation	C1:Propionamide:106.68;C10:Carbamidomethylation:1000.00	PEAKS PTM
R.Q(-17.03)MVQQFKR.E	Y	54.35	1046.5331	8	-1.0	524.2733	2	28.52	4	F4:1323	OB5931 H6 raw.raw	1.3333E5	2	2	112	119	Pyro-glu from Q	Q1:Pyro-glu from Q: 1000.00	PEAKS PTM
R.C(+57.02)(+28.03)C(+57.02)DELNEMENTQR.C	Y	53.62	1725.6757	13	0.1	863.8452	2	28.26	3	F3:1306	OB5930 H6 raw.raw	1.2726E4	2	2	79	91	Carbamidomethylation	C1:Carbamidomethylation:1000.00;C1:Ethylation:13.67;C2:Carbamidomethylation:1000.00	PEAKS PTM
R.IMGEQE(+21.98)QYDSYDIR.S	Y	52.44	1767.7385	14	1.6	884.8780	2	30.42	3	F3:1450	OB5930 H6 raw.raw	0	0	0	55	68		E6:Sodium adduct:12.81	PEAKS PTM
R.ELMNLPQQC(+57.02)NFR(+14.02).A	Y	46.74	1562.7334	12	1.5	782.3751	2	32.08	4	F4:1543	OB5931 H6 raw.raw	2.0814E5	2	2	120	131	Carbamidomethylation; Methylation(KR)	C9:Carbamidomethylation:1000.00;R12:Methylation(KR):1000.00	PEAKS PTM
**total 30 peptides**																			

Peptide	Uniq	-10lgP	Mass	Length	ppm	m/z	z	RT	Fraction	Scan	Source File	Area Raw in gel	#Feature	#Feature Raw in gel	Start	End	PTM	AScore	Found By
R.IMGEQEQ(+.98)YDSYDIR.S	Y	46.05	1746.7406	14	5.7	874.3826	2	40.55	4	F4:2044	OB5931 H6 raw.raw	0	0	0	55	68		Q7:Deamidation (N Q):0.00	PEAKS DB
R.QMVQQFK.R	Y	45.99	907.4586	7	-0.4	454.7364	2	26.12	3	F3:1199	OB5930 H6 raw.raw	9.8252E3	2	2	112	118			PEAKS DB
R.E(-18.01)LMNLPQQC(+57.02)NFR.A	Y	44.04	1530.7072	12	1.2	766.3618	2	35.00	3	F3:1722	OB5930 H6 raw.raw	0	0	0	120	131	Pyro-glu from E; Carbamidomethylation	E1:Pyro-glu from E:1000.00;C9:Carbamidomethylation:1000.00	PEAKS PTM
R.IMGEQEQYDSY(-18.01)DIR.S	Y	41.86	1727.7461	14	2.3	864.8823	2	31.01	3	F3:1485	OB5930 H6 raw.raw	0	0	0	55	68		Y11:Dehydration:0.00	PEAKS PTM
R.C(+57.02)M(+15.99)C(+57.02)EALQQIMENQC(+57.02)DR.L	Y	41.40	2100.8157	16	6.8	1051.4222	2	32.37	4	F4:1570	OB5931 H6 raw.raw	0	0	0	92	107	Carbamidomethylation	C1:Carbamidomethylation:1000.00;M2:Oxidation (M):43.13;C3:Carbamidomethylation:1000.00;C14:Carbamidomethylation:1000.00	PEAKS DB
R.VNLKPC(+57.02)EQHIM(+15.99)QR.I	Y	41.03	1667.8236	13	1.8	556.9495	3	28.32	4	F4:1320	OB5931 H6 raw.raw	3.1777E4	2	2	42	54	Carbamidomethylation; Oxidation (M)	C6:Carbamidomethylation:1000.00;M11:Oxidation (M):1000.00	PEAKS DB
R.IMGEQEQYDSYDIRSTR.S	Y	40.39	2089.9375	17	1.4	697.6541	3	30.22	4	F4:1428	OB5931 H6 raw.raw	2.3182E4	1	1	55	71			PEAKS DB
R.C(+57.02)M(+15.99)C(+57.02)EALQQIMENQC(+57.02)DRLQDR.Q	Y	38.06	2613.0862	20	-1.3	872.0349	3	33.74	4	F4:1642	OB5931 H6 raw.raw	2.8827E5	2	2	92	111	Carbamidomethylation; Oxidation (M)	C1:Carbamidomethylation:1000.00;M2:Oxidation (M):55.71;C3:Carbamidomethylation:1000.00;C14:Carbamidomethylation:1000.00	PEAKS DB
R.QVDRVNLKPC(+57.02)EQHIMQR.I	Y	36.67	2150.0837	17	-0.7	538.5278	4	33.49	3	F3:1625	OB5930 H6 raw.raw	2.7618E4	1	1	38	54	Carbamidomethylation	C10:Carbamidomethylation:1000.00	PEAKS DB
R.ELM(+15.99)NLPQQC(+57.02)NFR(+14.02).A	Y	35.50	1578.7283	12	0.4	790.3717	2	29.78	4	F4:1404	OB5931 H6 raw.raw	8.1416E3	1	1	120	131	Oxidation (M); Carbamidomethylation; Methylation(KR)	M3:Oxidation (M):1000.00;C9:Carbamidomethylation:1000.00;R12:Methylation(KR):1000.00	PEAKS PTM
R.C(+57.02)MC(+57.02)EALQ(+.98)QIMENQC(+57.02)DRLQDR.Q	Y	34.07	2598.0754	20	2.7	867.0348	3	39.74	3	F3:1998	OB5930 H6 raw.raw	0	0	0	92	111	Carbamidomethylation	C1:Carbamidomethylation:1000.00;C3:Carbamidomethylation:1000.00;Q7:Deamidation (NQ):0.00;C14:Carbamidomethylation:1000.00	PEAKS DB
R.IMGE(+21.98)QEQYDSYDIR.S	Y	33.97	1767.7385	14	0.1	884.8766	2	30.72	4	F4:1472	OB5931 H6 raw.raw	1.8041E4	1	1	55	68		E4:Sodium adduct:17.01	PEAKS PTM
R.IMGEQEQYD(-18.01)SYDIR.S	Y	33.03	1727.7461	14	-0.1	864.8802	2	31.20	4	F4:1499	OB5931 H6 raw.raw	0	0	0	55	68		D9:Dehydration:8.69	PEAKS PTM
**total 30 peptides**																			

A1E2B1|A1E2B1_ARAHY

back to list

| Protein Coverage | Supporting Peptides |

Protein Coverage:

66 C

1 MSSSLLFLTL AYLVLIERLT CHAQSHGDRI GECRLEHLSV MEPTKRVESE GGVAEFWDDK SQQLQCIGVT LIRYTIRPKG

C Carbamidomethylation (+57.02)
d Deamidation (NQ) (+0.98)
e Ethyl amino (+27.05)
o Oxidation (M) (+15.99)

109 C

81 LLLPFYTNAP RIHYILQGKG VMEIVVTGCR AMYRSSTKRG MMSSYSDEHQ KIQSIEQNDA VAVPSSSVHW IYNTGHSDLV

186 d 188 d 196 d

161 LFSLVDVANA DNQLDPTFRN FLLSGNGNGK EGEESNNNWF IKKKQREAQE GNVFSGLALE TLIGSFNVQR EIAEKVQGLK

241 DWRGSIILVK EGLDWLSPKE EEEEEGLEKR KKVMLMG

Supporting Peptides:

Peptide	Uniq	-10lgP	Mass	Length	ppm	m/z	z	RT	Fraction	Scan	Source File	Area Raw in gel	#Feature	#Feature Raw in gel	Start	End	PTM	AScore	Found By
R.VESEGGVAEFWDDKSQQLQC(+57.02)IGVTLIR.Y	Y	118.02	3063.4971	27	4.1	1022.1771	3	36.71	5	F5:1824	OB5932 H2 raw.raw	9.8845E6	5	5	47	73	Carbamidomethylation	C20:Carbamidomethylation:1000.00	PEAKS DB
K.GLLLPFYTNAPR.I	Y	103.14	1360.7502	12	3.3	681.3846	2	35.60	5	F5:1749	OB5932 H2 raw.raw	3.6865E6	3	3	80	91			PEAKS DB
K.GVMEIVVTGC(+57.02)R.A	Y	101.50	1219.6053	11	1.7	610.8110	2	31.32	5	F5:1506	OB5932 H2 raw.raw	9.381E5	3	3	100	110	Carbamidomethylation	C10:Carbamidomethylation:1000.00	PEAKS DB
K.RVESEGGVAEFWDDKSQQLQC(+57.02)IGVTLIR.Y	Y	93.84	3219.5981	28	2.3	1074.2091	3	35.76	6	F6:1794	OB5933 H2 raw.raw	2.9779E6	4	4	46	73	Carbamidomethylation	C21:Carbamidomethylation:1000.00	PEAKS DB
K.GVM(+15.99)EIVVTGC(+57.02)R.A	Y	80.01	1235.6002	11	2.0	618.8086	2	28.16	5	F5:1326	OB5932 H2 raw.raw	5.5468E3	1	1	100	110	Oxidation (M); Carbamidomethylation	M3:Oxidation (M):1000.00;C10:Carbamidomethylation:1000.00	PEAKS DB
R.NFLLSGN(+.98)GN(+.98)GKEGEESN.N	Y	75.03	1766.7594	17	0.2	884.3871	2	30.77	5	F5:1501	OB5932 H2 raw.raw	8.8395E4	1	1	180	196	Deamidation (NQ)	N7:Deamidation (NQ):96.06;N9:Deamidation (NQ):72.89	PEAKS DB
R.IHYILQGK.G	Y	74.99	970.5600	8	0.7	486.2876	2	27.62	5	F5:1288	OB5932 H2 raw.raw	3.1727E4	2	2	92	99			PEAKS DB
R.LEHLSVMEPTK.R	Y	72.49	1282.6591	11	-0.9	642.3362	2	27.79	6	F6:1323	OB5933 H2 raw.raw	1.5006E4	2	2	35	45			PEAKS DB
R.NFLLSGNGN(+.98)GKEGEESN.N	Y	69.37	1765.7754	17	-0.1	883.8949	2	29.97	1	F1:1326	OB5921 H2 raw.raw	8.6902E4	2	2	180	196		N9:Deamidation (NQ):39.76	PEAKS DB
R.IGEC(+57.02)RLEHLSVMEPTKR.V	Y	63.30	2054.0400	17	-1.6	514.5165	4	30.02	5	F5:1433	OB5932 H2 raw.raw	7.9524E4	3	3	30	46	Carbamidomethylation	C4:Carbamidomethylation:1000.00	PEAKS DB
R.IGEC(+57.02)RLEHLSVMEPTK.R	Y	61.10	1897.9390	16	0.1	633.6537	3	30.21	5	F5:1441	OB5932 H2 raw.raw	3.3681E5	3	3	30	45	Carbamidomethylation	C4:Carbamidomethylation:1000.00	PEAKS DB
R.NFLLSGN(+.98)GNGKEGEESN(+.98).N	Y	59.86	1766.7594	17	0.2	884.3871	2	30.77	5	F5:1462	OB5932 H2 raw.raw	1.261E5	2	2	180	196		N7:Deamidation (NQ):39.76;N17:Deamidation (NQ):47.16	PEAKS DB
R.LEHLSVMEPTKR.V	Y	58.37	1438.7603	12	0.4	720.3877	2	28.17	6	F6:1338	OB5933 H2 raw.raw	4.4747E3	1	1	35	46			PEAKS DB
R.NFLLSGNGNGKEGEESN(+.98).N	Y	57.94	1765.7754	17	-0.1	883.8949	2	29.97	1	F1:1310	OB5921 H2 raw.raw	9.4247E4	2	2	180	196		N17:Deamidation (NQ):23.01	PEAKS DB
R.VESEGGVAEFWDDKS(+27.05)QQLQC(+57.02)IGVTLIR.Y	Y	54.31	3090.5444	27	-9.7	1031.1787	3	35.92	1	F1:1673	OB5921 H2 raw.raw	1.3332E5	1	1	47	73	Ethyl amino; Carbamidomethylation	S15:Ethyl amino:119.64;C20:Carbamidomethylation:1000.00	PEAKS PTM

total 19 peptides

Peptide	Uniq	-10lgP	Mass	Length	ppm	m/z	z	RT	Fraction	Scan	Source File	Area Raw in gel	#Feature	#Feature Raw in gel	Start	End	PTM	AScore	Found By
R.NFLLSGNGN(+.98)GKEGEESN(+.98).N	Y	49.86	1766.7594	17	0.4	884.3873	2	30.59	6	F6:1477	OB5933 H2 raw.raw	8.6083E4	1	1	180	196	Deamidation (NQ)	N9:Deamidation (NQ):30.36;N17:Deamidation (NQ):65.92	PEAKS DB
Y.TIRPK(+149.03)GLLLPFYTNAPR.I	Y	47.37	2105.1606	17	-7.5	702.7222	3	34.98	1	F1:1620	OB5921 H2 raw.raw	1.195E5	1	1	75	91		K5:Benzyl isothiocyanate:4.73	PEAKS PTM
K.IQSIEQNDAVAVPSSSVHWIYNTGHSDLVLFSLVDVANADNQLDPTFR.N	Y	45.19	5281.5957	48	1.5	1321.4082	4	38.94	5	F5:1947	OB5932 H2 raw.raw	2.1676E6	2	2	132	179			PEAKS DB
Y.T(+149.03)IRPKGLLLPFYTNAPR.I	Y	42.83	2105.1606	17	-7.1	702.7225	3	34.97	5	F5:1719	OB5932 H2 raw.raw	0	0	0	75	91		T1:Benzyl isothiocyanate:5.74	PEAKS PTM
**total 19 peptides**																			

A0A0A6ZDT0|A0A0A6ZDT0_ARAHY

back to list

| Protein Coverage | Supporting Peptides |

Protein Coverage:

```
  1 MLVHYQIALF HLPRSSTTGL GIVEGLMTAV HSITATQKTV DGPSSKDWRG GRAASFNIIP SSTGAAKAVG KVLPSLNGKL
 81 TGMAFRVPTV DVSVVDLTVR LEKPATYDEI KQAIKEESEG KLKVILGYTE DDVVSTDFVG DSRSSIFDAK AGIALSKNFV
161 KIVSWYDNEW GYSTRVVDLV VHIAKQ
```

o Oxidation (M) (+15.99)

Supporting Peptides:

Peptide	Uniq	-10lgP	Mass	Length	ppm	m/z	z	RT	Fraction	Scan	Source File	Area Raw in gel	#Feature	#Feature Raw in gel	Start	End	PTM	AScore	Found By
K.IVSWYDNEWGYSTR.V	N	119.02	1774.7950	14	1.6	888.4062	2	33.83	11	F11:1692	OB5938 H3A raw.raw	2.0685E6	3	3	162	175			PEAKS DB
R.VPTVDVSVVDLTVR.L	N	109.01	1497.8402	14	0.8	749.9280	2	34.01	11	F11:1703	OB5938 H3A raw.raw	2.6396E6	3	3	87	100			PEAKS DB
R.VVDLVVHIAKQ	Y	88.97	1219.7289	11	0.6	610.8721	2	29.33	11	F11:1412	OB5938 H3A raw.raw	1.0958E6	6	6	176	186			PEAKS DB
R.AASFNIIPSSTGAAK.A	N	84.99	1433.7513	15	2.0	717.8844	2	31.01	10	F10:1503	OB5937 H3A raw.raw	5.133E6	5	5	53	67			PEAKS DB
K.LTGMAFRVPTVDVSVVDLTVR.L	N	83.77	2274.2405	21	-2.6	1138.1245	2	36.04	10	F10:1813	OB5937 H3A raw.raw	1.7435E6	4	4	80	100			PEAKS DB
K.LTGM(+15.99)AFRVPTVDVSVVDLTVR.L	N	80.37	2290.2354	21	-5.6	764.4148	3	36.83	9	F9:1833	OB5936 H3A raw.raw	1.375E6	4	4	80	100	Oxidation (M)	M4:Oxidation (M):1000.00	PEAKS DB
R.LEKPATYDEIK.Q	N	55.56	1305.6816	11	-1.7	653.8470	2	26.49	10	F10:1243	OB5937 H3A raw.raw	0	0	0	101	111			PEAKS DB
R.VPTVDVSVVDLTVRLEKPATYDEIK.Q	N	54.73	2785.5112	25	2.7	929.5135	3	34.75	11	F11:1737	OB5938 H3A raw.raw	2.5869E6	3	3	87	111			PEAKS DB
R.VVDLVVHIAK.Q	Y	49.58	1091.6703	10	0.4	546.8427	2	29.14	11	F11:1409	OB5938 H3A raw.raw	1.7498E4	2	2	176	185			PEAKS DB
K.LTGMAFRVPTVDVSVVDLTVRLEKPATYDEIK.Q	N	47.61	3561.9116	32	1.9	891.4869	4	36.22	11	F11:1821	OB5938 H3A raw.raw	1.7224E6	2	2	80	111			PEAKS DB
K.TVDGPSSKDWR.G	N	43.43	1246.5942	11	-1.7	624.3033	2	26.08	10	F10:1212	OB5937 H3A raw.raw	1.1675E4	2	2	39	49			PEAKS DB
K.LTGMAFR.V	N	35.18	794.4109	7	1.0	398.2131	2	30.69	9	F9:1449	OB5936 H3A raw.raw	9.0748E3	1	1	80	86			PEAKS DB
**total 12 peptides**																			

E9LFE9|E9LFE9_ARAHY

back to list

| Protein Coverage | Supporting Peptides |

Protein Coverage:

1 MGKPVIKPLV VLLLSLILVS ECAAIHSQKR RMGQR**VKLSL EECGELEKLE AIEPDYYR**IE AEGGVTESWN HTNKMLR**CAG**

81 **VALVR**RTVKP GGLVLPSYTN APQLMYYVQG SGIQGMTIFP SCPESFEEPE EAGQEYREQH QKVHEIR**GGD IIAIPAGIGY**

161 **WFYNNGDVPL VVVILLHTNN VANQLGTLPR** RFYIAGNTED EHGEGGREKS ISGR**NVFSGI SLNLLAQVFG VRVETAR**KIQ

241 GPDDKRKNIV MVGGRS

b Biotinylation (+226.08)
c Carbamidomethylation (+57.02)
n N-Succinimidyl-2-morpholine acetate (+127.06)

Supporting Peptides:

Peptide	Uniq	-10lgP	Mass	Length	ppm	m/z	z	RT	Fraction	Scan	Source File	Area Raw in gel	#Feature	#Feature Raw in gel	Start	End	PTM	AScore	Found By
R.NVFSGISLNLLAQVFGVR.V	Y	112.21	1933.0785	18	2.8	967.5492	2	43.21	7	F7:2172	OB5934 H3B raw.raw	1.5453E6	5	5	215	232			PEAKS DB
K.LSLEEC(+57.02)GELEKLEAIEPDYYR.I	Y	109.05	2555.2100	21	1.7	852.7454	3	34.79	7	F7:1692	OB5934 H3B raw.raw	1.2508E6	4	4	38	58	Carbamidomethylation	C6:Carbamidomethylation:1000.00	PEAKS DB
R.VKLSLEEC(+57.02)GELEKLEAIEPDYYR.I	Y	98.79	2782.3735	23	1.8	928.4668	3	34.24	7	F7:1649	OB5934 H3B raw.raw	7.0271E5	2	2	36	58	Carbamidomethylation	C8:Carbamidomethylation:1000.00	PEAKS DB
R.NVFSGISLNLLAQVFGVRVETAR.K	Y	80.79	2489.3755	23	3.0	830.8016	3	42.69	2	F2:1993	OB5925 H3B raw.raw	4.1048E6	4	4	215	237			PEAKS DB
R.C(+57.02)AGVALVR.R	Y	69.07	844.4589	8	1.7	423.2374	2	26.94	2	F2:1100	OB5925 H3B raw.raw	1.0611E3	1	1	78	85	Carbamidomethylation	C1:Carbamidomethylation:1000.00	PEAKS DB
N.V(+127.06)FSGISLNLLAQVFGVR.V	Y	53.35	1946.0989	17	-8.7	974.0482	2	41.55	2	F2:1934	OB5925 H3B raw.raw	1.8552E7	3	3	216	232	N-Succinimidyl-2-morpholine acetate	V1:N-Succinimidyl-2-morpholine acetate:1000.00	PEAKS PTM
R.GGDIIAIPAGIGYWFYNNGDVPLVVVILLHTNNVANQLGTLPR.R	Y	46.38	4573.4482	43	3.5	1525.4954	3	44.60	2	F2:2114	OB5925 H3B raw.raw	9.2311E5	3	3	148	190			PEAKS DB
R.NVFSGIS(+13.03)LNLLAQVFGVR.V	Y	44.51	1946.1101	18	-9.4	974.0532	2	47.20	7	F7:2410	OB5934 H3B raw.raw	0	0	0	215	232		S7:Michael addition with methylamine:16.65	PEAKS PTM
R.NVFSGIS(+14.02)LNLLAQVFGVR.V	Y	40.55	1947.0941	18	2.0	974.5563	2	47.47	2	F2:2277	OB5925 H3B raw.raw	0	0	0	215	232		S7:Methylation(others):15.62	PEAKS PTM
V.F(+226.08)SGISLNLLAQVFGVR.V	Y	32.77	1946.0447	16	2.4	974.0319	2	50.95	2	F2:2479	OB5925 H3B raw.raw	0	0	0	217	232	Biotinylation	F1:Biotinylation:1000.00	PEAKS PTM
**total 10 peptides**																			

Q647H1|Q647H1_ARAHY

back to list

| Protein Coverage | Supporting Peptides |

Protein Coverage:

1 MVIGPFRLSL CVCLVFLTSA CFGTRLEESF NECQLDRLNA LTPDNRIESQ GGITETWNSN HPELRCAGVT LLKRTIFPNG

d Diethylation (+56.06)

81 FHLPSYANYP QLIFIAQGNG VFGVSLPGCP VTYEEAESQS REDRRQRIVI KRESEQEQEQ QGDSHHKIYH FRQGHLLAIP
161 AGVPYWSFNY GNEPIVAITL LDTSNLDNQL DPSPRRFYLA GNPEEEHPET QQQQPQTRRR HGQHQQDEYG SQGEEEGNNV
241 LSGFSTQLLA HAFGVDEEIA RILQNPPEQT KDQIVRVEGG FRDVISPRWG EGKQYEDELE ERQRQPPRRD EQGKGYDYDD
321 DRRPRHRQDP YREGDEDDRR PRGSRQGQGR GYDDDDRRPG QYEEGEEDDR RPRRSSRPKR QGRRHDDDDR RADEDDRRGY
401 DDDERRPDED DRRGYDDDER RPDDDDRQGY DDDDRRPRWS SRPKGQGRNG VEETLCSPTL VEDIARPSR**A DFYNPAAGRI**
481 **SSANSLTFPI LRWFQLSAEH VLLYR**NGIYS PHWNNNANSI IYGLRGEGR**I QVVNSQGNAV FNGVLREGQI LLVPQNFAVG**
561 **K**QAGNEGFEY VAFKTADRAS PATSSKCLGE SPLMFSSMLL AFEIIKSVLS NTMETRPLWS LLMIPSMGLN VVINLNHNNN
d
641 AQVDSKNNDG SRLWWPSSII IK

Supporting Peptides:

Peptide	Uniq	-10lgP	Mass	Length	ppm	m/z	z	RT	Fraction	Scan	Source File	Area Raw in gel	#Feature	#Feature Raw in gel	Start	End	PTM	AScore	Found By
R.IQVVNSQGNAVFNGVLR.E	Y	125.58	1813.9799	17	2.0	907.9990	2	32.81	5	F5:1595	OB5932 H2 raw.raw	4.7427E5	2	2	530	546			PEAKS DB
R.WFQLSAEHVLLYR.N	Y	96.74	1660.8726	13	0.1	831.4436	2	34.98	1	F1:1614	OB5921 H2 raw.raw	2.1298E5	2	2	493	505			PEAKS DB
R.ADFYNPAAGR.I	Y	81.48	1080.4988	10	1.0	541.2572	2	28.15	1	F1:1213	OB5921 H2 raw.raw	4.2854E3	1	1	470	479			PEAKS DB
R.ISSANSLTFPILR.W	Y	79.81	1417.7928	13	1.5	709.9048	2	34.24	1	F1:1575	OB5921 H2 raw.raw	6.7566E5	3	3	480	492			PEAKS DB
R.IQVVNSQGN(+.98)AVFNGVLR.E	Y	73.39	1814.9639	17	0.5	908.4896	2	33.38	5	F5:1634	OB5932 H2 raw.raw	1.7282E4	1	1	530	546		N9:Deamidation (NQ):26.38	PEAKS DB
R.EGQILLVPQNFAVGK.Q	Y	64.50	1611.8984	15	2.3	806.9584	2	34.31	5	F5:1701	OB5932 H2 raw.raw	5.2907E5	3	3	547	561			PEAKS DB
R.EGQILLVPQNFAVGK(+56.06).Q	Y	36.48	1667.9609	15	2.1	834.9895	2	34.69	5	F5:1704	OB5932 H2 raw.raw	2.7965E6	2	2	547	561	Diethylation	K15:Diethylation:121.27	PEAKS PTM
total 7 peptides																			

A0A0F7GF62|A0A0F7GF62_ARAHY

back to list

| Protein Coverage | Supporting Peptides |

Protein Coverage:

2
a
1 M**ATLIAPSNH SSAEDAEALQ KAFKGWGADD KTIIAILGHR** NVHQRQQIRK AYEELHQEDL IKRLESEISG DFERAMYRWM
a

a Acetylation (Protein N-term) (+42.01)

81 LEPADRDAVL ANVAIRNGKK DLHVIAEIAC VLSAEELLAV RRAYRHRYKR **SLEEDVAANT TGHLR**QLLVG LVSSYRYEGD
161 EINARLAQSE ANILHETVKE KKGNYEEAIR ILTTR**SKTQL VATFNR**YRDE HAISISKK**LL DNQASDDFYK** ALHTAIRCIN
241 DHKKYYEKVL RNAIKKVGTD EDALSRVVVT RAEKDLRDIK ELYYKRNSVH LEDAVAKETS GDYKK**FLLTL LGKQD**

Supporting Peptides:

Peptide	Uniq	-10lgP	Mass	Length	ppm	m/z	z	RT	Fraction	Scan	Source File	Area Raw in gel	#Feature	#Feature Raw in gel	Start	End	PTM	AScore	Found By
R.SLEEDVAANTTGHLR.Q	Y	108.66	1611.7853	15	0.6	806.9004	2	29.32	10	F10:1409	OB5937 H3A raw.raw	2.1959E5	3	3	131	145			PEAKS DB
M.A(+42.01)TLIAPSNHSSAEDAEALQK.A	Y	96.71	2094.0229	20	1.7	1048.0205	2	30.83	10	F10:1507	OB5937 H3A raw.raw	1.9399E5	3	3	2	21	Acetylation (Protein N-term)	A1:Acetylation (Protein N-term):1000.00	PEAKS PTM
R.SKTQLVATFNR.Y	Y	81.35	1263.6935	11	0.6	632.8544	2	27.65	10	F10:1310	OB5937 H3A raw.raw	1.9511E3	1	1	196	206			PEAKS DB
K.FLLTLLGKQD	Y	73.91	1146.6648	10	0.9	574.3402	2	35.29	11	F11:1769	OB5938 H3A raw.raw	1.1511E6	2	2	306	315			PEAKS DB
K.AFKGWGADDKTIIAILGHR.N	Y	54.66	2068.1218	19	-0.4	518.0375	4	33.81	11	F11:1685	OB5938 H3A raw.raw	0	0	0	22	40			PEAKS DB
K.LLDNQASDDFYK.A	Y	41.68	1427.6569	12	0.8	714.8362	2	30.00	10	F10:1465	OB5937 H3A raw.raw	2.1343E4	2	2	219	230			PEAKS DB
total 6 peptides																			

B0YIU5|B0YIU5_ARAHY

back to list

| Protein Coverage | Supporting Peptides |

Protein Coverage:

47 d

1 MGVHTFEEES TSPVPPAKLF KATVVDGDEL TPKLIPAIQS IEIVEGNGGP GTVKKVTAVE DGKTSYVLHK IDAIDEATYT

d Deamidation (NQ) (+0.98)

81 YDYTISGGTG FQEILEKVSF KTKLEAADGG SKIKVSVTFH TKGDAPLPDE VHQDVKQKSQ GIFKAIEGYV LSN

Supporting Peptides:

Peptide	Uniq	-10lgP	Mass	Length	ppm	m/z	z	RT	Fraction	Scan	Source File	Area Raw in gel	#Feature	#Feature Raw in gel	Start	End	PTM	AScore	Found By
K.IDAIDEATYTYDYTISGGTGFQEILEK.V	Y	110.28	3012.4126	27	4.4	1507.2202	2	38.52	3	F3:1926	OB5930 H6 raw.raw	5.5055E4	1	1	71	97			PEAKS DB
K.LIPAIQSIEIVEGNGGPGTVK.K	Y	93.63	2091.1575	21	2.0	1046.5881	2	35.72	4	F4:1774	OB5931 H6 raw.raw	1.5174E5	2	2	34	54			PEAKS DB
K.LIPAIQSIEIVEGN(+.98)GGPGTVK.K	Y	83.95	2092.1416	21	3.9	1047.0822	2	36.03	4	F4:1784	OB5931 H6 raw.raw	5.1573E4	1	1	34	54	Deamidation (NQ)	N14:Deamidation (NQ):166.48	PEAKS DB
K.GDAPLPDEVHQDVK.Q	Y	60.93	1518.7314	14	3.9	760.3760	2	27.69	4	F4:1283	OB5931 H6 raw.raw	3.0914E3	2	2	123	136			PEAKS DB
K.ATVVDGDELTPK.L	Y	59.97	1243.6295	12	1.0	622.8226	2	28.00	4	F4:1307	OB5931 H6 raw.raw	5.1387E3	1	1	22	33			PEAKS DB
M.GVHTFEEESTSPVPPAK.L	Y	54.06	1810.8737	17	0.6	906.4446	2	28.77	4	F4:1359	OB5931 H6 raw.raw	4.7221E3	2	2	2	18			PEAKS DB
total 6 peptides																			

Q2PK12|Q2PK12_ARAHY

back to list

| Protein Coverage | Supporting Peptides |

Protein Coverage:

1 MANAASGMAV HDDCKLRFLE LKAKRTHRFI VFKIEENQKQ VIVEKLGEPA QGYEDFTACL PPNECRYAVY DFEYLTEGNV

C Carbamidomethylation (+57.02)

81 PKSRIFFIAW SPDTSRVRNK MIYASSKDRF KRELDGIQVE LQATDPTEMD LDVFKSRAN

Supporting Peptides:

Peptide	Uniq	-10lgP	Mass	Length	ppm	m/z	z	RT	Fraction	Scan	Source File	Area Raw in gel	#Feature	#Feature Raw in gel	Start	End	PTM	AScore	Found By
R.YAVYDFEYLTEGNVPK.S	Y	108.28	1906.8988	16	3.0	954.4595	2	35.51	3	F3:1754	OB5930 H6 raw.raw	3.2417E4	2	2	67	82			PEAKS DB
R.IFFIAWSPDTSR.V	Y	85.65	1438.7245	12	2.2	720.3711	2	37.18	4	F4:1851	OB5931 H6 raw.raw	5.3939E4	2	2	85	96			PEAKS DB
R.FIVFKIEENQK.Q	Y	67.92	1393.7605	11	2.4	697.8892	2	34.12	4	F4:1671	OB5931 H6 raw.raw	8.0867E3	2	2	29	39			PEAKS DB
K.LGEPAQGYEDFTAC(+57.02)LPPNEC(+57.02)R.Y	Y	50.89	2423.0522	21	-2.6	1212.5303	2	33.24	3	F3:1619	OB5930 H6 raw.raw	0	0	0	46	66	Carbamidomethylation	C14:Carbamidomethylation:1000.00;C20:Carbamidomethylation:1000.00	PEAKS DB
R.ELDGIQVELQATDPTEMDLDVFK.S	Y	49.35	2605.2468	23	-1.1	1303.6293	2	38.15	4	F4:1905	OB5931 H6 raw.raw	0	0	0	113	135			PEAKS DB
total 5 peptides																			

H6U596|H6U596_ARAHY

back to list

| Protein Coverage | Supporting Peptides |

Protein Coverage:

1 SPLFPRIFGH EAGGIVESVG EGVTHLKPGD HALPVFTGEC GECPHCKSEE SNMCDLLRIN TDRGVMIHDQ ESRFSIKGKP

81 IYHFVGTSTF SEYTVVHAGC VAKINPEAPL DKVCVLSCGI CTGLGATINV AKPKPGSSVA VFGLGAVGLA AAEGARISGA

161 SRIIGVDLVS SRFELAKKFG VNEFV

Supporting Peptides:

Peptide	Uniq	-10lgP	Mass	Length	ppm	m/z	z	RT	Fraction	Scan	Source File	Area Raw in gel	#Feature	#Feature Raw in gel	Start	End	PTM	AScore	Found By
R.IIGVDLVSSRFELAK.K	Y	107.11	1645.9402	15	1.1	823.9783	2	33.65	11	F11:1676	OB5938 H3A raw.raw	1.8835E7	6	6	163	177			PEAKS DB
R.IIGVDLVSSR.F	Y	72.92	1057.6132	10	0.2	529.8140	2	31.58	10	F10:1551	OB5937 H3A raw.raw	1.959E5	2	2	163	172			PEAKS DB
R.INTDRGVMIHDQESRFSIK.G	Y	67.78	2245.1272	19	0.2	562.2892	4	29.32	10	F10:1415	OB5937 H3A raw.raw	4.7416E4	1	1	59	77			PEAKS DB
K.PGSSVAVFGLGAVGLAAAEGAR.I	Y	52.36	1956.0428	22	-2.3	979.0265	2	37.95	9	F9:1901	OB5936 H3A raw.raw	1.1681E5	1	1	135	156			PEAKS DB
R.I(+57.02)IGVDLVSSRFELAK.K	Y	50.32	1702.9617	15	0.3	852.4884	2	36.07	9	F9:1782	OB5936 H3A raw.raw	0	0	0	163	177		I1:Carbamidomethylation (DHKE, X@N-term):4 1.53	PEAKS PTM
I.GVDLVSSRFELAK.K	Y	38.04	1419.7721	13	0.4	710.8936	2	33.84	10	F10:1685	OB5937 H3A raw.raw	3.3254E5	2	2	165	177			PEAKS DB
total 6 peptides																			

B4UW81|B4UW81_ARAHY

back to list

| Protein Coverage | Supporting Peptides |

Protein Coverage:

1 MASDTKKLKL YSYWRSSCSF RVRIALNLKE FLKLNPVGFV PVLLDGDLVL ADSLAIIMYL DDKYPQHPLL PSDIHKRAIN

b Beta-methylthiolation (ND) (+45.99)

81 FQATHIVSSS IQPLQNISFL NYIGEKVGPD EKLPWVQSVL RKGFTALEKL LKDHTGRYAT GDEIFLADVF LAPQLHAAFT

161 RFNIPMNEFP IMSRLHGNV

Supporting Peptides:

Peptide	Uniq	-10lgP	Mass	Length	ppm	m/z	z	RT	Fraction	Scan	Source File	Area Raw in gel	#Feature	#Feature Raw in gel	Start	End	PTM	AScore	Found By
K.VGPDEKLPWVQSVLR.K	Y	98.36	1721.9464	15	1.4	574.9902	3	34.05	7	F7:1636	OB5934 H3B raw.raw	1.7988E6	5	5	107	121			PEAKS DB
R.AINFQATHIVSSSIQPLQNISFLNYIGEK.V	Y	89.26	3231.6929	29	2.8	1078.2412	3	38.13	7	F7:1882	OB5934 H3B raw.raw	2.7951E6	4	4	78	106			PEAKS DB
R.YATGDEIFLADVFLAPQLHAAFTR.F	Y	76.78	2665.3540	24	2.2	889.4606	3	40.56	7	F7:2027	OB5934 H3B raw.raw	3.0891E6	4	4	138	161			PEAKS DB
R.FNIPMNEFPIMSR.L	Y	67.28	1594.7635	13	2.9	798.3914	2	35.91	2	F2:1610	OB5925 H3B raw.raw	1.1285E6	3	3	162	174			PEAKS DB
R.YATGD(+45.99)EIFLADVFLAPQLHAAFTR.F	Y	60.08	2711.3416	24	1.0	904.7887	3	40.37	7	F7:2016	OB5934 H3B raw.raw	1.2493E6	2	2	138	161	Beta-methylthiolation (ND)	D5:Beta-methylthiolation (N D):102.87	PEAKS PTM
K.VGPDEKLPWVQSVLRK.G	Y	33.26	1850.0414	16	-1.4	617.6869	3	32.38	2	F2:1404	OB5925 H3B raw.raw	5.764E4	1	1	107	122			PEAKS DB
total 6 peptides																			

D8KXZ8|D8KXZ8_ARAHY

back to list

| Protein Coverage | Supporting Peptides |

Protein Coverage:

1 MATTPFSGLQ TAMSRSCIPS SQRSNVMAFS GKSMVGSWNK LASVCHVSSV QPFQRVFTSS TIKFERIVTK AMSDSSQKSL

81 VSGLPIDLKG KRAFIAGVAD DNGYGWAIAK ALAAAGAQIL VGTWVPALNI FESSLRRGKF DESRVLPDGS LMEITKVYPL

161 DAVFDNPEDV PEDIKTNKRY AGSSKWTVQE VAESVKEDFG TIDILVHSLA NGPEVTKPLL ETSRNGYLAA LSASSYSYVS

241 LLKHFLPIMN PGGSSISLTY IASEKIIPGY GGGMSSAKAA LESDTRVLAF EAGRKRKIRV NTISAGPLRS RAAKAIGFID

321 MMIDYSIANA PLQKELSADE VGNSAAFLAS PLASAITGTV LYVDNGLNAM GVGVDSPVFK DLDIPKDQH

Supporting Peptides:

Peptide	Uniq	-10lgP	Mass	Length	ppm	m/z	z	RT	Fraction	Scan	Source File	Area Raw in gel	#Feature	#Feature Raw in gel	Start	End	PTM	AScore	Found By
K.AIGFIDMMIDYSIANAPLQK.E	N	100.82	2210.1116	20	3.4	1106.0668	2	40.60	11	F11:2085	OB5938 H3A raw.raw	0	0	0	315	334			PEAKS DB
K.VYPLDAVFDNPEDVPEDIKTNKR.Y	N	63.63	2673.3286	23	0.9	892.1176	3	33.08	11	F11:1637	OB5938 H3A raw.raw	5.7671E5	3	3	157	179			PEAKS DB
K.ALAAAGAQILVGTWVPALNIFESSLRR.G	Y	57.47	2823.5759	27	3.3	942.2024	3	42.17	9	F9:2142	OB5936 H3A raw.raw	0	0	0	111	137			PEAKS DB
R.VNTISAGPLR.S	N	55.49	1026.5822	10	1.4	514.2991	2	27.48	10	F10:1306	OB5937 H3A raw.raw	3.2787E3	1	1	300	309			PEAKS DB
K.ALAAAGAQILVGTWVPALNIFESSLR.R	Y	55.41	2667.4749	26	3.2	1334.7490	2	42.57	10	F10:2189	OB5937 H3A raw.raw	1.6879E4	2	2	111	136			PEAKS DB
K.SLVSGLPIDLK.G	N	39.53	1140.6754	11	-0.5	571.3447	2	34.19	10	F10:1708	OB5937 H3A raw.raw	2.8994E5	1	1	79	89			PEAKS DB

total 6 peptides

sp|Q6PSU2|CONG7_ARAHY

back to list

| Protein Coverage | Supporting Peptides |

Protein Coverage:

1 MAKLTILVAL ALFLLAAHAS ARQQWELQGD RRCQSQLERA NLRPCEQHLM QKIQRDEDSY GRDPYSPSQD PYSPSQDPDR

Carbamidomethylation (+57.02)
Oxidation (M) (+15.99)

81 RDPYSPSPYD RRGAGSSQHQ ERCCNELNEF ENNQRCMCEA LQQIMENQSD RLQGRQQEQQ FKRELRNLPQ QCGLRAPQRC

161 DLEVESGGRD RY

Supporting Peptides:

Peptide	Uniq	-10lgP	Mass	Length	ppm	m/z	z	RT	Fraction	Scan	Source File	Area Raw in gel	#Feature	#Feature Raw in gel	Start	End	PTM	AScore	Found By
R.C(+57.02)MC(+57.02)EALQQIMENQSDRLQGR.Q	Y	86.03	2466.0872	20	1.8	823.0378	3	33.67	2	F2:1478	OB5925 H3B raw.raw	6.5263E6	9	9	116	135	Carbamidomethylation	C1:Carbamidomethylation:1000.00;C3:Carbamidomethylation:1000.00	PEAKS DB
R.C(+57.02)C(+57.02)NELNEFENNQR.C	Y	77.43	1725.6835	13	0.8	863.8497	2	28.73	6	F6:1378	OB5933 H2 raw.raw	2.2936E5	3	3	103	115	Carbamidomethylation	C1:Carbamidomethylation:1000.00;C2:Carbamidomethylation:1000.00	PEAKS DB
R.ANLRPC(+57.02)EQHLMQK.I	Y	74.03	1623.7974	13	-0.3	542.2729	3	28.69	1	F1:1233	OB5921 H2 raw.raw	2.0501E5	3	3	40	52	Carbamidomethylation	C6:Carbamidomethylation:1000.00	PEAKS DB
R.C(+71.04)C(+57.02)NELNEFENNQR.C	Y	68.93	1739.6992	13	1.2	870.8580	2	28.91	5	F5:1352	OB5932 H2 raw.raw	1.8947E5	3	3	103	115	Carbamidomethylation	C1:Propionamide:11.06;C2:Carbamidomethylation:1000.00	PEAKS PTM
R.C(+57.02)M(+15.99)C(+57.02)EALQQIMENQSDRLQGR.Q	Y	59.67	2482.0820	20	2.0	828.3696	3	31.87	6	F6:1563	OB5933 H2 raw.raw	9.9293E5	4	4	116	135	Carbamidomethylation; Oxidation (M)	C1:Carbamidomethylation:1000.00;M2:Oxidation (M):73.16;C3:Carbamidomethylation:1000.00	PEAKS DB
R.C(+71.04)MC(+57.02)EALQQIMENQSDRLQGR.Q	Y	55.53	2480.1028	20	0.4	827.7086	3	33.68	1	F1:1538	OB5921 H2	8.4273E5	3	3	116	135	Carbamidomethylation	C1:Propionamide:32.28;C3:Carbamidomethyl	PEAKS PTM

total 12 peptides

Peptide	Uniq	-10lgP	Mass	Length	ppm	m/z	z	RT	Fraction	Scan	Source File	Area Raw in gel	#Feature	#Feature Raw in gel	Start	End	PTM	AScore	Found By
											raw.raw							ation:1000.00	
R.C(+57.02)M(+15.99)C(+57.02)EALQQIM(+15.99)ENQSDRLQGR.Q	Y	48.57	2498.0771	20	1.7	833.7010	3	29.28	6	F6:1401	OB5933 H2 raw.raw	1.0641E5	2	2	116	135	Carbamidomethylation; Oxidation (M)	C1:Carbamidomethylation:1000.00;M2:Oxidation (M):1000.00;C3:Carbamidomethylation:1000.00;M10:Oxidation (M):1000.00	PEAKS DB
R.C(+57.02)MC(+57.02)EALQQIM(+15.99)ENQSDRLQGR.Q	Y	47.95	2482.0820	20	1.4	828.3691	3	30.77	6	F6:1501	OB5933 H2 raw.raw	2.8999E5	2	2	116	135	Carbamidomethylation; Oxidation (M)	C1:Carbamidomethylation:1000.00;C3:Carbamidomethylation:1000.00;M10:Oxidation (M):139.69	PEAKS DB
R.C(+57.02)DLEVESGGRDRY	Y	46.09	1554.6732	13	1.2	778.3448	2	27.26	5	F5:1261	OB5932 H2 raw.raw	3.8274E4	2	2	160	172	Carbamidomethylation	C1:Carbamidomethylation:1000.00	PEAKS DB
R.NLPQQC(+57.02)GLR.A	Y	43.34	1084.5448	9	-0.6	543.2794	2	25.48	6	F6:1173	OB5933 H2 raw.raw	7.6965E4	2	2	147	155	Carbamidomethylation	C6:Carbamidomethylation:1000.00	PEAKS DB
Q.WELQGDRR.C	Y	36.22	1058.5258	8	-0.7	530.2698	2	27.08	5	F5:1254	OB5932 H2 raw.raw	3.4281E3	1	1	25	32			PEAKS DB
A.RQQWELQGDRR.C	Y	34.94	1470.7440	11	-0.2	491.2552	3	27.80	5	F5:1291	OB5932 H2 raw.raw	4.1565E4	1	1	22	32			PEAKS DB
**total 12 peptides**																			

E6Y9A9|E6Y9A9_ARAHY

back to list

| Protein Coverage | Supporting Peptides |

Protein Coverage:

```
  1 MATTPFSGLQ TAMSRSCIPS SQRSNVMAFS GKSMVGSWNK LASVCHVSSV QPFQRVFTSS TIKFERIVTK AMSDSSQKSL
 81 VSGLPIDLKG KRAFIAGVAD DNGYGWAIAK SLAAAGAQIL VGTWVPALNI FESSLRRGKF DESRVLPDGS LMEITKVYPL
161 DAVFDNPEDV PEDIKTNKRY AGSSKWTVQE VAESVKEDFG TIDILVHSLA NGPEVTKPLL ETSRNGYLAA LSASSYSYVS
241 LLKHFLPIMN PGGSSISLTY IASEKIIPGY GGGMSSAKAA LESDTRVLAF EAGRKRKIRV NTISAGPLRS RAAKAIGFID
321 MMIDYSIANA PLQKELSADE VGNSAAFLAS PLASAITGTV LYVDNGLNAM GVGVDSPVFK DLDIPKDQH
```

Supporting Peptides:

Peptide	Uniq	-10lgP	Mass	Length	ppm	m/z	z	RT	Fraction	Scan	Source File	Area Raw in gel	#Feature	#Feature Raw in gel	Start	End	PTM	AScore	Found By
K.AIGFIDMMIDYSIANAPLQK.E	N	100.82	2210.1116	20	3.4	1106.0668	2	40.60	11	F11:2085	OB5938 H3A raw.raw	0	0	0	315	334			PEAKS DB
K.VYPLDAVFDNPEDVPEDIKTNKR.Y	N	63.63	2673.3286	23	0.9	892.1176	3	33.08	11	F11:1637	OB5938 H3A raw.raw	5.7671E5	3	3	157	179			PEAKS DB
R.VNTISAGPLR.S	N	55.49	1026.5822	10	1.4	514.2991	2	27.48	10	F10:1306	OB5937 H3A raw.raw	3.2787E3	1	1	300	309			PEAKS DB
K.SLAAAGAQILVGTWVPALNIFESSLRR.G	Y	43.83	2839.5708	27	-2.5	947.5285	3	41.70	11	F11:2149	OB5938 H3A raw.raw	1.1751E4	1	1	111	137			PEAKS DB
K.SLVSGLPIDLK.G	N	39.53	1140.6754	11	-0.5	571.3447	2	34.19	10	F10:1708	OB5937 H3A raw.raw	2.8994E5	1	1	79	89			PEAKS DB
**total 5 peptides**																			

O20356|O20356_ARAHY

back to list

| Protein Coverage | Supporting Peptides |

Protein Coverage:

```
  1 VGFKAGVKDY KLTYYTPEYE TKDTDILAAF RVTPQPGVPP EEAGAAVAAE SSTGTWTTVW TDGLTSLDRY KGRCYNIEPV
 81 AGEENQYIAY VAYPLDLFEE GSVTNMFTSI VGNVFGFKAL RALRLEDLRI PISYIKTFQG PPHGIQVERD KLNKYGRPLL
                                                                211
161 GCTIKPKLGL SAKNYGRAVY ECLRGGLDFT KDDENVNSQP FMRWRDRFLF CAEAIFKSQA ETGEIKGHYL NATAGTCEEM
241 IKRAVFAREL GAPIVMHDYL TGGFTANTSL AHYCRDNGLL LHIHRAMHAV IDRQKNHGMH FRVLAKALRL SGGDHIHAGT
321 VVGKLEGERD ITLGFVDLLR DDFIEKDRSR GIYFTQDWVS LPGVLPVASG GIHVWHMPAL TEIFGDDSVL QFGGGTLGHP
                  417
401 WGNAPGAVAN RVALEACVQA RNEGRDLARE GNEIIREASK WSPELAAACE VWKAIKFEFP AMDTL
```

C Carbamidomethylation (+57.02)

Supporting Peptides:

Peptide	Uniq	-10lgP	Mass	Length	ppm	m/z	z	RT	Fraction	Scan	Source File	Area Raw in gel	#Feature	#Feature Raw in gel	Start	End	PTM	AScore	Found By
R.DITLGFVDLLRDDFIEKDR.S	Y	84.04	2279.1797	19	2.4	760.7357	3	38.30	11	F11:1951	OB5938 H3A raw.raw	2.0066E5	1	1	330	348			PEAKS DB
R.VALEAC(+57.02)VQAR.N	Y	78.81	1115.5757	10	3.3	558.7969	2	26.89	11	F11:1278	OB5938 H3A raw.raw	0	0	0	412	421	Carbamidomethylation	C6:Carbamidomethylation:1000.0 0	PEAKS DB
R.ALRLEDLRIPISYIK.T	Y	76.51	1799.0669	15	0.5	450.7742	4	34.01	11	F11:1706	OB5938 H3A raw.raw	1.0366E6	3	3	122	136			PEAKS DB
K.TFQGPPHGIQVER.D	Y	44.14	1464.7473	13	0.5	489.2566	3	30.19	9	F9:1435	OB5936 H3A raw.raw	2.0466E4	2	2	137	149			PEAKS DB
R.FLFC(+57.02)AEAIFK.S	Y	41.53	1244.6263	10	0.8	623.3209	2	36.22	11	F11:1836	OB5938 H3A raw.raw	8.051E4	1	1	208	217	Carbamidomethylation	C4:Carbamidomethylation:1000.0 0	PEAKS DB
R.DNGLLLHIHR.A	Y	33.91	1186.6571	10	0.2	396.5597	3	28.88	11	F11:1396	OB5938 H3A raw.raw	0	0	0	276	285			PEAKS DB

total 6 peptides

A0A075M981|A0A075M981_ARAHY

back to list

| Protein Coverage | Supporting Peptides |

Protein Coverage:

```
  1 FMLPQTETKA SVGFKAGVKD YKLTYYTPEY ETKDTDILAA FRVTPQPGVP PEEAGAAVAA ESSTGTWTTV WTDGLTSLDR
 81 YKGRCYNIEP VAGEENQYIA YVAYPLDLFE EGSVTNMFTS IVGNVFGFKA LRALRLEDLR IPISYIKTFQ GPPHGIQVER
                                                                        222
161 DKLNKYGRPL LGCTIKPKLG LSAKNYGRAV YECLRGGLDF TKDDENVNSQ PFMRWRDRFL FCAEAIFKSQ AETGEIKGHY
241 LNATAGTCEE MIKRAVFARG LGAPIVMHDY LTGGFTANTS LAHYCRDNGL LLHIHRAMHA VIDRQKNHGM HFRVLAKALR
321 LSGGDHIHAG TVVGKLEGER DITLGFVDLL RDDFIEKDRS RGIYFTQDWV SLPGVLPVAS GGIHVWHMPA LTEIFGDDSV
                              428
401 LQFGGGTLGH PWGNAPGAVA NRVALEACVQ ARNEGRDLAR EGNEIIREAS KWSPELAAAC EVWKAIKFE
```

C Carbamidomethylation (+57.02)

Supporting Peptides:

Peptide	Uniq	-10lgP	Mass	Length	ppm	m/z	z	RT	Fraction	Scan	Source File	Area Raw in gel	#Feature	#Feature Raw in gel	Start	End	PTM	AScore	Found By
R.DITLGFVDLLRDDFIEKDR.S	Y	84.04	2279.1797	19	2.4	760.7357	3	38.30	11	F11:1951	OB5938 H3A raw.raw	2.0066E5	1	1	341	359			PEAKS DB
R.VALEAC(+57.02)VQAR.N	Y	78.81	1115.5757	10	3.3	558.7969	2	26.89	11	F11:1278	OB5938 H3A raw.raw	0	0	0	423	432	Carbamidomethylation	C6:Carbamidomethylation:1000.0 0	PEAKS DB
R.ALRLEDLRIPISYIK.T	Y	76.51	1799.0669	15	0.5	450.7742	4	34.01	11	F11:1706	OB5938 H3A raw.raw	1.0366E6	3	3	133	147			PEAKS DB

total 6 peptides

Peptide	Uniq	-10lgP	Mass	Length	ppm	m/z	z	RT	Fraction	Scan	Source File	Area Raw in gel	#Feature	#Feature Raw in gel	Start	End	PTM	AScore	Found By
K.TFQGPPHGIQVER.D	Y	44.14	1464.7473	13	0.5	489.2566	3	30.19	9	F9:1435	OB5936 H3A raw.raw	2.0466E4	2	2	148	160			PEAKS DB
R.FLFC(+57.02)AEAIFK.S	Y	41.53	1244.6263	10	0.8	623.3209	2	36.22	11	F11:1836	OB5938 H3A raw.raw	8.051E4	1	1	219	228	Carbamidomethylation	C4:Carbamidomethylation:1000.0 0	PEAKS DB
R.DNGLLLHIHR.A	Y	33.91	1186.6571	10	0.2	396.5597	3	28.88	11	F11:1396	OB5938 H3A raw.raw	0	0	0	287	296			PEAKS DB
total 6 peptides																			

A0A191UJ50|A0A191UJ50_ARAHY

back to list

| Protein Coverage | Supporting Peptides |

Protein Coverage:

```
  1 MSPQTETKAS VGFKAGVKDY KLTYYTPEYE TKDTDILAAF RVTPQPGVPP EEAGAAVAAE SSTGTWTTVW TDGLTSLDRY
 81 KGRCYNIEPV AGEENQYIAY VAYPLDLFEE GSVTNMFTSI VGNVFGFKAL RALRLEDLRI PISYIKTFQG PPHGIQVERD
                                                                      221
                                                                       C
161 KLNKYGRPLL GCTIKPKLGL SAKNYGRAVY ECLRGGLDFT KDDENVNSQP FMRWRDRFLF CAEAIFKSQA ETGEIKGHYL
241 NATAGTCEEM IKRAVFAREL GAPIVMHDYL TGGFTANTSL AHYCRDNGLL LHIHRAMHAV IDRQKNHGMH FRVLAKALRL
321 SGGDHIHAGT VVGKLEGERD ITLGFVDLLR DDFIEKDRSR GIYFTQDWVS LPGVLPVASG GIHVWHMPAL TEIFGDDSVL
                                427
                                 C
401 QFGGGTLGHP WGNAPGAVAN RVALEACVQA RNEGRDLARE GNEIIREASK WSPELAAACE VWKAIKFEFP AMDTL
```

C Carbamidomethylation (+57.02)

Supporting Peptides:

Peptide	Uniq	-10lgP	Mass	Length	ppm	m/z	z	RT	Fraction	Scan	Source File	Area Raw in gel	#Feature	#Feature Raw in gel	Start	End	PTM	AScore	Found By
R.DITLGFVDLLRDDFIEKDR.S	Y	84.04	2279.1797	19	2.4	760.7357	3	38.30	11	F11:1951	OB5938 H3A raw.raw	2.0066E5	1	1	340	358			PEAKS DB
R.VALEAC(+57.02)VQAR.N	Y	78.81	1115.5757	10	3.3	558.7969	2	26.89	11	F11:1278	OB5938 H3A raw.raw	0	0	0	422	431	Carbamidomethylation	C6:Carbamidomethylation:1000.0 0	PEAKS DB
R.ALRLEDLRIPISYIK.T	Y	76.51	1799.0669	15	0.5	450.7742	4	34.01	11	F11:1706	OB5938 H3A raw.raw	1.0366E6	3	3	132	146			PEAKS DB
K.TFQGPPHGIQVER.D	Y	44.14	1464.7473	13	0.5	489.2566	3	30.19	9	F9:1435	OB5936 H3A raw.raw	2.0466E4	2	2	147	159			PEAKS DB
R.FLFC(+57.02)AEAIFK.S	Y	41.53	1244.6263	10	0.8	623.3209	2	36.22	11	F11:1836	OB5938 H3A raw.raw	8.051E4	1	1	218	227	Carbamidomethylation	C4:Carbamidomethylation:1000.0 0	PEAKS DB
R.DNGLLLHIHR.A	Y	33.91	1186.6571	10	0.2	396.5597	3	28.88	11	F11:1396	OB5938 H3A raw.raw	0	0	0	286	295			PEAKS DB
total 6 peptides																			

N1NKG9|N1NKG9_ARAHY

back to list

| Protein Coverage | Supporting Peptides |

Protein Coverage:

```
  1 MSQEQHRRPE ESDPIKYGDV FNVSGELAQK PVKPEDAAMM QSAETRVLGK TQPGGAAAAM QSAATLNEQA GLVGHHDVNE
 81 VAGDRGVTVT DMQAPGRRIV TESVGGQVVG QYVEPTPVQS SRASAVRESA ITIGEALEAT AQTVGSKPVE QSDAAAIQAA
161 EVRATGSNVI QPGGLAAMAQ SAVAFNAGCR NPRDKIKLAD ILTGATAKLP ADKAATHQDA EGVASAEARN NTGGTTPGGV
241 AASVAAAARL NENVNV
```

Supporting Peptides:

Peptide	Uniq	-10lgP	Mass	Length	ppm	m/z	z	RT	Fraction	Scan	Source File	Area Raw in gel	#Feature	#Feature Raw in gel	Start	End	PTM	AScore	Found By

Peptide	Uniq	-10lgP	Mass	Length	ppm	m/z	z	RT	Fraction	Scan	Source File	Area Raw in gel	#Feature	#Feature Raw in gel	Start	End	PTM	AScore	Found By
R.ESAITIGEALEATAQTVGSKPVEQSDAAAIQAAEVR.A	Y	89.79	3610.8325	36	1.7	1204.6201	3	35.85	11	F11:1809	OB5938 H3A raw.raw	2.358E6	4	4	128	163			PEAKS DB
K.LADILTGATAK.L	Y	84.03	1072.6128	11	0.5	537.3140	2	29.90	10	F10:1453	OB5937 H3A raw.raw	1.4089E5	3	3	198	208			PEAKS DB
R.IVTESVGGQVVGQYVEPTPVQSSR.A	Y	78.52	2515.2917	24	3.6	1258.6577	2	31.58	11	F11:1559	OB5938 H3A raw.raw	1.1935E5	2	2	99	122			PEAKS DB
R.NNTGGTTPGGVAASVAAAAR.L	Y	63.04	1741.8707	20	1.0	871.9435	2	32.21	9	F9:1555	OB5936 H3A raw.raw	9.5216E4	3	3	230	249			PEAKS DB
R.IVTESVGGQ(+.98)VVGQYVEPTPVQSSR.A	Y	52.37	2516.2759	24	9.6	1259.1573	2	31.77	7	F7:1521	OB5934 H3B raw.raw	9.01E3	1	1	99	122		Q9:Deamidation (NQ):48.43	PEAKS DB
R.NNTGGTTPGGVAASVAAAAR(+15.99).L	Y	36.10	1757.8656	20	2.7	879.9424	2	30.14	9	F9:1433	OB5936 H3A raw.raw	1.8033E4	1	1	230	249		R20:Oxidation or Hydroxylation: 20.78	PEAKS PTM
**total 6 peptides**																			

Q42515|Q42515_ARAHY

back to list

| **Protein Coverage** | **Supporting Peptides** |

Protein Coverage:

```
  1 MKFCNFFTFT LALIVVLQAL GASADDAGTI ITQPLYNEFL KHLTDSRCEA HGFYTYNAFV TAARAFPAFG TTGDDVTRKR
 81 ELAAFFGQTS HETTGGTTNA PDEFEWGYCF LREQTKEQHC DSTQAPCPAG KQYYGRGPIQ LTSNSNYQLA GQAIKADLIN
161 NPDLVATDAV ISFKTAIWFW MTPQGNKPSC HDVITNAWRP TATDSAAGRA PGYGVITNII NGGIECGKGA NTNSNNRIGF
241 YKRYCDILKI GYGSNLDCAN QQHF
```

c Carbamidomethylation (+57.02)

Supporting Peptides:

Peptide	Uniq	-10lgP	Mass	Length	ppm	m/z	z	RT	Fraction	Scan	Source File	Area Raw in gel	#Feature	#Feature Raw in gel	Start	End	PTM	AScore	Found By
K.ADLINNPDLVATDAVISFK.T	Y	96.44	2015.0575	19	2.8	1008.5388	2	36.32	8	F8:1776	OB5935 H3B raw.raw	3.6486E5	3	3	156	174			PEAKS DB
A.DDAGTIITQPLYNEFLK.H	Y	94.08	1936.9781	17	1.8	969.4980	2	37.02	7	F7:1823	OB5934 H3B raw.raw	3.7509E5	3	3	25	41			PEAKS DB
R.APGYGVITNIINGGIEC(+57.02)GK.G	Y	67.45	1931.9774	19	2.8	966.9987	2	35.96	8	F8:1755	OB5935 H3B raw.raw	1.6903E5	2	2	210	228	Carbamidomethylation	C17:Carbamidomethylation:1000.00	PEAKS DB
**total 3 peptides**																			

B4UW89|B4UW89_ARAHY

back to list

| **Protein Coverage** | **Supporting Peptides** |

Protein Coverage:

```
  1 MSMIPSVFGG RRSNILDPFS LDVWDPFQDI FSVAMSGPNA SASAREASAI ASTRVDWKET PEAHVFNVDL PGLKKEEVKV
                                                                  136
                                                                   d
 81 EVEDGRVLQI SGERSREQEQ KDDRWHRVER STGKFMRRFR LPENANMDEI RAAMENGVLT ITVPKVEEKK PEIKSIQISG
```

d Deamidation (NQ) (+0.98)

Supporting Peptides:

Peptide	Uniq	-10lgP	Mass	Length	ppm	m/z	z	RT	Fraction	Scan	Source File	Area Raw in gel	#Feature	#Feature Raw in gel	Start	End	PTM	AScore	Found By
R.AAMENGVLTITVPK.V	Y	86.90	1442.7803	14	1.7	722.3986	2	32.99	4	F4:1605	OB5931 H6 raw.raw	1.2216E4	2	2	132	145			PEAKS DB
R.LPENANMDEIR.A	Y	75.25	1300.6082	11	0.1	651.3114	2	28.51	3	F3:1341	OB5930 H6 raw.raw	9.9555E3	2	2	121	131			PEAKS DB
R.AAMEN(+.98)GVLTITVPK.V	Y	66.74	1443.7643	14	2.8	722.8914	2	33.41	4	F4:1632	OB5931 H6 raw.raw	0	0	0	132	145	Deamidation (NQ)	N5:Deamidation (NQ):1000.00	PEAKS DB
R.FRLPENANMDEIR.A	Y	45.65	1603.7776	13	1.5	802.8973	2	32.99	4	F4:1613	OB5931 H6 raw.raw	1.3562E4	2	2	119	131			PEAKS DB
R.VLQISGER.S	Y	40.10	900.5029	8	1.1	451.2592	2	25.82	6	F6:1202	OB5933 H2 raw.raw	1.3543E3	1	1	87	94			PEAKS DB
**total 5 peptides**																			

A0A0R4UXQ1|A0A0R4UXQ1_ARAHY

back to list

| **Protein Coverage** | **Supporting Peptides** |

Protein Coverage:

C Carbamidomethylation (+57.02)

```
  1 MEKAINRQKV LLHHLNPSSS NVDLSSSSSI FASVCLAGDS AAYQRTGVFG DDVVIVAAYR TAICKAKRGG FKDTPADDLL
 81 APVLKAVVEK TNLNPSEVGD IVVGSVLAPG ALRATECRMA AFYAGFPETV PVRTVNRQCS SGLQAVADVA ASIRAGFYEI
161 GIGAGLESMS TNAMGWDGDV NPKVKQFEQA RNCLLPMGVT SENVAQRFGV SRKEQDQAAV ESHRRAAAAT AAGKFKDEII
241 PVSTKIVDPK TGDETPVTIS VDDGIRPNAS VADLGKLKPV FKKDGSTTAG NSSQVSDGAG AVLLMKRSTA MQKGLPILGV
321 FRSFSAVGVD PAIMGVGPAA AIPVAVKAAG LELDDIDLFE INEAFASQFV YCRNKLGLDA EKINVNGGAM ALGHPLGATG
401 ARCTATLLHE MKRRGKDCRF GVISMCIGTG MGAAAVFERG DGVDELSNAR KVATNNLLSK DAR
```

Supporting Peptides:

Peptide	Uniq	-10lgP	Mass	Length	ppm	m/z	z	RT	Fraction	Scan	Source File	Area Raw in gel	#Feature	#Feature Raw in gel	Start	End	PTM	AScore	Found By
K.TNLNPSEVGDIVVGSVLAPGALR.A	Y	105.64	2277.2329	23	1.4	1139.6254	2	37.36	11	F11:1898	OB5938 H3A raw.raw	4.468E5	2	2	91	113			PEAKS DB
K.TGDETPVTISVDDGIRPNASVADLGK.L	Y	50.70	2626.3086	26	3.0	876.4461	3	32.33	10	F10:1599	OB5937 H3A raw.raw	4.6998E5	2	2	251	276			PEAKS DB
K.AAGLELDDIDLFEINEAFASQFVYC(+57.02)R.N	Y	49.27	3005.4116	26	5.1	1503.7207	2	42.64	9	F9:2172	OB5936 H3A raw.raw	2.4389E4	1	1	348	373	Carbamidomethylation	C25:Carbamidomethylation:1000.00	PEAKS DB
total 3 peptides																			

D4NXQ0|D4NXQ0_ARAHY

back to list

| Protein Coverage | Supporting Peptides |

Protein Coverage:

d Deamidation (NQ) (+0.98)

```
  1 MKSMRIHVLF LFSLLSTIPH YSYASVNDFC VADLKGPDSP SGYNCKPVSA VTADDFVYSG LVAGDTNNTF KAALTSAFVT
                     86
                     d
 81 DFPAVNGLGV SAARLDIAKG GSIPMHTHPG ATELLIMVEG EITAGFMTPF ALYSKTLKPG DVFVFPQGQL HFQVNSGKKK
161 ATAFLAFSSA NPGAQLLDLL LFGNALSSDI VAQTTFLDID QVKKLKARFG GKNLSKHMSM
```

Supporting Peptides:

Peptide	Uniq	-10lgP	Mass	Length	ppm	m/z	z	RT	Fraction	Scan	Source File	Area Raw in gel	#Feature	#Feature Raw in gel	Start	End	PTM	AScore	Found By
K.AALTSAFVTDFPAVNGLGVSAAR.L	Y	128.12	2234.1694	23	2.4	1118.0947	2	36.64	7	F7:1799	OB5934 H3B raw.raw	4.1064E5	3	3	72	94			PEAKS DB
K.AALTSAFVTDFPAVN(+.98)GLGVSAAR.L	Y	53.20	2235.1536	23	4.7	1118.5894	2	36.72	8	F8:1796	OB5935 H3B raw.raw	0	0	0	72	94	Deamidation (NQ)	N15:Deamidation (NQ):1000.00	PEAKS DB
total 2 peptides																			

A7LIS5|A7LIS5_ARAHY

back to list

| Protein Coverage | Supporting Peptides |

Protein Coverage:

d Deamidation (NQ) (+0.98)

```
  1 MKSMRIHVLF LFSLLSTIPH YSYASVNDFC VADLKGPDSP SGYNCKPVSA VTADDFVYSG LVAGDTNNTF KAALTSAFVT
                     86
                     d
 81 DFPAVNGLGV SAARLDIAKG GSIPMHTHPG ATELLIMVEG EITAGFMTPF ALYSKTLKPG DVFVFPQGQL HFQVNSGKKK
161 ATAFLAFSSA NPGAQLLDLL LFGNALSSDI VAQTTFLDID QVKKLKARFG GKNLNNHMSM
```

Supporting Peptides:

Peptide	Uniq	-10lgP	Mass	Length	ppm	m/z	z	RT	Fraction	Scan	Source File	Area Raw in gel	#Feature	#Feature Raw in gel	Start	End	PTM	AScore	Found By
K.AALTSAFVTDFPAVNGLGVSAAR.L	Y	128.12	2234.1694	23	2.4	1118.0947	2	36.64	7	F7:1799	OB5934 H3B raw.raw	4.1064E5	3	3	72	94			PEAKS DB
K.AALTSAFVTDFPAVN(+.98)GLGVSAAR.L	Y	53.20	2235.1536	23	4.7	1118.5894	2	36.72	8	F8:1796	OB5935 H3B raw.raw	0	0	0	72	94	Deamidation (NQ)	N15:Deamidation (NQ):1000.00	PEAKS DB
total 2 peptides																			

B4UWD5|B4UWD5_ARAHY

back to list

| Protein Coverage | Supporting Peptides |

Protein Coverage:

1 MARYDR**AITV FSPDGHLFQV EYALEAVR**KG NAAVGVRGTD NVVLGVEKKS TAKLQDSRTV RKIVNLDDHI ALACAGLKAD

81 ARVLINRARV ECQSHR**LTVE DPVTVEYITR** YIAGLHQSYT QSGGVRPFGL STLIVGF

Supporting Peptides:

Peptide	Uniq	-10lgP	Mass	Length	ppm	m/z	z	RT	Fraction	Scan	Source File	Area Raw in gel	#Feature	#Feature Raw in gel	Start	End	PTM	AScore	Found By
R.LTVEDPVTVEYITR.Y	Y	94.75	1633.8563	14	2.5	817.9375	2	33.34	8	F8:1599	OB5935 H3B raw.raw	9.0801E5	3	3	97	110			PEAKS DB
R.AITVFSPDGHLFQVEYALEAVR.K	Y	50.65	2461.2642	22	5.9	821.4335	3	38.74	2	F2:1769	OB5925 H3B raw.raw	2.6576E5	1	1	7	28			PEAKS DB
total 2 peptides																			

Q45W80|Q45W80_ARAHY

back to list

| Protein Coverage | Supporting Peptides |

Protein Coverage:

1 M**AEQTFIMIK PDGVQRGLVG EIISRFEK**KG FYLKGLKLIT VDRPFAERHY SDLSAKPFFN GLVEYIISGP VVAMVWEGKG

a Acetylation (Protein N-term) (+42.01)

81 VVATGRK**LIG ATNPLASEPG TIR**GDFAIDI GRNVIHGSDS VESATKEIAL WFPEGPANWQ SSLHSWIYE

Supporting Peptides:

Peptide	Uniq	-10lgP	Mass	Length	ppm	m/z	z	RT	Fraction	Scan	Source File	Area Raw in gel	#Feature	#Feature Raw in gel	Start	End	PTM	AScore	Found By
R.GLVGEIISRFEK.K	Y	66.28	1346.7557	12	0.4	674.3854	2	38.32	3	F3:1916	OB5930 H6 raw.raw	1.5957E4	1	1	17	28			PEAKS DB
M.A(+42.01)EQTFIMIKPDGVQR.G	Y	54.80	1773.9083	15	0.0	887.9614	2	34.01	3	F3:1657	OB5930 H6 raw.raw	2.1082E4	2	2	2	16	Acetylation (Protein N-term)	A1:Acetylation (Protein N-term):1000.00	PEAKS PTM
K.LIGATNPLASEPGTIR.G	Y	38.29	1608.8834	16	3.4	805.4517	2	31.83	3	F3:1539	OB5930 H6 raw.raw	6.2326E3	1	1	88	103			PEAKS DB
total 3 peptides																			

E5FHZ2|E5FHZ2_ARAHY

back to list

Prepared with PEAKS ™ (bioinfor.com)

| Protein Coverage | Supporting Peptides |

Protein Coverage:

1 MSQQQTPRRP QEEGQEHKDP IKYGDIFNVS GDLASEPIMP QDAALMQATE NQALGQTQKG GPASVMQSAA VTNVSAGLVG
81 RGDVSSVAKD QGICVLESKI GVNRVITESV GEQVVGEFVE PNVPMKTPGS ALDKDAITIG EALEATTLAP ASDKPVDQSD
161 AAAIQAAEMR ATGKNEIQPG GLAAVAQSAA TKNLRTMGDF QKTTLSDVLT DAKEKLPSDK **AVTREDAEGV IGAEIR**NKAD
241 MK**TTPGGVAA SLAAAATLNQ NK**SSS

Supporting Peptides:

Peptide	Uniq	-10lgP	Mass	Length	ppm	m/z	z	RT	Fraction	Scan	Source File	Area Raw in gel	#Feature	#Feature Raw in gel	Start	End	PTM	AScore	Found By
K.TTPGGVAASLAAAATLNQNK.S	Y	79.74	1854.9799	20	2.2	928.4993	2	33.30	2	F2:1460	OB5925 H3B raw.raw	6.4018E4	1	1	243	262			PEAKS DB
K.AVTREDAEGVIGAEIR.N	Y	33.63	1684.8744	16	1.9	843.4461	2	29.50	11	F11:1432	OB5938 H3A raw.raw	0	0	0	221	236			PEAKS DB
total 2 peptides																			

A0A290FZZ3|A0A290FZZ3_ARAHY

back to list

| Protein Coverage | Supporting Peptides |

Protein Coverage:

1 SDPSGMLSKW IRGTDCCTWP GLNCLFENKR VTSISIAGQP DQPNSFLSGT ISSSLSKLQF LDGIYFTNLR NISGPFPGFL
81 LNMPNLEYIY IENSQISGRI PDSFGNSTRK FGAFSFQGNR **LTGTVPSSLS LLTQLTQLK**L GDNLLTGAIP DGIRNLKNLT
161 YLSLQGNQLS GNIPDFFTSL KNLRILELSR NKFSGTIPAS IATLAPTLGY LELGHNSLSG KIPDFLGKMK ALDTLDLSSN
241 RFTGSVPQSF KNLTKIFNLD LSNNLLVDPF PEMNVKGIES LDLSNNNLHL GTIPKWVTSS PIIYSLKLAK CGIRMKLDDW
321 KPSETYFYDY IDLSGNDISG SAIGLLNRTD YLVGFWASGN KLKFDMGGLR IVEKLKY

Supporting Peptides:

Peptide	Uniq	-10lgP	Mass	Length	ppm	m/z	z	RT	Fraction	Scan	Source File	Area Raw in gel	#Feature	#Feature Raw in gel	Start	End	PTM	AScore	Found By
R.LTGTVPSSLSLLTQLTQLK.L	Y	84.25	1999.1565	19	-0.9	1000.5846	2	38.02	12	F12:1918	OB5939 H1 raw.raw	1.8555E5	1	1	121	139			PEAKS DB
total 1 peptides																			

N1NJN8|N1NJN8_ARAHY

back to list

| Protein Coverage | Supporting Peptides |

Protein Coverage:

1 MECVFGLVGK GFAIVVADTS AVHGILVHKT NEEKIMKLDS HKLMAASGEG GDRVQFTEYI LK**NVALYQFR** NGIPLTTSAA
81 ANFTRAELAA ALRKLVGVVF VQNPYAVNIL LAGYDNHTGP SLYYIDYLAT LHKVDKAAFG YASYFSLSIM DTHYHAGMSL
161 QEAIDLVDKC ITEIRSR**LVV APPNFVIK**IV DQHGARECAW RQSLPSASPT FIHN

Supporting Peptides:

Peptide	Uniq	-10lgP	Mass	Length	ppm	m/z	z	RT	Fraction	Scan	Source File	Area Raw in gel	#Feature	#Feature Raw in gel	Start	End	PTM	AScore	Found By
K.NVALYQFR.N	Y	68.32	1009.5345	8	-0.1	505.7745	2	30.58	5	F5:1470	OB5932 H2 raw.raw	1.2272E4	1	1	63	70			PEAKS DB
R.LVVAPPNFVIK.I	Y	56.67	1195.7328	11	0.7	598.8741	2	33.55	5	F5:1641	OB5932 H2 raw.raw	2.964E5	2	2	178	188			PEAKS DB
total 2 peptides																			

Q1PCR5|Q1PCR5_ARAHY

back to list

| Protein Coverage | Supporting Peptides |

Protein Coverage:

1 SDRSHAPPVV MAAAGFRLAD TFHTGASMGG YISSQSVKKI IEINPYMLGT MAGGAADCQF WHRNLGIKCR LHELANKRRI
81 SVTGASKLLA NILYSYRGMG LSVGTMIAGW DETGPGLYYV DSEGGRLKGT RFSVGSGSPY AYGILDSGYR YDLSVDEAVE
161 LGRRAIYHAT FRDGASGGVV SVYHVGPEGW KKWTGDDVGD LHYQYYPVVP STVEQEMAEA TGA

Supporting Peptides:

Peptide	Uniq	-10lgP	Mass	Length	ppm	m/z	z	RT	Fraction	Scan	Source File	Area Raw in gel	#Feature	#Feature Raw in gel	Start	End	PTM	AScore	Found By
K.LLANILYSYR.G	Y	95.82	1224.6866	10	0.8	613.3511	2	34.42	7	F7:1667	OB5934 H3B raw.raw	8.8119E5	5	5	88	97			PEAKS DB
**total 1 peptides**																			

Q06H31|Q06H31_ARAHY

back to list

| Protein Coverage | Supporting Peptides |

Protein Coverage:

1 MLVYQDLLTG DELLSDSFPY KEIENGILWE VEGKWVIQGA INVDIGANPS AEGGEEDESV DDQAVKVVDI VDTFRLQEQP
81 AFDKKQFVTY MKRYIKLLTA KLEPEQQEHF KKNIEGATKF LLSKLSDLQF FVGESMHDDG SLVFAYYKEG ATDPTFLYFA
161 HGLKEIKC

d Deamidation (NQ) (+0.98)
o Oxidation (M) (+15.99)

Supporting Peptides:

Peptide	Uniq	-10lgP	Mass	Length	ppm	m/z	z	RT	Fraction	Scan	Source File	Area Raw in gel	#Feature	#Feature Raw in gel	Start	End	PTM	AScore	Found By
MLVYQDLLTGDELLSDSFPYKEIENGILWEVEGK.W	Y	64.83	3942.9487	34	3.9	1315.3286	3	43.02	7	F7:2174	OB5934 H3B raw.raw	2.2514E5	1	1	1	34			PEAKS DB
M(+15.99)LVYQDLLTGDELLSDSFPYKEIENGILWEVEGK.W	Y	61.28	3958.9436	34	3.4	1320.6597	3	42.89	8	F8:2152	OB5935 H3B raw.raw	4.2504E5	2	2	1	34	Oxidation (M)	M1:Oxidation (M):1000.00	PEAKS DB
K.VVDIVDTFR.L	Y	58.97	1062.5709	9	-0.3	532.2926	2	32.96	8	F8:1559	OB5935 H3B raw.raw	1.3289E4	1	1	67	75			PEAKS DB
MLVYQDLLTGDELLSDSFPYKEIEN(+.98)GILWEVEGK.W	Y	35.73	3943.9329	34	3.6	1315.6562	3	40.52	6	F6:2071	OB5933 H2 raw.raw	0	0	0	1	34	Deamidation (NQ)	N25:Deamidation (NQ):59.09	PEAKS DB
**total 4 peptides**																			

Q06H23|Q06H23_ARAHY

back to list

| Protein Coverage | Supporting Peptides |

Protein Coverage:

1 MASKRILKEL KDLQKDPPTS CSAGPVAEDM FHWQATIMGP ADSPYTGGVF LVSIHFPPDY PFKPPKVAFR TKVFHPNINS
81 NGSICLDILK EQWSPALTIS KVLLSICSLL TDPNPDDPLV PEIAHMYKTD RAKYEATARS WTQKYAMG

c Carbamidomethylation (+57.02)
o Oxidation (M) (+15.99)

Supporting Peptides:

Peptide	Uniq	-10lgP	Mass	Length	ppm	m/z	z	RT	Fraction	Scan	Source File	Area Raw in gel	#Feature	#Feature Raw in gel	Start	End	PTM	AScore	Found By
K.VLLSIC(+57.02)SLLTDPNPDDPLVPEIAHM(+15.99)YK.T	Y	88.89	3065.5454	27	3.0	1022.8588	3	38.41	3	F3:1915	OB5930 H6 raw.raw	2.3781E4	1	1	102	128	Carbamidomethylation; Oxidation (M)	C6:Carbamidomethylation:1000.00;M25:Oxidation (M):1000.00	PEAKS DB
K.VLLSIC(+57.02)SLLTDPNPDDPLVPEIAHMYK.T	Y	60.27	3049.5503	27	2.0	1017.5261	3	39.46	4	F4:1971	OB5931 H6 raw.raw	7.298E4	1	1	102	128	Carbamidomethylation	C6:Carbamidomethylation:1000.00	PEAKS DB
**total 2 peptides**																			

Q1HDS7|Q1HDS7_ARAHY

back to list

| Protein Coverage | Supporting Peptides |

Protein Coverage:

1 MVKAVAVLSS SEGVSGTIQF SQEGNGPTTV TGNLAGLKPG LHGFHVHALG DTTNGCLSTG PHFNPNNKEH GAPEDENR**HA**

81 **GDLGNVNVGD DGTVSFSISD SQIPLSGPNS IVGRAVVVHA DPDDLGK**GGH ELSKSTGNAG GRVACGIIGL QG

Supporting Peptides:

Peptide	Uniq	-10lgP	Mass	Length	ppm	m/z	z	RT	Fraction	Scan	Source File	Area Raw in gel	#Feature	#Feature Raw in gel	Start	End	PTM	AScore	Found By
R.AVVVHADPDDLGK.G	Y	63.86	1334.6830	13	0.7	668.3492	2	27.55	4	F4:1279	OB5931 H6 raw.raw	7.1935E2	1	1	115	127			PEAKS DB
R.HAGDLGNVNVGDDGTVSFSISDSQIPLSGPNSIVGR.A	Y	40.27	3580.7393	36	4.0	1791.3840	2	35.84	4	F4:1773	OB5931 H6 raw.raw	1.9646E5	1	1	79	114			PEAKS DB
total 2 peptides																			

Q45W82|Q45W82_ARAHY

back to list

| Protein Coverage | Supporting Peptides |

Protein Coverage:

1 MVKAVAVLSS SEGVSGTIQF SQEGNGPTTV TGNLAGLKPG LHGFHVHALG DTTNGCLSTG PHFNPNNKEH GAPEDENR**HA**

81 **GDLGNVNVGD DGTVSFSISD SQIPLSGPNS IVGRAVVVHA DPDDLGK**GGH ELSKSTGNAG GGVACGIIGL QG

Supporting Peptides:

Peptide	Uniq	-10lgP	Mass	Length	ppm	m/z	z	RT	Fraction	Scan	Source File	Area Raw in gel	#Feature	#Feature Raw in gel	Start	End	PTM	AScore	Found By
R.AVVVHADPDDLGK.G	Y	63.86	1334.6830	13	0.7	668.3492	2	27.55	4	F4:1279	OB5931 H6 raw.raw	7.1935E2	1	1	115	127			PEAKS DB
R.HAGDLGNVNVGDDGTVSFSISDSQIPLSGPNSIVGR.A	Y	40.27	3580.7393	36	4.0	1791.3840	2	35.84	4	F4:1773	OB5931 H6 raw.raw	1.9646E5	1	1	79	114			PEAKS DB
total 2 peptides																			

K7PQ65|K7PQ65_ARAHY

back to list

| Protein Coverage | Supporting Peptides |

Protein Coverage:

1 MCMMGASVDP LVVGRVIGDV IDM**FVPSVGM SVYYGSK**HVT NGCDIKPSMA ISPPKVTLTG GNIHSLYTLV MTDPDAPSPS

81 EPTMREWLHW VVVDIPGGTN PTQGKEIVAY MGPRPPVGIH R**YILILFEQK** GVLGGVEQPA ARASFNTRYF ARQFNLGLPV

161 ATVYFNSQKE PASKRR

Supporting Peptides:

Peptide	Uniq	-10lgP	Mass	Length	ppm	m/z	z	RT	Fraction	Scan	Source File	Area Raw in gel	#Feature	#Feature Raw in gel	Start	End	PTM	AScore	Found By
R.YILILFEQK.G	Y	63.49	1165.6747	9	-3.3	583.8427	2	36.41	3	F3:1808	OB5930 H6 raw.raw	1.435E4	2	2	122	130			PEAKS DB
M.FVPSVGMSVYYGSK.H	Y	35.21	1519.7380	14	1.3	760.8773	2	32.86	3	F3:1596	OB5930 H6 raw.raw	0	0	0	24	37			PEAKS DB
total 2 peptides																			

B4UWB3|B4UWB3_ARAHY

back to list

| Protein Coverage | Supporting Peptides |

Protein Coverage:

1 MKYNPRVSSS RRKSRKAHFT APSSVRR**VLM SAPLTTDLR**S KYNVRSMPIR KDDEVQVVRG TYKGREGKVV QVYRRKWVIH

81 IERITREKVN GSTVNVGIHP SKVVITKLRL DKDRKSLLDR KAKGRAAADK EKGTKFAPED IMQTVD

Supporting Peptides:

Peptide	Uniq	-10lgP	Mass	Length	ppm	m/z	z	RT	Fraction	Scan	Source File	Area Raw in gel	#Feature	#Feature Raw in gel	Start	End	PTM	AScore	Found By
R.VLMSAPLTTDLR.S	Y	80.51	1315.7169	12	1.0	658.8664	2	32.24	1	F1:1455	OB5921 H2 raw.raw	0	0	0	28	39			PEAKS DB
total 1 peptides																			

Q647G5|Q647G5_ARAHY

back to list

| Protein Coverage | Supporting Peptides |

Protein Coverage:

1 MTDRTQPHTV QVHTTAGRFG DTAAGTNRYP DRGPSTSKVI AVITGLPIGG TLLLFAGLAL AGTLLGLAVT TPLFILFSPV
81 IVPAIIVVGL SVAGFLTSGA CGLTGLSSFS WVMNYIRQTH GSVPEQLEMA KHR**MADVAGY VGQK**TKDVGQ KTKEVGQEIQ
161 TKAQDSKRT

Supporting Peptides:

Peptide	Uniq	-10lgP	Mass	Length	ppm	m/z	z	RT	Fraction	Scan	Source File	Area Raw in gel	#Feature	#Feature Raw in gel	Start	End	PTM	AScore	Found By
R.MADVAGYVGQK.T	Y	79.23	1137.5488	11	0.8	569.7822	2	28.42	4	F4:1311	OB5931 H6 raw.raw	4.7315E4	2	2	134	144			PEAKS DB
total 1 peptides																			

Q647G4|Q647G4_ARAHY

back to list

| Protein Coverage | Supporting Peptides |

Protein Coverage:

1 MTDRTQPHAV QVHTTAGRFG DTAAGTNRYA DRGPSTSKVI AVITGLPIGG TLLLFAGLAL AGTLLGLAVT TPLFILFSPV
81 IVPATIVVGL SVAGFLTSGA CGLTGLSSFS WVMNYIRQTH GSVPEQLEMA KHR**MADVAGY VGQK**TKDVGQ

Supporting Peptides:

Peptide	Uniq	-10lgP	Mass	Length	ppm	m/z	z	RT	Fraction	Scan	Source File	Area Raw in gel	#Feature	#Feature Raw in gel	Start	End	PTM	AScore	Found By
R.MADVAGYVGQK.T	Y	79.23	1137.5488	11	0.8	569.7822	2	28.42	4	F4:1311	OB5931 H6 raw.raw	4.7315E4	2	2	134	144			PEAKS DB
total 1 peptides																			

B4UWC9|B4UWC9_ARAHY

back to list

| Protein Coverage | Supporting Peptides |

Protein Coverage:

1 MFKKFSFEDV SAQNQVKASV QRKIR**QSIAE EYPGLEPVLD DILPK**KSPLI VAKCQNHLNL VLVNNVPLFF SVRDGPYMPT
81 LRLLHQYPNI MKKLQVDRGA IRFVLSGANI MCPGLTSPGG ALDEEVE

Supporting Peptides:

Peptide	Uniq	-10lgP	Mass	Length	ppm	m/z	z	RT	Fraction	Scan	Source File	Area Raw in gel	#Feature	#Feature Raw in gel	Start	End	PTM	AScore	Found By
R.QSIAEEYPGLEPVLDDILPK.K	Y	67.57	2225.1467	20	1.4	1113.5822	2	38.37	5	F5:1928	OB5932 H2 raw.raw	1.231E5	1	1	26	45			PEAKS DB
total 1 peptides																			

B4UWB2|B4UWB2_ARAHY

back to list

| Protein Coverage | Supporting Peptides |

Protein Coverage:

1 MKSSSSVIAA MICFAMVTGM AASAAEPVLD VTGQKLRSGV K**YFILPVLR**G RGGGLTVGSS VNSTCPIYVL QDKLEVTRGT
81 PVTFTPSTPN KDGVILTSTD LNINSTSAPK CKESSVWRLL KVLSGVWFIS TDGVAGNPGV NTVVNWFKIE KDGKDYNLSF
161 CPSVCNCSTL CRALGIFTDS DGTKHLALSD QVPTFRSCSR RLN

Supporting Peptides:

Peptide	Uniq	-10lgP	Mass	Length	ppm	m/z	z	RT	Fraction	Scan	Source File	Area Raw in gel	#Feature	#Feature Raw in gel	Start	End	PTM	AScore	Found By
K.YFILPVLR.G	Y	65.65	1019.6168	8	0.3	510.8158	2	36.11	1	F1:1685	OB5921 H2 raw.raw	2.8581E5	2	2	42	49			PEAKS DB
total 1 peptides																			

A0A290GK88|A0A290GK88_ARAHY

back to list

| Protein Coverage | Supporting Peptides |

Protein Coverage:

1 GLDLPGPVDL PPSLFNLVDL DELLIFEMPN LKGQIPPQIS NLKKLTVLYL FSTNXSGPIP ESLAQIKTLT NINLAGNNLS
81 GPLPHLLPSL PSIRVIFADE NRISGPIPXS YGSFASXXLX TXTLSHNMLS GKIPAALKGL DAELLDLSWN KLEGDGSVLF
161 GAEKKTEEIT LAGNMLSFDI GKVEFGRNIT RLNLKHNRIX GKLPEQLTQL KQLTRFNVSY NQL

Supporting Peptides:

Peptide	Uniq	-10lgP	Mass	Length	ppm	m/z	z	RT	Fraction	Scan	Source File	Area Raw in gel	#Feature	#Feature Raw in gel	Start	End	PTM	AScore	Found By
K.GLDAELLDLSWNKLEGDGSVLFGAEK.K	Y	65.30	2775.3965	26	2.9	926.1421	3	39.81	9	F9:2010	OB5936 H3A raw.raw	2.2447E5	1	1	139	164			PEAKS DB
**total 1 peptides**																			

A0A0A1EXN0|A0A0A1EXN0_ARAHY

back to list

| Protein Coverage | Supporting Peptides |

Protein Coverage:

1 VFPSIVGRPR HTGVMVGMGQ KDAYVGDEAQ SKRGILTLKY PIEHGIVSNW DDMEKIWHHT FYNELRVAPE EHPVLLTEAP
81 LNPKANREKM TQIMFETFNV PAMYVAIQAV LSLYASGRTT GIVLDSGDGV SHTVPIYEGY ALPHAILRLD LAGRDLTDSL
161 MKILTERGYM FTTSAEREIV RDIKEKLAYV ALDYEQELET AKSSSSVEKN YELPDGQVIT IGAERFRCPE VLFQPSMIGM
241 EAAGIHETTY NSIMKCDVDI RKDLYGNIVL SGGSTMFPGI ADRMSKEITA LAPSSMKIKV VAPPERKYSV WIGGSILASL
321 STF

Supporting Peptides:

Peptide	Uniq	-10lgP	Mass	Length	ppm	m/z	z	RT	Fraction	Scan	Source File	Area Raw in gel	#Feature	#Feature Raw in gel	Start	End	PTM	AScore	Found By
R.TTGIVLDSGDGVSHTVPIYEGYALPHAILR.L	Y	65.01	3150.6350	30	1.6	1051.2206	3	36.10	9	F9:1789	OB5936 H3A raw.raw	7.7614E5	3	3	119	148			PEAKS DB
**total 1 peptides**																			

A0A0A1EUV7|A0A0A1EUV7_ARAHY

back to list

| Protein Coverage | Supporting Peptides |

Protein Coverage:

1 VFPSIVGRPR HTGVMVGMGQ KDAYVGDEAQ SKRGILTLKY PIEHGIVSNW DDMEKIWHHT FYNELRVAPE EHPVLLTEAP
81 LNPKANREKM TQIMFETFNA PAMHVAIQAV LSLYASGRTT GIVLDSGDGV SHTVPIYEGY ALPHAILRLD LAGRDLTDYL
161 MKILTERGYS FTTSAEREIV RDMKEKLSYI ALDYEQELET SKTSSAVEKS YELPDGQVIT IGAERFRCPE VLFQPSMIGM
241 EAAGIHETTY NSIMKCDVDI RKDLYGNIVL SGGTTMFPGI ADRMSKEISA LAPSSMKIKV VAPPERKYSV WIGGSILASL
321 STF

Supporting Peptides:

Peptide	Uniq	-10lgP	Mass	Length	ppm	m/z	z	RT	Fraction	Scan	Source File	Area Raw in gel	#Feature	#Feature Raw in gel	Start	End	PTM	AScore	Found By
R.TTGIVLDSGDGVSHTVPIYEGYALPHAILR.L	Y	65.01	3150.6350	30	1.6	1051.2206	3	36.10	9	F9:1789	OB5936 H3A raw.raw	7.7614E5	3	3	119	148			PEAKS DB
**total 1 peptides**																			

A0A0A1ESG0|A0A0A1ESG0_ARAHY

back to list

| Protein Coverage | Supporting Peptides |

Protein Coverage:

1 VFPSIVGRPR HTGVMVGMGQ KDAYVGDEAQ SKRGILTLKY PIEHGIVSNW DDMEKIWHHT FYNELRVAPE EHPVLLTEAP
81 LNPKANREKM TQIMFETFNV PAMYVAIQAV LSLYASGR**TT GIVLDSGDGV SHTVPIYEGY ALPHAILR**LD LAGRDLTDSL
161 MKILTERGYM FTTSAEREIV RDIKEKLAYV ALDYEQELET AKSSSAVEKN YELPDGQVIT IGAERFRCPE VLFQPSMIGM
241 EAAGIHETTY NSIMKCDVDI RKDLYGNIVL SGGSTMFPGI ADRMSKEITA LAPSSMKIKV VAPPERKYSV WIGGSILASL
321 STF

Supporting Peptides:

Peptide	Uniq	-10lgP	Mass	Length	ppm	m/z	z	RT	Fraction	Scan	Source File	Area Raw in gel	#Feature	#Feature Raw in gel	Start	End	PTM	AScore	Found By
R.TTGIVLDSGDGVSHTVPIYEGYALPHAILR.L	Y	65.01	3150.6350	30	1.6	1051.2206	3	36.10	9	F9:1789	OB5936 H3A raw.raw	7.7614E5	3	3	119	148			PEAKS DB
**total 1 peptides**																			

Q45W77|Q45W77_ARAHY

back to list

| Protein Coverage | Supporting Peptides |

Protein Coverage:

1 MANSNLPRRI IKETQR**LLSE PAPGISASPS EDNMR**YFNVM ILGPTQSPYE GGVFKLELFL PEEYPMAAPK VRFLTKIYHP
81 NIDKLGRICL DILKDKWSPA LQIRTVLLSI QALLSAPNPD DPLSENIAKH WKSNEAEAVE TAKEWTRLYA SGA

Supporting Peptides:

Peptide	Uniq	-10lgP	Mass	Length	ppm	m/z	z	RT	Fraction	Scan	Source File	Area Raw in gel	#Feature	#Feature Raw in gel	Start	End	PTM	AScore	Found By
R.LLSEPAPGISASPSEDNMR.Y	Y	60.66	1969.9414	19	2.5	985.9805	2	30.39	3	F3:1454	OB5930 H6 raw.raw	5.3162E3	1	1	17	35			PEAKS DB
**total 1 peptides**																			

F8UX79|F8UX79_ARAHY

back to list

| Protein Coverage | Supporting Peptides |

Protein Coverage:

76 d

1 MASATLSVAK PALQGNGGKG FSEFSGLRNS SSSYLPFSRK TSDDFHSIIS FQTSAVGSSG GYRKGVTEAK LK**VAINGFGR** d Deamidation (NQ) (+0.98)
81 IGRNFLRCWH GRKDSPLDVI AINDTGGVKQ ASHLLKYDST LGIFDADVKP VGEDAISVDG KVIKVVSNRN PANLPWKELG
161 VDLVIEGTGV FVDREGAGKH IQAGAKKVLI TAPGKGDIPT YVVGVNADAY SPDEPIISNA SCTTNCLAPF VKVLDQKFGI
241 IKGTMTTTHS YTGDQRLLDA SHRDLRRARA AALNIVPTST GAAKAVALVL PTLKGKLNGI ALRVPTPNVS VVDLVVQVSK
321 KTFAEEVNAA FRESAEKELN GILSVCDEPL VSVDFRCTDV SSTVDSSLTM VMGDDMVKVI AWYDNEWGYS QRVVDLADIV
401 ANSWK

Supporting Peptides:

Peptide	Uniq	-10lgP	Mass	Length	ppm	m/z	z	RT	Fraction	Scan	Source File	Area Raw in gel	#Feature	#Feature Raw in gel	Start	End	PTM	AScore	Found By
K.VAIN(+.98)GFGR.I	Y	57.87	833.4395	8	0.1	417.7271	2	30.08	10	F10:1489	OB5937 H3A raw.raw	6.5904E5	3	3	73	80	Deamidation (NQ)	N4:Deamidation (NQ):1000.0 0	PEAKS DB
K.VAINGFGR.I	Y	50.26	832.4555	8	2.0	417.2359	2	29.51	10	F10:1408	OB5937 H3A raw.raw	3.9665E5	3	3	73	80			PEAKS DB
**total 2 peptides**																			

B4UW57|B4UW57_ARAHY

back to list

| Protein Coverage | Supporting Peptides |

Protein Coverage:

1 MASSASAGAS ARVDLDGNPI KPLTICMIGA GGFIGSHLCE KLMSETQHKV LALDVYNDKI KHLLEPDNLP WHGRITFHRL
81 NIKHDSRLEG LIKMSDLTIN LAAICTPADY NTRPLDTIYS NFIDALPVVK YCSENNKR**LI HFSTCEVYGK** TIGSFLPKDS
161 PLRQDPAYYV LKEDESPCIF GSIEKQRWSY ACAKQLIERL IYAEGAENGM EFTNVRA

C Carbamidomethylation (+57.02)

Supporting Peptides:

Peptide	Uniq	-10lgP	Mass	Length	ppm	m/z	z	RT	Fraction	Scan	Source File	Area Raw in gel	#Feature	#Feature Raw in gel	Start	End	PTM	AScore	Found By
R.LIHFSTC(+57.02)EVYGK.T	Y	53.39	1452.7072	12	0.0	727.3608	2	29.33	11	F11:1430	OB5938 H3A raw.raw	3.2436E4	2	2	139	150	Carbamidomethylation	C7:Carbamidomethylation:1000.00	PEAKS DB
**total 1 peptides**																			

E9JSA9|E9JSA9_ARAHY

back to list

| Protein Coverage | Supporting Peptides |

Protein Coverage:

1 MALRLNPNPS QKLFLSPSSS SSSSSSFSLP QMASLRSPRF RMASTLRTGS KEVENLKKPF TPPREVHVQV THSMPPQKIE
81 IFKSLEGWAE ENILTLLKPV EKCWQPQDYL PEPSEDGFEE QVRELRARAK ELPDDYFVVL VGDMITEEAL PTYQTMLNTL
161 DGVRDETGAS LTSWAVWTRA WTAEENRHGD LLNKYLYLSG RVDLRQIEKT IQYLIGSGMD PRTENSPYLG FIYTSFQERA
241 TFISHGNTAR LAKEHGDMKL AQICGMIASD EKRHETAYTK IVEKLFEIDP DGTVMAFADM MRKKIAMPAH LMYDGRDDNL
321 FESYSAVAQR **IGVYTAKDYA DILEFLVGR**W KVADLTGLSG EGRKAQDYVC GLPPRIRRLE ERAQGRAKES PRLKFSWIYD
401 REVQL

Supporting Peptides:

Peptide	Uniq	-10lgP	Mass	Length	ppm	m/z	z	RT	Fraction	Scan	Source File	Area Raw in gel	#Feature	#Feature Raw in gel	Start	End	PTM	AScore	Found By
R.IGVYTAKDYADILEFLVGR.W	Y	44.46	2142.1360	19	1.5	1072.0769	2	39.60	10	F10:2024	OB5937 H3A raw.raw	3.8207E4	1	1	331	349			PEAKS DB
**total 1 peptides**																			

E9JSA8|E9JSA8_ARAHY

back to list

| Protein Coverage | Supporting Peptides |

Protein Coverage:

1 MALRLNPNPS QKLFLSPSSS SSSSSSSFSL PQMASLRSPR FRMASTLRTG SKEVENLKKP FTPPREVHVQ VTHSMPPQKI
81 EIFKSLEGWA EENILTLLKP VEKCWQPQDY LPEPSEDGFE EQVRELRARA KELPDDYFVV LVGDMITEEA LPTYQTMLNT
161 LDGVRDETGA SLTSWAVWTR AWTAEENRHG DLLNKYLYLS GRVDLRQIEK TIQYLIGSGM DPRTENSPYL GFIYTSFQER
241 ATFISHGNTA RLAKEHGDMK LAQICGMIAS DEKRHETAYT KIVEKLFEID PDGTVMAFAD MMRKKIAMPA HLMYDGRDDN
321 LFENYSAVAQ R**IGVYTAKDY ADILEFLVGR** WKVADLTGLS GEGRKAQDYV CGLPPRIRRL EERAQGRAKE SPRLKFSWIY
401 DREVQL

Supporting Peptides:

Peptide	Uniq	-10lgP	Mass	Length	ppm	m/z	z	RT	Fraction	Scan	Source File	Area Raw in gel	#Feature	#Feature Raw in gel	Start	End	PTM	AScore	Found By
R.IGVYTAKDYADILEFLVGR.W	Y	44.46	2142.1360	19	1.5	1072.0769	2	39.60	10	F10:2024	OB5937 H3A raw.raw	3.8207E4	1	1	332	350			PEAKS DB
**total 1 peptides**																			

B6ECZ0|B6ECZ0_ARAHY

back to list

| Protein Coverage | Supporting Peptides |

Protein Coverage:

1 MALRLNPNPS QKLFLSPSSS SSSSSSSFSL PQMASLRSPR FRMASTLRTG SKEVENLKKP FTPPREVHVQ VTHSMPPQKI
81 EIFKSLEGWA EENILTLLKP VGKCWQPQDY LPEPSEDGFE EQVRELRARA KELPDDYFVV LVGDMITEEA LPTYQTMLNT
161 LDGVRDETGA SLTSWAVWTR AWTAEENRHG DLLNKYLYLS GRVDLRQIEK TIQYLIGSGM DPRTENSPYL GFIYTSFQER
241 ATFISHGNTA RLAKEHGDMK LAQICGMIAS DEKRHETAYT KIVEKLFEID PDGTVMAFAD MMRKKIAMPA HLMYDGRDDN
321 LFENYSAVAQ R**IGVYTAKDY ADILEFLVGR** WKVADLTGLS GEGRKAQDYV CGLPPRIRRL EERAQGRAKE SPRLKFSWIY
401 DREVQL

Supporting Peptides:

Peptide	Uniq	-10lgP	Mass	Length	ppm	m/z	z	RT	Fraction	Scan	Source File	Area Raw in gel	#Feature	#Feature Raw in gel	Start	End	PTM	AScore	Found By
R.IGVYTAKDYADILEFLVGR.W	Y	44.46	2142.1360	19	1.5	1072.0769	2	39.60	10	F10:2024	OB5937 H3A raw.raw	3.8207E4	1	1	332	350			PEAKS DB
total 1 peptides																			

U3MF97|U3MF97_ARAHY

back to list

| Protein Coverage | Supporting Peptides |

Protein Coverage:

1 MGKTRGMGAA RKLKSHRRRQ RWADKSYKKS HLGNEWKKPF AGSSHAKGIV LEKIGIEAKQ PNSAIRKCAR VQLIKNGKK**I**
81 **AAFVPNDGCL NYIEENDEVL IAGFGR**KGHA VGDIPGVRFK VVKVSGVSLL ALFKEKKEKP RS

C Carbamidomethylation (+57.02)

Supporting Peptides:

Peptide	Uniq	-10lgP	Mass	Length	ppm	m/z	z	RT	Fraction	Scan	Source File	Area Raw in gel	#Feature	#Feature Raw in gel	Start	End	PTM	AScore	Found By
K.IAAFVPNDGC(+57.02)LNYIEENDEVLIAGFGR.K	Y	33.42	2995.4385	27	3.9	1498.7323	2	38.71	5	F5:1942	OB5932 H2 raw.raw	0	0	0	80	106	Carbamidomethylation	C10:Carbamidomethylation:1000.00	PEAKS DB
total 1 peptides																			

Peptide List

Summary

1. Notes

```
PEKAS PTM 37
ROASTED peanut extract files.
All roasted allergend bands fomr 1D SDS PAGE
```

2. Result Statistics

Figure 1. False discovery rate (FDR) curve. X axis is the number of peptides being kept. Y axis is the corresponding FDR.

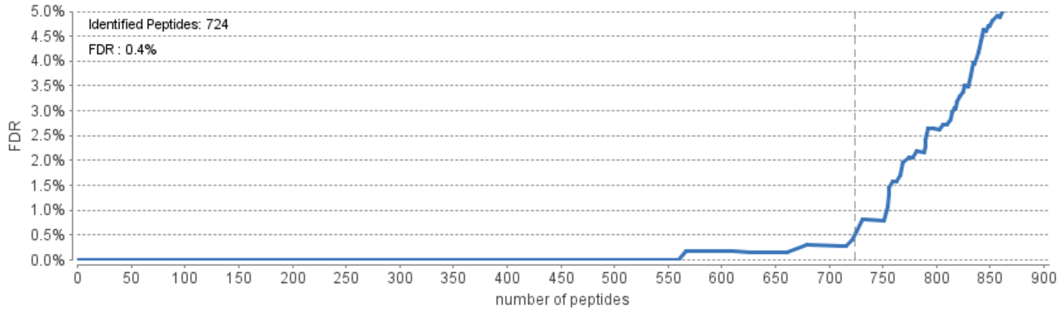

Figure 2. PSM score distribution. **(a)** Distribution of PEAKS peptide score; **(b)** Scatterplot of PEAKS peptide score versus precursor mass error.

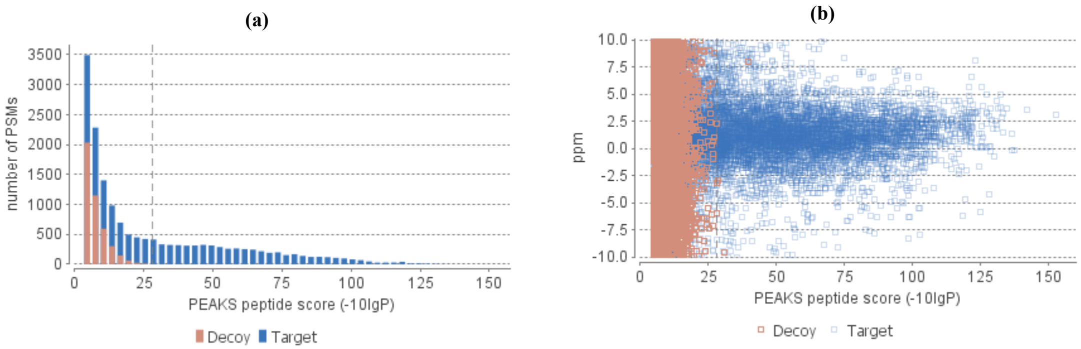

Table 1. Statistics of data.

	#Scans			#Features	Identified			#Peptides	#Sequences	#Proteins*		
	MS1	MS/MS	#Chimera		#PSMs	#Scans	#Features**			Groups	All	Top
Total	34006	24894	3326	39890	6083	5965	2378	724	367	34	52	46
Roasted in gel	34006	24894	3326	39890	6083	5965	2378	724	367	34	52	46

* proteins with significant peptides are used in counts.

** features are identified by DB search only.

Figure 3. Sample overlap for Proteins and Peptides (up to 8 samples). **(a)** All Proteins; **(b)** Top Proteins; **(c)** Peptides;

**(a)**	**(b)**	**(c)**
**Not applicable to only one sample**	**Not applicable to only one sample**	**Not applicable to only one sample**

Figure 4. Distribution of peptide feature detection. **(a)** Feature m/z distribution; **(b)** Feature RT distribution.

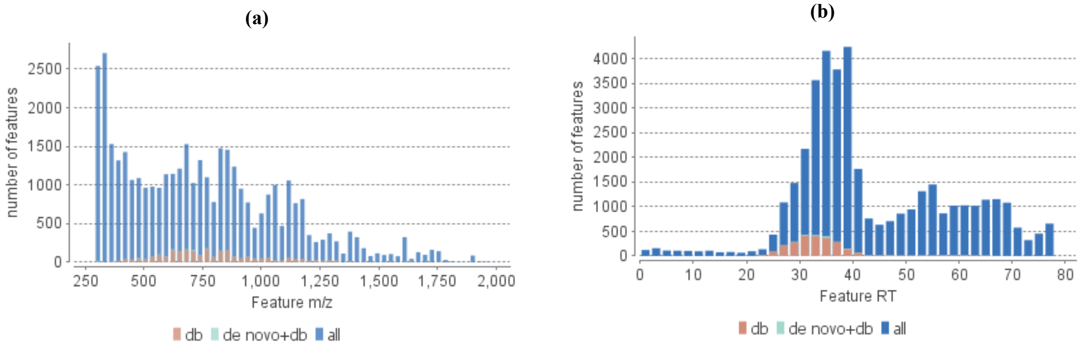

Figure 5. Distribution of identified peptide features. **(a)** Feature abundance distribution; **(b)** *De novo* sequencing validation.

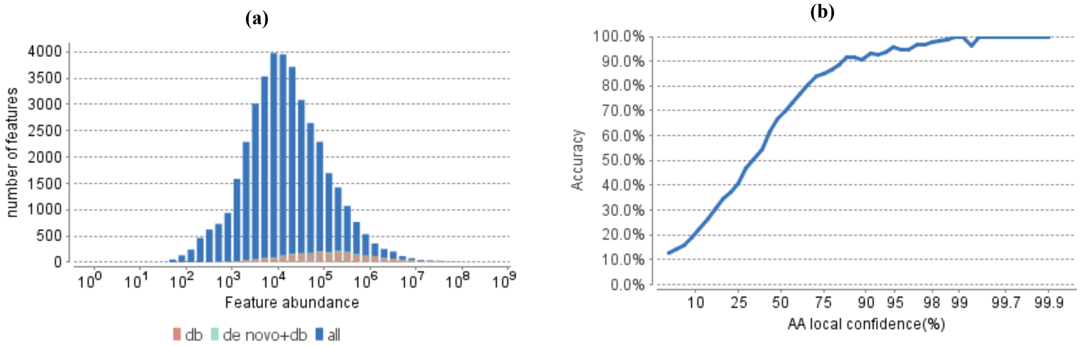

Table 2. Result filtration parameters.

Peptide -10lgP	≥28.3
PTM Ascore	≥20
Protein -10lgP	≥20
Proteins unique peptides	≥1
De novo score(%)	≥50%

Table 3. Statistics of filtered result.

FDR (Peptide-Spectrum Matches)	0.0%
FDR (Peptide Sequences)	0.4%
FDR (Protein Group)	2.9%
De Novo Only Spectra	185

Table 4. PTM profile.

Name	ΔMass	Position	#PSM	-10lgP	Abundance	AScore
Carbamidomethyl	57.02	C	863	136.41	3.83E6	1000.00
Oxidation	15.99	M	367	128.16	1.94E6	27.96
Deamidation	.98	NQ	246	152.35	9.54E5	180.29
Carbamidomethyl	57.02	DEHK,N-term	185	113.97	4.74E5	0.00
Oxidation	15.99	DKNPRY	43	62.00	3.32E5	55.92
Methylation(others)	14.02	DEHST	33	79.80	5.81E5	18.00
Pyro-glu from Q	-17.03	N-term	33	75.52	4.88E3	1000.00
Oxidation	15.99	HW	32	90.73	5.4E5	1000.00

Propionamide	71.04	C	29	73.23	1.33E4	11.06
Dehydration	-18.01	DSTY	26	67.70	1.91E5	40.00
Methylation(KR)	14.02	KR	21	103.68	3.31E6	1000.00
Dimethylation(KR)	28.03	KR	19	116.09	3.06E6	1000.00
Ethyl	28.03	D,N-term	15	66.95	8.8E5	42.22
Cation:Fe[II]	53.92	DE	14	48.78	2.33E5	154.98
Ammonia loss	-17.03	N	14	91.80	5.36E5	1000.00
Dethiomethyl	-48.00	M	12	88.05	1.37E6	14.02
Dihydroxy	31.99	FPRW	12	89.05	7.44E4	44.28
Sodium	21.98	DE	11	54.92	4.36E4	8.58
Formylation	27.99	N-term	10	84.60	7.41E5	98.75
SMA	127.06	N-term	9	50.76	8.68E5	1000.00
Pyro-glu from E	-18.01	N-term	7	58.23	7.73E4	1000.00
Amidine	41.03	K,N-term	7	48.20	1.55E4	96.48

3. Experiment Control

Figure 6. Precursor mass error of peptide-spectrum matches (PSM) in filtered result. **(a)** Distribution of precursor mass error in ppm; **(b)** Scatterplot of precursor m/z versus precursor mass error in ppm.

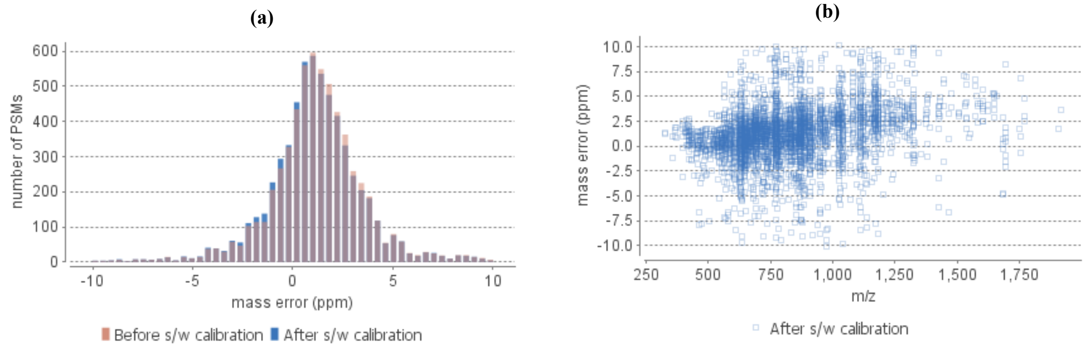

Table 5. Number of identified peptides in each sample by the number of missed cleavages.

Missed Cleavages	0	1	2	3	4+
Roasted in gel	385	268	71	0	0

4. Other Information

Table 6. Search parameters.
PEAKS Version: PEAKS Studio 10.6 build 20201015
Search Engine Name: PEAKS

Table 7. Instrument parameters.
Fractions: OB5922 H2 Ro.raw, OB5926 H3B Ro.raw, OB5942 H6 Ro.ra w, OB5943 H6 Ro.raw, OB5944 H6 Ro.raw, OB5945 H2 Ro.raw, OB59

Parent Mass Error Tolerance: 10.0 ppm
Fragment Mass Error Tolerance: 0.5 Da
Precursor Mass Search Type: monoisotopic
Enzyme: Trypsin
Max Missed Cleavages: 2
Digest Mode: Unspecific
Fixed Modifications:
Carbamidomethylation: 57.02
Variable Modifications:
Oxidation (M): 15.99
Deamidation (NQ): 0.98
Acetylation (K): 42.01
Acetylation (Protein N-term): 42.01
Acetylation (N-term): 42.01
Amidation: -0.98
Beta-methylthiolation: 45.99
Biotinylation: 226.08
and 303 more...
Max Variable PTM Per Peptide: 3
Database: Uniprot_Peanut-3818_Jul18
Taxon: All
Contaminant Database: contam MQ
Searched Entry: 1723
FDR Estimation: Enabled
De novo score(%) threshold: 15
Peptide hit threshold (-10logP): 30.0
Peaks run ID: 34
Merge Options: no merge
Precursor Options: corrected
Charge Options: no correction
Filter m/z: 200.0 - 2000.0
Filter Charge: 2 - 8
Process: true
Associate chimera: yes

46 H2 Ro.raw, OB5948 H3B Ro.raw, OB5949 H3B Ro.raw, OB5951 H3 A Ro.raw, OB5952 H3A Ro.raw, OB5953 H3A Ro.raw, OB5954 H1 Ro. raw, OB5955 H1 Ro.raw, OB5956 H1 Ro.raw
Ion Source: ESI(nano-spray)
Fragmentation Mode: CID, CAD(y and b ions)
MS Scan Mode: FT-ICR/Orbitrap
MS/MS Scan Mode: FT-ICR/Orbitrap

Protein List

Protein Accession Contains:
Protein Description Contains:
Protein Sample Area >=
Protein PTM Contains:

Protein Group	Protein ID	Accession	-10lgP	Coverage (%)	Coverage (%) Roasted in gel	Area Roasted in gel	#Peptides	#Unique	#Spec Roasted in gel	PTM	Avg. Mass	Description
**2**	7	sp|P43237|ALL11_ARAHY	565.06	63	63	1.0131E8	94	9	3063	Y	70283	Allergen Ara h 1, clone P17 OS=Arachis hypogaea OX=3818 PE=1 SV=1
**2**	6	B3IXL2|B3IXL2_ARAHY	565.06	63	63	1.0131E8	94	9	3063	Y	70283	Main allergen Ara h1 OS=Arachis hypogaea OX=3818 PE=2 SV=1
**2**	5	Q6PSU3|Q6PSU3_ARAHY	565.06	67	67	1.0131E8	94	9	3063	Y	66575	Conarachin (Fragment) OS=Arachis hypogaea OX=3818 PE=4 SV=1
**1**	3	N1NG13|N1NG13_ARAHY	560.56	62	62	2.424E7	91	8	3078	Y	71345	Seed storage protein Ara h1 OS=Arachis hypogaea OX=3818 GN=ARAX_AHF4 17E07-017 PE=4 SV=1
**1**	2	sp|P43238|ALL12_ARAHY	560.56	62	62	2.424E7	91	8	3078	Y	71345	Allergen Ara h 1, clone P41B OS=Arachis hypogaea OX=3818 PE=1 SV=1
**3**	8	Q6PSU6|Q6PSU6_ARAHY	506.79	68	68	2.2902E6	55	1	1557	Y	34133	Conarachin (Fragment) OS=Arachis hypogaea OX=3818 PE=2 SV=1
**4**	4	E5G076|E5G076_ARAHY	496.65	50	50	3.5399E6	60	1	1468	Y	70788	Ara h 1 allergen OS=Arachis hypogaea OX=3818 GN=ara h 1 PE=4 SV=1
**5**	11	A1DZF0|A1DZF0_ARAHY	471.62	75	75	1.2702E7	44	9	891	Y	60375	Arachin 6 OS=Arachis hypogaea OX=3818 PE=2 SV=1
**7**	9	Q647H3|Q647H3_ARAHY	466.93	68	68	6.9954E7	41	5	803	Y	61532	Arachin Ahy-2 OS=Arachis hypogaea OX=3818 PE=2 SV=1
**9**	13	Q9FZ11|Q9FZ11_ARAHY	462.45	61	61	3.0114E6	38	4	743	Y	60449	Gly1 OS=Arachis hypogaea OX=3818 GN=Gly1 PE=2 SV=1
**8**	10	B5TYU1|B5TYU1_ARAHY	456.31	73	73	2.0154E7	40	1	787	Y	60624	Arachin Arah3 isoform OS=Arachis hypogaea OX=3818 PE=1 SV=1
**10**	18	Q5I6T2|Q5I6T2_ARAHY	453.29	55	55	0	36	1	723	Y	60736	Arachin Ahy-4 OS=Arachis hypogaea OX=3818 PE=2 SV=1
**13**	19	Q6IWG5|Q6IWG5_ARAHY	432.31	59	59	1.7408E8	27	24	367	Y	58061	Glycinin (Fragment) OS=Arachis hypogaea OX=3818 PE=2 SV=1
**13**	20	Q0GM57|Q0GM57_ARAHY	432.31	59	59	1.7408E8	27	24	367	Y	58263	Iso-Ara h3 OS=Arachis hypogaea OX=3818 PE=2 SV=1

total 46 proteins

Protein Group	Protein ID	Accession	-10lgP	Coverage (%)	Coverage (%) Roasted in gel	Area Roasted in gel	#Peptides	#Unique	#Spec Roasted in gel	PTM	Avg. Mass	Description
**14**	29	O82580|O82580_ARAHY	388.18	37	37	3.3176E6	23	1	366	Y	58350	Glycinin (Fragment) OS=Arachis hypogaea OX=3818 GN=Arah3 PE=2 SV=1
**15**	25	E9LFE8|E9LFE8_ARAHY	379.35	82	82	2.5488E7	20	17	142	Y	28290	11S arachin (Fragment) OS=Arachis hypogaea OX=3818 PE=2 SV=1
**18**	28	sp|Q647H2|AHY3_ARAHY	337.21	38	38	9.6448E6	16	13	101	Y	54569	Arachin Ahy-3 OS=Arachis hypogaea OX=3818 PE=1 SV=1
**17**	26	Q38711|Q38711_ARAHY	334.78	85	85	3.3979E5	15	2	121	Y	29134	Galactose-binding lectin (Fragment) OS=Arachis hypogaea OX=3818 GN=lec PE=2 SV=1
**21**	27	A0A089ZXL7|A0A089ZXL7_ARAHY	316.72	77	77	2.7476E5	13	2	87	Y	29407	Peanut agglutinin variant OS=Arachis hypogaea OX=3818 PE=2 SV=1
**20**	30	A1E2B0|A1E2B0_ARAHY	302.05	69	69	1.2983E7	13	10	91	Y	33520	11S seed storage globulin B1 OS=Arachis hypogaea OX=3818 PE=2 SV=1
**23**	40	Q647H1|Q647H1_ARAHY	289.94	19	19	2.9236E6	10	10	52	Y	75933	Conarachin OS=Arachis hypogaea OX=3818 PE=2 SV=1
**12**	36	A5Z1R0|A5Z1R0_ARAHY	289.17	70	70	3.5713E7	14	14	374	Y	16920	Ara h 6 allergen OS=Arachis hypogaea OX=3818 GN=Ara h 6 PE=4 SV=1
**12**	35	sp|Q647G9|CONG_ARAHY	289.17	70	70	3.5713E7	14	14	374	Y	16920	Conglutin OS=Arachis hypogaea OX=3818 PE=1 SV=1
**25**	33	A1E2B1|A1E2B1_ARAHY	232.07	58	58	1.7932E6	8	8	22	Y	31268	11S seed storage globulin B2 OS=Arachis hypogaea OX=3818 PE=2 SV=1
**19**	44	sp|Q6PSU2|CONG7_ARAHY	194.74	46	46	5.2434E6	6	6	95	Y	20114	Conglutin-7 OS=Arachis hypogaea OX=3818 PE=1 SV=2
**24**	47	E9LFE9|E9LFE9_ARAHY	185.42	26	26	2.496E6	4	4	24	Y	28300	11S arachin OS=Arachis hypogaea OX=3818 PE=2 SV=1
**42**	32	A0A0A6ZDP1|A0A0A6ZDP1_ARAHY	128.27	11	11	1.1161E4	1	1	1	N	20101	Glyceraldehyde-3-phosphate dehydrogenase C2 OS=Arachis hypogaea OX=3818 PE=2 SV=1
**28**	62	E5FHZ1|E5FHZ1_ARAHY	127.84	27	27	9.4991E5	2	2	8	N	22636	Late embryogenesis abundant protein group 5 protein OS=Arachis hypogaea OX=3818 GN=LEA5-1 PE=2 SV=1
**28**	54	N1NKG9|N1NKG9_ARAHY	127.84	23	23	9.4991E5	2	2	8	N	26041	Seed maturation protein OS=Arachis hypogaea OX=3818 GN=ARAX_AHF417E07-003 PE=4 SV=1
**32**	2088	E5FHY1|E5FHY1_ARAHY	102.90	49	49	0	3	1	4	N	10140	Late embryogenesis abundant protein group 1 protein OS=Arachis hypogaea OX=3818 GN=LEA1-2 PE=2 SV=1
**30**	1390	E5FHY2|E5FHY2_ARAHY	102.36	47	47	1.1991E4	3	1	6	N	10652	Late embryogenesis abundant protein group 1 protein OS=Arachis hypogaea OX=3818 GN=LEA1-3 PE=2 SV=1
**37**	61	Q42515|Q42515_ARAHY	84.76	14	14	2.7447E4	2	2	2	N	28924	Chitinase (Class II) OS=Arachis hypogaea OX=3818 GN=chi2;2 PE=2 SV=1
**34**	133	B4UWB2|B4UWB2_ARAHY	83.50	4	4	3.6559E4	1	1	3	N	21637	Kunitz trypsin inhibitor 4 OS=Arachis hypogaea OX=3818 PE=2 SV=1
**35**	173	A7LIS5|A7LIS5_ARAHY	75.04	10	10	5.6415E3	1	1	2	N	23461	Oxalate oxidase OS=Arachis hypogaea OX=3818 GN=OxOxs PE=2 SV=1
**35**	161	D4NXQ0|D4NXQ0_ARAHY	75.04	10	10	5.6415E3	1	1	2	N	23448	Germin-like protein subfamily 3 member 3 OS=Arachis hypogaea OX=3818 GN=GLP1 PE=2 SV=1
**44**	63	H6U596|H6U596_ARAHY	54.03	8	8	6.1243E3	1	1	1	N	19370	Alcohol dehydrogenase (Fragment) OS=Arachis hypogaea OX=3818 PE=2 SV=1
**47**	436	A0A191UJ92|A0A191UJ92_ARAHY	38.97	8	8	6.2701E3	1	1	1	N	14064	50S ribosomal protein L20, chloroplastic OS=Arachis hypogaea OX=3818 GN=rpl20 PE=3 SV=1
**45**	74	E5FHZ2|E5FHZ2_ARAHY	38.10	8	8	5.6109E3	1	1	1	N	27370	Late embryogenesis abundant protein group 5 protein OS=Arachis hypogaea OX=3818 GN=LEA5-2 PE=2 SV=1
**52**	444	A0A075M4V7|A0A075M4V7_ARAHY	33.37	5	5	2.6266E4	1	1	1	N	16964	Ribulose bisphosphate carboxylase small chain (Fragment) OS=Arachis hypogaea OX=3818 GN=rbcS PE=3 SV=1
**52**	443	A0A075M514|A0A075M514_ARAHY	33.37	5	5	2.6266E4	1	1	1	N	16934	Ribulose bisphosphate carboxylase small chain (Fragment) OS=Arachis hypogaea OX=3818 GN=rbcS PE=3 SV=2
**52**	446	A0A075M9B7|A0A075M9B7_ARAHY	33.37	5	5	2.6266E4	1	1	1	N	18164	Ribulose bisphosphate carboxylase small chain (Fragment) OS=Arachis hypogaea OX=3818 GN=rbcS PE=3 SV=1
**52**	445	A0A075M3Q0|A0A075M3Q0_ARAHY	33.37	5	5	2.6266E4	1	1	1	N	18164	Ribulose bisphosphate carboxylase small chain (Fragment) OS=Arachis hypogaea OX=3818 GN=rbcS PE=3 SV=1
**52**	466	A0A075M506|A0A075M506_ARAHY	33.37	5	5	2.6266E4	1	1	1	N	18123	Ribulose bisphosphate carboxylase small chain (Fragment) OS=Arachis hypogaea OX=3818 GN=rbcS PE=3 SV=1
**52**	414	T2BAU5|T2BAU5_ARAHY	33.37	4	4	2.6266E4	1	1	1	N	20437	Ribulose bisphosphate carboxylase small chain OS=Arachis hypogaea OX=3818 PE=2 SV=1
**51**	440	V5T718|V5T718_ARAHY	32.27	5	5	1.384E4	1	1	1	N	35071	Putative MYB-related protein 27 OS=Arachis hypogaea OX=3818 GN=MYB27 PE=4 SV=1
**53**	192	V5T7W3|V5T7W3_ARAHY	30.31	4	4	1.5963E4	1	1	1	N	27579	Putative R2R3 MYB protein 3 OS=Arachis hypogaea OX=3818 GN=MYB3 PE=4 SV=1
**total 46 proteins**												

sp|P43237|ALL11_ARAHY

back to list

| Protein Coverage | Supporting Peptides |

Protein Coverage:

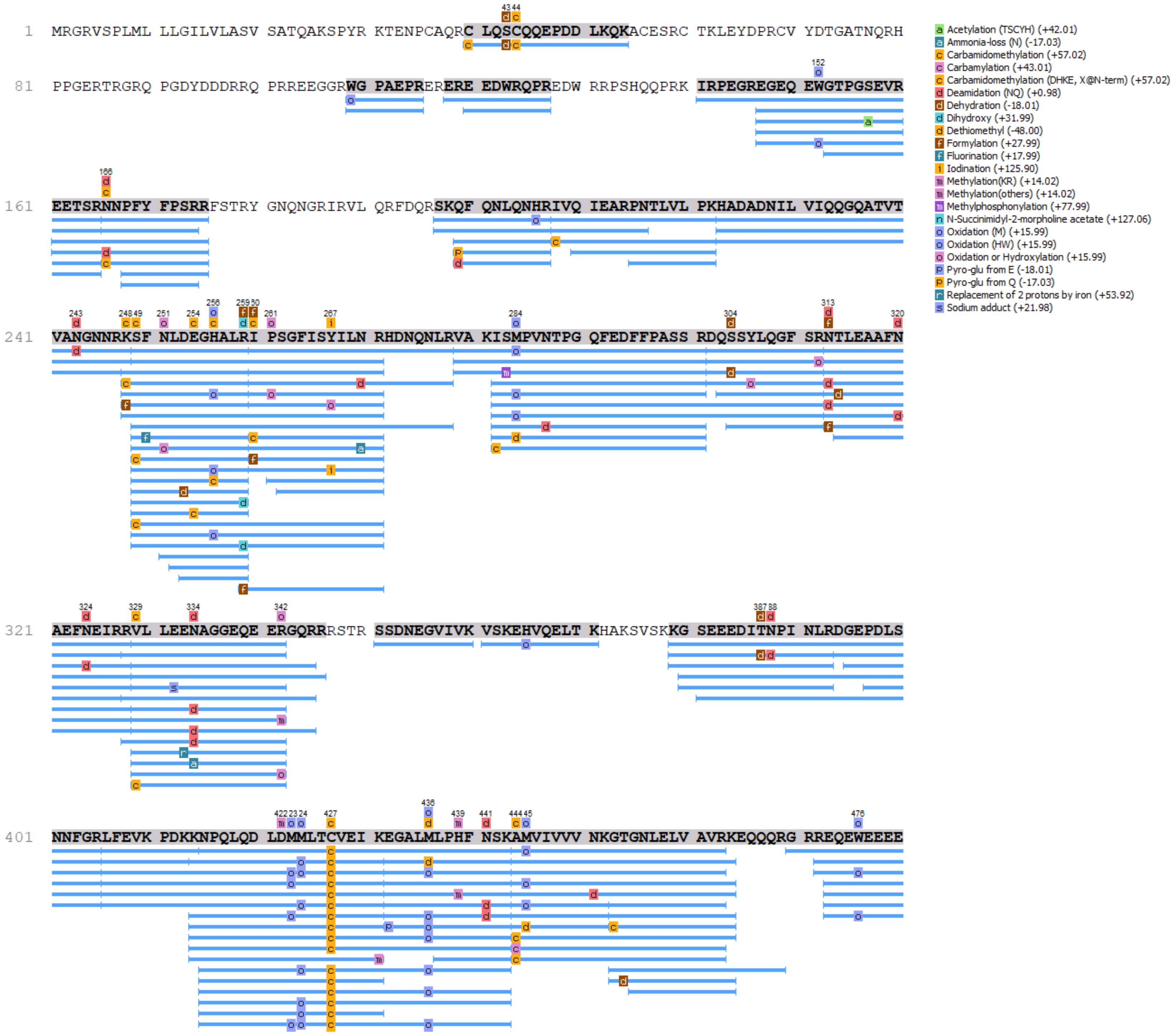
1 MRGRVSPLML LLGILVLASV SATQAKSPYR KTENPCAQRC LQSCQQEPDD LKQKACESRC TKLEYDPRCV YDTGATNQRH
81 PPGERTRGRQ PGDYDDDRRQ PRREEGGRWG PAEPRERERE EDWRQPREDW RRPSHQQPRK IRPEGREGEQ EWGTPGSEVR
161 EETSRNNPFY FPSRRFSTRY GNQNGRIRVL QRFDQRSKQF QNLQNHRIVQ IEARPNTLVL PKHADADNIL VIQQGQATVT
241 VANGNNRKSF NLDEGHALRI PSGFISYILN RHDNQNLRVA KISMPVNTPG QFEDFFPASS RDQSSYLQGF SRNTLEAAFN
321 AEFNEIRRVL LEENAGGEQE ERGQRRRSTR SSDNEGVIVK VSKEHVQELT KHAKSVSKKG SEEEDITNPI NLRDGEPDLS
401 NNFGRLFEVK PDKKNPQLQD LDMMLTCVEI KEGALMLPHF NSKAMVIVVV NKGTGNLELV AVRKEQQQRG RREQEWEEEE
a Acetylation (TSCYH) (+42.01)
a Ammonia-loss (N) (-17.03)
c Carbamidomethylation (+57.02)
c Carbamylation (+43.01)
c Carbamidomethylation (DHKE, X@N-term) (+57.02)
d Deamidation (NQ) (+0.98)
d Dehydration (-18.01)
d Dihydroxy (+31.99)
d Dethiomethyl (-48.00)
f Formylation (+27.99)
f Fluorination (+17.99)
i Iodination (+125.90)
m Methylation(KR) (+14.02)
m Methylation(others) (+14.02)
m Methylphosphonylation (+77.99)
n N-Succinimidyl-2-morpholine acetate (+127.06)
o Oxidation (M) (+15.99)
o Oxidation (HW) (+15.99)
o Oxidation or Hydroxylation (+15.99)
p Pyro-glu from E (-18.01)
p Pyro-glu from Q (-17.03)
r Replacement of 2 protons by iron (+53.92)
s Sodium adduct (+21.98)

Supporting Peptides:

481 **EDEEEEGSNR EVRR**YTARLK EGDVFIMPAA HPVAINASSE LHLLGFGINA ENNHR**IFLAG DKDNVIDQIE KQAKDLAFPG**

561 **SGEQVEKLIK** NQRESHFVSA RPQSQSPSSP EKEDQEEENQ GGKGPLLSIL KAFN

Peptide	Uniq	-10lgP	Mass	Length	ppm	m/z	z	RT	Fraction	Scan	Source File	Area Roasted in gel	#Feature	#Feature Roasted in gel	Start	End	PTM	AScore	Found By
K.KNPQLQDLDMMLTC(+57.02)VEIK.E	N	136.41	2175.0737	18	1.4	1088.5457	2	35.26	43	F43:1808	OB5956 H1 Ro.raw	1.4906E7	6	6	414	431	Carbamidomethylation	C14:Carbamidomethylation:1000.00	PEAKS DB
R.NTLEAAFNAEFNEIR.R	N	130.93	1737.8322	15	1.8	869.9249	2	35.34	38	F38:1714	OB5951 H3A Ro.raw	1.332E7	16	16	313	327			PEAKS DB
K.GSEEEDITNPINLRDGEPDLSNNFGR.L	N	129.76	2887.3220	26	-0.7	963.4473	3	32.67	43	F43:1660	OB5956 H1 Ro.raw	1.2475E7	11	11	380	405			PEAKS DB
K.NPQLQDLDMMLTC(+57.02)VEIK.E	N	128.40	2046.9788	17	0.2	1024.4968	2	37.11	43	F43:1936	OB5956 H1 Ro.raw	3.4643E7	10	10	415	431	Carbamidomethylation	C13:Carbamidomethylation:1000.00	PEAKS DB
K.NPQLQDLDM(+15.99)MLTC(+57.02)VEIK.E	N	128.16	2062.9736	17	-4.2	1032.4897	2	36.93	43	F43:1892	OB5956 H1 Ro.raw	3.3834E6	3	3	415	431	Oxidation (M); Carbamidomethylation	M9:Oxidation (M):30.46;C13:Carbamidomethylation:1000.00	PEAKS DB
K.NPQLQDLDMM(+15.99)LTC(+57.02)VEIK.E	N	127.13	2062.9736	17	5.0	1032.4993	2	34.71	43	F43:1780	OB5956 H1 Ro.raw	1.0665E7	4	4	415	431	Oxidation (M); Carbamidomethylation	M10:Oxidation (M):30.46;C13:Carbamidomethylation:1000.00	PEAKS DB
K.ISM(+15.99)PVNTPGQFEDFFPASSR.D	N	127.11	2242.0364	20	0.1	1122.0284	2	34.52	30	F30:1521	OB5926 H3B Ro.raw	2.1708E7	14	13	282	301	Oxidation (M)	M3:Oxidation (M):1000.00	PEAKS DB
R.EQEWEEEEEDEEEEGSNR.E	N	125.55	2280.8413	18	3.9	1141.4324	2	28.35	43	F43:1396	OB5956 H1 Ro.raw	4.3429E6	7	7	473	490			PEAKS DB
K.ISMPVNTPGQFEDFFPASSR.D	N	125.26	2226.0415	20	0.7	1114.0288	2	35.83	43	F43:1868	OB5956 H1 Ro.raw	8.8227E7	23	23	282	301			PEAKS DB
K.HADADNILVIQQGQATVTVAN(+.98)GNNRK.S	N	123.10	2747.3950	26	3.3	916.8087	3	29.62	43	F43:1472	OB5956 H1 Ro.raw	4.4266E6	11	11	223	248	Deamidation (NQ)	N21:Deamidation (NQ):31.91	PEAKS DB
K.NPQLQDLDM(+15.99)M(+15.99)LTC(+57.02)VEIK.E	N	121.59	2078.9688	17	6.6	1040.4985	2	33.82	43	F43:1726	OB5956 H1 Ro.raw	6.8984E4	2	2	415	431	Oxidation (M); Carbamidomethylation	M9:Oxidation (M):1000.00;M10:Oxidation (M):1000.00;C13:Carbamidomethylation:1000.00	PEAKS DB
K.SFNLDEGHALRIPSGFISYILNR.H	N	121.49	2618.3604	23	1.0	873.7949	3	37.51	41	F41:1949	OB5954 H1 Ro.raw	9.1637E7	28	28	249	271			PEAKS DB
K.KNPQLQDLDM(+15.99)MLTC(+57.02)VEIK.E	N	118.68	2191.0686	18	3.6	1096.5455	2	34.71	43	F43:1781	OB5956 H1 Ro.raw	1.3598E6	4	4	414	431	Oxidation (M); Carbamidomethylation	M10:Oxidation (M):27.96;C14:Carbamidomethylation:1000.00	PEAKS DB
K.AMVIVVVNKGTGNLELVAVR.K	N	114.87	2081.2031	20	-1.0	1041.6078	2	34.35	43	F43:1758	OB5956 H1 Ro.raw	3.8361E7	13	13	444	463			PEAKS DB
R.IFLAGDKDNVIDQIEK.Q	N	110.41	1816.9570	16	-1.1	909.4848	2	32.67	43	F43:1648	OB5956 H1 Ro.raw	4.2419E8	56	55	536	551			PEAKS DB

total 244 peptides

Peptide	Uniq	-10lgP	Mass	Length	ppm	m/z	z	RT	Fraction	Scan	Source File	Area Roasted in gel	#Feature	#Feature Roasted in gel	Start	End	PTM	AScore	Found By
K.KNPQLQDLDMM(+15.99)LTC(+57.02)VEIK.E	N	110.35	2191.0686	18	3.5	1096.5454	2	32.98	42	F42:1657	OB5955 H1 Ro.raw	1.6495E6	4	4	414	431	Oxidation (M); Carbamidomethylation	M11:Oxidation (M): 23.10;C14:Carbamidomethylation:1000.00	PEAKS DB
K.KGSEEEDITNPINLRDGEPDLSNNFGR.L	N	109.79	3015.4170	27	3.4	1006.1497	3	31.62	41	F41:1565	OB5954 H1 Ro.raw	1.1559E7	21	21	379	405			PEAKS DB
R.IPSGFISYILNR.H	N	109.18	1378.7609	12	2.5	690.3894	2	36.50	38	F38:1780	OB5951 H3A Ro.raw	4.4166E8	20	20	260	271			PEAKS DB
K.HADADNILVIQQGQATVTVANGNNRK.S	N	108.34	2746.4111	26	1.9	916.4794	3	29.61	43	F43:1451	OB5956 H1 Ro.raw	3.8404E6	5	5	223	248			PEAKS DB
R.VLLEENAGGEQEER.G	N	108.33	1571.7427	14	2.4	786.8805	2	26.83	42	F42:1293	OB5955 H1 Ro.raw	2.9858E7	10	10	329	342			PEAKS DB
R.KSFNLDEGHALR.I	N	108.28	1385.7051	12	0.6	462.9092	3	26.14	43	F43:1252	OB5956 H1 Ro.raw	5.3666E6	6	6	248	259			PEAKS DB
K.AMVIVVVNKGTGNLELVAVRK.E	N	107.96	2209.2981	21	1.1	737.4408	3	33.05	43	F43:1682	OB5956 H1 Ro.raw	1.2362E8	20	20	444	464			PEAKS DB
R.I(+57.02)PSGFISYILNR.H	N	107.77	1435.7823	12	-3.6	718.8959	2	36.92	43	F43:1901	OB5956 H1 Ro.raw	1.7559E7	7	7	260	271	Carbamidomethylation (DHKE, X@N-term)	I1:Carbamidomethylation (DHKE, X@N-term):1000.00	PEAKS PTM
K.KGSEEEDITNPINLR.D	N	107.76	1713.8533	15	2.8	857.9363	2	29.07	43	F43:1439	OB5956 H1 Ro.raw	8.6032E6	7	7	379	393			PEAKS DB
R.I(+57.02)FLAGDKDNVIDQIEK.Q	N	105.70	1873.9785	16	1.7	937.9982	2	32.48	43	F43:1653	OB5956 H1 Ro.raw	1.1722E7	5	5	536	551	Carbamidomethylation (DHKE, X@N-term)	I1:Carbamidomethylation (DHKE, X@N-term):90.91	PEAKS PTM
R.DQSSYLQGFSR.N	N	105.08	1286.5891	11	0.8	644.3024	2	30.81	40	F40:1431	OB5953 H3A Ro.raw	9.5783E7	31	31	302	312			PEAKS DB
R.V(+57.02)LLEENAGGEQEER.G	N	103.80	1628.7642	14	0.3	815.3896	2	27.08	43	F43:1322	OB5956 H1 Ro.raw	4.0193E5	3	3	329	342	Carbamidomethylation (DHKE, X@N-term)	V1:Carbamidomethylation (DHKE, X@N-term):49.37	PEAKS PTM
K.NPQLQDLDMMLTCVEIK(+14.02).E	N	103.68	2003.9730	17	-1.2	1002.9926	2	38.20	43	F43:1989	OB5956 H1 Ro.raw	5.9168E6	4	4	415	431	Methylation(KR)	K17:Methylation(KR):1000.00	PEAKS PTM
K.SFNLDEGHALR.I	N	103.18	1257.6101	11	-1.7	629.8113	2	28.00	43	F43:1362	OB5956 H1 Ro.raw	1.1584E8	33	31	249	259			PEAKS DB
K.AM(+15.99)VIVVVNKGTGNLELVAVR.K	N	103.14	2097.1980	20	1.6	700.0744	3	33.04	43	F43:1684	OB5956 H1 Ro.raw	2.3148E7	9	9	444	463	Oxidation (M)	M2:Oxidation (M):1000.00	PEAKS DB
R.DGEPDLSNNFGR.L	N	102.69	1319.5742	12	0.3	660.7946	2	29.24	43	F43:1495	OB5956 H1 Ro.raw	7.4875E6	3	3	394	405			PEAKS DB
K.DLAFPGSGEQVEK.L	N	100.93	1375.6619	13	-1.9	688.8369	2	30.34	43	F43:1508	OB5956 H1 Ro.raw	9.196E7	8	8	555	567			PEAKS DB

total 244 peptides

Peptide	Uniq	-10lgP	Mass	Length	ppm	m/z	z	RT	Fraction	Scan	Source File	Area Roasted in gel	#Feature	#Feature Roasted in gel	Start	End	PTM	AScore	Found By
K.NPQLQDLDM(+15.99)MLTC(+57.02)VEIKEGALMLPHFNSK.A	N	100.40	3387.6335	29	-0.6	1130.2178	3	37.70	41	F41:1939	OB5954 H1 Ro.raw	8.7706E7	6	6	415	443	Oxidation (M); Carbamidomethylation	M9:Oxidation (M):26.02;C13:Carbamidomethylation:1000.00	PEAKS DB
R.RVLLEENAGGEQEER.G	N	100.35	1727.8438	15	0.2	576.9553	3	25.30	42	F42:1185	OB5955 H1 Ro.raw	8.2964E5	6	6	328	342			PEAKS DB
R.EGEQEWGTPGSEVR.E	Y	100.27	1559.6852	14	0.8	780.8505	2	27.93	42	F42:1345	OB5955 H1 Ro.raw	3.381E6	3	3	147	160			PEAKS DB
K.NPQLQDLDMMLTC(+57.02)VEIKEGALMLPHFNSK.A	N	98.85	3371.6387	29	-2.7	843.9147	4	38.02	43	F43:1969	OB5956 H1 Ro.raw	1.5351E8	12	12	415	443	Carbamidomethylation	C13:Carbamidomethylation:1000.00	PEAKS DB
K.KNPQLQDLDMMLTC(+57.02)VEIKEGALMLPHFNSK.A	N	97.96	3499.7336	30	-3.9	875.9373	4	36.80	41	F41:1884	OB5954 H1 Ro.raw	6.677E6	4	4	414	443	Carbamidomethylation	C14:Carbamidomethylation:1000.00	PEAKS DB
R.VLLEEN(+.98)AGGEQEER.G	N	94.48	1572.7267	14	2.8	787.3728	2	27.46	43	F43:1340	OB5956 H1 Ro.raw	6.2395E5	3	3	329	342	Deamidation (NQ)	N6:Deamidation (NQ):121.37	PEAKS DB
R.IFLAGDKDNVIDQIEKQAK.D	N	94.35	2144.1477	19	-2.1	715.7217	3	32.42	42	F42:1625	OB5955 H1 Ro.raw	3.3984E6	9	9	536	554			PEAKS DB
R.KSFNLDEGHALRIPSGFISYILNR.H	N	93.78	2746.4553	24	-0.7	687.6206	4	36.29	43	F43:1876	OB5956 H1 Ro.raw	1.1783E6	4	4	248	271			PEAKS DB
R.REQEWEEEEEDEEEEGSNR.E	N	93.49	2436.9424	19	0.1	813.3215	3	26.72	43	F43:1292	OB5956 H1 Ro.raw	8.6025E5	7	7	472	490			PEAKS DB
R.EGEQEWGTPGSEVREETSR.N	Y	93.19	2161.9512	19	3.0	1081.9861	2	27.83	43	F43:1353	OB5956 H1 Ro.raw	3.9146E7	8	8	147	165			PEAKS DB
F.LAGDKDNVIDQIEK.Q	N	91.83	1556.8046	14	-6.3	779.4047	2	32.21	43	F43:1629	OB5956 H1 Ro.raw	5.4162E6	4	4	538	551			PEAKS DB
R.IPSGFISYILN(-17.03)R.H	N	91.80	1361.7343	12	-4.9	681.8711	2	38.02	43	F43:1980	OB5956 H1 Ro.raw	1.3387E6	3	3	260	271	Ammonia-loss (N)	N11:Ammonia-loss (N):1000.00	PEAKS PTM
K.AMVIVVVNK.G	N	91.04	971.5837	9	-0.5	486.7989	2	30.16	43	F43:1494	OB5956 H1 Ro.raw	7.1361E6	3	3	444	452			PEAKS DB
K.SFNLDEGH(+15.99)ALR.I	N	90.73	1273.6051	11	0.9	637.8104	2	30.16	43	F43:1507	OB5956 H1 Ro.raw	8.7681E5	2	2	249	259	Oxidation (HW)	H8:Oxidation (HW):1000.00	PEAKS PTM
R.NNPFYFPSRR.F	N	89.95	1296.6364	10	-2.5	649.3239	2	28.89	43	F43:1417	OB5956 H1 Ro.raw	1.3445E8	22	22	166	175			PEAKS DB
K.D(+57.02)LAFPGSGEQVEK.L	N	88.60	1432.6833	13	-0.1	717.3489	2	31.08	41	F41:1535	OB5954 H1 Ro.raw	9.7986E5	3	3	555	567	Carbamidomethylation (DHKE, X@N-term)	D1:Carbamidomethylation (DHKE, X@N-term):131.10	PEAKS PTM
K.SFNLDEGH(+57.02)ALR.I	N	88.30	1314.6316	11	-0.1	658.3230	2	28.22	41	F41:1349	OB5954 H1 Ro.raw	2.9996E6	3	3	249	259	Carbamidomethylation (DHKE, X@N-term)	H8:Carbamidomethylation (DHKE, X@N-term):30.36	PEAKS PTM
K.EGALMLPHFNSK.A	N	88.21	1342.6703	12	2.5	672.3441	2	30.67	43	F43:1529	OB5956 H1 Ro.raw	1.4087E7	5	5	432	443			PEAKS DB
K.NPQLQDLDMM(-48.00)LTC(+57.02)VEIK.E	N	88.05	1998.9755	17	1.9	1000.4969	2	32.85	43	F43:1670	OB5956 H1 Ro.raw	5.9032E6	3	3	415	431	Carbamidomethylation	M10:Dethiomethyl:14.02;C13:Carbamid	PEAKS PTM
**total 244 peptides**																			

Peptide	Uniq	-10lgP	Mass	Length	ppm	m/z	z	RT	Fraction	Scan	Source File	Area Roasted in gel	#Feature	#Feature Roasted in gel	Start	End	PTM	AScore	Found By
																		omethylation:1000.00	
K.GSEEEDITNPINLR.D	N	87.68	1585.7583	14	2.3	793.8882	2	31.16	42	F42:1548	OB5955 H1 Ro.raw	3.3709E6	3	3	380	393			PEAKS DB
R.GRREQEWEEEEEDEEEEGSNR.E	N	87.21	2650.0649	21	1.1	884.3632	3	26.73	41	F41:1280	OB5954 H1 Ro.raw	7.9956E5	6	6	470	490			PEAKS DB
R.NNPFYFPSR.R	N	86.35	1140.5352	9	-0.1	571.2748	2	31.76	43	F43:1593	OB5956 H1 Ro.raw	1.7218E7	11	11	166	174			PEAKS DB
K.S(+57.02)FNLDEGHALR.I	N	86.31	1314.6316	11	-0.5	658.3228	2	28.11	42	F42:1359	OB5955 H1 Ro.raw	2.6997E6	4	4	249	259	Carbamidomethylation (DHKE, X@N-term)	S1:Carbamidomethylation (DHKE, X@N-term):91.04	PEAKS PTM
I.PSGFISYILNR.H	N	85.97	1265.6768	11	-4.6	633.8427	2	36.75	43	F43:1894	OB5956 H1 Ro.raw	3.5986E5	2	2	261	271			PEAKS DB
R.IPSGFISYILNRHDNQNLR.V	N	84.75	2256.1763	19	6.5	753.0709	3	33.61	43	F43:1714	OB5956 H1 Ro.raw	6.1581E7	21	21	260	278			PEAKS DB
R.I(+27.99)FLAGDKDNVIDQIEK.Q	N	84.60	1844.9519	16	-2.1	923.4813	2	36.23	41	F41:1853	OB5954 H1 Ro.raw	1.9485E6	3	3	536	551	Formylation	I1:Formylation:98.75	PEAKS PTM
K.SFNLDEGH(+15.99)ALRIPSGFISYILNR.H	N	83.49	2634.3555	23	-4.0	879.1223	3	38.76	43	F43:2023	OB5956 H1 Ro.raw	8.8519E5	1	1	249	271	Oxidation (HW)	H8:Oxidation (HW):1000.00	PEAKS PTM
K.KNPQLQDLDMMLTC(+57.02)VEIKEGALM(+15.99)LPHFNSK.A	N	83.11	3515.7285	30	-1.9	879.9377	4	36.03	43	F43:1857	OB5956 H1 Ro.raw	6.6263E6	3	3	414	443	Carbamidomethylation; Oxidation (M)	C14:Carbamidomethylation:1000.00;M23:Oxidation (M):114.34	PEAKS DB
R.NTLEAAFNAEFNEIRR.V	N	82.99	1893.9332	16	-2.6	947.9714	2	34.11	42	F42:1711	OB5955 H1 Ro.raw	3.0959E8	36	35	313	328			PEAKS DB
K.A(+57.02)MVIVVVNKGTGNLELVAVR.K	N	82.14	2138.2246	20	-5.4	1070.1138	2	34.47	42	F42:1739	OB5955 H1 Ro.raw	4.4412E6	3	3	444	463	Carbamidomethylation (DHKE, X@N-term)	A1:Carbamidomethylation (DHKE, X@N-term):207.56	PEAKS PTM
K.DLAFPGSGEQVEKLIK.N	N	80.89	1729.9249	16	-1.6	865.9706	2	33.95	30	F30:1478	OB5926 H3B Ro.raw	3.9305E5	5	4	555	570			PEAKS DB
I.FLAGDKDNVIDQIEK.Q	N	80.64	1703.8729	15	-5.7	852.9388	2	32.22	43	F43:1635	OB5956 H1 Ro.raw	3.1343E5	3	3	537	551			PEAKS DB
K.EGALM(+15.99)LPHFNSK.A	N	80.48	1358.6653	12	1.1	680.3406	2	29.85	43	F43:1485	OB5956 H1 Ro.raw	3.0314E6	5	5	432	443	Oxidation (M)	M5:Oxidation (M):1000.00	PEAKS DB
K.GTGNLELVAVR.K	N	80.39	1127.6299	11	-1.3	564.8215	2	30.21	41	F41:1476	OB5954 H1 Ro.raw	2.4706E7	5	5	453	463			PEAKS DB
R.NTLEAAFN(+.98)AEFNEIRR.V	N	79.02	1894.9172	16	-2.2	948.4638	2	32.35	41	F41:1616	OB5954 H1 Ro.raw	1.3357E6	5	5	313	328	Deamidation (NQ)	N8:Deamidation (NQ):48.12	PEAKS DB
R.VLLEENAGGEQEER(+14.02).G	N	78.92	1585.7583	14	0.7	793.8870	2	27.86	41	F41:1339	OB5954 H1 Ro.raw	1.6341E5	3	3	329	342	Methylation(KR)	R14:Methylation(KR):1000.00	PEAKS PTM
K.GTGNLELVAVRK.E	N	78.87	1255.7249	12	0.7	628.8701	2	27.30	41	F41:1322	OB5954 H1 Ro.raw	2.29E7	10	10	453	464			PEAKS DB
K.QFQNLQNHR.I	N	78.35	1183.5846	9	0.0	592.7996	2	23.81	42	F42:1102	OB5955 H1 Ro.raw	1.5125E5	6	6	199	207			PEAKS DB

total 244 peptides

Peptide	Uniq	-10lgP	Mass	Length	ppm	m/z	z	RT	Fraction	Scan	Source File	Area Roasted in gel	#Feature	#Feature Roasted in gel	Start	End	PTM	AScore	Found By
K.NPQLQDLDMM(+15.99)LTC(+57.02)VEIKEGALMLPHFNSK.A	N	78.01	3387.6335	29	-0.8	1130.2175	3	36.42	41	F41:1862	OB5954 H1 Ro.raw	6.8935E7	6	6	415	443	Oxidation (M); Carbamidomethylation	M10:Oxidation (M):24.44;C13:Carbamidomethylation:1000.00	PEAKS DB
R.LFEVKPDKKNPQLQDLDMMLTC(+57.02)VEIK.E	N	77.87	3131.6069	26	-4.0	1044.8721	3	34.65	42	F42:1756	OB5955 H1 Ro.raw	5.0788E6	4	4	406	431	Carbamidomethylation	C22:Carbamidomethylation:1000.00	PEAKS DB
K.NPQLQDLDM(+15.99)MLTC(+57.02)VEIKEGALM(+15.99)LPHFNSK.A	N	77.10	3403.6284	29	1.1	851.9153	4	37.51	41	F41:1883	OB5954 H1 Ro.raw	7.3887E6	7	7	415	443	Oxidation (M); Carbamidomethylation	M9:Oxidation (M):27.96;C13:Carbamidomethylation:1000.00;M22:Oxidation (M):71.31	PEAKS DB
R.IFLAGDKDNVIDQIEKQ(+.98)AK.D	N	76.22	2145.1316	19	1.5	716.0522	3	33.08	42	F42:1661	OB5955 H1 Ro.raw	0	0	0	536	554	Deamidation (NQ)	Q17:Deamidation (NQ):48.83	PEAKS DB
R.EETSRN(+.98)NPFYFPSRR.F	N	75.62	1899.8864	15	-0.2	634.3026	3	28.39	41	F41:1381	OB5954 H1 Ro.raw	3.2976E5	2	2	161	175	Deamidation (NQ)	N6:Deamidation (NQ):33.98	PEAKS DB
L.AGDKDNVIDQIEK.Q	N	75.39	1443.7205	13	-3.7	722.8649	2	32.08	42	F42:1607	OB5955 H1 Ro.raw	3.3426E5	1	1	539	551			PEAKS DB
K.Q(-17.03)FQNLQNHR.I	N	75.35	1166.5581	9	-1.2	584.2856	2	28.00	43	F43:1365	OB5956 H1 Ro.raw	4.927E6	4	4	199	207	Pyro-glu from Q	Q1:Pyro-glu from Q:1000.00	PEAKS PTM
R.SSDNEGVIVK.V	N	75.32	1046.5244	10	-1.0	524.2690	2	23.58	41	F41:1103	OB5954 H1 Ro.raw	3.7568E5	3	3	351	360			PEAKS DB
R.VAKISMPVNTPGQFEDFFPASSR.D	N	74.27	2524.2419	23	-2.9	842.4188	3	34.29	42	F42:1735	OB5955 H1 Ro.raw	1.4483E6	3	3	279	301			PEAKS DB
K.NPQLQDLDM(+15.99)M(+15.99)LTC(+57.02)VEIKEGALMLPHFNSK.A	N	73.00	3403.6284	29	-3.9	1135.5457	3	35.83	43	F43:1849	OB5956 H1 Ro.raw	7.8068E6	5	5	415	443	Oxidation (M); Carbamidomethylation	M9:Oxidation (M):54.18;M10:Oxidation (M):63.99;C13:Carbamidomethylation:1000.00	PEAKS DB
K.KNPQLQDLDM(+15.99)MLTC(+57.02)VEIKEGALM(+15.99)LPHFNSK.A	N	72.71	3531.7234	30	-1.9	883.9364	4	35.86	41	F41:1821	OB5954 H1 Ro.raw	7.53E5	1	1	414	443	Carbamidomethylation; Oxidation (M)	M10:Oxidation (M):17.01;C14:Carbamidomethylation:1000.00;M23:Oxidation (M):85.25	PEAKS DB
R.VLLEENAGGEQEERGQR.R	N	72.44	1912.9238	17	0.6	957.4698	2	25.30	42	F42:1191	OB5955 H1 Ro.raw	3.1353E6	8	8	329	345			PEAKS DB
K.QAKDLAFPGSGEQVEK.L	N	72.39	1702.8525	16	1.2	568.6255	3	27.08	43	F43:1321	OB5956 H1 Ro.raw	2.8232E5	6	6	552	567			PEAKS DB
K.EGALMLPHFN(+.98)SK.A	N	71.37	1343.6543	12	2.6	672.8362	2	31.66	43	F43:1595	OB5956 H1 Ro.raw	1.0101E5	2	2	432	443	Deamidation (NQ)	N10:Deamidation (NQ):1000.00	PEAKS DB
R.EQEW(+15.99)EEEEEDEEEEGSNR.E	N	70.36	2296.8362	18	3.2	1149.4291	2	27.67	41	F41:1333	OB5954 H1 Ro.raw	1.024E6	3	3	473	490	Oxidation (HW)	W4:Oxidation (HW):1000.00	PEAKS PTM

total 244 peptides

Peptide	Uniq	-10lgP	Mass	Length	ppm	m/z	z	RT	Fraction	Scan	Source File	Area Roasted in gel	#Feature	#Feature Roasted in gel	Start	End	PTM	AScore	Found By
F.NLDEGHALR.I	N	70.14	1023.5097	9	-2.5	512.7609	2	28.00	43	F43:1370	OB5956 H1 Ro.raw	2.6181E6	3	3	251	259			PEAKS DB
R.N(+.98)TLEAAFNAEFNEIRR.V	N	69.73	1894.9172	16	0.4	948.4662	2	32.39	42	F42:1620	OB5955 H1 Ro.raw	2.6319E6	3	3	313	328	Deamidation (NQ)	N1:Deamidation (NQ):41.52	PEAKS DB
E.PDLSNNFGR.L	N	69.35	1018.4832	9	0.9	510.2493	2	31.57	43	F43:1584	OB5956 H1 Ro.raw	1.3026E6	4	4	397	405			PEAKS DB
R.K(+57.02)SFNLDEGHALR.I	N	69.20	1442.7266	12	-0.1	722.3705	2	26.25	42	F42:1250	OB5955 H1 Ro.raw	4.0798E5	6	6	248	259	Carbamidomethylation (DHKE, X@N-term)	K1:Carbamidomethylation (DHKE, X@N-term):76.17	PEAKS PTM
R.LFEVKPDKKNPQLQDLDMM(+15.99)LTC(+57.02)VEIK.E	N	69.17	3147.6018	26	2.2	787.9095	4	33.24	42	F42:1670	OB5955 H1 Ro.raw	2.0797E6	2	2	406	431	Oxidation (M); Carbamidomethylation	M19:Oxidation (M):40.00;C22:Carbamidomethylation:1000.00	PEAKS DB
K.SFNLDE(+57.02)GHALRIPSGFISYILNR.H	N	69.07	2675.3818	23	-1.9	892.7996	3	37.80	43	F43:1964	OB5956 H1 Ro.raw	0	0	0	249	271		E6:Carbamidomethylation (DHKE, X@N-term):0.00	PEAKS PTM
R.IFLAGDKDNVID(+57.02)QIEK.Q	N	69.05	1873.9785	16	-1.3	937.9953	2	32.42	42	F42:1608	OB5955 H1 Ro.raw	8.0339E6	1	1	536	551		D12:Carbamidomethylation (DHKE, X@N-term):15.85	PEAKS PTM
R.IVQIEARPNTLVLPK.H	Y	68.17	1690.0140	15	-1.0	846.0134	2	30.56	41	F41:1509	OB5954 H1 Ro.raw	4.6727E7	21	21	208	222			PEAKS DB
A.GDKDNVIDQIEK.Q	N	68.14	1372.6833	12	-7.0	687.3441	2	32.22	43	F43:1632	OB5956 H1 Ro.raw	1.1241E6	3	3	540	551			PEAKS DB
G.SEEEDITNPINLRDGEPDLSNNFGR.L	N	68.10	2830.3005	25	-1.6	944.4417	3	33.02	30	F30:1421	OB5926 H3B Ro.raw	1.3758E5	3	3	381	405			PEAKS DB
R.RVLLEEN(+.98)AGGEQEER.G	N	67.83	1728.8278	15	-0.8	577.2827	3	25.96	42	F42:1233	OB5955 H1 Ro.raw	1.359E4	2	2	328	342	Deamidation (NQ)	N7:Deamidation (NQ):77.53	PEAKS DB
R.DQS(-18.01)SYLQGFSR.N	N	67.70	1268.5785	11	0.2	635.2966	2	30.91	41	F41:1537	OB5954 H1 Ro.raw	6.4401E5	3	3	302	312	Dehydration	S3:Dehydration:40.00	PEAKS PTM
R.RVLLEENAGGEQEERGQR.R	N	67.67	2069.0249	18	1.9	690.6835	3	25.60	41	F41:1205	OB5954 H1 Ro.raw	1.8247E6	3	3	328	345			PEAKS DB
K.Q(-17.03)AKDLAFPGSGEQVEK.L	N	67.52	1685.8260	16	0.1	843.9204	2	29.98	43	F43:1503	OB5956 H1 Ro.raw	9.9003E4	3	3	552	567	Pyro-glu from Q	Q1:Pyro-glu from Q:1000.00	PEAKS PTM
K.NPQLQ(+.98)DLDMMLTC(+57.02)VEIK.E	N	67.25	2047.9629	17	1.5	1024.9902	2	44.46	43	F43:2348	OB5956 H1 Ro.raw	0	0	0	415	431	Carbamidomethylation	Q5:Deamidation (NQ):19.68;C13:Carbamidomethylation:1000.00	PEAKS DB
K.SFNLD(-18.01)EGHALR.I	N	66.88	1239.5996	11	2.9	620.8089	2	28.79	41	F41:1400	OB5954 H1 Ro.raw	0	0	0	249	259	Dehydration	D5:Dehydration:49.79	PEAKS PTM
K.AM(+15.99)VIVVVNK.G	N	66.42	987.5787	9	1.1	494.7972	2	27.11	41	F41:1283	OB5954 H1 Ro.raw	1.8879E6	3	3	444	452	Oxidation (M)	M2:Oxidation (M):1000.00	PEAKS DB
K.KNPQLQDLDMMLTCVEIK(+14.02).E	N	66.06	2132.0679	18	-0.6	1067.0405	2	36.31	41	F41:1858	OB5954 H1 Ro.raw	5.2356E5	2	2	414	431	Methylation(KR)	K18:Methylation(KR):130.57	PEAKS PTM
**total 244 peptides**																			

Peptide	Uniq	-10lgP	Mass	Length	ppm	m/z	z	RT	Fraction	Scan	Source File	Area Roasted in gel	#Feature	#Feature Roasted in gel	Start	End	PTM	AScore	Found By
R.EETSRNNPFYFPSRR.F	N	65.61	1898.9023	15	1.8	633.9759	3	27.75	42	F42:1339	OB5955 H1 Ro.raw	8.693E5	4	4	161	175			PEAKS DB
K.NPQLQDLDMMLTC(+57.02)VEIKEGALM(+15.99)LPHFNSK.A	N	64.76	3387.6335	29	-0.6	847.9152	4	37.63	43	F43:1915	OB5956 H1 Ro.raw	8.7706E7	6	6	415	443	Carbamidomethylation; Oxidation (M)	C13:Carbamidomethylation:1000.00;M22:Oxidation (M):82.05	PEAKS DB
R.IPSGFISY(+125.90)ILNR.H	N	64.64	1504.6575	12	-1.9	753.3346	2	38.39	43	F43:2001	OB5956 H1 Ro.raw	2.0334E5	2	2	260	271	Iodination	Y8:Iodination:1000.00	PEAKS PTM
R.IPSGFISYILN(+.98)RHDNQNLR.V	N	64.64	2257.1604	19	4.7	753.3976	3	34.29	42	F42:1740	OB5955 H1 Ro.raw	6.9901E5	2	2	260	278	Deamidation (NQ)	N11:Deamidation (NQ):36.24	PEAKS DB
R.WGPAEPR.E	Y	64.52	811.3976	7	1.8	406.7068	2	25.60	41	F41:1224	OB5954 H1 Ro.raw	1.6282E6	3	3	109	115			PEAKS DB
R.IVQIEARPN.T	Y	64.48	1038.5822	9	-0.5	520.2981	2	25.87	42	F42:1229	OB5955 H1 Ro.raw	4.4669E4	3	3	208	216			PEAKS DB
A.FPGSGEQVEK.L	N	64.10	1076.5138	10	-3.4	539.2623	2	30.29	42	F42:1495	OB5955 H1 Ro.raw	4.0412E5	3	3	558	567			PEAKS DB
K.ISMPVNTPGQFEDFFPASSRDQSSYLQGFSR.N	N	63.85	3494.6201	31	-0.9	1165.8796	3	36.01	43	F43:1851	OB5956 H1 Ro.raw	5.8723E6	6	6	282	312			PEAKS DB
R.LFEVKPDKK.N	N	63.79	1102.6385	9	0.6	552.3269	2	24.50	43	F43:1159	OB5956 H1 Ro.raw	1.1348E6	6	6	406	414			PEAKS DB
Q.SSYLQGFSR.N	N	63.16	1043.5035	9	1.4	522.7598	2	29.29	41	F41:1438	OB5954 H1 Ro.raw	6.2708E4	3	3	304	312			PEAKS DB
K.QFQNLQN(+.98)HR.I	N	63.09	1184.5686	9	5.4	593.2948	2	23.60	43	F43:1114	OB5956 H1 Ro.raw	8.0412E2	1	1	199	207		N7:Deamidation (NQ):0.00	PEAKS DB
K.EGALMLPH(+14.02)FNSK.A	N	63.06	1356.6860	12	-4.2	679.3475	2	30.85	43	F43:1554	OB5956 H1 Ro.raw	2.9707E5	3	3	432	443	Methylation(others)	H8:Methylation(others):65.81	PEAKS PTM
P.SGFISYILNR.H	N	63.04	1168.6240	10	1.5	585.3202	2	35.08	43	F43:1803	OB5956 H1 Ro.raw	2.9962E5	1	1	262	271			PEAKS DB
N.LDEGHALR.I	N	62.84	909.4668	8	0.0	455.7407	2	27.93	42	F42:1350	OB5955 H1 Ro.raw	1.8672E5	3	3	252	259			PEAKS DB
K.A(+57.02)MVIVVVNKGTGNLELVAVRK.E	N	62.52	2266.3196	21	0.7	1134.1678	2	32.98	42	F42:1660	OB5955 H1 Ro.raw	1.5099E7	6	6	444	464	Carbamidomethylation (DHKE, X@N-term)	A1:Carbamidomethylation (DHKE, X@N-term):139.69	PEAKS PTM
K.HADADNILVIQQGQATVTVANGN(+.98)NRK.S	N	62.31	2747.3950	26	-0.1	916.8055	3	30.70	41	F41:1517	OB5954 H1 Ro.raw	8.5956E3	1	1	223	248		N23:Deamidation (NQ):9.40	PEAKS DB
K.SFN(+15.99)LDEGHALR.I	N	62.00	1273.6051	11	0.0	637.8098	2	27.46	43	F43:1330	OB5956 H1 Ro.raw	9.622E5	3	3	249	259	Oxidation or Hydroxylation	N3:Oxidation or Hydroxylation:55.92	PEAKS PTM
R.IP(+15.99)SGFISYILNR.H	N	61.98	1394.7557	12	-0.8	698.3846	2	37.47	43	F43:1932	OB5956 H1 Ro.raw	3.2208E6	4	4	260	271	Oxidation or Hydroxylation	P2:Oxidation or Hydroxylation:39.25	PEAKS PTM
K.NPQLQDLDMM(+15.99)LTC(+57.02)VEIKEGALM(+15.99)LPHFNSK.A	N	61.19	3403.6284	29	0.3	851.9147	4	35.86	41	F41:1801	OB5954 H1 Ro.raw	3.4294E6	4	4	415	443	Oxidation (M); Carbamidomethylation	M10:Oxidation (M):21.94;C13:Carbamidomethylation:1000.00;M22:O	PEAKS DB

total 244 peptides

Peptide	Uniq	-10lgP	Mass	Length	ppm	m/z	z	RT	Fraction	Scan	Source File	Area Roasted in gel	#Feature	#Feature Roasted in gel	Start	End	PTM	AScore	Found By
																		xidation (M):61.50	
K.G(+57.02)TGNLELVAVRK.E	N	60.73	1312.7462	12	-0.4	657.3801	2	27.30	41	F41:1303	OB5954 H1 Ro.raw	3.6978E5	2	2	453	464	Carbamidomethylation (DHKE, X@N-term)	G1:Carbamidomethylation (DHKE, X@N-term):55.46	PEAKS PTM
R.EQEWEEEEEDEEEEGSNREVRR.Y	N	60.58	2821.1545	22	1.9	941.3939	3	26.71	43	F43:1297	OB5956 H1 Ro.raw	1.0274E6	4	4	473	494			PEAKS DB
K.NPQLQDLDM(+15.99)M(+15.99)LTC(+57.02)VEIKEGALM(+15.99)LPHFNSK.A	N	60.17	3419.6233	29	-0.9	855.9124	4	34.83	42	F42:1766	OB5955 H1 Ro.raw	1.2322E6	3	3	415	443	Oxidation (M); Carbamidomethylation	M9:Oxidation (M):1000.00;M10:Oxidation (M):1000.00;C13:Carbamidomethylation:1000.00;M22:Oxidation (M):1000.00	PEAKS DB
R.IPSGFISY(+15.99)ILNR.H	N	60.13	1394.7557	12	-0.7	698.3846	2	35.11	41	F41:1859	OB5954 H1 Ro.raw	3.4529E6	5	5	260	271	Oxidation or Hydroxylation	Y8:Oxidation or Hydroxylation:67.76	PEAKS PTM
L.DEGHALR.I	N	59.61	796.3828	7	2.5	399.1996	2	27.93	42	F42:1351	OB5955 H1 Ro.raw	9.3538E4	3	3	253	259			PEAKS DB
R.LFEVKPDKKNPQLQ(+.98)DLDMM(+15.99)LTC(+57.02)VEIK.E	N	59.38	3148.5857	26	7.4	788.1595	4	33.17	42	F42:1670	OB5955 H1 Ro.raw	3.9804E5	1	1	406	431	Oxidation (M); Carbamidomethylation	Q14:Deamidation (NQ):0.00;M19:Oxidation (M):23.10;C22:Carbamidomethylation:1000.00	PEAKS DB
R.VLLEEN(-17.03)AGGEQEER.G	N	59.22	1554.7162	14	1.2	778.3663	2	27.73	42	F42:1335	OB5955 H1 Ro.raw	0	0	0	329	342	Ammonia-loss (N)	N6:Ammonia-loss (N):1000.00	PEAKS PTM
K.SFNLDEGHALRIPSGFISYILNRHDNQNLR.V	N	59.07	3495.7759	30	1.5	700.1635	5	39.97	40	F40:1987	OB5953 H3A Ro.raw	2.0405E7	15	15	249	278			PEAKS DB
K.SFNLDEGHALR(+31.99)IPSGFISYILNR.H	N	58.88	2650.3503	23	0.1	884.4575	3	39.14	42	F42:2021	OB5955 H1 Ro.raw	5.6519E5	1	1	249	271	Dihydroxy	R11:Dihydroxy:46.57	PEAKS PTM
R.LFEVKPDK.K	N	58.71	974.5436	8	1.7	488.2799	2	25.30	42	F42:1183	OB5955 H1 Ro.raw	1.7315E6	6	6	406	413			PEAKS DB
K.GSEEEDITNPIN(+.98)LRDGEPDLSNNFGR.L	N	58.69	2888.3059	26	6.7	1445.1699	2	32.90	41	F41:1642	OB5954 H1 Ro.raw	1.7853E5	1	1	380	405		N12:Deamidation (NQ):12.33	PEAKS DB
K.SFNLDE(+57.02)GHALR.I	N	58.62	1314.6316	11	0.3	658.3232	2	27.83	43	F43:1458	OB5956 H1 Ro.raw	2.3371E6	2	2	249	259	Carbamidomethylation (DHKE, X@N-term)	E6:Carbamidomethylation (DHKE, X@N-term):27.25	PEAKS PTM
R.N(+57.02)NPFYFPSRR.F	N	58.56	1353.6577	10	1.1	677.8369	2	29.74	42	F42:1453	OB5955 H1 Ro.raw	9.24E5	3	3	166	175	Carbamidomethylation (DHKE, X@N-term)	N1:Carbamidomethylation (DHKE, X@N-term):1000.00	PEAKS PTM
R.IFLAGDKDNVIDQ(+.98)IEKQAK.D	N	58.20	2145.1316	19	1.4	716.0521	3	33.04	43	F43:1686	OB5956 H1 Ro.raw	1.2153E5	1	1	536	554	Deamidation (NQ)	Q13:Deamidation (NQ):25.1	PEAKS DB
**total 244 peptides**																			

Peptide	Uniq	-10lgP	Mass	Length	ppm	m/z	z	RT	Fraction	Scan	Source File	Area Roasted in gel	#Feature	#Feature Roasted in gel	Start	End	PTM	AScore	Found By
																		8	
R.VAKISM(+15.99)PVNTPGQFEDFFPASSR.D	N	57.67	2540.2368	23	2.1	847.7546	3	32.98	42	F42:1665	OB5955 H1 Ro.raw	1.5427E5	1	1	279	301	Oxidation (M)	M6:Oxidation (M):1000.00	PEAKS DB
K.G(+57.02)TGNLELVAVR.K	N	57.51	1184.6514	11	-2.7	593.3314	2	30.10	42	F42:1486	OB5955 H1 Ro.raw	1.7196E5	2	2	453	463		G1:Carbamidomethylation (DHKE, X@N-term):19.78	PEAKS PTM
R.IFLAGD(+57.02)KDNVIDQIEK.Q	N	57.43	1873.9785	16	1.7	625.6678	3	32.52	41	F41:1637	OB5954 H1 Ro.raw	2.7943E5	3	3	536	551		D6:Carbamidomethylation (DHKE, X@N-term):15.57	PEAKS PTM
R.IFLAGDK(+15.99)DNVIDQIEK.Q	N	57.39	1832.9519	16	-1.9	917.4814	2	31.99	41	F41:1600	OB5954 H1 Ro.raw	7.5922E5	2	2	536	551		K7:Oxidation or Hydroxylation:17.01	PEAKS PTM
N.PFYFPSRR.F	N	57.34	1068.5504	8	1.1	535.2831	2	28.95	41	F41:1414	OB5954 H1 Ro.raw	4.6082E6	5	5	168	175			PEAKS DB
R.K(+27.99)SFNLDEGHALR.I	N	57.29	1413.7001	12	1.4	707.8583	2	28.46	42	F42:1387	OB5955 H1 Ro.raw	2.9777E4	1	1	248	259	Formylation	K1:Formylation:1000.00	PEAKS PTM
R.LFEVKPDKKNPQLQDLDM(+15.99)MLTC(+57.02)VEIK.E	N	57.09	3147.6018	26	-1.9	787.9062	4	34.20	41	F41:1734	OB5954 H1 Ro.raw	2.4894E6	4	4	406	431	Oxidation (M); Carbamidomethylation	M18:Oxidation (M):33.98;C22:Carbamidomethylation:1000.00	PEAKS DB
K.SFNLDEGHALR(+31.99).I	N	55.96	1289.6000	11	-1.4	645.8063	2	30.41	43	F43:1520	OB5956 H1 Ro.raw	0	0	0	249	259	Dihydroxy	R11:Dihydroxy:119.59	PEAKS PTM
K.ISMPVN(+.98)TPGQFEDFFPASSR.D	N	55.55	2227.0254	20	6.6	1114.5273	2	36.77	41	F41:1885	OB5954 H1 Ro.raw	1.01E5	2	2	282	301	Deamidation (NQ)	N6:Deamidation (NQ):42.68	PEAKS DB
R.NTLEAAFNAEFN(+.98)EIRR.V	N	54.77	1894.9172	16	1.8	948.4676	2	33.04	43	F43:1685	OB5956 H1 Ro.raw	2.2573E6	5	5	313	328	Deamidation (NQ)	N12:Deamidation (NQ):39.02	PEAKS DB
V.QIEARPNTLVLPK.H	Y	54.45	1477.8616	13	-1.8	739.9368	2	30.56	41	F41:1513	OB5954 H1 Ro.raw	1.5146E5	3	3	210	222			PEAKS DB
K.S(+57.02)FNLDEGHALRIPSGFISYILNR.H	N	54.19	2675.3818	23	2.3	892.8033	3	39.35	38	F38:1901	OB5951 H3A Ro.raw	6.7035E5	1	1	249	271	Carbamidomethylation (DHKE, X@N-term)	S1:Carbamidomethylation (DHKE, X@N-term):63.97	PEAKS PTM
R.IFLAGDKDNVIDQIEK(+57.02)QAK.D	N	54.07	2201.1692	19	0.3	734.7306	3	32.48	43	F43:1657	OB5956 H1 Ro.raw	1.2122E6	3	3	536	554		K16:Carbamidomethylation (DHKE, X@N-term):11.12	PEAKS PTM
R.I(+27.99)PSGFISYILNR.H	N	53.77	1406.7557	12	0.7	704.3856	2	42.27	42	F42:2199	OB5955 H1 Ro.raw	0	0	0	260	271	Formylation	I1:Formylation:1000.00	PEAKS PTM
R.SKQFQNLQNHR.I	Y	53.65	1398.7117	11	0.4	467.2447	3	24.16	43	F43:1149	OB5956 H1 Ro.raw	1.1379E5	3	3	197	207			PEAKS DB
R.IPSGFISYILNRHDN(+.98)QNLR.V	N	53.60	2257.1604	19	-0.7	753.3936	3	34.26	41	F41:1732	OB5954 H1 Ro.raw	0	0	0	260	278		N15:Deamidation (NQ):0.00	PEAKS DB
K.HADADNILVIQQGQATVTVANGNNR.K	N	53.13	2618.3162	25	0.5	873.7798	3	30.34	38	F38:1426	OB5951 H3A Ro.raw	1.8654E3	1	1	223	247			PEAKS DB

total 244 peptides

Peptide	Uniq	-10lgP	Mass	Length	ppm	m/z	z	RT	Fraction	Scan	Source File	Area Roasted in gel	#Feature	#Feature Roasted in gel	Start	End	PTM	AScore	Found By
R.EGEQEWGTPGS(+42.01)EVR.E	Y	52.96	1601.6958	14	7.6	801.8613	2	27.46	43	F43:1334	OB5956 H1 Ro.raw	9.5213E4	3	3	147	160	Acetylation (TSCYH)	S11:Acetylation (TSCYH):32.97	PEAKS PTM
K.AM(+15.99)VIVVVNKGTGNLELVAVRK.E	N	52.95	2225.2930	21	-1.3	742.7706	3	32.42	42	F42:1596	OB5955 H1 Ro.raw	1.4247E6	3	3	444	464	Oxidation (M)	M2:Oxidation (M):1000.00	PEAKS DB
K.KNPQLQ(+.98)DLDMMLTC(+57.02)VEIKEGALMLPHFNSK.A	N	51.97	3500.7175	30	3.4	876.1896	4	36.93	43	F43:1903	OB5956 H1 Ro.raw	9.2646E6	2	2	414	443	Carbamidomethylation	Q6:Deamidation (NQ):14.04;C14:Carbamidomethylation:1000.00	PEAKS DB
R.IFLAGDKDNVID.Q	N	51.22	1318.6769	12	-0.9	660.3451	2	31.76	43	F43:1606	OB5956 H1 Ro.raw	1.1173E5	3	3	536	547			PEAKS DB
K.ISM(+15.99)PVNTPGQFEDFFPASSRDQSSYLQGFSR.N	N	50.25	3510.6150	31	0.3	1171.2126	3	35.35	36	F36:1707	OB5948 H3B Ro.raw	2.4799E6	5	5	282	312	Oxidation (M)	M3:Oxidation (M):1000.00	PEAKS DB
K.NPQLQDLDMM(+15.99)LTC(+57.02)VEIKEGALMLPHFN(+.98)SK.A	N	49.99	3388.6174	29	6.1	848.1668	4	36.39	43	F43:1880	OB5956 H1 Ro.raw	1.7975E6	1	1	415	443	Oxidation (M); Carbamidomethylation	M10:Oxidation (M):27.96;C13:Carbamidomethylation:1000.00;N27:Deamidation (NQ):18.27	PEAKS DB
R.N(-17.03)NPFYFPSRR.F	N	49.99	1279.6097	10	1.1	427.5443	3	28.89	43	F43:1434	OB5956 H1 Ro.raw	5.674E5	3	3	166	175		N1:Ammonia-loss (N):9.34	PEAKS PTM
R.EQEWEEEEEDEEEEGSNREVR.R	N	49.84	2665.0535	21	2.4	889.3605	3	27.88	41	F41:1353	OB5954 H1 Ro.raw	4.9786E4	1	1	473	493			PEAKS DB
T.GNLELVAVRK.E	N	49.53	1097.6556	10	0.5	549.8354	2	27.00	42	F42:1296	OB5955 H1 Ro.raw	3.4872E5	3	3	455	464			PEAKS DB
R.VAKIS(+77.99)MPVNTPGQFEDFFPASSR.D	N	49.16	2602.2290	23	3.0	1302.1257	2	38.06	42	F42:1958	OB5955 H1 Ro.raw	0	0	0	279	301	Methylphosphonylation	S5:Methylphosphonylation:56.99	PEAKS PTM
F.PGSGEQVEK.L	N	49.06	929.4454	9	-3.7	465.7282	2	30.34	43	F43:1524	OB5956 H1 Ro.raw	5.2705E4	1	1	559	567			PEAKS DB
K.AMVIVVVNK(+57.02)GTGNLELVAVRK.E	N	48.92	2266.3196	21	1.3	756.4481	3	33.23	43	F43:1673	OB5956 H1 Ro.raw	3.1193E6	1	1	444	464		K9:Carbamidomethylation (DHKE, X@N-term):8.78	PEAKS PTM
R.EEDWRQPR.E	N	48.87	1114.5155	8	1.8	558.2661	2	23.91	41	F41:1112	OB5954 H1 Ro.raw	1.6791E3	1	1	120	127			PEAKS DB
R.PNTLVLPK.H	N	48.84	880.5382	8	-2.9	441.2751	2	28.00	43	F43:1374	OB5956 H1 Ro.raw	1.9234E5	2	2	215	222			PEAKS DB
L.R(+27.99)IPSGFISYILNR.H	N	48.25	1562.8568	13	-2.0	782.4341	2	37.84	43	F43:1976	OB5956 H1 Ro.raw	1.5108E5	1	1	259	271	Formylation	R1:Formylation:1000.00	PEAKS PTM
R.EGEQEW(+15.99)GTPGSEVREETSR.N	Y	48.10	2177.9460	19	1.6	726.9905	3	26.51	43	F43:1296	OB5956 H1 Ro.raw	8.9341E6	3	3	147	165	Oxidation (HW)	W6:Oxidation (HW):1000.00	PEAKS PTM
R.I(+57.02)VQIEARPNTLVLPK.H	Y	46.89	1747.0355	15	-0.2	874.5248	2	30.98	42	F42:1537	OB5955 H1 Ro.raw	9.1688E5	3	3	208	222	Carbamidomethylation (DHKE, X@N-term)	I1:Carbamidomethylation (DHKE, X@N-term):69.71	PEAKS PTM

total 244 peptides

Peptide	Uniq	-10lgP	Mass	Length	ppm	m/z	z	RT	Fraction	Scan	Source File	Area Roasted in gel	#Feature	#Feature Roasted in gel	Start	End	PTM	AScore	Found By
R.N(+.98)TLEAAFNAEFNEIR.R	N	46.63	1738.8162	15	9.2	870.4233	2	44.36	42	F42:2303	OB5955 H1 Ro.raw	5.4416E3	1	1	313	327	Deamidation (NQ)	N1:Deamidation (NQ):39.47	PEAKS DB
R.VLLE(+21.98)ENAGGEQEER.G	N	46.37	1593.7246	14	-1.1	797.8687	2	26.71	43	F43:1300	OB5956 H1 Ro.raw	4.0134E5	3	3	329	342	Sodium adduct	E4:Sodium adduct:40.00	PEAKS PTM
N.PFYFPSR.R	N	46.32	912.4493	7	-0.1	457.2319	2	31.70	42	F42:1584	OB5955 H1 Ro.raw	2.4653E5	4	4	168	174			PEAKS DB
K.GTGNLELVAVRKEQQQR.G	N	46.31	1925.0442	17	1.4	642.6896	3	26.67	43	F43:1294	OB5956 H1 Ro.raw	2.1018E4	1	1	453	469			PEAKS DB
K.NPQLQDLD(+14.02)MMLTC(+57.02)VEIK.E	N	45.99	2060.9944	17	0.2	1031.5046	2	37.33	41	F41:1934	OB5954 H1 Ro.raw	2.7407E4	1	1	415	431	Methylation(others); Carbamidomethylation	D8:Methylation(others):42.68; C13:Carbamidomethylation:1000.00	PEAKS PTM
K.GTGNLE(+57.02)LVAVRK.E	N	45.75	1312.7462	12	-0.8	657.3799	2	27.17	42	F42:1295	OB5955 H1 Ro.raw	1.9882E5	1	1	453	464		E6:Carbamidomethylation (DHKE, X@N-term):0.00	PEAKS PTM
K.A(+43.01)MVIVVVNKGTGNLELVAVR.K	N	45.64	2124.2090	20	0.7	1063.1125	2	33.65	41	F41:1688	OB5954 H1 Ro.raw	8.0739E4	1	1	444	463	Carbamylation	A1:Carbamylation:161.20	PEAKS PTM
R.VLLEEN(+.98)AGGEQEERGQR.R	N	45.60	1913.9078	17	-1.6	638.9755	3	25.99	41	F41:1234	OB5954 H1 Ro.raw	0	0	0	329	345	Deamidation (NQ)	N6:Deamidation (NQ):28.79	PEAKS DB
K.KNPQLQDLDMM(+15.99)LTC(+57.02)VEIKEGALM(+15.99)LPHFNSK.A	N	45.45	3531.7234	30	-1.1	883.9372	4	35.80	42	F42:1822	OB5955 H1 Ro.raw	0	0	0	414	443	Carbamidomethylation; Oxidation (M)	M11:Oxidation (M):0.00;C14:Carbamidomethylation:1000.00;M23:Oxidation (M):45.87	PEAKS DB
R.LFEVKPDKKNPQLQDLDM(+15.99)M(+15.99)LTC(+57.02)VEIK.E	N	45.43	3163.5967	26	-2.8	791.9042	4	32.90	41	F41:1650	OB5954 H1 Ro.raw	2.0535E6	3	3	406	431	Oxidation (M); Carbamidomethylation	M18:Oxidation (M):1000.00; M19:Oxidation (M):1000.00; C22:Carbamidomethylation:1000.00	PEAKS DB
R.KSFNLDEGH(+15.99)ALR.I	N	45.40	1401.7001	12	3.9	701.8600	2	27.64	43	F43:1349	OB5956 H1 Ro.raw	6.6479E4	2	2	248	259	Oxidation (HW)	H9:Oxidation (HW):1000.00	PEAKS PTM
K.KNPQLQDLDM(+15.99)MLTC(+57.02)VEIKEGALMLPHFNSK.A	N	44.60	3515.7285	30	-1.9	879.9377	4	35.30	41	F41:1798	OB5954 H1 Ro.raw	6.4162E5	1	1	414	443	Carbamidomethylation	M10:Oxidation (M):14.02;C14:Carbamidomethylation:1000.00	PEAKS DB
R.DQSSYLQGFSR(+15.99).N	N	44.15	1302.5840	11	2.5	652.3009	2	30.79	43	F43:1544	OB5956 H1 Ro.raw	1.4308E5	3	3	302	312	Oxidation or Hydroxylation	R11:Oxidation or Hydroxylation:42.88	PEAKS PTM
K.Q(+.98)FQNLQNHR.I	N	43.70	1184.5686	9	4.6	593.2943	2	23.68	42	F42:1098	OB5955 H1 Ro.raw	0	0	0	199	207	Deamidation (NQ)	Q1:Deamidation (NQ):39.25	PEAKS DB
K.I(+57.02)SMPVNTPGQFEDFFPASSR.D	N	42.37	2283.0630	20	-0.7	1142.5380	2	36.01	43	F43:1860	OB5956 H1 Ro.raw	1.8515E5	1	1	282	301	Carbamidomethylation (DHKE, X@N-term)	I1:Carbamidomethylation (DHKE, X@N-term):50.99	PEAKS PTM

total 244 peptides

Peptide	Uniq	-10lgP	Mass	Length	ppm	m/z	z	RT	Fraction	Scan	Source File	Area Roasted in gel	#Feature	#Feature Roasted in gel	Start	End	PTM	AScore	Found By
K.ISM(-48.00)PVNTPGQFEDFFPASSR.D	N	42.09	2178.0381	20	2.8	727.0220	3	32.48	43	F43:1652	OB5956 H1 Ro.raw	5.8583E6	3	3	282	301	Dethiomethyl	M3:Dethiomethyl:1000.00	PEAKS PTM
R.SKQFQNLQNH(+15.99)R.I	Y	41.84	1414.7065	11	2.4	708.3622	2	24.12	42	F42:1124	OB5955 H1 Ro.raw	1.2986E3	1	1	197	207	Oxidation (HW)	H10:Oxidation (HW):1000.00	PEAKS PTM
K.KGSEEEDIT(-18.01)N(+.98)PINLR.D	N	41.27	1696.8268	15	2.3	566.6175	3	29.07	43	F43:1442	OB5956 H1 Ro.raw	3.8118E5	3	3	379	393	Dehydration; Deamidation (NQ)	T9:Dehydration:45.01;N10:Deamidation (NQ):59.10	PEAKS PTM
K.NPQLQDLDMM(+15.99)LTC(+57.02)VEIKEGALM(+15.99)LPHFN(+.98)SK.A	N	41.25	3404.6125	29	-0.4	1135.8777	3	36.69	42	F42:1878	OB5955 H1 Ro.raw	3.8423E5	1	1	415	443	Carbamidomethylation; Oxidation (M); Deamidation (NQ)	M10:Oxidation (M):0.00;C13:Carbamidomethylation:1000.00;M22:Oxidation (M):39.23;N27:Deamidation (NQ):69.52	PEAKS DB
R.REQEW(+15.99)EEEEEDEEEEGSNR.E	N	41.18	2452.9375	19	-0.6	818.6526	3	25.98	41	F41:1239	OB5954 H1 Ro.raw	2.4114E4	1	1	472	490	Oxidation (HW)	W5:Oxidation (HW):1000.00	PEAKS PTM
K.DLAFP(+15.99)GSGEQVEK.L	N	41.14	1391.6569	13	0.5	696.8361	2	29.36	42	F42:1432	OB5955 H1 Ro.raw	7.4528E5	3	3	555	567	Oxidation or Hydroxylation	P5:Oxidation or Hydroxylation:67.76	PEAKS PTM
K.AMVIVVVN(+.98)KGTGNLELVAVRK.E	N	41.03	2210.2820	21	1.9	1106.1504	2	34.35	42	F42:1720	OB5955 H1 Ro.raw	4.5718E5	1	1	444	464	Deamidation (NQ)	N8:Deamidation (NQ):28.79	PEAKS DB
K.EGALM(-48.00)LPHFNSK.A	N	40.53	1294.6670	12	0.5	432.5632	3	28.76	41	F41:1389	OB5954 H1 Ro.raw	2.208E6	2	2	432	443	Dethiomethyl	M5:Dethiomethyl:1000.00	PEAKS PTM
R.VLLEENAGGEQEERGQRR.R	N	40.17	2069.0249	18	1.0	518.2640	4	25.49	42	F42:1205	OB5955 H1 Ro.raw	2.0305E5	2	2	329	346			PEAKS DB
R.VLLEENAGGEQEER(+15.99).G	N	39.98	1587.7375	14	1.8	794.8775	2	26.70	41	F41:1275	OB5954 H1 Ro.raw	0	0	0	329	342	Oxidation or Hydroxylation	R14:Oxidation or Hydroxylation:20.88	PEAKS PTM
R.N(+27.99)TLEAAFNAEFNEIRR.V	N	39.68	1921.9282	16	-1.7	961.9697	2	37.51	41	F41:1908	OB5954 H1 Ro.raw	3.7767E4	1	1	313	328	Formylation	N1:Formylation:1000.00	PEAKS PTM
K.E(-18.01)GALM(+15.99)LPHFNSK.A	N	39.64	1340.6547	12	0.2	447.8923	3	30.21	41	F41:1479	OB5954 H1 Ro.raw	7.81E5	1	1	432	443	Pyro-glu from E; Oxidation (M)	E1:Pyro-glu from E:1000.00;M5:Oxidation (M):1000.00	PEAKS PTM
R.VLLEE(+53.92)NAGGEQEER.G	N	39.58	1625.6620	14	-1.0	542.8940	3	26.76	42	F42:1278	OB5955 H1 Ro.raw	0	0	0	329	342	Replacement of 2 protons by iron	E5:Replacement of 2 protons by iron:20.92	PEAKS PTM
D.GEPDLSNNFGR.L	N	39.46	1204.5472	11	3.0	603.2827	2	28.20	41	F41:1371	OB5954 H1 Ro.raw	1.5511E4	1	1	395	405			PEAKS DB
K.HADADNILVIQQGQ(+.98)ATVTVANGNNRK.S	N	39.33	2747.3950	26	2.6	916.8103	3	30.46	30	F30:1280	OB5926 H3B Ro.raw	0	0	0	223	248		Q14:Deamidation (NQ):5.07	PEAKS DB
K.NPQLQ(+.98)DLDMM(+15.99)LTC(+57.02)VEIKEGALM(+15.99)LPHFNSK.A	N	39.15	3404.6125	29	-1.8	852.1589	4	38.22	43	F43:1993	OB5956 H1 Ro.raw	7.6266E5	1	1	415	443	Deamidation (NQ); Carbamidomethylation	Q5:Deamidation (NQ):49.79;M10:Oxidation (M):0.00;C13:	PEAKS DB
**total 244 peptides**																			

Peptide	Uniq	-10lgP	Mass	Length	ppm	m/z	z	RT	Fraction	Scan	Source File	Area Roasted in gel	#Feature	#Feature Roasted in gel	Start	End	PTM	AScore	Found By
																		Carbamidomethylation:1000.00;M22:Oxidation (M):14.60	
K.N(+.98)PQLQDLDM(+15.99)MLTC(+57.02)VEIKEGALMLPHFNSK.A	N	38.83	3388.6174	29	8.2	1695.3300	2	37.72	41	F41:1941	OB5954 H1 Ro.raw	6.4698E5	1	1	415	443	Carbamidomethylation	N1:Deamidation (NQ):0.00;M9:Oxidation (M):0.00;C13:Carbamidomethylation:1000.00	PEAKS DB
K.D(+15.99)LAFPGSGEQVEK.L	N	38.81	1391.6569	13	0.5	696.8361	2	29.36	42	F42:1368	OB5955 H1 Ro.raw	2.5725E5	1	1	555	567	Oxidation or Hydroxylation	D1:Oxidation or Hydroxylation:77.13	PEAKS PTM
K.NPQ(+.98)LQDLDMMLTC(+57.02)VEIK.E	N	38.70	2047.9629	17	2.9	1024.9917	2	46.45	41	F41:2441	OB5954 H1 Ro.raw	0	0	0	415	431	Carbamidomethylation	Q3:Deamidation (NQ):14.04;C13:Carbamidomethylation:1000.00	PEAKS DB
R.IFLAGD(+15.99)KDNVIDQIEK.Q	N	38.52	1832.9519	16	1.9	917.4850	2	31.89	42	F42:1590	OB5955 H1 Ro.raw	4.6198E5	1	1	536	551		D6:Oxidation or Hydroxylation:12.60	PEAKS PTM
R.IFLAGDKDN(+.98)VIDQIEKQ(+.98)AK.D	N	37.85	2146.1157	19	9.8	716.3862	3	32.98	42	F42:1661	OB5955 H1 Ro.raw	8.6625E4	1	1	536	554		N9:Deamidation (NQ):13.83;Q17:Deamidation (NQ):17.65	PEAKS DB
K.DLAFPGSGE(+53.92)QVEK.L	N	37.31	1429.5812	13	-6.3	715.7933	2	30.34	43	F43:1518	OB5956 H1 Ro.raw	2.6492E5	1	1	555	567	Replacement of 2 protons by iron	E9:Replacement of 2 protons by iron:37.54	PEAKS PTM
R.W(+15.99)GPAEPR.E	Y	36.73	827.3926	7	0.0	414.7036	2	24.54	42	F42:1155	OB5955 H1 Ro.raw	3.7087E3	1	1	109	115	Oxidation (HW)	W1:Oxidation (HW):1000.00	PEAKS PTM
K.GT(-18.01)GNLELVAVRK.E	N	36.73	1237.7142	12	2.6	413.5797	3	27.00	42	F42:1287	OB5955 H1 Ro.raw	4.7714E5	1	1	453	464	Dehydration	T2:Dehydration:1000.00	PEAKS PTM
K.IRPEGREGEQEWGTPGSEVREETSR.N	Y	36.52	2870.3542	25	2.3	718.5975	4	27.83	43	F43:1355	OB5956 H1 Ro.raw	1.3647E5	1	1	141	165			PEAKS DB
R.NT(-18.01)LEAAFNAEFNEIRR.V	N	36.45	1875.9227	16	0.7	626.3153	3	34.16	43	F43:1739	OB5956 H1 Ro.raw	6.9172E6	2	2	313	328	Dehydration	T2:Dehydration:1000.00	PEAKS PTM
K.EGALM(+15.99)LPHFN(+.98)SK.A	N	36.41	1359.6493	12	2.8	680.8338	2	31.22	41	F41:1550	OB5954 H1 Ro.raw	0	0	0	432	443	Oxidation (M); Deamidation (NQ)	M5:Oxidation (M):1000.00;N10:Deamidation (NQ):1000.00	PEAKS DB
R.DQSSY(-18.01)LQGFSR.N	N	36.32	1268.5785	11	-0.6	635.2961	2	30.85	43	F43:1579	OB5956 H1 Ro.raw	2.378E5	1	1	302	312		Y5:Dehydration:0.00	PEAKS PTM
R.C(+57.02)LQS(-18.01)C(+57.02)Q(+.98)QEPDDLKQK.A	N	36.24	1858.8190	15	-0.1	930.4167	2	28.47	31	F31:1350	OB5942 H6 Ro.raw	0	0	0	40	54	Carbamidomethylation; Dehydration	C1:Carbamidomethylation:1000.00;S4:Dehydration:39.55;C5:Carbamidomethylation:10	PEAKS PTM
**total 244 peptides**																			

Peptide	Uniq	-10lgP	Mass	Length	ppm	m/z	z	RT	Fraction	Scan	Source File	Area Roasted in gel	#Feature	#Feature Roasted in gel	Start	End	PTM	AScore	Found By
																		00.00;Q6: Deamidation (NQ): 8.69	
M.LPHFNSK.A	N	36.02	841.4446	7	1.3	421.7301	2	30.63	42	F42:1510	OB5955 H1 Ro.raw	1.4673E6	2	2	437	443			PEAKS DB
K.NPQLQDLDM(-48.00)MLTC(+57.02)VEIK.E	N	35.81	1998.9755	17	6.7	667.3369	3	34.52	43	F43:1773	OB5956 H1 Ro.raw	8.4276E5	1	1	415	431	Carbamidomethylation	M9:Dethiomethyl:11.06;C13:Carbamidomethylation:1000.00	PEAKS PTM
K.SF(+17.99)NLDEGHALR.I	N	34.75	1275.6007	11	8.1	638.8128	2	26.62	42	F42:1267	OB5955 H1 Ro.raw	9.5828E3	1	1	249	259	Fluorination	F2:Fluorination:1000.00	PEAKS PTM
K.KNPQLQ(+.98)DLDMM(+15.99)LTC(+57.02)VEIKEGALMLPHFNSK.A	N	33.72	3516.7124	30	3.3	880.1883	4	35.48	41	F41:1798	OB5954 H1 Ro.raw	7.5658E5	1	1	414	443	Carbamidomethylation	Q6:Deamidation (NQ):18.55;M11:Oxidation (M):14.02;C14:Carbamidomethylation:1000.00	PEAKS DB
K.VSKEH(+15.99)VQELTK.H	N	33.71	1312.6986	11	1.8	657.3578	2	23.73	42	F42:1100	OB5955 H1 Ro.raw	1.3581E3	1	1	361	371	Oxidation (HW)	H5:Oxidation (HW):1000.00	PEAKS PTM
D.QSSYLQGFSR.N	N	33.26	1171.5621	10	2.1	586.7896	2	29.36	42	F42:1431	OB5955 H1 Ro.raw	3.3134E4	1	1	303	312			PEAKS DB
R.IFLAGDKDNVID(-18.01)QIEK.Q	N	33.09	1798.9464	16	0.7	900.4811	2	31.39	43	F43:1591	OB5956 H1 Ro.raw	6.7846E4	1	1	536	551		D12:Dehydration:13.16	PEAKS PTM
R.EREEDWRQPR.E	N	32.92	1399.6592	10	5.7	700.8409	2	24.05	43	F43:1141	OB5956 H1 Ro.raw	4.1173E4	1	1	118	127			PEAKS DB
D.NVIDQIEK.Q	N	32.64	957.5131	8	1.6	479.7646	2	28.19	42	F42:1367	OB5955 H1 Ro.raw	3.0062E3	1	1	544	551			PEAKS DB
K.AM(-48.00)VIVVVNK.G	N	32.49	923.5804	9	0.0	462.7975	2	25.00	43	F43:1192	OB5956 H1 Ro.raw	6.3177E3	1	1	444	452	Dethiomethyl	M2:Dethiomethyl:1000.00	PEAKS PTM
R.D(-18.01)QSSYLQGFSR.N	N	31.95	1268.5785	11	1.6	635.2975	2	29.92	43	F43:1489	OB5956 H1 Ro.raw	0	0	0	302	312		D1:Dehydration:0.00	PEAKS PTM
R.IFLAGDKD(-18.01)NVIDQIEK.Q	N	31.82	1798.9464	16	-0.9	600.6555	3	32.15	43	F43:1589	OB5956 H1 Ro.raw	3.5634E5	1	1	536	551	Dehydration	D8:Dehydration:30.83	PEAKS PTM
K.D(+53.92)LAFPGSGEQVEK.L	N	31.27	1429.5812	13	-4.3	715.7948	2	30.56	41	F41:1500	OB5954 H1 Ro.raw	3.7719E5	1	1	555	567		D1:Replacement of 2 protons by iron:0.00	PEAKS PTM
K.SFNLDE(+53.92)GHALR.I	N	31.27	1311.5294	11	1.0	438.1842	3	28.04	41	F41:1362	OB5954 H1 Ro.raw	5.8053E4	1	1	249	259		E6:Replacement of 2 protons by iron:16.90	PEAKS PTM
K.AM(+15.99)VIVVVNK(+57.02)GTGNLELVAVR.K	N	30.97	2154.2195	20	2.8	1078.1200	2	32.98	42	F42:1664	OB5955 H1 Ro.raw	2.0029E5	1	1	444	463	Oxidation (M)	M2:Oxidation (M):1000.00;K9:Carbamidomethylation (DHKE, X@N-term):0.00	PEAKS PTM
K.KNPQLQDLDMM(+15.99)LTC(+57.02)VEIKEGALMLPHFNSK.A	N	30.83	3515.7285	30	1.3	879.9406	4	35.25	42	F42:1790	OB5955 H1 Ro.raw	0	0	0	414	443	Carbamidomethylation	M11:Oxidation (M):12.28;C1	PEAKS DB
**total 244 peptides**																			

Peptide	Uniq	-10lgP	Mass	Length	ppm	m/z	z	RT	Fraction	Scan	Source File	Area Roasted in gel	#Feature	#Feature Roasted in gel	Start	End	PTM	AScore	Found By
																		4:Carbamidomethylation:1000.00	
R.DQSSY(+15.99)LQGFSR.N	N	30.72	1302.5840	11	1.6	652.3003	2	29.24	43	F43:1470	OB5956 H1 Ro.raw	6.7205E4	1	1	302	312	Oxidation or Hydroxylation	Y5:Oxidation or Hydroxylation:26.36	PEAKS PTM
K.SFNLDEGHALRIPSGFISYILNRHDNQN(+.98)LR.V	N	30.43	3496.7600	30	4.9	700.3627	5	37.51	39	F39:1925	OB5952 H3A Ro.raw	2.7478E5	1	1	249	278		N28:Deamidation (NQ):10.11	PEAKS DB
D.KDNVIDQIEK.Q	N	30.42	1200.6350	10	-0.3	601.3246	2	26.31	42	F42:1252	OB5955 H1 Ro.raw	0	0	0	542	551			PEAKS DB
N.TLEAAFNAEFNEIRR.V	N	30.42	1779.8904	15	3.5	890.9556	2	33.23	43	F43:1696	OB5956 H1 Ro.raw	1.5374E5	1	1	314	328			PEAKS DB
R.IPSGFISYILN(+15.99)R.H	N	30.30	1394.7557	12	-2.8	698.3832	2	36.42	41	F41:1875	OB5954 H1 Ro.raw	3.8045E5	1	1	260	271		N11:Oxidation or Hydroxylation:0.00	PEAKS PTM
W.GTPGSEVREETSR.N	Y	30.01	1403.6641	13	3.8	702.8420	2	27.77	42	F42:1332	OB5955 H1 Ro.raw	2.719E4	1	1	153	165			PEAKS DB
R.IFLAGDKD(-18.01)NV.I	N	30.00	1072.5553	10	0.7	537.2853	2	32.26	42	F42:1603	OB5955 H1 Ro.raw	2.1452E6	1	1	536	545	Dehydration	D8:Dehydration:45.01	PEAKS PTM
R.NTLEAAFNAEFN(+15.99)EIRR.V	N	29.99	1909.9282	16	0.9	955.9723	2	32.98	42	F42:1662	OB5955 H1 Ro.raw	2.366E6	2	2	313	328		N12:Oxidation or Hydroxylation:0.00	PEAKS PTM
R.IFLAGDKDNVIDQ(+.98)IEK.Q	N	29.73	1817.9410	16	7.7	909.9848	2	63.08	43	F43:3435	OB5956 H1 Ro.raw	3.1606E3	1	1	536	551		Q13:Deamidation (NQ):11.12	PEAKS DB
I.F(+127.06)LAGDKDNVIDQIEK.Q	N	29.59	1830.9363	15	0.7	916.4761	2	32.18	41	F41:1600	OB5954 H1 Ro.raw	6.5822E4	1	1	537	551	N-Succinimidyl-2-morpholine acetate	F1:N-Succinimidyl-2-morpholine acetate:28.79	PEAKS PTM
K.N(+.98)PQLQ(+.98)DLDMMLTC(+57.02)VEIKEGALM(+15.99)LPHFNSK.A	N	29.50	3389.6016	29	9.6	848.4158	4	37.65	43	F43:2047	OB5956 H1 Ro.raw	6.1505E6	1	1	415	443	Carbamidomethylation	N1:Deamidation (NQ):0.00;Q5:Deamidation (NQ):14.04;C13:Carbamidomethylation:1000.00;M22:Oxidation (M):0.00	PEAKS DB
R.NN(+.98)PFYFPSRR.F	N	29.19	1297.6204	10	9.3	649.8235	2	62.71	43	F43:3425	OB5956 H1 Ro.raw	0	0	0	166	175		N2:Deamidation (NQ):7.21	PEAKS DB
R.NTLEAAFNAEFN(+.98)EIR.R	N	29.07	1738.8162	15	9.2	870.4233	2	44.36	42	F42:2331	OB5955 H1 Ro.raw	5.4416E3	1	1	313	327		N12:Deamidation (NQ):0.00	PEAKS DB
K.KNPQ(+.98)LQ(+.98)DLDMM(+15.99)LTC(+57.02)VEIKEGALM(+15.99)LPHFNSK.A	N	28.74	3533.6914	30	9.7	884.4387	4	35.77	42	F42:1822	OB5955 H1 Ro.raw	2.7541E5	1	1	414	443	Carbamidomethylation; Oxidation (M)	Q4:Deamidation (NQ):8.22;Q6:Deamidation (NQ):5.55;M11:Oxidation (M):0.00;C14:Carbamidomethylation:1000.00;M23:Ox	PEAKS DB
**total 244 peptides**																			

Peptide	Uniq	-10lgP	Mass	Length	ppm	m/z	z	RT	Fraction	Scan	Source File	Area Roasted in gel	#Feature	#Feature Roasted in gel	Start	End	PTM	AScore	Found By
																		idation (M):38.29	
total 244 peptides																			

B3IXL2|B3IXL2_ARAHY

back to list

| Protein Coverage | Supporting Peptides |

Protein Coverage:

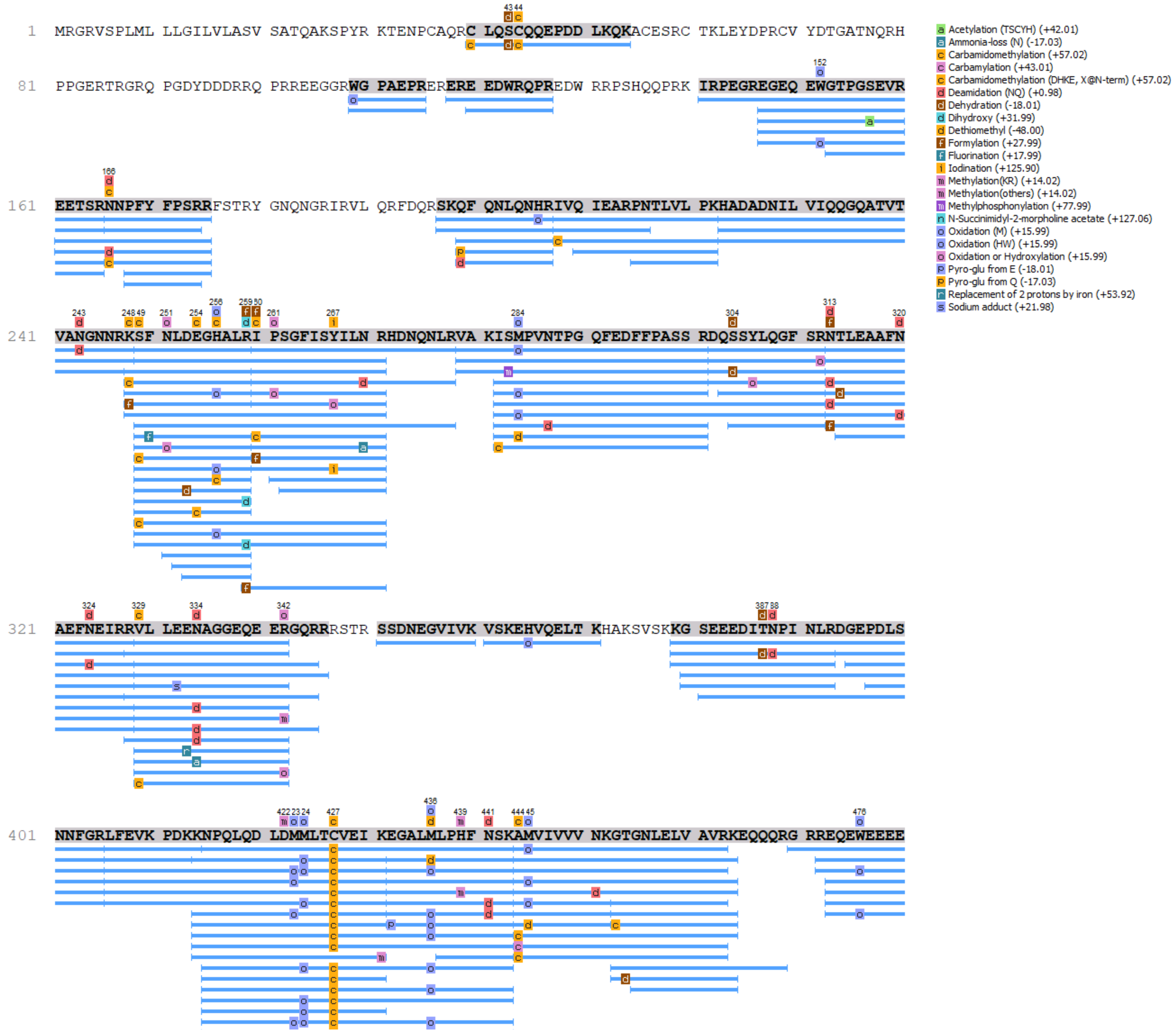
1 MRGRVSPLML LLGILVLASV SATQAKSPYR KTENPCAQRC LQSCQQEPDD LKQKACESRC TKLEYDPRCV YDTGATNQRH
81 PPGERTRGRQ PGDYDDDRRQ PRREEGGRWG PAEPRERERE EDWRQPREDW RRPSHQQPRK IRPEGREGEQ EWGTPGSEVR
161 EETSRNNPFY FPSRRFSTRY GNQNGRIRVL QRFDQRSKQF QNLQNHRIVQ IEARPNTLVL PKHADADNIL VIQQGQATVT
241 VANGNNRKSF NLDEGHALRI PSGFISYILN RHDNQNLRVA KISMPVNTPG QFEDFFPASS RDQSSYLQGF SRNTLEAAFN
321 AEFNEIRRVL LEENAGGEQE ERGQRRRSTR SSDNEGVIVK VSKEHVQELT KHAKSVSKKG SEEEDITNPI NLRDGEPDLS
401 NNFGRLFEVK PDKKNPQLQD LDMMLTCVEI KEGALMLPHF NSKAMVIVVV NKGTGNLELV AVRKEQQQRG RREQEWEEEE
Acetylation (TSCYH) (+42.01)
Ammonia-loss (N) (-17.03)
Carbamidomethylation (+57.02)
Carbamylation (+43.01)
Carbamidomethylation (DHKE, X@N-term) (+57.02)
Deamidation (NQ) (+0.98)
Dehydration (-18.01)
Dihydroxy (+31.99)
Dethiomethyl (-48.00)
Formylation (+27.99)
Fluorination (+17.99)
Iodination (+125.90)
Methylation(KR) (+14.02)
Methylation(others) (+14.02)
Methylphosphonylation (+77.99)
N-Succinimidyl-2-morpholine acetate (+127.06)
Oxidation (M) (+15.99)
Oxidation (HW) (+15.99)
Oxidation or Hydroxylation (+15.99)
Pyro-glu from E (-18.01)
Pyro-glu from Q (-17.03)
Replacement of 2 protons by iron (+53.92)
Sodium adduct (+21.98)

Supporting Peptides:

Peptide	Uniq	-10lgP	Mass	Length	ppm	m/z	z	RT	Fraction	Scan	Source File	Area Roasted in gel	#Feature	#Feature Roasted in gel	Start	End	PTM	AScore	Found By
K.KNPQLQDLDMMLTC(+57.02)VEIK.E	N	136.41	2175.0737	18	1.4	1088.5457	2	35.26	43	F43:1808	OB5956 H1 Ro.raw	1.4906E7	6	6	414	431	Carbamidomethylation	C14:Carbamidomethylation:1000.00	PEAKS DB
R.NTLEAAFNAEFNEIR.R	N	130.93	1737.8322	15	1.8	869.9249	2	35.34	38	F38:1714	OB5951 H3A Ro.raw	1.332E7	16	16	313	327			PEAKS DB
K.GSEEEDITNPINLRDGEPDLSNNFGR.L	N	129.76	2887.3220	26	-0.7	963.4473	3	32.67	43	F43:1660	OB5956 H1 Ro.raw	1.2475E7	11	11	380	405			PEAKS DB
K.NPQLQDLDMMLTC(+57.02)VEIK.E	N	128.40	2046.9788	17	0.2	1024.4968	2	37.11	43	F43:1936	OB5956 H1 Ro.raw	3.4643E7	10	10	415	431	Carbamidomethylation	C13:Carbamidomethylation:1000.00	PEAKS DB
K.NPQLQDLDM(+15.99)MLTC(+57.02)VEIK.E	N	128.16	2062.9736	17	-4.2	1032.4897	2	36.93	43	F43:1892	OB5956 H1 Ro.raw	3.3834E6	3	3	415	431	Oxidation (M); Carbamidomethylation	M9:Oxidation (M):30.46;C13:Carbamidomethylation:1000.00	PEAKS DB
K.NPQLQDLDMM(+15.99)LTC(+57.02)VEIK.E	N	127.13	2062.9736	17	5.0	1032.4993	2	34.71	43	F43:1780	OB5956 H1 Ro.raw	1.0665E7	4	4	415	431	Oxidation (M); Carbamidomethylation	M10:Oxidation (M):30.46;C13:Carbamidomethylation:1000.00	PEAKS DB
K.ISM(+15.99)PVNTPGQFEDFFPASSR.D	N	127.11	2242.0364	20	0.1	1122.0284	2	34.52	30	F30:1521	OB5926 H3B Ro.raw	2.1708E7	14	13	282	301	Oxidation (M)	M3:Oxidation (M):1000.00	PEAKS DB
R.EQEWEEEEEDEEEEGSNR.E	N	125.55	2280.8413	18	3.9	1141.4324	2	28.35	43	F43:1396	OB5956 H1 Ro.raw	4.3429E6	7	7	473	490			PEAKS DB
K.ISMPVNTPGQFEDFFPASSR.D	N	125.26	2226.0415	20	0.7	1114.0288	2	35.83	43	F43:1868	OB5956 H1 Ro.raw	8.8227E7	23	23	282	301			PEAKS DB
K.HADADNILVIQQGQATVTVAN(+.98)GNNRK.S	N	123.10	2747.3950	26	3.3	916.8087	3	29.62	43	F43:1472	OB5956 H1 Ro.raw	4.4266E6	11	11	223	248	Deamidation (NQ)	N21:Deamidation (NQ):31.91	PEAKS DB
K.NPQLQDLDM(+15.99)M(+15.99)LTC(+57.02)VEIK.E	N	121.59	2078.9688	17	6.6	1040.4985	2	33.82	43	F43:1726	OB5956 H1 Ro.raw	6.8984E4	2	2	415	431	Oxidation (M); Carbamidomethylation	M9:Oxidation (M):1000.00;M10:Oxidation (M):1000.00;C13:Carbamidomethylation:1000.00	PEAKS DB
K.SFNLDEGHALRIPSGFISYILNR.H	N	121.49	2618.3604	23	1.0	873.7949	3	37.51	41	F41:1949	OB5954 H1 Ro.raw	9.1637E7	28	28	249	271			PEAKS DB
K.KNPQLQDLDM(+15.99)MLTC(+57.02)VEIK.E	N	118.68	2191.0686	18	3.6	1096.5455	2	34.71	43	F43:1781	OB5956 H1 Ro.raw	1.3598E6	4	4	414	431	Oxidation (M); Carbamidomethylation	M10:Oxidation (M):27.96;C14:Carbamidomethylation:1000.00	PEAKS DB
K.AMVIVVVNKGTGNLELVAVR.K	N	114.87	2081.2031	20	-1.0	1041.6078	2	34.35	43	F43:1758	OB5956 H1 Ro.raw	3.8361E7	13	13	444	463			PEAKS DB
R.IFLAGDKDNVIDQIEK.Q	N	110.41	1816.9570	16	-1.1	909.4848	2	32.67	43	F43:1648	OB5956 H1 Ro.raw	4.2419E8	56	55	536	551			PEAKS DB

total 244 peptides

481 EDEEEEGSNR EVRRYTARLK EGDVFIMPAA HPVAINASSE LHLLGFGINA ENNHRIFLAG DKDNVIDQIE KQAKDLAFPG

561 SGEQVEKLIK NQRESHFVSA RPQSQSPSSP EKEDQEEENQ GGKGPLLSIL KAFN

Peptide	Uniq	-10lgP	Mass	Length	ppm	m/z	z	RT	Fraction	Scan	Source File	Area Roasted in gel	#Feature	#Feature Roasted in gel	Start	End	PTM	AScore	Found By
K.KNPQLQDLDMM(+15.99)LTC(+57.02)VEIK.E	N	110.35	2191.0686	18	3.5	1096.5454	2	32.98	42	F42:1657	OB5955 H1 Ro.raw	1.6495E6	4	4	414	431	Oxidation (M); Carbamidomethylation	M11:Oxidation (M):23.10;C14:Carbamidomethylation:1000.00	PEAKS DB
K.KGSEEEDITNPINLRDGEPDLSNNFGR.L	N	109.79	3015.4170	27	3.4	1006.1497	3	31.62	41	F41:1565	OB5954 H1 Ro.raw	1.1559E7	21	21	379	405			PEAKS DB
R.IPSGFISYILNR.H	N	109.18	1378.7609	12	2.5	690.3894	2	36.50	38	F38:1780	OB5951 H3A Ro.raw	4.4166E8	20	20	260	271			PEAKS DB
K.HADADNILVIQQGQATVTVANGNNRK.S	N	108.34	2746.4111	26	1.9	916.4794	3	29.61	43	F43:1451	OB5956 H1 Ro.raw	3.8404E6	5	5	223	248			PEAKS DB
R.VLLEENAGGEQEER.G	N	108.33	1571.7427	14	2.4	786.8805	2	26.83	42	F42:1293	OB5955 H1 Ro.raw	2.9858E7	10	10	329	342			PEAKS DB
R.KSFNLDEGHALR.I	N	108.28	1385.7051	12	0.6	462.9092	3	26.14	43	F43:1252	OB5956 H1 Ro.raw	5.3666E6	6	6	248	259			PEAKS DB
K.AMVIVVVNKGTGNLELVAVRK.E	N	107.96	2209.2981	21	1.1	737.4408	3	33.05	43	F43:1682	OB5956 H1 Ro.raw	1.2362E8	20	20	444	464			PEAKS DB
R.I(+57.02)PSGFISYILNR.H	N	107.77	1435.7823	12	-3.6	718.8959	2	36.92	43	F43:1901	OB5956 H1 Ro.raw	1.7559E7	7	7	260	271	Carbamidomethylation (DHKE, X@N-term)	I1:Carbamidomethylation (DHKE, X@N-term):1000.00	PEAKS PTM
K.KGSEEEDITNPINLR.D	N	107.76	1713.8533	15	2.8	857.9363	2	29.07	43	F43:1439	OB5956 H1 Ro.raw	8.6032E6	7	7	379	393			PEAKS DB
R.I(+57.02)FLAGDKDNVIDQIEK.Q	N	105.70	1873.9785	16	1.7	937.9982	2	32.48	43	F43:1653	OB5956 H1 Ro.raw	1.1722E7	5	5	536	551	Carbamidomethylation (DHKE, X@N-term)	I1:Carbamidomethylation (DHKE, X@N-term):90.91	PEAKS PTM
R.DQSSYLQGFSR.N	N	105.08	1286.5891	11	0.8	644.3024	2	30.81	40	F40:1431	OB5953 H3A Ro.raw	9.5783E7	31	31	302	312			PEAKS DB
R.V(+57.02)LLEENAGGEQEER.G	N	103.80	1628.7642	14	0.3	815.3896	2	27.08	43	F43:1322	OB5956 H1 Ro.raw	4.0193E5	3	3	329	342	Carbamidomethylation (DHKE, X@N-term)	V1:Carbamidomethylation (DHKE, X@N-term):49.37	PEAKS PTM
K.NPQLQDLDMMLTCVEIK(+14.02).E	N	103.68	2003.9730	17	-1.2	1002.9926	2	38.20	43	F43:1989	OB5956 H1 Ro.raw	5.9168E6	4	4	415	431	Methylation(KR)	K17:Methylation(KR):1000.00	PEAKS PTM
K.SFNLDEGHALR.I	N	103.18	1257.6101	11	-1.7	629.8113	2	28.00	43	F43:1362	OB5956 H1 Ro.raw	1.1584E8	33	31	249	259			PEAKS DB
K.AM(+15.99)VIVVVNKGTGNLELVAVR.K	N	103.14	2097.1980	20	1.6	700.0744	3	33.04	43	F43:1684	OB5956 H1 Ro.raw	2.3148E7	9	9	444	463	Oxidation (M)	M2:Oxidation (M):1000.00	PEAKS DB
R.DGEPDLSNNFGR.L	N	102.69	1319.5742	12	0.3	660.7946	2	29.24	43	F43:1495	OB5956 H1 Ro.raw	7.4875E6	3	3	394	405			PEAKS DB
K.DLAFPGSGEQVEK.L	N	100.93	1375.6619	13	-1.9	688.8369	2	30.34	43	F43:1508	OB5956 H1 Ro.raw	9.196E7	8	8	555	567			PEAKS DB
**total 244 peptides**																			

Peptide	Uniq	-10lgP	Mass	Length	ppm	m/z	z	RT	Fraction	Scan	Source File	Area Roasted in gel	#Feature	#Feature Roasted in gel	Start	End	PTM	AScore	Found By
K.NPQLQDLDM(+15.99)MLTC(+57.02)VEIKEGALMLPHFNSK.A	N	100.40	3387.6335	29	-0.6	1130.2178	3	37.70	41	F41:1939	OB5954 H1 Ro.raw	8.7706E7	6	6	415	443	Oxidation (M); Carbamidomethylation	M9:Oxidation (M):26.02;C13:Carbamidomethylation:1000.00	PEAKS DB
R.RVLLEENAGGEQEER.G	N	100.35	1727.8438	15	0.2	576.9553	3	25.30	42	F42:1185	OB5955 H1 Ro.raw	8.2964E5	6	6	328	342			PEAKS DB
R.EGEQEWGTPGSEVR.E	Y	100.27	1559.6852	14	0.8	780.8505	2	27.93	42	F42:1345	OB5955 H1 Ro.raw	3.381E6	3	3	147	160			PEAKS DB
K.NPQLQDLDMMLTC(+57.02)VEIKEGALMLPHFNSK.A	N	98.85	3371.6387	29	-2.7	843.9147	4	38.02	43	F43:1969	OB5956 H1 Ro.raw	1.5351E8	12	12	415	443	Carbamidomethylation	C13:Carbamidomethylation:1000.00	PEAKS DB
K.KNPQLQDLDMMLTC(+57.02)VEIKEGALMLPHFNSK.A	N	97.96	3499.7336	30	-3.9	875.9373	4	36.80	41	F41:1884	OB5954 H1 Ro.raw	6.677E6	4	4	414	443	Carbamidomethylation	C14:Carbamidomethylation:1000.00	PEAKS DB
R.VLLEEN(+.98)AGGEQEER.G	N	94.48	1572.7267	14	2.8	787.3728	2	27.46	43	F43:1340	OB5956 H1 Ro.raw	6.2395E5	3	3	329	342	Deamidation (NQ)	N6:Deamidation (NQ):121.37	PEAKS DB
R.IFLAGDKDNVIDQIEKQAK.D	N	94.35	2144.1477	19	-2.1	715.7217	3	32.42	42	F42:1625	OB5955 H1 Ro.raw	3.3984E6	9	9	536	554			PEAKS DB
R.KSFNLDEGHALRIPSGFISYILNR.H	N	93.78	2746.4553	24	-0.7	687.6206	4	36.29	43	F43:1876	OB5956 H1 Ro.raw	1.1783E6	4	4	248	271			PEAKS DB
R.REQEWEEEEEDEEEEGSNR.E	N	93.49	2436.9424	19	0.1	813.3215	3	26.72	43	F43:1292	OB5956 H1 Ro.raw	8.6025E5	7	7	472	490			PEAKS DB
R.EGEQEWGTPGSEVREETSR.N	Y	93.19	2161.9512	19	3.0	1081.9861	2	27.83	43	F43:1353	OB5956 H1 Ro.raw	3.9146E7	8	8	147	165			PEAKS DB
F.LAGDKDNVIDQIEK.Q	N	91.83	1556.8046	14	-6.3	779.4047	2	32.21	43	F43:1629	OB5956 H1 Ro.raw	5.4162E6	4	4	538	551			PEAKS DB
R.IPSGFISYILN(-17.03)R.H	N	91.80	1361.7343	12	-4.9	681.8711	2	38.02	43	F43:1980	OB5956 H1 Ro.raw	1.3387E6	3	3	260	271	Ammonia-loss (N)	N11:Ammonia-loss (N):1000.00	PEAKS PTM
K.AMVIVVVNK.G	N	91.04	971.5837	9	-0.5	486.7989	2	30.16	43	F43:1494	OB5956 H1 Ro.raw	7.1361E6	3	3	444	452			PEAKS DB
K.SFNLDEGH(+15.99)ALR.I	N	90.73	1273.6051	11	0.9	637.8104	2	30.16	43	F43:1507	OB5956 H1 Ro.raw	8.7681E5	2	2	249	259	Oxidation (HW)	H8:Oxidation (HW):1000.00	PEAKS PTM
R.NNPFYFPSRR.F	N	89.95	1296.6364	10	-2.5	649.3239	2	28.89	43	F43:1417	OB5956 H1 Ro.raw	1.3445E8	22	22	166	175			PEAKS DB
K.D(+57.02)LAFPGSGEQVEK.L	N	88.60	1432.6833	13	-0.1	717.3489	2	31.08	41	F41:1535	OB5954 H1 Ro.raw	9.7986E5	3	3	555	567	Carbamidomethylation (DHKE, X@N-term)	D1:Carbamidomethylation (DHKE, X@N-term):131.10	PEAKS PTM
K.SFNLDEGH(+57.02)ALR.I	N	88.30	1314.6316	11	-0.1	658.3230	2	28.22	41	F41:1349	OB5954 H1 Ro.raw	2.9996E6	3	3	249	259	Carbamidomethylation (DHKE, X@N-term)	H8:Carbamidomethylation (DHKE, X@N-term):30.36	PEAKS PTM
K.EGALMLPHFNSK.A	N	88.21	1342.6703	12	2.5	672.3441	2	30.67	43	F43:1529	OB5956 H1 Ro.raw	1.4087E7	5	5	432	443			PEAKS DB
K.NPQLQDLDMM(-48.00)LTC(+57.02)VEIK.E	N	88.05	1998.9755	17	1.9	1000.4969	2	32.85	43	F43:1670	OB5956 H1 Ro.raw	5.9032E6	3	3	415	431	Carbamidomethylation	M10:Dethiomethyl:14.02;C13:Carbamid	PEAKS PTM
**total 244 peptides**																			

Peptide	Uniq	-10lgP	Mass	Length	ppm	m/z	z	RT	Fraction	Scan	Source File	Area Roasted in gel	#Feature	#Feature Roasted in gel	Start	End	PTM	AScore	Found By
																		omethylation:1000.00	
K.GSEEEDITNPINLR.D	N	87.68	1585.7583	14	2.3	793.8882	2	31.16	42	F42:1548	OB5955 H1 Ro.raw	3.3709E6	3	3	380	393			PEAKS DB
R.GRREQEWEEEEEDEEEEGSNR.E	N	87.21	2650.0649	21	1.1	884.3632	3	26.73	41	F41:1280	OB5954 H1 Ro.raw	7.9956E5	6	6	470	490			PEAKS DB
R.NNPFYFPSR.R	N	86.35	1140.5352	9	-0.1	571.2748	2	31.76	43	F43:1593	OB5956 H1 Ro.raw	1.7218E7	11	11	166	174			PEAKS DB
K.S(+57.02)FNLDEGHALR.I	N	86.31	1314.6316	11	-0.5	658.3228	2	28.11	42	F42:1359	OB5955 H1 Ro.raw	2.6997E6	4	4	249	259	Carbamidomethylation (DHKE, X@N-term)	S1:Carbamidomethylation (DHKE, X@N-term):91.04	PEAKS PTM
I.PSGFISYILNR.H	N	85.97	1265.6768	11	-4.6	633.8427	2	36.75	43	F43:1894	OB5956 H1 Ro.raw	3.5986E5	2	2	261	271			PEAKS DB
R.IPSGFISYILNRHDNQNLR.V	N	84.75	2256.1763	19	6.5	753.0709	3	33.61	43	F43:1714	OB5956 H1 Ro.raw	6.1581E7	21	21	260	278			PEAKS DB
R.I(+27.99)FLAGDKDNVIDQIEK.Q	N	84.60	1844.9519	16	-2.1	923.4813	2	36.23	41	F41:1853	OB5954 H1 Ro.raw	1.9485E6	3	3	536	551	Formylation	I1:Formylation:98.75	PEAKS PTM
K.SFNLDEGH(+15.99)ALRIPSGFISYILNR.H	N	83.49	2634.3555	23	-4.0	879.1223	3	38.76	43	F43:2023	OB5956 H1 Ro.raw	8.8519E5	1	1	249	271	Oxidation (HW)	H8:Oxidation (HW):1000.00	PEAKS PTM
K.KNPQLQDLDMMLTC(+57.02)VEIKEGALM(+15.99)LPHFNSK.A	N	83.11	3515.7285	30	-1.9	879.9377	4	36.03	43	F43:1857	OB5956 H1 Ro.raw	6.6263E6	3	3	414	443	Carbamidomethylation; Oxidation (M)	C14:Carbamidomethylation:1000.00;M23:Oxidation (M):114.34	PEAKS DB
R.NTLEAAFNAEFNEIRR.V	N	82.99	1893.9332	16	-2.6	947.9714	2	34.11	42	F42:1711	OB5955 H1 Ro.raw	3.0959E8	36	35	313	328			PEAKS DB
K.A(+57.02)MVIVVVNKGTGNLELVAVR.K	N	82.14	2138.2246	20	-5.4	1070.1138	2	34.47	42	F42:1739	OB5955 H1 Ro.raw	4.4412E6	3	3	444	463	Carbamidomethylation (DHKE, X@N-term)	A1:Carbamidomethylation (DHKE, X@N-term):207.56	PEAKS PTM
K.DLAFPGSGEQVEKLIK.N	N	80.89	1729.9249	16	-1.6	865.9706	2	33.95	30	F30:1478	OB5926 H3B Ro.raw	3.9305E5	5	4	555	570			PEAKS DB
I.FLAGDKDNVIDQIEK.Q	N	80.64	1703.8729	15	-5.7	852.9388	2	32.22	43	F43:1635	OB5956 H1 Ro.raw	3.1343E5	3	3	537	551			PEAKS DB
K.EGALM(+15.99)LPHFNSK.A	N	80.48	1358.6653	12	1.1	680.3406	2	29.85	43	F43:1485	OB5956 H1 Ro.raw	3.0314E6	5	5	432	443	Oxidation (M)	M5:Oxidation (M):1000.00	PEAKS DB
K.GTGNLELVAVR.K	N	80.39	1127.6299	11	-1.3	564.8215	2	30.21	41	F41:1476	OB5954 H1 Ro.raw	2.4706E7	5	5	453	463			PEAKS DB
R.NTLEAAFN(+.98)AEFNEIRR.V	N	79.02	1894.9172	16	-2.2	948.4638	2	32.35	41	F41:1616	OB5954 H1 Ro.raw	1.3357E6	5	5	313	328	Deamidation (NQ)	N8:Deamidation (NQ):48.12	PEAKS DB
R.VLLEENAGGEQEER(+14.02).G	N	78.92	1585.7583	14	0.7	793.8870	2	27.86	41	F41:1339	OB5954 H1 Ro.raw	1.6341E5	3	3	329	342	Methylation(KR)	R14:Methylation(KR):1000.00	PEAKS PTM
K.GTGNLELVAVRK.E	N	78.87	1255.7249	12	0.7	628.8701	2	27.30	41	F41:1322	OB5954 H1 Ro.raw	2.29E7	10	10	453	464			PEAKS DB
K.QFQNLQNHR.I	N	78.35	1183.5846	9	0.0	592.7996	2	23.81	42	F42:1102	OB5955 H1 Ro.raw	1.5125E5	6	6	199	207			PEAKS DB
**total 244 peptides**																			

Peptide	Uniq	-10lgP	Mass	Length	ppm	m/z	z	RT	Fraction	Scan	Source File	Area Roasted in gel	#Feature	#Feature Roasted in gel	Start	End	PTM	AScore	Found By
K.NPQLQDLDMM(+15.99)LTC(+57.02)VEIKEGALMLPHFNSK.A	N	78.01	3387.6335	29	-0.8	1130.2175	3	36.42	41	F41:1862	OB5954 H1 Ro.raw	6.8935E7	6	6	415	443	Oxidation (M); Carbamidomethylation	M10:Oxidation (M):24.44;C13:Carbamidomethylation:1000.00	PEAKS DB
R.LFEVKPDKKNPQLQDLDMMLTC(+57.02)VEIK.E	N	77.87	3131.6069	26	-4.0	1044.8721	3	34.65	42	F42:1756	OB5955 H1 Ro.raw	5.0788E6	4	4	406	431	Carbamidomethylation	C22:Carbamidomethylation:1000.00	PEAKS DB
K.NPQLQDLDM(+15.99)MLTC(+57.02)VEIKEGALM(+15.99)LPHFNSK.A	N	77.10	3403.6284	29	1.1	851.9153	4	37.51	41	F41:1883	OB5954 H1 Ro.raw	7.3887E6	7	7	415	443	Oxidation (M); Carbamidomethylation	M9:Oxidation (M):27.96;C13:Carbamidomethylation:1000.00;M22:Oxidation (M):71.31	PEAKS DB
R.IFLAGDKDNVIDQIEKQ(+.98)AK.D	N	76.22	2145.1316	19	1.5	716.0522	3	33.08	42	F42:1661	OB5955 H1 Ro.raw	0	0	0	536	554	Deamidation (NQ)	Q17:Deamidation (NQ):48.83	PEAKS DB
R.EETSRN(+.98)NPFYFPSRR.F	N	75.62	1899.8864	15	-0.2	634.3026	3	28.39	41	F41:1381	OB5954 H1 Ro.raw	3.2976E5	2	2	161	175	Deamidation (NQ)	N6:Deamidation (NQ):33.98	PEAKS DB
L.AGDKDNVIDQIEK.Q	N	75.39	1443.7205	13	-3.7	722.8649	2	32.08	42	F42:1607	OB5955 H1 Ro.raw	3.3426E5	1	1	539	551			PEAKS DB
K.Q(-17.03)FQNLQNHR.I	N	75.35	1166.5581	9	-1.2	584.2856	2	28.00	43	F43:1365	OB5956 H1 Ro.raw	4.927E6	4	4	199	207	Pyro-glu from Q	Q1:Pyro-glu from Q:1000.00	PEAKS PTM
R.SSDNEGVIVK.V	N	75.32	1046.5244	10	-1.0	524.2690	2	23.58	41	F41:1103	OB5954 H1 Ro.raw	3.7568E5	3	3	351	360			PEAKS DB
R.VAKISMPVNTPGQFEDFFPASSR.D	N	74.27	2524.2419	23	-2.9	842.4188	3	34.29	42	F42:1735	OB5955 H1 Ro.raw	1.4483E6	3	3	279	301			PEAKS DB
K.NPQLQDLDM(+15.99)M(+15.99)LTC(+57.02)VEIKEGALMLPHFNSK.A	N	73.00	3403.6284	29	-3.9	1135.5457	3	35.83	43	F43:1849	OB5956 H1 Ro.raw	7.8068E6	5	5	415	443	Oxidation (M); Carbamidomethylation	M9:Oxidation (M):54.18;M10:Oxidation (M):63.99;C13:Carbamidomethylation:1000.00	PEAKS DB
K.KNPQLQDLDM(+15.99)MLTC(+57.02)VEIKEGALM(+15.99)LPHFNSK.A	N	72.71	3531.7234	30	-1.9	883.9364	4	35.86	41	F41:1821	OB5954 H1 Ro.raw	7.53E5	1	1	414	443	Carbamidomethylation; Oxidation (M)	M10:Oxidation (M):17.01;C14:Carbamidomethylation:1000.00;M23:Oxidation (M):85.25	PEAKS DB
R.VLLEENAGGEQEERGQR.R	N	72.44	1912.9238	17	0.6	957.4698	2	25.30	42	F42:1191	OB5955 H1 Ro.raw	3.1353E6	8	8	329	345			PEAKS DB
K.QAKDLAFPGSGEQVEK.L	N	72.39	1702.8525	16	1.2	568.6255	3	27.08	43	F43:1321	OB5956 H1 Ro.raw	2.8232E5	6	6	552	567			PEAKS DB
K.EGALMLPHFN(+.98)SK.A	N	71.37	1343.6543	12	2.6	672.8362	2	31.66	43	F43:1595	OB5956 H1 Ro.raw	1.0101E5	2	2	432	443	Deamidation (NQ)	N10:Deamidation (NQ):1000.00	PEAKS DB
R.EQEW(+15.99)EEEEEDEEEEGSNR.E	N	70.36	2296.8362	18	3.2	1149.4291	2	27.67	41	F41:1333	OB5954 H1 Ro.raw	1.024E6	3	3	473	490	Oxidation (HW)	W4:Oxidation (HW):1000.00	PEAKS PTM

total 244 peptides

Peptide	Uniq	-10lgP	Mass	Length	ppm	m/z	z	RT	Fraction	Scan	Source File	Area Roasted in gel	#Feature	#Feature Roasted in gel	Start	End	PTM	AScore	Found By
F.NLDEGHALR.I	N	70.14	1023.5097	9	-2.5	512.7609	2	28.00	43	F43:1370	OB5956 H1 Ro.raw	2.6181E6	3	3	251	259			PEAKS DB
R.N(+.98)TLEAAFNAEFNEIRR.V	N	69.73	1894.9172	16	0.4	948.4662	2	32.39	42	F42:1620	OB5955 H1 Ro.raw	2.6319E6	3	3	313	328	Deamidation (NQ)	N1:Deamidation (NQ):41.52	PEAKS DB
E.PDLSNNFGR.L	N	69.35	1018.4832	9	0.9	510.2493	2	31.57	43	F43:1584	OB5956 H1 Ro.raw	1.3026E6	4	4	397	405			PEAKS DB
R.K(+57.02)SFNLDEGHALR.I	N	69.20	1442.7266	12	-0.1	722.3705	2	26.25	42	F42:1250	OB5955 H1 Ro.raw	4.0798E5	6	6	248	259	Carbamidomethylation (DHKE, X@N-term)	K1:Carbamidomethylation (DHKE, X@N-term):76.17	PEAKS PTM
R.LFEVKPDKKNPQLQDLDMM(+15.99)LTC(+57.02)VEIK.E	N	69.17	3147.6018	26	2.2	787.9095	4	33.24	42	F42:1670	OB5955 H1 Ro.raw	2.0797E6	2	2	406	431	Oxidation (M); Carbamidomethylation	M19:Oxidation (M):40.00;C22:Carbamidomethylation:1000.00	PEAKS DB
K.SFNLDE(+57.02)GHALRIPSGFISYILNR.H	N	69.07	2675.3818	23	-1.9	892.7996	3	37.80	43	F43:1964	OB5956 H1 Ro.raw	0	0	0	249	271		E6:Carbamidomethylation (DHKE, X@N-term):0.00	PEAKS PTM
R.IFLAGDKDNVID(+57.02)QIEK.Q	N	69.05	1873.9785	16	-1.3	937.9953	2	32.42	42	F42:1608	OB5955 H1 Ro.raw	8.0339E6	1	1	536	551		D12:Carbamidomethylation (DHKE, X@N-term):15.85	PEAKS PTM
R.IVQIEARPNTLVLPK.H	Y	68.17	1690.0140	15	-1.0	846.0134	2	30.56	41	F41:1509	OB5954 H1 Ro.raw	4.6727E7	21	21	208	222			PEAKS DB
A.GDKDNVIDQIEK.Q	N	68.14	1372.6833	12	-7.0	687.3441	2	32.22	43	F43:1632	OB5956 H1 Ro.raw	1.1241E6	3	3	540	551			PEAKS DB
G.SEEEDITNPINLRDGEPDLSNNFGR.L	N	68.10	2830.3005	25	-1.6	944.4417	3	33.02	30	F30:1421	OB5926 H3B Ro.raw	1.3758E5	3	3	381	405			PEAKS DB
R.RVLLEEN(+.98)AGGEQEER.G	N	67.83	1728.8278	15	-0.8	577.2827	3	25.96	42	F42:1233	OB5955 H1 Ro.raw	1.359E4	2	2	328	342	Deamidation (NQ)	N7:Deamidation (NQ):77.53	PEAKS DB
R.DQS(-18.01)SYLQGFSR.N	N	67.70	1268.5785	11	0.2	635.2966	2	30.91	41	F41:1537	OB5954 H1 Ro.raw	6.4401E5	3	3	302	312	Dehydration	S3:Dehydration:40.00	PEAKS PTM
R.RVLLEENAGGEQEERGQR.R	N	67.67	2069.0249	18	1.9	690.6835	3	25.60	41	F41:1205	OB5954 H1 Ro.raw	1.8247E6	3	3	328	345			PEAKS DB
K.Q(-17.03)AKDLAFPGSGEQVEK.L	N	67.52	1685.8260	16	0.1	843.9204	2	29.98	43	F43:1503	OB5956 H1 Ro.raw	9.9003E4	3	3	552	567	Pyro-glu from Q	Q1:Pyro-glu from Q:1000.00	PEAKS PTM
K.NPQLQ(+.98)DLDMMLTC(+57.02)VEIK.E	N	67.25	2047.9629	17	1.5	1024.9902	2	44.46	43	F43:2348	OB5956 H1 Ro.raw	0	0	0	415	431	Carbamidomethylation	Q5:Deamidation (NQ):19.68;C13:Carbamidomethylation:1000.00	PEAKS DB
K.SFNLD(-18.01)EGHALR.I	N	66.88	1239.5996	11	2.9	620.8089	2	28.79	41	F41:1400	OB5954 H1 Ro.raw	0	0	0	249	259	Dehydration	D5:Dehydration:49.79	PEAKS PTM
K.AM(+15.99)VIVVVNK.G	N	66.42	987.5787	9	1.1	494.7972	2	27.11	41	F41:1283	OB5954 H1 Ro.raw	1.8879E6	3	3	444	452	Oxidation (M)	M2:Oxidation (M):1000.00	PEAKS DB
K.KNPQLQDLDMMLTCVEIK(+14.02).E	N	66.06	2132.0679	18	-0.6	1067.0405	2	36.31	41	F41:1858	OB5954 H1 Ro.raw	5.2356E5	2	2	414	431	Methylation(KR)	K18:Methylation(KR):130.57	PEAKS PTM

total 244 peptides

Peptide	Uniq	-10lgP	Mass	Length	ppm	m/z	z	RT	Fraction	Scan	Source File	Area Roasted in gel	#Feature	#Feature Roasted in gel	Start	End	PTM	AScore	Found By
R.EETSRNNPFYFPSRR.F	N	65.61	1898.9023	15	1.8	633.9759	3	27.75	42	F42:1339	OB5955 H1 Ro.raw	8.693E5	4	4	161	175			PEAKS DB
K.NPQLQDLDMMLTC(+57.02)VEIKEGALM(+15.99)LPHFNSK.A	N	64.76	3387.6335	29	-0.6	847.9152	4	37.63	43	F43:1915	OB5956 H1 Ro.raw	8.7706E7	6	6	415	443	Carbamidomethylation; Oxidation (M)	C13:Carbamidomethylation:1000.00;M22:Oxidation (M):82.05	PEAKS DB
R.IPSGFISY(+125.90)ILNR.H	N	64.64	1504.6575	12	-1.9	753.3346	2	38.39	43	F43:2001	OB5956 H1 Ro.raw	2.0334E5	2	2	260	271	Iodination	Y8:Iodination:1000.00	PEAKS PTM
R.IPSGFISYILN(+.98)RHDNQNLR.V	N	64.64	2257.1604	19	4.7	753.3976	3	34.29	42	F42:1740	OB5955 H1 Ro.raw	6.9901E5	2	2	260	278	Deamidation (NQ)	N11:Deamidation (NQ):36.24	PEAKS DB
R.WGPAEPR.E	Y	64.52	811.3976	7	1.8	406.7068	2	25.60	41	F41:1224	OB5954 H1 Ro.raw	1.6282E6	3	3	109	115			PEAKS DB
R.IVQIEARPN.T	Y	64.48	1038.5822	9	-0.5	520.2981	2	25.87	42	F42:1229	OB5955 H1 Ro.raw	4.4669E4	3	3	208	216			PEAKS DB
A.FPGSGEQVEK.L	N	64.10	1076.5138	10	-3.4	539.2623	2	30.29	42	F42:1495	OB5955 H1 Ro.raw	4.0412E5	3	3	558	567			PEAKS DB
K.ISMPVNTPGQFEDFFPASSRDQSSYLQGFSR.N	N	63.85	3494.6201	31	-0.9	1165.8796	3	36.01	43	F43:1851	OB5956 H1 Ro.raw	5.8723E6	6	6	282	312			PEAKS DB
R.LFEVKPDKK.N	N	63.79	1102.6385	9	0.6	552.3269	2	24.50	43	F43:1159	OB5956 H1 Ro.raw	1.1348E6	6	6	406	414			PEAKS DB
Q.SSYLQGFSR.N	N	63.16	1043.5035	9	1.4	522.7598	2	29.29	41	F41:1438	OB5954 H1 Ro.raw	6.2708E4	3	3	304	312			PEAKS DB
K.QFQNLQN(+.98)HR.I	N	63.09	1184.5686	9	5.4	593.2948	2	23.60	43	F43:1114	OB5956 H1 Ro.raw	8.0412E2	1	1	199	207		N7:Deamidation (NQ):0.00	PEAKS DB
K.EGALMLPH(+14.02)FNSK.A	N	63.06	1356.6860	12	-4.2	679.3475	2	30.85	43	F43:1554	OB5956 H1 Ro.raw	2.9707E5	3	3	432	443	Methylation(others)	H8:Methylation(others):65.81	PEAKS PTM
P.SGFISYILNR.H	N	63.04	1168.6240	10	1.5	585.3202	2	35.08	43	F43:1803	OB5956 H1 Ro.raw	2.9962E5	1	1	262	271			PEAKS DB
N.LDEGHALR.I	N	62.84	909.4668	8	0.0	455.7407	2	27.93	42	F42:1350	OB5955 H1 Ro.raw	1.8672E5	3	3	252	259			PEAKS DB
K.A(+57.02)MVIVVVNKGTGNLELVAVRK.E	N	62.52	2266.3196	21	0.7	1134.1678	2	32.98	42	F42:1660	OB5955 H1 Ro.raw	1.5099E7	6	6	444	464	Carbamidomethylation (DHKE, X@N-term)	A1:Carbamidomethylation (DHKE, X@N-term):139.69	PEAKS PTM
K.HADADNILVIQQGQATVTVANGN(+.98)NRK.S	N	62.31	2747.3950	26	-0.1	916.8055	3	30.70	41	F41:1517	OB5954 H1 Ro.raw	8.5956E3	1	1	223	248		N23:Deamidation (NQ):9.40	PEAKS DB
K.SFN(+15.99)LDEGHALR.I	N	62.00	1273.6051	11	0.0	637.8098	2	27.46	43	F43:1330	OB5956 H1 Ro.raw	9.622E5	3	3	249	259	Oxidation or Hydroxylation	N3:Oxidation or Hydroxylation:55.92	PEAKS PTM
R.IP(+15.99)SGFISYILNR.H	N	61.98	1394.7557	12	-0.8	698.3846	2	37.47	43	F43:1932	OB5956 H1 Ro.raw	3.2208E6	4	4	260	271	Oxidation or Hydroxylation	P2:Oxidation or Hydroxylation:39.25	PEAKS PTM
K.NPQLQDLDMM(+15.99)LTC(+57.02)VEIKEGALM(+15.99)LPHFNSK.A	N	61.19	3403.6284	29	0.3	851.9147	4	35.86	41	F41:1801	OB5954 H1 Ro.raw	3.4294E6	4	4	415	443	Oxidation (M); Carbamidomethylation	M10:Oxidation (M):21.94;C13:Carbamidomethylation:1000.00;M22:O	PEAKS DB
**total 244 peptides**																			

Peptide	Uniq	-10lgP	Mass	Length	ppm	m/z	z	RT	Fraction	Scan	Source File	Area Roasted in gel	#Feature	#Feature Roasted in gel	Start	End	PTM	AScore	Found By
																		xidation (M):61.50	
K.G(+57.02)TGNLELVAVRK.E	N	60.73	1312.7462	12	-0.4	657.3801	2	27.30	41	F41:1303	OB5954 H1 Ro.raw	3.6978E5	2	2	453	464	Carbamidomethylation (DHKE, X@N-term)	G1:Carbamidomethylation (DHKE, X@N-term):55.46	PEAKS PTM
R.EQEWEEEEEDEEEEGSNREVRR.Y	N	60.58	2821.1545	22	1.9	941.3939	3	26.71	43	F43:1297	OB5956 H1 Ro.raw	1.0274E6	4	4	473	494			PEAKS DB
K.NPQLQDLDM(+15.99)M(+15.99)LTC(+57.02)VEIKEGALM(+15.99)LPHFNSK.A	N	60.17	3419.6233	29	-0.9	855.9124	4	34.83	42	F42:1766	OB5955 H1 Ro.raw	1.2322E6	3	3	415	443	Oxidation (M); Carbamidomethylation	M9:Oxidation (M):1000.00;M10:Oxidation (M):1000.00;C13:Carbamidomethylation:1000.00;M22:Oxidation (M):1000.00	PEAKS DB
R.IPSGFISY(+15.99)ILNR.H	N	60.13	1394.7557	12	-0.7	698.3846	2	35.11	41	F41:1859	OB5954 H1 Ro.raw	3.4529E6	5	5	260	271	Oxidation or Hydroxylation	Y8:Oxidation or Hydroxylation:67.76	PEAKS PTM
L.DEGHALR.I	N	59.61	796.3828	7	2.5	399.1996	2	27.93	42	F42:1351	OB5955 H1 Ro.raw	9.3538E4	3	3	253	259			PEAKS DB
R.LFEVKPDKKNPQLQ(+.98)DLDMM(+15.99)LTC(+57.02)VEIK.E	N	59.38	3148.5857	26	7.4	788.1595	4	33.17	42	F42:1670	OB5955 H1 Ro.raw	3.9804E5	1	1	406	431	Oxidation (M); Carbamidomethylation	Q14:Deamidation (NQ):0.00;M19:Oxidation (M):23.10;C22:Carbamidomethylation:1000.00	PEAKS DB
R.VLLEEN(-17.03)AGGEQEER.G	N	59.22	1554.7162	14	1.2	778.3663	2	27.73	42	F42:1335	OB5955 H1 Ro.raw	0	0	0	329	342	Ammonia-loss (N)	N6:Ammonia-loss (N):1000.00	PEAKS PTM
K.SFNLDEGHALRIPSGFISYILNRHDNQNLR.V	N	59.07	3495.7759	30	1.5	700.1635	5	39.97	40	F40:1987	OB5953 H3A Ro.raw	2.0405E7	15	15	249	278			PEAKS DB
K.SFNLDEGHALR(+31.99)IPSGFISYILNR.H	N	58.88	2650.3503	23	0.1	884.4575	3	39.14	42	F42:2021	OB5955 H1 Ro.raw	5.6519E5	1	1	249	271	Dihydroxy	R11:Dihydroxy:46.57	PEAKS PTM
R.LFEVKPDK.K	N	58.71	974.5436	8	1.7	488.2799	2	25.30	42	F42:1183	OB5955 H1 Ro.raw	1.7315E6	6	6	406	413			PEAKS DB
K.GSEEEDITNPIN(+.98)LRDGEPDLSNNFGR.L	N	58.69	2888.3059	26	6.7	1445.1699	2	32.90	41	F41:1642	OB5954 H1 Ro.raw	1.7853E5	1	1	380	405		N12:Deamidation (NQ):12.33	PEAKS DB
K.SFNLDE(+57.02)GHALR.I	N	58.62	1314.6316	11	0.3	658.3232	2	27.83	43	F43:1458	OB5956 H1 Ro.raw	2.3371E6	2	2	249	259	Carbamidomethylation (DHKE, X@N-term)	E6:Carbamidomethylation (DHKE, X@N-term):27.25	PEAKS PTM
R.N(+57.02)NPFYFPSRR.F	N	58.56	1353.6577	10	1.1	677.8369	2	29.74	42	F42:1453	OB5955 H1 Ro.raw	9.24E5	3	3	166	175	Carbamidomethylation (DHKE, X@N-term)	N1:Carbamidomethylation (DHKE, X@N-term):1000.00	PEAKS PTM
R.IFLAGDKDNVIDQ(+.98)IEKQAK.D	N	58.20	2145.1316	19	1.4	716.0521	3	33.04	43	F43:1686	OB5956 H1 Ro.raw	1.2153E5	1	1	536	554	Deamidation (NQ)	Q13:Deamidation (NQ):25.1	PEAKS DB

total 244 peptides

Peptide	Uniq	-10lgP	Mass	Length	ppm	m/z	z	RT	Fraction	Scan	Source File	Area Roasted in gel	#Feature	#Feature Roasted in gel	Start	End	PTM	AScore	Found By
																		8	
R.VAKISM(+15.99)PVNTPGQFEDFFPASSR.D	N	57.67	2540.2368	23	2.1	847.7546	3	32.98	42	F42:1665	OB5955 H1 Ro.raw	1.5427E5	1	1	279	301	Oxidation (M)	M6:Oxidation (M):1000.00	PEAKS DB
K.G(+57.02)TGNLELVAVR.K	N	57.51	1184.6514	11	-2.7	593.3314	2	30.10	42	F42:1486	OB5955 H1 Ro.raw	1.7196E5	2	2	453	463		G1:Carbamidomethylation (DHKE, X@N-term):19.78	PEAKS PTM
R.IFLAGD(+57.02)KDNVIDQIEK.Q	N	57.43	1873.9785	16	1.7	625.6678	3	32.52	41	F41:1637	OB5954 H1 Ro.raw	2.7943E5	3	3	536	551		D6:Carbamidomethylation (DHKE, X@N-term):15.57	PEAKS PTM
R.IFLAGDK(+15.99)DNVIDQIEK.Q	N	57.39	1832.9519	16	-1.9	917.4814	2	31.99	41	F41:1600	OB5954 H1 Ro.raw	7.5922E5	2	2	536	551		K7:Oxidation or Hydroxylation:17.01	PEAKS PTM
N.PFYFPSRR.F	N	57.34	1068.5504	8	1.1	535.2831	2	28.95	41	F41:1414	OB5954 H1 Ro.raw	4.6082E6	5	5	168	175			PEAKS DB
R.K(+27.99)SFNLDEGHALR.I	N	57.29	1413.7001	12	1.4	707.8583	2	28.46	42	F42:1387	OB5955 H1 Ro.raw	2.9777E4	1	1	248	259	Formylation	K1:Formylation:1000.00	PEAKS PTM
R.LFEVKPDKKNPQLQDLDM(+15.99)MLTC(+57.02)VEIK.E	N	57.09	3147.6018	26	-1.9	787.9062	4	34.20	41	F41:1734	OB5954 H1 Ro.raw	2.4894E6	4	4	406	431	Oxidation (M); Carbamidomethylation	M18:Oxidation (M):33.98;C22:Carbamidomethylation:1000.00	PEAKS DB
K.SFNLDEGHALR(+31.99).I	N	55.96	1289.6000	11	-1.4	645.8063	2	30.41	43	F43:1520	OB5956 H1 Ro.raw	0	0	0	249	259	Dihydroxy	R11:Dihydroxy:119.59	PEAKS PTM
K.ISMPVN(+.98)TPGQFEDFFPASSR.D	N	55.55	2227.0254	20	6.6	1114.5273	2	36.77	41	F41:1885	OB5954 H1 Ro.raw	1.01E5	2	2	282	301	Deamidation (NQ)	N6:Deamidation (NQ):42.68	PEAKS DB
R.NTLEAAFNAEFN(+.98)EIRR.V	N	54.77	1894.9172	16	1.8	948.4676	2	33.04	43	F43:1685	OB5956 H1 Ro.raw	2.2573E6	5	5	313	328	Deamidation (NQ)	N12:Deamidation (NQ):39.02	PEAKS DB
V.QIEARPNTLVLPK.H	Y	54.45	1477.8616	13	-1.8	739.9368	2	30.56	41	F41:1513	OB5954 H1 Ro.raw	1.5146E5	3	3	210	222			PEAKS DB
K.S(+57.02)FNLDEGHALRIPSGFISYILNR.H	N	54.19	2675.3818	23	2.3	892.8033	3	39.35	38	F38:1901	OB5951 H3A Ro.raw	6.7035E5	1	1	249	271	Carbamidomethylation (DHKE, X@N-term)	S1:Carbamidomethylation (DHKE, X@N-term):63.97	PEAKS PTM
R.IFLAGDKDNVIDQIEK(+57.02)QAK.D	N	54.07	2201.1692	19	0.3	734.7306	3	32.48	43	F43:1657	OB5956 H1 Ro.raw	1.2122E6	3	3	536	554		K16:Carbamidomethylation (DHKE, X@N-term):11.12	PEAKS PTM
R.I(+27.99)PSGFISYILNR.H	N	53.77	1406.7557	12	0.7	704.3856	2	42.27	42	F42:2199	OB5955 H1 Ro.raw	0	0	0	260	271	Formylation	I1:Formylation:1000.00	PEAKS PTM
R.SKQFQNLQNHR.I	Y	53.65	1398.7117	11	0.4	467.2447	3	24.16	43	F43:1149	OB5956 H1 Ro.raw	1.1379E5	3	3	197	207			PEAKS DB
R.IPSGFISYILNRHDN(+.98)QNLR.V	N	53.60	2257.1604	19	-0.7	753.3936	3	34.26	41	F41:1732	OB5954 H1 Ro.raw	0	0	0	260	278		N15:Deamidation (NQ):0.00	PEAKS DB
K.HADADNILVIQQGQATVTVANGNNR.K	N	53.13	2618.3162	25	0.5	873.7798	3	30.34	38	F38:1426	OB5951 H3A Ro.raw	1.8654E3	1	1	223	247			PEAKS DB

total 244 peptides

Peptide	Uniq	-10lgP	Mass	Length	ppm	m/z	z	RT	Fraction	Scan	Source File	Area Roasted in gel	#Feature	#Feature Roasted in gel	Start	End	PTM	AScore	Found By
R.EGEQEWGTPGS(+42.01)EVR.E	Y	52.96	1601.6958	14	7.6	801.8613	2	27.46	43	F43:1334	OB5956H1Ro.raw	9.5213E4	3	3	147	160	Acetylation (TSCYH)	S11:Acetylation (TSCYH):32.97	PEAKS PTM
K.AM(+15.99)VIVVVNKGTGNLELVAVRK.E	N	52.95	2225.2930	21	-1.3	742.7706	3	32.42	42	F42:1596	OB5955H1Ro.raw	1.4247E6	3	3	444	464	Oxidation (M)	M2:Oxidation (M):1000.00	PEAKS DB
K.KNPQLQ(+.98)DLDMMLTC(+57.02)VEIKEGALMLPHFNSK.A	N	51.97	3500.7175	30	3.4	876.1896	4	36.93	43	F43:1903	OB5956H1Ro.raw	9.2646E6	2	2	414	443	Carbamidomethylation	Q6:Deamidation (NQ):14.04;C14:Carbamidomethylation:1000.00	PEAKS DB
R.IFLAGDKDNVID.Q	N	51.22	1318.6769	12	-0.9	660.3451	2	31.76	43	F43:1606	OB5956H1Ro.raw	1.1173E5	3	3	536	547			PEAKS DB
K.ISM(+15.99)PVNTPGQFEDFFPASSRDQSSYLQGFSR.N	N	50.25	3510.6150	31	0.3	1171.2126	3	35.35	36	F36:1707	OB5948H3BRo.raw	2.4799E6	5	5	282	312	Oxidation (M)	M3:Oxidation (M):1000.00	PEAKS DB
K.NPQLQDLDMM(+15.99)LTC(+57.02)VEIKEGALMLPHFN(+.98)SK.A	N	49.99	3388.6174	29	6.1	848.1668	4	36.39	43	F43:1880	OB5956H1Ro.raw	1.7975E6	1	1	415	443	Oxidation (M); Carbamidomethylation	M10:Oxidation (M):27.96;C13:Carbamidomethylation:1000.00;N27:Deamidation (NQ):18.27	PEAKS DB
R.N(-17.03)NPFYFPSRR.F	N	49.99	1279.6097	10	1.1	427.5443	3	28.89	43	F43:1434	OB5956H1Ro.raw	5.674E5	3	3	166	175		N1:Ammonia-loss (N):9.34	PEAKS PTM
R.EQEWEEEEDEEEEGSNREVR.R	N	49.84	2665.0535	21	2.4	889.3605	3	27.88	41	F41:1353	OB5954H1Ro.raw	4.9786E4	1	1	473	493			PEAKS DB
T.GNLELVAVRK.E	N	49.53	1097.6556	10	0.5	549.8354	2	27.00	42	F42:1296	OB5955H1Ro.raw	3.4872E5	3	3	455	464			PEAKS DB
R.VAKIS(+77.99)MPVNTPGQFEDFFPASSR.D	N	49.16	2602.2290	23	3.0	1302.1257	2	38.06	42	F42:1958	OB5955H1Ro.raw	0	0	0	279	301	Methylphosphonylation	S5:Methylphosphonylation:56.99	PEAKS PTM
F.PGSGEQVEK.L	N	49.06	929.4454	9	-3.7	465.7282	2	30.34	43	F43:1524	OB5956H1Ro.raw	5.2705E4	1	1	559	567			PEAKS DB
K.AMVIVVVNK(+57.02)GTGNLELVAVRK.E	N	48.92	2266.3196	21	1.3	756.4481	3	33.23	43	F43:1673	OB5956H1Ro.raw	3.1193E6	1	1	444	464		K9:Carbamidomethylation (DHKE, X@N-term):8.78	PEAKS PTM
R.EEDWRQPR.E	N	48.87	1114.5155	8	1.8	558.2661	2	23.91	41	F41:1112	OB5954H1Ro.raw	1.6791E3	1	1	120	127			PEAKS DB
R.PNTLVLPK.H	N	48.84	880.5382	8	-2.9	441.2751	2	28.00	43	F43:1374	OB5956H1Ro.raw	1.9234E5	2	2	215	222			PEAKS DB
L.R(+27.99)IPSGFISYILNR.H	N	48.25	1562.8568	13	-2.0	782.4341	2	37.84	43	F43:1976	OB5956H1Ro.raw	1.5108E5	1	1	259	271	Formylation	R1:Formylation:1000.00	PEAKS PTM
R.EGEQEW(+15.99)GTPGSEVREETSR.N	Y	48.10	2177.9460	19	1.6	726.9905	3	26.51	43	F43:1296	OB5956H1Ro.raw	8.9341E6	3	3	147	165	Oxidation (HW)	W6:Oxidation (HW):1000.00	PEAKS PTM
R.I(+57.02)VQIEARPNTLVLPK.H	Y	46.89	1747.0355	15	-0.2	874.5248	2	30.98	42	F42:1537	OB5955H1Ro.raw	9.1688E5	3	3	208	222	Carbamidomethylation (DHKE, X@N-term)	I1:Carbamidomethylation (DHKE, X@N-term):69.71	PEAKS PTM

total 244 peptides

Peptide	Uniq	-10lgP	Mass	Length	ppm	m/z	z	RT	Fraction	Scan	Source File	Area Roasted in gel	#Feature	#Feature Roasted in gel	Start	End	PTM	AScore	Found By
R.N(+.98)TLEAAFNAEFNEIR.R	N	46.63	1738.8162	15	9.2	870.4233	2	44.36	42	F42:2303	OB5955 H1 Ro.raw	5.4416E3	1	1	313	327	Deamidation (NQ)	N1:Deamidation (NQ):39.47	PEAKS DB
R.VLLE(+21.98)ENAGGEQEER.G	N	46.37	1593.7246	14	-1.1	797.8687	2	26.71	43	F43:1300	OB5956 H1 Ro.raw	4.0134E5	3	3	329	342	Sodium adduct	E4:Sodium adduct: 40.00	PEAKS PTM
N.PFYFPSR.R	N	46.32	912.4493	7	-0.1	457.2319	2	31.70	42	F42:1584	OB5955 H1 Ro.raw	2.4653E5	4	4	168	174			PEAKS DB
K.GTGNLELVAVRKEQQQR.G	N	46.31	1925.0442	17	1.4	642.6896	3	26.67	43	F43:1294	OB5956 H1 Ro.raw	2.1018E4	1	1	453	469			PEAKS DB
K.NPQLQDLD(+14.02)MMLTC(+57.02)VEIK.E	N	45.99	2060.9944	17	0.2	1031.5046	2	37.33	41	F41:1934	OB5954 H1 Ro.raw	2.7407E4	1	1	415	431	Methylation(others); Carbamidomethylation	D8:Methylation(others):42.68; C13:Carbamidomethylation:1000.00	PEAKS PTM
K.GTGNLE(+57.02)LVAVRK.E	N	45.75	1312.7462	12	-0.8	657.3799	2	27.17	42	F42:1295	OB5955 H1 Ro.raw	1.9882E5	1	1	453	464		E6:Carbamidomethylation (DHKE, X@N-term):0.00	PEAKS PTM
K.A(+43.01)MVIVVVNKGTGNLELVAVR.K	N	45.64	2124.2090	20	0.7	1063.1125	2	33.65	41	F41:1688	OB5954 H1 Ro.raw	8.0739E4	1	1	444	463	Carbamylation	A1:Carbamylation: 161.20	PEAKS PTM
R.VLLEEN(+.98)AGGEQEERGQR.R	N	45.60	1913.9078	17	-1.6	638.9755	3	25.99	41	F41:1234	OB5954 H1 Ro.raw	0	0	0	329	345	Deamidation (NQ)	N6:Deamidation (NQ):28.79	PEAKS DB
K.KNPQLQDLDMM(+15.99)LTC(+57.02)VEIKEGALM(+15.99)LPHFNSK.A	N	45.45	3531.7234	30	-1.1	883.9372	4	35.80	42	F42:1822	OB5955 H1 Ro.raw	0	0	0	414	443	Carbamidomethylation; Oxidation (M)	M11:Oxidation (M): 0.00;C14:Carbamidomethylation:1000.00;M23:Oxidation (M):45.87	PEAKS DB
R.LFEVKPDKKNPQLQDLDM(+15.99)M(+15.99)LTC(+57.02)VEIK.E	N	45.43	3163.5967	26	-2.8	791.9042	4	32.90	41	F41:1650	OB5954 H1 Ro.raw	2.0535E6	3	3	406	431	Oxidation (M); Carbamidomethylation	M18:Oxidation (M): 1000.00; M19:Oxidation (M): 1000.00; C22:Carbamidomethylation:1000.00	PEAKS DB
R.KSFNLDEGH(+15.99)ALR.I	N	45.40	1401.7001	12	3.9	701.8600	2	27.64	43	F43:1349	OB5956 H1 Ro.raw	6.6479E4	2	2	248	259	Oxidation (HW)	H9:Oxidation (HW): 1000.00	PEAKS PTM
K.KNPQLQDLDM(+15.99)MLTC(+57.02)VEIKEGALMLPHFNSK.A	N	44.60	3515.7285	30	-1.9	879.9377	4	35.30	41	F41:1798	OB5954 H1 Ro.raw	6.4162E5	1	1	414	443	Carbamidomethylation	M10:Oxidation (M): 14.02;C14:Carbamidomethylation:1000.00	PEAKS DB
R.DQSSYLQGFSR(+15.99).N	N	44.15	1302.5840	11	2.5	652.3009	2	30.79	43	F43:1544	OB5956 H1 Ro.raw	1.4308E5	3	3	302	312	Oxidation or Hydroxylation	R11:Oxidation or Hydroxylation:42.88	PEAKS PTM
K.Q(+.98)FQNLQNHR.I	N	43.70	1184.5686	9	4.6	593.2943	2	23.68	42	F42:1098	OB5955 H1 Ro.raw	0	0	0	199	207	Deamidation (NQ)	Q1:Deamidation (NQ):39.25	PEAKS DB
K.I(+57.02)SMPVNTPGQFEDFFPASSR.D	N	42.37	2283.0630	20	-0.7	1142.5380	2	36.01	43	F43:1860	OB5956 H1 Ro.raw	1.8515E5	1	1	282	301	Carbamidomethylation (DHKE, X@N-term)	I1:Carbamidomethylation (DHKE, X@N-term):50.99	PEAKS PTM

total 244 peptides

Peptide	Uniq	-10lgP	Mass	Length	ppm	m/z	z	RT	Fraction	Scan	Source File	Area Roasted in gel	#Feature	#Feature Roasted in gel	Start	End	PTM	AScore	Found By
K.ISM(-48.00)PVNTPGQFEDFFPASSR.D	N	42.09	2178.0381	20	2.8	727.0220	3	32.48	43	F43:1652	OB5956H1Ro.raw	5.8583E6	3	3	282	301	Dethiomethyl	M3:Dethiomethyl:1000.00	PEAKS PTM
R.SKQFQNLQNH(+15.99)R.I	Y	41.84	1414.7065	11	2.4	708.3622	2	24.12	42	F42:1124	OB5955H1Ro.raw	1.2986E3	1	1	197	207	Oxidation (HW)	H10:Oxidation (HW):1000.00	PEAKS PTM
K.KGSEEEDIT(-18.01)N(+.98)PINLR.D	N	41.27	1696.8268	15	2.3	566.6175	3	29.07	43	F43:1442	OB5956H1Ro.raw	3.8118E5	3	3	379	393	Dehydration; Deamidation (NQ)	T9:Dehydration:45.01;N10:Deamidation (NQ):59.10	PEAKS PTM
K.NPQLQDLDMM(+15.99)LTC(+57.02)VEIKEGALM(+15.99)LPHFN(+.98)SK.A	N	41.25	3404.6125	29	-0.4	1135.8777	3	36.69	42	F42:1878	OB5955H1Ro.raw	3.8423E5	1	1	415	443	Carbamidomethylation; Oxidation (M); Deamidation (NQ)	M10:Oxidation (M):0.00;C13:Carbamidomethylation:1000.00;M22:Oxidation (M):39.23;N27:Deamidation (NQ):69.52	PEAKS DB
R.REQEW(+15.99)EEEEEDEEEEGSNR.E	N	41.18	2452.9375	19	-0.6	818.6526	3	25.98	41	F41:1239	OB5954H1Ro.raw	2.4114E4	1	1	472	490	Oxidation (HW)	W5:Oxidation (HW):1000.00	PEAKS PTM
K.DLAFP(+15.99)GSGEQVEK.L	N	41.14	1391.6569	13	0.5	696.8361	2	29.36	42	F42:1432	OB5955H1Ro.raw	7.4528E5	3	3	555	567	Oxidation or Hydroxylation	P5:Oxidation or Hydroxylation:67.76	PEAKS PTM
K.AMVIVVVN(+.98)KGTGNLELVAVRK.E	N	41.03	2210.2820	21	1.9	1106.1504	2	34.35	42	F42:1720	OB5955H1Ro.raw	4.5718E5	1	1	444	464	Deamidation (NQ)	N8:Deamidation (NQ):28.79	PEAKS DB
K.EGALM(-48.00)LPHFNSK.A	N	40.53	1294.6670	12	0.5	432.5632	3	28.76	41	F41:1389	OB5954H1Ro.raw	2.208E6	2	2	432	443	Dethiomethyl	M5:Dethiomethyl:1000.00	PEAKS PTM
R.VLLEENAGGEQEERGQRR.R	N	40.17	2069.0249	18	1.0	518.2640	4	25.49	42	F42:1205	OB5955H1Ro.raw	2.0305E5	2	2	329	346			PEAKS DB
R.VLLEENAGGEQEER(+15.99).G	N	39.98	1587.7375	14	1.8	794.8775	2	26.70	41	F41:1275	OB5954H1Ro.raw	0	0	0	329	342	Oxidation or Hydroxylation	R14:Oxidation or Hydroxylation:20.88	PEAKS PTM
R.N(+27.99)TLEAAFNAEFNEIRR.V	N	39.68	1921.9282	16	-1.7	961.9697	2	37.51	41	F41:1908	OB5954H1Ro.raw	3.7767E4	1	1	313	328	Formylation	N1:Formylation:1000.00	PEAKS PTM
K.E(-18.01)GALM(+15.99)LPHFNSK.A	N	39.64	1340.6547	12	0.2	447.8923	3	30.21	41	F41:1479	OB5954H1Ro.raw	7.81E5	1	1	432	443	Pyro-glu from E; Oxidation (M)	E1:Pyro-glu from E:1000.00;M5:Oxidation (M):1000.00	PEAKS PTM
R.VLLEE(+53.92)NAGGEQEER.G	N	39.58	1625.6620	14	-1.0	542.8940	3	26.76	42	F42:1278	OB5955H1Ro.raw	0	0	0	329	342	Replacement of 2 protons by iron	E5:Replacement of 2 protons by iron:20.92	PEAKS PTM
D.GEPDLSNNFGR.L	N	39.46	1204.5472	11	3.0	603.2827	2	28.20	41	F41:1371	OB5954H1Ro.raw	1.5511E4	1	1	395	405			PEAKS DB
K.HADADNILVIQQGQ(+.98)ATVTVANGNNRK.S	N	39.33	2747.3950	26	2.6	916.8103	3	30.46	30	F30:1280	OB5926H3BRo.raw	0	0	0	223	248		Q14:Deamidation (NQ):5.07	PEAKS DB
K.NPQLQ(+.98)DLDMM(+15.99)LTC(+57.02)VEIKEGALM(+15.99)LPHFNSK.A	N	39.15	3404.6125	29	-1.8	852.1589	4	38.22	43	F43:1993	OB5956H1Ro.raw	7.6266E5	1	1	415	443	Deamidation (NQ); Carbamidomethylation	Q5:Deamidation (NQ):49.79;M10:Oxidation (M):0.00;C13:	PEAKS DB
**total 244 peptides**																			

Peptide	Uniq	-10lgP	Mass	Length	ppm	m/z	z	RT	Fraction	Scan	Source File	Area Roasted in gel	#Feature	#Feature Roasted in gel	Start	End	PTM	AScore	Found By
																		Carbamidomethylation:1000.00;M22:Oxidation (M):14.60	
K.N(+.98)PQLQDLDM(+15.99)MLTC(+57.02)VEIKEGALMLPHFNSK.A	N	38.83	3388.6174	29	8.2	1695.3300	2	37.72	41	F41:1941	OB5954 H1 Ro.raw	6.4698E5	1	1	415	443	Carbamidomethylation	N1:Deamidation (NQ):0.00;M9:Oxidation (M):0.00;C13:Carbamidomethylation:1000.00	PEAKS DB
K.D(+15.99)LAFPGSGEQVEK.L	N	38.81	1391.6569	13	0.5	696.8361	2	29.36	42	F42:1368	OB5955 H1 Ro.raw	2.5725E5	1	1	555	567	Oxidation or Hydroxylation	D1:Oxidation or Hydroxylation:77.13	PEAKS PTM
K.NPQ(+.98)LQDLDMMLTC(+57.02)VEIK.E	N	38.70	2047.9629	17	2.9	1024.9917	2	46.45	41	F41:2441	OB5954 H1 Ro.raw	0	0	0	415	431	Carbamidomethylation	Q3:Deamidation (NQ):14.04;C13:Carbamidomethylation:1000.00	PEAKS DB
R.IFLAGD(+15.99)KDNVIDQIEK.Q	N	38.52	1832.9519	16	1.9	917.4850	2	31.89	42	F42:1590	OB5955 H1 Ro.raw	4.6198E5	1	1	536	551		D6:Oxidation or Hydroxylation:12.60	PEAKS PTM
R.IFLAGDKDN(+.98)VIDQIEKQ(+.98)AK.D	N	37.85	2146.1157	19	9.8	716.3862	3	32.98	42	F42:1661	OB5955 H1 Ro.raw	8.6625E4	1	1	536	554		N9:Deamidation (NQ):13.83;Q17:Deamidation (NQ):17.65	PEAKS DB
K.DLAFPGSGE(+53.92)QVEK.L	N	37.31	1429.5812	13	-6.3	715.7933	2	30.34	43	F43:1518	OB5956 H1 Ro.raw	2.6492E5	1	1	555	567	Replacement of 2 protons by iron	E9:Replacement of 2 protons by iron:37.54	PEAKS PTM
R.W(+15.99)GPAEPR.E	Y	36.73	827.3926	7	0.0	414.7036	2	24.54	42	F42:1155	OB5955 H1 Ro.raw	3.7087E3	1	1	109	115	Oxidation (HW)	W1:Oxidation (HW):1000.00	PEAKS PTM
K.GT(-18.01)GNLELVAVRK.E	N	36.73	1237.7142	12	2.6	413.5797	3	27.00	42	F42:1287	OB5955 H1 Ro.raw	4.7714E5	1	1	453	464	Dehydration	T2:Dehydration:1000.00	PEAKS PTM
K.IRPEGREGEQEWGTPGSEVREETSR.N	Y	36.52	2870.3542	25	2.3	718.5975	4	27.83	43	F43:1355	OB5956 H1 Ro.raw	1.3647E5	1	1	141	165			PEAKS DB
R.NT(-18.01)LEAAFNAEFNEIRR.V	N	36.45	1875.9227	16	0.7	626.3153	3	34.16	43	F43:1739	OB5956 H1 Ro.raw	6.9172E6	2	2	313	328	Dehydration	T2:Dehydration:1000.00	PEAKS PTM
K.EGALM(+15.99)LPHFN(+.98)SK.A	N	36.41	1359.6493	12	2.8	680.8338	2	31.22	41	F41:1550	OB5954 H1 Ro.raw	0	0	0	432	443	Oxidation (M); Deamidation (NQ)	M5:Oxidation (M):1000.00;N10:Deamidation (NQ):1000.00	PEAKS DB
R.DQSSY(-18.01)LQGFSR.N	N	36.32	1268.5785	11	-0.6	635.2961	2	30.85	43	F43:1579	OB5956 H1 Ro.raw	2.378E5	1	1	302	312		Y5:Dehydration:0.00	PEAKS PTM
R.C(+57.02)LQS(-18.01)C(+57.02)Q(+.98)QEPDDLKQK.A	N	36.24	1858.8190	15	-0.1	930.4167	2	28.47	31	F31:1350	OB5942 H6 Ro.raw	0	0	0	40	54	Carbamidomethylation; Dehydration	C1:Carbamidomethylation:1000.00;S4:Dehydration:39.55;C5:Carbamidomethylation:10	PEAKS PTM
**total 244 peptides**																			

Peptide	Uniq	-10lgP	Mass	Length	ppm	m/z	z	RT	Fraction	Scan	Source File	Area Roasted in gel	#Feature	#Feature Roasted in gel	Start	End	PTM	AScore	Found By
																		00.00;Q6:Deamidation (NQ):8.69	
M.LPHFNSK.A	N	36.02	841.4446	7	1.3	421.7301	2	30.63	42	F42:1510	OB5955H1Ro.raw	1.4673E6	2	2	437	443			PEAKS DB
K.NPQLQDLDM(-48.00)MLTC(+57.02)VEIK.E	N	35.81	1998.9755	17	6.7	667.3369	3	34.52	43	F43:1773	OB5956H1Ro.raw	8.4276E5	1	1	415	431	Carbamidomethylation	M9:Dethiomethyl:11.06;C13:Carbamidomethylation:1000.00	PEAKS PTM
K.SF(+17.99)NLDEGHALR.I	N	34.75	1275.6007	11	8.1	638.8128	2	26.62	42	F42:1267	OB5955H1Ro.raw	9.5828E3	1	1	249	259	Fluorination	F2:Fluorination:1000.00	PEAKS PTM
K.KNPQLQ(+.98)DLDMM(+15.99)LTC(+57.02)VEIKEGALMLPHFNSK.A	N	33.72	3516.7124	30	3.3	880.1883	4	35.48	41	F41:1798	OB5954H1Ro.raw	7.5658E5	1	1	414	443	Carbamidomethylation	Q6:Deamidation (NQ):18.55;M11:Oxidation (M):14.02;C14:Carbamidomethylation:1000.00	PEAKS DB
K.VSKEH(+15.99)VQELTK.H	N	33.71	1312.6986	11	1.8	657.3578	2	23.73	42	F42:1100	OB5955H1Ro.raw	1.3581E3	1	1	361	371	Oxidation (HW)	H5:Oxidation (HW):1000.00	PEAKS PTM
D.QSSYLQGFSR.N	N	33.26	1171.5621	10	2.1	586.7896	2	29.36	42	F42:1431	OB5955H1Ro.raw	3.3134E4	1	1	303	312			PEAKS DB
R.IFLAGDKDNVID(-18.01)QIEK.Q	N	33.09	1798.9464	16	0.7	900.4811	2	31.39	43	F43:1591	OB5956H1Ro.raw	6.7846E4	1	1	536	551		D12:Dehydration:13.16	PEAKS PTM
R.EREEDWRQPR.E	N	32.92	1399.6592	10	5.7	700.8409	2	24.05	43	F43:1141	OB5956H1Ro.raw	4.1173E4	1	1	118	127			PEAKS DB
D.NVIDQIEK.Q	N	32.64	957.5131	8	1.6	479.7646	2	28.19	42	F42:1367	OB5955H1Ro.raw	3.0062E3	1	1	544	551			PEAKS DB
K.AM(-48.00)VIVVVNK.G	N	32.49	923.5804	9	0.0	462.7975	2	25.00	43	F43:1192	OB5956H1Ro.raw	6.3177E3	1	1	444	452	Dethiomethyl	M2:Dethiomethyl:1000.00	PEAKS PTM
R.D(-18.01)QSSYLQGFSR.N	N	31.95	1268.5785	11	1.6	635.2975	2	29.92	43	F43:1489	OB5956H1Ro.raw	0	0	0	302	312		D1:Dehydration:0.00	PEAKS PTM
R.IFLAGDKD(-18.01)NVIDQIEK.Q	N	31.82	1798.9464	16	-0.9	600.6555	3	32.15	43	F43:1589	OB5956H1Ro.raw	3.5634E5	1	1	536	551	Dehydration	D8:Dehydration:30.83	PEAKS PTM
K.D(+53.92)LAFPGSGEQVEK.L	N	31.27	1429.5812	13	-4.3	715.7948	2	30.56	41	F41:1500	OB5954H1Ro.raw	3.7719E5	1	1	555	567		D1:Replacement of 2 protons by iron:0.00	PEAKS PTM
K.SFNLDE(+53.92)GHALR.I	N	31.27	1311.5294	11	1.0	438.1842	3	28.04	41	F41:1362	OB5954H1Ro.raw	5.8053E4	1	1	249	259		E6:Replacement of 2 protons by iron:16.90	PEAKS PTM
K.AM(+15.99)VIVVVNK(+57.02)GTGNLELVAVR.K	N	30.97	2154.2195	20	2.8	1078.1200	2	32.98	42	F42:1664	OB5955H1Ro.raw	2.0029E5	1	1	444	463	Oxidation (M)	M2:Oxidation (M):1000.00;K9:Carbamidomethylation (DHKE, X@N-term):0.00	PEAKS PTM
K.KNPQLQDLDMM(+15.99)LTC(+57.02)VEIKEGALMLPHFNSK.A	N	30.83	3515.7285	30	1.3	879.9406	4	35.25	42	F42:1790	OB5955H1Ro.raw	0	0	0	414	443	Carbamidomethylation	M11:Oxidation (M):12.28;C1	PEAKS DB
**total 244 peptides**																			

Peptide	Uniq	-10lgP	Mass	Length	ppm	m/z	z	RT	Fraction	Scan	Source File	Area Roasted in gel	#Feature	#Feature Roasted in gel	Start	End	PTM	AScore	Found By
																		4:Carbamidomethylation:1000.00	
R.DQSSY(+15.99)LQGFSR.N	N	30.72	1302.5840	11	1.6	652.3003	2	29.24	43	F43:1470	OB5956 H1 Ro.raw	6.7205E4	1	1	302	312	Oxidation or Hydroxylation	Y5:Oxidation or Hydroxylation:26.36	PEAKS PTM
K.SFNLDEGHALRIPSGFISYILNRHDNQN(+.98)LR.V	N	30.43	3496.7600	30	4.9	700.3627	5	37.51	39	F39:1925	OB5952 H3A Ro.raw	2.7478E5	1	1	249	278		N28:Deamidation (NQ):10.11	PEAKS DB
D.KDNVIDQIEK.Q	N	30.42	1200.6350	10	-0.3	601.3246	2	26.31	42	F42:1252	OB5955 H1 Ro.raw	0	0	0	542	551			PEAKS DB
N.TLEAAFNAEFNEIRR.V	N	30.42	1779.8904	15	3.5	890.9556	2	33.23	43	F43:1696	OB5956 H1 Ro.raw	1.5374E5	1	1	314	328			PEAKS DB
R.IPSGFISYILN(+15.99)R.H	N	30.30	1394.7557	12	-2.8	698.3832	2	36.42	41	F41:1875	OB5954 H1 Ro.raw	3.8045E5	1	1	260	271		N11:Oxidation or Hydroxylation:0.00	PEAKS PTM
W.GTPGSEVREETSR.N	Y	30.01	1403.6641	13	3.8	702.8420	2	27.77	42	F42:1332	OB5955 H1 Ro.raw	2.719E4	1	1	153	165			PEAKS DB
R.IFLAGDKD(-18.01)NV.I	N	30.00	1072.5553	10	0.7	537.2853	2	32.26	42	F42:1603	OB5955 H1 Ro.raw	2.1452E6	1	1	536	545	Dehydration	D8:Dehydration:45.01	PEAKS PTM
R.NTLEAAFNAEFN(+15.99)EIRR.V	N	29.99	1909.9282	16	0.9	955.9723	2	32.98	42	F42:1662	OB5955 H1 Ro.raw	2.366E6	2	2	313	328		N12:Oxidation or Hydroxylation:0.00	PEAKS PTM
R.IFLAGDKDNVIDQ(+.98)IEK.Q	N	29.73	1817.9410	16	7.7	909.9848	2	63.08	43	F43:3435	OB5956 H1 Ro.raw	3.1606E3	1	1	536	551		Q13:Deamidation (NQ):11.12	PEAKS DB
I.F(+127.06)LAGDKDNVIDQIEK.Q	N	29.59	1830.9363	15	0.7	916.4761	2	32.18	41	F41:1600	OB5954 H1 Ro.raw	6.5822E4	1	1	537	551	N-Succinimidyl-2-morpholine acetate	F1:N-Succinimidyl-2-morpholine acetate:28.79	PEAKS PTM
K.N(+.98)PQLQ(+.98)DLDMMLTC(+57.02)VEIKEGALM(+15.99)LPHFNSK.A	N	29.50	3389.6016	29	9.6	848.4158	4	37.65	43	F43:2047	OB5956 H1 Ro.raw	6.1505E6	1	1	415	443	Carbamidomethylation	N1:Deamidation (NQ):0.00;Q5:Deamidation (NQ):14.04;C13:Carbamidomethylation:1000.00;M22:Oxidation (M):0.00	PEAKS DB
R.NN(+.98)PFYFPSRR.F	N	29.19	1297.6204	10	9.3	649.8235	2	62.71	43	F43:3425	OB5956 H1 Ro.raw	0	0	0	166	175		N2:Deamidation (NQ):7.21	PEAKS DB
R.NTLEAAFNAEFN(+.98)EIR.R	N	29.07	1738.8162	15	9.2	870.4233	2	44.36	42	F42:2331	OB5955 H1 Ro.raw	5.4416E3	1	1	313	327		N12:Deamidation (NQ):0.00	PEAKS DB
K.KNPQ(+.98)LQ(+.98)DLDMM(+15.99)LTC(+57.02)VEIKEGALM(+15.99)LPHFNSK.A	N	28.74	3533.6914	30	9.7	884.4387	4	35.77	42	F42:1822	OB5955 H1 Ro.raw	2.7541E5	1	1	414	443	Carbamidomethylation; Oxidation (M)	Q4:Deamidation (NQ):8.22;Q6:Deamidation (NQ):5.55;M11:Oxidation (M):0.00;C14:Carbamidomethylation:1000.00;M23:Ox	PEAKS DB
**total 244 peptides**																			

Peptide	Uniq	-10lgP	Mass	Length	ppm	m/z	z	RT	Fraction	Scan	Source File	Area Roasted in gel	#Feature	#Feature Roasted in gel	Start	End	PTM	AScore	Found By
																		idation (M):38.29	
total 244 peptides																			

Q6PSU3|Q6PSU3_ARAHY

back to list

| Protein Coverage | Supporting Peptides |

Protein Coverage:

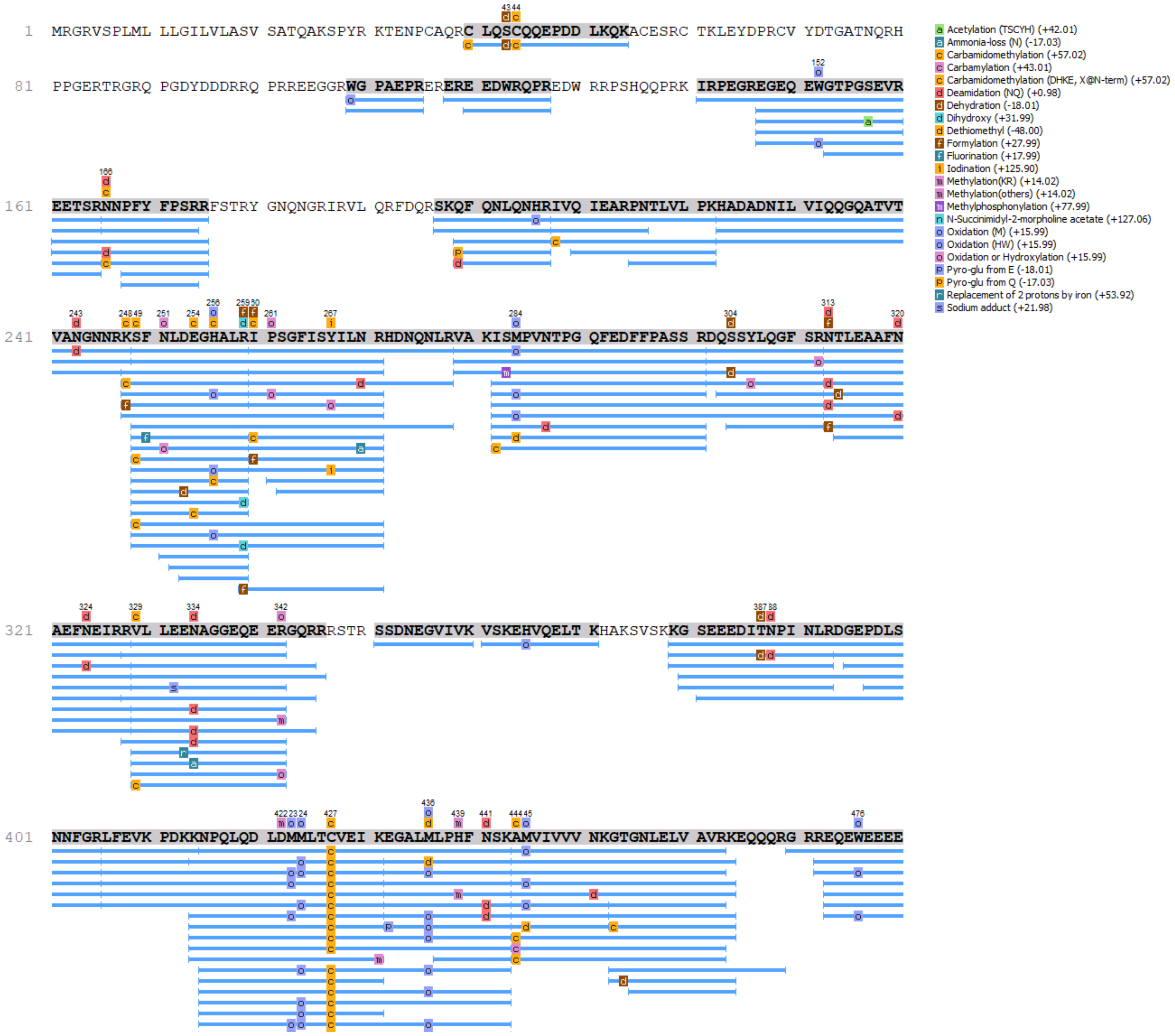
1 MRGRVSPLML LLGILVLASV SATQAKSPYR KTENPCAQRC LQSCQQEPDD LKQKACESRC TKLEYDPRCV YDTGATNQRH
81 PPGERTRGRQ PGDYDDDRRQ PRREEGGRWG PAEPRERERE EDWRQPREDW RRPSHQQPRK IRPEGREGEQ EWGTPGSEVR
161 EETSRNNPFY FPSRRFSTRY GNQNGRIRVL QRFDQRSKQF QNLQNHRIVQ IEARPNTLVL PKHADADNIL VIQQGQATVT
241 VANGNNRKSF NLDEGHALRI PSGFISYILN RHDNQNLRVA KISMPVNTPG QFEDFFPASS RDQSSYLQGF SRNTLEAAFN
321 AEFNEIRRVL LEENAGGEQE ERGQRRRSTR SSDNEGVIVK VSKEHVQELT KHAKSVSKKG SEEEDITNPI NLRDGEPDLS
401 NNFGRLFEVK PDKKNPQLQD LDMMLTCVEI KEGALMLPHF NSKAMVIVVV NKGTGNLELV AVRKEQQQRG RREQEWEEEE
a Acetylation (TSCYH) (+42.01)
a Ammonia-loss (N) (-17.03)
c Carbamidomethylation (+57.02)
c Carbamylation (+43.01)
c Carbamidomethylation (DHKE, X@N-term) (+57.02)
d Deamidation (NQ) (+0.98)
d Dehydration (-18.01)
d Dihydroxy (+31.99)
d Dethiomethyl (-48.00)
f Formylation (+27.99)
f Fluorination (+17.99)
i Iodination (+125.90)
m Methylation(KR) (+14.02)
m Methylation(others) (+14.02)
m Methylphosphonylation (+77.99)
n N-Succinimidyl-2-morpholine acetate (+127.06)
o Oxidation (M) (+15.99)
o Oxidation (HW) (+15.99)
o Oxidation or Hydroxylation (+15.99)
p Pyro-glu from E (-18.01)
p Pyro-glu from Q (-17.03)
r Replacement of 2 protons by iron (+53.92)
s Sodium adduct (+21.98)

Supporting Peptides:

Peptide	Uniq	-10lgP	Mass	Length	ppm	m/z	z	RT	Fraction	Scan	Source File	Area Roasted in gel	#Feature	#Feature Roasted in gel	Start	End	PTM	AScore	Found By
K.KNPQLQDLDMMLTC(+57.02)VEIK.E	N	136.41	2175.0737	18	1.4	1088.5457	2	35.26	43	F43:1808	OB5956 H1 Ro.raw	1.4906E7	6	6	414	431	Carbamidomethylation	C14:Carbamidomethylation:1000.00	PEAKS DB
R.NTLEAAFNAEFNEIR.R	N	130.93	1737.8322	15	1.8	869.9249	2	35.34	38	F38:1714	OB5951 H3A Ro.raw	1.332E7	16	16	313	327			PEAKS DB
K.GSEEEDITNPINLRDGEPDLSNNFGR.L	N	129.76	2887.3220	26	-0.7	963.4473	3	32.67	43	F43:1660	OB5956 H1 Ro.raw	1.2475E7	11	11	380	405			PEAKS DB
K.NPQLQDLDMMLTC(+57.02)VEIK.E	N	128.40	2046.9788	17	0.2	1024.4968	2	37.11	43	F43:1936	OB5956 H1 Ro.raw	3.4643E7	10	10	415	431	Carbamidomethylation	C13:Carbamidomethylation:1000.00	PEAKS DB
K.NPQLQDLDM(+15.99)MLTC(+57.02)VEIK.E	N	128.16	2062.9736	17	-4.2	1032.4897	2	36.93	43	F43:1892	OB5956 H1 Ro.raw	3.3834E6	3	3	415	431	Oxidation (M); Carbamidomethylation	M9:Oxidation (M):30.46;C13:Carbamidomethylation:1000.00	PEAKS DB
K.NPQLQDLDMM(+15.99)LTC(+57.02)VEIK.E	N	127.13	2062.9736	17	5.0	1032.4993	2	34.71	43	F43:1780	OB5956 H1 Ro.raw	1.0665E7	4	4	415	431	Oxidation (M); Carbamidomethylation	M10:Oxidation (M):30.46;C13:Carbamidomethylation:1000.00	PEAKS DB
K.ISM(+15.99)PVNTPGQFEDFFPASSR.D	N	127.11	2242.0364	20	0.1	1122.0284	2	34.52	30	F30:1521	OB5926 H3B Ro.raw	2.1708E7	14	13	282	301	Oxidation (M)	M3:Oxidation (M):1000.00	PEAKS DB
R.EQEWEEEEEDEEEEGSNR.E	N	125.55	2280.8413	18	3.9	1141.4324	2	28.35	43	F43:1396	OB5956 H1 Ro.raw	4.3429E6	7	7	473	490			PEAKS DB
K.ISMPVNTPGQFEDFFPASSR.D	N	125.26	2226.0415	20	0.7	1114.0288	2	35.83	43	F43:1868	OB5956 H1 Ro.raw	8.8227E7	23	23	282	301			PEAKS DB
K.HADADNILVIQQGQATVTVAN(+.98)GNNRK.S	N	123.10	2747.3950	26	3.3	916.8087	3	29.62	43	F43:1472	OB5956 H1 Ro.raw	4.4266E6	11	11	223	248	Deamidation (NQ)	N21:Deamidation (NQ):31.91	PEAKS DB
K.NPQLQDLDM(+15.99)M(+15.99)LTC(+57.02)VEIK.E	N	121.59	2078.9688	17	6.6	1040.4985	2	33.82	43	F43:1726	OB5956 H1 Ro.raw	6.8984E4	2	2	415	431	Oxidation (M); Carbamidomethylation	M9:Oxidation (M):1000.00;M10:Oxidation (M):1000.00;C13:Carbamidomethylation:1000.00	PEAKS DB
K.SFNLDEGHALRIPSGFISYILNR.H	N	121.49	2618.3604	23	1.0	873.7949	3	37.51	41	F41:1949	OB5954 H1 Ro.raw	9.1637E7	28	28	249	271			PEAKS DB
K.KNPQLQDLDM(+15.99)MLTC(+57.02)VEIK.E	N	118.68	2191.0686	18	3.6	1096.5455	2	34.71	43	F43:1781	OB5956 H1 Ro.raw	1.3598E6	4	4	414	431	Oxidation (M); Carbamidomethylation	M10:Oxidation (M):27.96;C14:Carbamidomethylation:1000.00	PEAKS DB
K.AMVIVVVNKGTGNLELVAVR.K	N	114.87	2081.2031	20	-1.0	1041.6078	2	34.35	43	F43:1758	OB5956 H1 Ro.raw	3.8361E7	13	13	444	463			PEAKS DB
R.IFLAGDKDNVIDQIEK.Q	N	110.41	1816.9570	16	-1.1	909.4848	2	32.67	43	F43:1648	OB5956 H1 Ro.raw	4.2419E8	56	55	536	551			PEAKS DB

total 244 peptides

481 EDEEEEGSNR EVRRYTARLK EGDVFIMPAA HPVAINASSE LHLLGFGINA ENNHRIFLAG DKDNVIDQIE KQAKDLAFPG

561 SGEQVEKLIK NQRESHFVSA

Peptide	Uniq	-10lgP	Mass	Length	ppm	m/z	z	RT	Fraction	Scan	Source File	Area Roasted in gel	#Feature	#Feature Roasted in gel	Start	End	PTM	AScore	Found By
K.KNPQLQDLDMM(+15.99)LTC(+57.02)VEIK.E	N	110.35	2191.0686	18	3.5	1096.5454	2	32.98	42	F42:1657	OB5955 H1 Ro.raw	1.6495E6	4	4	414	431	Oxidation (M); Carbamidomethylation	M11:Oxidation (M):23.10;C14:Carbamidomethylation:1000.00	PEAKS DB
K.KGSEEEDITNPINLRDGEPDLSNNFGR.L	N	109.79	3015.4170	27	3.4	1006.1497	3	31.62	41	F41:1565	OB5954 H1 Ro.raw	1.1559E7	21	21	379	405			PEAKS DB
R.IPSGFISYILNR.H	N	109.18	1378.7609	12	2.5	690.3894	2	36.50	38	F38:1780	OB5951 H3A Ro.raw	4.4166E8	20	20	260	271			PEAKS DB
K.HADADNILVIQQGQATVTVANGNNRK.S	N	108.34	2746.4111	26	1.9	916.4794	3	29.61	43	F43:1451	OB5956 H1 Ro.raw	3.8404E6	5	5	223	248			PEAKS DB
R.VLLEENAGGEQEER.G	N	108.33	1571.7427	14	2.4	786.8805	2	26.83	42	F42:1293	OB5955 H1 Ro.raw	2.9858E7	10	10	329	342			PEAKS DB
R.KSFNLDEGHALR.I	N	108.28	1385.7051	12	0.6	462.9092	3	26.14	43	F43:1252	OB5956 H1 Ro.raw	5.3666E6	6	6	248	259			PEAKS DB
K.AMVIVVVNKGTGNLELVAVRK.E	N	107.96	2209.2981	21	1.1	737.4408	3	33.05	43	F43:1682	OB5956 H1 Ro.raw	1.2362E8	20	20	444	464			PEAKS DB
R.I(+57.02)PSGFISYILNR.H	N	107.77	1435.7823	12	-3.6	718.8959	2	36.92	43	F43:1901	OB5956 H1 Ro.raw	1.7559E7	7	7	260	271	Carbamidomethylation (DHKE, X@N-term)	I1:Carbamidomethylation (DHKE, X@N-term):1000.00	PEAKS PTM
K.KGSEEEDITNPINLR.D	N	107.76	1713.8533	15	2.8	857.9363	2	29.07	43	F43:1439	OB5956 H1 Ro.raw	8.6032E6	7	7	379	393			PEAKS DB
R.I(+57.02)FLAGDKDNVIDQIEK.Q	N	105.70	1873.9785	16	1.7	937.9982	2	32.48	43	F43:1653	OB5956 H1 Ro.raw	1.1722E7	5	5	536	551	Carbamidomethylation (DHKE, X@N-term)	I1:Carbamidomethylation (DHKE, X@N-term):90.91	PEAKS PTM
R.DQSSYLQGFSR.N	N	105.08	1286.5891	11	0.8	644.3024	2	30.81	40	F40:1431	OB5953 H3A Ro.raw	9.5783E7	31	31	302	312			PEAKS DB
R.V(+57.02)LLEENAGGEQEER.G	N	103.80	1628.7642	14	0.3	815.3896	2	27.08	43	F43:1322	OB5956 H1 Ro.raw	4.0193E5	3	3	329	342	Carbamidomethylation (DHKE, X@N-term)	V1:Carbamidomethylation (DHKE, X@N-term):49.37	PEAKS PTM
K.NPQLQDLDMMLTCVEIK(+14.02).E	N	103.68	2003.9730	17	-1.2	1002.9926	2	38.20	43	F43:1989	OB5956 H1 Ro.raw	5.9168E6	4	4	415	431	Methylation(KR)	K17:Methylation(KR):1000.00	PEAKS PTM
K.SFNLDEGHALR.I	N	103.18	1257.6101	11	-1.7	629.8113	2	28.00	43	F43:1362	OB5956 H1 Ro.raw	1.1584E8	33	31	249	259			PEAKS DB
K.AM(+15.99)VIVVVNKGTGNLELVAVR.K	N	103.14	2097.1980	20	1.6	700.0744	3	33.04	43	F43:1684	OB5956 H1 Ro.raw	2.3148E7	9	9	444	463	Oxidation (M)	M2:Oxidation (M):1000.00	PEAKS DB
R.DGEPDLSNNFGR.L	N	102.69	1319.5742	12	0.3	660.7946	2	29.24	43	F43:1495	OB5956 H1 Ro.raw	7.4875E6	3	3	394	405			PEAKS DB
K.DLAFPGSGEQVEK.L	N	100.93	1375.6619	13	-1.9	688.8369	2	30.34	43	F43:1508	OB5956 H1 Ro.raw	9.196E7	8	8	555	567			PEAKS DB

total 244 peptides

Peptide	Uniq	-10lgP	Mass	Length	ppm	m/z	z	RT	Fraction	Scan	Source File	Area Roasted in gel	#Feature	#Feature Roasted in gel	Start	End	PTM	AScore	Found By
K.NPQLQDLDM(+15.99)MLTC(+57.02)VEIKEGALMLPHFNSK.A	N	100.40	3387.6335	29	-0.6	1130.2178	3	37.70	41	F41:1939	OB5954 H1 Ro.raw	8.7706E7	6	6	415	443	Oxidation (M); Carbamidomethylation	M9:Oxidation (M):26.02;C13:Carbamidomethylation:1000.00	PEAKS DB
R.RVLLEENAGGEQEER.G	N	100.35	1727.8438	15	0.2	576.9553	3	25.30	42	F42:1185	OB5955 H1 Ro.raw	8.2964E5	6	6	328	342			PEAKS DB
R.EGEQEWGTPGSEVR.E	Y	100.27	1559.6852	14	0.8	780.8505	2	27.93	42	F42:1345	OB5955 H1 Ro.raw	3.381E6	3	3	147	160			PEAKS DB
K.NPQLQDLDMMLTC(+57.02)VEIKEGALMLPHFNSK.A	N	98.85	3371.6387	29	-2.7	843.9147	4	38.02	43	F43:1969	OB5956 H1 Ro.raw	1.5351E8	12	12	415	443	Carbamidomethylation	C13:Carbamidomethylation:1000.00	PEAKS DB
K.KNPQLQDLDMMLTC(+57.02)VEIKEGALMLPHFNSK.A	N	97.96	3499.7336	30	-3.9	875.9373	4	36.80	41	F41:1884	OB5954 H1 Ro.raw	6.677E6	4	4	414	443	Carbamidomethylation	C14:Carbamidomethylation:1000.00	PEAKS DB
R.VLLEEN(+.98)AGGEQEER.G	N	94.48	1572.7267	14	2.8	787.3728	2	27.46	43	F43:1340	OB5956 H1 Ro.raw	6.2395E5	3	3	329	342	Deamidation (NQ)	N6:Deamidation (NQ):121.37	PEAKS DB
R.IFLAGDKDNVIDQIEKQAK.D	N	94.35	2144.1477	19	-2.1	715.7217	3	32.42	42	F42:1625	OB5955 H1 Ro.raw	3.3984E6	9	9	536	554			PEAKS DB
R.KSFNLDEGHALRIPSGFISYILNR.H	N	93.78	2746.4553	24	-0.7	687.6206	4	36.29	43	F43:1876	OB5956 H1 Ro.raw	1.1783E6	4	4	248	271			PEAKS DB
R.REQEWEEEEEDEEEEGSNR.E	N	93.49	2436.9424	19	0.1	813.3215	3	26.72	43	F43:1292	OB5956 H1 Ro.raw	8.6025E5	7	7	472	490			PEAKS DB
R.EGEQEWGTPGSEVREETSR.N	Y	93.19	2161.9512	19	3.0	1081.9861	2	27.83	43	F43:1353	OB5956 H1 Ro.raw	3.9146E7	8	8	147	165			PEAKS DB
F.LAGDKDNVIDQIEK.Q	N	91.83	1556.8046	14	-6.3	779.4047	2	32.21	43	F43:1629	OB5956 H1 Ro.raw	5.4162E6	4	4	538	551			PEAKS DB
R.IPSGFISYILN(-17.03)R.H	N	91.80	1361.7343	12	-4.9	681.8711	2	38.02	43	F43:1980	OB5956 H1 Ro.raw	1.3387E6	3	3	260	271	Ammonia-loss (N)	N11:Ammonia-loss (N):1000.00	PEAKS PTM
K.AMVIVVVNK.G	N	91.04	971.5837	9	-0.5	486.7989	2	30.16	43	F43:1494	OB5956 H1 Ro.raw	7.1361E6	3	3	444	452			PEAKS DB
K.SFNLDEGH(+15.99)ALR.I	N	90.73	1273.6051	11	0.9	637.8104	2	30.16	43	F43:1507	OB5956 H1 Ro.raw	8.7681E5	2	2	249	259	Oxidation (HW)	H8:Oxidation (HW):1000.00	PEAKS PTM
R.NNPFYFPSRR.F	N	89.95	1296.6364	10	-2.5	649.3239	2	28.89	43	F43:1417	OB5956 H1 Ro.raw	1.3445E8	22	22	166	175			PEAKS DB
K.D(+57.02)LAFPGSGEQVEK.L	N	88.60	1432.6833	13	-0.1	717.3489	2	31.08	41	F41:1535	OB5954 H1 Ro.raw	9.7986E5	3	3	555	567	Carbamidomethylation (DHKE, X@N-term)	D1:Carbamidomethylation (DHKE, X@N-term):131.10	PEAKS PTM
K.SFNLDEGH(+57.02)ALR.I	N	88.30	1314.6316	11	-0.1	658.3230	2	28.22	41	F41:1349	OB5954 H1 Ro.raw	2.9996E6	3	3	249	259	Carbamidomethylation (DHKE, X@N-term)	H8:Carbamidomethylation (DHKE, X@N-term):30.36	PEAKS PTM
K.EGALMLPHFNSK.A	N	88.21	1342.6703	12	2.5	672.3441	2	30.67	43	F43:1529	OB5956 H1 Ro.raw	1.4087E7	5	5	432	443			PEAKS DB
K.NPQLQDLDMM(-48.00)LTC(+57.02)VEIK.E	N	88.05	1998.9755	17	1.9	1000.4969	2	32.85	43	F43:1670	OB5956 H1 Ro.raw	5.9032E6	3	3	415	431	Carbamidomethylation	M10:Dethiomethyl:14.02;C13:Carbamid	PEAKS PTM
**total 244 peptides**																			

Peptide	Uniq	-10lgP	Mass	Length	ppm	m/z	z	RT	Fraction	Scan	Source File	Area Roasted in gel	#Feature	#Feature Roasted in gel	Start	End	PTM	AScore	Found By
																		omethylation:1000.00	
K.GSEEEDITNPINLR.D	N	87.68	1585.7583	14	2.3	793.8882	2	31.16	42	F42:1548	OB5955 H1 Ro.raw	3.3709E6	3	3	380	393			PEAKS DB
R.GRREQEWEEEEEDEEEEGSNR.E	N	87.21	2650.0649	21	1.1	884.3632	3	26.73	41	F41:1280	OB5954 H1 Ro.raw	7.9956E5	6	6	470	490			PEAKS DB
R.NNPFYFPSR.R	N	86.35	1140.5352	9	-0.1	571.2748	2	31.76	43	F43:1593	OB5956 H1 Ro.raw	1.7218E7	11	11	166	174			PEAKS DB
K.S(+57.02)FNLDEGHALR.I	N	86.31	1314.6316	11	-0.5	658.3228	2	28.11	42	F42:1359	OB5955 H1 Ro.raw	2.6997E6	4	4	249	259	Carbamidomethylation (DHKE, X@N-term)	S1:Carbamidomethylation (DHKE, X@N-term):91.04	PEAKS PTM
I.PSGFISYILNR.H	N	85.97	1265.6768	11	-4.6	633.8427	2	36.75	43	F43:1894	OB5956 H1 Ro.raw	3.5986E5	2	2	261	271			PEAKS DB
R.IPSGFISYILNRHDNQNLR.V	N	84.75	2256.1763	19	6.5	753.0709	3	33.61	43	F43:1714	OB5956 H1 Ro.raw	6.1581E7	21	21	260	278			PEAKS DB
R.I(+27.99)FLAGDKDNVIDQIEK.Q	N	84.60	1844.9519	16	-2.1	923.4813	2	36.23	41	F41:1853	OB5954 H1 Ro.raw	1.9485E6	3	3	536	551	Formylation	I1:Formylation:98.75	PEAKS PTM
K.SFNLDEGH(+15.99)ALRIPSGFISYILNR.H	N	83.49	2634.3555	23	-4.0	879.1223	3	38.76	43	F43:2023	OB5956 H1 Ro.raw	8.8519E5	1	1	249	271	Oxidation (HW)	H8:Oxidation (HW):1000.00	PEAKS PTM
K.KNPQLQDLDMMLTC(+57.02)VEIKEGALM(+15.99)LPHFNSK.A	N	83.11	3515.7285	30	-1.9	879.9377	4	36.03	43	F43:1857	OB5956 H1 Ro.raw	6.6263E6	3	3	414	443	Carbamidomethylation; Oxidation (M)	C14:Carbamidomethylation:1000.00;M23:Oxidation (M):114.34	PEAKS DB
R.NTLEAAFNAEFNEIRR.V	N	82.99	1893.9332	16	-2.6	947.9714	2	34.11	42	F42:1711	OB5955 H1 Ro.raw	3.0959E8	36	35	313	328			PEAKS DB
K.A(+57.02)MVIVVVNKGTGNLELVAVR.K	N	82.14	2138.2246	20	-5.4	1070.1138	2	34.47	42	F42:1739	OB5955 H1 Ro.raw	4.4412E6	3	3	444	463	Carbamidomethylation (DHKE, X@N-term)	A1:Carbamidomethylation (DHKE, X@N-term):207.56	PEAKS PTM
K.DLAFPGSGEQVEKLIK.N	N	80.89	1729.9249	16	-1.6	865.9706	2	33.95	30	F30:1478	OB5926 H3B Ro.raw	3.9305E5	5	4	555	570			PEAKS DB
I.FLAGDKDNVIDQIEK.Q	N	80.64	1703.8729	15	-5.7	852.9388	2	32.22	43	F43:1635	OB5956 H1 Ro.raw	3.1343E5	3	3	537	551			PEAKS DB
K.EGALM(+15.99)LPHFNSK.A	N	80.48	1358.6653	12	1.1	680.3406	2	29.85	43	F43:1485	OB5956 H1 Ro.raw	3.0314E6	5	5	432	443	Oxidation (M)	M5:Oxidation (M):1000.00	PEAKS DB
K.GTGNLELVAVR.K	N	80.39	1127.6299	11	-1.3	564.8215	2	30.21	41	F41:1476	OB5954 H1 Ro.raw	2.4706E7	5	5	453	463			PEAKS DB
R.NTLEAAFN(+.98)AEFNEIRR.V	N	79.02	1894.9172	16	-2.2	948.4638	2	32.35	41	F41:1616	OB5954 H1 Ro.raw	1.3357E6	5	5	313	328	Deamidation (NQ)	N8:Deamidation (NQ):48.12	PEAKS DB
R.VLLEENAGGEQEER(+14.02).G	N	78.92	1585.7583	14	0.7	793.8870	2	27.86	41	F41:1339	OB5954 H1 Ro.raw	1.6341E5	3	3	329	342	Methylation(KR)	R14:Methylation(KR):1000.00	PEAKS PTM
K.GTGNLELVAVRK.E	N	78.87	1255.7249	12	0.7	628.8701	2	27.30	41	F41:1322	OB5954 H1 Ro.raw	2.29E7	10	10	453	464			PEAKS DB
K.QFQNLQNHR.I	N	78.35	1183.5846	9	0.0	592.7996	2	23.81	42	F42:1102	OB5955 H1 Ro.raw	1.5125E5	6	6	199	207			PEAKS DB

total 244 peptides

Peptide	Uniq	-10lgP	Mass	Length	ppm	m/z	z	RT	Fraction	Scan	Source File	Area Roasted in gel	#Feature	#Feature Roasted in gel	Start	End	PTM	AScore	Found By
K.NPQLQDLDMM(+15.99)LTC(+57.02)VEIKEGALMLPHFNSK.A	N	78.01	3387.6335	29	-0.8	1130.2175	3	36.42	41	F41:1862	OB5954H1Ro.raw	6.8935E7	6	6	415	443	Oxidation (M); Carbamidomethylation	M10:Oxidation (M):24.44;C13:Carbamidomethylation:1000.00	PEAKS DB
R.LFEVKPDKKNPQLQDLDMMLTC(+57.02)VEIK.E	N	77.87	3131.6069	26	-4.0	1044.8721	3	34.65	42	F42:1756	OB5955H1Ro.raw	5.0788E6	4	4	406	431	Carbamidomethylation	C22:Carbamidomethylation:1000.00	PEAKS DB
K.NPQLQDLDM(+15.99)MLTC(+57.02)VEIKEGALM(+15.99)LPHFNSK.A	N	77.10	3403.6284	29	1.1	851.9153	4	37.51	41	F41:1883	OB5954H1Ro.raw	7.3887E6	7	7	415	443	Oxidation (M); Carbamidomethylation	M9:Oxidation (M):27.96;C13:Carbamidomethylation:1000.00;M22:Oxidation (M):71.31	PEAKS DB
R.IFLAGDKDNVIDQIEKQ(+.98)AK.D	N	76.22	2145.1316	19	1.5	716.0522	3	33.08	42	F42:1661	OB5955H1Ro.raw	0	0	0	536	554	Deamidation (NQ)	Q17:Deamidation (NQ):48.83	PEAKS DB
R.EETSRN(+.98)NPFYFPSRR.F	N	75.62	1899.8864	15	-0.2	634.3026	3	28.39	41	F41:1381	OB5954H1Ro.raw	3.2976E5	2	2	161	175	Deamidation (NQ)	N6:Deamidation (NQ):33.98	PEAKS DB
L.AGDKDNVIDQIEK.Q	N	75.39	1443.7205	13	-3.7	722.8649	2	32.08	42	F42:1607	OB5955H1Ro.raw	3.3426E5	1	1	539	551			PEAKS DB
K.Q(-17.03)FQNLQNHR.I	N	75.35	1166.5581	9	-1.2	584.2856	2	28.00	43	F43:1365	OB5956H1Ro.raw	4.927E6	4	4	199	207	Pyro-glu from Q	Q1:Pyro-glu from Q:1000.00	PEAKS PTM
R.SSDNEGVIVK.V	N	75.32	1046.5244	10	-1.0	524.2690	2	23.58	41	F41:1103	OB5954H1Ro.raw	3.7568E5	3	3	351	360			PEAKS DB
R.VAKISMPVNTPGQFEDFFPASSR.D	N	74.27	2524.2419	23	-2.9	842.4188	3	34.29	42	F42:1735	OB5955H1Ro.raw	1.4483E6	3	3	279	301			PEAKS DB
K.NPQLQDLDM(+15.99)M(+15.99)LTC(+57.02)VEIKEGALMLPHFNSK.A	N	73.00	3403.6284	29	-3.9	1135.5457	3	35.83	43	F43:1849	OB5956H1Ro.raw	7.8068E6	5	5	415	443	Oxidation (M); Carbamidomethylation	M9:Oxidation (M):54.18;M10:Oxidation (M):63.99;C13:Carbamidomethylation:1000.00	PEAKS DB
K.KNPQLQDLDM(+15.99)MLTC(+57.02)VEIKEGALM(+15.99)LPHFNSK.A	N	72.71	3531.7234	30	-1.9	883.9364	4	35.86	41	F41:1821	OB5954H1Ro.raw	7.53E5	1	1	414	443	Carbamidomethylation; Oxidation (M)	M10:Oxidation (M):17.01;C14:Carbamidomethylation:1000.00;M23:Oxidation (M):85.25	PEAKS DB
R.VLLEENAGGEQEERGQR.R	N	72.44	1912.9238	17	0.6	957.4698	2	25.30	42	F42:1191	OB5955H1Ro.raw	3.1353E6	8	8	329	345			PEAKS DB
K.QAKDLAFPGSGEQVEK.L	N	72.39	1702.8525	16	1.2	568.6255	3	27.08	43	F43:1321	OB5956H1Ro.raw	2.8232E5	6	6	552	567			PEAKS DB
K.EGALMLPHFN(+.98)SK.A	N	71.37	1343.6543	12	2.6	672.8362	2	31.66	43	F43:1595	OB5956H1Ro.raw	1.0101E5	2	2	432	443	Deamidation (NQ)	N10:Deamidation (NQ):1000.00	PEAKS DB
R.EQEW(+15.99)EEEEEDEEEEGSNR.E	N	70.36	2296.8362	18	3.2	1149.4291	2	27.67	41	F41:1333	OB5954H1Ro.raw	1.024E6	3	3	473	490	Oxidation (HW)	W4:Oxidation (HW):1000.00	PEAKS PTM

total 244 peptides

Peptide	Uniq	-10lgP	Mass	Length	ppm	m/z	z	RT	Fraction	Scan	Source File	Area Roasted in gel	#Feature	#Feature Roasted in gel	Start	End	PTM	AScore	Found By
F.NLDEGHALR.I	N	70.14	1023.5097	9	-2.5	512.7609	2	28.00	43	F43:1370	OB5956 H1 Ro.raw	2.6181E6	3	3	251	259			PEAKS DB
R.N(+.98)TLEAAFNAEFNEIRR.V	N	69.73	1894.9172	16	0.4	948.4662	2	32.39	42	F42:1620	OB5955 H1 Ro.raw	2.6319E6	3	3	313	328	Deamidation (NQ)	N1:Deamidation (NQ):41.52	PEAKS DB
E.PDLSNNFGR.L	N	69.35	1018.4832	9	0.9	510.2493	2	31.57	43	F43:1584	OB5956 H1 Ro.raw	1.3026E6	4	4	397	405			PEAKS DB
R.K(+57.02)SFNLDEGHALR.I	N	69.20	1442.7266	12	-0.1	722.3705	2	26.25	42	F42:1250	OB5955 H1 Ro.raw	4.0798E5	6	6	248	259	Carbamidomethylation (DHKE, X@N-term)	K1:Carbamidomethylation (DHKE, X@N-term):76.17	PEAKS PTM
R.LFEVKPDKKNPQLQDLDMM(+15.99)LTC(+57.02)VEIK.E	N	69.17	3147.6018	26	2.2	787.9095	4	33.24	42	F42:1670	OB5955 H1 Ro.raw	2.0797E6	2	2	406	431	Oxidation (M); Carbamidomethylation	M19:Oxidation (M):40.00;C22:Carbamidomethylation:1000.00	PEAKS DB
K.SFNLDE(+57.02)GHALRIPSGFISYILNR.H	N	69.07	2675.3818	23	-1.9	892.7996	3	37.80	43	F43:1964	OB5956 H1 Ro.raw	0	0	0	249	271		E6:Carbamidomethylation (DHKE, X@N-term):0.00	PEAKS PTM
R.IFLAGDKDNVID(+57.02)QIEK.Q	N	69.05	1873.9785	16	-1.3	937.9953	2	32.42	42	F42:1608	OB5955 H1 Ro.raw	8.0339E6	1	1	536	551		D12:Carbamidomethylation (DHKE, X@N-term):15.85	PEAKS PTM
R.IVQIEARPNTLVLPK.H	Y	68.17	1690.0140	15	-1.0	846.0134	2	30.56	41	F41:1509	OB5954 H1 Ro.raw	4.6727E7	21	21	208	222			PEAKS DB
A.GDKDNVIDQIEK.Q	N	68.14	1372.6833	12	-7.0	687.3441	2	32.22	43	F43:1632	OB5956 H1 Ro.raw	1.1241E6	3	3	540	551			PEAKS DB
G.SEEEDITNPINLRDGEPDLSNNFGR.L	N	68.10	2830.3005	25	-1.6	944.4417	3	33.02	30	F30:1421	OB5926 H3B Ro.raw	1.3758E5	3	3	381	405			PEAKS DB
R.RVLLEEN(+.98)AGGEQEER.G	N	67.83	1728.8278	15	-0.8	577.2827	3	25.96	42	F42:1233	OB5955 H1 Ro.raw	1.359E4	2	2	328	342	Deamidation (NQ)	N7:Deamidation (NQ):77.53	PEAKS DB
R.DQS(-18.01)SYLQGFSR.N	N	67.70	1268.5785	11	0.2	635.2966	2	30.91	41	F41:1537	OB5954 H1 Ro.raw	6.4401E5	3	3	302	312	Dehydration	S3:Dehydration:40.00	PEAKS PTM
R.RVLLEENAGGEQEERGQR.R	N	67.67	2069.0249	18	1.9	690.6835	3	25.60	41	F41:1205	OB5954 H1 Ro.raw	1.8247E6	3	3	328	345			PEAKS DB
K.Q(-17.03)AKDLAFPGSGEQVEK.L	N	67.52	1685.8260	16	0.1	843.9204	2	29.98	43	F43:1503	OB5956 H1 Ro.raw	9.9003E4	3	3	552	567	Pyro-glu from Q	Q1:Pyro-glu from Q:1000.00	PEAKS PTM
K.NPQLQ(+.98)DLDMMLTC(+57.02)VEIK.E	N	67.25	2047.9629	17	1.5	1024.9902	2	44.46	43	F43:2348	OB5956 H1 Ro.raw	0	0	0	415	431	Carbamidomethylation	Q5:Deamidation (NQ):19.68;C13:Carbamidomethylation:1000.00	PEAKS DB
K.SFNLD(-18.01)EGHALR.I	N	66.88	1239.5996	11	2.9	620.8089	2	28.79	41	F41:1400	OB5954 H1 Ro.raw	0	0	0	249	259	Dehydration	D5:Dehydration:49.79	PEAKS PTM
K.AM(+15.99)VIVVVNK.G	N	66.42	987.5787	9	1.1	494.7972	2	27.11	41	F41:1283	OB5954 H1 Ro.raw	1.8879E6	3	3	444	452	Oxidation (M)	M2:Oxidation (M):1000.00	PEAKS DB
K.KNPQLQDLDMMLTCVEIK(+14.02).E	N	66.06	2132.0679	18	-0.6	1067.0405	2	36.31	41	F41:1858	OB5954 H1 Ro.raw	5.2356E5	2	2	414	431	Methylation(KR)	K18:Methylation(KR):130.57	PEAKS PTM

total 244 peptides

Peptide	Uniq	-10lgP	Mass	Length	ppm	m/z	z	RT	Fraction	Scan	Source File	Area Roasted in gel	#Feature	#Feature Roasted in gel	Start	End	PTM	AScore	Found By
R.EETSRNNPFYFPSRR.F	N	65.61	1898.9023	15	1.8	633.9759	3	27.75	42	F42:1339	OB5955 H1 Ro.raw	8.693E5	4	4	161	175			PEAKS DB
K.NPQLQDLDMMLTC(+57.02)VEIKEGALM(+15.99)LPHFNSK.A	N	64.76	3387.6335	29	-0.6	847.9152	4	37.63	43	F43:1915	OB5956 H1 Ro.raw	8.7706E7	6	6	415	443	Carbamidomethylation; Oxidation (M)	C13:Carbamidomethylation:1000.00;M22:Oxidation (M):82.05	PEAKS DB
R.IPSGFISY(+125.90)ILNR.H	N	64.64	1504.6575	12	-1.9	753.3346	2	38.39	43	F43:2001	OB5956 H1 Ro.raw	2.0334E5	2	2	260	271	Iodination	Y8:Iodination:1000.00	PEAKS PTM
R.IPSGFISYILN(+.98)RHDNQNLR.V	N	64.64	2257.1604	19	4.7	753.3976	3	34.29	42	F42:1740	OB5955 H1 Ro.raw	6.9901E5	2	2	260	278	Deamidation (NQ)	N11:Deamidation (NQ):36.24	PEAKS DB
R.WGPAEPR.E	Y	64.52	811.3976	7	1.8	406.7068	2	25.60	41	F41:1224	OB5954 H1 Ro.raw	1.6282E6	3	3	109	115			PEAKS DB
R.IVQIEARPN.T	Y	64.48	1038.5822	9	-0.5	520.2981	2	25.87	42	F42:1229	OB5955 H1 Ro.raw	4.4669E4	3	3	208	216			PEAKS DB
A.FPGSGEQVEK.L	N	64.10	1076.5138	10	-3.4	539.2623	2	30.29	42	F42:1495	OB5955 H1 Ro.raw	4.0412E5	3	3	558	567			PEAKS DB
K.ISMPVNTPGQFEDFFPASSRDQSSYLQGFSR.N	N	63.85	3494.6201	31	-0.9	1165.8796	3	36.01	43	F43:1851	OB5956 H1 Ro.raw	5.8723E6	6	6	282	312			PEAKS DB
R.LFEVKPDKK.N	N	63.79	1102.6385	9	0.6	552.3269	2	24.50	43	F43:1159	OB5956 H1 Ro.raw	1.1348E6	6	6	406	414			PEAKS DB
Q.SSYLQGFSR.N	N	63.16	1043.5035	9	1.4	522.7598	2	29.29	41	F41:1438	OB5954 H1 Ro.raw	6.2708E4	3	3	304	312			PEAKS DB
K.QFQNLQN(+.98)HR.I	N	63.09	1184.5686	9	5.4	593.2948	2	23.60	43	F43:1114	OB5956 H1 Ro.raw	8.0412E2	1	1	199	207		N7:Deamidation (NQ):0.00	PEAKS DB
K.EGALMLPH(+14.02)FNSK.A	N	63.06	1356.6860	12	-4.2	679.3475	2	30.85	43	F43:1554	OB5956 H1 Ro.raw	2.9707E5	3	3	432	443	Methylation(others)	H8:Methylation(others):65.81	PEAKS PTM
P.SGFISYILNR.H	N	63.04	1168.6240	10	1.5	585.3202	2	35.08	43	F43:1803	OB5956 H1 Ro.raw	2.9962E5	1	1	262	271			PEAKS DB
N.LDEGHALR.I	N	62.84	909.4668	8	0.0	455.7407	2	27.93	42	F42:1350	OB5955 H1 Ro.raw	1.8672E5	3	3	252	259			PEAKS DB
K.A(+57.02)MVIVVVNKGTGNLELVAVRK.E	N	62.52	2266.3196	21	0.7	1134.1678	2	32.98	42	F42:1660	OB5955 H1 Ro.raw	1.5099E7	6	6	444	464	Carbamidomethylation (DHKE, X@N-term)	A1:Carbamidomethylation (DHKE, X@N-term):139.69	PEAKS PTM
K.HADADNILVIQQGQATVTVANGN(+.98)NRK.S	N	62.31	2747.3950	26	-0.1	916.8055	3	30.70	41	F41:1517	OB5954 H1 Ro.raw	8.5956E3	1	1	223	248		N23:Deamidation (NQ):9.40	PEAKS DB
K.SFN(+15.99)LDEGHALR.I	N	62.00	1273.6051	11	0.0	637.8098	2	27.46	43	F43:1330	OB5956 H1 Ro.raw	9.622E5	3	3	249	259	Oxidation or Hydroxylation	N3:Oxidation or Hydroxylation:55.92	PEAKS PTM
R.IP(+15.99)SGFISYILNR.H	N	61.98	1394.7557	12	-0.8	698.3846	2	37.47	43	F43:1932	OB5956 H1 Ro.raw	3.2208E6	4	4	260	271	Oxidation or Hydroxylation	P2:Oxidation or Hydroxylation:39.25	PEAKS PTM
K.NPQLQDLDMM(+15.99)LTC(+57.02)VEIKEGALM(+15.99)LPHFNSK.A	N	61.19	3403.6284	29	0.3	851.9147	4	35.86	41	F41:1801	OB5954 H1 Ro.raw	3.4294E6	4	4	415	443	Oxidation (M); Carbamidomethylation	M10:Oxidation (M):21.94;C13:Carbamidomethylation:1000.00;M22:O	PEAKS DB
**total 244 peptides**																			

Peptide	Uniq	-10lgP	Mass	Length	ppm	m/z	z	RT	Fraction	Scan	Source File	Area Roasted in gel	#Feature	#Feature Roasted in gel	Start	End	PTM	AScore	Found By
																		xidation (M):61.50	
K.G(+57.02)TGNLELVAVRK.E	N	60.73	1312.7462	12	-0.4	657.3801	2	27.30	41	F41:1303	OB5954 H1 Ro.raw	3.6978E5	2	2	453	464	Carbamidomethylation (DHKE, X@N-term)	G1:Carbamidomethylation (DHKE, X@N-term):55.46	PEAKS PTM
R.EQEWEEEEEDEEEEGSNREVRR.Y	N	60.58	2821.1545	22	1.9	941.3939	3	26.71	43	F43:1297	OB5956 H1 Ro.raw	1.0274E6	4	4	473	494			PEAKS DB
K.NPQLQDLDM(+15.99)M(+15.99)LTC(+57.02)VEIKEGALM(+15.99)LPHFNSK.A	N	60.17	3419.6233	29	-0.9	855.9124	4	34.83	42	F42:1766	OB5955 H1 Ro.raw	1.2322E6	3	3	415	443	Oxidation (M); Carbamidomethylation	M9:Oxidation (M):1000.00;M10:Oxidation (M):1000.00;C13:Carbamidomethylation:1000.00;M22:Oxidation (M):1000.00	PEAKS DB
R.IPSGFISY(+15.99)ILNR.H	N	60.13	1394.7557	12	-0.7	698.3846	2	35.11	41	F41:1859	OB5954 H1 Ro.raw	3.4529E6	5	5	260	271	Oxidation or Hydroxylation	Y8:Oxidation or Hydroxylation:67.76	PEAKS PTM
L.DEGHALR.I	N	59.61	796.3828	7	2.5	399.1996	2	27.93	42	F42:1351	OB5955 H1 Ro.raw	9.3538E4	3	3	253	259			PEAKS DB
R.LFEVKPDKKNPQLQ(+.98)DLDMM(+15.99)LTC(+57.02)VEIK.E	N	59.38	3148.5857	26	7.4	788.1595	4	33.17	42	F42:1670	OB5955 H1 Ro.raw	3.9804E5	1	1	406	431	Oxidation (M); Carbamidomethylation	Q14:Deamidation (NQ):0.00;M19:Oxidation (M):23.10;C22:Carbamidomethylation:1000.00	PEAKS DB
R.VLLEEN(-17.03)AGGEQEER.G	N	59.22	1554.7162	14	1.2	778.3663	2	27.73	42	F42:1335	OB5955 H1 Ro.raw	0	0	0	329	342	Ammonia-loss (N)	N6:Ammonia-loss (N):1000.00	PEAKS PTM
K.SFNLDEGHALRIPSGFISYILNRHDNQNLR.V	N	59.07	3495.7759	30	1.5	700.1635	5	39.97	40	F40:1987	OB5953 H3A Ro.raw	2.0405E7	15	15	249	278			PEAKS DB
K.SFNLDEGHALR(+31.99)IPSGFISYILNR.H	N	58.88	2650.3503	23	0.1	884.4575	3	39.14	42	F42:2021	OB5955 H1 Ro.raw	5.6519E5	1	1	249	271	Dihydroxy	R11:Dihydroxy:46.57	PEAKS PTM
R.LFEVKPDK.K	N	58.71	974.5436	8	1.7	488.2799	2	25.30	42	F42:1183	OB5955 H1 Ro.raw	1.7315E6	6	6	406	413			PEAKS DB
K.GSEEEDITNPIN(+.98)LRDGEPDLSNNFGR.L	N	58.69	2888.3059	26	6.7	1445.1699	2	32.90	41	F41:1642	OB5954 H1 Ro.raw	1.7853E5	1	1	380	405		N12:Deamidation (NQ):12.33	PEAKS DB
K.SFNLDE(+57.02)GHALR.I	N	58.62	1314.6316	11	0.3	658.3232	2	27.83	43	F43:1458	OB5956 H1 Ro.raw	2.3371E6	2	2	249	259	Carbamidomethylation (DHKE, X@N-term)	E6:Carbamidomethylation (DHKE, X@N-term):27.25	PEAKS PTM
R.N(+57.02)NPFYFPSRR.F	N	58.56	1353.6577	10	1.1	677.8369	2	29.74	42	F42:1453	OB5955 H1 Ro.raw	9.24E5	3	3	166	175	Carbamidomethylation (DHKE, X@N-term)	N1:Carbamidomethylation (DHKE, X@N-term):1000.00	PEAKS PTM
R.IFLAGDKDNVIDQ(+.98)IEKQAK.D	N	58.20	2145.1316	19	1.4	716.0521	3	33.04	43	F43:1686	OB5956 H1 Ro.raw	1.2153E5	1	1	536	554	Deamidation (NQ)	Q13:Deamidation (NQ):25.1	PEAKS DB

total 244 peptides

Peptide	Uniq	-10lgP	Mass	Length	ppm	m/z	z	RT	Fraction	Scan	Source File	Area Roasted in gel	#Feature	#Feature Roasted in gel	Start	End	PTM	AScore	Found By
																		8	
R.VAKISM(+15.99)PVNTPGQFEDFFPASSR.D	N	57.67	2540.2368	23	2.1	847.7546	3	32.98	42	F42:1665	OB5955 H1 Ro.raw	1.5427E5	1	1	279	301	Oxidation (M)	M6:Oxidation (M):1000.00	PEAKS DB
K.G(+57.02)TGNLELVAVR.K	N	57.51	1184.6514	11	-2.7	593.3314	2	30.10	42	F42:1486	OB5955 H1 Ro.raw	1.7196E5	2	2	453	463		G1:Carbamidomethylation (DHKE, X@N-term):19.78	PEAKS PTM
R.IFLAGD(+57.02)KDNVIDQIEK.Q	N	57.43	1873.9785	16	1.7	625.6678	3	32.52	41	F41:1637	OB5954 H1 Ro.raw	2.7943E5	3	3	536	551		D6:Carbamidomethylation (DHKE, X@N-term):15.57	PEAKS PTM
R.IFLAGDK(+15.99)DNVIDQIEK.Q	N	57.39	1832.9519	16	-1.9	917.4814	2	31.99	41	F41:1600	OB5954 H1 Ro.raw	7.5922E5	2	2	536	551		K7:Oxidation or Hydroxylation:17.01	PEAKS PTM
N.PFYFPSRR.F	N	57.34	1068.5504	8	1.1	535.2831	2	28.95	41	F41:1414	OB5954 H1 Ro.raw	4.6082E6	5	5	168	175			PEAKS DB
R.K(+27.99)SFNLDEGHALR.I	N	57.29	1413.7001	12	1.4	707.8583	2	28.46	42	F42:1387	OB5955 H1 Ro.raw	2.9777E4	1	1	248	259	Formylation	K1:Formylation:1000.00	PEAKS PTM
R.LFEVKPDKKNPQLQDLDM(+15.99)MLTC(+57.02)VEIK.E	N	57.09	3147.6018	26	-1.9	787.9062	4	34.20	41	F41:1734	OB5954 H1 Ro.raw	2.4894E6	4	4	406	431	Oxidation (M); Carbamidomethylation	M18:Oxidation (M):33.98;C22:Carbamidomethylation:1000.00	PEAKS DB
K.SFNLDEGHALR(+31.99).I	N	55.96	1289.6000	11	-1.4	645.8063	2	30.41	43	F43:1520	OB5956 H1 Ro.raw	0	0	0	249	259	Dihydroxy	R11:Dihydroxy:119.59	PEAKS PTM
K.ISMPVN(+.98)TPGQFEDFFPASSR.D	N	55.55	2227.0254	20	6.6	1114.5273	2	36.77	41	F41:1885	OB5954 H1 Ro.raw	1.01E5	2	2	282	301	Deamidation (NQ)	N6:Deamidation (NQ):42.68	PEAKS DB
R.NTLEAAFNAEFN(+.98)EIRR.V	N	54.77	1894.9172	16	1.8	948.4676	2	33.04	43	F43:1685	OB5956 H1 Ro.raw	2.2573E6	5	5	313	328	Deamidation (NQ)	N12:Deamidation (NQ):39.02	PEAKS DB
V.QIEARPNTLVLPK.H	Y	54.45	1477.8616	13	-1.8	739.9368	2	30.56	41	F41:1513	OB5954 H1 Ro.raw	1.5146E5	3	3	210	222			PEAKS DB
K.S(+57.02)FNLDEGHALRIPSGFISYILNR.H	N	54.19	2675.3818	23	2.3	892.8033	3	39.35	38	F38:1901	OB5951 H3A Ro.raw	6.7035E5	1	1	249	271	Carbamidomethylation (DHKE, X@N-term)	S1:Carbamidomethylation (DHKE, X@N-term):63.97	PEAKS PTM
R.IFLAGDKDNVIDQIEK(+57.02)QAK.D	N	54.07	2201.1692	19	0.3	734.7306	3	32.48	43	F43:1657	OB5956 H1 Ro.raw	1.2122E6	3	3	536	554		K16:Carbamidomethylation (DHKE, X@N-term):11.12	PEAKS PTM
R.I(+27.99)PSGFISYILNR.H	N	53.77	1406.7557	12	0.7	704.3856	2	42.27	42	F42:2199	OB5955 H1 Ro.raw	0	0	0	260	271	Formylation	I1:Formylation:1000.00	PEAKS PTM
R.SKQFQNLQNHR.I	Y	53.65	1398.7117	11	0.4	467.2447	3	24.16	43	F43:1149	OB5956 H1 Ro.raw	1.1379E5	3	3	197	207			PEAKS DB
R.IPSGFISYILNRHDN(+.98)QNLR.V	N	53.60	2257.1604	19	-0.7	753.3936	3	34.26	41	F41:1732	OB5954 H1 Ro.raw	0	0	0	260	278		N15:Deamidation (NQ):0.00	PEAKS DB
K.HADADNILVIQQGQATVTVANGNNR.K	N	53.13	2618.3162	25	0.5	873.7798	3	30.34	38	F38:1426	OB5951 H3A Ro.raw	1.8654E3	1	1	223	247			PEAKS DB

total 244 peptides

Peptide	Uniq	-10lgP	Mass	Length	ppm	m/z	z	RT	Fraction	Scan	Source File	Area Roasted in gel	#Feature	#Feature Roasted in gel	Start	End	PTM	AScore	Found By
R.EGEQEWGTPGS(+42.01)EVR.E	Y	52.96	1601.6958	14	7.6	801.8613	2	27.46	43	F43:1334	OB5956 H1 Ro.raw	9.5213E4	3	3	147	160	Acetylation (TSCYH)	S11:Acetylation (TSCYH):32.97	PEAKS PTM
K.AM(+15.99)VIVVVNKGTGNLELVAVRK.E	N	52.95	2225.2930	21	-1.3	742.7706	3	32.42	42	F42:1596	OB5955 H1 Ro.raw	1.4247E6	3	3	444	464	Oxidation (M)	M2:Oxidation (M):1000.00	PEAKS DB
K.KNPQLQ(+.98)DLDMMLTC(+57.02)VEIKEGALMLPHFNSK.A	N	51.97	3500.7175	30	3.4	876.1896	4	36.93	43	F43:1903	OB5956 H1 Ro.raw	9.2646E6	2	2	414	443	Carbamidomethylation	Q6:Deamidation (NQ):14.04;C14:Carbamidomethylation:1000.00	PEAKS DB
R.IFLAGDKDNVID.Q	N	51.22	1318.6769	12	-0.9	660.3451	2	31.76	43	F43:1606	OB5956 H1 Ro.raw	1.1173E5	3	3	536	547			PEAKS DB
K.ISM(+15.99)PVNTPGQFEDFFPASSRDQSSYLQGFSR.N	N	50.25	3510.6150	31	0.3	1171.2126	3	35.35	36	F36:1707	OB5948 H3B Ro.raw	2.4799E6	5	5	282	312	Oxidation (M)	M3:Oxidation (M):1000.00	PEAKS DB
K.NPQLQDLDMM(+15.99)LTC(+57.02)VEIKEGALMLPHFN(+.98)SK.A	N	49.99	3388.6174	29	6.1	848.1668	4	36.39	43	F43:1880	OB5956 H1 Ro.raw	1.7975E6	1	1	415	443	Oxidation (M); Carbamidomethylation	M10:Oxidation (M):27.96;C13:Carbamidomethylation:1000.00;N27:Deamidation (NQ):18.27	PEAKS DB
R.N(-17.03)NPFYFPSRR.F	N	49.99	1279.6097	10	1.1	427.5443	3	28.89	43	F43:1434	OB5956 H1 Ro.raw	5.674E5	3	3	166	175		N1:Ammonia-loss (N):9.34	PEAKS PTM
R.EQEWEEEEDEEEEGSNREVR.R	N	49.84	2665.0535	21	2.4	889.3605	3	27.88	41	F41:1353	OB5954 H1 Ro.raw	4.9786E4	1	1	473	493			PEAKS DB
T.GNLELVAVRK.E	N	49.53	1097.6556	10	0.5	549.8354	2	27.00	42	F42:1296	OB5955 H1 Ro.raw	3.4872E5	3	3	455	464			PEAKS DB
R.VAKIS(+77.99)MPVNTPGQFEDFFPASSR.D	N	49.16	2602.2290	23	3.0	1302.1257	2	38.06	42	F42:1958	OB5955 H1 Ro.raw	0	0	0	279	301	Methylphosphonylation	S5:Methylphosphonylation:56.99	PEAKS PTM
F.PGSGEQVEK.L	N	49.06	929.4454	9	-3.7	465.7282	2	30.34	43	F43:1524	OB5956 H1 Ro.raw	5.2705E4	1	1	559	567			PEAKS DB
K.AMVIVVVNK(+57.02)GTGNLELVAVRK.E	N	48.92	2266.3196	21	1.3	756.4481	3	33.23	43	F43:1673	OB5956 H1 Ro.raw	3.1193E6	1	1	444	464		K9:Carbamidomethylation (DHKE, X@N-term):8.78	PEAKS PTM
R.EEDWRQPR.E	N	48.87	1114.5155	8	1.8	558.2661	2	23.91	41	F41:1112	OB5954 H1 Ro.raw	1.6791E3	1	1	120	127			PEAKS DB
R.PNTLVLPK.H	N	48.84	880.5382	8	-2.9	441.2751	2	28.00	43	F43:1374	OB5956 H1 Ro.raw	1.9234E5	2	2	215	222			PEAKS DB
L.R(+27.99)IPSGFISYILNR.H	N	48.25	1562.8568	13	-2.0	782.4341	2	37.84	43	F43:1976	OB5956 H1 Ro.raw	1.5108E5	1	1	259	271	Formylation	R1:Formylation:1000.00	PEAKS PTM
R.EGEQEW(+15.99)GTPGSEVREETSR.N	Y	48.10	2177.9460	19	1.6	726.9905	3	26.51	43	F43:1296	OB5956 H1 Ro.raw	8.9341E6	3	3	147	165	Oxidation (HW)	W6:Oxidation (HW):1000.00	PEAKS PTM
R.I(+57.02)VQIEARPNTLVLPK.H	Y	46.89	1747.0355	15	-0.2	874.5248	2	30.98	42	F42:1537	OB5955 H1 Ro.raw	9.1688E5	3	3	208	222	Carbamidomethylation (DHKE, X@N-term)	I1:Carbamidomethylation (DHKE, X@N-term):69.71	PEAKS PTM
**total 244 peptides**																			

Peptide	Uniq	-10lgP	Mass	Length	ppm	m/z	z	RT	Fraction	Scan	Source File	Area Roasted in gel	#Feature	#Feature Roasted in gel	Start	End	PTM	AScore	Found By
R.N(+.98)TLEAAFNAEFNEIR.R	N	46.63	1738.8162	15	9.2	870.4233	2	44.36	42	F42:2303	OB5955 H1 Ro.raw	5.4416E3	1	1	313	327	Deamidation (NQ)	N1:Deamidation (NQ):39.47	PEAKS DB
R.VLLE(+21.98)ENAGGEQEER.G	N	46.37	1593.7246	14	-1.1	797.8687	2	26.71	43	F43:1300	OB5956 H1 Ro.raw	4.0134E5	3	3	329	342	Sodium adduct	E4:Sodium adduct: 40.00	PEAKS PTM
N.PFYFPSR.R	N	46.32	912.4493	7	-0.1	457.2319	2	31.70	42	F42:1584	OB5955 H1 Ro.raw	2.4653E5	4	4	168	174			PEAKS DB
K.GTGNLELVAVRKEQQQR.G	N	46.31	1925.0442	17	1.4	642.6896	3	26.67	43	F43:1294	OB5956 H1 Ro.raw	2.1018E4	1	1	453	469			PEAKS DB
K.NPQLQDLD(+14.02)MMLTC(+57.02)VEIK.E	N	45.99	2060.9944	17	0.2	1031.5046	2	37.33	41	F41:1934	OB5954 H1 Ro.raw	2.7407E4	1	1	415	431	Methylation(others); Carbamidomethylation	D8:Methylation(others):42.68; C13:Carbamidomethylation:1000.00	PEAKS PTM
K.GTGNLE(+57.02)LVAVRK.E	N	45.75	1312.7462	12	-0.8	657.3799	2	27.17	42	F42:1295	OB5955 H1 Ro.raw	1.9882E5	1	1	453	464		E6:Carbamidomethylation (DHKE, X@N-term):0.00	PEAKS PTM
K.A(+43.01)MVIVVVNKGTGNLELVAVR.K	N	45.64	2124.2090	20	0.7	1063.1125	2	33.65	41	F41:1688	OB5954 H1 Ro.raw	8.0739E4	1	1	444	463	Carbamylation	A1:Carbamylation: 161.20	PEAKS PTM
R.VLLEEN(+.98)AGGEQEERGQR.R	N	45.60	1913.9078	17	-1.6	638.9755	3	25.99	41	F41:1234	OB5954 H1 Ro.raw	0	0	0	329	345	Deamidation (NQ)	N6:Deamidation (NQ):28.79	PEAKS DB
K.KNPQLQDLDMM(+15.99)LTC(+57.02)VEIKEGALM(+15.99)LPHFNSK.A	N	45.45	3531.7234	30	-1.1	883.9372	4	35.80	42	F42:1822	OB5955 H1 Ro.raw	0	0	0	414	443	Carbamidomethylation; Oxidation (M)	M11:Oxidation (M): 0.00;C14: Carbamidomethylation:1000.00;M23:Oxidation (M):45.87	PEAKS DB
R.LFEVKPDKKNPQLQDLDM(+15.99)M(+15.99)LTC(+57.02)VEIK.E	N	45.43	3163.5967	26	-2.8	791.9042	4	32.90	41	F41:1650	OB5954 H1 Ro.raw	2.0535E6	3	3	406	431	Oxidation (M); Carbamidomethylation	M18:Oxidation (M): 1000.00; M19:Oxidation (M): 1000.00; C22:Carbamidomethylation:1000.00	PEAKS DB
R.KSFNLDEGH(+15.99)ALR.I	N	45.40	1401.7001	12	3.9	701.8600	2	27.64	43	F43:1349	OB5956 H1 Ro.raw	6.6479E4	2	2	248	259	Oxidation (HW)	H9:Oxidation (HW): 1000.00	PEAKS PTM
K.KNPQLQDLDM(+15.99)MLTC(+57.02)VEIKEGALMLPHFNSK.A	N	44.60	3515.7285	30	-1.9	879.9377	4	35.30	41	F41:1798	OB5954 H1 Ro.raw	6.4162E5	1	1	414	443	Carbamidomethylation	M10:Oxidation (M): 14.02;C14:Carbamidomethylation:1000.00	PEAKS DB
R.DQSSYLQGFSR(+15.99).N	N	44.15	1302.5840	11	2.5	652.3009	2	30.79	43	F43:1544	OB5956 H1 Ro.raw	1.4308E5	3	3	302	312	Oxidation or Hydroxylation	R11:Oxidation or Hydroxylation:42.88	PEAKS PTM
K.Q(+.98)FQNLQNHR.I	N	43.70	1184.5686	9	4.6	593.2943	2	23.68	42	F42:1098	OB5955 H1 Ro.raw	0	0	0	199	207	Deamidation (NQ)	Q1:Deamidation (NQ):39.25	PEAKS DB
K.I(+57.02)SMPVNTPGQFEDFFPASSR.D	N	42.37	2283.0630	20	-0.7	1142.5380	2	36.01	43	F43:1860	OB5956 H1 Ro.raw	1.8515E5	1	1	282	301	Carbamidomethylation (DHKE, X@N-term)	I1:Carbamidomethylation (DHKE, X@N-term):50.99	PEAKS PTM

total 244 peptides

Peptide	Uniq	-10lgP	Mass	Length	ppm	m/z	z	RT	Fraction	Scan	Source File	Area Roasted in gel	#Feature	#Feature Roasted in gel	Start	End	PTM	AScore	Found By
K.ISM(-48.00)PVNTPGQFEDFFPASSR.D	N	42.09	2178.0381	20	2.8	727.0220	3	32.48	43	F43:1652	OB5956 H1 Ro.raw	5.8583E6	3	3	282	301	Dethiomethyl	M3:Dethiomethyl:1000.00	PEAKS PTM
R.SKQFQNLQNH(+15.99)R.I	Y	41.84	1414.7065	11	2.4	708.3622	2	24.12	42	F42:1124	OB5955 H1 Ro.raw	1.2986E3	1	1	197	207	Oxidation (HW)	H10:Oxidation (HW):1000.00	PEAKS PTM
K.KGSEEEDIT(-18.01)N(+.98)PINLR.D	N	41.27	1696.8268	15	2.3	566.6175	3	29.07	43	F43:1442	OB5956 H1 Ro.raw	3.8118E5	3	3	379	393	Dehydration; Deamidation (NQ)	T9:Dehydration:45.01;N10:Deamidation (NQ):59.10	PEAKS PTM
K.NPQLQDLDMM(+15.99)LTC(+57.02)VEIKEGALM(+15.99)LPHFN(+.98)SK.A	N	41.25	3404.6125	29	-0.4	1135.8777	3	36.69	42	F42:1878	OB5955 H1 Ro.raw	3.8423E5	1	1	415	443	Carbamidomethylation; Oxidation (M); Deamidation (NQ)	M10:Oxidation (M):0.00;C13:Carbamidomethylation:1000.00;M22:Oxidation (M):39.23;N27:Deamidation (NQ):69.52	PEAKS DB
R.REQEW(+15.99)EEEEEDEEEEGSNR.E	N	41.18	2452.9375	19	-0.6	818.6526	3	25.98	41	F41:1239	OB5954 H1 Ro.raw	2.4114E4	1	1	472	490	Oxidation (HW)	W5:Oxidation (HW):1000.00	PEAKS PTM
K.DLAFP(+15.99)GSGEQVEK.L	N	41.14	1391.6569	13	0.5	696.8361	2	29.36	42	F42:1432	OB5955 H1 Ro.raw	7.4528E5	3	3	555	567	Oxidation or Hydroxylation	P5:Oxidation or Hydroxylation:67.76	PEAKS PTM
K.AMVIVVVN(+.98)KGTGNLELVAVRK.E	N	41.03	2210.2820	21	1.9	1106.1504	2	34.35	42	F42:1720	OB5955 H1 Ro.raw	4.5718E5	1	1	444	464	Deamidation (NQ)	N8:Deamidation (NQ):28.79	PEAKS DB
K.EGALM(-48.00)LPHFNSK.A	N	40.53	1294.6670	12	0.5	432.5632	3	28.76	41	F41:1389	OB5954 H1 Ro.raw	2.208E6	2	2	432	443	Dethiomethyl	M5:Dethiomethyl:1000.00	PEAKS PTM
R.VLLEENAGGEQEERGQRR.R	N	40.17	2069.0249	18	1.0	518.2640	4	25.49	42	F42:1205	OB5955 H1 Ro.raw	2.0305E5	2	2	329	346			PEAKS DB
R.VLLEENAGGEQEER(+15.99).G	N	39.98	1587.7375	14	1.8	794.8775	2	26.70	41	F41:1275	OB5954 H1 Ro.raw	0	0	0	329	342	Oxidation or Hydroxylation	R14:Oxidation or Hydroxylation:20.88	PEAKS PTM
R.N(+27.99)TLEAAFNAEFNEIRR.V	N	39.68	1921.9282	16	-1.7	961.9697	2	37.51	41	F41:1908	OB5954 H1 Ro.raw	3.7767E4	1	1	313	328	Formylation	N1:Formylation:1000.00	PEAKS PTM
K.E(-18.01)GALM(+15.99)LPHFNSK.A	N	39.64	1340.6547	12	0.2	447.8923	3	30.21	41	F41:1479	OB5954 H1 Ro.raw	7.81E5	1	1	432	443	Pyro-glu from E; Oxidation (M)	E1:Pyro-glu from E:1000.00;M5:Oxidation (M):1000.00	PEAKS PTM
R.VLLEE(+53.92)NAGGEQEER.G	N	39.58	1625.6620	14	-1.0	542.8940	3	26.76	42	F42:1278	OB5955 H1 Ro.raw	0	0	0	329	342	Replacement of 2 protons by iron	E5:Replacement of 2 protons by iron:20.92	PEAKS PTM
D.GEPDLSNNFGR.L	N	39.46	1204.5472	11	3.0	603.2827	2	28.20	41	F41:1371	OB5954 H1 Ro.raw	1.5511E4	1	1	395	405			PEAKS DB
K.HADADNILVIQQGQ(+.98)ATVTVANGNNRK.S	N	39.33	2747.3950	26	2.6	916.8103	3	30.46	30	F30:1280	OB5926 H3B Ro.raw	0	0	0	223	248		Q14:Deamidation (NQ):5.07	PEAKS DB
K.NPQLQ(+.98)DLDMM(+15.99)LTC(+57.02)VEIKEGALM(+15.99)LPHFNSK.A	N	39.15	3404.6125	29	-1.8	852.1589	4	38.22	43	F43:1993	OB5956 H1 Ro.raw	7.6266E5	1	1	415	443	Deamidation (NQ); Carbamidomethylation	Q5:Deamidation (NQ):49.79;M10:Oxidation (M):0.00;C13:	PEAKS DB
**total 244 peptides**																			

Peptide	Uniq	-10lgP	Mass	Length	ppm	m/z	z	RT	Fraction	Scan	Source File	Area Roasted in gel	#Feature	#Feature Roasted in gel	Start	End	PTM	AScore	Found By
																		Carbamidomethylation:1000.00;M22:Oxidation (M):14.60	
K.N(+.98)PQLQDLDM(+15.99)MLTC(+57.02)VEIKEGALMLPHFNSK.A	N	38.83	3388.6174	29	8.2	1695.3300	2	37.72	41	F41:1941	OB5954H1Ro.raw	6.4698E5	1	1	415	443	Carbamidomethylation	N1:Deamidation (NQ):0.00;M9:Oxidation (M):0.00;C13:Carbamidomethylation:1000.00	PEAKS DB
K.D(+15.99)LAFPGSGEQVEK.L	N	38.81	1391.6569	13	0.5	696.8361	2	29.36	42	F42:1368	OB5955H1Ro.raw	2.5725E5	1	1	555	567	Oxidation or Hydroxylation	D1:Oxidation or Hydroxylation:77.13	PEAKS PTM
K.NPQ(+.98)LQDLDMMLTC(+57.02)VEIK.E	N	38.70	2047.9629	17	2.9	1024.9917	2	46.45	41	F41:2441	OB5954H1Ro.raw	0	0	0	415	431	Carbamidomethylation	Q3:Deamidation (NQ):14.04;C13:Carbamidomethylation:1000.00	PEAKS DB
R.IFLAGD(+15.99)KDNVIDQIEK.Q	N	38.52	1832.9519	16	1.9	917.4850	2	31.89	42	F42:1590	OB5955H1Ro.raw	4.6198E5	1	1	536	551		D6:Oxidation or Hydroxylation:12.60	PEAKS PTM
R.IFLAGDKDN(+.98)VIDQIEKQ(+.98)AK.D	N	37.85	2146.1157	19	9.8	716.3862	3	32.98	42	F42:1661	OB5955H1Ro.raw	8.6625E4	1	1	536	554		N9:Deamidation (NQ):13.83;Q17:Deamidation (NQ):17.65	PEAKS DB
K.DLAFPGSGE(+53.92)QVEK.L	N	37.31	1429.5812	13	-6.3	715.7933	2	30.34	43	F43:1518	OB5956H1Ro.raw	2.6492E5	1	1	555	567	Replacement of 2 protons by iron	E9:Replacement of 2 protons by iron:37.54	PEAKS PTM
R.W(+15.99)GPAEPR.E	Y	36.73	827.3926	7	0.0	414.7036	2	24.54	42	F42:1155	OB5955H1Ro.raw	3.7087E3	1	1	109	115	Oxidation (HW)	W1:Oxidation (HW):1000.00	PEAKS PTM
K.GT(-18.01)GNLELVAVRK.E	N	36.73	1237.7142	12	2.6	413.5797	3	27.00	42	F42:1287	OB5955H1Ro.raw	4.7714E5	1	1	453	464	Dehydration	T2:Dehydration:1000.00	PEAKS PTM
K.IRPEGREGEQEWGTPGSEVREETSR.N	Y	36.52	2870.3542	25	2.3	718.5975	4	27.83	43	F43:1355	OB5956H1Ro.raw	1.3647E5	1	1	141	165			PEAKS DB
R.NT(-18.01)LEAAFNAEFNEIRR.V	N	36.45	1875.9227	16	0.7	626.3153	3	34.16	43	F43:1739	OB5956H1Ro.raw	6.9172E6	2	2	313	328	Dehydration	T2:Dehydration:1000.00	PEAKS PTM
K.EGALM(+15.99)LPHFN(+.98)SK.A	N	36.41	1359.6493	12	2.8	680.8338	2	31.22	41	F41:1550	OB5954H1Ro.raw	0	0	0	432	443	Oxidation (M); Deamidation (NQ)	M5:Oxidation (M):1000.00;N10:Deamidation (NQ):1000.00	PEAKS DB
R.DQSSY(-18.01)LQGFSR.N	N	36.32	1268.5785	11	-0.6	635.2961	2	30.85	43	F43:1579	OB5956H1Ro.raw	2.378E5	1	1	302	312		Y5:Dehydration:0.00	PEAKS PTM
R.C(+57.02)LQS(-18.01)C(+57.02)Q(+.98)QEPDDLKQK.A	N	36.24	1858.8190	15	-0.1	930.4167	2	28.47	31	F31:1350	OB5942H6Ro.raw	0	0	0	40	54	Carbamidomethylation; Dehydration	C1:Carbamidomethylation:1000.00;S4:Dehydration:39.55;C5:Carbamidomethylation:10	PEAKS PTM

total 244 peptides

Peptide	Uniq	-10lgP	Mass	Length	ppm	m/z	z	RT	Fraction	Scan	Source File	Area Roasted in gel	#Feature	#Feature Roasted in gel	Start	End	PTM	AScore	Found By
																		00.00;Q6: Deamidation (NQ): 8.69	
M.LPHFNSK.A	N	36.02	841.4446	7	1.3	421.7301	2	30.63	42	F42:1510	OB5955 H1 Ro.raw	1.4673E6	2	2	437	443			PEAKS DB
K.NPQLQDLDM(-48.00)MLTC(+57.02)VEIK.E	N	35.81	1998.9755	17	6.7	667.3369	3	34.52	43	F43:1773	OB5956 H1 Ro.raw	8.4276E5	1	1	415	431	Carbamidomethylation	M9:Dethiomethyl:11.06;C13:Carbamidomethylation:1000.00	PEAKS PTM
K.SF(+17.99)NLDEGHALR.I	N	34.75	1275.6007	11	8.1	638.8128	2	26.62	42	F42:1267	OB5955 H1 Ro.raw	9.5828E3	1	1	249	259	Fluorination	F2:Fluorination:1000.00	PEAKS PTM
K.KNPQLQ(+.98)DLDMM(+15.99)LTC(+57.02)VEIKEGALMLPHFNSK.A	N	33.72	3516.7124	30	3.3	880.1883	4	35.48	41	F41:1798	OB5954 H1 Ro.raw	7.5658E5	1	1	414	443	Carbamidomethylation	Q6:Deamidation (NQ):18.55;M11:Oxidation (M):14.02;C14:Carbamidomethylation:1000.00	PEAKS DB
K.VSKEH(+15.99)VQELTK.H	N	33.71	1312.6986	11	1.8	657.3578	2	23.73	42	F42:1100	OB5955 H1 Ro.raw	1.3581E3	1	1	361	371	Oxidation (HW)	H5:Oxidation (HW):1000.00	PEAKS PTM
D.QSSYLQGFSR.N	N	33.26	1171.5621	10	2.1	586.7896	2	29.36	42	F42:1431	OB5955 H1 Ro.raw	3.3134E4	1	1	303	312			PEAKS DB
R.IFLAGDKDNVID(-18.01)QIEK.Q	N	33.09	1798.9464	16	0.7	900.4811	2	31.39	43	F43:1591	OB5956 H1 Ro.raw	6.7846E4	1	1	536	551		D12:Dehydration:13.16	PEAKS PTM
R.EREEDWRQPR.E	N	32.92	1399.6592	10	5.7	700.8409	2	24.05	43	F43:1141	OB5956 H1 Ro.raw	4.1173E4	1	1	118	127			PEAKS DB
D.NVIDQIEK.Q	N	32.64	957.5131	8	1.6	479.7646	2	28.19	42	F42:1367	OB5955 H1 Ro.raw	3.0062E3	1	1	544	551			PEAKS DB
K.AM(-48.00)VIVVVNK.G	N	32.49	923.5804	9	0.0	462.7975	2	25.00	43	F43:1192	OB5956 H1 Ro.raw	6.3177E3	1	1	444	452	Dethiomethyl	M2:Dethiomethyl:1000.00	PEAKS PTM
R.D(-18.01)QSSYLQGFSR.N	N	31.95	1268.5785	11	1.6	635.2975	2	29.92	43	F43:1489	OB5956 H1 Ro.raw	0	0	0	302	312		D1:Dehydration:0.00	PEAKS PTM
R.IFLAGDKD(-18.01)NVIDQIEK.Q	N	31.82	1798.9464	16	-0.9	600.6555	3	32.15	43	F43:1589	OB5956 H1 Ro.raw	3.5634E5	1	1	536	551	Dehydration	D8:Dehydration:30.83	PEAKS PTM
K.D(+53.92)LAFPGSGEQVEK.L	N	31.27	1429.5812	13	-4.3	715.7948	2	30.56	41	F41:1500	OB5954 H1 Ro.raw	3.7719E5	1	1	555	567		D1:Replacement of 2 protons by iron:0.00	PEAKS PTM
K.SFNLDE(+53.92)GHALR.I	N	31.27	1311.5294	11	1.0	438.1842	3	28.04	41	F41:1362	OB5954 H1 Ro.raw	5.8053E4	1	1	249	259		E6:Replacement of 2 protons by iron:16.90	PEAKS PTM
K.AM(+15.99)VIVVVNK(+57.02)GTGNLELVAVR.K	N	30.97	2154.2195	20	2.8	1078.1200	2	32.98	42	F42:1664	OB5955 H1 Ro.raw	2.0029E5	1	1	444	463	Oxidation (M)	M2:Oxidation (M):1000.00;K9:Carbamidomethylation (DHKE, X@N-term):0.00	PEAKS PTM
K.KNPQLQDLDMM(+15.99)LTC(+57.02)VEIKEGALMLPHFNSK.A	N	30.83	3515.7285	30	1.3	879.9406	4	35.25	42	F42:1790	OB5955 H1 Ro.raw	0	0	0	414	443	Carbamidomethylation	M11:Oxidation (M):12.28;C1	PEAKS DB
**total 244 peptides**																			

Peptide	Uniq	-10lgP	Mass	Length	ppm	m/z	z	RT	Fraction	Scan	Source File	Area Roasted in gel	#Feature	#Feature Roasted in gel	Start	End	PTM	AScore	Found By
																		4:Carbamidomethylation:1000.00	
R.DQSSY(+15.99)LQGFSR.N	N	30.72	1302.5840	11	1.6	652.3003	2	29.24	43	F43:1470	OB5956 H1 Ro.raw	6.7205E4	1	1	302	312	Oxidation or Hydroxylation	Y5:Oxidation or Hydroxylation:26.36	PEAKS PTM
K.SFNLDEGHALRIPSGFISYILNRHDNQN(+.98)LR.V	N	30.43	3496.7600	30	4.9	700.3627	5	37.51	39	F39:1925	OB5952 H3A Ro.raw	2.7478E5	1	1	249	278		N28:Deamidation (NQ):10.11	PEAKS DB
D.KDNVIDQIEK.Q	N	30.42	1200.6350	10	-0.3	601.3246	2	26.31	42	F42:1252	OB5955 H1 Ro.raw	0	0	0	542	551			PEAKS DB
N.TLEAAFNAEFNEIRR.V	N	30.42	1779.8904	15	3.5	890.9556	2	33.23	43	F43:1696	OB5956 H1 Ro.raw	1.5374E5	1	1	314	328			PEAKS DB
R.IPSGFISYILN(+15.99)R.H	N	30.30	1394.7557	12	-2.8	698.3832	2	36.42	41	F41:1875	OB5954 H1 Ro.raw	3.8045E5	1	1	260	271		N11:Oxidation or Hydroxylation:0.00	PEAKS PTM
W.GTPGSEVREETSR.N	Y	30.01	1403.6641	13	3.8	702.8420	2	27.77	42	F42:1332	OB5955 H1 Ro.raw	2.719E4	1	1	153	165			PEAKS DB
R.IFLAGDKD(-18.01)NV.I	N	30.00	1072.5553	10	0.7	537.2853	2	32.26	42	F42:1603	OB5955 H1 Ro.raw	2.1452E6	1	1	536	545	Dehydration	D8:Dehydration:45.01	PEAKS PTM
R.NTLEAAFNAEFN(+15.99)EIRR.V	N	29.99	1909.9282	16	0.9	955.9723	2	32.98	42	F42:1662	OB5955 H1 Ro.raw	2.366E6	2	2	313	328		N12:Oxidation or Hydroxylation:0.00	PEAKS PTM
R.IFLAGDKDNVIDQ(+.98)IEK.Q	N	29.73	1817.9410	16	7.7	909.9848	2	63.08	43	F43:3435	OB5956 H1 Ro.raw	3.1606E3	1	1	536	551		Q13:Deamidation (NQ):11.12	PEAKS DB
I.F(+127.06)LAGDKDNVIDQIEK.Q	N	29.59	1830.9363	15	0.7	916.4761	2	32.18	41	F41:1600	OB5954 H1 Ro.raw	6.5822E4	1	1	537	551	N-Succinimidyl-2-morpholine acetate	F1:N-Succinimidyl-2-morpholine acetate:28.79	PEAKS PTM
K.N(+.98)PQLQ(+.98)DLDMMLTC(+57.02)VEIKEGALM(+15.99)LPHFNSK.A	N	29.50	3389.6016	29	9.6	848.4158	4	37.65	43	F43:2047	OB5956 H1 Ro.raw	6.1505E6	1	1	415	443	Carbamidomethylation	N1:Deamidation (NQ):0.00;Q5:Deamidation (NQ):14.04;C13:Carbamidomethylation:1000.00;M22:Oxidation (M):0.00	PEAKS DB
R.NN(+.98)PFYFPSRR.F	N	29.19	1297.6204	10	9.3	649.8235	2	62.71	43	F43:3425	OB5956 H1 Ro.raw	0	0	0	166	175		N2:Deamidation (NQ):7.21	PEAKS DB
R.NTLEAAFNAEFN(+.98)EIR.R	N	29.07	1738.8162	15	9.2	870.4233	2	44.36	42	F42:2331	OB5955 H1 Ro.raw	5.4416E3	1	1	313	327		N12:Deamidation (NQ):0.00	PEAKS DB
K.KNPQ(+.98)LQ(+.98)DLDMM(+15.99)LTC(+57.02)VEIKEGALM(+15.99)LPHFNSK.A	N	28.74	3533.6914	30	9.7	884.4387	4	35.77	42	F42:1822	OB5955 H1 Ro.raw	2.7541E5	1	1	414	443	Carbamidomethylation; Oxidation (M)	Q4:Deamidation (NQ):8.22;Q6:Deamidation (NQ):5.55;M11:Oxidation (M):0.00;C14:Carbamidomethylation:1000.00;M23:Ox	PEAKS DB
**total 244 peptides**																			

Peptide	Uniq	-10lgP	Mass	Length	ppm	m/z	z	RT	Fraction	Scan	Source File	Area Roasted in gel	#Feature	#Feature Roasted in gel	Start	End	PTM	AScore	Found By
																		idation (M):38.29	
total 244 peptides																			

N1NG13|N1NG13_ARAHY

back to list

| Protein Coverage | Supporting Peptides |

Protein Coverage:

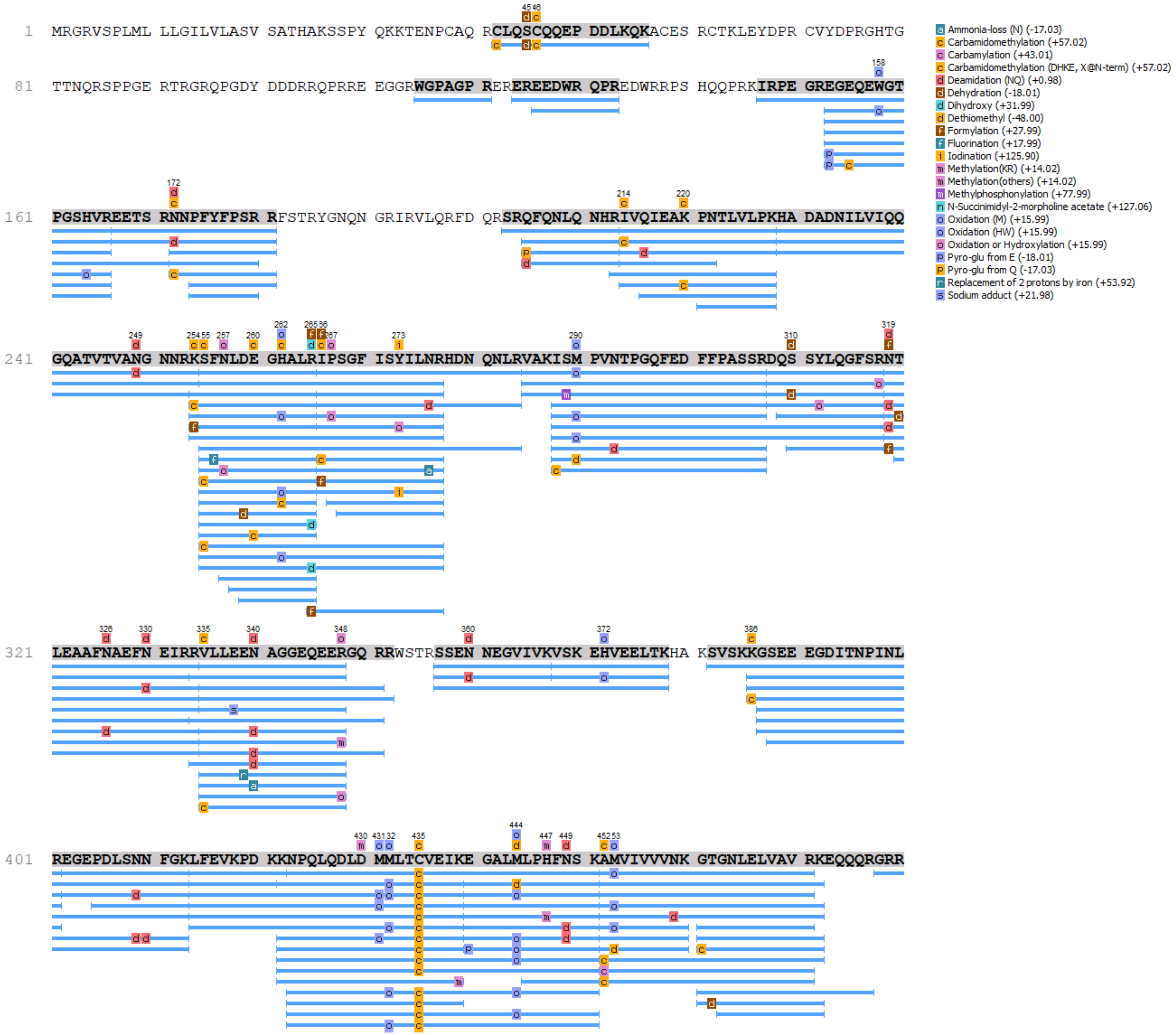
1 MRGRVSPLML LLGILVLASV SATHAKSSPY QKKTENPCAQ RCLQSCQQEP DDLKQKACES RCTKLEYDPR CVYDPRGHTG
81 TTNQRSPPGE RTRGRQPGDY DDDRRQPRRE EGGRWGPAGP REREREEDWR QPREDWRRPS HQQPRKIRPE GREGEQEWGT
161 PGSHVREETS RNNPFYFPSR RFSTRYGNQN GRIRVLQRFD QRSRQFQNLQ NHRIVQIEAK PNTLVLPKHA DADNILVIQQ
241 GQATVTVANG NNRKSFNLDE GHALRIPSGF ISYILNRHDN QNLRVAKISM PVNTPGQFED FFPASSRDQS SYLQGFSRNT
321 LEAAFNAEFN EIRRVLLEEN AGGEQEERGQ RRWSTRSSEN NEGVIVKVSK EHVEELTKHA KSVSKKGSEE EGDITNPINL
401 REGEPDLSNN FGKLFEVKPD KKNPQLQDLD MMLTCVEIKE GALMLPHFNS KAMVIVVVNK GTGNLELVAV RKEQQQRGRR
a Ammonia-loss (N) (-17.03)
c Carbamidomethylation (+57.02)
c Carbamylation (+43.01)
c Carbamidomethylation (DHKE, X@N-term) (+57.02)
d Deamidation (NQ) (+0.98)
d Dehydration (-18.01)
d Dihydroxy (+31.99)
d Dethiomethyl (-48.00)
f Formylation (+27.99)
f Fluorination (+17.99)
i Iodination (+125.90)
m Methylation(KR) (+14.02)
m Methylation(others) (+14.02)
m Methylphosphonylation (+77.99)
n N-Succinimidyl-2-morpholine acetate (+127.06)
o Oxidation (M) (+15.99)
o Oxidation (HW) (+15.99)
o Oxidation or Hydroxylation (+15.99)
p Pyro-glu from E (-18.01)
p Pyro-glu from Q (-17.03)
r Replacement of 2 protons by iron (+53.92)
s Sodium adduct (+21.98)

Supporting Peptides:

Peptide	Uniq	-10lgP	Mass	Length	ppm	m/z	z	RT	Fraction	Scan	Source File	Area Roasted in gel	#Feature	#Feature Roasted in gel	Start	End	PTM	AScore	Found By
K.KNPQLQDLDMMLTC(+57.02)VEIK.E	N	136.41	2175.0737	18	1.4	1088.5457	2	35.26	43	F43:1808	OB5956 H1 Ro.raw	1.4906E7	6	6	422	439	Carbamidomethylation	C14:Carbamidomethylation:1000.00	PEAKS DB
K.KGSEEEG[illegible]	Y	132.44	3058.4478	28	2.9	1020.4928	3	30.98	42	F42:1535	OB5955 H1 Ro.raw	5.2193E6	11	11	386	413			PEAKS DB
R.NTLEAAFNAEFNEIR.R	N	130.93	1737.8322	15	1.8	869.9249	2	35.34	38	F38:1714	OB5951 H3A Ro.raw	1.332E7	16	16	319	333			PEAKS DB
K.NPQLQDLDMMLTC(+57.02)VEIK.E	N	128.40	2046.9788	17	0.2	1024.4968	2	37.11	43	F43:1936	OB5956 H1 Ro.raw	3.4643E7	10	10	423	439	Carbamidomethylation	C13:Carbamidomethylation:1000.00	PEAKS DB
K.NPQLQDLDM(+15.99)MLTC(+57.02)VEIK.E	N	128.16	2062.9736	17	-4.2	1032.4897	2	36.93	43	F43:1892	OB5956 H1 Ro.raw	3.3834E6	3	3	423	439	Oxidation (M); Carbamidomethylation	M9:Oxidation (M):30.46;C13:Carbamidomethylation:1000.00	PEAKS DB
K.NPQLQDLDMM(+15.99)LTC(+57.02)VEIK.E	N	127.13	2062.9736	17	5.0	1032.4993	2	34.71	43	F43:1780	OB5956 H1 Ro.raw	1.0665E7	4	4	423	439	Oxidation (M); Carbamidomethylation	M10:Oxidation (M):30.46;C13:Carbamidomethylation:1000.00	PEAKS DB
K.ISM(+15.99)PVNTPGQFEDFFPASSR.D	N	127.11	2242.0364	20	0.1	1122.0284	2	34.52	30	F30:1521	OB5926 H3B Ro.raw	2.1708E7	14	13	288	307	Oxidation (M)	M3:Oxidation (M):1000.00	PEAKS DB
K.ISMPVNTPGQFEDFFPASSR.D	N	125.26	2226.0415	20	0.7	1114.0288	2	35.83	43	F43:1868	OB5956 H1 Ro.raw	8.8227E7	23	23	288	307			PEAKS DB
K.HADADNILVIQQGQATVTVAN(+.98)GNNRK.S	N	123.10	2747.3950	26	3.3	916.8087	3	29.62	43	F43:1472	OB5956 H1 Ro.raw	4.4266E6	11	11	229	254	Deamidation (NQ)	N21:Deamidation (NQ):31.91	PEAKS DB
K.NPQLQDLDM(+15.99)M(+15.99)LTC(+57.02)VEIK.E	N	121.59	2078.9688	17	6.6	1040.4985	2	33.82	43	F43:1726	OB5956 H1 Ro.raw	6.8984E4	2	2	423	439	Oxidation (M); Carbamidomethylation	M9:Oxidation (M):1000.00;M10:Oxidation (M):1000.00;C13:Carbamidomethylation:1000.00	PEAKS DB
K.SFNLDEGHALRIPSGFISYILNR.H	N	121.49	2618.3604	23	1.0	873.7949	3	37.51	41	F41:1949	OB5954 H1 Ro.raw	9.1637E7	28	28	255	277			PEAKS DB
K.KNPQLQDLDM(+15.99)MLTC(+57.02)VEIK.E	N	118.68	2191.0686	18	3.6	1096.5455	2	34.71	43	F43:1781	OB5956 H1 Ro.raw	1.3598E6	4	4	422	439	Oxidation (M); Carbamidomethylation	M10:Oxidation (M):27.96;C14:Carbamidomethylation:1000.00	PEAKS DB
K.KGSEEEGDITNPINLR.E	N	117.89	1770.8748	16	0.6	886.4452	2	28.91	43	F43:1429	OB5956 H1 Ro.raw	3.1542E7	8	8	386	401			PEAKS DB
K.AMVIVVVNKGTGNLELVAVR.K	N	114.87	2081.2031	20	-1.0	1041.6078	2	34.35	43	F43:1758	OB5956 H1 Ro.raw	3.8361E7	13	13	452	471			PEAKS DB
R.IFLAGDKDNVIDQIEK.Q	N	110.41	1816.9570	16	-1.1	909.4848	2	32.67	43	F43:1648	OB5956 H1 Ro.raw	4.2419E8	56	55	541	556			PEAKS DB

total 250 peptides

481 EEEEEDEEEE EGSNREVRRY TARLKEGDVF IMPAAHPVAI NASSEEHLLG FGINAENNHR IFLAGDKDNV IDQIEKQAKD

561 LAFPGSGEQV EKLIKNQKES HFVSARPQSQ SQSPSSPEKE SPEKEDQEEE NQGGKGPLLS ILKAFN

Peptide	Uniq	-10lgP	Mass	Length	ppm	m/z	z	RT	Fraction	Scan	Source File	Area Roasted in gel	#Feature	#Feature Roasted in gel	Start	End	PTM	AScore	Found By
K.KNPQLQDLDMM(+15.99)LTC(+57.02)VEIK.E	N	110.35	2191.0686	18	3.5	1096.5454	2	32.98	42	F42:1657	OB5955 H1 Ro.raw	1.6495E6	4	4	422	439	Oxidation (M); Carbamidomethylation	M11:Oxidation (M):23.10;C14:Carbamidomethylation:1000.00	PEAKS DB
R.IPSGFISYILNR.H	N	109.18	1378.7609	12	2.5	690.3894	2	36.50	38	F38:1780	OB5951 H3A Ro.raw	4.4166E8	20	20	266	277			PEAKS DB
K.HADADNILVIQQGQATVTVANGNNRK.S	N	108.34	2746.4111	26	1.9	916.4794	3	29.61	43	F43:1451	OB5956 H1 Ro.raw	3.8404E6	5	5	229	254			PEAKS DB
R.VLLEENAGGEQEER.G	N	108.33	1571.7427	14	2.4	786.8805	2	26.83	42	F42:1293	OB5955 H1 Ro.raw	2.9858E7	10	10	335	348			PEAKS DB
R.KSFNLDEGHALR.I	N	108.28	1385.7051	12	0.6	462.9092	3	26.14	43	F43:1252	OB5956 H1 Ro.raw	5.3666E6	6	6	254	265			PEAKS DB
K.GSEEEGDITNPINLR.E	N	108.06	1642.7798	15	-0.5	822.3967	2	30.80	42	F42:1519	OB5955 H1 Ro.raw	1.1767E7	3	3	387	401			PEAKS DB
K.AMVIVVVNKGTGNLELVAVRK.E	N	107.96	2209.2981	21	1.1	737.4408	3	33.05	43	F43:1682	OB5956 H1 Ro.raw	1.2362E8	20	20	452	472			PEAKS DB
R.I(+57.02)PSGFISYILNR.H	N	107.77	1435.7823	12	-3.6	718.8959	2	36.92	43	F43:1901	OB5956 H1 Ro.raw	1.7559E7	7	7	266	277	Carbamidomethylation (DHKE, X@N-term)	I1:Carbamidomethylation (DHKE, X@N-term):1000.00	PEAKS PTM
R.I(+57.02)FLAGDKDNVIDQIEK.Q	N	105.70	1873.9785	16	1.7	937.9982	2	32.48	43	F43:1653	OB5956 H1 Ro.raw	1.1722E7	5	5	541	556	Carbamidomethylation (DHKE, X@N-term)	I1:Carbamidomethylation (DHKE, X@N-term):90.91	PEAKS PTM
R.DQSSYLQGFSR.N	N	105.08	1286.5891	11	0.8	644.3024	2	30.81	40	F40:1431	OB5953 H3A Ro.raw	9.5783E7	31	31	308	318			PEAKS DB
R.EGEQEWGTPGSHVR.E	N	104.71	1567.7015	14	2.6	784.8601	2	25.68	42	F42:1206	OB5955 H1 Ro.raw	3.4943E6	6	6	153	166			PEAKS DB
R.V(+57.02)LLEENAGGEQEER.G	N	103.80	1628.7642	14	0.3	815.3896	2	27.08	43	F43:1322	OB5956 H1 Ro.raw	4.0193E5	3	3	335	348	Carbamidomethylation (DHKE, X@N-term)	V1:Carbamidomethylation (DHKE, X@N-term):49.37	PEAKS PTM
K.NPQLQDLDMMLTCVEIK(+14.02).E	N	103.68	2003.9730	17	-1.2	1002.9926	2	38.20	43	F43:1989	OB5956 H1 Ro.raw	5.9168E6	4	4	423	439	Methylation(KR)	K17:Methylation(KR):1000.00	PEAKS PTM
K.SFNLDEGHALR.I	N	103.18	1257.6101	11	-1.7	629.8113	2	28.00	43	F43:1362	OB5956 H1 Ro.raw	1.1584E8	33	31	255	265			PEAKS DB
K.AM(+15.99)VIVVVNKGTGNLELVAVR.K	N	103.14	2097.1980	20	1.6	700.0744	3	33.04	43	F43:1684	OB5956 H1 Ro.raw	2.3148E7	9	9	452	471	Oxidation (M)	M2:Oxidation (M):1000.00	PEAKS DB
K.DLAFPGSGEQVEK.L	N	100.93	1375.6619	13	-1.9	688.8369	2	30.34	43	F43:1508	OB5956 H1 Ro.raw	9.196E7	8	8	560	572			PEAKS DB
K.NPQLQDLDM(+15.99)MLTC(+57.02)VEIKEGALMLPHFNSK.A	N	100.40	3387.6335	29	-0.6	1130.2178	3	37.70	41	F41:1939	OB5954 H1 Ro.raw	8.7706E7	6	6	423	451	Oxidation (M); Carbamidomethylation	M9:Oxidation (M):26.02;C13:Carbamidomethylation:1000.00	PEAKS DB
**total 250 peptides**																			

Peptide	Uniq	-10lgP	Mass	Length	ppm	m/z	z	RT	Fraction	Scan	Source File	Area Roasted in gel	#Feature	#Feature Roasted in gel	Start	End	PTM	AScore	Found By
R.RVLLEENAGGEQEER.G	N	100.35	1727.8438	15	0.2	576.9553	3	25.30	42	F42:1185	OB5955 H1 Ro.raw	8.2964E5	6	6	334	348			PEAKS DB
K.NPQLQDLDMMLTC(+57.02)VEIKEGALMLPHFNSK.A	N	98.85	3371.6387	29	-2.7	843.9147	4	38.02	43	F43:1969	OB5956 H1 Ro.raw	1.5351E8	12	12	423	451	Carbamidomethylation	C13:Carbamidomethylation:1000.00	PEAKS DB
R.EGEPDLSNNFGK.L	Y	98.79	1305.5836	12	-0.8	653.7986	2	28.17	43	F43:1363	OB5956 H1 Ro.raw	1.5024E7	3	3	402	413			PEAKS DB
K.GSEEEGDITNPINLREGEPDLSNNFGK.L	Y	98.63	2930.3530	27	-8.3	977.7835	3	32.31	43	F43:1637	OB5956 H1 Ro.raw	2.5702E6	8	8	387	413			PEAKS DB
K.KNPQLQDLDMMLTC(+57.02)VEIKEGALMLPHFNSK.A	N	97.96	3499.7336	30	-3.9	875.9373	4	36.80	41	F41:1884	OB5954 H1 Ro.raw	6.677E6	4	4	422	451	Carbamidomethylation	C14:Carbamidomethylation:1000.00	PEAKS DB
R.VLLEEN(+.98)AGGEQEER.G	N	94.48	1572.7267	14	2.8	787.3728	2	27.46	43	F43:1340	OB5956 H1 Ro.raw	6.2395E5	3	3	335	348	Deamidation (NQ)	N6:Deamidation (NQ):121.37	PEAKS DB
R.IFLAGDKDNVIDQIEKQAK.D	N	94.35	2144.1477	19	-2.1	715.7217	3	32.42	42	F42:1625	OB5955 H1 Ro.raw	3.3984E6	9	9	541	559			PEAKS DB
R.KSFNLDEGHALRIPSGFISYILNR.H	N	93.78	2746.4553	24	-0.7	687.6206	4	36.29	43	F43:1876	OB5956 H1 Ro.raw	1.1783E6	4	4	254	277			PEAKS DB
F.LAGDKDNVIDQIEK.Q	N	91.83	1556.8046	14	-6.3	779.4047	2	32.21	43	F43:1629	OB5956 H1 Ro.raw	5.4162E6	4	4	543	556			PEAKS DB
R.IPSGFISYILN(-17.03)R.H	N	91.80	1361.7343	12	-4.9	681.8711	2	38.02	43	F43:1980	OB5956 H1 Ro.raw	1.3387E6	3	3	266	277	Ammonia-loss (N)	N11:Ammonia-loss (N):1000.00	PEAKS PTM
K.AMVIVVVNK.G	N	91.04	971.5837	9	-0.5	486.7989	2	30.16	43	F43:1494	OB5956 H1 Ro.raw	7.1361E6	3	3	452	460			PEAKS DB
K.SFNLDEGH(+15.99)ALR.I	N	90.73	1273.6051	11	0.9	637.8104	2	30.16	43	F43:1507	OB5956 H1 Ro.raw	8.7681E5	2	2	255	265	Oxidation (HW)	H8:Oxidation (HW):1000.00	PEAKS PTM
R.NNPFYFPSRR.F	N	89.95	1296.6364	10	-2.5	649.3239	2	28.89	43	F43:1417	OB5956 H1 Ro.raw	1.3445E8	22	22	172	181			PEAKS DB
K.D(+57.02)LAFPGSGEQVEK.L	N	88.60	1432.6833	13	-0.1	717.3489	2	31.08	41	F41:1535	OB5954 H1 Ro.raw	9.7986E5	3	3	560	572	Carbamidomethylation (DHKE, X@N-term)	D1:Carbamidomethylation (DHKE, X@N-term):131.10	PEAKS PTM
K.SFNLDEGH(+57.02)ALR.I	N	88.30	1314.6316	11	-0.1	658.3230	2	28.22	41	F41:1349	OB5954 H1 Ro.raw	2.9996E6	3	3	255	265	Carbamidomethylation (DHKE, X@N-term)	H8:Carbamidomethylation (DHKE, X@N-term):30.36	PEAKS PTM
R.IVQIEAKPNTLVLPK.H	N	88.27	1662.0079	15	0.9	832.0120	2	30.16	43	F43:1505	OB5956 H1 Ro.raw	2.8901E7	31	31	214	228			PEAKS DB
K.EGALMLPHFNSK.A	N	88.21	1342.6703	12	2.5	672.3441	2	30.67	43	F43:1529	OB5956 H1 Ro.raw	1.4087E7	5	5	440	451			PEAKS DB
K.NPQLQDLDMM(-48.00)LTC(+57.02)VEIK.E	N	88.05	1998.9755	17	1.9	1000.4969	2	32.85	43	F43:1670	OB5956 H1 Ro.raw	5.9032E6	3	3	423	439	Carbamidomethylation	M10:Dethiomethyl:14.02;C13:Carbamidomethylation:1000.00	PEAKS PTM
R.NNPFYFPSR.R	N	86.35	1140.5352	9	-0.1	571.2748	2	31.76	43	F43:1593	OB5956 H1 Ro.raw	1.7218E7	11	11	172	180			PEAKS DB

total 250 peptides

Peptide	Uniq	-10lgP	Mass	Length	ppm	m/z	z	RT	Fraction	Scan	Source File	Area Roasted in gel	#Feature	#Feature Roasted in gel	Start	End	PTM	AScore	Found By
K.S(+57.02)FNLDEGHALR.I	N	86.31	1314.6316	11	-0.5	658.3228	2	28.11	42	F42:1359	OB5955 H1 Ro.raw	2.6997E6	4	4	255	265	Carbamidomethylation (DHKE, X@N-term)	S1:Carbamidomethylation (DHKE, X@N-term):91.04	PEAKS PTM
I.PSGFISYILNR.H	N	85.97	1265.6768	11	-4.6	633.8427	2	36.75	43	F43:1894	OB5956 H1 Ro.raw	3.5986E5	2	2	267	277			PEAKS DB
R.SSENNEGVIVK.V	Y	85.30	1174.5830	11	0.1	588.2988	2	23.40	43	F43:1111	OB5956 H1 Ro.raw	5.1333E5	3	3	357	367			PEAKS DB
R.IPSGFISYILNRHDNQNLR.V	N	84.75	2256.1763	19	6.5	753.0709	3	33.61	43	F43:1714	OB5956 H1 Ro.raw	6.1581E7	21	21	266	284			PEAKS DB
R.I(+27.99)FLAGDKDNVIDQIEK.Q	N	84.60	1844.9519	16	-2.1	923.4813	2	36.23	41	F41:1853	OB5954 H1 Ro.raw	1.9485E6	3	3	541	556	Formylation	I1:Formylation:98.75	PEAKS PTM
R.EGEQEWGTPGSHVREETSR.N	N	83.87	2169.9675	19	2.2	724.3314	3	26.51	43	F43:1287	OB5956 H1 Ro.raw	5.5466E5	3	3	153	171			PEAKS DB
K.SFNLDEGH(+15.99)ALRIPSGFISYILNR.H	N	83.49	2634.3555	23	-4.0	879.1223	3	38.76	43	F43:2023	OB5956 H1 Ro.raw	8.8519E5	1	1	255	277	Oxidation (HW)	H8:Oxidation (HW):1000.00	PEAKS PTM
K.KNPQLQDLDMMLTC(+57.02)VEIKEGALM(+15.99)LPHFNSK.A	N	83.11	3515.7285	30	-1.9	879.9377	4	36.03	43	F43:1857	OB5956 H1 Ro.raw	6.6263E6	3	3	422	451	Carbamidomethylation; Oxidation (M)	C14:Carbamidomethylation:1000.00;M23:Oxidation (M):114.34	PEAKS DB
R.NTLEAAFNAEFNEIRR.V	N	82.99	1893.9332	16	-2.6	947.9714	2	34.11	42	F42:1711	OB5955 H1 Ro.raw	3.0959E8	36	35	319	334			PEAKS DB
K.A(+57.02)MVIVVVNKGTGNLELVAVR.K	N	82.14	2138.2246	20	-5.4	1070.1138	2	34.47	42	F42:1739	OB5955 H1 Ro.raw	4.4412E6	3	3	452	471	Carbamidomethylation (DHKE, X@N-term)	A1:Carbamidomethylation (DHKE, X@N-term):207.56	PEAKS PTM
K.DLAFPGSGEQVEKLIK.N	N	80.89	1729.9249	16	-1.6	865.9706	2	33.95	30	F30:1478	OB5926 H3B Ro.raw	3.9305E5	5	4	560	575			PEAKS DB
I.FLAGDKDNVIDQIEK.Q	N	80.64	1703.8729	15	-5.7	852.9388	2	32.22	43	F43:1635	OB5956 H1 Ro.raw	3.1343E5	3	3	542	556			PEAKS DB
K.EGALM(+15.99)LPHFNSK.A	N	80.48	1358.6653	12	1.1	680.3406	2	29.85	43	F43:1485	OB5956 H1 Ro.raw	3.0314E6	5	5	440	451	Oxidation (M)	M5:Oxidation (M):1000.00	PEAKS DB
K.GTGNLELVAVR.K	N	80.39	1127.6299	11	-1.3	564.8215	2	30.21	41	F41:1476	OB5954 H1 Ro.raw	2.4706E7	5	5	461	471			PEAKS DB
R.NTLEAAFN(+.98)AEFNEIRR.V	N	79.02	1894.9172	16	-2.2	948.4638	2	32.35	41	F41:1616	OB5954 H1 Ro.raw	1.3357E6	5	5	319	334	Deamidation (NQ)	N8:Deamidation (NQ):48.12	PEAKS DB
R.VLLEENAGGEQEER(+14.02).G	N	78.92	1585.7583	14	0.7	793.8870	2	27.86	41	F41:1339	OB5954 H1 Ro.raw	1.6341E5	3	3	335	348	Methylation(KR)	R14:Methylation(KR):1000.00	PEAKS PTM
K.GTGNLELVAVRK.E	N	78.87	1255.7249	12	0.7	628.8701	2	27.30	41	F41:1322	OB5954 H1 Ro.raw	2.29E7	10	10	461	472			PEAKS DB
R.QFQNLQNHR.I	N	78.35	1183.5846	9	0.0	592.7996	2	23.81	42	F42:1102	OB5955 H1 Ro.raw	1.5125E5	6	6	205	213			PEAKS DB
K.K(+57.02)GSEEEGDITNPINLR.E	N	78.20	1827.8962	16	-0.5	914.9550	2	28.83	42	F42:1409	OB5955 H1 Ro.raw	1.3158E5	2	2	386	401	Carbamidomethylation (DHKE, X@N-term)	K1:Carbamidomethylation (DHKE, X@N-term):32.46	PEAKS PTM
**total 250 peptides**																			

Peptide	Uniq	-10lgP	Mass	Length	ppm	m/z	z	RT	Fraction	Scan	Source File	Area Roasted in gel	#Feature	#Feature Roasted in gel	Start	End	PTM	AScore	Found By
K.NPQLQDLDMM(+15.99)LTC(+57.02)VEIKEGALMLPHFNSK.A	N	78.01	3387.6335	29	-0.8	1130.2175	3	36.42	41	F41:1862	OB5954 H1 Ro.raw	6.8935E7	6	6	423	451	Oxidation (M); Carbamidomethylation	M10:Oxidation (M): 24.44;C13:Carbamidomethylation:1000.00	PEAKS DB
K.LFEVKPDKKNPQLQDLDMMLTC(+57.02)VEIK.E	N	77.87	3131.6069	26	-4.0	1044.8721	3	34.65	42	F42:1756	OB5955 H1 Ro.raw	5.0788E6	4	4	414	439	Carbamidomethylation	C22:Carbamidomethylation:1000.00	PEAKS DB
K.NPQLQDLDM(+15.99)MLTC(+57.02)VEIKEGALM(+15.99)LPHFNSK.A	N	77.10	3403.6284	29	1.1	851.9153	4	37.51	41	F41:1883	OB5954 H1 Ro.raw	7.3887E6	7	7	423	451	Oxidation (M); Carbamidomethylation	M9:Oxidation (M):27.96;C13:Carbamidomethylation:1000.00;M22:Oxidation (M):71.31	PEAKS DB
R.IFLAGDKDNVIDQIEKQ(+.98)AK.D	N	76.22	2145.1316	19	1.5	716.0522	3	33.08	42	F42:1661	OB5955 H1 Ro.raw	0	0	0	541	559	Deamidation (NQ)	Q17:Deamidation (NQ):48.83	PEAKS DB
R.EETSRN(+.98)NPFYFPSRR.F	N	75.62	1899.8864	15	-0.2	634.3026	3	28.39	41	F41:1381	OB5954 H1 Ro.raw	3.2976E5	2	2	167	181	Deamidation (NQ)	N6:Deamidation (NQ):33.98	PEAKS DB
L.AGDKDNVIDQIEK.Q	N	75.39	1443.7205	13	-3.7	722.8649	2	32.08	42	F42:1607	OB5955 H1 Ro.raw	3.3426E5	1	1	544	556			PEAKS DB
R.Q(-17.03)FQNLQNHR.I	N	75.35	1166.5581	9	-1.2	584.2856	2	28.00	43	F43:1365	OB5956 H1 Ro.raw	4.927E6	4	4	205	213	Pyro-glu from Q	Q1:Pyro-glu from Q: 1000.00	PEAKS PTM
R.VAKISMPVNTPGQFEDFFPASSR.D	N	74.27	2524.2419	23	-2.9	842.4188	3	34.29	42	F42:1735	OB5955 H1 Ro.raw	1.4483E6	3	3	285	307			PEAKS DB
K.NPQLQDLDM(+15.99)M(+15.99)LTC(+57.02)VEIKEGALMLPHFNSK.A	N	73.00	3403.6284	29	-3.9	1135.5457	3	35.83	43	F43:1849	OB5956 H1 Ro.raw	7.8068E6	5	5	423	451	Oxidation (M); Carbamidomethylation	M9:Oxidation (M):54.18;M10:Oxidation (M):63.99;C13:Carbamidomethylation:1000.00	PEAKS DB
K.KNPQLQDLDM(+15.99)MLTC(+57.02)VEIKEGALM(+15.99)LPHFNSK.A	N	72.71	3531.7234	30	-1.9	883.9364	4	35.86	41	F41:1821	OB5954 H1 Ro.raw	7.53E5	1	1	422	451	Carbamidomethylation; Oxidation (M)	M10:Oxidation (M): 17.01;C14:Carbamidomethylation:1000.00;M23:Oxidation (M):85.25	PEAKS DB
R.VLLEENAGGEQEERGQR.R	N	72.44	1912.9238	17	0.6	957.4698	2	25.30	42	F42:1191	OB5955 H1 Ro.raw	3.1353E6	8	8	335	351			PEAKS DB
K.VSKEHVEELTK.H	N	72.42	1297.6877	11	-0.3	649.8510	2	24.21	41	F41:1125	OB5954 H1 Ro.raw	2.2246E5	5	5	368	378			PEAKS DB
K.QAKDLAFPGSGEQVEK.L	N	72.39	1702.8525	16	1.2	568.6255	3	27.08	43	F43:1321	OB5956 H1 Ro.raw	2.8232E5	6	6	557	572			PEAKS DB
K.EGALMLPHFN(+.98)SK.A	N	71.37	1343.6543	12	2.6	672.8362	2	31.66	43	F43:1595	OB5956 H1 Ro.raw	1.0101E5	2	2	440	451	Deamidation (NQ)	N10:Deamidation (NQ):1000.00	PEAKS DB
F.NLDEGHALR.I	N	70.14	1023.5097	9	-2.5	512.7609	2	28.00	43	F43:1370	OB5956 H1 Ro.raw	2.6181E6	3	3	257	265			PEAKS DB
**total 250 peptides**																			

Peptide	Uniq	-10lgP	Mass	Length	ppm	m/z	z	RT	Fraction	Scan	Source File	Area Roasted in gel	#Feature	#Feature Roasted in gel	Start	End	PTM	AScore	Found By
R.N(+.98)TLEAAFNAEFNEIRR.V	N	69.73	1894.9172	16	0.4	948.4662	2	32.39	42	F42:1620	OB5955 H1 Ro.raw	2.6319E6	3	3	319	334	Deamidation (NQ)	N1:Deamidation (NQ):41.52	PEAKS DB
R.EGEPDLSN(+.98)NFGK.L	Y	69.72	1306.5676	12	-0.5	654.2908	2	28.90	43	F43:1436	OB5956 H1 Ro.raw	4.1115E5	3	3	402	413	Deamidation (NQ)	N8:Deamidation (NQ):30.46	PEAKS DB
R.K(+57.02)SFNLDEGHALR.I	N	69.20	1442.7266	12	-0.1	722.3705	2	26.25	42	F42:1250	OB5955 H1 Ro.raw	4.0798E5	6	6	254	265	Carbamidomethylation (DHKE, X@N-term)	K1:Carbamidomethylation (DHKE, X@N-term):76.17	PEAKS PTM
K.LFEVKPDKKNPQLQDLDMM(+15.99)LTC(+57.02)VEIK.E	N	69.17	3147.6018	26	2.2	787.9095	4	33.24	42	F42:1670	OB5955 H1 Ro.raw	2.0797E6	2	2	414	439	Oxidation (M); Carbamidomethylation	M19:Oxidation (M):40.00;C22:Carbamidomethylation:1000.00	PEAKS DB
K.SFNLDE(+57.02)GHALRIPSGFISYILNR.H	N	69.07	2675.3818	23	-1.9	892.7996	3	37.80	43	F43:1964	OB5956 H1 Ro.raw	0	0	0	255	277		E6:Carbamidomethylation (DHKE, X@N-term):0.00	PEAKS PTM
R.IFLAGDKDNVID(+57.02)QIEK.Q	N	69.05	1873.9785	16	-1.3	937.9953	2	32.42	42	F42:1608	OB5955 H1 Ro.raw	8.0339E6	1	1	541	556		D12:Carbamidomethylation (DHKE, X@N-term):15.85	PEAKS PTM
R.EGEQEWGTPGSH(+15.99)VR.E	N	68.71	1583.6964	14	0.8	792.8561	2	27.64	43	F43:1358	OB5956 H1 Ro.raw	1.1836E5	4	4	153	166	Oxidation (HW)	H12:Oxidation (HW):95.51	PEAKS PTM
A.GDKDNVIDQIEK.Q	N	68.14	1372.6833	12	-7.0	687.3441	2	32.22	43	F43:1632	OB5956 H1 Ro.raw	1.1241E6	3	3	545	556			PEAKS DB
R.I(+57.02)VQIEAKPNTLVLPK.H	N	68.01	1719.0294	15	-3.7	860.5188	2	30.87	43	F43:1547	OB5956 H1 Ro.raw	7.3731E5	5	4	214	228	Carbamidomethylation (DHKE, X@N-term)	I1:Carbamidomethylation (DHKE, X@N-term):109.97	PEAKS PTM
R.RVLLEEN(+.98)AGGEQEER.G	N	67.83	1728.8278	15	-0.8	577.2827	3	25.96	42	F42:1233	OB5955 H1 Ro.raw	1.359E4	2	2	334	348	Deamidation (NQ)	N7:Deamidation (NQ):77.53	PEAKS DB
R.DQS(-18.01)SYLQGFSR.N	N	67.70	1268.5785	11	0.2	635.2966	2	30.91	41	F41:1537	OB5954 H1 Ro.raw	6.4401E5	3	3	308	318	Dehydration	S3:Dehydration:40.00	PEAKS PTM
R.RVLLEENAGGEQEERGQR.R	N	67.67	2069.0249	18	1.9	690.6835	3	25.60	41	F41:1205	OB5954 H1 Ro.raw	1.8247E6	3	3	334	351			PEAKS DB
K.Q(-17.03)AKDLAFPGSGEQVEK.L	N	67.52	1685.8260	16	0.1	843.9204	2	29.98	43	F43:1503	OB5956 H1 Ro.raw	9.9003E4	3	3	557	572	Pyro-glu from Q	Q1:Pyro-glu from Q:1000.00	PEAKS PTM
K.NPQLQ(+.98)DLDMMLTC(+57.02)VEIK.E	N	67.25	2047.9629	17	1.5	1024.9902	2	44.46	43	F43:2348	OB5956 H1 Ro.raw	0	0	0	423	439	Carbamidomethylation	Q5:Deamidation (NQ):19.68;C13:Carbamidomethylation:1000.00	PEAKS DB
R.WGPAGPR.E	N	66.93	739.3765	7	1.0	370.6959	2	25.22	41	F41:1191	OB5954 H1 Ro.raw	3.1676E5	3	3	115	121			PEAKS DB
K.SFNLD(-18.01)EGHALR.I	N	66.88	1239.5996	11	2.9	620.8089	2	28.79	41	F41:1400	OB5954 H1 Ro.raw	0	0	0	255	265	Dehydration	D5:Dehydration:49.79	PEAKS PTM
K.AM(+15.99)VIVVVNK.G	N	66.42	987.5787	9	1.1	494.7972	2	27.11	41	F41:1283	OB5954 H1 Ro.raw	1.8879E6	3	3	452	460	Oxidation (M)	M2:Oxidation (M):1000.00	PEAKS DB
**total 250 peptides**																			

Peptide	Uniq	-10lgP	Mass	Length	ppm	m/z	z	RT	Fraction	Scan	Source File	Area Roasted in gel	#Feature	#Feature Roasted in gel	Start	End	PTM	AScore	Found By
K.KNPQLQDLDMMLTCVEIK(+14.02).E	N	66.06	2132.0679	18	-0.6	1067.0405	2	36.31	41	F41:1858	OB5954 H1 Ro.raw	5.2356E5	2	2	422	439	Methylation(KR)	K18:Methylation(KR):130.57	PEAKS PTM
R.EETSRNNPFYFPSRR.F	N	65.61	1898.9023	15	1.8	633.9759	3	27.75	42	F42:1339	OB5955 H1 Ro.raw	8.693E5	4	4	167	181			PEAKS DB
K.NPQLQDLDMMLTC(+57.02)VEIKEGALM(+15.99)LPHFNSK.A	N	64.76	3387.6335	29	-0.6	847.9152	4	37.63	43	F43:1915	OB5956 H1 Ro.raw	8.7706E7	6	6	423	451	Carbamidomethylation; Oxidation (M)	C13:Carbamidomethylation:1000.00;M22:Oxidation (M):82.05	PEAKS DB
R.IPSGFISY(+125.90)ILNR.H	N	64.64	1504.6575	12	-1.9	753.3346	2	38.39	43	F43:2001	OB5956 H1 Ro.raw	2.0334E5	2	2	266	277	Iodination	Y8:Iodination:1000.00	PEAKS PTM
R.IPSGFISYILN(+.98)RHDNQNLR.V	N	64.64	2257.1604	19	4.7	753.3976	3	34.29	42	F42:1740	OB5955 H1 Ro.raw	6.9901E5	2	2	266	284	Deamidation (NQ)	N11:Deamidation (NQ):36.24	PEAKS DB
A.FPGSGEQVEK.L	N	64.10	1076.5138	10	-3.4	539.2623	2	30.29	42	F42:1495	OB5955 H1 Ro.raw	4.0412E5	3	3	563	572			PEAKS DB
R.SRQFQNLQNHR.I	N	63.98	1426.7178	11	0.3	476.5800	3	24.16	43	F43:1148	OB5956 H1 Ro.raw	5.7314E4	3	3	203	213			PEAKS DB
K.ISMPVNTPGQFEDFFPASSRDQSSYLQGFSR.N	N	63.85	3494.6201	31	-0.9	1165.8796	3	36.01	43	F43:1851	OB5956 H1 Ro.raw	5.8723E6	6	6	288	318			PEAKS DB
K.LFEVKPDKK.N	N	63.79	1102.6385	9	0.6	552.3269	2	24.50	43	F43:1159	OB5956 H1 Ro.raw	1.1348E6	6	6	414	422			PEAKS DB
R.SSEN(+.98)NEGVIVK.V	Y	63.52	1175.5670	11	0.2	588.7909	2	24.46	41	F41:1140	OB5954 H1 Ro.raw	5.8916E4	4	4	357	367	Deamidation (NQ)	N4:Deamidation (NQ):33.98	PEAKS DB
Q.SSYLQGFSR.N	N	63.16	1043.5035	9	1.4	522.7598	2	29.29	41	F41:1438	OB5954 H1 Ro.raw	6.2708E4	3	3	310	318			PEAKS DB
R.QFQNLQN(+.98)HR.I	N	63.09	1184.5686	9	5.4	593.2948	2	23.60	43	F43:1114	OB5956 H1 Ro.raw	8.0412E2	1	1	205	213		N7:Deamidation (NQ):0.00	PEAKS DB
K.EGALMLPH(+14.02)FNSK.A	N	63.06	1356.6860	12	-4.2	679.3475	2	30.85	43	F43:1554	OB5956 H1 Ro.raw	2.9707E5	3	3	440	451	Methylation(others)	H8:Methylation(others):65.81	PEAKS PTM
P.SGFISYILNR.H	N	63.04	1168.6240	10	1.5	585.3202	2	35.08	43	F43:1803	OB5956 H1 Ro.raw	2.9962E5	1	1	268	277			PEAKS DB
N.LDEGHALR.I	N	62.84	909.4668	8	0.0	455.7407	2	27.93	42	F42:1350	OB5955 H1 Ro.raw	1.8672E5	3	3	258	265			PEAKS DB
G.SEEEGDITNPINLREGEPDLSNNFGK.L	Y	62.76	2873.3315	26	-0.4	958.7865	3	32.44	30	F30:1400	OB5926 H3B Ro.raw	1.9904E4	1	1	388	413			PEAKS DB
K.A(+57.02)MVIVVVNKGTGNLELVAVRK.E	N	62.52	2266.3196	21	0.7	1134.1678	2	32.98	42	F42:1660	OB5955 H1 Ro.raw	1.5099E7	6	6	452	472	Carbamidomethylation (DHKE, X@N-term)	A1:Carbamidomethylation (DHKE, X@N-term):139.69	PEAKS PTM
K.HADADNILVIQQGQATVTVANGN(+.98)NRK.S	N	62.31	2747.3950	26	-0.1	916.8055	3	30.70	41	F41:1517	OB5954 H1 Ro.raw	8.5956E3	1	1	229	254		N23:Deamidation (NQ):9.40	PEAKS DB
K.SFN(+15.99)LDEGHALR.I	N	62.00	1273.6051	11	0.0	637.8098	2	27.46	43	F43:1330	OB5956 H1 Ro.raw	9.622E5	3	3	255	265	Oxidation or Hydroxylation	N3:Oxidation or Hydroxylation:55.92	PEAKS PTM
R.IP(+15.99)SGFISYILNR.H	N	61.98	1394.7557	12	-0.8	698.3846	2	37.47	43	F43:1932	OB5956 H1 Ro.raw	3.2208E6	4	4	266	277	Oxidation or Hydroxylation	P2:Oxidation or Hydroxylation:39.25	PEAKS PTM

total 250 peptides

Peptide	Uniq	-10lgP	Mass	Length	ppm	m/z	z	RT	Fraction	Scan	Source File	Area Roasted in gel	#Feature	#Feature Roasted in gel	Start	End	PTM	AScore	Found By
K.NPQLQDLDMM(+15.99)LTC(+57.02)VEIKEGALM(+15.99)LPHFNSK.A	N	61.19	3403.6284	29	0.3	851.9147	4	35.86	41	F41:1801	OB5954 H1 Ro.raw	3.4294E6	4	4	423	451	Oxidation (M); Carbamidomethylation	M10:Oxidation (M):21.94;C13:Carbamidomethylation:1000.00;M22:Oxidation (M):61.50	PEAKS DB
K.G(+57.02)TGNLELVAVRK.E	N	60.73	1312.7462	12	-0.4	657.3801	2	27.30	41	F41:1303	OB5954 H1 Ro.raw	3.6978E5	2	2	461	472	Carbamidomethylation (DHKE, X@N-term)	G1:Carbamidomethylation (DHKE, X@N-term):55.46	PEAKS PTM
K.NPQLQDLDM(+15.99)M(+15.99)LTC(+57.02)VEIKEGALM(+15.99)LPHFNSK.A	N	60.17	3419.6233	29	-0.9	855.9124	4	34.83	42	F42:1766	OB5955 H1 Ro.raw	1.2322E6	3	3	423	451	Oxidation (M); Carbamidomethylation	M9:Oxidation (M):1000.00;M10:Oxidation (M):1000.00;C13:Carbamidomethylation:1000.00;M22:Oxidation (M):1000.00	PEAKS DB
R.IPSGFISY(+15.99)ILNR.H	N	60.13	1394.7557	12	-0.7	698.3846	2	35.11	41	F41:1859	OB5954 H1 Ro.raw	3.4529E6	5	5	266	277	Oxidation or Hydroxylation	Y8:Oxidation or Hydroxylation:67.76	PEAKS PTM
L.DEGHALR.I	N	59.61	796.3828	7	2.5	399.1996	2	27.93	42	F42:1351	OB5955 H1 Ro.raw	9.3538E4	3	3	259	265			PEAKS DB
K.LFEVKPDKKNPQLQ(+.98)DLDMM(+15.99)LTC(+57.02)VEIK.E	N	59.38	3148.5857	26	7.4	788.1595	4	33.17	42	F42:1670	OB5955 H1 Ro.raw	3.9804E5	1	1	414	439	Oxidation (M); Carbamidomethylation	Q14:Deamidation (NQ):0.00;M19:Oxidation (M):23.10;C22:Carbamidomethylation:1000.00	PEAKS DB
R.VLLEEN(-17.03)AGGEQEER.G	N	59.22	1554.7162	14	1.2	778.3663	2	27.73	42	F42:1335	OB5955 H1 Ro.raw	0	0	0	335	348	Ammonia-loss (N)	N6:Ammonia-loss (N):1000.00	PEAKS PTM
K.SFNLDEGHALRIPSGFISYILNRHDNQNLR.V	N	59.07	3495.7759	30	1.5	700.1635	5	39.97	40	F40:1987	OB5953 H3A Ro.raw	2.0405E7	15	15	255	284			PEAKS DB
K.GSE(+57.02)EEGDITNPINLR.E	N	58.95	1699.8013	15	-3.0	850.9054	2	30.85	43	F43:1556	OB5956 H1 Ro.raw	4.2816E4	1	1	387	401		E3:Carbamidomethylation (DHKE, X@N-term):0.00	PEAKS PTM
K.SFNLDEGHALR(+31.99)IPSGFISYILNR.H	N	58.88	2650.3503	23	0.1	884.4575	3	39.14	42	F42:2021	OB5955 H1 Ro.raw	5.6519E5	1	1	255	277	Dihydroxy	R11:Dihydroxy:46.57	PEAKS PTM
K.LFEVKPDK.K	N	58.71	974.5436	8	1.7	488.2799	2	25.30	42	F42:1183	OB5955 H1 Ro.raw	1.7315E6	6	6	414	421			PEAKS DB
K.SFNLDE(+57.02)GHALR.I	N	58.62	1314.6316	11	0.3	658.3232	2	27.83	43	F43:1458	OB5956 H1 Ro.raw	2.3371E6	2	2	255	265	Carbamidomethylation (DHKE, X@N-term)	E6:Carbamidomethylation (DHKE, X@N-term):27.25	PEAKS PTM
R.N(+57.02)NPFYFPSRR.F	N	58.56	1353.6577	10	1.1	677.8369	2	29.74	42	F42:1453	OB5955 H1 Ro.raw	9.24E5	3	3	172	181	Carbamidomethylation (DHKE, X@N-term)	N1:Carbamidomethylation (DHKE, X@N	PEAKS PTM
**total 250 peptides**																			

Peptide	Uniq	-10lgP	Mass	Length	ppm	m/z	z	RT	Fraction	Scan	Source File	Area Roasted in gel	#Feature	#Feature Roasted in gel	Start	End	PTM	AScore	Found By
																		-term):1000.00	
R.E(-18.01)GE(+57.02)QEWGTPGSHVR.E	N	58.23	1606.7124	14	0.2	804.3636	2	27.56	42	F42:1323	OB5955 H1 Ro.raw	2.2302E5	3	3	153	166	Pyro-glu from E; Carbamidomethylation (DHKE, X@N-term)	E1:Pyro-glu from E:1000.00;E3:Carbamidomethylation (DHKE, X@N-term):60.92	PEAKS PTM
R.IFLAGDKDNVIDQ(+.98)IEKQAK.D	N	58.20	2145.1316	19	1.4	716.0521	3	33.04	43	F43:1686	OB5956 H1 Ro.raw	1.2153E5	1	1	541	559	Deamidation (NQ)	Q13:Deamidation (NQ):25.18	PEAKS DB
R.VAKISM(+15.99)PVNTPGQFEDFFPASSR.D	N	57.67	2540.2368	23	2.1	847.7546	3	32.98	42	F42:1665	OB5955 H1 Ro.raw	1.5427E5	1	1	285	307	Oxidation (M)	M6:Oxidation (M):1000.00	PEAKS DB
K.G(+57.02)TGNLELVAVR.K	N	57.51	1184.6514	11	-2.7	593.3314	2	30.10	42	F42:1486	OB5955 H1 Ro.raw	1.7196E5	2	2	461	471		G1:Carbamidomethylation (DHKE, X@N-term):19.78	PEAKS PTM
R.IFLAGD(+57.02)KDNVIDQIEK.Q	N	57.43	1873.9785	16	1.7	625.6678	3	32.52	41	F41:1637	OB5954 H1 Ro.raw	2.7943E5	3	3	541	556		D6:Carbamidomethylation (DHKE, X@N-term):15.57	PEAKS PTM
R.IFLAGDK(+15.99)DNVIDQIEK.Q	N	57.39	1832.9519	16	-1.9	917.4814	2	31.99	41	F41:1600	OB5954 H1 Ro.raw	7.5922E5	2	2	541	556		K7:Oxidation or Hydroxylation:17.01	PEAKS PTM
N.PFYFPSRR.F	N	57.34	1068.5504	8	1.1	535.2831	2	28.95	41	F41:1414	OB5954 H1 Ro.raw	4.6082E6	5	5	174	181			PEAKS DB
R.K(+27.99)SFNLDEGHALR.I	N	57.29	1413.7001	12	1.4	707.8583	2	28.46	42	F42:1387	OB5955 H1 Ro.raw	2.9777E4	1	1	254	265	Formylation	K1:Formylation:1000.00	PEAKS PTM
K.SVSKKGSEEEGDITNPINLR.E	N	57.26	2172.1023	20	0.9	725.0420	3	27.48	41	F41:1325	OB5954 H1 Ro.raw	1.6504E5	2	2	382	401			PEAKS DB
K.LFEVKPDKKNPQLQDLDM(+15.99)MLTC(+57.02)VEIK.E	N	57.09	3147.6018	26	-1.9	787.9062	4	34.20	41	F41:1734	OB5954 H1 Ro.raw	2.4894E6	4	4	414	439	Oxidation (M); Carbamidomethylation	M18:Oxidation (M):33.98;C22:Carbamidomethylation:1000.00	PEAKS DB
K.IRPEGREGEQEWGTPGSHVR.E	N	56.62	2276.1045	20	0.5	570.0337	4	27.56	42	F42:1329	OB5955 H1 Ro.raw	8.9716E4	2	2	147	166			PEAKS DB
K.SFNLDEGHALR(+31.99).I	N	55.96	1289.6000	11	-1.4	645.8063	2	30.41	43	F43:1520	OB5956 H1 Ro.raw	0	0	0	255	265	Dihydroxy	R11:Dihydroxy:119.59	PEAKS PTM
K.ISMPVN(+.98)TPGQFEDFFPASSR.D	N	55.55	2227.0254	20	6.6	1114.5273	2	36.77	41	F41:1885	OB5954 H1 Ro.raw	1.01E5	2	2	288	307	Deamidation (NQ)	N6:Deamidation (NQ):42.68	PEAKS DB
R.NTLEAAFNAEFN(+.98)EIRR.V	N	54.77	1894.9172	16	1.8	948.4676	2	33.04	43	F43:1685	OB5956 H1 Ro.raw	2.2573E6	5	5	319	334	Deamidation (NQ)	N12:Deamidation (NQ):39.02	PEAKS DB
K.S(+57.02)FNLDEGHALRIPSGFISYILNR.H	N	54.19	2675.3818	23	2.3	892.8033	3	39.35	38	F38:1901	OB5951 H3A Ro.raw	6.7035E5	1	1	255	277	Carbamidomethylation (DHKE, X@N-term)	S1:Carbamidomethylation (DHKE, X@N-term):63.97	PEAKS PTM
R.IFLAGDKDNVIDQIEK(+57.02)QAK.D	N	54.07	2201.1692	19	0.3	734.7306	3	32.48	43	F43:1657	OB5956 H1 Ro.raw	1.2122E6	3	3	541	559		K16:Carbamidomethylation (DHKE, X	PEAKS PTM

total 250 peptides

Peptide	Uniq	-10lgP	Mass	Length	ppm	m/z	z	RT	Fraction	Scan	Source File	Area Roasted in gel	#Feature	#Feature Roasted in gel	Start	End	PTM	AScore	Found By
																		@N-term):11.12	
R.I(+27.99)PSGFISYILNR.H	N	53.77	1406.7557	12	0.7	704.3856	2	42.27	42	F42:2199	OB5955 H1 Ro.raw	0	0	0	266	277	Formylation	I1:Formylation:1000.00	PEAKS PTM
R.IPSGFISYILNRHDN(+.98)QNLR.V	N	53.60	2257.1604	19	-0.7	753.3936	3	34.26	41	F41:1732	OB5954 H1 Ro.raw	0	0	0	266	284		N15:Deamidation (NQ):0.00	PEAKS DB
K.HADADNILVIQQGQATVTVANGNNR.K	N	53.13	2618.3162	25	0.5	873.7798	3	30.34	38	F38:1426	OB5951 H3A Ro.raw	1.8654E3	1	1	229	253			PEAKS DB
R.IVQIEAK(+57.02)PNTLVLPK.H	N	53.06	1719.0294	15	-1.6	860.5206	2	30.29	43	F43:1512	OB5956 H1 Ro.raw	2.587E5	1	1	214	228	Carbamidomethylation (DHKE, X@N-term)	K7:Carbamidomethylation (DHKE, X@N-term):25.69	PEAKS PTM
K.AM(+15.99)VIVVVNKGTGNLELVAVRK.E	N	52.95	2225.2930	21	-1.3	742.7706	3	32.42	42	F42:1596	OB5955 H1 Ro.raw	1.4247E6	3	3	452	472	Oxidation (M)	M2:Oxidation (M):1000.00	PEAKS DB
R.IVQIEAKPN.T	N	52.62	1010.5760	9	-0.3	506.2951	2	25.40	42	F42:1199	OB5955 H1 Ro.raw	0	0	0	214	222			PEAKS DB
K.KNPQLQ(+.98)DLDMMLTC(+57.02)VEIKEGALMLPHFNSK.A	N	51.97	3500.7175	30	3.4	876.1896	4	36.93	43	F43:1903	OB5956 H1 Ro.raw	9.2646E6	2	2	422	451	Carbamidomethylation	Q6:Deamidation (NQ):14.04;C14:Carbamidomethylation:1000.00	PEAKS DB
R.E(-18.01)GEQEWGTPGSHVR.E	N	51.90	1549.6909	14	0.7	775.8533	2	27.75	42	F42:1342	OB5955 H1 Ro.raw	3.4987E5	3	3	153	166	Pyro-glu from E	E1:Pyro-glu from E:1000.00	PEAKS PTM
R.IFLAGDKDNVID.Q	N	51.22	1318.6769	12	-0.9	660.3451	2	31.76	43	F43:1606	OB5956 H1 Ro.raw	1.1173E5	3	3	541	552			PEAKS DB
K.ISM(+15.99)PVNTPGQFEDFFPASSRDQSSYLQGFSR.N	N	50.25	3510.6150	31	0.3	1171.2126	3	35.35	36	F36:1707	OB5948 H3B Ro.raw	2.4799E6	5	5	288	318	Oxidation (M)	M3:Oxidation (M):1000.00	PEAKS DB
K.NPQLQDLDMM(+15.99)LTC(+57.02)VEIKEGALMLPHFN(+.98)SK.A	N	49.99	3388.6174	29	6.1	848.1668	4	36.39	43	F43:1880	OB5956 H1 Ro.raw	1.7975E6	1	1	423	451	Oxidation (M); Carbamidomethylation	M10:Oxidation (M):27.96;C13:Carbamidomethylation:1000.00;N27:Deamidation (NQ):18.27	PEAKS DB
R.N(-17.03)NPFYFPSRR.F	N	49.99	1279.6097	10	1.1	427.5443	3	28.89	43	F43:1434	OB5956 H1 Ro.raw	5.674E5	3	3	172	181		N1:Ammonia-loss (N):9.34	PEAKS PTM
T.GNLELVAVRK.E	N	49.53	1097.6556	10	0.5	549.8354	2	27.00	42	F42:1296	OB5955 H1 Ro.raw	3.4872E5	3	3	463	472			PEAKS DB
R.VAKIS(+77.99)MPVNTPGQFEDFFPASSR.D	N	49.16	2602.2290	23	3.0	1302.1257	2	38.06	42	F42:1958	OB5955 H1 Ro.raw	0	0	0	285	307	Methylphosphonylation	S5:Methylphosphonylation:56.99	PEAKS PTM
F.PGSGEQVEK.L	N	49.06	929.4454	9	-3.7	465.7282	2	30.34	43	F43:1524	OB5956 H1 Ro.raw	5.2705E4	1	1	564	572			PEAKS DB
K.AMVIVVVNK(+57.02)GTGNLELVAVRK.E	N	48.92	2266.3196	21	1.3	756.4481	3	33.23	43	F43:1673	OB5956 H1 Ro.raw	3.1193E6	1	1	452	472		K9:Carbamidomethylation (DHKE, X@N-term):8.78	PEAKS PTM
R.EEDWRQPR.E	N	48.87	1114.5155	8	1.8	558.2661	2	23.91	41	F41:1112	OB5954 H1 Ro.raw	1.6791E3	1	1	126	133			PEAKS DB
**total 250 peptides**																			

Peptide	Uniq	-10lgP	Mass	Length	ppm	m/z	z	RT	Fraction	Scan	Source File	Area Roasted in gel	#Feature	#Feature Roasted in gel	Start	End	PTM	AScore	Found By
K.PNTLVLPK.H	N	48.84	880.5382	8	-2.9	441.2751	2	28.00	43	F43:1374	OB5956 H1 Ro.raw	1.9234E5	2	2	221	228			PEAKS DB
L.R(+27.99)IPSGFISYILNR.H	N	48.25	1562.8568	13	-2.0	782.4341	2	37.84	43	F43:1976	OB5956 H1 Ro.raw	1.5108E5	1	1	265	277	Formylation	R1:Formylation:1000.00	PEAKS PTM
R.EGEQEW(+15.99)GTPGSHVR.E	N	47.73	1583.6964	14	-0.6	792.8550	2	25.22	41	F41:1196	OB5954 H1 Ro.raw	2.0714E4	1	1	153	166	Oxidation (HW)	W6:Oxidation (HW):72.73	PEAKS PTM
V.QIEAKPNTLVLPK.H	N	47.49	1449.8555	13	-3.8	725.9323	2	30.34	43	F43:1513	OB5956 H1 Ro.raw	4.7367E4	1	1	216	228			PEAKS DB
K.GSEEEGDITNPINLREGEPDLSN(+.98)N(+.98)FGK.L	Y	47.23	2932.3210	27	4.0	978.4515	3	32.30	43	F43:1637	OB5956 H1 Ro.raw	3.6476E5	1	1	387	413	Deamidation (NQ)	N23:Deamidation (NQ):55.39;N24:Deamidation (NQ):48.34	PEAKS DB
R.N(+.98)TLEAAFNAEFNEIR.R	N	46.63	1738.8162	15	9.2	870.4233	2	44.36	42	F42:2303	OB5955 H1 Ro.raw	5.4416E3	1	1	319	333	Deamidation (NQ)	N1:Deamidation (NQ):39.47	PEAKS DB
R.VLLE(+21.98)ENAGGEQEER.G	N	46.37	1593.7246	14	-1.1	797.8687	2	26.71	43	F43:1300	OB5956 H1 Ro.raw	4.0134E5	3	3	335	348	Sodium adduct	E4:Sodium adduct:40.00	PEAKS PTM
N.PFYFPSR.R	N	46.32	912.4493	7	-0.1	457.2319	2	31.70	42	F42:1584	OB5955 H1 Ro.raw	2.4653E5	4	4	174	180			PEAKS DB
K.GTGNLELVAVRKEQQQR.G	N	46.31	1925.0442	17	1.4	642.6896	3	26.67	43	F43:1294	OB5956 H1 Ro.raw	2.1018E4	1	1	461	477			PEAKS DB
K.NPQLQDLD(+14.02)MMLTC(+57.02)VEIK.E	N	45.99	2060.9944	17	0.2	1031.5046	2	37.33	41	F41:1934	OB5954 H1 Ro.raw	2.7407E4	1	1	423	439	Methylation(others); Carbamidomethylation	D8:Methylation(others):42.68; C13:Carbamidomethylation:1000.00	PEAKS PTM
K.VSKEH(+15.99)VEELTK.H	N	45.89	1313.6826	11	0.8	657.8491	2	24.35	43	F43:1164	OB5956 H1 Ro.raw	1.7904E4	3	3	368	378	Oxidation (HW)	H5:Oxidation (HW):1000.00	PEAKS PTM
R.SSENNEGVIVKVSKEHVEELTK.H	Y	45.89	2454.2603	22	1.5	819.0952	3	30.81	39	F39:1445	OB5952 H3A Ro.raw	1.1749E4	1	1	357	378			PEAKS DB
K.GTGNLE(+57.02)LVAVRK.E	N	45.75	1312.7462	12	-0.8	657.3799	2	27.17	42	F42:1295	OB5955 H1 Ro.raw	1.9882E5	1	1	461	472		E6:Carbamidomethylation (DHKE, X@N-term):0.00	PEAKS PTM
K.A(+43.01)MVIVVVNKGTGNLELVAVR.K	N	45.64	2124.2090	20	0.7	1063.1125	2	33.65	41	F41:1688	OB5954 H1 Ro.raw	8.0739E4	1	1	452	471	Carbamylation	A1:Carbamylation:161.20	PEAKS PTM
R.VLLEEN(+.98)AGGEQEERGQR.R	N	45.60	1913.9078	17	-1.6	638.9755	3	25.99	41	F41:1234	OB5954 H1 Ro.raw	0	0	0	335	351	Deamidation (NQ)	N6:Deamidation (NQ):28.79	PEAKS DB
K.KNPQLQDLDMM(+15.99)LTC(+57.02)VEIKEGALM(+15.99)LPHFNSK.A	N	45.45	3531.7234	30	-1.1	883.9372	4	35.80	42	F42:1822	OB5955 H1 Ro.raw	0	0	0	422	451	Carbamidomethylation; Oxidation (M)	M11:Oxidation (M):0.00;C14:Carbamidomethylation:1000.00;M23:Oxidation (M):45.87	PEAKS DB
K.LFEVKPDKKNPQLQDLDM(+15.99)M(+15.99)LTC(+57.02)VEIK.E	N	45.43	3163.5967	26	-2.8	791.9042	4	32.90	41	F41:1650	OB5954 H1 Ro.raw	2.0535E6	3	3	414	439	Oxidation (M); Carbamidomethylation	M18:Oxidation (M):1000.00; M19:Oxidation (M):1000.00; C22:Carb	PEAKS DB

total 250 peptides

Peptide	Uniq	-10lgP	Mass	Length	ppm	m/z	z	RT	Fraction	Scan	Source File	Area Roasted in gel	#Feature	#Feature Roasted in gel	Start	End	PTM	AScore	Found By
																		amidomethylation:1000.00	
R.KSFNLDEGH(+15.99)ALR.I	N	45.40	1401.7001	12	3.9	701.8600	2	27.64	43	F43:1349	OB5956 H1 Ro.raw	6.6479E4	2	2	254	265	Oxidation (HW)	H9:Oxidation (HW): 1000.00	PEAKS PTM
K.KNPQLQDLDM(+15.99)MLTC(+57.02)VEIKEGALMLPHFNSK.A	N	44.60	3515.7285	30	-1.9	879.9377	4	35.30	41	F41:1798	OB5954 H1 Ro.raw	6.4162E5	1	1	422	451	Carbamidomethylation	M10:Oxidation (M): 14.02;C14:Carbamidomethylation:1000.00	PEAKS DB
R.DQSSYLQGFSR(+15.99).N	N	44.15	1302.5840	11	2.5	652.3009	2	30.79	43	F43:1544	OB5956 H1 Ro.raw	1.4308E5	3	3	308	318	Oxidation or Hydroxylation	R11:Oxidation or Hydroxylation:42.88	PEAKS PTM
R.Q(+.98)FQNLQNHR.I	N	43.70	1184.5686	9	4.6	593.2943	2	23.68	42	F42:1098	OB5955 H1 Ro.raw	0	0	0	205	213	Deamidation (NQ)	Q1:Deamidation (NQ):39.25	PEAKS DB
K.I(+57.02)SMPVNTPGQFEDFFPASSR.D	N	42.37	2283.0630	20	-0.7	1142.5380	2	36.01	43	F43:1860	OB5956 H1 Ro.raw	1.8515E5	1	1	288	307	Carbamidomethylation (DHKE, X@N-term)	I1:Carbamidomethylation (DHKE, X@N-term):50.99	PEAKS PTM
K.ISM(-48.00)PVNTPGQFEDFFPASSR.D	N	42.09	2178.0381	20	2.8	727.0220	3	32.48	43	F43:1652	OB5956 H1 Ro.raw	5.8583E6	3	3	288	307	Dethiomethyl	M3:Dethiomethyl:1000.00	PEAKS PTM
K.NPQLQDLDMM(+15.99)LTC(+57.02)VEIKEGALM(+15.99)LPHFN(+.98)SK.A	N	41.25	3404.6125	29	-0.4	1135.8777	3	36.69	42	F42:1878	OB5955 H1 Ro.raw	3.8423E5	1	1	423	451	Carbamidomethylation; Oxidation (M); Deamidation (NQ)	M10:Oxidation (M): 0.00;C13:Carbamidomethylation:1000.00;M22:Oxidation (M):39.23;N27:Deamidation (NQ):69.52	PEAKS DB
K.DLAFP(+15.99)GSGEQVEK.L	N	41.14	1391.6569	13	0.5	696.8361	2	29.36	42	F42:1432	OB5955 H1 Ro.raw	7.4528E5	3	3	560	572	Oxidation or Hydroxylation	P5:Oxidation or Hydroxylation:67.76	PEAKS PTM
K.AMVIVVVN(+.98)KGTGNLELVAVRK.E	N	41.03	2210.2820	21	1.9	1106.1504	2	34.35	42	F42:1720	OB5955 H1 Ro.raw	4.5718E5	1	1	452	472	Deamidation (NQ)	N8:Deamidation (NQ):28.79	PEAKS DB
K.EGALM(-48.00)LPHFNSK.A	N	40.53	1294.6670	12	0.5	432.5632	3	28.76	41	F41:1389	OB5954 H1 Ro.raw	2.208E6	2	2	440	451	Dethiomethyl	M5:Dethiomethyl:1000.00	PEAKS PTM
R.IVQ(+.98)IEAKPNTLVLPK.H	N	40.35	1662.9919	15	8.1	832.5100	2	30.29	42	F42:1514	OB5955 H1 Ro.raw	3.5627E6	1	1	214	228	Deamidation (NQ)	Q3:Deamidation (NQ):22.65	PEAKS DB
R.VLLEENAGGEQEERGQRR.W	N	40.17	2069.0249	18	1.0	518.2640	4	25.49	42	F42:1205	OB5955 H1 Ro.raw	2.0305E5	2	2	335	352			PEAKS DB
R.VLLEENAGGEQEER(+15.99).G	N	39.98	1587.7375	14	1.8	794.8775	2	26.70	41	F41:1275	OB5954 H1 Ro.raw	0	0	0	335	348	Oxidation or Hydroxylation	R14:Oxidation or Hydroxylation:20.88	PEAKS PTM
R.N(+27.99)TLEAAFNAEFNEIRR.V	N	39.68	1921.9282	16	-1.7	961.9697	2	37.51	41	F41:1908	OB5954 H1 Ro.raw	3.7767E4	1	1	319	334	Formylation	N1:Formylation:1000.00	PEAKS PTM
K.E(-18.01)GALM(+15.99)LPHFNSK.A	N	39.64	1340.6547	12	0.2	447.8923	3	30.21	41	F41:1479	OB5954 H1 Ro.raw	7.81E5	1	1	440	451	Pyro-glu from E; Oxidation (M)	E1:Pyro-glu from E: 1000.00; M5:Oxidation (M):1000.00	PEAKS PTM

total 250 peptides

Peptide	Uniq	-10lgP	Mass	Length	ppm	m/z	z	RT	Fraction	Scan	Source File	Area Roasted in gel	#Feature	#Feature Roasted in gel	Start	End	PTM	AScore	Found By
R.VLLEE(+53.92)NAGGEQEER.G	N	39.58	1625.6620	14	-1.0	542.8940	3	26.76	42	F42:1278	OB5955 H1 Ro.raw	0	0	0	335	348	Replacement of 2 protons by iron	E5:Replacement of 2 protons by iron:20.92	PEAKS PTM
K.HADADNILVIQQGQ(+.98)ATVTVANGNNRK.S	N	39.33	2747.3950	26	2.6	916.8103	3	30.46	30	F30:1280	OB5926 H3B Ro.raw	0	0	0	229	254		Q14:Deamidation (NQ):5.07	PEAKS DB
K.NPQLQ(+.98)DLDMM(+15.99)LTC(+57.02)VEIKEGALM(+15.99)LPHFNSK.A	N	39.15	3404.6125	29	-1.8	852.1589	4	38.22	43	F43:1993	OB5956 H1 Ro.raw	7.6266E5	1	1	423	451	Deamidation (NQ); Carbamidomethylation	Q5:Deamidation (NQ):49.79;M10:Oxidation (M):0.00;C13:Carbamidomethylation:1000.00;M22:Oxidation (M):14.60	PEAKS DB
K.N(+.98)PQLQDLDM(+15.99)MLTC(+57.02)VEIKEGALMLPHFNSK.A	N	38.83	3388.6174	29	8.2	1695.3300	2	37.72	41	F41:1941	OB5954 H1 Ro.raw	6.4698E5	1	1	423	451	Carbamidomethylation	N1:Deamidation (NQ):0.00;M9:Oxidation (M):0.00;C13:Carbamidomethylation:1000.00	PEAKS DB
K.D(+15.99)LAFPGSGEQVEK.L	N	38.81	1391.6569	13	0.5	696.8361	2	29.36	42	F42:1368	OB5955 H1 Ro.raw	2.5725E5	1	1	560	572	Oxidation or Hydroxylation	D1:Oxidation or Hydroxylation:77.13	PEAKS PTM
K.NPQ(+.98)LQDLDMMLTC(+57.02)VEIK.E	N	38.70	2047.9629	17	2.9	1024.9917	2	46.45	41	F41:2441	OB5954 H1 Ro.raw	0	0	0	423	439	Carbamidomethylation	Q3:Deamidation (NQ):14.04;C13:Carbamidomethylation:1000.00	PEAKS DB
R.IFLAGD(+15.99)KDNVIDQIEK.Q	N	38.52	1832.9519	16	1.9	917.4850	2	31.89	42	F42:1590	OB5955 H1 Ro.raw	4.6198E5	1	1	541	556		D6:Oxidation or Hydroxylation:12.60	PEAKS PTM
R.IFLAGDKDN(+.98)VIDQIEKQ(+.98)AK.D	N	37.85	2146.1157	19	9.8	716.3862	3	32.98	42	F42:1661	OB5955 H1 Ro.raw	8.6625E4	1	1	541	559		N9:Deamidation (NQ):13.83;Q17:Deamidation (NQ):17.65	PEAKS DB
K.DLAFPGSGE(+53.92)QVEK.L	N	37.31	1429.5812	13	-6.3	715.7933	2	30.34	43	F43:1518	OB5956 H1 Ro.raw	2.6492E5	1	1	560	572	Replacement of 2 protons by iron	E9:Replacement of 2 protons by iron:37.54	PEAKS PTM
K.GT(-18.01)GNLELVAVRK.E	N	36.73	1237.7142	12	2.6	413.5797	3	27.00	42	F42:1287	OB5955 H1 Ro.raw	4.7714E5	1	1	461	472	Dehydration	T2:Dehydration:1000.00	PEAKS PTM
R.NT(-18.01)LEAAFNAEFNEIRR.V	N	36.45	1875.9227	16	0.7	626.3153	3	34.16	43	F43:1739	OB5956 H1 Ro.raw	6.9172E6	2	2	319	334	Dehydration	T2:Dehydration:1000.00	PEAKS PTM
K.EGALM(+15.99)LPHFN(+.98)SK.A	N	36.41	1359.6493	12	2.8	680.8338	2	31.22	41	F41:1550	OB5954 H1 Ro.raw	0	0	0	440	451	Oxidation (M); Deamidation (NQ)	M5:Oxidation (M):1000.00;N10:Deamidation (NQ):1000.00	PEAKS DB
R.GRREEEEDEDEEEEGSNR.E	Y	36.40	2192.8689	18	0.0	731.9636	3	23.90	41	F41:1110	OB5954 H1 Ro.raw	1.2738E4	2	2	478	495			PEAKS DB

total 250 peptides

Peptide	Uniq	-10lgP	Mass	Length	ppm	m/z	z	RT	Fraction	Scan	Source File	Area Roasted in gel	#Feature	#Feature Roasted in gel	Start	End	PTM	AScore	Found By
R.DQSSY(-18.01)LQGFSR.N	N	36.32	1268.5785	11	-0.6	635.2961	2	30.85	43	F43:1579	OB5956 H1 Ro.raw	2.378E5	1	1	308	318		Y5:Dehydration:0.00	PEAKS PTM
R.C(+57.02)LQS(-18.01)C(+57.02)Q(+.98)QEPDDLKQK.A	N	36.24	1858.8190	15	-0.1	930.4167	2	28.47	31	F31:1350	OB5942 H6 Ro.raw	0	0	0	42	56	Carbamidomethylation; Dehydration	C1:Carbamidomethylation:1000.00;S4:Dehydration:39.55;C5:Carbamidomethylation:1000.00;Q6:Deamidation (NQ):8.69	PEAKS PTM
M.LPHFNSK.A	N	36.02	841.4446	7	1.3	421.7301	2	30.63	42	F42:1510	OB5955 H1 Ro.raw	1.4673E6	2	2	445	451			PEAKS DB
E.PDLSNNFGK.L	Y	35.93	990.4771	9	-1.0	496.2453	2	28.09	42	F42:1365	OB5955 H1 Ro.raw	3.4068E4	1	1	405	413			PEAKS DB
K.NPQLQDLDM(-48.00)MLTC(+57.02)VEIK.E	N	35.81	1998.9755	17	6.7	667.3369	3	34.52	43	F43:1773	OB5956 H1 Ro.raw	8.4276E5	1	1	423	439	Carbamidomethylation	M9:Dethiomethyl:11.06;C13:Carbamidomethylation:1000.00	PEAKS PTM
K.SF(+17.99)NLDEGHALR.I	N	34.75	1275.6007	11	8.1	638.8128	2	26.62	42	F42:1267	OB5955 H1 Ro.raw	9.5828E3	1	1	255	265	Fluorination	F2:Fluorination:1000.00	PEAKS PTM
K.KNPQLQ(+.98)DLDMM(+15.99)LTC(+57.02)VEIKEGALMLPHFNSK.A	N	33.72	3516.7124	30	3.3	880.1883	4	35.48	41	F41:1798	OB5954 H1 Ro.raw	7.5658E5	1	1	422	451	Carbamidomethylation	Q6:Deamidation (NQ):18.55;M11:Oxidation (M):14.02;C14:Carbamidomethylation:1000.00	PEAKS DB
D.QSSYLQGFSR.N	N	33.26	1171.5621	10	2.1	586.7896	2	29.36	42	F42:1431	OB5955 H1 Ro.raw	3.3134E4	1	1	309	318			PEAKS DB
R.IVQIEAKP(+15.99)NTLVLPK.H	N	33.15	1678.0028	15	-0.7	840.0081	2	30.35	41	F41:1495	OB5954 H1 Ro.raw	0	0	0	214	228		P8:Oxidation or Hydroxylation:9.34	PEAKS PTM
R.IFLAGDKDNVID(-18.01)QIEK.Q	N	33.09	1798.9464	16	0.7	900.4811	2	31.39	43	F43:1591	OB5956 H1 Ro.raw	6.7846E4	1	1	541	556		D12:Dehydration:13.16	PEAKS PTM
R.EREEDWRQPR.E	N	32.92	1399.6592	10	5.7	700.8409	2	24.05	43	F43:1141	OB5956 H1 Ro.raw	4.1173E4	1	1	124	133			PEAKS DB
D.NVIDQIEK.Q	N	32.64	957.5131	8	1.6	479.7646	2	28.19	42	F42:1367	OB5955 H1 Ro.raw	3.0062E3	1	1	549	556			PEAKS DB
K.AM(-48.00)VIVVVNK.G	N	32.49	923.5804	9	0.0	462.7975	2	25.00	43	F43:1192	OB5956 H1 Ro.raw	6.3177E3	1	1	452	460	Dethiomethyl	M2:Dethiomethyl:1000.00	PEAKS PTM
R.D(-18.01)QSSYLQGFSR.N	N	31.95	1268.5785	11	1.6	635.2975	2	29.92	43	F43:1489	OB5956 H1 Ro.raw	0	0	0	308	318		D1:Dehydration:0.00	PEAKS PTM
R.IFLAGDKD(-18.01)NVIDQIEK.Q	N	31.82	1798.9464	16	-0.9	600.6555	3	32.15	43	F43:1589	OB5956 H1 Ro.raw	3.5634E5	1	1	541	556	Dehydration	D8:Dehydration:30.83	PEAKS PTM
K.D(+53.92)LAFPGSGEQVEK.L	N	31.27	1429.5812	13	-4.3	715.7948	2	30.56	41	F41:1500	OB5954 H1 Ro.raw	3.7719E5	1	1	560	572		D1:Replacement of 2 protons by iron:0.00	PEAKS PTM
**total 250 peptides**																			

Peptide	Uniq	-10lgP	Mass	Length	ppm	m/z	z	RT	Fraction	Scan	Source File	Area Roasted in gel	#Feature	#Feature Roasted in gel	Start	End	PTM	AScore	Found By
K.SFNLDE(+53.92)GHALR.I	N	31.27	1311.5294	11	1.0	438.1842	3	28.04	41	F41:1362	OB5954 H1 Ro.raw	5.8053E4	1	1	255	265		E6:Replacement of 2 protons by iron:16.90	PEAKS PTM
K.KGSEEEGDITNPINLREGEPDLSN(+.98)NFGK.L	Y	31.18	3059.4319	28	2.8	765.8674	4	31.36	36	F36:1485	OB5948 H3B Ro.raw	0	0	0	386	413		N24:Deamidation (NQ):0.00	PEAKS DB
K.AM(+15.99)VIVVVNK(+57.02)GTGNLELVAVR.K	N	30.97	2154.2195	20	2.8	1078.1200	2	32.98	42	F42:1664	OB5955 H1 Ro.raw	2.0029E5	1	1	452	471	Oxidation (M)	M2:Oxidation (M):1000.00;K9:Carbamidomethylation (DHKE, X@N-term):0.00	PEAKS PTM
K.KNPQLQDLDMM(+15.99)LTC(+57.02)VEIKEGALMLPHFNSK.A	N	30.83	3515.7285	30	1.3	879.9406	4	35.25	42	F42:1790	OB5955 H1 Ro.raw	0	0	0	422	451	Carbamidomethylation	M11:Oxidation (M):12.28;C14:Carbamidomethylation:1000.00	PEAKS DB
K.KGSEEEGDITNPINLREGEPDLSNN(+.98)FGK.L	Y	30.79	3059.4319	28	8.8	765.8720	4	31.05	38	F38:1466	OB5951 H3A Ro.raw	0	0	0	386	413		N25:Deamidation (NQ):0.00	PEAKS DB
R.DQSSY(+15.99)LQGFSR.N	N	30.72	1302.5840	11	1.6	652.3003	2	29.24	43	F43:1470	OB5956 H1 Ro.raw	6.7205E4	1	1	308	318	Oxidation or Hydroxylation	Y5:Oxidation or Hydroxylation:26.36	PEAKS PTM
K.SFNLDEGHALRIPSGFISYILNRHDNQN(+.98)LR.V	N	30.43	3496.7600	30	4.9	700.3627	5	37.51	39	F39:1925	OB5952 H3A Ro.raw	2.7478E5	1	1	255	284		N28:Deamidation (NQ):10.11	PEAKS DB
D.KDNVIDQIEK.Q	N	30.42	1200.6350	10	-0.3	601.3246	2	26.31	42	F42:1252	OB5955 H1 Ro.raw	0	0	0	547	556			PEAKS DB
N.TLEAAFNAEFNEIRR.V	N	30.42	1779.8904	15	3.5	890.9556	2	33.23	43	F43:1696	OB5956 H1 Ro.raw	1.5374E5	1	1	320	334			PEAKS DB
R.IPSGFISYILN(+15.99)R.H	N	30.30	1394.7557	12	-2.8	698.3832	2	36.42	41	F41:1875	OB5954 H1 Ro.raw	3.8045E5	1	1	266	277		N11:Oxidation or Hydroxylation:0.00	PEAKS PTM
R.IFLAGDKD(-18.01)NV.I	N	30.00	1072.5553	10	0.7	537.2853	2	32.26	42	F42:1603	OB5955 H1 Ro.raw	2.1452E6	1	1	541	550	Dehydration	D8:Dehydration:45.01	PEAKS PTM
R.NTLEAAFNAEFN(+15.99)EIRR.V	N	29.99	1909.9282	16	0.9	955.9723	2	32.98	42	F42:1662	OB5955 H1 Ro.raw	2.366E6	2	2	319	334		N12:Oxidation or Hydroxylation:0.00	PEAKS PTM
R.IFLAGDKDNVIDQ(+.98)IEK.Q	N	29.73	1817.9410	16	7.7	909.9848	2	63.08	43	F43:3435	OB5956 H1 Ro.raw	3.1606E3	1	1	541	556		Q13:Deamidation (NQ):11.12	PEAKS DB
K.V(+57.02)SKEHVEELTK.H	N	29.60	1354.7092	11	-1.7	678.3607	2	24.05	42	F42:1120	OB5955 H1 Ro.raw	0	0	0	368	378		V1:Carbamidomethylation (DHKE, X@N-term):0.00	PEAKS PTM
I.F(+127.06)LAGDKDNVIDQIEK.Q	N	29.59	1830.9363	15	0.7	916.4761	2	32.18	41	F41:1600	OB5954 H1 Ro.raw	6.5822E4	1	1	542	556	N-Succinimidyl-2-morpholine acetate	F1:N-Succinimidyl-2-morpholine acetate:28.79	PEAKS PTM
K.N(+.98)PQLQ(+.98)DLDMMLTC(+57.02)VEIKEGALM(+15.99)LPHFNSK.A	N	29.50	3389.6016	29	9.6	848.4158	4	37.65	43	F43:2047	OB5956 H1 Ro.raw	6.1505E6	1	1	423	451	Carbamidomethylation	N1:Deamidation (NQ):0.00;Q5:Deamidation (NQ):14.04;C13:Carb	PEAKS DB
**total 250 peptides**																			

Peptide	Uniq	-10lgP	Mass	Length	ppm	m/z	z	RT	Fraction	Scan	Source File	Area Roasted in gel	#Feature	#Feature Roasted in gel	Start	End	PTM	AScore	Found By
																		amidomethylation:1000.00;M22:Oxidation (M):0.00	
H.RIVQIEAKPNTLVLPK.H	N	29.34	1818.1090	16	0.6	607.0439	3	29.55	42	F42:1454	OB5955H1Ro.raw	7.8202E4	1	1	213	228			PEAKS DB
R.NN(+.98)PFYFPSRR.F	N	29.19	1297.6204	10	9.3	649.8235	2	62.71	43	F43:3425	OB5956H1Ro.raw	0	0	0	172	181		N2:Deamidation (NQ):7.21	PEAKS DB
R.NTLEAAFNAEFN(+.98)EIR.R	N	29.07	1738.8162	15	9.2	870.4233	2	44.36	42	F42:2331	OB5955H1Ro.raw	5.4416E3	1	1	319	333		N12:Deamidation (NQ):0.00	PEAKS DB
R.EGE(+21.98)QEWGTPGSHVR.E	N	28.92	1589.6835	14	1.4	795.8501	2	25.83	41	F41:1231	OB5954H1Ro.raw	3.6144E3	1	1	153	166		E3:Sodium adduct:9.74	PEAKS PTM
K.KNPQ(+.98)LQ(+.98)DLDMM(+15.99)LTC(+57.02)VEIKEGALM(+15.99)LPHFNSK.A	N	28.74	3533.6914	30	9.7	884.4387	4	35.77	42	F42:1822	OB5955H1Ro.raw	2.7541E5	1	1	422	451	Carbamidomethylation; Oxidation (M)	Q4:Deamidation (NQ):8.22;Q6:Deamidation (NQ):5.55;M11:Oxidation (M):0.00;C14:Carbamidomethylation:1000.00;M23:Oxidation (M):38.29	PEAKS DB
**total 250 peptides**																			

sp|P43238|ALL12_ARAHY

back to list

| Protein Coverage | Supporting Peptides |

Protein Coverage:

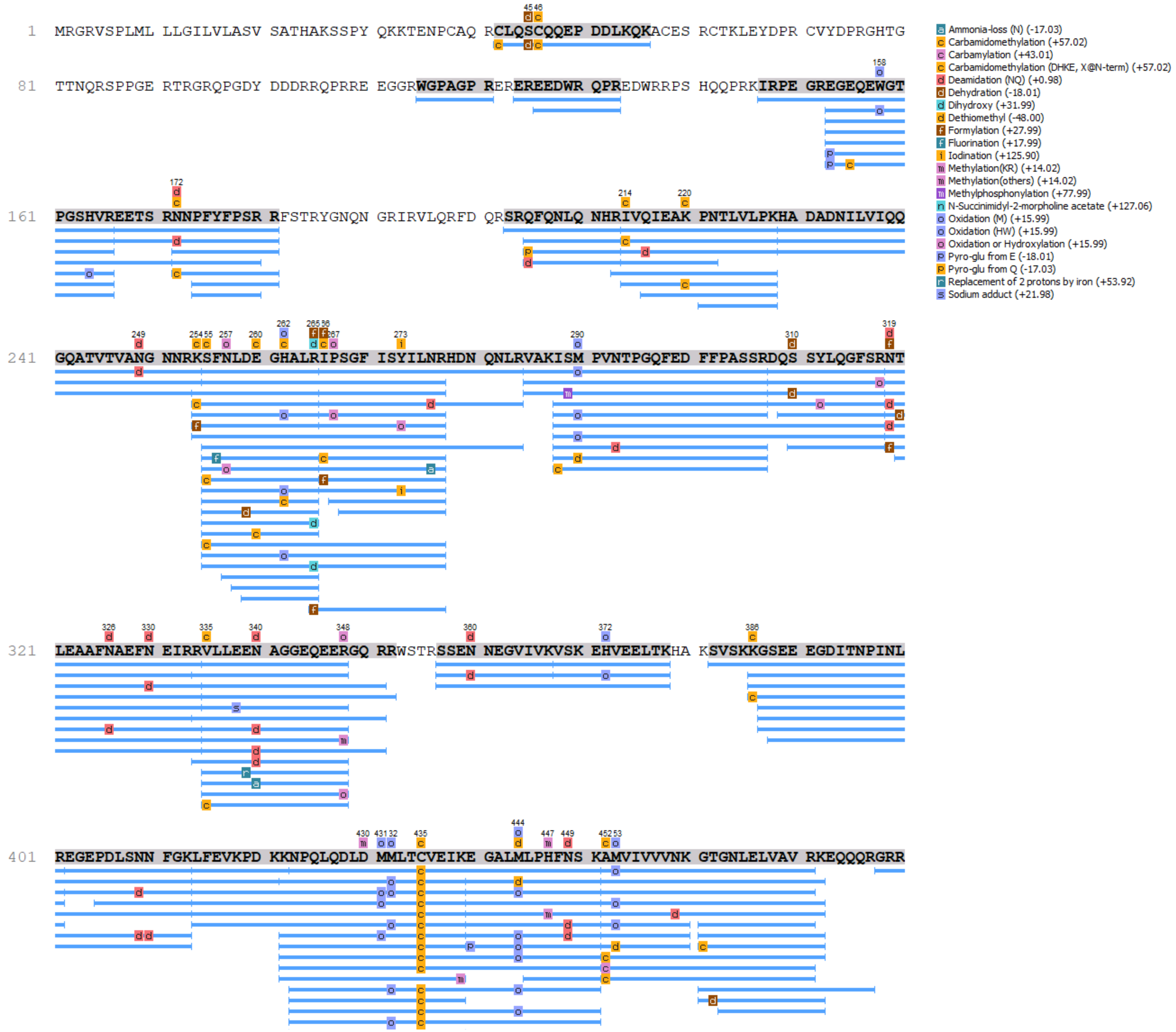
1 MRGRVSPLML LLGILVLASV SATHAKSSPY QKKTENPCAQ RCLQSCQQEP DDLKQKACES RCTKLEYDPR CVYDPRGHTG
81 TTNQRSPPGE RTRGRQPGDY DDDRRQPRRE EGGRWGPAGP REREREEDWR QPREDWRRPS HQQPRKIRPE GREGEQEWGT
161 PGSHVREETS RNNPFYFPSR RFSTRYGNQN GRIRVLQRFD QRSRQFQNLQ NHRIVQIEAK PNTLVLPKHA DADNILVIQQ
241 GQATVTVANG NNRKSFNLDE GHALRIPSGF ISYILNRHDN QNLRVAKISM PVNTPGQFED FFPASSRDQS SYLQGFSRNT
321 LEAAFNAEFN EIRRVLLEEN AGGEQEERGQ RRWSTRSSEN NEGVIVKVSK EHVEELTKHA KSVSKKGSEE EGDITNPINL
401 REGEPDLSNN FGKLFEVKPD KKNPQLQDLD MMLTCVEIKE GALMLPHFNS KAMVIVVVNK GTGNLELVAV RKEQQQRGRR
a Ammonia-loss (N) (-17.03)
c Carbamidomethylation (+57.02)
c Carbamylation (+43.01)
c Carbamidomethylation (DHKE, X@N-term) (+57.02)
d Deamidation (NQ) (+0.98)
d Dehydration (-18.01)
d Dihydroxy (+31.99)
d Dethiomethyl (-48.00)
f Formylation (+27.99)
f Fluorination (+17.99)
i Iodination (+125.90)
m Methylation(KR) (+14.02)
m Methylation(others) (+14.02)
m Methylphosphonylation (+77.99)
n N-Succinimidyl-2-morpholine acetate (+127.06)
o Oxidation (M) (+15.99)
o Oxidation (HW) (+15.99)
o Oxidation or Hydroxylation (+15.99)
p Pyro-glu from E (-18.01)
p Pyro-glu from Q (-17.03)
r Replacement of 2 protons by iron (+53.92)
s Sodium adduct (+21.98)

Supporting Peptides:

Peptide	Uniq	-10lgP	Mass	Length	ppm	m/z	z	RT	Fraction	Scan	Source File	Area Roasted in gel	#Feature	#Feature Roasted in gel	Start	End	PTM	AScore	Found By
K.KNPQLQDLDMMLTC(+57.02)VEIK.E	N	136.41	2175.0737	18	1.4	1088.5457	2	35.26	43	F43:1808	OB5956 H1 Ro.raw	1.4906E7	6	6	422	439	Carbamidomethylation	C14:Carbamidomethylation:1000.00	PEAKS DB
K.KGSEEEG[illegible]	Y	132.44	3058.4478	28	2.9	1020.4928	3	30.98	42	F42:1535	OB5955 H1 Ro.raw	5.2193E6	11	11	386	413			PEAKS DB
R.NTLEAAFNAEFNEIR.R	N	130.93	1737.8322	15	1.8	869.9249	2	35.34	38	F38:1714	OB5951 H3A Ro.raw	1.332E7	16	16	319	333			PEAKS DB
K.NPQLQDLDMMLTC(+57.02)VEIK.E	N	128.40	2046.9788	17	0.2	1024.4968	2	37.11	43	F43:1936	OB5956 H1 Ro.raw	3.4643E7	10	10	423	439	Carbamidomethylation	C13:Carbamidomethylation:1000.00	PEAKS DB
K.NPQLQDLDM(+15.99)MLTC(+57.02)VEIK.E	N	128.16	2062.9736	17	-4.2	1032.4897	2	36.93	43	F43:1892	OB5956 H1 Ro.raw	3.3834E6	3	3	423	439	Oxidation (M); Carbamidomethylation	M9:Oxidation (M):30.46;C13:Carbamidomethylation:1000.00	PEAKS DB
K.NPQLQDLDMM(+15.99)LTC(+57.02)VEIK.E	N	127.13	2062.9736	17	5.0	1032.4993	2	34.71	43	F43:1780	OB5956 H1 Ro.raw	1.0665E7	4	4	423	439	Oxidation (M); Carbamidomethylation	M10:Oxidation (M):30.46;C13:Carbamidomethylation:1000.00	PEAKS DB
K.ISM(+15.99)PVNTPGQFEDFFPASSR.D	N	127.11	2242.0364	20	0.1	1122.0284	2	34.52	30	F30:1521	OB5926 H3B Ro.raw	2.1708E7	14	13	288	307	Oxidation (M)	M3:Oxidation (M):1000.00	PEAKS DB
K.ISMPVNTPGQFEDFFPASSR.D	N	125.26	2226.0415	20	0.7	1114.0288	2	35.83	43	F43:1868	OB5956 H1 Ro.raw	8.8227E7	23	23	288	307			PEAKS DB
K.HADADNILVIQQGQATVTVAN(+.98)GNNRK.S	N	123.10	2747.3950	26	3.3	916.8087	3	29.62	43	F43:1472	OB5956 H1 Ro.raw	4.4266E6	11	11	229	254	Deamidation (NQ)	N21:Deamidation (NQ):31.91	PEAKS DB
K.NPQLQDLDM(+15.99)M(+15.99)LTC(+57.02)VEIK.E	N	121.59	2078.9688	17	6.6	1040.4985	2	33.82	43	F43:1726	OB5956 H1 Ro.raw	6.8984E4	2	2	423	439	Oxidation (M); Carbamidomethylation	M9:Oxidation (M):1000.00;M10:Oxidation (M):1000.00;C13:Carbamidomethylation:1000.00	PEAKS DB
K.SFNLDEGHALRIPSGFISYILNR.H	N	121.49	2618.3604	23	1.0	873.7949	3	37.51	41	F41:1949	OB5954 H1 Ro.raw	9.1637E7	28	28	255	277			PEAKS DB
K.KNPQLQDLDM(+15.99)MLTC(+57.02)VEIK.E	N	118.68	2191.0686	18	3.6	1096.5455	2	34.71	43	F43:1781	OB5956 H1 Ro.raw	1.3598E6	4	4	422	439	Oxidation (M); Carbamidomethylation	M10:Oxidation (M):27.96;C14:Carbamidomethylation:1000.00	PEAKS DB
K.KGSEEEGDITNPINLR.E	N	117.89	1770.8748	16	0.6	886.4452	2	28.91	43	F43:1429	OB5956 H1 Ro.raw	3.1542E7	8	8	386	401			PEAKS DB
K.AMVIVVVNKGTGNLELVAVR.K	N	114.87	2081.2031	20	-1.0	1041.6078	2	34.35	43	F43:1758	OB5956 H1 Ro.raw	3.8361E7	13	13	452	471			PEAKS DB
R.IFLAGDKDNVIDQIEK.Q	N	110.41	1816.9570	16	-1.1	909.4848	2	32.67	43	F43:1648	OB5956 H1 Ro.raw	4.2419E8	56	55	541	556			PEAKS DB

total 250 peptides

481 **EEEEEDEDEEE EGSNR**EVRRY TARLKEGDVF IMPAAHPVAI NASSEEHLLG FGINAENNHR **IFLAGDKDNV IDQIEKQAKD**

561 **LAFPGSGEQV EKLIK**NQKES HFVSARPQSQ SQSPSSPEKE SPEKEDQEEE NQGGKGPLLS ILKAFN

Peptide	Uniq	-10lgP	Mass	Length	ppm	m/z	z	RT	Fraction	Scan	Source File	Area Roasted in gel	#Feature	#Feature Roasted in gel	Start	End	PTM	AScore	Found By
K.KNPQLQDLDMM(+15.99)LTC(+57.02)VEIK.E	N	110.35	2191.0686	18	3.5	1096.5454	2	32.98	42	F42:1657	OB5955 H1 Ro.raw	1.6495E6	4	4	422	439	Oxidation (M); Carbamidomethylation	M11:Oxidation (M):23.10;C14:Carbamidomethylation:1000.00	PEAKS DB
R.IPSGFISYILNR.H	N	109.18	1378.7609	12	2.5	690.3894	2	36.50	38	F38:1780	OB5951 H3A Ro.raw	4.4166E8	20	20	266	277			PEAKS DB
K.HADADNILVIQQGQATVTVANGNNRK.S	N	108.34	2746.4111	26	1.9	916.4794	3	29.61	43	F43:1451	OB5956 H1 Ro.raw	3.8404E6	5	5	229	254			PEAKS DB
R.VLLEENAGGEQEER.G	N	108.33	1571.7427	14	2.4	786.8805	2	26.83	42	F42:1293	OB5955 H1 Ro.raw	2.9858E7	10	10	335	348			PEAKS DB
R.KSFNLDEGHALR.I	N	108.28	1385.7051	12	0.6	462.9092	3	26.14	43	F43:1252	OB5956 H1 Ro.raw	5.3666E6	6	6	254	265			PEAKS DB
K.GSEEEGDITNPINLR.E	N	108.06	1642.7798	15	-0.5	822.3967	2	30.80	42	F42:1519	OB5955 H1 Ro.raw	1.1767E7	3	3	387	401			PEAKS DB
K.AMVIVVVNKGTGNLELVAVRK.E	N	107.96	2209.2981	21	1.1	737.4408	3	33.05	43	F43:1682	OB5956 H1 Ro.raw	1.2362E8	20	20	452	472			PEAKS DB
R.I(+57.02)PSGFISYILNR.H	N	107.77	1435.7823	12	-3.6	718.8959	2	36.92	43	F43:1901	OB5956 H1 Ro.raw	1.7559E7	7	7	266	277	Carbamidomethylation (DHKE, X@N-term)	I1:Carbamidomethylation (DHKE, X@N-term):1000.00	PEAKS PTM
R.I(+57.02)FLAGDKDNVIDQIEK.Q	N	105.70	1873.9785	16	1.7	937.9982	2	32.48	43	F43:1653	OB5956 H1 Ro.raw	1.1722E7	5	5	541	556	Carbamidomethylation (DHKE, X@N-term)	I1:Carbamidomethylation (DHKE, X@N-term):90.91	PEAKS PTM
R.DQSSYLQGFSR.N	N	105.08	1286.5891	11	0.8	644.3024	2	30.81	40	F40:1431	OB5953 H3A Ro.raw	9.5783E7	31	31	308	318			PEAKS DB
R.EGEQEWGTPGSHVR.E	N	104.71	1567.7015	14	2.6	784.8601	2	25.68	42	F42:1206	OB5955 H1 Ro.raw	3.4943E6	6	6	153	166			PEAKS DB
R.V(+57.02)LLEENAGGEQEER.G	N	103.80	1628.7642	14	0.3	815.3896	2	27.08	43	F43:1322	OB5956 H1 Ro.raw	4.0193E5	3	3	335	348	Carbamidomethylation (DHKE, X@N-term)	V1:Carbamidomethylation (DHKE, X@N-term):49.37	PEAKS PTM
K.NPQLQDLDMMLTCVEIK(+14.02).E	N	103.68	2003.9730	17	-1.2	1002.9926	2	38.20	43	F43:1989	OB5956 H1 Ro.raw	5.9168E6	4	4	423	439	Methylation(KR)	K17:Methylation(KR):1000.00	PEAKS PTM
K.SFNLDEGHALR.I	N	103.18	1257.6101	11	-1.7	629.8113	2	28.00	43	F43:1362	OB5956 H1 Ro.raw	1.1584E8	33	31	255	265			PEAKS DB
K.AM(+15.99)VIVVVNKGTGNLELVAVR.K	N	103.14	2097.1980	20	1.6	700.0744	3	33.04	43	F43:1684	OB5956 H1 Ro.raw	2.3148E7	9	9	452	471	Oxidation (M)	M2:Oxidation (M):1000.00	PEAKS DB
K.DLAFPGSGEQVEK.L	N	100.93	1375.6619	13	-1.9	688.8369	2	30.34	43	F43:1508	OB5956 H1 Ro.raw	9.196E7	8	8	560	572			PEAKS DB
K.NPQLQDLDM(+15.99)MLTC(+57.02)VEIKEGALMLPHFNSK.A	N	100.40	3387.6335	29	-0.6	1130.2178	3	37.70	41	F41:1939	OB5954 H1 Ro.raw	8.7706E7	6	6	423	451	Oxidation (M); Carbamidomethylation	M9:Oxidation (M):26.02;C13:Carbamidomethylation:1000.00	PEAKS DB
**total 250 peptides**																			

Peptide	Uniq	-10lgP	Mass	Length	ppm	m/z	z	RT	Fraction	Scan	Source File	Area Roasted in gel	#Feature	#Feature Roasted in gel	Start	End	PTM	AScore	Found By
R.RVLLEENAGGEQEER.G	N	100.35	1727.8438	15	0.2	576.9553	3	25.30	42	F42:1185	OB5955 H1 Ro.raw	8.2964E5	6	6	334	348			PEAKS DB
K.NPQLQDLDMMLTC(+57.02)VEIKEGALMLPHFNSK.A	N	98.85	3371.6387	29	-2.7	843.9147	4	38.02	43	F43:1969	OB5956 H1 Ro.raw	1.5351E8	12	12	423	451	Carbamidomethylation	C13:Carbamidomethylation:1000.00	PEAKS DB
R.EGEPDLSNNFGK.L	Y	98.79	1305.5836	12	-0.8	653.7986	2	28.17	43	F43:1363	OB5956 H1 Ro.raw	1.5024E7	3	3	402	413			PEAKS DB
K.GSEEEGDITNPINLREGEPDLSNNFGK.L	Y	98.63	2930.3530	27	-8.3	977.7835	3	32.31	43	F43:1637	OB5956 H1 Ro.raw	2.5702E6	8	8	387	413			PEAKS DB
K.KNPQLQDLDMMLTC(+57.02)VEIKEGALMLPHFNSK.A	N	97.96	3499.7336	30	-3.9	875.9373	4	36.80	41	F41:1884	OB5954 H1 Ro.raw	6.677E6	4	4	422	451	Carbamidomethylation	C14:Carbamidomethylation:1000.00	PEAKS DB
R.VLLEEN(+.98)AGGEQEER.G	N	94.48	1572.7267	14	2.8	787.3728	2	27.46	43	F43:1340	OB5956 H1 Ro.raw	6.2395E5	3	3	335	348	Deamidation (NQ)	N6:Deamidation (NQ):121.37	PEAKS DB
R.IFLAGDKDNVIDQIEKQAK.D	N	94.35	2144.1477	19	-2.1	715.7217	3	32.42	42	F42:1625	OB5955 H1 Ro.raw	3.3984E6	9	9	541	559			PEAKS DB
R.KSFNLDEGHALRIPSGFISYILNR.H	N	93.78	2746.4553	24	-0.7	687.6206	4	36.29	43	F43:1876	OB5956 H1 Ro.raw	1.1783E6	4	4	254	277			PEAKS DB
F.LAGDKDNVIDQIEK.Q	N	91.83	1556.8046	14	-6.3	779.4047	2	32.21	43	F43:1629	OB5956 H1 Ro.raw	5.4162E6	4	4	543	556			PEAKS DB
R.IPSGFISYILN(-17.03)R.H	N	91.80	1361.7343	12	-4.9	681.8711	2	38.02	43	F43:1980	OB5956 H1 Ro.raw	1.3387E6	3	3	266	277	Ammonia-loss (N)	N11:Ammonia-loss (N):1000.00	PEAKS PTM
K.AMVIVVVNK.G	N	91.04	971.5837	9	-0.5	486.7989	2	30.16	43	F43:1494	OB5956 H1 Ro.raw	7.1361E6	3	3	452	460			PEAKS DB
K.SFNLDEGH(+15.99)ALR.I	N	90.73	1273.6051	11	0.9	637.8104	2	30.16	43	F43:1507	OB5956 H1 Ro.raw	8.7681E5	2	2	255	265	Oxidation (HW)	H8:Oxidation (HW):1000.00	PEAKS PTM
R.NNPFYFPSRR.F	N	89.95	1296.6364	10	-2.5	649.3239	2	28.89	43	F43:1417	OB5956 H1 Ro.raw	1.3445E8	22	22	172	181			PEAKS DB
K.D(+57.02)LAFPGSGEQVEK.L	N	88.60	1432.6833	13	-0.1	717.3489	2	31.08	41	F41:1535	OB5954 H1 Ro.raw	9.7986E5	3	3	560	572	Carbamidomethylation (DHKE, X@N-term)	D1:Carbamidomethylation (DHKE, X@N-term):131.10	PEAKS PTM
K.SFNLDEGH(+57.02)ALR.I	N	88.30	1314.6316	11	-0.1	658.3230	2	28.22	41	F41:1349	OB5954 H1 Ro.raw	2.9996E6	3	3	255	265	Carbamidomethylation (DHKE, X@N-term)	H8:Carbamidomethylation (DHKE, X@N-term):30.36	PEAKS PTM
R.IVQIEAKPNTLVLPK.H	N	88.27	1662.0079	15	0.9	832.0120	2	30.16	43	F43:1505	OB5956 H1 Ro.raw	2.8901E7	31	31	214	228			PEAKS DB
K.EGALMLPHFNSK.A	N	88.21	1342.6703	12	2.5	672.3441	2	30.67	43	F43:1529	OB5956 H1 Ro.raw	1.4087E7	5	5	440	451			PEAKS DB
K.NPQLQDLDMM(-48.00)LTC(+57.02)VEIK.E	N	88.05	1998.9755	17	1.9	1000.4969	2	32.85	43	F43:1670	OB5956 H1 Ro.raw	5.9032E6	3	3	423	439	Carbamidomethylation	M10:Dethiomethyl:14.02;C13:Carbamidomethylation:1000.00	PEAKS PTM
R.NNPFYFPSR.R	N	86.35	1140.5352	9	-0.1	571.2748	2	31.76	43	F43:1593	OB5956 H1 Ro.raw	1.7218E7	11	11	172	180			PEAKS DB

total 250 peptides

Peptide	Uniq	-10lgP	Mass	Length	ppm	m/z	z	RT	Fraction	Scan	Source File	Area Roasted in gel	#Feature	#Feature Roasted in gel	Start	End	PTM	AScore	Found By
K.S(+57.02)FNLDEGHALR.I	N	86.31	1314.6316	11	-0.5	658.3228	2	28.11	42	F42:1359	OB5955 H1 Ro.raw	2.6997E6	4	4	255	265	Carbamidomethylation (DHKE, X@N-term)	S1:Carbamidomethylation (DHKE, X@N-term):91.04	PEAKS PTM
I.PSGFISYILNR.H	N	85.97	1265.6768	11	-4.6	633.8427	2	36.75	43	F43:1894	OB5956 H1 Ro.raw	3.5986E5	2	2	267	277			PEAKS DB
R.SSENNEGVIVK.V	Y	85.30	1174.5830	11	0.1	588.2988	2	23.40	43	F43:1111	OB5956 H1 Ro.raw	5.1333E5	3	3	357	367			PEAKS DB
R.IPSGFISYILNRHDNQNLR.V	N	84.75	2256.1763	19	6.5	753.0709	3	33.61	43	F43:1714	OB5956 H1 Ro.raw	6.1581E7	21	21	266	284			PEAKS DB
R.I(+27.99)FLAGDKDNVIDQIEK.Q	N	84.60	1844.9519	16	-2.1	923.4813	2	36.23	41	F41:1853	OB5954 H1 Ro.raw	1.9485E6	3	3	541	556	Formylation	I1:Formylation:98.75	PEAKS PTM
R.EGEQEWGTPGSHVREETSR.N	N	83.87	2169.9675	19	2.2	724.3314	3	26.51	43	F43:1287	OB5956 H1 Ro.raw	5.5466E5	3	3	153	171			PEAKS DB
K.SFNLDEGH(+15.99)ALRIPSGFISYILNR.H	N	83.49	2634.3555	23	-4.0	879.1223	3	38.76	43	F43:2023	OB5956 H1 Ro.raw	8.8519E5	1	1	255	277	Oxidation (HW)	H8:Oxidation (HW):1000.00	PEAKS PTM
K.KNPQLQDLDMMLTC(+57.02)VEIKEGALM(+15.99)LPHFNSK.A	N	83.11	3515.7285	30	-1.9	879.9377	4	36.03	43	F43:1857	OB5956 H1 Ro.raw	6.6263E6	3	3	422	451	Carbamidomethylation; Oxidation (M)	C14:Carbamidomethylation:1000.00;M23:Oxidation (M):114.34	PEAKS DB
R.NTLEAAFNAEFNEIRR.V	N	82.99	1893.9332	16	-2.6	947.9714	2	34.11	42	F42:1711	OB5955 H1 Ro.raw	3.0959E8	36	35	319	334			PEAKS DB
K.A(+57.02)MVIVVVNKGTGNLELVAVR.K	N	82.14	2138.2246	20	-5.4	1070.1138	2	34.47	42	F42:1739	OB5955 H1 Ro.raw	4.4412E6	3	3	452	471	Carbamidomethylation (DHKE, X@N-term)	A1:Carbamidomethylation (DHKE, X@N-term):207.56	PEAKS PTM
K.DLAFPGSGEQVEKLIK.N	N	80.89	1729.9249	16	-1.6	865.9706	2	33.95	30	F30:1478	OB5926 H3B Ro.raw	3.9305E5	5	4	560	575			PEAKS DB
I.FLAGDKDNVIDQIEK.Q	N	80.64	1703.8729	15	-5.7	852.9388	2	32.22	43	F43:1635	OB5956 H1 Ro.raw	3.1343E5	3	3	542	556			PEAKS DB
K.EGALM(+15.99)LPHFNSK.A	N	80.48	1358.6653	12	1.1	680.3406	2	29.85	43	F43:1485	OB5956 H1 Ro.raw	3.0314E6	5	5	440	451	Oxidation (M)	M5:Oxidation (M):1000.00	PEAKS DB
K.GTGNLELVAVR.K	N	80.39	1127.6299	11	-1.3	564.8215	2	30.21	41	F41:1476	OB5954 H1 Ro.raw	2.4706E7	5	5	461	471			PEAKS DB
R.NTLEAAFN(+.98)AEFNEIRR.V	N	79.02	1894.9172	16	-2.2	948.4638	2	32.35	41	F41:1616	OB5954 H1 Ro.raw	1.3357E6	5	5	319	334	Deamidation (NQ)	N8:Deamidation (NQ):48.12	PEAKS DB
R.VLLEENAGGEQEER(+14.02).G	N	78.92	1585.7583	14	0.7	793.8870	2	27.86	41	F41:1339	OB5954 H1 Ro.raw	1.6341E5	3	3	335	348	Methylation(KR)	R14:Methylation(KR):1000.00	PEAKS PTM
K.GTGNLELVAVRK.E	N	78.87	1255.7249	12	0.7	628.8701	2	27.30	41	F41:1322	OB5954 H1 Ro.raw	2.29E7	10	10	461	472			PEAKS DB
R.QFQNLQNHR.I	N	78.35	1183.5846	9	0.0	592.7996	2	23.81	42	F42:1102	OB5955 H1 Ro.raw	1.5125E5	6	6	205	213			PEAKS DB
K.K(+57.02)GSEEEGDITNPINLR.E	N	78.20	1827.8962	16	-0.5	914.9550	2	28.83	42	F42:1409	OB5955 H1 Ro.raw	1.3158E5	2	2	386	401	Carbamidomethylation (DHKE, X@N-term)	K1:Carbamidomethylation (DHKE, X@N-term):32.46	PEAKS PTM

total 250 peptides

Peptide	Uniq	-10lgP	Mass	Length	ppm	m/z	z	RT	Fraction	Scan	Source File	Area Roasted in gel	#Feature	#Feature Roasted in gel	Start	End	PTM	AScore	Found By
K.NPQLQDLDMM(+15.99)LTC(+57.02)VEIKEGALMLPHFNSK.A	N	78.01	3387.6335	29	-0.8	1130.2175	3	36.42	41	F41:1862	OB5954 H1 Ro.raw	6.8935E7	6	6	423	451	Oxidation (M); Carbamidomethylation	M10:Oxidation (M):24.44;C13:Carbamidomethylation:1000.00	PEAKS DB
K.LFEVKPDKKNPQLQDLDMMLTC(+57.02)VEIK.E	N	77.87	3131.6069	26	-4.0	1044.8721	3	34.65	42	F42:1756	OB5955 H1 Ro.raw	5.0788E6	4	4	414	439	Carbamidomethylation	C22:Carbamidomethylation:1000.00	PEAKS DB
K.NPQLQDLDM(+15.99)MLTC(+57.02)VEIKEGALM(+15.99)LPHFNSK.A	N	77.10	3403.6284	29	1.1	851.9153	4	37.51	41	F41:1883	OB5954 H1 Ro.raw	7.3887E6	7	7	423	451	Oxidation (M); Carbamidomethylation	M9:Oxidation (M):27.96;C13:Carbamidomethylation:1000.00;M22:Oxidation (M):71.31	PEAKS DB
R.IFLAGDKDNVIDQIEKQ(+.98)AK.D	N	76.22	2145.1316	19	1.5	716.0522	3	33.08	42	F42:1661	OB5955 H1 Ro.raw	0	0	0	541	559	Deamidation (NQ)	Q17:Deamidation (NQ):48.83	PEAKS DB
R.EETSRN(+.98)NPFYFPSRR.F	N	75.62	1899.8864	15	-0.2	634.3026	3	28.39	41	F41:1381	OB5954 H1 Ro.raw	3.2976E5	2	2	167	181	Deamidation (NQ)	N6:Deamidation (NQ):33.98	PEAKS DB
L.AGDKDNVIDQIEK.Q	N	75.39	1443.7205	13	-3.7	722.8649	2	32.08	42	F42:1607	OB5955 H1 Ro.raw	3.3426E5	1	1	544	556			PEAKS DB
R.Q(-17.03)FQNLQNHR.I	N	75.35	1166.5581	9	-1.2	584.2856	2	28.00	43	F43:1365	OB5956 H1 Ro.raw	4.927E6	4	4	205	213	Pyro-glu from Q	Q1:Pyro-glu from Q:1000.00	PEAKS PTM
R.VAKISMPVNTPGQFEDFFPASSR.D	N	74.27	2524.2419	23	-2.9	842.4188	3	34.29	42	F42:1735	OB5955 H1 Ro.raw	1.4483E6	3	3	285	307			PEAKS DB
K.NPQLQDLDM(+15.99)M(+15.99)LTC(+57.02)VEIKEGALMLPHFNSK.A	N	73.00	3403.6284	29	-3.9	1135.5457	3	35.83	43	F43:1849	OB5956 H1 Ro.raw	7.8068E6	5	5	423	451	Oxidation (M); Carbamidomethylation	M9:Oxidation (M):54.18;M10:Oxidation (M):63.99;C13:Carbamidomethylation:1000.00	PEAKS DB
K.KNPQLQDLDM(+15.99)MLTC(+57.02)VEIKEGALM(+15.99)LPHFNSK.A	N	72.71	3531.7234	30	-1.9	883.9364	4	35.86	41	F41:1821	OB5954 H1 Ro.raw	7.53E5	1	1	422	451	Carbamidomethylation; Oxidation (M)	M10:Oxidation (M):17.01;C14:Carbamidomethylation:1000.00;M23:Oxidation (M):85.25	PEAKS DB
R.VLLEENAGGEQEERGQR.R	N	72.44	1912.9238	17	0.6	957.4698	2	25.30	42	F42:1191	OB5955 H1 Ro.raw	3.1353E6	8	8	335	351			PEAKS DB
K.VSKEHVEELTK.H	N	72.42	1297.6877	11	-0.3	649.8510	2	24.21	41	F41:1125	OB5954 H1 Ro.raw	2.2246E5	5	5	368	378			PEAKS DB
K.QAKDLAFPGSGEQVEK.L	N	72.39	1702.8525	16	1.2	568.6255	3	27.08	43	F43:1321	OB5956 H1 Ro.raw	2.8232E5	6	6	557	572			PEAKS DB
K.EGALMLPHFN(+.98)SK.A	N	71.37	1343.6543	12	2.6	672.8362	2	31.66	43	F43:1595	OB5956 H1 Ro.raw	1.0101E5	2	2	440	451	Deamidation (NQ)	N10:Deamidation (NQ):1000.00	PEAKS DB
F.NLDEGHALR.I	N	70.14	1023.5097	9	-2.5	512.7609	2	28.00	43	F43:1370	OB5956 H1 Ro.raw	2.6181E6	3	3	257	265			PEAKS DB

total 250 peptides

Peptide	Uniq	-10lgP	Mass	Length	ppm	m/z	z	RT	Fraction	Scan	Source File	Area Roasted in gel	#Feature	#Feature Roasted in gel	Start	End	PTM	AScore	Found By
R.N(+.98)TLEAAFNAEFNEIRR.V	N	69.73	1894.9172	16	0.4	948.4662	2	32.39	42	F42:1620	OB5955 H1 Ro.raw	2.6319E6	3	3	319	334	Deamidation (NQ)	N1:Deamidation (NQ):41.52	PEAKS DB
R.EGEPDLSN(+.98)NFGK.L	Y	69.72	1306.5676	12	-0.5	654.2908	2	28.90	43	F43:1436	OB5956 H1 Ro.raw	4.1115E5	3	3	402	413	Deamidation (NQ)	N8:Deamidation (NQ):30.46	PEAKS DB
R.K(+57.02)SFNLDEGHALR.I	N	69.20	1442.7266	12	-0.1	722.3705	2	26.25	42	F42:1250	OB5955 H1 Ro.raw	4.0798E5	6	6	254	265	Carbamidomethylation (DHKE, X@N-term)	K1:Carbamidomethylation (DHKE, X@N-term):76.17	PEAKS PTM
K.LFEVKPDKKNPQLQDLDMM(+15.99)LTC(+57.02)VEIK.E	N	69.17	3147.6018	26	2.2	787.9095	4	33.24	42	F42:1670	OB5955 H1 Ro.raw	2.0797E6	2	2	414	439	Oxidation (M); Carbamidomethylation	M19:Oxidation (M):40.00;C22:Carbamidomethylation:1000.00	PEAKS DB
K.SFNLDE(+57.02)GHALRIPSGFISYILNR.H	N	69.07	2675.3818	23	-1.9	892.7996	3	37.80	43	F43:1964	OB5956 H1 Ro.raw	0	0	0	255	277		E6:Carbamidomethylation (DHKE, X@N-term):0.00	PEAKS PTM
R.IFLAGDKDNVID(+57.02)QIEK.Q	N	69.05	1873.9785	16	-1.3	937.9953	2	32.42	42	F42:1608	OB5955 H1 Ro.raw	8.0339E6	1	1	541	556		D12:Carbamidomethylation (DHKE, X@N-term):15.85	PEAKS PTM
R.EGEQEWGTPGSH(+15.99)VR.E	N	68.71	1583.6964	14	0.8	792.8561	2	27.64	43	F43:1358	OB5956 H1 Ro.raw	1.1836E5	4	4	153	166	Oxidation (HW)	H12:Oxidation (HW):95.51	PEAKS PTM
A.GDKDNVIDQIEK.Q	N	68.14	1372.6833	12	-7.0	687.3441	2	32.22	43	F43:1632	OB5956 H1 Ro.raw	1.1241E6	3	3	545	556			PEAKS DB
R.I(+57.02)VQIEAKPNTLVLPK.H	N	68.01	1719.0294	15	-3.7	860.5188	2	30.87	43	F43:1547	OB5956 H1 Ro.raw	7.3731E5	5	4	214	228	Carbamidomethylation (DHKE, X@N-term)	I1:Carbamidomethylation (DHKE, X@N-term):109.97	PEAKS PTM
R.RVLLEEN(+.98)AGGEQEER.G	N	67.83	1728.8278	15	-0.8	577.2827	3	25.96	42	F42:1233	OB5955 H1 Ro.raw	1.359E4	2	2	334	348	Deamidation (NQ)	N7:Deamidation (NQ):77.53	PEAKS DB
R.DQS(-18.01)SYLQGFSR.N	N	67.70	1268.5785	11	0.2	635.2966	2	30.91	41	F41:1537	OB5954 H1 Ro.raw	6.4401E5	3	3	308	318	Dehydration	S3:Dehydration:40.00	PEAKS PTM
R.RVLLEENAGGEQEERGQR.R	N	67.67	2069.0249	18	1.9	690.6835	3	25.60	41	F41:1205	OB5954 H1 Ro.raw	1.8247E6	3	3	334	351			PEAKS DB
K.Q(-17.03)AKDLAFPGSGEQVEK.L	N	67.52	1685.8260	16	0.1	843.9204	2	29.98	43	F43:1503	OB5956 H1 Ro.raw	9.9003E4	3	3	557	572	Pyro-glu from Q	Q1:Pyro-glu from Q:1000.00	PEAKS PTM
K.NPQLQ(+.98)DLDMMLTC(+57.02)VEIK.E	N	67.25	2047.9629	17	1.5	1024.9902	2	44.46	43	F43:2348	OB5956 H1 Ro.raw	0	0	0	423	439	Carbamidomethylation	Q5:Deamidation (NQ):19.68;C13:Carbamidomethylation:1000.00	PEAKS DB
R.WGPAGPR.E	N	66.93	739.3765	7	1.0	370.6959	2	25.22	41	F41:1191	OB5954 H1 Ro.raw	3.1676E5	3	3	115	121			PEAKS DB
K.SFNLD(-18.01)EGHALR.I	N	66.88	1239.5996	11	2.9	620.8089	2	28.79	41	F41:1400	OB5954 H1 Ro.raw	0	0	0	255	265	Dehydration	D5:Dehydration:49.79	PEAKS PTM
K.AM(+15.99)VIVVVNK.G	N	66.42	987.5787	9	1.1	494.7972	2	27.11	41	F41:1283	OB5954 H1 Ro.raw	1.8879E6	3	3	452	460	Oxidation (M)	M2:Oxidation (M):1000.00	PEAKS DB

total 250 peptides

Peptide	Uniq	-10lgP	Mass	Length	ppm	m/z	z	RT	Fraction	Scan	Source File	Area Roasted in gel	#Feature	#Feature Roasted in gel	Start	End	PTM	AScore	Found By
K.KNPQLQDLDMMLTCVEIK(+14.02).E	N	66.06	2132.0679	18	-0.6	1067.0405	2	36.31	41	F41:1858	OB5954 H1 Ro.raw	5.2356E5	2	2	422	439	Methylation(KR)	K18:Methylation(KR):130.57	PEAKS PTM
R.EETSRNNPFYFPSRR.F	N	65.61	1898.9023	15	1.8	633.9759	3	27.75	42	F42:1339	OB5955 H1 Ro.raw	8.693E5	4	4	167	181			PEAKS DB
K.NPQLQDLDMMLTC(+57.02)VEIKEGALM(+15.99)LPHFNSK.A	N	64.76	3387.6335	29	-0.6	847.9152	4	37.63	43	F43:1915	OB5956 H1 Ro.raw	8.7706E7	6	6	423	451	Carbamidomethylation; Oxidation (M)	C13:Carbamidomethylation:1000.00;M22:Oxidation (M):82.05	PEAKS DB
R.IPSGFISY(+125.90)ILNR.H	N	64.64	1504.6575	12	-1.9	753.3346	2	38.39	43	F43:2001	OB5956 H1 Ro.raw	2.0334E5	2	2	266	277	Iodination	Y8:Iodination:1000.00	PEAKS PTM
R.IPSGFISYILN(+.98)RHDNQNLR.V	N	64.64	2257.1604	19	4.7	753.3976	3	34.29	42	F42:1740	OB5955 H1 Ro.raw	6.9901E5	2	2	266	284	Deamidation (NQ)	N11:Deamidation (NQ):36.24	PEAKS DB
A.FPGSGEQVEK.L	N	64.10	1076.5138	10	-3.4	539.2623	2	30.29	42	F42:1495	OB5955 H1 Ro.raw	4.0412E5	3	3	563	572			PEAKS DB
R.SRQFQNLQNHR.I	N	63.98	1426.7178	11	0.3	476.5800	3	24.16	43	F43:1148	OB5956 H1 Ro.raw	5.7314E4	3	3	203	213			PEAKS DB
K.ISMPVNTPGQFEDFFPASSRDQSSYLQGFSR.N	N	63.85	3494.6201	31	-0.9	1165.8796	3	36.01	43	F43:1851	OB5956 H1 Ro.raw	5.8723E6	6	6	288	318			PEAKS DB
K.LFEVKPDKK.N	N	63.79	1102.6385	9	0.6	552.3269	2	24.50	43	F43:1159	OB5956 H1 Ro.raw	1.1348E6	6	6	414	422			PEAKS DB
R.SSEN(+.98)NEGVIVK.V	Y	63.52	1175.5670	11	0.2	588.7909	2	24.46	41	F41:1140	OB5954 H1 Ro.raw	5.8916E4	4	4	357	367	Deamidation (NQ)	N4:Deamidation (NQ):33.98	PEAKS DB
Q.SSYLQGFSR.N	N	63.16	1043.5035	9	1.4	522.7598	2	29.29	41	F41:1438	OB5954 H1 Ro.raw	6.2708E4	3	3	310	318			PEAKS DB
R.QFQNLQN(+.98)HR.I	N	63.09	1184.5686	9	5.4	593.2948	2	23.60	43	F43:1114	OB5956 H1 Ro.raw	8.0412E2	1	1	205	213		N7:Deamidation (NQ):0.00	PEAKS DB
K.EGALMLPH(+14.02)FNSK.A	N	63.06	1356.6860	12	-4.2	679.3475	2	30.85	43	F43:1554	OB5956 H1 Ro.raw	2.9707E5	3	3	440	451	Methylation(others)	H8:Methylation(others):65.81	PEAKS PTM
P.SGFISYILNR.H	N	63.04	1168.6240	10	1.5	585.3202	2	35.08	43	F43:1803	OB5956 H1 Ro.raw	2.9962E5	1	1	268	277			PEAKS DB
N.LDEGHALR.I	N	62.84	909.4668	8	0.0	455.7407	2	27.93	42	F42:1350	OB5955 H1 Ro.raw	1.8672E5	3	3	258	265			PEAKS DB
G.SEEEGDITNPINLREGEPDLSNNFGK.L	Y	62.76	2873.3315	26	-0.4	958.7865	3	32.44	30	F30:1400	OB5926 H3B Ro.raw	1.9904E4	1	1	388	413			PEAKS DB
K.A(+57.02)MVIVVVNKGTGNLELVAVRK.E	N	62.52	2266.3196	21	0.7	1134.1678	2	32.98	42	F42:1660	OB5955 H1 Ro.raw	1.5099E7	6	6	452	472	Carbamidomethylation (DHKE, X@N-term)	A1:Carbamidomethylation (DHKE, X@N-term):139.69	PEAKS PTM
K.HADADNILVIQQGQATVTVANGN(+.98)NRK.S	N	62.31	2747.3950	26	-0.1	916.8055	3	30.70	41	F41:1517	OB5954 H1 Ro.raw	8.5956E3	1	1	229	254		N23:Deamidation (NQ):9.40	PEAKS DB
K.SFN(+15.99)LDEGHALR.I	N	62.00	1273.6051	11	0.0	637.8098	2	27.46	43	F43:1330	OB5956 H1 Ro.raw	9.622E5	3	3	255	265	Oxidation or Hydroxylation	N3:Oxidation or Hydroxylation:55.92	PEAKS PTM
R.IP(+15.99)SGFISYILNR.H	N	61.98	1394.7557	12	-0.8	698.3846	2	37.47	43	F43:1932	OB5956 H1 Ro.raw	3.2208E6	4	4	266	277	Oxidation or Hydroxylation	P2:Oxidation or Hydroxylation:39.25	PEAKS PTM

total 250 peptides

Peptide	Uniq	-10lgP	Mass	Length	ppm	m/z	z	RT	Fraction	Scan	Source File	Area Roasted in gel	#Feature	#Feature Roasted in gel	Start	End	PTM	AScore	Found By
K.NPQLQDLDMM(+15.99)LTC(+57.02)VEIKEGALM(+15.99)LPHFNSK.A	N	61.19	3403.6284	29	0.3	851.9147	4	35.86	41	F41:1801	OB5954 H1 Ro.raw	3.4294E6	4	4	423	451	Oxidation (M); Carbamidomethylation	M10:Oxidation (M):21.94;C13:Carbamidomethylation:1000.00;M22:Oxidation (M):61.50	PEAKS DB
K.G(+57.02)TGNLELVAVRK.E	N	60.73	1312.7462	12	-0.4	657.3801	2	27.30	41	F41:1303	OB5954 H1 Ro.raw	3.6978E5	2	2	461	472	Carbamidomethylation (DHKE, X@N-term)	G1:Carbamidomethylation (DHKE, X@N-term):55.46	PEAKS PTM
K.NPQLQDLDM(+15.99)M(+15.99)LTC(+57.02)VEIKEGALM(+15.99)LPHFNSK.A	N	60.17	3419.6233	29	-0.9	855.9124	4	34.83	42	F42:1766	OB5955 H1 Ro.raw	1.2322E6	3	3	423	451	Oxidation (M); Carbamidomethylation	M9:Oxidation (M):1000.00;M10:Oxidation (M):1000.00;C13:Carbamidomethylation:1000.00;M22:Oxidation (M):1000.00	PEAKS DB
R.IPSGFISY(+15.99)ILNR.H	N	60.13	1394.7557	12	-0.7	698.3846	2	35.11	41	F41:1859	OB5954 H1 Ro.raw	3.4529E6	5	5	266	277	Oxidation or Hydroxylation	Y8:Oxidation or Hydroxylation:67.76	PEAKS PTM
L.DEGHALR.I	N	59.61	796.3828	7	2.5	399.1996	2	27.93	42	F42:1351	OB5955 H1 Ro.raw	9.3538E4	3	3	259	265			PEAKS DB
K.LFEVKPDKKNPQLQ(+.98)DLDMM(+15.99)LTC(+57.02)VEIK.E	N	59.38	3148.5857	26	7.4	788.1595	4	33.17	42	F42:1670	OB5955 H1 Ro.raw	3.9804E5	1	1	414	439	Oxidation (M); Carbamidomethylation	Q14:Deamidation (NQ):0.00;M19:Oxidation (M):23.10;C22:Carbamidomethylation:1000.00	PEAKS DB
R.VLLEEN(-17.03)AGGEQEER.G	N	59.22	1554.7162	14	1.2	778.3663	2	27.73	42	F42:1335	OB5955 H1 Ro.raw	0	0	0	335	348	Ammonia-loss (N)	N6:Ammonia-loss (N):1000.00	PEAKS PTM
K.SFNLDEGHALRIPSGFISYILNRHDNQNLR.V	N	59.07	3495.7759	30	1.5	700.1635	5	39.97	40	F40:1987	OB5953 H3A Ro.raw	2.0405E7	15	15	255	284			PEAKS DB
K.GSE(+57.02)EEGDITNPINLR.E	N	58.95	1699.8013	15	-3.0	850.9054	2	30.85	43	F43:1556	OB5956 H1 Ro.raw	4.2816E4	1	1	387	401		E3:Carbamidomethylation (DHKE, X@N-term):0.00	PEAKS PTM
K.SFNLDEGHALR(+31.99)IPSGFISYILNR.H	N	58.88	2650.3503	23	0.1	884.4575	3	39.14	42	F42:2021	OB5955 H1 Ro.raw	5.6519E5	1	1	255	277	Dihydroxy	R11:Dihydroxy:46.57	PEAKS PTM
K.LFEVKPDK.K	N	58.71	974.5436	8	1.7	488.2799	2	25.30	42	F42:1183	OB5955 H1 Ro.raw	1.7315E6	6	6	414	421			PEAKS DB
K.SFNLDE(+57.02)GHALR.I	N	58.62	1314.6316	11	0.3	658.3232	2	27.83	43	F43:1458	OB5956 H1 Ro.raw	2.3371E6	2	2	255	265	Carbamidomethylation (DHKE, X@N-term)	E6:Carbamidomethylation (DHKE, X@N-term):27.25	PEAKS PTM
R.N(+57.02)NPFYFPSRR.F	N	58.56	1353.6577	10	1.1	677.8369	2	29.74	42	F42:1453	OB5955 H1 Ro.raw	9.24E5	3	3	172	181	Carbamidomethylation (DHKE, X@N-term)	N1:Carbamidomethylation (DHKE, X@N	PEAKS PTM
**total 250 peptides**																			

Peptide	Uniq	-10lgP	Mass	Length	ppm	m/z	z	RT	Fraction	Scan	Source File	Area Roasted in gel	#Feature	#Feature Roasted in gel	Start	End	PTM	AScore	Found By
																		-term):1000.00	
R.E(-18.01)GE(+57.02)QEWGTPGSHVR.E	N	58.23	1606.7124	14	0.2	804.3636	2	27.56	42	F42:1323	OB5955 H1 Ro.raw	2.2302E5	3	3	153	166	Pyro-glu from E; Carbamidomethylation (DHKE, X@N-term)	E1:Pyro-glu from E:1000.00;E3:Carbamidomethylation (DHKE, X@N-term):60.92	PEAKS PTM
R.IFLAGDKDNVIDQ(+.98)IEKQAK.D	N	58.20	2145.1316	19	1.4	716.0521	3	33.04	43	F43:1686	OB5956 H1 Ro.raw	1.2153E5	1	1	541	559	Deamidation (NQ)	Q13:Deamidation (NQ):25.18	PEAKS DB
R.VAKISM(+15.99)PVNTPGQFEDFFPASSR.D	N	57.67	2540.2368	23	2.1	847.7546	3	32.98	42	F42:1665	OB5955 H1 Ro.raw	1.5427E5	1	1	285	307	Oxidation (M)	M6:Oxidation (M):1000.00	PEAKS DB
K.G(+57.02)TGNLELVAVR.K	N	57.51	1184.6514	11	-2.7	593.3314	2	30.10	42	F42:1486	OB5955 H1 Ro.raw	1.7196E5	2	2	461	471		G1:Carbamidomethylation (DHKE, X@N-term):19.78	PEAKS PTM
R.IFLAGD(+57.02)KDNVIDQIEK.Q	N	57.43	1873.9785	16	1.7	625.6678	3	32.52	41	F41:1637	OB5954 H1 Ro.raw	2.7943E5	3	3	541	556		D6:Carbamidomethylation (DHKE, X@N-term):15.57	PEAKS PTM
R.IFLAGDK(+15.99)DNVIDQIEK.Q	N	57.39	1832.9519	16	-1.9	917.4814	2	31.99	41	F41:1600	OB5954 H1 Ro.raw	7.5922E5	2	2	541	556		K7:Oxidation or Hydroxylation:17.01	PEAKS PTM
N.PFYFPSRR.F	N	57.34	1068.5504	8	1.1	535.2831	2	28.95	41	F41:1414	OB5954 H1 Ro.raw	4.6082E6	5	5	174	181			PEAKS DB
R.K(+27.99)SFNLDEGHALR.I	N	57.29	1413.7001	12	1.4	707.8583	2	28.46	42	F42:1387	OB5955 H1 Ro.raw	2.9777E4	1	1	254	265	Formylation	K1:Formylation:1000.00	PEAKS PTM
K.SVSKKGSEEEGDITNPINLR.E	N	57.26	2172.1023	20	0.9	725.0420	3	27.48	41	F41:1325	OB5954 H1 Ro.raw	1.6504E5	2	2	382	401			PEAKS DB
K.LFEVKPDKKNPQLQDLDM(+15.99)MLTC(+57.02)VEIK.E	N	57.09	3147.6018	26	-1.9	787.9062	4	34.20	41	F41:1734	OB5954 H1 Ro.raw	2.4894E6	4	4	414	439	Oxidation (M); Carbamidomethylation	M18:Oxidation (M):33.98;C22:Carbamidomethylation:1000.00	PEAKS DB
K.IRPEGREGEQEWGTPGSHVR.E	N	56.62	2276.1045	20	0.5	570.0337	4	27.56	42	F42:1329	OB5955 H1 Ro.raw	8.9716E4	2	2	147	166			PEAKS DB
K.SFNLDEGHALR(+31.99).I	N	55.96	1289.6000	11	-1.4	645.8063	2	30.41	43	F43:1520	OB5956 H1 Ro.raw	0	0	0	255	265	Dihydroxy	R11:Dihydroxy:119.59	PEAKS PTM
K.ISMPVN(+.98)TPGQFEDFFPASSR.D	N	55.55	2227.0254	20	6.6	1114.5273	2	36.77	41	F41:1885	OB5954 H1 Ro.raw	1.01E5	2	2	288	307	Deamidation (NQ)	N6:Deamidation (NQ):42.68	PEAKS DB
R.NTLEAAFNAEFN(+.98)EIRR.V	N	54.77	1894.9172	16	1.8	948.4676	2	33.04	43	F43:1685	OB5956 H1 Ro.raw	2.2573E6	5	5	319	334	Deamidation (NQ)	N12:Deamidation (NQ):39.02	PEAKS DB
K.S(+57.02)FNLDEGHALRIPSGFISYILNR.H	N	54.19	2675.3818	23	2.3	892.8033	3	39.35	38	F38:1901	OB5951 H3A Ro.raw	6.7035E5	1	1	255	277	Carbamidomethylation (DHKE, X@N-term)	S1:Carbamidomethylation (DHKE, X@N-term):63.97	PEAKS PTM
R.IFLAGDKDNVIDQIEK(+57.02)QAK.D	N	54.07	2201.1692	19	0.3	734.7306	3	32.48	43	F43:1657	OB5956 H1 Ro.raw	1.2122E6	3	3	541	559		K16:Carbamidomethylation (DHKE, X	PEAKS PTM

total 250 peptides

Peptide	Uniq	-10lgP	Mass	Length	ppm	m/z	z	RT	Fraction	Scan	Source File	Area Roasted in gel	#Feature	#Feature Roasted in gel	Start	End	PTM	AScore	Found By
																		@N-term):11.12	
R.I(+27.99)PSGFISYILNR.H	N	53.77	1406.7557	12	0.7	704.3856	2	42.27	42	F42:2199	OB5955 H1 Ro.raw	0	0	0	266	277	Formylation	I1:Formylation:1000.00	PEAKS PTM
R.IPSGFISYILNRHDN(+.98)QNLR.V	N	53.60	2257.1604	19	-0.7	753.3936	3	34.26	41	F41:1732	OB5954 H1 Ro.raw	0	0	0	266	284		N15:Deamidation (NQ):0.00	PEAKS DB
K.HADADNILVIQQGQATVTVANGNNR.K	N	53.13	2618.3162	25	0.5	873.7798	3	30.34	38	F38:1426	OB5951 H3A Ro.raw	1.8654E3	1	1	229	253			PEAKS DB
R.IVQIEAK(+57.02)PNTLVLPK.H	N	53.06	1719.0294	15	-1.6	860.5206	2	30.29	43	F43:1512	OB5956 H1 Ro.raw	2.587E5	1	1	214	228	Carbamidomethylation (DHKE, X@N-term)	K7:Carbamidomethylation (DHKE, X@N-term):25.69	PEAKS PTM
K.AM(+15.99)VIVVVNKGTGNLELVAVRK.E	N	52.95	2225.2930	21	-1.3	742.7706	3	32.42	42	F42:1596	OB5955 H1 Ro.raw	1.4247E6	3	3	452	472	Oxidation (M)	M2:Oxidation (M):1000.00	PEAKS DB
R.IVQIEAKPN.T	N	52.62	1010.5760	9	-0.3	506.2951	2	25.40	42	F42:1199	OB5955 H1 Ro.raw	0	0	0	214	222			PEAKS DB
K.KNPQLQ(+.98)DLDMMLTC(+57.02)VEIKEGALMLPHFNSK.A	N	51.97	3500.7175	30	3.4	876.1896	4	36.93	43	F43:1903	OB5956 H1 Ro.raw	9.2646E6	2	2	422	451	Carbamidomethylation	Q6:Deamidation (NQ):14.04;C14:Carbamidomethylation:1000.00	PEAKS DB
R.E(-18.01)GEQEWGTPGSHVR.E	N	51.90	1549.6909	14	0.7	775.8533	2	27.75	42	F42:1342	OB5955 H1 Ro.raw	3.4987E5	3	3	153	166	Pyro-glu from E	E1:Pyro-glu from E:1000.00	PEAKS PTM
R.IFLAGDKDNVID.Q	N	51.22	1318.6769	12	-0.9	660.3451	2	31.76	43	F43:1606	OB5956 H1 Ro.raw	1.1173E5	3	3	541	552			PEAKS DB
K.ISM(+15.99)PVNTPGQFEDFFPASSRDQSSYLQGFSR.N	N	50.25	3510.6150	31	0.3	1171.2126	3	35.35	36	F36:1707	OB5948 H3B Ro.raw	2.4799E6	5	5	288	318	Oxidation (M)	M3:Oxidation (M):1000.00	PEAKS DB
K.NPQLQDLDMM(+15.99)LTC(+57.02)VEIKEGALMLPHFN(+.98)SK.A	N	49.99	3388.6174	29	6.1	848.1668	4	36.39	43	F43:1880	OB5956 H1 Ro.raw	1.7975E6	1	1	423	451	Oxidation (M); Carbamidomethylation	M10:Oxidation (M):27.96;C13:Carbamidomethylation:1000.00;N27:Deamidation (NQ):18.27	PEAKS DB
R.N(-17.03)NPFYFPSRR.F	N	49.99	1279.6097	10	1.1	427.5443	3	28.89	43	F43:1434	OB5956 H1 Ro.raw	5.674E5	3	3	172	181		N1:Ammonia-loss (N):9.34	PEAKS PTM
T.GNLELVAVRK.E	N	49.53	1097.6556	10	0.5	549.8354	2	27.00	42	F42:1296	OB5955 H1 Ro.raw	3.4872E5	3	3	463	472			PEAKS DB
R.VAKIS(+77.99)MPVNTPGQFEDFFPASSR.D	N	49.16	2602.2290	23	3.0	1302.1257	2	38.06	42	F42:1958	OB5955 H1 Ro.raw	0	0	0	285	307	Methylphosphonylation	S5:Methylphosphonylation:56.99	PEAKS PTM
F.PGSGEQVEK.L	N	49.06	929.4454	9	-3.7	465.7282	2	30.34	43	F43:1524	OB5956 H1 Ro.raw	5.2705E4	1	1	564	572			PEAKS DB
K.AMVIVVVNK(+57.02)GTGNLELVAVRK.E	N	48.92	2266.3196	21	1.3	756.4481	3	33.23	43	F43:1673	OB5956 H1 Ro.raw	3.1193E6	1	1	452	472		K9:Carbamidomethylation (DHKE, X@N-term):8.78	PEAKS PTM
R.EEDWRQPR.E	N	48.87	1114.5155	8	1.8	558.2661	2	23.91	41	F41:1112	OB5954 H1 Ro.raw	1.6791E3	1	1	126	133			PEAKS DB

total 250 peptides

Peptide	Uniq	-10lgP	Mass	Length	ppm	m/z	z	RT	Fraction	Scan	Source File	Area Roasted in gel	#Feature	#Feature Roasted in gel	Start	End	PTM	AScore	Found By
K.PNTLVLPK.H	N	48.84	880.5382	8	-2.9	441.2751	2	28.00	43	F43:1374	OB5956 H1 Ro.raw	1.9234E5	2	2	221	228			PEAKS DB
L.R(+27.99)IPSGFISYILNR.H	N	48.25	1562.8568	13	-2.0	782.4341	2	37.84	43	F43:1976	OB5956 H1 Ro.raw	1.5108E5	1	1	265	277	Formylation	R1:Formylation:1000.00	PEAKS PTM
R.EGEQEW(+15.99)GTPGSHVR.E	N	47.73	1583.6964	14	-0.6	792.8550	2	25.22	41	F41:1196	OB5954 H1 Ro.raw	2.0714E4	1	1	153	166	Oxidation (HW)	W6:Oxidation (HW):72.73	PEAKS PTM
V.QIEAKPNTLVLPK.H	N	47.49	1449.8555	13	-3.8	725.9323	2	30.34	43	F43:1513	OB5956 H1 Ro.raw	4.7367E4	1	1	216	228			PEAKS DB
K.GSEEEGDITNPINLREGEPDLSN(+.98)N(+.98)FGK.L	Y	47.23	2932.3210	27	4.0	978.4515	3	32.30	43	F43:1637	OB5956 H1 Ro.raw	3.6476E5	1	1	387	413	Deamidation (NQ)	N23:Deamidation (NQ):55.39;N24:Deamidation (NQ):48.34	PEAKS DB
R.N(+.98)TLEAAFNAEFNEIR.R	N	46.63	1738.8162	15	9.2	870.4233	2	44.36	42	F42:2303	OB5955 H1 Ro.raw	5.4416E3	1	1	319	333	Deamidation (NQ)	N1:Deamidation (NQ):39.47	PEAKS DB
R.VLLE(+21.98)ENAGGEQEER.G	N	46.37	1593.7246	14	-1.1	797.8687	2	26.71	43	F43:1300	OB5956 H1 Ro.raw	4.0134E5	3	3	335	348	Sodium adduct	E4:Sodium adduct:40.00	PEAKS PTM
N.PFYFPSR.R	N	46.32	912.4493	7	-0.1	457.2319	2	31.70	42	F42:1584	OB5955 H1 Ro.raw	2.4653E5	4	4	174	180			PEAKS DB
K.GTGNLELVAVRKEQQQR.G	N	46.31	1925.0442	17	1.4	642.6896	3	26.67	43	F43:1294	OB5956 H1 Ro.raw	2.1018E4	1	1	461	477			PEAKS DB
K.NPQLQDLD(+14.02)MMLTC(+57.02)VEIK.E	N	45.99	2060.9944	17	0.2	1031.5046	2	37.33	41	F41:1934	OB5954 H1 Ro.raw	2.7407E4	1	1	423	439	Methylation(others); Carbamidomethylation	D8:Methylation(others):42.68; C13:Carbamidomethylation:1000.00	PEAKS PTM
K.VSKEH(+15.99)VEELTK.H	N	45.89	1313.6826	11	0.8	657.8491	2	24.35	43	F43:1164	OB5956 H1 Ro.raw	1.7904E4	3	3	368	378	Oxidation (HW)	H5:Oxidation (HW): 1000.00	PEAKS PTM
R.SSENNEGVIVKVSKEHVEELTK.H	Y	45.89	2454.2603	22	1.5	819.0952	3	30.81	39	F39:1445	OB5952 H3A Ro.raw	1.1749E4	1	1	357	378			PEAKS DB
K.GTGNLE(+57.02)LVAVRK.E	N	45.75	1312.7462	12	-0.8	657.3799	2	27.17	42	F42:1295	OB5955 H1 Ro.raw	1.9882E5	1	1	461	472		E6:Carbamidomethylation (DHKE, X@N-term):0.00	PEAKS PTM
K.A(+43.01)MVIVVVNKGTGNLELVAVR.K	N	45.64	2124.2090	20	0.7	1063.1125	2	33.65	41	F41:1688	OB5954 H1 Ro.raw	8.0739E4	1	1	452	471	Carbamylation	A1:Carbamylation: 161.20	PEAKS PTM
R.VLLEEN(+.98)AGGEQEERGQR.R	N	45.60	1913.9078	17	-1.6	638.9755	3	25.99	41	F41:1234	OB5954 H1 Ro.raw	0	0	0	335	351	Deamidation (NQ)	N6:Deamidation (NQ):28.79	PEAKS DB
K.KNPQLQDLDMM(+15.99)LTC(+57.02)VEIKEGALM(+15.99)LPHFNSK.A	N	45.45	3531.7234	30	-1.1	883.9372	4	35.80	42	F42:1822	OB5955 H1 Ro.raw	0	0	0	422	451	Carbamidomethylation; Oxidation (M)	M11:Oxidation (M): 0.00;C14: Carbamidomethylation:1000.00;M23:Oxidation (M):45.87	PEAKS DB
K.LFEVKPDKKNPQLQDLDM(+15.99)M(+15.99)LTC(+57.02)VEIK.E	N	45.43	3163.5967	26	-2.8	791.9042	4	32.90	41	F41:1650	OB5954 H1 Ro.raw	2.0535E6	3	3	414	439	Oxidation (M); Carbamidomethylation	M18:Oxidation (M): 1000.00; M19:Oxidation (M): 1000.00; C22:Carb	PEAKS DB

total 250 peptides

Peptide	Uniq	-10lgP	Mass	Length	ppm	m/z	z	RT	Fraction	Scan	Source File	Area Roasted in gel	#Feature	#Feature Roasted in gel	Start	End	PTM	AScore	Found By
																		amidomethylation:1000.00	
R.KSFNLDEGH(+15.99)ALR.I	N	45.40	1401.7001	12	3.9	701.8600	2	27.64	43	F43:1349	OB5956 H1 Ro.raw	6.6479E4	2	2	254	265	Oxidation (HW)	H9:Oxidation (HW): 1000.00	PEAKS PTM
K.KNPQLQDLDM(+15.99)MLTC(+57.02)VEIKEGALMLPHFNSK.A	N	44.60	3515.7285	30	-1.9	879.9377	4	35.30	41	F41:1798	OB5954 H1 Ro.raw	6.4162E5	1	1	422	451	Carbamidomethylation	M10:Oxidation (M): 14.02;C14:Carbamidomethylation:1000.00	PEAKS DB
R.DQSSYLQGFSR(+15.99).N	N	44.15	1302.5840	11	2.5	652.3009	2	30.79	43	F43:1544	OB5956 H1 Ro.raw	1.4308E5	3	3	308	318	Oxidation or Hydroxylation	R11:Oxidation or Hydroxylation:42.88	PEAKS PTM
R.Q(+.98)FQNLQNHR.I	N	43.70	1184.5686	9	4.6	593.2943	2	23.68	42	F42:1098	OB5955 H1 Ro.raw	0	0	0	205	213	Deamidation (NQ)	Q1:Deamidation (NQ):39.25	PEAKS DB
K.I(+57.02)SMPVNTPGQFEDFFPASSR.D	N	42.37	2283.0630	20	-0.7	1142.5380	2	36.01	43	F43:1860	OB5956 H1 Ro.raw	1.8515E5	1	1	288	307	Carbamidomethylation (DHKE, X@N-term)	I1:Carbamidomethylation (DHKE, X@N-term):50.99	PEAKS PTM
K.ISM(-48.00)PVNTPGQFEDFFPASSR.D	N	42.09	2178.0381	20	2.8	727.0220	3	32.48	43	F43:1652	OB5956 H1 Ro.raw	5.8583E6	3	3	288	307	Dethiomethyl	M3:Dethiomethyl:1000.00	PEAKS PTM
K.NPQLQDLDMM(+15.99)LTC(+57.02)VEIKEGALM(+15.99)LPHFN(+.98)SK.A	N	41.25	3404.6125	29	-0.4	1135.8777	3	36.69	42	F42:1878	OB5955 H1 Ro.raw	3.8423E5	1	1	423	451	Carbamidomethylation; Oxidation (M); Deamidation (NQ)	M10:Oxidation (M): 0.00;C13:Carbamidomethylation:1000.00;M22:Oxidation (M):39.23;N27:Deamidation (NQ):69.52	PEAKS DB
K.DLAFP(+15.99)GSGEQVEK.L	N	41.14	1391.6569	13	0.5	696.8361	2	29.36	42	F42:1432	OB5955 H1 Ro.raw	7.4528E5	3	3	560	572	Oxidation or Hydroxylation	P5:Oxidation or Hydroxylation:67.76	PEAKS PTM
K.AMVIVVVN(+.98)KGTGNLELVAVRK.E	N	41.03	2210.2820	21	1.9	1106.1504	2	34.35	42	F42:1720	OB5955 H1 Ro.raw	4.5718E5	1	1	452	472	Deamidation (NQ)	N8:Deamidation (NQ):28.79	PEAKS DB
K.EGALM(-48.00)LPHFNSK.A	N	40.53	1294.6670	12	0.5	432.5632	3	28.76	41	F41:1389	OB5954 H1 Ro.raw	2.208E6	2	2	440	451	Dethiomethyl	M5:Dethiomethyl:1000.00	PEAKS PTM
R.IVQ(+.98)IEAKPNTLVLPK.H	N	40.35	1662.9919	15	8.1	832.5100	2	30.29	42	F42:1514	OB5955 H1 Ro.raw	3.5627E6	1	1	214	228	Deamidation (NQ)	Q3:Deamidation (NQ):22.65	PEAKS DB
R.VLLEENAGGEQEERGQRR.W	N	40.17	2069.0249	18	1.0	518.2640	4	25.49	42	F42:1205	OB5955 H1 Ro.raw	2.0305E5	2	2	335	352			PEAKS DB
R.VLLEENAGGEQEER(+15.99).G	N	39.98	1587.7375	14	1.8	794.8775	2	26.70	41	F41:1275	OB5954 H1 Ro.raw	0	0	0	335	348	Oxidation or Hydroxylation	R14:Oxidation or Hydroxylation:20.88	PEAKS PTM
R.N(+27.99)TLEAAFNAEFNEIRR.V	N	39.68	1921.9282	16	-1.7	961.9697	2	37.51	41	F41:1908	OB5954 H1 Ro.raw	3.7767E4	1	1	319	334	Formylation	N1:Formylation:1000.00	PEAKS PTM
K.E(-18.01)GALM(+15.99)LPHFNSK.A	N	39.64	1340.6547	12	0.2	447.8923	3	30.21	41	F41:1479	OB5954 H1 Ro.raw	7.81E5	1	1	440	451	Pyro-glu from E; Oxidation (M)	E1:Pyro-glu from E: 1000.00; M5:Oxidation (M):1000.00	PEAKS PTM

total 250 peptides

Peptide	Uniq	-10lgP	Mass	Length	ppm	m/z	z	RT	Fraction	Scan	Source File	Area Roasted in gel	#Feature	#Feature Roasted in gel	Start	End	PTM	AScore	Found By
R.VLLEE(+53.92)NAGGEQEER.G	N	39.58	1625.6620	14	-1.0	542.8940	3	26.76	42	F42:1278	OB5955H1Ro.raw	0	0	0	335	348	Replacement of 2 protons by iron	E5:Replacement of 2 protons by iron:20.92	PEAKS PTM
K.HADADNILVIQQGQ(+.98)ATVTVANGNNRK.S	N	39.33	2747.3950	26	2.6	916.8103	3	30.46	30	F30:1280	OB5926H3BRo.raw	0	0	0	229	254		Q14:Deamidation (NQ):5.07	PEAKS DB
K.NPQLQ(+.98)DLDMM(+15.99)LTC(+57.02)VEIKEGALM(+15.99)LPHFNSK.A	N	39.15	3404.6125	29	-1.8	852.1589	4	38.22	43	F43:1993	OB5956H1Ro.raw	7.6266E5	1	1	423	451	Deamidation (NQ); Carbamidomethylation	Q5:Deamidation (NQ):49.79;M10:Oxidation (M):0.00;C13:Carbamidomethylation:1000.00;M22:Oxidation (M):14.60	PEAKS DB
K.N(+.98)PQLQDLDM(+15.99)MLTC(+57.02)VEIKEGALMLPHFNSK.A	N	38.83	3388.6174	29	8.2	1695.3300	2	37.72	41	F41:1941	OB5954H1Ro.raw	6.4698E5	1	1	423	451	Carbamidomethylation	N1:Deamidation (NQ):0.00;M9:Oxidation (M):0.00;C13:Carbamidomethylation:1000.00	PEAKS DB
K.D(+15.99)LAFPGSGEQVEK.L	N	38.81	1391.6569	13	0.5	696.8361	2	29.36	42	F42:1368	OB5955H1Ro.raw	2.5725E5	1	1	560	572	Oxidation or Hydroxylation	D1:Oxidation or Hydroxylation:77.13	PEAKS PTM
K.NPQ(+.98)LQDLDMMLTC(+57.02)VEIK.E	N	38.70	2047.9629	17	2.9	1024.9917	2	46.45	41	F41:2441	OB5954H1Ro.raw	0	0	0	423	439	Carbamidomethylation	Q3:Deamidation (NQ):14.04;C13:Carbamidomethylation:1000.00	PEAKS DB
R.IFLAGD(+15.99)KDNVIDQIEK.Q	N	38.52	1832.9519	16	1.9	917.4850	2	31.89	42	F42:1590	OB5955H1Ro.raw	4.6198E5	1	1	541	556		D6:Oxidation or Hydroxylation:12.60	PEAKS PTM
R.IFLAGDKDN(+.98)VIDQIEKQ(+.98)AK.D	N	37.85	2146.1157	19	9.8	716.3862	3	32.98	42	F42:1661	OB5955H1Ro.raw	8.6625E4	1	1	541	559		N9:Deamidation (NQ):13.83;Q17:Deamidation (NQ):17.65	PEAKS DB
K.DLAFPGSGE(+53.92)QVEK.L	N	37.31	1429.5812	13	-6.3	715.7933	2	30.34	43	F43:1518	OB5956H1Ro.raw	2.6492E5	1	1	560	572	Replacement of 2 protons by iron	E9:Replacement of 2 protons by iron:37.54	PEAKS PTM
K.GT(-18.01)GNLELVAVRK.E	N	36.73	1237.7142	12	2.6	413.5797	3	27.00	42	F42:1287	OB5955H1Ro.raw	4.7714E5	1	1	461	472	Dehydration	T2:Dehydration:1000.00	PEAKS PTM
R.NT(-18.01)LEAAFNAEFNEIRR.V	N	36.45	1875.9227	16	0.7	626.3153	3	34.16	43	F43:1739	OB5956H1Ro.raw	6.9172E6	2	2	319	334	Dehydration	T2:Dehydration:1000.00	PEAKS PTM
K.EGALM(+15.99)LPHFN(+.98)SK.A	N	36.41	1359.6493	12	2.8	680.8338	2	31.22	41	F41:1550	OB5954H1Ro.raw	0	0	0	440	451	Oxidation (M); Deamidation (NQ)	M5:Oxidation (M):1000.00;N10:Deamidation (NQ):1000.00	PEAKS DB
R.GRREEEEDEDEEEEGSNR.E	Y	36.40	2192.8689	18	0.0	731.9636	3	23.90	41	F41:1110	OB5954H1Ro.raw	1.2738E4	2	2	478	495			PEAKS DB

total 250 peptides

Peptide	Uniq	-10lgP	Mass	Length	ppm	m/z	z	RT	Fraction	Scan	Source File	Area Roasted in gel	#Feature	#Feature Roasted in gel	Start	End	PTM	AScore	Found By
R.DQSSY(-18.01)LQGFSR.N	N	36.32	1268.5785	11	-0.6	635.2961	2	30.85	43	F43:1579	OB5956H1Ro.raw	2.378E5	1	1	308	318		Y5:Dehydration:0.00	PEAKS PTM
R.C(+57.02)LQS(-18.01)C(+57.02)Q(+.98)QEPDDLKQK.A	N	36.24	1858.8190	15	-0.1	930.4167	2	28.47	31	F31:1350	OB5942H6Ro.raw	0	0	0	42	56	Carbamidomethylation; Dehydration	C1:Carbamidomethylation:1000.00;S4:Dehydration:39.55;C5:Carbamidomethylation:1000.00;Q6:Deamidation (NQ):8.69	PEAKS PTM
M.LPHFNSK.A	N	36.02	841.4446	7	1.3	421.7301	2	30.63	42	F42:1510	OB5955H1Ro.raw	1.4673E6	2	2	445	451			PEAKS DB
E.PDLSNNFGK.L	Y	35.93	990.4771	9	-1.0	496.2453	2	28.09	42	F42:1365	OB5955H1Ro.raw	3.4068E4	1	1	405	413			PEAKS DB
K.NPQLQDLDM(-48.00)MLTC(+57.02)VEIK.E	N	35.81	1998.9755	17	6.7	667.3369	3	34.52	43	F43:1773	OB5956H1Ro.raw	8.4276E5	1	1	423	439	Carbamidomethylation	M9:Dethiomethyl:11.06;C13:Carbamidomethylation:1000.00	PEAKS PTM
K.SF(+17.99)NLDEGHALR.I	N	34.75	1275.6007	11	8.1	638.8128	2	26.62	42	F42:1267	OB5955H1Ro.raw	9.5828E3	1	1	255	265	Fluorination	F2:Fluorination:1000.00	PEAKS PTM
K.KNPQLQ(+.98)DLDMM(+15.99)LTC(+57.02)VEIKEGALMLPHFNSK.A	N	33.72	3516.7124	30	3.3	880.1883	4	35.48	41	F41:1798	OB5954H1Ro.raw	7.5658E5	1	1	422	451	Carbamidomethylation	Q6:Deamidation (NQ):18.55;M11:Oxidation (M):14.02;C14:Carbamidomethylation:1000.00	PEAKS DB
D.QSSYLQGFSR.N	N	33.26	1171.5621	10	2.1	586.7896	2	29.36	42	F42:1431	OB5955H1Ro.raw	3.3134E4	1	1	309	318			PEAKS DB
R.IVQIEAKP(+15.99)NTLVLPK.H	N	33.15	1678.0028	15	-0.7	840.0081	2	30.35	41	F41:1495	OB5954H1Ro.raw	0	0	0	214	228		P8:Oxidation or Hydroxylation:9.34	PEAKS PTM
R.IFLAGDKDNVID(-18.01)QIEK.Q	N	33.09	1798.9464	16	0.7	900.4811	2	31.39	43	F43:1591	OB5956H1Ro.raw	6.7846E4	1	1	541	556		D12:Dehydration:13.16	PEAKS PTM
R.EREEDWRQPR.E	N	32.92	1399.6592	10	5.7	700.8409	2	24.05	43	F43:1141	OB5956H1Ro.raw	4.1173E4	1	1	124	133			PEAKS DB
D.NVIDQIEK.Q	N	32.64	957.5131	8	1.6	479.7646	2	28.19	42	F42:1367	OB5955H1Ro.raw	3.0062E3	1	1	549	556			PEAKS DB
K.AM(-48.00)VIVVVNK.G	N	32.49	923.5804	9	0.0	462.7975	2	25.00	43	F43:1192	OB5956H1Ro.raw	6.3177E3	1	1	452	460	Dethiomethyl	M2:Dethiomethyl:1000.00	PEAKS PTM
R.D(-18.01)QSSYLQGFSR.N	N	31.95	1268.5785	11	1.6	635.2975	2	29.92	43	F43:1489	OB5956H1Ro.raw	0	0	0	308	318		D1:Dehydration:0.00	PEAKS PTM
R.IFLAGDKD(-18.01)NVIDQIEK.Q	N	31.82	1798.9464	16	-0.9	600.6555	3	32.15	43	F43:1589	OB5956H1Ro.raw	3.5634E5	1	1	541	556	Dehydration	D8:Dehydration:30.83	PEAKS PTM
K.D(+53.92)LAFPGSGEQVEK.L	N	31.27	1429.5812	13	-4.3	715.7948	2	30.56	41	F41:1500	OB5954H1Ro.raw	3.7719E5	1	1	560	572		D1:Replacement of 2 protons by iron:0.00	PEAKS PTM

total 250 peptides

Peptide	Uniq	-10lgP	Mass	Length	ppm	m/z	z	RT	Fraction	Scan	Source File	Area Roasted in gel	#Feature	#Feature Roasted in gel	Start	End	PTM	AScore	Found By
K.SFNLDE(+53.92)GHALR.I	N	31.27	1311.5294	11	1.0	438.1842	3	28.04	41	F41:1362	OB5954 H1 Ro.raw	5.8053E4	1	1	255	265		E6:Replacement of 2 protons by iron:16.90	PEAKS PTM
K.KGSEEEGDITNPINLREGEPDLSN(+.98)NFGK.L	Y	31.18	3059.4319	28	2.8	765.8674	4	31.36	36	F36:1485	OB5948 H3B Ro.raw	0	0	0	386	413		N24:Deamidation (NQ):0.00	PEAKS DB
K.AM(+15.99)VIVVVNK(+57.02)GTGNLELVAVR.K	N	30.97	2154.2195	20	2.8	1078.1200	2	32.98	42	F42:1664	OB5955 H1 Ro.raw	2.0029E5	1	1	452	471	Oxidation (M)	M2:Oxidation (M):1000.00;K9:Carbamidomethylation (DHKE, X@N-term):0.00	PEAKS PTM
K.KNPQLQDLDMM(+15.99)LTC(+57.02)VEIKEGALMLPHFNSK.A	N	30.83	3515.7285	30	1.3	879.9406	4	35.25	42	F42:1790	OB5955 H1 Ro.raw	0	0	0	422	451	Carbamidomethylation	M11:Oxidation (M):12.28;C14:Carbamidomethylation:1000.00	PEAKS DB
K.KGSEEEGDITNPINLREGEPDLSNN(+.98)FGK.L	Y	30.79	3059.4319	28	8.8	765.8720	4	31.05	38	F38:1466	OB5951 H3A Ro.raw	0	0	0	386	413		N25:Deamidation (NQ):0.00	PEAKS DB
R.DQSSY(+15.99)LQGFSR.N	N	30.72	1302.5840	11	1.6	652.3003	2	29.24	43	F43:1470	OB5956 H1 Ro.raw	6.7205E4	1	1	308	318	Oxidation or Hydroxylation	Y5:Oxidation or Hydroxylation:26.36	PEAKS PTM
K.SFNLDEGHALRIPSGFISYILNRHDNQN(+.98)LR.V	N	30.43	3496.7600	30	4.9	700.3627	5	37.51	39	F39:1925	OB5952 H3A Ro.raw	2.7478E5	1	1	255	284		N28:Deamidation (NQ):10.11	PEAKS DB
D.KDNVIDQIEK.Q	N	30.42	1200.6350	10	-0.3	601.3246	2	26.31	42	F42:1252	OB5955 H1 Ro.raw	0	0	0	547	556			PEAKS DB
N.TLEAAFNAEFNEIRR.V	N	30.42	1779.8904	15	3.5	890.9556	2	33.23	43	F43:1696	OB5956 H1 Ro.raw	1.5374E5	1	1	320	334			PEAKS DB
R.IPSGFISYILN(+15.99)R.H	N	30.30	1394.7557	12	-2.8	698.3832	2	36.42	41	F41:1875	OB5954 H1 Ro.raw	3.8045E5	1	1	266	277		N11:Oxidation or Hydroxylation:0.00	PEAKS PTM
R.IFLAGDKD(-18.01)NV.I	N	30.00	1072.5553	10	0.7	537.2853	2	32.26	42	F42:1603	OB5955 H1 Ro.raw	2.1452E6	1	1	541	550	Dehydration	D8:Dehydration:45.01	PEAKS PTM
R.NTLEAAFNAEFN(+15.99)EIRR.V	N	29.99	1909.9282	16	0.9	955.9723	2	32.98	42	F42:1662	OB5955 H1 Ro.raw	2.366E6	2	2	319	334		N12:Oxidation or Hydroxylation:0.00	PEAKS PTM
R.IFLAGDKDNVIDQ(+.98)IEK.Q	N	29.73	1817.9410	16	7.7	909.9848	2	63.08	43	F43:3435	OB5956 H1 Ro.raw	3.1606E3	1	1	541	556		Q13:Deamidation (NQ):11.12	PEAKS DB
K.V(+57.02)SKEHVEELTK.H	N	29.60	1354.7092	11	-1.7	678.3607	2	24.05	42	F42:1120	OB5955 H1 Ro.raw	0	0	0	368	378		V1:Carbamidomethylation (DHKE, X@N-term):0.00	PEAKS PTM
I.F(+127.06)LAGDKDNVIDQIEK.Q	N	29.59	1830.9363	15	0.7	916.4761	2	32.18	41	F41:1600	OB5954 H1 Ro.raw	6.5822E4	1	1	542	556	N-Succinimidyl-2-morpholine acetate	F1:N-Succinimidyl-2-morpholine acetate:28.79	PEAKS PTM
K.N(+.98)PQLQ(+.98)DLDMMLTC(+57.02)VEIKEGALM(+15.99)LPHFNSK.A	N	29.50	3389.6016	29	9.6	848.4158	4	37.65	43	F43:2047	OB5956 H1 Ro.raw	6.1505E6	1	1	423	451	Carbamidomethylation	N1:Deamidation (NQ):0.00;Q5:Deamidation (NQ):14.04;C13:Carb	PEAKS DB
**total 250 peptides**																			

Peptide	Uniq	-10lgP	Mass	Length	ppm	m/z	z	RT	Fraction	Scan	Source File	Area Roasted in gel	#Feature	#Feature Roasted in gel	Start	End	PTM	AScore	Found By
																		amidomethylation:1000.00;M22:Oxidation (M):0.00	
H.RIVQIEAKPNTLVLPK.H	N	29.34	1818.1090	16	0.6	607.0439	3	29.55	42	F42:1454	OB5955H1Ro.raw	7.8202E4	1	1	213	228			PEAKS DB
R.NN(+.98)PFYFPSRR.F	N	29.19	1297.6204	10	9.3	649.8235	2	62.71	43	F43:3425	OB5956H1Ro.raw	0	0	0	172	181		N2:Deamidation (NQ):7.21	PEAKS DB
R.NTLEAAFNAEFN(+.98)EIR.R	N	29.07	1738.8162	15	9.2	870.4233	2	44.36	42	F42:2331	OB5955H1Ro.raw	5.4416E3	1	1	319	333		N12:Deamidation (NQ):0.00	PEAKS DB
R.EGE(+21.98)QEWGTPGSHVR.E	N	28.92	1589.6835	14	1.4	795.8501	2	25.83	41	F41:1231	OB5954H1Ro.raw	3.6144E3	1	1	153	166		E3:Sodium adduct:9.74	PEAKS PTM
K.KNPQ(+.98)LQ(+.98)DLDMM(+15.99)LTC(+57.02)VEIKEGALM(+15.99)LPHFNSK.A	N	28.74	3533.6914	30	9.7	884.4387	4	35.77	42	F42:1822	OB5955H1Ro.raw	2.7541E5	1	1	422	451	Carbamidomethylation; Oxidation (M)	Q4:Deamidation (NQ):8.22;Q6:Deamidation (NQ):5.55;M11:Oxidation (M):0.00;C14:Carbamidomethylation:1000.00;M23:Oxidation (M):38.29	PEAKS DB
**total 250 peptides**																			

Q6PSU6|Q6PSU6_ARAHY

back to list

| Protein Coverage | Supporting Peptides |

Protein Coverage:

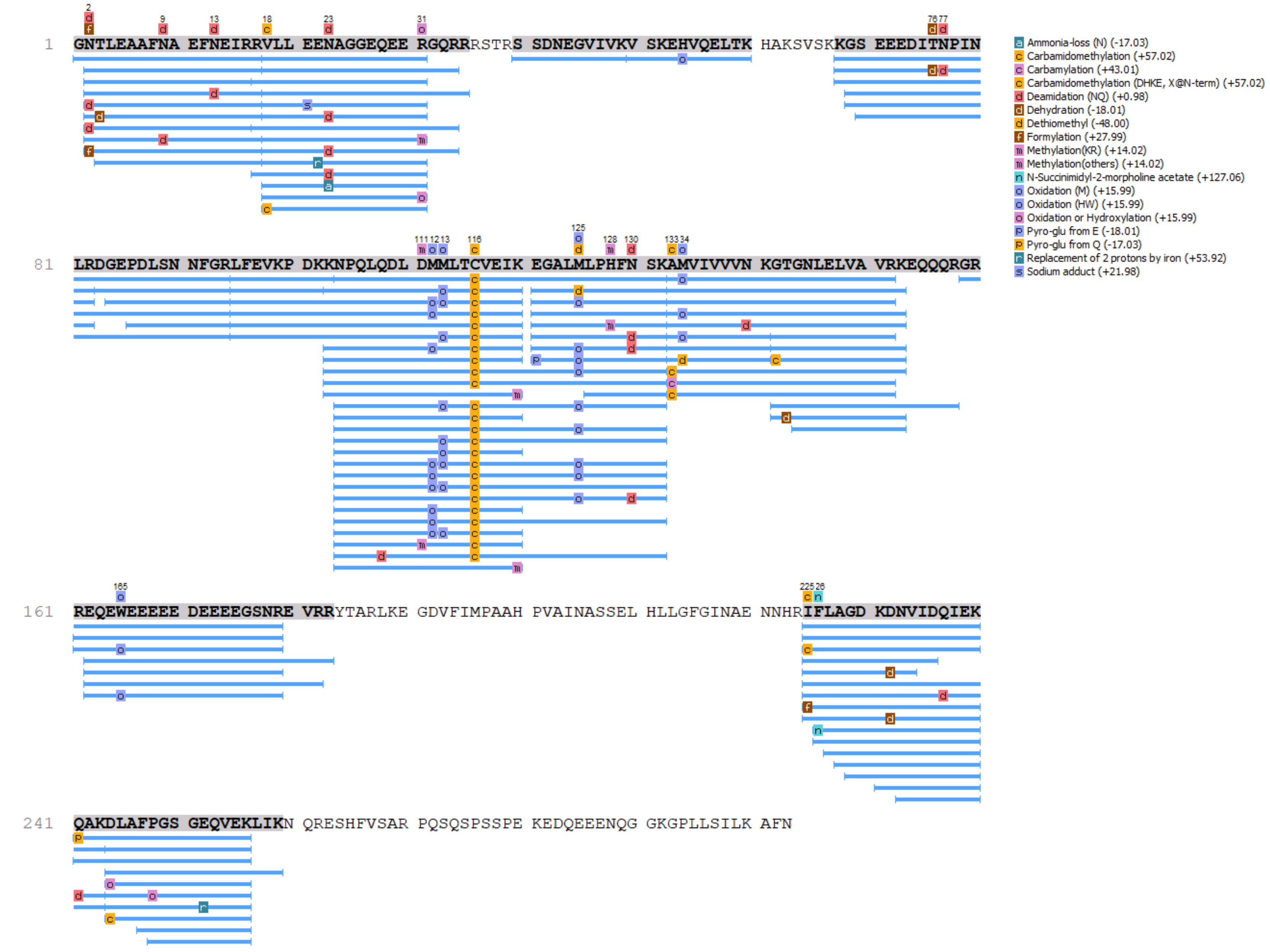

Supporting Peptides:

Peptide	Uniq	-10lgP	Mass	Length	ppm	m/z	z	RT	Fraction	Scan	Source File	Area Roasted in gel	#Feature	#Feature Roasted in gel	Start	End	PTM	AScore	Found By
K.KNPQLQDLDMMLTC(+57.02)VEIK.E	N	136.41	2175.0737	18	1.4	1088.5457	2	35.26	43	F43:1808	OB5956 H1 Ro.raw	1.4906E7	6	6	103	120	Carbamidomethylation	C14:Carbamidomethylation:1	PEAKS DB
**total 152 peptides**																			

Peptide	Uniq	-10lgP	Mass	Length	ppm	m/z	z	RT	Fraction	Scan	Source File	Area Roasted in gel	#Feature	#Feature Roasted in gel	Start	End	PTM	AScore	Found By
																		000.00	
G.NTLEAAFNAEFNEIR.R	N	130.93	1737.8322	15	1.8	869.9249	2	35.34	38	F38:1714	OB5951 H3A Ro.raw	1.332E7	16	16	2	16			PEAKS DB
K.GSEEEDITNPINLRDGEPDLSNNFGR.L	N	129.76	2887.3220	26	-0.7	963.4473	3	32.67	43	F43:1660	OB5956 H1 Ro.raw	1.2475E7	11	11	69	94			PEAKS DB
K.NPQLQDLDMMLTC(+57.02)VEIK.E	N	128.40	2046.9788	17	0.2	1024.4968	2	37.11	43	F43:1936	OB5956 H1 Ro.raw	3.4643E7	10	10	104	120	Carbamidomethylation	C13:Carbamidomethylation:1000.00	PEAKS DB
K.NPQLQDLDM(+15.99)MLTC(+57.02)VEIK.E	N	128.16	2062.9736	17	-4.2	1032.4897	2	36.93	43	F43:1892	OB5956 H1 Ro.raw	3.3834E6	3	3	104	120	Oxidation (M); Carbamidomethylation	M9:Oxidation (M):30.46;C13:Carbamidomethylation:1000.00	PEAKS DB
K.NPQLQDLDMM(+15.99)LTC(+57.02)VEIK.E	N	127.13	2062.9736	17	5.0	1032.4993	2	34.71	43	F43:1780	OB5956 H1 Ro.raw	1.0665E7	4	4	104	120	Oxidation (M); Carbamidomethylation	M10:Oxidation (M):30.46;C13:Carbamidomethylation:1000.00	PEAKS DB
R.EQEWEEEEEDEEEEGSNR.E	N	125.55	2280.8413	18	3.9	1141.4324	2	28.35	43	F43:1396	OB5956 H1 Ro.raw	4.3429E6	7	7	162	179			PEAKS DB
K.NPQLQDLDM(+15.99)M(+15.99)LTC(+57.02)VEIK.E	N	121.59	2078.9688	17	6.6	1040.4985	2	33.82	43	F43:1726	OB5956 H1 Ro.raw	6.8984E4	2	2	104	120	Oxidation (M); Carbamidomethylation	M9:Oxidation (M):1000.00;M10:Oxidation (M):1000.00;C13:Carbamidomethylation:1000.00	PEAKS DB
K.KNPQLQDLDM(+15.99)MLTC(+57.02)VEIK.E	N	118.68	2191.0686	18	3.6	1096.5455	2	34.71	43	F43:1781	OB5956 H1 Ro.raw	1.3598E6	4	4	103	120	Oxidation (M); Carbamidomethylation	M10:Oxidation (M):27.96;C14:Carbamidomethylation:1000.00	PEAKS DB
K.AMVIVVVNKGTGNLELVAVR.K	N	114.87	2081.2031	20	-1.0	1041.6078	2	34.35	43	F43:1758	OB5956 H1 Ro.raw	3.8361E7	13	13	133	152			PEAKS DB
R.IFLAGDKDNVIDQIEK.Q	N	110.41	1816.9570	16	-1.1	909.4848	2	32.67	43	F43:1648	OB5956 H1 Ro.raw	4.2419E8	56	55	225	240			PEAKS DB
K.KNPQLQDLDMM(+15.99)LTC(+57.02)VEIK.E	N	110.35	2191.0686	18	3.5	1096.5454	2	32.98	42	F42:1657	OB5955 H1 Ro.raw	1.6495E6	4	4	103	120	Oxidation (M); Carbamidomethylation	M11:Oxidation (M):23.10;C14:Carbamidomethylation:1000.00	PEAKS DB
K.KGSEEEDITNPINLRDGEPDLSNNFGR.L	N	109.79	3015.4170	27	3.4	1006.1497	3	31.62	41	F41:1565	OB5954 H1 Ro.raw	1.1559E7	21	21	68	94			PEAKS DB
R.VLLEENAGGEQEER.G	N	108.33	1571.7427	14	2.4	786.8805	2	26.83	42	F42:1293	OB5955 H1 Ro.raw	2.9858E7	10	10	18	31			PEAKS DB
K.AMVIVVVNKGTGNLELVAVRK.E	N	107.96	2209.2981	21	1.1	737.4408	3	33.05	43	F43:1682	OB5956 H1 Ro.raw	1.2362E8	20	20	133	153			PEAKS DB
K.KGSEEEDITNPINLR.D	N	107.76	1713.8533	15	2.8	857.9363	2	29.07	43	F43:1439	OB5956 H1 Ro.raw	8.6032E6	7	7	68	82			PEAKS DB
R.I(+57.02)FLAGDKDNVIDQIEK.Q	N	105.70	1873.9785	16	1.7	937.9982	2	32.48	43	F43:1653	OB5956 H1	1.1722E7	5	5	225	240	Carbamidomethylation (DHKE, X@N-term)	I1:Carbamidometh	PEAKS PTM
**total 152 peptides**																			

Peptide	Uniq	-10lgP	Mass	Length	ppm	m/z	z	RT	Fraction	Scan	Source File	Area Roasted in gel	#Feature	#Feature Roasted in gel	Start	End	PTM	AScore	Found By
											Ro.raw							ylation (DHKE, X@N-term):90.91	
R.V(+57.02)LLEENAGGEQEER.G	N	103.80	1628.7642	14	0.3	815.3896	2	27.08	43	F43:1322	OB5956H1 Ro.raw	4.0193E5	3	3	18	31	Carbamidomethylation (DHKE, X@N-term)	V1:Carbamidomethylation (DHKE, X@N-term):49.37	PEAKS PTM
K.NPQLQDLDMMLTCVEIK(+14.02).E	N	103.68	2003.9730	17	-1.2	1002.9926	2	38.20	43	F43:1989	OB5956H1 Ro.raw	5.9168E6	4	4	104	120	Methylation(KR)	K17:Methylation(KR):1000.00	PEAKS PTM
K.AM(+15.99)VIVVVNKGTGNLELVAVR.K	N	103.14	2097.1980	20	1.6	700.0744	3	33.04	43	F43:1684	OB5956H1 Ro.raw	2.3148E7	9	9	133	152	Oxidation (M)	M2:Oxidation (M):1000.00	PEAKS DB
R.DGEPDLSNNFGR.L	N	102.69	1319.5742	12	0.3	660.7946	2	29.24	43	F43:1495	OB5956H1 Ro.raw	7.4875E6	3	3	83	94			PEAKS DB
K.DLAFPGSGEQVEK.L	N	100.93	1375.6619	13	-1.9	688.8369	2	30.34	43	F43:1508	OB5956H1 Ro.raw	9.196E7	8	8	244	256			PEAKS DB
K.NPQLQDLDM(+15.99)MLTC(+57.02)VEIKEGALMLPHFNSK.A	N	100.40	3387.6335	29	-0.6	1130.2178	3	37.70	41	F41:1939	OB5954H1 Ro.raw	8.7706E7	6	6	104	132	Oxidation (M); Carbamidomethylation	M9:Oxidation (M):26.02;C13:Carbamidomethylation:1000.00	PEAKS DB
R.RVLLEENAGGEQEER.G	N	100.35	1727.8438	15	0.2	576.9553	3	25.30	42	F42:1185	OB5955H1 Ro.raw	8.2964E5	6	6	17	31			PEAKS DB
K.NPQLQDLDMMLTC(+57.02)VEIKEGALMLPHFNSK.A	N	98.85	3371.6387	29	-2.7	843.9147	4	38.02	43	F43:1969	OB5956H1 Ro.raw	1.5351E8	12	12	104	132	Carbamidomethylation	C13:Carbamidomethylation:1000.00	PEAKS DB
K.KNPQLQDLDMMLTC(+57.02)VEIKEGALMLPHFNSK.A	N	97.96	3499.7336	30	-3.9	875.9373	4	36.80	41	F41:1884	OB5954H1 Ro.raw	6.677E6	4	4	103	132	Carbamidomethylation	C14:Carbamidomethylation:1000.00	PEAKS DB
R.VLLEEN(+.98)AGGEQEER.G	N	94.48	1572.7267	14	2.8	787.3728	2	27.46	43	F43:1340	OB5956H1 Ro.raw	6.2395E5	3	3	18	31	Deamidation (NQ)	N6:Deamidation (NQ):121.37	PEAKS DB
R.IFLAGDKDNVIDQIEKQAK.D	N	94.35	2144.1477	19	-2.1	715.7217	3	32.42	42	F42:1625	OB5955H1 Ro.raw	3.3984E6	9	9	225	243			PEAKS DB
R.REQEWEEEEEDEEEEGSNR.E	N	93.49	2436.9424	19	0.1	813.3215	3	26.72	43	F43:1292	OB5956H1 Ro.raw	8.6025E5	7	7	161	179			PEAKS DB
F.LAGDKDNVIDQIEK.Q	N	91.83	1556.8046	14	-6.3	779.4047	2	32.21	43	F43:1629	OB5956H1 Ro.raw	5.4162E6	4	4	227	240			PEAKS DB
K.AMVIVVVNK.G	N	91.04	971.5837	9	-0.5	486.7989	2	30.16	43	F43:1494	OB5956H1 Ro.raw	7.1361E6	3	3	133	141			PEAKS DB
K.D(+57.02)LAFPGSGEQVEK.L	N	88.60	1432.6833	13	-0.1	717.3489	2	31.08	41	F41:1535	OB5954H1 Ro.raw	9.7986E5	3	3	244	256	Carbamidomethylation (DHKE, X@N-term)	D1:Carbamidomethylation (DHKE, X@N-term):131.10	PEAKS PTM
K.EGALMLPHFNSK.A	N	88.21	1342.6703	12	2.5	672.3441	2	30.67	43	F43:1529	OB5956H1 Ro.raw	1.4087E7	5	5	121	132			PEAKS DB
K.NPQLQDLDMM(-48.00)LTC(+57.02)VEIK.E	N	88.05	1998.9755	17	1.9	1000.4969	2	32.85	43	F43:1670	OB5956H1 Ro.raw	5.9032E6	3	3	104	120	Carbamidomethylation	M10:Dethiomethyl:14.02;C13:Carbamidomethylation:1000.00	PEAKS PTM

total 152 peptides

Peptide	Uniq	-10lgP	Mass	Length	ppm	m/z	z	RT	Fraction	Scan	Source File	Area Roasted in gel	#Feature	#Feature Roasted in gel	Start	End	PTM	AScore	Found By
K.GSEEEDITNPINLR.D	N	87.68	1585.7583	14	2.3	793.8882	2	31.16	42	F42:1548	OB5955 H1 Ro.raw	3.3709E6	3	3	69	82			PEAKS DB
R.GRREQEWEEEEEDEEEEGSNR.E	N	87.21	2650.0649	21	1.1	884.3632	3	26.73	41	F41:1280	OB5954 H1 Ro.raw	7.9956E5	6	6	159	179			PEAKS DB
GNTLEAAFNAEFNEIRR.V	Y	86.80	1950.9547	17	3.7	976.4882	2	35.08	43	F43:1794	OB5956 H1 Ro.raw	2.2902E6	8	8	1	17			PEAKS DB
R.I(+27.99)FLAGDKDNVIDQIEK.Q	N	84.60	1844.9519	16	-2.1	923.4813	2	36.23	41	F41:1853	OB5954 H1 Ro.raw	1.9485E6	3	3	225	240	Formylation	I1:Formylation:98.75	PEAKS PTM
K.KNPQLQDLDMMLTC(+57.02)VEIKEGALM(+15.99)LPHFNSK.A	N	83.11	3515.7285	30	-1.9	879.9377	4	36.03	43	F43:1857	OB5956 H1 Ro.raw	6.6263E6	3	3	103	132	Carbamidomethylation; Oxidation (M)	C14:Carbamidomethylation:1000.00;M23:Oxidation (M):114.34	PEAKS DB
G.NTLEAAFNAEFNEIRR.V	N	82.99	1893.9332	16	-2.6	947.9714	2	34.11	42	F42:1711	OB5955 H1 Ro.raw	3.0959E8	36	35	2	17			PEAKS DB
K.A(+57.02)MVIVVVNKGTGNLELVAVR.K	N	82.14	2138.2246	20	-5.4	1070.1138	2	34.47	42	F42:1739	OB5955 H1 Ro.raw	4.4412E6	3	3	133	152	Carbamidomethylation (DHKE, X@N-term)	A1:Carbamidomethylation (DHKE, X@N-term):207.56	PEAKS PTM
K.DLAFPGSGEQVEKLIK.N	N	80.89	1729.9249	16	-1.6	865.9706	2	33.95	30	F30:1478	OB5926 H3B Ro.raw	3.9305E5	5	4	244	259			PEAKS DB
I.FLAGDKDNVIDQIEK.Q	N	80.64	1703.8729	15	-5.7	852.9388	2	32.22	43	F43:1635	OB5956 H1 Ro.raw	3.1343E5	3	3	226	240			PEAKS DB
K.EGALM(+15.99)LPHFNSK.A	N	80.48	1358.6653	12	1.1	680.3406	2	29.85	43	F43:1485	OB5956 H1 Ro.raw	3.0314E6	5	5	121	132	Oxidation (M)	M5:Oxidation (M):1000.00	PEAKS DB
K.GTGNLELVAVR.K	N	80.39	1127.6299	11	-1.3	564.8215	2	30.21	41	F41:1476	OB5954 H1 Ro.raw	2.4706E7	5	5	142	152			PEAKS DB
G.NTLEAAFN(+.98)AEFNEIRR.V	N	79.02	1894.9172	16	-2.2	948.4638	2	32.35	41	F41:1616	OB5954 H1 Ro.raw	1.3357E6	5	5	2	17	Deamidation (NQ)	N8:Deamidation (NQ):48.12	PEAKS DB
R.VLLEENAGGEQEER(+14.02).G	N	78.92	1585.7583	14	0.7	793.8870	2	27.86	41	F41:1339	OB5954 H1 Ro.raw	1.6341E5	3	3	18	31	Methylation(KR)	R14:Methylation(KR):1000.00	PEAKS PTM
K.GTGNLELVAVRK.E	N	78.87	1255.7249	12	0.7	628.8701	2	27.30	41	F41:1322	OB5954 H1 Ro.raw	2.29E7	10	10	142	153			PEAKS DB
K.NPQLQDLDMM(+15.99)LTC(+57.02)VEIKEGALMLPHFNSK.A	N	78.01	3387.6335	29	-0.8	1130.2175	3	36.42	41	F41:1862	OB5954 H1 Ro.raw	6.8935E7	6	6	104	132	Oxidation (M); Carbamidomethylation	M10:Oxidation (M):24.44;C13:Carbamidomethylation:1000.00	PEAKS DB
R.LFEVKPDKKNPQLQDLDMMLTC(+57.02)VEIK.E	N	77.87	3131.6069	26	-4.0	1044.8721	3	34.65	42	F42:1756	OB5955 H1 Ro.raw	5.0788E6	4	4	95	120	Carbamidomethylation	C22:Carbamidomethylation:1000.00	PEAKS DB
K.NPQLQDLDM(+15.99)MLTC(+57.02)VEIKEGALM(+15.99)LPHFNSK.A	N	77.10	3403.6284	29	1.1	851.9153	4	37.51	41	F41:1883	OB5954 H1 Ro.raw	7.3887E6	7	7	104	132	Oxidation (M); Carbamidomethylation	M9:Oxidation (M):27.96;C13:Carbamidomethylation:1000.00;M22:Oxidation (M):71.31	PEAKS DB
**total 152 peptides**																			

Peptide	Uniq	-10lgP	Mass	Length	ppm	m/z	z	RT	Fraction	Scan	Source File	Area Roasted in gel	#Feature	#Feature Roasted in gel	Start	End	PTM	AScore	Found By
R.IFLAGDKDNVIDQIEKQ(+.98)AK.D	N	76.22	2145.1316	19	1.5	716.0522	3	33.08	42	F42:1661	OB5955 H1 Ro.raw	0	0	0	225	243	Deamidation (NQ)	Q17:Deamidation (NQ):48.83	PEAKS DB
L.AGDKDNVIDQIEK.Q	N	75.39	1443.7205	13	-3.7	722.8649	2	32.08	42	F42:1607	OB5955 H1 Ro.raw	3.3426E5	1	1	228	240			PEAKS DB
R.SSDNEGVIVK.V	N	75.32	1046.5244	10	-1.0	524.2690	2	23.58	41	F41:1103	OB5954 H1 Ro.raw	3.7568E5	3	3	40	49			PEAKS DB
K.NPQLQDLDM(+15.99)M(+15.99)LTC(+57.02)VEIKEGALMLPHFNSK.A	N	73.00	3403.6284	29	-3.9	1135.5457	3	35.83	43	F43:1849	OB5956 H1 Ro.raw	7.8068E6	5	5	104	132	Oxidation (M); Carbamidomethylation	M9:Oxidation (M):54.18;M10:Oxidation (M):63.99;C13:Carbamidomethylation:1000.00	PEAKS DB
K.KNPQLQDLDM(+15.99)MLTC(+57.02)VEIKEGALM(+15.99)LPHFNSK.A	N	72.71	3531.7234	30	-1.9	883.9364	4	35.86	41	F41:1821	OB5954 H1 Ro.raw	7.53E5	1	1	103	132	Carbamidomethylation; Oxidation (M)	M10:Oxidation (M):17.01;C14:Carbamidomethylation:1000.00;M23:Oxidation (M):85.25	PEAKS DB
R.VLLEENAGGEQEERGQR.R	N	72.44	1912.9238	17	0.6	957.4698	2	25.30	42	F42:1191	OB5955 H1 Ro.raw	3.1353E6	8	8	18	34			PEAKS DB
K.QAKDLAFPGSGEQVEK.L	N	72.39	1702.8525	16	1.2	568.6255	3	27.08	43	F43:1321	OB5956 H1 Ro.raw	2.8232E5	6	6	241	256			PEAKS DB
K.EGALMLPHFN(+.98)SK.A	N	71.37	1343.6543	12	2.6	672.8362	2	31.66	43	F43:1595	OB5956 H1 Ro.raw	1.0101E5	2	2	121	132	Deamidation (NQ)	N10:Deamidation (NQ):1000.00	PEAKS DB
R.EQEW(+15.99)EEEEEDEEEEGSNR.E	N	70.36	2296.8362	18	3.2	1149.4291	2	27.67	41	F41:1333	OB5954 H1 Ro.raw	1.024E6	3	3	162	179	Oxidation (HW)	W4:Oxidation (HW):1000.00	PEAKS PTM
G.N(+.98)TLEAAFNAEFNEIRR.V	N	69.73	1894.9172	16	0.4	948.4662	2	32.39	42	F42:1620	OB5955 H1 Ro.raw	2.6319E6	3	3	2	17	Deamidation (NQ)	N1:Deamidation (NQ):41.52	PEAKS DB
E.PDLSNNFGR.L	N	69.35	1018.4832	9	0.9	510.2493	2	31.57	43	F43:1584	OB5956 H1 Ro.raw	1.3026E6	4	4	86	94			PEAKS DB
R.LFEVKPDKKNPQLQDLDMM(+15.99)LTC(+57.02)VEIK.E	N	69.17	3147.6018	26	2.2	787.9095	4	33.24	42	F42:1670	OB5955 H1 Ro.raw	2.0797E6	2	2	95	120	Oxidation (M); Carbamidomethylation	M19:Oxidation (M):40.00;C22:Carbamidomethylation:1000.00	PEAKS DB
R.IFLAGDKDNVID(+57.02)QIEK.Q	N	69.05	1873.9785	16	-1.3	937.9953	2	32.42	42	F42:1608	OB5955 H1 Ro.raw	8.0339E6	1	1	225	240		D12:Carbamidomethylation (DHKE, X @N-term):15.85	PEAKS PTM
A.GDKDNVIDQIEK.Q	N	68.14	1372.6833	12	-7.0	687.3441	2	32.22	43	F43:1632	OB5956 H1 Ro.raw	1.1241E6	3	3	229	240			PEAKS DB
G.SEEEDITNPINLRDGEPDLSNNFGR.L	N	68.10	2830.3005	25	-1.6	944.4417	3	33.02	30	F30:1421	OB5926 H3B Ro.raw	1.3758E5	3	3	70	94			PEAKS DB
R.RVLLEEN(+.98)AGGEQEER.G	N	67.83	1728.8278	15	-0.8	577.2827	3	25.96	42	F42:1233	OB5955 H1 Ro.raw	1.359E4	2	2	17	31	Deamidation (NQ)	N7:Deamidation (NQ):77.53	PEAKS DB
**total 152 peptides**																			

Peptide	Uniq	-10lgP	Mass	Length	ppm	m/z	z	RT	Fraction	Scan	Source File	Area Roasted in gel	#Feature	#Feature Roasted in gel	Start	End	PTM	AScore	Found By
R.RVLLEENAGGEQEERGQR.R	N	67.67	2069.0249	18	1.9	690.6835	3	25.60	41	F41:1205	OB5954 H1 Ro.raw	1.8247E6	3	3	17	34			PEAKS DB
K.Q(-17.03)AKDLAFPGSGEQVEK.L	N	67.52	1685.8260	16	0.1	843.9204	2	29.98	43	F43:1503	OB5956 H1 Ro.raw	9.9003E4	3	3	241	256	Pyro-glu from Q	Q1:Pyro-glu from Q: 1000.00	PEAKS PTM
K.NPQLQ(+.98)DLDMMLTC(+57.02)VEIK.E	N	67.25	2047.9629	17	1.5	1024.9902	2	44.46	43	F43:2348	OB5956 H1 Ro.raw	0	0	0	104	120	Carbamidomethylation	Q5:Deamidation (NQ):19.68; C13:Carbamidomethylation:1000.00	PEAKS DB
K.AM(+15.99)VIVVVNK.G	N	66.42	987.5787	9	1.1	494.7972	2	27.11	41	F41:1283	OB5954 H1 Ro.raw	1.8879E6	3	3	133	141	Oxidation (M)	M2:Oxidation (M):1000.00	PEAKS DB
K.KNPQLQDLDMMLTCVEIK(+14.02).E	N	66.06	2132.0679	18	-0.6	1067.0405	2	36.31	41	F41:1858	OB5954 H1 Ro.raw	5.2356E5	2	2	103	120	Methylation(KR)	K18:Methylation(KR):130.57	PEAKS PTM
K.NPQLQDLDMMLTC(+57.02)VEIKEGALM(+15.99)LPHFNSK.A	N	64.76	3387.6335	29	-0.6	847.9152	4	37.63	43	F43:1915	OB5956 H1 Ro.raw	8.7706E7	6	6	104	132	Carbamidomethylation; Oxidation (M)	C13:Carbamidomethylation:1000.00;M22:Oxidation (M):82.05	PEAKS DB
A.FPGSGEQVEK.L	N	64.10	1076.5138	10	-3.4	539.2623	2	30.29	42	F42:1495	OB5955 H1 Ro.raw	4.0412E5	3	3	247	256			PEAKS DB
R.LFEVKPDKK.N	N	63.79	1102.6385	9	0.6	552.3269	2	24.50	43	F43:1159	OB5956 H1 Ro.raw	1.1348E6	6	6	95	103			PEAKS DB
K.EGALMLPH(+14.02)FNSK.A	N	63.06	1356.6860	12	-4.2	679.3475	2	30.85	43	F43:1554	OB5956 H1 Ro.raw	2.9707E5	3	3	121	132	Methylation(others)	H8:Methylation(others):65.81	PEAKS PTM
K.A(+57.02)MVIVVVNKGTGNLELVAVRK.E	N	62.52	2266.3196	21	0.7	1134.1678	2	32.98	42	F42:1660	OB5955 H1 Ro.raw	1.5099E7	6	6	133	153	Carbamidomethylation (DHKE, X@N-term)	A1:Carbamidomethylation (DHKE, X@N-term):139.69	PEAKS PTM
K.NPQLQDLDMM(+15.99)LTC(+57.02)VEIKEGALM(+15.99)LPHFNSK.A	N	61.19	3403.6284	29	0.3	851.9147	4	35.86	41	F41:1801	OB5954 H1 Ro.raw	3.4294E6	4	4	104	132	Oxidation (M); Carbamidomethylation	M10:Oxidation (M): 21.94;C13:Carbamidomethylation:1000.00;M22:Oxidation (M):61.50	PEAKS DB
K.G(+57.02)TGNLELVAVRK.E	N	60.73	1312.7462	12	-0.4	657.3801	2	27.30	41	F41:1303	OB5954 H1 Ro.raw	3.6978E5	2	2	142	153	Carbamidomethylation (DHKE, X@N-term)	G1:Carbamidomethylation (DHKE, X@N-term):55.46	PEAKS PTM
R.EQEWEEEEEDEEEEGSNREVRR.Y	N	60.58	2821.1545	22	1.9	941.3939	3	26.71	43	F43:1297	OB5956 H1 Ro.raw	1.0274E6	4	4	162	183			PEAKS DB
K.NPQLQDLDM(+15.99)M(+15.99)LTC(+57.02)VEIKEGALM(+15.99)LPHFNSK.A	N	60.17	3419.6233	29	-0.9	855.9124	4	34.83	42	F42:1766	OB5955 H1 Ro.raw	1.2322E6	3	3	104	132	Oxidation (M); Carbamidomethylation	M9:Oxidation (M):1000.00;M10:Oxidation (M):1000.00;C13:Carbamidomethylation:1000.00;M22:Oxidation (M):1000.00	PEAKS DB

total 152 peptides

Peptide	Uniq	-10lgP	Mass	Length	ppm	m/z	z	RT	Fraction	Scan	Source File	Area Roasted in gel	#Feature	#Feature Roasted in gel	Start	End	PTM	AScore	Found By
R.LFEVKPDKKNPQLQ(+.98)DLDMM(+15.99)LTC(+57.02)VEIK.E	N	59.38	3148.5857	26	7.4	788.1595	4	33.17	42	F42:1670	OB5955 H1 Ro.raw	3.9804E5	1	1	95	120	Oxidation (M); Carbamidomethylation	Q14:Deamidation (NQ):0.00;M19:Oxidation (M):23.10;C22:Carbamidomethylation:1000.00	PEAKS DB
R.VLLEEN(-17.03)AGGEQEER.G	N	59.22	1554.7162	14	1.2	778.3663	2	27.73	42	F42:1335	OB5955 H1 Ro.raw	0	0	0	18	31	Ammonia-loss (N)	N6:Ammonia-loss (N):1000.00	PEAKS PTM
R.LFEVKPDK.K	N	58.71	974.5436	8	1.7	488.2799	2	25.30	42	F42:1183	OB5955 H1 Ro.raw	1.7315E6	6	6	95	102			PEAKS DB
K.GSEEEDITNPIN(+.98)LRDGEPDLSNNFGR.L	N	58.69	2888.3059	26	6.7	1445.1699	2	32.90	41	F41:1642	OB5954 H1 Ro.raw	1.7853E5	1	1	69	94		N12:Deamidation (NQ):12.33	PEAKS DB
R.IFLAGDKDNVIDQ(+.98)IEKQAK.D	N	58.20	2145.1316	19	1.4	716.0521	3	33.04	43	F43:1686	OB5956 H1 Ro.raw	1.2153E5	1	1	225	243	Deamidation (NQ)	Q13:Deamidation (NQ):25.18	PEAKS DB
K.G(+57.02)TGNLELVAVR.K	N	57.51	1184.6514	11	-2.7	593.3314	2	30.10	42	F42:1486	OB5955 H1 Ro.raw	1.7196E5	2	2	142	152		G1:Carbamidomethylation (DHKE, X@N-term):19.78	PEAKS PTM
R.IFLAGD(+57.02)KDNVIDQIEK.Q	N	57.43	1873.9785	16	1.7	625.6678	3	32.52	41	F41:1637	OB5954 H1 Ro.raw	2.7943E5	3	3	225	240		D6:Carbamidomethylation (DHKE, X@N-term):15.57	PEAKS PTM
R.IFLAGDK(+15.99)DNVIDQIEK.Q	N	57.39	1832.9519	16	-1.9	917.4814	2	31.99	41	F41:1600	OB5954 H1 Ro.raw	7.5922E5	2	2	225	240		K7:Oxidation or Hydroxylation:17.01	PEAKS PTM
R.LFEVKPDKKNPQLQDLDM(+15.99)MLTC(+57.02)VEIK.E	N	57.09	3147.6018	26	-1.9	787.9062	4	34.20	41	F41:1734	OB5954 H1 Ro.raw	2.4894E6	4	4	95	120	Oxidation (M); Carbamidomethylation	M18:Oxidation (M):33.98;C22:Carbamidomethylation:1000.00	PEAKS DB
G.NTLEAAFNAEFN(+.98)EIRR.V	N	54.77	1894.9172	16	1.8	948.4676	2	33.04	43	F43:1685	OB5956 H1 Ro.raw	2.2573E6	5	5	2	17	Deamidation (NQ)	N12:Deamidation (NQ):39.02	PEAKS DB
R.IFLAGDKDNVIDQIEK(+57.02)QAK.D	N	54.07	2201.1692	19	0.3	734.7306	3	32.48	43	F43:1657	OB5956 H1 Ro.raw	1.2122E6	3	3	225	243		K16:Carbamidomethylation (DHKE, X@N-term):11.12	PEAKS PTM
K.AM(+15.99)VIVVVNKGTGNLELVAVRK.E	N	52.95	2225.2930	21	-1.3	742.7706	3	32.42	42	F42:1596	OB5955 H1 Ro.raw	1.4247E6	3	3	133	153	Oxidation (M)	M2:Oxidation (M):1000.00	PEAKS DB
K.KNPQLQ(+.98)DLDMMLTC(+57.02)VEIKEGALMLPHFNSK.A	N	51.97	3500.7175	30	3.4	876.1896	4	36.93	43	F43:1903	OB5956 H1 Ro.raw	9.2646E6	2	2	103	132	Carbamidomethylation	Q6:Deamidation (NQ):14.04;C14:Carbamidomethylation:1000.00	PEAKS DB
R.IFLAGDKDNVID.Q	N	51.22	1318.6769	12	-0.9	660.3451	2	31.76	43	F43:1606	OB5956 H1 Ro.raw	1.1173E5	3	3	225	236			PEAKS DB
**total 152 peptides**																			

Peptide	Uniq	-10lgP	Mass	Length	ppm	m/z	z	RT	Fraction	Scan	Source File	Area Roasted in gel	#Feature	#Feature Roasted in gel	Start	End	PTM	AScore	Found By
K.NPQLQDLDMM(+15.99)LTC(+57.02)VEIKEGALMLPHFN(+.98)SK.A	N	49.99	3388.6174	29	6.1	848.1668	4	36.39	43	F43:1880	OB5956 H1 Ro.raw	1.7975E6	1	1	104	132	Oxidation (M); Carbamidomethylation	M10:Oxidation (M):27.96;C13:Carbamidomethylation:1000.00;N27:Deamidation (NQ):18.27	PEAKS DB
R.EQEWEEEEEDEEEEGSNREVR.R	N	49.84	2665.0535	21	2.4	889.3605	3	27.88	41	F41:1353	OB5954 H1 Ro.raw	4.9786E4	1	1	162	182			PEAKS DB
T.GNLELVAVRK.E	N	49.53	1097.6556	10	0.5	549.8354	2	27.00	42	F42:1296	OB5955 H1 Ro.raw	3.4872E5	3	3	144	153			PEAKS DB
F.PGSGEQVEK.L	N	49.06	929.4454	9	-3.7	465.7282	2	30.34	43	F43:1524	OB5956 H1 Ro.raw	5.2705E4	1	1	248	256			PEAKS DB
K.AMVIVVVNK(+57.02)GTGNLELVAVRK.E	N	48.92	2266.3196	21	1.3	756.4481	3	33.23	43	F43:1673	OB5956 H1 Ro.raw	3.1193E6	1	1	133	153		K9:Carbamidomethylation (DHKE, X@N-term):8.78	PEAKS PTM
G.N(+.98)TLEAAFNAEFNEIR.R	N	46.63	1738.8162	15	9.2	870.4233	2	44.36	42	F42:2303	OB5955 H1 Ro.raw	5.4416E3	1	1	2	16	Deamidation (NQ)	N1:Deamidation (NQ):39.47	PEAKS DB
R.VLLE(+21.98)ENAGGEQEER.G	N	46.37	1593.7246	14	-1.1	797.8687	2	26.71	43	F43:1300	OB5956 H1 Ro.raw	4.0134E5	3	3	18	31	Sodium adduct	E4:Sodium adduct:40.00	PEAKS PTM
K.GTGNLELVAVRKEQQQR.G	N	46.31	1925.0442	17	1.4	642.6896	3	26.67	43	F43:1294	OB5956 H1 Ro.raw	2.1018E4	1	1	142	158			PEAKS DB
K.NPQLQDLD(+14.02)MMLTC(+57.02)VEIK.E	N	45.99	2060.9944	17	0.2	1031.5046	2	37.33	41	F41:1934	OB5954 H1 Ro.raw	2.7407E4	1	1	104	120	Methylation(others); Carbamidomethylation	D8:Methylation(others):42.68;C13:Carbamidomethylation:1000.00	PEAKS PTM
K.GTGNLE(+57.02)LVAVRK.E	N	45.75	1312.7462	12	-0.8	657.3799	2	27.17	42	F42:1295	OB5955 H1 Ro.raw	1.9882E5	1	1	142	153		E6:Carbamidomethylation (DHKE, X@N-term):0.00	PEAKS PTM
K.A(+43.01)MVIVVVNKGTGNLELVAVR.K	N	45.64	2124.2090	20	0.7	1063.1125	2	33.65	41	F41:1688	OB5954 H1 Ro.raw	8.0739E4	1	1	133	152	Carbamylation	A1:Carbamylation:161.20	PEAKS PTM
R.VLLEEN(+.98)AGGEQEERGQR.R	N	45.60	1913.9078	17	-1.6	638.9755	3	25.99	41	F41:1234	OB5954 H1 Ro.raw	0	0	0	18	34	Deamidation (NQ)	N6:Deamidation (NQ):28.79	PEAKS DB
K.KNPQLQDLDMM(+15.99)LTC(+57.02)VEIKEGALM(+15.99)LPHFNSK.A	N	45.45	3531.7234	30	-1.1	883.9372	4	35.80	42	F42:1822	OB5955 H1 Ro.raw	0	0	0	103	132	Carbamidomethylation; Oxidation (M)	M11:Oxidation (M):0.00;C14:Carbamidomethylation:1000.00;M23:Oxidation (M):45.87	PEAKS DB
R.LFEVKPDKKNPQLQDLDM(+15.99)M(+15.99)LTC(+57.02)VEIK.E	N	45.43	3163.5967	26	-2.8	791.9042	4	32.90	41	F41:1650	OB5954 H1 Ro.raw	2.0535E6	3	3	95	120	Oxidation (M); Carbamidomethylation	M18:Oxidation (M):1000.00;M19:Oxidation (M):1000.00;C22:Carbamidomethylation:1000.00	PEAKS DB

total 152 peptides

Peptide	Uniq	-10lgP	Mass	Length	ppm	m/z	z	RT	Fraction	Scan	Source File	Area Roasted in gel	#Feature	#Feature Roasted in gel	Start	End	PTM	AScore	Found By
K.KNPQLQDLDM(+15.99)MLTC(+57.02)VEIKEGALMLPHFNSK.A	N	44.60	3515.7285	30	-1.9	879.9377	4	35.30	41	F41:1798	OB5954 H1 Ro.raw	6.4162E5	1	1	103	132	Carbamidomethylation	M10:Oxidation (M):14.02;C14:Carbamidomethylation:1000.00	PEAKS DB
K.KGSEEEDIT(-18.01)N(+.98)PINLR.D	N	41.27	1696.8268	15	2.3	566.6175	3	29.07	43	F43:1442	OB5956 H1 Ro.raw	3.8118E5	3	3	68	82	Dehydration; Deamidation (NQ)	T9:Dehydration:45.01;N10:Deamidation (NQ):59.10	PEAKS PTM
K.NPQLQDLDMM(+15.99)LTC(+57.02)VEIKEGALM(+15.99)LPHFN(+.98)SK.A	N	41.25	3404.6125	29	-0.4	1135.8777	3	36.69	42	F42:1878	OB5955 H1 Ro.raw	3.8423E5	1	1	104	132	Carbamidomethylation; Oxidation (M); Deamidation (NQ)	M10:Oxidation (M):0.00;C13:Carbamidomethylation:1000.00;M22:Oxidation (M):39.23;N27:Deamidation (NQ):69.52	PEAKS DB
R.REQEW(+15.99)EEEEEDEEEEGSNR.E	N	41.18	2452.9375	19	-0.6	818.6526	3	25.98	41	F41:1239	OB5954 H1 Ro.raw	2.4114E4	1	1	161	179	Oxidation (HW)	W5:Oxidation (HW):1000.00	PEAKS PTM
K.DLAFP(+15.99)GSGEQVEK.L	N	41.14	1391.6569	13	0.5	696.8361	2	29.36	42	F42:1432	OB5955 H1 Ro.raw	7.4528E5	3	3	244	256	Oxidation or Hydroxylation	P5:Oxidation or Hydroxylation:67.76	PEAKS PTM
K.AMVIVVVN(+.98)KGTGNLELVAVRK.E	N	41.03	2210.2820	21	1.9	1106.1504	2	34.35	42	F42:1720	OB5955 H1 Ro.raw	4.5718E5	1	1	133	153	Deamidation (NQ)	N8:Deamidation (NQ):28.79	PEAKS DB
K.EGALM(-48.00)LPHFNSK.A	N	40.53	1294.6670	12	0.5	432.5632	3	28.76	41	F41:1389	OB5954 H1 Ro.raw	2.208E6	2	2	121	132	Dethiomethyl	M5:Dethiomethyl:1000.00	PEAKS PTM
R.VLLEENAGGEQEERGQRR.R	N	40.17	2069.0249	18	1.0	518.2640	4	25.49	42	F42:1205	OB5955 H1 Ro.raw	2.0305E5	2	2	18	35			PEAKS DB
R.VLLEENAGGEQEER(+15.99).G	N	39.98	1587.7375	14	1.8	794.8775	2	26.70	41	F41:1275	OB5954 H1 Ro.raw	0	0	0	18	31	Oxidation or Hydroxylation	R14:Oxidation or Hydroxylation:20.88	PEAKS PTM
G.N(+27.99)TLEAAFNAEFNEIRR.V	N	39.68	1921.9282	16	-1.7	961.9697	2	37.51	41	F41:1908	OB5954 H1 Ro.raw	3.7767E4	1	1	2	17	Formylation	N1:Formylation:1000.00	PEAKS PTM
K.E(-18.01)GALM(+15.99)LPHFNSK.A	N	39.64	1340.6547	12	0.2	447.8923	3	30.21	41	F41:1479	OB5954 H1 Ro.raw	7.81E5	1	1	121	132	Pyro-glu from E; Oxidation (M)	E1:Pyro-glu from E:1000.00;M5:Oxidation (M):1000.00	PEAKS PTM
R.VLLEE(+53.92)NAGGEQEER.G	N	39.58	1625.6620	14	-1.0	542.8940	3	26.76	42	F42:1278	OB5955 H1 Ro.raw	0	0	0	18	31	Replacement of 2 protons by iron	E5:Replacement of 2 protons by iron:20.92	PEAKS PTM
D.GEPDLSNNFGR.L	N	39.46	1204.5472	11	3.0	603.2827	2	28.20	41	F41:1371	OB5954 H1 Ro.raw	1.5511E4	1	1	84	94			PEAKS DB
K.NPQLQ(+.98)DLDMM(+15.99)LTC(+57.02)VEIKEGALM(+15.99)LPHFNSK.A	N	39.15	3404.6125	29	-1.8	852.1589	4	38.22	43	F43:1993	OB5956 H1 Ro.raw	7.6266E5	1	1	104	132	Deamidation (NQ); Carbamidomethylation	Q5:Deamidation (NQ):49.79;M10:Oxidation (M):0.00;C13:Carbamidomethylation:1000.00;M22:O	PEAKS DB

total 152 peptides

Peptide	Uniq	-10lgP	Mass	Length	ppm	m/z	z	RT	Fraction	Scan	Source File	Area Roasted in gel	#Feature	#Feature Roasted in gel	Start	End	PTM	AScore	Found By
																		xidation (M):14.60	
K.N(+.98)PQLQDLDM(+15.99)MLTC(+57.02)VEIKEGALMLPHFNSK.A	N	38.83	3388.6174	29	8.2	1695.3300	2	37.72	41	F41:1941	OB5954 H1 Ro.raw	6.4698E5	1	1	104	132	Carbamidomethylation	N1:Deamidation (NQ):0.00;M9:Oxidation (M):0.00;C13:Carbamidomethylation:1000.00	PEAKS DB
K.D(+15.99)LAFPGSGEQVEK.L	N	38.81	1391.6569	13	0.5	696.8361	2	29.36	42	F42:1368	OB5955 H1 Ro.raw	2.5725E5	1	1	244	256	Oxidation or Hydroxylation	D1:Oxidation or Hydroxylation:77.13	PEAKS PTM
K.NPQ(+.98)LQDLDMMLTC(+57.02)VEIK.E	N	38.70	2047.9629	17	2.9	1024.9917	2	46.45	41	F41:2441	OB5954 H1 Ro.raw	0	0	0	104	120	Carbamidomethylation	Q3:Deamidation (NQ):14.04;C13:Carbamidomethylation:1000.00	PEAKS DB
R.IFLAGD(+15.99)KDNVIDQIEK.Q	N	38.52	1832.9519	16	1.9	917.4850	2	31.89	42	F42:1590	OB5955 H1 Ro.raw	4.6198E5	1	1	225	240		D6:Oxidation or Hydroxylation:12.60	PEAKS PTM
R.IFLAGDKDN(+.98)VIDQIEKQ(+.98)AK.D	N	37.85	2146.1157	19	9.8	716.3862	3	32.98	42	F42:1661	OB5955 H1 Ro.raw	8.6625E4	1	1	225	243		N9:Deamidation (NQ):13.83;Q17:Deamidation (NQ):17.65	PEAKS DB
K.DLAFPGSGE(+53.92)QVEK.L	N	37.31	1429.5812	13	-6.3	715.7933	2	30.34	43	F43:1518	OB5956 H1 Ro.raw	2.6492E5	1	1	244	256	Replacement of 2 protons by iron	E9:Replacement of 2 protons by iron:37.54	PEAKS PTM
K.GT(-18.01)GNLELVAVRK.E	N	36.73	1237.7142	12	2.6	413.5797	3	27.00	42	F42:1287	OB5955 H1 Ro.raw	4.7714E5	1	1	142	153	Dehydration	T2:Dehydration:1000.00	PEAKS PTM
G.NT(-18.01)LEAAFNAEFNEIRR.V	N	36.45	1875.9227	16	0.7	626.3153	3	34.16	43	F43:1739	OB5956 H1 Ro.raw	6.9172E6	2	2	2	17	Dehydration	T2:Dehydration:1000.00	PEAKS PTM
K.EGALM(+15.99)LPHFN(+.98)SK.A	N	36.41	1359.6493	12	2.8	680.8338	2	31.22	41	F41:1550	OB5954 H1 Ro.raw	0	0	0	121	132	Oxidation (M); Deamidation (NQ)	M5:Oxidation (M):1000.00;N10:Deamidation (NQ):1000.00	PEAKS DB
M.LPHFNSK.A	N	36.02	841.4446	7	1.3	421.7301	2	30.63	42	F42:1510	OB5955 H1 Ro.raw	1.4673E6	2	2	126	132			PEAKS DB
K.NPQLQDLDM(-48.00)MLTC(+57.02)VEIK.E	N	35.81	1998.9755	17	6.7	667.3369	3	34.52	43	F43:1773	OB5956 H1 Ro.raw	8.4276E5	1	1	104	120	Carbamidomethylation	M9:Dethiomethyl:11.06;C13:Carbamidomethylation:1000.00	PEAKS PTM
K.KNPQLQ(+.98)DLDMM(+15.99)LTC(+57.02)VEIKEGALMLPHFNSK.A	N	33.72	3516.7124	30	3.3	880.1883	4	35.48	41	F41:1798	OB5954 H1 Ro.raw	7.5658E5	1	1	103	132	Carbamidomethylation	Q6:Deamidation (NQ):18.55;M11:Oxidation (M):14.02;C14:Carbamidomethylation:1000.00	PEAKS DB
K.VSKEH(+15.99)VQELTK.H	N	33.71	1312.6986	11	1.8	657.3578	2	23.73	42	F42:1100	OB5955 H1 Ro.raw	1.3581E3	1	1	50	60	Oxidation (HW)	H5:Oxidation (HW):1000.00	PEAKS PTM
**total 152 peptides**																			

Peptide	Uniq	-10lgP	Mass	Length	ppm	m/z	z	RT	Fraction	Scan	Source File	Area Roasted in gel	#Feature	#Feature Roasted in gel	Start	End	PTM	AScore	Found By
R.IFLAGDKDNVID(-18.01)QIEK.Q	N	33.09	1798.9464	16	0.7	900.4811	2	31.39	43	F43:1591	OB5956H1Ro.raw	6.7846E4	1	1	225	240		D12:Dehydration:13.16	PEAKS PTM
D.NVIDQIEK.Q	N	32.64	957.5131	8	1.6	479.7646	2	28.19	42	F42:1367	OB5955H1Ro.raw	3.0062E3	1	1	233	240			PEAKS DB
K.AM(-48.00)VIVVVNK.G	N	32.49	923.5804	9	0.0	462.7975	2	25.00	43	F43:1192	OB5956H1Ro.raw	6.3177E3	1	1	133	141	Dethiomethyl	M2:Dethiomethyl:1000.00	PEAKS PTM
R.IFLAGDKD(-18.01)NVIDQIEK.Q	N	31.82	1798.9464	16	-0.9	600.6555	3	32.15	43	F43:1589	OB5956H1Ro.raw	3.5634E5	1	1	225	240	Dehydration	D8:Dehydration:30.83	PEAKS PTM
K.D(+53.92)LAFPGSGEQVEK.L	N	31.27	1429.5812	13	-4.3	715.7948	2	30.56	41	F41:1500	OB5954H1Ro.raw	3.7719E5	1	1	244	256		D1:Replacement of 2 protons by iron:0.00	PEAKS PTM
K.AM(+15.99)VIVVVNK(+57.02)GTGNLELVAVR.K	N	30.97	2154.2195	20	2.8	1078.1200	2	32.98	42	F42:1664	OB5955H1Ro.raw	2.0029E5	1	1	133	152	Oxidation (M)	M2:Oxidation (M):1000.00;K9:Carbamidomethylation (DHKE, X@N-term):0.00	PEAKS PTM
K.KNPQLQDLDMM(+15.99)LTC(+57.02)VEIKEGALMLPHFNSK.A	N	30.83	3515.7285	30	1.3	879.9406	4	35.25	42	F42:1790	OB5955H1Ro.raw	0	0	0	103	132	Carbamidomethylation	M11:Oxidation (M):12.28;C14:Carbamidomethylation:1000.00	PEAKS DB
D.KDNVIDQIEK.Q	N	30.42	1200.6350	10	-0.3	601.3246	2	26.31	42	F42:1252	OB5955H1Ro.raw	0	0	0	231	240			PEAKS DB
N.TLEAAFNAEFNEIRR.V	N	30.42	1779.8904	15	3.5	890.9556	2	33.23	43	F43:1696	OB5956H1Ro.raw	1.5374E5	1	1	3	17			PEAKS DB
R.IFLAGDKD(-18.01)NV.I	N	30.00	1072.5553	10	0.7	537.2853	2	32.26	42	F42:1603	OB5955H1Ro.raw	2.1452E6	1	1	225	234	Dehydration	D8:Dehydration:45.01	PEAKS PTM
G.NTLEAAFNAEFN(+15.99)EIRR.V	N	29.99	1909.9282	16	0.9	955.9723	2	32.98	42	F42:1662	OB5955H1Ro.raw	2.366E6	2	2	2	17		N12:Oxidation or Hydroxylation:0.00	PEAKS PTM
R.IFLAGDKDNVIDQ(+.98)IEK.Q	N	29.73	1817.9410	16	7.7	909.9848	2	63.08	43	F43:3435	OB5956H1Ro.raw	3.1606E3	1	1	225	240		Q13:Deamidation (NQ):11.12	PEAKS DB
I.F(+127.06)LAGDKDNVIDQIEK.Q	N	29.59	1830.9363	15	0.7	916.4761	2	32.18	41	F41:1600	OB5954H1Ro.raw	6.5822E4	1	1	226	240	N-Succinimidyl-2-morpholine acetate	F1:N-Succinimidyl-2-morpholine acetate:28.79	PEAKS PTM
K.N(+.98)PQLQ(+.98)DLDMMLTC(+57.02)VEIKEGALM(+15.99)LPHFNSK.A	N	29.50	3389.6016	29	9.6	848.4158	4	37.65	43	F43:2047	OB5956H1Ro.raw	6.1505E6	1	1	104	132	Carbamidomethylation	N1:Deamidation (NQ):0.00;Q5:Deamidation (NQ):14.04;C13:Carbamidomethylation:1000.00;M22:Oxidation (M):0.00	PEAKS DB
G.NTLEAAFNAEFN(+.98)EIR.R	N	29.07	1738.8162	15	9.2	870.4233	2	44.36	42	F42:2331	OB5955H1Ro.raw	5.4416E3	1	1	2	16		N12:Deamidation (NQ):0.00	PEAKS DB

total 152 peptides

Peptide	Uniq	-10lgP	Mass	Length	ppm	m/z	z	RT	Fraction	Scan	Source File	Area Roasted in gel	#Feature	#Feature Roasted in gel	Start	End	PTM	AScore	Found By
K.KNPQ(+.98)LQ(+.98)DLDMM(+15.99)LTC(+57.02)VEIKEGALM(+15.99)LPHFNSK.A	N	28.74	3533.6914	30	9.7	884.4387	4	35.77	42	F42:1822	OB5955 H1 Ro.raw	2.7541E5	1	1	103	132	Carbamidomethylation; Oxidation (M)	Q4:Deamidation (NQ):8.22;Q6:Deamidation (NQ):5.55;M11:Oxidation (M):0.00;C14:Carbamidomethylation:1000.00;M23:Oxidation (M):38.29	PEAKS DB
total 152 peptides																			

E5G076|E5G076_ARAHY

back to list

| Protein Coverage | Supporting Peptides |

Protein Coverage:

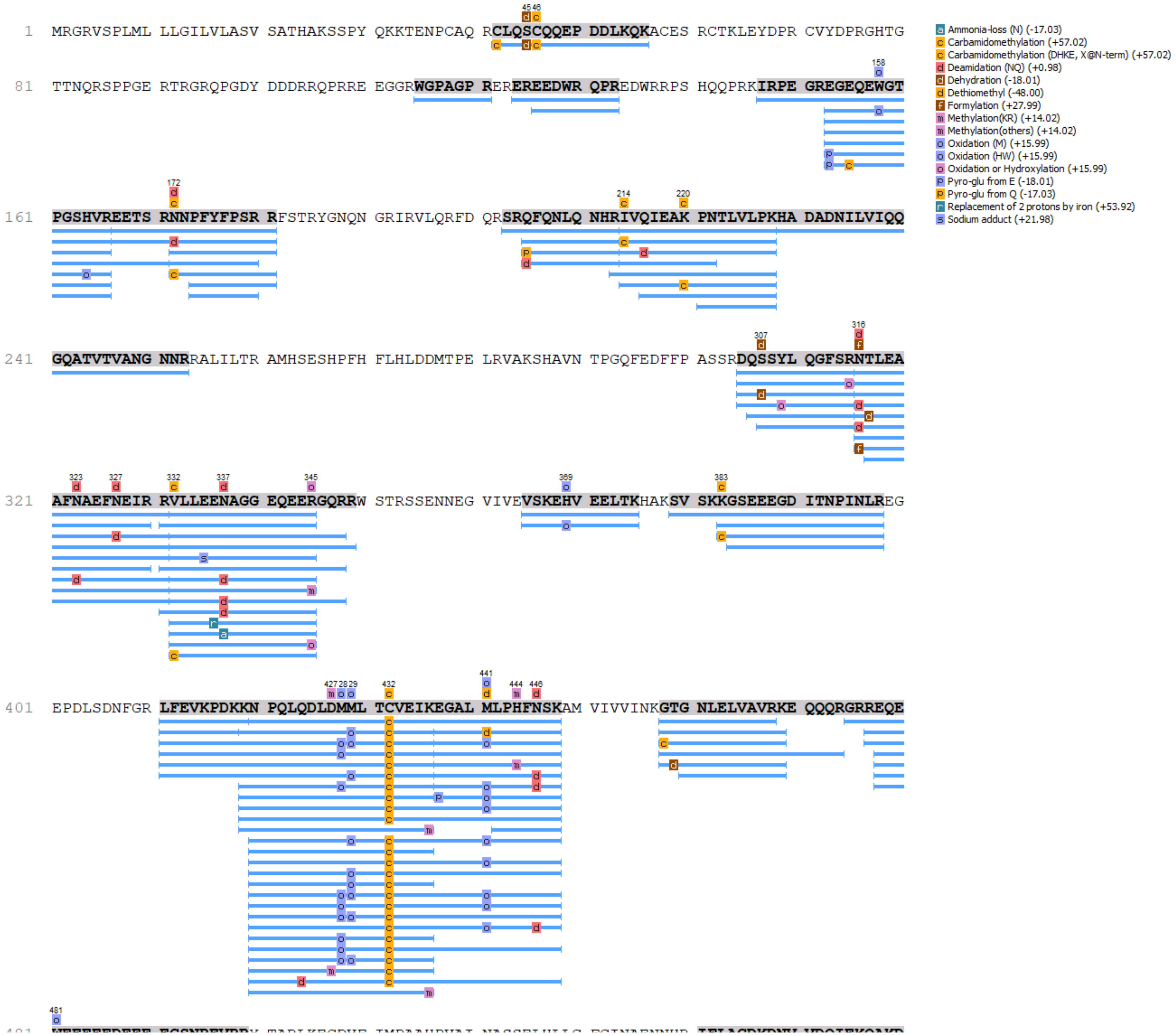
1 MRGRVSPLML LLGILVLASV SATHAKSSPY QKKTENPCAQ RCLQSCQQEP DDLKQKACES RCTKLEYDPR CVYDPRGHTG
81 TTNQRSPPGE RTRGRQPGDY DDDRRQPRRE EGGRWGPAGP REREREEDWR QPREDWRRPS HQQPRKIRPE GREGEQEWGT
161 PGSHVREETS RNNPFYFPSR RFSTRYGNQN GRIRVLQRFD QRSRQFQNLQ NHRIVQIEAK PNTLVLPKHA DADNILVIQQ
241 GQATVTVANG NNRRALILTR AMHSESHPFH FLHLDDMTPE LRVAKSHAVN TPGQFEDFFP ASSRDQSSYL QGFSRNTLEA
321 AFNAEFNEIR RVLLEENAGG EQEERGQRRW STRSSENNEG VIVEVSKEHV EELTKHAKSV SKKGSEEEGD ITNPINLREG
401 EPDLSDNFGR LFEVKPDKKN PQLQDLDMML TCVEIKEGAL MLPHFNSKAM VIVVINKGTG NLELVAVRKE QQQRGRREQE
Ammonia-loss (N) (-17.03)
Carbamidomethylation (+57.02)
Carbamidomethylation (DHKE, X@N-term) (+57.02)
Deamidation (NQ) (+0.98)
Dehydration (-18.01)
Dethiomethyl (-48.00)
Formylation (+27.99)
Methylation(KR) (+14.02)
Methylation(others) (+14.02)
Oxidation (M) (+15.99)
Oxidation (HW) (+15.99)
Oxidation or Hydroxylation (+15.99)
Pyro-glu from E (-18.01)
Pyro-glu from Q (-17.03)
Replacement of 2 protons by iron (+53.92)
Sodium adduct (+21.98)

[illegible]

Supporting Peptides:

561 LAFPGSGEQV EKLIKNQRES HFVSARPQSQ SPSSPEKEDQ EEENQGGKGP LLSILKAFN

Peptide	Uniq	-10lgP	Mass	Length	ppm	m/z	z	RT	Fraction	Scan	Source File	Area Roasted in gel	#Feature	#Feature Roasted in gel	Start	End	PTM	AScore	Found By
K.KNPQLQDLDMMLTC(+57.02)VEIK.E	N	136.41	2175.0737	18	1.4	1088.5457	2	35.26	43	F43:1808	OB5956 H1 Ro.raw	1.4906E7	6	6	419	436	Carbamidomethylation	C14:Carbamidomethylation:1000.00	PEAKS DB
R.NTLEAAFNAEFNEIR.R	N	130.93	1737.8322	15	1.8	869.9249	2	35.34	38	F38:1714	OB5951 H3A Ro.raw	1.332E7	16	16	316	330			PEAKS DB
K.NPQLQDLDMMLTC(+57.02)VEIK.E	N	128.40	2046.9788	17	0.2	1024.4968	2	37.11	43	F43:1936	OB5956 H1 Ro.raw	3.4643E7	10	10	420	436	Carbamidomethylation	C13:Carbamidomethylation:1000.00	PEAKS DB
K.NPQLQDLDM(+15.99)MLTC(+57.02)VEIK.E	N	128.16	2062.9736	17	-4.2	1032.4897	2	36.93	43	F43:1892	OB5956 H1 Ro.raw	3.3834E6	3	3	420	436	Oxidation (M); Carbamidomethylation	M9:Oxidation (M):30.46;C13:Carbamidomethylation:1000.00	PEAKS DB
K.NPQLQDLDMM(+15.99)LTC(+57.02)VEIK.E	N	127.13	2062.9736	17	5.0	1032.4993	2	34.71	43	F43:1780	OB5956 H1 Ro.raw	1.0665E7	4	4	420	436	Oxidation (M); Carbamidomethylation	M10:Oxidation (M):30.46;C13:Carbamidomethylation:1000.00	PEAKS DB
R.EQEWEEEEEDEEEEGSNR.E	N	125.55	2280.8413	18	3.9	1141.4324	2	28.35	43	F43:1396	OB5956 H1 Ro.raw	4.3429E6	7	7	478	495			PEAKS DB
K.NPQLQDLDM(+15.99)M(+15.99)LTC(+57.02)VEIK.E	N	121.59	2078.9688	17	6.6	1040.4985	2	33.82	43	F43:1726	OB5956 H1 Ro.raw	6.8984E4	2	2	420	436	Oxidation (M); Carbamidomethylation	M9:Oxidation (M):1000.00;M10:Oxidation (M):1000.00;C13:Carbamidomethylation:1000.00	PEAKS DB
K.KNPQLQDLDM(+15.99)MLTC(+57.02)VEIK.E	N	118.68	2191.0686	18	3.6	1096.5455	2	34.71	43	F43:1781	OB5956 H1 Ro.raw	1.3598E6	4	4	419	436	Oxidation (M); Carbamidomethylation	M10:Oxidation (M):27.96;C14:Carbamidomethylation:1000.00	PEAKS DB
K.KGSEEEGDITNPINLR.E	N	117.89	1770.8748	16	0.6	886.4452	2	28.91	43	F43:1429	OB5956 H1 Ro.raw	3.1542E7	8	8	383	398			PEAKS DB
K.KNPQLQDLDMM(+15.99)LTC(+57.02)VEIK.E	N	110.35	2191.0686	18	3.5	1096.5454	2	32.98	42	F42:1657	OB5955 H1 Ro.raw	1.6495E6	4	4	419	436	Oxidation (M); Carbamidomethylation	M11:Oxidation (M):23.10;C14:Carbamidomethylation:1000.00	PEAKS DB
R.VLLEENAGGEQEER.G	N	108.33	1571.7427	14	2.4	786.8805	2	26.83	42	F42:1293	OB5955 H1 Ro.raw	2.9858E7	10	10	332	345			PEAKS DB
K.GSEEEGDITNPINLR.E	N	108.06	1642.7798	15	-0.5	822.3967	2	30.80	42	F42:1519	OB5955 H1 Ro.raw	1.1767E7	3	3	384	398			PEAKS DB
R.DQSSYLQGFSR.N	N	105.08	1286.5891	11	0.8	644.3024	2	30.81	40	F40:1431	OB5953 H3A Ro.raw	9.5783E7	31	31	305	315			PEAKS DB
R.EGEQEWGTPGSHVR.E	N	104.71	1567.7015	14	2.6	784.8601	2	25.68	42	F42:1206	OB5955 H1 Ro.raw	3.4943E6	6	6	153	166			PEAKS DB
R.V(+57.02)LLEENAGGEQEER.G	N	103.80	1628.7642	14	0.3	815.3896	2	27.08	43	F43:1322	OB5956 H1 Ro.raw	4.0193E5	3	3	332	345	Carbamidomethylation (DHKE, X@N-term)	V1:Carbamidomethylation (D	PEAKS PTM

total 156 peptides

Peptide	Uniq	-10lgP	Mass	Length	ppm	m/z	z	RT	Fraction	Scan	Source File	Area Roasted in gel	#Feature	#Feature Roasted in gel	Start	End	PTM	AScore	Found By
																		HKE, X@N -term):4 9.37	
K.NPQLQDLDMMLTCVEIK(+14.02).E	N	103.68	2003.9730	17	-1.2	1002.9926	2	38.20	43	F43:1989	OB5956 H1 Ro.raw	5.9168E6	4	4	420	436	Methylation(KR)	K17:Meth ylation(K R):1000.0 0	PEAKS PTM
K.DLAFPGSGEQVEK.L	N	100.93	1375.6619	13	-1.9	688.8369	2	30.34	43	F43:1508	OB5956 H1 Ro.raw	9.196E7	8	8	560	572			PEAKS DB
K.NPQLQDLDM(+15.99)MLTC(+57.02)VEIKEGALMLPHFNSK.A	N	100.40	3387.6335	29	-0.6	1130.2178	3	37.70	41	F41:1939	OB5954 H1 Ro.raw	8.7706E7	6	6	420	448	Oxidation (M); Carbamidomethylation	M9:Oxidat ion (M):2 6.02;C13: Carbamid omethylat ion:1000. 00	PEAKS DB
R.RVLLEENAGGEQEER.G	N	100.35	1727.8438	15	0.2	576.9553	3	25.30	42	F42:1185	OB5955 H1 Ro.raw	8.2964E5	6	6	331	345			PEAKS DB
K.NPQLQDLDMMLTC(+57.02)VEIKEGALMLPHFNSK.A	N	98.85	3371.6387	29	-2.7	843.9147	4	38.02	43	F43:1969	OB5956 H1 Ro.raw	1.5351E8	12	12	420	448	Carbamidomethylation	C13:Carb amidomet hylation:1 000.00	PEAKS DB
K.KNPQLQDLDMMLTC(+57.02)VEIKEGALMLPHFNSK.A	N	97.96	3499.7336	30	-3.9	875.9373	4	36.80	41	F41:1884	OB5954 H1 Ro.raw	6.677E6	4	4	419	448	Carbamidomethylation	C14:Carb amidomet hylation:1 000.00	PEAKS DB
R.VLLEEN(+.98)AGGEQEER.G	N	94.48	1572.7267	14	2.8	787.3728	2	27.46	43	F43:1340	OB5956 H1 Ro.raw	6.2395E5	3	3	332	345	Deamidation (NQ)	N6:Deami dation (N Q):121.37	PEAKS DB
R.REQEWEEEEEDEEEEGSNR.E	N	93.49	2436.9424	19	0.1	813.3215	3	26.72	43	F43:1292	OB5956 H1 Ro.raw	8.6025E5	7	7	477	495			PEAKS DB
R.NNPFYFPSRR.F	N	89.95	1296.6364	10	-2.5	649.3239	2	28.89	43	F43:1417	OB5956 H1 Ro.raw	1.3445E8	22	22	172	181			PEAKS DB
K.D(+57.02)LAFPGSGEQVEK.L	N	88.60	1432.6833	13	-0.1	717.3489	2	31.08	41	F41:1535	OB5954 H1 Ro.raw	9.7986E5	3	3	560	572	Carbamidomethylation (DHKE, X@N-term)	D1:Carba midometh ylation (D HKE, X@N -term):13 1.10	PEAKS PTM
R.IVQIEAKPNTLVLPK.H	N	88.27	1662.0079	15	0.9	832.0120	2	30.16	43	F43:1505	OB5956 H1 Ro.raw	2.8901E7	31	31	214	228			PEAKS DB
K.EGALMLPHFNSK.A	N	88.21	1342.6703	12	2.5	672.3441	2	30.67	43	F43:1529	OB5956 H1 Ro.raw	1.4087E7	5	5	437	448			PEAKS DB
K.NPQLQDLDMM(-48.00)LTC(+57.02)VEIK.E	N	88.05	1998.9755	17	1.9	1000.4969	2	32.85	43	F43:1670	OB5956 H1 Ro.raw	5.9032E6	3	3	420	436	Carbamidomethylation	M10:Dethi omethyl:1 4.02;C13: Carbamid omethylat ion:1000. 00	PEAKS PTM
R.GRREQEWEEEEEDEEEEGSNR.E	N	87.21	2650.0649	21	1.1	884.3632	3	26.73	41	F41:1280	OB5954 H1 Ro.raw	7.9956E5	6	6	475	495			PEAKS DB
R.NNPFYFPSR.R	N	86.35	1140.5352	9	-0.1	571.2748	2	31.76	43	F43:1593	OB5956 H1 Ro.raw	1.7218E7	11	11	172	180			PEAKS DB
R.EGEQEWGTPGSHVREETSR.N	N	83.87	2169.9675	19	2.2	724.3314	3	26.51	43	F43:1287	OB5956 H1 Ro.raw	5.5466E5	3	3	153	171			PEAKS DB
K.KNPQLQDLDMMLTC(+57.02)VEIKEGALM(+15.99)LPHFNSK.A	N	83.11	3515.7285	30	-1.9	879.9377	4	36.03	43	F43:1857	OB5956 H1 Ro.raw	6.6263E6	3	3	419	448	Carbamidomethylation; Oxidation (M)	C14:Carb amidomet hylation:1 000.00;M 23:Oxidati on (M):11 4.34	PEAKS DB
**total 156 peptides**																			

Peptide	Uniq	-10lgP	Mass	Length	ppm	m/z	z	RT	Fraction	Scan	Source File	Area Roasted in gel	#Feature	#Feature Roasted in gel	Start	End	PTM	AScore	Found By
R.NTLEAAFNAEFNEIRR.V	N	82.99	1893.9332	16	-2.6	947.9714	2	34.11	42	F42:1711	OB5955 H1 Ro.raw	3.0959E8	36	35	316	331			PEAKS DB
K.DLAFPGSGEQVEKLIK.N	N	80.89	1729.9249	16	-1.6	865.9706	2	33.95	30	F30:1478	OB5926 H3B Ro.raw	3.9305E5	5	4	560	575			PEAKS DB
K.EGALM(+15.99)LPHFNSK.A	N	80.48	1358.6653	12	1.1	680.3406	2	29.85	43	F43:1485	OB5956 H1 Ro.raw	3.0314E6	5	5	437	448	Oxidation (M)	M5:Oxidation (M):1000.00	PEAKS DB
K.GTGNLELVAVR.K	N	80.39	1127.6299	11	-1.3	564.8215	2	30.21	41	F41:1476	OB5954 H1 Ro.raw	2.4706E7	5	5	458	468			PEAKS DB
R.NTLEAAFN(+.98)AEFNEIRR.V	N	79.02	1894.9172	16	-2.2	948.4638	2	32.35	41	F41:1616	OB5954 H1 Ro.raw	1.3357E6	5	5	316	331	Deamidation (NQ)	N8:Deamidation (NQ):48.12	PEAKS DB
R.VLLEENAGGEQEER(+14.02).G	N	78.92	1585.7583	14	0.7	793.8870	2	27.86	41	F41:1339	OB5954 H1 Ro.raw	1.6341E5	3	3	332	345	Methylation(KR)	R14:Methylation(KR):1000.00	PEAKS PTM
K.GTGNLELVAVRK.E	N	78.87	1255.7249	12	0.7	628.8701	2	27.30	41	F41:1322	OB5954 H1 Ro.raw	2.29E7	10	10	458	469			PEAKS DB
R.QFQNLQNHR.I	N	78.35	1183.5846	9	0.0	592.7996	2	23.81	42	F42:1102	OB5955 H1 Ro.raw	1.5125E5	6	6	205	213			PEAKS DB
K.K(+57.02)GSEEEGDITNPINLR.E	N	78.20	1827.8962	16	-0.5	914.9550	2	28.83	42	F42:1409	OB5955 H1 Ro.raw	1.3158E5	2	2	383	398	Carbamidomethylation (DHKE, X@N-term)	K1:Carbamidomethylation (DHKE, X@N-term):32.46	PEAKS PTM
K.NPQLQDLDMM(+15.99)LTC(+57.02)VEIKEGALMLPHFNSK.A	N	78.01	3387.6335	29	-0.8	1130.2175	3	36.42	41	F41:1862	OB5954 H1 Ro.raw	6.8935E7	6	6	420	448	Oxidation (M); Carbamidomethylation	M10:Oxidation (M):24.44;C13:Carbamidomethylation:1000.00	PEAKS DB
R.LFEVKPDKKNPQLQDLDMMLTC(+57.02)VEIK.E	N	77.87	3131.6069	26	-4.0	1044.8721	3	34.65	42	F42:1756	OB5955 H1 Ro.raw	5.0788E6	4	4	411	436	Carbamidomethylation	C22:Carbamidomethylation:1000.00	PEAKS DB
K.NPQLQDLDM(+15.99)MLTC(+57.02)VEIKEGALM(+15.99)LPHFNSK.A	N	77.10	3403.6284	29	1.1	851.9153	4	37.51	41	F41:1883	OB5954 H1 Ro.raw	7.3887E6	7	7	420	448	Oxidation (M); Carbamidomethylation	M9:Oxidation (M):27.96;C13:Carbamidomethylation:1000.00;M22:Oxidation (M):71.31	PEAKS DB
R.EETSRN(+.98)NPFYFPSRR.F	N	75.62	1899.8864	15	-0.2	634.3026	3	28.39	41	F41:1381	OB5954 H1 Ro.raw	3.2976E5	2	2	167	181	Deamidation (NQ)	N6:Deamidation (NQ):33.98	PEAKS DB
R.Q(-17.03)FQNLQNHR.I	N	75.35	1166.5581	9	-1.2	584.2856	2	28.00	43	F43:1365	OB5956 H1 Ro.raw	4.927E6	4	4	205	213	Pyro-glu from Q	Q1:Pyro-glu from Q:1000.00	PEAKS PTM
K.NPQLQDLDM(+15.99)M(+15.99)LTC(+57.02)VEIKEGALMLPHFNSK.A	N	73.00	3403.6284	29	-3.9	1135.5457	3	35.83	43	F43:1849	OB5956 H1 Ro.raw	7.8068E6	5	5	420	448	Oxidation (M); Carbamidomethylation	M9:Oxidation (M):54.18;M10:Oxidation (M):63.99;C13:Carbamidomethylation:1000.00	PEAKS DB
K.KNPQLQDLDM(+15.99)MLTC(+57.02)VEIKEGALM(+15.99)LPHFNSK.A	N	72.71	3531.7234	30	-1.9	883.9364	4	35.86	41	F41:1821	OB5954 H1 Ro.raw	7.53E5	1	1	419	448	Carbamidomethylation; Oxidation (M)	M10:Oxidation (M):17.01;C14:Carbamidomethylation:1000.	PEAKS DB

total 156 peptides

Peptide	Uniq	-10lgP	Mass	Length	ppm	m/z	z	RT	Fraction	Scan	Source File	Area Roasted in gel	#Feature	#Feature Roasted in gel	Start	End	PTM	AScore	Found By
																		00;M23:Oxidation (M):85.25	
R.VLLEENAGGEQEERGQR.R	N	72.44	1912.9238	17	0.6	957.4698	2	25.30	42	F42:1191	OB5955H1Ro.raw	3.1353E6	8	8	332	348			PEAKS DB
E.VSKEHVEELTK.H	N	72.42	1297.6877	11	-0.3	649.8510	2	24.21	41	F41:1125	OB5954H1Ro.raw	2.2246E5	5	5	365	375			PEAKS DB
K.QAKDLAFPGSGEQVEK.L	N	72.39	1702.8525	16	1.2	568.6255	3	27.08	43	F43:1321	OB5956H1Ro.raw	2.8232E5	6	6	557	572			PEAKS DB
K.EGALMLPHFN(+.98)SK.A	N	71.37	1343.6543	12	2.6	672.8362	2	31.66	43	F43:1595	OB5956H1Ro.raw	1.0101E5	2	2	437	448	Deamidation (NQ)	N10:Deamidation (NQ):1000.00	PEAKS DB
R.EQEW(+15.99)EEEEEDEEEEGSNR.E	N	70.36	2296.8362	18	3.2	1149.4291	2	27.67	41	F41:1333	OB5954H1Ro.raw	1.024E6	3	3	478	495	Oxidation (HW)	W4:Oxidation (HW):1000.00	PEAKS PTM
R.N(+.98)TLEAAFNAEFNEIRR.V	N	69.73	1894.9172	16	0.4	948.4662	2	32.39	42	F42:1620	OB5955H1Ro.raw	2.6319E6	3	3	316	331	Deamidation (NQ)	N1:Deamidation (NQ):41.52	PEAKS DB
R.LFEVKPDKKNPQLQDLDMM(+15.99)LTC(+57.02)VEIK.E	N	69.17	3147.6018	26	2.2	787.9095	4	33.24	42	F42:1670	OB5955H1Ro.raw	2.0797E6	2	2	411	436	Oxidation (M); Carbamidomethylation	M19:Oxidation (M):40.00;C22:Carbamidomethylation:1000.00	PEAKS DB
R.EGEQEWGTPGSH(+15.99)VR.E	N	68.71	1583.6964	14	0.8	792.8561	2	27.64	43	F43:1358	OB5956H1Ro.raw	1.1836E5	4	4	153	166	Oxidation (HW)	H12:Oxidation (HW):95.51	PEAKS PTM
R.I(+57.02)VQIEAKPNTLVLPK.H	N	68.01	1719.0294	15	-3.7	860.5188	2	30.87	43	F43:1547	OB5956H1Ro.raw	7.3731E5	5	4	214	228	Carbamidomethylation (DHKE, X@N-term)	I1:Carbamidomethylation (DHKE, X@N-term):109.97	PEAKS PTM
R.RVLLEEN(+.98)AGGEQEER.G	N	67.83	1728.8278	15	-0.8	577.2827	3	25.96	42	F42:1233	OB5955H1Ro.raw	1.359E4	2	2	331	345	Deamidation (NQ)	N7:Deamidation (NQ):77.53	PEAKS DB
R.DQS(-18.01)SYLQGFSR.N	N	67.70	1268.5785	11	0.2	635.2966	2	30.91	41	F41:1537	OB5954H1Ro.raw	6.4401E5	3	3	305	315	Dehydration	S3:Dehydration:40.00	PEAKS PTM
R.RVLLEENAGGEQEERGQR.R	N	67.67	2069.0249	18	1.9	690.6835	3	25.60	41	F41:1205	OB5954H1Ro.raw	1.8247E6	3	3	331	348			PEAKS DB
K.Q(-17.03)AKDLAFPGSGEQVEK.L	N	67.52	1685.8260	16	0.1	843.9204	2	29.98	43	F43:1503	OB5956H1Ro.raw	9.9003E4	3	3	557	572	Pyro-glu from Q	Q1:Pyro-glu from Q:1000.00	PEAKS PTM
K.NPQLQ(+.98)DLDMMLTC(+57.02)VEIK.E	N	67.25	2047.9629	17	1.5	1024.9902	2	44.46	43	F43:2348	OB5956H1Ro.raw	0	0	0	420	436	Carbamidomethylation	Q5:Deamidation (NQ):19.68;C13:Carbamidomethylation:1000.00	PEAKS DB
R.WGPAGPR.E	N	66.93	739.3765	7	1.0	370.6959	2	25.22	41	F41:1191	OB5954H1Ro.raw	3.1676E5	3	3	115	121			PEAKS DB
K.KNPQLQDLDMMLTCVEIK(+14.02).E	N	66.06	2132.0679	18	-0.6	1067.0405	2	36.31	41	F41:1858	OB5954H1Ro.raw	5.2356E5	2	2	419	436	Methylation(KR)	K18:Methylation(KR):130.57	PEAKS PTM
R.EETSRNNPFYFPSRR.F	N	65.61	1898.9023	15	1.8	633.9759	3	27.75	42	F42:1339	OB5955H1Ro.raw	8.693E5	4	4	167	181			PEAKS DB
K.NPQLQDLDMMLTC(+57.02)VEIKEGALM(+15.99)LPHFNSK.A	N	64.76	3387.6335	29	-0.6	847.9152	4	37.63	43	F43:1915	OB5956H1Ro.raw	8.7706E7	6	6	420	448	Carbamidomethylation; Oxidation (M)	C13:Carbamidomethylation:1000.00;M	PEAKS DB

total 156 peptides

Peptide	Uniq	-10lgP	Mass	Length	ppm	m/z	z	RT	Fraction	Scan	Source File	Area Roasted in gel	#Feature	#Feature Roasted in gel	Start	End	PTM	AScore	Found By
																		22:Oxidation (M):82.05	
A.FPGSGEQVEK.L	N	64.10	1076.5138	10	-3.4	539.2623	2	30.29	42	F42:1495	OB5955 H1 Ro.raw	4.0412E5	3	3	563	572			PEAKS DB
R.SRQFQNLQNHR.I	N	63.98	1426.7178	11	0.3	476.5800	3	24.16	43	F43:1148	OB5956 H1 Ro.raw	5.7314E4	3	3	203	213			PEAKS DB
R.LFEVKPDKK.N	N	63.79	1102.6385	9	0.6	552.3269	2	24.50	43	F43:1159	OB5956 H1 Ro.raw	1.1348E6	6	6	411	419			PEAKS DB
Q.SSYLQGFSR.N	N	63.16	1043.5035	9	1.4	522.7598	2	29.29	41	F41:1438	OB5954 H1 Ro.raw	6.2708E4	3	3	307	315			PEAKS DB
R.QFQNLQN(+.98)HR.I	N	63.09	1184.5686	9	5.4	593.2948	2	23.60	43	F43:1114	OB5956 H1 Ro.raw	8.0412E2	1	1	205	213		N7:Deamidation (NQ):0.00	PEAKS DB
K.EGALMLPH(+14.02)FNSK.A	N	63.06	1356.6860	12	-4.2	679.3475	2	30.85	43	F43:1554	OB5956 H1 Ro.raw	2.9707E5	3	3	437	448	Methylation(others)	H8:Methylation(others):65.81	PEAKS PTM
K.NPQLQDLDMM(+15.99)LTC(+57.02)VEIKEGALM(+15.99)LPHFNSK.A	N	61.19	3403.6284	29	0.3	851.9147	4	35.86	41	F41:1801	OB5954 H1 Ro.raw	3.4294E6	4	4	420	448	Oxidation (M); Carbamidomethylation	M10:Oxidation (M):21.94;C13:Carbamidomethylation:1000.00;M22:Oxidation (M):61.50	PEAKS DB
K.G(+57.02)TGNLELVAVRK.E	N	60.73	1312.7462	12	-0.4	657.3801	2	27.30	41	F41:1303	OB5954 H1 Ro.raw	3.6978E5	2	2	458	469	Carbamidomethylation (DHKE, X@N-term)	G1:Carbamidomethylation (DHKE, X@N-term):55.46	PEAKS PTM
R.EQEWEEEEEDEEEEGSNREVRR.Y	N	60.58	2821.1545	22	1.9	941.3939	3	26.71	43	F43:1297	OB5956 H1 Ro.raw	1.0274E6	4	4	478	499			PEAKS DB
K.NPQLQDLDM(+15.99)M(+15.99)LTC(+57.02)VEIKEGALM(+15.99)LPHFNSK.A	N	60.17	3419.6233	29	-0.9	855.9124	4	34.83	42	F42:1766	OB5955 H1 Ro.raw	1.2322E6	3	3	420	448	Oxidation (M); Carbamidomethylation	M9:Oxidation (M):1000.00;M10:Oxidation (M):1000.00;C13:Carbamidomethylation:1000.00;M22:Oxidation (M):1000.00	PEAKS DB
R.LFEVKPDKKNPQLQ(+.98)DLDMM(+15.99)LTC(+57.02)VEIK.E	N	59.38	3148.5857	26	7.4	788.1595	4	33.17	42	F42:1670	OB5955 H1 Ro.raw	3.9804E5	1	1	411	436	Oxidation (M); Carbamidomethylation	Q14:Deamidation (NQ):0.00;M19:Oxidation (M):23.10;C22:Carbamidomethylation:1000.00	PEAKS DB
R.VLLEEN(-17.03)AGGEQEER.G	N	59.22	1554.7162	14	1.2	778.3663	2	27.73	42	F42:1335	OB5955 H1 Ro.raw	0	0	0	332	345	Ammonia-loss (N)	N6:Ammonia-loss (N):1000.00	PEAKS PTM
K.GSE(+57.02)EEGDITNPINLR.E	N	58.95	1699.8013	15	-3.0	850.9054	2	30.85	43	F43:1556	OB5956 H1 Ro.raw	4.2816E4	1	1	384	398		E3:Carbamidomethylation (DHKE, X@N-term):0.00	PEAKS PTM
**total 156 peptides**																			

Peptide	Uniq	-10lgP	Mass	Length	ppm	m/z	z	RT	Fraction	Scan	Source File	Area Roasted in gel	#Feature	#Feature Roasted in gel	Start	End	PTM	AScore	Found By
R.LFEVKPDK.K	N	58.71	974.5436	8	1.7	488.2799	2	25.30	42	F42:1183	OB5955 H1 Ro.raw	1.7315E6	6	6	411	418			PEAKS DB
R.N(+57.02)NPFYFPSRR.F	N	58.56	1353.6577	10	1.1	677.8369	2	29.74	42	F42:1453	OB5955 H1 Ro.raw	9.24E5	3	3	172	181	Carbamidomethylation (DHKE, X@N-term)	N1:Carbamidomethylation (DHKE, X@N-term):1000.00	PEAKS PTM
R.E(-18.01)GE(+57.02)QEWGTPGSHVR.E	N	58.23	1606.7124	14	0.2	804.3636	2	27.56	42	F42:1323	OB5955 H1 Ro.raw	2.2302E5	3	3	153	166	Pyro-glu from E; Carbamidomethylation (DHKE, X@N-term)	E1:Pyro-glu from E:1000.00;E3:Carbamidomethylation (DHKE, X@N-term):60.92	PEAKS PTM
K.G(+57.02)TGNLELVAVR.K	N	57.51	1184.6514	11	-2.7	593.3314	2	30.10	42	F42:1486	OB5955 H1 Ro.raw	1.7196E5	2	2	458	468		G1:Carbamidomethylation (DHKE, X@N-term):19.78	PEAKS PTM
N.PFYFPSRR.F	N	57.34	1068.5504	8	1.1	535.2831	2	28.95	41	F41:1414	OB5954 H1 Ro.raw	4.6082E6	5	5	174	181			PEAKS DB
K.SVSKKGSEEEGDITNPINLR.E	N	57.26	2172.1023	20	0.9	725.0420	3	27.48	41	F41:1325	OB5954 H1 Ro.raw	1.6504E5	2	2	379	398			PEAKS DB
R.LFEVKPDKKNPQLQDLDM(+15.99)MLTC(+57.02)VEIK.E	N	57.09	3147.6018	26	-1.9	787.9062	4	34.20	41	F41:1734	OB5954 H1 Ro.raw	2.4894E6	4	4	411	436	Oxidation (M); Carbamidomethylation	M18:Oxidation (M):33.98;C22:Carbamidomethylation:1000.00	PEAKS DB
K.IRPEGREGEQEWGTPGSHVR.E	N	56.62	2276.1045	20	0.5	570.0337	4	27.56	42	F42:1329	OB5955 H1 Ro.raw	8.9716E4	2	2	147	166			PEAKS DB
R.NTLEAAFNAEFN(+.98)EIRR.V	N	54.77	1894.9172	16	1.8	948.4676	2	33.04	43	F43:1685	OB5956 H1 Ro.raw	2.2573E6	5	5	316	331	Deamidation (NQ)	N12:Deamidation (NQ):39.02	PEAKS DB
K.HADADNILVIQQGQATVTVANGNNR.R	N	53.13	2618.3162	25	0.5	873.7798	3	30.34	38	F38:1426	OB5951 H3A Ro.raw	1.8654E3	1	1	229	253			PEAKS DB
R.IVQIEAK(+57.02)PNTLVLPK.H	N	53.06	1719.0294	15	-1.6	860.5206	2	30.29	43	F43:1512	OB5956 H1 Ro.raw	2.587E5	1	1	214	228	Carbamidomethylation (DHKE, X@N-term)	K7:Carbamidomethylation (DHKE, X@N-term):25.69	PEAKS PTM
R.IVQIEAKPN.T	N	52.62	1010.5760	9	-0.3	506.2951	2	25.40	42	F42:1199	OB5955 H1 Ro.raw	0	0	0	214	222			PEAKS DB
K.KNPQLQ(+.98)DLDMMLTC(+57.02)VEIKEGALMLPHFNSK.A	N	51.97	3500.7175	30	3.4	876.1896	4	36.93	43	F43:1903	OB5956 H1 Ro.raw	9.2646E6	2	2	419	448	Carbamidomethylation	Q6:Deamidation (NQ):14.04;C14:Carbamidomethylation:1000.00	PEAKS DB
R.E(-18.01)GEQEWGTPGSHVR.E	N	51.90	1549.6909	14	0.7	775.8533	2	27.75	42	F42:1342	OB5955 H1 Ro.raw	3.4987E5	3	3	153	166	Pyro-glu from E	E1:Pyro-glu from E:1000.00	PEAKS PTM
K.NPQLQDLDMM(+15.99)LTC(+57.02)VEIKEGALMLPHFN(+.98)SK.A	N	49.99	3388.6174	29	6.1	848.1668	4	36.39	43	F43:1880	OB5956 H1 Ro.raw	1.7975E6	1	1	420	448	Oxidation (M); Carbamidomethylation	M10:Oxidation (M):27.96;C13:Carbamidomethylation:1000.00;N27:Deamidatio	PEAKS DB
**total 156 peptides**																			

Peptide	Uniq	-10lgP	Mass	Length	ppm	m/z	z	RT	Fraction	Scan	Source File	Area Roasted in gel	#Feature	#Feature Roasted in gel	Start	End	PTM	AScore	Found By
																		n (NQ):18.27	
R.N(-17.03)NPFYFPSRR.F	N	49.99	1279.6097	10	1.1	427.5443	3	28.89	43	F43:1434	OB5956 H1 Ro.raw	5.674E5	3	3	172	181		N1:Ammonia-loss (N):9.34	PEAKS PTM
R.EQEWEEEEEDEEEEGSNREVR.R	N	49.84	2665.0535	21	2.4	889.3605	3	27.88	41	F41:1353	OB5954 H1 Ro.raw	4.9786E4	1	1	478	498			PEAKS DB
T.GNLELVAVRK.E	N	49.53	1097.6556	10	0.5	549.8354	2	27.00	42	F42:1296	OB5955 H1 Ro.raw	3.4872E5	3	3	460	469			PEAKS DB
F.PGSGEQVEK.L	N	49.06	929.4454	9	-3.7	465.7282	2	30.34	43	F43:1524	OB5956 H1 Ro.raw	5.2705E4	1	1	564	572			PEAKS DB
R.EEDWRQPR.E	N	48.87	1114.5155	8	1.8	558.2661	2	23.91	41	F41:1112	OB5954 H1 Ro.raw	1.6791E3	1	1	126	133			PEAKS DB
K.PNTLVLPK.H	N	48.84	880.5382	8	-2.9	441.2751	2	28.00	43	F43:1374	OB5956 H1 Ro.raw	1.9234E5	2	2	221	228			PEAKS DB
R.EGEQEW(+15.99)GTPGSHVR.E	N	47.73	1583.6964	14	-0.6	792.8550	2	25.22	41	F41:1196	OB5954 H1 Ro.raw	2.0714E4	1	1	153	166	Oxidation (HW)	W6:Oxidation (HW):72.73	PEAKS PTM
V.QIEAKPNTLVLPK.H	N	47.49	1449.8555	13	-3.8	725.9323	2	30.34	43	F43:1513	OB5956 H1 Ro.raw	4.7367E4	1	1	216	228			PEAKS DB
R.N(+.98)TLEAAFNAEFNEIR.R	N	46.63	1738.8162	15	9.2	870.4233	2	44.36	42	F42:2303	OB5955 H1 Ro.raw	5.4416E3	1	1	316	330	Deamidation (NQ)	N1:Deamidation (NQ):39.47	PEAKS DB
R.VLLE(+21.98)ENAGGEQEER.G	N	46.37	1593.7246	14	-1.1	797.8687	2	26.71	43	F43:1300	OB5956 H1 Ro.raw	4.0134E5	3	3	332	345	Sodium adduct	E4:Sodium adduct:40.00	PEAKS PTM
N.PFYFPSR.R	N	46.32	912.4493	7	-0.1	457.2319	2	31.70	42	F42:1584	OB5955 H1 Ro.raw	2.4653E5	4	4	174	180			PEAKS DB
K.GTGNLELVAVRKEQQQR.G	N	46.31	1925.0442	17	1.4	642.6896	3	26.67	43	F43:1294	OB5956 H1 Ro.raw	2.1018E4	1	1	458	474			PEAKS DB
K.NPQLQDLD(+14.02)MMLTC(+57.02)VEIK.E	N	45.99	2060.9944	17	0.2	1031.5046	2	37.33	41	F41:1934	OB5954 H1 Ro.raw	2.7407E4	1	1	420	436	Methylation(others); Carbamidomethylation	D8:Methylation(others):42.68; C13:Carbamidomethylation:1000.00	PEAKS PTM
E.VSKEH(+15.99)VEELTK.H	N	45.89	1313.6826	11	0.8	657.8491	2	24.35	43	F43:1164	OB5956 H1 Ro.raw	1.7904E4	3	3	365	375	Oxidation (HW)	H5:Oxidation (HW):1000.00	PEAKS PTM
K.GTGNLE(+57.02)LVAVRK.E	N	45.75	1312.7462	12	-0.8	657.3799	2	27.17	42	F42:1295	OB5955 H1 Ro.raw	1.9882E5	1	1	458	469		E6:Carbamidomethylation (DHKE, X@N-term):0.00	PEAKS PTM
R.VLLEEN(+.98)AGGEQEERGQR.R	N	45.60	1913.9078	17	-1.6	638.9755	3	25.99	41	F41:1234	OB5954 H1 Ro.raw	0	0	0	332	348	Deamidation (NQ)	N6:Deamidation (NQ):28.79	PEAKS DB
K.KNPQLQDLDMM(+15.99)LTC(+57.02)VEIKEGALM(+15.99)LPHFNSK.A	N	45.45	3531.7234	30	-1.1	883.9372	4	35.80	42	F42:1822	OB5955 H1 Ro.raw	0	0	0	419	448	Carbamidomethylation; Oxidation (M)	M11:Oxidation (M):0.00;C14:Carbamidomethylation:1000.00;M23:Oxidation (M):45.87	PEAKS DB
R.LFEVKPDKKNPQLQDLDM(+15.99)M(+15.99)LTC(+57.02)VEIK.E	N	45.43	3163.5967	26	-2.8	791.9042	4	32.90	41	F41:1650	OB5954 H1 Ro.raw	2.0535E6	3	3	411	436	Oxidation (M); Carbamidomethylation	M18:Oxidation (M):1000.00; M19:Oxidation (M):	PEAKS DB
**total 156 peptides**																			

Peptide	Uniq	-10lgP	Mass	Length	ppm	m/z	z	RT	Fraction	Scan	Source File	Area Roasted in gel	#Feature	#Feature Roasted in gel	Start	End	PTM	AScore	Found By
																		1000.00;C22:Carbamidomethylation:1000.00	
K.KNPQLQDLDM(+15.99)MLTC(+57.02)VEIKEGALMLPHFNSK.A	N	44.60	3515.7285	30	-1.9	879.9377	4	35.30	41	F41:1798	OB5954H1Ro.raw	6.4162E5	1	1	419	448	Carbamidomethylation	M10:Oxidation (M):14.02;C14:Carbamidomethylation:1000.00	PEAKS DB
R.DQSSYLQGFSR(+15.99).N	N	44.15	1302.5840	11	2.5	652.3009	2	30.79	43	F43:1544	OB5956H1Ro.raw	1.4308E5	3	3	305	315	Oxidation or Hydroxylation	R11:Oxidation or Hydroxylation:42.88	PEAKS PTM
R.Q(+.98)FQNLQNHR.I	N	43.70	1184.5686	9	4.6	593.2943	2	23.68	42	F42:1098	OB5955H1Ro.raw	0	0	0	205	213	Deamidation (NQ)	Q1:Deamidation (NQ):39.25	PEAKS DB
K.NPQLQDLDMM(+15.99)LTC(+57.02)VEIKEGALM(+15.99)LPHFN(+.98)SK.A	N	41.25	3404.6125	29	-0.4	1135.8777	3	36.69	42	F42:1878	OB5955H1Ro.raw	3.8423E5	1	1	420	448	Carbamidomethylation; Oxidation (M); Deamidation (NQ)	M10:Oxidation (M):0.00;C13:Carbamidomethylation:1000.00;M22:Oxidation (M):39.23;N27:Deamidation (NQ):69.52	PEAKS DB
R.IFLAGDKDNVVD(+17.03)QIEK.Q	Y	41.19	1819.9679	16	-3.9	607.6609	3	32.67	43	F43:1692	OB5956H1Ro.raw	3.5399E6	2	2	541	556		D12:Replacement of proton with ammonium ion:16.65	PEAKS PTM
R.REQEW(+15.99)EEEEEDEEEEGSNR.E	N	41.18	2452.9375	19	-0.6	818.6526	3	25.98	41	F41:1239	OB5954H1Ro.raw	2.4114E4	1	1	477	495	Oxidation (HW)	W5:Oxidation (HW):1000.00	PEAKS PTM
K.DLAFP(+15.99)GSGEQVEK.L	N	41.14	1391.6569	13	0.5	696.8361	2	29.36	42	F42:1432	OB5955H1Ro.raw	7.4528E5	3	3	560	572	Oxidation or Hydroxylation	P5:Oxidation or Hydroxylation:67.76	PEAKS PTM
K.EGALM(-48.00)LPHFNSK.A	N	40.53	1294.6670	12	0.5	432.5632	3	28.76	41	F41:1389	OB5954H1Ro.raw	2.208E6	2	2	437	448	Dethiomethyl	M5:Dethiomethyl:1000.00	PEAKS PTM
R.IVQ(+.98)IEAKPNTLVLPK.H	N	40.35	1662.9919	15	8.1	832.5100	2	30.29	42	F42:1514	OB5955H1Ro.raw	3.5627E6	1	1	214	228	Deamidation (NQ)	Q3:Deamidation (NQ):22.65	PEAKS DB
R.VLLEENAGGEQEERGQRR.W	N	40.17	2069.0249	18	1.0	518.2640	4	25.49	42	F42:1205	OB5955H1Ro.raw	2.0305E5	2	2	332	349			PEAKS DB
R.VLLEENAGGEQEER(+15.99).G	N	39.98	1587.7375	14	1.8	794.8775	2	26.70	41	F41:1275	OB5954H1Ro.raw	0	0	0	332	345	Oxidation or Hydroxylation	R14:Oxidation or Hydroxylation:20.88	PEAKS PTM
R.N(+27.99)TLEAAFNAEFNEIRR.V	N	39.68	1921.9282	16	-1.7	961.9697	2	37.51	41	F41:1908	OB5954H1Ro.raw	3.7767E4	1	1	316	331	Formylation	N1:Formylation:1000.00	PEAKS PTM
K.E(-18.01)GALM(+15.99)LPHFNSK.A	N	39.64	1340.6547	12	0.2	447.8923	3	30.21	41	F41:1479	OB5954H1Ro.raw	7.81E5	1	1	437	448	Pyro-glu from E; Oxidation (M)	E1:Pyro-glu from E:1000.00;M5:Oxidation (M):1000.00	PEAKS PTM
R.VLLEE(+53.92)NAGGEQEER.G	N	39.58	1625.6620	14	-1.0	542.8940	3	26.76	42	F42:1278	OB5955H1Ro.raw	0	0	0	332	345	Replacement of 2 protons by iron	E5:Replacement of 2 protons by iron:20.92	PEAKS PTM

total 156 peptides

Peptide	Uniq	-10lgP	Mass	Length	ppm	m/z	z	RT	Fraction	Scan	Source File	Area Roasted in gel	#Feature	#Feature Roasted in gel	Start	End	PTM	AScore	Found By
K.NPQLQ(+.98)DLDMM(+15.99)LTC(+57.02)VEIKEGALM(+15.99)LPHFNSK.A	N	39.15	3404.6125	29	-1.8	852.1589	4	38.22	43	F43:1993	OB5956 H1 Ro.raw	7.6266E5	1	1	420	448	Deamidation (NQ); Carbamidomethylation	Q5:Deamidation (NQ):49.79;M10:Oxidation (M):0.00;C13:Carbamidomethylation:1000.00;M22:Oxidation (M):14.60	PEAKS DB
K.N(+.98)PQLQDLDM(+15.99)MLTC(+57.02)VEIKEGALMLPHFNSK.A	N	38.83	3388.6174	29	8.2	1695.3300	2	37.72	41	F41:1941	OB5954 H1 Ro.raw	6.4698E5	1	1	420	448	Carbamidomethylation	N1:Deamidation (NQ):0.00;M9:Oxidation (M):0.00;C13:Carbamidomethylation:1000.00	PEAKS DB
K.D(+15.99)LAFPGSGEQVEK.L	N	38.81	1391.6569	13	0.5	696.8361	2	29.36	42	F42:1368	OB5955 H1 Ro.raw	2.5725E5	1	1	560	572	Oxidation or Hydroxylation	D1:Oxidation or Hydroxylation:77.13	PEAKS PTM
K.NPQ(+.98)LQDLDMMLTC(+57.02)VEIK.E	N	38.70	2047.9629	17	2.9	1024.9917	2	46.45	41	F41:2441	OB5954 H1 Ro.raw	0	0	0	420	436	Carbamidomethylation	Q3:Deamidation (NQ):14.04;C13:Carbamidomethylation:1000.00	PEAKS DB
K.DLAFPGSGE(+53.92)QVEK.L	N	37.31	1429.5812	13	-6.3	715.7933	2	30.34	43	F43:1518	OB5956 H1 Ro.raw	2.6492E5	1	1	560	572	Replacement of 2 protons by iron	E9:Replacement of 2 protons by iron:37.54	PEAKS PTM
K.GT(-18.01)GNLELVAVRK.E	N	36.73	1237.7142	12	2.6	413.5797	3	27.00	42	F42:1287	OB5955 H1 Ro.raw	4.7714E5	1	1	458	469	Dehydration	T2:Dehydration:1000.00	PEAKS PTM
R.NT(-18.01)LEAAFNAEFNEIRR.V	N	36.45	1875.9227	16	0.7	626.3153	3	34.16	43	F43:1739	OB5956 H1 Ro.raw	6.9172E6	2	2	316	331	Dehydration	T2:Dehydration:1000.00	PEAKS PTM
K.EGALM(+15.99)LPHFN(+.98)SK.A	N	36.41	1359.6493	12	2.8	680.8338	2	31.22	41	F41:1550	OB5954 H1 Ro.raw	0	0	0	437	448	Oxidation (M); Deamidation (NQ)	M5:Oxidation (M):1000.00;N10:Deamidation (NQ):1000.00	PEAKS DB
R.DQSSY(-18.01)LQGFSR.N	N	36.32	1268.5785	11	-0.6	635.2961	2	30.85	43	F43:1579	OB5956 H1 Ro.raw	2.378E5	1	1	305	315		Y5:Dehydration:0.00	PEAKS PTM
R.C(+57.02)LQS(-18.01)C(+57.02)Q(+.98)QEPDDLKQK.A	N	36.24	1858.8190	15	-0.1	930.4167	2	28.47	31	F31:1350	OB5942 H6 Ro.raw	0	0	0	42	56	Carbamidomethylation; Dehydration	C1:Carbamidomethylation:1000.00;S4:Dehydration:39.55;C5:Carbamidomethylation:1000.00;Q6:Deamidation (NQ):8.69	PEAKS PTM
M.LPHFNSK.A	N	36.02	841.4446	7	1.3	421.7301	2	30.63	42	F42:1510	OB5955 H1 Ro.raw	1.4673E6	2	2	442	448			PEAKS DB
K.NPQLQDLDM(-48.00)MLTC(+57.02)VEIK.E	N	35.81	1998.9755	17	6.7	667.3369	3	34.52	43	F43:1773	OB5956 H1 Ro.raw	8.4276E5	1	1	420	436	Carbamidomethylation	M9:Dethiomethyl:11.06;C13:Carbamidomethylation:1000.	PEAKS PTM
**total 156 peptides**																			

Peptide	Uniq	-10lgP	Mass	Length	ppm	m/z	z	RT	Fraction	Scan	Source File	Area Roasted in gel	#Feature	#Feature Roasted in gel	Start	End	PTM	AScore	Found By
																		00	
K.KNPQLQ(+.98)DLDMM(+15.99)LTC(+57.02)VEIKEGALMLPHFNSK.A	N	33.72	3516.7124	30	3.3	880.1883	4	35.48	41	F41:1798	OB5954 H1 Ro.raw	7.5658E5	1	1	419	448	Carbamidomethylation	Q6:Deamidation (NQ):18.55; M11:Oxidation (M): 14.02;C14:Carbamidomethylation:1000.00	PEAKS DB
D.QSSYLQGFSR.N	N	33.26	1171.5621	10	2.1	586.7896	2	29.36	42	F42:1431	OB5955 H1 Ro.raw	3.3134E4	1	1	306	315			PEAKS DB
R.IVQIEAKP(+15.99)NTLVLPK.H	N	33.15	1678.0028	15	-0.7	840.0081	2	30.35	41	F41:1495	OB5954 H1 Ro.raw	0	0	0	214	228		P8:Oxidation or Hydroxylation:9.34	PEAKS PTM
R.EREEDWRQPR.E	N	32.92	1399.6592	10	5.7	700.8409	2	24.05	43	F43:1141	OB5956 H1 Ro.raw	4.1173E4	1	1	124	133			PEAKS DB
R.D(-18.01)QSSYLQGFSR.N	N	31.95	1268.5785	11	1.6	635.2975	2	29.92	43	F43:1489	OB5956 H1 Ro.raw	0	0	0	305	315		D1:Dehydration:0.00	PEAKS PTM
K.D(+53.92)LAFPGSGEQVEK.L	N	31.27	1429.5812	13	-4.3	715.7948	2	30.56	41	F41:1500	OB5954 H1 Ro.raw	3.7719E5	1	1	560	572		D1:Replacement of 2 protons by iron:0.00	PEAKS PTM
K.KNPQLQDLDMM(+15.99)LTC(+57.02)VEIKEGALMLPHFNSK.A	N	30.83	3515.7285	30	1.3	879.9406	4	35.25	42	F42:1790	OB5955 H1 Ro.raw	0	0	0	419	448	Carbamidomethylation	M11:Oxidation (M): 12.28;C14:Carbamidomethylation:1000.00	PEAKS DB
R.DQSSY(+15.99)LQGFSR.N	N	30.72	1302.5840	11	1.6	652.3003	2	29.24	43	F43:1470	OB5956 H1 Ro.raw	6.7205E4	1	1	305	315	Oxidation or Hydroxylation	Y5:Oxidation or Hydroxylation:26.36	PEAKS PTM
N.TLEAAFNAEFNEIRR.V	N	30.42	1779.8904	15	3.5	890.9556	2	33.23	43	F43:1696	OB5956 H1 Ro.raw	1.5374E5	1	1	317	331			PEAKS DB
R.IFLAGDKD(-18.01)NV.V	N	30.00	1072.5553	10	0.7	537.2853	2	32.26	42	F42:1603	OB5955 H1 Ro.raw	2.1452E6	1	1	541	550	Dehydration	D8:Dehydration:45.01	PEAKS PTM
R.NTLEAAFNAEFN(+15.99)EIRR.V	N	29.99	1909.9282	16	0.9	955.9723	2	32.98	42	F42:1662	OB5955 H1 Ro.raw	2.366E6	2	2	316	331		N12:Oxidation or Hydroxylation:0.00	PEAKS PTM
E.V(+57.02)SKEHVEELTK.H	N	29.60	1354.7092	11	-1.7	678.3607	2	24.05	42	F42:1120	OB5955 H1 Ro.raw	0	0	0	365	375		V1:Carbamidomethylation (DHKE, X@N-term):0.00	PEAKS PTM
K.N(+.98)PQLQ(+.98)DLDMMLTC(+57.02)VEIKEGALM(+15.99)LPHFNSK.A	N	29.50	3389.6016	29	9.6	848.4158	4	37.65	43	F43:2047	OB5956 H1 Ro.raw	6.1505E6	1	1	420	448	Carbamidomethylation	N1:Deamidation (NQ):0.00;Q5:Deamidation (NQ):14.04; C13:Carbamidomethylation:1000.00;M22:Oxidation (M):0.00	PEAKS DB
H.RIVQIEAKPNTLVLPK.H	N	29.34	1818.1090	16	0.6	607.0439	3	29.55	42	F42:1454	OB5955 H1 Ro.raw	7.8202E4	1	1	213	228			PEAKS DB
**total 156 peptides**																			

Peptide	Uniq	-10lgP	Mass	Length	ppm	m/z	z	RT	Fraction	Scan	Source File	Area Roasted in gel	#Feature	#Feature Roasted in gel	Start	End	PTM	AScore	Found By
R.NN(+.98)PFYFPSRR.F	N	29.19	1297.6204	10	9.3	649.8235	2	62.71	43	F43:3425	OB5956 H1 Ro.raw	0	0	0	172	181		N2:Deamidation (NQ):7.21	PEAKS DB
R.NTLEAAFNAEFN(+.98)EIR.R	N	29.07	1738.8162	15	9.2	870.4233	2	44.36	42	F42:2331	OB5955 H1 Ro.raw	5.4416E3	1	1	316	330		N12:Deamidation (NQ):0.00	PEAKS DB
R.EGE(+21.98)QEWGTPGSHVR.E	N	28.92	1589.6835	14	1.4	795.8501	2	25.83	41	F41:1231	OB5954 H1 Ro.raw	3.6144E3	1	1	153	166		E3:Sodium adduct: 9.74	PEAKS PTM
K.KNPQ(+.98)LQ(+.98)DLDMM(+15.99)LTC(+57.02)VEIKEGALM(+15.99)LPHFNSK.A	N	28.74	3533.6914	30	9.7	884.4387	4	35.77	42	F42:1822	OB5955 H1 Ro.raw	2.7541E5	1	1	419	448	Carbamidomethylation; Oxidation (M)	Q4:Deamidation (NQ):8.22;Q6:Deamidation (NQ):5.55;M11:Oxidation (M):0.00;C14:Carbamidomethylation:1000.00;M23:Oxidation (M):38.29	PEAKS DB
**total 156 peptides**																			

A1DZF0|A1DZF0_ARAHY

back to list

| Protein Coverage | Supporting Peptides |

Protein Coverage:

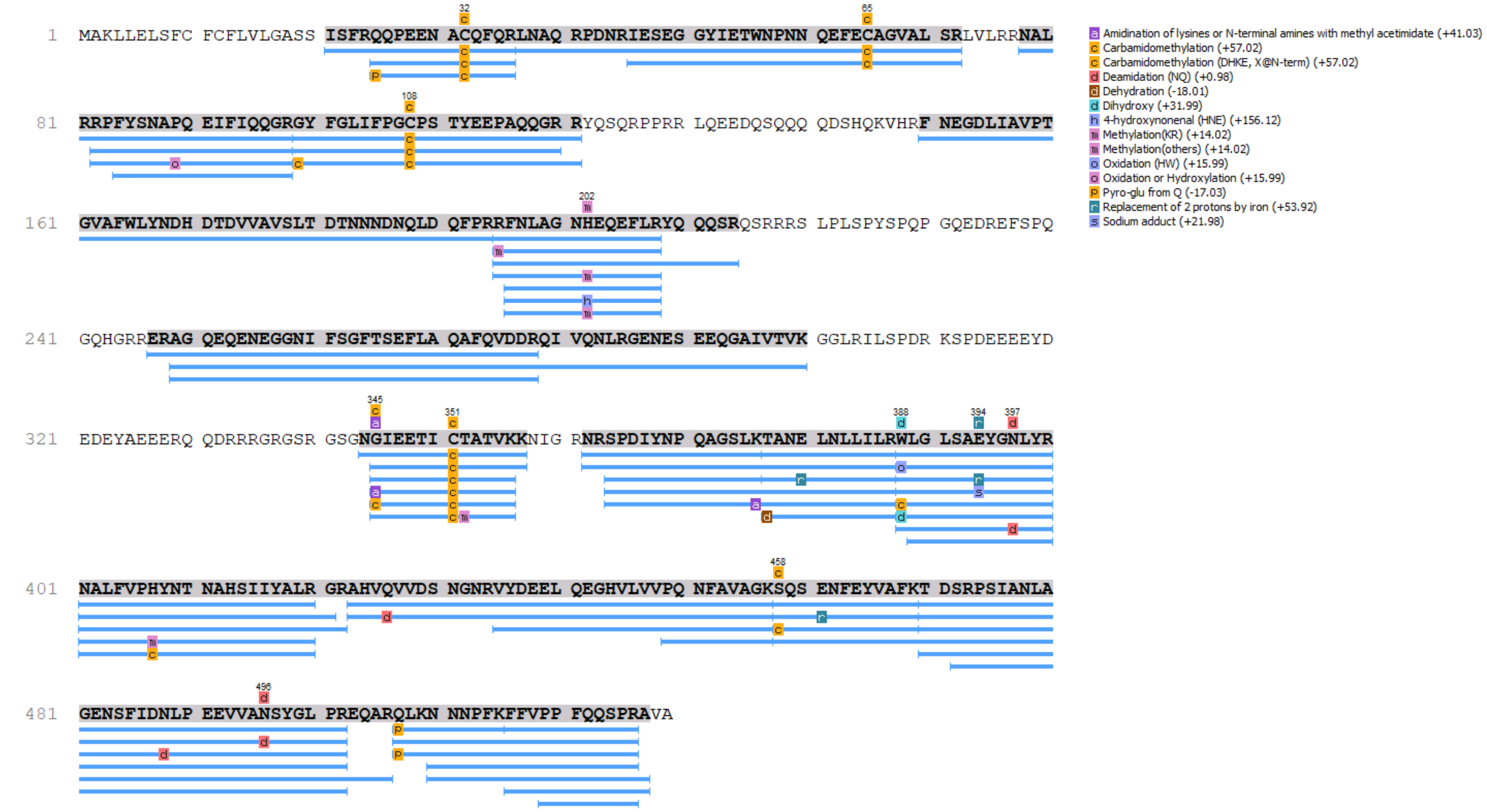

Supporting Peptides:

Peptide	Uniq	-10lgP	Mass	Length	ppm	m/z	z	RT	Fraction	Scan	Source File	Area Roasted in gel	#Feature	#Feature Roasted in gel	Start	End	PTM	AScore	Found By
R.NALFVPHYNTNAHSIIYALR.G	N	134.27	2313.2019	20	3.7	1157.6125	2	33.79	29	F29:1486	OB5922 H2 Ro.raw	5.1511E7	23	23	401	420			PEAKS DB
R.GYFGLIFPGC(+57.02)PSTYEEPAQQGR.R	N	127.83	2473.1372	22	3.0	1237.5796	2	36.11	38	F38:1750	OB5951 H3A Ro.raw	2.5988E6	9	9	99	120	Carbamidomethylation	C10:Carbamidomethylation: 1000.00	PEAKS DB
R.SPDIYNPQAGSLKTANELNLLILR.W	N	125.09	2639.4282	24	2.1	880.8185	3	35.72	35	F35:1738	OB5946 H2 Ro.raw	6.3847E6	8	7	364	387			PEAKS DB
R.WLGLSAEYGNLYR.N	N	121.81	1540.7673	13	2.8	771.3931	2	34.72	34	F34:1730	OB5945 H2 Ro.raw	5.9147E7	35	35	388	400			PEAKS DB
R.NRSPDIYNPQAGSLKTANELNLLILR.W	N	119.99	2909.5723	26	2.3	970.8669	3	34.91	29	F29:1560	OB5922 H2 Ro.raw	3.3615E6	6	6	362	387			PEAKS DB

total 97 peptides

Peptide	Uniq	-10lgP	Mass	Length	ppm	m/z	z	RT	Fraction	Scan	Source File	Area Roasted in gel	#Feature	#Feature Roasted in gel	Start	End	PTM	AScore	Found By
K.TDSRPSIANLAGENSFIDNLPEEVVANSYGLPR.E	N	117.95	3544.7434	33	2.8	1182.5917	3	39.03	29	F29:1808	OB5922 H2 Ro.raw	6.0445E7	20	20	470	502			PEAKS DB
K.SQSENFEYVAFK.T	N	116.27	1447.6619	12	3.0	724.8404	2	31.51	34	F34:1498	OB5945 H2 Ro.raw	1.8189E7	10	10	458	469			PEAKS DB
R.RPFYSNAPQEIFIQQGR.G	N	110.90	2050.0383	17	3.2	684.3556	3	31.52	38	F38:1483	OB5951 H3A Ro.raw	9.1631E6	15	15	82	98			PEAKS DB
K.FFVPPFQQSPR.A	Y	102.82	1348.6927	11	2.2	675.3551	2	33.42	29	F29:1469	OB5922 H2 Ro.raw	3.3748E6	6	6	516	526			PEAKS DB
R.NALRRPFYSNAPQEIFIQQGR.G	N	97.93	2504.3037	21	1.0	835.7760	3	32.71	40	F40:1535	OB5953 H3A Ro.raw	6.3111E6	14	13	78	98			PEAKS DB
R.NRSPDIYNPQAGSLK.T	N	96.54	1658.8376	15	0.1	830.4261	2	27.65	32	F32:1291	OB5943 H6 Ro.raw	8.0621E5	12	12	362	376			PEAKS DB
K.FFVPPFQQSPRA.V	Y	95.20	1419.7299	12	1.7	710.8734	2	33.01	34	F34:1599	OB5945 H2 Ro.raw	1.6137E6	4	4	516	527			PEAKS DB
N.GIEETIC(+57.02)TATVK.K	N	95.04	1320.6595	12	-0.2	661.3369	2	29.91	29	F29:1260	OB5922 H2 Ro.raw	2.5094E5	6	6	345	356	Carbamidomethylation	C7:Carbamidomethylation:1000.00	PEAKS DB
R.SPDIYNPQAGSLK.T	N	92.16	1388.6936	13	1.7	695.3552	2	28.58	34	F34:1323	OB5945 H2 Ro.raw	1.33E6	6	6	364	376			PEAKS DB
R.FNLAGNHEQEFLR.Y	N	91.88	1573.7637	13	4.3	787.8925	2	30.57	38	F38:1440	OB5951 H3A Ro.raw	1.243E5	7	7	196	208			PEAKS DB
K.NNNPFKFFVPPFQQSPR.A	Y	91.78	2063.0376	17	1.8	688.6877	3	35.64	29	F29:1615	OB5922 H2 Ro.raw	2.4453E5	5	5	510	526			PEAKS DB
R.VYDEELQEGHVLVVPQNFAVAGK.S	N	90.91	2540.2910	23	1.4	847.7721	3	33.42	29	F29:1478	OB5922 H2 Ro.raw	1.9062E6	10	10	435	457			PEAKS DB
K.TDSRPSIANLAGENSFIDNLPEEVVAN(+.98)SYGLPR.E	N	88.11	3545.7273	33	1.8	1182.9185	3	36.63	34	F34:1807	OB5945 H2 Ro.raw	3.983E3	1	1	470	502	Deamidation (NQ)	N27:Deamidation (NQ):103.10	PEAKS DB
R.RFNLAGNHEQEFLR.Y	N	87.38	1729.8647	14	1.5	577.6297	3	31.36	40	F40:1478	OB5953 H3A Ro.raw	1.8294E6	7	7	195	208			PEAKS DB
N.GIEETIC(+57.02)TATVKK.N	N	82.16	1448.7545	13	0.7	725.3850	2	27.45	31	F31:1292	OB5942 H6 Ro.raw	4.6237E5	5	5	345	357	Carbamidomethylation	C7:Carbamidomethylation:1000.00	PEAKS DB
R.NALFVPH(+14.02)YNTNAHSIIYALR.G	N	79.80	2327.2175	20	0.7	776.7470	3	34.91	29	F29:1561	OB5922 H2 Ro.raw	1.3103E6	3	3	401	420	Methylation(others)	H7:Methylation(others):22.45	PEAKS PTM
R.AHVQVVDSNGNRVYDEELQEGHVLVVPQNFAVAGK.S	N	77.75	3816.9182	35	3.6	955.2402	4	32.32	35	F35:1544	OB5946 H2 Ro.raw	8.2893E6	8	8	423	457			PEAKS DB
R.GYFGLIFPGC(+57.02)PSTYEEPAQQGRR.Y	N	76.83	2629.2383	23	2.4	877.4221	3	35.18	40	F40:1676	OB5953 H3A Ro.raw	5.6622E6	9	9	99	121	Carbamidomethylation	C10:Carbamidomethylation:1000.00	PEAKS DB
K.TANELNLLILR.W	N	76.42	1268.7452	11	0.6	635.3802	2	35.27	29	F29:1655	OB5922 H2 Ro.raw	2.1461E7	10	10	377	387			PEAKS DB
R.Q(-17.03)QPEENAC(+57.02)QFQR.L	N	75.52	1516.6365	12	2.5	759.3274	2	28.03	32	F32:1319	OB5943 H6 Ro.raw	7.279E3	2	2	25	36	Pyro-glu from Q; Carbamidomethylation	Q1:Pyro-glu from Q:1000.00;C8:Carbamidomethylation:1000.00	PEAKS PTM
V.PQNFAVAGK.S	N	75.51	930.4923	9	1.0	466.2539	2	32.32	35	F35:1542	OB5946 H2 Ro.raw	3.3444E6	3	3	449	457			PEAKS DB
R.IESEGGYIETWNPNNQEFEC(+57.02)AGVALSR.L	N	74.31	3069.3774	27	1.4	1024.1345	3	34.50	31	F31:1703	OB5942 H6 Ro.raw	1.0361E4	1	1	46	72	Carbamidomethylation	C20:Carbamidomethylation:1000.00	PEAKS DB

total 97 peptides

Peptide	Uniq	-10lgP	Mass	Length	ppm	m/z	z	RT	Fraction	Scan	Source File	Area Roasted in gel	#Feature	#Feature Roasted in gel	Start	End	PTM	AScore	Found By
R.QQPEENAC(+57.02)QFQR.L	N	73.76	1533.6630	12	1.7	767.8400	2	25.24	32	F32:1157	OB5943 H6 Ro.raw	3.7544E4	3	3	25	36	Carbamidomethylation	C8:Carbamidomethylation:1000.00	PEAKS DB
R.QLKNNNPFKFFVPPFQQSPR.A	Y	70.66	2432.2751	20	0.9	609.0766	4	34.45	34	F34:1679	OB5945 H2 Ro.raw	6.0459E5	3	3	507	526			PEAKS DB
R.AGQEQENEGGNIFSGFTSEFLAQAFQVDDR.Q	Y	69.87	3290.4751	30	5.3	1646.2535	2	40.50	36	F36:2017	OB5948 H3B Ro.raw	5.1868E4	1	1	249	278			PEAKS DB
K.S(+57.02)QSENFEYVAFK.T	N	65.89	1504.6833	12	0.0	753.3489	2	31.77	35	F35:1521	OB5946 H2 Ro.raw	4.3047E5	3	3	458	469	Carbamidomethylation (DHKE, X@N-term)	S1:Carbamidomethylation (DHKE, X@N-term):56.34	PEAKS PTM
N.G(+57.02)IEETIC(+57.02)TATVK.K	N	64.33	1377.6809	12	2.0	689.8491	2	30.19	29	F29:1285	OB5922 H2 Ro.raw	5.5841E3	1	1	345	356	Carbamidomethylation (DHKE, X@N-term); Carbamidomethylation	G1:Carbamidomethylation (DHKE, X@N-term):30.83;C7:Carbamidomethylation:1000.00	PEAKS PTM
R.AHVQVVDSN(+.98)GNRVYDEELQEGHVLVVPQNFAVAGK.S	N	64.02	3817.9023	35	5.0	955.4877	4	33.04	29	F29:1438	OB5922 H2 Ro.raw	1.4175E6	2	2	423	457		N9:Deamidation (NQ):9.40	PEAKS DB
R.NALFVPH(+57.02)YNTNAHSIIYALR.G	N	63.66	2370.2231	20	0.1	791.0817	3	34.03	35	F35:1631	OB5946 H2 Ro.raw	8.9747E5	1	1	401	420	Carbamidomethylation (DHKE, X@N-term)	H7:Carbamidomethylation (DHKE, X@N-term):20.35	PEAKS PTM
R.RFNLAGNHEQEFLRYQQQSR.Q	N	62.22	2520.2371	20	-2.3	631.0667	4	34.12	30	F30:1496	OB5926 H3B Ro.raw	7.5688E4	2	2	195	214			PEAKS DB
R.LNAQRPDNRIESEGGYIETWNPNNQEFEC(+57.02)AGVALSR.L	N	60.14	4133.9248	36	2.4	1034.4910	4	33.87	33	F33:1670	OB5944 H6 Ro.raw	1.5556E6	3	3	37	72	Carbamidomethylation	C29:Carbamidomethylation:1000.00	PEAKS DB
R.GYFGLIFPGC(+57.02)PS(+14.02)TYEEPAQQGR.R	N	59.71	2487.1528	22	2.2	1244.5864	2	36.43	32	F32:1817	OB5943 H6 Ro.raw	7.7963E4	3	3	99	120	Carbamidomethylation	C10:Carbamidomethylation:1000.00;S12:Methylation(others):9.34	PEAKS PTM
R.W(+31.99)LGLSAEYGNLYR.N	N	59.45	1572.7572	13	-1.6	787.3846	2	33.64	35	F35:1624	OB5946 H2 Ro.raw	8.5976E4	2	2	388	400	Dihydroxy	W1:Dihydroxy:62.93	PEAKS PTM
P.FYSNAPQEIFIQQGR.G	N	59.30	1796.8845	15	-1.2	899.4484	2	32.27	31	F31:1574	OB5942 H6 Ro.raw	0	0	0	84	98			PEAKS DB
K.FFVPPFQQSP(+15.99)R.A	Y	58.77	1364.6876	11	0.9	683.3517	2	33.23	29	F29:1459	OB5922 H2 Ro.raw	1.167E6	2	2	516	526		P10:Oxidation or Hydroxylation:12.28	PEAKS PTM
R.FNEGDLIAVPTGVAFWLYNDHDTDVVAVSLTDTNNNDNQLDQFPR.R	N	58.15	5049.3691	45	0.7	1684.1357	3	40.24	30	F30:1843	OB5926 H3B Ro.raw	9.8654E5	1	1	150	194			PEAKS DB
R.GYFGLIFPGC(+57.02)PST(+14.02)YEEPAQQGR.R	N	58.14	2487.1528	22	2.8	1244.5872	2	36.17	31	F31:1801	OB5942 H6 Ro.raw	2.5405E4	2	2	99	120	Carbamidomethylation	C10:Carbamidomethylation:1000.00;T13:Methylation(others):14.02	PEAKS PTM
R.W(+57.02)LGLSAEYGNLYR.N	N	57.46	1597.7888	13	0.6	799.9022	2	35.09	34	F34:1725	OB5945 H2 Ro.raw	1.848E4	1	1	388	400	Carbamidomethylation (DHKE, X@N-term)	W1:Carbamidomethylation (DHKE, X@N-term):62.19	PEAKS PTM
R.Q(-17.03)LKNNNPFK.F	N	56.41	1084.5665	9	1.6	543.2914	2	28.75	34	F34:1346	OB5945 H2 Ro.raw	1.0606E4	2	2	507	515	Pyro-glu from Q	Q1:Pyro-glu from Q:1000.00	PEAKS PTM
K.SQSENFE(+21.98)YVAFK.T	N	54.92	1469.6438	12	-1.1	735.8284	2	31.92	29	F29:1388	OB5922 H2 Ro.raw	6.4574E4	2	2	458	469		E7:Sodium adduct:14.84	PEAKS PTM
R.AHVQVVDSNGN(+.98)RVYDEELQEGHVLVVPQNFAVAGK.S	N	54.09	3817.9023	35	3.2	955.4859	4	32.51	34	F34:1567	OB5945 H2 Ro.raw	7.3088E4	3	3	423	457		N11:Deamidation (NQ):8.22	PEAKS DB

total 97 peptides

Peptide	Uniq	-10lgP	Mass	Length	ppm	m/z	z	RT	Fraction	Scan	Source File	Area Roasted in gel	#Feature	#Feature Roasted in gel	Start	End	PTM	AScore	Found By
K.SQS(-18.01)ENFEYVAFK.T	N	53.31	1429.6514	12	1.9	715.8344	2	31.92	29	F29:1380	OB5922 H2 Ro.raw	3.0777E4	1	1	458	469		S3:Dehydration:14.04	PEAKS PTM
R.IESEGGYIETWNPNNQ(+.98)EFEC(+57.02)AGVALSR.L	N	52.73	3070.3613	27	6.0	1024.4672	3	34.92	32	F32:1736	OB5943 H6 Ro.raw	6.6817E3	1	1	46	72	Carbamidomethylation	Q16:Deamidation (NQ):0.00; C20:Carbamidomethylation:1000.00	PEAKS DB
K.NNNPFKFFVPPFQQSPRA.V	Y	51.92	2134.0747	18	0.0	712.3655	3	35.15	35	F35:1723	OB5946 H2 Ro.raw	8.836E4	2	2	510	527			PEAKS DB
K.SQSE(+21.98)NFEYVAFK.T	N	51.89	1469.6438	12	-0.6	735.8287	2	31.51	34	F34:1513	OB5945 H2 Ro.raw	2.7649E4	1	1	458	469		E4:Sodium adduct:0.00	PEAKS PTM
R.VYDEELQEGHVLVVPQN(+.98)FAVAGK.S	N	51.23	2541.2751	23	9.5	1271.6569	2	32.92	31	F31:1618	OB5942 H6 Ro.raw	3.629E3	1	1	435	457		N17:Deamidation (NQ):14.02	PEAKS DB
R.S(+57.02)PDIYNPQAGSLK.T	N	50.29	1445.7150	13	5.7	723.8689	2	28.94	34	F34:1350	OB5945 H2 Ro.raw	6.4489E3	2	2	364	376		S1:Carbamidomethylation (DHKE, X@N-term):14.04	PEAKS PTM
K.TANE(+53.92)LNLLILR.W	N	48.78	1322.6646	11	-1.4	662.3386	2	35.27	29	F29:1590	OB5922 H2 Ro.raw	2.333E5	1	1	377	387	Replacement of 2 protons by iron	E4:Replacement of 2 protons by iron:154.98	PEAKS PTM
R.N(+57.02)ALFVPHYNTNAHSIIYALR.G	N	48.76	2370.2231	20	-0.1	791.0816	3	34.53	29	F29:1557	OB5922 H2 Ro.raw	3.2966E6	1	1	401	420		N1:Carbamidomethylation (DHKE, X@N-term):10.82	PEAKS PTM
R.WLGLSAE(+53.92)YGNLYR.N	N	48.21	1594.6866	13	0.4	532.5697	3	35.09	29	F29:1579	OB5922 H2 Ro.raw	6.0447E5	3	3	388	400	Replacement of 2 protons by iron	E7:Replacement of 2 protons by iron:123.05	PEAKS PTM
K.TDSRPSIANLAGENSFIDN(+.98)LPEEVVANSYGLPR.E	N	47.95	3545.7273	33	5.5	1182.9229	3	37.60	29	F29:1724	OB5922 H2 Ro.raw	0	0	0	470	502	Deamidation (NQ)	N19:Deamidation (NQ):20.10	PEAKS DB
K.TDSRPSIANLAGENSFIDNLPEEVVANSYGLPREQAR.Q	N	47.12	4028.9827	37	2.2	1008.2552	4	36.41	34	F34:1777	OB5945 H2 Ro.raw	1.9797E6	2	2	470	506			PEAKS DB
V.PPFQQSPR.A	Y	46.41	955.4875	8	-0.2	478.7510	2	35.15	35	F35:1720	OB5946 H2 Ro.raw	1.8433E5	2	2	519	526			PEAKS DB
K.SQSENFEYVAFKTDSRPSIANLAGENSFIDNLPEEVVANSYGLPR.E	N	45.95	4974.3945	45	3.1	1244.6097	4	37.99	35	F35:1870	OB5946 H2 Ro.raw	6.2475E6	4	4	458	502			PEAKS DB
R.SPD(+57.02)IYNPQAGSLK.T	N	45.16	1445.7150	13	2.2	723.8663	2	29.51	29	F29:1244	OB5922 H2 Ro.raw	0	0	0	364	376		D3:Carbamidomethylation (DHKE, X@N-term):0.00	PEAKS PTM
R.NALFVPHYNTNAHSIIYALRG.R	N	44.84	2370.2231	21	1.2	791.0826	3	33.77	34	F34:1624	OB5945 H2 Ro.raw	4.2894E6	2	2	401	421			PEAKS DB
N.GIEETIC(+57.02)T(+14.02)ATVK.K	N	42.70	1334.6752	12	3.2	668.3470	2	29.62	34	F34:1395	OB5945 H2 Ro.raw	7.3781E3	1	1	345	356	Carbamidomethylation; Methylation(others)	C7:Carbamidomethylation:1000.00;T8:Methylation(others):28.36	PEAKS PTM
K.TDSRPSIANLAGEN(+.98)SFIDNLPEEVVANSYGLPR.E	N	42.55	3545.7273	33	9.2	1182.9272	3	48.53	34	F34:2493	OB5945 H2 Ro.raw	0	0	0	470	502		N14:Deamidation (NQ):0.00	PEAKS DB
K.SQSE(+53.92)NFEYVAFK.T	N	42.20	1501.5812	12	-2.3	751.7961	2	31.58	35	F35:1507	OB5946 H2 Ro.raw	9.4575E4	1	1	458	469	Replacement of 2 protons by iron	E4:Replacement of 2 protons by iron:34.87	PEAKS PTM
D.SRPSIANLAGENSFIDNLPEEVVANSYGLPR.E	N	42.12	3328.6687	31	3.9	1110.5679	3	37.36	34	F34:1841	OB5945 H2 Ro.raw	3.0619E5	2	2	472	502			PEAKS DB
R.FNLAGNH(+14.02)EQEFLR.Y	N	40.38	1587.7793	13	0.7	530.2674	3	30.81	39	F39:1450	OB5952 H3A Ro.raw	1.3642E5	3	3	196	208	Methylation(others)	H7:Methylation(others):33.98	PEAKS PTM
R.AHVQVVDSNGNRVYDEELQ(+.98)EGHVLVVPQNFAVAGK.S	N	40.20	3817.9023	35	7.9	955.4904	4	33.43	35	F35:1615	OB5946 H2 Ro.raw	0	0	0	423	457		Q19:Deamidation (NQ):4.91	PEAKS DB

total 97 peptides

Peptide	Uniq	-10lgP	Mass	Length	ppm	m/z	z	RT	Fraction	Scan	Source File	Area Roasted in gel	#Feature	#Feature Roasted in gel	Start	End	PTM	AScore	Found By
R.AGQEQENEGGNIFSGFTSEFLAQAFQVDDRQIVQNLRGENESEEQGAIVTVK.G	Y	39.44	5712.7202	52	4.9	1429.1943	4	39.90	37	F37:1989	OB5949 H3B Ro.raw	4.1738E6	1	1	249	300			PEAKS DB
R.AHVQVVDSN(+.98)GN(+.98)RVYDEELQEGHVLVVPQNFAVAGK.S	N	39.19	3818.8862	35	8.6	955.7371	4	34.33	32	F32:1692	OB5943 H6 Ro.raw	0	0	0	423	457		N9:Deamidation (NQ):0.00;N11:Deamidation (NQ):0.00	PEAKS DB
K.SQSEN(+.98)FEYVAFKTDSRPSIANLAGENSFIDNLPEEVVANSYGLPR.E	N	38.92	4975.3789	45	2.8	1244.8555	4	39.75	34	F34:1987	OB5945 H2 Ro.raw	0	0	0	458	502		N5:Deamidation (NQ):0.00	PEAKS DB
R.RFNLAGNH(+14.02)EQEFLR.Y	N	38.64	1743.8805	14	0.5	582.3011	3	31.14	38	F38:1453	OB5951 H3A Ro.raw	7.3646E5	3	3	195	208	Methylation(others)	H8:Methylation(others):40.00	PEAKS PTM
R.AHVQ(+.98)VVDSNGNRVYDEELQEGHVLVVPQNFAVAGK.S	N	38.57	3817.9023	35	0.9	955.4838	4	33.12	31	F31:1624	OB5942 H6 Ro.raw	4.5408E4	1	1	423	457	Deamidation (NQ)	Q4:Deamidation (NQ):21.59	PEAKS DB
W.LGLSAEYGNLYR.N	N	38.37	1354.6881	12	3.5	678.3537	2	31.68	31	F31:1539	OB5942 H6 Ro.raw	0	0	0	389	400			PEAKS DB
R.ERAGQEQENEGGNIFSGFTSEFLAQAFQVDDR.Q	Y	38.12	3575.6189	32	1.7	1192.8823	3	39.35	36	F36:1955	OB5948 H3B Ro.raw	5.3613E4	1	1	247	278			PEAKS DB
S.ISFRQQPEENAC(+57.02)QFQR.L	N	37.74	2036.9486	16	0.8	679.9907	3	28.84	36	F36:1329	OB5948 H3B Ro.raw	3.1194E4	4	4	21	36	Carbamidomethylation	C12:Carbamidomethylation:1000.00	PEAKS DB
K.FFVPPFQQSPR(+15.99)A.V	Y	37.64	1435.7247	12	2.2	718.8712	2	33.04	29	F29:1458	OB5922 H2 Ro.raw	6.9299E5	1	1	516	527		R11:Oxidation or Hydroxylation:12.28	PEAKS PTM
R.G(+57.02)YFGLIFPGC(+57.02)PSTYEEPAQQGRR.Y	N	37.54	2686.2598	23	1.6	896.4286	3	35.57	39	F39:1718	OB5952 H3A Ro.raw	5.4933E4	1	1	99	121	Carbamidomethylation (DHKE, X@N-term); Carbamidomethylation	G1:Carbamidomethylation (DHKE, X@N-term):130.55;C10:Carbamidomethylation:1000.00	PEAKS PTM
R.Q(-17.03)LKNNNPFKFFVPPFQQSPR.A	Y	37.42	2415.2488	20	1.8	806.0917	3	36.41	34	F34:1798	OB5945 H2 Ro.raw	1.5157E5	1	1	507	526	Pyro-glu from Q	Q1:Pyro-glu from Q:1000.00	PEAKS PTM
N.G(+41.03)IEETIC(+57.02)TATVK.K	N	36.93	1361.6860	12	0.7	681.8508	2	29.66	34	F34:1390	OB5945 H2 Ro.raw	1.6824E4	1	1	345	356	Amidination of lysines or N-terminal amines with methyl acetimidate; Carbamidomethylation	G1:Amidination of lysines or N-terminal amines with methyl acetimidate:96.48;C7:Carbamidomethylation:1000.00	PEAKS PTM
R.NALFVPHYNTNAHSIIYALRGR.A	N	36.04	2526.3245	22	0.6	843.1160	3	33.60	29	F29:1492	OB5922 H2 Ro.raw	7.6168E5	2	2	401	422			PEAKS DB
R.R(+14.02)FNLAGNHEQEFLR.Y	N	34.57	1743.8805	14	0.9	582.3013	3	31.38	39	F39:1472	OB5952 H3A Ro.raw	2.9356E5	2	2	195	208	Methylation(KR)	R1:Methylation (KR):67.58	PEAKS PTM
K.FFVPPFQQSP(+15.99)RA.V	Y	34.52	1435.7247	12	1.0	718.8704	2	32.88	35	F35:1577	OB5946 H2 Ro.raw	3.008E5	2	2	516	527		P10:Oxidation or Hydroxylation:0.00	PEAKS PTM
N.GIEETIC(+57.02)T(+14.02)ATVKK.N	N	34.37	1462.7701	13	0.9	732.3930	2	27.73	31	F31:1307	OB5942 H6 Ro.raw	3.1361E3	1	1	345	357	Carbamidomethylation	C7:Carbamidomethylation:1000.00;T8:Methylation(others):19.16	PEAKS PTM
R.WLGLSAEYGN(+.98)LYR.N	N	33.97	1541.7513	13	5.4	771.8871	2	54.24	29	F29:2679	OB5922 H2 Ro.raw	0	0	0	388	400	Deamidation (NQ)	N10:Deamidation (NQ):1000.00	PEAKS DB
G.NGIEETIC(+57.02)TATVKK.N	N	33.56	1562.7974	14	0.1	782.4060	2	29.85	34	F34:1417	OB5945 H2 Ro.raw	8.2899E3	2	2	344	357	Carbamidomethylation	C8:Carbamidomethylation:1000.00	PEAKS DB
R.RPFYSNAP(+15.99)QEIFIQQGR.G	N	33.55	2066.0332	17	0.2	689.6852	3	31.94	40	F40:1497	OB5953 H3A Ro.raw	4.3282E4	1	1	82	98	Oxidation or Hydroxylation	P8:Oxidation or Hydroxylation:32.28	PEAKS PTM

total 97 peptides

Peptide	Uniq	-10lgP	Mass	Length	ppm	m/z	z	RT	Fraction	Scan	Source File	Area Roasted in gel	#Feature	#Feature Roasted in gel	Start	End	PTM	AScore	Found By
R.AHVQ(+.98)VVDSNGNRVYDEELQEGHVLVVPQ(+.98)NFAVAGK.S	N	33.38	3818.8862	35	8.9	955.7373	4	33.12	31	F31:1624	OB5942 H6 Ro.raw	3.8034E4	1	1	423	457		Q4:Deamidation (NQ):8.81;Q28:Deamidation (NQ):12.28	PEAKS DB
R.FNLAGNH(+156.12)EQEFLR.Y	N	33.01	1729.8788	13	-7.6	577.6292	3	31.13	38	F38:1444	OB5951 H3A Ro.raw	7.0376E5	2	2	196	208	4-hydroxynonenal (HNE)	H7:4-hydroxynonenal (HNE): 1000.00	PEAKS PTM
R.N(+.98)ALFVPHYNTNAHSIIYALR.G	N	32.68	2314.1858	20	7.3	772.4082	3	50.66	29	F29:2450	OB5922 H2 Ro.raw	1.0573E4	1	1	401	420		N1:Deamidation (NQ):0.00	PEAKS DB
R.IESEGGYIETWN(+.98)PNNQEFEC(+57.02)AGVALSR.L	N	32.58	3070.3613	27	6.9	1536.1985	2	34.73	32	F32:1729	OB5943 H6 Ro.raw	2.6575E4	1	1	46	72	Carbamidomethylation	N12:Deamidation (NQ):0.00; C20:Carbamidomethylation: 1000.00	PEAKS DB
K.T(-18.01)ANELNLLILR.W	N	31.74	1250.7346	11	2.2	626.3760	2	35.27	29	F29:1600	OB5922 H2 Ro.raw	1.738E4	1	1	377	387	Dehydration	T1:Dehydration:1000.00	PEAKS PTM
R.SPDIYNPQAGSLK(+41.03)TANELNLLILR.W	N	31.55	2680.4548	24	0.2	894.4924	3	36.03	34	F34:1771	OB5945 H2 Ro.raw	3.6458E4	1	1	364	387	Amidination of lysines or N-terminal amines with methyl acetimidate	K13:Amidination of lysines or N-terminal amines with methyl acetimidate:54.40	PEAKS PTM
R.WLGLSAE(+21.98)YGNLYR.N	N	30.00	1562.7494	13	-8.4	782.3754	2	35.27	29	F29:1583	OB5922 H2 Ro.raw	5.0047E4	2	2	388	400	Sodium adduct	E7:Sodium adduct:42.78	PEAKS PTM
R.LNAQRPDNRIESEGGYIETWNPNN(+.98)QEFEC(+57.02)AGVALSR.L	N	29.77	4134.9087	36	4.7	1379.3167	3	33.85	33	F33:1671	OB5944 H6 Ro.raw	8.0788E4	1	1	37	72	Carbamidomethylation	N24:Deamidation (NQ):0.00; C29:Carbamidomethylation: 1000.00	PEAKS DB
K.TDSRPSIAN(+.98)LAGENSFIDNLPEEVVANSYGLPR.E	N	29.47	3545.7273	33	8.7	1182.9266	3	48.17	29	F29:2399	OB5922 H2 Ro.raw	7.2607E4	1	1	470	502		N9:Deamidation (NQ):0.00	PEAKS DB
N.GIEET(+14.02)IC(+57.02)TATVKK.N	N	29.08	1462.7701	13	-1.4	732.3914	2	28.11	32	F32:1329	OB5943 H6 Ro.raw	2.1469E3	1	1	345	357	Carbamidomethylation	T5:Methylation (others):9.08; C7:Carbamidomethylation:1000.00	PEAKS PTM
R.W(+15.99)LGLSAEYGNLYR.N	N	28.45	1556.7623	13	-0.8	779.3878	2	34.26	31	F31:1698	OB5942 H6 Ro.raw	3.8864E3	1	1	388	400	Oxidation (HW)	W1:Oxidation (HW):1000.00	PEAKS PTM
**total 97 peptides**																			

Q647H3|Q647H3_ARAHY

back to list

| Protein Coverage | Supporting Peptides |

Protein Coverage:

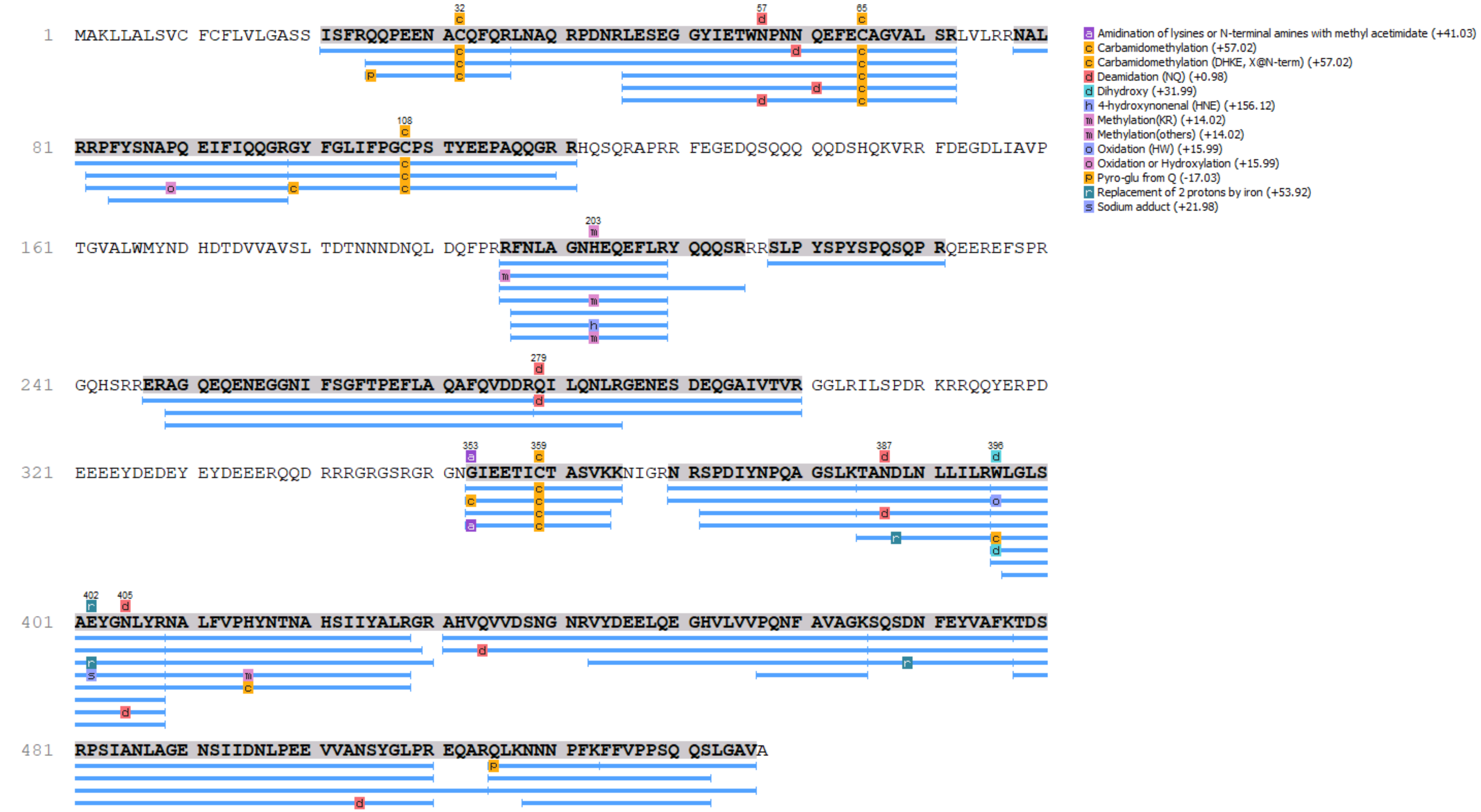

Supporting Peptides:

Peptide	Uniq	-10lgP	Mass	Length	ppm	m/z	z	RT	Fraction	Scan	Source File	Area Roasted in gel	#Feature	#Feature Roasted in gel	Start	End	PTM	AScore	Found By
R.SPDIYNPQAGSLKTANDLNLLILR.W	N	136.50	2625.4126	24	-0.1	876.1447	3	35.61	29	F29:1607	OB5922 H2 Ro.raw	1.276E6	3	3	372	395			PEAKS DB
R.NALFVPHYNTNAHSIIYALR.G	N	134.27	2313.2019	20	3.7	1157.6125	2	33.79	29	F29:1486	OB5922 H2 Ro.raw	5.1511E7	23	23	409	428			PEAKS DB
R.GYFGLIFPGC(+57.02)PSTYEEPAQQGR.R	N	127.83	2473.1372	22	3.0	1237.5796	2	36.11	38	F38:1750	OB5951 H3A Ro.raw	2.5988E6	9	9	99	120	Carbamidomethylation	C10:Carbamidomethylation:1000.00	PEAKS DB
K.TDSRPSIANLAGENSIIDNLPEEVVANSYGLPR.E	Y	125.30	3510.7590	33	5.7	1171.2670	3	37.70	29	F29:1764	OB5922 H2 Ro.raw	6.4659E7	19	19	478	510			PEAKS DB
R.WLGLSAEYGNLYR.N	N	121.81	1540.7673	13	2.8	771.3931	2	34.72	34	F34:1730	OB5945 H2 Ro.raw	5.9147E7	35	35	396	408			PEAKS DB
**total 83 peptides**																			

Peptide	Uniq	-10lgP	Mass	Length	ppm	m/z	z	RT	Fraction	Scan	Source File	Area Roasted in gel	#Feature	#Feature Roasted in gel	Start	End	PTM	AScore	Found By
R.NRSPDIYNPQAGSLKTANDLNLLILR.W	N	120.82	2895.5566	26	1.6	966.1943	3	34.35	29	F29:1530	OB5922 H2 Ro.raw	2.2791E6	6	6	370	395			PEAKS DB
K.SQSDNFEYVAFK.T	N	114.21	1433.6462	12	2.6	717.8323	2	31.76	35	F35:1518	OB5946 H2 Ro.raw	5.4437E6	6	6	466	477			PEAKS DB
R.RPFYSNAPQEIFIQQGR.G	N	110.90	2050.0383	17	3.2	684.3556	3	31.52	38	F38:1483	OB5951 H3A Ro.raw	9.1631E6	15	15	82	98			PEAKS DB
R.NALRRPFYSNAPQEIFIQQGR.G	N	97.93	2504.3037	21	1.0	835.7760	3	32.71	40	F40:1535	OB5953 H3A Ro.raw	6.3111E6	14	13	78	98			PEAKS DB
R.NRSPDIYNPQAGSLK.T	N	96.54	1658.8376	15	0.1	830.4261	2	27.65	32	F32:1291	OB5943 H6 Ro.raw	8.0621E5	12	12	370	384			PEAKS DB
R.SPDIYNPQAGSLK.T	N	92.16	1388.6936	13	1.7	695.3552	2	28.58	34	F34:1323	OB5945 H2 Ro.raw	1.33E6	6	6	372	384			PEAKS DB
R.FNLAGNHEQEFLR.Y	N	91.88	1573.7637	13	4.3	787.8925	2	30.57	38	F38:1440	OB5951 H3A Ro.raw	1.243E5	7	7	197	209			PEAKS DB
R.VYDEELQEGHVLVVPQNFAVAGK.S	N	90.91	2540.2910	23	1.4	847.7721	3	33.42	29	F29:1478	OB5922 H2 Ro.raw	1.9062E6	10	10	443	465			PEAKS DB
N.GIEETIC(+57.02)TASVK.K	N	90.29	1306.6438	12	0.6	654.3296	2	28.94	34	F34:1356	OB5945 H2 Ro.raw	1.977E5	6	6	353	364	Carbamidomethylation	C7:Carbamidomethylation:1000.00	PEAKS DB
R.RFNLAGNHEQEFLR.Y	N	87.38	1729.8647	14	1.5	577.6297	3	31.36	40	F40:1478	OB5953 H3A Ro.raw	1.8294E6	7	7	196	209			PEAKS DB
R.NALFVPH(+14.02)YNTNAHSIIYALR.G	N	79.80	2327.2175	20	0.7	776.7470	3	34.91	29	F29:1561	OB5922 H2 Ro.raw	1.3103E6	3	3	409	428	Methylation(others)	H7:Methylation(others):22.45	PEAKS PTM
R.AHVQVVDSNGNRVYDEELQEGHVLVVPQNFAVAGK.S	N	77.75	3816.9182	35	3.6	955.2402	4	32.32	35	F35:1544	OB5946 H2 Ro.raw	8.2893E6	8	8	431	465			PEAKS DB
R.GYFGLIFPGC(+57.02)PSTYEEPAQQGRR.H	N	76.83	2629.2383	23	2.4	877.4221	3	35.18	40	F40:1676	OB5953 H3A Ro.raw	5.6622E6	9	9	99	121	Carbamidomethylation	C10:Carbamidomethylation:1000.00	PEAKS DB
R.AGQEQENEGGNIFSGFTPEFLAQAFQVDDR.Q	N	76.51	3300.4958	30	5.3	1651.2639	2	39.94	40	F40:1970	OB5953 H3A Ro.raw	0	0	0	249	278			PEAKS DB
N.GIEETIC(+57.02)TASVKK.N	N	76.18	1434.7388	13	0.7	718.3771	2	26.91	33	F33:1250	OB5944 H6 Ro.raw	3.5109E5	8	8	353	365	Carbamidomethylation	C7:Carbamidomethylation:1000.00	PEAKS DB
R.Q(-17.03)QPEENAC(+57.02)QFQR.L	N	75.52	1516.6365	12	2.5	759.3274	2	28.03	32	F32:1319	OB5943 H6 Ro.raw	7.279E3	2	2	25	36	Pyro-glu from Q; Carbamidomethylation	Q1:Pyro-glu from Q:1000.00;C8:Carbamidomethylation:1000.00	PEAKS PTM
V.PQNFAVAGK.S	N	75.51	930.4923	9	1.0	466.2539	2	32.32	35	F35:1542	OB5946 H2 Ro.raw	3.3444E6	3	3	457	465			PEAKS DB
R.LESEGGYIETWNPNNQEFEC(+57.02)AGVALSR.L	N	74.31	3069.3774	27	1.4	1024.1345	3	34.50	31	F31:1703	OB5942 H6 Ro.raw	1.0361E4	1	1	46	72	Carbamidomethylation	C20:Carbamidomethylation:1000.00	PEAKS DB
K.TANDLNLLILR.W	N	73.94	1254.7296	11	1.8	628.3732	2	34.79	35	F35:1750	OB5946 H2 Ro.raw	1.0437E7	10	10	385	395			PEAKS DB
R.QQPEENAC(+57.02)QFQR.L	N	73.76	1533.6630	12	1.7	767.8400	2	25.24	32	F32:1157	OB5943 H6 Ro.raw	3.7544E4	3	3	25	36	Carbamidomethylation	C8:Carbamidomethylation:1000.00	PEAKS DB
R.QILQNLRGENESDEQGAIVTVR.G	N	71.90	2468.2620	22	-1.5	823.7600	3	30.32	38	F38:1424	OB5951 H3A Ro.raw	3.9221E4	1	1	279	300			PEAKS DB
R.S(+57.02)PDIYNPQAGSLKTANDLNLLILR.W	N	64.15	2682.4341	24	1.5	895.1533	3	35.28	34	F34:1735	OB5945 H2 Ro.raw	9.5441E4	1	1	372	395		S1:Carbamidomethylation (DHKE, X @N-term):14.04	PEAKS PTM
**total 83 peptides**																			

Peptide	Uniq	-10lgP	Mass	Length	ppm	m/z	z	RT	Fraction	Scan	Source File	Area Roasted in gel	#Feature	#Feature Roasted in gel	Start	End	PTM	AScore	Found By
R.AHVQVVDSN(+.98)GNRVYDEELQEGHVLVVPQNFAVAGK.S	N	64.02	3817.9023	35	5.0	955.4877	4	33.04	29	F29:1438	OB5922 H2 Ro.raw	1.4175E6	2	2	431	465		N9:Deamidation (NQ):9.40	PEAKS DB
R.NALFVPH(+57.02)YNTNAHSIIYALR.G	N	63.66	2370.2231	20	0.1	791.0817	3	34.03	35	F35:1631	OB5946 H2 Ro.raw	8.9747E5	1	1	409	428	Carbamidomethylation (DHKE, X@N-term)	H7:Carbamidomethylation (DHKE, X@N-term):20.35	PEAKS PTM
R.RFNLAGNHEQEFLRYQQQSR.R	N	62.22	2520.2371	20	-2.3	631.0667	4	34.12	30	F30:1496	OB5926 H3B Ro.raw	7.5688E4	2	2	196	215			PEAKS DB
R.LNAQRPDNRLESEGGYIETWNPNNQEFEC(+57.02)AGVALSR.L	N	60.14	4133.9248	36	2.4	1034.4910	4	33.87	33	F33:1670	OB5944 H6 Ro.raw	1.5556E6	3	3	37	72	Carbamidomethylation	C29:Carbamidomethylation:1000.00	PEAKS DB
R.GYFGLIFPGC(+57.02)PS(+14.02)TYEEPAQQGR.R	N	59.71	2487.1528	22	2.2	1244.5864	2	36.43	32	F32:1817	OB5943 H6 Ro.raw	7.7963E4	3	3	99	120	Carbamidomethylation	C10:Carbamidomethylation:1000.00;S12:Methylation(others):9.34	PEAKS PTM
R.W(+31.99)LGLSAEYGNLYR.N	N	59.45	1572.7572	13	-1.6	787.3846	2	33.64	35	F35:1624	OB5946 H2 Ro.raw	8.5976E4	2	2	396	408	Dihydroxy	W1:Dihydroxy:62.93	PEAKS PTM
P.FYSNAPQEIFIQQGR.G	N	59.30	1796.8845	15	-1.2	899.4484	2	32.27	31	F31:1574	OB5942 H6 Ro.raw	0	0	0	84	98			PEAKS DB
R.GYFGLIFPGC(+57.02)PST(+14.02)YEEPAQQGR.R	N	58.14	2487.1528	22	2.8	1244.5872	2	36.17	31	F31:1801	OB5942 H6 Ro.raw	2.5405E4	2	2	99	120	Carbamidomethylation	C10:Carbamidomethylation:1000.00;T13:Methylation(others):14.02	PEAKS PTM
R.W(+57.02)LGLSAEYGNLYR.N	N	57.46	1597.7888	13	0.6	799.9022	2	35.09	34	F34:1725	OB5945 H2 Ro.raw	1.848E4	1	1	396	408	Carbamidomethylation (DHKE, X@N-term)	W1:Carbamidomethylation (DHKE, X@N-term):62.19	PEAKS PTM
R.Q(-17.03)LKNNNPFK.F	N	56.41	1084.5665	9	1.6	543.2914	2	28.75	34	F34:1346	OB5945 H2 Ro.raw	1.0606E4	2	2	515	523	Pyro-glu from Q	Q1:Pyro-glu from Q:1000.00	PEAKS PTM
R.ERAGQEQENEGGNIFSGFTPEFLAQAFQVDDR.Q	N	54.92	3585.6396	32	-0.3	1196.2201	3	38.71	40	F40:1900	OB5953 H3A Ro.raw	0	0	0	247	278			PEAKS DB
R.AHVQVVDSNGN(+.98)RVYDEELQEGHVLVVPQNFAVAGK.S	N	54.09	3817.9023	35	3.2	955.4859	4	32.51	34	F34:1567	OB5945 H2 Ro.raw	7.3088E4	3	3	431	465		N11:Deamidation (NQ):8.22	PEAKS DB
R.LESEGGYIETWNPNNQ(+.98)EFEC(+57.02)AGVALSR.L	N	52.73	3070.3613	27	6.0	1024.4672	3	34.92	32	F32:1736	OB5943 H6 Ro.raw	6.6817E3	1	1	46	72	Deamidation (NQ); Carbamidomethylation	Q16:Deamidation (NQ):1000.00;C20:Carbamidomethylation:1000.00	PEAKS DB
R.ERAGQEQEN(+.98)EGGNIFSGFTPEFLAQAFQVDDR.Q	N	51.36	3586.6235	32	1.3	1196.5500	3	38.82	40	F40:1900	OB5953 H3A Ro.raw	1.0134E5	1	1	247	278		N9:Deamidation (NQ):0.00	PEAKS DB
R.VYDEELQEGHVLVVPQN(+.98)FAVAGK.S	N	51.23	2541.2751	23	9.5	1271.6569	2	32.92	31	F31:1618	OB5942 H6 Ro.raw	3.629E3	1	1	443	465		N17:Deamidation (NQ):14.02	PEAKS DB
K.TAN(+.98)DLNLLILR.W	N	50.73	1255.7136	11	2.6	628.8657	2	35.22	31	F31:1745	OB5942 H6 Ro.raw	0	0	0	385	395	Deamidation (NQ)	N3:Deamidation (NQ):68.31	PEAKS DB
R.S(+57.02)PDIYNPQAGSLK.T	N	50.29	1445.7150	13	5.7	723.8689	2	28.94	34	F34:1350	OB5945 H2 Ro.raw	6.4489E3	2	2	372	384		S1:Carbamidomethylation (DHKE, X@N-term):14.04	PEAKS PTM
R.N(+57.02)ALFVPHYNTNAHSIIYALR.G	N	48.76	2370.2231	20	-0.1	791.0816	3	34.53	29	F29:1557	OB5922 H2 Ro.raw	3.2966E6	1	1	409	428		N1:Carbamidomethylation (DHKE, X@N-term):10.82	PEAKS PTM
R.WLGLSAE(+53.92)YGNLYR.N	N	48.21	1594.6866	13	0.4	532.5697	3	35.09	29	F29:1579	OB5922 H2 Ro.raw	6.0447E5	3	3	396	408	Replacement of 2 protons by iron	E7:Replacement of 2 protons by iron:123.05	PEAKS PTM
N.G(+41.03)IEETIC(+57.02)TASVK.K	N	48.20	1347.6704	12	0.3	674.8427	2	29.12	34	F34:1370	OB5945 H2 Ro.raw	1.5496E4	1	1	353	364	Amidination of lysines or N-terminal amines with methyl acetimidate; Carbamidomethylation	G1:Amidination of lysines or N-terminal amines with methyl acetimidate:96.48;C7:Carbamidomethylation:1000.00	PEAKS PTM
R.AGQEQEN(+.98)EGGNIFSGFTPEFLAQAFQVDDR.Q	N	47.49	3301.4800	30	5.2	1651.7559	2	39.74	38	F38:1968	OB5951 H3A Ro.raw	2.9773E5	2	2	249	278		N7:Deamidation (NQ):0.00	PEAKS DB

total 83 peptides

Peptide	Uniq	-10lgP	Mass	Length	ppm	m/z	z	RT	Fraction	Scan	Source File	Area Roasted in gel	#Feature	#Feature Roasted in gel	Start	End	PTM	AScore	Found By
R.SPD(+57.02)IYNPQAGSLK.T	N	45.16	1445.7150	13	2.2	723.8663	2	29.51	29	F29:1244	OB5922 H2 Ro.raw	0	0	0	372	384		D3:Carbamidomethylation (DHKE, X@N-term):0.00	PEAKS PTM
R.NALFVPHYNTNAHSIIYALRG.R	N	44.84	2370.2231	21	1.2	791.0826	3	33.77	34	F34:1624	OB5945 H2 Ro.raw	4.2894E6	2	2	409	429			PEAKS DB
R.QLKNNNPFKFFVPPSQQSLGAV.A	Y	43.05	2459.2961	22	3.6	820.7756	3	33.50	29	F29:1470	OB5922 H2 Ro.raw	5.0433E5	1	1	515	536			PEAKS DB
K.SQSDNFEYVAFKTDSRPSIANLAGENSIIDNLPEEVVANSYGLPR.E	Y	42.72	4926.3945	45	-0.7	1232.6051	4	37.16	34	F34:1856	OB5945 H2 Ro.raw	4.0502E6	2	2	466	510			PEAKS DB
R.QLKNNNPFKFFVPPSQQS.L	N	42.33	2119.0850	18	1.9	707.3703	3	33.27	35	F35:1604	OB5946 H2 Ro.raw	6.7737E4	1	1	515	532			PEAKS DB
R.Q(+.98)ILQNLRGENESDEQGAIVTVR.G	N	41.79	2469.2458	22	-2.8	824.0869	3	30.49	40	F40:1426	OB5953 H3A Ro.raw	0	0	0	279	300	Deamidation (NQ)	Q1:Deamidation (NQ):28.70	PEAKS DB
K.NNNPFKFFVPPSQQS.L	N	41.33	1749.8474	15	1.0	875.9319	2	34.91	29	F29:1573	OB5922 H2 Ro.raw	4.7342E4	1	1	518	532			PEAKS DB
N.G(+57.02)IEETIC(+57.02)TASVKK.N	N	41.05	1491.7603	13	8.7	746.8939	2	26.96	31	F31:1261	OB5942 H6 Ro.raw	3.2429E4	4	4	353	365	Carbamidomethylation (DHKE, X@N-term); Carbamidomethylation	G1:Carbamidomethylation (DHKE, X@N-term):32.28; C7:Carbamidomethylation:1000.00	PEAKS PTM
R.FNLAGNH(+14.02)EQEFLR.Y	N	40.38	1587.7793	13	0.7	530.2674	3	30.81	39	F39:1450	OB5952 H3A Ro.raw	1.3642E5	3	3	197	209	Methylation(others)	H7:Methylation(others):33.98	PEAKS PTM
K.SQSD(+53.92)NFEYVAFK.T	N	40.31	1487.5656	12	-2.8	744.7880	2	32.03	29	F29:1395	OB5922 H2 Ro.raw	0	0	0	466	477	Replacement of 2 protons by iron	D4:Replacement of 2 protons by iron:20.50	PEAKS PTM
R.AHVQVVDSNGNRVYDEELQ(+.98)EGHVLVVPQNFAVAGK.S	N	40.20	3817.9023	35	7.9	955.4904	4	33.43	35	F35:1615	OB5946 H2 Ro.raw	0	0	0	431	465		Q19:Deamidation (NQ):4.91	PEAKS DB
R.AHVQVVDSN(+.98)GN(+.98)RVYDEELQEGHVLVVPQNFAVAGK.S	N	39.19	3818.8862	35	8.6	955.7371	4	34.33	32	F32:1692	OB5943 H6 Ro.raw	0	0	0	431	465		N9:Deamidation (NQ):0.00;N11:Deamidation (NQ):0.00	PEAKS DB
K.TAND(+53.92)LNLLILR.W	N	38.76	1308.6488	11	0.2	655.3318	2	35.09	29	F29:1584	OB5922 H2 Ro.raw	8.7715E4	1	1	385	395	Replacement of 2 protons by iron	D4:Replacement of 2 protons by iron:55.73	PEAKS PTM
R.RFNLAGNH(+14.02)EQEFLR.Y	N	38.64	1743.8805	14	0.5	582.3011	3	31.14	38	F38:1453	OB5951 H3A Ro.raw	7.3646E5	3	3	196	209	Methylation(others)	H8:Methylation(others):40.00	PEAKS PTM
R.AHVQ(+.98)VVDSNGNRVYDEELQEGHVLVVPQNFAVAGK.S	N	38.57	3817.9023	35	0.9	955.4838	4	33.12	31	F31:1624	OB5942 H6 Ro.raw	4.5408E4	1	1	431	465	Deamidation (NQ)	Q4:Deamidation (NQ):21.59	PEAKS DB
W.LGLSAEYGNLYR.N	N	38.37	1354.6881	12	3.5	678.3537	2	31.68	31	F31:1539	OB5942 H6 Ro.raw	0	0	0	397	408			PEAKS DB
S.ISFRQQPEENAC(+57.02)QFQR.L	N	37.74	2036.9486	16	0.8	679.9907	3	28.84	36	F36:1329	OB5948 H3B Ro.raw	3.1194E4	4	4	21	36	Carbamidomethylation	C12:Carbamidomethylation:1000.00	PEAKS DB
R.G(+57.02)YFGLIFPGC(+57.02)PSTYEEPAQQGRR.H	N	37.54	2686.2598	23	1.6	896.4286	3	35.57	39	F39:1718	OB5952 H3A Ro.raw	5.4933E4	1	1	99	121	Carbamidomethylation (DHKE, X@N-term); Carbamidomethylation	G1:Carbamidomethylation (DHKE, X@N-term):130.55;C10:Carbamidomethylation:1000.00	PEAKS PTM
K.TDSRPSIANLAGEN(+.98)SIIDNLPEEVVANSYGLPR.E	Y	36.75	3511.7429	33	8.6	1171.5983	3	48.17	29	F29:2321	OB5922 H2 Ro.raw	6.9222E4	3	3	478	510		N14:Deamidation (NQ):10.19	PEAKS DB
K.FFVPPSQQSLGAV.A	Y	36.25	1375.7135	13	9.3	688.8704	2	30.71	31	F31:1587	OB5942 H6 Ro.raw	8.8364E4	1	1	524	536			PEAKS DB
R.SLPYSPYSP(+31.99)QSQPR.Q	N	36.19	1637.7686	14	3.2	819.8942	2	27.07	38	F38:1250	OB5951 H3A Ro.raw	6.6096E3	1	1	218	231		P9:Dihydroxy:9.42	PEAKS PTM

total 83 peptides

Peptide	Uniq	-10lgP	Mass	Length	ppm	m/z	z	RT	Fraction	Scan	Source File	Area Roasted in gel	#Feature	#Feature Roasted in gel	Start	End	PTM	AScore	Found By
R.NALFVPHYNTNAHSIIYALRGR.A	N	36.04	2526.3245	22	0.6	843.1160	3	33.60	29	F29:1492	OB5922 H2 Ro.raw	7.6168E5	2	2	409	430			PEAKS DB
K.TDSRPSIANLAGENSIIDNLPEEVVAN(+.98)SYGLPR.E	Y	35.07	3511.7429	33	5.1	1171.5942	3	46.13	34	F34:2348	OB5945 H2 Ro.raw	4.9547E4	2	2	478	510	Deamidation (NQ)	N27:Deamidation (NQ):43.76	PEAKS DB
R.R(+14.02)FNLAGNHEQEFLR.Y	N	34.57	1743.8805	14	0.9	582.3013	3	31.38	39	F39:1472	OB5952 H3A Ro.raw	2.9356E5	2	2	196	209	Methylation(KR)	R1:Methylation(KR):67.58	PEAKS PTM
R.AGQEQENEGGNIFSGFTPEFLAQAFQVDDRQILQNLR.G	N	34.03	4166.0093	37	1.9	1389.6797	3	40.12	38	F38:1986	OB5951 H3A Ro.raw	6.2075E4	1	1	249	285			PEAKS DB
R.WLGLSAEYGN(+.98)LYR.N	N	33.97	1541.7513	13	5.4	771.8871	2	54.24	29	F29:2679	OB5922 H2 Ro.raw	0	0	0	396	408	Deamidation (NQ)	N10:Deamidation (NQ):1000.00	PEAKS DB
R.RPFYSNAP(+15.99)QEIFIQQGR.G	N	33.55	2066.0332	17	0.2	689.6852	3	31.94	40	F40:1497	OB5953 H3A Ro.raw	4.3282E4	1	1	82	98	Oxidation or Hydroxylation	P8:Oxidation or Hydroxylation:32.28	PEAKS PTM
R.AHVQ(+.98)VVDSNGNRVYDEELQEGHVLVVPQ(+.98)NFAVAGK.S	N	33.38	3818.8862	35	8.9	955.7373	4	33.12	31	F31:1624	OB5942 H6 Ro.raw	3.8034E4	1	1	431	465		Q4:Deamidation (NQ):8.81;Q28:Deamidation (NQ):12.28	PEAKS DB
R.FNLAGNH(+156.12)EQEFLR.Y	N	33.01	1729.8788	13	-7.6	577.6292	3	31.13	38	F38:1444	OB5951 H3A Ro.raw	7.0376E5	2	2	197	209	4-hydroxynonenal (HNE)	H7:4-hydroxynonenal (HNE):1000.00	PEAKS PTM
R.N(+.98)ALFVPHYNTNAHSIIYALR.G	N	32.68	2314.1858	20	7.3	772.4082	3	50.66	29	F29:2450	OB5922 H2 Ro.raw	1.0573E4	1	1	409	428		N1:Deamidation (NQ):0.00	PEAKS DB
R.LESEGGYIETWN(+.98)PNNQEFEC(+57.02)AGVALSR.L	N	32.58	3070.3613	27	6.9	1536.1985	2	34.73	32	F32:1729	OB5943 H6 Ro.raw	2.6575E4	1	1	46	72	Deamidation (NQ); Carbamidomethylation	N12:Deamidation (NQ):1000.00;C20:Carbamidomethylation:1000.00	PEAKS DB
K.TDSRPSIANLAGENSIIDNLPEEVVANSYGLPREQAR.Q	Y	30.77	3994.9983	37	2.4	999.7593	4	35.84	34	F34:1758	OB5945 H2 Ro.raw	5.332E5	1	1	478	514			PEAKS DB
R.WLGLSAE(+21.98)YGNLYR.N	N	30.00	1562.7494	13	-8.4	782.3754	2	35.27	29	F29:1583	OB5922 H2 Ro.raw	5.0047E4	2	2	396	408	Sodium adduct	E7:Sodium adduct:42.78	PEAKS PTM
R.LNAQRPDNRLESEGGYIETWNPNN(+.98)QEFEC(+57.02)AGVALSR.L	N	29.77	4134.9087	36	4.7	1379.3167	3	33.85	33	F33:1671	OB5944 H6 Ro.raw	8.0788E4	1	1	37	72	Deamidation (NQ); Carbamidomethylation	N24:Deamidation (NQ):1000.00;C29:Carbamidomethylation:1000.00	PEAKS DB
R.W(+15.99)LGLSAEYGNLYR.N	N	28.45	1556.7623	13	-0.8	779.3878	2	34.26	31	F31:1698	OB5942 H6 Ro.raw	3.8864E3	1	1	396	408	Oxidation (HW)	W1:Oxidation (HW):1000.00	PEAKS PTM

total 83 peptides

Q9FZ11|Q9FZ11_ARAHY

back to list

| Protein Coverage | Supporting Peptides |

Protein Coverage:

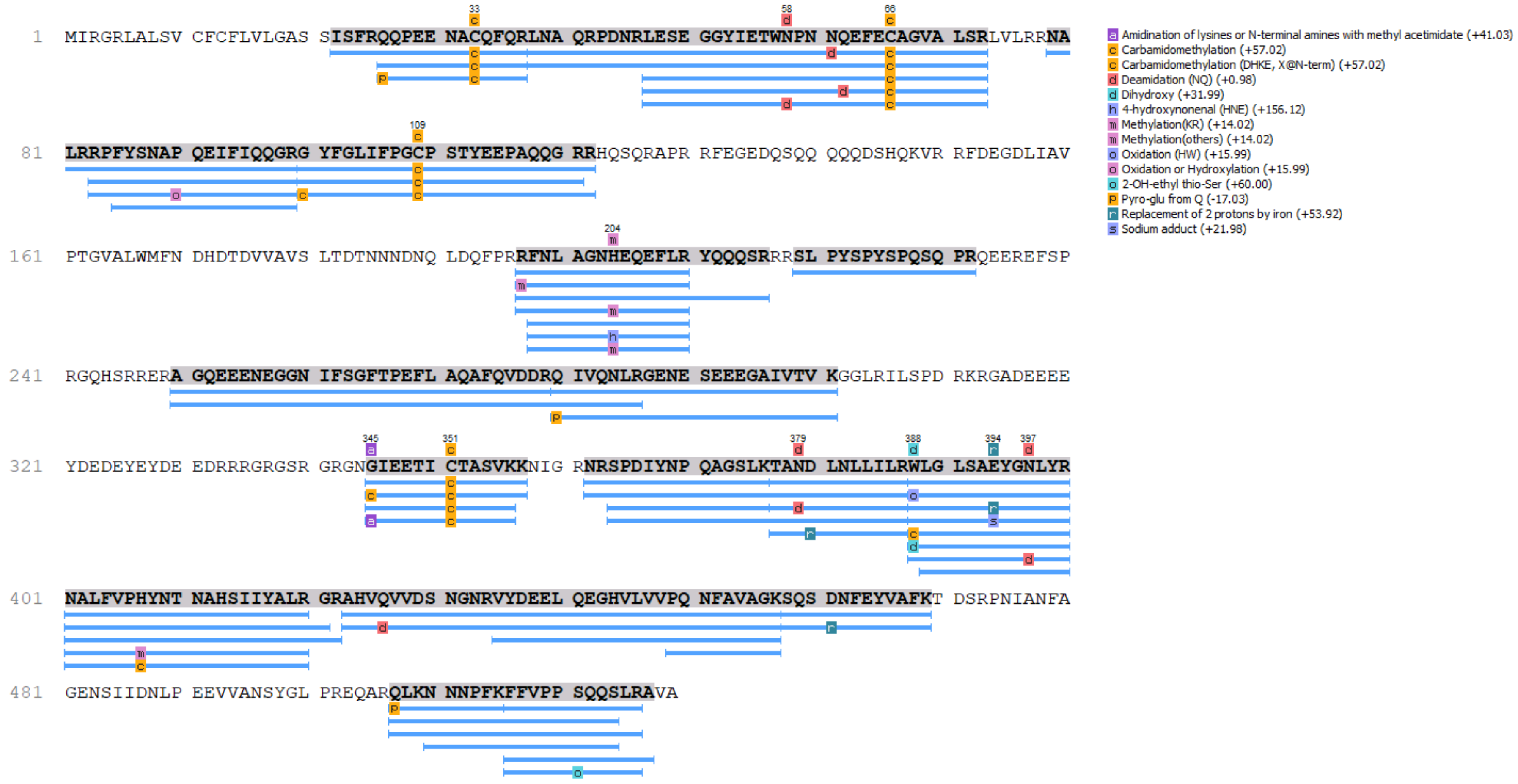

Supporting Peptides:

Peptide	Uniq	-10lgP	Mass	Length	ppm	m/z	z	RT	Fraction	Scan	Source File	Area Roasted in gel	#Feature	#Feature Roasted in gel	Start	End	PTM	AScore	Found By
R.SPDIYNPQAGSLKTANDLNLLILR.W	N	136.50	2625.4126	24	-0.1	876.1447	3	35.61	29	F29:1607	OB5922 H2 Ro.raw	1.276E6	3	3	364	387			PEAKS DB
R.NALFVPHYNTNAHSIIYALR.G	N	134.27	2313.2019	20	3.7	1157.6125	2	33.79	29	F29:1486	OB5922 H2 Ro.raw	5.1511E7	23	23	401	420			PEAKS DB
R.GYFGLIFPGC(+57.02)PSTYEEPAQQGR.R	N	127.83	2473.1372	22	3.0	1237.5796	2	36.11	38	F38:1750	OB5951 H3A Ro.raw	2.5988E6	9	9	100	121	Carbamidomethylation	C10:Carbamidomethylation:1000.00	PEAKS DB
R.WLGLSAEYGNLYR.N	N	121.81	1540.7673	13	2.8	771.3931	2	34.72	34	F34:1730	OB5945 H2 Ro.raw	5.9147E7	35	35	388	400			PEAKS DB
R.NRSPDIYNPQAGSLKTANDLNLLILR.W	N	120.82	2895.5566	26	1.6	966.1943	3	34.35	29	F29:1530	OB5922 H2 Ro.raw	2.2791E6	6	6	362	387			PEAKS DB
K.SQSDNFEYVAFK.T	N	114.21	1433.6462	12	2.6	717.8323	2	31.76	35	F35:1518	OB5946 H2 Ro.raw	5.4437E6	6	6	458	469			PEAKS DB

total 79 peptides

Peptide	Uniq	-10lgP	Mass	Length	ppm	m/z	z	RT	Fraction	Scan	Source File	Area Roasted in gel	#Feature	#Feature Roasted in gel	Start	End	PTM	AScore	Found By
R.RPFYSNAPQEIFIQQGR.G	N	110.90	2050.0383	17	3.2	684.3556	3	31.52	38	F38:1483	OB5951 H3A Ro.raw	9.1631E6	15	15	83	99			PEAKS DB
K.FFVPPSQQSLRA.V	Y	98.38	1375.7247	12	2.6	688.8715	2	30.85	35	F35:1464	OB5946 H2 Ro.raw	1.6951E6	6	6	516	527			PEAKS DB
R.NALRRPFYSNAPQEIFIQQGR.G	N	97.93	2504.3037	21	1.0	835.7760	3	32.71	40	F40:1535	OB5953 H3A Ro.raw	6.3111E6	14	13	79	99			PEAKS DB
R.NRSPDIYNPQAGSLK.T	N	96.54	1658.8376	15	0.1	830.4261	2	27.65	32	F32:1291	OB5943 H6 Ro.raw	8.0621E5	12	12	362	376			PEAKS DB
R.SPDIYNPQAGSLK.T	N	92.16	1388.6936	13	1.7	695.3552	2	28.58	34	F34:1323	OB5945 H2 Ro.raw	1.33E6	6	6	364	376			PEAKS DB
R.FNLAGNHEQEFLR.Y	N	91.88	1573.7637	13	4.3	787.8925	2	30.57	38	F38:1440	OB5951 H3A Ro.raw	1.243E5	7	7	198	210			PEAKS DB
R.VYDEELQEGHVLVVPQNFAVAGK.S	N	90.91	2540.2910	23	1.4	847.7721	3	33.42	29	F29:1478	OB5922 H2 Ro.raw	1.9062E6	10	10	435	457			PEAKS DB
N.GIEETIC(+57.02)TASVK.K	N	90.29	1306.6438	12	0.6	654.3296	2	28.94	34	F34:1356	OB5945 H2 Ro.raw	1.977E5	6	6	345	356	Carbamidomethylation	C7:Carbamidomethylation:1000.00	PEAKS DB
R.RFNLAGNHEQEFLR.Y	N	87.38	1729.8647	14	1.5	577.6297	3	31.36	40	F40:1478	OB5953 H3A Ro.raw	1.8294E6	7	7	197	210			PEAKS DB
K.FFVPPSQQSLR.A	Y	86.75	1304.6876	11	0.7	653.3516	2	30.28	35	F35:1421	OB5946 H2 Ro.raw	6.0578E5	6	6	516	526			PEAKS DB
R.QIVQNLRGENESEEEGAIVTVK.G	Y	85.48	2441.2397	22	-0.8	814.7532	3	30.43	39	F39:1429	OB5952 H3A Ro.raw	3.5103E4	2	2	280	301			PEAKS DB
R.NALFVPH(+14.02)YNTNAHSIIYALR.G	N	79.80	2327.2175	20	0.7	776.7470	3	34.91	29	F29:1561	OB5922 H2 Ro.raw	1.3103E6	3	3	401	420	Methylation(others)	H7:Methylation(others):22.45	PEAKS PTM
R.QLKNNNPFKFFVPPSQQSLR.A	Y	78.28	2388.2703	20	2.2	797.0991	3	33.23	29	F29:1468	OB5922 H2 Ro.raw	1.3469E5	1	1	507	526			PEAKS DB
R.AHVQVVDSNGNRVYDEELQEGHVLVVPQNFAVAGK.S	N	77.75	3816.9182	35	3.6	955.2402	4	32.32	35	F35:1544	OB5946 H2 Ro.raw	8.2893E6	8	8	423	457			PEAKS DB
R.GYFGLIFPGC(+57.02)PSTYEEPAQQGRR.H	N	76.83	2629.2383	23	2.4	877.4221	3	35.18	40	F40:1676	OB5953 H3A Ro.raw	5.6622E6	9	9	100	122	Carbamidomethylation	C10:Carbamidomethylation:1000.00	PEAKS DB
N.GIEETIC(+57.02)TASVKK.N	N	76.18	1434.7388	13	0.7	718.3771	2	26.91	33	F33:1250	OB5944 H6 Ro.raw	3.5109E5	8	8	345	357	Carbamidomethylation	C7:Carbamidomethylation:1000.00	PEAKS DB
R.Q(-17.03)QPEENAC(+57.02)QFQR.L	N	75.52	1516.6365	12	2.5	759.3274	2	28.03	32	F32:1319	OB5943 H6 Ro.raw	7.279E3	2	2	26	37	Pyro-glu from Q; Carbamidomethylation	Q1:Pyro-glu from Q:1000.00;C8:Carbamidomethylation:1000.00	PEAKS PTM
V.PQNFAVAGK.S	N	75.51	930.4923	9	1.0	466.2539	2	32.32	35	F35:1542	OB5946 H2 Ro.raw	3.3444E6	3	3	449	457			PEAKS DB
R.LESEGGYIETWNPNNQEFEC(+57.02)AGVALSR.L	N	74.31	3069.3774	27	1.4	1024.1345	3	34.50	31	F31:1703	OB5942 H6 Ro.raw	1.0361E4	1	1	47	73	Carbamidomethylation	C20:Carbamidomethylation:1000.00	PEAKS DB
K.TANDLNLLILR.W	N	73.94	1254.7296	11	1.8	628.3732	2	34.79	35	F35:1750	OB5946 H2 Ro.raw	1.0437E7	10	10	377	387			PEAKS DB
R.QQPEENAC(+57.02)QFQR.L	N	73.76	1533.6630	12	1.7	767.8400	2	25.24	32	F32:1157	OB5943 H6 Ro.raw	3.7544E4	3	3	26	37	Carbamidomethylation	C8:Carbamidomethylation:1000.00	PEAKS DB
R.S(+57.02)PDIYNPQAGSLKTANDLNLLILR.W	N	64.15	2682.4341	24	1.5	895.1533	3	35.28	34	F34:1735	OB5945 H2 Ro.raw	9.5441E4	1	1	364	387		S1:Carbamidomethylation (DHKE, X@N-term):14.04	PEAKS PTM

total 79 peptides

Peptide	Uniq	-10lgP	Mass	Length	ppm	m/z	z	RT	Fraction	Scan	Source File	Area Roasted in gel	#Feature	#Feature Roasted in gel	Start	End	PTM	AScore	Found By
R.AHVQVVDSN(+.98)GNRVYDEELQEGHVLVVPQNFAVAGK.S	N	64.02	3817.9023	35	5.0	955.4877	4	33.04	29	F29:1438	OB5922 H2 Ro.raw	1.4175E6	2	2	423	457		N9:Deamidation (NQ):9.40	PEAKS DB
R.NALFVPH(+57.02)YNTNAHSIIYALR.G	N	63.66	2370.2231	20	0.1	791.0817	3	34.03	35	F35:1631	OB5946 H2 Ro.raw	8.9747E5	1	1	401	420	Carbamidomethylation (DHKE, X@N-term)	H7:Carbamidomethylation (DHKE, X@N-term):20.35	PEAKS PTM
R.RFNLAGNHEQEFLRYQQQSR.R	N	62.22	2520.2371	20	-2.3	631.0667	4	34.12	30	F30:1496	OB5926 H3B Ro.raw	7.5688E4	2	2	197	216			PEAKS DB
R.LNAQRPDNRLESEGGYIETWNPNNQEFEC(+57.02)AGVALSR.L	N	60.14	4133.9248	36	2.4	1034.4910	4	33.87	33	F33:1670	OB5944 H6 Ro.raw	1.5556E6	3	3	38	73	Carbamidomethylation	C29:Carbamidomethylation:1000.00	PEAKS DB
R.GYFGLIFPGC(+57.02)PS(+14.02)TYEEPAQQGR.R	N	59.71	2487.1528	22	2.2	1244.5864	2	36.43	32	F32:1817	OB5943 H6 Ro.raw	7.7963E4	3	3	100	121	Carbamidomethylation	C10:Carbamidomethylation:1000.00;S12:Methylation(others):9.34	PEAKS PTM
R.W(+31.99)LGLSAEYGNLYR.N	N	59.45	1572.7572	13	-1.6	787.3846	2	33.64	35	F35:1624	OB5946 H2 Ro.raw	8.5976E4	2	2	388	400	Dihydroxy	W1:Dihydroxy:62.93	PEAKS PTM
P.FYSNAPQEIFIQQGR.G	N	59.30	1796.8845	15	-1.2	899.4484	2	32.27	31	F31:1574	OB5942 H6 Ro.raw	0	0	0	85	99			PEAKS DB
K.FFVPPSQQ(+.98)SLR.A	Y	58.48	1305.6716	11	2.9	653.8450	2	30.71	31	F31:1484	OB5942 H6 Ro.raw	2.0502E5	3	3	516	526		Q8:Deamidation (NQ):12.79	PEAKS DB
R.GYFGLIFPGC(+57.02)PST(+14.02)YEEPAQQGR.R	N	58.14	2487.1528	22	2.8	1244.5872	2	36.17	31	F31:1801	OB5942 H6 Ro.raw	2.5405E4	2	2	100	121	Carbamidomethylation	C10:Carbamidomethylation:1000.00;T13:Methylation (others):14.02	PEAKS PTM
R.W(+57.02)LGLSAEYGNLYR.N	N	57.46	1597.7888	13	0.6	799.9022	2	35.09	34	F34:1725	OB5945 H2 Ro.raw	1.848E4	1	1	388	400	Carbamidomethylation (DHKE, X@N-term)	W1:Carbamidomethylation (DHKE, X@N-term):62.19	PEAKS PTM
R.Q(-17.03)LKNNNPFK.F	N	56.41	1084.5665	9	1.6	543.2914	2	28.75	34	F34:1346	OB5945 H2 Ro.raw	1.0606E4	2	2	507	515	Pyro-glu from Q	Q1:Pyro-glu from Q:1000.00	PEAKS PTM
R.AHVQVVDSNGN(+.98)RVYDEELQEGHVLVVPQNFAVAGK.S	N	54.09	3817.9023	35	3.2	955.4859	4	32.51	34	F34:1567	OB5945 H2 Ro.raw	7.3088E4	3	3	423	457		N11:Deamidation (NQ):8.22	PEAKS DB
R.Q(+.98)IVQNLRGENESEEEGAIVTVK.G	Y	53.37	2442.2236	22	8.7	815.0889	3	30.23	40	F40:1412	OB5953 H3A Ro.raw	2.1426E4	1	1	280	301		Q1:Deamidation (NQ):16.03	PEAKS DB
K.FFVPPS(+60.00)QQSLR.A	Y	52.88	1364.6910	11	-1.4	683.3518	2	32.82	34	F34:1584	OB5945 H2 Ro.raw	3.1431E5	1	1	516	526	2-OH-ethyl thio-Ser	S6:2-OH-ethyl thio-Ser:91.37	PEAKS PTM
R.LESEGGYIETWNPNNQ(+.98)EFEC(+57.02)AGVALSR.L	N	52.73	3070.3613	27	6.0	1024.4672	3	34.92	32	F32:1736	OB5943 H6 Ro.raw	6.6817E3	1	1	47	73	Deamidation (NQ); Carbamidomethylation	Q16:Deamidation (NQ):1000.00;C20:Carbamidomethylation:1000.00	PEAKS DB
R.Q(-17.03)IVQNLRGENESEEEGAIVTVK.G	Y	52.08	2424.2131	22	4.4	1213.1191	2	31.72	38	F38:1505	OB5951 H3A Ro.raw	0	0	0	280	301	Pyro-glu from Q	Q1:Pyro-glu from Q:1000.00	PEAKS PTM
R.VYDEELQEGHVLVVPQN(+.98)FAVAGK.S	N	51.23	2541.2751	23	9.5	1271.6569	2	32.92	31	F31:1618	OB5942 H6 Ro.raw	3.629E3	1	1	435	457		N17:Deamidation (NQ):14.02	PEAKS DB
K.TAN(+.98)DLNLLILR.W	N	50.73	1255.7136	11	2.6	628.8657	2	35.22	31	F31:1745	OB5942 H6 Ro.raw	0	0	0	377	387	Deamidation (NQ)	N3:Deamidation (NQ):68.31	PEAKS DB
R.AGQEEENEGGNIFSGFTPEFLAQAFQVDDR.Q	N	50.48	3301.4800	30	4.2	1651.7543	2	40.18	39	F39:1990	OB5952 H3A Ro.raw	2.7256E5	1	1	250	279			PEAKS DB
R.S(+57.02)PDIYNPQAGSLK.T	N	50.29	1445.7150	13	5.7	723.8689	2	28.94	34	F34:1350	OB5945 H2 Ro.raw	6.4489E3	2	2	364	376		S1:Carbamidomethylation (DHKE, X@N-term):14.04	PEAKS PTM
R.N(+57.02)ALFVPHYNTNAHSIIYALR.G	N	48.76	2370.2231	20	-0.1	791.0816	3	34.53	29	F29:1557	OB5922 H2 Ro.raw	3.2966E6	1	1	401	420		N1:Carbamidomethylation (DHKE, X@N-term):10.82	PEAKS PTM
R.WLGLSAE(+53.92)YGNLYR.N	N	48.21	1594.6866	13	0.4	532.5697	3	35.09	29	F29:1579	OB5922 H2 Ro.raw	6.0447E5	3	3	388	400	Replacement of 2 protons by iron	E7:Replacement of 2 protons by iron:123.05	PEAKS PTM

total 79 peptides

Peptide	Uniq	-10lgP	Mass	Length	ppm	m/z	z	RT	Fraction	Scan	Source File	Area Roasted in gel	#Feature	#Feature Roasted in gel	Start	End	PTM	AScore	Found By
N.G(+41.03)IEETIC(+57.02)TASVK.K	N	48.20	1347.6704	12	0.3	674.8427	2	29.12	34	F34:1370	OB5945 H2 Ro.raw	1.5496E4	1	1	345	356	Amidination of lysines or N-terminal amines with methyl acetimidate; Carbamidomethylation	G1:Amidination of lysines or N-terminal amines with methyl acetimidate:96.48;C7:Carbamidomethylation:1000.00	PEAKS PTM
R.SPD(+57.02)IYNPQAGSLK.T	N	45.16	1445.7150	13	2.2	723.8663	2	29.51	29	F29:1244	OB5922 H2 Ro.raw	0	0	0	364	376		D3:Carbamidomethylation (DHKE, X@N-term):0.00	PEAKS PTM
R.NALFVPHYNTNAHSIIYALRG.R	N	44.84	2370.2231	21	1.2	791.0826	3	33.77	34	F34:1624	OB5945 H2 Ro.raw	4.2894E6	2	2	401	421			PEAKS DB
R.QLKNNNPFKFFVPPSQQS.L	N	42.33	2119.0850	18	1.9	707.3703	3	33.27	35	F35:1604	OB5946 H2 Ro.raw	6.7737E4	1	1	507	524			PEAKS DB
K.NNNPFKFFVPPSQQS.L	N	41.33	1749.8474	15	1.0	875.9319	2	34.91	29	F29:1573	OB5922 H2 Ro.raw	4.7342E4	1	1	510	524			PEAKS DB
N.G(+57.02)IEETIC(+57.02)TASVKK.N	N	41.05	1491.7603	13	8.7	746.8939	2	26.96	31	F31:1261	OB5942 H6 Ro.raw	3.2429E4	4	4	345	357	Carbamidomethylation (DHKE, X@N-term); Carbamidomethylation	G1:Carbamidomethylation (DHKE, X@N-term):32.28;C7:Carbamidomethylation:1000.00	PEAKS PTM
R.FNLAGNH(+14.02)EQEFLR.Y	N	40.38	1587.7793	13	0.7	530.2674	3	30.81	39	F39:1450	OB5952 H3A Ro.raw	1.3642E5	3	3	198	210	Methylation(others)	H7:Methylation(others):33.98	PEAKS PTM
K.SQSD(+53.92)NFEYVAFK.T	N	40.31	1487.5656	12	-2.8	744.7880	2	32.03	29	F29:1395	OB5922 H2 Ro.raw	0	0	0	458	469	Replacement of 2 protons by iron	D4:Replacement of 2 protons by iron:20.50	PEAKS PTM
R.AHVQVVDSNGNRVYDEELQ(+.98)EGHVLVVPQNFAVAGK.S	N	40.20	3817.9023	35	7.9	955.4904	4	33.43	35	F35:1615	OB5946 H2 Ro.raw	0	0	0	423	457		Q19:Deamidation (NQ):4.91	PEAKS DB
R.AHVQVVDSN(+.98)GN(+.98)RVYDEELQEGHVLVVPQNFAVAGK.S	N	39.19	3818.8862	35	8.6	955.7371	4	34.33	32	F32:1692	OB5943 H6 Ro.raw	0	0	0	423	457		N9:Deamidation (NQ):0.00;N11:Deamidation (NQ):0.00	PEAKS DB
K.TAND(+53.92)LNLLILR.W	N	38.76	1308.6488	11	0.2	655.3318	2	35.09	29	F29:1584	OB5922 H2 Ro.raw	8.7715E4	1	1	377	387	Replacement of 2 protons by iron	D4:Replacement of 2 protons by iron:55.73	PEAKS PTM
R.RFNLAGNH(+14.02)EQEFLR.Y	N	38.64	1743.8805	14	0.5	582.3011	3	31.14	38	F38:1453	OB5951 H3A Ro.raw	7.3646E5	3	3	197	210	Methylation(others)	H8:Methylation(others):40.00	PEAKS PTM
R.AHVQ(+.98)VVDSNGNRVYDEELQEGHVLVVPQNFAVAGK.S	N	38.57	3817.9023	35	0.9	955.4838	4	33.12	31	F31:1624	OB5942 H6 Ro.raw	4.5408E4	1	1	423	457	Deamidation (NQ)	Q4:Deamidation (NQ):21.59	PEAKS DB
W.LGLSAEYGNLYR.N	N	38.37	1354.6881	12	3.5	678.3537	2	31.68	31	F31:1539	OB5942 H6 Ro.raw	0	0	0	389	400			PEAKS DB
S.ISFRQQPEENAC(+57.02)QFQR.L	N	37.74	2036.9486	16	0.8	679.9907	3	28.84	36	F36:1329	OB5948 H3B Ro.raw	3.1194E4	4	4	22	37	Carbamidomethylation	C12:Carbamidomethylation:1000.00	PEAKS DB
R.G(+57.02)YFGLIFPGC(+57.02)PSTYEEPAQQGRR.H	N	37.54	2686.2598	23	1.6	896.4286	3	35.57	39	F39:1718	OB5952 H3A Ro.raw	5.4933E4	1	1	100	122	Carbamidomethylation (DHKE, X@N-term); Carbamidomethylation	G1:Carbamidomethylation (DHKE, X@N-term):130.55;C10:Carbamidomethylation:1000.00	PEAKS PTM
R.SLPYSPYSP(+31.99)QSQPR.Q	N	36.19	1637.7686	14	3.2	819.8942	2	27.07	38	F38:1250	OB5951 H3A Ro.raw	6.6096E3	1	1	219	232		P9:Dihydroxy:9.42	PEAKS PTM
R.NALFVPHYNTNAHSIIYALRGR.A	N	36.04	2526.3245	22	0.6	843.1160	3	33.60	29	F29:1492	OB5922 H2 Ro.raw	7.6168E5	2	2	401	422			PEAKS DB
R.R(+14.02)FNLAGNHEQEFLR.Y	N	34.57	1743.8805	14	0.9	582.3013	3	31.38	39	F39:1472	OB5952 H3A Ro.raw	2.9356E5	2	2	197	210	Methylation(KR)	R1:Methylation(KR):67.58	PEAKS PTM
R.WLGLSAEYGN(+.98)LYR.N	N	33.97	1541.7513	13	5.4	771.8871	2	54.24	29	F29:2679	OB5922 H2 Ro.raw	0	0	0	388	400	Deamidation (NQ)	N10:Deamidation (NQ):1000.00	PEAKS DB

total 79 peptides

Peptide	Uniq	-10lgP	Mass	Length	ppm	m/z	z	RT	Fraction	Scan	Source File	Area Roasted in gel	#Feature	#Feature Roasted in gel	Start	End	PTM	AScore	Found By
R.RPFYSNAP(+15.99)QEIFIQQGR.G	N	33.55	2066.0332	17	0.2	689.6852	3	31.94	40	F40:1497	OB5953 H3A Ro.raw	4.3282E4	1	1	83	99	Oxidation or Hydroxylation	P8:Oxidation or Hydroxylation:32.28	PEAKS PTM
R.AHVQ(+.98)VVDSNGNRVYDEELQEGHVLVVPQ(+.98)NFAVAGK.S	N	33.38	3818.8862	35	8.9	955.7373	4	33.12	31	F31:1624	OB5942 H6 Ro.raw	3.8034E4	1	1	423	457		Q4:Deamidation (NQ):8.81;Q28:Deamidation (NQ):12.28	PEAKS DB
R.FNLAGNH(+156.12)EQEFLR.Y	N	33.01	1729.8788	13	-7.6	577.6292	3	31.13	38	F38:1444	OB5951 H3A Ro.raw	7.0376E5	2	2	198	210	4-hydroxynonenal (HNE)	H7:4-hydroxynonenal (HNE):1000.00	PEAKS PTM
R.N(+.98)ALFVPHYNTNAHSIIYALR.G	N	32.68	2314.1858	20	7.3	772.4082	3	50.66	29	F29:2450	OB5922 H2 Ro.raw	1.0573E4	1	1	401	420		N1:Deamidation (NQ):0.00	PEAKS DB
R.LESEGGYIETWN(+.98)PNNQEFEC(+57.02)AGVALSR.L	N	32.58	3070.3613	27	6.9	1536.1985	2	34.73	32	F32:1729	OB5943 H6 Ro.raw	2.6575E4	1	1	47	73	Deamidation (NQ); Carbamidomethylation	N12:Deamidation (NQ):1000.00;C20:Carbamidomethylation:1000.00	PEAKS DB
R.AGQEEENEGGNIFSGFTPEFLAQAFQVDDRQIVQNLR.G	N	32.18	4152.9775	37	5.2	1385.3403	3	39.54	38	F38:1958	OB5951 H3A Ro.raw	1.056E5	1	1	250	286			PEAKS DB
R.WLGLSAE(+21.98)YGNLYR.N	N	30.00	1562.7494	13	-8.4	782.3754	2	35.27	29	F29:1583	OB5922 H2 Ro.raw	5.0047E4	2	2	388	400	Sodium adduct	E7:Sodium adduct:42.78	PEAKS PTM
R.LNAQRPDNRLESEGGYIETWNPNN(+.98)QEFEC(+57.02)AGVALSR.L	N	29.77	4134.9087	36	4.7	1379.3167	3	33.85	33	F33:1671	OB5944 H6 Ro.raw	8.0788E4	1	1	38	73	Deamidation (NQ); Carbamidomethylation	N24:Deamidation (NQ):1000.00;C29:Carbamidomethylation:1000.00	PEAKS DB
R.W(+15.99)LGLSAEYGNLYR.N	N	28.45	1556.7623	13	-0.8	779.3878	2	34.26	31	F31:1698	OB5942 H6 Ro.raw	3.8864E3	1	1	388	400	Oxidation (HW)	W1:Oxidation (HW):1000.00	PEAKS PTM
**total 79 peptides**																			

B5TYU1|B5TYU1_ARAHY

back to list

| Protein Coverage | Supporting Peptides |

Protein Coverage:

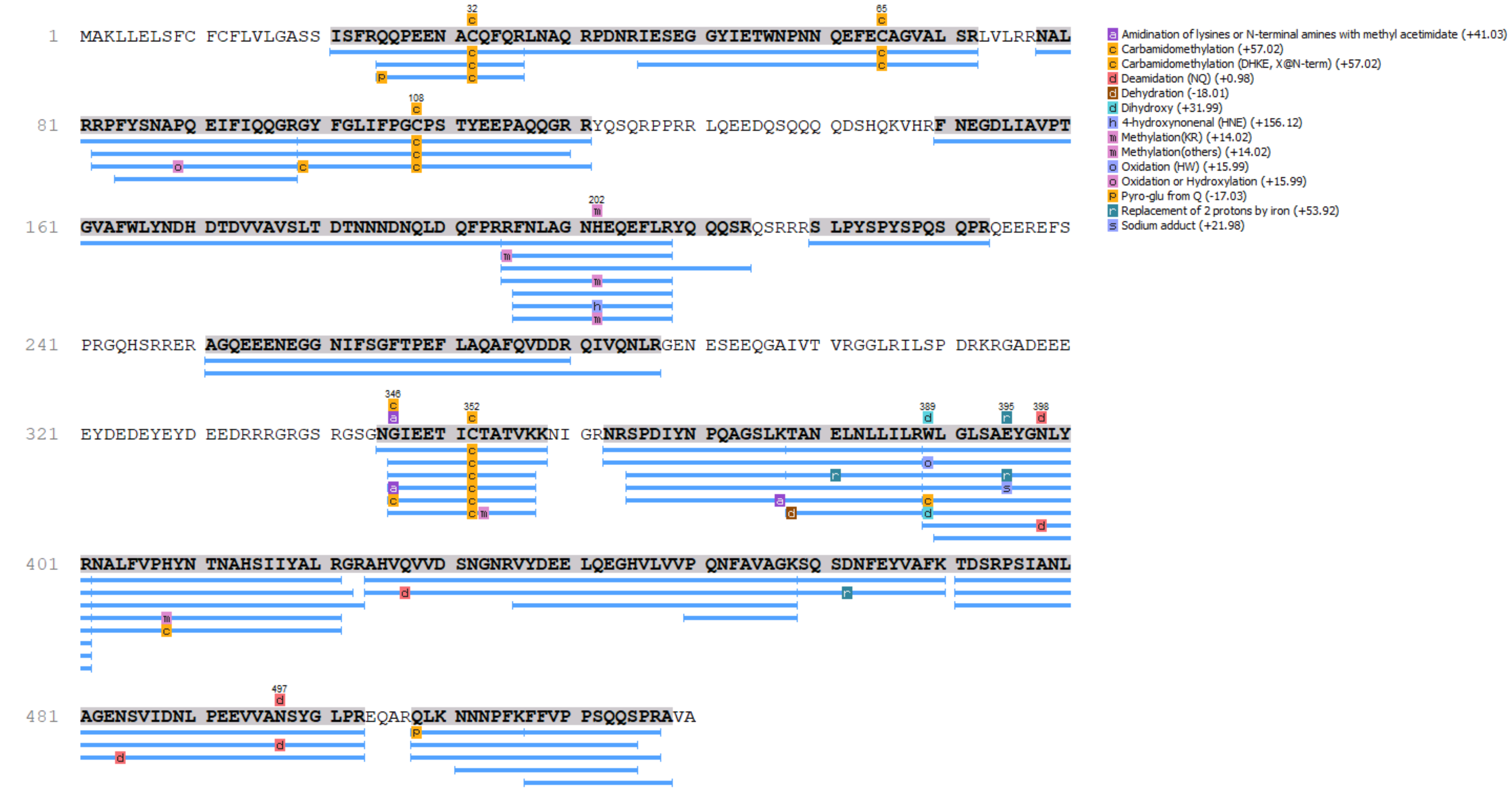

Supporting Peptides:

Peptide	Uniq	-10lgP	Mass	Length	ppm	m/z	z	RT	Fraction	Scan	Source File	Area Roasted in gel	#Feature	#Feature Roasted in gel	Start	End	PTM	AScore	Found By
R.NALFVPHYNTNAHSIIYALR.G	N	134.27	2313.2019	20	3.7	1157.6125	2	33.79	29	F29:1486	OB5922 H2 Ro.raw	5.1511E7	23	23	402	421			PEAKS DB
R.GYFGLIFPGC(+57.02)PSTYEEPAQQGR.R	N	127.83	2473.1372	22	3.0	1237.5796	2	36.11	38	F38:1750	OB5951 H3A Ro.raw	2.5988E6	9	9	99	120	Carbamidomethylation	C10:Carbamidomethylation:1000.00	PEAKS DB
R.SPDIYNPQAGSLKTANELNLLILR.W	N	125.09	2639.4282	24	2.1	880.8185	3	35.72	35	F35:1738	OB5946 H2 Ro.raw	6.3847E6	8	7	365	388			PEAKS DB
R.WLGLSAEYGNLYR.N	N	121.81	1540.7673	13	2.8	771.3931	2	34.72	34	F34:1730	OB5945 H2 Ro.raw	5.9147E7	35	35	389	401			PEAKS DB
R.NRSPDIYNPQAGSLKTANELNLLILR.W	N	119.99	2909.5723	26	2.3	970.8669	3	34.91	29	F29:1560	OB5922 H2 Ro.raw	3.3615E6	6	6	363	388			PEAKS DB
K.SQSDNFEYVAFK.T	N	114.21	1433.6462	12	2.6	717.8323	2	31.76	35	F35:1518	OB5946 H2 Ro.raw	5.4437E6	6	6	459	470			PEAKS DB

total 83 peptides

Peptide	Uniq	-10lgP	Mass	Length	ppm	m/z	z	RT	Fraction	Scan	Source File	Area Roasted in gel	#Feature	#Feature Roasted in gel	Start	End	PTM	AScore	Found By
R.RPFYSNAPQEIFIQQGR.G	N	110.90	2050.0383	17	3.2	684.3556	3	31.52	38	F38:1483	OB5951 H3A Ro.raw	9.1631E6	15	15	82	98			PEAKS DB
K.TDSRPSIANLAGENSVIDNLPEEVVANSYGLPR.E	Y	107.61	3496.7434	33	2.2	1166.5909	3	36.86	35	F35:1804	OB5946 H2 Ro.raw	1.9989E7	11	11	471	503			PEAKS DB
R.NALRRPFYSNAPQEIFIQQGR.G	N	97.93	2504.3037	21	1.0	835.7760	3	32.71	40	F40:1535	OB5953 H3A Ro.raw	6.3111E6	14	13	78	98			PEAKS DB
R.NRSPDIYNPQAGSLK.T	N	96.54	1658.8376	15	0.1	830.4261	2	27.65	32	F32:1291	OB5943 H6 Ro.raw	8.0621E5	12	12	363	377			PEAKS DB
N.GIEETIC(+57.02)TATVK.K	N	95.04	1320.6595	12	-0.2	661.3369	2	29.91	29	F29:1260	OB5922 H2 Ro.raw	2.5094E5	6	6	346	357	Carbamidomethylation	C7:Carbamidomethylation:1000.00	PEAKS DB
R.SPDIYNPQAGSLK.T	N	92.16	1388.6936	13	1.7	695.3552	2	28.58	34	F34:1323	OB5945 H2 Ro.raw	1.33E6	6	6	365	377			PEAKS DB
R.FNLAGNHEQEFLR.Y	N	91.88	1573.7637	13	4.3	787.8925	2	30.57	38	F38:1440	OB5951 H3A Ro.raw	1.243E5	7	7	196	208			PEAKS DB
R.VYDEELQEGHVLVVPQNFAVAGK.S	N	90.91	2540.2910	23	1.4	847.7721	3	33.42	29	F29:1478	OB5922 H2 Ro.raw	1.9062E6	10	10	436	458			PEAKS DB
R.RFNLAGNHEQEFLR.Y	N	87.38	1729.8647	14	1.5	577.6297	3	31.36	40	F40:1478	OB5953 H3A Ro.raw	1.8294E6	7	7	195	208			PEAKS DB
N.GIEETIC(+57.02)TATVKK.N	N	82.16	1448.7545	13	0.7	725.3850	2	27.45	31	F31:1292	OB5942 H6 Ro.raw	4.6237E5	5	5	346	358	Carbamidomethylation	C7:Carbamidomethylation:1000.00	PEAKS DB
R.NALFVPH(+14.02)YNTNAHSIIYALR.G	N	79.80	2327.2175	20	0.7	776.7470	3	34.91	29	F29:1561	OB5922 H2 Ro.raw	1.3103E6	3	3	402	421	Methylation(others)	H7:Methylation(others):22.45	PEAKS PTM
R.AHVQVVDSNGNRVYDEELQEGHVLVVPQNFAVAGK.S	N	77.75	3816.9182	35	3.6	955.2402	4	32.32	35	F35:1544	OB5946 H2 Ro.raw	8.2893E6	8	8	424	458			PEAKS DB
R.GYFGLIFPGC(+57.02)PSTYEEPAQQGRR.Y	N	76.83	2629.2383	23	2.4	877.4221	3	35.18	40	F40:1676	OB5953 H3A Ro.raw	5.6622E6	9	9	99	121	Carbamidomethylation	C10:Carbamidomethylation:1000.00	PEAKS DB
K.TANELNLLILR.W	N	76.42	1268.7452	11	0.6	635.3802	2	35.27	29	F29:1655	OB5922 H2 Ro.raw	2.1461E7	10	10	378	388			PEAKS DB
R.Q(-17.03)QPEENAC(+57.02)QFQR.L	N	75.52	1516.6365	12	2.5	759.3274	2	28.03	32	F32:1319	OB5943 H6 Ro.raw	7.279E3	2	2	25	36	Pyro-glu from Q; Carbamidomethylation	Q1:Pyro-glu from Q:1000.00;C8:Carbamidomethylation:1000.00	PEAKS PTM
V.PQNFAVAGK.S	N	75.51	930.4923	9	1.0	466.2539	2	32.32	35	F35:1542	OB5946 H2 Ro.raw	3.3444E6	3	3	450	458			PEAKS DB
K.TDSRPSIANLAGENSVIDNLPEEVVAN(+.98)SYGLPR.E	Y	74.76	3497.7273	33	1.1	1166.9176	3	35.87	34	F34:1763	OB5945 H2 Ro.raw	0	0	0	471	503	Deamidation (NQ)	N27:Deamidation (NQ):83.90	PEAKS DB
R.IESEGGYIETWNPNNQEFEC(+57.02)AGVALSR.L	N	74.31	3069.3774	27	1.4	1024.1345	3	34.50	31	F31:1703	OB5942 H6 Ro.raw	1.0361E4	1	1	46	72	Carbamidomethylation	C20:Carbamidomethylation:1000.00	PEAKS DB
R.QQPEENAC(+57.02)QFQR.L	N	73.76	1533.6630	12	1.7	767.8400	2	25.24	32	F32:1157	OB5943 H6 Ro.raw	3.7544E4	3	3	25	36	Carbamidomethylation	C8:Carbamidomethylation:1000.00	PEAKS DB
K.FFVPPSQQSPR.A	N	69.38	1288.6564	11	0.2	645.3356	2	28.58	32	F32:1346	OB5943 H6 Ro.raw	2.1108E5	5	5	517	527			PEAKS DB
N.G(+57.02)IEETIC(+57.02)TATVK.K	N	64.33	1377.6809	12	2.0	689.8491	2	30.19	29	F29:1285	OB5922 H2 Ro.raw	5.5841E3	1	1	346	357	Carbamidomethylation (DHKE, X@N-term); Carbamidomethylation	G1:Carbamidomethylation (DHKE, X@N-term):30.83;C7:Carbamidomethylation:1000.00	PEAKS PTM

total 83 peptides

Peptide	Uniq	-10lgP	Mass	Length	ppm	m/z	z	RT	Fraction	Scan	Source File	Area Roasted in gel	#Feature	#Feature Roasted in gel	Start	End	PTM	AScore	Found By
R.AHVQVVDSN(+.98)GNRVYDEELQEGHVLVVPQNFAVAGK.S	N	64.02	3817.9023	35	5.0	955.4877	4	33.04	29	F29:1438	OB5922 H2 Ro.raw	1.4175E6	2	2	424	458		N9:Deamidation (NQ):9.40	PEAKS DB
R.NALFVPH(+57.02)YNTNAHSIIYALR.G	N	63.66	2370.2231	20	0.1	791.0817	3	34.03	35	F35:1631	OB5946 H2 Ro.raw	8.9747E5	1	1	402	421	Carbamidomethylation (DHKE, X@N-term)	H7:Carbamidomethylation (DHKE, X@N-term):20.35	PEAKS PTM
K.TDSRPSIANLAGEN(+.98)SVIDNLPEEVVANSYGLPR.E	Y	63.57	3497.7273	33	9.1	1166.9270	3	36.70	36	F36:1801	OB5948 H3B Ro.raw	1.1598E5	2	2	471	503	Deamidation (NQ)	N14:Deamidation (NQ):39.44	PEAKS DB
R.RFNLAGNHEQEFLRYQQQSR.Q	N	62.22	2520.2371	20	-2.3	631.0667	4	34.12	30	F30:1496	OB5926 H3B Ro.raw	7.5688E4	2	2	195	214			PEAKS DB
R.LNAQRPDNRIESEGGYIETWNPNNQEFEC(+57.02)AGVALSR.L	N	60.14	4133.9248	36	2.4	1034.4910	4	33.87	33	F33:1670	OB5944 H6 Ro.raw	1.5556E6	3	3	37	72	Carbamidomethylation	C29:Carbamidomethylation:1000.00	PEAKS DB
R.GYFGLIFPGC(+57.02)PS(+14.02)TYEEPAQQGR.R	N	59.71	2487.1528	22	2.2	1244.5864	2	36.43	32	F32:1817	OB5943 H6 Ro.raw	7.7963E4	3	3	99	120	Carbamidomethylation	C10:Carbamidomethylation:1000.00;S12:Methylation(others):9.34	PEAKS PTM
R.W(+31.99)LGLSAEYGNLYR.N	N	59.45	1572.7572	13	-1.6	787.3846	2	33.64	35	F35:1624	OB5946 H2 Ro.raw	8.5976E4	2	2	389	401	Dihydroxy	W1:Dihydroxy:62.93	PEAKS PTM
P.FYSNAPQEIFIQQGR.G	N	59.30	1796.8845	15	-1.2	899.4484	2	32.27	31	F31:1574	OB5942 H6 Ro.raw	0	0	0	84	98			PEAKS DB
R.FNEGDLIAVPTGVAFWLYNDHDTDVVAVSLTDTNNNDNQLDQFPR.R	N	58.15	5049.3691	45	0.7	1684.1357	3	40.24	30	F30:1843	OB5926 H3B Ro.raw	9.8654E5	1	1	150	194			PEAKS DB
R.GYFGLIFPGC(+57.02)PST(+14.02)YEEPAQQGR.R	N	58.14	2487.1528	22	2.8	1244.5872	2	36.17	31	F31:1801	OB5942 H6 Ro.raw	2.5405E4	2	2	99	120	Carbamidomethylation	C10:Carbamidomethylation:1000.00;T13:Methylation(others):14.02	PEAKS PTM
R.W(+57.02)LGLSAEYGNLYR.N	N	57.46	1597.7888	13	0.6	799.9022	2	35.09	34	F34:1725	OB5945 H2 Ro.raw	1.848E4	1	1	389	401	Carbamidomethylation (DHKE, X@N-term)	W1:Carbamidomethylation (DHKE, X@N-term):62.19	PEAKS PTM
R.Q(-17.03)LKNNNPFK.F	N	56.41	1084.5665	9	1.6	543.2914	2	28.75	34	F34:1346	OB5945 H2 Ro.raw	1.0606E4	2	2	508	516	Pyro-glu from Q	Q1:Pyro-glu from Q:1000.00	PEAKS PTM
R.AHVQVVDSNGN(+.98)RVYDEELQEGHVLVVPQNFAVAGK.S	N	54.09	3817.9023	35	3.2	955.4859	4	32.51	34	F34:1567	OB5945 H2 Ro.raw	7.3088E4	3	3	424	458		N11:Deamidation (NQ):8.22	PEAKS DB
R.IESEGGYIETWNPNNQ(+.98)EFEC(+57.02)AGVALSR.L	N	52.73	3070.3613	27	6.0	1024.4672	3	34.92	32	F32:1736	OB5943 H6 Ro.raw	6.6817E3	1	1	46	72	Carbamidomethylation	Q16:Deamidation (NQ):0.00;C20:Carbamidomethylation:1000.00	PEAKS DB
K.FFVPPSQQSPRA.V	N	51.84	1359.6935	12	1.1	680.8547	2	29.14	32	F32:1381	OB5943 H6 Ro.raw	3.8841E4	2	2	517	528			PEAKS DB
R.VYDEELQEGHVLVVPQN(+.98)FAVAGK.S	N	51.23	2541.2751	23	9.5	1271.6569	2	32.92	31	F31:1618	OB5942 H6 Ro.raw	3.629E3	1	1	436	458		N17:Deamidation (NQ):14.02	PEAKS DB
R.AGQEEENEGGNIFSGFTPEFLAQAFQVDDR.Q	N	50.48	3301.4800	30	4.2	1651.7543	2	40.18	39	F39:1990	OB5952 H3A Ro.raw	2.7256E5	1	1	251	280			PEAKS DB
R.S(+57.02)PDIYNPQAGSLK.T	N	50.29	1445.7150	13	5.7	723.8689	2	28.94	34	F34:1350	OB5945 H2 Ro.raw	6.4489E3	2	2	365	377		S1:Carbamidomethylation (DHKE, X@N-term):14.04	PEAKS PTM
K.TANE(+53.92)LNLLILR.W	N	48.78	1322.6646	11	-1.4	662.3386	2	35.27	29	F29:1590	OB5922 H2 Ro.raw	2.333E5	1	1	378	388	Replacement of 2 protons by iron	E4:Replacement of 2 protons by iron:154.98	PEAKS PTM
R.N(+57.02)ALFVPHYNTNAHSIIYALR.G	N	48.76	2370.2231	20	-0.1	791.0816	3	34.53	29	F29:1557	OB5922 H2 Ro.raw	3.2966E6	1	1	402	421		N1:Carbamidomethylation (DHKE, X@N-term):10.82	PEAKS PTM
R.WLGLSAE(+53.92)YGNLYR.N	N	48.21	1594.6866	13	0.4	532.5697	3	35.09	29	F29:1579	OB5922 H2 Ro.raw	6.0447E5	3	3	389	401	Replacement of 2 protons by iron	E7:Replacement of 2 protons by iron:123.05	PEAKS PTM
R.SPD(+57.02)IYNPQAGSLK.T	N	45.16	1445.7150	13	2.2	723.8663	2	29.51	29	F29:1244	OB5922 H2 Ro.raw	0	0	0	365	377		D3:Carbamidomethylation (DHKE, X@N-term):0.00	PEAKS PTM

total 83 peptides

Peptide	Uniq	-10lgP	Mass	Length	ppm	m/z	z	RT	Fraction	Scan	Source File	Area Roasted in gel	#Feature	#Feature Roasted in gel	Start	End	PTM	AScore	Found By
R.NALFVPHYNTNAHSIIYALRG.R	N	44.84	2370.2231	21	1.2	791.0826	3	33.77	34	F34:1624	OB5945 H2 Ro.raw	4.2894E6	2	2	402	422			PEAKS DB
N.GIEETIC(+57.02)T(+14.02)ATVK.K	N	42.70	1334.6752	12	3.2	668.3470	2	29.62	34	F34:1395	OB5945 H2 Ro.raw	7.3781E3	1	1	346	357	Carbamidomethylation; Methylation(others)	C7:Carbamidomethylation:1000.00;T8:Methylation (others):28.36	PEAKS PTM
R.QLKNNNPFKFFVPPSQQS.P	N	42.33	2119.0850	18	1.9	707.3703	3	33.27	35	F35:1604	OB5946 H2 Ro.raw	6.7737E4	1	1	508	525			PEAKS DB
K.NNNPFKFFVPPSQQS.P	N	41.33	1749.8474	15	1.0	875.9319	2	34.91	29	F29:1573	OB5922 H2 Ro.raw	4.7342E4	1	1	511	525			PEAKS DB
R.FNLAGNH(+14.02)EQEFLR.Y	N	40.38	1587.7793	13	0.7	530.2674	3	30.81	39	F39:1450	OB5952 H3A Ro.raw	1.3642E5	3	3	196	208	Methylation(others)	H7:Methylation(others):33.98	PEAKS PTM
K.SQSD(+53.92)NFEYVAFK.T	N	40.31	1487.5656	12	-2.8	744.7880	2	32.03	29	F29:1395	OB5922 H2 Ro.raw	0	0	0	459	470	Replacement of 2 protons by iron	D4:Replacement of 2 protons by iron:20.50	PEAKS PTM
R.AHVQVVDSNGNRVYDEELQ(+.98)EGHVLVVPQNFAVAGK.S	N	40.20	3817.9023	35	7.9	955.4904	4	33.43	35	F35:1615	OB5946 H2 Ro.raw	0	0	0	424	458		Q19:Deamidation (NQ):4.91	PEAKS DB
R.AHVQVVDSN(+.98)GN(+.98)RVYDEELQEGHVLVVPQNFAVAGK.S	N	39.19	3818.8862	35	8.6	955.7371	4	34.33	32	F32:1692	OB5943 H6 Ro.raw	0	0	0	424	458		N9:Deamidation (NQ):0.00;N11:Deamidation (NQ):0.00	PEAKS DB
R.RFNLAGNH(+14.02)EQEFLR.Y	N	38.64	1743.8805	14	0.5	582.3011	3	31.14	38	F38:1453	OB5951 H3A Ro.raw	7.3646E5	3	3	195	208	Methylation(others)	H8:Methylation(others):40.00	PEAKS PTM
R.AHVQ(+.98)VVDSNGNRVYDEELQEGHVLVVPQNFAVAGK.S	N	38.57	3817.9023	35	0.9	955.4838	4	33.12	31	F31:1624	OB5942 H6 Ro.raw	4.5408E4	1	1	424	458	Deamidation (NQ)	Q4:Deamidation (NQ):21.59	PEAKS DB
W.LGLSAEYGNLYR.N	N	38.37	1354.6881	12	3.5	678.3537	2	31.68	31	F31:1539	OB5942 H6 Ro.raw	0	0	0	390	401			PEAKS DB
S.ISFRQQPEENAC(+57.02)QFQR.L	N	37.74	2036.9486	16	0.8	679.9907	3	28.84	36	F36:1329	OB5948 H3B Ro.raw	3.1194E4	4	4	21	36	Carbamidomethylation	C12:Carbamidomethylation:1000.00	PEAKS DB
R.G(+57.02)YFGLIFPGC(+57.02)PSTYEEPAQQGRR.Y	N	37.54	2686.2598	23	1.6	896.4286	3	35.57	39	F39:1718	OB5952 H3A Ro.raw	5.4933E4	1	1	99	121	Carbamidomethylation (DHKE, X@N-term); Carbamidomethylation	G1:Carbamidomethylation (DHKE, X@N-term):130.55;C10:Carbamidomethylation:1000.00	PEAKS PTM
N.G(+41.03)IEETIC(+57.02)TATVK.K	N	36.93	1361.6860	12	0.7	681.8508	2	29.66	34	F34:1390	OB5945 H2 Ro.raw	1.6824E4	1	1	346	357	Amidination of lysines or N-terminal amines with methyl acetimidate; Carbamidomethylation	G1:Amidination of lysines or N-terminal amines with methyl acetimidate:96.48;C7:Carbamidomethylation:1000.00	PEAKS PTM
R.SLPYSPYSP(+31.99)QSQPR.Q	N	36.19	1637.7686	14	3.2	819.8942	2	27.07	38	F38:1250	OB5951 H3A Ro.raw	6.6096E3	1	1	220	233		P9:Dihydroxy:9.42	PEAKS PTM
R.NALFVPHYNTNAHSIIYALRGR.A	N	36.04	2526.3245	22	0.6	843.1160	3	33.60	29	F29:1492	OB5922 H2 Ro.raw	7.6168E5	2	2	402	423			PEAKS DB
R.R(+14.02)FNLAGNHEQEFLR.Y	N	34.57	1743.8805	14	0.9	582.3013	3	31.38	39	F39:1472	OB5952 H3A Ro.raw	2.9356E5	2	2	195	208	Methylation(KR)	R1:Methylation(KR):67.58	PEAKS PTM
N.GIEETIC(+57.02)T(+14.02)ATVKK.N	N	34.37	1462.7701	13	0.9	732.3930	2	27.73	31	F31:1307	OB5942 H6 Ro.raw	3.1361E3	1	1	346	358	Carbamidomethylation	C7:Carbamidomethylation:1000.00;T8:Methylation (others):19.16	PEAKS PTM
K.TDSRPSIANLAGENSVIDN(+.98)LPEEVVANSYGLPR.E	Y	34.18	3497.7273	33	4.8	1166.9220	3	37.42	37	F37:1844	OB5949 H3B Ro.raw	4.9402E4	1	1	471	503		N19:Deamidation (NQ):0.00	PEAKS DB
R.WLGLSAEYGN(+.98)LYR.N	N	33.97	1541.7513	13	5.4	771.8871	2	54.24	29	F29:2679	OB5922 H2 Ro.raw	0	0	0	389	401	Deamidation (NQ)	N10:Deamidation (NQ):1000.00	PEAKS DB

total 83 peptides

Peptide	Uniq	-10lgP	Mass	Length	ppm	m/z	z	RT	Fraction	Scan	Source File	Area Roasted in gel	#Feature	#Feature Roasted in gel	Start	End	PTM	AScore	Found By
G.NGIEETIC(+57.02)TATVKK.N	N	33.56	1562.7974	14	0.1	782.4060	2	29.85	34	F34:1417	OB5945 H2 Ro.raw	8.2899E3	2	2	345	358	Carbamidomethylation	C8:Carbamidome thylation:1000.00	PEAKS DB
R.RPFYSNAP(+15.99)QEIFIQQGR.G	N	33.55	2066.0332	17	0.2	689.6852	3	31.94	40	F40:1497	OB5953 H3A Ro.raw	4.3282E4	1	1	82	98	Oxidation or Hydroxylation	P8:Oxidation or H ydroxylation:32.2 8	PEAKS PTM
R.AHVQ(+.98)VVDSNGNRVYDEELQEGHVLVVPQ(+.98)NFAVAGK.S	N	33.38	3818.8862	35	8.9	955.7373	4	33.12	31	F31:1624	OB5942 H6 Ro.raw	3.8034E4	1	1	424	458		Q4:Deamidation (NQ):8.81;Q28:D eamidation (NQ): 12.28	PEAKS DB
R.FNLAGNH(+156.12)EQEFLR.Y	N	33.01	1729.8788	13	-7.6	577.6292	3	31.13	38	F38:1444	OB5951 H3A Ro.raw	7.0376E5	2	2	196	208	4-hydroxynonenal (HNE)	H7:4-hydroxynon enal (HNE):1000. 00	PEAKS PTM
R.N(+.98)ALFVPHYNTNAHSIIYALR.G	N	32.68	2314.1858	20	7.3	772.4082	3	50.66	29	F29:2450	OB5922 H2 Ro.raw	1.0573E4	1	1	402	421		N1:Deamidation (NQ):0.00	PEAKS DB
R.IESEGGYIETWN(+.98)PNNQEFEC(+57.02)AGVALSR.L	N	32.58	3070.3613	27	6.9	1536.1985	2	34.73	32	F32:1729	OB5943 H6 Ro.raw	2.6575E4	1	1	46	72	Carbamidomethylation	N12:Deamidation (NQ):0.00;C20:C arbamidomethyla tion:1000.00	PEAKS DB
R.AGQEEENEGGNIFSGFTPEFLAQAFQVDDRQIVQNLR.G	N	32.18	4152.9775	37	5.2	1385.3403	3	39.54	38	F38:1958	OB5951 H3A Ro.raw	1.056E5	1	1	251	287			PEAKS DB
K.T(-18.01)ANELNLLILR.W	N	31.74	1250.7346	11	2.2	626.3760	2	35.27	29	F29:1600	OB5922 H2 Ro.raw	1.738E4	1	1	378	388	Dehydration	T1:Dehydration:1 000.00	PEAKS PTM
R.QLKNNNPFKFFVPPSQQSPR.A	N	31.56	2372.2388	20	0.5	791.7539	3	33.17	29	F29:1463	OB5922 H2 Ro.raw	0	0	0	508	527			PEAKS DB
R.SPDIYNPQAGSLK(+41.03)TANELNLLILR.W	N	31.55	2680.4548	24	0.2	894.4924	3	36.03	34	F34:1771	OB5945 H2 Ro.raw	3.6458E4	1	1	365	388	Amidination of lysines or N-terminal amines with methyl acetimidate	K13:Amidination of lysines or N-ter minal amines wit h methyl acetimid ate:54.40	PEAKS PTM
R.WLGLSAE(+21.98)YGNLYR.N	N	30.00	1562.7494	13	-8.4	782.3754	2	35.27	29	F29:1583	OB5922 H2 Ro.raw	5.0047E4	2	2	389	401	Sodium adduct	E7:Sodium adduc t:42.78	PEAKS PTM
R.LNAQRPDNRIESEGGYIETWNPNN(+.98)QEFEC(+57.02)AGVALSR.L	N	29.77	4134.9087	36	4.7	1379.3167	3	33.85	33	F33:1671	OB5944 H6 Ro.raw	8.0788E4	1	1	37	72	Carbamidomethylation	N24:Deamidation (NQ):0.00;C29:C arbamidomethyla tion:1000.00	PEAKS DB
N.GIEET(+14.02)IC(+57.02)TATVKK.N	N	29.08	1462.7701	13	-1.4	732.3914	2	28.11	32	F32:1329	OB5943 H6 Ro.raw	2.1469E3	1	1	346	358	Carbamidomethylation	T5:Methylation(ot hers):9.08;C7:Ca rbamidomethylati on:1000.00	PEAKS PTM
R.W(+15.99)LGLSAEYGNLYR.N	N	28.45	1556.7623	13	-0.8	779.3878	2	34.26	31	F31:1698	OB5942 H6 Ro.raw	3.8864E3	1	1	389	401	Oxidation (HW)	W1:Oxidation (H W):1000.00	PEAKS PTM
**total 83 peptides**																			

Q5I6T2|Q5I6T2_ARAHY

back to list

| Protein Coverage | Supporting Peptides |

Protein Coverage:

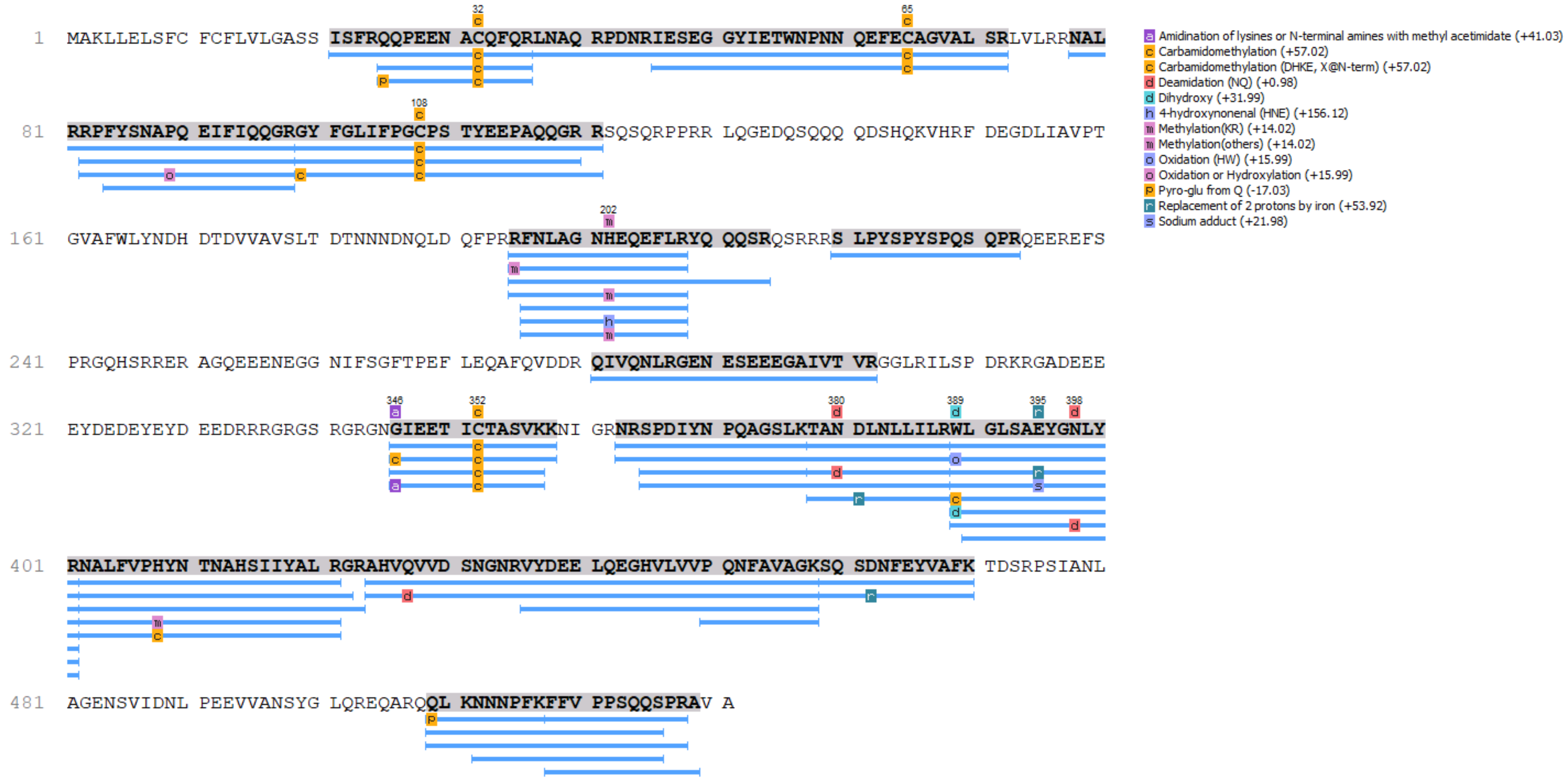

Supporting Peptides:

Peptide	Uniq	-10lgP	Mass	Length	ppm	m/z	z	RT	Fraction	Scan	Source File	Area Roasted in gel	#Feature	#Feature Roasted in gel	Start	End	PTM	AScore	Found By
R.SPDIYNPQAGSLKTANDLNLLILR.W	N	136.50	2625.4126	24	-0.1	876.1447	3	35.61	29	F29:1607	OB5922 H2 Ro.raw	1.276E6	3	3	365	388			PEAKS DB
R.NALFVPHYNTNAHSIIYALR.G	N	134.27	2313.2019	20	3.7	1157.6125	2	33.79	29	F29:1486	OB5922 H2 Ro.raw	5.1511E7	23	23	402	421			PEAKS DB
R.GYFGLIFPGC(+57.02)PSTYEEPAQQGR.R	N	127.83	2473.1372	22	3.0	1237.5796	2	36.11	38	F38:1750	OB5951 H3A Ro.raw	2.5988E6	9	9	99	120	Carbamidomethylation	C10:Carbamidomethylation:1000.00	PEAKS DB
R.WLGLSAEYGNLYR.N	N	121.81	1540.7673	13	2.8	771.3931	2	34.72	34	F34:1730	OB5945 H2 Ro.raw	5.9147E7	35	35	389	401			PEAKS DB
R.NRSPDIYNPQAGSLKTANDLNLLILR.W	N	120.82	2895.5566	26	1.6	966.1943	3	34.35	29	F29:1530	OB5922 H2 Ro.raw	2.2791E6	6	6	363	388			PEAKS DB
K.SQSDNFEYVAFK.T	N	114.21	1433.6462	12	2.6	717.8323	2	31.76	35	F35:1518	OB5946 H2 Ro.raw	5.4437E6	6	6	459	470			PEAKS DB
R.RPFYSNAPQEIFIQQGR.G	N	110.90	2050.0383	17	3.2	684.3556	3	31.52	38	F38:1483	OB5951 H3A Ro.raw	9.1631E6	15	15	82	98			PEAKS DB

total 73 peptides

Peptide	Uniq	-10lgP	Mass	Length	ppm	m/z	z	RT	Fraction	Scan	Source File	Area Roasted in gel	#Feature	#Feature Roasted in gel	Start	End	PTM	AScore	Found By
R.NALRRPFYSNAPQEIFIQQGR.G	N	97.93	2504.3037	21	1.0	835.7760	3	32.71	40	F40:1535	OB5953 H3A Ro.raw	6.3111E6	14	13	78	98			PEAKS DB
R.NRSPDIYNPQAGSLK.T	N	96.54	1658.8376	15	0.1	830.4261	2	27.65	32	F32:1291	OB5943 H6 Ro.raw	8.0621E5	12	12	363	377			PEAKS DB
R.SPDIYNPQAGSLK.T	N	92.16	1388.6936	13	1.7	695.3552	2	28.58	34	F34:1323	OB5945 H2 Ro.raw	1.33E6	6	6	365	377			PEAKS DB
R.FNLAGNHEQEFLR.Y	N	91.88	1573.7637	13	4.3	787.8925	2	30.57	38	F38:1440	OB5951 H3A Ro.raw	1.243E5	7	7	196	208			PEAKS DB
R.VYDEELQEGHVLVVPQNFAVAGK.S	N	90.91	2540.2910	23	1.4	847.7721	3	33.42	29	F29:1478	OB5922 H2 Ro.raw	1.9062E6	10	10	436	458			PEAKS DB
N.GIEETIC(+57.02)TASVK.K	N	90.29	1306.6438	12	0.6	654.3296	2	28.94	34	F34:1356	OB5945 H2 Ro.raw	1.977E5	6	6	346	357	Carbamidomethylation	C7:Carbamidomethylation:1000.00	PEAKS DB
R.RFNLAGNHEQEFLR.Y	N	87.38	1729.8647	14	1.5	577.6297	3	31.36	40	F40:1478	OB5953 H3A Ro.raw	1.8294E6	7	7	195	208			PEAKS DB
R.NALFVPH(+14.02)YNTNAHSIIYALR.G	N	79.80	2327.2175	20	0.7	776.7470	3	34.91	29	F29:1561	OB5922 H2 Ro.raw	1.3103E6	3	3	402	421	Methylation(others)	H7:Methylation(others):22.45	PEAKS PTM
R.AHVQVVDSNGNRVYDEELQEGHVLVVPQNFAVAGK.S	N	77.75	3816.9182	35	3.6	955.2402	4	32.32	35	F35:1544	OB5946 H2 Ro.raw	8.2893E6	8	8	424	458			PEAKS DB
R.GYFGLIFPGC(+57.02)PSTYEEPAQQGRR.S	N	76.83	2629.2383	23	2.4	877.4221	3	35.18	40	F40:1676	OB5953 H3A Ro.raw	5.6622E6	9	9	99	121	Carbamidomethylation	C10:Carbamidomethylation:1000.00	PEAKS DB
N.GIEETIC(+57.02)TASVKK.N	N	76.18	1434.7388	13	0.7	718.3771	2	26.91	33	F33:1250	OB5944 H6 Ro.raw	3.5109E5	8	8	346	358	Carbamidomethylation	C7:Carbamidomethylation:1000.00	PEAKS DB
R.Q(-17.03)QPEENAC(+57.02)QFQR.L	N	75.52	1516.6365	12	2.5	759.3274	2	28.03	32	F32:1319	OB5943 H6 Ro.raw	7.279E3	2	2	25	36	Pyro-glu from Q; Carbamidomethylation	Q1:Pyro-glu from Q:1000.00;C8:Carbamidomethylation:1000.00	PEAKS PTM
V.PQNFAVAGK.S	N	75.51	930.4923	9	1.0	466.2539	2	32.32	35	F35:1542	OB5946 H2 Ro.raw	3.3444E6	3	3	450	458			PEAKS DB
R.IESEGGYIETWNPNNQEFEC(+57.02)AGVALSR.L	N	74.31	3069.3774	27	1.4	1024.1345	3	34.50	31	F31:1703	OB5942 H6 Ro.raw	1.0361E4	1	1	46	72	Carbamidomethylation	C20:Carbamidomethylation:1000.00	PEAKS DB
K.TANDLNLLILR.W	N	73.94	1254.7296	11	1.8	628.3732	2	34.79	35	F35:1750	OB5946 H2 Ro.raw	1.0437E7	10	10	378	388			PEAKS DB
R.QQPEENAC(+57.02)QFQR.L	N	73.76	1533.6630	12	1.7	767.8400	2	25.24	32	F32:1157	OB5943 H6 Ro.raw	3.7544E4	3	3	25	36	Carbamidomethylation	C8:Carbamidomethylation:1000.00	PEAKS DB
K.FFVPPSQQSPR.A	N	69.38	1288.6564	11	0.2	645.3356	2	28.58	32	F32:1346	OB5943 H6 Ro.raw	2.1108E5	5	5	518	528			PEAKS DB
R.S(+57.02)PDIYNPQAGSLKTANDLNLLILR.W	N	64.15	2682.4341	24	1.5	895.1533	3	35.28	34	F34:1735	OB5945 H2 Ro.raw	9.5441E4	1	1	365	388		S1:Carbamidomethylation (DHKE, X@N-term):14.04	PEAKS PTM
R.AHVQVVDSN(+.98)GNRVYDEELQEGHVLVVPQNFAVAGK.S	N	64.02	3817.9023	35	5.0	955.4877	4	33.04	29	F29:1438	OB5922 H2 Ro.raw	1.4175E6	2	2	424	458		N9:Deamidation (NQ):9.40	PEAKS DB
R.NALFVPH(+57.02)YNTNAHSIIYALR.G	N	63.66	2370.2231	20	0.1	791.0817	3	34.03	35	F35:1631	OB5946 H2 Ro.raw	8.9747E5	1	1	402	421	Carbamidomethylation (DHKE, X@N-term)	H7:Carbamidomethylation (DHKE, X@N-term):20.35	PEAKS PTM
R.RFNLAGNHEQEFLRYQQQSR.Q	N	62.22	2520.2371	20	-2.3	631.0667	4	34.12	30	F30:1496	OB5926 H3B Ro.raw	7.5688E4	2	2	195	214			PEAKS DB
R.LNAQRPDNRIESEGGYIETWNPNNQEFEC(+57.02)AGVALSR.L	N	60.14	4133.9248	36	2.4	1034.4910	4	33.87	33	F33:1670	OB5944 H6 Ro.raw	1.5556E6	3	3	37	72	Carbamidomethylation	C29:Carbamidomethylation:1000.00	PEAKS DB
**total 73 peptides**																			

Peptide	Uniq	-10lgP	Mass	Length	ppm	m/z	z	RT	Fraction	Scan	Source File	Area Roasted in gel	#Feature	#Feature Roasted in gel	Start	End	PTM	AScore	Found By
R.GYFGLIFPGC(+57.02)PS(+14.02)TYEEPAQQGR.R	N	59.71	2487.1528	22	2.2	1244.5864	2	36.43	32	F32:1817	OB5943 H6 Ro.raw	7.7963E4	3	3	99	120	Carbamidomethylation	C10:Carbamidomethylation:1000.00;S12:Methylation (others):9.34	PEAKS PTM
R.W(+31.99)LGLSAEYGNLYR.N	N	59.45	1572.7572	13	-1.6	787.3846	2	33.64	35	F35:1624	OB5946 H2 Ro.raw	8.5976E4	2	2	389	401	Dihydroxy	W1:Dihydroxy:62.93	PEAKS PTM
P.FYSNAPQEIFIQQGR.G	N	59.30	1796.8845	15	-1.2	899.4484	2	32.27	31	F31:1574	OB5942 H6 Ro.raw	0	0	0	84	98			PEAKS DB
R.GYFGLIFPGC(+57.02)PST(+14.02)YEEPAQQGR.R	N	58.14	2487.1528	22	2.8	1244.5872	2	36.17	31	F31:1801	OB5942 H6 Ro.raw	2.5405E4	2	2	99	120	Carbamidomethylation	C10:Carbamidomethylation:1000.00;T13:Methylation (others):14.02	PEAKS PTM
R.W(+57.02)LGLSAEYGNLYR.N	N	57.46	1597.7888	13	0.6	799.9022	2	35.09	34	F34:1725	OB5945 H2 Ro.raw	1.848E4	1	1	389	401	Carbamidomethylation (DHKE, X@N-term)	W1:Carbamidomethylation (DHKE, X@N-term):62.19	PEAKS PTM
Q.Q(-17.03)LKNNNPFK.F	N	56.41	1084.5665	9	1.6	543.2914	2	28.75	34	F34:1346	OB5945 H2 Ro.raw	1.0606E4	2	2	509	517	Pyro-glu from Q	Q1:Pyro-glu from Q:1000.00	PEAKS PTM
R.AHVQVVDSNGN(+.98)RVYDEELQEGHVLVVPQNFAVAGK.S	N	54.09	3817.9023	35	3.2	955.4859	4	32.51	34	F34:1567	OB5945 H2 Ro.raw	7.3088E4	3	3	424	458		N11:Deamidation (NQ):8.22	PEAKS DB
R.IESEGGYIETWNPNNQ(+.98)EFEC(+57.02)AGVALSR.L	N	52.73	3070.3613	27	6.0	1024.4672	3	34.92	32	F32:1736	OB5943 H6 Ro.raw	6.6817E3	1	1	46	72	Carbamidomethylation	Q16:Deamidation (NQ):0.00;C20:Carbamidomethylation:1000.00	PEAKS DB
K.FFVPPSQQSPRA.V	N	51.84	1359.6935	12	1.1	680.8547	2	29.14	32	F32:1381	OB5943 H6 Ro.raw	3.8841E4	2	2	518	529			PEAKS DB
R.VYDEELQEGHVLVVPQN(+.98)FAVAGK.S	N	51.23	2541.2751	23	9.5	1271.6569	2	32.92	31	F31:1618	OB5942 H6 Ro.raw	3.629E3	1	1	436	458		N17:Deamidation (NQ):14.02	PEAKS DB
K.TAN(+.98)DLNLLILR.W	N	50.73	1255.7136	11	2.6	628.8657	2	35.22	31	F31:1745	OB5942 H6 Ro.raw	0	0	0	378	388	Deamidation (NQ)	N3:Deamidation (NQ):68.31	PEAKS DB
R.S(+57.02)PDIYNPQAGSLK.T	N	50.29	1445.7150	13	5.7	723.8689	2	28.94	34	F34:1350	OB5945 H2 Ro.raw	6.4489E3	2	2	365	377		S1:Carbamidomethylation (DHKE, X@N-term):14.04	PEAKS PTM
R.N(+57.02)ALFVPHYNTNAHSIIYALR.G	N	48.76	2370.2231	20	-0.1	791.0816	3	34.53	29	F29:1557	OB5922 H2 Ro.raw	3.2966E6	1	1	402	421		N1:Carbamidomethylation (DHKE, X@N-term):10.82	PEAKS PTM
R.WLGLSAE(+53.92)YGNLYR.N	N	48.21	1594.6866	13	0.4	532.5697	3	35.09	29	F29:1579	OB5922 H2 Ro.raw	6.0447E5	3	3	389	401	Replacement of 2 protons by iron	E7:Replacement of 2 protons by iron:123.05	PEAKS PTM
N.G(+41.03)IEETIC(+57.02)TASVK.K	N	48.20	1347.6704	12	0.3	674.8427	2	29.12	34	F34:1370	OB5945 H2 Ro.raw	1.5496E4	1	1	346	357	Amidination of lysines or N-terminal amines with methyl acetimidate; Carbamidomethylation	G1:Amidination of lysines or N-terminal amines with methyl acetimidate:96.48;C7:Carbamidomethylation:1000.00	PEAKS PTM
R.SPD(+57.02)IYNPQAGSLK.T	N	45.16	1445.7150	13	2.2	723.8663	2	29.51	29	F29:1244	OB5922 H2 Ro.raw	0	0	0	365	377		D3:Carbamidomethylation (DHKE, X@N-term):0.00	PEAKS PTM
R.NALFVPHYNTNAHSIIYALRG.R	N	44.84	2370.2231	21	1.2	791.0826	3	33.77	34	F34:1624	OB5945 H2 Ro.raw	4.2894E6	2	2	402	422			PEAKS DB
Q.QLKNNNPFKFFVPPSQQS.P	N	42.33	2119.0850	18	1.9	707.3703	3	33.27	35	F35:1604	OB5946 H2 Ro.raw	6.7737E4	1	1	509	526			PEAKS DB
K.NNNPFKFFVPPSQQS.P	N	41.33	1749.8474	15	1.0	875.9319	2	34.91	29	F29:1573	OB5922 H2 Ro.raw	4.7342E4	1	1	512	526			PEAKS DB
N.G(+57.02)IEETIC(+57.02)TASVKK.N	N	41.05	1491.7603	13	8.7	746.8939	2	26.96	31	F31:1261	OB5942 H6 Ro.raw	3.2429E4	4	4	346	358	Carbamidomethylation (DHKE, X@N-term); Carbamidomethylation	G1:Carbamidomethylation (DHKE, X@N-term):32.28;C7:Carbamidomethylation:1000.00	PEAKS PTM

total 73 peptides

Peptide	Uniq	-10lgP	Mass	Length	ppm	m/z	z	RT	Fraction	Scan	Source File	Area Roasted in gel	#Feature	#Feature Roasted in gel	Start	End	PTM	AScore	Found By
R.FNLAGNH(+14.02)EQEFLR.Y	N	40.38	1587.7793	13	0.7	530.2674	3	30.81	39	F39:1450	OB5952 H3A Ro.raw	1.3642E5	3	3	196	208	Methylation(others)	H7:Methylation(others):33.98	PEAKS PTM
K.SQSD(+53.92)NFEYVAFK.T	N	40.31	1487.5656	12	-2.8	744.7880	2	32.03	29	F29:1395	OB5922 H2 Ro.raw	0	0	0	459	470	Replacement of 2 protons by iron	D4:Replacement of 2 protons by iron:20.50	PEAKS PTM
R.AHVQVVDSNGNRVYDEELQ(+.98)EGHVLVVPQNFAVAGK.S	N	40.20	3817.9023	35	7.9	955.4904	4	33.43	35	F35:1615	OB5946 H2 Ro.raw	0	0	0	424	458		Q19:Deamidation (NQ):4.91	PEAKS DB
R.AHVQVVDSN(+.98)GN(+.98)RVYDEELQEGHVLVVPQNFAVAGK.S	N	39.19	3818.8862	35	8.6	955.7371	4	34.33	32	F32:1692	OB5943 H6 Ro.raw	0	0	0	424	458		N9:Deamidation (NQ):0.00;N11:Deamidation (NQ):0.00	PEAKS DB
K.TAND(+53.92)LNLLILR.W	N	38.76	1308.6488	11	0.2	655.3318	2	35.09	29	F29:1584	OB5922 H2 Ro.raw	8.7715E4	1	1	378	388	Replacement of 2 protons by iron	D4:Replacement of 2 protons by iron:55.73	PEAKS PTM
R.RFNLAGNH(+14.02)EQEFLR.Y	N	38.64	1743.8805	14	0.5	582.3011	3	31.14	38	F38:1453	OB5951 H3A Ro.raw	7.3646E5	3	3	195	208	Methylation(others)	H8:Methylation(others):40.00	PEAKS PTM
R.AHVQ(+.98)VVDSNGNRVYDEELQEGHVLVVPQNFAVAGK.S	N	38.57	3817.9023	35	0.9	955.4838	4	33.12	31	F31:1624	OB5942 H6 Ro.raw	4.5408E4	1	1	424	458	Deamidation (NQ)	Q4:Deamidation (NQ):21.59	PEAKS DB
W.LGLSAEYGNLYR.N	N	38.37	1354.6881	12	3.5	678.3537	2	31.68	31	F31:1539	OB5942 H6 Ro.raw	0	0	0	390	401			PEAKS DB
S.ISFRQQPEENAC(+57.02)QFQR.L	N	37.74	2036.9486	16	0.8	679.9907	3	28.84	36	F36:1329	OB5948 H3B Ro.raw	3.1194E4	4	4	21	36	Carbamidomethylation	C12:Carbamidomethylation:1000.00	PEAKS DB
R.G(+57.02)YFGLIFPGC(+57.02)PSTYEEPAQQGRR.S	N	37.54	2686.2598	23	1.6	896.4286	3	35.57	39	F39:1718	OB5952 H3A Ro.raw	5.4933E4	1	1	99	121	Carbamidomethylation (DHKE, X@N-term); Carbamidomethylation	G1:Carbamidomethylation (DHKE, X@N-term):130.55;C10:Carbamidomethylation:1000.00	PEAKS PTM
R.SLPYSPYSP(+31.99)QSQPR.Q	N	36.19	1637.7686	14	3.2	819.8942	2	27.07	38	F38:1250	OB5951 H3A Ro.raw	6.6096E3	1	1	220	233		P9:Dihydroxy:9.42	PEAKS PTM
R.NALFVPHYNTNAHSIIYALRGR.A	N	36.04	2526.3245	22	0.6	843.1160	3	33.60	29	F29:1492	OB5922 H2 Ro.raw	7.6168E5	2	2	402	423			PEAKS DB
R.R(+14.02)FNLAGNHEQEFLR.Y	N	34.57	1743.8805	14	0.9	582.3013	3	31.38	39	F39:1472	OB5952 H3A Ro.raw	2.9356E5	2	2	195	208	Methylation(KR)	R1:Methylation(KR):67.58	PEAKS PTM
R.WLGLSAEYGN(+.98)LYR.N	N	33.97	1541.7513	13	5.4	771.8871	2	54.24	29	F29:2679	OB5922 H2 Ro.raw	0	0	0	389	401	Deamidation (NQ)	N10:Deamidation (NQ):1000.00	PEAKS DB
R.RPFYSNAP(+15.99)QEIFIQQGR.G	N	33.55	2066.0332	17	0.2	689.6852	3	31.94	40	F40:1497	OB5953 H3A Ro.raw	4.3282E4	1	1	82	98	Oxidation or Hydroxylation	P8:Oxidation or Hydroxylation:32.28	PEAKS PTM
R.AHVQ(+.98)VVDSNGNRVYDEELQEGHVLVVPQ(+.98)NFAVAGK.S	N	33.38	3818.8862	35	8.9	955.7373	4	33.12	31	F31:1624	OB5942 H6 Ro.raw	3.8034E4	1	1	424	458		Q4:Deamidation (NQ):8.81;Q28:Deamidation (NQ):12.28	PEAKS DB
R.FNLAGNH(+156.12)EQEFLR.Y	N	33.01	1729.8788	13	-7.6	577.6292	3	31.13	38	F38:1444	OB5951 H3A Ro.raw	7.0376E5	2	2	196	208	4-hydroxynonenal (HNE)	H7:4-hydroxynonenal (HNE):1000.00	PEAKS PTM
R.N(+.98)ALFVPHYNTNAHSIIYALR.G	N	32.68	2314.1858	20	7.3	772.4082	3	50.66	29	F29:2450	OB5922 H2 Ro.raw	1.0573E4	1	1	402	421		N1:Deamidation (NQ):0.00	PEAKS DB
R.IESEGGYIETWN(+.98)PNNQEFEC(+57.02)AGVALSR.L	N	32.58	3070.3613	27	6.9	1536.1985	2	34.73	32	F32:1729	OB5943 H6 Ro.raw	2.6575E4	1	1	46	72	Carbamidomethylation	N12:Deamidation (NQ):0.00;C20:Carbamidomethylation:1000.00	PEAKS DB
R.QIVQNLRGENESEEEGAIVTVR.G	Y	31.60	2469.2458	22	7.0	824.0950	3	30.09	38	F38:1410	OB5951 H3A Ro.raw	0	0	0	281	302			PEAKS DB
Q.QLKNNNPFKFFVPPSQQSPR.A	N	31.56	2372.2388	20	0.5	791.7539	3	33.17	29	F29:1463	OB5922 H2 Ro.raw	0	0	0	509	528			PEAKS DB

total 73 peptides

Peptide	Uniq	-10lgP	Mass	Length	ppm	m/z	z	RT	Fraction	Scan	Source File	Area Roasted in gel	#Feature	#Feature Roasted in gel	Start	End	PTM	AScore	Found By
R.WLGLSAE(+21.98)YGNLYR.N	N	30.00	1562.7494	13	-8.4	782.3754	2	35.27	29	F29:1583	OB5922 H2 Ro.raw	5.0047E4	2	2	389	401	Sodium adduct	E7:Sodium adduct:42.78	PEAKS PTM
R.LNAQRPDNRIESEGGYIETWNPNN(+.98)QEFEC(+57.02)AGVALSR.L	N	29.77	4134.9087	36	4.7	1379.3167	3	33.85	33	F33:1671	OB5944 H6 Ro.raw	8.0788E4	1	1	37	72	Carbamidomethylation	N24:Deamidation (NQ):0.00;C29:Carbamidomethylation:1000.00	PEAKS DB
R.W(+15.99)LGLSAEYGNLYR.N	N	28.45	1556.7623	13	-0.8	779.3878	2	34.26	31	F31:1698	OB5942 H6 Ro.raw	3.8864E3	1	1	389	401	Oxidation (HW)	W1:Oxidation (HW):1000.00	PEAKS PTM
**total 73 peptides**																			

Q6IWG5|Q6IWG5_ARAHY

back to list

| Protein Coverage | Supporting Peptides |

Protein Coverage:

1 KLLALSLCFC VLVLGASSVT FRQGGEENEC QFQRLNAQRP DNRIESEGGY IETWNPNNQE FQCAGVALSR TVLRRNALRR

81 PFYSNAPLEI YVQQGSGYFG LIFPGCPSTY EEPAQEGRRY QSQKPSRRFQ VGQDDPSQQQ QDSHQKVHRF DEGDLIAVPT

161 GVAFWMYNDE DTDVVTVTLS DTSSIHNQLD QFPRRFYLAG NQEQEFLRYQ QQQGSRPHYR QISPRVRGDE QENEGSNIFS

241 GFAQEFLQHA FQVDRQTVEN LRGENEREEQ GAIVTVKGGL RILSPDEEDE SSRSPPSRRE EFDEDRSRPQ QRGKYDENRR

321 GYKNGIEETI CSASVKKNLG RSSNPDIYNP QAGSLRSVNE LDLPILGWLG LSAQHGTIYR NAMFVPHYTL NAHTIVVALN

401 GRAHVQVVDS NGNRVYDEEL QEGHVLVVPQ NFAVAAKAQS ENYEYLAFKT DSRPSIANLA GENSIIDNLP EEVVANSYRL

481 PREQARQLKN NNPFKFFVPP FDHQSMREVA

(Modification markers: 63 c; 325 a; 331 c; 368 d; 383 o; 400 d; 438 c; 476 d)

- a Amidination of lysines or N-terminal amines with methyl acetimidate (+41.03)
- c Carbamidomethylation (+57.02)
- c Carbamidomethylation (DHKE, X@N-term) (+57.02)
- d Deamidation (NQ) (+0.98)
- d Dihydroxy (+31.99)
- o Oxidation (M) (+15.99)
- p Pyro-glu from Q (-17.03)
- s Sodium adduct (+21.98)

Supporting Peptides:

Peptide	Uniq	-10lgP	Mass	Length	ppm	m/z	z	RT	Fraction	Scan	Source File	Area Roasted in gel	#Feature	#Feature Roasted in gel	Start	End	PTM	AScore	Found By
R.NAMFVPHYTLNAHTIVVALN(+.98)GR.A	Y	152.35	2438.2529	22	3.0	1220.1375	2	33.96	34	F34:1643	OB5945 H2 Ro.raw	9.2221E6	5	5	381	402	Deamidation (NQ)	N20:Deamidation (NQ):180.29	PEAKS DB
R.NAMFVPHYTLNAHTIVVALNGR.A	Y	141.80	2437.2688	22	3.5	1219.6460	2	34.54	34	F34:1687	OB5945 H2 Ro.raw	7.7824E6	7	7	381	402			PEAKS DB
R.SVNELDLPILGWLGLSAQHGTIYR.N	Y	132.10	2651.4070	24	3.8	1326.7158	2	39.91	35	F35:1991	OB5946 H2 Ro.raw	6.4841E7	30	30	357	380			PEAKS DB
**total 54 peptides**																			

Peptide	Uniq	-10lgP	Mass	Length	ppm	m/z	z	RT	Fraction	Scan	Source File	Area Roasted in gel	#Feature	#Feature Roasted in gel	Start	End	PTM	AScore	Found By
K.TDSRPSIANLAGENSIIDNLPEEVVANSYR.L	Y	132.04	3243.6006	30	5.0	1082.2129	3	36.29	35	F35:1782	OB5946 H2 Ro.raw	1.3497E7	15	15	450	479			PEAKS DB
R.NAM(+15.99)FVPHYTLNAHTIVVALNGR.A	Y	115.24	2453.2637	22	1.4	818.7629	3	34.16	29	F29:1519	OB5922 H2 Ro.raw	2.32E6	3	3	381	402	Oxidation (M)	M3:Oxidation (M):1000.00	PEAKS DB
R.S(+57.02)VNELDLPILGWLGLSAQHGTIYR.N	Y	113.97	2708.4285	24	-0.8	1355.2205	2	40.55	29	F29:1898	OB5922 H2 Ro.raw	4.7421E5	1	1	357	380		S1:Carbamidomethylation (DHKE, X@N-term): 0.00	PEAKS PTM
R.SSNPDIYNPQAGSLR.S	Y	113.20	1617.7747	15	0.7	809.8952	2	29.12	29	F29:1205	OB5922 H2 Ro.raw	1.3276E6	6	6	342	356			PEAKS DB
K.AQSENYEYLAFK.T	Y	111.58	1461.6776	12	0.6	731.8465	2	31.92	29	F29:1369	OB5922 H2 Ro.raw	6.5899E6	6	6	438	449			PEAKS DB
L.PILGWLGLSAQHGTIYR.N	Y	107.51	1881.0261	17	0.7	941.5210	2	40.57	29	F29:1889	OB5922 H2 Ro.raw	2.9616E5	2	2	364	380			PEAKS DB
R.FYLAGNQEQEFLR.Y	Y	105.48	1613.7837	13	0.1	807.8992	2	32.67	38	F38:1561	OB5951 H3A Ro.raw	5.1792E4	4	4	196	208			PEAKS DB
R.NAM(+15.99)FVPHYTLNAHTIVVALN(+.98)GR.A	Y	100.76	2454.2478	22	0.3	819.0901	3	32.82	34	F34:1580	OB5945 H2 Ro.raw	1.0956E6	4	4	381	402	Oxidation (M); Deamidation (NQ)	M3:Oxidation (M):1000.00;N20:Deamidation (NQ):104.25	PEAKS DB
R.AHVQVVDSNGN(+.98)RVYDEELQEGHVLVVPQNFAVAAK.A	Y	91.52	3831.9180	35	1.5	1278.3152	3	32.86	29	F29:1448	OB5922 H2 Ro.raw	3.2728E6	4	4	403	437		N11:Deamidation (NQ):15.73	PEAKS DB
K.TDSRPSIANLAGENSIIDNLPEEVVANSYRLPR.E	Y	90.25	3609.8386	33	3.4	903.4700	4	37.52	29	F29:1753	OB5922 H2 Ro.raw	2.6255E7	6	6	450	482			PEAKS DB
R.AHVQVVDSN(+.98)GNRVYDEELQEGHVLVVPQNFAVAAK.A	Y	89.16	3831.9180	35	4.4	1278.3188	3	32.26	34	F34:1553	OB5945 H2 Ro.raw	5.1124E6	4	4	403	437	Deamidation (NQ)	N9:Deamidation (NQ):39.44	PEAKS DB
R.SVNELDLPILGW(+31.99)LGLSAQHGTIYR.N	Y	89.05	2683.3970	24	2.4	1342.7090	2	39.79	29	F29:1852	OB5922 H2 Ro.raw	7.442E4	1	1	357	380	Dihydroxy	W12:Dihydroxy: 44.28	PEAKS PTM
K.A(+57.02)QSENYEYLAFK.T	Y	86.48	1518.6990	12	-0.7	760.3562	2	31.51	34	F34:1505	OB5945 H2 Ro.raw	1.9478E5	3	3	438	449	Carbamidomethylation (DHKE, X@N-term)	A1:Carbamidomethylation (DHKE, X@N-term):56.34	PEAKS PTM
K.FFVPPFDHQSMRE.V	Y	78.58	1635.7504	13	0.3	818.8827	2	32.86	29	F29:1440	OB5922 H2 Ro.raw	2.6869E6	5	5	496	508			PEAKS DB
R.LNAQRPDNRIESEGGYIETWNPNNQEFQC(+57.02)AGVALSR.T	Y	78.42	4132.9409	36	3.9	1034.2466	4	33.25	38	F38:1571	OB5951 H3A Ro.raw	2.0581E7	9	9	35	70	Carbamidomethylation	C29:Carbamidomethylation:1000.00	PEAKS DB
N.GIEETIC(+57.02)SASVK.K	Y	75.28	1292.6282	12	0.9	647.3220	2	29.13	34	F34:1363	OB5945 H2 Ro.raw	6.9435E4	3	3	325	336	Carbamidomethylation	C7:Carbamidomethylation:1000.00	PEAKS DB
K.TDSRPSIANLAGENSIIDNLPEEVVAN(+.98)SYR.L	Y	75.20	3244.5847	30	1.7	1082.5374	3	35.58	34	F34:1746	OB5945 H2 Ro.raw	0	0	0	450	479	Deamidation (NQ)	N27:Deamidation (NQ):78.75	PEAKS DB
R.NAM(+15.99)FVPHYTLN(+.98)AHTIVVALNGR.A	Y	66.50	2454.2478	22	1.2	819.0908	3	33.30	34	F34:1613	OB5945 H2 Ro.raw	0	0	0	381	402	Oxidation (M)	M3:Oxidation (M):1000.00;N11:Deamidation (NQ):0.00	PEAKS DB
R.VYDEELQEGHVLVVPQNFAVAAK.A	Y	66.30	2554.3066	23	0.5	852.4432	3	33.26	35	F35:1608	OB5946 H2 Ro.raw	2.0809E4	1	1	415	437			PEAKS DB
N.GIEETIC(+57.02)SASVKK.N	Y	64.42	1420.7231	13	1.3	711.3698	2	28.13	29	F29:1149	OB5922 H2 Ro.raw	1.9909E5	4	4	325	337	Carbamidomethylation	C7:Carbamidomethylation:1000.00	PEAKS DB
R.RFYLAGNQEQEFLR.Y	Y	63.90	1769.8849	14	-1.3	590.9681	3	31.33	38	F38:1486	OB5951 H3A Ro.raw	6.7764E4	3	3	195	208			PEAKS DB

total 54 peptides

Peptide	Uniq	-10lgP	Mass	Length	ppm	m/z	z	RT	Fraction	Scan	Source File	Area Roasted in gel	#Feature	#Feature Roasted in gel	Start	End	PTM	AScore	Found By
R.AHVQVVDSN(+.98)GN(+.98)RVYDEELQEGHVLVVPQNFAVAAK.A	Y	62.76	3832.9019	35	7.9	767.5937	5	32.26	34	F34:1556	OB5945 H2 Ro.raw	3.9346E5	1	1	403	437		N9:Deamidation (NQ):5.83;N11:Deamidation (NQ):8.28	PEAKS DB
R.N(+.98)AMFVPHYTLNAHTIVVALNGR.A	Y	60.88	2438.2529	22	3.0	1220.1375	2	33.96	34	F34:1680	OB5945 H2 Ro.raw	9.5447E5	1	1	381	402	Deamidation (NQ)	N1:Deamidation (NQ):63.03	PEAKS DB
D.LPILGWLGLSAQHGTIYR.N	Y	60.53	1994.1101	18	2.4	998.0647	2	40.55	29	F29:1901	OB5922 H2 Ro.raw	1.1908E5	1	1	363	380			PEAKS DB
V.PQNFAVAAK.A	N	59.75	944.5079	9	0.9	473.2617	2	32.86	29	F29:1447	OB5922 H2 Ro.raw	2.0461E6	3	3	429	437			PEAKS DB
K.FFVPPFDHQSM(+15.99)RE.V	Y	57.98	1651.7452	13	1.7	826.8813	2	32.49	29	F29:1425	OB5922 H2 Ro.raw	3.6985E5	3	3	496	508	Oxidation (M)	M11:Oxidation (M):1000.00	PEAKS DB
R.Q(-17.03)LKNNNPFK.F	N	56.41	1084.5665	9	1.6	543.2914	2	28.75	34	F34:1346	OB5945 H2 Ro.raw	1.0606E4	2	2	487	495	Pyro-glu from Q	Q1:Pyro-glu from Q:1000.00	PEAKS PTM
R.VRGDEQENEGSNIFSGFAQEFLQHAFQVDR.Q	Y	55.37	3453.5974	30	4.1	864.4102	4	39.78	40	F40:1952	OB5953 H3A Ro.raw	3.9334E5	2	2	226	255			PEAKS DB
R.RPFYSNAPLEIYVQQGSGYFGLIFPGC(+57.02)PSTYEEPAQEGR.R	Y	54.12	4424.0845	39	5.3	1475.7100	3	38.84	39	F39:1912	OB5952 H3A Ro.raw	1.0194E6	2	2	80	118	Carbamidomethylation	C27:Carbamidomethylation:1000.00	PEAKS DB
R.NAMFVPHYTLNAH(+57.02)TIVVALNGR.A	Y	50.87	2494.2903	22	-3.1	832.4348	3	34.84	34	F34:1702	OB5945 H2 Ro.raw	0	0	0	381	402		H13:Carbamidomethylation (DHKE, X@N-term):5.30	PEAKS PTM
K.AQSENYEYLAFKTDSRPSIANLAGENSIIDNLPEEVVANSYR.L	Y	48.94	4687.2676	42	3.1	1172.8278	4	36.79	34	F34:1810	OB5945 H2 Ro.raw	2.0852E6	1	1	438	479			PEAKS DB
R.RPFYSNAPLEIYVQQGSGYFGLIFPGC(+57.02)PSTYEEPAQEGRR.Y	Y	48.23	4580.1860	40	7.2	1146.0620	4	37.82	38	F38:1860	OB5951 H3A Ro.raw	1.9768E5	1	1	80	119	Carbamidomethylation	C27:Carbamidomethylation:1000.00	PEAKS DB
K.NNNPFKFFVPPFDHQSMRE.V	Y	46.05	2350.0952	19	1.4	784.3734	3	34.54	34	F34:1693	OB5945 H2 Ro.raw	4.2082E5	2	2	490	508			PEAKS DB
R.SVN(+.98)ELDLPILGWLGLSAQHGTIYR.N	Y	45.95	2652.3911	24	9.1	1327.2150	2	46.79	29	F29:2254	OB5922 H2 Ro.raw	0	0	0	357	380	Deamidation (NQ)	N3:Deamidation (NQ):57.31	PEAKS DB
R.AHVQ(+.98)VVDSNGNRVYDEELQEGHVLVVPQNFAVAAK.A	Y	45.08	3831.9180	35	2.7	767.3929	5	32.86	29	F29:1451	OB5922 H2 Ro.raw	3.9416E5	1	1	403	437		Q4:Deamidation (NQ):0.00	PEAKS DB
K.FFVPPFDHQSM.R	Y	43.41	1350.6067	11	0.3	676.3108	2	34.60	35	F35:1680	OB5946 H2 Ro.raw	2.36E5	3	3	496	506			PEAKS DB
K.TDSRPSIANLAGENSIIDNLPEEVVAN(+.98)SYRLPR.E	Y	39.68	3610.8225	33	5.4	1204.6213	3	35.38	34	F34:1734	OB5945 H2 Ro.raw	0	0	0	450	482	Deamidation (NQ)	N27:Deamidation (NQ):32.94	PEAKS DB
R.NALRRPFYSNAPLEIYVQQGSGYFGLIFPGC(+57.02)PSTYEEPAQEGR.R	Y	39.53	4878.3501	43	2.7	1220.5981	4	38.66	40	F40:1897	OB5953 H3A Ro.raw	5.9202E5	2	2	76	118	Carbamidomethylation	C31:Carbamidomethylation:1000.00	PEAKS DB
K.FFVPPFDHQS.M	Y	38.94	1219.5662	10	1.5	610.7913	2	33.58	34	F34:1616	OB5945 H2 Ro.raw	1.6091E5	1	1	496	505			PEAKS DB
N.G(+57.02)IEETIC(+57.02)SASVKK.N	Y	37.63	1477.7446	13	-0.5	739.8792	2	28.58	29	F29:1182	OB5922 H2 Ro.raw	1.8561E4	2	2	325	337	Carbamidomethylation (DHKE, X@N-term); Carbamidomethylation	G1:Carbamidomethylation (DHKE, X@N-term):22.85;C7:Carbamidomethylation:1000.00	PEAKS PTM
R.NAMFVPHYTLNAH(+57.02)TIVVALN(+.98)GR.A	Y	36.75	2495.2744	22	0.0	832.7654	3	33.96	34	F34:1660	OB5945 H2 Ro.raw	1.2865E5	1	1	381	402	Deamidation (NQ)	H13:Carbamidomethylation (DHKE, X@N-term):0.00;N20:Deamidation (NQ):43.52	PEAKS PTM

total 54 peptides

Peptide	Uniq	-10lgP	Mass	Length	ppm	m/z	z	RT	Fraction	Scan	Source File	Area Roasted in gel	#Feature	#Feature Roasted in gel	Start	End	PTM	AScore	Found By
R.RPFYSNAPLEIYVQQGSGYFGLIFPGC(+57.02)PSTYEEPAQ(+.98)EGRR.Y	Y	36.17	4581.1699	40	8.8	1146.3098	4	38.27	39	F39:1881	OB5952 H3A Ro.raw	4.8906E5	2	2	80	119	Carbamidomethylation	C27:Carbamidomethylation:1000.00;Q36:Deamidation (NQ):12.58	PEAKS DB
R.SSNPD(+21.98)IYNPQAGSLR.S	Y	34.15	1639.7566	15	4.2	820.8890	2	29.21	29	F29:1226	OB5922 H2 Ro.raw	0	0	0	342	356	Sodium adduct	D5:Sodium adduct:43.70	PEAKS PTM
K.FFVPPFDHQSM(+15.99).R	Y	33.46	1366.6016	11	1.6	684.3091	2	33.79	29	F29:1494	OB5922 H2 Ro.raw	5.6927E4	1	1	496	506	Oxidation (M)	M11:Oxidation (M):1000.00	PEAKS DB
R.IESEGGYIETWNPNNQEFQC(+57.02)AGVALSR.T	N	30.17	3068.3933	27	-0.9	1023.8041	3	34.37	33	F33:1699	OB5944 H6 Ro.raw	0	0	0	44	70	Carbamidomethylation	C20:Carbamidomethylation:1000.00	PEAKS DB
R.S(+57.02)SNPDIYNPQAGSLR.S	Y	29.73	1674.7961	15	7.9	838.4119	2	29.48	29	F29:1242	OB5922 H2 Ro.raw	0	0	0	342	356		S1:Carbamidomethylation (DHKE, X@N-term):11.12	PEAKS PTM
N.G(+41.03)IEETIC(+57.02)SASVK.K	Y	29.70	1333.6548	12	0.4	667.8350	2	29.26	35	F35:1372	OB5946 H2 Ro.raw	8.2083E3	1	1	325	336	Amidination of lysines or N-terminal amines with methyl acetimidate; Carbamidomethylation	G1:Amidination of lysines or N-terminal amines with methyl acetimidate:71.87;C7:Carbamidomethylation:1000.00	PEAKS PTM
R.LNAQRPDNRIESEGGYIETWN(+.98)PNNQEFQC(+57.02)AGVALSR.T	Y	29.65	4133.9248	36	-0.7	1034.4878	4	43.56	39	F39:2183	OB5952 H3A Ro.raw	0	0	0	35	70	Carbamidomethylation	N21:Deamidation (NQ):7.58;C29:Carbamidomethylation:1000.00	PEAKS DB
K.TDSRPSIAN(+.98)LAGEN(+.98)SIIDNLPEEVVANSYR.L	Y	29.26	3245.5686	30	4.2	1082.8680	3	47.69	29	F29:2306	OB5922 H2 Ro.raw	0	0	0	450	479		N9:Deamidation (NQ):12.20;N14:Deamidation (NQ):10.19	PEAKS DB
K.AQSENYE(+21.98)YLAFK.T	Y	28.80	1483.6595	12	-1.5	742.8359	2	31.58	35	F35:1513	OB5946 H2 Ro.raw	8.2984E3	1	1	438	449		E7:Sodium adduct:0.00	PEAKS PTM
R.LNAQRPDN(+.98)RIESEGGYIETWNPNNQEFQC(+57.02)AGVALSR.T	Y	28.37	4133.9248	36	5.5	1034.4941	4	34.29	31	F31:1691	OB5942 H6 Ro.raw	0	0	0	35	70	Carbamidomethylation	N8:Deamidation (NQ):0.00;C29:Carbamidomethylation:1000.00	PEAKS DB
**total 54 peptides**																			

Q0GM57|Q0GM57_ARAHY

back to list

| Protein Coverage | Supporting Peptides |

Protein Coverage:

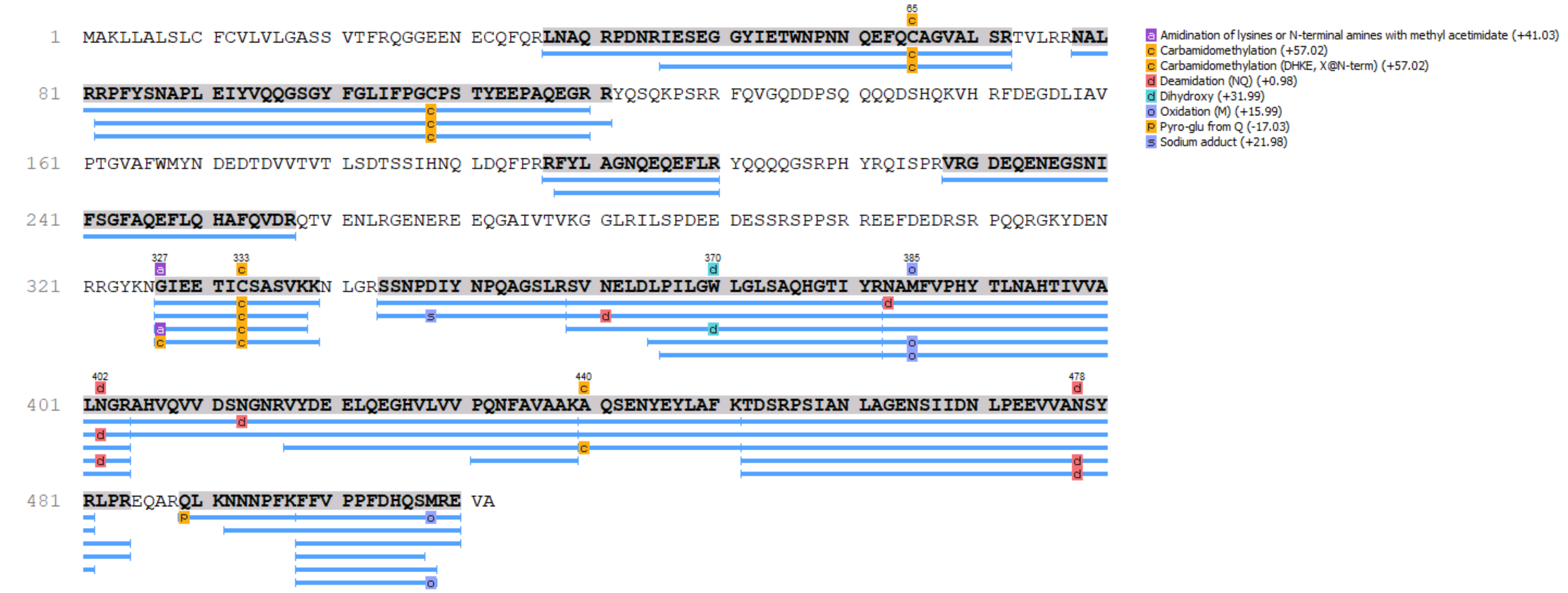

Supporting Peptides:

Peptide	Uniq	-10lgP	Mass	Length	ppm	m/z	z	RT	Fraction	Scan	Source File	Area Roasted in gel	#Feature	#Feature Roasted in gel	Start	End	PTM	AScore	Found By
R.NAMFVPHYTLNAHTIVVALN(+.98)GR.A	Y	152.35	2438.2529	22	3.0	1220.1375	2	33.96	34	F34:1643	OB5945 H2 Ro.raw	9.2221E6	5	5	383	404	Deamidation (NQ)	N20:Deamidation (NQ):180.29	PEAKS DB
R.NAMFVPHYTLNAHTIVVALNGR.A	Y	141.80	2437.2688	22	3.5	1219.6460	2	34.54	34	F34:1687	OB5945 H2 Ro.raw	7.7824E6	7	7	383	404			PEAKS DB
R.SVNELDLPILGWLGLSAQHGTIYR.N	Y	132.10	2651.4070	24	3.8	1326.7158	2	39.91	35	F35:1991	OB5946 H2 Ro.raw	6.4841E7	30	30	359	382			PEAKS DB
K.TDSRPSIANLAGENSIIDNLPEEVVANSYR.L	Y	132.04	3243.6006	30	5.0	1082.2129	3	36.29	35	F35:1782	OB5946 H2 Ro.raw	1.3497E7	15	15	452	481			PEAKS DB
R.NAM(+15.99)FVPHYTLNAHTIVVALNGR.A	Y	115.24	2453.2637	22	1.4	818.7629	3	34.16	29	F29:1519	OB5922 H2 Ro.raw	2.32E6	3	3	383	404	Oxidation (M)	M3:Oxidation (M):1000.00	PEAKS DB
R.S(+57.02)VNELDLPILGWLGLSAQHGTIYR.N	Y	113.97	2708.4285	24	-0.8	1355.2205	2	40.55	29	F29:1898	OB5922 H2 Ro.raw	4.7421E5	1	1	359	382		S1:Carbamidomethylation (DHKE, X@N-term): 0.00	PEAKS PTM
R.SSNPDIYNPQAGSLR.S	Y	113.20	1617.7747	15	0.7	809.8952	2	29.12	29	F29:1205	OB5922 H2 Ro.raw	1.3276E6	6	6	344	358			PEAKS DB
K.AQSENYEYLAFK.T	Y	111.58	1461.6776	12	0.6	731.8465	2	31.92	29	F29:1369	OB5922 H2 Ro.raw	6.5899E6	6	6	440	451			PEAKS DB
L.PILGWLGLSAQHGTIYR.N	Y	107.51	1881.0261	17	0.7	941.5210	2	40.57	29	F29:1889	OB5922 H2 Ro.raw	2.9616E5	2	2	366	382			PEAKS DB
R.FYLAGNQEQEFLR.Y	Y	105.48	1613.7837	13	0.1	807.8992	2	32.67	38	F38:1561	OB5951 H3A Ro.raw	5.1792E4	4	4	198	210			PEAKS DB

total 54 peptides

Peptide	Uniq	-10lgP	Mass	Length	ppm	m/z	z	RT	Fraction	Scan	Source File	Area Roasted in gel	#Feature	#Feature Roasted in gel	Start	End	PTM	AScore	Found By
R.NAM(+15.99)FVPHYTLNAHTIVVALN(+.98)GR.A	Y	100.76	2454.2478	22	0.3	819.0901	3	32.82	34	F34:1580	OB5945 H2 Ro.raw	1.0956E6	4	4	383	404	Oxidation (M); Deamidation (NQ)	M3:Oxidation (M):1000.00;N20:Deamidation (NQ):104.25	PEAKS DB
R.AHVQVVDSNGN(+.98)RVYDEELQEGHVLVVPQNFAVAAK.A	Y	91.52	3831.9180	35	1.5	1278.3152	3	32.86	29	F29:1448	OB5922 H2 Ro.raw	3.2728E6	4	4	405	439		N11:Deamidation (NQ):15.73	PEAKS DB
K.TDSRPSIANLAGENSIIDNLPEEVVANSYRLPR.E	Y	90.25	3609.8386	33	3.4	903.4700	4	37.52	29	F29:1753	OB5922 H2 Ro.raw	2.6255E7	6	6	452	484			PEAKS DB
R.AHVQVVDSN(+.98)GNRVYDEELQEGHVLVVPQNFAVAAK.A	Y	89.16	3831.9180	35	4.4	1278.3188	3	32.26	34	F34:1553	OB5945 H2 Ro.raw	5.1124E6	4	4	405	439	Deamidation (NQ)	N9:Deamidation (NQ):39.44	PEAKS DB
R.SVNELDLPILGW(+31.99)LGLSAQHGTIYR.N	Y	89.05	2683.3970	24	2.4	1342.7090	2	39.79	29	F29:1852	OB5922 H2 Ro.raw	7.442E4	1	1	359	382	Dihydroxy	W12:Dihydroxy:44.28	PEAKS PTM
K.A(+57.02)QSENYEYLAFK.T	Y	86.48	1518.6990	12	-0.7	760.3562	2	31.51	34	F34:1505	OB5945 H2 Ro.raw	1.9478E5	3	3	440	451	Carbamidomethylation (DHKE, X@N-term)	A1:Carbamidomethylation (DHKE, X@N-term):56.34	PEAKS PTM
K.FFVPPFDHQSMRE.V	Y	78.58	1635.7504	13	0.3	818.8827	2	32.86	29	F29:1440	OB5922 H2 Ro.raw	2.6869E6	5	5	498	510			PEAKS DB
R.LNAQRPDNRIESEGGYIETWNPNNQEFQC(+57.02)AGVALSR.T	Y	78.42	4132.9409	36	3.9	1034.2466	4	33.25	38	F38:1571	OB5951 H3A Ro.raw	2.0581E7	9	9	37	72	Carbamidomethylation	C29:Carbamidomethylation:1000.00	PEAKS DB
N.GIEETIC(+57.02)SASVK.K	Y	75.28	1292.6282	12	0.9	647.3220	2	29.13	34	F34:1363	OB5945 H2 Ro.raw	6.9435E4	3	3	327	338	Carbamidomethylation	C7:Carbamidomethylation:1000.00	PEAKS DB
K.TDSRPSIANLAGENSIIDNLPEEVVAN(+.98)SYR.L	Y	75.20	3244.5847	30	1.7	1082.5374	3	35.58	34	F34:1746	OB5945 H2 Ro.raw	0	0	0	452	481	Deamidation (NQ)	N27:Deamidation (NQ):78.75	PEAKS DB
R.NAM(+15.99)FVPHYTLN(+.98)AHTIVVALNGR.A	Y	66.50	2454.2478	22	1.2	819.0908	3	33.30	34	F34:1613	OB5945 H2 Ro.raw	0	0	0	383	404	Oxidation (M)	M3:Oxidation (M):1000.00;N11:Deamidation (NQ):0.00	PEAKS DB
R.VYDEELQEGHVLVVPQNFAVAAK.A	Y	66.30	2554.3066	23	0.5	852.4432	3	33.26	35	F35:1608	OB5946 H2 Ro.raw	2.0809E4	1	1	417	439			PEAKS DB
N.GIEETIC(+57.02)SASVKK.N	Y	64.42	1420.7231	13	1.3	711.3698	2	28.13	29	F29:1149	OB5922 H2 Ro.raw	1.9909E5	4	4	327	339	Carbamidomethylation	C7:Carbamidomethylation:1000.00	PEAKS DB
R.RFYLAGNQEQEFLR.Y	Y	63.90	1769.8849	14	-1.3	590.9681	3	31.33	38	F38:1486	OB5951 H3A Ro.raw	6.7764E4	3	3	197	210			PEAKS DB
R.AHVQVVDSN(+.98)GN(+.98)RVYDEELQEGHVLVVPQNFAVAAK.A	Y	62.76	3832.9019	35	7.9	767.5937	5	32.26	34	F34:1556	OB5945 H2 Ro.raw	3.9346E5	1	1	405	439		N9:Deamidation (NQ):5.83;N11:Deamidation (NQ):8.28	PEAKS DB
R.N(+.98)AMFVPHYTLNAHTIVVALNGR.A	Y	60.88	2438.2529	22	3.0	1220.1375	2	33.96	34	F34:1680	OB5945 H2 Ro.raw	9.5447E5	1	1	383	404	Deamidation (NQ)	N1:Deamidation (NQ):63.03	PEAKS DB
D.LPILGWLGLSAQHGTIYR.N	Y	60.53	1994.1101	18	2.4	998.0647	2	40.55	29	F29:1901	OB5922 H2 Ro.raw	1.1908E5	1	1	365	382			PEAKS DB
V.PQNFAVAAK.A	N	59.75	944.5079	9	0.9	473.2617	2	32.86	29	F29:1447	OB5922 H2 Ro.raw	2.0461E6	3	3	431	439			PEAKS DB
K.FFVPPFDHQSM(+15.99)RE.V	Y	57.98	1651.7452	13	1.7	826.8813	2	32.49	29	F29:1425	OB5922 H2 Ro.raw	3.6985E5	3	3	498	510	Oxidation (M)	M11:Oxidation (M):1000.00	PEAKS DB
R.Q(-17.03)LKNNNPFK.F	N	56.41	1084.5665	9	1.6	543.2914	2	28.75	34	F34:1346	OB5945 H2 Ro.raw	1.0606E4	2	2	489	497	Pyro-glu from Q	Q1:Pyro-glu from Q:1000.00	PEAKS PTM
R.VRGDEQENEGSNIFSGFAQEFLQHAFQVDR.Q	Y	55.37	3453.5974	30	4.1	864.4102	4	39.78	40	F40:1952	OB5953 H3A Ro.raw	3.9334E5	2	2	228	257			PEAKS DB

total 54 peptides

Peptide	Uniq	-10lgP	Mass	Length	ppm	m/z	z	RT	Fraction	Scan	Source File	Area Roasted in gel	#Feature	#Feature Roasted in gel	Start	End	PTM	AScore	Found By
R.RPFYSNAPLEIYVQQGSGYFGLIFPGC(+57.02)PSTYEEPAQEGR.R	Y	54.12	4424.0845	39	5.3	1475.7100	3	38.84	39	F39:1912	OB5952 H3A Ro.raw	1.0194E6	2	2	82	120	Carbamidomethylation	C27:Carbamido methylation:100 0.00	PEAKS DB
R.NAMFVPHYTLNAH(+57.02)TIVVALNGR.A	Y	50.87	2494.2903	22	-3.1	832.4348	3	34.84	34	F34:1702	OB5945 H2 Ro.raw	0	0	0	383	404		H13:Carbamido methylation (DH KE, X@N-term): 5.30	PEAKS PTM
K.AQSENYEYLAFKTDSRPSIANLAGENSIIDNLPEEVVANSYR.L	Y	48.94	4687.2676	42	3.1	1172.8278	4	36.79	34	F34:1810	OB5945 H2 Ro.raw	2.0852E6	1	1	440	481			PEAKS DB
R.RPFYSNAPLEIYVQQGSGYFGLIFPGC(+57.02)PSTYEEPAQEGRR.Y	Y	48.23	4580.1860	40	7.2	1146.0620	4	37.82	38	F38:1860	OB5951 H3A Ro.raw	1.9768E5	1	1	82	121	Carbamidomethylation	C27:Carbamido methylation:100 0.00	PEAKS DB
K.NNNPFKFFVPPFDHQSMRE.V	Y	46.05	2350.0952	19	1.4	784.3734	3	34.54	34	F34:1693	OB5945 H2 Ro.raw	4.2082E5	2	2	492	510			PEAKS DB
R.SVN(+.98)ELDLPILGWLGLSAQHGTIYR.N	Y	45.95	2652.3911	24	9.1	1327.2150	2	46.79	29	F29:2254	OB5922 H2 Ro.raw	0	0	0	359	382	Deamidation (NQ)	N3:Deamidation (NQ):57.31	PEAKS DB
R.AHVQ(+.98)VVDSNGNRVYDEELQEGHVLVVPQNFAVAAK.A	Y	45.08	3831.9180	35	2.7	767.3929	5	32.86	29	F29:1451	OB5922 H2 Ro.raw	3.9416E5	1	1	405	439		Q4:Deamidation (NQ):0.00	PEAKS DB
K.FFVPPFDHQSM.R	Y	43.41	1350.6067	11	0.3	676.3108	2	34.60	35	F35:1680	OB5946 H2 Ro.raw	2.36E5	3	3	498	508			PEAKS DB
K.TDSRPSIANLAGENSIIDNLPEEVVAN(+.98)SYRLPR.E	Y	39.68	3610.8225	33	5.4	1204.6213	3	35.38	34	F34:1734	OB5945 H2 Ro.raw	0	0	0	452	484	Deamidation (NQ)	N27:Deamidatio n (NQ):32.94	PEAKS DB
R.NALRRPFYSNAPLEIYVQQGSGYFGLIFPGC(+57.02)PSTYEEPAQEGR.R	Y	39.53	4878.3501	43	2.7	1220.5981	4	38.66	40	F40:1897	OB5953 H3A Ro.raw	5.9202E5	2	2	78	120	Carbamidomethylation	C31:Carbamido methylation:100 0.00	PEAKS DB
K.FFVPPFDHQS.M	Y	38.94	1219.5662	10	1.5	610.7913	2	33.58	34	F34:1616	OB5945 H2 Ro.raw	1.6091E5	1	1	498	507			PEAKS DB
N.G(+57.02)IEETIC(+57.02)SASVKK.N	Y	37.63	1477.7446	13	-0.5	739.8792	2	28.58	29	F29:1182	OB5922 H2 Ro.raw	1.8561E4	2	2	327	339	Carbamidomethylation (DHKE, X@N-term); Carbamidomethylation	G1:Carbamidom ethylation (DHK E, X@N-term):2 2.85;C7:Carbam idomethylation: 1000.00	PEAKS PTM
R.NAMFVPHYTLNAH(+57.02)TIVVALN(+.98)GR.A	Y	36.75	2495.2744	22	0.0	832.7654	3	33.96	34	F34:1660	OB5945 H2 Ro.raw	1.2865E5	1	1	383	404	Deamidation (NQ)	H13:Carbamido methylation (DH KE, X@N-term): 0.00;N20:Deami dation (NQ):43. 52	PEAKS PTM
R.RPFYSNAPLEIYVQQGSGYFGLIFPGC(+57.02)PSTYEEPAQ(+.98)EGRR.Y	Y	36.17	4581.1699	40	8.8	1146.3098	4	38.27	39	F39:1881	OB5952 H3A Ro.raw	4.8906E5	2	2	82	121	Carbamidomethylation	C27:Carbamido methylation:100 0.00;Q36:Deami dation (NQ):12. 58	PEAKS DB
R.SSNPD(+21.98)IYNPQAGSLR.S	Y	34.15	1639.7566	15	4.2	820.8890	2	29.21	29	F29:1226	OB5922 H2 Ro.raw	0	0	0	344	358	Sodium adduct	D5:Sodium add uct:43.70	PEAKS PTM
K.FFVPPFDHQSM(+15.99).R	Y	33.46	1366.6016	11	1.6	684.3091	2	33.79	29	F29:1494	OB5922 H2 Ro.raw	5.6927E4	1	1	498	508	Oxidation (M)	M11:Oxidation (M):1000.00	PEAKS DB
R.IESEGGYIETWNPNNQEFQC(+57.02)AGVALSR.T	N	30.17	3068.3933	27	-0.9	1023.8041	3	34.37	33	F33:1699	OB5944 H6 Ro.raw	0	0	0	46	72	Carbamidomethylation	C20:Carbamido methylation:100 0.00	PEAKS DB
R.S(+57.02)SNPDIYNPQAGSLR.S	Y	29.73	1674.7961	15	7.9	838.4119	2	29.48	29	F29:1242	OB5922 H2 Ro.raw	0	0	0	344	358		S1:Carbamidom ethylation (DHK E, X@N-term):1 1.12	PEAKS PTM
N.G(+41.03)IEETIC(+57.02)SASVK.K	Y	29.70	1333.6548	12	0.4	667.8350	2	29.26	35	F35:1372	OB5946 H2 Ro.raw	8.2083E3	1	1	327	338	Amidination of lysines or N-terminal amines with methyl acetimidate; Carbamidomethylation	G1:Amidination of lysines or N-t erminal amines with methyl acet imidate:71.87;C 7:Carbamidome thylation:1000.0	PEAKS PTM
**total 54 peptides**																			

Peptide	Uniq	-10lgP	Mass	Length	ppm	m/z	z	RT	Fraction	Scan	Source File	Area Roasted in gel	#Feature	#Feature Roasted in gel	Start	End	PTM	AScore	Found By
																		0	
R.LNAQRPDNRIESEGGYIETWN(+.98)PNNQEFQC(+57.02)AGVALSR.T	Y	29.65	4133.9248	36	-0.7	1034.4878	4	43.56	39	F39:2183	OB5952 H3A Ro.raw	0	0	0	37	72	Carbamidomethylation	N21:Deamidation (NQ):7.58;C29:Carbamidomethylation:1000.00	PEAKS DB
K.TDSRPSIAN(+.98)LAGEN(+.98)SIIDNLPEEVVANSYR.L	Y	29.26	3245.5686	30	4.2	1082.8680	3	47.69	29	F29:2306	OB5922 H2 Ro.raw	0	0	0	452	481		N9:Deamidation (NQ):12.20;N14:Deamidation (NQ):10.19	PEAKS DB
K.AQSENYE(+21.98)YLAFK.T	Y	28.80	1483.6595	12	-1.5	742.8359	2	31.58	35	F35:1513	OB5946 H2 Ro.raw	8.2984E3	1	1	440	451		E7:Sodium adduct:0.00	PEAKS PTM
R.LNAQRPDN(+.98)RIESEGGYIETWNPNNQEFQC(+57.02)AGVALSR.T	Y	28.37	4133.9248	36	5.5	1034.4941	4	34.29	31	F31:1691	OB5942 H6 Ro.raw	0	0	0	37	72	Carbamidomethylation	N8:Deamidation (NQ):0.00;C29:Carbamidomethylation:1000.00	PEAKS DB
**total 54 peptides**																			

O82580|O82580_ARAHY

back to list

| Protein Coverage | Supporting Peptides |

Protein Coverage:

```
  1 RQQPEENACQ FQRLNAQRPD NRIESEGGYI ETWNPNNQEF ECAGVALSRL VLRRNALRRP FYSNAPQEIF IQQGRGYFGL
 81 IFPGCPRHYE EPHTQGRRSQ SQRPPRRLQG EDQSQQQRDS HQKVHRFDEG DLIAVPTGVA FWLYNDHDTD VVAVSLTDTN
161 NNDNQLDQFP RRFNLAGNTE QEFLRYQQQS RQSRRRSLPY SPYSPQSQPR QEEREFSPRG QHSRRERAGQ EEENEGGNIF
241 SGFTPEFLEQ AFQVDDRQIV QNLRGETESE EEGAIVTVRG GLRILSPDRK RRADEEEEYD EDEYEYDEED RRRGRGSRGR
321 GNGIEETICT ASAKKNIGRN RSPDIYNPQA GSLKTANDLN LLILRWLGPS AEYGNLYRNA LFVAHYNTNA HSIIYRLRGR
401 AHVQVVDSNG NRVYDEELQE GHVLVVPQNF AVAGKSQSEN FEYVAFKTDS RPSIANLAGE NSVIDNLPEE VVANSYGLQR
481 EQARQLKNNN PFKFFVPPSQ QSPRAVA
```

c Carbamidomethylation (+57.02)
c Carbamidomethylation (DHKE, X@N-term) (+57.02)
d Deamidation (NQ) (+0.98)
e Ethylation (+28.03)
o Oxidation or Hydroxylation (+15.99)
p Pyro-glu from Q (-17.03)
r Replacement of 2 protons by iron (+53.92)

Supporting Peptides:

Peptide	Uniq	-10lgP	Mass	Length	ppm	m/z	z	RT	Fraction	Scan	Source File	Area Roasted in gel	#Feature	#Feature Roasted in gel	Start	End	PTM	AScore	Found By
R.SPDIYNPQAGSLKTANDLNLLILR.W	N	136.50	2625.4126	24	-0.1	876.1447	3	35.61	29	F29:1607	OB5922 H2 Ro.raw	1.276E6	3	3	342	365			PEAKS DB
R.NRSPDIYNPQAGSLKTANDLNLLILR.W	N	120.82	2895.5566	26	1.6	966.1943	3	34.35	29	F29:1530	OB5922 H2 Ro.raw	2.2791E6	6	6	340	365			PEAKS DB
**total 45 peptides**																			

Peptide	Uniq	-10lgP	Mass	Length	ppm	m/z	z	RT	Fraction	Scan	Source File	Area Roasted in gel	#Feature	#Feature Roasted in gel	Start	End	PTM	AScore	Found By
K.SQSENFEYVAFK.T	N	116.27	1447.6619	12	3.0	724.8404	2	31.51	34	F34:1498	OB5945 H2 Ro.raw	1.8189E7	10	10	436	447			PEAKS DB
R.RPFYSNAPQEIFIQQGR.G	N	110.90	2050.0383	17	3.2	684.3556	3	31.52	38	F38:1483	OB5951 H3A Ro.raw	9.1631E6	15	15	59	75			PEAKS DB
R.NALRRPFYSNAPQEIFIQQGR.G	N	97.93	2504.3037	21	1.0	835.7760	3	32.71	40	F40:1535	OB5953 H3A Ro.raw	6.3111E6	14	13	55	75			PEAKS DB
R.NRSPDIYNPQAGSLK.T	N	96.54	1658.8376	15	0.1	830.4261	2	27.65	32	F32:1291	OB5943 H6 Ro.raw	8.0621E5	12	12	340	354			PEAKS DB
R.SPDIYNPQAGSLK.T	N	92.16	1388.6936	13	1.7	695.3552	2	28.58	34	F34:1323	OB5945 H2 Ro.raw	1.33E6	6	6	342	354			PEAKS DB
R.VYDEELQEGHVLVVPQNFAVAGK.S	N	90.91	2540.2910	23	1.4	847.7721	3	33.42	29	F29:1478	OB5922 H2 Ro.raw	1.9062E6	10	10	413	435			PEAKS DB
R.AHVQVVDSNGNRVYDEELQEGHVLVVPQNFAVAGK.S	N	77.75	3816.9182	35	3.6	955.2402	4	32.32	35	F35:1544	OB5946 H2 Ro.raw	8.2893E6	8	8	401	435			PEAKS DB
R.Q(-17.03)QPEENAC(+57.02)QFQR.L	N	75.52	1516.6365	12	2.5	759.3274	2	28.03	32	F32:1319	OB5943 H6 Ro.raw	7.279E3	2	2	2	13	Pyro-glu from Q; Carbamidomethylation	Q1:Pyro-glu from Q:1000.00;C8:Carbamidomethylation:1000.00	PEAKS PTM
V.PQNFAVAGK.S	N	75.51	930.4923	9	1.0	466.2539	2	32.32	35	F35:1542	OB5946 H2 Ro.raw	3.3444E6	3	3	427	435			PEAKS DB
R.IESEGGYIETWNPNNQEFEC(+57.02)AGVALSR.L	N	74.31	3069.3774	27	1.4	1024.1345	3	34.50	31	F31:1703	OB5942 H6 Ro.raw	1.0361E4	1	1	23	49	Carbamidomethylation	C20:Carbamidomethylation:1000.00	PEAKS DB
K.TANDLNLLILR.W	N	73.94	1254.7296	11	1.8	628.3732	2	34.79	35	F35:1750	OB5946 H2 Ro.raw	1.0437E7	10	10	355	365			PEAKS DB
R.QQPEENAC(+57.02)QFQR.L	N	73.76	1533.6630	12	1.7	767.8400	2	25.24	32	F32:1157	OB5943 H6 Ro.raw	3.7544E4	3	3	2	13	Carbamidomethylation	C8:Carbamidomethylation:1000.00	PEAKS DB
K.FFVPPSQQSPR.A	N	69.38	1288.6564	11	0.2	645.3356	2	28.58	32	F32:1346	OB5943 H6 Ro.raw	2.1108E5	5	5	494	504			PEAKS DB
R.W(+28.03)LGPSAEYGNLYR.N	Y	66.95	1552.7673	13	1.8	777.3923	2	35.72	35	F35:1749	OB5946 H2 Ro.raw	3.3176E6	6	6	366	378	Ethylation	W1:Ethylation:51.01	PEAKS PTM
K.S(+57.02)QSENFEYVAFK.T	N	65.89	1504.6833	12	0.0	753.3489	2	31.77	35	F35:1521	OB5946 H2 Ro.raw	4.3047E5	3	3	436	447	Carbamidomethylation (DHKE, X@N-term)	S1:Carbamidomethylation (DHKE, X@N-term):56.34	PEAKS PTM
R.S(+57.02)PDIYNPQAGSLKTANDLNLLILR.W	N	64.15	2682.4341	24	1.5	895.1533	3	35.28	34	F34:1735	OB5945 H2 Ro.raw	9.5441E4	1	1	342	365		S1:Carbamidomethylation (DHKE, X@N-term):14.04	PEAKS PTM
R.AHVQVVDSN(+.98)GNRVYDEELQEGHVLVVPQNFAVAGK.S	N	64.02	3817.9023	35	5.0	955.4877	4	33.04	29	F29:1438	OB5922 H2 Ro.raw	1.4175E6	2	2	401	435		N9:Deamidation (NQ):9.40	PEAKS DB
R.LNAQRPDNRIESEGGYIETWNPNNQEFEC(+57.02)AGVALSR.L	N	60.14	4133.9248	36	2.4	1034.4910	4	33.87	33	F33:1670	OB5944 H6 Ro.raw	1.5556E6	3	3	14	49	Carbamidomethylation	C29:Carbamidomethylation:1000.00	PEAKS DB
P.FYSNAPQEIFIQQGR.G	N	59.30	1796.8845	15	-1.2	899.4484	2	32.27	31	F31:1574	OB5942 H6 Ro.raw	0	0	0	61	75			PEAKS DB
R.Q(-17.03)LKNNNPFK.F	N	56.41	1084.5665	9	1.6	543.2914	2	28.75	34	F34:1346	OB5945 H2 Ro.raw	1.0606E4	2	2	485	493	Pyro-glu from Q	Q1:Pyro-glu from Q:1000.00	PEAKS PTM
K.SQSENFE(+21.98)YVAFK.T	N	54.92	1469.6438	12	-1.1	735.8284	2	31.92	29	F29:1388	OB5922 H2 Ro.raw	6.4574E4	2	2	436	447		E7:Sodium adduct:14.84	PEAKS PTM
R.AHVQVVDSNGN(+.98)RVYDEELQEGHVLVVPQNFAVAGK.S	N	54.09	3817.9023	35	3.2	955.4859	4	32.51	34	F34:1567	OB5945 H2 Ro.raw	7.3088E4	3	3	401	435		N11:Deamidation (NQ):8.22	PEAKS DB

total 45 peptides

Peptide	Uniq	-10lgP	Mass	Length	ppm	m/z	z	RT	Fraction	Scan	Source File	Area Roasted in gel	#Feature	#Feature Roasted in gel	Start	End	PTM	AScore	Found By
K.SQS(-18.01)ENFEYVAFK.T	N	53.31	1429.6514	12	1.9	715.8344	2	31.92	29	F29:1380	OB5922 H2 Ro.raw	3.0777E4	1	1	436	447		S3:Dehydration:14.04	PEAKS PTM
R.IESEGGYIETWNPNNQ(+.98)EFEC(+57.02)AGVALSR.L	N	52.73	3070.3613	27	6.0	1024.4672	3	34.92	32	F32:1736	OB5943 H6 Ro.raw	6.6817E3	1	1	23	49	Carbamidomethylation	Q16:Deamidation (NQ):0.00;C20:Carbamidomethylation:1000.00	PEAKS DB
K.SQSE(+21.98)NFEYVAFK.T	N	51.89	1469.6438	12	-0.6	735.8287	2	31.51	34	F34:1513	OB5945 H2 Ro.raw	2.7649E4	1	1	436	447		E4:Sodium adduct:0.00	PEAKS PTM
K.FFVPPSQQSPRA.V	N	51.84	1359.6935	12	1.1	680.8547	2	29.14	32	F32:1381	OB5943 H6 Ro.raw	3.8841E4	2	2	494	505			PEAKS DB
R.VYDEELQEGHVLVVPQN(+.98)FAVAGK.S	N	51.23	2541.2751	23	9.5	1271.6569	2	32.92	31	F31:1618	OB5942 H6 Ro.raw	3.629E3	1	1	413	435		N17:Deamidation (NQ):14.02	PEAKS DB
K.TAN(+.98)DLNLLILR.W	N	50.73	1255.7136	11	2.6	628.8657	2	35.22	31	F31:1745	OB5942 H6 Ro.raw	0	0	0	355	365	Deamidation (NQ)	N3:Deamidation (NQ):68.31	PEAKS DB
R.S(+57.02)PDIYNPQAGSLK.T	N	50.29	1445.7150	13	5.7	723.8689	2	28.94	34	F34:1350	OB5945 H2 Ro.raw	6.4489E3	2	2	342	354		S1:Carbamidomethylation (DHKE, X@N-term):14.04	PEAKS PTM
R.SPD(+57.02)IYNPQAGSLK.T	N	45.16	1445.7150	13	2.2	723.8663	2	29.51	29	F29:1244	OB5922 H2 Ro.raw	0	0	0	342	354		D3:Carbamidomethylation (DHKE, X@N-term):0.00	PEAKS PTM
R.QLKNNNPFKFFVPPSQQS.P	N	42.33	2119.0850	18	1.9	707.3703	3	33.27	35	F35:1604	OB5946 H2 Ro.raw	6.7737E4	1	1	485	502			PEAKS DB
K.SQSE(+53.92)NFEYVAFK.T	N	42.20	1501.5812	12	-2.3	751.7961	2	31.58	35	F35:1507	OB5946 H2 Ro.raw	9.4575E4	1	1	436	447	Replacement of 2 protons by iron	E4:Replacement of 2 protons by iron:34.87	PEAKS PTM
K.NNNPFKFFVPPSQQS.P	N	41.33	1749.8474	15	1.0	875.9319	2	34.91	29	F29:1573	OB5922 H2 Ro.raw	4.7342E4	1	1	488	502			PEAKS DB
R.AHVQVVDSNGNRVYDEELQ(+.98)EGHVLVVPQNFAVAGK.S	N	40.20	3817.9023	35	7.9	955.4904	4	33.43	35	F35:1615	OB5946 H2 Ro.raw	0	0	0	401	435		Q19:Deamidation (NQ):4.91	PEAKS DB
R.AHVQVVDSN(+.98)GN(+.98)RVYDEELQEGHVLVVPQNFAVAGK.S	N	39.19	3818.8862	35	8.6	955.7371	4	34.33	32	F32:1692	OB5943 H6 Ro.raw	0	0	0	401	435		N9:Deamidation (NQ):0.00;N11:Deamidation (NQ):0.00	PEAKS DB
K.TAND(+53.92)LNLLILR.W	N	38.76	1308.6488	11	0.2	655.3318	2	35.09	29	F29:1584	OB5922 H2 Ro.raw	8.7715E4	1	1	355	365	Replacement of 2 protons by iron	D4:Replacement of 2 protons by iron:55.73	PEAKS PTM
R.AHVQ(+.98)VVDSNGNRVYDEELQEGHVLVVPQNFAVAGK.S	N	38.57	3817.9023	35	0.9	955.4838	4	33.12	31	F31:1624	OB5942 H6 Ro.raw	4.5408E4	1	1	401	435	Deamidation (NQ)	Q4:Deamidation (NQ):21.59	PEAKS DB
R.SLPYSPYSP(+31.99)QSQPR.Q	N	36.19	1637.7686	14	3.2	819.8942	2	27.07	38	F38:1250	OB5951 H3A Ro.raw	6.6096E3	1	1	197	210		P9:Dihydroxy:9.42	PEAKS PTM
R.RPFYSNAP(+15.99)QEIFIQQGR.G	N	33.55	2066.0332	17	0.2	689.6852	3	31.94	40	F40:1497	OB5953 H3A Ro.raw	4.3282E4	1	1	59	75	Oxidation or Hydroxylation	P8:Oxidation or Hydroxylation:32.28	PEAKS PTM
R.AHVQ(+.98)VVDSNGNRVYDEELQEGHVLVVPQ(+.98)NFAVAGK.S	N	33.38	3818.8862	35	8.9	955.7373	4	33.12	31	F31:1624	OB5942 H6 Ro.raw	3.8034E4	1	1	401	435		Q4:Deamidation (NQ):8.81;Q28:Deamidation (NQ):12.28	PEAKS DB
R.IESEGGYIETWN(+.98)PNNQEFEC(+57.02)AGVALSR.L	N	32.58	3070.3613	27	6.9	1536.1985	2	34.73	32	F32:1729	OB5943 H6 Ro.raw	2.6575E4	1	1	23	49	Carbamidomethylation	N12:Deamidation (NQ):0.00;C20:Carbamidomethylation:1000.00	PEAKS DB
R.QLKNNNPFKFFVPPSQQSPR.A	N	31.56	2372.2388	20	0.5	791.7539	3	33.17	29	F29:1463	OB5922 H2 Ro.raw	0	0	0	485	504			PEAKS DB
R.LNAQRPDNRIESEGGYIETWNPNN(+.98)QEFEC(+57.02)AGVALSR.L	N	29.77	4134.9087	36	4.7	1379.3167	3	33.85	33	F33:1671	OB5944 H6 Ro.raw	8.0788E4	1	1	14	49	Carbamidomethylation	N24:Deamidation (NQ):0.00;C29:Carbamidomethylation:1000.00	PEAKS DB
**total 45 peptides**																			

E9LFE8|E9LFE8_ARAHY

back to list

| Protein Coverage | Supporting Peptides |

Protein Coverage:

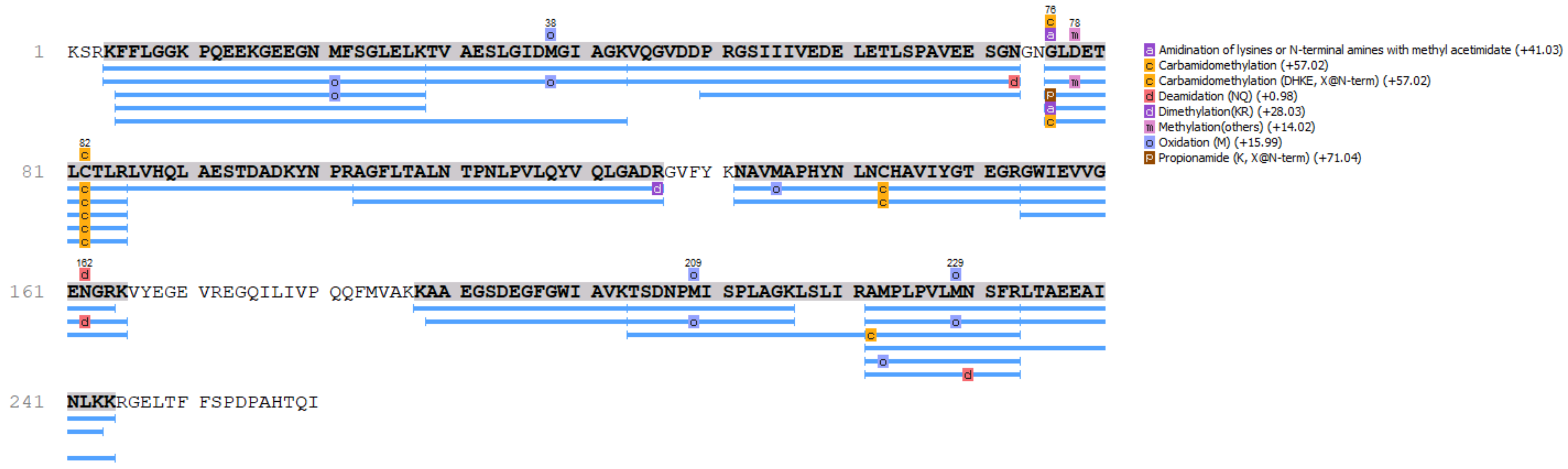

Supporting Peptides:

Peptide	Uniq	-10lgP	Mass	Length	ppm	m/z	z	RT	Fraction	Scan	Source File	Area Roasted in gel	#Feature	#Feature Roasted in gel	Start	End	PTM	AScore	Found By
K.KAAEGSDEGFGWIAVK.T	Y	124.05	1663.8206	16	2.8	832.9199	2	32.11	29	F29:1402	OB5922 H2 Ro.raw	5.9962E5	5	5	188	203			PEAKS DB
K.AAEGSDEGFGWIAVK.T	Y	123.90	1535.7256	15	3.4	768.8727	2	33.97	29	F29:1512	OB5922 H2 Ro.raw	4.1372E6	7	7	189	203			PEAKS DB
R.AGFLTALNTPNLPVLQYVQLGADR(+28.03).G	Y	116.09	2598.4170	24	4.1	1300.2211	2	40.36	29	F29:1874	OB5922 H2 Ro.raw	8.9656E6	11	11	103	126	Dimethylation(KR)	R24:Dimethylation(KR): 1000.00	PEAKS PTM
K.TVAESLGIDMGIAGK.V	Y	112.09	1460.7544	15	-1.6	731.3852	2	33.39	30	F30:1455	OB5926 H3B Ro.raw	1.9292E5	4	4	29	43			PEAKS DB
R.AMPLPVLMNSFR.L	Y	107.77	1374.7152	12	1.6	688.3660	2	36.22	34	F34:1784	OB5945 H2 Ro.raw	4.2293E6	6	6	222	233			PEAKS DB
R.AMPLPVLM(+15.99)NSFR.L	Y	101.69	1390.7101	12	2.1	696.3638	2	34.40	29	F29:1535	OB5922 H2 Ro.raw	4.2221E5	2	2	222	233	Oxidation (M)	M8:Oxidation (M):146.6 2	PEAKS DB
R.GWIEVVGENGR.K	Y	99.75	1214.6044	11	0.0	608.3094	2	31.76	35	F35:1523	OB5946 H2 Ro.raw	6.5203E4	2	2	154	164			PEAKS DB
K.TSDNPMISPLAGK.L	Y	97.69	1329.6598	13	1.5	665.8381	2	30.34	29	F29:1281	OB5922 H2 Ro.raw	4.6762E5	5	5	204	216			PEAKS DB
R.KFFLGGKPQEEKGEEGNMFSGLELK.T	N	95.61	2798.3948	25	-0.5	933.8075	3	36.62	30	F30:1634	OB5926 H3B Ro.raw	2.112E5	1	1	4	28			PEAKS DB
K.TVAESLGIDM(+15.99)GIAGK.V	Y	91.50	1476.7494	15	-2.7	739.3818	2	30.86	30	F30:1309	OB5926 H3B Ro.raw	3.516E3	1	1	29	43	Oxidation (M)	M10:Oxidation (M):100 0.00	PEAKS DB
N.GLDETLC(+57.02)TLR.L	Y	89.29	1176.5808	10	0.9	589.2982	2	30.78	34	F34:1456	OB5945 H2 Ro.raw	4.2059E5	3	3	76	85	Carbamidomethylation	C7:Carbamidomethylati on:1000.00	PEAKS DB

total 36 peptides

Peptide	Uniq	-10lgP	Mass	Length	ppm	m/z	z	RT	Fraction	Scan	Source File	Area Roasted in gel	#Feature	#Feature Roasted in gel	Start	End	PTM	AScore	Found By
K.NAVMAPHYNLNC(+57.02)HAVIYGTEGR.G	Y	85.03	2486.1582	22	1.2	829.7277	3	31.22	35	F35:1485	OB5946 H2 Ro.raw	3.5159E5	2	2	132	153	Carbamidomethylation	C12:Carbamidomethylation:1000.00	PEAKS DB
K.FFLGGKPQEEKGEEGNMFSGLELK.T	N	83.26	2670.2998	24	-0.7	891.1088	3	36.43	30	F30:1612	OB5926 H3B Ro.raw	1.0891E6	5	5	5	28			PEAKS DB
K.VQGVDDPRGSIIIVEDELETLSPAVEESGN.G	Y	78.33	3166.5518	30	4.3	1584.2899	2	39.16	36	F36:1927	OB5948 H3B Ro.raw	1.8789E6	3	3	44	73			PEAKS DB
K.TSDNPM(+15.99)ISPLAGK.L	Y	77.31	1345.6548	13	-1.4	673.8337	2	27.49	34	F34:1267	OB5945 H2 Ro.raw	8.6931E3	3	3	204	216	Oxidation (M)	M6:Oxidation (M):1000.00	PEAKS DB
K.TSDNPMISPLAGKLSLIR.A	Y	76.06	1912.0452	18	-0.1	638.3556	3	34.16	29	F29:1525	OB5922 H2 Ro.raw	1.9762E5	2	2	204	221			PEAKS DB
R.LTAEEAINLK.K	Y	71.99	1100.6077	10	1.8	551.3121	2	29.67	34	F34:1400	OB5945 H2 Ro.raw	2.6983E4	3	3	234	243			PEAKS DB
R.GWIEVVGEN(+.98)GRK.V	Y	65.81	1343.6833	12	0.3	672.8492	2	30.41	34	F34:1444	OB5945 H2 Ro.raw	1.4478E4	1	1	154	165	Deamidation (NQ)	N9:Deamidation (NQ):1000.00	PEAKS DB
N.G(+57.02)LDETLC(+57.02)TLR.L	Y	64.83	1233.6023	10	2.6	617.8100	2	30.88	34	F34:1470	OB5945 H2 Ro.raw	0	0	0	76	85	Carbamidomethylation (DHKE, X@N-term); Carbamidomethylation	G1:Carbamidomethylation (DHKE, X@N-term): 26.31;C7:Carbamidomethylation:1000.00	PEAKS PTM
R.GWIEVVGENGRK.V	Y	64.09	1342.6993	12	0.6	672.3574	2	30.50	35	F35:1442	OB5946 H2 Ro.raw	0	0	0	154	165			PEAKS DB
R.AGFLTALNTPNLPVLQYVQLGAD(+28.03)R.G	Y	63.41	2598.4170	24	-1.1	867.1453	3	39.63	33	F33:2004	OB5944 H6 Ro.raw	3.6606E4	2	2	103	126		D23:Ethylation:8.14	PEAKS PTM
R.LVHQLAESTDADKYNPR.A	N	61.22	1955.9701	17	-6.1	978.9863	2	30.10	29	F29:1292	OB5922 H2 Ro.raw	8.9618E4	4	4	86	102			PEAKS DB
N.GLD(+14.02)ETLC(+57.02)TLR.L	Y	59.74	1190.5966	10	1.5	596.3065	2	30.96	34	F34:1503	OB5945 H2 Ro.raw	1.792E6	3	3	76	85	Methylation(others); Carbamidomethylation	D3:Methylation(others): 33.98;C7:Carbamidomethylation:1000.00	PEAKS PTM
R.LTAEEAINLKK.R	Y	59.42	1228.7026	11	1.1	615.3593	2	28.04	29	F29:1144	OB5922 H2 Ro.raw	4.2354E4	3	3	234	244			PEAKS DB
K.FFLGGKPQEEKGEEGNM(+15.99)FSGLELK.T	N	58.55	2686.2947	24	-1.0	896.4402	3	34.90	30	F30:1542	OB5926 H3B Ro.raw	3.6375E5	2	2	5	28	Oxidation (M)	M17:Oxidation (M):1000.00	PEAKS DB
R.A(+57.02)MPLPVLMNSFR.L	Y	56.31	1431.7367	12	1.5	716.8767	2	36.95	29	F29:1692	OB5922 H2 Ro.raw	1.0591E5	3	3	222	233	Carbamidomethylation (DHKE, X@N-term)	A1:Carbamidomethylation (DHKE, X@N-term): 1000.00	PEAKS PTM
N.G(+71.04)LDETLC(+57.02)TLR.L	Y	53.84	1247.6179	10	1.2	624.8170	2	31.22	35	F35:1488	OB5946 H2 Ro.raw	4.2493E4	2	2	76	85	Propionamide (K, X@N-term); Carbamidomethylation	G1:Propionamide (K, X@N-term):1000.00;C7: Carbamidomethylation: 1000.00	PEAKS PTM
K.NAVM(+15.99)APHYNLNC(+57.02)HAVIYGTEGR.G	Y	52.74	2502.1533	22	1.1	835.0593	3	30.66	35	F35:1454	OB5946 H2 Ro.raw	9.2873E4	3	3	132	153	Oxidation (M); Carbamidomethylation	M4:Oxidation (M):1000.00;C12:Carbamidomethylation:1000.00	PEAKS DB
K.VQGVDDPRGSIIIVEDELETLSPAVEESGN(+.98).G	Y	52.14	3167.5356	30	9.3	1056.8623	3	39.51	37	F37:1974	OB5949 H3B Ro.raw	3.4995E4	1	1	44	73	Deamidation (NQ)	N30:Deamidation (NQ): 53.58	PEAKS DB
R.AM(+15.99)PLPVLMNSFR.L	Y	46.50	1390.7101	12	3.1	696.3645	2	34.72	29	F29:1571	OB5922 H2 Ro.raw	5.1224E5	2	2	222	233	Oxidation (M)	M2:Oxidation (M):71.27	PEAKS DB
D.PRGSIIIVEDELETLSPAVEESGN.G	Y	45.80	2553.2810	24	2.7	1277.6512	2	38.18	37	F37:1893	OB5949 H3B Ro.raw	1.8676E5	2	2	50	73			PEAKS DB
R.AMPLPVLMN(+.98)SFR.L	Y	45.50	1375.6992	12	1.7	688.8580	2	36.11	29	F29:1637	OB5922 H2 Ro.raw	0	0	0	222	233	Deamidation (NQ)	N9:Deamidation (NQ):1000.00	PEAKS DB
R.AMPLPVLMNSFRLTAEEAINLKK.R	Y	36.82	2585.4072	23	2.5	862.8118	3	39.79	29	F29:1844	OB5922 H2 Ro.raw	4.4272E5	1	1	222	244			PEAKS DB

total 36 peptides

Peptide	Uniq	-10lgP	Mass	Length	ppm	m/z	z	RT	Fraction	Scan	Source File	Area Roasted in gel	#Feature	#Feature Roasted in gel	Start	End	PTM	AScore	Found By
R.KFFLGGKPQEEKGEEGNM(+15.99)FSGLELK.T	N	35.69	2814.3896	25	1.4	939.1409	3	35.47	30	F30:1579	OB5926 H3B Ro.raw	8.019E4	1	1	4	28	Oxidation (M)	M18:Oxidation (M):100 0.00	PEAKS DB
N.G(+41.03)LDETLC(+57.02)TLR.L	Y	31.53	1217.6074	10	0.9	609.8115	2	31.33	34	F34:1490	OB5945 H2 Ro.raw	2.7231E4	1	1	76	85	Amidination of lysines or N-terminal amines with methyl acetimidate; Carbamidomethylation	G1:Amidination of lysines or N-terminal amines with methyl acetimidate:1000.00;C7:Carbamidomethylation:1000.00	PEAKS PTM
K.FFLGGKPQEEKGEEGNMFSGLELKTVAESLGIDMGIAGK.V	Y	29.96	4113.0439	39	-1.9	1029.2689	4	40.43	30	F30:1855	OB5926 H3B Ro.raw	1.8985E5	1	1	5	43			PEAKS DB

total 36 peptides

sp|Q647H2|AHY3_ARAHY

back to list

| Protein Coverage | Supporting Peptides |

Protein Coverage:

1 MAKLLALSVC FCFLVLGASS VTFRQQGEEN ECQFQRLNAQ RPDNCIESEG GYIETWNPNN QEFQCAGVAL SRFVLRRNAL
81 RRPFYSNAPQ EIFIYQGSGY FGLIFPGCPG TFEEPIQGSE QFQRPSRHFQ GQDQSQRPLD THQKVHGFRE GDLIAVPHGV
161 AFWIYNDQDT DVVAISVLHT NSLHNQLDQF PRRFNLAGKQ EQEFLRYQQR SGRQSPKGEE QEQEQENEGG NVFSGFSTEF
241 LSHGFQVNED IVRNLRGENE REEQGAIVTV KGGLSILVPP EWRQSYQQPG RGDKDFNNGI EETICTATVK MNIGKSTSAD
321 IYNPQAGSVR TVNELDLPIL NRLGLSAEYG SIHRDAMFVP HYNMNANSMI YALHGGAHVQ VVDCNGNRVF DEELQEGQSL
401 VVPQNFAVAA KSQSEHFLYV AFKTNSRASI SNLAGKNSYM WNLPEDVVAN SYGLQYEQAR QLKNNNPFTF LVPPQDSQMI
481 RTVA

- a Amidination of lysines or N-terminal amines with methyl acetimidate (+41.03)
- c Carbamidomethylation (+57.02)
- c Carbamidomethylation (DHKE, X@N-term) (+57.02)
- m Methylation(others) (+14.02)
- o Oxidation (M) (+15.99)

Supporting Peptides:

Peptide	Uniq	-10lgP	Mass	Length	ppm	m/z	z	RT	Fraction	Scan	Source File	Area Roasted in gel	#Feature	#Feature Roasted in gel	Start	End	PTM	AScore	Found By
K.NSYMWNLPEDVVANSYGLQYEQAR.Q	Y	127.00	2846.2969	24	3.2	1424.1603	2	37.32	29	F29:1709	OB5922 H2 Ro.raw	9.5352E5	4	4	437	460			PEAKS DB
K.SQSEHFLYVAFK.T	Y	121.04	1454.7194	12	1.5	728.3680	2	32.30	29	F29:1412	OB5922 H2 Ro.raw	2.2354E6	9	9	412	423			PEAKS DB
R.TVNELDLPILNR.L	Y	103.51	1395.7721	12	2.8	698.8953	2	34.72	29	F29:1555	OB5922 H2 Ro.raw	4.6895E6	10	10	331	342			PEAKS DB
K.NSYM(+15.99)WNLPEDVVANSYGLQYEQAR.Q	Y	96.60	2862.2917	24	4.0	1432.1589	2	36.20	29	F29:1647	OB5922 H2 Ro.raw	5.9092E4	2	2	437	460	Oxidation (M)	M4:Oxidation (M):1000.00	PEAKS DB
K.SQSEHFLYVAFKTNSR.A	Y	95.50	1912.9431	16	1.0	638.6556	3	31.92	29	F29:1392	OB5922 H2 Ro.raw	1.6409E5	3	3	412	427			PEAKS DB
N.GIEETIC(+57.02)TATVK.M	N	95.04	1320.6595	12	-0.2	661.3369	2	29.91	29	F29:1260	OB5922 H2 Ro.raw	2.5094E5	6	6	299	310	Carbamidomethylation	C7:Carbamidomethylation: 1000.00	PEAKS DB

total 22 peptides

Peptide	Uniq	-10lgP	Mass	Length	ppm	m/z	z	RT	Fraction	Scan	Source File	Area Roasted in gel	#Feature	#Feature Roasted in gel	Start	End	PTM	AScore	Found By
R.ASISNLAGKNSYMWNLPEDVVANSYGLQYEQAR.Q	Y	93.33	3687.7627	33	7.0	1230.2701	3	36.76	29	F29:1683	OB5922 H2 Ro.raw	3.0022E5	3	3	428	460			PEAKS DB
R.LGLSAEYGSIHR.D	Y	93.12	1301.6727	12	0.5	651.8440	2	29.26	35	F35:1369	OB5946 H2 Ro.raw	2.0752E5	6	6	343	354			PEAKS DB
K.STSADIYNPQAGSVR.T	Y	76.60	1564.7482	15	-0.5	783.3810	2	27.48	32	F32:1285	OB5943 H6 Ro.raw	6.6305E4	6	6	316	330			PEAKS DB
R.VFDEELQEGQSLVVPQNFAVAAK.S	Y	67.53	2517.2751	23	4.7	1259.6508	2	35.09	34	F34:1723	OB5945 H2 Ro.raw	7.46E4	3	3	389	411			PEAKS DB
N.G(+57.02)IEETIC(+57.02)TATVK.M	N	64.33	1377.6809	12	2.0	689.8491	2	30.19	29	F29:1285	OB5922 H2 Ro.raw	5.5841E3	1	1	299	310	Carbamidomethylation (DHKE, X@N-term); Carbamidomethylation	G1:Carbamidomethylation (DHKE, X@N-term):30.83; C7:Carbamidomethylation: 1000.00	PEAKS PTM
V.PQNFAVAAK.S	N	59.75	944.5079	9	0.9	473.2617	2	32.86	29	F29:1447	OB5922 H2 Ro.raw	2.0461E6	3	3	403	411			PEAKS DB
R.RFNLAGKQEQEFLR.Y	Y	57.44	1734.9165	14	0.4	579.3130	3	31.51	34	F34:1507	OB5945 H2 Ro.raw	2.9508E5	4	4	193	206			PEAKS DB
R.FNLAGKQEQEFLR.Y	Y	56.13	1578.8154	13	-2.4	790.4151	2	31.75	30	F30:1357	OB5926 H3B Ro.raw	0	0	0	194	206			PEAKS DB
K.S(+57.02)QSEHFLYVAFK.T	Y	49.11	1511.7408	12	0.6	756.8782	2	32.49	29	F29:1431	OB5922 H2 Ro.raw	4.3132E4	1	1	412	423		S1:Carbamidomethylation (DHKE, X@N-term):9.42	PEAKS PTM
R.QLKNNNPFTFLVPPQDSQMIRT.V	Y	46.30	2587.3218	22	0.9	863.4487	3	35.15	35	F35:1717	OB5946 H2 Ro.raw	5.4546E5	2	2	461	482			PEAKS DB
K.N(+.98)SYMWNLPEDVVANSYGLQYEQAR.Q	Y	46.12	2847.2810	24	9.4	1424.6611	2	39.12	35	F35:1945	OB5946 H2 Ro.raw	0	0	0	437	460		N1:Deamidation (NQ):7.38	PEAKS DB
N.GIEETIC(+57.02)T(+14.02)ATVK.M	N	42.70	1334.6752	12	3.2	668.3470	2	29.62	34	F34:1395	OB5945 H2 Ro.raw	7.3781E3	1	1	299	310	Carbamidomethylation; Methylation(others)	C7:Carbamidomethylation: 1000.00;T8:Methylation(ot hers):28.36	PEAKS PTM
K.NNNPFTFLVPPQDSQMIRT.V	Y	39.74	2218.0840	19	2.9	1110.0525	2	37.02	29	F29:1690	OB5922 H2 Ro.raw	0	0	0	464	482			PEAKS DB
N.G(+41.03)IEETIC(+57.02)TATVK.M	N	36.93	1361.6860	12	0.7	681.8508	2	29.66	34	F34:1390	OB5945 H2 Ro.raw	1.6824E4	1	1	299	310	Amidination of lysines or N-terminal amines with methyl acetimidate; Carbamidomethylation	G1:Amidination of lysines or N-terminal amines with methyl acetimidate:96.48; C7:Carbamidomethylation: 1000.00	PEAKS PTM
K.NNNPFTFLVPPQDSQMIR.T	Y	32.90	2117.0364	18	2.7	1059.5283	2	36.76	29	F29:1684	OB5922 H2 Ro.raw	1.089E4	1	1	464	481			PEAKS DB
C.IESEGGYIETWNPNNQEFQC(+57.02)AGVALSR.F	N	30.17	3068.3933	27	-0.9	1023.8041	3	34.37	33	F33:1699	OB5944 H6 Ro.raw	0	0	0	46	72	Carbamidomethylation	C20:Carbamidomethylatio n:1000.00	PEAKS DB
**total 22 peptides**																			

Q38711|Q38711_ARAHY

back to list

| Protein Coverage | Supporting Peptides |

Protein Coverage:

71

1 KPFCVFLTFF LLLAASKKVN SAETVSFNFN SFSEGNPAIN FQGDVTVLSN GNIQLTNLNK VNSVGRVLYA MPVRIWSSAT

81 GNVASFLTSF SFEMKDIKDY DPADGIIFFI APEDTQIPAG SIGGGTLGVS DTKGAGHFVG VEFDTYSNSE YNDPPTDHVG

225

161 IDVNSVDSVK TVPWNSVSGA VVQVTVIYDS STKTLSVAVT NENGDITTIA QVVDLKAKLP ERVKFGFSAS GSLGGRQIHL

241 IRSWSFTSTL ITTTRRSIDN NEKKIMNMAT A

c Carbamidomethylation (DHKE, X@N-term) (+57.02)
d Deamidation (NQ) (+0.98)
o Oxidation (M) (+15.99)

Supporting Peptides:

Peptide	Uniq	-10lgP	Mass	Length	ppm	m/z	z	RT	Fraction	Scan	Source File	Area Roasted in gel	#Feature	#Feature Roasted in gel	Start	End	PTM	AScore	Found By
R.SWSFTSTLITTTR.R	N	125.73	1499.7620	13	1.0	750.8890	2	34.40	36	F36:1663	OB5948 H3B Ro.raw	1.0426E6	6	6	243	255			PEAKS DB
R.IWSSATGNVASFLTSFSFEMK.D	N	120.82	2309.1038	21	1.3	1155.5636	2	40.24	30	F30:1850	OB5926 H3B Ro.raw	5.3682E5	3	3	75	95			PEAKS DB
K.FGFSASGSLGGR.Q	N	101.25	1141.5515	12	-1.4	571.7837	2	30.73	30	F30:1279	OB5926 H3B Ro.raw	4.8005E5	3	3	225	236			PEAKS DB
K.TLSVAVTNENGDITTIAQVVDLK.A	Y	96.60	2400.2747	23	1.9	1201.1469	2	35.93	36	F36:1752	OB5948 H3B Ro.raw	2.3058E5	4	4	194	216			PEAKS DB
R.IWSSATGNVASFLTSFSFEM(+15.99)K.D	N	96.28	2325.0986	21	1.5	1163.5583	2	38.94	36	F36:1921	OB5948 H3B Ro.raw	4.3252E4	1	1	75	95	Oxidation (M)	M20:Oxidation (M):1000.00	PEAKS DB
D.PPTDHVGIDVNSVDSVK.T	N	90.80	1777.8846	17	-1.8	593.6359	3	30.26	30	F30:1269	OB5926 H3B Ro.raw	1.0613E6	9	9	154	170			PEAKS DB
K.GAGHFVGVEFDTYSNSEYND.P	N	84.78	2206.9080	20	2.6	1104.4641	2	32.88	36	F36:1577	OB5948 H3B Ro.raw	3.4015E5	3	3	134	153			PEAKS DB
K.TVPWNSVSGAVVQVTVIYDSSTK.T	Y	82.78	2436.2537	23	2.3	1219.1370	2	37.45	36	F36:1834	OB5948 H3B Ro.raw	1.092E5	3	3	171	193			PEAKS DB
K.DIKDYDPADGIIFFIAPEDTQIPAGSIGGGTLGVSDTK.G	N	82.25	3892.9258	38	3.0	1298.6531	3	38.97	36	F36:1923	OB5948 H3B Ro.raw	1.1326E6	3	3	96	133			PEAKS DB
R.VKFGFSASGSLGGR.Q	N	79.59	1368.7150	14	-2.5	685.3648	2	31.03	30	F30:1308	OB5926 H3B Ro.raw	2.1689E4	3	3	223	236			PEAKS DB
R.VLYAMPVR.I	N	77.27	947.5262	8	0.2	474.7705	2	29.80	36	F36:1400	OB5948 H3B Ro.raw	5.0266E5	3	3	67	74			PEAKS DB
K.F(+57.02)GFSASGSLGGR.Q	N	67.36	1198.5730	12	4.0	600.2977	2	30.99	30	F30:1312	OB5926 H3B Ro.raw	0	0	0	225	236	Carbamidomethylation (DHKE, X@N-term)	F1:Carbamidomethylation (DHKE, X@N-term):1000.00	PEAKS PTM
K.GAGHFVGVEFDTYSNSEYNDPPTDHVGIDVNSVDSVK.T	N	64.62	3966.7820	37	3.2	992.7060	4	33.64	36	F36:1609	OB5948 H3B Ro.raw	3.9201E6	5	5	134	170			PEAKS DB

total 23 peptides

Peptide	Uniq	-10lgP	Mass	Length	ppm	m/z	z	RT	Fraction	Scan	Source File	Area Roasted in gel	#Feature	#Feature Roasted in gel	Start	End	PTM	AScore	Found By
R.VLYAM(+15.99)PVR.I	N	53.97	963.5212	8	-2.7	482.7678	2	27.52	30	F30:1124	OB5926 H3B Ro.raw	1.3575E4	3	3	67	74	Oxidation (M)	M5:Oxidation (M):1000.00	PEAKS DB
R.SWSFTSTLITTTRR.S	N	53.42	1655.8632	14	0.3	828.9391	2	33.07	36	F36:1575	OB5948 H3B Ro.raw	1.4241E6	4	4	243	256			PEAKS DB
K.FGFSASGSLGGRQIHLIR.S	N	45.69	1902.0223	18	-1.7	635.0153	3	35.47	30	F30:1558	OB5926 H3B Ro.raw	1.7216E5	2	2	225	242			PEAKS DB
D.PPTDHVGIDVN(+.98)SVDSVK.T	N	45.05	1778.8687	17	2.5	593.9665	3	29.71	30	F30:1235	OB5926 H3B Ro.raw	0	0	0	154	170	Deamidation (NQ)	N11:Deamidation (NQ):1000.00	PEAKS DB
R.IWSSATGNVASFLTSFSFEM(+15.99)KDIKDYDPADGIIFFIAPEDTQIPAGSIGGGTLGVSDTK.G	N	44.89	6200.0137	59	1.2	1551.0165	4	41.20	30	F30:1895	OB5926 H3B Ro.raw	7.8846E5	1	1	75	133	Oxidation (M)	M20:Oxidation (M):1000.00	PEAKS DB
R.IWSSATGNVASFLTSFSFEMKDIKDYDPADGIIFFIAPEDTQIPAGSIGGGTLGVSDTK.G	N	41.67	6184.0190	59	0.9	1547.0173	4	42.35	30	F30:1960	OB5926 H3B Ro.raw	8.2055E5	1	1	75	133			PEAKS DB
S.AETVSFNFNSFSEGNPAINFQGDVTVLSNGNIQLTNLNKVNSVGR.V	N	40.66	4812.3740	45	4.5	1605.1392	3	40.87	37	F37:2044	OB5949 H3B Ro.raw	5.6245E6	3	3	22	66			PEAKS DB
R.SW(+31.99)SFTSTLITTTR.R	N	40.52	1531.7518	13	0.9	766.8839	2	33.45	36	F36:1617	OB5948 H3B Ro.raw	3.3337E3	1	1	243	255		W2:Dihydroxy:17.01	PEAKS PTM
D.P(+57.02)PTDHVGIDVNSVDSVK.T	N	29.71	1834.9061	17	-0.9	918.4595	2	30.51	37	F37:1440	OB5949 H3B Ro.raw	0	0	0	154	170		P1:Carbamidomethylation (DHKE, X@N-term):19.05	PEAKS PTM
S.AETVSFNFNSFSEGNPAINFQGDVTVLSNGNIQLTNLN(+.98)KVNSVGR.V	N	29.65	4813.3584	45	8.6	1204.3572	4	38.44	37	F37:1904	OB5949 H3B Ro.raw	0	0	0	22	66		N38:Deamidation (NQ):0.00	PEAKS DB
**total 23 peptides**																			

A0A089ZXL7|A0A089ZXL7_ARAHY

back to list

| Protein Coverage | Supporting Peptides |

Protein Coverage:

```
73
1   MKPFCVFLTF FLLLAASSKK VNSAETVSFN FNSFSEGNPA INFQGDVTVL SNGNIQLTNL NKVNSVGRVL YAMPVRIWSS
81  ATGNVASFLT SFSFEMKDIK DYDPADGIIF FIAPEDTQIP AGSIGGGTLG VSDTKGAGYF VGVEFDTYSN SEYNDPPTDH
161 VGIDVNSVDS VKTVPWNSVS GAVVKVTVIY DSSTKTLSVA VTNDNGDITT IAQVVDLKAK LPERVKFGFS ASGSRAGRQI
241 HLIRSWSFTS TLITTTRRSI DNNEKKIMNM ASA
```

d Deamidation (NQ) (+0.98)
o Oxidation (M) (+15.99)

Supporting Peptides:

Peptide	Uniq	-10lgP	Mass	Length	ppm	m/z	z	RT	Fraction	Scan	Source File	Area Roasted	#Feature	#Feature Roasted in	Start	End	PTM	AScore	Found By

												in gel		gel					
R.SWSFTSTLITTTR.R	N	125.73	1499.7620	13	1.0	750.8890	2	34.40	36	F36:1663	OB5948 H3B Ro.raw	1.0426E6	6	6	245	257			PEAKS DB
R.IWSSATGNVASFLTSFSFEMK.D	N	120.82	2309.1038	21	1.3	1155.5636	2	40.24	30	F30:1850	OB5926 H3B Ro.raw	5.3682E5	3	3	77	97			PEAKS DB
K.TLSVAVTNDNGDITTIAQVVDLK.A	N	109.79	2386.2590	23	0.5	1194.1405	2	36.05	30	F30:1608	OB5926 H3B Ro.raw	7.7468E5	3	3	196	218			PEAKS DB
R.IWSSATGNVASFLTSFSFEM(+15.99)K.D	N	96.28	2325.0986	21	1.5	1163.5583	2	38.94	36	F36:1921	OB5948 H3B Ro.raw	4.3252E4	1	1	77	97	Oxidation (M)	M20:Oxidation (M):1000.00	PEAKS DB
D.PPTDHVGIDVNSVDSVK.T	N	90.80	1777.8846	17	-1.8	593.6359	3	30.26	30	F30:1269	OB5926 H3B Ro.raw	1.0613E6	9	9	156	172			PEAKS DB
K.TVPWNSVSGAVVK.V	N	82.61	1342.7245	13	1.4	672.3705	2	30.95	36	F36:1462	OB5948 H3B Ro.raw	1.4738E5	4	4	173	185			PEAKS DB
K.DIKDYDPADGIIFFIAPEDTQIPAGSIGGGTLGVSDTK.G	N	82.25	3892.9258	38	3.0	1298.6531	3	38.97	36	F36:1923	OB5948 H3B Ro.raw	1.1326E6	3	3	98	135			PEAKS DB
R.VLYAMPVR.I	N	77.27	947.5262	8	0.2	474.7705	2	29.80	36	F36:1400	OB5948 H3B Ro.raw	5.0266E5	3	3	69	76			PEAKS DB
K.FGFSASGSR.A	Y	57.37	914.4246	9	0.9	458.2212	2	27.95	30	F30:1148	OB5926 H3B Ro.raw	0	0	0	227	235			PEAKS DB
K.GAGYFVGVEFDTYSNSEYNDPPTDHVGIDVNSVDSVK.T	Y	54.56	3992.7864	37	-0.9	1331.9382	3	35.47	30	F30:1682	OB5926 H3B Ro.raw	6.5603E4	1	1	136	172			PEAKS DB
R.VLYAM(+15.99)PVR.I	N	53.97	963.5212	8	-2.7	482.7678	2	27.52	30	F30:1124	OB5926 H3B Ro.raw	1.3575E4	3	3	69	76	Oxidation (M)	M5:Oxidation (M):1000.00	PEAKS DB
R.SWSFTSTLITTTRR.S	N	53.42	1655.8632	14	0.3	828.9391	2	33.07	36	F36:1575	OB5948 H3B Ro.raw	1.4241E6	4	4	245	258			PEAKS DB
K.TLSVAVTNDNGDITTIAQVVDLKAK.L	N	50.12	2585.3911	25	0.9	862.8051	3	34.40	36	F36:1665	OB5948 H3B Ro.raw	1.5947E4	1	1	196	220			PEAKS DB
K.TLSVAVTNDN(+.98)GDITTIAQVVDLK.A	N	48.85	2387.2432	23	-0.8	1194.6310	2	36.59	30	F30:1638	OB5926 H3B Ro.raw	0	0	0	196	218		N10:Deamidation (NQ): 8.22	PEAKS DB
D.PPTDHVGIDVN(+.98)SVDSVK.T	N	45.05	1778.8687	17	2.5	593.9665	3	29.71	30	F30:1235	OB5926 H3B Ro.raw	0	0	0	156	172	Deamidation (NQ)	N11:Deamidation (NQ):1000.00	PEAKS DB
R.IWSSATGNVASFLTSFSFEM(+15.99)KDIKDYDPADGIIFFIAPEDTQIPAGSIGGGTLGVSDTK.G	N	44.89	6200.0137	59	1.2	1551.0165	4	41.20	30	F30:1895	OB5926 H3B Ro.raw	7.8846E5	1	1	77	135	Oxidation (M)	M20:Oxidation (M):1000.00	PEAKS DB
R.IWSSATGNVASFLTSFSFEMKDIKDYDPADGIIFFIAPEDTQIPAGSIGGGTLGVSDTK.G	N	41.67	6184.0190	59	0.9	1547.0173	4	42.35	30	F30:1960	OB5926 H3B Ro.raw	8.2055E5	1	1	77	135			PEAKS DB
S.AETVSFNFNSFSEGNPAINFQGDVTVLSNGNIQLTNLNKVNSVGR.V	N	40.66	4812.3740	45	4.5	1605.1392	3	40.87	37	F37:2044	OB5949 H3B Ro.raw	5.6245E6	3	3	24	68			PEAKS DB
R.SW(+31.99)SFTSTLITTTR.R	N	40.52	1531.7518	13	0.9	766.8839	2	33.45	36	F36:1617	OB5948 H3B Ro.raw	3.3337E3	1	1	245	257		W2:Dihydroxy:17.01	PEAKS PTM
K.GAGYFVGVEFDTYSN(+.98)SEYNDPPTDHVGIDVNSVDSVK.T	Y	37.65	3993.7703	37	7.3	1332.2772	3	35.85	30	F30:1577	OB5926 H3B Ro.raw	2.0916E5	1	1	136	172	Deamidation (NQ)	N15:Deamidation (NQ):25.70	PEAKS DB
D.P(+57.02)PTDHVGIDVNSVDSVK.T	N	29.71	1834.9061	17	-0.9	918.4595	2	30.51	37	F37:1440	OB5949 H3B Ro.raw	0	0	0	156	172		P1:Carbamidomethylation (DHKE, X @N-term):19.05	PEAKS PTM
S.AETVSFNFNSFSEGNPAINFQGDVTVLSNGNIQLTNLN(+.98)KVNSVGR.V	N	29.65	4813.3584	45	8.6	1204.3572	4	38.44	37	F37:1904	OB5949 H3B Ro.raw	0	0	0	24	68		N38:Deamidation (NQ): 0.00	PEAKS DB
**total 22 peptides**																			

A1E2B0|A1E2B0_ARAHY

back to list

Prepared with PEAKS ™ (bioinfor.com)

| Protein Coverage | Supporting Peptides |

Protein Coverage:

62

1 MANSLRLAFL VLFQSSLALS LAHRYPTKCS FDKLVALEPT KRVESEGGFT EYWDSKNDQF QCVGVSALRY SIKPKGLLLP

81 HYINAPRLQY VLQGTGILET VVPGCPETFR EQTRHGDQHQ KIHATREGDV IVVPTGSAQW IYNSGETDMV IFSVIDSANE

204

161 DNQLDLKVRK FFLGGKPQEE KGEEGNMFSG LELKTVAESL DIDMGIAGKV QGVDDPRGSM IIVEDELETL SPAVEESGNG

241 NGLIETVCTL RLVHQLAEST DADKYNPRAG FLTALTPLTF PSFNMSNLAL IDVFSTECCD GATLH

c Carbamidomethylation (+57.02)
c Carbamidomethylation (DHKE, X@N-term) (+57.02)
d Deamidation (NQ) (+0.98)
o Oxidation (M) (+15.99)

Supporting Peptides:

Peptide	Uniq	-10lgP	Mass	Length	ppm	m/z	z	RT	Fraction	Scan	Source File	Area Roasted in gel	#Feature	#Feature Roasted in gel	Start	End	PTM	AScore	Found By
K.TVAESLDIDMGIAGK.V	Y	117.87	1518.7599	15	-1.7	760.3879	2	33.39	30	F30:1446	OB5926 H3B Ro.raw	8.1424E4	2	2	195	209			PEAKS DB
K.GLLLPHYINAPR.L	Y	106.43	1362.7771	12	0.2	682.3977	2	33.21	30	F30:1443	OB5926 H3B Ro.raw	2.2835E6	7	7	76	87			PEAKS DB
R.KFFLGGKPQEEKGEEGNMFSGLELK.T	N	95.61	2798.3948	25	-0.5	933.8075	3	36.62	30	F30:1634	OB5926 H3B Ro.raw	2.112E5	1	1	170	194			PEAKS DB
R.LQYVLQGTGILETVVPGC(+57.02)PETFR.E	Y	93.70	2576.3308	23	3.4	1289.1771	2	36.71	36	F36:1795	OB5948 H3B Ro.raw	6.8807E4	2	2	88	110	Carbamidomethylation	C18:Carbamidomethylation:1000.00	PEAKS DB
K.TVAESLDIDM(+15.99)GIAGK.V	Y	93.12	1534.7549	15	0.7	768.3872	2	30.86	30	F30:1307	OB5926 H3B Ro.raw	5.6446E3	1	1	195	209	Oxidation (M)	M10:Oxidation (M):1000.00	PEAKS DB
R.LQYVLQGTGILETVVPGC(+57.02)PETFREQTR.H	Y	83.46	3090.5808	27	4.5	1031.2056	3	35.54	36	F36:1773	OB5948 H3B Ro.raw	8.339E6	5	5	88	114	Carbamidomethylation	C18:Carbamidomethylation:1000.00	PEAKS DB
K.FFLGGKPQEEKGEEGNMFSGLELK.T	N	83.26	2670.2998	24	-0.7	891.1088	3	36.43	30	F30:1612	OB5926 H3B Ro.raw	1.0891E6	5	5	171	194			PEAKS DB
R.VESEGGFTEYWDSKNDQFQC(+57.02)VGVSALR.Y	Y	82.89	3107.3931	27	2.8	1036.8079	3	34.76	37	F37:1692	OB5949 H3B Ro.raw	9.1132E5	3	3	43	69	Carbamidomethylation	C20:Carbamidomethylation:1000.00	PEAKS DB
K.RVESEGGFTEYWDSKNDQFQC(+57.02)VGVSALR.Y	Y	73.88	3263.4941	28	-0.3	1088.8411	3	34.90	30	F30:1540	OB5926 H3B Ro.raw	7.5628E4	1	1	42	69	Carbamidomethylation	C21:Carbamidomethylation:1000.00	PEAKS DB
K.G(+57.02)LLLPHYINAPR.L	Y	69.30	1419.7986	12	0.9	710.9072	2	32.70	36	F36:1554	OB5948 H3B Ro.raw	1.9861E5	3	3	76	87	Carbamidomethylation (DHKE, X@N-term)	G1:Carbamidomethylation (DHKE, X@N-term):71.64	PEAKS PTM
R.LVHQLAESTDADKYNPR.A	N	61.22	1955.9701	17	-6.1	978.9863	2	30.10	29	F29:1292	OB5922 H2 Ro.raw	8.9618E4	4	4	252	268			PEAKS DB
R.EGDVIVVPTGSAQWIYNSGETDMVIFSVIDSANEDNQLDLKVR.K	Y	59.34	4723.2964	43	3.0	1575.4441	3	40.31	36	F36:1999	OB5948 H3B Ro.raw	4.2652E5	2	2	127	169			PEAKS DB
K.FFLGGKPQEEKGEEGNM(+15.99)FSGLELK.T	N	58.55	2686.2947	24	-1.0	896.4402	3	34.90	30	F30:1542	OB5926 H3B Ro.raw	3.6375E5	2	2	171	194	Oxidation (M)	M17:Oxidation (M):1000.00	PEAKS DB
K.VQGVDDPRGSMIIVEDELETLSPAVEESGN.G	Y	57.35	3184.5081	30	4.2	1593.2681	2	38.96	37	F37:1934	OB5949 H3B Ro.raw	0	0	0	210	239			PEAKS DB

total 21 peptides

Peptide	Uniq	-10lgP	Mass	Length	ppm	m/z	z	RT	Fraction	Scan	Source File	Area Roasted in gel	#Feature	#Feature Roasted in gel	Start	End	PTM	AScore	Found By
K.C(+57.02)SFDKLVALEPTK.R	Y	41.84	1506.7751	13	1.4	754.3959	2	31.21	36	F36:1476	OB5948 H3B Ro.raw	0	0	0	29	41	Carbamidomethylation	C1:Carbamidomethylation:1000.00	PEAKS DB
R.KFFLGGKPQEEKGEEGNM(+15.99)FSGLELK.T	N	35.69	2814.3896	25	1.4	939.1409	3	35.47	30	F30:1579	OB5926 H3B Ro.raw	8.019E4	1	1	170	194	Oxidation (M)	M18:Oxidation (M):1000.00	PEAKS DB
R.EGDVIVVPTGSAQ(+.98)WIYNSGETDMVIFSVIDSANEDNQLDLKVR.K	Y	34.54	4724.2803	43	5.0	1575.7793	3	40.63	30	F30:1862	OB5926 H3B Ro.raw	2.2969E5	1	1	127	169	Deamidation (NQ)	Q13:Deamidation (NQ):34.15	PEAKS DB
L.PHYINAPR.L	Y	33.58	966.5035	8	-2.2	484.2592	2	33.21	30	F30:1450	OB5926 H3B Ro.raw	3.5979E4	2	2	80	87			PEAKS DB
R.LQYVLQGTGILETVVPGC(+57.02)PETFREQ(+.98)TR.H	Y	31.85	3091.5647	27	3.3	1031.5322	3	43.45	36	F36:2184	OB5948 H3B Ro.raw	0	0	0	88	114	Carbamidomethylation; Deamidation (NQ)	C18:Carbamidomethylation:1000.00;Q25:Deamidation (NQ):42.82	PEAKS DB
R.LQYVLQGTGILETVVPGC(+57.02)PETFR(+14.02)EQTR.H	Y	29.44	3104.5964	27	2.9	1035.8757	3	35.54	36	F36:1791	OB5948 H3B Ro.raw	3.2709E5	1	1	88	114	Carbamidomethylation	C18:Carbamidomethylation:1000.00;R23:Methylation(KR):11.01	PEAKS PTM
R.LQYVLQGTGILETVVPGCPETFRE(+57.02)QTR.H	Y	28.85	3090.5808	27	6.1	1031.2098	3	48.29	30	F30:2312	OB5926 H3B Ro.raw	0	0	0	88	114		E24:Carbamidomethylation (DHKE, X@N-term):6.65	PEAKS PTM

total 21 peptides

Q647H1|Q647H1_ARAHY

back to list

| Protein Coverage | Supporting Peptides |

Protein Coverage:

```
  1 MVIGPFRLSL CVCLVFLTSA CFGTRLEESF NECQLDRLNA LTPDNRIESQ GGITETWNSN HPELRCAGVT LLKRTIFPNG
 81 FHLPSYANYP QLIFIAQGNG VFGVSLPGCP VTYEEAESQS REDRRQRIVI KRESEQEQEQ QGDSHHKIYH FRQGHLLAIP
161 AGVPYWSFNY GNEPIVAITL LDTSNLDNQL DPSPRRFYLA GNPEEEHPET QQQQPQTRRR HGQHQQDEYG SQGEEEGNNV
241 LSGFSTQLLA HAFGVDEEIA RILQNPPEQT KDQIVRVEGG FRDVISPRWG EGKQYEDELE ERQRQPPRRD EQGKGYDYDD
321 DRRPRHRQDP YREGDEDDRR PRGSRQGQGR GYDDDDRRPG QYEEGEEDDR RPRRSSRPKR QGRRHDDDDR RADEDDRRGY
                                                        456
401 DDDERRPDED DRRGYDDDER RPDDDDRQGY DDDDRRPRWS SRPKGQGRNG VEETLCSPTL VEDIARPSRA DFYNPAAGRI
                                     506                                       542
481 SSANSLTFPI LRWFQLSAEH VLLYRNGIYS PHWNNNANSI IYGLRGEGRI QVVNSQGNAV FNGVLREGQI LLVPQNFAVG
561 KQAGNEGFEY VAFKTADRAS PATSSKCLGE SPLMFSSMLL AFEIIKSVLS NTMETRPLWS LLMIPSMGLN VVINLNHNNN
641 AQVDSKNNDG SRLWWPSSII IK
```

c Carbamidomethylation (+57.02)
d Deamidation (NQ) (+0.98)
d Diethylation (+56.06)

Supporting Peptides:

Peptide	Uniq	-10lgP	Mass	Length	ppm	m/z	z	RT	Fraction	Scan	Source File	Area Roasted in gel	#Feature	#Feature Roasted in gel	Start	End	PTM	AScore	Found By
R.IQVVNSQGNAVFNGVLR.E	Y	130.47	1813.9799	17	2.5	907.9995	2	33.04	29	F29:1457	OB5922 H2 Ro.raw	4.3436E5	6	6	530	546			PEAKS DB
R.WFQLSAEHVLLYR.N	Y	114.22	1660.8726	13	0.2	831.4437	2	34.54	34	F34:1689	OB5945 H2 Ro.raw	5.8119E5	9	9	493	505			PEAKS DB

total 15 peptides

Peptide	Uniq	-10lgP	Mass	Length	ppm	m/z	z	RT	Fraction	Scan	Source File	Area Roasted in gel	#Feature	#Feature Roasted in gel	Start	End	PTM	AScore	Found By
R.IQVVNSQGNAVFN(+.98)GVLR.E	Y	112.05	1814.9639	17	0.5	908.4896	2	32.42	33	F33:1586	OB5944 H6 Ro.raw	0	0	0	530	546	Deamidation (NQ)	N13:Deamidation (NQ):77.51	PEAKS DB
R.ISSANSLTFPILR.W	Y	100.20	1417.7928	13	0.5	709.9041	2	34.35	34	F34:1668	OB5945 H2 Ro.raw	5.7598E5	6	6	480	492			PEAKS DB
R.EGQILLVPQNFAVGK.Q	Y	83.62	1611.8984	15	1.4	806.9576	2	34.05	33	F33:1681	OB5944 H6 Ro.raw	4.8642E5	6	6	547	561			PEAKS DB
K.QAGNEGFEYVAFK.T	Y	78.82	1458.6779	13	0.7	730.3467	2	32.31	29	F29:1403	OB5922 H2 Ro.raw	2.1182E5	2	2	562	574			PEAKS DB
R.IQ(+.98)VVNSQGNAVFNGVLR.E	Y	73.16	1814.9639	17	2.4	908.4914	2	32.79	32	F32:1603	OB5943 H6 Ro.raw	0	0	0	530	546	Deamidation (NQ)	Q2:Deamidation (NQ):37.78	PEAKS DB
R.ADFYNPAAGR.I	Y	71.58	1080.4988	10	-0.6	541.2563	2	28.20	34	F34:1311	OB5945 H2 Ro.raw	3.6064E4	6	6	470	479			PEAKS DB
R.IQVVNSQGNAVFNGVLREGQILLVPQNFAVGK.Q	Y	55.28	3407.8677	32	5.1	1136.9690	3	38.18	35	F35:1888	OB5946 H2 Ro.raw	3.4122E4	1	1	530	561			PEAKS DB
R.IQVVN(+.98)SQGNAVFNGVLR.E	Y	45.93	1814.9639	17	1.6	908.4907	2	33.23	33	F33:1633	OB5944 H6 Ro.raw	0	0	0	530	546		N5:Deamidation (NQ):0.00	PEAKS DB
N.GVEETLC(+57.02)SPTLVEDIARPSR.A	Y	41.24	2228.1106	20	2.1	743.7124	3	32.69	35	F35:1580	OB5946 H2 Ro.raw	1.5861E5	2	2	450	469	Carbamidomethylation	C7:Carbamidomethylation: 1000.00	PEAKS DB
R.N(+.98)GIYSPHWNNNANSIIYGLR.G	Y	39.54	2303.1084	20	4.1	768.7132	3	34.70	33	F33:1718	OB5944 H6 Ro.raw	0	0	0	506	525	Deamidation (NQ)	N1:Deamidation (NQ):32.94	PEAKS DB
R.EGQILLVPQNFAVGK(+56.06).Q	Y	36.77	1667.9609	15	0.8	834.9884	2	34.91	29	F29:1568	OB5922 H2 Ro.raw	4.0125E5	1	1	547	561	Diethylation	K15:Diethylation:121.40	PEAKS PTM
R.N(+.98)GIYSPHWNNNAN(+.98)SIIYGLR.G	Y	29.76	2304.0923	20	8.5	769.0446	3	34.92	32	F32:1731	OB5943 H6 Ro.raw	3.732E3	1	1	506	525		N1:Deamidation (NQ):17.32;N13:Deamidation (NQ): 6.63	PEAKS DB
R.N(+.98)GIYSPHWNNNANSIIYGLRGEGR.I	Y	28.99	2702.2949	24	1.9	901.7739	3	33.89	29	F29:1505	OB5922 H2 Ro.raw	0	0	0	506	529	Deamidation (NQ)	N1:Deamidation (NQ):47.84	PEAKS DB
total 15 peptides																			

A5Z1R0|A5Z1R0_ARAHY

back to list

| Protein Coverage | Supporting Peptides |

Protein Coverage:

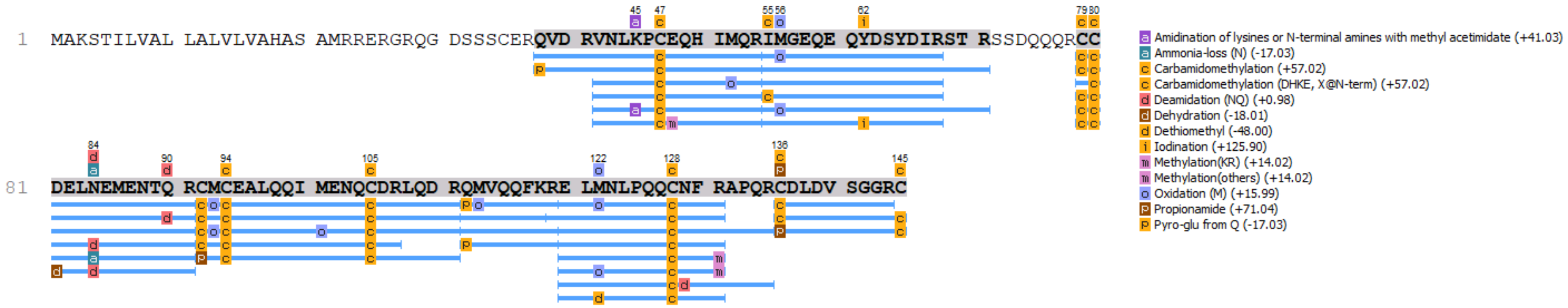

Supporting Peptides:

Peptide	Uniq	-10lgP	Mass	Length	ppm	m/z	z	RT	Fraction	Scan	Source File	Area Roasted in gel	#Feature	#Feature Roasted in gel	Start	End	PTM	AScore	Found By
R.IMGEQEQYDSYDIR.S	Y	107.91	1745.7566	14	3.7	873.8888	2	30.09	33	F33:1427	OB5944 H6 Ro.raw	1.44E7	14	14	55	68			PEAKS DB
R.IM(+15.99)GEQEQYDSYDIR.S	Y	105.99	1761.7516	14	1.3	881.8842	2	28.67	33	F33:1354	OB5944 H6 Ro.raw	2.9749E6	8	8	55	68	Oxidation (M)	M2:Oxidation (M):100 0.00	PEAKS DB
total 46 peptides																			

Peptide	Uniq	-10lgP	Mass	Length	ppm	m/z	z	RT	Fraction	Scan	Source File	Area Roasted in gel	#Feature	#Feature Roasted in gel	Start	End	PTM	AScore	Found By
R.ELMNLPQQC(+57.02)NFR.A	Y	100.23	1548.7177	12	1.9	775.3676	2	31.56	29	F29:1364	OB5922 H2 Ro.raw	8.4271E6	16	16	120	131	Carbamidomethylation	C9:Carbamidomethylation:1000.00	PEAKS DB
R.I(+57.02)MGEQEQYDSYDIR.S	Y	99.36	1802.7781	14	1.9	902.3981	2	30.45	32	F32:1466	OB5943 H6 Ro.raw	1.078E5	3	3	55	68	Carbamidomethylation (DHKE, X@N-term)	I1:Carbamidomethylation (DHKE, X@N-term):49.43	PEAKS PTM
R.C(+57.02)MC(+57.02)EALQQIMENQC(+57.02)DR.L	Y	96.38	2084.8206	16	2.3	1043.4199	2	33.49	31	F31:1648	OB5942 H6 Ro.raw	3.1431E4	2	2	92	107	Carbamidomethylation	C1:Carbamidomethylation:1000.00;C3:Carbamidomethylation:1000.00;C14:Carbamidomethylation:1000.00	PEAKS DB
R.ELM(+15.99)NLPQQC(+57.02)NFR.A	Y	91.99	1564.7126	12	-0.6	783.3632	2	28.85	33	F33:1365	OB5944 H6 Ro.raw	9.5551E5	5	5	120	131	Oxidation (M); Carbamidomethylation	M3:Oxidation (M):1000.00;C9:Carbamidomethylation:1000.00	PEAKS DB
R.C(+57.02)C(+57.02)DELNEMENTQR.C	Y	90.44	1697.6444	13	0.6	849.8300	2	27.44	31	F31:1278	OB5942 H6 Ro.raw	3.0478E5	3	3	79	91	Carbamidomethylation	C1:Carbamidomethylation:1000.00;C2:Carbamidomethylation:1000.00	PEAKS DB
R.C(+57.02)C(+57.02)DELNEMENTQ(+.98)R.C	Y	85.19	1698.6284	13	0.2	850.3217	2	28.19	31	F31:1324	OB5942 H6 Ro.raw	3.601E5	3	3	79	91	Carbamidomethylation; Deamidation (NQ)	C1:Carbamidomethylation:1000.00;C2:Carbamidomethylation:1000.00;Q12:Deamidation (NQ):60.92	PEAKS DB
R.VNLKPC(+57.02)EQHIMQR.I	Y	77.65	1651.8286	13	0.5	551.6171	3	28.40	32	F32:1356	OB5943 H6 Ro.raw	1.0332E6	5	5	42	54	Carbamidomethylation	C6:Carbamidomethylation:1000.00	PEAKS DB
R.C(+57.02)MC(+57.02)EALQQIMENQC(+57.02)DRLQDR.Q	Y	76.01	2597.0913	20	3.1	866.7070	3	33.49	31	F31:1646	OB5942 H6 Ro.raw	3.1154E6	12	12	92	111	Carbamidomethylation	C1:Carbamidomethylation:1000.00;C3:Carbamidomethylation:1000.00;C14:Carbamidomethylation:1000.00	PEAKS DB
R.Q(-17.03)VDRVNLKPC(+57.02)EQHIMQR.I	Y	74.55	2133.0571	17	1.2	534.2722	4	30.44	32	F32:1454	OB5943 H6 Ro.raw	8.5431E5	5	5	38	54	Pyro-glu from Q; Carbamidomethylation	Q1:Pyro-glu from Q:1000.00;C10:	PEAKS PTM
**total 46 peptides**																			

Peptide	Uniq	-10lgP	Mass	Length	ppm	m/z	z	RT	Fraction	Scan	Source File	Area Roasted in gel	#Feature	#Feature Roasted in gel	Start	End	PTM	AScore	Found By
																		Carbamidomethylation:1000.00	
R.QVDRVNLKPC(+57.02)EQHIMQR.I	Y	71.69	2150.0837	17	0.0	538.5282	4	29.70	32	F32:1432	OB5943 H6 Ro.raw	4.1805E5	4	4	38	54	Carbamidomethylation	C10:Carbamidomethylation:1000.00	PEAKS DB
R.C(+57.02)DLDVSGGRC(+57.02)	Y	70.42	1137.4543	10	-0.5	569.7342	2	25.28	32	F32:1158	OB5943 H6 Ro.raw	4.867E4	3	3	136	145	Carbamidomethylation	C1:Carbamidomethylation:1000.00;C10:Carbamidomethylation:1000.00	PEAKS DB
R.C(+71.04)DLDVSGGRC(+57.02)	Y	64.73	1151.4700	10	0.9	576.7428	2	25.64	32	F32:1183	OB5943 H6 Ro.raw	1.3899E4	3	3	136	145	Propionamide; Carbamidomethylation	C1:Propionamide:156.52;C10:Carbamidomethylation:1000.00	PEAKS PTM
R.C(+71.04)C(+57.02)DELNEMENTQR.C	Y	63.47	1711.6600	13	1.0	856.8381	2	27.64	31	F31:1283	OB5942 H6 Ro.raw	2.8007E5	3	3	79	91	Carbamidomethylation	C1:Propionamide:10.11; C2:Carbamidomethylation:1000.00	PEAKS PTM
R.C(+57.02)DLDVSGGR.C	Y	63.35	977.4236	9	-0.5	489.7189	2	24.23	33	F33:1121	OB5944 H6 Ro.raw	8.8623E2	1	1	136	144	Carbamidomethylation	C1:Carbamidomethylation:1000.00	PEAKS DB
R.C(+57.02)M(+15.99)C(+57.02)EALQQIMENQC(+57.02)DRLQDR.Q	Y	60.71	2613.0862	20	1.4	872.0372	3	31.19	32	F32:1576	OB5943 H6 Ro.raw	5.7636E5	5	5	92	111	Carbamidomethylation; Oxidation (M)	C1:Carbamidomethylation:1000.00;M2:Oxidation (M):89.48;C3:Carbamidomethylation:1000.00;C14:Carbamidomethylation:1000.00	PEAKS DB
R.Q(-17.03)MVQQFKR.E	Y	60.42	1046.5331	8	0.0	524.2738	2	27.84	32	F32:1322	OB5943 H6 Ro.raw	3.5192E5	3	3	112	119	Pyro-glu from Q	Q1:Pyro-glu from Q:1000.00	PEAKS PTM
R.C(+71.04)MC(+57.02)EALQQIMENQC(+57.02)DRLQDR.Q	Y	60.30	2611.1069	20	1.1	871.3772	3	33.49	31	F31:1647	OB5942 H6 Ro.raw	6.2487E5	2	2	92	111	Propionamide; Carbamidomethylation	C1:Propionamide:20.41; C3:Carbamidomethylation:1000.00;C14:Carbamidomethylation:1000.00	PEAKS PTM
R.C(+57.02)(+28.03)C(+57.02)DELNEMENTQR.C	Y	57.63	1725.6757	13	1.0	863.8460	2	28.03	32	F32:1316	OB5943 H6 Ro.raw	1.6949E4	1	1	79	91	Carbamidomethylation	C1:Carbamidomethylation:1000.00;C1:Eth	PEAKS PTM
**total 46 peptides**																			

Peptide	Uniq	-10lgP	Mass	Length	ppm	m/z	z	RT	Fraction	Scan	Source File	Area Roasted in gel	#Feature	#Feature Roasted in gel	Start	End	PTM	AScore	Found By
																		ylation:18.53;C2:Carbamidomethylation:1000.00	
R.ELMNLPQQC(+57.02)NFRAPQR.C	Y	56.72	2000.9673	16	0.5	667.9967	3	30.28	35	F35:1430	OB5946H2 Ro.raw	1.0432E5	5	5	120	135	Carbamidomethylation	C9:Carbamidomethylation:1000.00	PEAKS DB
R.ELMNLPQQC(+57.02)NF(+31.99)R.A	Y	49.69	1580.7075	12	-9.5	791.3535	2	31.67	31	F31:1538	OB5942H6 Ro.raw	0	0	0	120	131	Carbamidomethylation	C9:Carbamidomethylation:1000.00;F11:Dihydroxy:0.00	PEAKS PTM
R.ELMNLPQQC(+57.02)NFR(+14.02).A	Y	49.60	1562.7334	12	0.3	782.3742	2	31.27	33	F33:1519	OB5944H6 Ro.raw	2.0625E5	3	3	120	131	Carbamidomethylation; Methylation(KR)	C9:Carbamidomethylation:1000.00;R12:Methylation(KR):1000.00	PEAKS PTM
R.C(+57.02)C(+57.02)DELN(+.98)EMENTQR.C	Y	49.50	1698.6284	13	-1.6	850.3201	2	29.09	31	F31:1387	OB5942H6 Ro.raw	0	0	0	79	91	Carbamidomethylation; Deamidation (NQ)	C1:Carbamidomethylation:1000.00;C2:Carbamidomethylation:1000.00;N6:Deamidation (NQ):31.56	PEAKS DB
R.IM(+15.99)GE(+57.02)QEQYDSYDIR.S	Y	48.79	1818.7729	14	0.3	910.3940	2	29.25	32	F32:1394	OB5943H6 Ro.raw	2.5217E3	1	1	55	68	Oxidation (M)	M2:Oxidation (M):1000.00;E4:Carbamidomethylation (DHKE, X@N-term):0.00	PEAKS PTM
R.ELM(-48.00)NLPQQC(+57.02)NFR.A	Y	48.30	1500.7144	12	-0.1	501.2454	3	27.84	32	F32:1311	OB5943H6 Ro.raw	2.9967E5	2	2	120	131	Dethiomethyl; Carbamidomethylation	M3:Dethiomethyl:1000.00;C9:Carbamidomethylation:1000.00	PEAKS PTM
R.QMVQQFK.R	Y	48.24	907.4586	7	-0.6	454.7363	2	25.61	33	F33:1183	OB5944H6 Ro.raw	8.0264E3	2	2	112	118			PEAKS DB
R.ELMNLPQQC(+57.02)N(+.98)FRAPQR.C	Y	47.04	2001.9513	16	1.0	668.3250	3	30.88	32	F32:1490	OB5943H6 Ro.raw	0	0	0	120	135	Carbamidomethylation; Deamidation (NQ)	C9:Carbamidomethylation:1000.00;N10:Deamidation (NQ):24.24	PEAKS DB
R.C(+57.02)C(+57.02)DELN(-17.03)EMENTQR.C	Y	44.87	1680.6178	13	1.0	841.3170	2	32.29	32	F32:1575	OB5943H6 Ro.raw	3.9042E4	2	2	79	91	Carbamidomethylation; Ammonia-loss (N)	C1:Carbamidomethylation:1000.00;C2:Carbamidomethylation:1000.00;N6:Am	PEAKS PTM
**total 46 peptides**																			

Peptide	Uniq	-10lgP	Mass	Length	ppm	m/z	z	RT	Fraction	Scan	Source File	Area Roasted in gel	#Feature	#Feature Roasted in gel	Start	End	PTM	AScore	Found By
																		monia-loss (N):115.18	
R.IMGEQEQYDSYDIRSTR.S	Y	44.57	2089.9375	17	2.0	697.6545	3	28.94	31	F31:1381	OB5942H6Ro.raw	4.9618E4	2	2	55	71			PEAKS DB
R.C(+57.02)M(+15.99)C(+57.02)EALQQIM(+15.99)ENQC(+57.02)DRLQDR.Q	Y	43.42	2629.0811	20	2.0	877.3694	3	29.88	32	F32:1425	OB5943H6Ro.raw	7.9642E3	1	1	92	111	Carbamidomethylation; Oxidation (M)	C1:Carbamidomethylation:1000.00;M2:Oxidation (M):1000.00;C3:Carbamidomethylation:1000.00;M10:Oxidation (M):1000.00;C14:Carbamidomethylation:1000.00	PEAKS DB
R.VNLKPC(+57.02)EQHIM(+15.99)QR.I	Y	43.11	1667.8236	13	0.6	556.9488	3	26.91	31	F31:1258	OB5942H6Ro.raw	2.1143E4	1	1	42	54	Carbamidomethylation; Oxidation (M)	C6:Carbamidomethylation:1000.00;M11:Oxidation (M):1000.00	PEAKS DB
K.RELMNLPQQC(+57.02)NFR.A	Y	42.73	1704.8188	13	0.8	569.2807	3	29.89	31	F31:1434	OB5942H6Ro.raw	0	0	0	119	131	Carbamidomethylation	C10:Carbamidomethylation:1000.00	PEAKS DB
R.VNLK(+41.03)PC(+57.02)EQHIMQR.I	Y	39.06	1692.8552	13	3.3	565.2942	3	29.51	32	F32:1408	OB5943H6Ro.raw	4.1722E3	1	1	42	54	Amidination of lysines or N-terminal amines with methyl acetimidate; Carbamidomethylation	K4:Amidination of lysines or N-terminal amines with methyl acetimidate:38.16;C6:Carbamidomethylation:1000.00	PEAKS PTM
R.IMGE(+17.03)QEQYDSYDIR.S	Y	37.70	1762.7832	14	-4.0	882.3953	2	30.83	31	F31:1488	OB5942H6Ro.raw	0	0	0	55	68		E4:Replacement of proton with ammonium ion:18.53	PEAKS PTM
R.IMGEQEQY(-18.01)DSYDIR.S	Y	36.74	1727.7461	14	3.4	864.8833	2	30.78	32	F32:1484	OB5943H6Ro.raw	0	0	0	55	68		Y8:Dehydration:0.00	PEAKS PTM
R.IM(+15.99)GEQEQYDSYDIRSTR.S	Y	35.70	2105.9324	17	-1.9	702.9834	3	27.45	31	F31:1288	OB5942H6Ro.raw	2.3858E3	1	1	55	71	Oxidation (M)	M2:Oxidation (M):1000.00	PEAKS DB
R.ELM(+15.99)NLPQQC(+57.02)NFR(+14.02).A	Y	35.08	1578.7283	12	1.8	790.3729	2	29.10	33	F33:1390	OB5944H6Ro.raw	0	0	0	120	131	Oxidation (M); Carbamidomethylation; Methylation(KR)	M3:Oxidation (M):1000.00;C9:Carbamidomethylation:1000.00;R12:Methyl	PEAKS PTM

total 46 peptides

Peptide	Uniq	-10lgP	Mass	Length	ppm	m/z	z	RT	Fraction	Scan	Source File	Area Roasted in gel	#Feature	#Feature Roasted in gel	Start	End	PTM	AScore	Found By
																		ation(KR):1000.00	
R.QMVQQFKR.E	Y	34.38	1063.5597	8	0.9	532.7876	2	26.84	32	F32:1239	OB5943H6Ro.raw	1.9987E4	2	2	112	119			PEAKS DB
R.C(+57.02)C(+57.02)D(-18.01)ELN(+.98)EMENTQR.C	Y	33.99	1680.6178	13	1.7	841.3176	2	31.98	31	F31:1599	OB5942H6Ro.raw	2.4089E4	1	1	79	91	Carbamidomethylation; Dehydration; Deamidation (NQ)	C1:Carbamidomethylation:1000.00;C2:Carbamidomethylation:1000.00;D3:Dehydration:62.90;N6:Deamidation (NQ):83.17	PEAKS PTM
R.I(+57.02)M(+15.99)GEQEQYDSYDIR.S	Y	33.09	1818.7729	14	0.8	910.3945	2	28.84	31	F31:1372	OB5942H6Ro.raw	0	0	0	55	68	Oxidation (M)	I1:Carbamidomethylation (DHKE, X@N-term):17.32;M2:Oxidation (M):1000.00	PEAKS PTM
R.IMGEQE(+53.92)QYDSYDIR.S	Y	32.96	1799.6759	14	-2.3	600.8979	3	30.20	32	F32:1450	OB5943H6Ro.raw	0	0	0	55	68		E6:Replacement of 2 protons by iron:14.63	PEAKS PTM
R.C(+57.02)MC(+57.02)EALQQ(+.98)IMENQC(+57.02)DRLQDR.Q	Y	32.01	2598.0754	20	-6.2	867.0270	3	33.99	36	F36:1638	OB5948H3BRo.raw	0	0	0	92	111	Carbamidomethylation	C1:Carbamidomethylation:1000.00;C3:Carbamidomethylation:1000.00;Q8:Deamidation (NQ):0.00;C14:Carbamidomethylation:1000.00	PEAKS DB
R.VNLKPC(+57.02)E(+14.02)QHIMQR.I	Y	31.29	1665.8444	13	-0.4	556.2885	3	28.77	32	F32:1372	OB5943H6Ro.raw	2.0373E4	1	1	42	54	Carbamidomethylation; Methylation(others)	C6:Carbamidomethylation:1000.00;E7:Methylation(others):55.92	PEAKS PTM
R.Q(-17.03)M(+15.99)VQQFKR.E	Y	31.12	1062.5281	8	3.0	532.2729	2	25.67	32	F32:1189	OB5943H6Ro.raw	0	0	0	112	119	Pyro-glu from Q; Oxidation (M)	Q1:Pyro-glu from Q:1000.00;M2:Oxidation (M):1000.00	PEAKS PTM
R.IMGEQEQY(+125.90)DSYDIR.S	Y	29.13	1871.6533	14	2.0	936.8358	2	32.29	32	F32:1560	OB5943H6Ro.raw	6.88E3	1	1	55	68	Iodination	Y8:Iodination:41.59	PEAKS PTM
**total 46 peptides**																			

sp|Q647G9|CONG_ARAHY

back to list

| Protein Coverage | Supporting Peptides |

Protein Coverage:

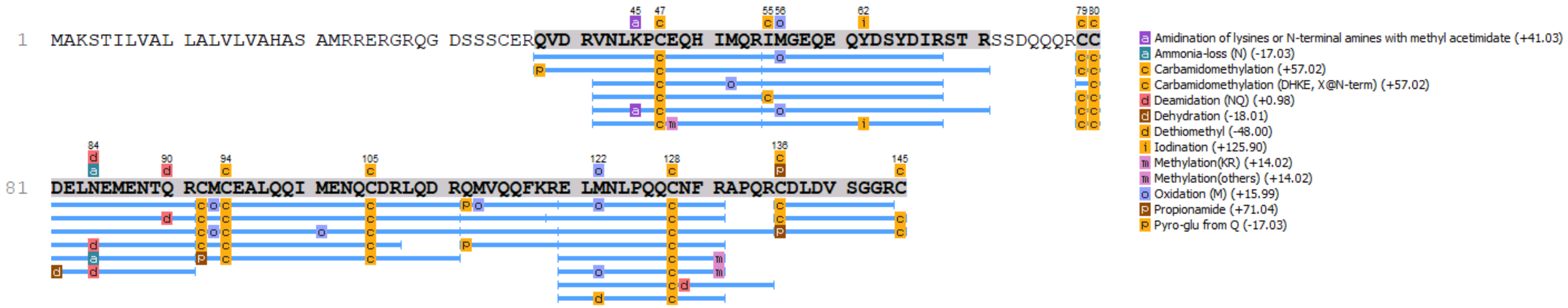

Supporting Peptides:

Peptide	Uniq	-10lgP	Mass	Length	ppm	m/z	z	RT	Fraction	Scan	Source File	Area Roasted in gel	#Feature	#Feature Roasted in gel	Start	End	PTM	AScore	Found By
R.IMGEQEQYDSYDIR.S	Y	107.91	1745.7566	14	3.7	873.8888	2	30.09	33	F33:1427	OB5944 H6 Ro.raw	1.44E7	14	14	55	68			PEAKS DB
R.IM(+15.99)GEQEQYDSYDIR.S	Y	105.99	1761.7516	14	1.3	881.8842	2	28.67	33	F33:1354	OB5944 H6 Ro.raw	2.9749E6	8	8	55	68	Oxidation (M)	M2:Oxidation (M):1000.00	PEAKS DB
R.ELMNLPQQC(+57.02)NFR.A	Y	100.23	1548.7177	12	1.9	775.3676	2	31.56	29	F29:1364	OB5922 H2 Ro.raw	8.4271E6	16	16	120	131	Carbamidomethylation	C9:Carbamidomethylation:1000.00	PEAKS DB
R.I(+57.02)MGEQEQYDSYDIR.S	Y	99.36	1802.7781	14	1.9	902.3981	2	30.45	32	F32:1466	OB5943 H6 Ro.raw	1.078E5	3	3	55	68	Carbamidomethylation (DHKE, X@N-term)	I1:Carbamidomethylation (DHKE, X@N-term):49.43	PEAKS PTM
R.C(+57.02)MC(+57.02)EALQQIMENQC(+57.02)DR.L	Y	96.38	2084.8206	16	2.3	1043.4199	2	33.49	31	F31:1648	OB5942 H6 Ro.raw	3.1431E4	2	2	92	107	Carbamidomethylation	C1:Carbamidomethylation:1000.00;C3:Carbamidomethylation:1000.00;C14:Carbamidomethylation:1000.00	PEAKS DB
R.ELM(+15.99)NLPQQC(+57.02)NFR.A	Y	91.99	1564.7126	12	-0.6	783.3632	2	28.85	33	F33:1365	OB5944 H6 Ro.raw	9.5551E5	5	5	120	131	Oxidation (M); Carbamidomethylation	M3:Oxidation (M):1000.00; C9:Carbamidomethylation:1000.00	PEAKS DB
R.C(+57.02)C(+57.02)DELNEMENTQR.C	Y	90.44	1697.6444	13	0.6	849.8300	2	27.44	31	F31:1278	OB5942 H6 Ro.raw	3.0478E5	3	3	79	91	Carbamidomethylation	C1:Carbamidomethylation:1000.00;C2:C	PEAKS DB

total 46 peptides

Peptide	Uniq	-10lgP	Mass	Length	ppm	m/z	z	RT	Fraction	Scan	Source File	Area Roasted in gel	#Feature	#Feature Roasted in gel	Start	End	PTM	AScore	Found By
																		arbamidomethylation:1000.00	
R.C(+57.02)C(+57.02)DELNEMENTQ(+.98)R.C	Y	85.19	1698.6284	13	0.2	850.3217	2	28.19	31	F31:1324	OB5942H6Ro.raw	3.601E5	3	3	79	91	Carbamidomethylation; Deamidation (NQ)	C1:Carbamidomethylation:1000.00;C2:Carbamidomethylation:1000.00;Q12:Deamidation (NQ):60.92	PEAKS DB
R.VNLKPC(+57.02)EQHIMQR.I	Y	77.65	1651.8286	13	0.5	551.6171	3	28.40	32	F32:1356	OB5943H6Ro.raw	1.0332E6	5	5	42	54	Carbamidomethylation	C6:Carbamidomethylation:1000.00	PEAKS DB
R.C(+57.02)MC(+57.02)EALQQIMENQC(+57.02)DRLQDR.Q	Y	76.01	2597.0913	20	3.1	866.7070	3	33.49	31	F31:1646	OB5942H6Ro.raw	3.1154E6	12	12	92	111	Carbamidomethylation	C1:Carbamidomethylation:1000.00;C3:Carbamidomethylation:1000.00;C14:Carbamidomethylation:1000.00	PEAKS DB
R.Q(-17.03)VDRVNLKPC(+57.02)EQHIMQR.I	Y	74.55	2133.0571	17	1.2	534.2722	4	30.44	32	F32:1454	OB5943H6Ro.raw	8.5431E5	5	5	38	54	Pyro-glu from Q; Carbamidomethylation	Q1:Pyro-glu from Q:1000.00;C10:Carbamidomethylation:1000.00	PEAKS PTM
R.QVDRVNLKPC(+57.02)EQHIMQR.I	Y	71.69	2150.0837	17	0.0	538.5282	4	29.70	32	F32:1432	OB5943H6Ro.raw	4.1805E5	4	4	38	54	Carbamidomethylation	C10:Carbamidomethylation:1000.00	PEAKS DB
R.C(+57.02)DLDVSGGRC(+57.02)	Y	70.42	1137.4543	10	-0.5	569.7342	2	25.28	32	F32:1158	OB5943H6Ro.raw	4.867E4	3	3	136	145	Carbamidomethylation	C1:Carbamidomethylation:1000.00;C10:Carbamidomethylation:1000.00	PEAKS DB
R.C(+71.04)DLDVSGGRC(+57.02)	Y	64.73	1151.4700	10	0.9	576.7428	2	25.64	32	F32:1183	OB5943H6Ro.raw	1.3899E4	3	3	136	145	Propionamide; Carbamidomethylation	C1:Propionamide:156.52;C10:Carbamidomethylation:1000.00	PEAKS PTM

total 46 peptides

Peptide	Uniq	-10lgP	Mass	Length	ppm	m/z	z	RT	Fraction	Scan	Source File	Area Roasted in gel	#Feature	#Feature Roasted in gel	Start	End	PTM	AScore	Found By
R.C(+71.04)C(+57.02)DELNEMENTQR.C	Y	63.47	1711.6600	13	1.0	856.8381	2	27.64	31	F31:1283	OB5942 H6 Ro.raw	2.8007E5	3	3	79	91	Carbamidomethylation	C1:Propionamide:10.11;C2:Carbamidomethylation:1000.00	PEAKS PTM
R.C(+57.02)DLDVSGGR.C	Y	63.35	977.4236	9	-0.5	489.7189	2	24.23	33	F33:1121	OB5944 H6 Ro.raw	8.8623E2	1	1	136	144	Carbamidomethylation	C1:Carbamidomethylation:1000.00	PEAKS DB
R.C(+57.02)M(+15.99)C(+57.02)EALQQIMENQC(+57.02)DRLQDR.Q	Y	60.71	2613.0862	20	1.4	872.0372	3	31.19	32	F32:1576	OB5943 H6 Ro.raw	5.7636E5	5	5	92	111	Carbamidomethylation; Oxidation (M)	C1:Carbamidomethylation:1000.00;M2:Oxidation (M):89.48;C3:Carbamidomethylation:1000.00;C14:Carbamidomethylation:1000.00	PEAKS DB
R.Q(-17.03)MVQQFKR.E	Y	60.42	1046.5331	8	0.0	524.2738	2	27.84	32	F32:1322	OB5943 H6 Ro.raw	3.5192E5	3	3	112	119	Pyro-glu from Q	Q1:Pyro-glu from Q:1000.00	PEAKS PTM
R.C(+71.04)MC(+57.02)EALQQIMENQC(+57.02)DRLQDR.Q	Y	60.30	2611.1069	20	1.1	871.3772	3	33.49	31	F31:1647	OB5942 H6 Ro.raw	6.2487E5	2	2	92	111	Propionamide; Carbamidomethylation	C1:Propionamide:20.41;C3:Carbamidomethylation:1000.00;C14:Carbamidomethylation:1000.00	PEAKS PTM
R.C(+57.02)(+28.03)C(+57.02)DELNEMENTQR.C	Y	57.63	1725.6757	13	1.0	863.8460	2	28.03	32	F32:1316	OB5943 H6 Ro.raw	1.6949E4	1	1	79	91	Carbamidomethylation	C1:Carbamidomethylation:1000.00;C1:Ethylation:18.53;C2:Carbamidomethylation:1000.00	PEAKS PTM
R.ELMNLPQQC(+57.02)NFRAPQR.C	Y	56.72	2000.9673	16	0.5	667.9967	3	30.28	35	F35:1430	OB5946 H2 Ro.raw	1.0432E5	5	5	120	135	Carbamidomethylation	C9:Carbamidomethylation:1000.00	PEAKS DB
R.ELMNLPQQC(+57.02)NF(+31.99)R.A	Y	49.69	1580.7075	12	-9.5	791.3535	2	31.67	31	F31:1538	OB5942 H6 Ro.raw	0	0	0	120	131	Carbamidomethylation	C9:Carbamidomethylation:1000.00;F11:	PEAKS PTM

total 46 peptides

Peptide	Uniq	-10lgP	Mass	Length	ppm	m/z	z	RT	Fraction	Scan	Source File	Area Roasted in gel	#Feature	#Feature Roasted in gel	Start	End	PTM	AScore	Found By
																		Dihydroxy:0.00	
R.ELMNLPQQC(+57.02)NFR(+14.02).A	Y	49.60	1562.7334	12	0.3	782.3742	2	31.27	33	F33:1519	OB5944 H6 Ro.raw	2.0625E5	3	3	120	131	Carbamidomethylation; Methylation(KR)	C9:Carbamidomethylation:1000.00;R12:Methylation(KR):1000.00	PEAKS PTM
R.C(+57.02)C(+57.02)DELN(+.98)EMENTQR.C	Y	49.50	1698.6284	13	-1.6	850.3201	2	29.09	31	F31:1387	OB5942 H6 Ro.raw	0	0	0	79	91	Carbamidomethylation; Deamidation (NQ)	C1:Carbamidomethylation:1000.00;C2:Carbamidomethylation:1000.00;N6:Deamidation (NQ):31.56	PEAKS DB
R.IM(+15.99)GE(+57.02)QEQYDSYDIR.S	Y	48.79	1818.7729	14	0.3	910.3940	2	29.25	32	F32:1394	OB5943 H6 Ro.raw	2.5217E3	1	1	55	68	Oxidation (M)	M2:Oxidation (M):1000.00;E4:Carbamidomethylation (DHKE, X@N-term):0.00	PEAKS PTM
R.ELM(-48.00)NLPQQC(+57.02)NFR.A	Y	48.30	1500.7144	12	-0.1	501.2454	3	27.84	32	F32:1311	OB5943 H6 Ro.raw	2.9967E5	2	2	120	131	Dethiomethyl; Carbamidomethylation	M3:Dethiomethyl:1000.00;C9:Carbamidomethylation:1000.00	PEAKS PTM
R.QMVQQFK.R	Y	48.24	907.4586	7	-0.6	454.7363	2	25.61	33	F33:1183	OB5944 H6 Ro.raw	8.0264E3	2	2	112	118			PEAKS DB
R.ELMNLPQQC(+57.02)N(+.98)FRAPQR.C	Y	47.04	2001.9513	16	1.0	668.3250	3	30.88	32	F32:1490	OB5943 H6 Ro.raw	0	0	0	120	135	Carbamidomethylation; Deamidation (NQ)	C9:Carbamidomethylation:1000.00;N10:Deamidation (NQ):24.24	PEAKS DB
R.C(+57.02)C(+57.02)DELN(-17.03)EMENTQR.C	Y	44.87	1680.6178	13	1.0	841.3170	2	32.29	32	F32:1575	OB5943 H6 Ro.raw	3.9042E4	2	2	79	91	Carbamidomethylation; Ammonia-loss (N)	C1:Carbamidomethylation:1000.00;C2:Carbamidomethylation:1000.00;N6:Ammonia-loss (N):115.18	PEAKS PTM

total 46 peptides

Peptide	Uniq	-10lgP	Mass	Length	ppm	m/z	z	RT	Fraction	Scan	Source File	Area Roasted in gel	#Feature	#Feature Roasted in gel	Start	End	PTM	AScore	Found By
R.IMGEQEQYDSYDIRSTR.S	Y	44.57	2089.9375	17	2.0	697.6545	3	28.94	31	F31:1381	OB5942 H6 Ro.raw	4.9618E4	2	2	55	71			PEAKS DB
R.C(+57.02)M(+15.99)C(+57.02)EALQQIM(+15.99)ENQC(+57.02)DRLQDR.Q	Y	43.42	2629.0811	20	2.0	877.3694	3	29.88	32	F32:1425	OB5943 H6 Ro.raw	7.9642E3	1	1	92	111	Carbamidomethylation; Oxidation (M)	C1:Carbamidomethylation:1000.00;M2:Oxidation (M):1000.00;C3:Carbamidomethylation:1000.00;M10:Oxidation (M):1000.00;C14:Carbamidomethylation:1000.00	PEAKS DB
R.VNLKPC(+57.02)EQHIM(+15.99)QR.I	Y	43.11	1667.8236	13	0.6	556.9488	3	26.91	31	F31:1258	OB5942 H6 Ro.raw	2.1143E4	1	1	42	54	Carbamidomethylation; Oxidation (M)	C6:Carbamidomethylation:1000.00;M11:Oxidation (M):1000.00	PEAKS DB
K.RELMNLPQQC(+57.02)NFR.A	Y	42.73	1704.8188	13	0.8	569.2807	3	29.89	31	F31:1434	OB5942 H6 Ro.raw	0	0	0	119	131	Carbamidomethylation	C10:Carbamidomethylation:1000.00	PEAKS DB
R.VNLK(+41.03)PC(+57.02)EQHIMQR.I	Y	39.06	1692.8552	13	3.3	565.2942	3	29.51	32	F32:1408	OB5943 H6 Ro.raw	4.1722E3	1	1	42	54	Amidination of lysines or N-terminal amines with methyl acetimidate; Carbamidomethylation	K4:Amidination of lysines or N-terminal amines with methyl acetimidate:38.16;C6:Carbamidomethylation:1000.00	PEAKS PTM
R.IMGE(+17.03)QEQYDSYDIR.S	Y	37.70	1762.7832	14	-4.0	882.3953	2	30.83	31	F31:1488	OB5942 H6 Ro.raw	0	0	0	55	68		E4:Replacement of proton with ammonium ion:18.53	PEAKS PTM
R.IMGEQEQY(-18.01)DSYDIR.S	Y	36.74	1727.7461	14	3.4	864.8833	2	30.78	32	F32:1484	OB5943 H6 Ro.raw	0	0	0	55	68		Y8:Dehydration:0.00	PEAKS PTM
R.IM(+15.99)GEQEQYDSYDIRSTR.S	Y	35.70	2105.9324	17	-1.9	702.9834	3	27.45	31	F31:1288	OB5942 H6 Ro.raw	2.3858E3	1	1	55	71	Oxidation (M)	M2:Oxidation (M):1000.00	PEAKS DB
R.ELM(+15.99)NLPQQC(+57.02)NFR(+14.02).A	Y	35.08	1578.7283	12	1.8	790.3729	2	29.10	33	F33:1390	OB5944 H6 Ro.raw	0	0	0	120	131	Oxidation (M); Carbamidomethylation; Methylation(KR)	M3:Oxidation (M):10	PEAKS PTM
**total 46 peptides**																			

Peptide	Uniq	-10lgP	Mass	Length	ppm	m/z	z	RT	Fraction	Scan	Source File	Area Roasted in gel	#Feature	#Feature Roasted in gel	Start	End	PTM	AScore	Found By
																		00.00;C9:Carbamidomethylation:1000.00;R12:Methylation(KR):1000.00	
R.QMVQQFKR.E	Y	34.38	1063.5597	8	0.9	532.7876	2	26.84	32	F32:1239	OB5943H6Ro.raw	1.9987E4	2	2	112	119			PEAKS DB
R.C(+57.02)C(+57.02)D(-18.01)ELN(+.98)EMENTQR.C	Y	33.99	1680.6178	13	1.7	841.3176	2	31.98	31	F31:1599	OB5942H6Ro.raw	2.4089E4	1	1	79	91	Carbamidomethylation; Dehydration; Deamidation (NQ)	C1:Carbamidomethylation:1000.00;C2:Carbamidomethylation:1000.00;D3:Dehydration:62.90;N6:Deamidation(NQ):83.17	PEAKS PTM
R.I(+57.02)M(+15.99)GEQEQYDSYDIR.S	Y	33.09	1818.7729	14	0.8	910.3945	2	28.84	31	F31:1372	OB5942H6Ro.raw	0	0	0	55	68	Oxidation (M)	I1:Carbamidomethylation(DHKE,X@N-term):17.32;M2:Oxidation(M):1000.00	PEAKS PTM
R.IMGEQE(+53.92)QYDSYDIR.S	Y	32.96	1799.6759	14	-2.3	600.8979	3	30.20	32	F32:1450	OB5943H6Ro.raw	0	0	0	55	68		E6:Replacement of 2 protons by iron:14.63	PEAKS PTM
R.C(+57.02)MC(+57.02)EALQQ(+.98)IMENQC(+57.02)DRLQDR.Q	Y	32.01	2598.0754	20	-6.2	867.0270	3	33.99	36	F36:1638	OB5948H3BRo.raw	0	0	0	92	111	Carbamidomethylation	C1:Carbamidomethylation:1000.00;C3:Carbamidomethylation:1000.00;Q8:Deamidation (NQ):0.00;C14:Carbamidomethylation:1000.00	PEAKS DB
R.VNLKPC(+57.02)E(+14.02)QHIMQR.I	Y	31.29	1665.8444	13	-0.4	556.2885	3	28.77	32	F32:1372	OB5943H6Ro.raw	2.0373E4	1	1	42	54	Carbamidomethylation; Methylation(others)	C6:Carbamidomethylation:1000.00;E7:M	PEAKS PTM
**total 46 peptides**																			

Peptide	Uniq	-10lgP	Mass	Length	ppm	m/z	z	RT	Fraction	Scan	Source File	Area Roasted in gel	#Feature	#Feature Roasted in gel	Start	End	PTM	AScore	Found By
																		ethylation(others):55.92	
R.Q(-17.03)M(+15.99)VQQFKR.E	Y	31.12	1062.5281	8	3.0	532.2729	2	25.67	32	F32:1189	OB5943 H6 Ro.raw	0	0	0	112	119	Pyro-glu from Q; Oxidation (M)	Q1:Pyro-glu from Q:1000.00;M2:Oxidation (M):1000.00	PEAKS PTM
R.IMGEQEQY(+125.90)DSYDIR.S	Y	29.13	1871.6533	14	2.0	936.8358	2	32.29	32	F32:1560	OB5943 H6 Ro.raw	6.88E3	1	1	55	68	Iodination	Y8:Iodination: 41.59	PEAKS PTM
**total 46 peptides**																			

A1E2B1|A1E2B1_ARAHY

back to list

| Protein Coverage | Supporting Peptides |

Protein Coverage:

1 MSSSLLFLTL AYLVLIERLT CHAQSHGDRI GECRLEHLSV MEPTKRVESE GGVAEFWDDK SQQLQCIGVT LIRYTIRPKG

81 LLLPFYTNAP RIHYILQGKG VMEIVVTGCR AMYRSSTKRG MMSSYSDEHQ KIQSIEQNDA VAVPSSSVHW IYNTGHSDLV

161 LFSLVDVANA DNQLDPTFRN FLLSGNGNGK EGEESNNNWF IKKKQREAQE GNVFSGLALE TLIGSFNVQR EIAEKVQGLK

241 DWRGSIILVK EGLDWLSPKE EEEEGLEKR KKVMLMG

b Benzyl isothiocyanate (+149.03)
c Carbamidomethylation (+57.02)
c Carbamidomethylation (DHKE, X@N-term) (+57.02)
d Deamidation (NQ) (+0.98)

Supporting Peptides:

Peptide	Uniq	-10lgP	Mass	Length	ppm	m/z	z	RT	Fraction	Scan	Source File	Area Roasted in gel	#Feature	#Feature Roasted in gel	Start	End	PTM	AScore	Found By
K.GLLLPFYTNAPR.I	Y	109.84	1360.7502	12	2.1	681.3839	2	35.28	34	F34:1731	OB5945 H2 Ro.raw	8.7006E5	3	3	80	91			PEAKS DB
K.GVMEIVVTGC(+57.02)R.A	Y	101.91	1219.6053	11	0.3	610.8101	2	31.33	34	F34:1490	OB5945 H2 Ro.raw	1.3757E5	2	2	100	110	Carbamidomethylation	C10:Carbamidomethylation:1000.00	PEAKS DB
R.VESEGGVAEFWDDKSQQLQC(+57.02)IGVTLIR.Y	Y	79.49	3063.4971	27	2.5	1022.1755	3	37.32	29	F29:1703	OB5922 H2 Ro.raw	5.5365E5	3	3	47	73	Carbamidomethylation	C20:Carbamidomethylation:1000.00	PEAKS DB
R.EAQEGNVFSGLALETLIGSFNVQR.E	Y	78.67	2578.3027	24	3.8	1290.1636	2	40.50	36	F36:2018	OB5948 H3B Ro.raw	1.1946E5	3	3	207	230			PEAKS DB
Y.TIRPK(+149.03)GLLLPFYTNAPR.I	Y	42.74	2105.1606	17	-7.1	702.7225	3	34.93	34	F34:1700	OB5945 H2 Ro.raw	5.1304E4	1	1	75	91	Benzyl isothiocyanate	K5:Benzyl isothiocyanate:22.37	PEAKS PTM
K.G(+57.02)LLLPFYTNAPR.I	Y	42.29	1417.7717	12	3.7	709.8958	2	35.99	29	F29:1630	OB5922 H2 Ro.raw	0	0	0	80	91	Carbamidomethylation (DHKE, X@N-term)	G1:Carbamidomethylation (DHKE, X@N-term):10	PEAKS PTM
**total 9 peptides**																			

Peptide	Uniq	-10lgP	Mass	Length	ppm	m/z	z	RT	Fraction	Scan	Source File	Area Roasted in gel	#Feature	#Feature Roasted in gel	Start	End	PTM	AScore	Found By
																		00.00	
R.NFLLSGN(+.98)GNGKEGEESN.N	Y	41.96	1765.7754	17	2.2	883.8969	2	30.89	29	F29:1327	OB5922 H2 Ro.raw	0	0	0	180	196		N7:Deamidation (NQ):18.53	PEAKS DB
R.IGEC(+57.02)RLEHLSVMEPTK.R	Y	40.45	1897.9390	16	1.1	633.6543	3	31.15	34	F34:1494	OB5945 H2 Ro.raw	1.9364E4	1	1	30	45	Carbamidomethylation	C4:Carbamidomethylation:1000.00	PEAKS DB
K.IQSIEQNDAVAVPSSSVHWIYNTGHSDLVLFSLVDVAN(+.98)ADNQLDPTFR.N	Y	30.21	5282.5796	48	8.2	1321.6630	4	39.07	34	F34:1954	OB5945 H2 Ro.raw	4.1788E4	1	1	132	179	Deamidation (NQ)	N38:Deamidation (NQ):22.45	PEAKS DB

total 9 peptides

sp|Q6PSU2|CONG7_ARAHY

back to list

| Protein Coverage | Supporting Peptides |

Protein Coverage:

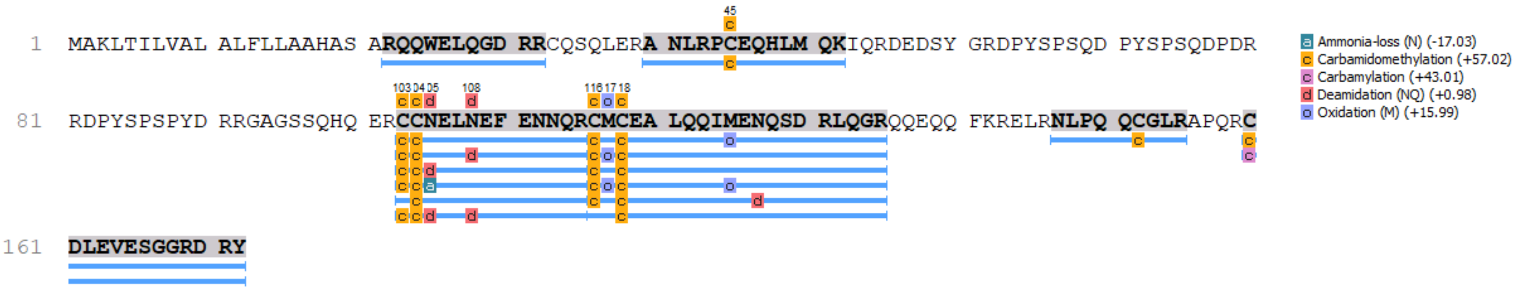

Supporting Peptides:

Peptide	Uniq	-10lgP	Mass	Length	ppm	m/z	z	RT	Fraction	Scan	Source File	Area Roasted in gel	#Feature	#Feature Roasted in gel	Start	End	PTM	AScore	Found By
R.C(+57.02)MC(+57.02)EALQQIMENQSDRLQGR.Q	Y	95.41	2466.0872	20	1.5	823.0375	3	33.97	29	F29:1513	OB5922 H2 Ro.raw	3.4972E6	10	10	116	135	Carbamidomethylation	C1:Carbamidomethylation:1000.00;C3:Carbamidomethylation:1000.00	PEAKS DB
R.C(+57.02)C(+57.02)NELNEFENNQR.C	Y	83.07	1725.6835	13	0.3	863.8493	2	28.42	31	F31:1347	OB5942 H6 Ro.raw	1.2521E5	6	6	103	115	Carbamidomethylation	C1:Carbamidomethylation:1000.00;C2:Carbamidomethylation:1000.00	PEAKS DB
R.C(+71.04)C(+57.02)NELNEFENNQR.C	Y	73.23	1739.6992	13	1.9	870.8585	2	28.96	32	F32:1370	OB5943 H6 Ro.raw	6.1256E4	7	7	103	115	Carbamidomethylation	C1:Propionamide:17.01;C2:Carbamidomethylation:100	PEAKS PTM

total 18 peptides

Peptide	Uniq	-10lgP	Mass	Length	ppm	m/z	z	RT	Fraction	Scan	Source File	Area Roasted in gel	#Feature	#Feature Roasted in gel	Start	End	PTM	AScore	Found By
																		0.00	
R.C(+57.02)M(+15.99)C(+57.02)EALQQIMENQSDRLQGR.Q	Y	70.68	2482.0820	20	2.7	828.3702	3	32.38	29	F29:1416	OB5922 H2 Ro.raw	6.6021E5	6	6	116	135	Carbamidomethylation; Oxidation (M)	C1:Carbamidomethylation:1000.00;M2:Oxidation (M):113.22; C3:Carbamidomethylation:1000.00	PEAKS DB
R.ANLRPC(+57.02)EQHLMQK.I	Y	69.33	1623.7974	13	2.9	542.2747	3	27.48	33	F33:1292	OB5944 H6 Ro.raw	1.3154E4	2	2	40	52	Carbamidomethylation	C6:Carbamidomethylation:1000.00	PEAKS DB
R.C(+57.02)MC(+57.02)EALQQIM(+15.99)ENQSDRLQGR.Q	Y	68.85	2482.0820	20	2.2	828.3698	3	31.37	29	F29:1354	OB5922 H2 Ro.raw	5.4499E5	7	7	116	135	Carbamidomethylation; Oxidation (M)	C1:Carbamidomethylation:1000.00;C3:Carbamidomethylation:1000.00;M10:Oxidation (M):99.62	PEAKS DB
R.C(+57.02)DLEVESGGRDRY	Y	66.83	1554.6732	13	-0.6	778.3434	2	27.14	32	F32:1269	OB5943 H6 Ro.raw	1.1586E4	3	3	160	172	Carbamidomethylation	C1:Carbamidomethylation:1000.00	PEAKS DB
R.C(+57.02)C(+57.02)N(+.98)ELNEFENNQR.C	Y	60.97	1726.6675	13	-3.2	864.3383	2	28.65	32	F32:1358	OB5943 H6 Ro.raw	0	0	0	103	115	Carbamidomethylation; Deamidation (NQ)	C1:Carbamidomethylation:1000.00;C2:Carbamidomethylation:1000.00;N3:Deamidation (NQ):22.69	PEAKS DB
R.C(+57.02)C(+57.02)N(-17.03)ELNEFENNQR.C	Y	50.14	1708.6570	13	1.1	855.3367	2	32.90	32	F32:1610	OB5943 H6 Ro.raw	1.9004E4	2	2	103	115	Carbamidomethylation; Ammonia-loss (N)	C1:Carbamidomethylation:1000.00;C2:Carbamidomethylation:1000.00;N3:Ammonia-loss (N):67.21	PEAKS PTM
R.NLPQQC(+57.02)GLR.A	Y	50.09	1084.5448	9	-1.6	543.2788	2	25.64	32	F32:1178	OB5943 H6 Ro.raw	1.8503E4	3	3	147	155	Carbamidomethylation	C6:Carbamidomethylation:1000.00	PEAKS DB
R.C(+57.02)C(+57.02)NELN(+.98)EFENNQR.C	Y	49.85	1726.6675	13	-3.5	864.3380	2	28.36	33	F33:1346	OB5944 H6	0	0	0	103	115	Carbamidomethylation; Deamidation (NQ)	C1:Car bamido	PEAKS DB
**total 18 peptides**																			

Peptide	Uniq	-10lgP	Mass	Length	ppm	m/z	z	RT	Fraction	Scan	Source File	Area Roasted in gel	#Feature	#Feature Roasted in gel	Start	End	PTM	AScore	Found By
											Ro.raw							methylation:1000.00;C2:Carbamidomethylation:1000.00;N6:Deamidation (NQ):26.38	
R.C(+71.04)MC(+57.02)EALQQIMENQSDRLQGR.Q	Y	47.99	2480.1028	20	0.2	827.7084	3	33.64	35	F35:1632	OB5946 H2 Ro.raw	1.3204E5	1	1	116	135	Carbamidomethylation	C1:Propionamide:11.10;C3:Carbamidomethylation:1000.00	PEAKS PTM
R.C(+57.02)M(+15.99)C(+57.02)EALQQIM(+15.99)ENQSDRLQGR.Q	Y	47.72	2498.0771	20	1.5	833.7009	3	30.07	37	F37:1419	OB5949 H3B Ro.raw	1.3961E5	5	5	116	135	Carbamidomethylation; Oxidation (M)	C1:Carbamidomethylation:1000.00;M2:Oxidation (M):1000.00;C3:Carbamidomethylation:1000.00;M10:Oxidation (M):1000.00	PEAKS DB
A.RQQWELQGDRR.C	Y	47.67	1470.7440	11	-0.4	491.2551	3	29.55	29	F29:1249	OB5922 H2 Ro.raw	2.0657E4	3	3	22	32			PEAKS DB
R.C(+57.02)MC(+57.02)EALQQIMEN(+.98)QSDRLQGR.Q	Y	44.70	2467.0713	20	2.3	1234.5490	2	34.84	30	F30:1538	OB5926 H3B Ro.raw	0	0	0	116	135	Carbamidomethylation; Deamidation (NQ)	C1:Carbamidomethylation:1000.00;C3:Carbamidomethylation:1000.00;N12:Deamidation (NQ):27.42	PEAKS DB
R.C(+57.02)C(+57.02)NELN(-17.03)EFENNQR.C	Y	41.93	1708.6570	13	0.8	855.3364	2	32.73	36	F36:1565	OB5948 H3B Ro.raw	0	0	0	103	115	Carbamidomethylation	C1:Carbamidomethylation:1000.00;C2:Carbamidomethylation:1000.00;N6:Ammonia-loss (N):0.00	PEAKS PTM
R.C(+57.02)C(+57.02)N(+.98)ELN(+.98)EFENNQR.C	Y	31.03	1727.6515	13	8.5	864.8404	2	28.91	31	F31:1376	OB5942 H6 Ro.raw	0	0	0	103	115	Carbamidomethylation; Deamidation (NQ)	C1:Carbamidomethyl	PEAKS DB

total 18 peptides

Peptide	Uniq	-10lgP	Mass	Length	ppm	m/z	z	RT	Fraction	Scan	Source File	Area Roasted in gel	#Feature	#Feature Roasted in gel	Start	End	PTM	AScore	Found By
																		ation:1000.00;C2:Carbamidomethylation:1000.00;N3:Deamidation (NQ):28.25;N6:Deamidation (NQ):42.78	
R.C(+43.01)DLEVESGGRDRY	Y	28.80	1540.6576	13	1.4	771.3372	2	26.82	33	F33:1254	OB5944 H6 Ro.raw	0	0	0	160	172	Carbamylation	C1:Carbamylation:1000.00	PEAKS PTM
**total 18 peptides**																			

E9LFE9|E9LFE9_ARAHY

back to list

| Protein Coverage | Supporting Peptides |

Protein Coverage:

1 MGKPVIKPLV VLLLSLILVS ECAAIHSQKR RMGQRVKLSL EECGELEKLE AIEPDYYRIE AEGGVTESWN HTNKMLRCAG
81 VALVRRTVKP GGLVLPSYTN APQLMYYVQG SGIQGMTIFP SCPESFEEPE EAGQEYREQH QKVHEIRGGD IIAIPAGIGY
161 WFYNNGDVPL VVVILLHTNN VANQLGTLPR RFYIAGNTED EHGEGGREKS ISGRNVFSGI SLNLLAQVFG VRVETARKIQ
241 GPDDKRKNIV MVGGRS

d Deamidation (NQ) (+0.98)
n N-Succinimidyl-2-morpholine acetate (+127.06)

216

Supporting Peptides:

Peptide	Uniq	-10lgP	Mass	Length	ppm	m/z	z	RT	Fraction	Scan	Source File	Area Roasted in gel	#Feature	#Feature Roasted in gel	Start	End	PTM	AScore	Found By
R.NVFSGISLNLLAQVFGVR.V	Y	119.29	1933.0785	18	2.9	967.5493	2	42.42	36	F36:2125	OB5948 H3B Ro.raw	1.2301E5	3	3	215	232			PEAKS DB
R.NVFSGISLNLLAQVFGVRVETAR.K	Y	83.20	2489.3755	23	0.1	830.8013	3	41.58	30	F30:1917	OB5926 H3B Ro.raw	9.2573E5	4	4	215	237			PEAKS DB
N.V(+127.06)FSGISLNLLAQVFGVR.V	Y	50.76	1946.0989	17	-8.5	974.0485	2	40.88	36	F36:2037	OB5948 H3B Ro.raw	1.4115E6	3	3	216	232	N-Succinimidyl-2-morpholine acetate	V1:N-Succinimidyl-2-morpholine acetate:1000.00	PEAKS PTM
R.GGDIIAIPAGIGYWFYN(+.98)NGDVPLVVVILLHTNNVANQLGTLPR.R	Y	30.43	4574.4326	43	7.0	1525.8328	3	44.05	30	F30:2059	OB5926 H3B Ro.raw	3.5735E4	1	1	148	190	Deamidation (NQ)	N17:Deamidation (NQ):30.46	PEAKS DB
**total 4 peptides**																			

A0A0A6ZDP1|A0A0A6ZDP1_ARAHY

back to list

| Protein Coverage | Supporting Peptides |

Protein Coverage:

1 MLVHYQIALF HLPRSSTTGL GIVEGLMTTV HSITATQKTV DGPSSKDWRG GRAASFNIIP SSTGAAKAVG KVLPSLNGKL
81 TGMAFRVPTV DVSVVDLTVR LEKPATYDEI KQAIKEESEG KLK**GILGYTE DDVVSTDFVG DSR**SSIFDAK AGIALSKNFV
161 KIVSWYDNEW GYSTRVVDLI VHIAKQ

Supporting Peptides:

Peptide	Uniq	-10lgP	Mass	Length	ppm	m/z	z	RT	Fraction	Scan	Source File	Area Roasted in gel	#Feature	#Feature Roasted in gel	Start	End	PTM	AScore	Found By
K.GILGYTEDDVVSTDFVGDSR.S	Y	128.27	2143.9910	20	-0.3	1073.0024	2	34.39	38	F38:1661	OB5951 H3A Ro.raw	1.1161E4	1	1	124	143			PEAKS DB
**total 1 peptides**																			

E5FHZ1|E5FHZ1_ARAHY

back to list

| Protein Coverage | Supporting Peptides |

Protein Coverage:

1 MSQEQHRRPE ESDPIKYGDV FNVSGELAQK PVKPEDAAMM QSAETRVLGK TQPGGAAAAM QSAATLNEQA GLVGHHDVNE
81 VAGDRGVTVT DMQAPGRR**IV TESVGGQVVG QYVEPTPVQS SR**ASAVR**ESA ITIGEALEAT AQTVGSKPVE QSDAAAIQAA**
161 **EVR**ATGSNVI QPGGLAAMAQ SAVAFNAGCR NPRDKIKLAD ILTGATAKLP ADKAATHQGC

Supporting Peptides:

Peptide	Uniq	-10lgP	Mass	Length	ppm	m/z	z	RT	Fraction	Scan	Source File	Area Roasted in gel	#Feature	#Feature Roasted in gel	Start	End	PTM	AScore	Found By
R.IVTESVGGQVVGQYVEPTPVQSSR.A	Y	92.62	2515.2917	24	3.8	1258.6580	2	31.22	38	F38:1476	OB5951 H3A Ro.raw	3.0299E4	1	1	99	122			PEAKS DB
R.ESAITIGEALEATAQTVGSKPVEQSDAAAIQAAEVR.A	Y	70.43	3610.8325	36	2.4	1204.6210	3	35.92	38	F38:1740	OB5951 H3A Ro.raw	8.9254E5	3	3	128	163			PEAKS DB
R.IVTESVGGQVVGQYVEPTPVQ(+.98)SSR.A	Y	33.87	2516.2759	24	9.4	1259.1571	2	31.76	39	F39:1505	OB5952 H3A Ro.raw	2.7069E4	1	1	99	122		Q21:Deamidation (NQ):0.00	PEAKS DB
**total 3 peptides**																			

N1NKG9|N1NKG9_ARAHY

back to list

| Protein Coverage | Supporting Peptides |

Protein Coverage:

1 MSQEQHRRPE ESDPIKYGDV FNVSGELAQK PVKPEDAAMM QSAETRVLGK TQPGGAAAAM QSAATLNEQA GLVGHHDVNE
81 VAGDRGVTVT DMQAPGRR**IV TESVGGQVVG QYVEPTPVQS SR**ASAVR**ESA ITIGEALEAT AQTVGSKPVE QSDAAAIQAA**
161 **EVR**ATGSNVI QPGGLAAMAQ SAVAFNAGCR NPRDKIKLAD ILTGATAKLP ADKAATHQDA EGVASAEARN NTGGTTPGGV
241 AASVAAAARL NENVNV

Supporting Peptides:

Peptide	Uniq	-10lgP	Mass	Length	ppm	m/z	z	RT	Fraction	Scan	Source File	Area Roasted in gel	#Feature	#Feature Roasted in gel	Start	End	PTM	AScore	Found By
R.IVTESVGGQVVGQYVEPTPVQSSR.A	Y	92.62	2515.2917	24	3.8	1258.6580	2	31.22	38	F38:1476	OB5951 H3A Ro.raw	3.0299E4	1	1	99	122			PEAKS DB
R.ESAITIGEALEATAQTVGSKPVEQSDAAAIQAAEVR.A	Y	70.43	3610.8325	36	2.4	1204.6210	3	35.92	38	F38:1740	OB5951 H3A Ro.raw	8.9254E5	3	3	128	163			PEAKS DB
**total 3 peptides**																			

Peptide	Uniq	-10lgP	Mass	Length	ppm	m/z	z	RT	Fraction	Scan	Source File	Area Roasted in gel	#Feature	#Feature Roasted in gel	Start	End	PTM	AScore	Found By
R.IVTESVGGQVVGQYVEPTPVQ(+.98)SSR.A	Y	33.87	2516.2759	24	9.4	1259.1571	2	31.76	39	F39:1505	OB5952 H3A Ro.raw	2.7069E4	1	1	99	122		Q21:Deamidation (NQ):0.00	PEAKS DB
total 3 peptides																			

E5FHY1|E5FHY1_ARAHY

back to list

| Protein Coverage | Supporting Peptides |

Protein Coverage:

1 MASKQRQDLN EKAKQGETVV PGGTGGKTLE AQEHLAEGRS KGGQTRKEQL GTEGYQEMGR KGGLSTMEKS GGERAEEEGV

81 EIDESKFATK NQNK

Supporting Peptides:

Peptide	Uniq	-10lgP	Mass	Length	ppm	m/z	z	RT	Fraction	Scan	Source File	Area Roasted in gel	#Feature	#Feature Roasted in gel	Start	End	PTM	AScore	Found By
K.EQLGTEGYQEMGR.K	N	61.85	1496.6565	13	0.6	749.3359	2	27.83	33	F33:1314	OB5944 H6 Ro.raw	0	0	0	48	60			PEAKS DB
K.SGGERAEEEGVEIDESKFATK.N	N	55.33	2267.0552	21	1.0	756.6931	3	28.68	32	F32:1360	OB5943 H6 Ro.raw	1.288E4	2	2	70	90			PEAKS DB
K.TLEAQEHLAEGR.S	Y	40.13	1352.6685	12	2.1	677.3429	2	26.59	32	F32:1236	OB5943 H6 Ro.raw	0	0	0	28	39			PEAKS DB
total 3 peptides																			

E5FHY2|E5FHY2_ARAHY

back to list

| Protein Coverage | Supporting Peptides |

Protein Coverage:

1 MASKQQKQEL DERAKQGETV VPGGTGGKSL EAQEHLAEGR SKGGQTRREQ LGTEGYQEMG RKGGFSTMEK SGGERAEEEG

81 VEIDESKFVT KKPKQVVP

Supporting Peptides:

Peptide	Uniq	-10lgP	Mass	Length	ppm	m/z	z	RT	Fraction	Scan	Source File	Area Roasted in gel	#Feature	#Feature Roasted in gel	Start	End	PTM	AScore	Found By
R.EQLGTEGYQEMGR.K	N	61.85	1496.6565	13	0.6	749.3359	2	27.83	33	F33:1314	OB5944 H6 Ro.raw	0	0	0	49	61			PEAKS DB
K.SLEAQEHLAEGR.S	N	50.96	1338.6527	12	-0.2	670.3335	2	26.22	31	F31:1219	OB5942 H6 Ro.raw	3.9095E3	2	2	29	40			PEAKS DB
K.SGGERAEEEGVEIDESKFVTK.K	Y	45.08	2295.0867	21	0.9	766.0369	3	29.51	32	F32:1403	OB5943 H6 Ro.raw	1.1991E4	1	1	71	91			PEAKS DB
total 3 peptides																			

Q42515|Q42515_ARAHY

back to list

| Protein Coverage | Supporting Peptides |

Protein Coverage:

1 MKFCNFFTFT LALIVVLQAL GASADDAGTI ITQPLYNEFL KHLTDSRCEA HGFYTYNAFV TAARAFPAFG TTGDDVTRKR

81 ELAAFFGQTS HETTGGTTNA PDEFEWGYCF LREQTKEQHC DSTQAPCPAG KQYYGRGPIQ LTSNSNYQLA GQAIKADLIN

161 NPDLVATDAV ISFKTAIWFW MTPQGNKPSC HDVITNAWRP TATDSAAGRA PGYGVITNII NGGIECGKGA NTNSNNRIGF

241 YKRYCDILKI GYGSNLDCAN QQHF

Supporting Peptides:

Peptide	Uniq	-10lgP	Mass	Length	ppm	m/z	z	RT	Fraction	Scan	Source File	Area Roasted in gel	#Feature	#Feature Roasted in gel	Start	End	PTM	AScore	Found By
A.DDAGTIITQPLYNEFLK.H	Y	57.78	1936.9781	17	2.4	969.4987	2	37.23	37	F37:1836	OB5949 H3B Ro.raw	2.7447E4	1	1	25	41			PEAKS DB
R.GPIQLTSNSNYQLAGQAIK.A	Y	53.96	2002.0483	19	5.5	1002.0396	2	31.80	30	F30:1360	OB5926 H3B Ro.raw	0	0	0	137	155			PEAKS DB
total 2 peptides																			

B4UWB2|B4UWB2_ARAHY

back to list

| Protein Coverage | Supporting Peptides |

Protein Coverage:

```
  1  MKSSSSVIAA MICFAMVTGM AASAAEPVLD VTGQKLRSGV KYFILPVLRG RGGGLTVGSS VNSTCPIYVL QDKLEVTRGT
 81  PVTFTPSTPN KDGVILTSTD LNINSTSAPK CKESSVWRLL KVLSGVWFIS TDGVAGNPGV NTVVNWFKIE KDGKDYNLSF
161  CPSVCNCSTL CRALGIFTDS DGTKHLALSD QVPTFRSCSR RLN
```

Supporting Peptides:

Peptide	Uniq	-10lgP	Mass	Length	ppm	m/z	z	RT	Fraction	Scan	Source File	Area Roasted in gel	#Feature	#Feature Roasted in gel	Start	End	PTM	AScore	Found By
K.YFILPVLR.G	Y	83.50	1019.6168	8	0.0	510.8157	2	36.39	29	F29:1656	OB5922 H2 Ro.raw	3.6559E4	2	2	42	49			PEAKS DB
total 1 peptides																			

A7LIS5|A7LIS5_ARAHY

back to list

| Protein Coverage | Supporting Peptides |

Protein Coverage:

```
  1  MKSMRIHVLF LFSLLSTIPH YSYASVNDFC VADLKGPDSP SGYNCKPVSA VTADDFVYSG LVAGDTNNTF KAALTSAFVT
 81  DFPAVNGLGV SAARLDIAKG GSIPMHTHPG ATELLIMVEG EITAGFMTPF ALYSKTLKPG DVFVFPQGQL HFQVNSGKKK
161  ATAFLAFSSA NPGAQLLDLL LFGNALSSDI VAQTTFLDID QVKKLKARFG GKNLNNHMSM
```

Supporting Peptides:

Peptide	Uniq	-10lgP	Mass	Length	ppm	m/z	z	RT	Fraction	Scan	Source File	Area Roasted in gel	#Feature	#Feature Roasted in gel	Start	End	PTM	AScore	Found By
K.AALTSAFVTDFPAVNGLGVSAAR.L	Y	75.04	2234.1694	23	-0.7	1118.0941	2	36.71	30	F30:1645	OB5926 H3B Ro.raw	5.6415E3	1	1	72	94			PEAKS DB
total 1 peptides																			

D4NXQ0|D4NXQ0_ARAHY

back to list

| Protein Coverage | Supporting Peptides |

Protein Coverage:

```
  1  MKSMRIHVLF LFSLLSTIPH YSYASVNDFC VADLKGPDSP SGYNCKPVSA VTADDFVYSG LVAGDTNNTF KAALTSAFVT
 81  DFPAVNGLGV SAARLDIAKG GSIPMHTHPG ATELLIMVEG EITAGFMTPF ALYSKTLKPG DVFVFPQGQL HFQVNSGKKK
161  ATAFLAFSSA NPGAQLLDLL LFGNALSSDI VAQTTFLDID QVKKLKARFG GKNLSKHMSM
```

Supporting Peptides:

Peptide	Uniq	-10lgP	Mass	Length	ppm	m/z	z	RT	Fraction	Scan	Source File	Area Roasted in gel	#Feature	#Feature Roasted in gel	Start	End	PTM	AScore	Found By

Peptide	Uniq	-10lgP	Mass	Length	ppm	m/z	z	RT	Fraction	Scan	Source File	Area Roasted in gel	#Feature	#Feature Roasted in gel	Start	End	PTM	AScore	Found By
K.AALTSAFVTDFPAVNGLGVSAAR.L	Y	75.04	2234.1694	23	-0.7	1118.0941	2	36.71	30	F30:1645	OB5926 H3B Ro.raw	5.6415E3	1	1	72	94			PEAKS DB
**total 1 peptides**																			

H6U596|H6U596_ARAHY

back to list

| Protein Coverage | Supporting Peptides |

Protein Coverage:

```
  1   SPLFPRIFGH EAGGIVESVG EGVTHLKPGD HALPVFTGEC GECPHCKSEE SNMCDLLRIN TDRGVMIHDQ ESRFSIKGKP
 81   IYHFVGTSTF SEYTVVHAGC VAKINPEAPL DKVCVLSCGI CTGLGATINV AKPKPGSSVA VFGLGAVGLA AAEGARISGA
161   SRIIGVDLVS SRFELAKKFG VNEFV
```

Supporting Peptides:

Peptide	Uniq	-10lgP	Mass	Length	ppm	m/z	z	RT	Fraction	Scan	Source File	Area Roasted in gel	#Feature	#Feature Roasted in gel	Start	End	PTM	AScore	Found By
R.IIGVDLVSSRFELAK.K	Y	54.03	1645.9402	15	2.4	823.9794	2	34.15	39	F39:1640	OB5952 H3A Ro.raw	6.1243E3	1	1	163	177			PEAKS DB
**total 1 peptides**																			

A0A191UJ92|A0A191UJ92_ARAHY

back to list

| Protein Coverage | Supporting Peptides |

Protein Coverage:

```
  1   MTRIKRGYIA RKRRTKIRLF TSSFRGAHSR LTRTISQQKI KALVSANRDR DRKKRGFRGL WIHRINAIIG KNKKKQYNRY
 81   SNLLYNMYKG QLLLNRKIVA QMGILKGNCL LMIANEIIT
```

Supporting Peptides:

Peptide	Uniq	-10lgP	Mass	Length	ppm	m/z	z	RT	Fraction	Scan	Source File	Area Roasted in gel	#Feature	#Feature Roasted in gel	Start	End	PTM	AScore	Found By
K.ALVSANRDR.D	Y	38.97	1000.5414	9	-7.6	501.2742	2	31.38	32	F32:1526	OB5943 H6 Ro.raw	6.2701E3	1	1	42	50			PEAKS DB
**total 1 peptides**																			

E5FHZ2|E5FHZ2_ARAHY

back to list

| Protein Coverage | Supporting Peptides |

Protein Coverage:

```
  1   MSQQQTPRRP QEEGQEHKDP IKYGDIFNVS GDLASEPIMP QDAALMQATE NQALGQTQKG GPASVMQSAA VTNVSAGLVG
 81   RGDVSSVAKD QGICVLESKI GVNRVITESV GEQVVGEFVE PNVPMKTPGS ALDKDAITIG EALEATTLAP ASDKPVDQSD
161   AAAIQAAEMR ATGKNEIQPG GLAAVAQSAA TKNLRTMGDF QKTTLSDVLT DAKEKLPSDK AVTREDAEGV IGAEIRNKAD
241   MKTTPGGVAA SLAAAATLNQ NKSSS
```

Supporting Peptides:

Peptide	Uniq	-10lgP	Mass	Length	ppm	m/z	z	RT	Fraction	Scan	Source File	Area Roasted in gel	#Feature	#Feature Roasted in gel	Start	End	PTM	AScore	Found By
K.TTPGGVAASLAAAATLNQNK.S	Y	38.10	1854.9799	20	2.9	928.4999	2	33.28	40	F40:1592	OB5953 H3A Ro.raw	5.6109E3	1	1	243	262			PEAKS DB
**total 1 peptides**																			

A0A075M4V7|A0A075M4V7_ARAHY

back to list

| Protein Coverage | Supporting Peptides |

Protein Coverage:

1 PAFTTVNRAS PAQANMVAPF SGLKSLGGFP VTRKTNDITS VANNGGRVQC MQVWPPLGKK KFETLSYLPD LNEEQLR**KEV**

81 **DYLLR**KGWVP CLEFEVEYGF PYRENHRSPG YYDGRYWTMW KLPMFGCTDS GQVIKELQEA KAAYPKAFV

Supporting Peptides:

Peptide	Uniq	-10lgP	Mass	Length	ppm	m/z	z	RT	Fraction	Scan	Source File	Area Roasted in gel	#Feature	#Feature Roasted in gel	Start	End	PTM	AScore	Found By
R.KEVDYLLR.K	Y	33.37	1034.5760	8	0.2	518.2954	2	31.00	39	F39:1454	OB5952 H3A Ro.raw	2.6266E4	1	1	78	85			PEAKS DB
total 1 peptides																			

A0A075M514|A0A075M514_ARAHY

back to list

| Protein Coverage | Supporting Peptides |

Protein Coverage:

1 PAFTAVNRAS PAQANMVAPF SGLKSLGGFP VTRKTNDITS VANNGGRVQC MQVWPPLGKK KFETLSYLPD LNEEQLR**KEV**

81 **DYLLR**KGWVP CLEFEVEYGF PYRENHRSPG YYDGRYWTMW KLPMFGCTDS GQVIKELQEA KAAYPKAFV

Supporting Peptides:

Peptide	Uniq	-10lgP	Mass	Length	ppm	m/z	z	RT	Fraction	Scan	Source File	Area Roasted in gel	#Feature	#Feature Roasted in gel	Start	End	PTM	AScore	Found By
R.KEVDYLLR.K	Y	33.37	1034.5760	8	0.2	518.2954	2	31.00	39	F39:1454	OB5952 H3A Ro.raw	2.6266E4	1	1	78	85			PEAKS DB
total 1 peptides																			

A0A075M9B7|A0A075M9B7_ARAHY

back to list

| Protein Coverage | Supporting Peptides |

Protein Coverage:

1 PAFTAVNRAS PAQANMVAPF SGLKSLGGFP VTRKTNDITS VTNNGGRVQC MQVWPPLGKK KFETLSYLPD LNEEQLR**KEV**

81 **DYLLR**KGWVP CLEFEVEYGF PYRENHRSPG YYDGRYWTMW KLPMFGCTDS GQVIKELQEA KAAYPKAFVR IIGFDNVRQ

Supporting Peptides:

Peptide	Uniq	-10lgP	Mass	Length	ppm	m/z	z	RT	Fraction	Scan	Source File	Area Roasted in gel	#Feature	#Feature Roasted in gel	Start	End	PTM	AScore	Found By
R.KEVDYLLR.K	Y	33.37	1034.5760	8	0.2	518.2954	2	31.00	39	F39:1454	OB5952 H3A Ro.raw	2.6266E4	1	1	78	85			PEAKS DB
total 1 peptides																			

A0A075M3Q0|A0A075M3Q0_ARAHY

back to list

| Protein Coverage | Supporting Peptides |

Protein Coverage:

1 PAFTTVNRAS PAQANMVAPF SGLKSLGGFP VTRKTNDITS VANNGGRVQC MQVWPPLGKK KFETLSYLPD LNEEQLR**KEV**

81 **DYLLR**KGWVP CLEFEVEYGF PYRENHRSPG YYDGRYWTMW KLPMFGCTDS GQVIKELQEA KAAYPKAFVR IIGFDNVRQ

Supporting Peptides:

Peptide	Uniq	-10lgP	Mass	Length	ppm	m/z	z	RT	Fraction	Scan	Source File	Area Roasted in gel	#Feature	#Feature Roasted in gel	Start	End	PTM	AScore	Found By
total 1 peptides																			

Peptide	Uniq	-10lgP	Mass	Length	ppm	m/z	z	RT	Fraction	Scan	Source File	Area Roasted in gel	#Feature	#Feature Roasted in gel	Start	End	PTM	AScore	Found By
R.KEVDYLLR.K	Y	33.37	1034.5760	8	0.2	518.2954	2	31.00	39	F39:1454	OB5952 H3A Ro.raw	2.6266E4	1	1	78	85			PEAKS DB
total 1 peptides																			

A0A075M506|A0A075M506_ARAHY

back to list

| Protein Coverage | Supporting Peptides |

Protein Coverage:

1 PAVTTVSRAS PAQANMVAPF TGLKSLAGFP VTRKANNDIT SVANNGGRVQ CMQVWPPVGK KKFETLSYLP DLTDEQLR**KE**

81 **VDYLLR**KGWV PCLEFELEHG FVYRENHRSP GYYDGRYWTM WKLPMFGCTD SGQVIKELQE AKAAYPSAFI RIIGFDNVRQ

Supporting Peptides:

Peptide	Uniq	-10lgP	Mass	Length	ppm	m/z	z	RT	Fraction	Scan	Source File	Area Roasted in gel	#Feature	#Feature Roasted in gel	Start	End	PTM	AScore	Found By
R.KEVDYLLR.K	Y	33.37	1034.5760	8	0.2	518.2954	2	31.00	39	F39:1454	OB5952 H3A Ro.raw	2.6266E4	1	1	79	86			PEAKS DB
total 1 peptides																			

T2BAU5|T2BAU5_ARAHY

back to list

| Protein Coverage | Supporting Peptides |

Protein Coverage:

1 MAISMISSPA VTTVSRASPA QANMVAPFTG LKSLAGFPVT RKANNDITSV ANNGGRVQCM QVWPPVGKKK FETLSYLPDL

81 TDEQLR**KEVD YLLR**KGWVPC LEFEVGYGFP YRENHRSPGY YDGRYWTMWK LPMFGCTDSG QVIKELQEAK AAYPKAFVRI

161 IGFDNVRQVQ CISFIAYKPG GY

Supporting Peptides:

Peptide	Uniq	-10lgP	Mass	Length	ppm	m/z	z	RT	Fraction	Scan	Source File	Area Roasted in gel	#Feature	#Feature Roasted in gel	Start	End	PTM	AScore	Found By
R.KEVDYLLR.K	Y	33.37	1034.5760	8	0.2	518.2954	2	31.00	39	F39:1454	OB5952 H3A Ro.raw	2.6266E4	1	1	87	94			PEAKS DB
total 1 peptides																			

V5T718|V5T718_ARAHY

back to list

| Protein Coverage | Supporting Peptides |

Protein Coverage:

1 MVSKNPNPPE GFYLDPTGAA LPGLAPFASV TAAATATAVS APNSAEDAAK KIRKPYTITK SRENWTEPEH DKFLEALQLF

81 DRDWKKIGAF VGSKTVIQIR SHAQKYFIKV QKSGTSEHLP PPRPKRKAAH PYPQKASKNA PLLSQLSESF QSSSALLESG

161 YVLKHDPSTM LKTPIMNTVV PSWSNSTLQT TNLLHVPKVN DPSGSSESTP RVQPGDESNG QGNSSFPLRV LPDFTQVYSF

241 IGSVFDPNTT DHLHKLK**NMD RIDVETVLLL MR**NLSVNLTS PDFEDHRKVL STYEVELDTD DYINADILQD DQLKSAAT

Supporting Peptides:

Peptide	Uniq	-10lgP	Mass	Length	ppm	m/z	z	RT	Fraction	Scan	Source File	Area Roasted in gel	#Feature	#Feature Roasted in gel	Start	End	PTM	AScore	Found By
K.NMDRIDVETVLLLMR.N	Y	32.27	1816.9539	15	3.3	909.4872	2	64.32	41	F41:3492	OB5954 H1 Ro.raw	1.384E4	1	1	258	272			PEAKS DB
total 1 peptides																			

V5T7W3|V5T7W3_ARAHY

back to list

| Protein Coverage | Supporting Peptides |

Protein Coverage:

```
  1  MDRVKGPWSP EEDETLRRLV GAHGPRNWTI ISKSIPGRSG KSCRLRWCNQ LSPEVEHRAF TPEEDDIIVR AHAEFGNKWA
 81  TIARLLNGRT DNAVKNHWNS TLKRKCRSSA SDAVTVAVYP DAQPLKRSAS VGPCHVTTPS SPSGSELSDP GLPSLSAPGS
161  DPRTLLSLSL PGSSGSGAGS DSIKDGLGSG LSPDPDPAQM FSPEFLAMMQ EMVRKEVRNY MSMVEENGVR MQQTEAIRNG
241  VMKRMGIRNG IER
```

Supporting Peptides:

Peptide	Uniq	-10lgP	Mass	Length	ppm	m/z	z	RT	Fraction	Scan	Source File	Area Roasted in gel	#Feature	#Feature Roasted in gel	Start	End	PTM	AScore	Found By
R.TLLSLSLPGSS.G	Y	30.31	1073.5968	11	-0.3	537.8055	2	29.85	34	F34:1402	OB5945 H2 Ro.raw	1.5963E4	1	1	164	174			PEAKS DB
**total 1 peptides**																			

Peptide List
