# Supplementary material for: Proteomic Profiling of Major Peanut Allergens and Their Post-Translational Modifications Affected by Roasting
Source: Foods. 2022 Dec 9;11(24):3993. doi: 10.3390/foods11243993 (PMC9778155; doi:10.3390/foods11243993)
Supplement: Supplementary file 1 [file foods-11-03993-s001.zip › Supplementary material.pdf]

Supplementary online material

Proteomic profiling of major peanut allergens and their post-translational modifications affected by roasting

Teodora Đukić[1], Katarina Smiljanić[1], Jelena Mihailović[1], Ivana Prodić[2], Danijela Apostolović[3], Shu-Hua Liu[4], Michelle M. Epstein[4], Marianne van Hage[3], Dragana Stanić-Vučinić[1], Tanja Ćirković Veličković[1,5,6,7,*]

[1]University of Belgrade - Faculty of Chemistry, Department of Biochemistry and Centre of Excellence for Molecular Food Sciences, Studentski Trg 12-14, 11000, Belgrade, Serbia
[2]University of Belgrade - Faculty of Chemistry, Innovation Centre Ltd. Studentski Trg 12-14, 11000, Belgrade, Serbia
[3]Department of Medicine Solna, Division of Immunology and Allergy, Karolinska Institutet and Karolinska University Hospital, SE-171 76 Stockholm, Sweden
[4]Medical University of Vienna Department of Dermatology, Experimental Allergy Laboratory Waehringer Guertel 18-20, room 7H.9.11 1090 Vienna, Austria
[5]Ghent University Global Campus, Incheon, South Korea, Yeonsu-gu, Songdo 1(il)-dong, 송도문화로 119
[6]Faculty of Bioscience Engineering, Ghent University, Coupure Links 653, 9000, Ghent, Belgium
[7]Serbian Academy of Sciences and Arts, Belgrade, Serbia
[*]Correspondence: tcirkov@chem.bg.ac.rs; tanja.velickovic@ghent.ac.kr; Tel.: +381113336608

Materials and Methods

Patient's cohort and ethics statement

Sera from 10 Swedish peanut-sensitised patients with IgE levels to whole peanut extract (range 11–415 kU_A/L; median 57 kU_A/L), Ara h 1 (range <0.1–96 kU_A/L; median 7.8 kU_A/L), Ara h 2 (range 0.14–192 kU_A/L; median 30 kU_A/L) and Ara h 3 (range <0.1–52 kU_A/L; median 1.6 kU_A/L) (Phadia/Thermo Fisher Scientific, Uppsala, Sweden), were selected at the Department of Clinical Immunology, Karolinska University Hospital, Stockholm (Table S1). Data were processed and stored according to the principles of the Declaration of Helsinki. The local ethics committee approved the study of Karolinska Institute (No. 20112085-314 and 2016/1348-32), and all experiments were per relevant guidelines and regulations. Sample collection was blinded.

Table S1. IgE levels of peanut-sensitised patients determined by ImmunoCAP in kU_A/L

Patient's ID	Whole peanut extract	rAra h 1	rAra h 2	rAra h 3
1	415	96	192	52
2	11	<0.10	5	<0.10
3	65	12.4	36	6.4
4	48	14	20	2.6
5	34	2.6	24	0.66
6	152	2.4	78	<0.20
7	218	92	68	34
8	225	66	63	3.9
9	23	0.19	0.24	0.58
10	11	3.2	0.14	<0.10

Peanut thermal treatment and extract preparation

Preparation of the insoluble fractions of raw and roasted peanuts was done as described in Prodić et al., 2018 [21]. Peanuts were ground in a blender and defatted by lipid extraction with petroleum ether (1:1, w/v). The peanut powder obtained was air-dried overnight. 30 mg of defatted and non-defatted peanut flour was mixed with Laemmli buffer overnight to extract the full peanut protein profile. The suspension was centrifuged at 13000 rpm for 5 minutes, and the supernatant was analysed on 14% SDS-PAGE. Also, another portion of proteins was extracted by suspending 10 g of peanut powder in 100 mL of phosphate-buffered saline (PBS, 1/10, v/v) with a protease inhibitor cocktail (P2714, Sigma-Aldrich, Germany), providing extract (~100 mL) and pellet (~10 g). Later, the extract was clarified by mixing with dichloroethylene (1:2, v/v) and centrifuged at 13 000 rpm for 5 minutes. The organic layer, after clarification of the extract, was discarded. Pellets were subjected to extraction with a denaturing buffer (25 mL; 7M urea, 2M Thiourea, 2% CHAPS, 1% Triton X-100 in 200 mM $NaHCO_3$, pH 8.0). Both extract and pellet extracts were resolved by SDS-PAGE, according to the volume. Volumes were adjusted with the Laemmli buffer to a final concentration of 1x. The procedure was repeated for raw and roasted peanuts in the same way.

Identification of major peanut allergens and their post-translational modifications (PTMs)

Peanut proteins were identified using the PEAKS XPro platform (Bioinformatics Solutions Inc., Canada). To identify peanut proteins, signature MS/MS spectra were searched using the PEAKS DB and PTM algorithms against a database consisting of UniProtKB (http://www.uniprot.org/) *Arachis hypogaea* entries (taxon ID 3818, 98981 sequences, accessed 18/10/2019), and contamination database comprised of common Repository of Adventitious Protein entries (http://www.thegpm.org/) (116 sequences, accessed 18/10/2019). Carbamidomethylation (Cys) was fixed, while oxidation (Met), deamidation (Gln, Asn), acetylation (*N*-terminus), and hydroxylation (Pro) were considered variable modifications. Post-translational modifications (PTMs) of peanut proteins were identified from an available Unimod database list of 313 naturally occurring PTM items using the PEAKS PTM algorithm. Two trypsin-missed cleavages were allowed with non-specific cleavage on one end of the peptide. Only proteins with the following criteria were considered: protein score ($-10 \times \log P$) ≥ 20, unique peptides ≥ 2, and false discovery rate for peptide-spectrum matches $< 1\%$. Up to five variable modifications were allowed per peptide. Confident PTM identification was ensured by setting the AScore to at least 50. Ascore calculates an ambiguity score of -10*log10P, where P is the probability value, which indicates the likelihood that the peptide is matched by chance.

Post-translational modification detection, PTM pattern and profiling employment by PEAKS Studio X pro

After the PEAKS DB (database search) with only a few variable modifications elected, the PEAKS PTM search was done to get the most out of mass spectrometry data when modifications are concerned. An Open PTMs search was performed with 313 post-translational and chemical modifications checked as variable modifications. Upon manual inspection of results, several modifications were filtered out because they are considered artefacts (e.g. sodium adducts, iodination, dethiomethyl, carbamoylation on N-term of amino

acids if it follows the trypsin cleavage, etc.) or are a result of sample preparation (e.g. propionamide, dethiomethylation, modifications of terminal residues). Carbamoylation was considered because samples were prepared through in-gel methodology without adding urea. To gain insight into patterns that modifications form along the amino acid sequence, acquired files (.raw) were organised as fractions of 2 groups (for each allergen gel band) – raw and roasted, each consisting of three technical replicates in-gel prepared samples (from raw and roasted kernels, respectively). This strategy results in an overview of all modifications present on peanut proteins and gives insight into pattern differences arising from roasting peanut kernels.

After the determination of PTM patterns, PTM profiling was done. PTM profiling provides a relative ratio of the modified peptide to the unmodified peptide between two samples. With this approach, we can conclude what AA sites are modified compared to the same AA sites within the specified peptide sequence in the other sample. In addition, 100% identical protein peanut isoforms were manually filtered out after the BLAST analysis in both samples.

2D spot quantification by Image Master 2DE Platinum 7.0 software

2D PAGE of raw and roasted peanut extracts was performed in 3-10 NL pH range with 250 µg applied per strip and resolved in reducing conditions in the second dimension (Figure 1c). Protein spots were detected, matched, and quantified between the two gels using Image Master 2DE Platinum 7.0 software (GE Healthcare, USA) (Figure S1). Spot detection algorithm quantifies spots by computing the amount of protein present in each spot as a product of its intensity and area and by combining them into volume (Figure S2). Spot volumes - product of pixel intensities and the spot boundary (area), are expressed with background subtracted. The volume of a spot (Vol) is calculated as the volume above the spot outline, which is situated at 75% of the spot height (as measured from the peak of each spot). The background is subtracted on a spot-specific basis by excluding the lowest 10th percentile pixel value on the spot boundary from all other pixel values within the spot boundary. Each of the studied allergens is represented by several proteoforms i.e. several spots (see Figure 1c).

Results

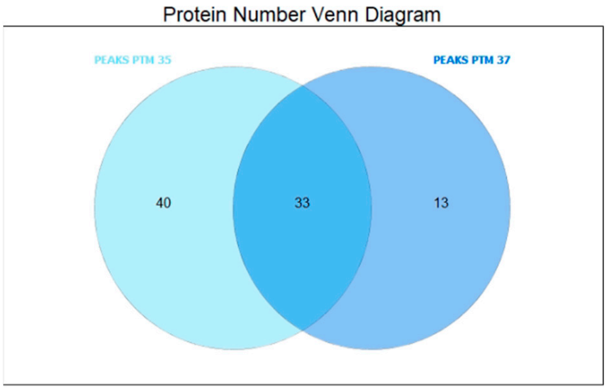

Figure S1. Venn diagram of all raw and roasted peanut gel bands that were processed in MudPIT manner, showing the number of unique proteins found in each sample, including the shared ones.

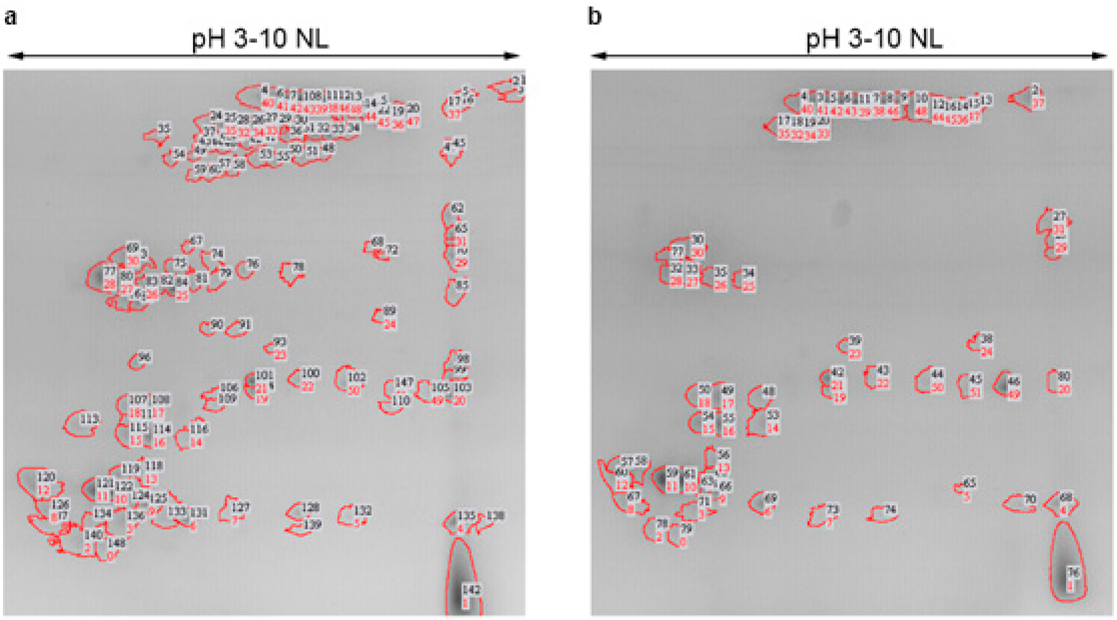

Figure S2. 2D SDS-PAGE protein profiles of raw (a) and roasted (b) peanut extracts with spots and spot matches numbered. Peanut extracts (250 µg per strip) were resolved in 3-10 non-linear (NL) pH range on 13 cm IPG strips and subsequently on 14% PAA in reducing conditions. Spots were detected by Image Master 2D Platinum 7.0 (GE Life sciences, USA) and outlined in red, each spot ID is shown black, and match IDs are shown in red.

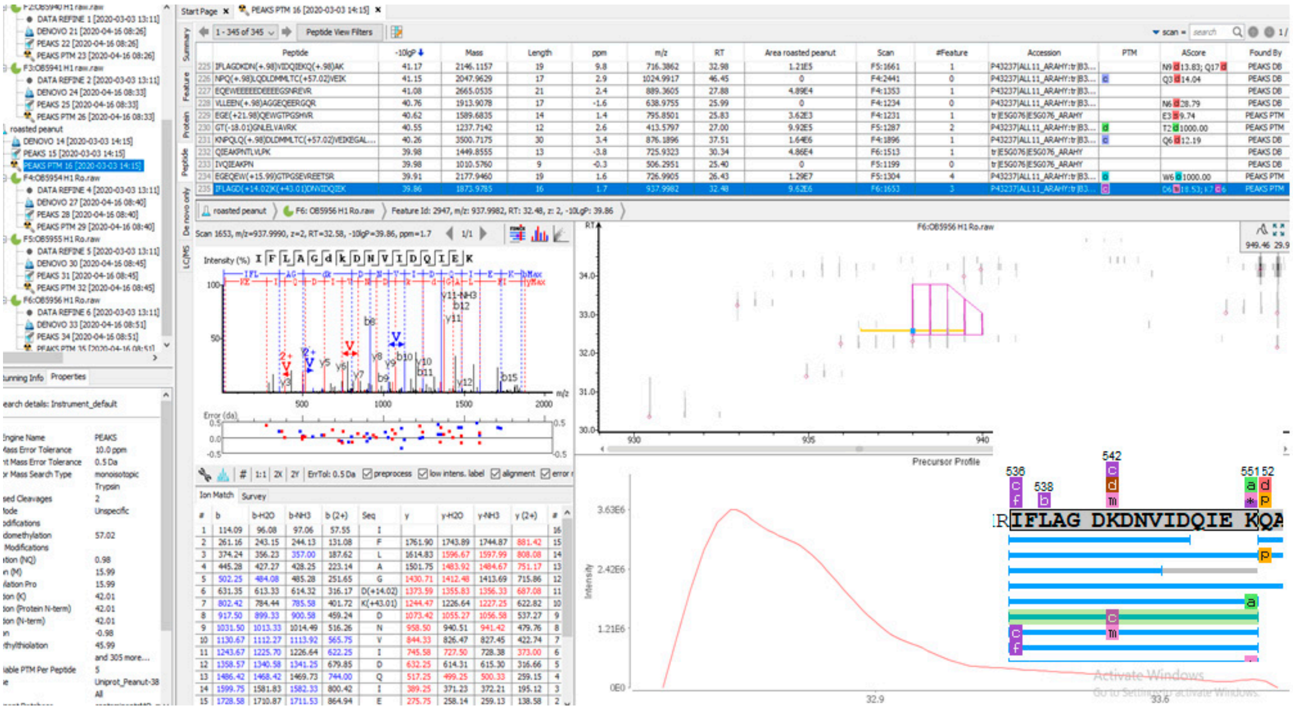

Figure S3. Overview of MS2 spectrum of the peptide with K542 carbamoylation modification on Ara h 1 isoform E5G076, containing a confident modification with AScore of 62.7 in roasted PE (XIC area non-normalised 9.62E6). The same peptide exists in the raw Ara h 1, with AScore 57.6 and a non-normalised XIC area of 4.01E6.

References

21. Prodić, I.; Stanić-Vučinić; D., Apostolović D.; Mihailović, J.; Radibratović, M.; Radosavljević, J.; Burazer, L.; Milčić, M.; Smiljanić, K.; van Hage, M.; Ćirkovic Veličković, T. Influence of peanut matrix on stability of allergens in gastric-simulated digesta: 2S albumins are main contributors to the IgE reactivity of short digestion-resistant peptides. *Clin. Exp. Allergy*. **2018**, 48, 731-740. https://doi.org/10.1111/cea.13113
